# Supplementary figures and images for: Pharmacophagy in green lacewings (Neuroptera: Chrysopidae: Chrysopa spp.)?
Source: PeerJ. 2016 Jan 18;4:e1564. doi: 10.7717/peerj.1564 (PMC4727961; doi:10.7717/peerj.1564)

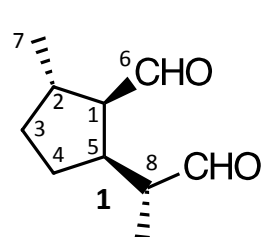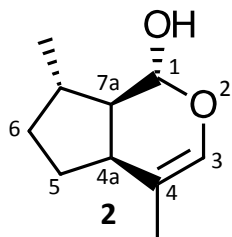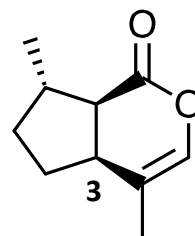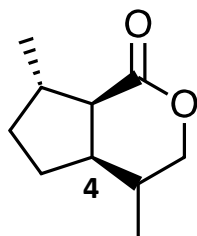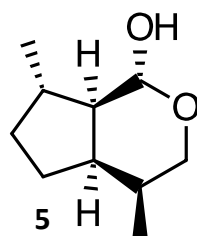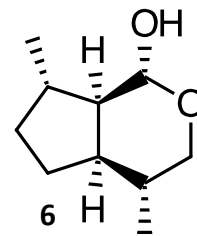

Supplement: Figure S1 — 1: (1R,2S,5R, 8R)-iridodial, 2: (1R,4S,4aR,7S,7aR)-dihydronepetalactol, 3: (4aS,7S,7aR)-nepetalactone, 4: dihydronepetalactone, 5: (1R,4S,4aR,7S,7aR)-dihydronepetalactol, 6: (1R,4R,4aR,7S,7aR[i])-dihydronepetalactol. [file peerj-04-1564-s001.pdf]

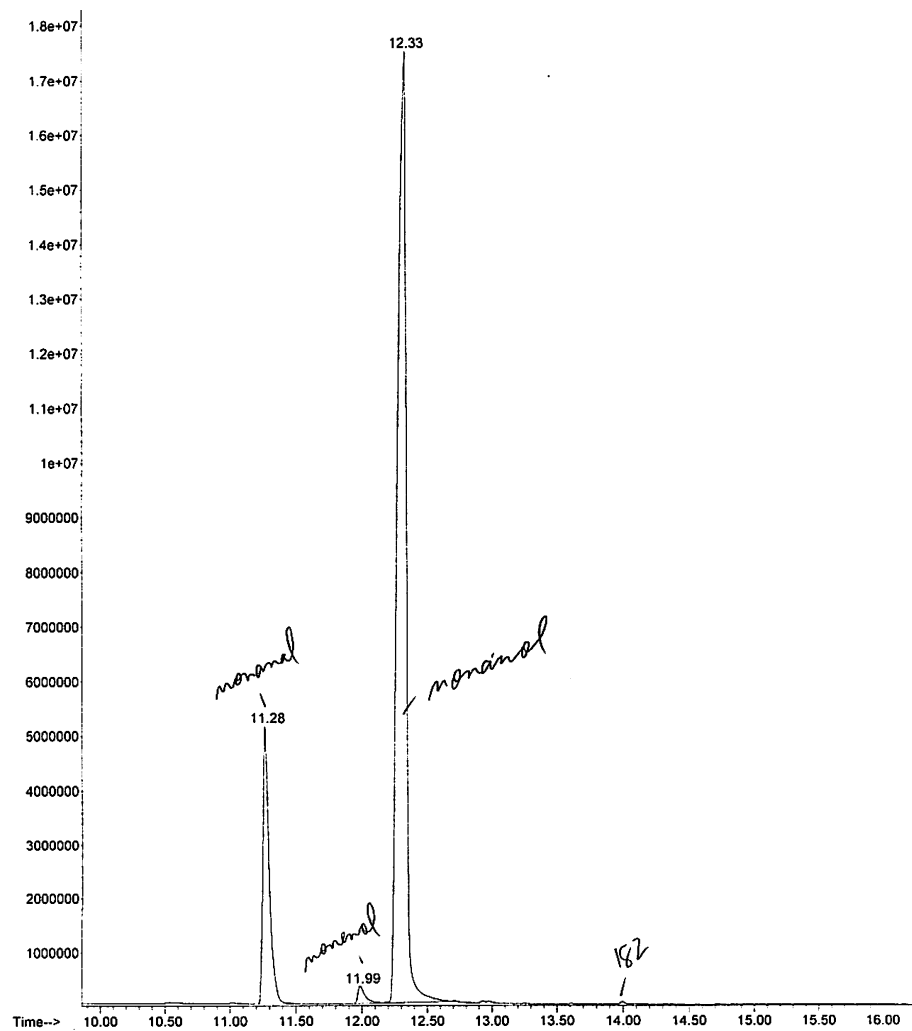

Supplement: Figure S2 — The antennae of eight 1–5-day-old males were removed, and a pooled extract of these males was prepared 13 days later for GC-MS analysis; no iridodial was detected. [file peerj-04-1564-s002.pdf]

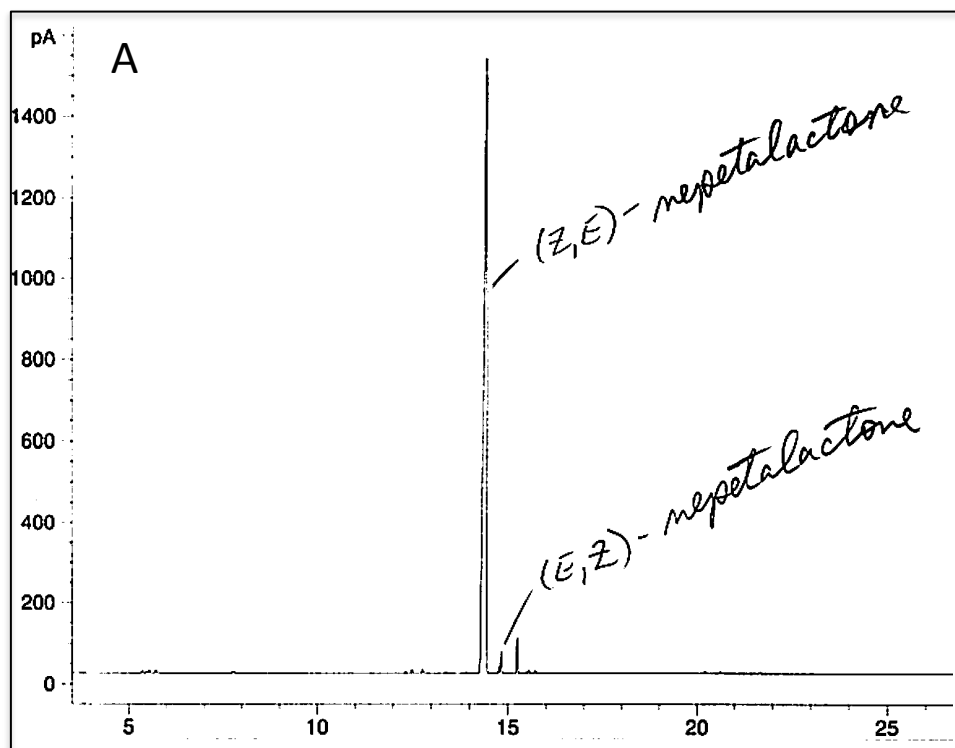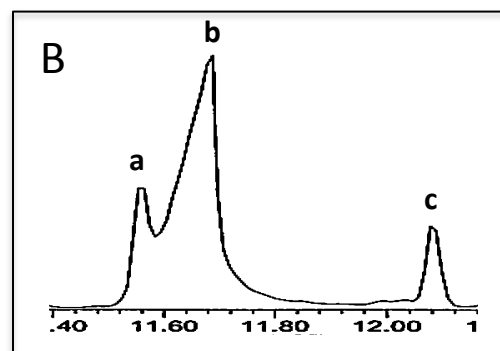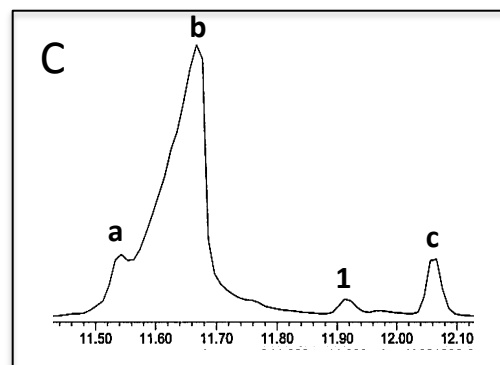

Supplement: Figure S3 — (A) Filter paper extract of N. cataria foliage showing 4aS,7S,7aR(Z,E)-nepetalactone (3) and 4aS,7S,7aS(E,Z)-nepetalactone. (B & C) The 11–12 min range of C. oculata extracts, respectively; a: decanal, b: nonanoic acid, 1: (1R, 2S, 5R, 8R)-iridodial, and c: tridecane. [file peerj-04-1564-s003.pdf]

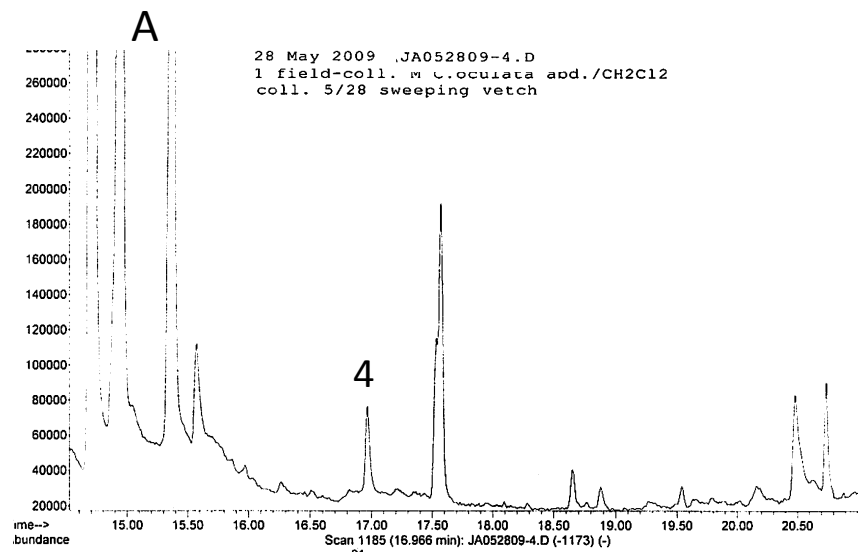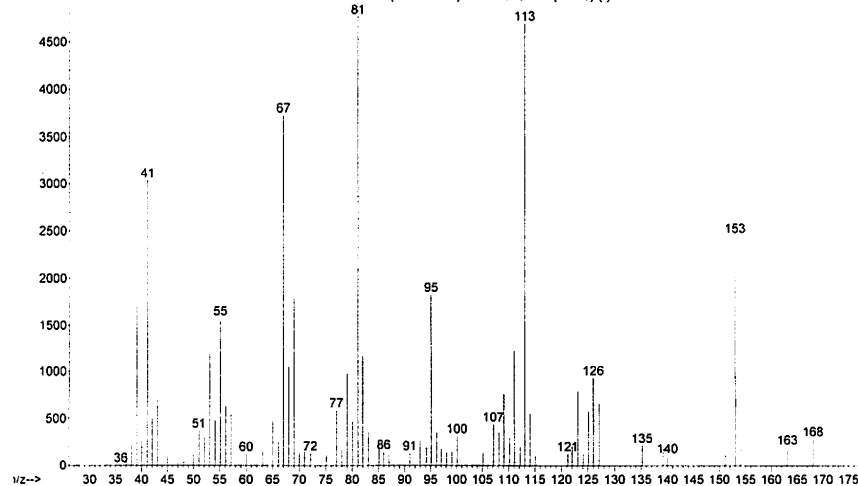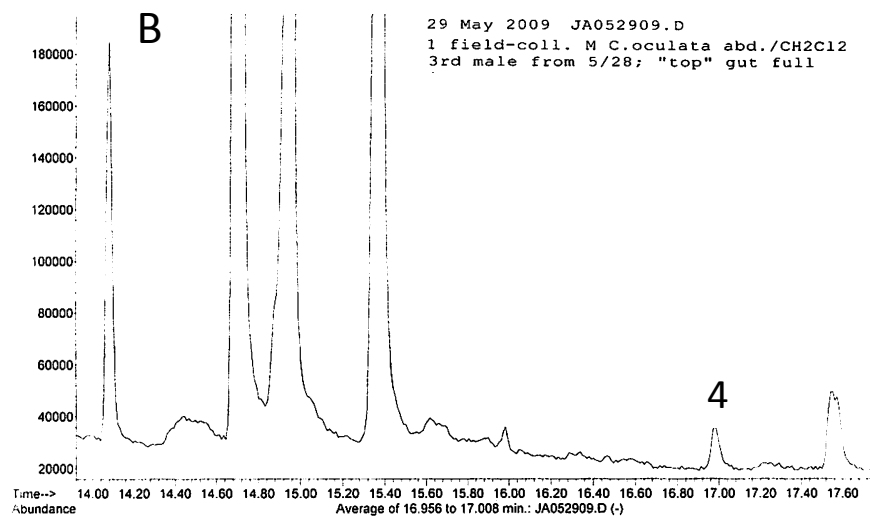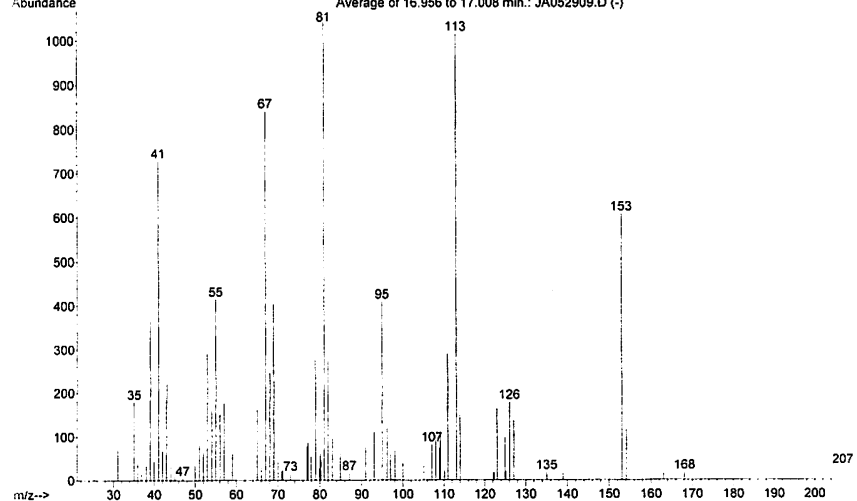

Supplement: Figure S4 — Compound 4, dihydronepetalactone (column, 30 m HP-5); conditions described in text. [file peerj-04-1564-s004.pdf]

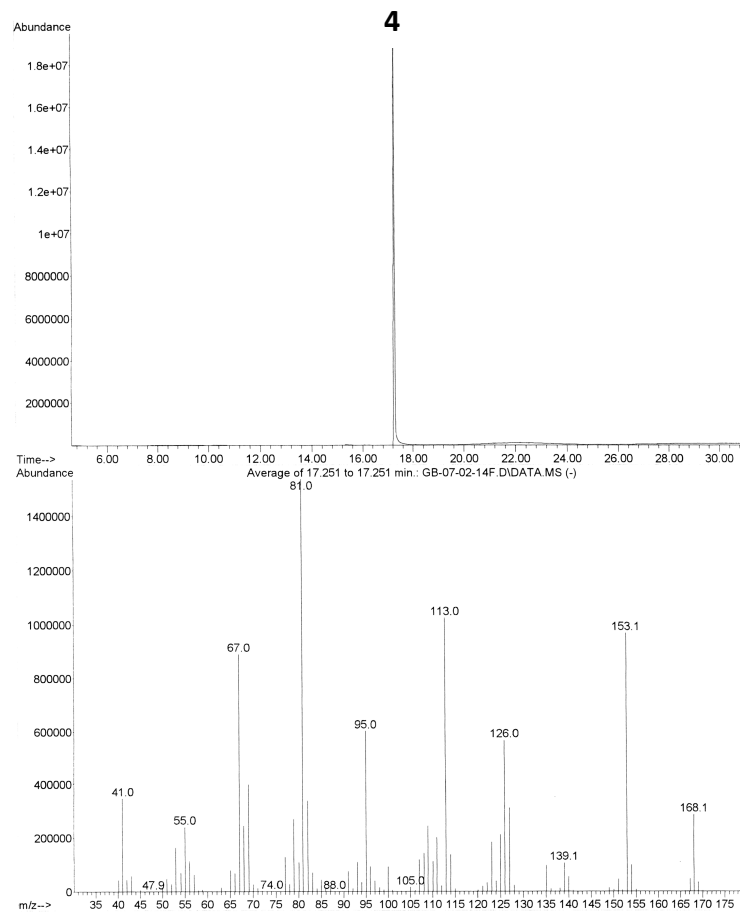

Supplement: Figure S5 — Analyzed on an HP 6890N GC coupled in series with an HP 5973 mass selective detector using a 30m DB-5 capillary column (250 µm ×0.25 µm film thickness; Agilent Technologies, Wilmington, DE, USA), 50 °C for 5 min, to 280 °C at 10 °C/min, hold 3 min. [file peerj-04-1564-s005.pdf]
