# Supplementary material for: Pharmacophagy in green lacewings (Neuroptera: Chrysopidae: Chrysopa spp.)?
Source: PeerJ. 2016 Jan 18;4:e1564. doi: 10.7717/peerj.1564 (PMC4727961; doi:10.7717/peerj.1564)

inj. remaining 1 ul of sample of field coll. male C. oc
ulata caught 5/15 sweeping (/10ul CH2Cl2), HP-5.

===

Injection Date : 8/12/2008 10:57:34 AM
Sample Name : rerun JA-05191.D Location : -
Acq. Operator : Aldrich Inj : 1
Inj Volume : Manually
Method : C:\HPCHEM\1\METHODS\DBLESS08.M
Last changed : 8/12/2008 10:03:36 AM by Aldrich
(modified after loading)
6/11/08; editing new method for desired output

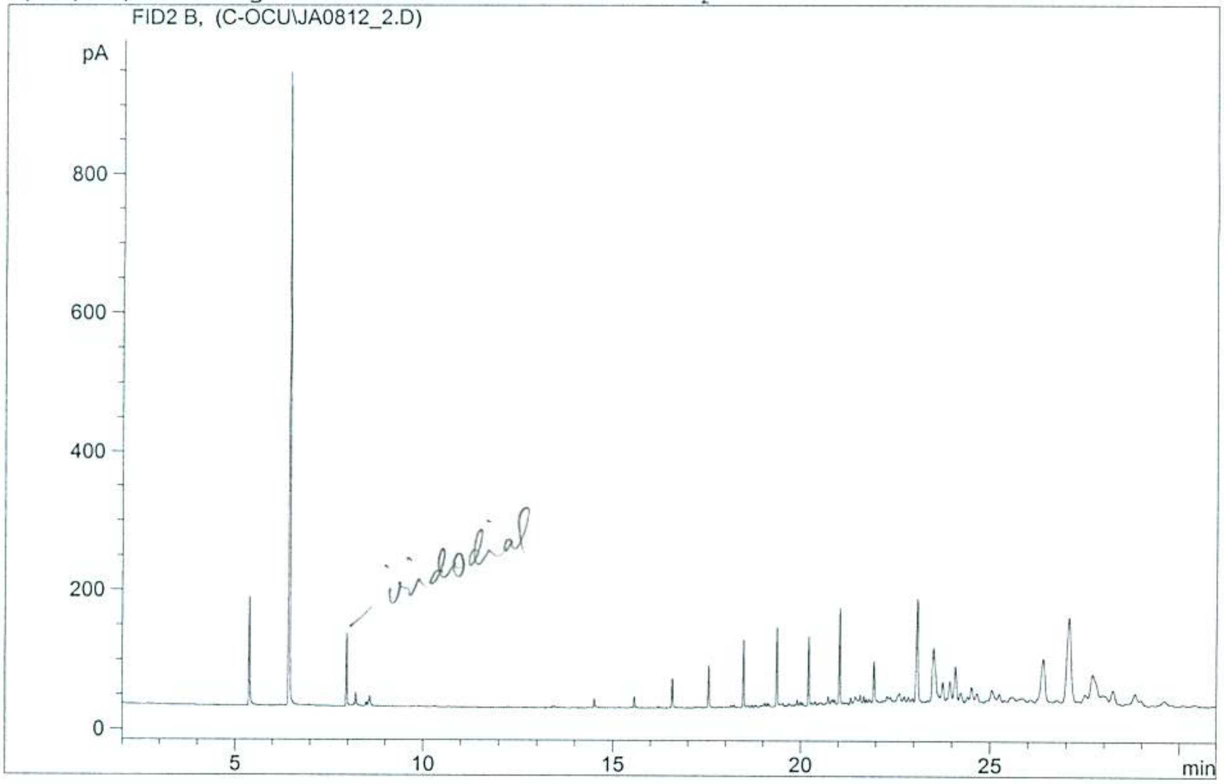

1 ♂ from sweep on 5/15/08 - w/ iridodial

===

Area Percent Report

===

Sorted By : Signal
Multiplier : 1.0000
Dilution : 1.0000

Signal 1: FID2 B,

Peak #	RetTime [min]	Type	Width [min]	Area [pA*s]	Height [pA]	Area %
1	0.478	PP	0.0153	215.09013	188.03360	0.00353
2	0.542	VV S	0.0476	1.68119e6	5.88398e5	27.61665
3	0.636	VV S	0.1157	4.39637e6	6.33365e5	72.21863
4	1.421	VV S	0.0161	286.27216	295.67578	0.00470
5	1.441	VB S	0.0262	335.81180	213.74525	0.00552
6	1.660	PV	0.0174	8.85842	7.72985	0.00015
7	1.701	VV	0.0300	34.42138	15.47402	0.00057
8	1.758	VV	0.0223	34.71598	23.38529	0.00057
9	1.798	VV	0.0292	47.67987	22.98003	0.00078
10	1.858	VV	0.0230	17.74743	11.50542	0.00029
11	1.899	VB	0.0303	42.04175	19.40183	0.00069
12	5.372	PB	0.0314	311.34625	155.55124	0.00511
13	6.458	BB	0.0307	2004.98535	909.92169	0.03294

Peak #	RetTime [min]	Type	Width [min]	Area [pA*s]	Height [pA]	Area %
14	7.978	PB	0.0291	189.00882	104.39306	0.00310
15	8.181	BV	0.0206	4.05992	3.03682	6.669e-5
16	8.220	VP	0.0295	37.47970	18.59586	0.00062
17	8.501	PV	0.0327	13.90286	6.31112	0.00023
18	8.563	VV	0.0235	11.72160	7.82198	0.00019
19	8.597	VB	0.0319	32.89018	14.83930	0.00054
20	13.408	PB	0.0349	3.92071	1.63795	6.440e-5
21	14.513	PP	0.0308	25.35539	12.45461	0.00042
22	15.570	BB	0.0311	30.96771	15.60660	0.00051
23	16.210	BP	0.0382	4.77683	1.90976	7.847e-5
24	16.579	BB	0.0317	86.13635	42.46165	0.00141
25	17.547	BB	0.0307	117.64736	60.36224	0.00193
26	18.135	BV	0.0283	4.26028	2.44751	6.998e-5
27	18.218	VB	0.0394	9.57677	3.44327	0.00016
28	18.475	BP	0.0299	180.03954	95.77506	0.00296
29	18.618	PP	0.0251	2.30270	1.48336	3.783e-5
30	18.700	PB	0.0295	5.27294	2.85605	8.662e-5
31	18.803	BB	0.0519	8.26395	2.35871	0.00014
32	19.035	BV	0.0507	22.21611	6.06362	0.00036
33	19.115	VV	0.0314	11.40747	5.46066	0.00019
34	19.159	VP	0.0333	7.07858	3.13902	0.00012
35	19.365	BV	0.0326	229.44980	113.28062	0.00377
36	19.521	VB	0.0807	28.75328	4.49201	0.00047
37	19.680	PB	0.0436	10.35132	3.48757	0.00017
38	19.805	PP	0.0268	2.39397	1.56595	3.933e-5
39	19.905	VV	0.0353	21.21113	9.05450	0.00035
40	19.994	VV	0.0418	16.97497	6.65406	0.00028
41	20.056	VP	0.0321	9.91246	4.79894	0.00016
42	20.222	BV	0.0319	203.57118	99.34179	0.00334
43	20.300	VV	0.0383	10.70943	3.99226	0.00018
44	20.397	VV	0.0494	22.30564	6.28283	0.00037
45	20.521	VV	0.0876	29.86813	4.43275	0.00049
46	20.640	VV	0.0309	6.09885	2.74579	0.00010
47	20.739	VV	0.0466	41.50782	12.87020	0.00068
48	20.821	VV	0.0272	13.37910	7.02367	0.00022
49	20.857	VV	0.0343	20.34077	8.70614	0.00033
50	20.918	VV	0.0387	20.87193	7.92235	0.00034
51	21.046	VB	0.0330	295.99847	138.17804	0.00486
52	21.190	BP	0.0666	16.39768	3.26484	0.00027
53	21.337	VV	0.0431	27.78979	10.10280	0.00046
54	21.457	VV	0.0644	54.11434	11.19195	0.00089
55	21.593	VV	0.0516	51.82933	13.86185	0.00085
56	21.685	VV	0.0389	30.41992	12.29220	0.00050
57	21.756	VV	0.0394	19.29253	7.40761	0.00032
58	21.835	VV	0.0396	23.72516	8.24353	0.00039
59	21.956	VV	0.0471	188.35709	60.87270	0.00309
60	22.160	VB	0.0832	23.41425	3.68126	0.00038
61	22.303	BV	0.0591	43.71392	10.39295	0.00072
62	22.391	VB	0.0652	40.60858	8.58578	0.00067
63	22.629	BV	0.0824	87.38718	13.88397	0.00144
64	22.752	VV	0.0548	35.19067	9.37312	0.00058
65	22.864	VV	0.0532	27.99697	8.11604	0.00046
66	22.972	VV	0.0435	18.97262	6.06318	0.00031
67	23.096	VB	0.0497	487.04370	146.93196	0.00800
68	23.372	PV	0.0382	6.09370	2.28100	0.00010
69	23.525	VV	0.0889	489.74039	77.36690	0.00804
70	23.767	VV	0.0644	126.69332	29.29813	0.00208
71	23.957	VV	0.0730	146.57333	29.97460	0.00241
72	24.100	VV	0.0612	221.25066	51.36860	0.00363
73	24.235	VP	0.0739	69.52882	13.76554	0.00114
74	24.417	VV	0.0635	34.71703	8.02268	0.00057
75	24.524	VV	0.0679	102.59321	22.21782	0.00169
76	24.657	VB	0.0692	73.34789	12.92538	0.00120
77	25.059	PV	0.1000	135.27133	17.54196	0.00222
78	25.253	VB	0.0799	67.38235	11.75325	0.00111

— viridodial

Peak #	RetTime [min]	Type	Width [min]	Area [pA*s]	Height [pA]	Area %
79	25.607	BB	0.1974	120.62022	7.27543	0.00198
80	26.057	PP	0.0719	22.78115	3.91389	0.00037
81	26.402	VB	0.1015	472.37653	62.27951	0.00776
82	27.083	PP	0.1028	918.86835	120.67532	0.01509
83	27.513	PV	0.0785	54.36015	9.26413	0.00089
84	27.702	VV	0.1209	358.92969	35.52689	0.00590
85	28.244	PP	0.0702	93.61933	16.25072	0.00154
86	28.832	BB	0.1316	157.59207	15.24018	0.00259
87	29.614	BB	0.0998	48.14132	5.86359	0.00079

Totals : 6.08759e6 1.22536e6

Results obtained with enhanced integrator!

===

*** End of Report ***

inj. 1ul abdominal cuticle extract of 5 lab-reared male
s 20-29-days-old without access to plants; fed pea aphi
d, Sitotroga eggs & honey water, & dissected 8/13 (5 ma
les /40ul CH2Cl2 conc. to ca. 5ul), HP-5.

===

```
Injection Date  : 8/13/2008 12:16:15 PM
Sample Name     : lab 5M C.ocu abd                  Location :  -
Acq. Operator   : Aldrich                                Inj :  1
                                                  Inj Volume : Manually
Method          : C:\HPCHEM\1\METHODS\DBLESS08.M
Last changed    : 8/12/2008 11:55:44 AM by Aldrich
6/11/08; editing new method for desired output
```

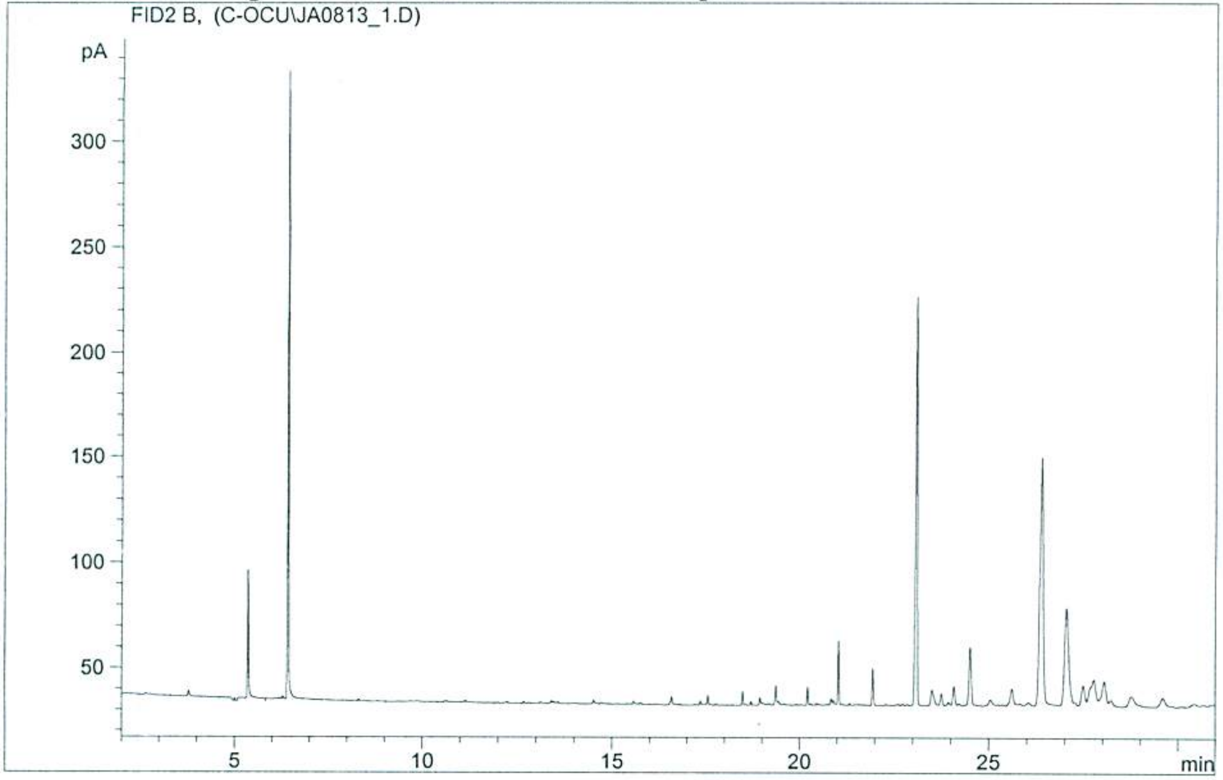

===

Area Percent Report

===

```
Sorted By        :      Signal
Multiplier       :      1.0000
Dilution         :      1.0000
```

Signal 1: FID2 B,

Peak #	RetTime [min]	Type	Width [min]	Area [pA*s]	Height [pA]	Area %
1	0.467	PP	0.0146	11.00343	11.05655	0.00042
2	0.533	VV S	0.0158	1.49228e5	1.78474e5	5.64316
3	0.580	VV S	0.1004	2.49193e6	4.13768e5	94.23391
4	1.460	VB S	0.0150	94.79893	105.41685	0.00358
5	1.607	BV	0.0346	3.69842	1.82972	0.00014
6	1.705	VV	0.0500	13.28734	3.69150	0.00050
7	1.808	VB	0.0391	8.82340	3.31357	0.00033
8	3.779	BB	0.0424	7.50409	2.69936	0.00028
9	5.000	PP	0.0199	1.56996	1.31328	5.937e-5
10	5.350	PB	0.0319	124.22678	60.51726	0.00470
11	6.423	PB	0.0291	560.39868	295.85358	0.02119
12	14.517	PP	0.0367	3.98305	1.67948	0.00015

5 lab ♂♂ abd – w/o
[illegible]

Peak #	RetTime [min]	Type	Width [min]	Area [pA*s]	Height [pA]	Area %
13	15.573	PB	0.0323	2.98141	1.43181	0.00011
14	16.582	BB	0.0399	9.70355	3.54346	0.00037
15	17.346	PB	0.0380	4.34248	1.80657	0.00016
16	17.548	BB	0.0316	8.73558	4.31165	0.00033
17	18.474	PB	0.0305	12.21688	6.34637	0.00046
18	18.698	PP	0.0316	3.95342	1.95121	0.00015
19	18.937	PB	0.0366	8.24157	3.24932	0.00031
20	19.363	PB	0.0374	21.85039	8.65924	0.00083
21	20.219	PP	0.0305	16.78219	8.69974	0.00063
22	20.841	PV	0.0381	7.63631	3.05681	0.00029
23	20.905	VB	0.0408	6.59770	2.34588	0.00025
24	21.042	BB	0.0321	61.60423	29.81875	0.00233
25	21.952	BB	0.0384	42.66174	17.52662	0.00161
26	23.097	BB	0.0503	635.12579	193.42932	0.02402
27	23.514	PB	0.0719	40.07027	7.20589	0.00152
28	23.759	BP	0.0534	18.85434	5.44812	0.00071
29	24.090	PP	0.0583	31.83170	8.57640	0.00120
30	24.514	BP	0.0645	111.57770	27.38947	0.00422
31	25.609	BB	0.0803	39.63595	7.41917	0.00150
32	26.385	BB	0.0864	692.35138	116.42314	0.02618
33	27.046	BB	0.1021	337.83240	45.27579	0.01278
34	27.486	PV	0.0771	59.37449	9.47800	0.00225
35	27.771	VV	0.1364	135.91484	12.25323	0.00514
36	28.044	VB	0.1226	111.49355	11.55581	0.00422
Totals :				2.64441e6	5.93267e5	

Results obtained with enhanced integrator!

==

*** End of Report ***

File :D:\DATA\Aldrich\JA-09\JA071010-2.D
Operator : Aldrich
Acquired : 10 Jul 2009 17:18 using AcqMethod JA-WAX09.M
Instrument : Instrument #1
Sample Name: 10 lab male 2wk-old fed nepetalactol 7/1-10
Misc Info : JA071009-1 & here C.oculata; rev.diss order
Vial Number: 1

emerged: 06/15-17/09 & fed 7/1 & diss. 7/10 } 23-25 days old

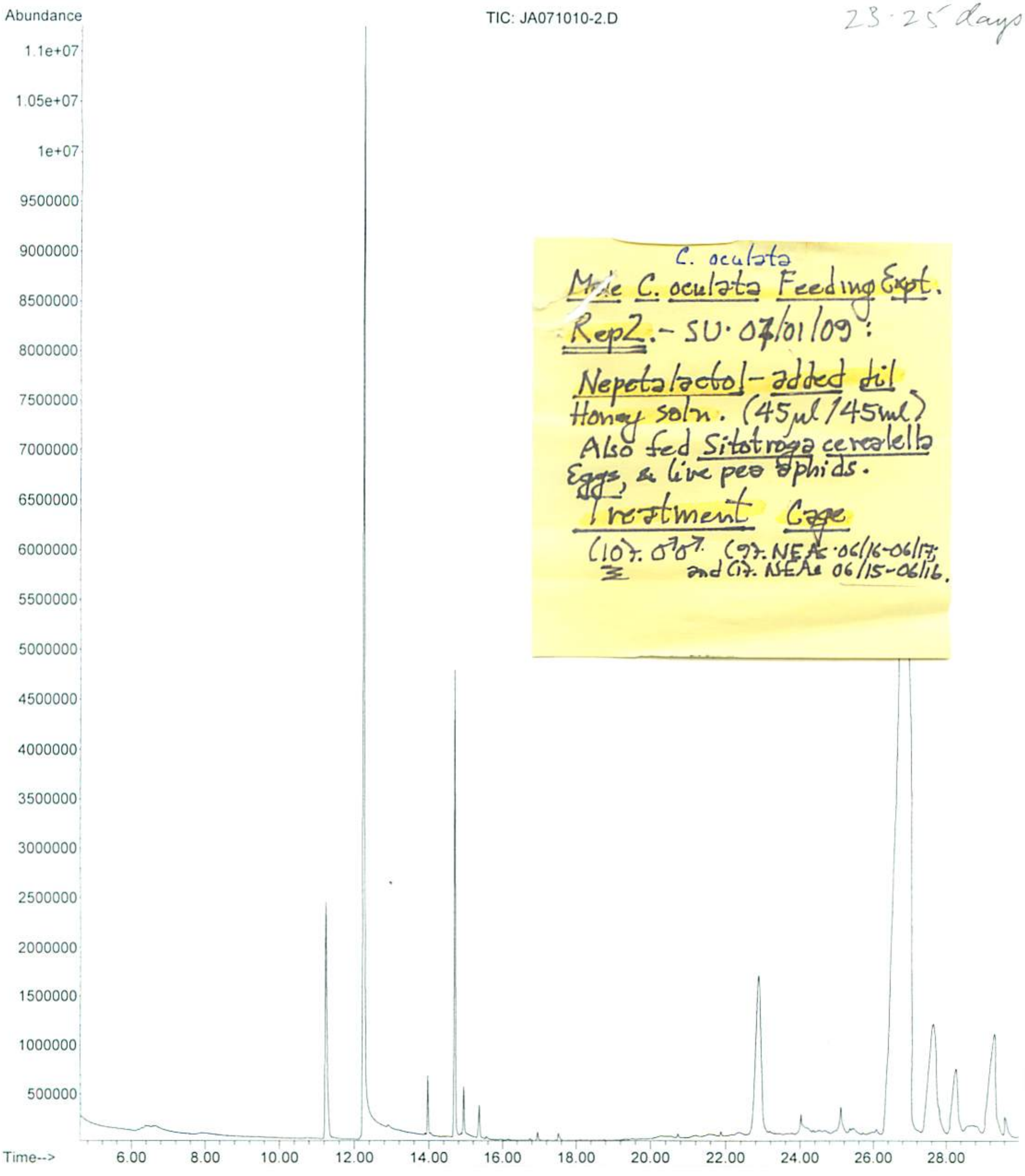

C. oculata
Male C. oculata Feeding Expt.
Rep2. - SU. 07/01/09:
Nepetalactol - added dil
Honey soln. (45µl/45ml)
Also fed Sitotroga cerealella
Eggs, & live pea aphids.
Treatment Cage
(10). ♂♂. (9). NEA 06/16-06/17;
and (1). NEA 06/15-06/16.

File :D:\DATA\ALDRICH\JA-09\Snapshot\JA071010-2.D
Operator : Aldrich
Acquired : 10 Jul 2009 17:18 using AcqMethod JA-WAX09.M
Instrument : Instrument #1
Sample Name: 10 lab male 2wk-old fed nepetalactol 7/1-10
Misc Info : JA071009-1 & here C.oculata; rev.diss order
Vial Number: 1

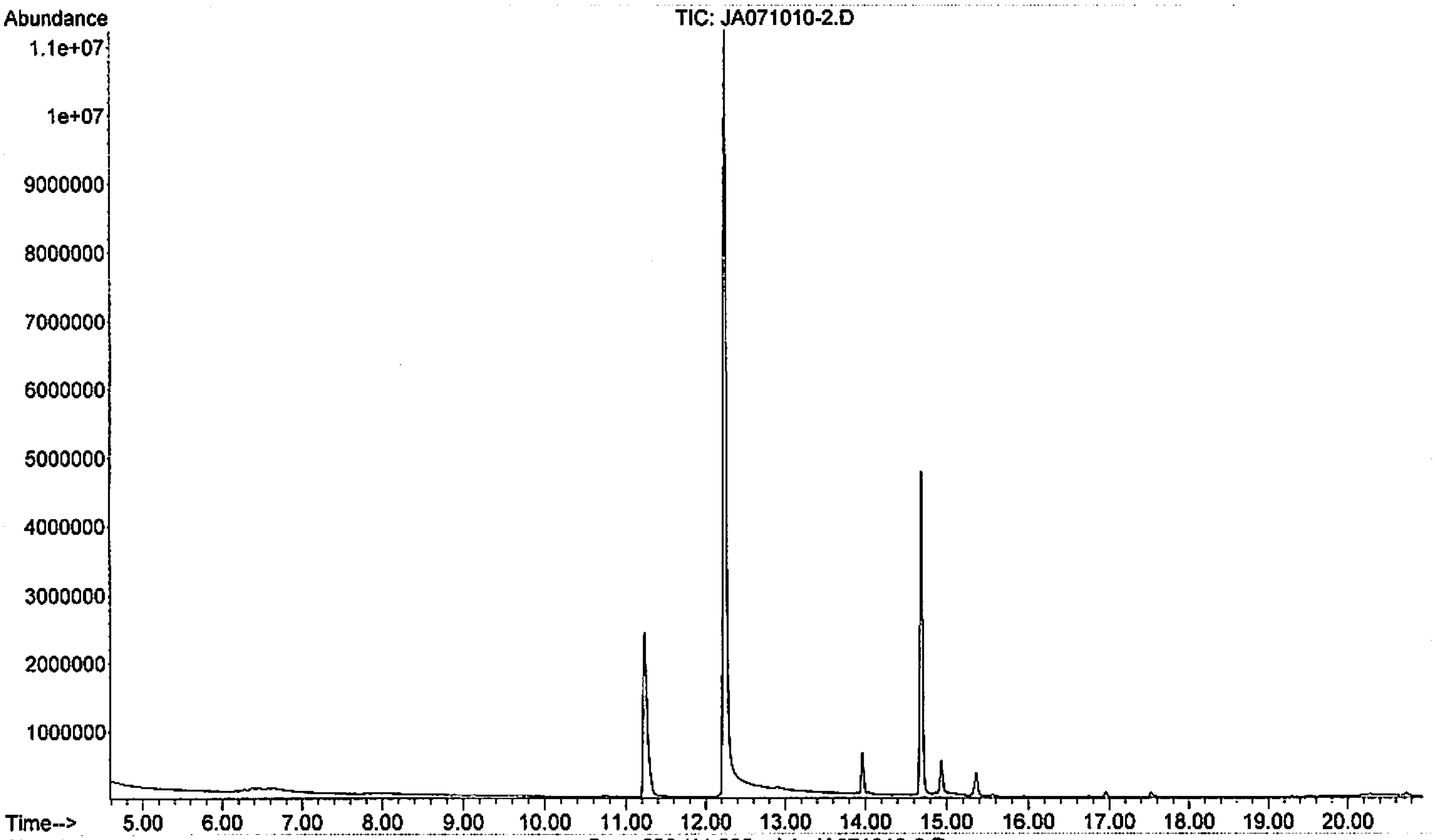

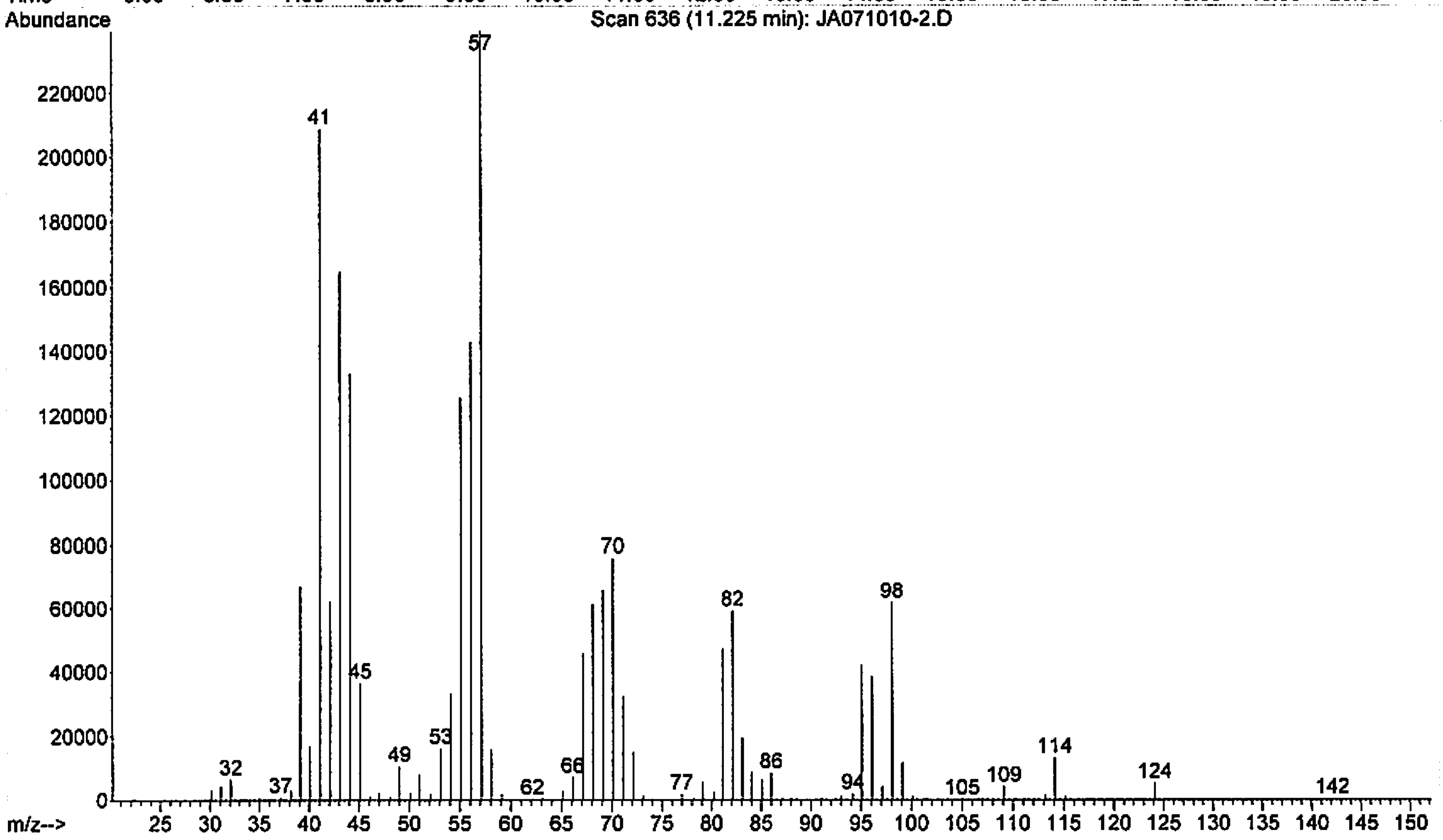

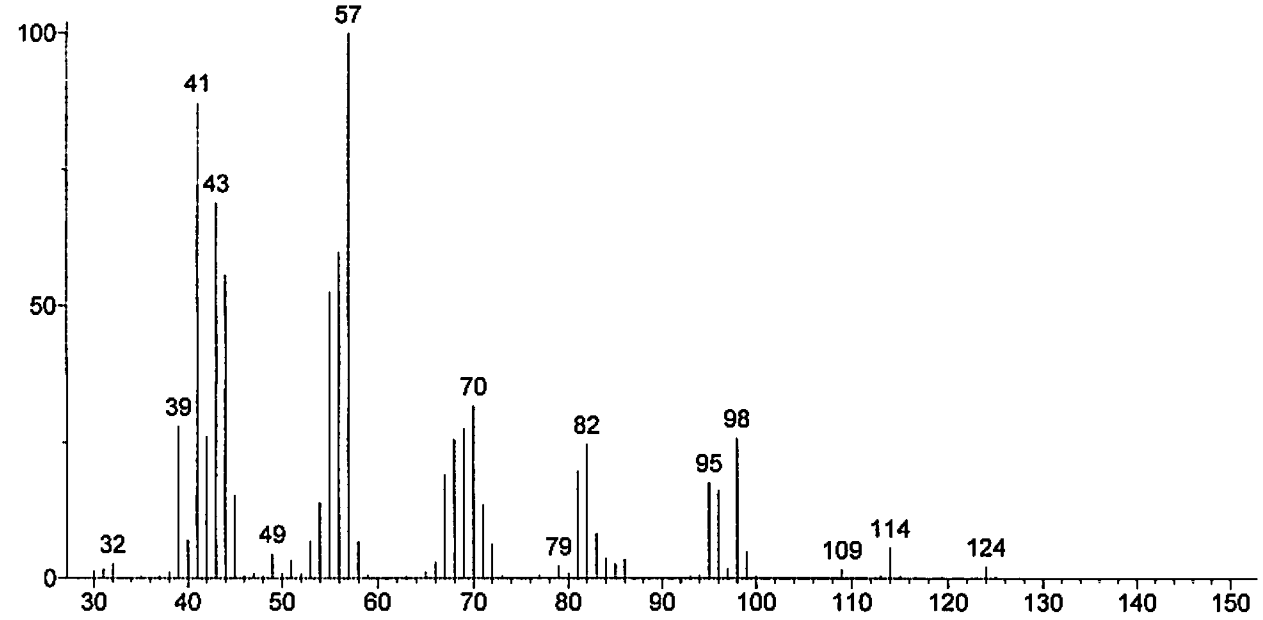

(Text File) Scan 636 (11.225 min): JA071010-2.D

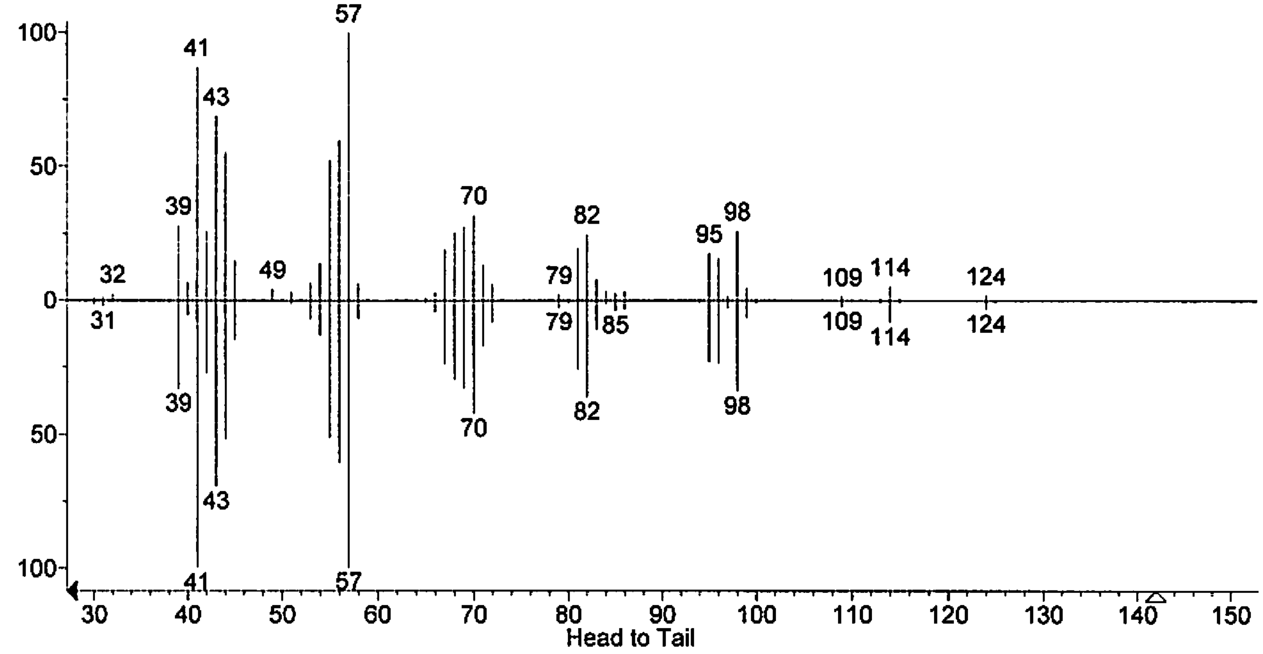

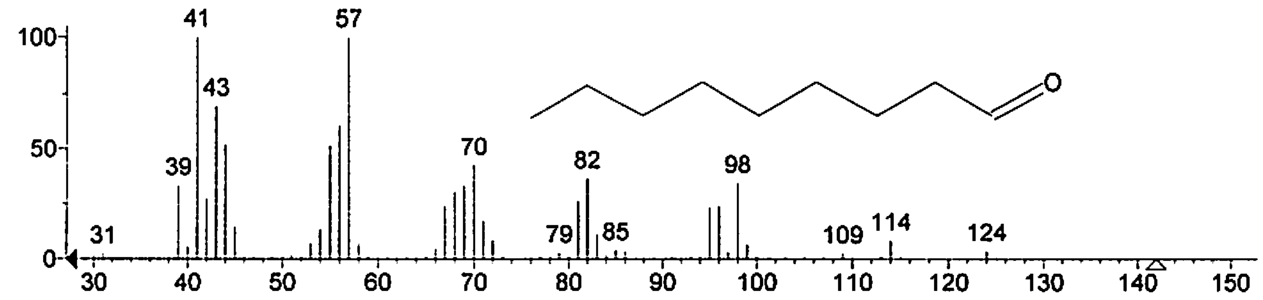

(replib) Nonanal

```
File        :D:\DATA\ALDRICH\JA-09\Snapshot\JA071010-2.D
Operator    : Aldrich
Acquired    : 10 Jul 2009  17:18     using AcqMethod JA-WAX09.M
Instrument  :    Instrument #1
Sample Name: 10 lab male 2wk-old fed nepetalactol 7/1-10
Misc Info   : JA071009-1 & here C.oculata; rev.diss order
Vial Number: 1
```

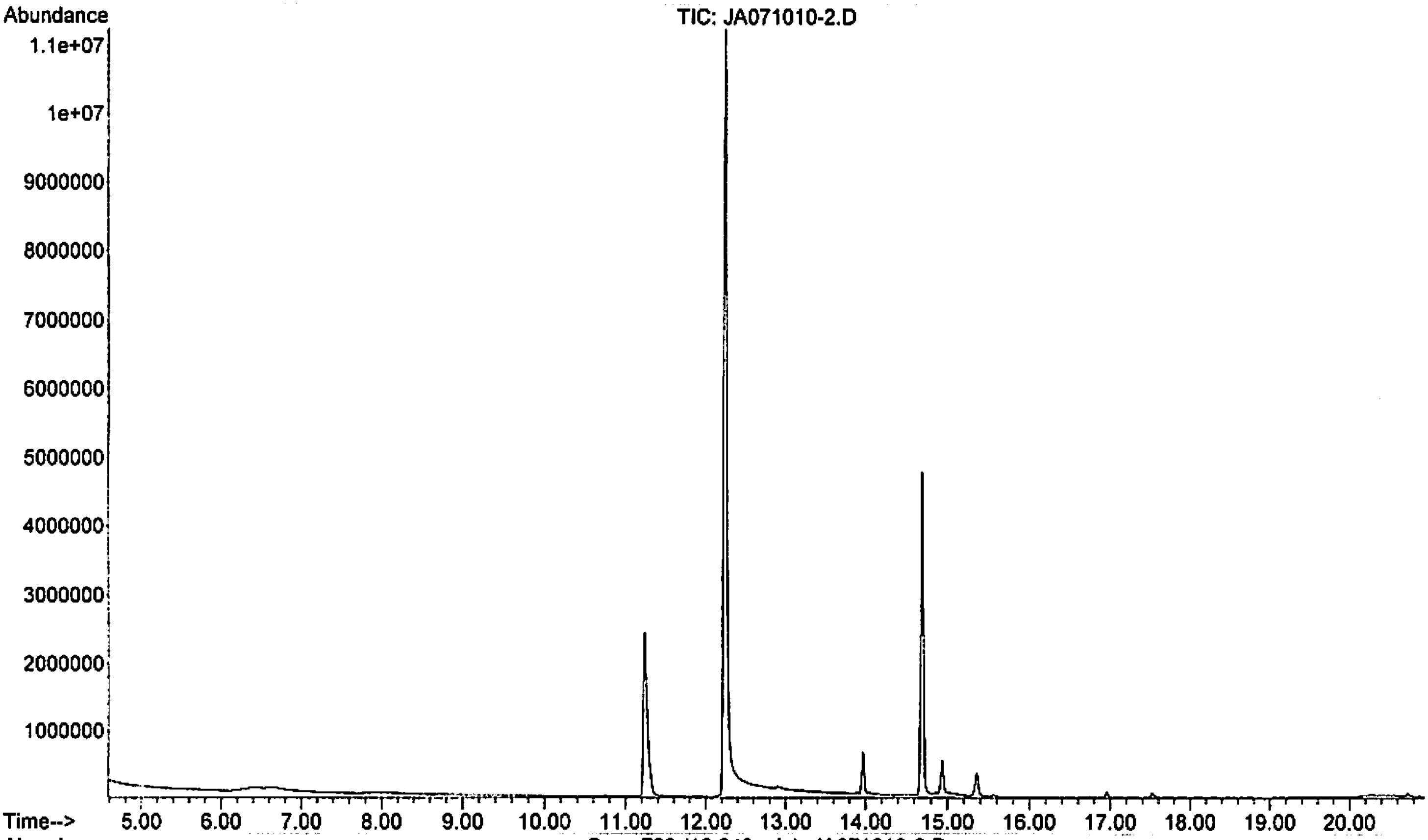

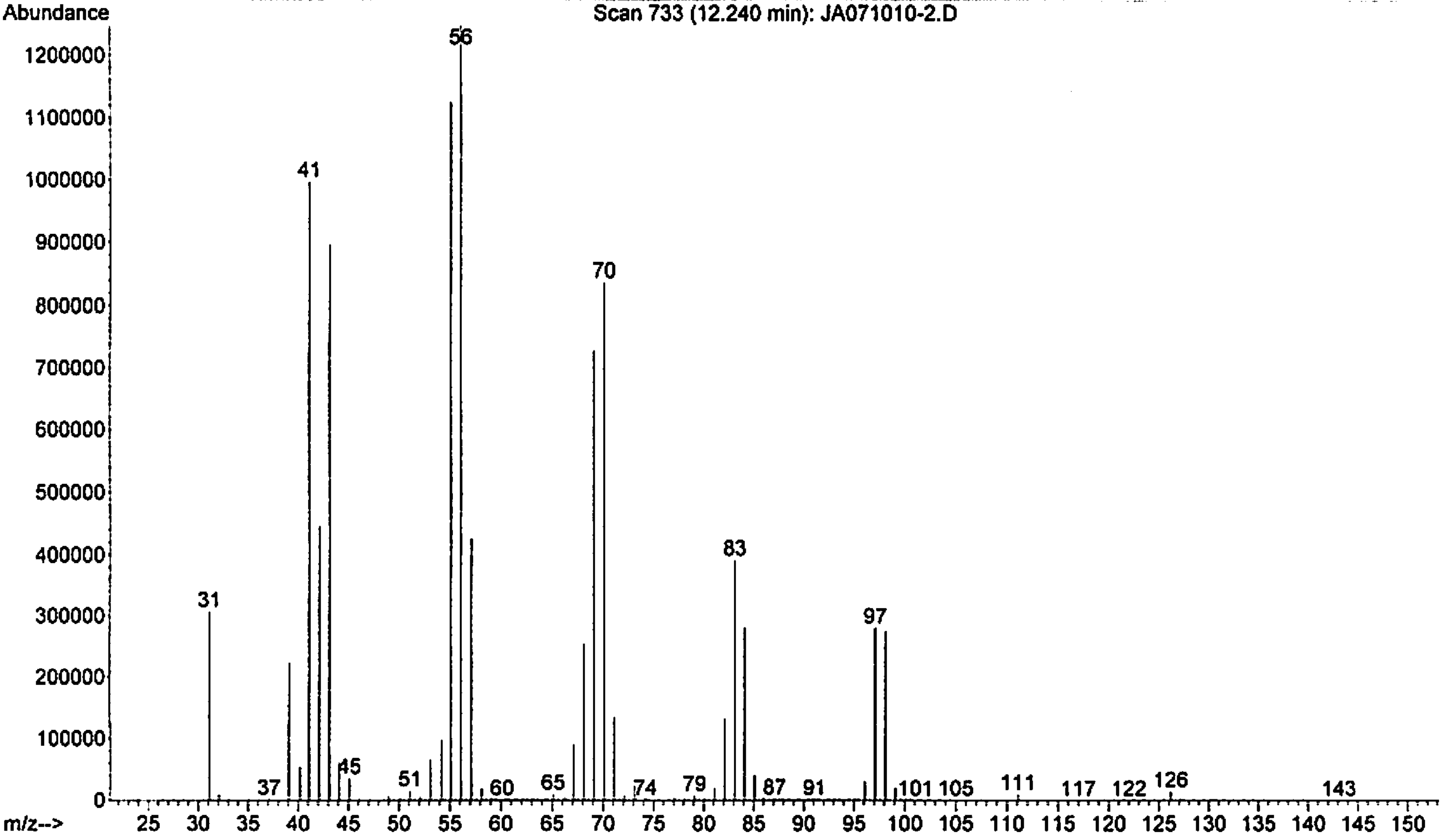

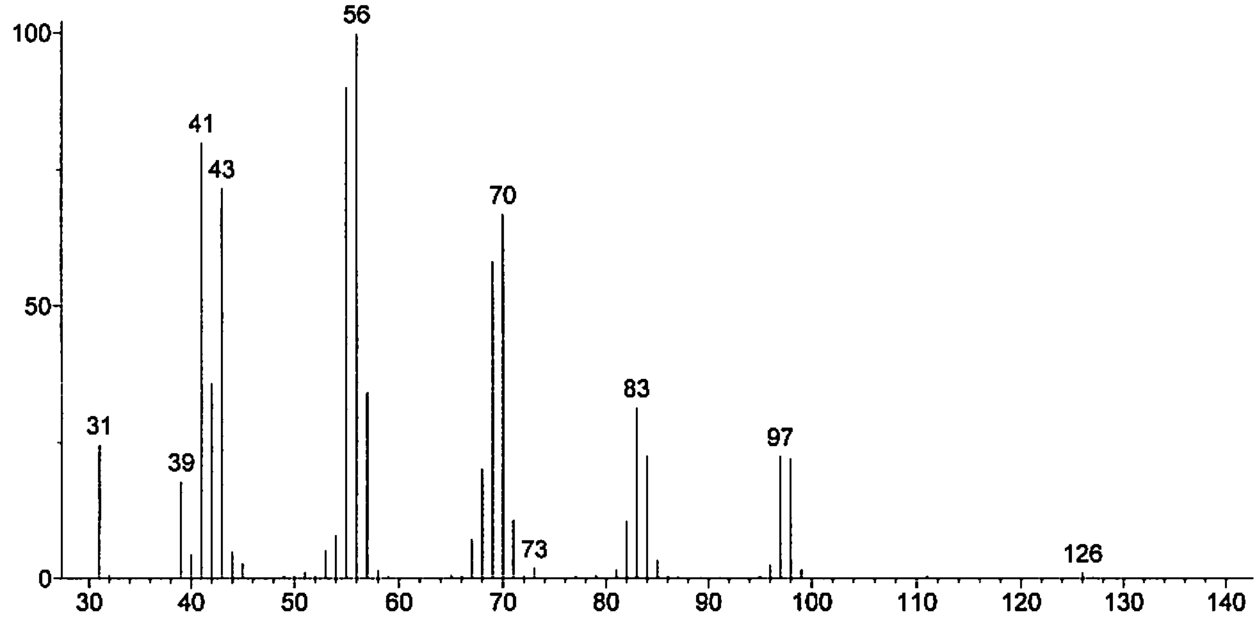

(Text File) Scan 733 (12.240 min): JA071010-2.D

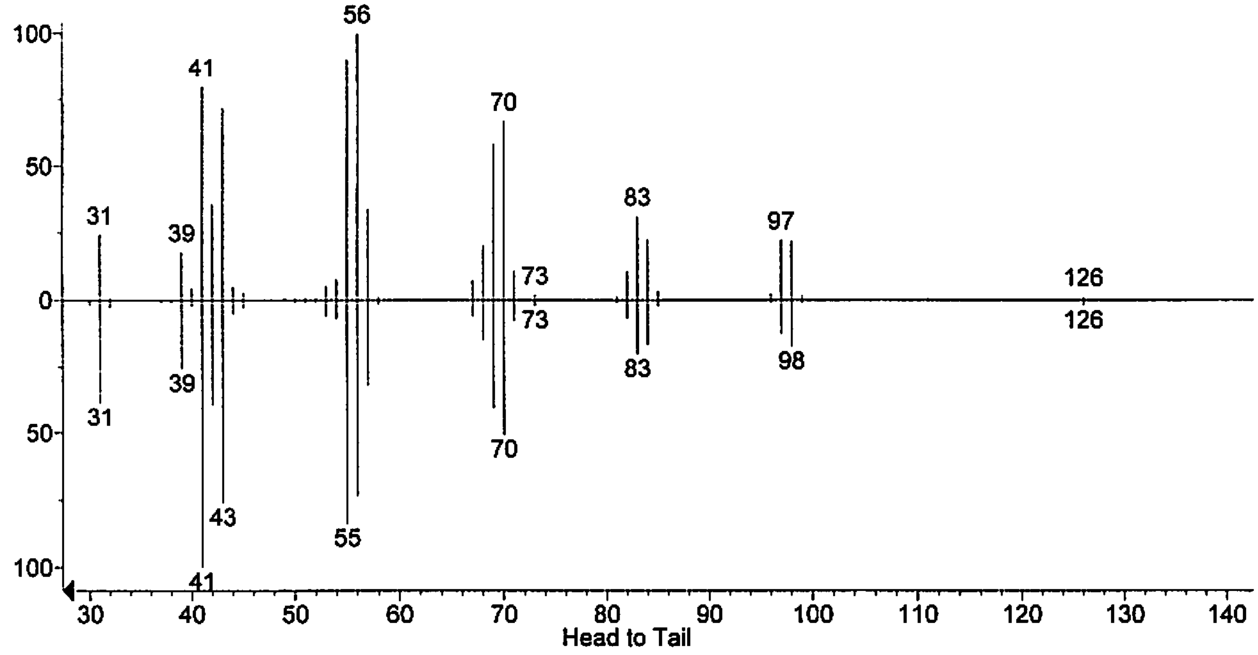

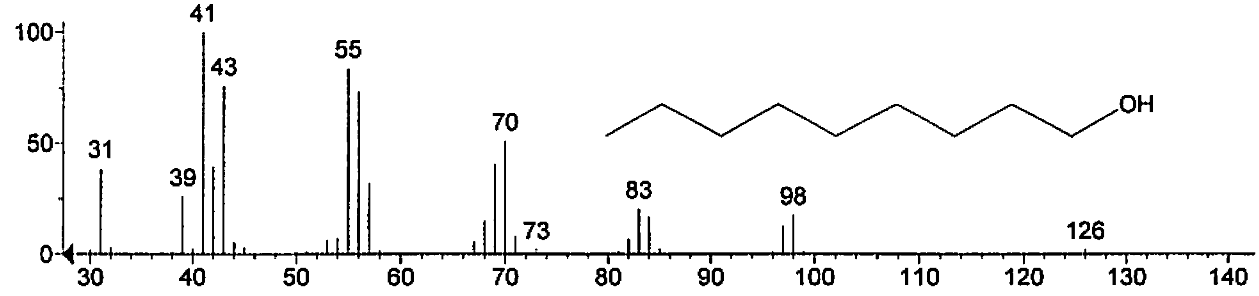

(replib) 1-Nonanol

File :D:\DATA\ALDRICH\JA-09\Snapshot\JA071010-2.D
Operator : Aldrich
Acquired : 10 Jul 2009 17:18 using AcqMethod JA-WAX09.M
Instrument : Instrument #1
Sample Name: 10 lab male 2wk-old fed nepetalactol 7/1-10
Misc Info : JA071009-1 & here C.oculata; rev.diss order
Vial Number: 1

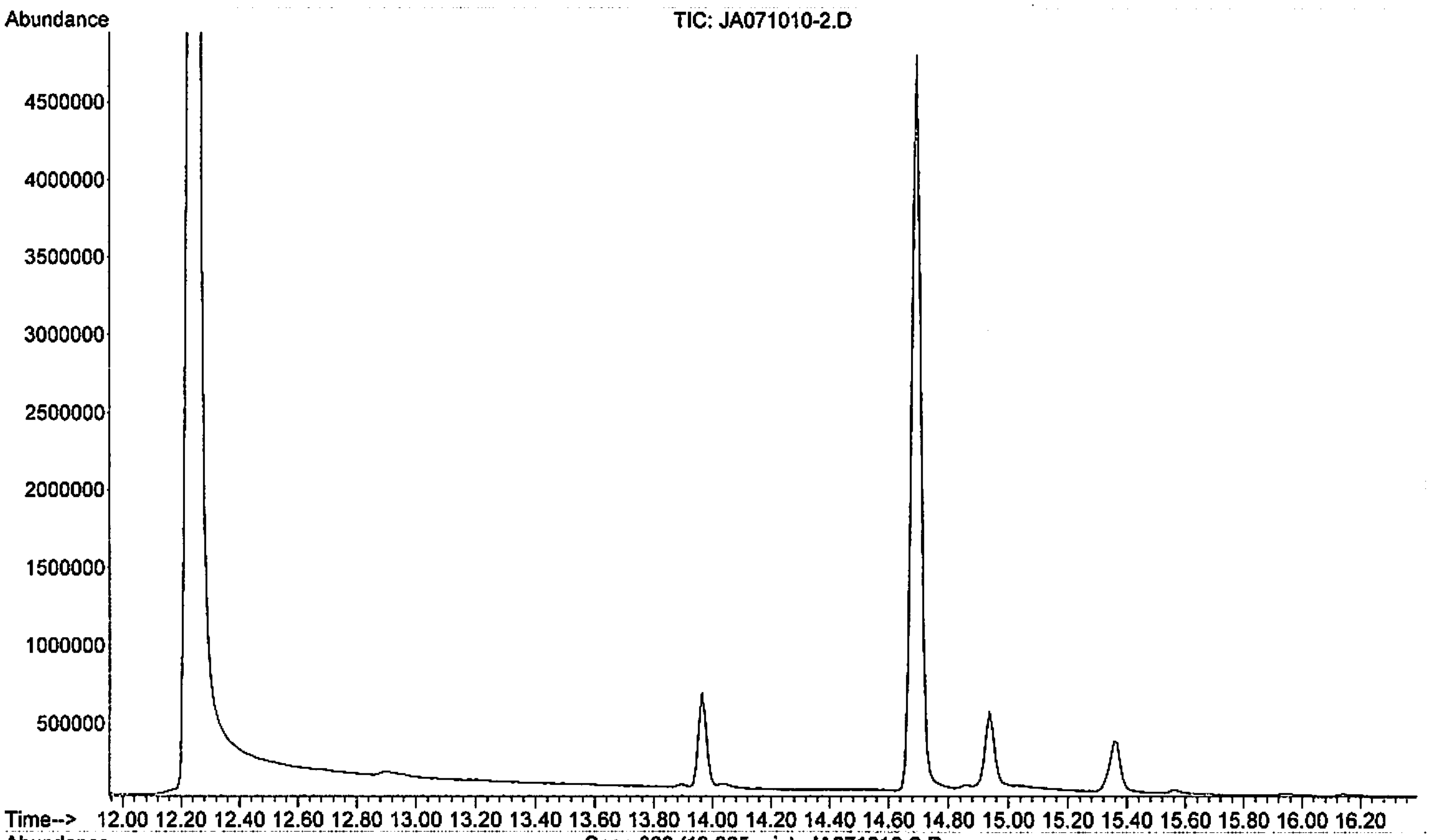

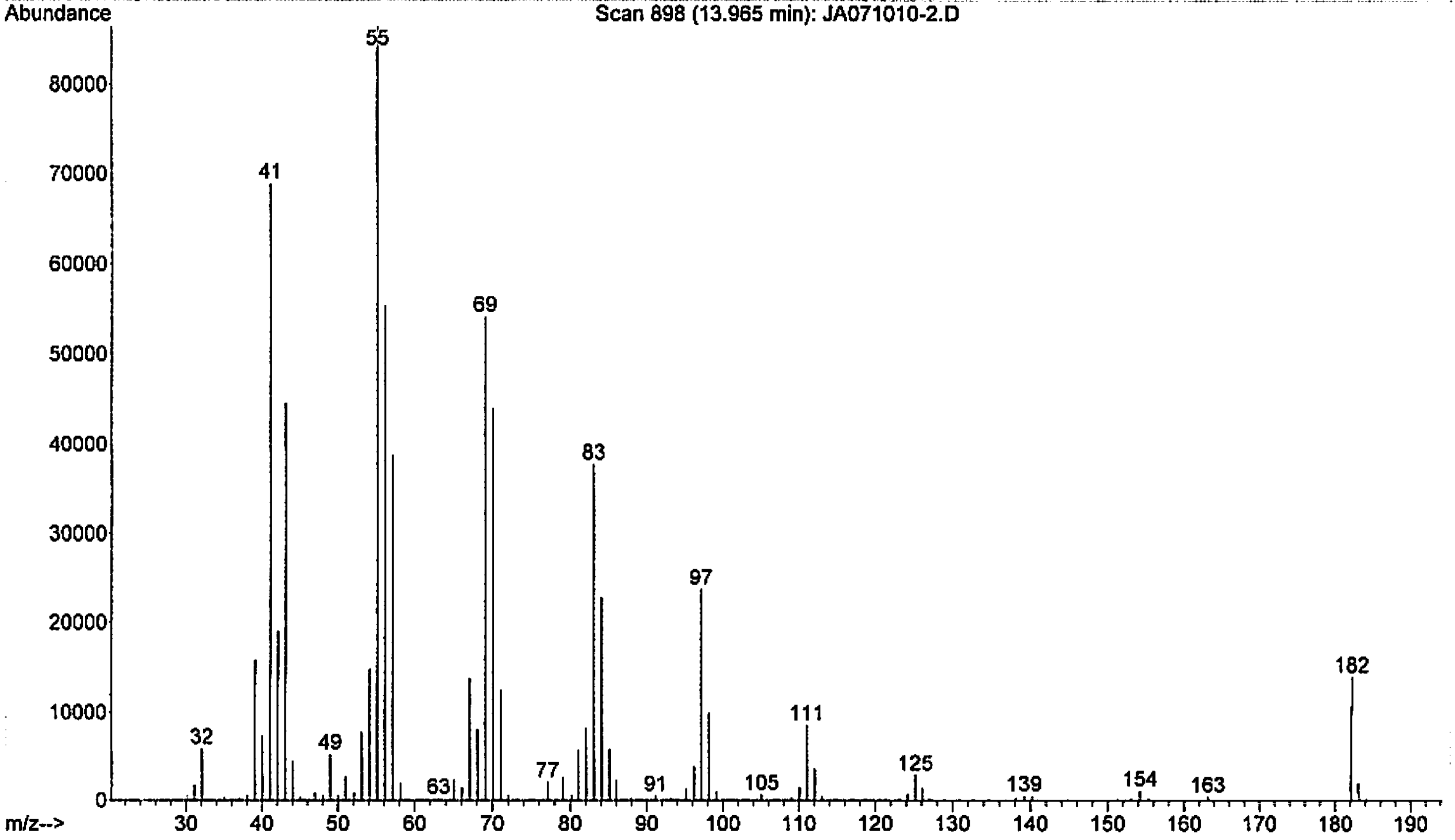

```
File          :D:\DATA\ALDRICH\JA-09\Snapshot\JA071010-2.D
Operator      : Aldrich
Acquired      : 10 Jul 2009  17:18     using AcqMethod JA-WAX09.M
Instrument :     Instrument #1
Sample Name: 10 lab male 2wk-old fed nepetalactol 7/1-10
Misc Info   : JA071009-1 & here C.oculata; rev.diss order
Vial Number: 1
```

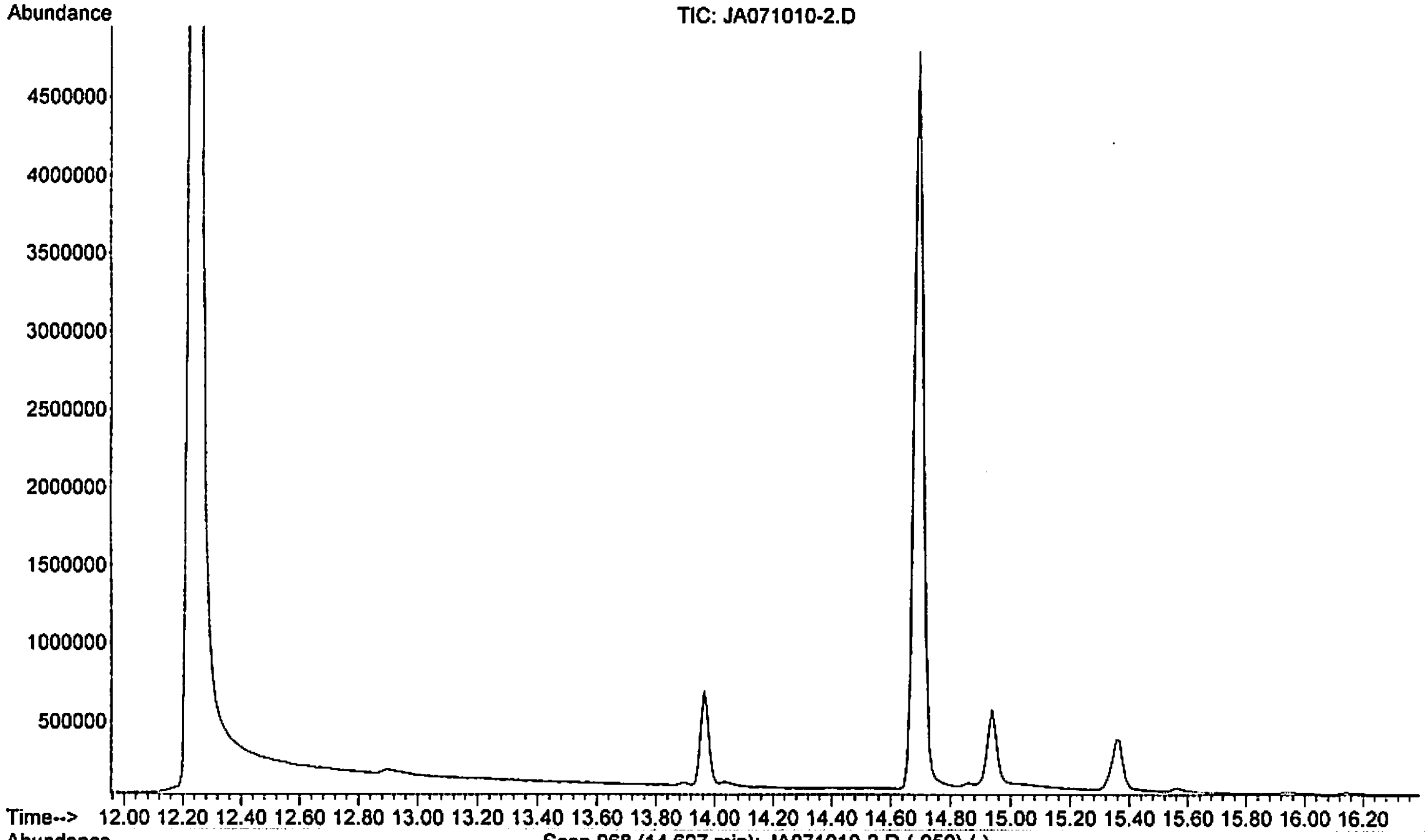

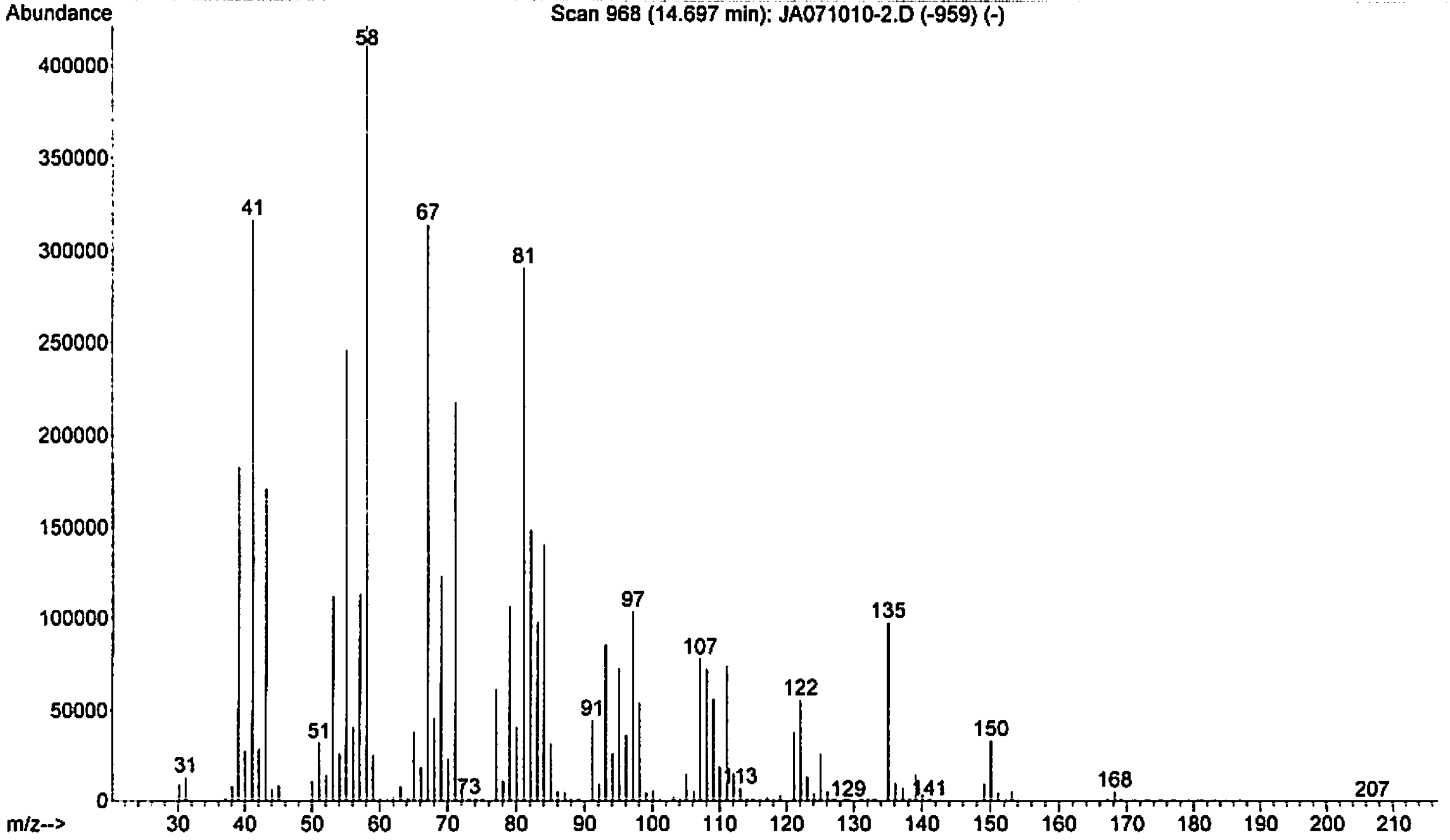

```
File        :D:\DATA\ALDRICH\JA-09\Snapshot\JA071010-2.D
Operator    : Aldrich
Acquired    : 10 Jul 2009  17:18     using AcqMethod JA-WAX09.M
Instrument  :    Instrument #1
Sample Name: 10 lab male 2wk-old fed nepetalactol 7/1-10
Misc Info   : JA071009-1 & here C.oculata; rev.diss order
Vial Number: 1
```

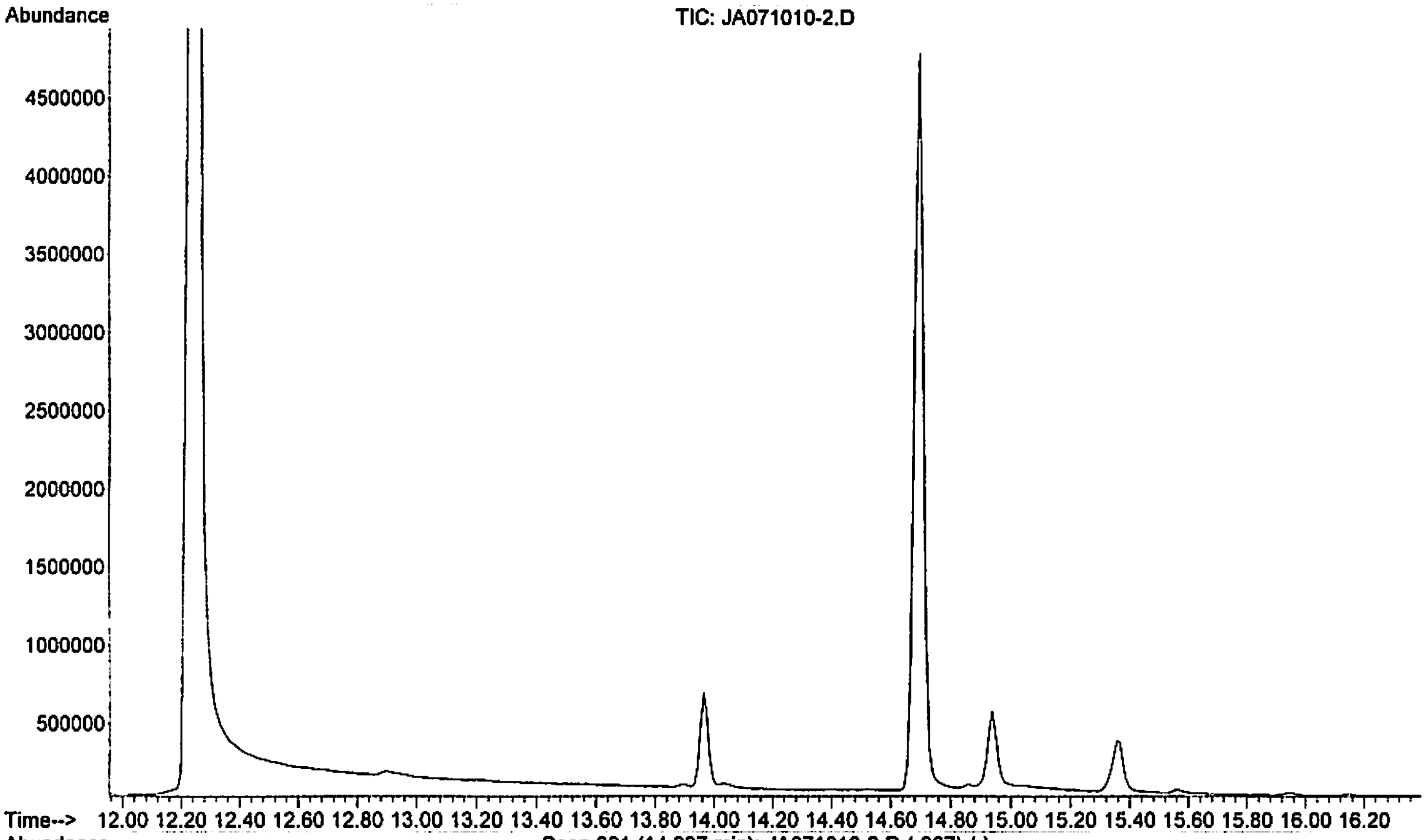

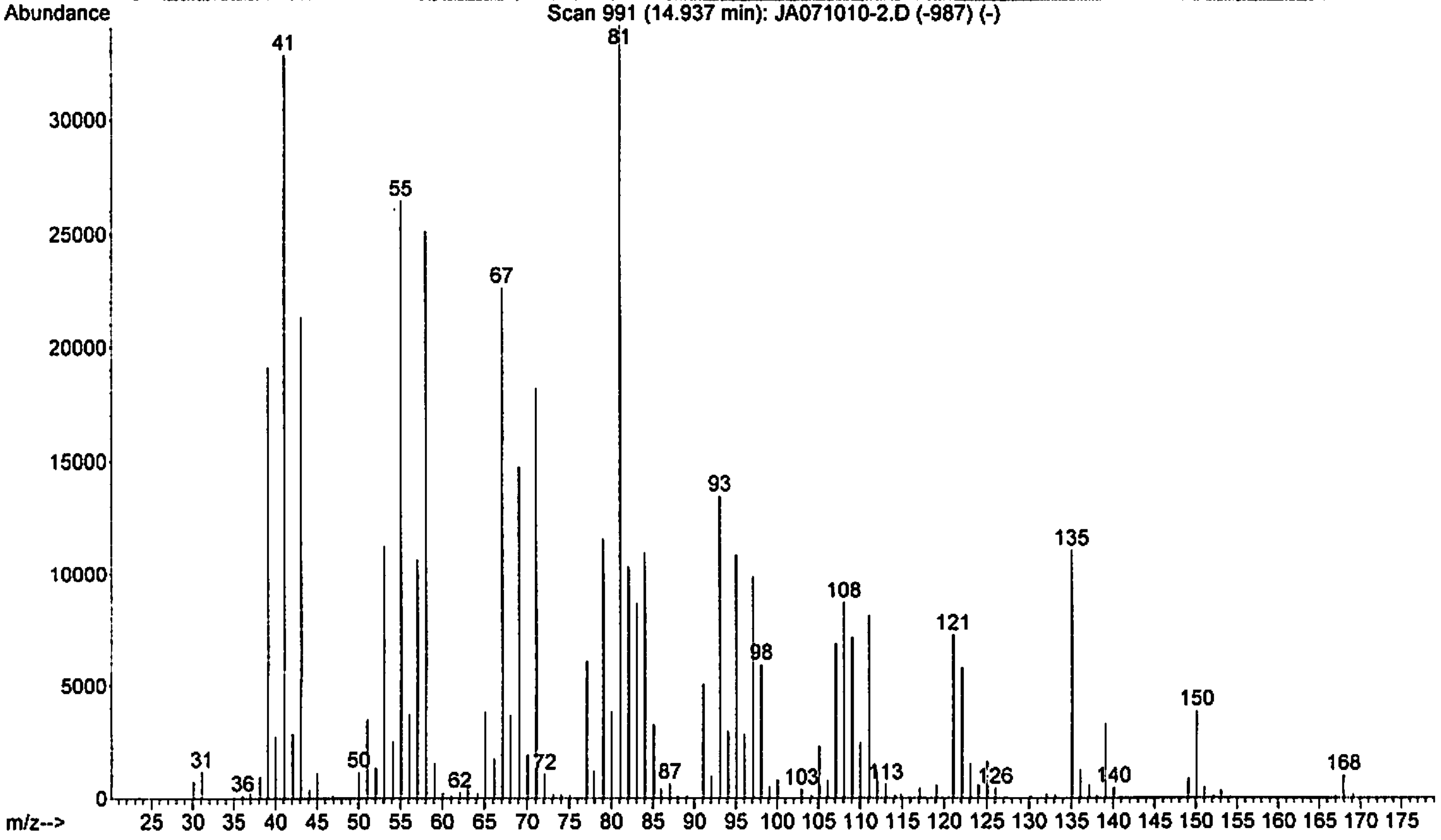

```
File         :D:\DATA\ALDRICH\JA-09\Snapshot\JA071010-2.D
Operator     : Aldrich
Acquired     : 10 Jul 2009  17:18      using AcqMethod JA-WAX09.M
Instrument :      Instrument #1
Sample Name: 10 lab male 2wk-old fed nepetalactol 7/1-10
Misc Info    : JA071009-1 & here C.oculata; rev.diss order
Vial Number: 1
```

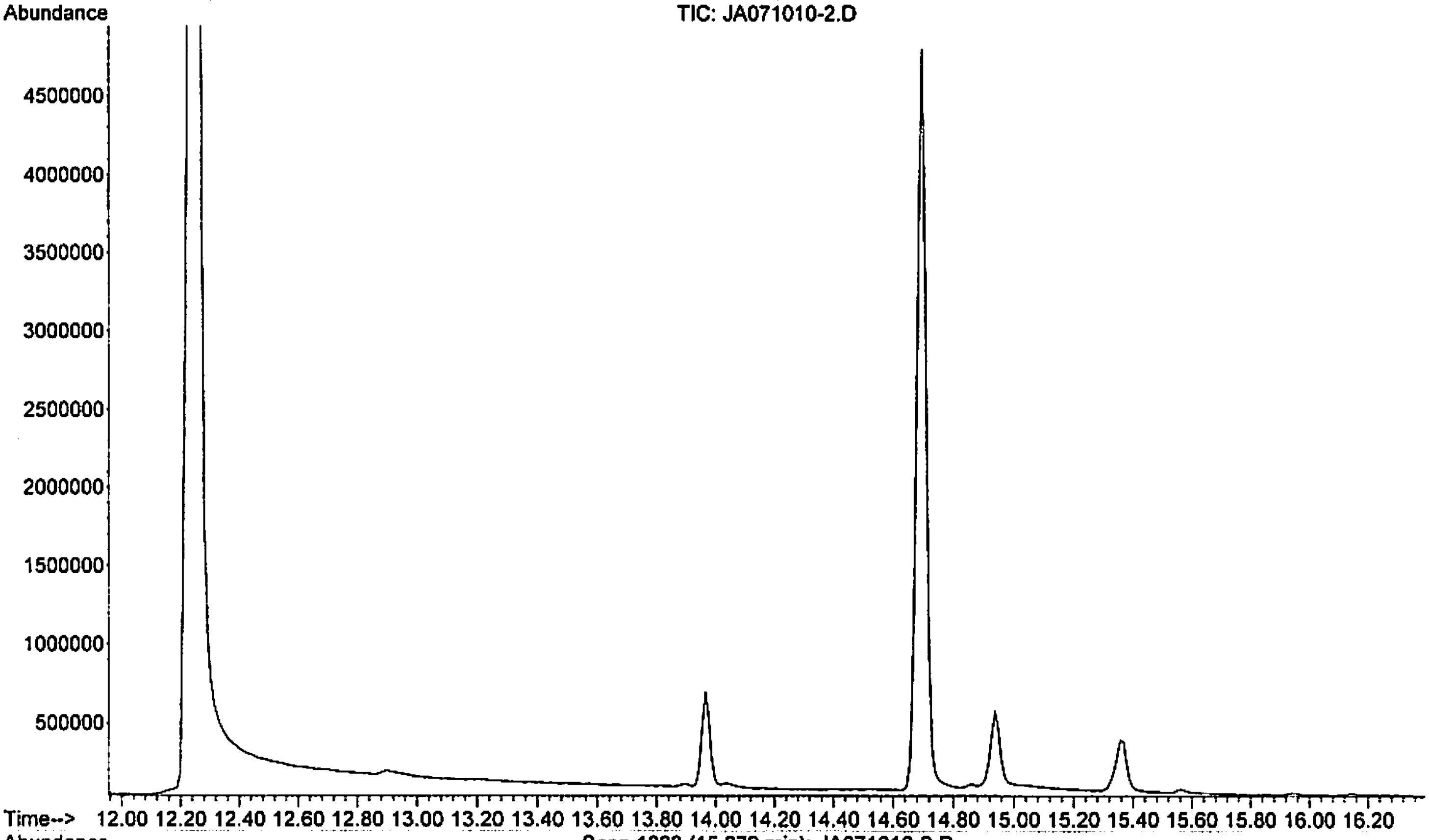

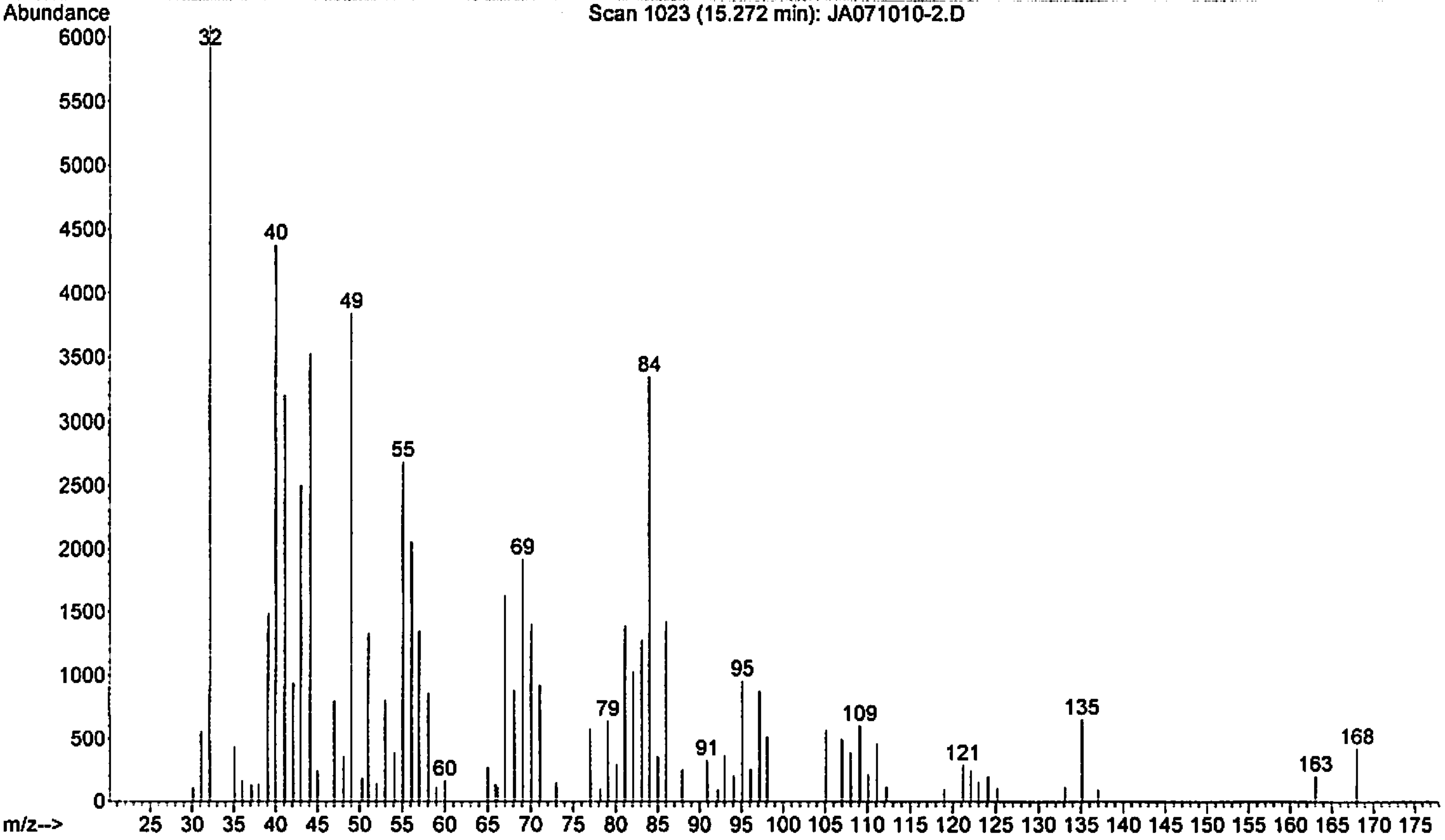

File :D:\DATA\ALDRICH\JA-09\Snapshot\JA071010-2.D
Operator : Aldrich
Acquired : 10 Jul 2009 17:18 using AcqMethod JA-WAX09.M
Instrument : Instrument #1
Sample Name: 10 lab male 2wk-old fed nepetalactol 7/1-10
Misc Info : JA071009-1 & here C.oculata; rev.diss order
Vial Number: 1

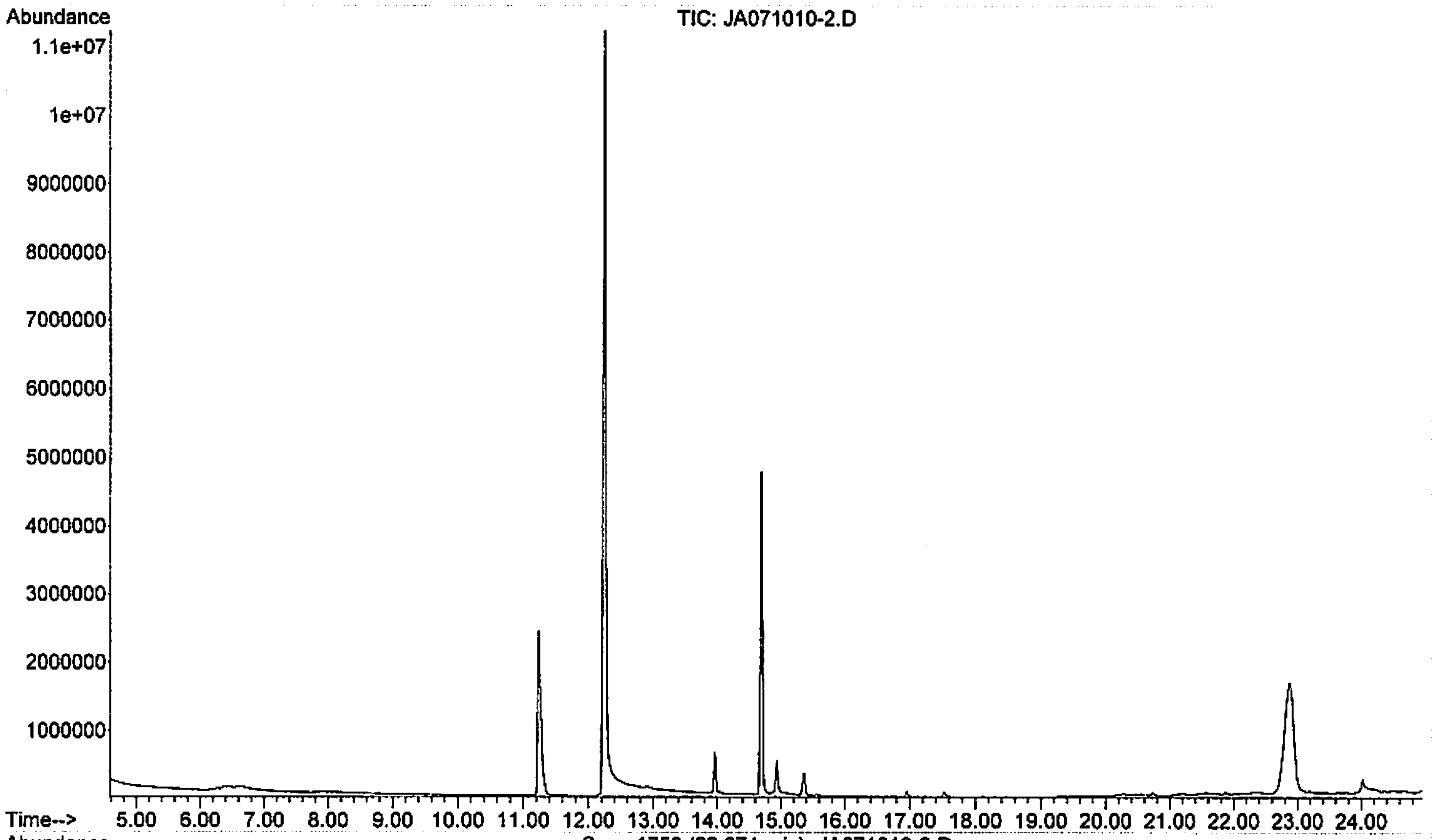

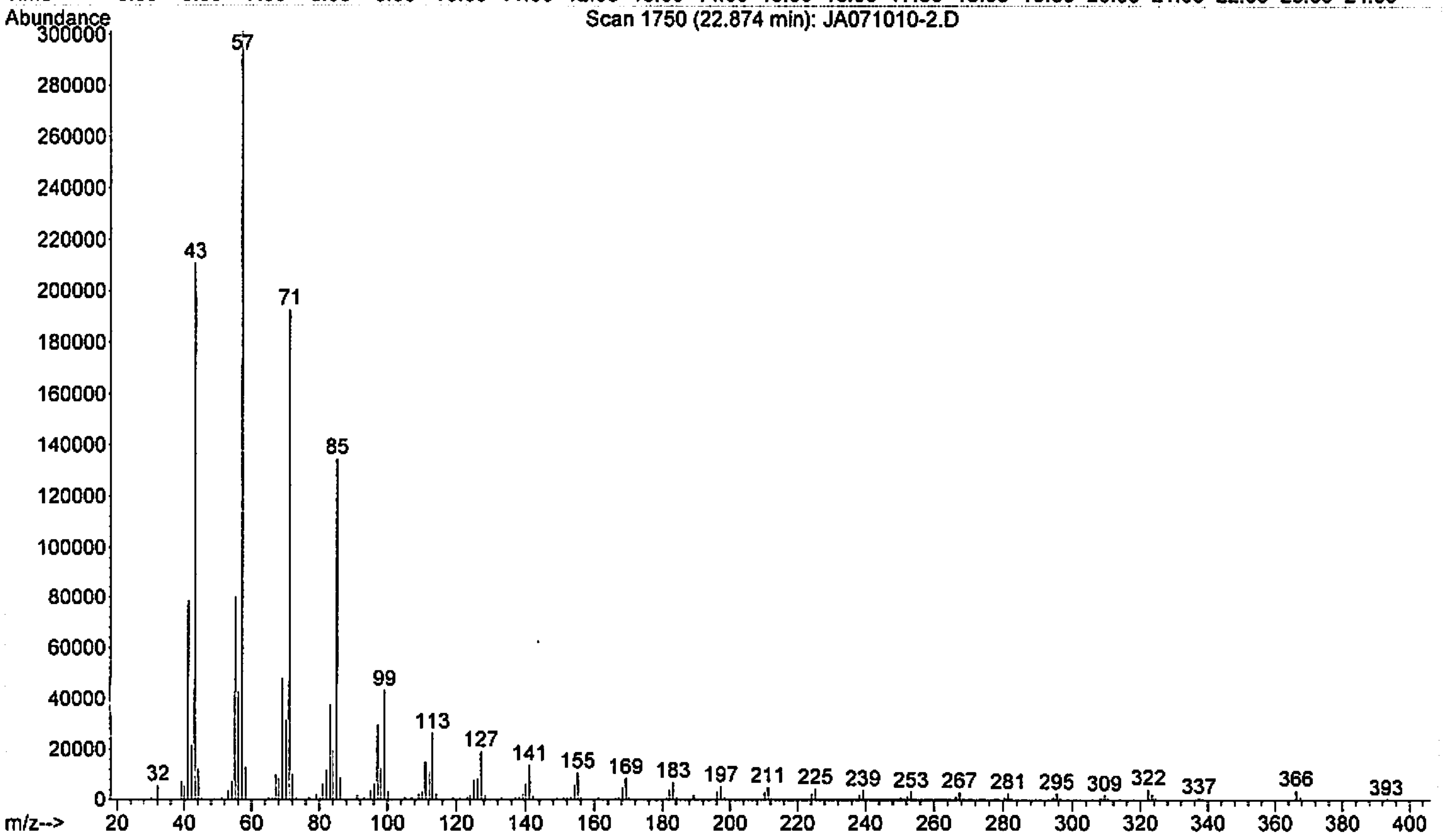

```
File        :D:\DATA\ALDRICH\JA-09\Snapshot\JA071010-2.D
Operator    : Aldrich
Acquired    : 10 Jul 2009  17:18     using AcqMethod JA-WAX09.M
Instrument :     Instrument #1
Sample Name: 10 lab male 2wk-old fed nepetalactol 7/1-10
Misc Info   : JA071009-1 & here C.oculata; rev.diss order
Vial Number: 1
```

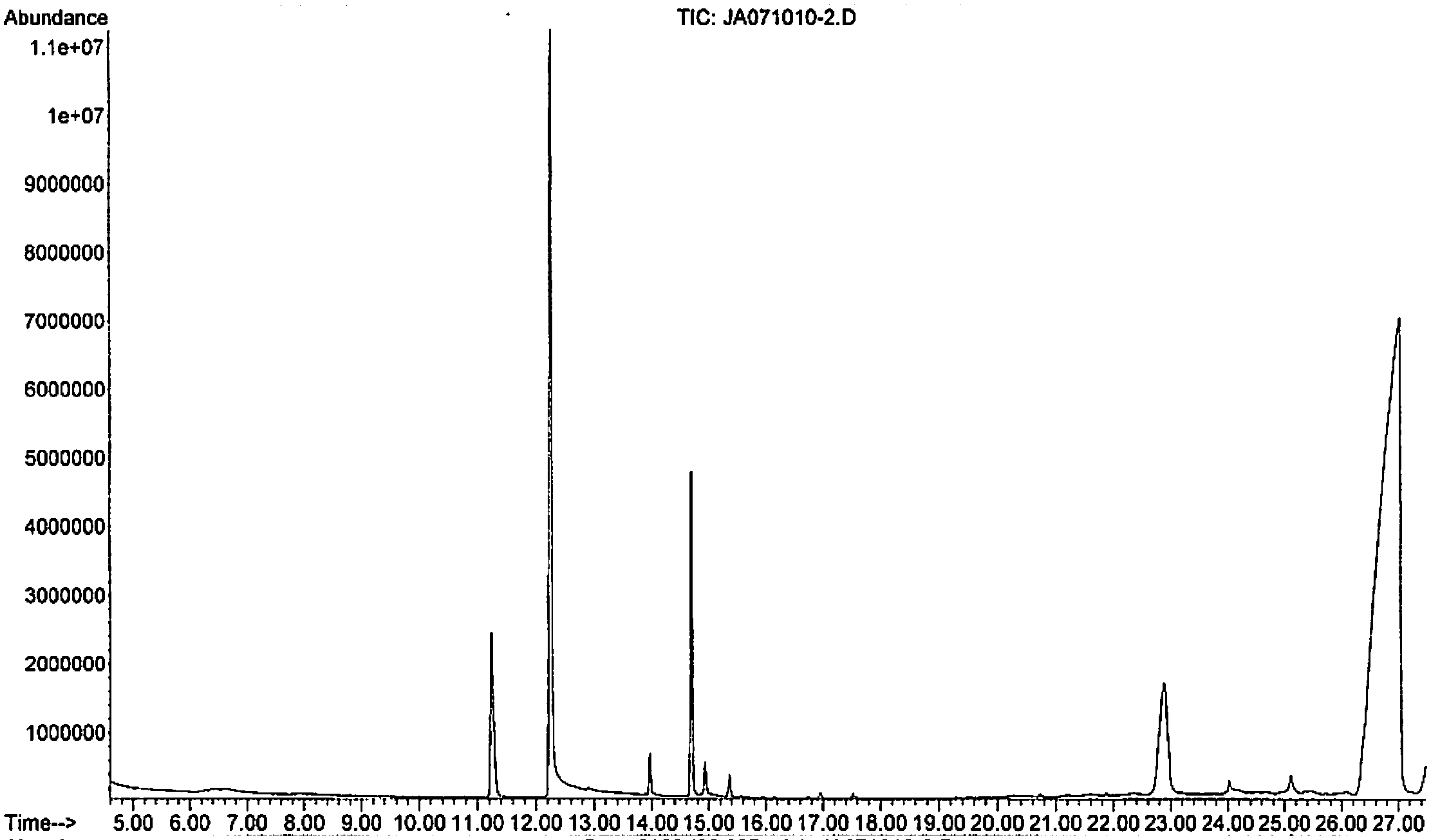

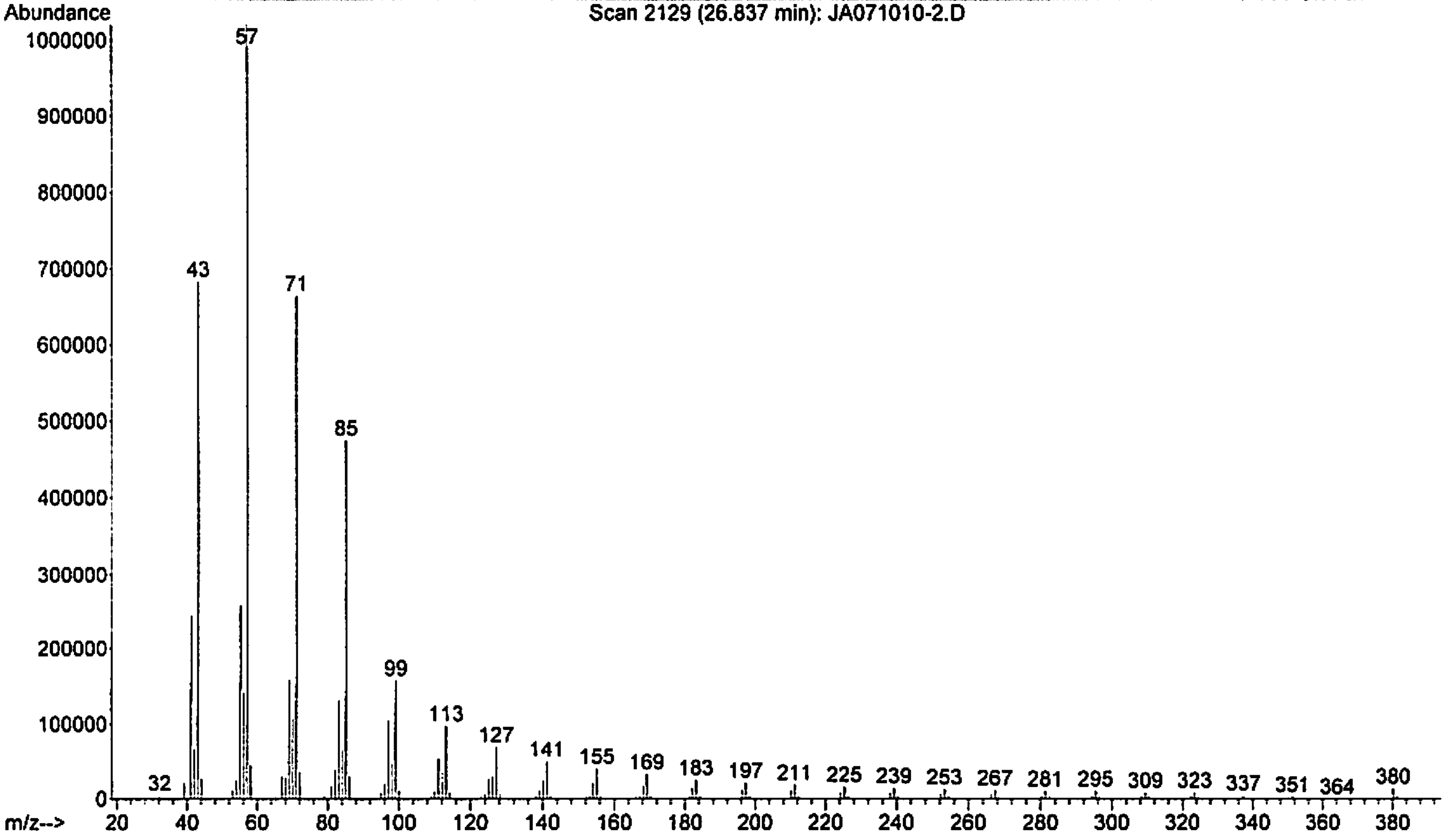

File :D:\DATA\ALDRICH\JA-09\Snapshot\JA071010-2.D
Operator : Aldrich
Acquired : 10 Jul 2009 17:18 using AcqMethod JA-WAX09.M
Instrument : Instrument #1
Sample Name: 10 lab male 2wk-old fed nepetalactol 7/1-10
Misc Info : JA071009-1 & here C.oculata; rev.diss order
Vial Number: 1

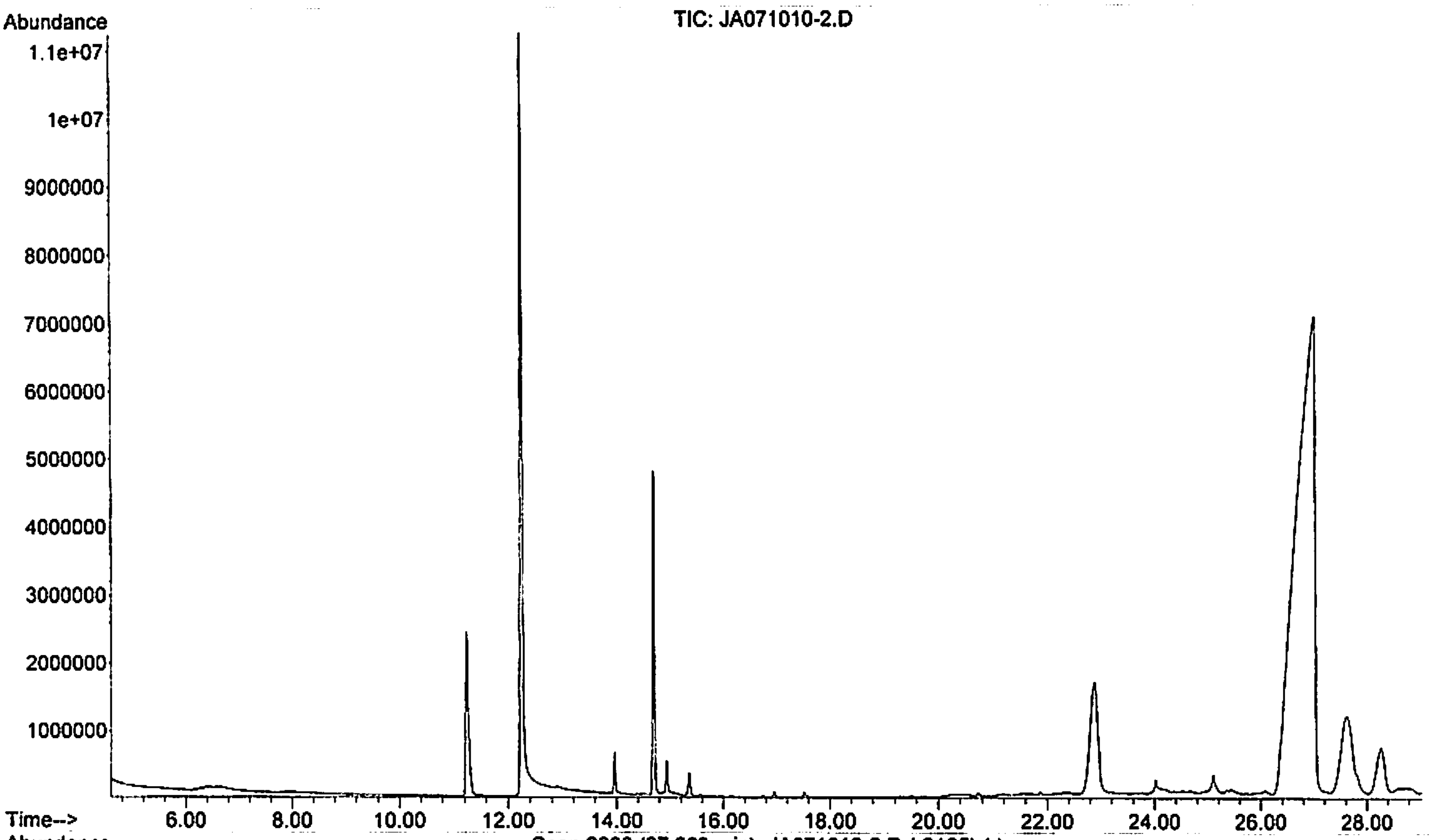

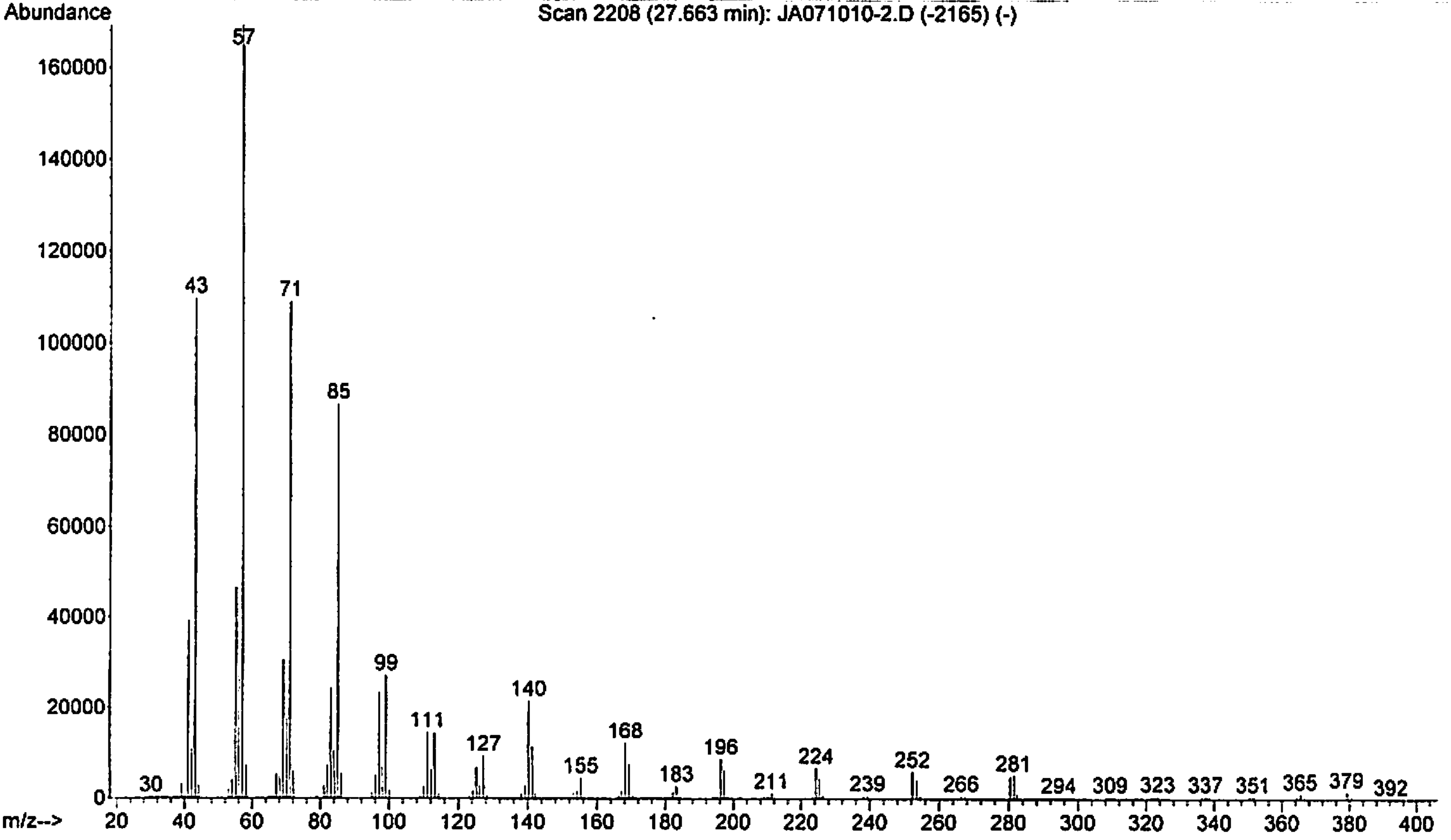

```
File        :D:\DATA\ALDRICH\JA-09\Snapshot\JA071010-2.D
Operator    : Aldrich
Acquired    : 10 Jul 2009  17:18     using AcqMethod JA-WAX09.M
Instrument  :    Instrument #1
Sample Name: 10 lab male 2wk-old fed nepetalactol 7/1-10
Misc Info   : JA071009-1 & here C.oculata; rev.diss order
Vial Number: 1
```

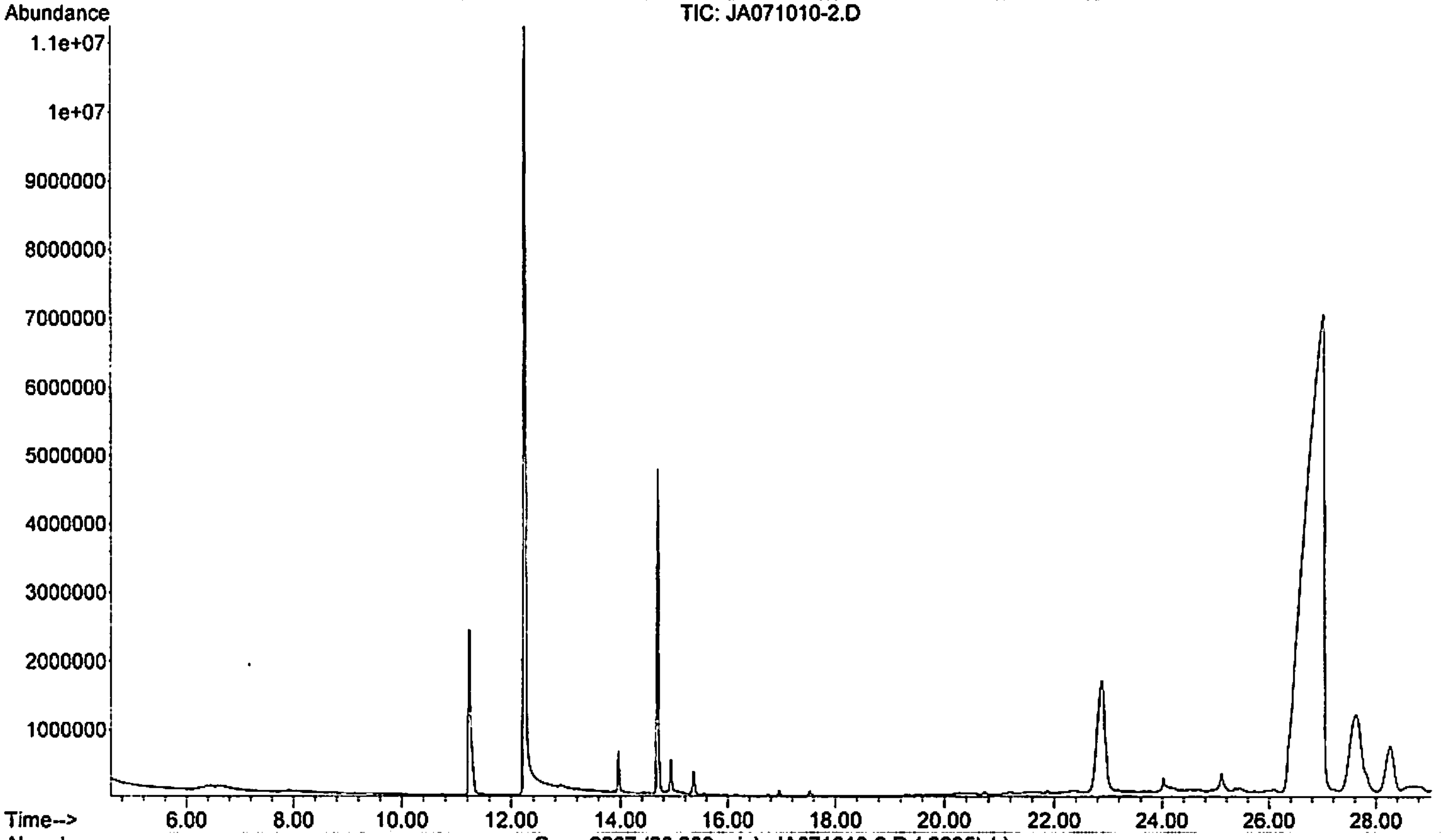

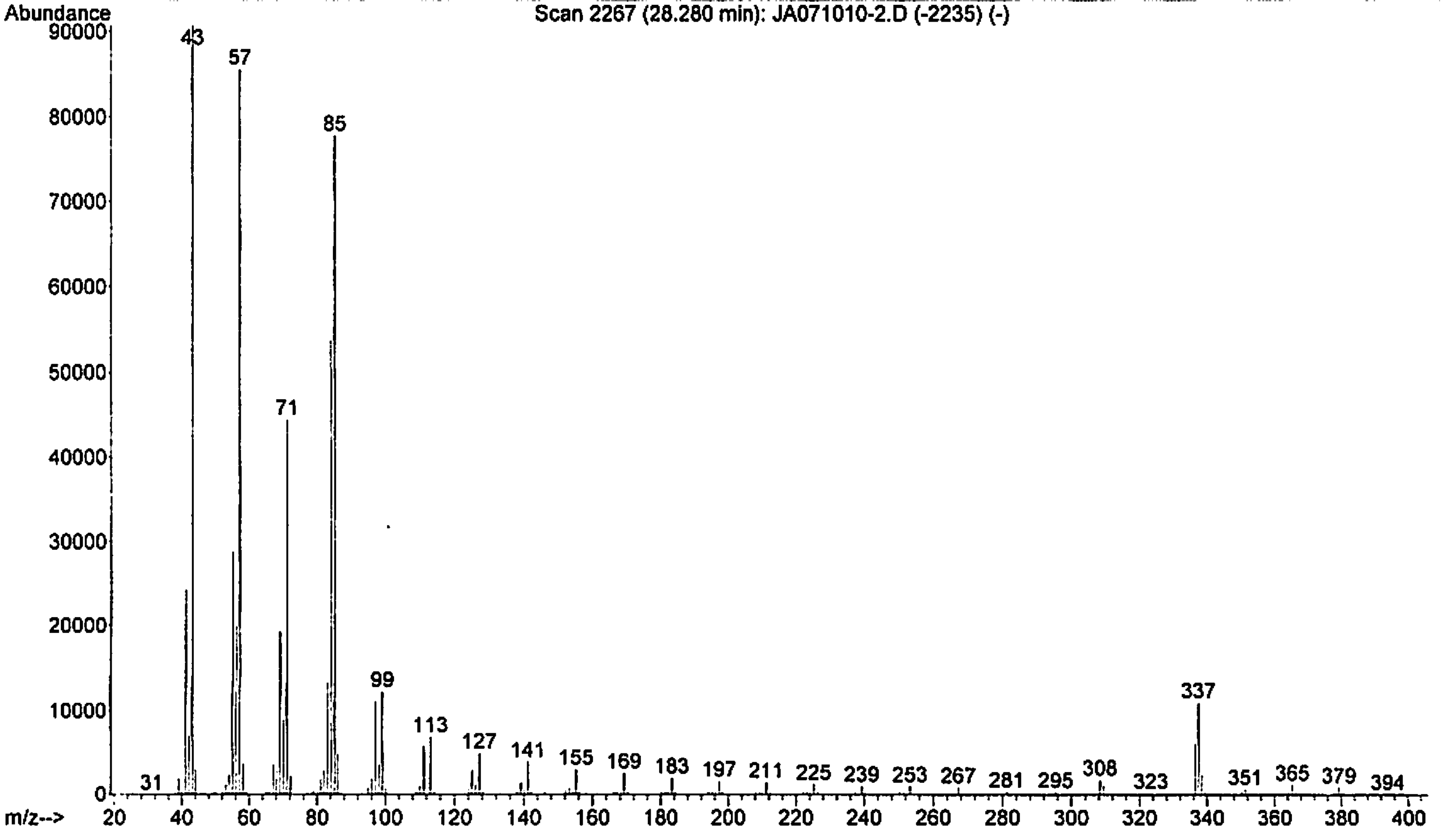

Supplement: Data S1 [file peerj-04-1564-s006.pdf]
