# Supplementary material for: Pharmacophagy in green lacewings (Neuroptera: Chrysopidae: Chrysopa spp.)?
Source: PeerJ. 2016 Jan 18;4:e1564. doi: 10.7717/peerj.1564 (PMC4727961; doi:10.7717/peerj.1564)

Area Percent Report

Data Path : D:\DATA\Aldrich\JA-09\
Data File : JA032409-1.D
Acq On : 24 Mar 2009 14:43
Operator : Aldrich
Sample : 8 male C.oculata 1-5-d-old antennectomized
Misc : dissected 13 days later/5ul CH2Cl2 (Sig #1); (Sig #2)
ALS Vial : 1 Sample Multiplier: 1

Integration Parameters: AUTOINT1.E
Integrator: ChemStation 6890 Scale Mode: Large solvent peaks clipped

Method : C:\MSDCHEM\1\METHODS\JA-XLB09.M

Signal : TIC

peak #	R.T. min	first scan	max scan	last scan	PK TY	peak height	corr. area	corr. % max.	% of total
1	11.279	680	689	715	BB	4993761	168644611	21.30%	17.330%
2	11.991	748	757	773	BV	327611	12697454	1.60%	1.305%
3	12.336	773	790	824	VV	17052181	791811124	100.00%	81.366%

Sum of corrected areas: 973153189

Signal : JA032409-1.D\TST1A.CH

peak #	R.T. min	Start min	End min	PK TY	peak height	corr. area	corr. % max.	% of total
1	4.250	4.057	4.437	BV	9449	756272	1.00%	0.053%
2	4.750	4.437	4.970	PV	946821	76002112	100.00%	5.297%
3	5.250	4.970	5.463	PV	94662	7596640	10.00%	0.529%
4	5.750	5.463	5.937	PV	9449	756272	1.00%	0.053%
5	6.250	5.937	6.470	PV	946822	76002112	100.00%	5.297%
6	6.750	6.470	6.963	PV	94662	7596640	10.00%	0.529%
7	7.250	6.963	7.437	PV	9449	756272	1.00%	0.053%
8	7.750	7.437	7.970	PV	946822	76002112	100.00%	5.297%
9	8.250	7.970	8.463	PV	94662	7596640	10.00%	0.529%
10	8.750	8.463	8.937	PV	9449	756272	1.00%	0.053%
11	9.250	8.937	9.470	PV	946822	76002112	100.00%	5.297%
12	9.750	9.470	9.963	PV	94662	7596640	10.00%	0.529%
13	10.250	9.963	10.437	PV	9449	756272	1.00%	0.053%
14	10.750	10.437	10.970	PV	946822	76002112	100.00%	5.297%
15	11.250	10.970	11.463	PV	94662	7596640	10.00%	0.529%
16	11.750	11.463	11.937	PV	9449	756272	1.00%	0.053%
17	12.250	11.937	12.470	PV	946822	76002112	100.00%	5.297%
18	12.750	12.470	12.963	PV	94662	7596640	10.00%	0.529%
19	13.250	12.963	13.437	PV	9449	756272	1.00%	0.053%
20	13.750	13.437	13.970	PV	946822	76002112	100.00%	5.297%
21	14.250	13.970	14.463	PV	94662	7596640	10.00%	0.529%
22	14.750	14.463	14.937	PV	9449	756272	1.00%	0.053%
23	15.250	14.937	15.470	PV	946822	76002112	100.00%	5.297%
24	15.750	15.470	15.963	PV	94662	7596640	10.00%	0.529%
25	16.250	15.963	16.437	PV	9449	756272	1.00%	0.053%
26	16.750	16.437	16.970	PV	946822	76002112	100.00%	5.297%
27	17.250	16.970	17.463	PV	94662	7596640	10.00%	0.529%
28	17.750	17.463	17.937	PV	9449	756272	1.00%	0.053%
29	18.250	17.937	18.470	PV	946822	76002112	100.00%	5.297%
30	18.750	18.470	18.963	PV	94662	7596640	10.00%	0.529%
31	19.250	18.963	19.437	PV	9449	756272	1.00%	0.053%

32	19.750	19.437	19.970	PV	946822	76002112	100.00%	5.297%
33	20.250	19.970	20.463	PV	94662	7596640	10.00%	0.529%
34	20.750	20.463	20.937	PV	9449	756272	1.00%	0.053%
35	21.250	20.937	21.470	PV	946822	76002112	100.00%	5.297%
36	21.750	21.470	21.963	PV	94662	7596640	10.00%	0.529%
37	22.250	21.963	22.437	PV	9449	756272	1.00%	0.053%
38	22.750	22.437	22.970	PV	946822	76002112	100.00%	5.297%
39	23.250	22.970	23.463	PV	94662	7596640	10.00%	0.529%
40	23.750	23.463	23.937	PV	9449	756272	1.00%	0.053%
41	24.250	23.937	24.470	PV	946822	76002112	100.00%	5.297%
42	24.750	24.470	24.963	PV	94662	7596640	10.00%	0.529%
43	25.250	24.963	25.437	PV	9449	756272	1.00%	0.053%
44	25.750	25.437	25.970	PV	946822	76002112	100.00%	5.297%
45	26.250	25.970	26.463	PV	94662	7596640	10.00%	0.529%
46	26.750	26.463	26.937	PV	9449	756272	1.00%	0.053%
47	27.250	26.937	27.470	PV	946822	76002112	100.00%	5.297%
48	27.750	27.470	27.963	PV	94662	7596640	10.00%	0.529%
49	28.250	27.963	28.437	PV	9449	756272	1.00%	0.053%
50	28.750	28.437	28.970	PV	946822	76002112	100.00%	5.297%
51	29.250	28.970	29.463	PV	94662	7596640	10.00%	0.529%
52	29.750	29.463	29.968	PBA	9449	756272	1.00%	0.053%

Sum of corrected areas: 1434791680

[illegible]A-XLB09.M Fri Jul 24 15:12:46 2009

```
File       :D:\DATA\Aldrich\JA-09\JA032409-1.D
Operator   : Aldrich
Acquired   : 24 Mar 2009  14:43     using AcqMethod JA-WAX08.M
Instrument :   Instrument #1
Sample Name: 8 male C.oculata 1-5-d-old antennectomized
Misc Info  : dissected 13 days later/5ul CH2Cl2
Vial Number: 1
```

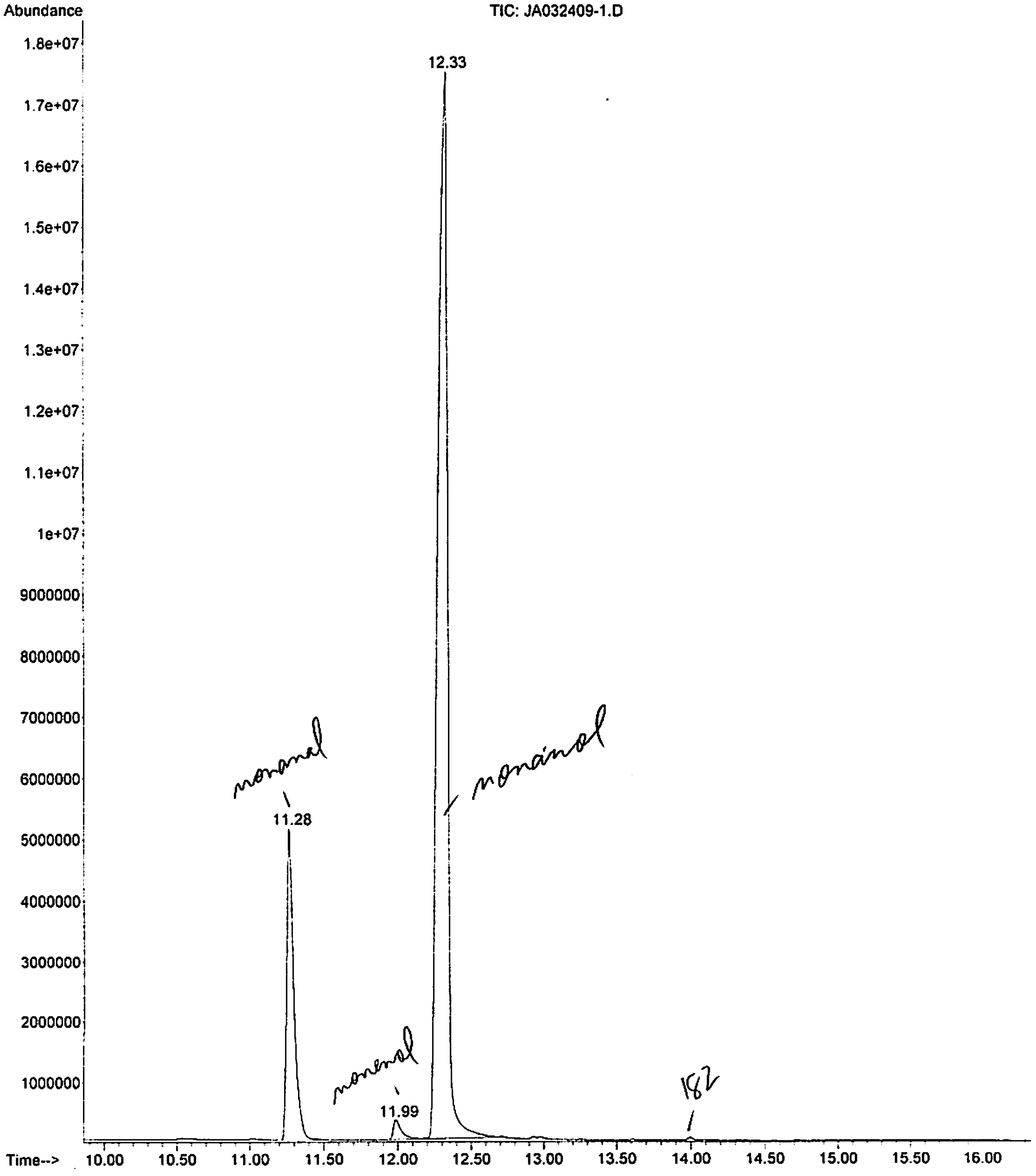

Supplement: Data S2 [file peerj-04-1564-s007.pdf]
