# Supplementary material for: Pharmacophagy in green lacewings (Neuroptera: Chrysopidae: Chrysopa spp.)?
Source: PeerJ. 2016 Jan 18;4:e1564. doi: 10.7717/peerj.1564 (PMC4727961; doi:10.7717/peerj.1564)

inj. 1.5ul Nepeta cataria 2nd leaf extract (/50ul CH2Cl2), DB-5. Note: From plant grown in our greenhouse; filter paper rubbing.

===

Injection Date	: 5/28/2008 2:01:16 PM		
Sample Name	: Nepeta cataria	Vial :	-
Acq. Operator	: Aldrich	Inj :	1
		Inj Volume :	Manually
Method	: C:\HPCHEM\1\METHODS\DBLESS1.M		
Last changed	: 2/29/2008 10:02:00 AM by Aldrich		

2/29/07; editing new method for desired output

FID1 A, (NEPETA\JA-05282.D)

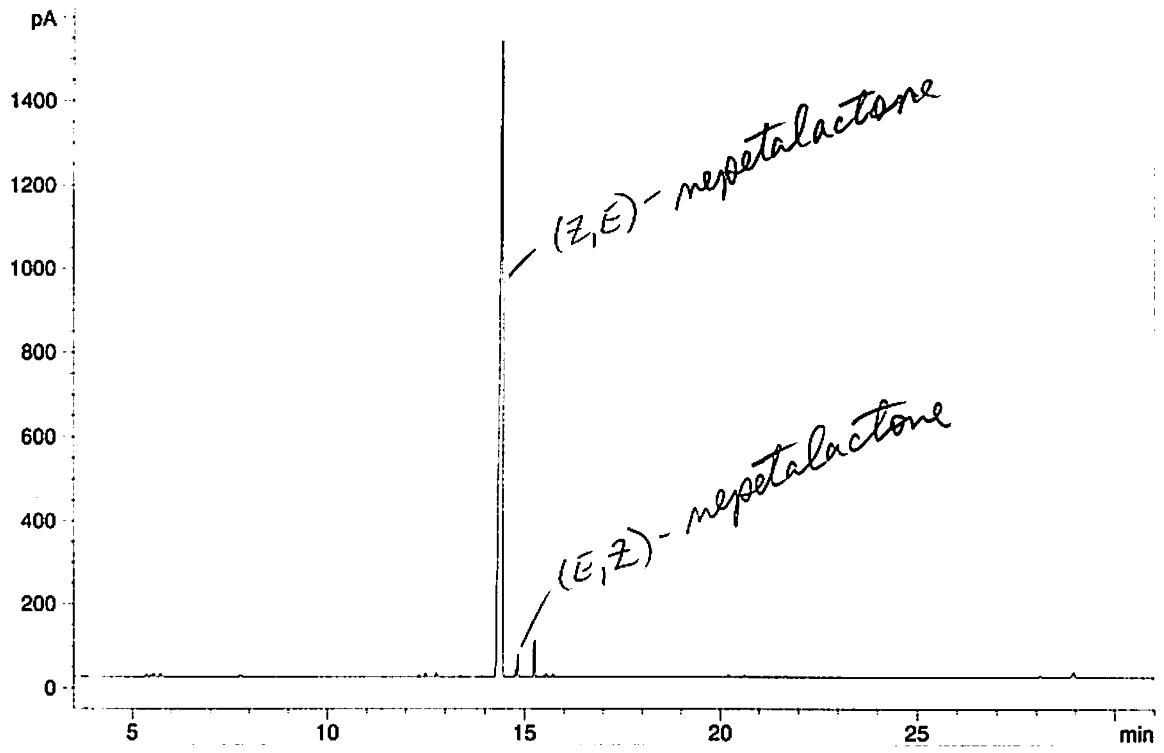

===

Area Percent Report

===

Sorted By	:	Signal
Multiplier	:	1.0000
Dilution	:	1.0000

Signal 1: FID1 A,

Peak #	RetTime [min]	Type	Width [min]	Area [pA*s]	Height [pA]	Area %
1	5.356	PB	0.0240	8.78574	5.68110	0.10085
2	5.470	PV	0.0243	6.94077	4.41408	0.07967
3	5.543	VB	0.0231	11.74346	7.55296	0.13480
4	5.716	PB	0.0248	12.14510	7.53389	0.13941
5	7.764	PP	0.0254	4.34185	2.61343	0.04984
6	12.329	PB	0.0298	5.45318	2.78975	0.06259
7	12.492	BB	0.0288	17.78157	9.52591	0.20410
8	12.770	BB	0.0273	18.38568	10.07146	0.21104
9	14.440	BB	0.0668	8208.72363	1524.32458	94.22245
10	14.768	BV	0.0282	29.38062	16.17481	0.33724
11	14.832	VB	0.0290	100.39045	53.29138	1.15231
12	15.251	BB	0.0298	163.45575	87.29059	1.87620
13	15.445	BV	0.0281	3.77907	2.08474	0.04338
14	15.494	VV	0.0269	3.75397	2.19825	0.04309
15	15.550	VP	0.0307	15.74674	8.10833	0.18075
16	15.724	BB	0.0303	13.53114	7.07726	0.15531

Peak #	RetTime [min]	Type	Width [min]	Area [pA*s]	Height [pA]	Area %
17	17.368	PB	0.0325	4.66588	2.22459	0.05356
18	20.209	BB	0.0288	9.97267	5.34009	0.11447
19	20.616	PB	0.0321	8.66736	4.19147	0.09949
20	20.691	BB	0.0318	3.88665	1.90400	0.04461
21	21.675	PB	0.0348	7.00172	3.16382	0.08037
22	28.941	BB	0.0701	53.53487	11.55540	0.61449

Totals : 8712.06786 1779.11192

Results obtained with enhanced integrator!

===

*** End of Report ***

IIBB1 Area Percent Report

Data Path : D:\Aldrich\JA-08\
Data File : JA081308-1.D
Acq On : 13 Aug 2008 14:49
Operator :
Sample : 5 lab C.oculata males 20-29-days-old,no plant
Misc : GC run JA0813_1.D; /abd. cuticle/CH2Cl2
ALS Vial : 1 Sample Multiplier: 1

Integration Parameters: autoint1.e
Integrator: ChemStation

Method : C:\msdchem\1\METHODS\Episesquithugene.M
Title :

Signal : TIC: JA081308-1.D\data.ms

peak #	R.T. min	first scan	max scan	last scan	PK TY	peak height	corr. area	corr. % max.	% of total
1	9.197	472	498	515	BB	22329243	651136710	30.81%	21.367%
X 2	9.930	562	570	585	BV	2121796	59621277	2.82%	1.956%
3	10.286	585	604	633	VV	41616730	2113187716	100.00%	69.345%
4	11.564	720	729	732	BV 2	785635	18315625	0.87%	0.601%
5	11.686	732	741	762	VB	1669640	79383677	3.76%	2.605%
6	12.085	765	780	785	BV	526286	10947517	0.52%	0.359%
7	12.423	797	813	819	BB	693433	12703882	0.60%	0.417%
8	12.976	855	867	871	BV 3	1112077	36947248	1.75%	1.212%
9	13.063	871	875	899	VV 2	1611734	65125221	3.08%	2.137%

Sum of corrected areas: 3047368873

Episesquithugene.M Fri Jul 24 14:33:36 2009

```
File        :D:\Aldrich\JA-08\JA081308-1.D
Operator    :
Acquired    : 13 Aug 2008  14:49     using AcqMethod JA-50-280LESS.M
Instrument  :   Buba
Sample Name: 5 lab C.oculata males 20-29-days-old,no plant
Misc Info   : GC run JA0813_1.D; /abd. cuticle/CH2Cl2
Vial Number: 1
```

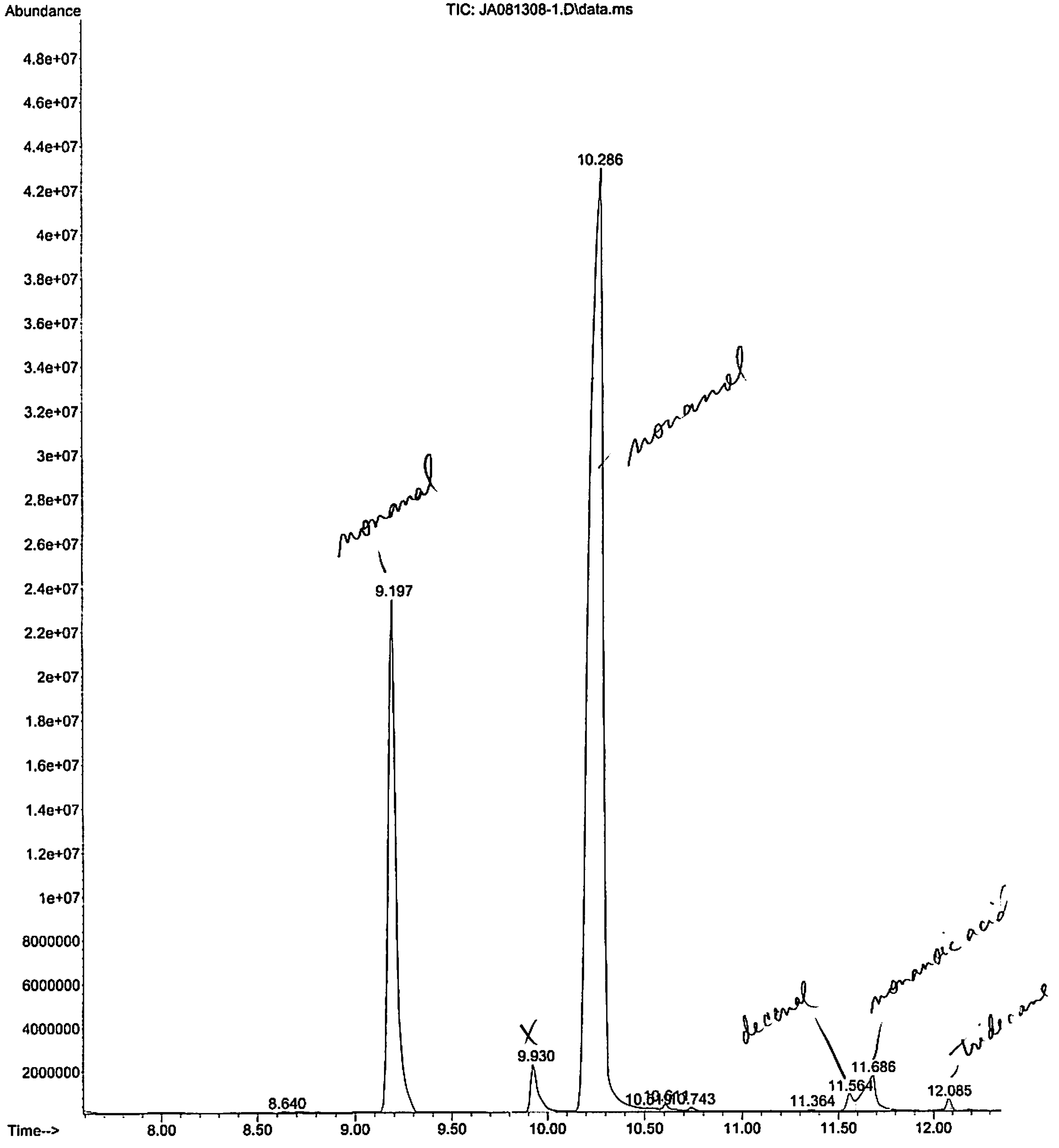

```
File       :D:\Aldrich\JA081208-3.D
Operator   :
Acquired   : 12 Aug 2008  16:43     using AcqMethod JA-50-280LESS.M
Instrument :   Buba
Sample Name: 5 lab-reared C.oculata males w/ catnip; abd.
Misc Info  : GC run JA0812_4.D; /CH2Cl2
Vial Number: 1
```

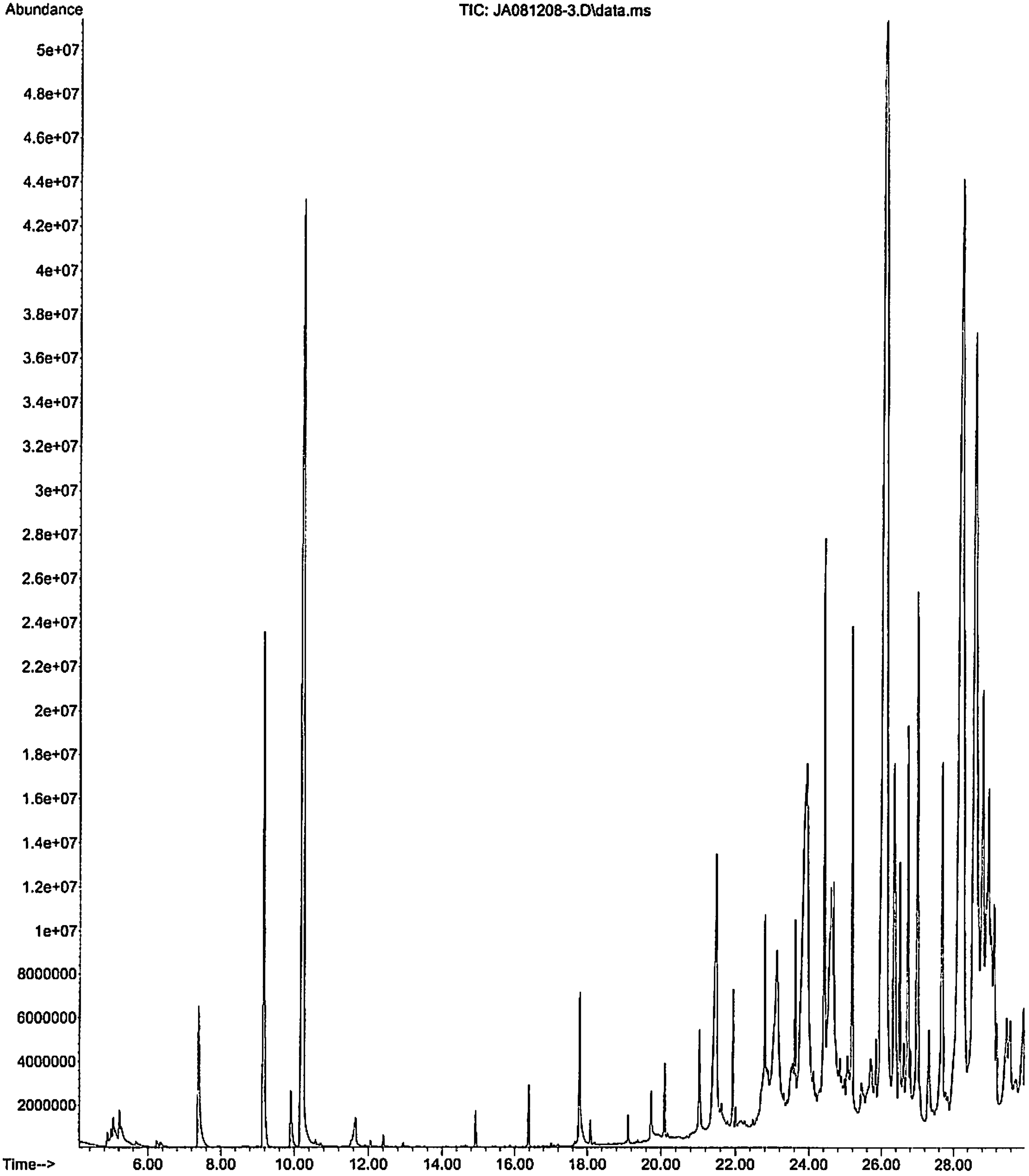

```
File        :D:\Aldrich\JA081208-3.D
Operator    :
Acquired    : 12 Aug 2008  16:43     using AcqMethod JA-50-280LESS.M
Instrument :    Buba
Sample Name: 5 lab-reared C.oculata males w/ catnip; abd.
Misc Info   : GC run JA0812_4.D; /CH2Cl2
Vial Number: 1
```

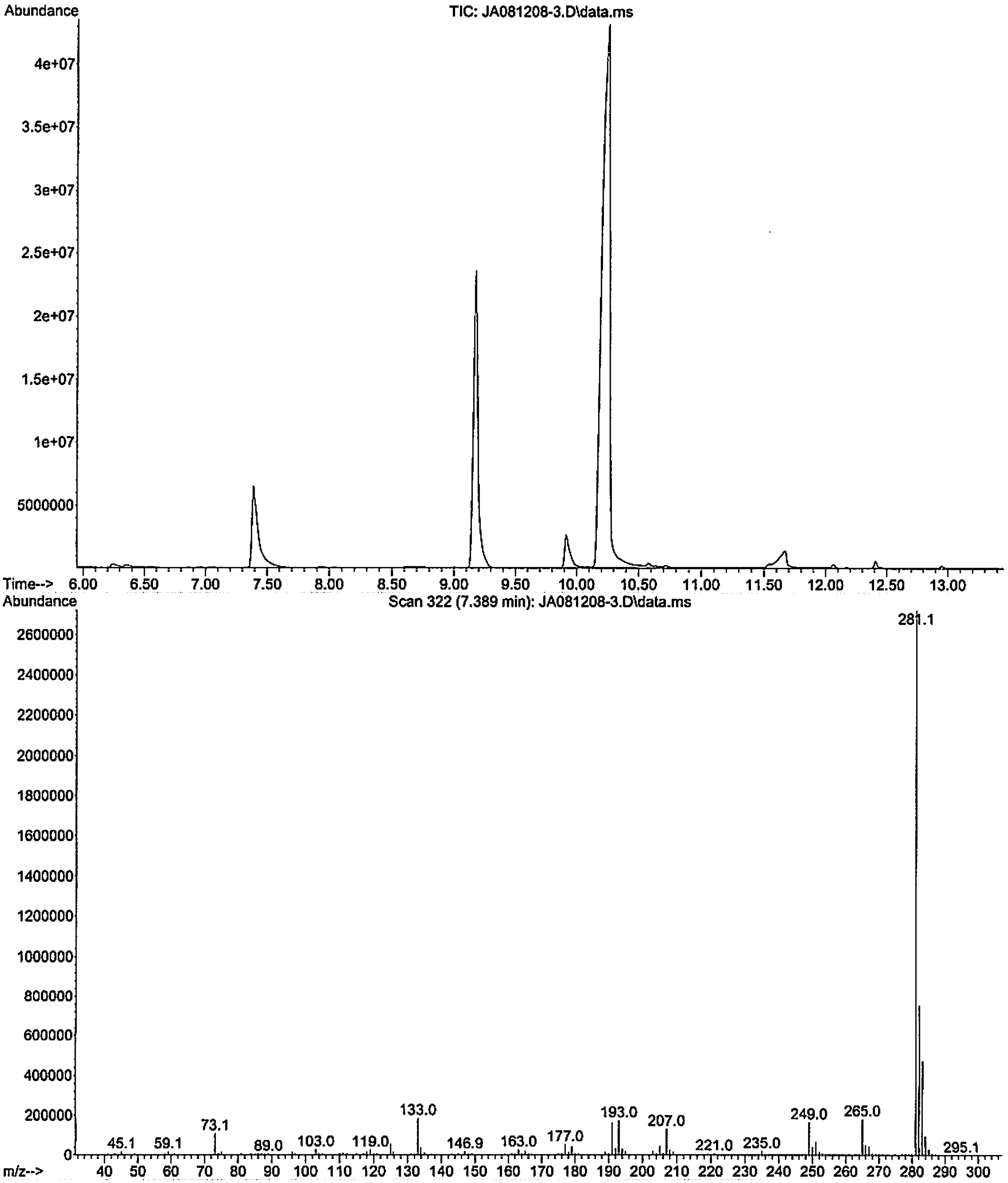

```
File        :D:\Aldrich\JA081208-3.D
Operator    :
Acquired    : 12 Aug 2008  16:43     using AcqMethod JA-50-280LESS.M
Instrument  :    Buba
Sample Name: 5 lab-reared C.oculata males w/ catnip; abd.
Misc Info   : GC run JA0812_4.D; /CH2Cl2
Vial Number: 1
```

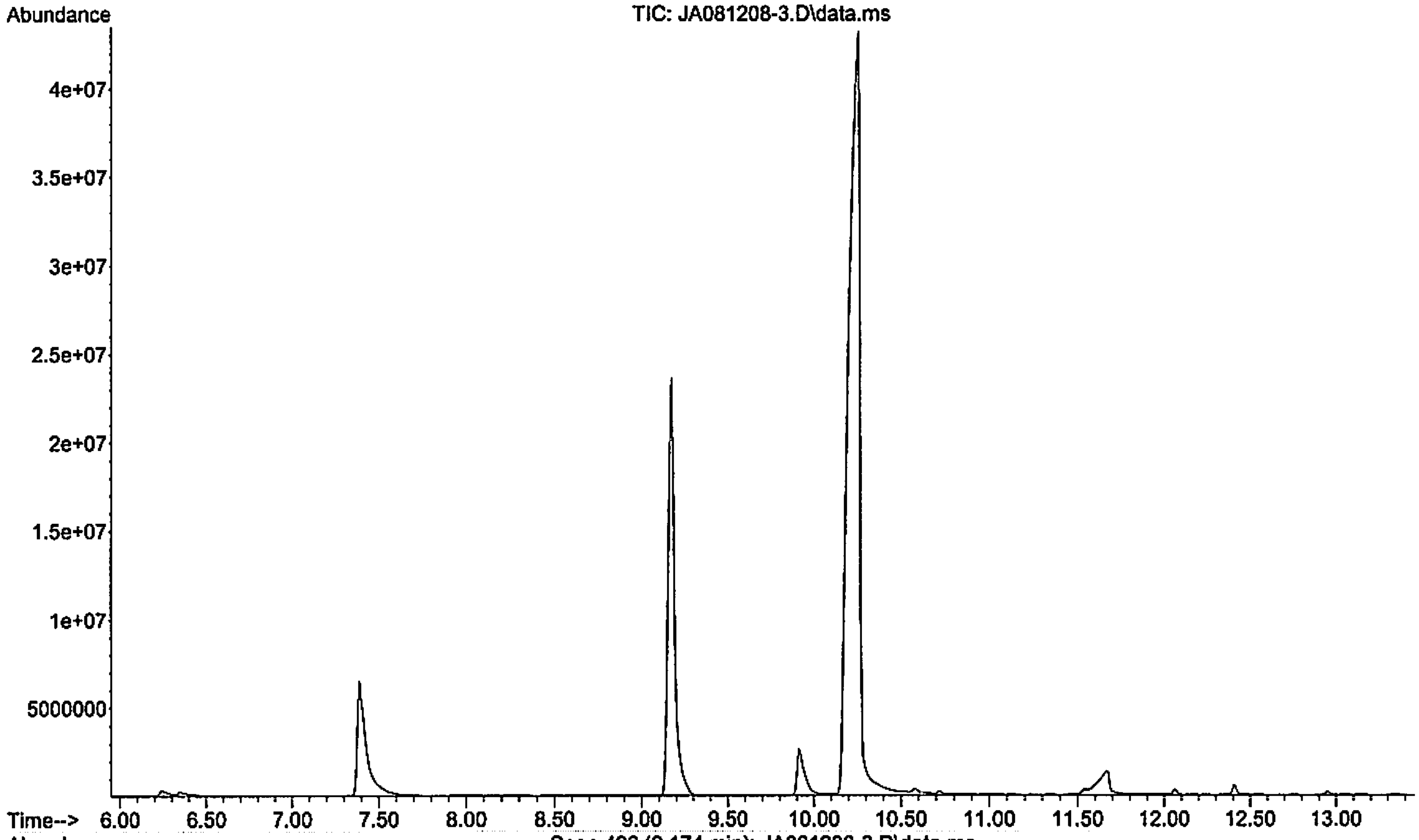

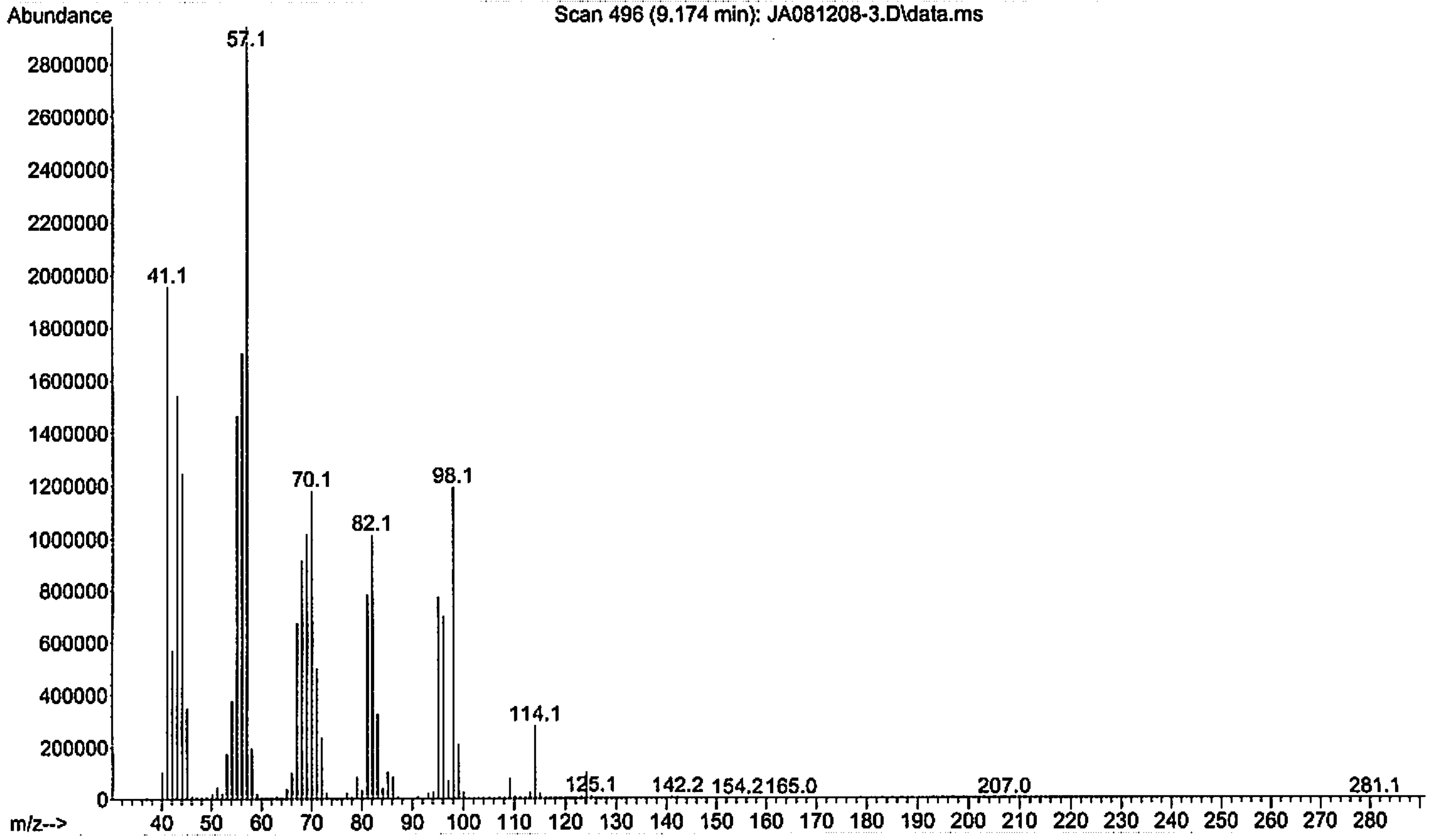

File   :D:\Aldrich\JA081208-3.D
Operator   :
Acquired   : 12 Aug 2008 16:43   using AcqMethod JA-50-280LESS.M
Instrument :   Buba
Sample Name: 5 lab-reared C.oculata males w/ catnip; abd.
Misc Info   : GC run JA0812_4.D; /CH2Cl2
Vial Number: 1

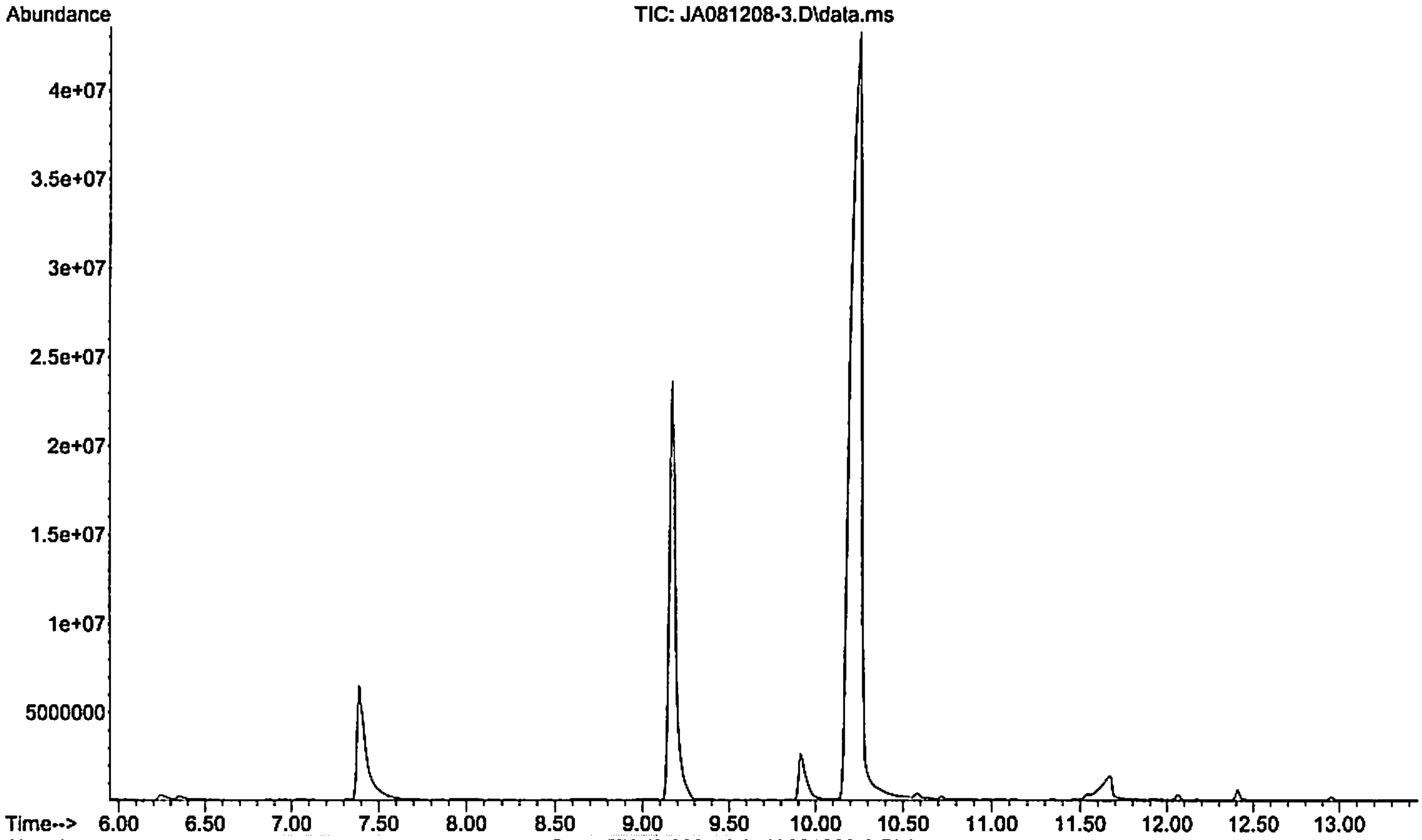

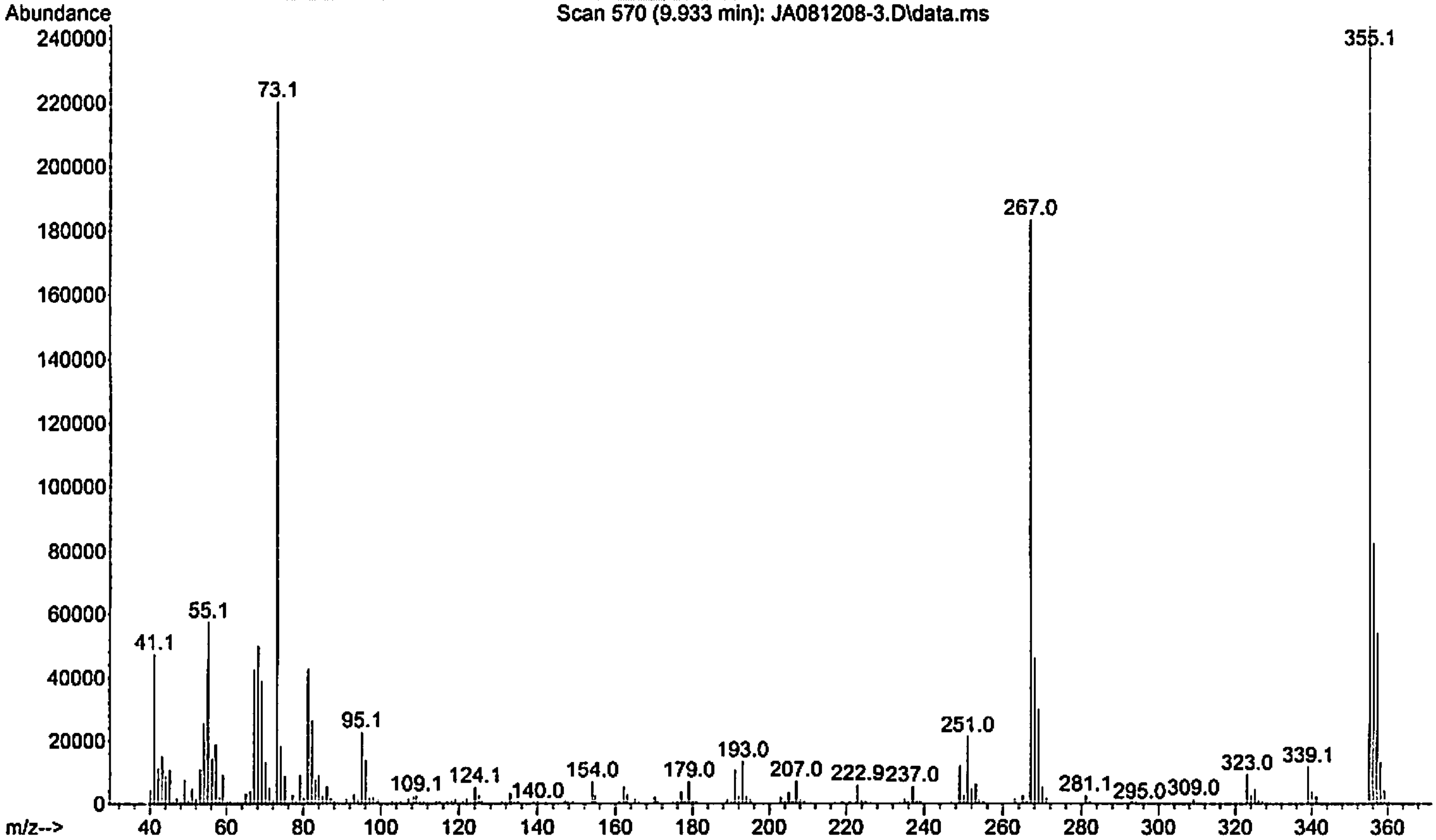

File   :D:\Aldrich\JA081208-3.D
Operator   :
Acquired   : 12 Aug 2008 16:43   using AcqMethod JA-50-280LESS.M
Instrument :   Buba
Sample Name: 5 lab-reared C.oculata males w/ catnip; abd.
Misc Info : GC run JA0812_4.D; /CH2Cl2
Vial Number: 1

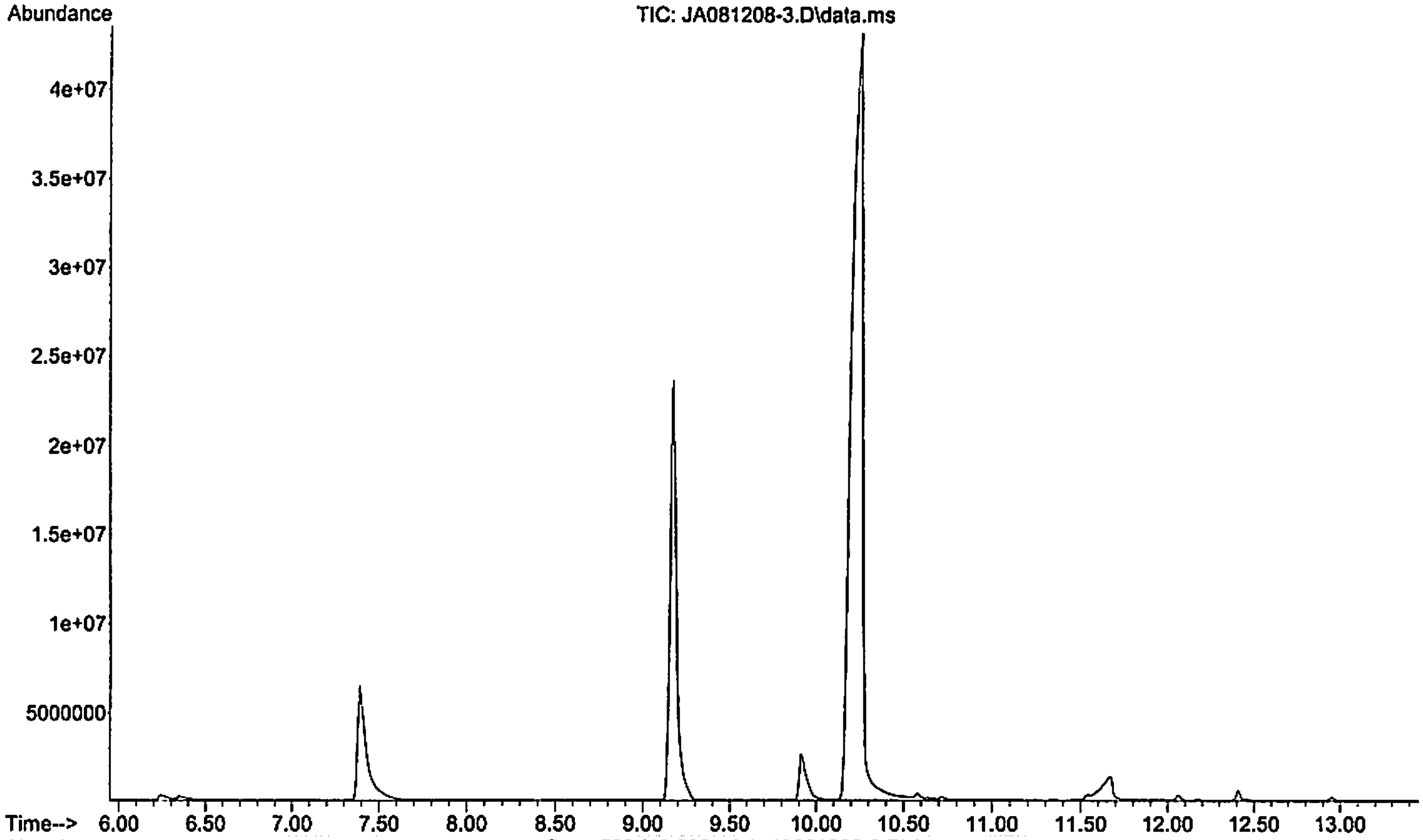

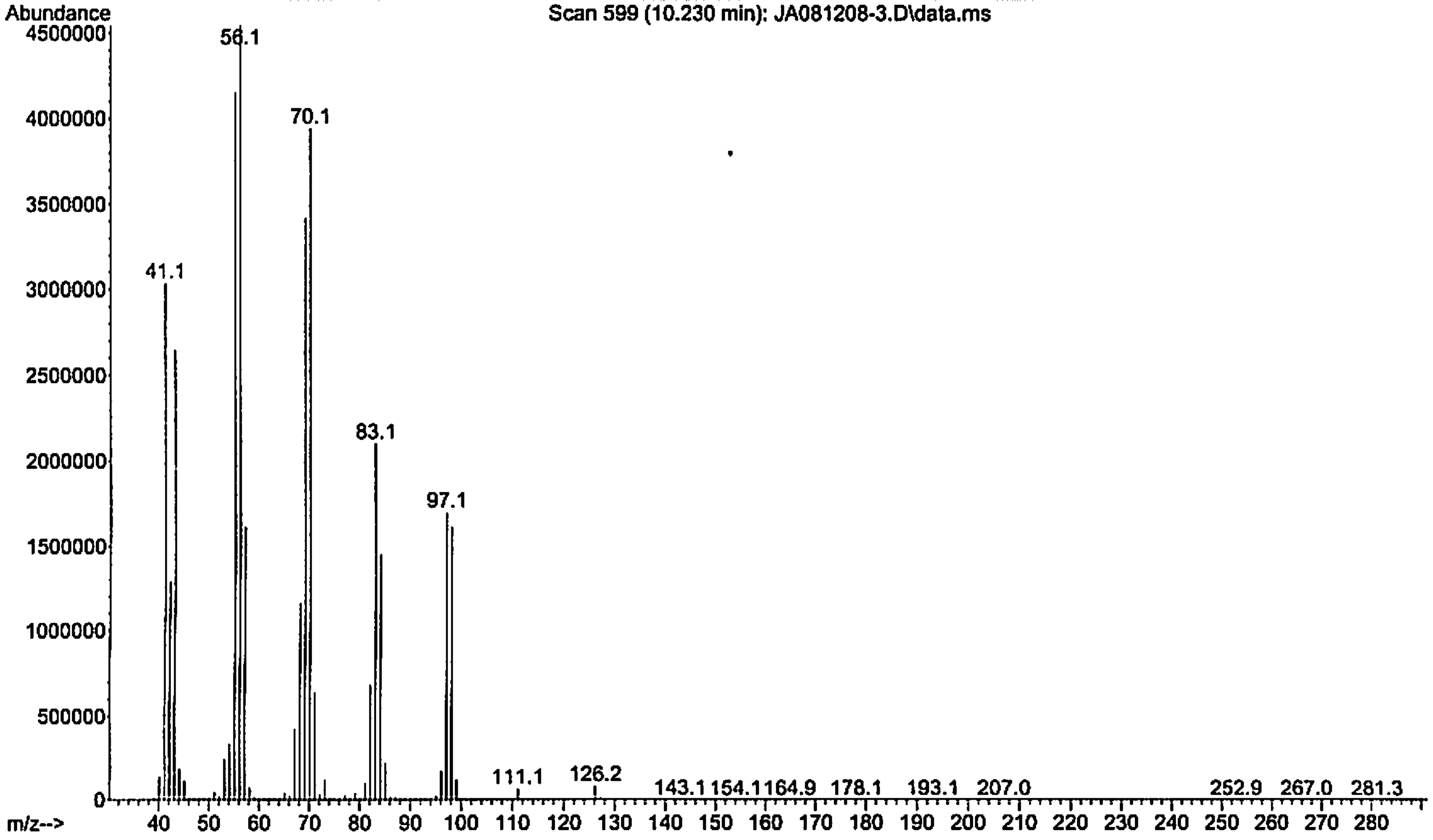

```
File        :D:\Aldrich\JA081208-3.D
Operator    :
Acquired    : 12 Aug 2008  16:43     using AcqMethod JA-50-280LESS.M
Instrument :    Buba
Sample Name: 5 lab-reared C.oculata males w/ catnip; abd.
Misc Info   : GC run JA0812_4.D; /CH2Cl2
Vial Number: 1
```

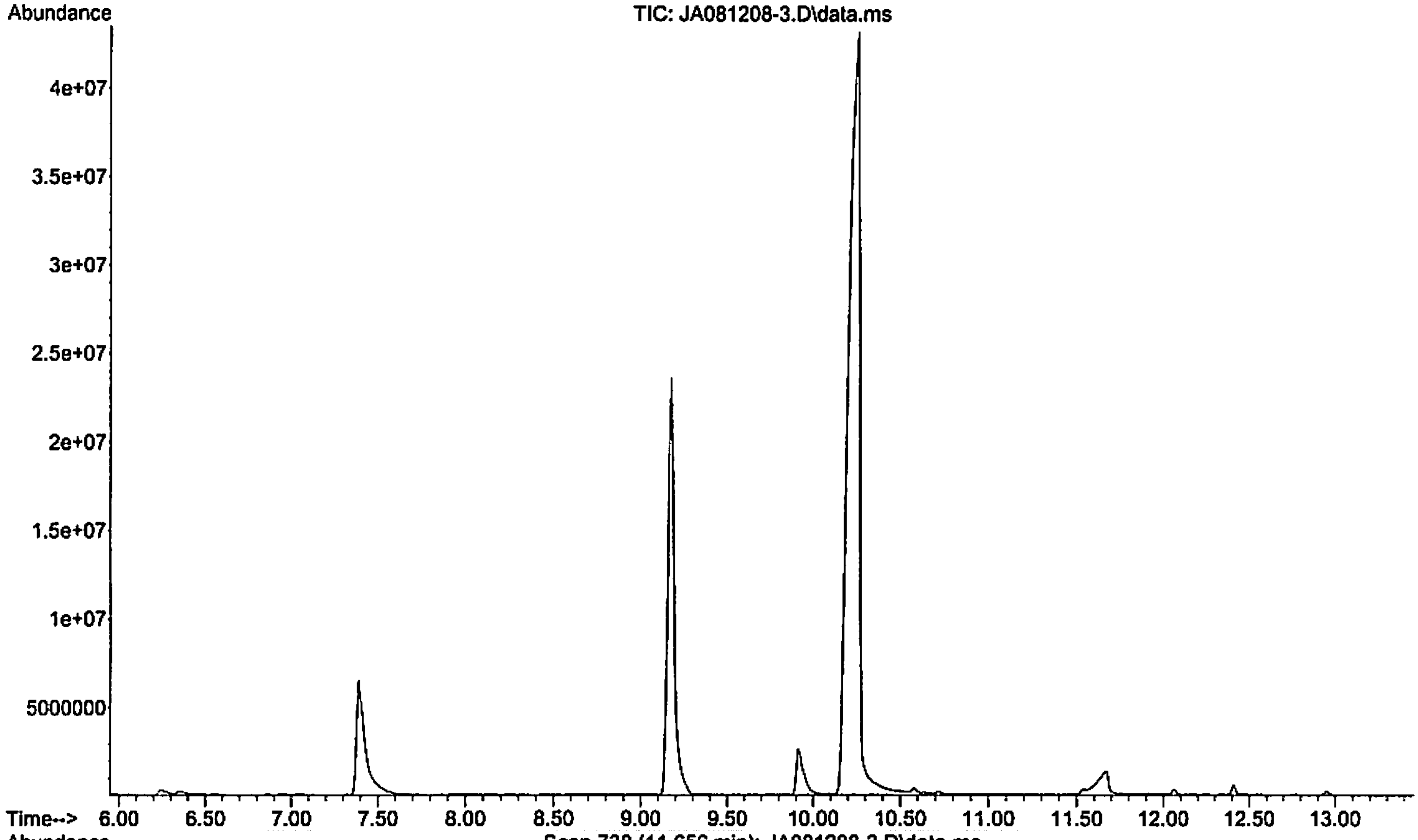

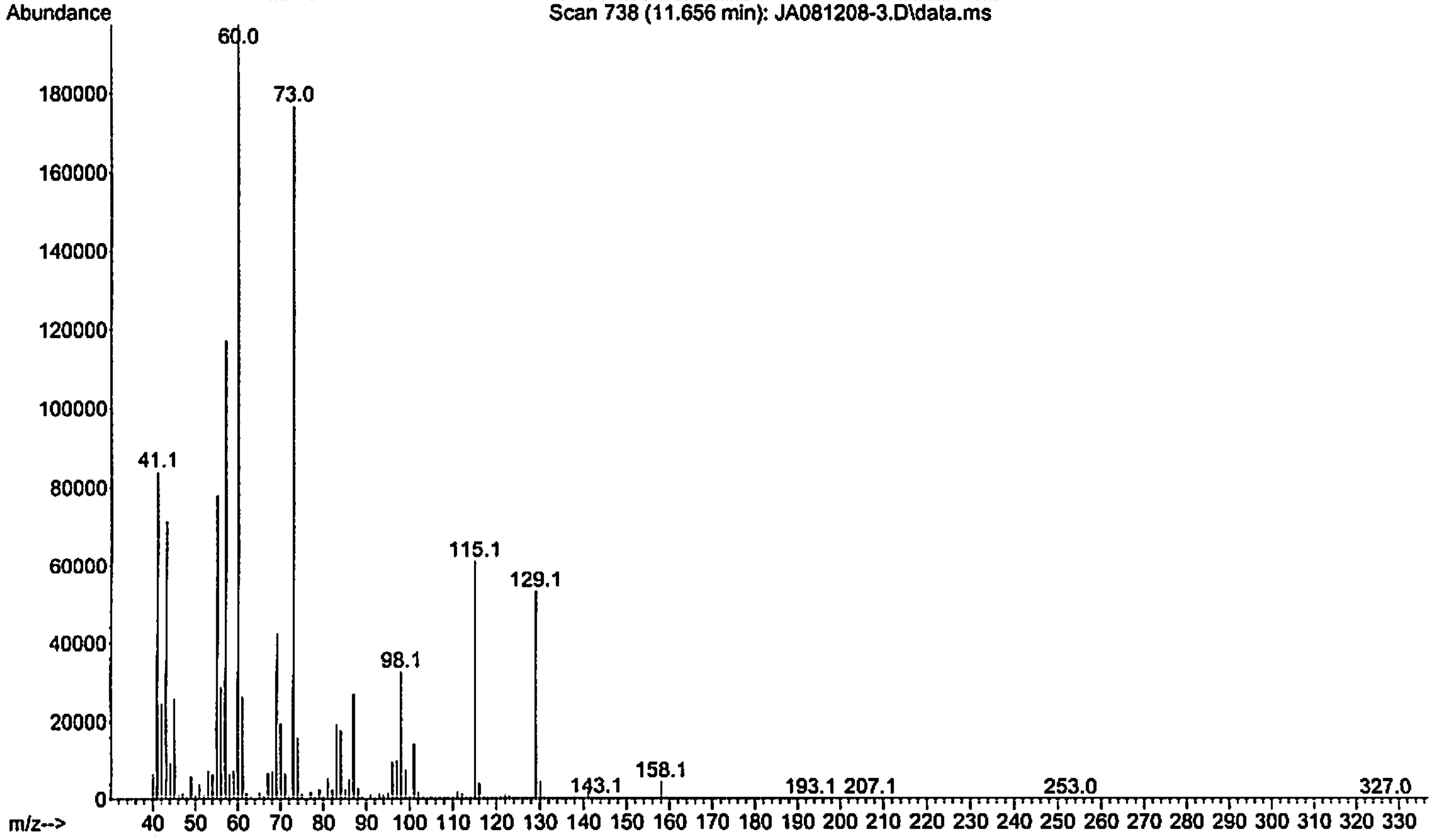

```
File        :D:\Aldrich\JA081208-3.D
Operator    :
Acquired    : 12 Aug 2008  16:43     using AcqMethod JA-50-280LESS.M
Instrument :    Buba
Sample Name: 5 lab-reared C.oculata males w/ catnip; abd.
Misc Info   : GC run JA0812_4.D; /CH2Cl2
Vial Number: 1
```

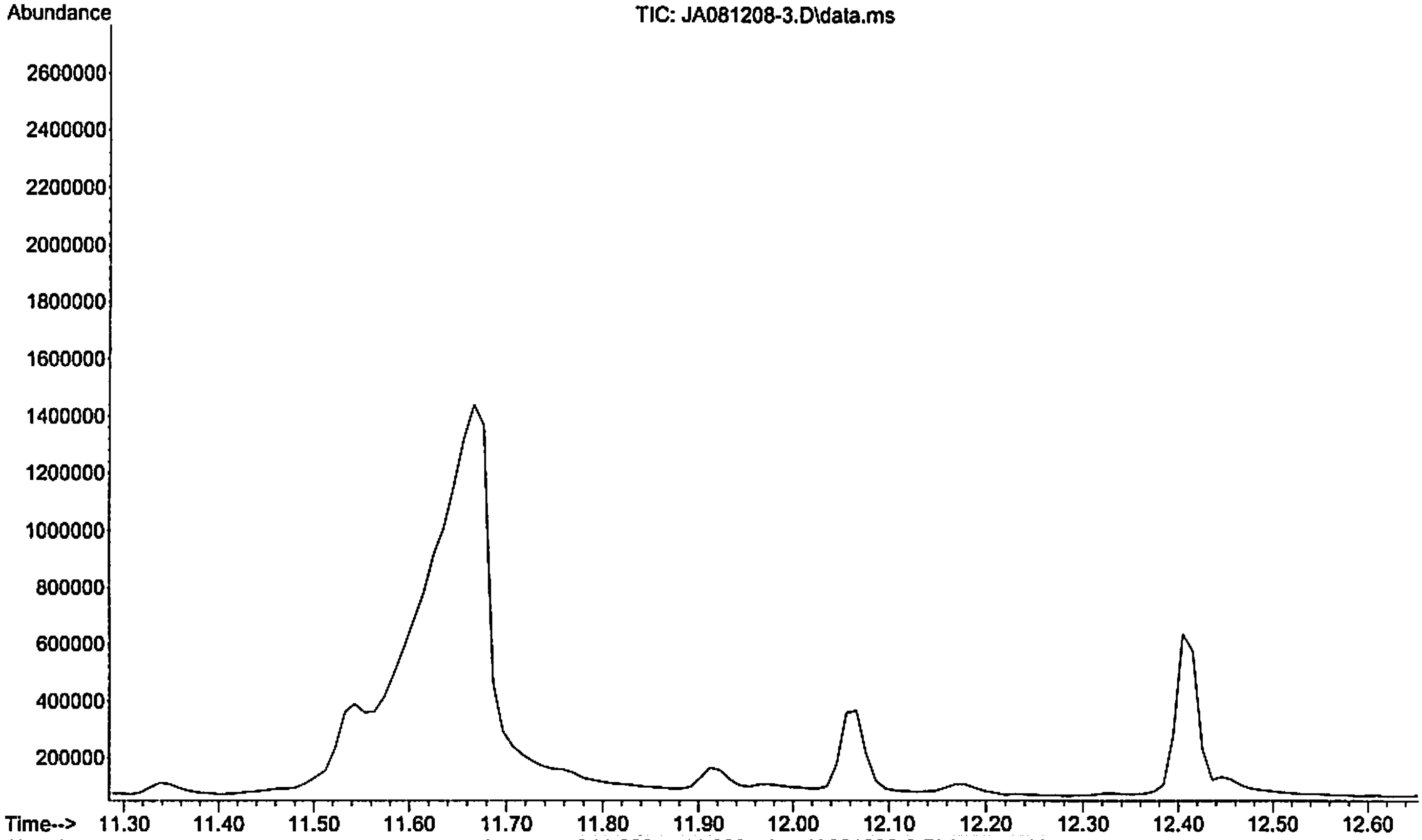

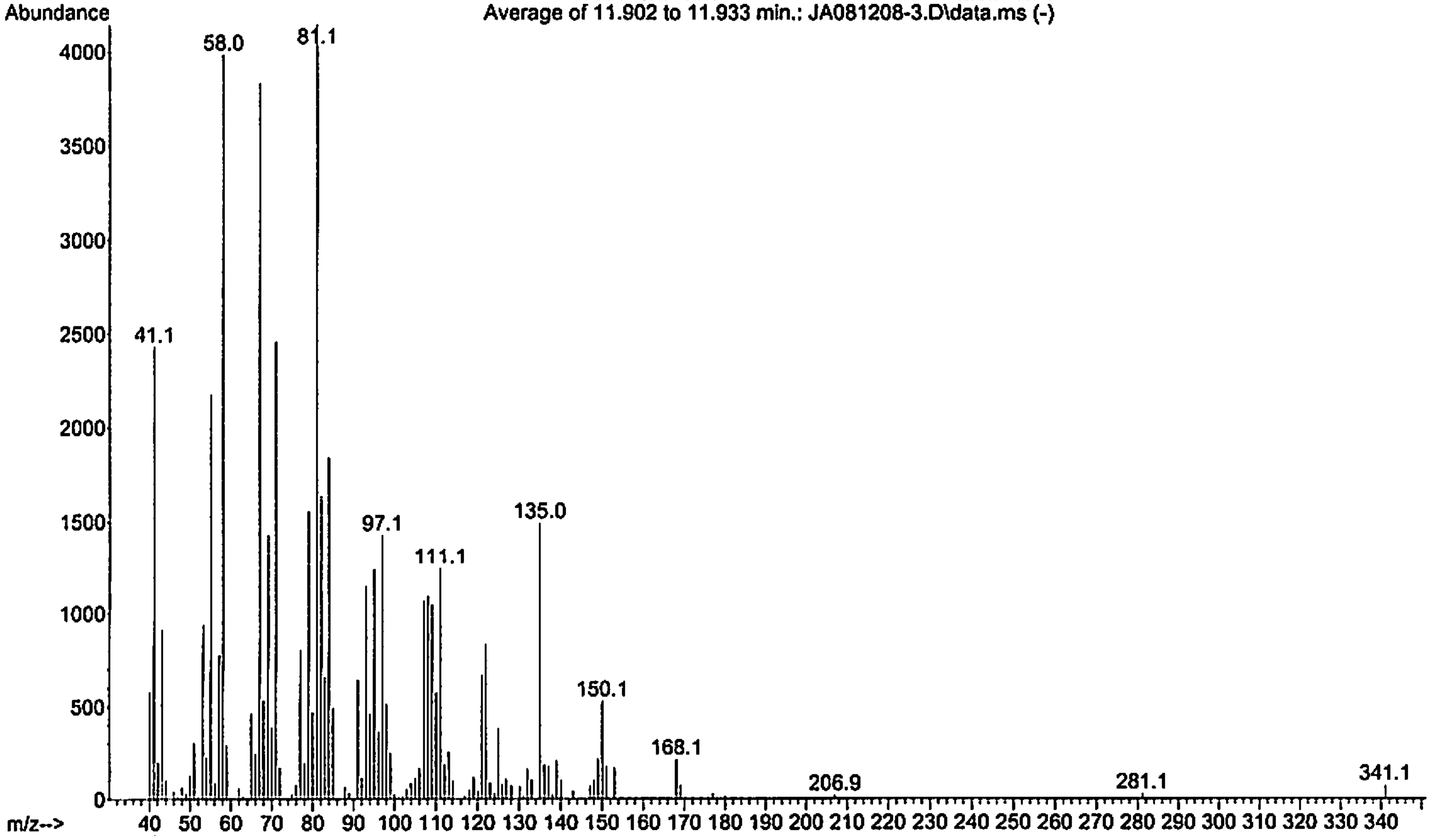

```
File        :D:\Aldrich\JA081208-3.D
Operator    :
Acquired    : 12 Aug 2008  16:43     using AcqMethod JA-50-280LESS.M
Instrument :    Buba
Sample Name: 5 lab-reared C.oculata males w/ catnip; abd.
Misc Info   : GC run JA0812_4.D; /CH2Cl2
Vial Number: 1
```

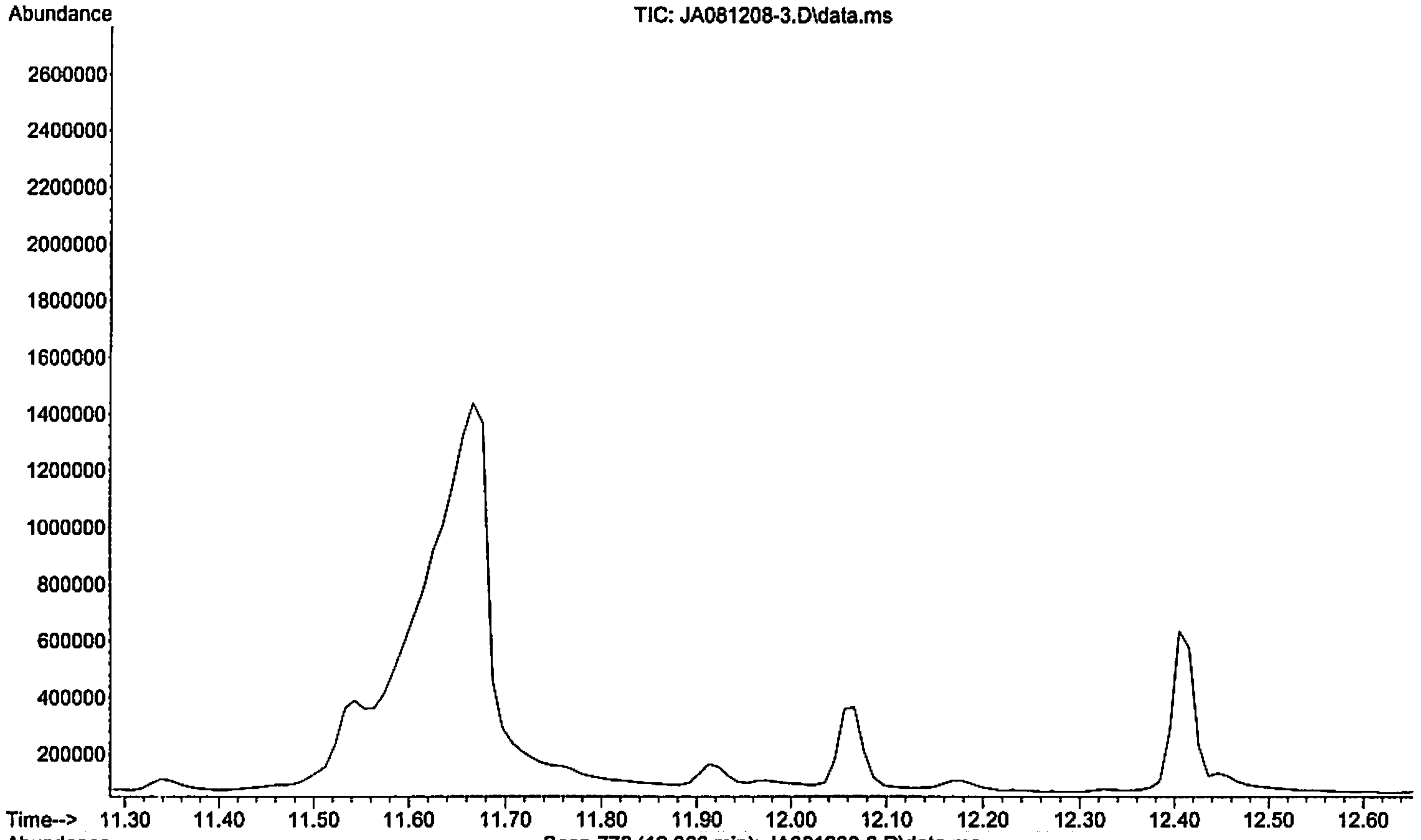

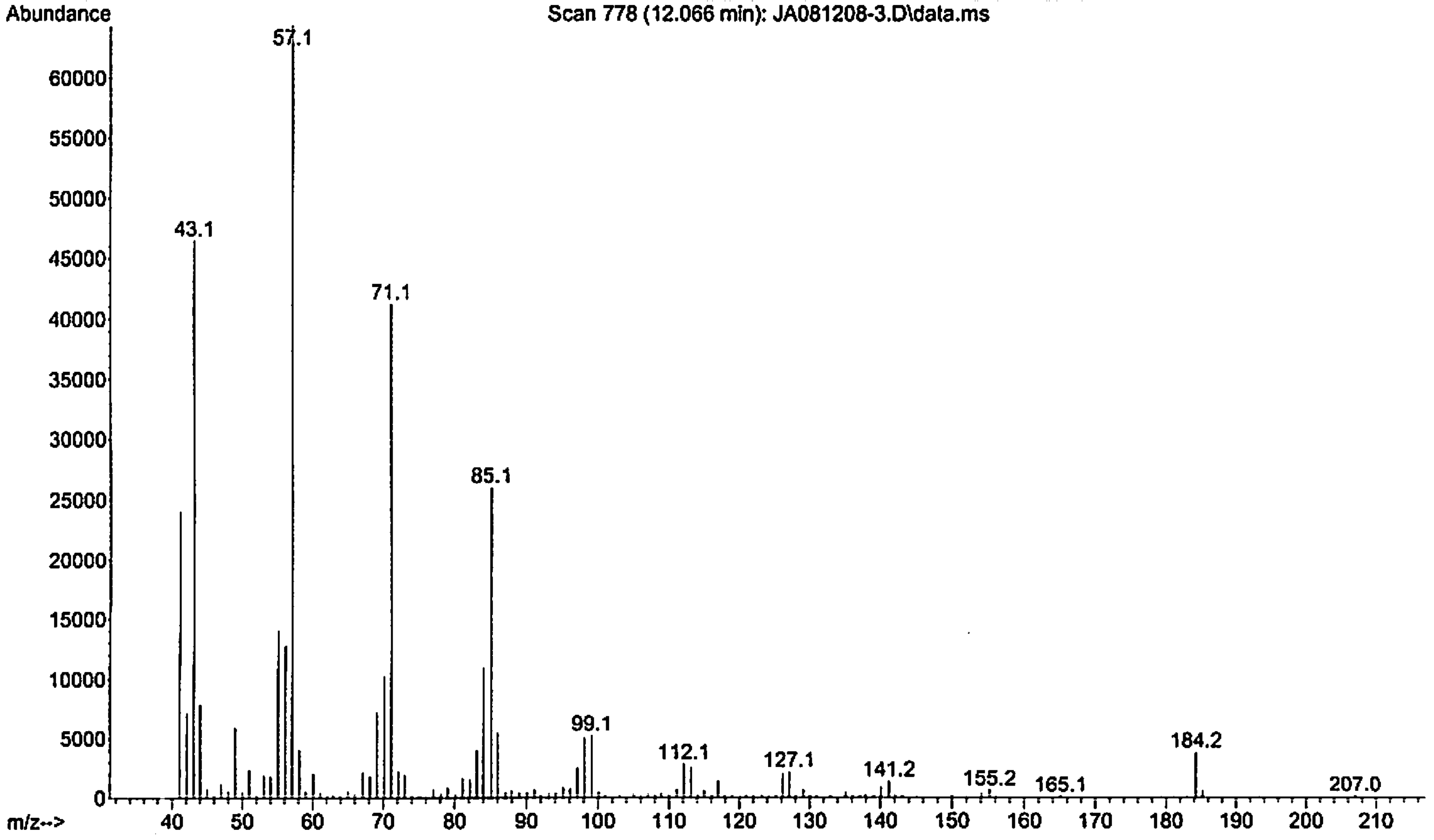

```
File        :D:\Aldrich\JA081208-3.D
Operator    :
Acquired    : 12 Aug 2008  16:43     using AcqMethod JA-50-280LESS.M
Instrument :    Buba
Sample Name: 5 lab-reared C.oculata males w/ catnip; abd.
Misc Info   : GC run JA0812_4.D; /CH2Cl2
Vial Number: 1
```

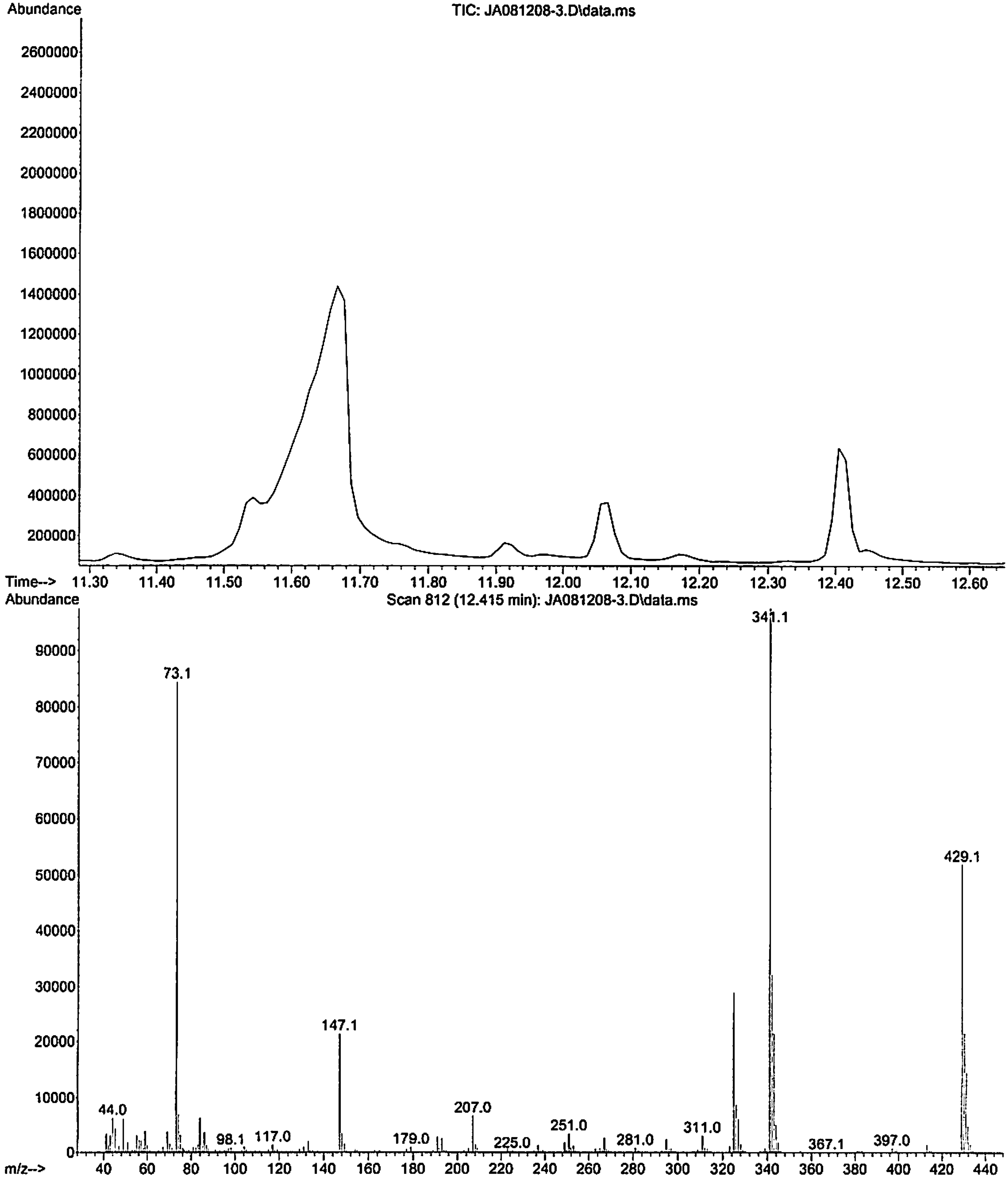

Supplement: Data S3 [file peerj-04-1564-s008.pdf]
