# Supplementary material for: Pharmacophagy in green lacewings (Neuroptera: Chrysopidae: Chrysopa spp.)?
Source: PeerJ. 2016 Jan 18;4:e1564. doi: 10.7717/peerj.1564 (PMC4727961; doi:10.7717/peerj.1564)

File : D:\DATA\Aldrich\JA-09\JA052809-4.D
Operator : Aldrich
Acquired : 28 May 2009 16:03 using AcqMethod JA-WAX08.M
Instrument : Instrument #1
Sample Name: 1 field-coll. M C.oculata abd./CH2Cl2
Misc Info : coll. 5/28 sweeping vetch; second male today
Vial Number: 1

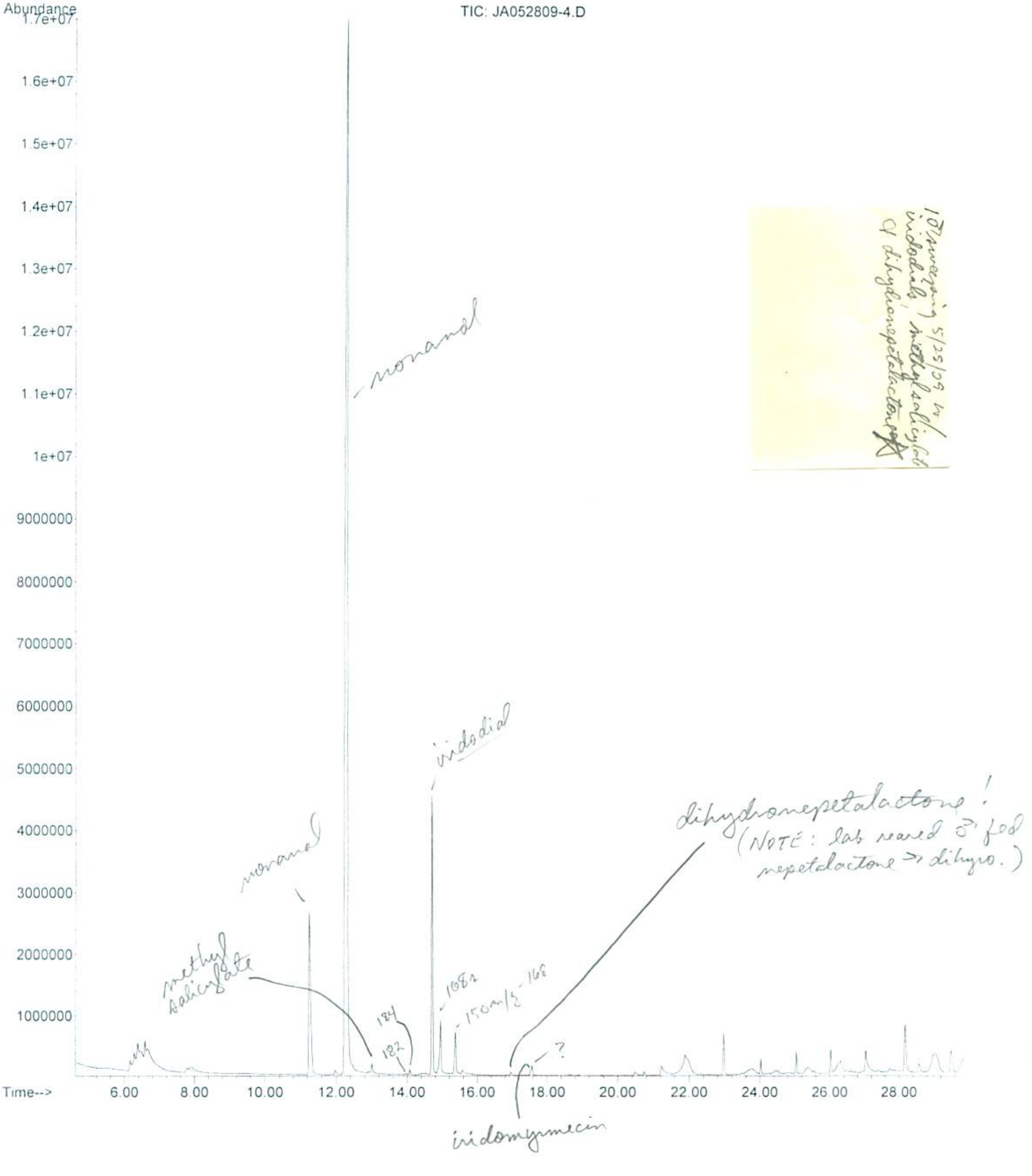

[illegible]e :D:\DATA\ALDRICH\JA-09\Snapshot\JA052809-4.D
[illegible]rator : Aldrich
[illegible]ired : 28 May 2009 16:03 using AcqMethod JA-WAX08.M
[illegible]rument : Instrument #1
[illegible]le Name: 1 field-coll. M C.oculata abd./CH2Cl2
[illegible] Info : coll. 5/28 sweeping vetch; second male today
[illegible] Number: 1

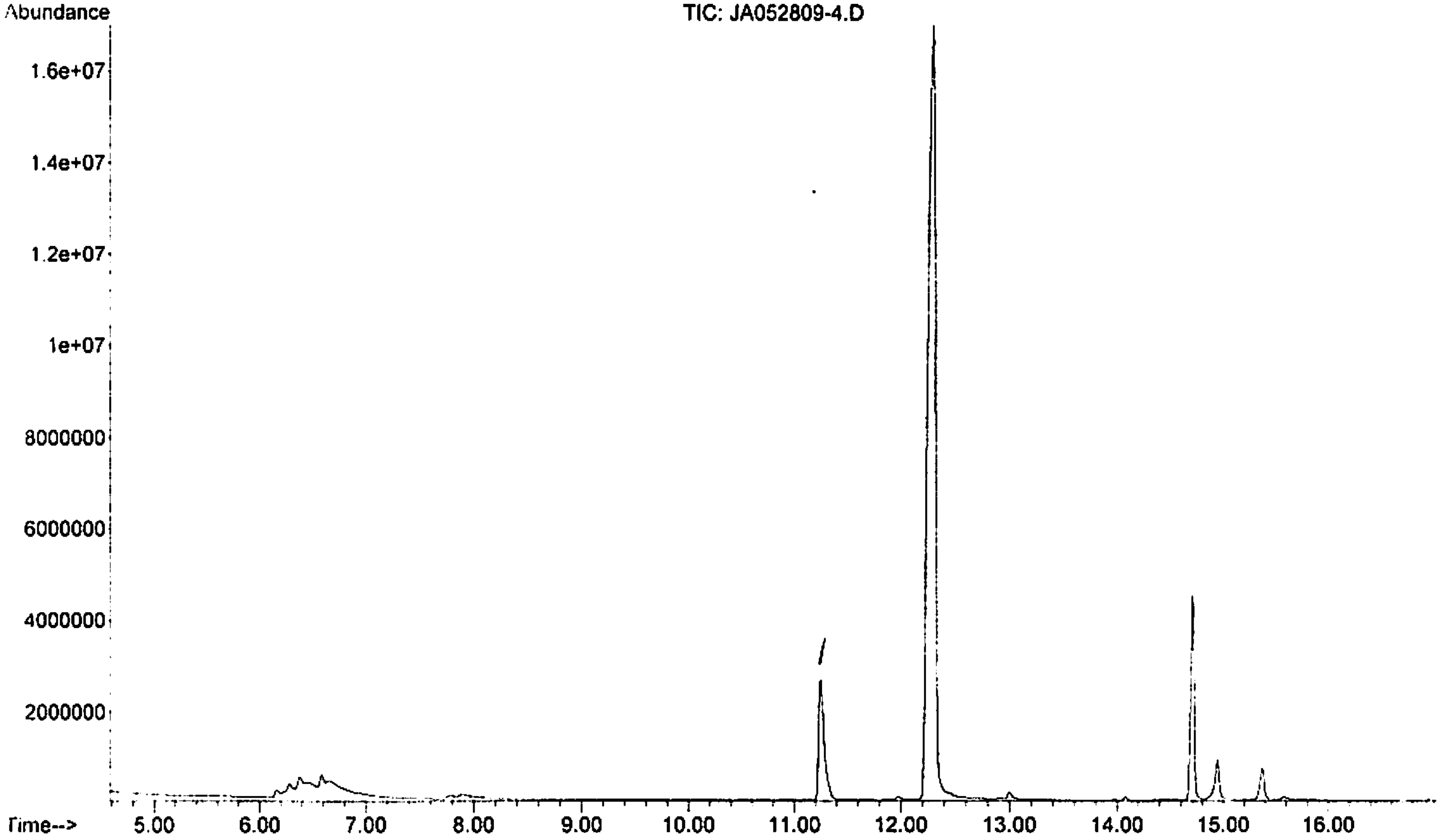

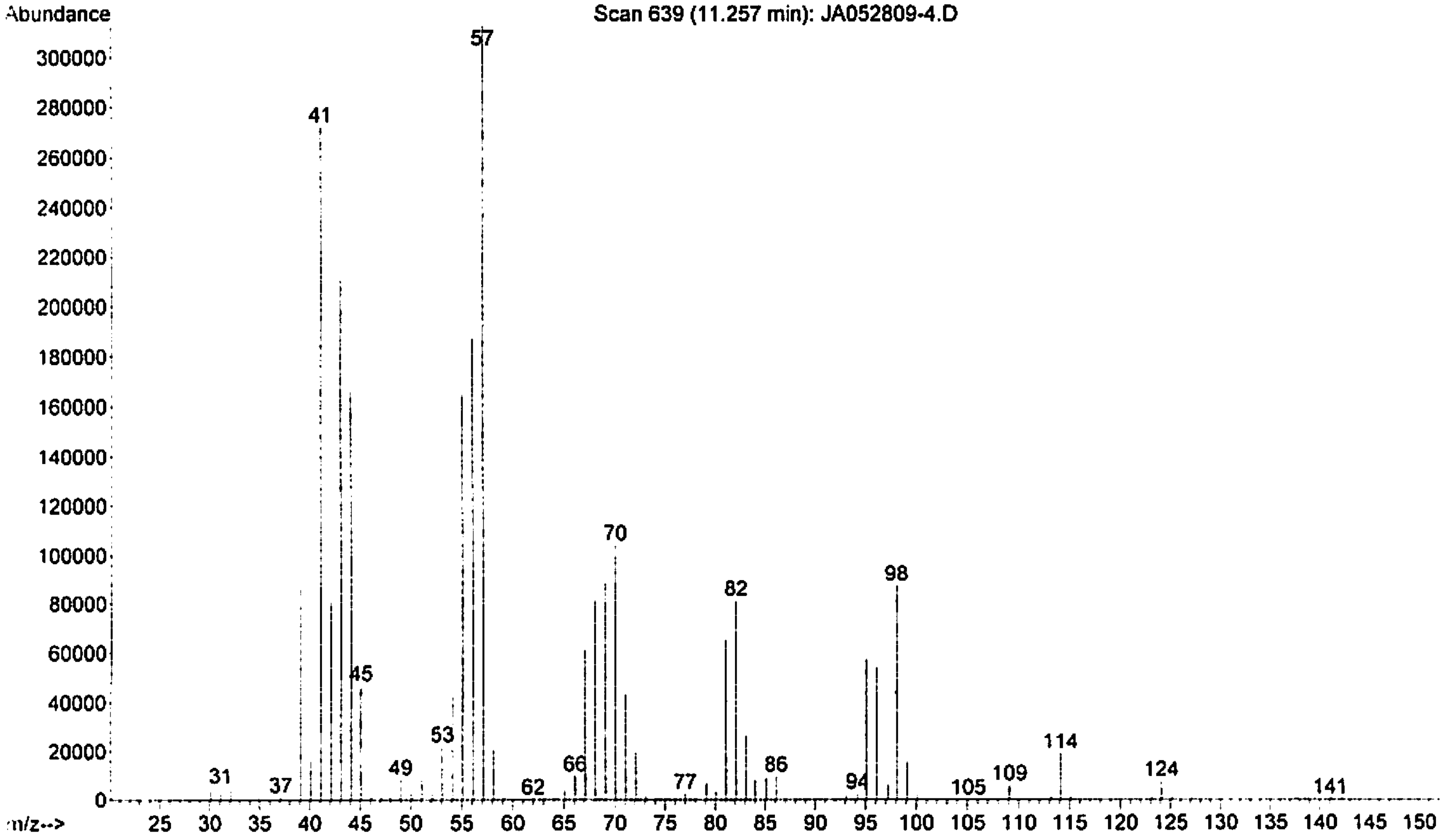

[illegible] :D:\DATA\ALDRICH\JA-09\Snapshot\JA052809-4.D
[illegible]rator : Aldrich
[illegible]uired : 28 May 2009 16:03 using AcqMethod JA-WAX08.M
[illegible]trument : Instrument #1
[illegible]ple Name: 1 field-coll. M C.oculata abd./CH2Cl2
[illegible] Info : coll. 5/28 sweeping vetch; second male today
[illegible] Number: 1

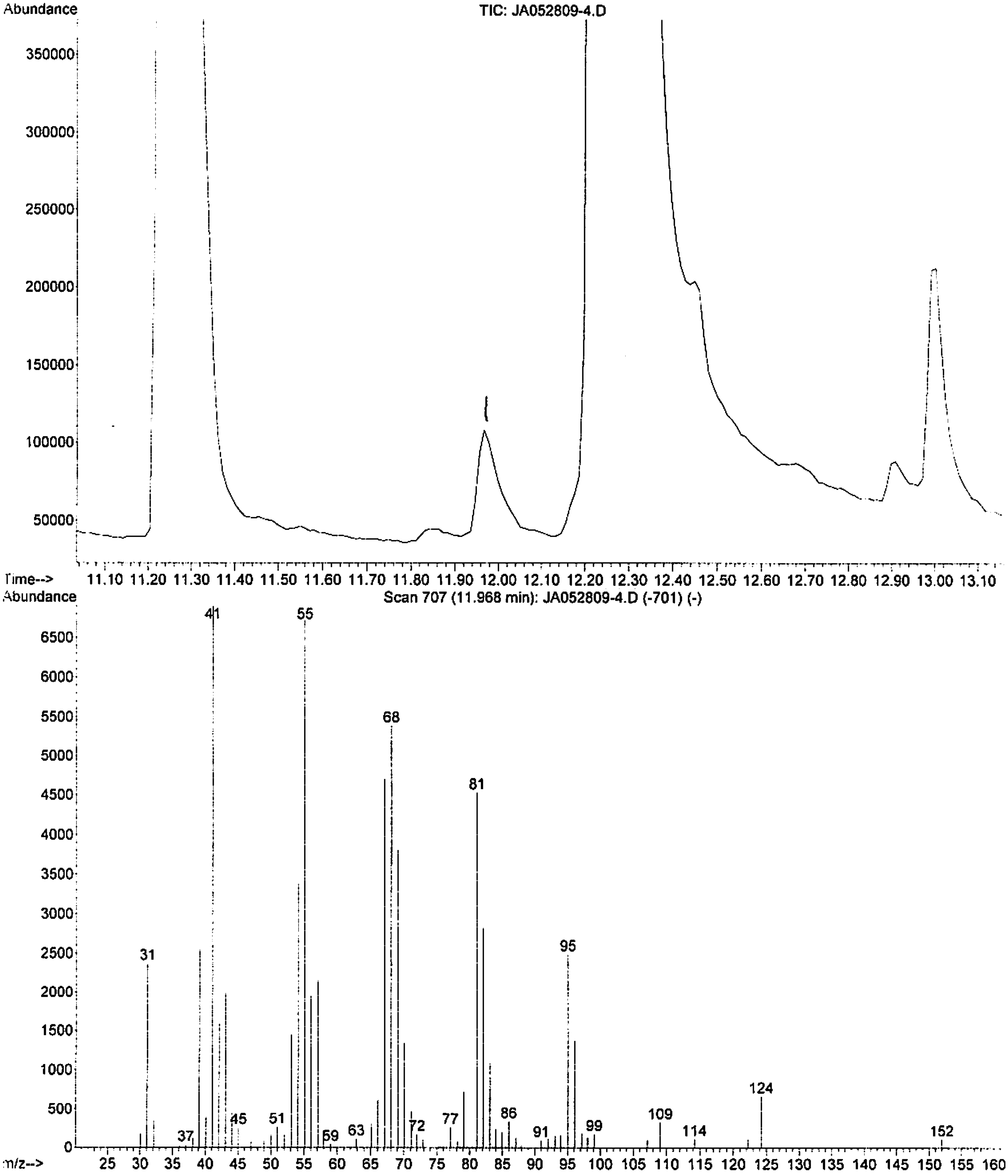

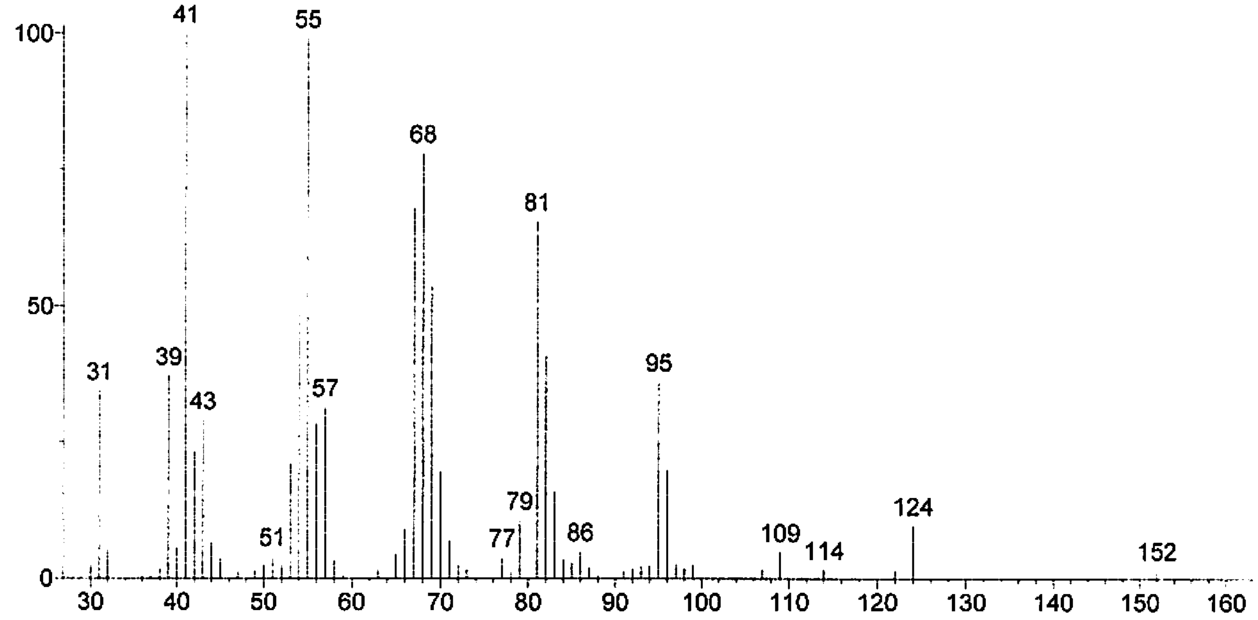

(Text File) Scan 707 (11.968 min): JA052809-4.D (-701)

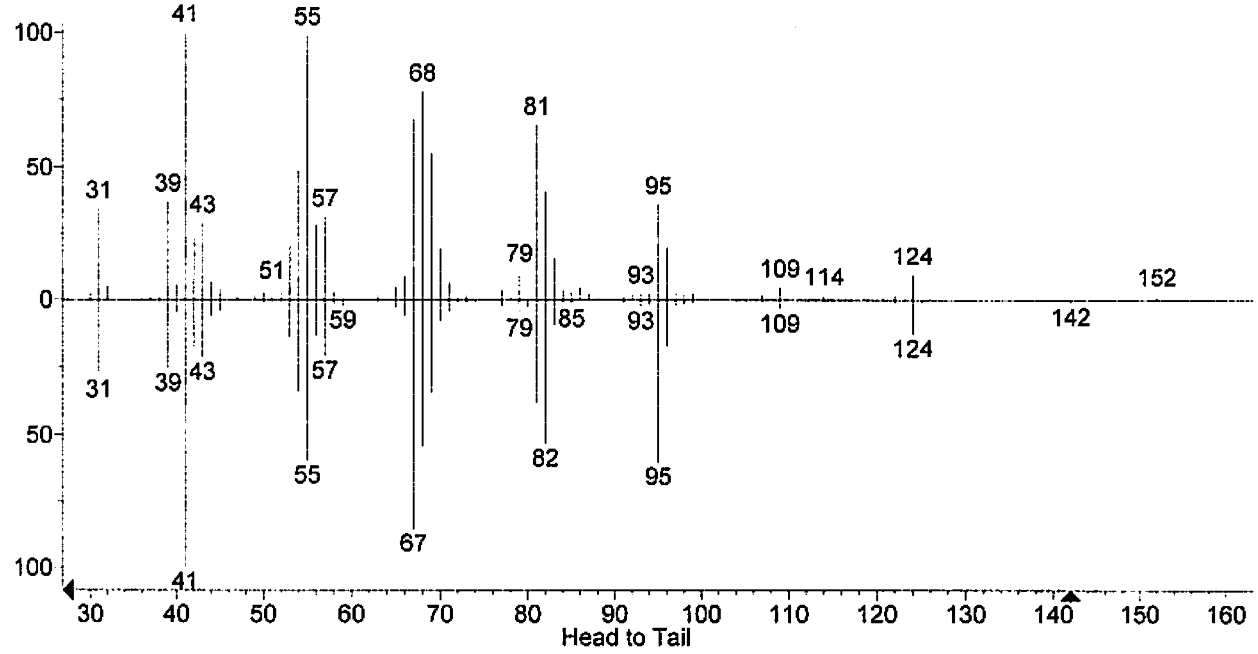

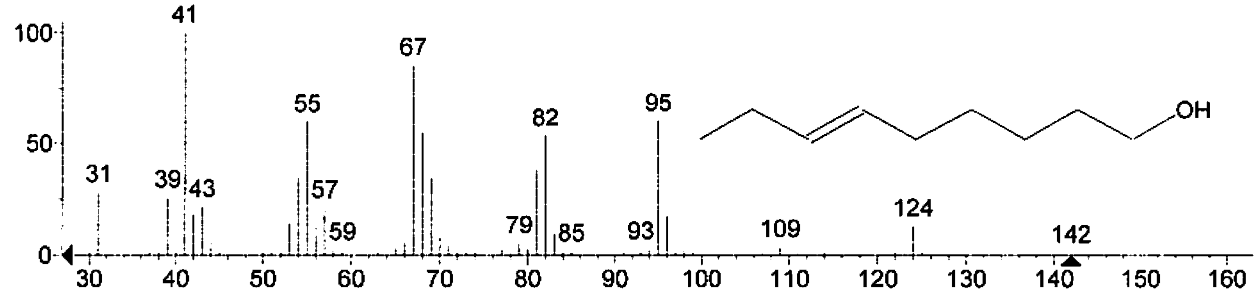

(mainlib) 6-Nonen-1-ol, (E)-

```
File        :D:\DATA\ALDRICH\JA-09\Snapshot\JA052809-4.D
Operator    : Aldrich
Acquired    : 28 May 2009  16:03     using AcqMethod JA-WAX08.M
Instrument  :     Instrument #1
Sample Name: 1 field-coll. M C.oculata abd./CH2Cl2
Misc Info   : coll. 5/28 sweeping vetch; second male today
Vial Number: 1
```

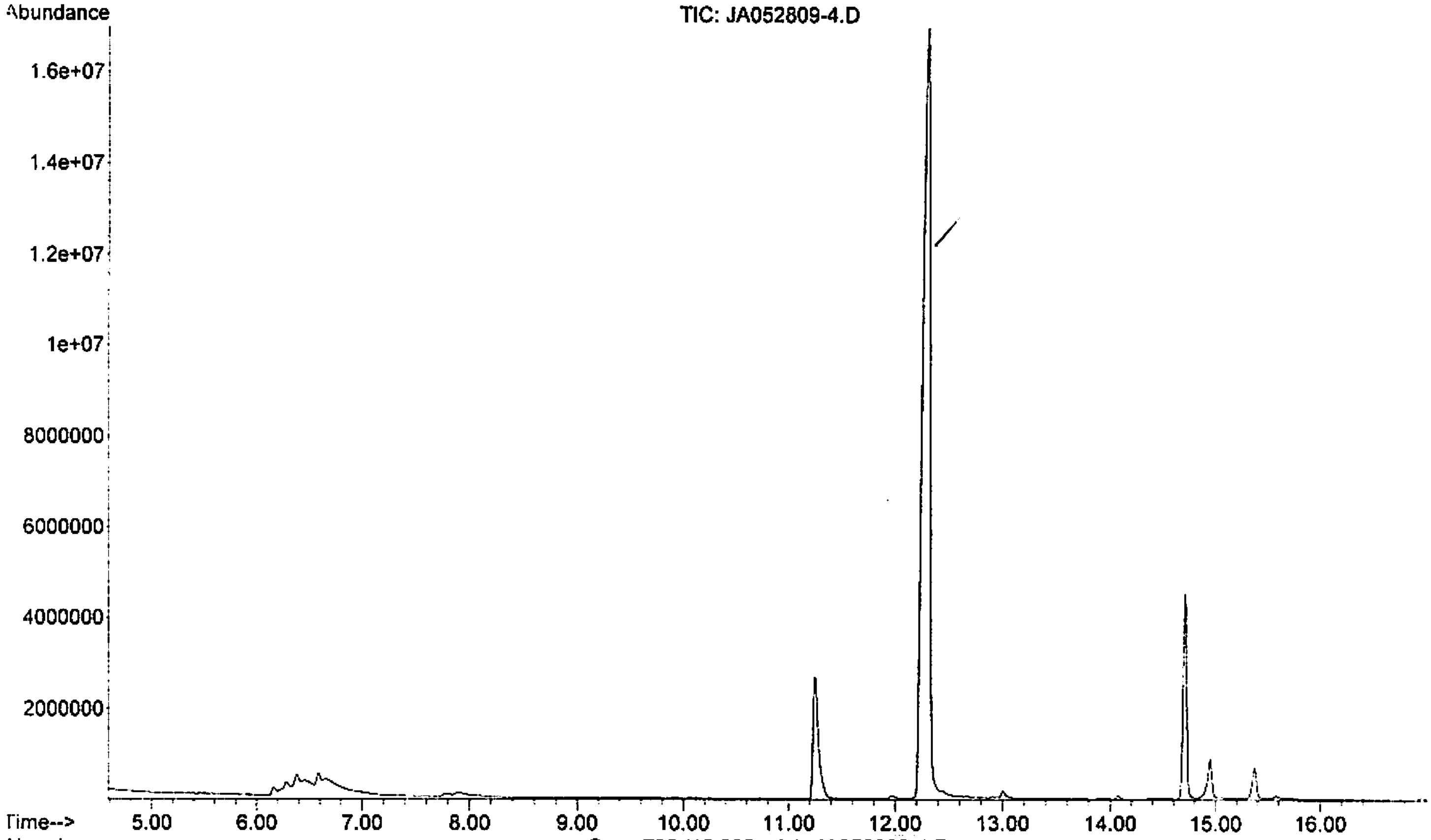

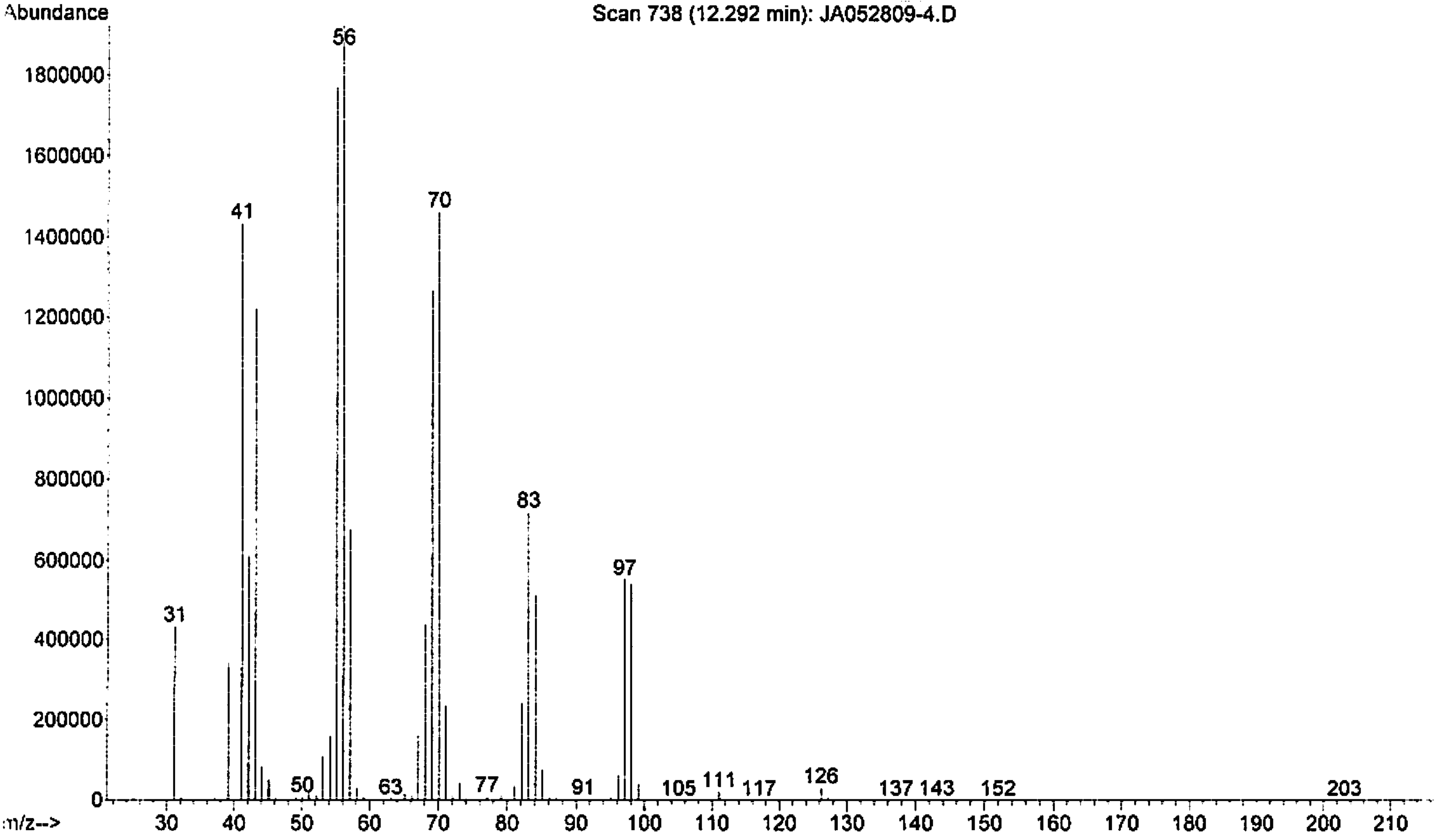

[illegible] :D:\DATA\ALDRICH\JA-09\Snapshot\JA052809-4.D
[illegible]ator : Aldrich
[illegible]ired : 28 May 2009 16:03 using AcqMethod JA-WAX08.M
[illegible]rument : Instrument #1
[illegible]le Name: 1 field-coll. M C.oculata abd./CH2Cl2
[illegible] Info : coll. 5/28 sweeping vetch; second male today
[illegible] Number: 1

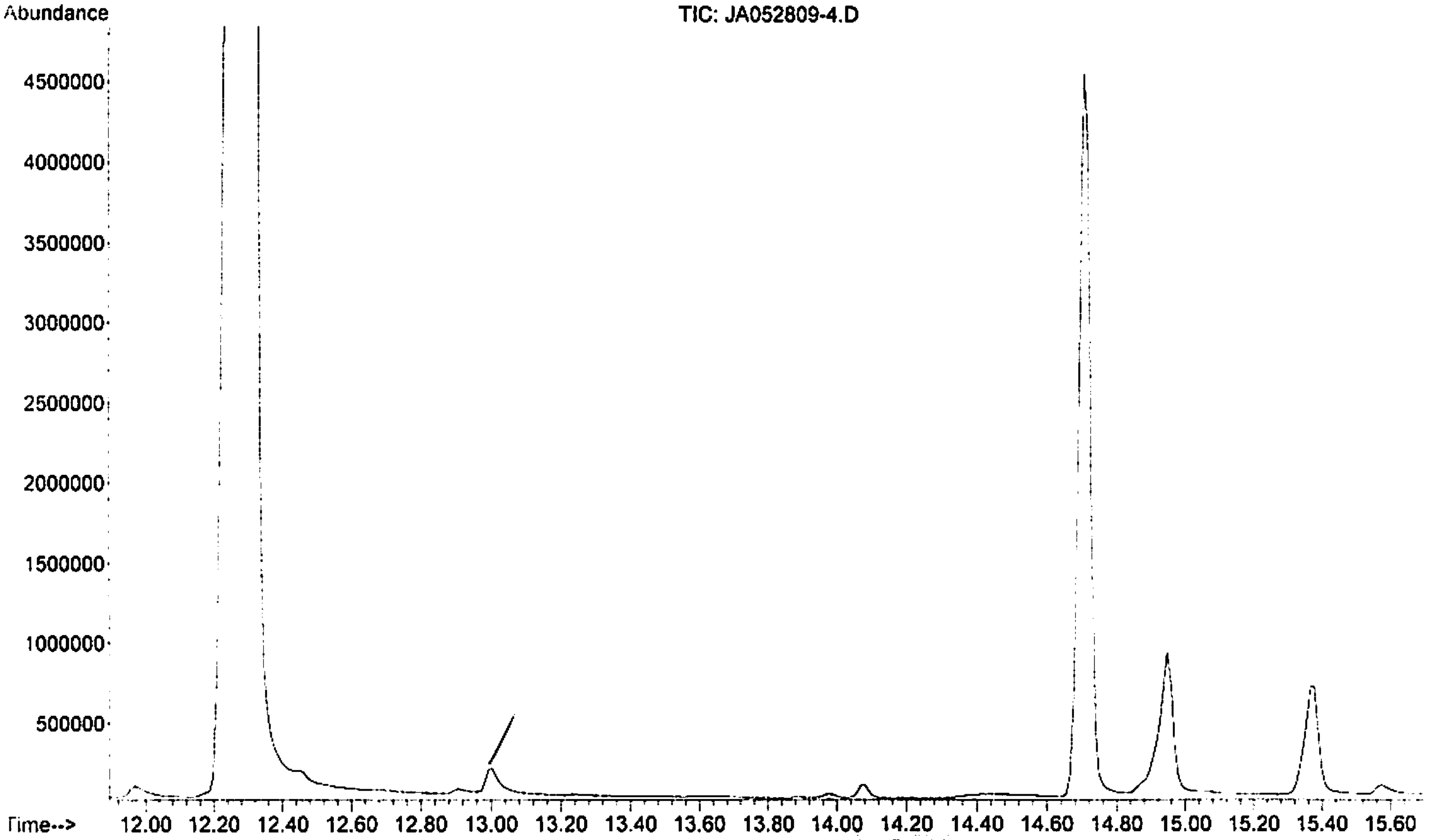

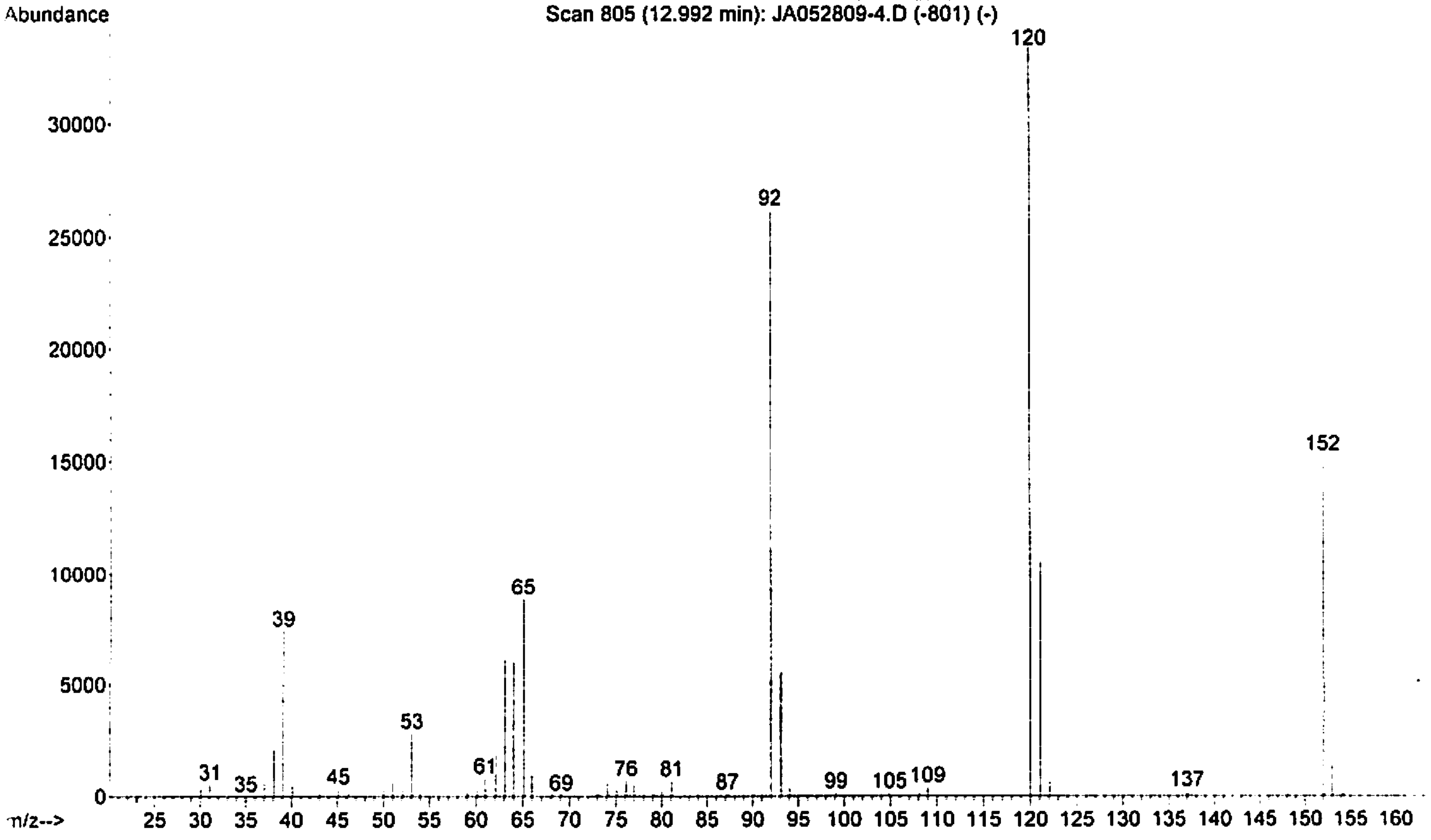

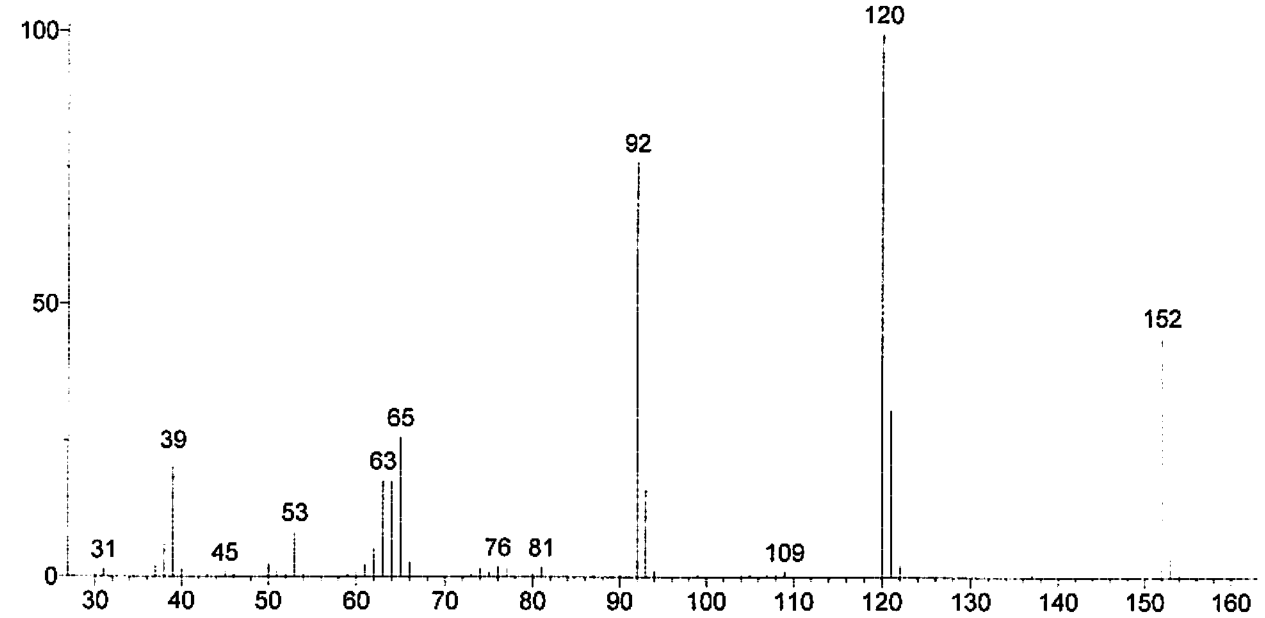

(Text File) Scan 805 (12.992 min): JA052809-4.D (-801)

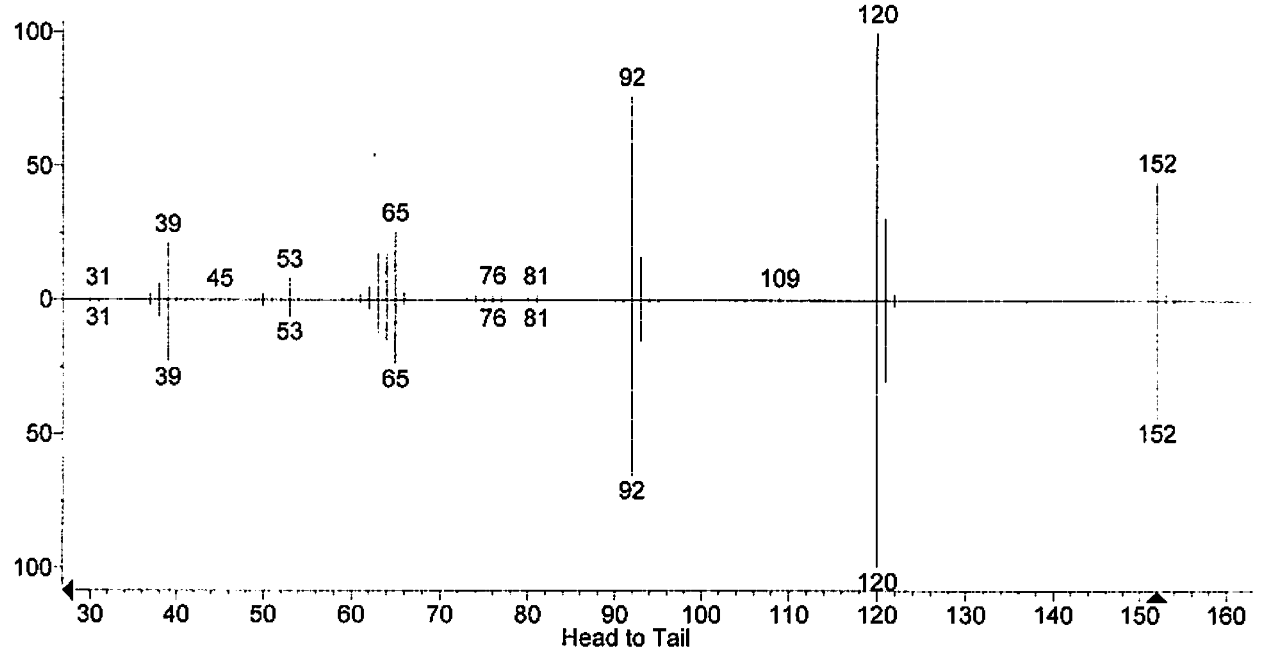

Head to Tail

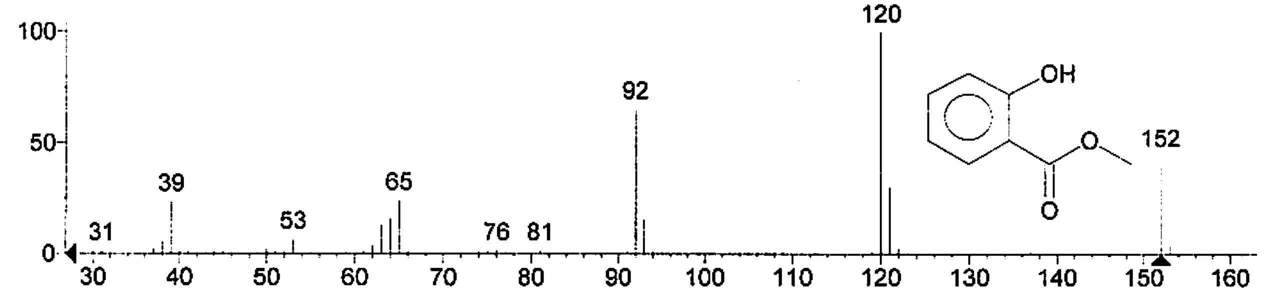

(mainlib) Methyl Salicylate

[illegible] :D:\DATA\ALDRICH\JA-09\Snapshot\JA052809-4.D
[illegible]rator : Aldrich
[illegible]ired : 28 May 2009 16:03 using AcqMethod JA-WAX08.M
[illegible]rument : Instrument #1
[illegible]le Name: 1 field-coll. M C.oculata abd./CH2Cl2
[illegible] Info : coll. 5/28 sweeping vetch; second male today
[illegible] Number: 1

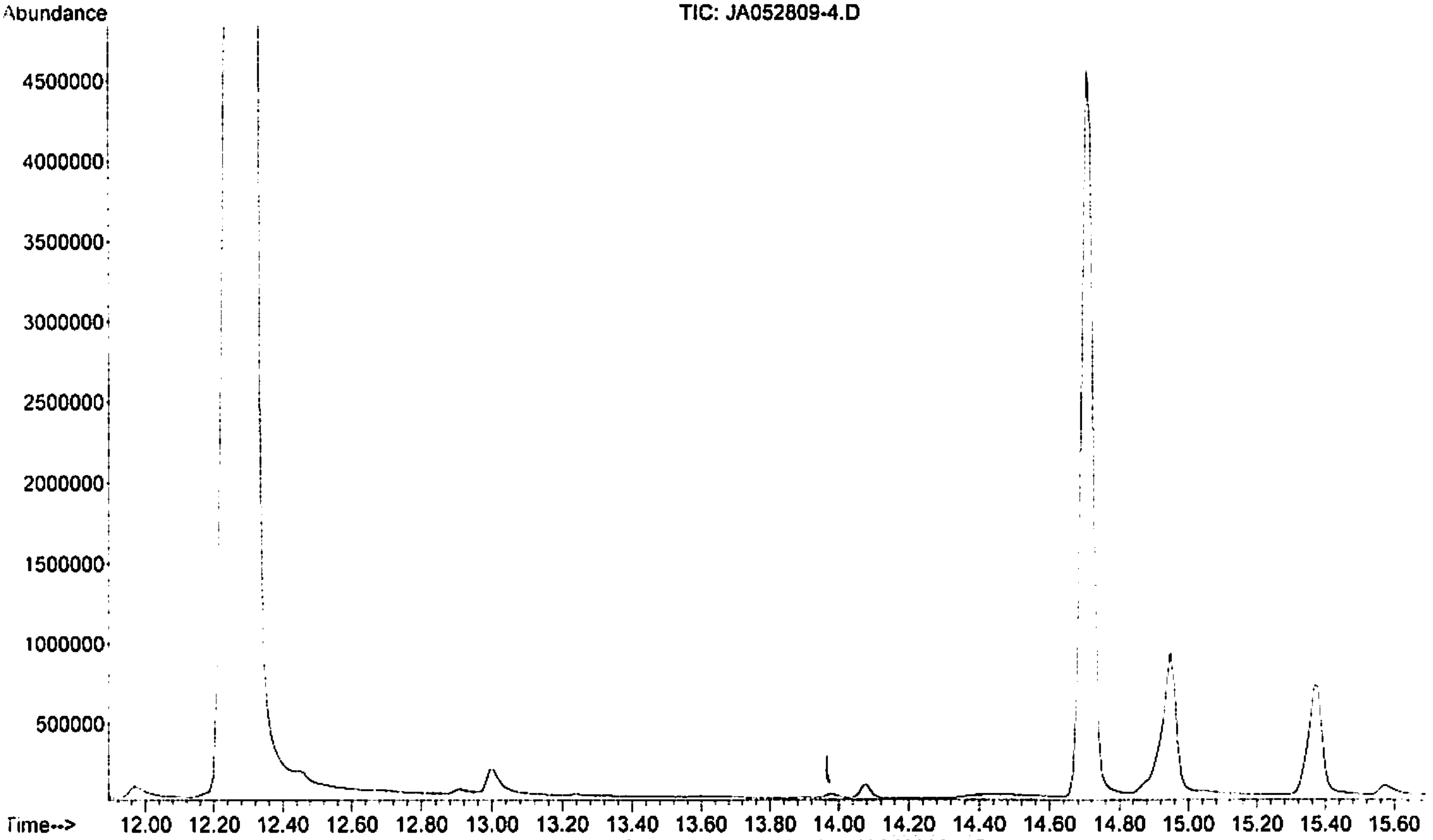

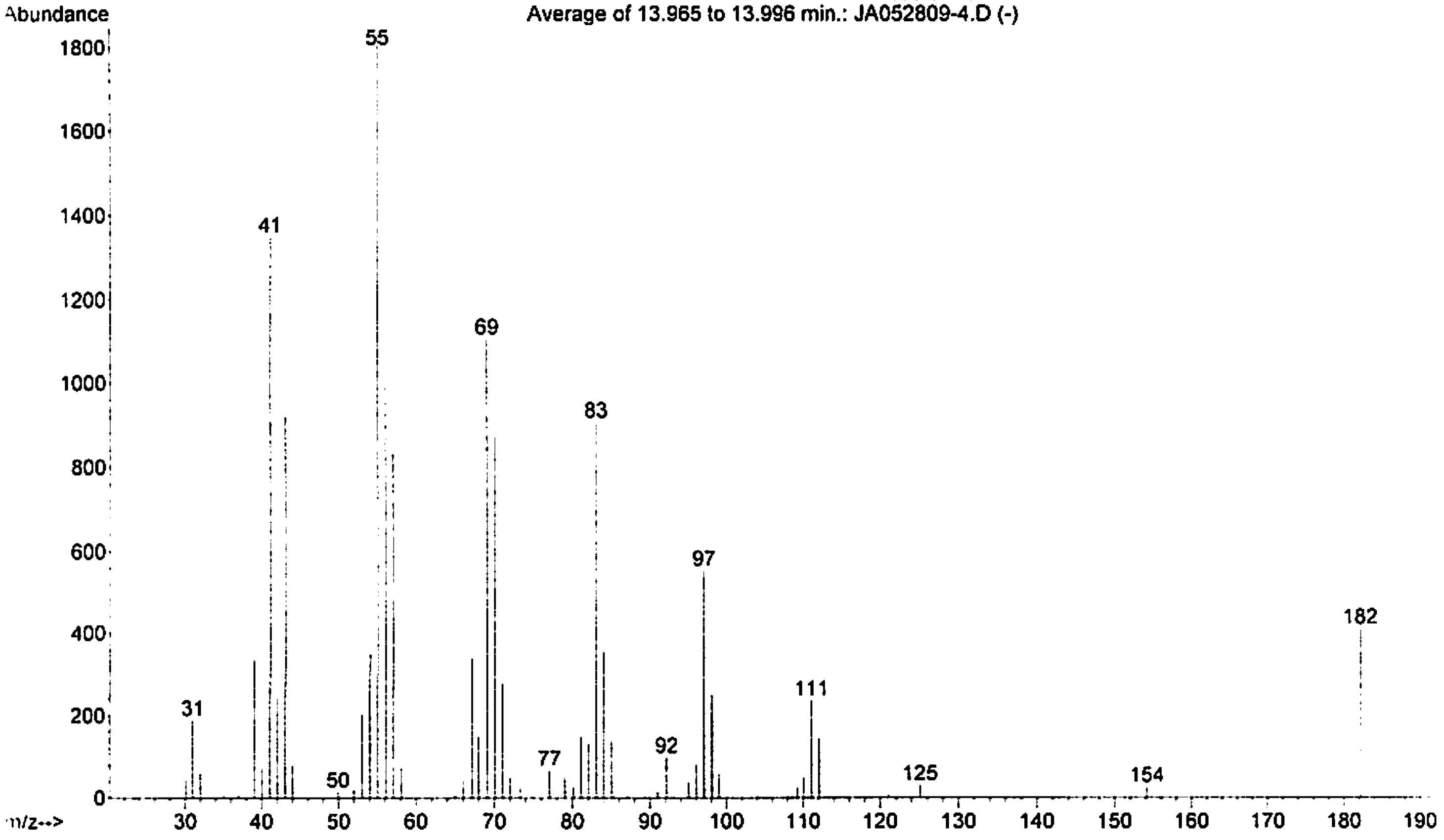

[illegible] :D:\DATA\ALDRICH\JA-09\Snapshot\JA052809-4.D
[illegible]ator : Aldrich
[illegible]ired : 28 May 2009 16:03 using AcqMethod JA-WAX08.M
[illegible]rument : Instrument #1
[illegible]ple Name: 1 field-coll. M C.oculata abd./CH2Cl2
[illegible] Info : coll. 5/28 sweeping vetch; second male today
[illegible] Number: 1

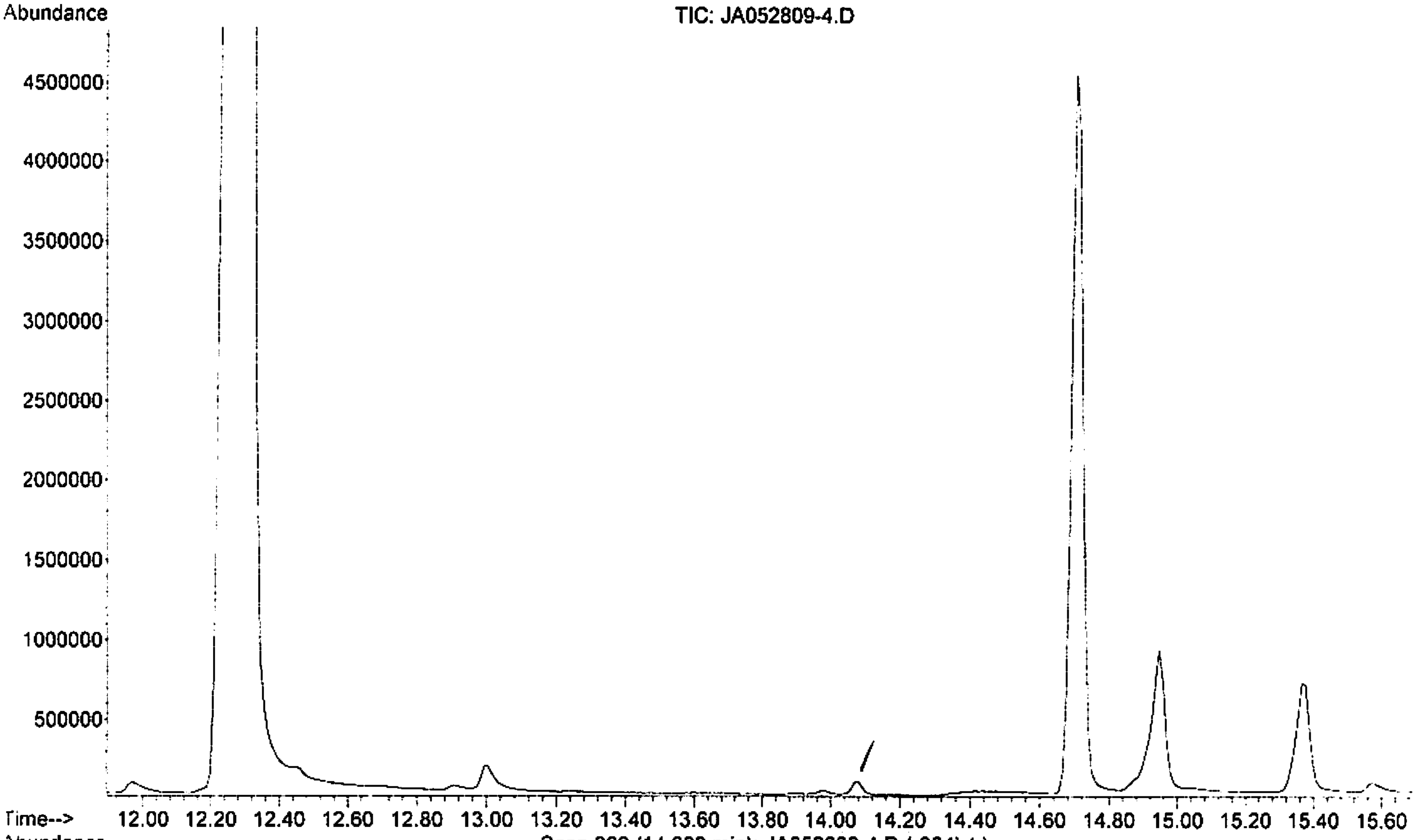

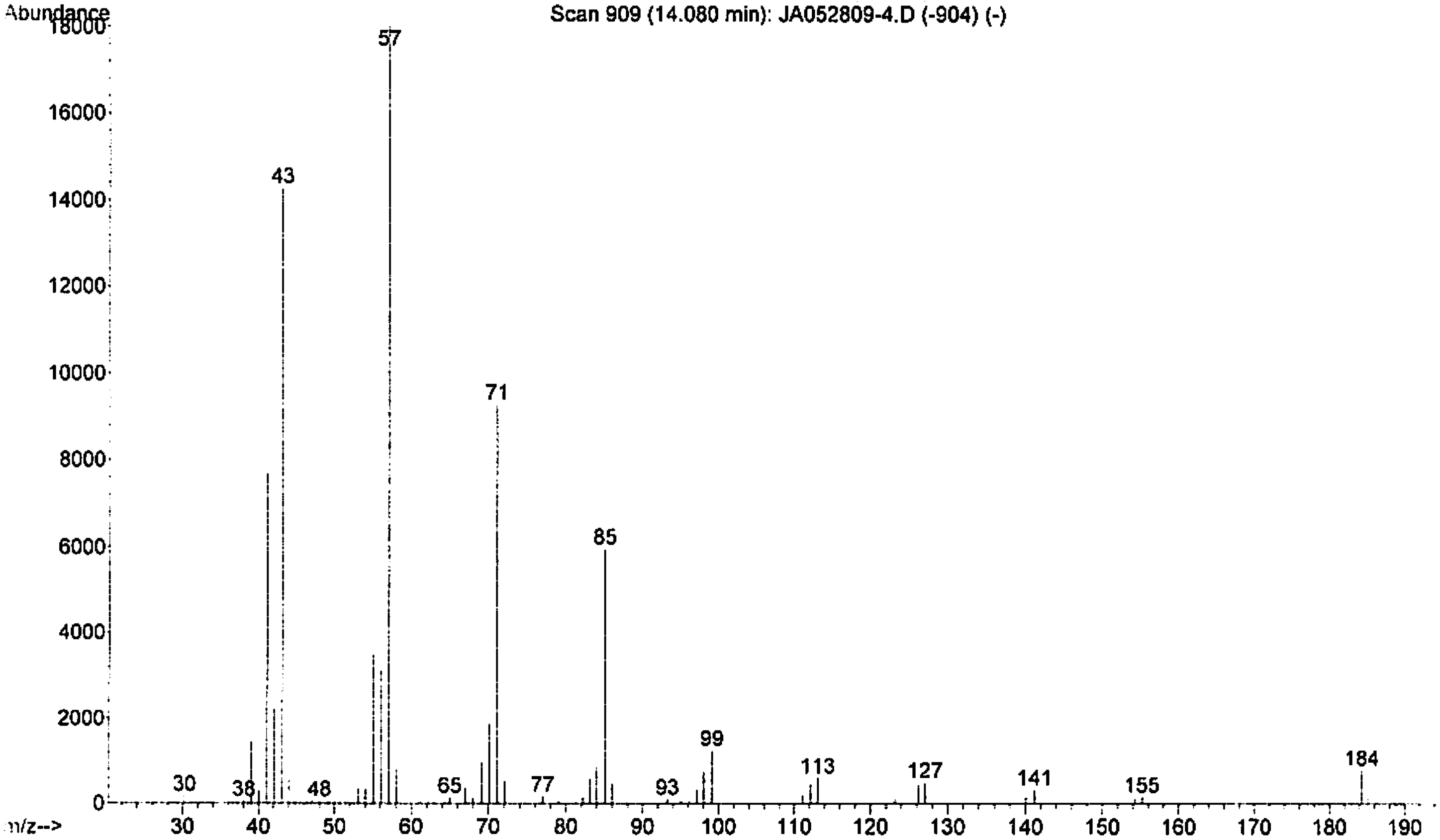

[illegible] :D:\DATA\ALDRICH\JA-09\Snapshot\JA052809-4.D
[illegible]rator : Aldrich
[illegible]uired : 28 May 2009 16:03 using AcqMethod JA-WAX08.M
[illegible]rument : Instrument #1
[illegible]ple Name: 1 field-coll. M C.oculata abd./CH2Cl2
[illegible] Info : coll. 5/28 sweeping vetch; second male today
[illegible] Number: 1

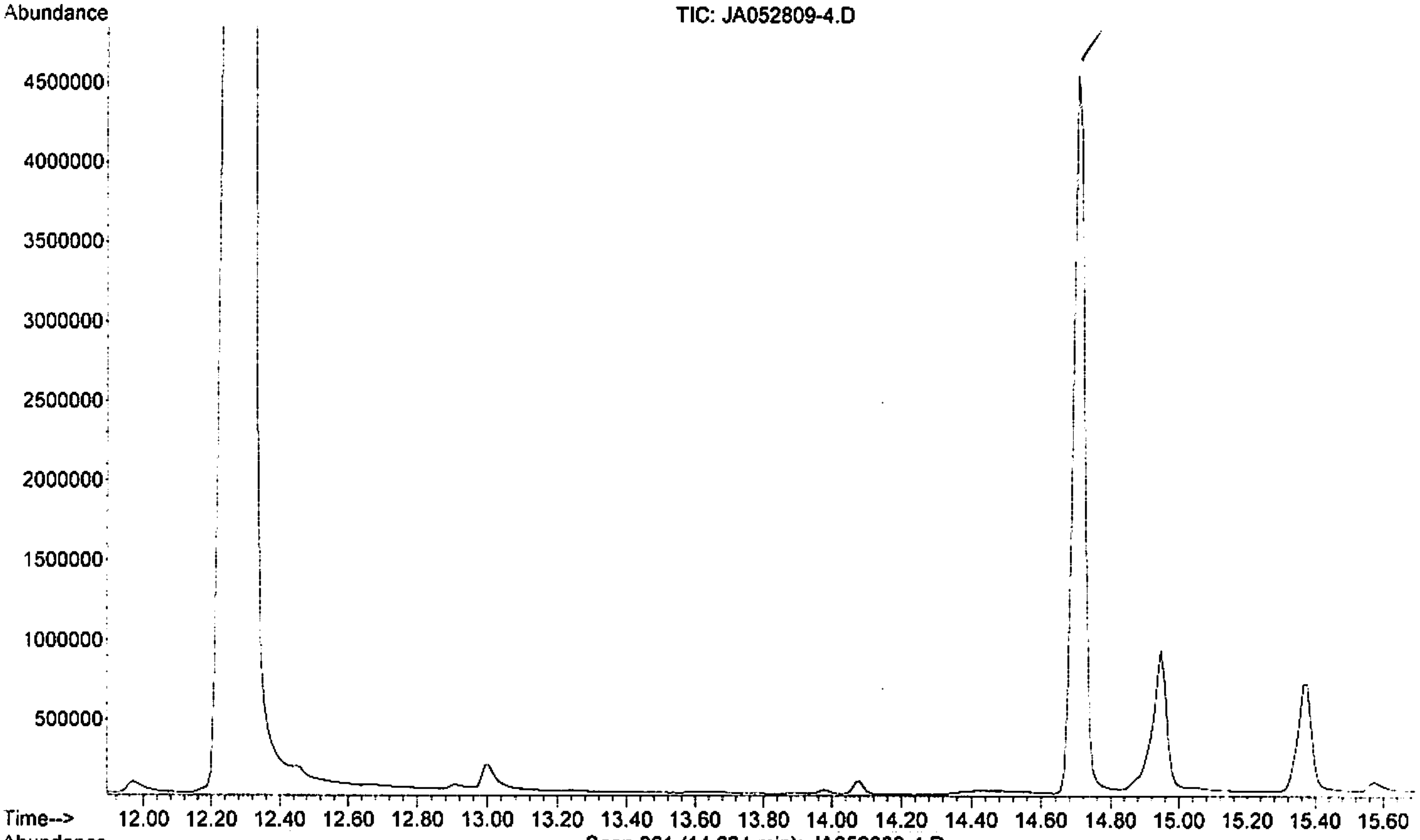

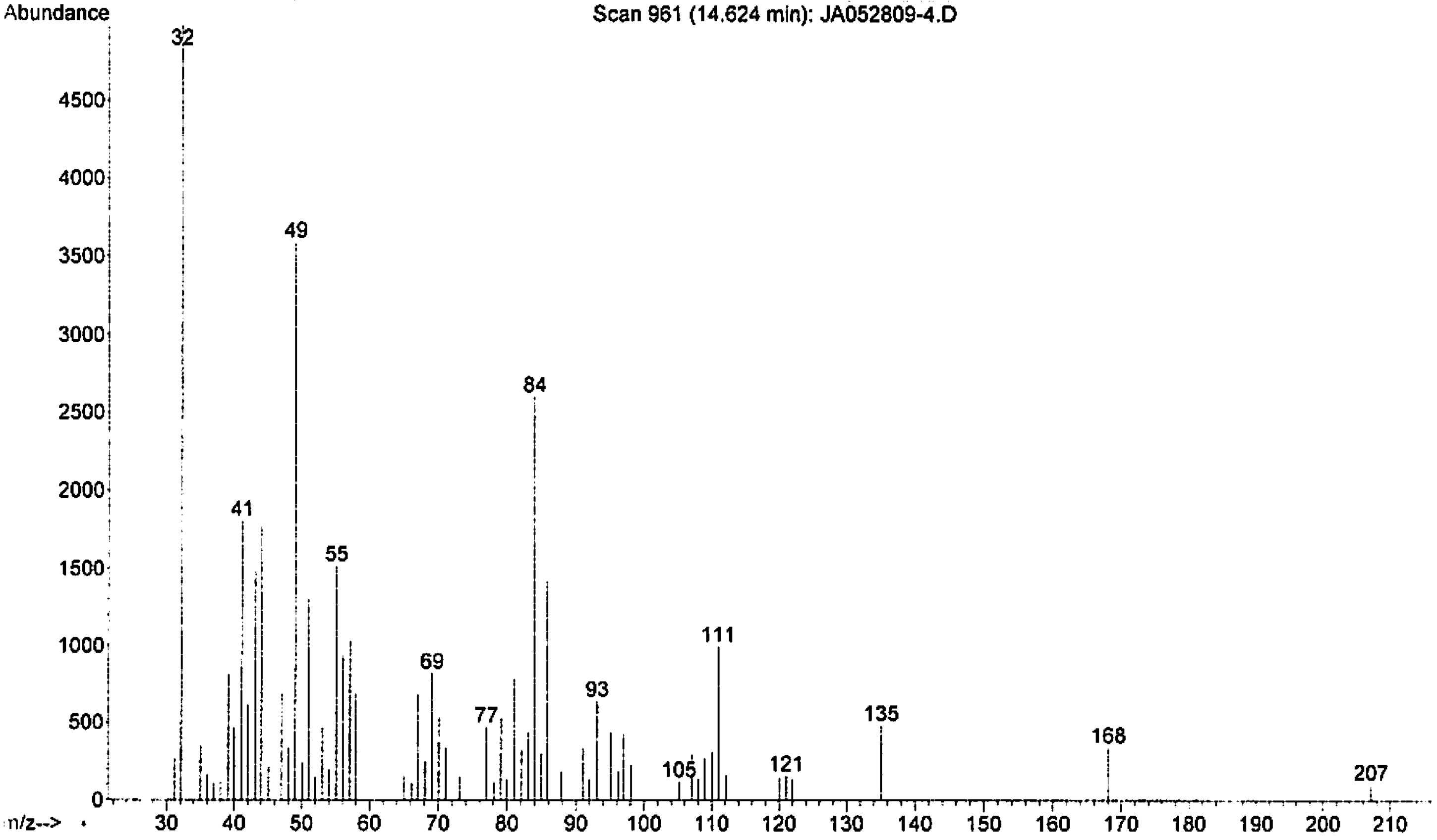

[illegible]e' :D:\DATA\ALDRICH\JA-09\Snapshot\JA052809-4.D
[illegible]rator : Aldrich
[illegible]uired : 28 May 2009 16:03 using AcqMethod JA-WAX08.M
[illegible]trument : Instrument #1
[illegible]ple Name: 1 field-coll. M C.oculata abd./CH2Cl2
[illegible] Info : coll. 5/28 sweeping vetch; second male today
[illegible]al Number: 1

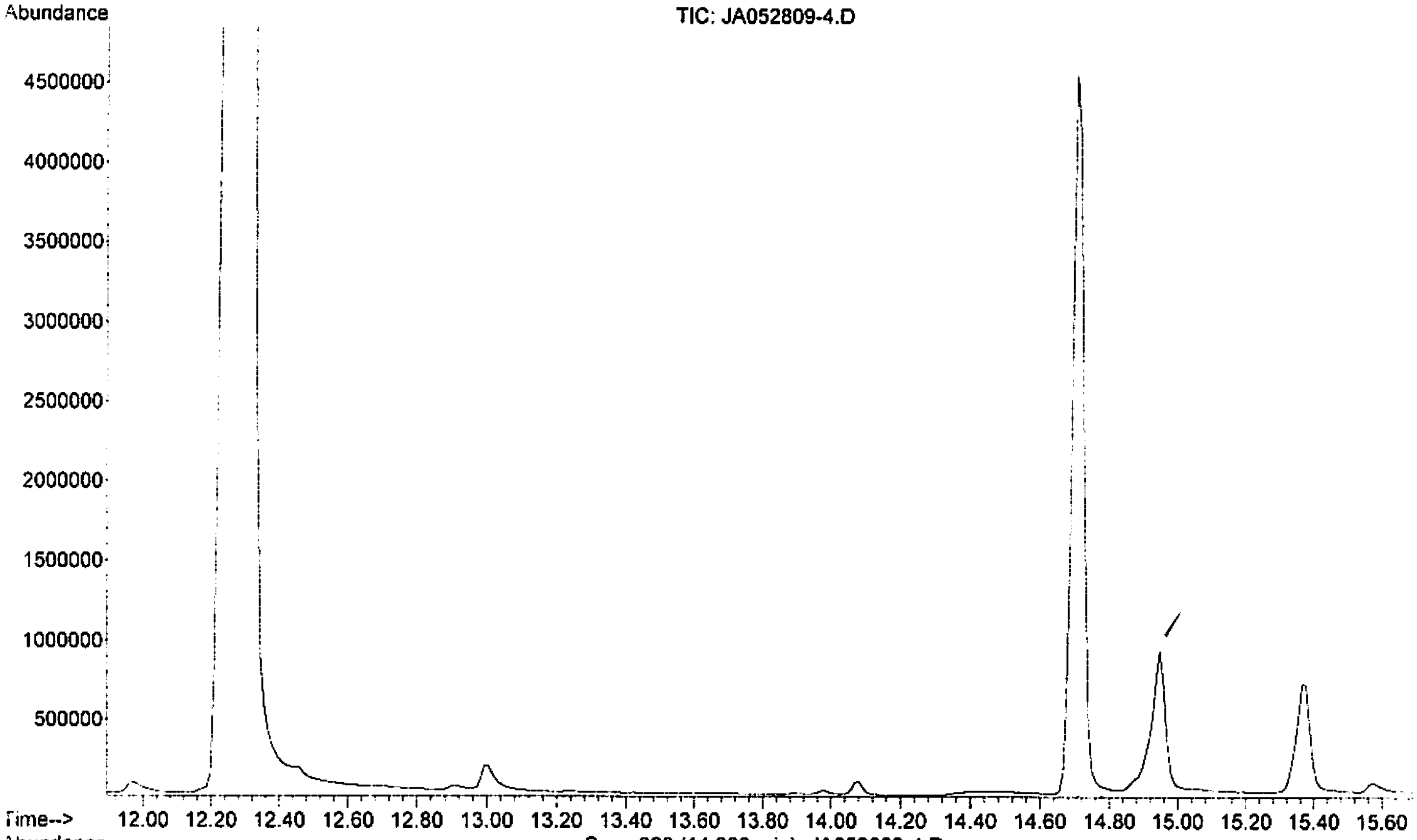

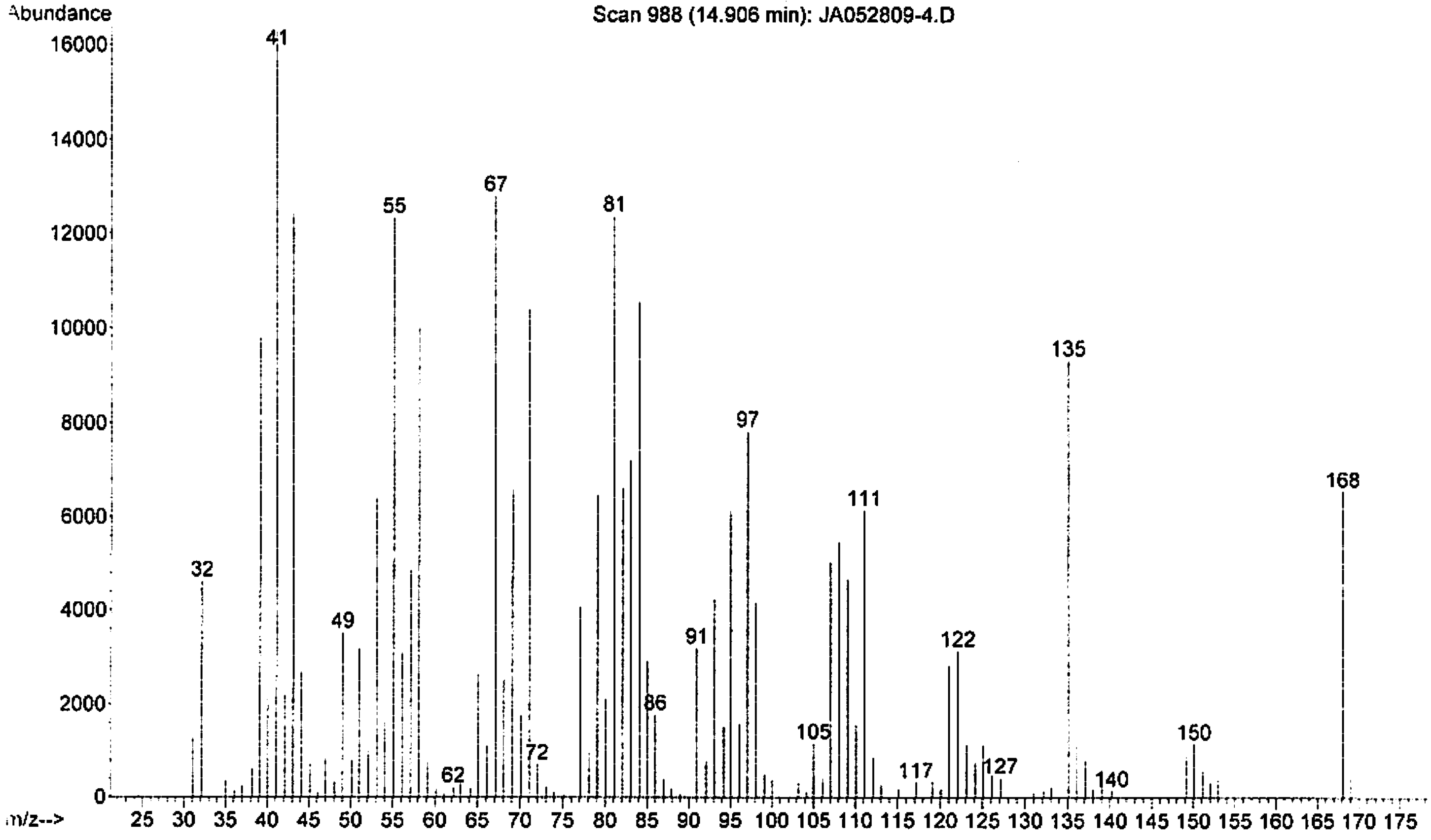

[illegible] :D:\DATA\ALDRICH\JA-09\Snapshot\JA052809-4.D
[illegible]ator : Aldrich
[illegible]ired : 28 May 2009 16:03 using AcqMethod JA-WAX08.M
[illegible]rument : Instrument #1
[illegible]le Name: 1 field-coll. M C.oculata abd./CH2Cl2
[illegible] Info : coll. 5/28 sweeping vetch; second male today
[illegible] Number: 1

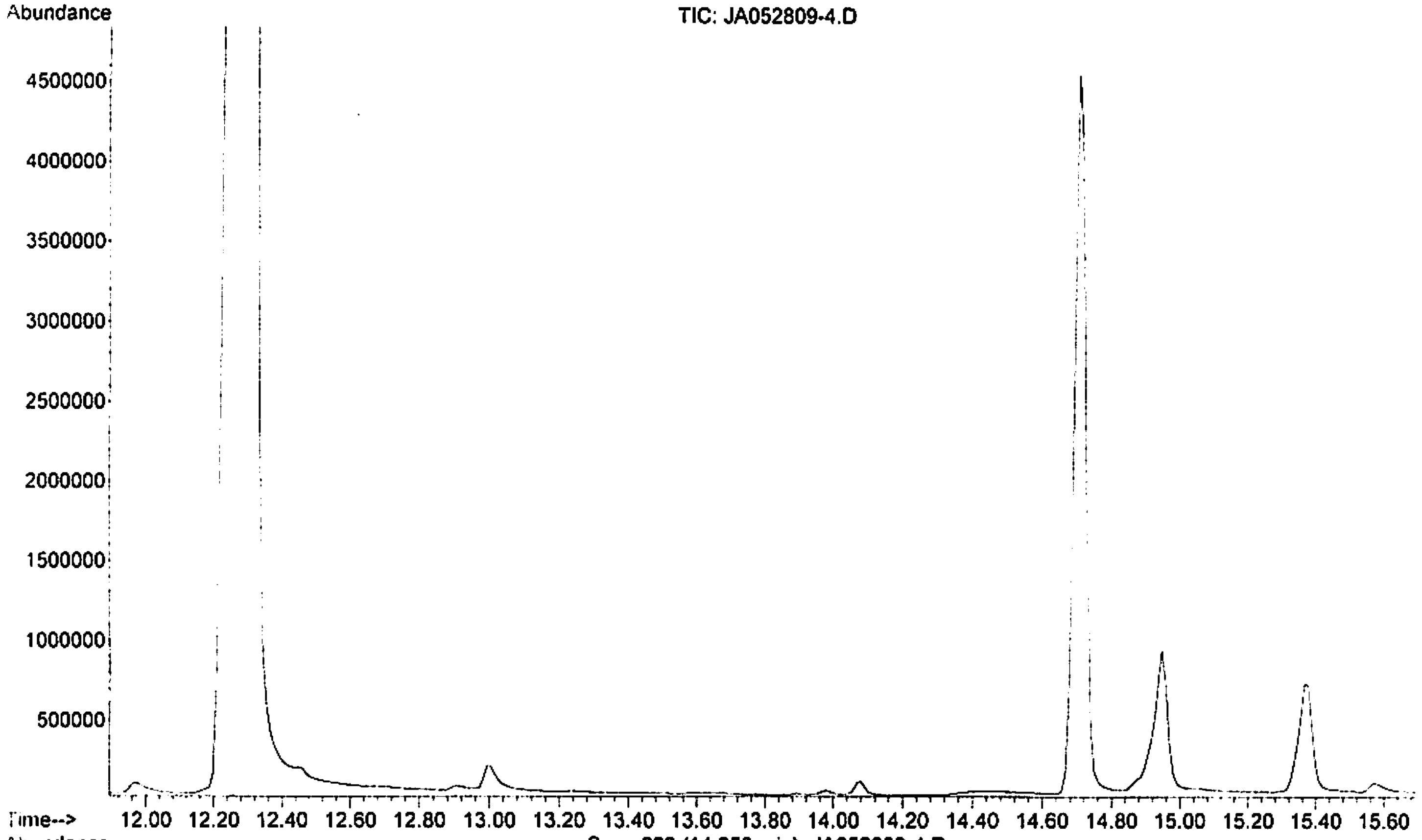

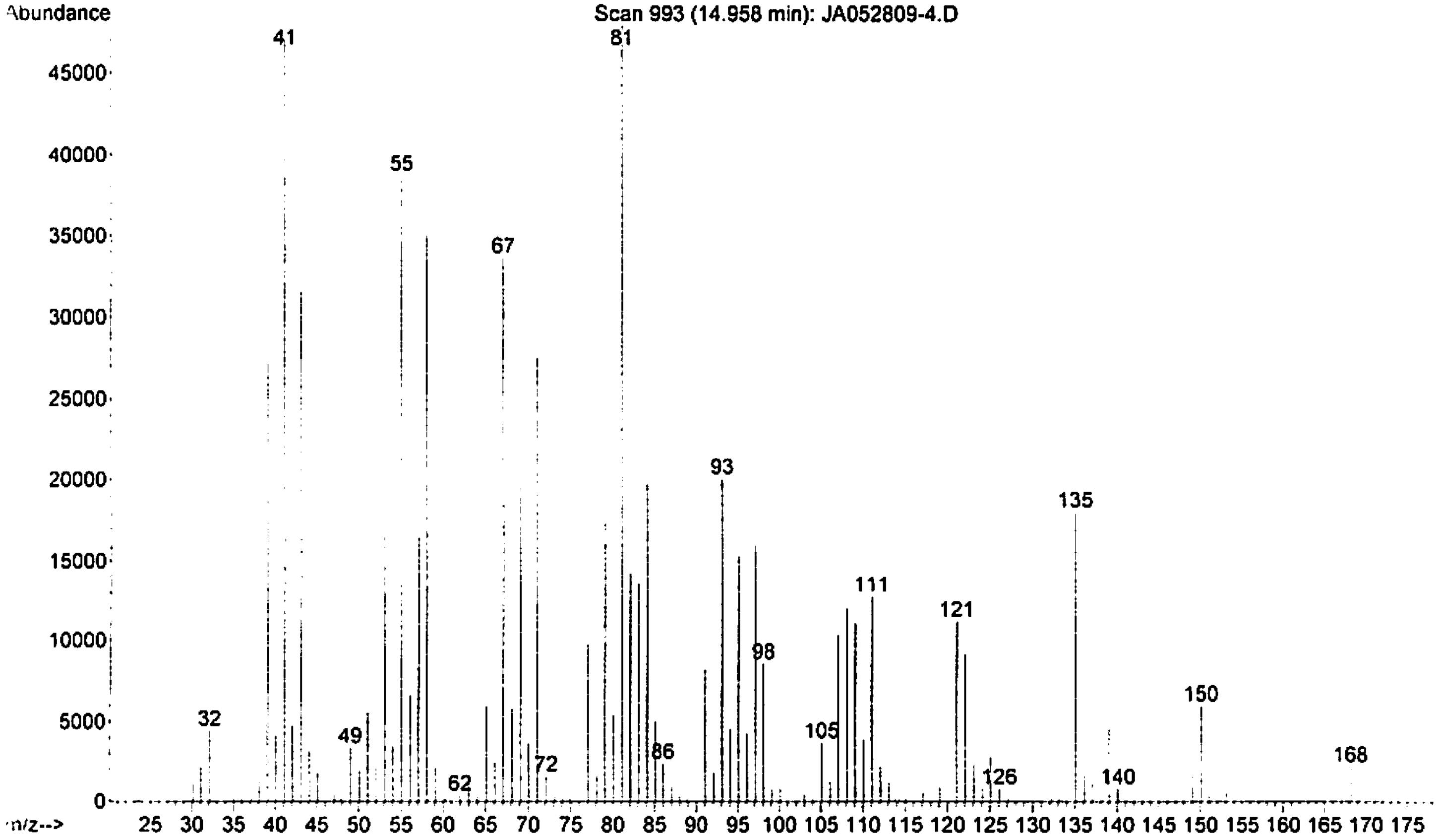

[illegible] :D:\DATA\ALDRICH\JA-09\Snapshot\JA052809-4.D
[illegible]rator : Aldrich
[illegible]uired : 28 May 2009 16:03 using AcqMethod JA-WAX08.M
[illegible]trument : Instrument #1
[illegible]le Name: 1 field-coll. M C.oculata abd./CH2Cl2
[illegible] Info : coll. 5/28 sweeping vetch; second male today
[illegible] Number: 1

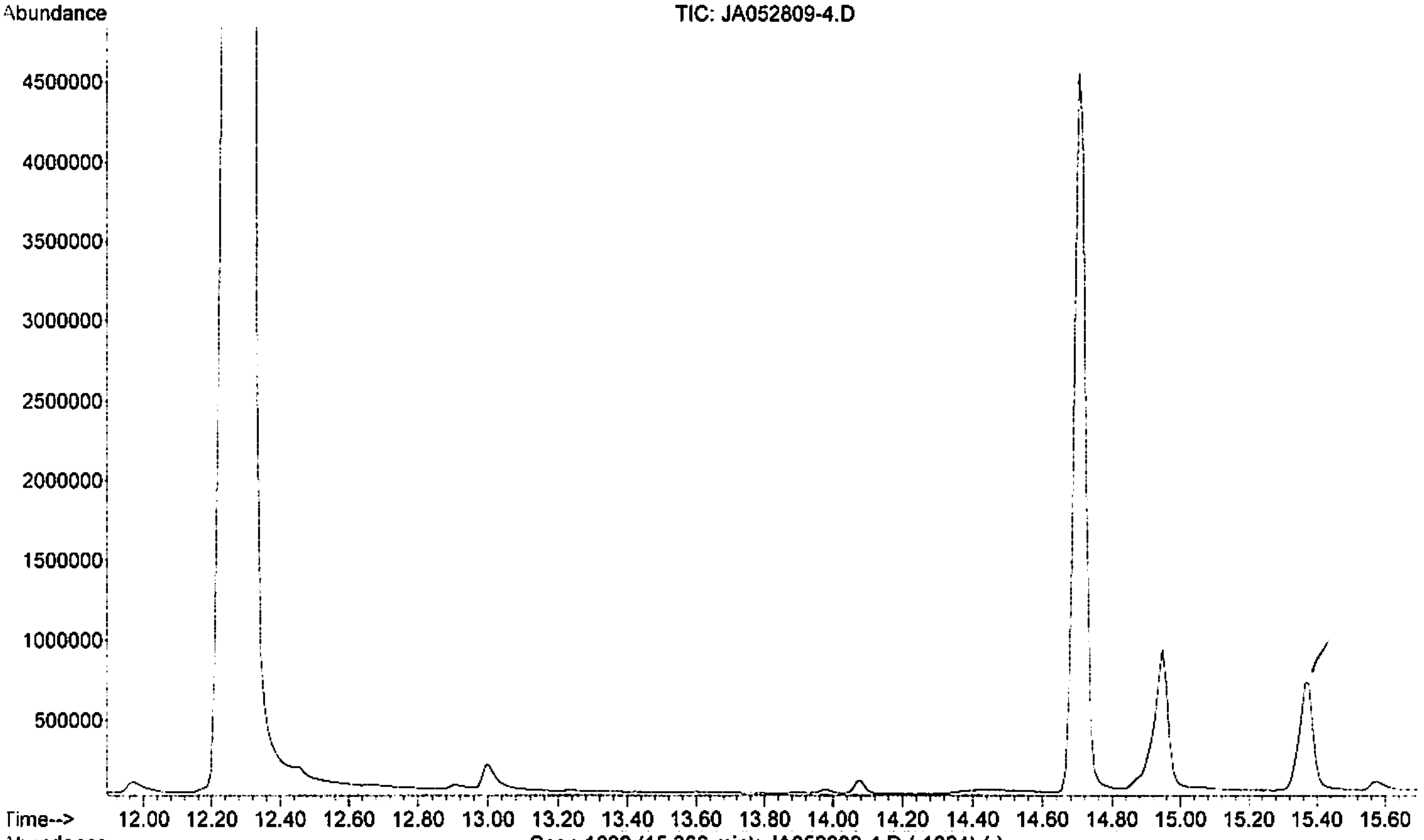

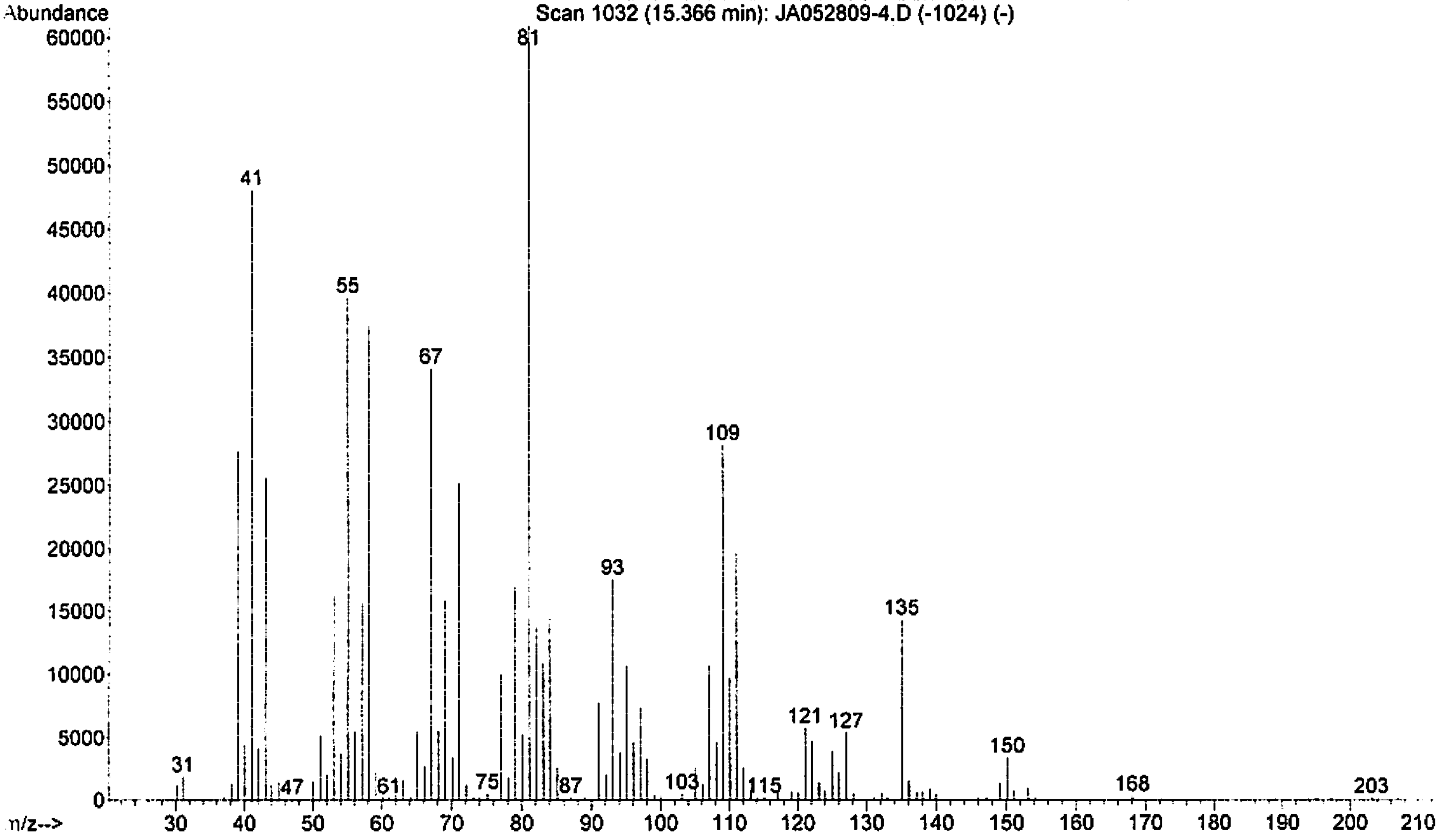

:D:\DATA\ALDRICH\JA-09\Snapshot\JA052809-4.D
ator : Aldrich
ired : 28 May 2009 16:03 using AcqMethod JA-WAX08.M
rument : Instrument #1
le Name: 1 field-coll. M C.oculata abd./CH2Cl2
: Info : coll. 5/28 sweeping vetch; second male today
Number: 1

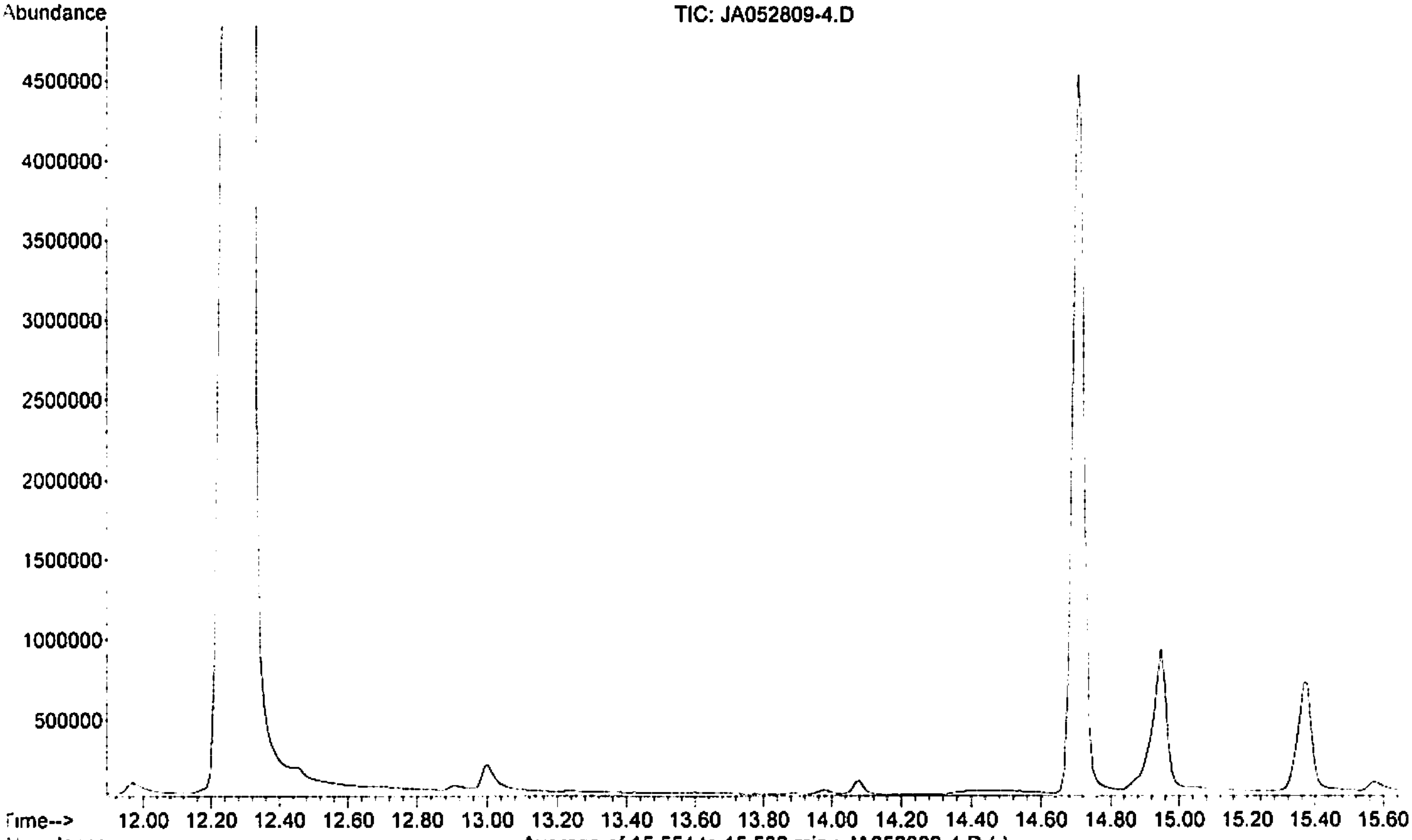

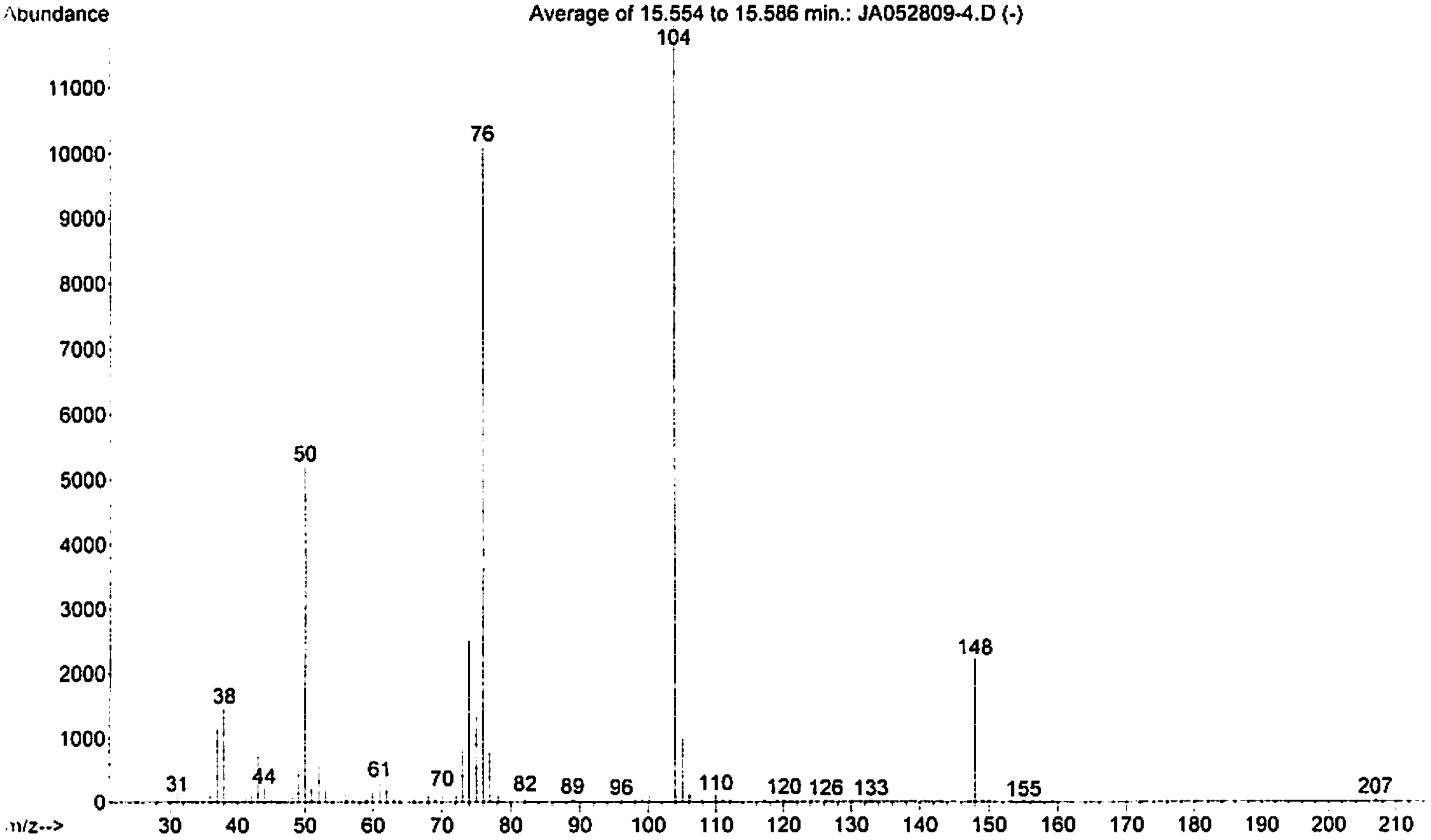

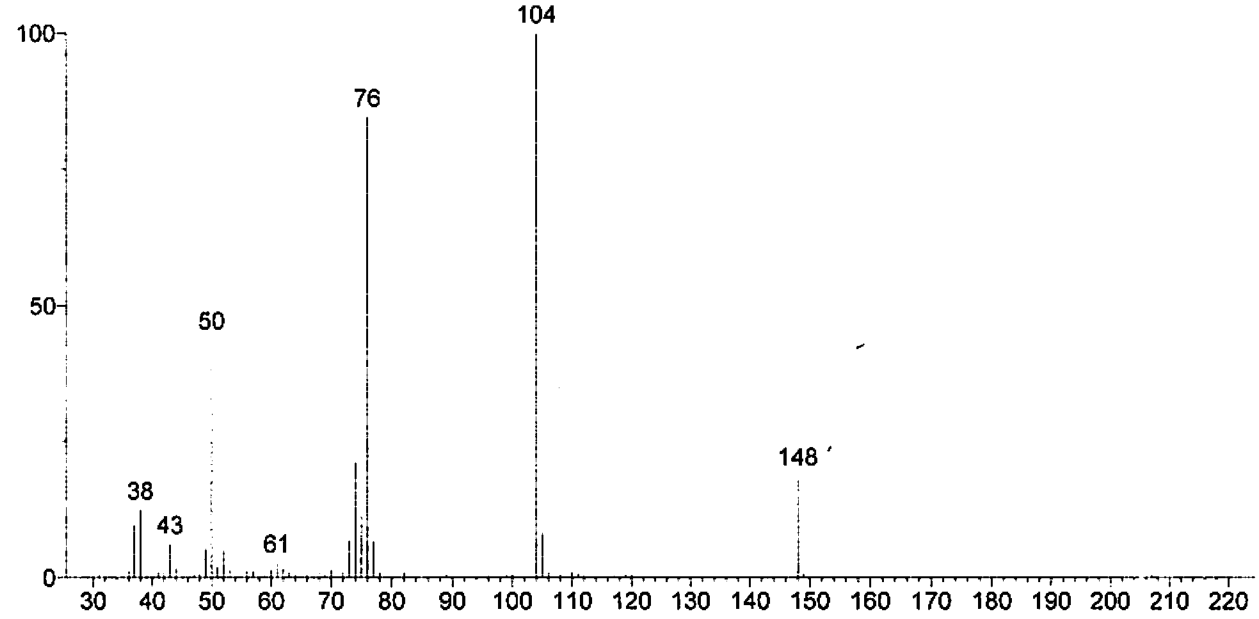

(Text File) Average of 15.554 to 15.586 min.: JA052809-4.D

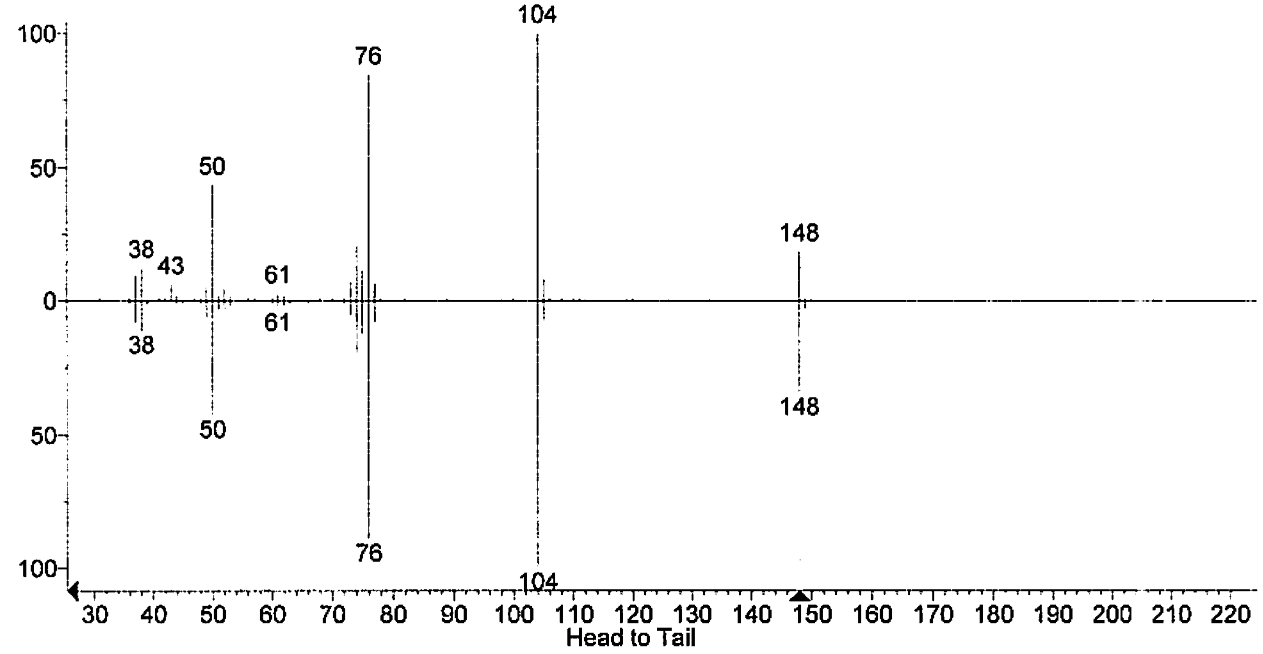

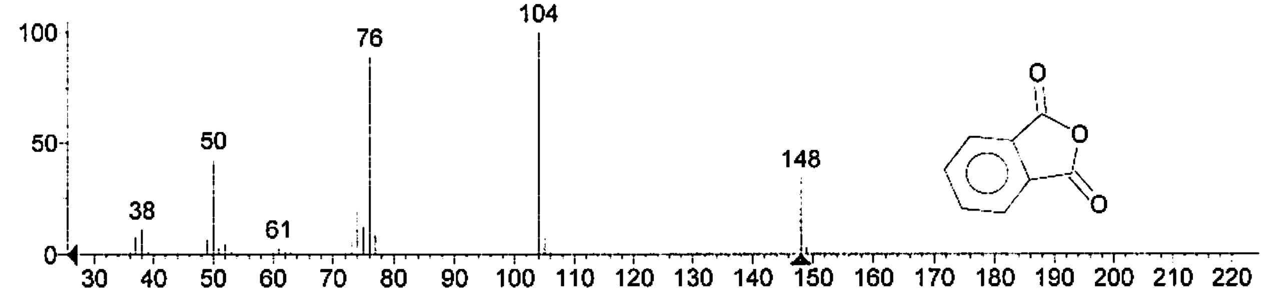

(mainlib) Phthalic anhydride

:D:\DATA\ALDRICH\JA-09\Snapshot\JA052809-4.D
[illegible]ator : Aldrich
[illegible]ired : 28 May 2009 16:03 using AcqMethod JA-WAX08.M
[illegible]rument : Instrument #1
[illegible]le Name: 1 field-coll. M C.oculata abd./CH2Cl2
[illegible] Info : coll. 5/28 sweeping vetch; second male today
[illegible] Number: 1

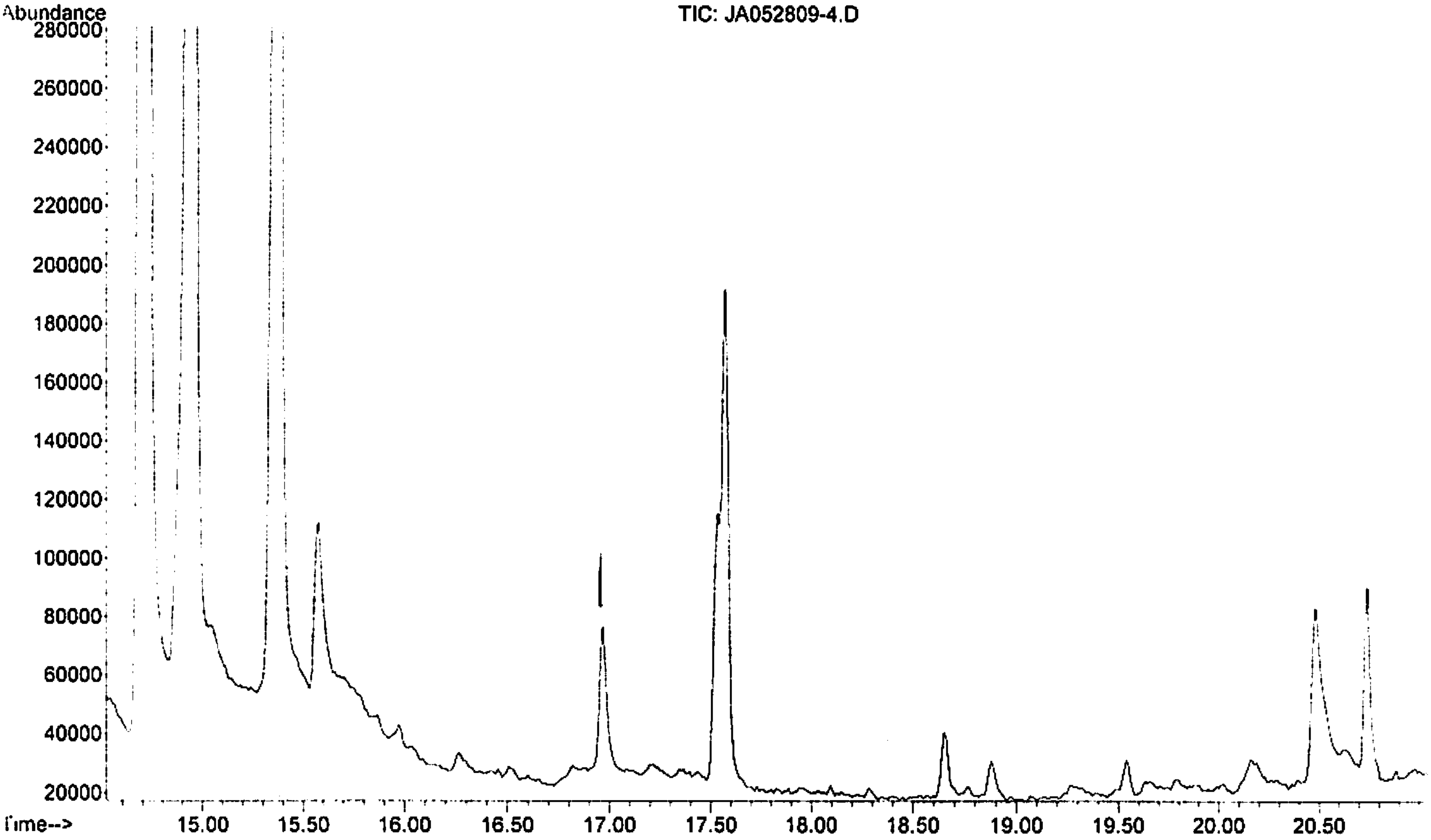

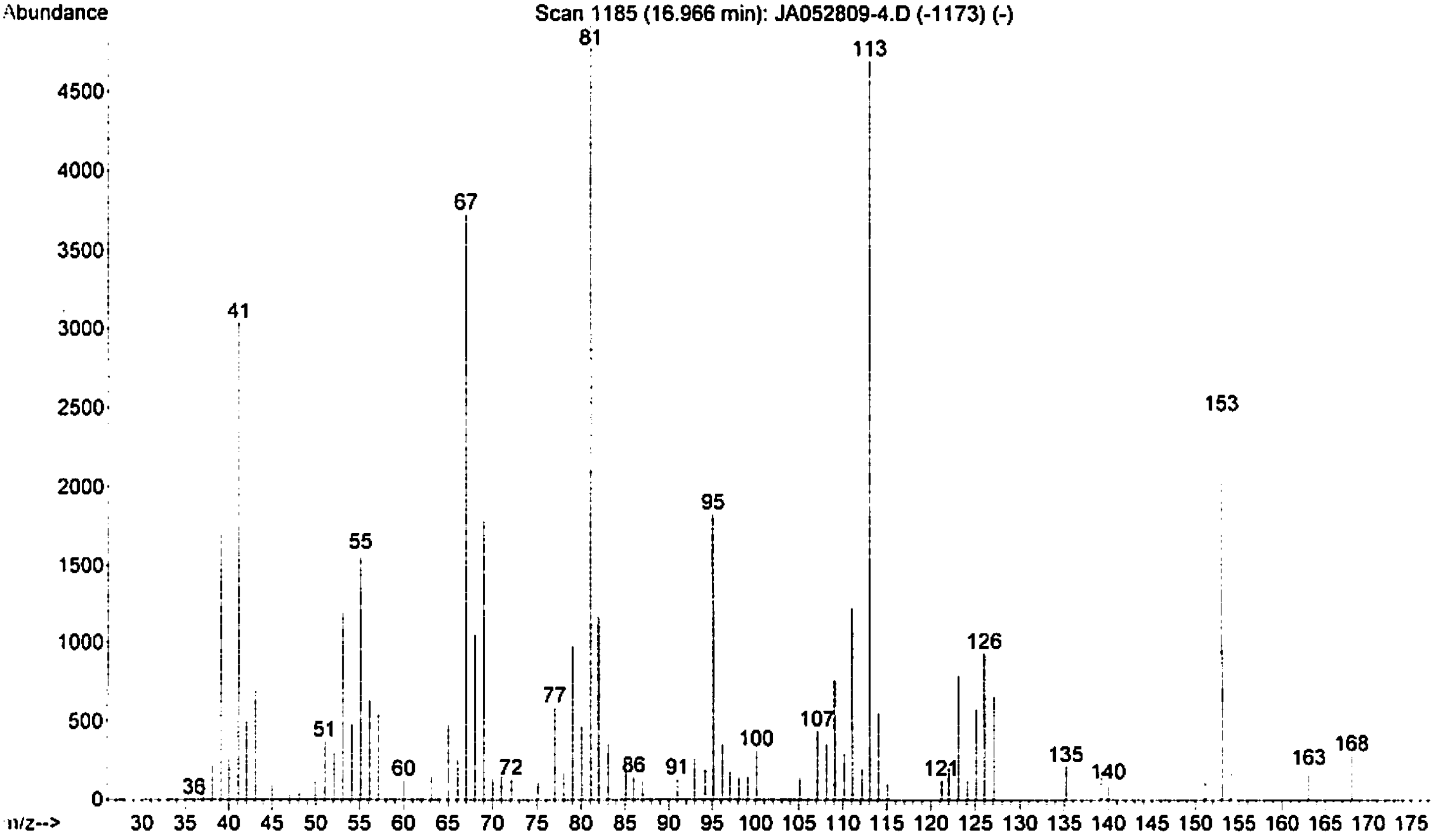

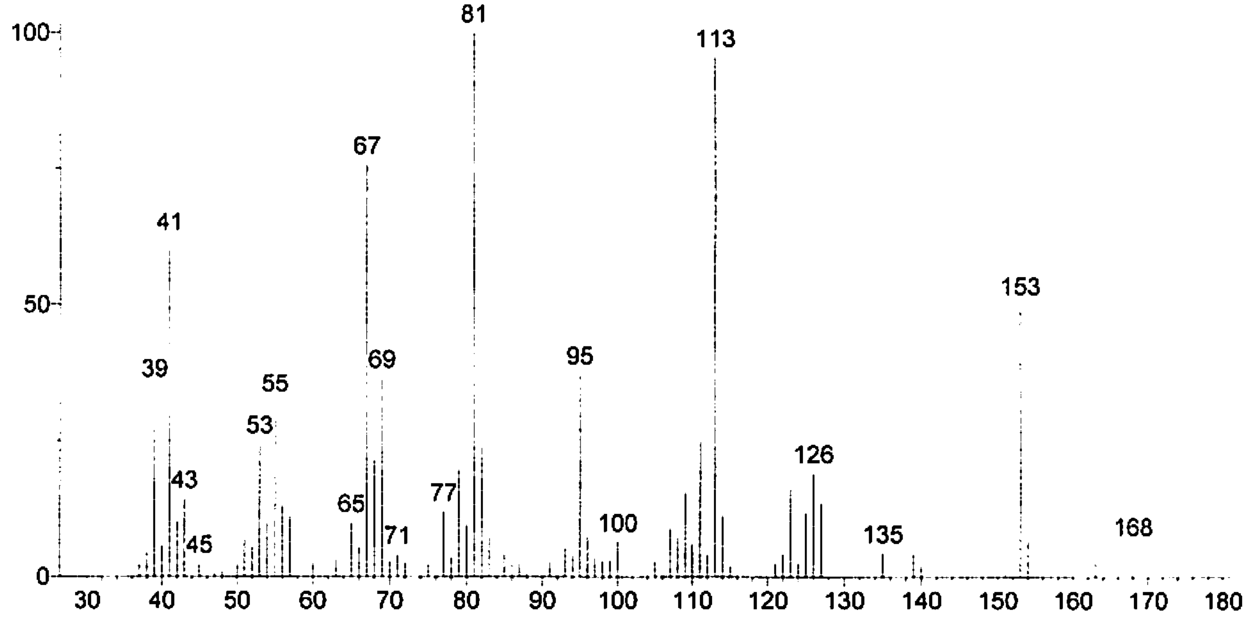

(Text File) Scan 1185 (16.966 min): JA052809-4.D (-1173)

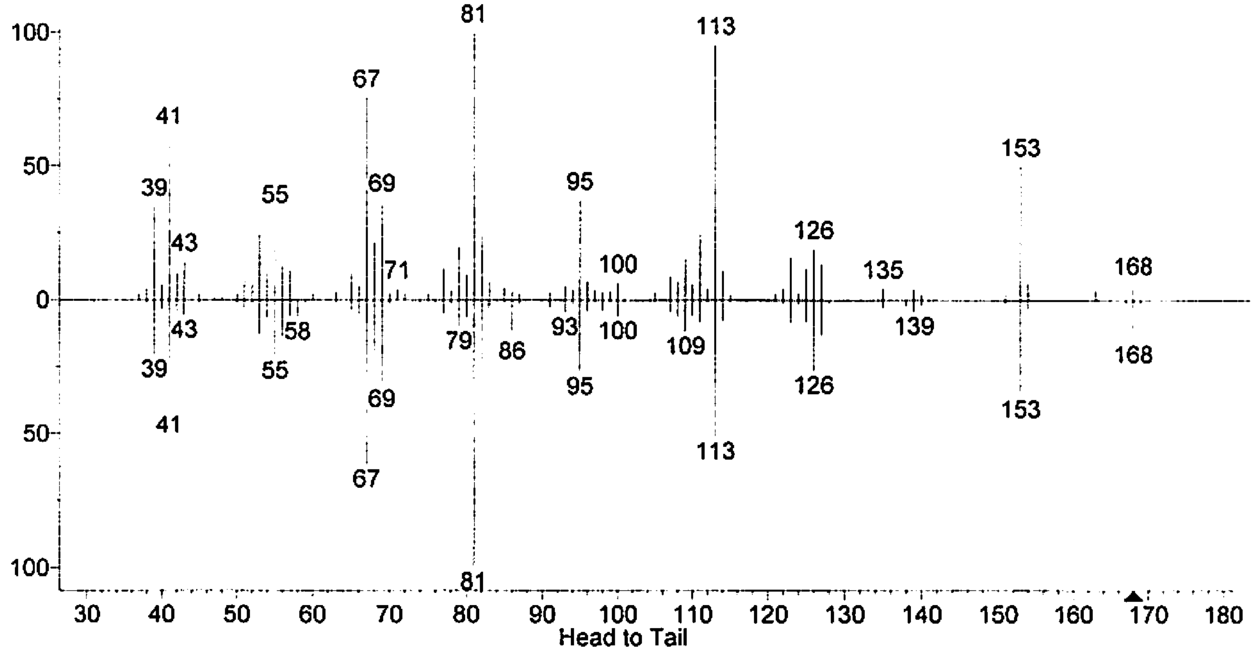

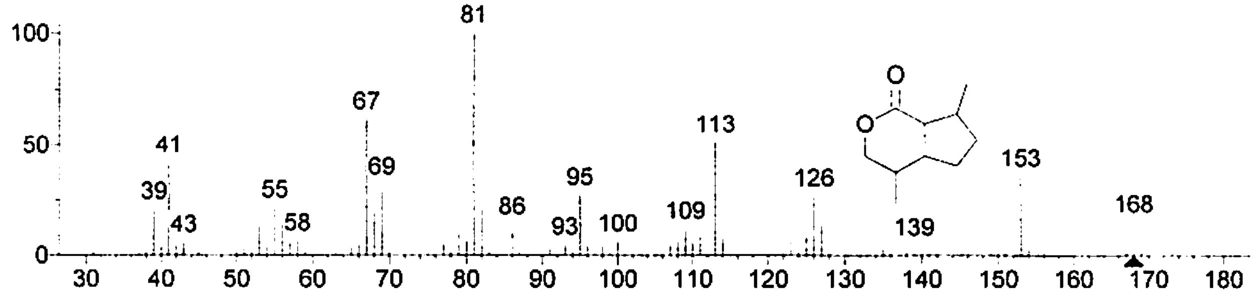

(mainlib) Cyclopenta[c]pyran-1(3H)-one, hexahydro-4,7-dimethyl-, (4.alpha.,4a.alpha.,7.alpha.,7a.alpha.)-

[illegible] : D:\DATA\ALDRICH\JA-09\Snapshot\JA052809-4.D
[illegible]ator : Aldrich
[illegible]ired : 28 May 2009 16:03 using AcqMethod JA-WAX08.M
[illegible]rument : Instrument #1
[illegible]le Name: 1 field-coll. M C.oculata abd./CH2Cl2
[illegible] Info : coll. 5/28 sweeping vetch; second male today
[illegible] Number: 1

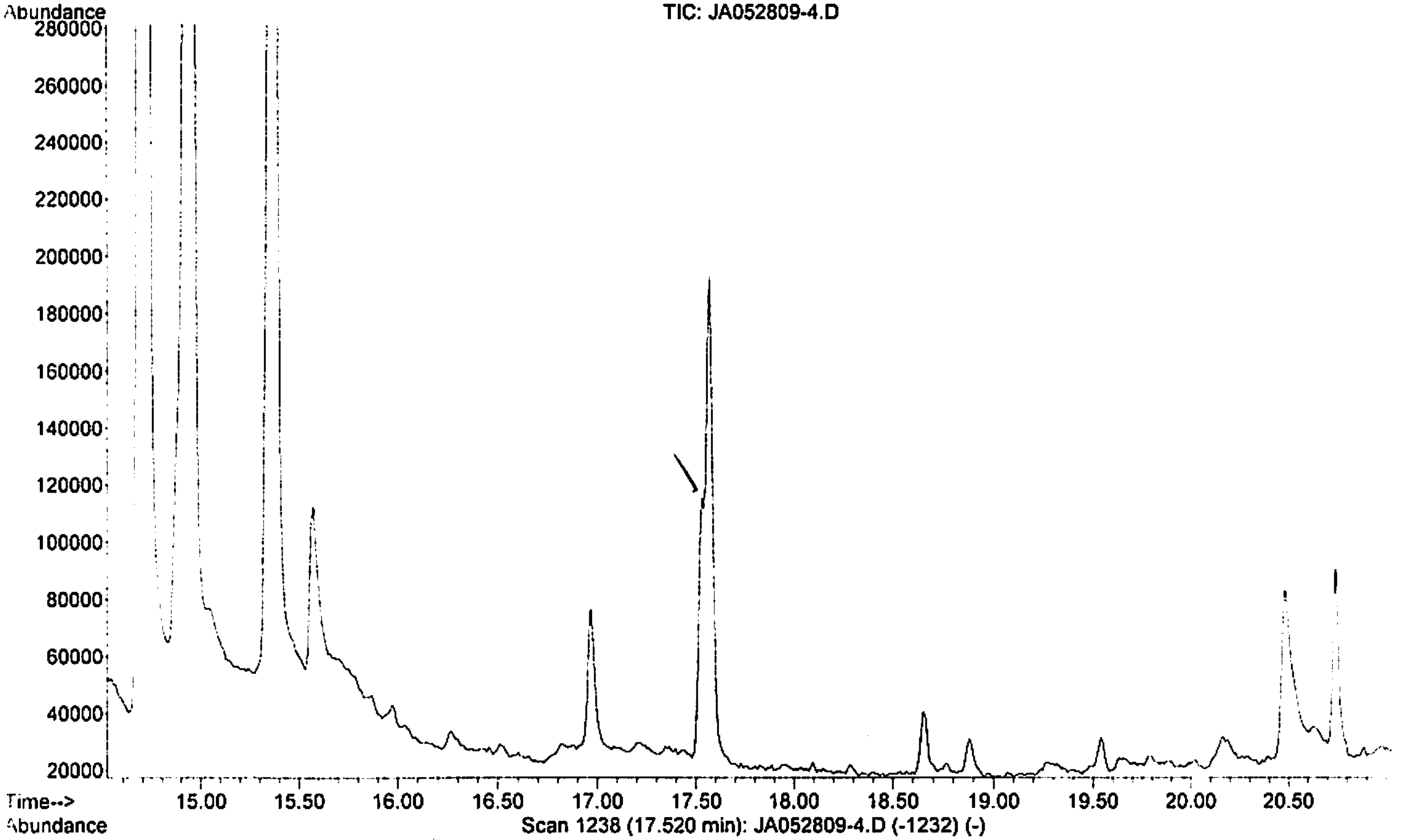

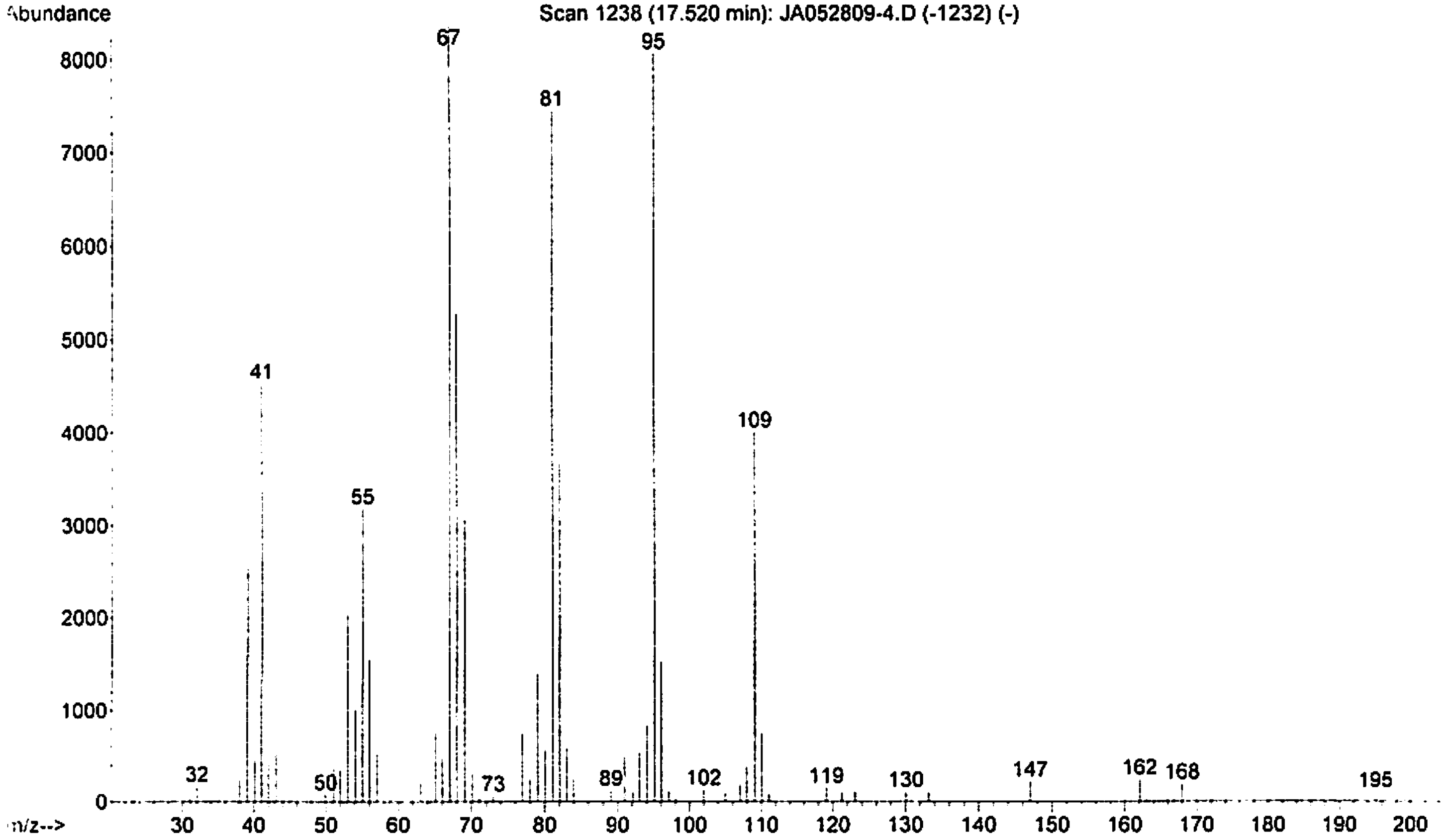

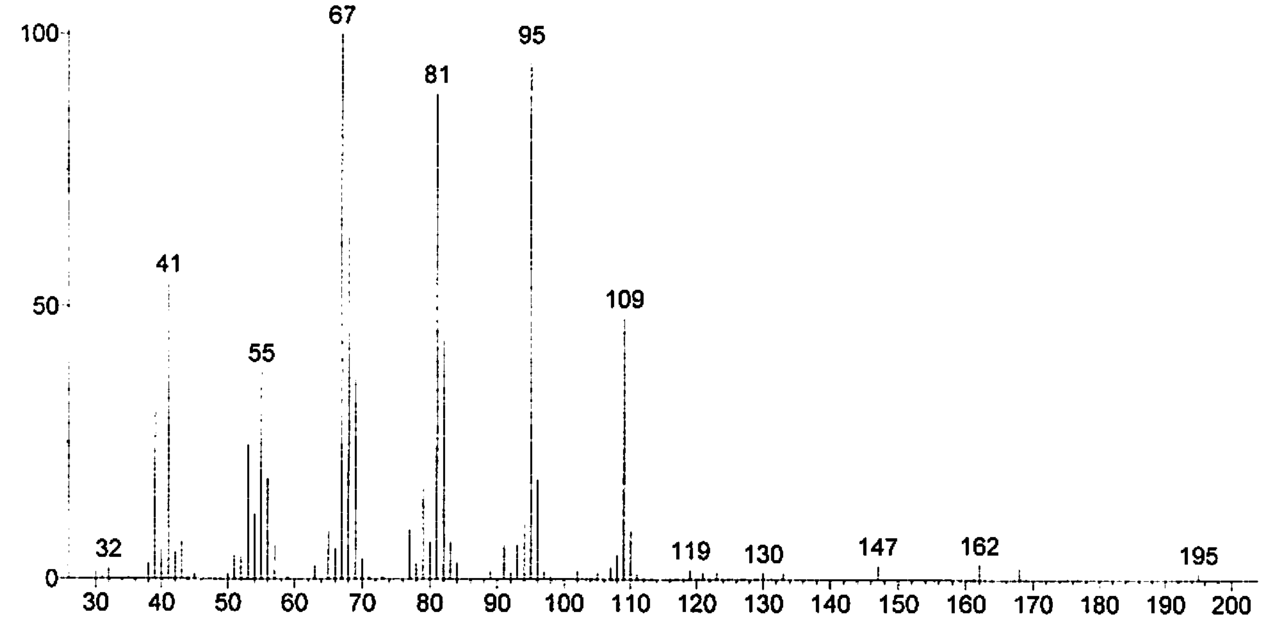

(Text File) Scan 1238 (17.520 min): JA052809-4.D (-1232)

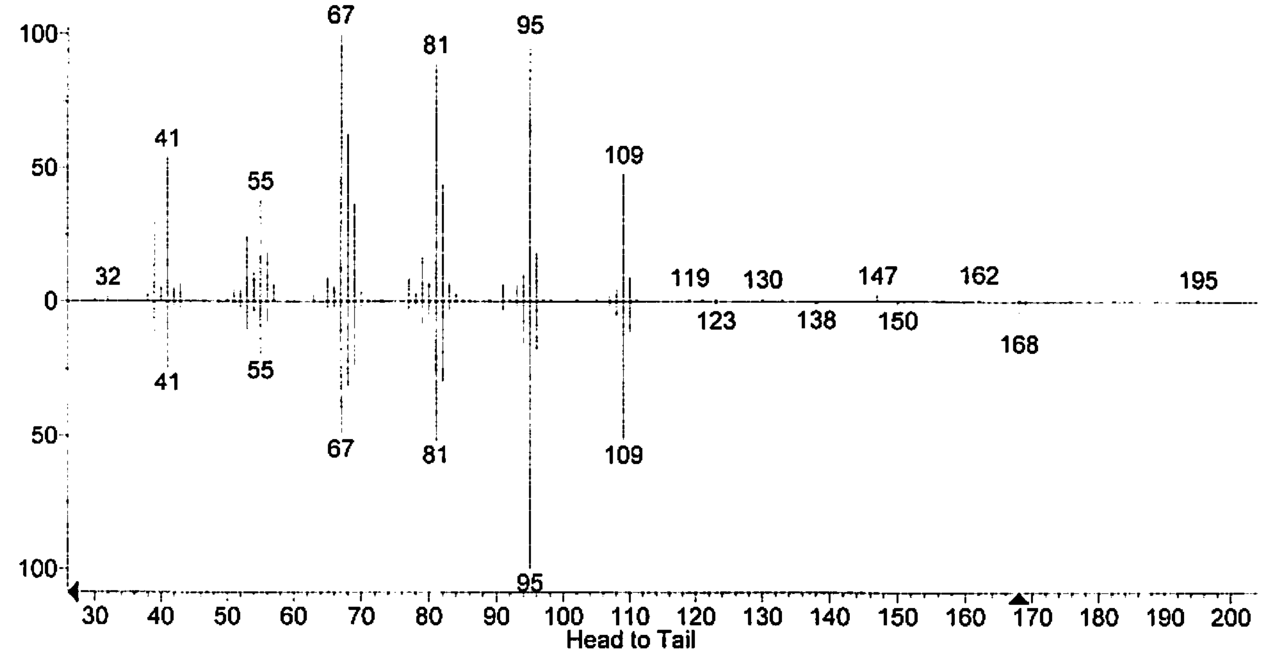

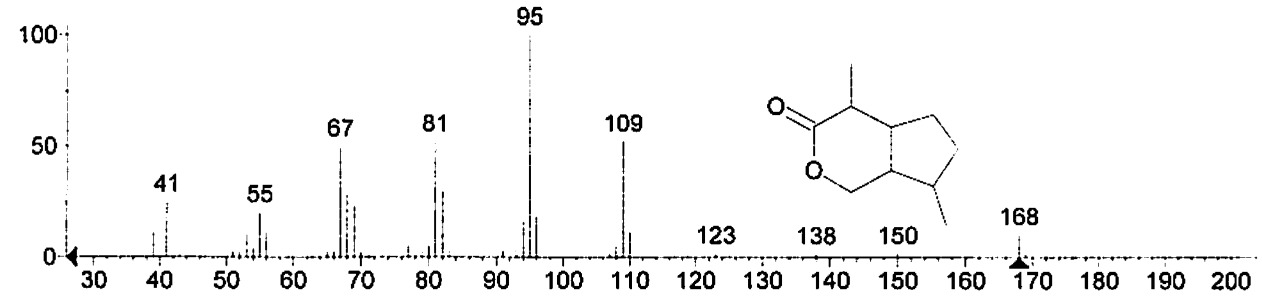

(replib) Iridomyrmecin

```
 e          :D:\DATA\ALDRICH\JA-09\Snapshot\JA052809-4.D
 erator     : Aldrich
 quired     : 28 May 2009  16:03      using AcqMethod JA-WAX08.M
 strument   :     Instrument #1
 mple Name: 1 field-coll. M C.oculata abd./CH2Cl2
  sc Info   : coll. 5/28 sweeping vetch; second male today
  al Number: 1
```

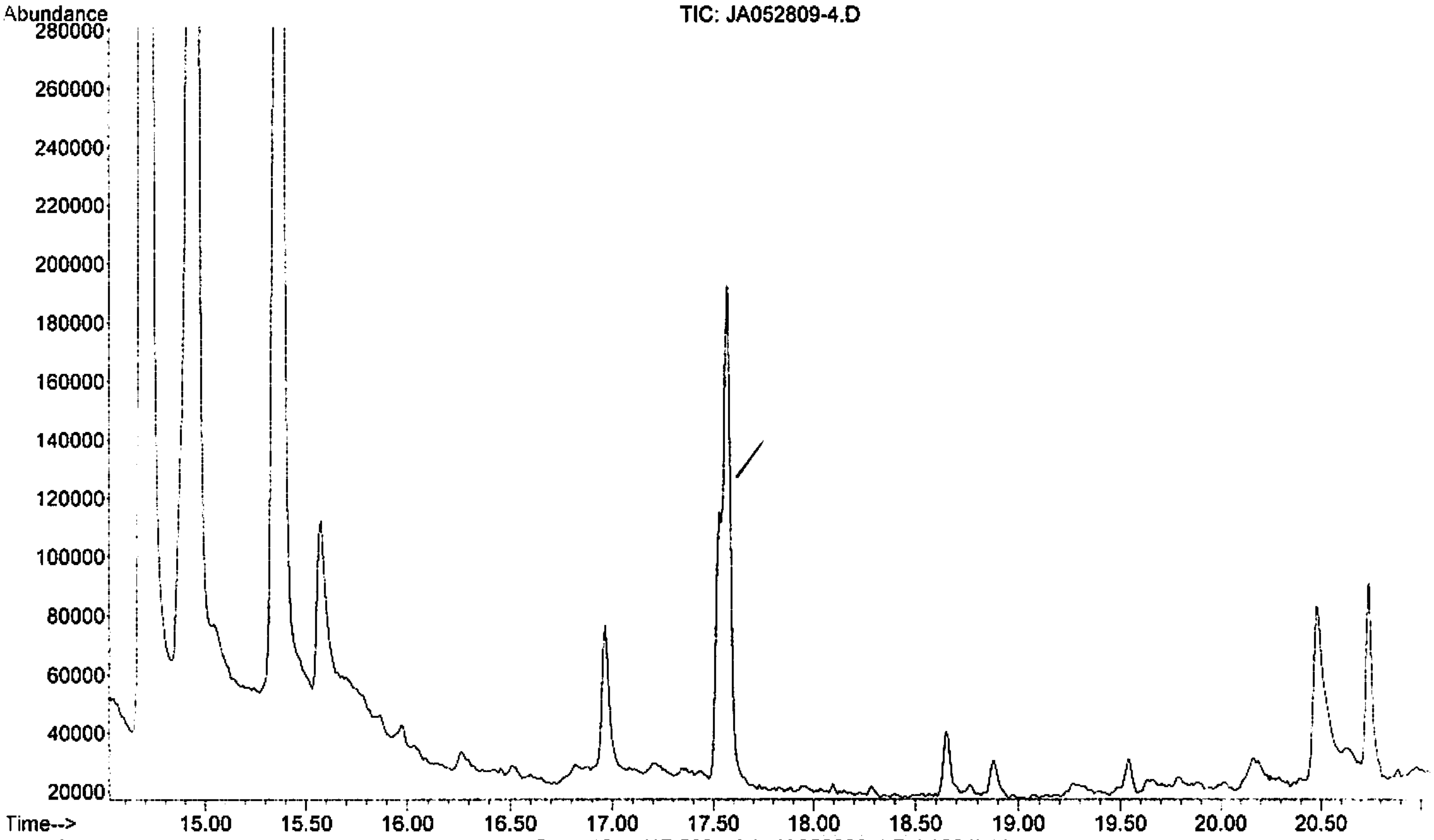

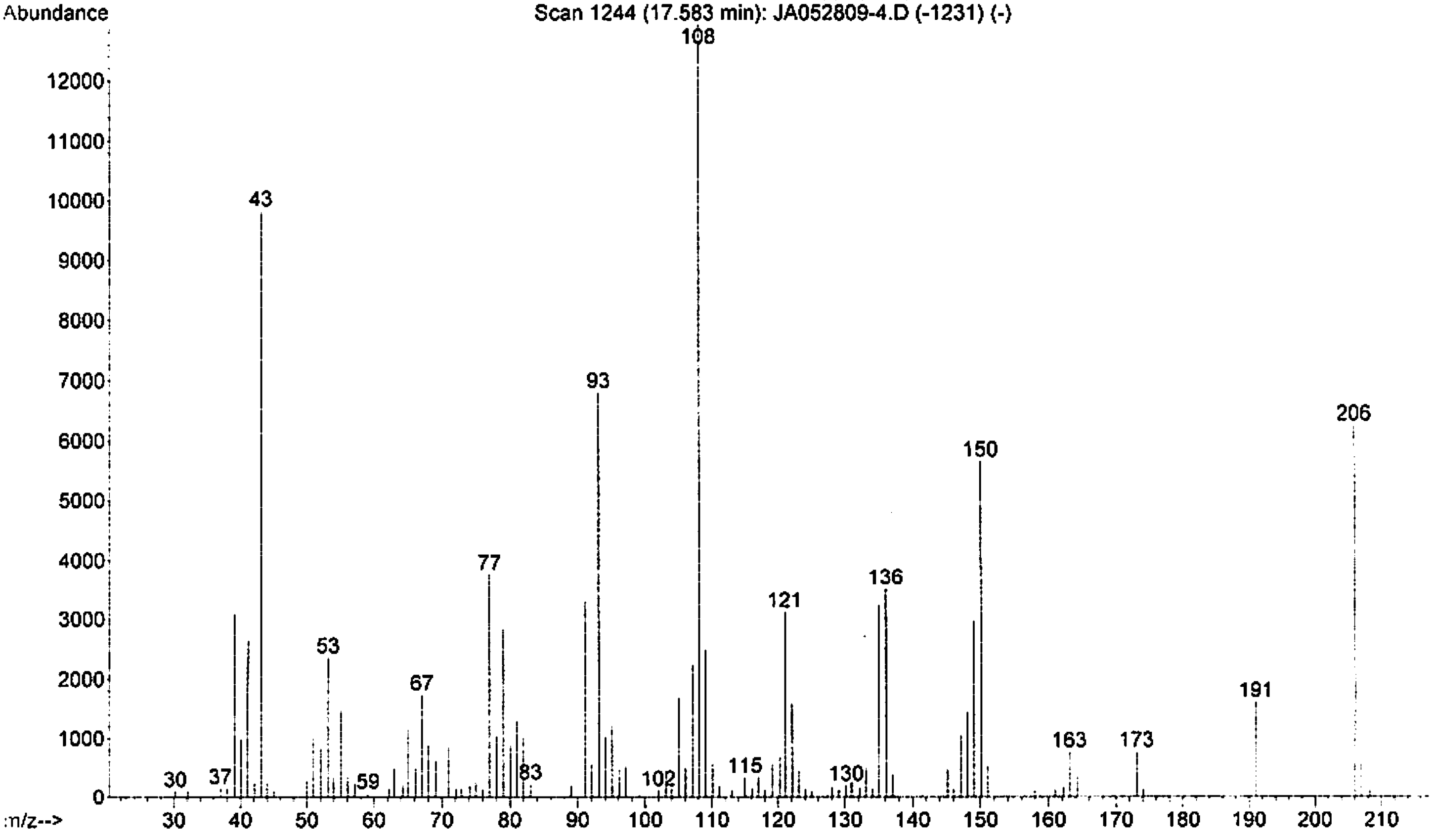

:D:\DATA\ALDRICH\JA-09\Snapshot\JA052809-4.D
[illegible]ator : Aldrich
[illegible]ired : 28 May 2009 16:03 using AcqMethod JA-WAX08.M
[illegible]rument : Instrument #1
[illegible]ple Name: 1 field-coll. M C.oculata abd./CH2Cl2
[illegible] Info : coll. 5/28 sweeping vetch; second male today
[illegible] Number: 1

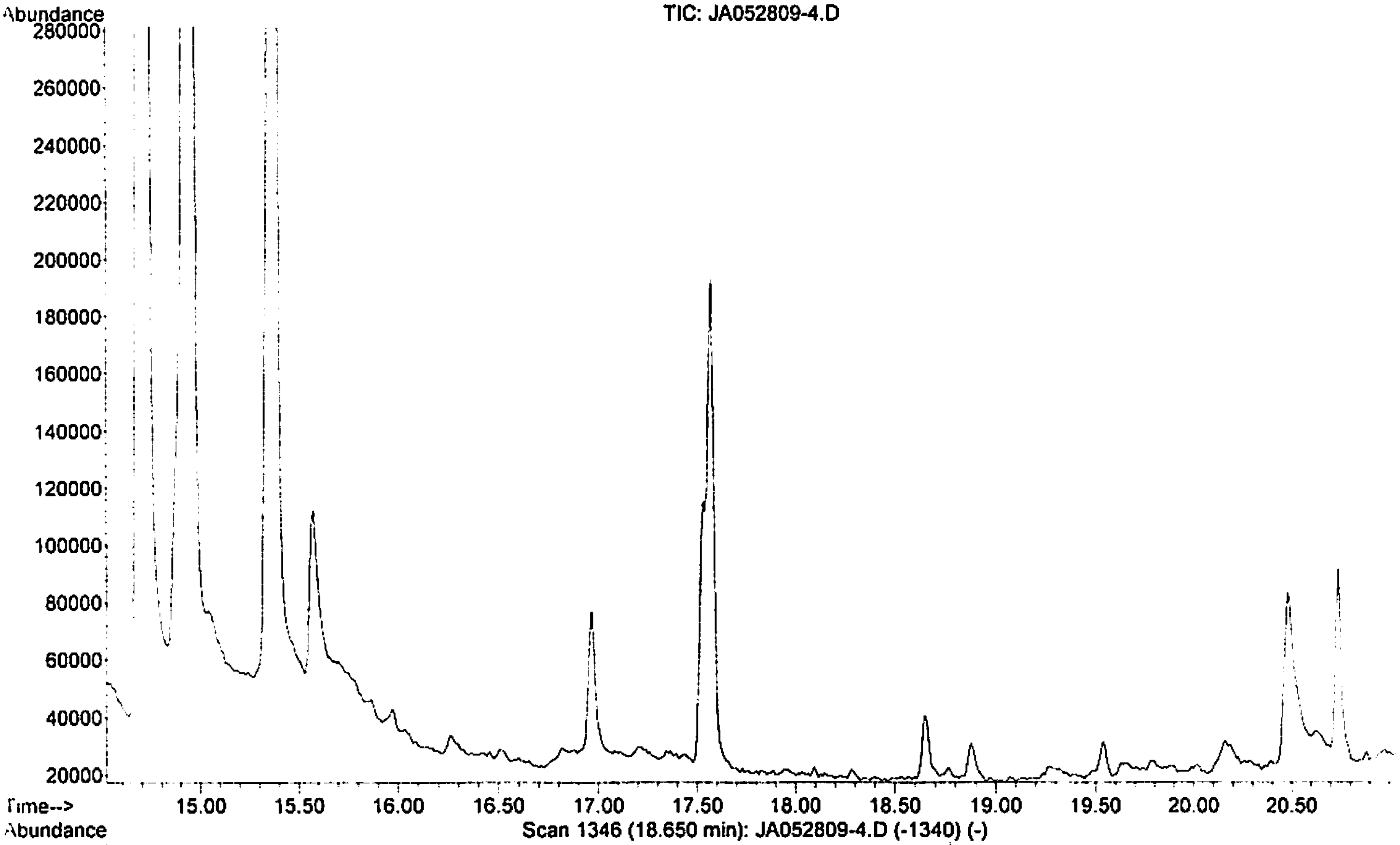

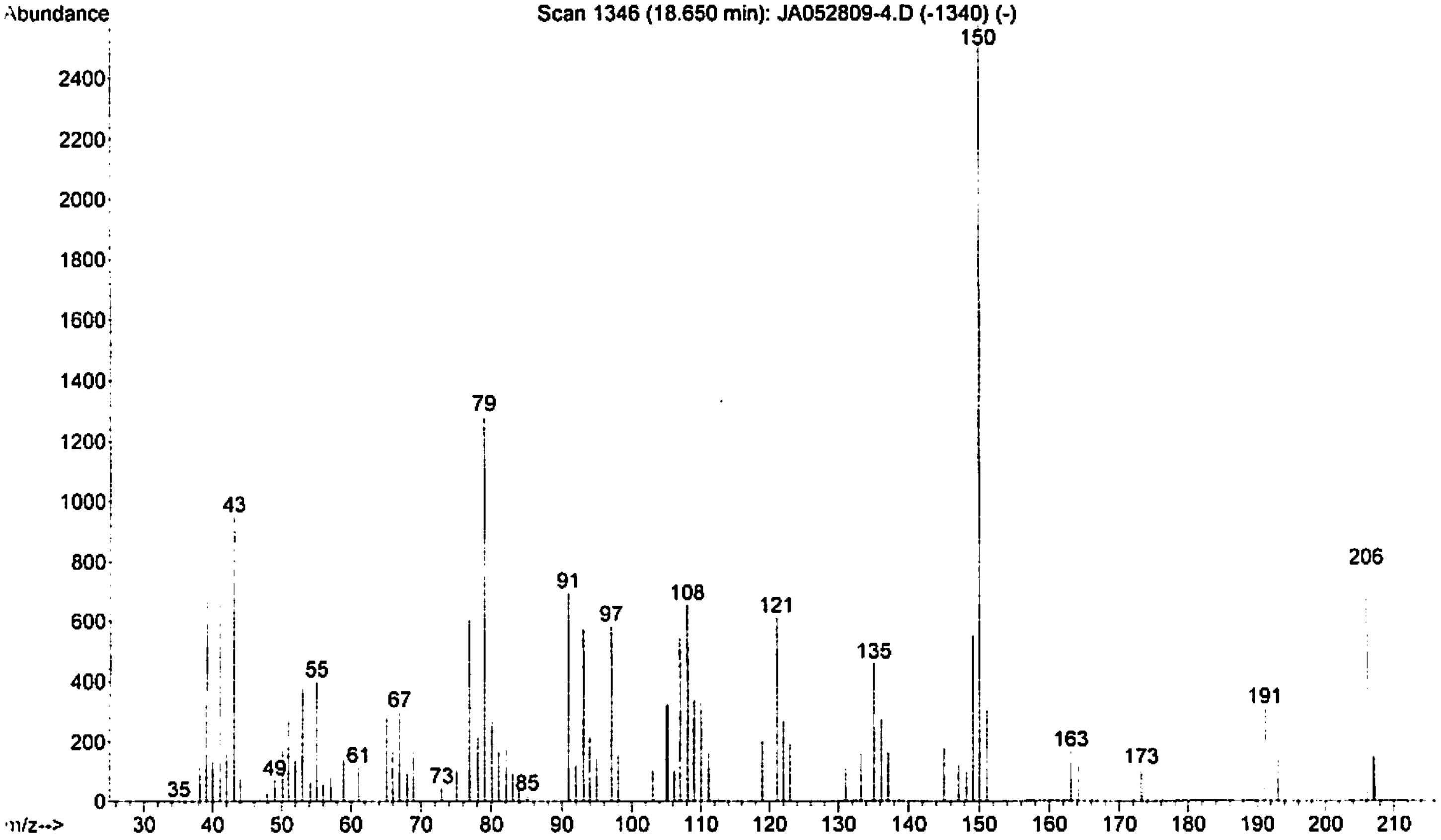

:D:\DATA\ALDRICH\JA-09\Snapshot\JA052809-4.D
rator : Aldrich
ired : 28 May 2009 16:03 using AcqMethod JA-WAX08.M
trument : Instrument #1
le Name: 1 field-coll. M C.oculata abd./CH2Cl2
Info : coll. 5/28 sweeping vetch; second male today
Number: 1

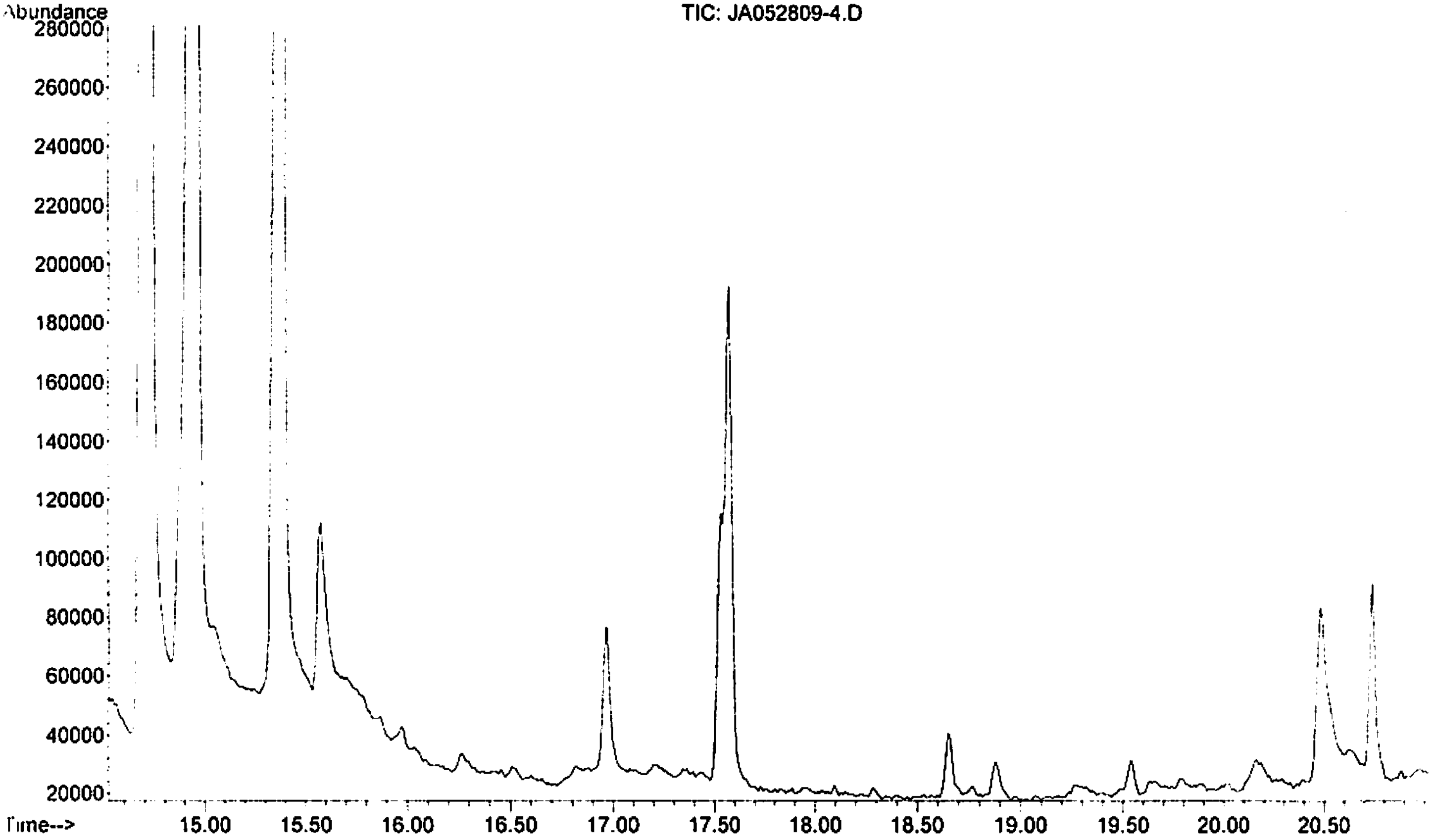

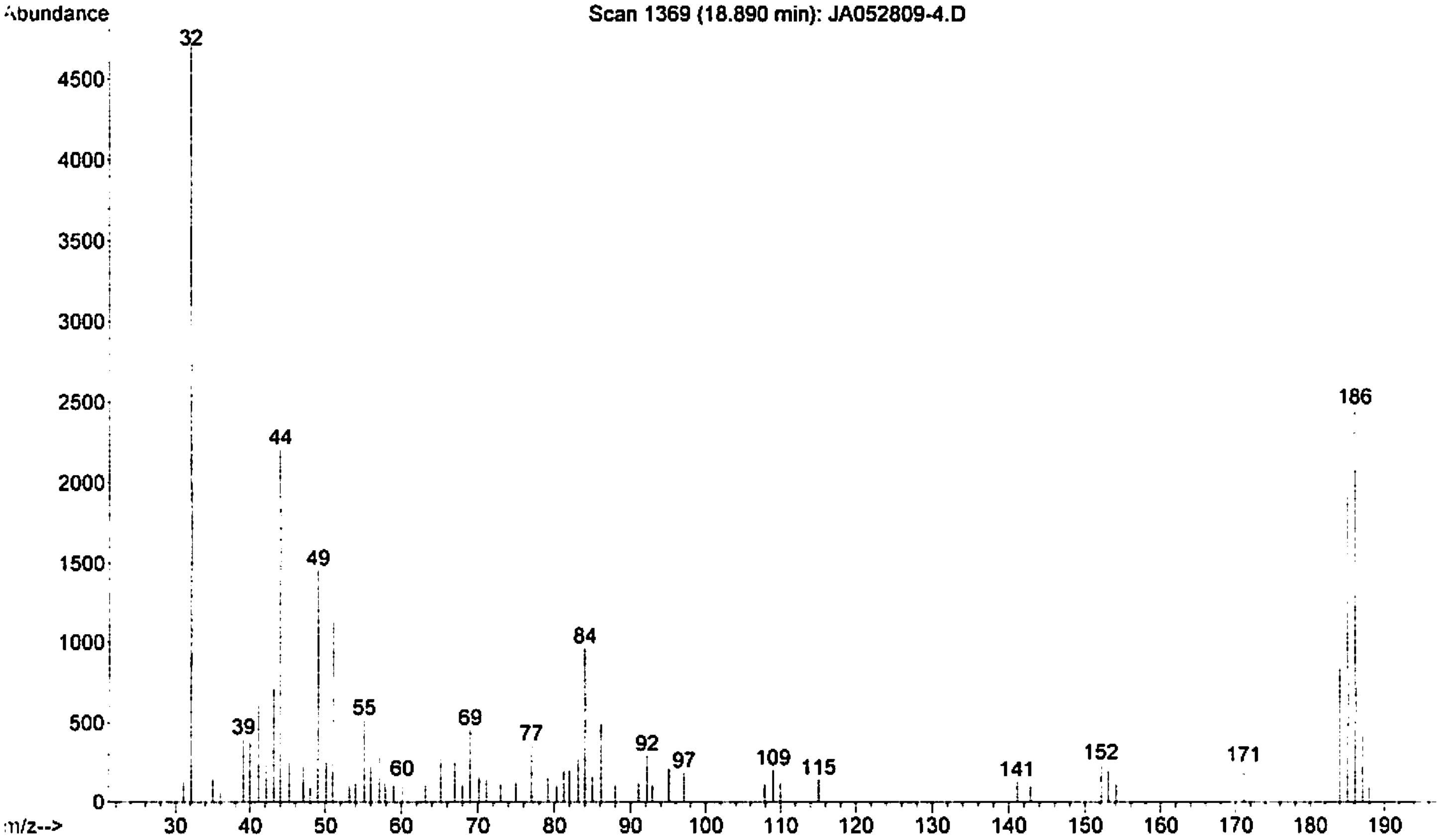

:D:\DATA\ALDRICH\JA-09\Snapshot\JA052809-4.D
rator : Aldrich
uired : 28 May 2009 16:03 using AcqMethod JA-WAX08.M
trument : Instrument #1
le Name: 1 field-coll. M C.oculata abd./CH2C12
Info : coll. 5/28 sweeping vetch; second male today
Number: 1

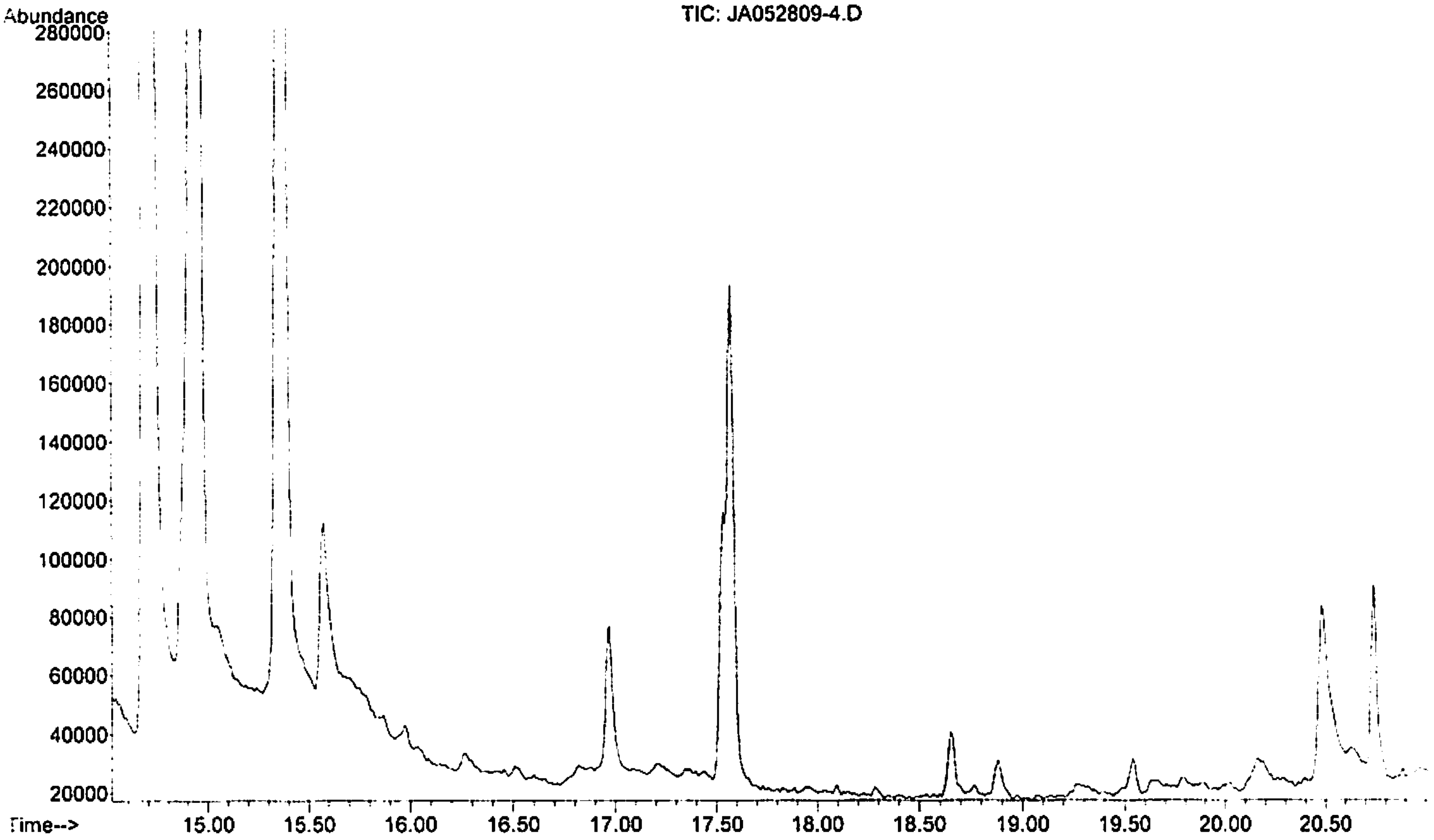

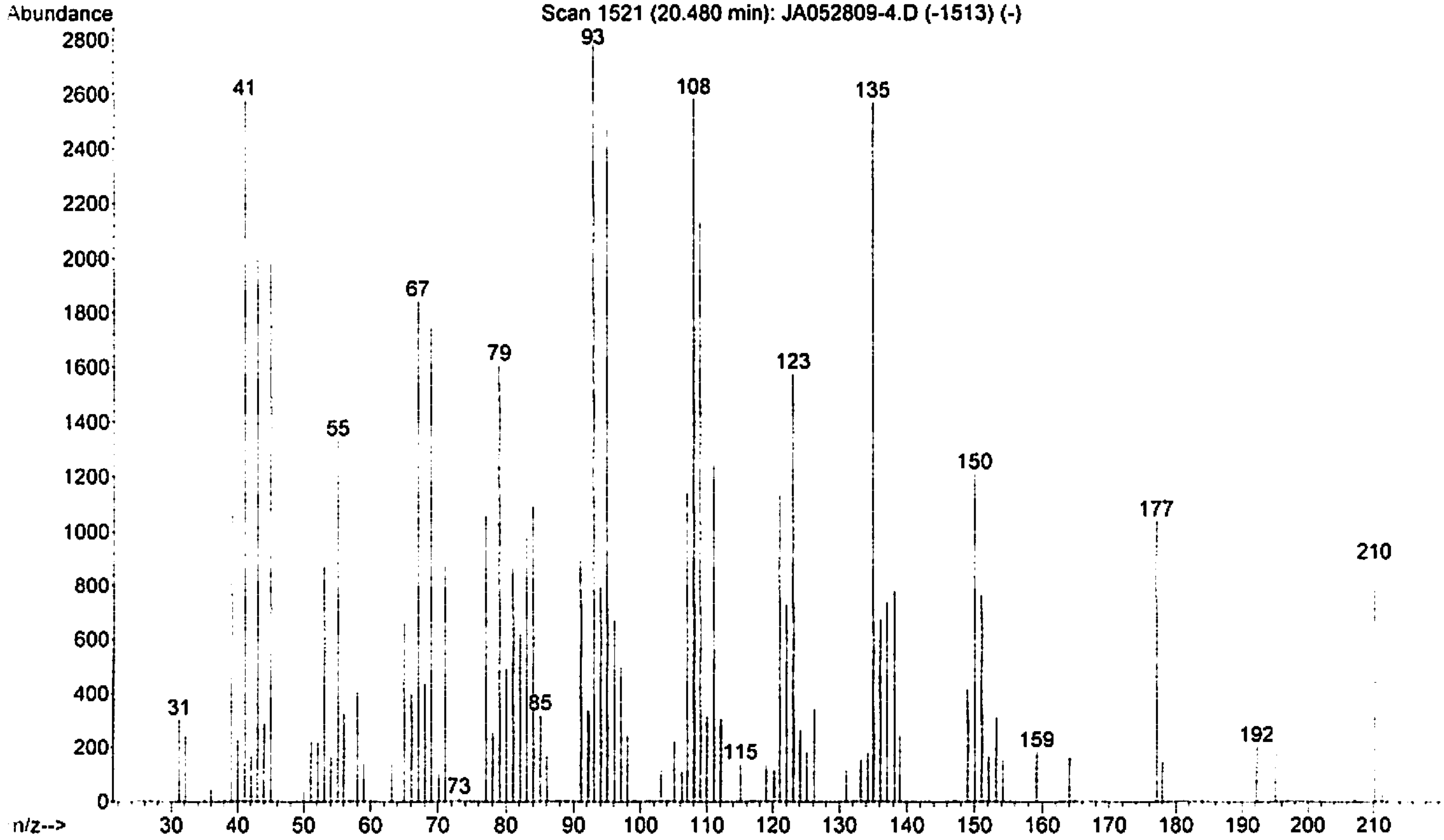

:D:\DATA\ALDRICH\JA-09\Snapshot\JA052809-4.D
[illegible]rator : Aldrich
[illegible]ired : 28 May 2009 16:03 using AcqMethod JA-WAX08.M
[illegible]rument : Instrument #1
[illegible]le Name: 1 field-coll. M C.oculata abd./CH2Cl2
[illegible] Info : coll. 5/28 sweeping vetch; second male today
[illegible] Number: 1

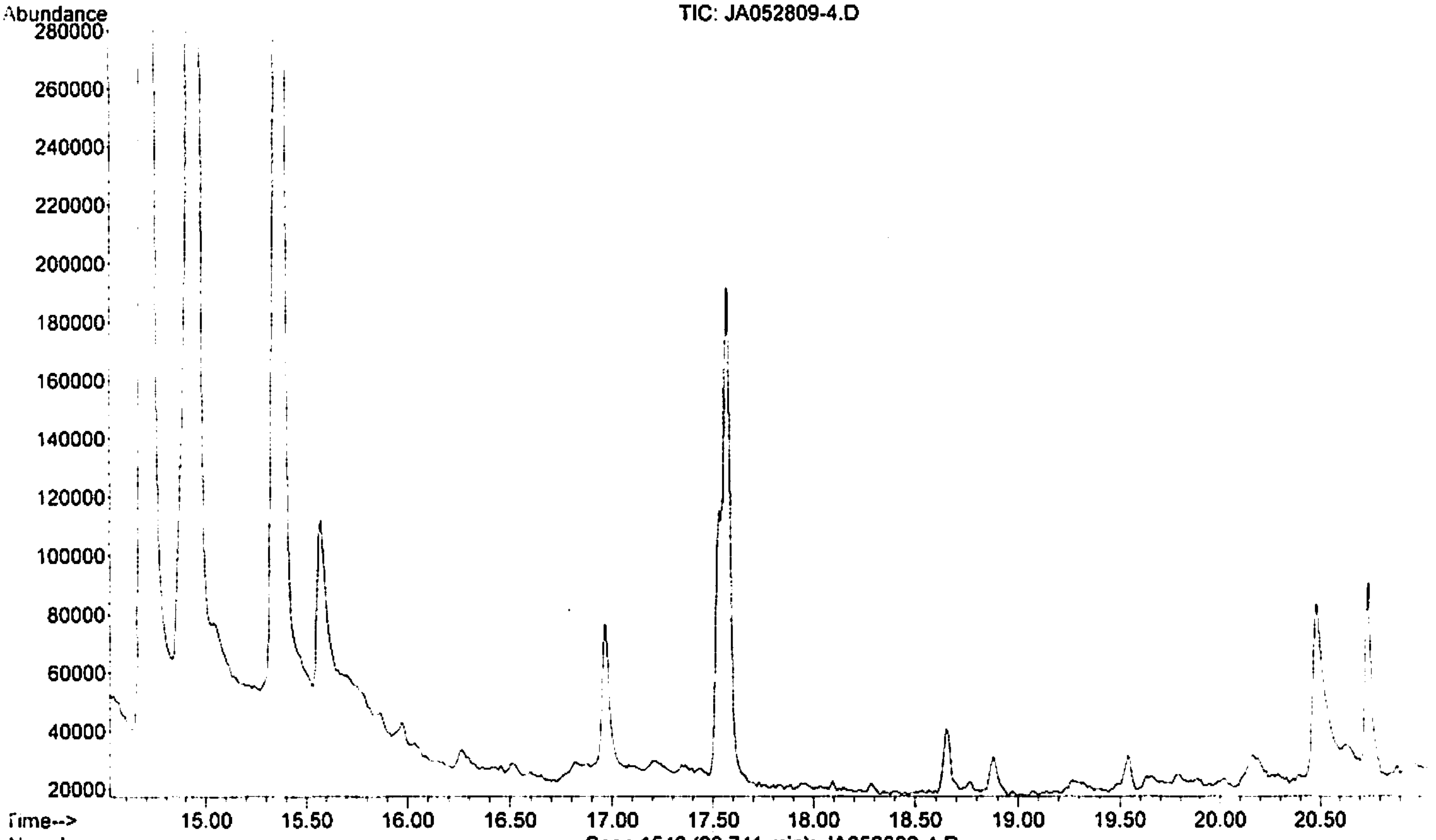

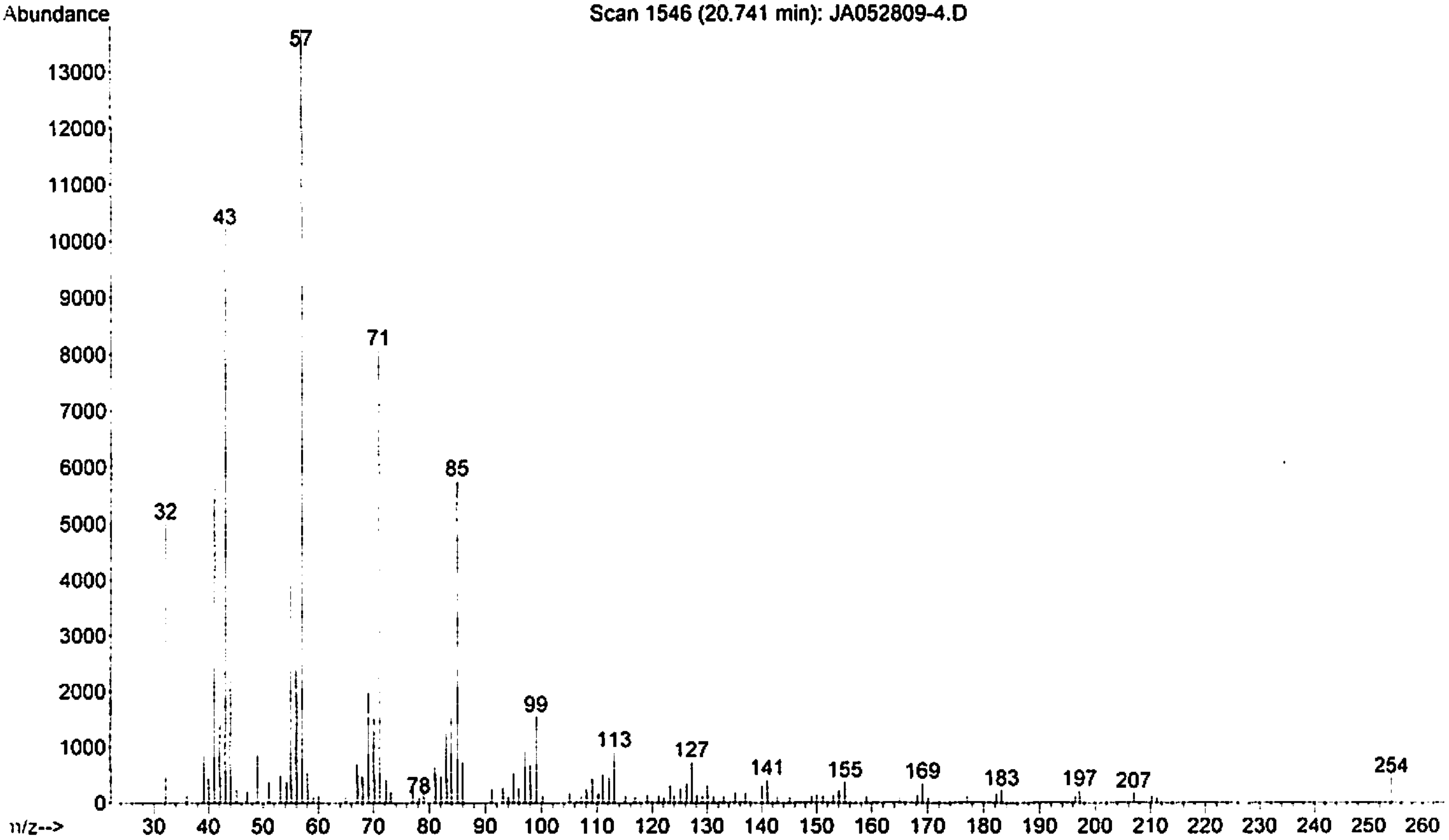

File : D:\DATA\Aldrich\JA-09\JA052909-1.D
Operator : Aldrich
Acquired : 29 May 2009 9:32 using AcqMethod JA-WAX08.M
Instrument : Instrument #1
Sample Name: 1 field-coll. M C.oculata abd./CH2Cl2
Misc Info : 3rd male from 5/28; "top" gut full w/ yellow
Vial Number: 1

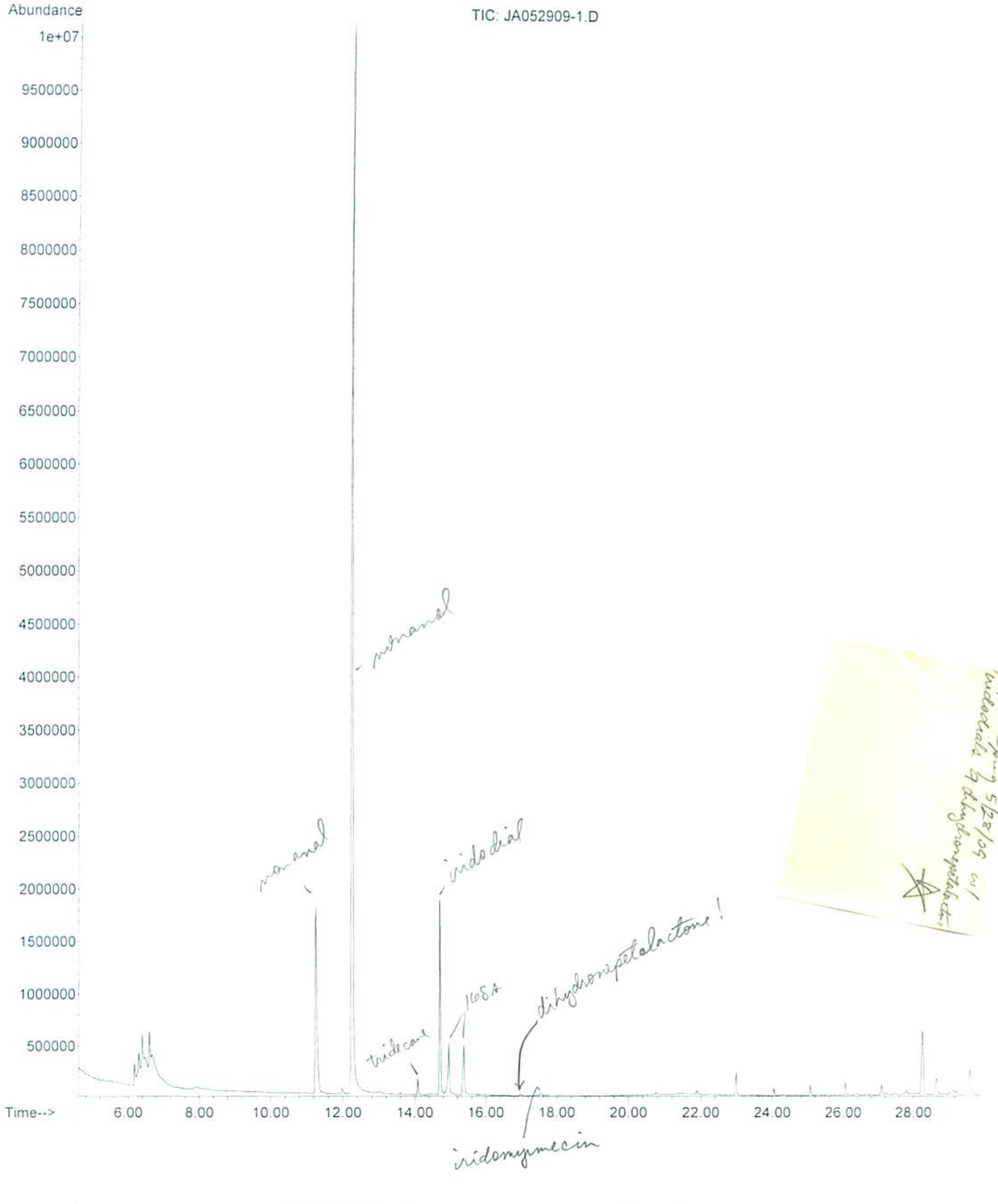

1 O. [illegible] 5/28/09 w/ iridodials & dihydronepetalact.

File :D:\DATA\ALDRICH\JA-09\Snapshot\JA052909.D
Operator : Aldrich
Acquired : 29 May 2009 9:32 using AcqMethod JA-WAX08.M
Instrument : Instrument #1
Sample Name: 1 field-coll. M C.oculata abd./CH2Cl2
Misc Info : 3rd male from 5/28; "top" gut full w/ yellow
Vial Number: 1

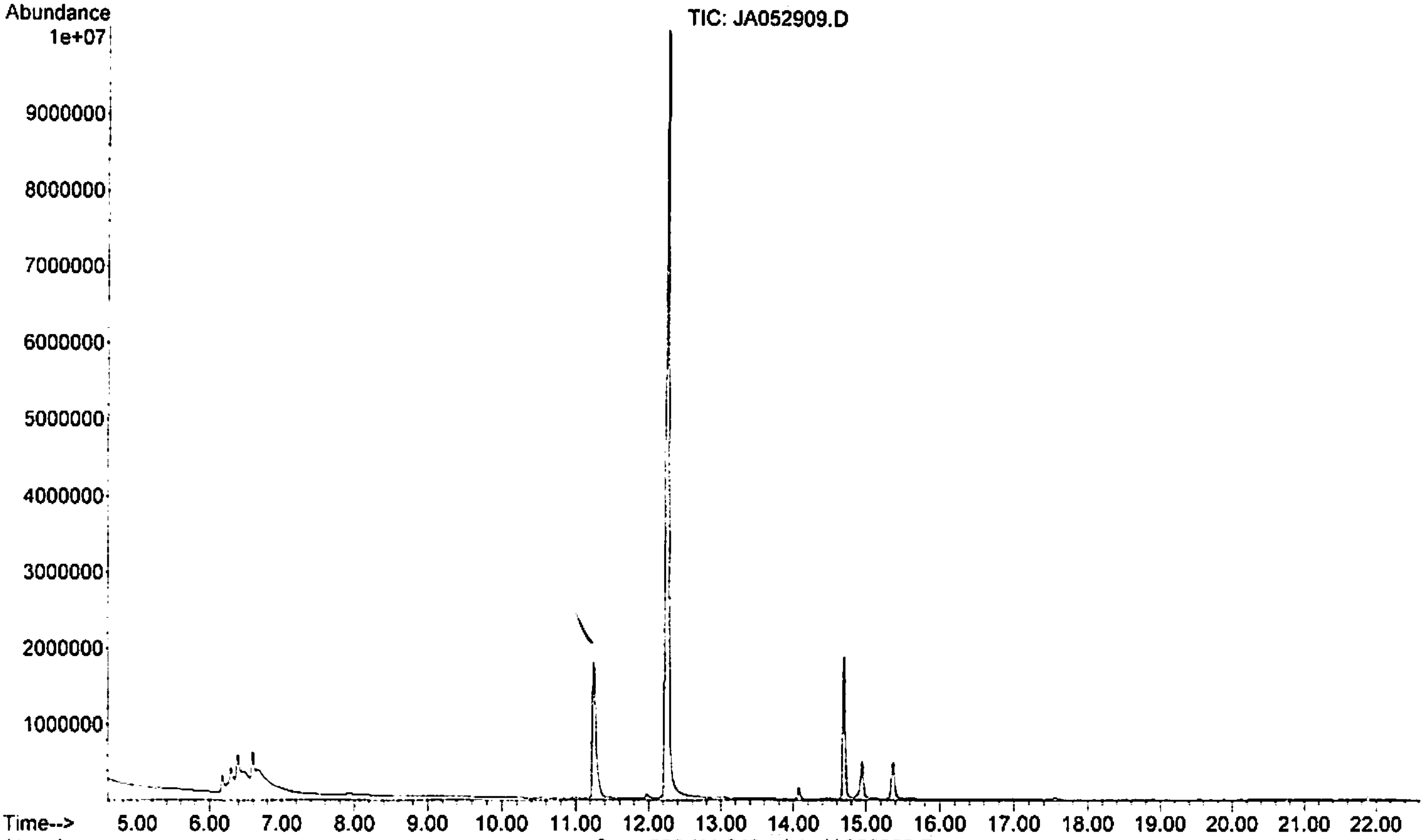

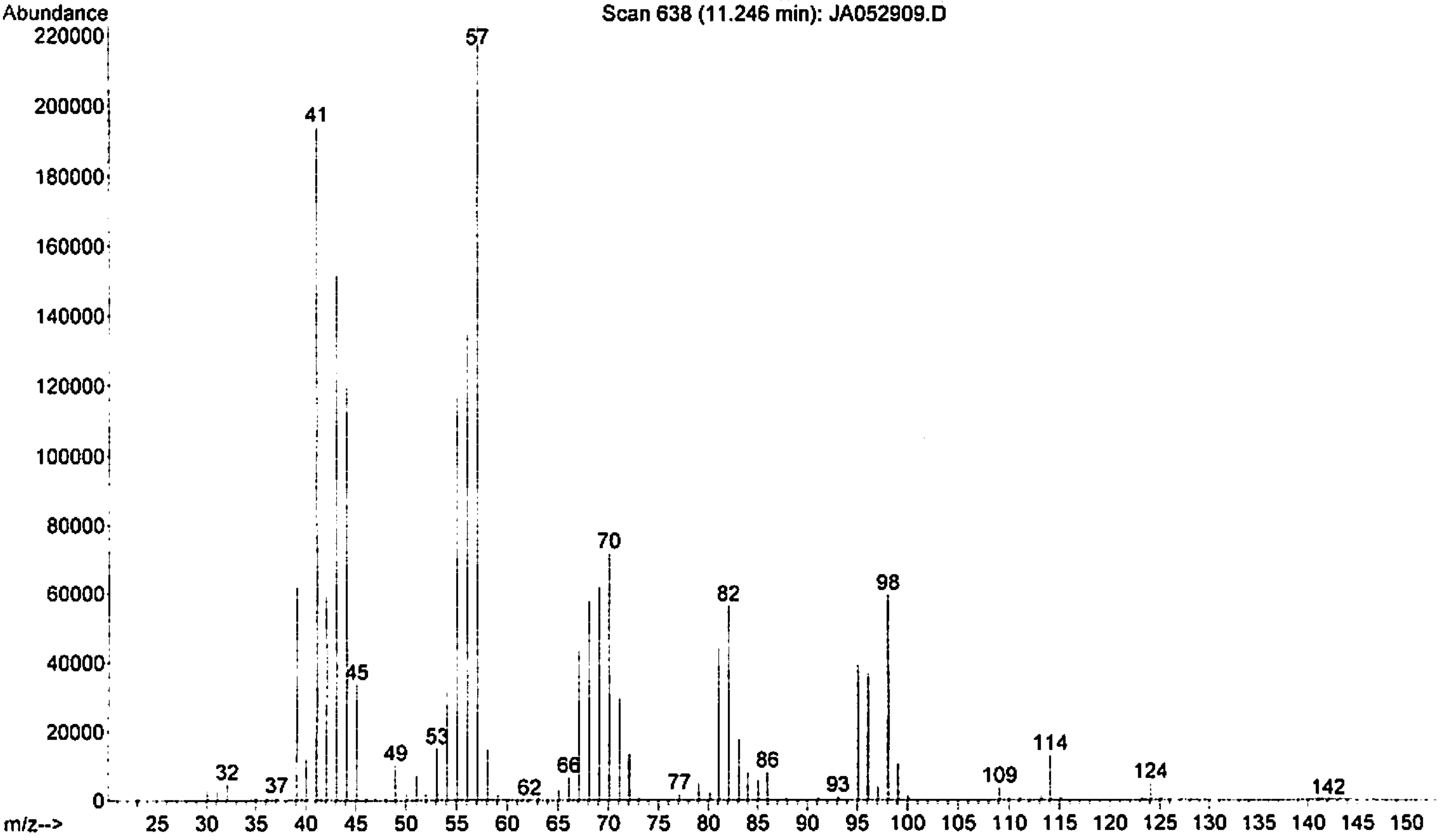

[illegible]e :D:\DATA\ALDRICH\JA-09\Snapshot\JA052909.D
[illegible]erator : Aldrich
[illegible]quired : 29 May 2009 9:32 using AcqMethod JA-WAX08.M
[illegible]strument : Instrument #1
[illegible]mple Name: 1 field-coll. M C.oculata abd./CH2Cl2
[illegible] Info : 3rd male from 5/28; "top" gut full w/ yellow
[illegible]al Number: 1

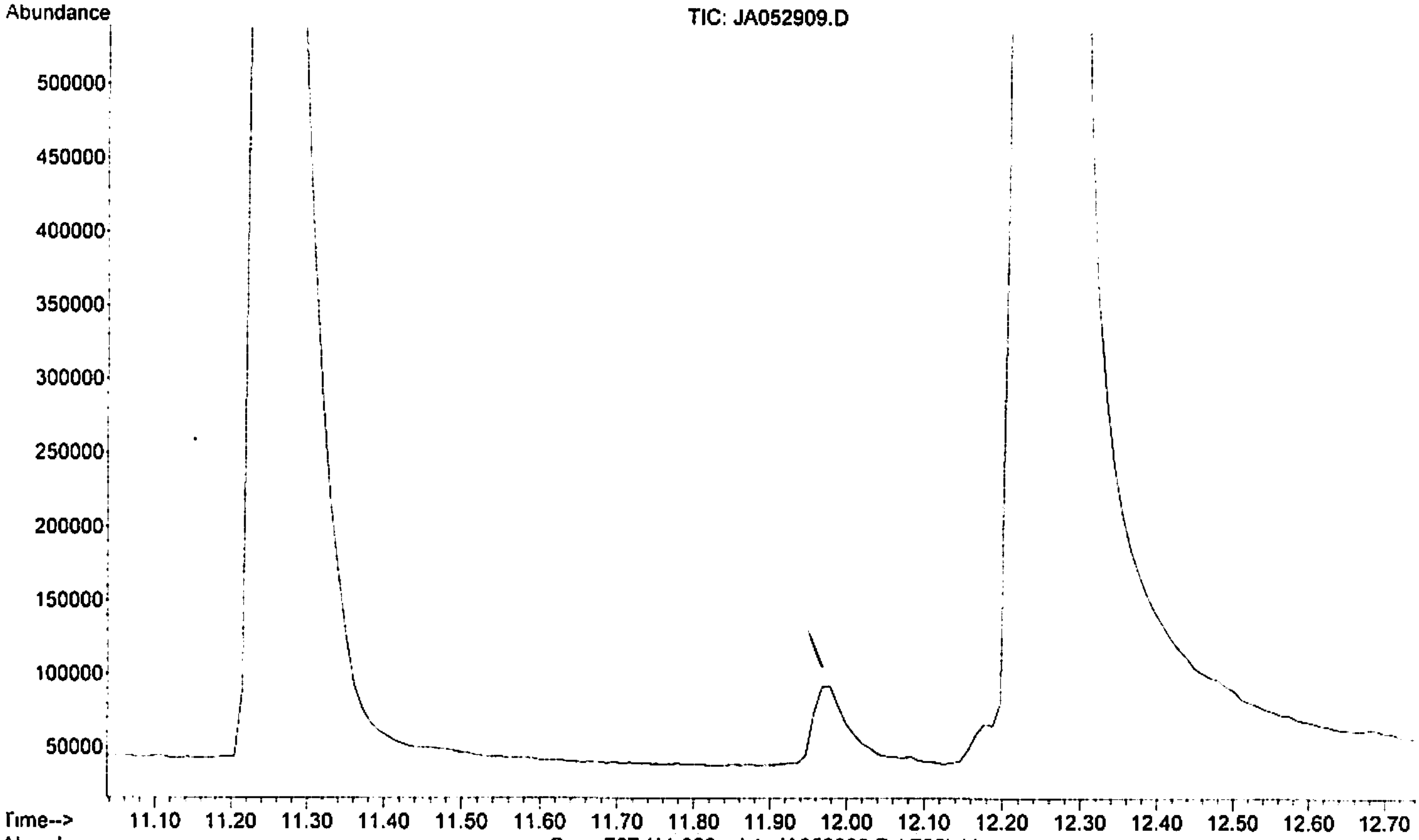

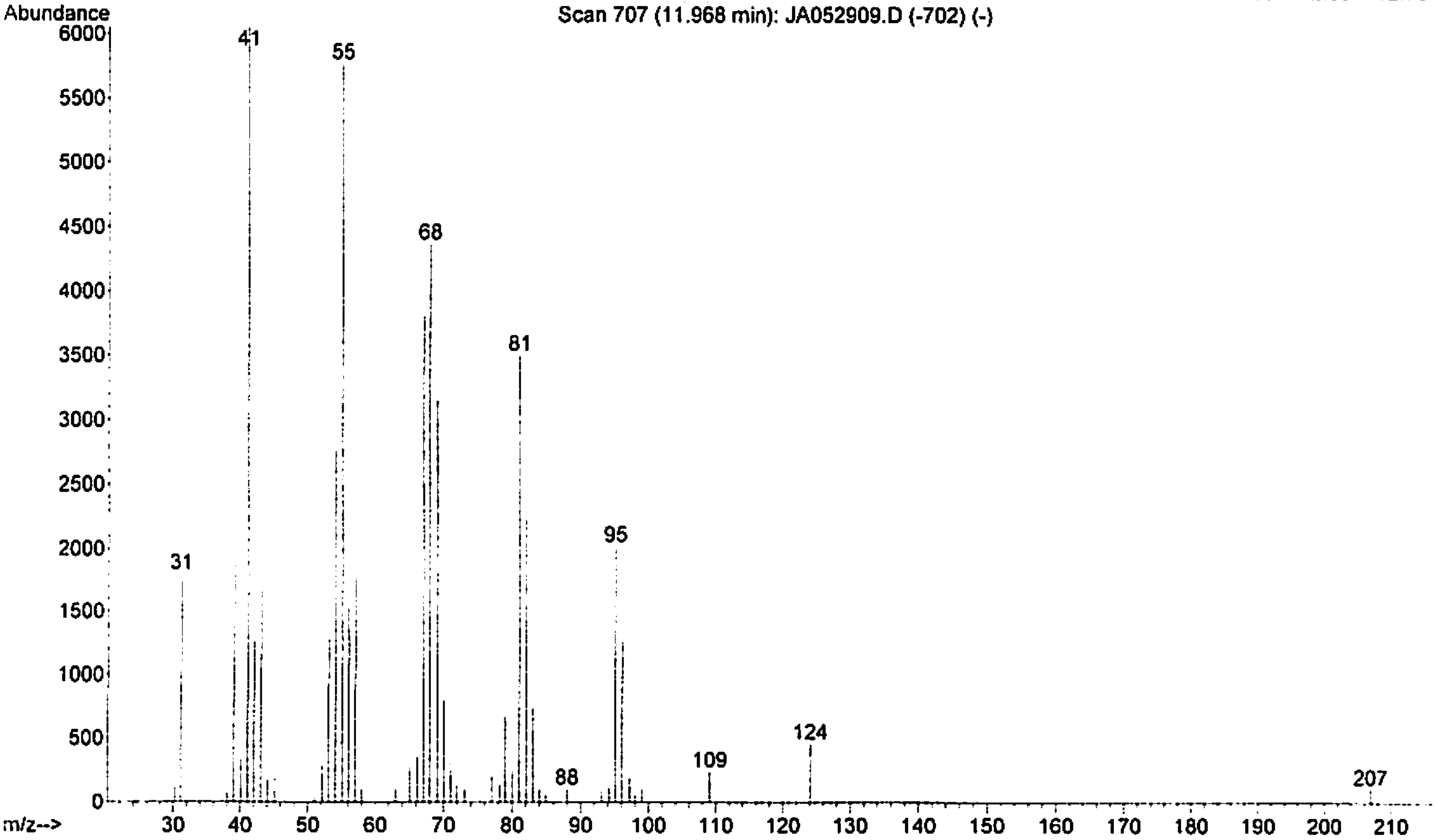

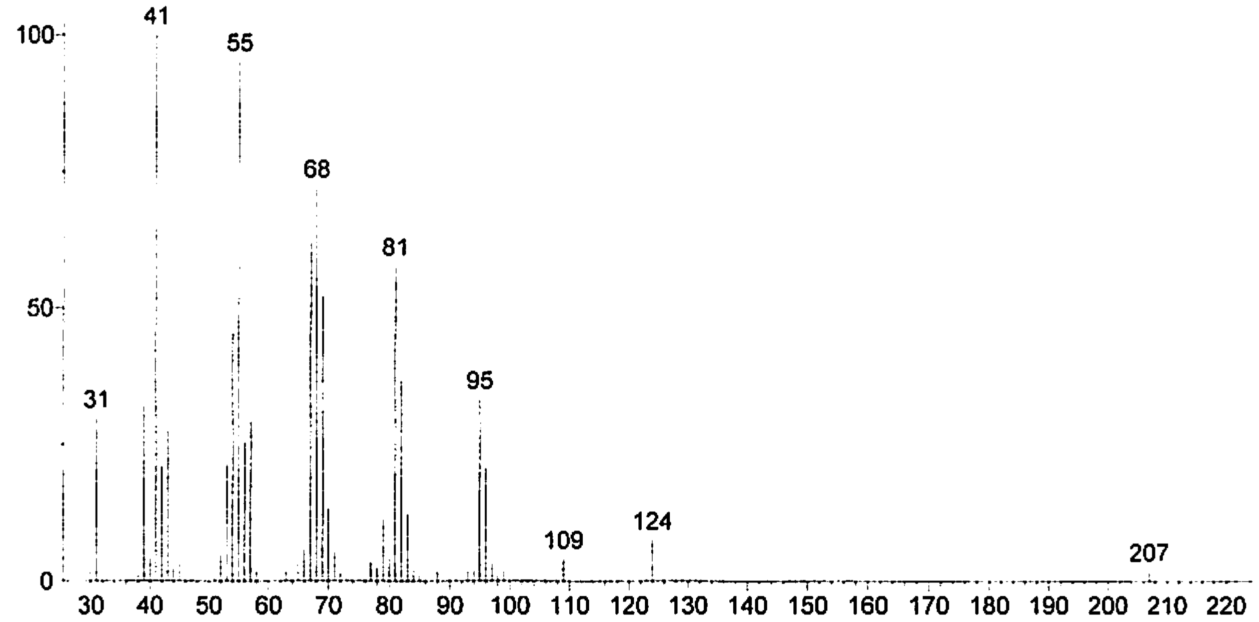

(Text File) Scan 707 (11.968 min): JA052909.D (-702)

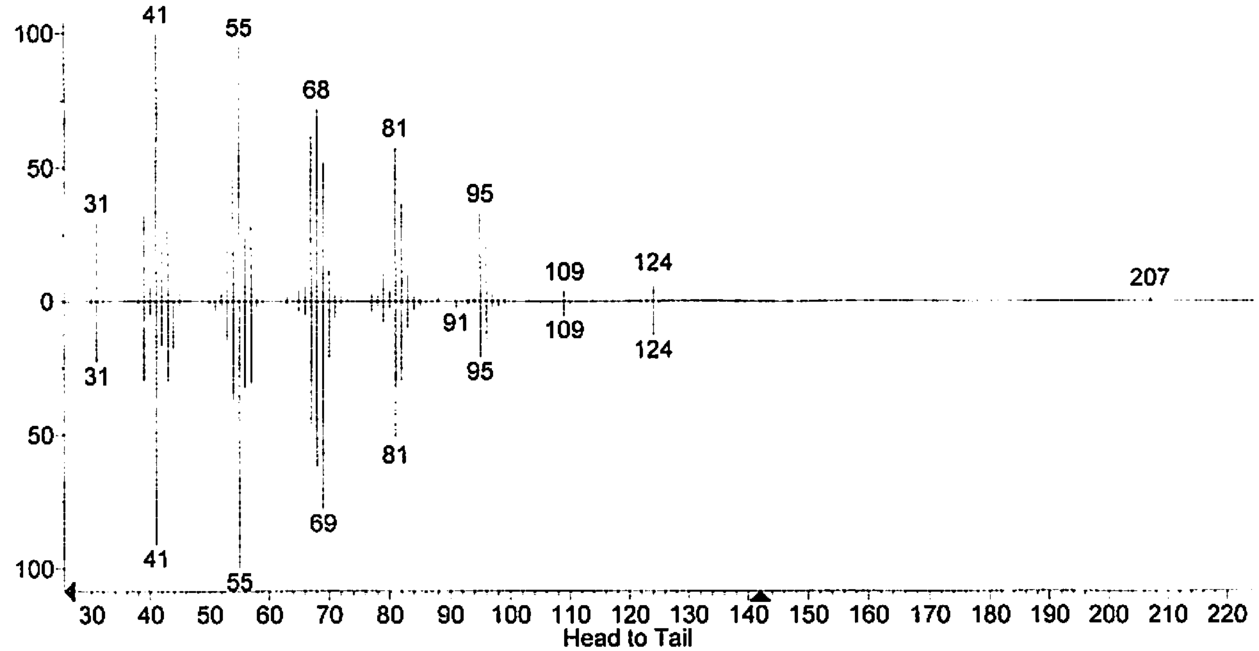

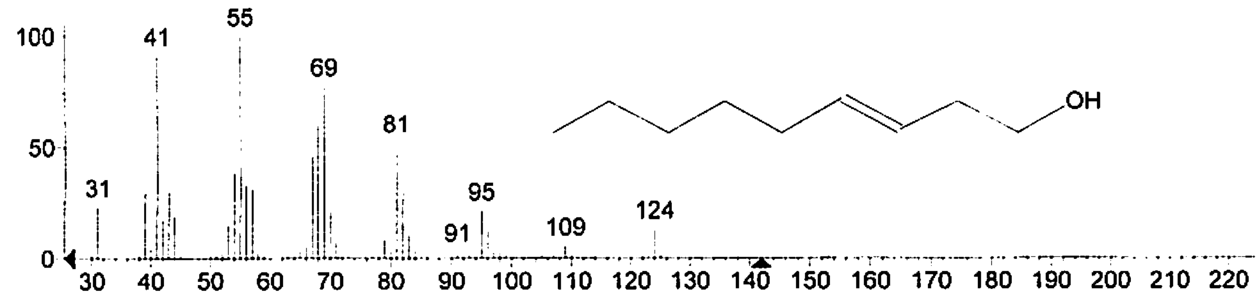

(mainlib) 3-Nonen-1-ol, (E)-

e :D:\DATA\ALDRICH\JA-09\Snapshot\JA052909.D
rator : Aldrich
quired : 29 May 2009 9:32 using AcqMethod JA-WAX08.M
rument : Instrument #1
ple Name: 1 field-coll. M C.oculata abd./CH2C12
Info : 3rd male from 5/28; "top" gut full w/ yellow
Number: 1

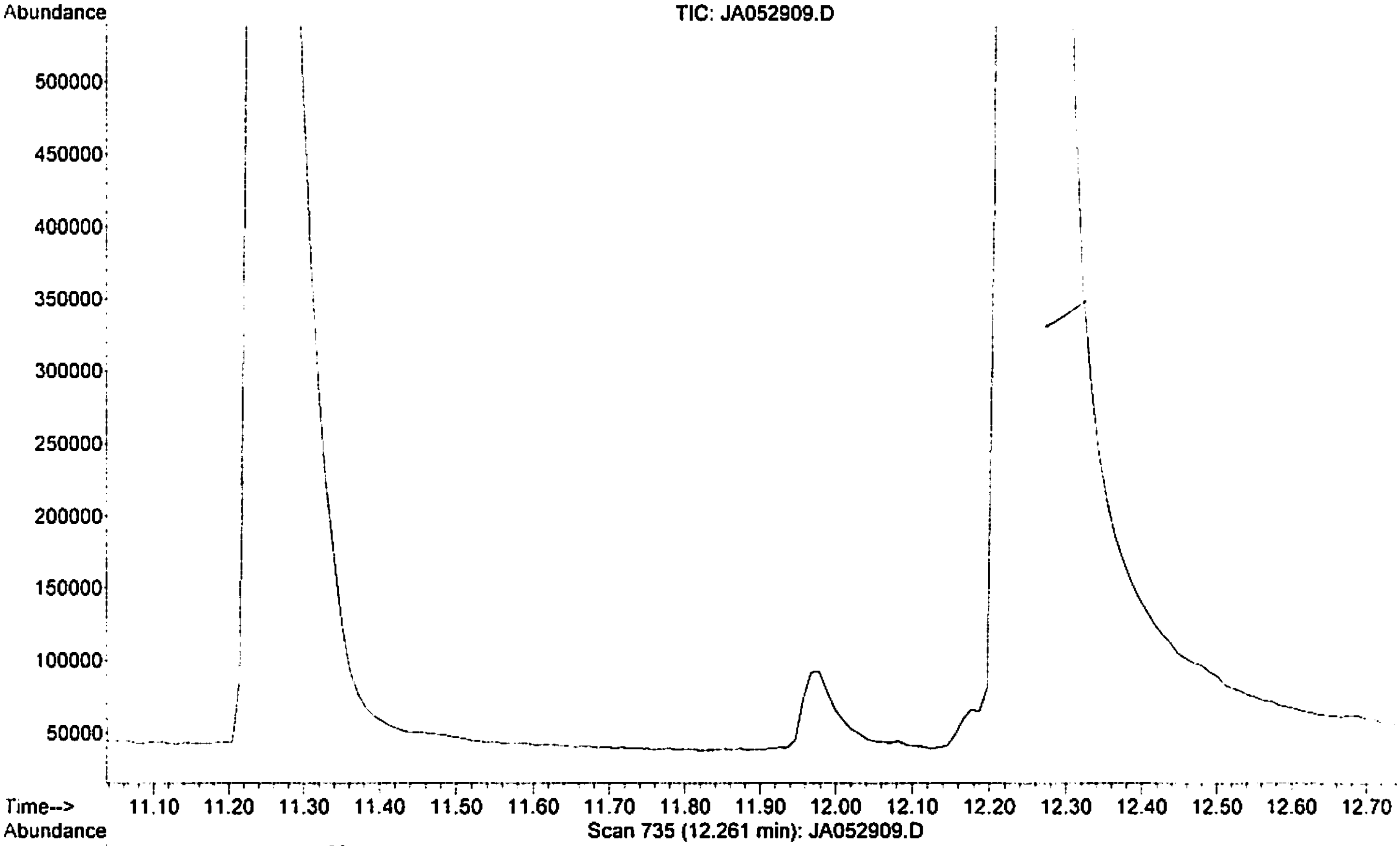

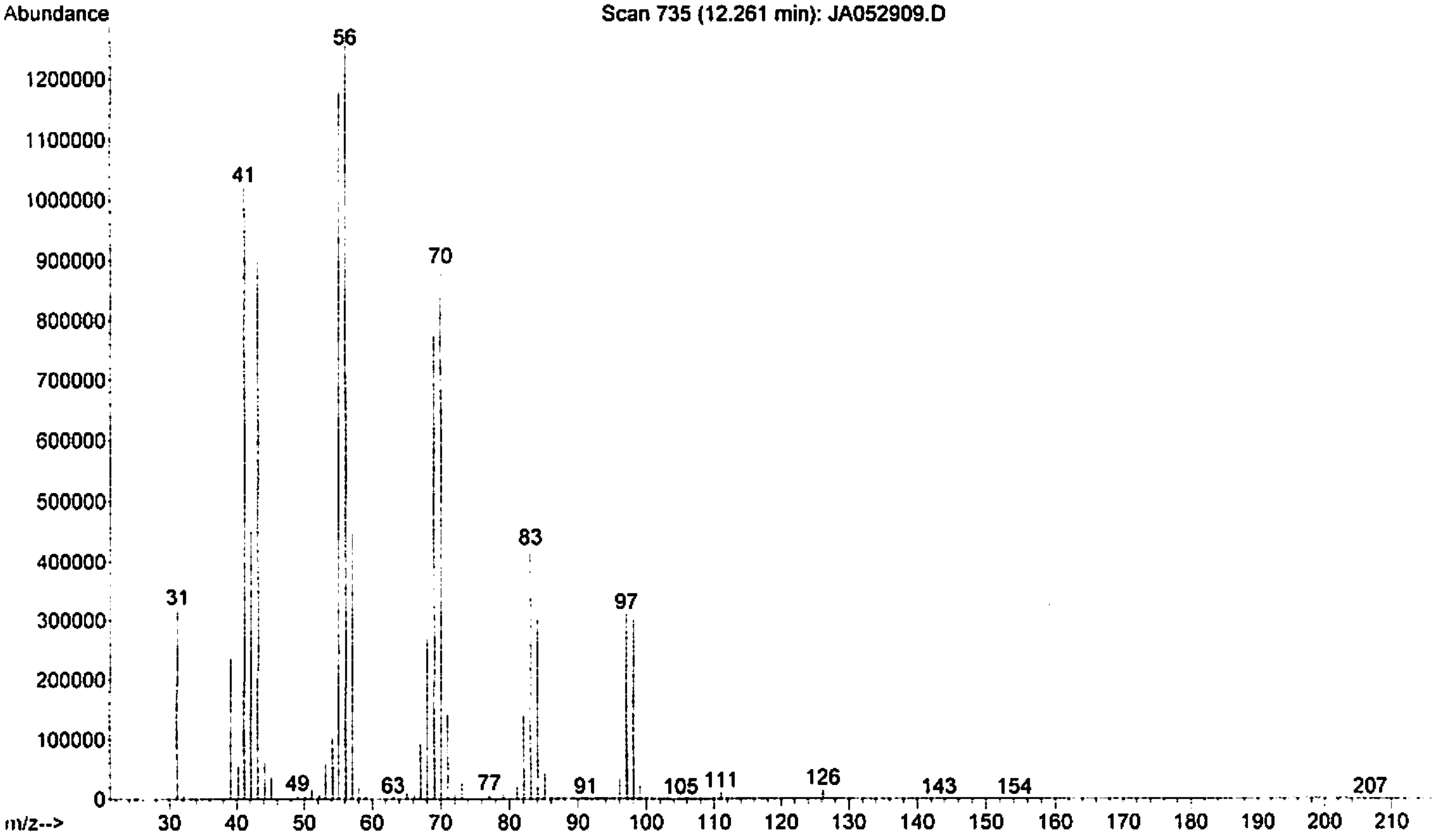

:D:\DATA\ALDRICH\JA-09\Snapshot\JA052909.D
[illegible]rator : Aldrich
[illegible]ired : 29 May 2009 9:32 using AcqMethod JA-WAX08.M
[illegible]rument : Instrument #1
[illegible]ple Name: 1 field-coll. M C.oculata abd./CH2Cl2
[illegible] Info : 3rd male from 5/28; "top" gut full w/ yellow
[illegible] Number: 1

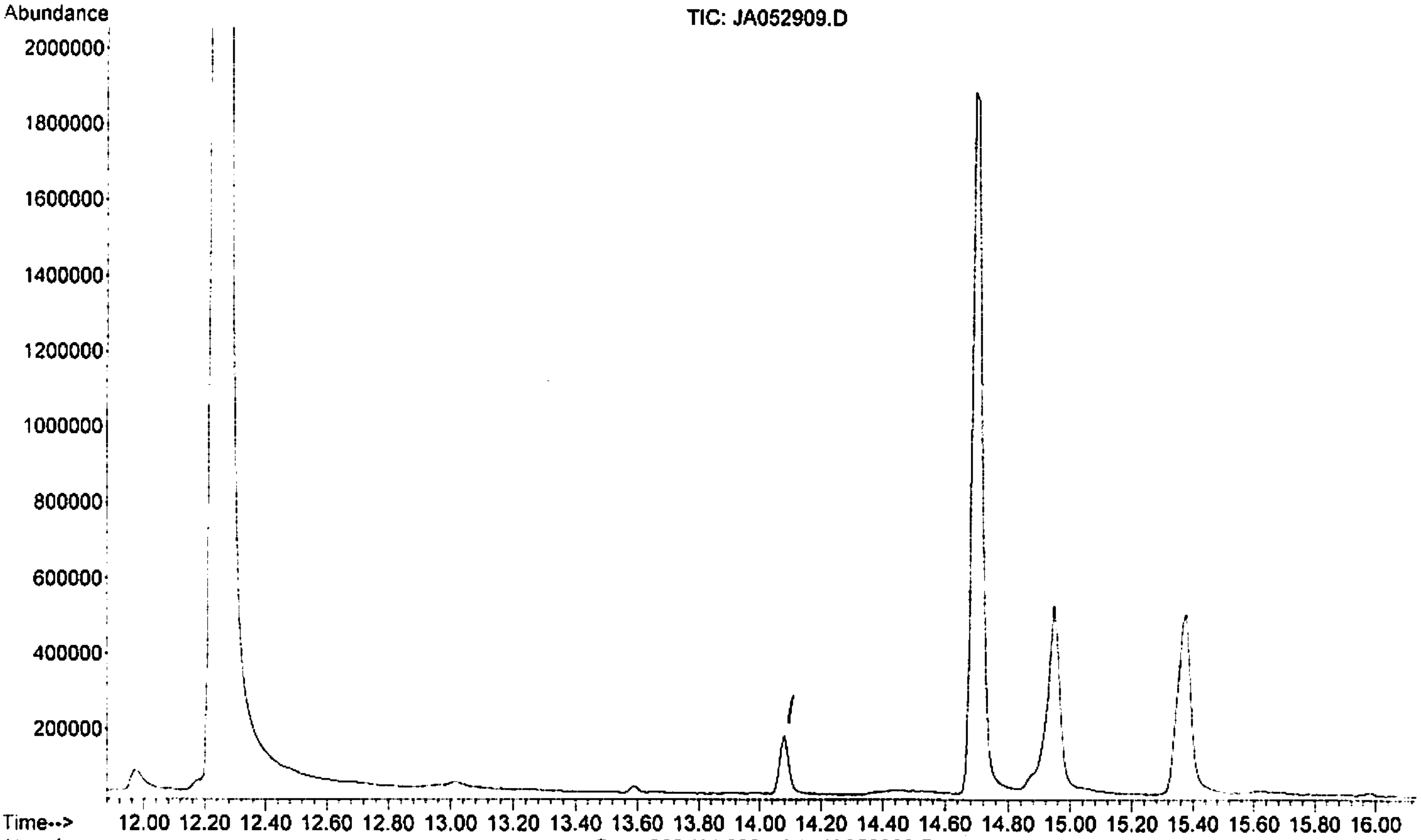

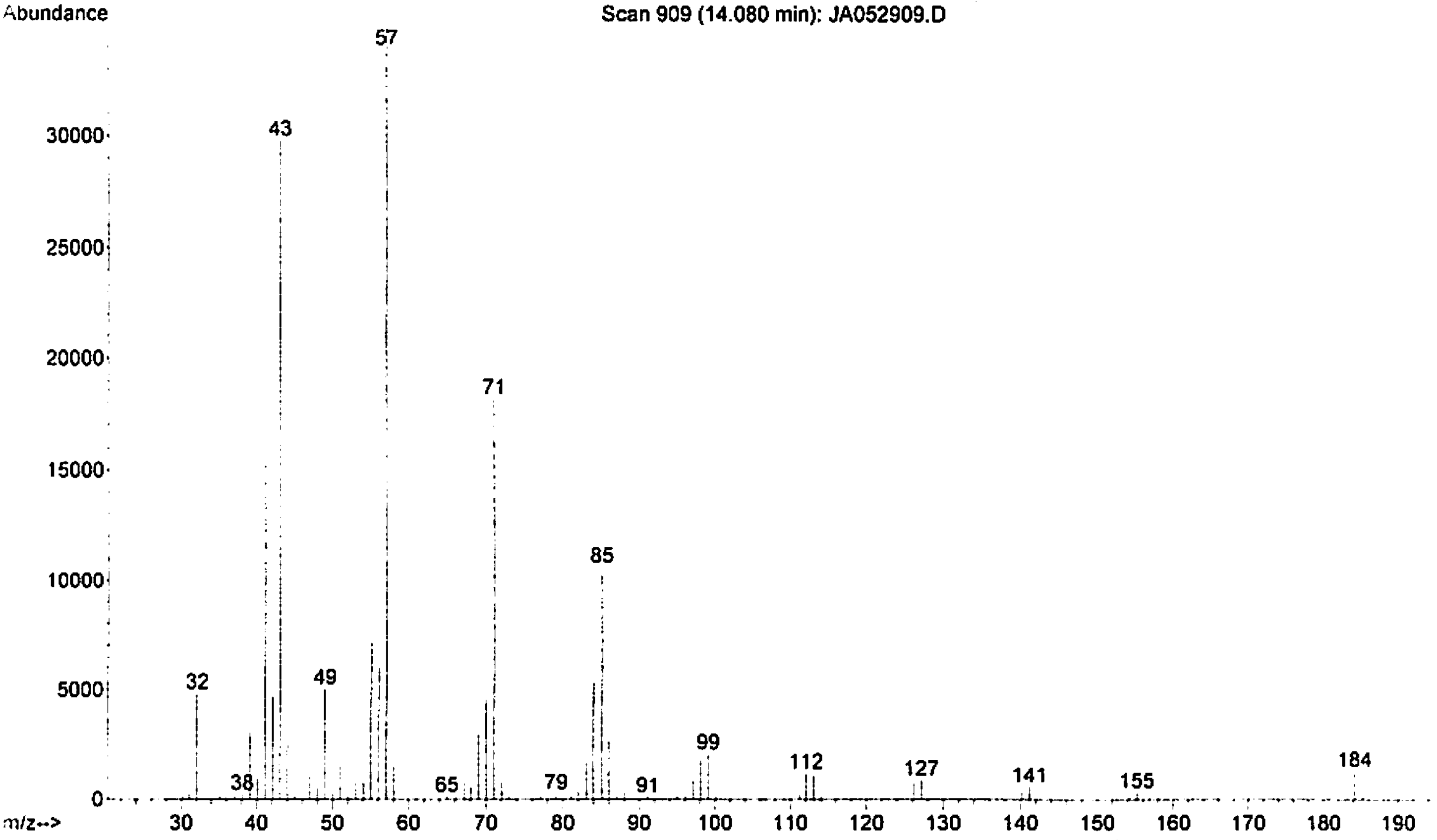

:D:\DATA\ALDRICH\JA-09\Snapshot\JA052909.D
ator : Aldrich
ired : 29 May 2009 9:32 using AcqMethod JA-WAX08.M
rument : Instrument #1
ple Name: 1 field-coll. M C.oculata abd./CH2C12
Info : 3rd male from 5/28; "top" gut full w/ yellow
Number: 1

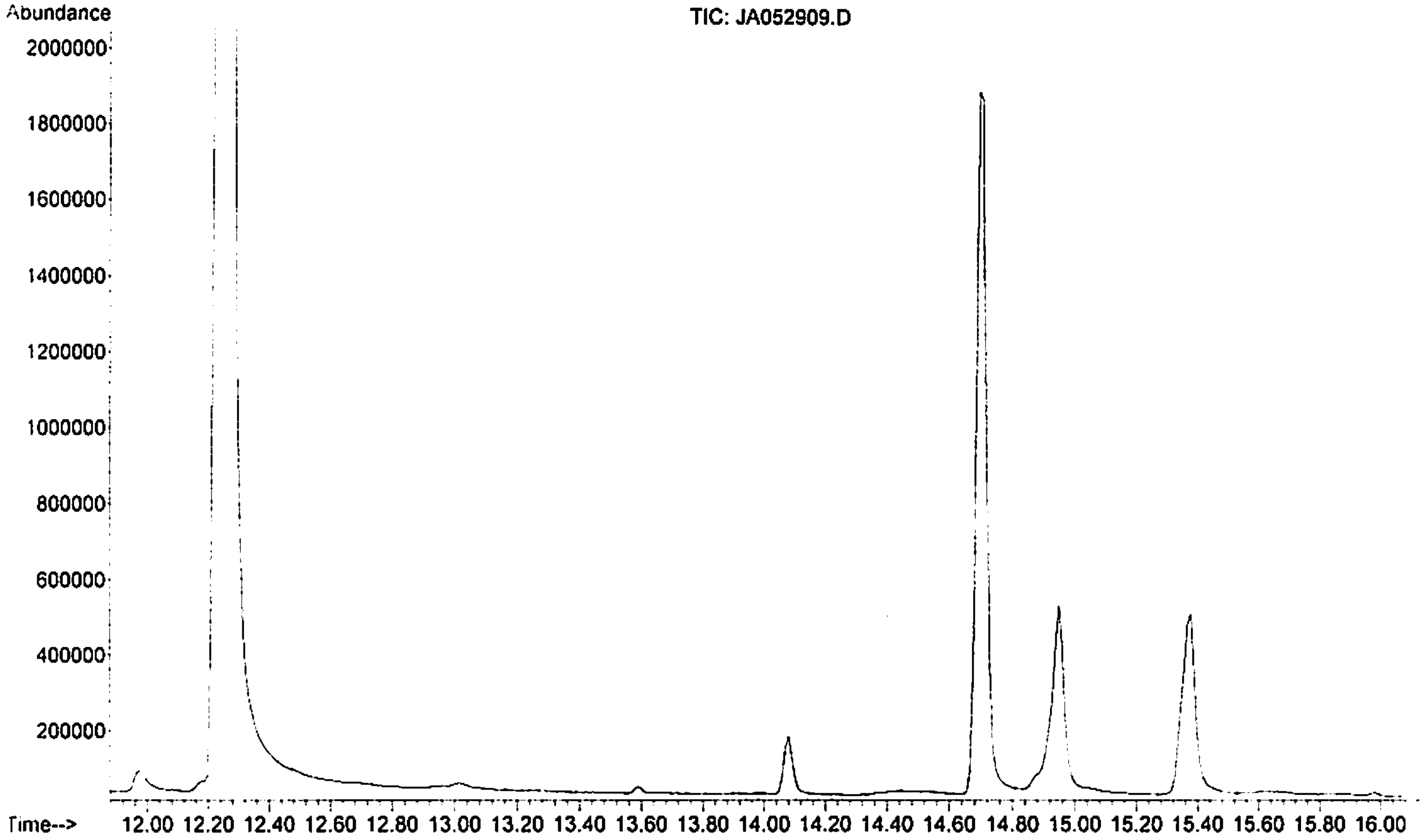

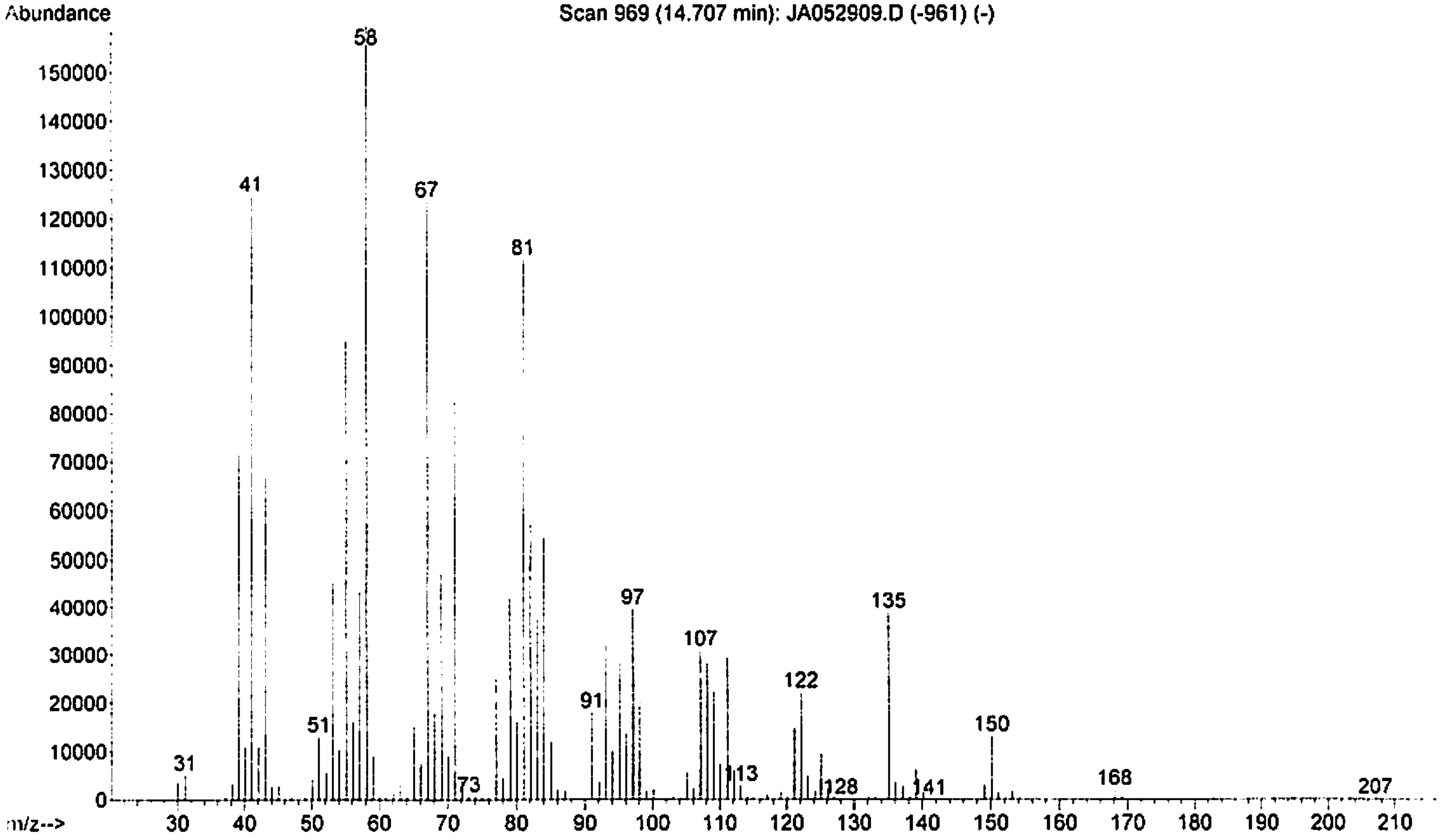

[illegible] :D:\DATA\ALDRICH\JA-09\Snapshot\JA052909.D
[illegible]rator : Aldrich
[illegible]uired : 29 May 2009 9:32 using AcqMethod JA-WAX08.M
[illegible]rument : Instrument #1
[illegible]ple Name: 1 field-coll. M C.oculata abd./CH2Cl2
[illegible] Info : 3rd male from 5/28; "top" gut full w/ yellow
[illegible] Number: 1

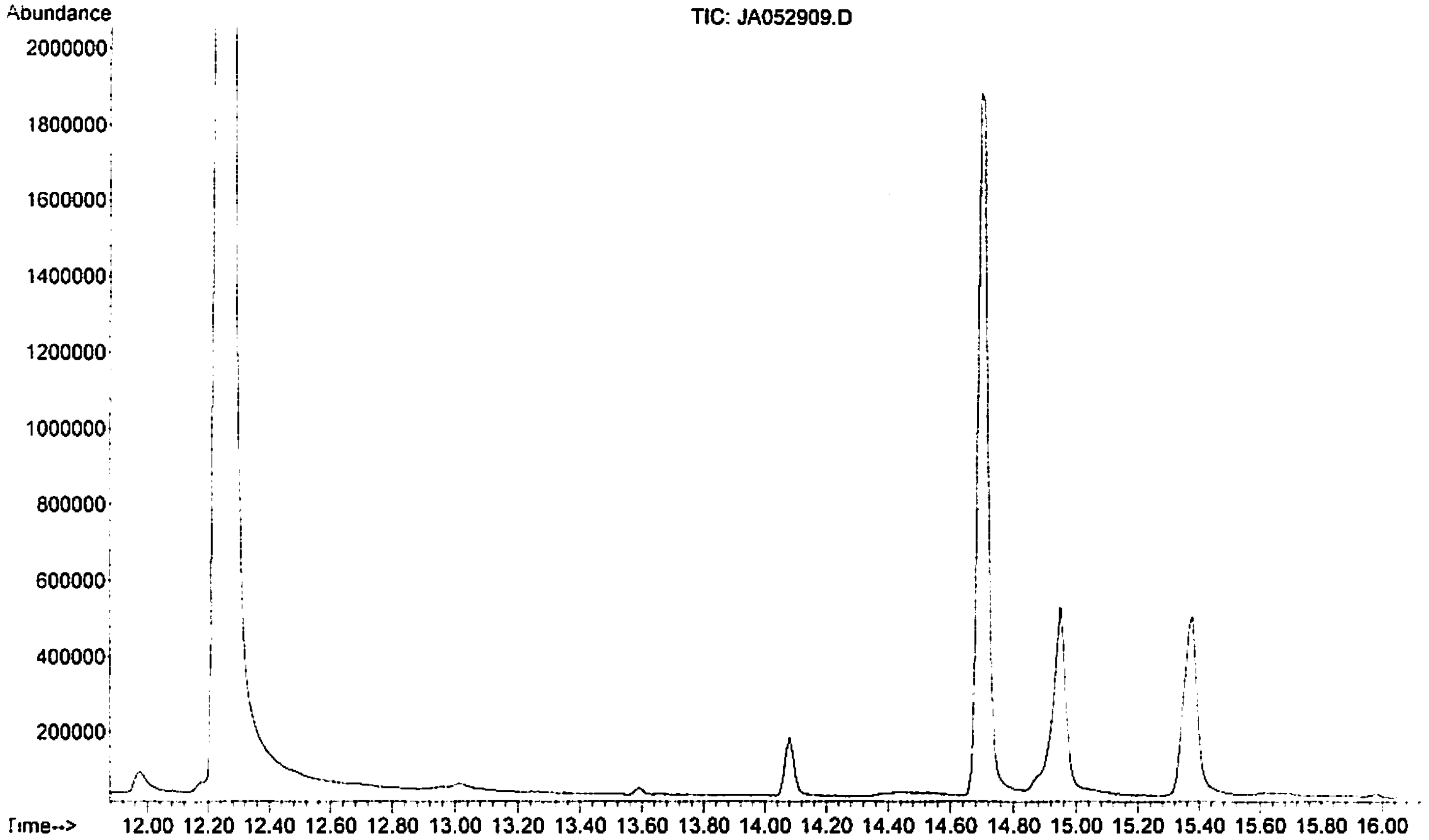

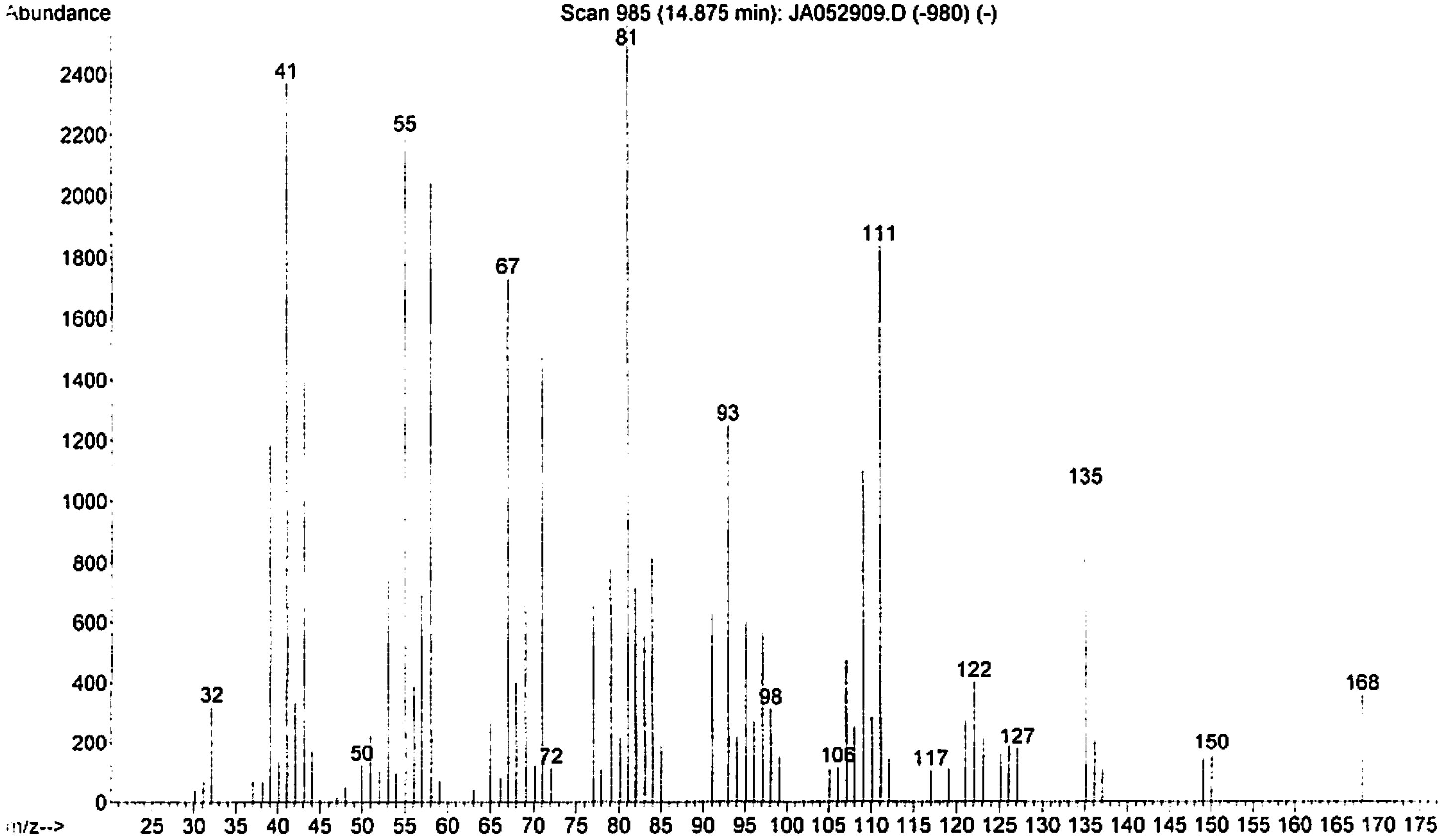

[illegible] :D:\DATA\ALDRICH\JA-09\Snapshot\JA052909.D
[illegible]ator : Aldrich
[illegible]ired : 29 May 2009 9:32 using AcqMethod JA-WAX08.M
[illegible]rument : Instrument #1
[illegible]ple Name: 1 field-coll. M C.oculata abd./CH2Cl2
[illegible] Info : 3rd male from 5/28; "top" gut full w/ yellow
[illegible] Number: 1

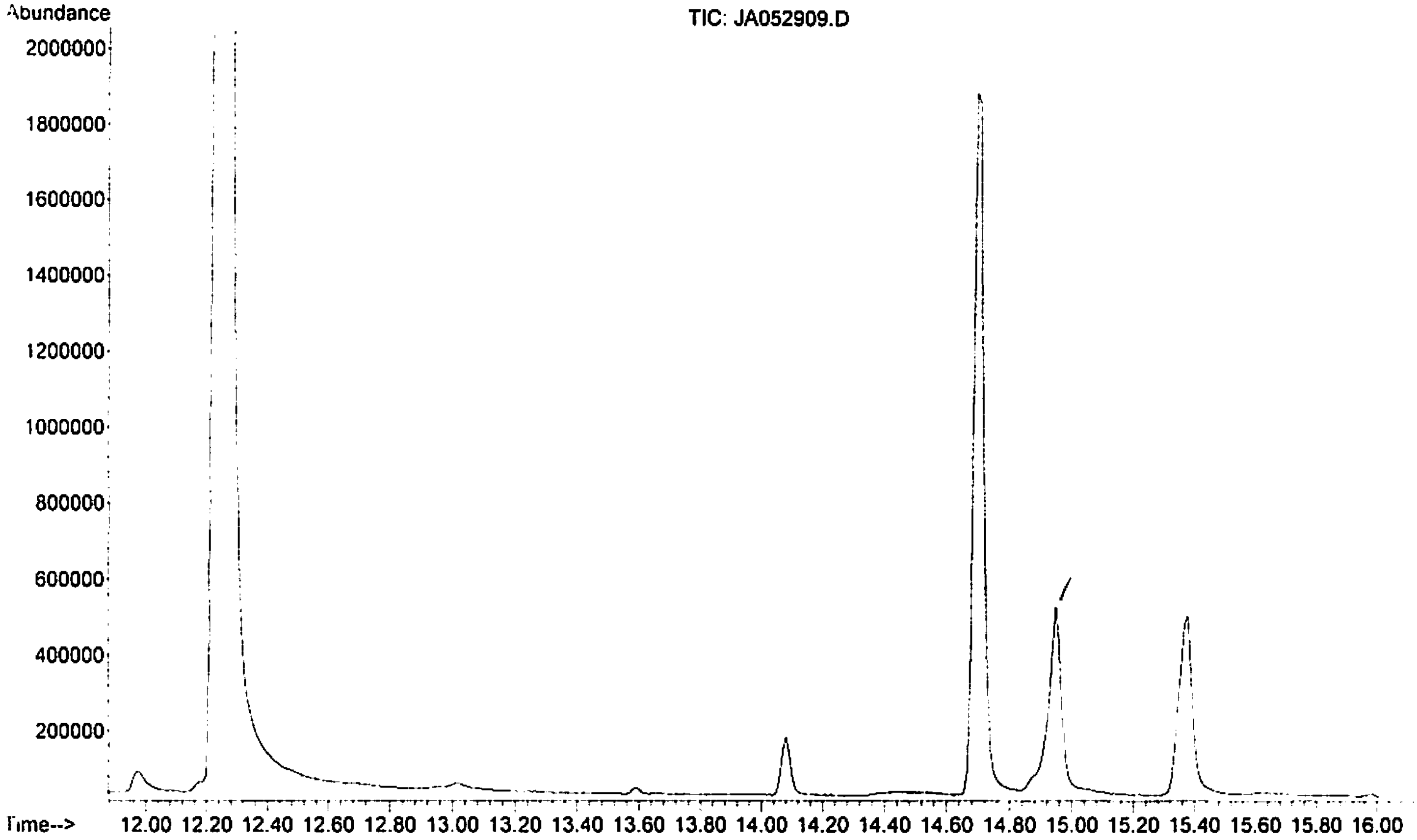

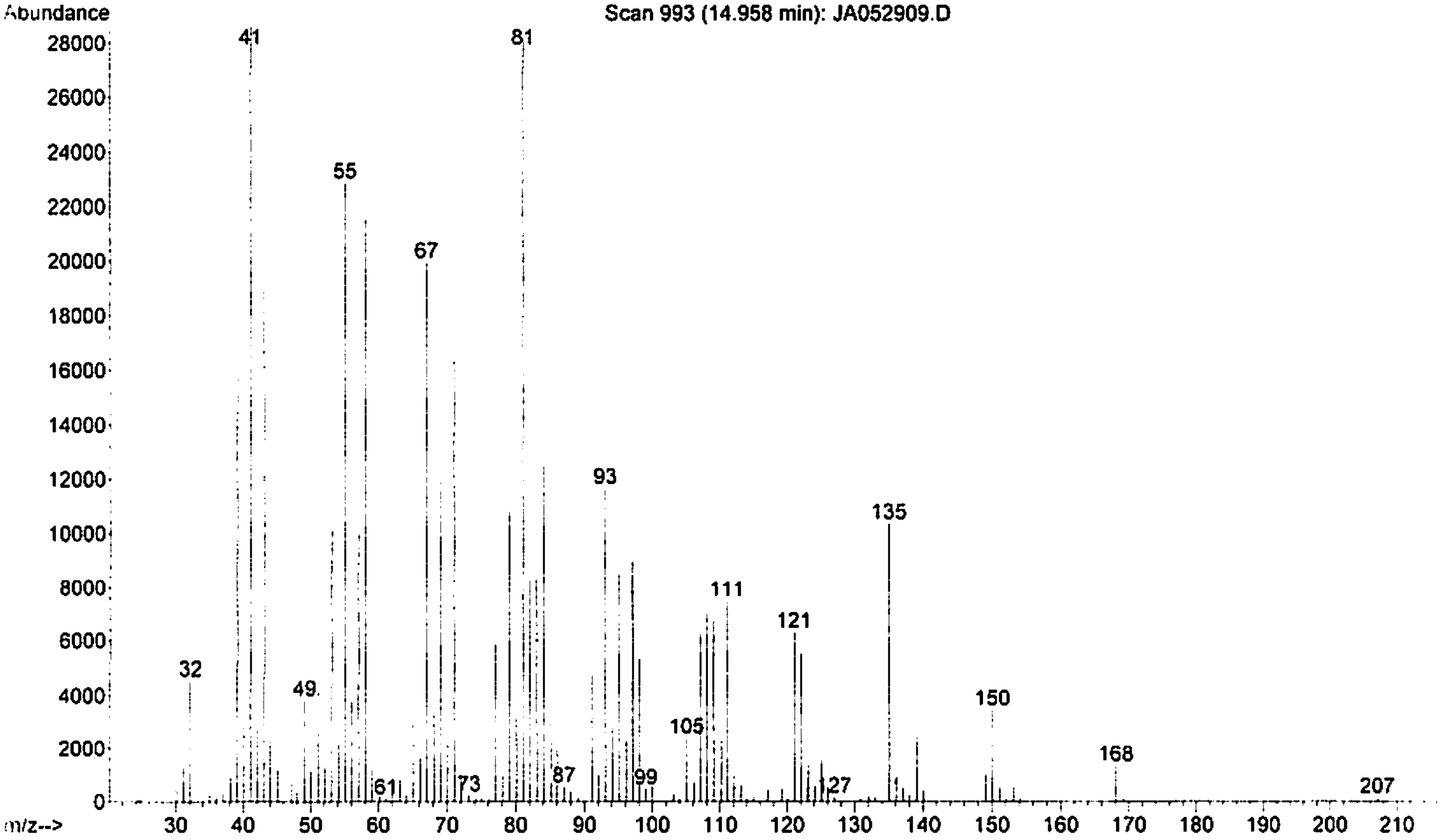

:D:\DATA\ALDRICH\JA-09\Snapshot\JA052909.D
ator : Aldrich
ired : 29 May 2009 9:32 using AcqMethod JA-WAX08.M
rument : Instrument #1
ple Name: 1 field-coll. M C.oculata abd./CH2Cl2
Info : 3rd male from 5/28; "top" gut full w/ yellow
Number: 1

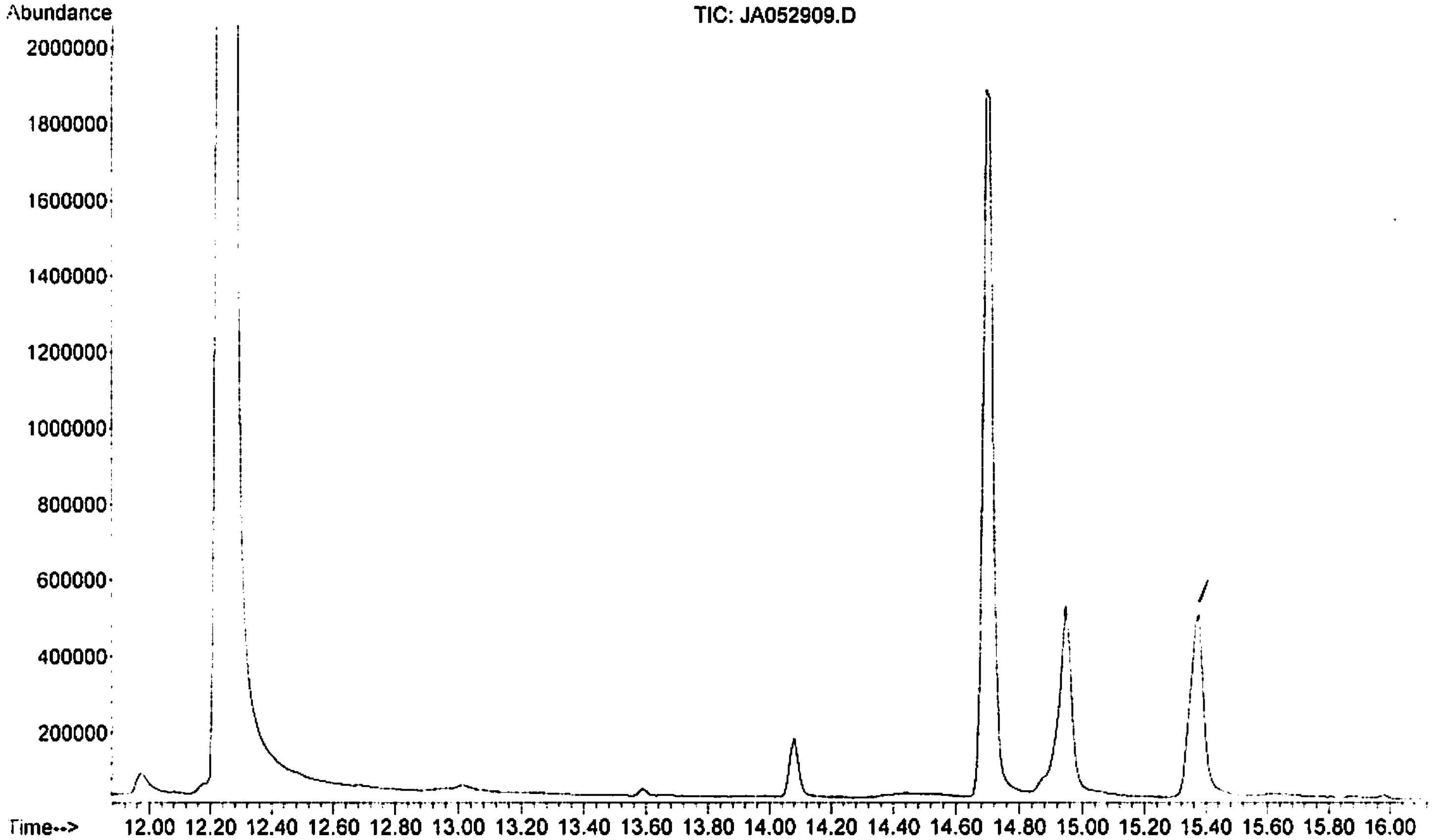

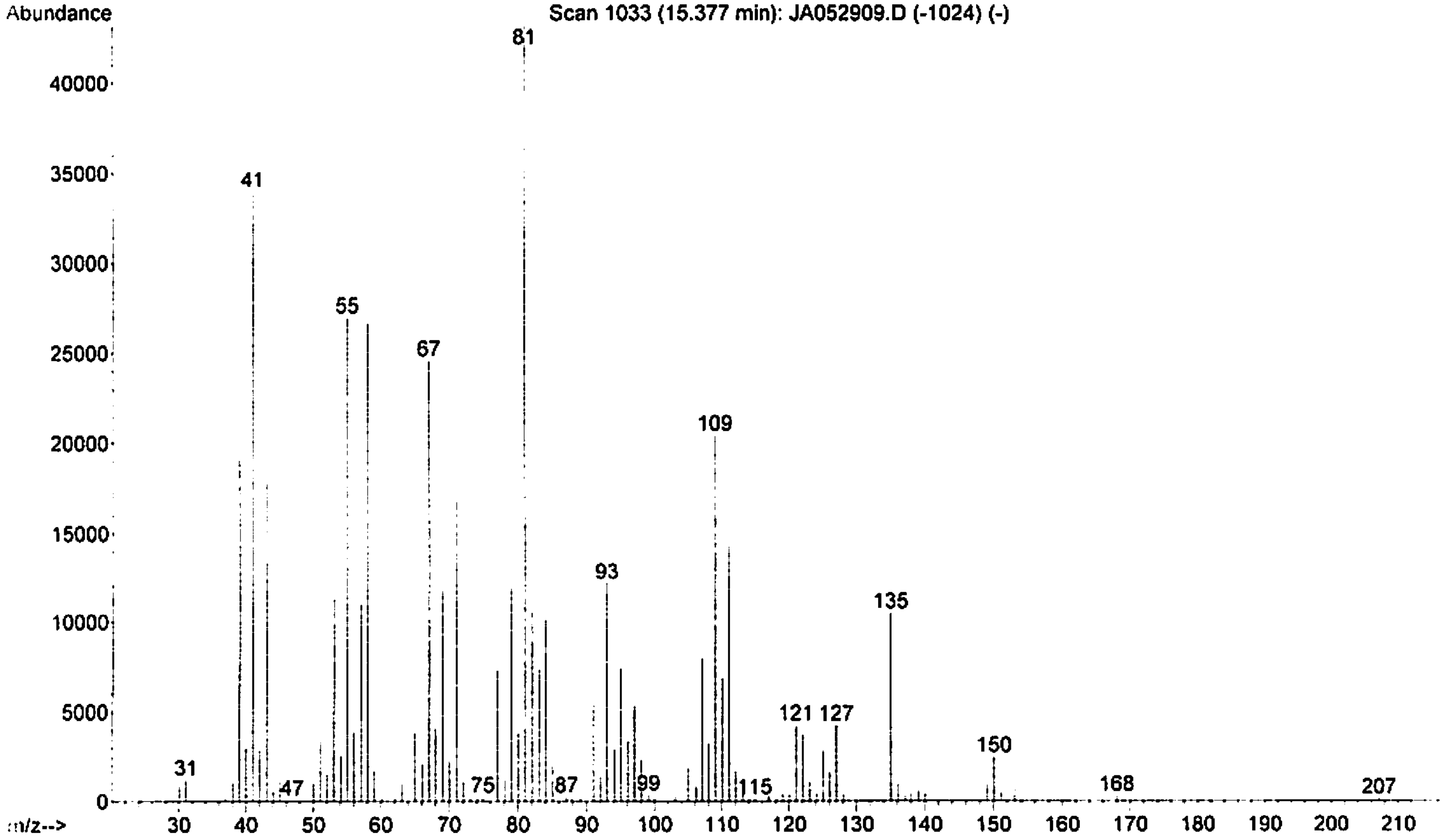

: D:\DATA\ALDRICH\JA-09\Snapshot\JA052909.D
rator : Aldrich
ired : 29 May 2009 9:32 using AcqMethod JA-WAX08.M
rument : Instrument #1
ple Name: 1 field-coll. M C.oculata abd./CH2Cl2
Info : 3rd male from 5/28; "top" gut full w/ yellow
Number: 1

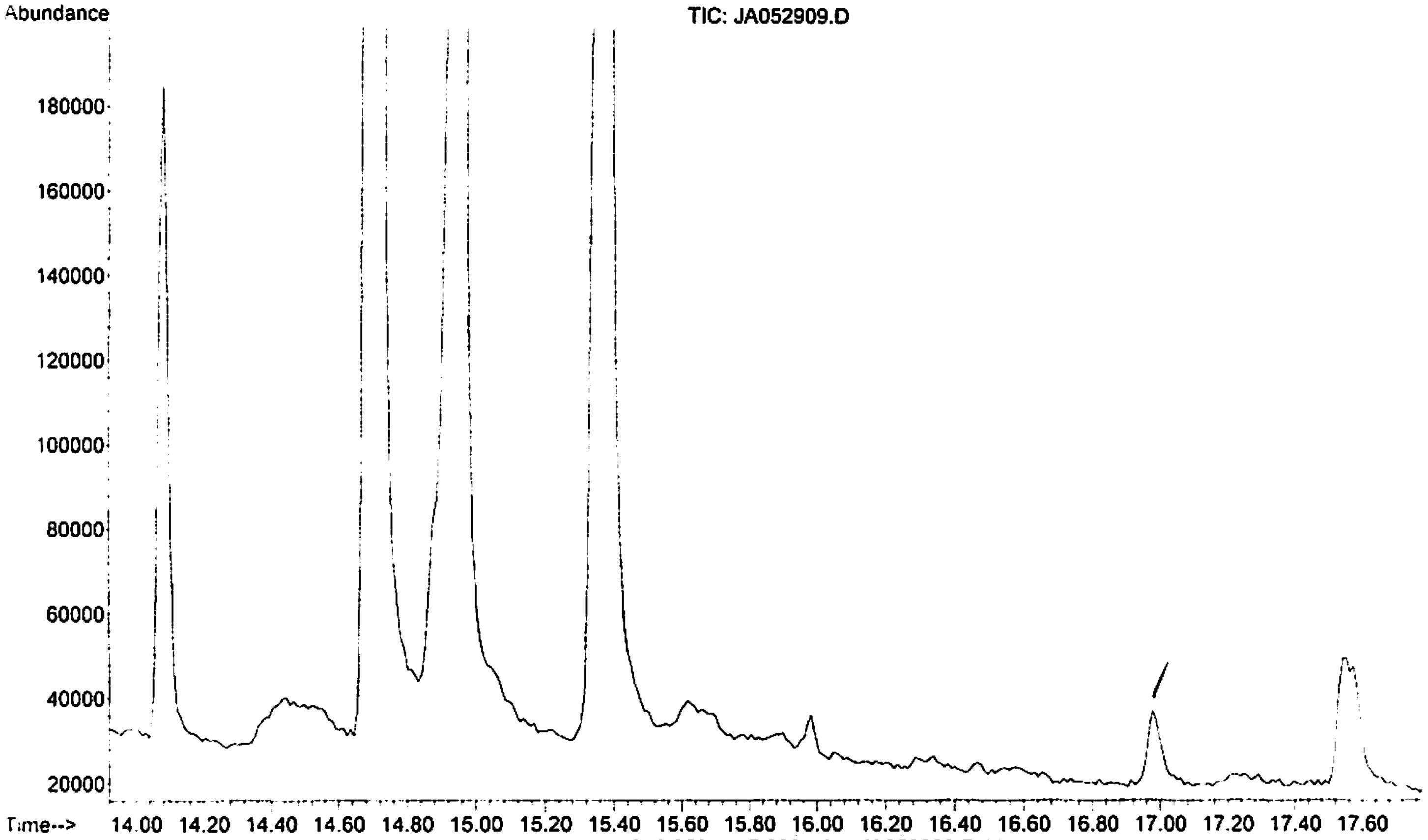

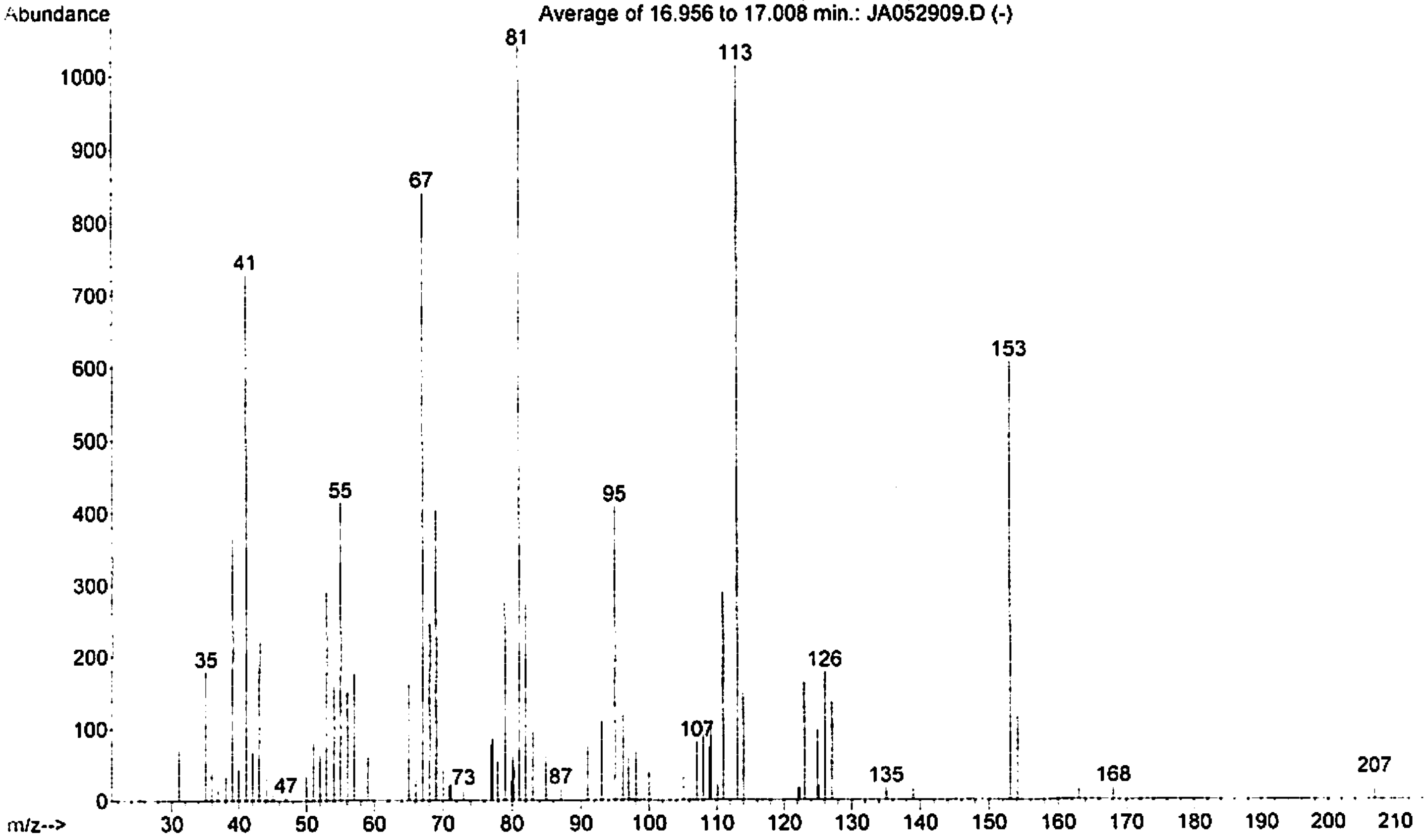

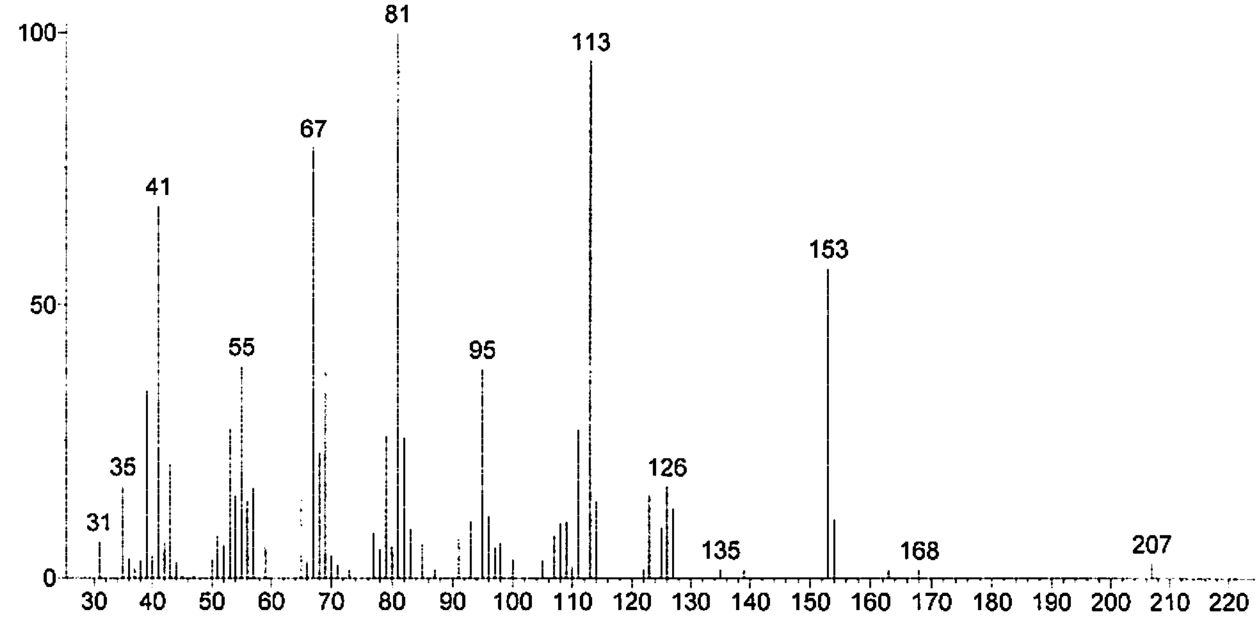

(Text File) Average of 16.956 to 17.008 min.: JA052909.D

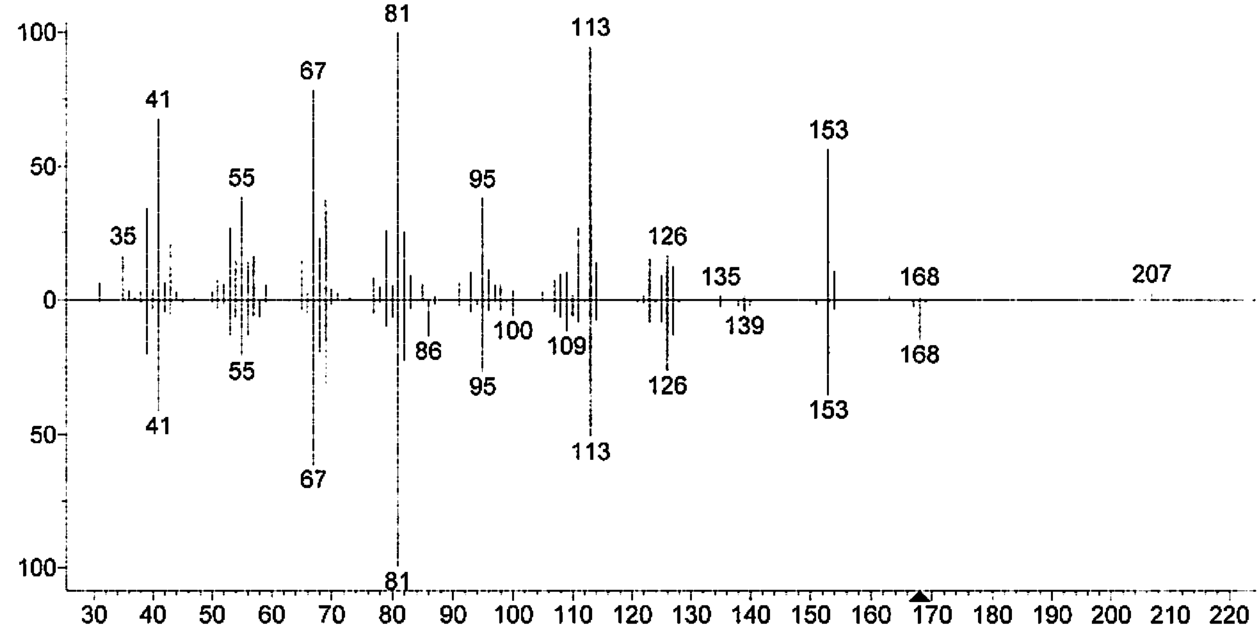

Head to Tail

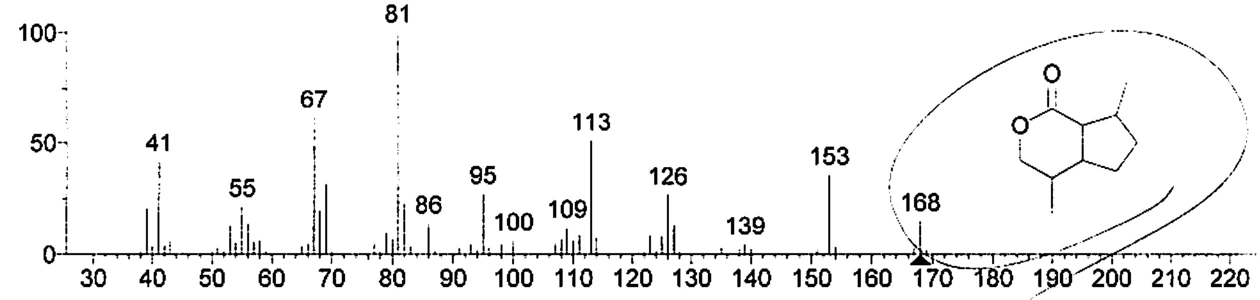

(mainlib) Cyclopenta[c]pyran-1(3H)-one, hexahydro-4,7-dimethyl-, (4.alpha.,4a.alpha.,7.alpha.,7a.alpha.)-

[illegible]e :D:\DATA\ALDRICH\JA-09\Snapshot\JA052909.D
[illegible]rator : Aldrich
[illegible]quired : 29 May 2009 9:32 using AcqMethod JA-WAX08.M
[illegible]rument : Instrument #1
[illegible]ple Name: 1 field-coll. M C.oculata abd./CH2Cl2
[illegible] Info : 3rd male from 5/28; "top" gut full w/ yellow
[illegible] Number: 1

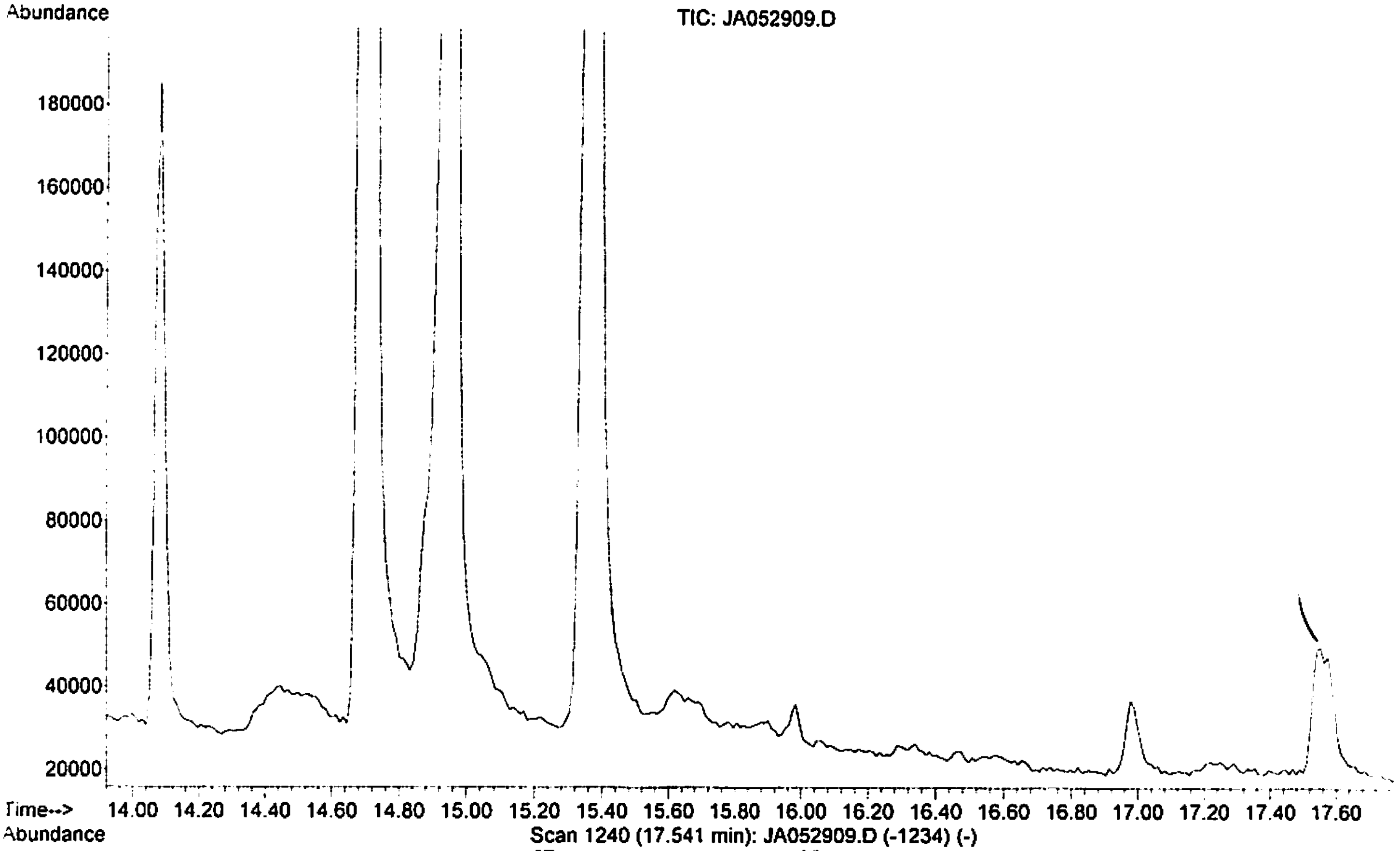

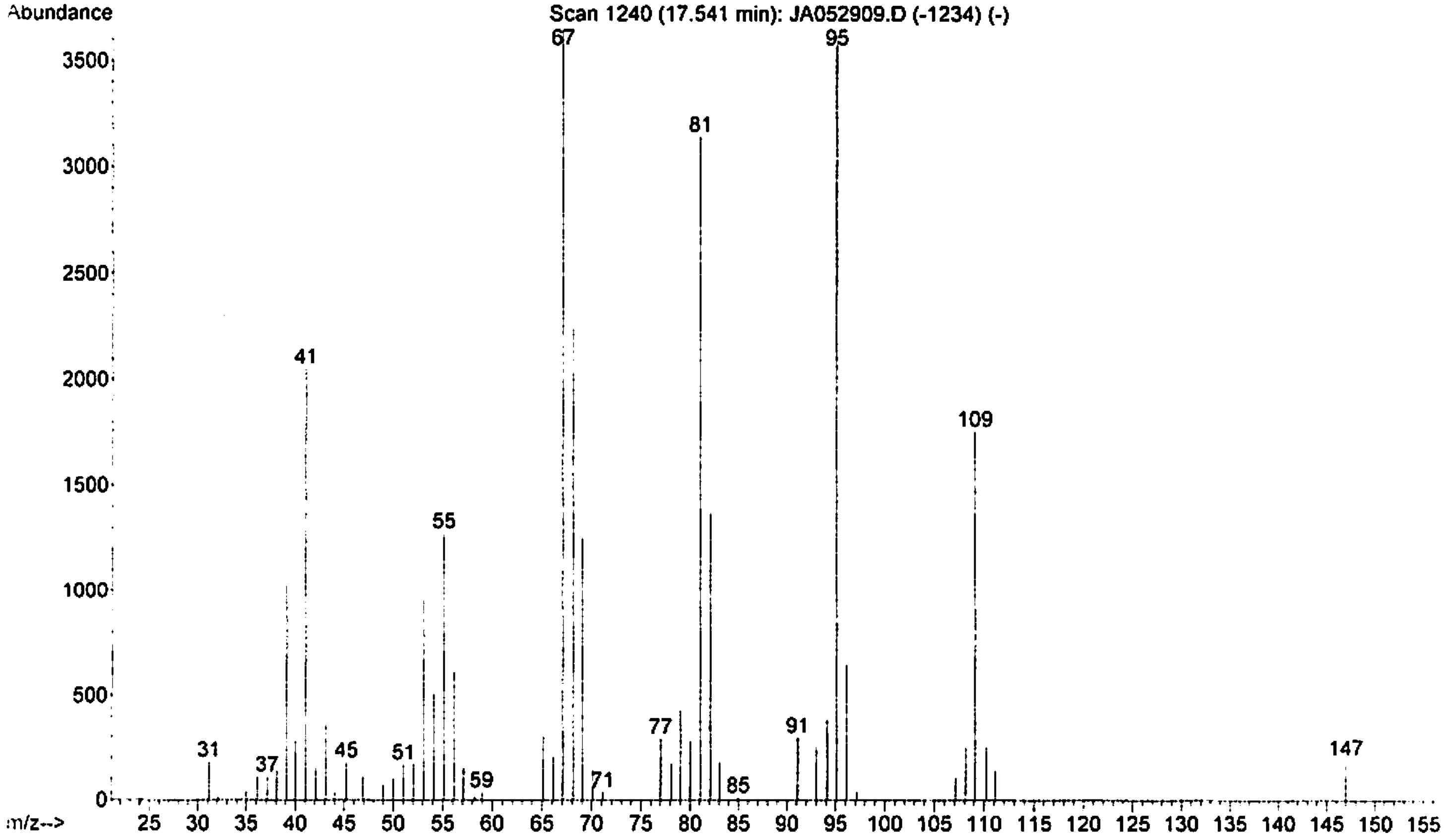

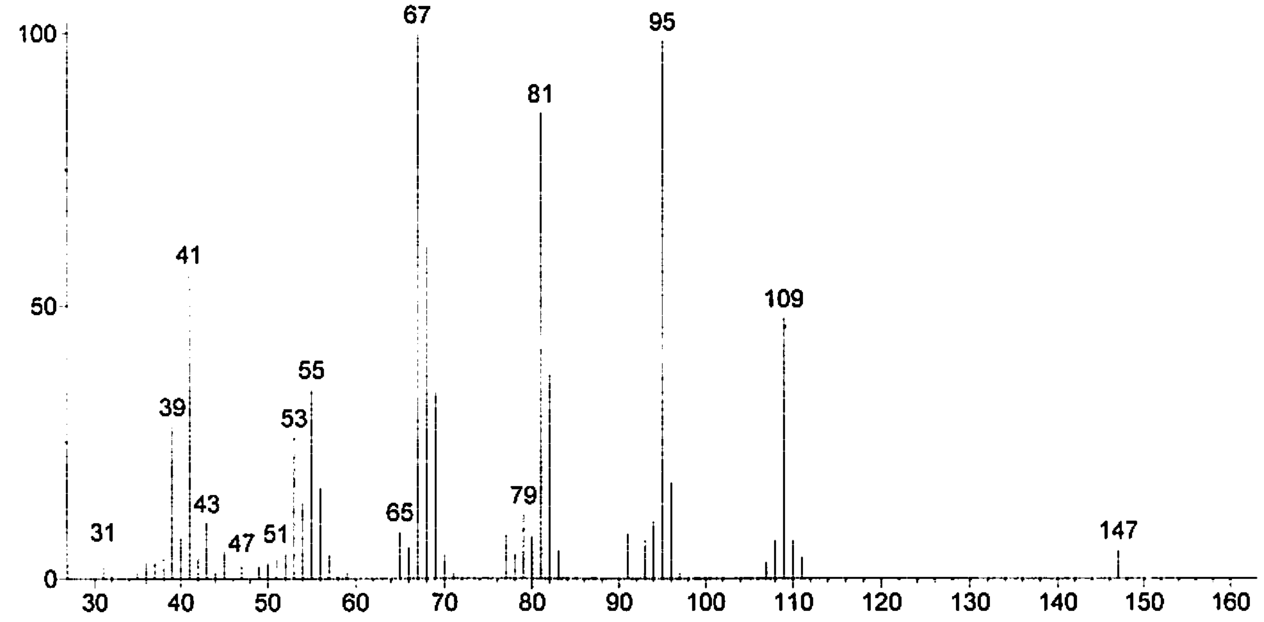

(Text File) Scan 1240 (17.541 min): JA052909.D (-1234)

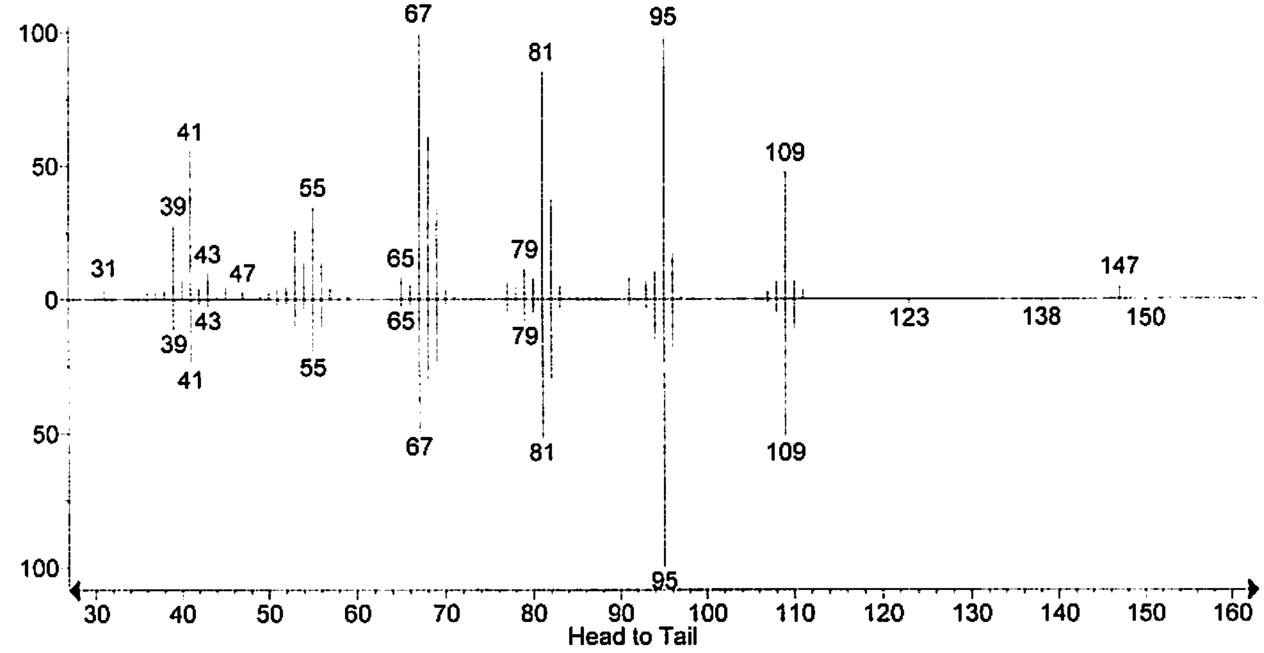

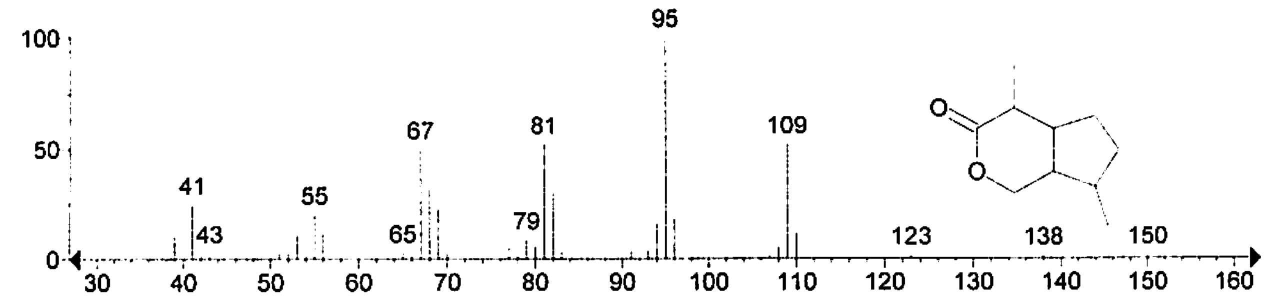

(replib) Iridomyrmecin

[illegible] :D:\DATA\ALDRICH\JA-09\Snapshot\JA052909.D
[illegible]ator : Aldrich
[illegible]ired : 29 May 2009 9:32 using AcqMethod JA-WAX08.M
[illegible]rument : Instrument #1
[illegible]ple Name: 1 field-coll. M C.oculata abd./CH2Cl2
[illegible] Info : 3rd male from 5/28; "top" gut full w/ yellow
[illegible] Number: 1

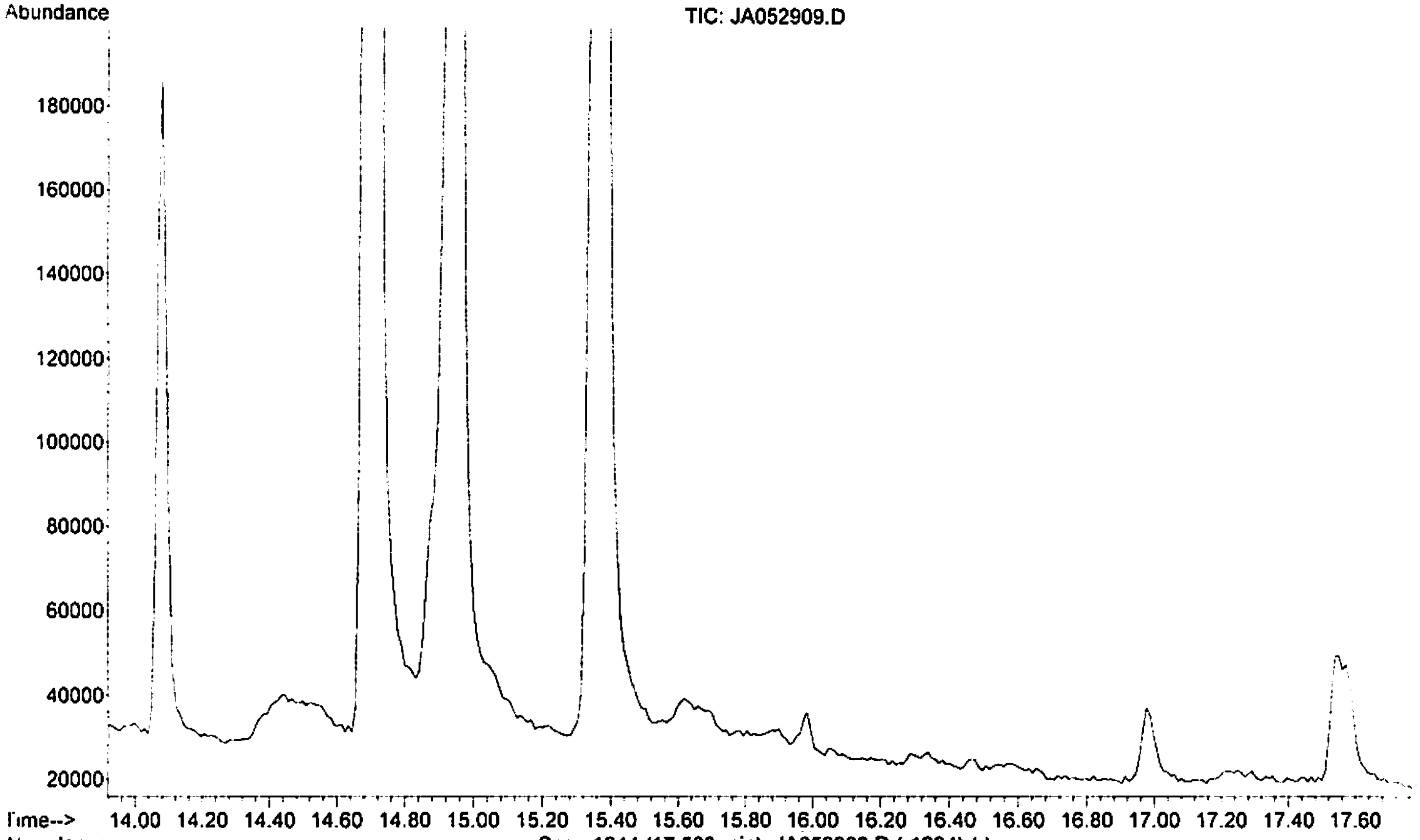

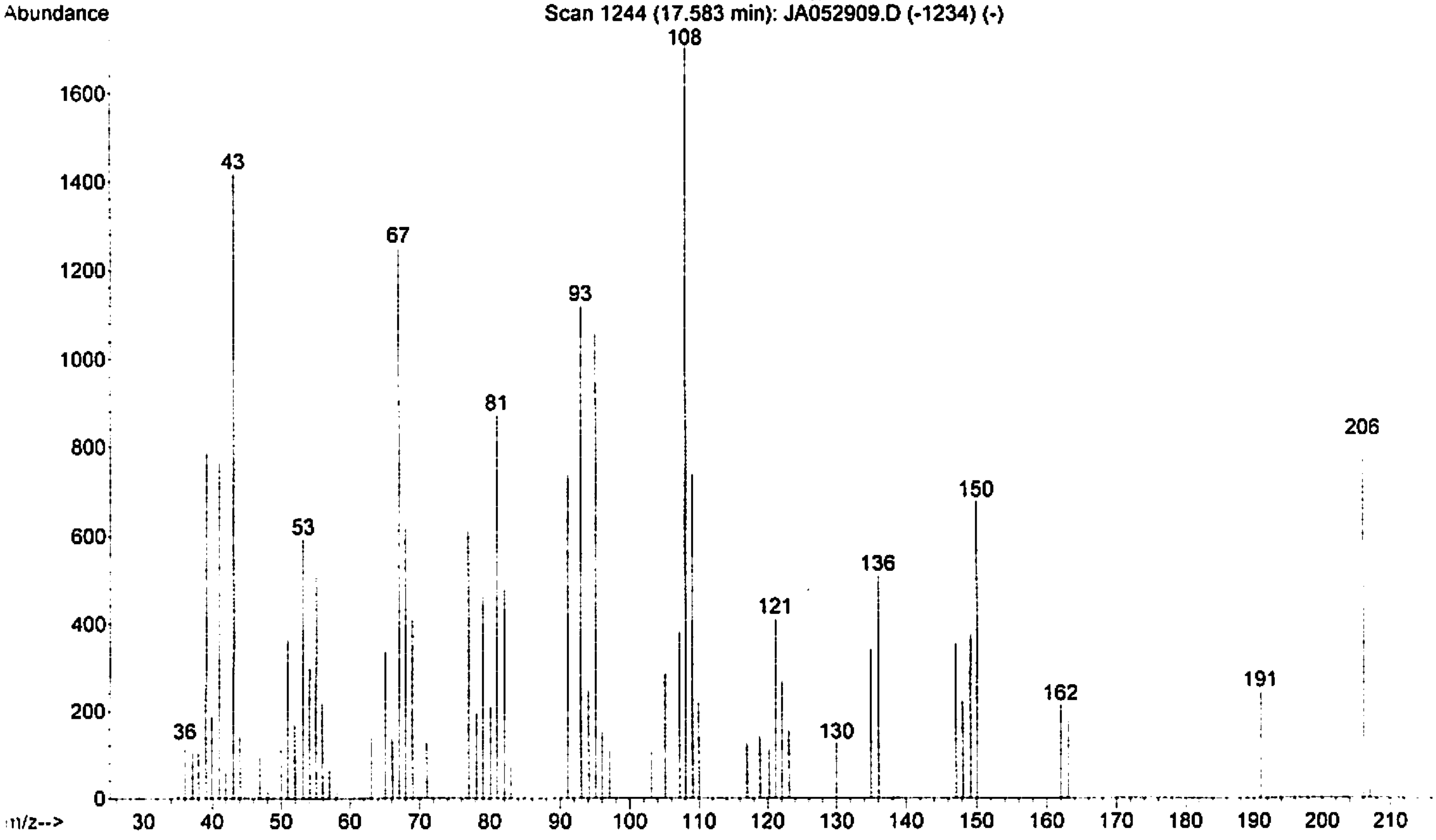

[illegible]e :D:\DATA\ALDRICH\JA-09\Snapshot\JA052909.D
[illegible]erator : Aldrich
[illegible]uired : 29 May 2009 9:32 using AcqMethod JA-WAX08.M
[illegible]strument : Instrument #1
[illegible]ple Name: 1 field-coll. M C.oculata abd./CH2Cl2
[illegible] Info : 3rd male from 5/28; "top" gut full w/ yellow
[illegible]al Number: 1

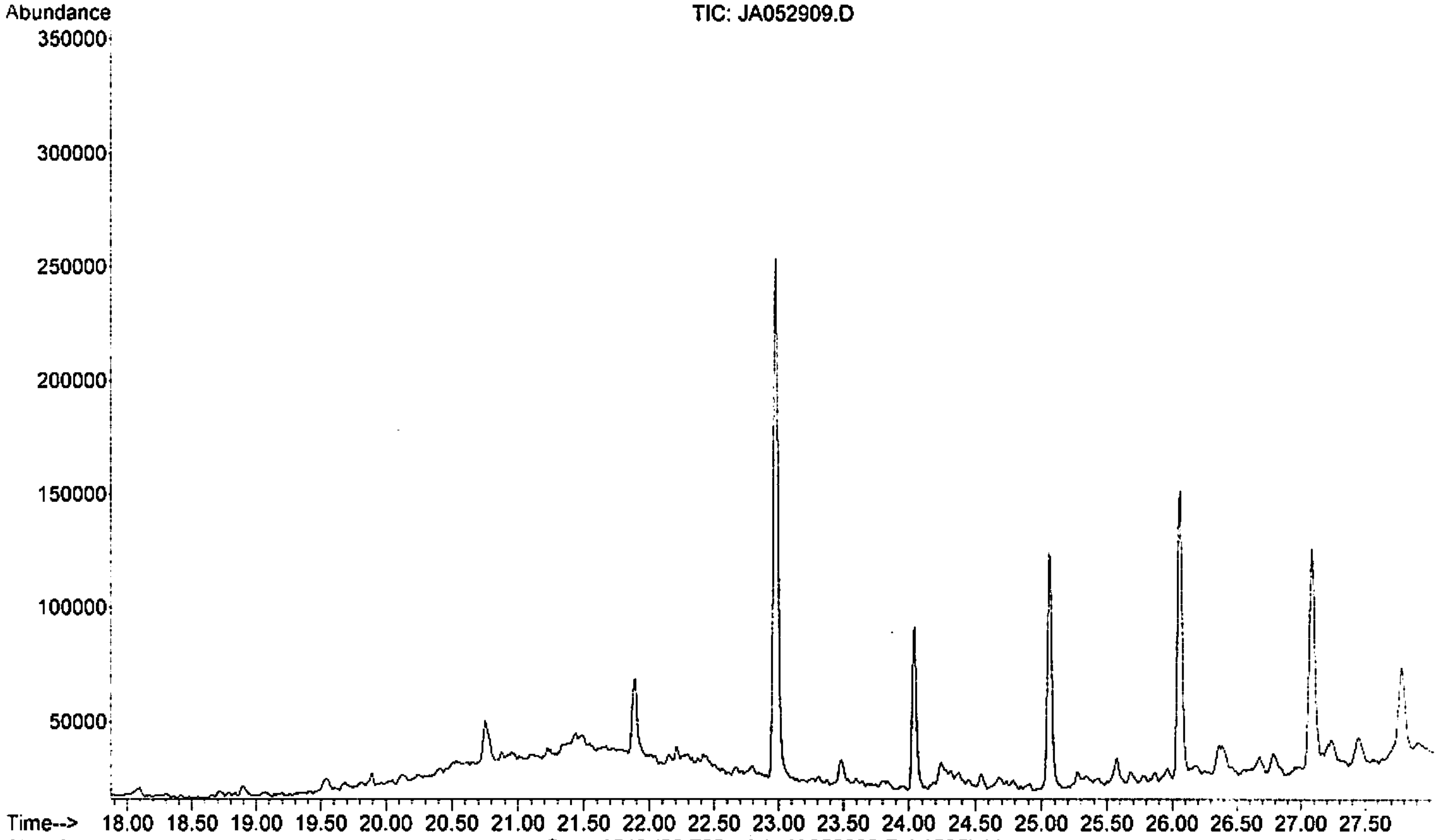

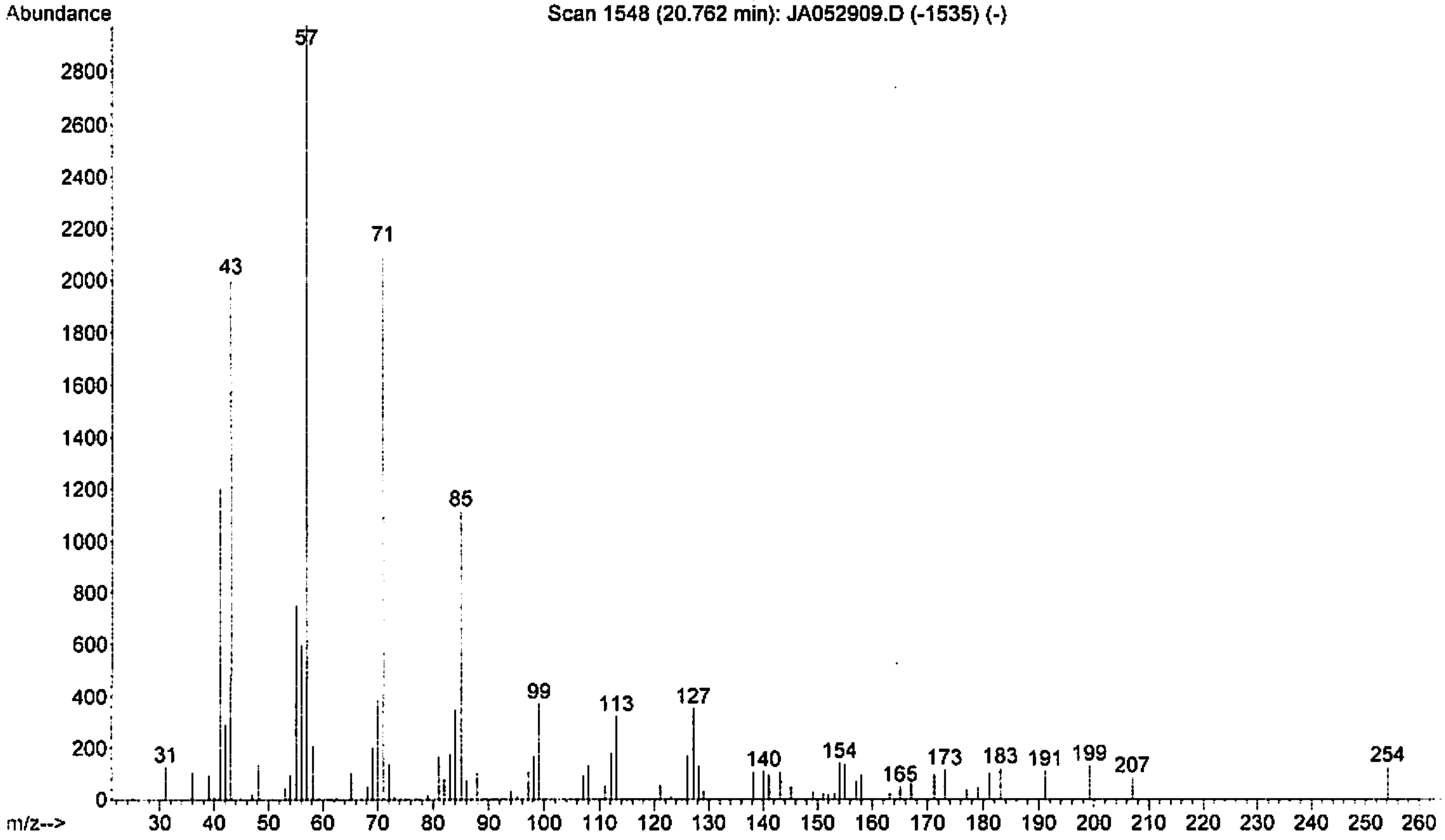

:D:\DATA\ALDRICH\JA-09\Snapshot\JA052909.D
rator : Aldrich
ired : 29 May 2009 9:32 using AcqMethod JA-WAX08.M
rument : Instrument #1
ple Name: 1 field-coll. M C.oculata abd./CH2Cl2
Info : 3rd male from 5/28; "top" gut full w/ yellow
Number: 1

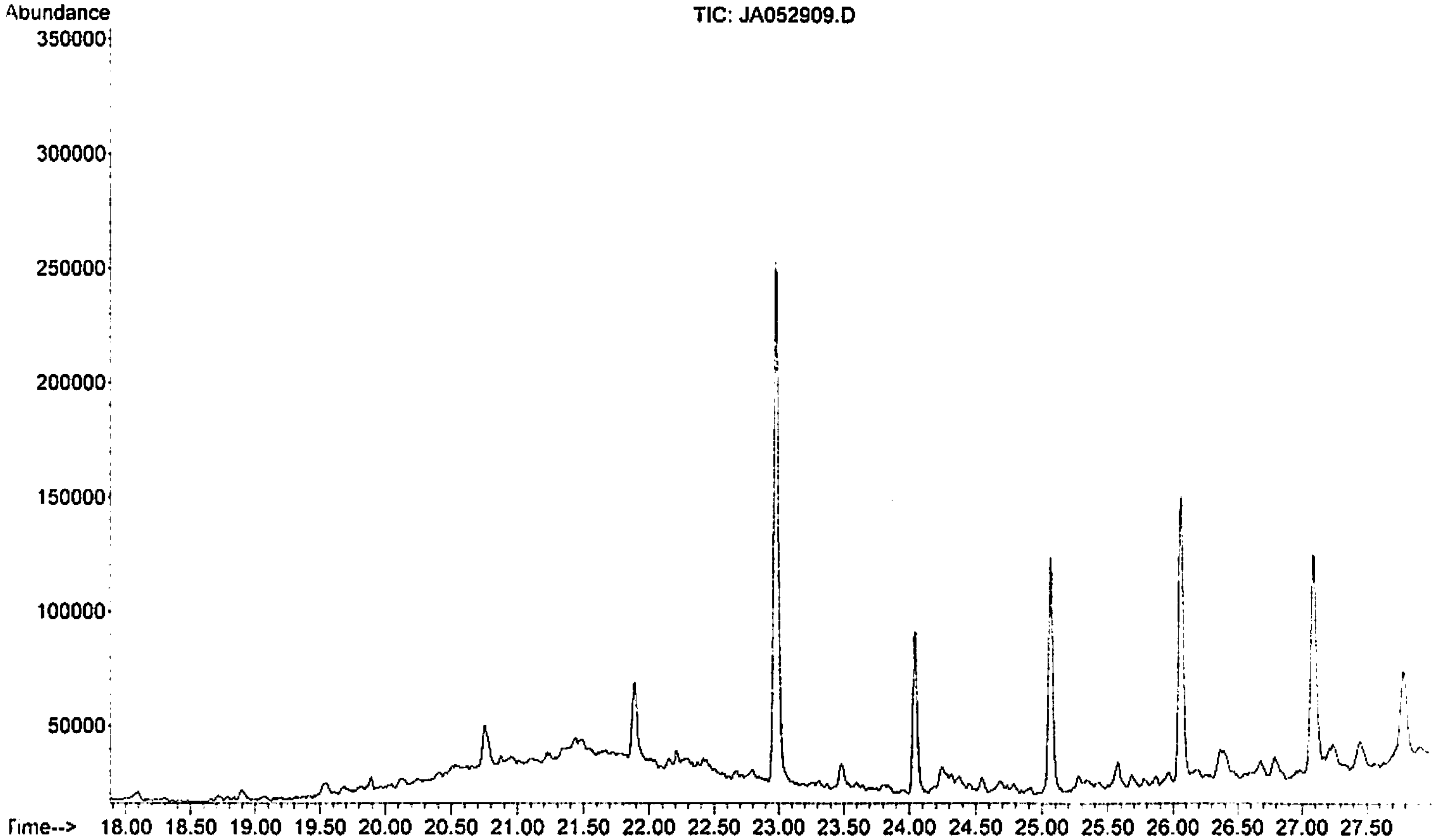

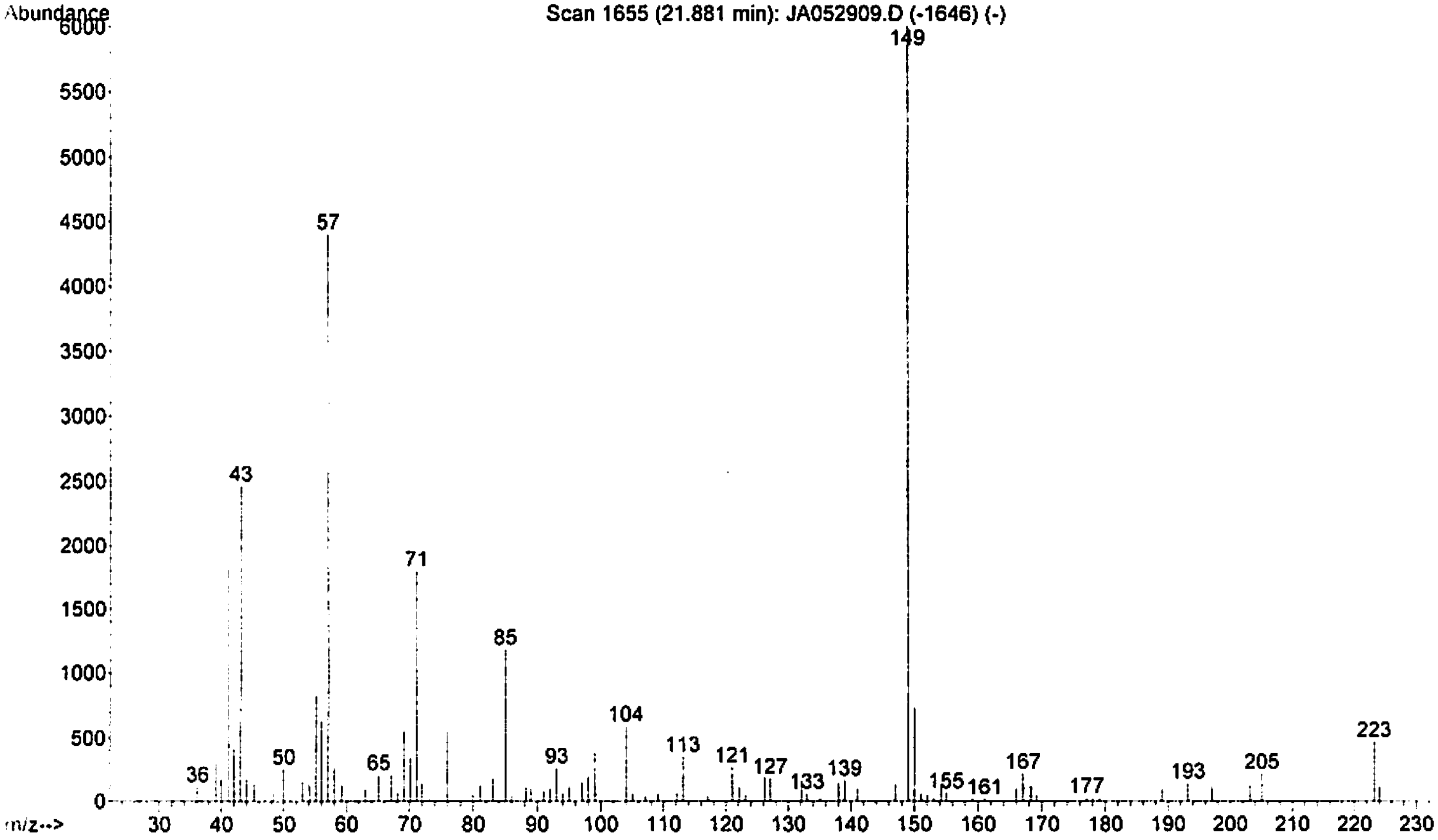

:D:\DATA\ALDRICH\JA-09\Snapshot\JA052909.D
ator : Aldrich
uired : 29 May 2009 9:32 using AcqMethod JA-WAX08.M
rument : Instrument #1
le Name: 1 field-coll. M C.oculata abd./CH2Cl2
Info : 3rd male from 5/28; "top" gut full w/ yellow
. Number: 1

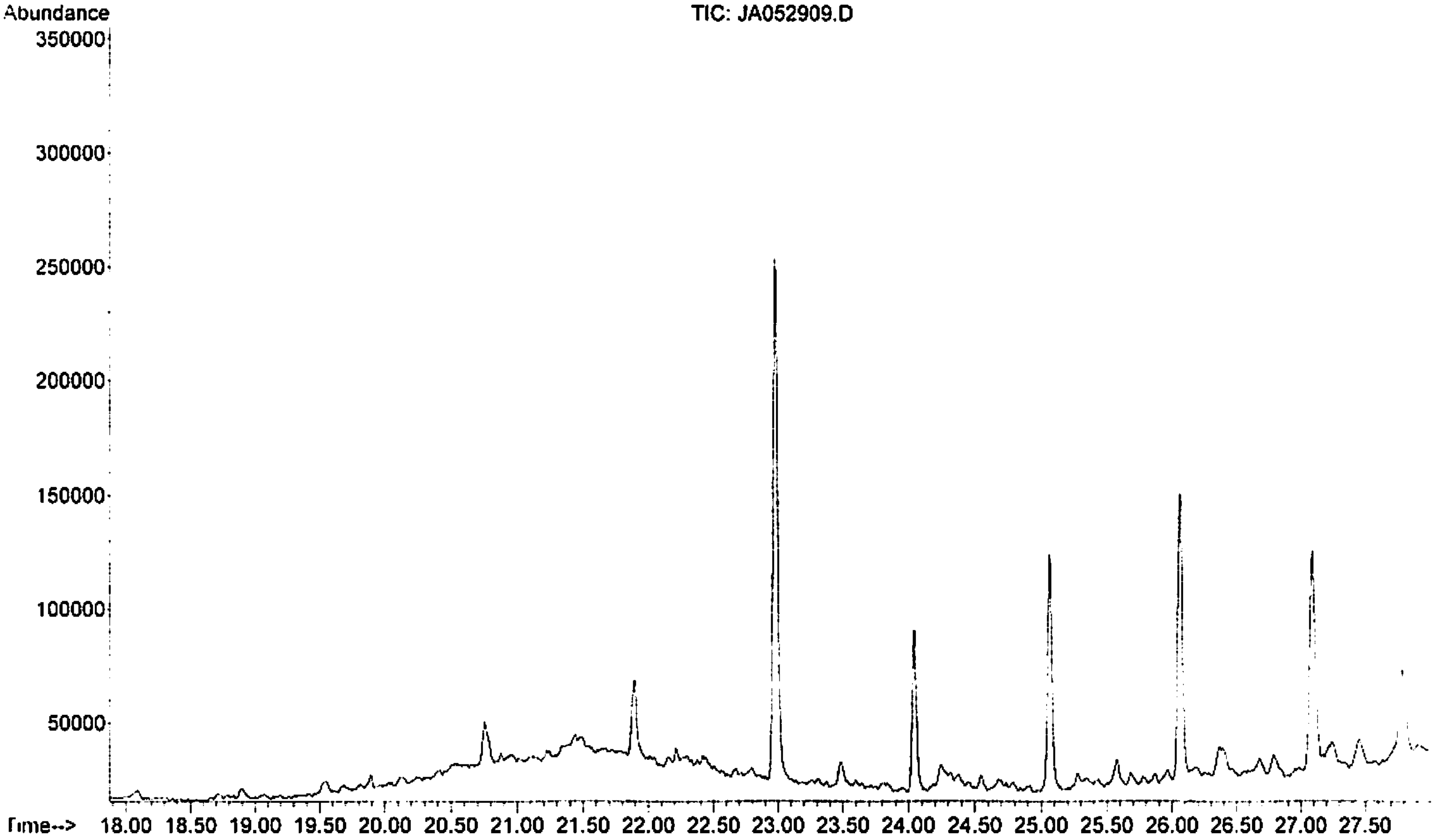

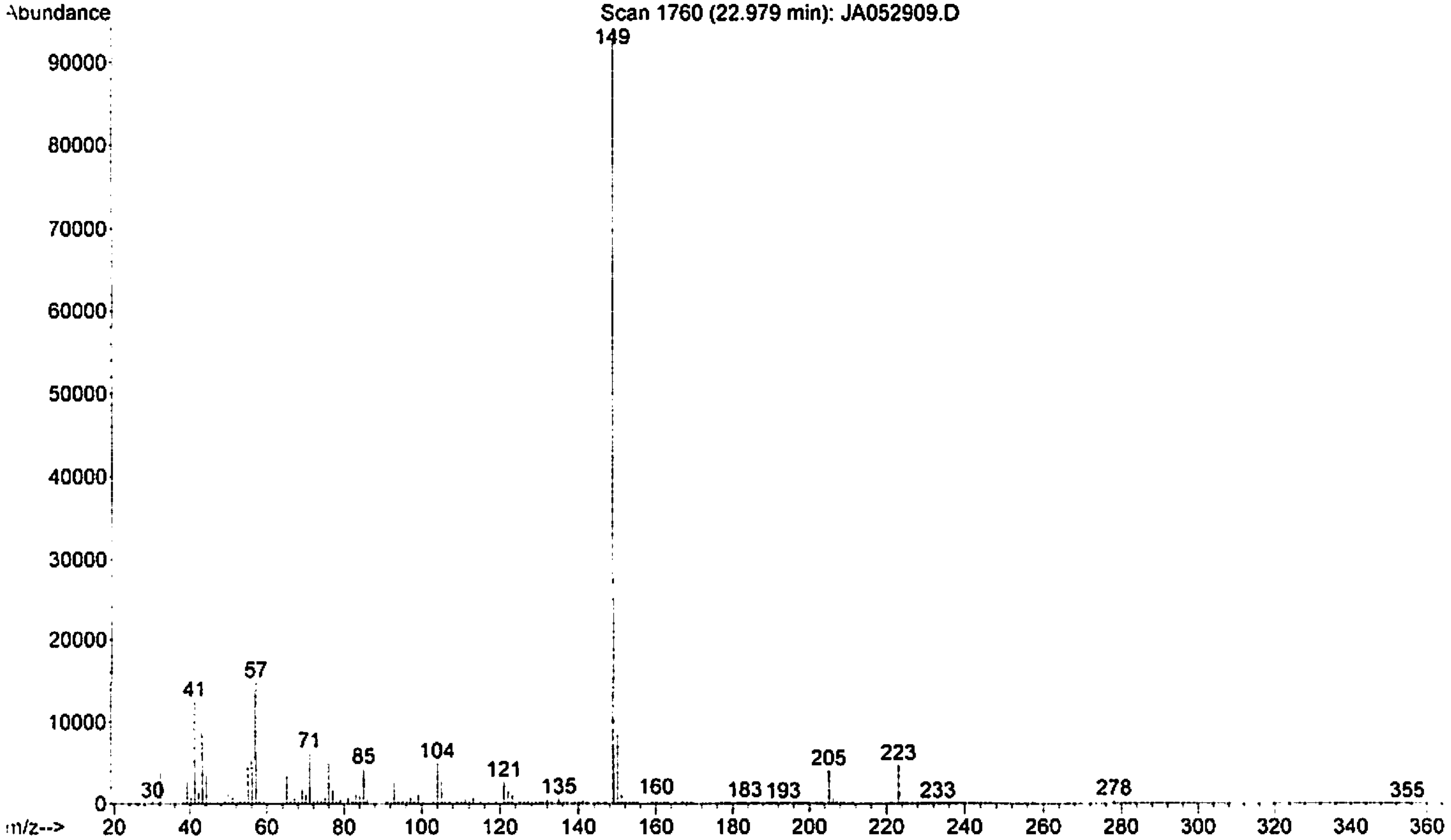

[illegible] :D:\DATA\ALDRICH\JA-09\Snapshot\JA052909.D
[illegible]ator : Aldrich
[illegible]ired : 29 May 2009 9:32 using AcqMethod JA-WAX08.M
[illegible]rument : Instrument #1
[illegible]le Name: 1 field-coll. M C.oculata abd./CH2Cl2
[illegible] Info : 3rd male from 5/28; "top" gut full w/ yellow
[illegible] Number: 1

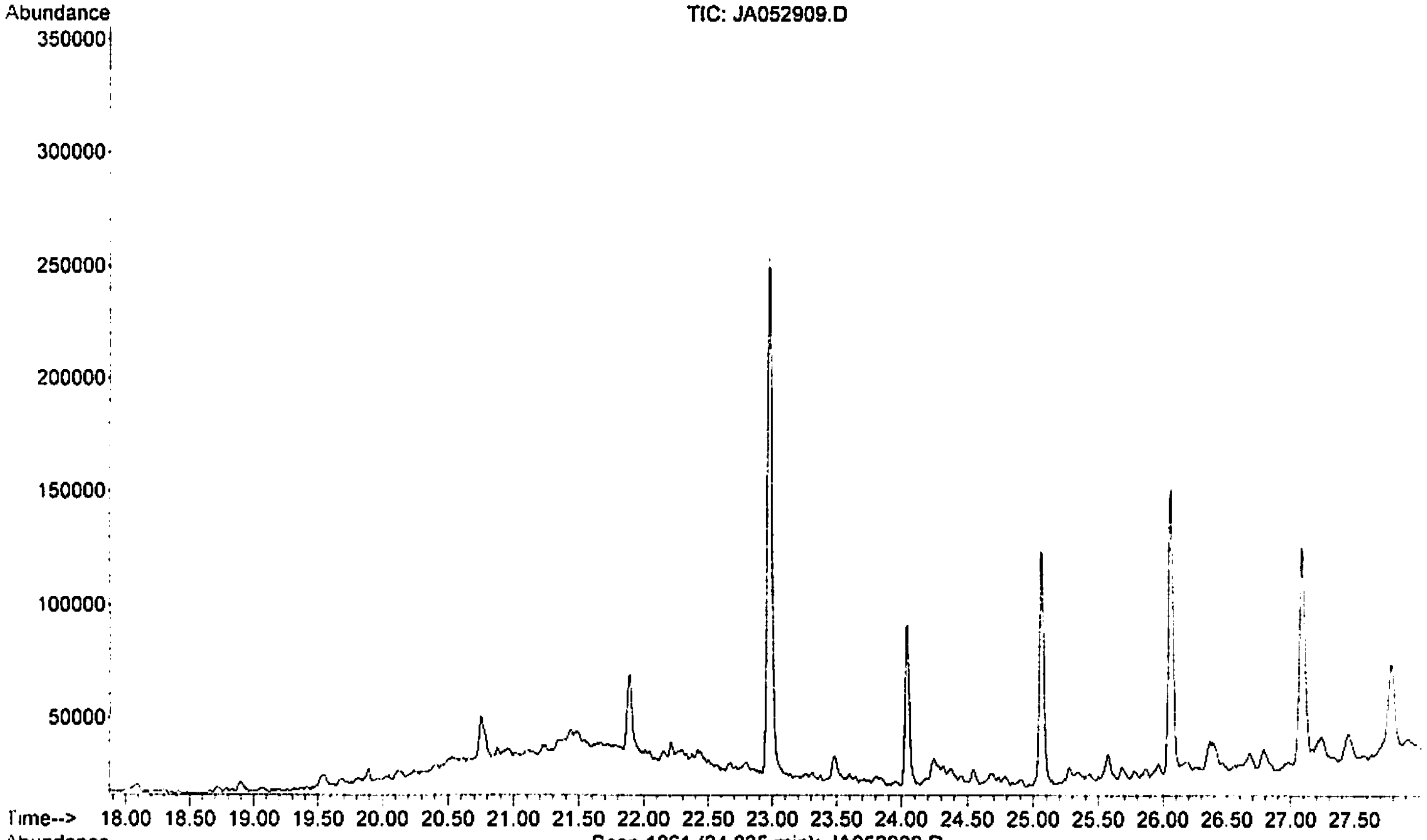

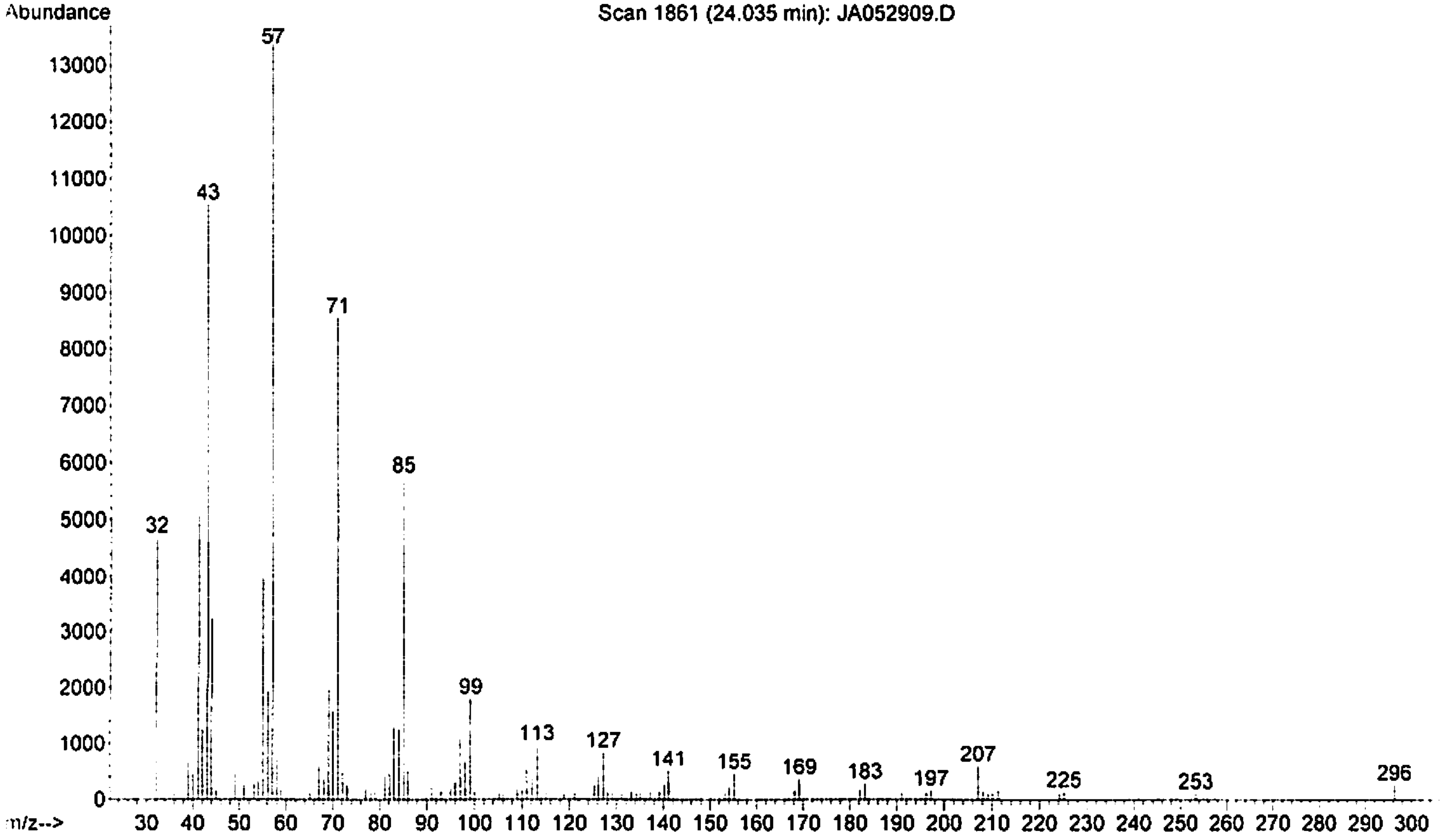

[illegible]:D:\DATA\ALDRICH\JA-09\Snapshot\JA052909.D
[illegible]ator : Aldrich
[illegible]ired : 29 May 2009 9:32 using AcqMethod JA-WAX08.M
[illegible]rument : Instrument #1
[illegible]ple Name: 1 field-coll. M C.oculata abd./CH2Cl2
[illegible] Info : 3rd male from 5/28; "top" gut full w/ yellow
[illegible] Number: 1

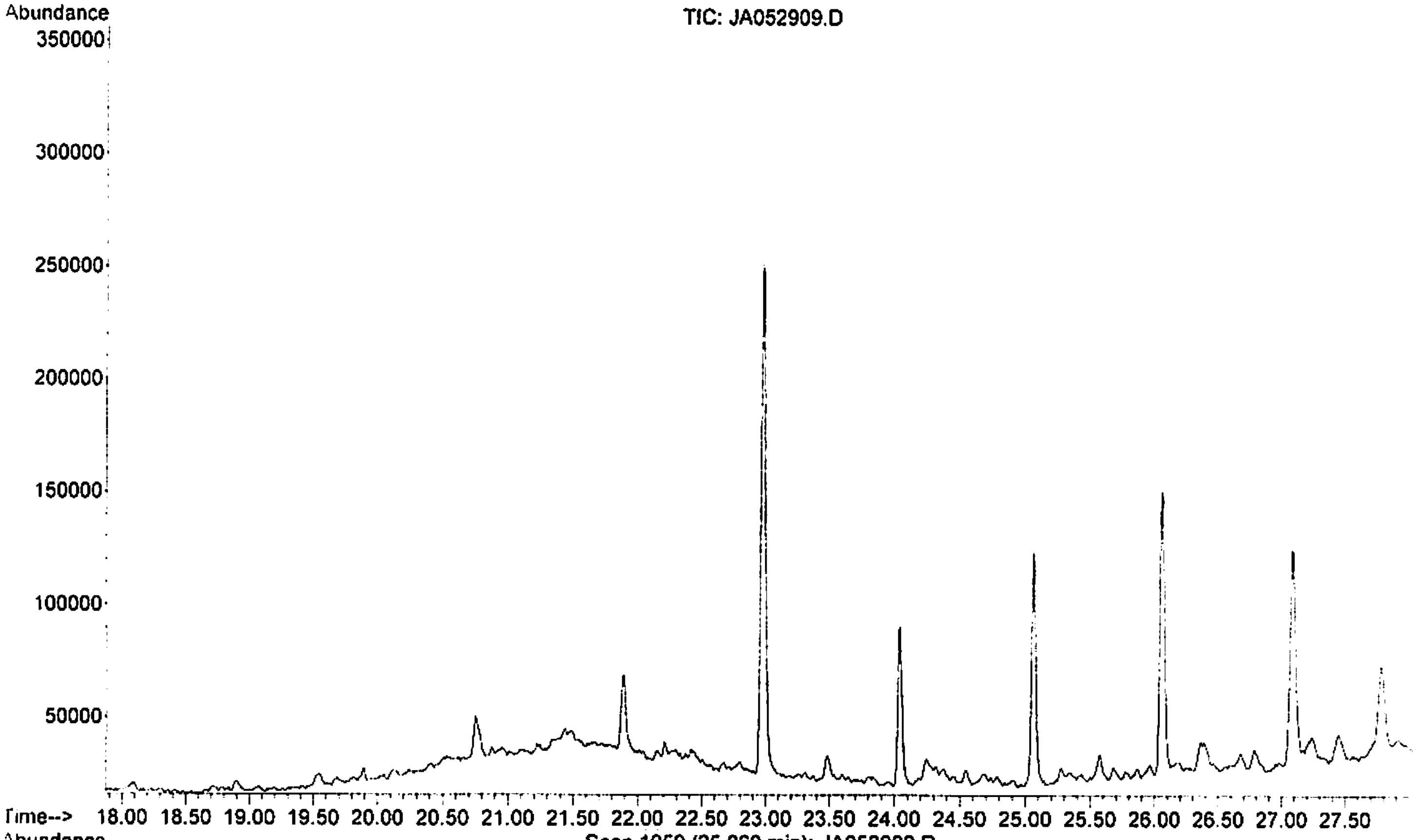

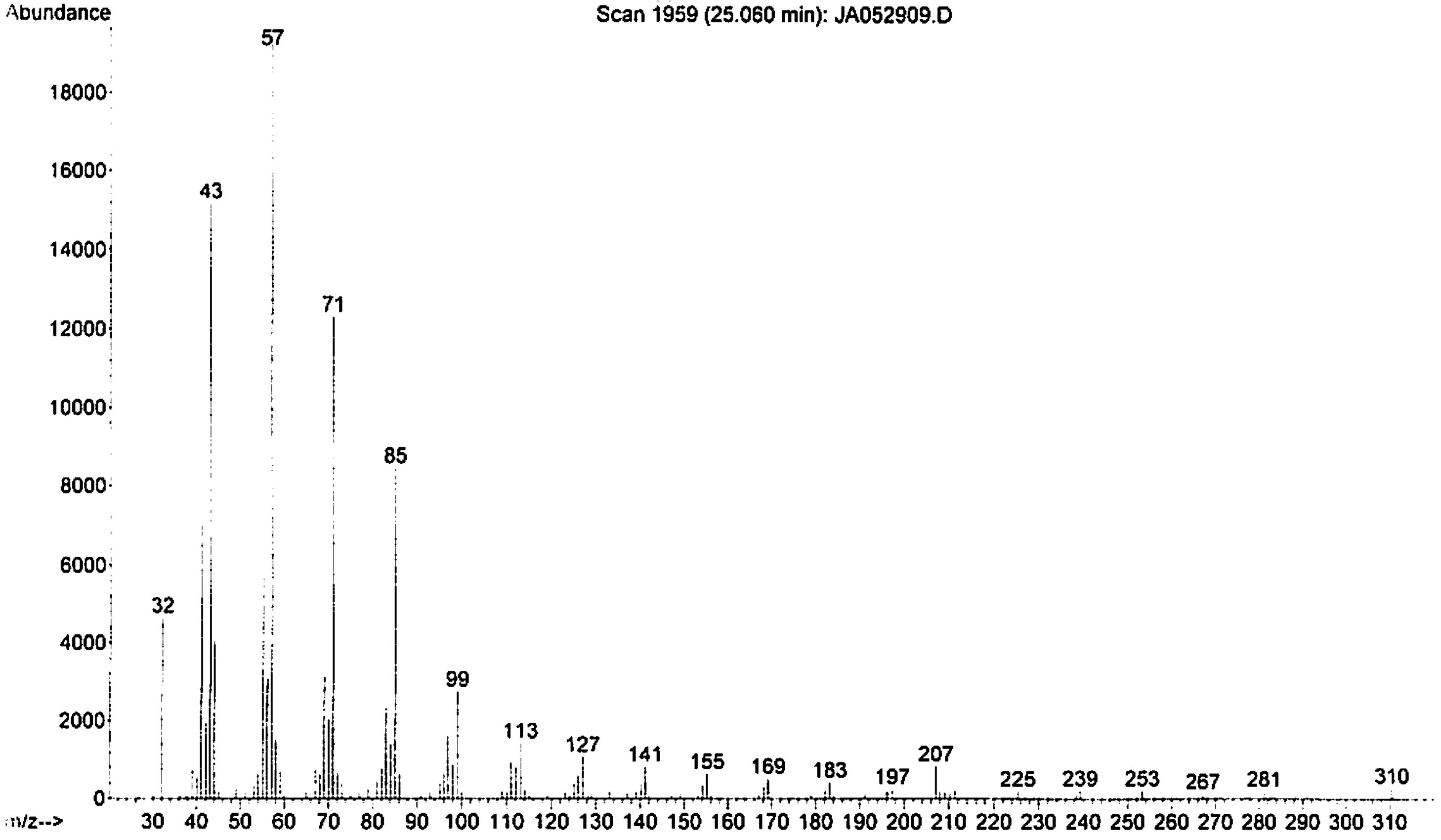

:D:\DATA\ALDRICH\JA-09\Snapshot\JA052909.D
[illegible]rator : Aldrich
[illegible]ired : 29 May 2009 9:32 using AcqMethod JA-WAX08.M
[illegible]rument : Instrument #1
[illegible]le Name: 1 field-coll. M C.oculata abd./CH2Cl2
[illegible] Info : 3rd male from 5/28; "top" gut full w/ yellow
[illegible] Number: 1

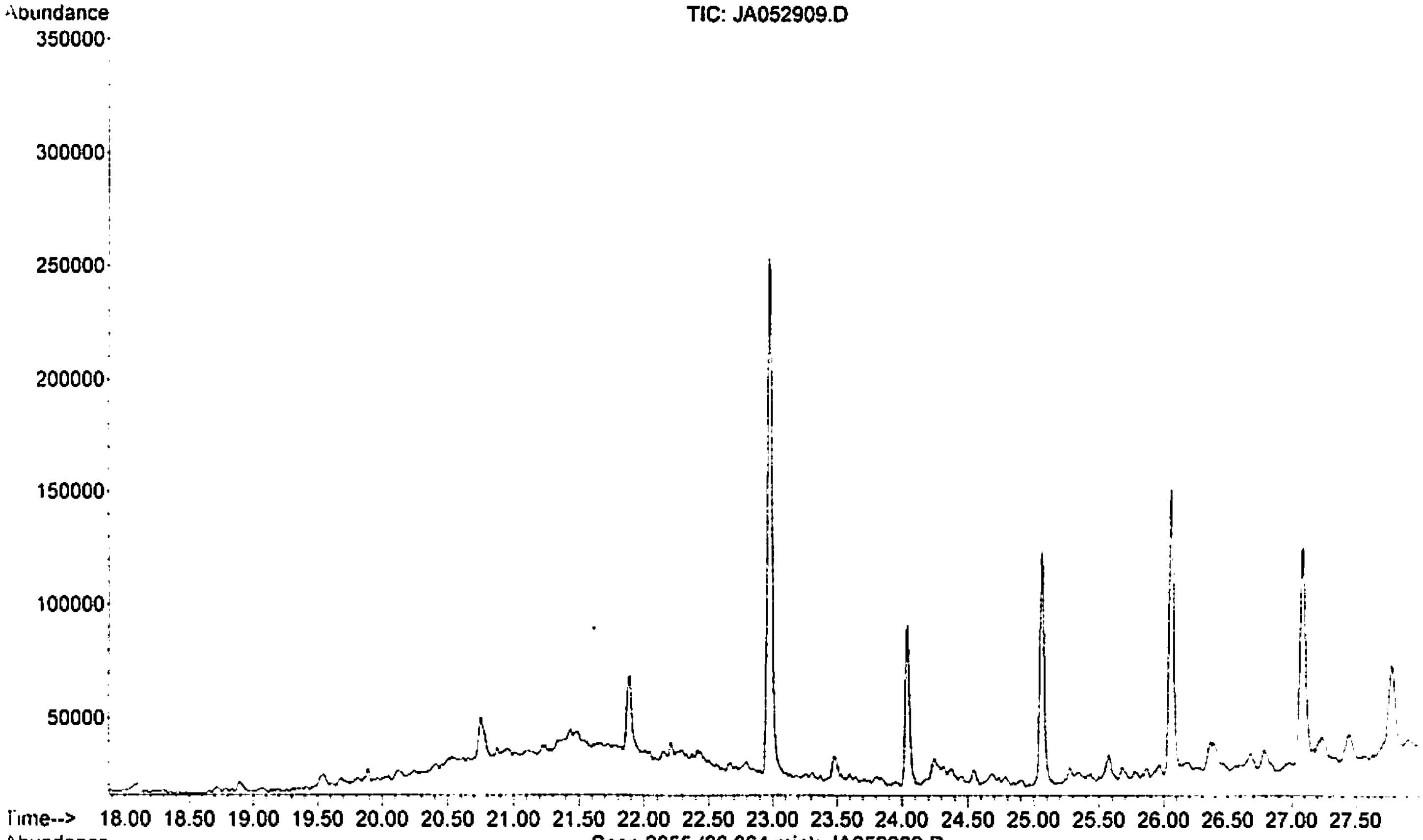

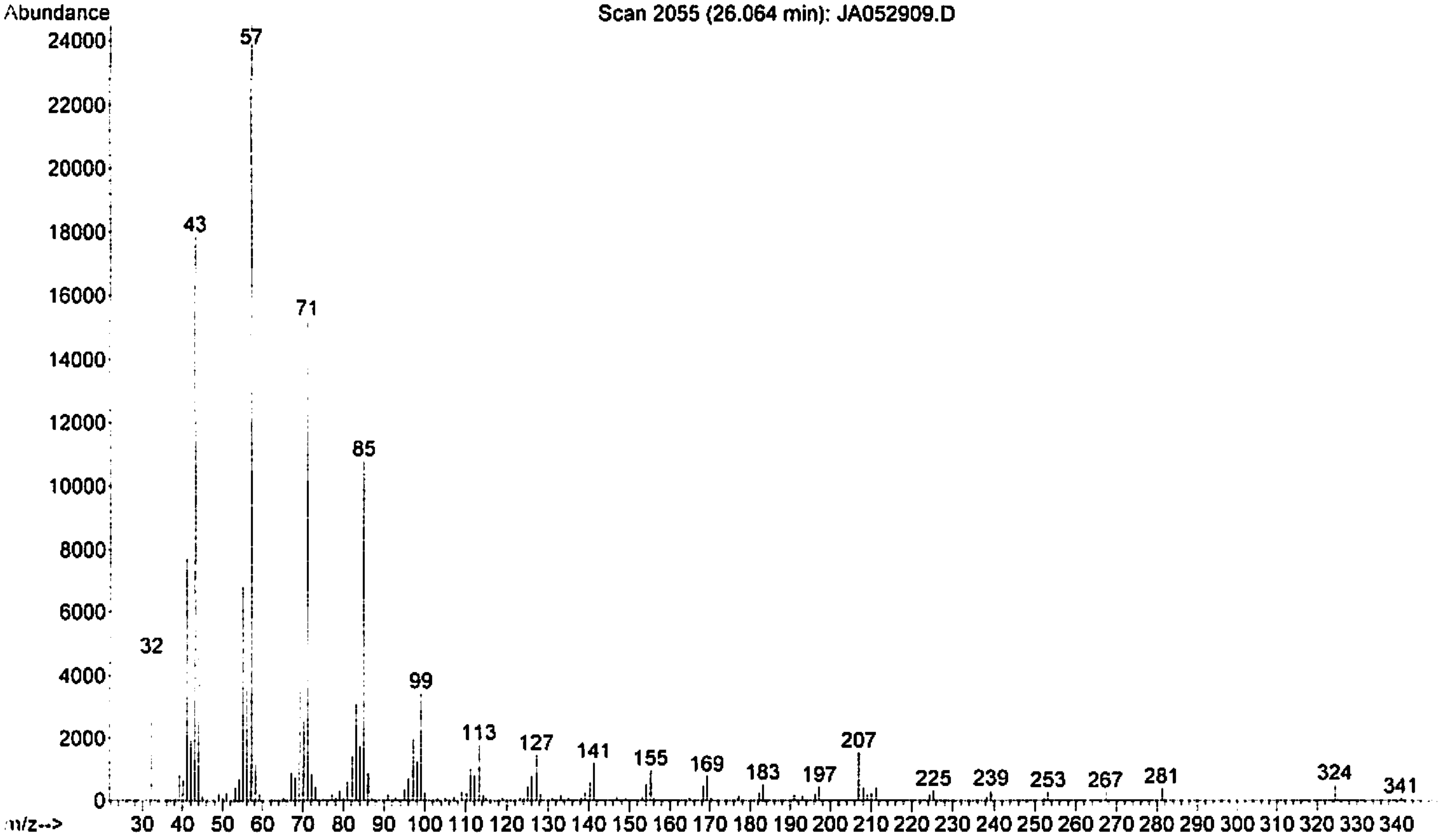

:D:\DATA\ALDRICH\JA-09\Snapshot\JA052909.D
[illegible]ator : Aldrich
[illegible]ired : 29 May 2009 9:32 using AcqMethod JA-WAX08.M
[illegible]rument : Instrument #1
[illegible]le Name: 1 field-coll. M C.oculata abd./CH2Cl2
[illegible] Info : 3rd male from 5/28; "top" gut full w/ yellow
[illegible] Number: 1

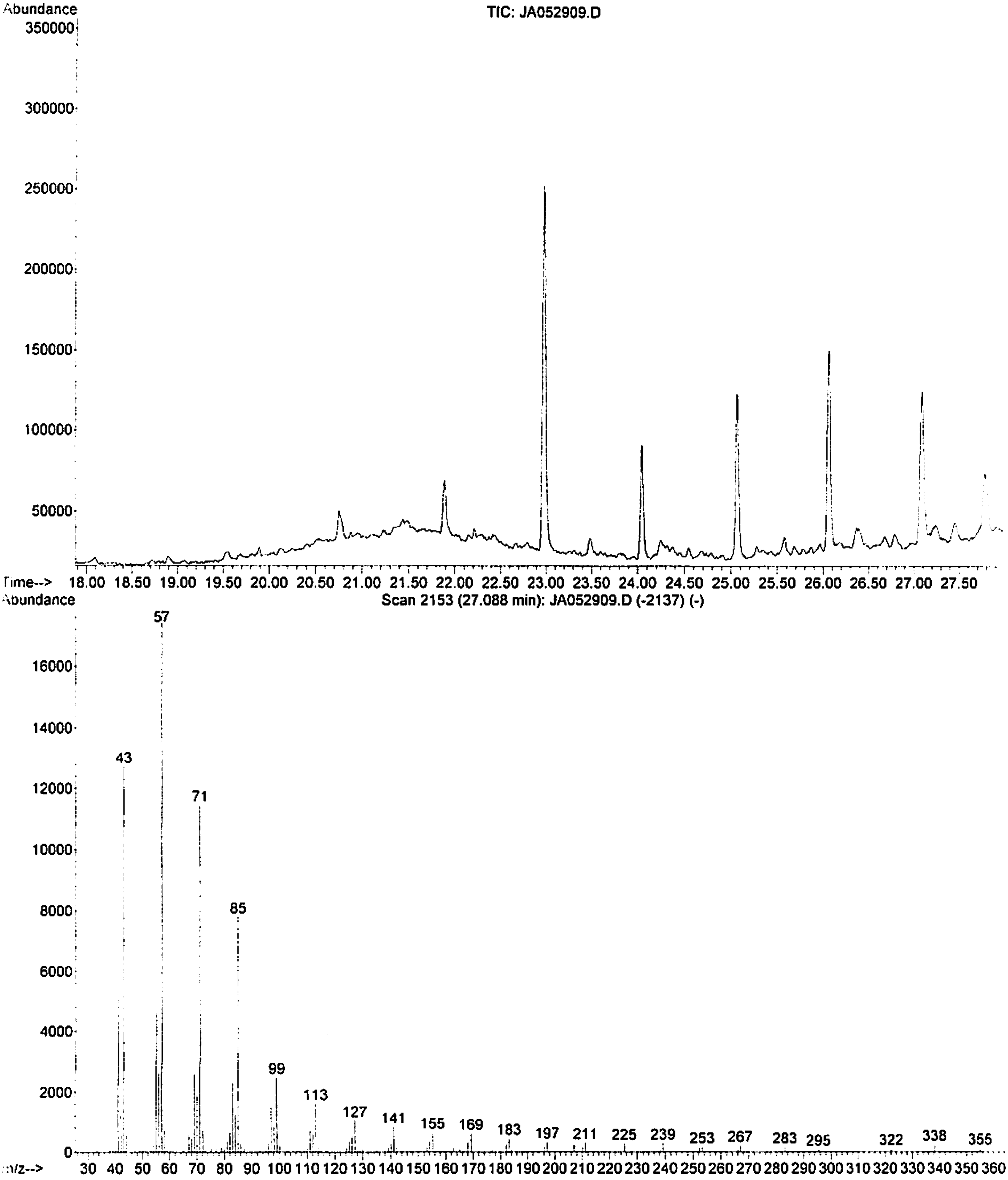

[illegible]e :D:\DATA\ALDRICH\JA-09\Snapshot\JA052909.D
[illegible]rator : Aldrich
[illegible]ired : 29 May 2009 9:32 using AcqMethod JA-WAX08.M
[illegible]rument : Instrument #1
[illegible]ple Name: 1 field-coll. M C.oculata abd./CH2Cl2
[illegible] Info : 3rd male from 5/28; "top" gut full w/ yellow
[illegible] Number: 1

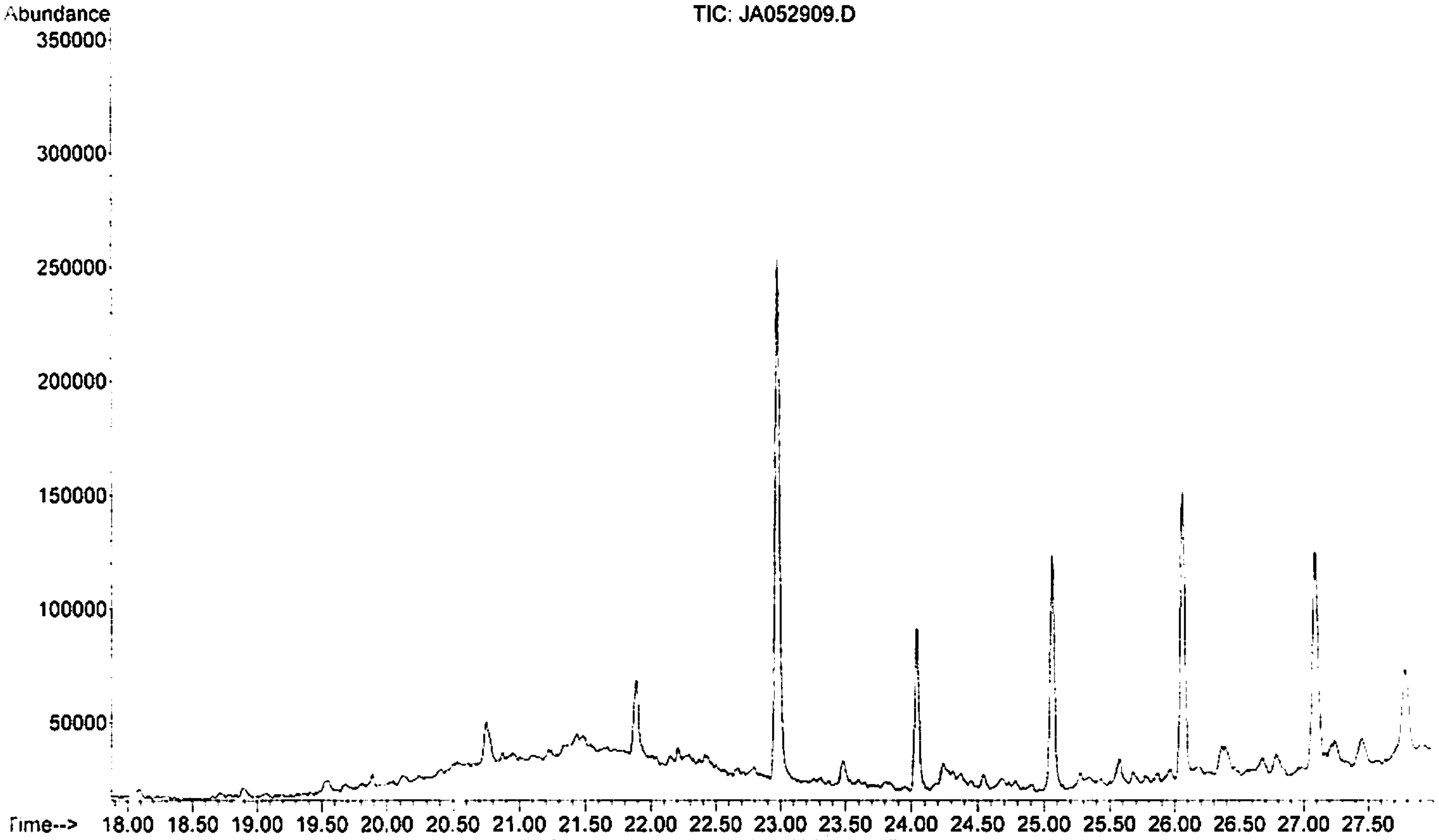

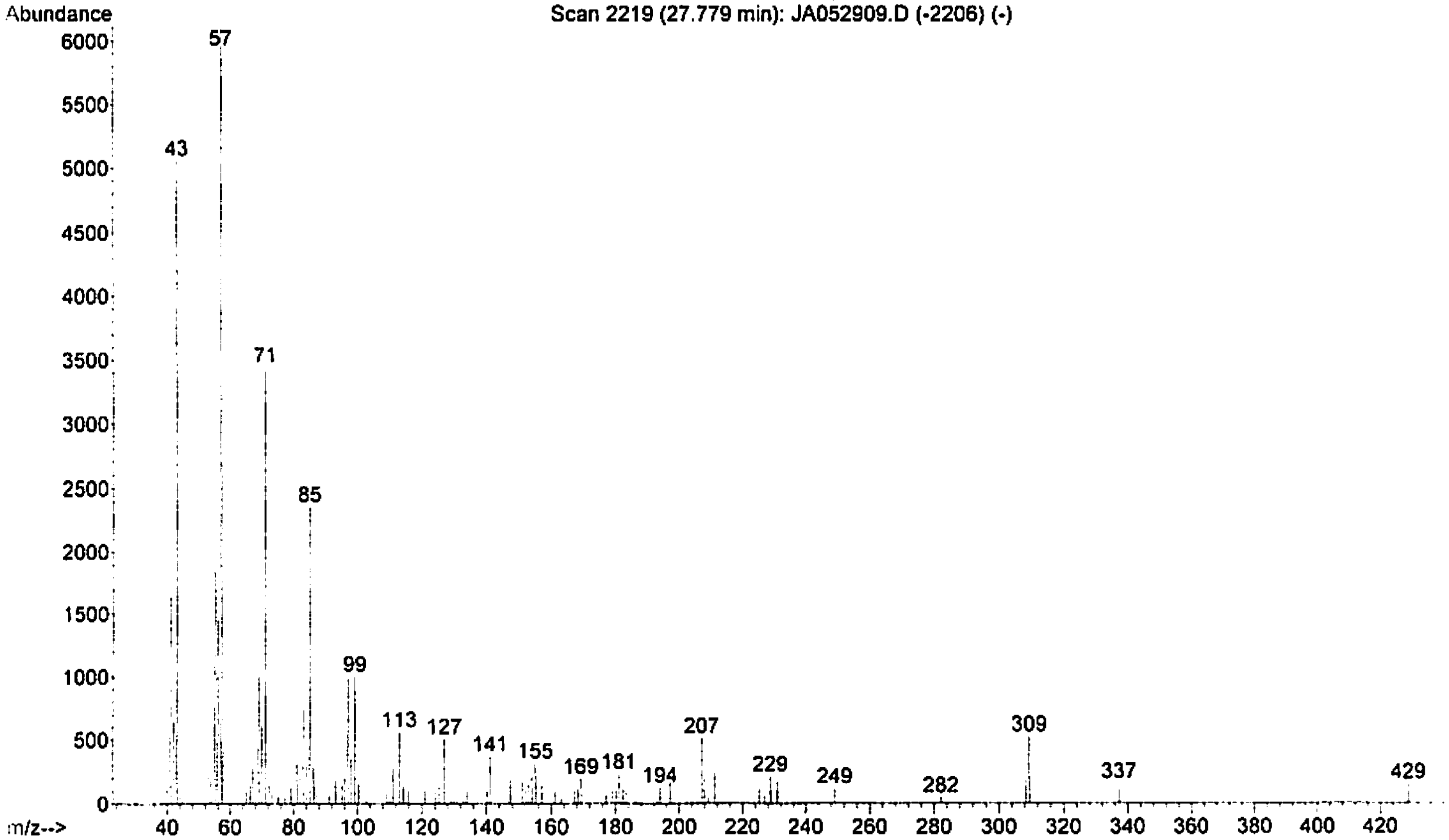

Supplement: Data S4 [file peerj-04-1564-s009.pdf]
