# Supplementary material for: Pharmacophagy in green lacewings (Neuroptera: Chrysopidae: Chrysopa spp.)?
Source: PeerJ. 2016 Jan 18;4:e1564. doi: 10.7717/peerj.1564 (PMC4727961; doi:10.7717/peerj.1564)

File   :D:\Ganga\GB-07-02-14F.D
Operator   : GANGA
Acquired   : 2 Jul 2014 13:15   using AcqMethod TLBACKSPLIT20.M
Instrument :   Instrument #1
Sample Name: GB-02-33
Misc Info : test tube 175
Vial Number: 1

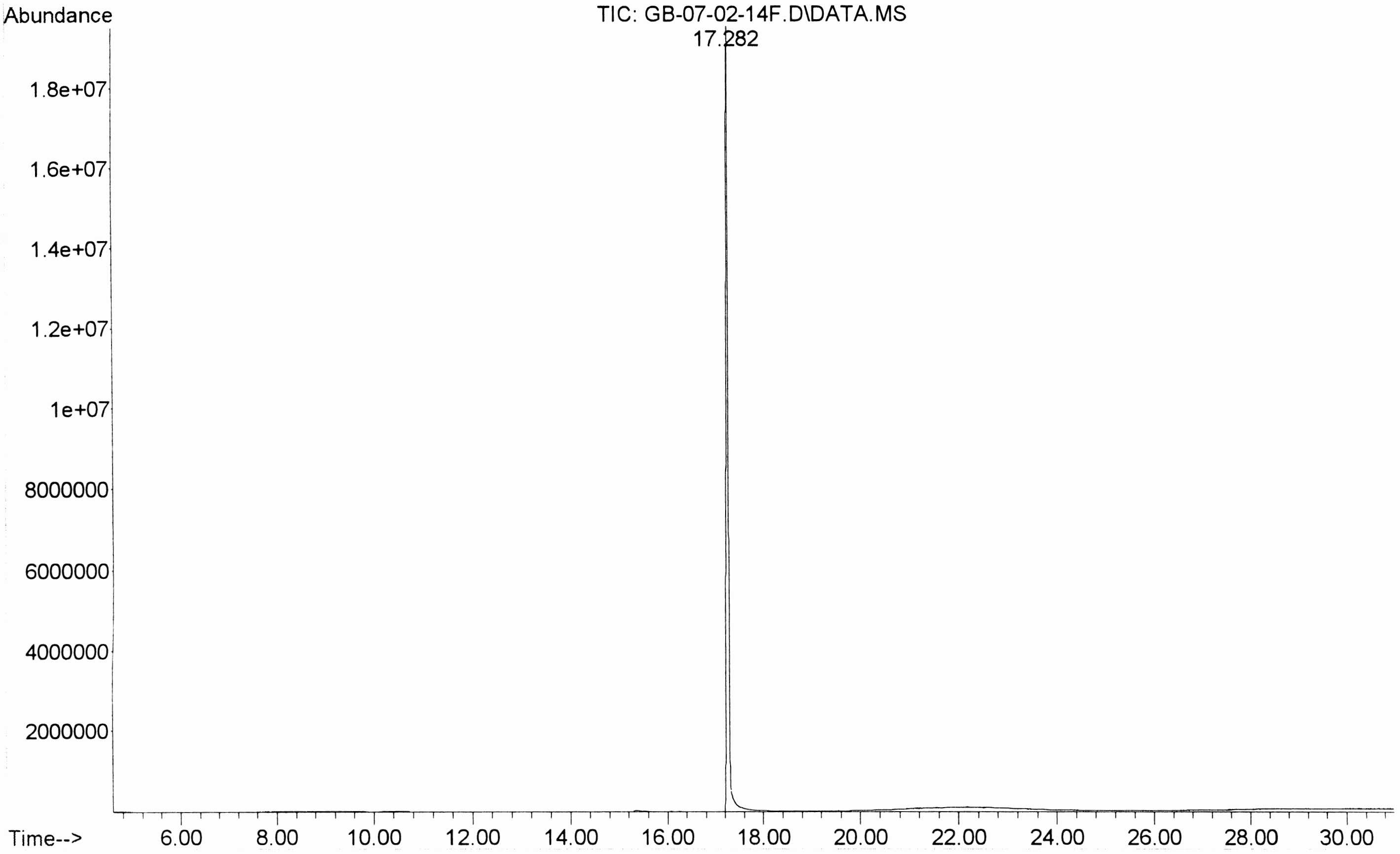

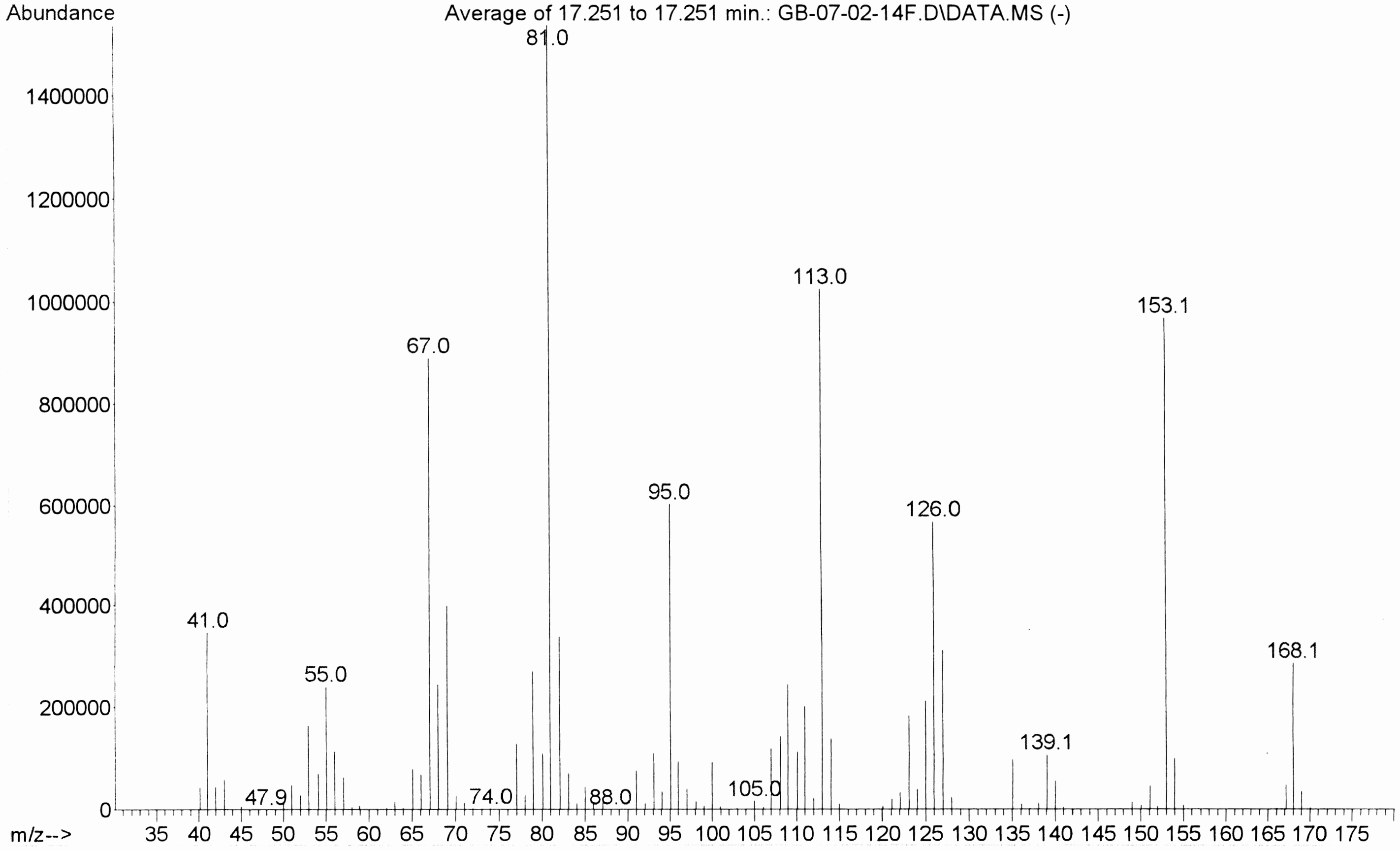

Supplement: Data S5 [file peerj-04-1564-s010.pdf]
