# Supplementary material for: Pharmacophagy in green lacewings (Neuroptera: Chrysopidae: Chrysopa spp.)?
Source: PeerJ. 2016 Jan 18;4:e1564. doi: 10.7717/peerj.1564 (PMC4727961; doi:10.7717/peerj.1564)

```
File        :D:\DATA\JA-08\JA0514-1.D
Operator    : Aldrich
Acquired    : 14 May 2008   14:42      using AcqMethod JA-BACK.M
Instrument  :    Instrument #1
Sample Name: male C. oculata (coll. 5/13) abdominal cut.
Misc Info   : GC run JA-05143.D; /CH2Cl2; trt IR+SK trap
Vial Number: 1
```

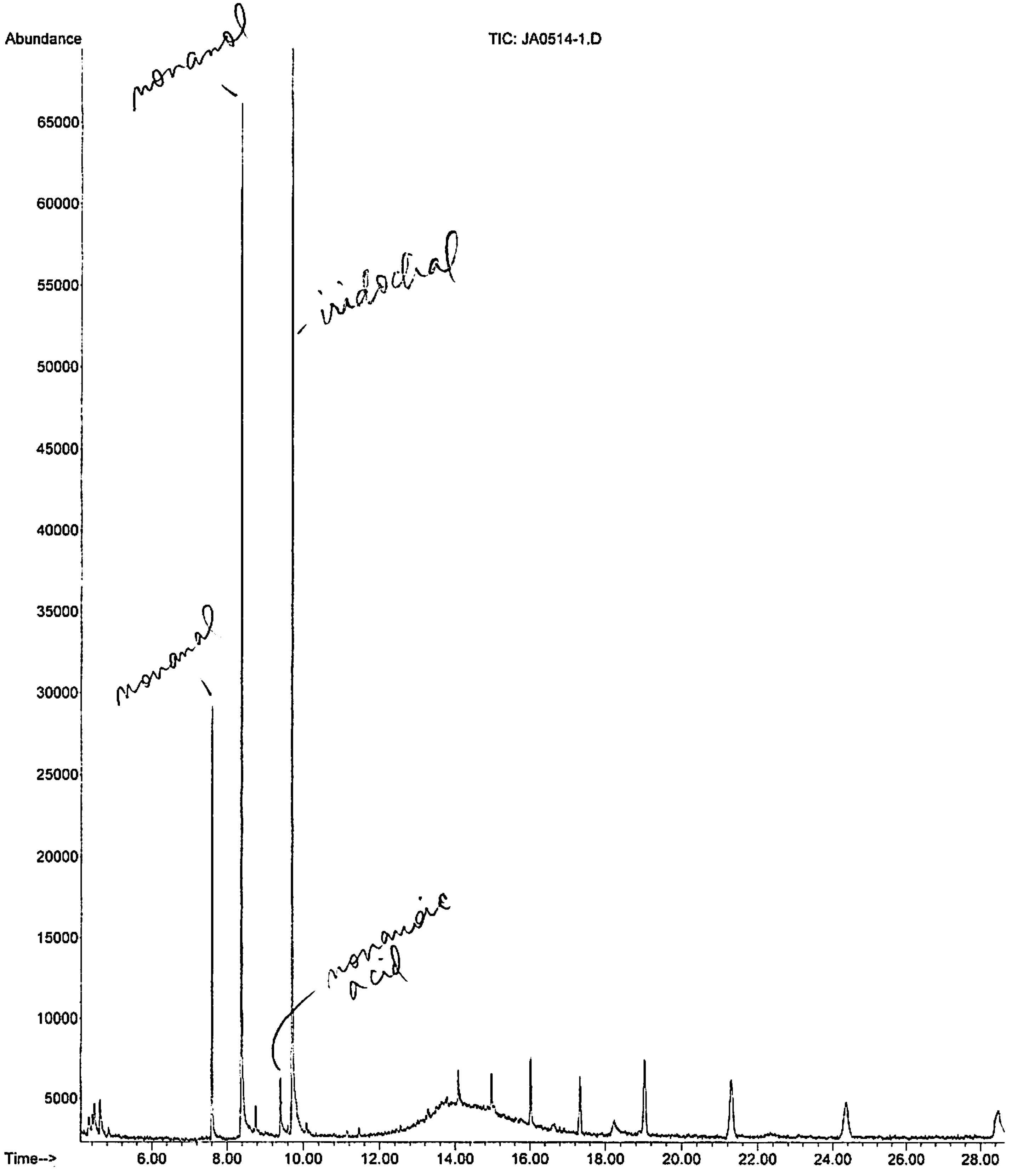

```
File        :D:\DATA\JA-08\Snapshot\JA0514-1.D
Operator    : Aldrich
Acquired    : 14 May 2008  14:42     using AcqMethod JA-BACK.M
Instrument  :    Instrument #1
Sample Name: male C. oculata (coll. 5/13) abdominal cut.
Misc Info   : GC run JA-05143.D; /CH2Cl2; trt IR+SK trap
Vial Number: 1
```

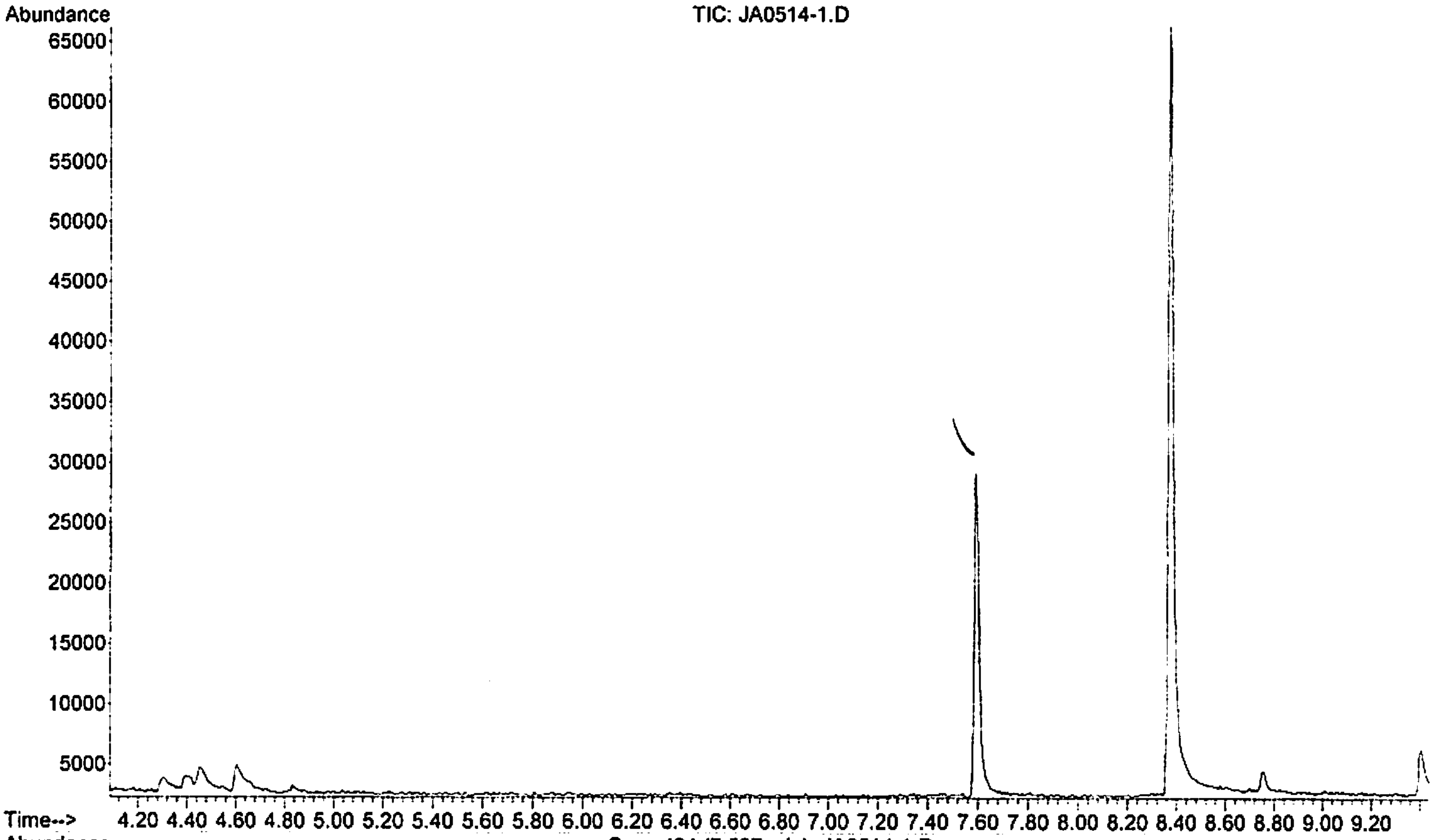

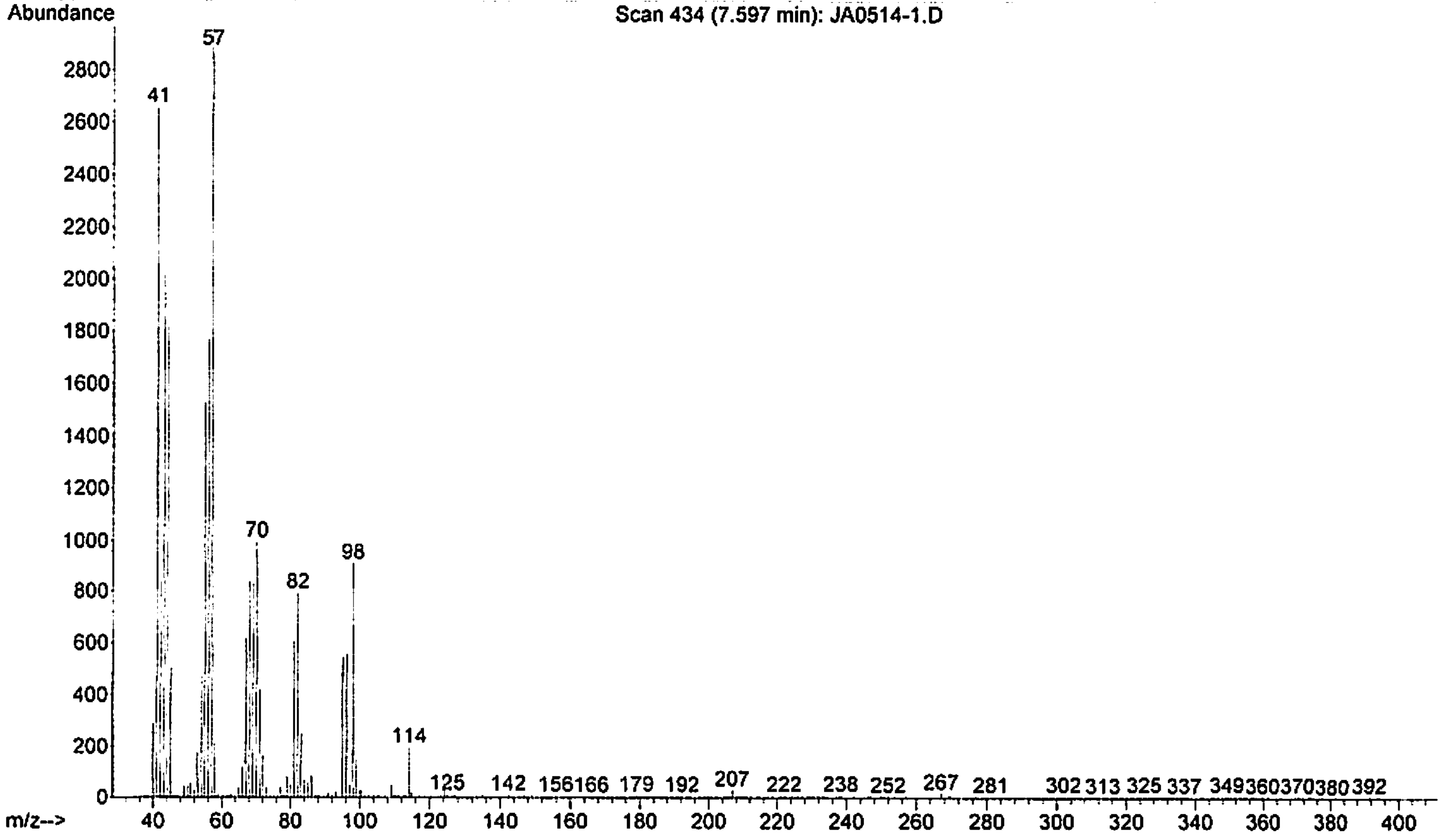

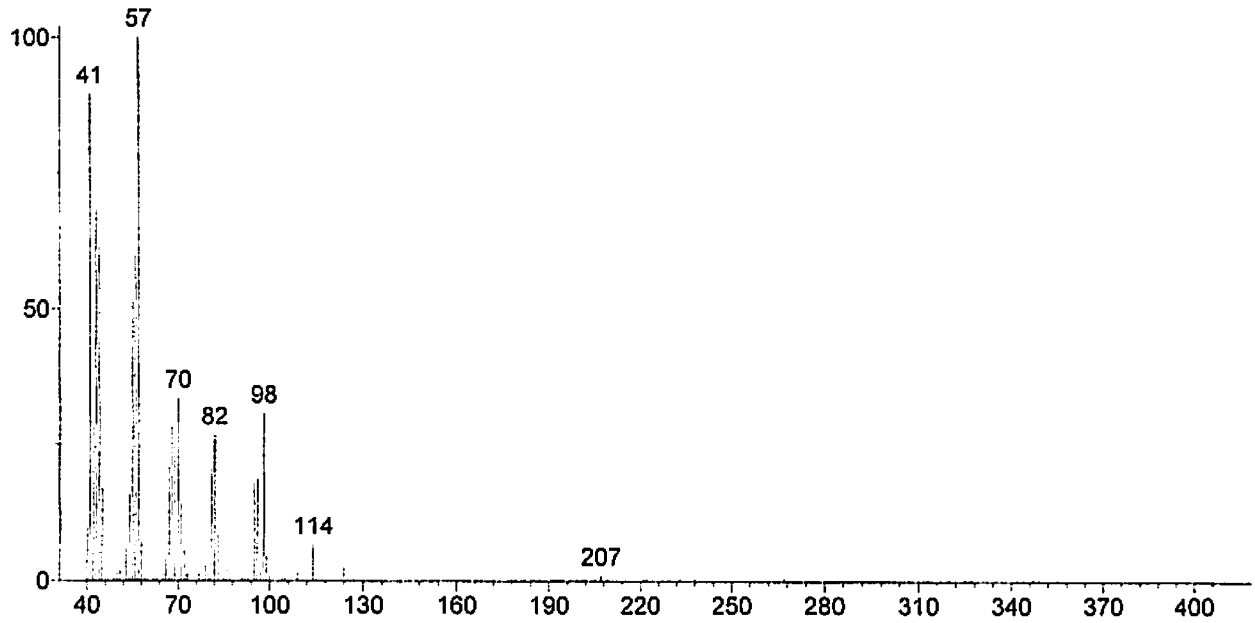

(Text File) Scan 434 (7.597 min): JA0514-1.D

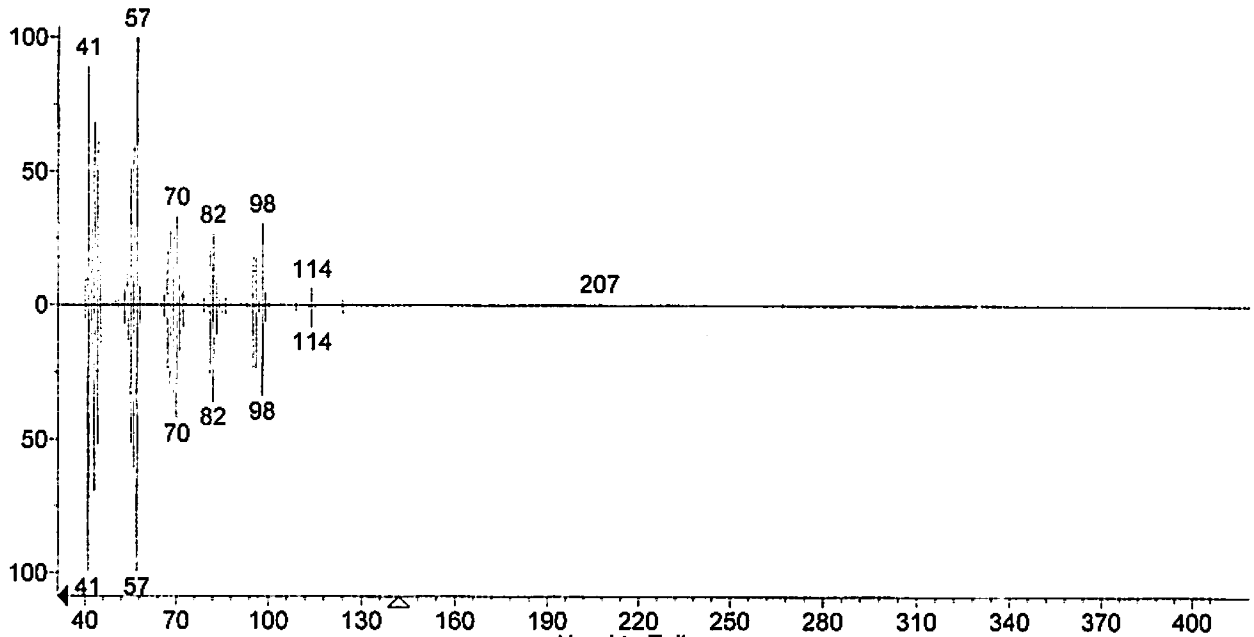

Head to Tail

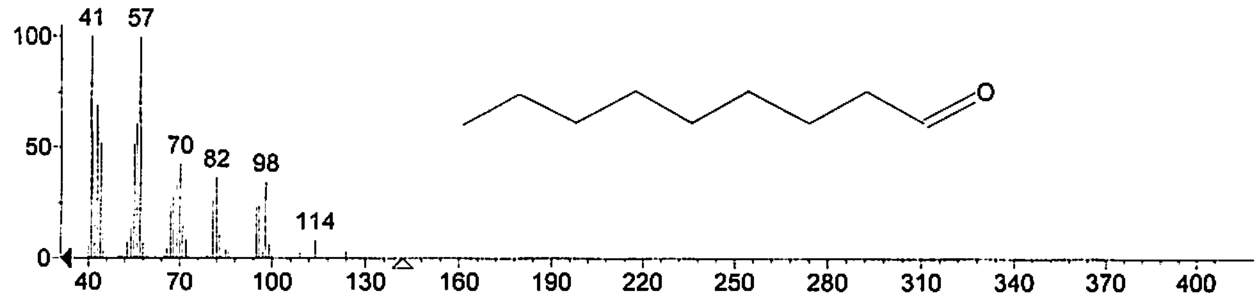

(replib) Nonanal

```
File        :D:\DATA\JA-08\Snapshot\JA0514-1.D
Operator    : Aldrich
Acquired    : 14 May 2008  14:42     using AcqMethod JA-BACK.M
Instrument  :    Instrument #1
Sample Name: male C. oculata (coll. 5/13) abdominal cut.
Misc Info   : GC run JA-05143.D; /CH2Cl2; trt IR+SK trap
Vial Number: 1
```

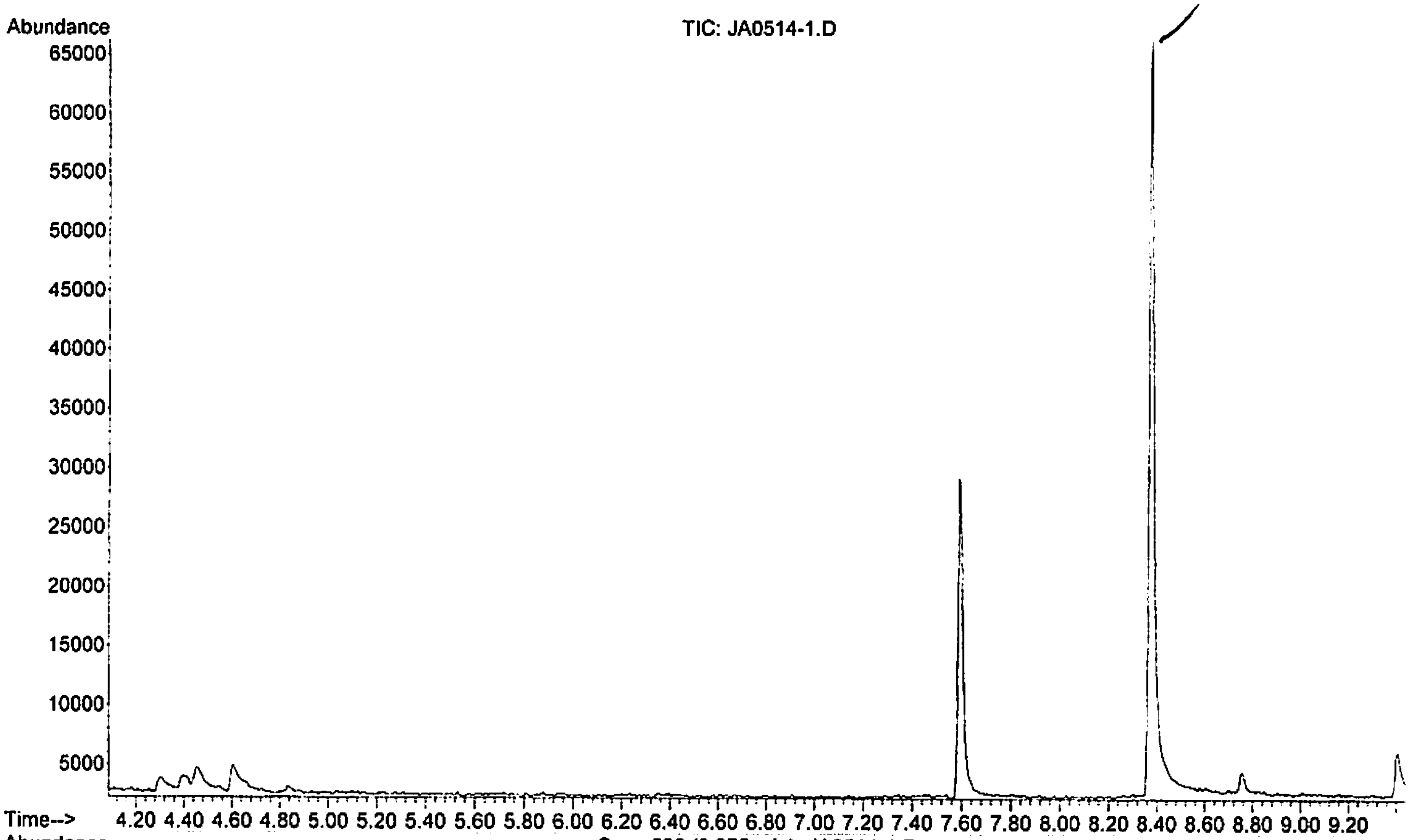

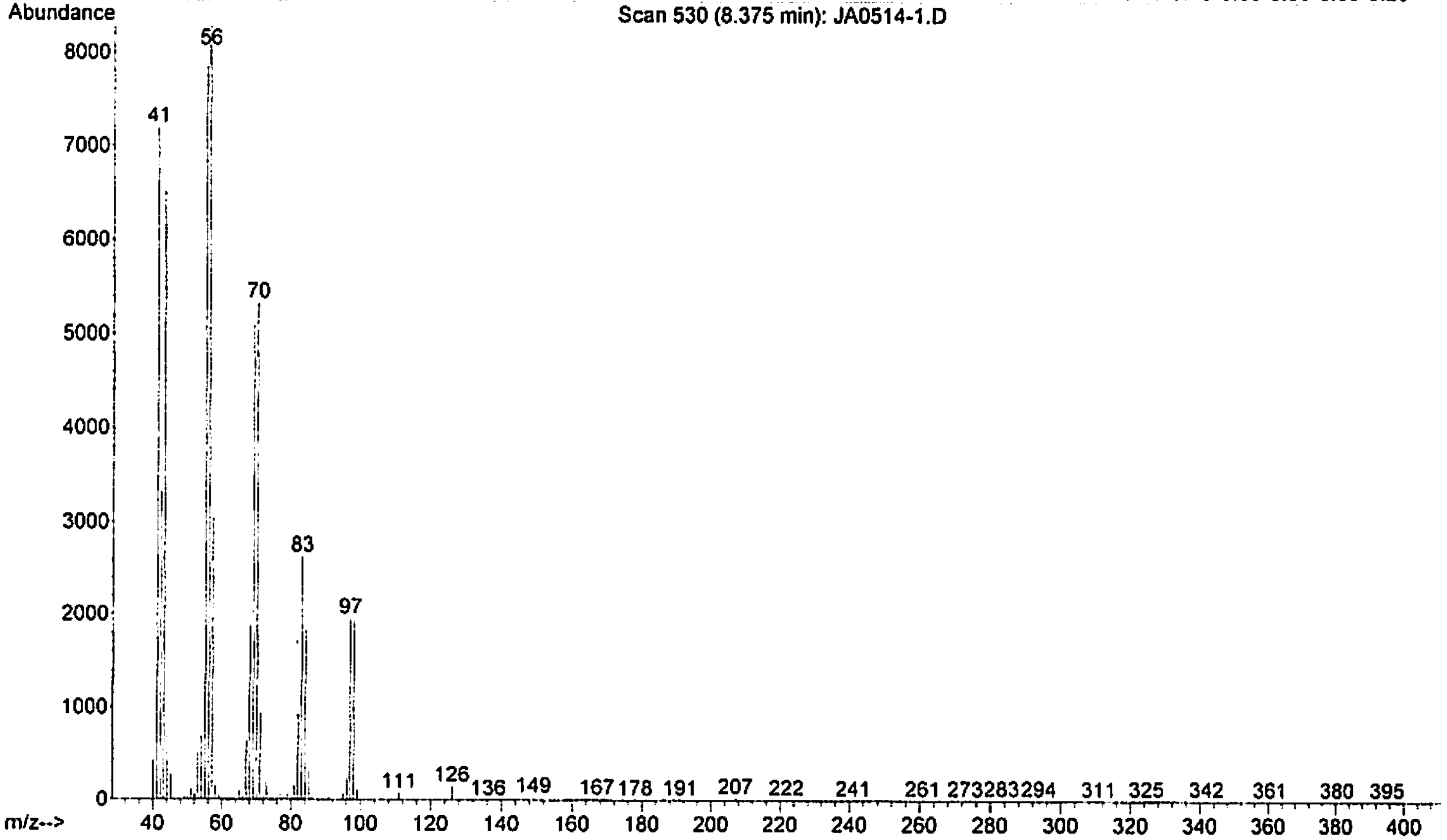

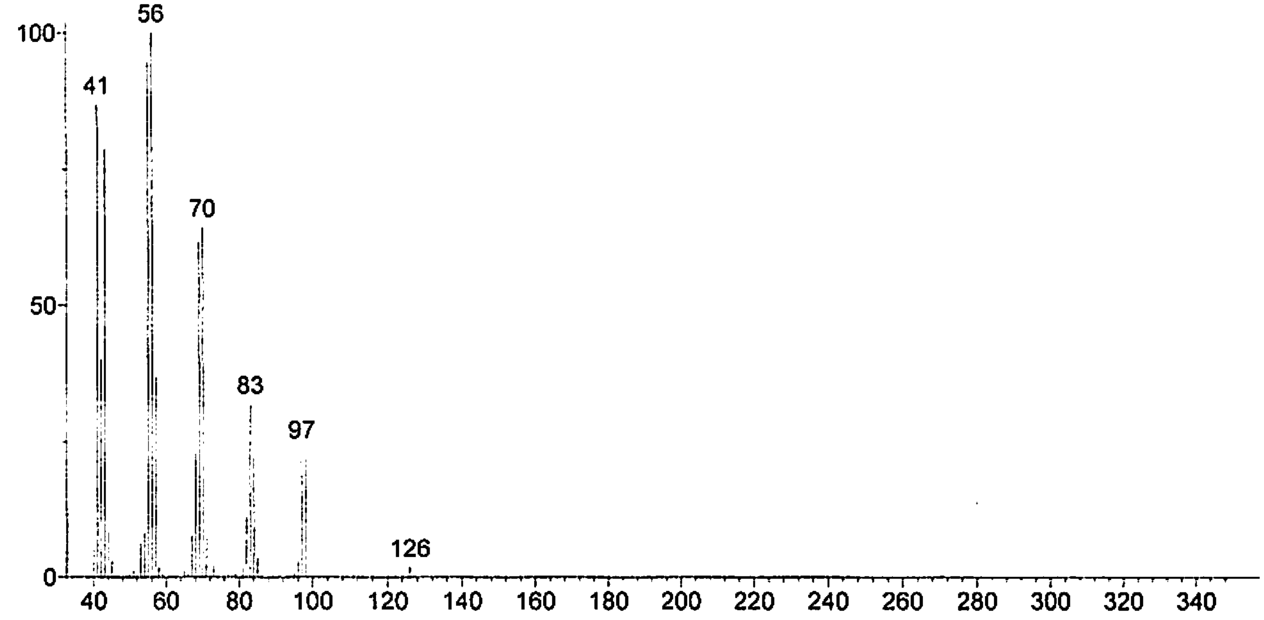

(Text File) Scan 530 (8.375 min): JA0514-1.D

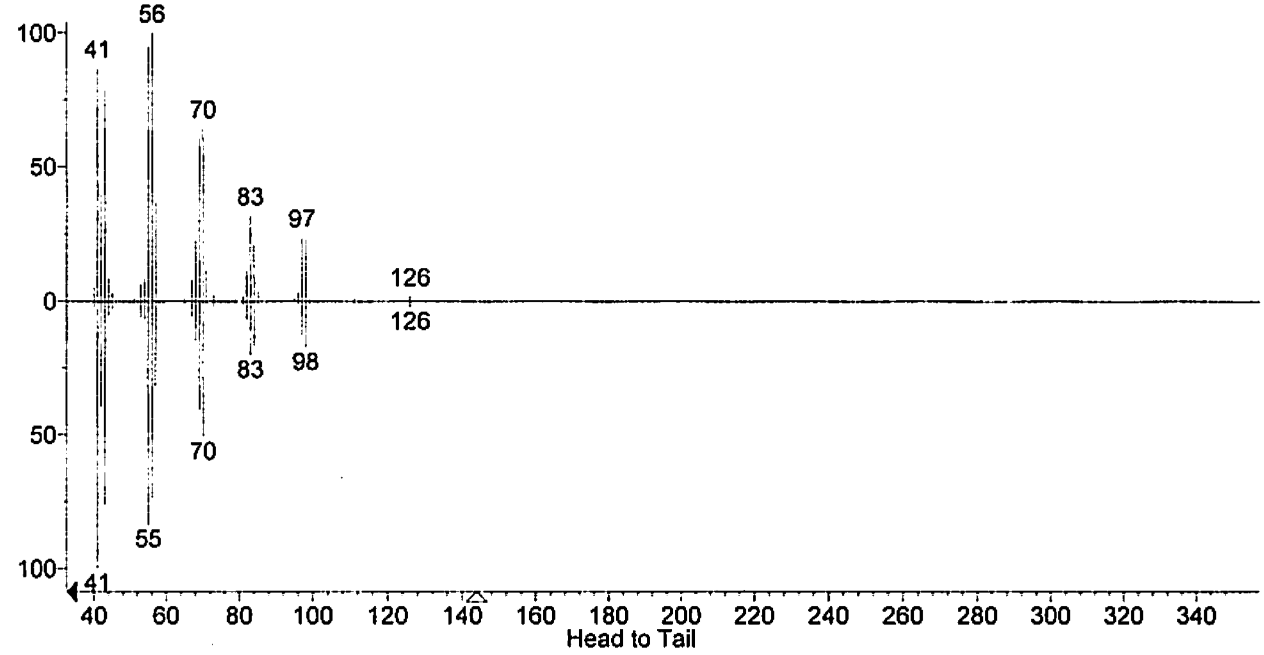

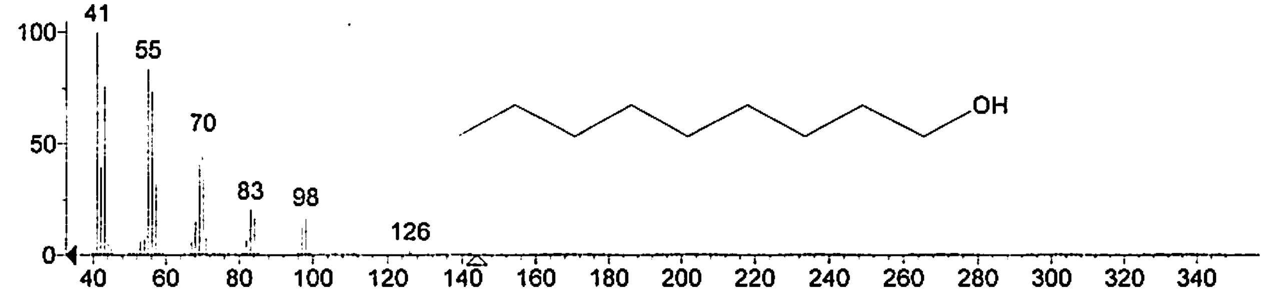

(replib) 1-Nonanol

File   :D:\DATA\JA-08\Snapshot\JA0514-1.D
Operator   : Aldrich
Acquired   : 14 May 2008 14:42   using AcqMethod JA-BACK.M
Instrument :   Instrument #1
Sample Name: male C. oculata (coll. 5/13) abdominal cut.
Misc Info   : GC run JA-05143.D; /CH2Cl2; trt IR+SK trap
Vial Number: 1

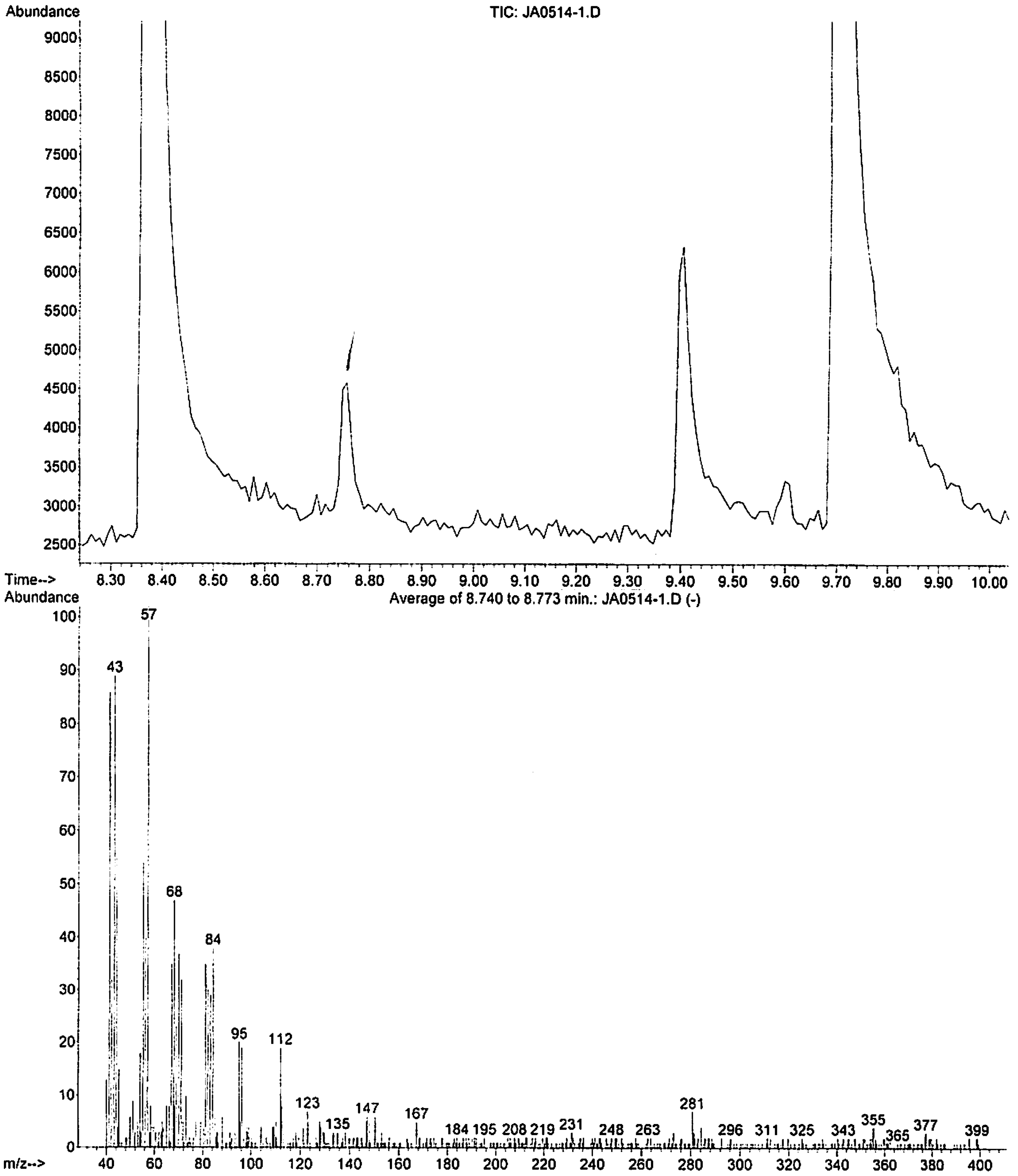

File :D:\DATA\JA-08\Snapshot\JA0514-1.D
Operator : Aldrich
Acquired : 14 May 2008 14:42 using AcqMethod JA-BACK.M
Instrument : Instrument #1
Sample Name: male C. oculata (coll. 5/13) abdominal cut.
Misc Info : GC run JA-05143.D; /CH2Cl2; trt IR+SK trap
Vial Number: 1

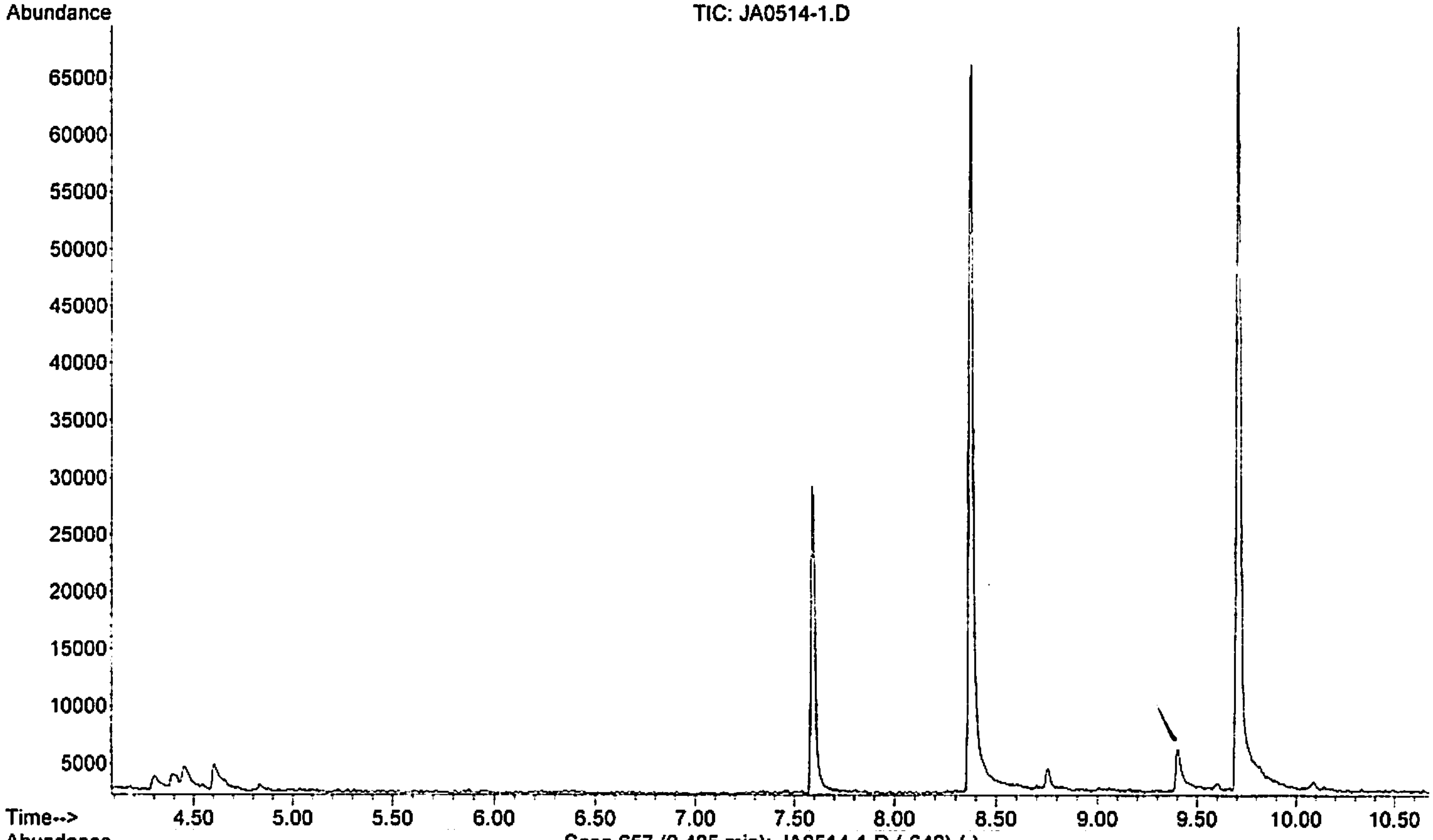

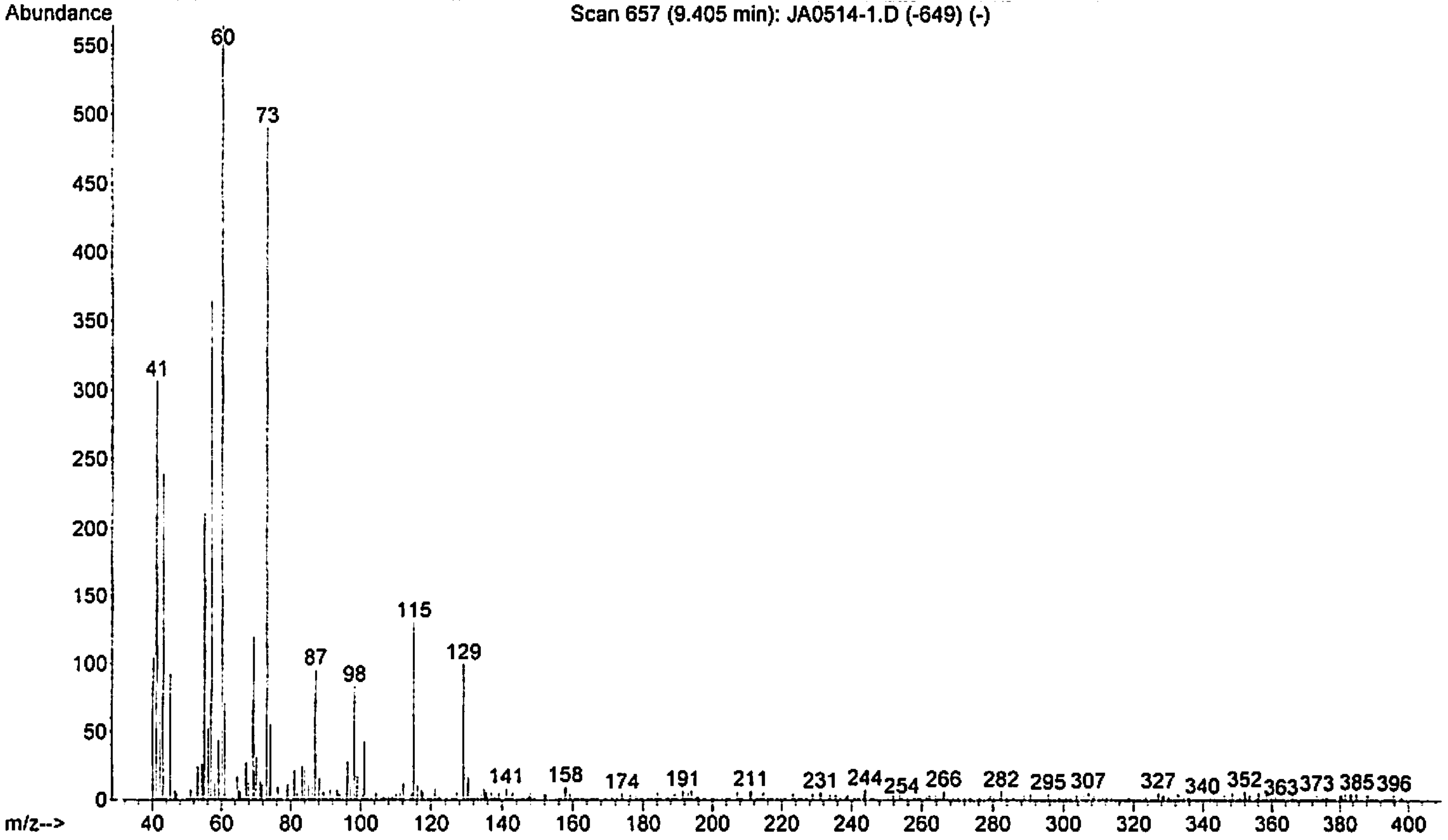

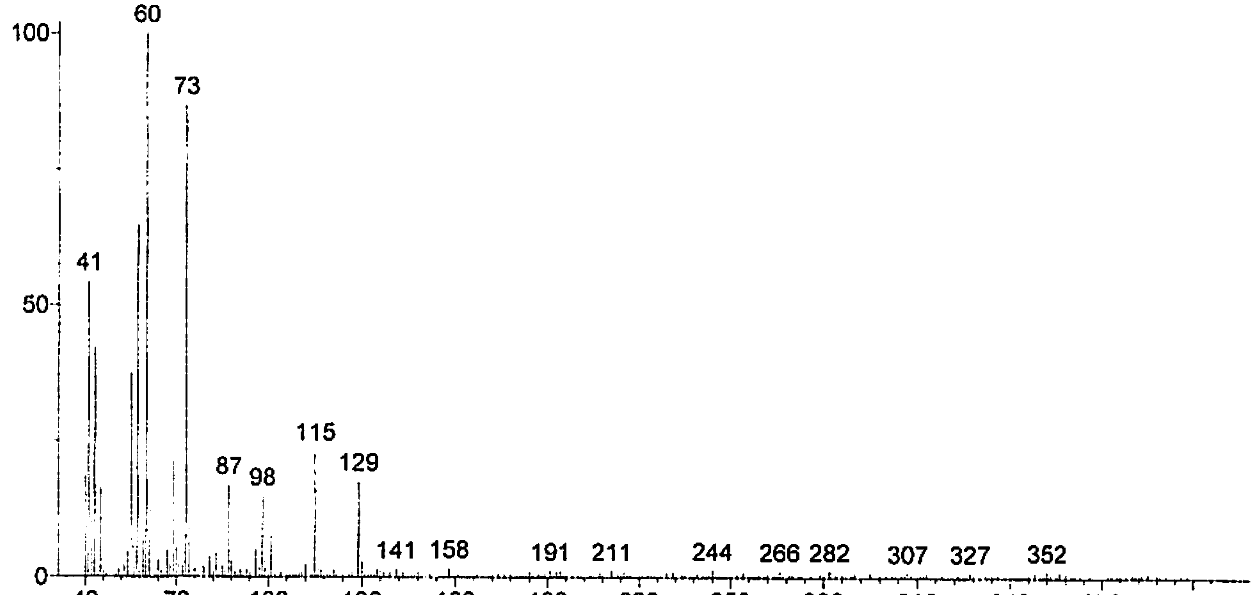

(Text File) Scan 657 (9.405 min): JA0514-1.D (-649)

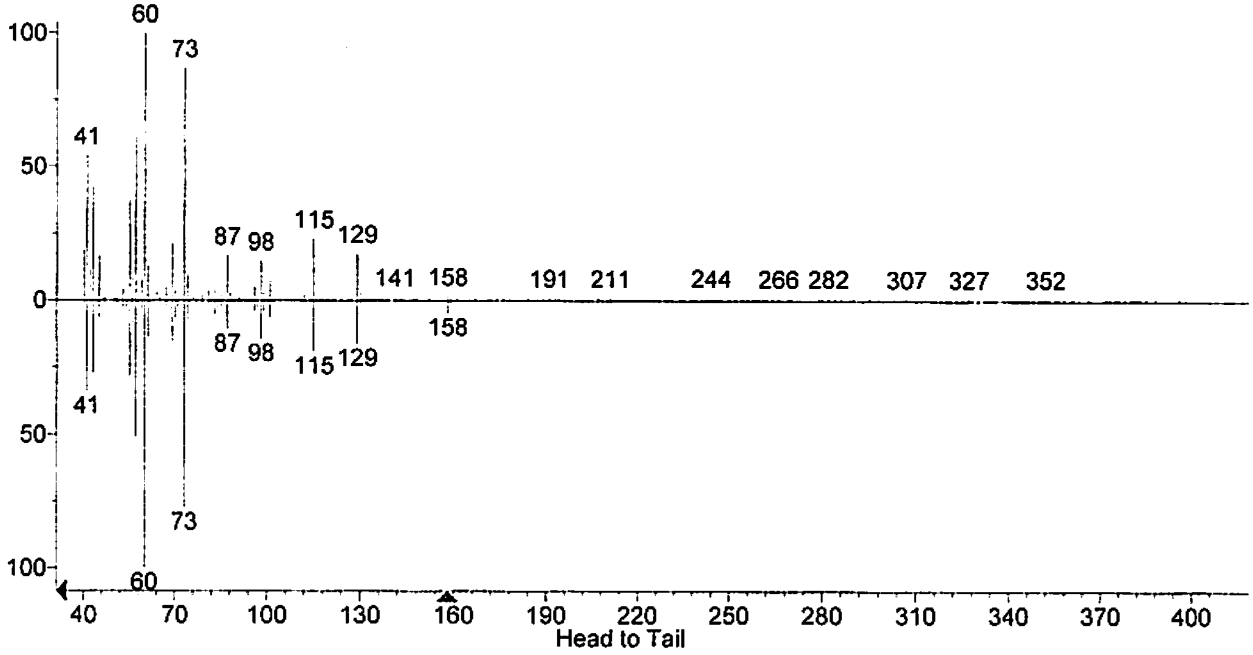

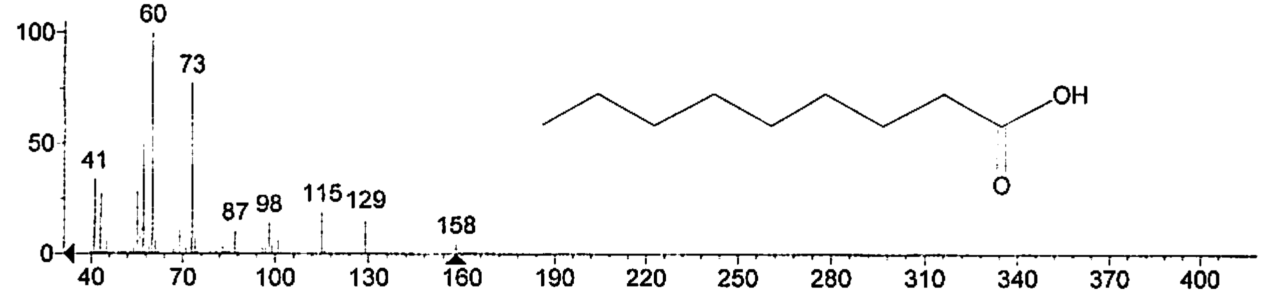

(mainlib) Nonanoic acid

File :D:\DATA\JA-08\Snapshot\JA0514-1.D
Operator : Aldrich
Acquired : 14 May 2008 14:42 using AcqMethod JA-BACK.M
Instrument : Instrument #1
Sample Name: male C. oculata (coll. 5/13) abdominal cut.
Misc Info : GC run JA-05143.D; /CH2Cl2; trt IR+SK trap
Vial Number: 1

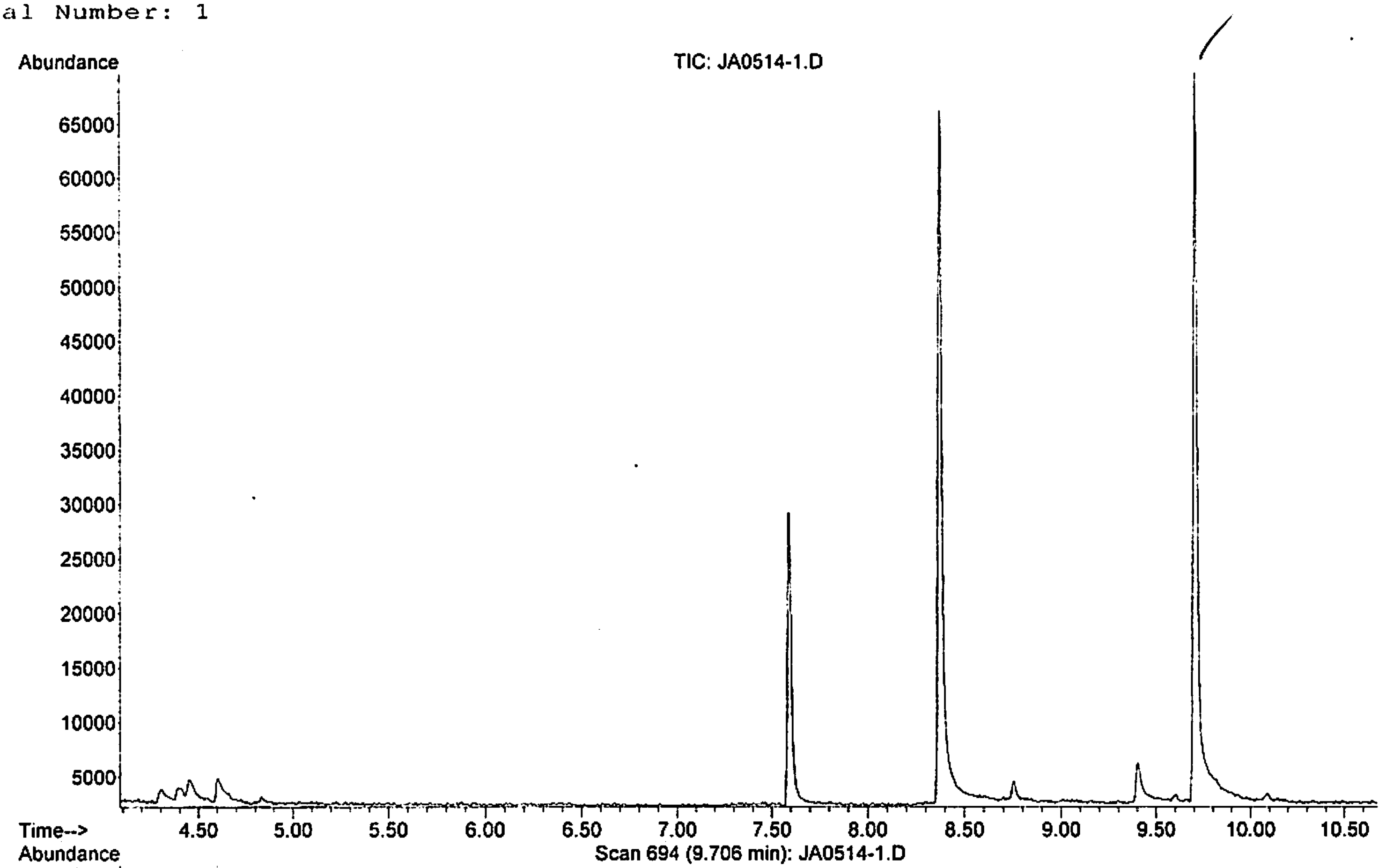

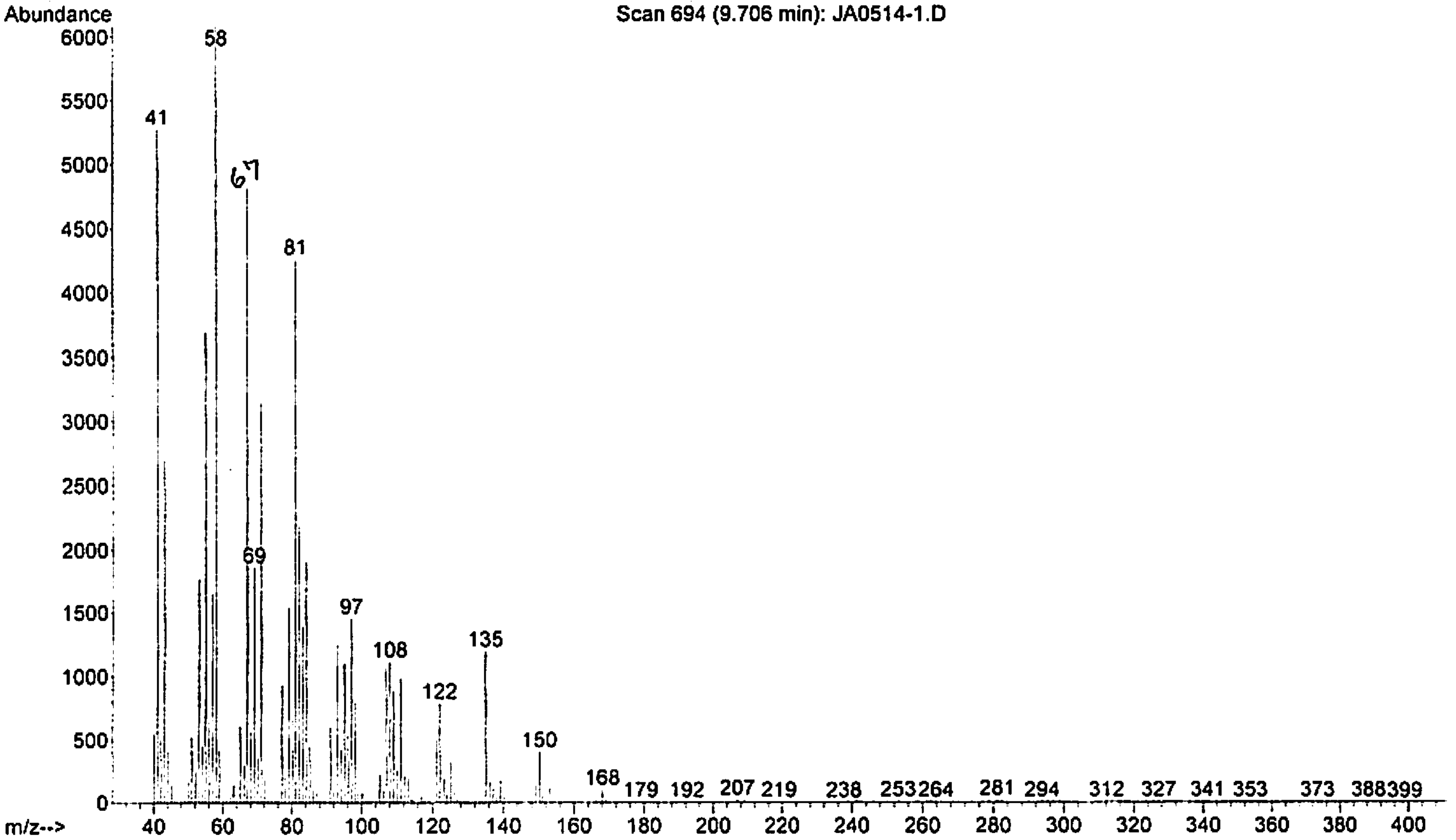

```
File        :D:\DATA\Aldrich\JA-09\JA051509-2.D
Operator    : Aldrich
Acquired    : 15 May 2009  13:47      using AcqMethod JA-WAX08.M
Instrument :    Instrument #1
Sample Name: 4 field coll. male C.oculata abd/2ul CH2Cl2
Misc Info   : Ed coll. sweeping vetch, 5/13&14; fed in lab
Vial Number: 1
```

♂ from sweeping 5/13-14/09
w/ MS + iridodial

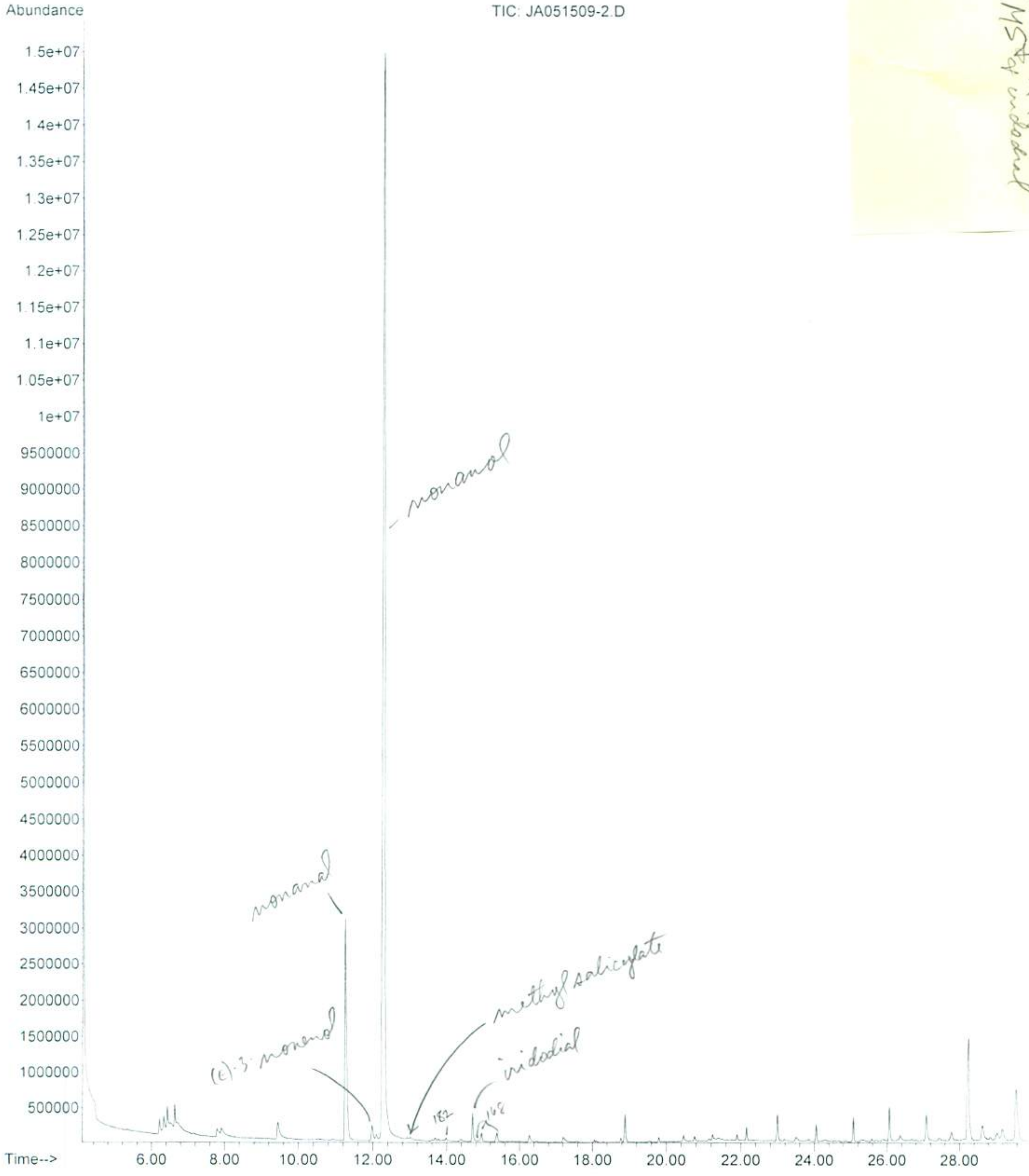

```
File        :D:\DATA\Aldrich\JA-09\JA051509-1.D
Operator    : Aldrich
Acquired    : 15 May 2009  11:19     using AcqMethod JA-WAX08.M
Instrument  :   Instrument #1
Sample Name: frozen Z,E-nepetalactol (0.5ug/ul CH2Cl2)
Misc Info   : test of GC-MS after not using for awhile...
Vial Number: 1
```

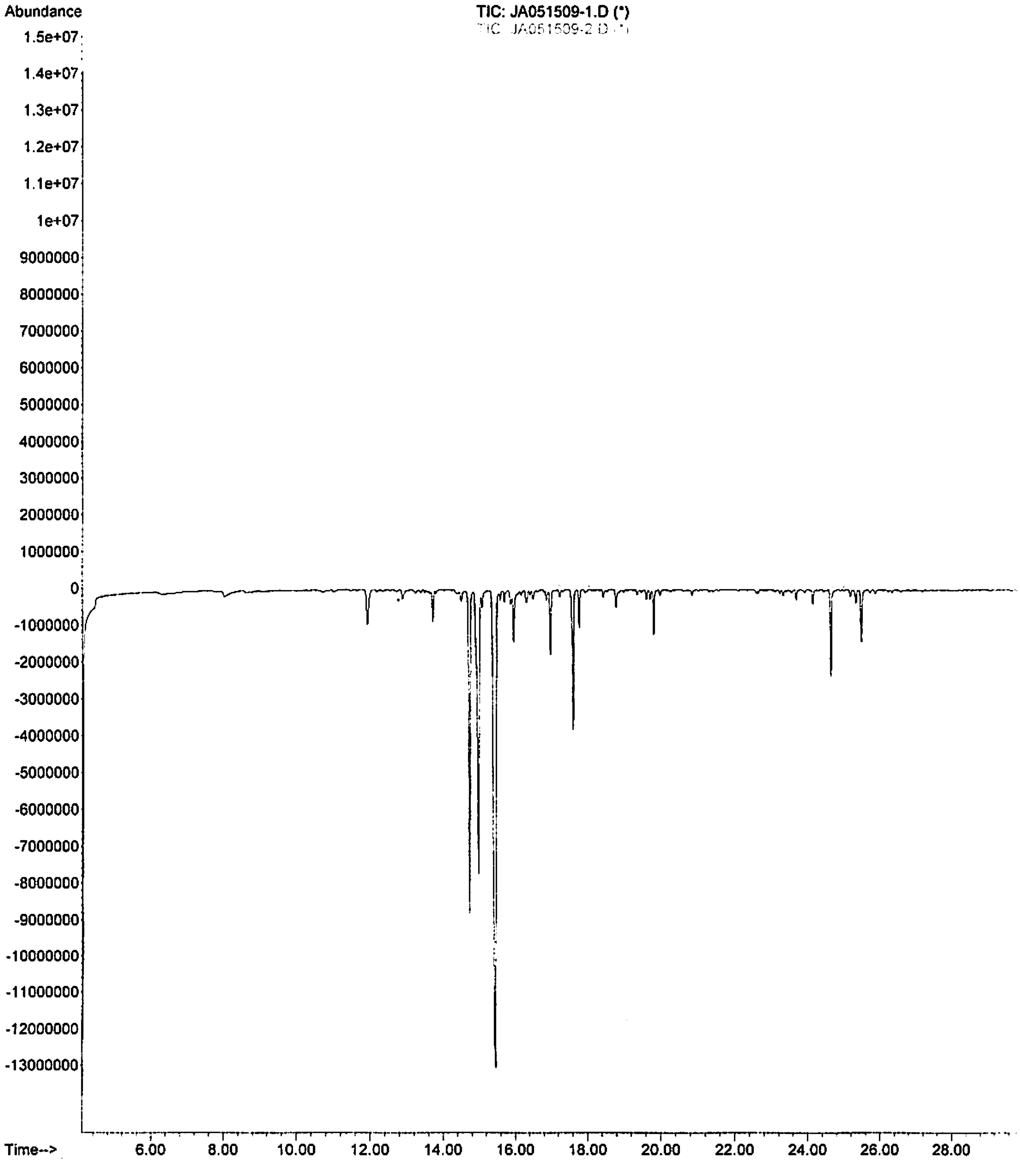

File   :D:\DATA\Aldrich\JA-09\JA051509-2.D
Operator   : Aldrich
Acquired   : 15 May 2009 13:47   using AcqMethod JA-WAX08.M
Instrument :   Instrument #1
Sample Name: 4 field coll. male C.oculata abd/2ul CH2Cl2
Misc Info   : Ed coll. sweeping vetch, 5/13&14; fed in lab
Vial Number: 1

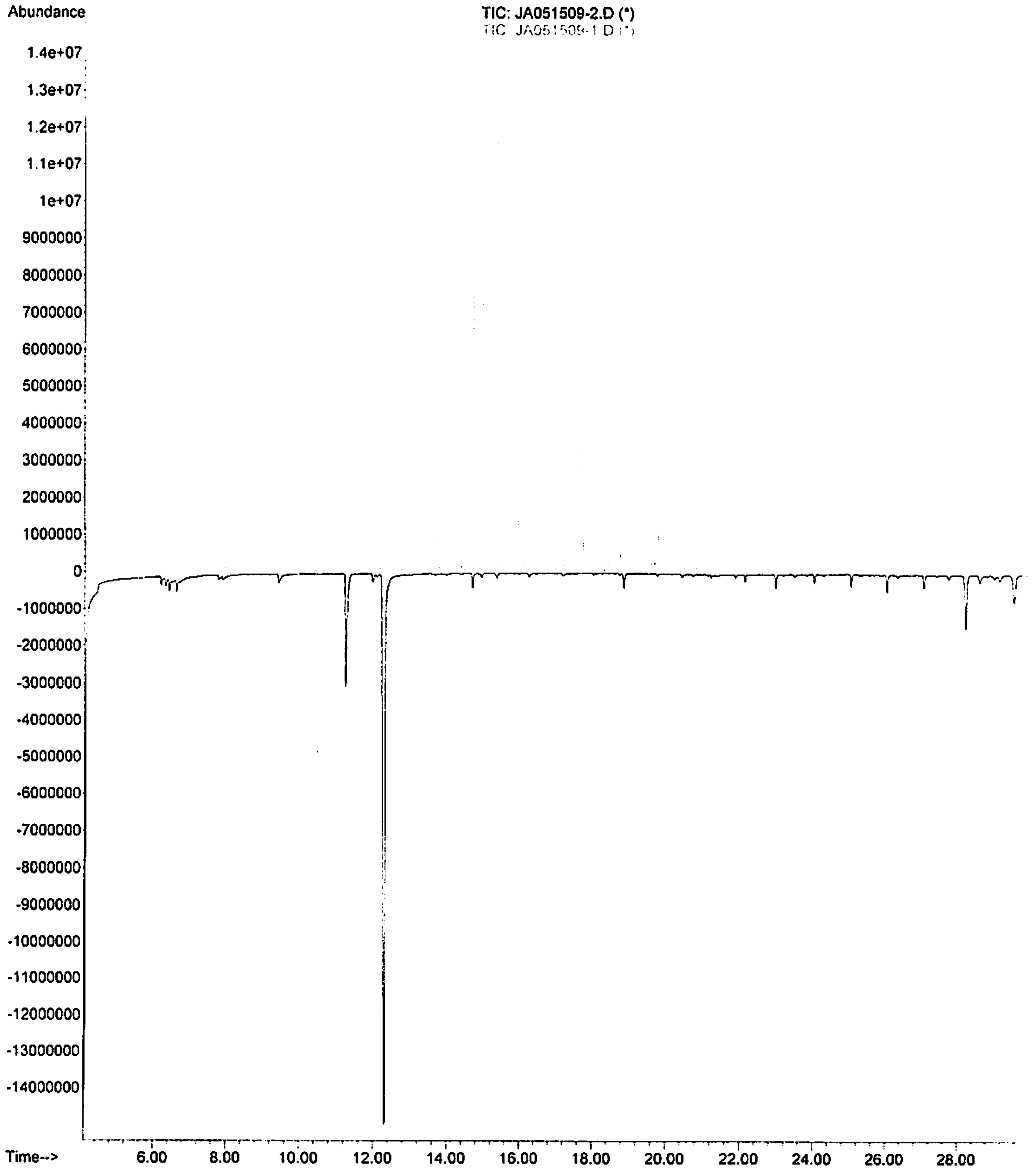

File : D:\DATA\ALDRICH\JA-09\Snapshot\JA051509-2.D
Operator : Aldrich
Acquired : 15 May 2009 13:47 using AcqMethod JA-WAX08.M
Instrument : Instrument #1
Sample Name: 4 field coll. male C.oculata abd/2ul CH2Cl2
Misc Info : Ed coll. sweeping vetch, 5/13&14; fed in lab
Vial Number: 1

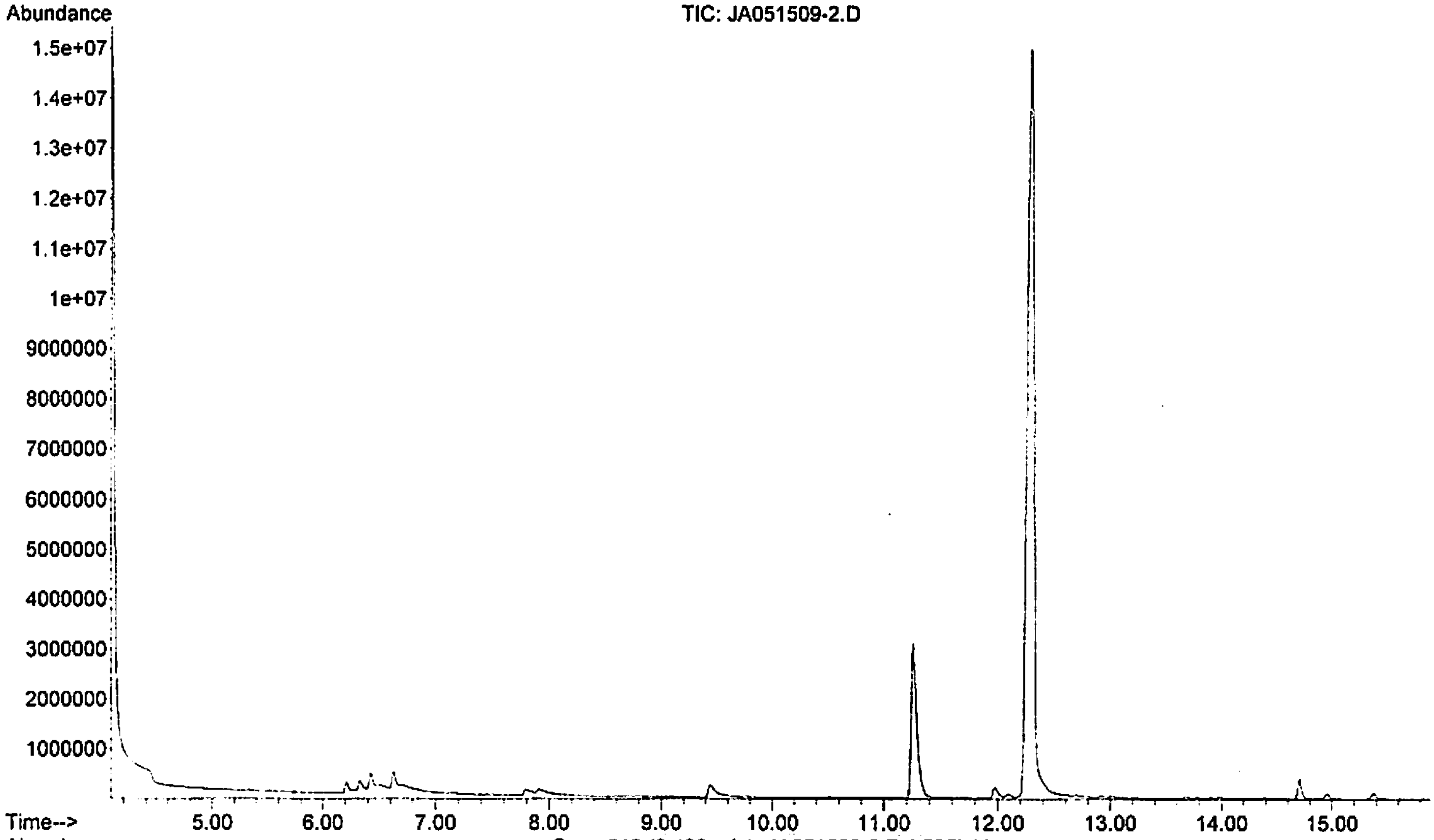

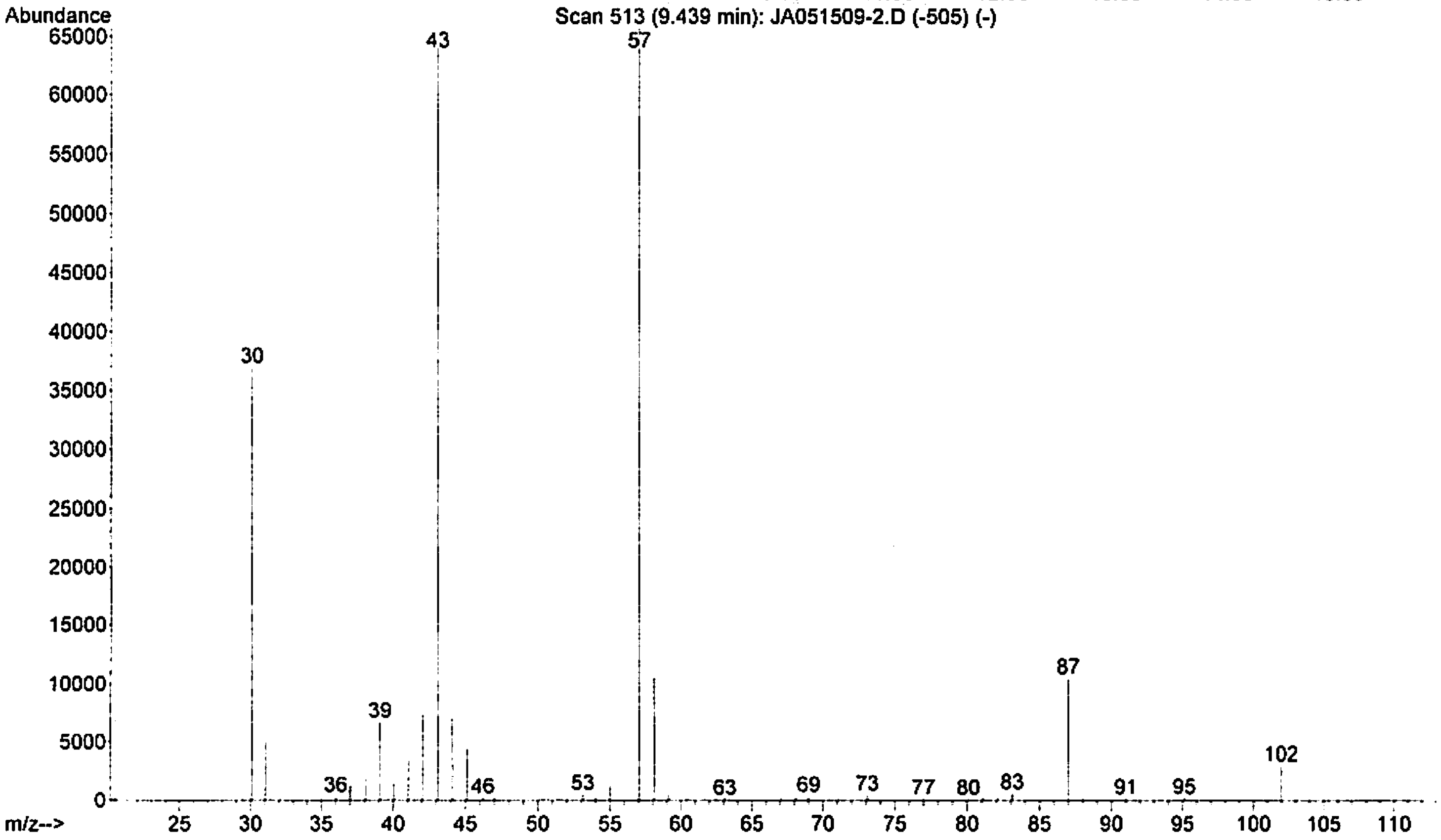

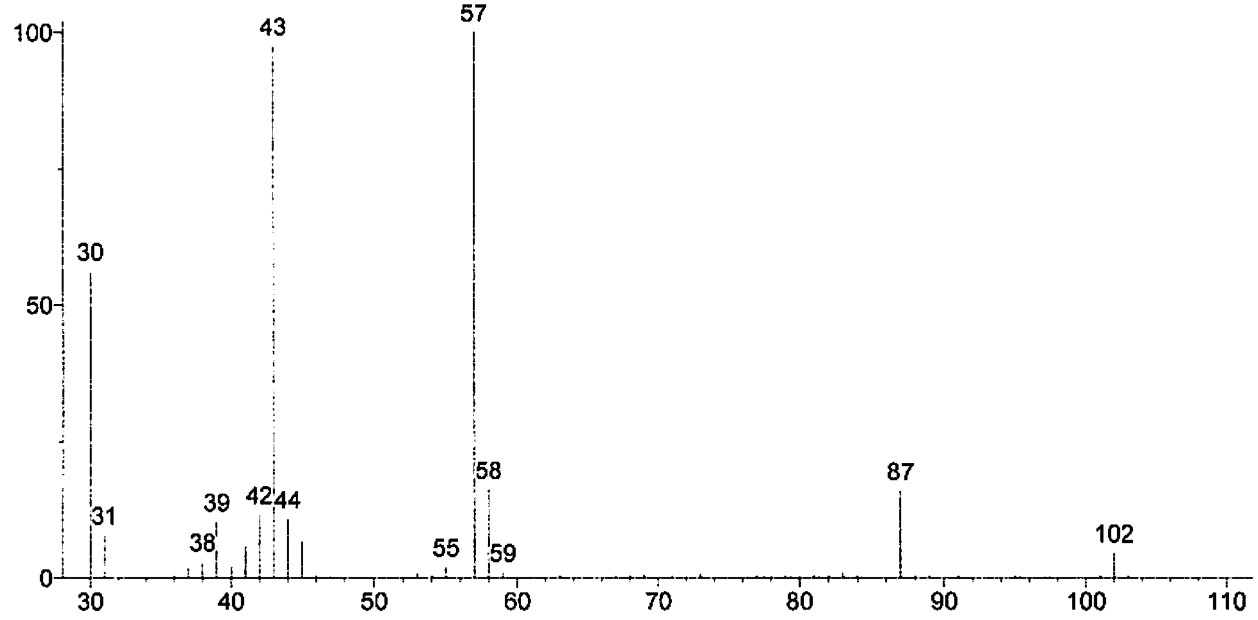

(Text File) Scan 513 (9.439 min): JA051509-2.D (-505)

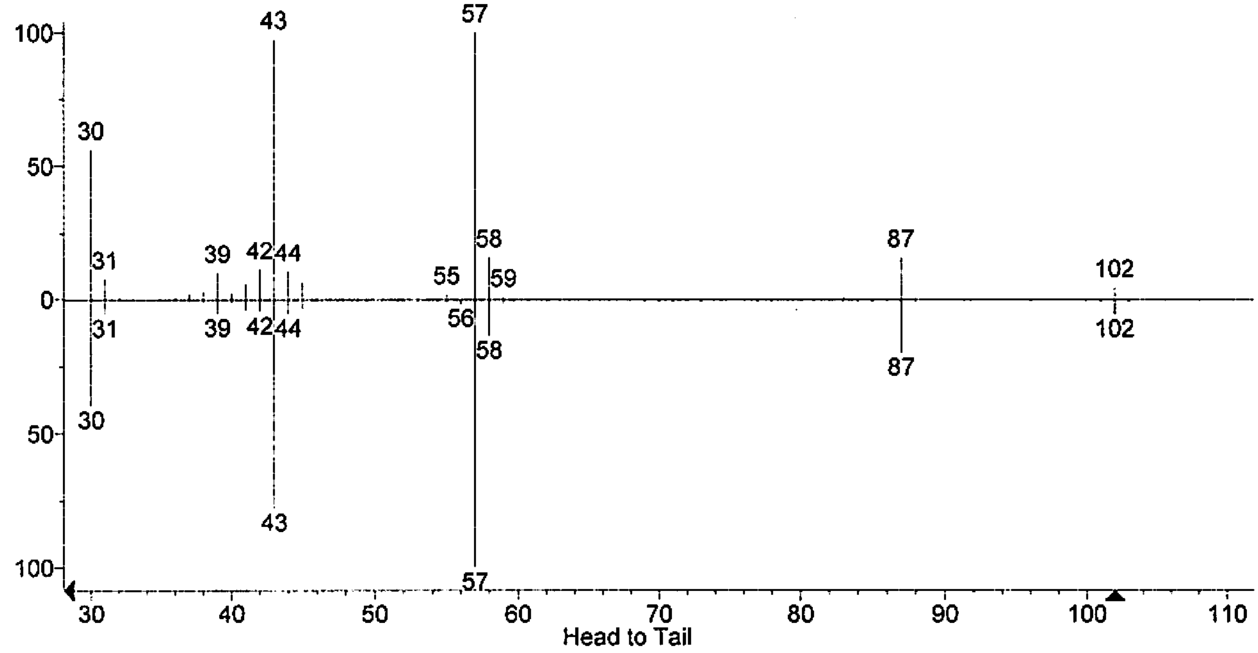

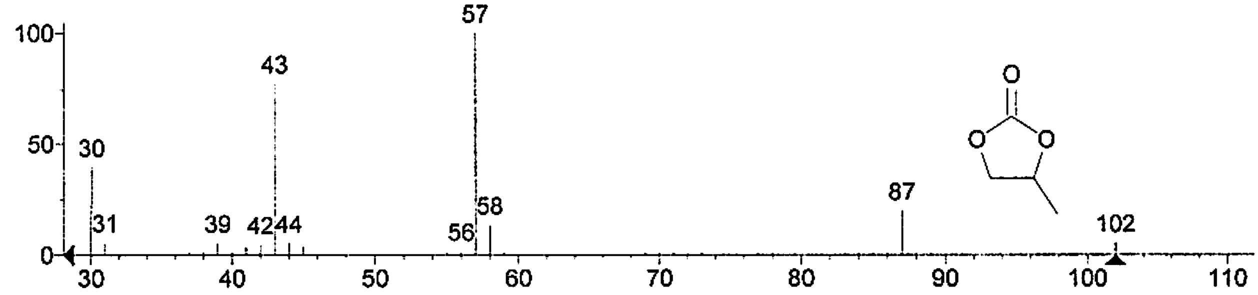

(mainlib) Propylene Carbonate

File : D:\DATA\ALDRICH\JA-09\Snapshot\JA051509-2.D
Operator : Aldrich
Acquired : 15 May 2009 13:47 using AcqMethod JA-WAX08.M
Instrument : Instrument #1
Sample Name: 4 field coll. male C.oculata abd/2ul CH2Cl2
Misc Info : Ed coll. sweeping vetch, 5/13&14; fed in lab
Vial Number: 1

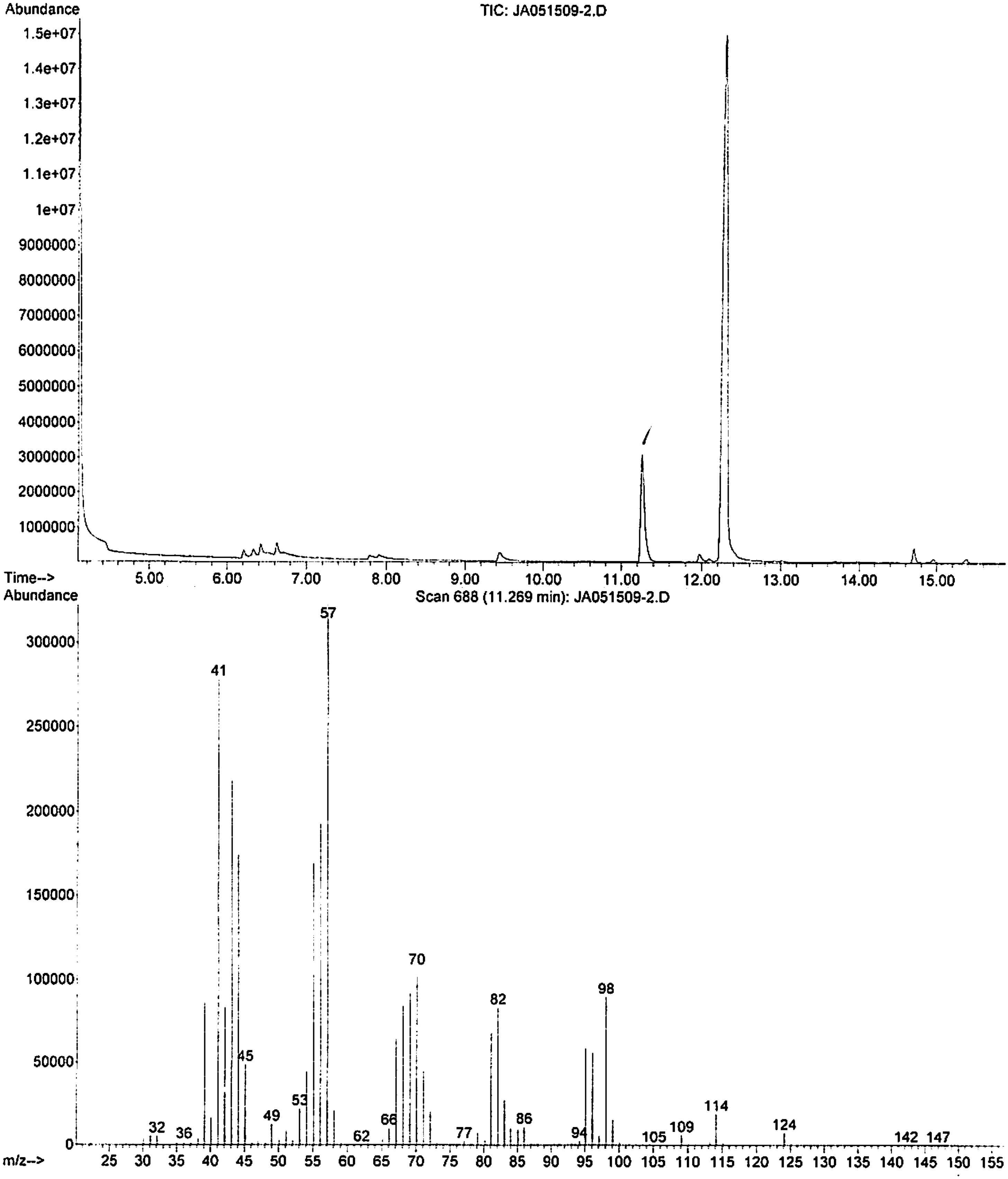

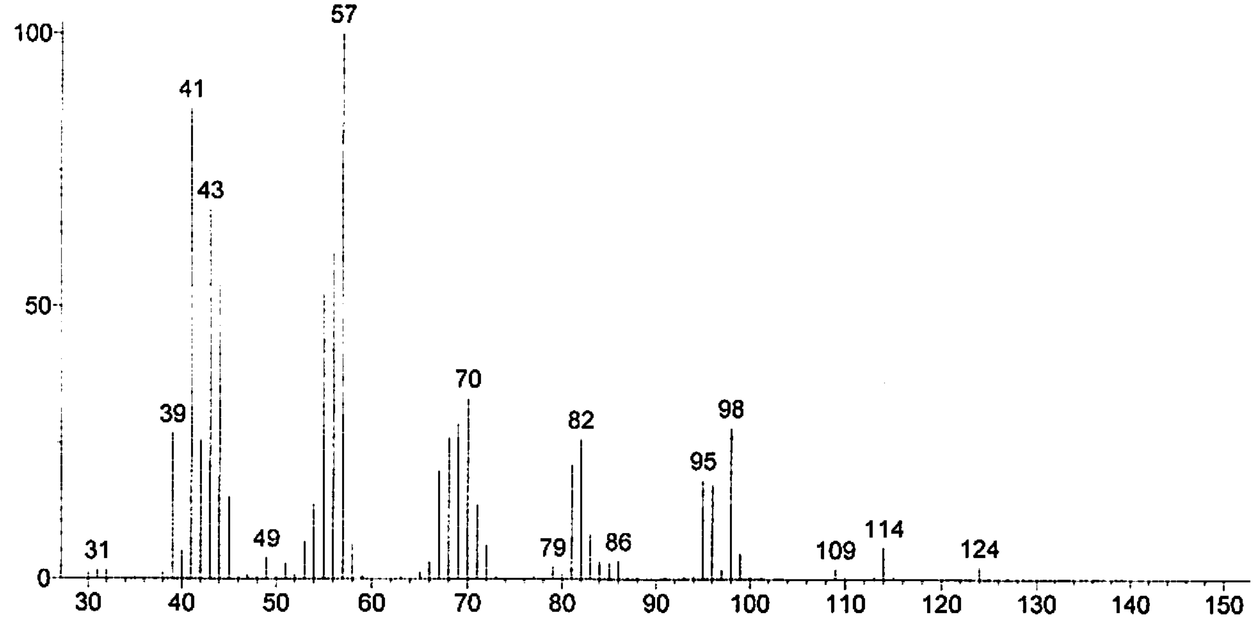

(Text File) Scan 688 (11.269 min): JA051509-2.D

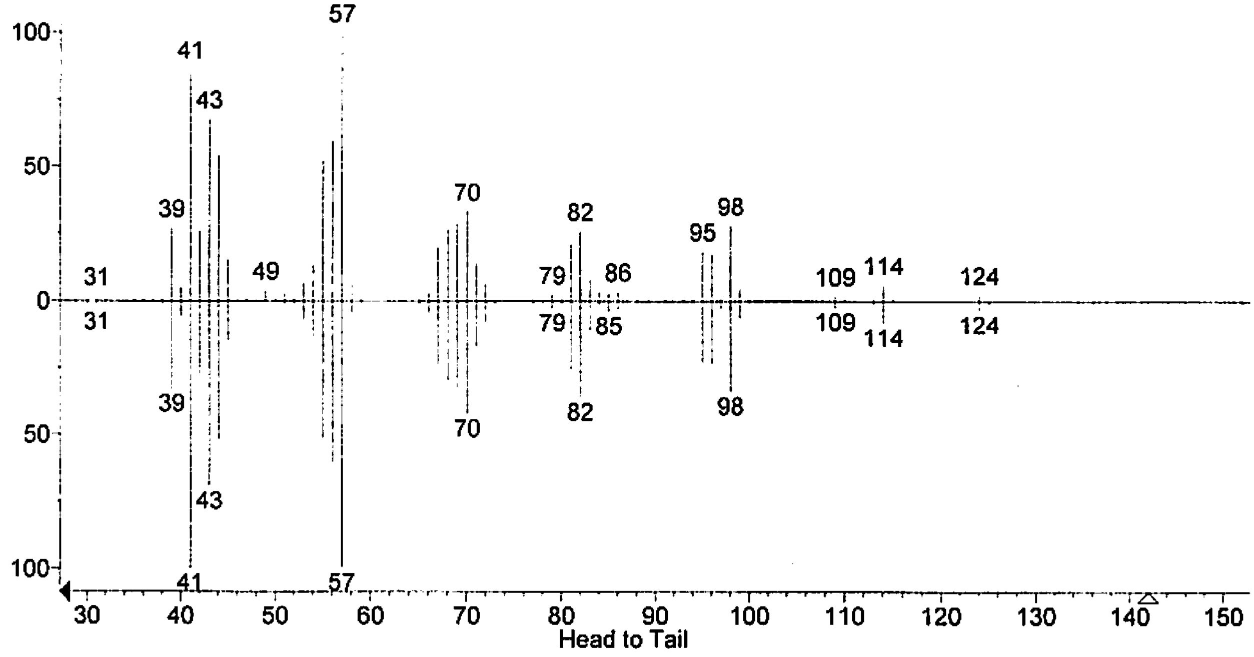

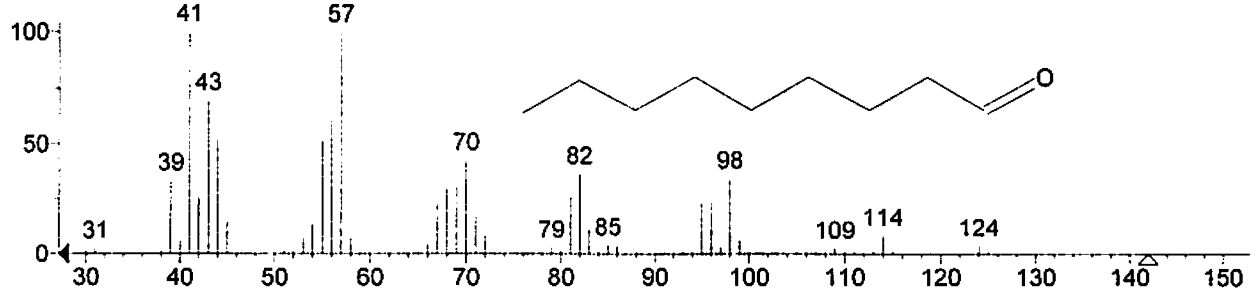

(replib) Nonanal

```
File      :D:\DATA\ALDRICH\JA-09\Snapshot\JA051509-2.D
Operator   : Aldrich
Acquired   : 15 May 2009  13:47     using AcqMethod JA-WAX08.M
Instrument :    Instrument #1
Sample Name: 4 field coll. male C.oculata abd/2ul CH2Cl2
Misc Info  : Ed coll. sweeping vetch, 5/13&14; fed in lab
Vial Number: 1
```

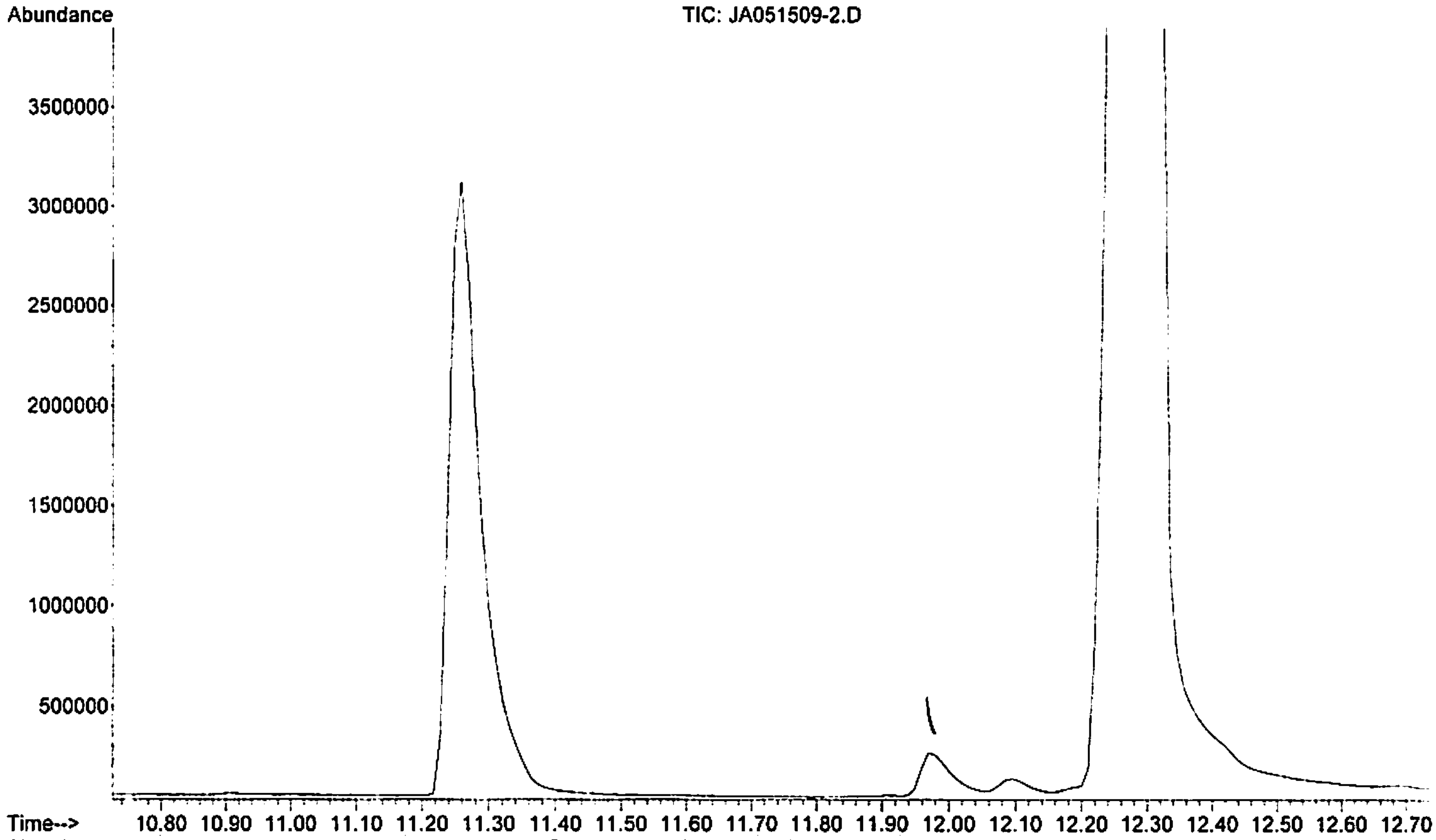

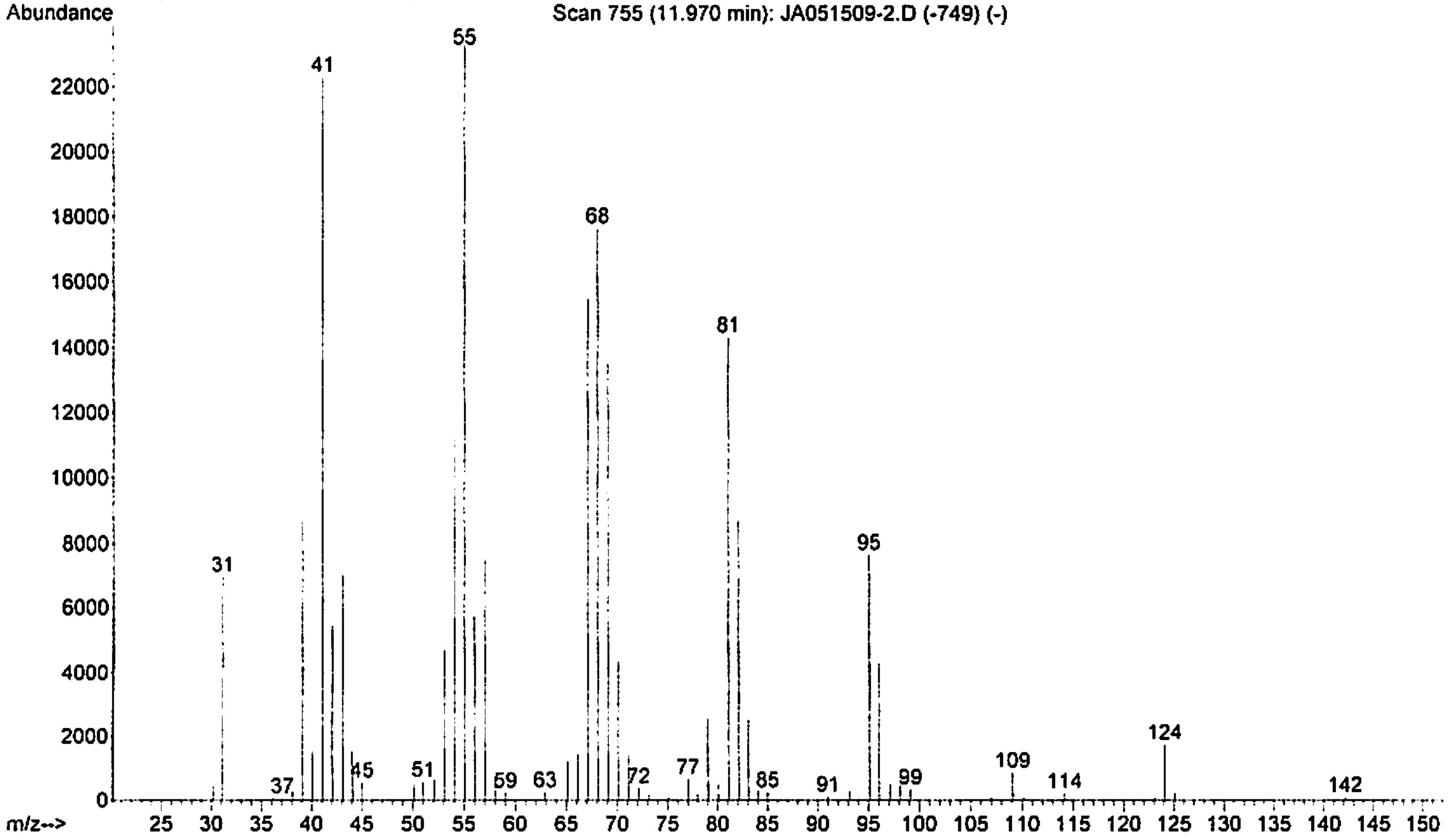

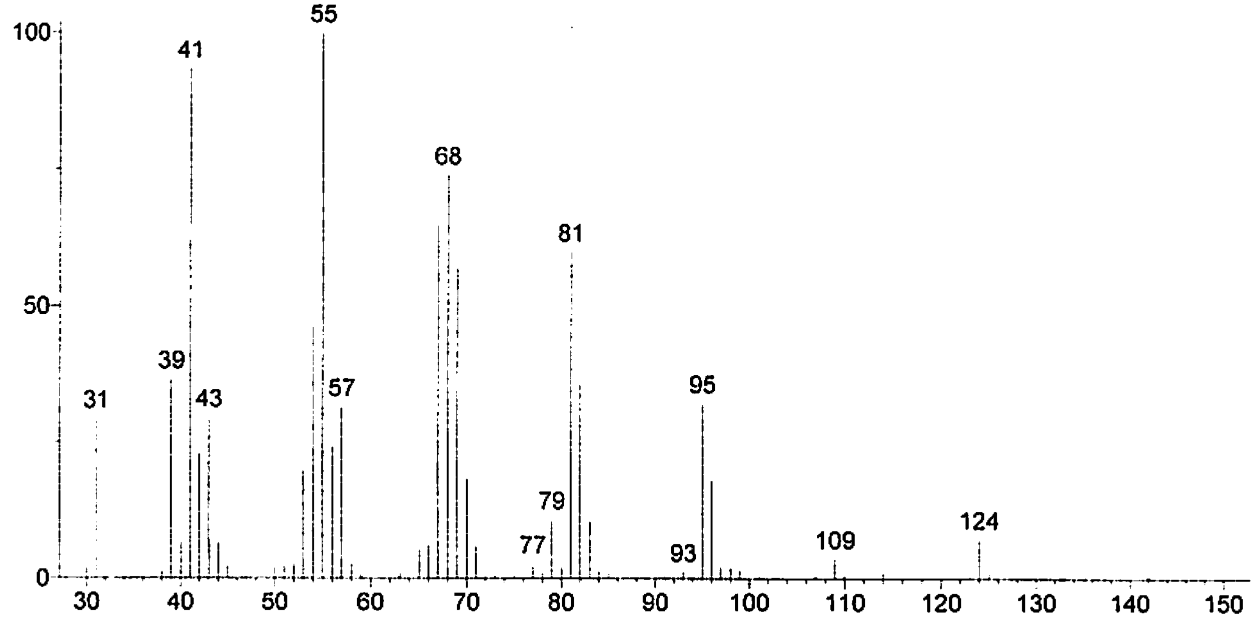

(Text File) Scan 755 (11.970 min): JA051509-2.D (-749)

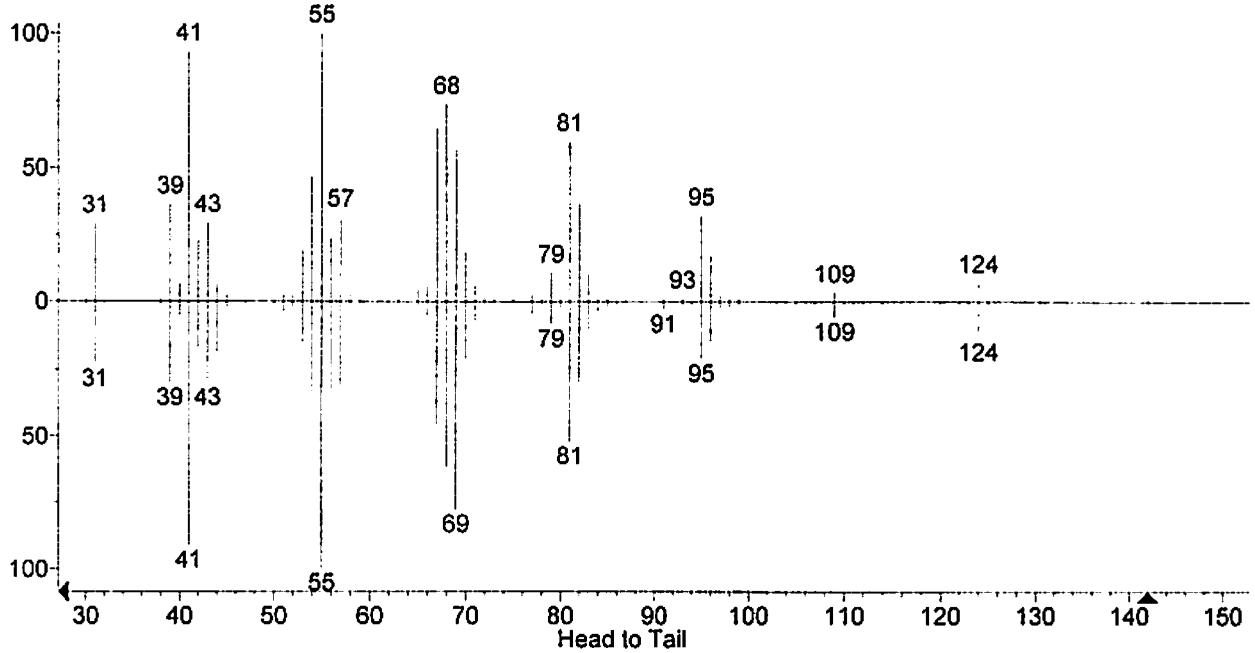

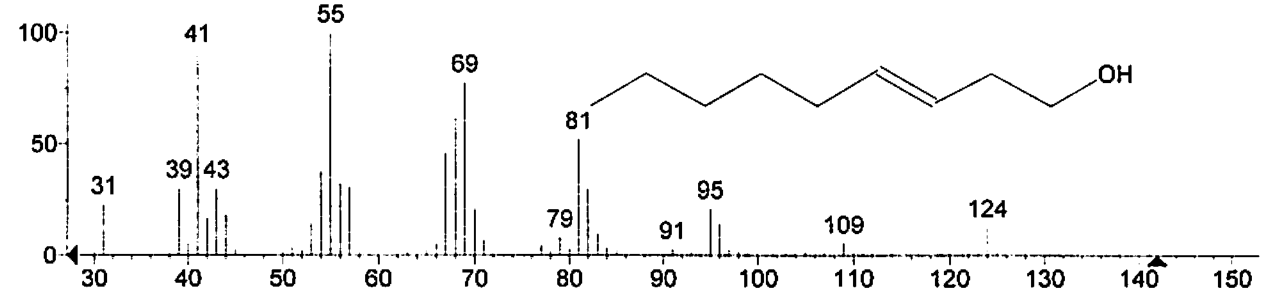

(mainlib) 3-Nonen-1-ol, (E)-

[illegible]e :D:\DATA\ALDRICH\JA-09\Snapshot\JA051509-2.D
[illegible]erator : Aldrich
[illegible]quired : 15 May 2009 13:47 using AcqMethod JA-WAX08.M
[illegible]strument : Instrument #1
[illegible]mple Name: 4 field coll. male C.oculata abd/2ul CH2Cl2
[illegible]sc Info : Ed coll. sweeping vetch, 5/13&14; fed in lab
[illegible]al Number: 1

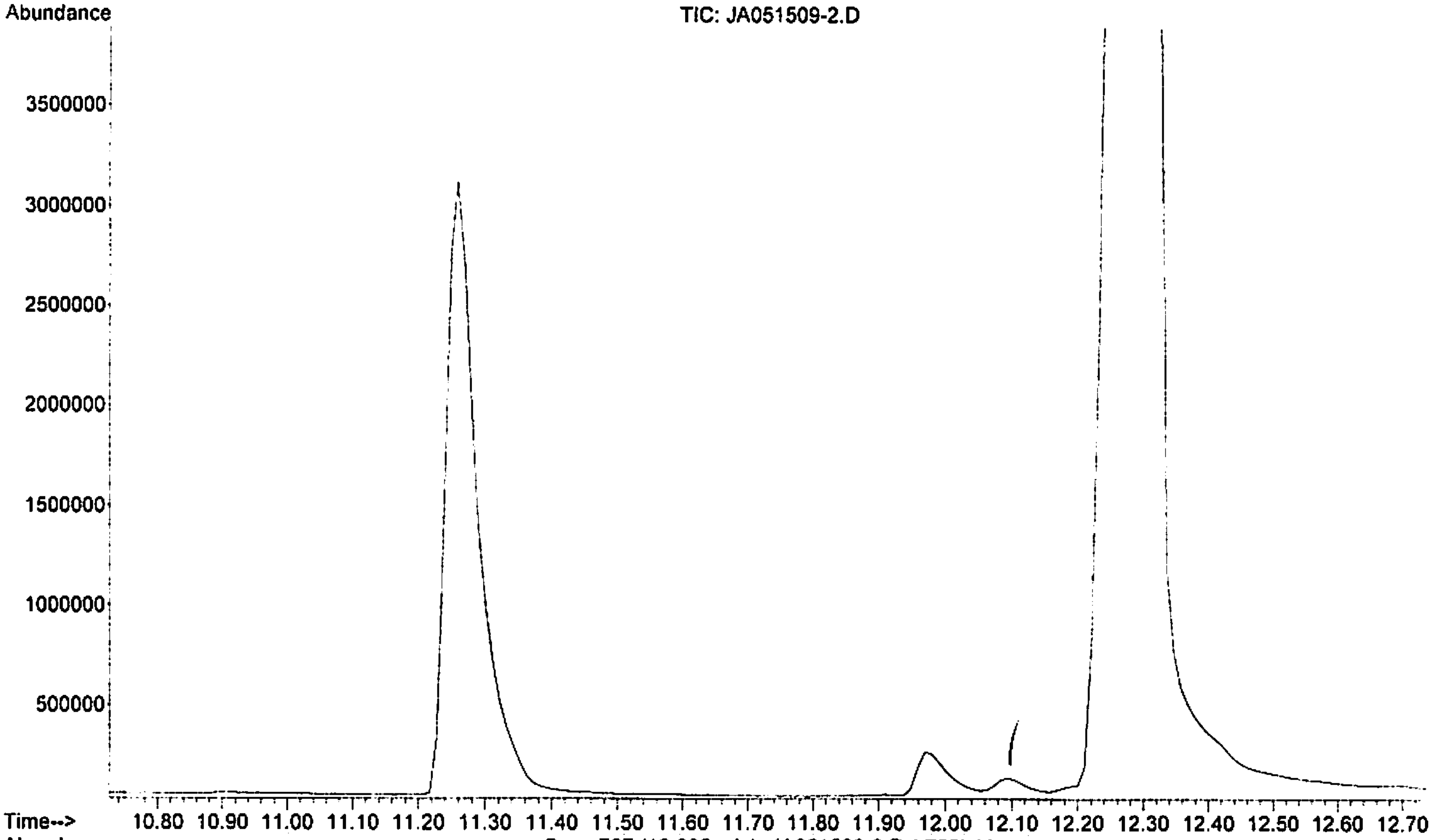

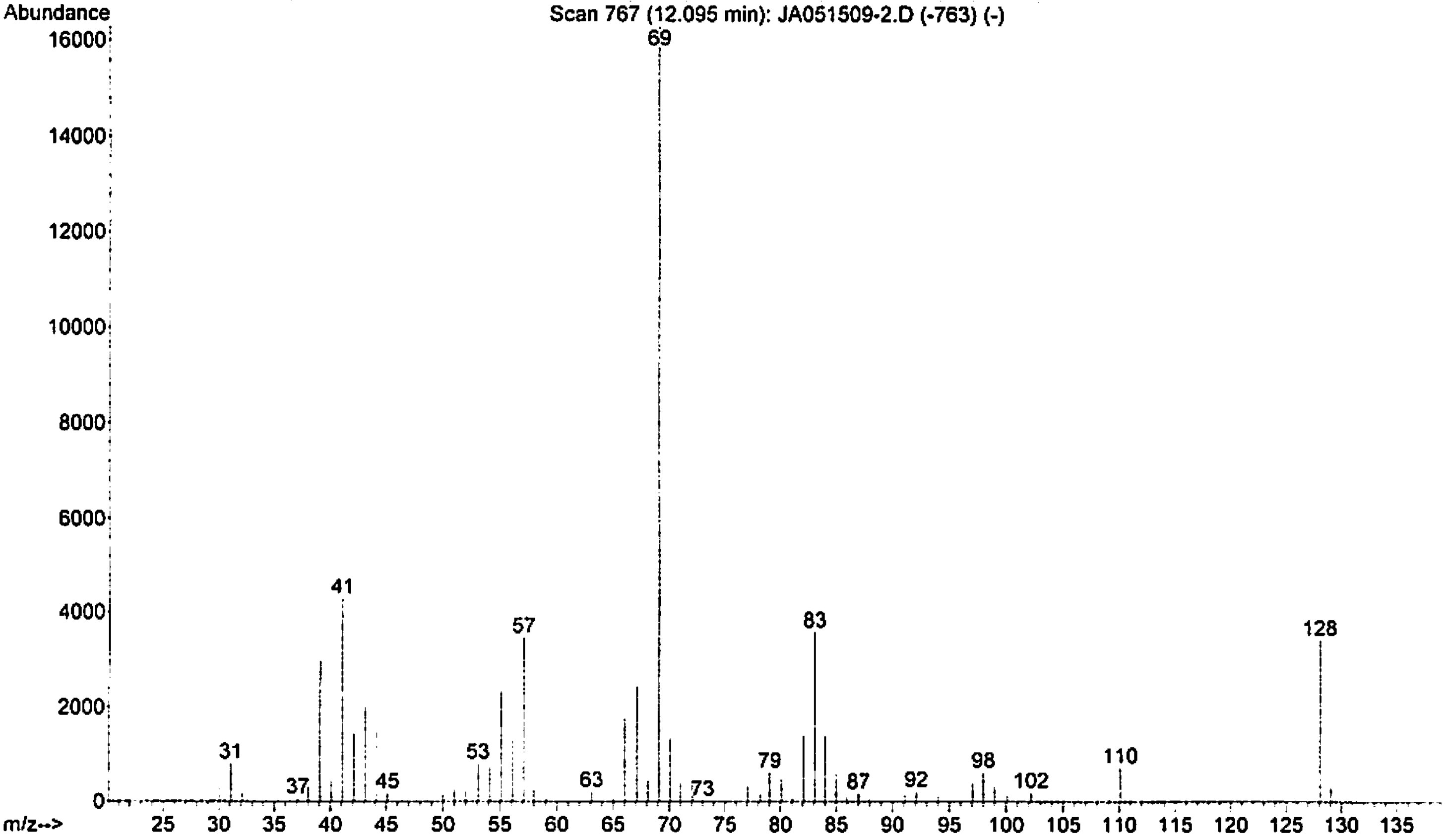

```
File       :D:\DATA\ALDRICH\JA-09\Snapshot\JA051509-2.D
Operator   : Aldrich
Acquired   : 15 May 2009  13:47     using AcqMethod JA-WAX08.M
Instrument :     Instrument #1
Sample Name: 4 field coll. male C.oculata abd/2ul CH2Cl2
Misc Info  : Ed coll. sweeping vetch, 5/13&14; fed in lab
Vial Number: 1
```

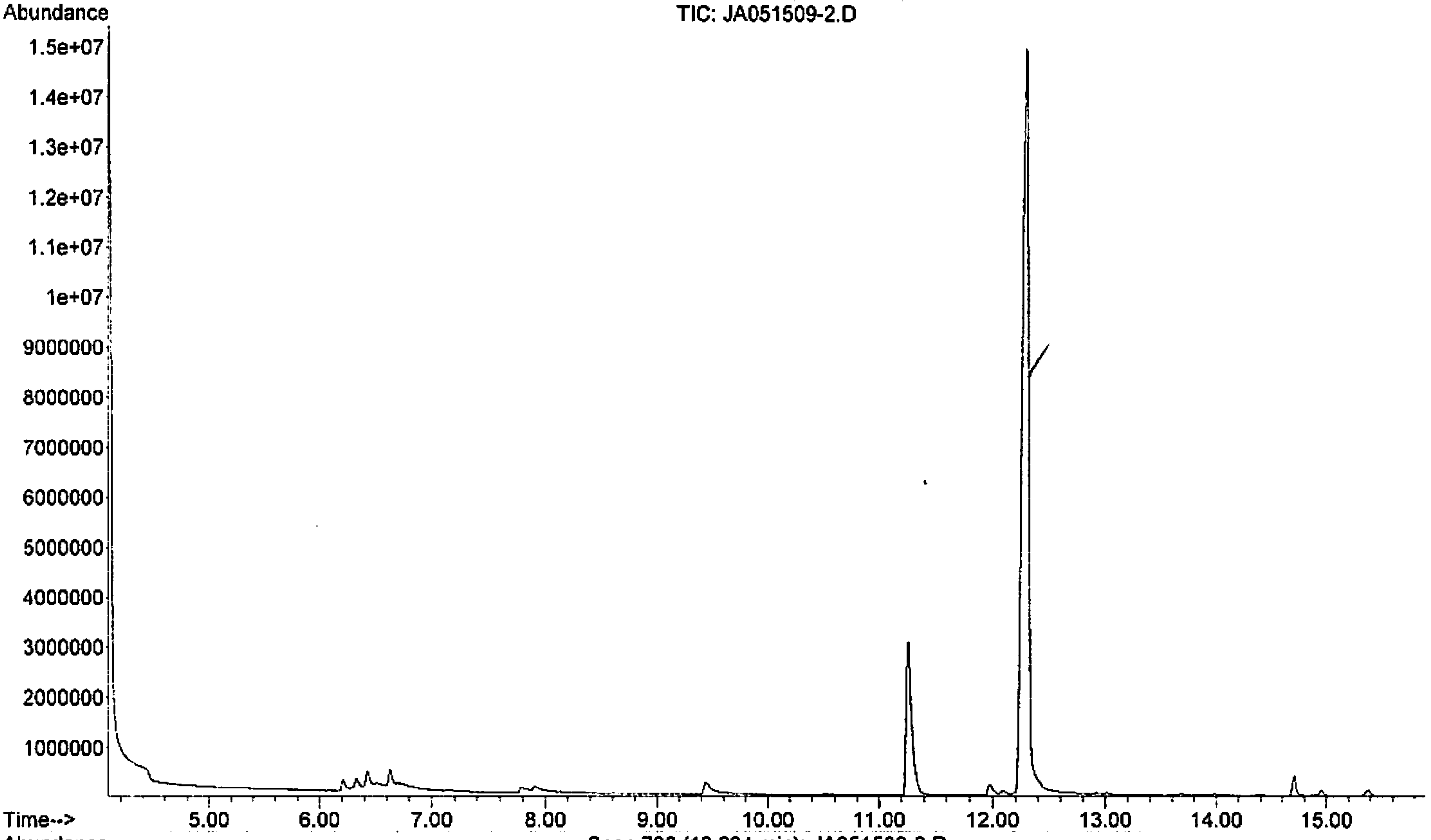

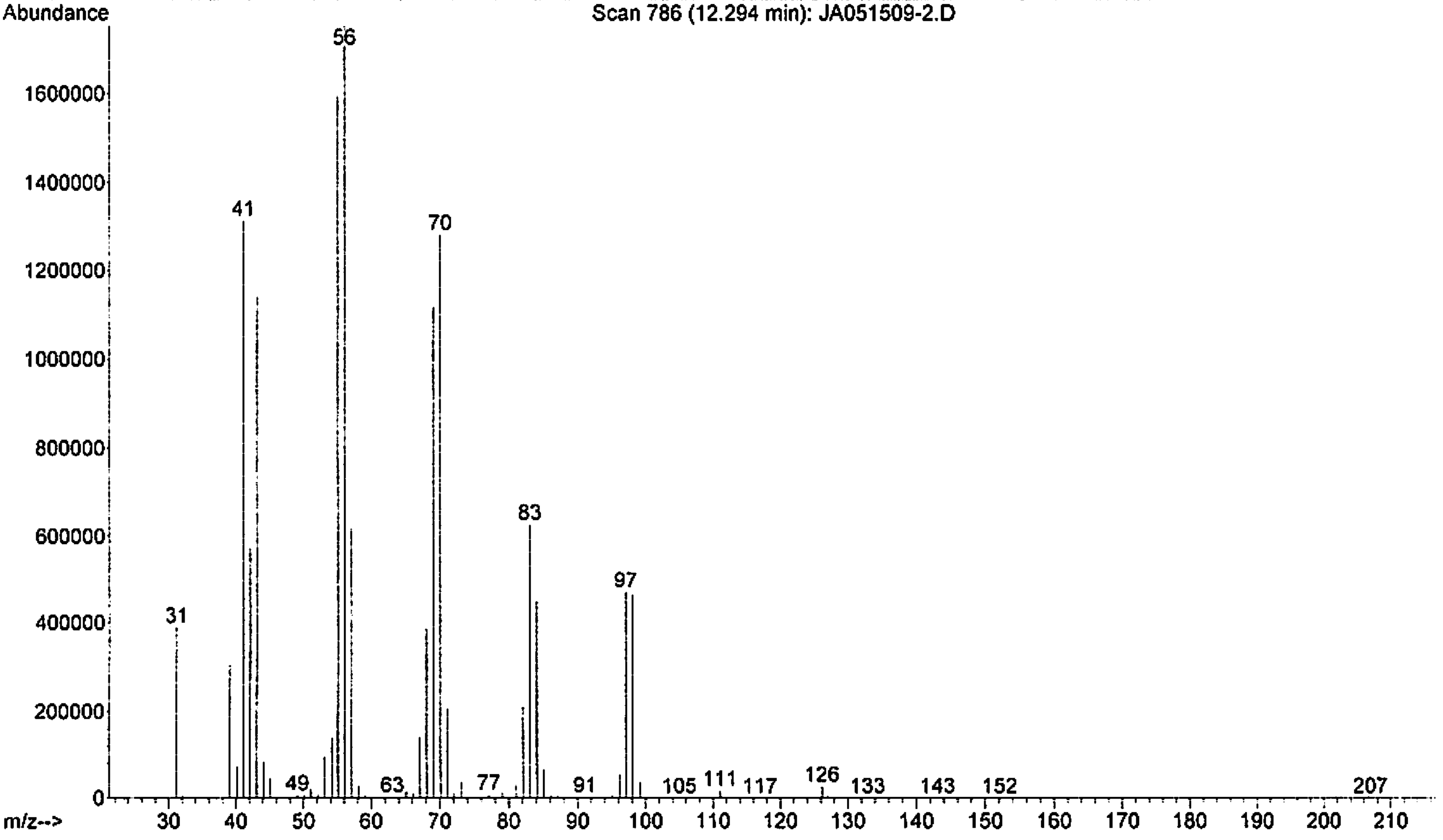

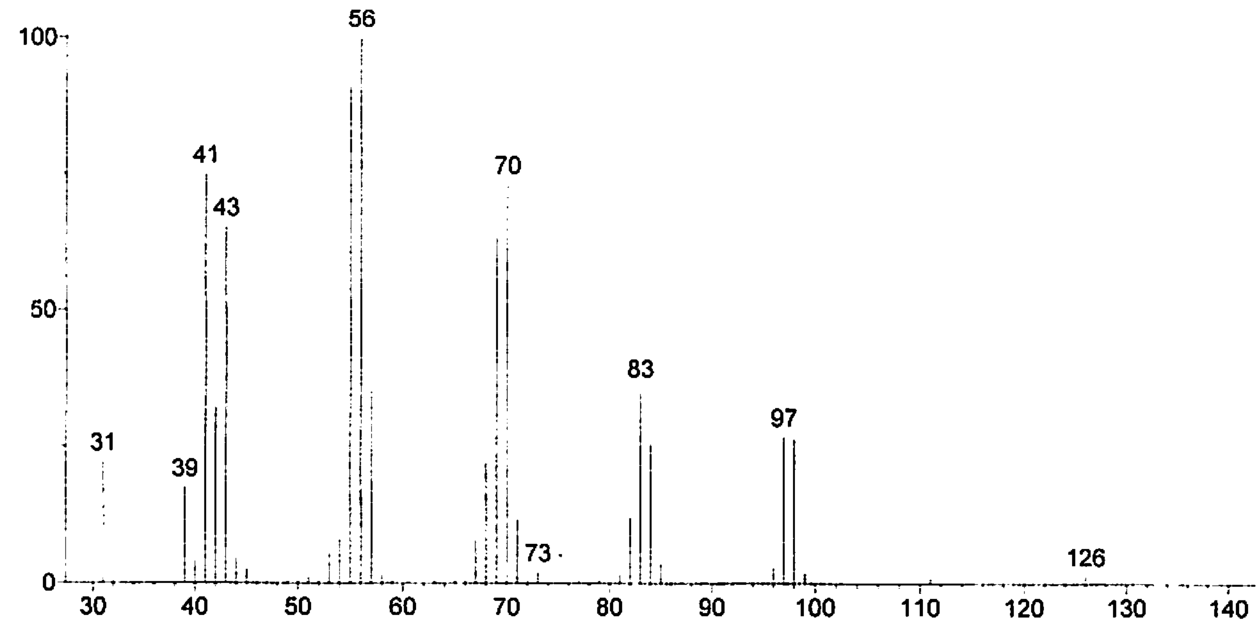

(Text File) Scan 786 (12.294 min): JA051509-2.D

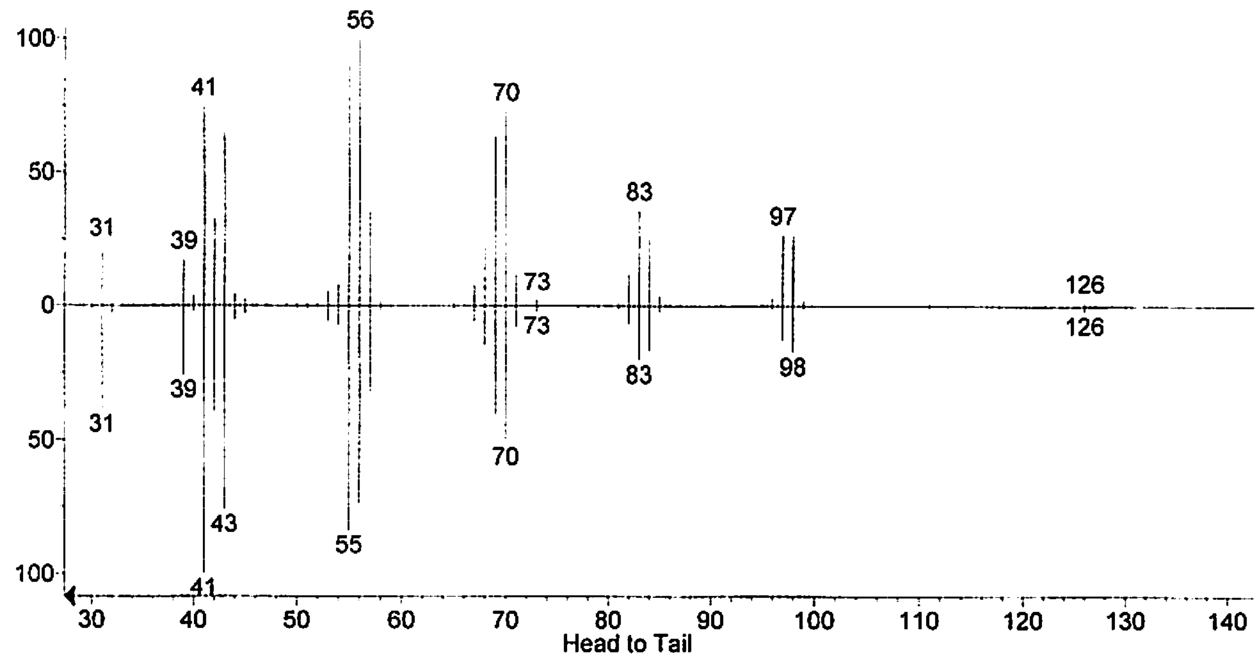

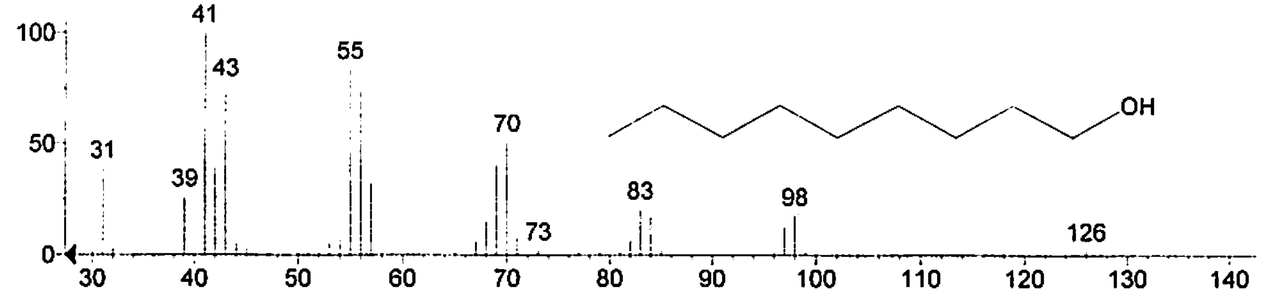

(replib) 1-Nonanol

File :D:\DATA\ALDRICH\JA-09\Snapshot\JA051509-2.D
Operator : Aldrich
Acquired : 15 May 2009 13:47 using AcqMethod JA-WAX08.M
Instrument : Instrument #1
Sample Name: 4 field coll. male C.oculata abd/2ul CH2Cl2
Misc Info : Ed coll. sweeping vetch, 5/13&14; fed in lab
Vial Number: 1

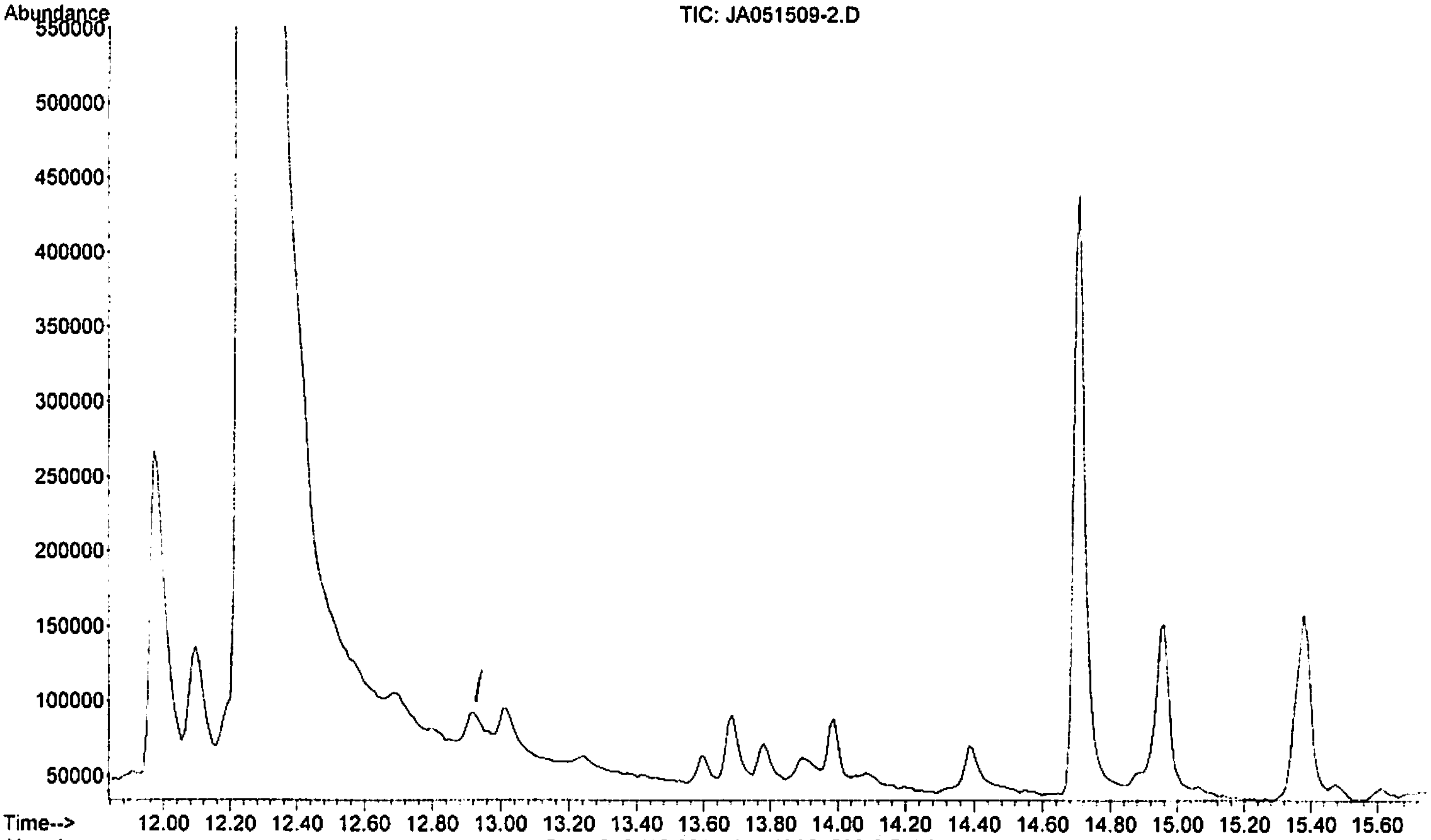

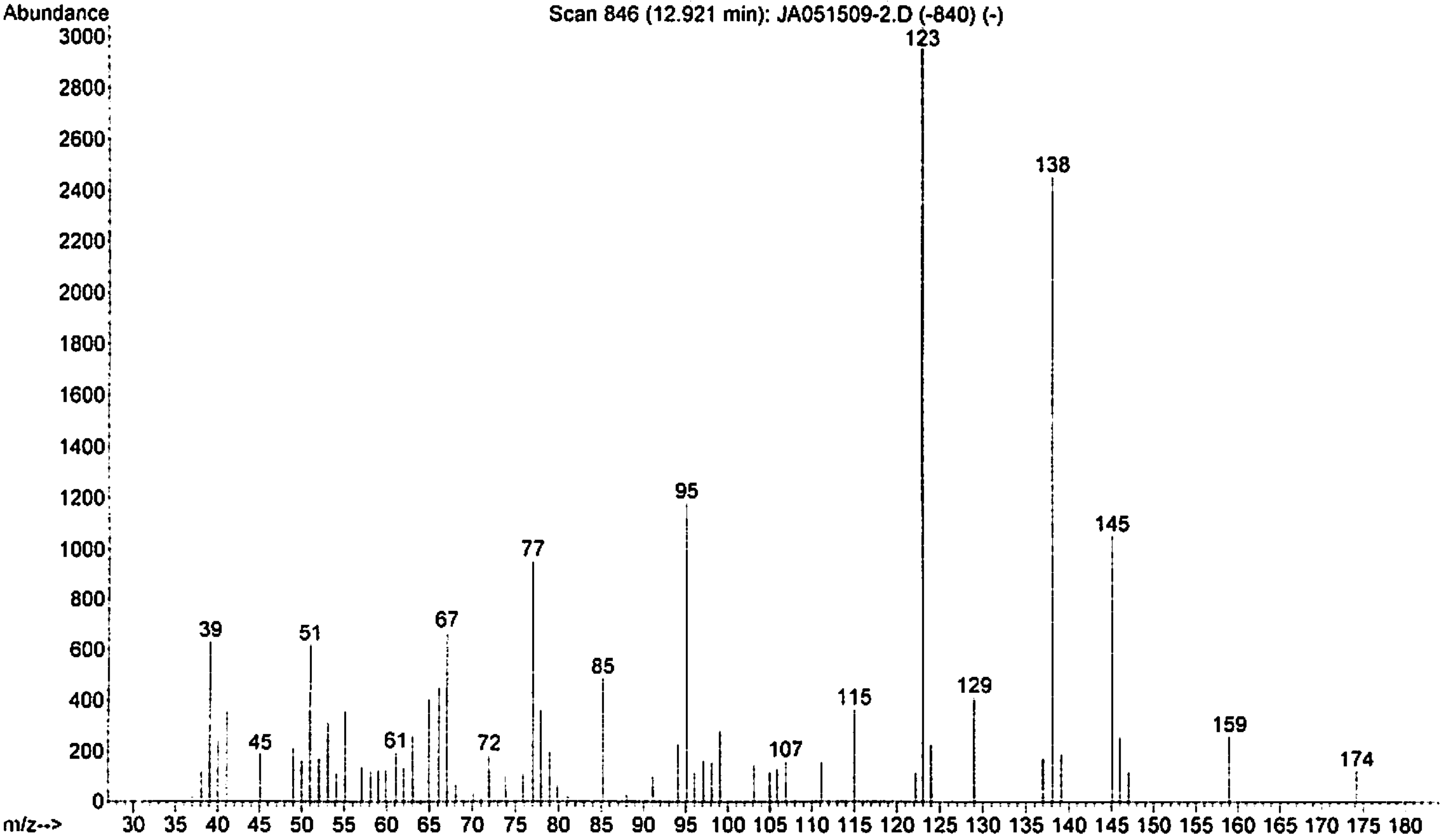

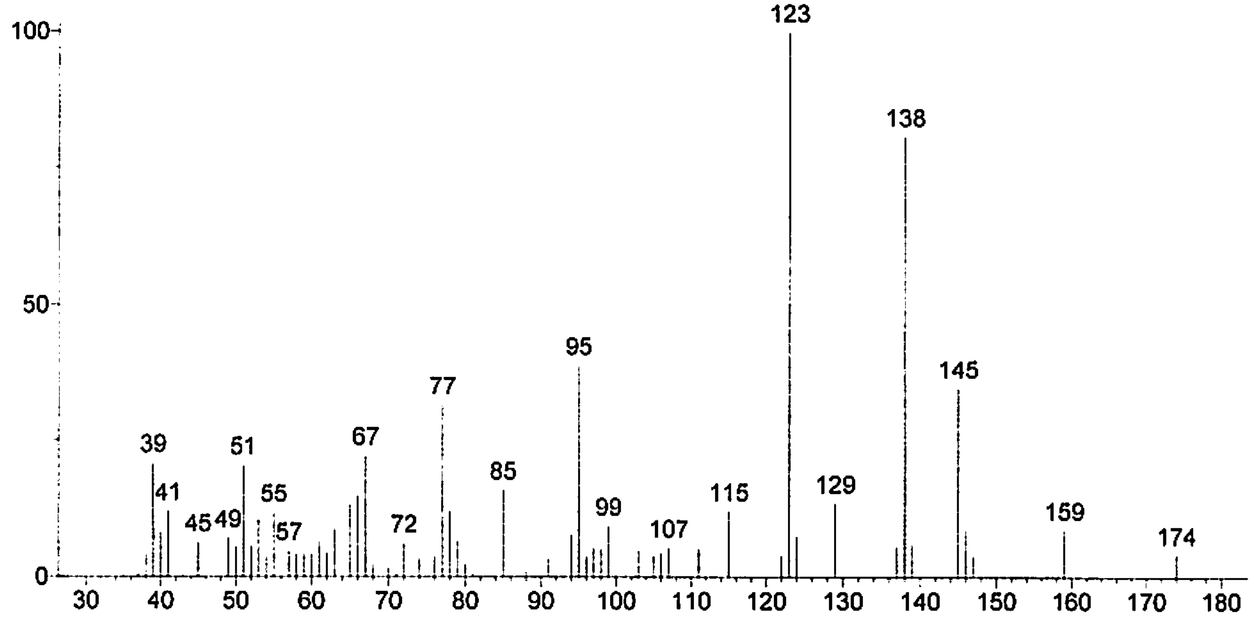

(Text File) Scan 846 (12.921 min): JA051509-2.D (-840)

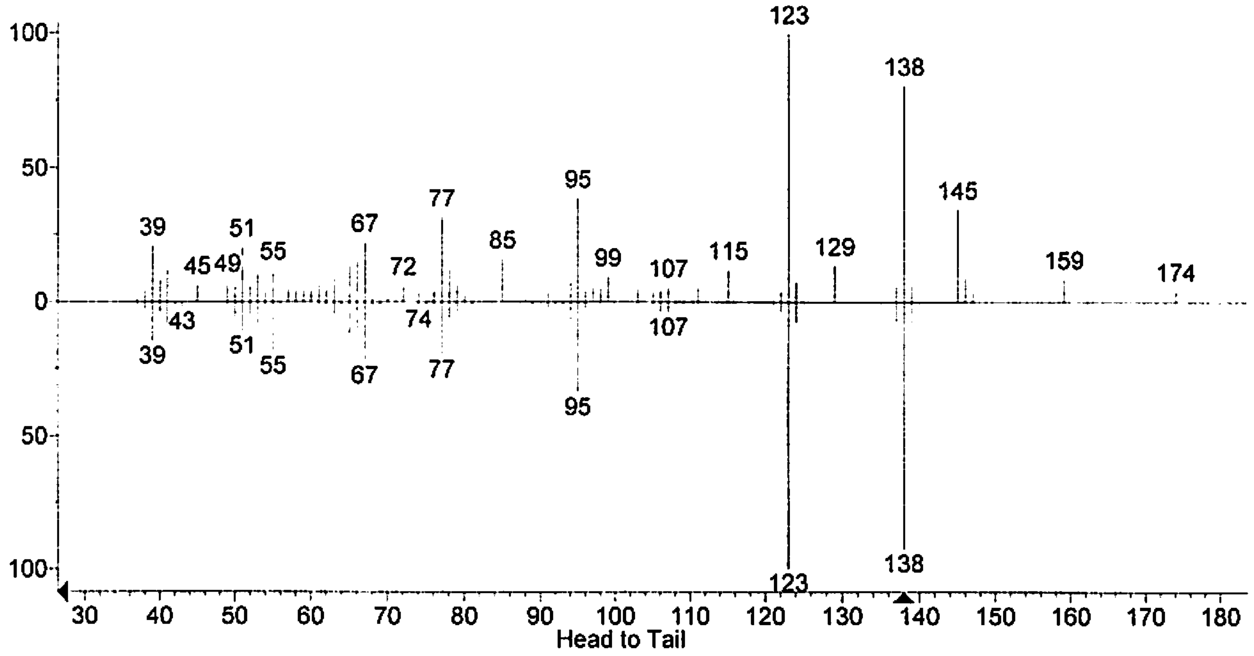

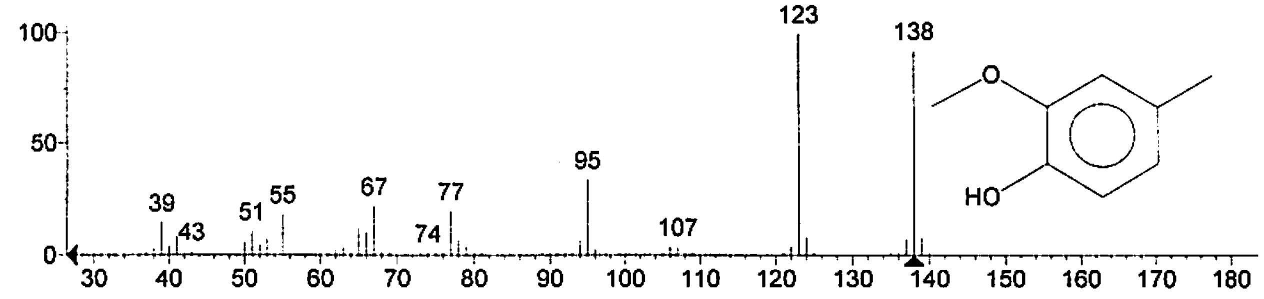

(replib) Phenol, 2-methoxy-4-methyl-

File :D:\DATA\ALDRICH\JA-09\Snapshot\JA051509-2.D
Operator : Aldrich
Acquired : 15 May 2009 13:47 using AcqMethod JA-WAX08.M
Instrument : Instrument #1
Sample Name: 4 field coll. male C.oculata abd/2ul CH2Cl2
Misc Info : Ed coll. sweeping vetch, 5/13&14; fed in lab
Vial Number: 1

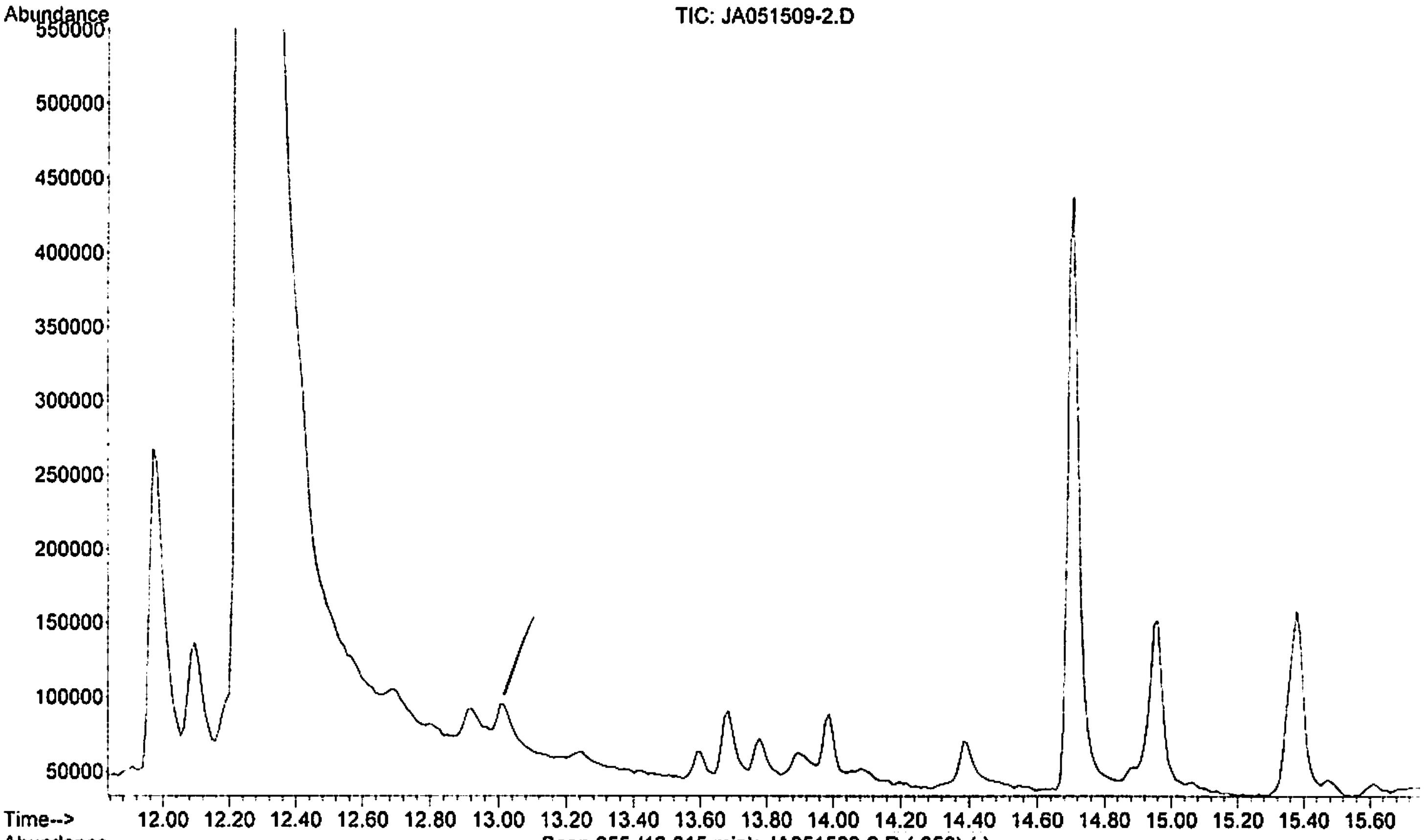

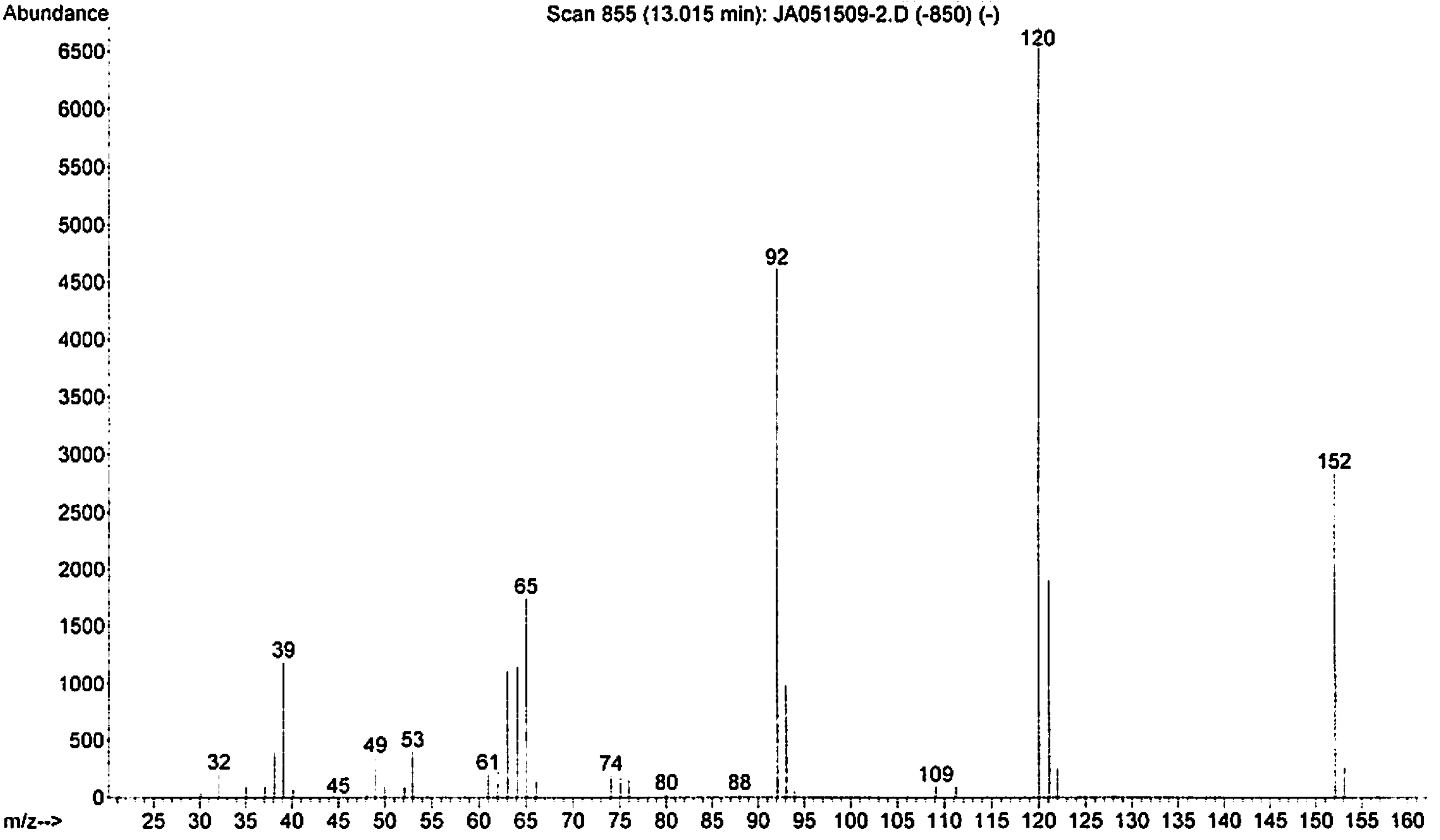

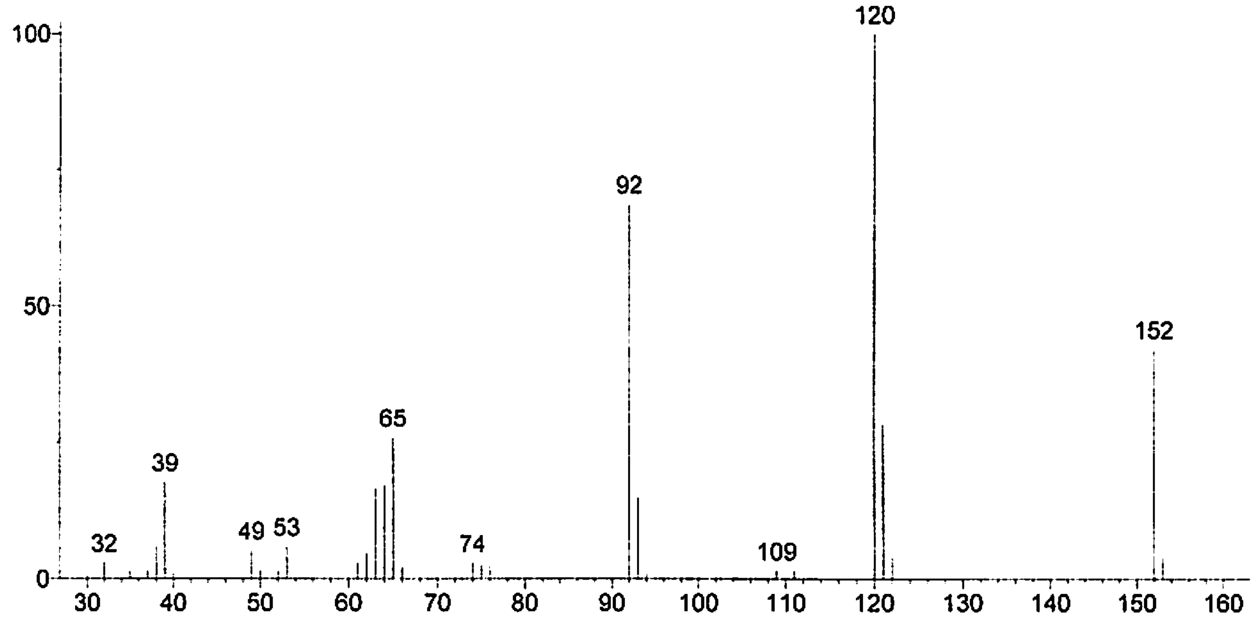

(Text File) Scan 855 (13.015 min): JA051509-2.D (-850)

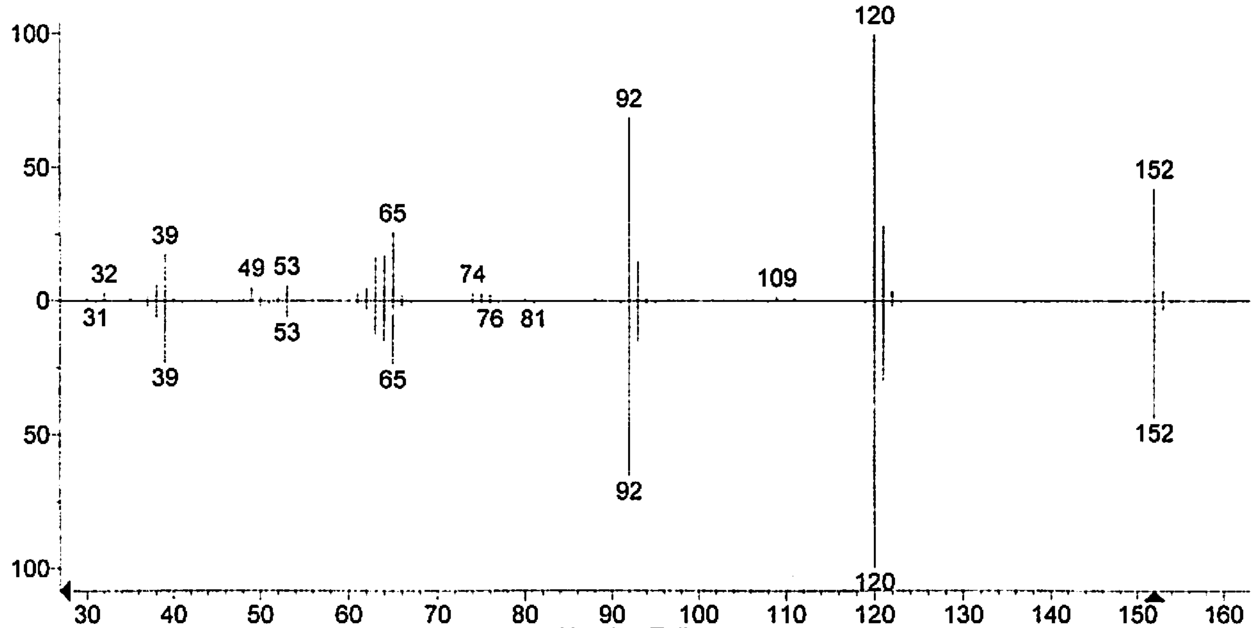

Head to Tail

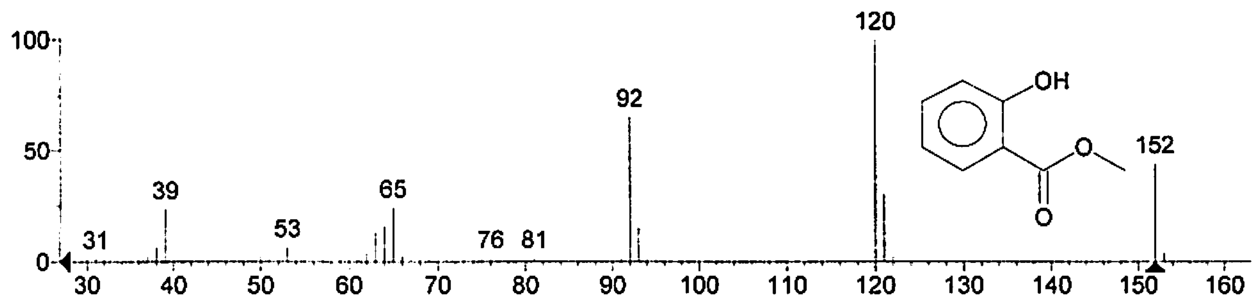

(mainlib) Methyl Salicylate

[illegible]e : D:\DATA\ALDRICH\JA-09\Snapshot\JA051509-2.D
[illegible]rator : Aldrich
[illegible]uired : 15 May 2009 13:47 using AcqMethod JA-WAX08.M
[illegible]trument : Instrument #1
[illegible]ple Name: 4 field coll. male C.oculata abd/2ul CH2Cl2
[illegible]c Info : Ed coll. sweeping vetch, 5/13&14; fed in lab
[illegible]l Number: 1

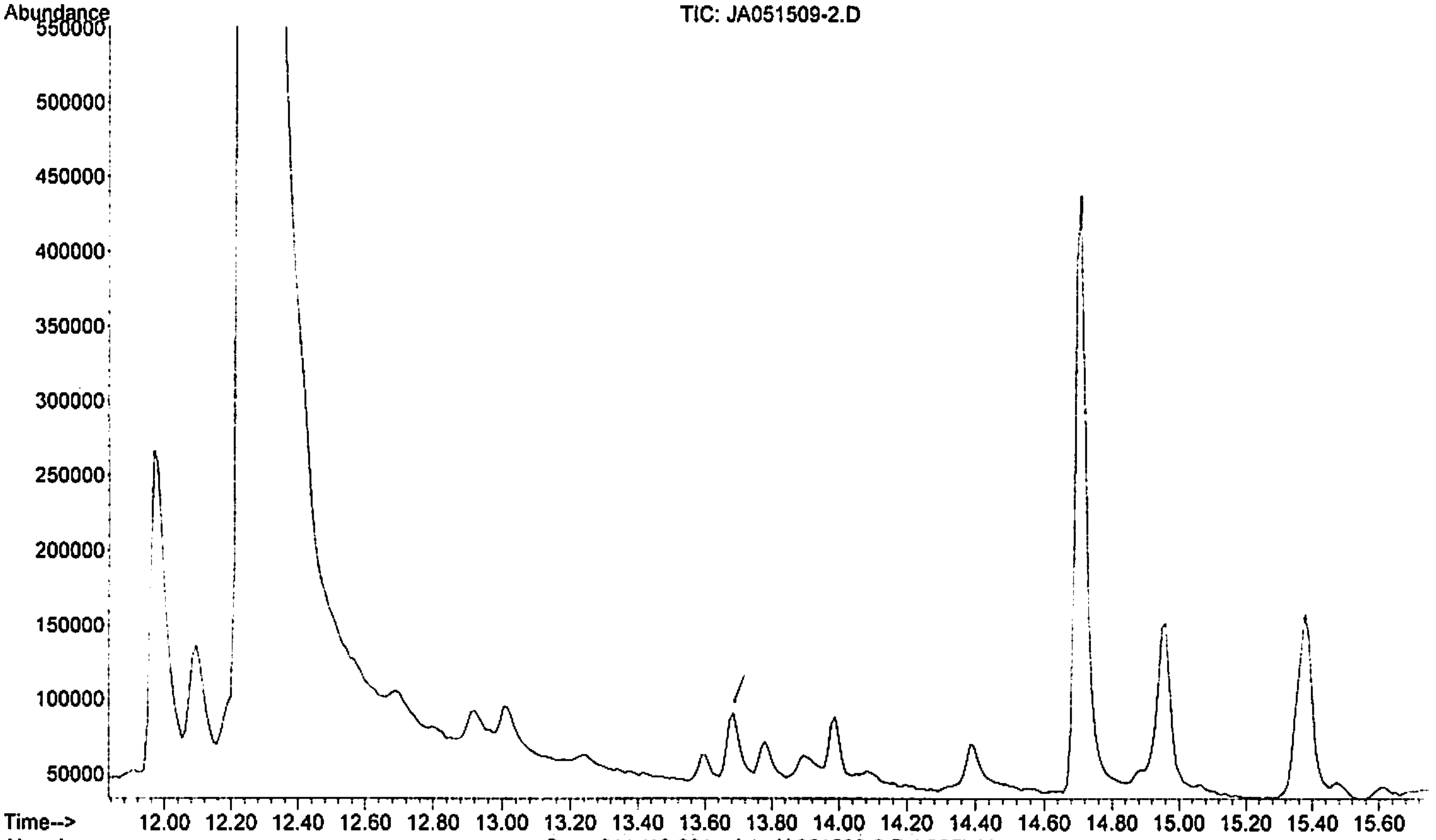

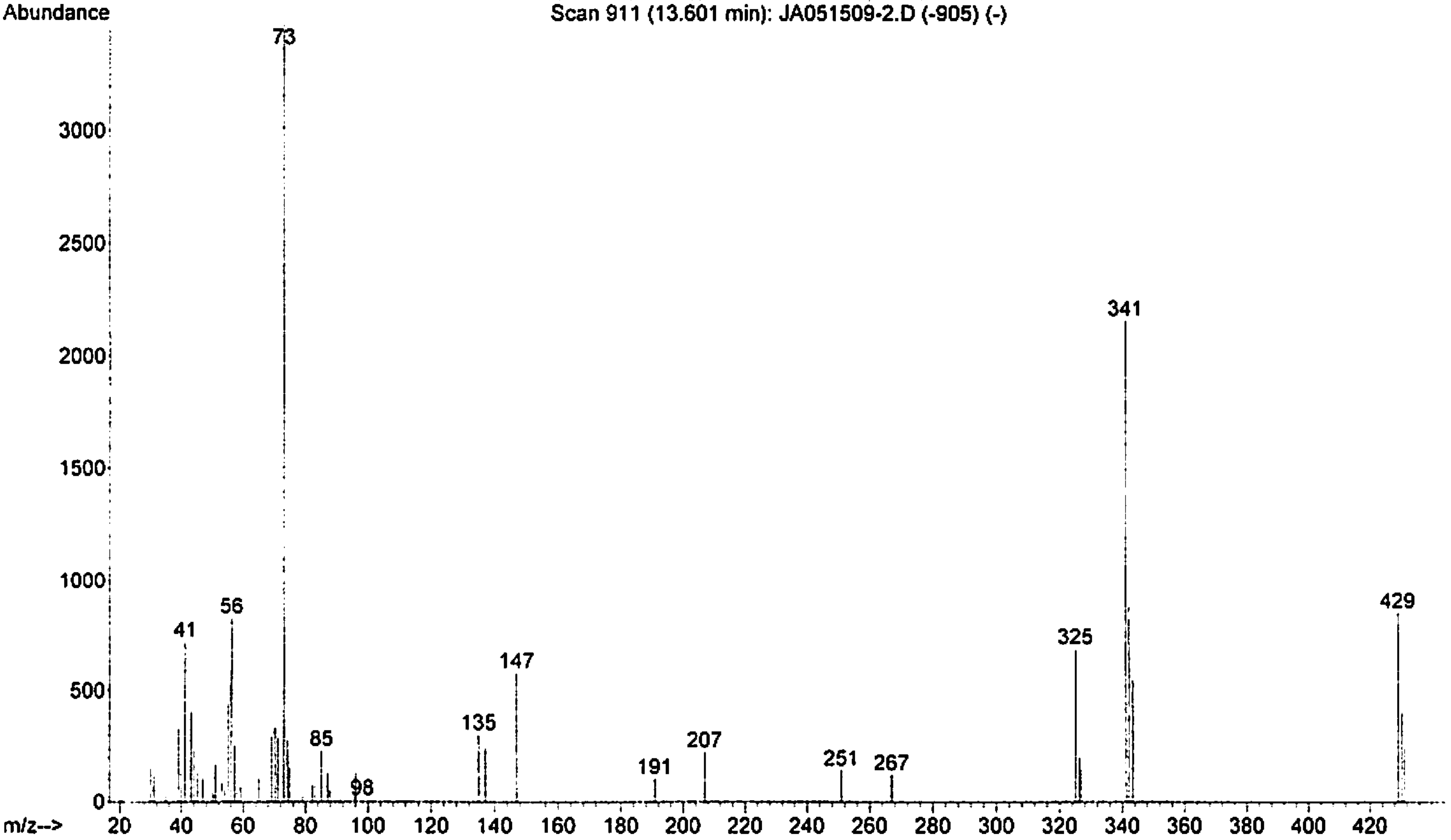

File : D:\DATA\ALDRICH\JA-09\Snapshot\JA051509-2.D
Operator : Aldrich
Acquired : 15 May 2009 13:47 using AcqMethod JA-WAX08.M
Instrument : Instrument #1
Sample Name: 4 field coll. male C.oculata abd/2ul CH2Cl2
Misc Info : Ed coll. sweeping vetch, 5/13&14; fed in lab
Vial Number: 1

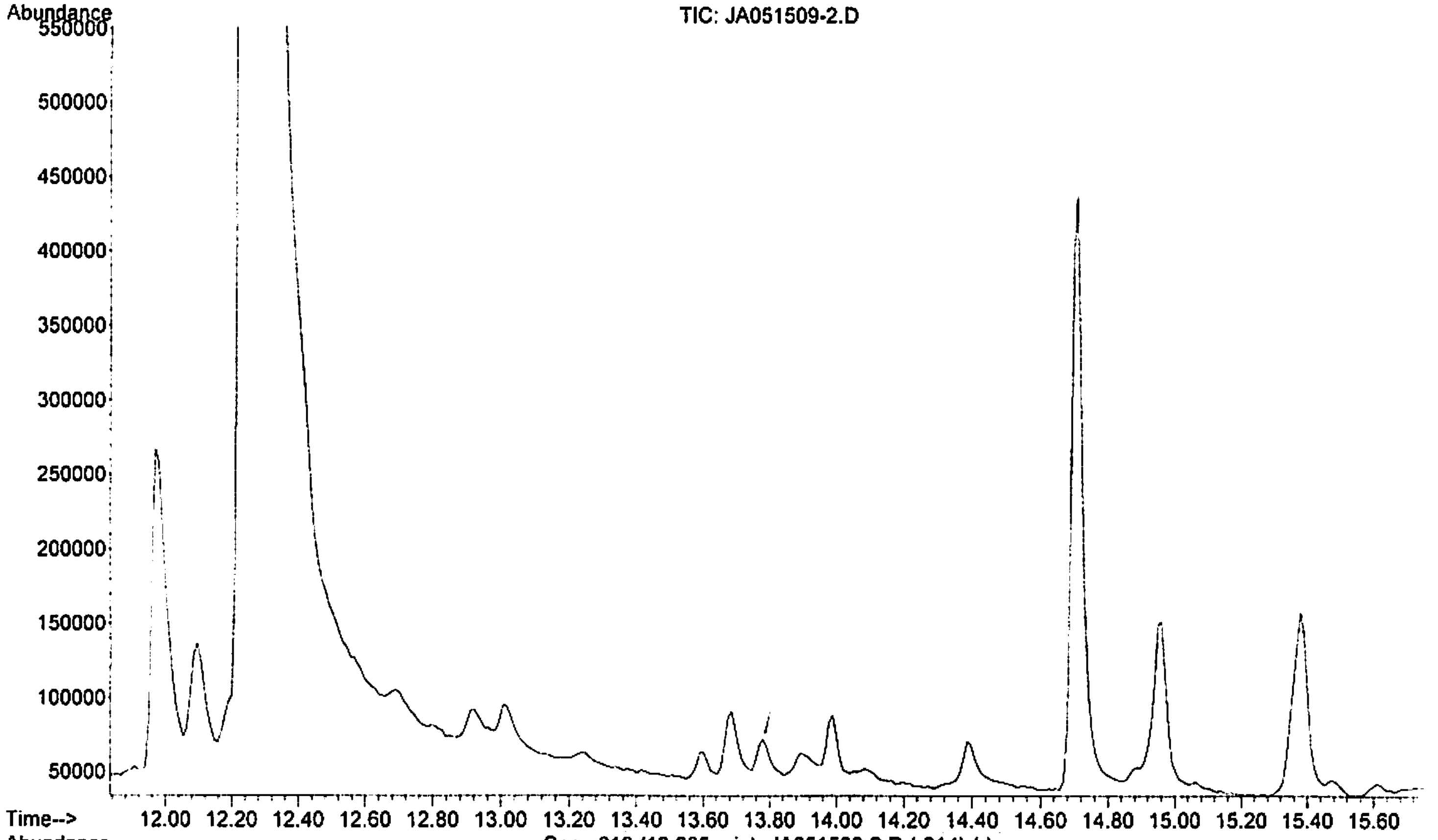

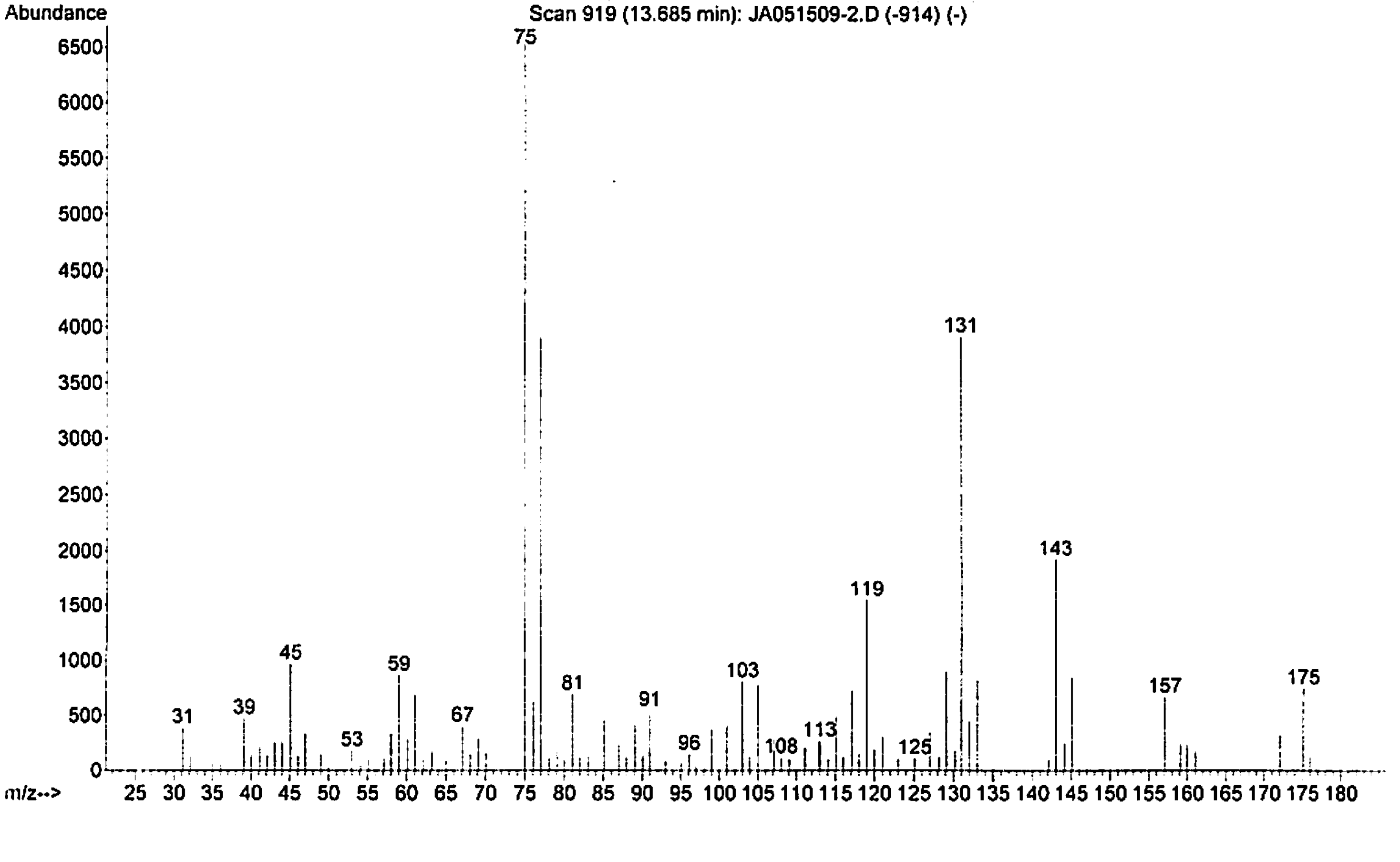

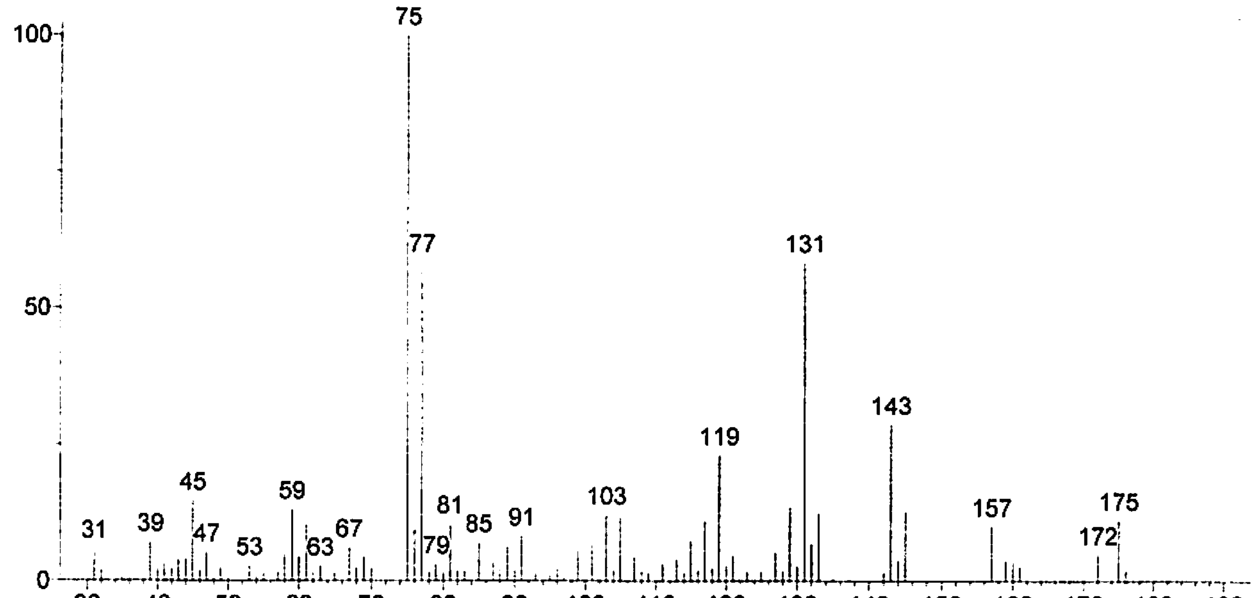

(Text File) Scan 919 (13.685 min): JA051509-2.D (-914)

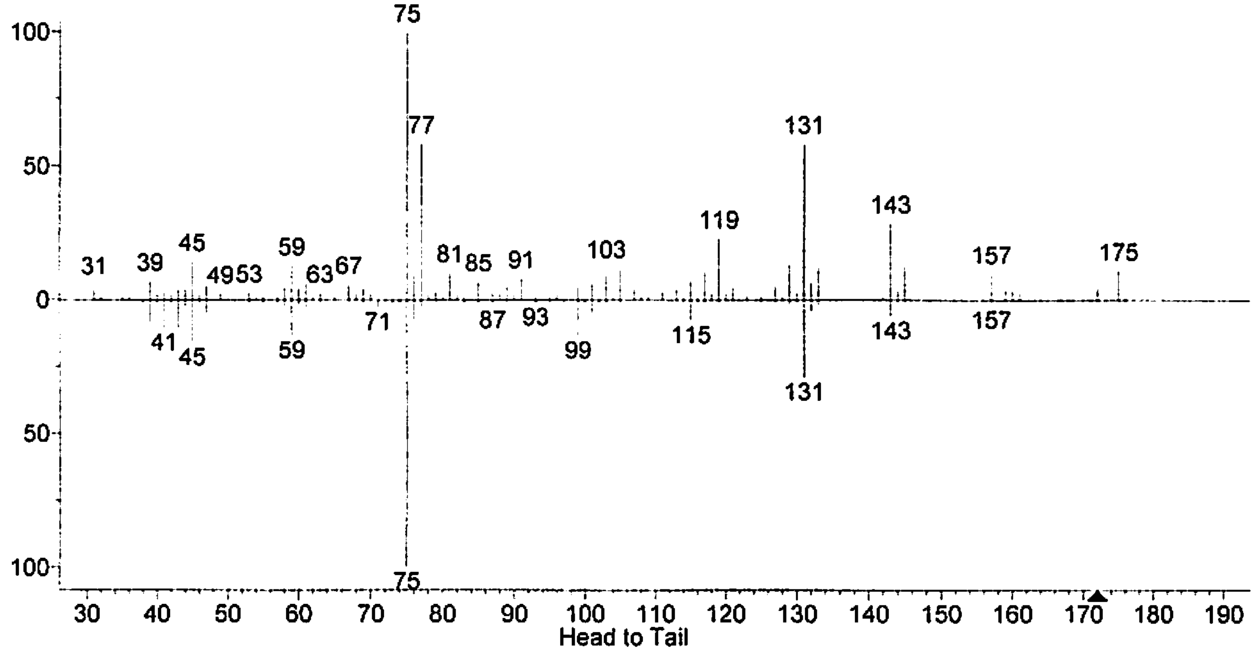

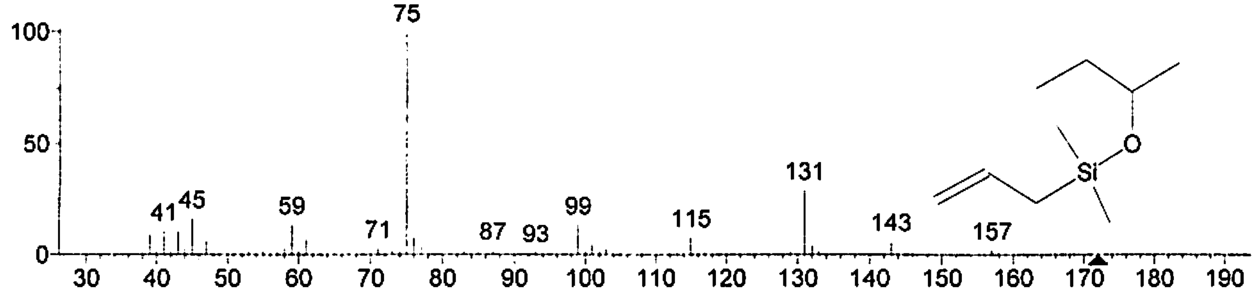

(mainlib) Allyl(2-butoxy)dimethylsilane

File : D:\DATA\ALDRICH\JA-09\Snapshot\JA051509-2.D
Operator : Aldrich
Acquired : 15 May 2009 13:47 using AcqMethod JA-WAX08.M
Instrument : Instrument #1
Sample Name: 4 field coll. male C.oculata abd/2ul CH2Cl2
Misc Info : Ed coll. sweeping vetch, 5/13&14; fed in lab
Vial Number: 1

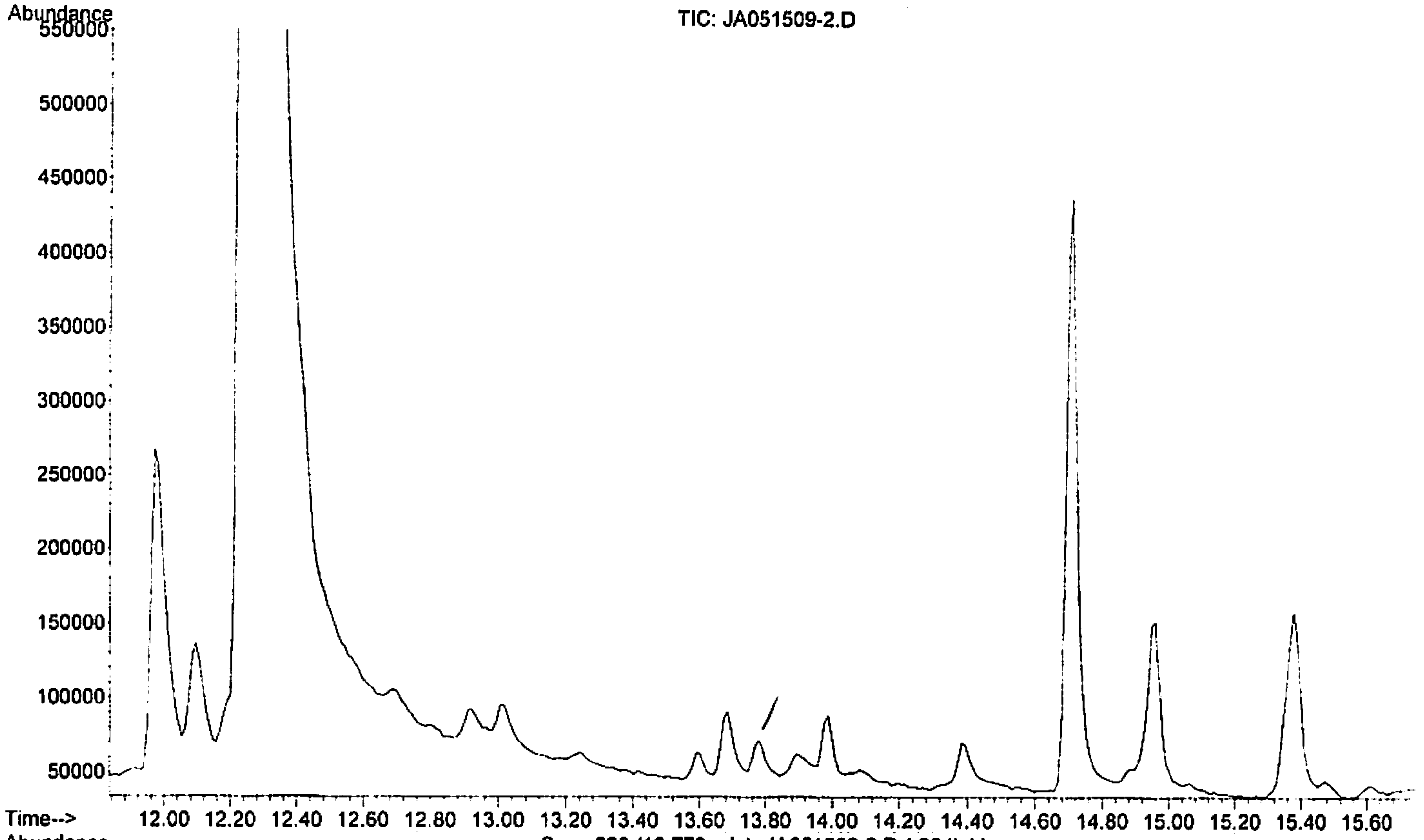

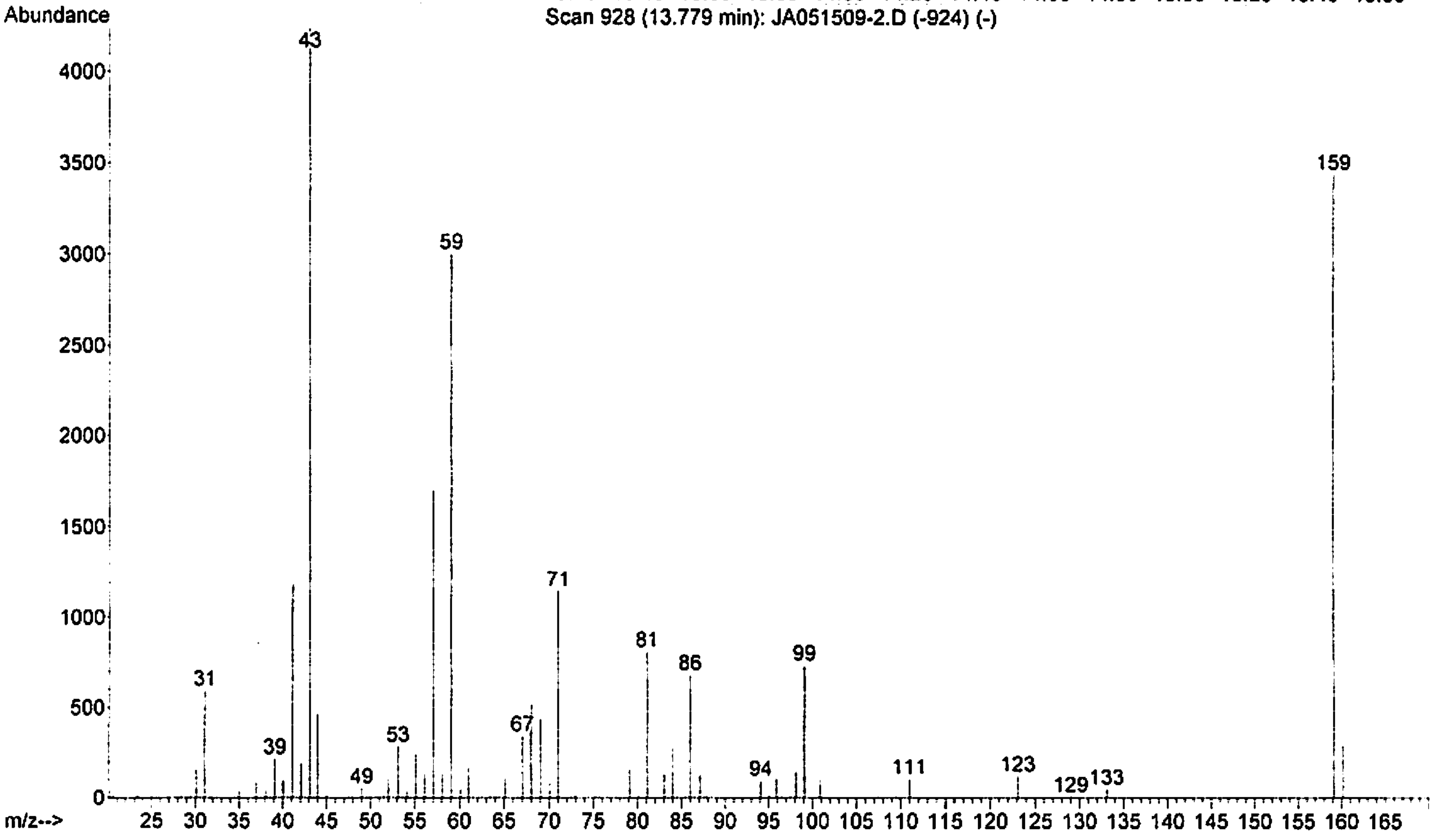

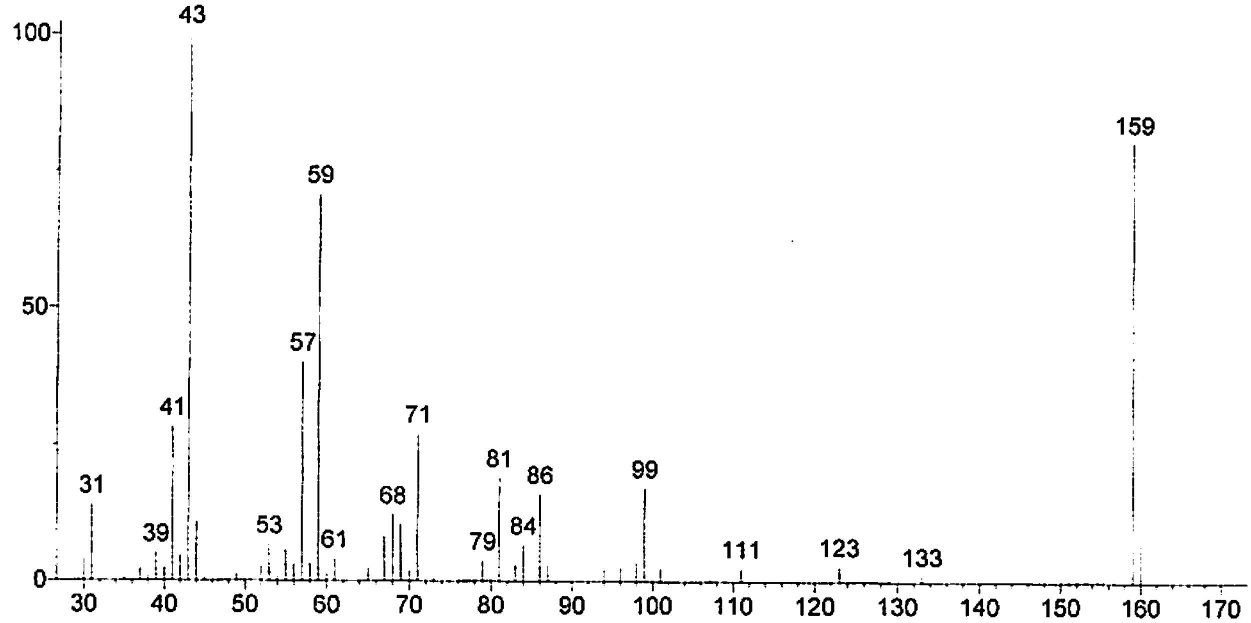

(Text File) Scan 928 (13.779 min): JA051509-2.D (-924)

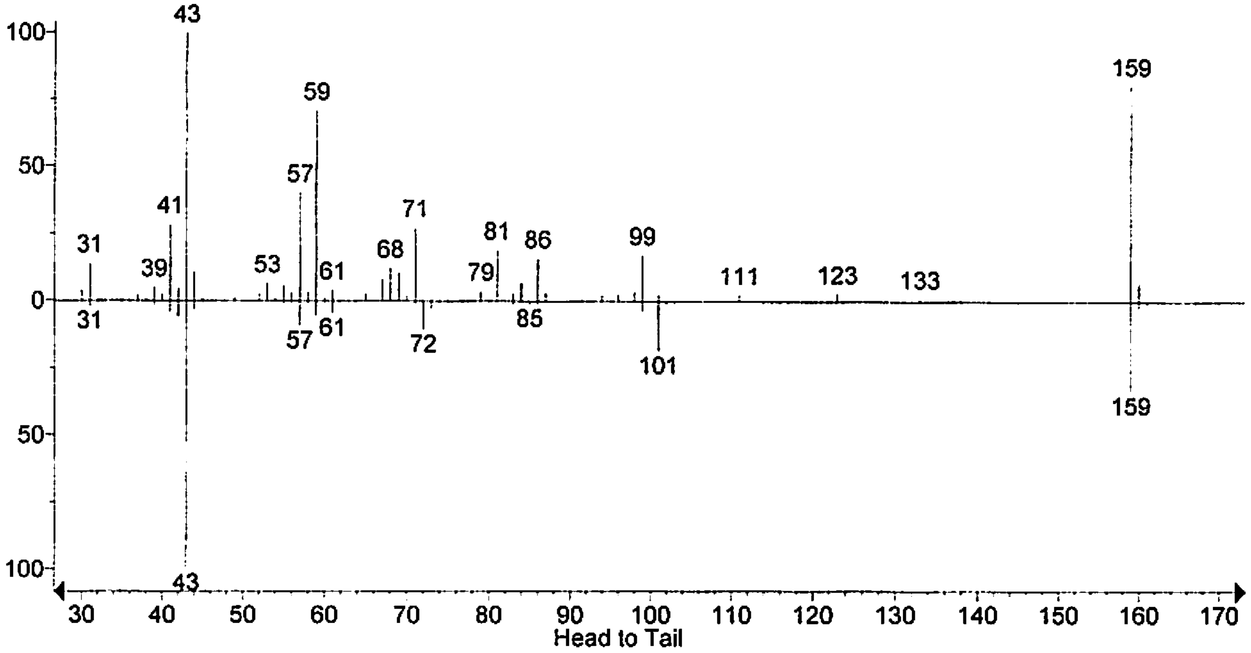

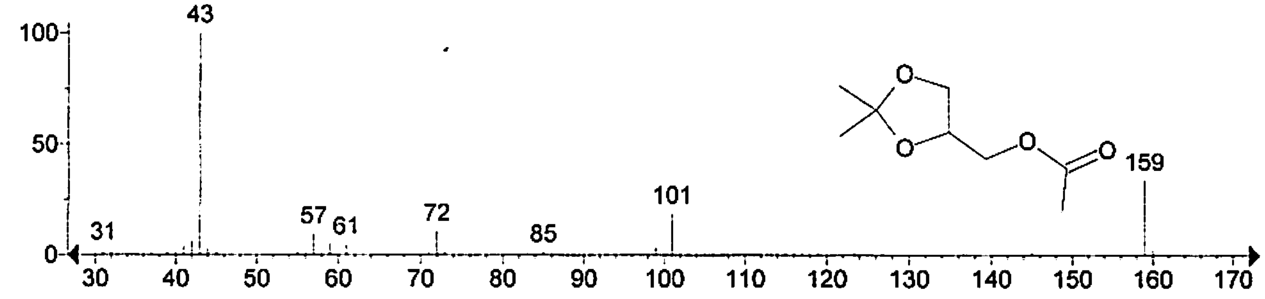

(mainlib) 1,3-Dioxolane-4-methanol, 2,2-dimethyl-, acetate

```
[illegible]e       :D:\DATA\ALDRICH\JA-09\Snapshot\JA051509-2.D
[illegible]rator   : Aldrich
[illegible]uired   : 15 May 2009  13:47     using AcqMethod JA-WAX08.M
[illegible]trument :     Instrument #1
[illegible]ple Name: 4 field coll. male C.oculata abd/2ul CH2Cl2
[illegible]c Info  : Ed coll. sweeping vetch, 5/13&14; fed in lab
[illegible]l Number: 1
```

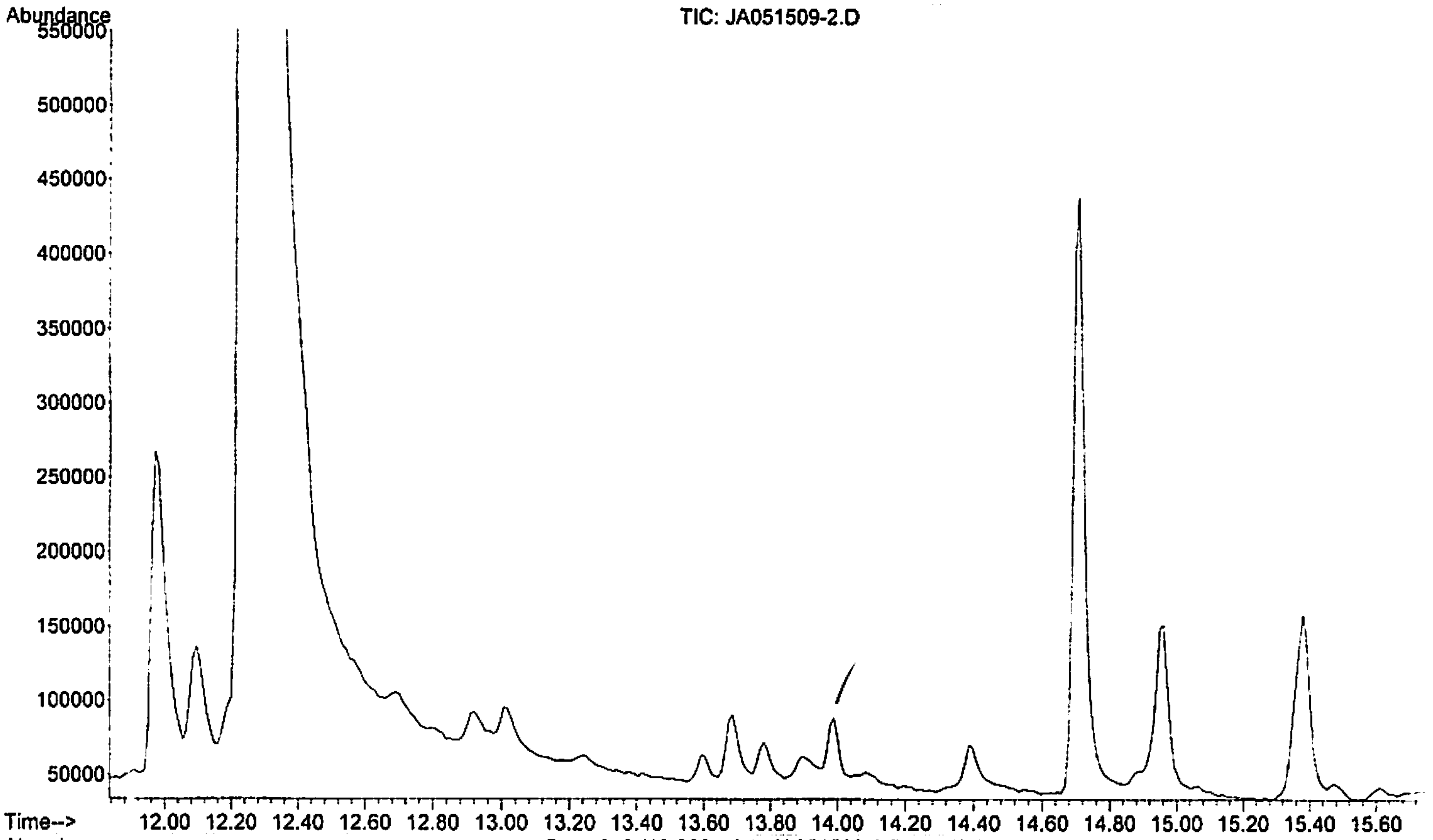

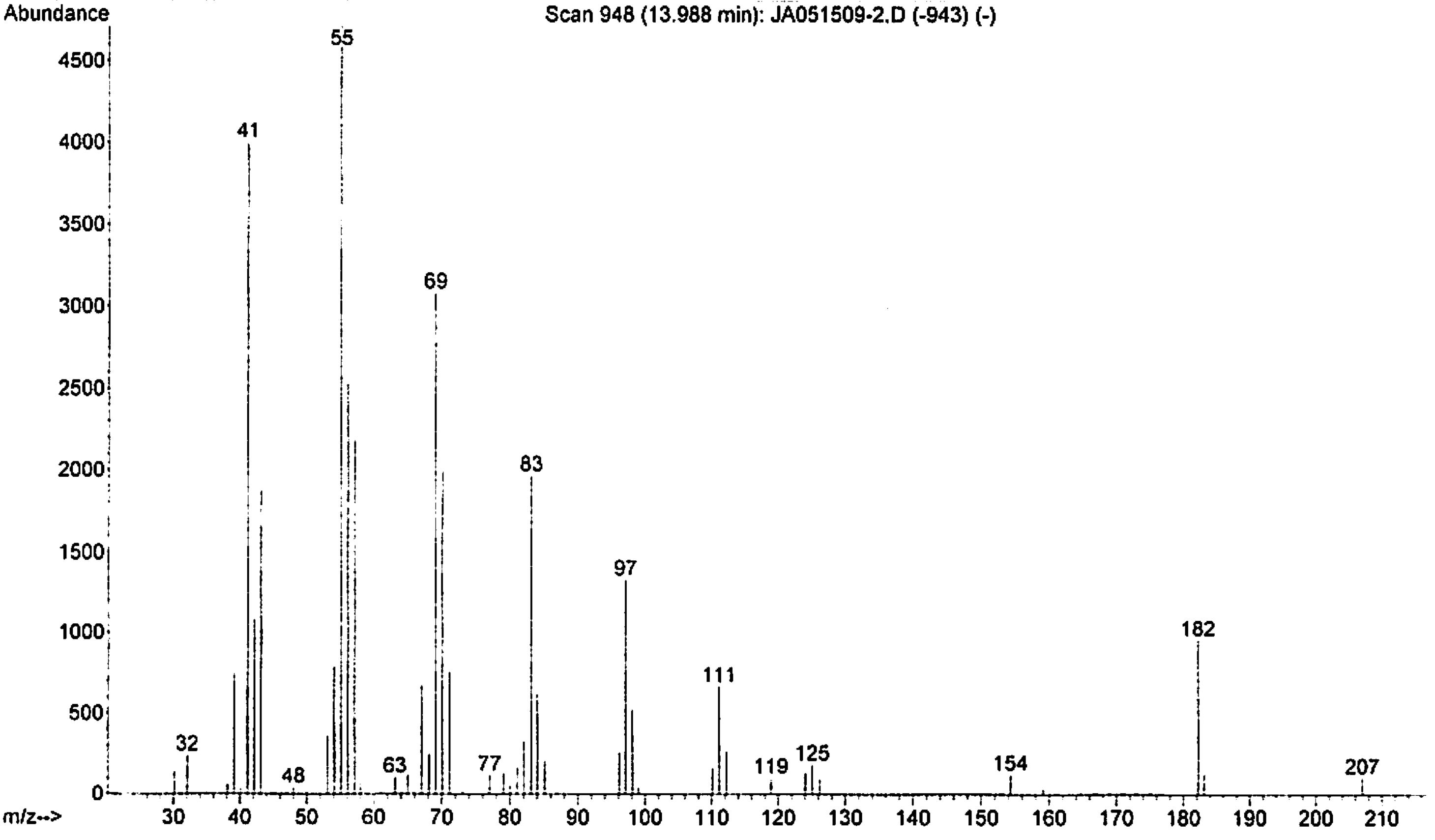

[illegible] :D:\DATA\ALDRICH\JA-09\Snapshot\JA051509-2.D
[illegible]rator : Aldrich
[illegible]quired : 15 May 2009 13:47 using AcqMethod JA-WAX08.M
[illegible]strument : Instrument #1
[illegible]mple Name: 4 field coll. male C.oculata abd/2ul CH2Cl2
[illegible]sc Info : Ed coll. sweeping vetch, 5/13&14; fed in lab
[illegible]l Number: 1

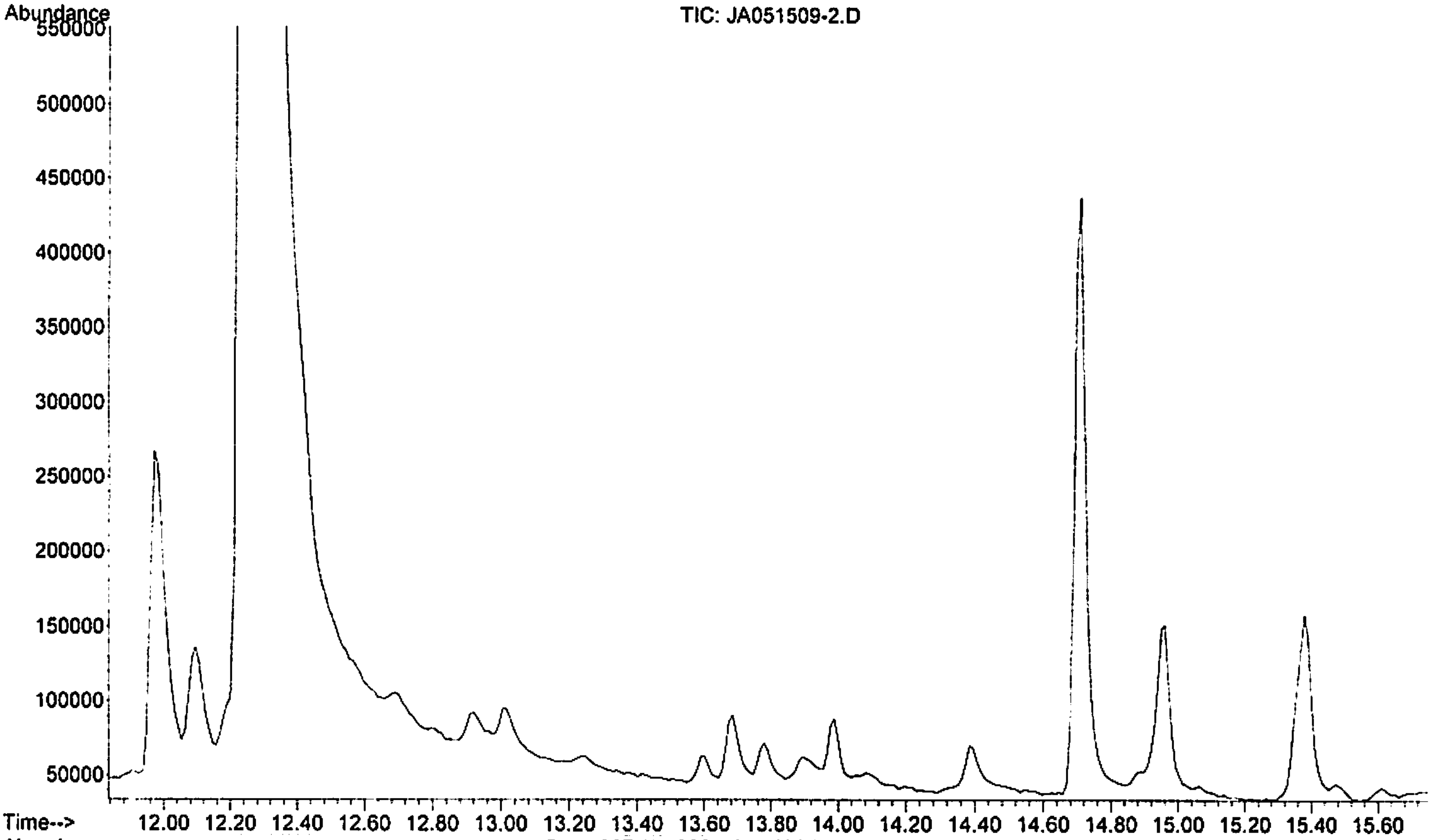

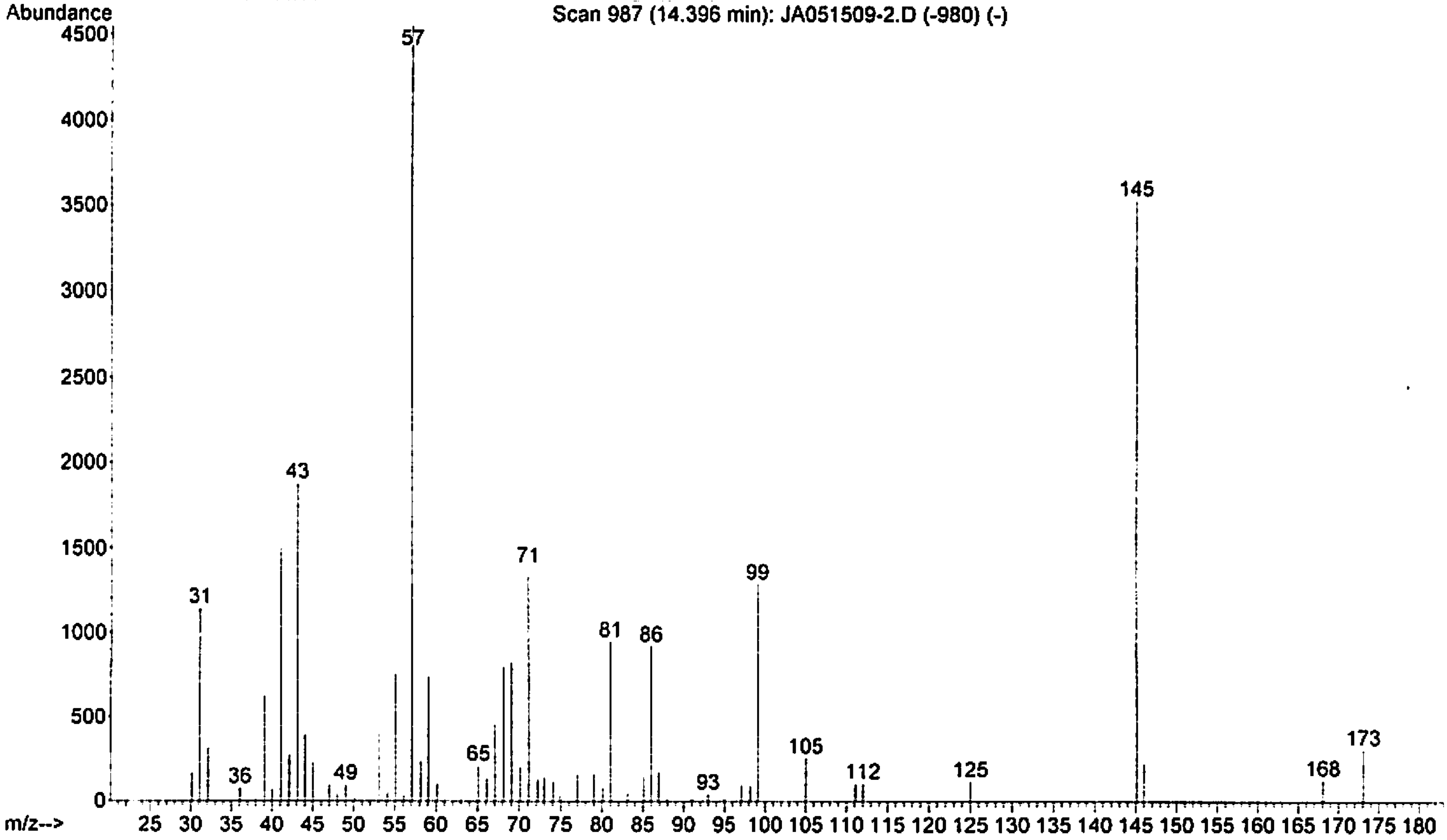

```
File        :D:\DATA\ALDRICH\JA-09\Snapshot\JA051509-2.D
Operator    : Aldrich
Acquired    : 15 May 2009  13:47     using AcqMethod JA-WAX08.M
Instrument  :    Instrument #1
Sample Name: 4 field coll. male C.oculata abd/2ul CH2C12
Misc Info   : Ed coll. sweeping vetch, 5/13&14; fed in lab
Vial Number: 1
```

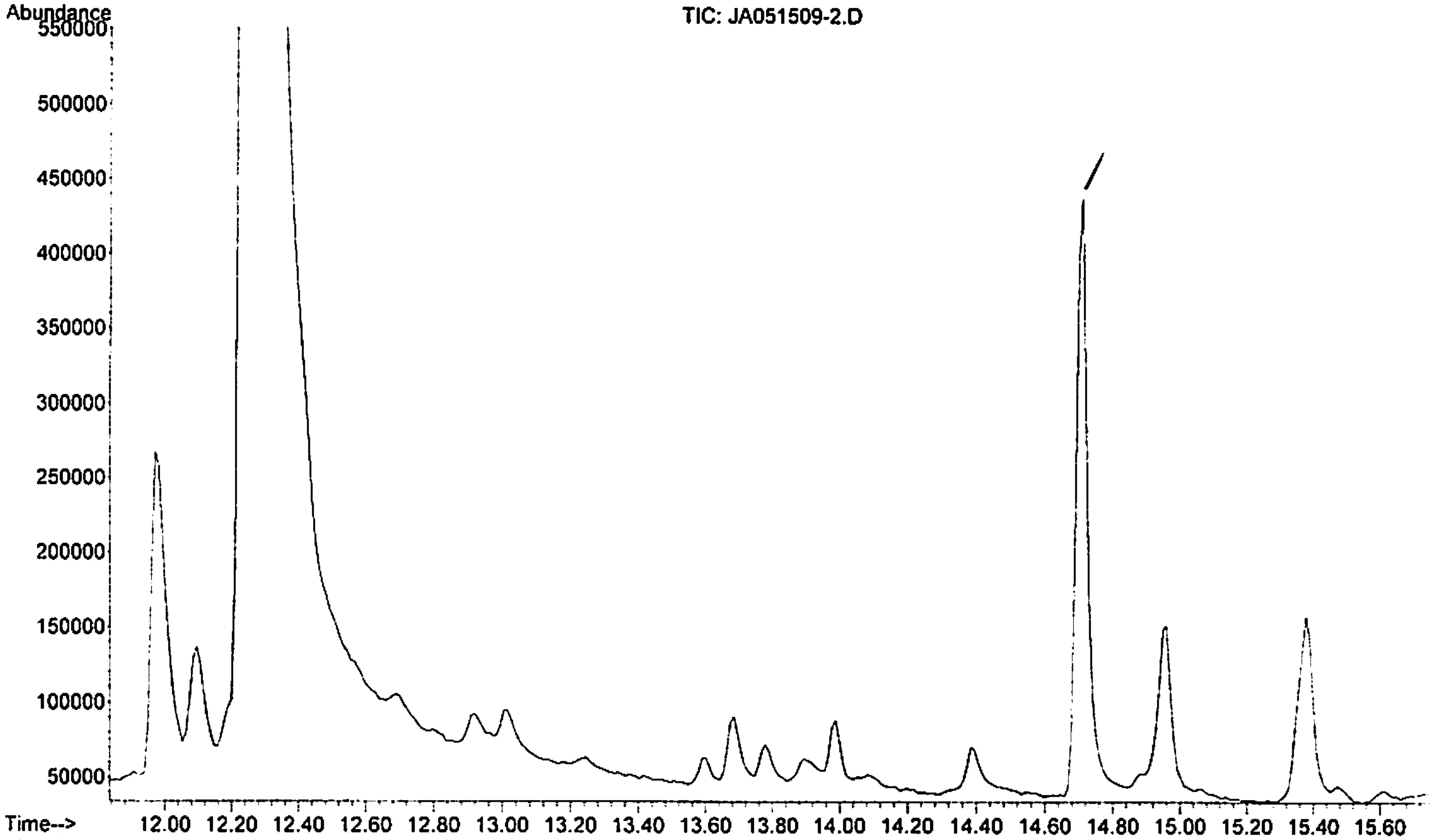

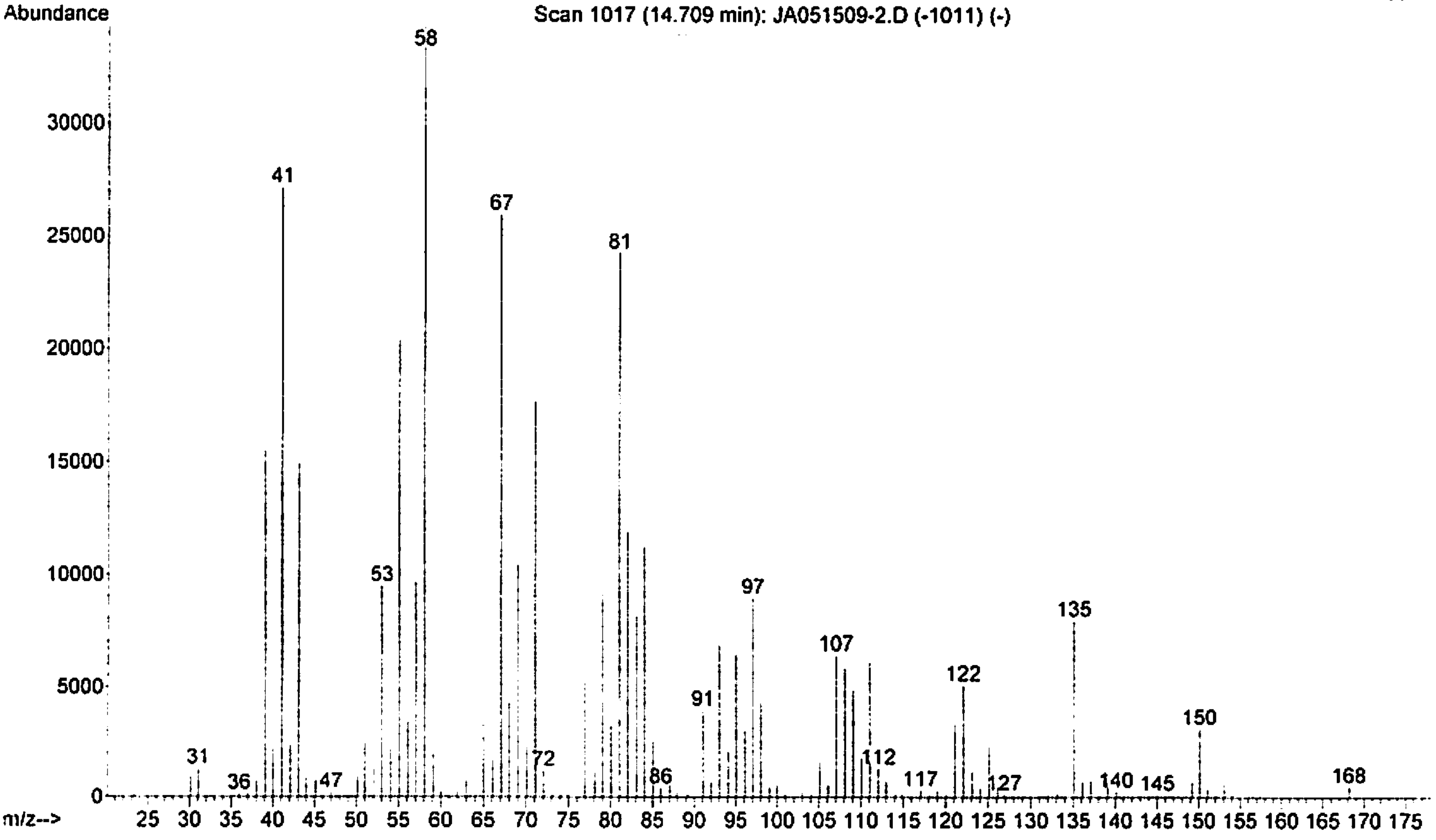

.e : D:\DATA\ALDRICH\JA-09\Snapshot\JA051509-2.D
rator : Aldrich
uired : 15 May 2009 13:47 using AcqMethod JA-WAX08.M
strument : Instrument #1
mple Name: 4 field coll. male C.oculata abd/2ul CH2Cl2
c Info : Ed coll. sweeping vetch, 5/13&14; fed in lab
l Number: 1

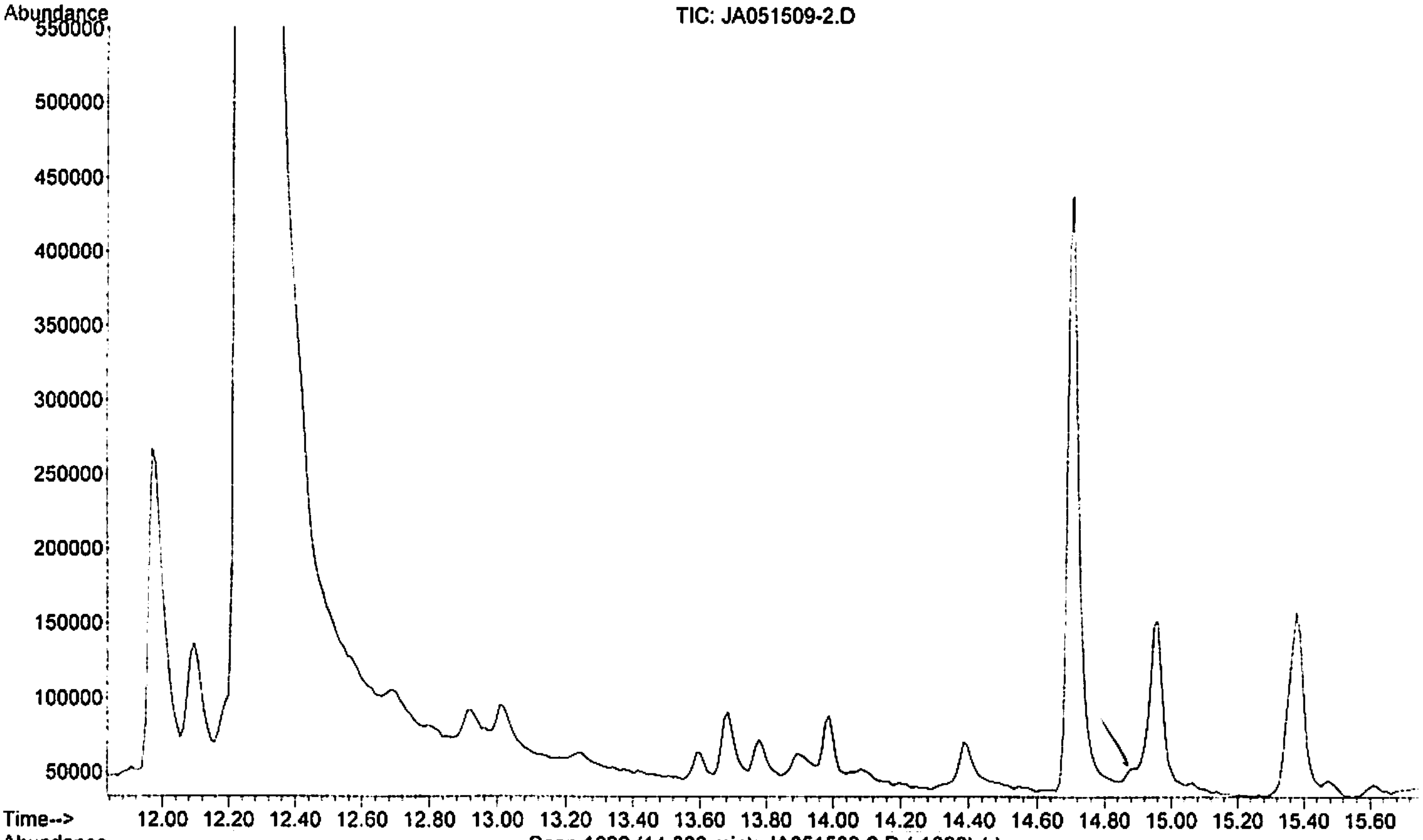

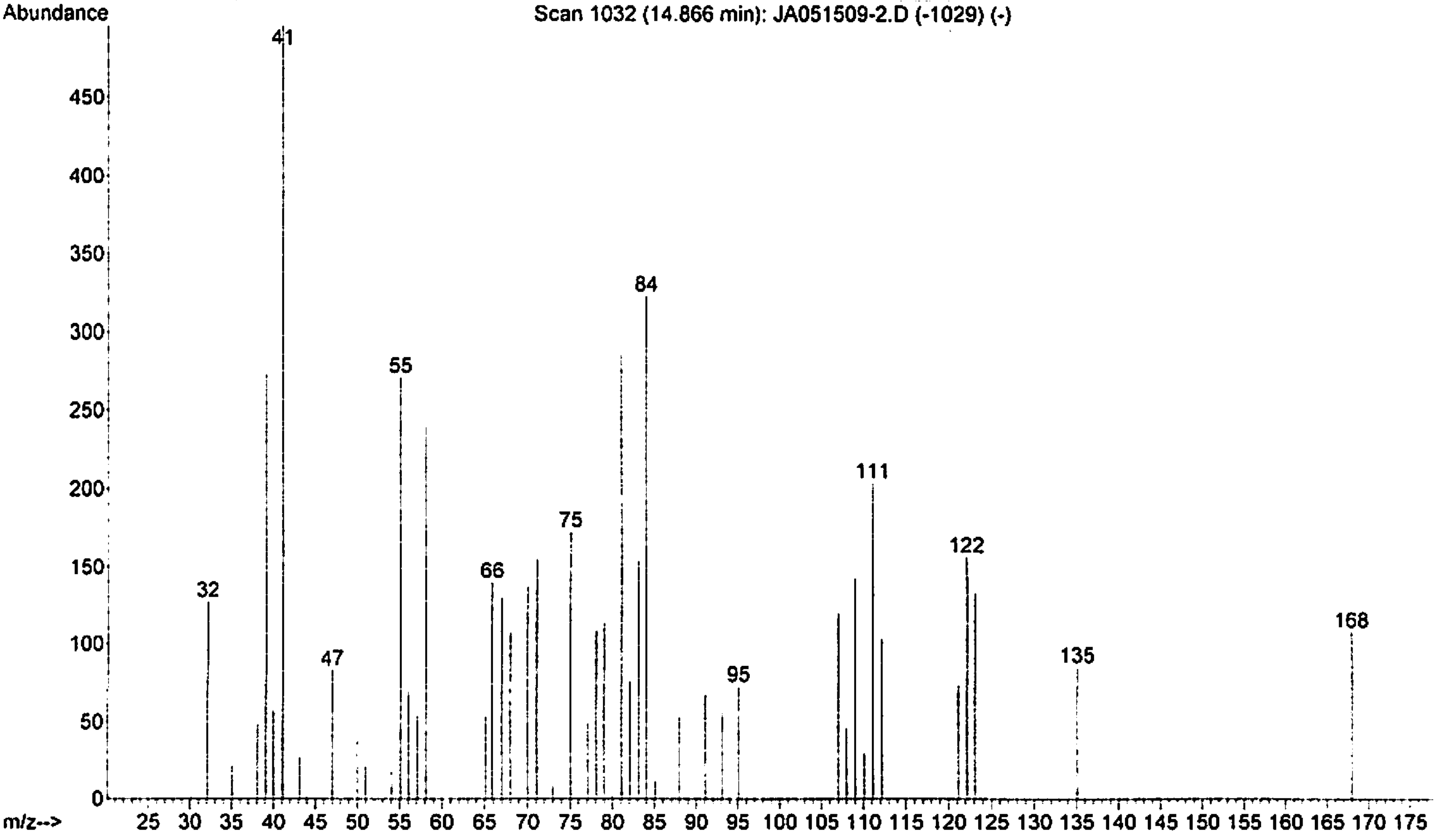

```
[illegible]e        :D:\DATA\ALDRICH\JA-09\Snapshot\JA051509-2.D
[illegible]rator    : Aldrich
[illegible]quired   : 15 May 2009   13:47      using AcqMethod JA-WAX08.M
[illegible]trument  :     Instrument #1
[illegible]ple Name: 4 field coll. male C.oculata abd/2ul CH2Cl2
[illegible]c Info   : Ed coll. sweeping vetch, 5/13&14; fed in lab
[illegible]l Number: 1
```

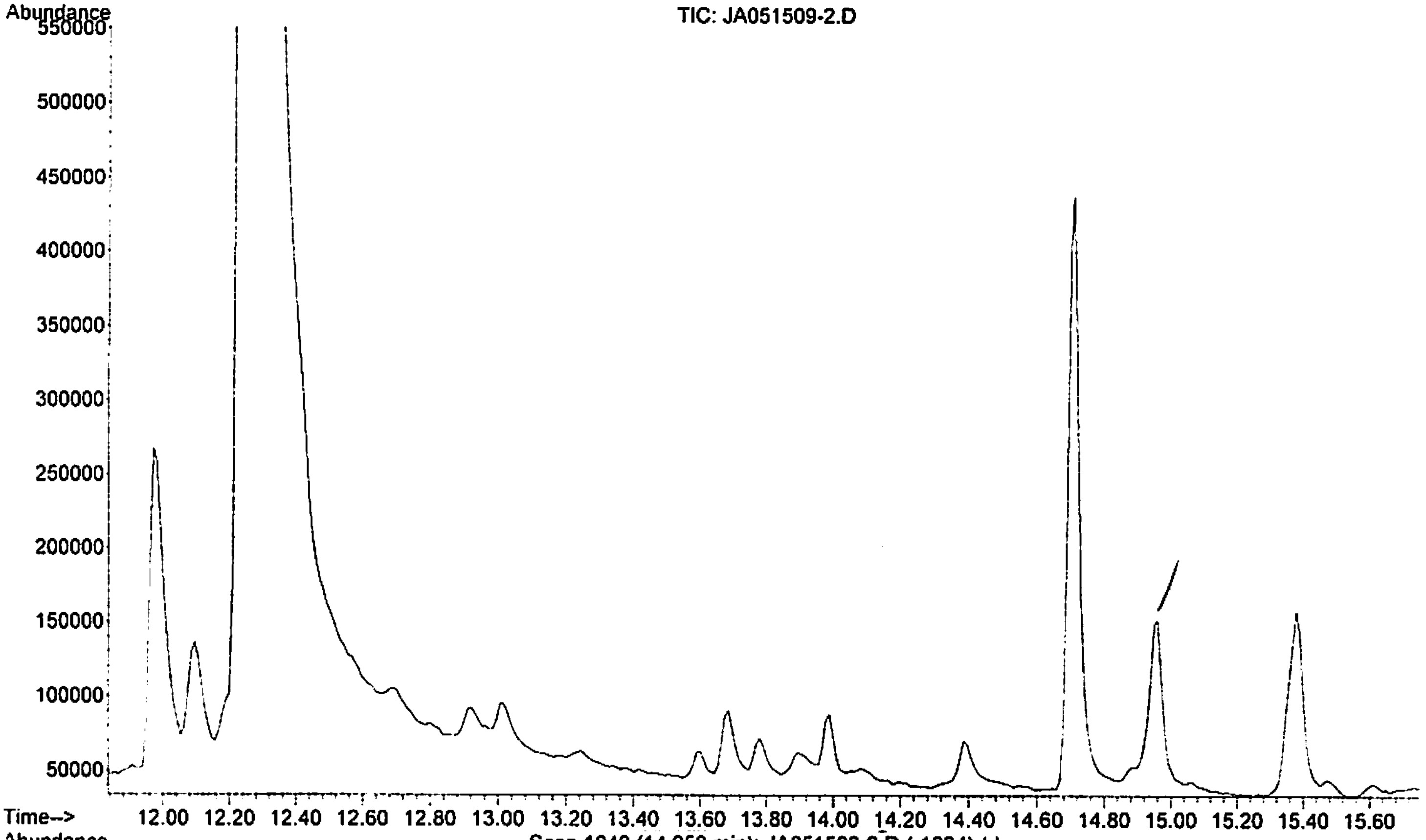

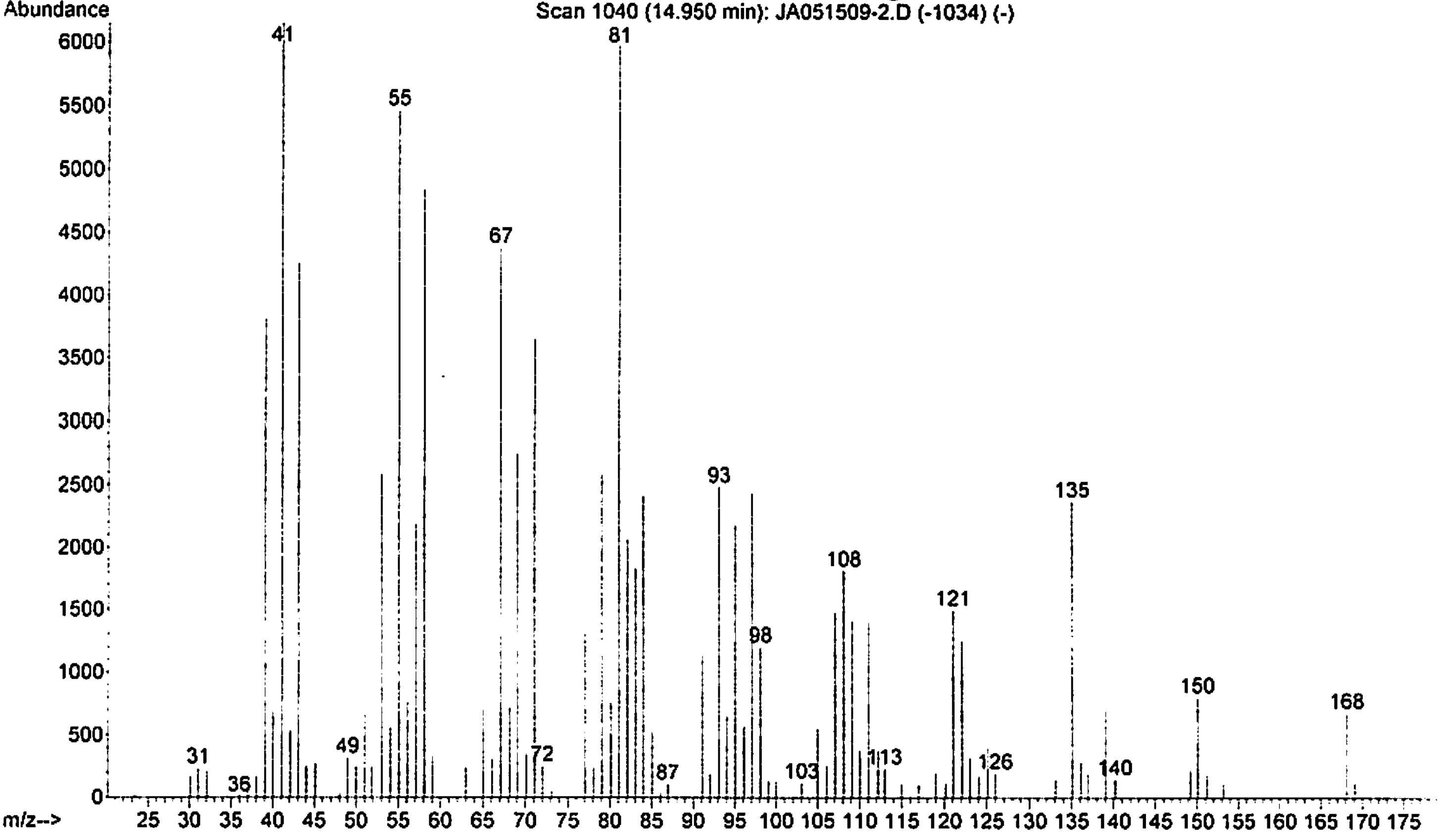

[illegible]e [illegible] : D:\DATA\ALDRICH\JA-09\Snapshot\JA051509-2.D
[illegible]rator : Aldrich
[illegible]uired : 15 May 2009 13:47 using AcqMethod JA-WAX08.M
[illegible]trument : Instrument #1
[illegible]ple Name: 4 field coll. male C.oculata abd/2ul CH2Cl2
[illegible] Info : Ed coll. sweeping vetch, 5/13&14; fed in lab
[illegible]l Number: 1

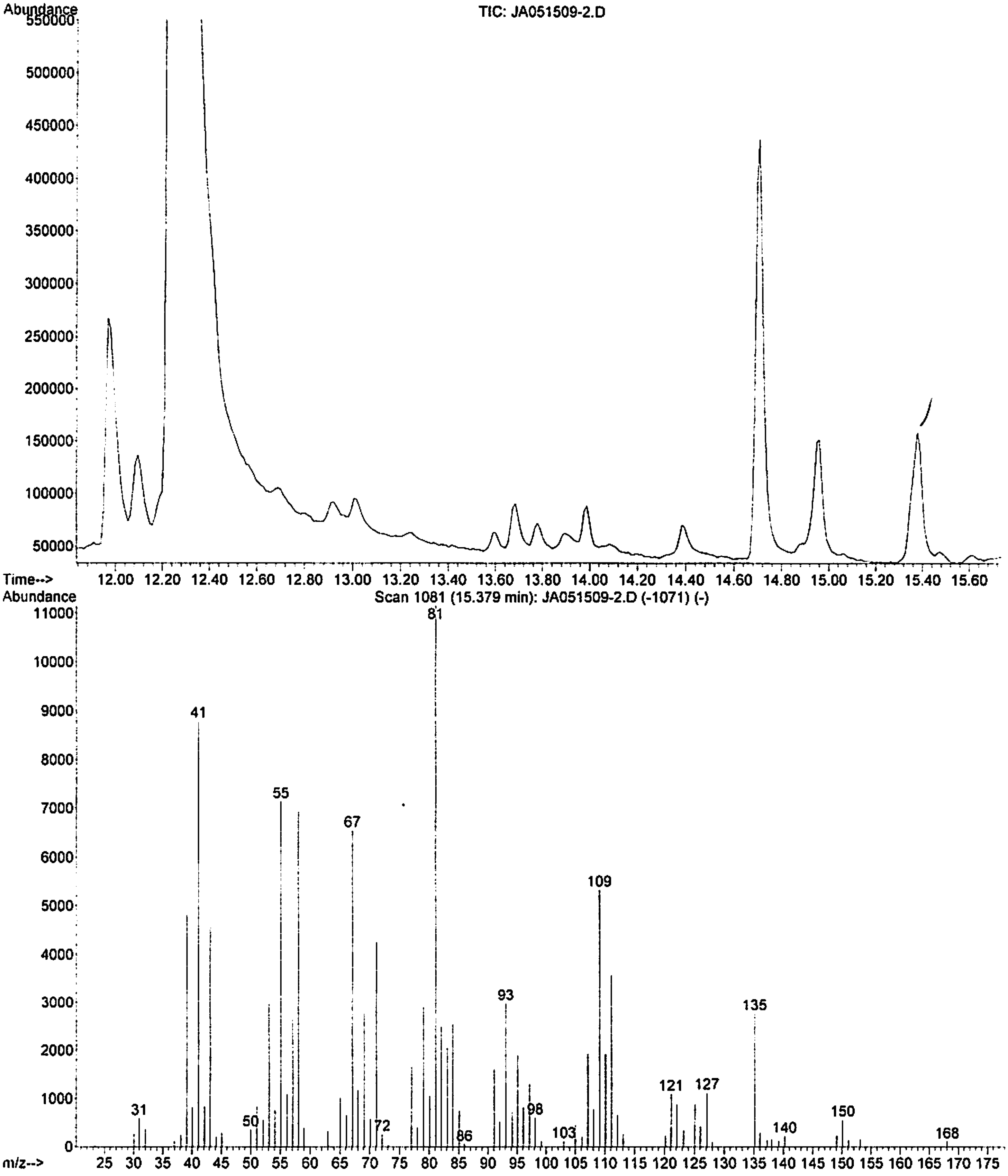

:D:\DATA\ALDRICH\JA-09\Snapshot\JA051509-2.D
rator : Aldrich
uired : 15 May 2009 13:47 using AcqMethod JA-WAX08.M
trument : Instrument #1
ple Name: 4 field coll. male C.oculata abd/2ul CH2Cl2
c Info : Ed coll. sweeping vetch, 5/13&14; fed in lab
l Number: 1

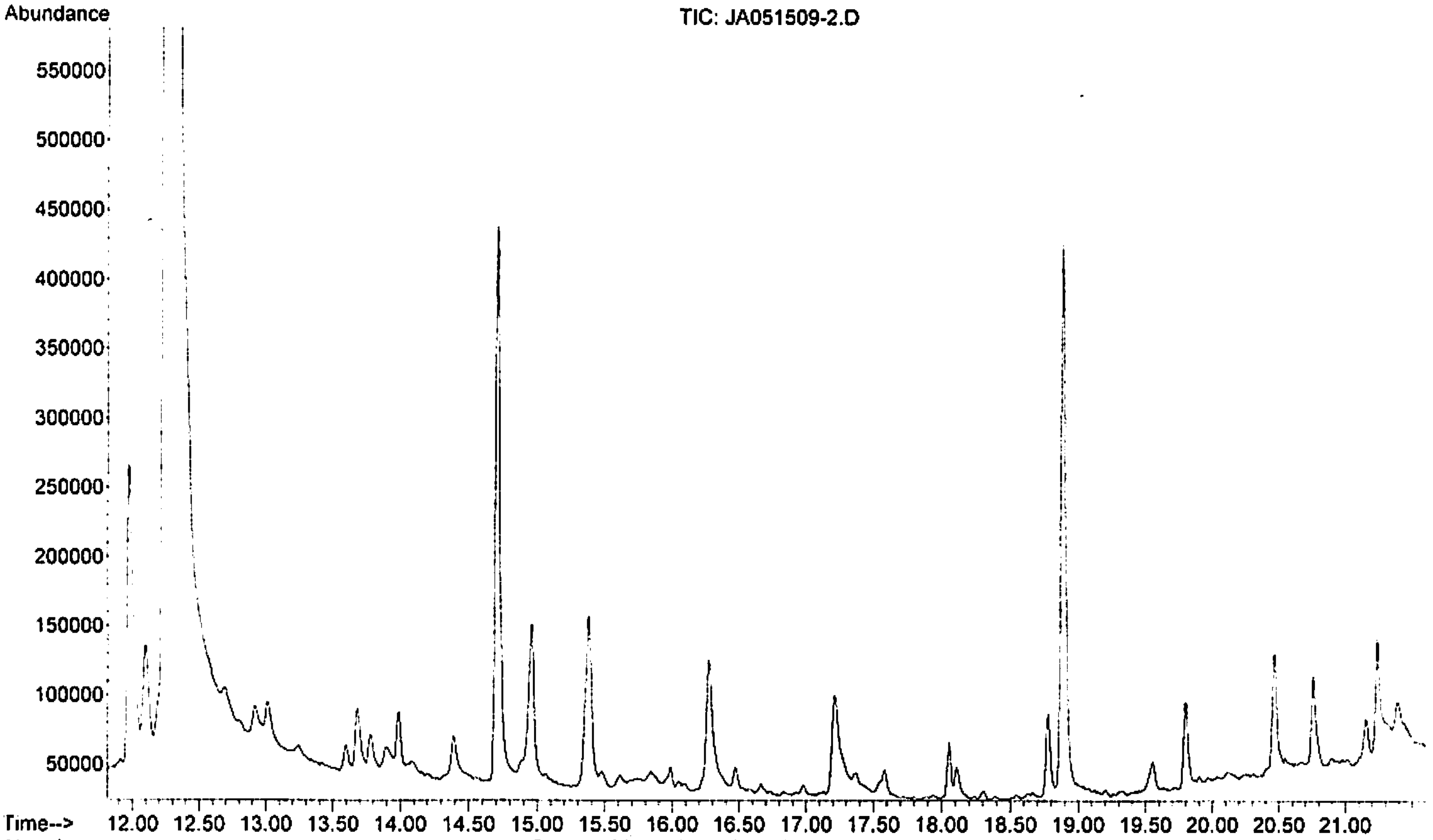

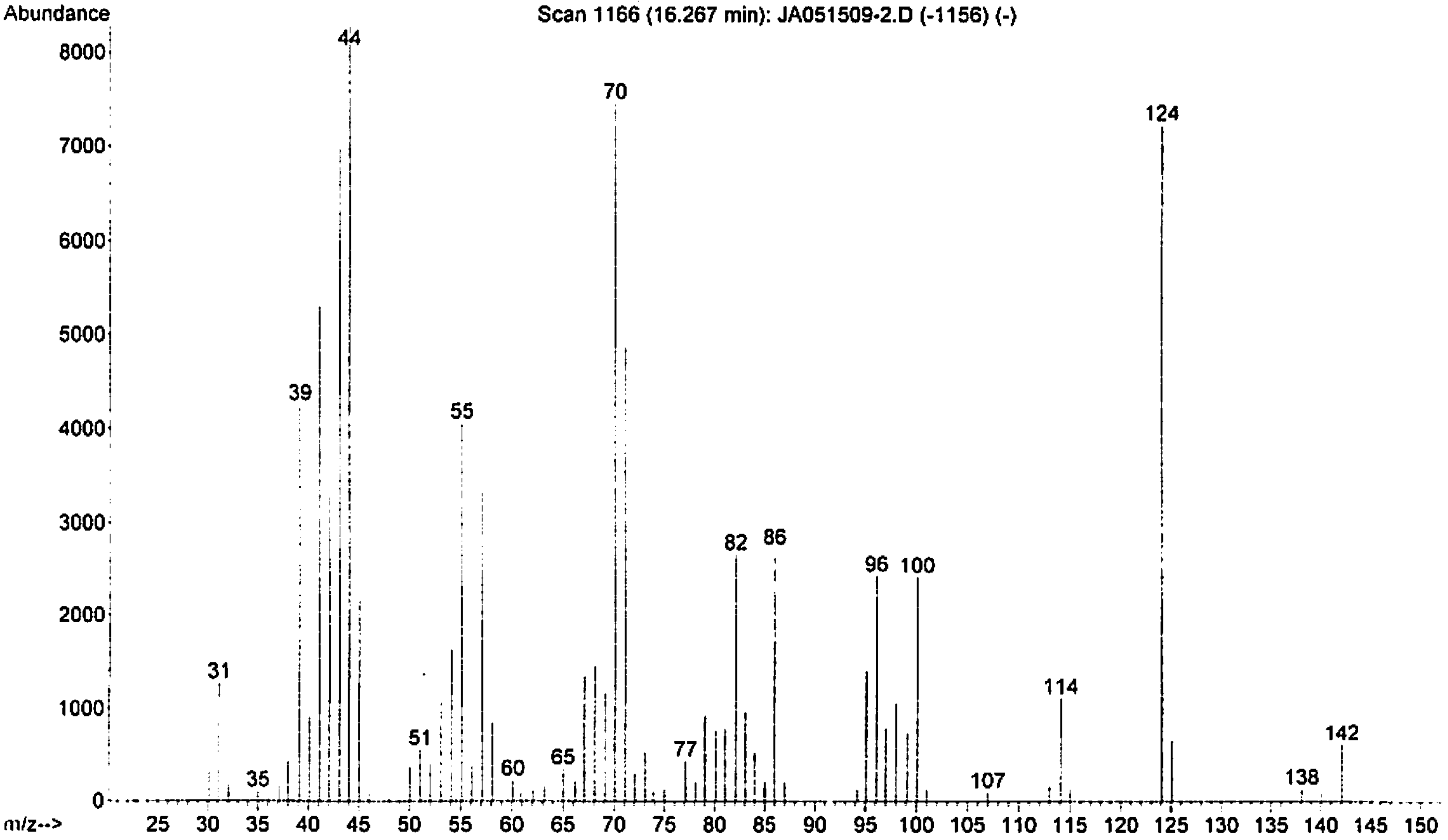

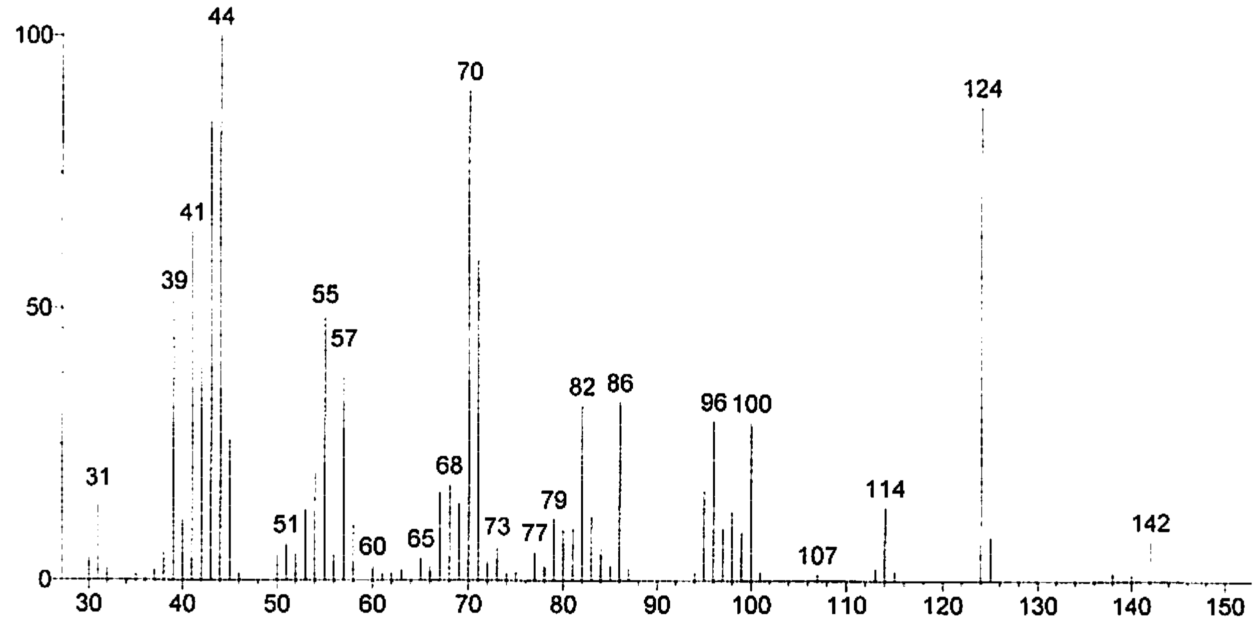

(Text File) Scan 1166 (16.267 min): JA051509-2.D (-1156)

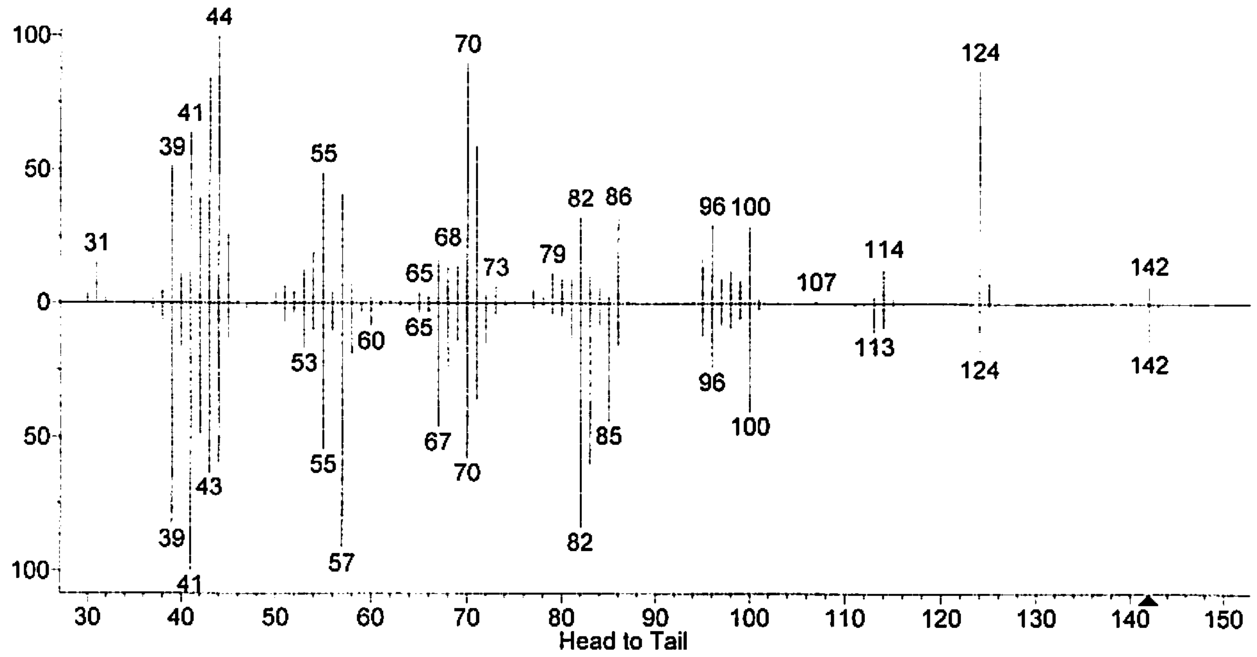

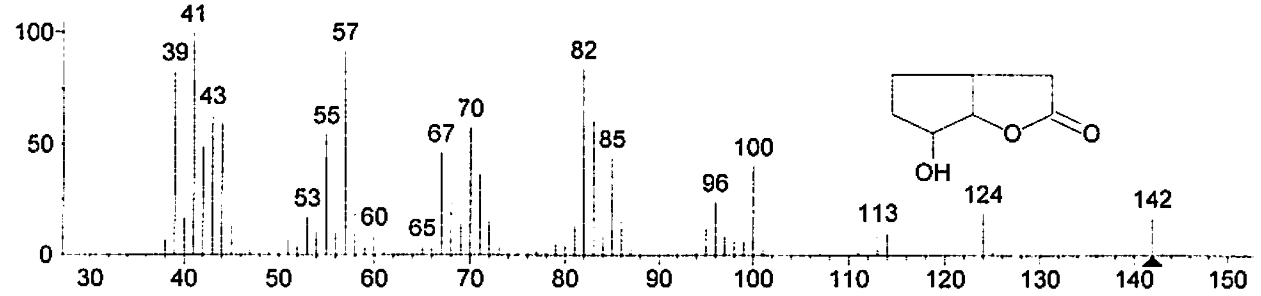

(mainlib) 6-Hydroxyhexahydrocyclopenta[b]furan-2-one

```
[illegible]     :D:\DATA\ALDRICH\JA-09\Snapshot\JA051509-2.D
[illegible]rator    : Aldrich
[illegible]ired     : 15 May 2009  13:47      using AcqMethod JA-WAX08.M
[illegible]rument :     Instrument #1
[illegible]ple Name: 4 field coll. male C.oculata abd/2ul CH2Cl2
[illegible] Info   : Ed coll. sweeping vetch, 5/13&14; fed in lab
[illegible]l Number: 1
```

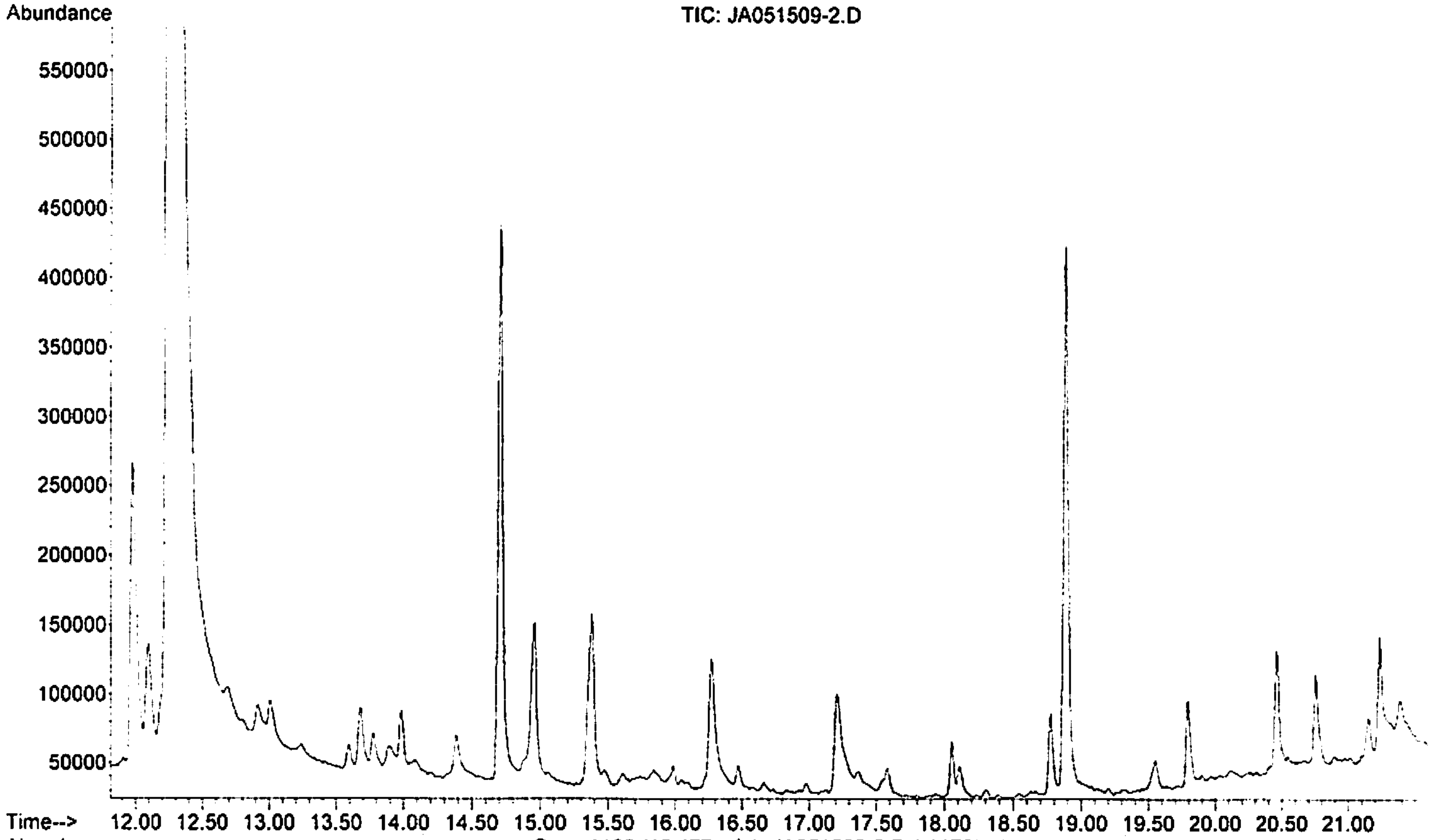

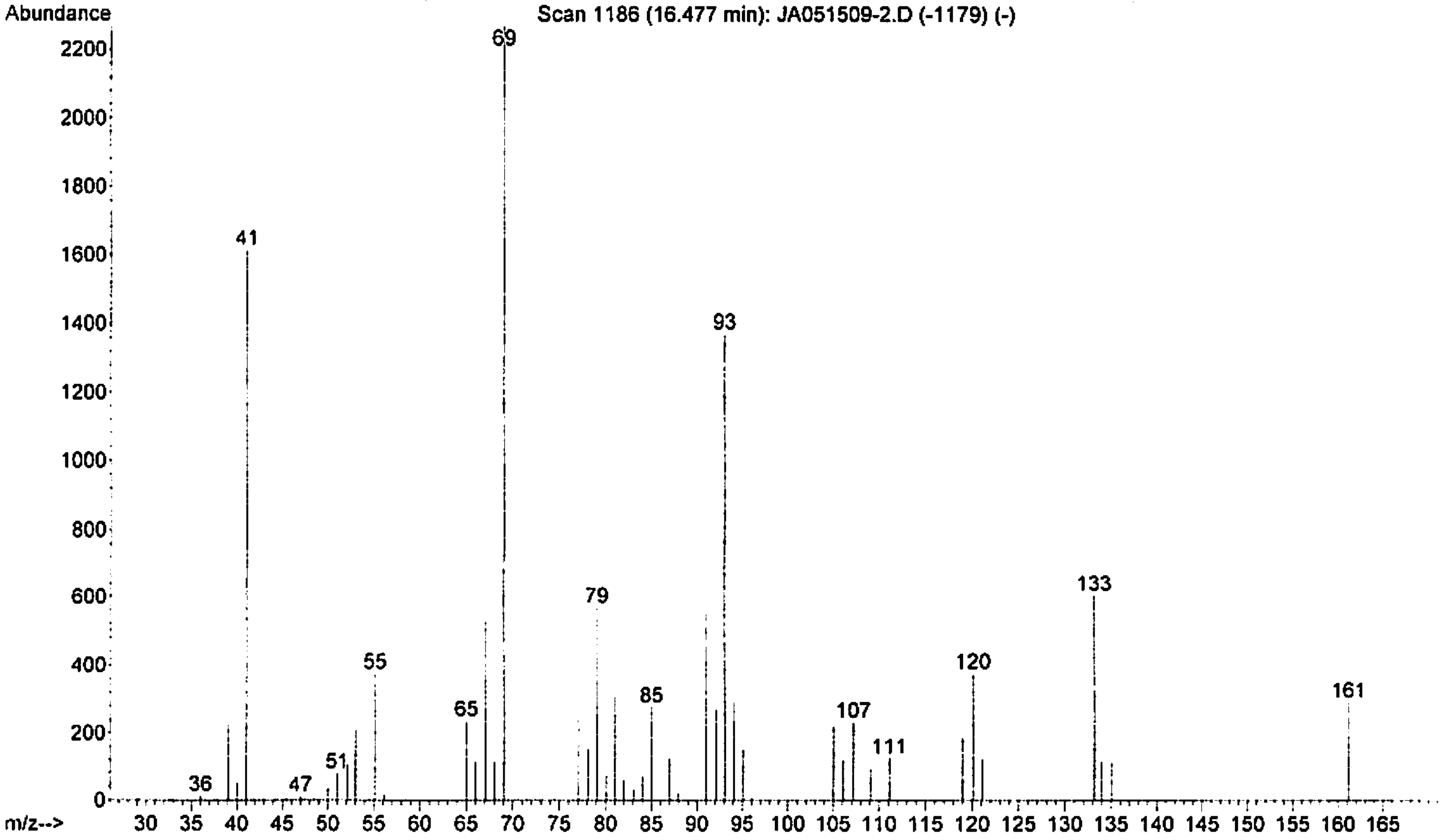

[illegible] :D:\DATA\Aldrich\JA-09\JA051509-2.D
[illegible]rator : Aldrich
[illegible]uired : 15 May 2009 13:47 using AcqMethod JA-WAX08.M
[illegible]trument : Instrument #1
[illegible]ple Name: 4 field coll. male C.oculata abd/2ul CH2Cl2
[illegible] Info : Ed coll. sweeping vetch, 5/13&14; fed in lab
[illegible] Number: 1

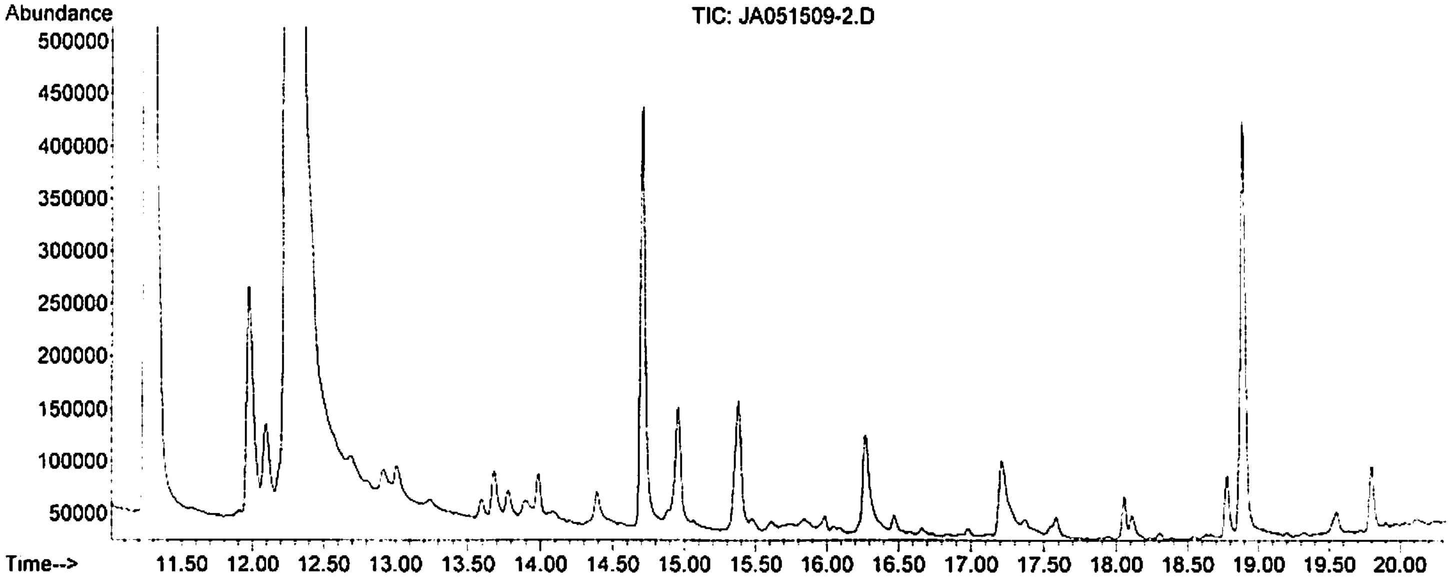

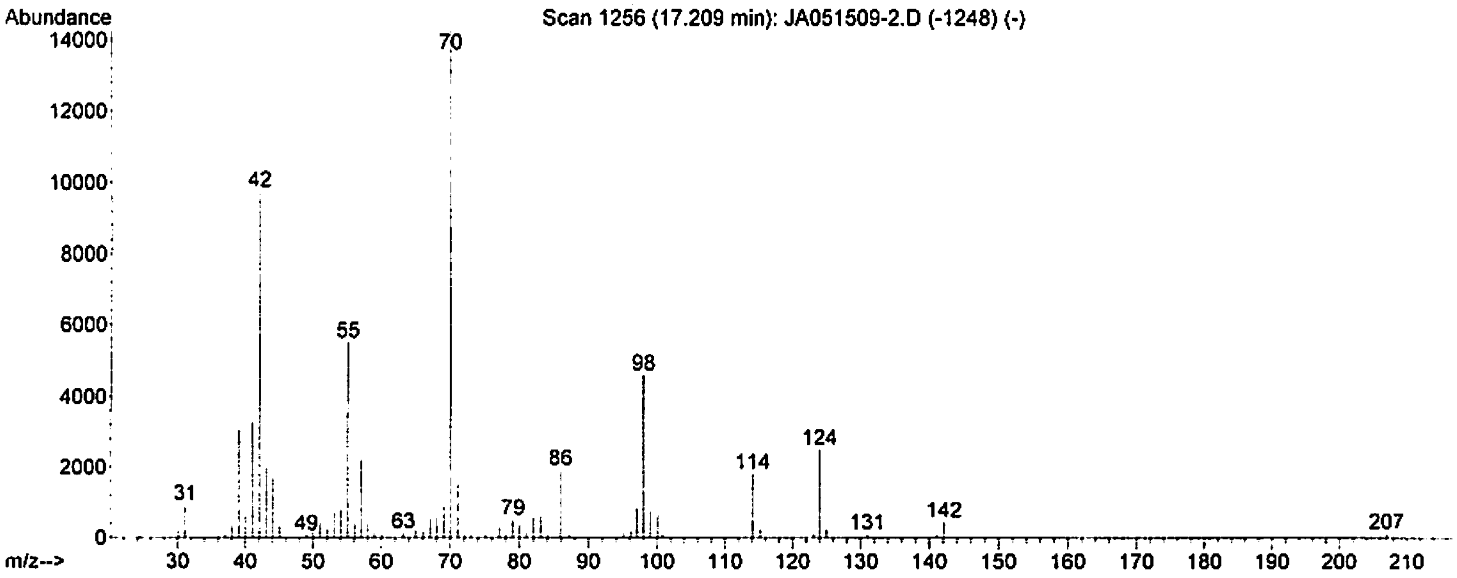

.e   :D:\DATA\Aldrich\JA-09\JA051509-2.D
rator   : Aldrich
uired   : 15 May 2009 13:47   using AcqMethod JA-WAX08.M
trument :   Instrument #1
ple Name: 4 field coll. male C.oculata abd/2ul CH2Cl2
 Info   : Ed coll. sweeping vetch, 5/13&14; fed in lab
l Number: 1

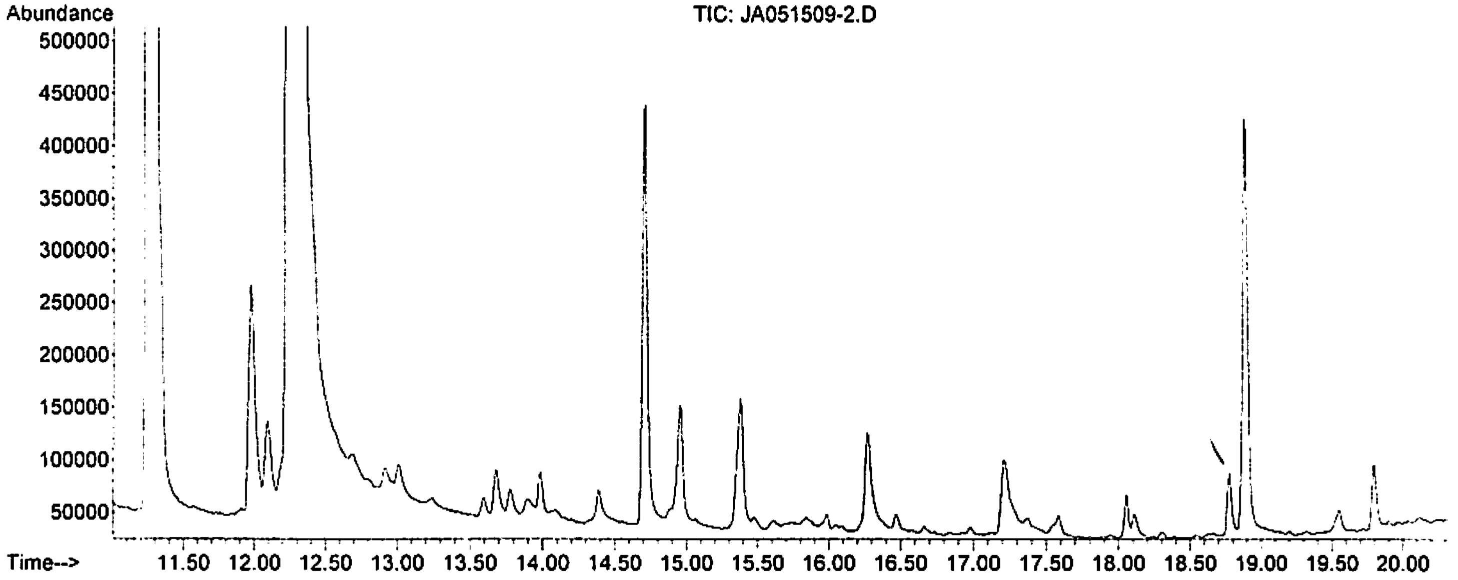

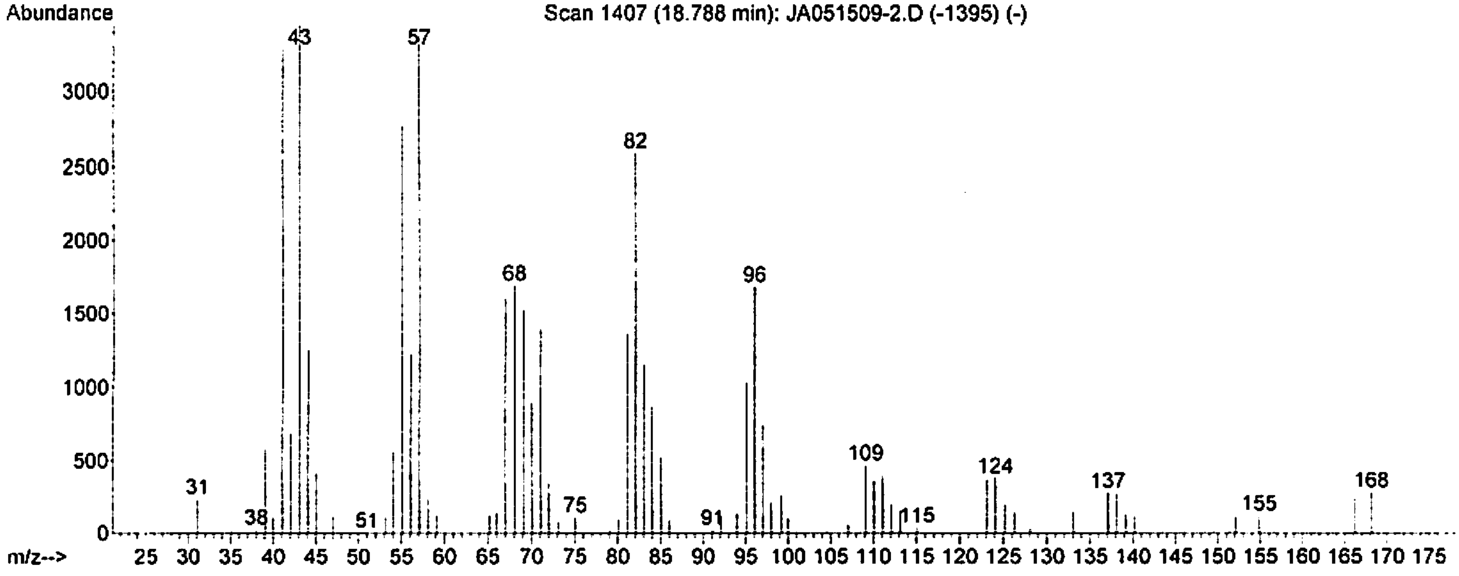

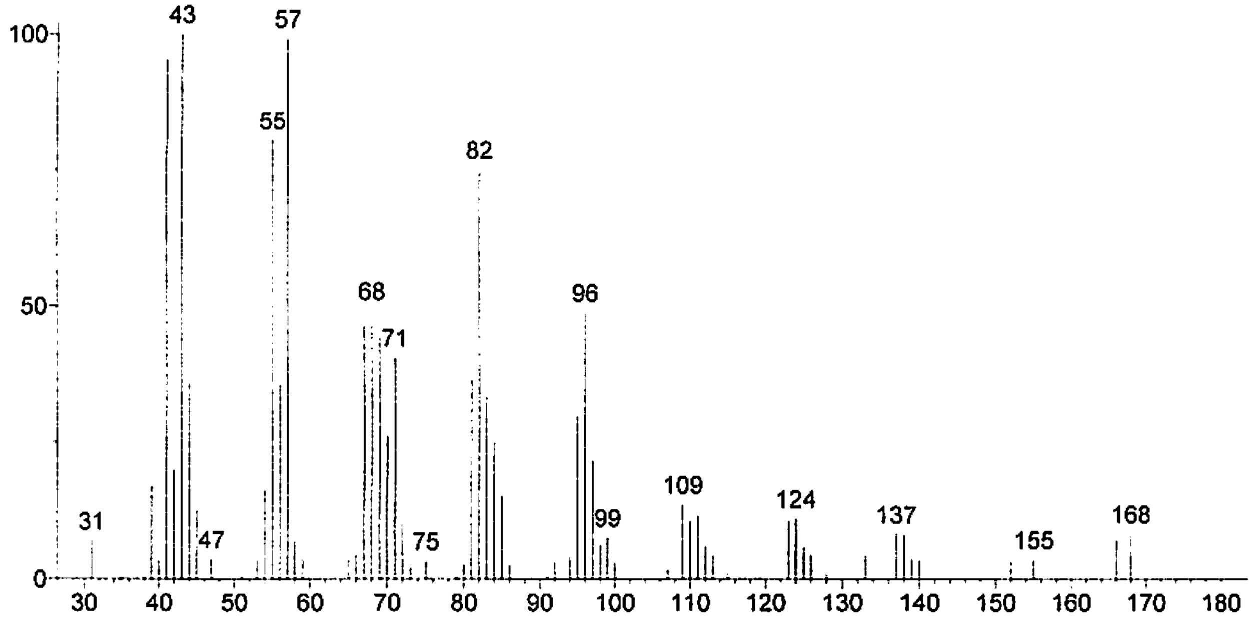

(Text File) Scan 1407 (18.788 min): JA051509-2.D (-1395)

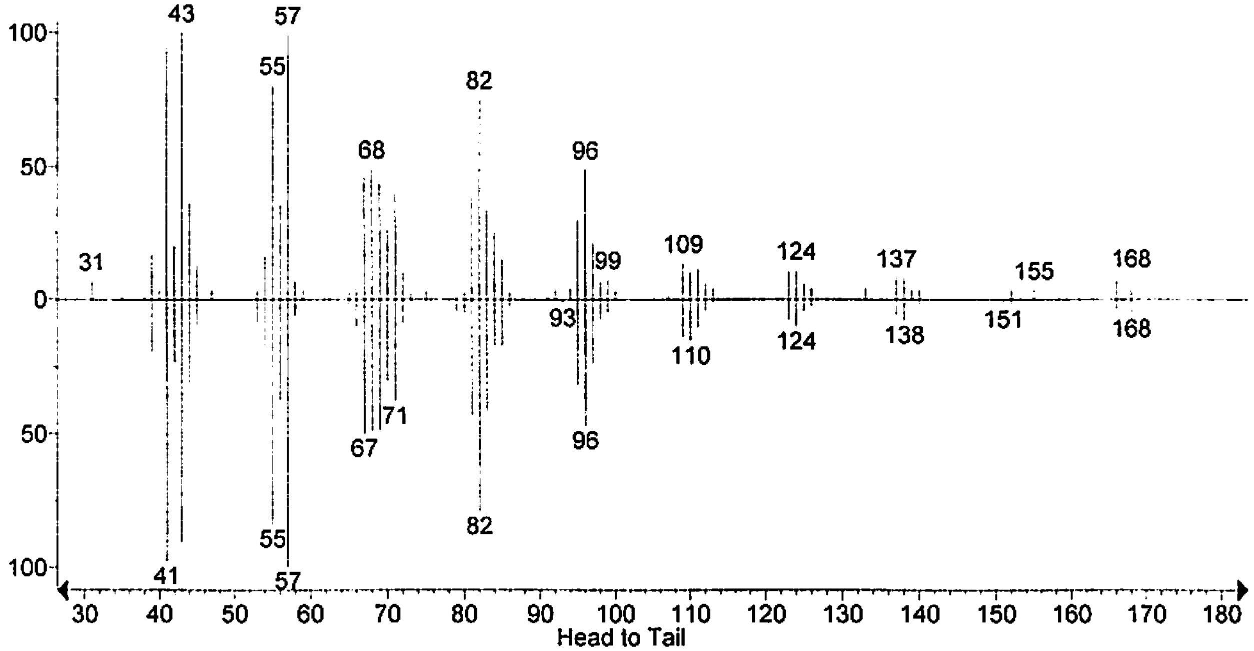

Head to Tail

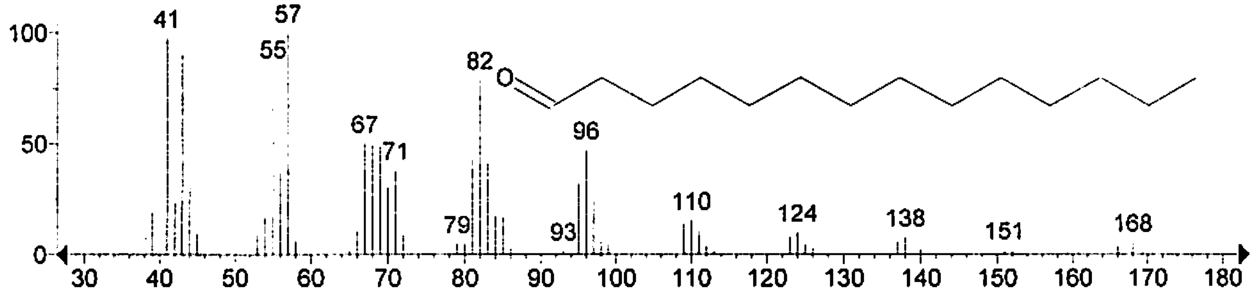

(replib) Tetradecanal

```
[illegible]e        :D:\DATA\Aldrich\JA-09\JA051509-2.D
[illegible]rator    : Aldrich
[illegible]uired    : 15 May 2009  13:47     using AcqMethod JA-WAX08.M
[illegible]trument :    Instrument #1
[illegible]ple Name: 4 field coll. male C.oculata abd/2ul CH2Cl2
[illegible]c Info   : Ed coll. sweeping vetch, 5/13&14; fed in lab
[illegible]l Number: 1
```

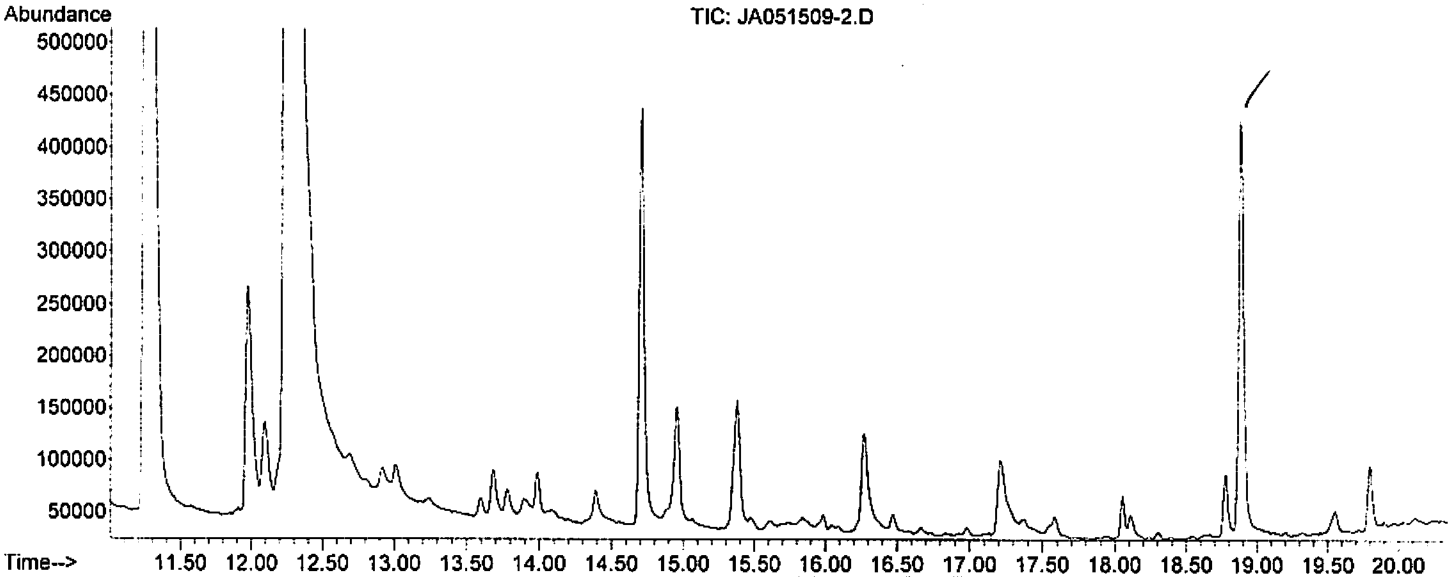

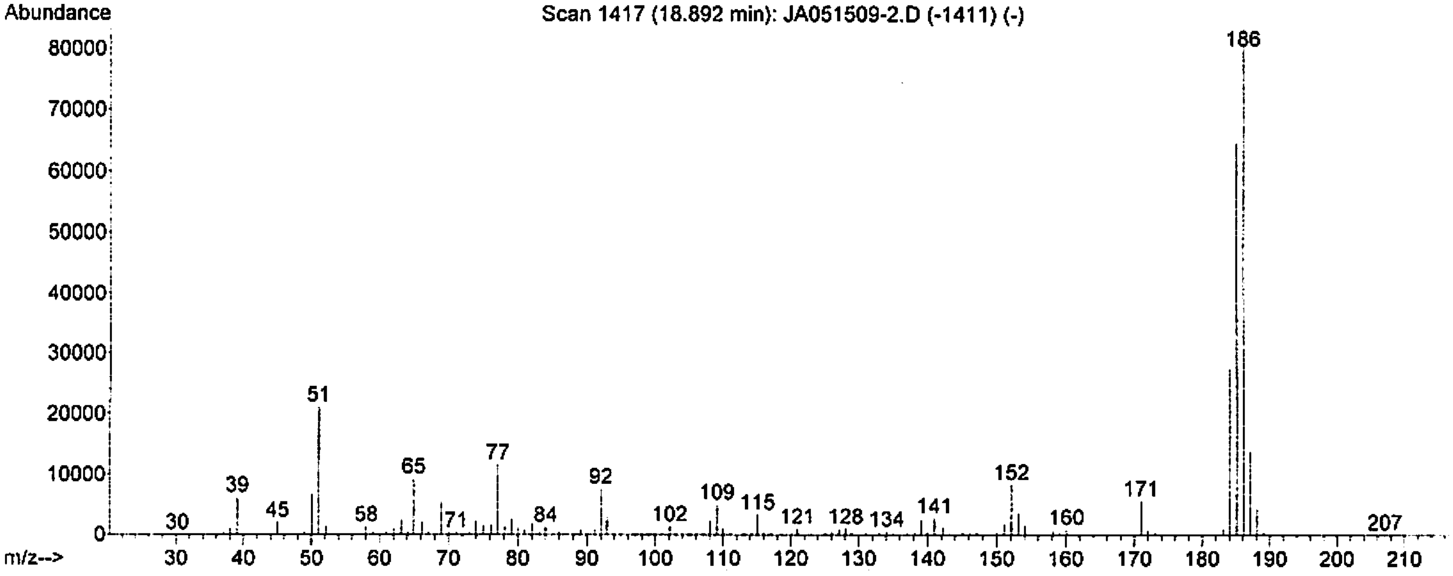

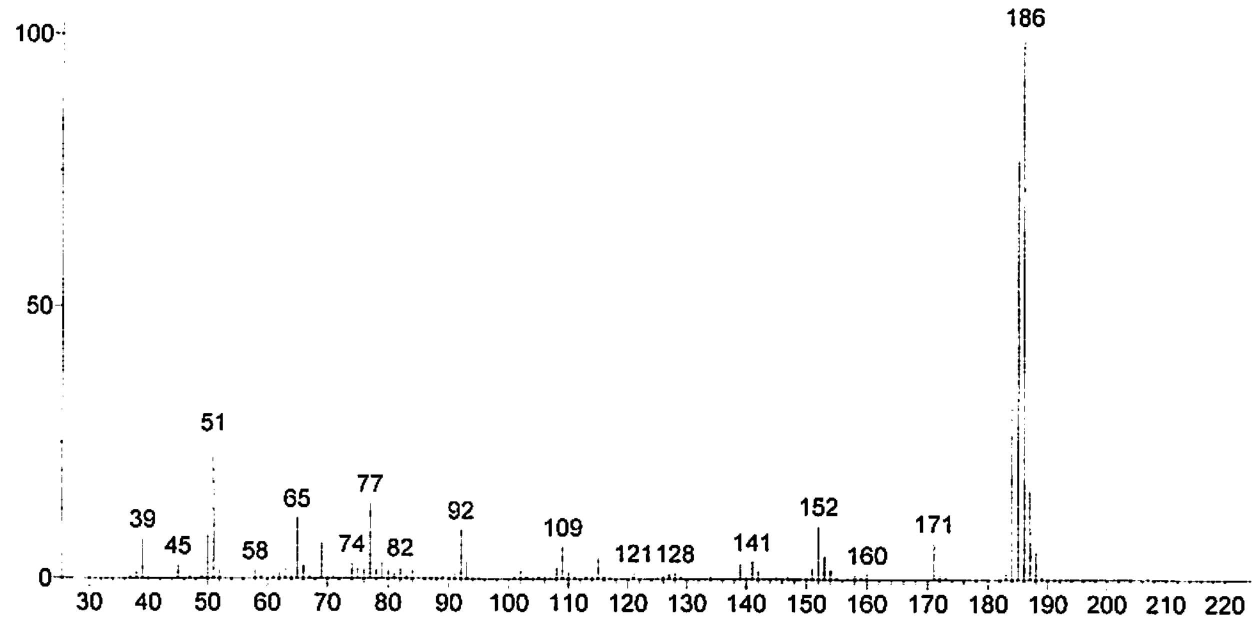

(Text File) Scan 1417 (18.892 min): JA051509-2.D (-1411)

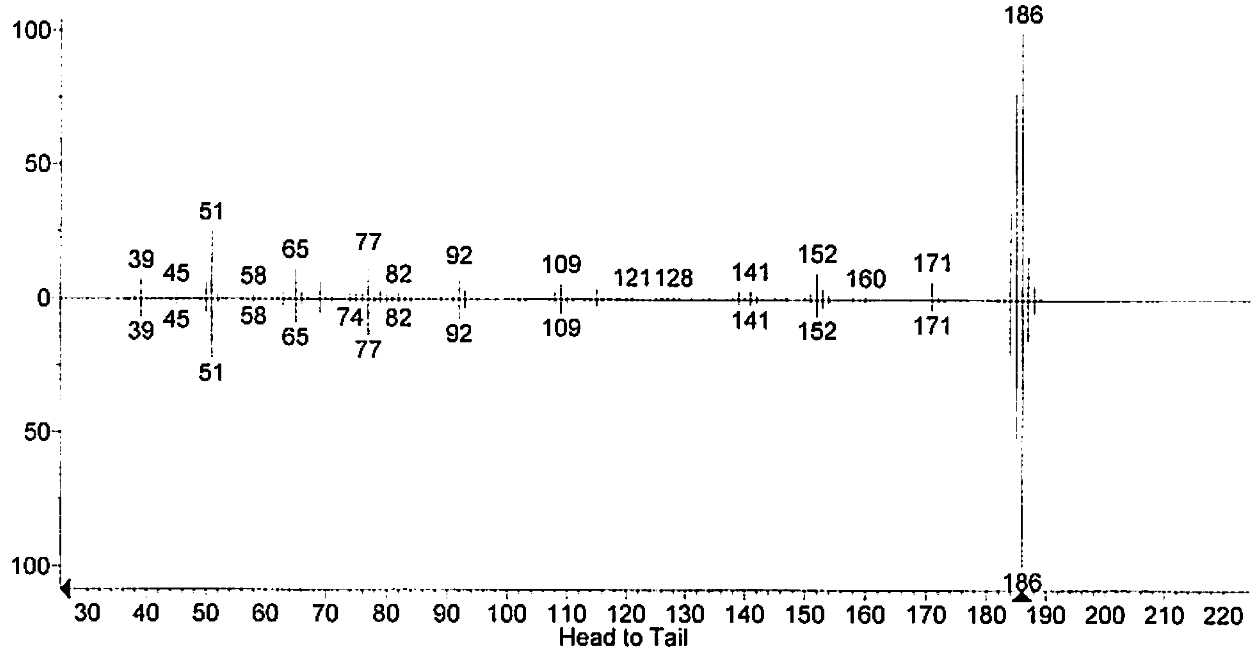

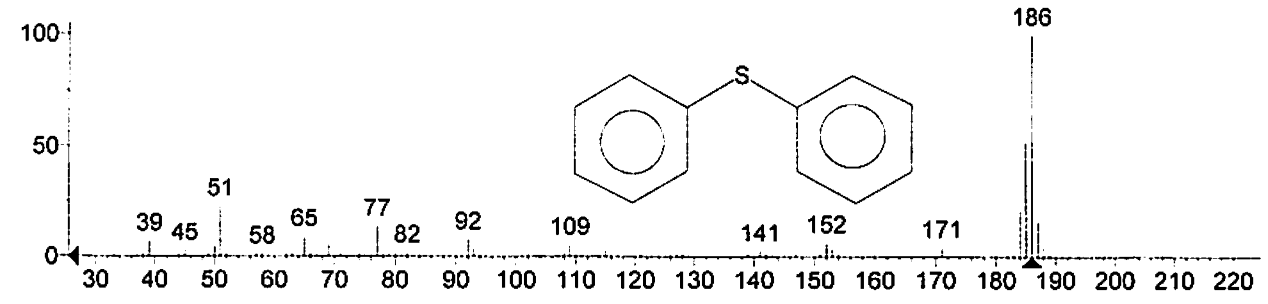

(replib) Diphenyl sulfide

e : D:\DATA\Aldrich\JA-09\JA051509-2.D
rator : Aldrich
uired : 15 May 2009 13:47 using AcqMethod JA-WAX08.M
trument : Instrument #1
ple Name: 4 field coll. male C.oculata abd/2ul CH2Cl2
c Info : Ed coll. sweeping vetch, 5/13&14; fed in lab
l Number: 1

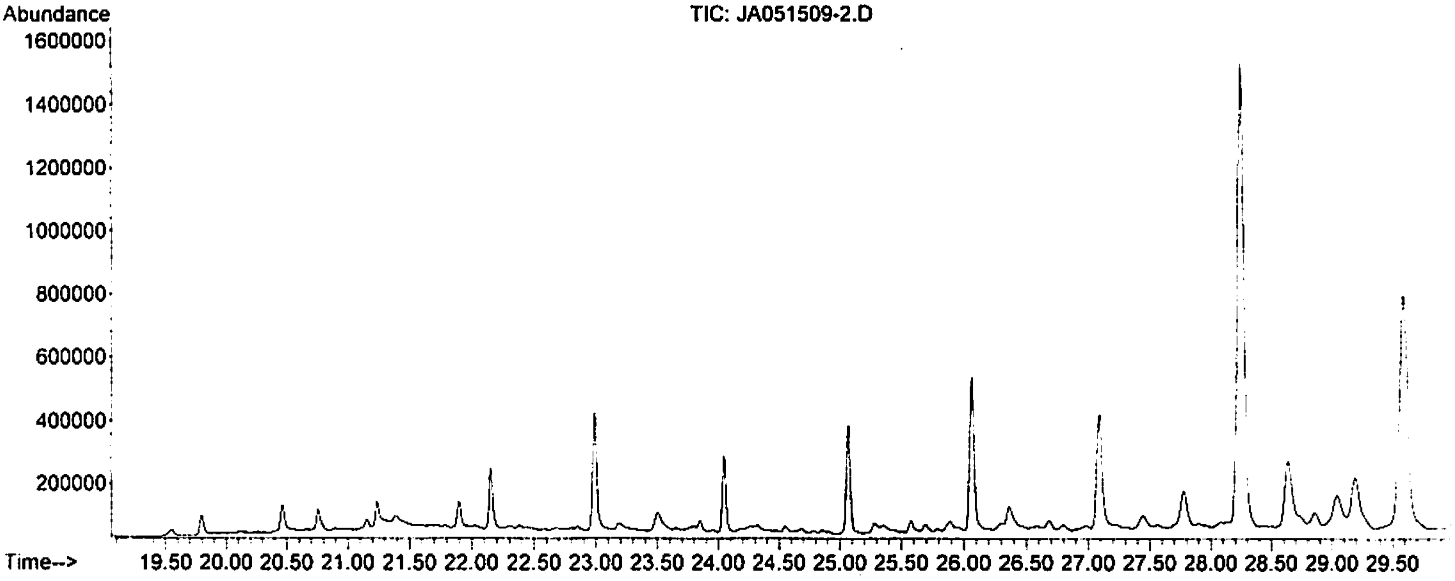

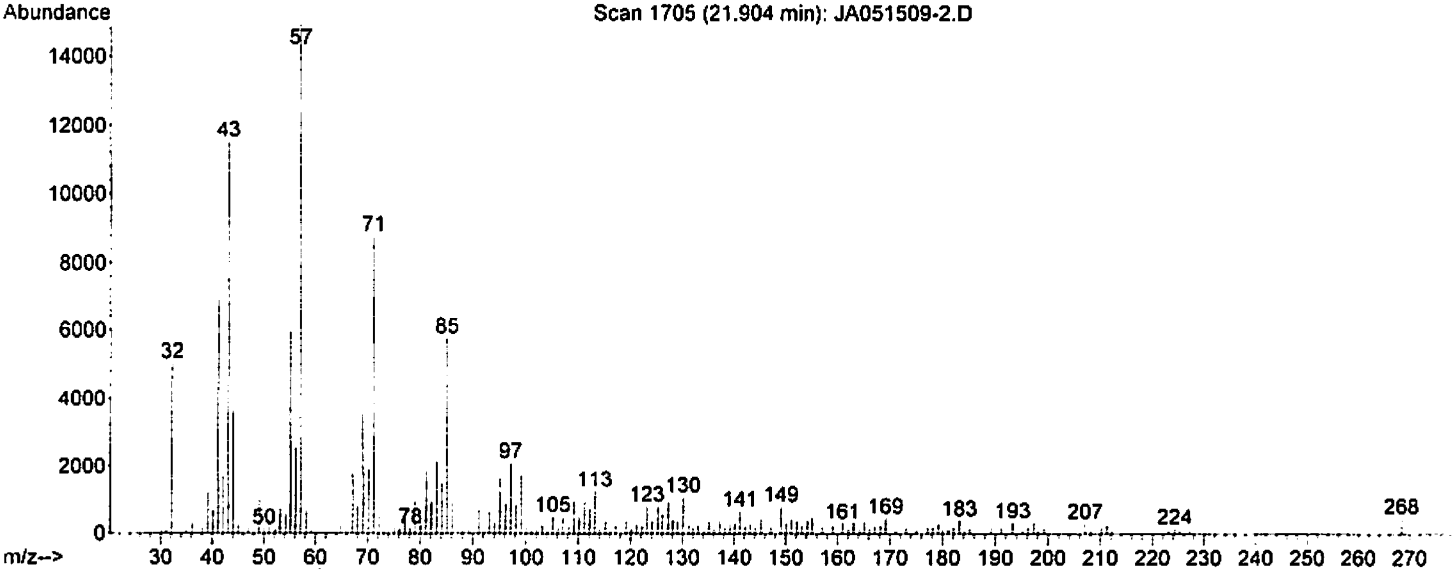

e • :D:\DATA\Aldrich\JA-09\JA051509-2.D
rator : Aldrich
uired : 15 May 2009 13:47 using AcqMethod JA-WAX08.M
trument : Instrument #1
ple Name: 4 field coll. male C.oculata abd/2ul CH2Cl2
Info : Ed coll. sweeping vetch, 5/13&14; fed in lab
l Number: 1

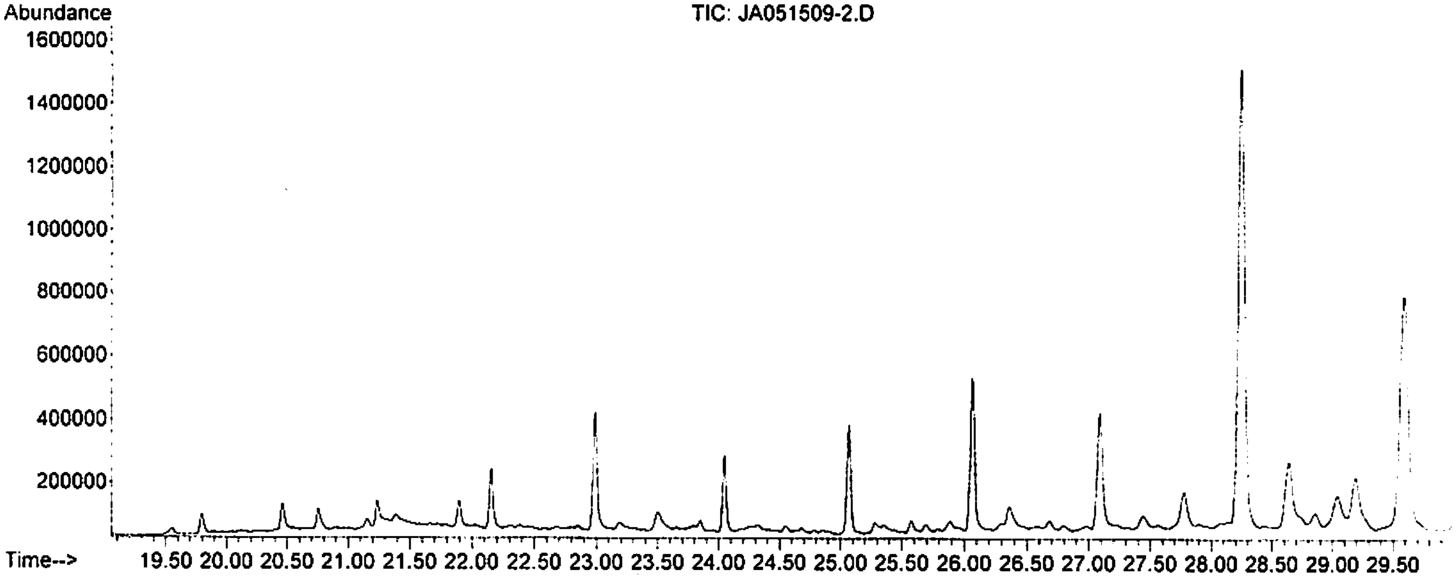

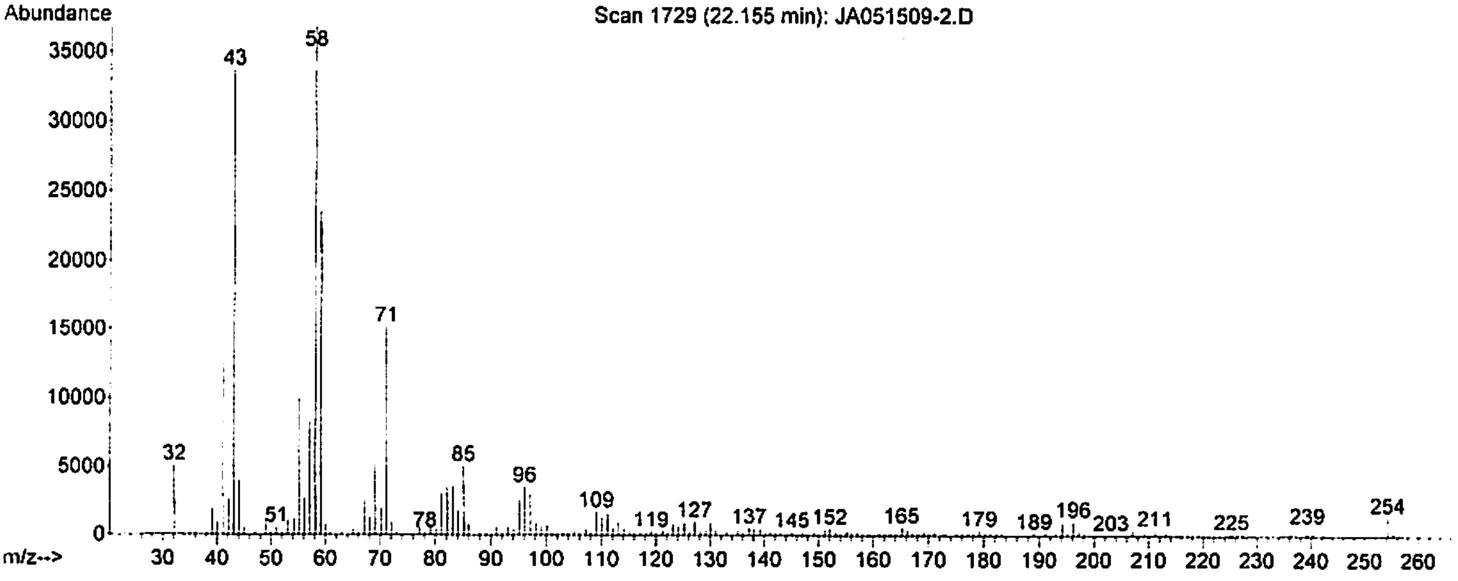

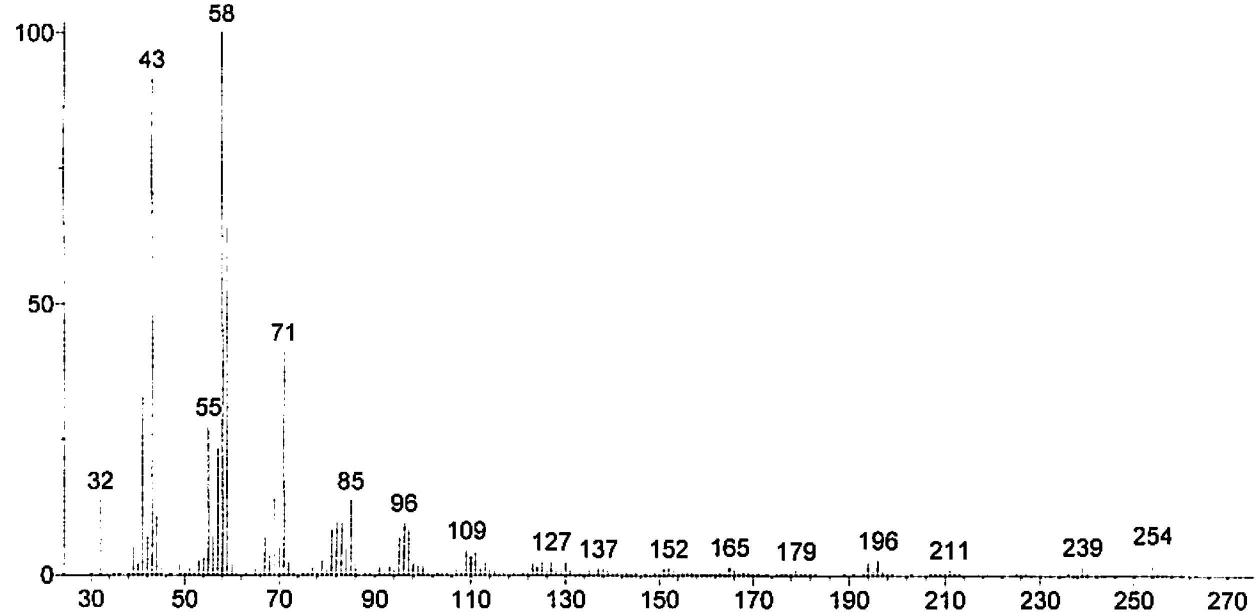

(Text File) Scan 1729 (22.155 min): JA051509-2.D

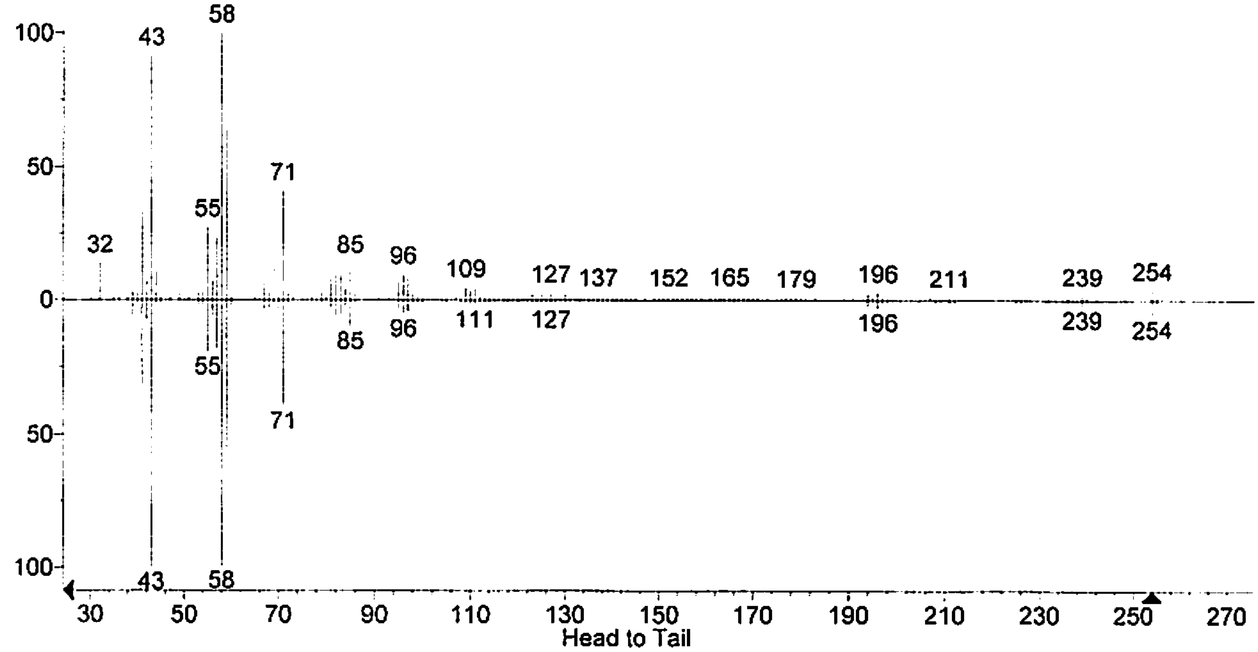

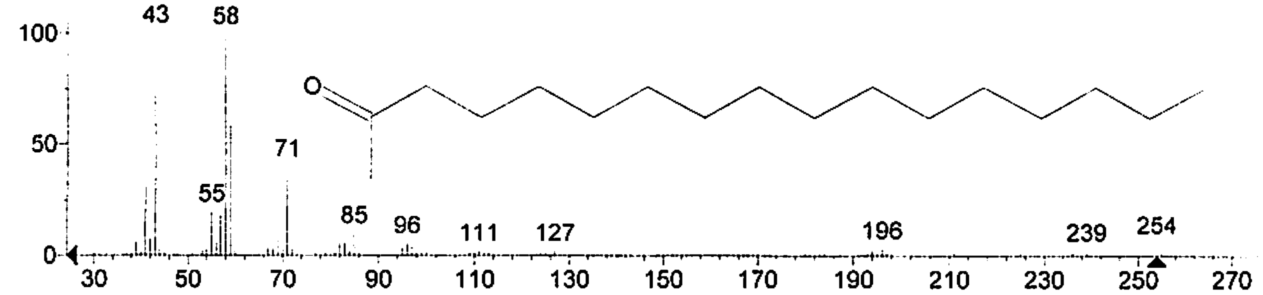

(replib) 2-Heptadecanone

e :D:\DATA\Aldrich\JA-09\JA051509-2.D
rator : Aldrich
uired : 15 May 2009 13:47 using AcqMethod JA-WAX08.M
trument : Instrument #1
ple Name: 4 field coll. male C.oculata abd/2ul CH2C12
c Info : Ed coll. sweeping vetch, 5/13&14; fed in lab
l Number: 1

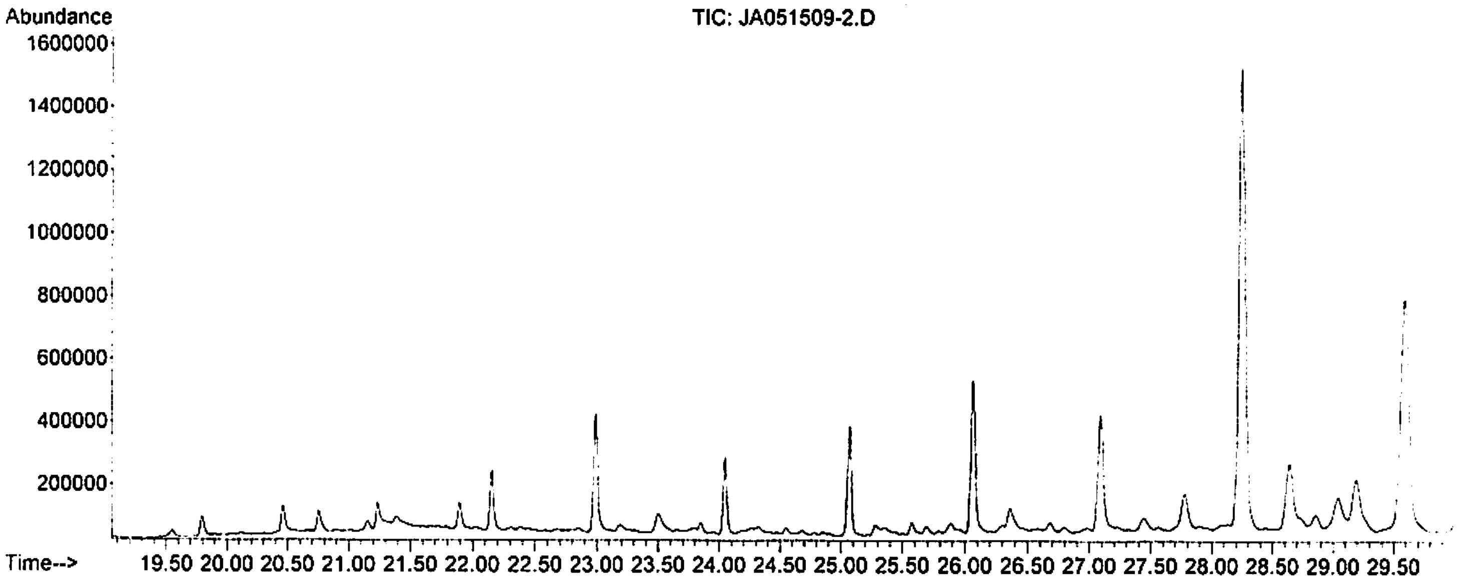

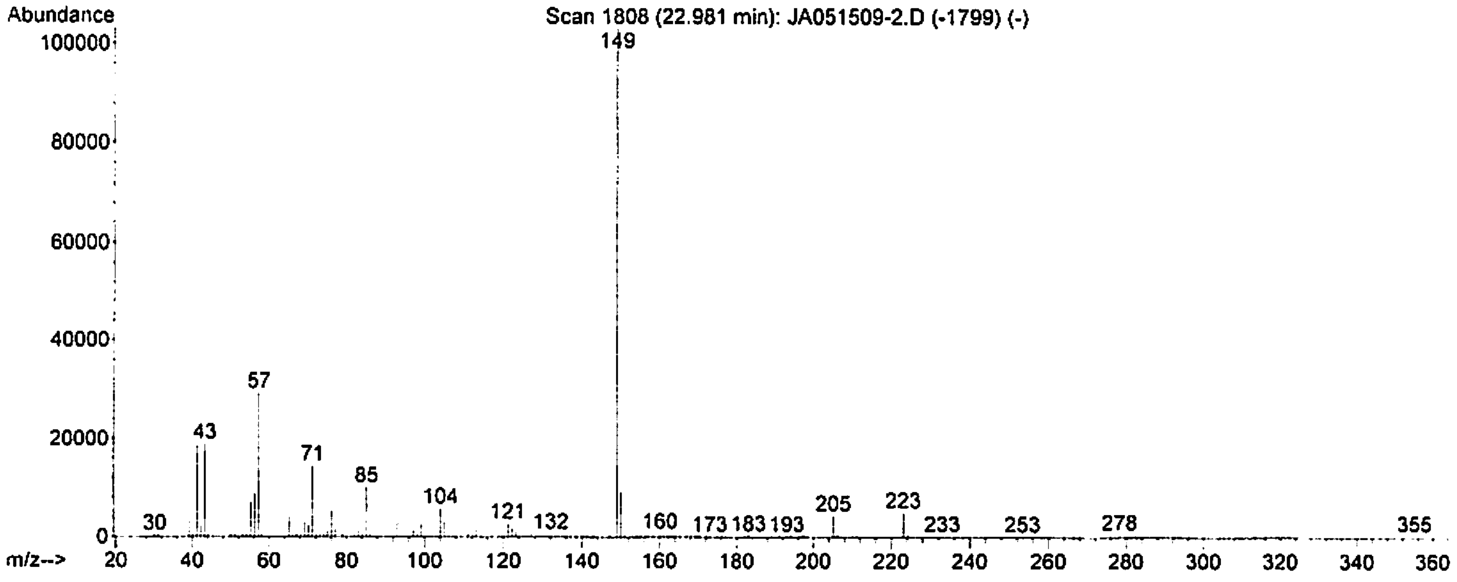

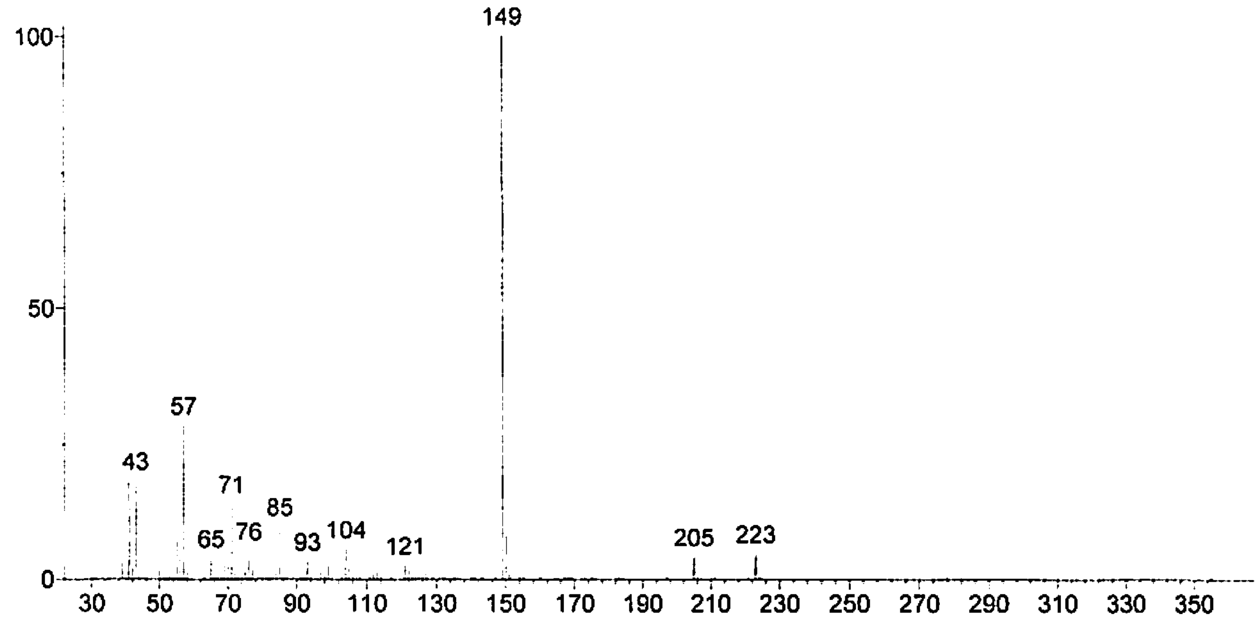

(Text File) Scan 1808 (22.981 min): JA051509-2.D (-1799)

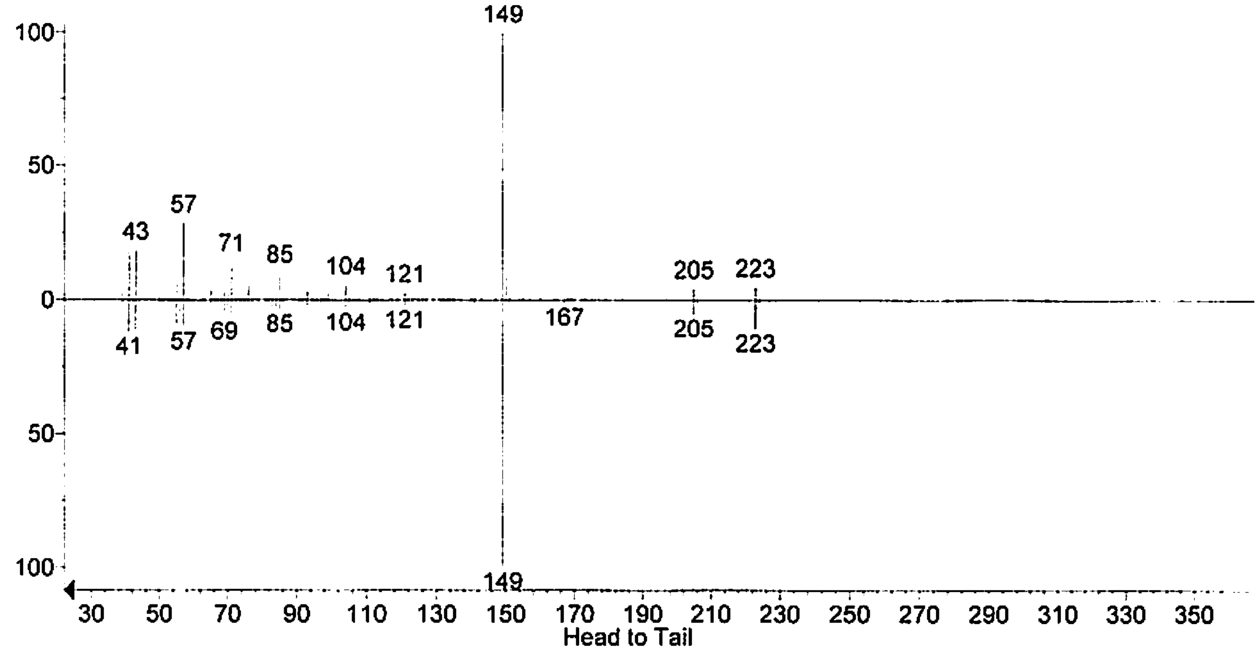

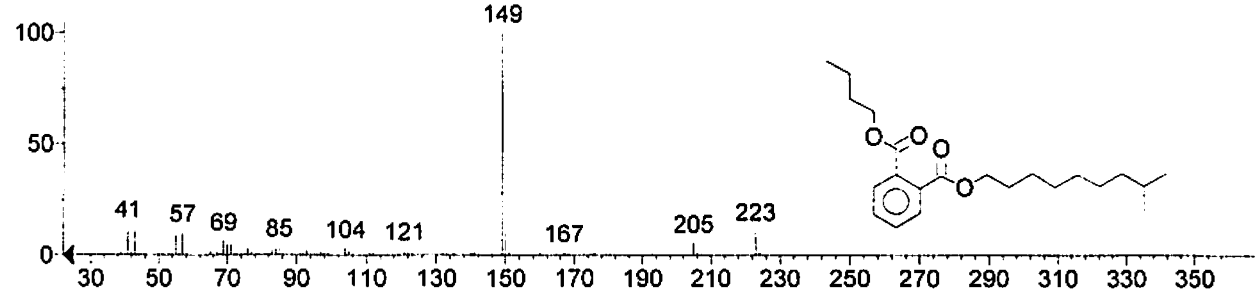

(replib) 1,2-Benzenedicarboxylic acid, butyl 8-methylnonyl ester

:D:\DATA\Aldrich\JA-09\JA051509-2.D
rator : Aldrich
uired : 15 May 2009 13:47 using AcqMethod JA-WAX08.M
trument : Instrument #1
ple Name: 4 field coll. male C.oculata abd/2ul CH2Cl2
Info : Ed coll. sweeping vetch, 5/13&14; fed in lab
l Number: 1

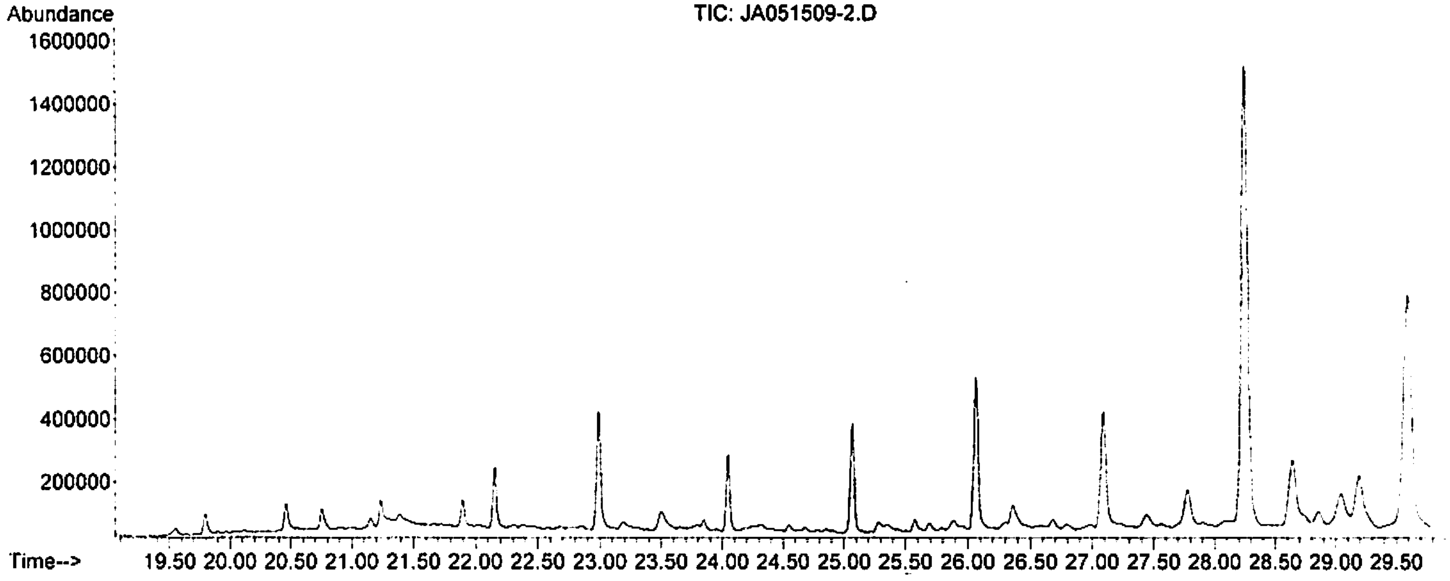

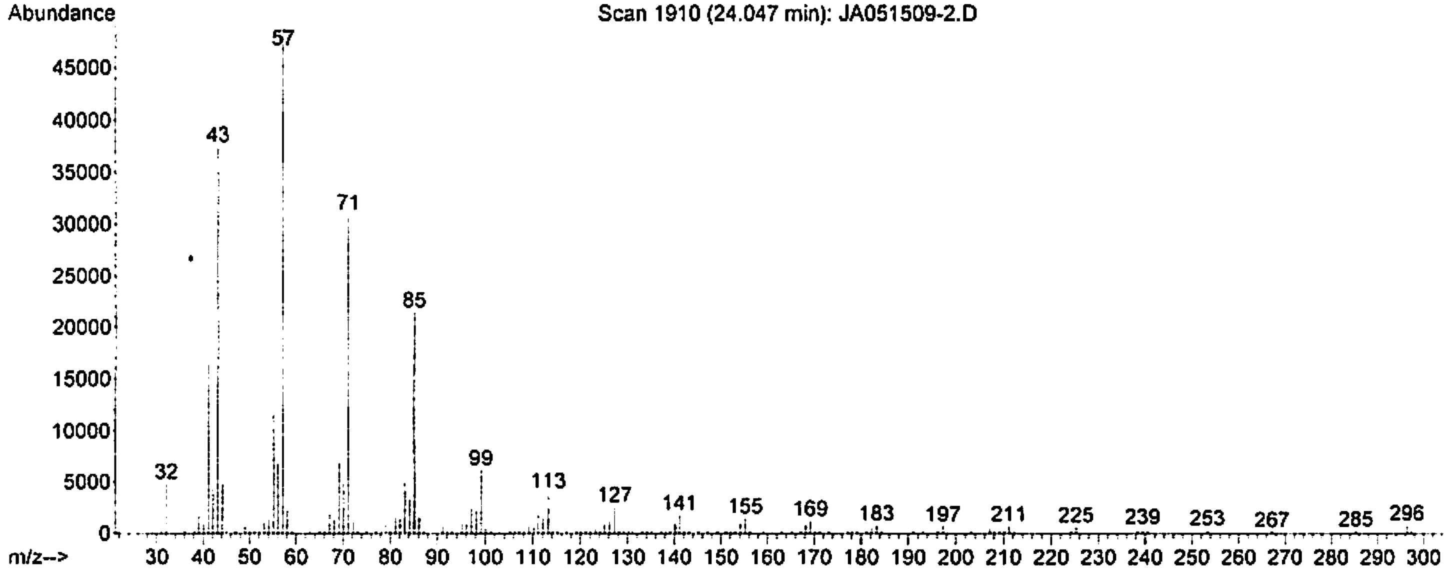

e :D:\DATA\Aldrich\JA-09\JA051509-2.D
rator : Aldrich
uired : 15 May 2009 13:47 using AcqMethod JA-WAX08.M
trument : Instrument #1
ple Name: 4 field coll. male C.oculata abd/2ul CH2Cl2
c Info : Ed coll. sweeping vetch, 5/13&14; fed in lab
l Number: 1

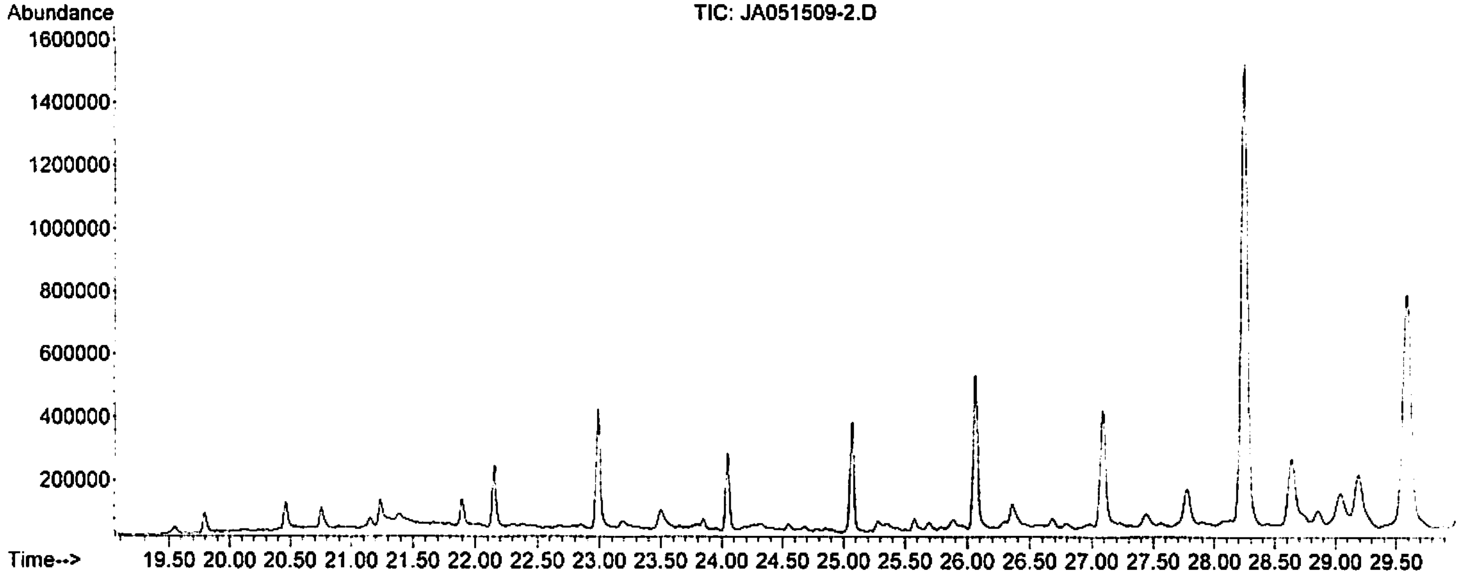

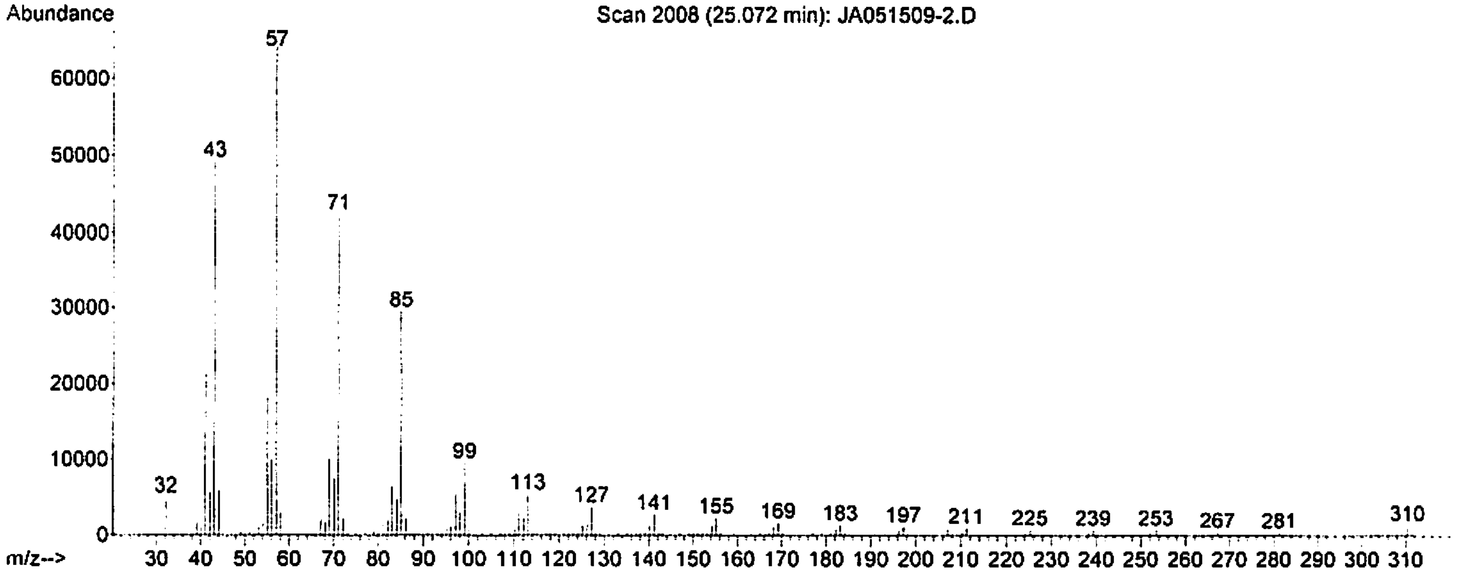

```
e        :D:\DATA\Aldrich\JA-09\JA051509-2.D
rator    : Aldrich
uired    : 15 May 2009  13:47     using AcqMethod JA-WAX08.M
trument  :     Instrument #1
ple Name: 4 field coll. male C.oculata abd/2ul CH2Cl2
c Info   : Ed coll. sweeping vetch, 5/13&14; fed in lab
l Number: 1
```

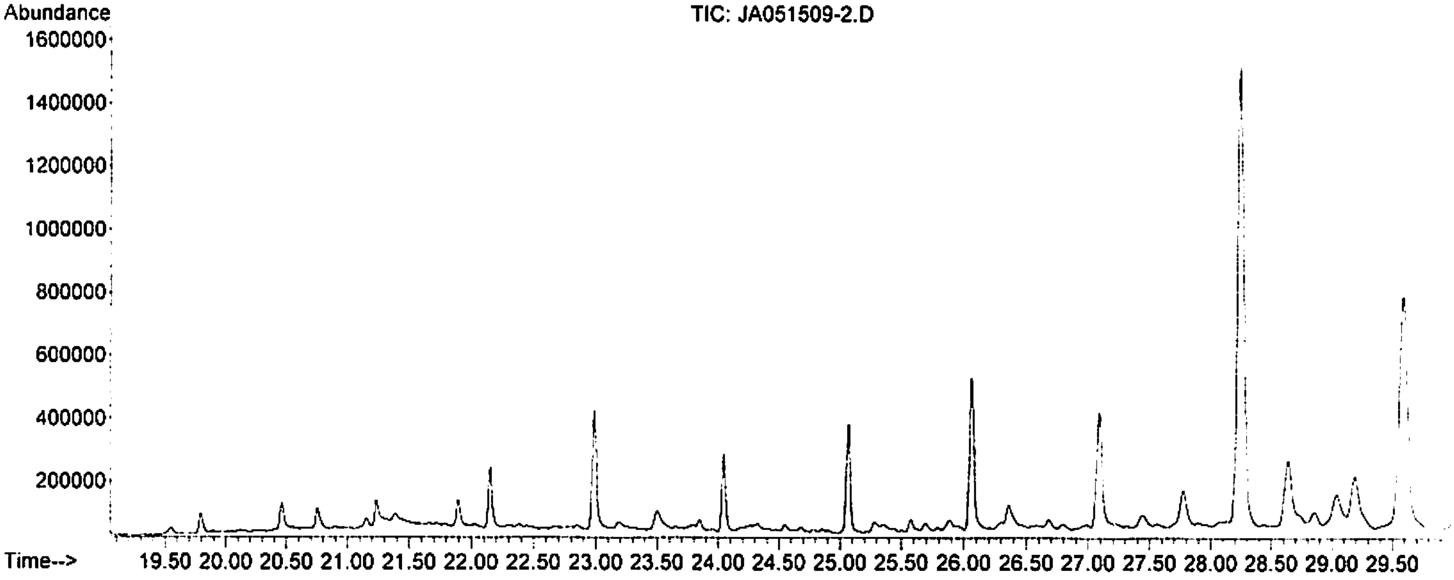

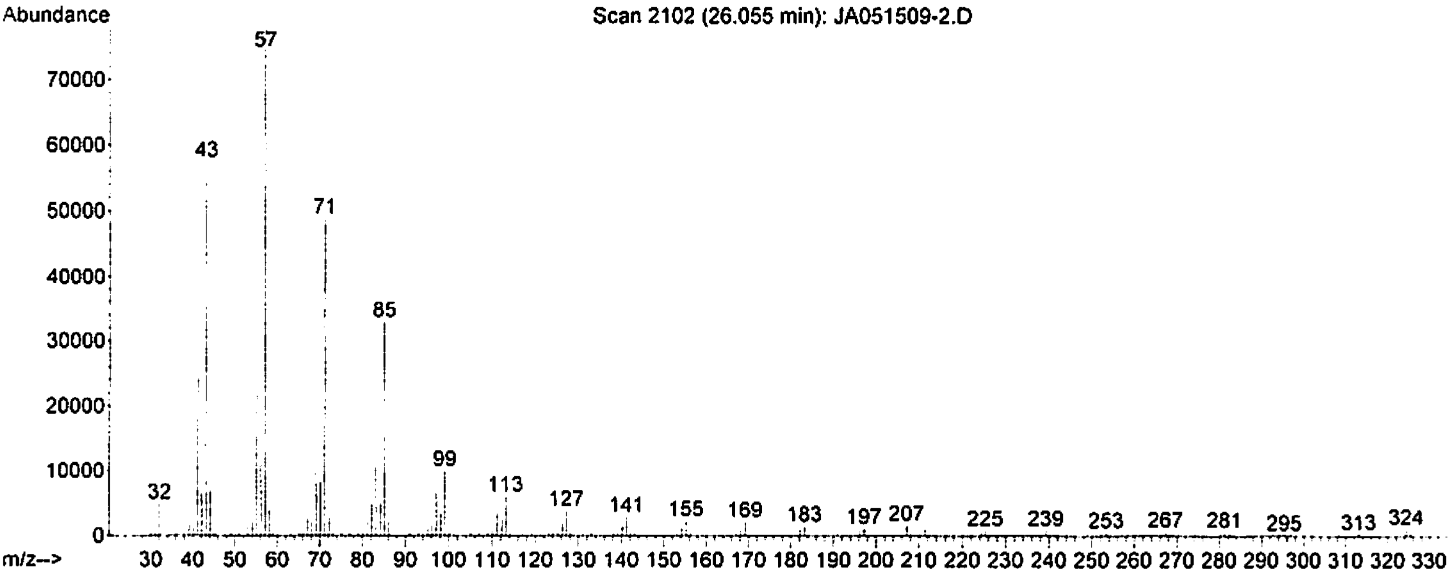

e • :D:\DATA\Aldrich\JA-09\JA051509-2.D
rator : Aldrich
aired : 15 May 2009 13:47 using AcqMethod JA-WAX08.M
trument : Instrument #1
ple Name: 4 field coll. male C.oculata abd/2ul CH2Cl2
Info : Ed coll. sweeping vetch, 5/13&14; fed in lab
Number: 1

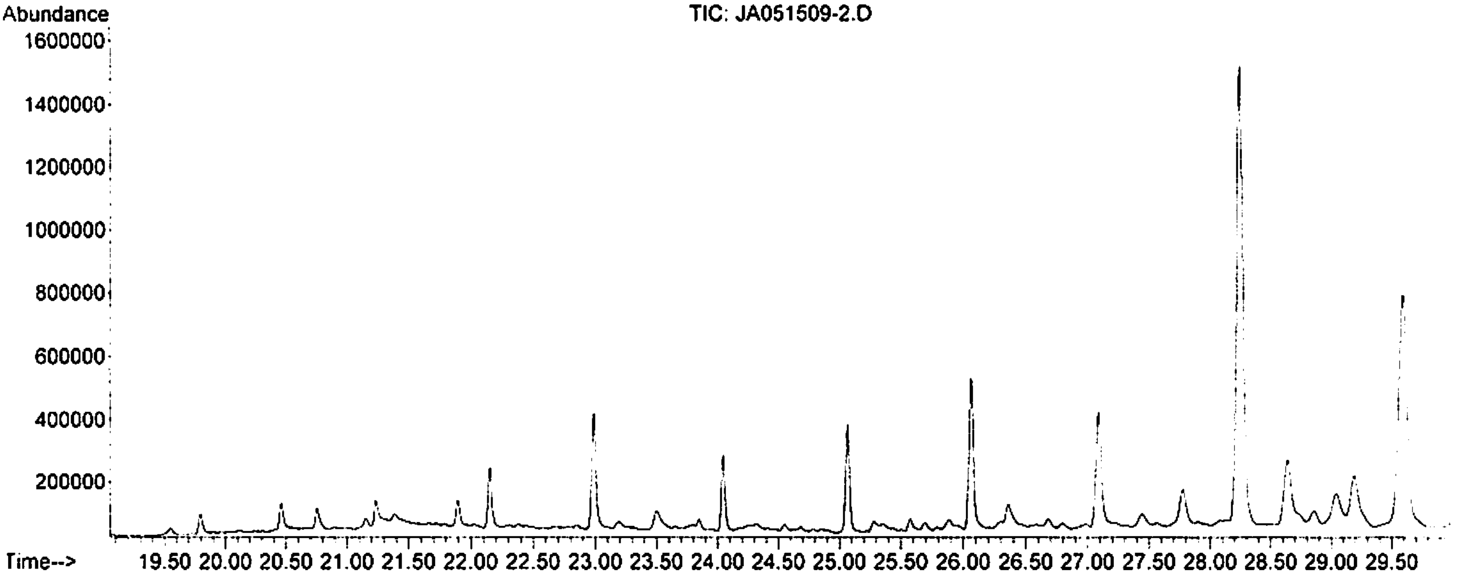

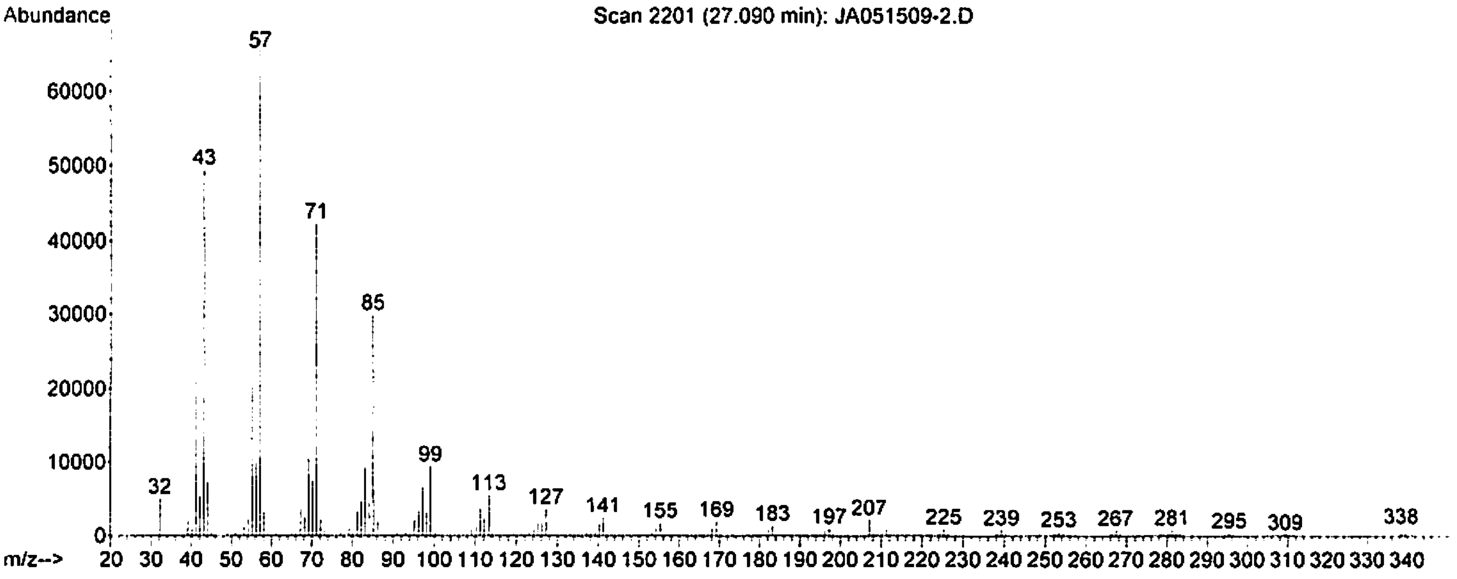

e • :D:\DATA\Aldrich\JA-09\JA051509-2.D
rator : Aldrich
aired : 15 May 2009 13:47 using AcqMethod JA-WAX08.M
trument : Instrument #1
ple Name: 4 field coll. male C.oculata abd/2ul CH2Cl2
c Info : Ed coll. sweeping vetch, 5/13&14; fed in lab
l Number: 1

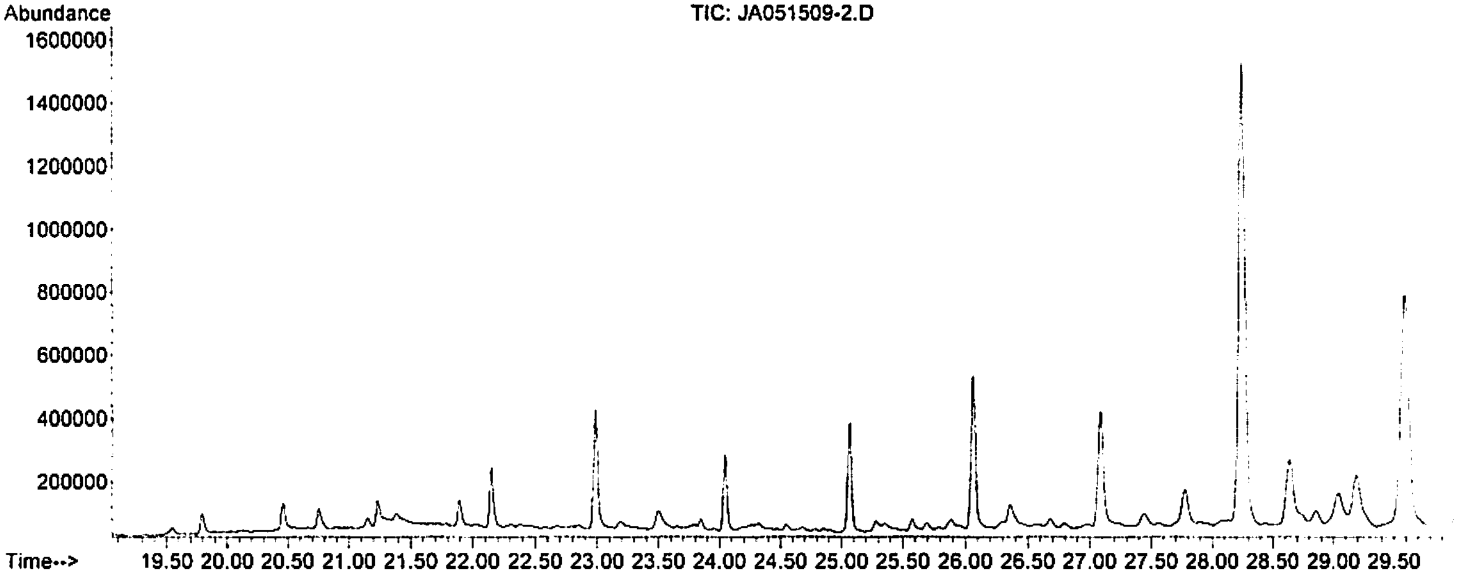

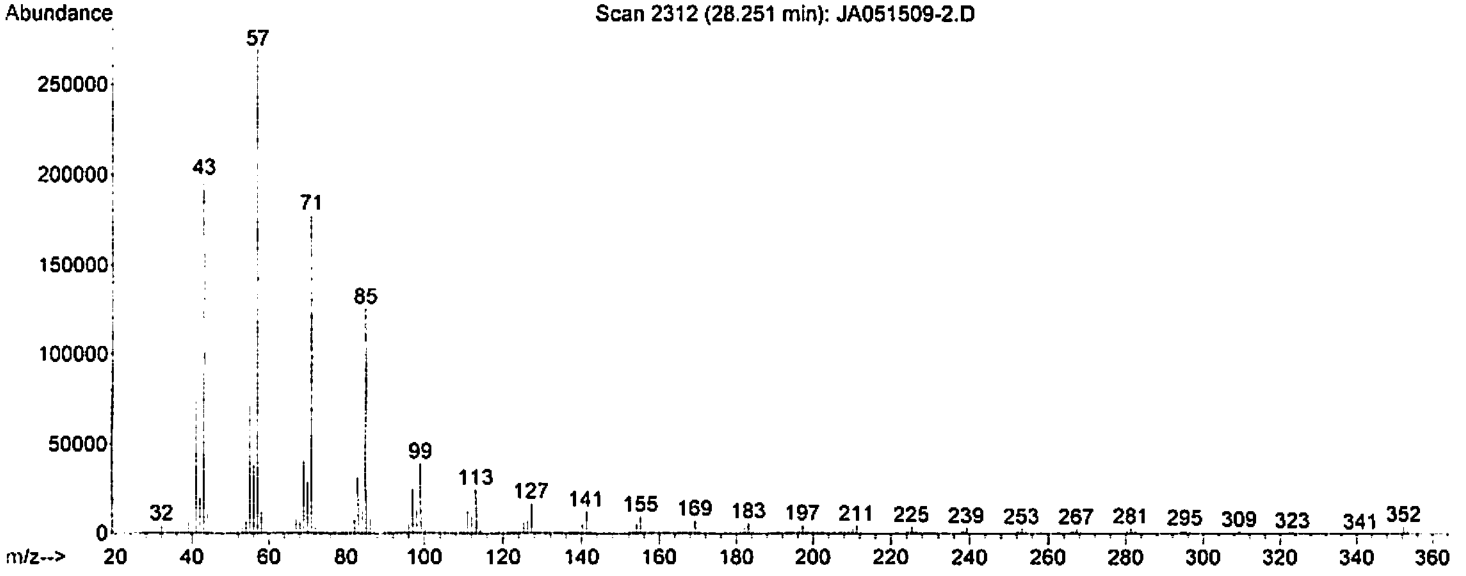

```
e °      :D:\DATA\Aldrich\JA-09\JA051509-2.D
rator    : Aldrich
Aired    : 15 May 2009   13:47      using AcqMethod JA-WAX08.M
trument :      Instrument #1
ple Name: 4 field coll. male C.oculata abd/2ul CH2Cl2
c Info   : Ed coll. sweeping vetch, 5/13&14; fed in lab
l Number: 1
```

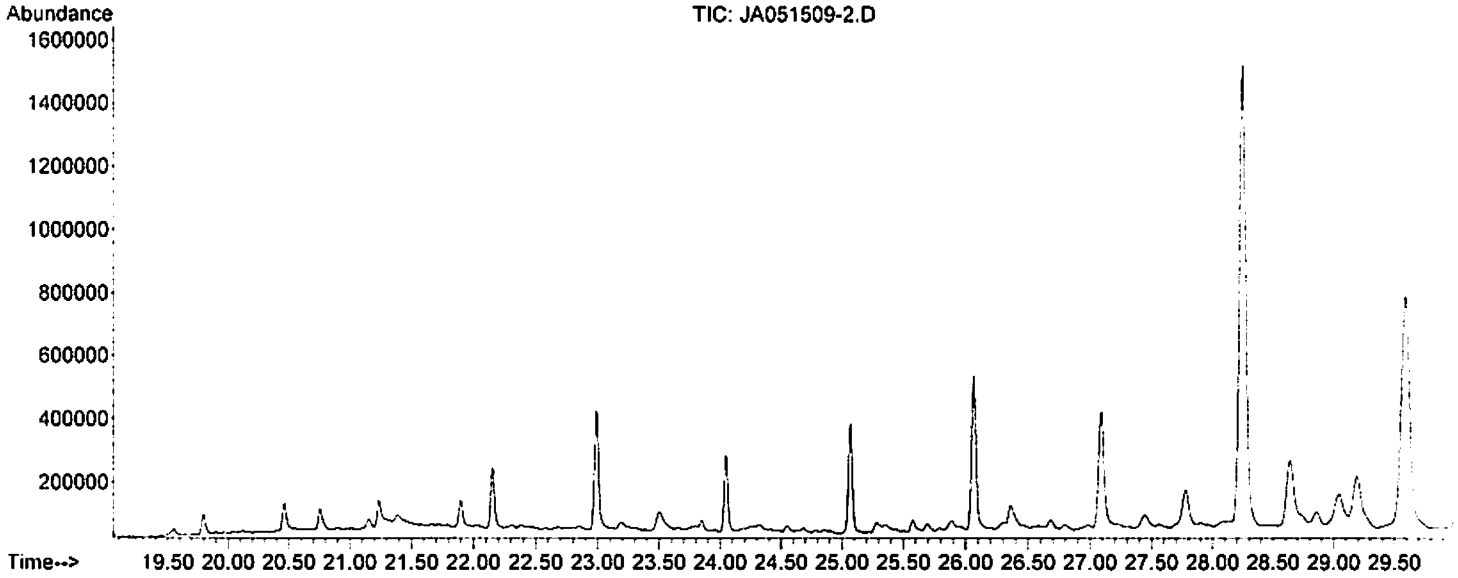

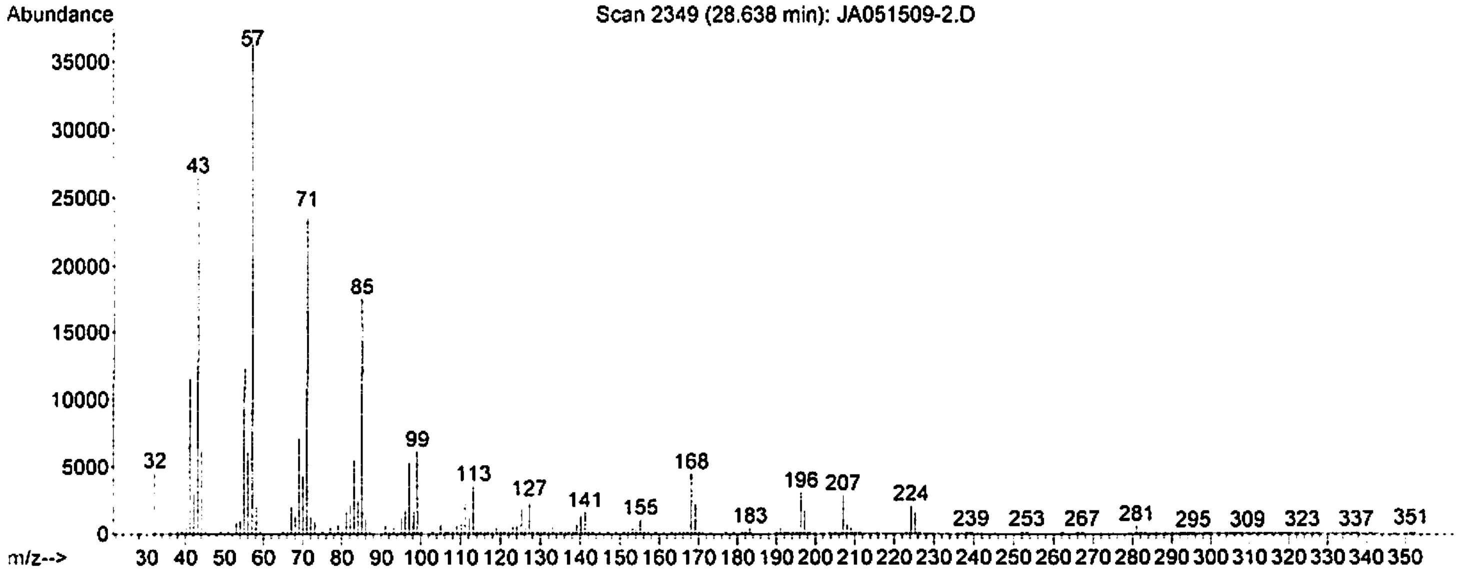

e o :D:\DATA\Aldrich\JA-09\JA051509-2.D
rator : Aldrich
ired : 15 May 2009 13:47 using AcqMethod JA-WAX08.M
trument : Instrument #1
ple Name: 4 field coll. male C.oculata abd/2ul CH2Cl2
c Info : Ed coll. sweeping vetch, 5/13&14; fed in lab
l Number: 1

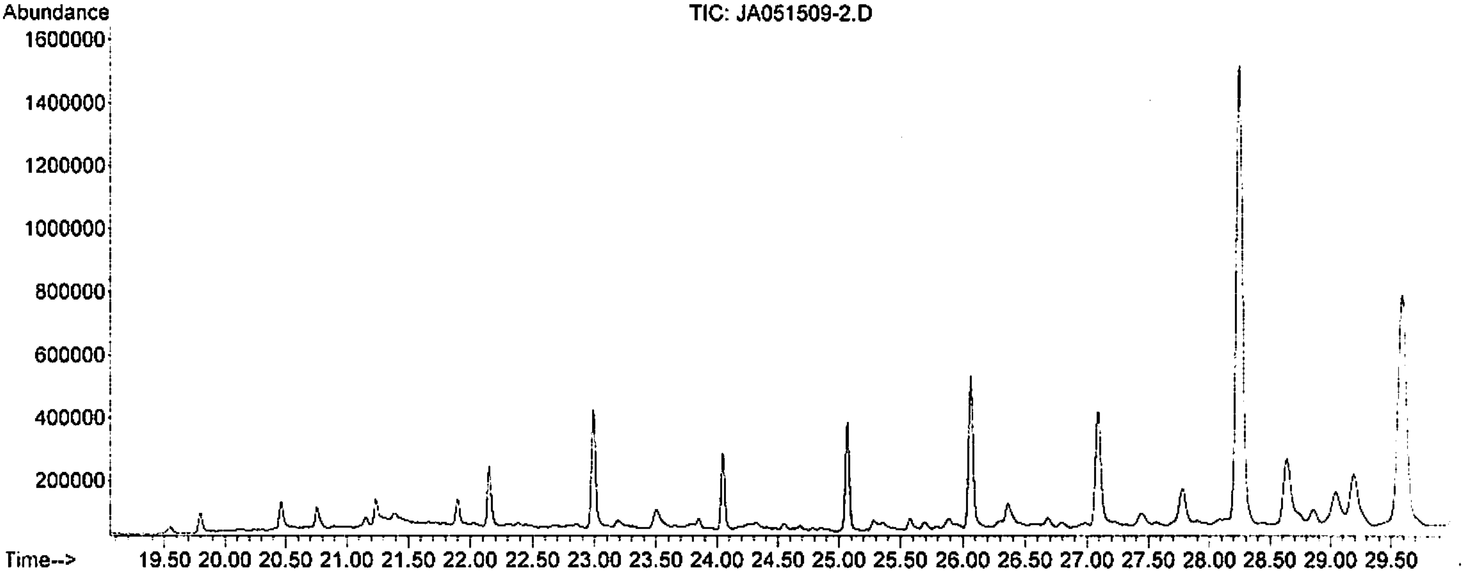

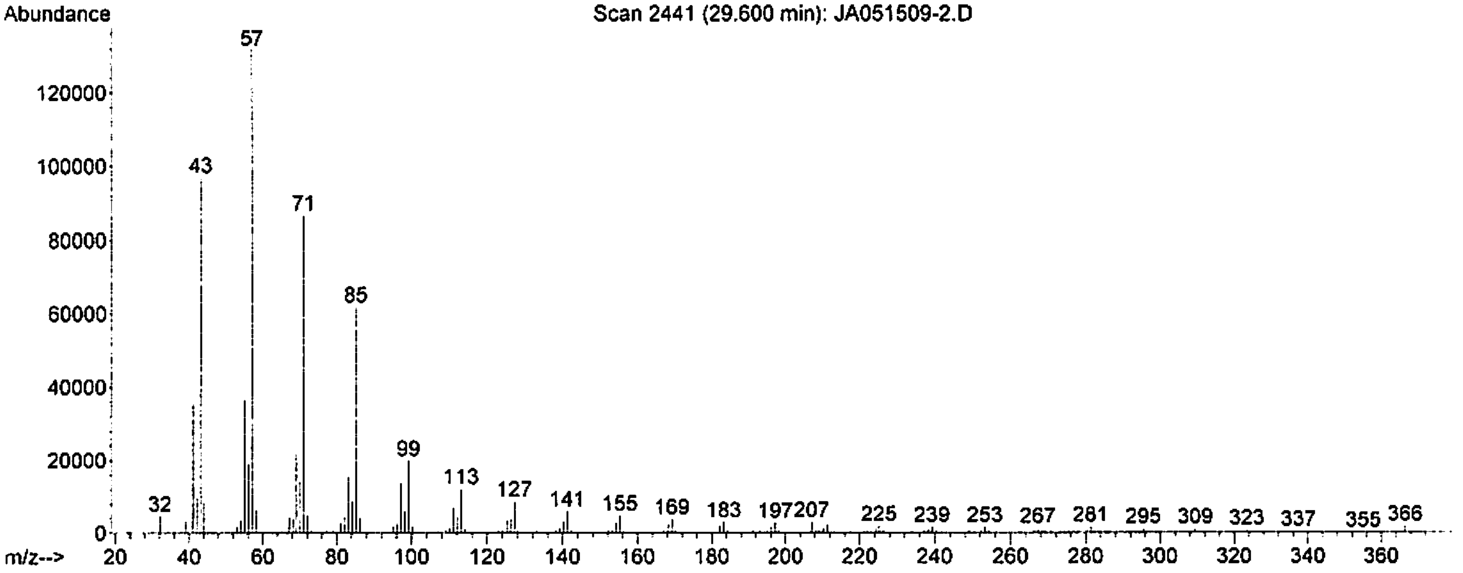

File   :D:\DATA\Aldrich\JA-09\JA051909-3.D
Operator   : Aldrich
Acquired   : 19 May 2009 14:31   using AcqMethod JA-WAX08.M
Instrument :   Instrument #1
Sample Name: 2 field-coll. male C.oculata abd./2.5ul CH2Cl
Misc Info : DB-WAX; coll. 5/18 by Ed; fed only honey
Vial Number: 1

287 std injecting 5/19/09 -
w/ iridodial

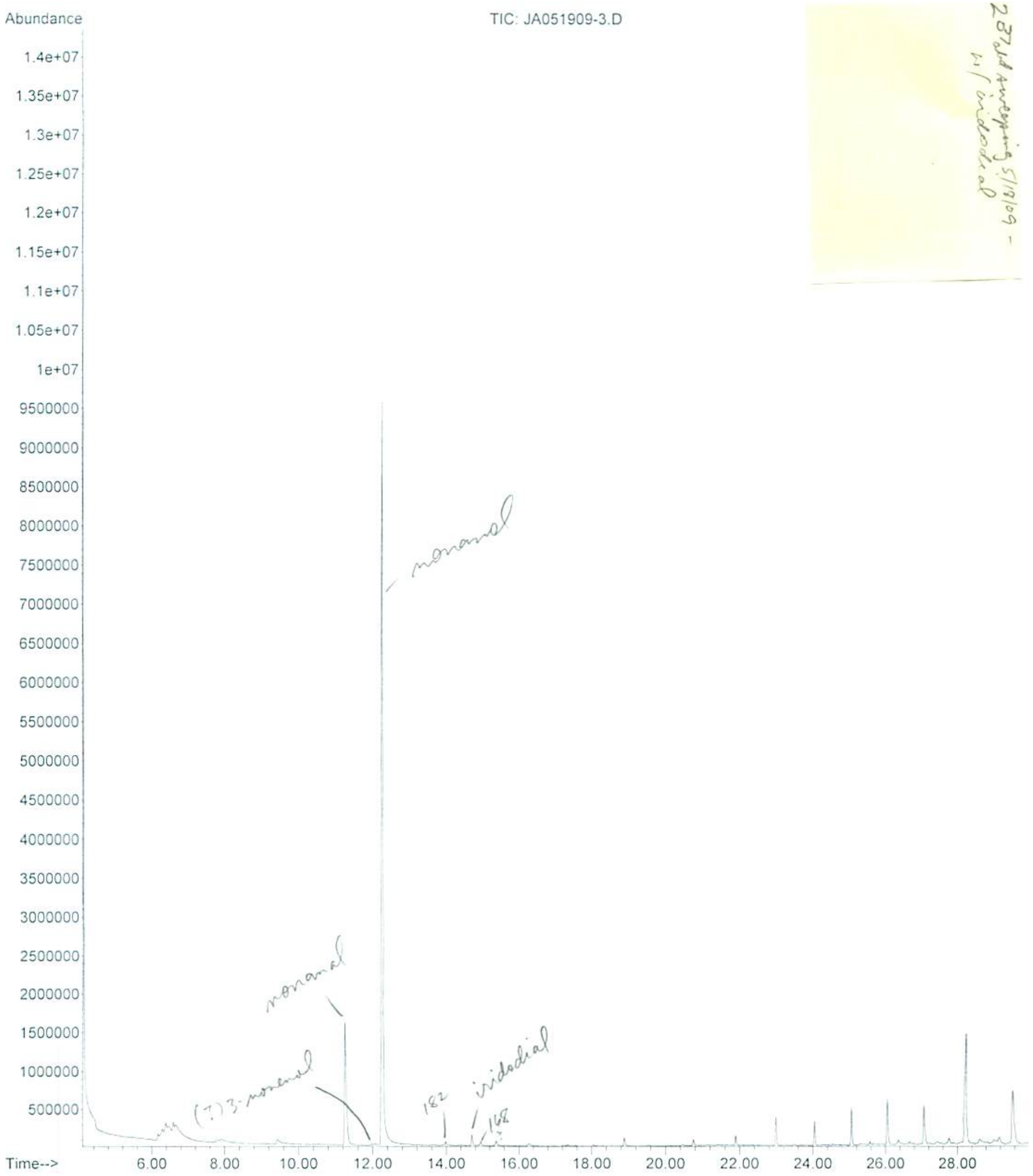

[illegible]le : :D:\DATA\ALDRICH\JA-09\Snapshot\JA051909-3.D
[illegible]erator : Aldrich
[illegible]quired : 19 May 2009 14:31 using AcqMethod JA-WAX08.M
[illegible]strument : Instrument #1
[illegible]mple Name: 2 field-coll. male C.oculata abd./2.5ul CH2Cl
[illegible]c Info : DB-WAX; coll. 5/18 by Ed; fed only honey
[illegible]l Number: 1

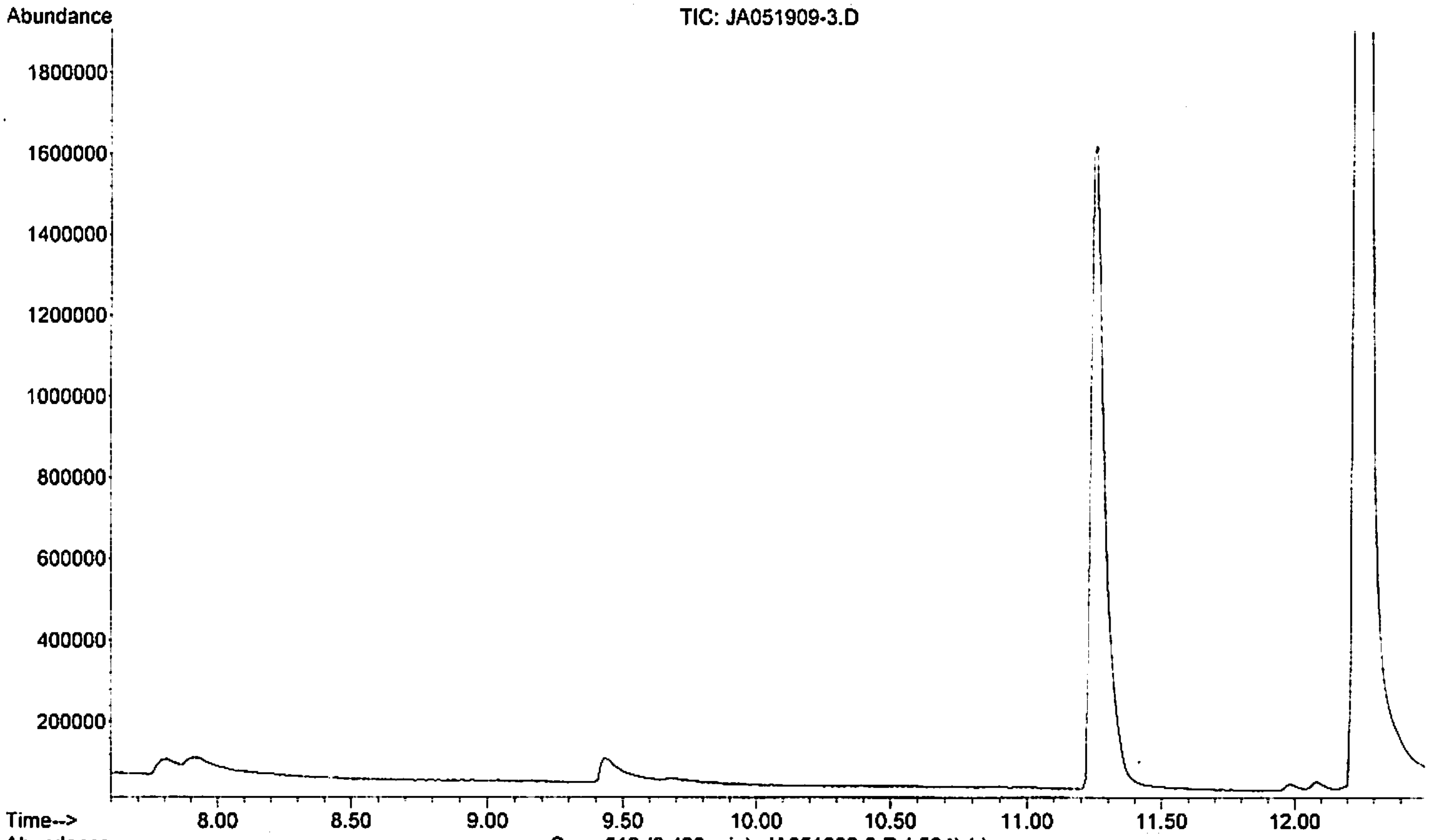

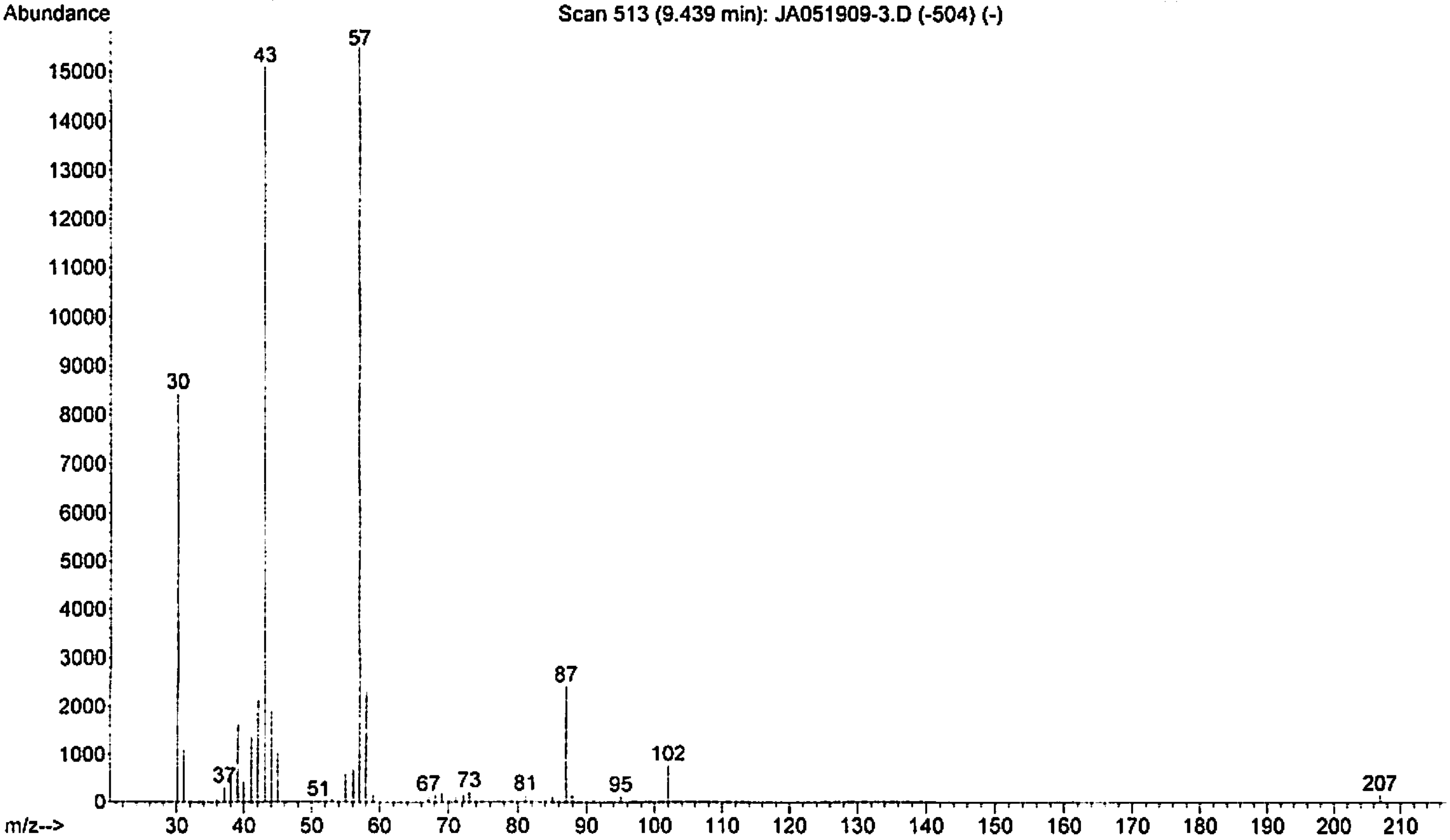

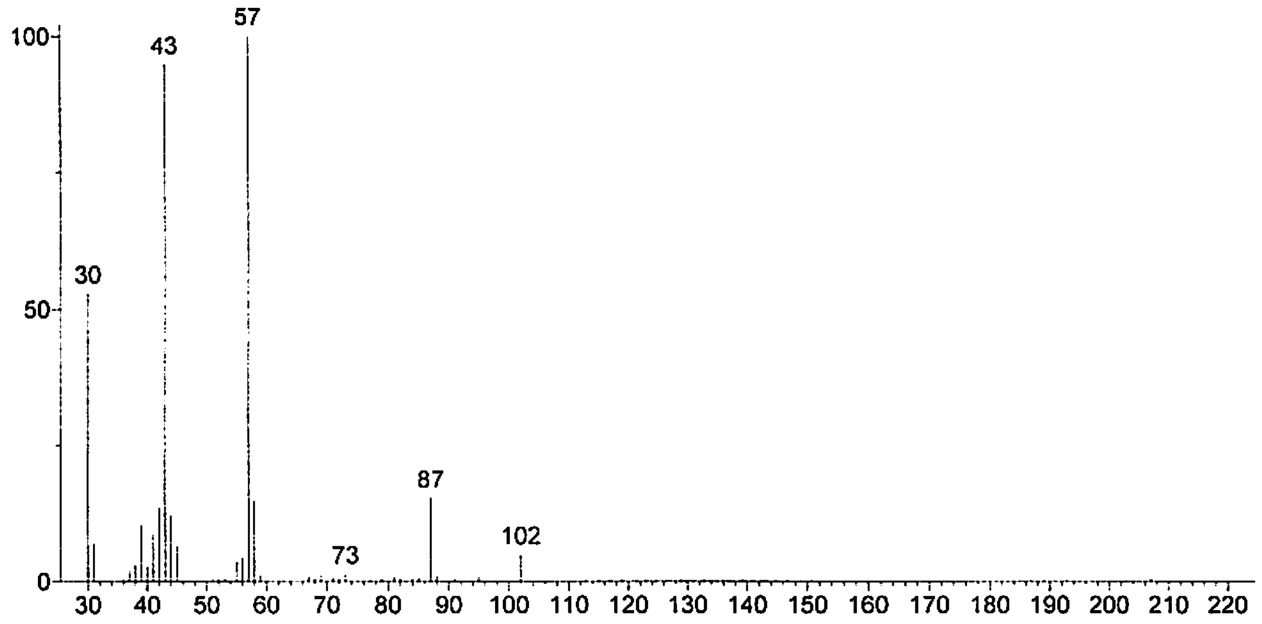

(Text File) Scan 513 (9.439 min): JA051909-3.D (-504)

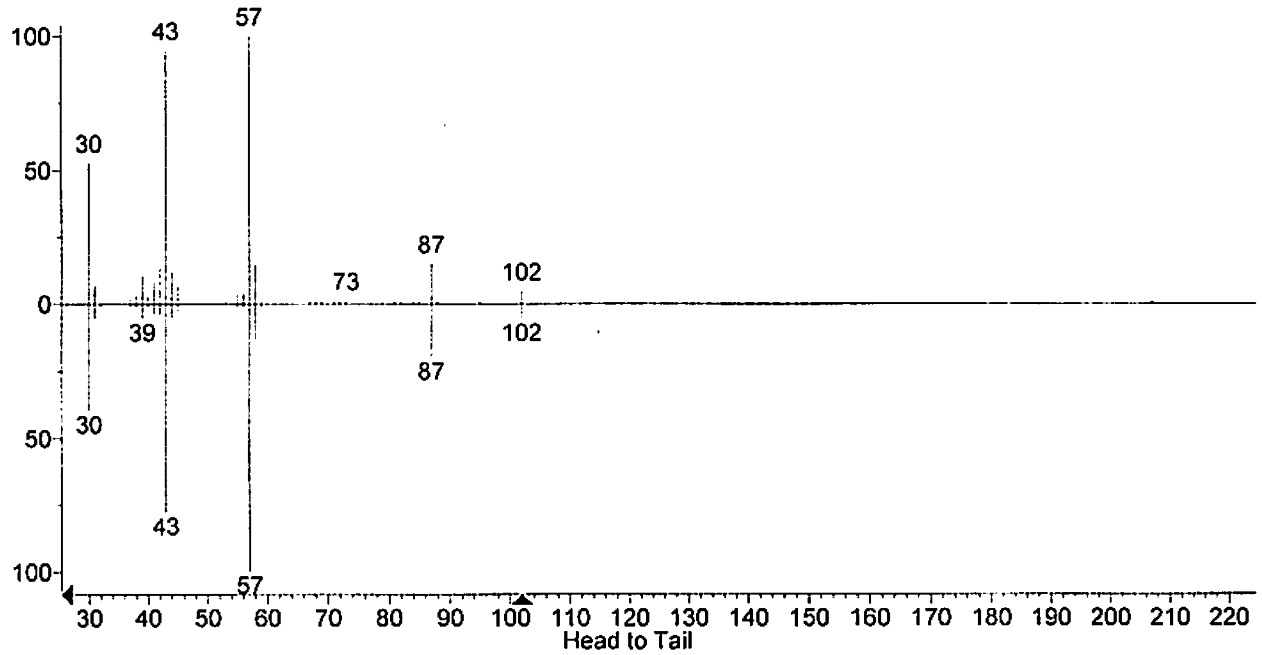

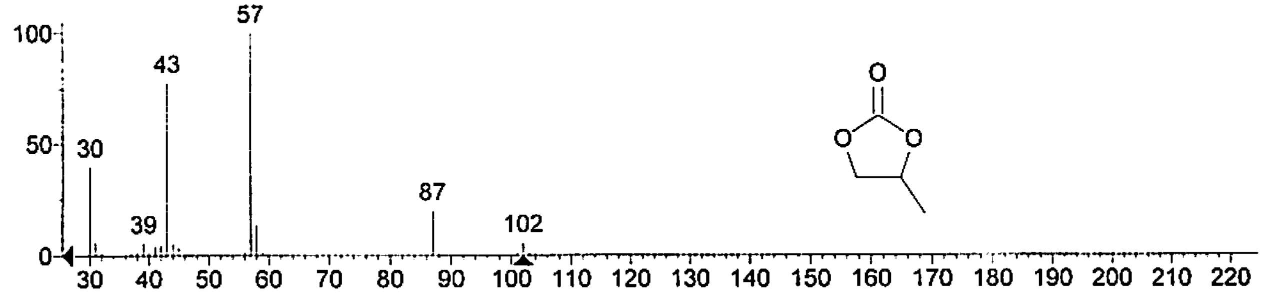

(mainlib) Propylene Carbonate

[illegible]le : D:\DATA\ALDRICH\JA-09\Snapshot\JA051909-3.D
[illegible]erator : Aldrich
[illegible]quired : 19 May 2009 14:31 using AcqMethod JA-WAX08.M
[illegible]strument : Instrument #1
[illegible]mple Name: 2 field-coll. male C.oculata abd./2.5ul CH2Cl
[illegible]sc Info : DB-WAX; coll. 5/18 by Ed; fed only honey
[illegible]al Number: 1

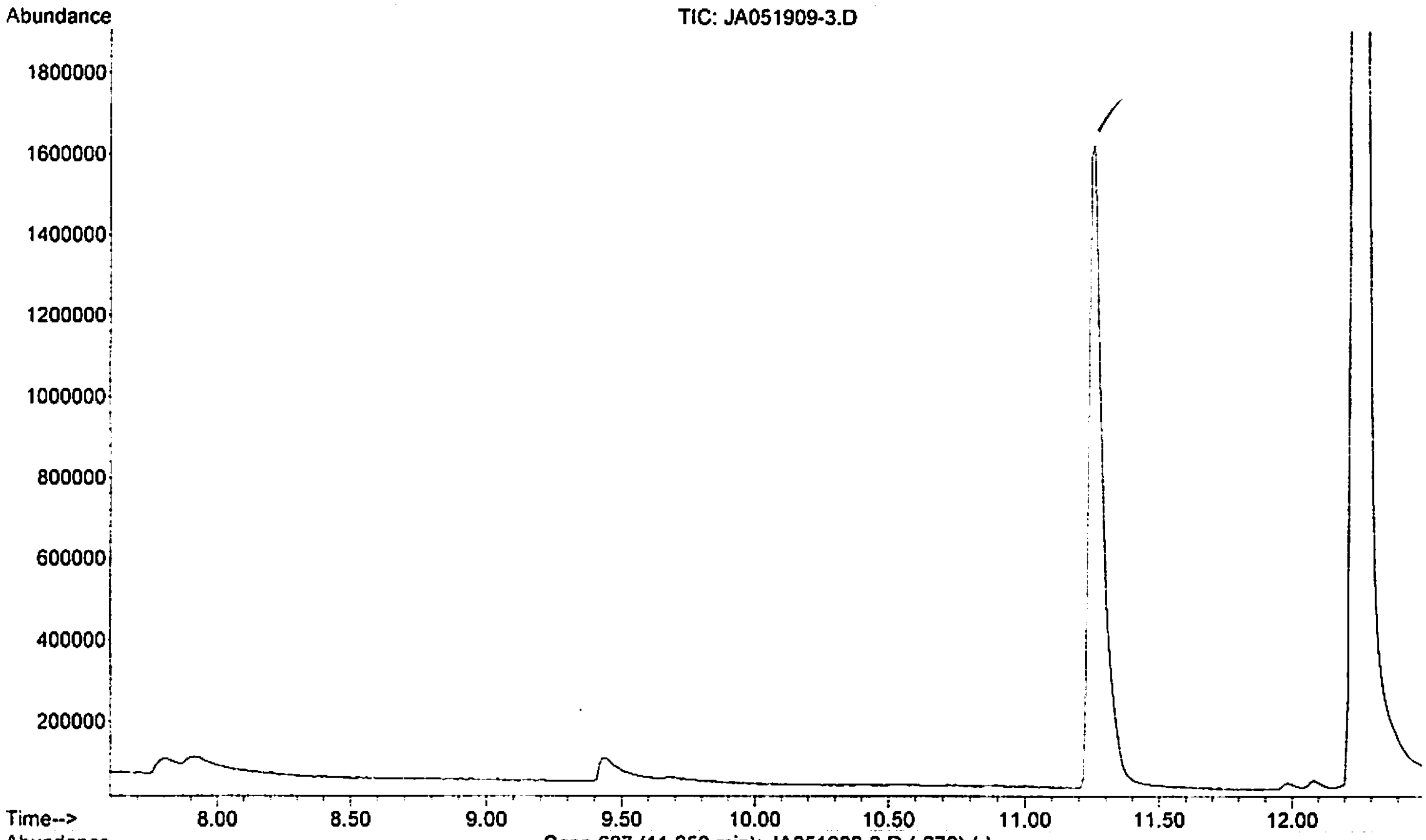

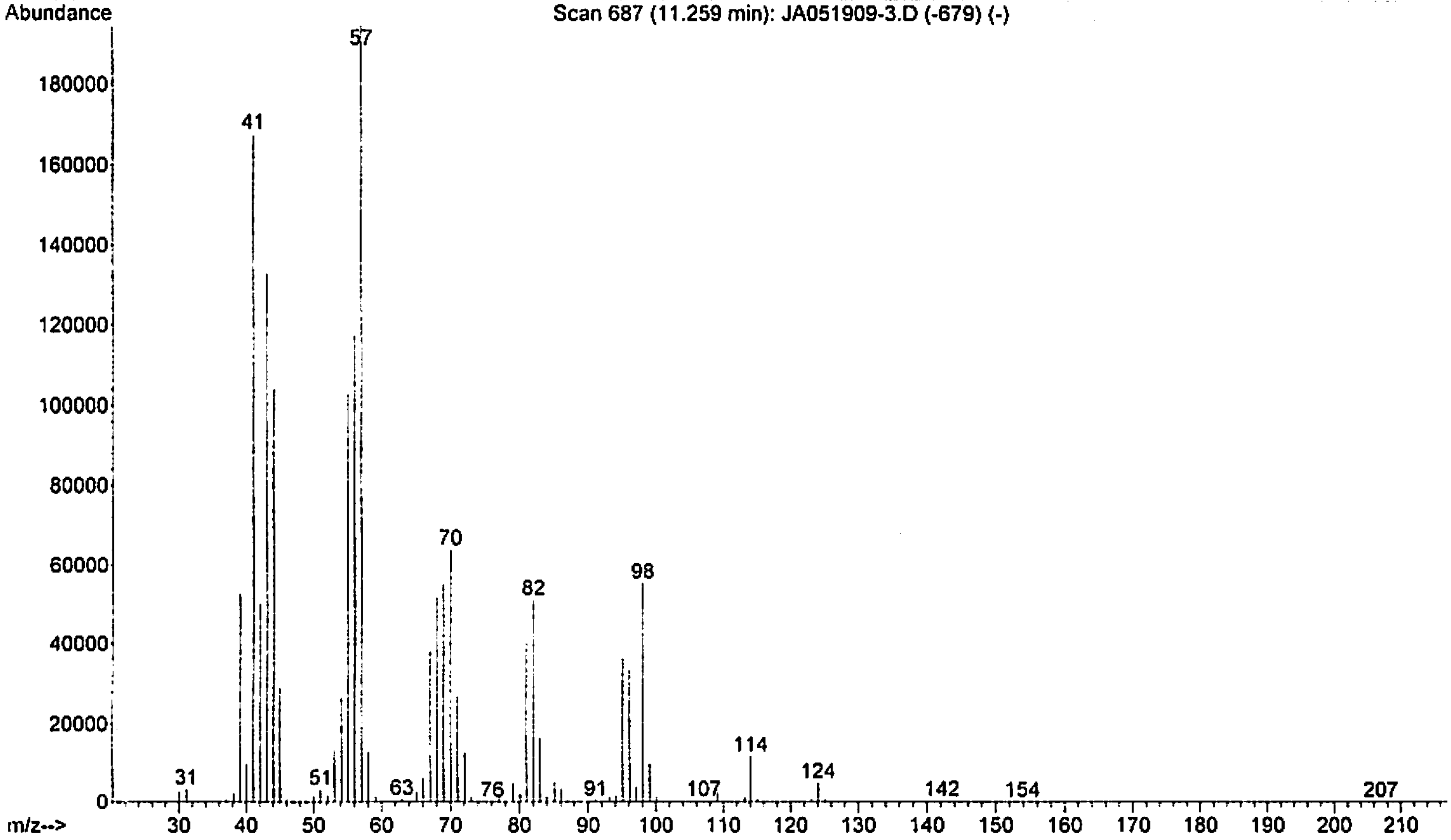

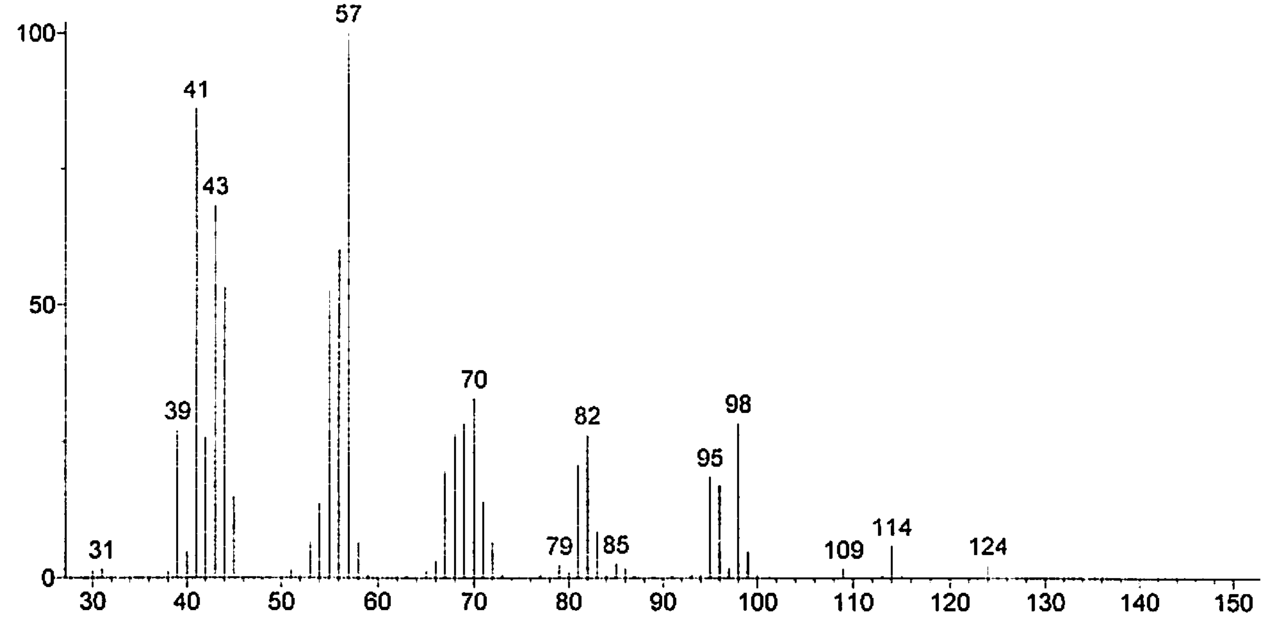

(Text File) Scan 687 (11.259 min): JA051909-3.D (-679)

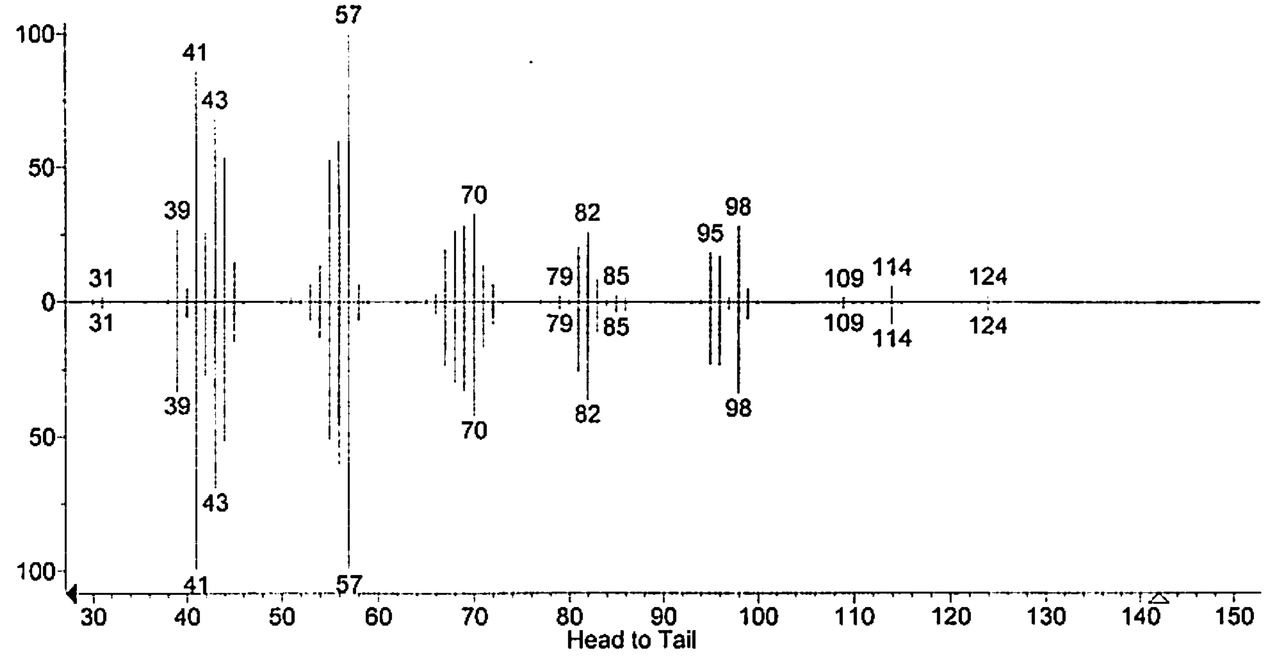

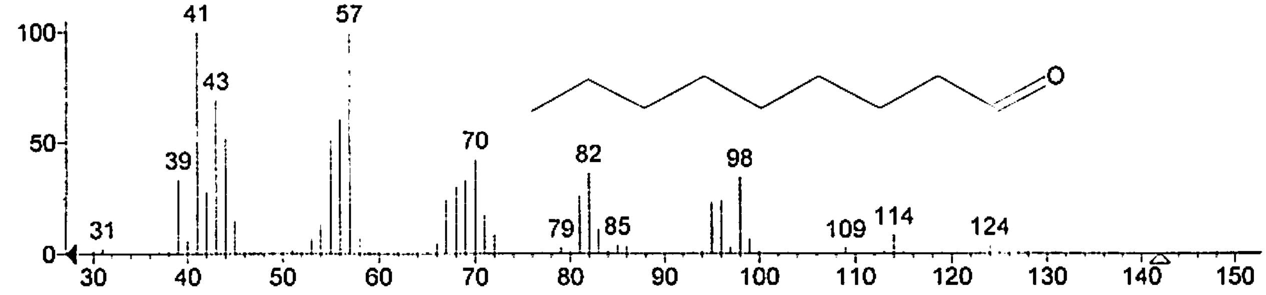

(replib) Nonanal

...e :D:\DATA\ALDRICH\JA-09\Snapshot\JA051909-3.D
...rator : Aldrich
...quired : 19 May 2009 14:31 using AcqMethod JA-WAX08.M
...strument : Instrument #1
...mple Name: 2 field-coll. male C.oculata abd./2.5ul CH2Cl
...sc Info : DB-WAX; coll. 5/18 by Ed; fed only honey
...al Number: 1

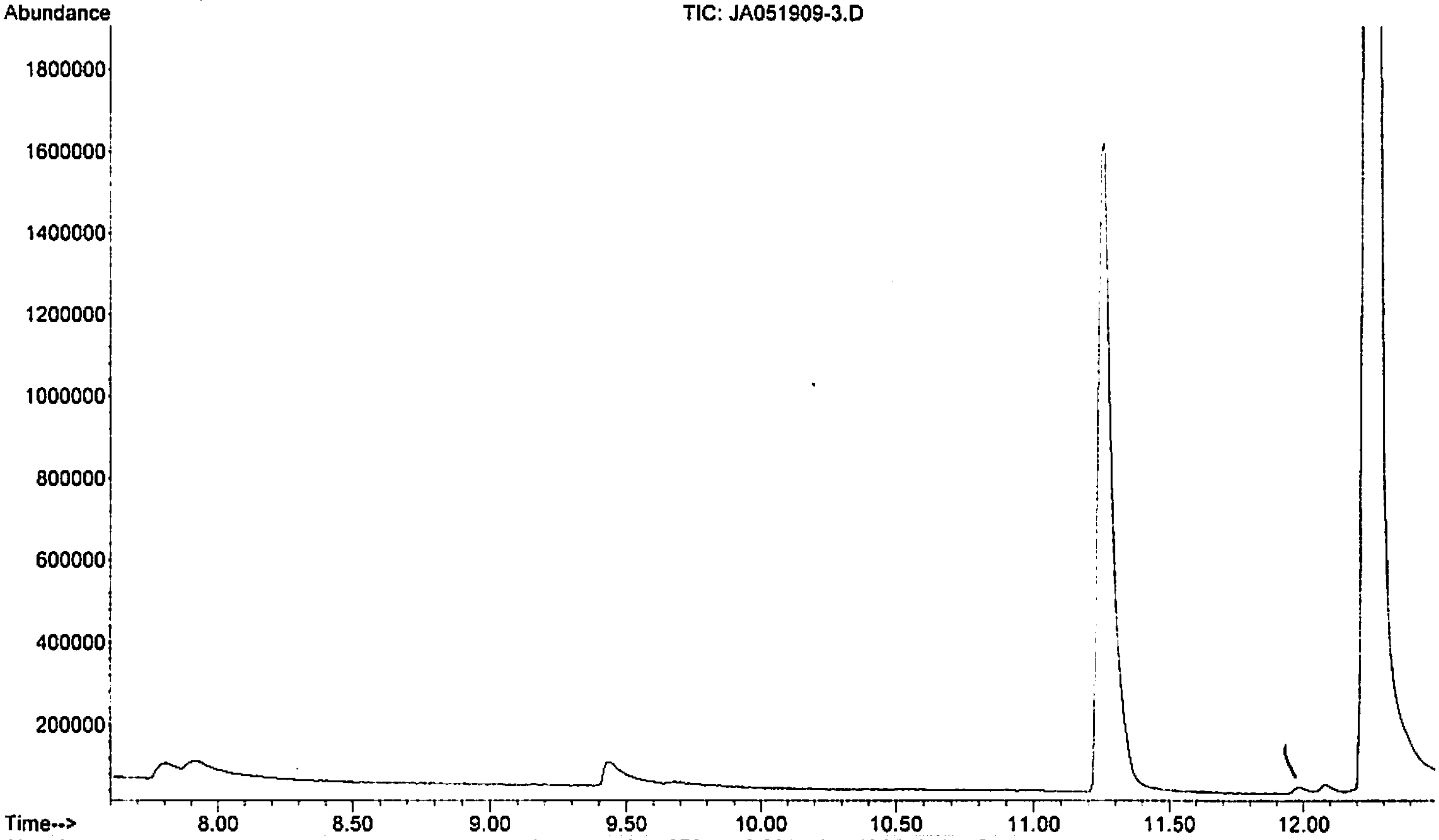

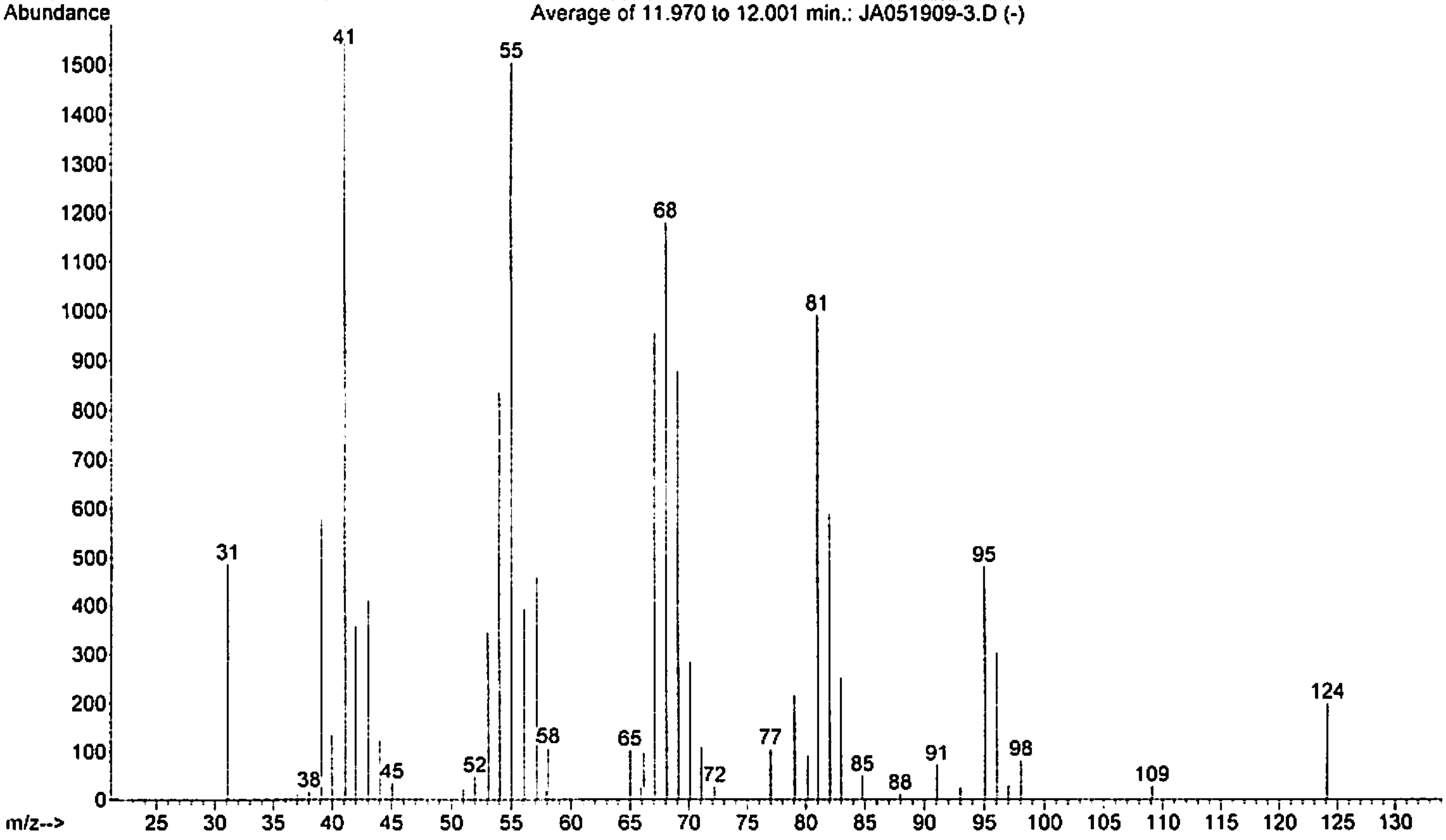

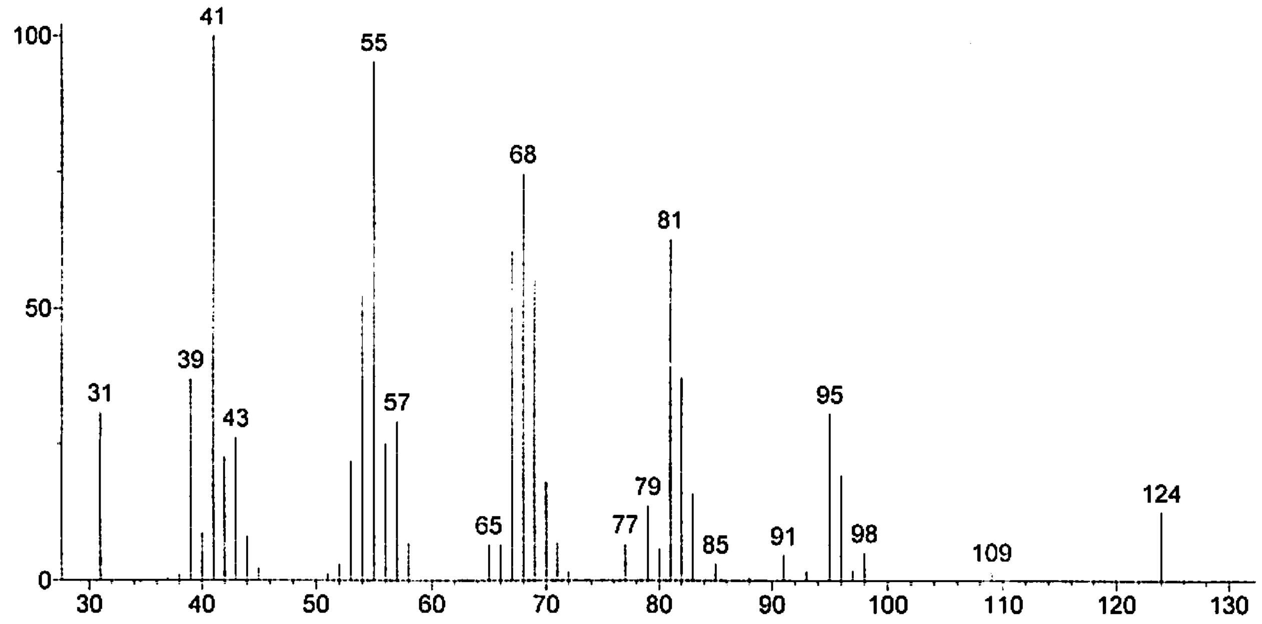

(Text File) Average of 11.970 to 12.001 min.: JA051909-3.D

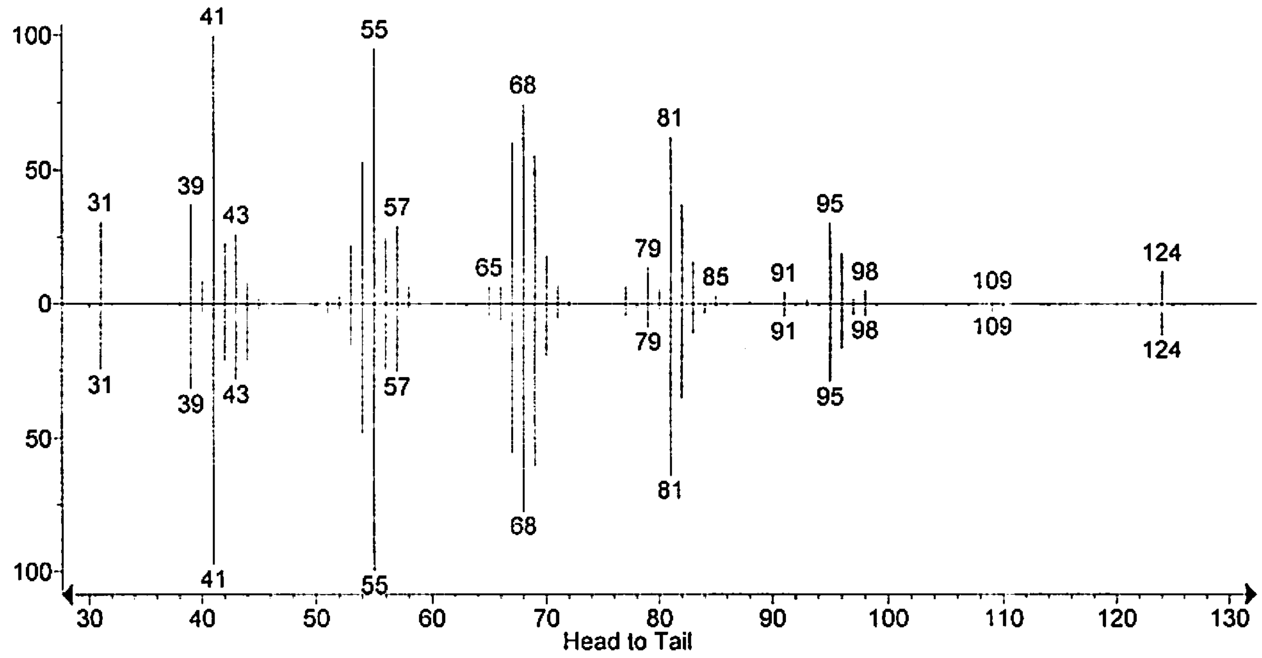

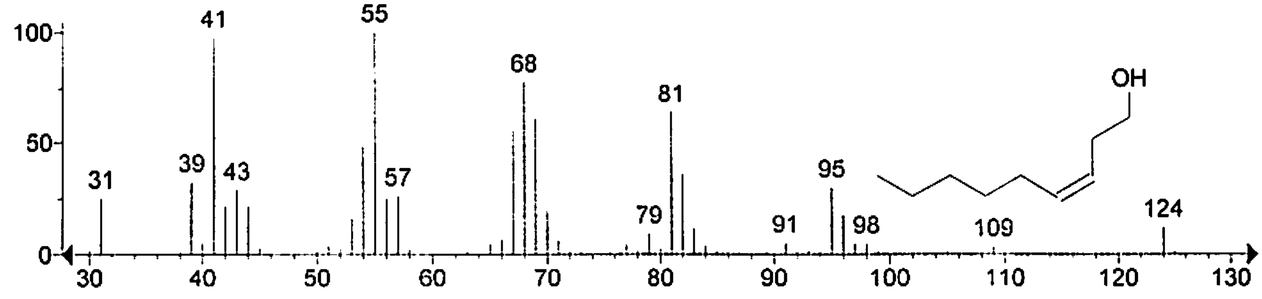

(replib) 3-Nonen-1-ol, (Z)-

[illegible]e : D:\DATA\ALDRICH\JA-09\Snapshot\JA051909-3.D
[illegible]erator : Aldrich
[illegible]quired : 19 May 2009 14:31 using AcqMethod JA-WAX08.M
[illegible]strument : Instrument #1
[illegible]mple Name: 2 field-coll. male C.oculata abd./2.5ul CH2Cl
[illegible]sc Info : DB-WAX; coll. 5/18 by Ed; fed only honey
[illegible]al Number: 1

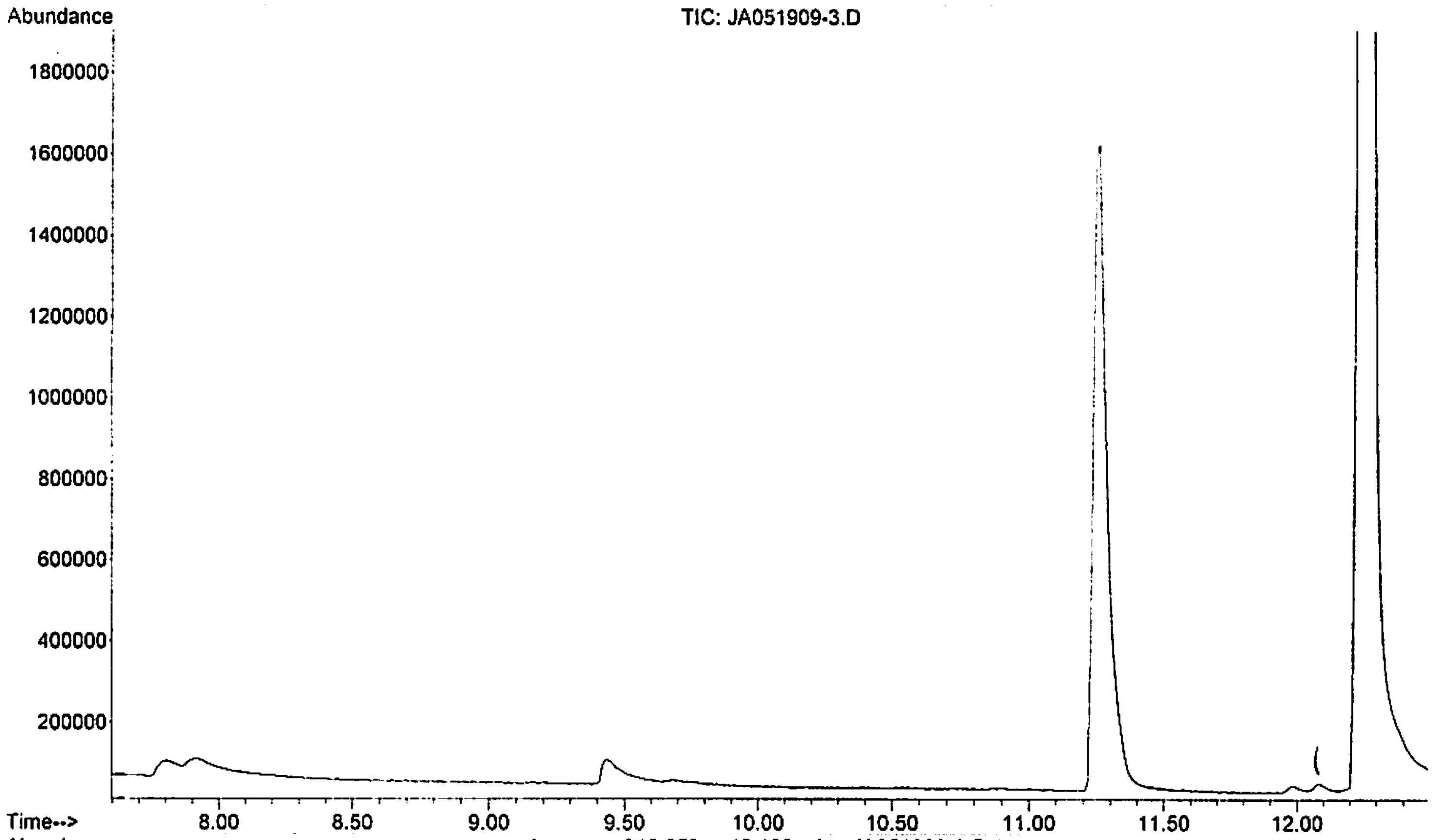

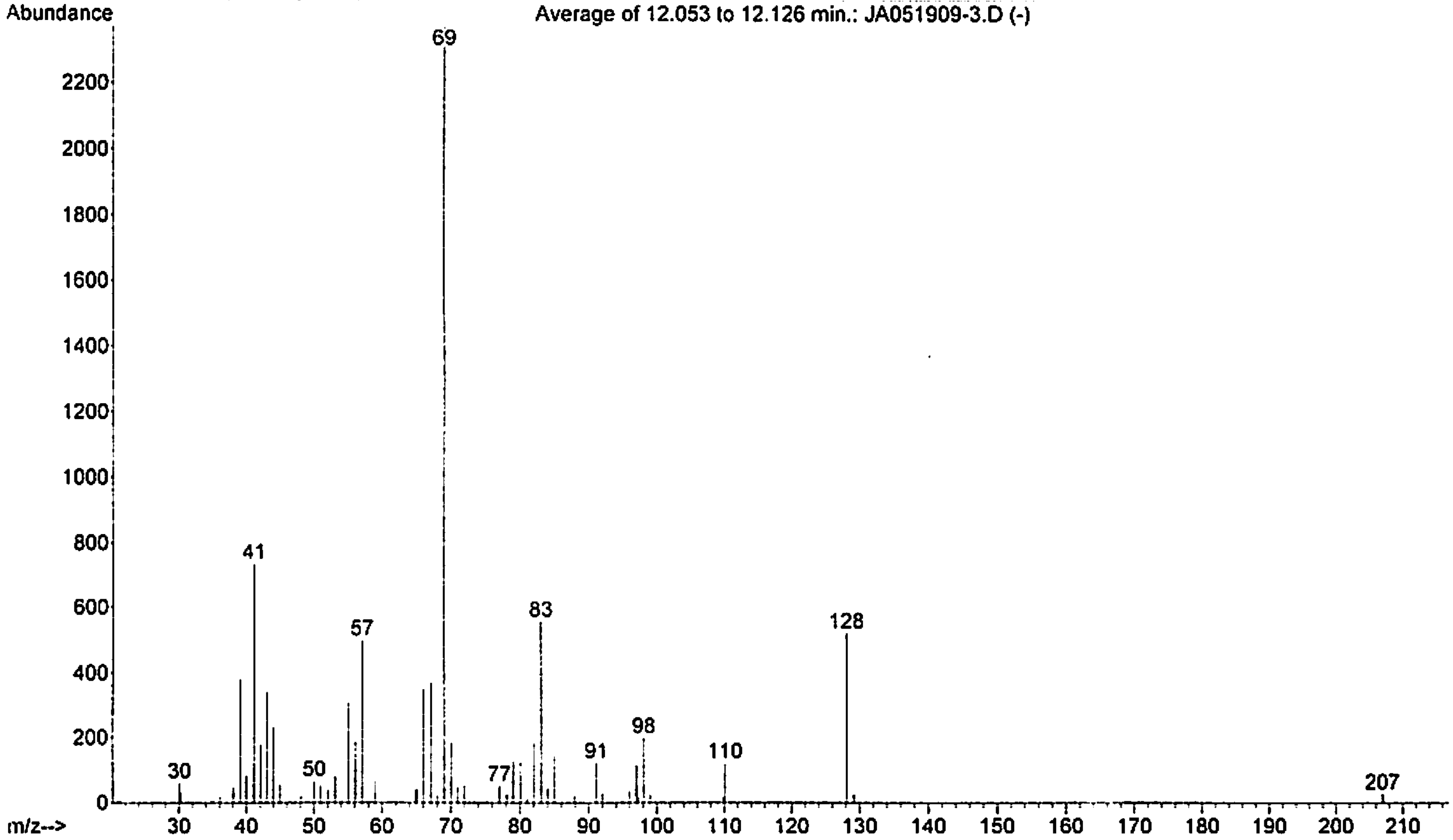

File :D:\DATA\ALDRICH\JA-09\Snapshot\JA051909-3.D
Operator : Aldrich
Acquired : 19 May 2009 14:31 using AcqMethod JA-WAX08.M
Instrument : Instrument #1
Sample Name: 2 field-coll. male C.oculata abd./2.5ul CH2Cl
Misc Info : DB-WAX; coll. 5/18 by Ed; fed only honey
Vial Number: 1

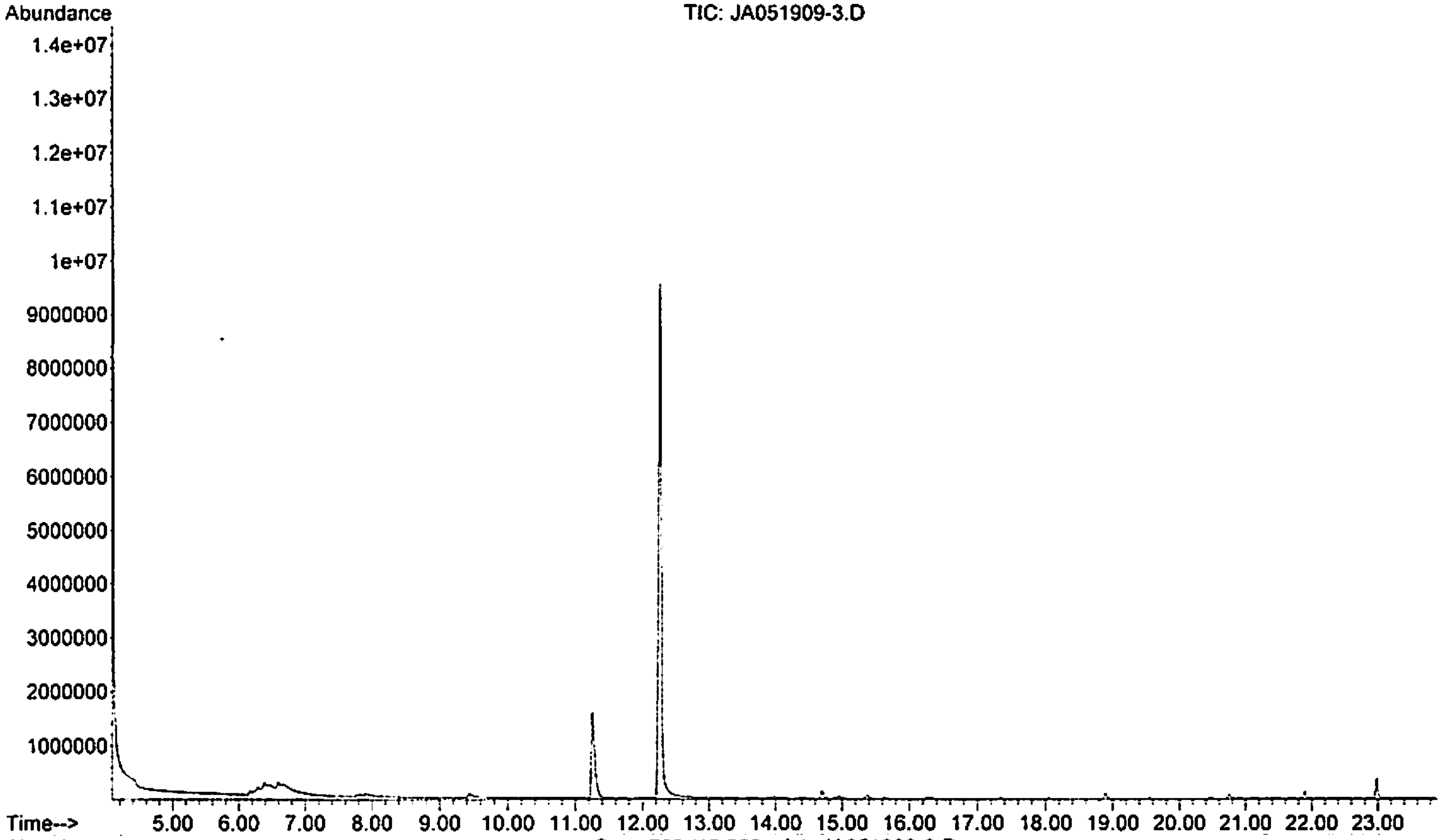

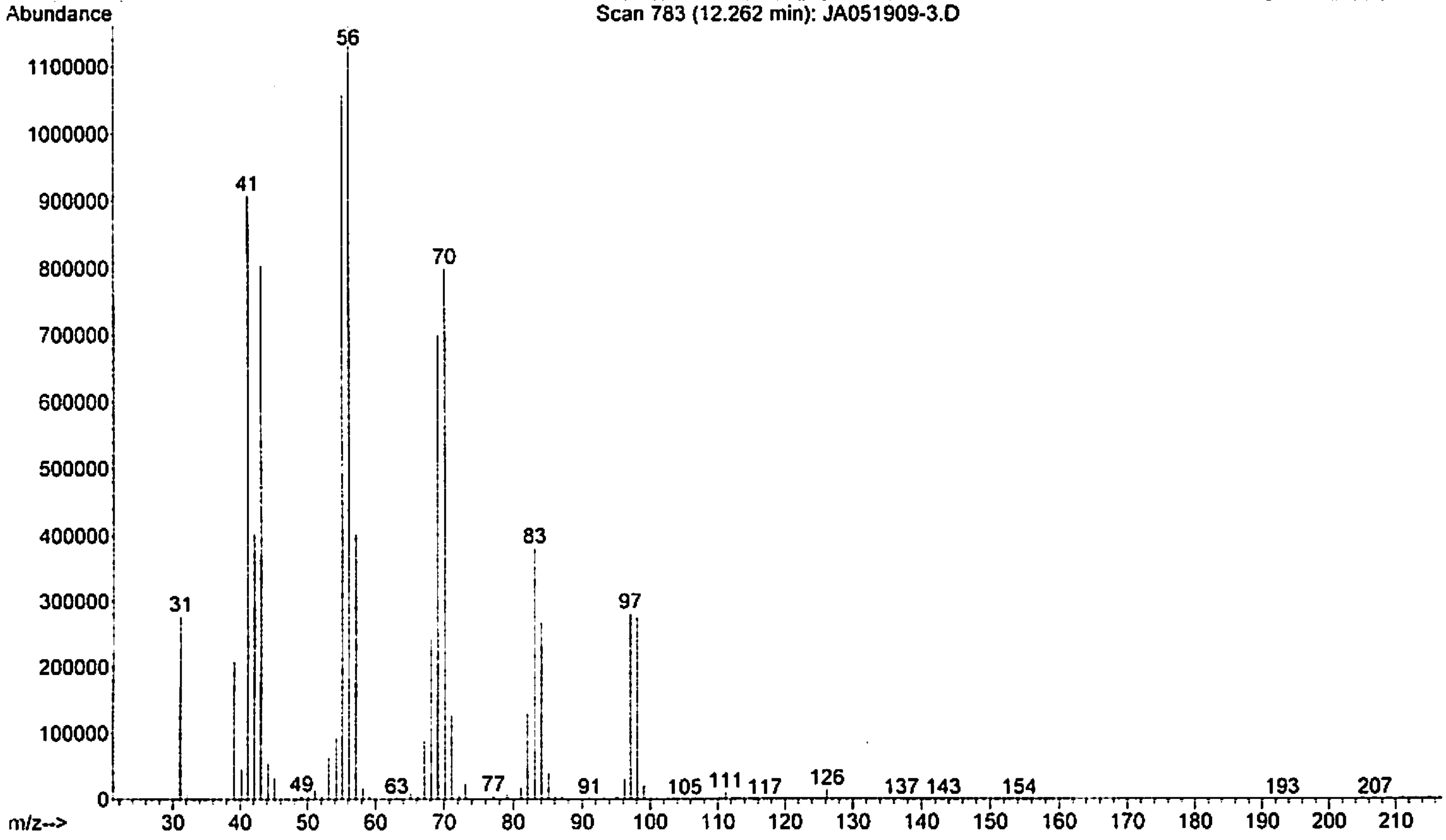

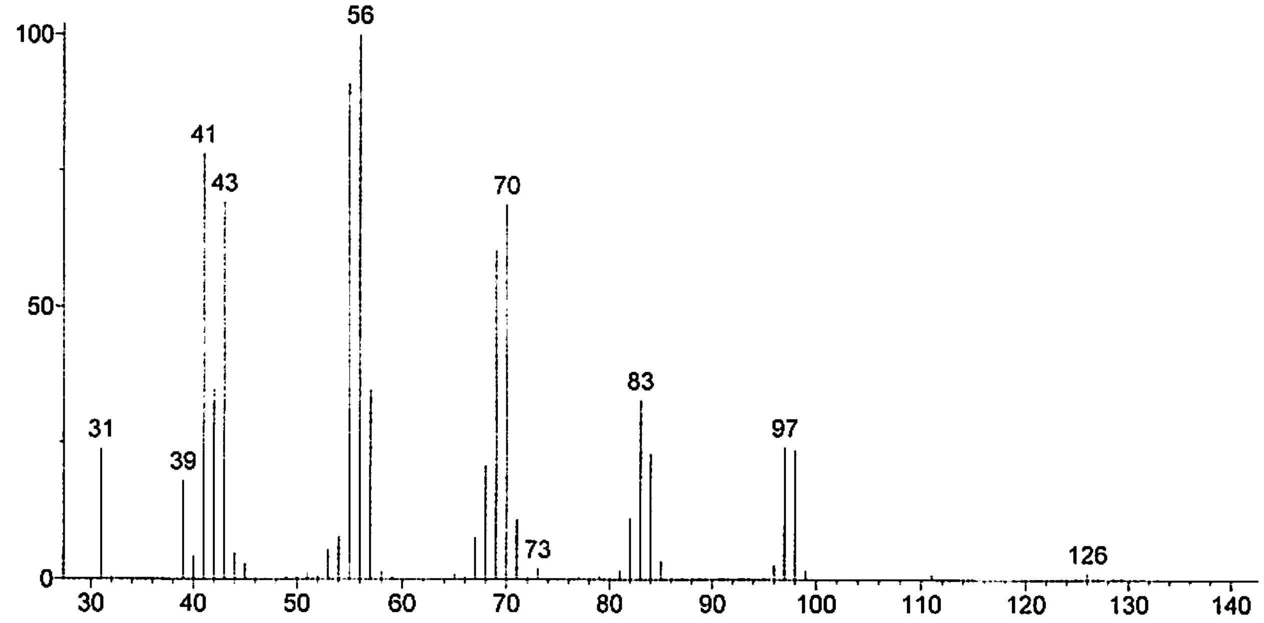

(Text File) Scan 783 (12.262 min): JA051909-3.D

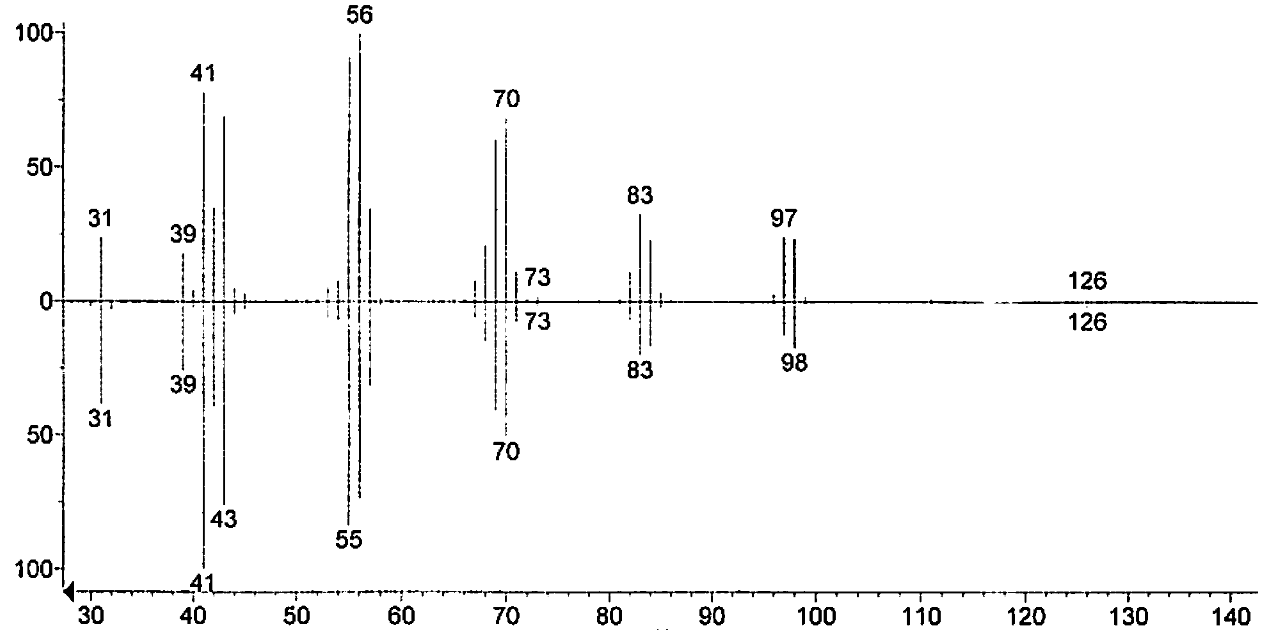

Head to Tail

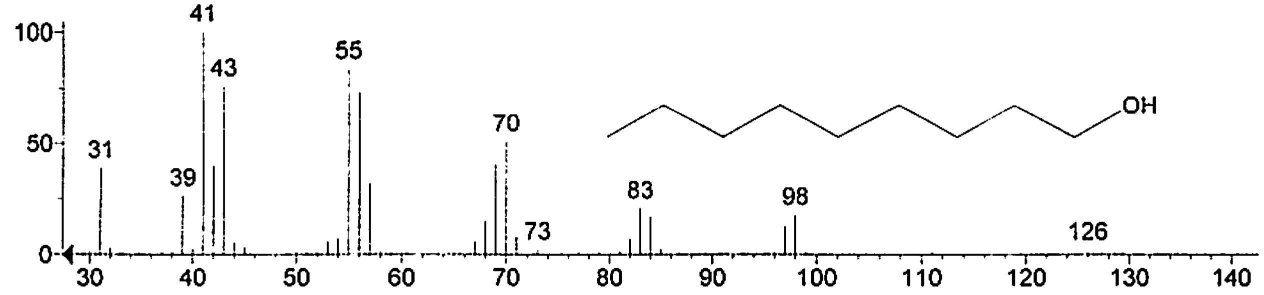

(replib) 1-Nonanol

File : D:\DATA\ALDRICH\JA-09\Snapshot\JA051909-3.D
Operator : Aldrich
Acquired : 19 May 2009 14:31 using AcqMethod JA-WAX08.M
Instrument : Instrument #1
Sample Name: 2 field-coll. male C.oculata abd./2.5ul CH2Cl
Misc Info : DB-WAX; coll. 5/18 by Ed; fed only honey
Vial Number: 1

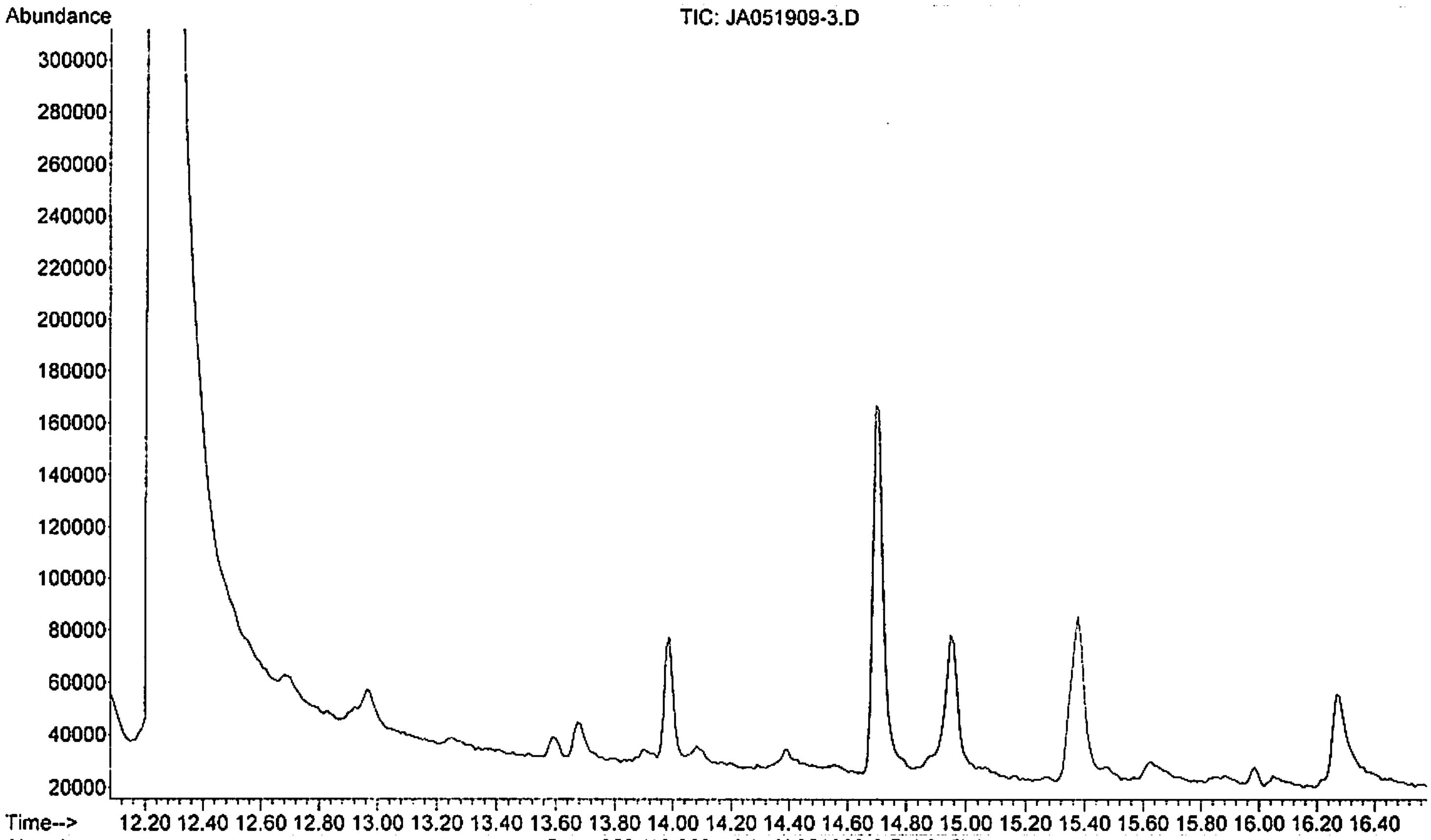

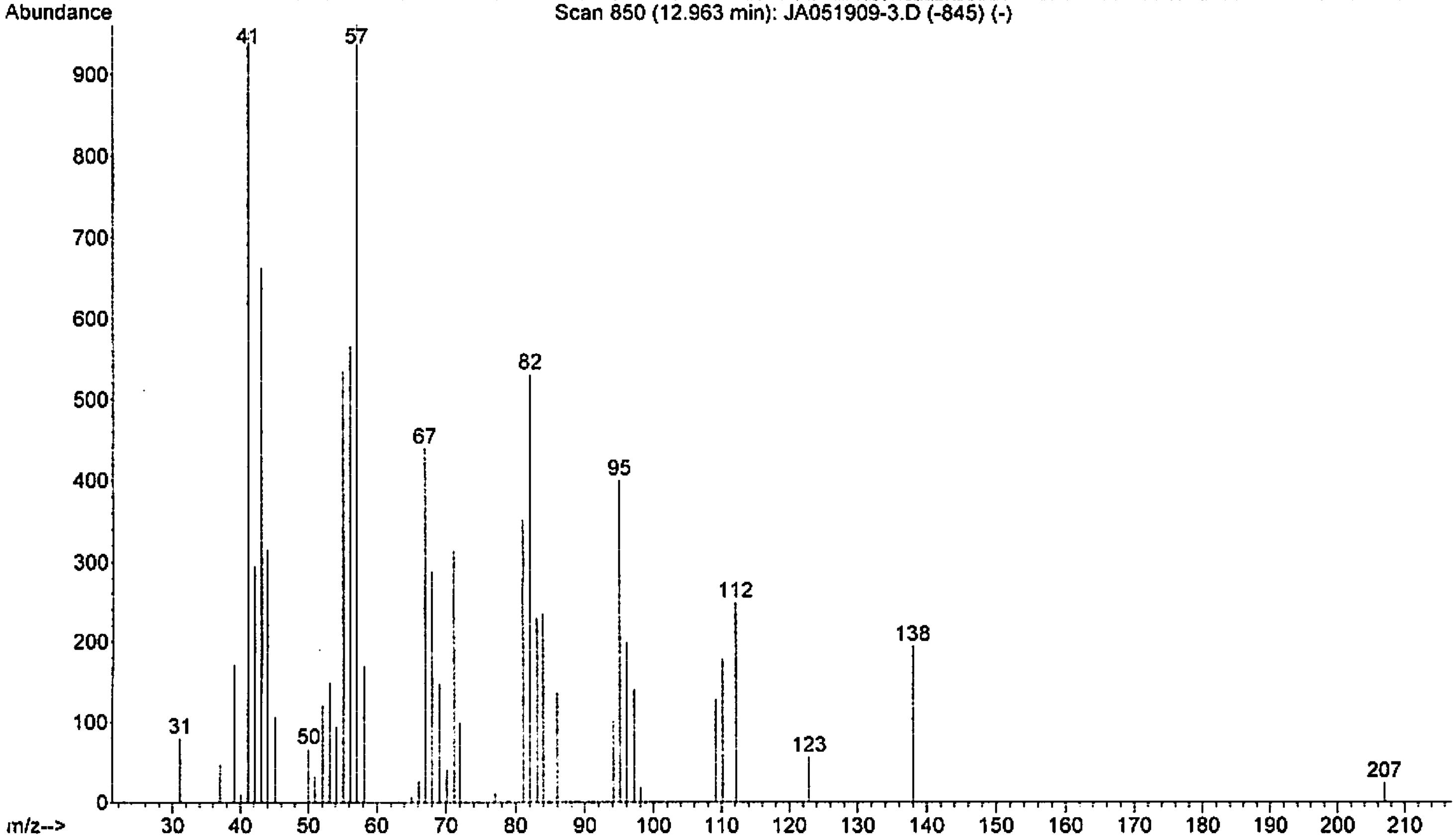

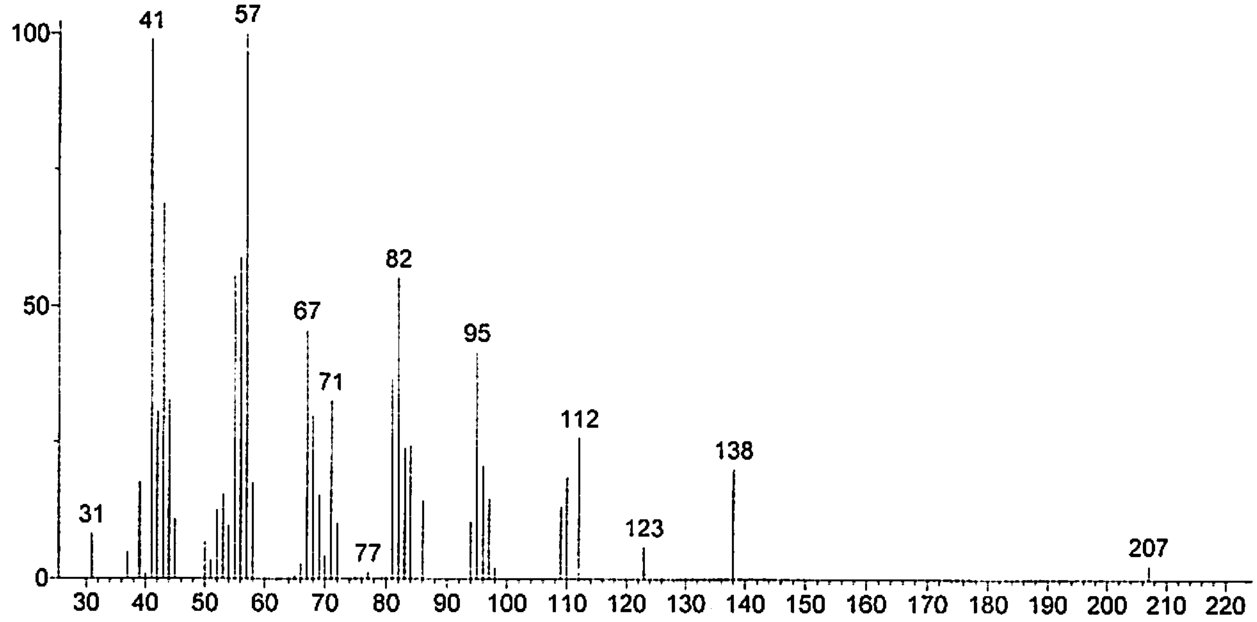

(Text File) Scan 850 (12.963 min): JA051909-3.D (-845)

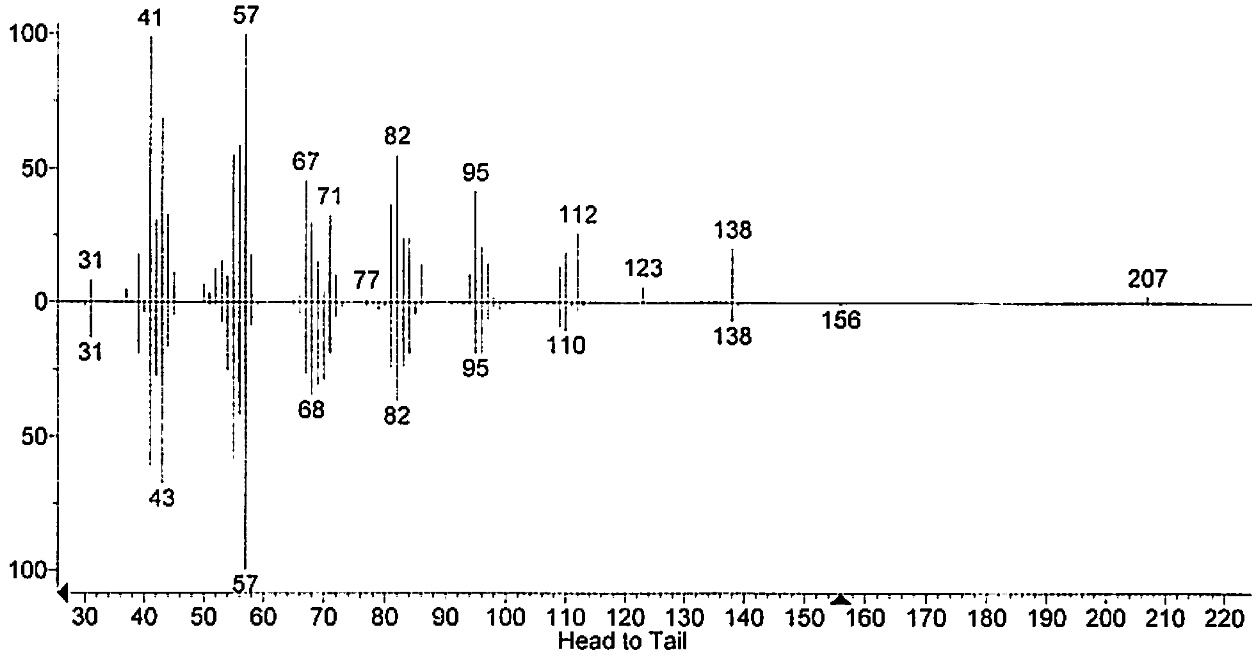

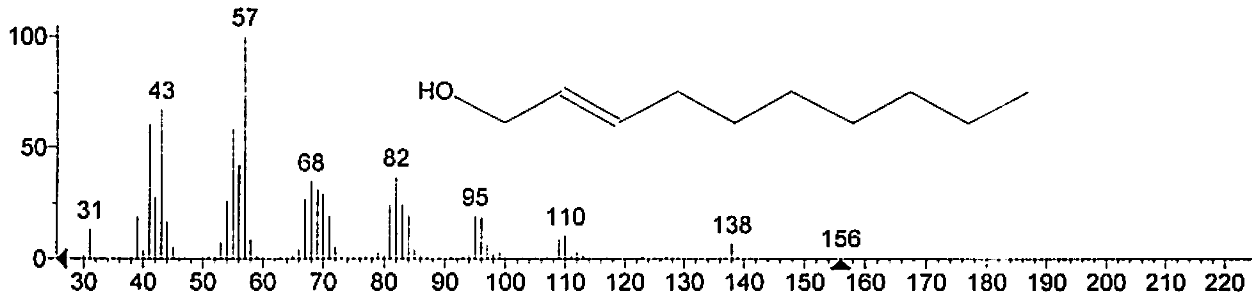

(replib) 2-Decen-1-ol, (E)-

```
[illegible]e..        :D:\DATA\ALDRICH\JA-09\Snapshot\JA051909-3.D
[illegible]erator     : Aldrich
[illegible]quired     : 19 May 2009  14:31      using AcqMethod JA-WAX08.M
[illegible]strument  :     Instrument #1
[illegible]mple Name: 2 field-coll. male C.oculata abd./2.5ul CH2Cl
[illegible]sc Info    : DB-WAX; coll. 5/18 by Ed; fed only honey
[illegible]al Number: 1
```

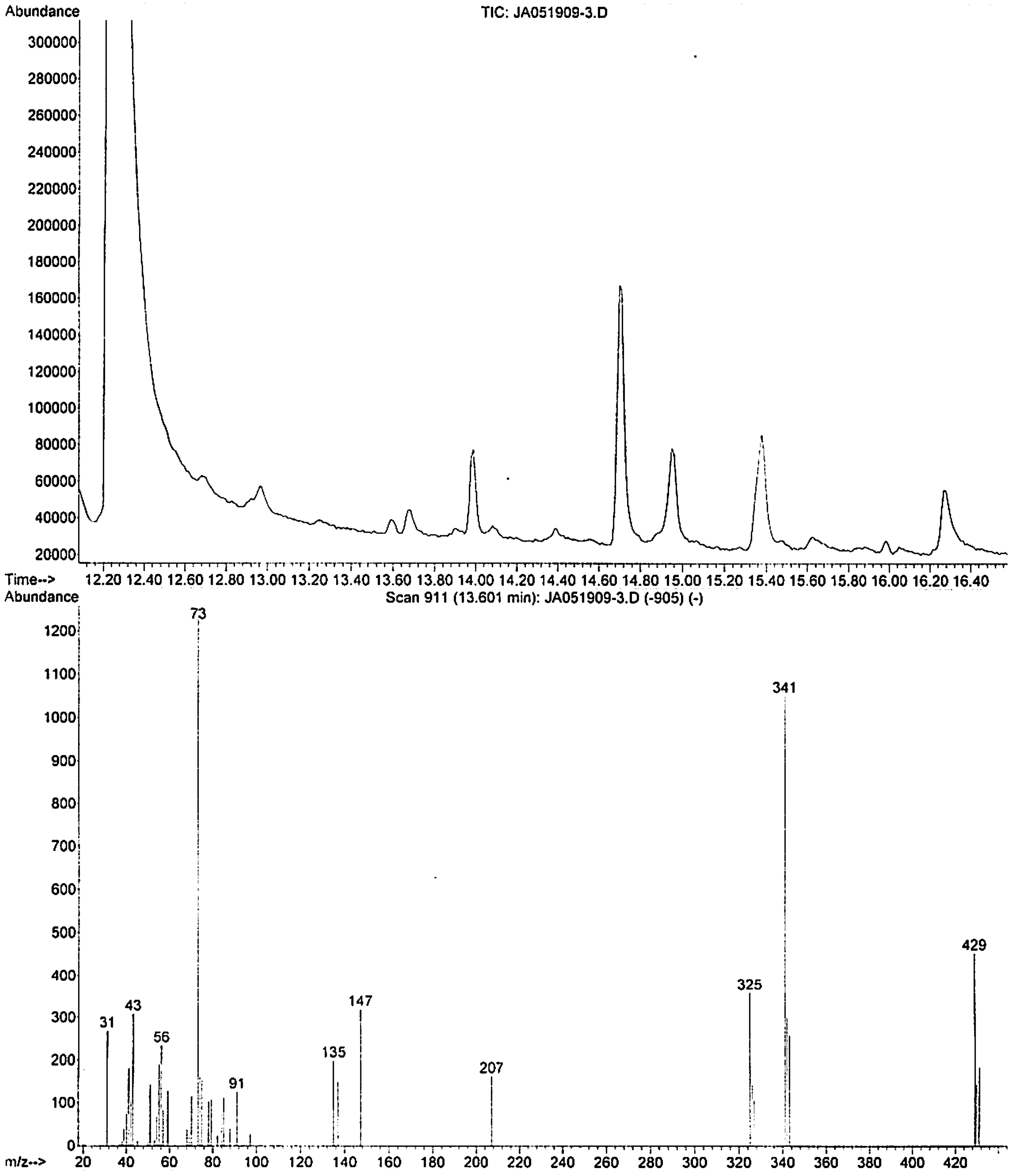

```
File        :D:\DATA\ALDRICH\JA-09\Snapshot\JA051909-3.D
Operator    : Aldrich
Acquired    : 19 May 2009  14:31      using AcqMethod JA-WAX08.M
Instrument :     Instrument #1
Sample Name: 2 field-coll. male C.oculata abd./2.5ul CH2Cl
Misc Info   : DB-WAX; coll. 5/18 by Ed; fed only honey
Vial Number: 1
```

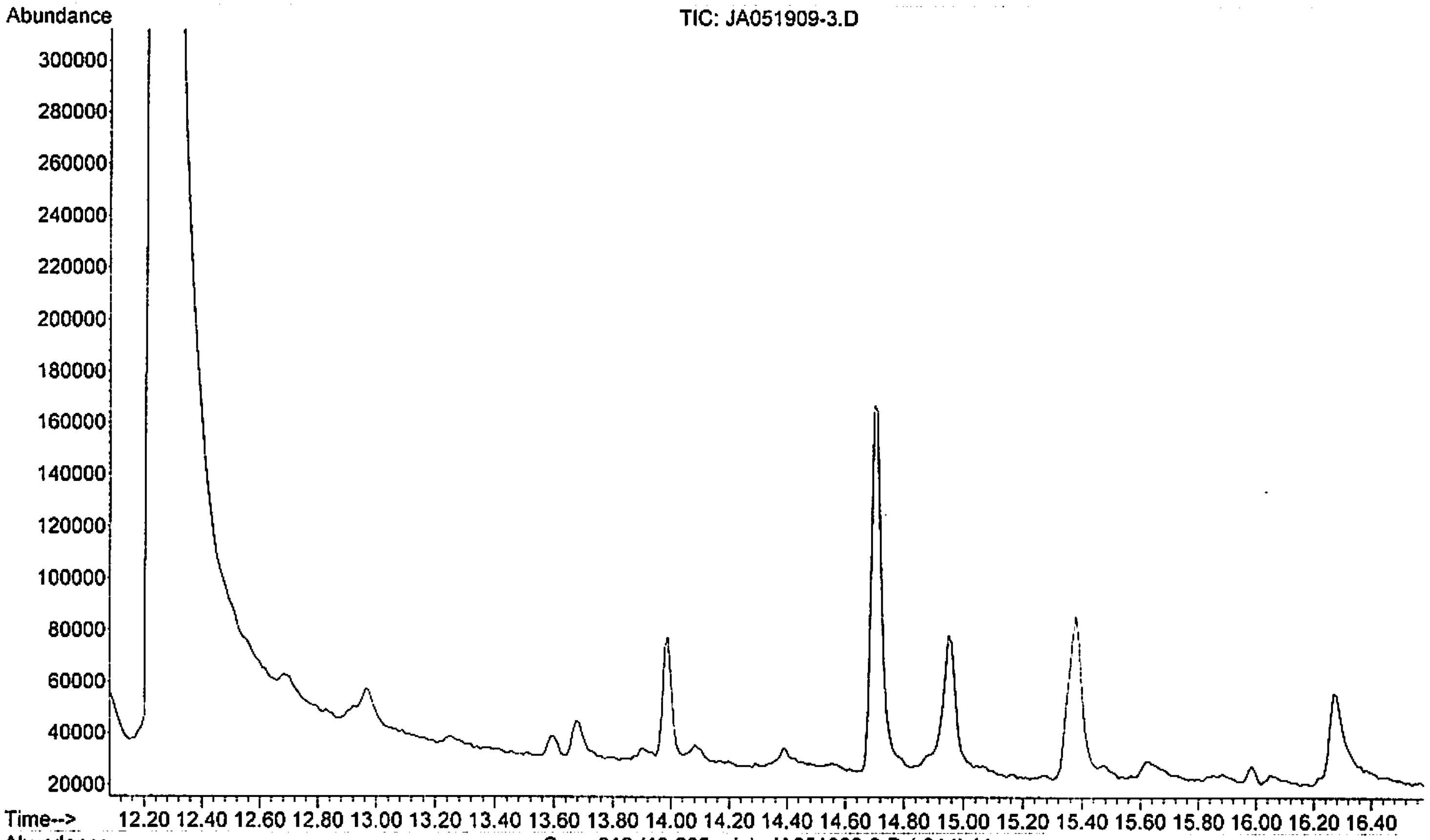

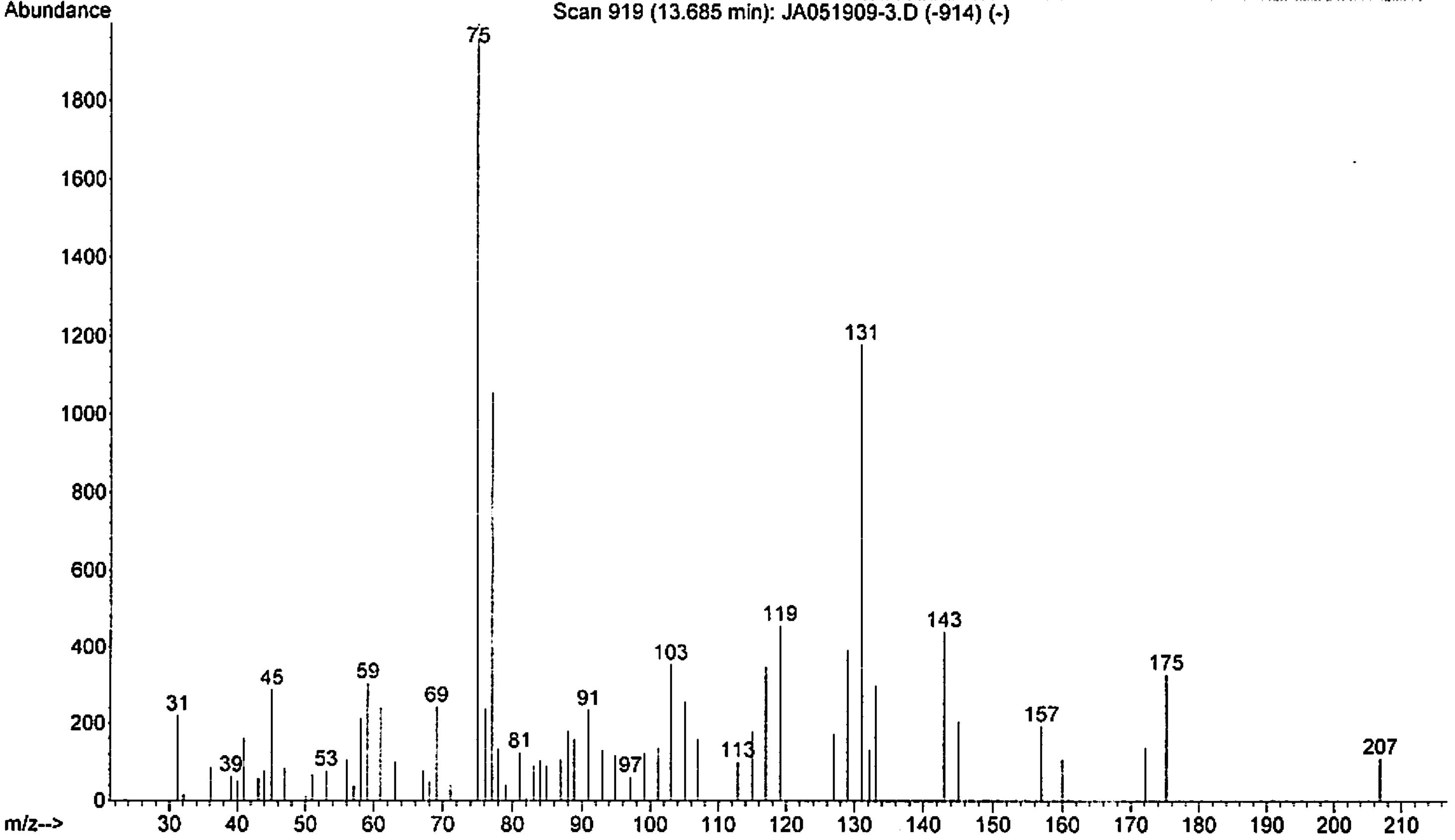

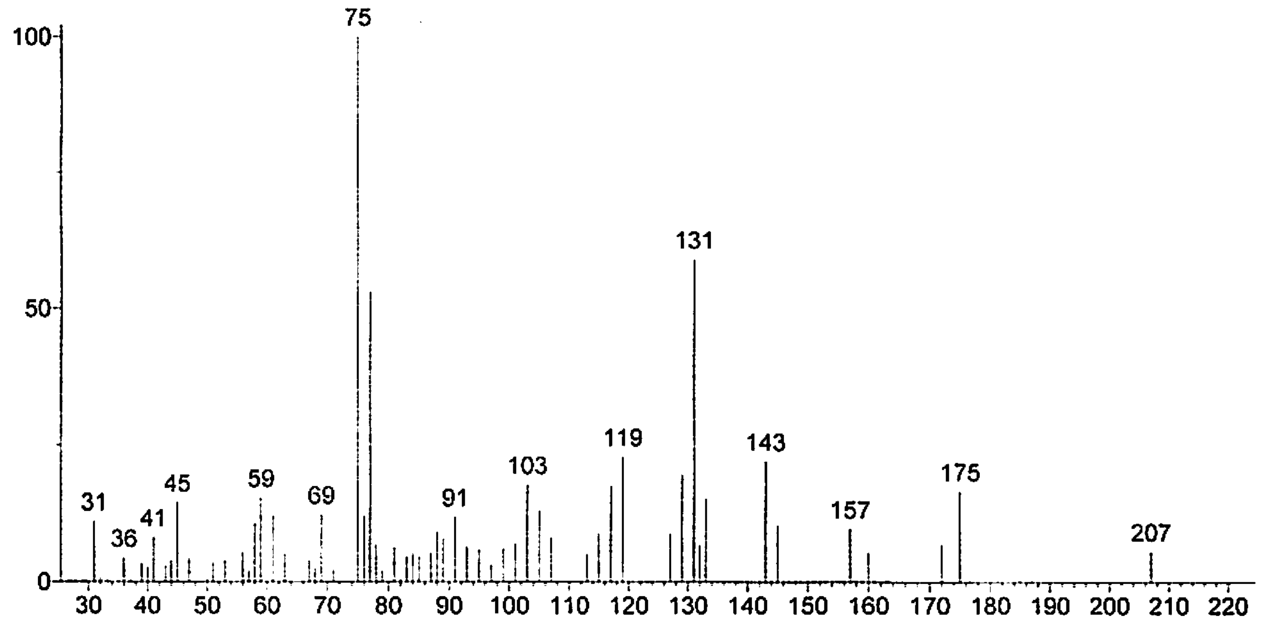

(Text File) Scan 919 (13.685 min): JA051909-3.D (-914)

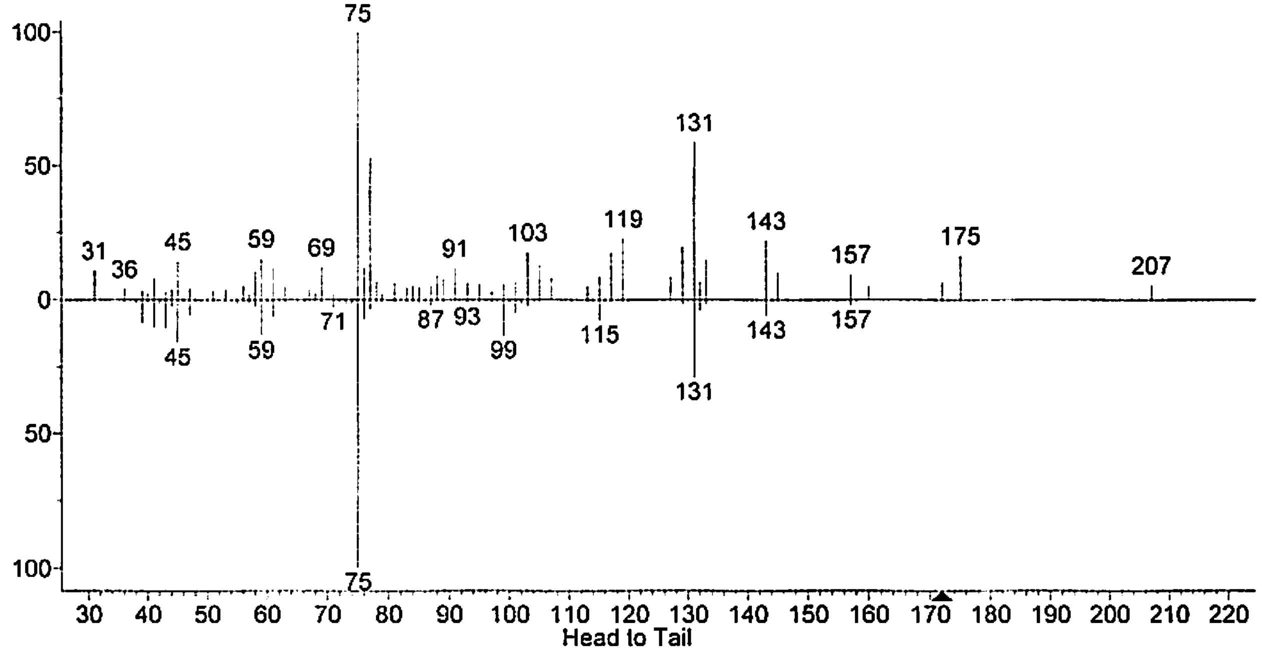

Head to Tail

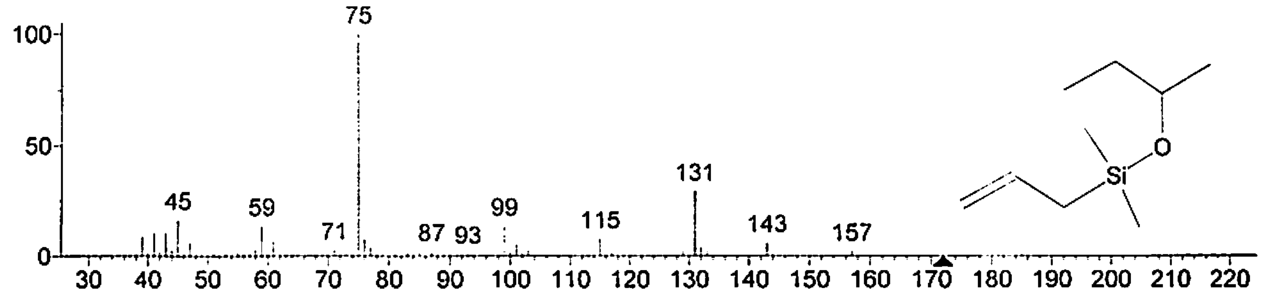

(mainlib) Allyl(2-butoxy)dimethylsilane

```
[illegible]le..      :D:\DATA\ALDRICH\JA-09\Snapshot\JA051909-3.D
[illegible]erator    : Aldrich
[illegible]quired    : 19 May 2009  14:31     using AcqMethod JA-WAX08.M
[illegible]strument :     Instrument #1
[illegible]mple Name: 2 field-coll. male C.oculata abd./2.5ul CH2Cl
[illegible]sc Info  : DB-WAX; coll. 5/18 by Ed; fed only honey
[illegible]al Number: 1
```

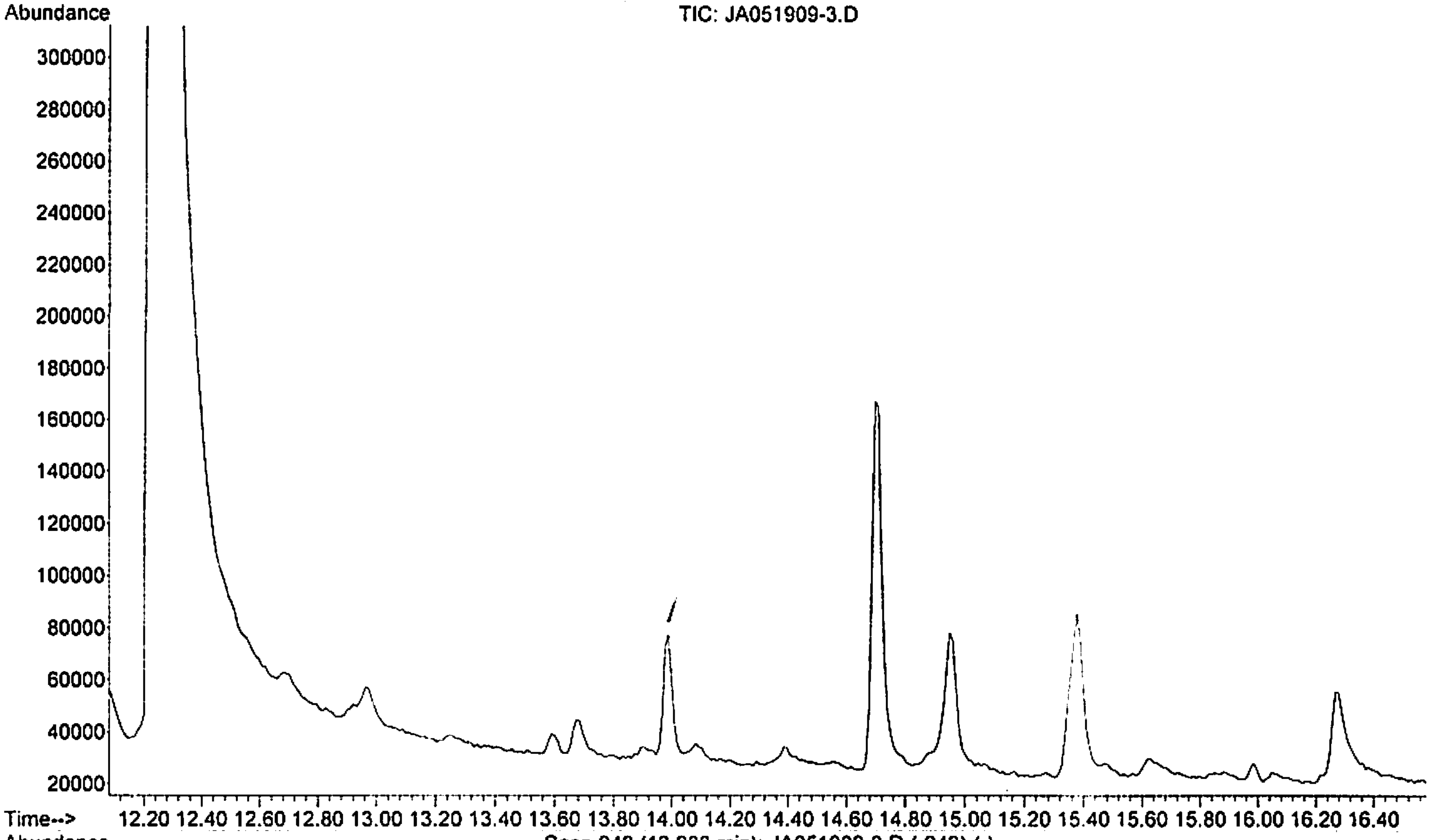

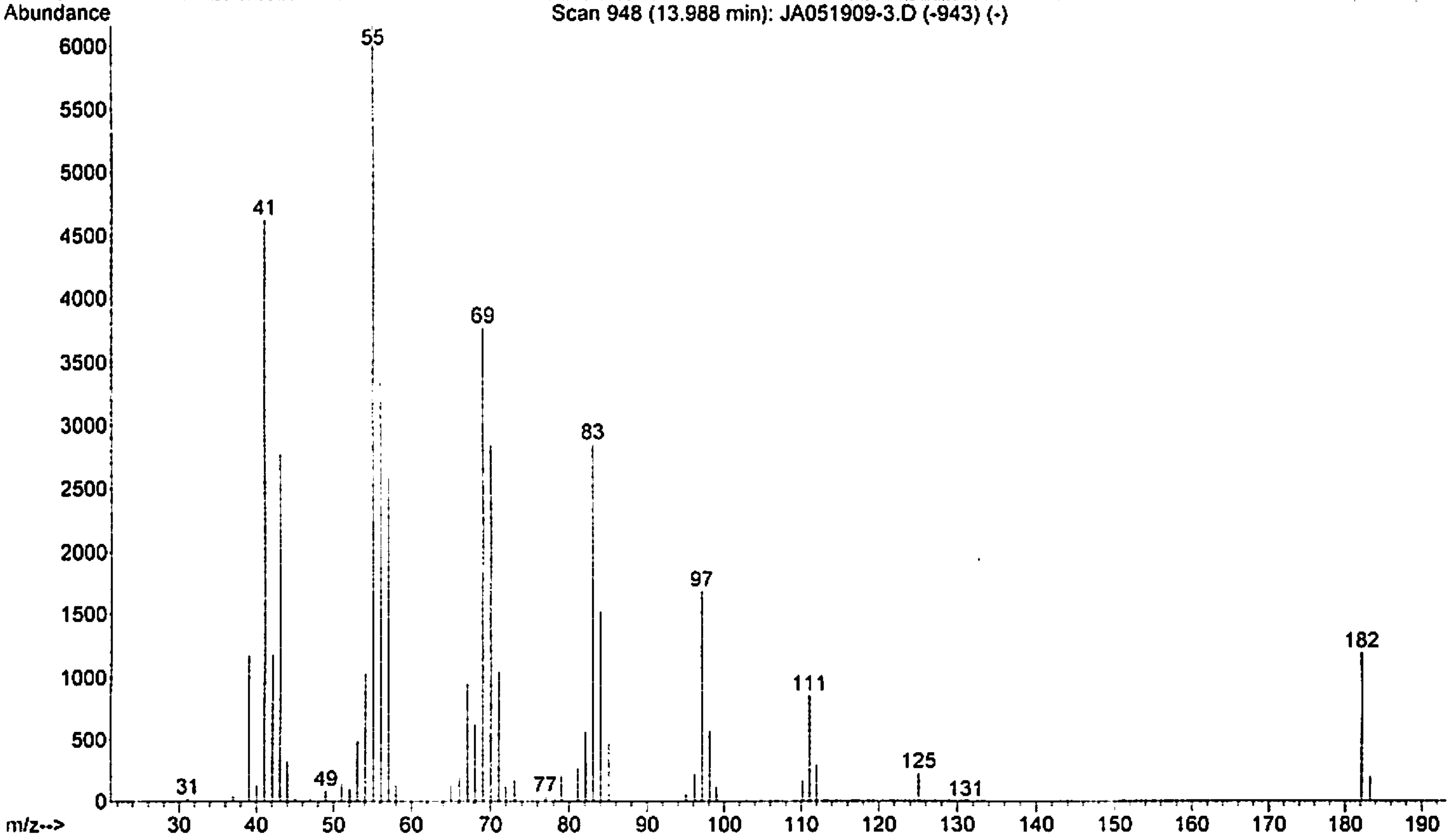

```
File      :D:\DATA\ALDRICH\JA-09\Snapshot\JA051909-3.D
Operator  : Aldrich
Acquired  : 19 May 2009   14:31     using AcqMethod JA-WAX08.M
Instrument :     Instrument #1
Sample Name: 2 field-coll. male C.oculata abd./2.5ul CH2Cl
Misc Info  : DB-WAX; coll. 5/18 by Ed; fed only honey
Vial Number: 1
```

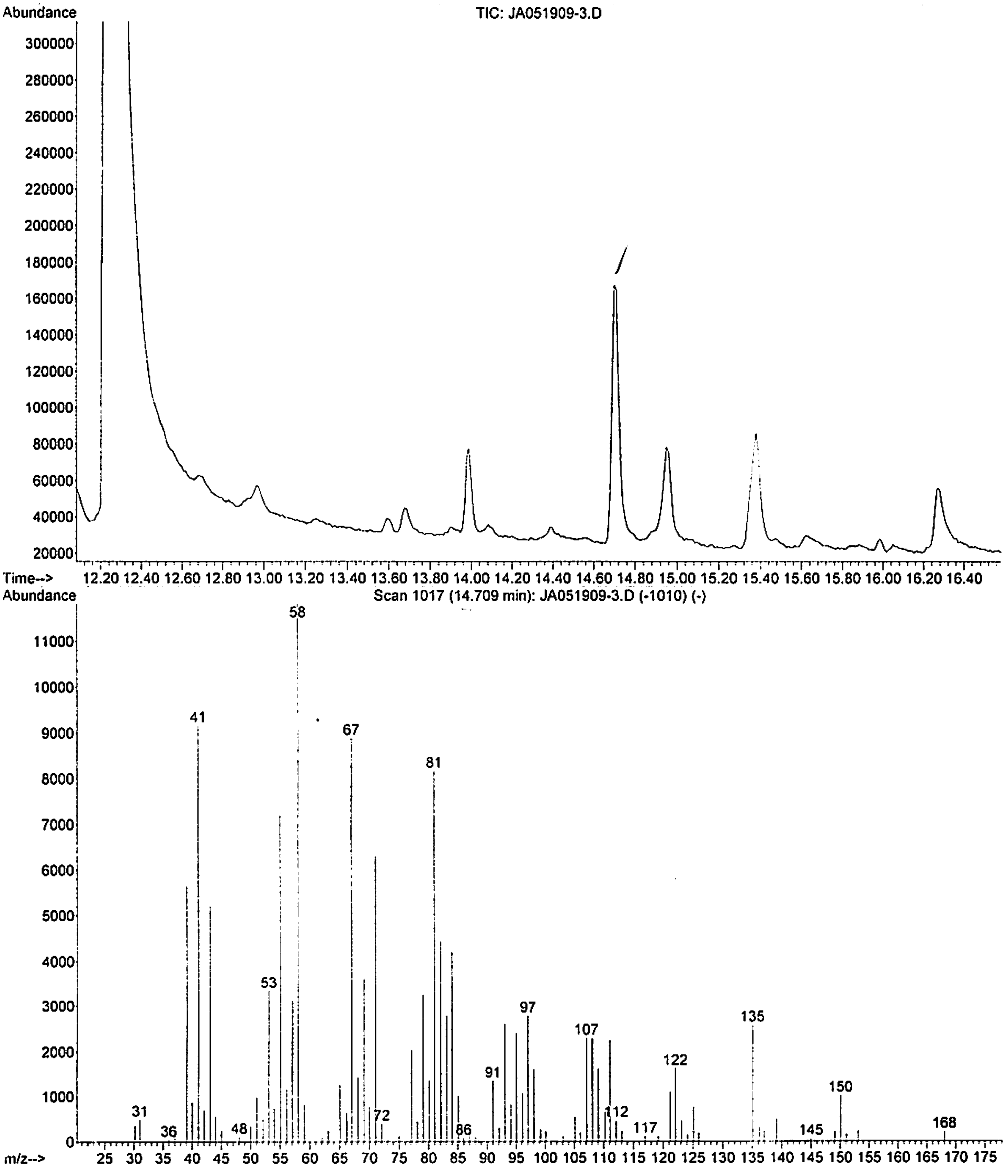

File : D:\DATA\ALDRICH\JA-09\Snapshot\JA051909-3.D
Operator : Aldrich
Acquired : 19 May 2009 14:31 using AcqMethod JA-WAX08.M
Instrument : Instrument #1
Sample Name: 2 field-coll. male C.oculata abd./2.5ul CH2Cl
Misc Info : DB-WAX; coll. 5/18 by Ed; fed only honey
Vial Number: 1

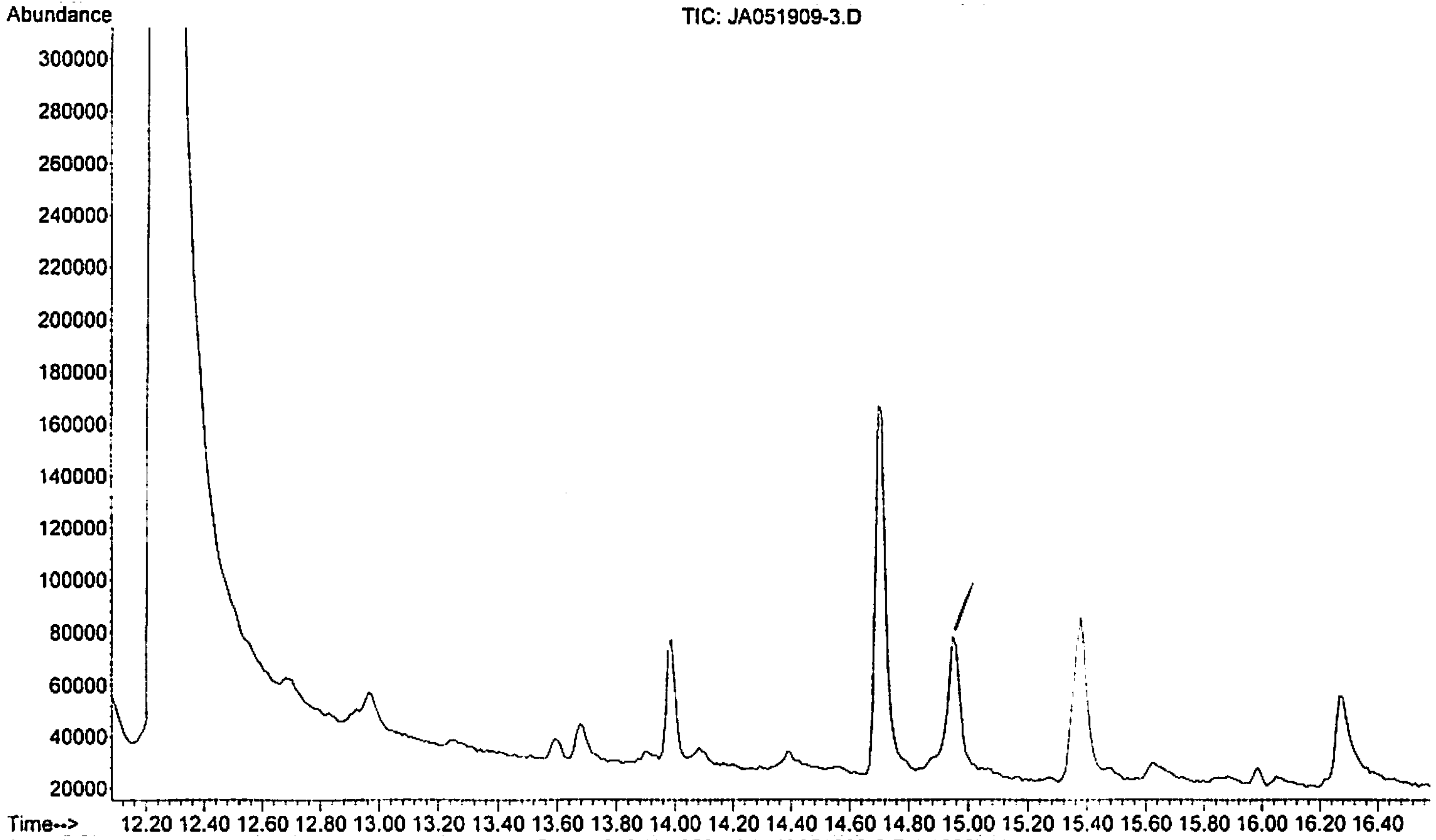

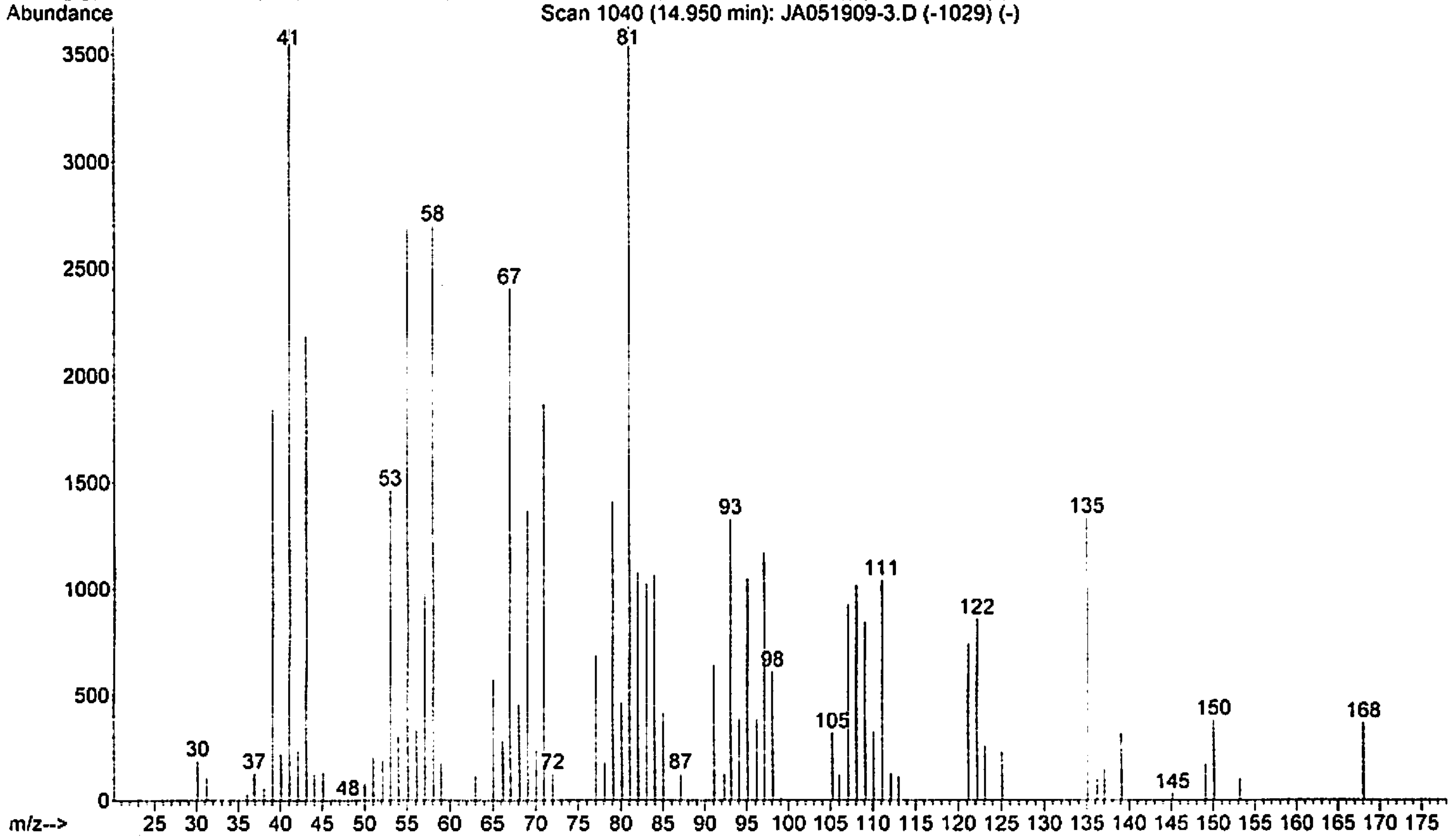

File : D:\DATA\ALDRICH\JA-09\Snapshot\JA051909-3.D
Operator : Aldrich
Acquired : 19 May 2009 14:31 using AcqMethod JA-WAX08.M
Instrument : Instrument #1
Sample Name: 2 field-coll. male C.oculata abd./2.5ul CH2Cl
Misc Info : DB-WAX; coll. 5/18 by Ed; fed only honey
Vial Number: 1

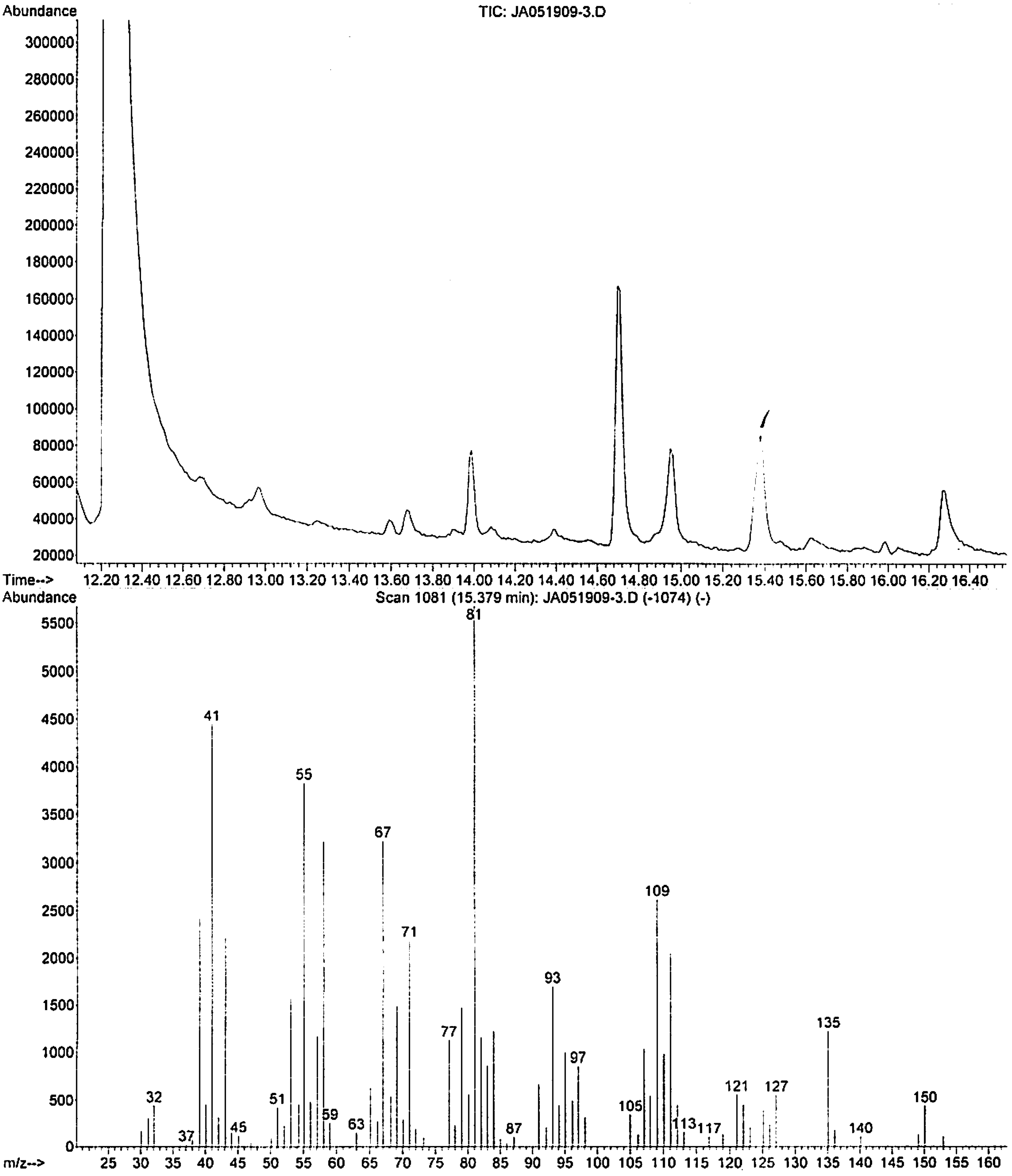

File : D:\DATA\ALDRICH\JA-09\Snapshot\JA051909-3.D
Operator : Aldrich
Acquired : 19 May 2009 14:31 using AcqMethod JA-WAX08.M
Instrument : Instrument #1
Sample Name: 2 field-coll. male C.oculata abd./2.5ul CH2Cl
Misc Info : DB-WAX; coll. 5/18 by Ed; fed only honey
Vial Number: 1

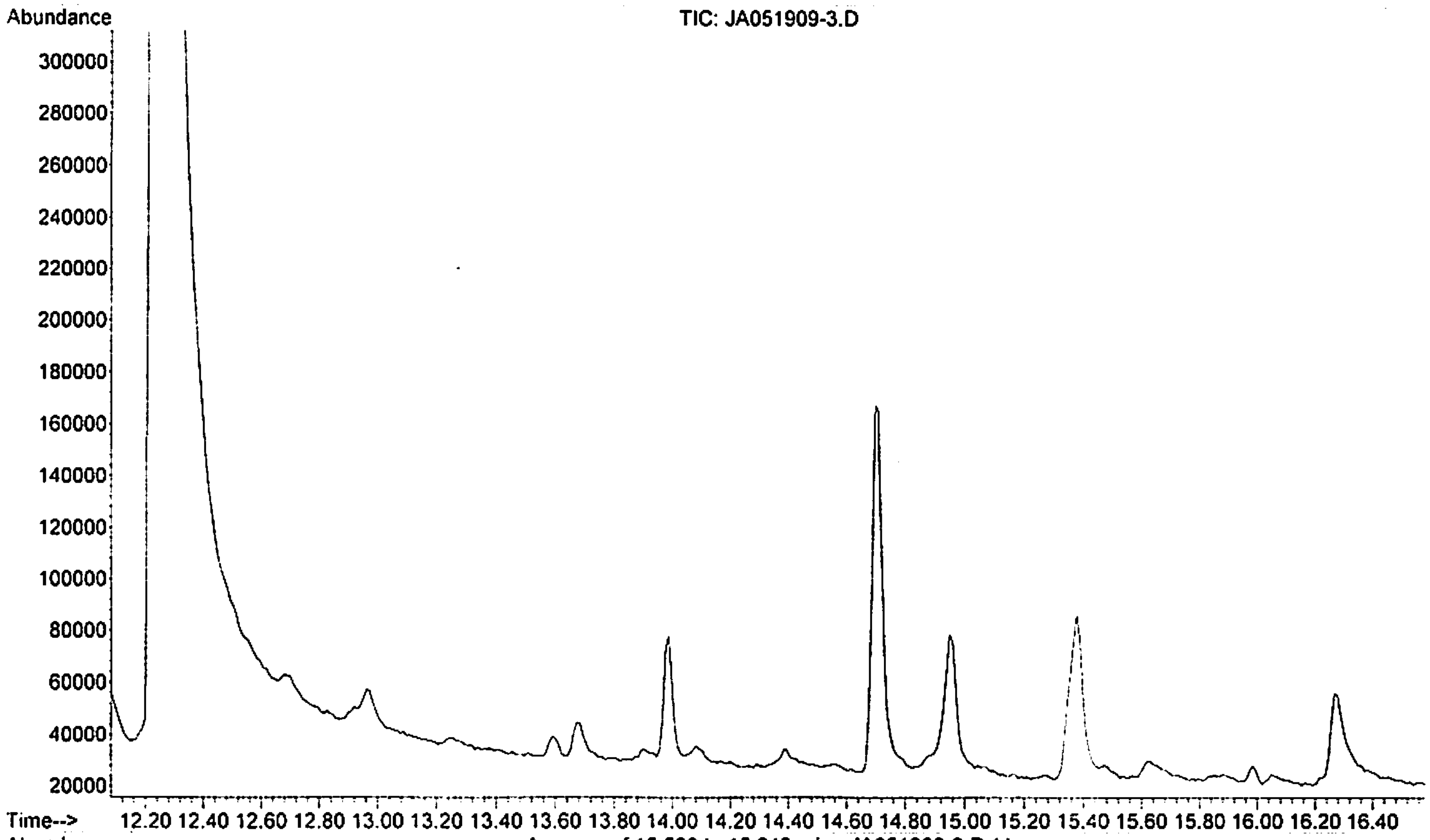

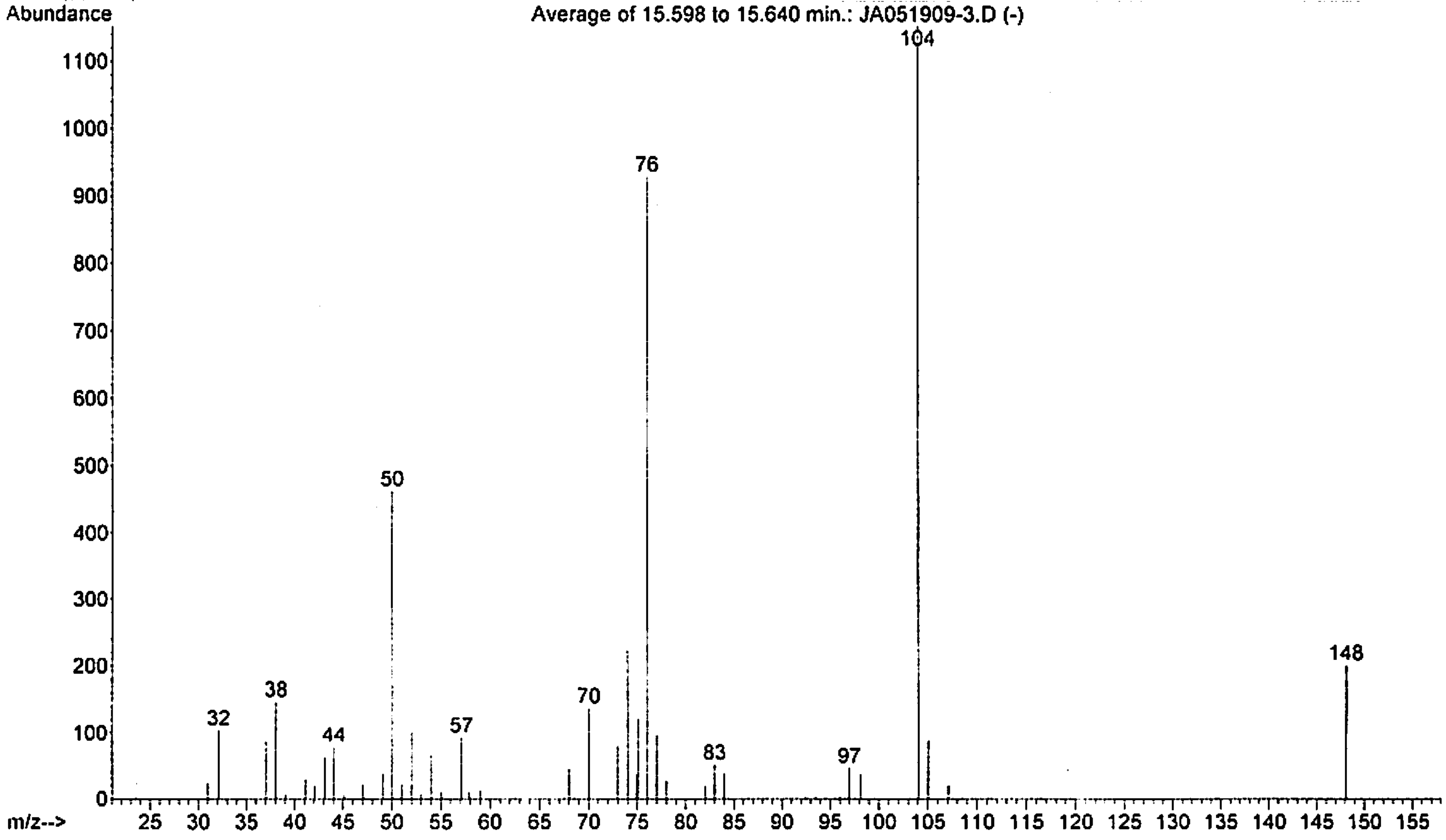

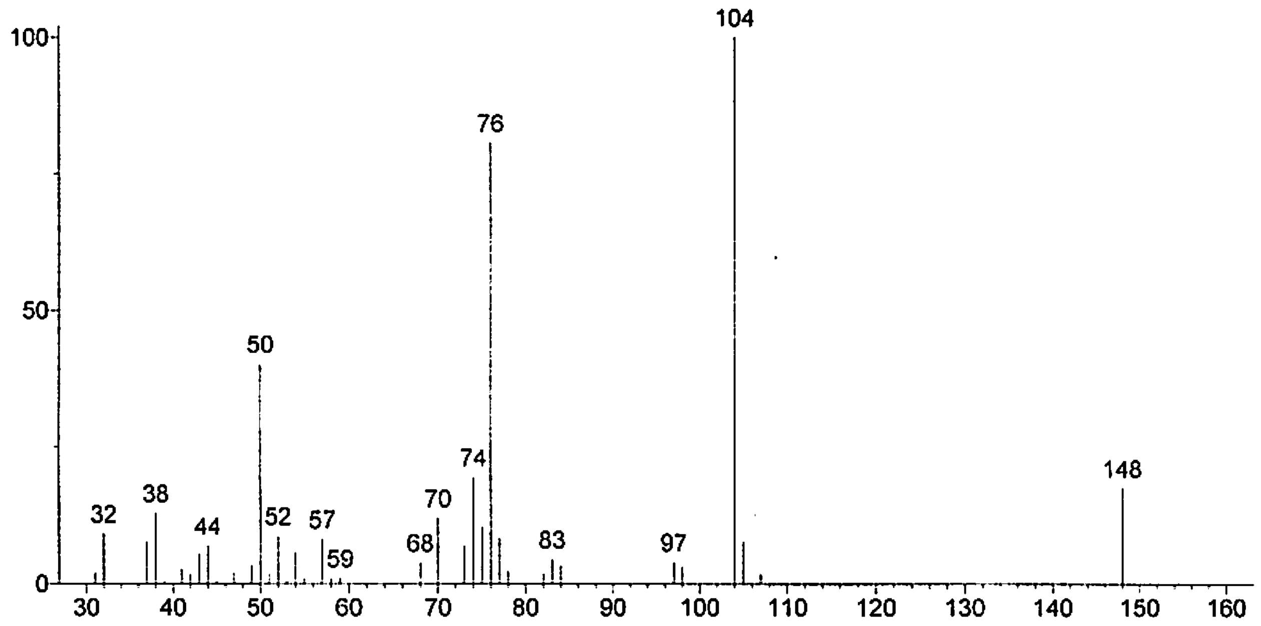

(Text File) Average of 15.598 to 15.640 min.: JA051909-3.D

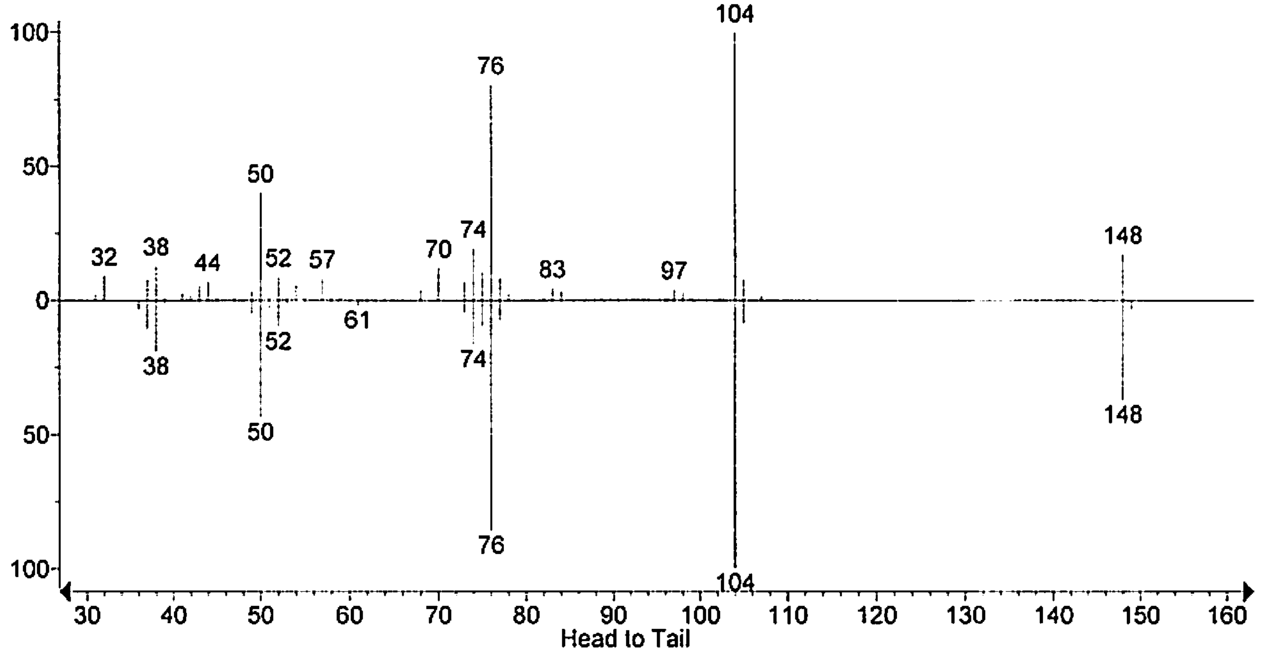

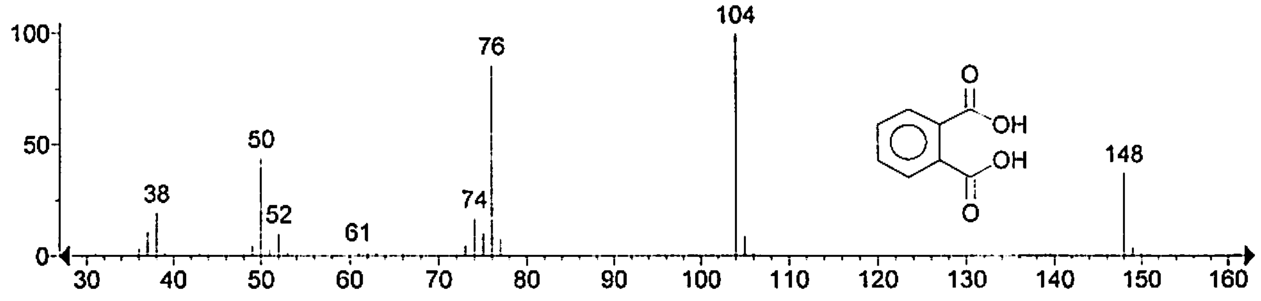

(mainlib) 1,2-Benzenedicarboxylic acid

File : D:\DATA\ALDRICH\JA-09\Snapshot\JA051909-3.D
Operator : Aldrich
Acquired : 19 May 2009 14:31 using AcqMethod JA-WAX08.M
Instrument : Instrument #1
Sample Name: 2 field-coll. male C.oculata abd./2.5ul CH2Cl
Misc Info : DB-WAX; coll. 5/18 by Ed; fed only honey
Vial Number: 1

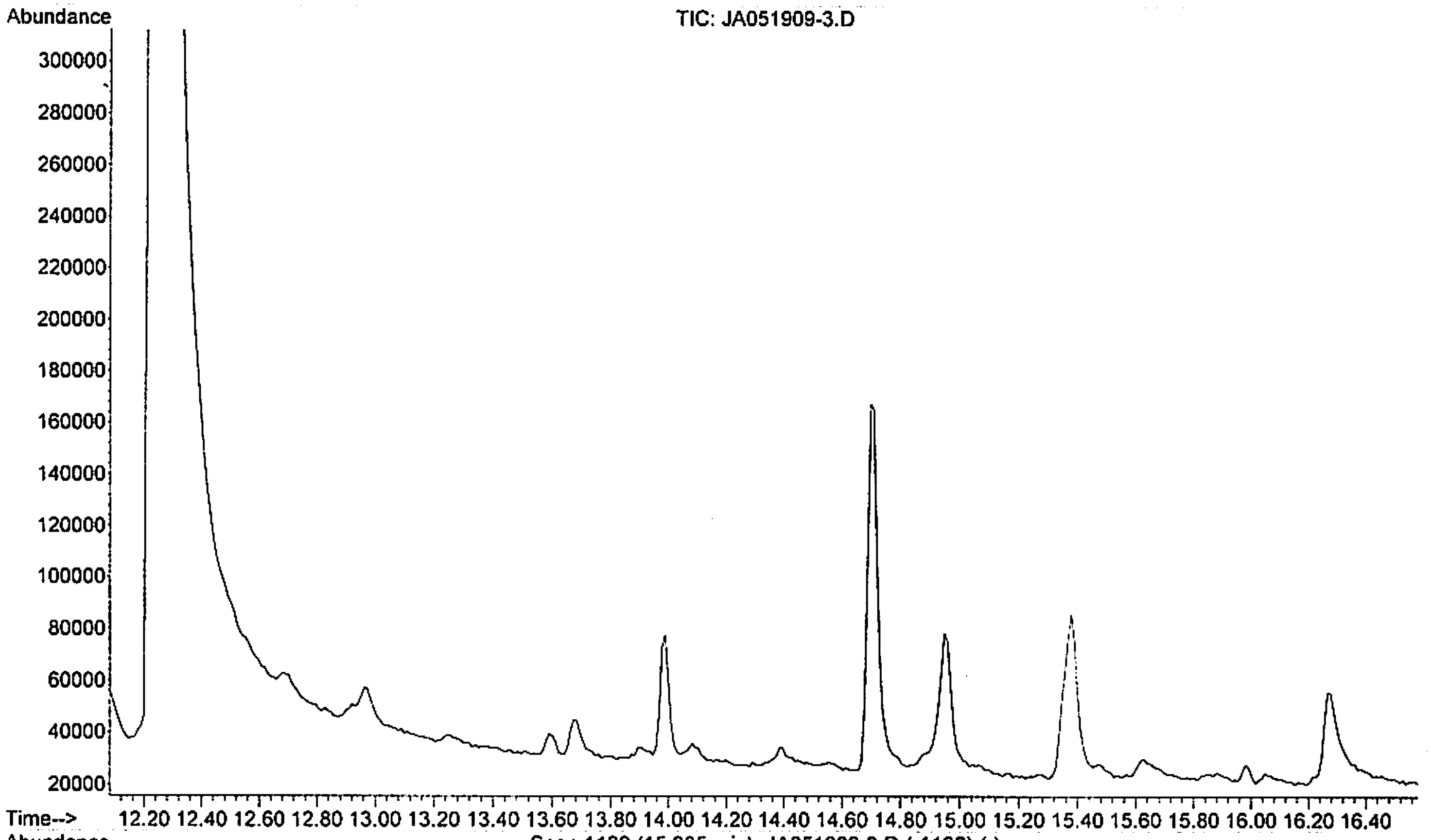

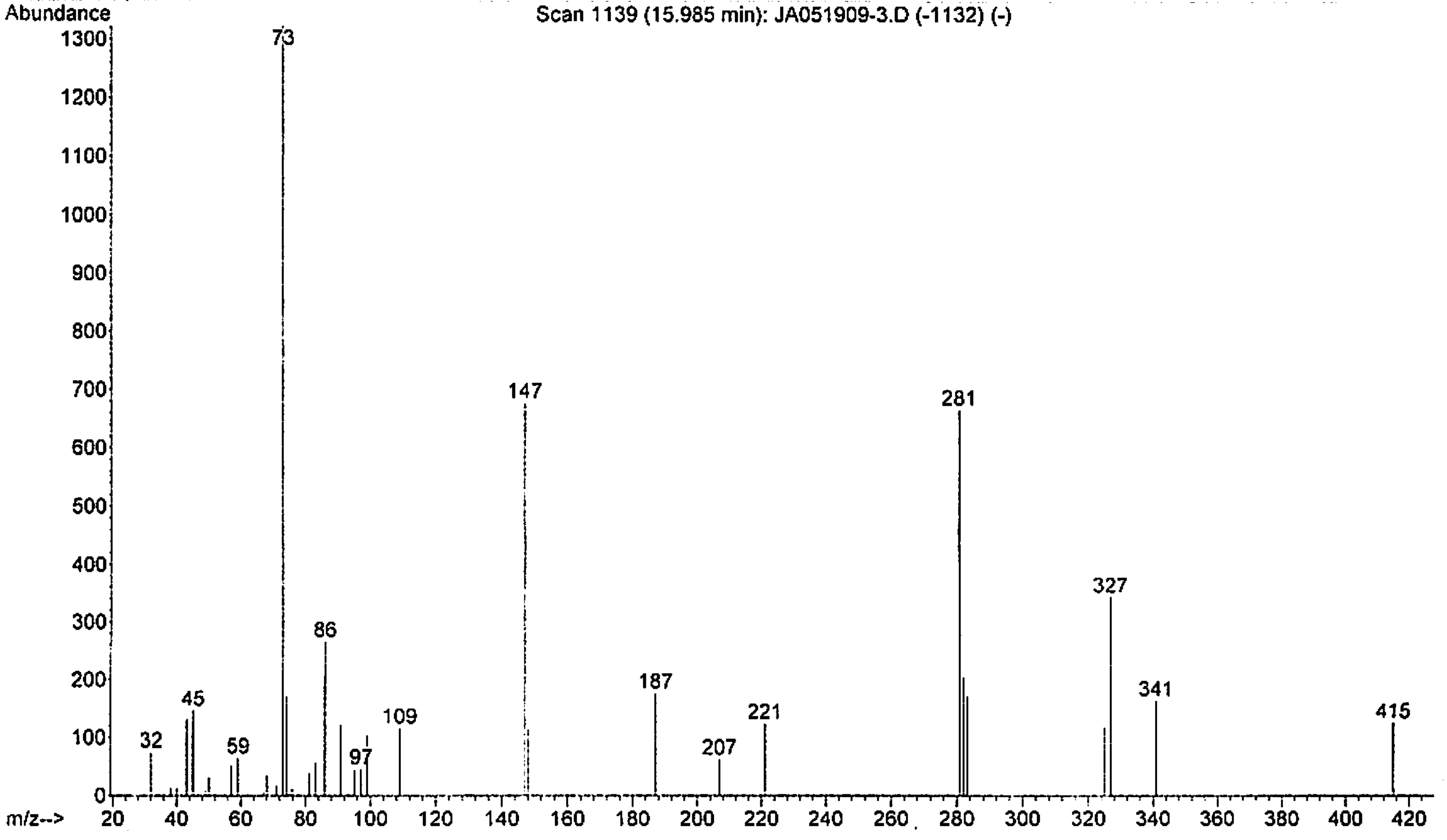

```
[illegible]le      :D:\DATA\ALDRICH\JA-09\Snapshot\JA051909-3.D
[illegible]erator   : Aldrich
[illegible]quired   : 19 May 2009  14:31     using AcqMethod JA-WAX08.M
[illegible]strument :    Instrument #1
[illegible]mple Name: 2 field-coll. male C.oculata abd./2.5ul CH2Cl
[illegible]sc Info  : DB-WAX; coll. 5/18 by Ed; fed only honey
[illegible]al Number: 1
```

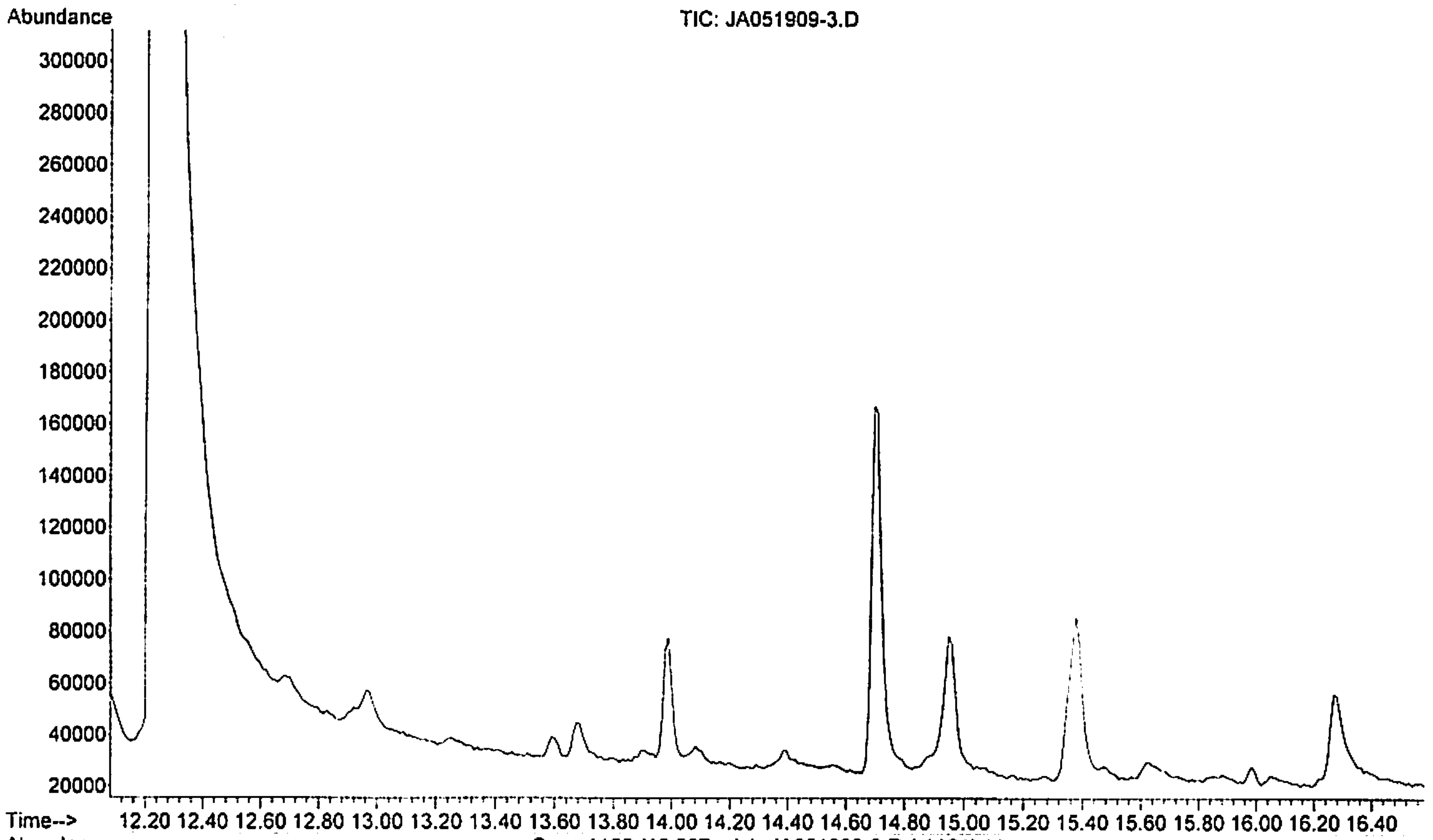

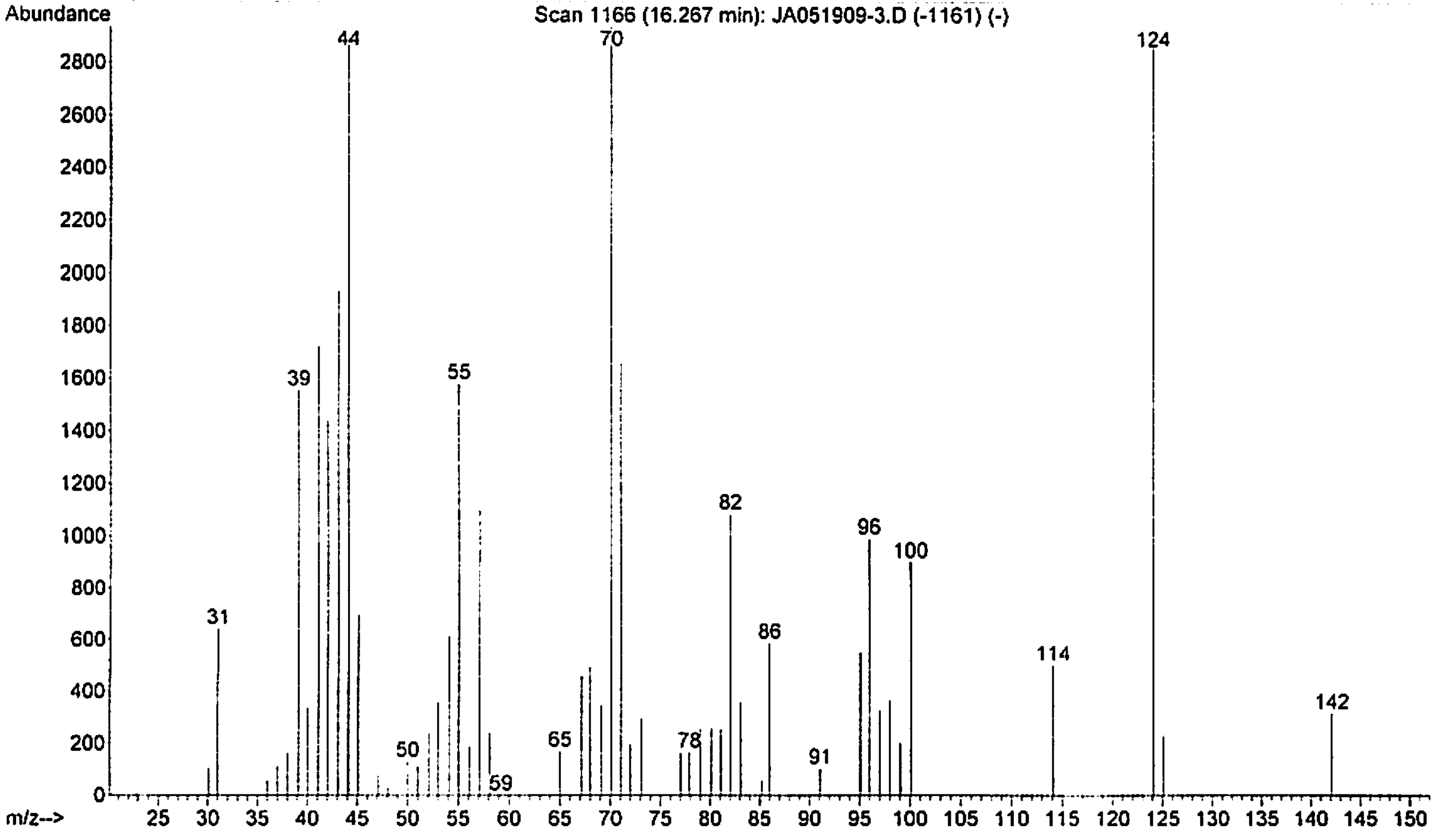

File :D:\DATA\ALDRICH\JA-09\Snapshot\JA051909-3.D
Operator : Aldrich
Acquired : 19 May 2009 14:31 using AcqMethod JA-WAX08.M
Instrument : Instrument #1
Sample Name: 2 field-coll. male C.oculata abd./2.5ul CH2Cl
Misc Info : DB-WAX; coll. 5/18 by Ed; fed only honey
Vial Number: 1

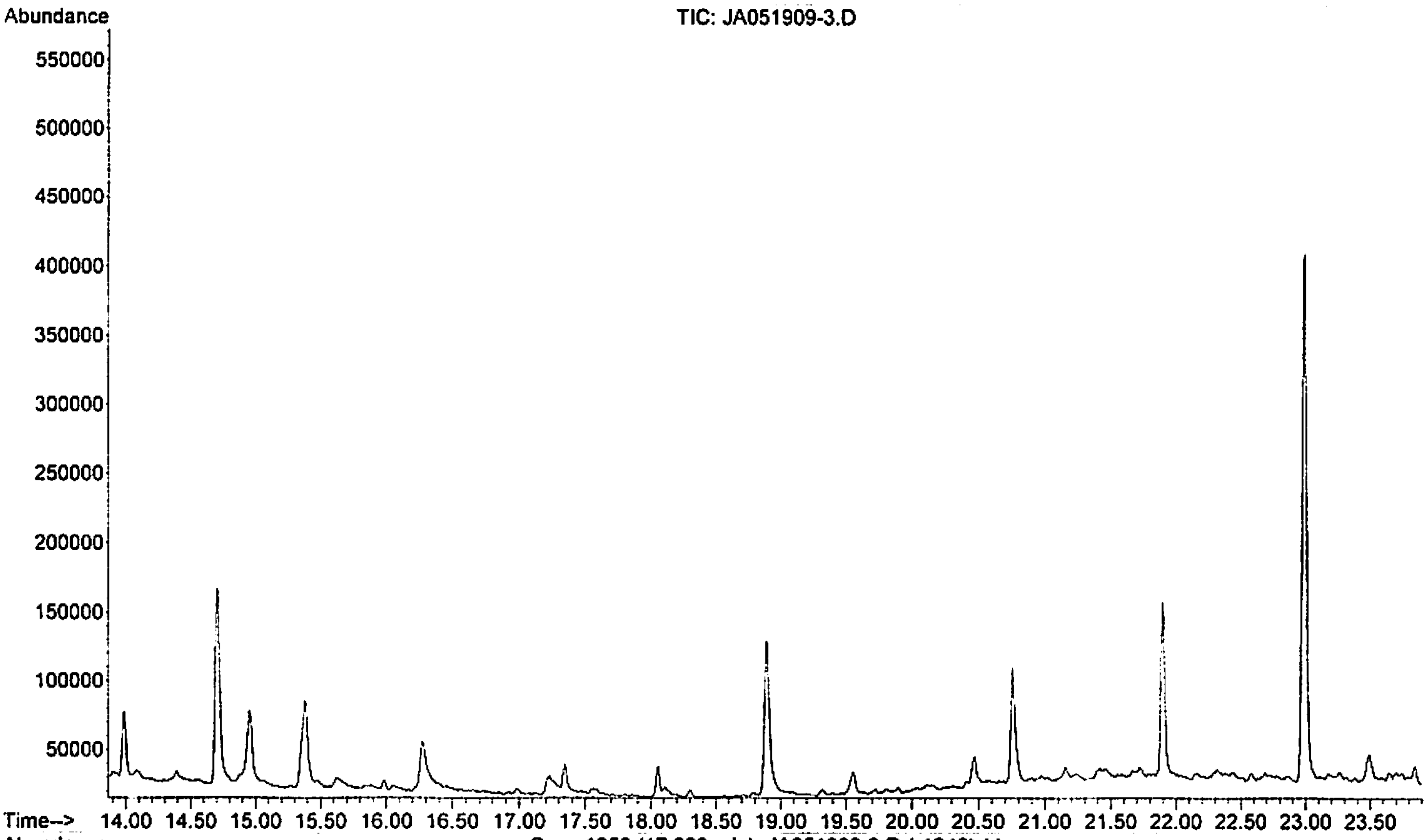

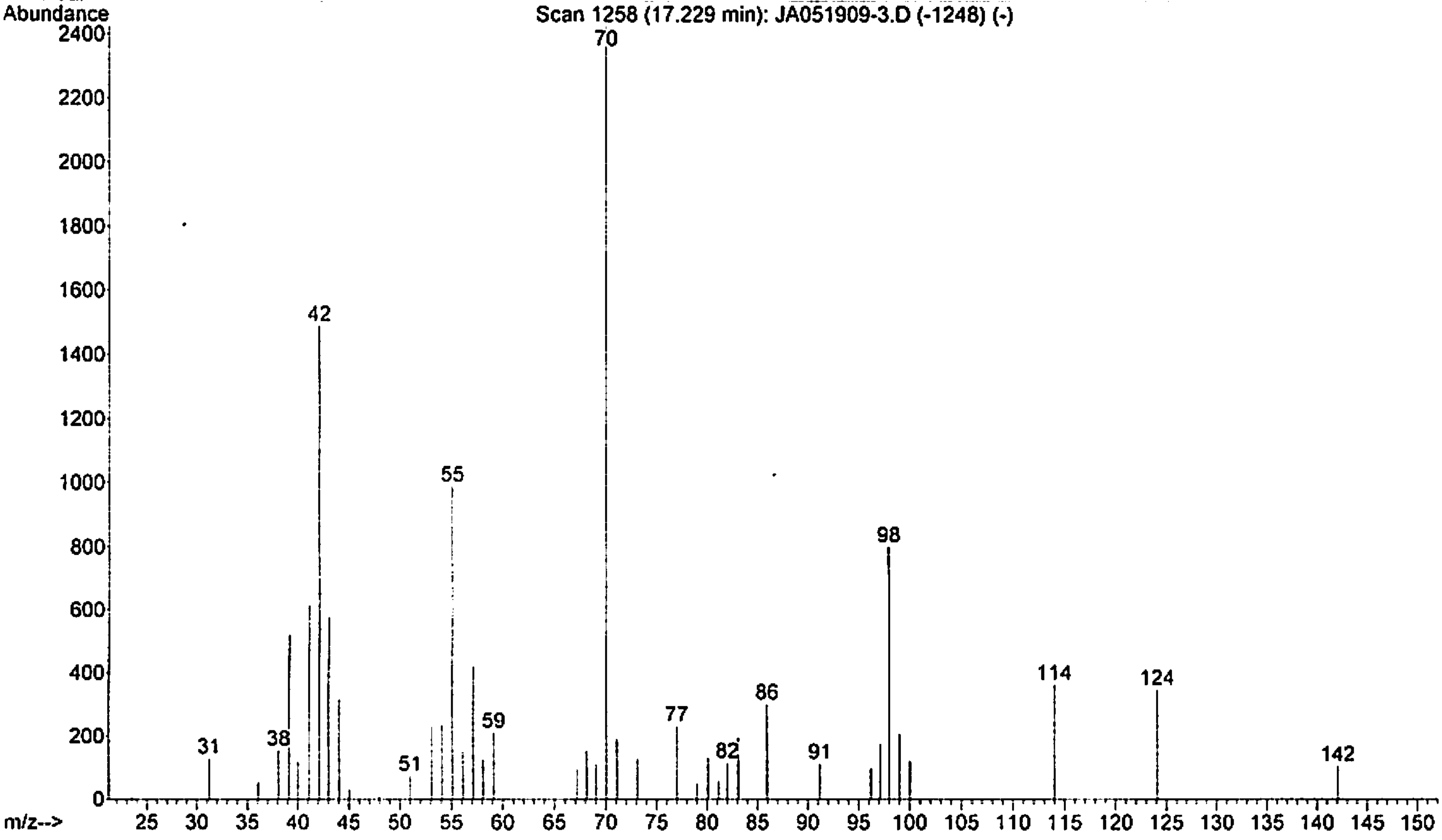

```
File    :D:\DATA\ALDRICH\JA-09\Snapshot\JA051909-3.D
Operator    : Aldrich
Acquired    : 19 May 2009  14:31     using AcqMethod JA-WAX08.M
Instrument :     Instrument #1
Sample Name: 2 field-coll. male C.oculata abd./2.5ul CH2Cl
Misc Info  : DB-WAX; coll. 5/18 by Ed; fed only honey
Vial Number: 1
```

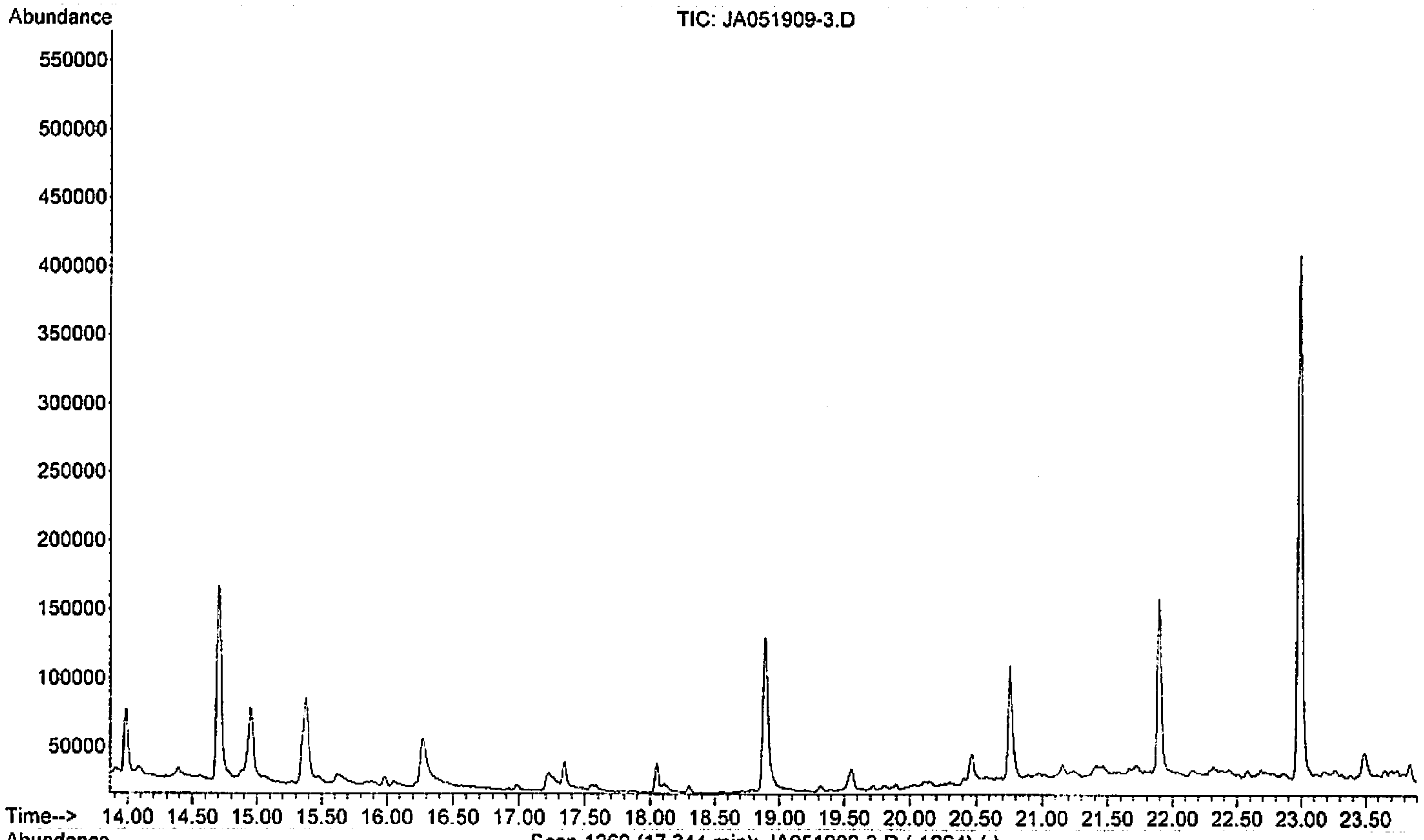

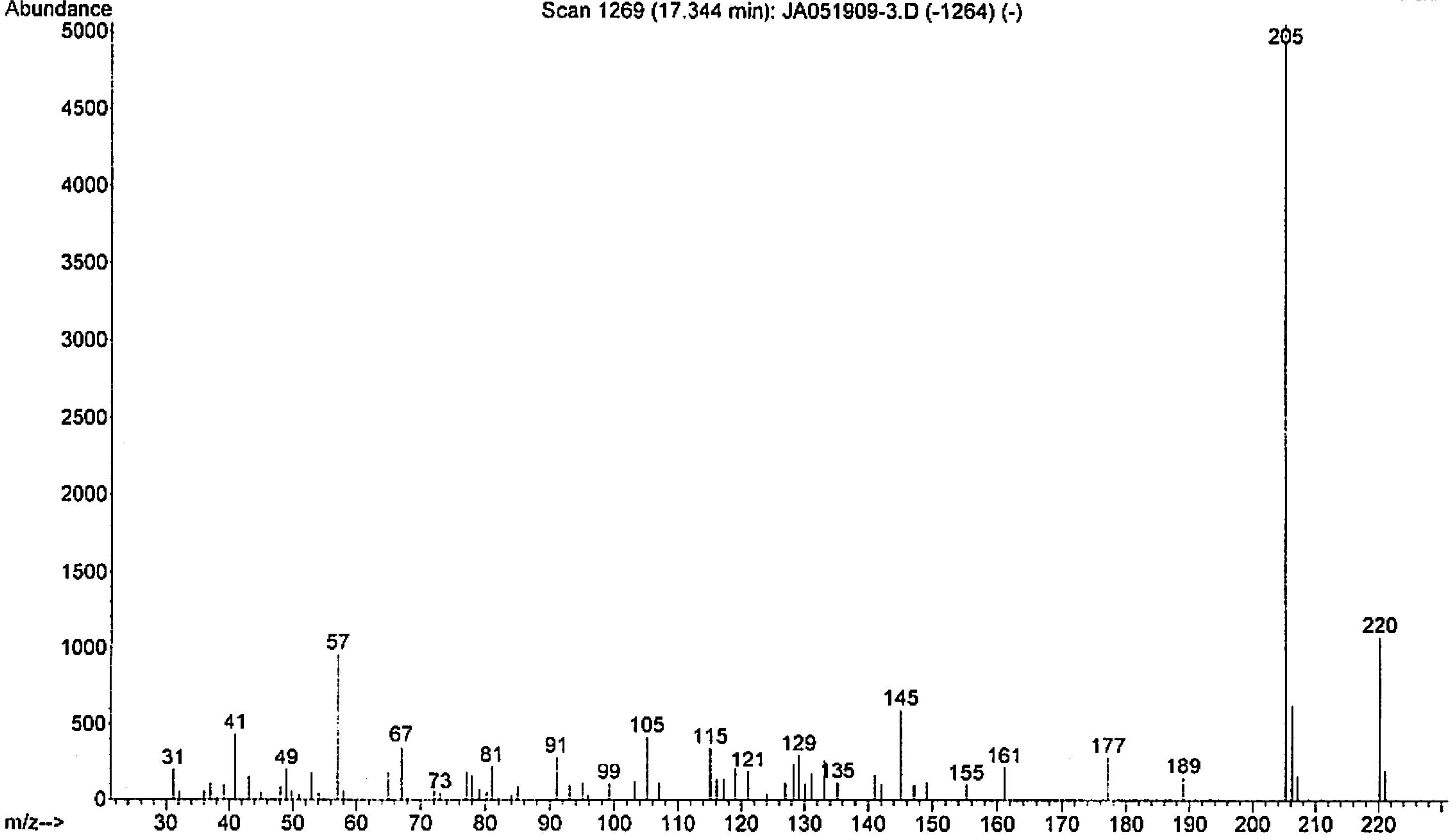

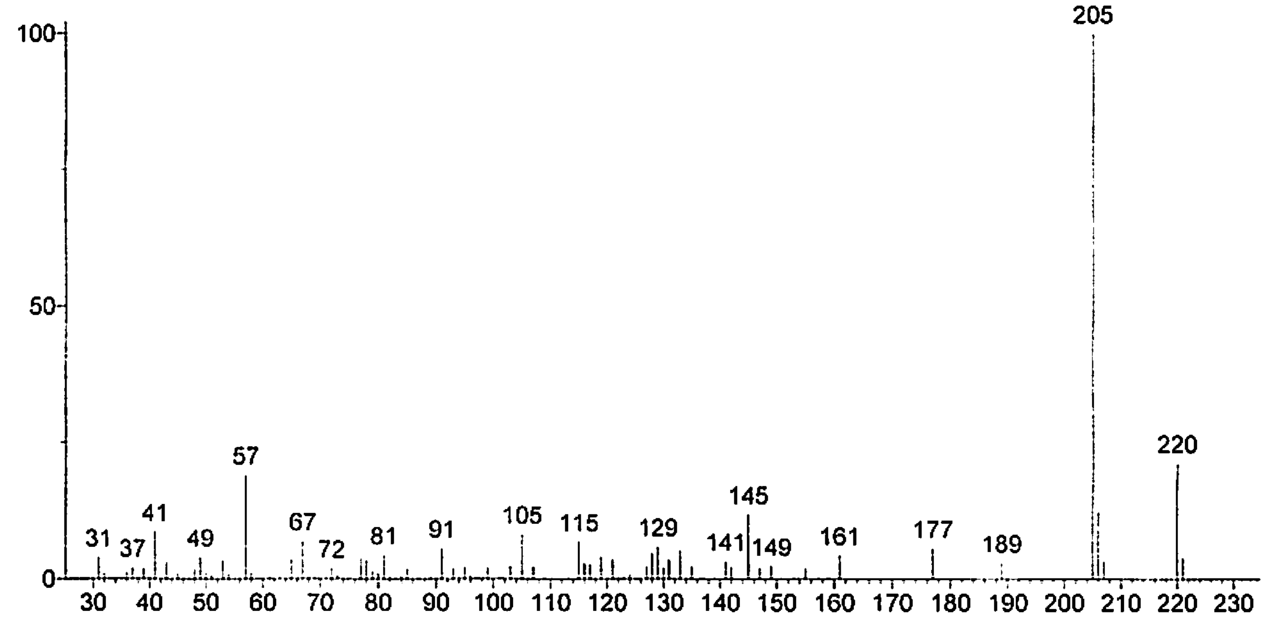

(Text File) Scan 1269 (17.344 min): JA051909-3.D (-1264)

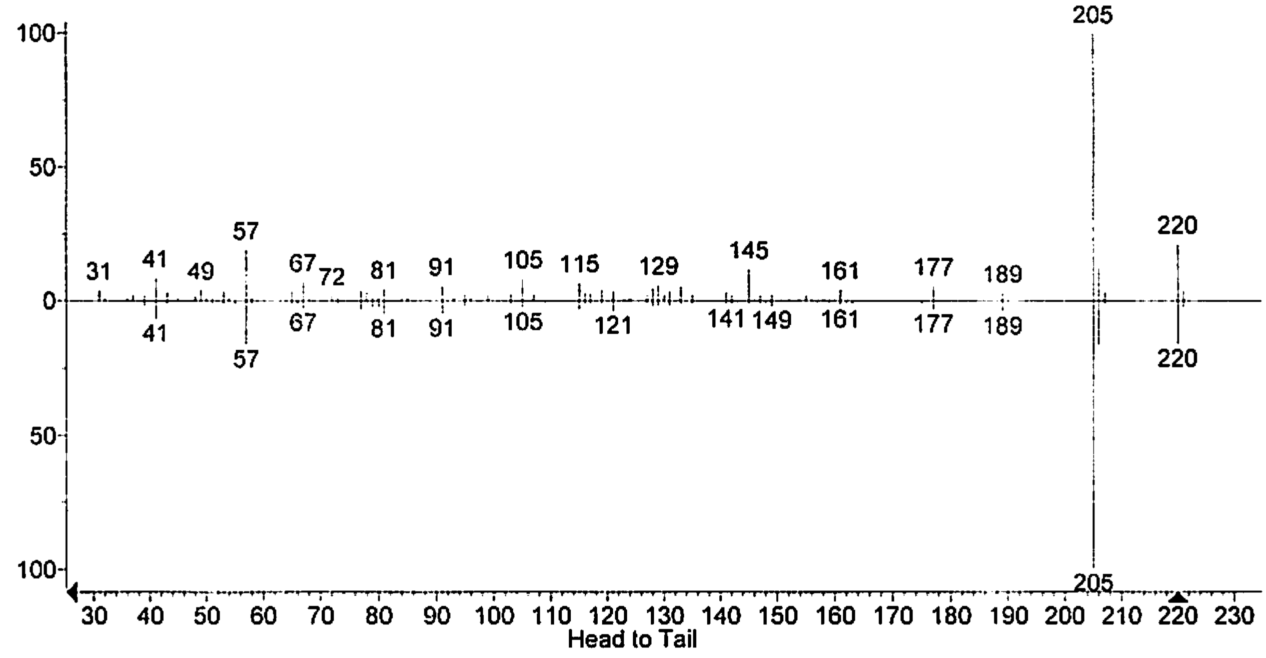

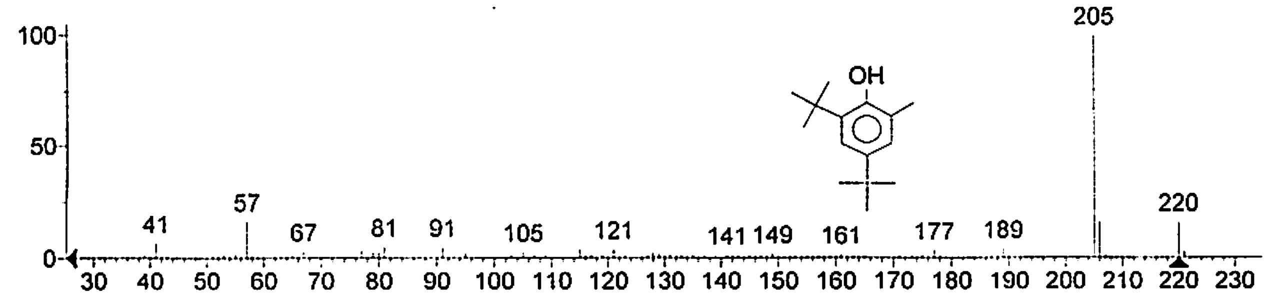

(mainlib) Phenol, 4,6-di(1,1-dimethylethyl)-2-methyl-

File : D:\DATA\ALDRICH\JA-09\Snapshot\JA051909-3.D
Operator : Aldrich
Acquired : 19 May 2009 14:31 using AcqMethod JA-WAX08.M
Instrument : Instrument #1
Sample Name: 2 field-coll. male C.oculata abd./2.5ul CH2Cl
Misc Info : DB-WAX; coll. 5/18 by Ed; fed only honey
Vial Number: 1

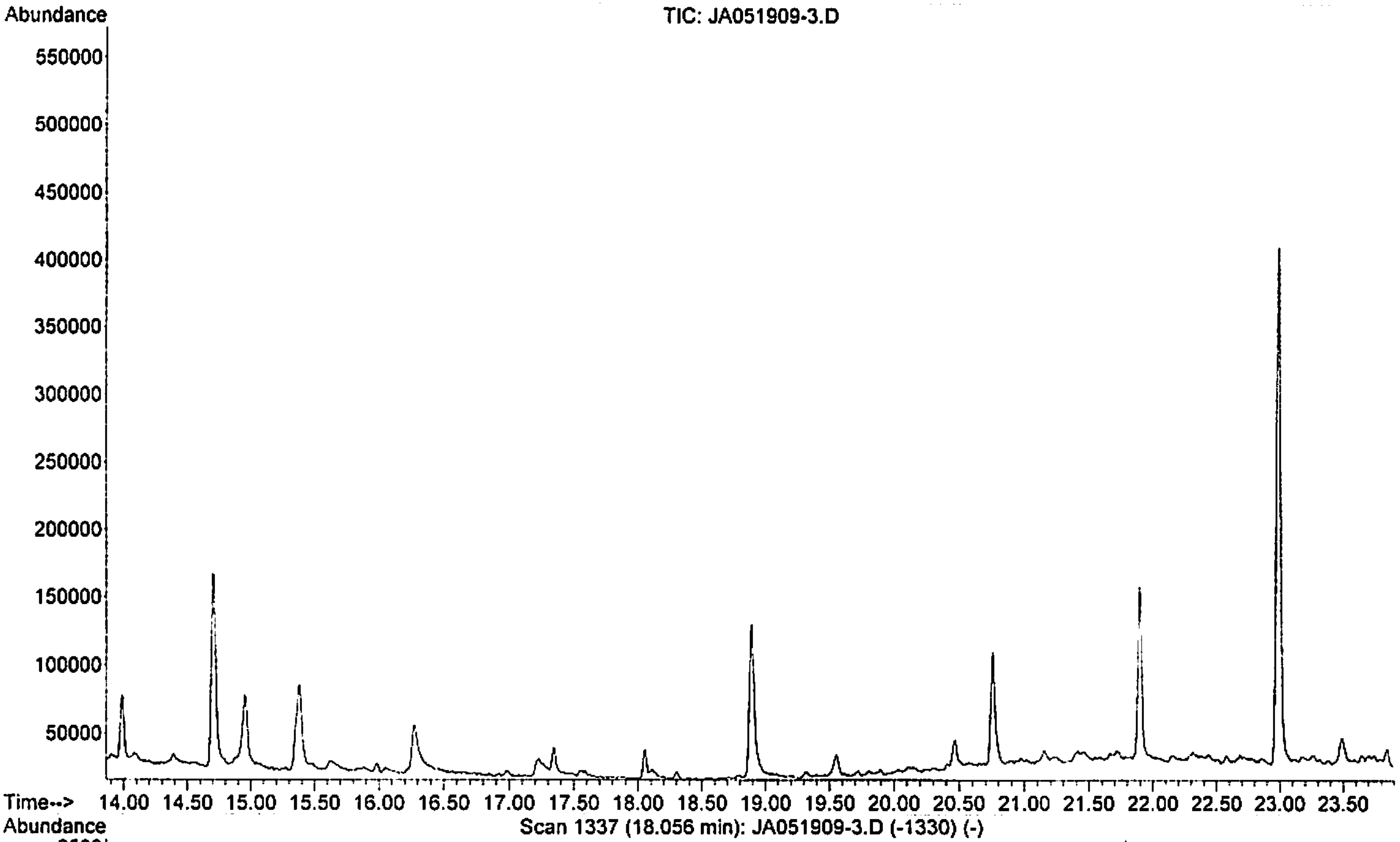

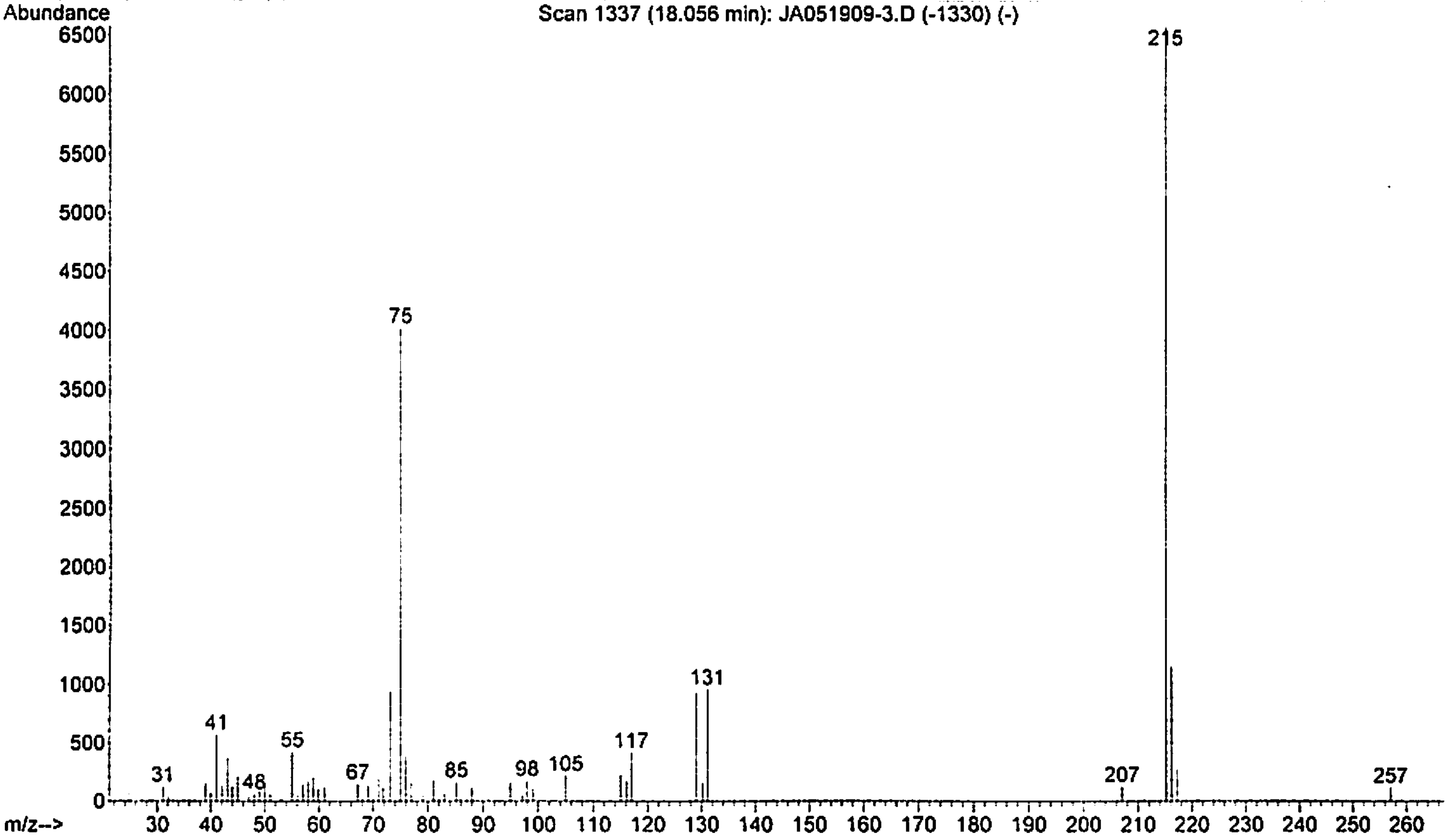

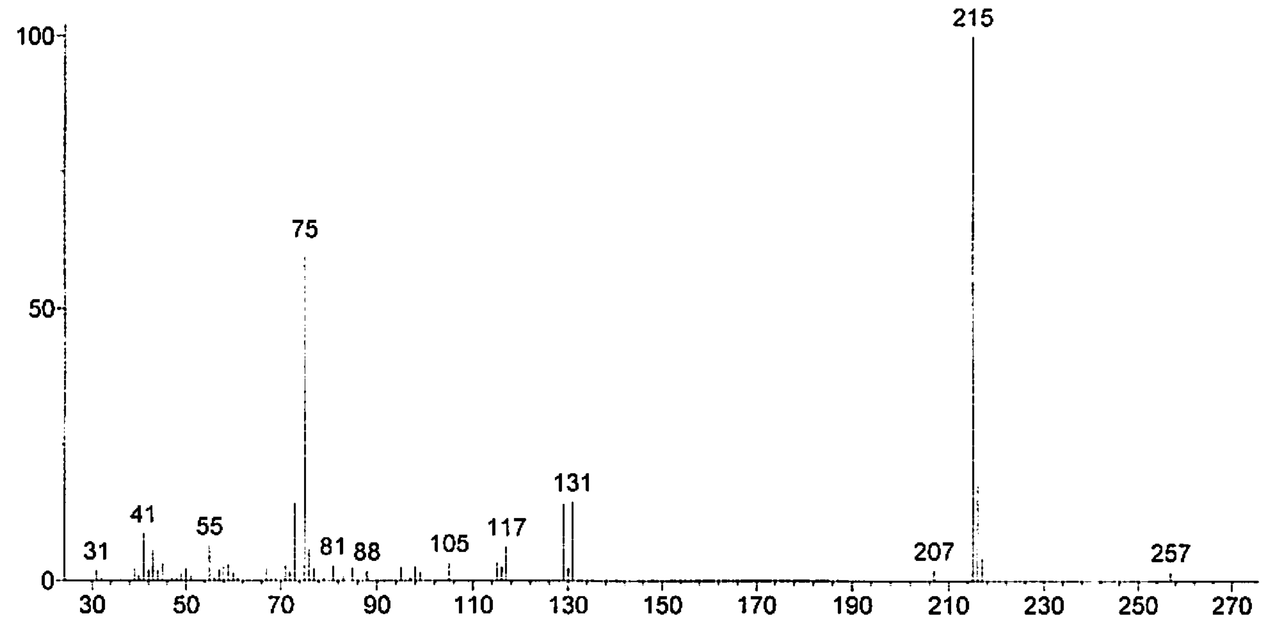

(Text File) Scan 1337 (18.056 min): JA051909-3.D (-1330)

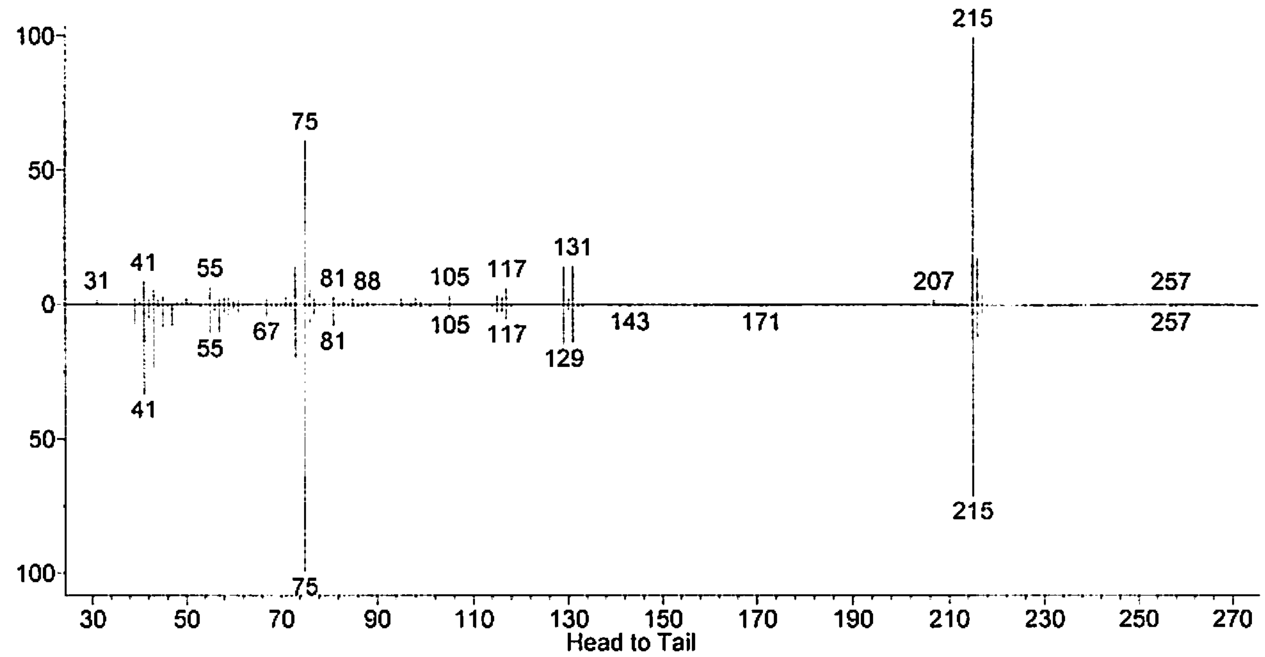

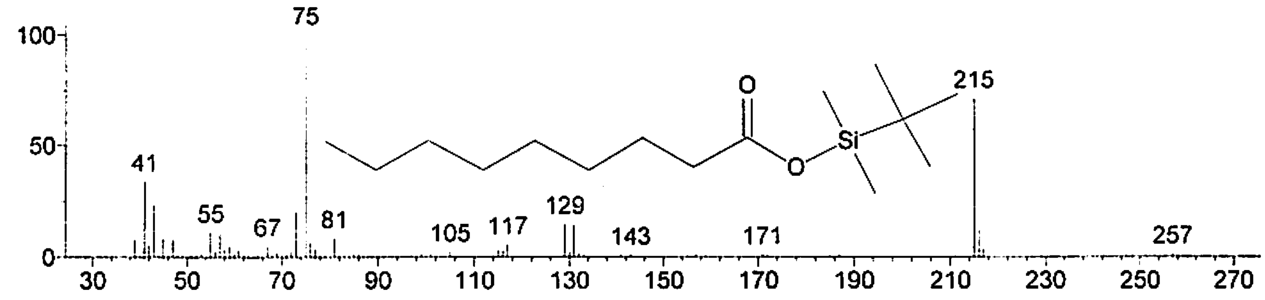

(mainlib) Nonanoic acid, tert-butyldimethylsilyl ester

File : D:\DATA\ALDRICH\JA-09\Snapshot\JA051909-3.D
Operator : Aldrich
Acquired : 19 May 2009 14:31 using AcqMethod JA-WAX08.M
Instrument : Instrument #1
Sample Name: 2 field-coll. male C.oculata abd./2.5ul CH2Cl
Misc Info : DB-WAX; coll. 5/18 by Ed; fed only honey
Vial Number: 1

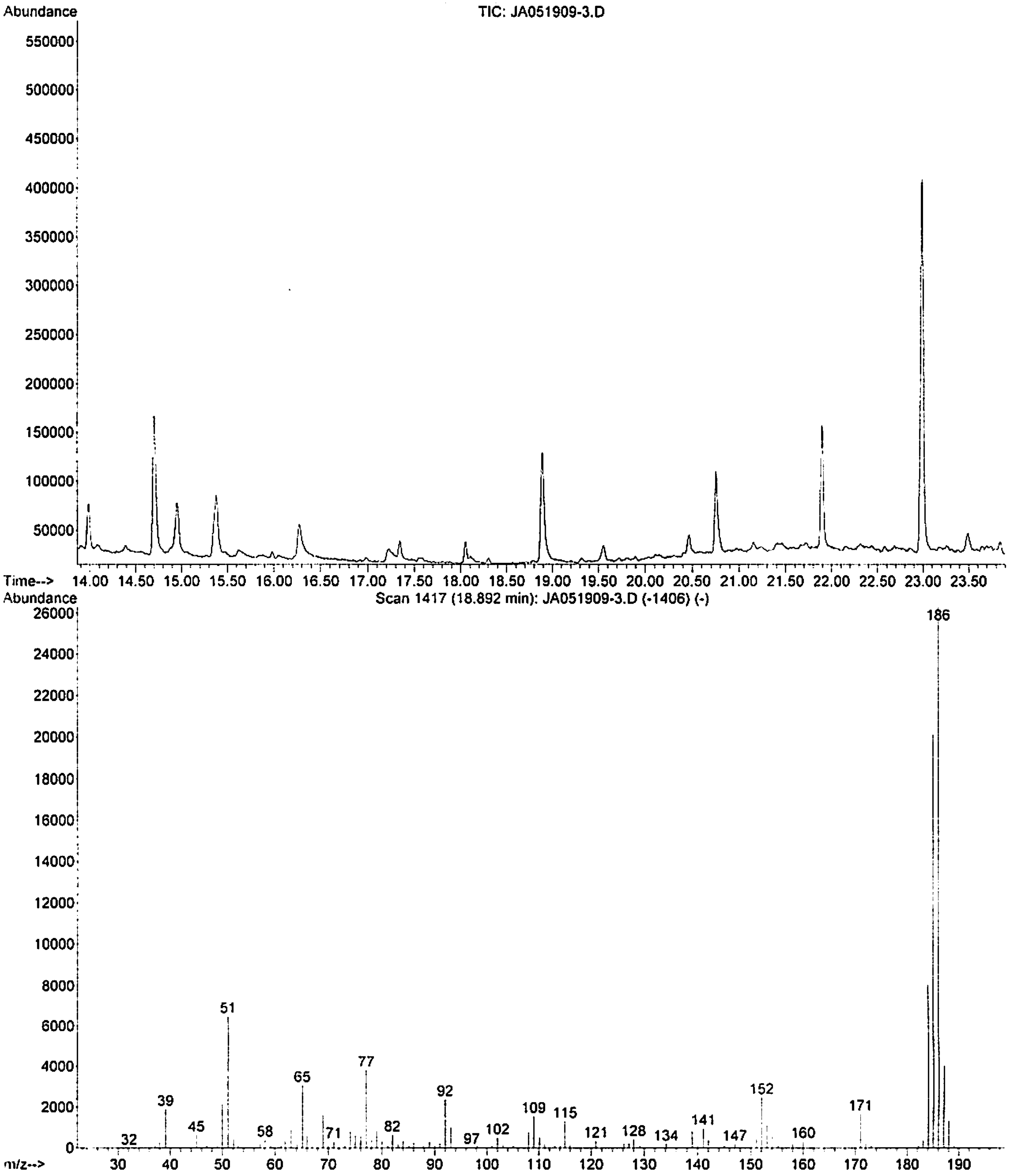

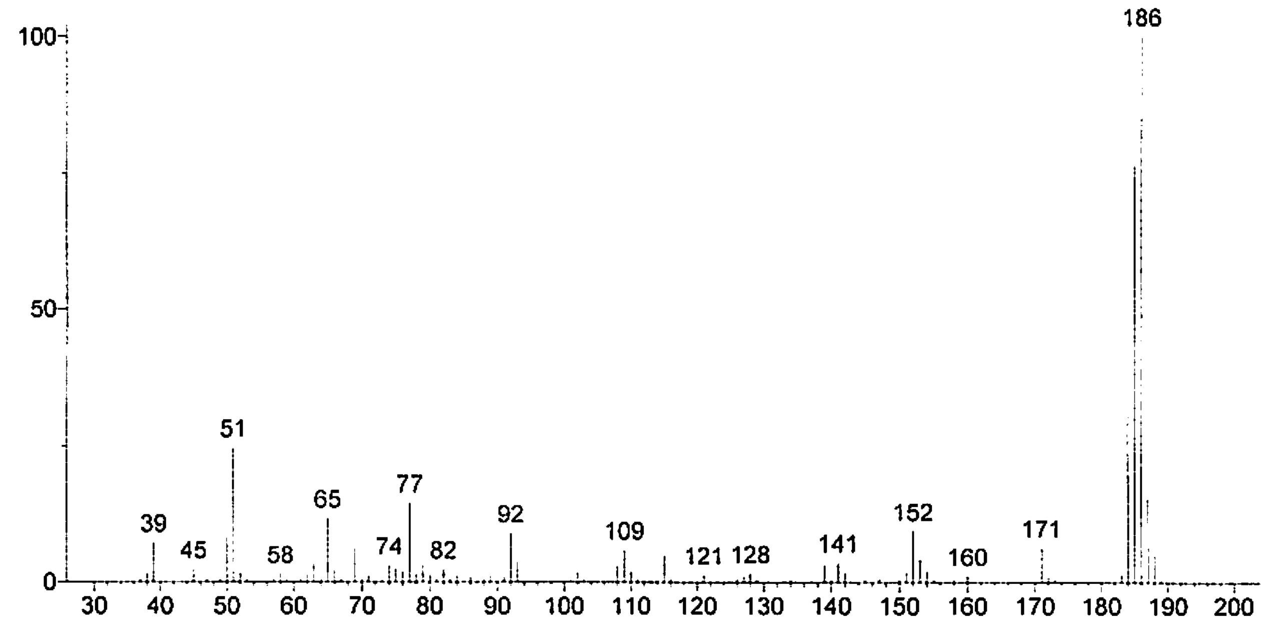

(Text File) Scan 1417 (18.892 min): JA051909-3.D (-1406)

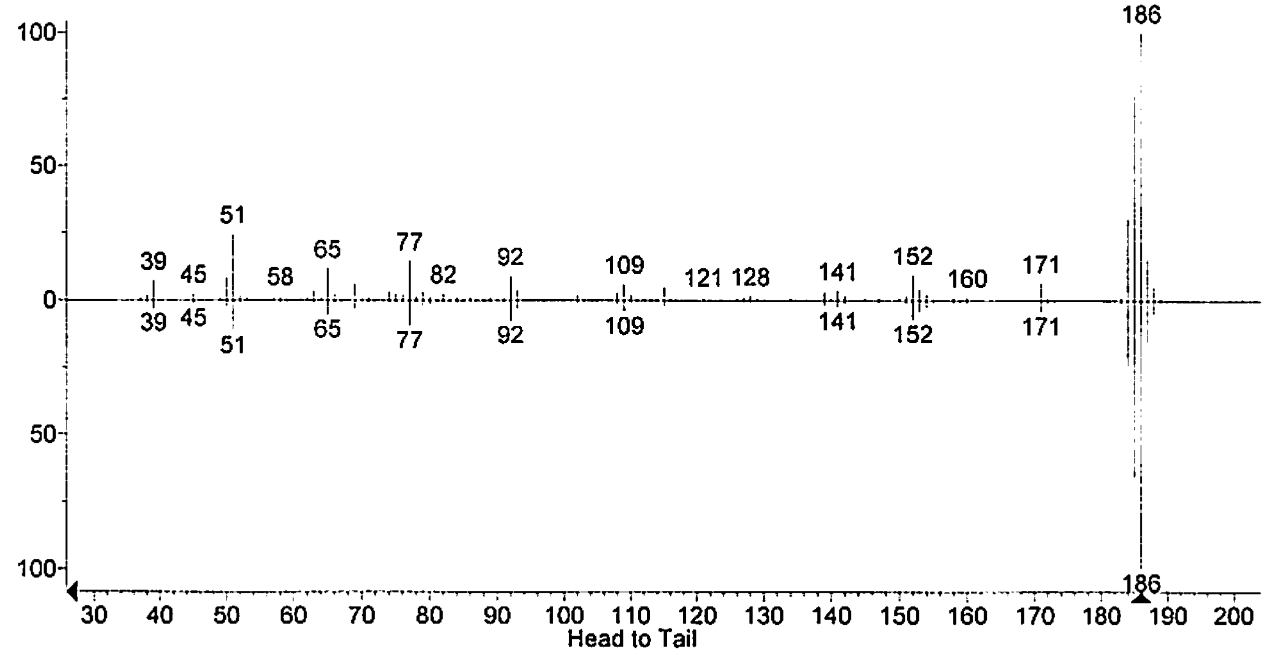

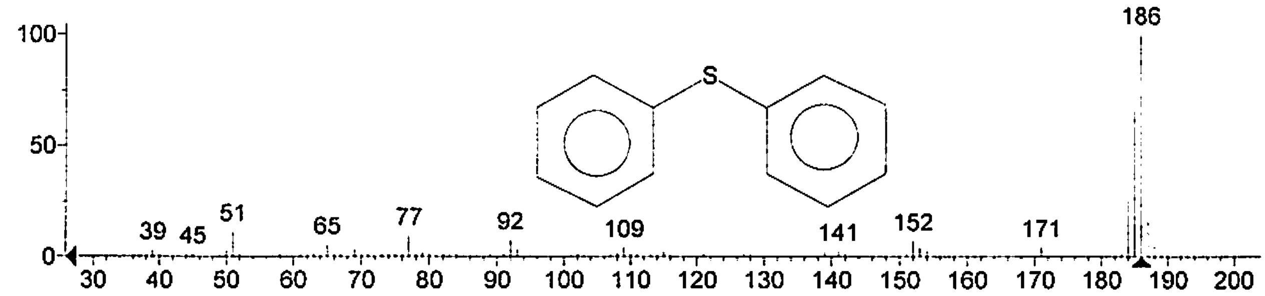

(mainlib) Diphenyl sulfide

[illegible] :D:\DATA\ALDRICH\JA-09\Snapshot\JA051909-3.D
[illegible]rator : Aldrich
[illegible]ired : 19 May 2009 14:31 using AcqMethod JA-WAX08.M
[illegible]strument : Instrument #1
[illegible]ple Name: 2 field-coll. male C.oculata abd./2.5ul CH2Cl
[illegible]sc Info : DB-WAX; coll. 5/18 by Ed; fed only honey
[illegible]al Number: 1

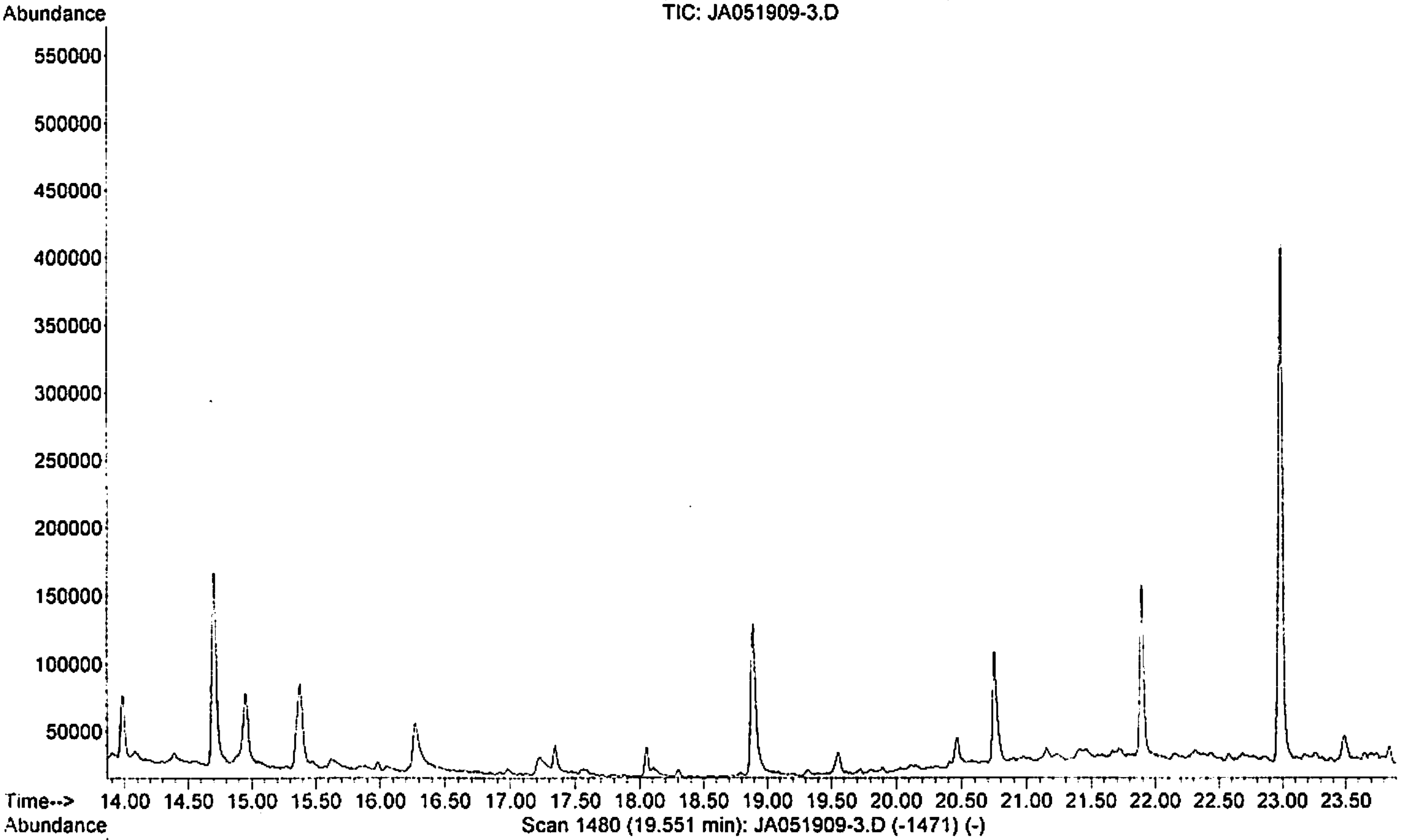

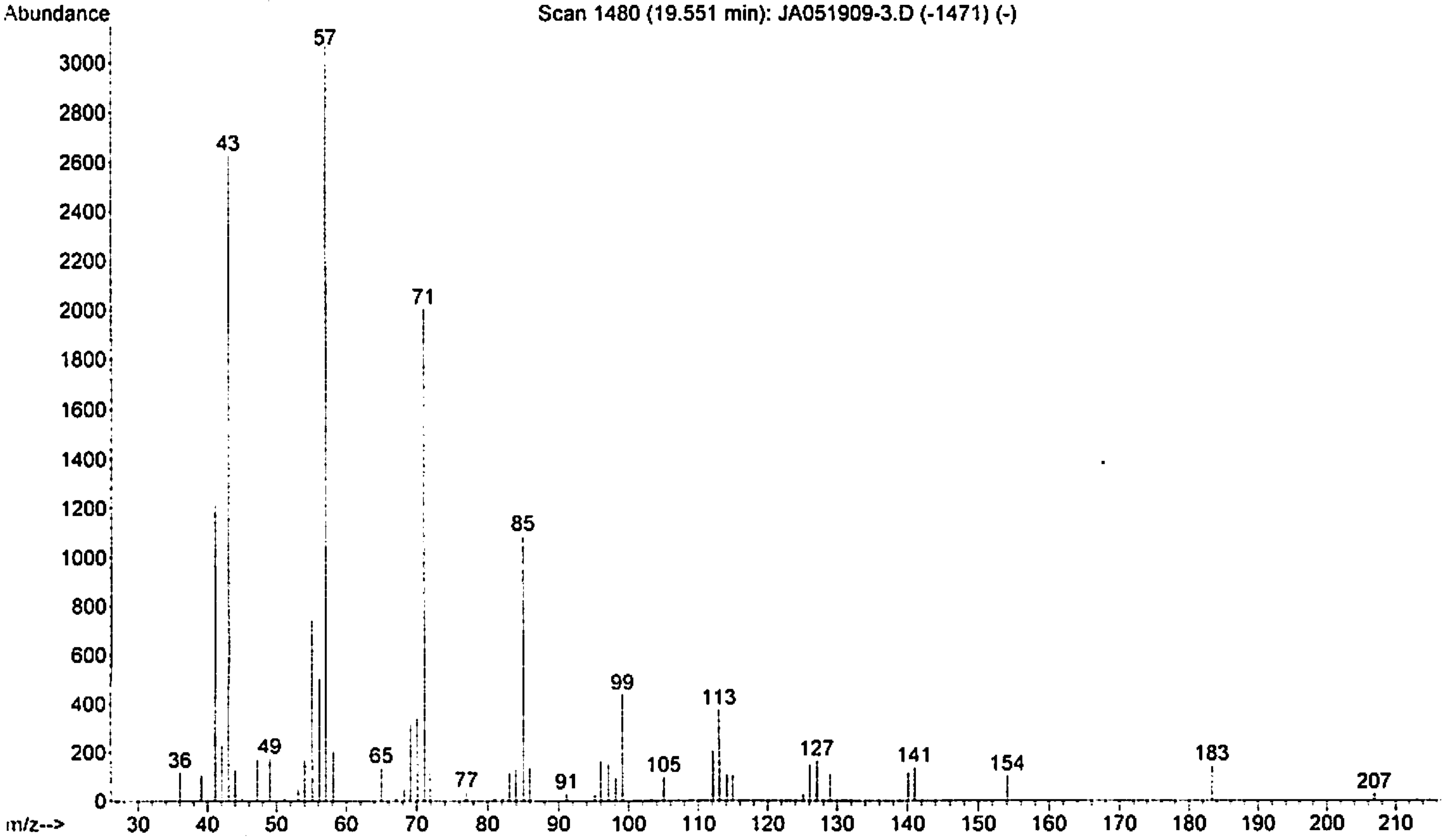

[illegible] :D:\DATA\ALDRICH\JA-09\Snapshot\JA051909-3.D
[illegible]erator : Aldrich
[illegible]quired : 19 May 2009 14:31 using AcqMethod JA-WAX08.M
[illegible]trument : Instrument #1
[illegible]mple Name: 2 field-coll. male C.oculata abd./2.5ul CH2Cl
[illegible]sc Info : DB-WAX; coll. 5/18 by Ed; fed only honey
[illegible]al Number: 1

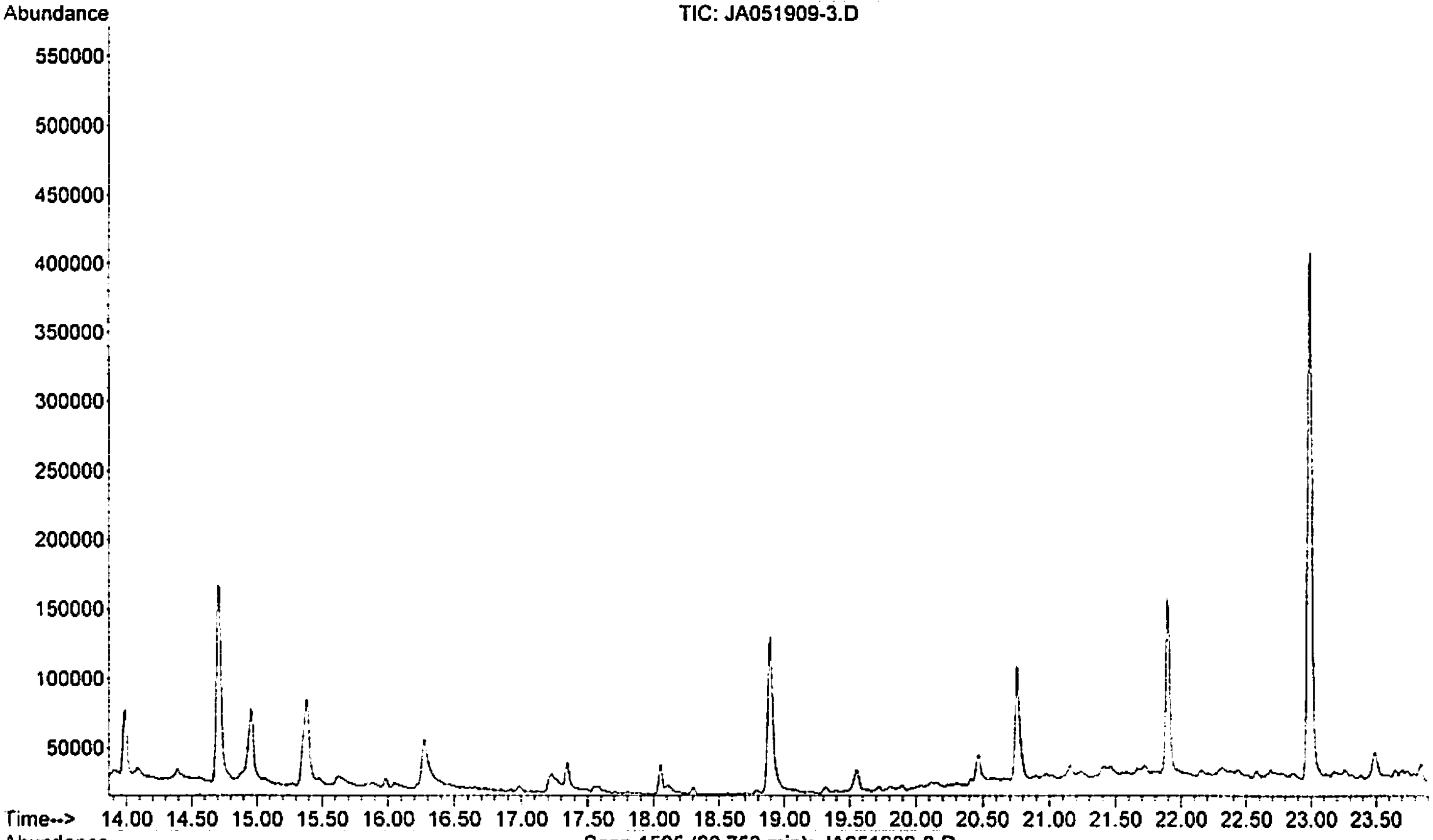

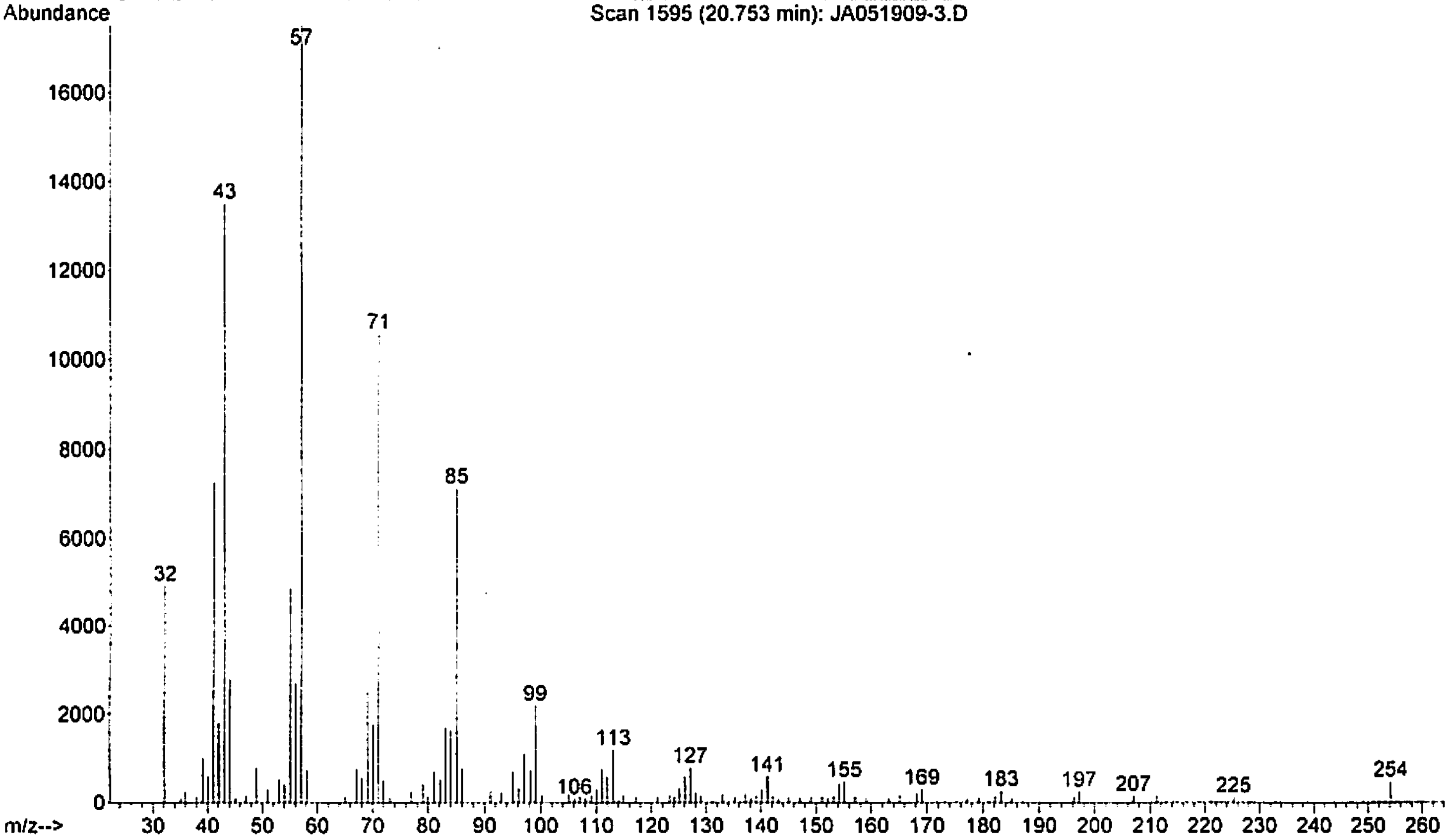

e • :D:\DATA\ALDRICH\JA-09\Snapshot\JA051909-3.D
erator : Aldrich
quired : 19 May 2009 14:31 using AcqMethod JA-WAX08.M
strument : Instrument #1
ple Name: 2 field-coll. male C.oculata abd./2.5ul CH2Cl
sc Info : DB-WAX; coll. 5/18 by Ed; fed only honey
al Number: 1

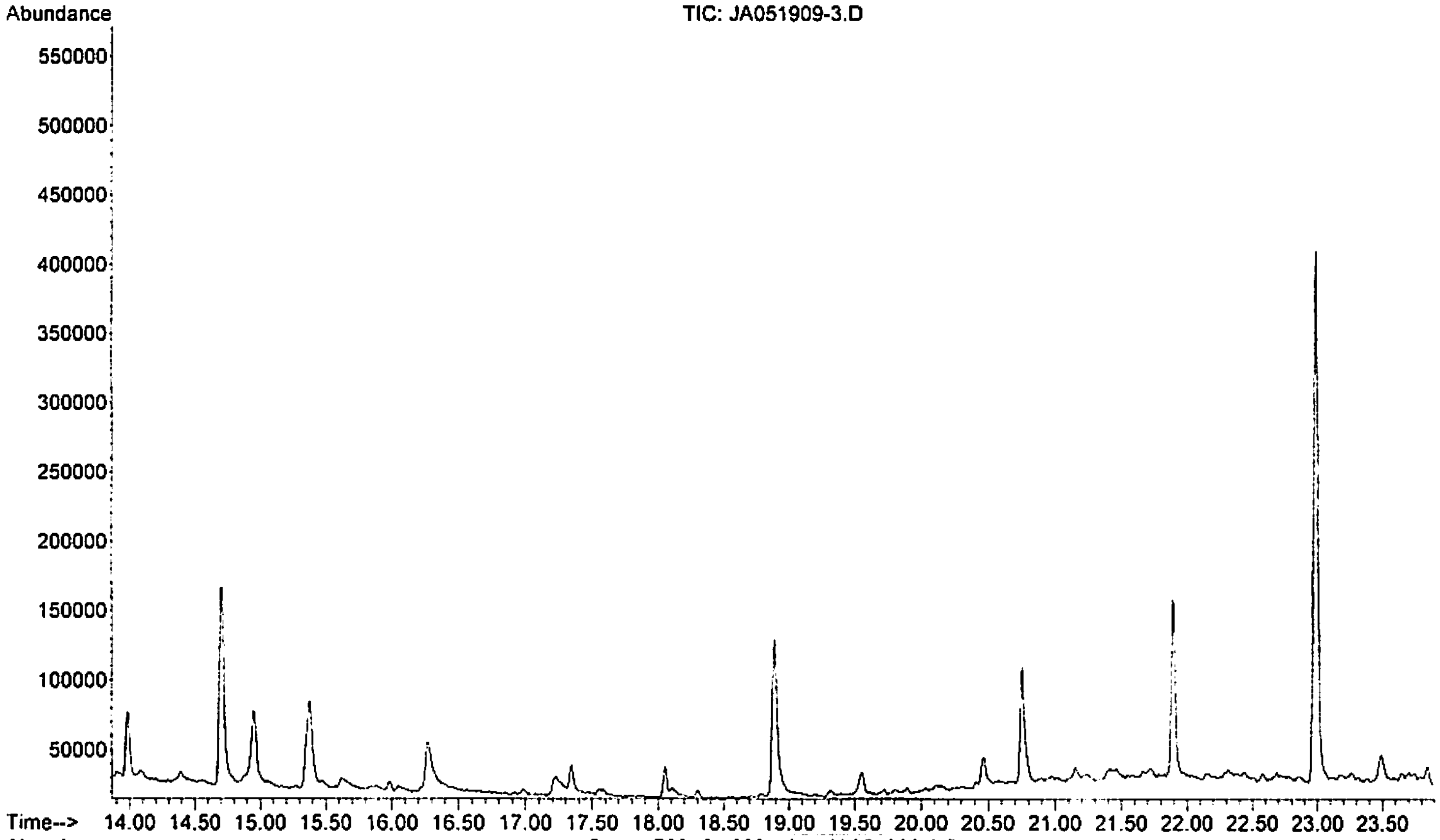

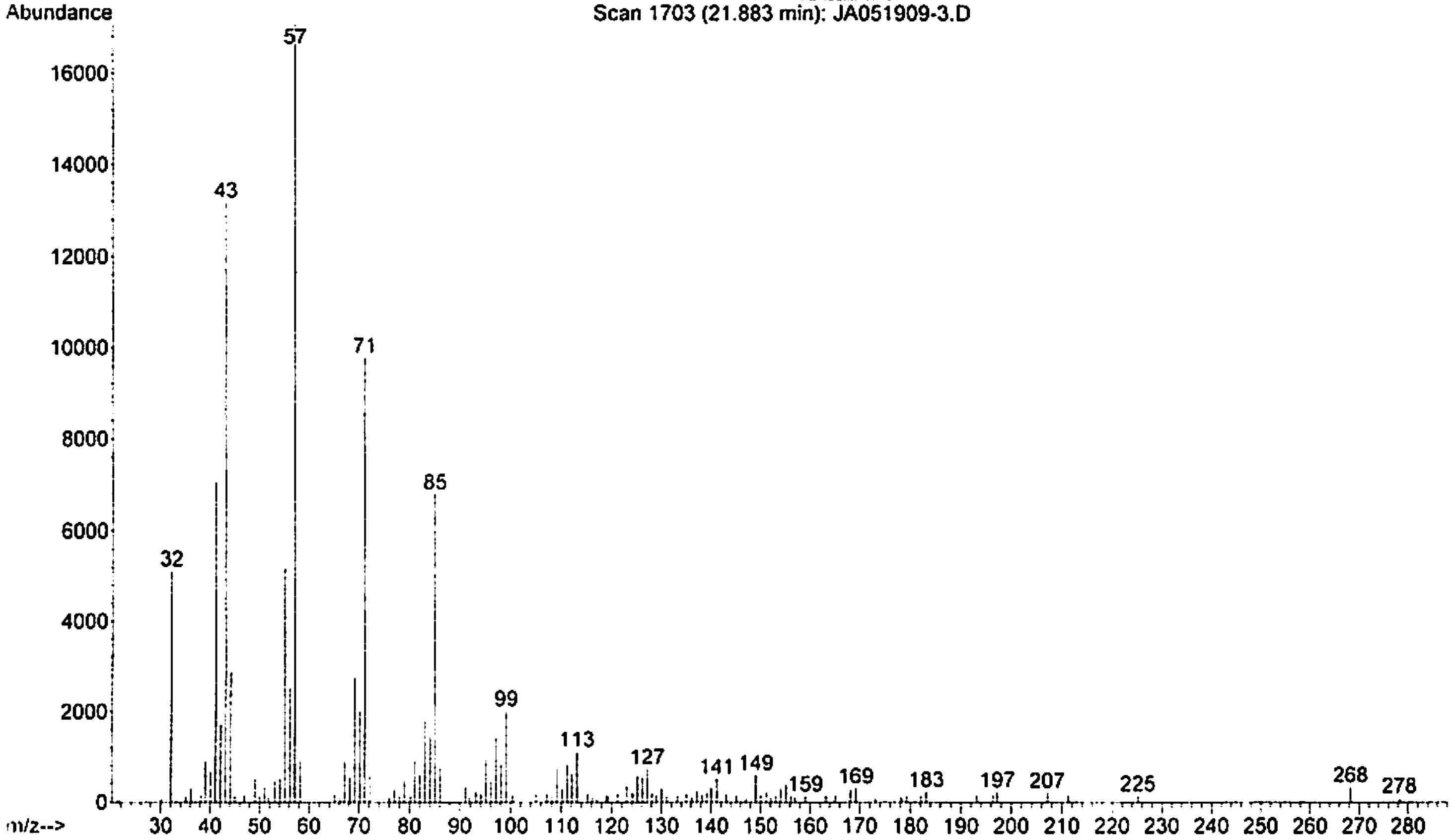

[illegible] :D:\DATA\ALDRICH\JA-09\Snapshot\JA051909-3.D
[illegible]rator : Aldrich
[illegible]ired : 19 May 2009 14:31 using AcqMethod JA-WAX08.M
[illegible]trument : Instrument #1
[illegible]ple Name: 2 field-coll. male C.oculata abd./2.5ul CH2Cl
[illegible] Info : DB-WAX; coll. 5/18 by Ed; fed only honey
[illegible] Number: 1

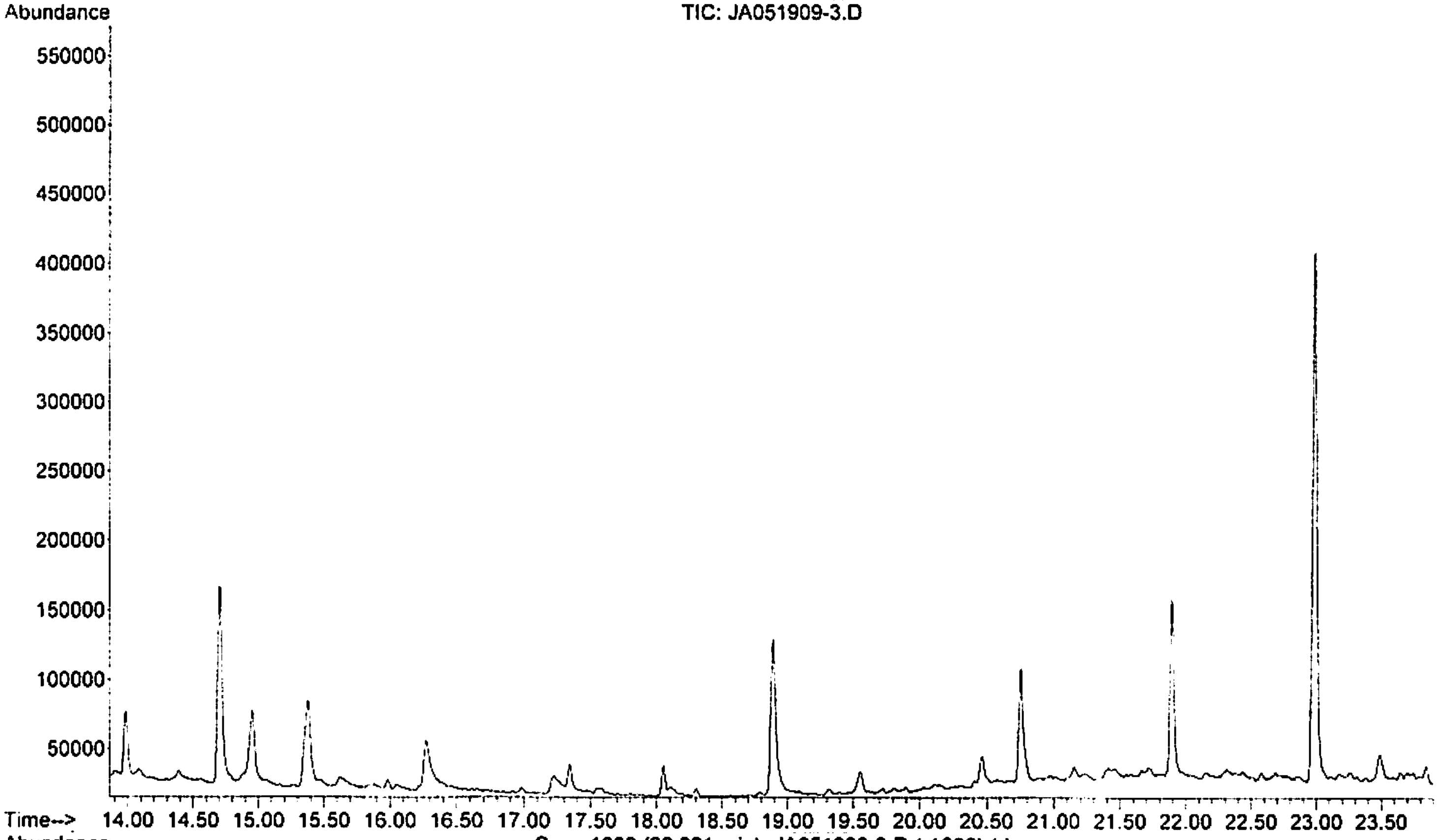

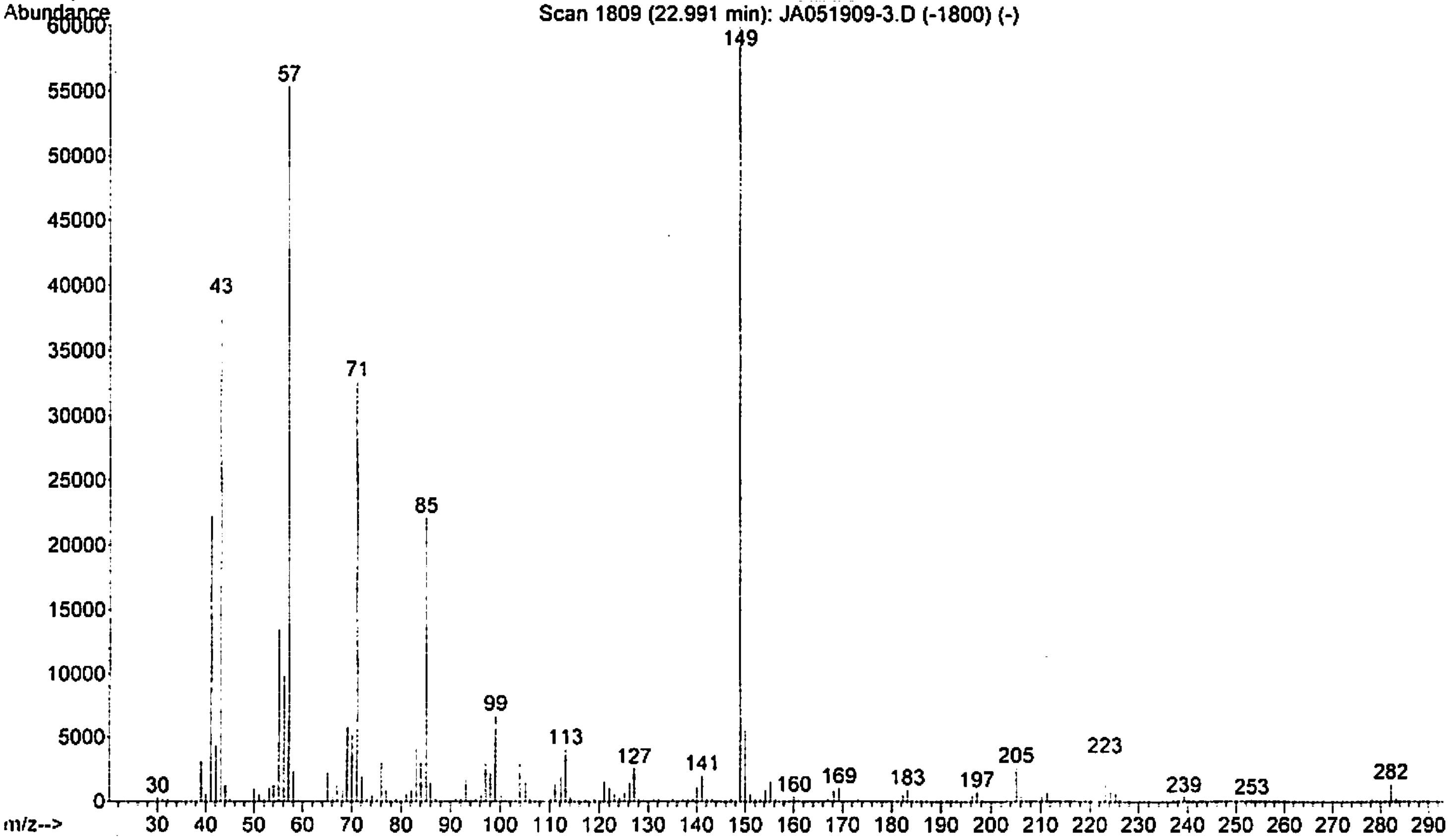

[illegible] : D:\DATA\ALDRICH\JA-09\Snapshot\JA051909-3.D
[illegible]rator : Aldrich
[illegible]ired : 19 May 2009 14:31 using AcqMethod JA-WAX08.M
[illegible]trument : Instrument #1
[illegible]ple Name: 2 field-coll. male C.oculata abd./2.5ul CH2Cl
[illegible]c Info : DB-WAX; coll. 5/18 by Ed; fed only honey
[illegible]l Number: 1

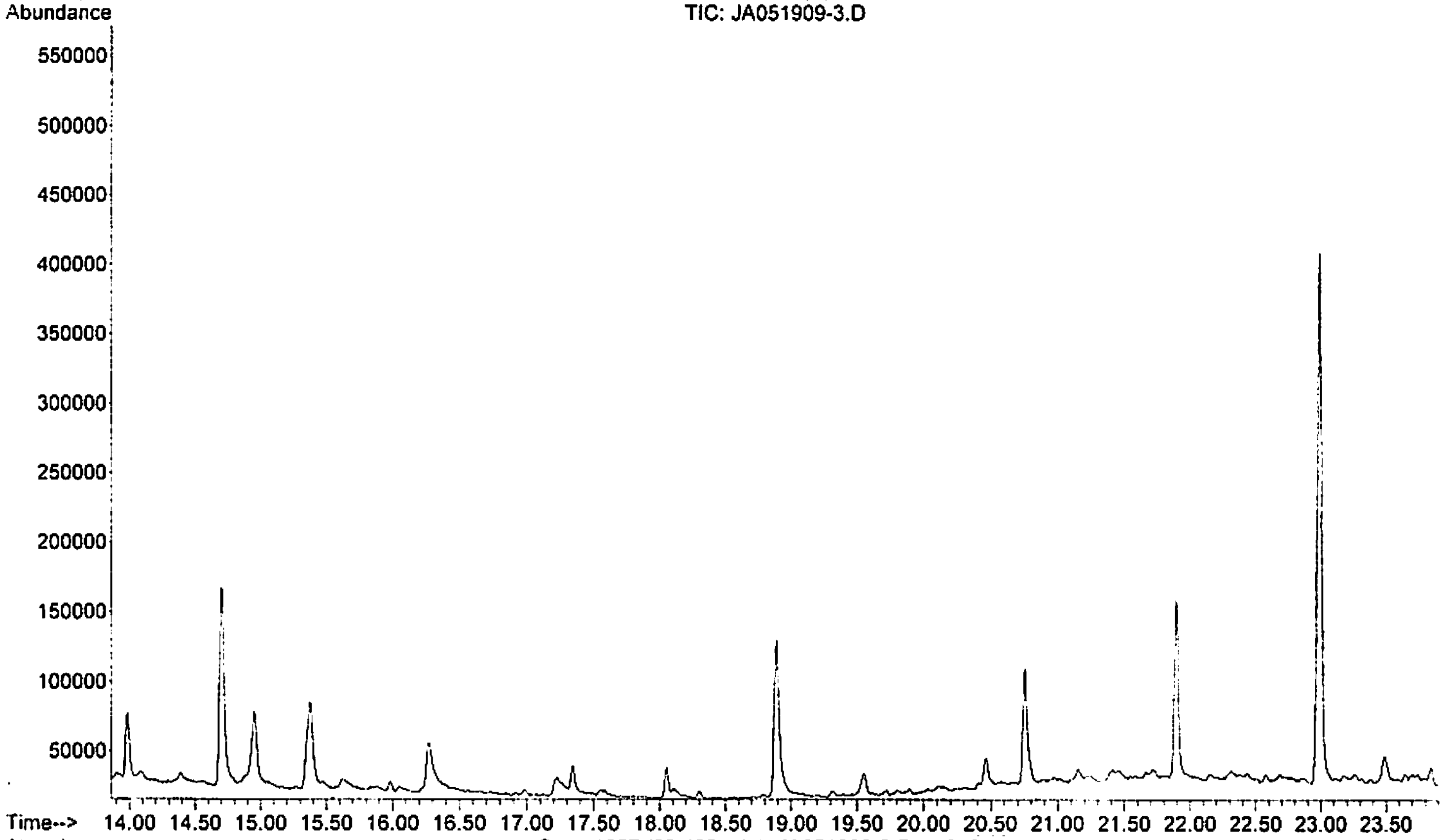

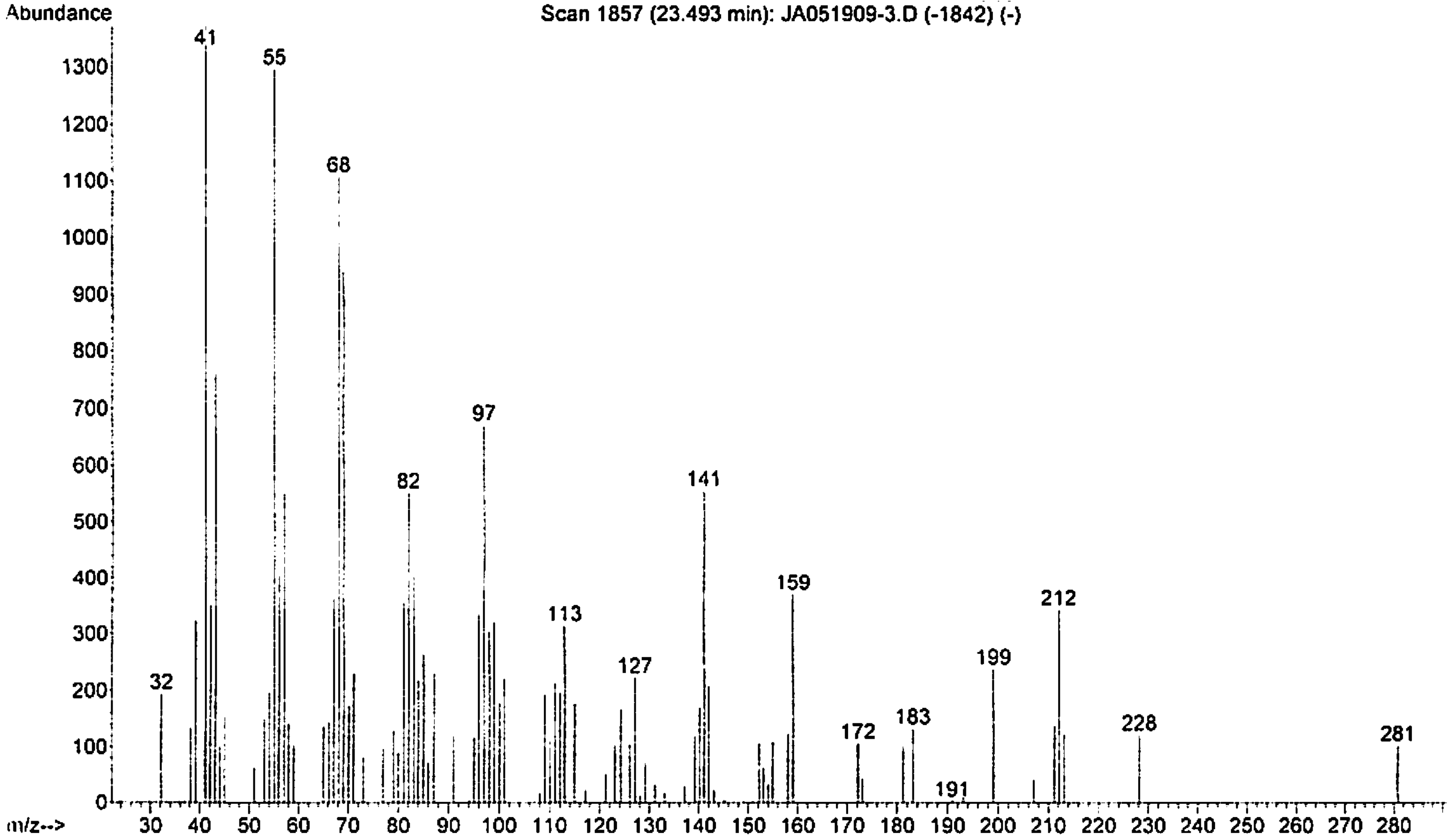

[illegible] :D:\DATA\Aldrich\JA-09\JA051909-3.D
[illegible]rator : Aldrich
[illegible]uired : 19 May 2009 14:31 using AcqMethod JA-WAX08.M
[illegible]trument : Instrument #1
[illegible]ple Name: 2 field-coll. male C.oculata abd./2.5ul CH2Cl
[illegible] Info : DB-WAX; coll. 5/18 by Ed; fed only honey
[illegible]l Number: 1

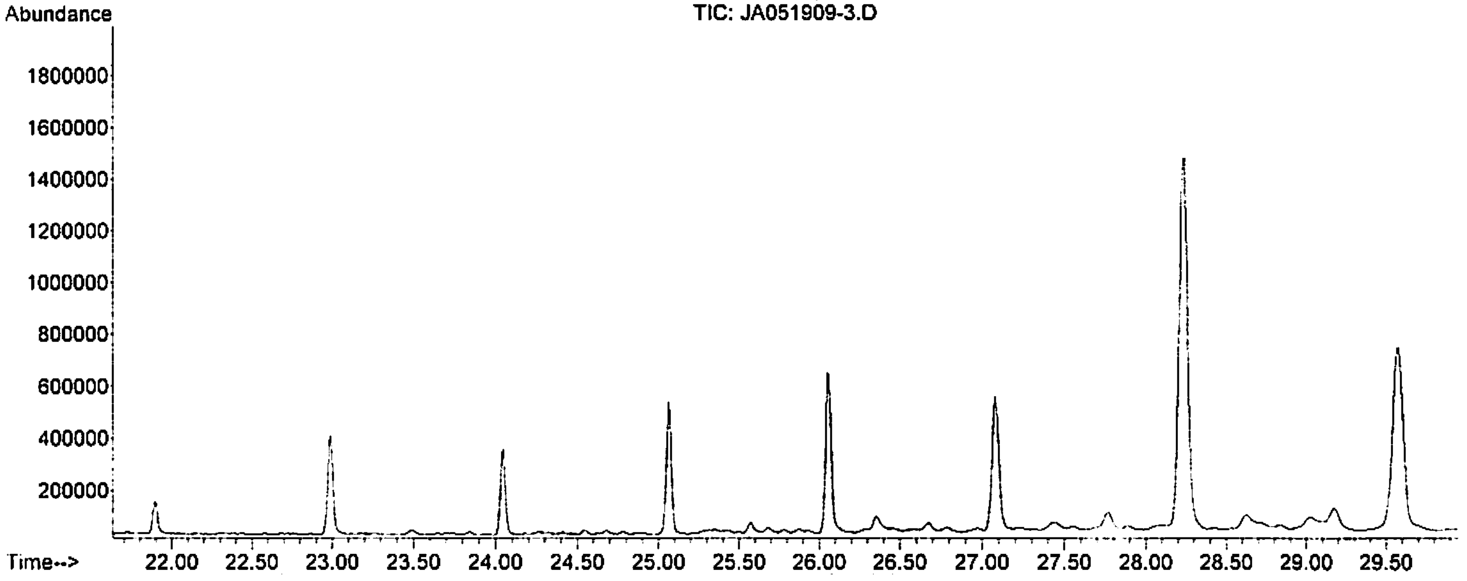

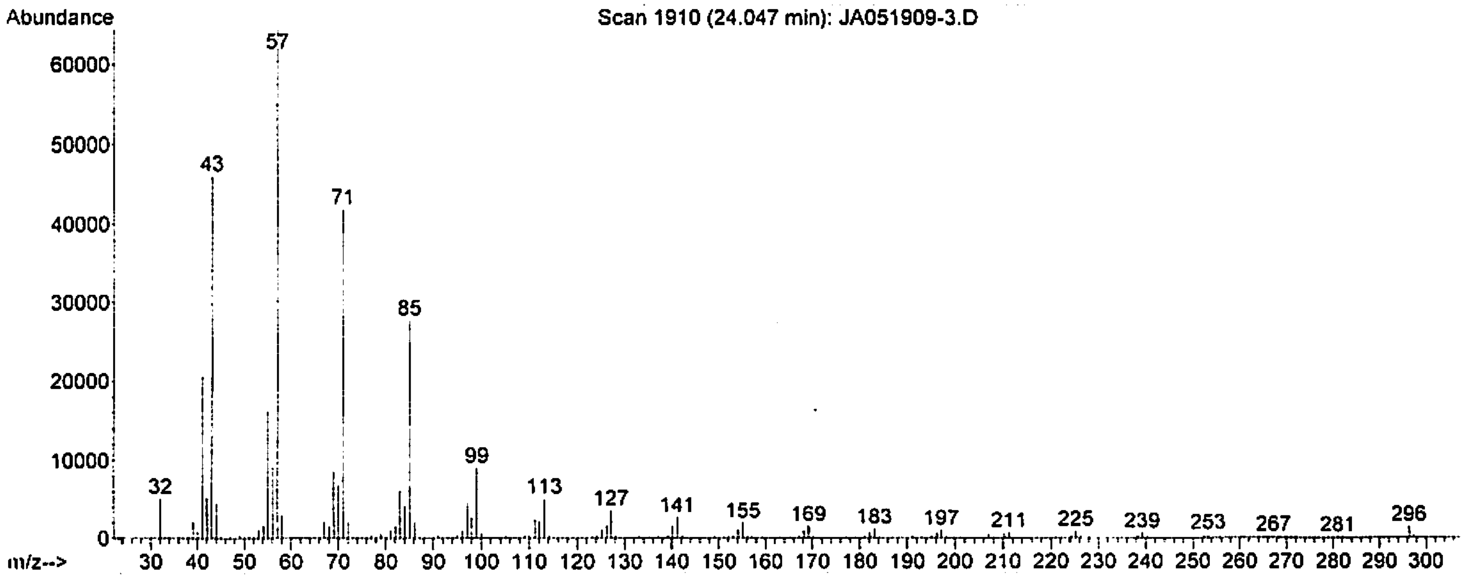

[illegible] :D:\DATA\Aldrich\JA-09\JA051909-3.D
[illegible]erator : Aldrich
[illegible]quired : 19 May 2009 14:31 using AcqMethod JA-WAX08.M
[illegible]trument : Instrument #1
[illegible]ple Name: 2 field-coll. male C.oculata abd./2.5ul CH2Cl
[illegible] Info : DB-WAX; coll. 5/18 by Ed; fed only honey
[illegible] Number: 1

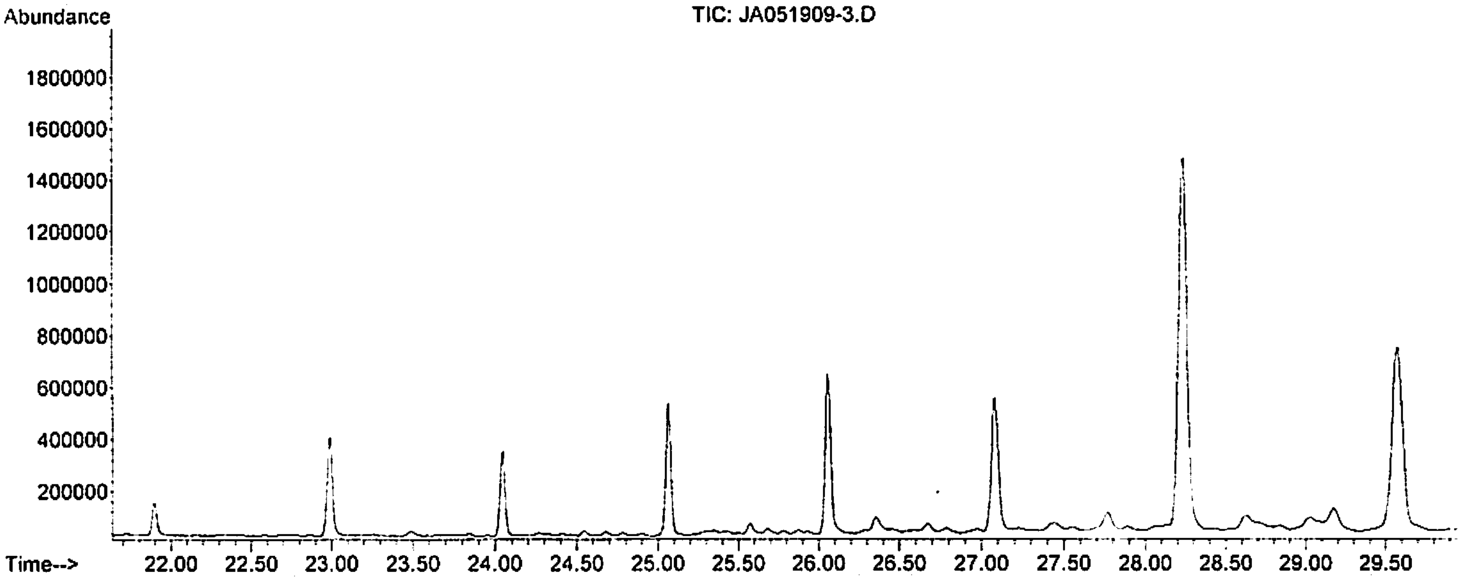

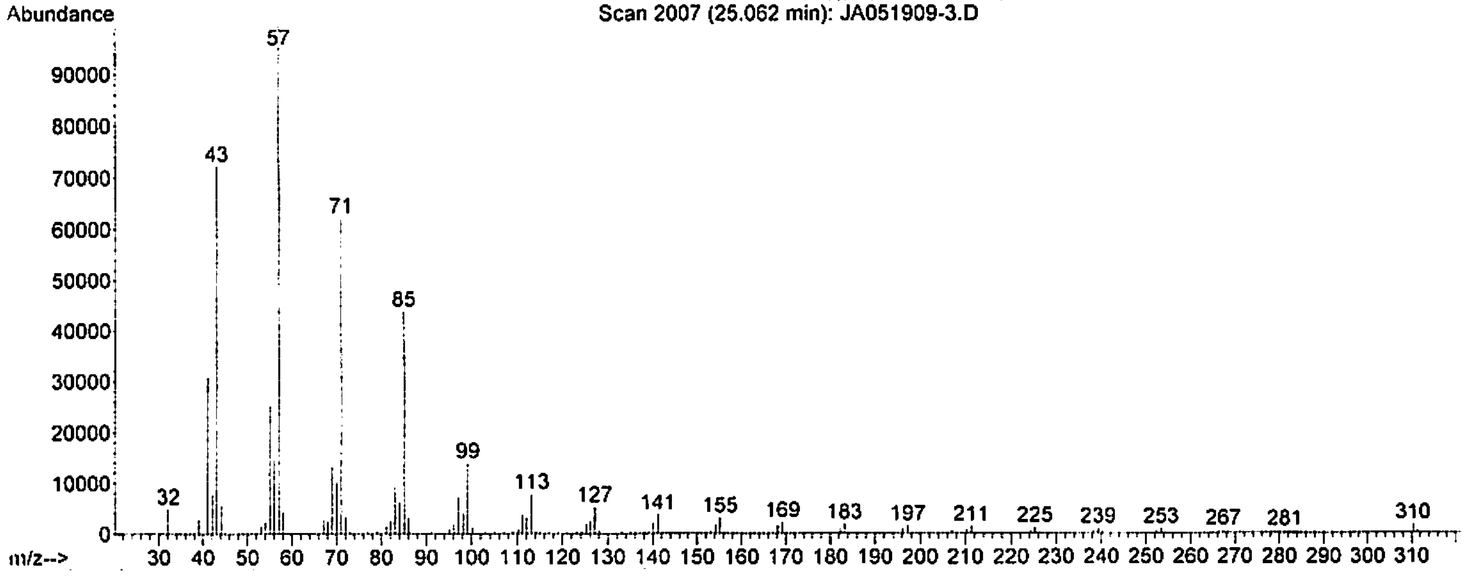

```
le           :D:\DATA\Aldrich\JA-09\JA051909-3.D
erator     : Aldrich
quired     : 19 May 2009  14:31     using AcqMethod JA-WAX08.M
strument :     Instrument #1
mple Name: 2 field-coll. male C.oculata abd./2.5ul CH2Cl
sc Info    : DB-WAX; coll. 5/18 by Ed; fed only honey
al Number: 1
```

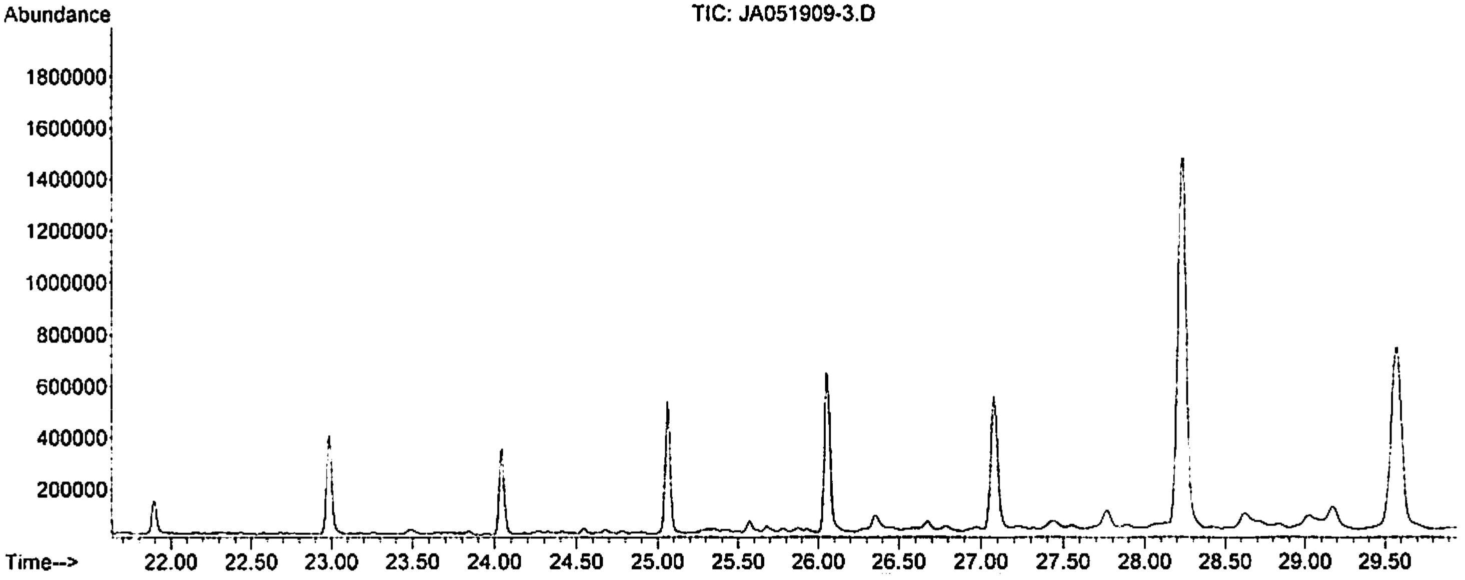

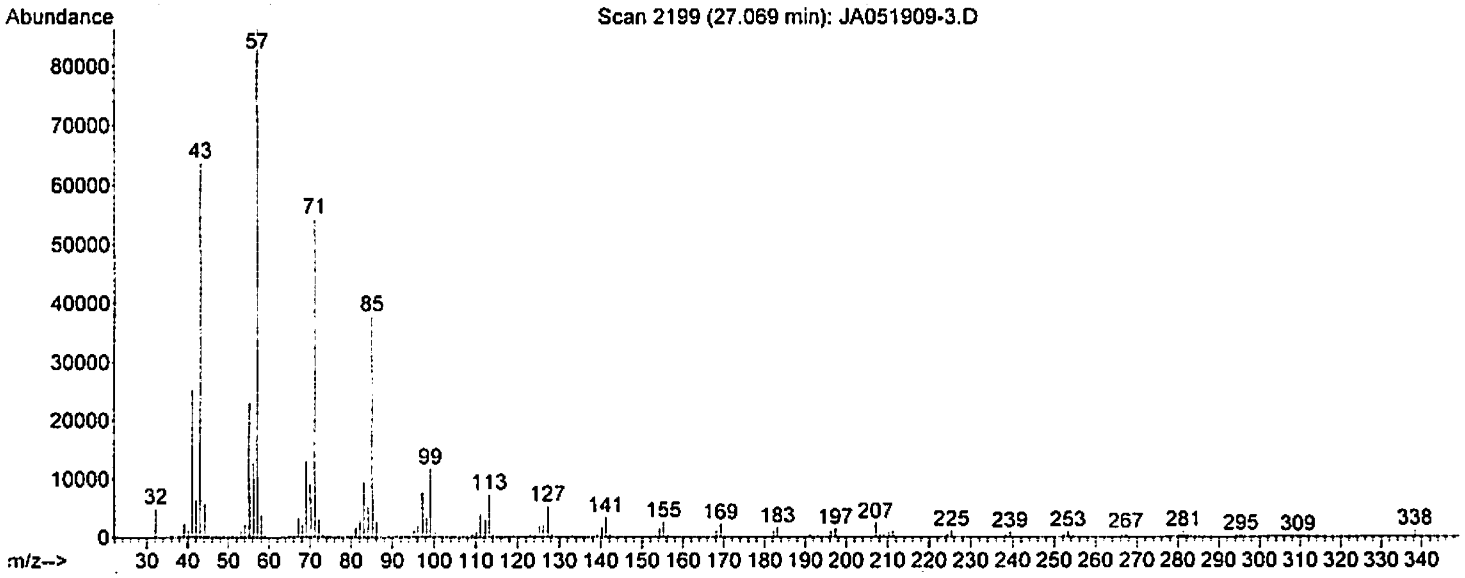

.le N : D:\DATA\Aldrich\JA-09\JA051909-3.D
perator : Aldrich
cquired : 19 May 2009 14:31 using AcqMethod JA-WAX08.M
nstrument : Instrument #1
ample Name: 2 field-coll. male C.oculata abd./2.5ul CH2Cl
isc Info : DB-WAX; coll. 5/18 by Ed; fed only honey
ial Number: 1

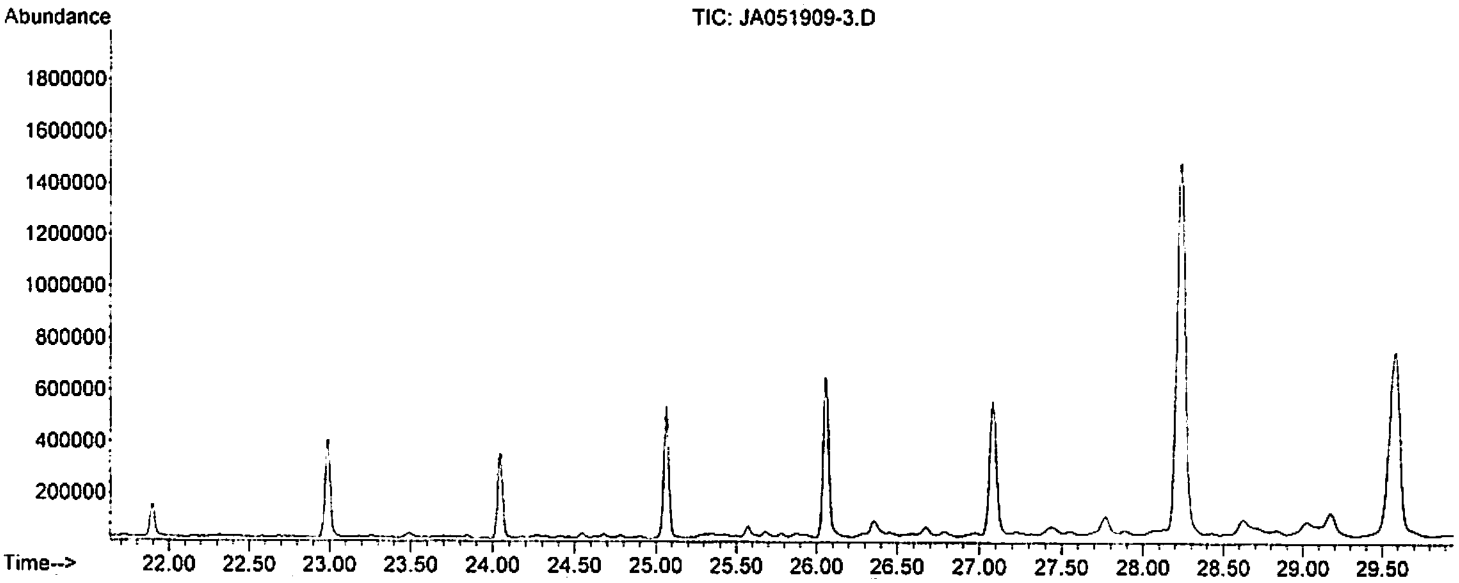

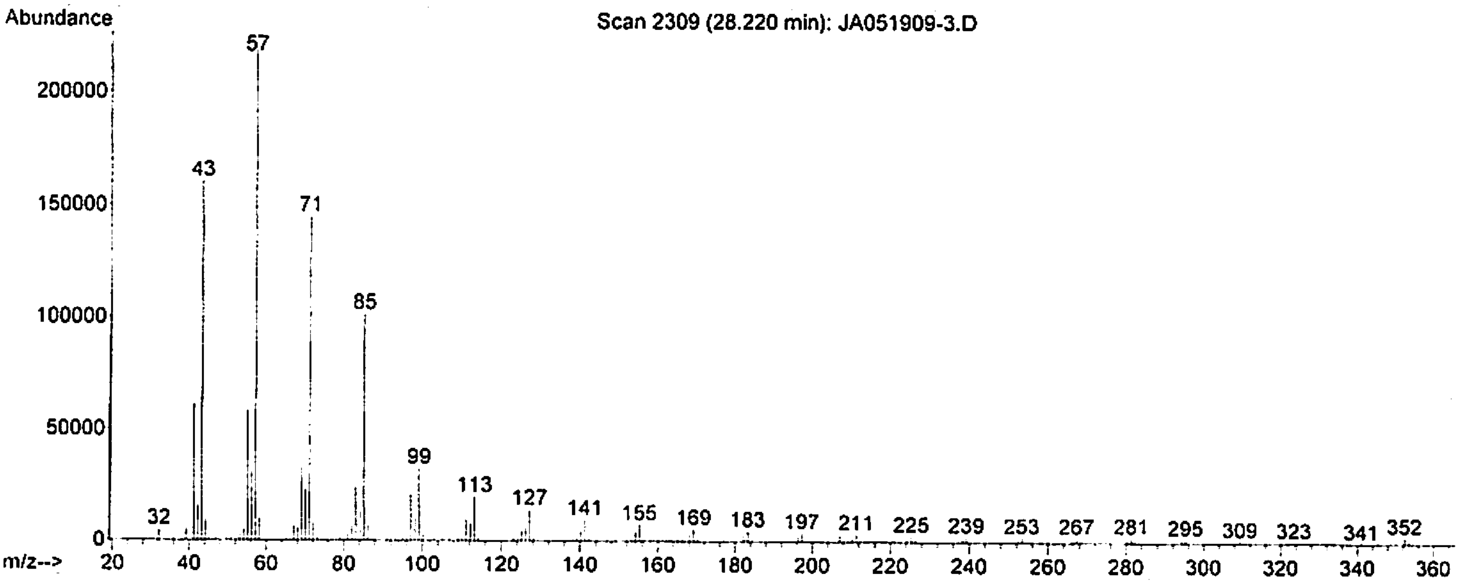

[illegible]le : D:\DATA\Aldrich\JA-09\JA051909-3.D
[illegible]erator : Aldrich
[illegible]quired : 19 May 2009 14:31 using AcqMethod JA-WAX08.M
[illegible]strument : Instrument #1
[illegible]mple Name: 2 field-coll. male C.oculata abd./2.5ul CH2Cl
[illegible]sc Info : DB-WAX; coll. 5/18 by Ed; fed only honey
[illegible]al Number: 1

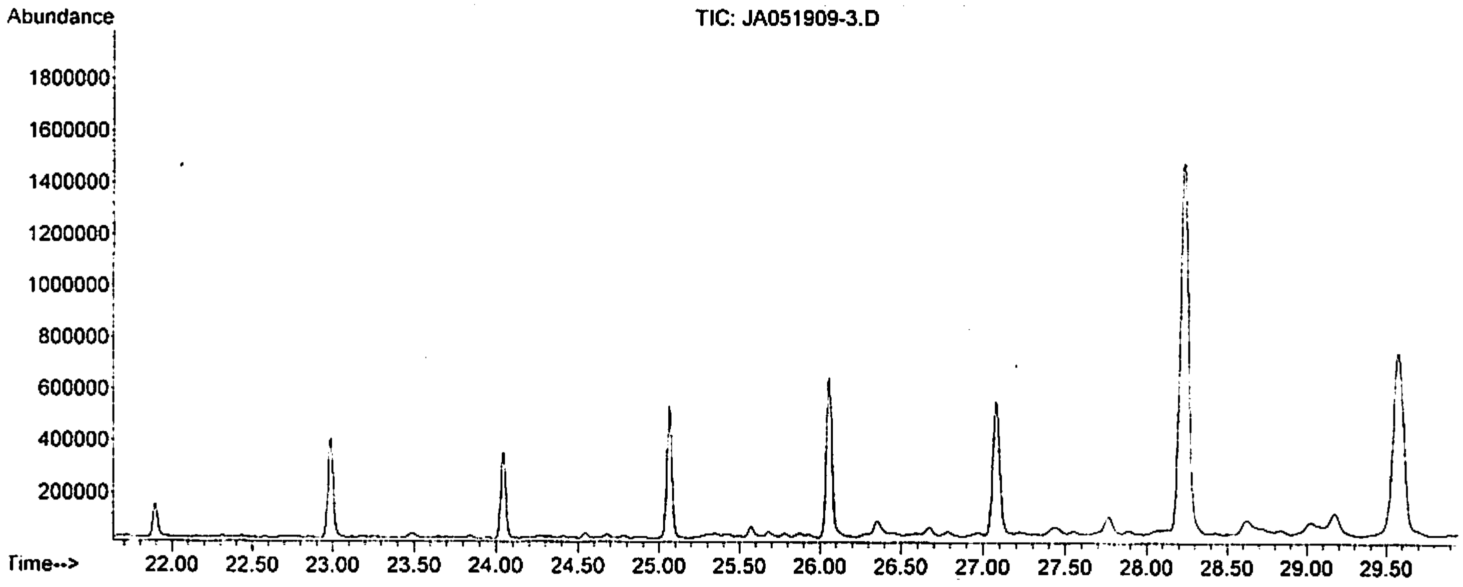

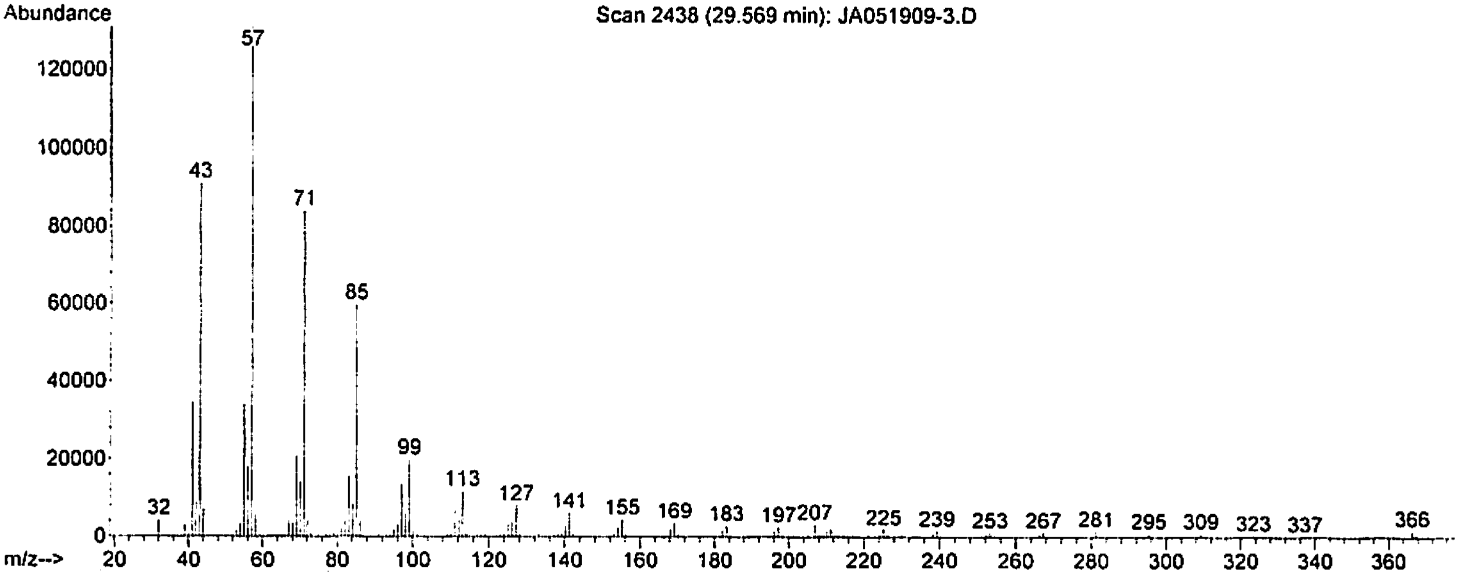

```
File        :D:\DATA\Aldrich\JA-09\JA052209-3.D
Operator    : Aldrich
Acquired    : 22 May 2009  15:55     using AcqMethod JA-WAX08.M
Instrument  :   Instrument #1
Sample Name: 1 field-coll. male C.oculata abd./CH2Cl2
Misc Info   : sweeping vetch 5/22 am; dissected
Vial Number: 1
```

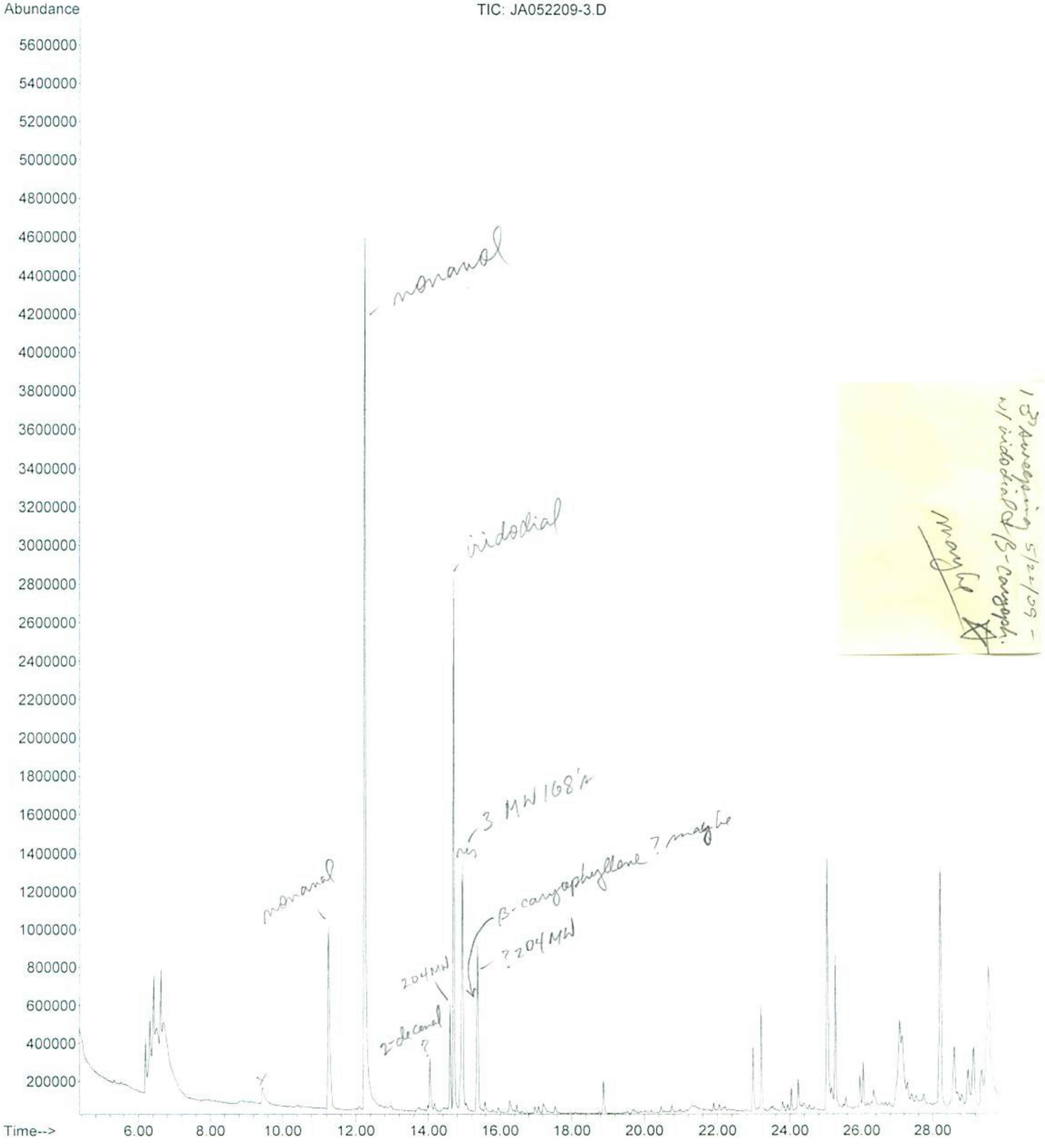

```
File         :D:\DATA\Aldrich\JA-09\JA052209-3.D
Operator     : Aldrich
Acquired     : 22 May 2009  15:55     using AcqMethod JA-WAX08.M
Instrument :    Instrument #1
Sample Name: 1 field-coll. male C.oculata abd./CH2Cl2
Misc Info  : sweeping vetch 5/22 am; dissected
Vial Number: 1
```

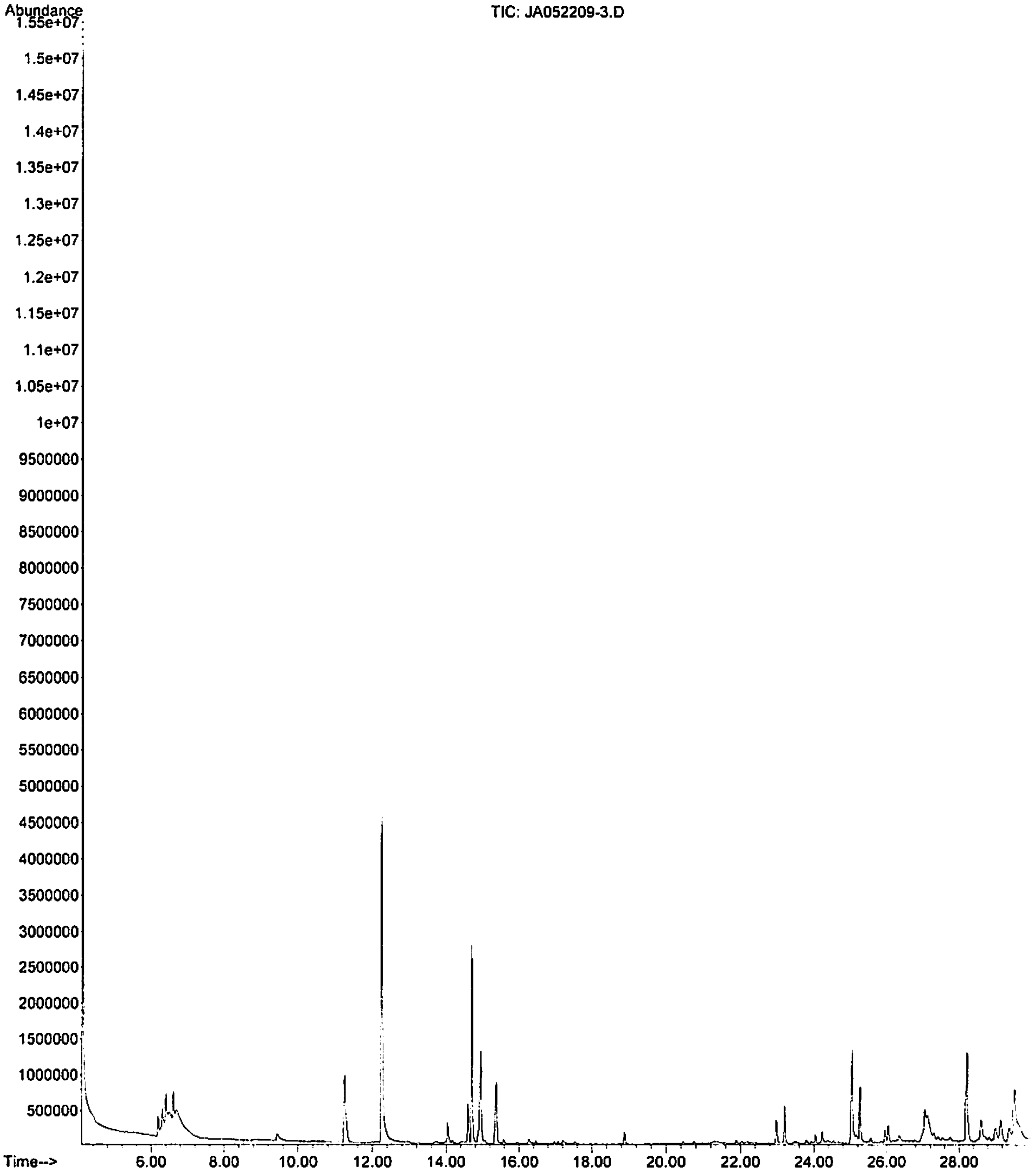

[illegible] : D:\DATA\ALDRICH\JA-09\Snapshot\JA052209-3.D
[illegible]erator : Aldrich
[illegible]quired : 22 May 2009 15:55 using AcqMethod JA-WAX08.M
[illegible]strument : Instrument #1
[illegible]ple Name: 1 field-coll. male C.oculata abd./CH2Cl2
[illegible]sc Info : sweeping vetch 5/22 am; dissected
[illegible]al Number: 1

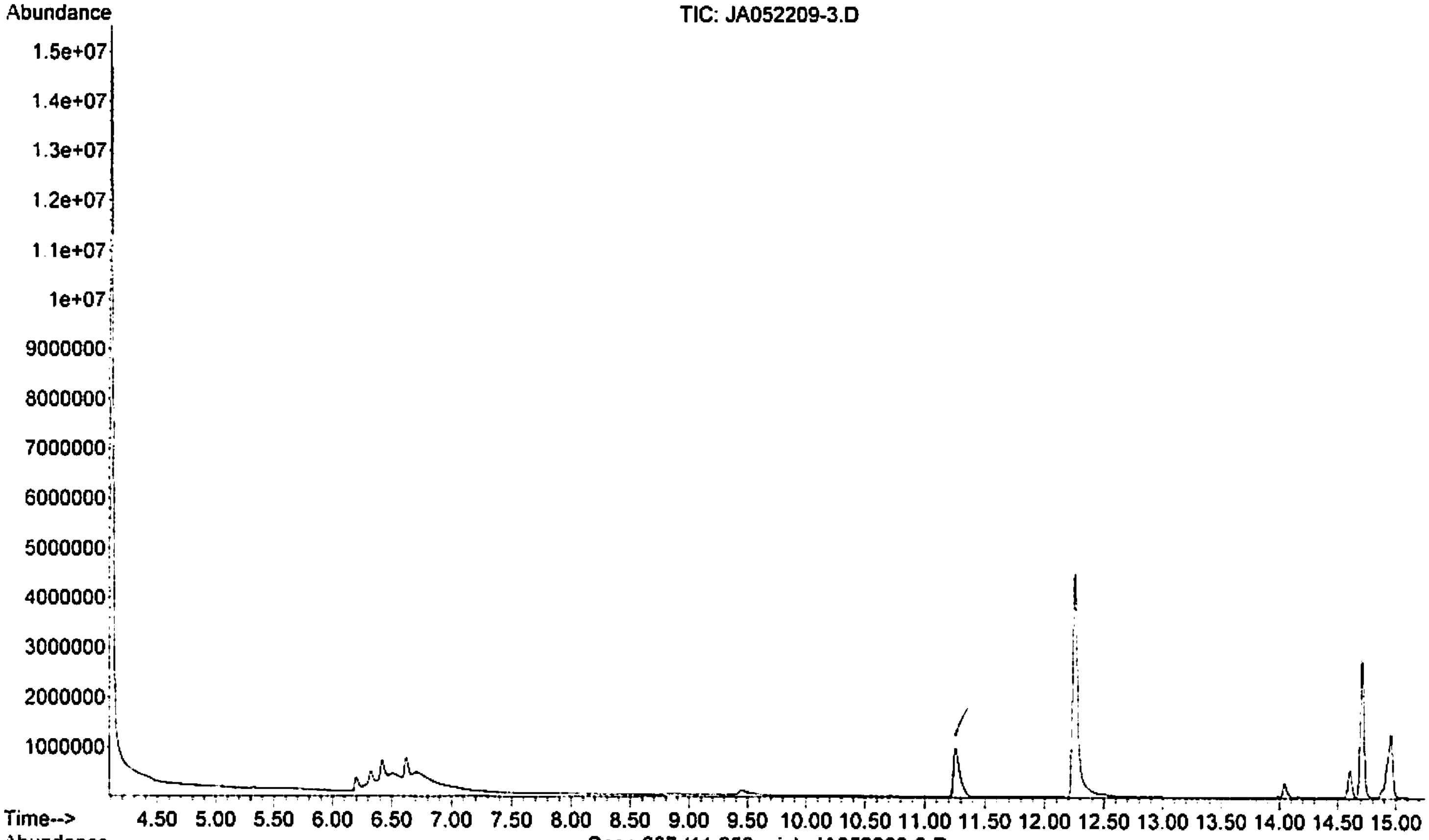

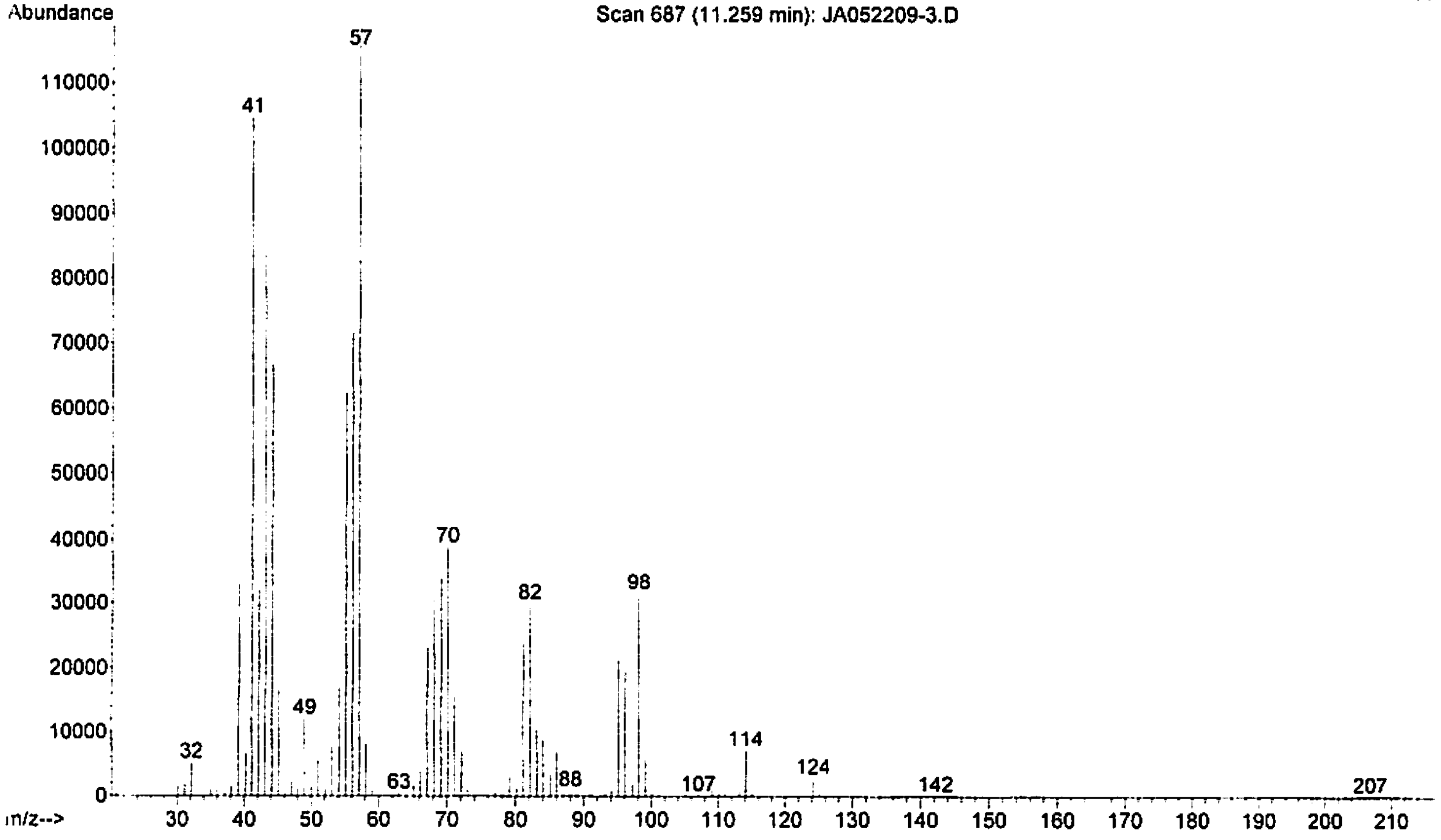

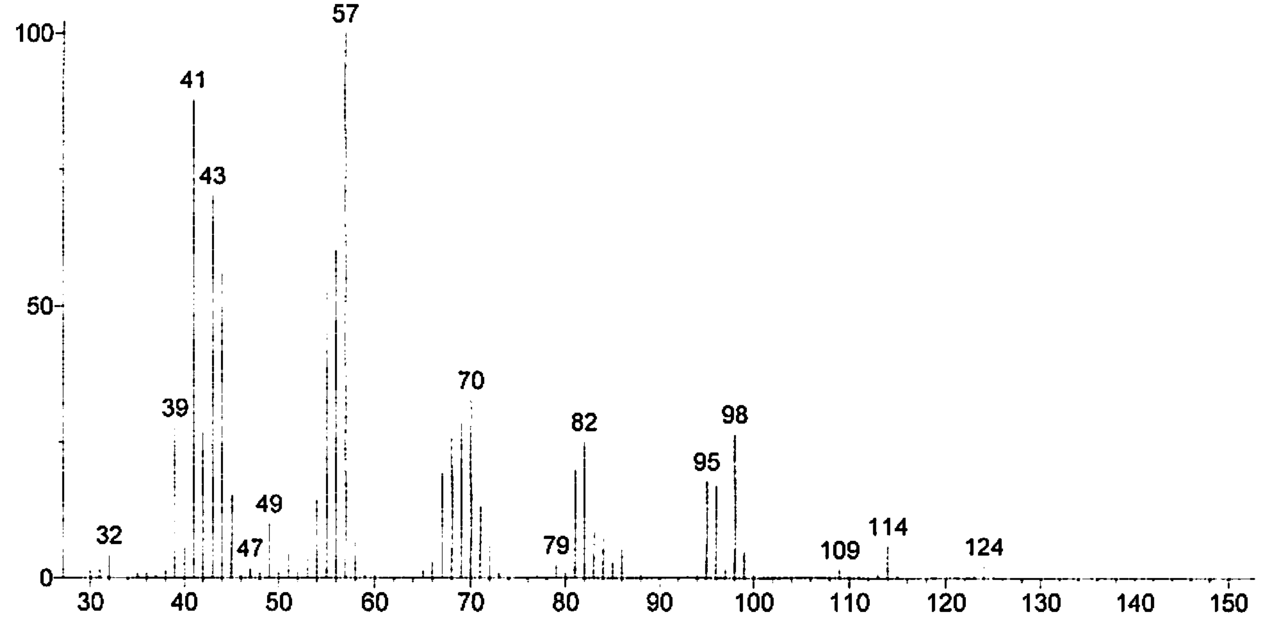

(Text File) Scan 687 (11.259 min): JA052209-3.D

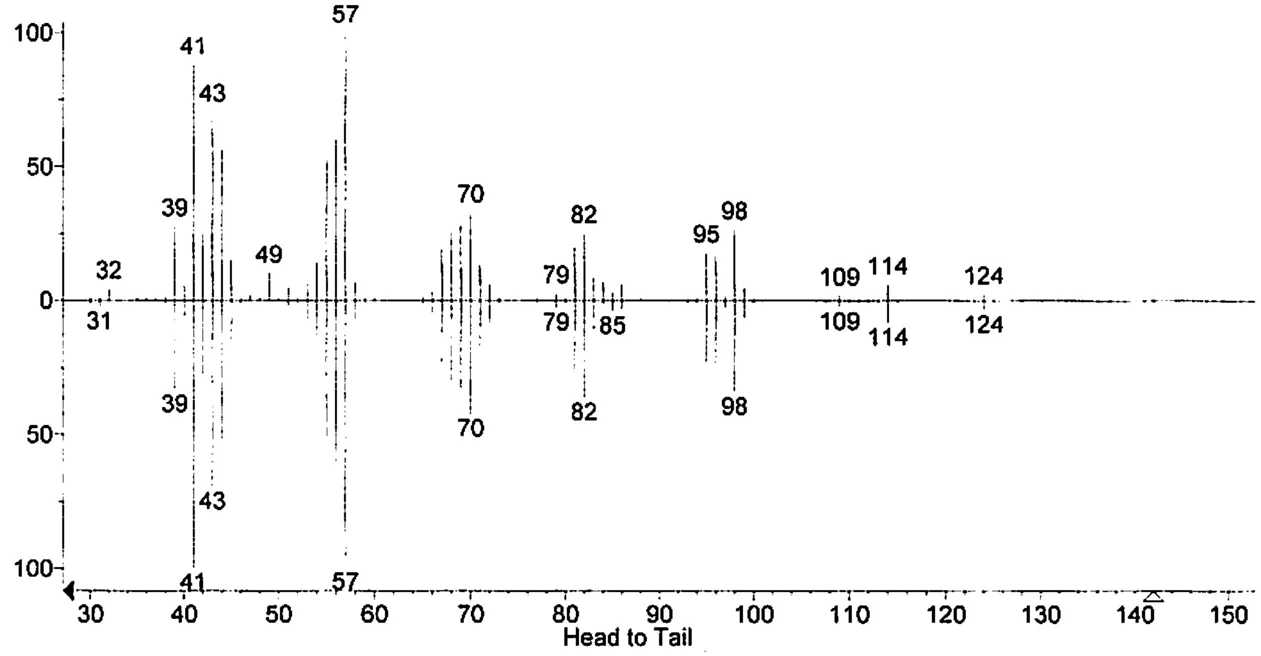

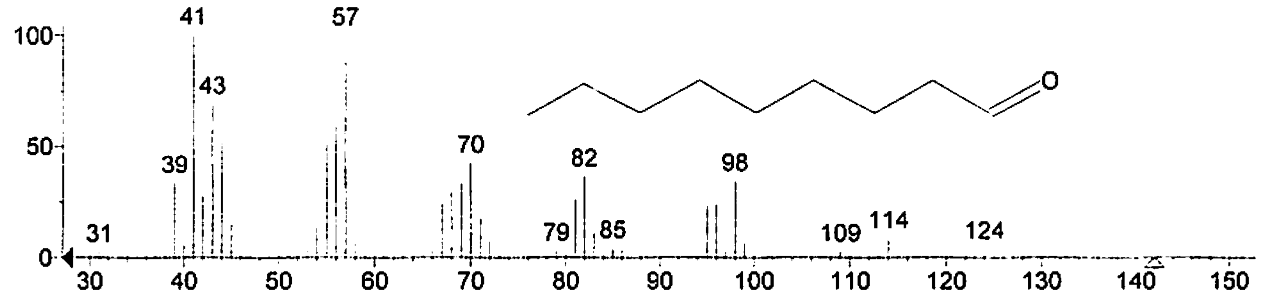

(replib) Nonanal

[illegible]e :D:\DATA\ALDRICH\JA-09\Snapshot\JA052209-3.D
[illegible]rator : Aldrich
[illegible]uired : 22 May 2009 15:55 using AcqMethod JA-WAX08.M
[illegible]trument : Instrument #1
[illegible]ple Name: 1 field-coll. male C.oculata abd./CH2Cl2
[illegible] Info : sweeping vetch 5/22 am; dissected
[illegible]l Number: 1

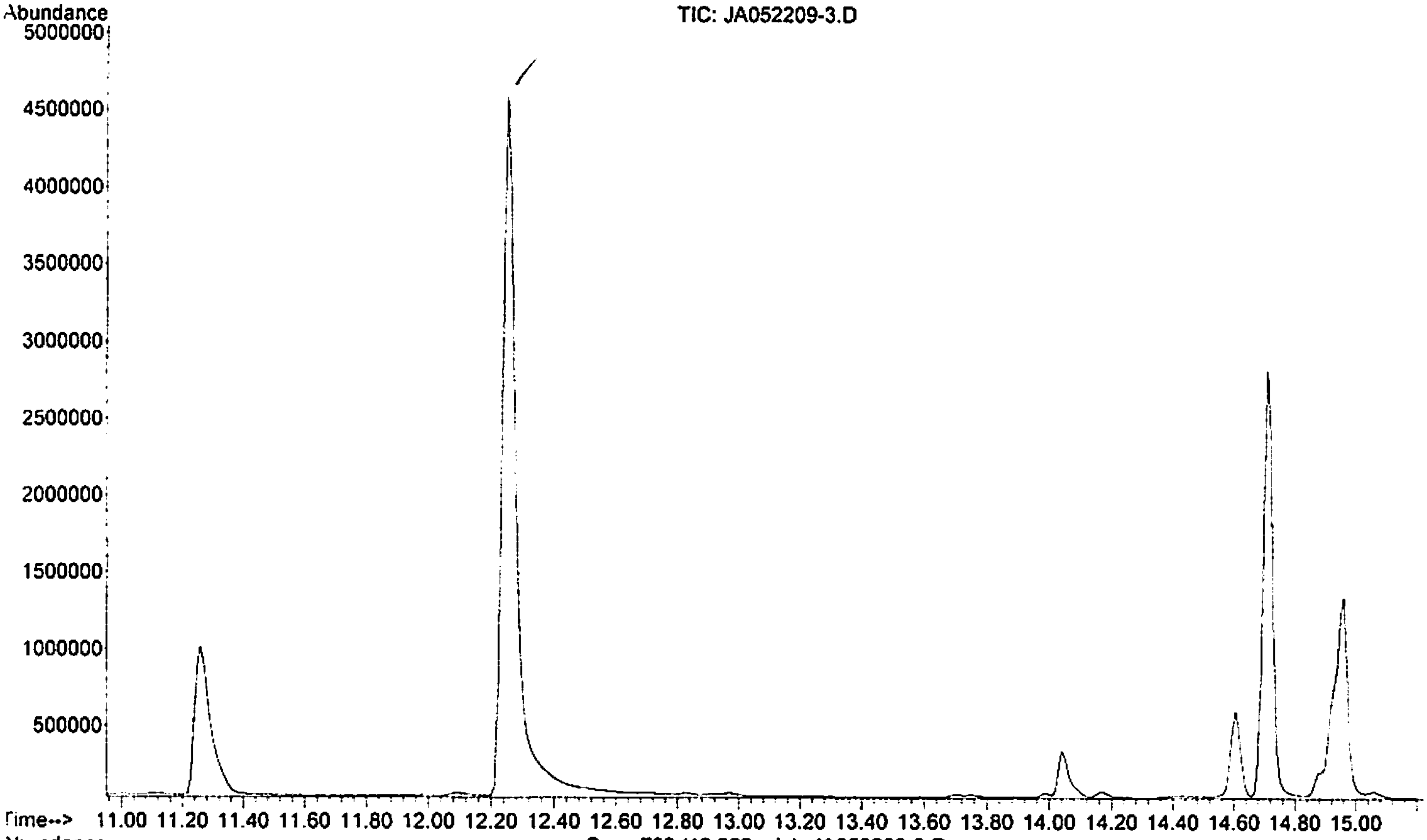

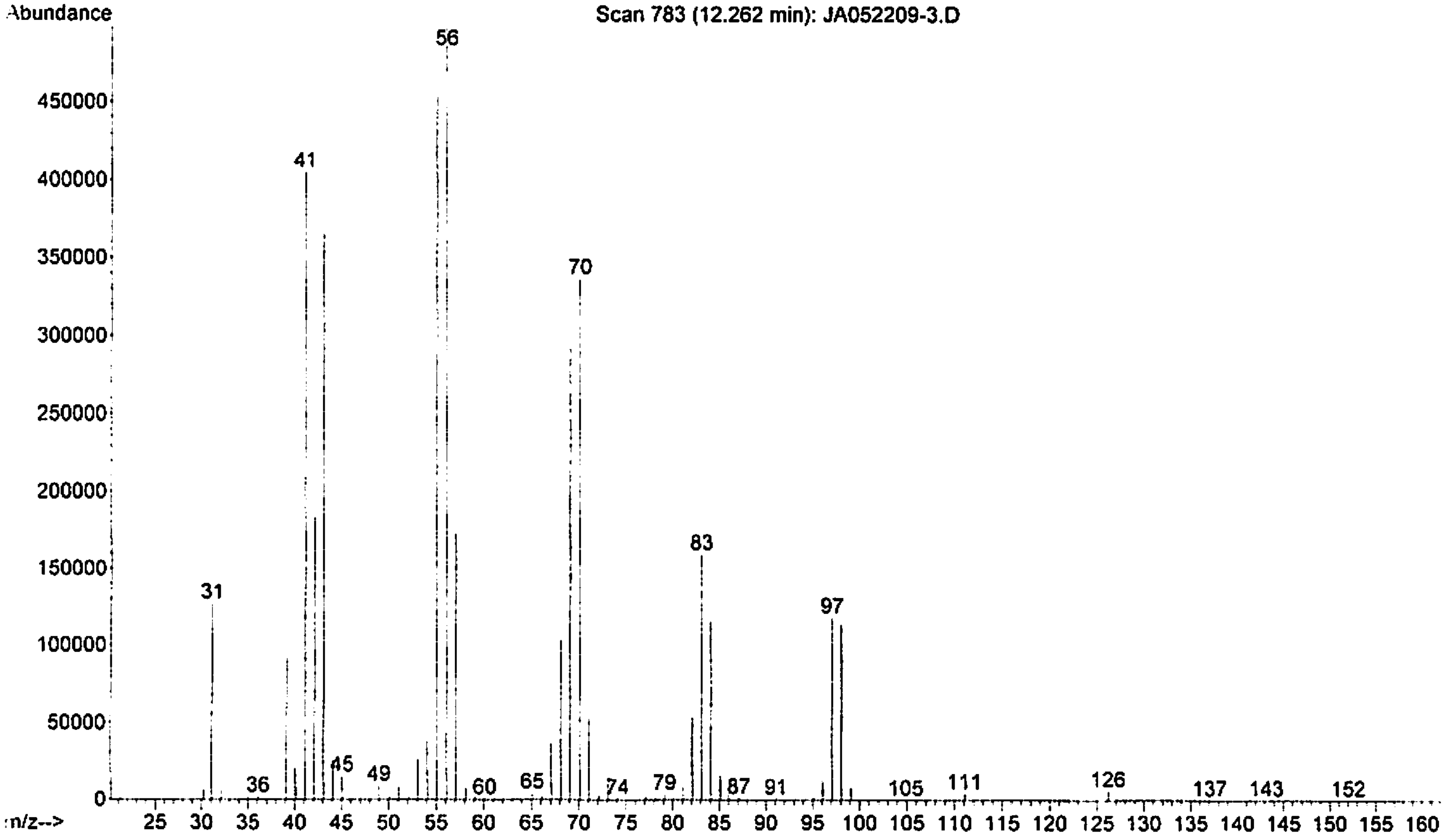

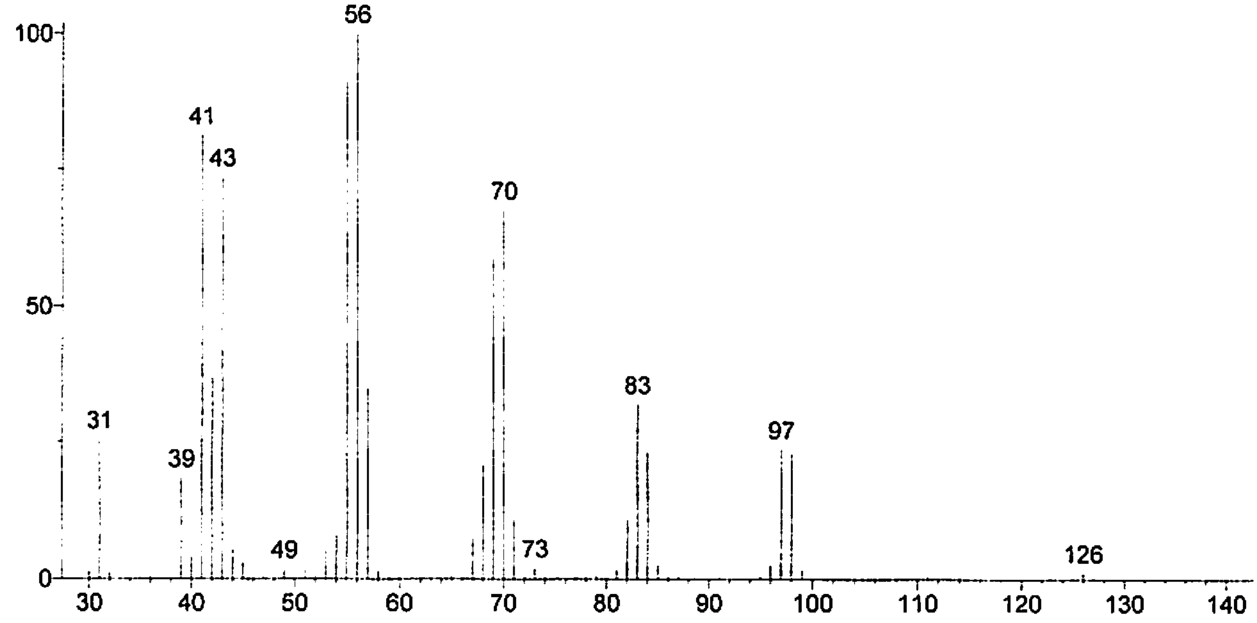

(Text File) Scan 783 (12.262 min): JA052209-3.D

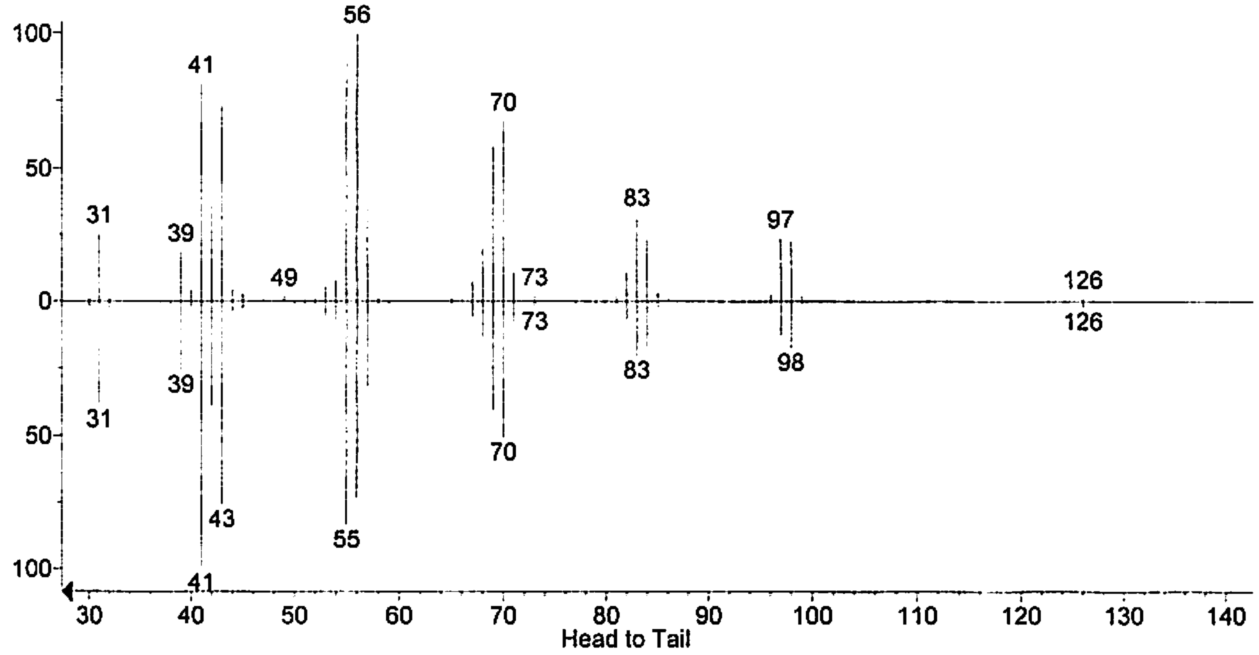

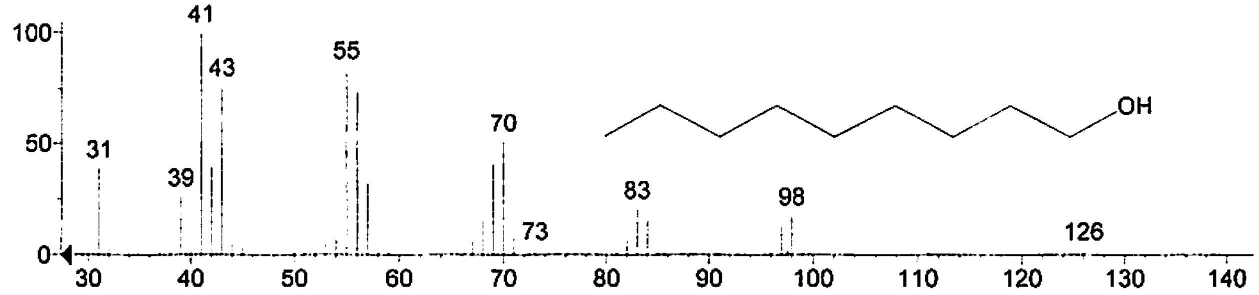

(replib) 1-Nonanol

[illegible] :D:\DATA\ALDRICH\JA-09\Snapshot\JA052209-3.D
[illegible]rator : Aldrich
[illegible]uired : 22 May 2009 15:55 using AcqMethod JA-WAX08.M
[illegible]trument : Instrument #1
[illegible]ple Name: 1 field-coll. male C.oculata abd./CH2Cl2
[illegible] Info : sweeping vetch 5/22 am; dissected
[illegible] Number: 1

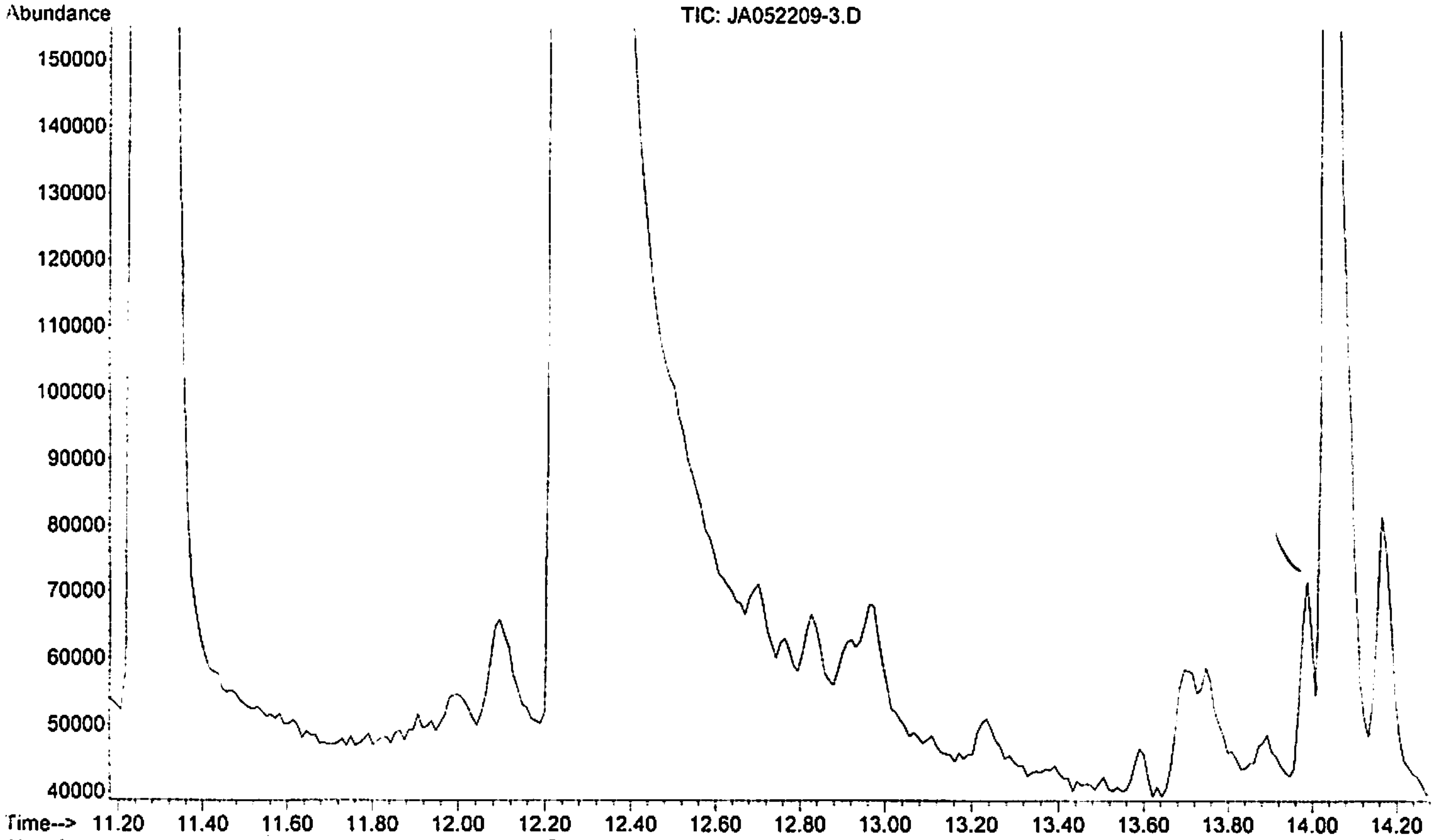

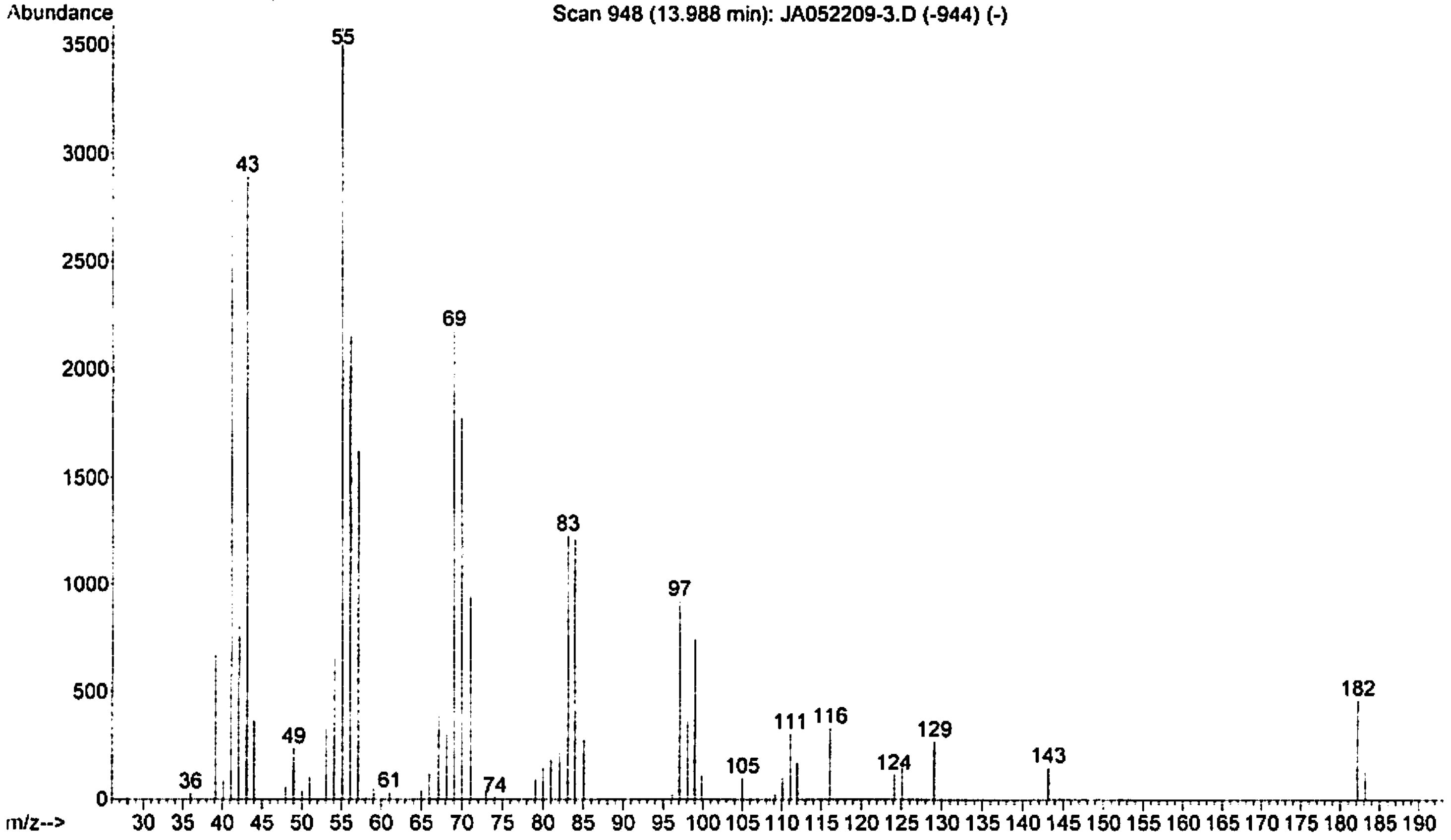

[illegible] :D:\DATA\ALDRICH\JA-09\Snapshot\JA052209-3.D
[illegible]rator : Aldrich
[illegible]quired : 22 May 2009 15:55 using AcqMethod JA-WAX08.M
[illegible]strument : Instrument #1
[illegible]ple Name: 1 field-coll. male C.oculata abd./CH2Cl2
[illegible] Info : sweeping vetch 5/22 am; dissected
[illegible]l Number: 1

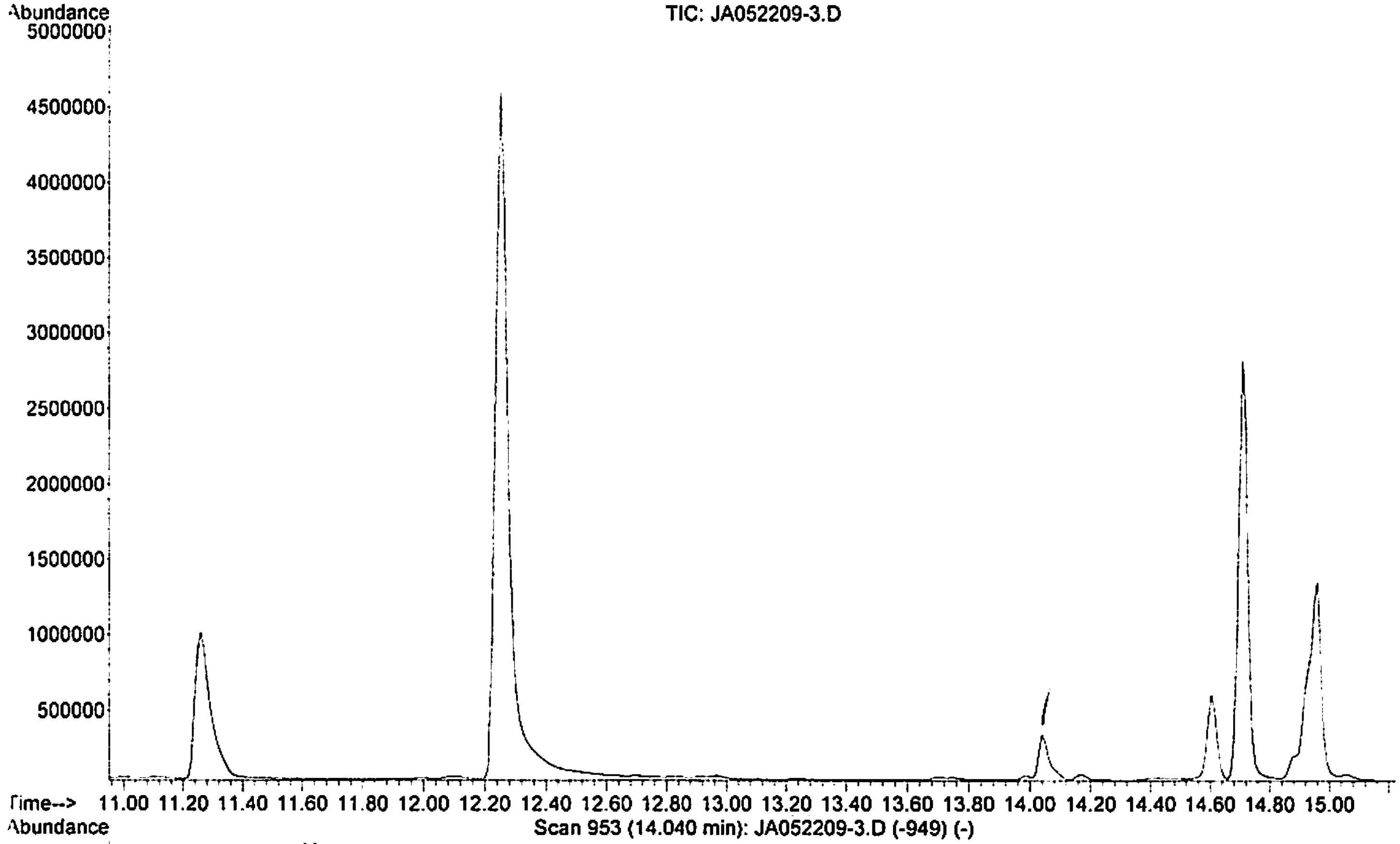

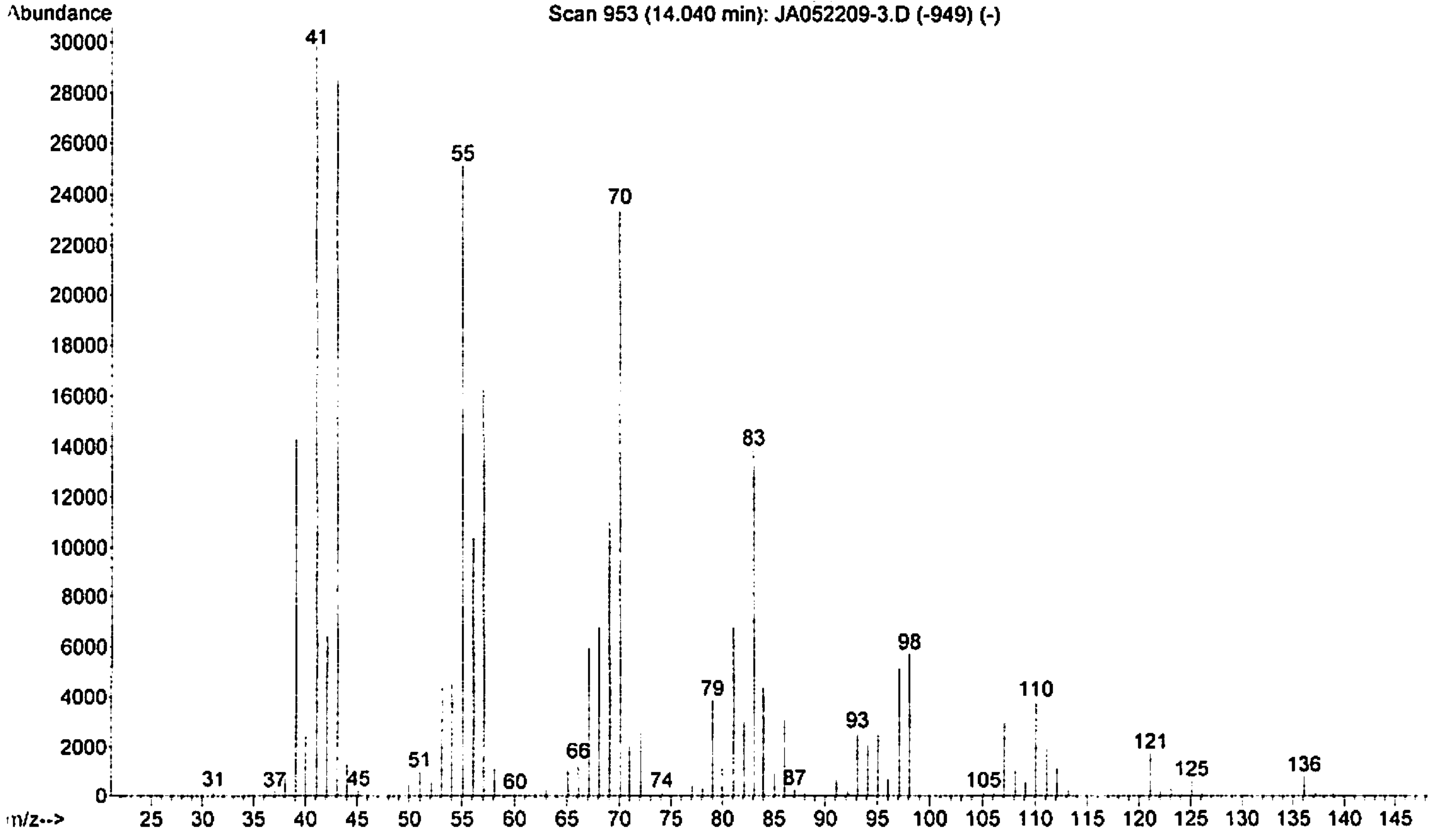

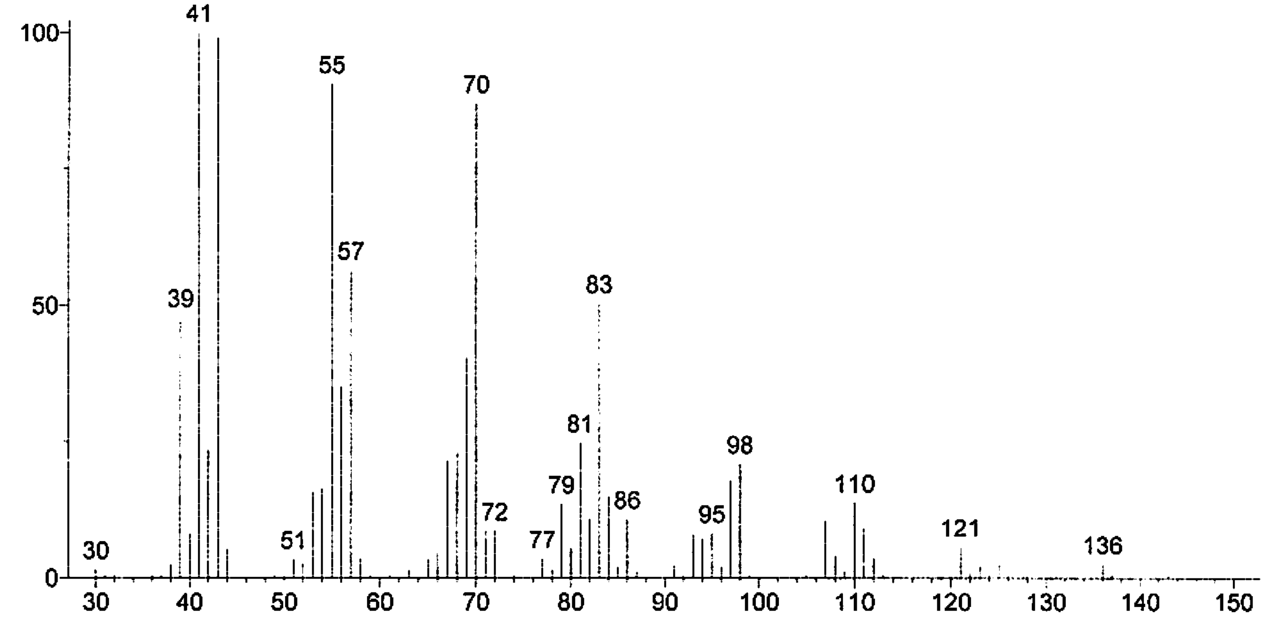

(Text File) Scan 954 (14.051 min): JA052209-3.D (-950)

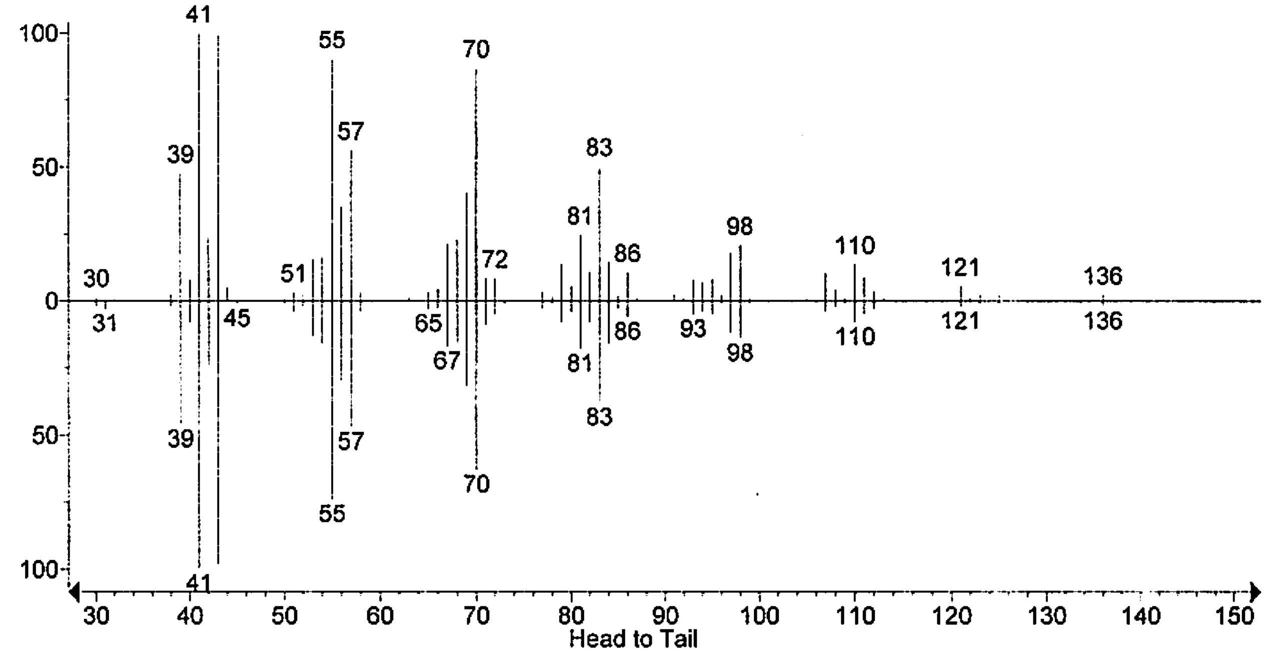

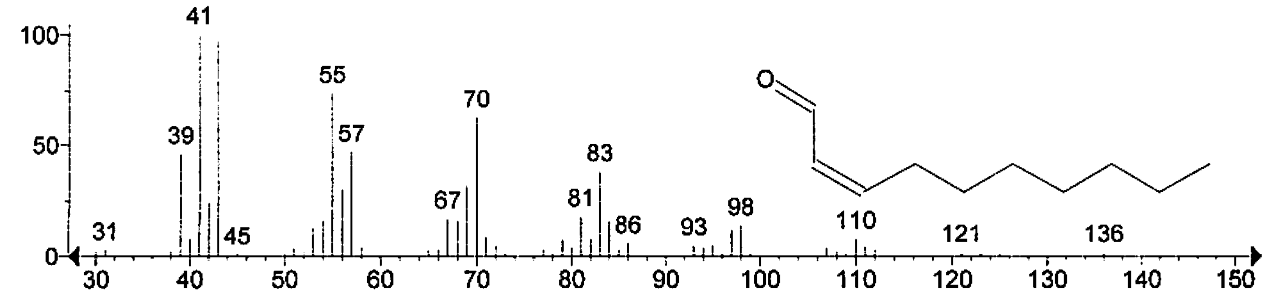

(mainlib) 2-Decenal, (Z)-

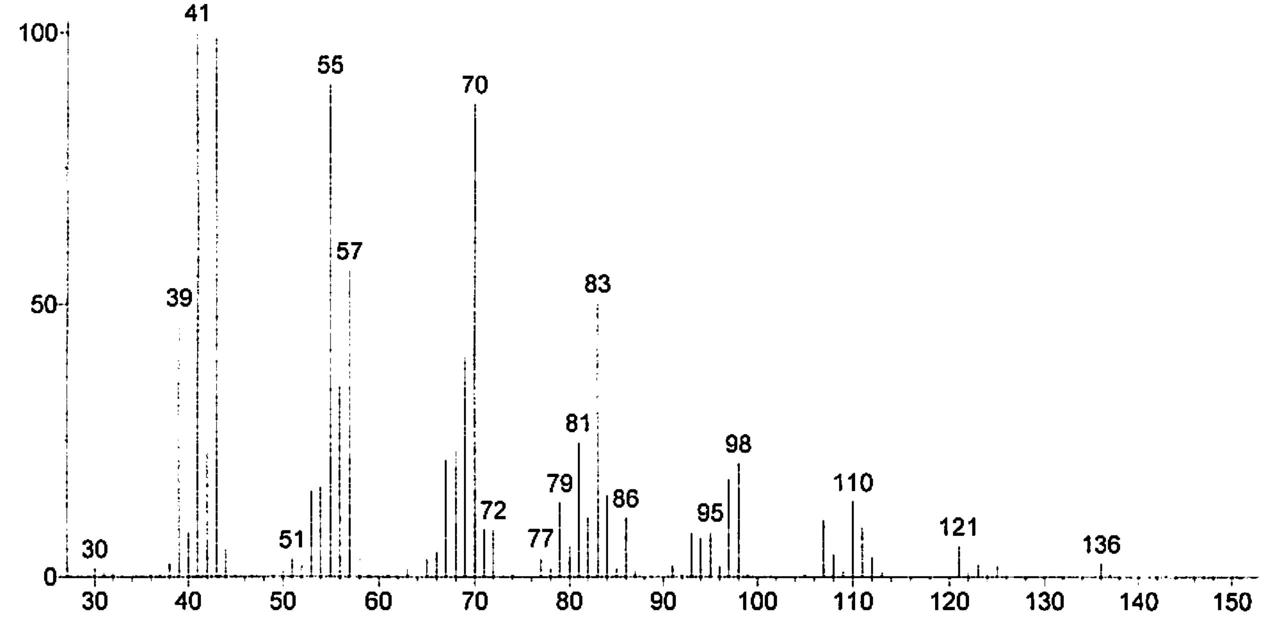

(Text File) Scan 954 (14.051 min): JA052209-3.D (-950)

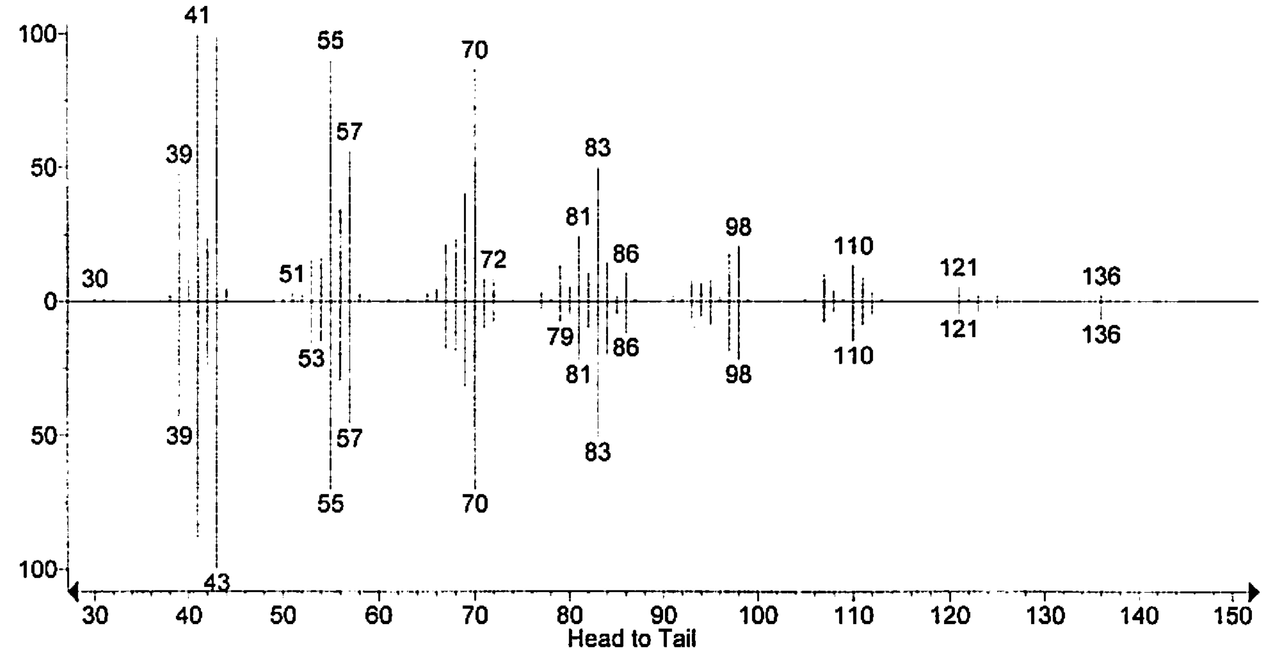

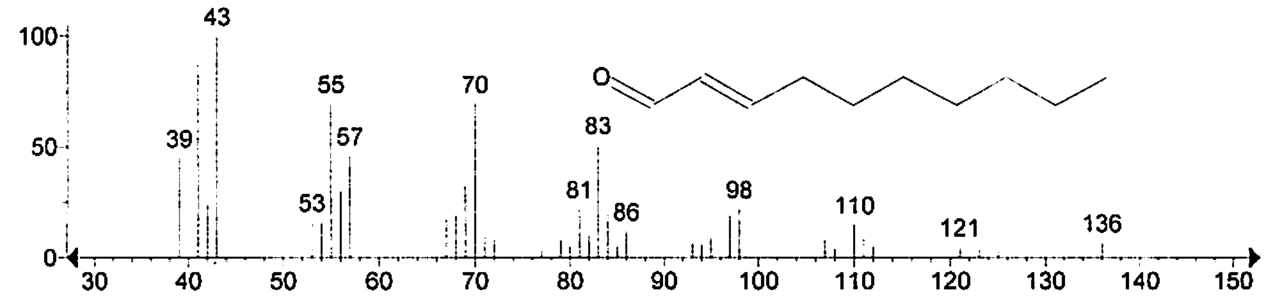

(mainlib) 2-Decenal, (E)-

[illegible] :D:\DATA\ALDRICH\JA-09\Snapshot\JA052209-3.D
erator : Aldrich
quired : 22 May 2009 15:55 using AcqMethod JA-WAX08.M
strument : Instrument #1
mple Name: 1 field-coll. male C.oculata abd./CH2Cl2
sc Info : sweeping vetch 5/22 am; dissected
al Number: 1

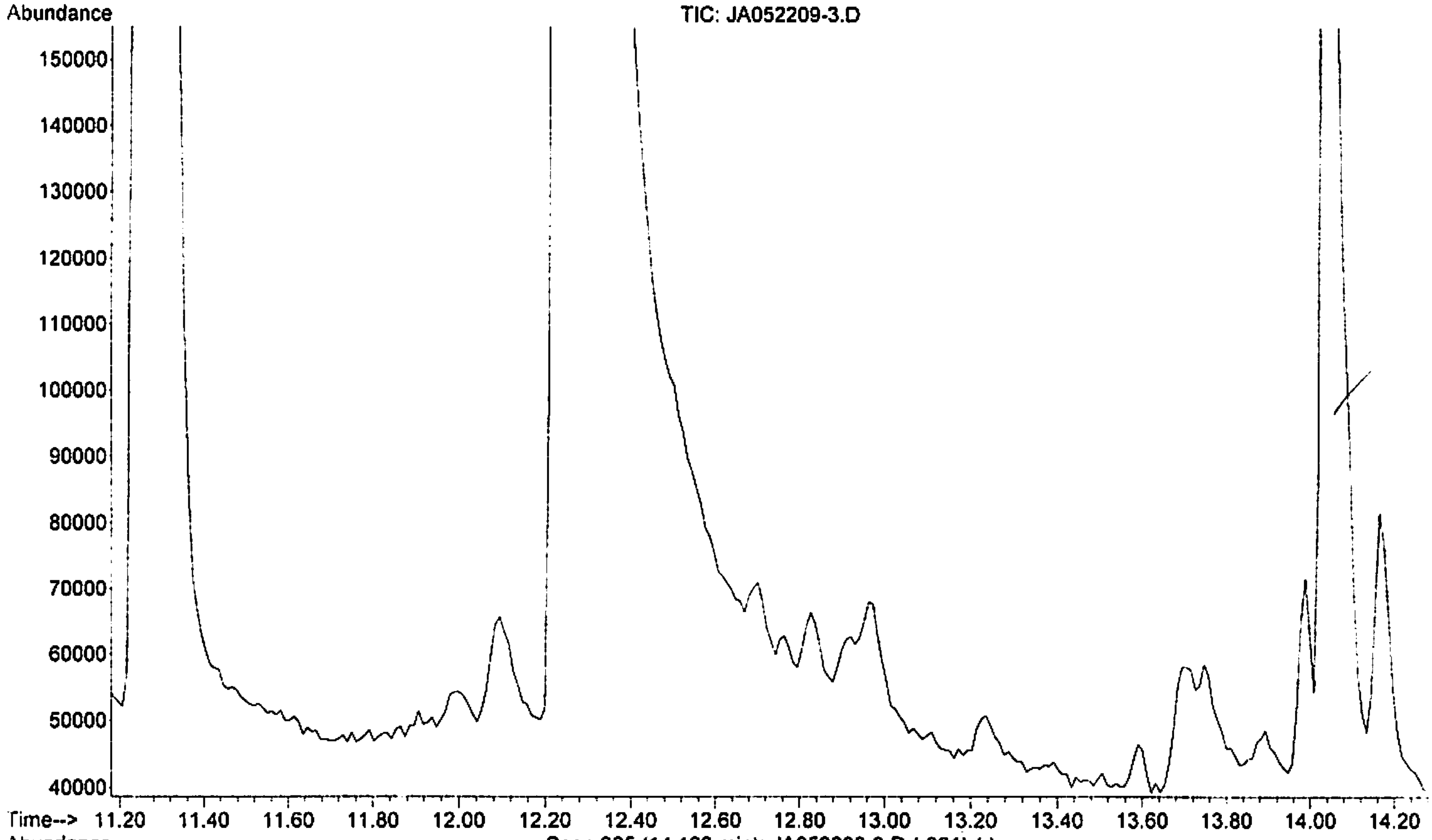

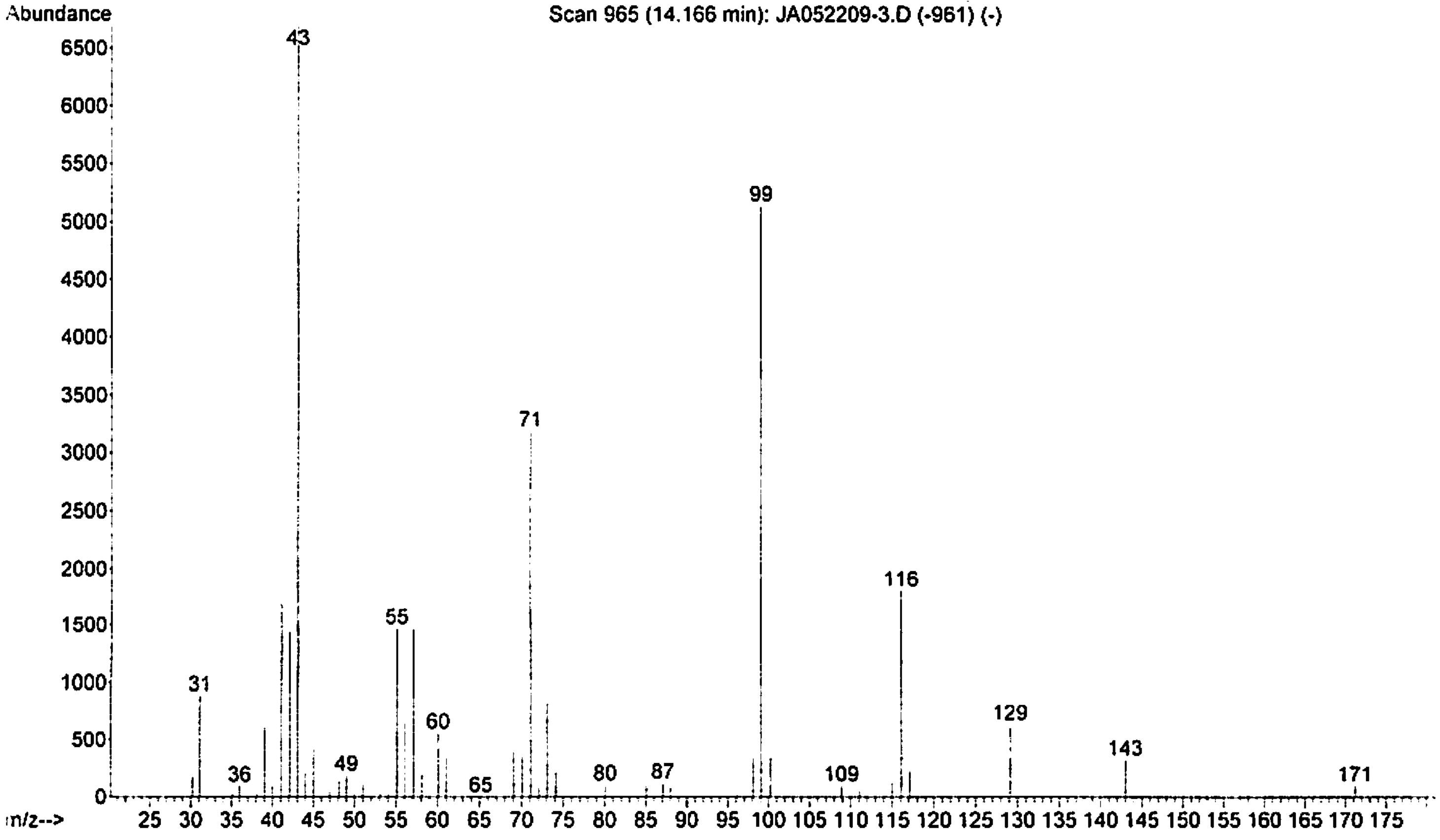

[illegible] :D:\DATA\ALDRICH\JA-09\Snapshot\JA052209-3.D
[illegible]rator : Aldrich
[illegible]quired : 22 May 2009 15:55 using AcqMethod JA-WAX08.M
[illegible]strument : Instrument #1
[illegible]nple Name: 1 field-coll. male C.oculata abd./CH2Cl2
[illegible]sc Info : sweeping vetch 5/22 am; dissected
[illegible]l Number: 1

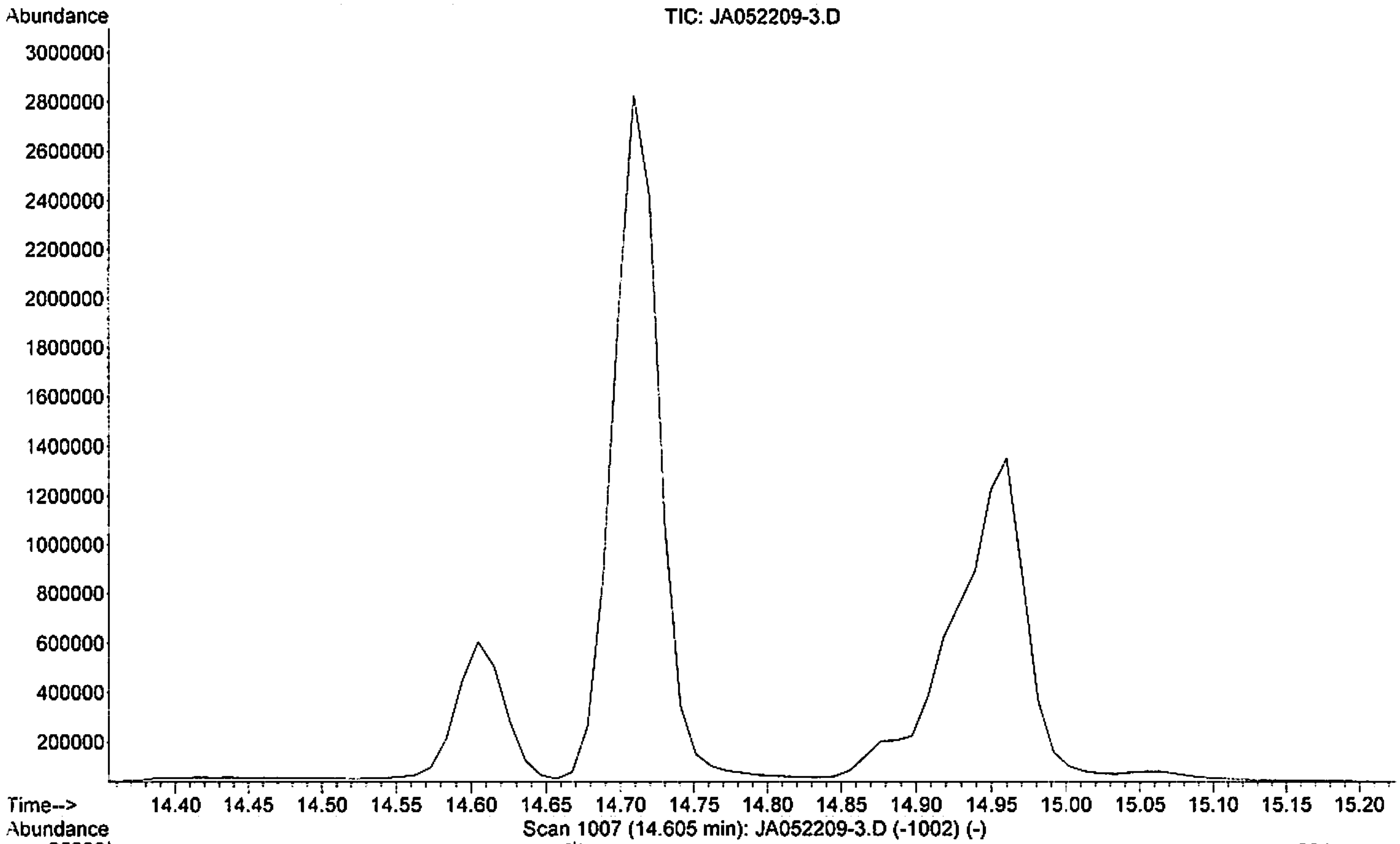

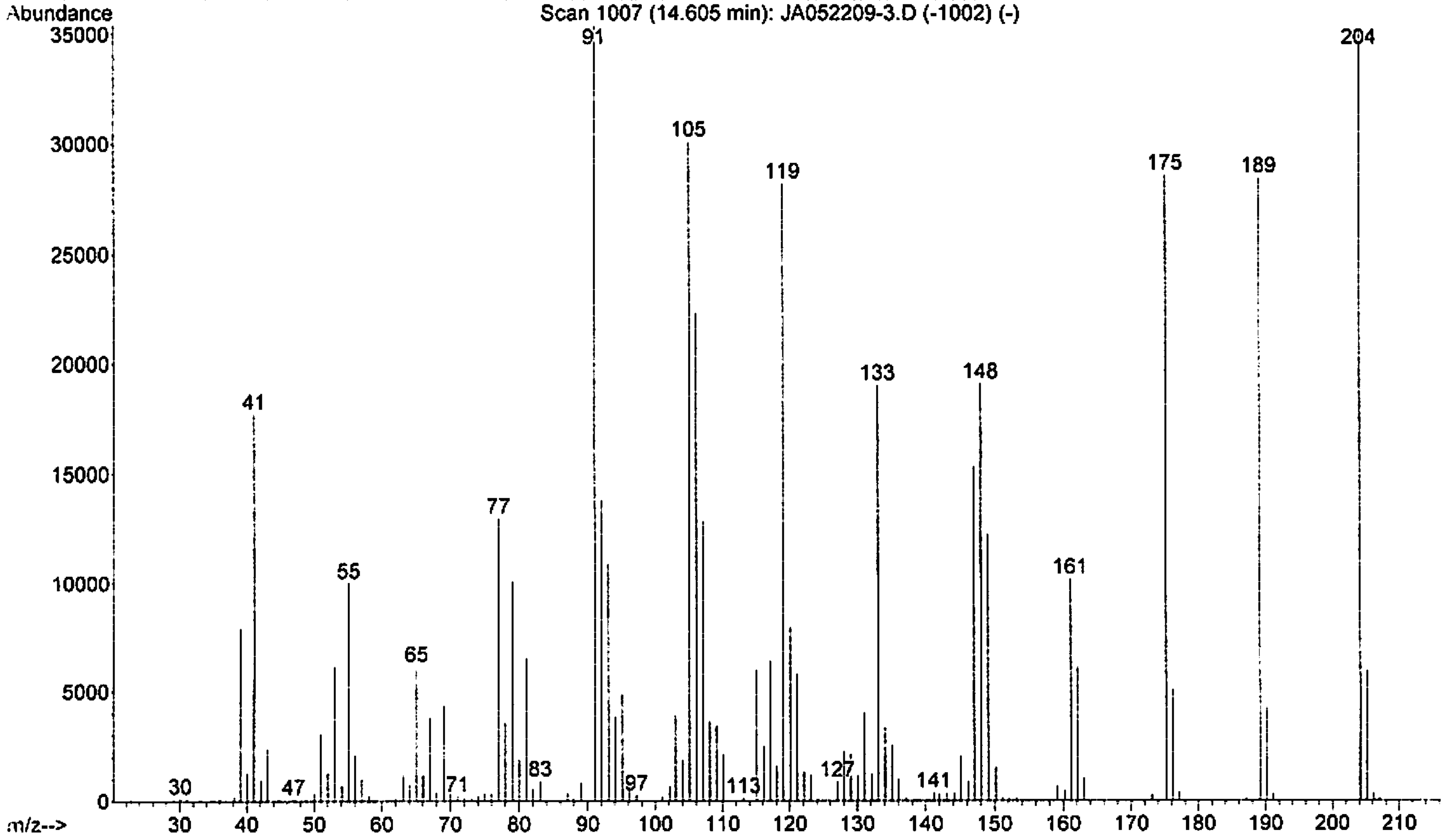

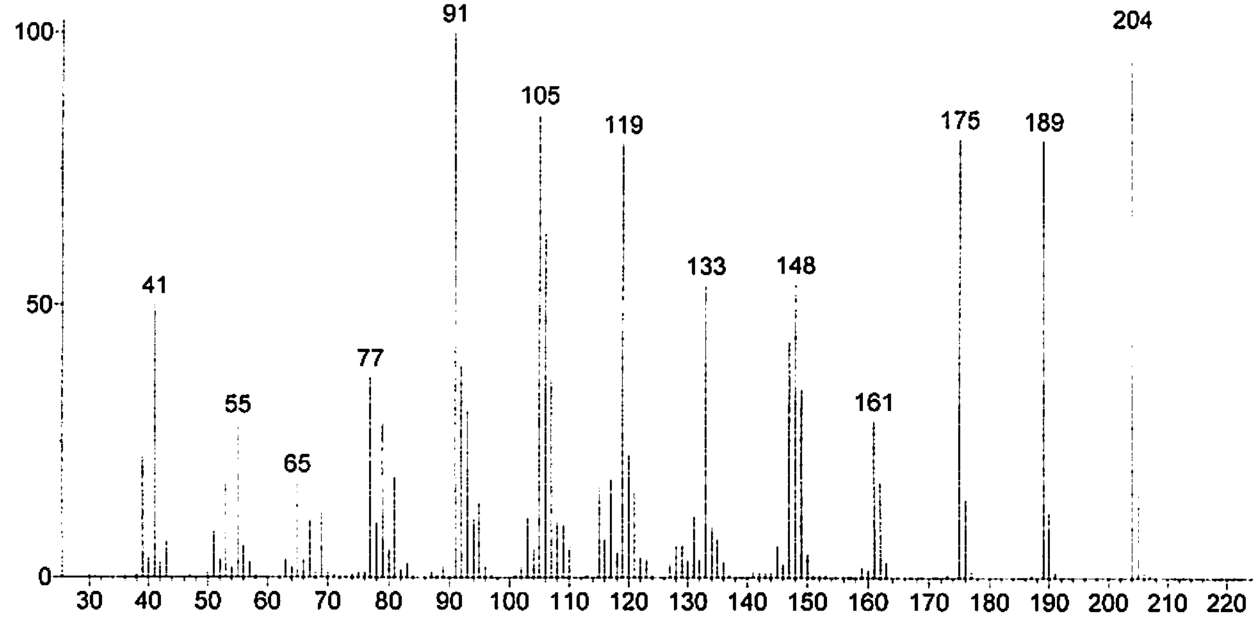

(Text File) Scan 1007 (14.605 min): JA052209-3.D (-1002)

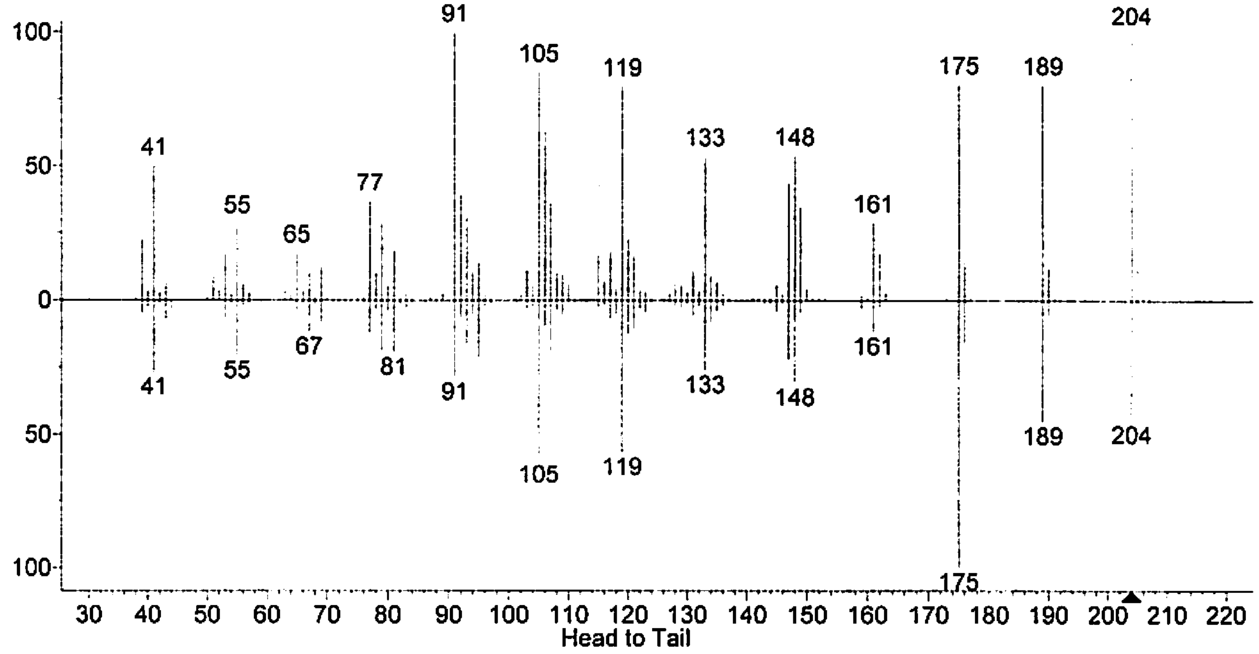

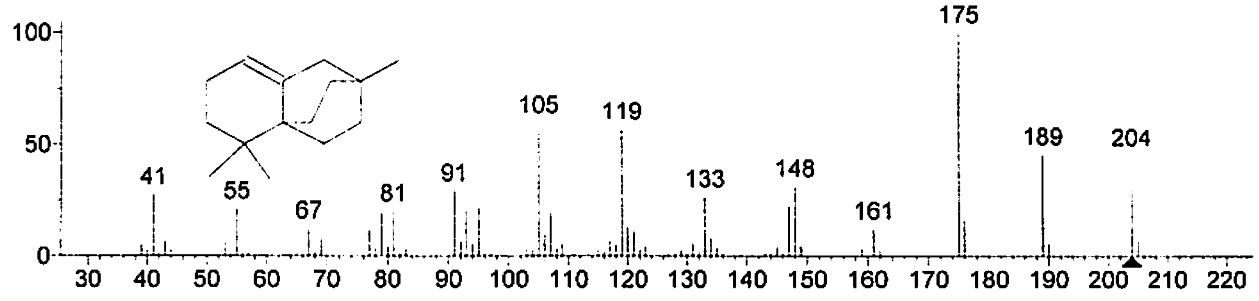

(mainlib) 2H-2,4a-Ethanonaphthalene, 1,3,4,5,6,7-hexahydro-2,5,5-trimethyl-

e :D:\DATA\ALDRICH\JA-09\Snapshot\JA052209-3.D
rator : Aldrich
uired : 22 May 2009 15:55 using AcqMethod JA-WAX08.M
rument : Instrument #1
ple Name: 1 field-coll. male C.oculata abd./CH2Cl2
Info : sweeping vetch 5/22 am; dissected
l Number: 1

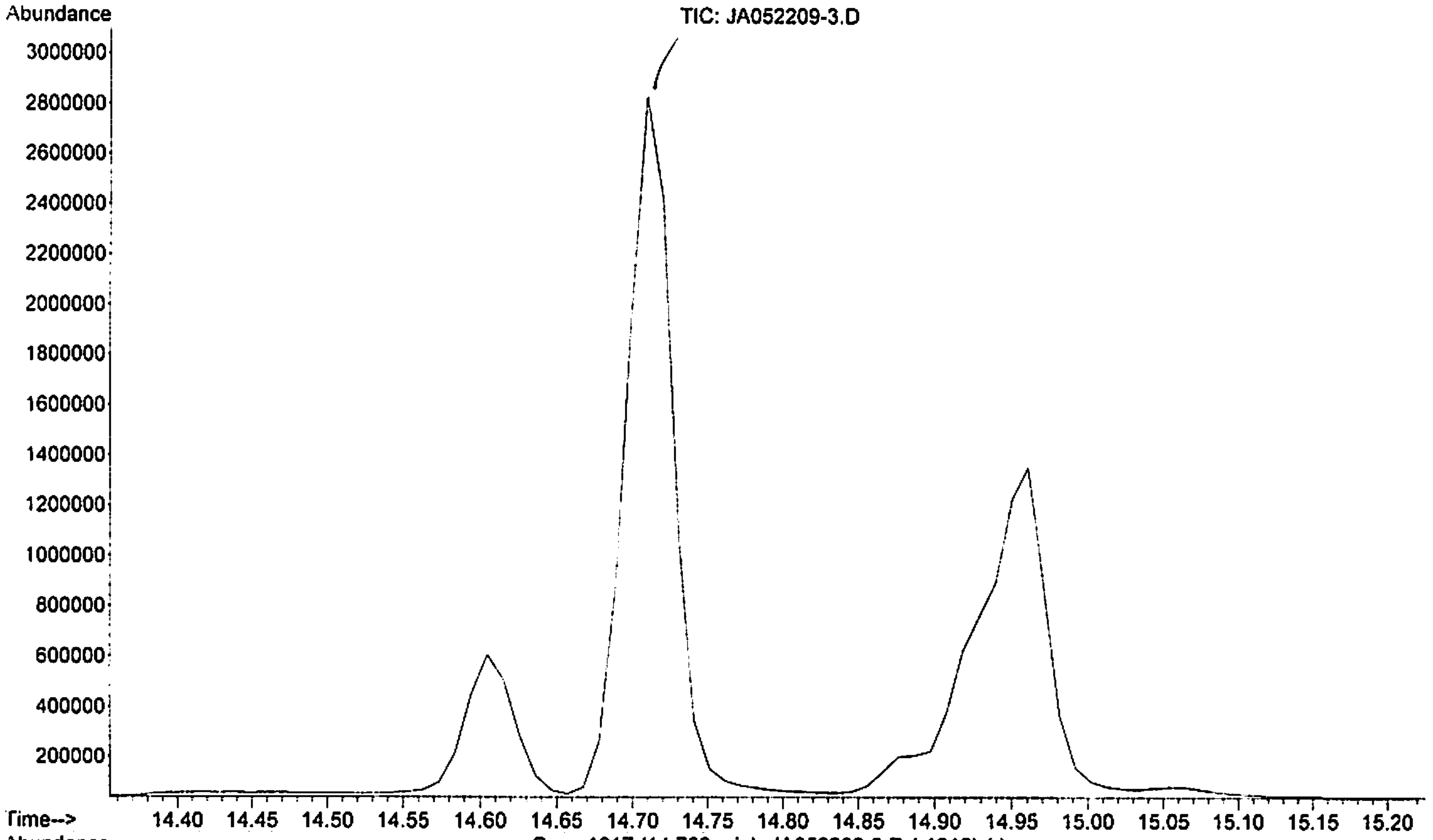

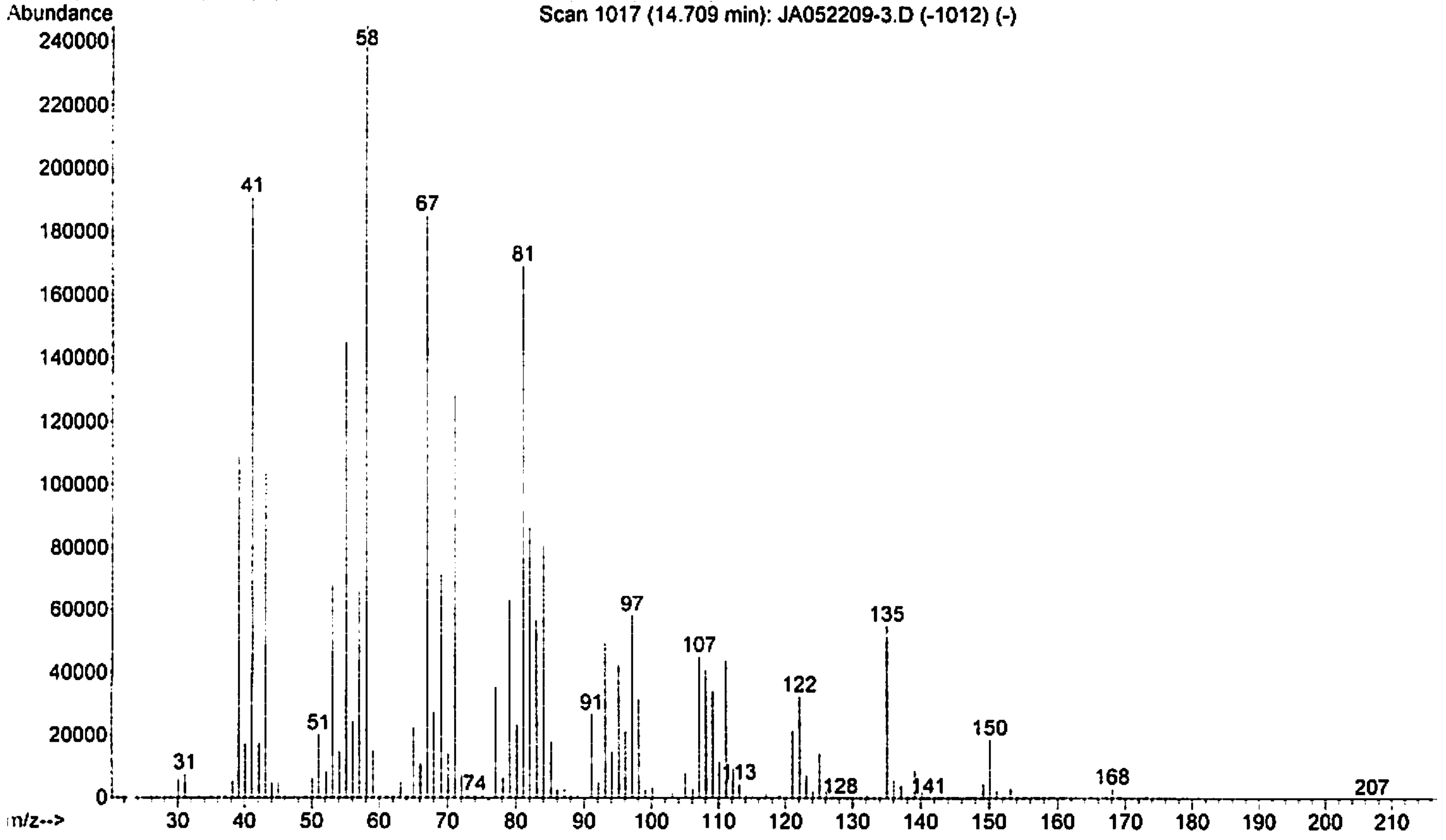

e :D:\DATA\ALDRICH\JA-09\Snapshot\JA052209-3.D
erator : Aldrich
quired : 22 May 2009 15:55 using AcqMethod JA-WAX08.M
strument : Instrument #1
mple Name: 1 field-coll. male C.oculata abd./CH2Cl2
sc Info : sweeping vetch 5/22 am; dissected
al Number: 1

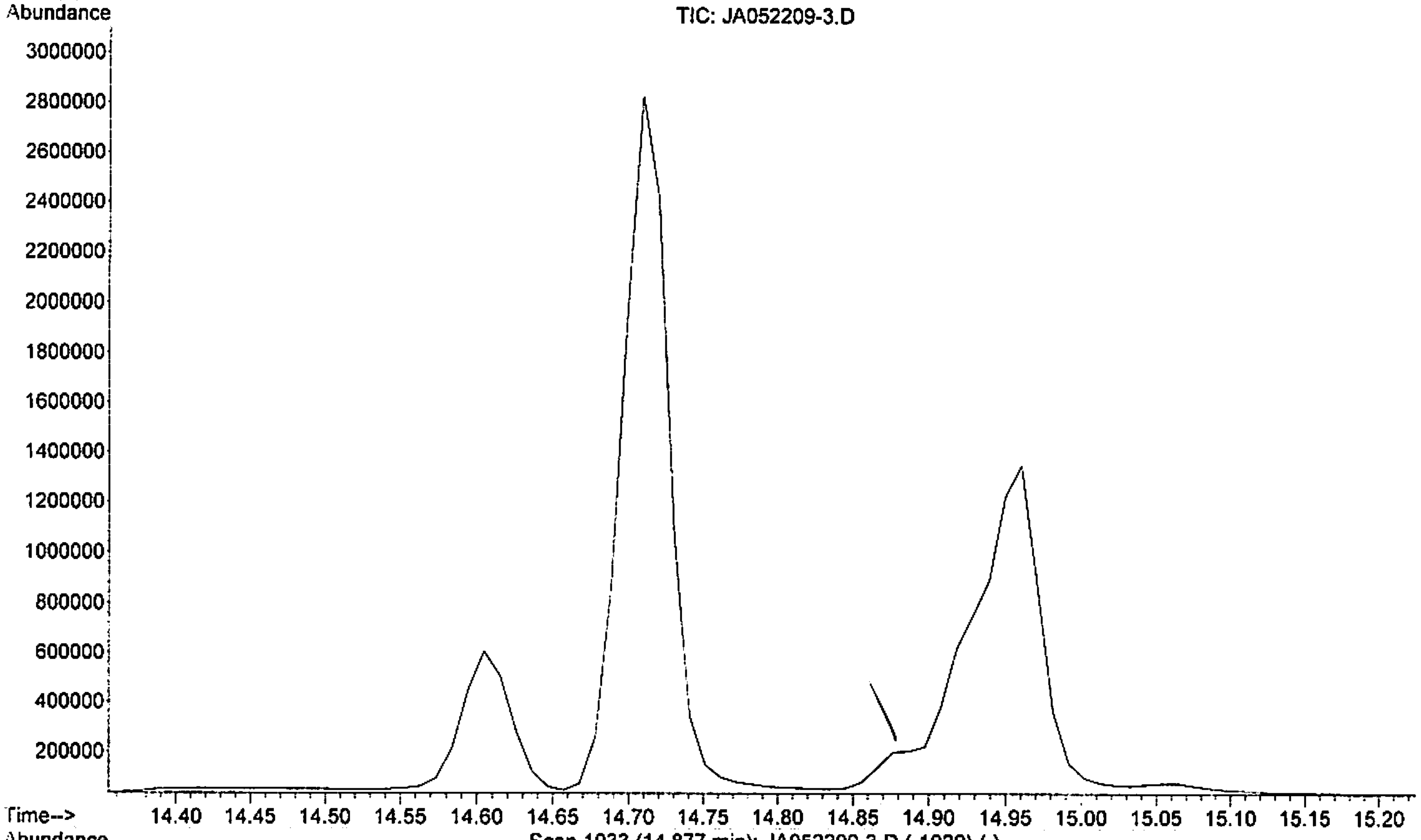

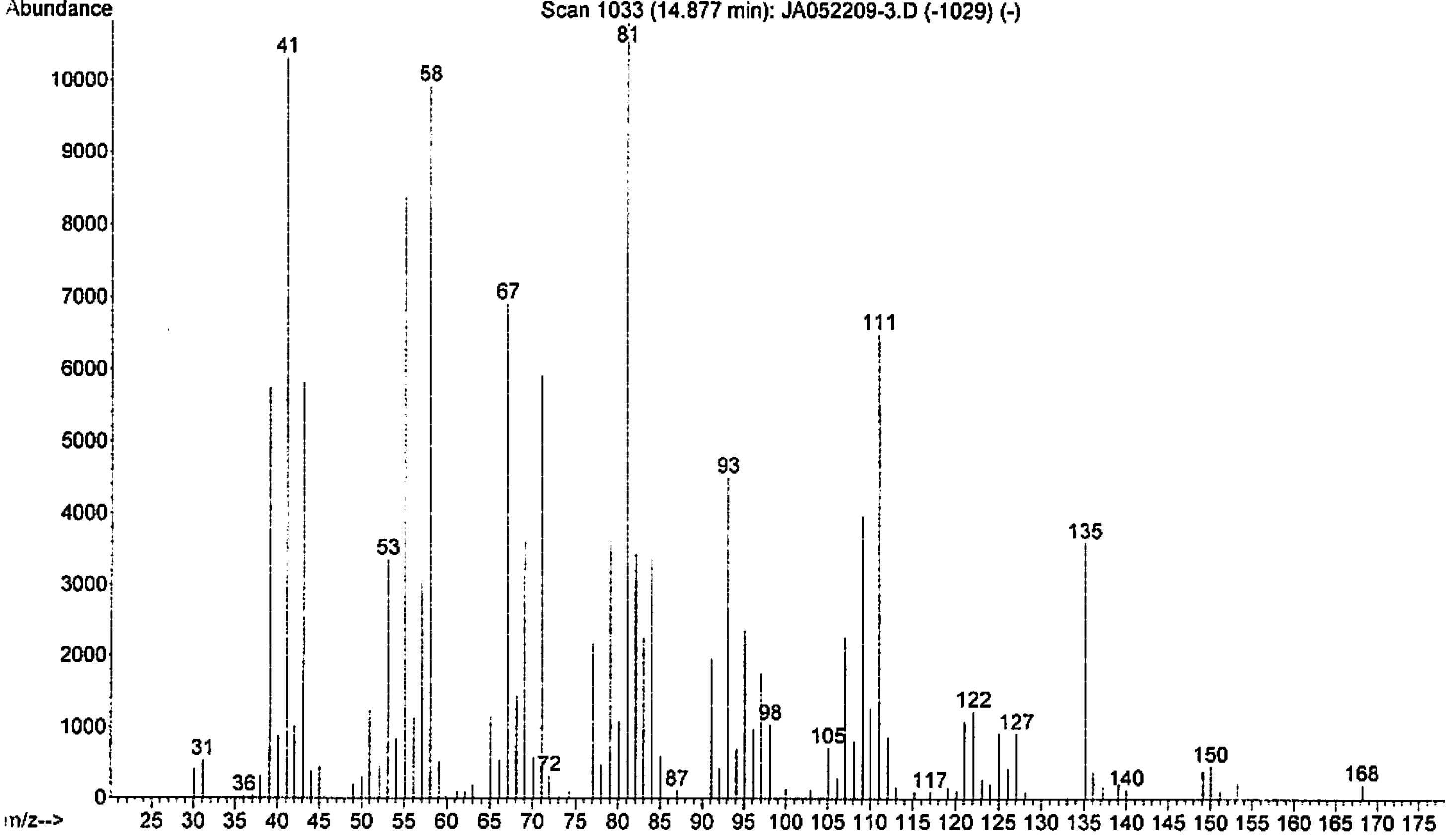

:D:\DATA\ALDRICH\JA-09\Snapshot\JA052209-3.D
[illegible]rator : Aldrich
[illegible]uired : 22 May 2009 15:55 using AcqMethod JA-WAX08.M
[illegible]strument : Instrument #1
[illegible]ple Name: 1 field-coll. male C.oculata abd./CH2Cl2
[illegible] Info : sweeping vetch 5/22 am; dissected
[illegible]al Number: 1

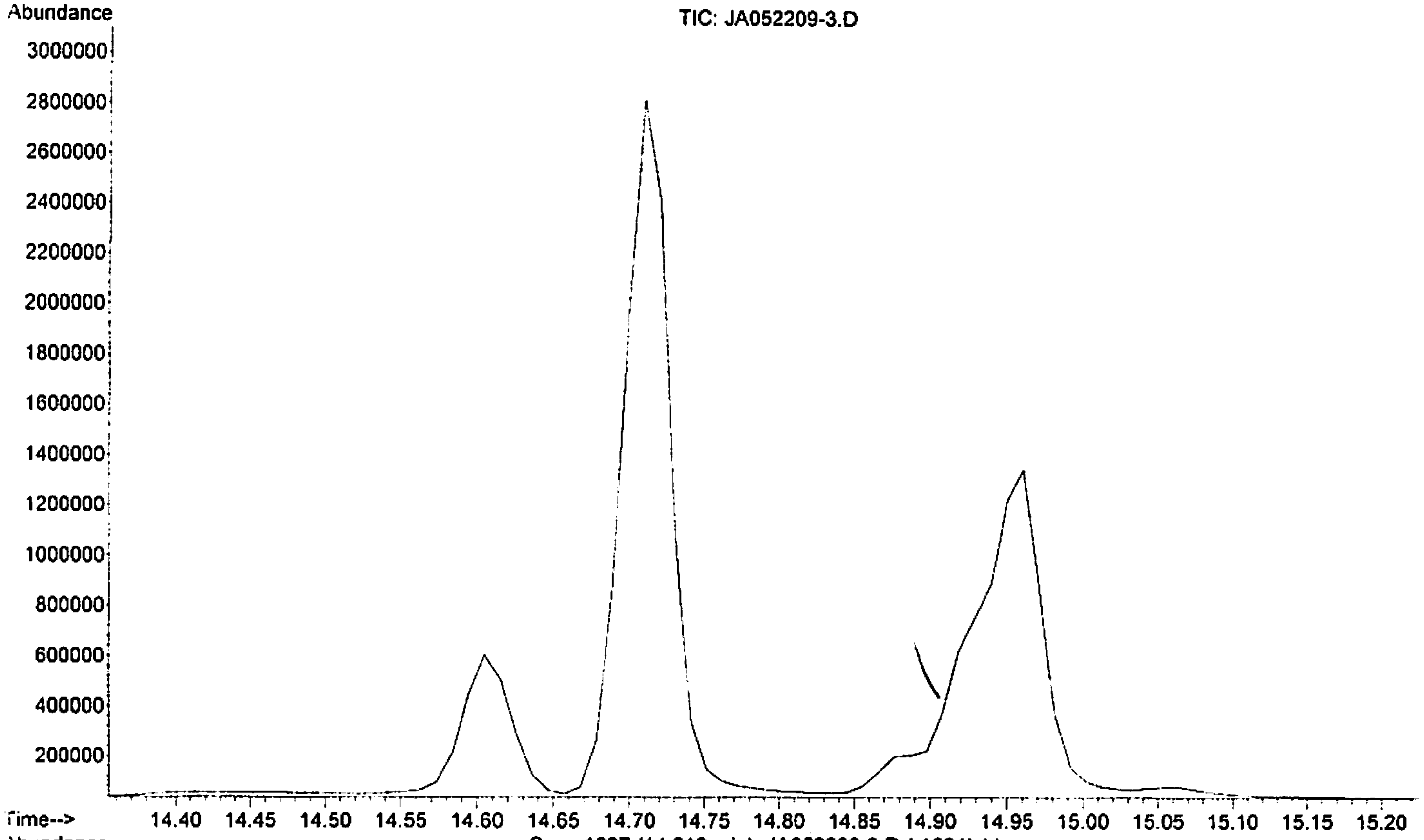

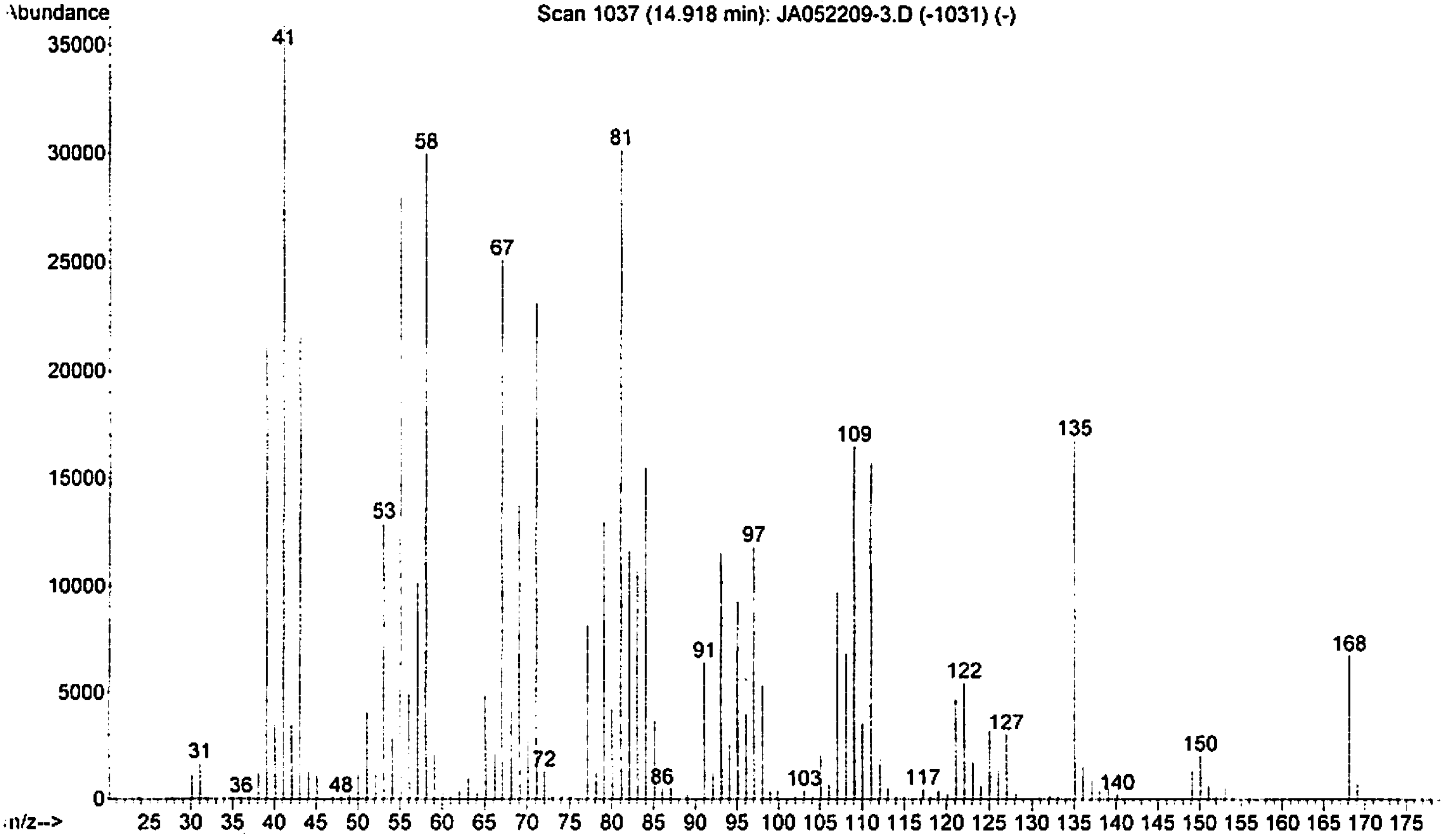

[illegible]e :D:\DATA\ALDRICH\JA-09\Snapshot\JA052209-3.D
[illegible]erator : Aldrich
[illegible]quired : 22 May 2009 15:55 using AcqMethod JA-WAX08.M
[illegible]strument : Instrument #1
[illegible]ple Name: 1 field-coll. male C.oculata abd./CH2Cl2
[illegible] Info : sweeping vetch 5/22 am; dissected
[illegible]al Number: 1

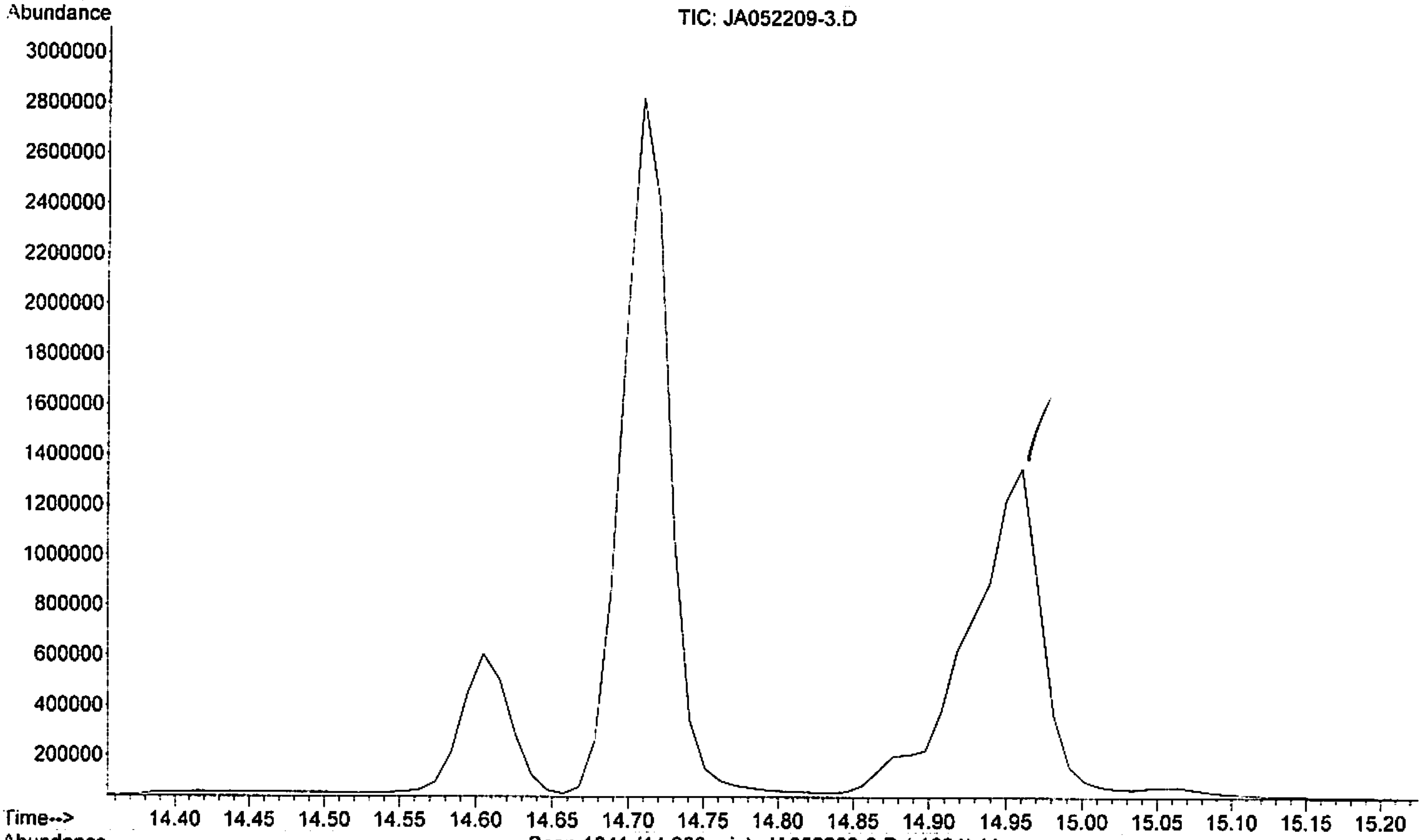

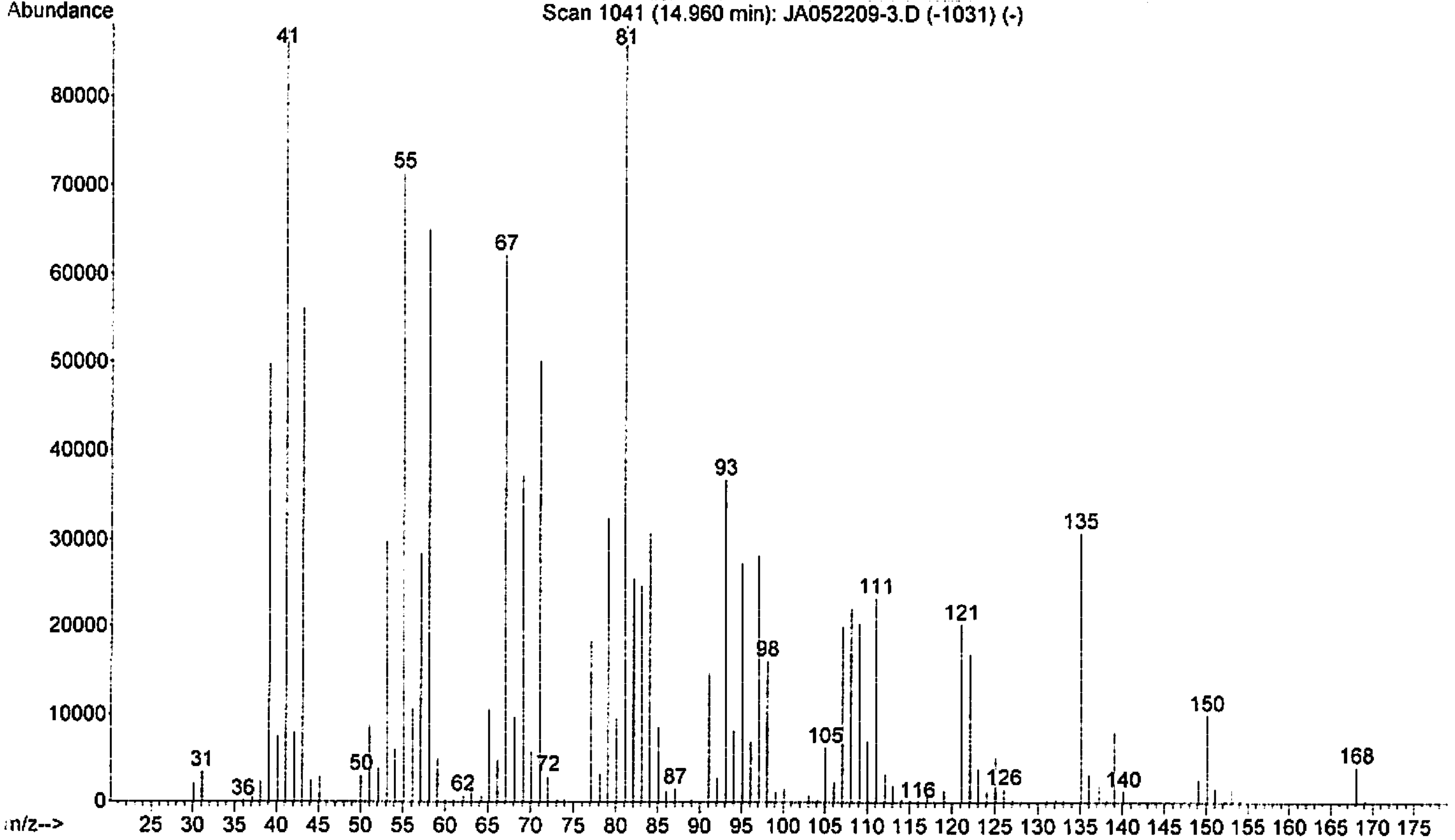

[illegible] :D:\DATA\ALDRICH\JA-09\Snapshot\JA052209-3.D
Operator : Aldrich
Acquired : 22 May 2009 15:55 using AcqMethod JA-WAX08.M
Instrument : Instrument #1
Sample Name: 1 field-coll. male C.oculata abd./CH2Cl2
Misc Info : sweeping vetch 5/22 am; dissected
Vial Number: 1

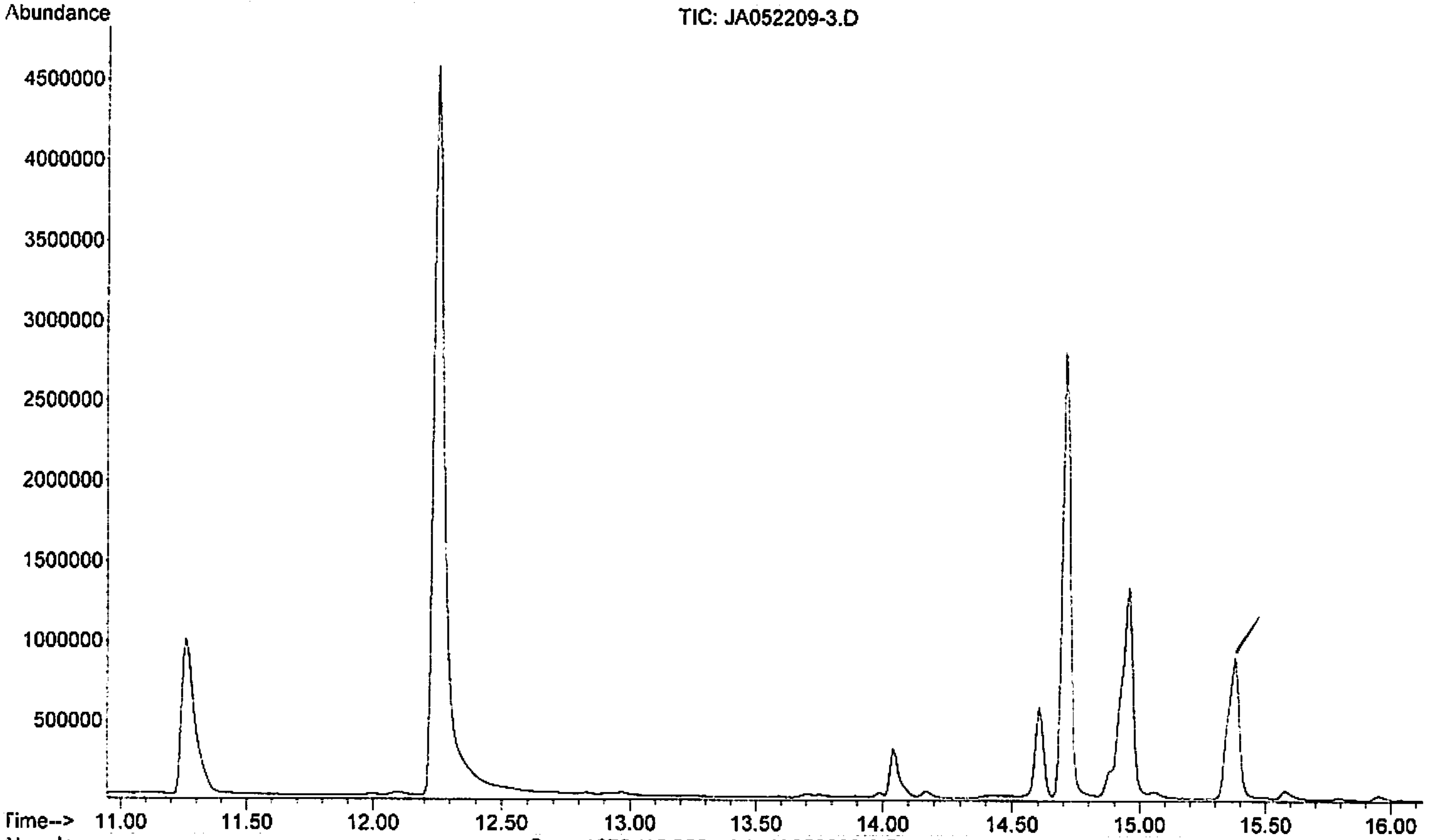

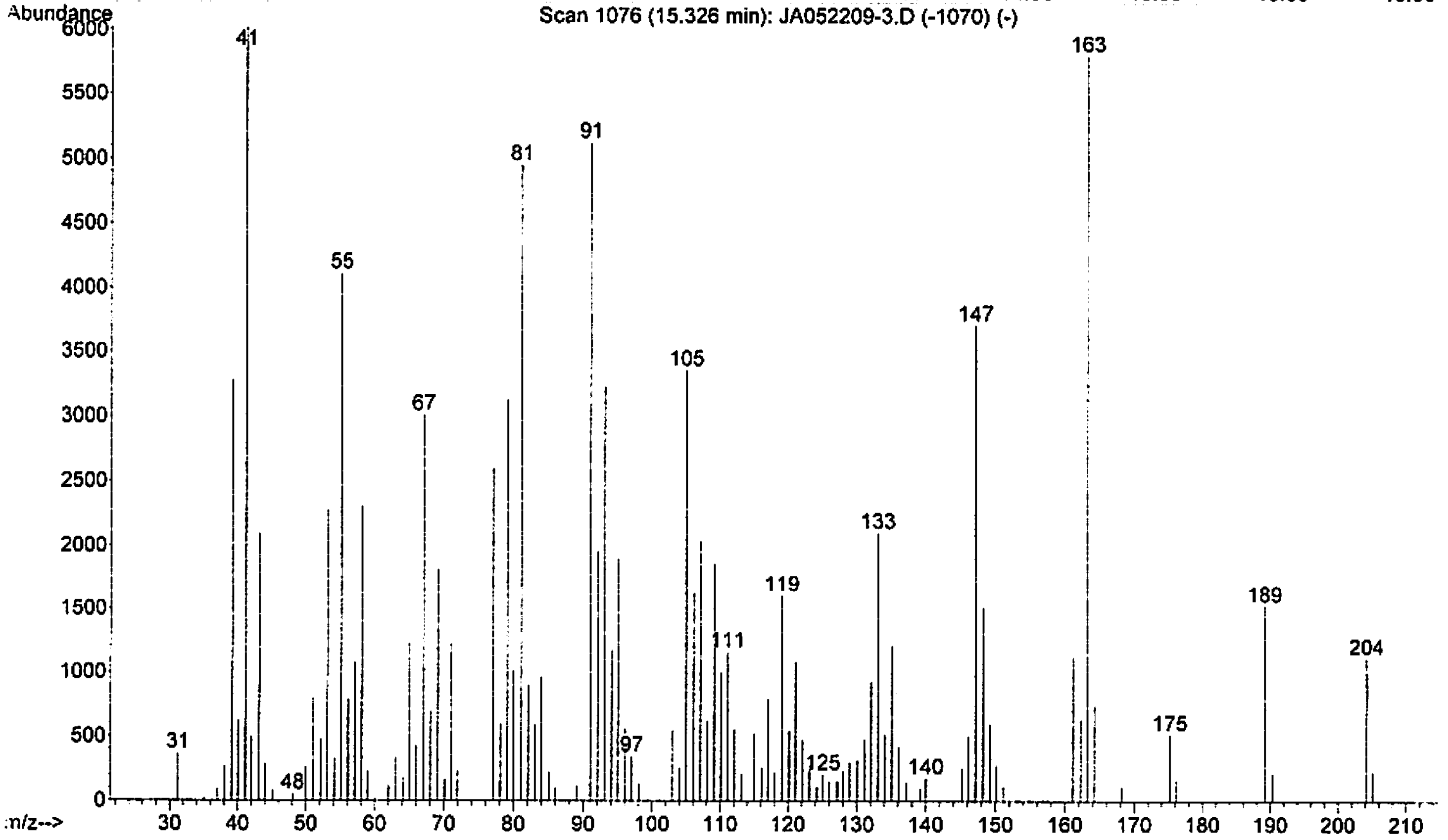

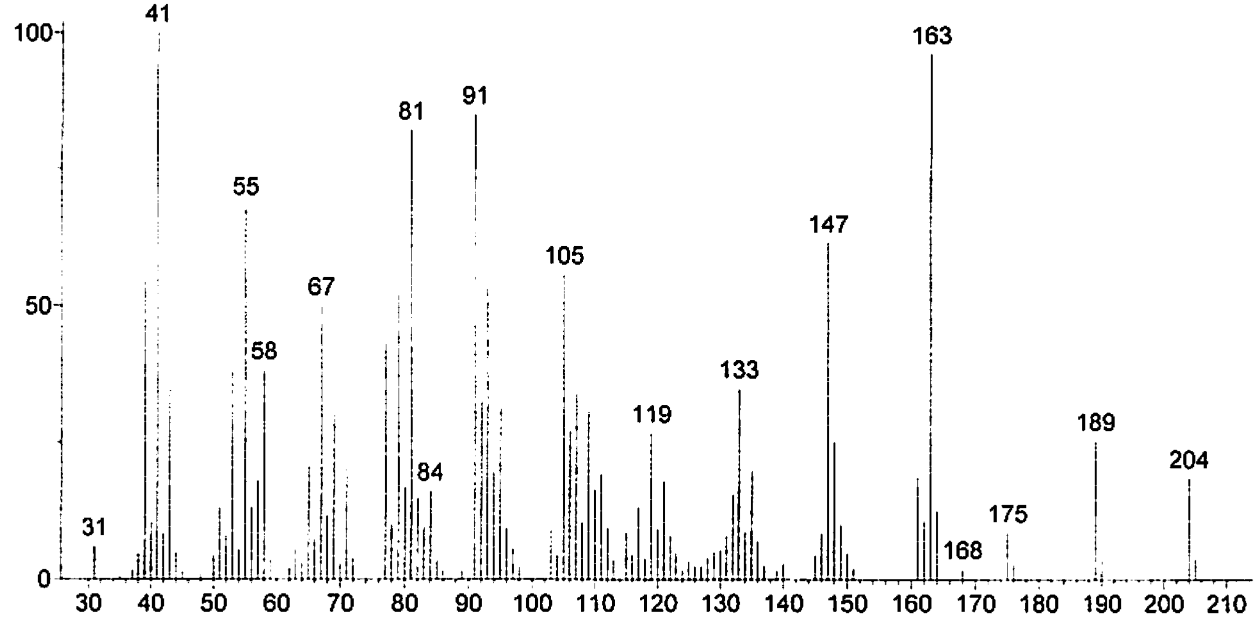

(Text File) Scan 1076 (15.326 min): JA052209-3.D (-1070)

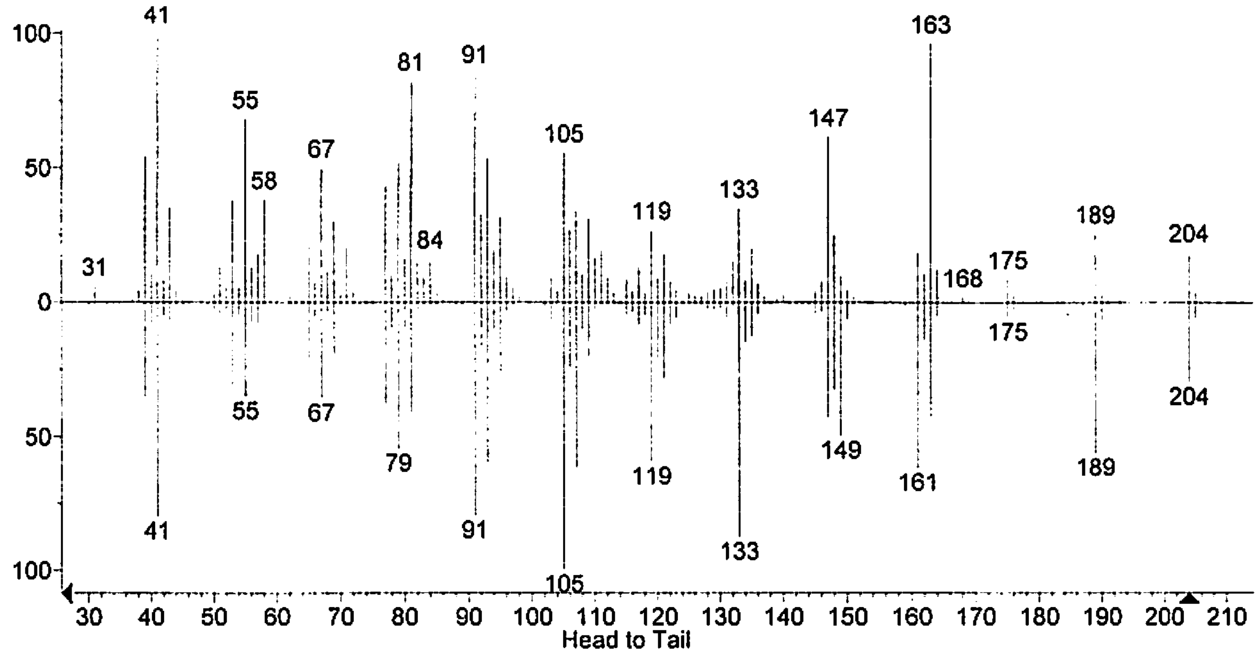

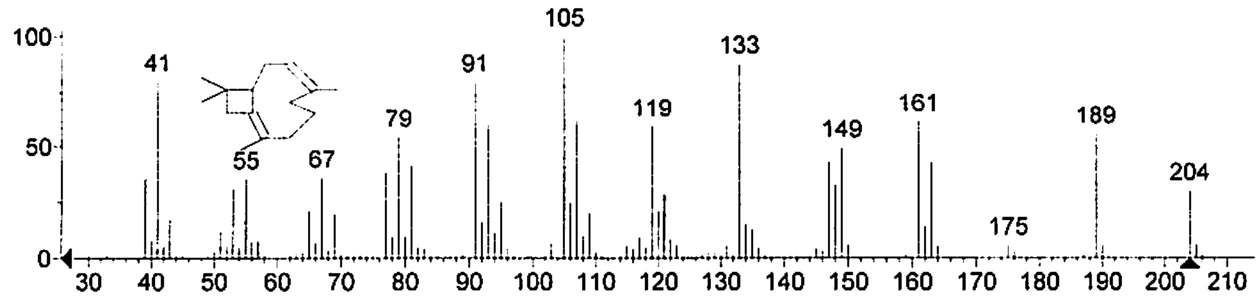

(mainlib) Caryophyllene-(I1)

[illegible]e : D:\DATA\ALDRICH\JA-09\Snapshot\JA052209-3.D
[illegible]perator : Aldrich
[illegible]quired : 22 May 2009 15:55 using AcqMethod JA-WAX08.M
[illegible]strument : Instrument #1
[illegible]mple Name: 1 field-coll. male C.oculata abd./CH2Cl2
[illegible] Info : sweeping vetch 5/22 am; dissected
[illegible] Number: 1

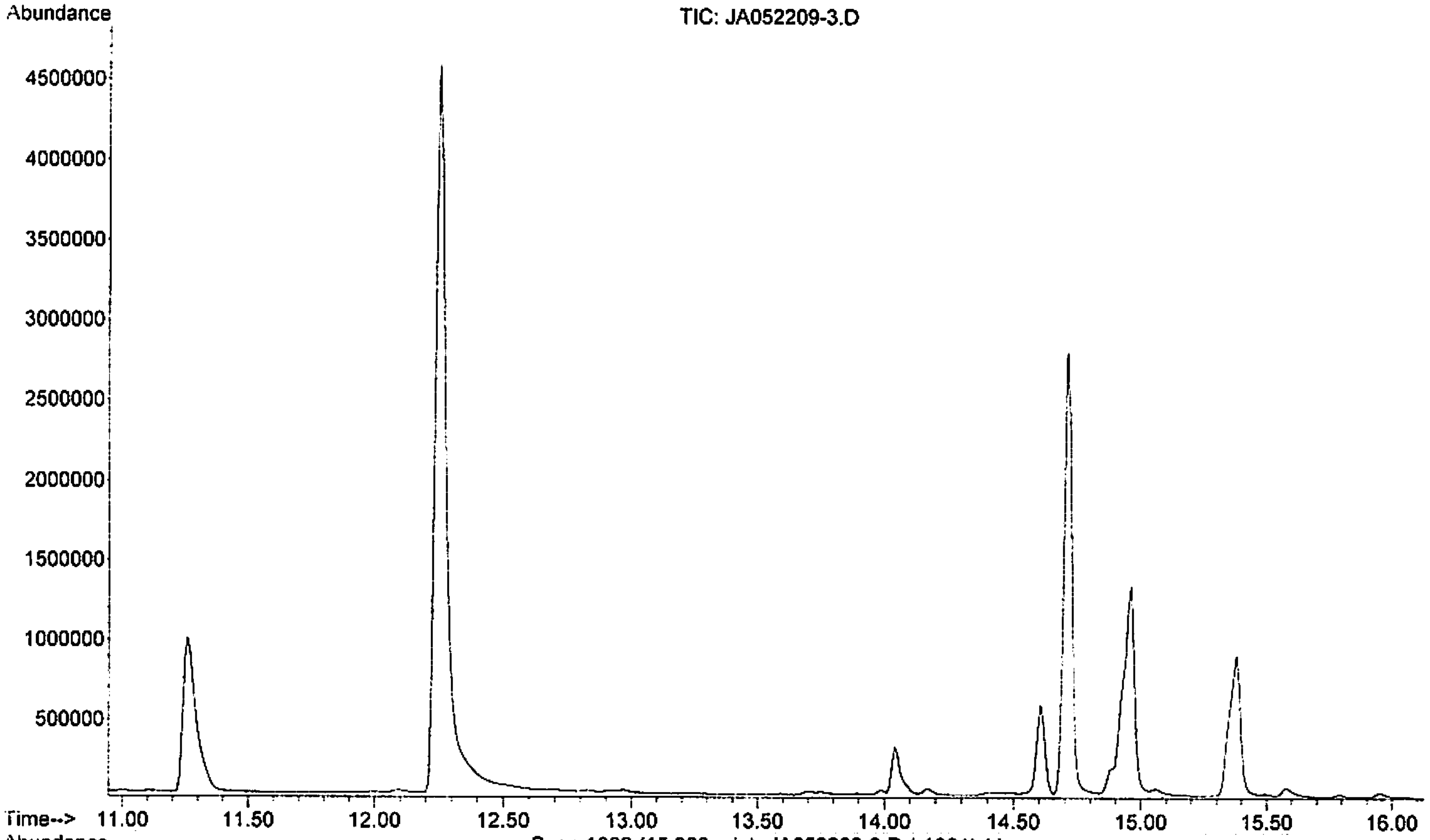

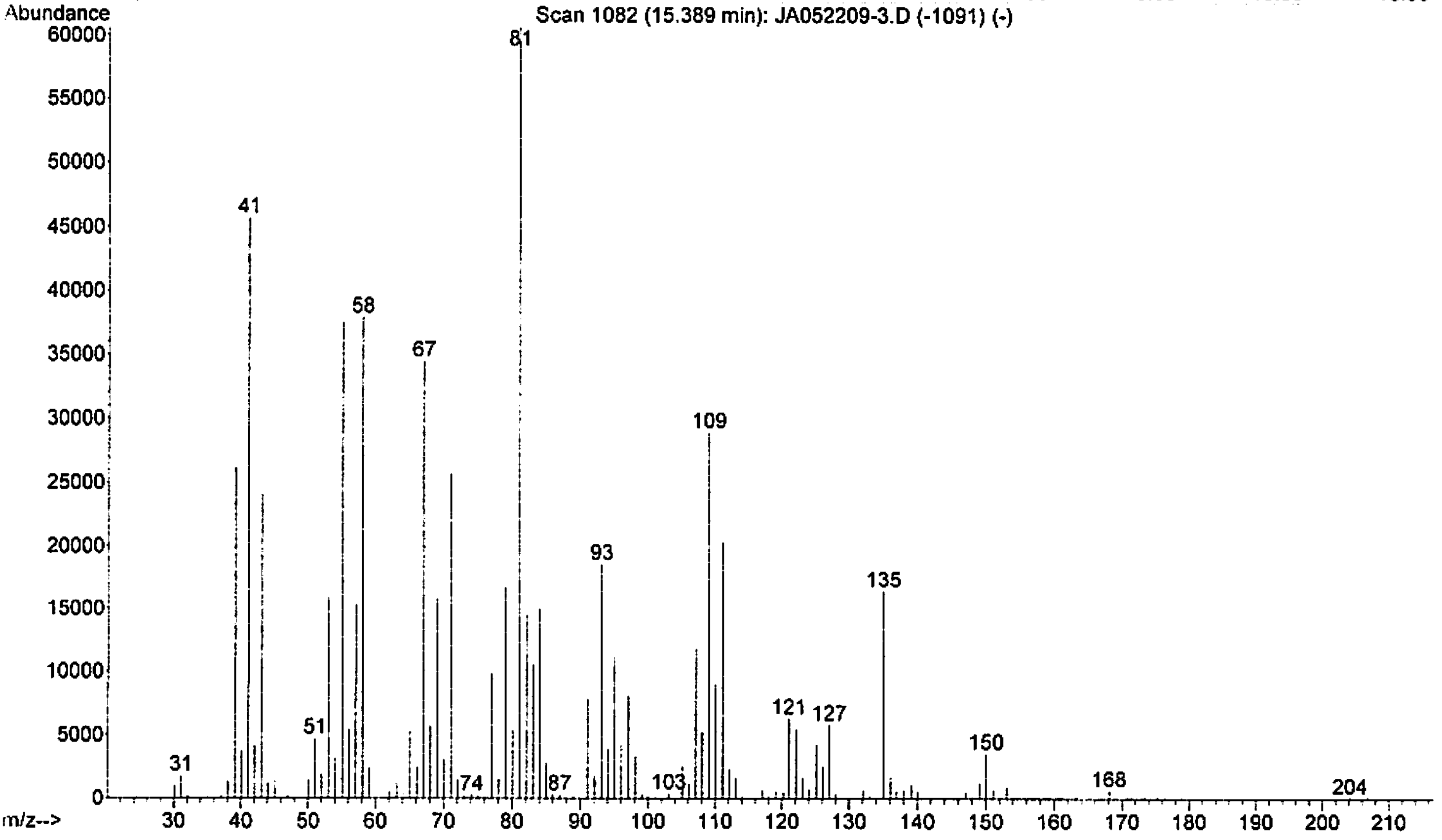

```
[illegible]e        :D:\DATA\ALDRICH\JA-09\Snapshot\JA052209-3.D
[illegible]rator    : Aldrich
[illegible]quired   : 22 May 2009  15:55     using AcqMethod JA-WAX08.M
[illegible]strument :     Instrument #1
[illegible]ple Name: 1 field-coll. male C.oculata abd./CH2Cl2
[illegible] Info    : sweeping vetch 5/22 am; dissected
[illegible]l Number: 1
```

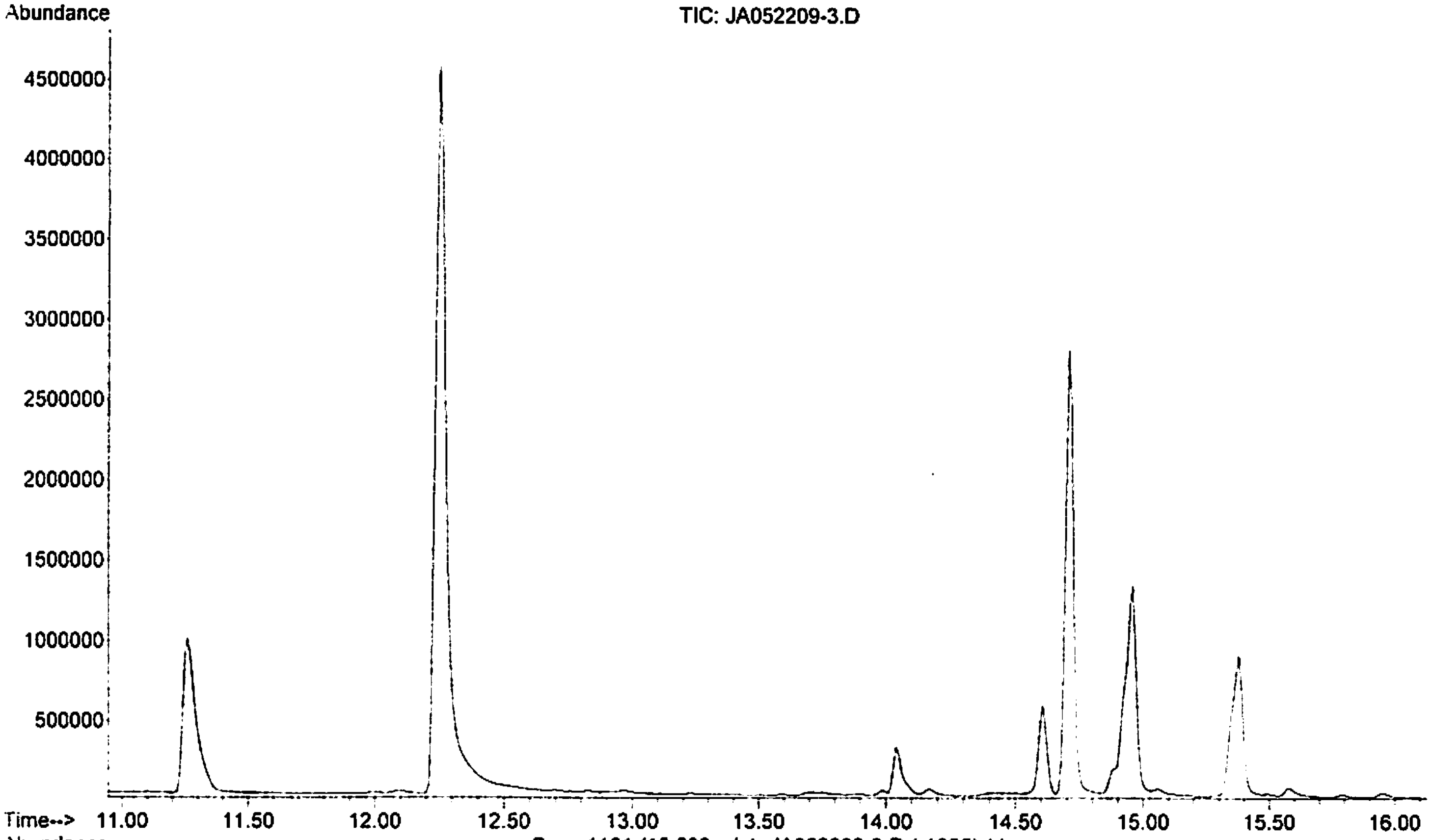

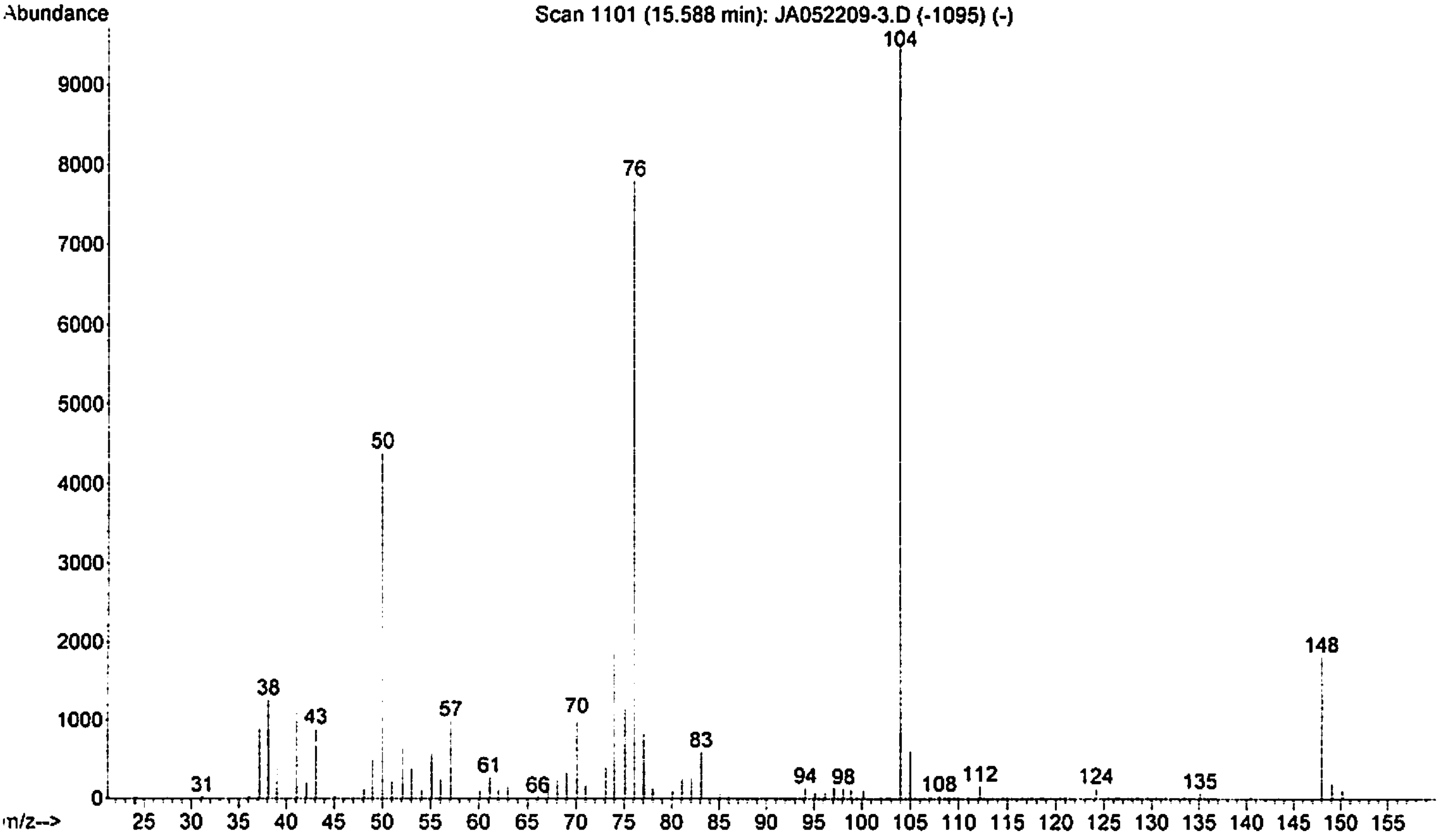

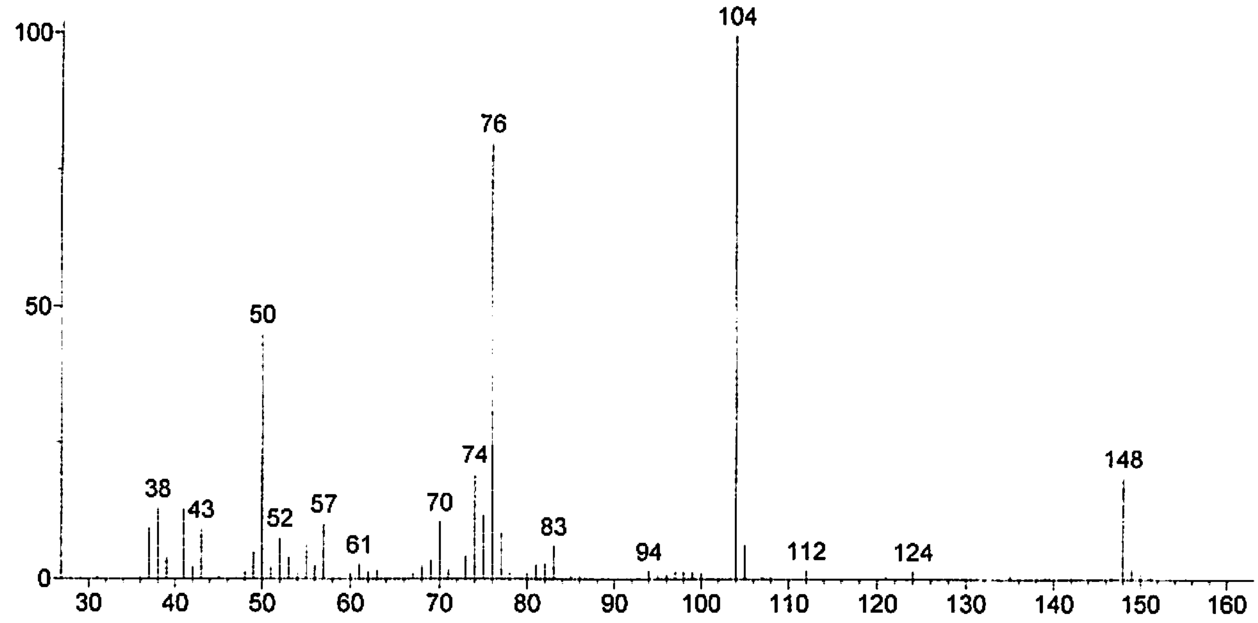

(Text File) Scan 1101 (15.588 min): JA052209-3.D (-1095)

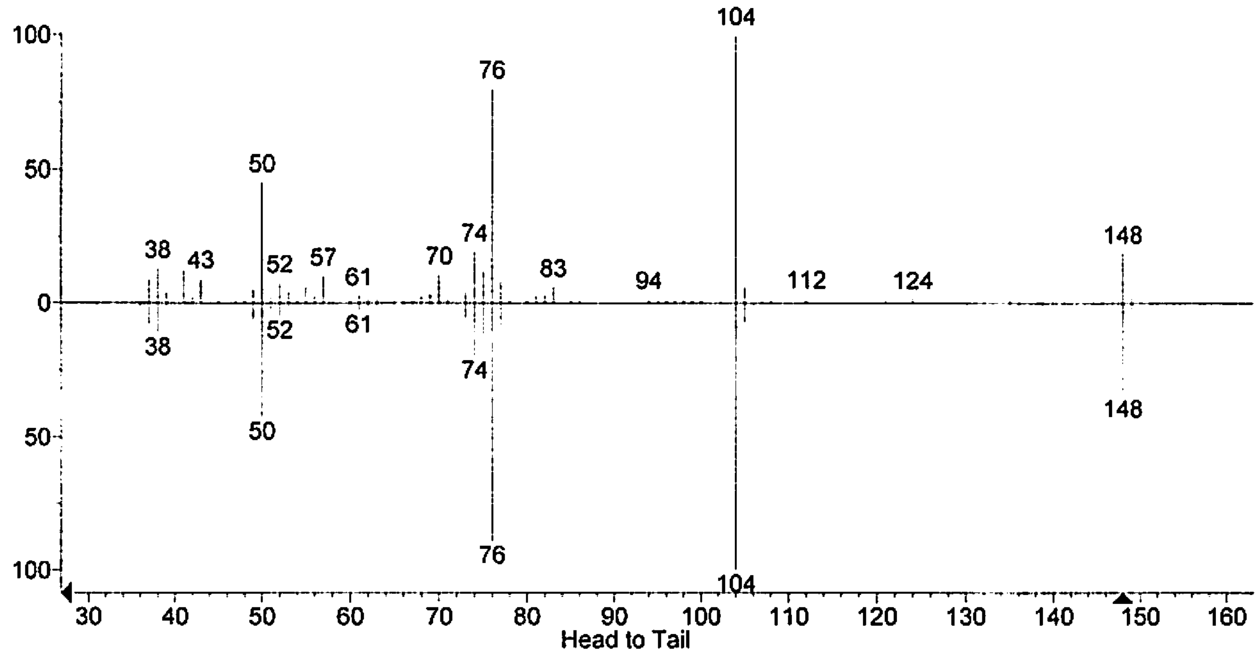

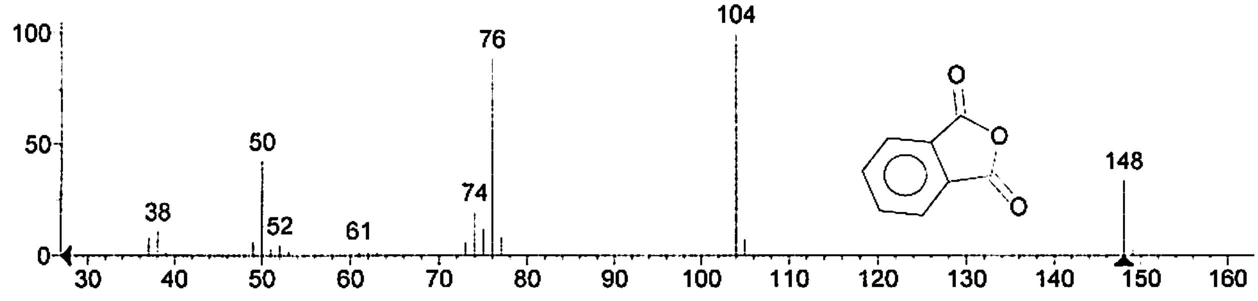

(mainlib) Phthalic anhydride

[illegible]e :D:\DATA\ALDRICH\JA-09\Snapshot\JA052209-3.D
[illegible]erator : Aldrich
[illegible]quired : 22 May 2009 15:55 using AcqMethod JA-WAX08.M
[illegible]strument : Instrument #1
[illegible]ple Name: 1 field-coll. male C.oculata abd./CH2Cl2
[illegible]sc Info : sweeping vetch 5/22 am; dissected
[illegible]al Number: 1

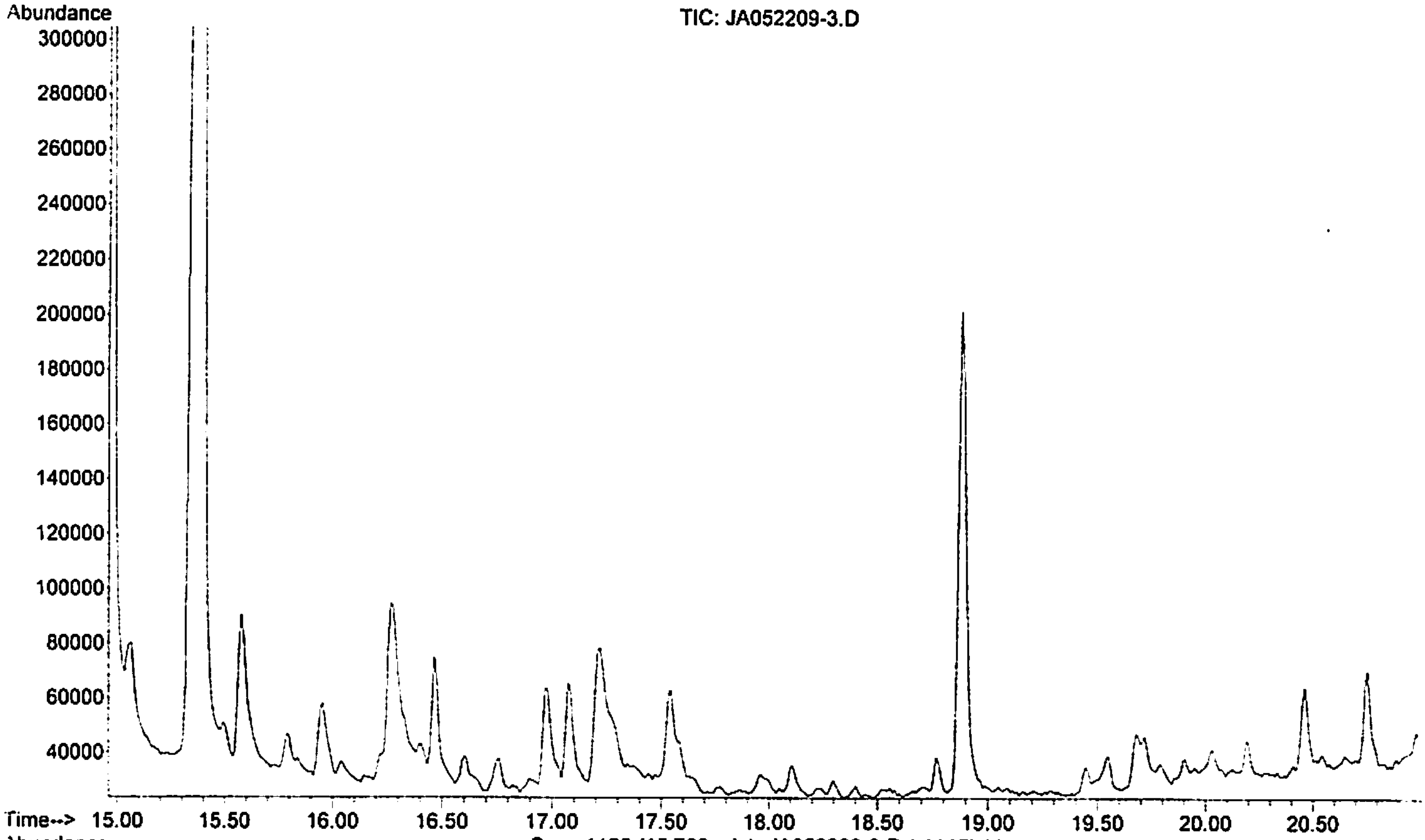

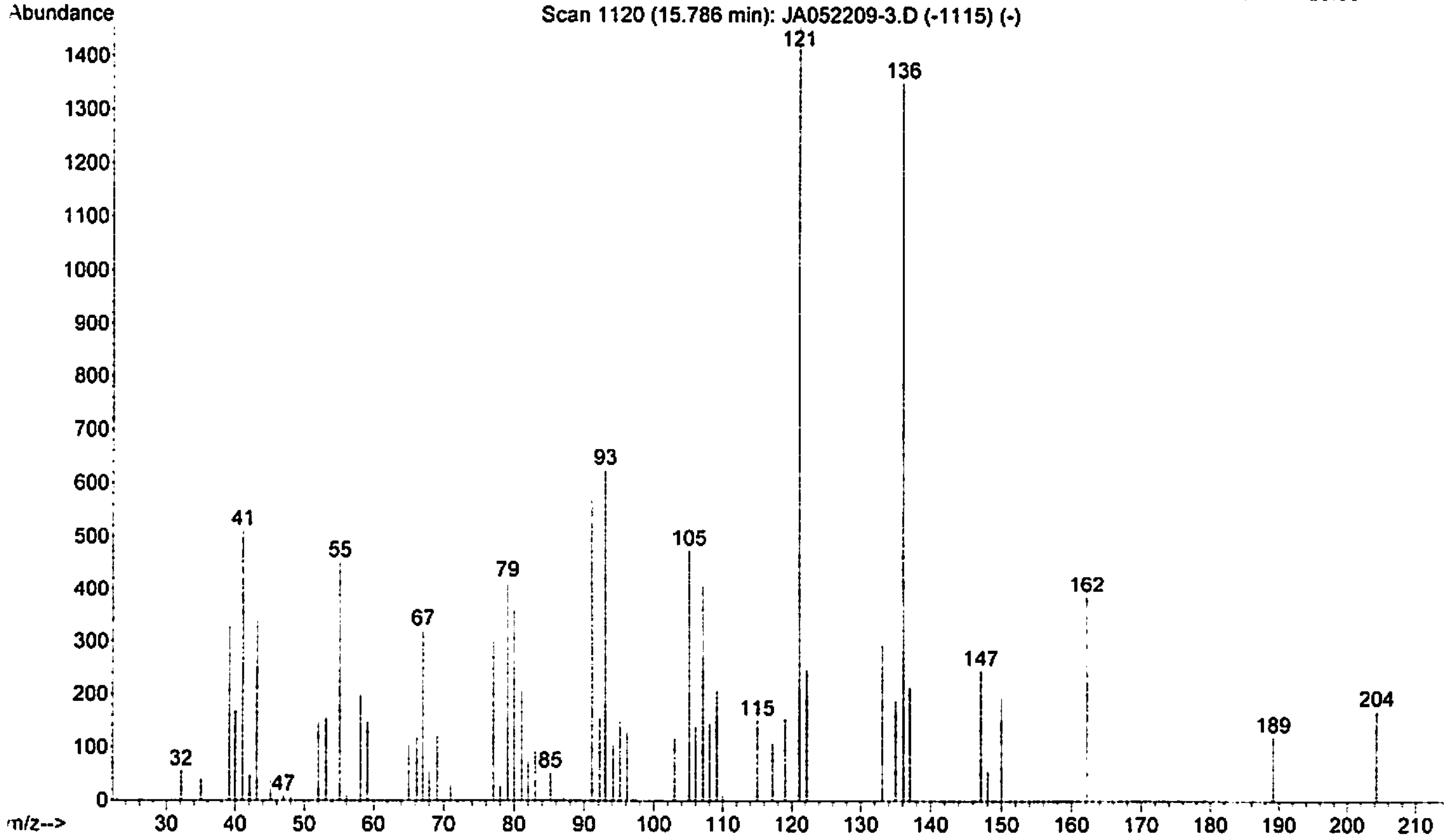

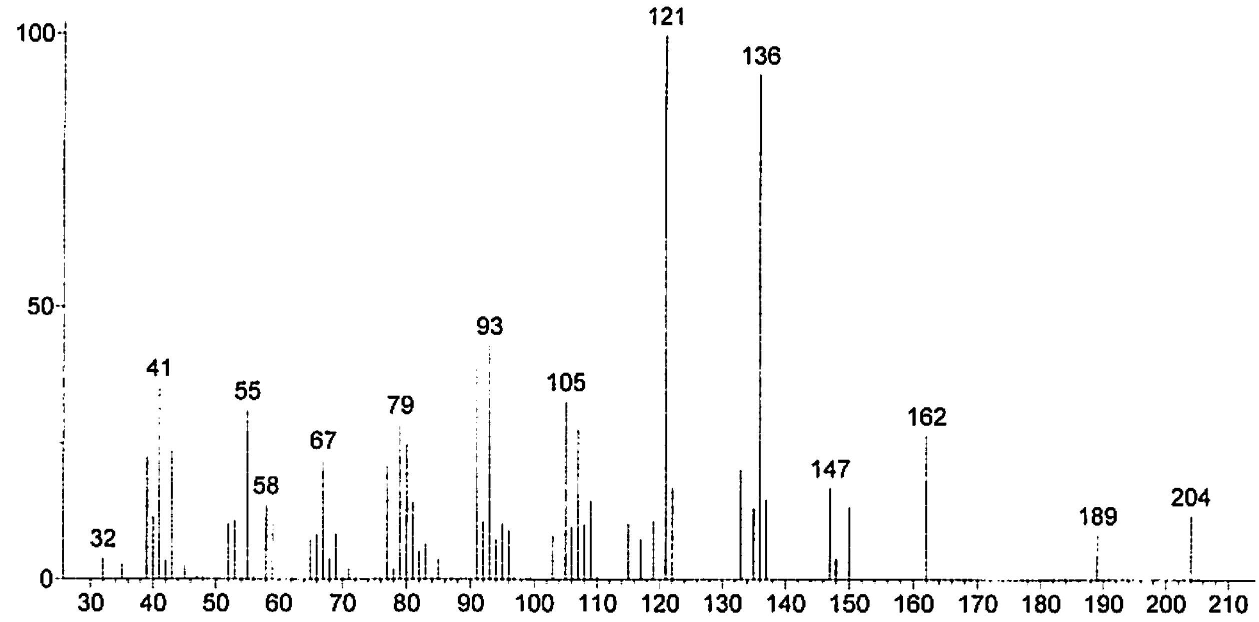

(Text File) Scan 1120 (15.786 min): JA052209-3.D (-1115)

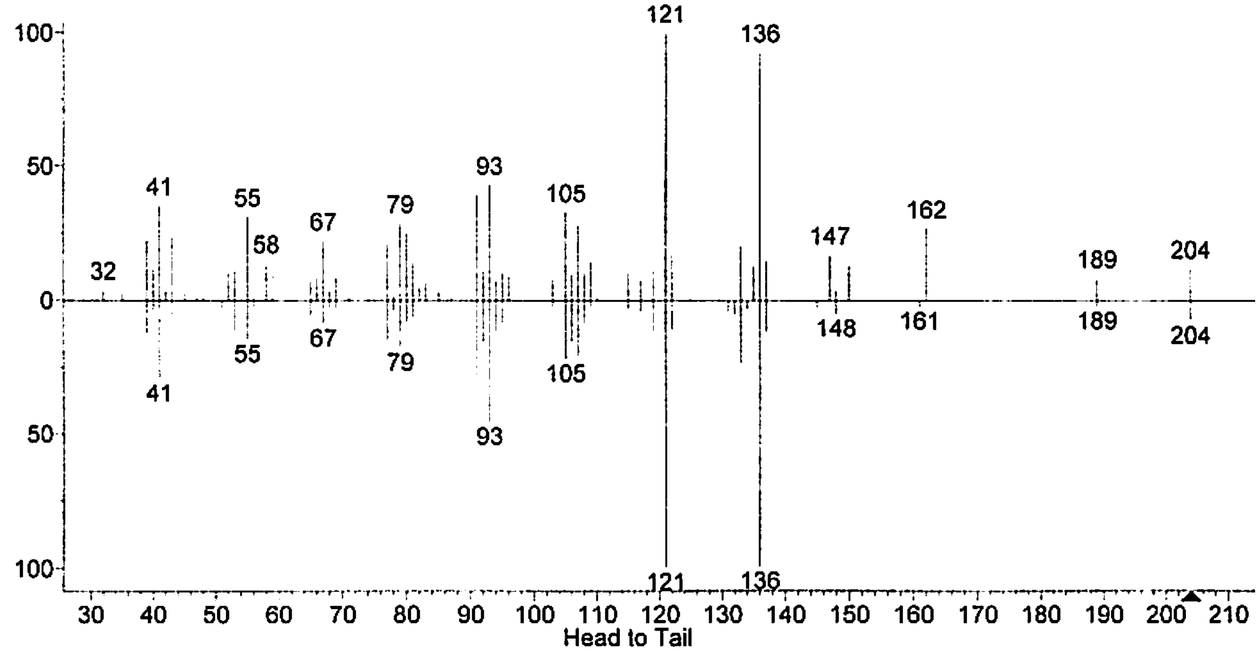

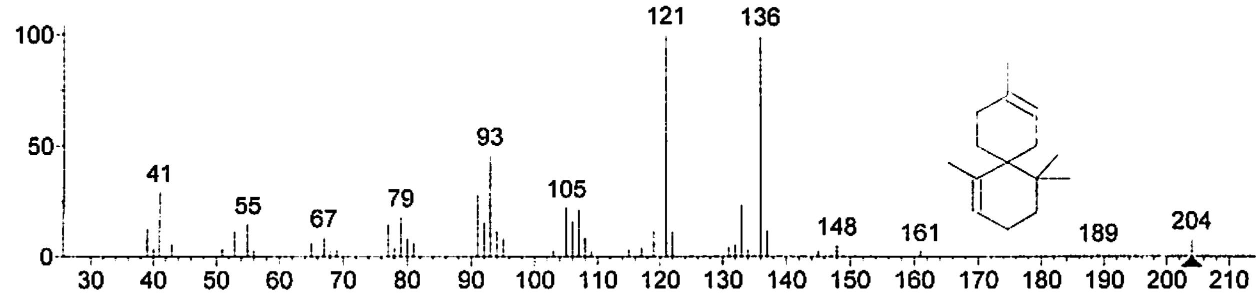

(replib) Spiro[5.5]undeca-1,8-diene, 1,5,5,9-tetramethyl-, (R)-

[illegible]e : D:\DATA\ALDRICH\JA-09\Snapshot\JA052209-3.D
[illegible]rator : Aldrich
[illegible]uired : 22 May 2009 15:55 using AcqMethod JA-WAX08.M
[illegible]trument : Instrument #1
[illegible]ple Name: 1 field-coll. male C.oculata abd./CH2Cl2
[illegible] Info : sweeping vetch 5/22 am; dissected
[illegible] Number: 1

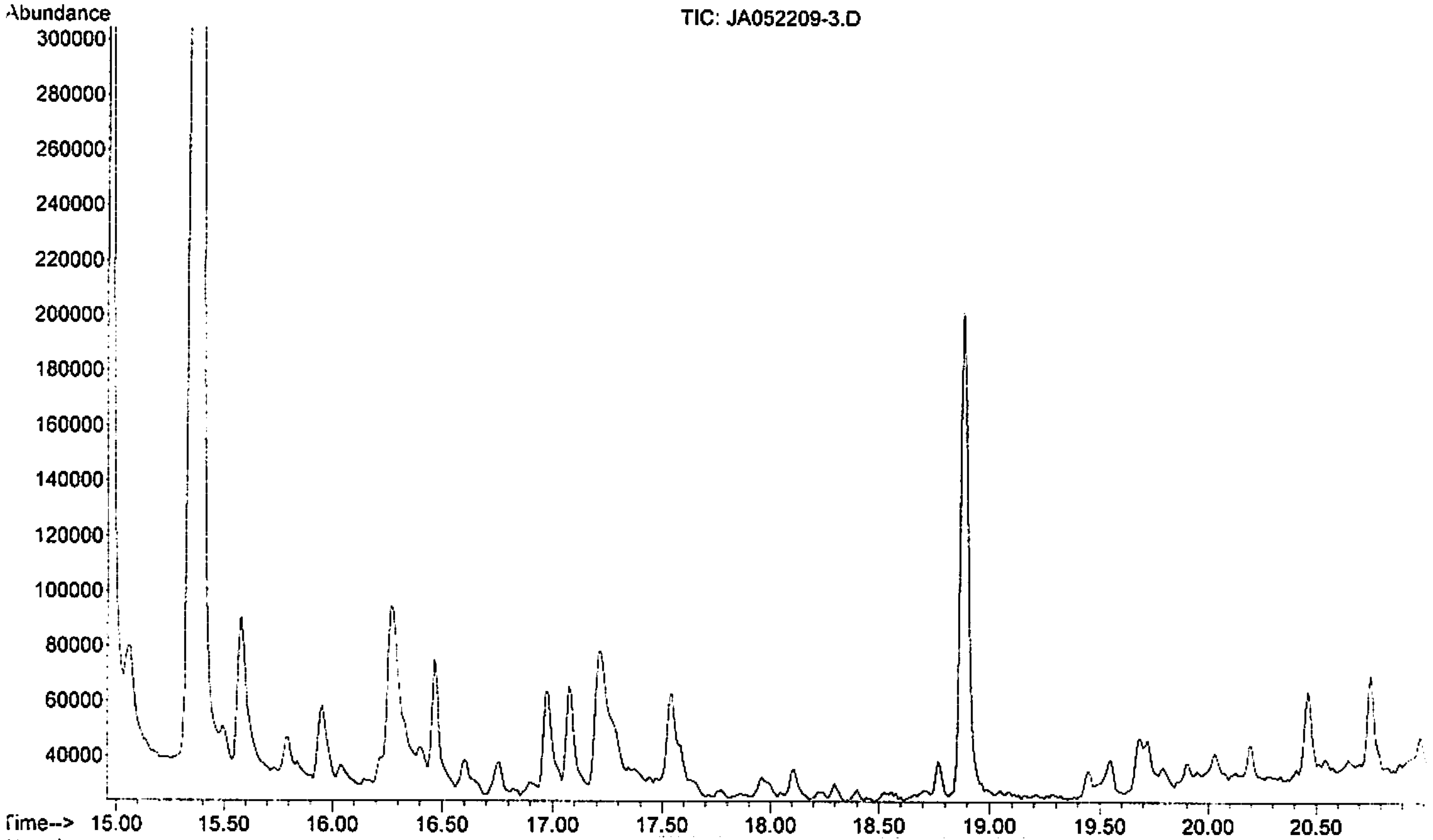

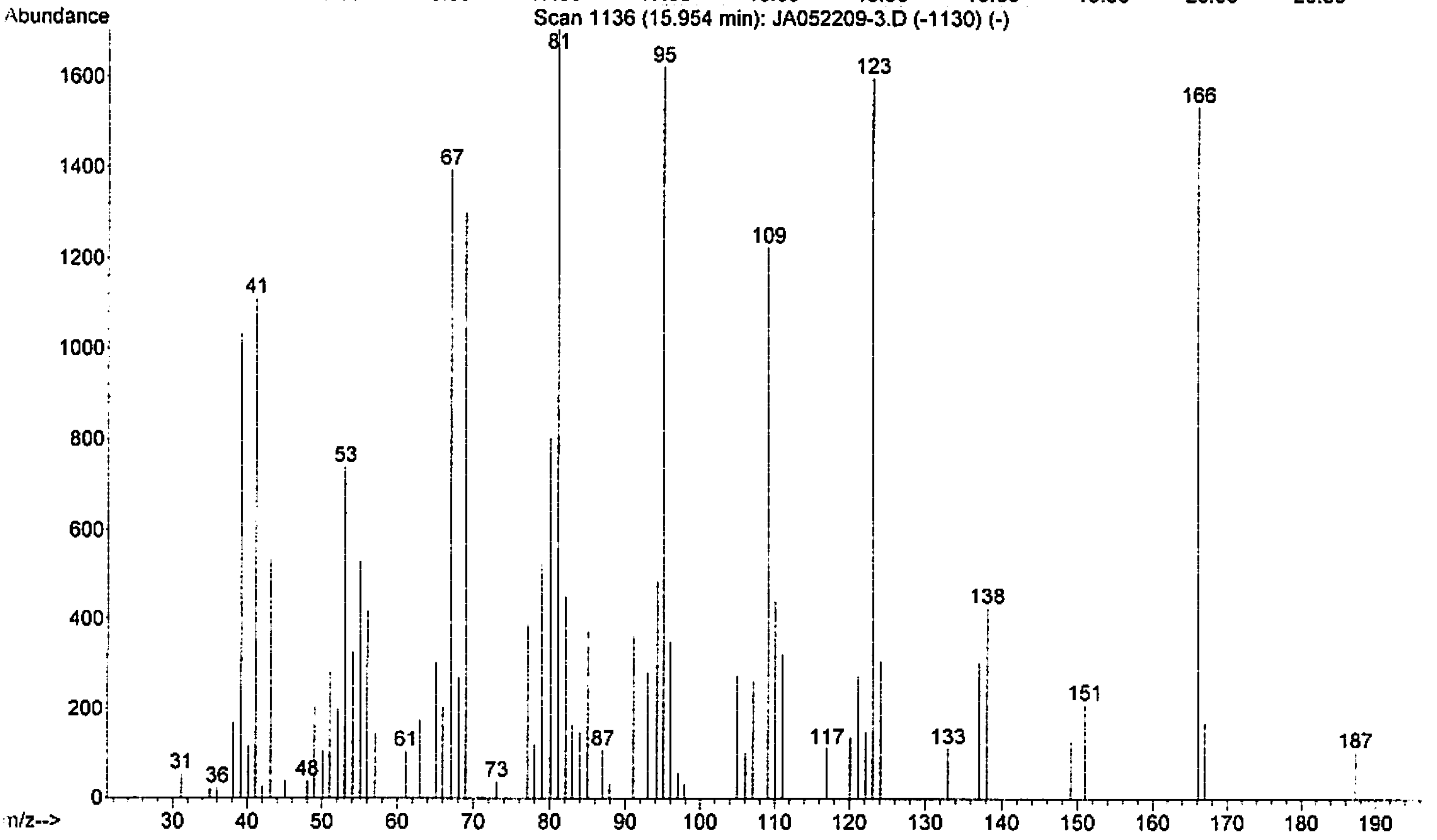

[illegible] :D:\DATA\ALDRICH\JA-09\Snapshot\JA052209-3.D
[illegible]ator : Aldrich
[illegible]ired : 22 May 2009 15:55 using AcqMethod JA-WAX08.M
[illegible]rument : Instrument #1
[illegible]le Name: 1 field-coll. male C.oculata abd./CH2Cl2
[illegible] Info : sweeping vetch 5/22 am; dissected
[illegible] Number: 1

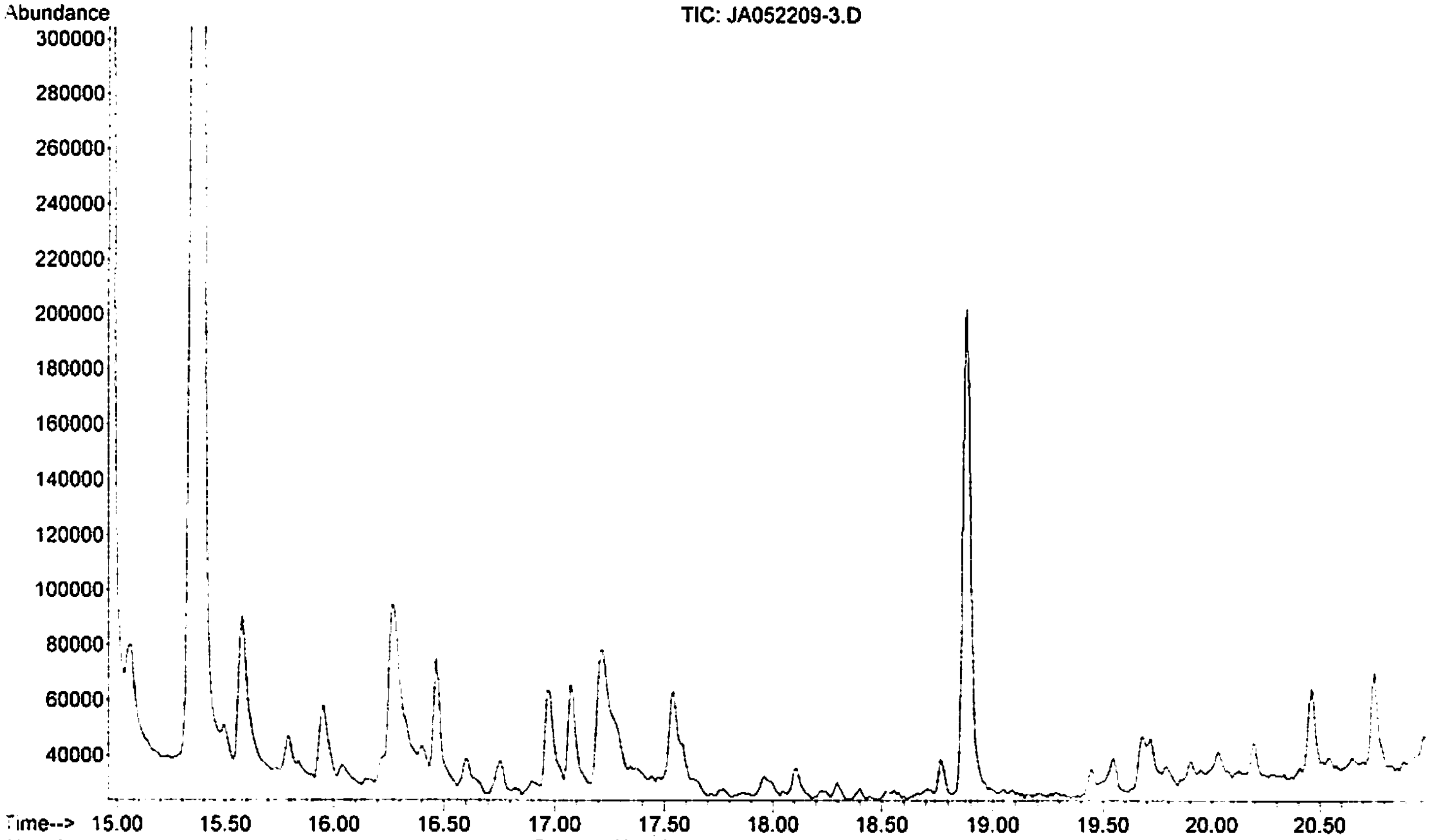

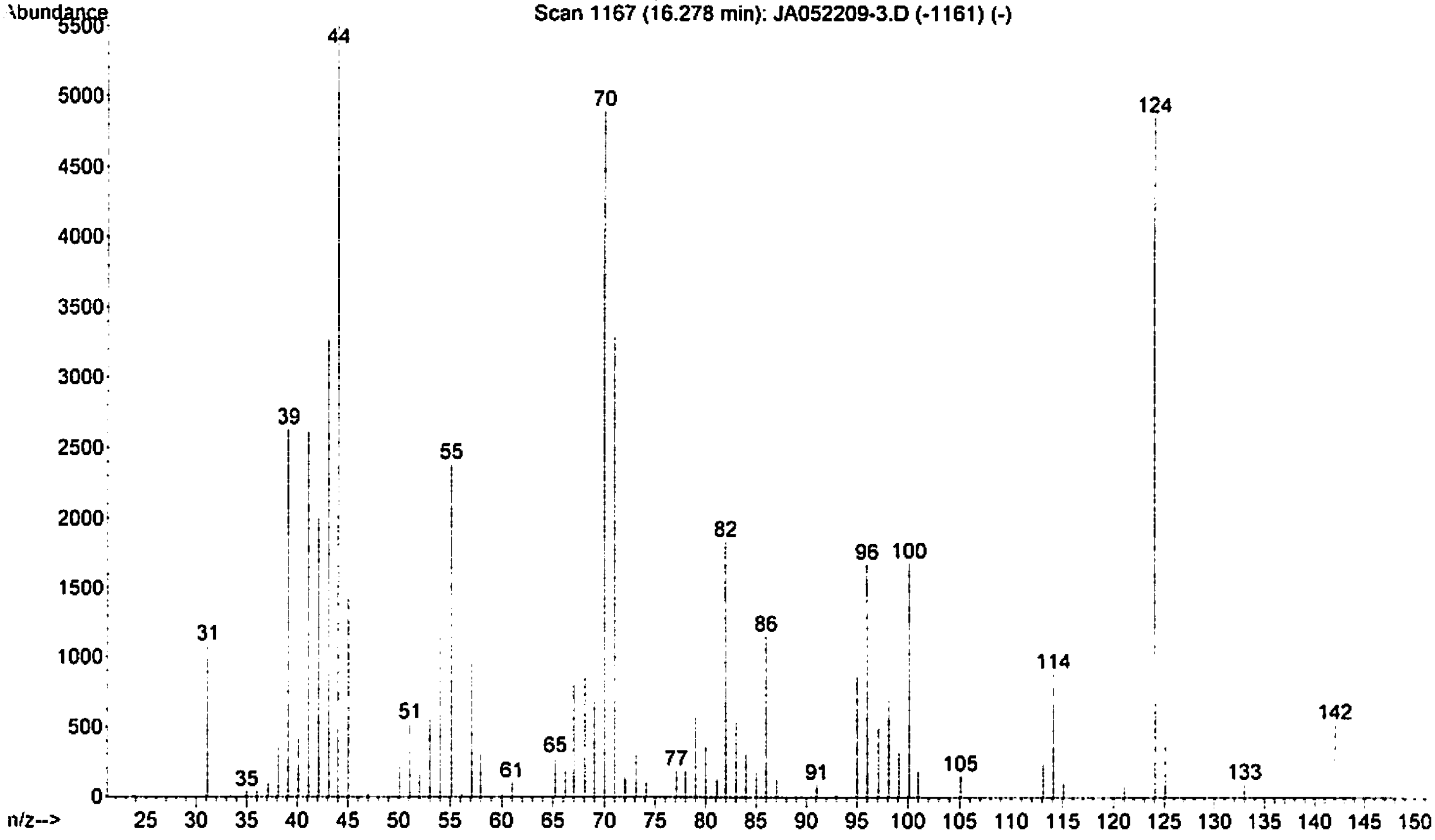

: D:\DATA\ALDRICH\JA-09\Snapshot\JA052209-3.D
[illegible]ator : Aldrich
[illegible]uired : 22 May 2009 15:55 using AcqMethod JA-WAX08.M
[illegible]rument : Instrument #1
[illegible]ple Name: 1 field-coll. male C.oculata abd./CH2C12
[illegible] Info : sweeping vetch 5/22 am; dissected
[illegible]l Number: 1

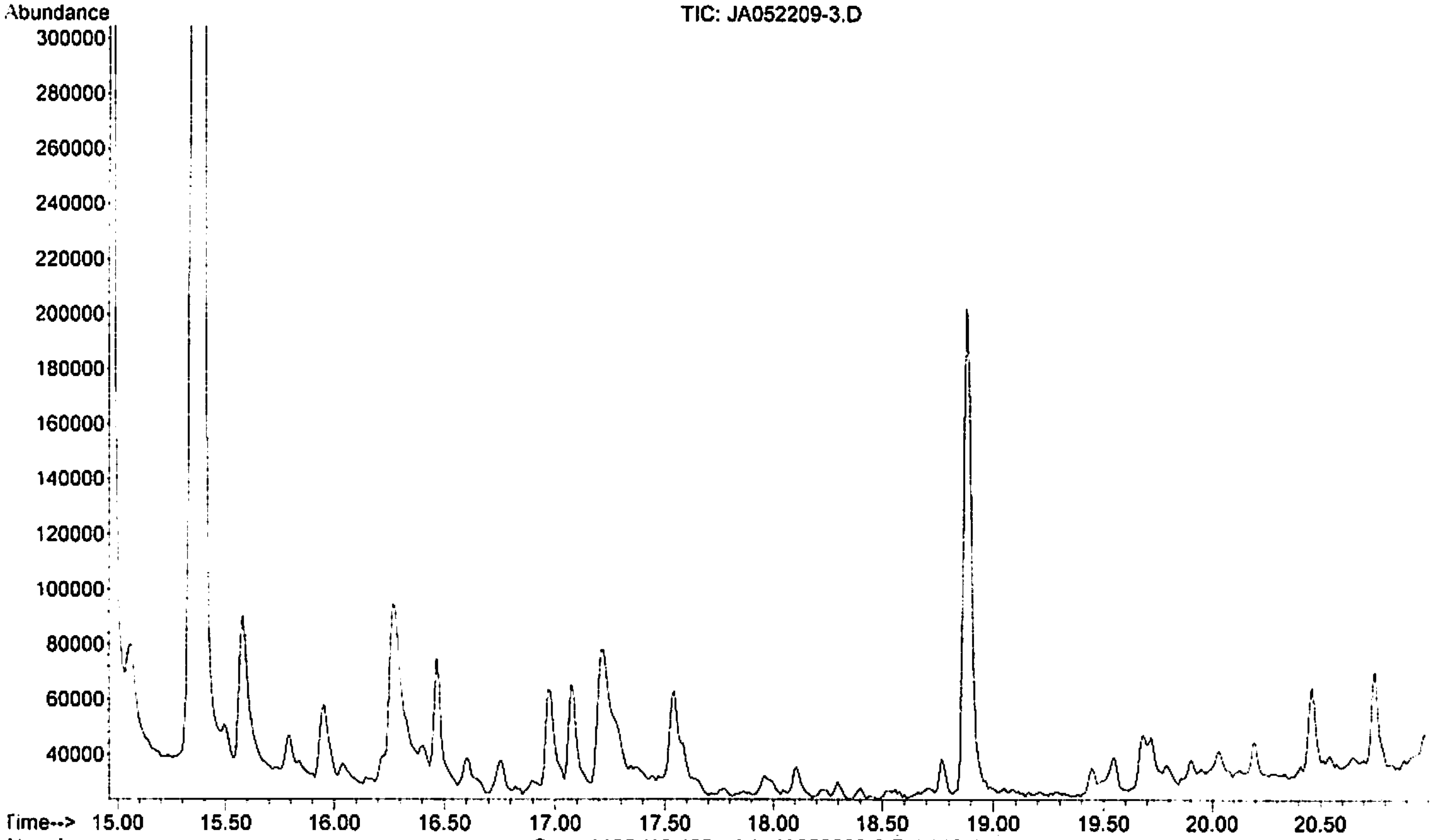

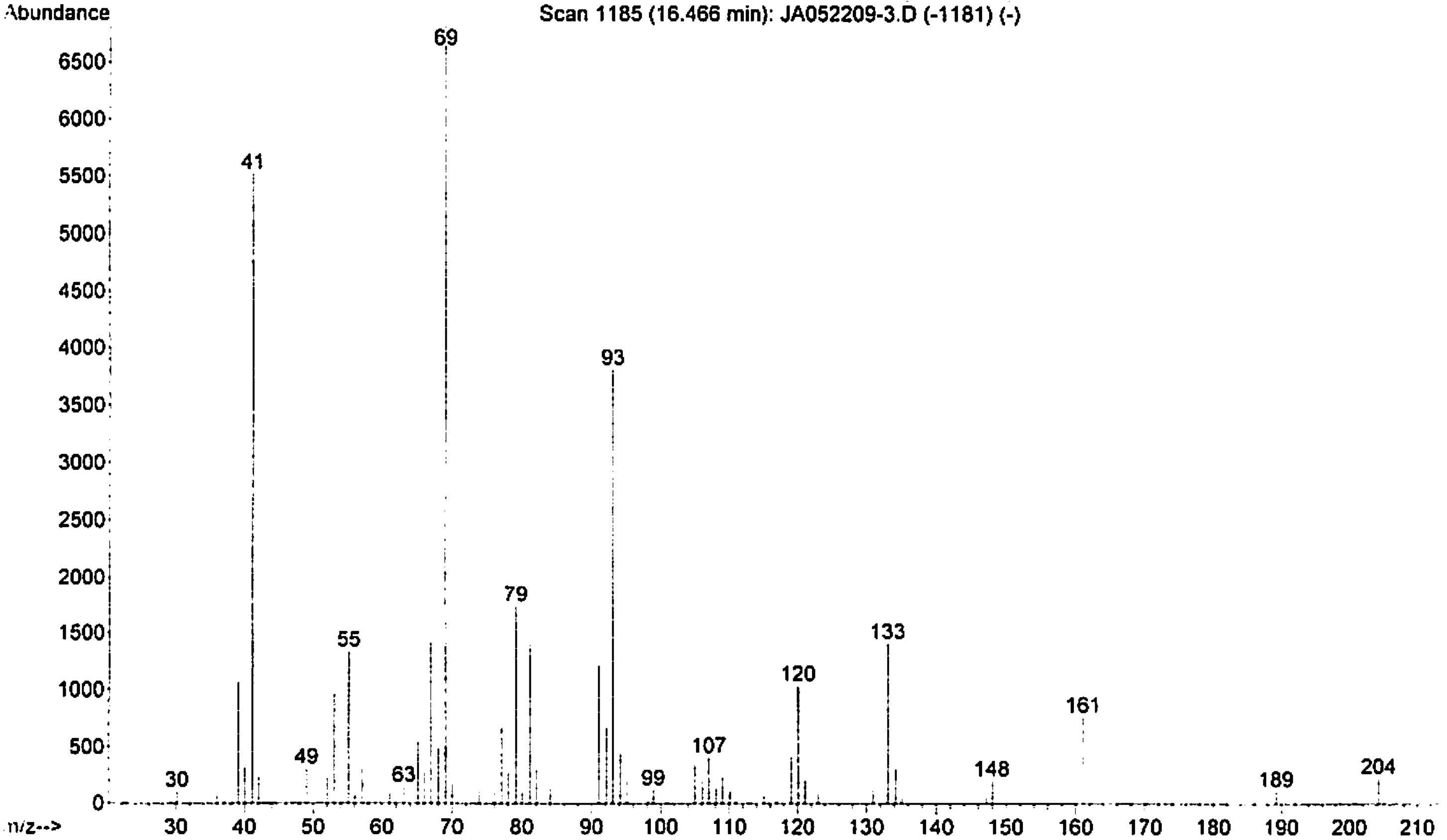

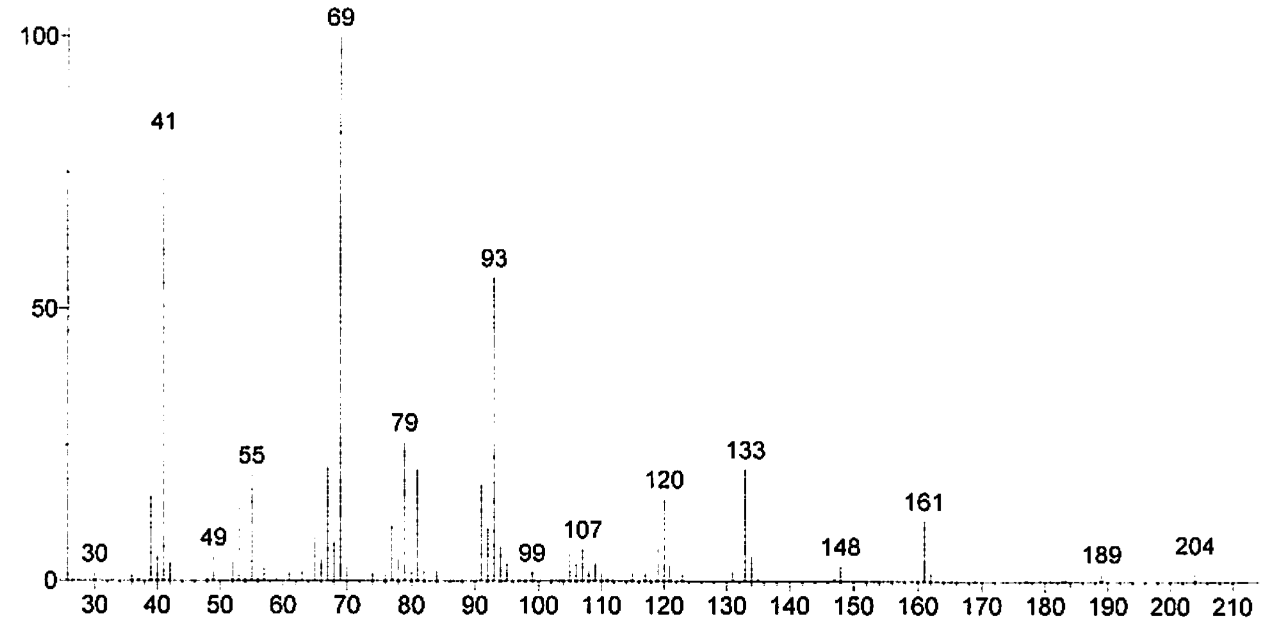

(Text File) Scan 1185 (16.466 min): JA052209-3.D (-1181)

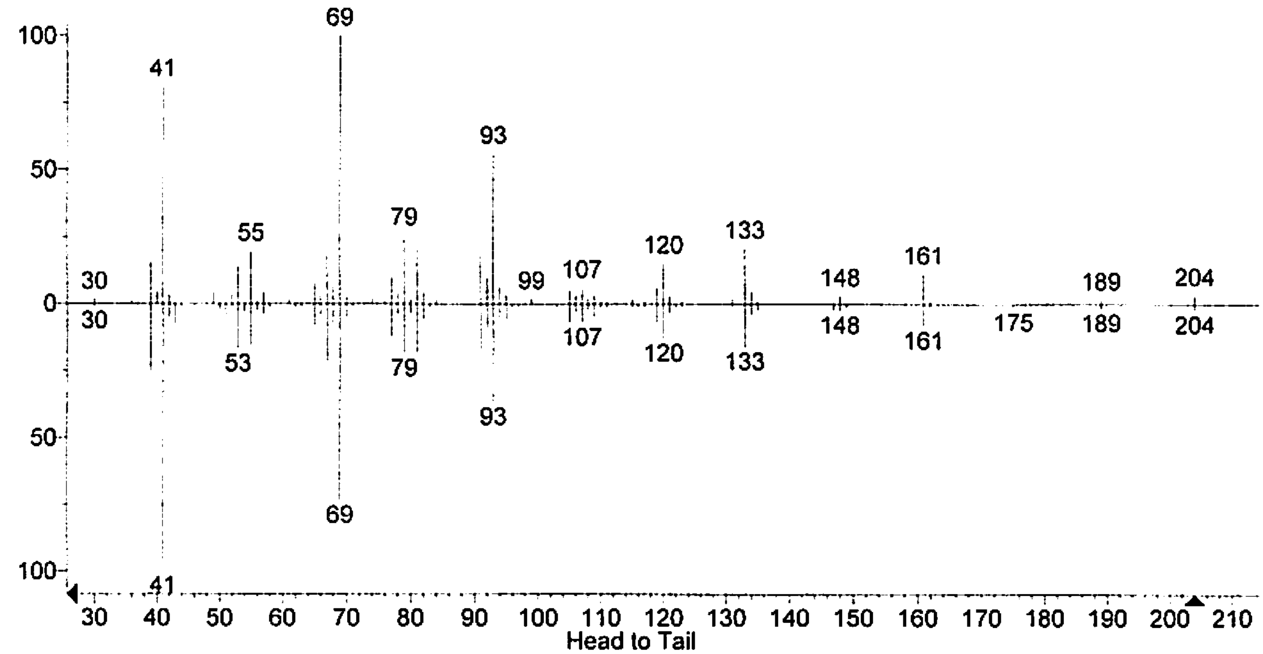

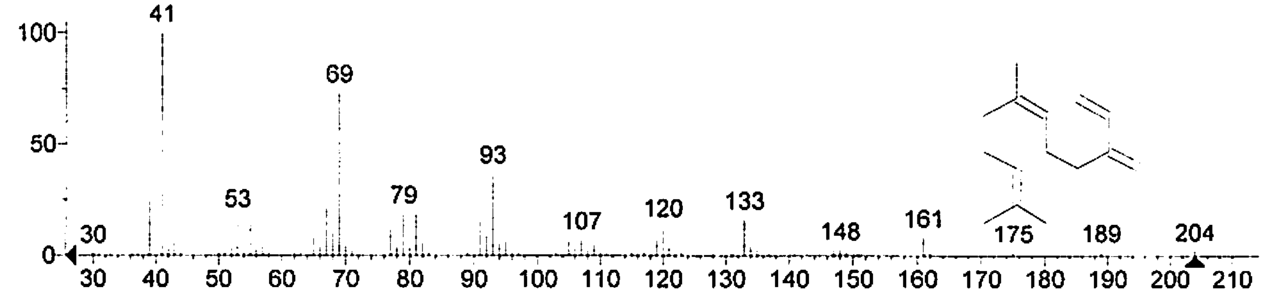

(replib) 1,6,10-Dodecatriene, 7,11-dimethyl-3-methylene-, (Z)-

[illegible] :D:\DATA\ALDRICH\JA-09\Snapshot\JA052209-3.D
[illegible]rator : Aldrich
[illegible]ired : 22 May 2009 15:55 using AcqMethod JA-WAX08.M
[illegible]rument : Instrument #1
[illegible]le Name: 1 field-coll. male C.oculata abd./CH2Cl2
[illegible] Info : sweeping vetch 5/22 am; dissected
[illegible] Number: 1

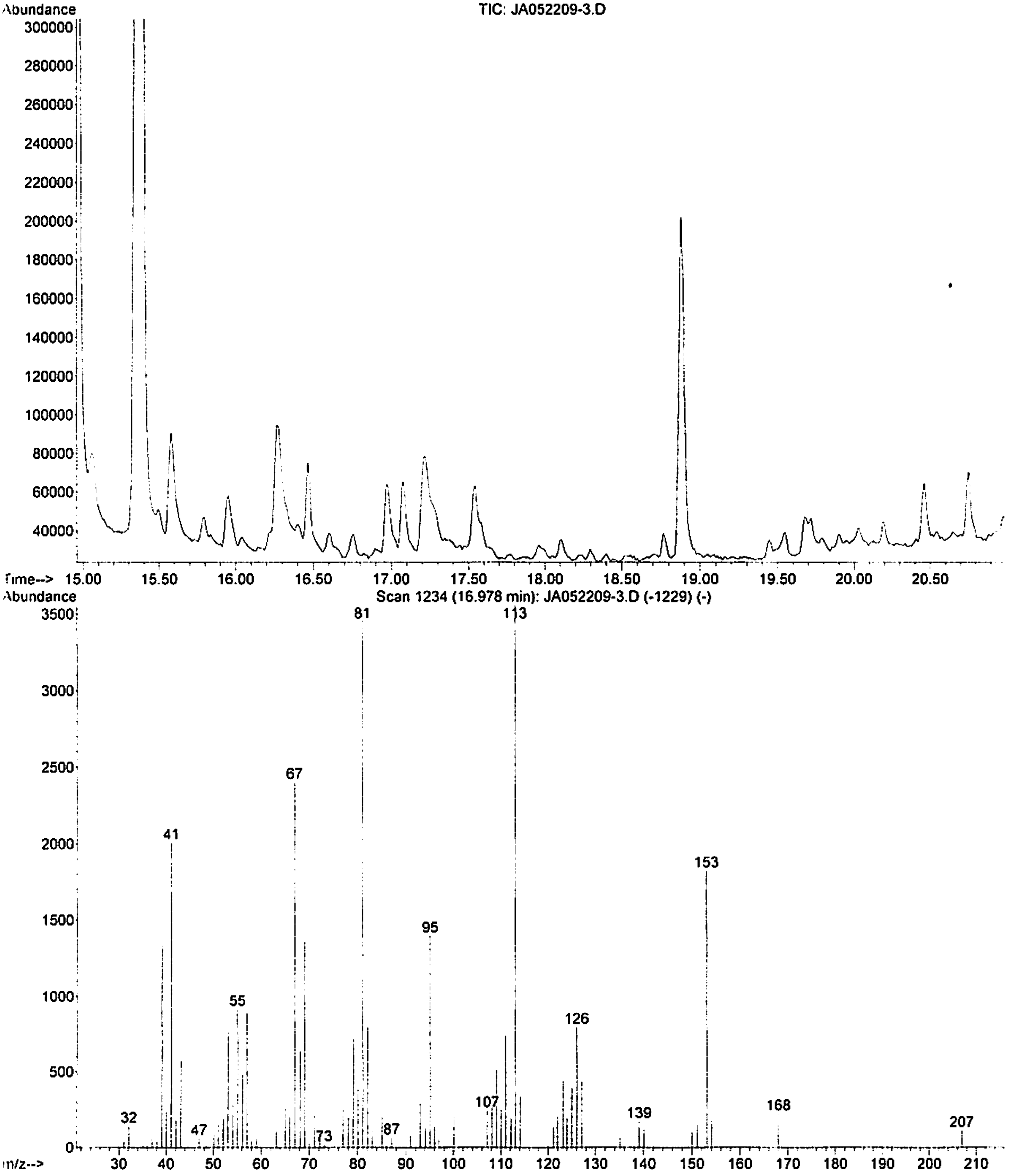

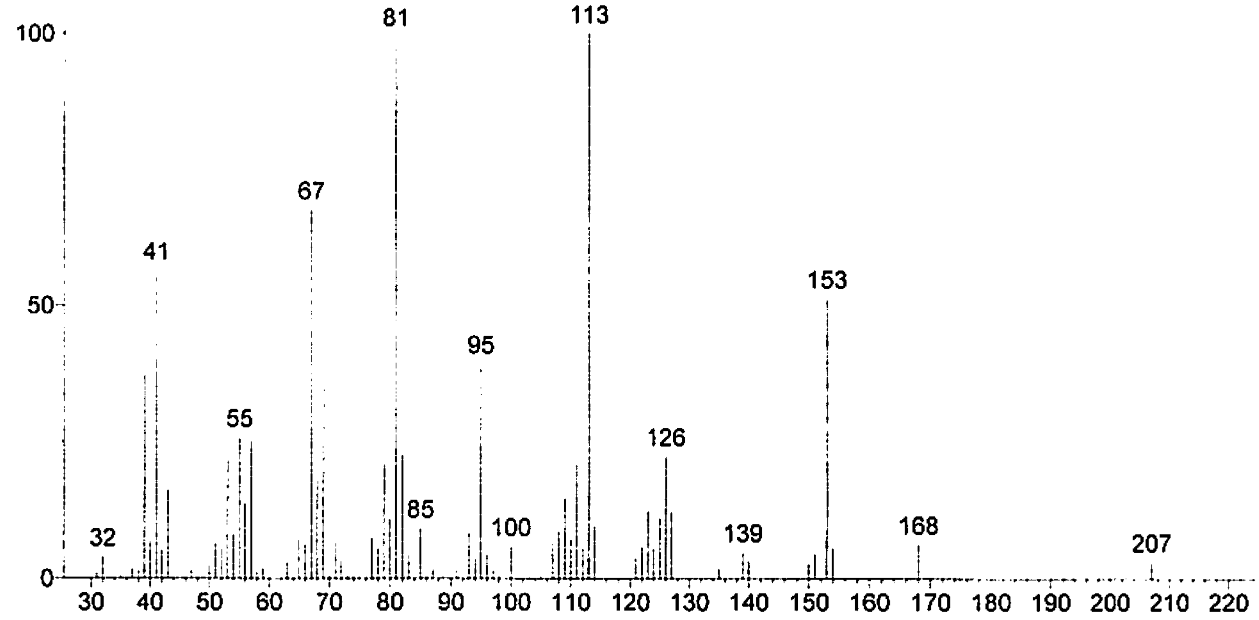

(Text File) Scan 1234 (16.978 min): JA052209-3.D (-1229)

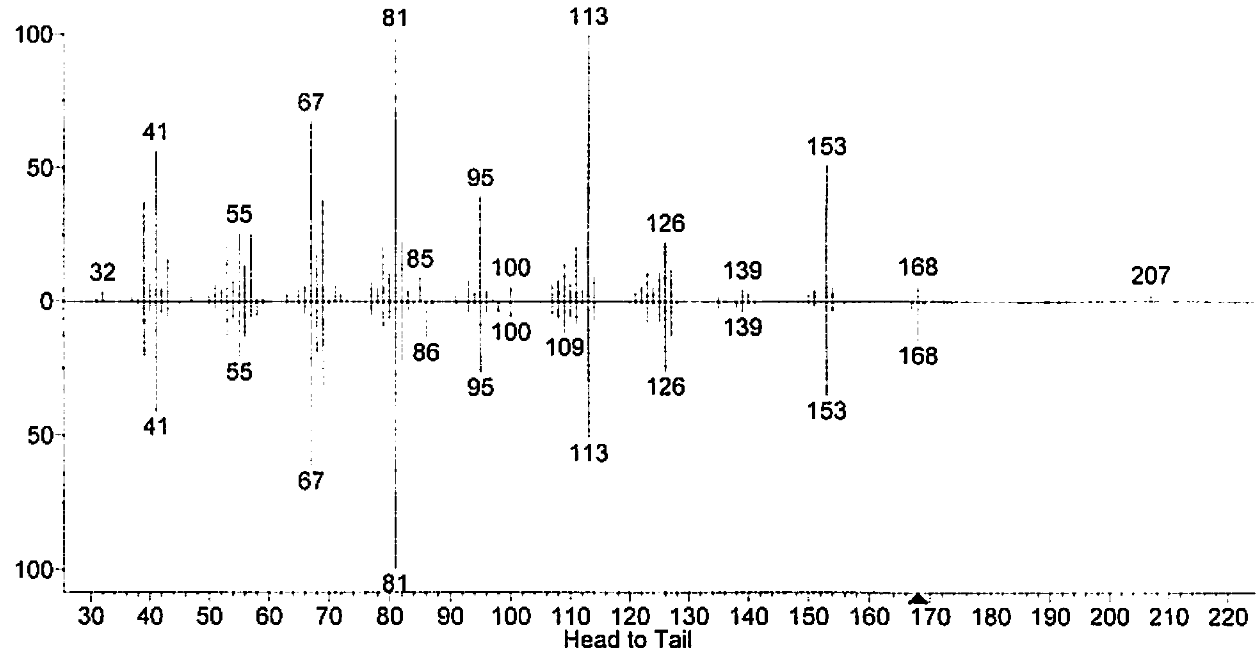

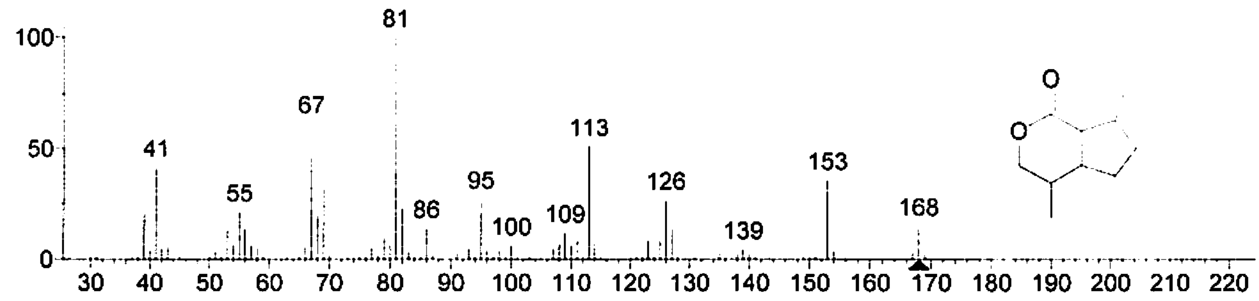

(mainlib) Cyclopenta[c]pyran-1(3H)-one, hexahydro-4,7-dimethyl-, (4.alpha.,4a.alpha.,7.alpha.,7a.alpha.)-

[illegible] :D:\DATA\ALDRICH\JA-09\Snapshot\JA052209-3.D
[illegible]ator : Aldrich
[illegible]ired : 22 May 2009 15:55 using AcqMethod JA-WAX08.M
[illegible]rument : Instrument #1
[illegible]ple Name: 1 field-coll. male C.oculata abd./CH2Cl2
[illegible] Info : sweeping vetch 5/22 am; dissected
[illegible]l Number: 1

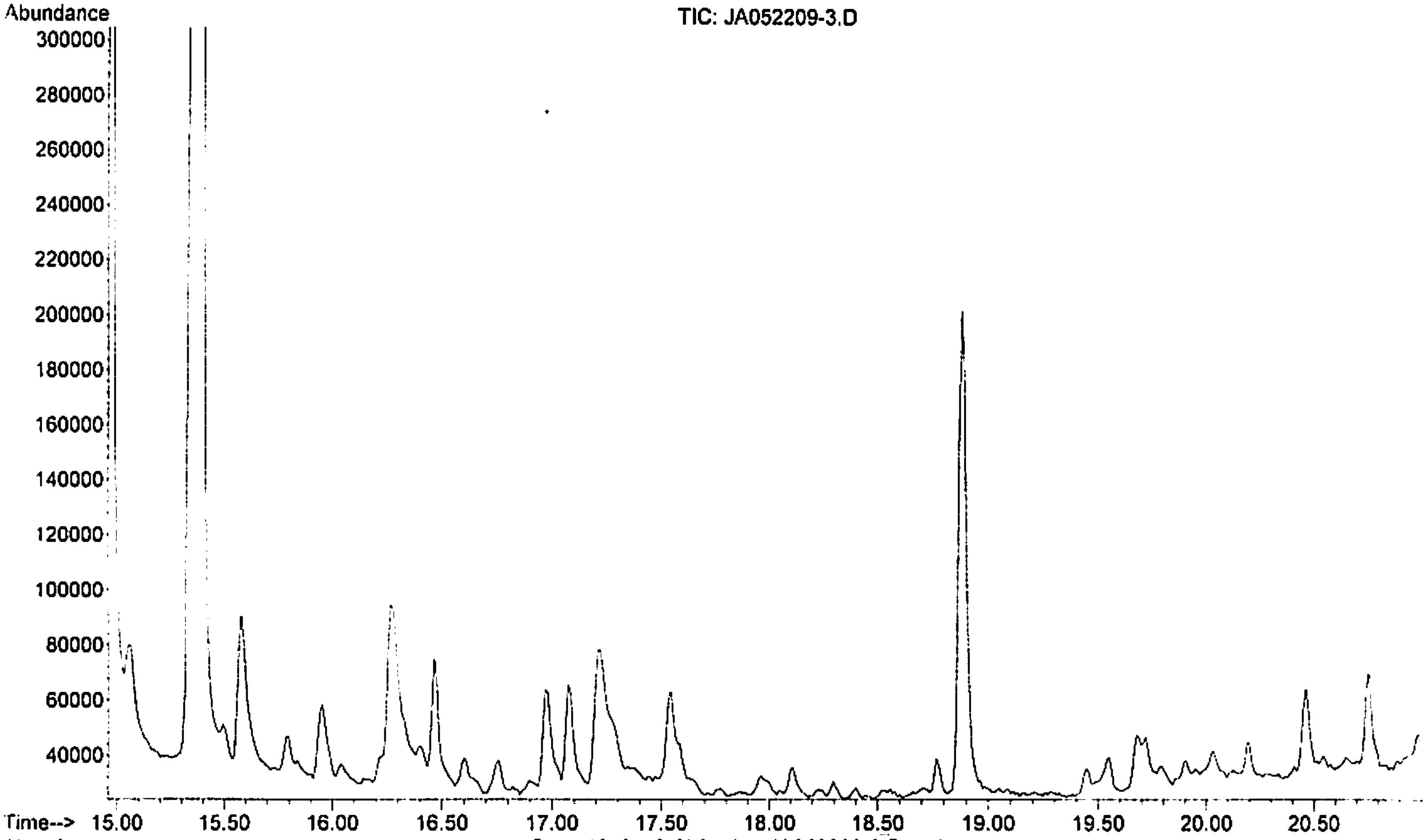

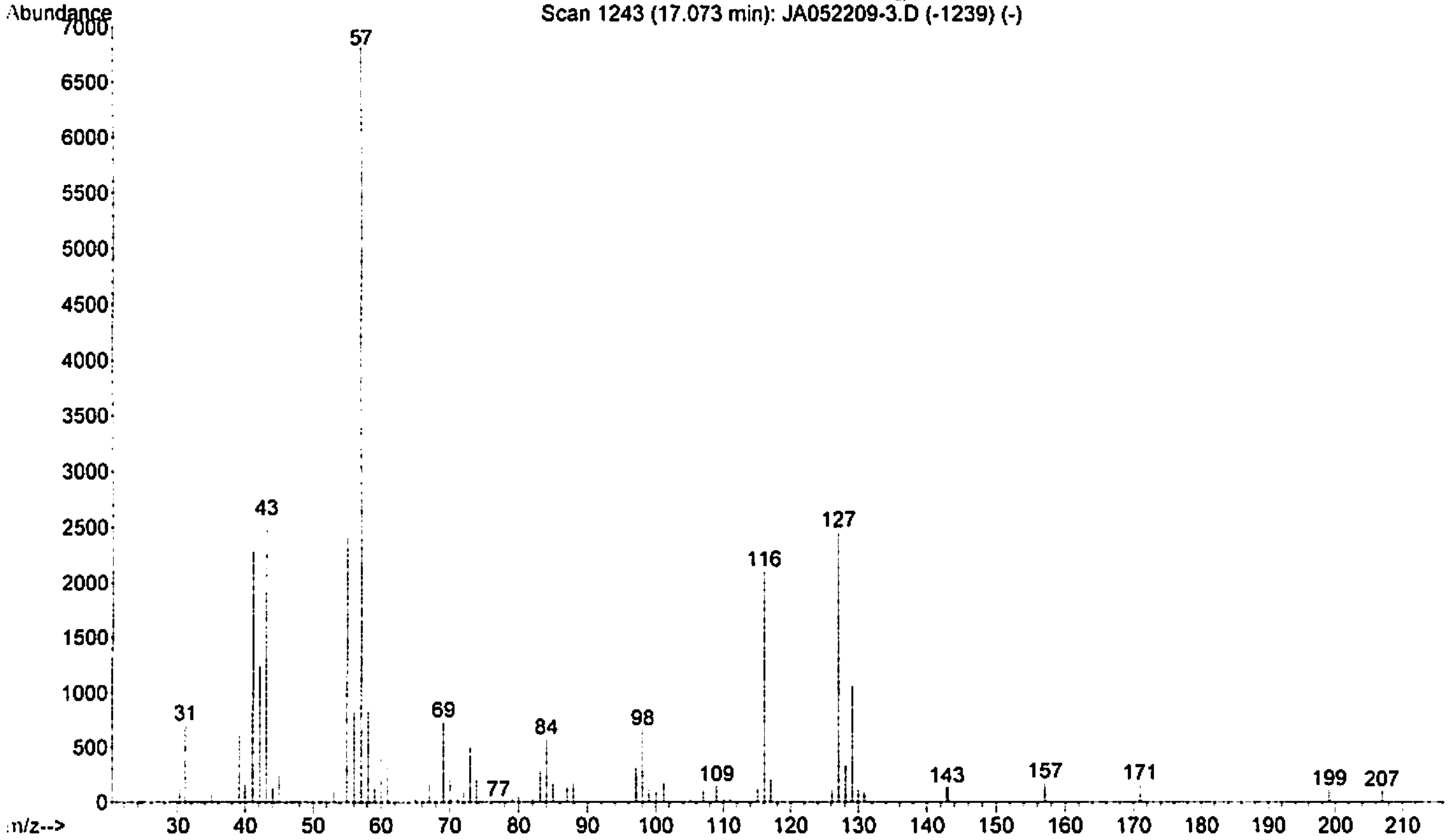

[illegible]e :D:\DATA\ALDRICH\JA-09\Snapshot\JA052209-3.D
[illegible]rator : Aldrich
[illegible]uired : 22 May 2009 15:55 using AcqMethod JA-WAX08.M
[illegible]strument : Instrument #1
[illegible]ple Name: 1 field-coll. male C.oculata abd./CH2Cl2
[illegible] Info : sweeping vetch 5/22 am; dissected
[illegible] Number: 1

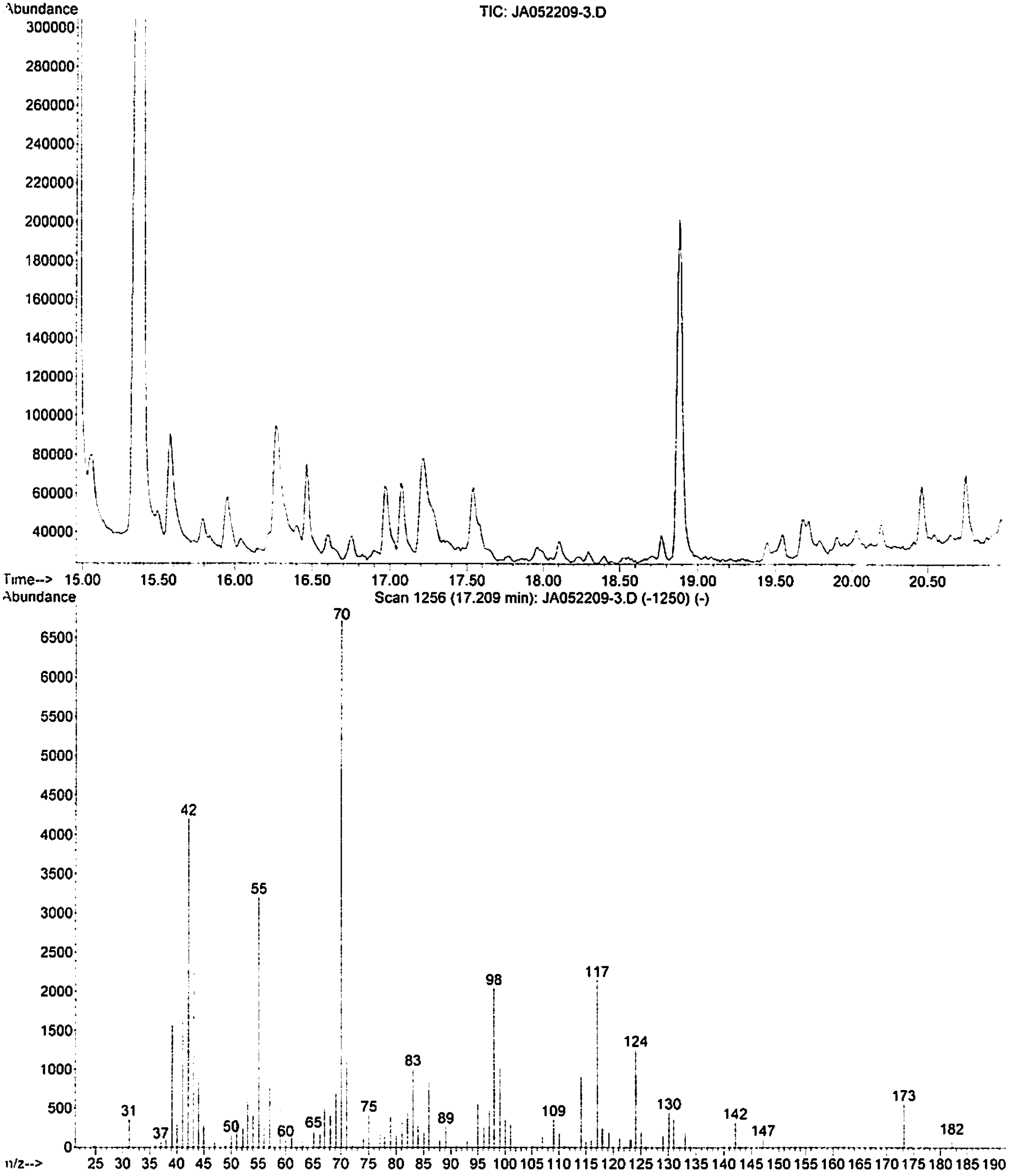

:D:\DATA\ALDRICH\JA-09\Snapshot\JA052209-3.D
ator : Aldrich
ired : 22 May 2009 15:55 using AcqMethod JA-WAX08.M
rument : Instrument #1
le Name: 1 field-coll. male C.oculata abd./CH2Cl2
: Info : sweeping vetch 5/22 am; dissected
Number: 1

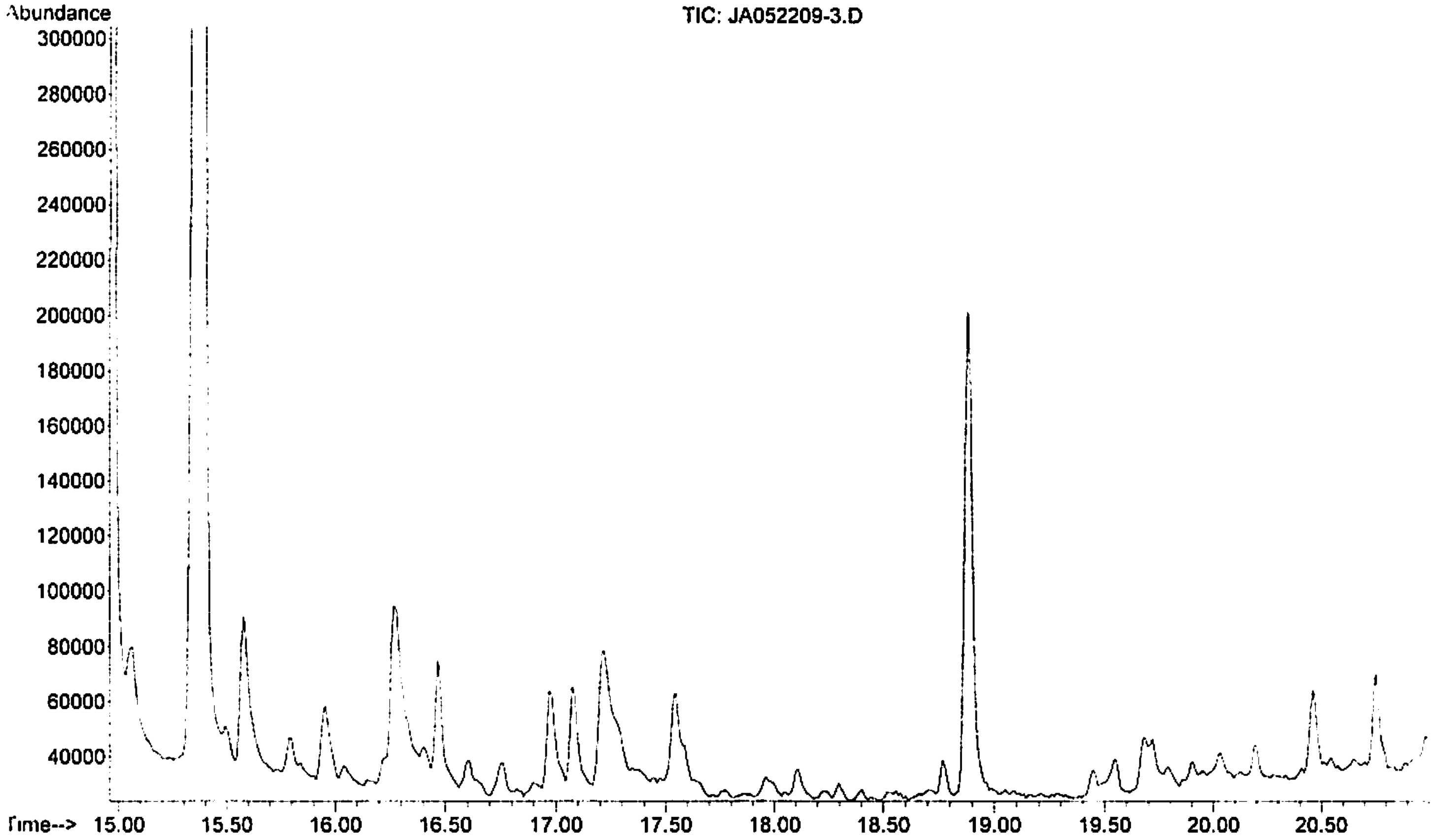

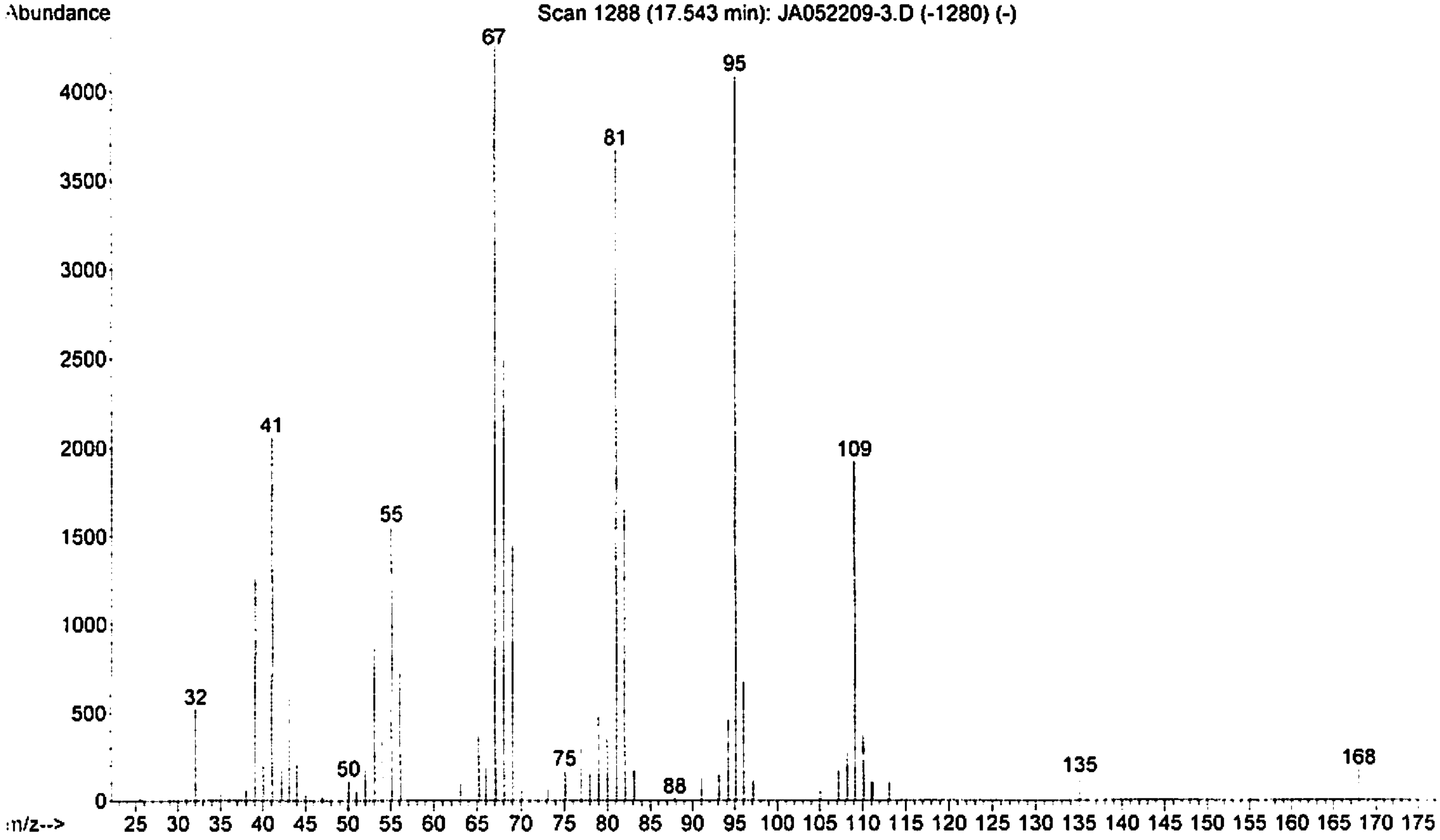

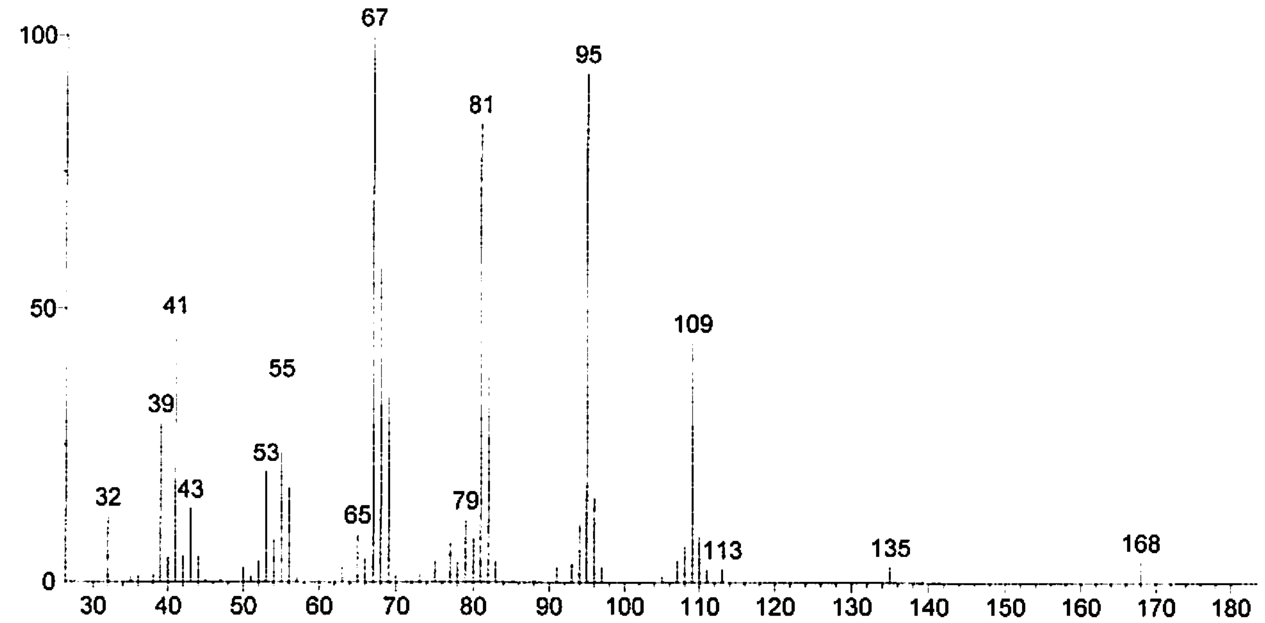

(Text File) Scan 1288 (17.543 min): JA052209-3.D (-1280)

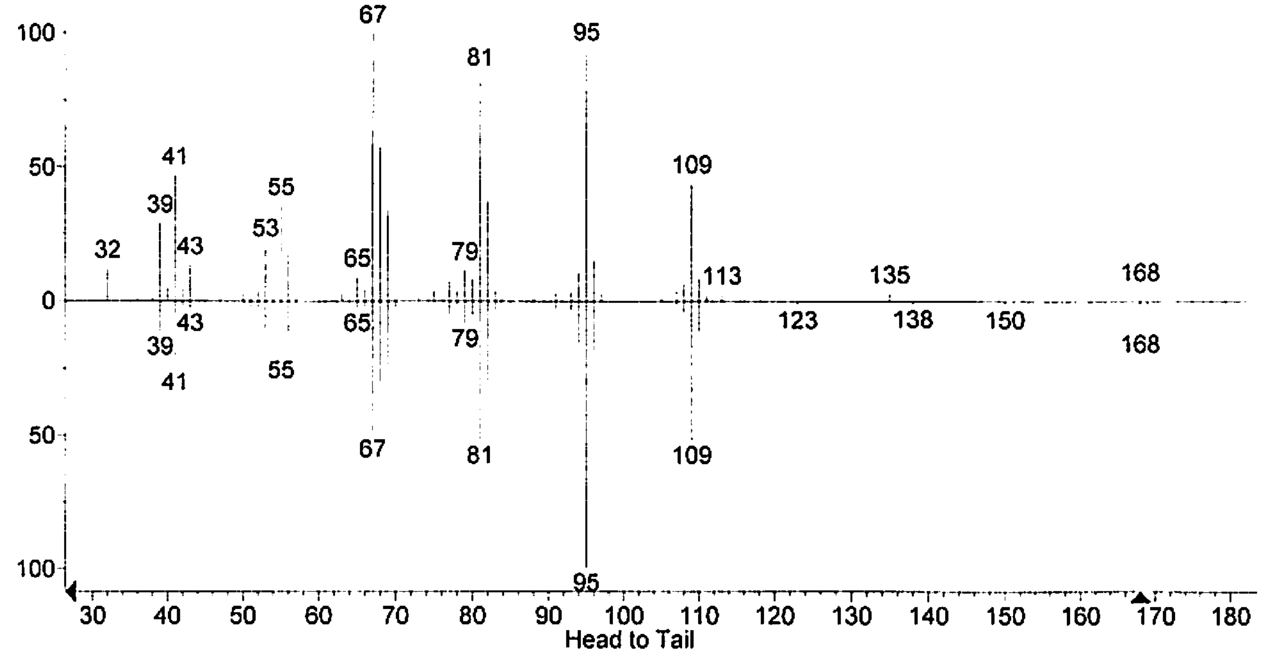

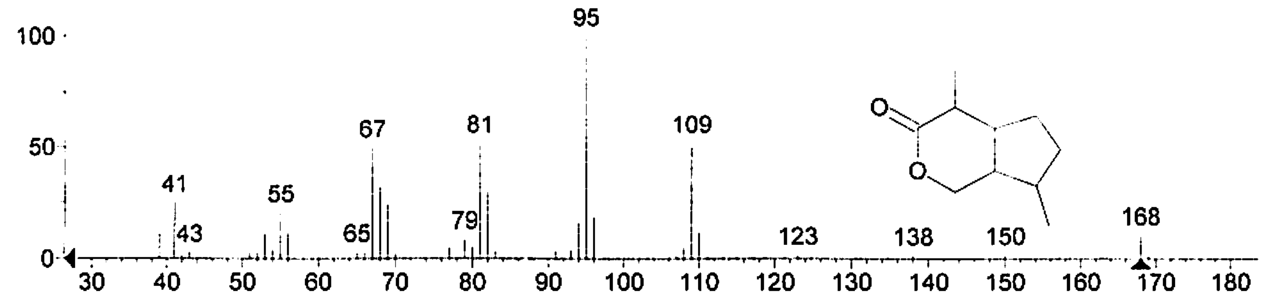

(replib) Iridomyrmecin

[illegible] :D:\DATA\ALDRICH\JA-09\Snapshot\JA052209-3.D
[illegible]ator : Aldrich
[illegible]ired : 22 May 2009 15:55 using AcqMethod JA-WAX08.M
[illegible]rument : Instrument #1
[illegible]ple Name: 1 field-coll. male C.oculata abd./CH2Cl2
[illegible] Info : sweeping vetch 5/22 am; dissected
[illegible] Number: 1

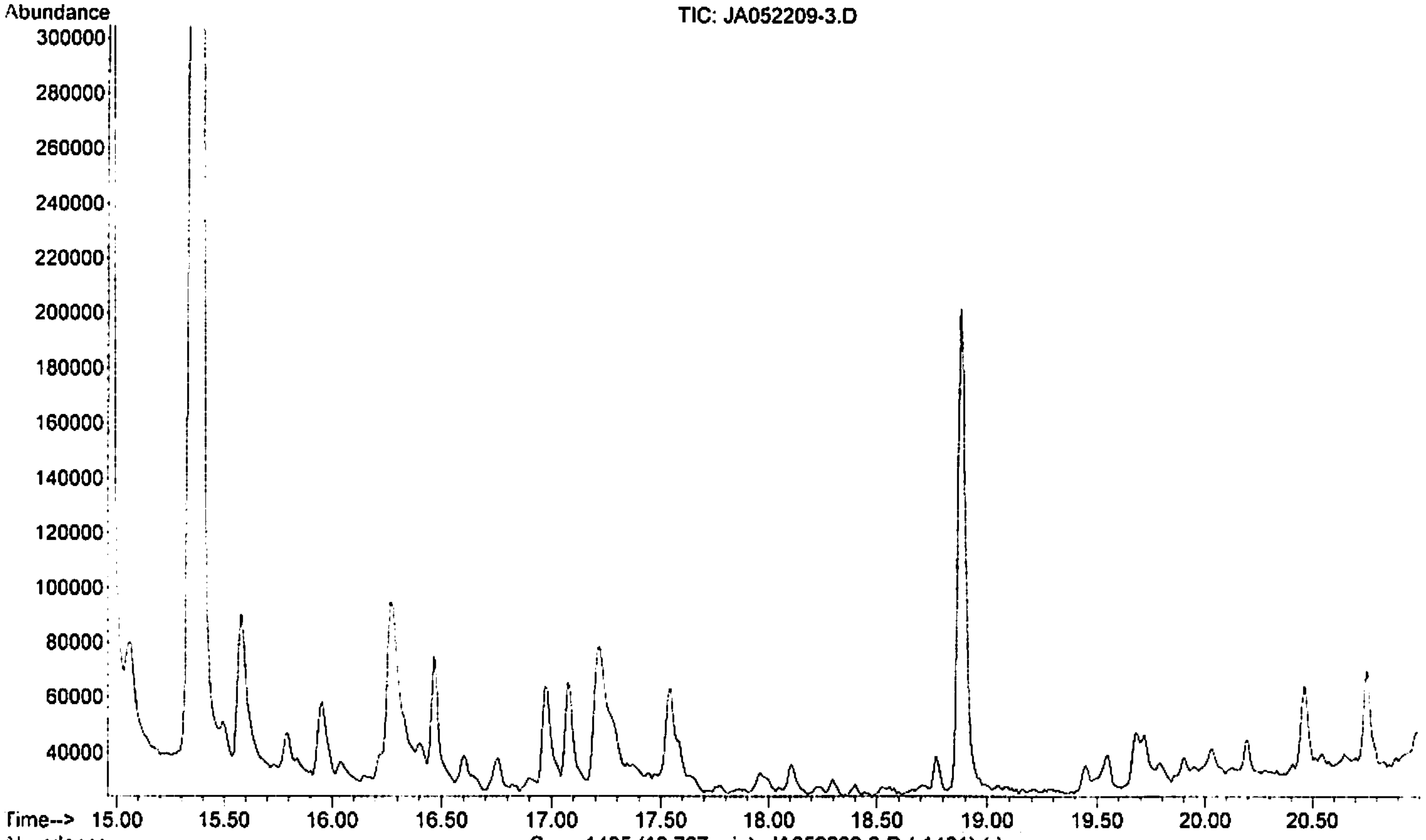

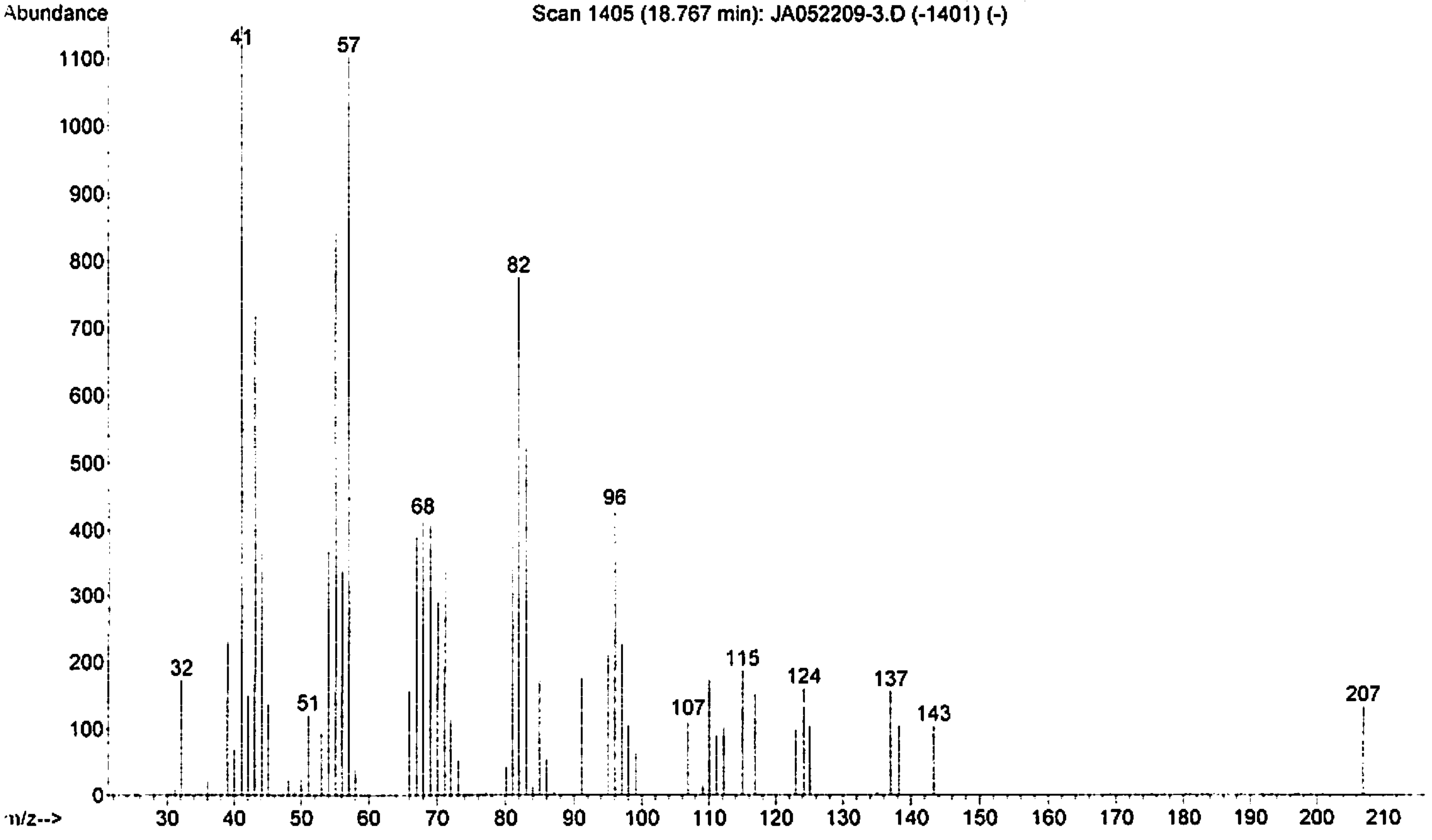

e :D:\DATA\ALDRICH\JA-09\Snapshot\JA052209-3.D
rator : Aldrich
uired : 22 May 2009 15:55 using AcqMethod JA-WAX08.M
strument : Instrument #1
ple Name: 1 field-coll. male C.oculata abd./CH2Cl2
c Info : sweeping vetch 5/22 am; dissected
al Number: 1

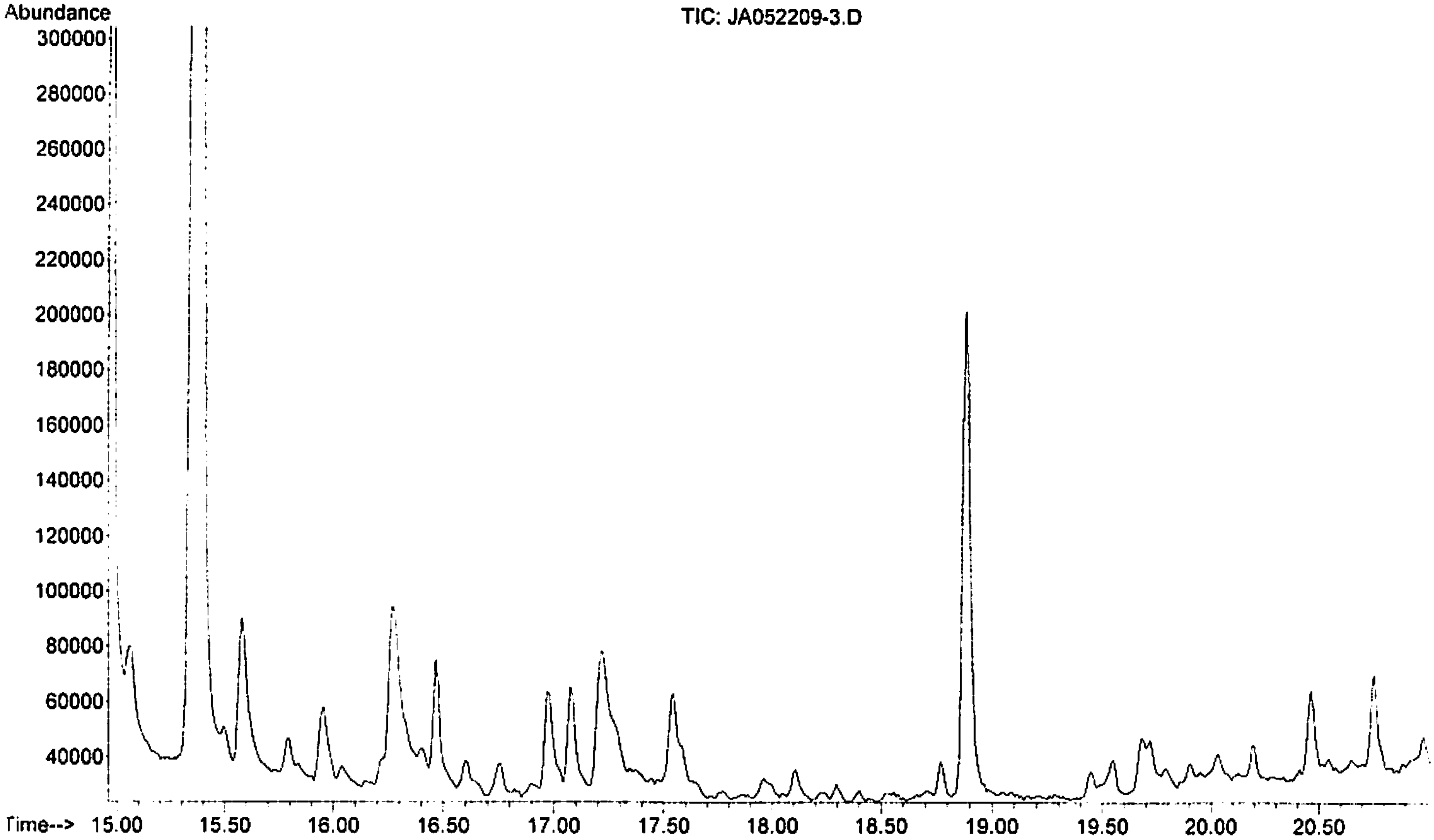

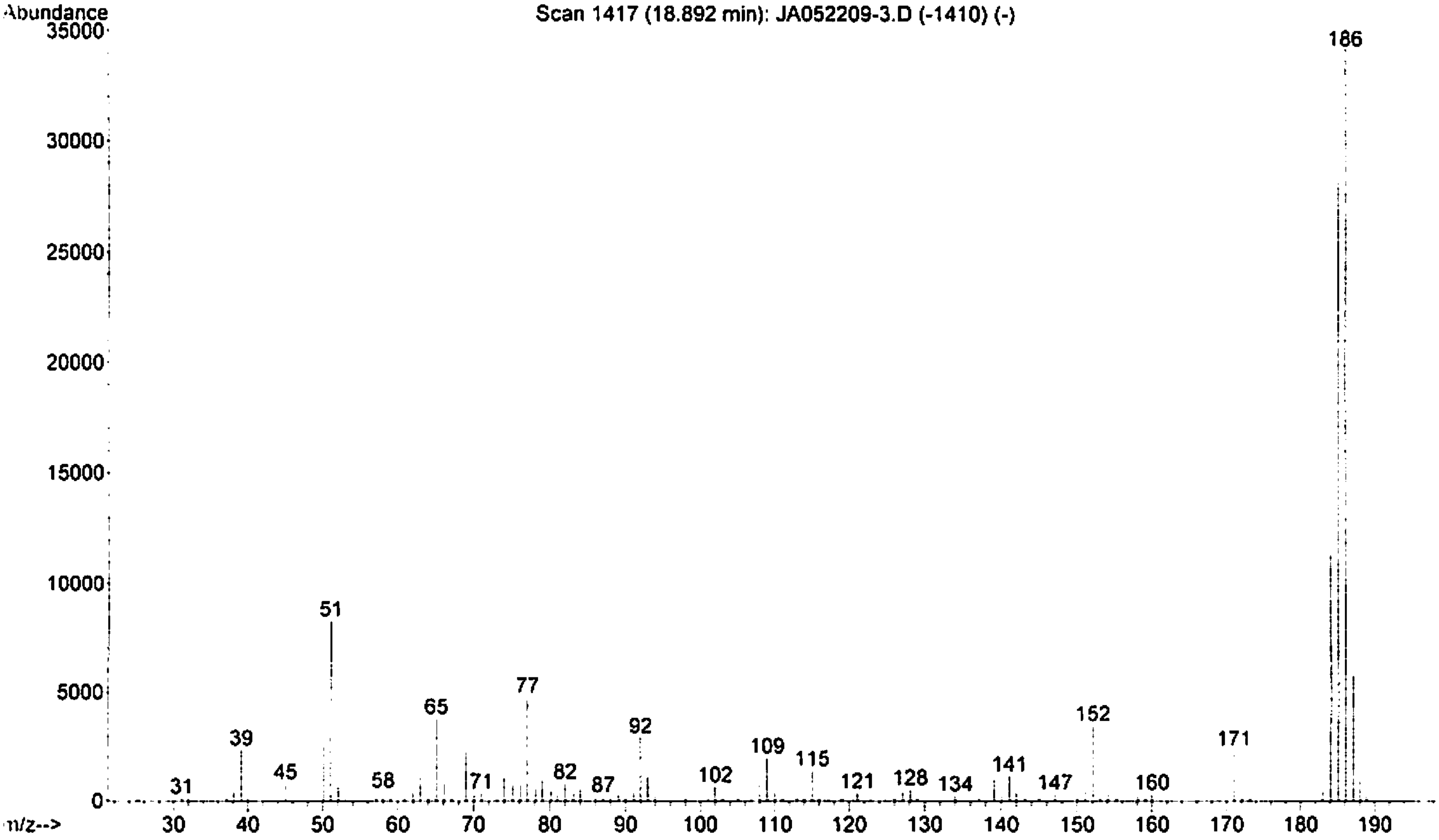

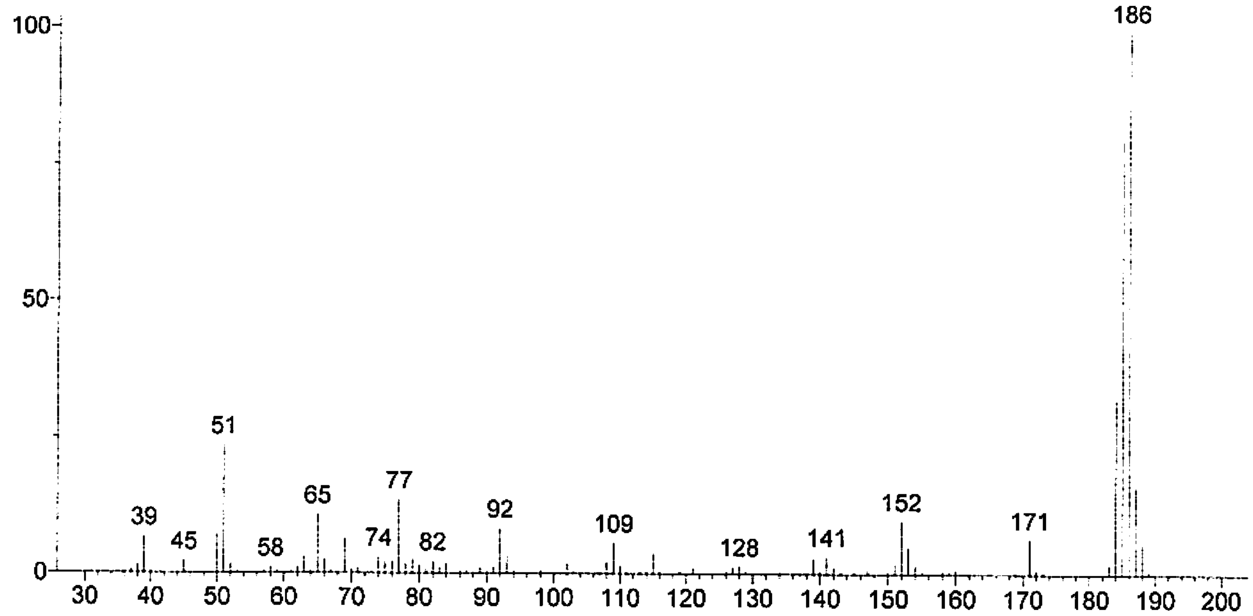

(Text File) Scan 1417 (18.892 min): JA052209-3.D (-1410)

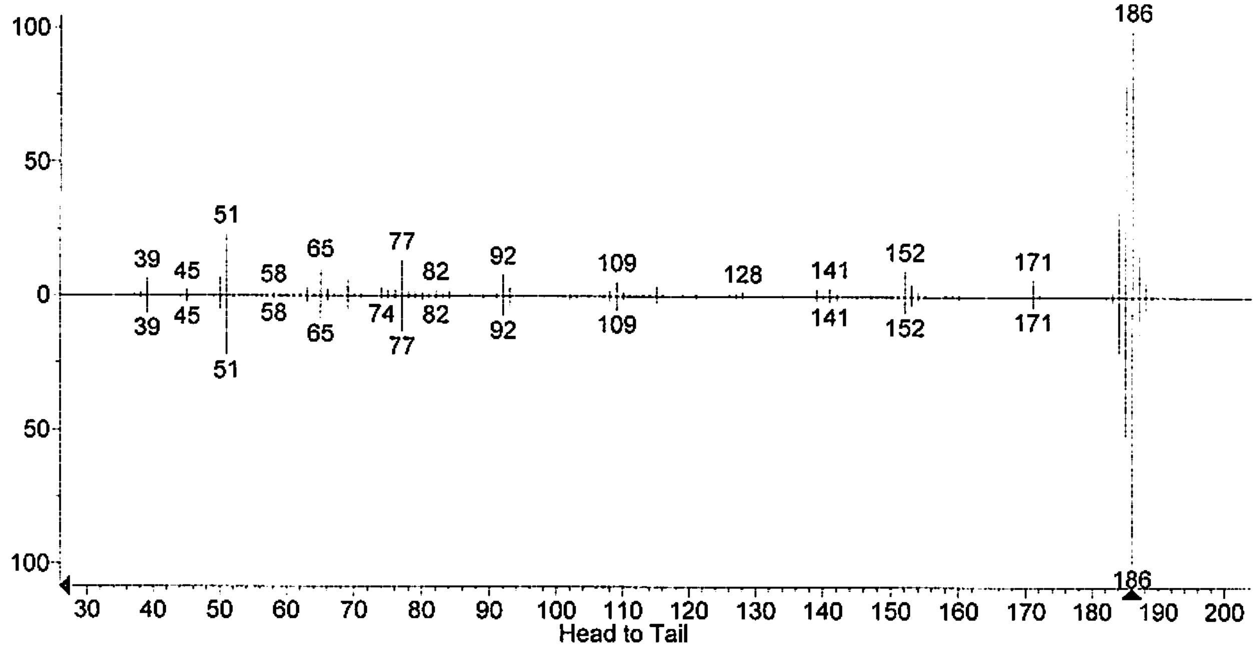

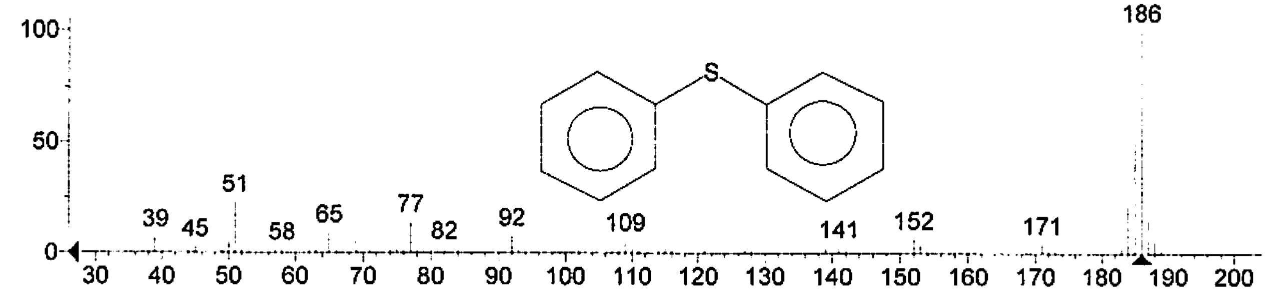

(replib) Diphenyl sulfide

[illegible] :D:\DATA\ALDRICH\JA-09\Snapshot\JA052209-3.D
[illegible]rator : Aldrich
[illegible]uired : 22 May 2009 15:55 using AcqMethod JA-WAX08.M
[illegible]rument : Instrument #1
[illegible]le Name: 1 field-coll. male C.oculata abd./CH2Cl2
[illegible] Info : sweeping vetch 5/22 am; dissected
[illegible] Number: 1

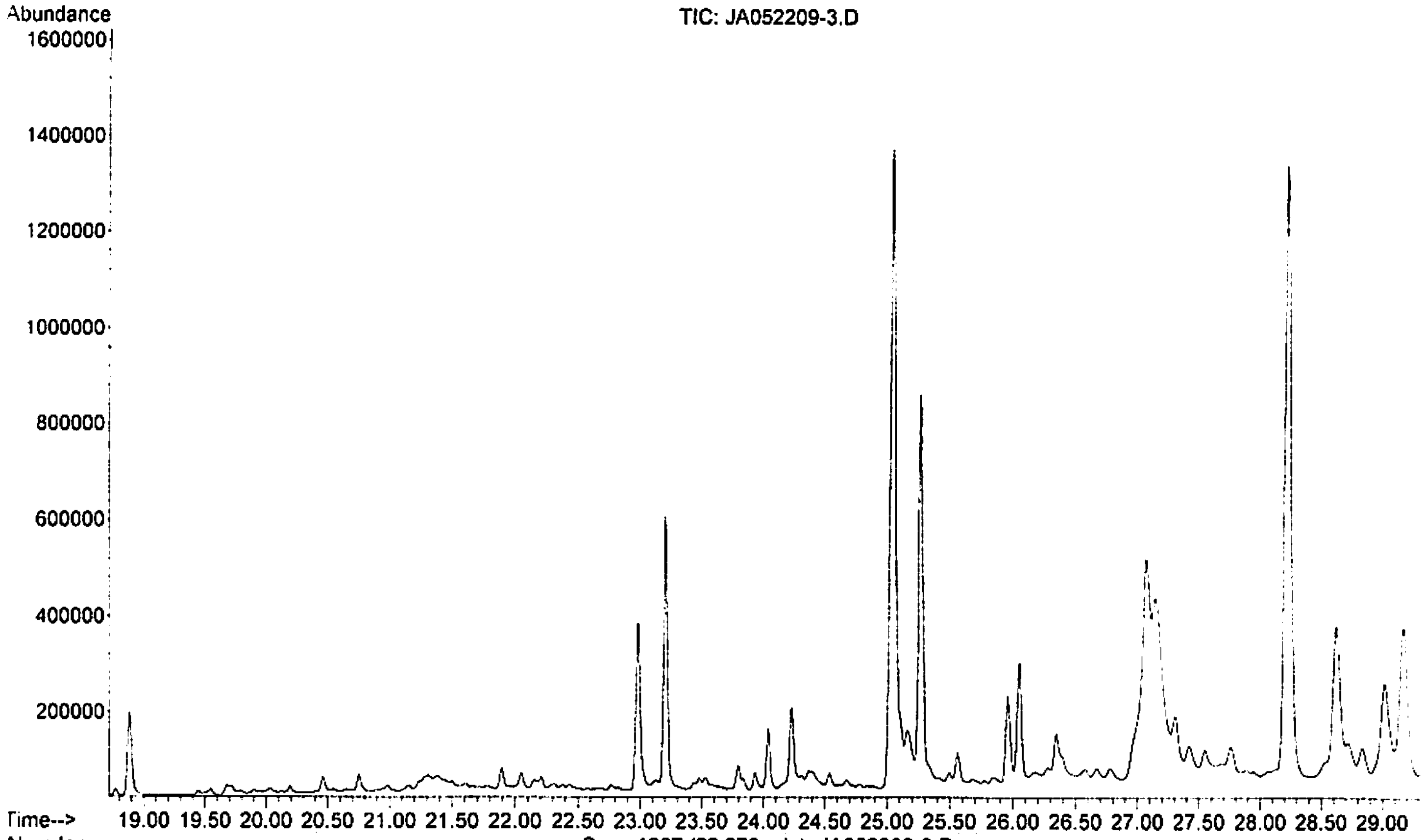

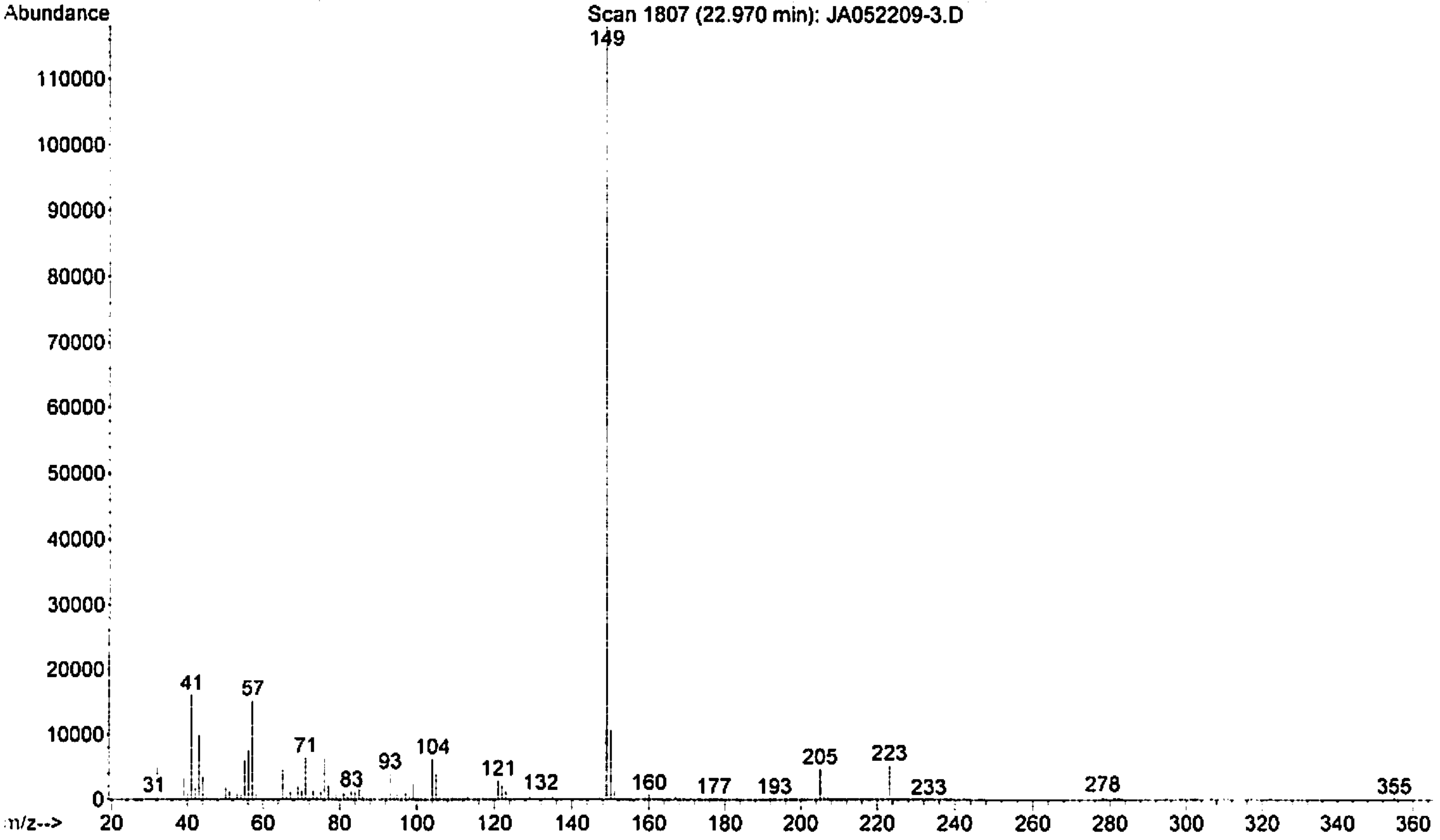

[illegible] :D:\DATA\ALDRICH\JA-09\Snapshot\JA052209-3.D
[illegible]rator : Aldrich
[illegible]ired : 22 May 2009 15:55 using AcqMethod JA-WAX08.M
[illegible]rument : Instrument #1
[illegible]ple Name: 1 field-coll. male C.oculata abd./CH2Cl2
[illegible] Info : sweeping vetch 5/22 am; dissected
[illegible]l Number: 1

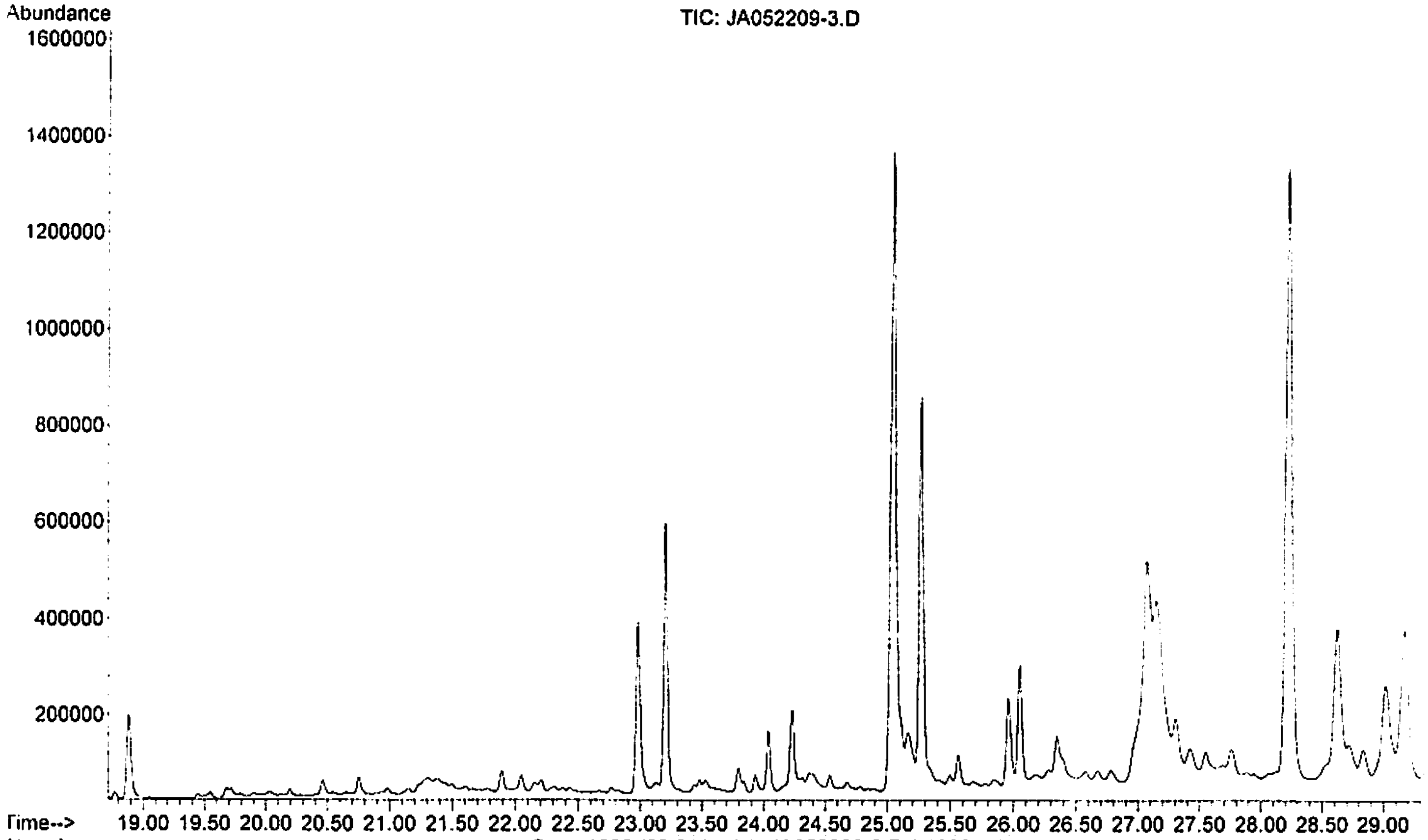

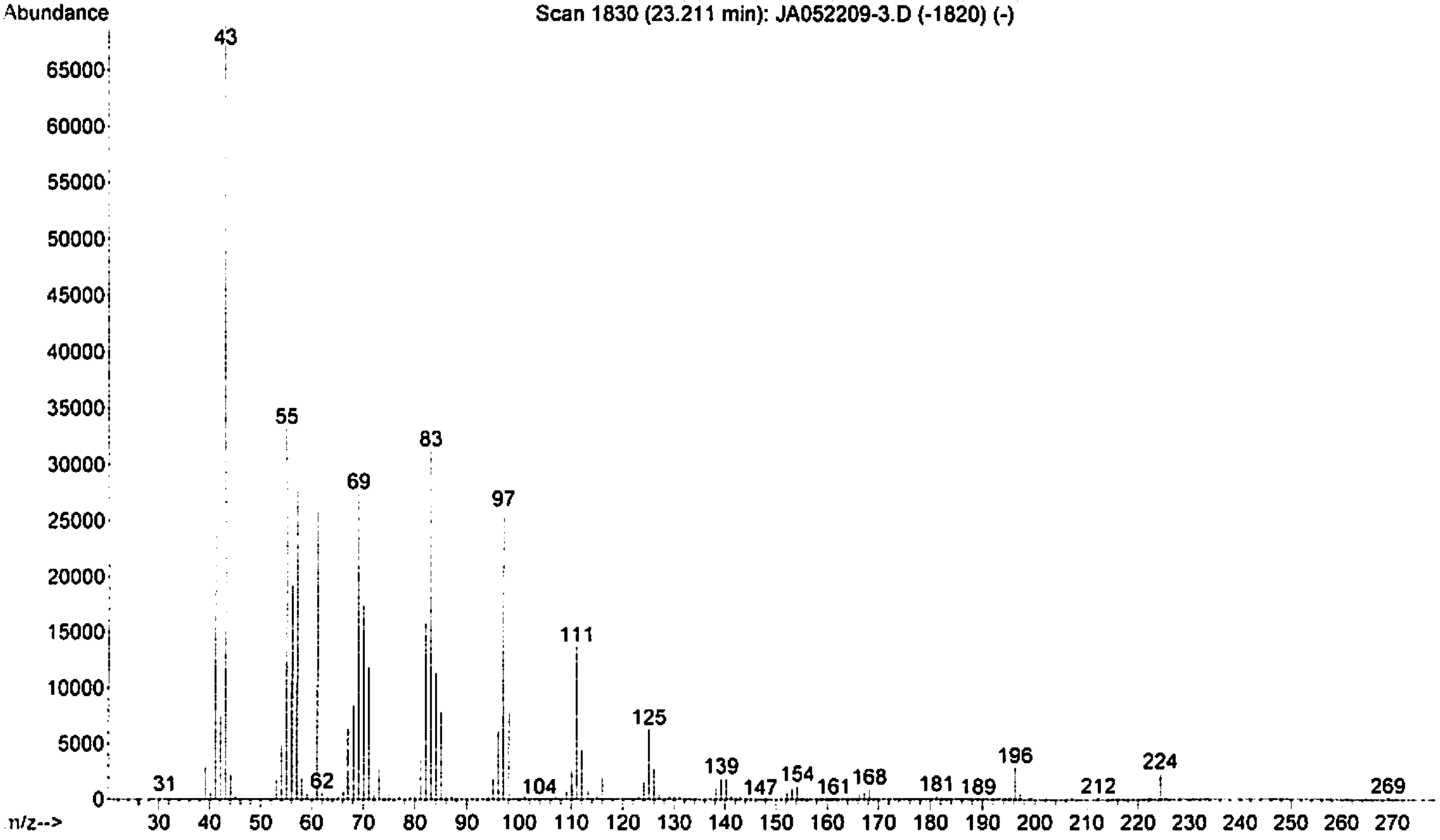

[illegible] :D:\DATA\Aldrich\JA-09\JA052209-3.D
[illegible]ator : Aldrich
[illegible]ired : 22 May 2009 15:55 using AcqMethod JA-WAX08.M
[illegible]trument : Instrument #1
[illegible]ple Name: 1 field-coll. male C.oculata abd./CH2Cl2
[illegible] Info : sweeping vetch 5/22 am; dissected
[illegible]l Number: 1

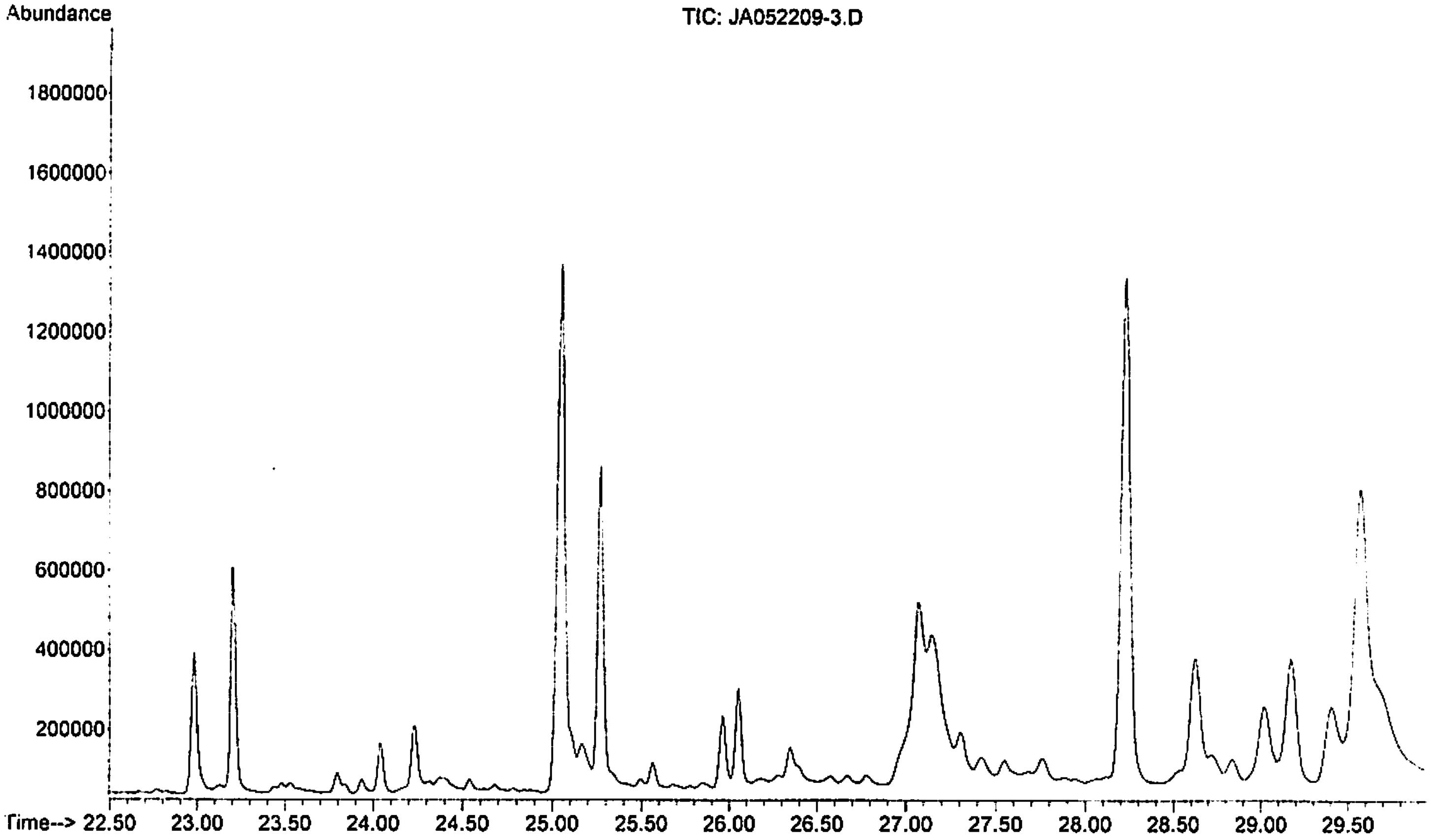

[illegible]e . :D:\DATA\Aldrich\JA-09\JA052209-3.D
[illegible]rator : Aldrich
[illegible]quired : 22 May 2009 15:55 using AcqMethod JA-WAX08.M
[illegible]strument : Instrument #1
[illegible]ple Name: 1 field-coll. male C.oculata abd./CH2Cl2
[illegible]sc Info : sweeping vetch 5/22 am; dissected
[illegible]al Number: 1

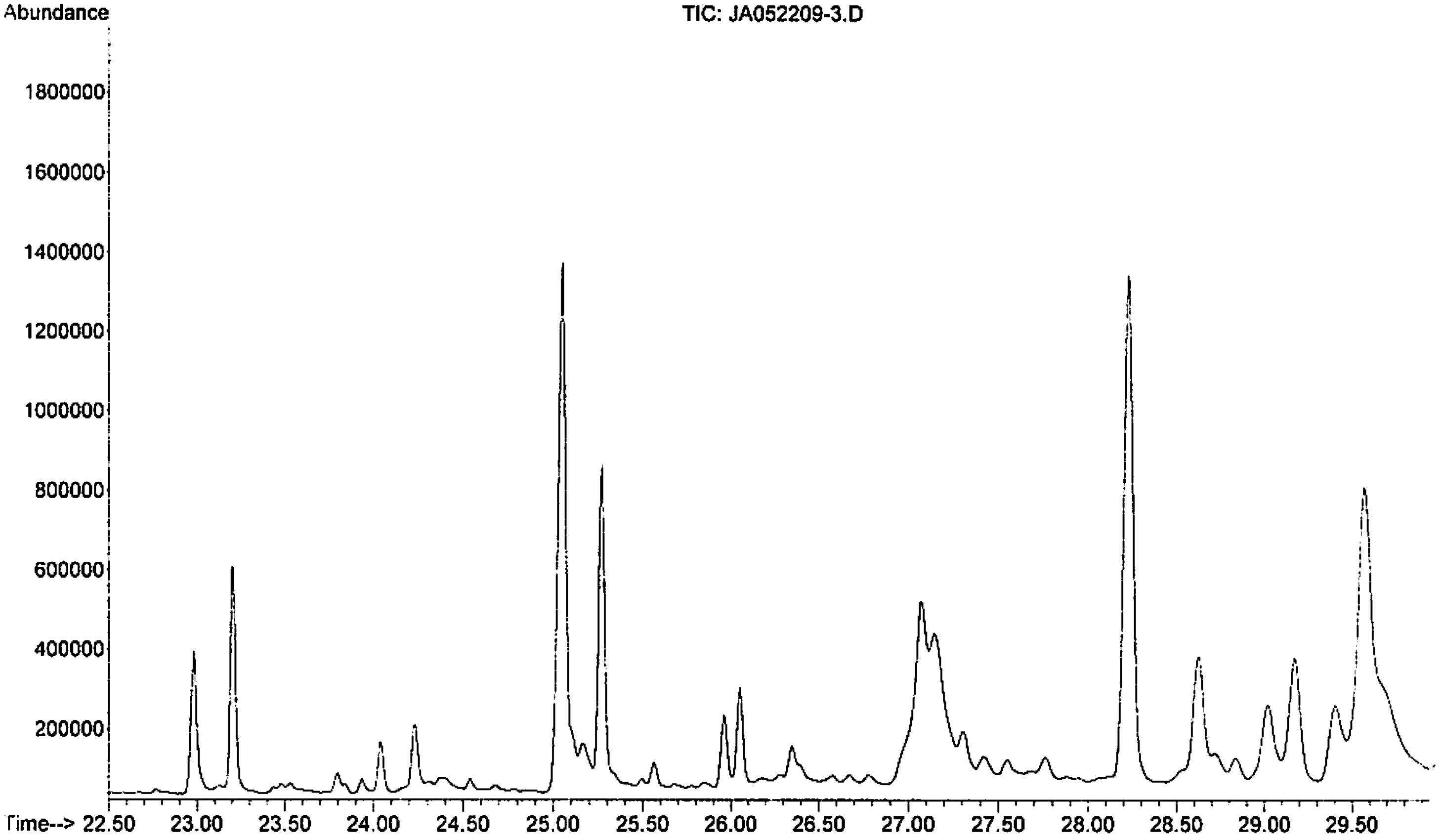

File   :D:\DATA\Aldrich\JA-09\JA052809-3.D
Operator   : Aldrich
Acquired   : 28 May 2009 15:24   using AcqMethod JA-WAX08.M
Instrument :   Instrument #1
Sample Name: 1 field-coll. M C.oculata abd./CH2Cl2
Misc Info   : coll. 5/28 sweeping vetch; squashed
Vial Number: 1

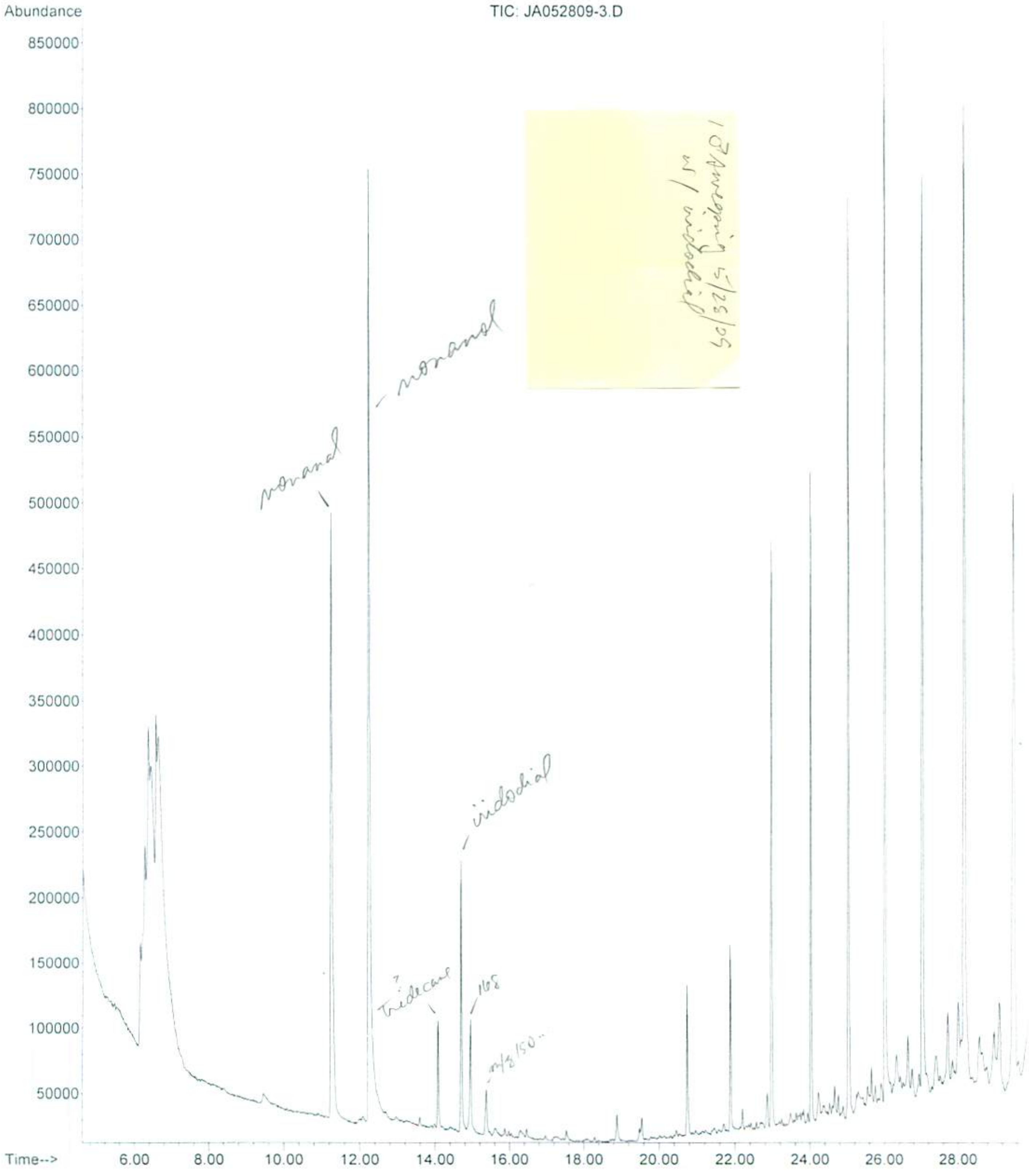

```
File         :D:\DATA\ALDRICH\JA-09\Snapshot\JA052809-3.D
Operator     : Aldrich
Acquired     : 28 May 2009  15:24      using AcqMethod JA-WAX08.M
Instrument :     Instrument #1
Sample Name: 1 field-coll. M C.oculata abd./CH2Cl2
Misc Info   : coll. 5/28 sweeping vetch; squashed
Vial Number: 1
```

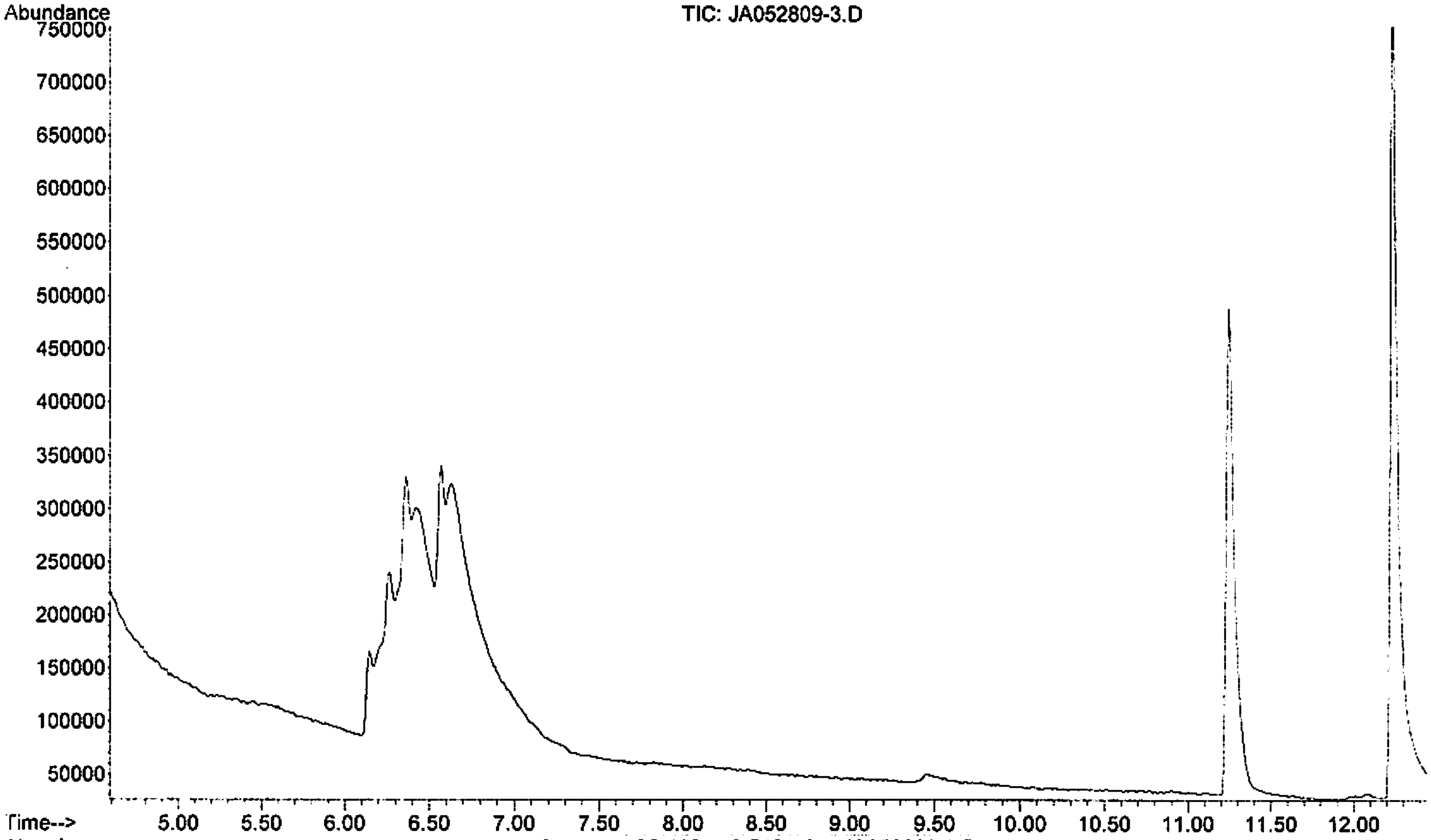

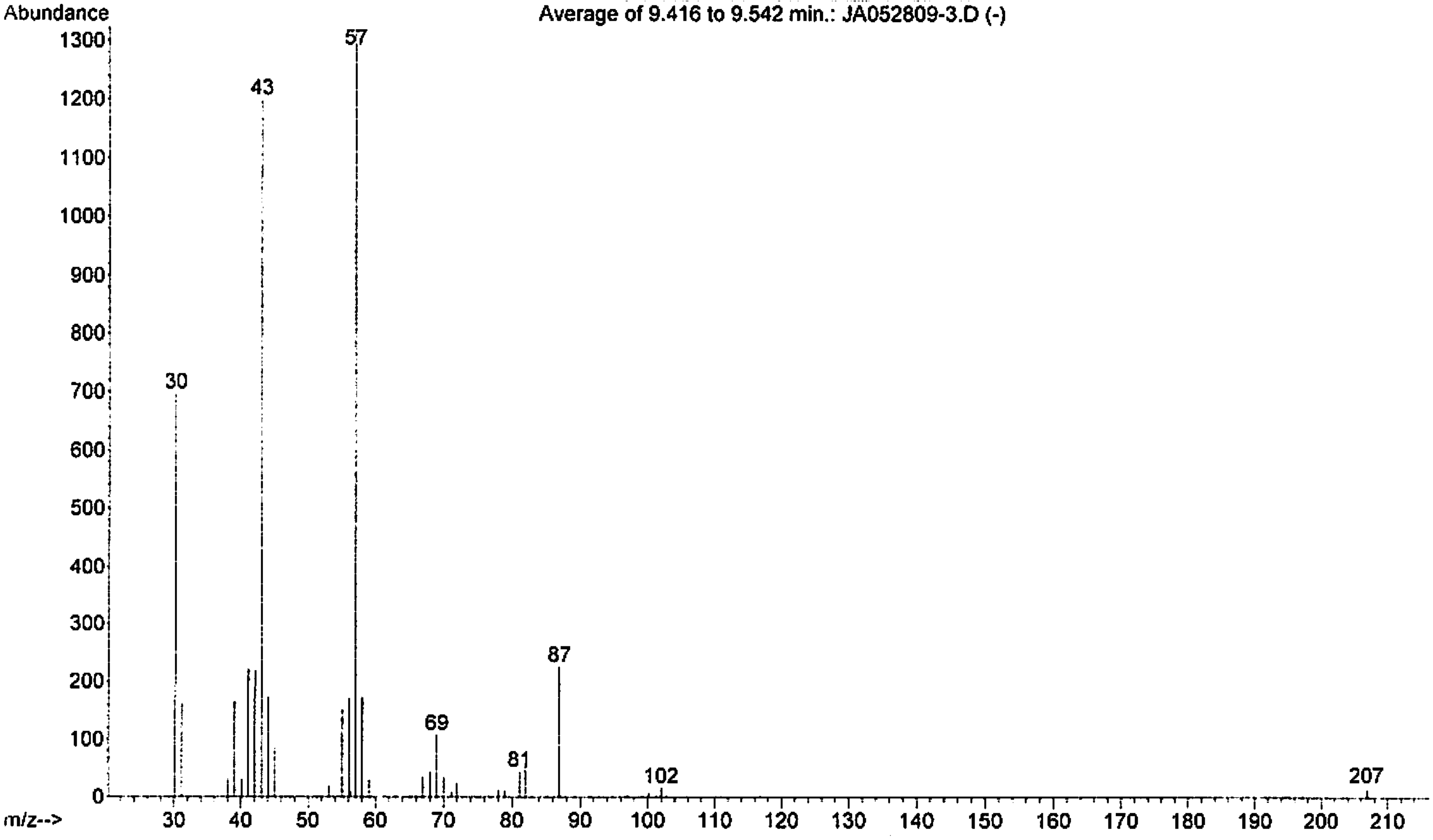

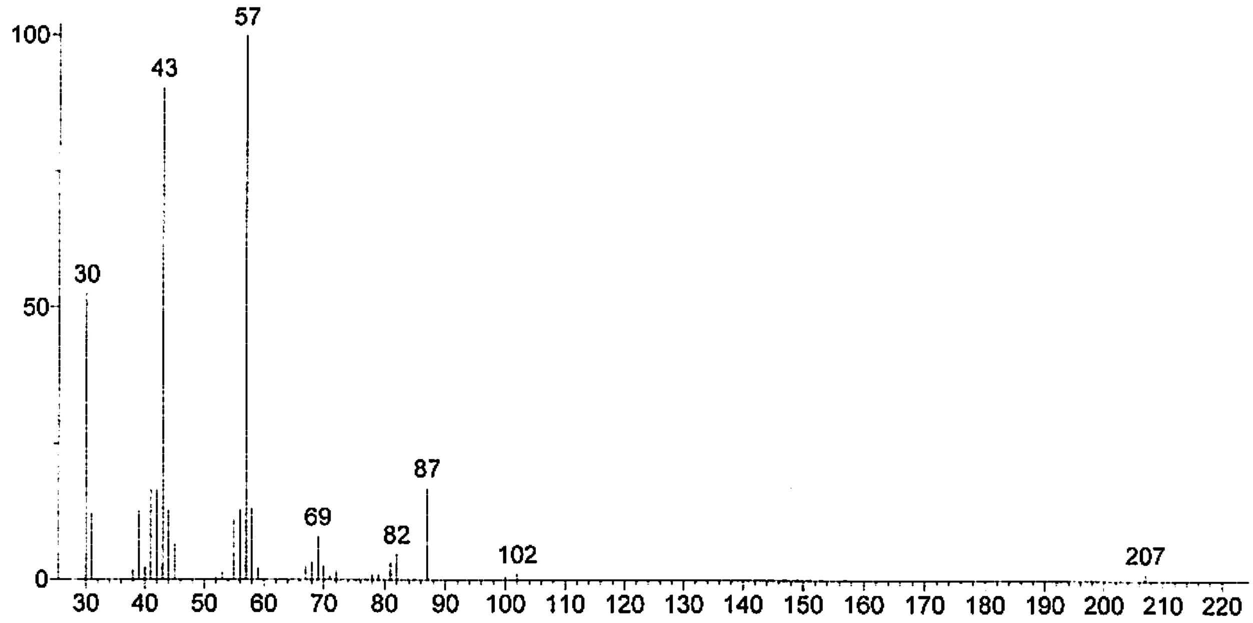

(Text File) Average of 9.416 to 9.542 min.: JA052809-3.D

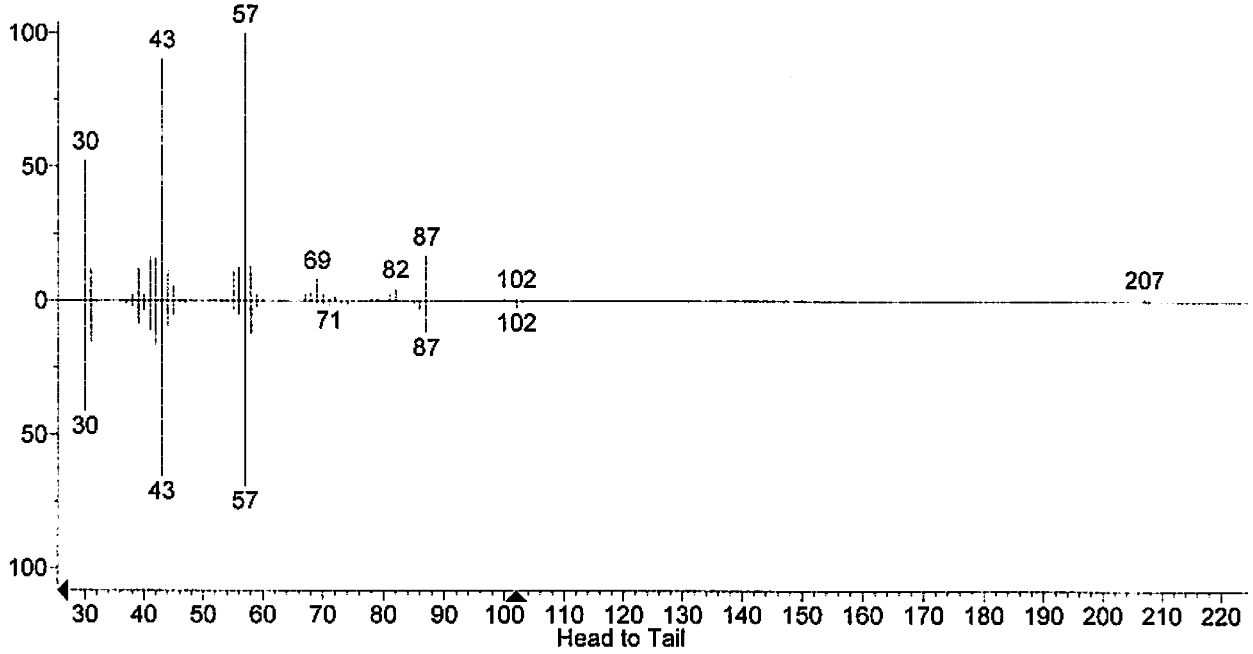

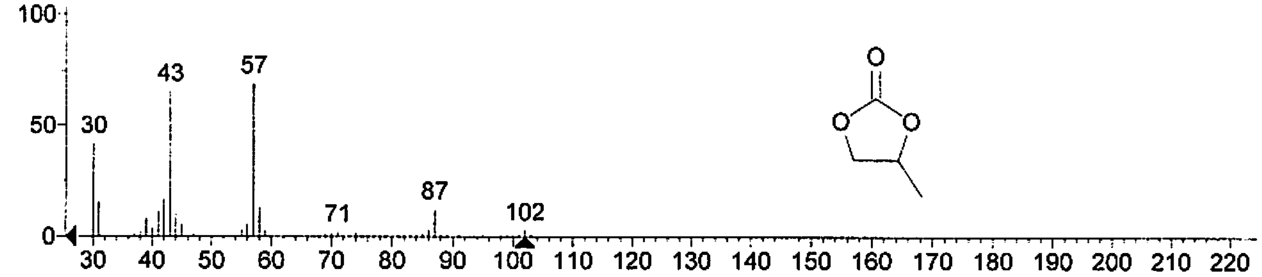

(replib) Propylene Carbonate

[illegible] :D:\DATA\ALDRICH\JA-09\Snapshot\JA052809-3.D
[illegible]rator : Aldrich
[illegible]uired : 28 May 2009 15:24 using AcqMethod JA-WAX08.M
[illegible]rument : Instrument #1
[illegible]ple Name: 1 field-coll. M C.oculata abd./CH2Cl2
[illegible] Info : coll. 5/28 sweeping vetch; squashed
[illegible] Number: 1

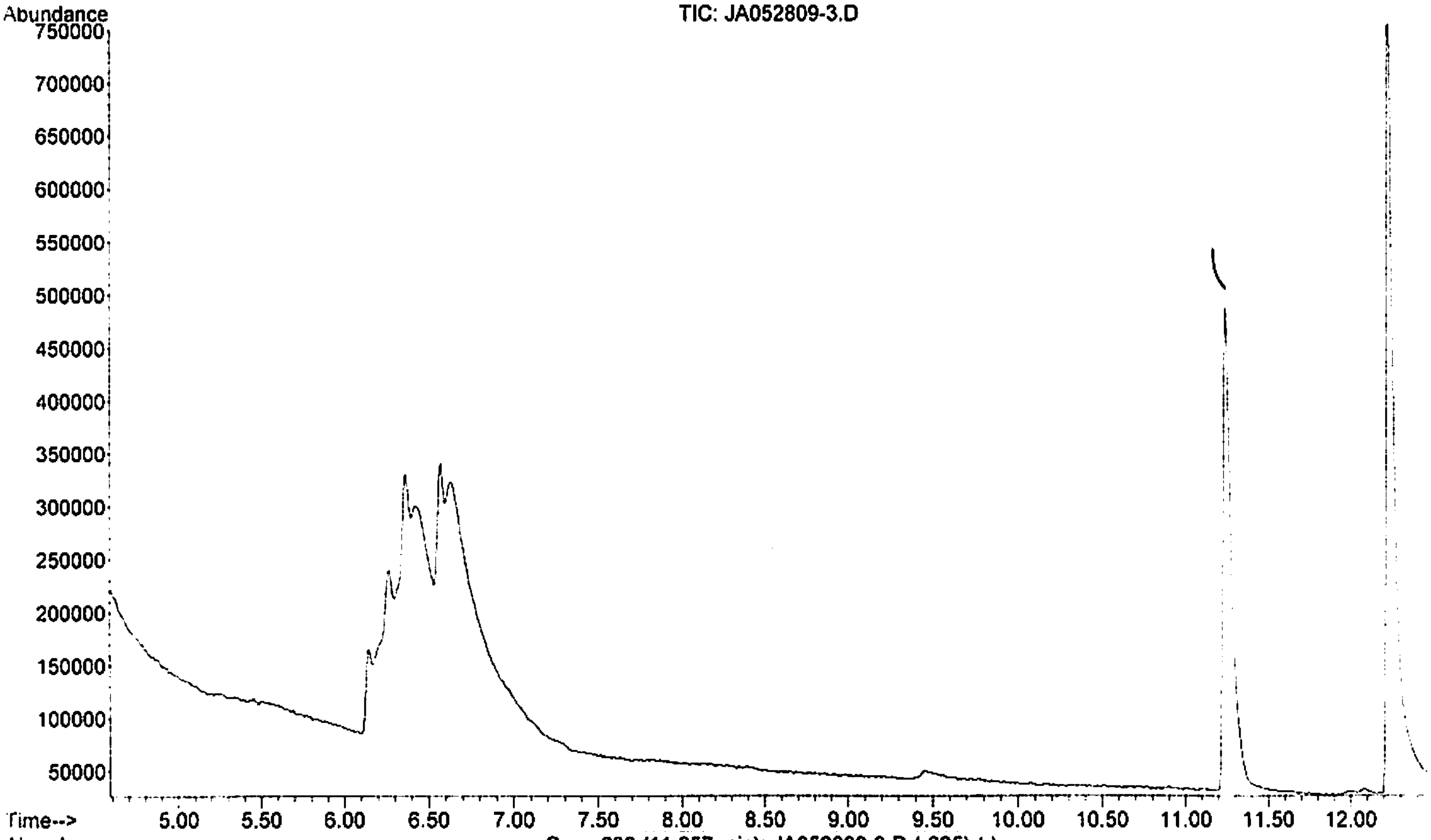

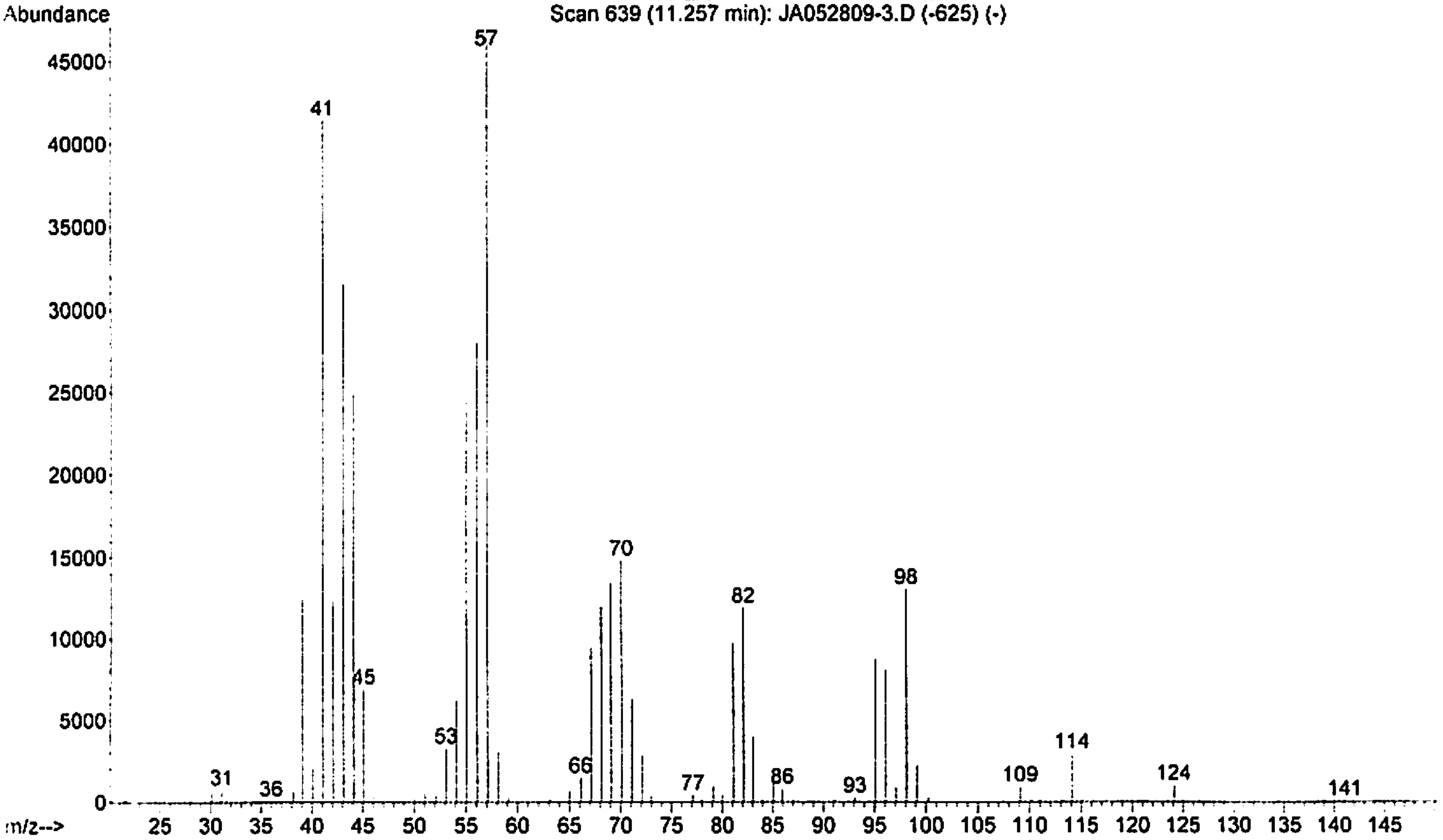

[illegible]e :D:\DATA\ALDRICH\JA-09\Snapshot\JA052809-3.D
[illegible]erator : Aldrich
[illegible]quired : 28 May 2009 15:24 using AcqMethod JA-WAX08.M
[illegible]strument : Instrument #1
[illegible]ple Name: 1 field-coll. M C.oculata abd./CH2Cl2
[illegible] Info : coll. 5/28 sweeping vetch; squashed
[illegible]al Number: 1

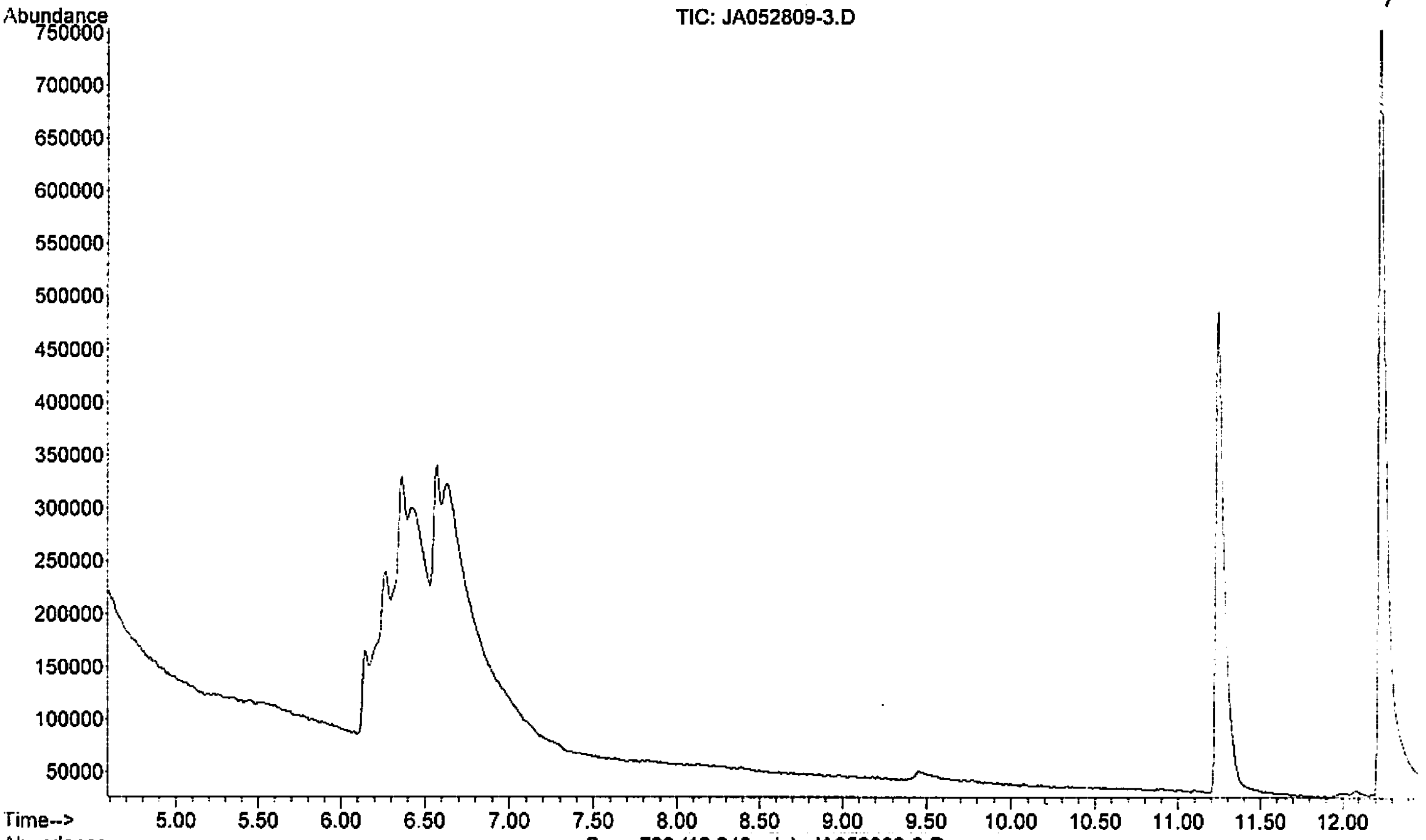

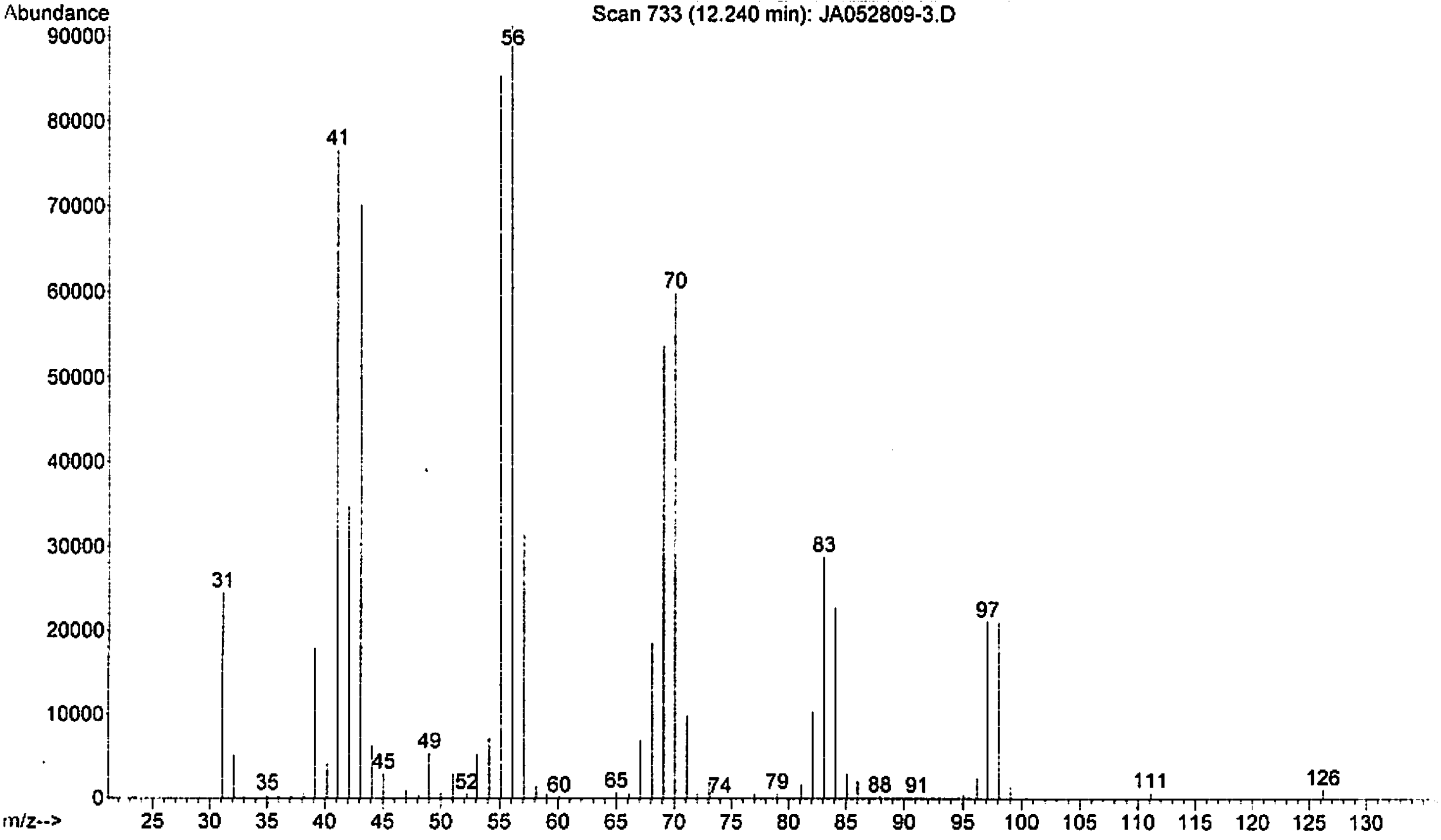

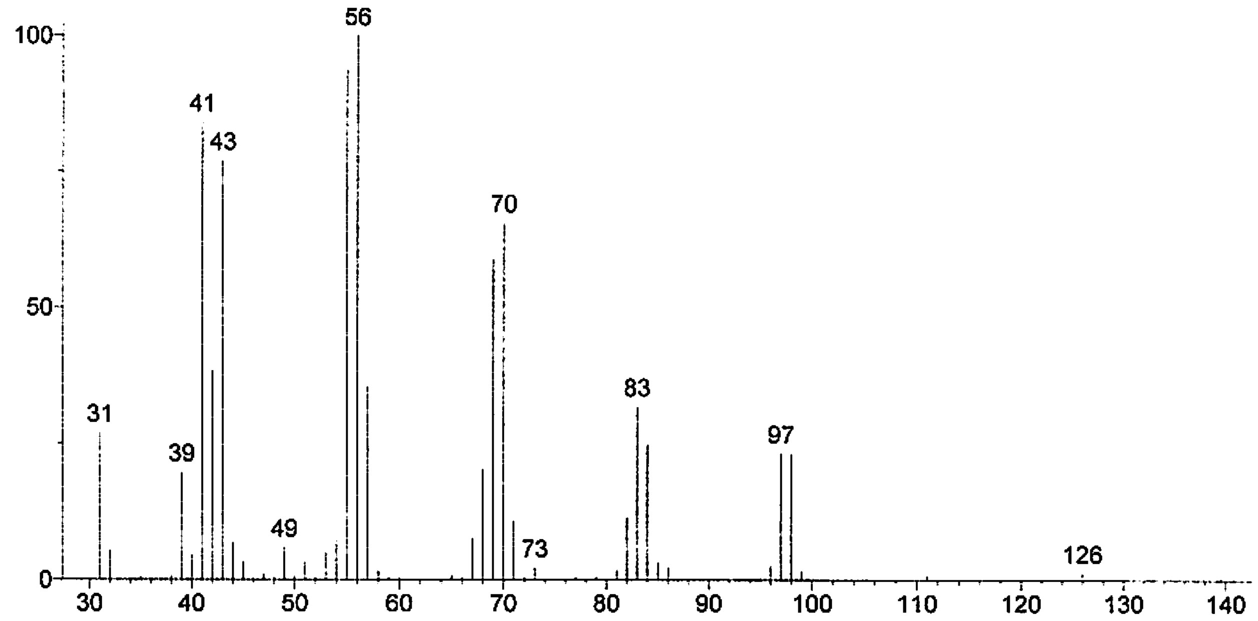

(Text File) Scan 733 (12.240 min): JA052809-3.D

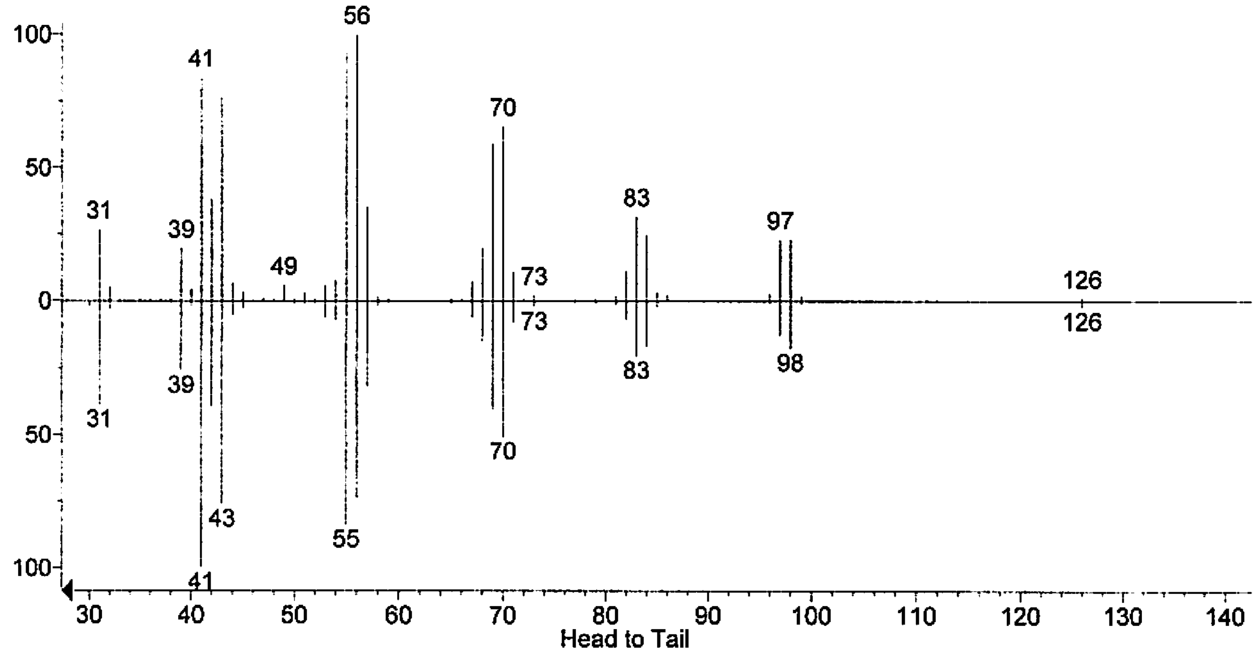

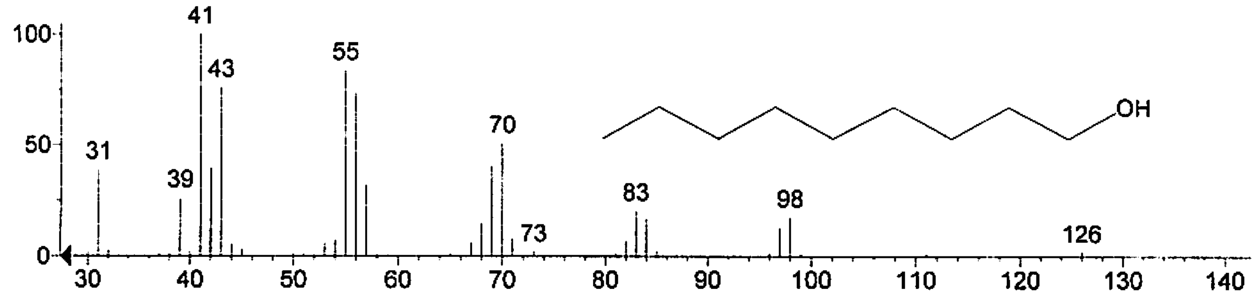

(replib) 1-Nonanol

```
File        :D:\DATA\ALDRICH\JA-09\Snapshot\JA052809-3.D
Operator    : Aldrich
Acquired    : 28 May 2009  15:24     using AcqMethod JA-WAX08.M
Instrument  :    Instrument #1
Sample Name: 1 field-coll. M C.oculata abd./CH2Cl2
Misc Info   : coll. 5/28 sweeping vetch; squashed
Vial Number: 1
```

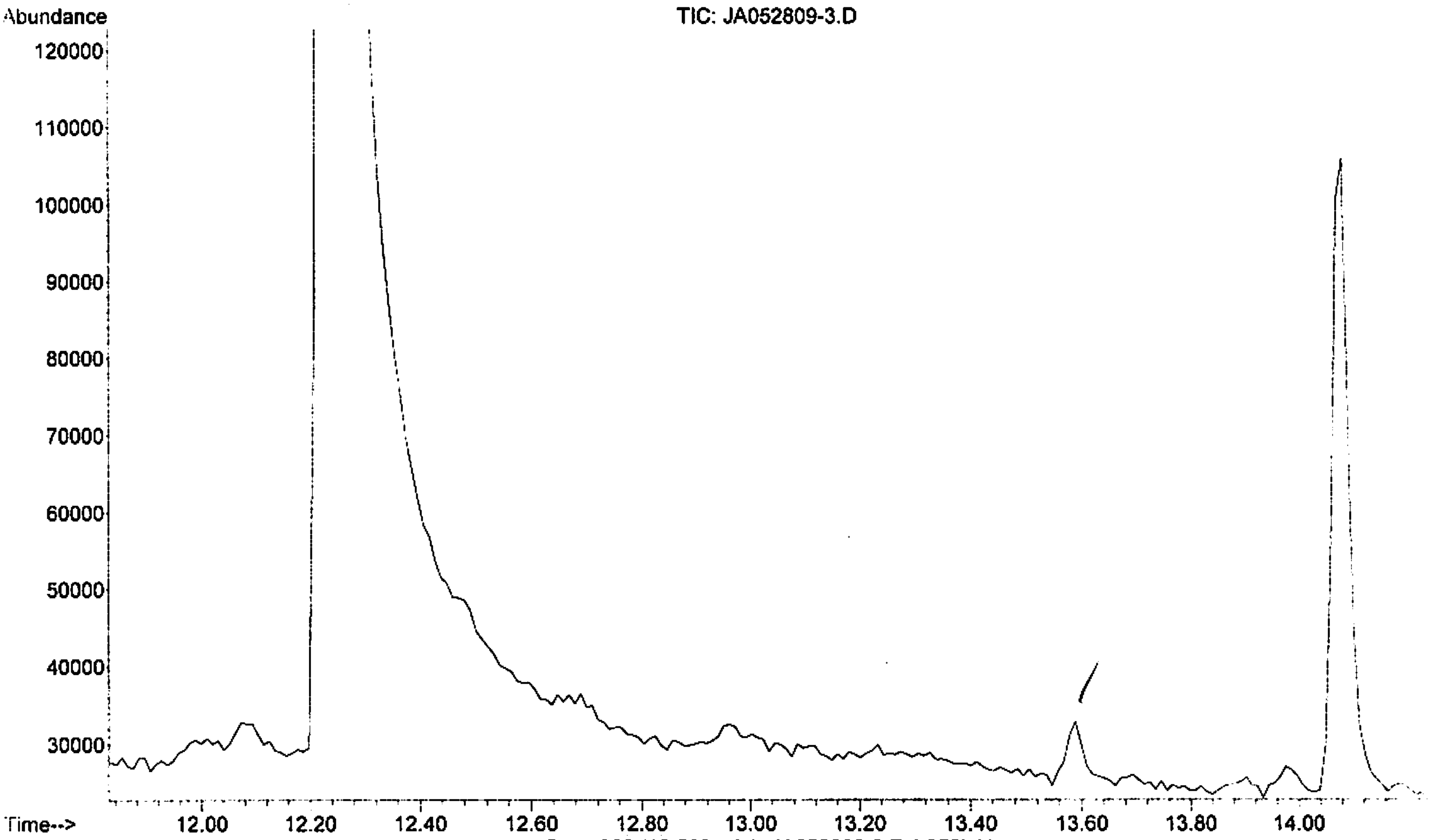

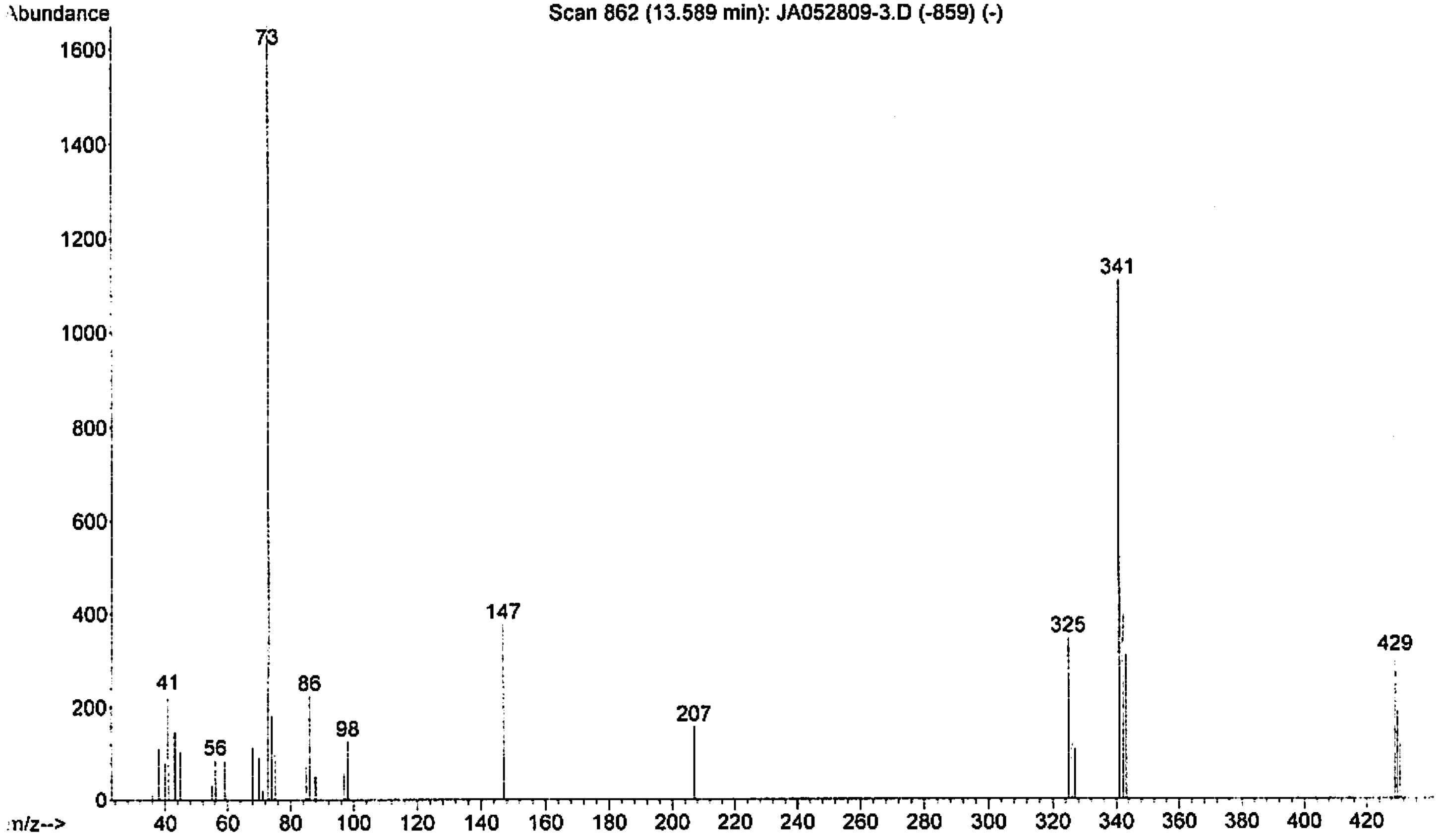

[illegible] :D:\DATA\ALDRICH\JA-09\Snapshot\JA052809-3.D
[illegible]rator : Aldrich
[illegible]uired : 28 May 2009 15:24 using AcqMethod JA-WAX08.M
[illegible]rument : Instrument #1
[illegible]ple Name: 1 field-coll. M C.oculata abd./CH2Cl2
[illegible] Info : coll. 5/28 sweeping vetch; squashed
[illegible] Number: 1

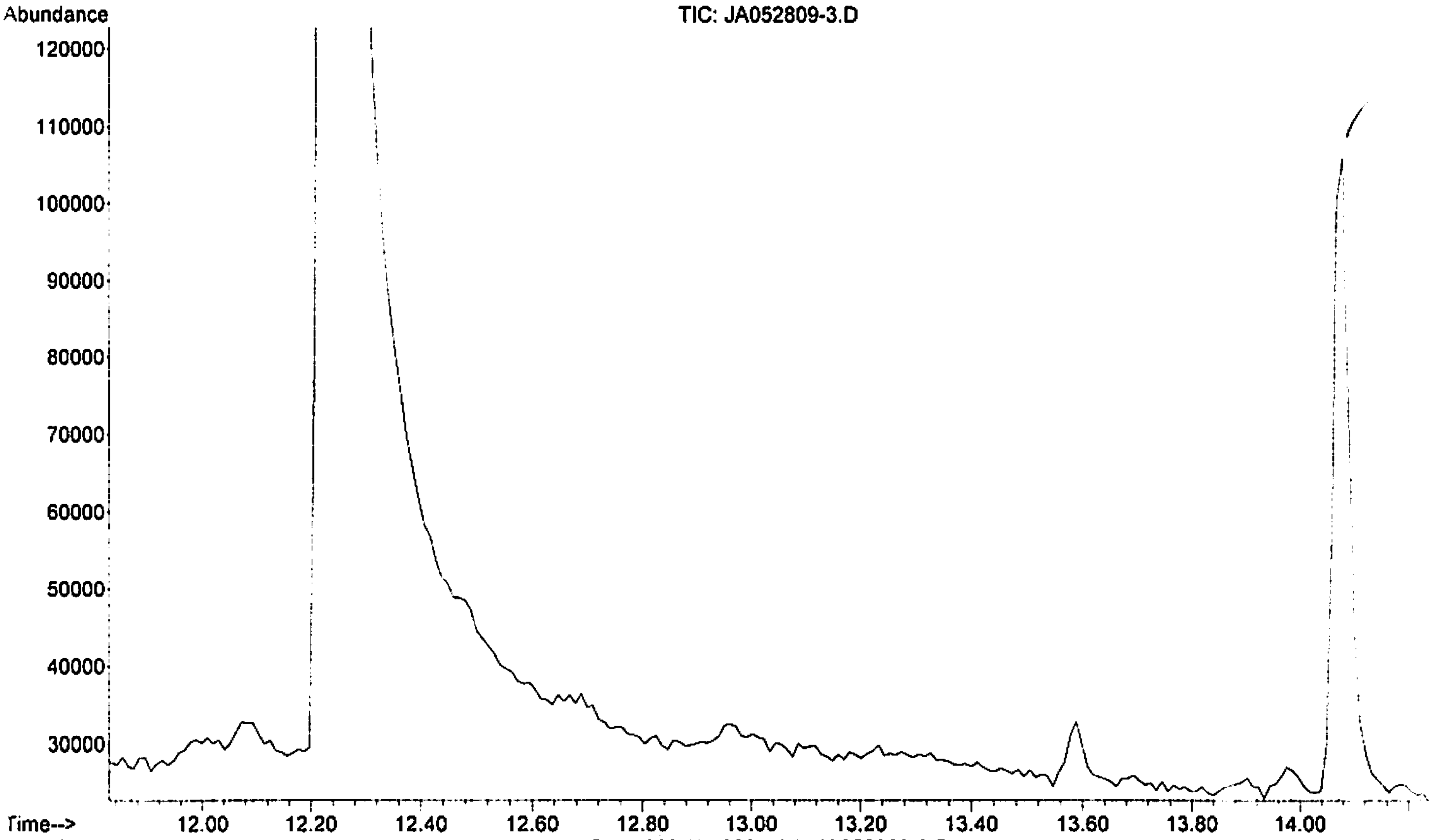

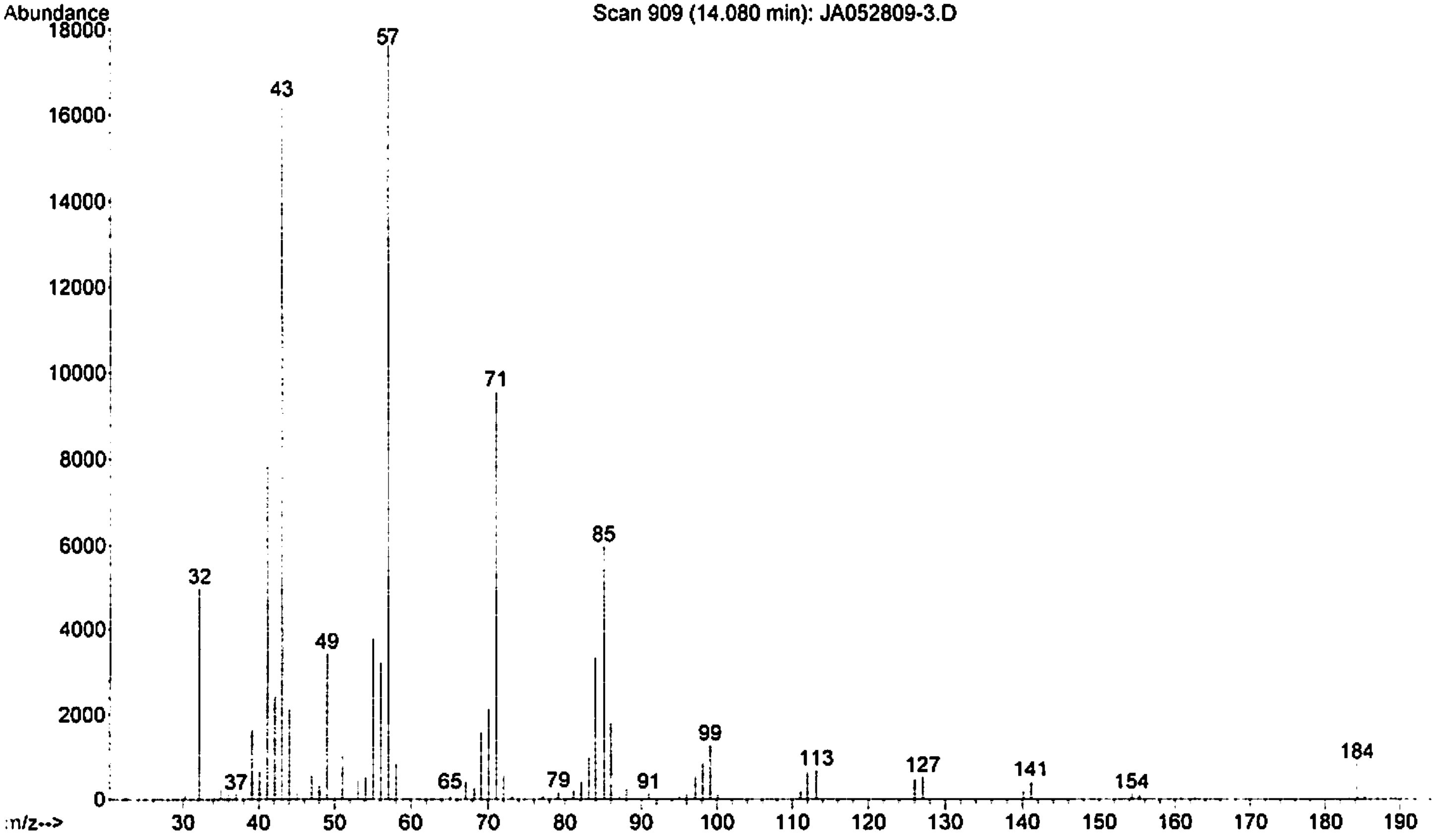

[illegible]e :D:\DATA\ALDRICH\JA-09\Snapshot\JA052809-3.D
[illegible]erator : Aldrich
[illegible]quired : 28 May 2009 15:24 using AcqMethod JA-WAX08.M
[illegible]strument : Instrument #1
[illegible]ple Name: 1 field-coll. M C.oculata abd./CH2Cl2
[illegible] Info : coll. 5/28 sweeping vetch; squashed
[illegible]al Number: 1

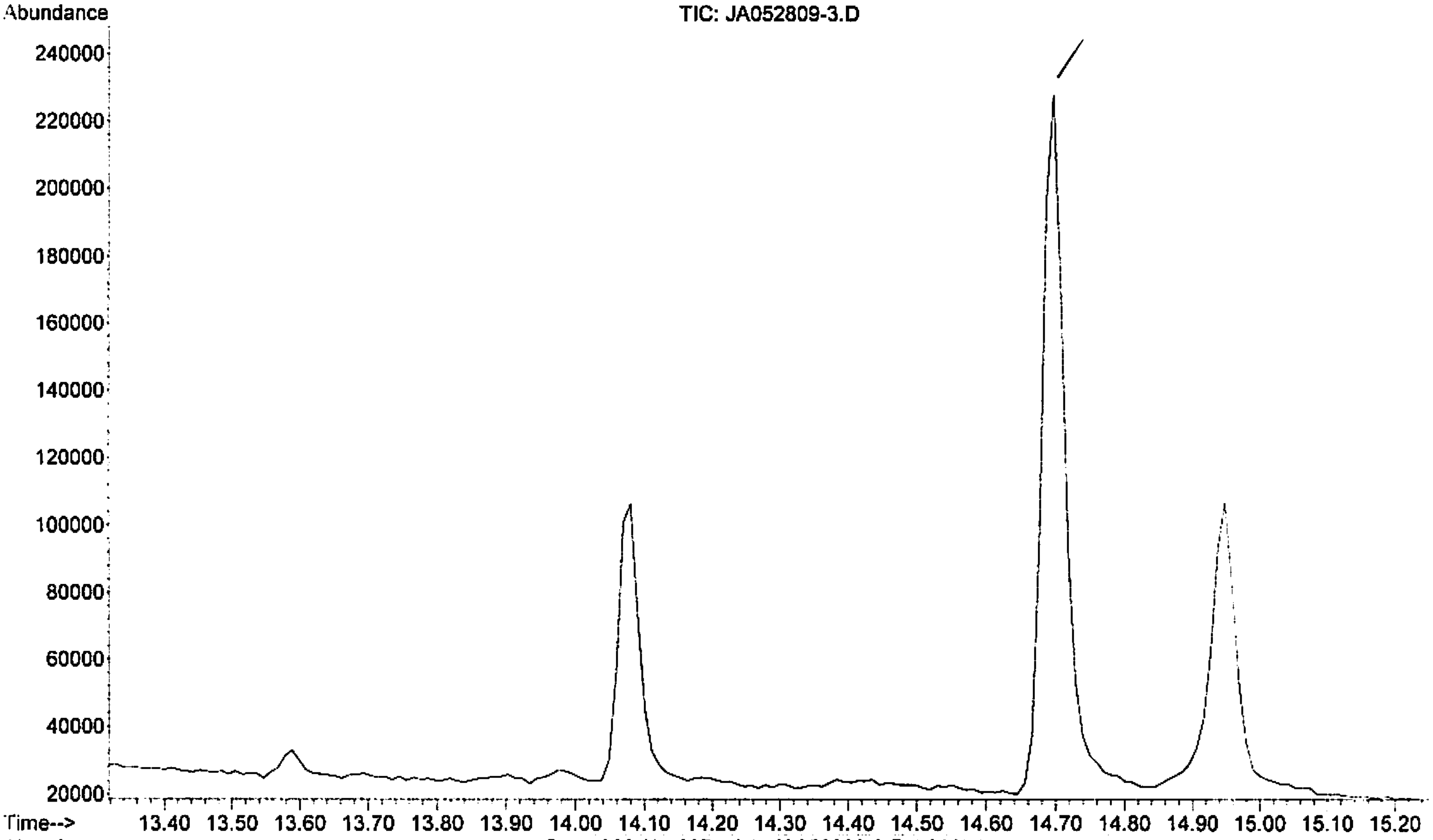

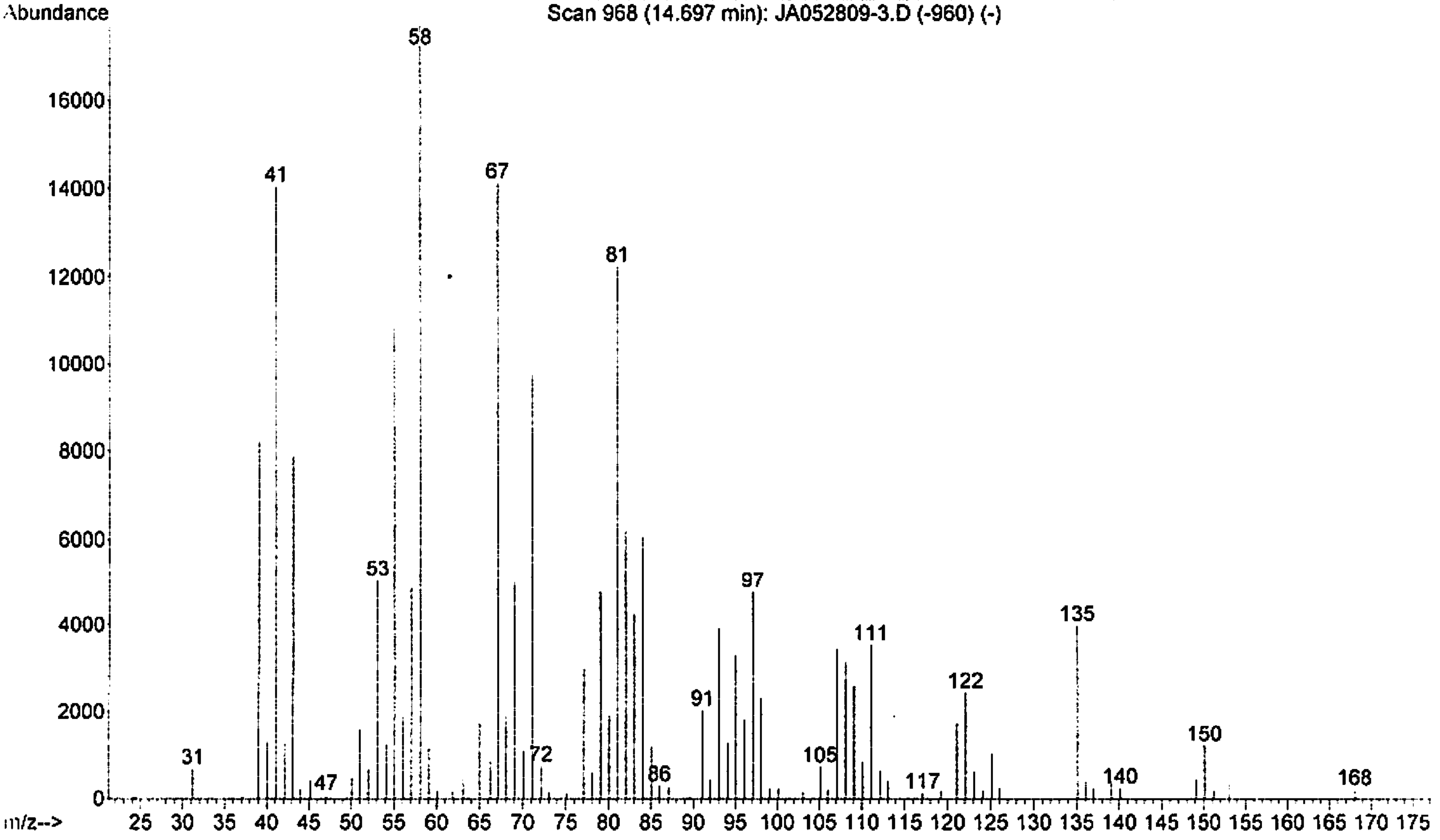

[illegible]e :D:\DATA\ALDRICH\JA-09\Snapshot\JA052809-3.D
[illegible]rator : Aldrich
[illegible]ired : 28 May 2009 15:24 using AcqMethod JA-WAX08.M
[illegible]rument : Instrument #1
[illegible]le Name: 1 field-coll. M C.oculata abd./CH2Cl2
[illegible] Info : coll. 5/28 sweeping vetch; squashed
[illegible] Number: 1

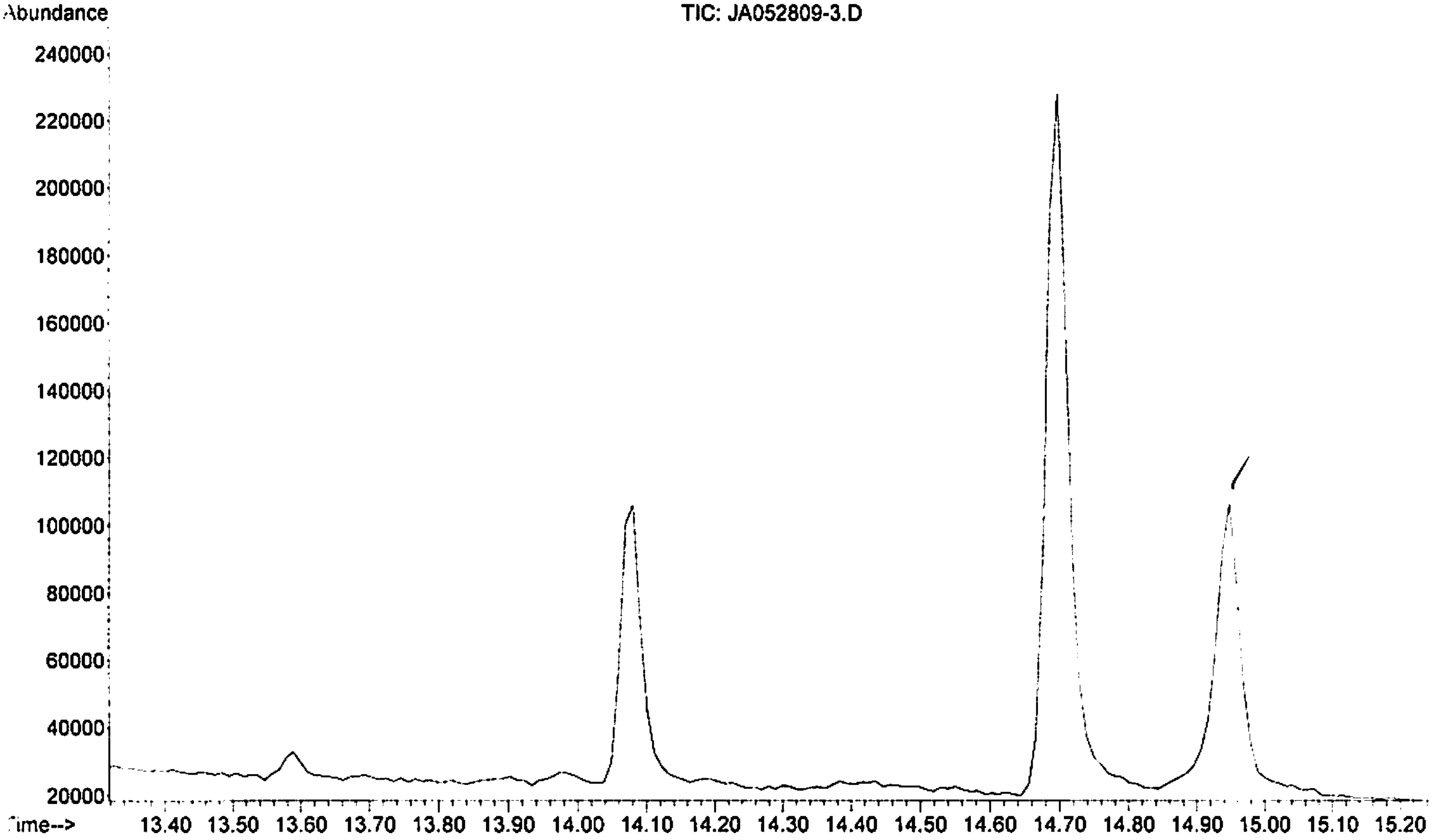

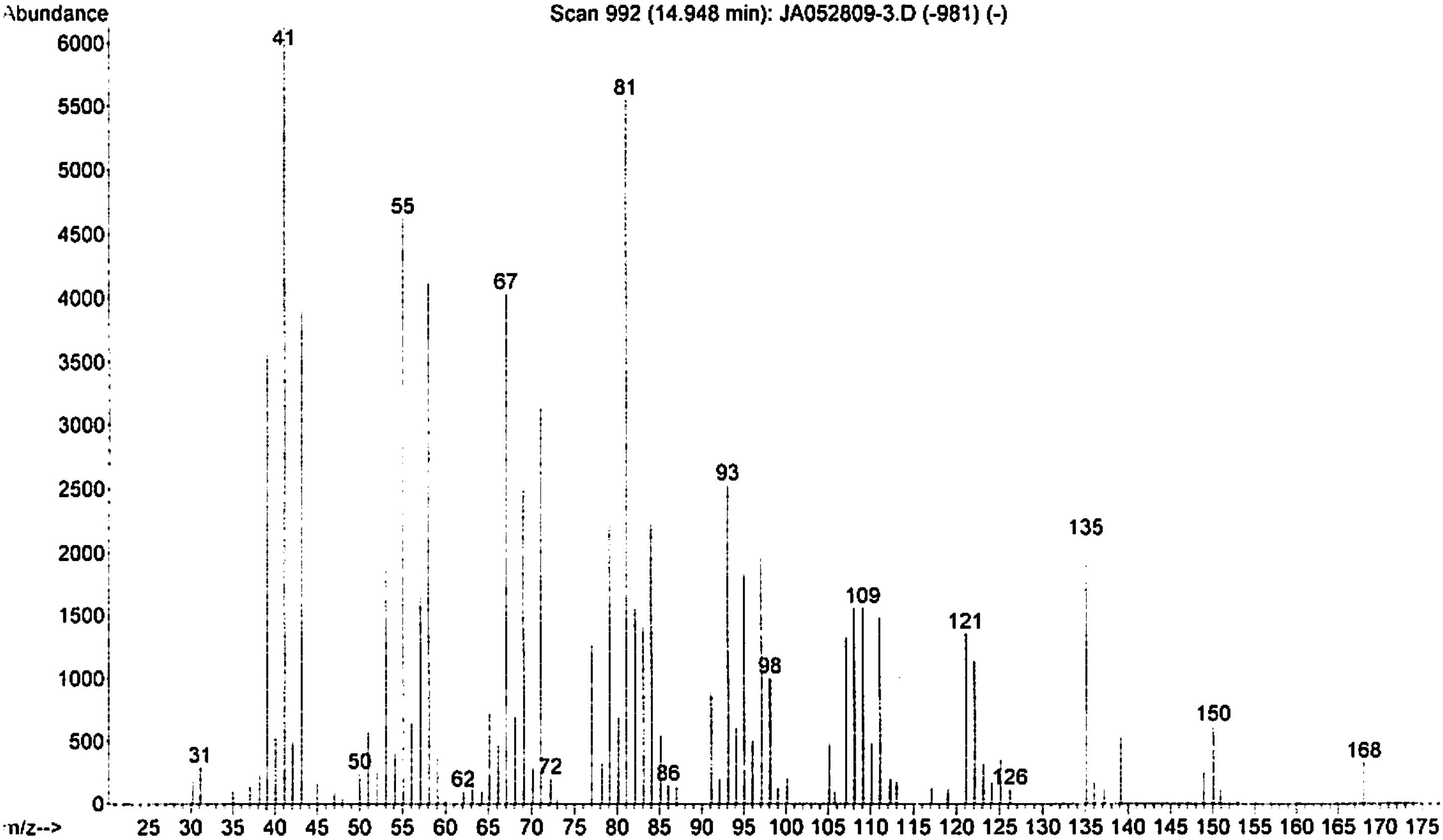

[illegible] :D:\DATA\ALDRICH\JA-09\Snapshot\JA052809-3.D
[illegible]rator : Aldrich
[illegible]ired : 28 May 2009 15:24 using AcqMethod JA-WAX08.M
[illegible]rument : Instrument #1
[illegible]le Name: 1 field-coll. M C.oculata abd./CH2Cl2
[illegible] Info : coll. 5/28 sweeping vetch; squashed
[illegible] Number: 1

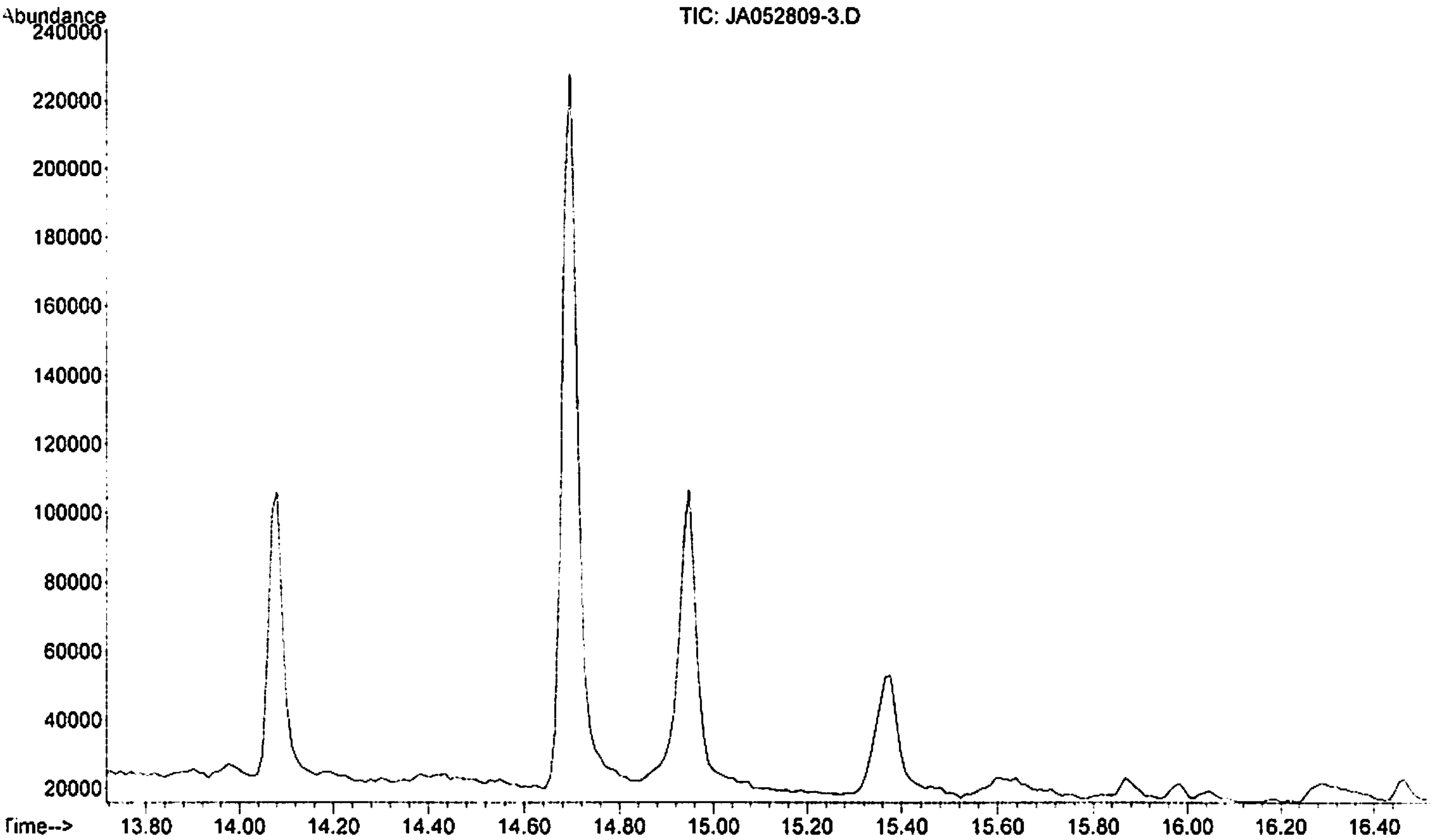

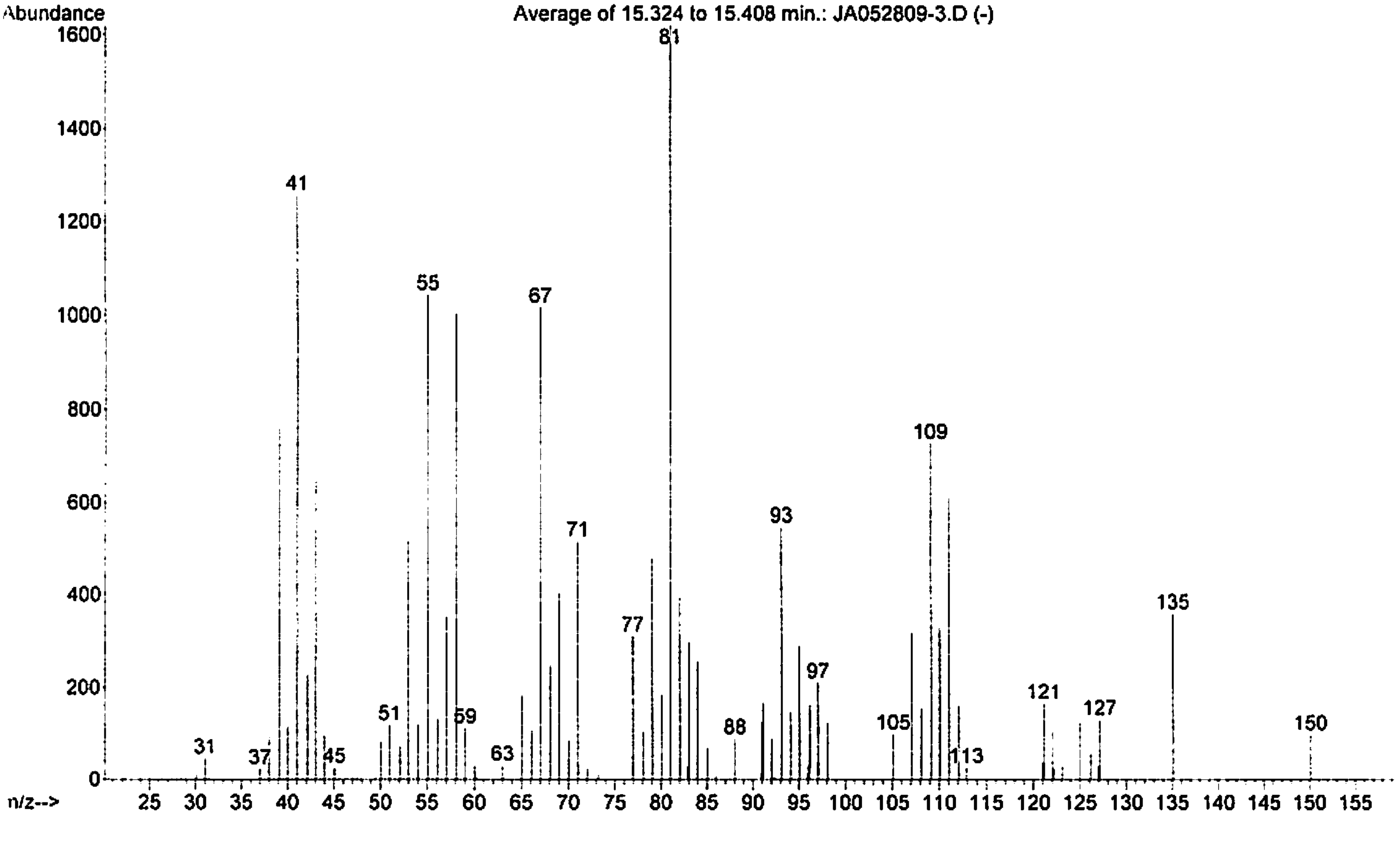

[illegible]e :D:\DATA\ALDRICH\JA-09\Snapshot\JA052809-3.D
[illegible]erator : Aldrich
[illegible]quired : 28 May 2009 15:24 using AcqMethod JA-WAX08.M
[illegible]strument : Instrument #1
[illegible]mple Name: 1 field-coll. M C.oculata abd./CH2Cl2
[illegible] Info : coll. 5/28 sweeping vetch; squashed
[illegible] Number: 1

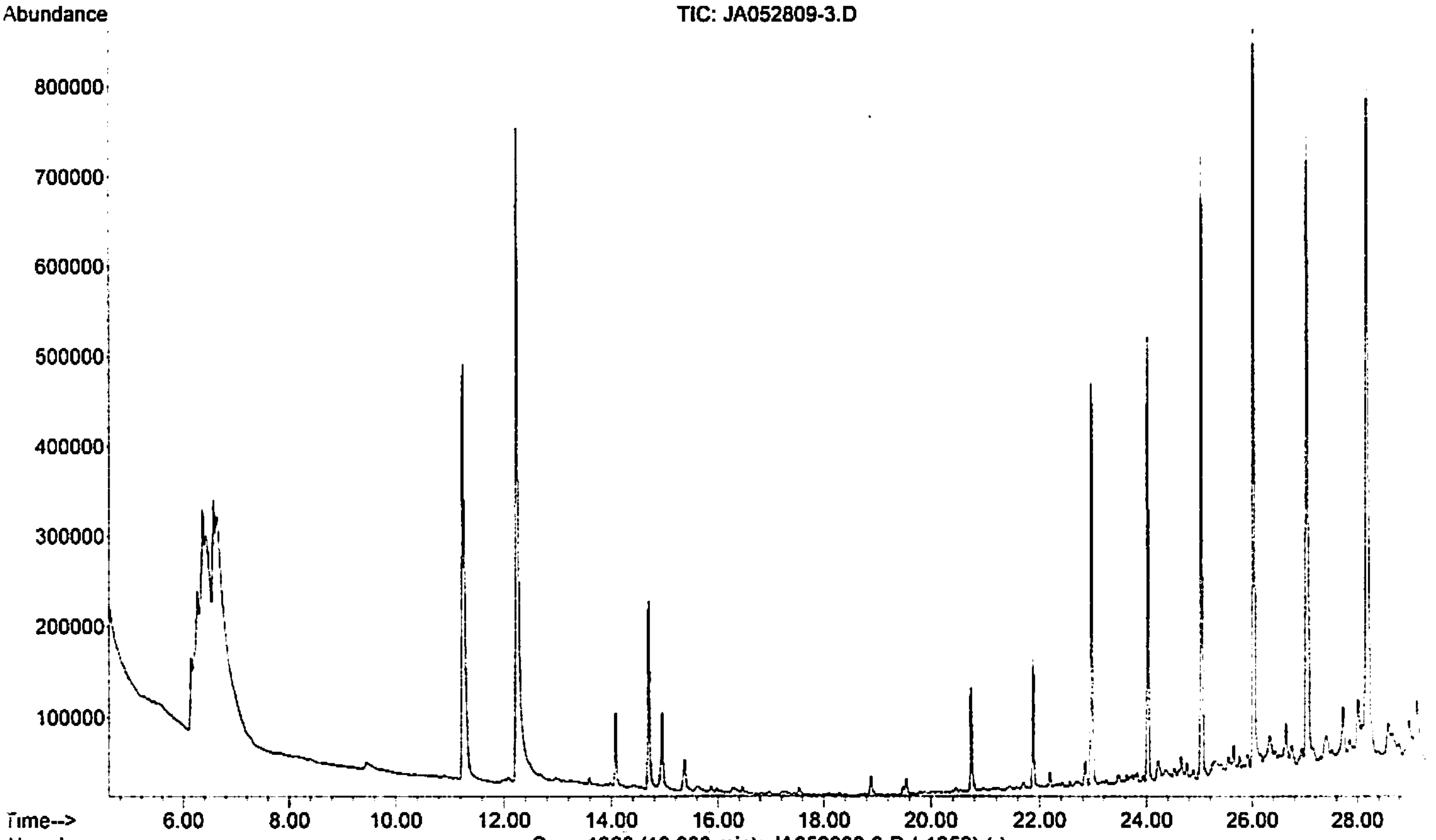

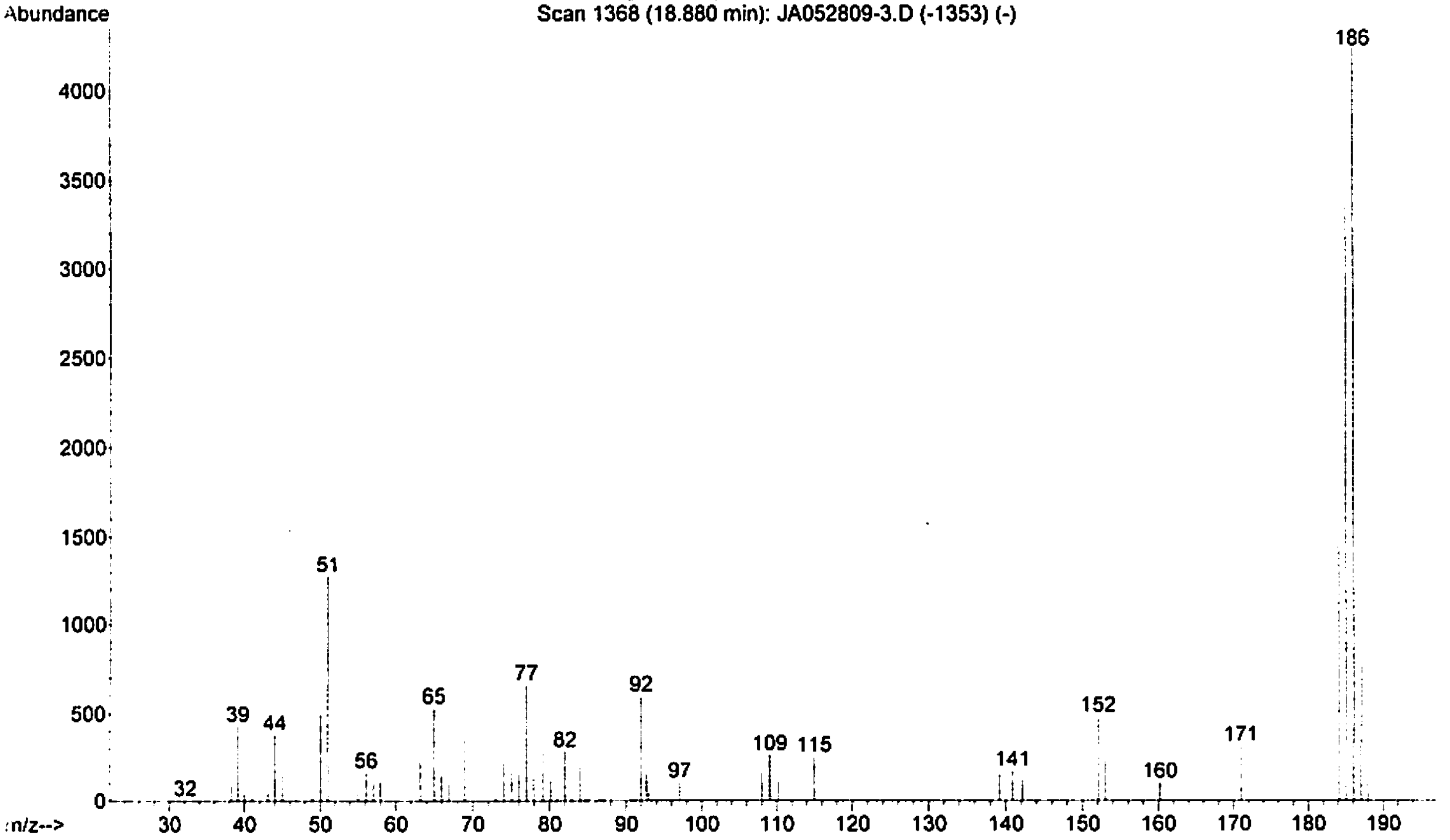

```
[illegible]      :D:\DATA\ALDRICH\JA-09\Snapshot\JA052809-3.D
[illegible]rator    : Aldrich
[illegible]quired   : 28 May 2009  15:24     using AcqMethod JA-WAX08.M
[illegible]rument :     Instrument #1
[illegible]ple Name: 1 field-coll. M C.oculata abd./CH2Cl2
[illegible] Info   : coll. 5/28 sweeping vetch; squashed
[illegible] Number: 1
```

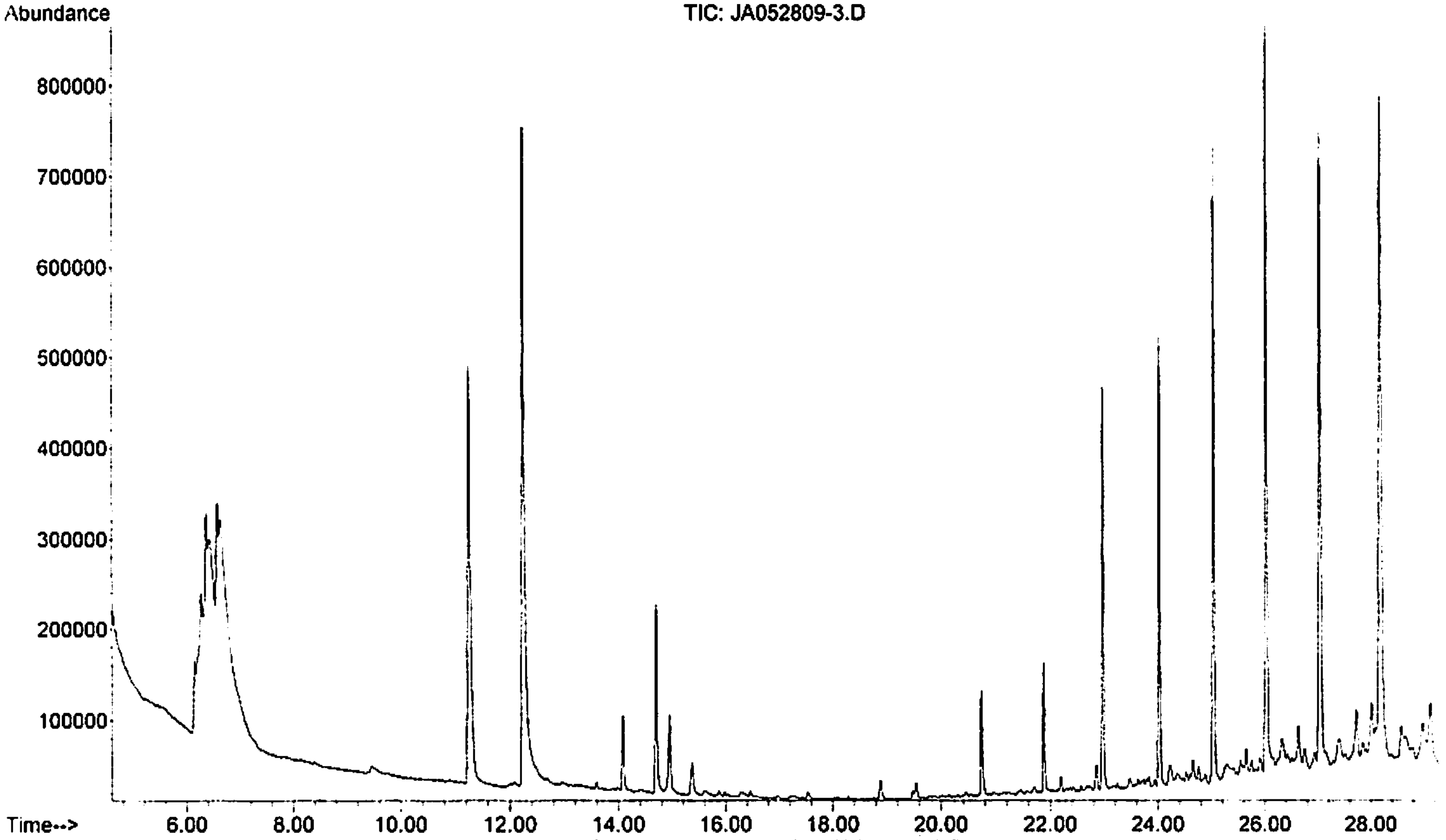

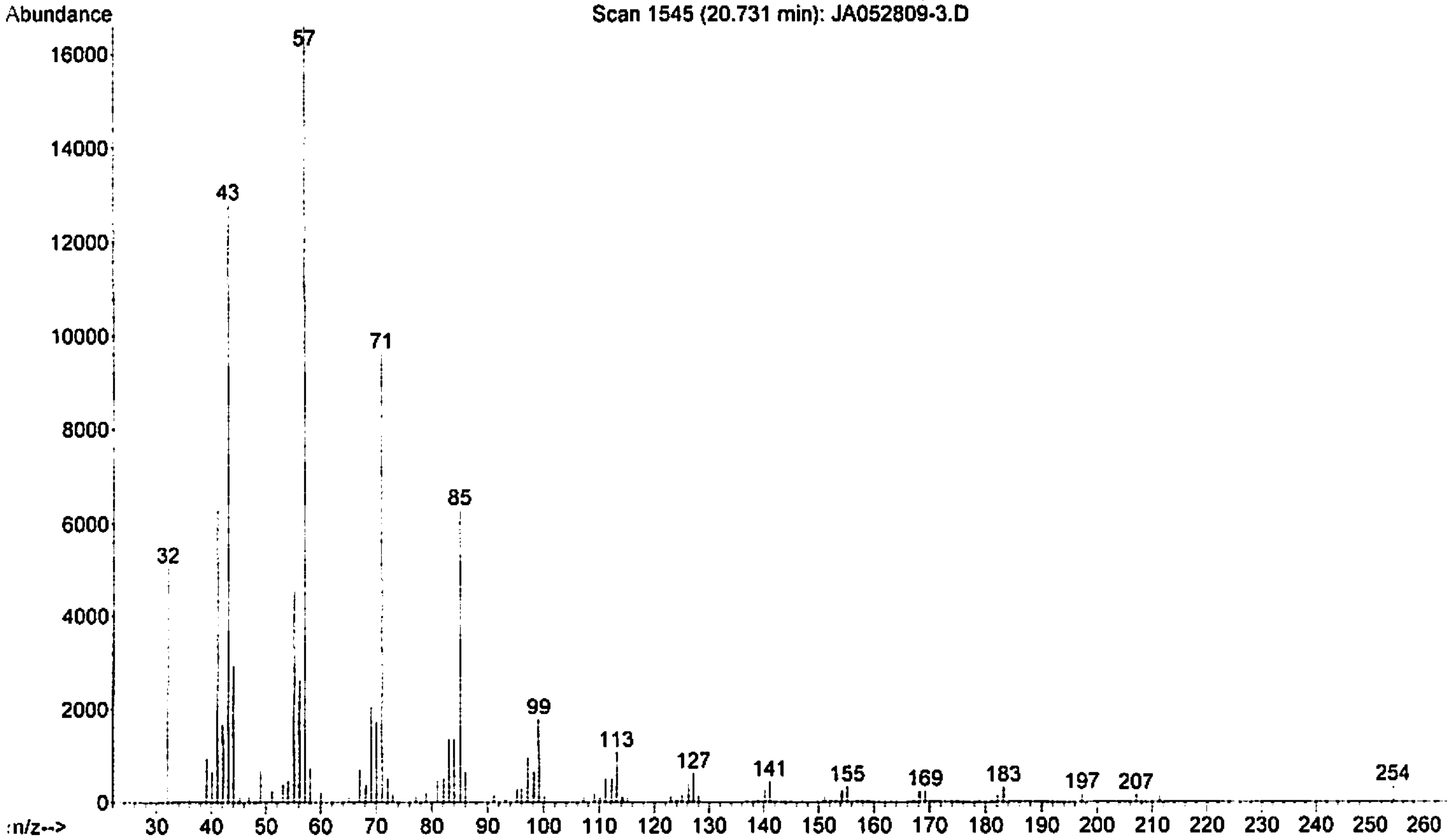

```
File         :D:\DATA\Aldrich\JA-09\JA052809-4.D
Operator     : Aldrich
Acquired     : 28 May 2009  16:03     using AcqMethod JA-WAX08.M
Instrument :    Instrument #1
Sample Name: 1 field-coll. M C.oculata abd./CH2Cl2
Misc Info   : coll. 5/28 sweeping vetch; second male today
Vial Number: 1
```

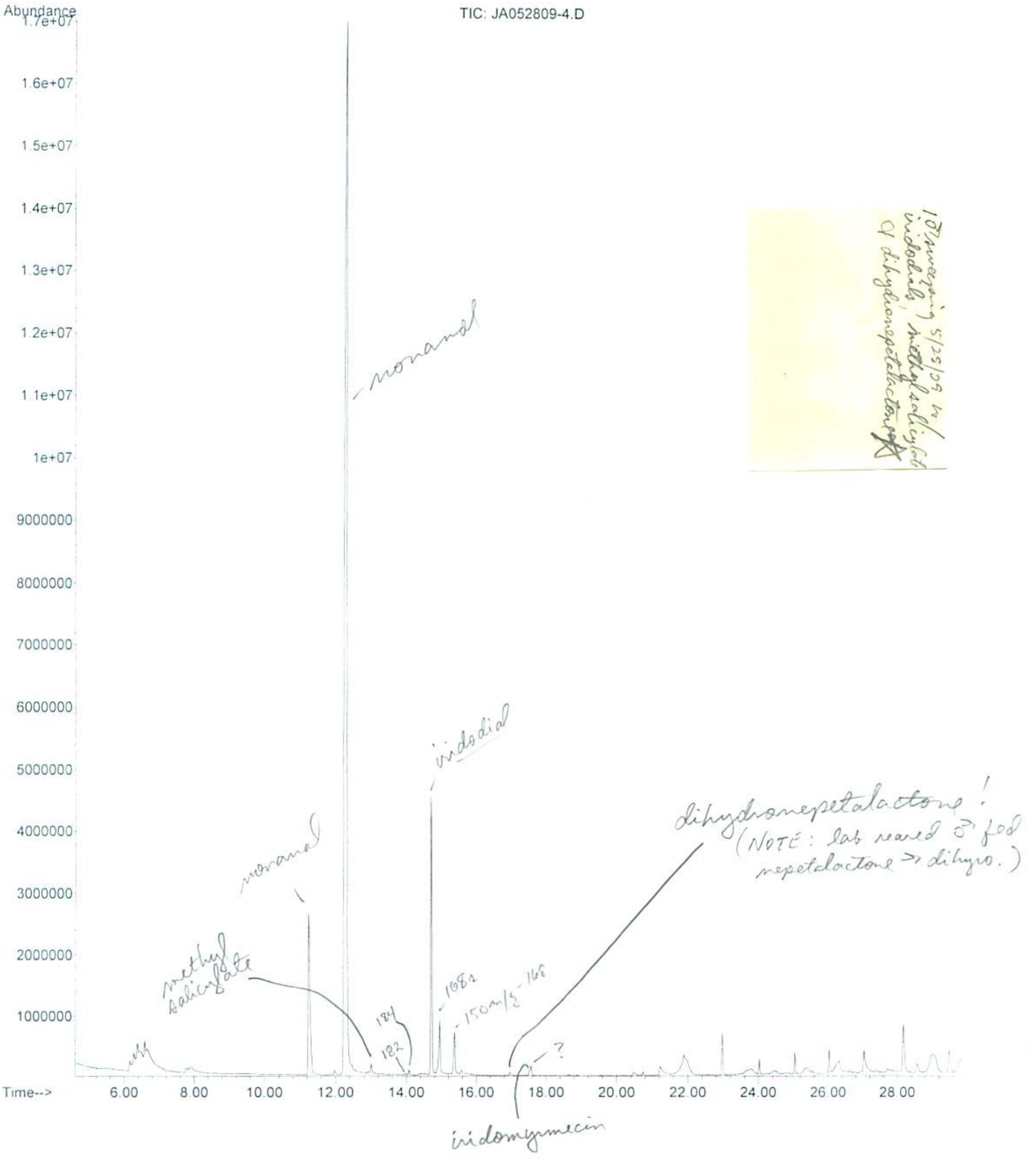

[illegible]e : D:\DATA\ALDRICH\JA-09\Snapshot\JA052809-4.D
[illegible]rator : Aldrich
[illegible]ired : 28 May 2009 16:03 using AcqMethod JA-WAX08.M
[illegible]rument : Instrument #1
[illegible]le Name: 1 field-coll. M C.oculata abd./CH2Cl2
[illegible] Info : coll. 5/28 sweeping vetch; second male today
[illegible] Number: 1

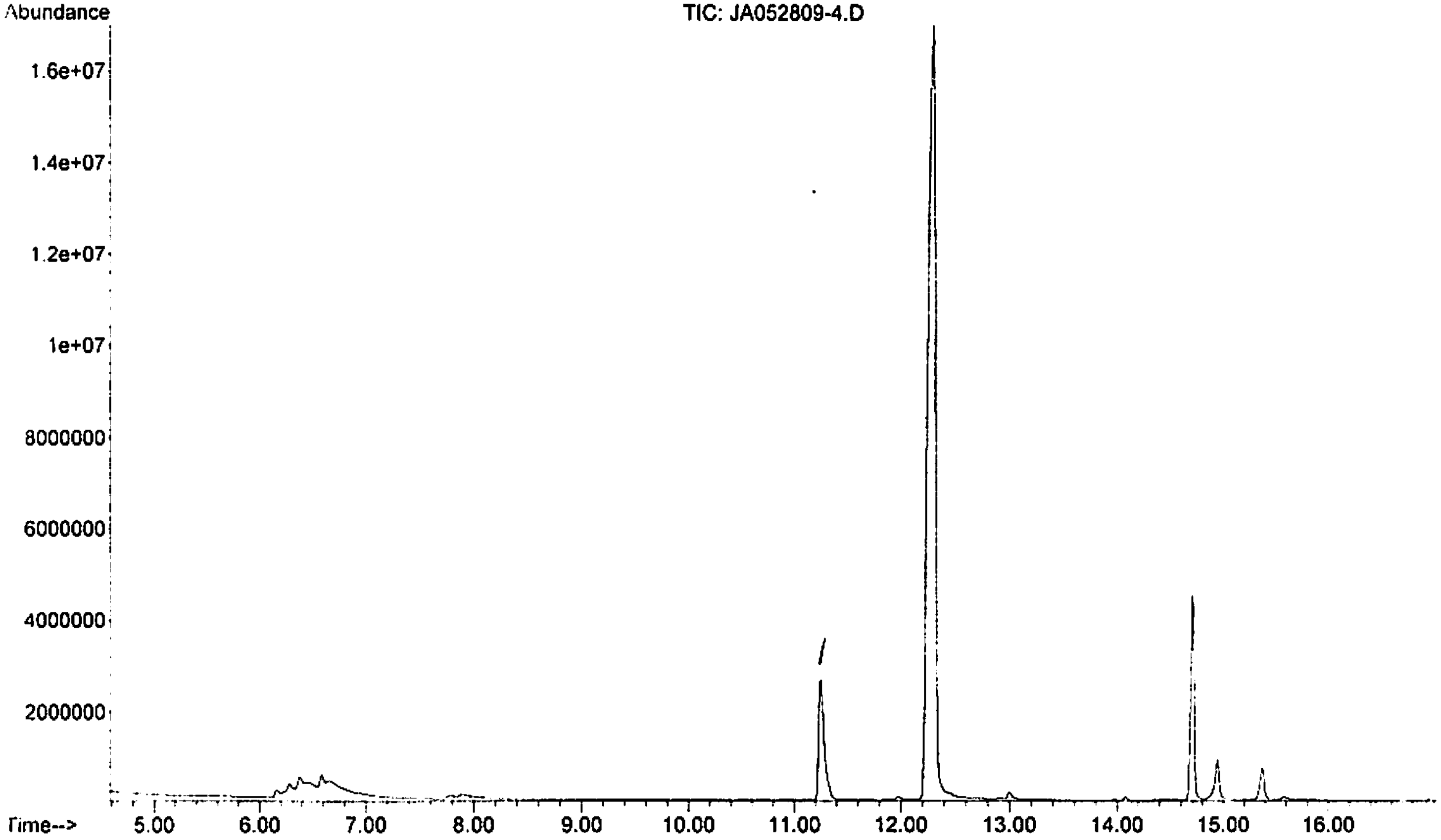

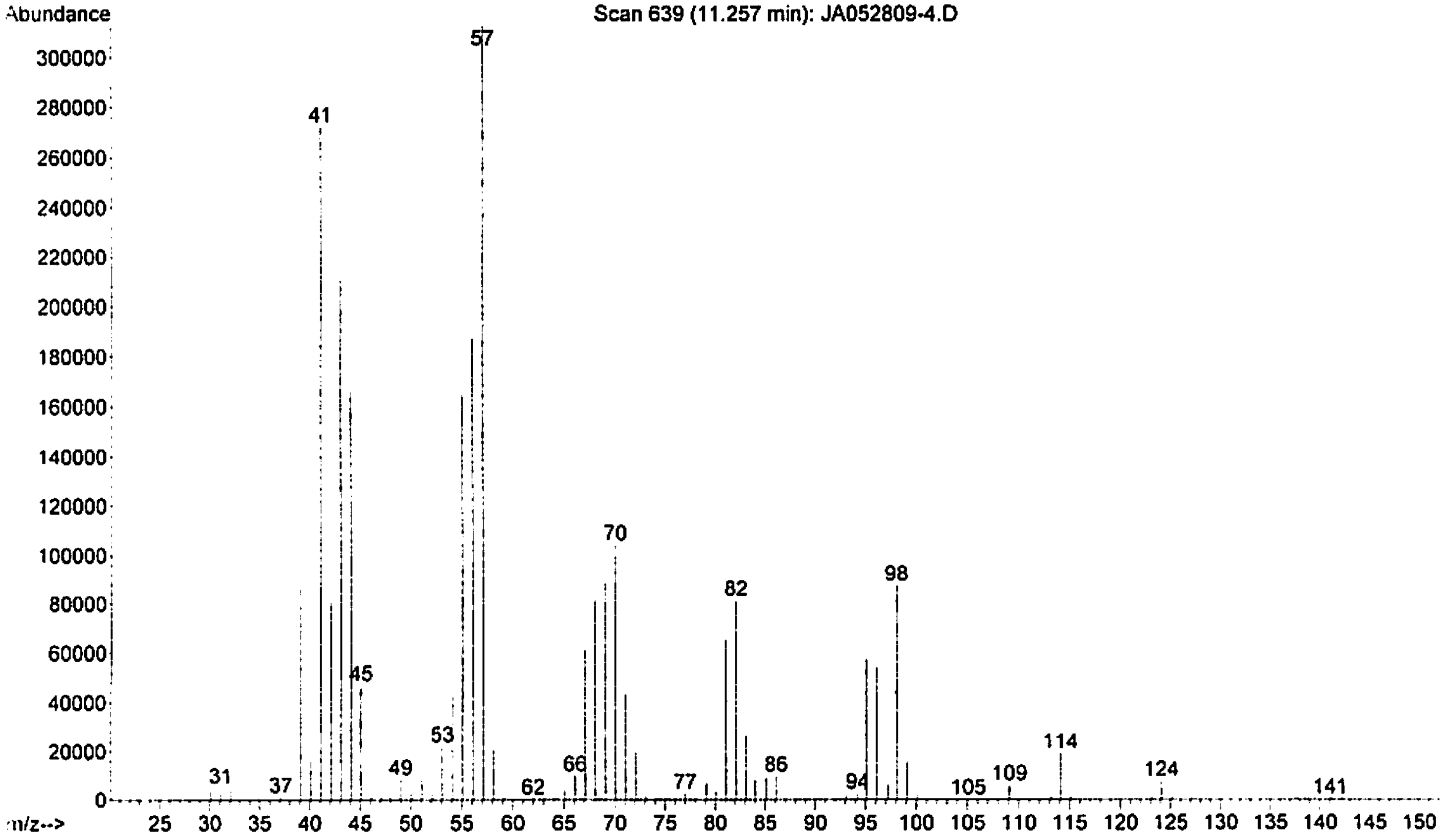

e :D:\DATA\ALDRICH\JA-09\Snapshot\JA052809-4.D
ator : Aldrich
ired : 28 May 2009 16:03 using AcqMethod JA-WAX08.M
rument : Instrument #1
ple Name: 1 field-coll. M C.oculata abd./CH2Cl2
Info : coll. 5/28 sweeping vetch; second male today
Number: 1

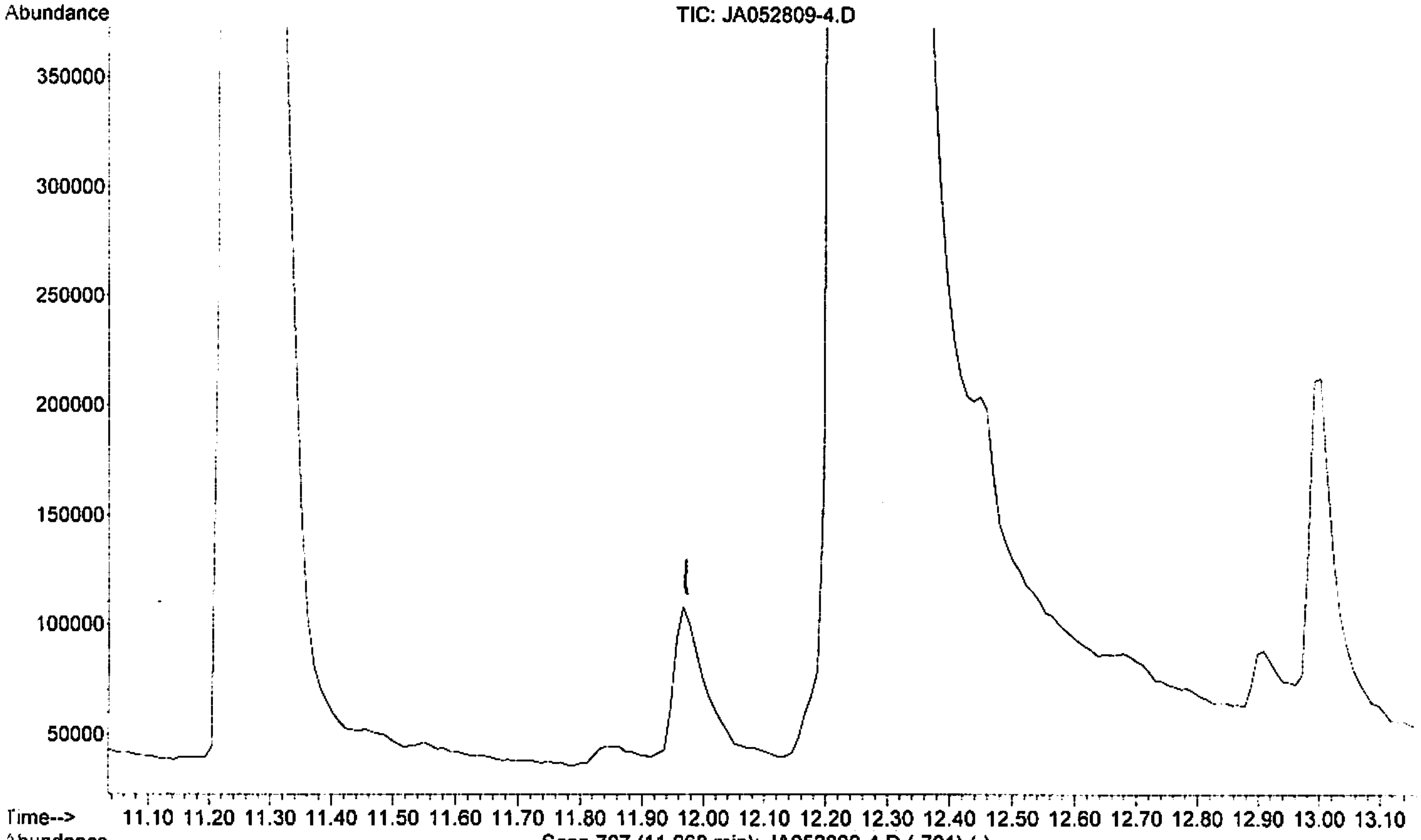

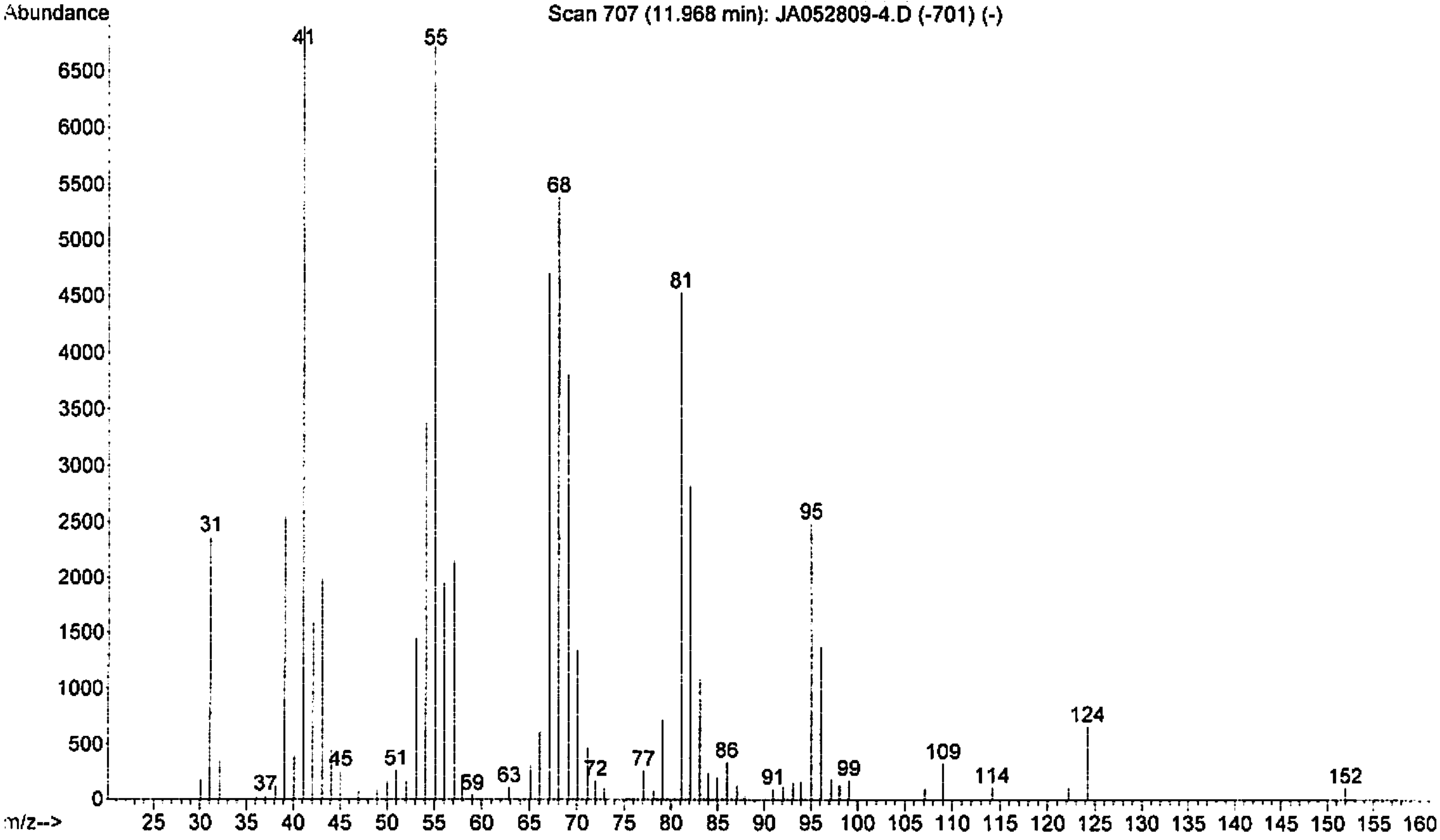

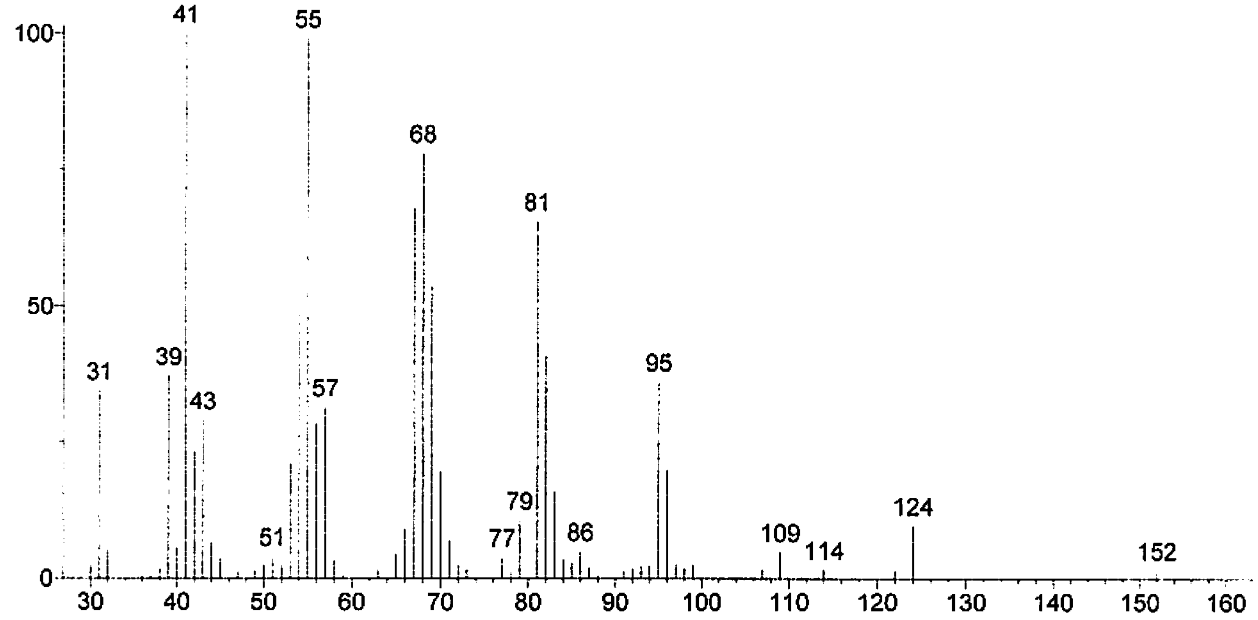

(Text File) Scan 707 (11.968 min): JA052809-4.D (-701)

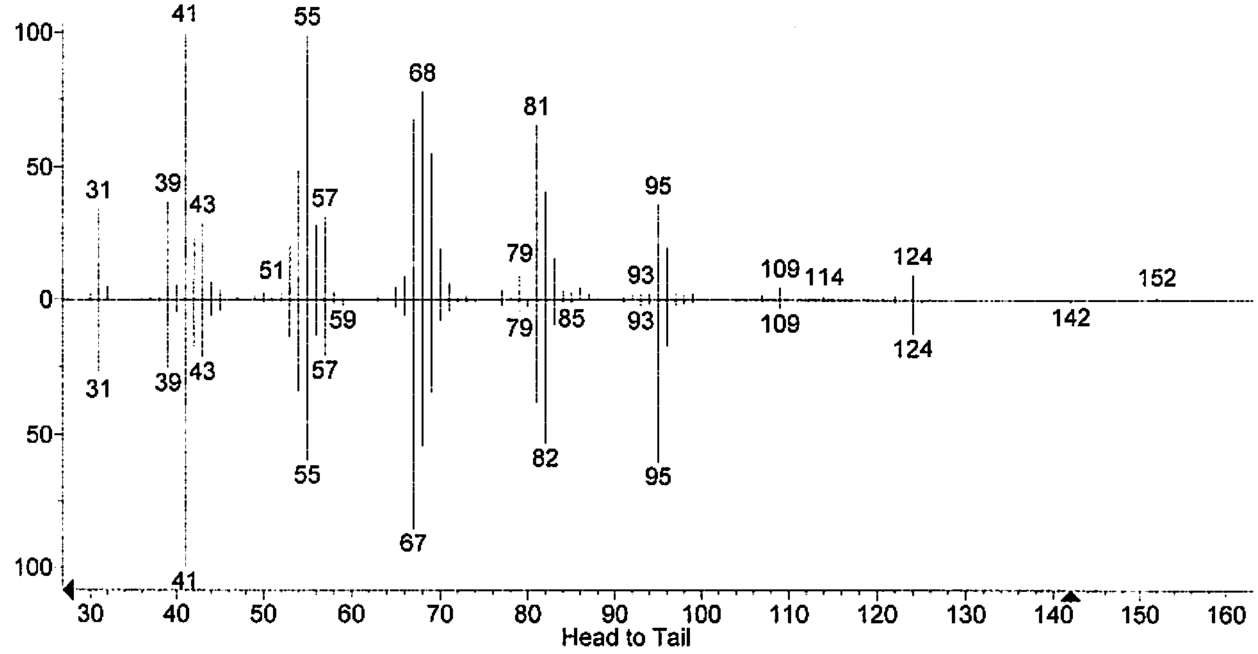

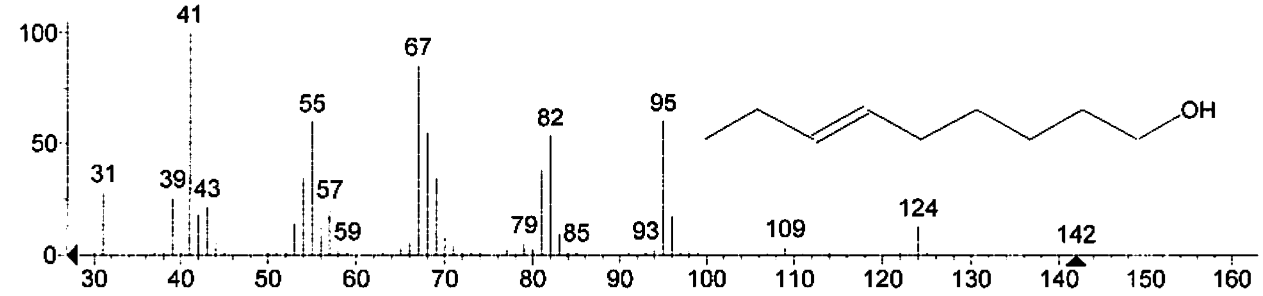

(mainlib) 6-Nonen-1-ol, (E)-

```
[illegible]e'        :D:\DATA\ALDRICH\JA-09\Snapshot\JA052809-4.D
[illegible]erator    : Aldrich
[illegible]quired    : 28 May 2009  16:03     using AcqMethod JA-WAX08.M
[illegible]strument :     Instrument #1
[illegible]ple Name: 1 field-coll. M C.oculata abd./CH2Cl2
[illegible]sc Info   : coll. 5/28 sweeping vetch; second male today
[illegible]al Number: 1
```

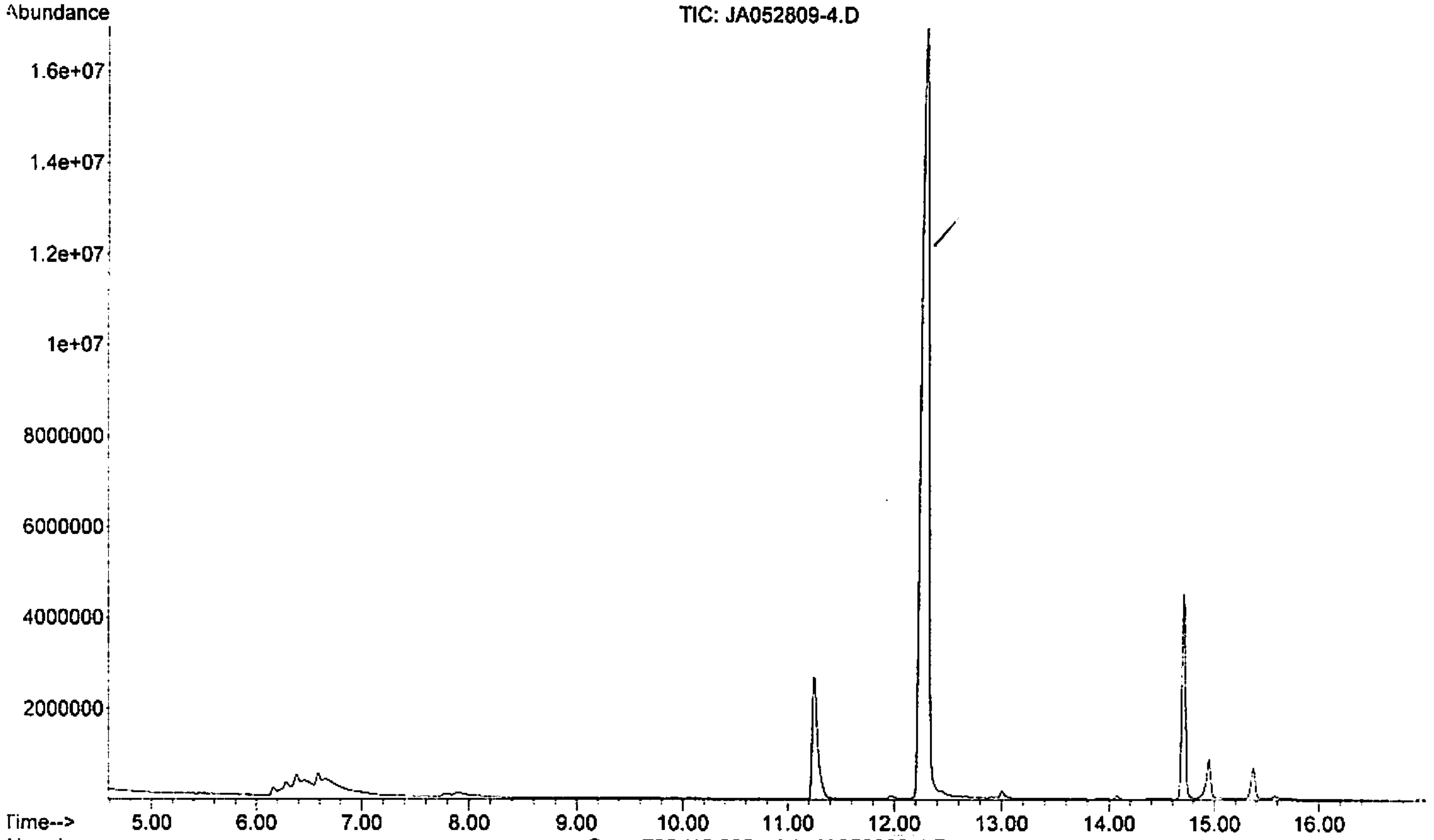

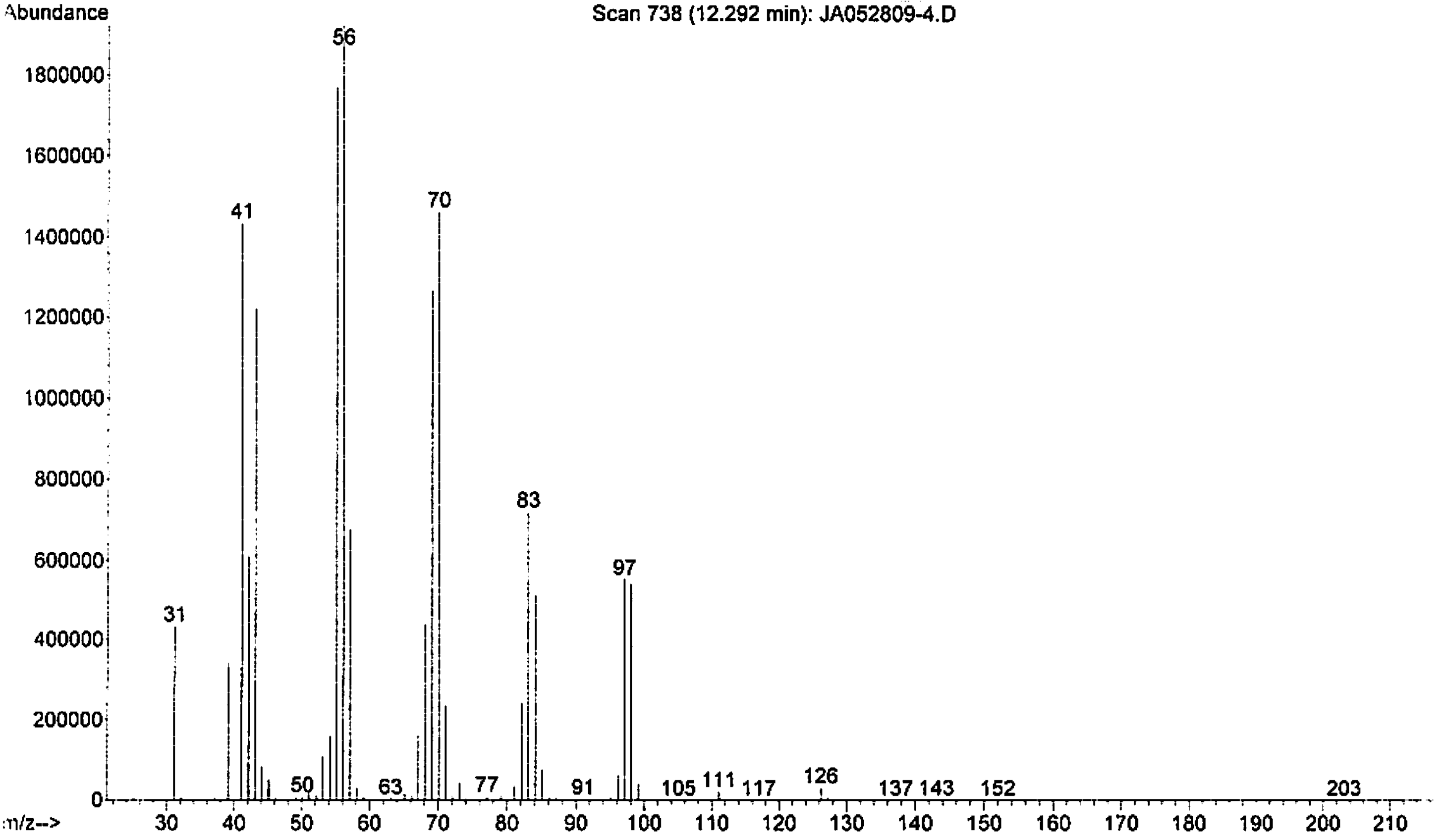

[illegible] :D:\DATA\ALDRICH\JA-09\Snapshot\JA052809-4.D
[illegible]rator : Aldrich
[illegible]ired : 28 May 2009 16:03 using AcqMethod JA-WAX08.M
[illegible]rument : Instrument #1
[illegible]le Name: 1 field-coll. M C.oculata abd./CH2Cl2
[illegible] Info : coll. 5/28 sweeping vetch; second male today
[illegible] Number: 1

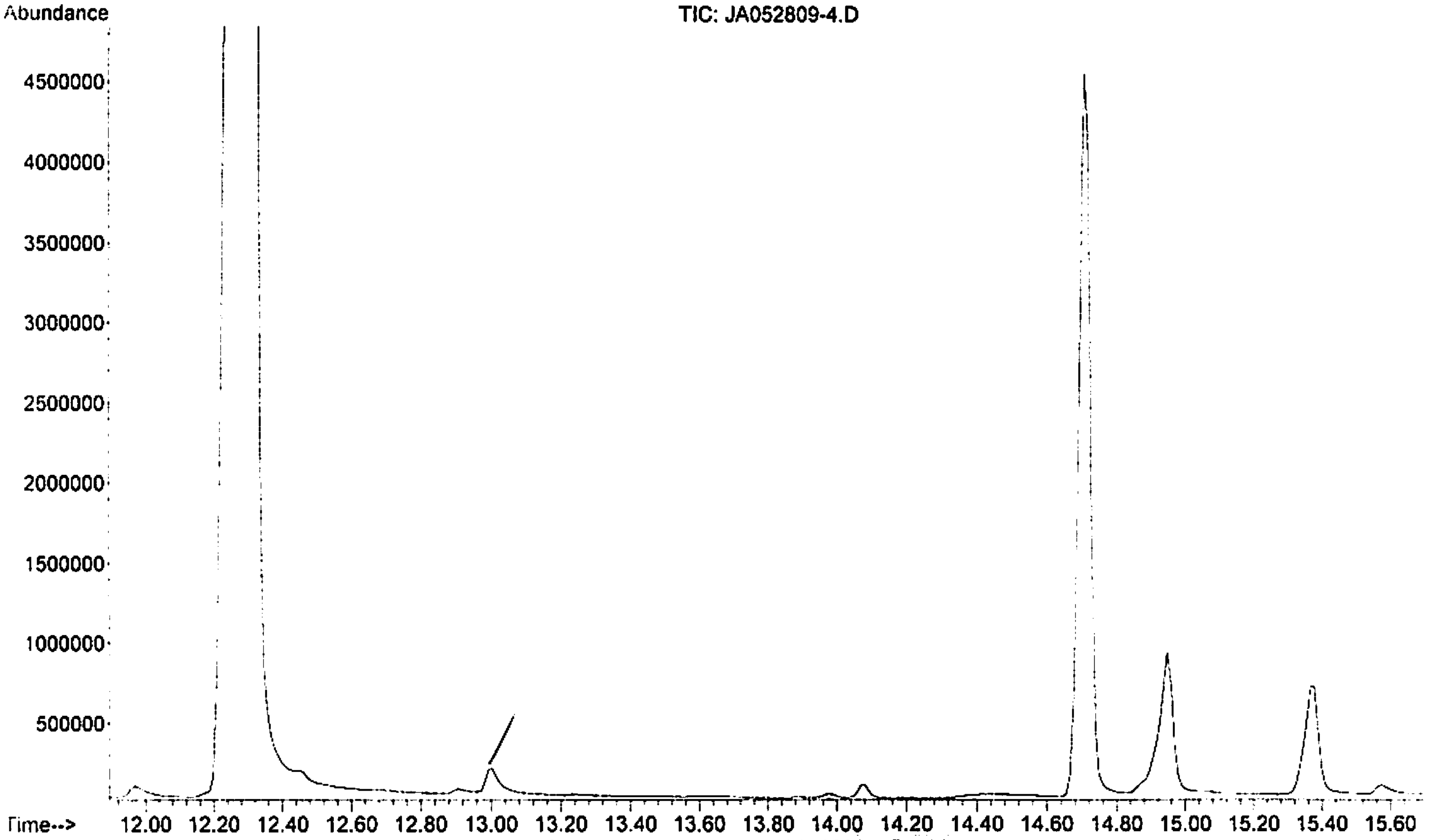

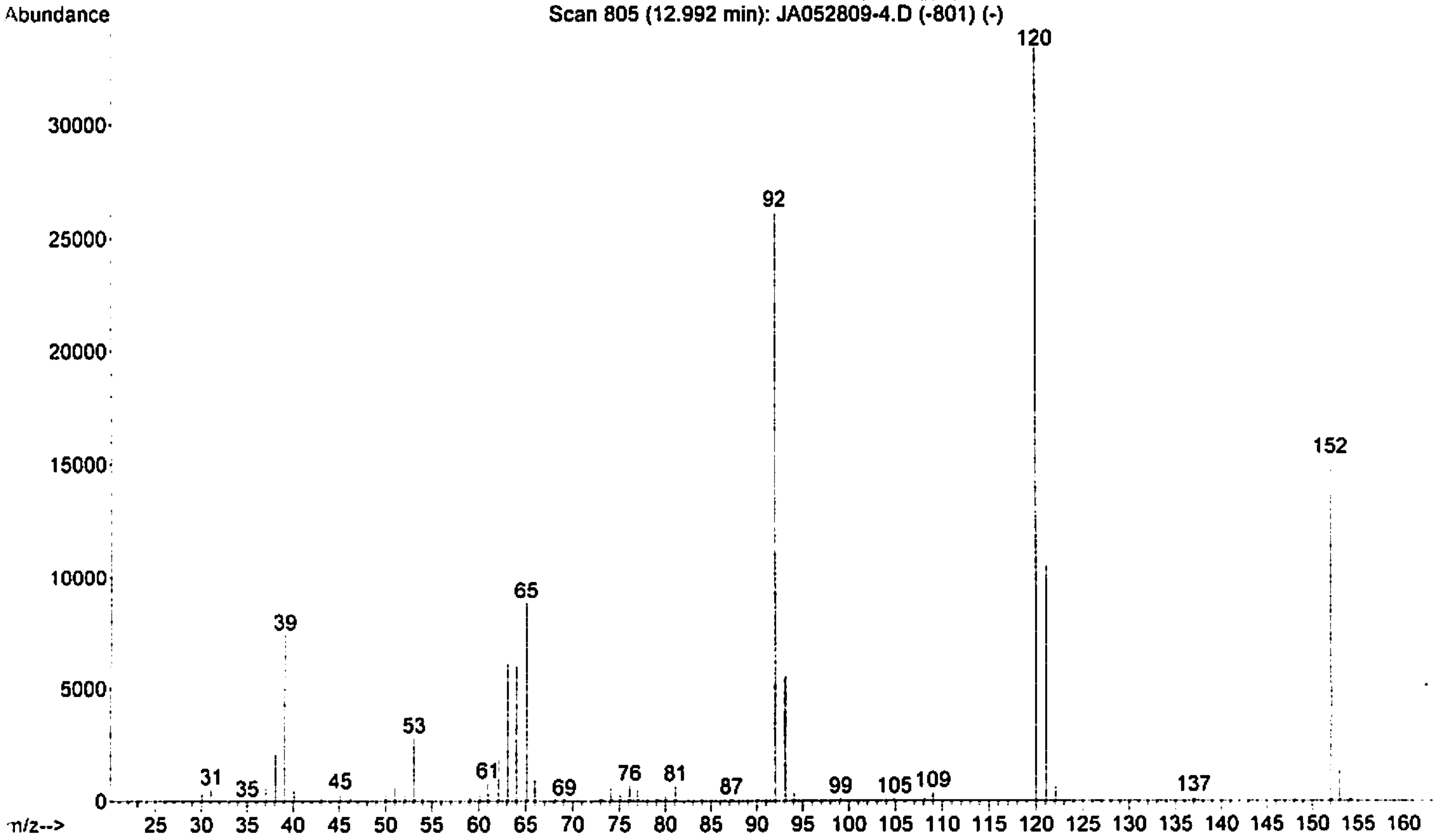

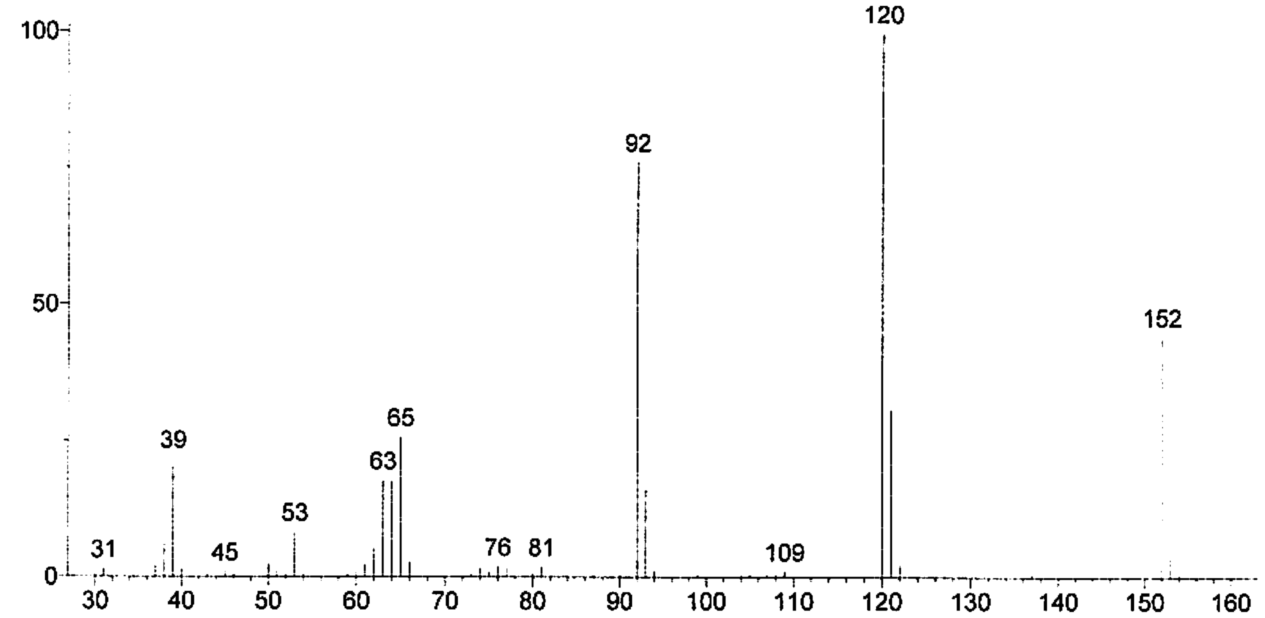

(Text File) Scan 805 (12.992 min): JA052809-4.D (-801)

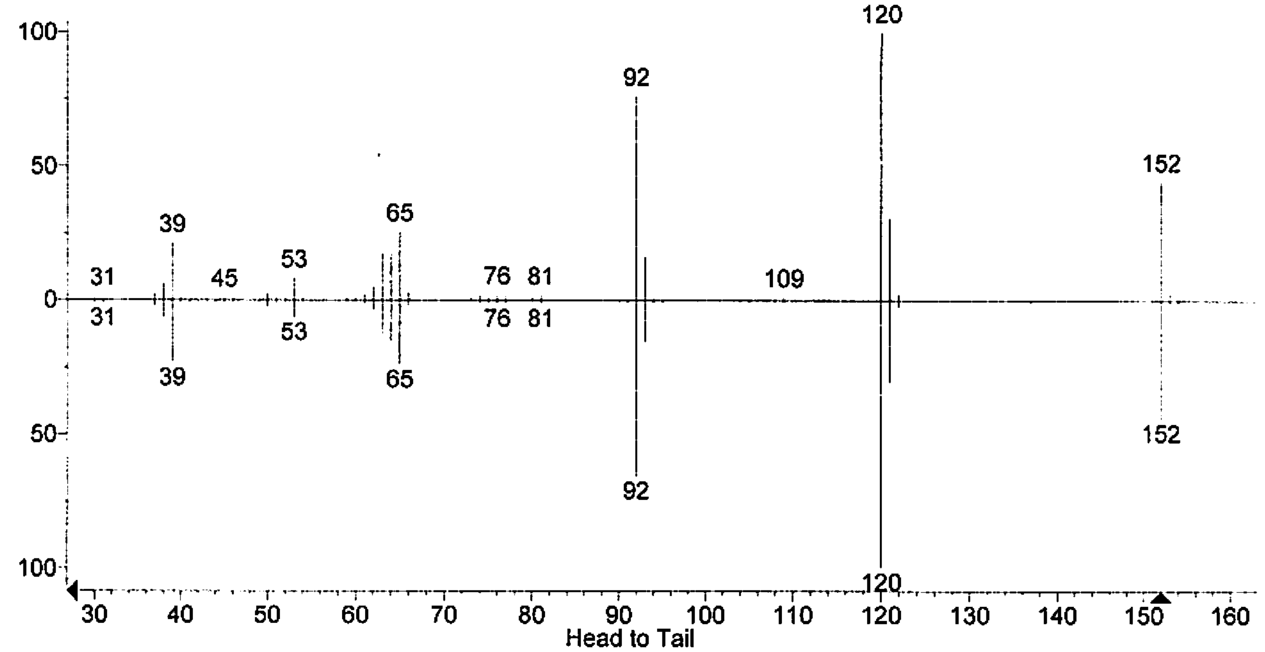

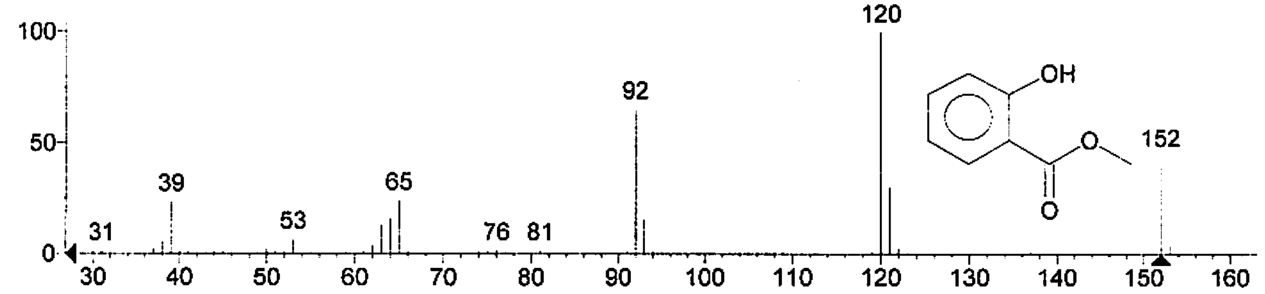

(mainlib) Methyl Salicylate

[illegible] :D:\DATA\ALDRICH\JA-09\Snapshot\JA052809-4.D
[illegible]rator : Aldrich
[illegible]ired : 28 May 2009 16:03 using AcqMethod JA-WAX08.M
[illegible]rument : Instrument #1
[illegible]le Name: 1 field-coll. M C.oculata abd./CH2Cl2
[illegible] Info : coll. 5/28 sweeping vetch; second male today
[illegible] Number: 1

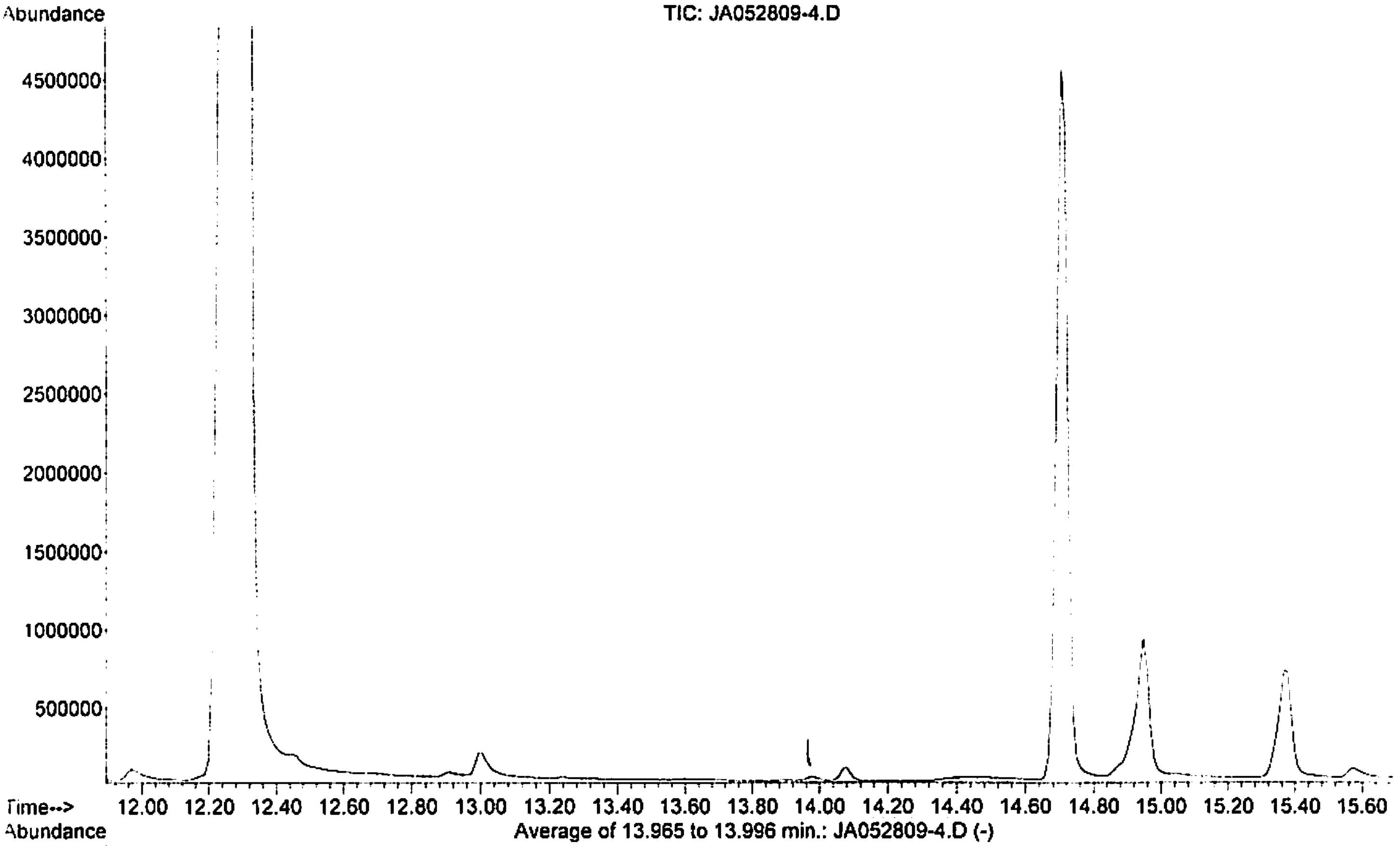

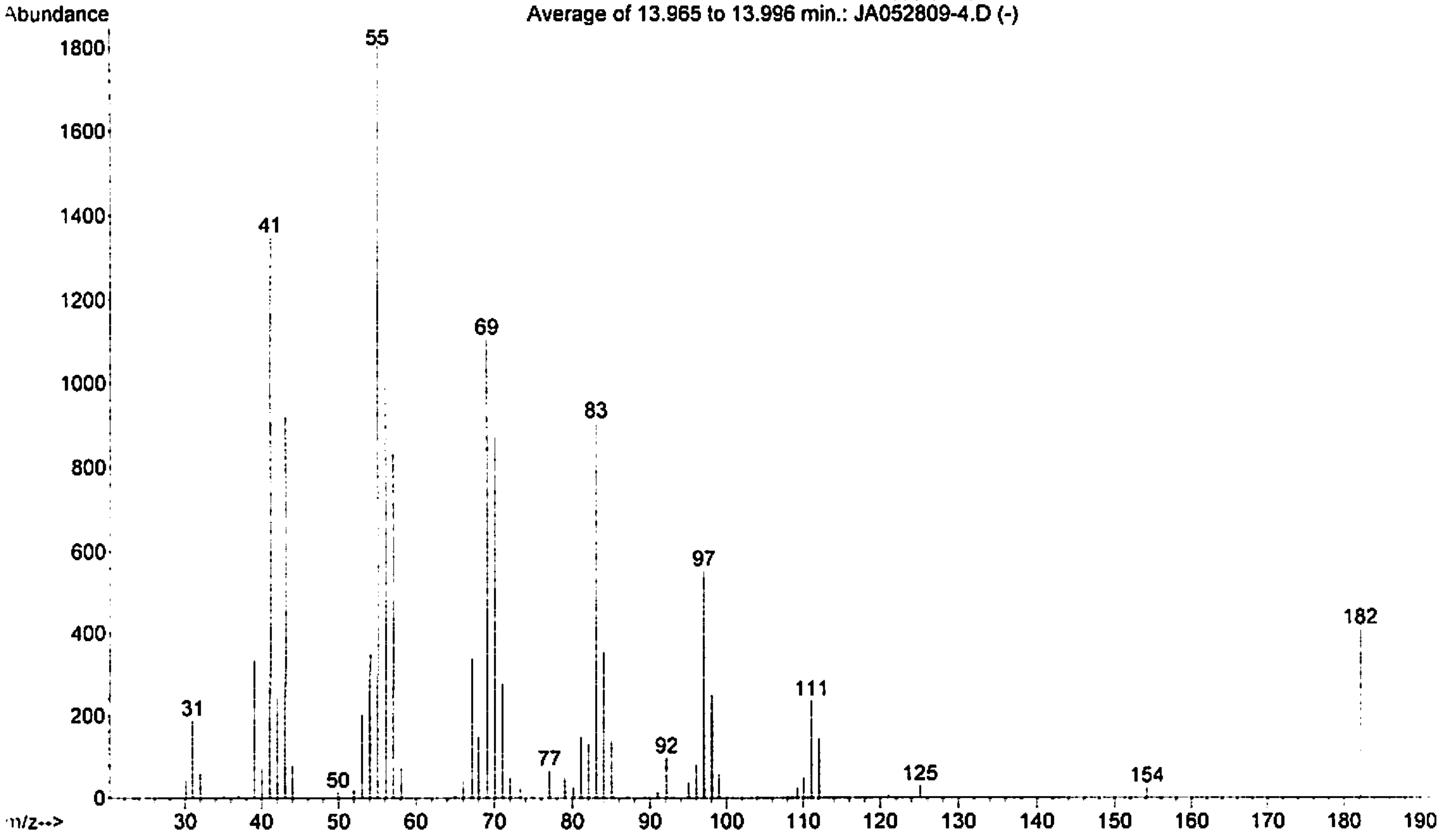

[illegible] :D:\DATA\ALDRICH\JA-09\Snapshot\JA052809-4.D
[illegible]ator : Aldrich
[illegible]ired : 28 May 2009 16:03 using AcqMethod JA-WAX08.M
[illegible]rument : Instrument #1
[illegible]ple Name: 1 field-coll. M C.oculata abd./CH2Cl2
[illegible] Info : coll. 5/28 sweeping vetch; second male today
[illegible] Number: 1

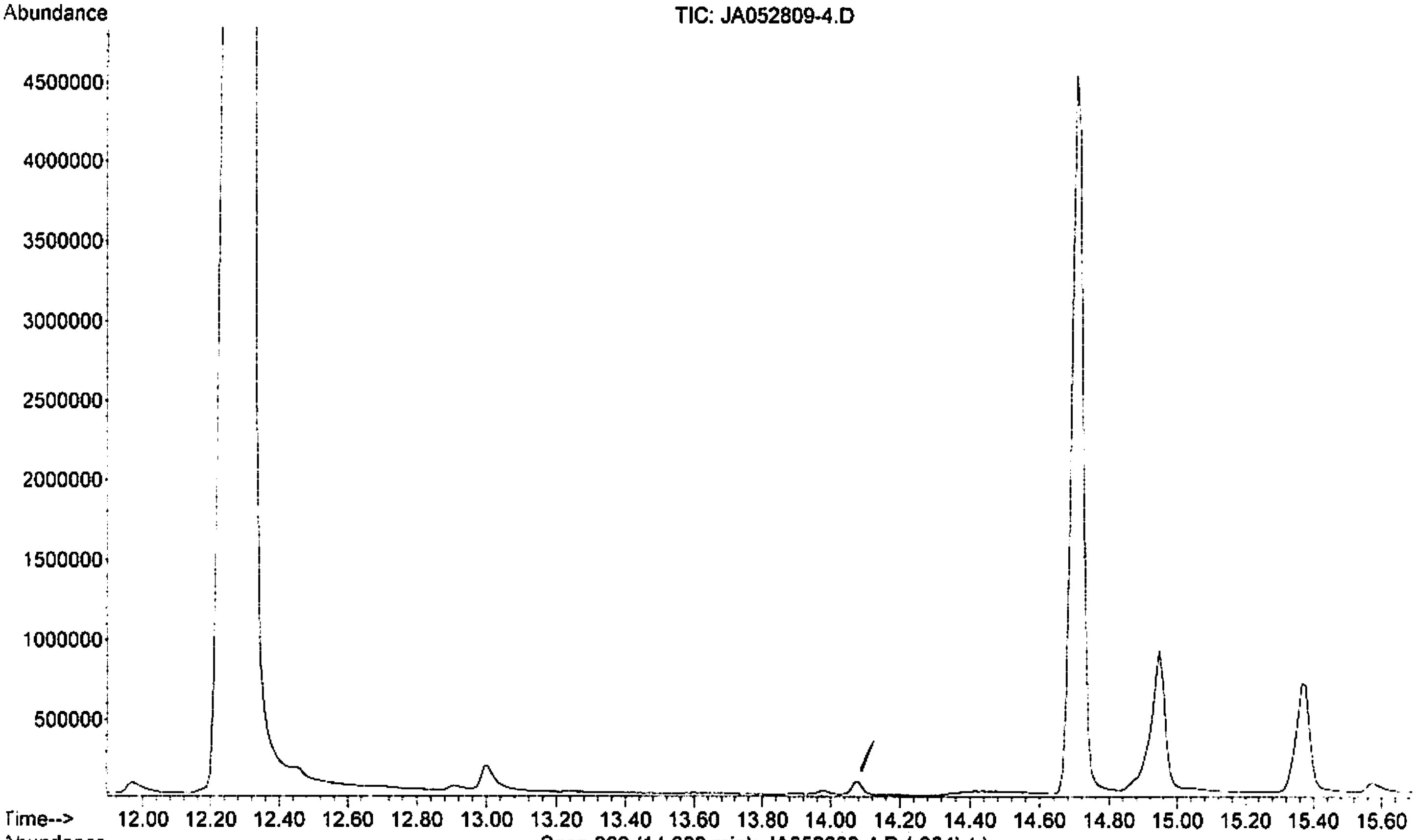

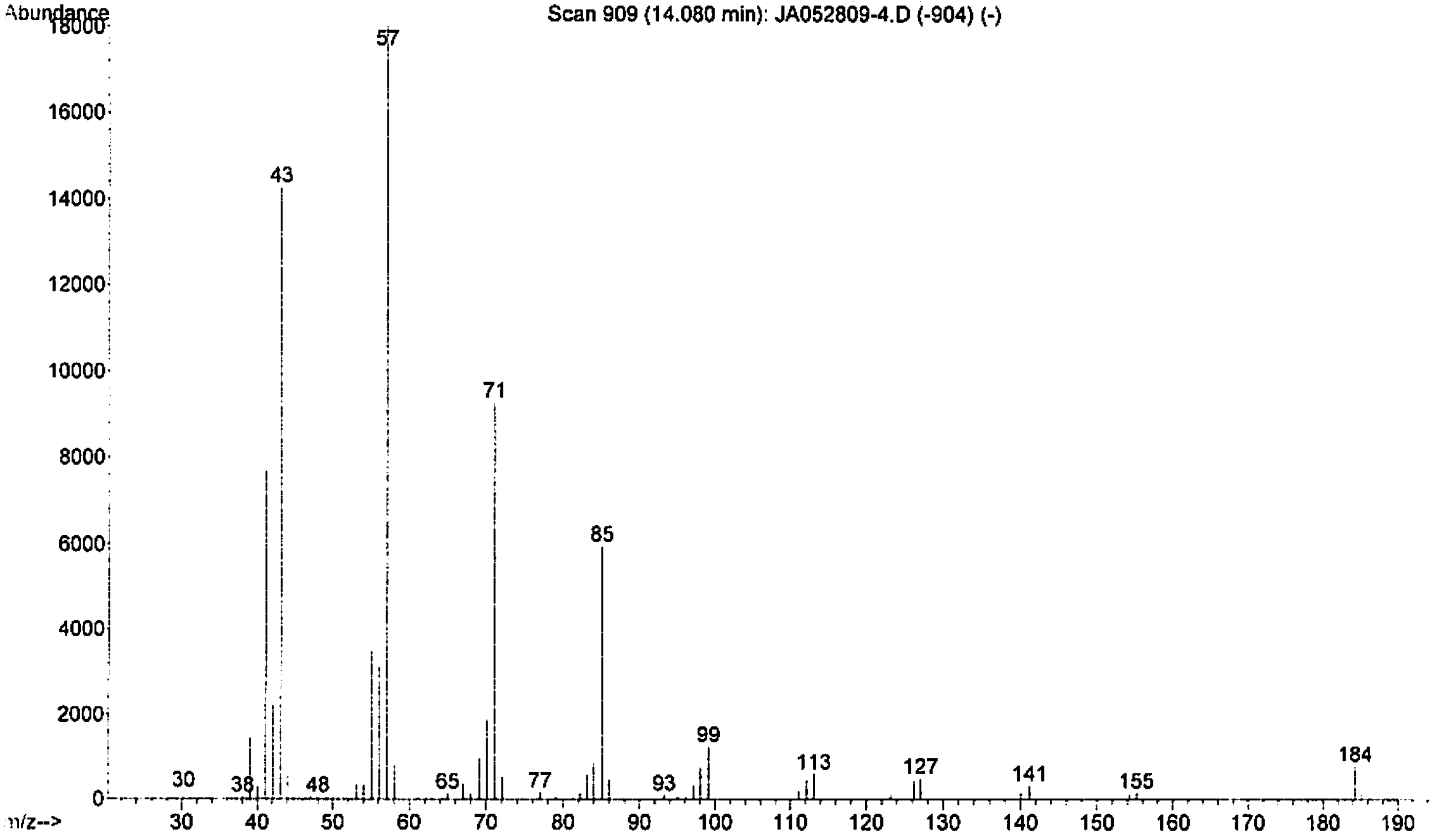

[illegible] :D:\DATA\ALDRICH\JA-09\Snapshot\JA052809-4.D
[illegible]rator : Aldrich
[illegible]uired : 28 May 2009 16:03 using AcqMethod JA-WAX08.M
[illegible]rument : Instrument #1
[illegible]le Name: 1 field-coll. M C.oculata abd./CH2Cl2
[illegible] Info : coll. 5/28 sweeping vetch; second male today
[illegible] Number: 1

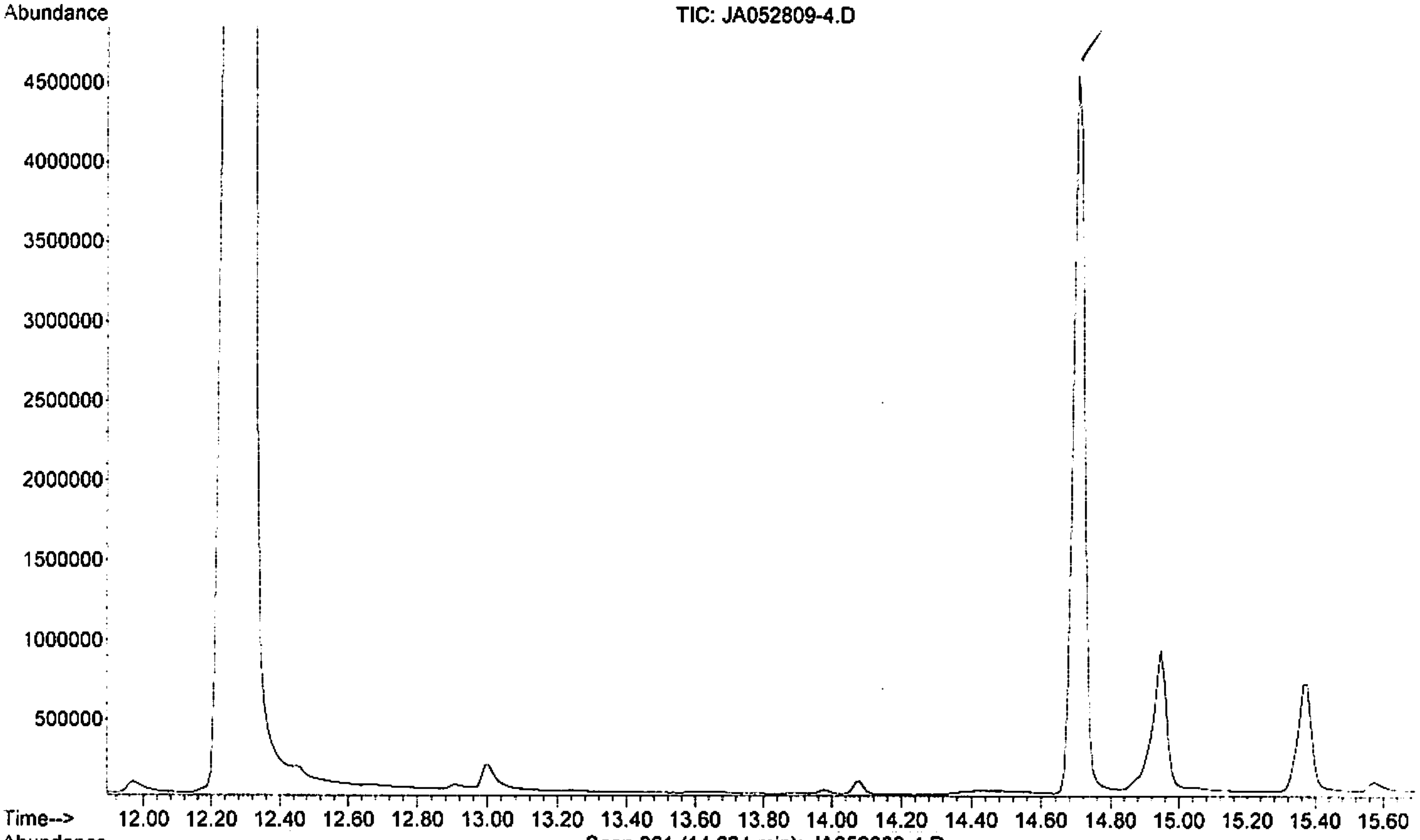

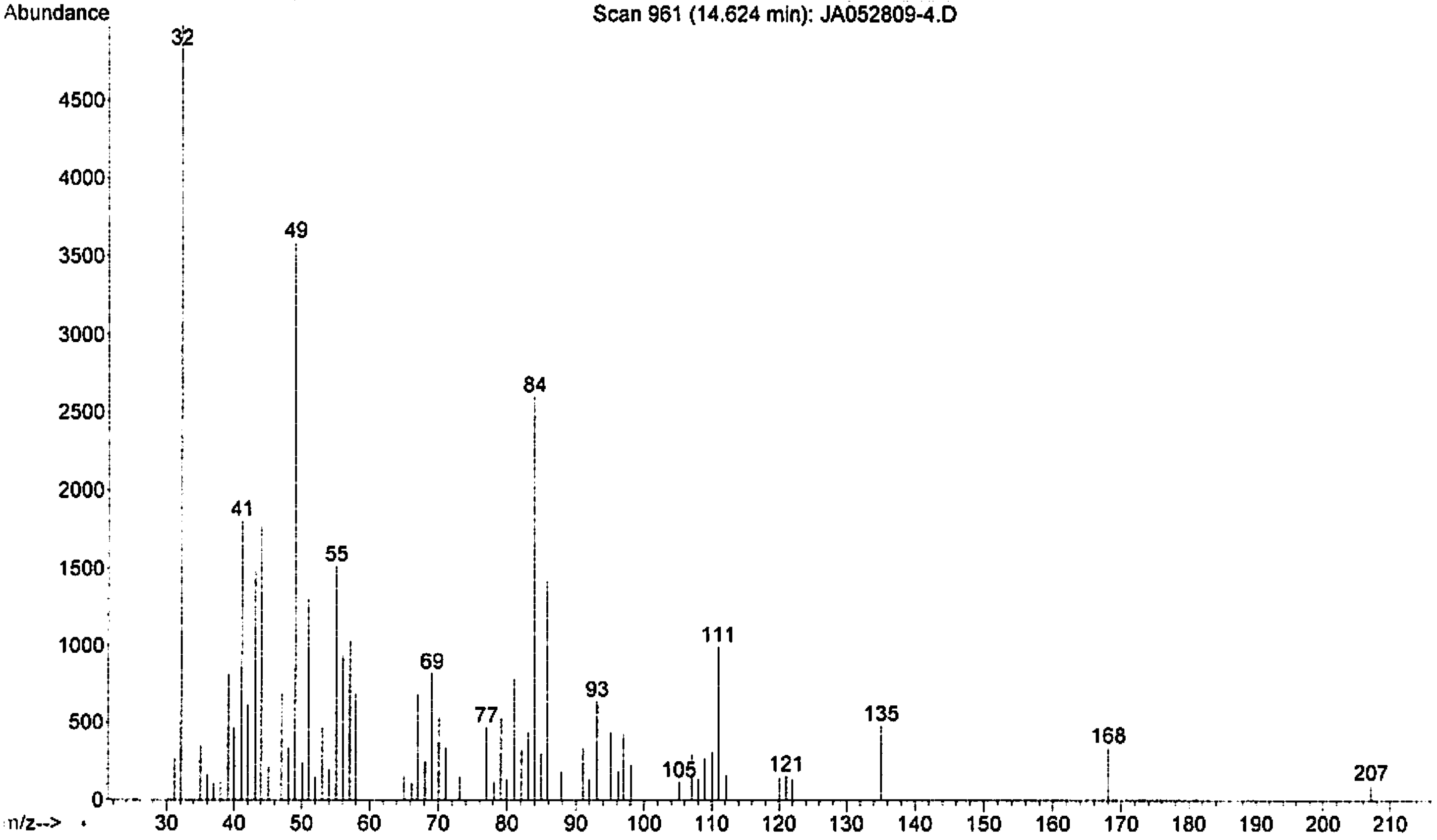

[illegible]e' :D:\DATA\ALDRICH\JA-09\Snapshot\JA052809-4.D
[illegible]erator : Aldrich
[illegible]quired : 28 May 2009 16:03 using AcqMethod JA-WAX08.M
[illegible]strument : Instrument #1
[illegible]mple Name: 1 field-coll. M C.oculata abd./CH2Cl2
[illegible] Info : coll. 5/28 sweeping vetch; second male today
[illegible]al Number: 1

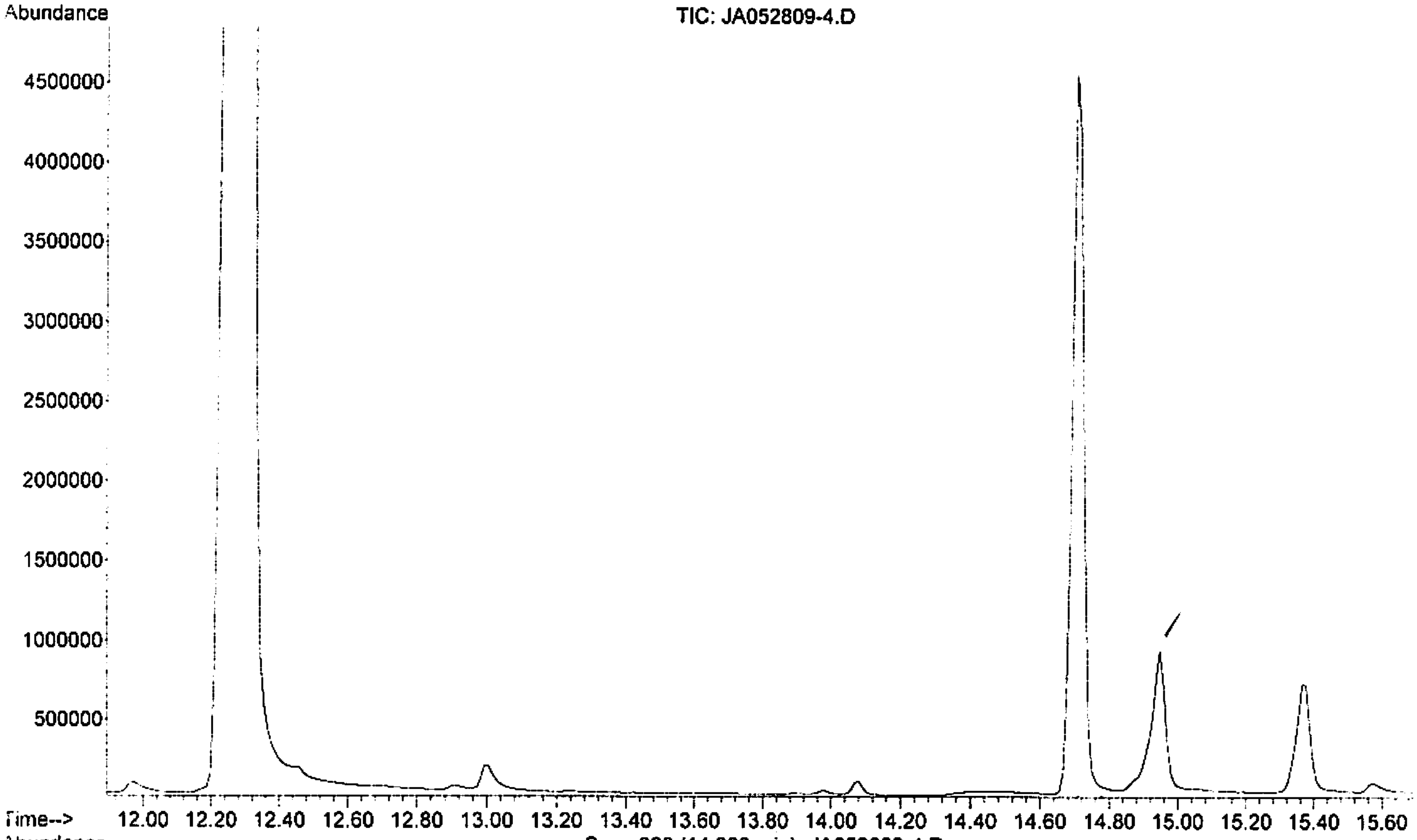

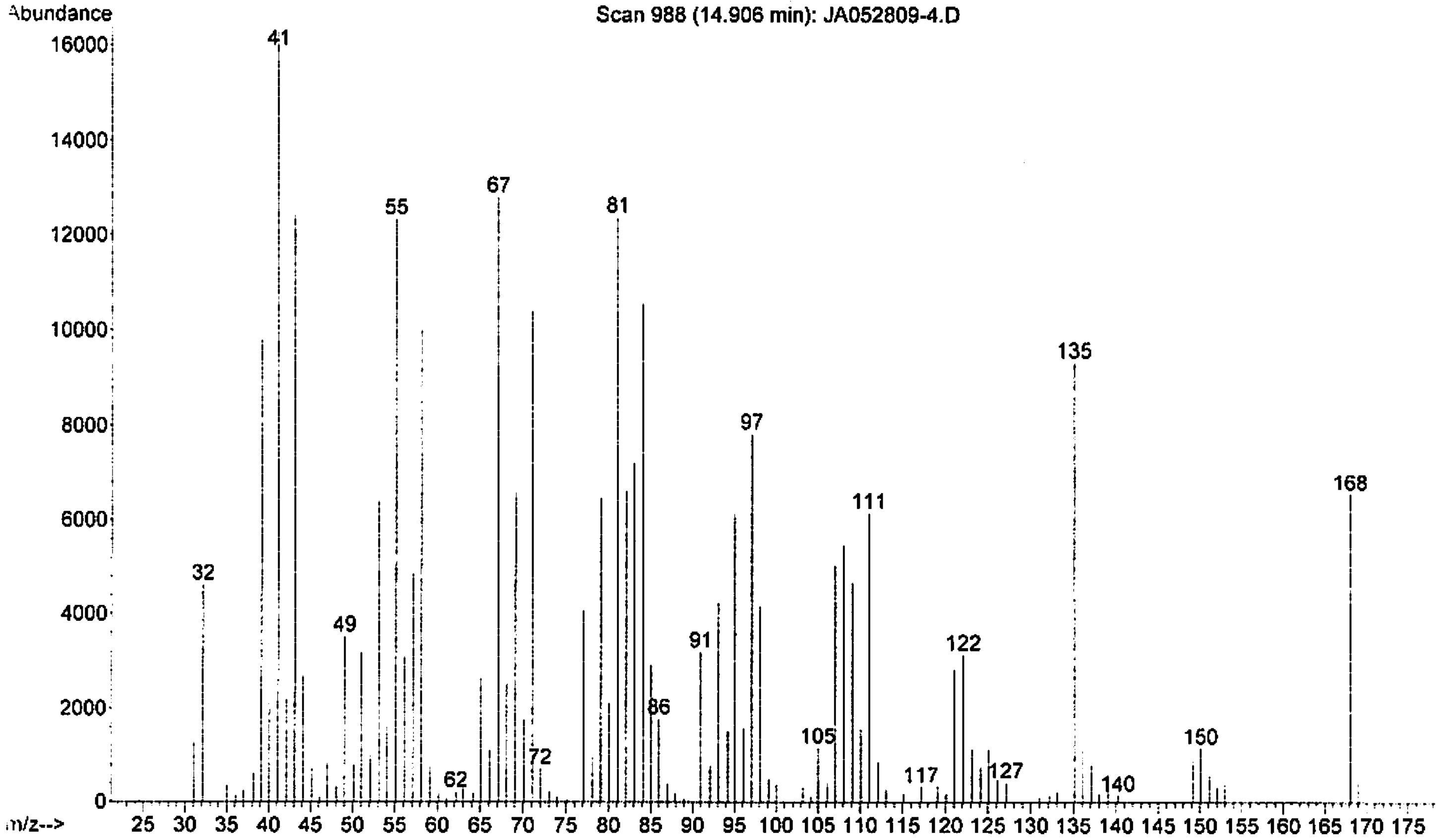

[illegible] :D:\DATA\ALDRICH\JA-09\Snapshot\JA052809-4.D
[illegible]ator : Aldrich
[illegible]ired : 28 May 2009 16:03 using AcqMethod JA-WAX08.M
[illegible]rument : Instrument #1
[illegible]le Name: 1 field-coll. M C.oculata abd./CH2Cl2
[illegible] Info : coll. 5/28 sweeping vetch; second male today
[illegible] Number: 1

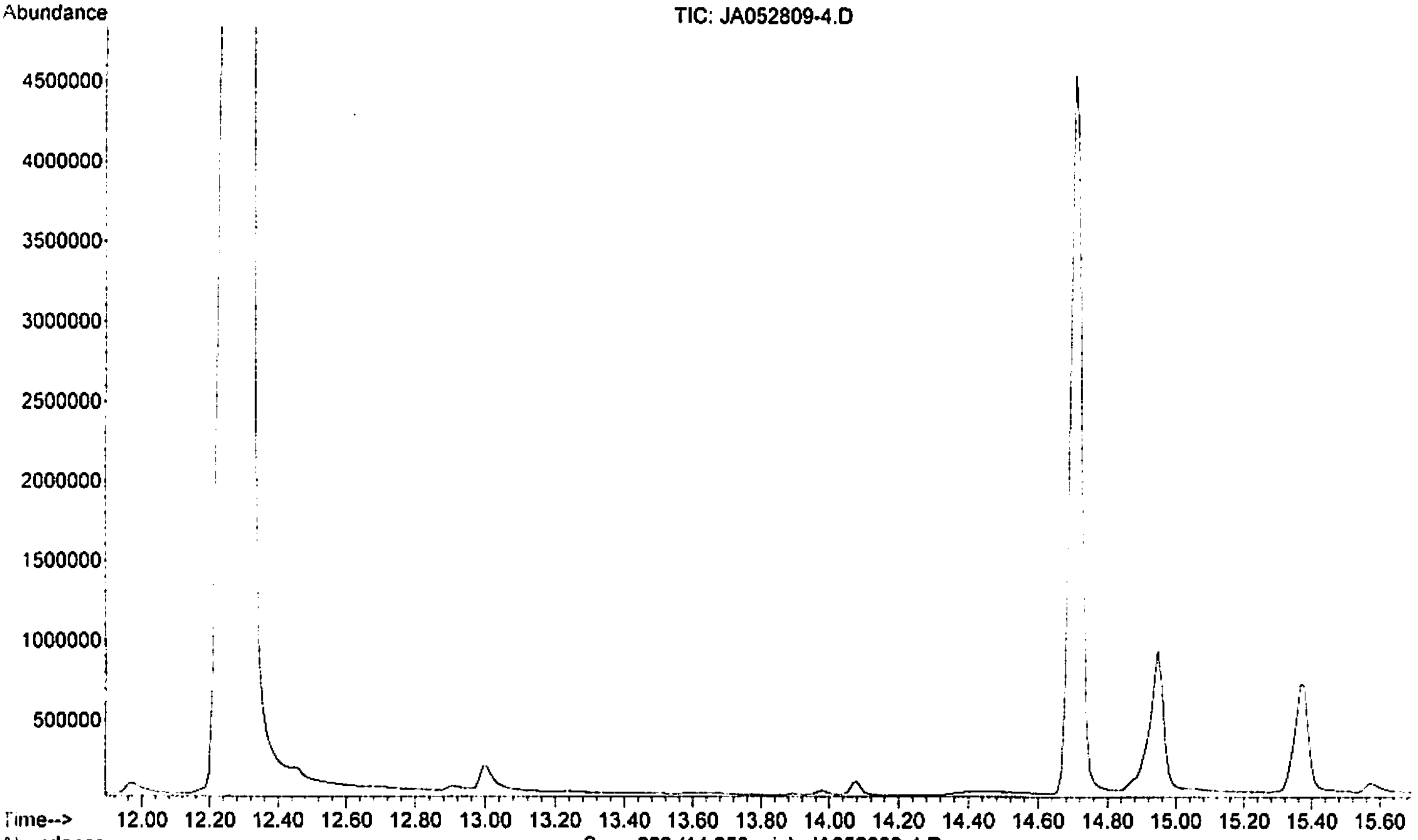

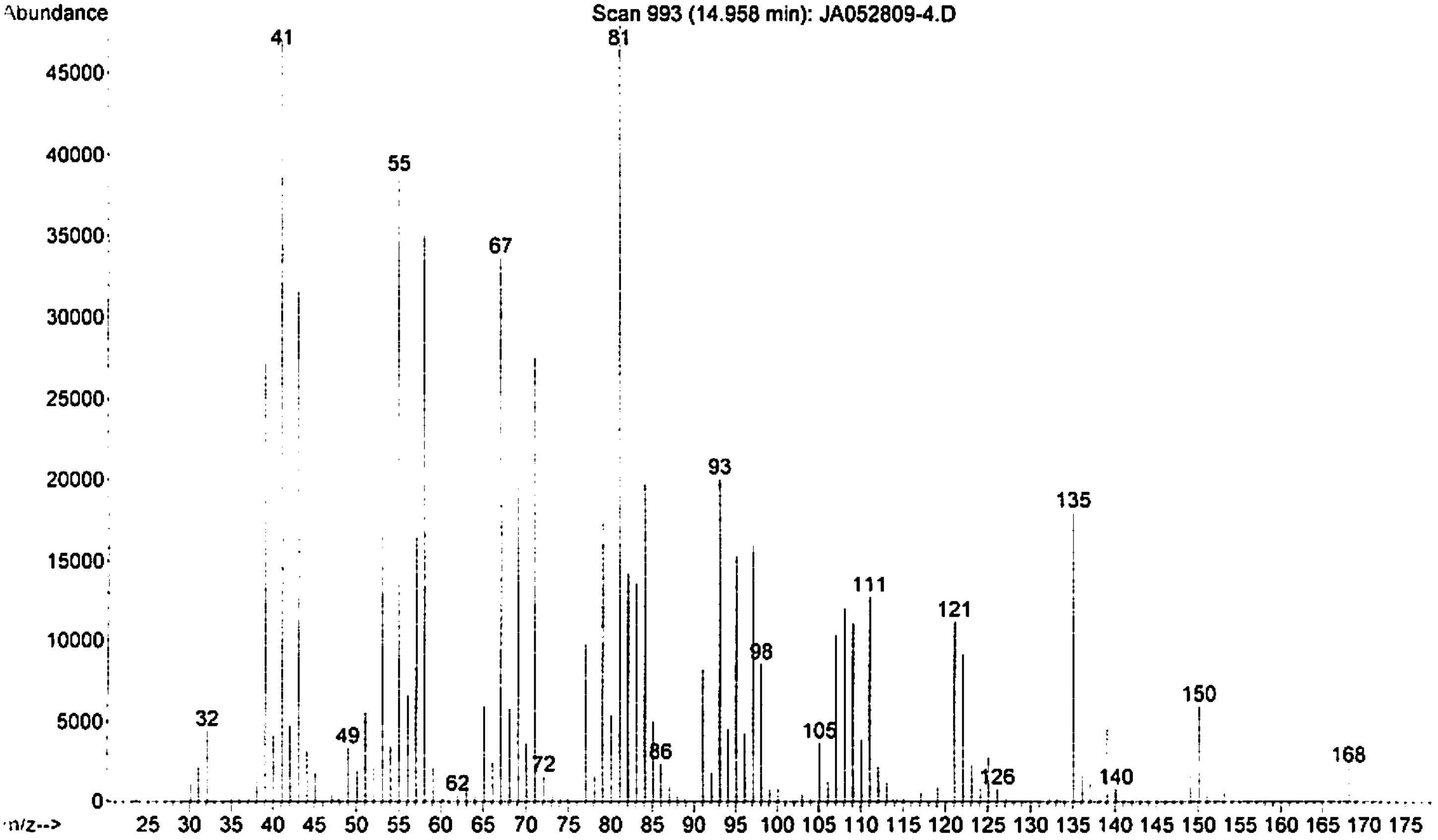

[illegible] :D:\DATA\ALDRICH\JA-09\Snapshot\JA052809-4.D
[illegible]rator : Aldrich
[illegible]uired : 28 May 2009 16:03 using AcqMethod JA-WAX08.M
[illegible]rument : Instrument #1
[illegible]le Name: 1 field-coll. M C.oculata abd./CH2Cl2
[illegible] Info : coll. 5/28 sweeping vetch; second male today
[illegible] Number: 1

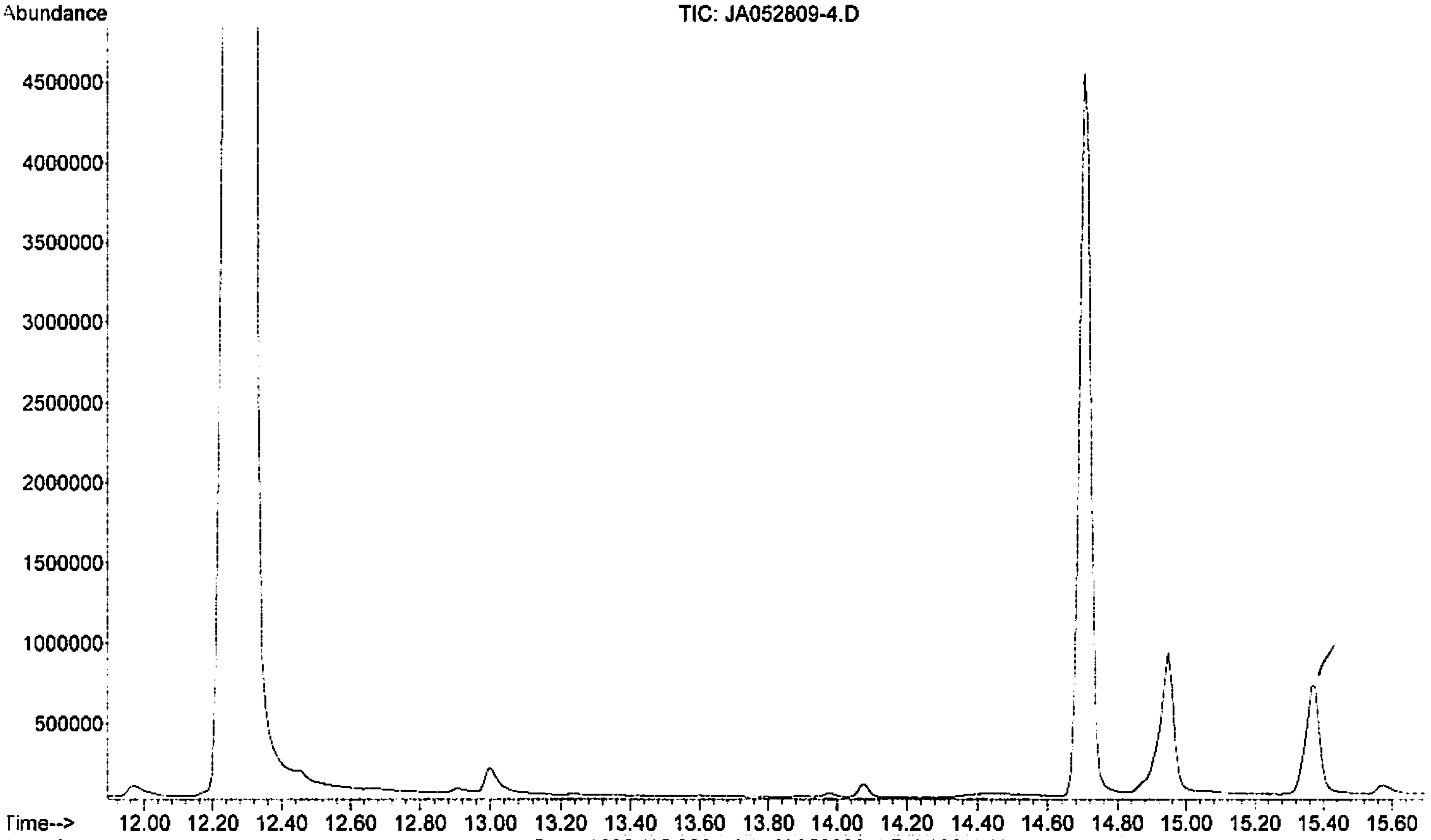

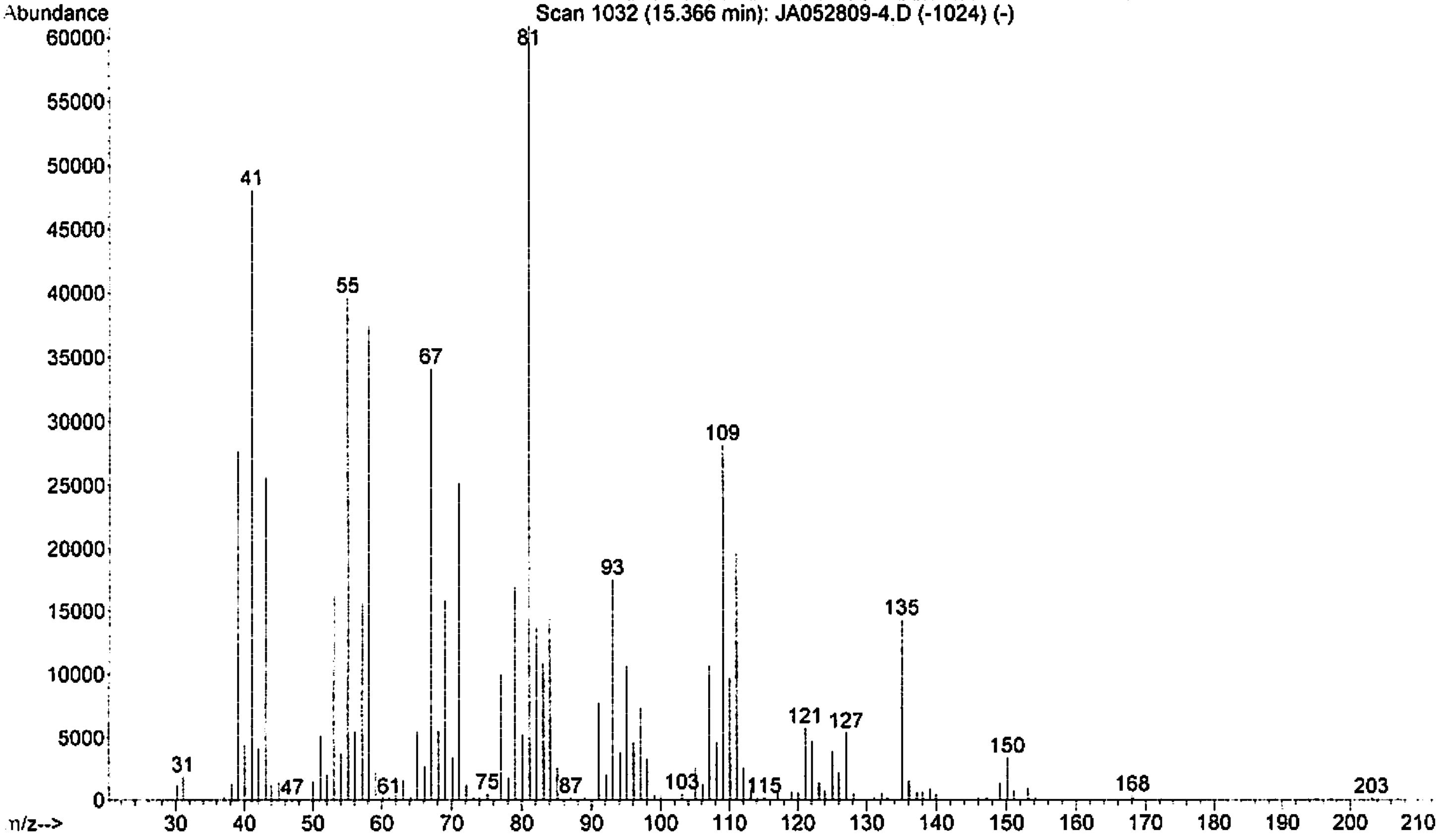

:D:\DATA\ALDRICH\JA-09\Snapshot\JA052809-4.D
[illegible]ator : Aldrich
[illegible]ired : 28 May 2009 16:03 using AcqMethod JA-WAX08.M
[illegible]rument : Instrument #1
[illegible]le Name: 1 field-coll. M C.oculata abd./CH2Cl2
[illegible] Info : coll. 5/28 sweeping vetch; second male today
[illegible] Number: 1

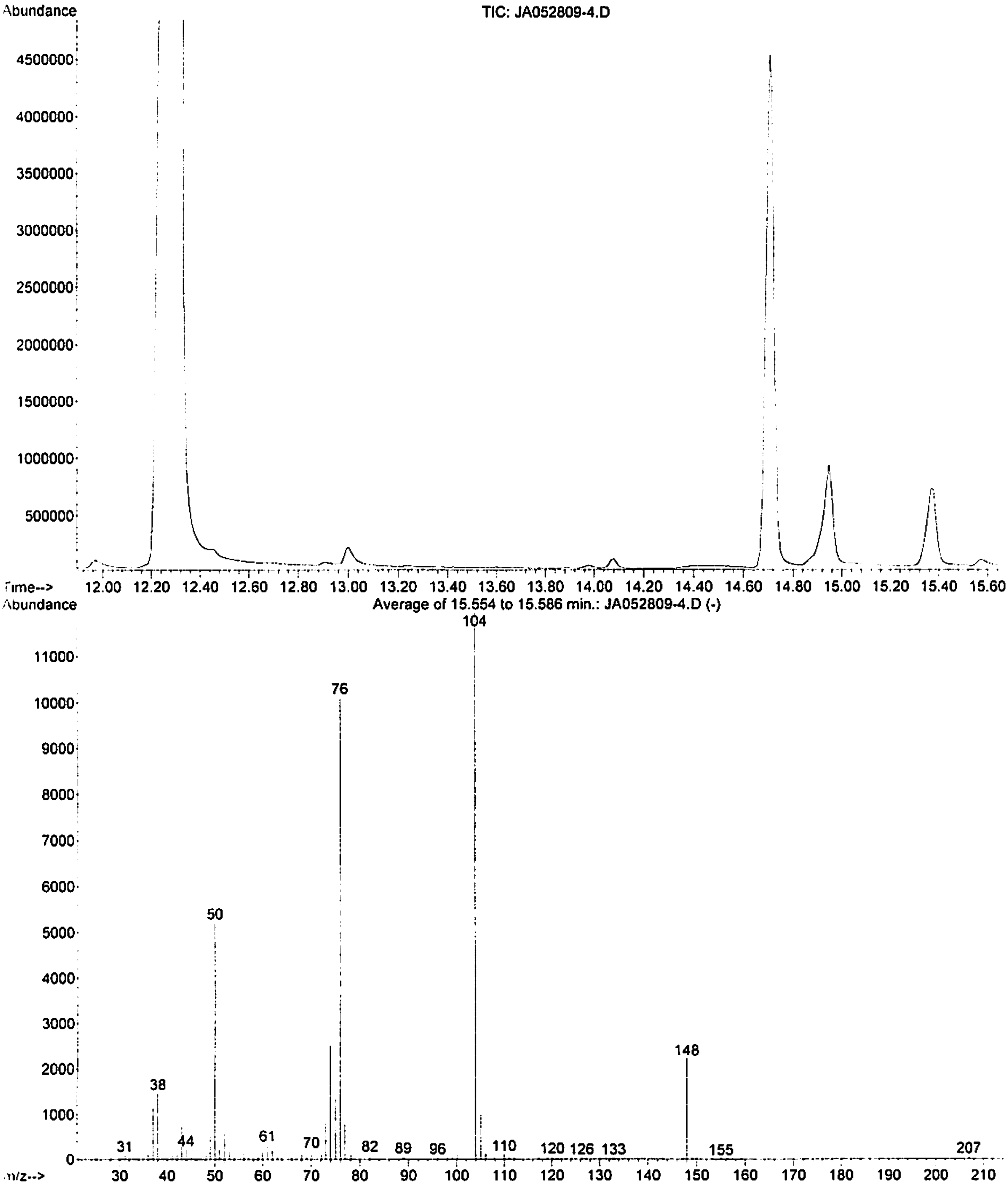

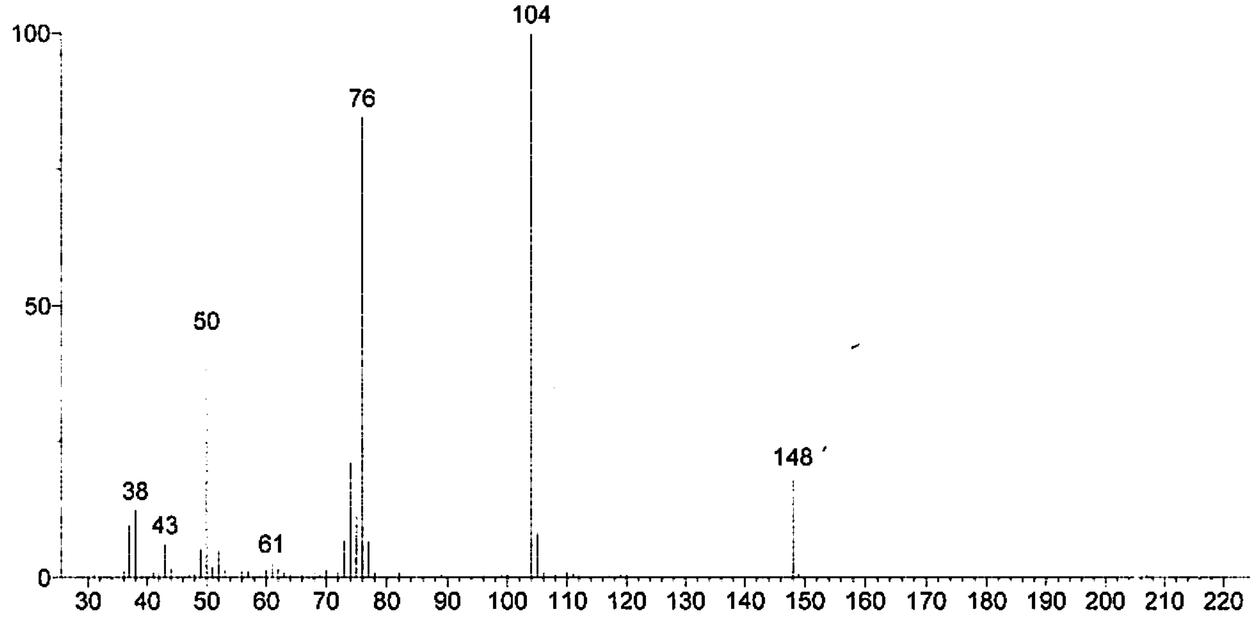

(Text File) Average of 15.554 to 15.586 min.: JA052809-4.D

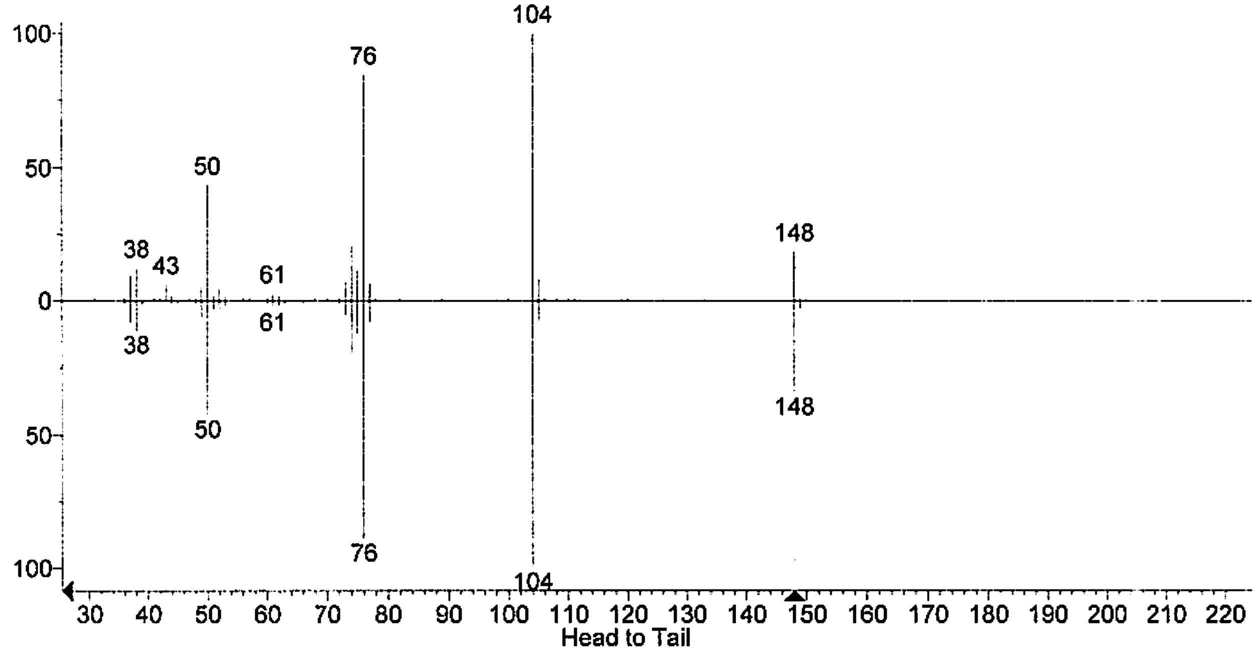

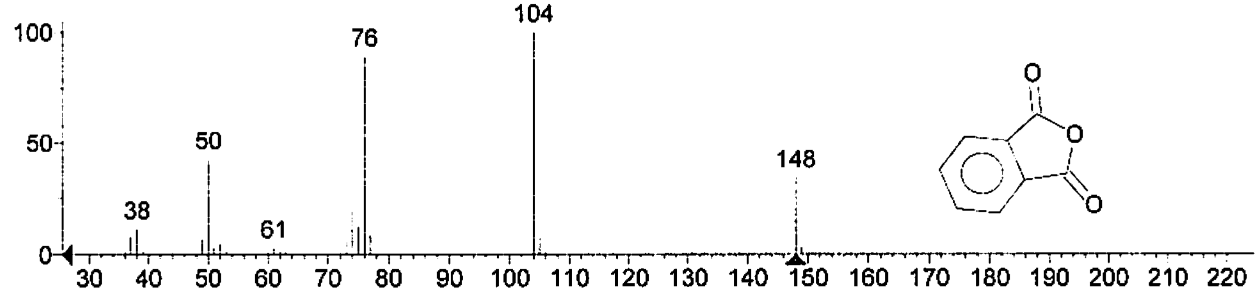

(mainlib) Phthalic anhydride

:D:\DATA\ALDRICH\JA-09\Snapshot\JA052809-4.D
[illegible]ator : Aldrich
[illegible]ired : 28 May 2009 16:03 using AcqMethod JA-WAX08.M
[illegible]rument : Instrument #1
[illegible]le Name: 1 field-coll. M C.oculata abd./CH2Cl2
[illegible] Info : coll. 5/28 sweeping vetch; second male today
[illegible] Number: 1

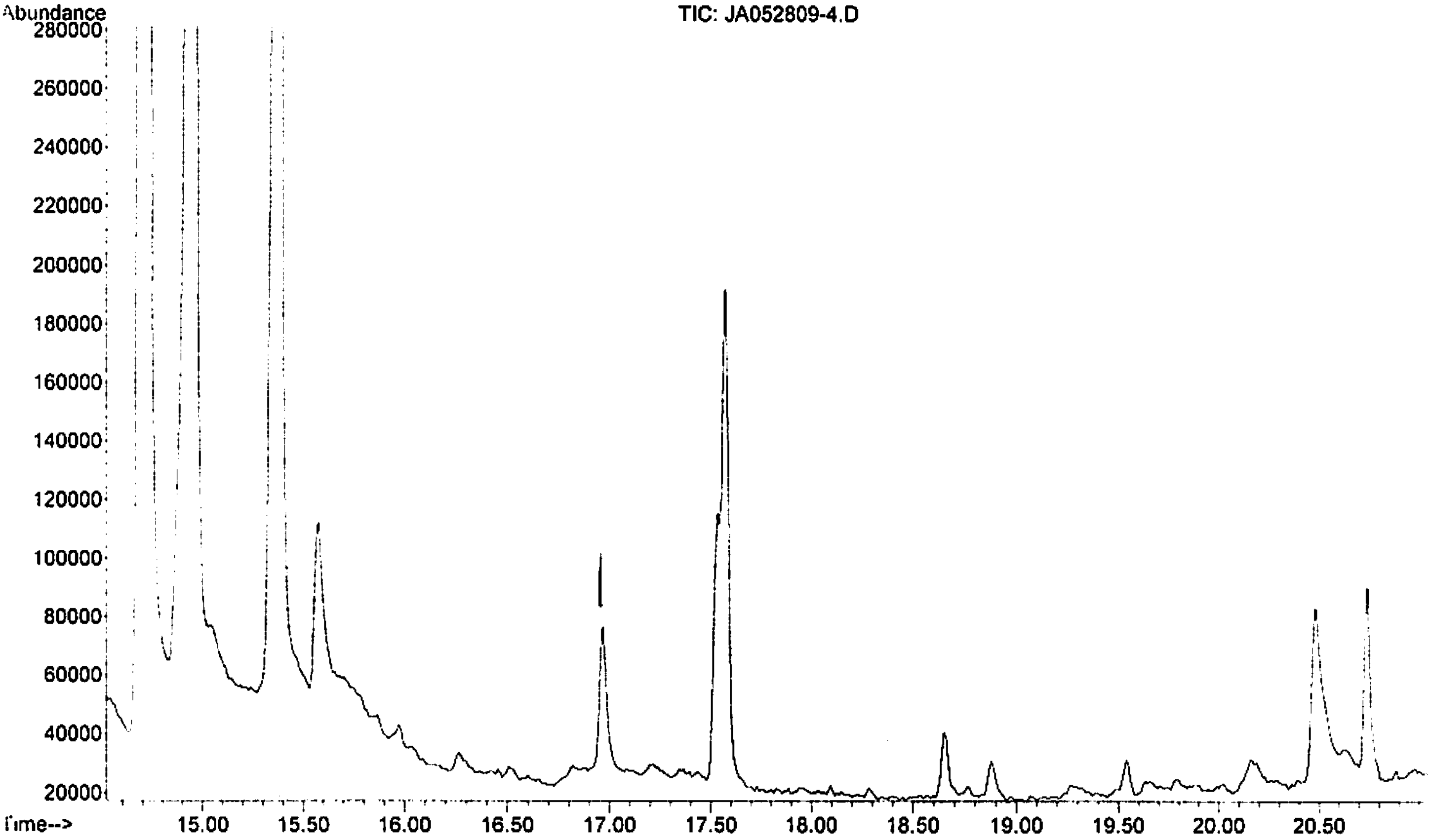

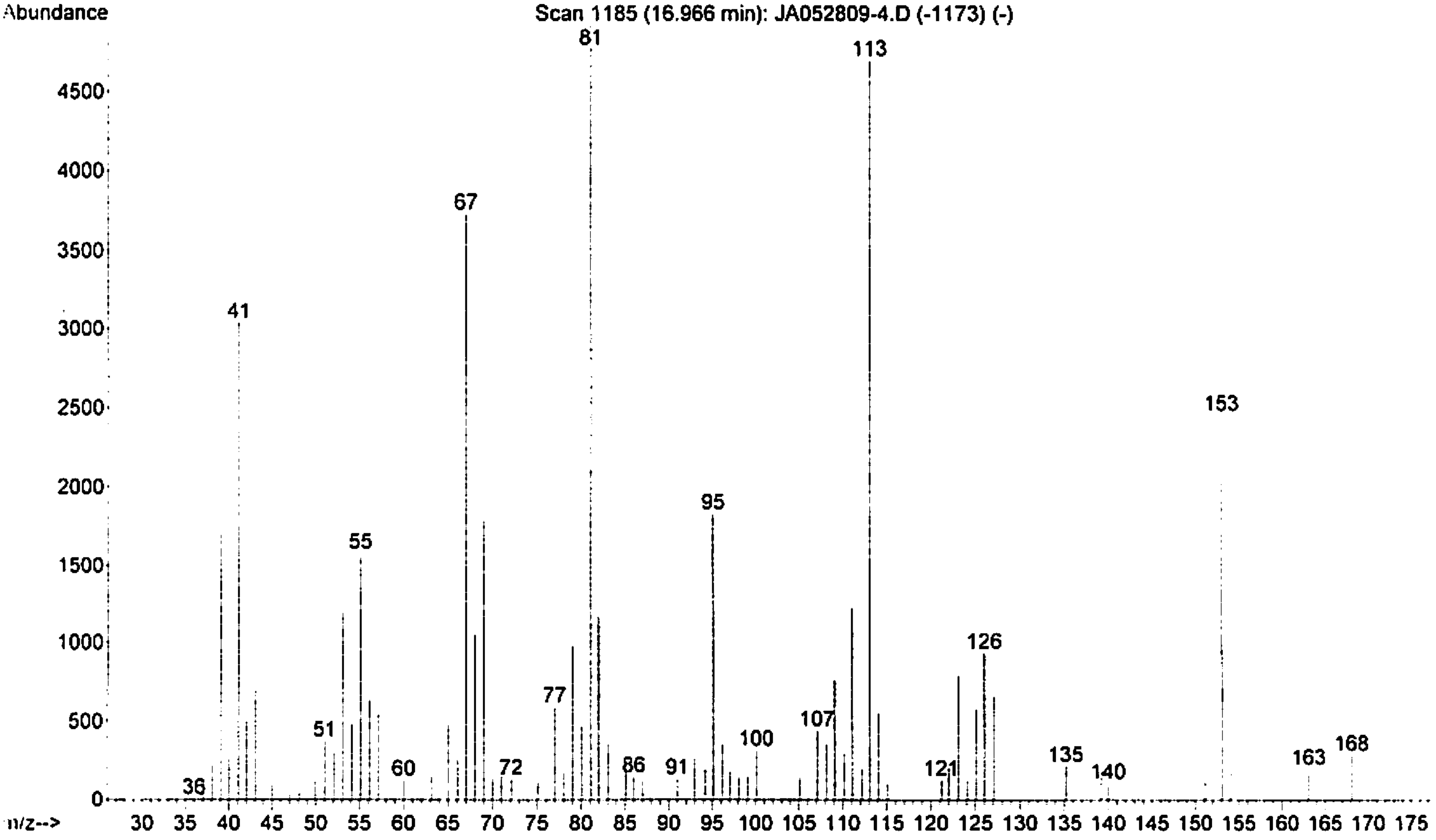

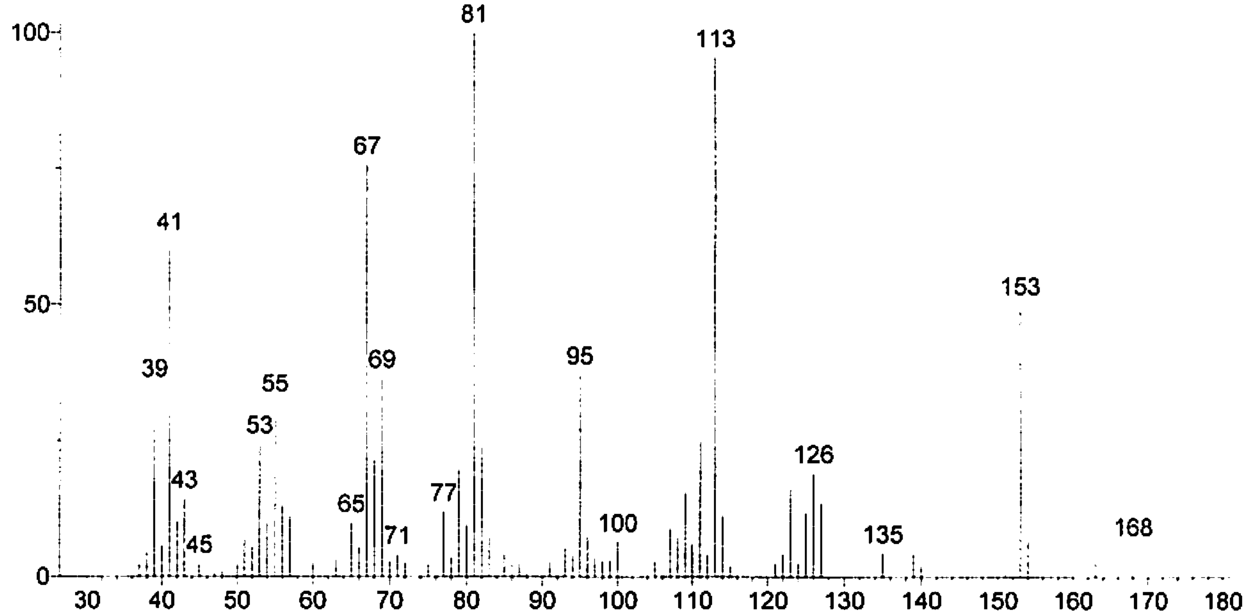

(Text File) Scan 1185 (16.966 min): JA052809-4.D (-1173)

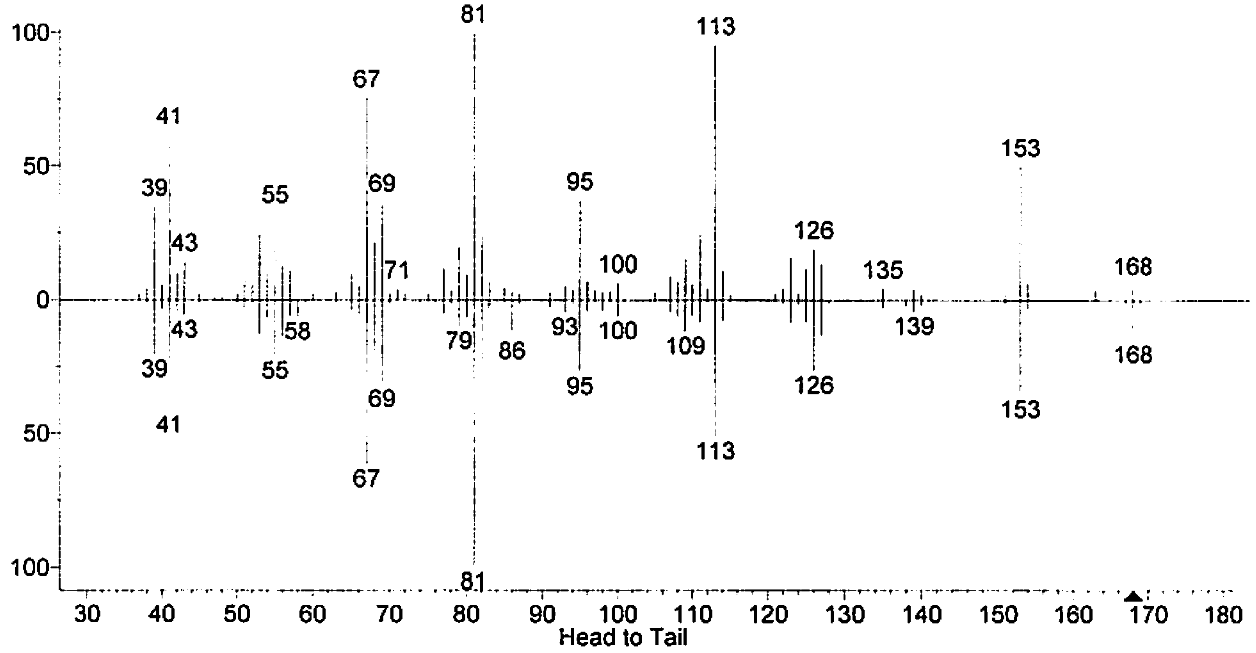

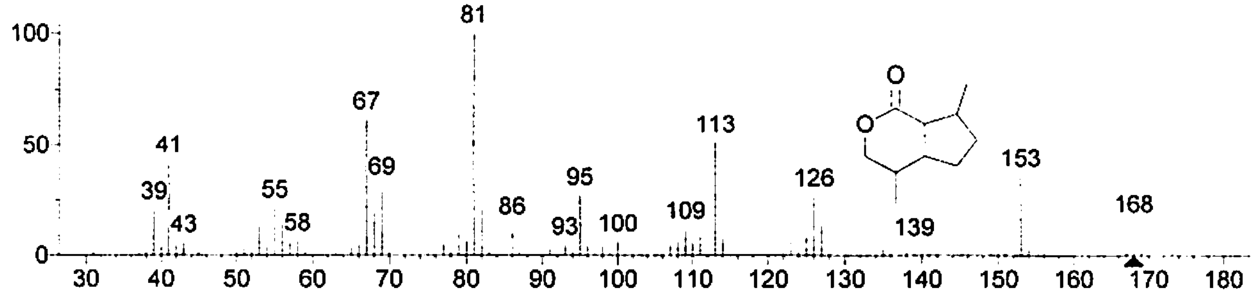

(mainlib) Cyclopenta[c]pyran-1(3H)-one, hexahydro-4,7-dimethyl-, (4.alpha.,4a.alpha.,7.alpha.,7a.alpha.)-

[illegible] :D:\DATA\ALDRICH\JA-09\Snapshot\JA052809-4.D
[illegible]ator : Aldrich
[illegible]ired : 28 May 2009 16:03 using AcqMethod JA-WAX08.M
[illegible]rument : Instrument #1
[illegible]le Name: 1 field-coll. M C.oculata abd./CH2C12
[illegible] Info : coll. 5/28 sweeping vetch; second male today
[illegible] Number: 1

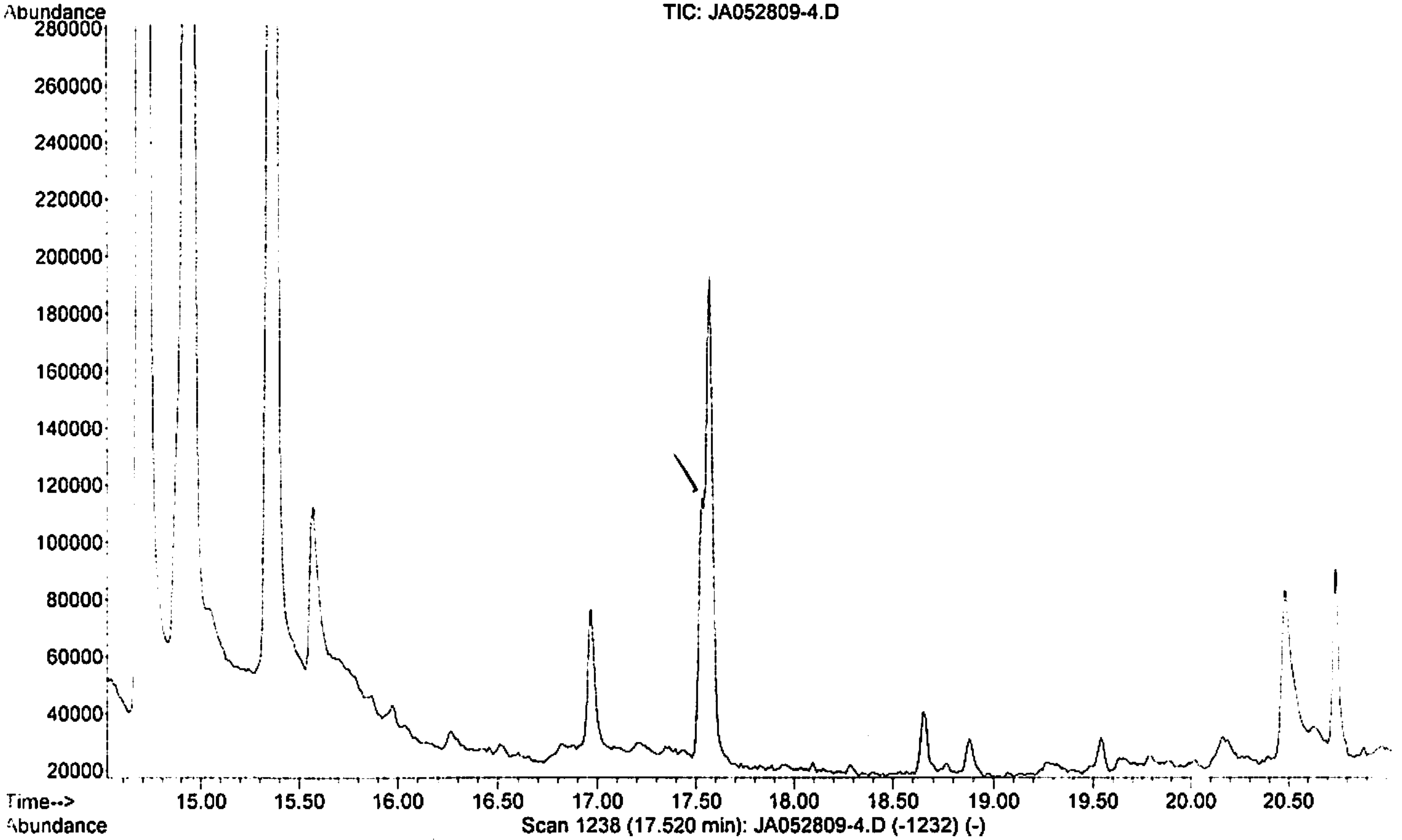

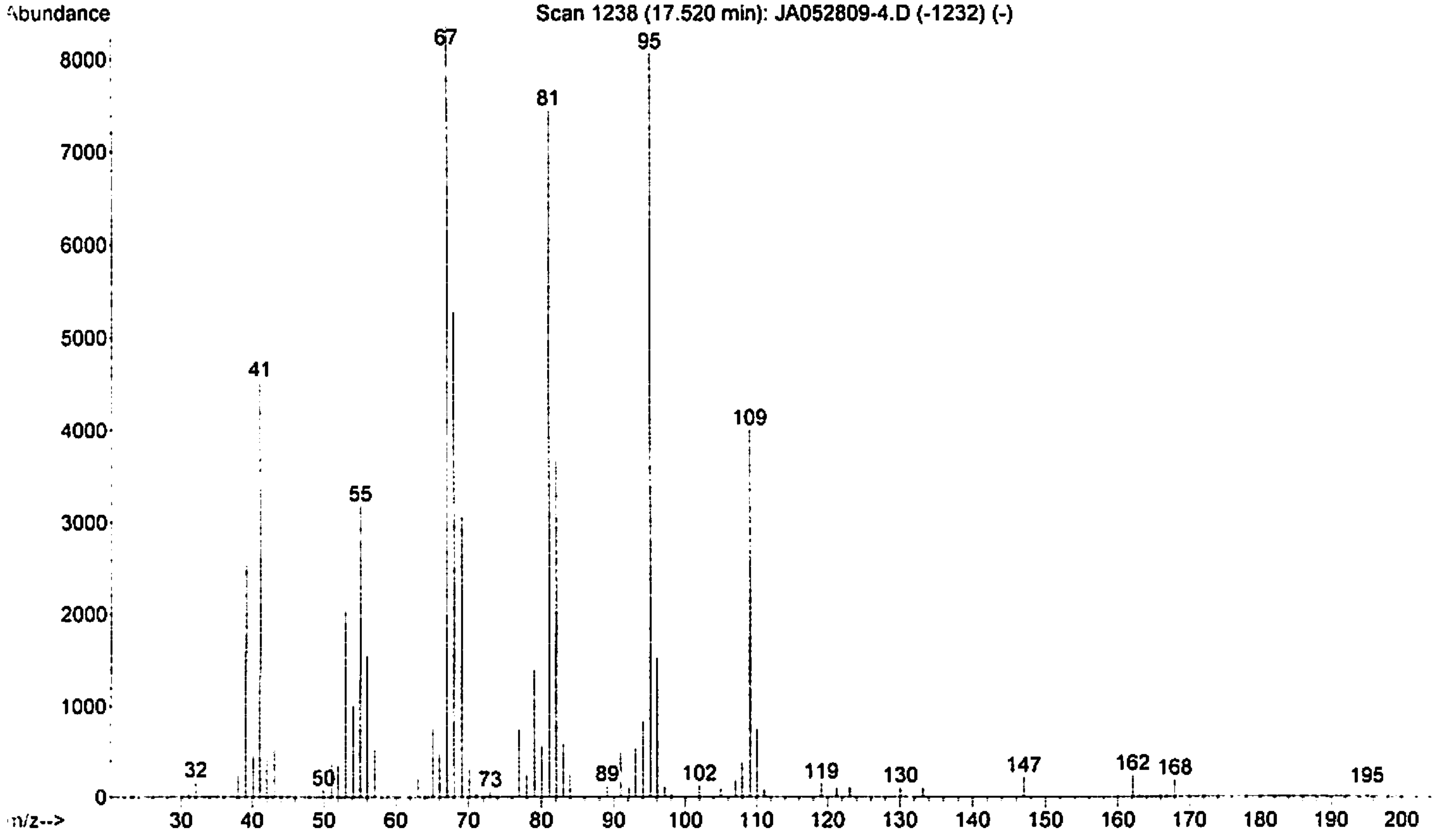

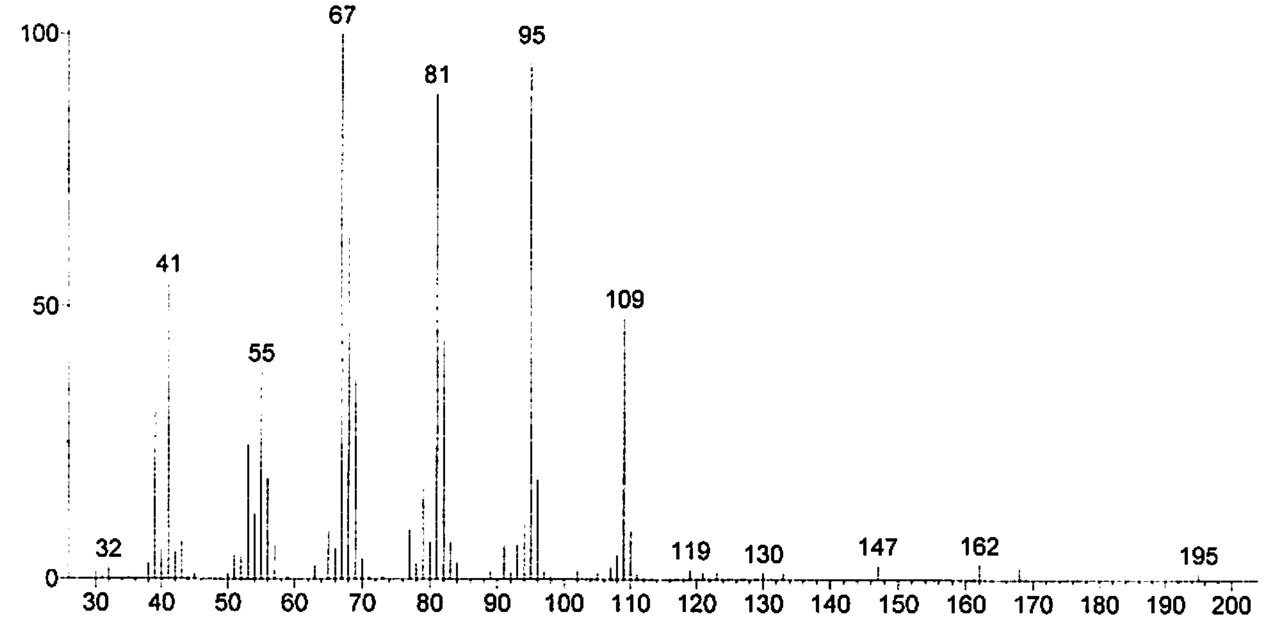

(Text File) Scan 1238 (17.520 min): JA052809-4.D (-1232)

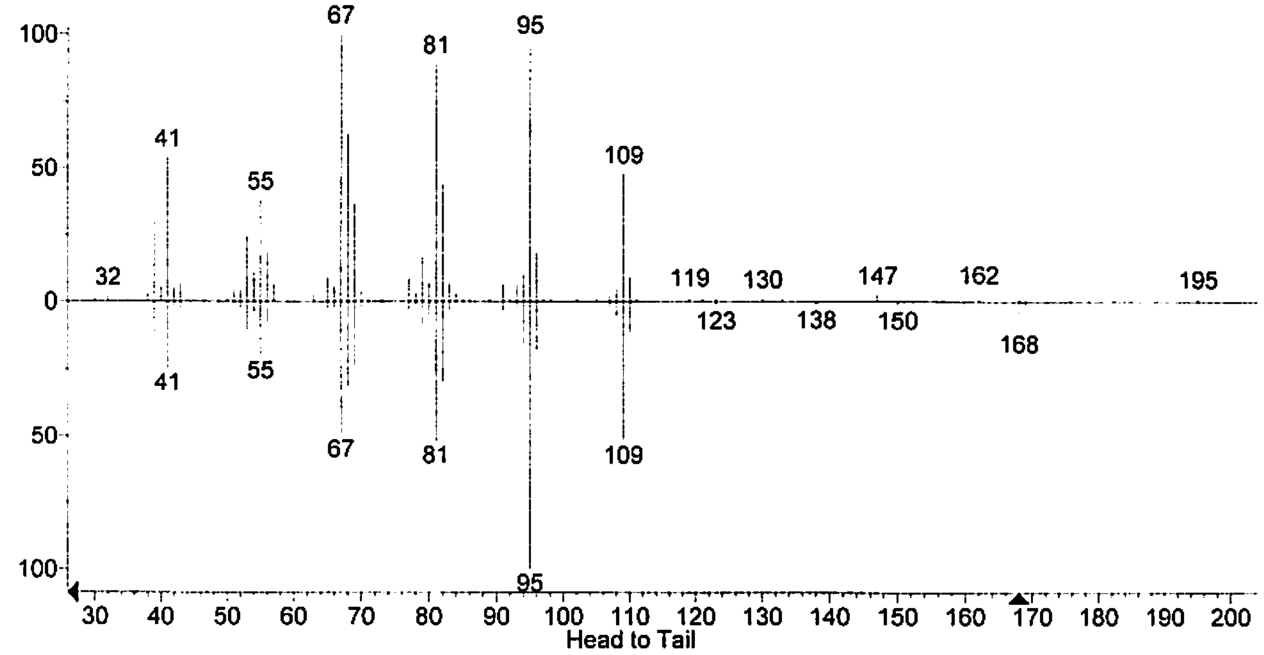

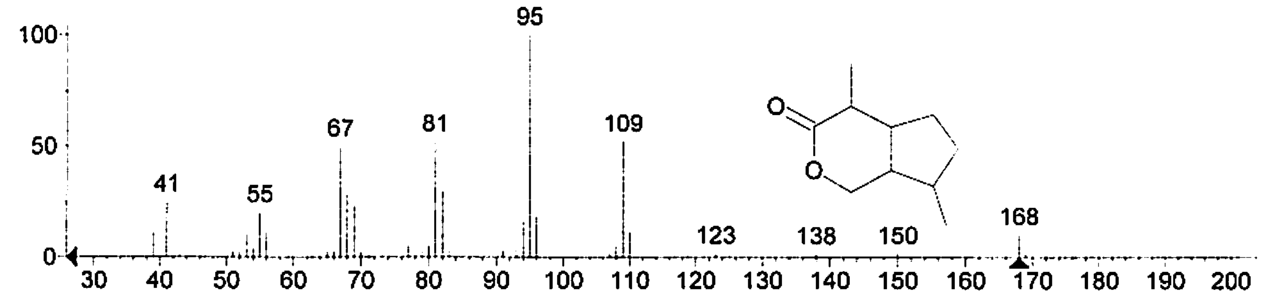

(replib) Iridomyrmecin

```
[illegible]e          :D:\DATA\ALDRICH\JA-09\Snapshot\JA052809-4.D
[illegible]erator     : Aldrich
[illegible]quired     : 28 May 2009   16:03      using AcqMethod JA-WAX08.M
[illegible]strument   :     Instrument #1
[illegible]mple Name: 1 field-coll. M C.oculata abd./CH2Cl2
[illegible] Info      : coll. 5/28 sweeping vetch; second male today
[illegible] Number: 1
```

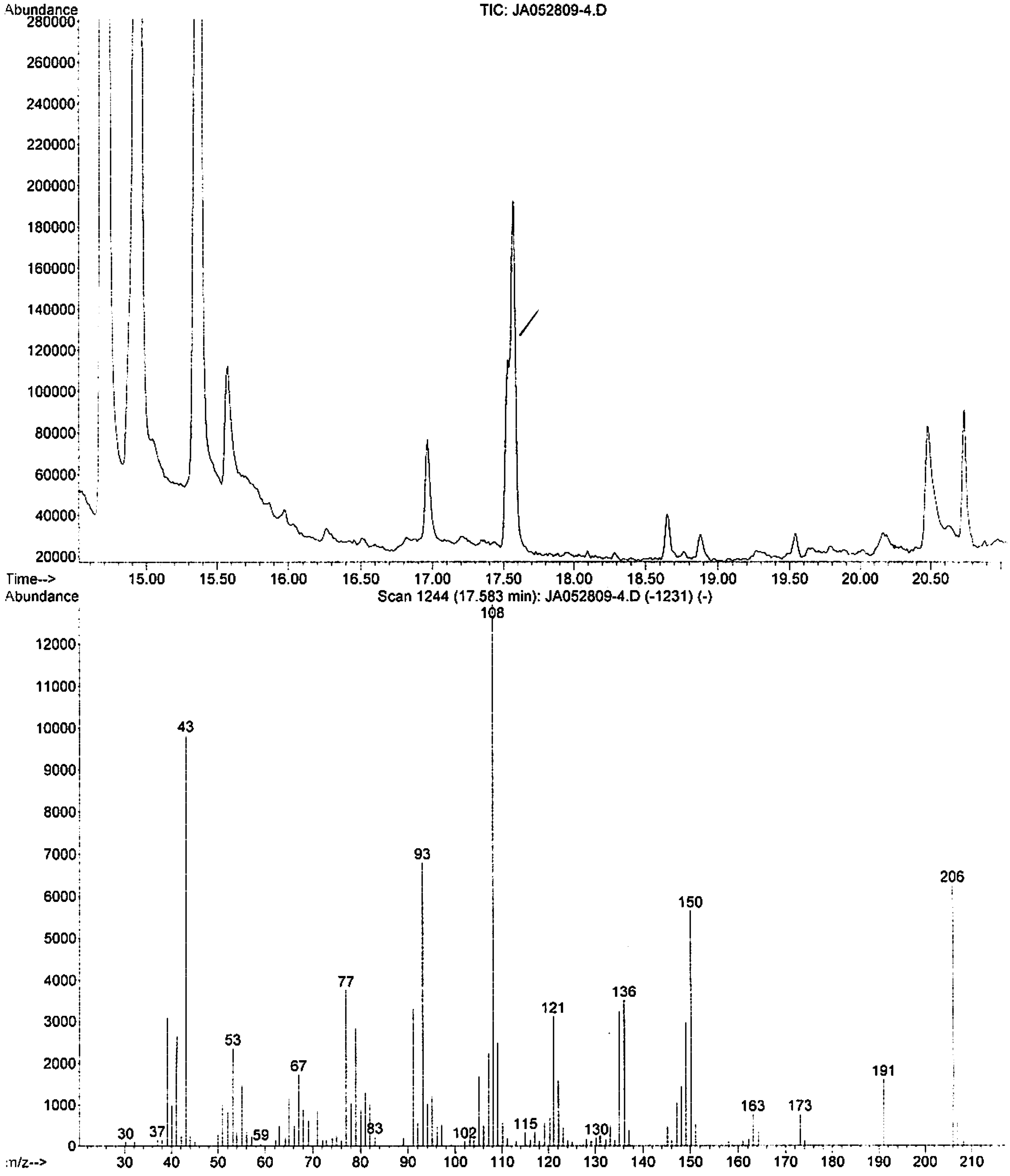

[illegible] :D:\DATA\ALDRICH\JA-09\Snapshot\JA052809-4.D
[illegible]ator : Aldrich
[illegible]ired : 28 May 2009 16:03 using AcqMethod JA-WAX08.M
[illegible]rument : Instrument #1
[illegible]ple Name: 1 field-coll. M C.oculata abd./CH2Cl2
[illegible] Info : coll. 5/28 sweeping vetch; second male today
[illegible] Number: 1

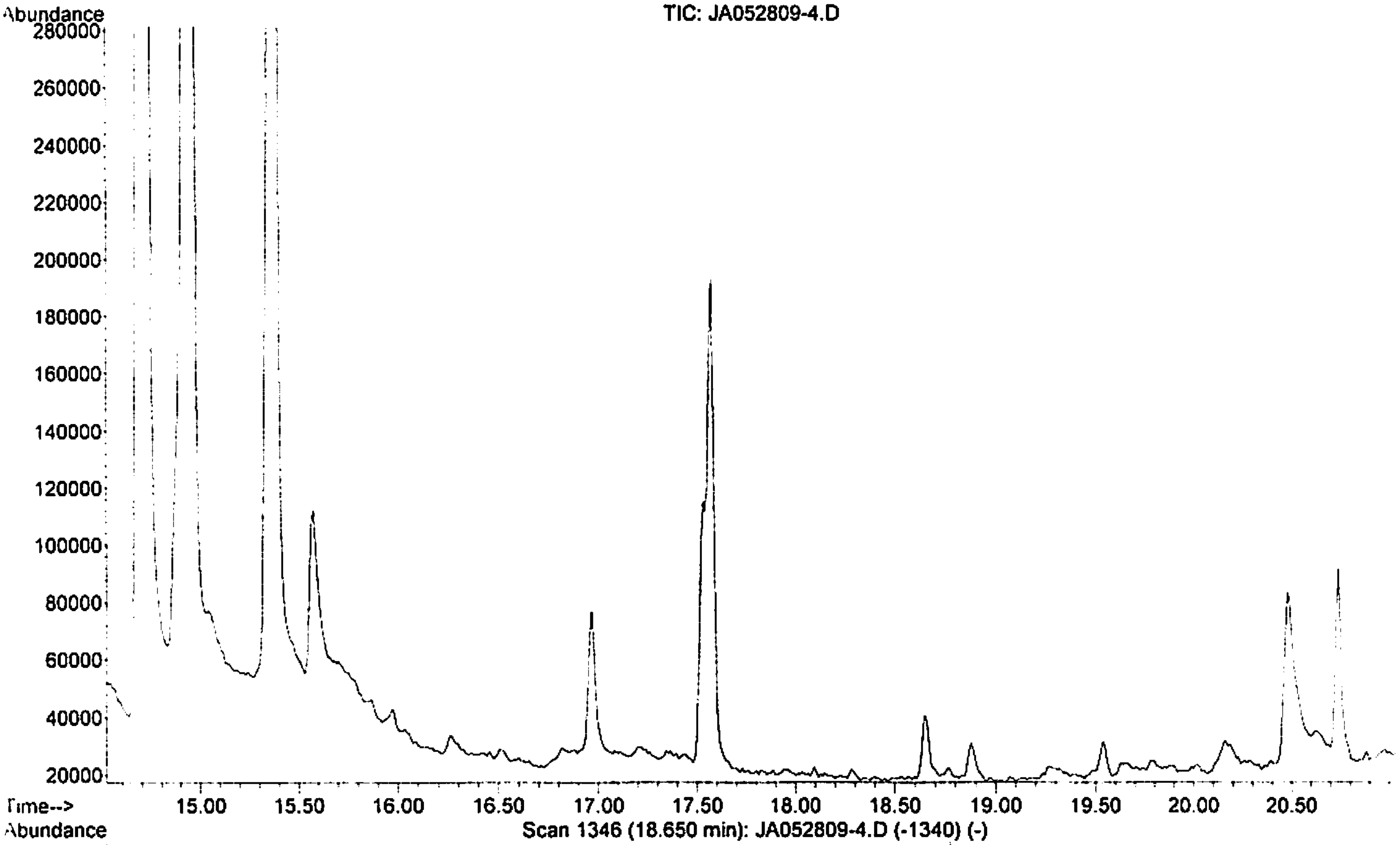

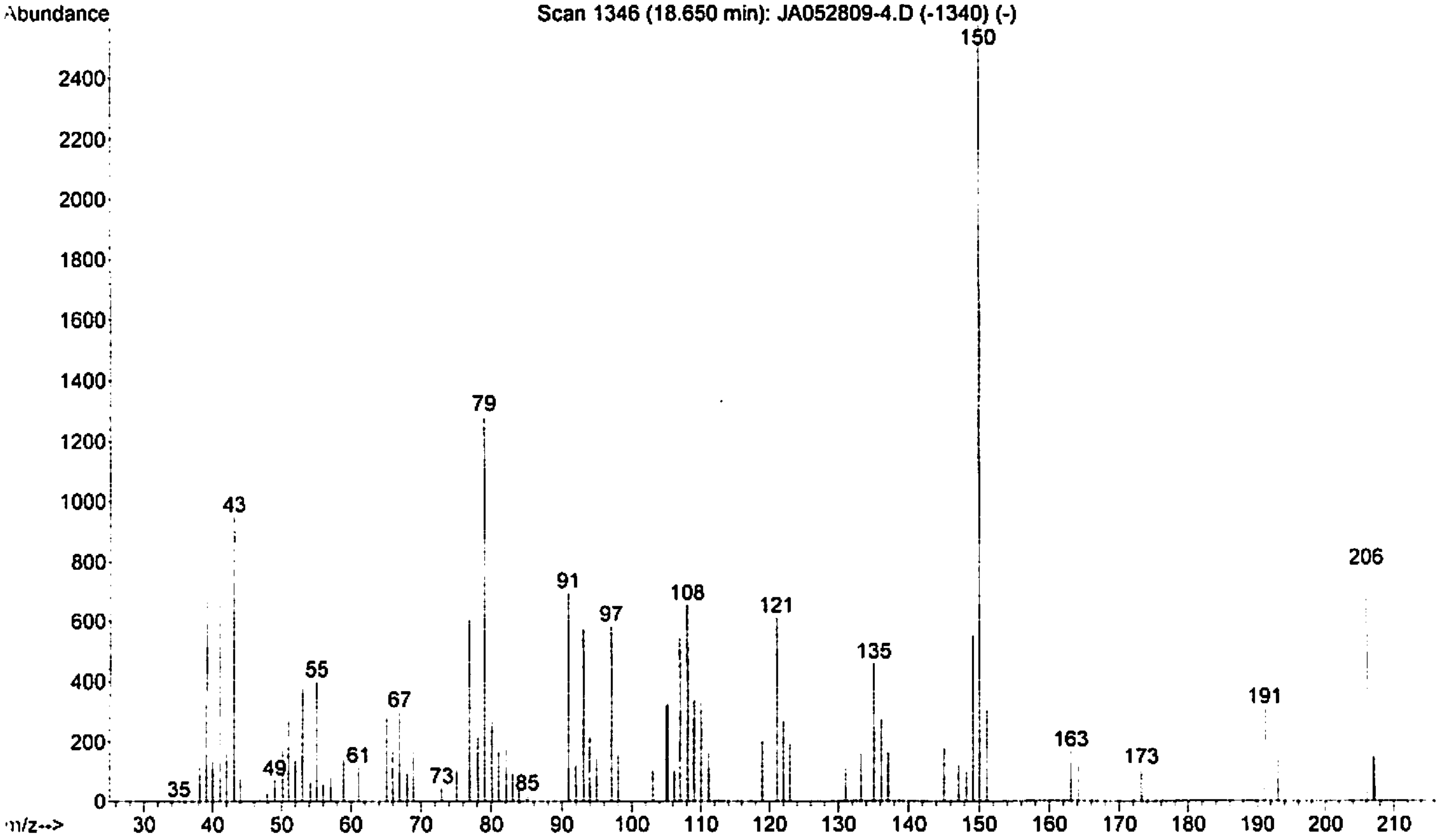

:D:\DATA\ALDRICH\JA-09\Snapshot\JA052809-4.D
[illegible]rator : Aldrich
[illegible]uired : 28 May 2009 16:03 using AcqMethod JA-WAX08.M
[illegible]strument : Instrument #1
[illegible]le Name: 1 field-coll. M C.oculata abd./CH2Cl2
[illegible] Info : coll. 5/28 sweeping vetch; second male today
[illegible] Number: 1

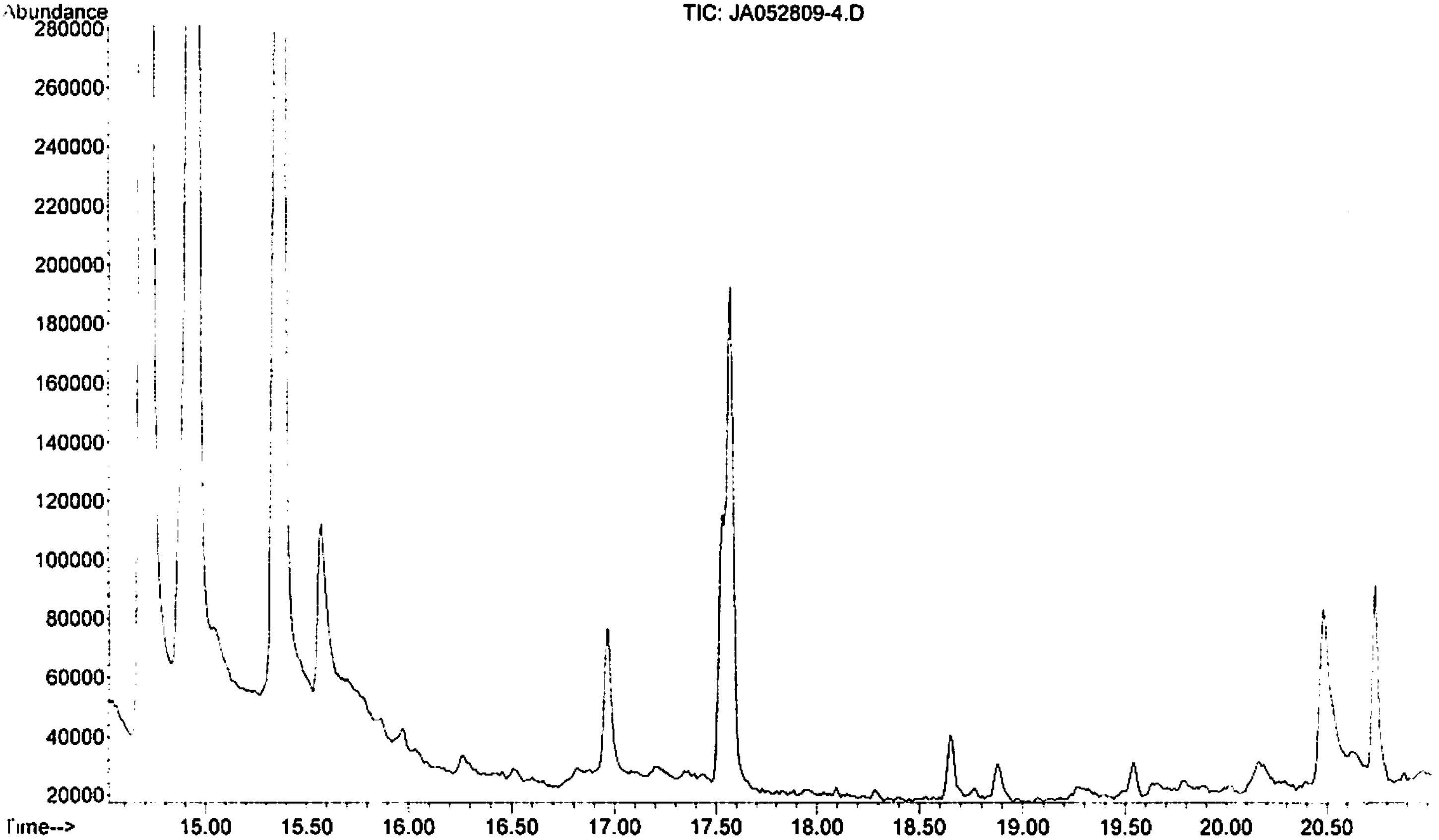

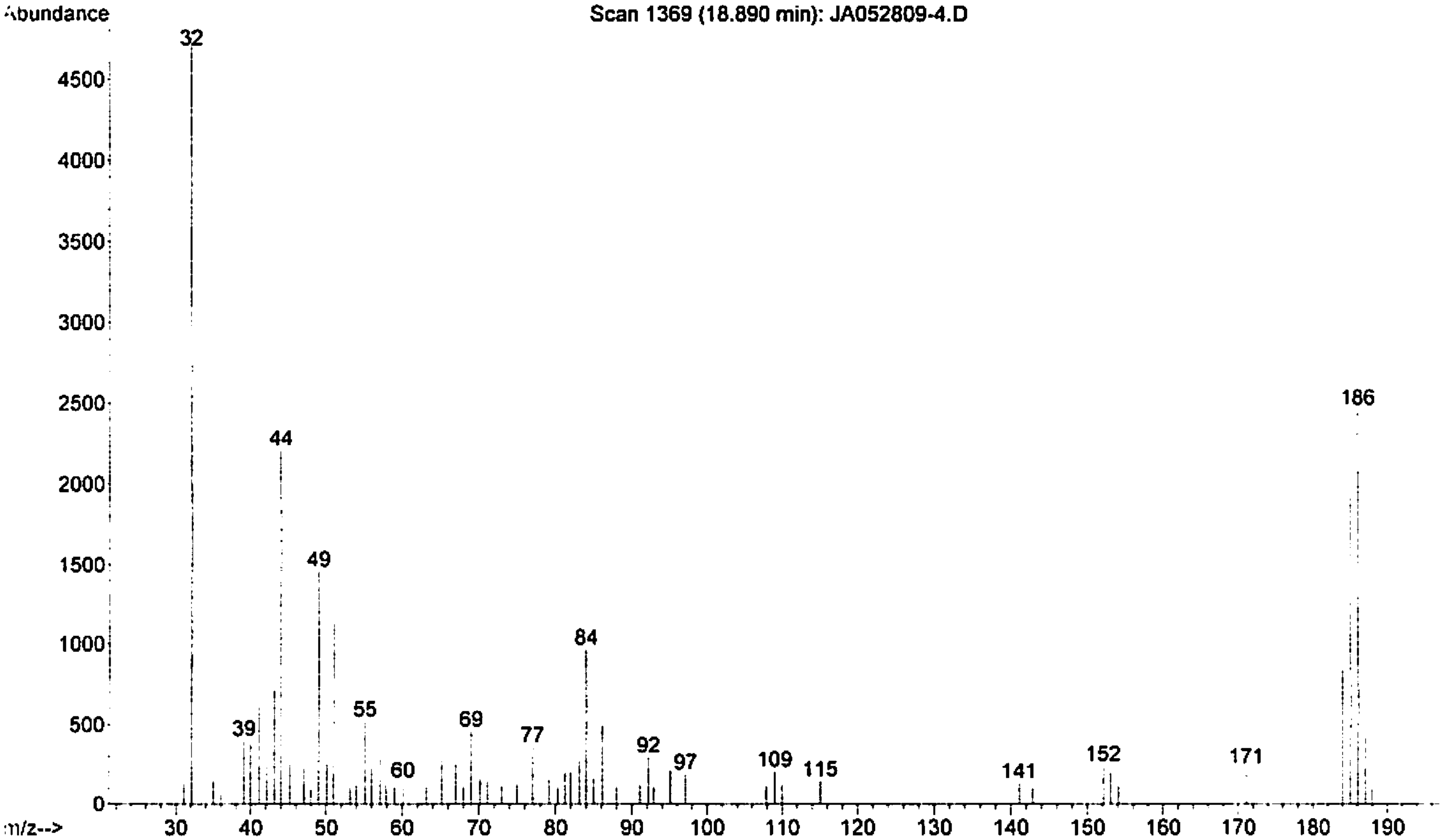

[illegible] :D:\DATA\ALDRICH\JA-09\Snapshot\JA052809-4.D
[illegible]rator : Aldrich
[illegible]ired : 28 May 2009 16:03 using AcqMethod JA-WAX08.M
[illegible]trument : Instrument #1
[illegible]le Name: 1 field-coll. M C.oculata abd./CH2Cl2
[illegible] Info : coll. 5/28 sweeping vetch; second male today
[illegible] Number: 1

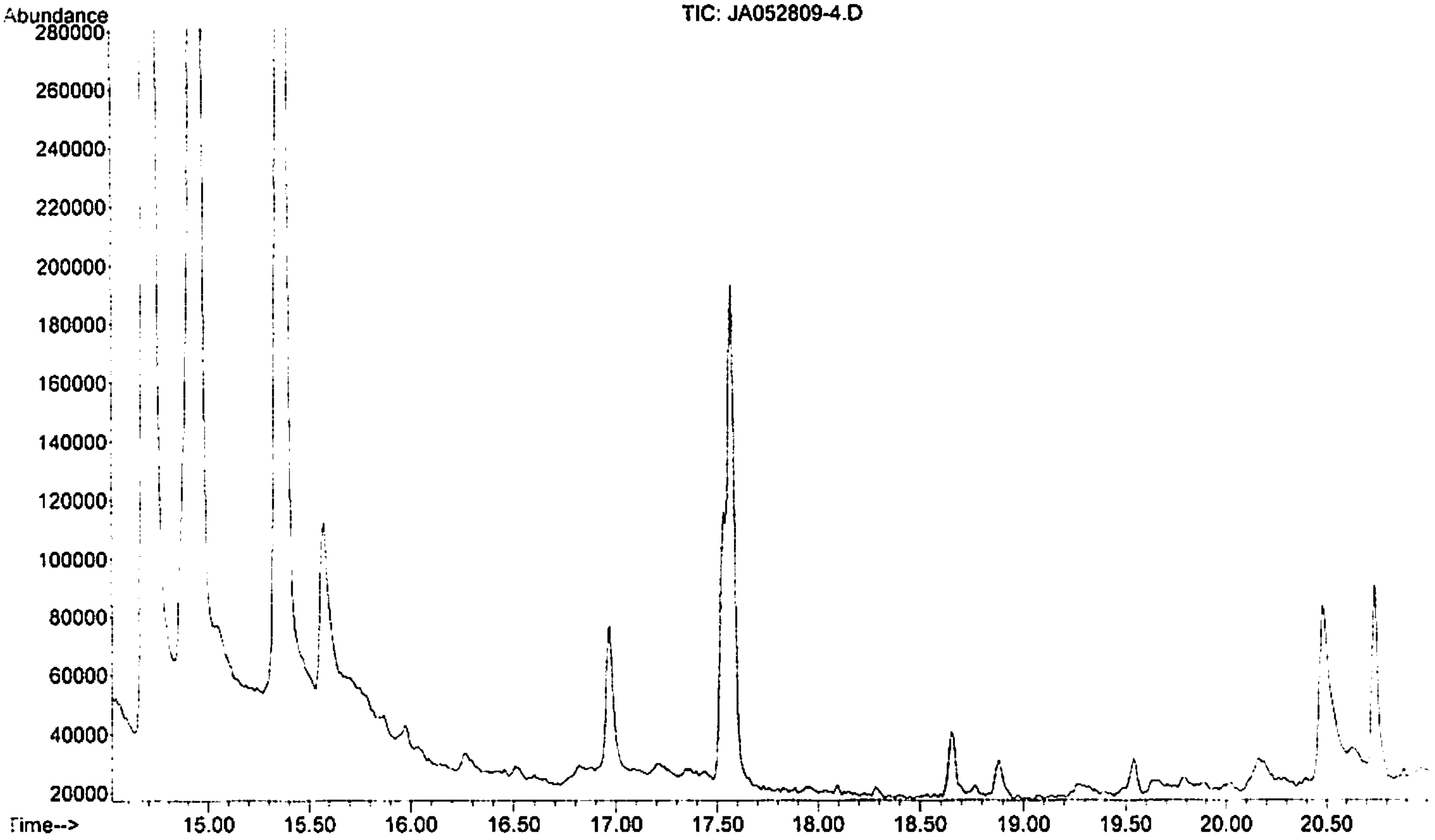

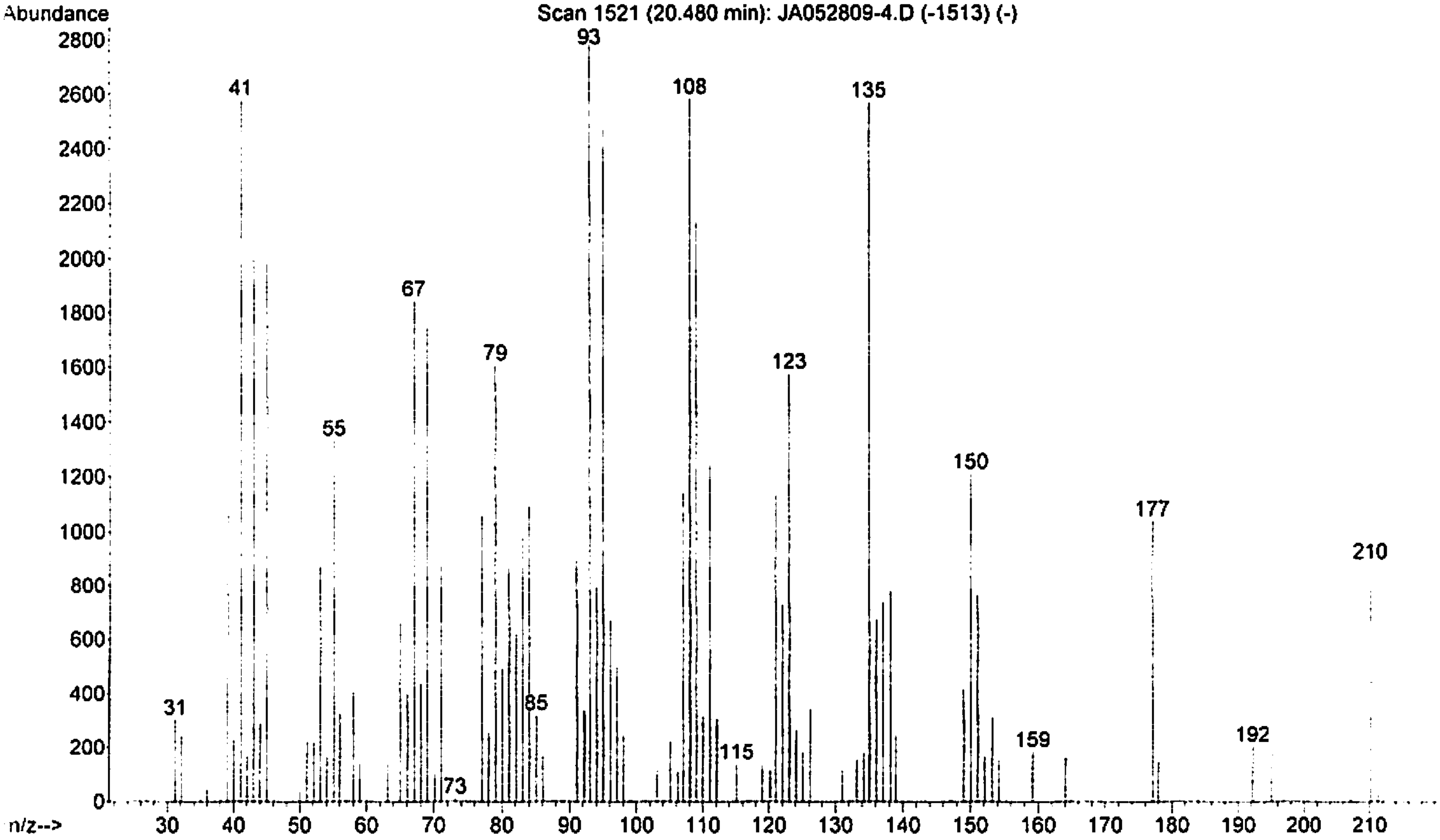

:D:\DATA\ALDRICH\JA-09\Snapshot\JA052809-4.D
[illegible]rator : Aldrich
[illegible]ired : 28 May 2009 16:03 using AcqMethod JA-WAX08.M
[illegible]rument : Instrument #1
[illegible]ple Name: 1 field-coll. M C.oculata abd./CH2Cl2
[illegible] Info : coll. 5/28 sweeping vetch; second male today
[illegible] Number: 1

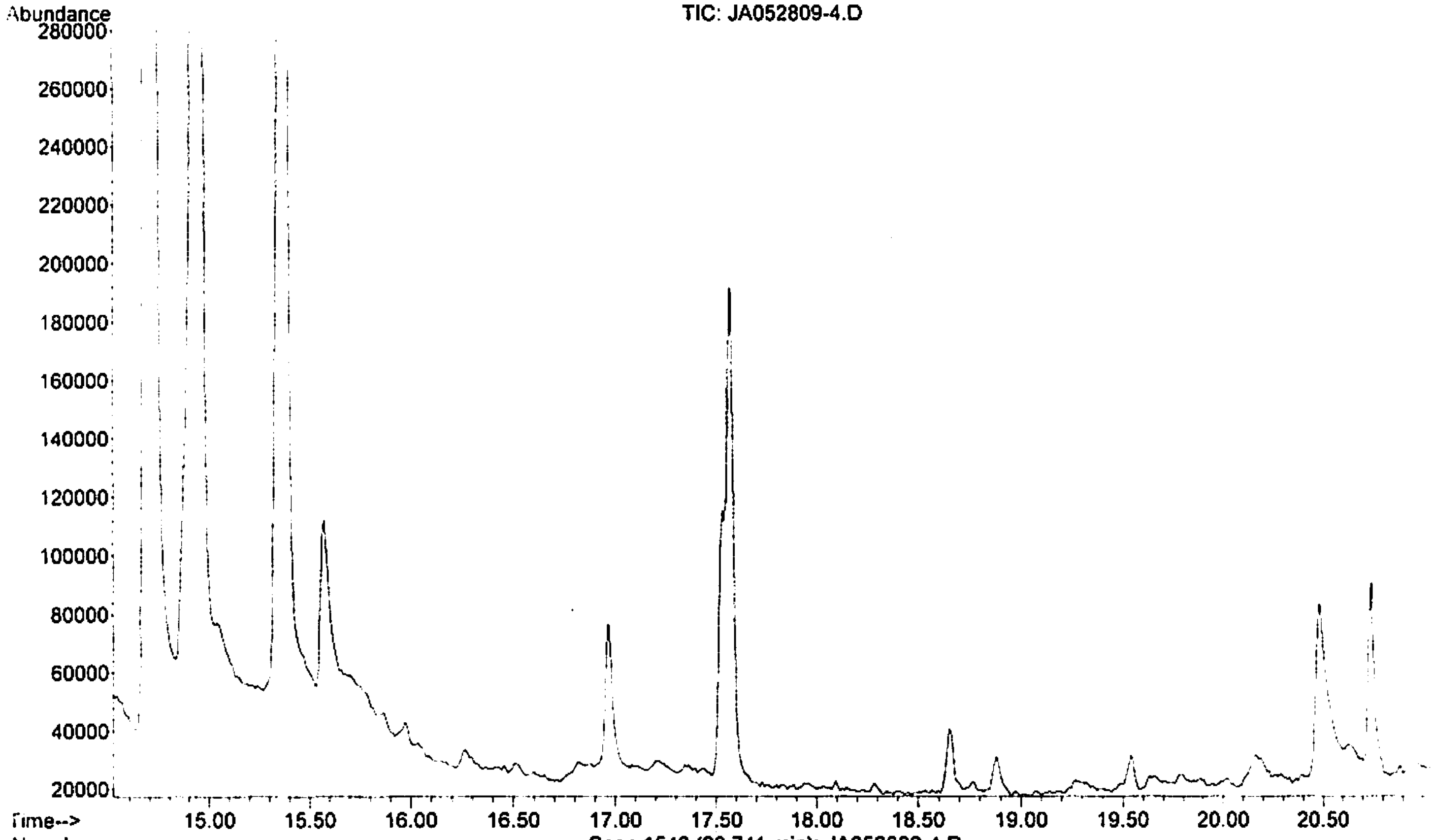

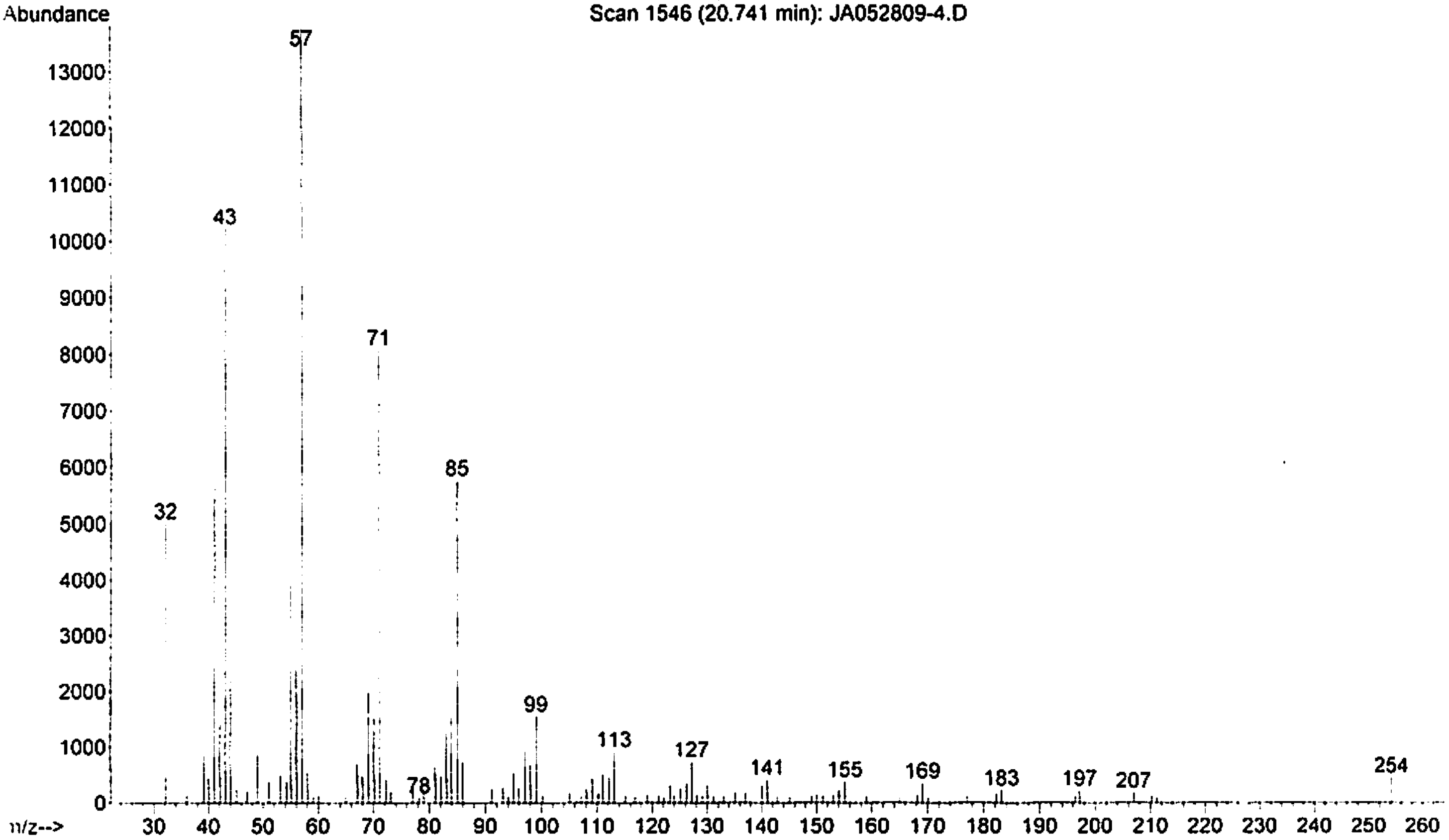

File   :D:\DATA\Aldrich\JA-09\JA060209-1.D
Operator   : Aldrich
Acquired   : 2 Jun 2009 10:47   using AcqMethod JA-WAX08.M
Instrument :   Instrument #1
Sample Name: 1 field-coll. M C.oculata abd./CH2Cl2
Misc Info   : coll. 6/1; 1st of two; top gut full
Vial Number: 1

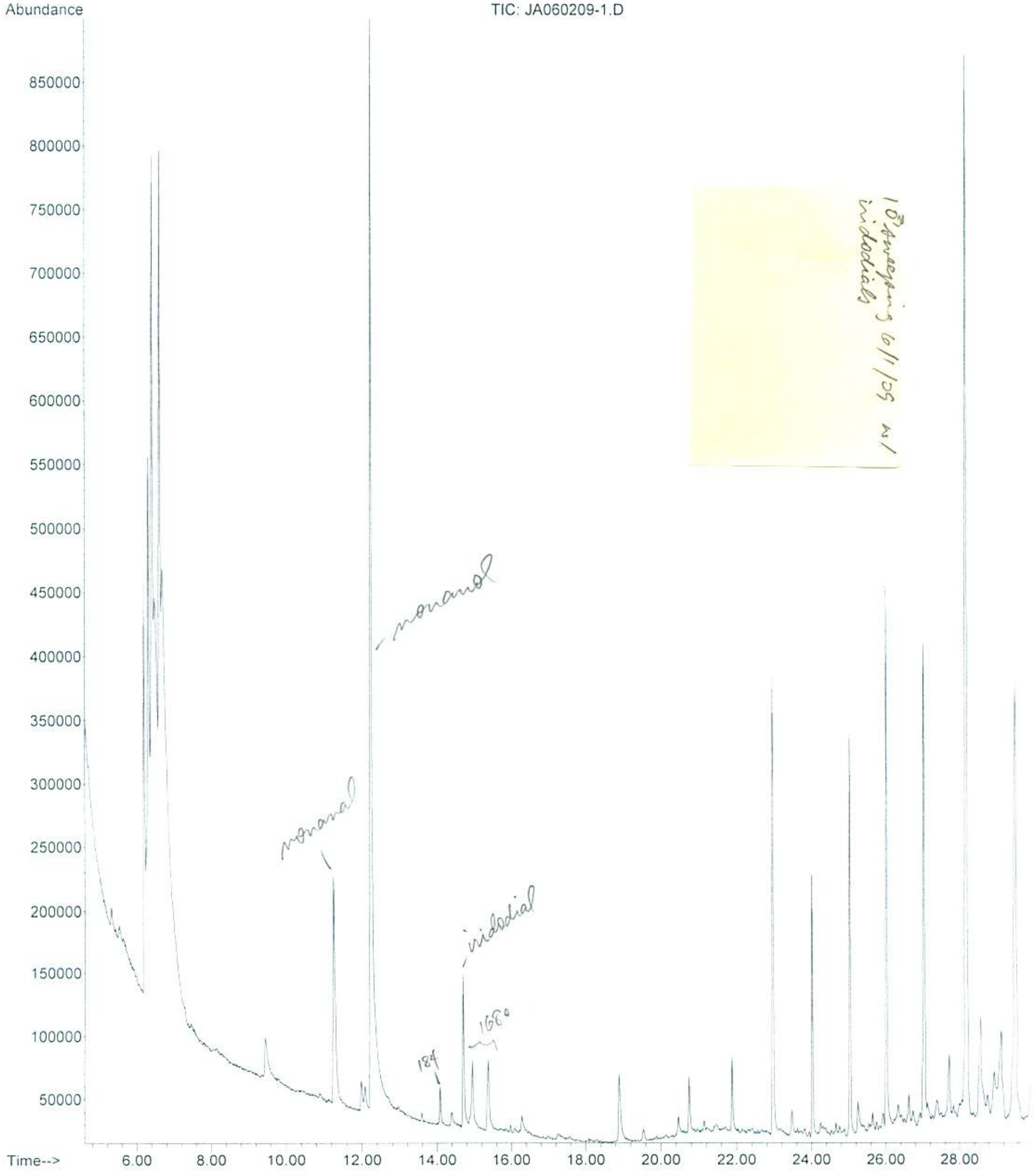

```
File          :D:\DATA\ALDRICH\JA-09\Snapshot\JA060209-1.D
Operator      : Aldrich
Acquired      :  2 Jun 2009  10:47     using AcqMethod JA-WAX08.M
Instrument    :     Instrument #1
Sample Name: 1 field-coll. M C.oculata abd./CH2Cl2
Misc Info     : coll. 6/1; 1st of two; top gut full
Vial Number: 1
```

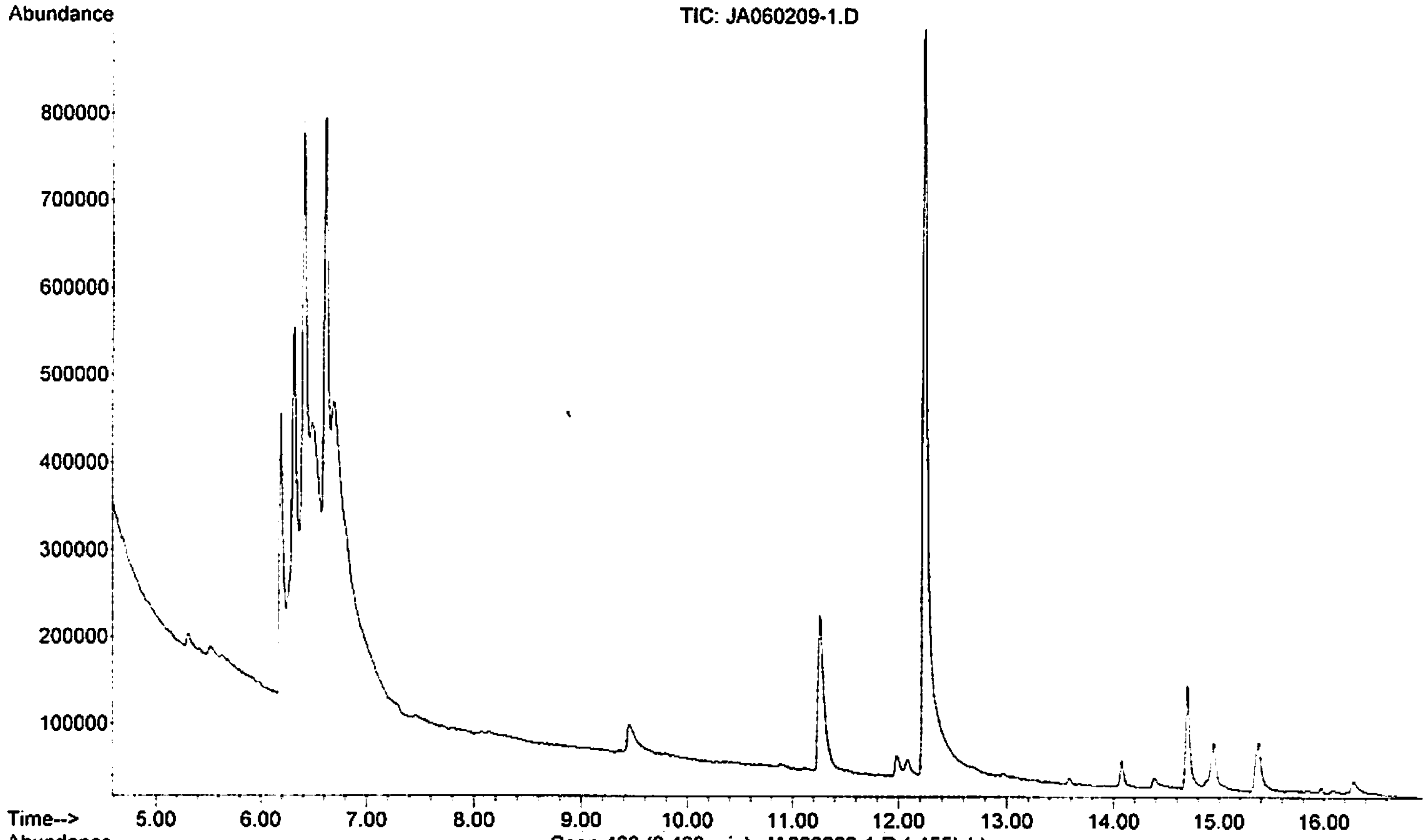

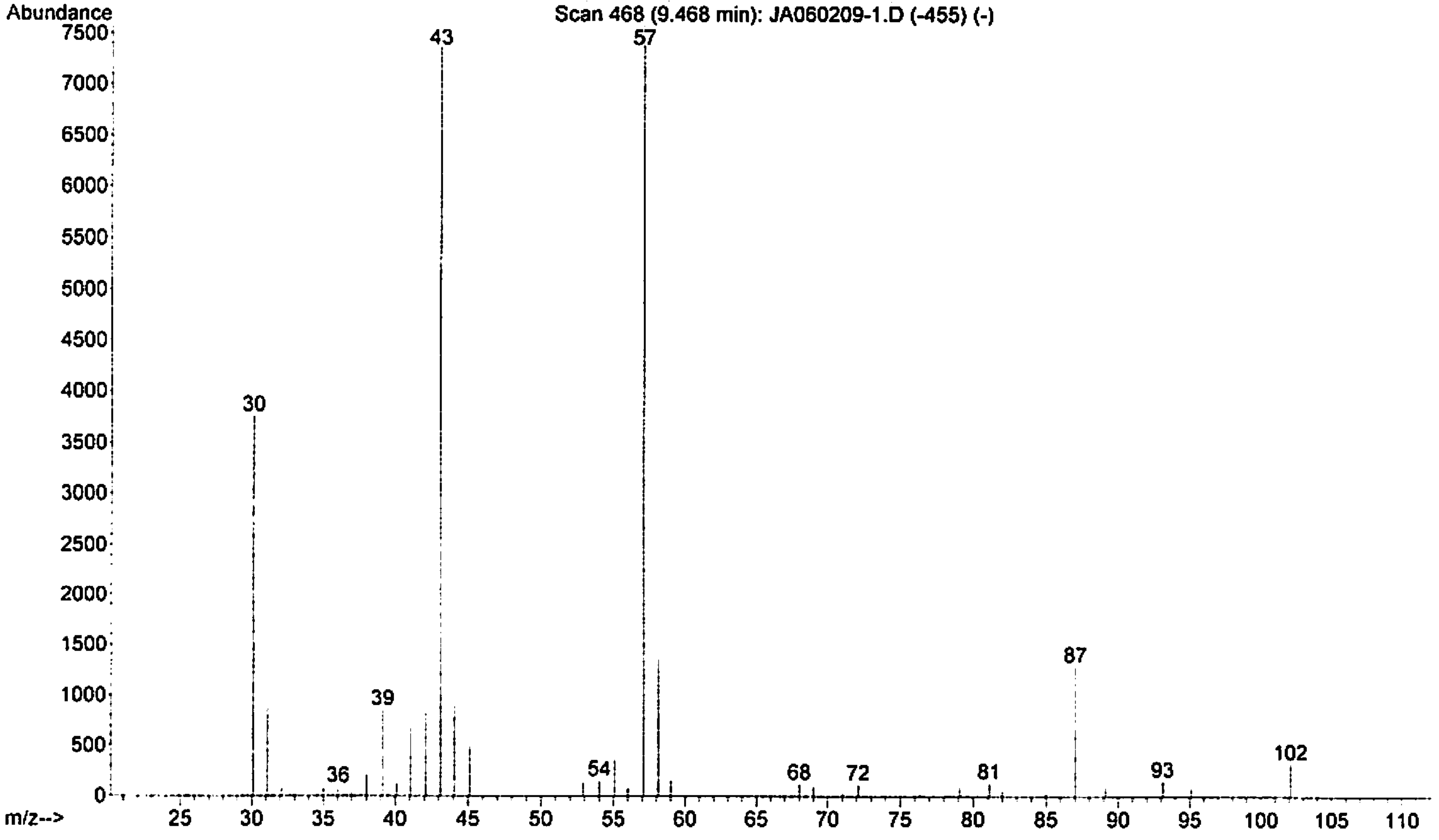

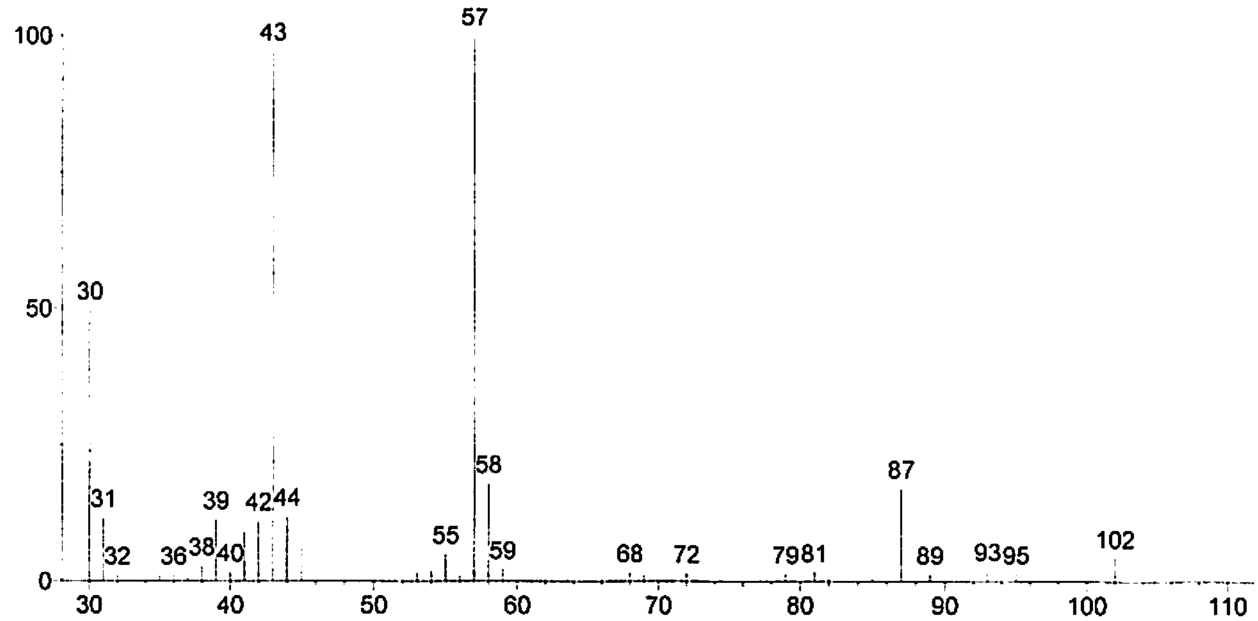

(Text File) Scan 468 (9.468 min): JA060209-1.D (-455)

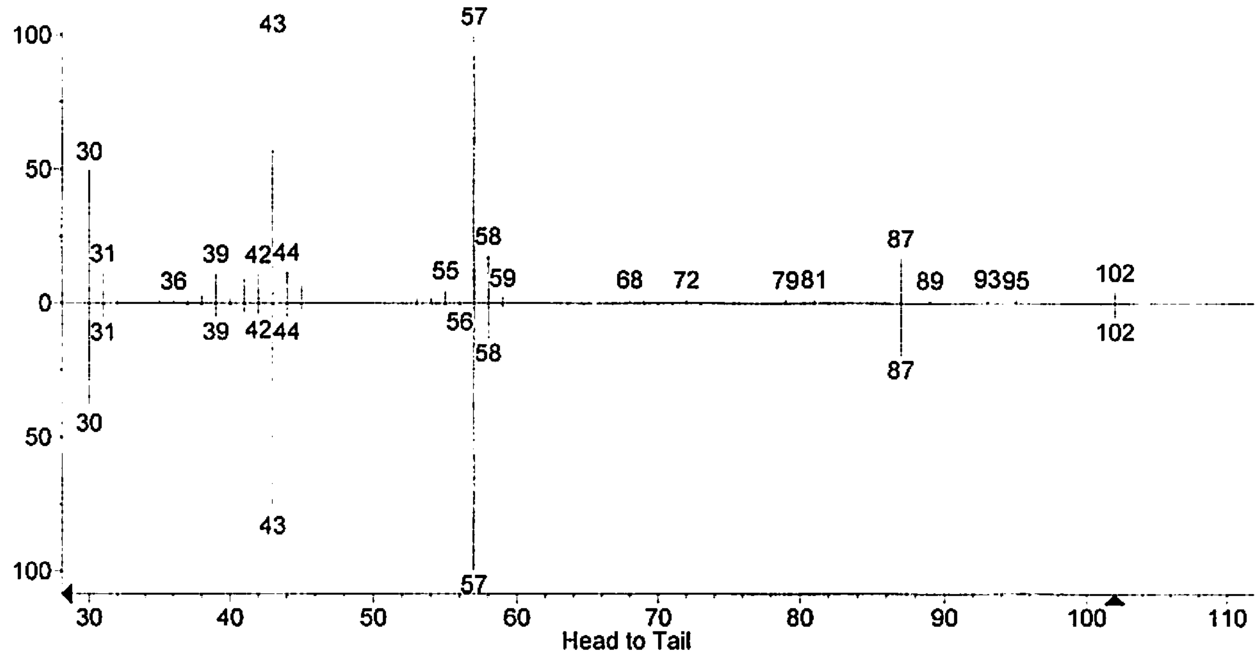

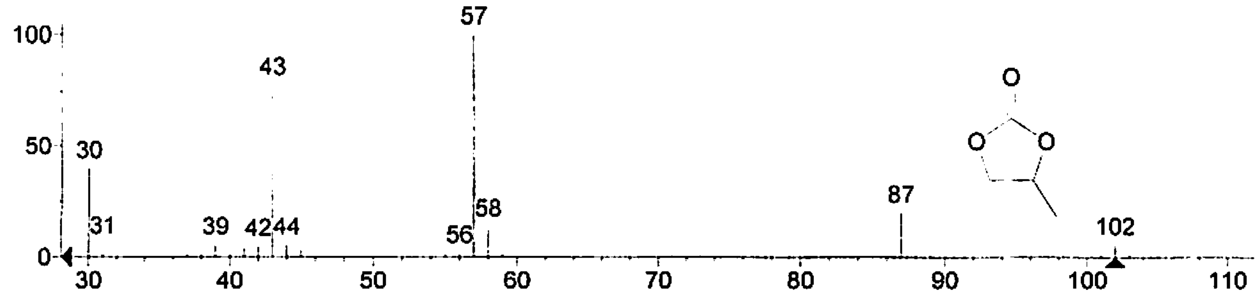

(mainlib) Propylene Carbonate

File :D:\DATA\ALDRICH\JA-09\Snapshot\JA060209-1.D
Operator : Aldrich
Acquired : 2 Jun 2009 10:47 using AcqMethod JA-WAX08.M
Instrument : Instrument #1
Sample Name: 1 field-coll. M C.oculata abd./CH2Cl2
Misc Info : coll. 6/1; 1st of two; top gut full
Vial Number: 1

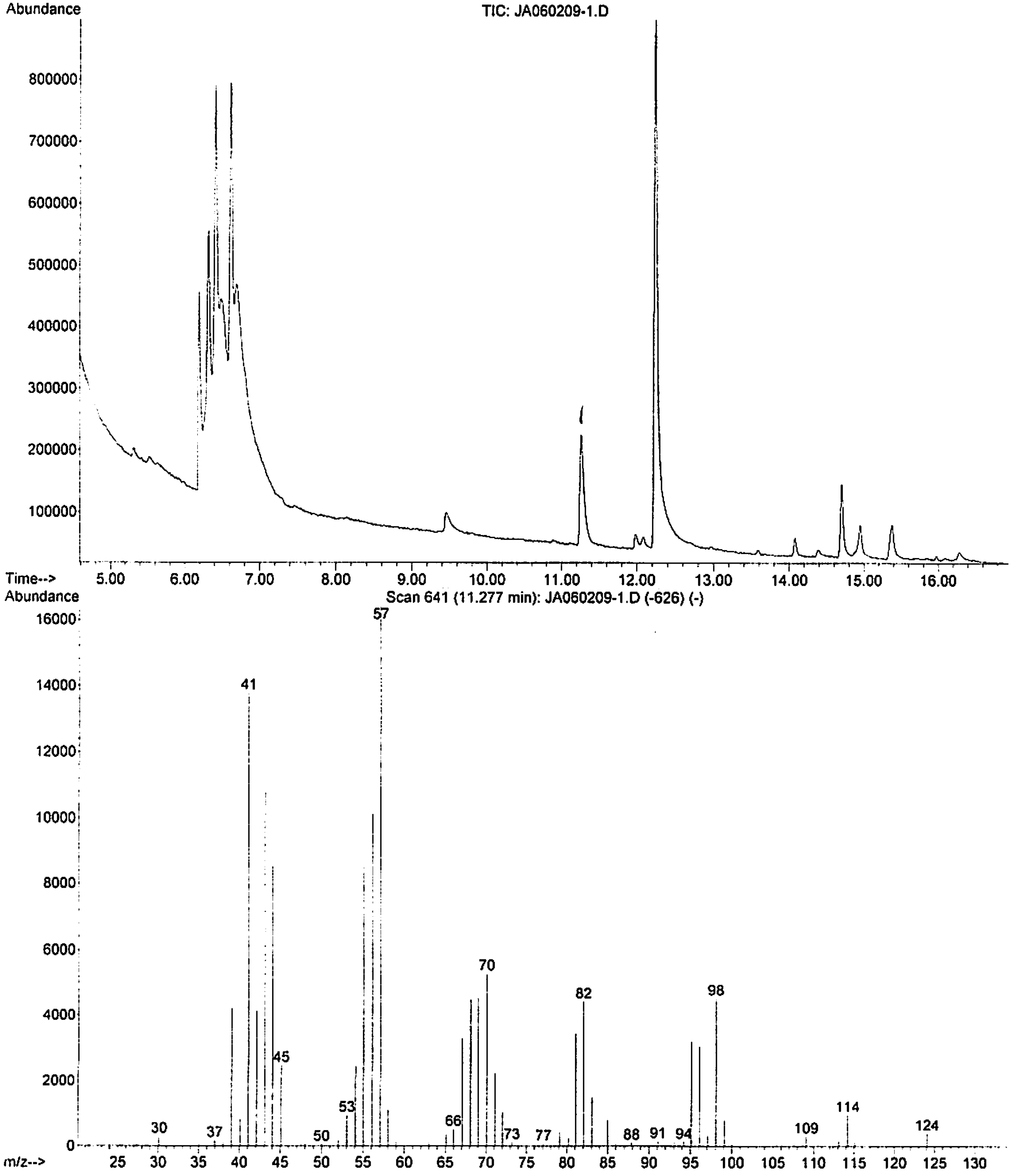

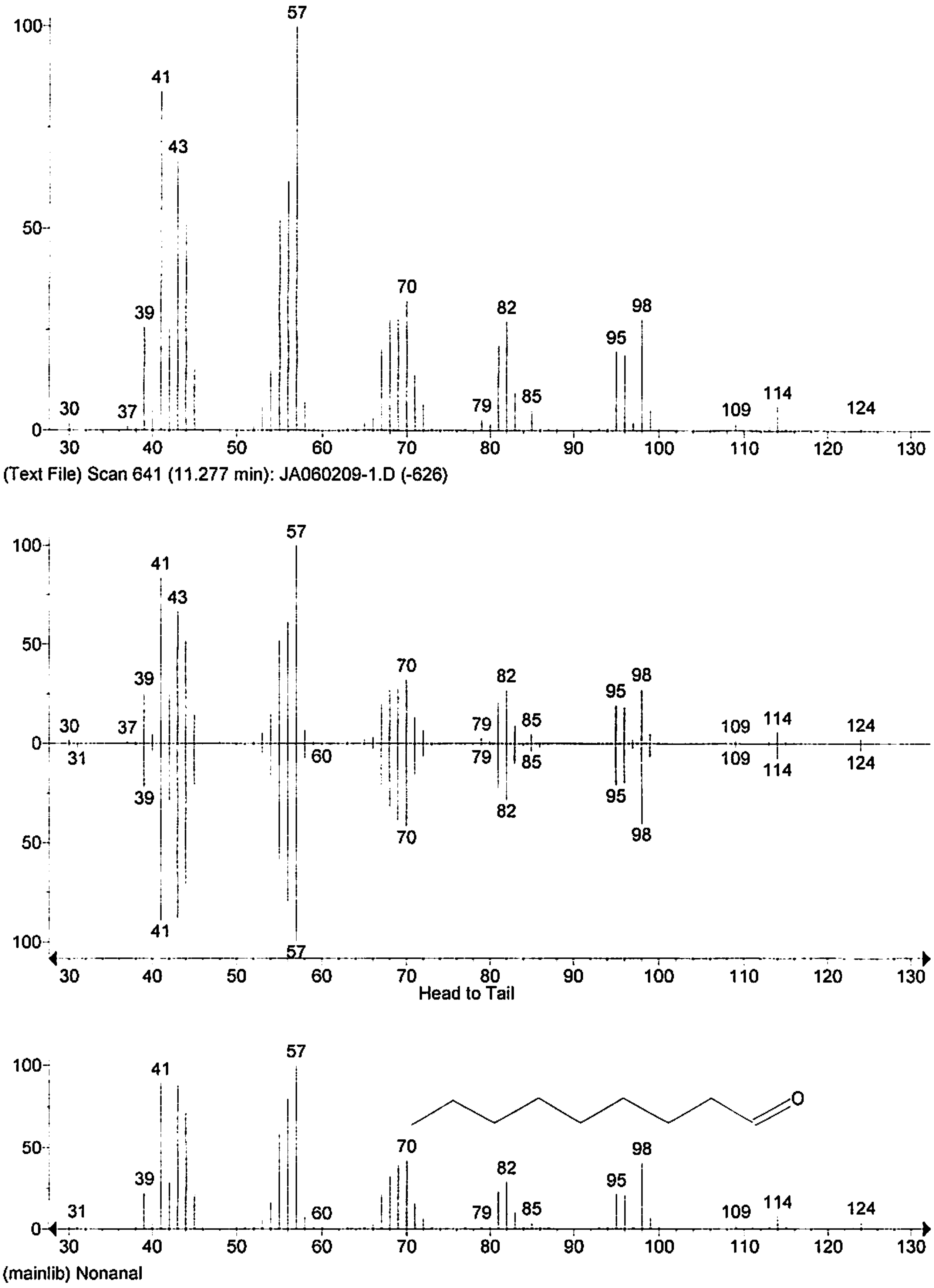

(Text File) Scan 641 (11.277 min): JA060209-1.D (-626)
Head to Tail
(mainlib) Nonanal

File : D:\DATA\ALDRICH\JA-09\Snapshot\JA060209-1.D
Operator : Aldrich
Acquired : 2 Jun 2009 10:47 using AcqMethod JA-WAX08.M
Instrument : Instrument #1
Sample Name: 1 field-coll. M C.oculata abd./CH2Cl2
Misc Info : coll. 6/1; 1st of two; top gut full
Vial Number: 1

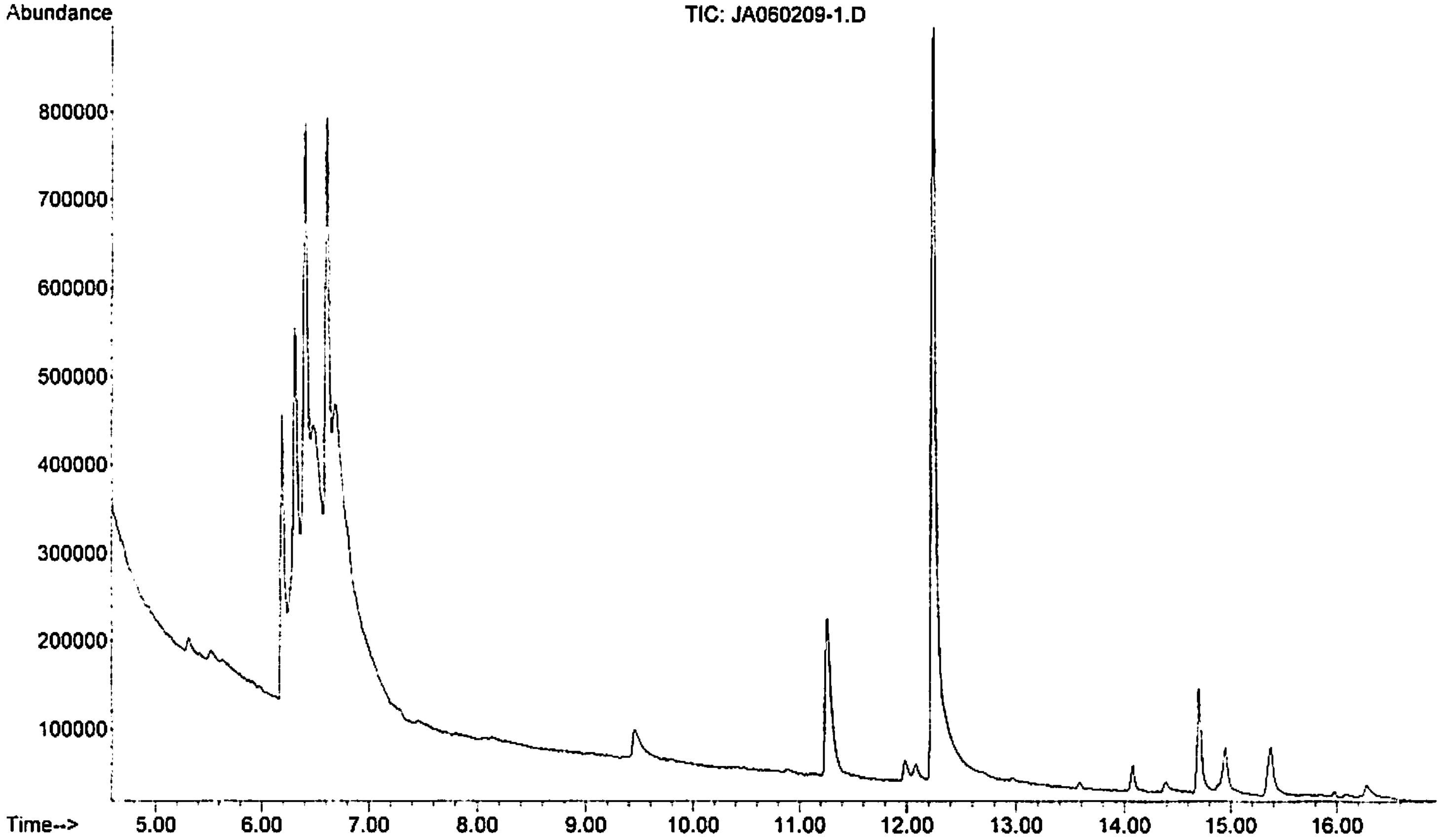

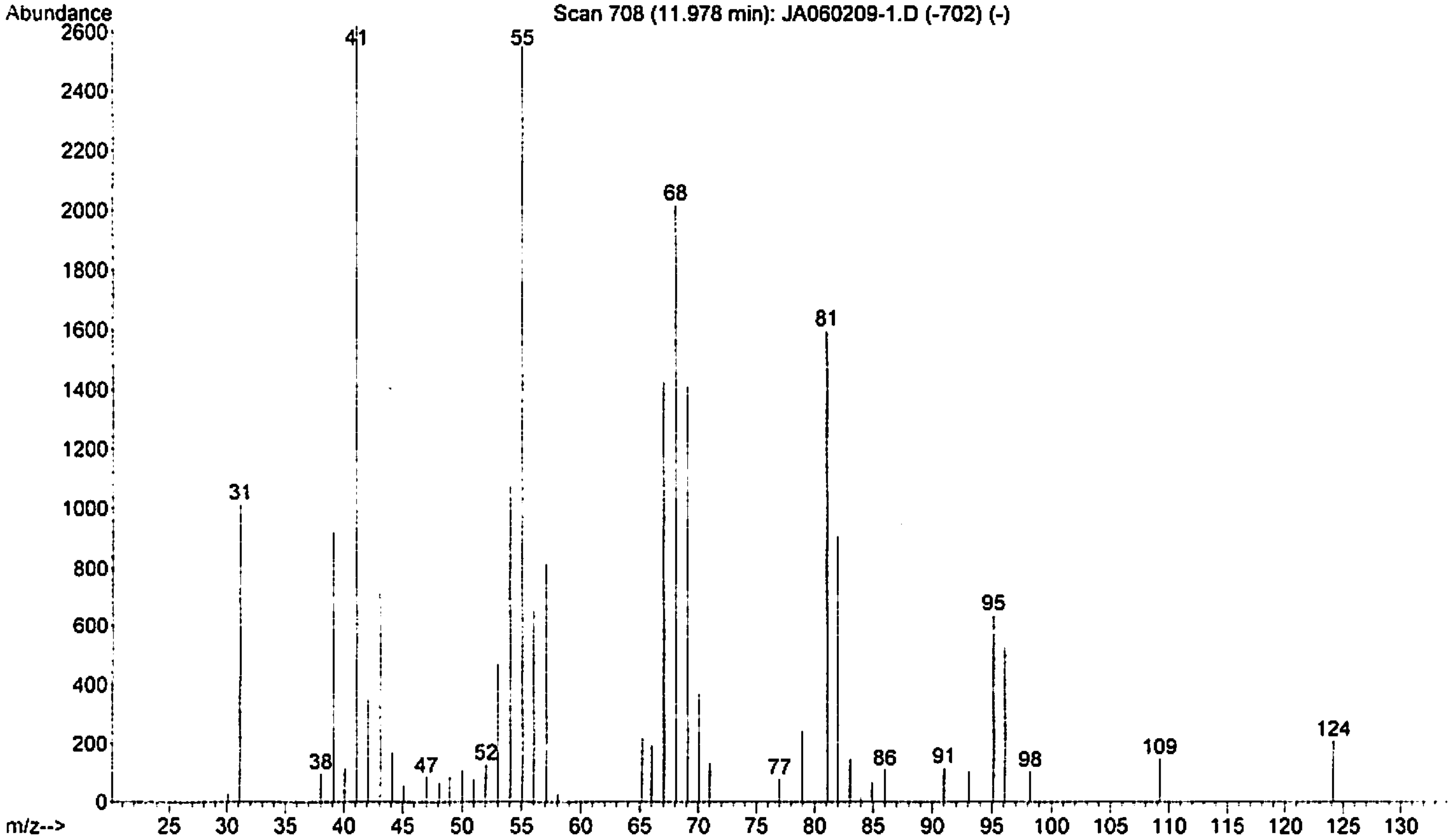

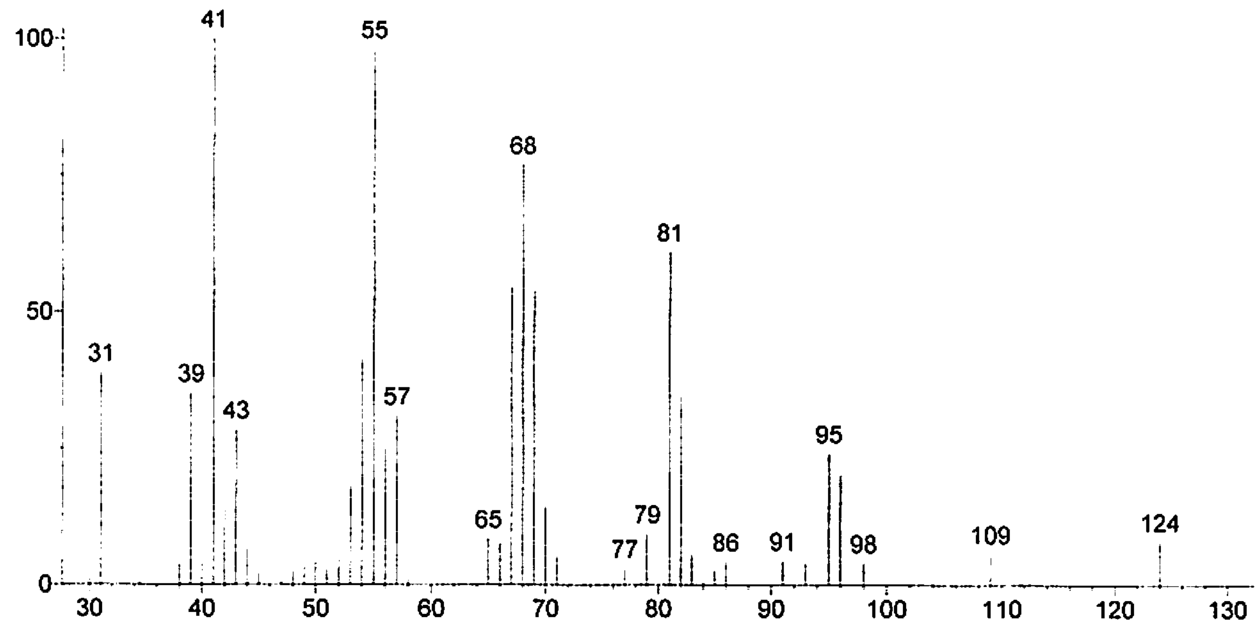

(Text File) Scan 708 (11.978 min): JA060209-1.D (-702)

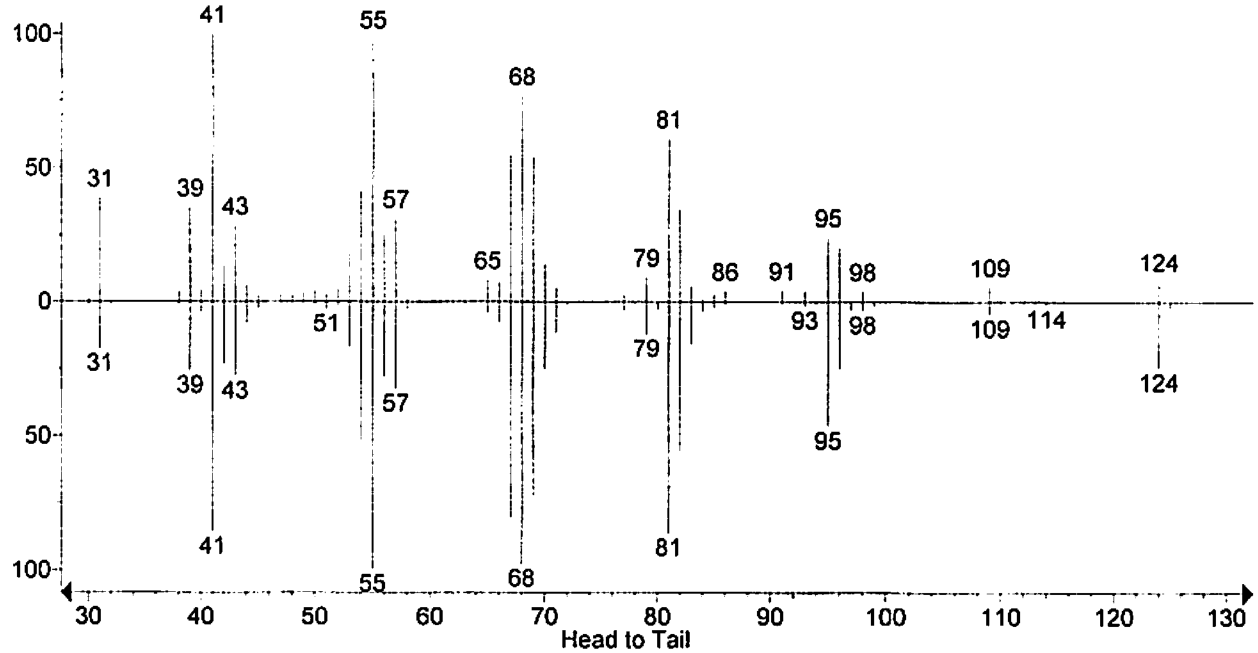

Head to Tail

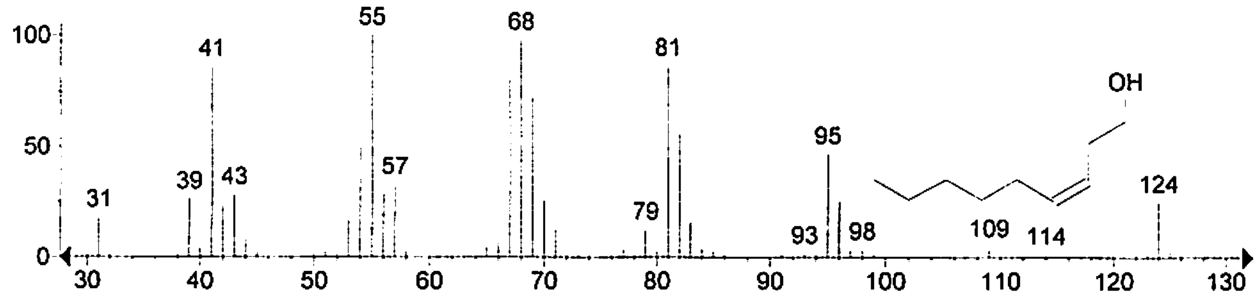

(mainlib) 3-Nonen-1-ol, (Z)-

File   : D:\DATA\ALDRICH\JA-09\Snapshot\JA060209-1.D
Operator   : Aldrich
Acquired   : 2 Jun 2009 10:47   using AcqMethod JA-WAX08.M
Instrument :   Instrument #1
Sample Name: 1 field-coll. M C.oculata abd./CH2Cl2
Misc Info   : coll. 6/1; 1st of two; top gut full
Vial Number: 1

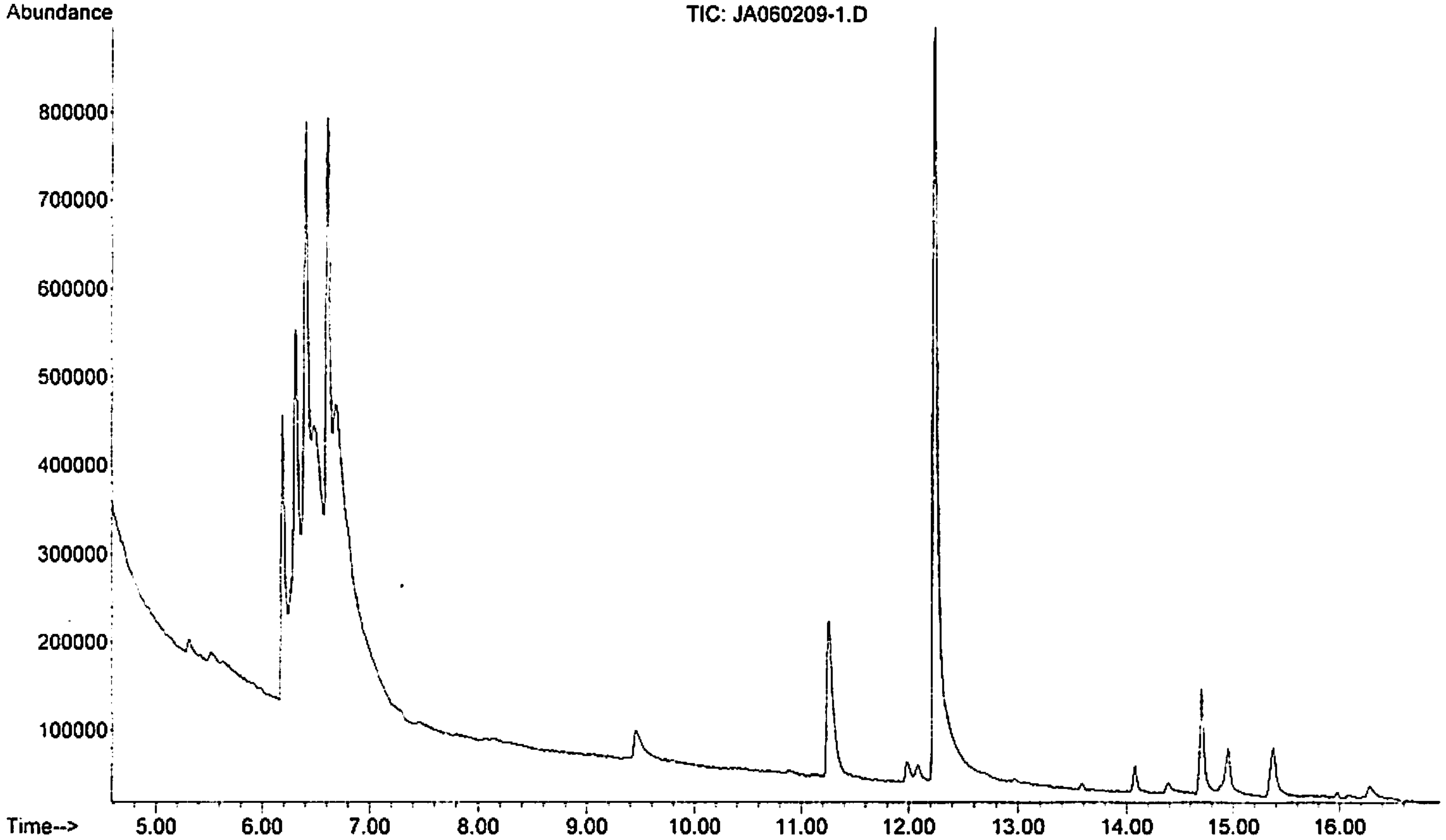

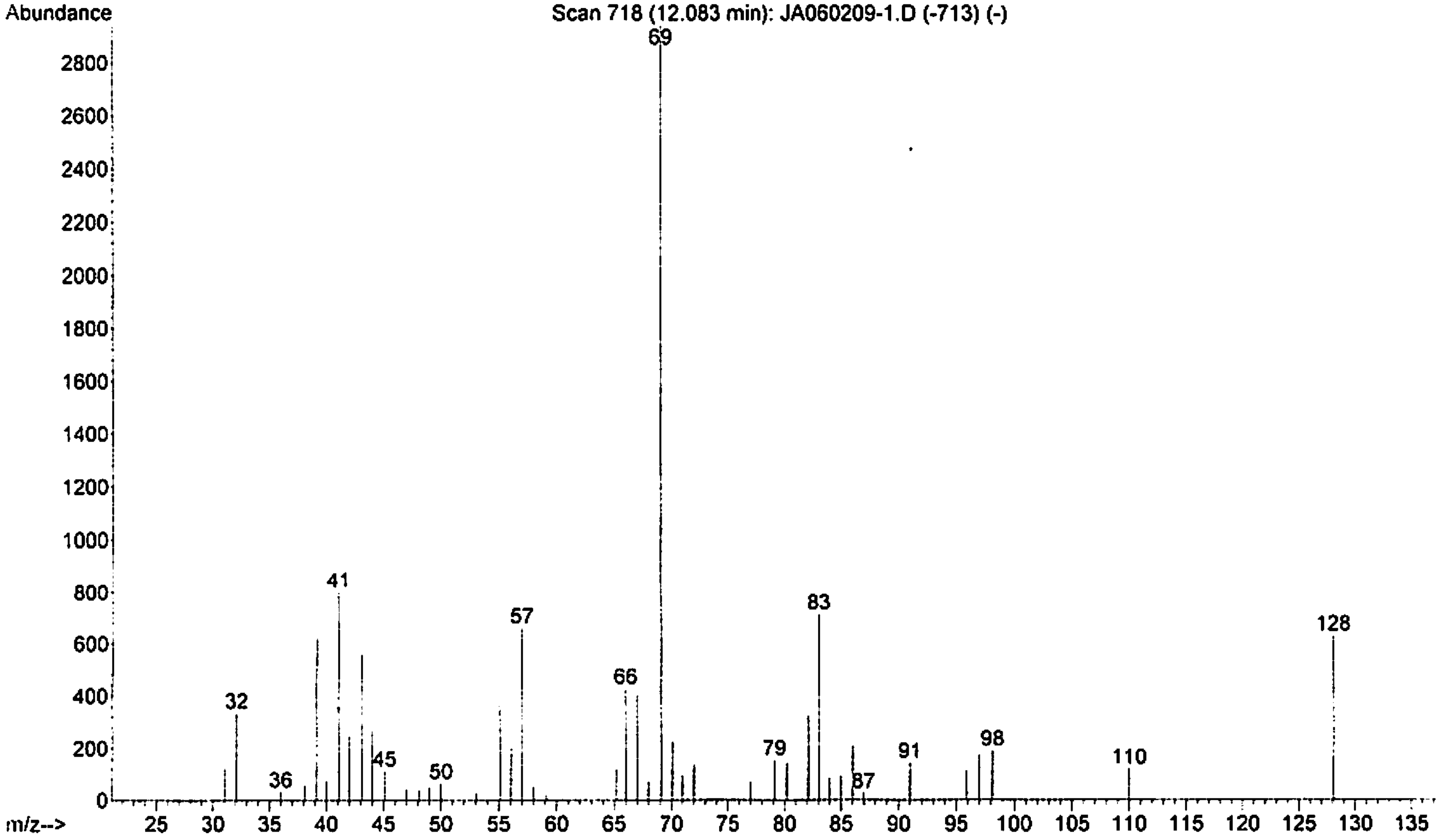

File :D:\DATA\ALDRICH\JA-09\Snapshot\JA060209-1.D
Operator : Aldrich
Acquired : 2 Jun 2009 10:47 using AcqMethod JA-WAX08.M
Instrument : Instrument #1
Sample Name: 1 field-coll. M C.oculata abd./CH2Cl2
Misc Info : coll. 6/1; 1st of two; top gut full
Vial Number: 1

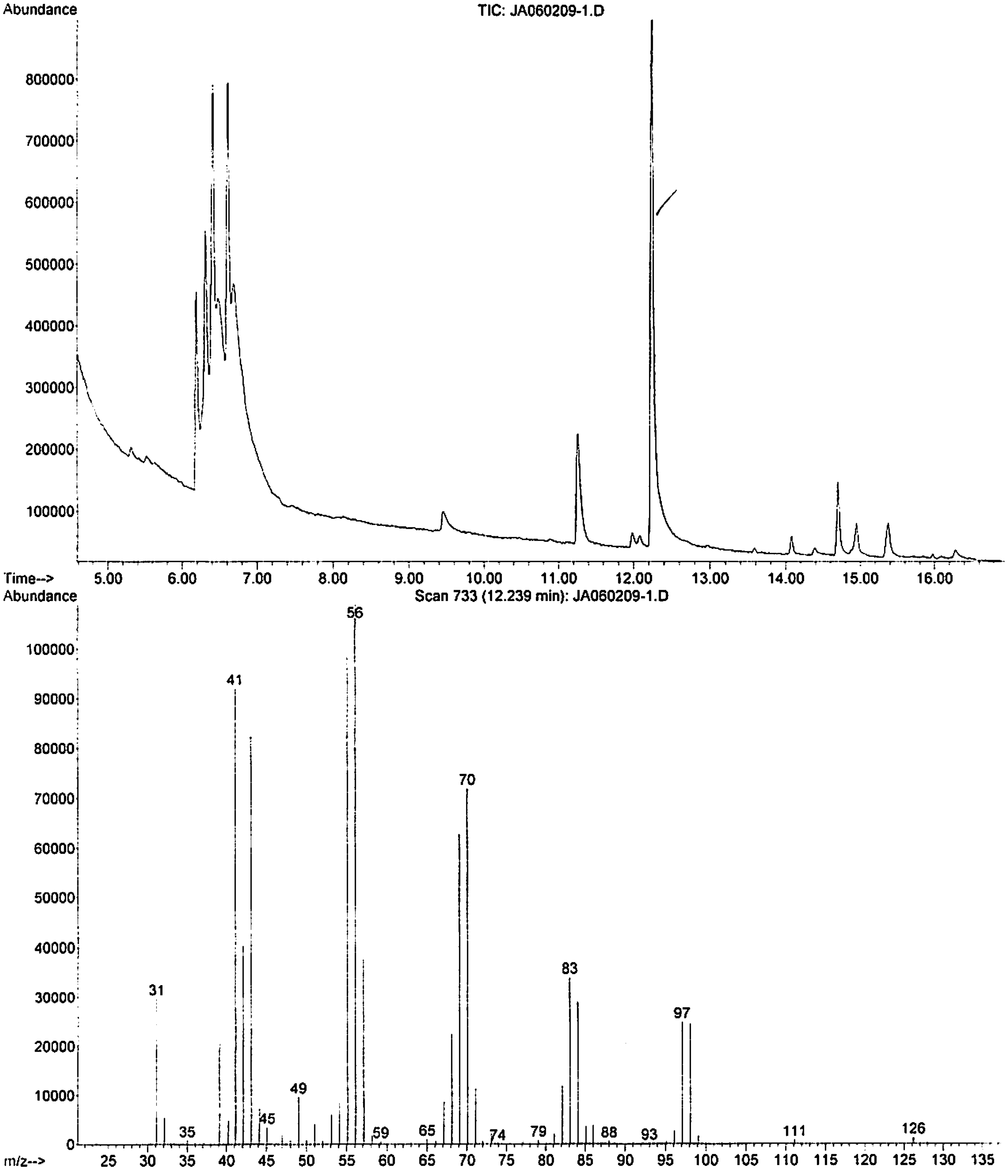

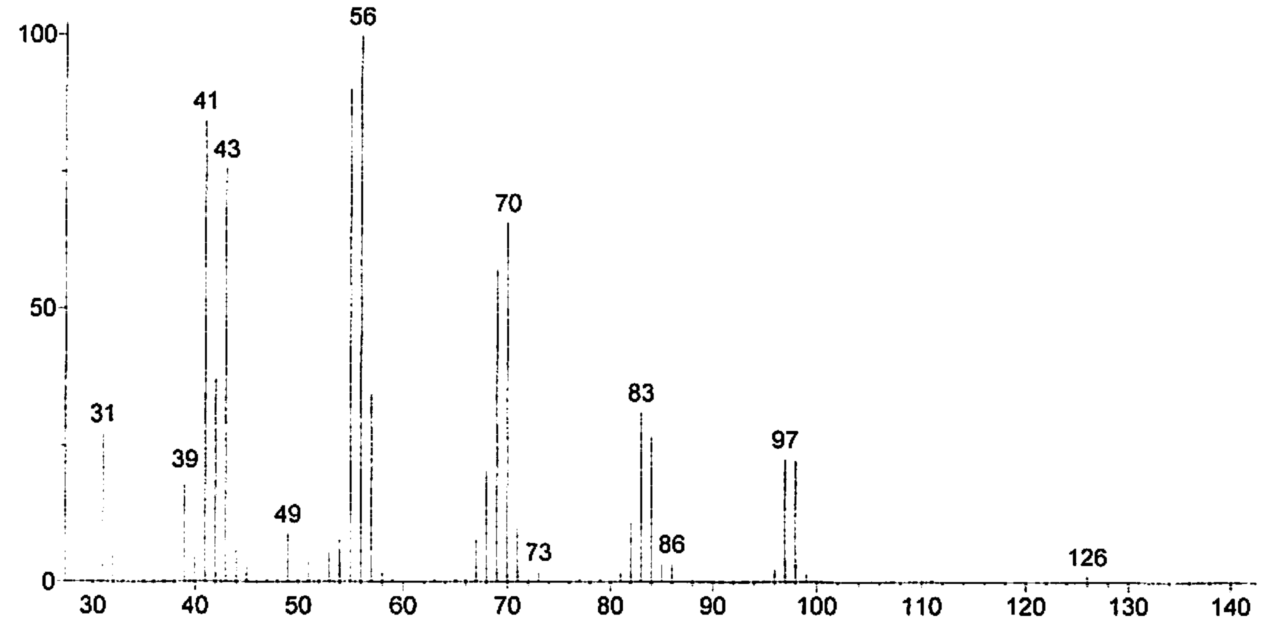

(Text File) Scan 733 (12.239 min): JA060209-1.D

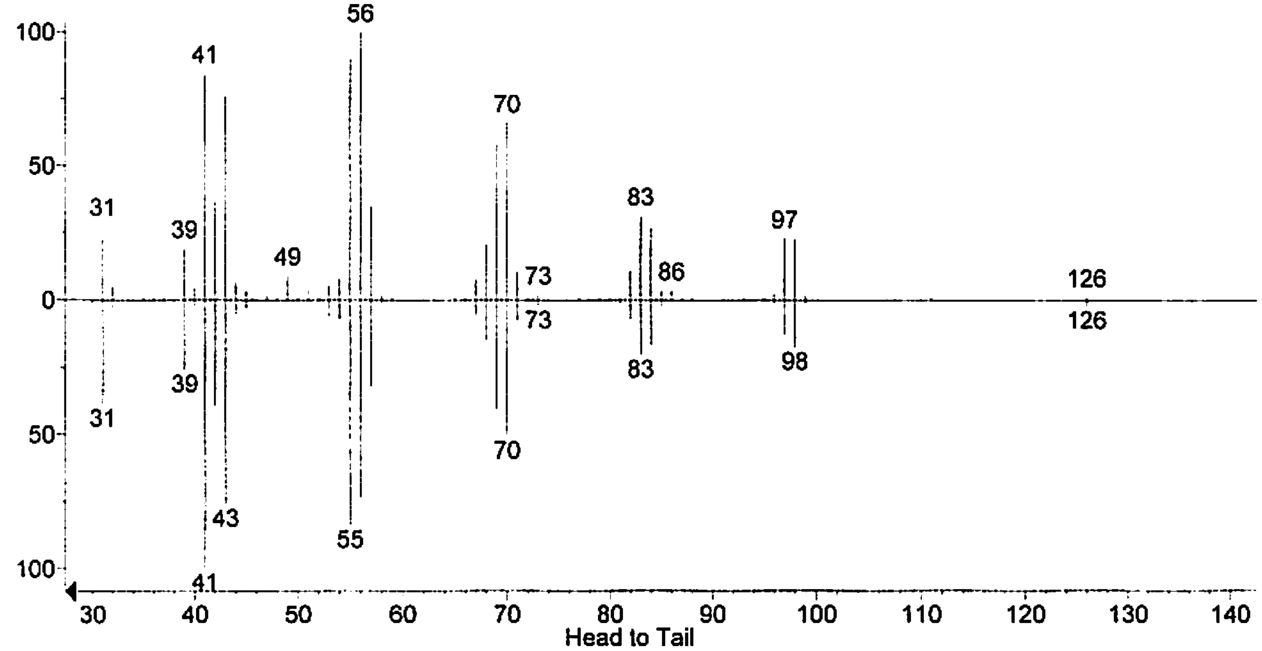

Head to Tail

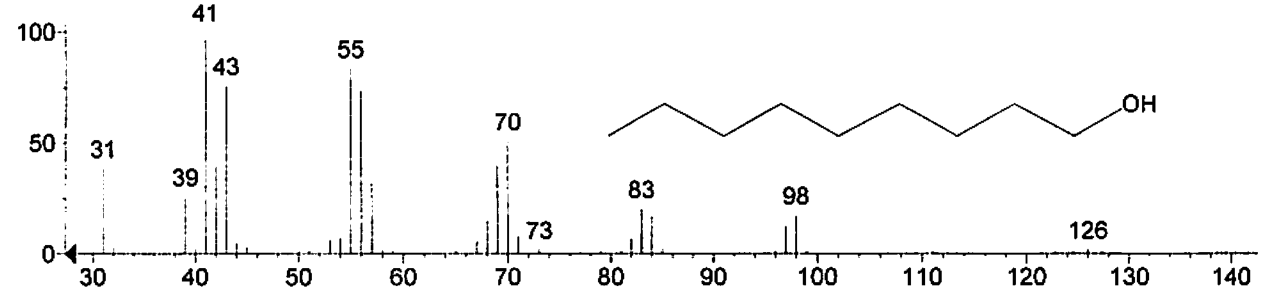

(replib) 1-Nonanol

File :D:\DATA\ALDRICH\JA-09\Snapshot\JA060209-1.D
Operator : Aldrich
Acquired : 2 Jun 2009 10:47 using AcqMethod JA-WAX08.M
Instrument : Instrument #1
Sample Name: 1 field-coll. M C.oculata abd./CH2Cl2
Misc Info : coll. 6/1; 1st of two; top gut full
Vial Number: 1

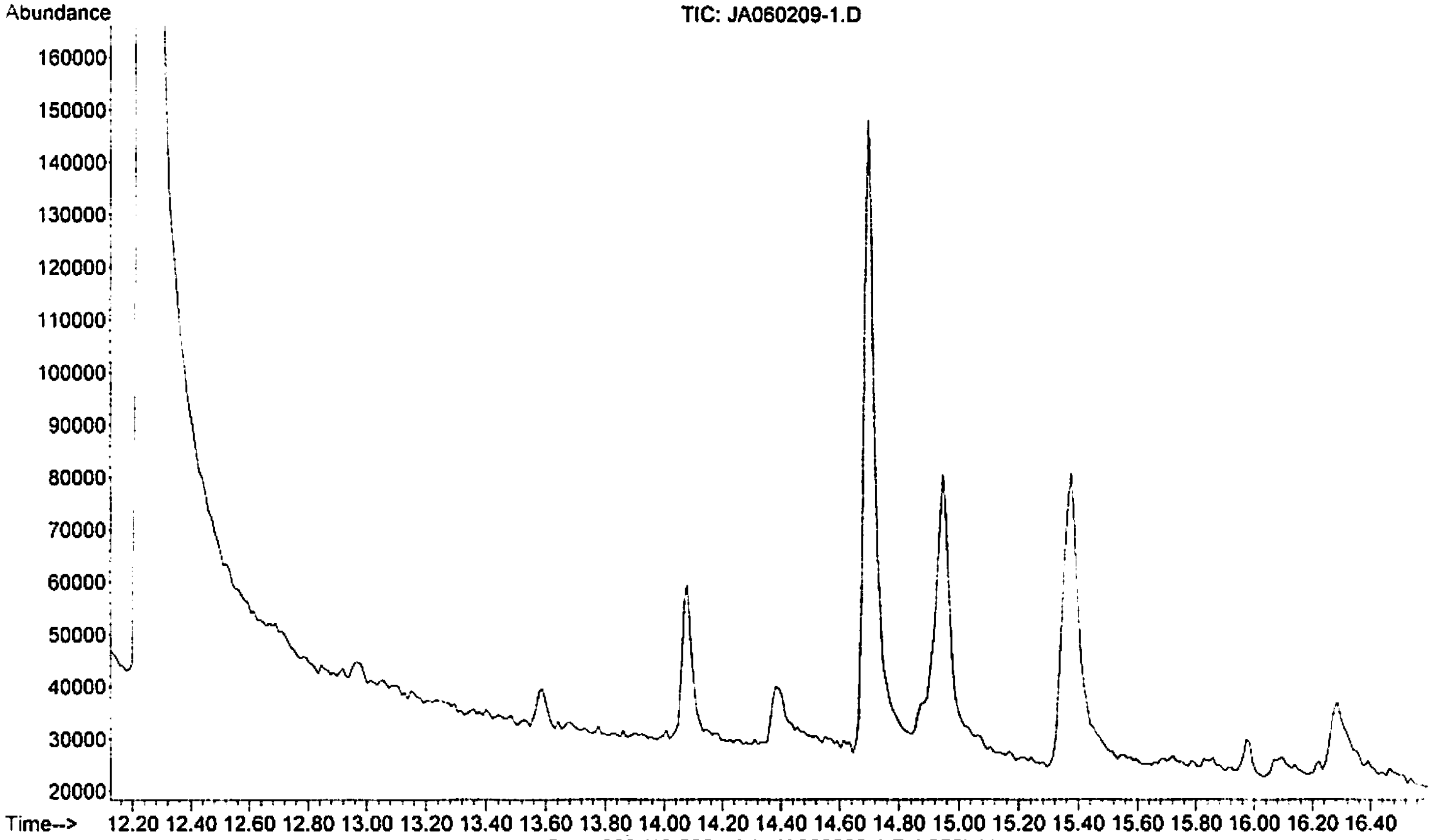

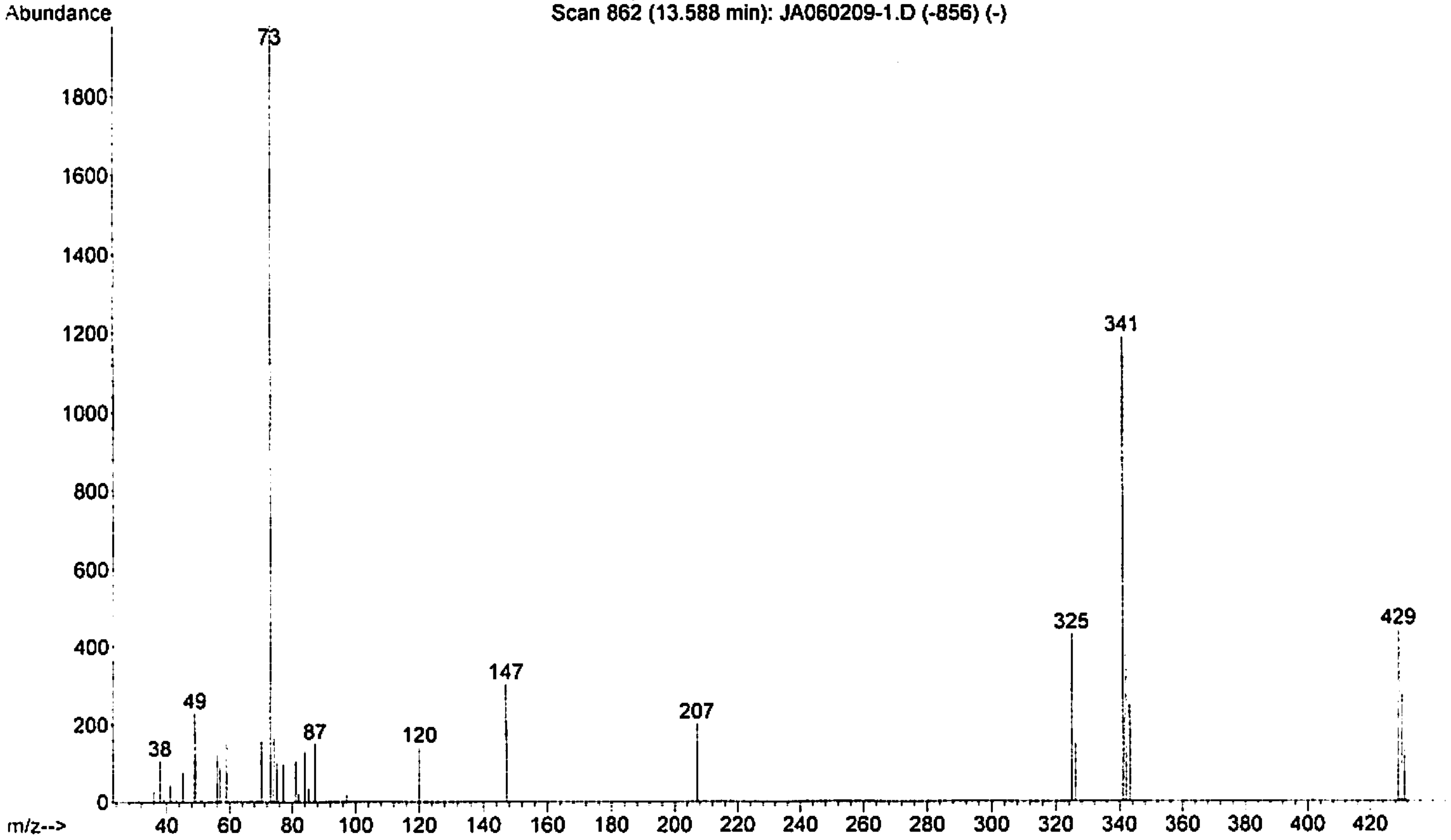

[illegible]e. :D:\DATA\ALDRICH\JA-09\Snapshot\JA060209-1.D
[illegible]rator : Aldrich
[illegible]quired : 2 Jun 2009 10:47 using AcqMethod JA-WAX08.M
[illegible]strument : Instrument #1
[illegible]ple Name: 1 field-coll. M C.oculata abd./CH2Cl2
[illegible] Info : coll. 6/1; 1st of two; top gut full
[illegible] Number: 1

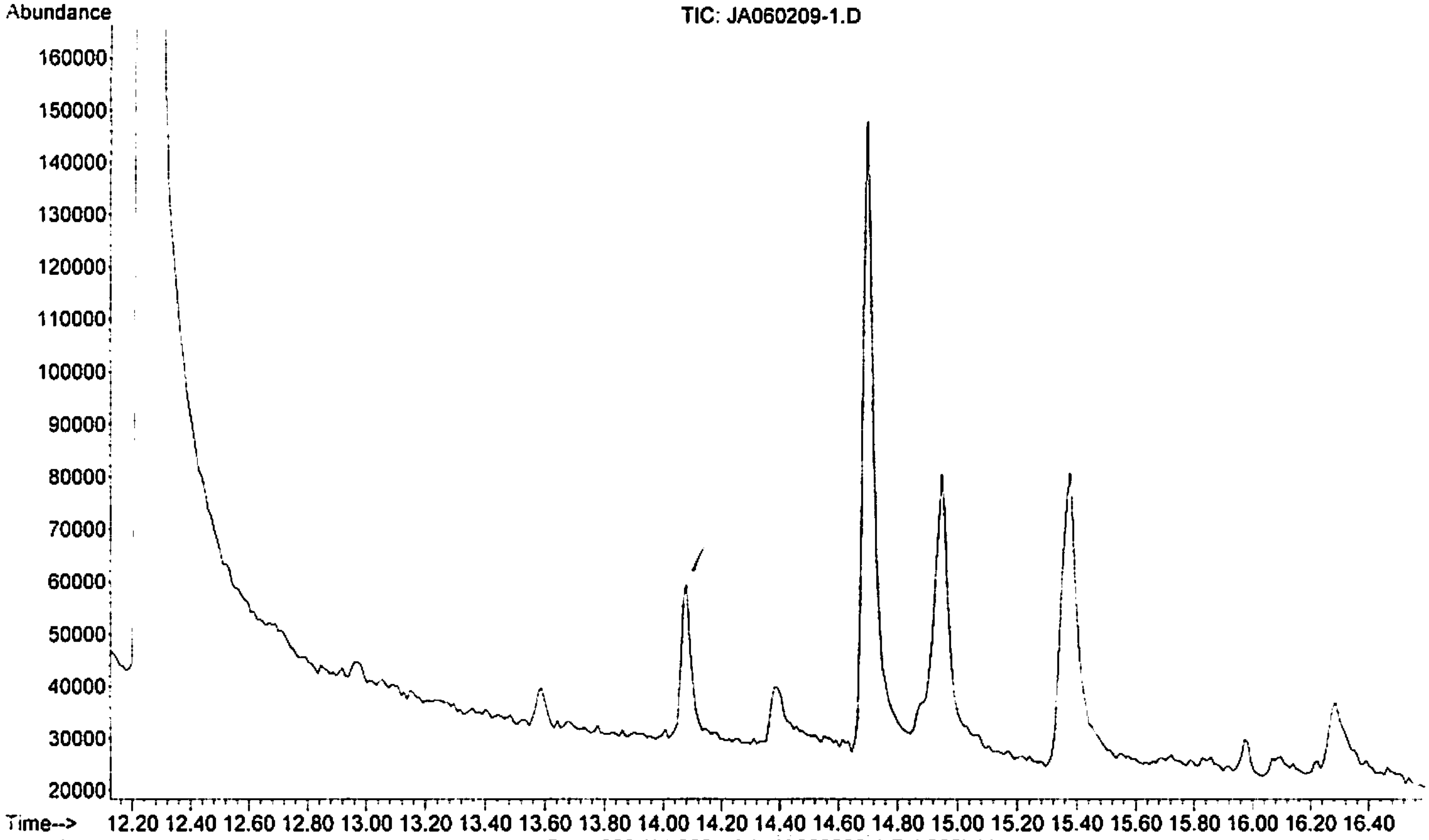

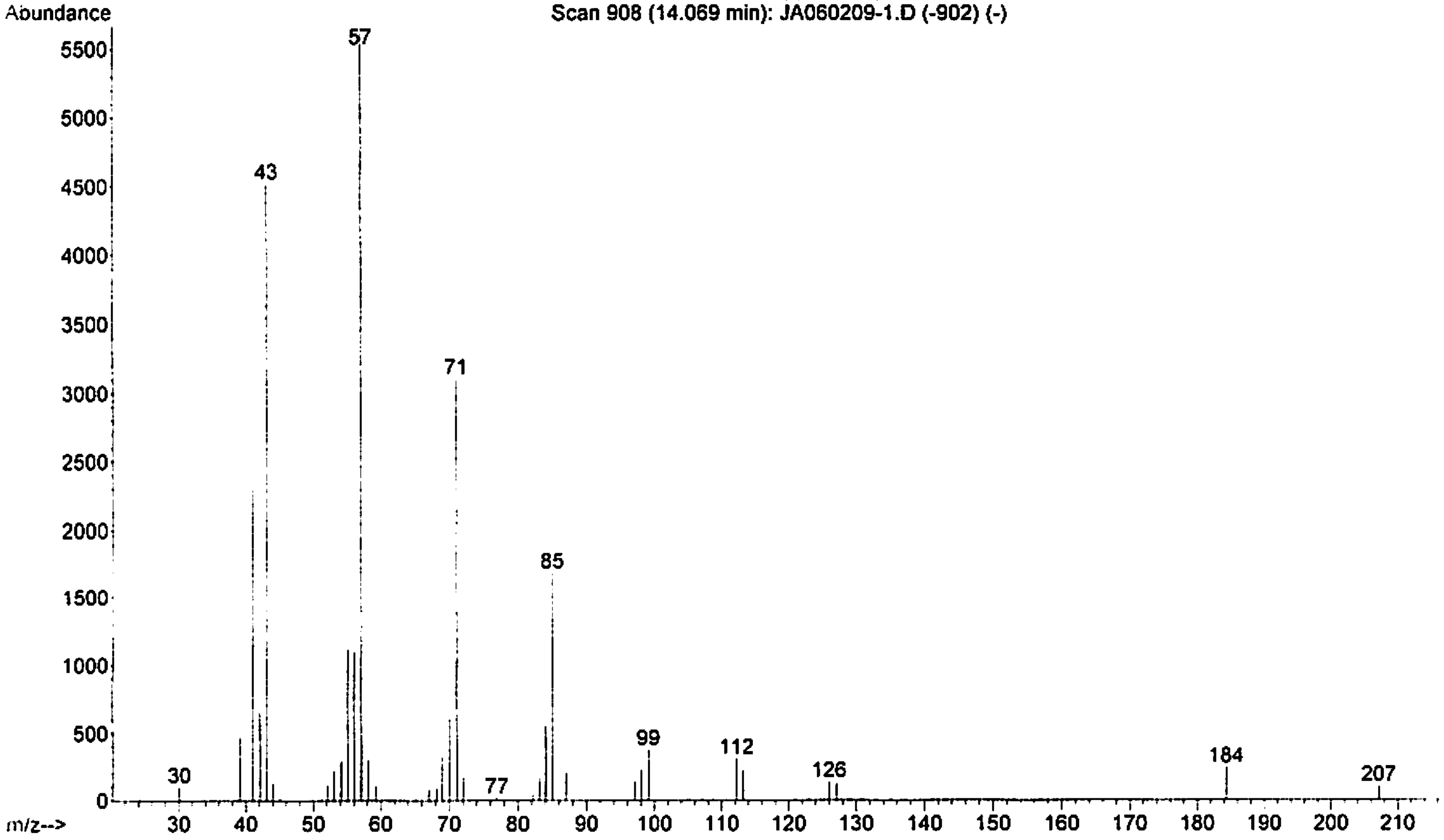

```
[illegible]      :D:\DATA\ALDRICH\JA-09\Snapshot\JA060209-1.D
[illegible]rator    : Aldrich
[illegible]uired    :  2 Jun 2009   10:47      using AcqMethod JA-WAX08.M
[illegible]trument :     Instrument #1
[illegible]ple Name: 1 field-coll. M C.oculata abd./CH2Cl2
[illegible] Info    : coll. 6/1; 1st of two; top gut full
[illegible] Number: 1
```

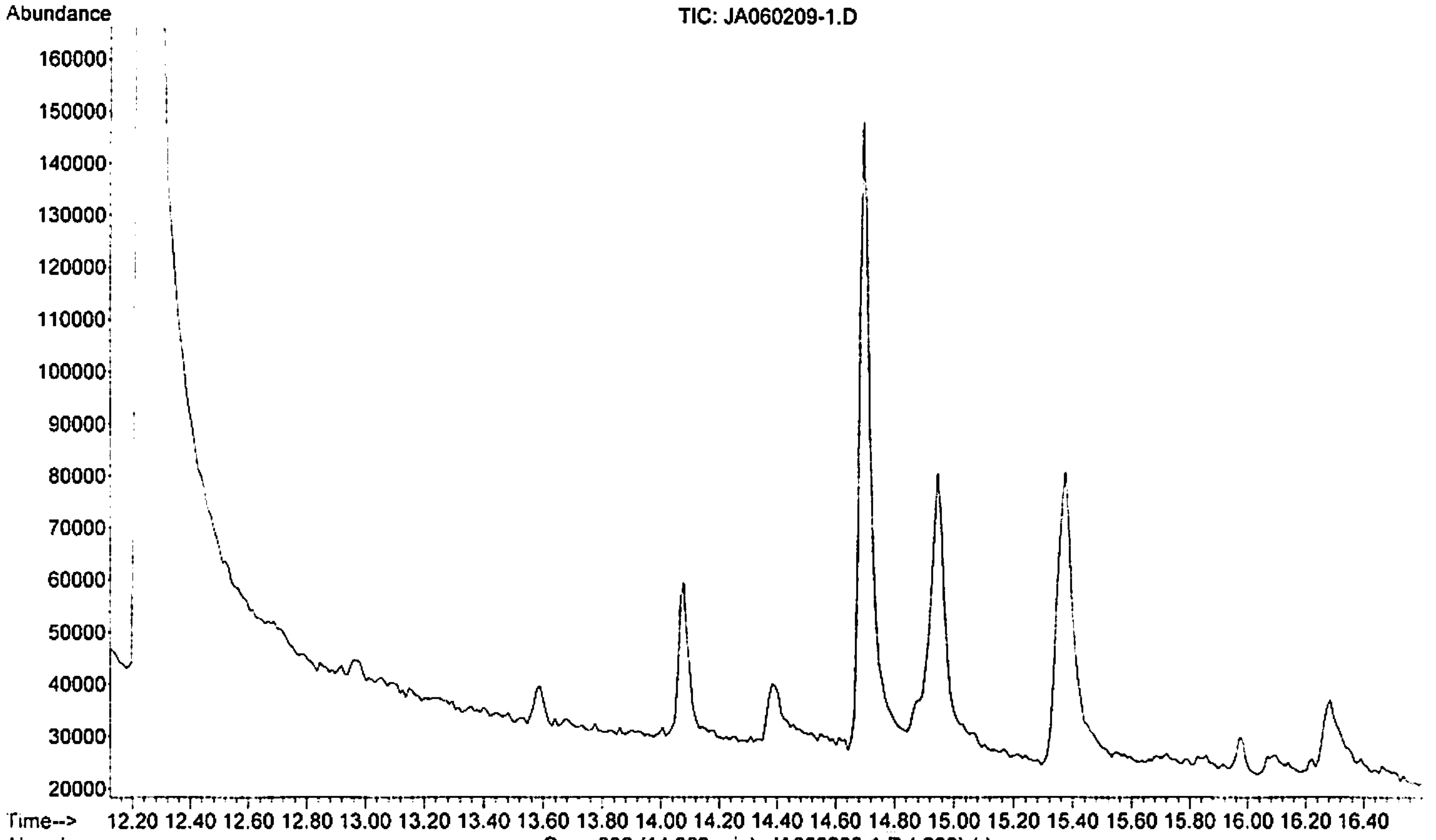

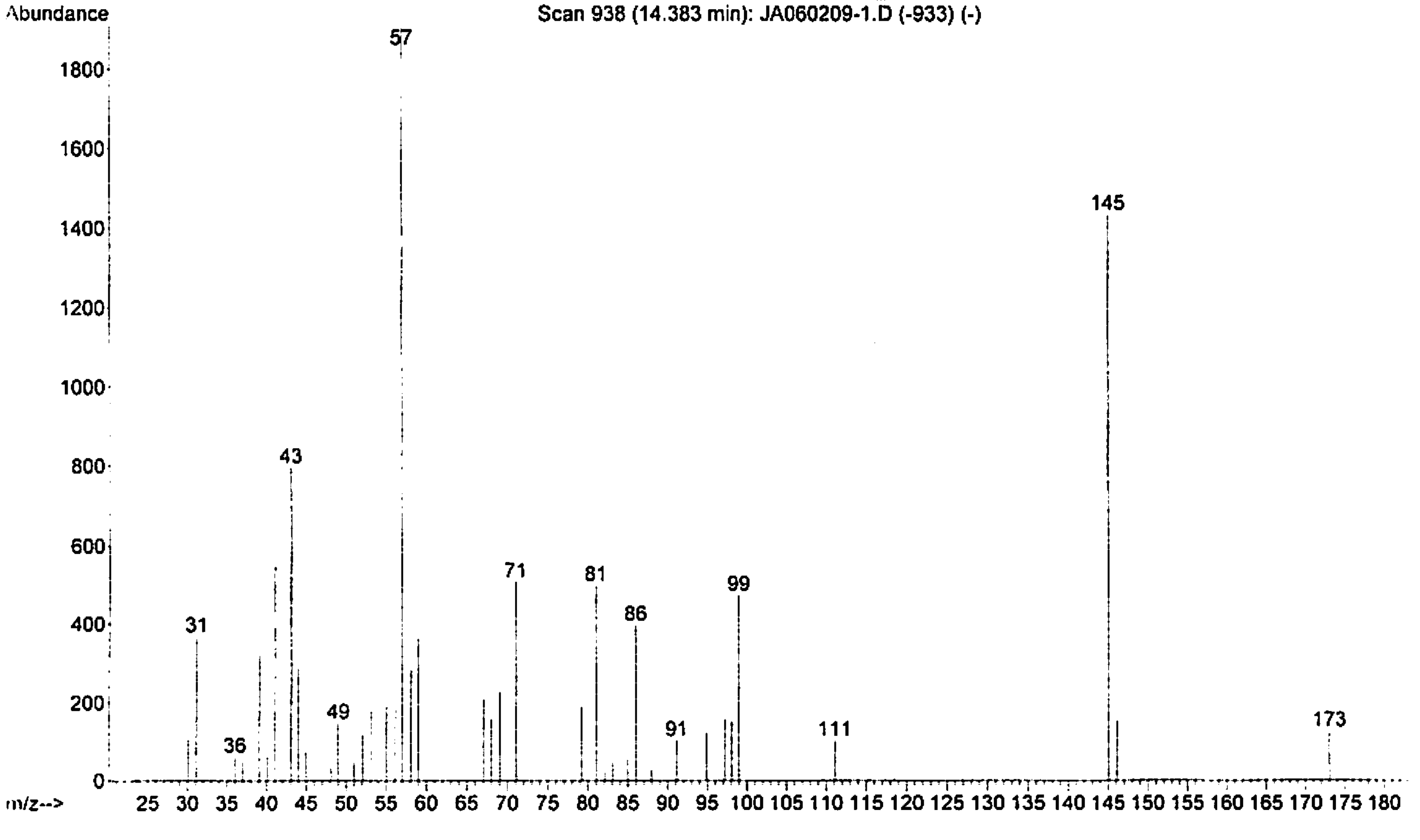

File :D:\DATA\ALDRICH\JA-09\Snapshot\JA060209-1.D
Operator : Aldrich
Acquired : 2 Jun 2009 10:47 using AcqMethod JA-WAX08.M
Instrument : Instrument #1
Sample Name: 1 field-coll. M C.oculata abd./CH2Cl2
Misc Info : coll. 6/1; 1st of two; top gut full
Vial Number: 1

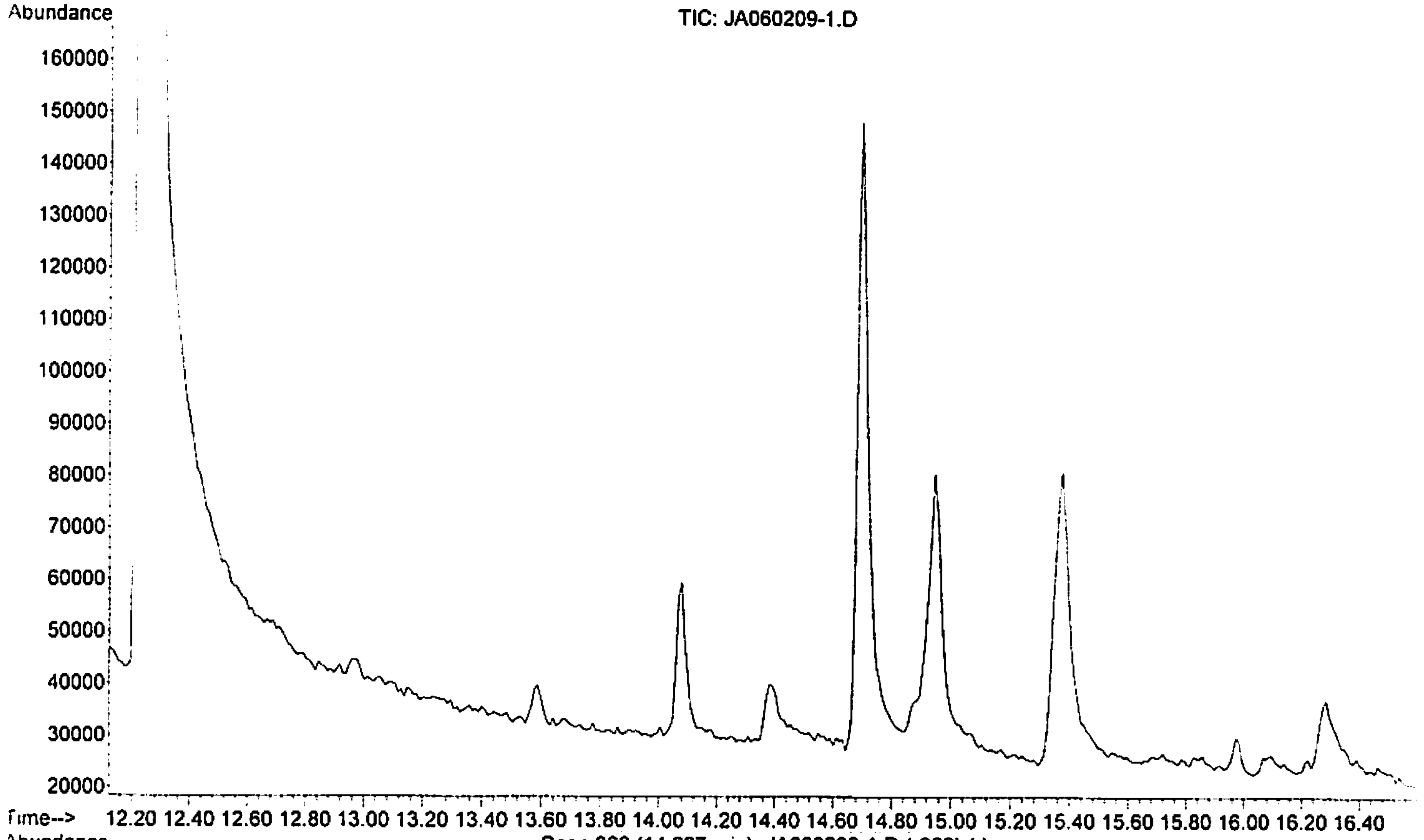

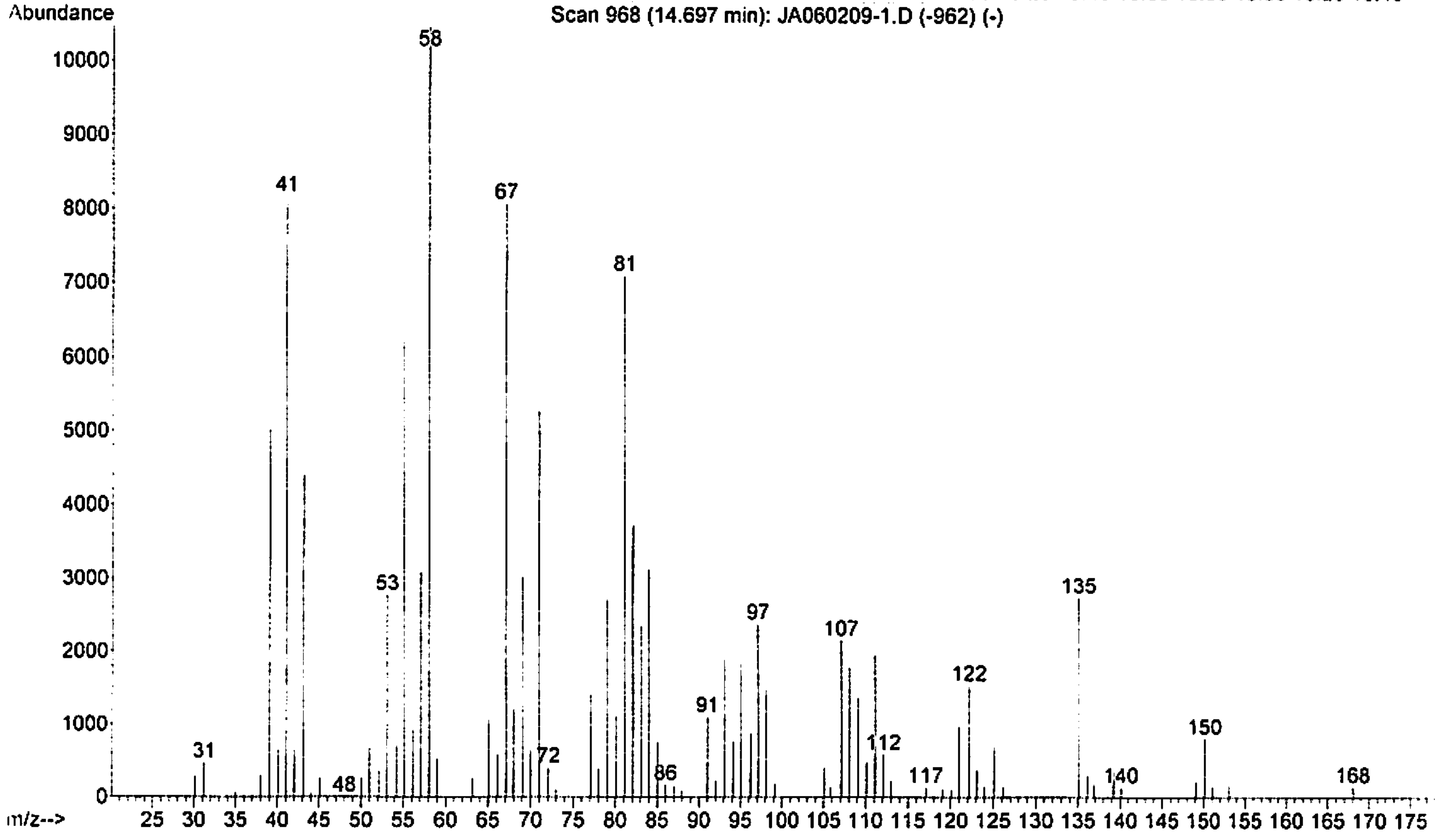

File :D:\DATA\ALDRICH\JA-09\Snapshot\JA060209-1.D
Operator : Aldrich
Acquired : 2 Jun 2009 10:47 using AcqMethod JA-WAX08.M
Instrument : Instrument #1
Sample Name: 1 field-coll. M C.oculata abd./CH2Cl2
Misc Info : coll. 6/1; 1st of two; top gut full
Vial Number: 1

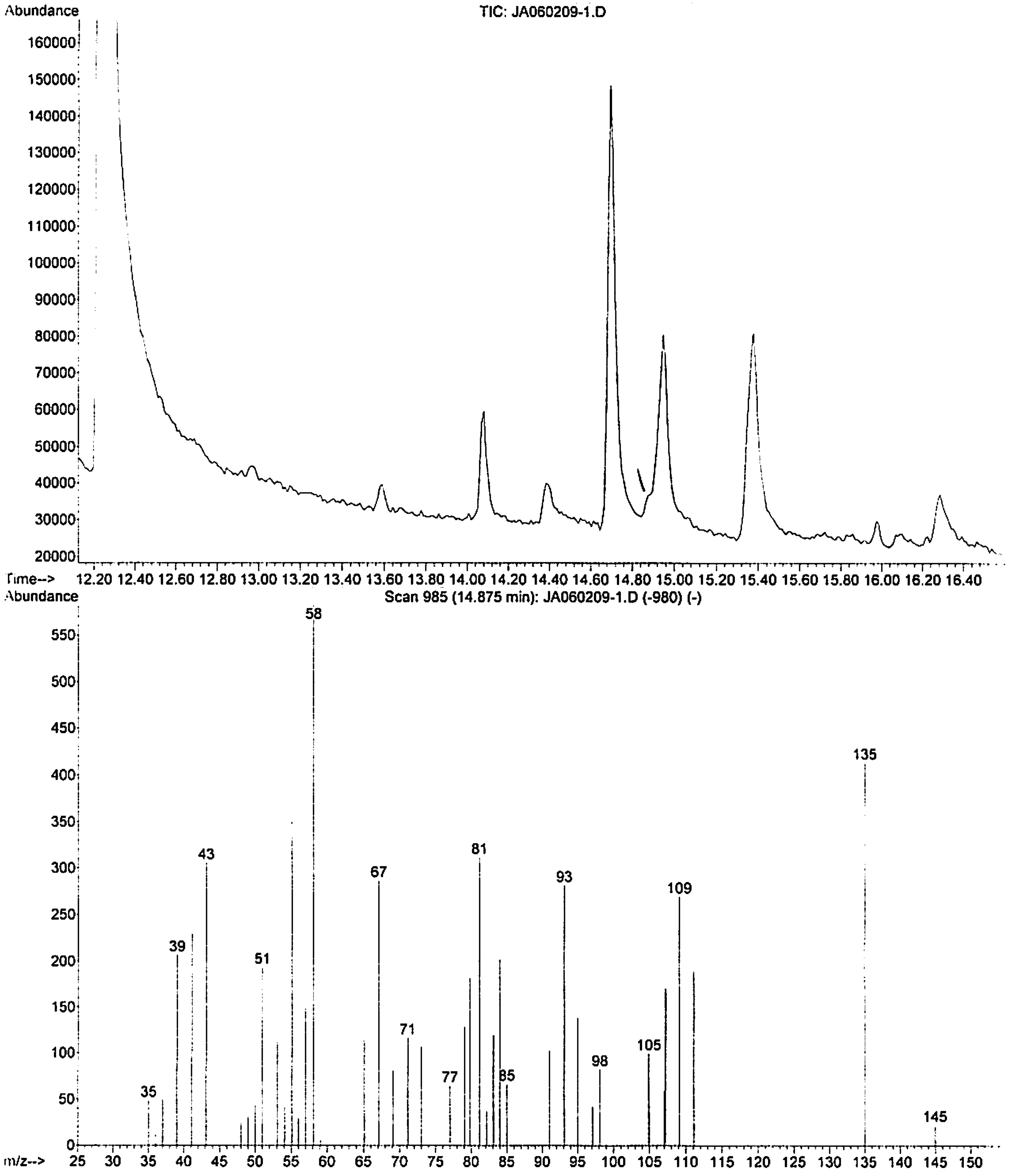

[illegible] : D:\DATA\ALDRICH\JA-09\Snapshot\JA060209-1.D
[illegible]rator : Aldrich
[illegible]ired : 2 Jun 2009 10:47 using AcqMethod JA-WAX08.M
[illegible]rument : Instrument #1
[illegible]le Name: 1 field-coll. M C.oculata abd./CH2Cl2
[illegible] Info : coll. 6/1; 1st of two; top gut full
[illegible] Number: 1

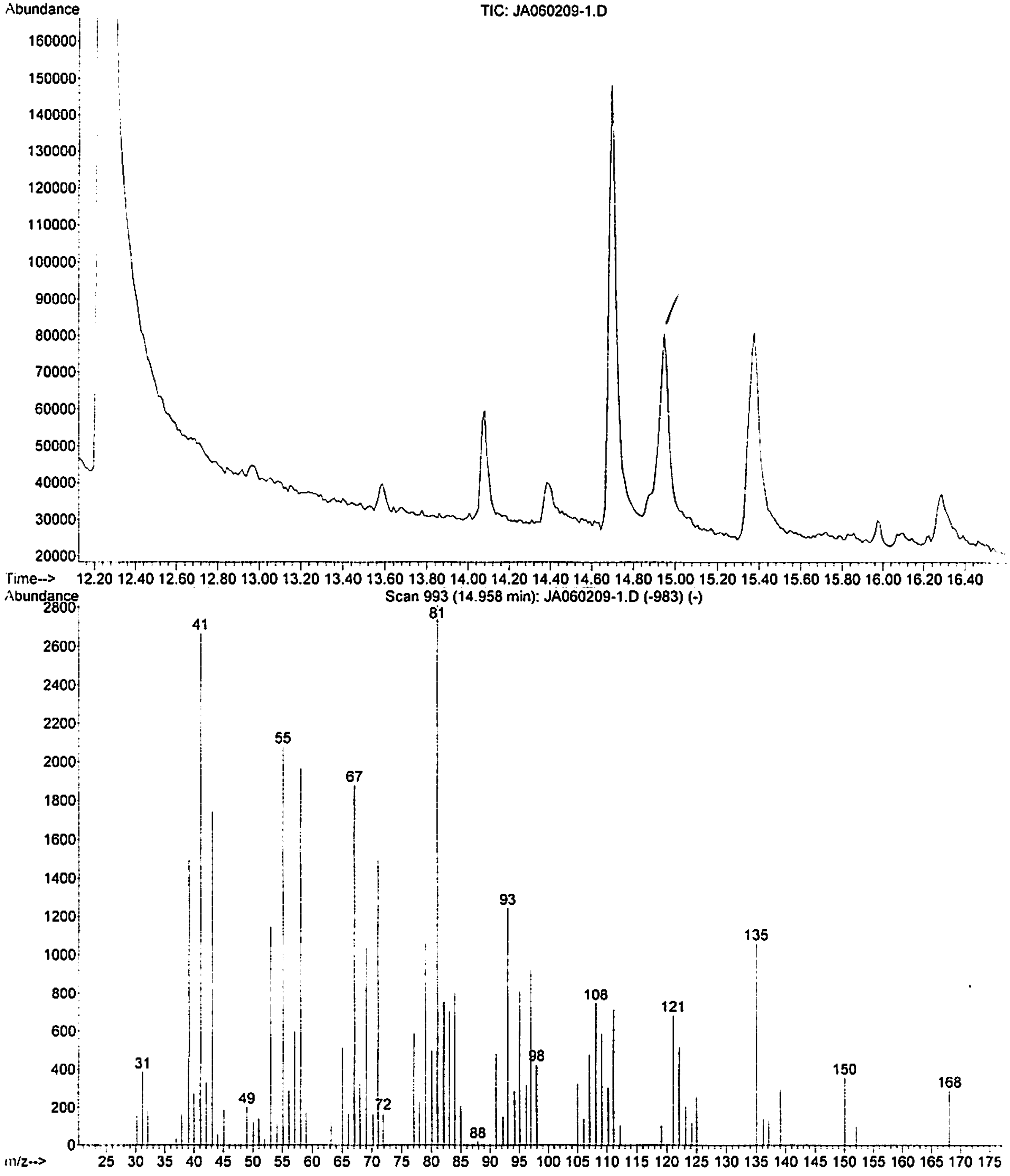

[illegible] :D:\DATA\ALDRICH\JA-09\Snapshot\JA060209-1.D
[illegible]rator : Aldrich
[illegible]uired : 2 Jun 2009 10:47 using AcqMethod JA-WAX08.M
[illegible]rument : Instrument #1
[illegible]ple Name: 1 field-coll. M C.oculata abd./CH2Cl2
[illegible] Info : coll. 6/1; 1st of two; top gut full
[illegible] Number: 1

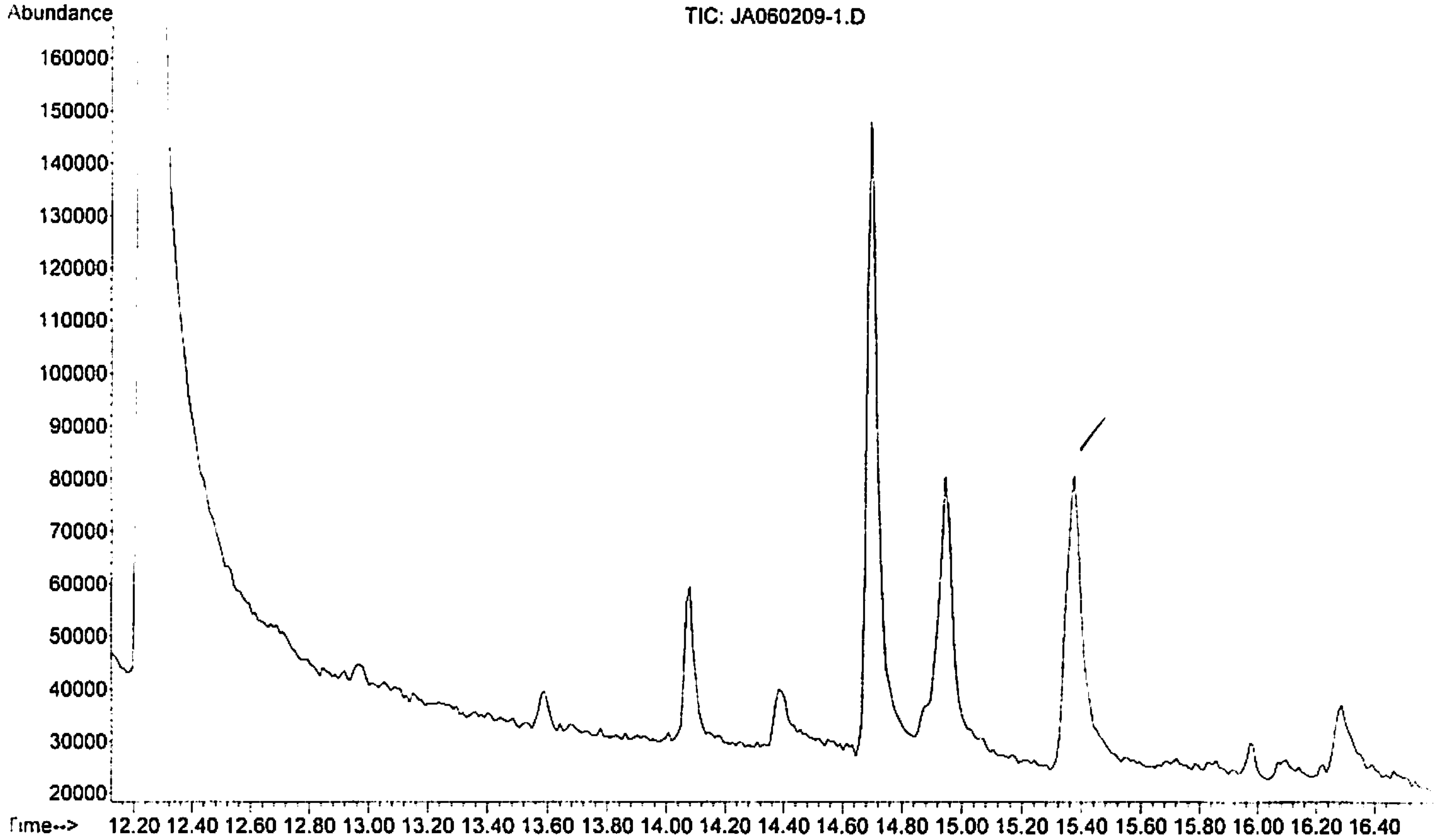

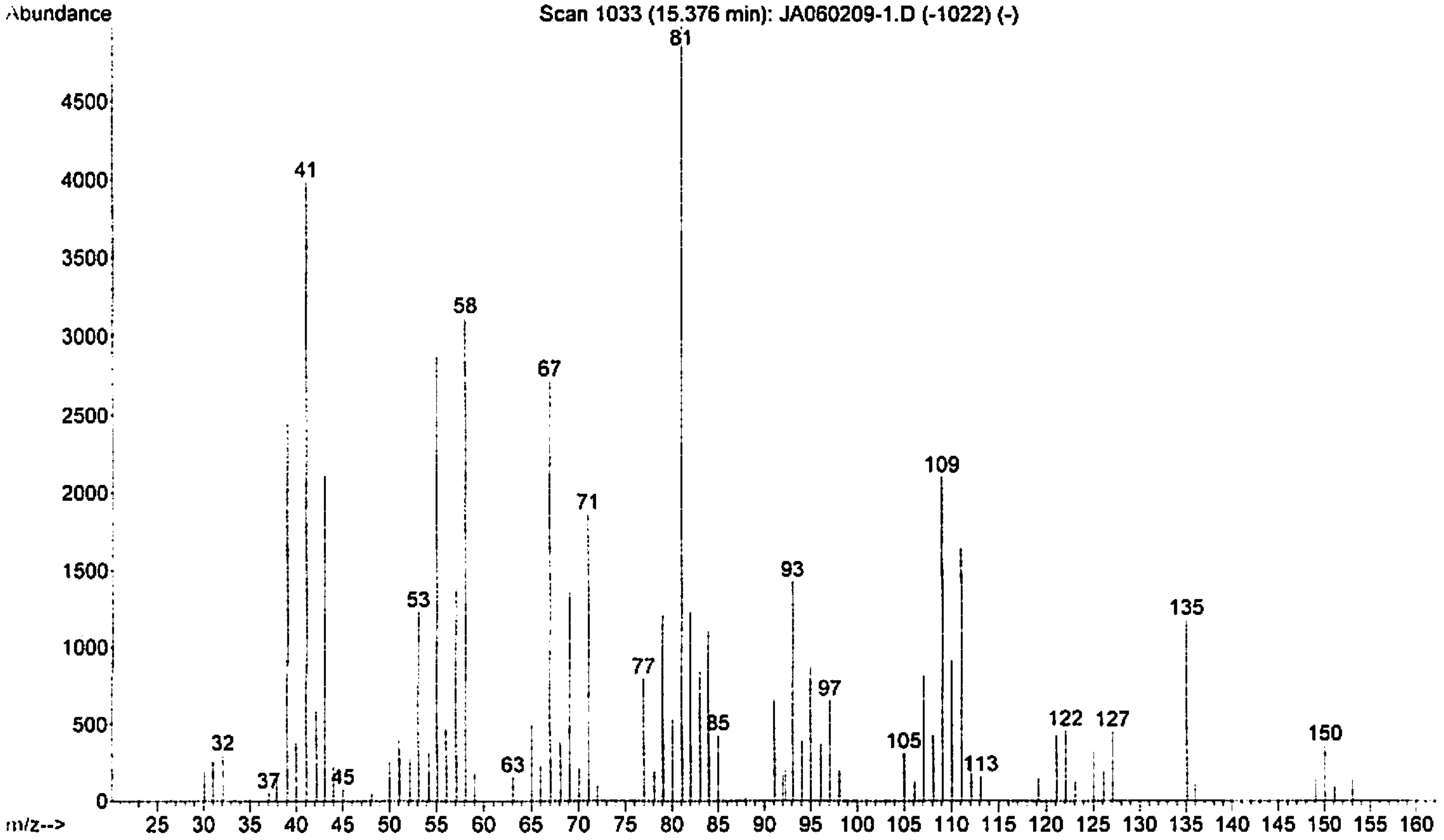

File   :D:\DATA\ALDRICH\JA-09\Snapshot\JA060209-1.D
Operator   : Aldrich
Acquired   : 2 Jun 2009   10:47   using AcqMethod JA-WAX08.M
Instrument :   Instrument #1
Sample Name: 1 field-coll. M C.oculata abd./CH2Cl2
Misc Info   : coll. 6/1; 1st of two; top gut full
Vial Number: 1

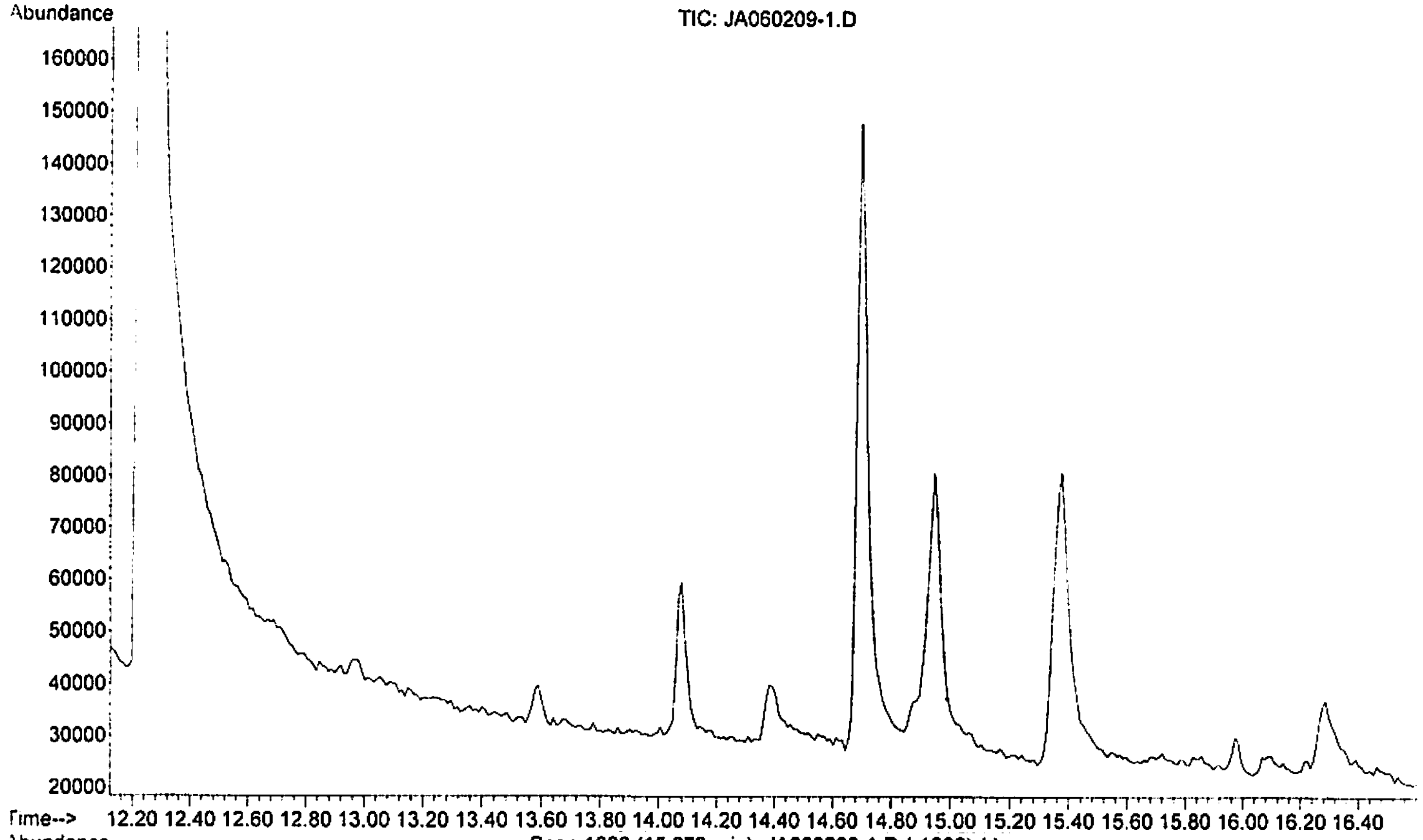

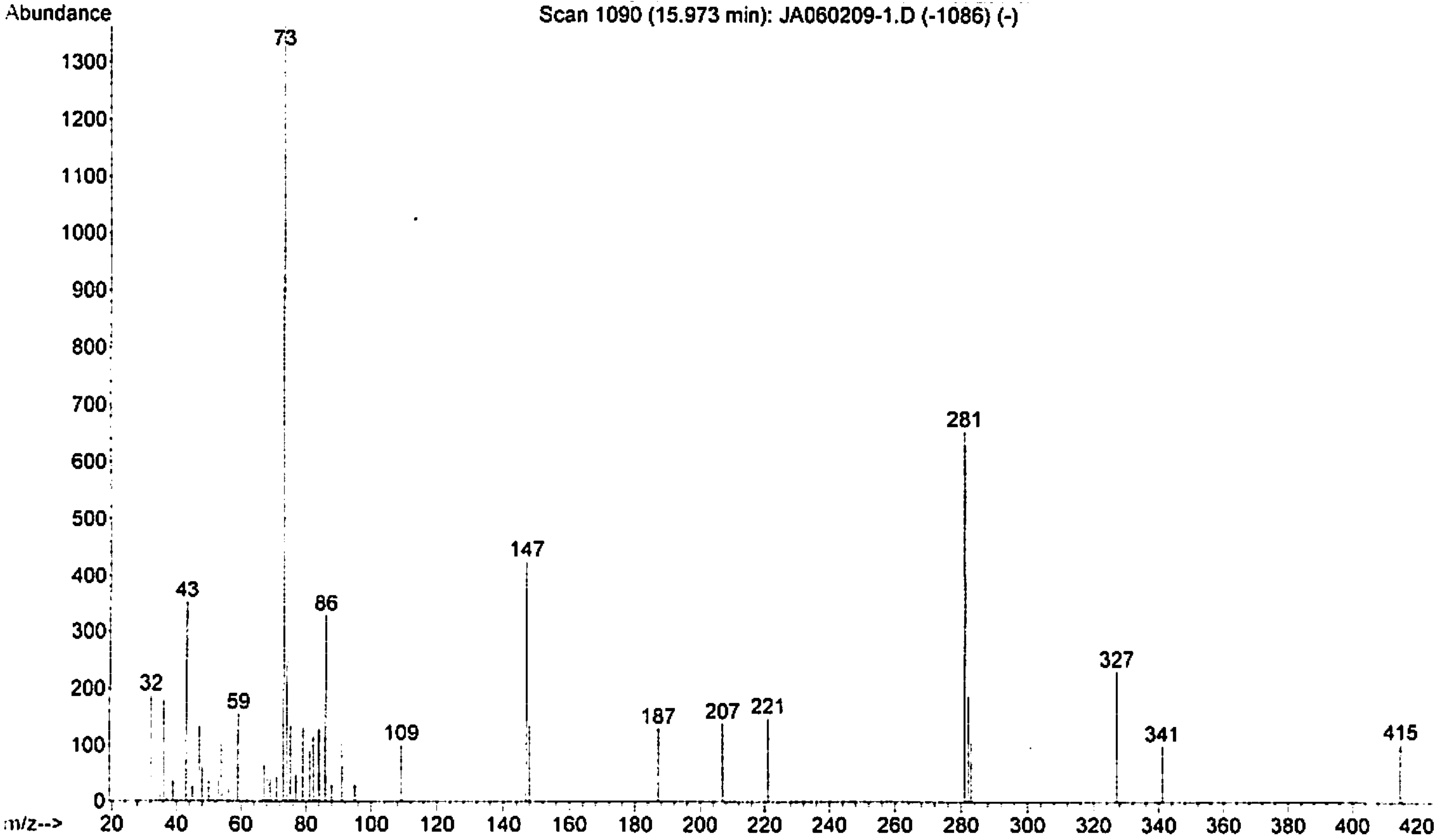

[illegible] : D:\DATA\ALDRICH\JA-09\Snapshot\JA060209-1.D
[illegible]rator : Aldrich
[illegible]quired : 2 Jun 2009 10:47 using AcqMethod JA-WAX08.M
[illegible]rument : Instrument #1
[illegible]ple Name: 1 field-coll. M C.oculata abd./CH2Cl2
[illegible] Info : coll. 6/1; 1st of two; top gut full
[illegible] Number: 1

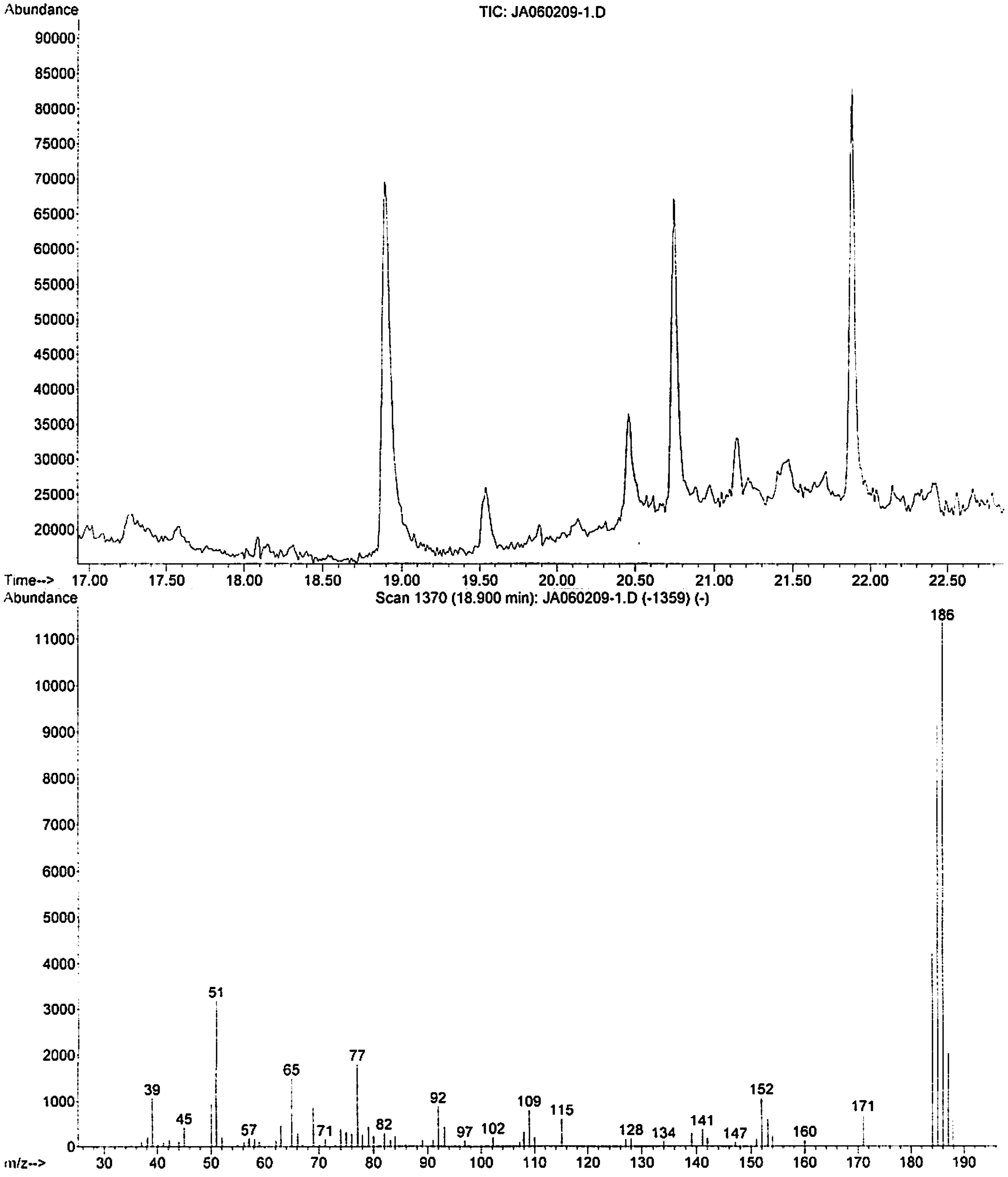

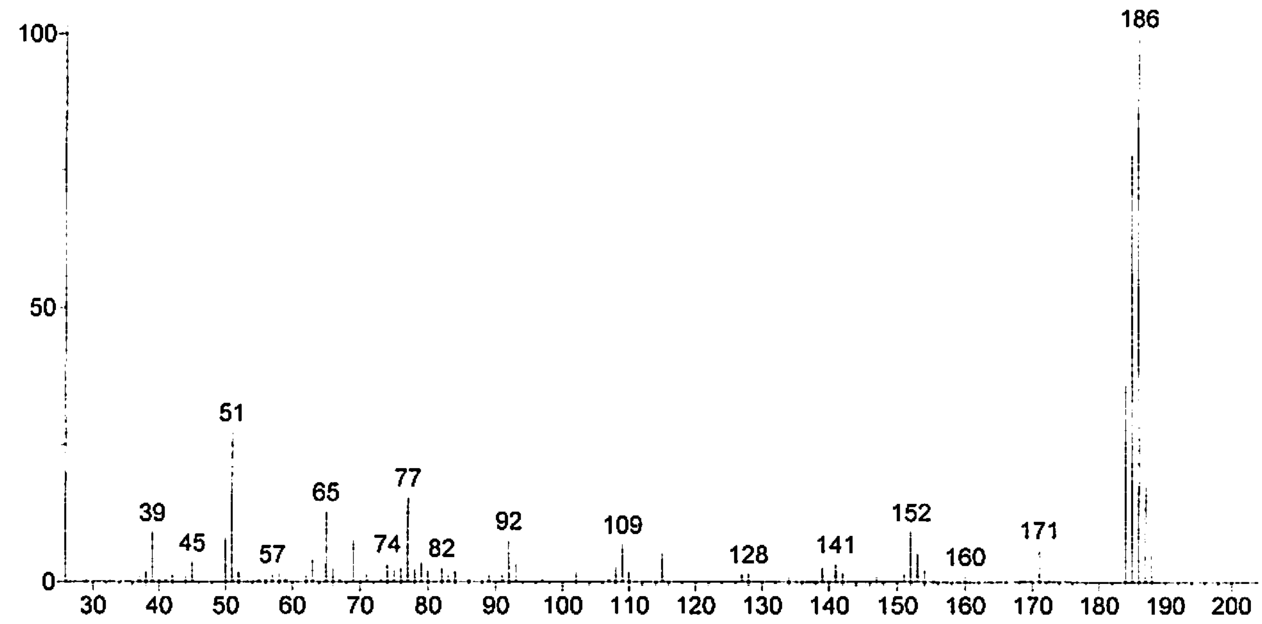

(Text File) Scan 1370 (18.900 min): JA060209-1.D (-1359)

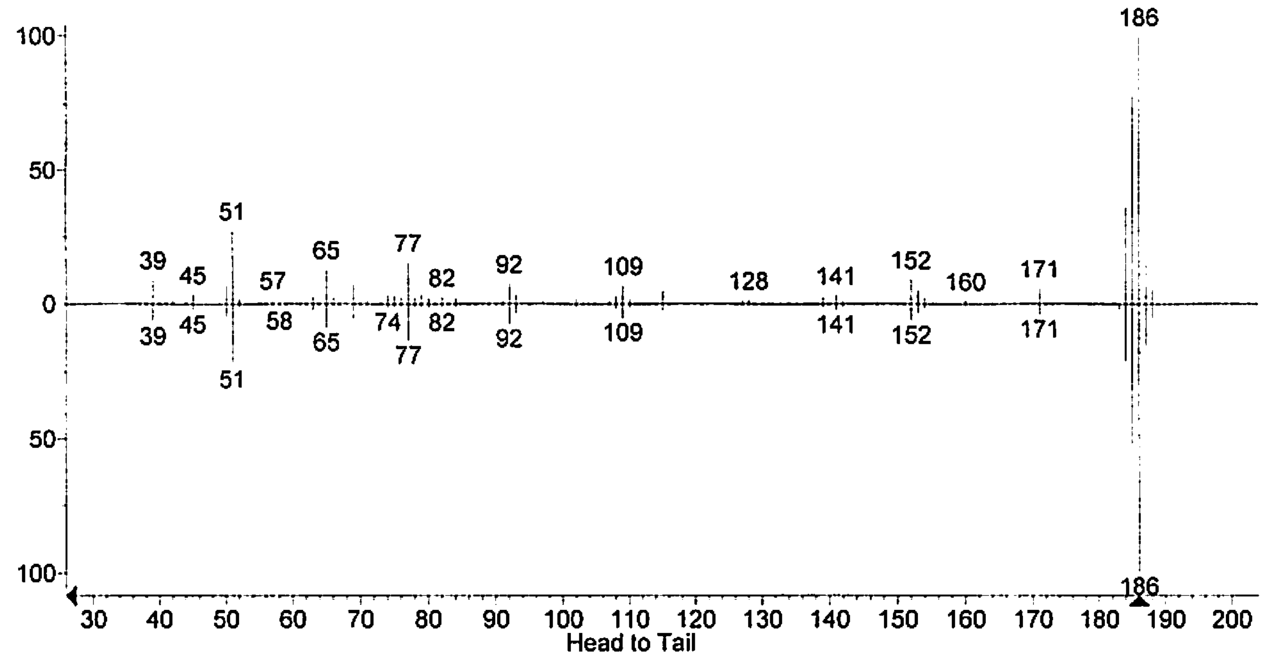

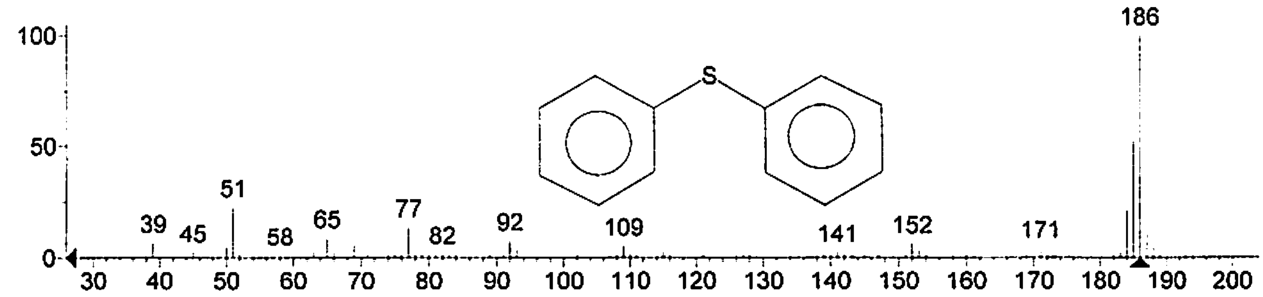

(replib) Diphenyl sulfide

File :D:\DATA\ALDRICH\JA-09\Snapshot\JA060209-1.D
Operator : Aldrich
Acquired : 2 Jun 2009 10:47 using AcqMethod JA-WAX08.M
Instrument : Instrument #1
Sample Name: 1 field-coll. M C.oculata abd./CH2Cl2
Misc Info : coll. 6/1; 1st of two; top gut full
Vial Number: 1

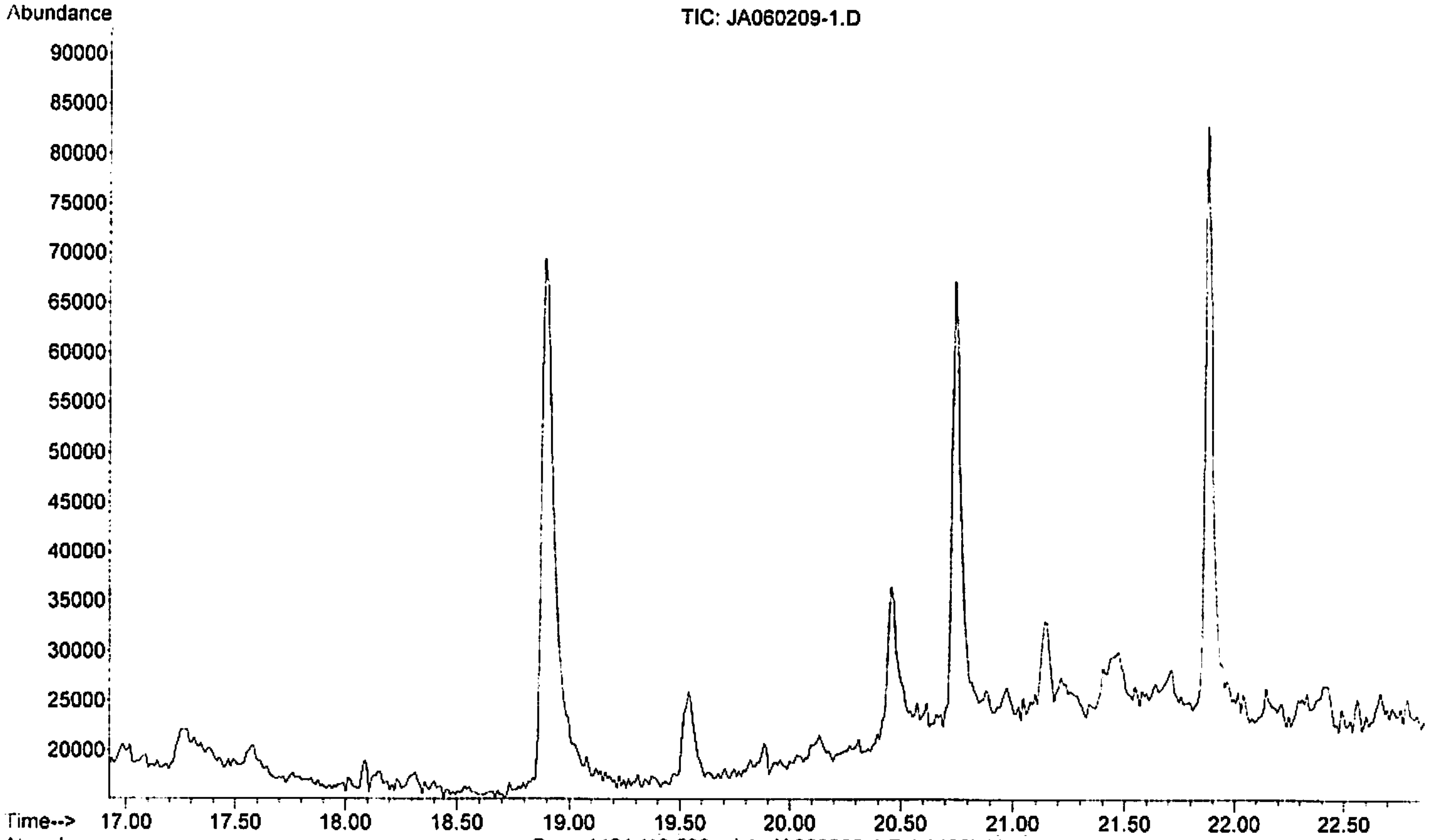

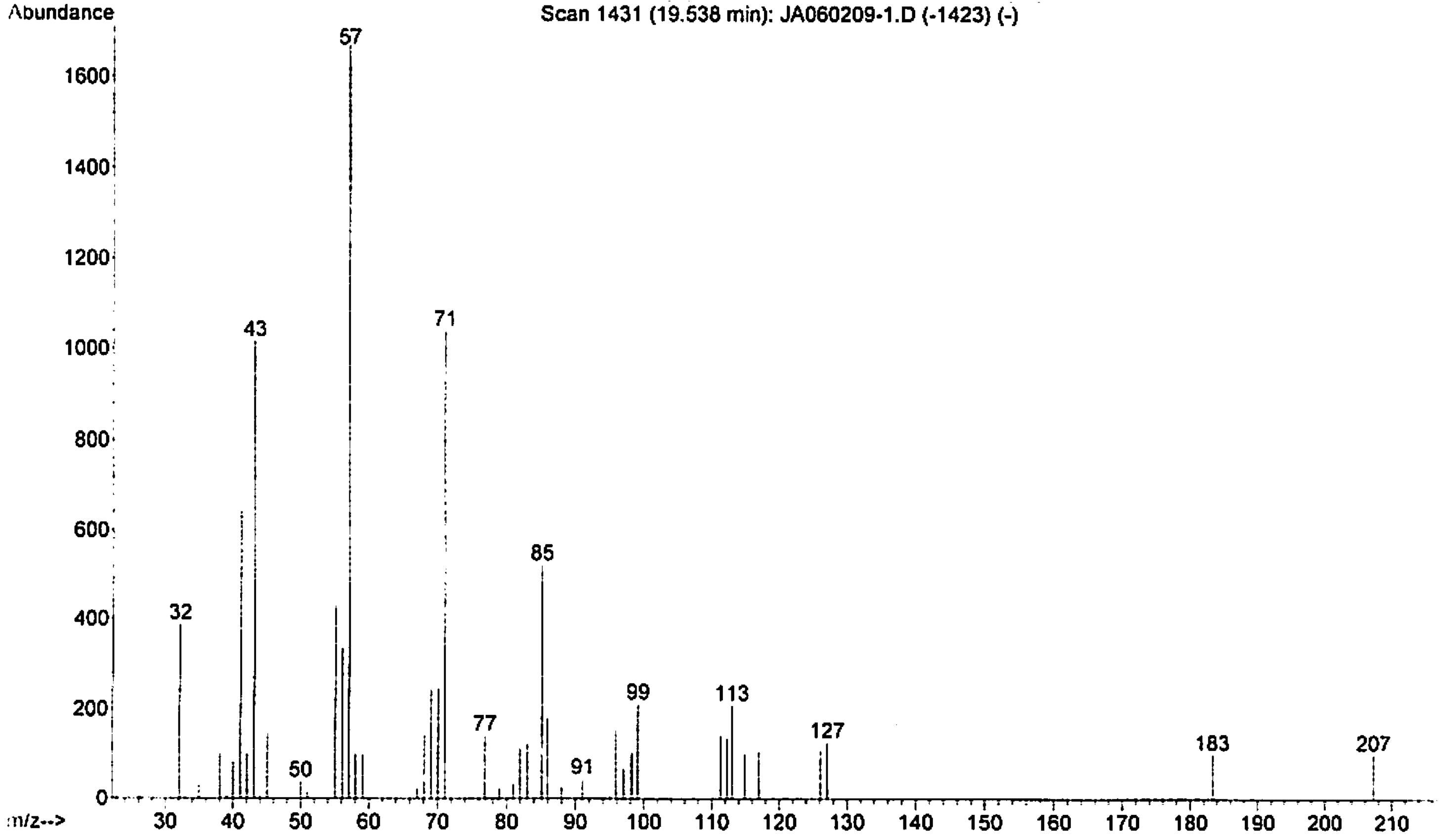

[illegible] :D:\DATA\ALDRICH\JA-09\Snapshot\JA060209-1.D
[illegible]rator : Aldrich
[illegible]ired : 2 Jun 2009 10:47 using AcqMethod JA-WAX08.M
[illegible]rument : Instrument #1
[illegible]ple Name: 1 field-coll. M C.oculata abd./CH2Cl2
[illegible] Info : coll. 6/1; 1st of two; top gut full
[illegible] Number: 1

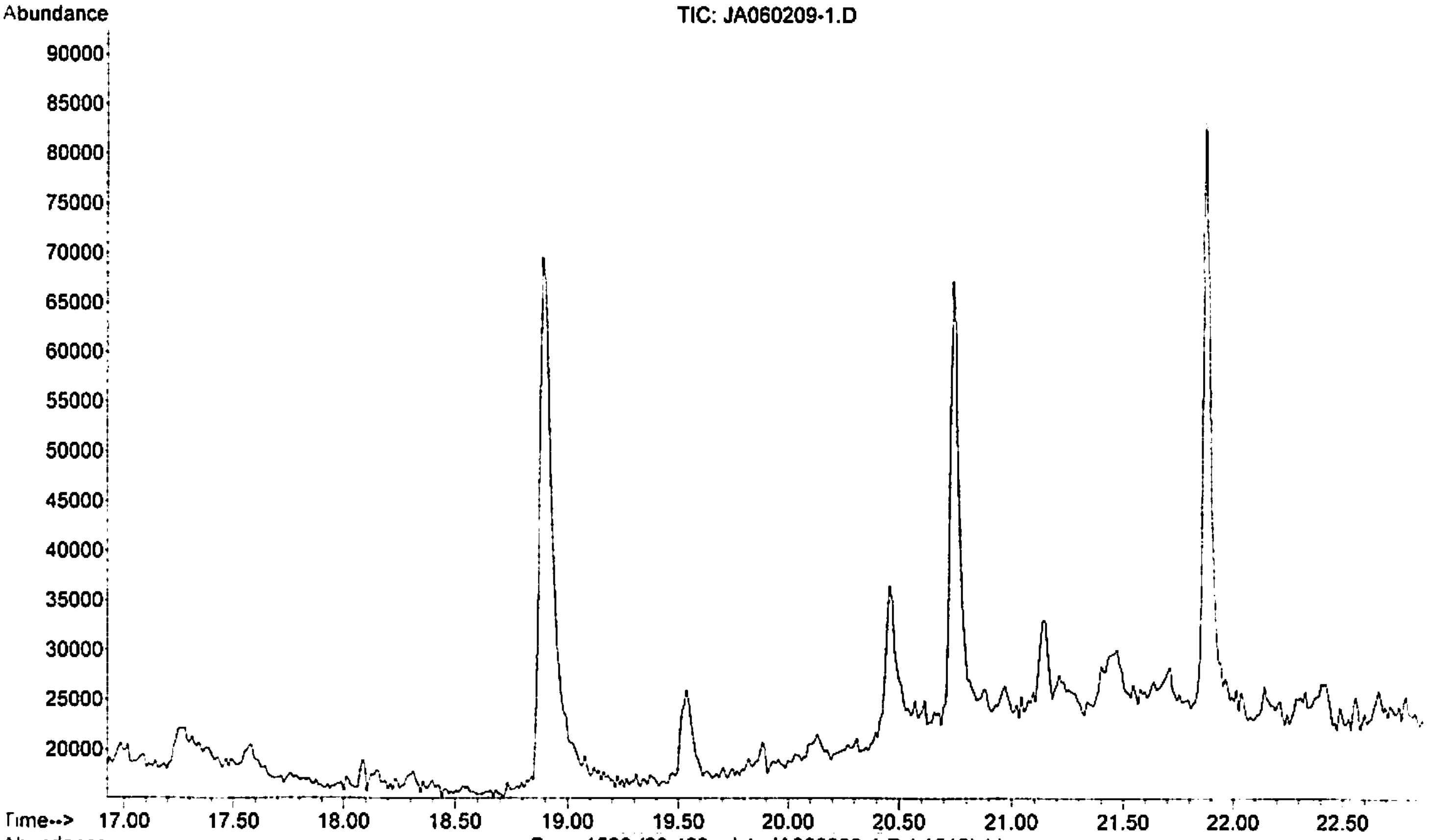

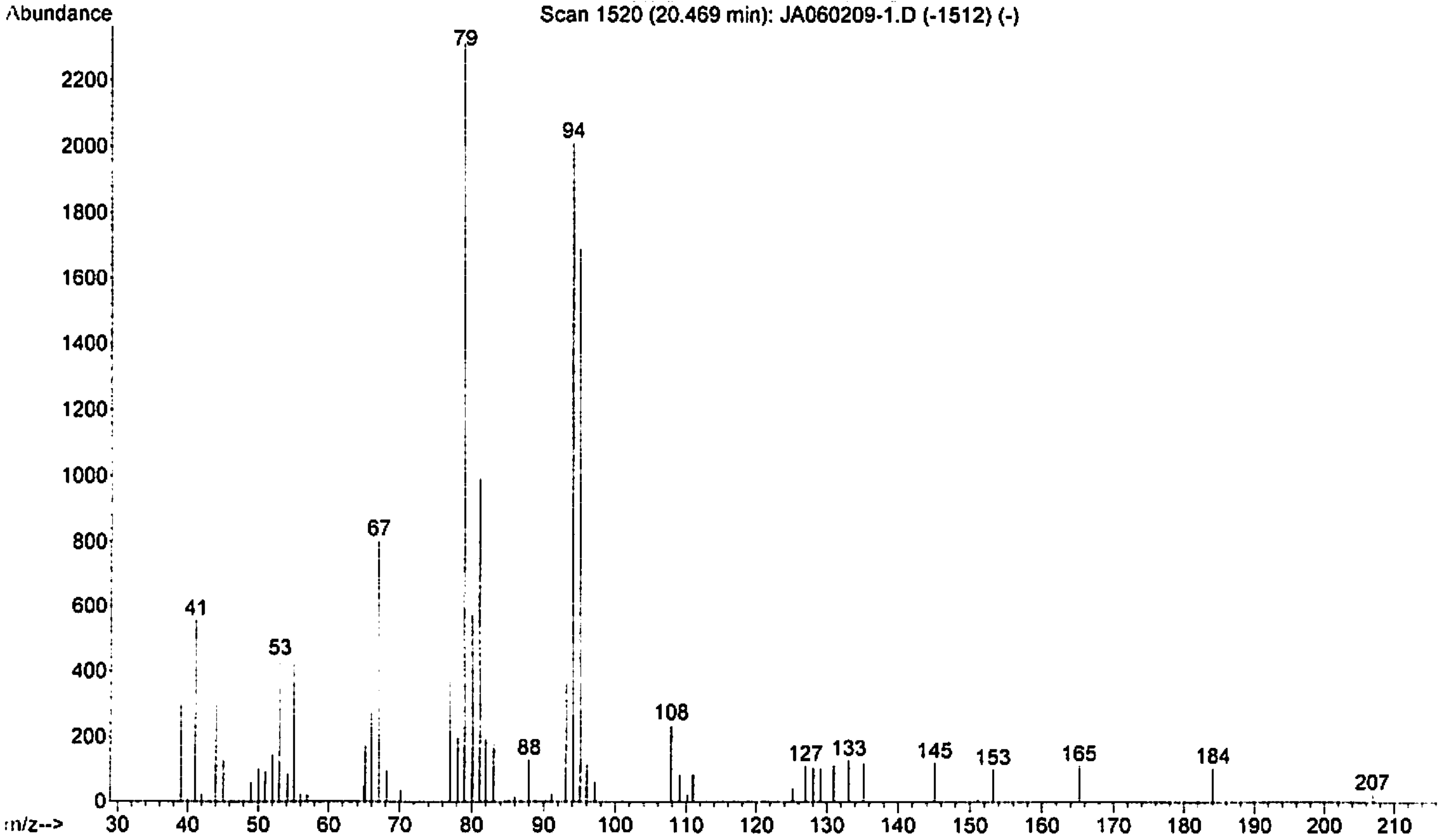

[illegible] :D:\DATA\ALDRICH\JA-09\Snapshot\JA060209-1.D
[illegible]ator : Aldrich
[illegible]uired : 2 Jun 2009 10:47 using AcqMethod JA-WAX08.M
[illegible]rument : Instrument #1
[illegible]le Name: 1 field-coll. M C.oculata abd./CH2Cl2
[illegible] Info : coll. 6/1; 1st of two; top gut full
[illegible] Number: 1

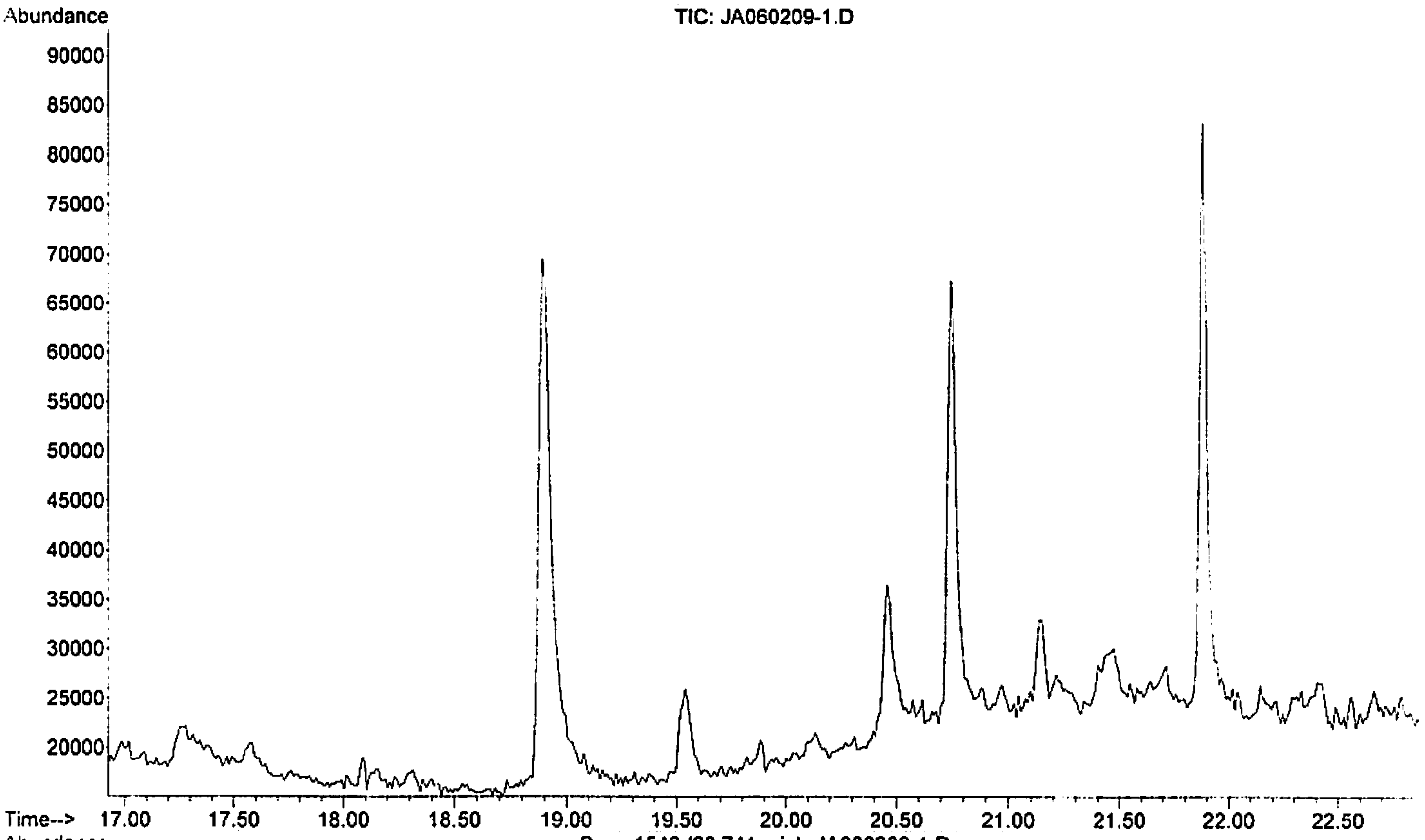

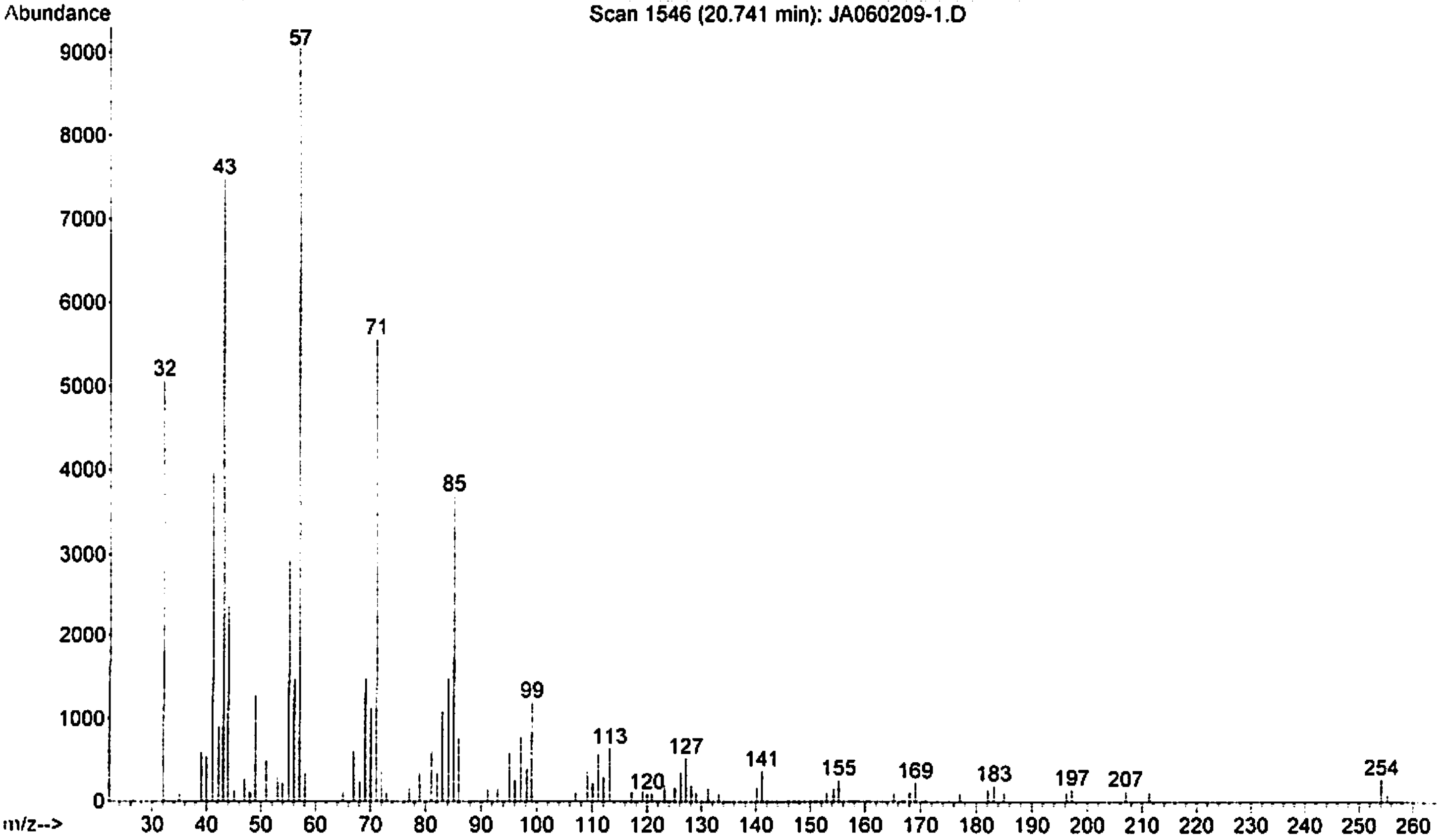

File : D:\DATA\ALDRICH\JA-09\Snapshot\JA060209-1.D
Operator : Aldrich
Acquired : 2 Jun 2009 10:47 using AcqMethod JA-WAX08.M
Instrument : Instrument #1
Sample Name: 1 field-coll. M C.oculata abd./CH2Cl2
Misc Info : coll. 6/1; 1st of two; top gut full
Vial Number: 1

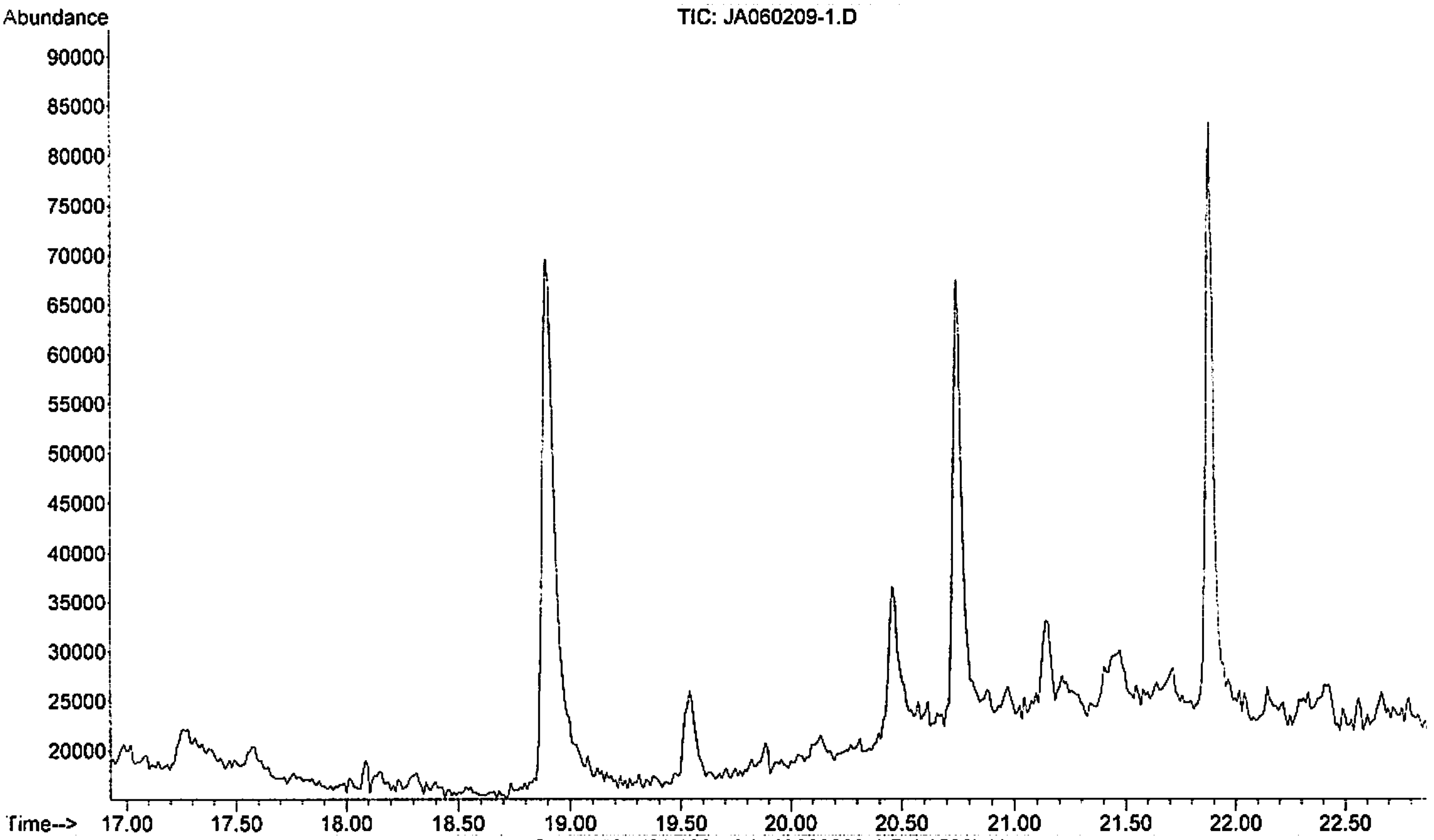

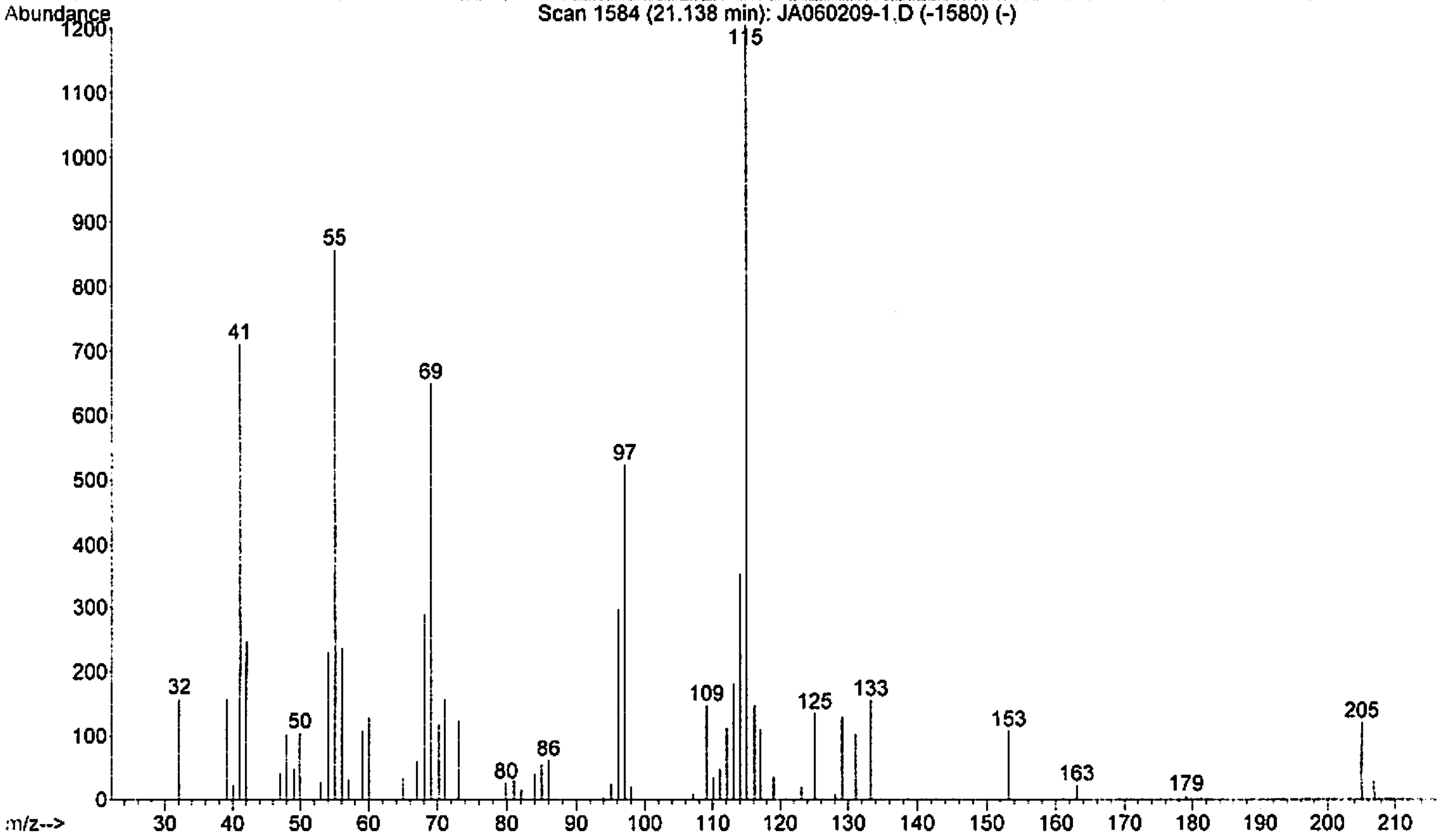

[illegible]e . :D:\DATA\ALDRICH\JA-09\Snapshot\JA060209-1.D
[illegible]rator : Aldrich
[illegible]ired : 2 Jun 2009 10:47 using AcqMethod JA-WAX08.M
[illegible]rument : Instrument #1
[illegible]le Name: 1 field-coll. M C.oculata abd./CH2Cl2
[illegible] Info : coll. 6/1; 1st of two; top gut full
[illegible] Number: 1

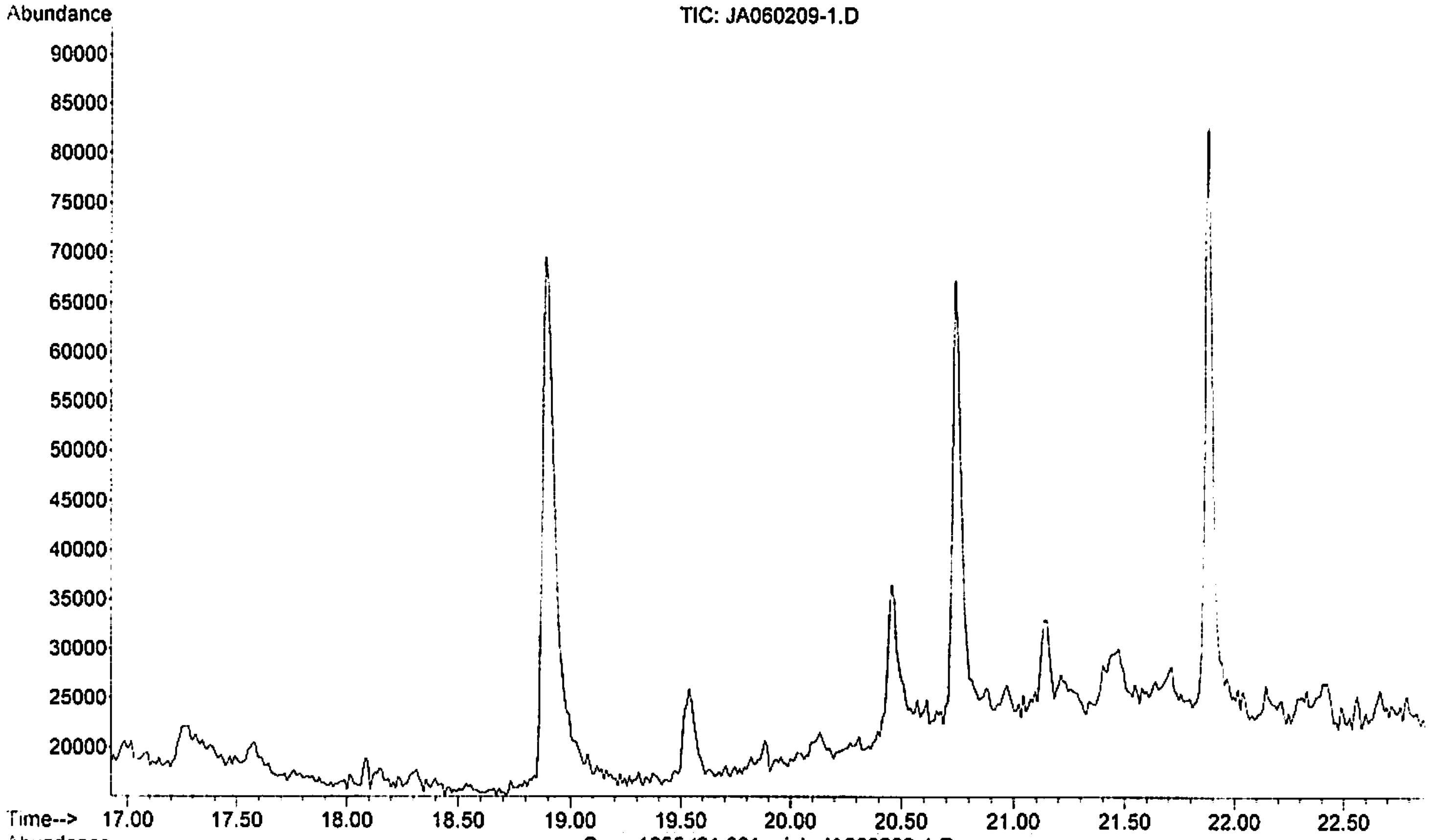

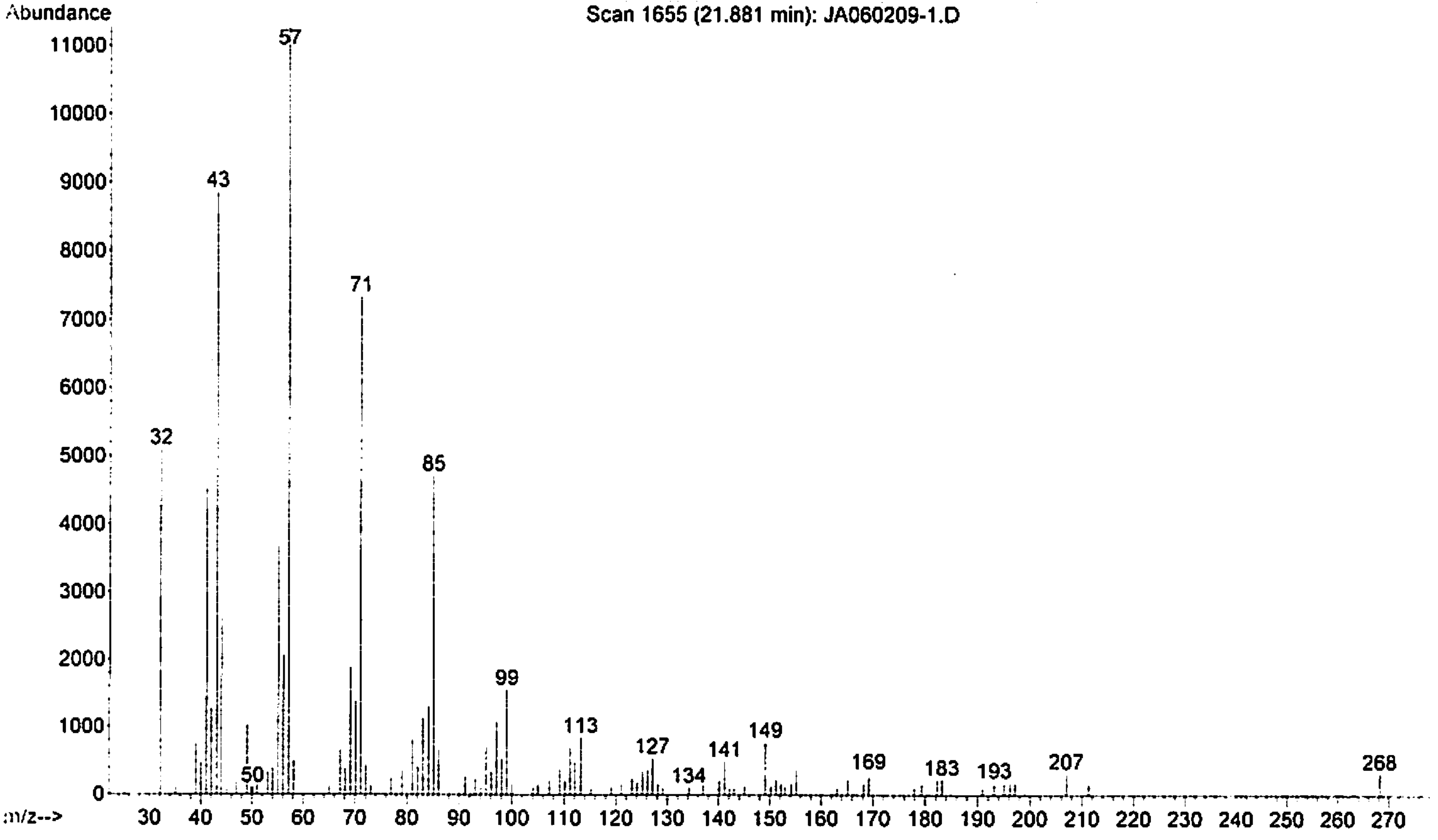

[illegible] :D:\DATA\ALDRICH\JA-09\Snapshot\JA060209-1.D
[illegible]rator : Aldrich
[illegible]uired : 2 Jun 2009 10:47 using AcqMethod JA-WAX08.M
[illegible]rument : Instrument #1
[illegible]ple Name: 1 field-coll. M C.oculata abd./CH2Cl2
[illegible] Info : coll. 6/1; 1st of two; top gut full
[illegible] Number: 1

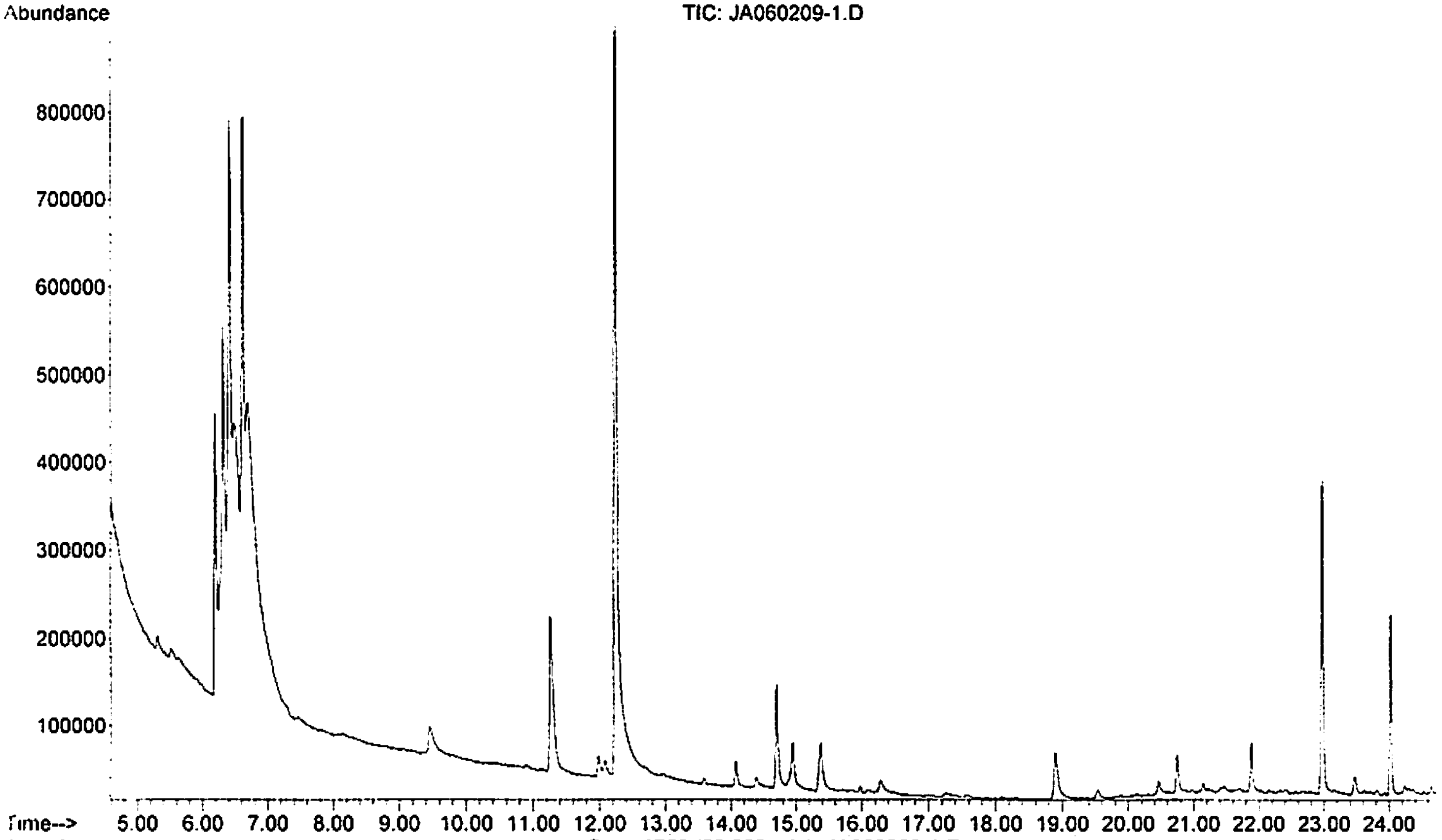

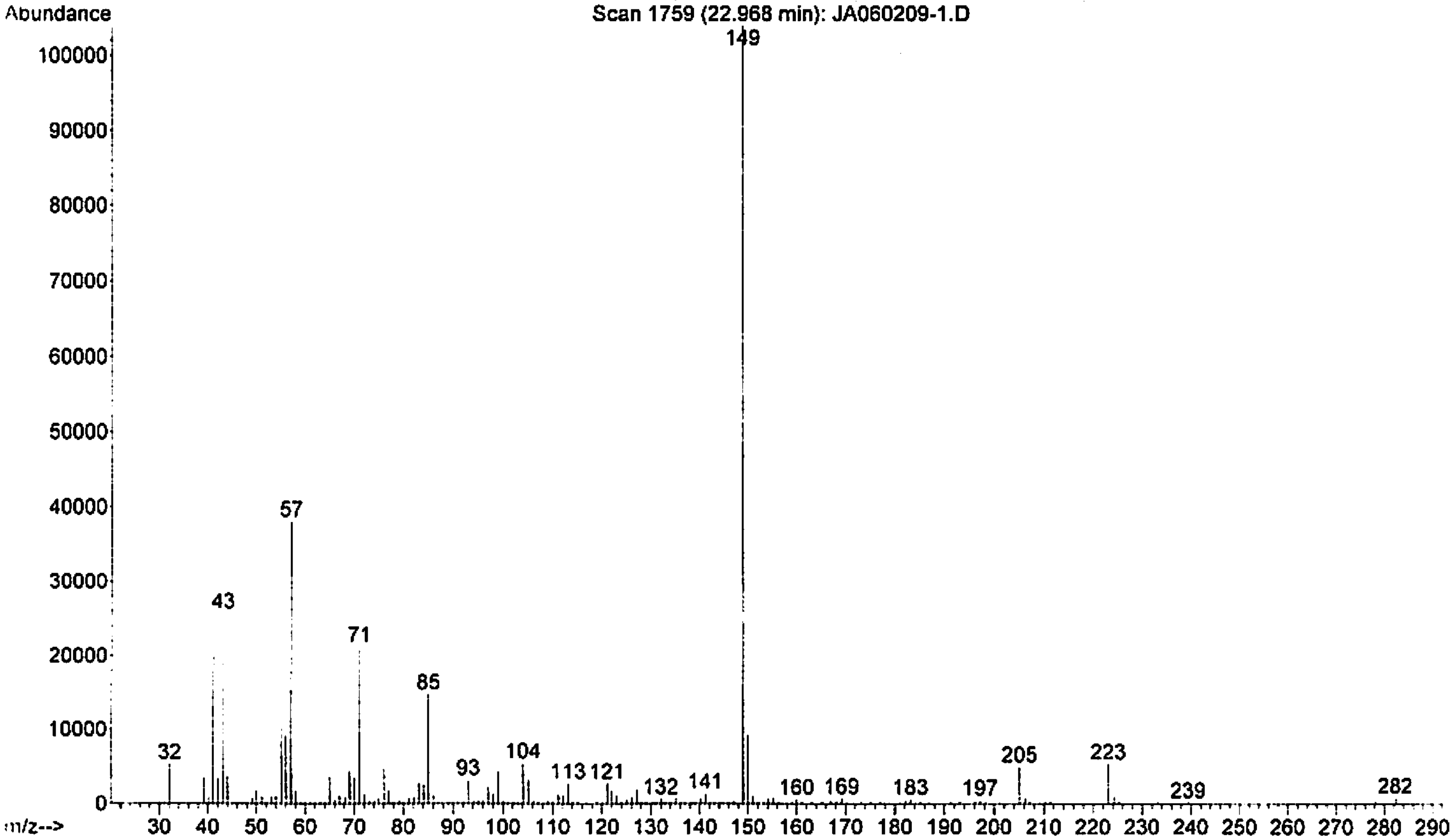

[illegible] :D:\DATA\ALDRICH\JA-09\Snapshot\JA060209-1.D
[illegible]ator : Aldrich
[illegible]ired : 2 Jun 2009 10:47 using AcqMethod JA-WAX08.M
[illegible]rument : Instrument #1
[illegible]le Name: 1 field-coll. M C.oculata abd./CH2Cl2
[illegible] Info : coll. 6/1; 1st of two; top gut full
[illegible] Number: 1

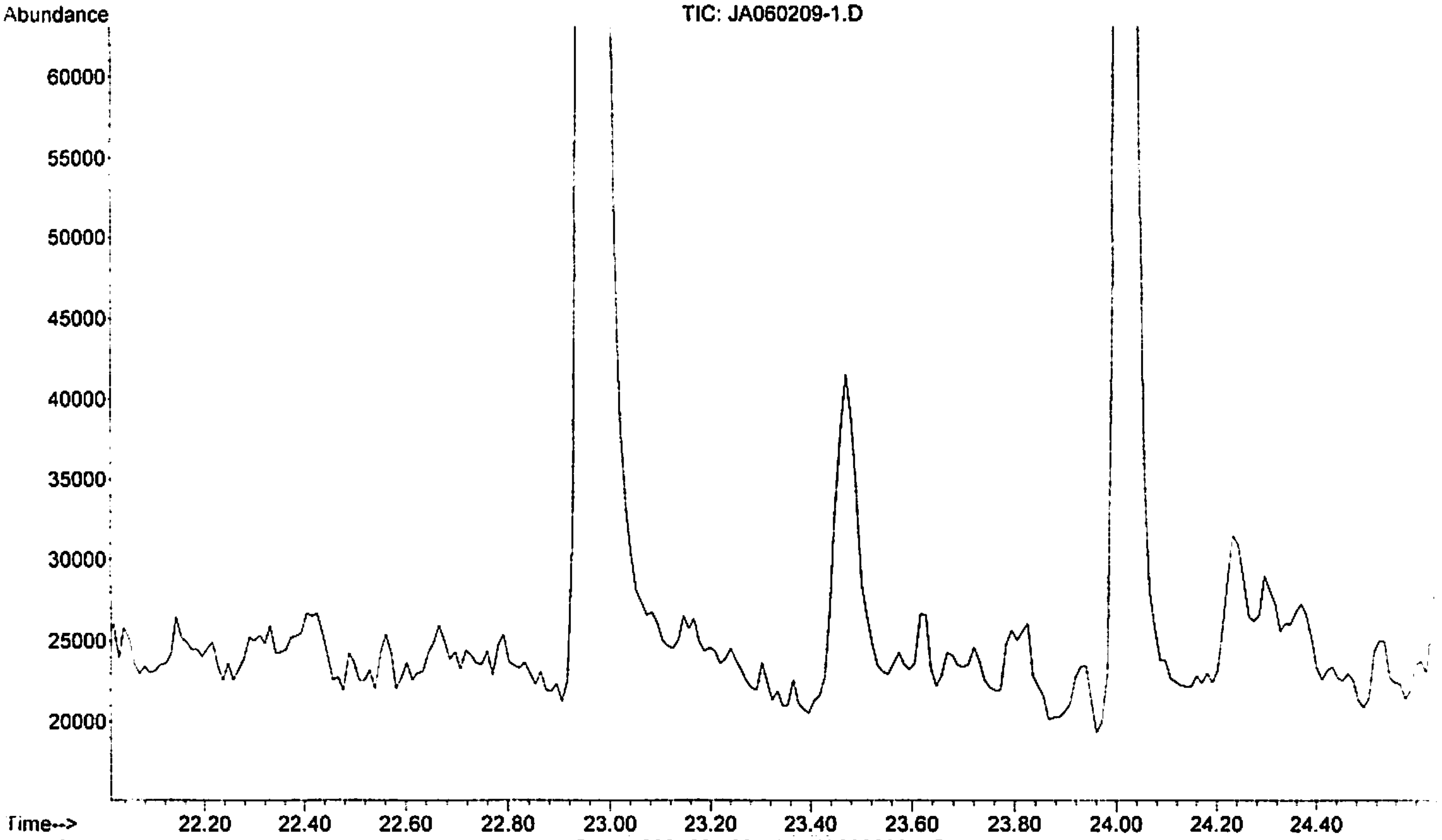

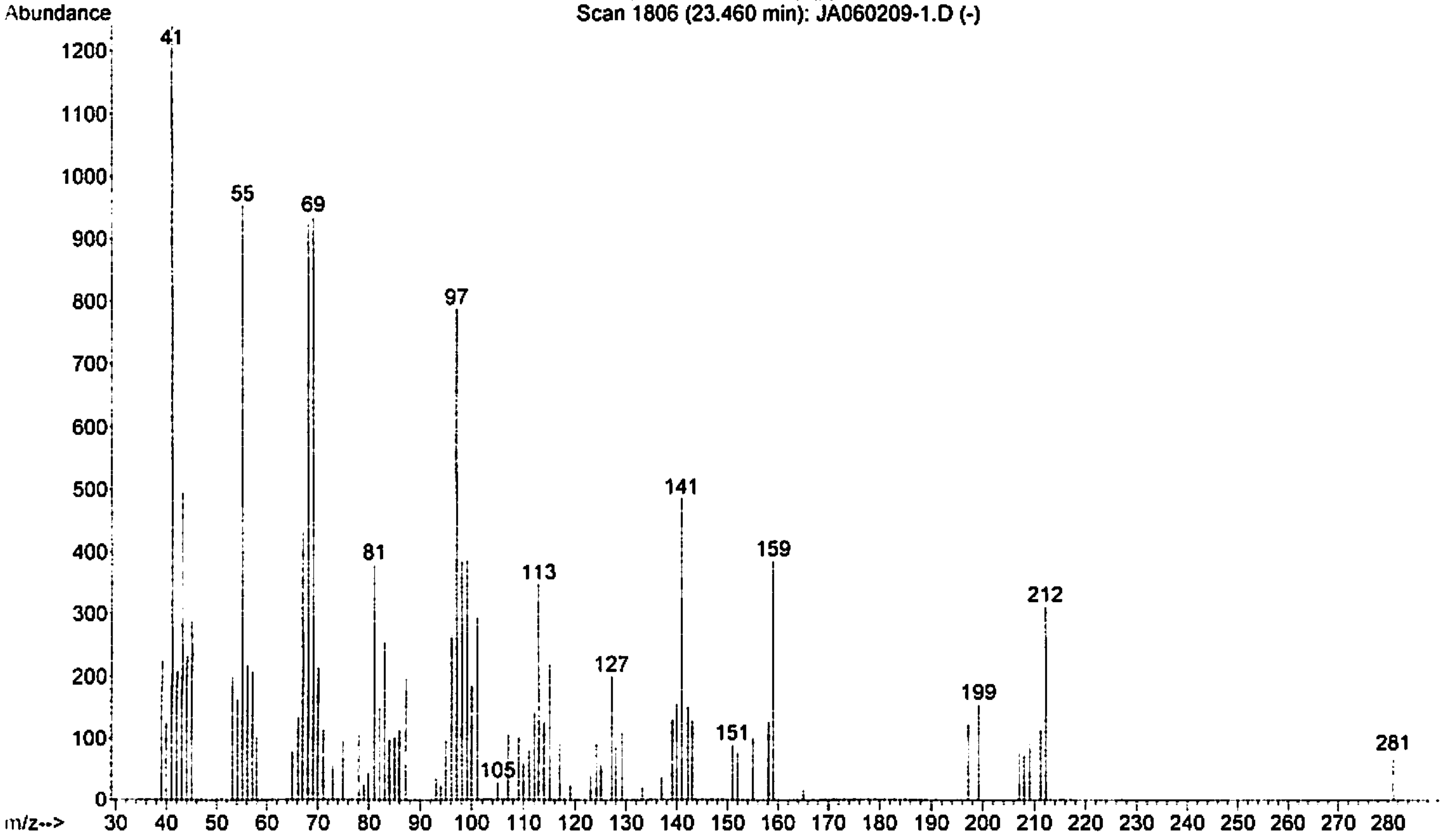

[illegible] :D:\DATA\ALDRICH\JA-09\Snapshot\JA060209-1.D
[illegible]ator : Aldrich
[illegible]ired : 2 Jun 2009 10:47 using AcqMethod JA-WAX08.M
[illegible]rument : Instrument #1
[illegible]le Name: 1 field-coll. M C.oculata abd./CH2Cl2
[illegible] Info : coll. 6/1; 1st of two; top gut full
[illegible] Number: 1

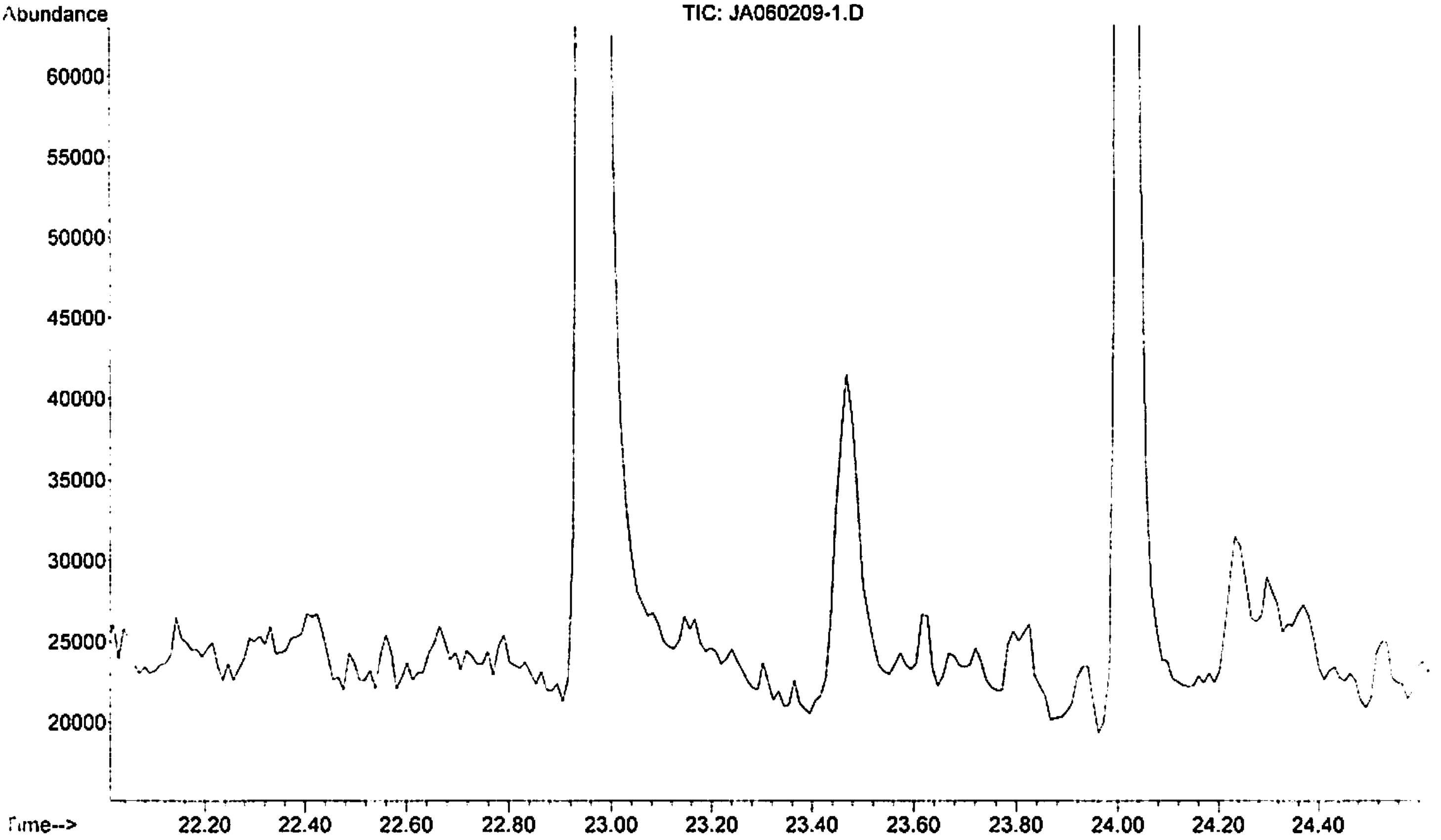

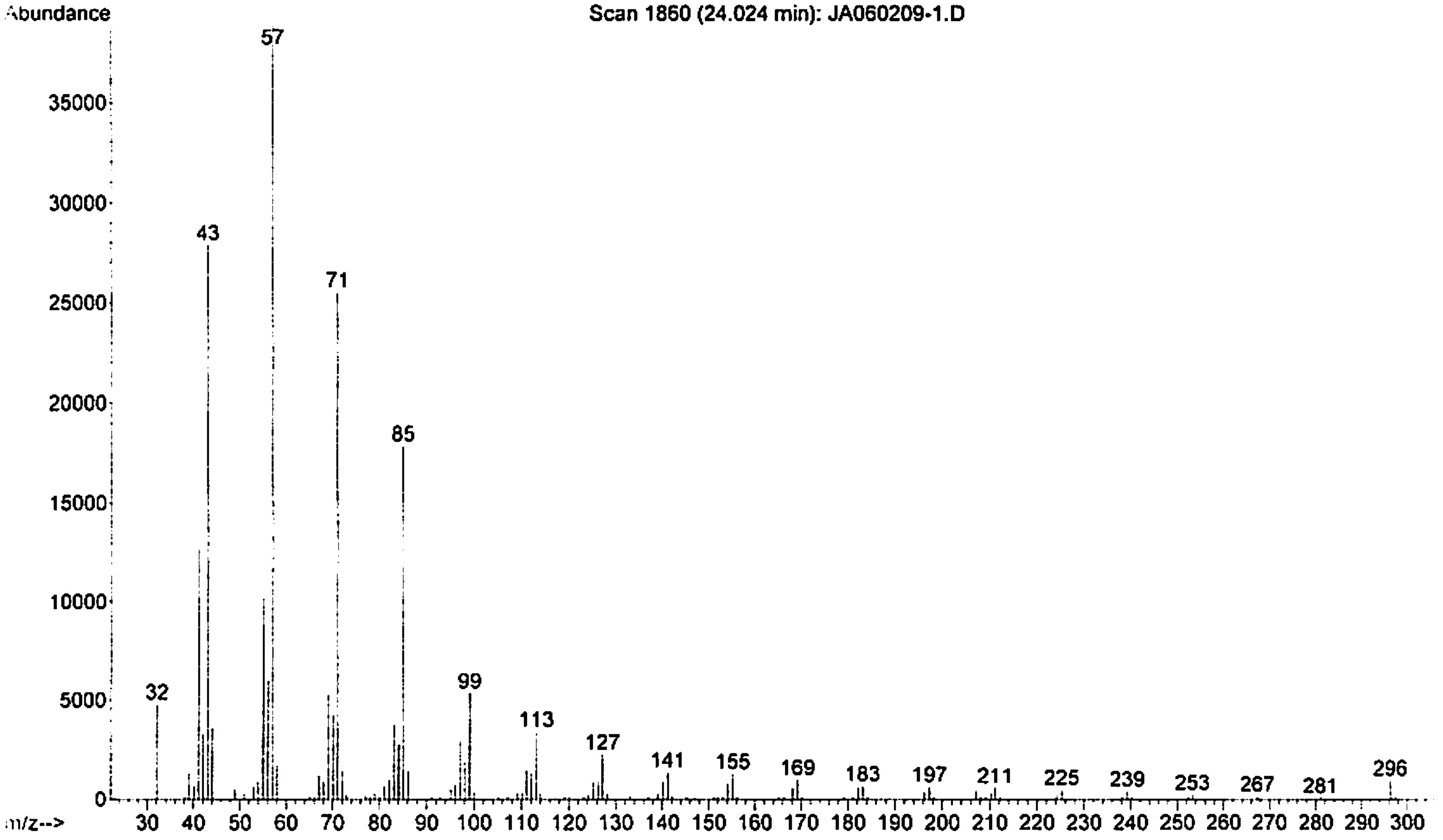

[illegible] : D:\DATA\ALDRICH\JA-09\Snapshot\JA060209-1.D
[illegible]rator : Aldrich
[illegible]ired : 2 Jun 2009 10:47 using AcqMethod JA-WAX08.M
[illegible]rument : Instrument #1
[illegible]le Name: 1 field-coll. M C.oculata abd./CH2Cl2
[illegible] Info : coll. 6/1; 1st of two; top gut full
[illegible] Number: 1

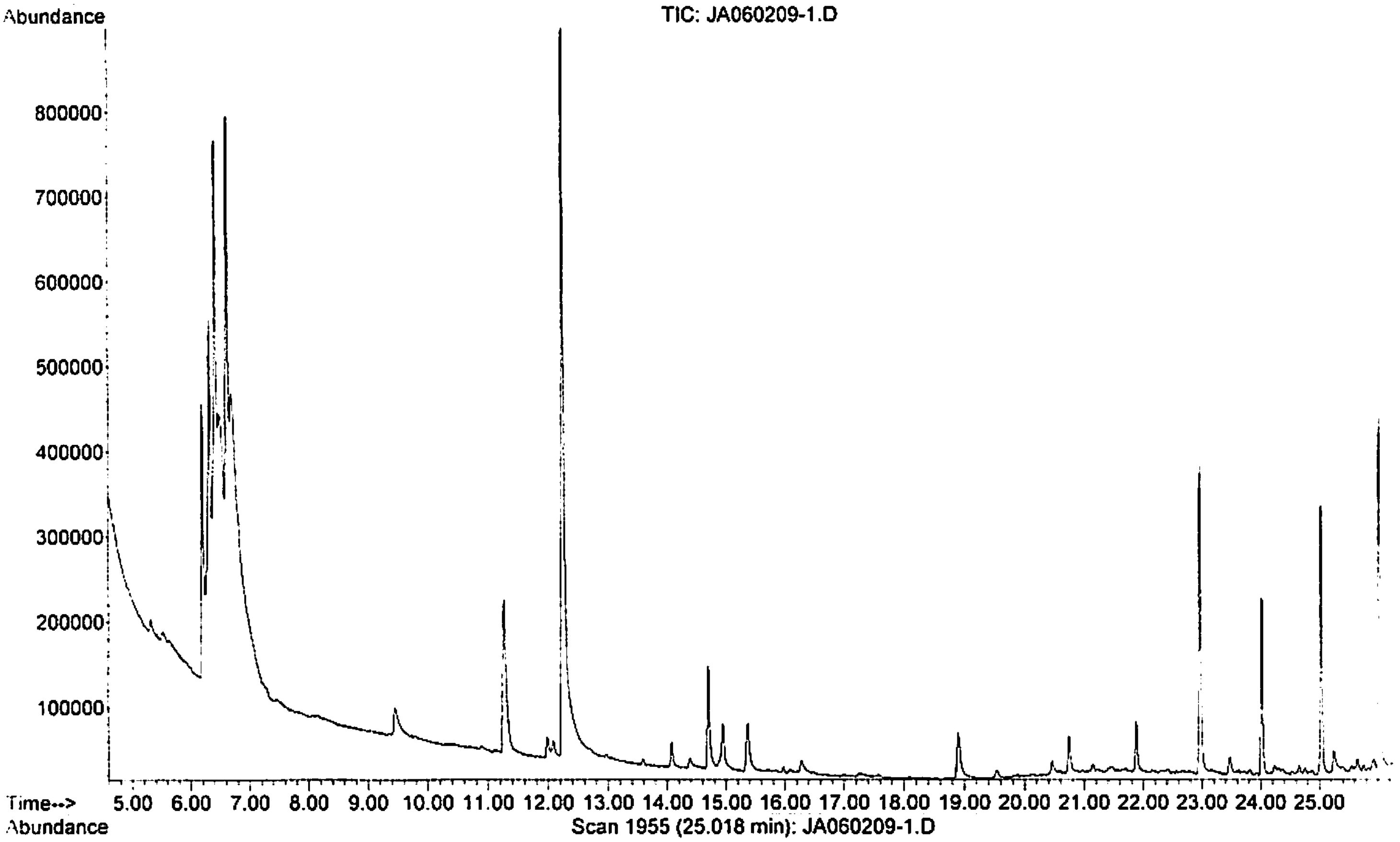

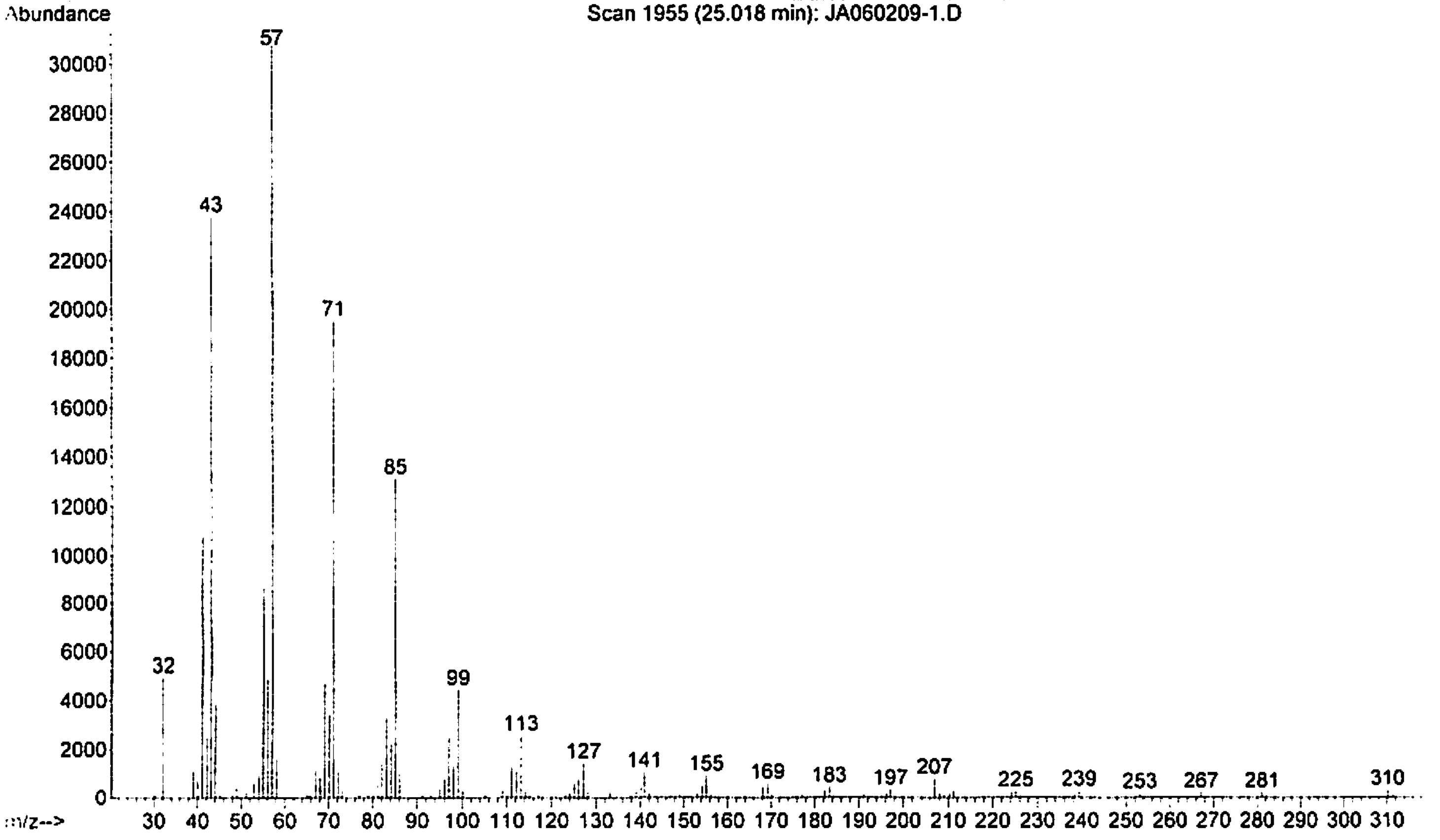

[illegible] :D:\DATA\ALDRICH\JA-09\Snapshot\JA060209-1.D
[illegible]ator : Aldrich
[illegible]ired : 2 Jun 2009 10:47 using AcqMethod JA-WAX08.M
[illegible]rument : Instrument #1
[illegible]ple Name: 1 field-coll. M C.oculata abd./CH2C12
[illegible] Info : coll. 6/1; 1st of two; top gut full
[illegible] Number: 1

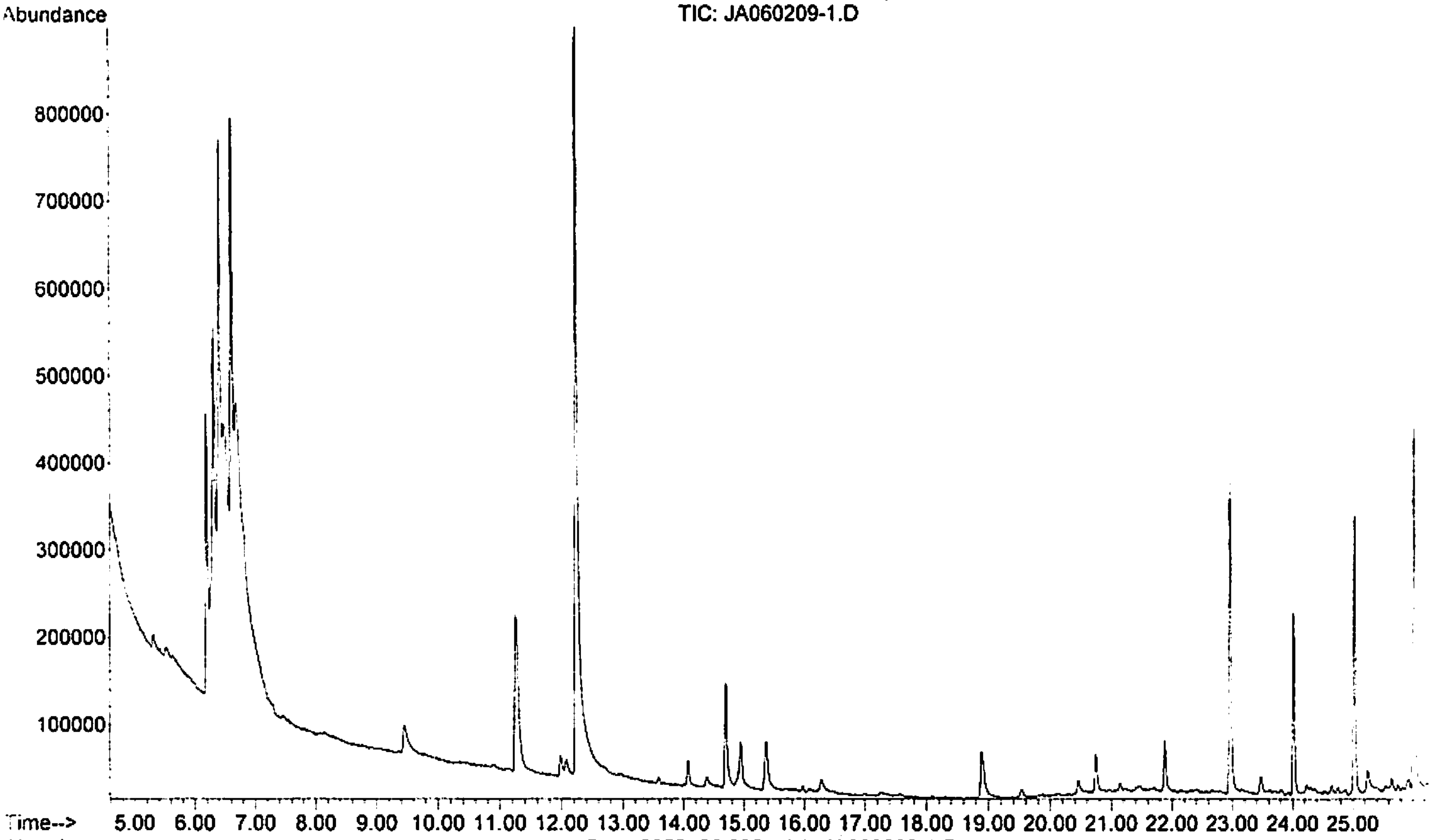

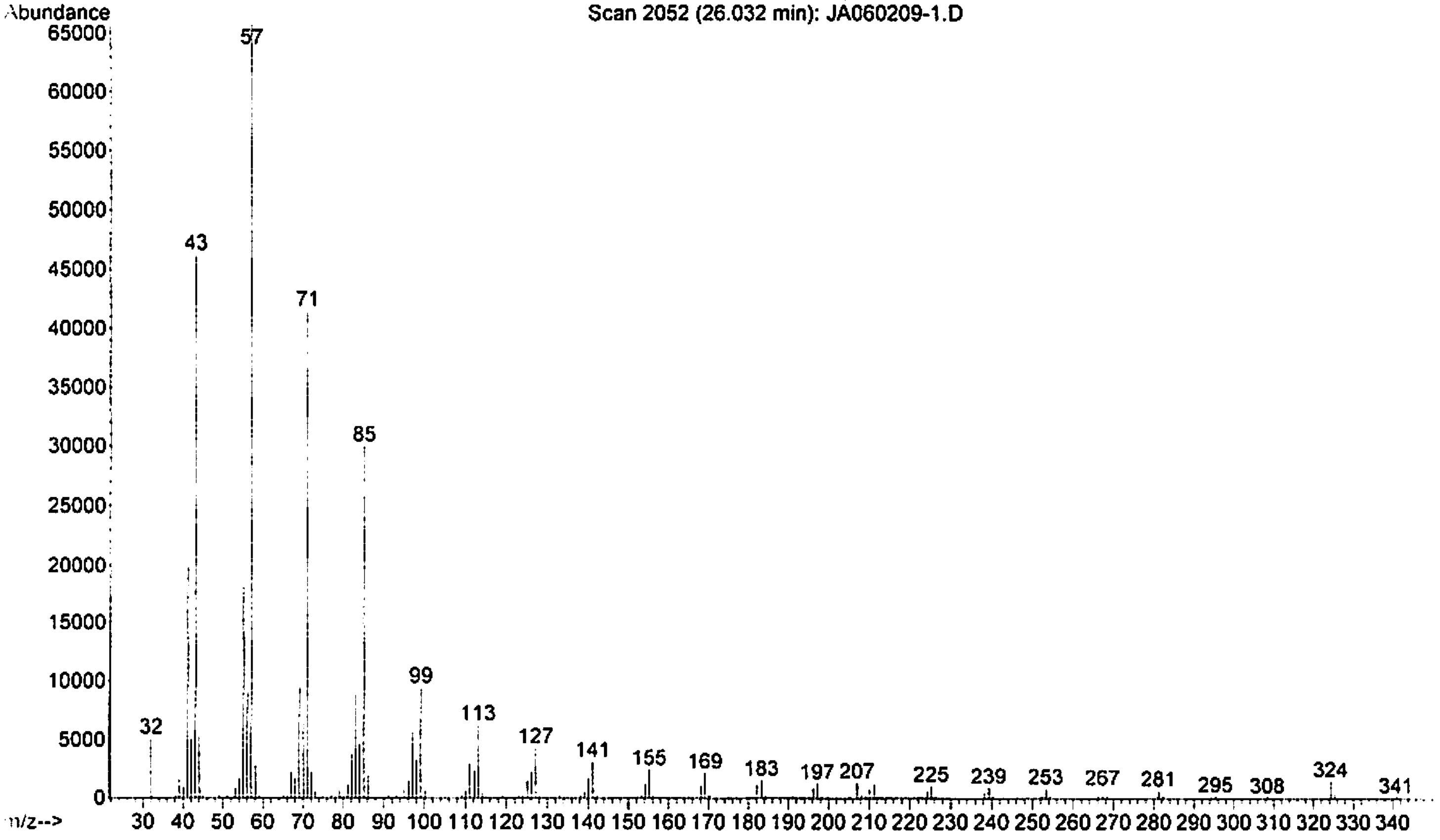

[illegible] :D:\DATA\ALDRICH\JA-09\Snapshot\JA060209-1.D
[illegible]erator : Aldrich
[illegible]uired : 2 Jun 2009 10:47 using AcqMethod JA-WAX08.M
[illegible]rument : Instrument #1
[illegible]ple Name: 1 field-coll. M C.oculata abd./CH2Cl2
[illegible] Info : coll. 6/1; 1st of two; top gut full
[illegible] Number: 1

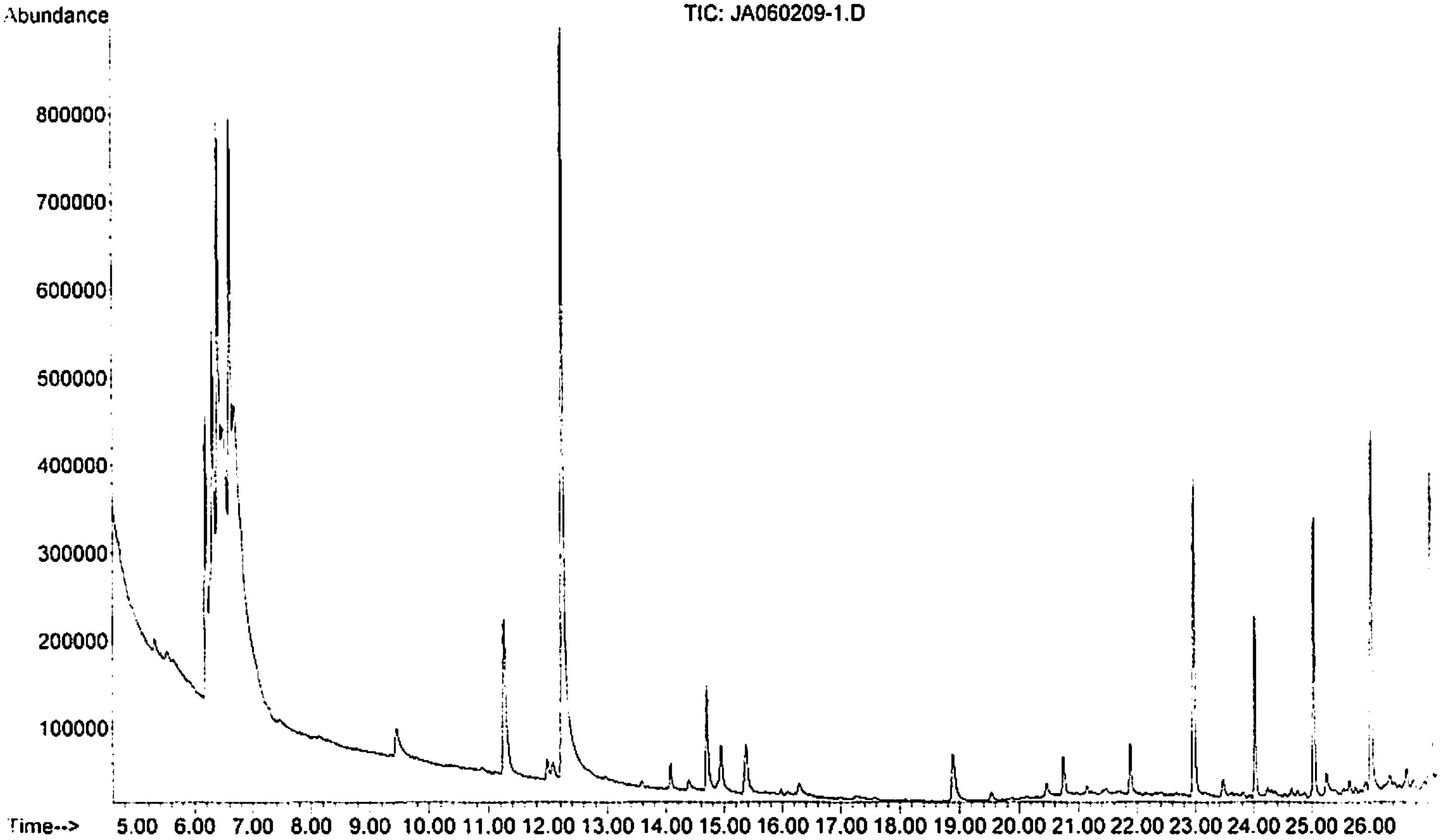

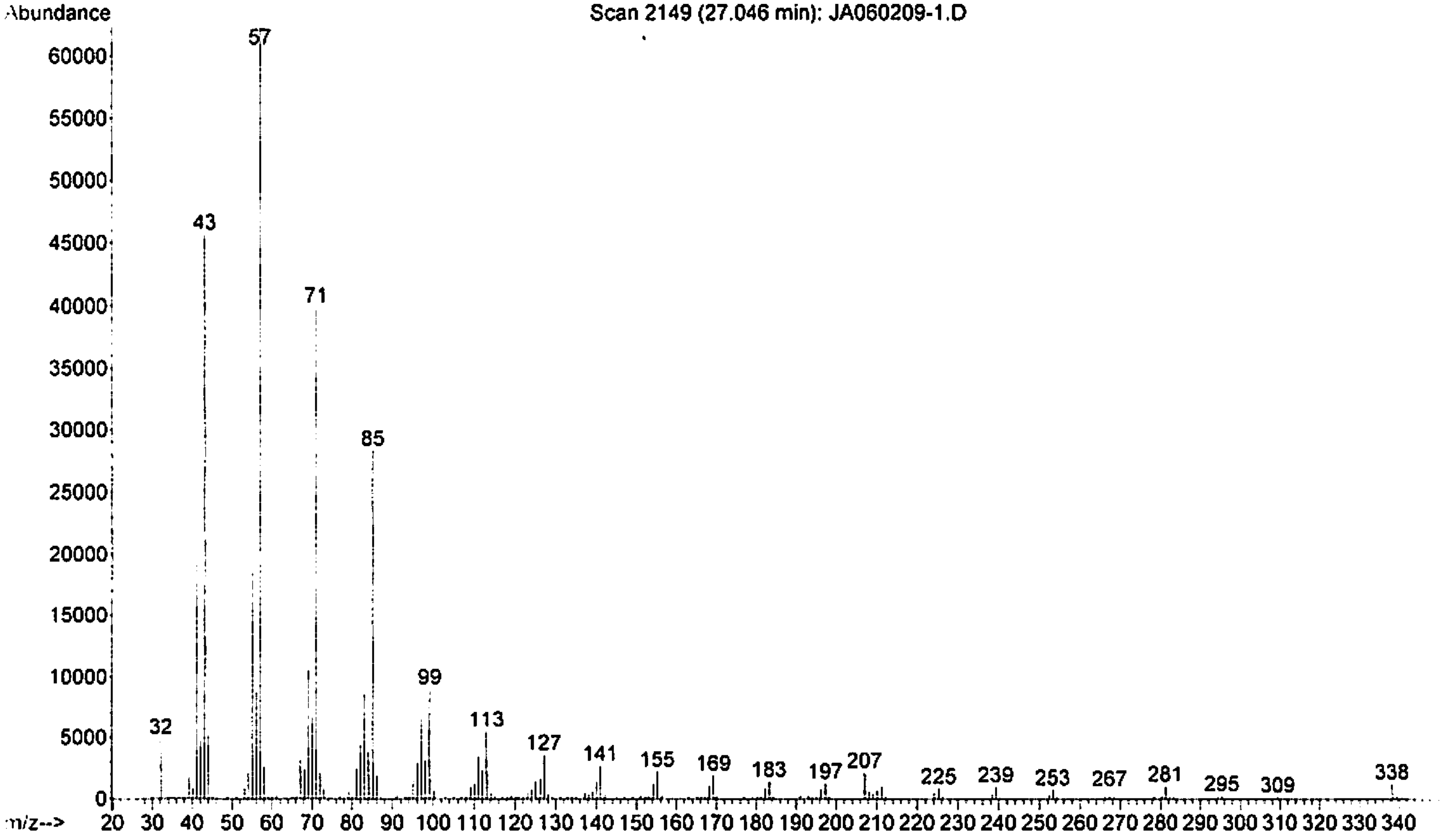

File : D:\DATA\Aldrich\JA-09\JA060209-1.D
Operator : Aldrich
Acquired : 2 Jun 2009 10:47 using AcqMethod JA-WAX08.M
Instrument : Instrument #1
Sample Name: 1 field-coll. M C.oculata abd./CH2Cl2
Misc Info : coll. 6/1; 1st of two; top gut full
Vial Number: 1

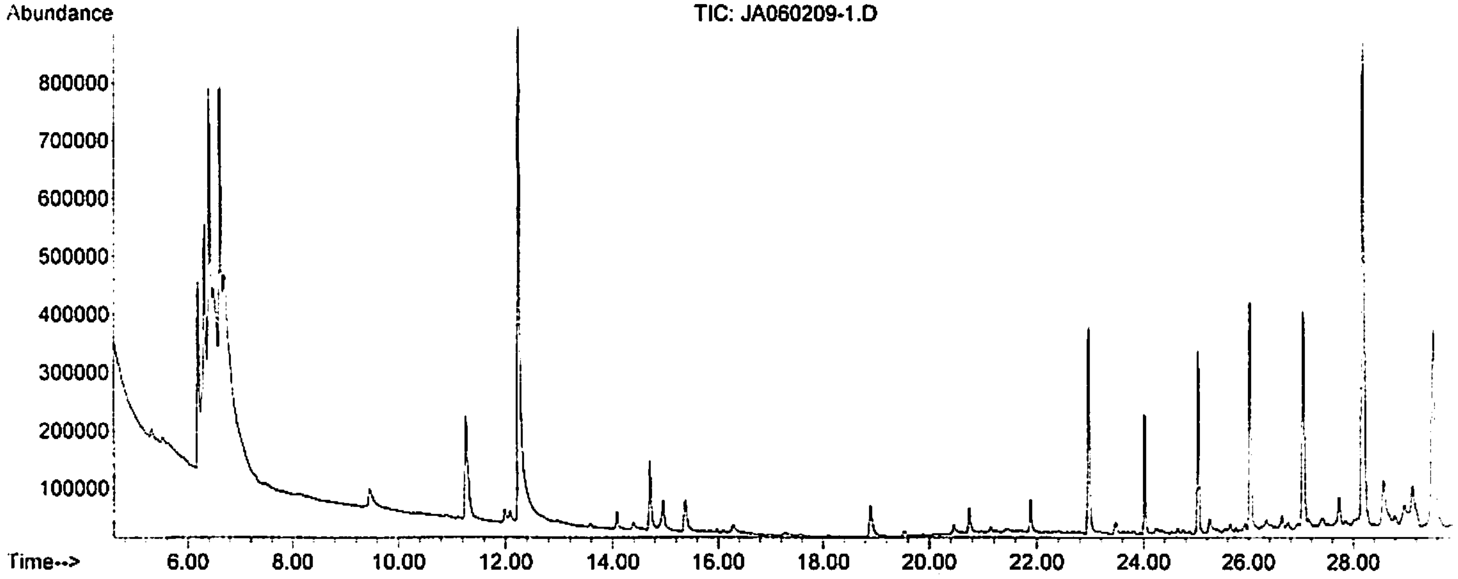

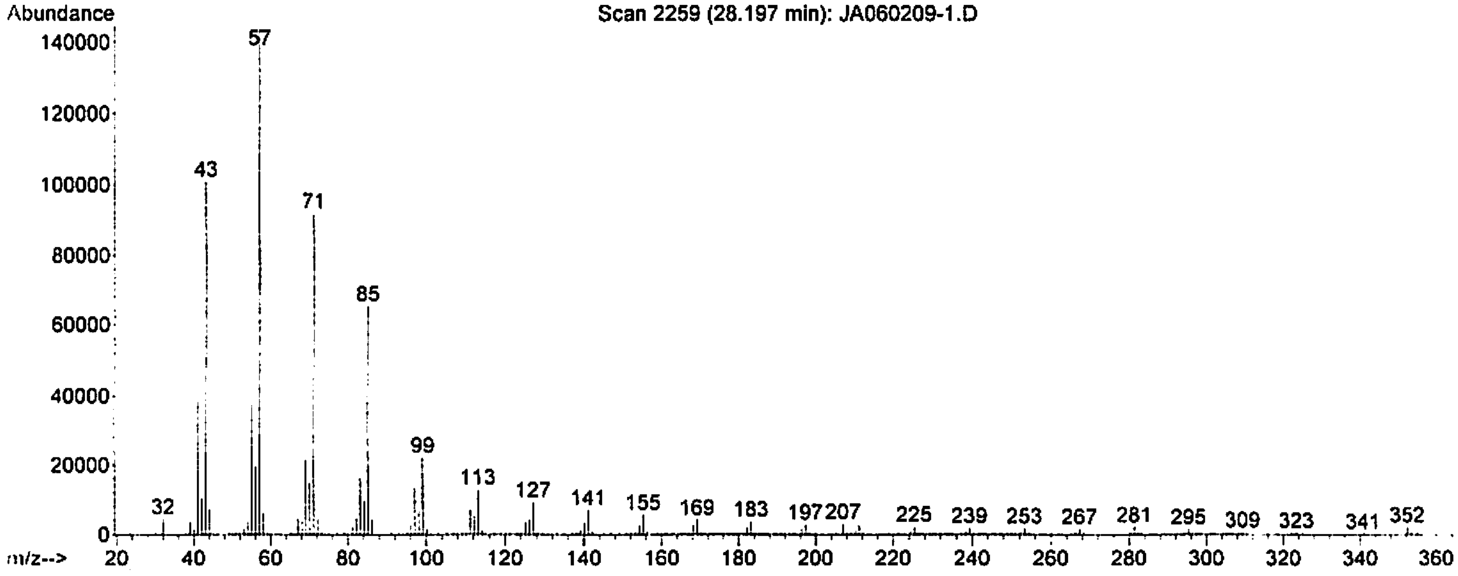

[illegible] :D:\DATA\Aldrich\JA-09\JA060209-1.D
[illegible]rator : Aldrich
[illegible]uired : 2 Jun 2009 10:47 using AcqMethod JA-WAX08.M
[illegible]rument : Instrument #1
[illegible]ple Name: 1 field-coll. M C.oculata abd./CH2Cl2
[illegible] Info : coll. 6/1; 1st of two; top gut full
[illegible] Number: 1

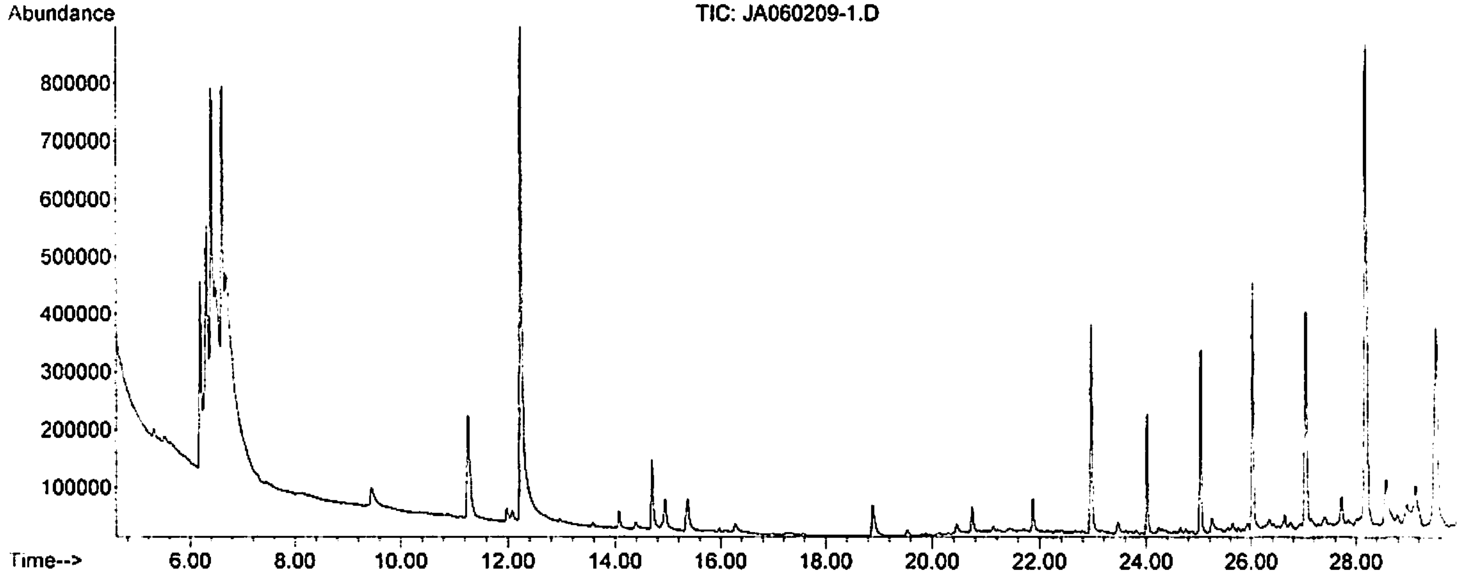

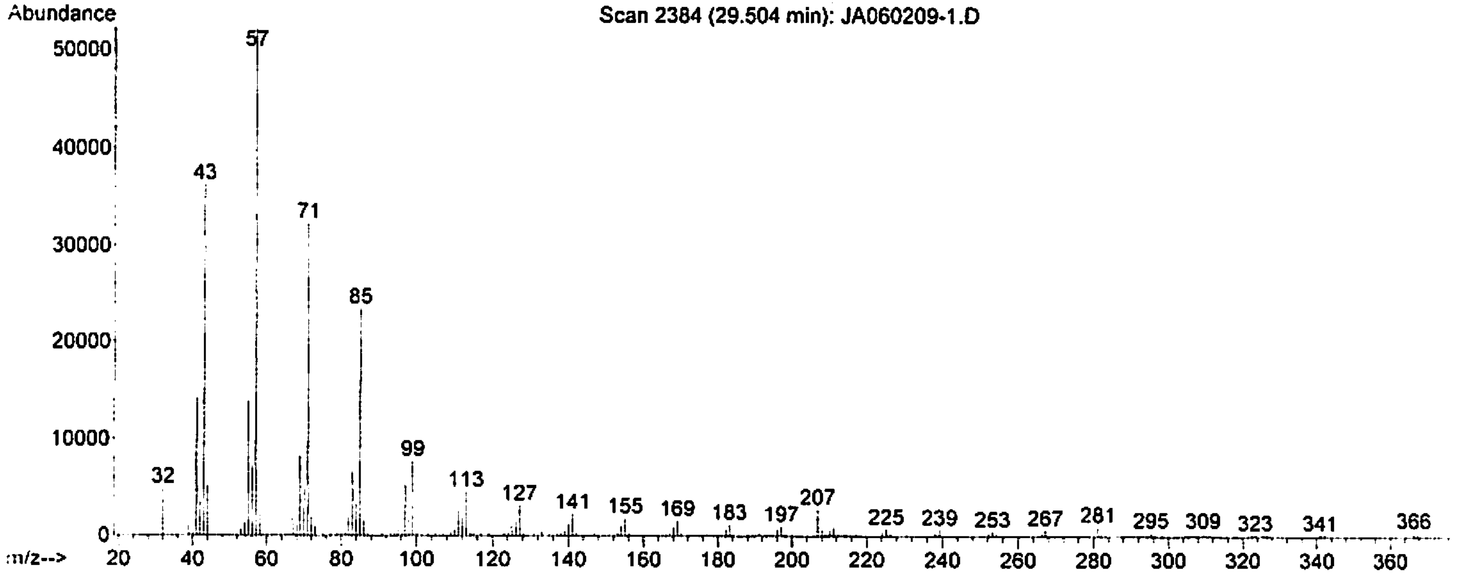

```
File          :D:\DATA\Aldrich\JA-09\JA060209-2.D
Operator      : Aldrich
Acquired      :  2 Jun 2009  11:30      using AcqMethod JA-WAX08.M
Instrument :     Instrument #1
Sample Name: 1 field-coll. M C.oculata abd./CH2Cl2
Misc Info   : coll. 6/1; 2nd of two; top gut full
Vial Number: 1
```

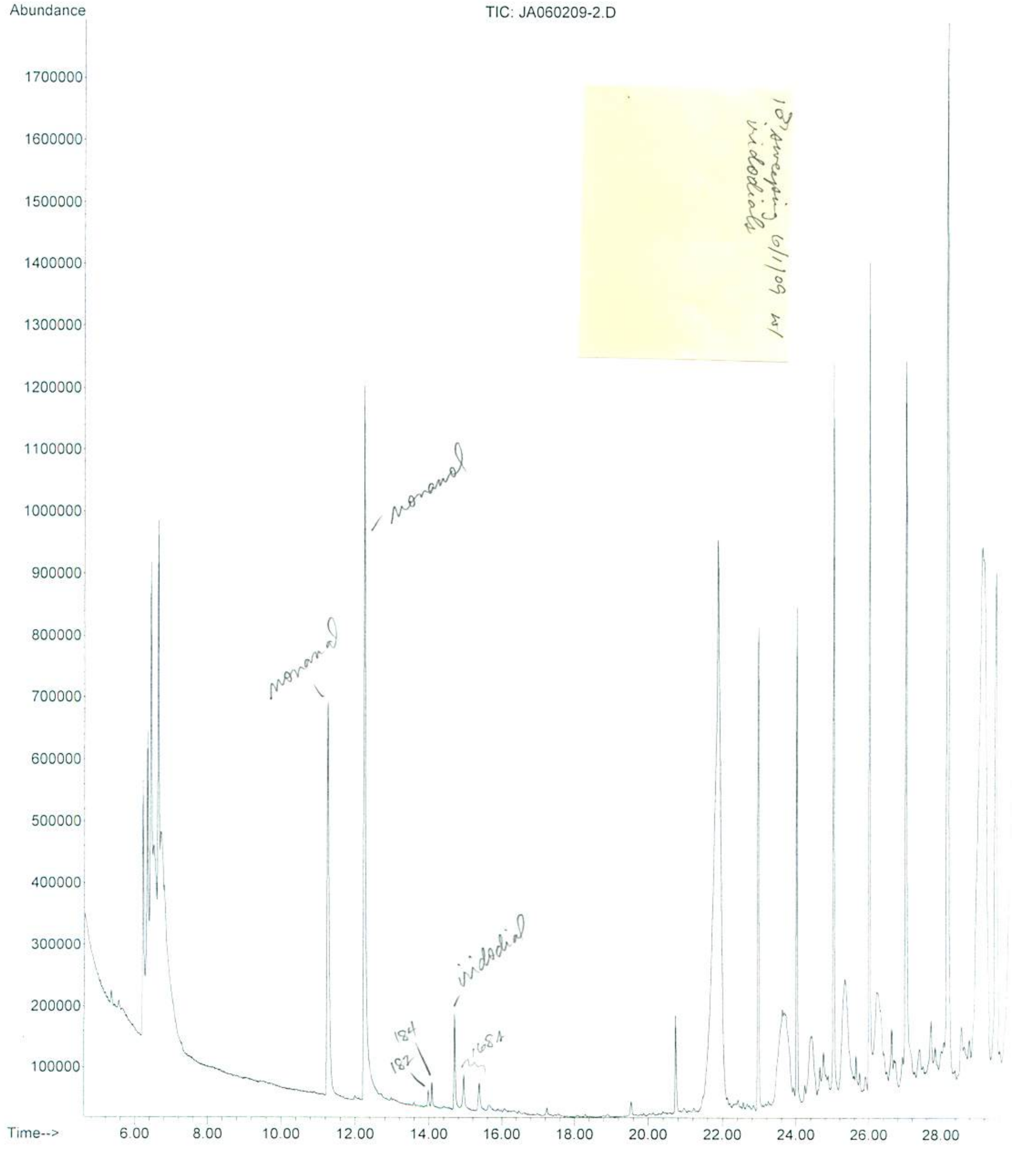

```
File        :D:\DATA\Aldrich\JA-09\JA060209-2.D
Operator    : Aldrich
Acquired    :  2 Jun 2009  11:30     using AcqMethod JA-WAX08.M
Instrument :    Instrument #1
Sample Name: 1 field-coll. M C.oculata abd./CH2Cl2
Misc Info   : coll. 6/1; 2nd of two; top gut full
Vial Number: 1
```

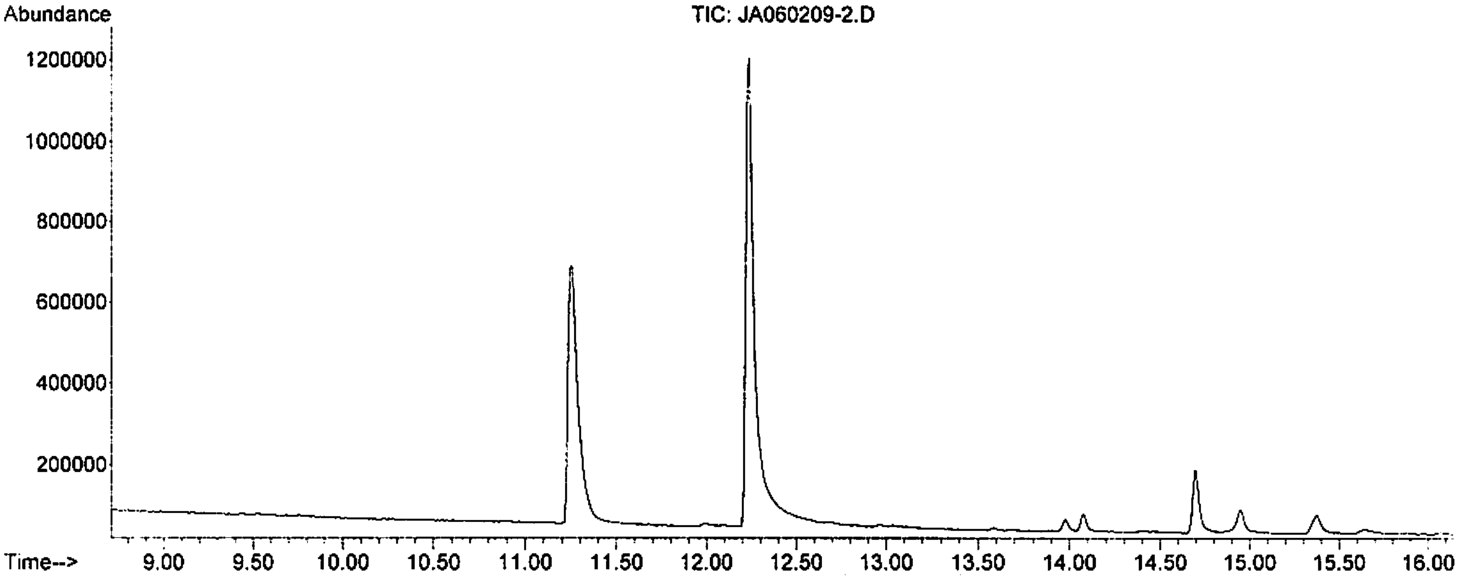

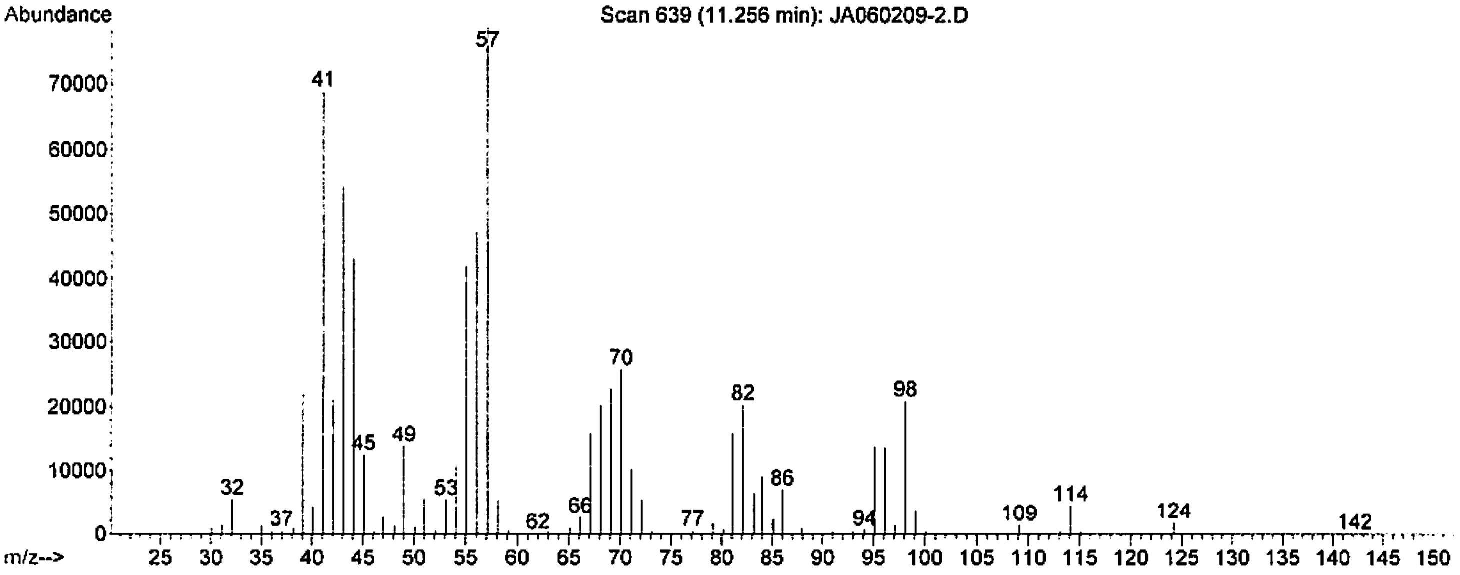

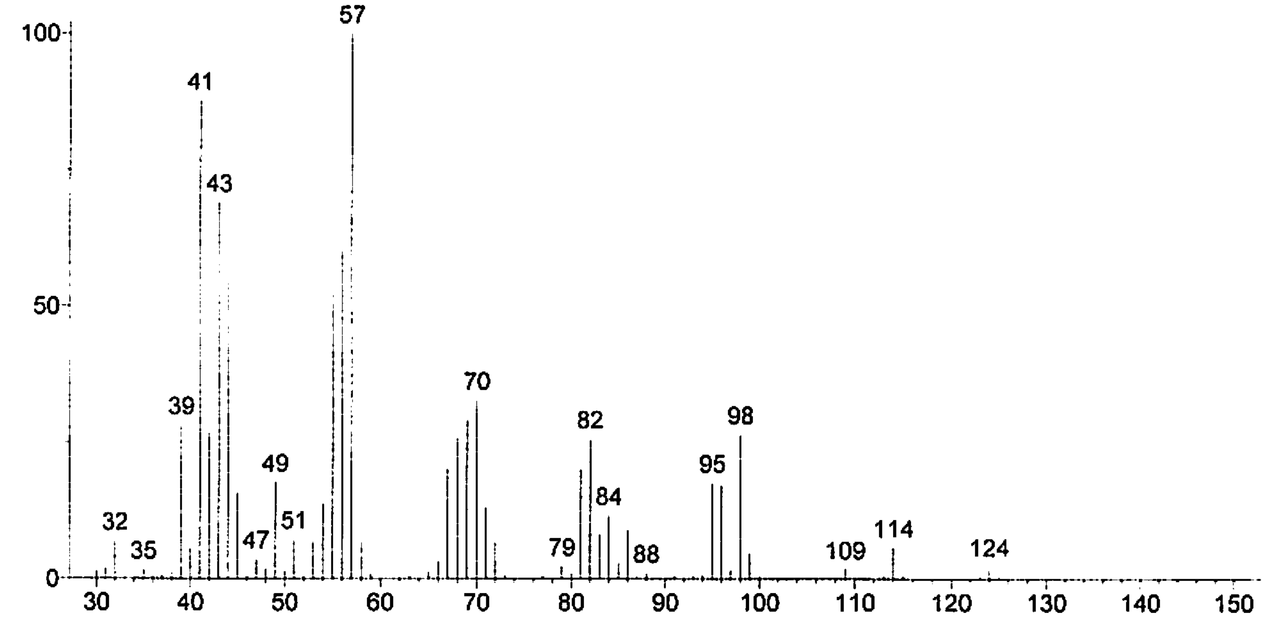

(Text File) Scan 639 (11.256 min): JA060209-2.D

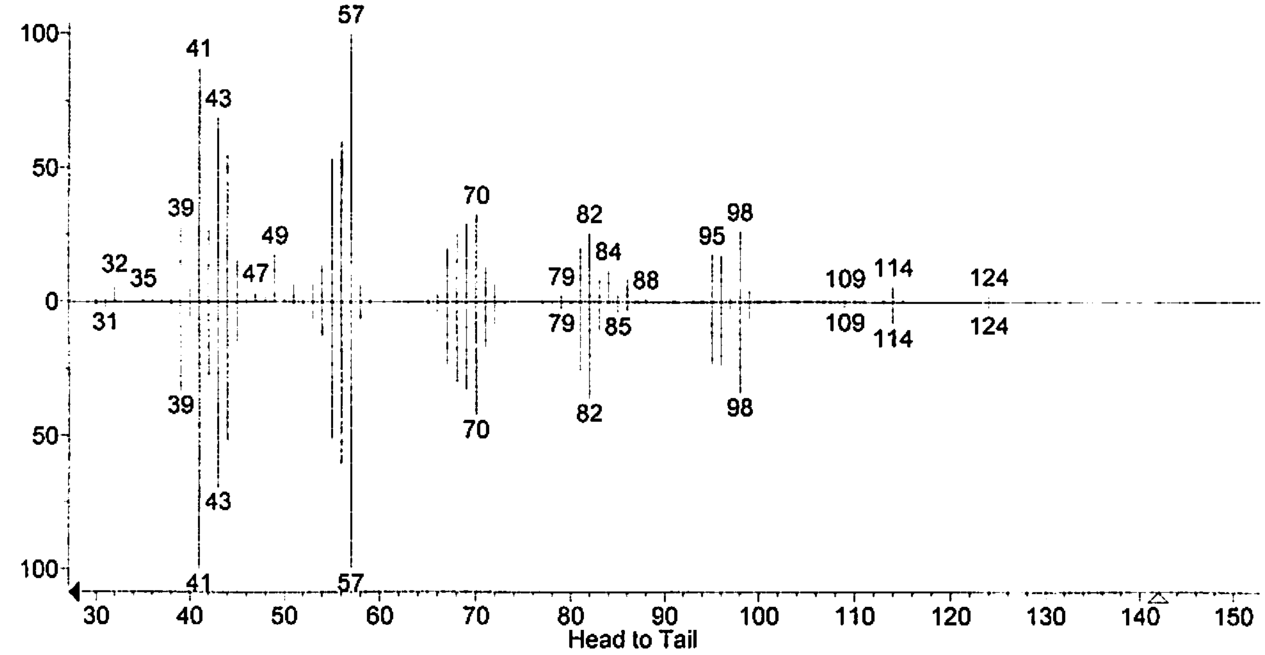

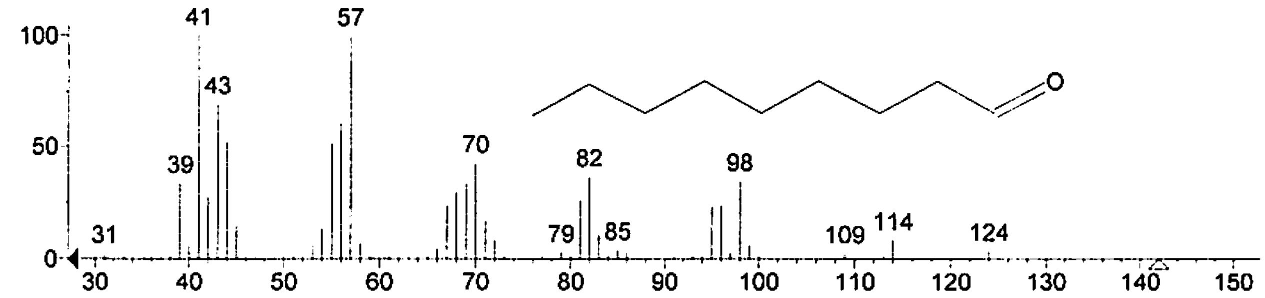

(replib) Nonanal

```
File        :D:\DATA\Aldrich\JA-09\JA060209-2.D
Operator    : Aldrich
Acquired    :  2 Jun 2009  11:30      using AcqMethod JA-WAX08.M
[illegible]rument :     Instrument #1
[illegible]ple Name: 1 field-coll. M C.oculata abd./CH2Cl2
[illegible] Info   : coll. 6/1; 2nd of two; top gut full
[illegible] Number: 1
```

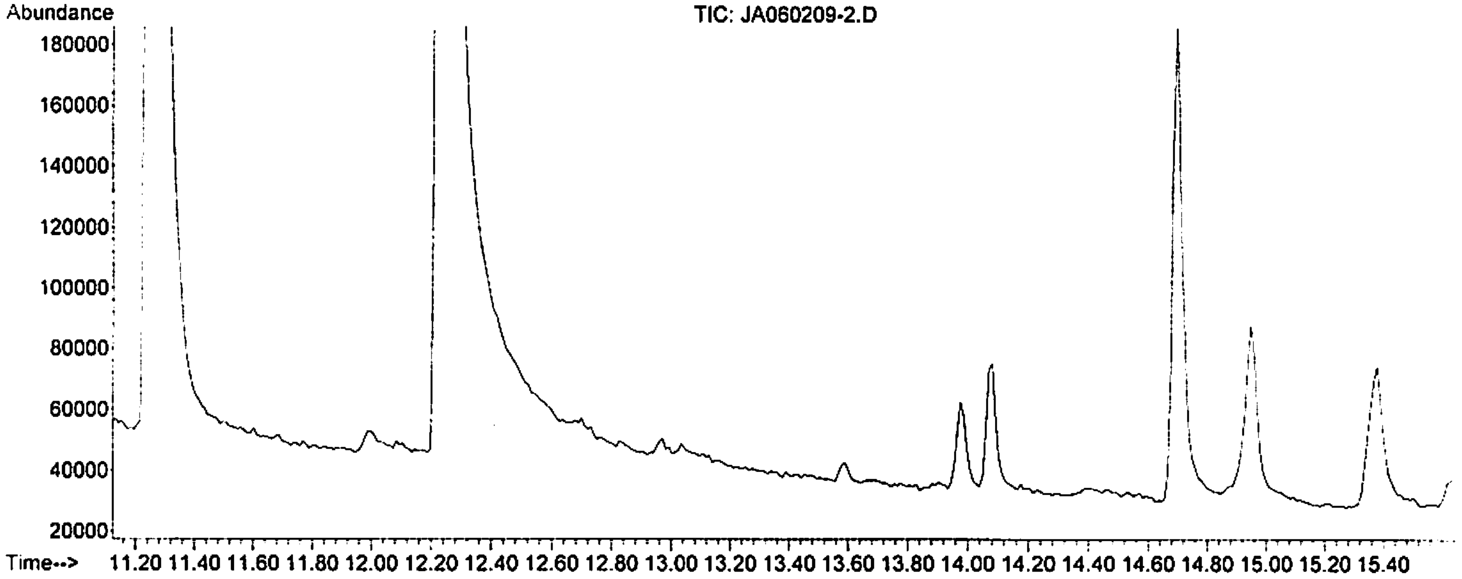

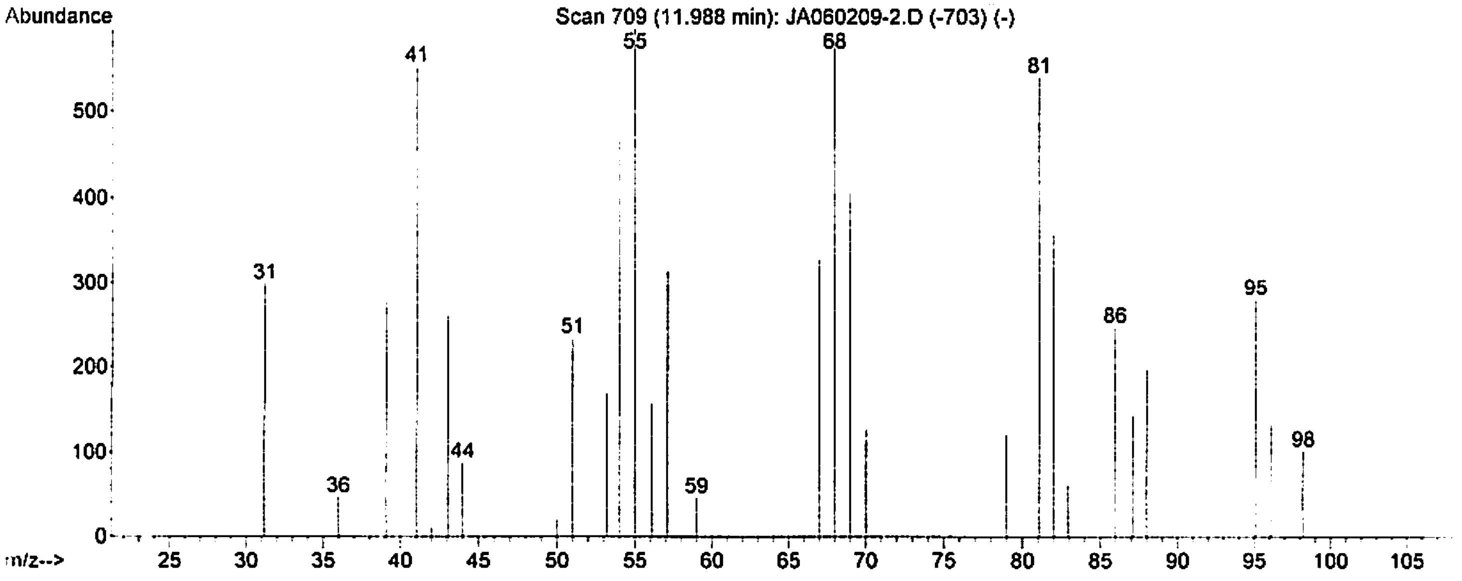

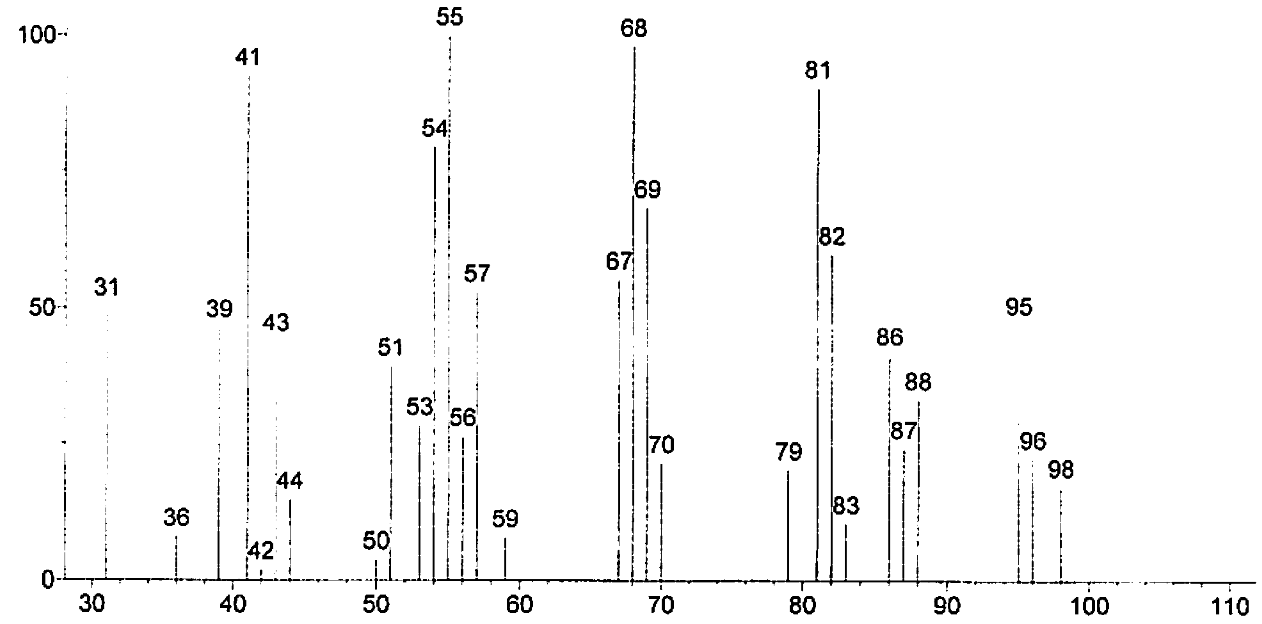

(Text File) Scan 709 (11.988 min): JA060209-2.D (-703)

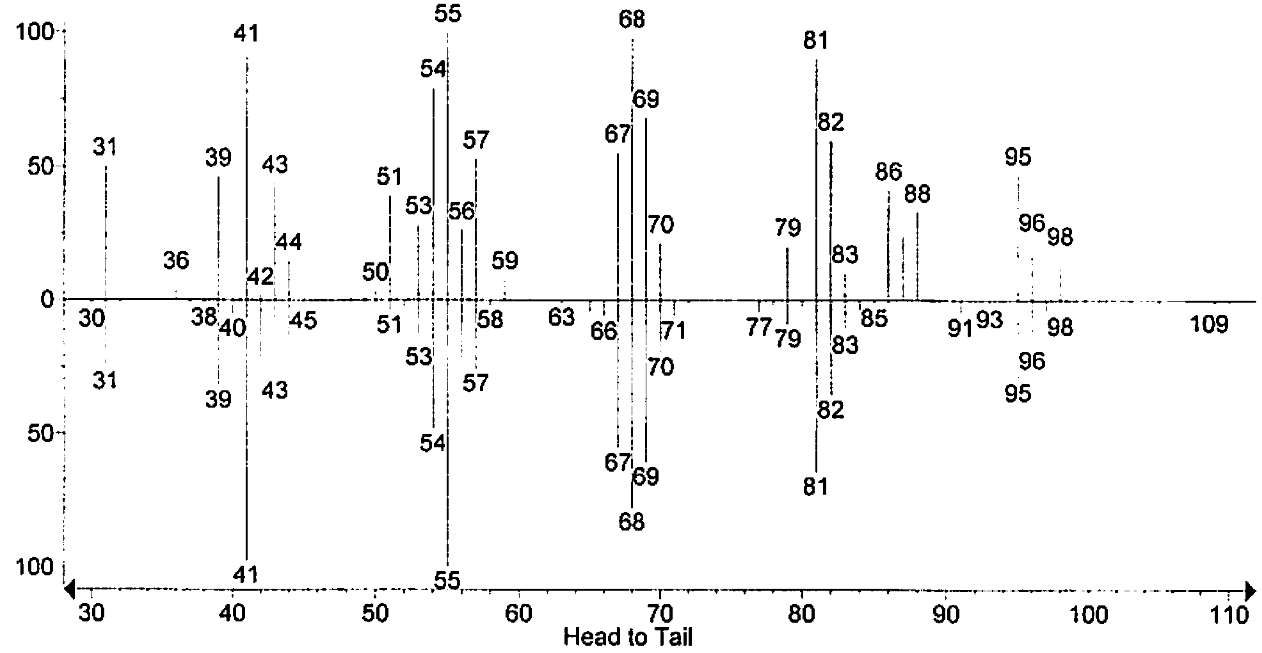

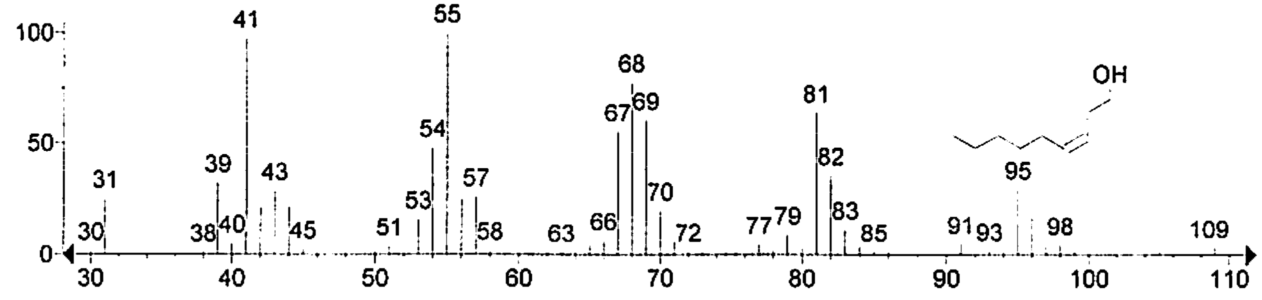

(replib) 3-Nonen-1-ol, (Z)-

[illegible] :D:\DATA\Aldrich\JA-09\JA060209-2.D
[illegible]ator : Aldrich
[illegible]ired : 2 Jun 2009 11:30 using AcqMethod JA-WAX08.M
[illegible]rument : Instrument #1
[illegible]le Name: 1 field-coll. M C.oculata abd./CH2Cl2
[illegible] Info : coll. 6/1; 2nd of two; top gut full
[illegible] Number: 1

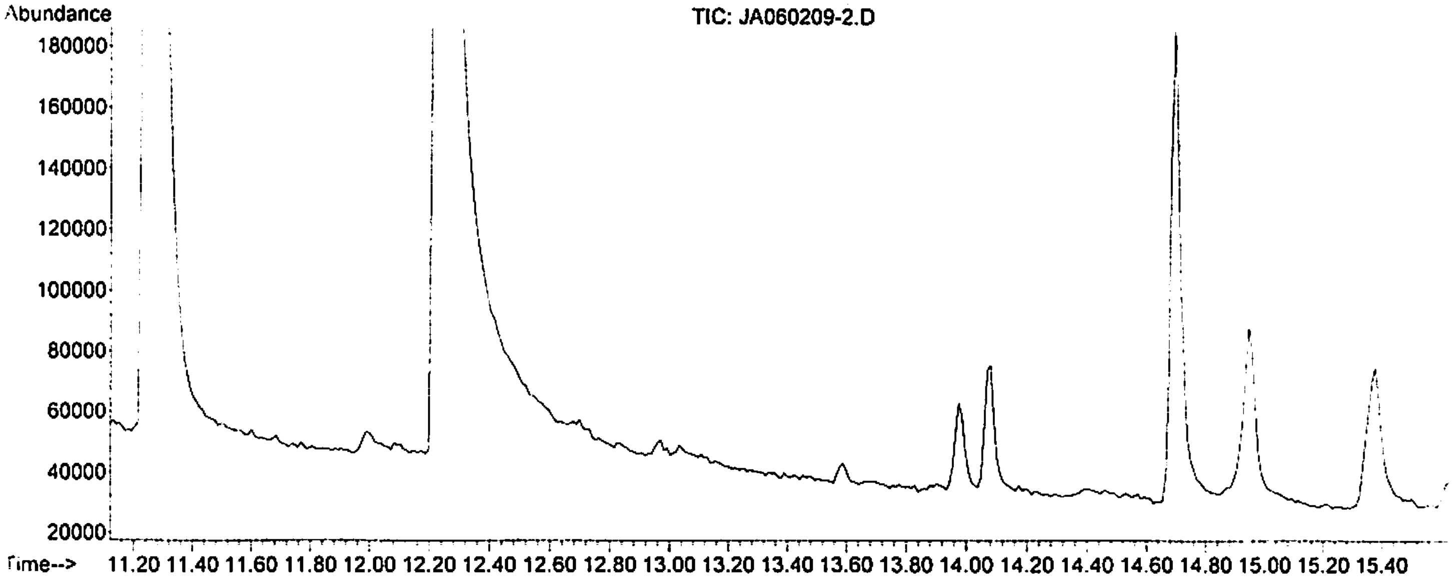

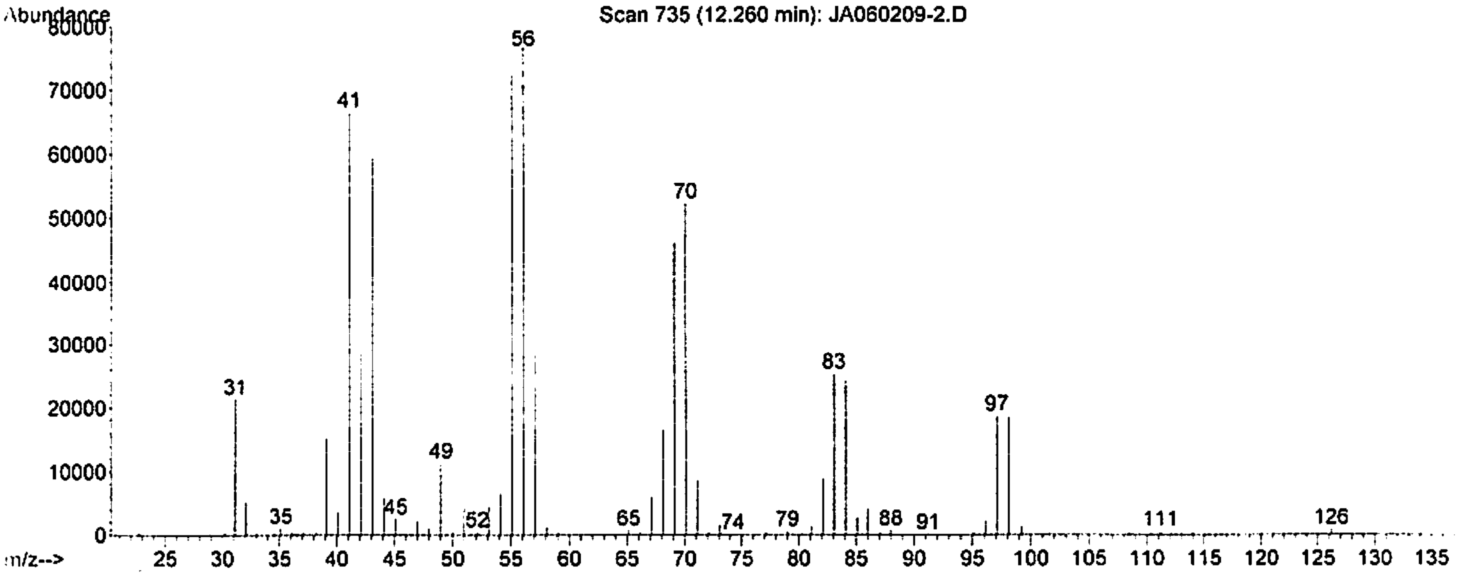

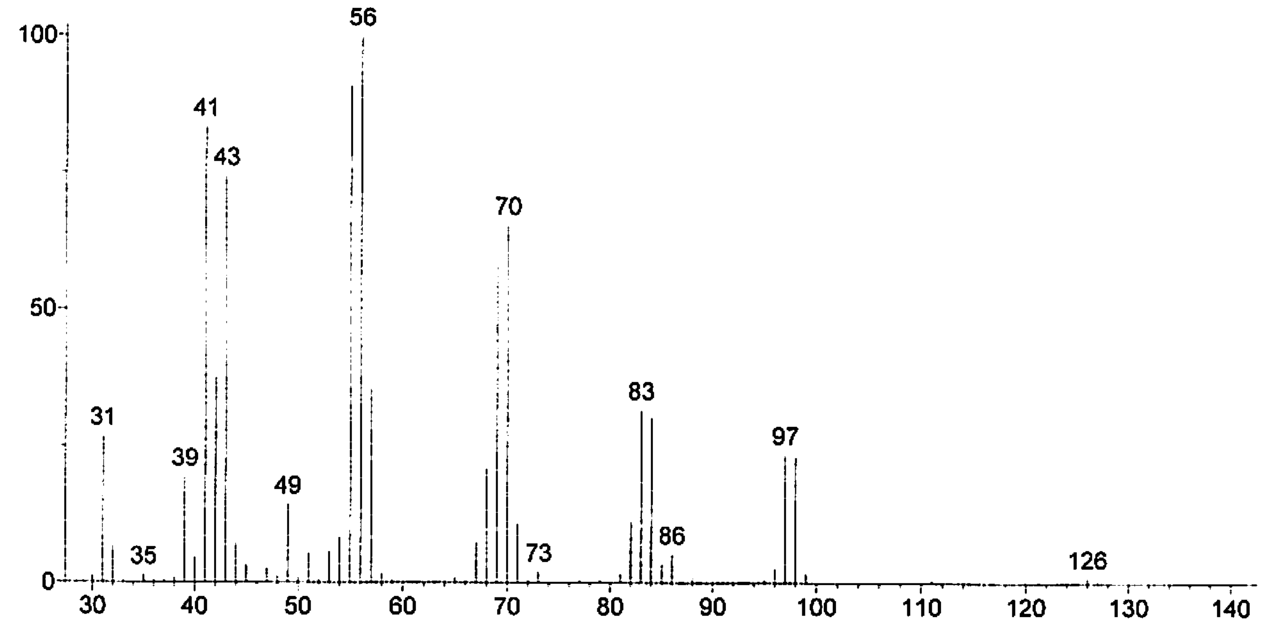

(Text File) Scan 735 (12.260 min): JA060209-2.D

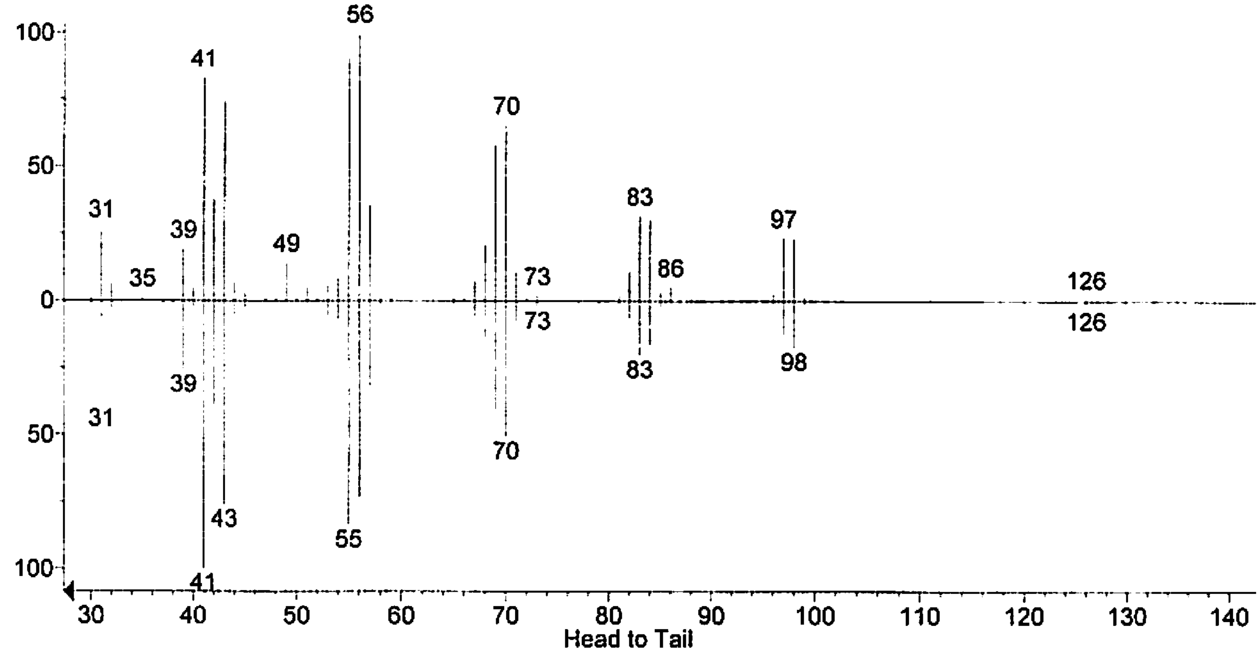

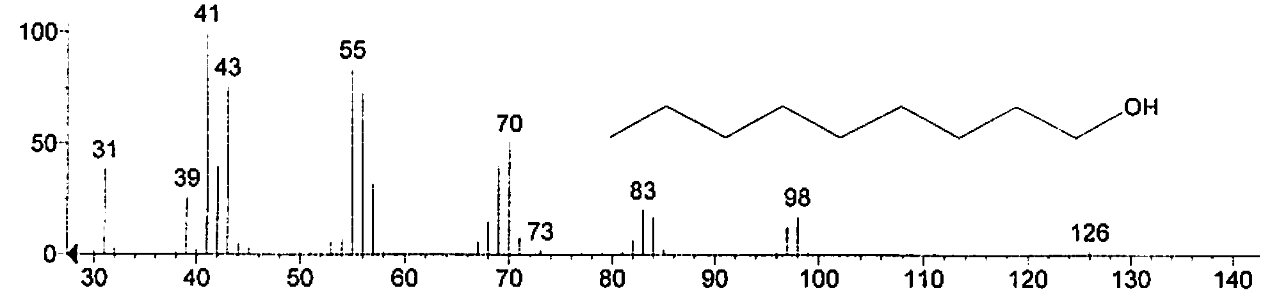

(replib) 1-Nonanol

```
File       :D:\DATA\Aldrich\JA-09\JA060209-2.D
Operator   : Aldrich
Acquired   :  2 Jun 2009  11:30     using AcqMethod JA-WAX08.M
Instrument :    Instrument #1
Sample Name: 1 field-coll. M C.oculata abd./CH2Cl2
Misc Info  : coll. 6/1; 2nd of two; top gut full
Vial Number: 1
```

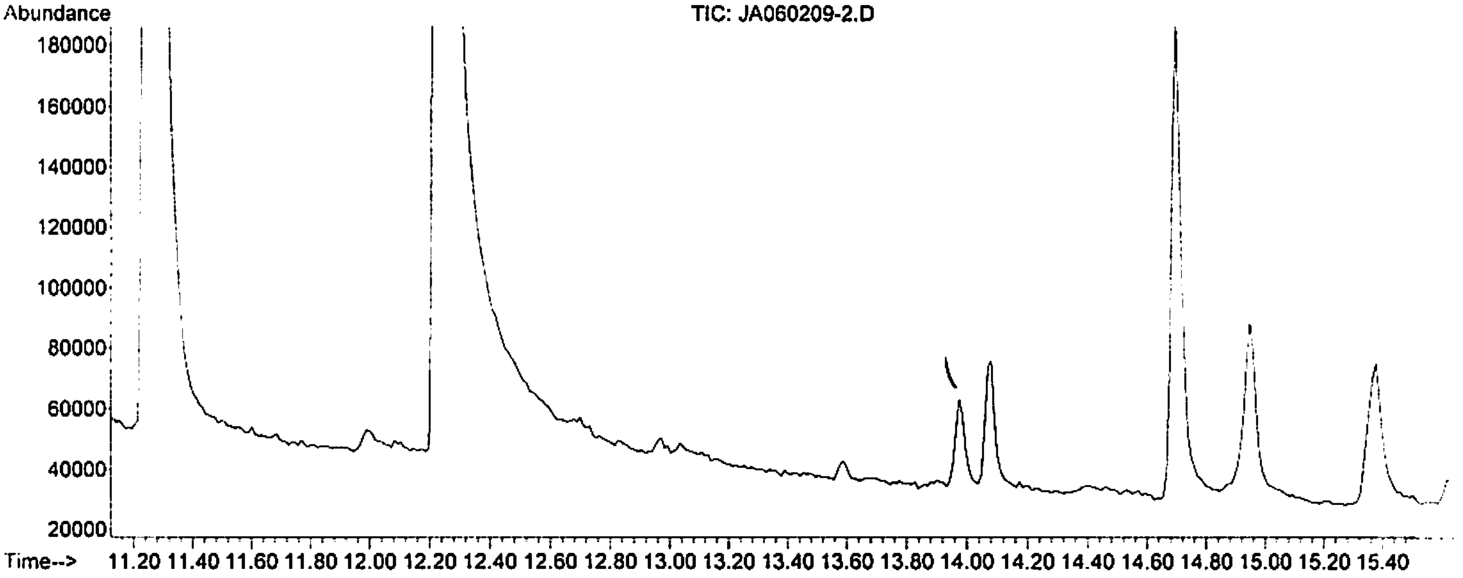

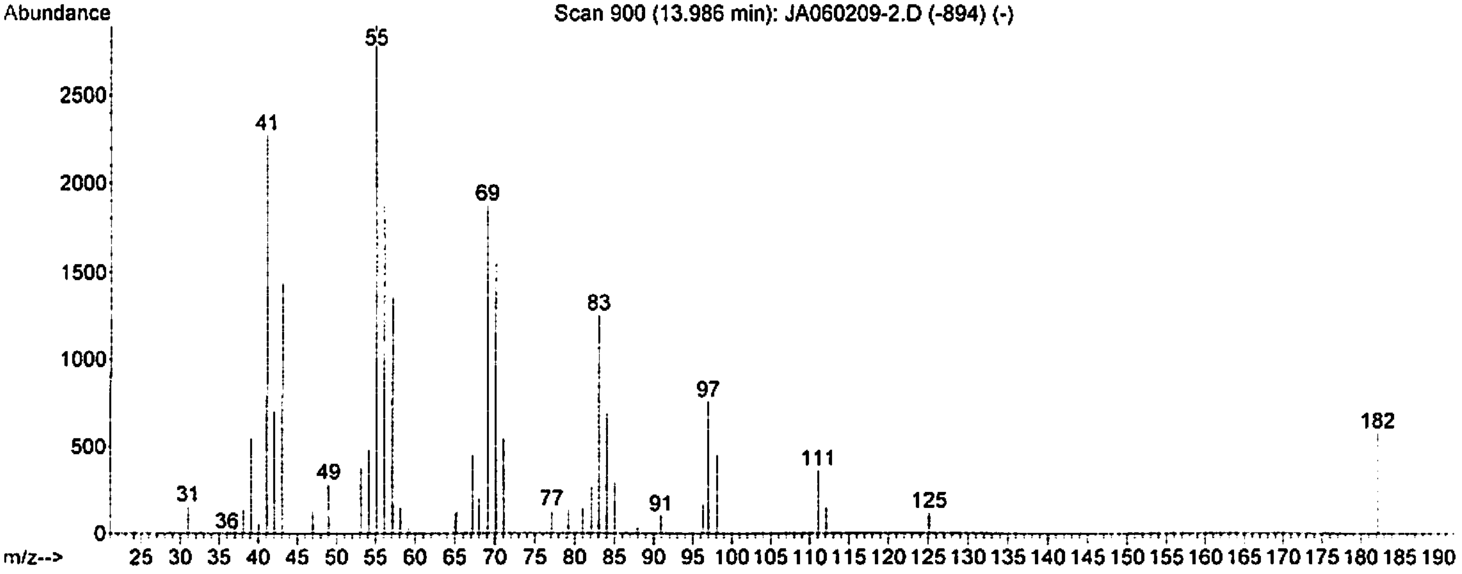

[illegible] :D:\DATA\Aldrich\JA-09\JA060209-2.D
[illegible]rator : Aldrich
[illegible]uired : 2 Jun 2009 11:30 using AcqMethod JA-WAX08.M
[illegible]rument : Instrument #1
[illegible]le Name: 1 field-coll. M C.oculata abd./CH2Cl2
[illegible] Info : coll. 6/1; 2nd of two; top gut full
[illegible] Number: 1

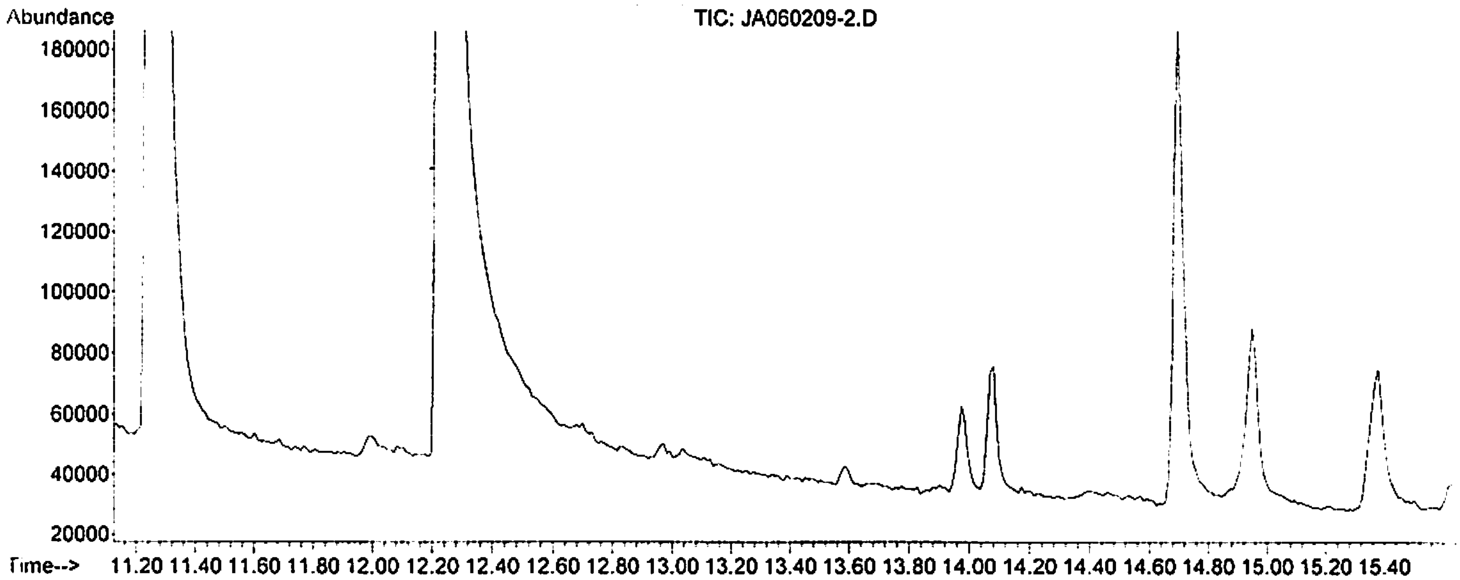

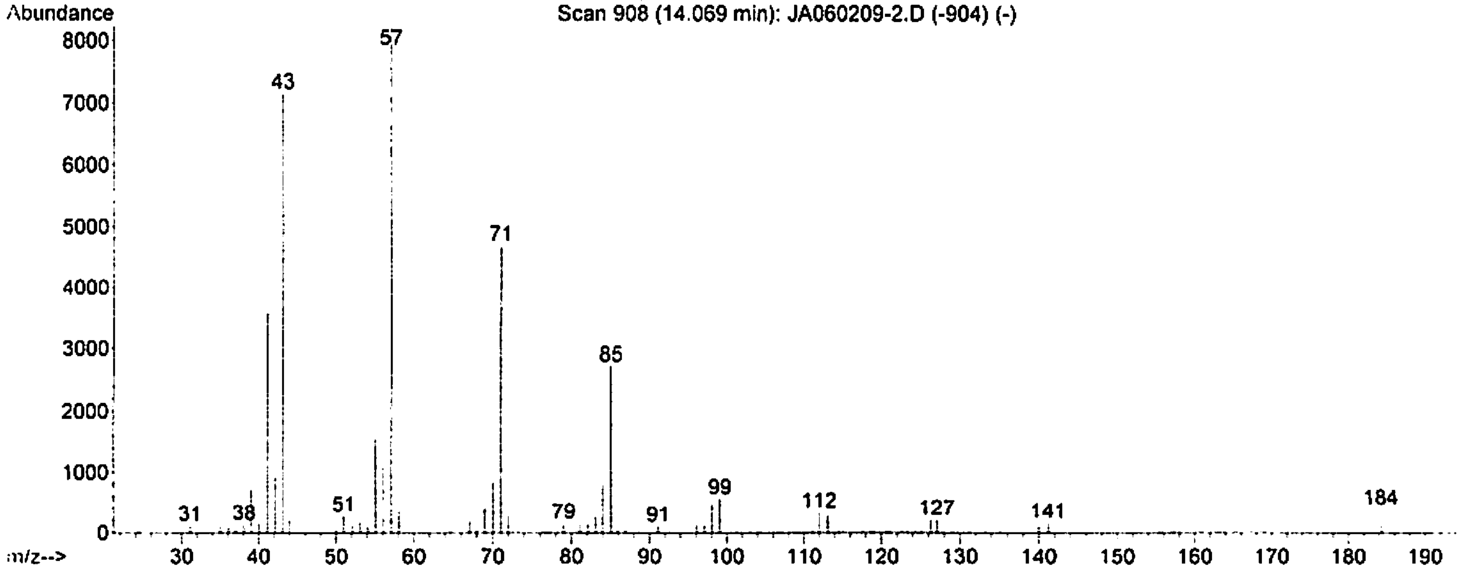

[illegible] :D:\DATA\Aldrich\JA-09\JA060209-2.D
[illegible]rator : Aldrich
[illegible]ired : 2 Jun 2009 11:30 using AcqMethod JA-WAX08.M
[illegible]rument : Instrument #1
[illegible]le Name: 1 field-coll. M C.oculata abd./CH2Cl2
[illegible] Info : coll. 6/1; 2nd of two; top gut full
[illegible] Number: 1

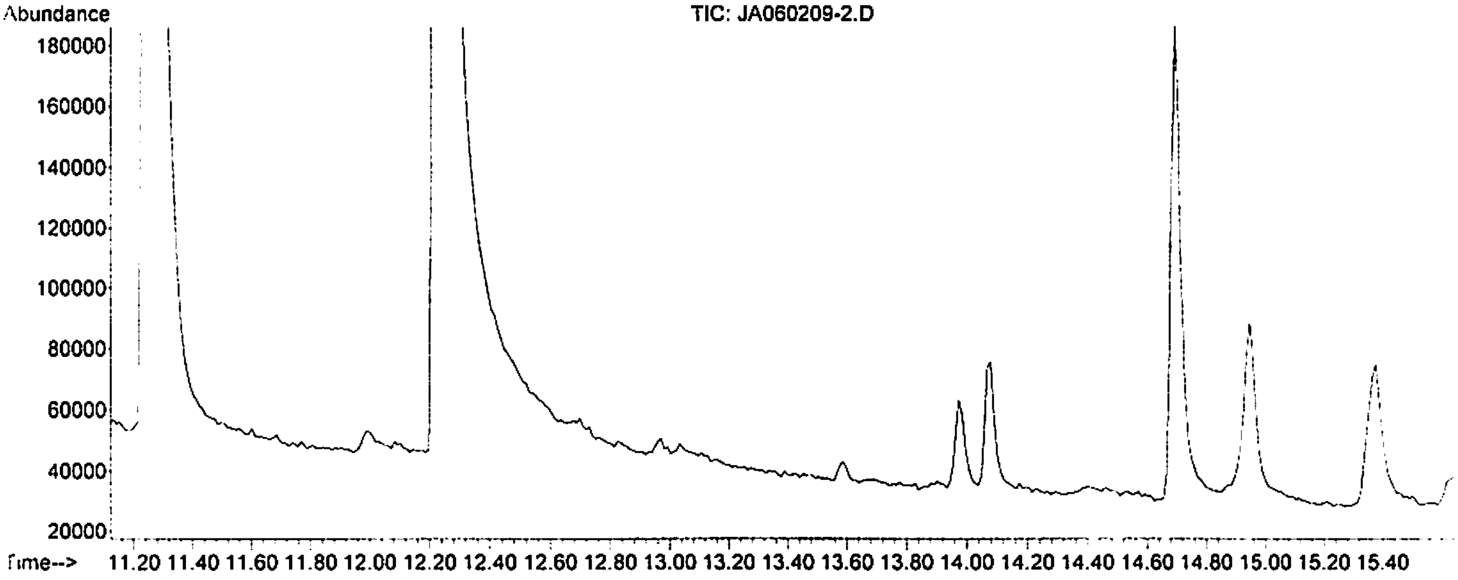

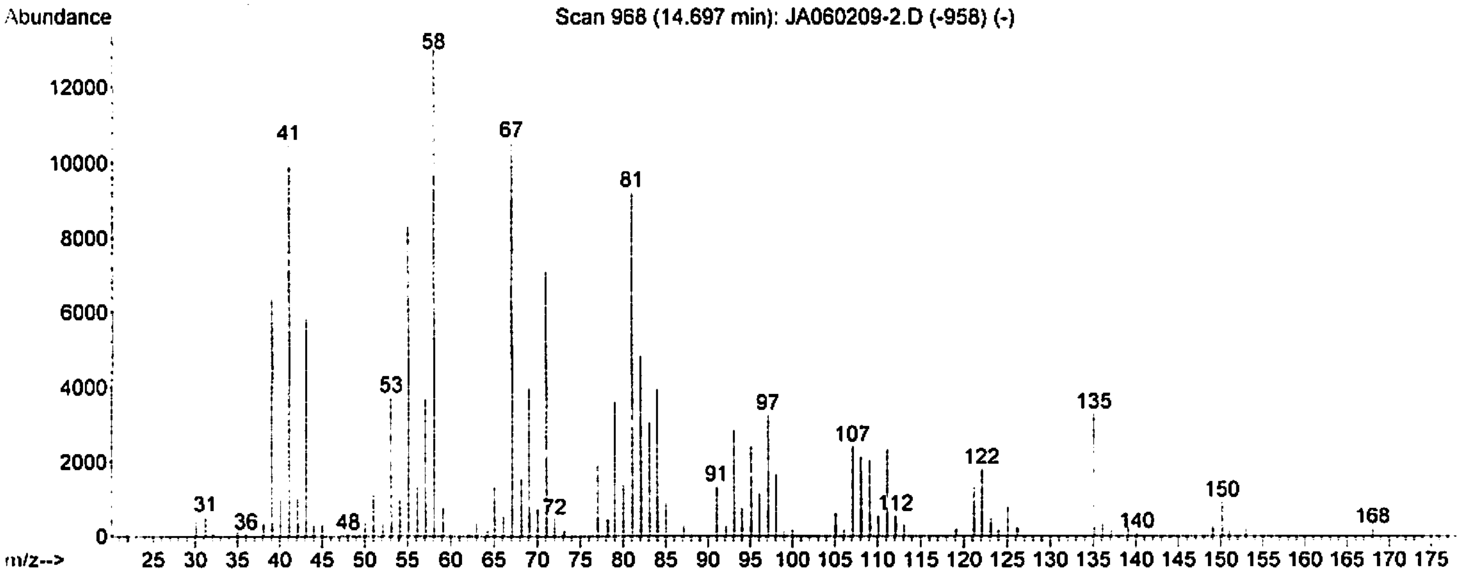

[illegible] :D:\DATA\Aldrich\JA-09\JA060209-2.D
[illegible]ator : Aldrich
[illegible]uired : 2 Jun 2009 11:30 using AcqMethod JA-WAX08.M
[illegible]rument : Instrument #1
[illegible]le Name: 1 field-coll. M C.oculata abd./CH2Cl2
[illegible] Info : coll. 6/1; 2nd of two; top gut full
[illegible] Number: 1

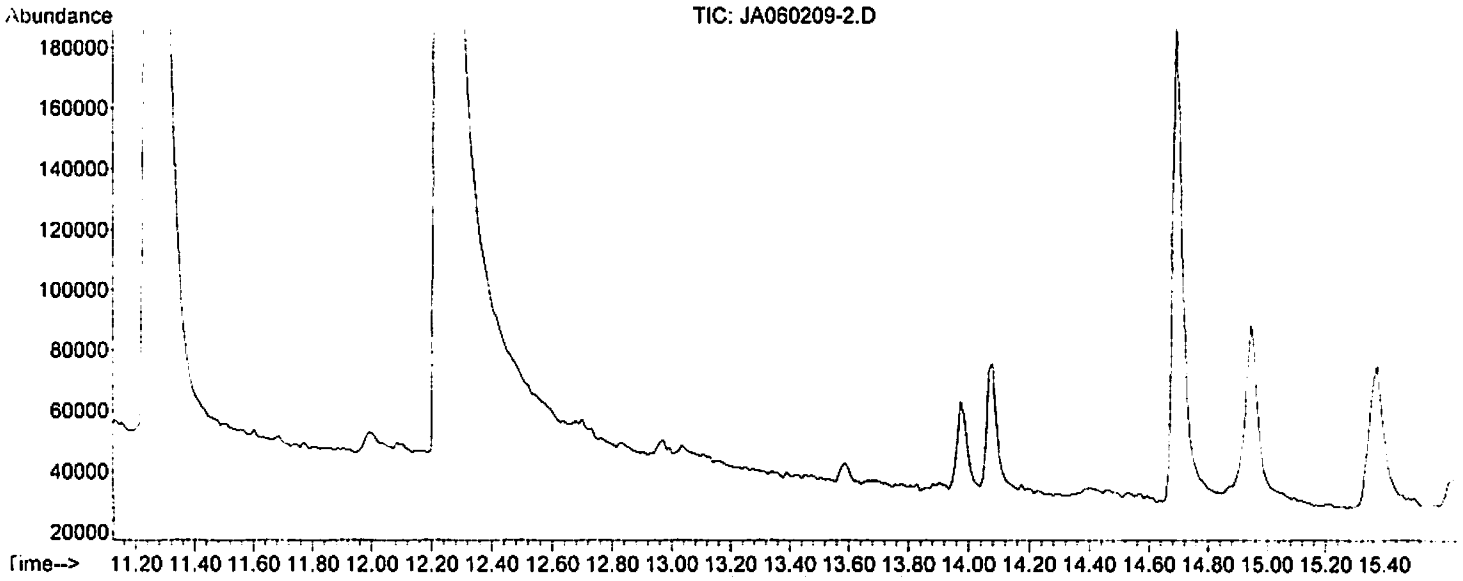

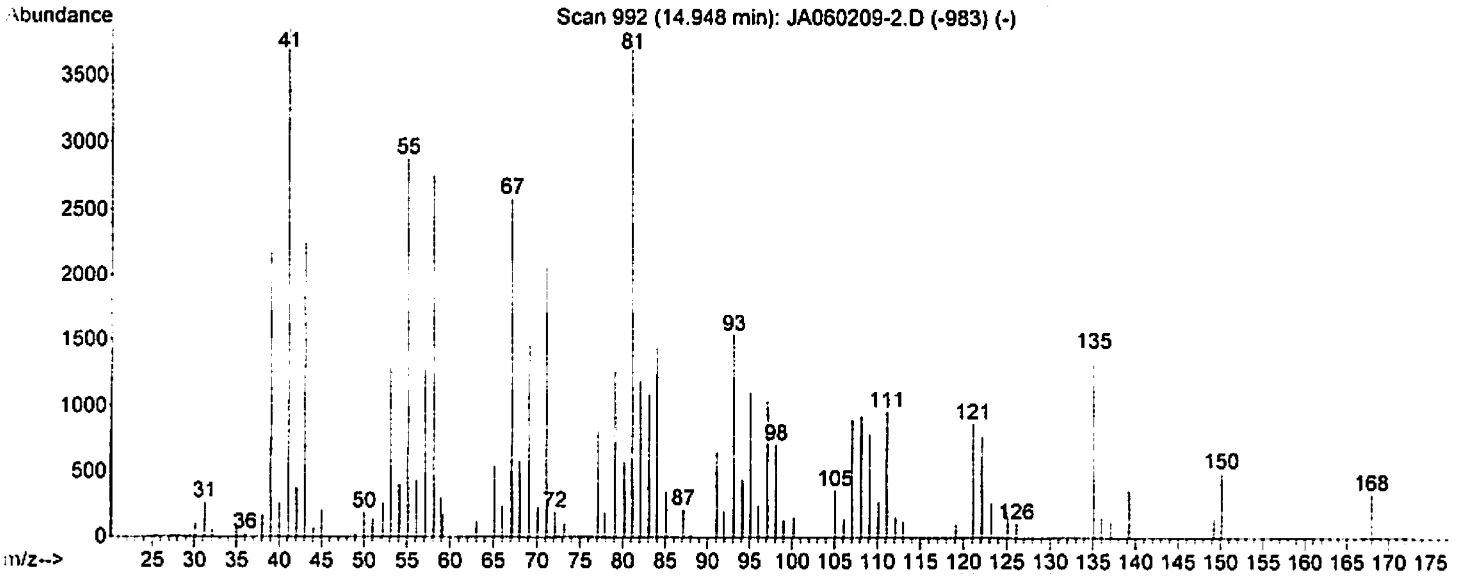

[illegible] :D:\DATA\Aldrich\JA-09\JA060209-2.D
Operator : Aldrich
Acquired : 2 Jun 2009 11:30 using AcqMethod JA-WAX08.M
Instrument : Instrument #1
Sample Name: 1 field-coll. M C.oculata abd./CH2Cl2
Misc Info : coll. 6/1; 2nd of two; top gut full
Vial Number: 1

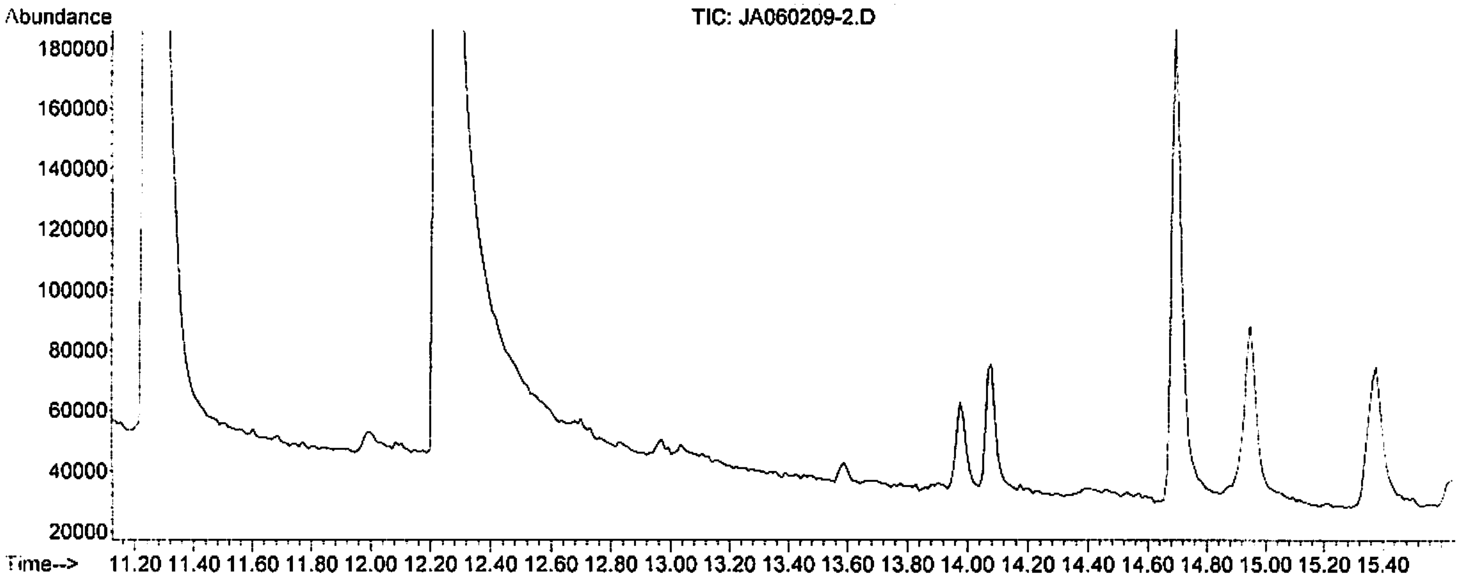

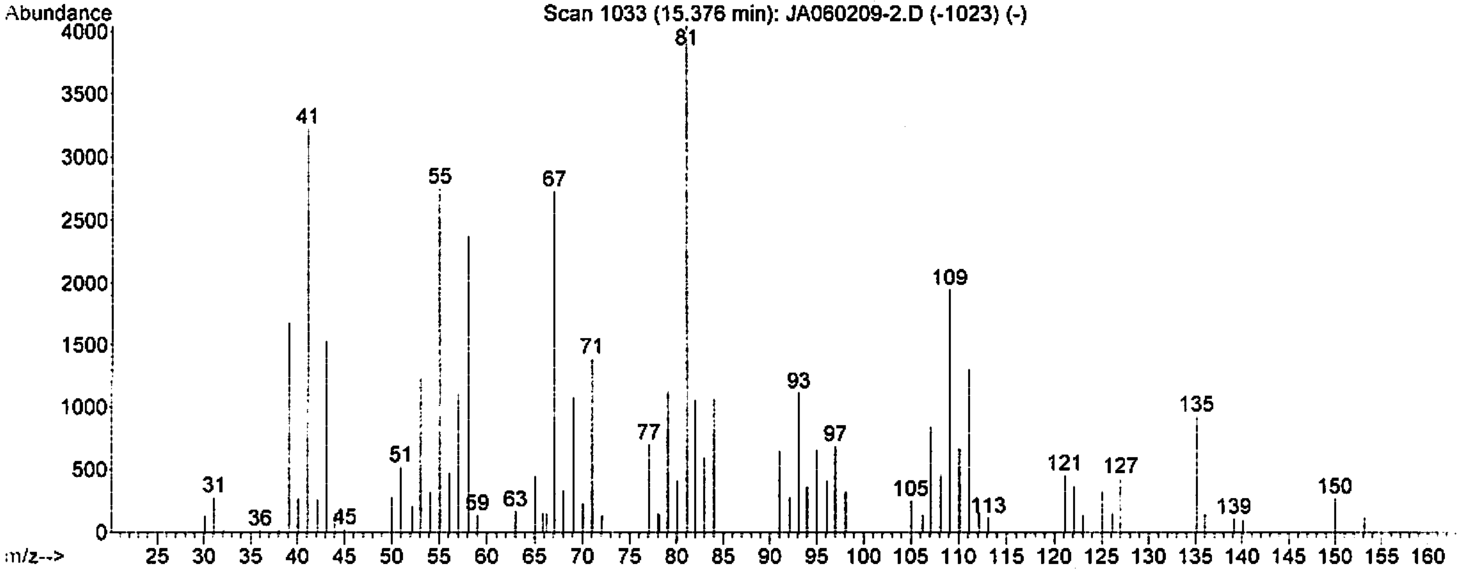

```
File        :D:\DATA\Aldrich\JA-09\JA010509-3.D
Operator    : Aldrich
Acquired    :  5 Jan 2009  15:23     using AcqMethod JA-WAX08.M
Instrument  :   Instrument #1
Sample Name: 5 male C.oculata abd.sternites/CH2Cl2
Misc Info   : honey water only; control for JA010509-2
Vial Number: 1
```

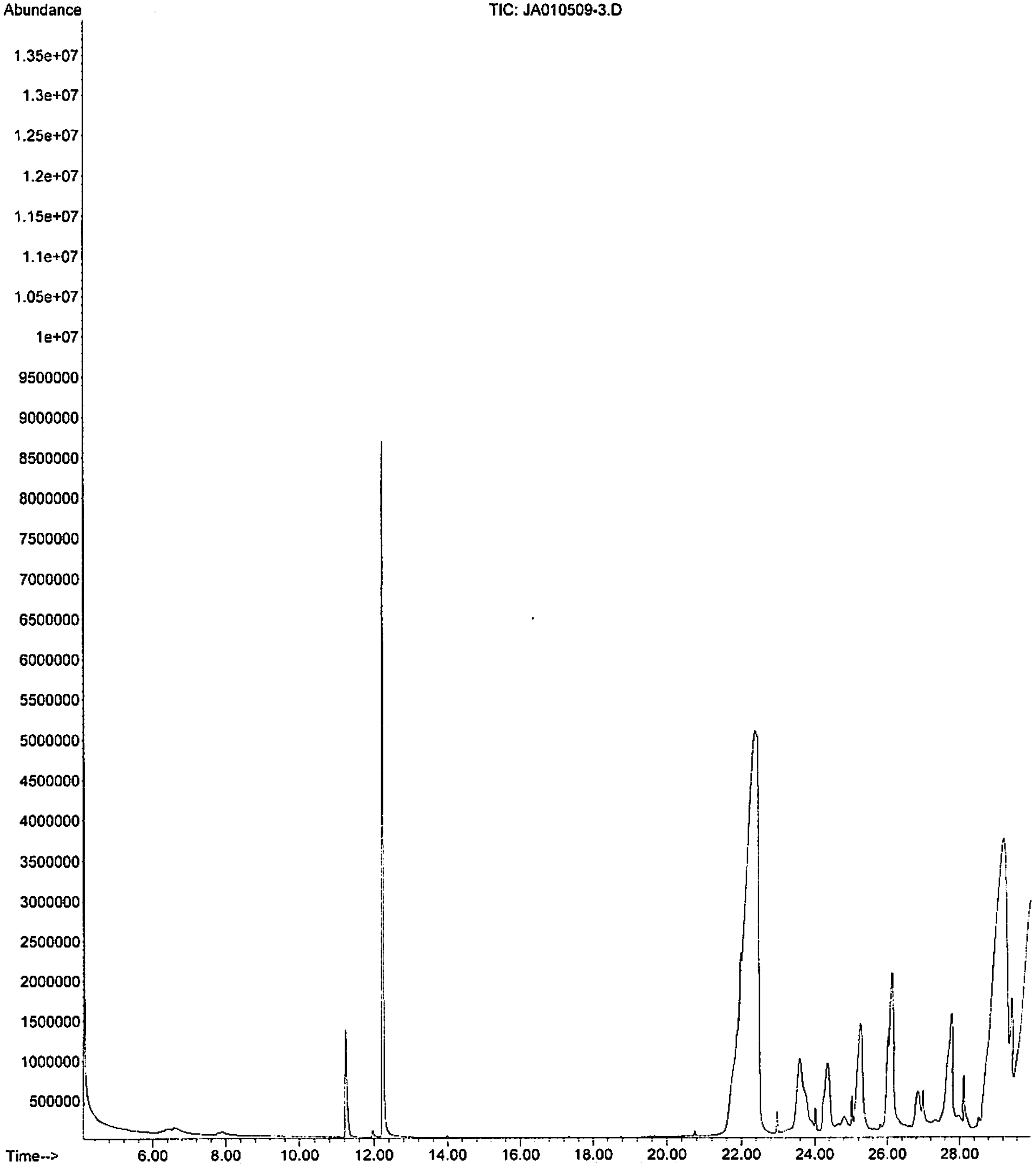

File : D:\DATA\Aldrich\JA-09\JA010509-3.D
Operator : Aldrich
Acquired : 5 Jan 2009 15:23 using AcqMethod JA-WAX08.M
Instrument : Instrument #1
Sample Name: 5 male C.oculata abd.sternites/CH2Cl2
Misc Info : honey water only; control for JA010509-2
Vial Number: 1

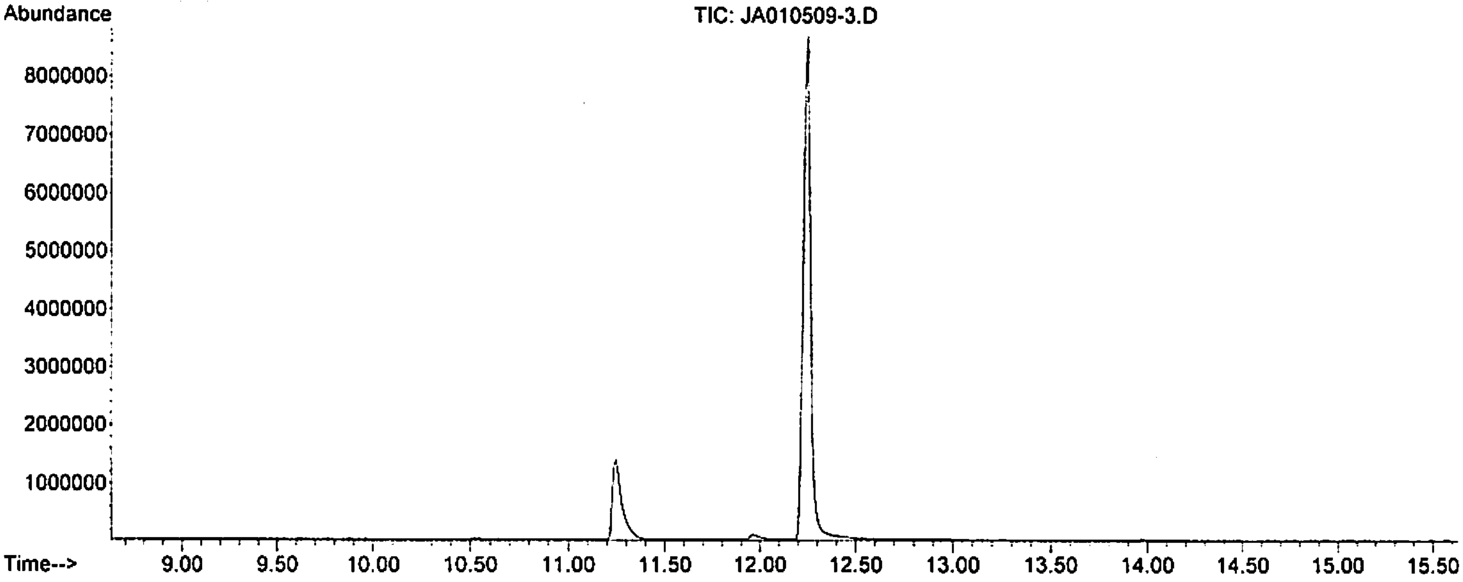

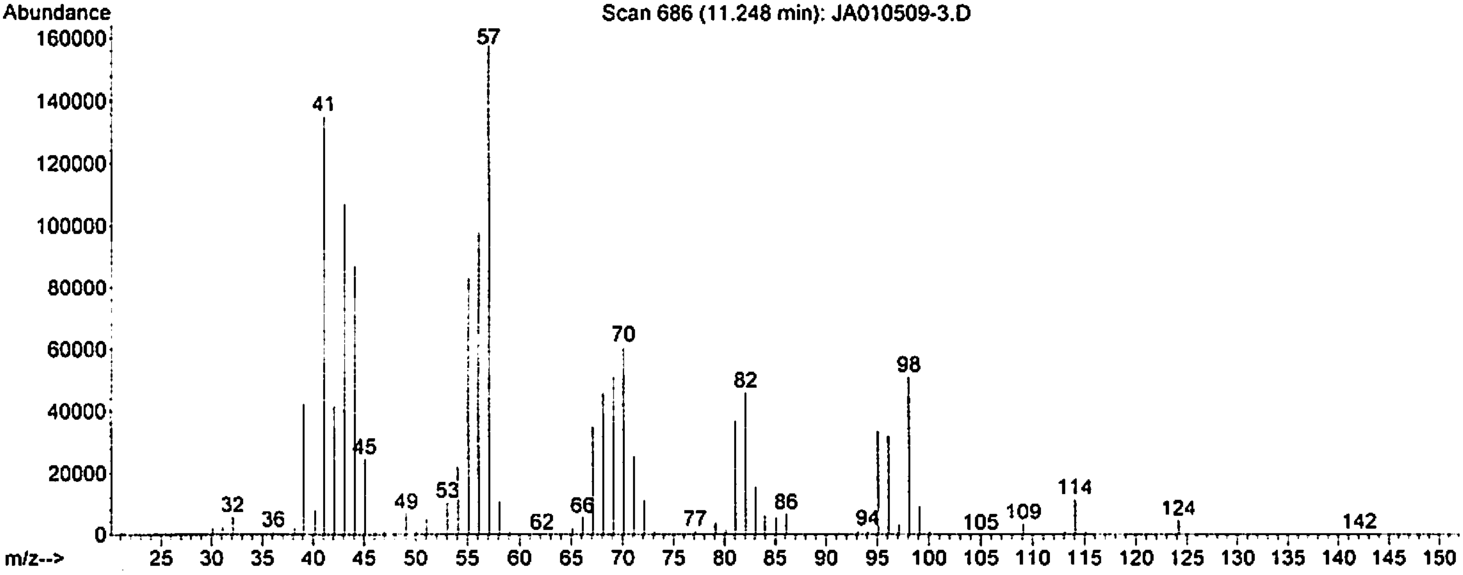

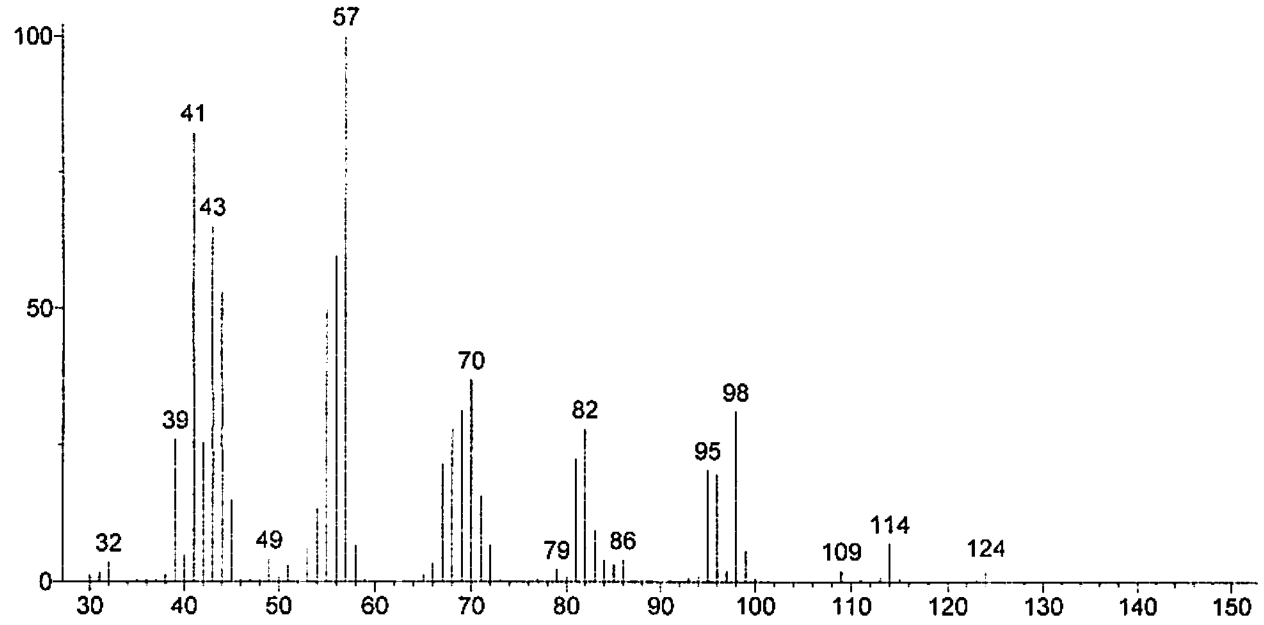

(Text File) Scan 686 (11.248 min): JA010509-3.D

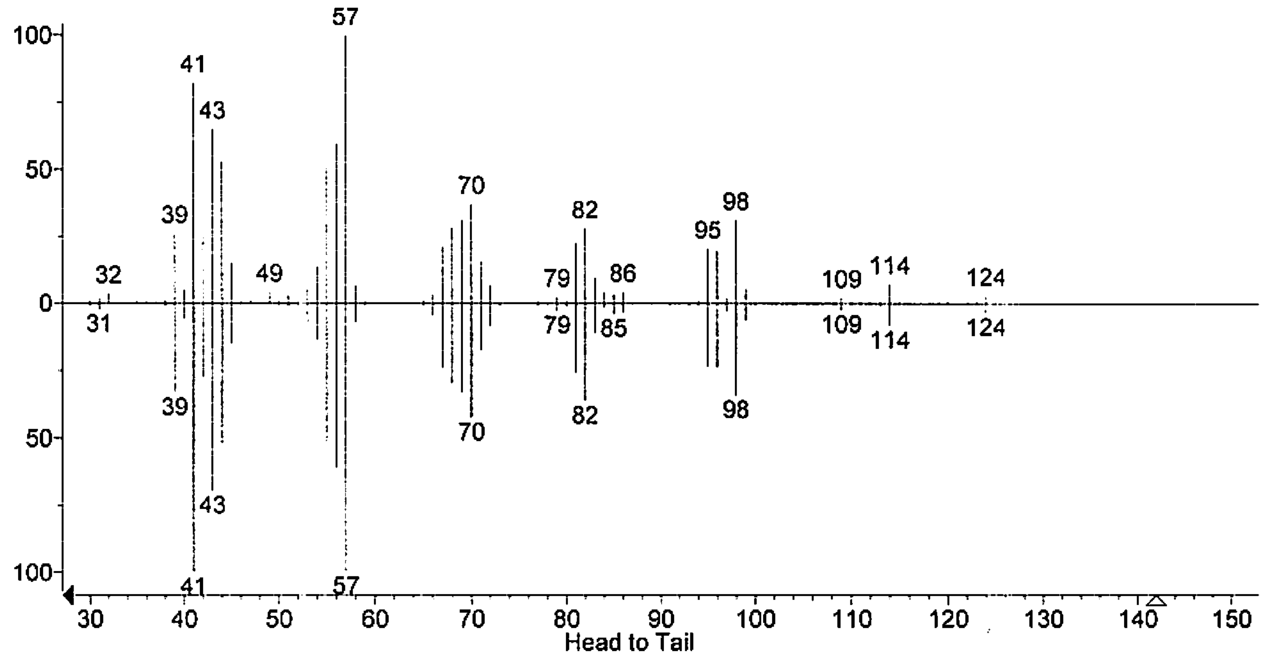

Head to Tail

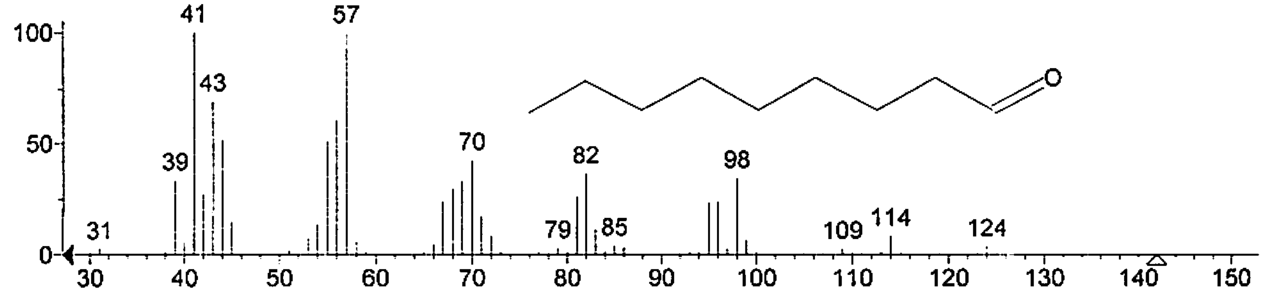

(replib) Nonanal

File : D:\DATA\Aldrich\JA-09\JA010509-3.D
Operator : Aldrich
Acquired : 5 Jan 2009 15:23 using AcqMethod JA-WAX08.M
Instrument : Instrument #1
Sample Name: 5 male C.oculata abd.sternites/CH2Cl2
Misc Info : honey water only; control for JA010509-2
Vial Number: 1

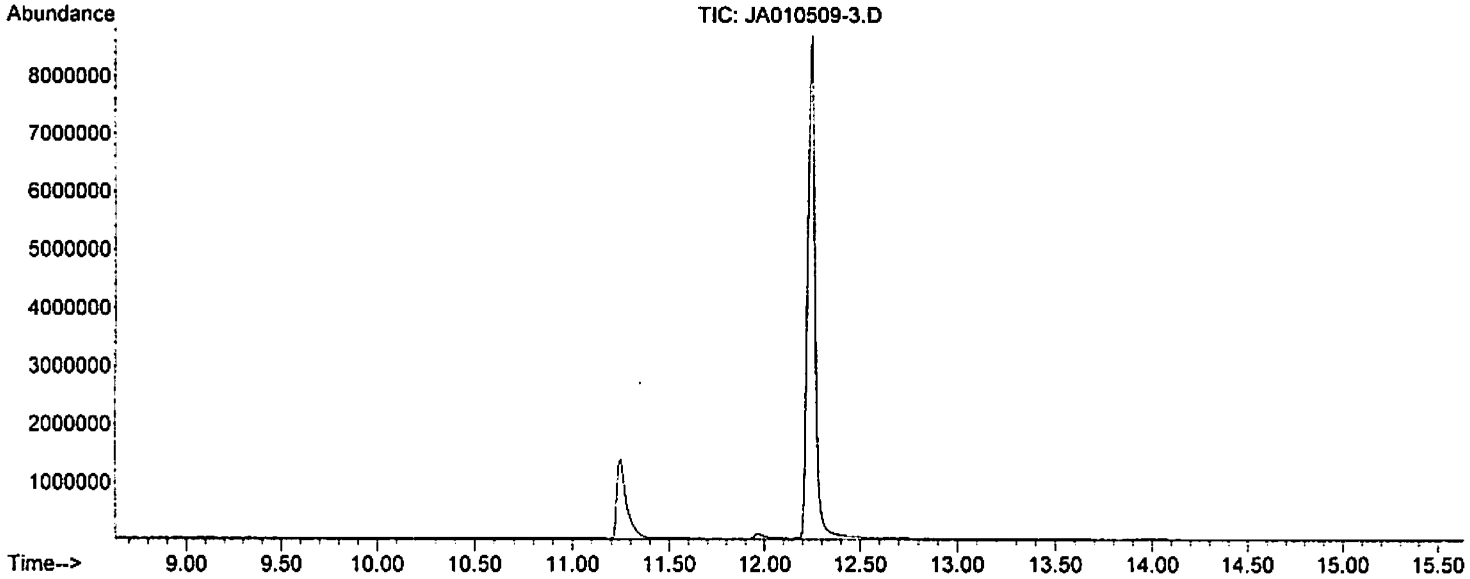

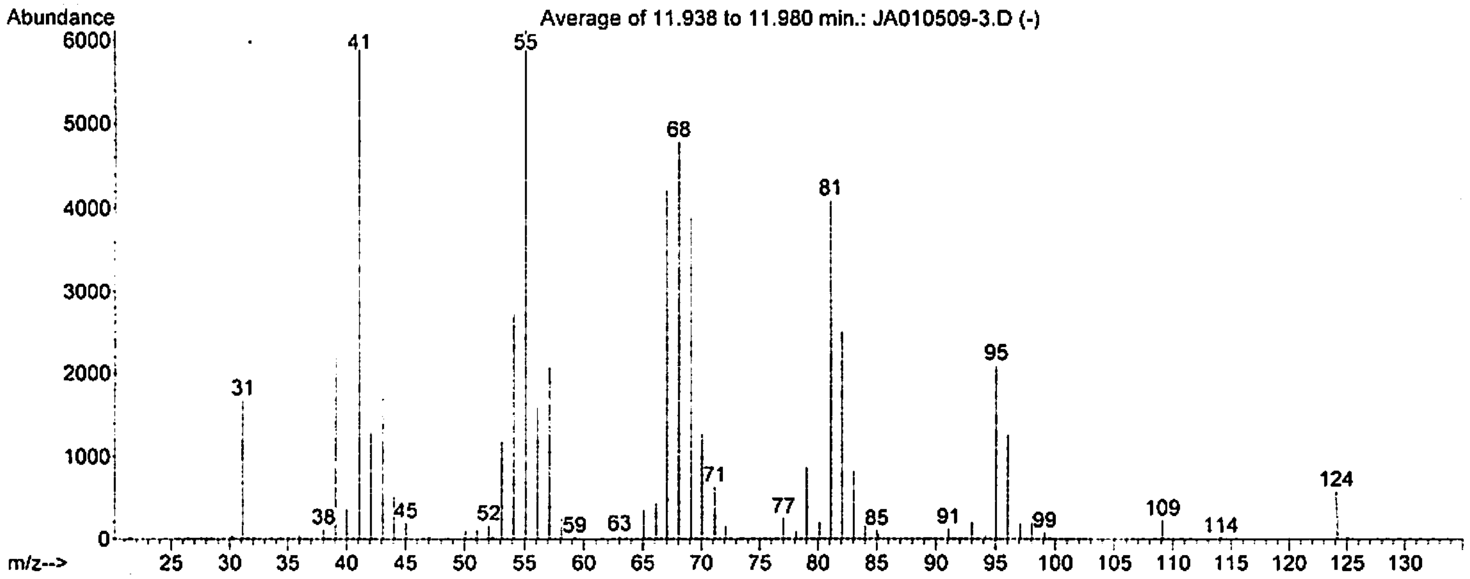

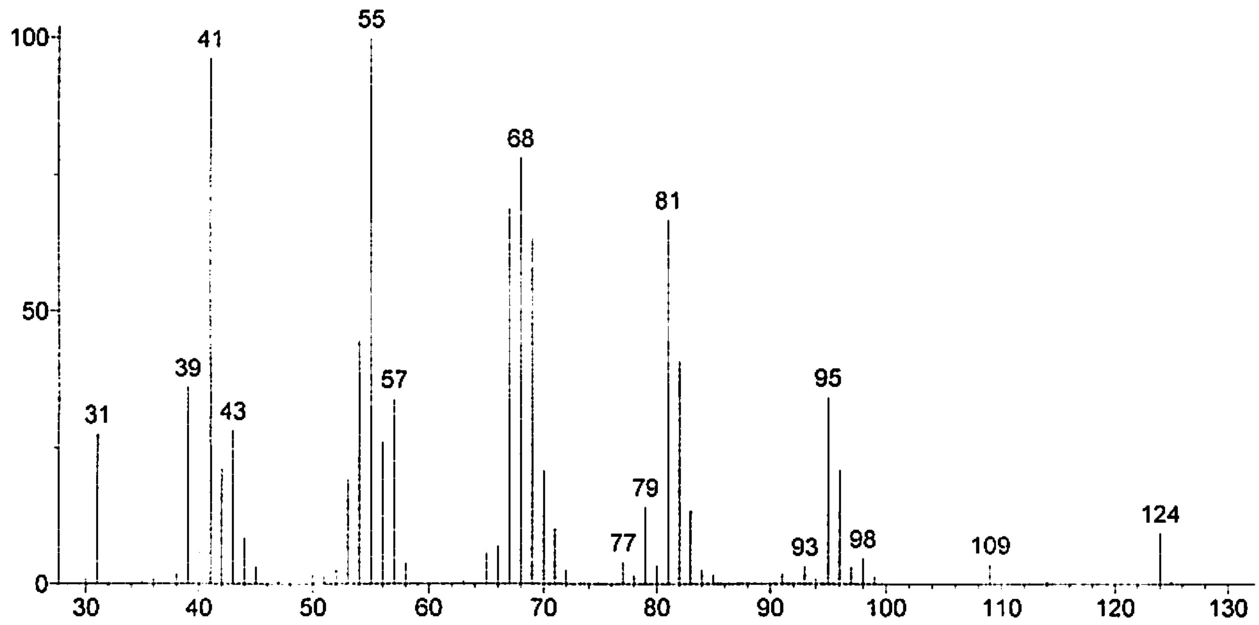

(Text File) Average of 11.938 to 11.980 min.: JA010509-3.D

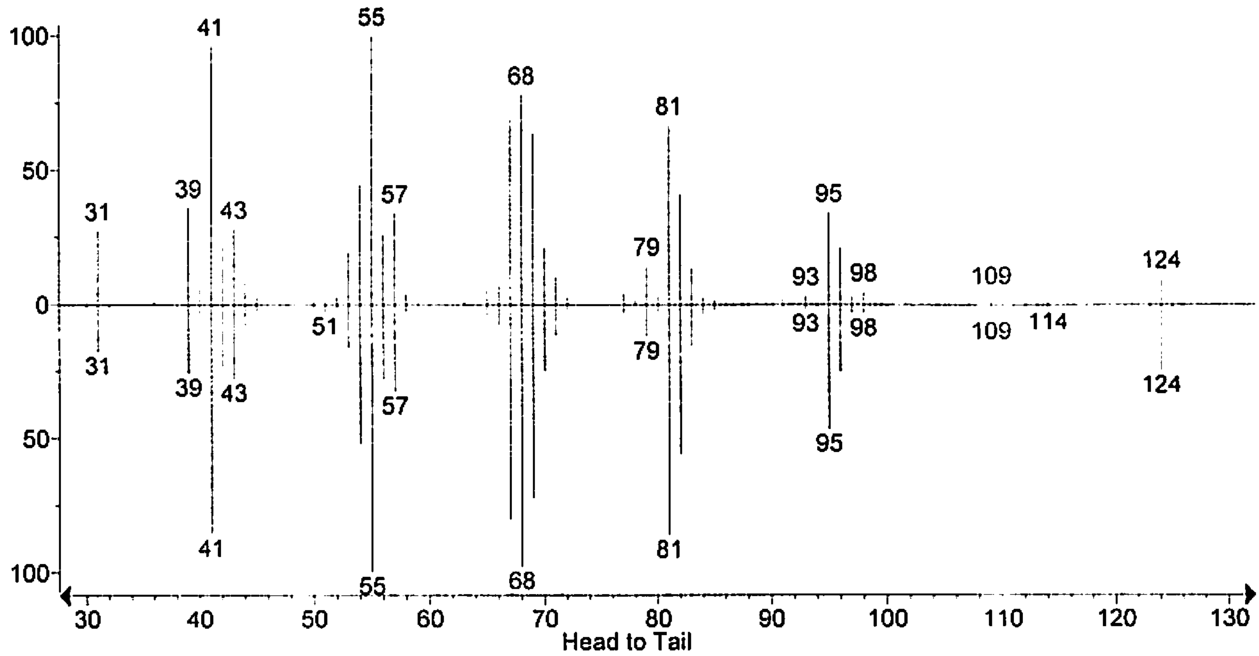

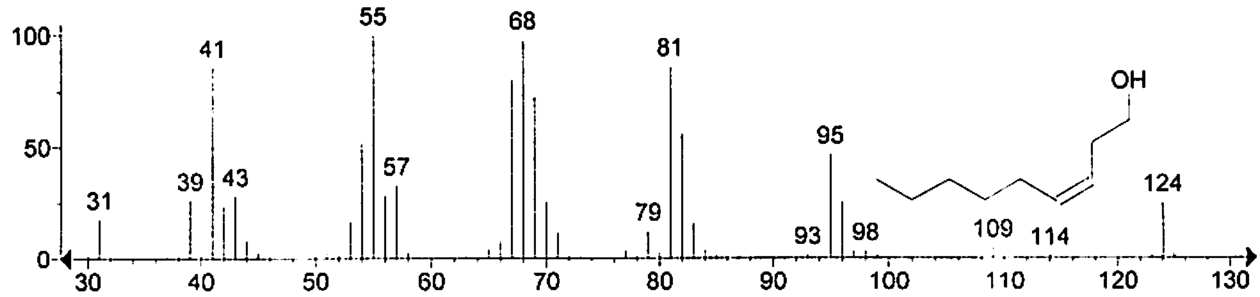

(mainlib) 3-Nonen-1-ol, (Z)-

```
File     :D:\DATA\Aldrich\JA-09\JA010509-3.D
Operator : Aldrich
Acquired :  5 Jan 2009  15:23    using AcqMethod JA-WAX08.M
Instrument :    Instrument #1
Sample Name: 5 male C.oculata abd.sternites/CH2Cl2
Misc Info  : honey water only; control for JA010509-2
Vial Number: 1
```

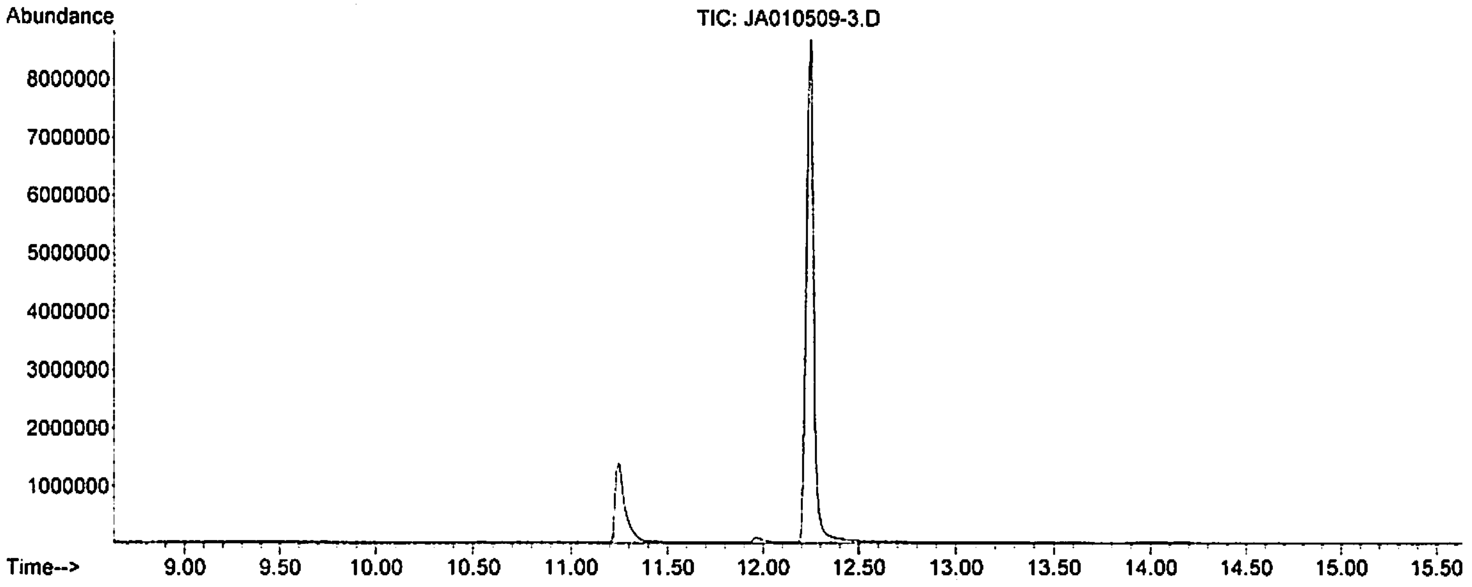

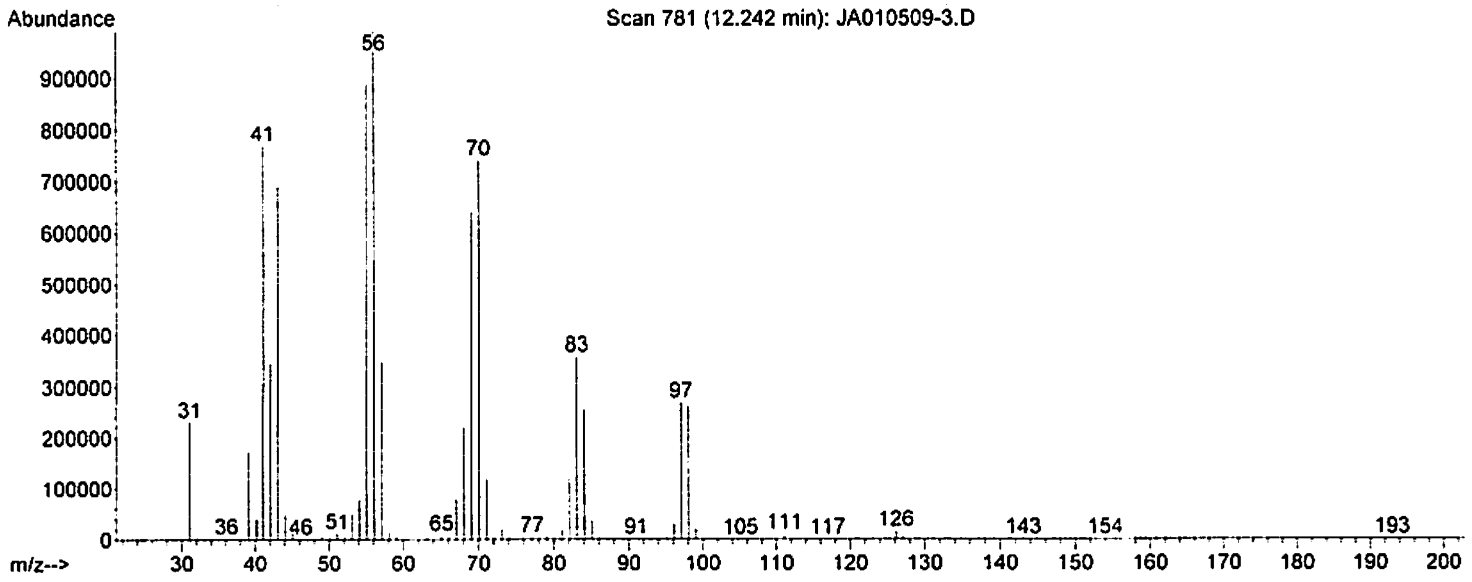

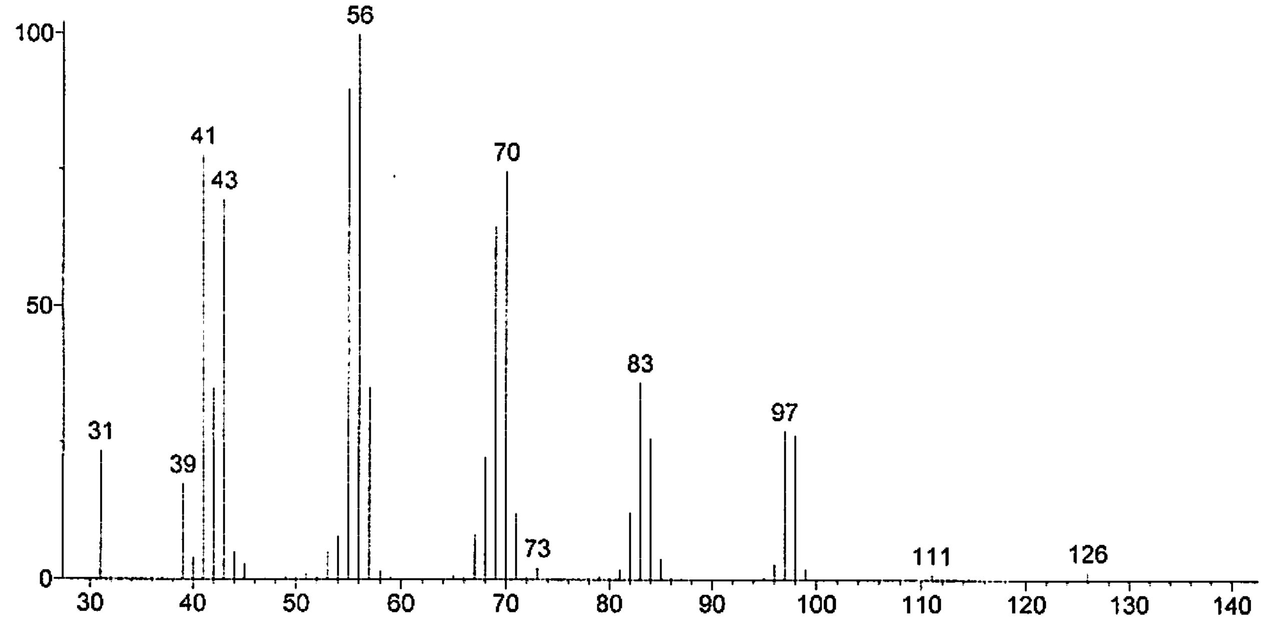

(Text File) Scan 781 (12.242 min): JA010509-3.D

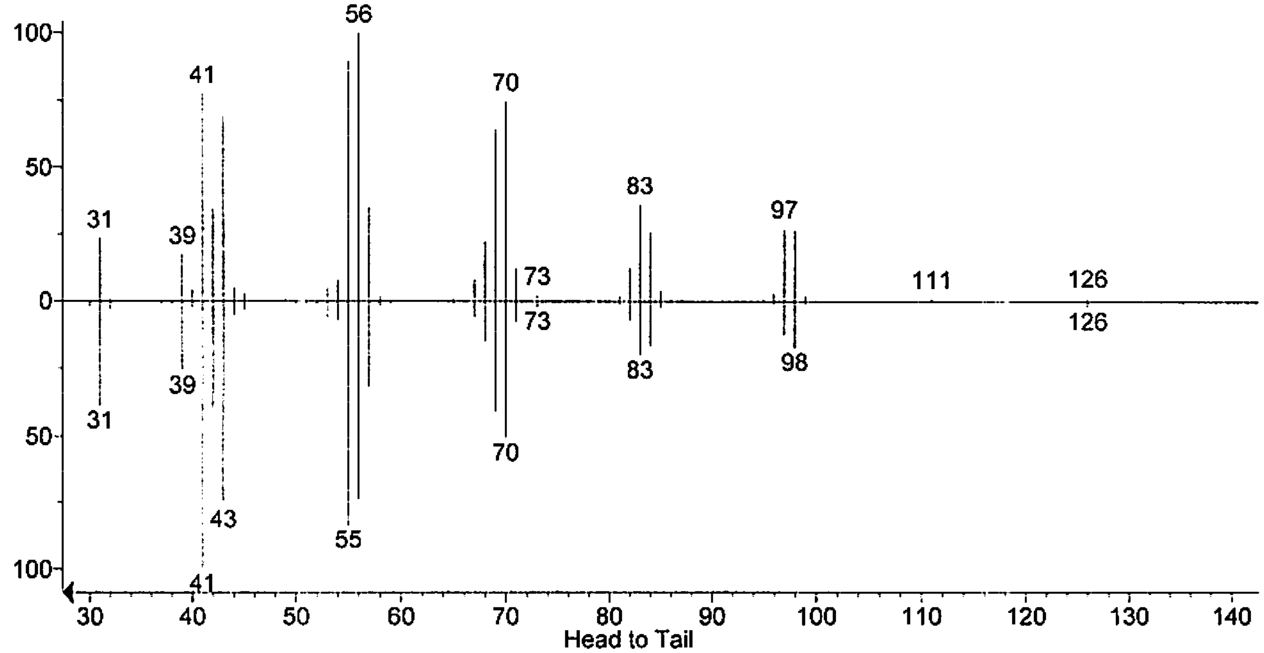

Head to Tail

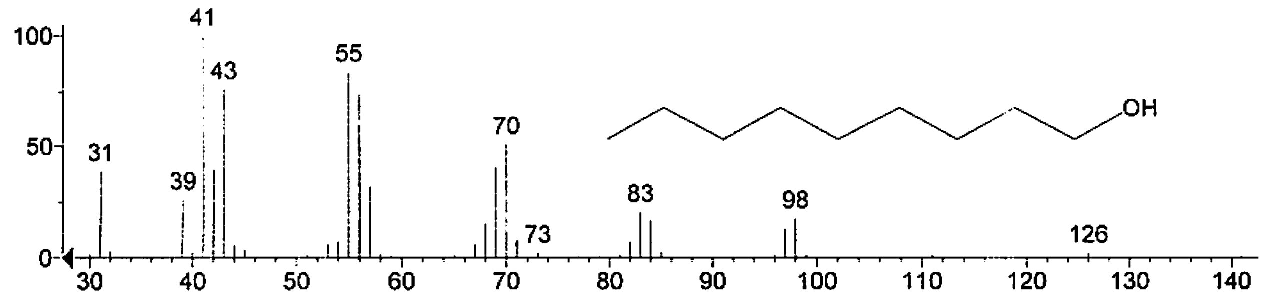

(replib) 1-Nonanol

```
File          :D:\DATA\Aldrich\JA-09\JA010509-3.D
Operator      : Aldrich
Acquired      :  5 Jan 2009  15:23     using AcqMethod JA-WAX08.M
Instrument    :    Instrument #1
Sample Name: 5 male C.oculata abd.sternites/CH2Cl2
Misc Info     : honey water only; control for JA010509-2
Vial Number: 1
```

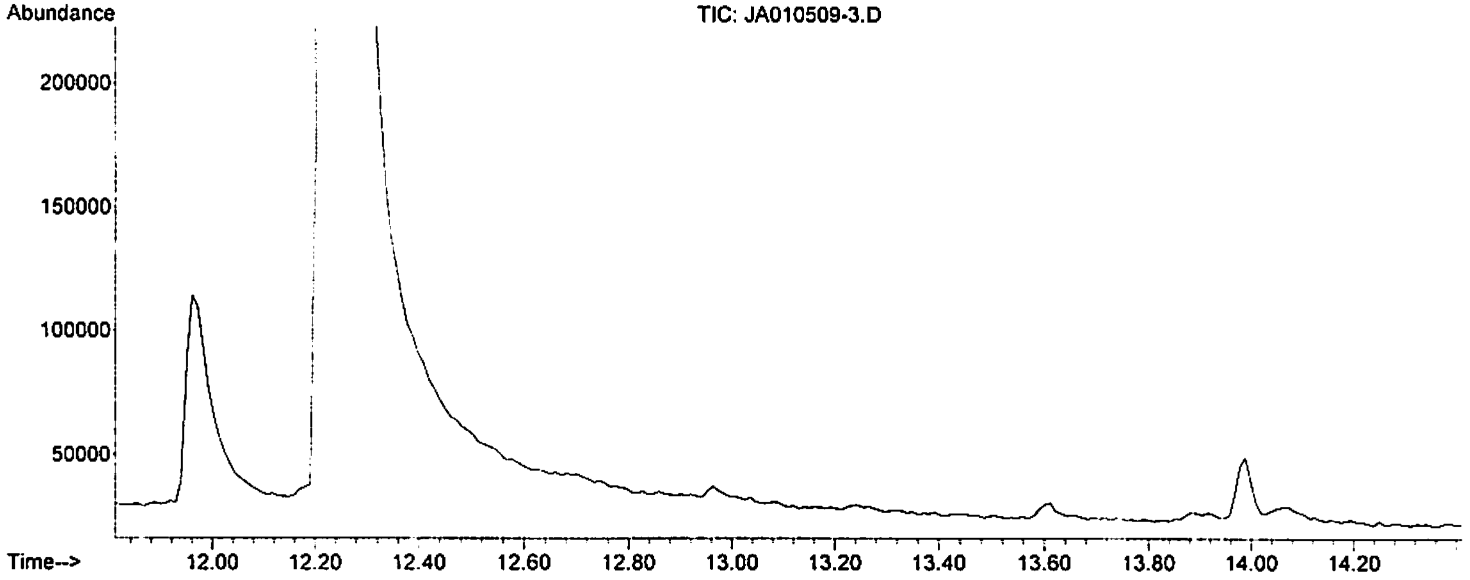

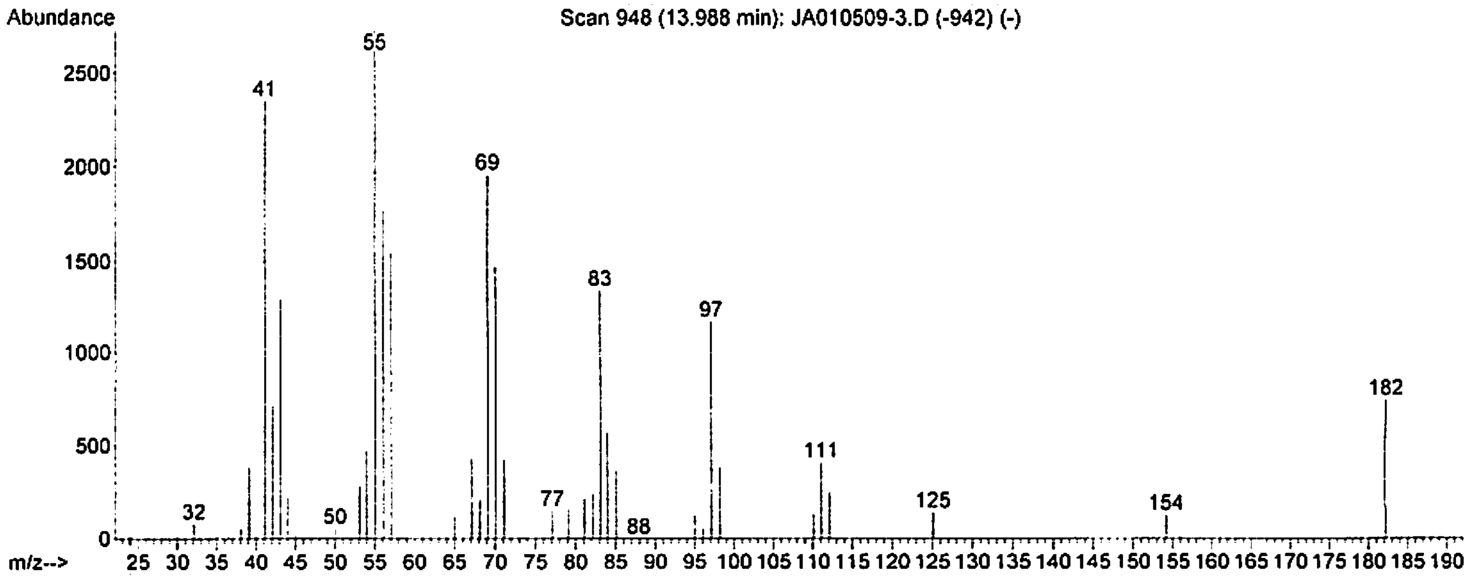

inj. 1.0 ul abdominal cuticle extract of 8 lab-reared C
hrysopa oculata males (3-8 days old, fed aphids; /ca. 4
0ul conc. to 10 ul CH2Cl2), Kamal's HP-5.

===

Injection Date : 6/27/2008 12:13:31 PM
Sample Name : 8M C.ocu. lab-ab Location : -
Acq. Operator : Aldrich Inj : 1
Inj Volume : Manually
Method : C:\HPCHEM\1\METHODS\DBLESS08.M
Last changed : 6/12/2008 10:49:14 AM by Aldrich
6/11/08; editing new method for desired output

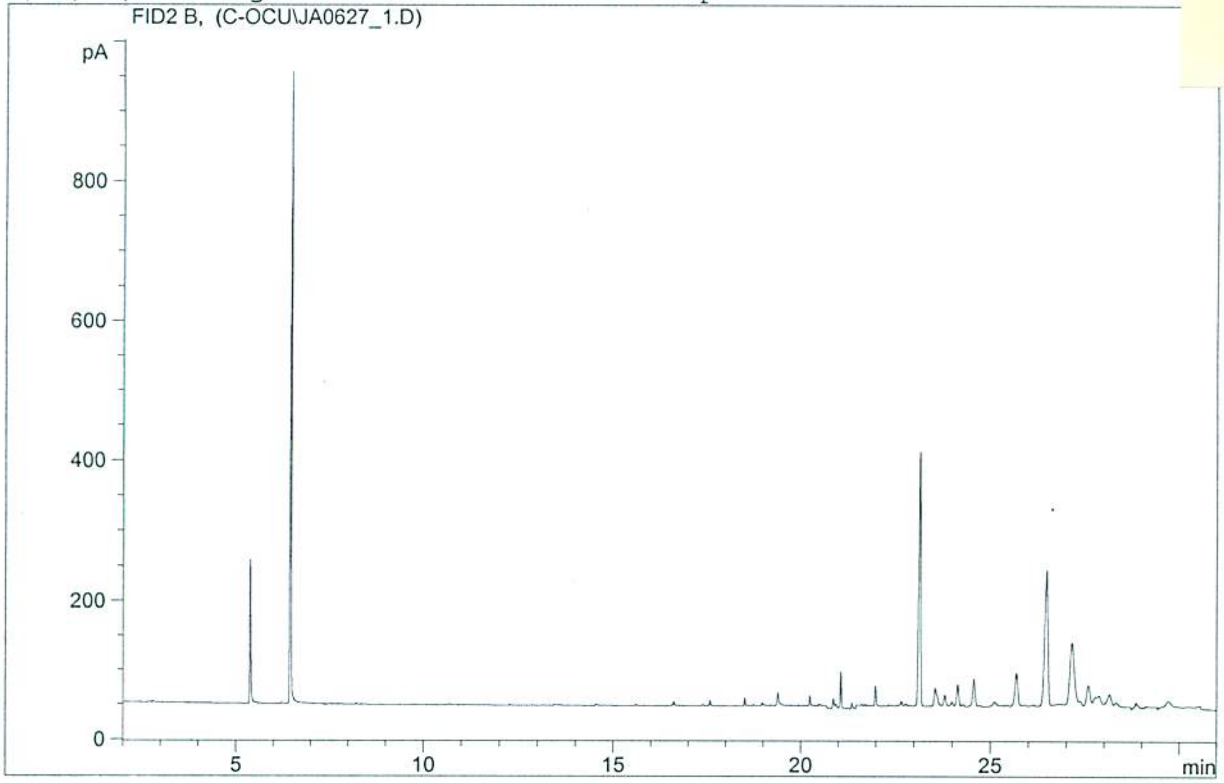

===

Area Percent Report

===

Sorted By : Signal
Multiplier : 1.0000
Dilution : 1.0000

Signal 1: FID2 B,

Peak #	RetTime [min]	Type	Width [min]	Area [pA*s]	Height [pA]	Area %
1	0.468	PP	0.0153	19.08689	16.70625	0.00068
2	0.536	VV S	0.0130	1.48424e5	1.73817e5	5.26365
3	0.587	VV S	0.1042	2.64396e6	4.22708e5	93.76427
4	1.020	VV S	0.1350	1.60639e4	1983.48303	0.56968
5	1.248	VV S	0.0566	1895.44934	558.52875	0.06722
6	1.317	VV S	0.0457	1043.36963	380.88409	0.03700
7	1.378	VB S	0.0554	800.23853	240.84387	0.02838
8	1.633	BV	0.0308	5.47331	2.68390	0.00019
9	1.730	VV	0.0541	19.75575	4.88995	0.00070
10	1.836	VB	0.0374	9.93249	3.94312	0.00035
11	5.378	PB	0.0319	422.01617	206.22372	0.01497
12	6.471	BB	0.0317	1992.33899	904.04736	0.07066
13	8.227	BP	0.0328	3.16840	1.55117	0.00011

Peak #	RetTime [min]	Type	Width [min]	Area [pA*s]	Height [pA]	Area %
14	14.544	PP	0.0373	4.96667	1.97668	0.00018
15	15.599	PB	0.0332	4.52217	2.09559	0.00016
16	16.608	BB	0.0364	15.92690	6.11684	0.00056
17	17.374	PB	0.0345	4.22127	1.79359	0.00015
18	17.574	BB	0.0324	16.02617	7.66727	0.00057
19	18.499	BP	0.0305	22.61930	11.72717	0.00080
20	18.966	PB	0.0425	10.40165	3.85482	0.00037
21	19.385	PB	0.0451	59.36433	18.67942	0.00211
22	20.245	PP	0.0311	29.33456	14.79286	0.00104
23	20.488	PB	0.0390	5.92741	2.30839	0.00021
24	20.868	PV	0.0365	35.49558	14.01465	0.00126
25	20.938	VV	0.0430	18.74686	6.24640	0.00066
26	21.069	VP	0.0338	111.73968	52.65177	0.00396
27	21.362	PP	0.0356	17.95033	7.86454	0.00064
28	21.622	BV	0.0566	15.92291	3.90649	0.00056
29	21.716	VB	0.0562	10.64588	2.68697	0.00038
30	21.987	BB	0.0385	72.99472	28.87706	0.00259
31	22.670	BB	0.0456	17.96630	6.06047	0.00064
32	22.789	BP	0.0424	6.77294	2.68524	0.00024
33	23.158	BB	0.0503	1290.22253	364.34610	0.04576
34	23.558	BB	0.0753	136.87875	25.24579	0.00485
35	23.812	PB	0.0537	56.08517	15.34171	0.00199
36	23.995	BV	0.0596	21.83828	5.58980	0.00077
37	24.151	VV	0.0602	122.58260	30.96257	0.00435
38	24.575	BP	0.0628	158.11914	38.61266	0.00561
39	25.113	BB	0.0788	36.01327	6.20126	0.00128
40	25.690	BP	0.0831	245.88568	46.93348	0.00872
41	26.486	BP	0.0852	1139.94482	194.97992	0.04043
42	27.157	BP	0.1146	776.19067	89.98493	0.02753
43	27.589	BV	0.0903	185.88461	29.59532	0.00659
44	27.869	VV	0.1502	175.78506	14.73474	0.00623
45	28.151	VV	0.0997	142.76718	17.40252	0.00506
46	28.316	VB	0.0908	39.83866	5.47827	0.00141
47	28.863	PB	0.0662	41.06844	7.70412	0.00146
48	29.724	BB	0.1280	85.34355	8.44015	0.00303
Totals :				2.81979e6	6.01926e5	

Results obtained with enhanced integrator!

==

*** End of Report ***

```
File        :D:\ALDRICH\Snapshot\JA062708-2.D
Operator    : Aldrich
Acquired    : 27 Jun 2008  12:39     using AcqMethod JA-50-280LESS.M
Instrument  :   Buba
Sample Name: 8 lab-reared C.oculata male abdomen/CH2Cl2
Misc Info   : GC run JA0627_1.D;fed aphids, 3-8 days old
Vial Number: 1
```

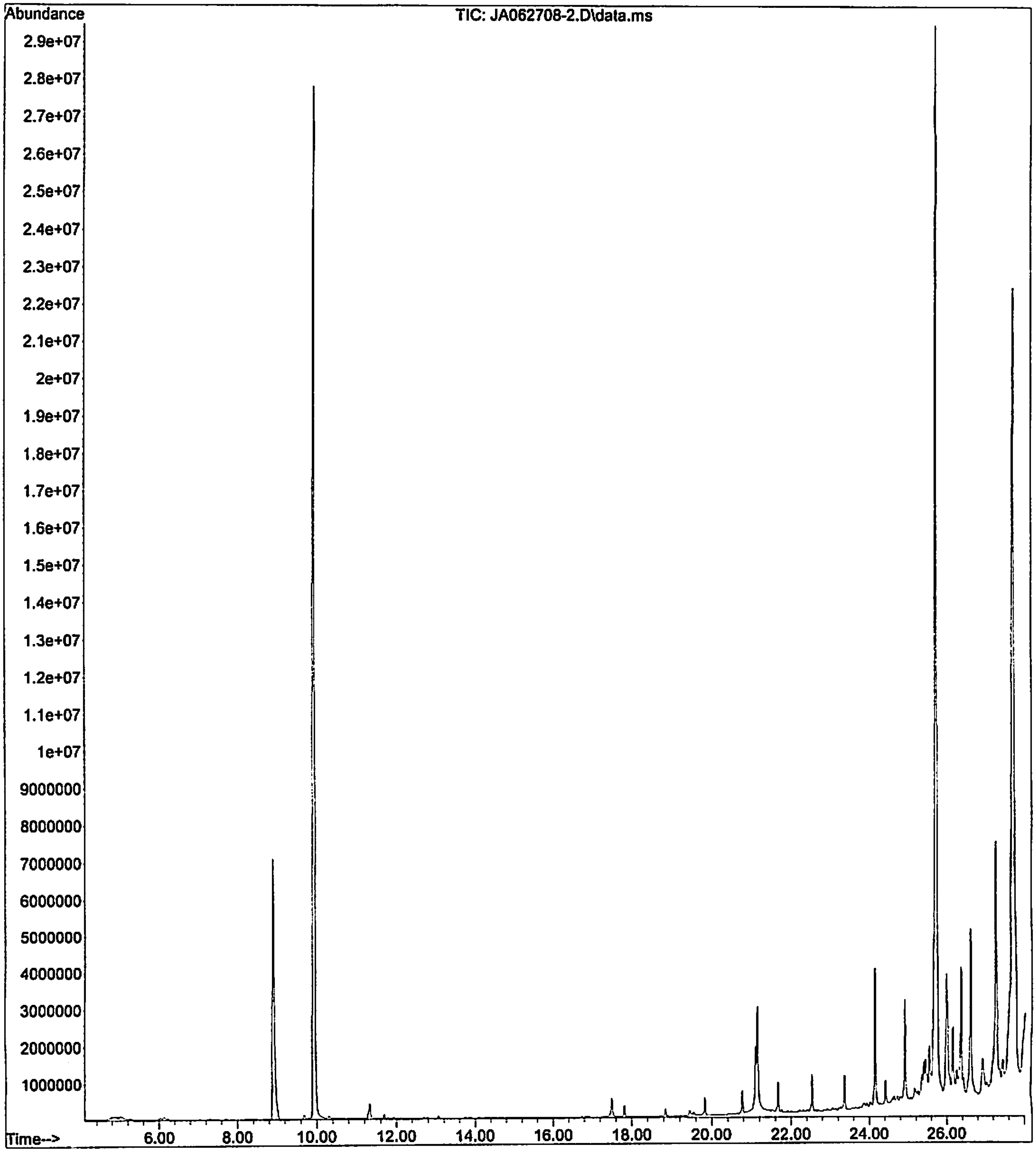

```
File         :D:\ALDRICH\Snapshot\JA062708-2.D
Operator     : Aldrich
Acquired     : 27 Jun 2008  12:39     using AcqMethod JA-50-280LESS.M
Instrument :    Buba
Sample Name: 8 lab-reared C.oculata male abdomen/CH2Cl2
Misc Info  : GC run JA0627_1.D;fed aphids, 3-8 days old
Vial Number: 1
```

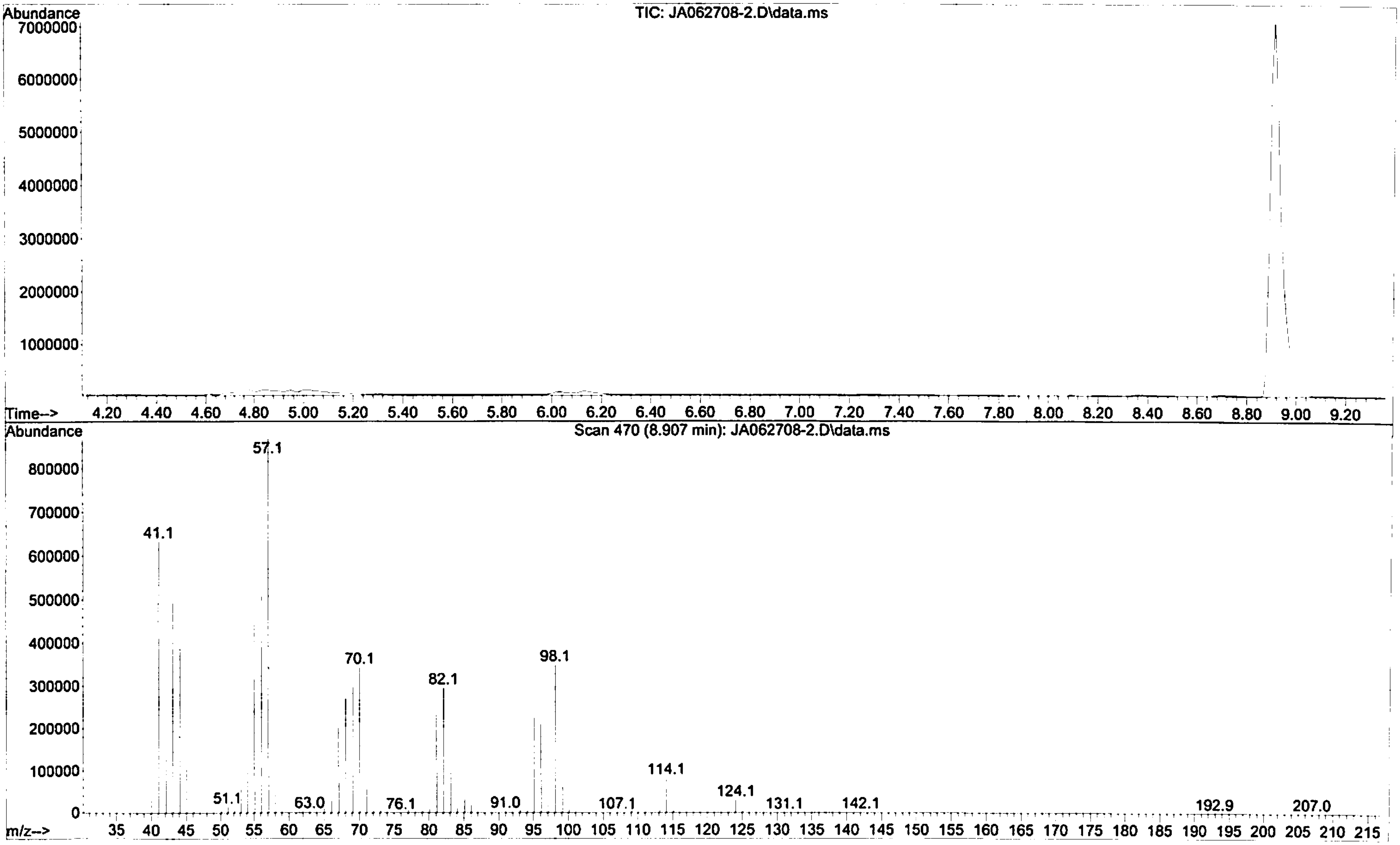

```
Library Searched : D:\DATABASE\W8N05ST.L
Quality          : 91
ID               : NONANAL $ PELARGONALDEHYDE $ 1-NONALDEHYDE $ 1-NONANAL $ 1-NONYL ALDEHYDE $ AI3
                   -04859 $ ALDEHYDE C-9 $ BRN 1236701 $ C-9 ALDEHYDE $ CCRIS 664 $ EINECS 204-688
                   -5 $ FEMA NO. 2782 $ HSDB 7229 $ N-NONALDEHYDE $ N-NONAN-1-AL $ N-NONANAL $ N-N
                   ONYLALDEHYDE $ NCI
```

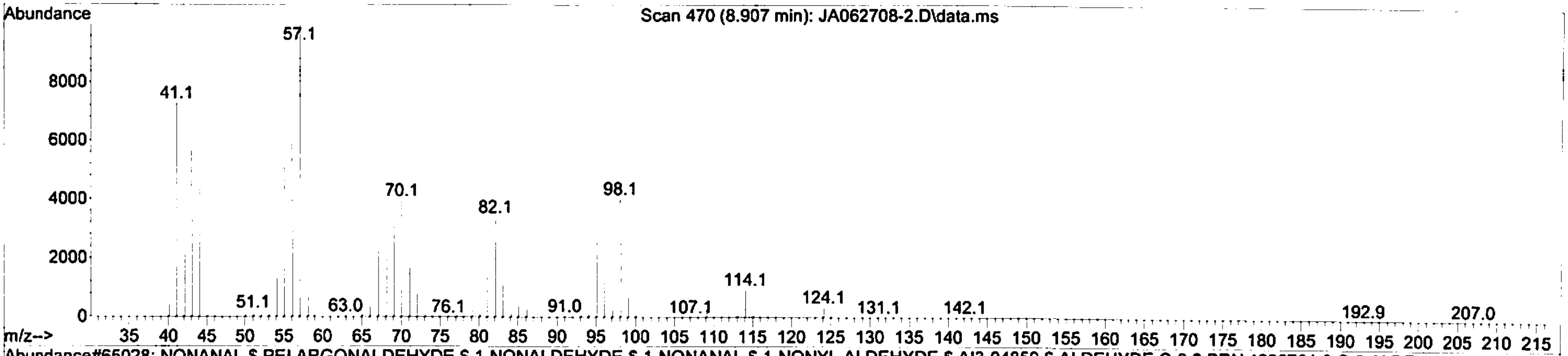

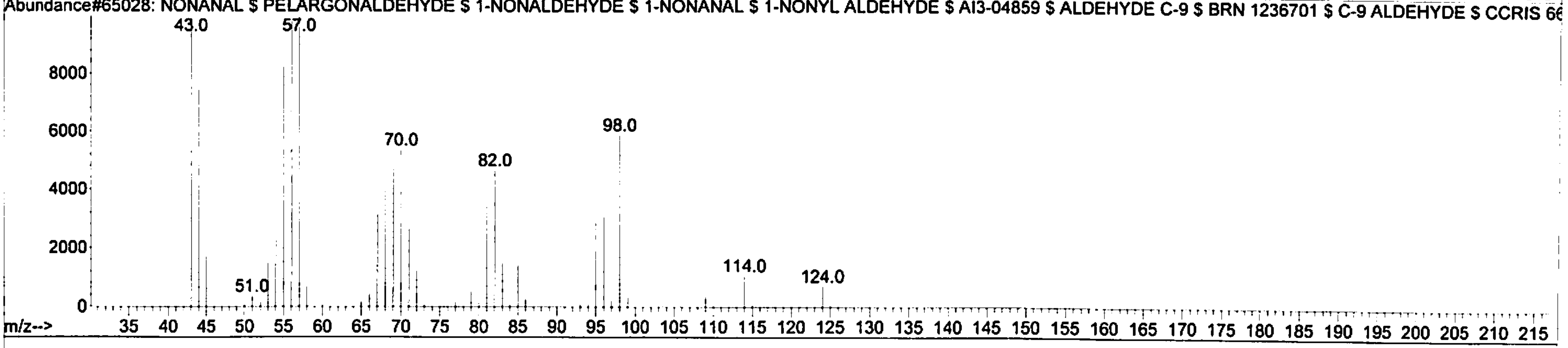

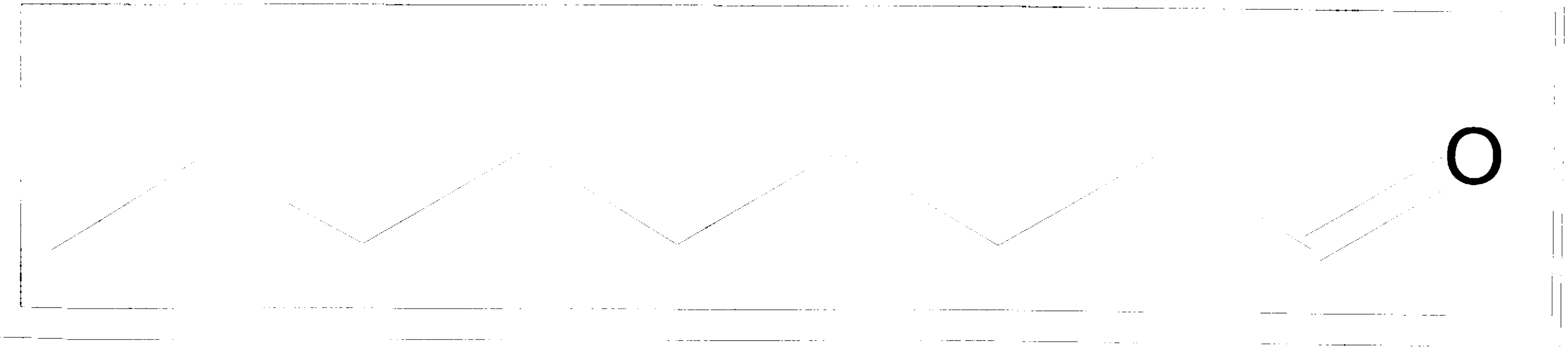

File :D:\ALDRICH\Snapshot\JA062708-2.D
Operator : Aldrich
Acquired : 27 Jun 2008 12:39 using AcqMethod JA-50-280LESS.M
Instrument : Buba
Sample Name: 8 lab-reared C.oculata male abdomen/CH2Cl2
Misc Info : GC run JA0627_1.D;fed aphids, 3-8 days old
Vial Number: 1

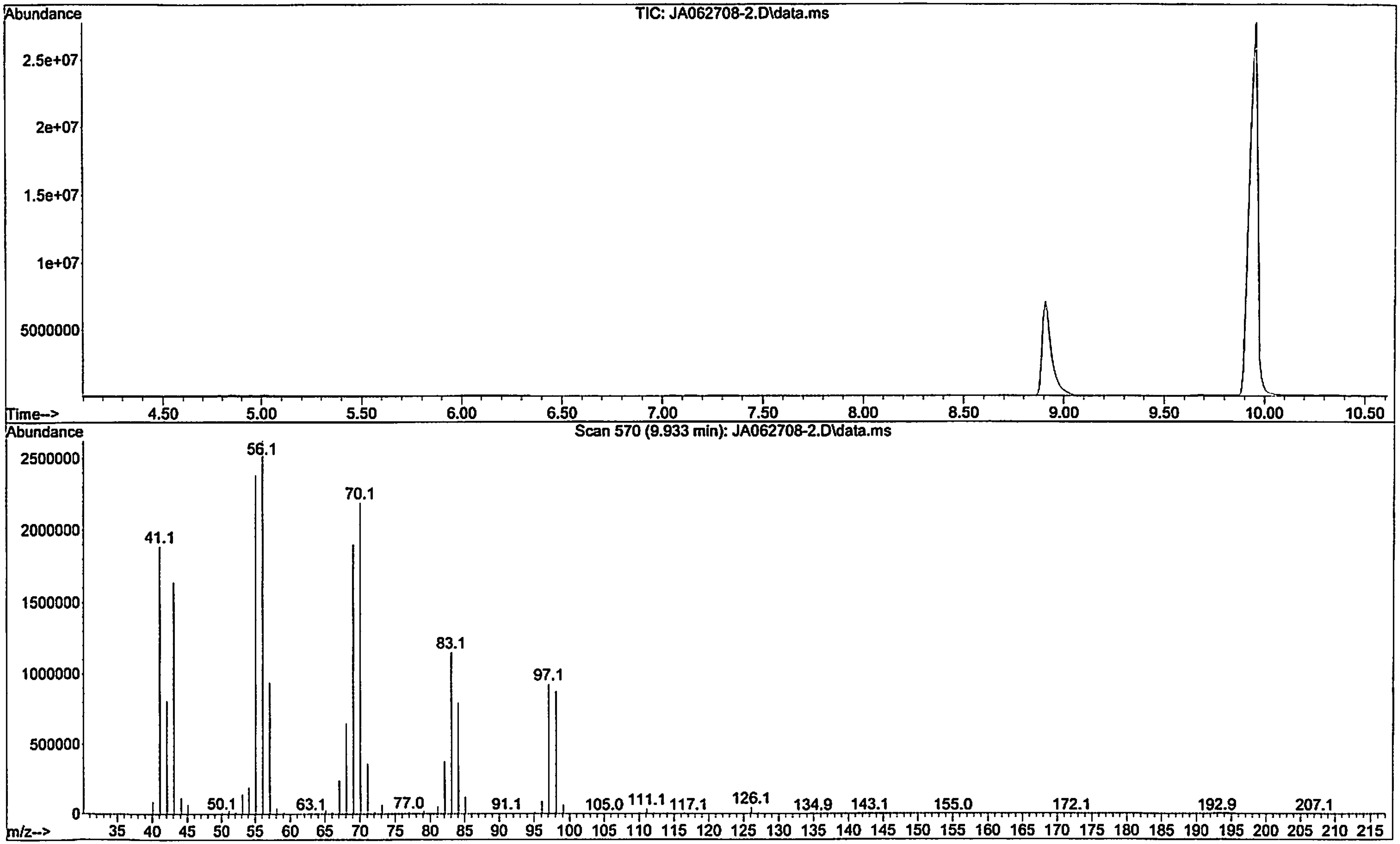

Library Searched : D:\DATABASE\W8N05ST.L
Quality : 91
ID : 1-NONANOL $ NONANOL $ NONAN-1-OL $ 1-HYDROXYNONANE $ 1-NONANO $ AI3-03962 $ ALCOHOL C-9 $ BRN 0969213 $ C9 ALCOHOL $ EINECS 205-583-7 $ FATTY ALCOHOL(C9) $ FEMA NO. 2789 $ HSDB 5145 $ N-NONAN-1-OL $ N-NONANOL $ N-NONYL ALCOHOL $ NONALOL $ NONANOL-(1) $ NO

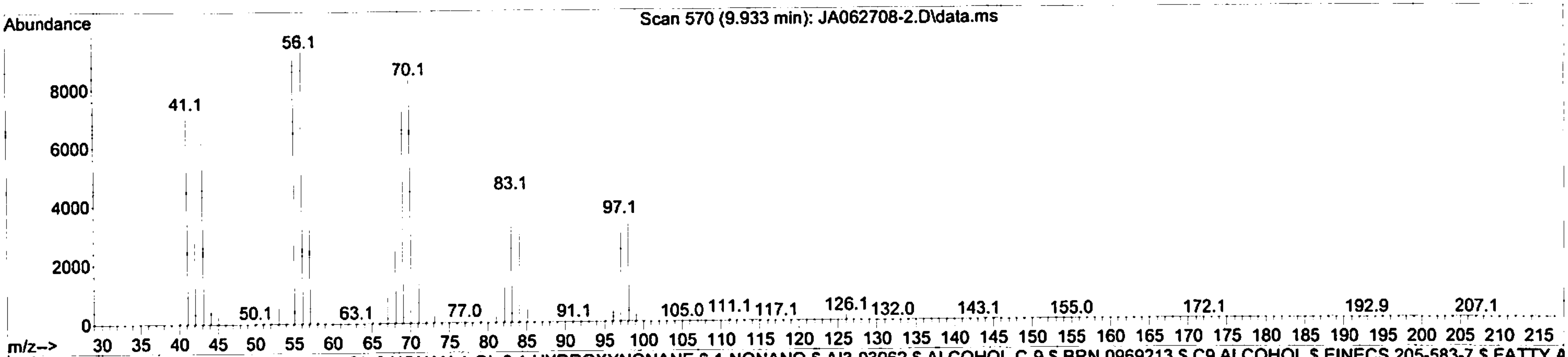

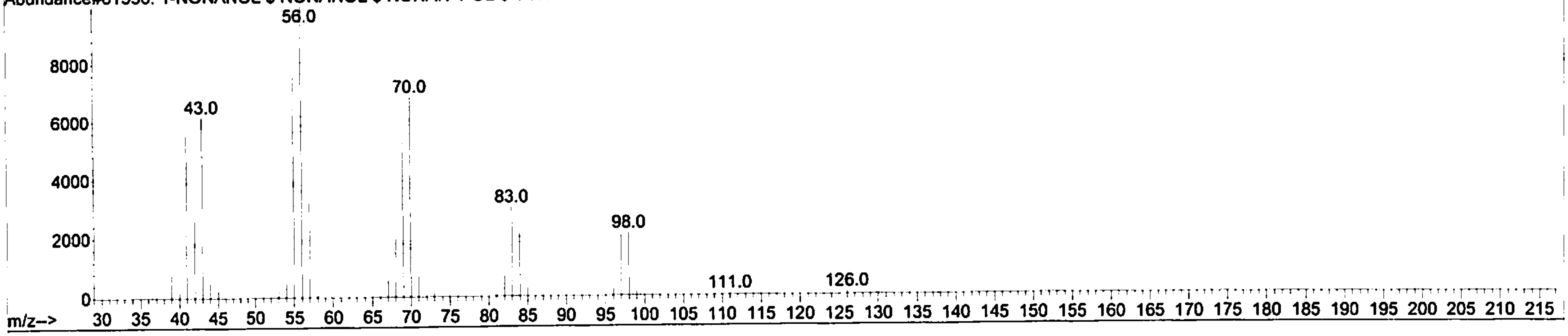

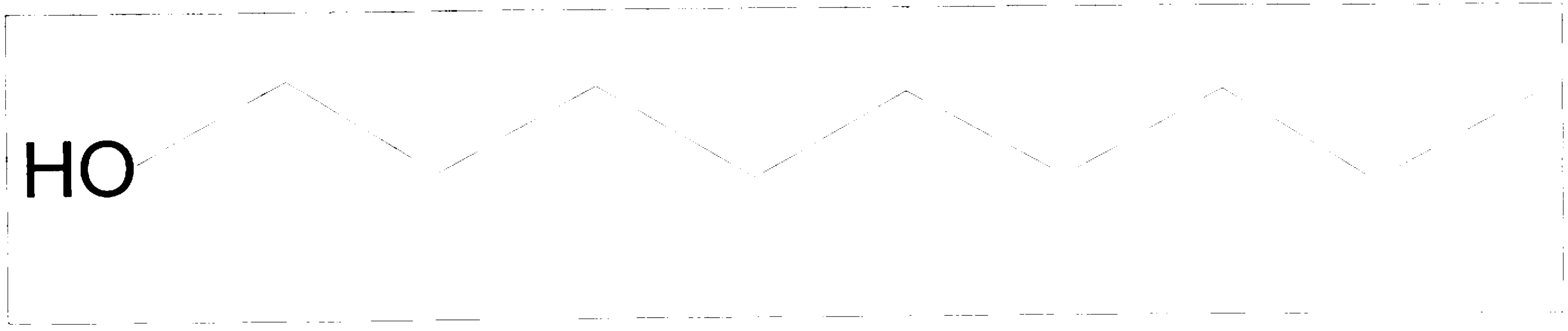

```
File          :D:\ALDRICH\Snapshot\JA062708-2.D
Operator      : Aldrich
Acquired      : 27 Jun 2008  12:39     using AcqMethod JA-50-280LESS.M
Instrument    :     Buba
Sample Name: 8 lab-reared C.oculata male abdomen/CH2Cl2
Misc Info     : GC run JA0627_1.D;fed aphids, 3-8 days old
Vial Number: 1
```

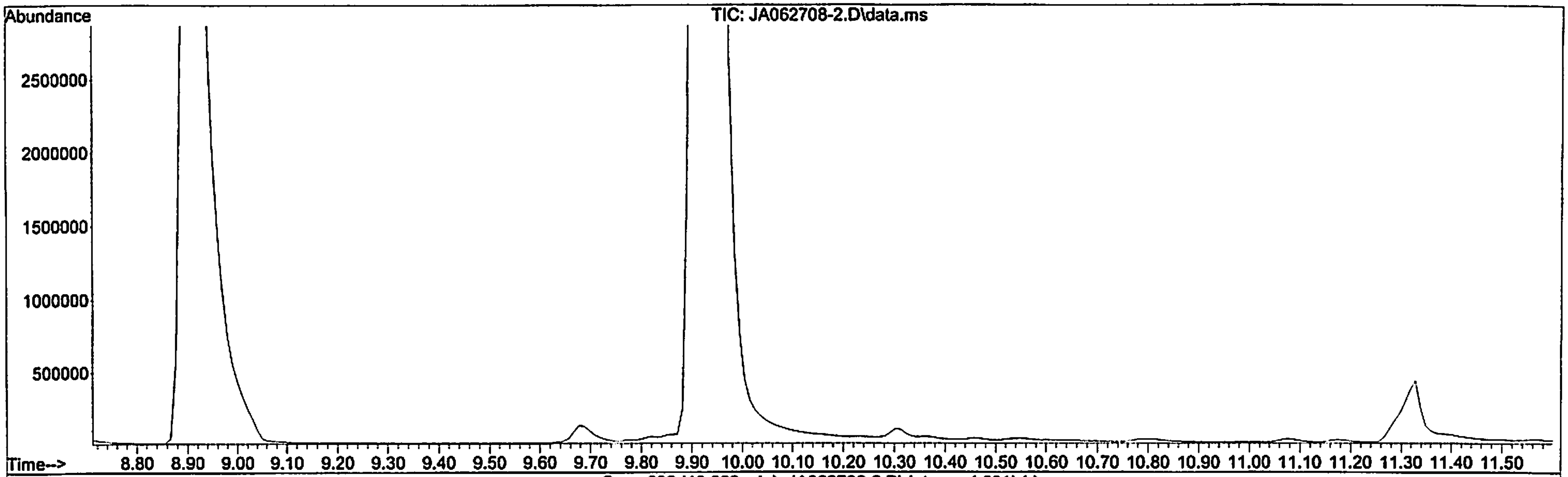

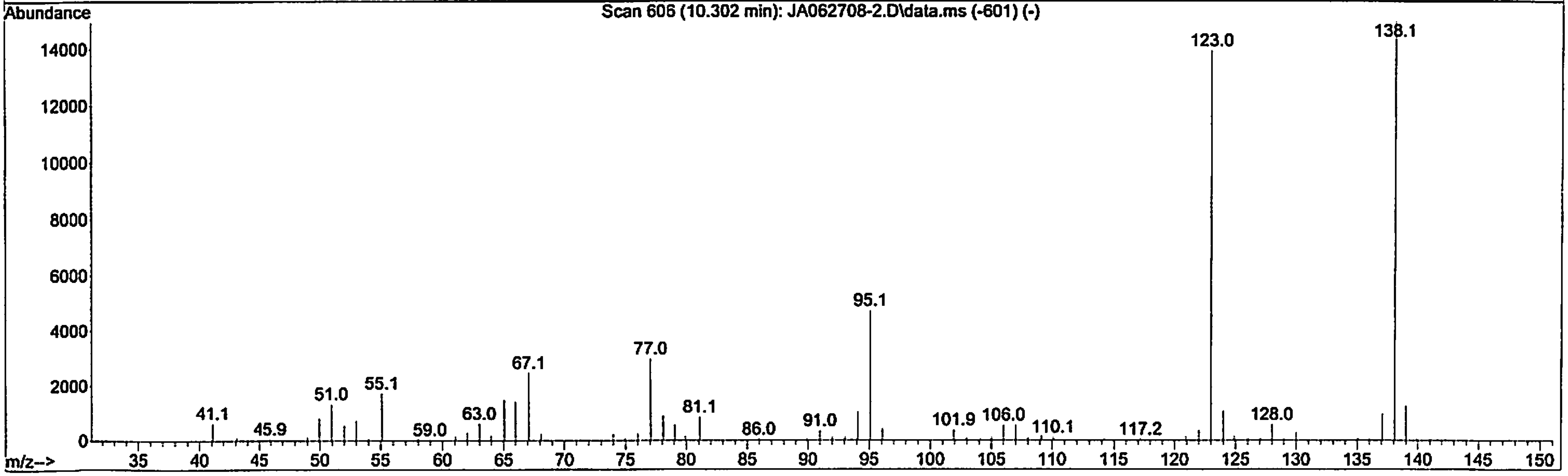

Library Searched : D:\DATABASE\W8N05ST.L
Quality : 95
ID : Phenol, 2-methoxy-4-methyl- $ p-Cresol, 2-methoxy- $ p-Creosol $ p-Methylguaiacol $ Creosol $ Homoguaiacol $ 2-Methoxy-p-cresol $ 2-Methoxy-4-cresol $ 2-Methoxy-4-methylphenol $ 3-Methoxy-4-hydroxytoluene $ 4-Hydroxy-3-methoxytoluene $ 4-Methyl-2-methoxyp

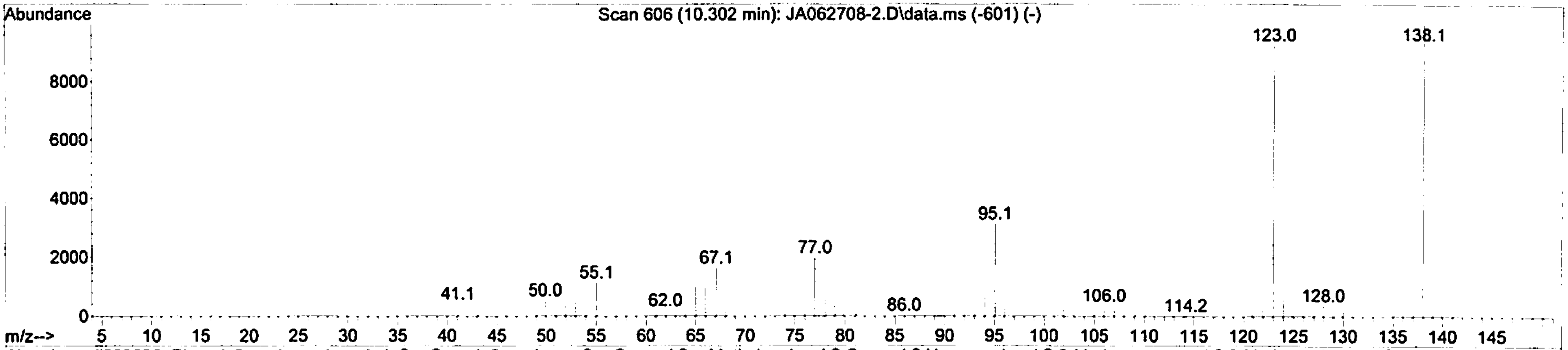

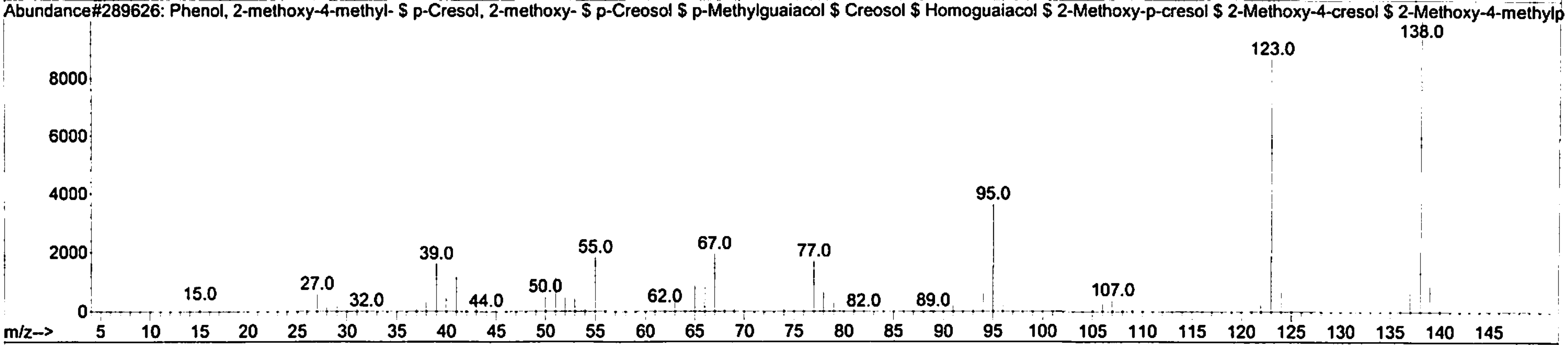

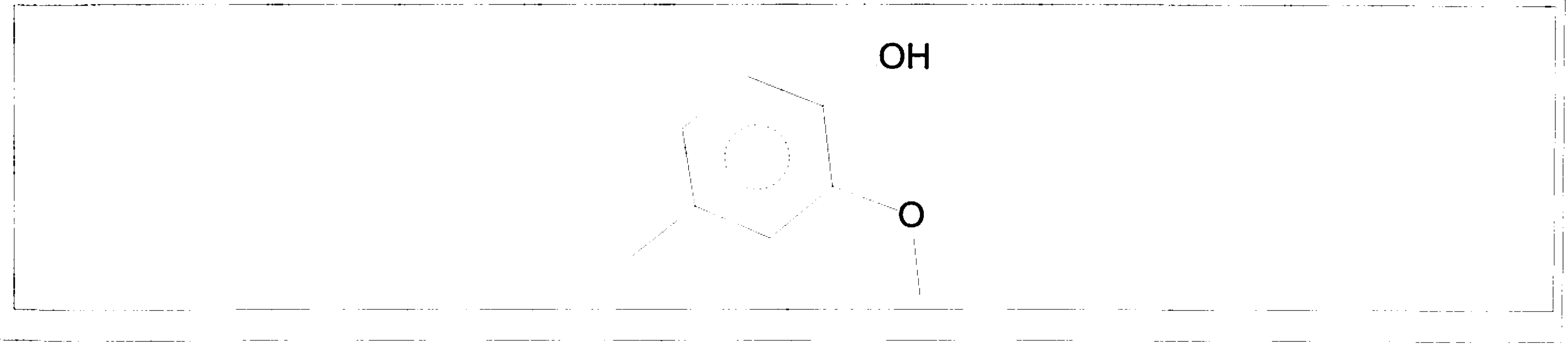

```
File          :D:\ALDRICH\Snapshot\JA062708-2.D
Operator      : Aldrich
Acquired      : 27 Jun 2008  12:39      using AcqMethod JA-50-280LESS.M
Instrument    :    Buba
Sample Name: 8 lab-reared C.oculata male abdomen/CH2Cl2
Misc Info     : GC run JA0627_1.D;fed aphids, 3-8 days old
Vial Number: 1
```

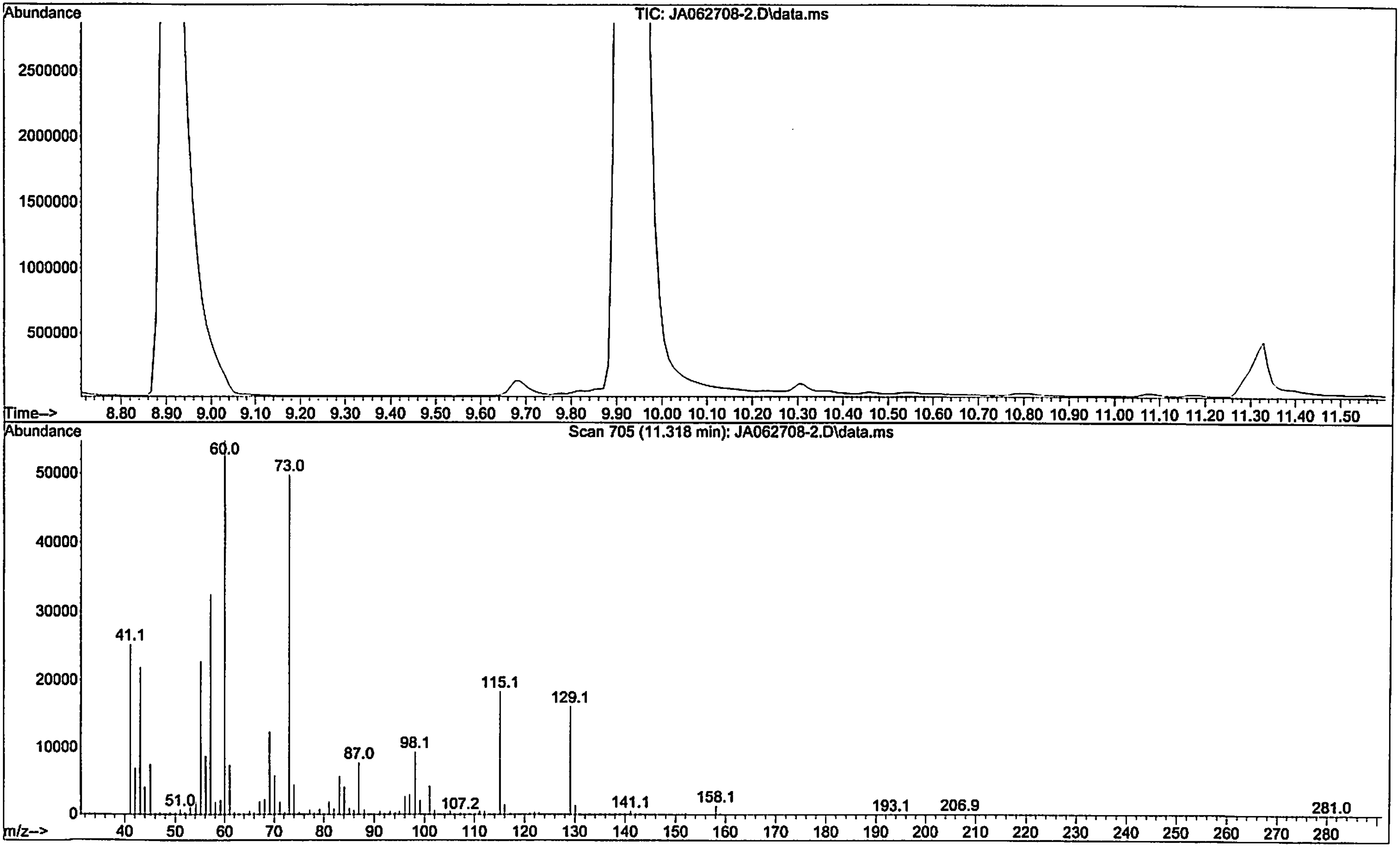

Library Searched : D:\DATABASE\W8N05ST.L
Quality : 95
ID : NONANOIC ACID $ CALCIUM PELARGONATE $ PELARGONIC ACID $ POTASSIUM PELARGONATE $ 1-NONANOIC ACID $ 1-OCTANECARBOXYIC ACID $ 1-OCTANECARBOXYLIC ACID $ AI3-04164 $ AIDS-017605 $ BRN 1752351 $ CADMIUM NONAN-1-OATE $ CADMIUM PELARGONATE $ CALCIUM NONAN-1-OATE

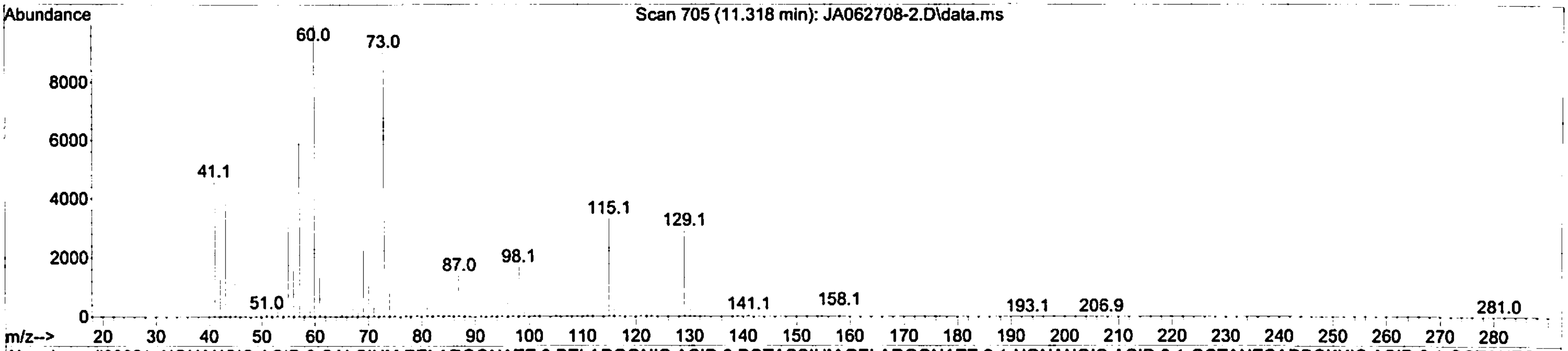

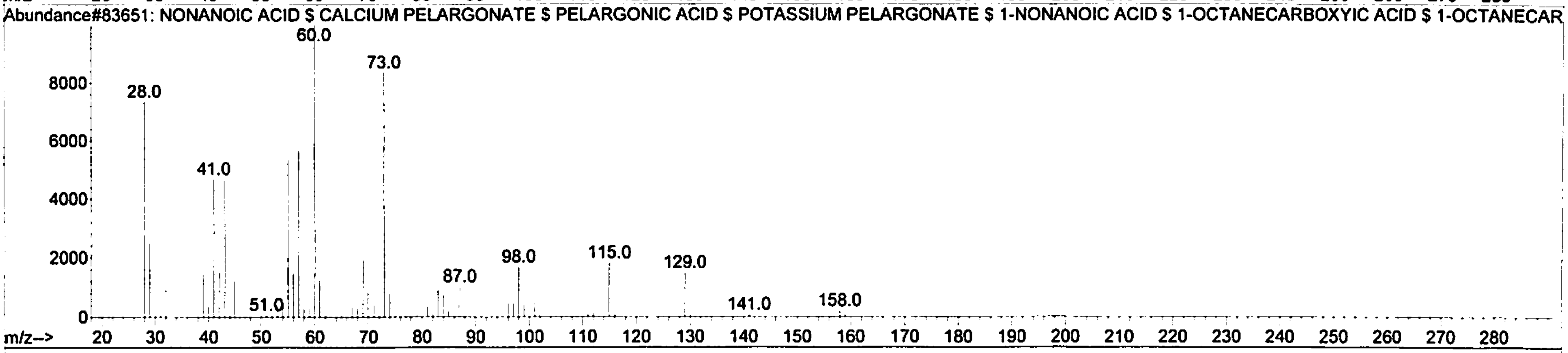

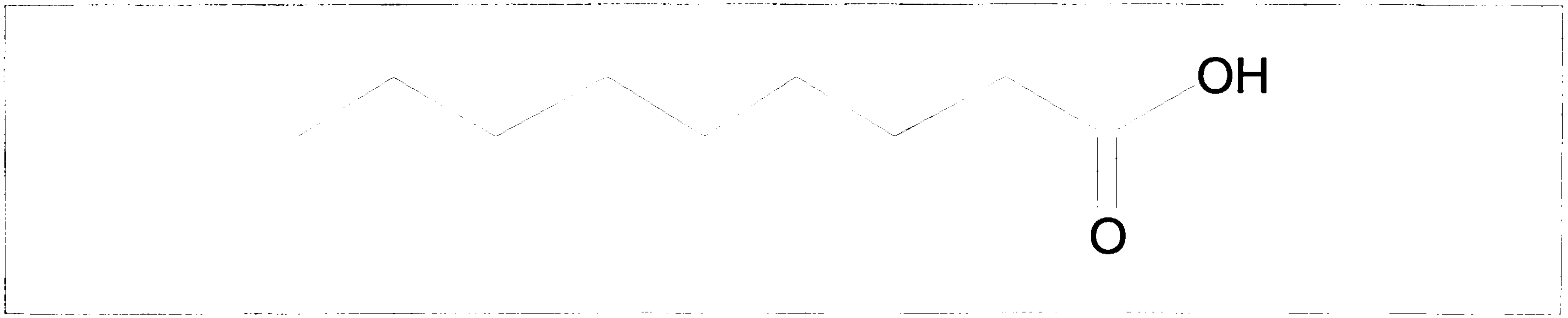

```
File        :D:\ALDRICH\Snapshot\JA062708-2.D
Operator    : Aldrich
Acquired    : 27 Jun 2008  12:39     using AcqMethod JA-50-280LESS.M
Instrument  :    Buba
Sample Name: 8 lab-reared C.oculata male abdomen/CH2Cl2
Misc Info   : GC run JA0627_1.D;fed aphids, 3-8 days old
Vial Number: 1
```

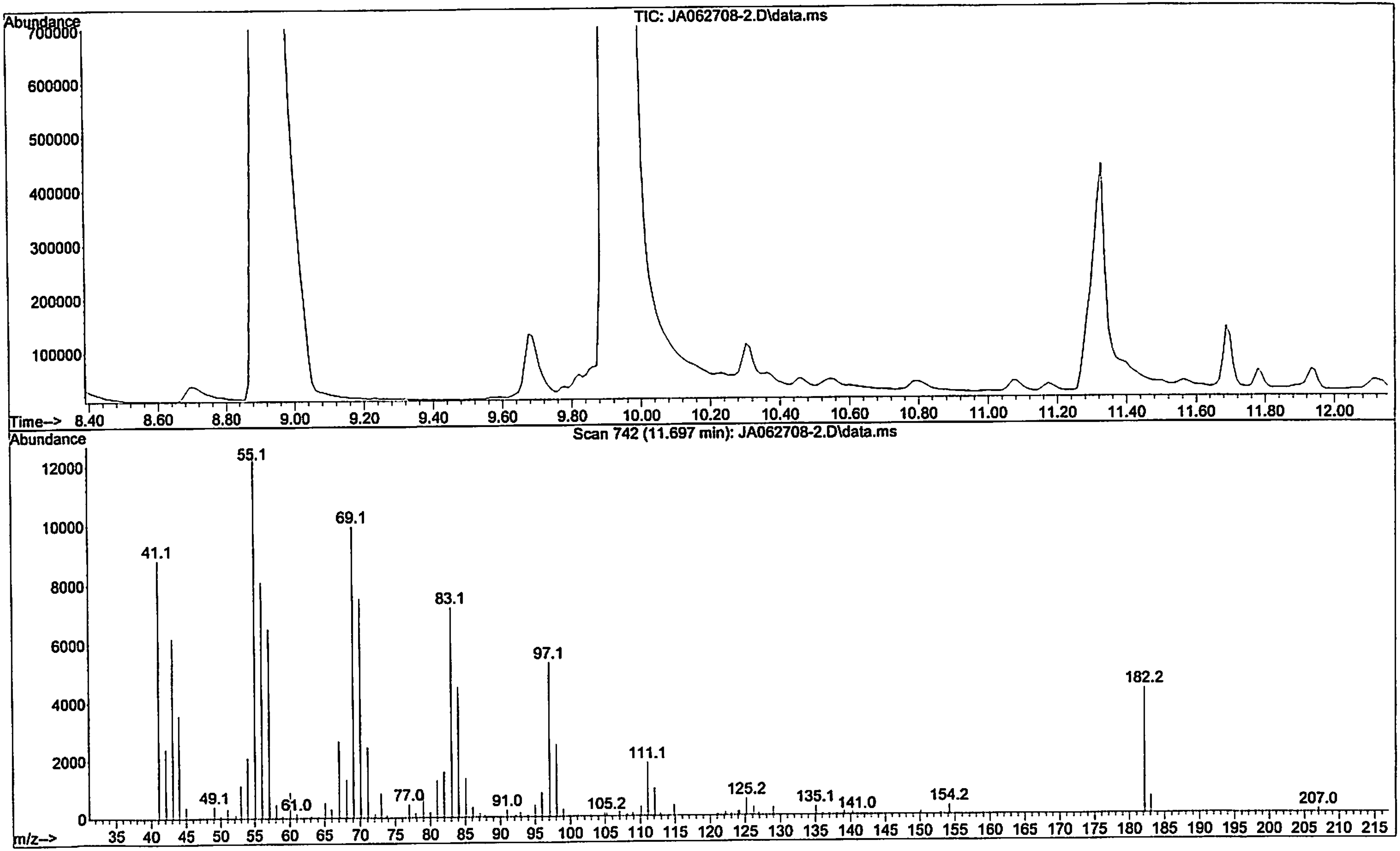

```
File        :D:\ALDRICH\Snapshot\JA062708-2.D
Operator    : Aldrich
Acquired    : 27 Jun 2008  12:39      using AcqMethod JA-50-280LESS.M
Instrument  :    Buba
Sample Name: 8 lab-reared C.oculata male abdomen/CH2Cl2
Misc Info   : GC run JA0627_1.D;fed aphids, 3-8 days old
Vial Number: 1
```

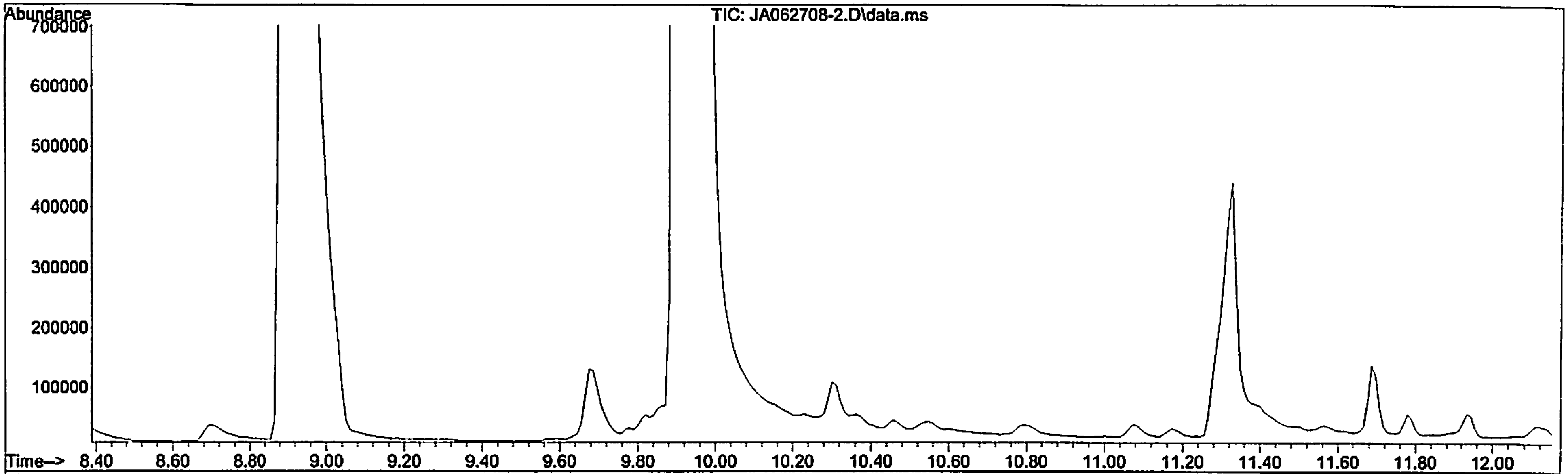

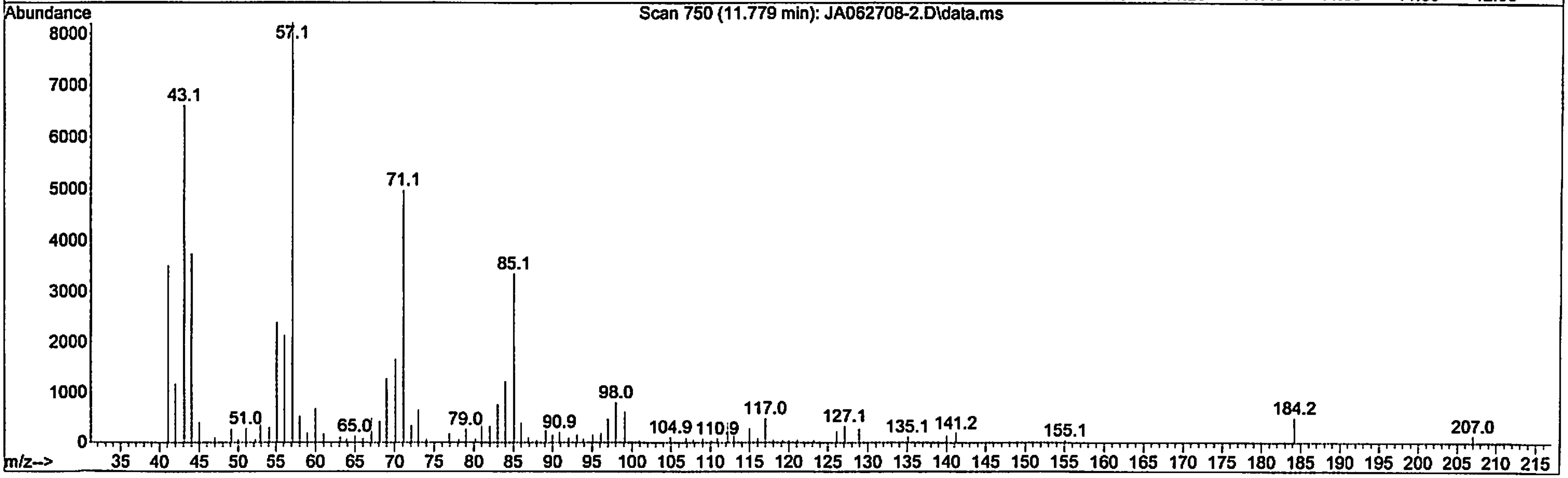

File :D:\ALDRICH\Snapshot\JA062708-2.D
Operator : Aldrich
Acquired : 27 Jun 2008 12:39 using AcqMethod JA-50-280LESS.M
Instrument : Buba
Sample Name: 8 lab-reared C.oculata male abdomen/CH2Cl2
Misc Info : GC run JA0627_1.D;fed aphids, 3-8 days old
Vial Number: 1

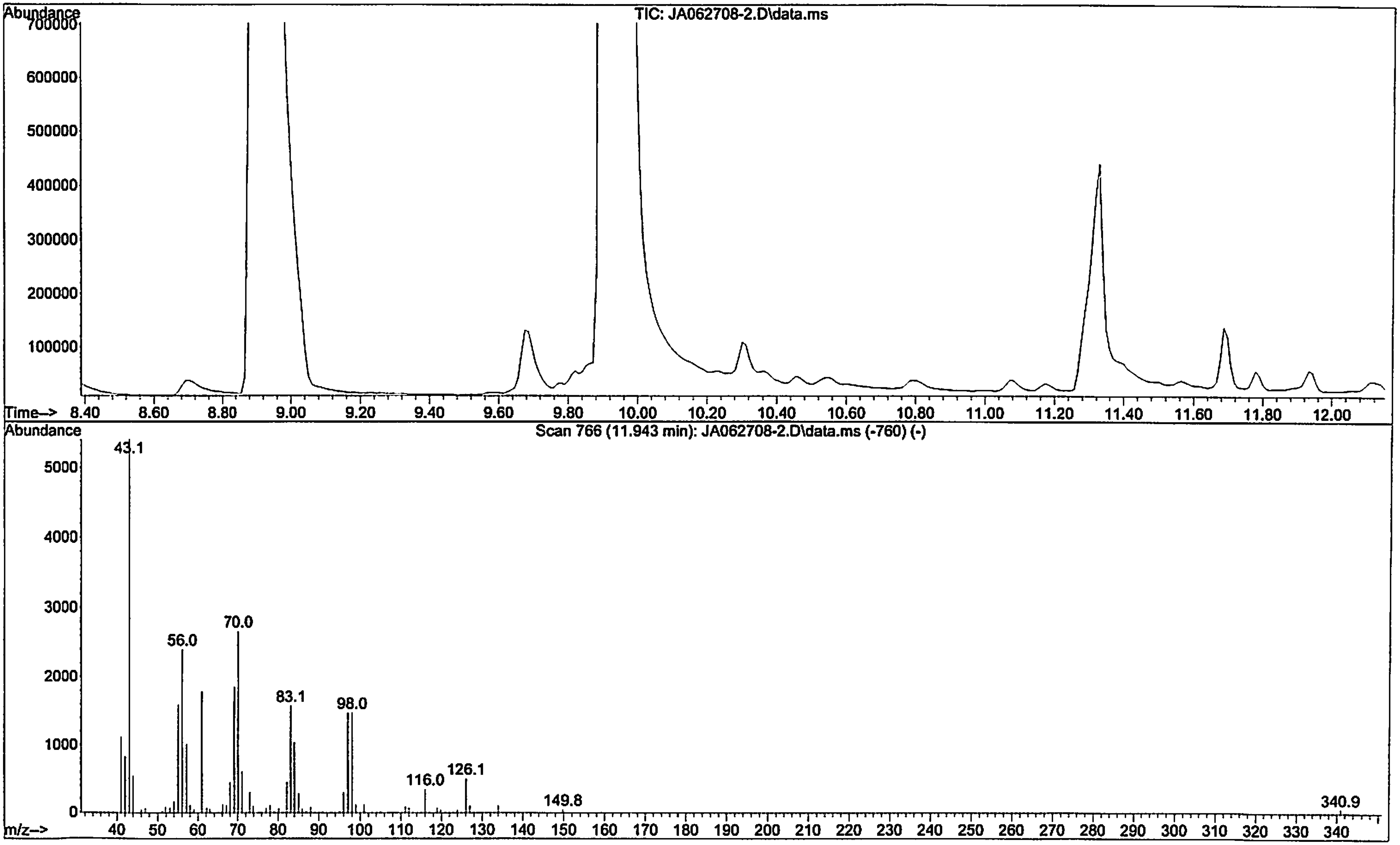

Library Searched : D:\DATABASE\W8N05ST.L
Quality : 80
ID : ACETIC ACID, NONYL ESTER $ NONYL ACETATE $ 1-ACETOXYNONANE $ 1-NONYL ACETATE $ ACETATE C-9 $ ACETIC ACID N-NONYL ESTER $ AI3-11583 $ EINECS 205-585-8 $ FEMA NO. 2788 $ N-NONANYL ACETATE $ N-NONYL ACETATE $ N-NONYL ETHANOATE $ NONANOL ACETATE $ NONYL ALCOH

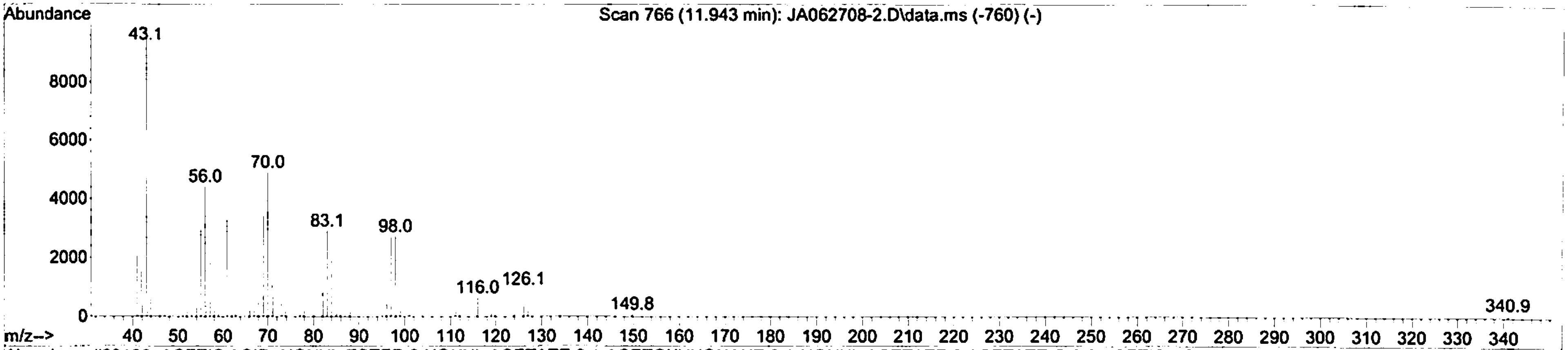

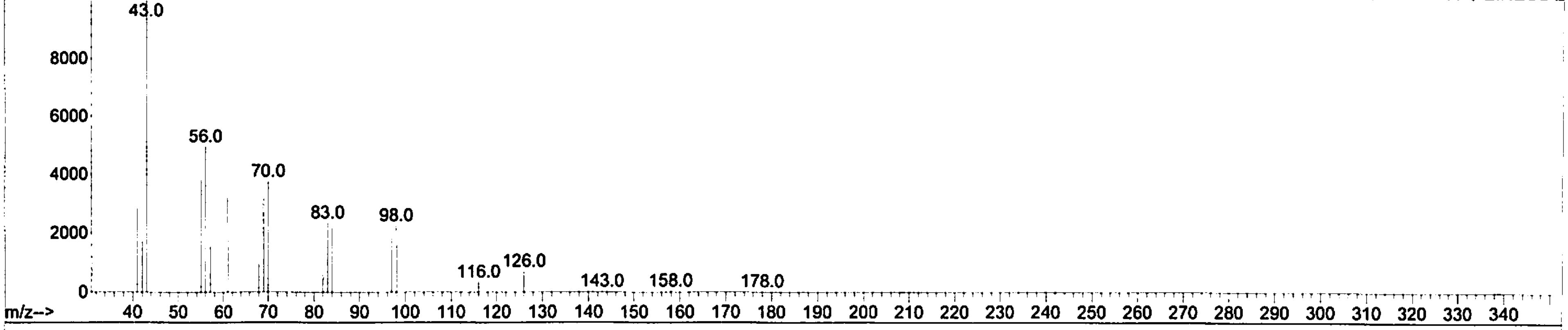

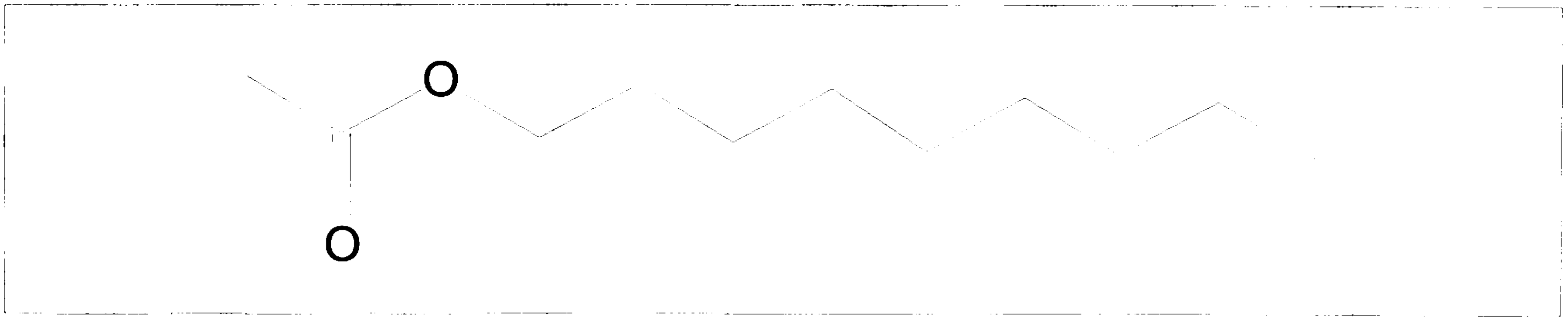

File   :D:\ALDRICH\Snapshot\JA062708-2.D
Operator   : Aldrich
Acquired   : 27 Jun 2008 12:39   using AcqMethod JA-50-280LESS.M
Instrument   :   Buba
Sample Name: 8 lab-reared C.oculata male abdomen/CH2Cl2
Misc Info   : GC run JA0627_1.D;fed aphids, 3-8 days old
Vial Number: 1

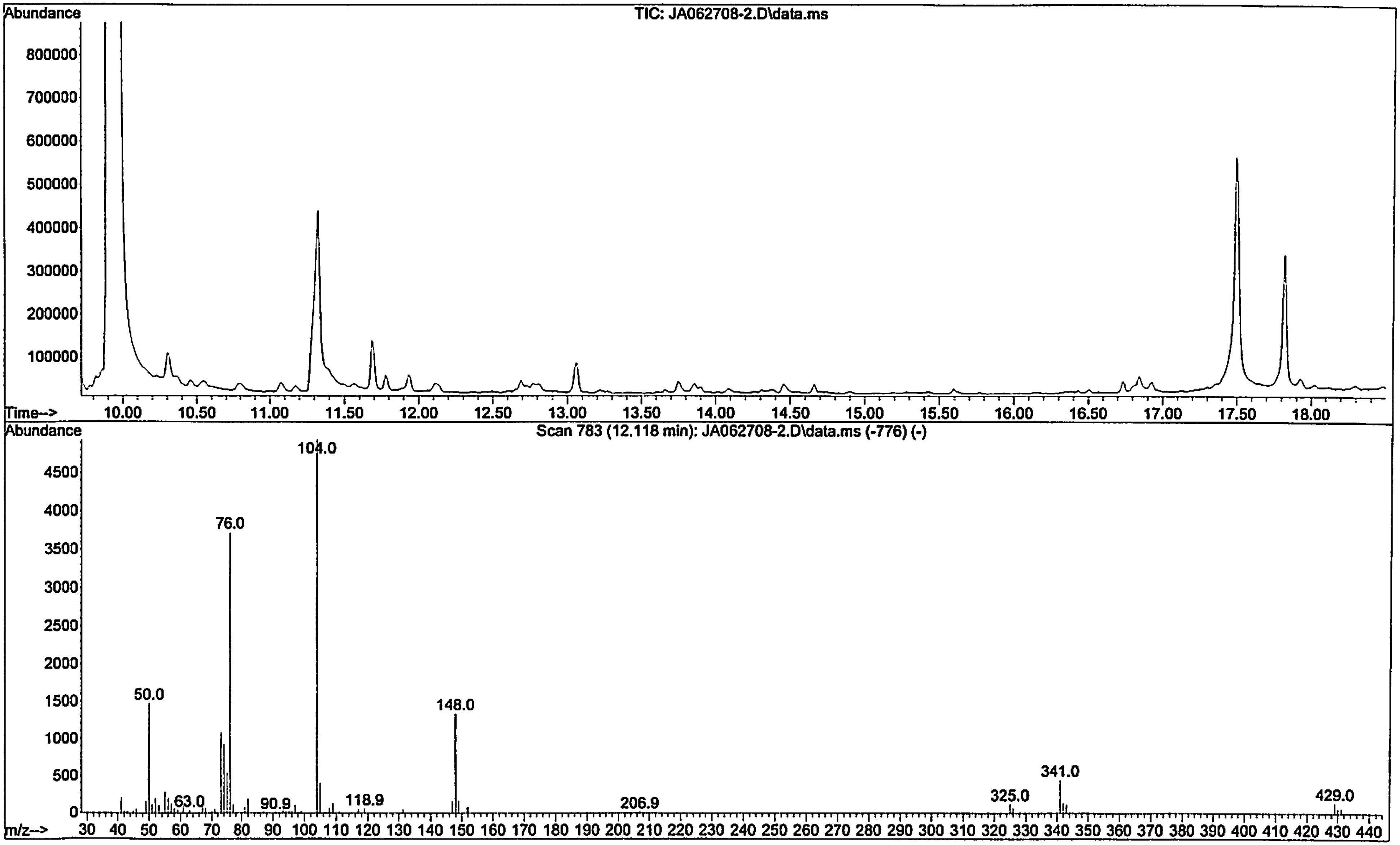

Library Searched : D:\DATABASE\W8N05ST.L
Quality : 90
ID : 1,3-ISOBENZOFURANDIONE $ ISOBENZOFURAN-1,3-DIONE $ 2-BENZOFURAN-1,3-DIONE # $ 1, 2-BENZENEDICARBOXYLIC ANHYDRIDE $ 1, 3-ISOBENZOFURANDIONE $ 1,2-BENZENEDICARBOXYLIC ACID ANHYDRIDE $ 1,2-BENZENEDICARBOXYLIC ANHYDRIDE $ 1,3-DIOXOPHTHALAN $ 1,3-ISOBENZOFURAN

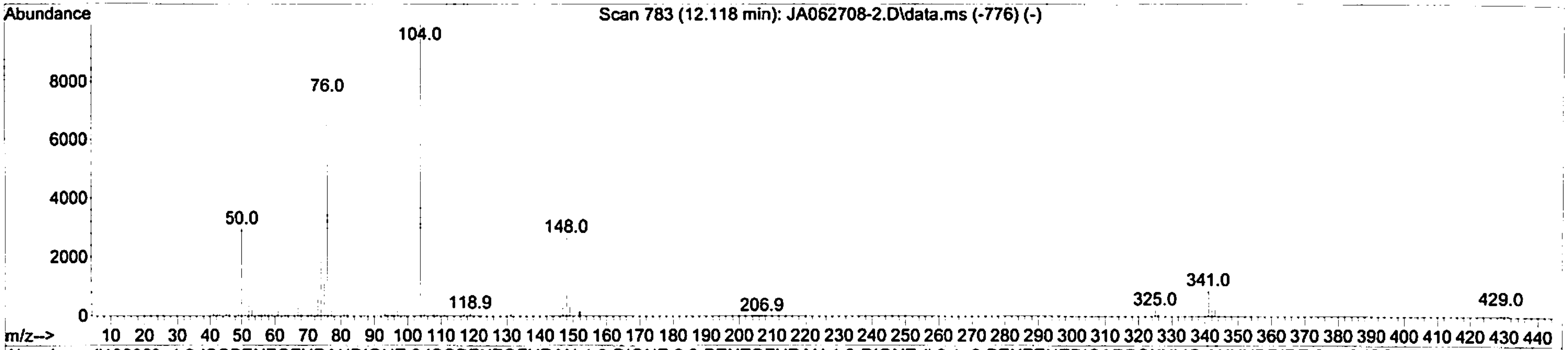

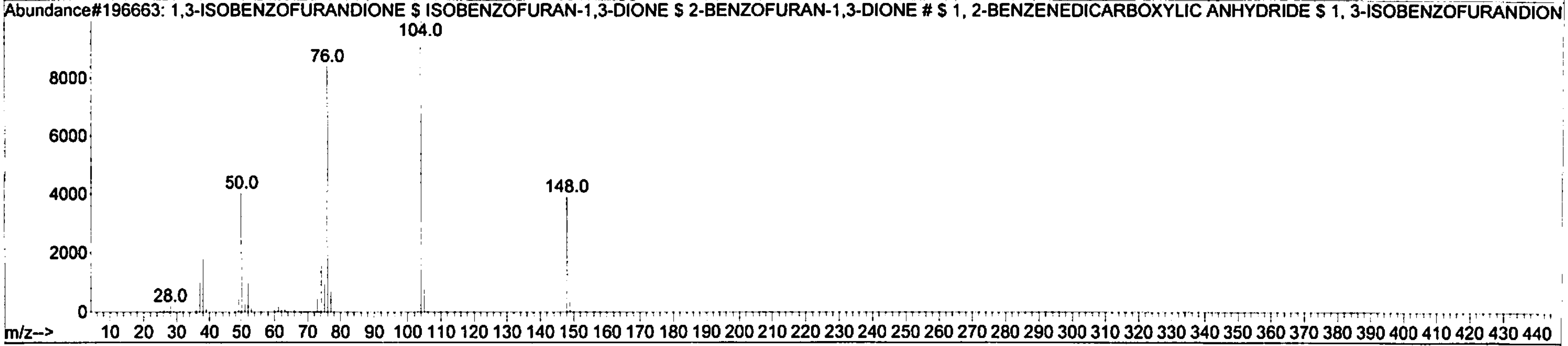

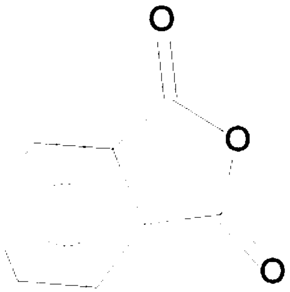

```
File        :D:\ALDRICH\Snapshot\JA062708-2.D
Operator    : Aldrich
Acquired    : 27 Jun 2008  12:39      using AcqMethod JA-50-280LESS.M
Instrument  :    Buba
Sample Name: 8 lab-reared C.oculata male abdomen/CH2Cl2
Misc Info   : GC run JA0627_1.D;fed aphids, 3-8 days old
Vial Number: 1
```

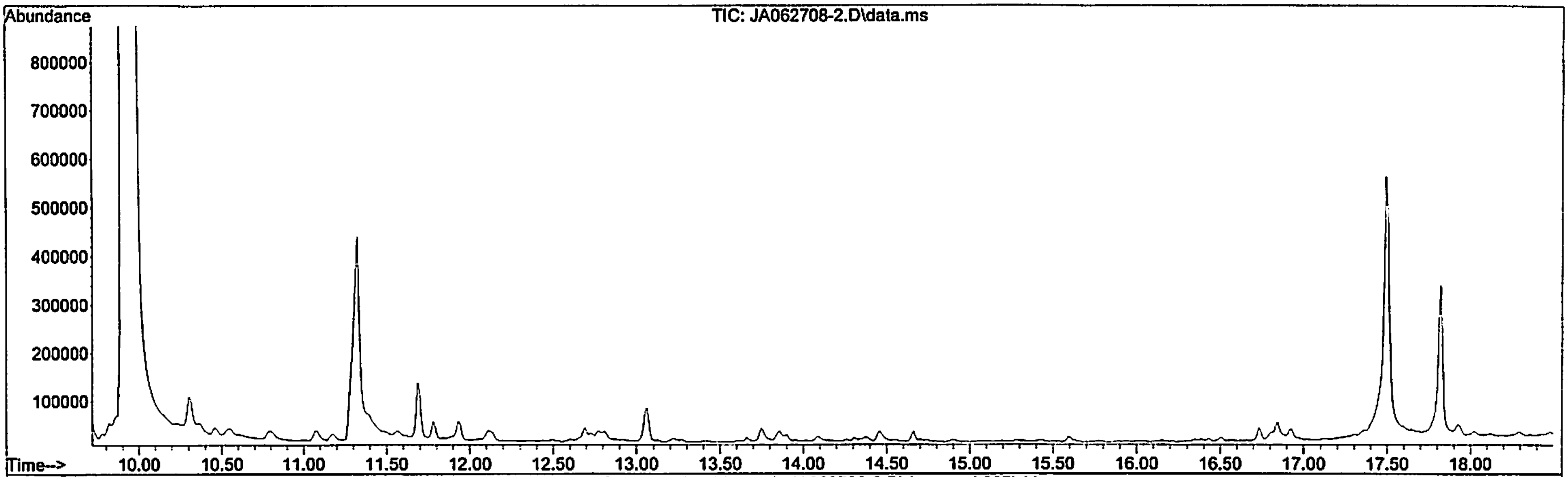

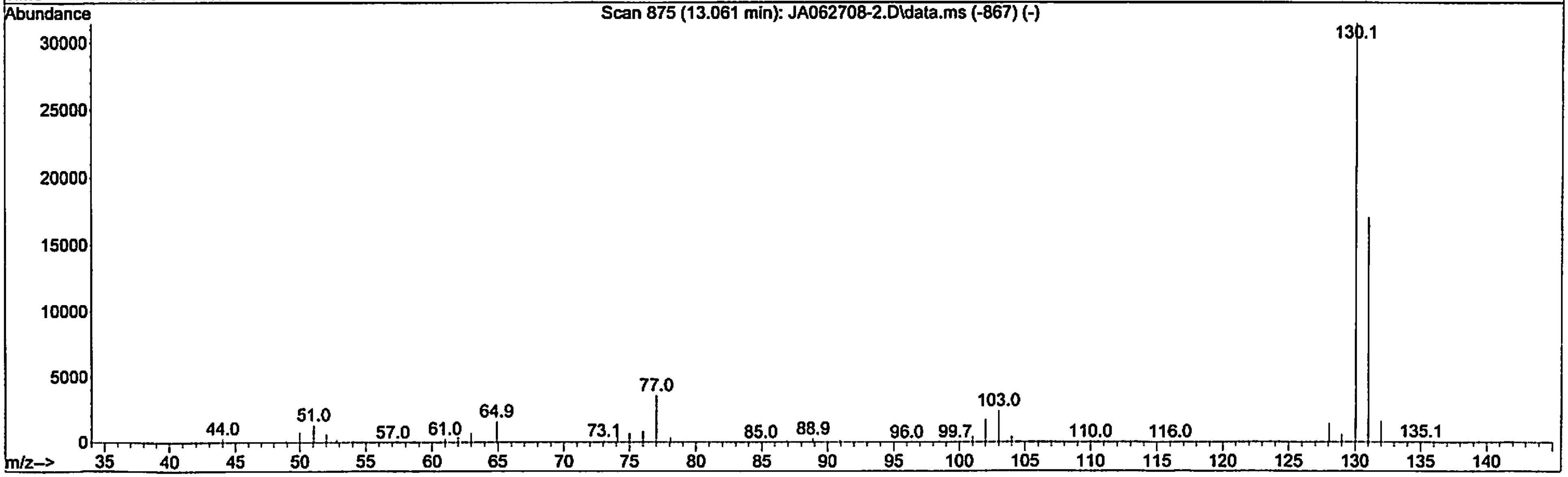

Library Searched : D:\DATABASE\W8N05ST.L
Quality : 95
ID : 1H-Indole, 3-methyl- $ Indole, 3-methyl- $ .beta.-Methylindole $ Scatole $ Skatol $ Skatole $ 3-Methylindole $ 3-MI $ 3-Methyl-1H-indole

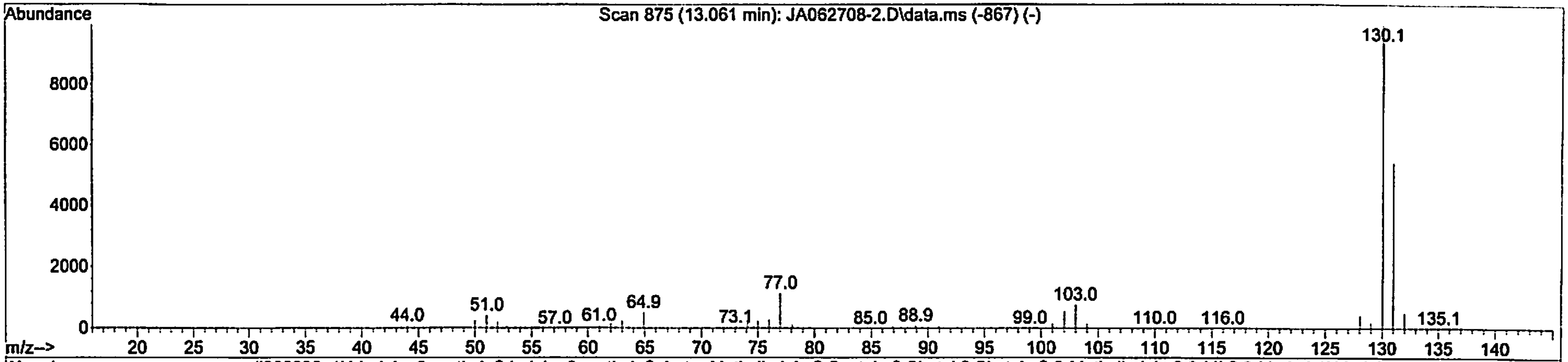

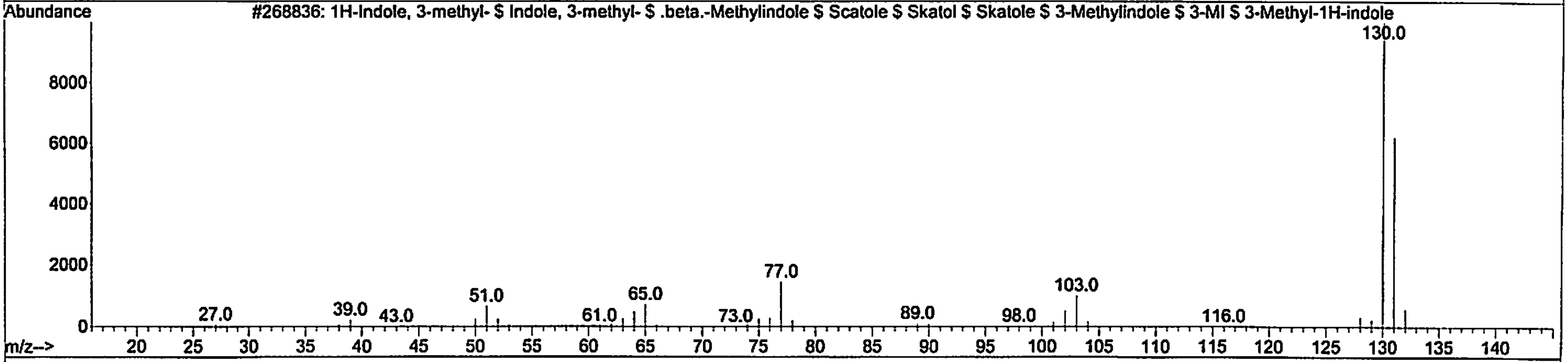

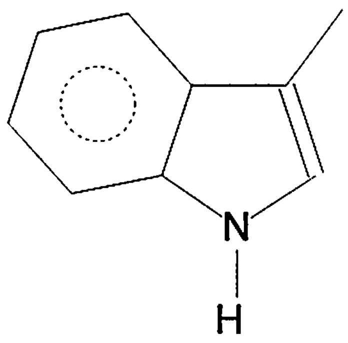

File   :D:\ALDRICH\Snapshot\JA062708-2.D
Operator   : Aldrich
Acquired   : 27 Jun 2008 12:39   using AcqMethod JA-50-280LESS.M
Instrument :   Buba
Sample Name: 8 lab-reared C.oculata male abdomen/CH2Cl2
Misc Info   : GC run JA0627_1.D;fed aphids, 3-8 days old
Vial Number: 1

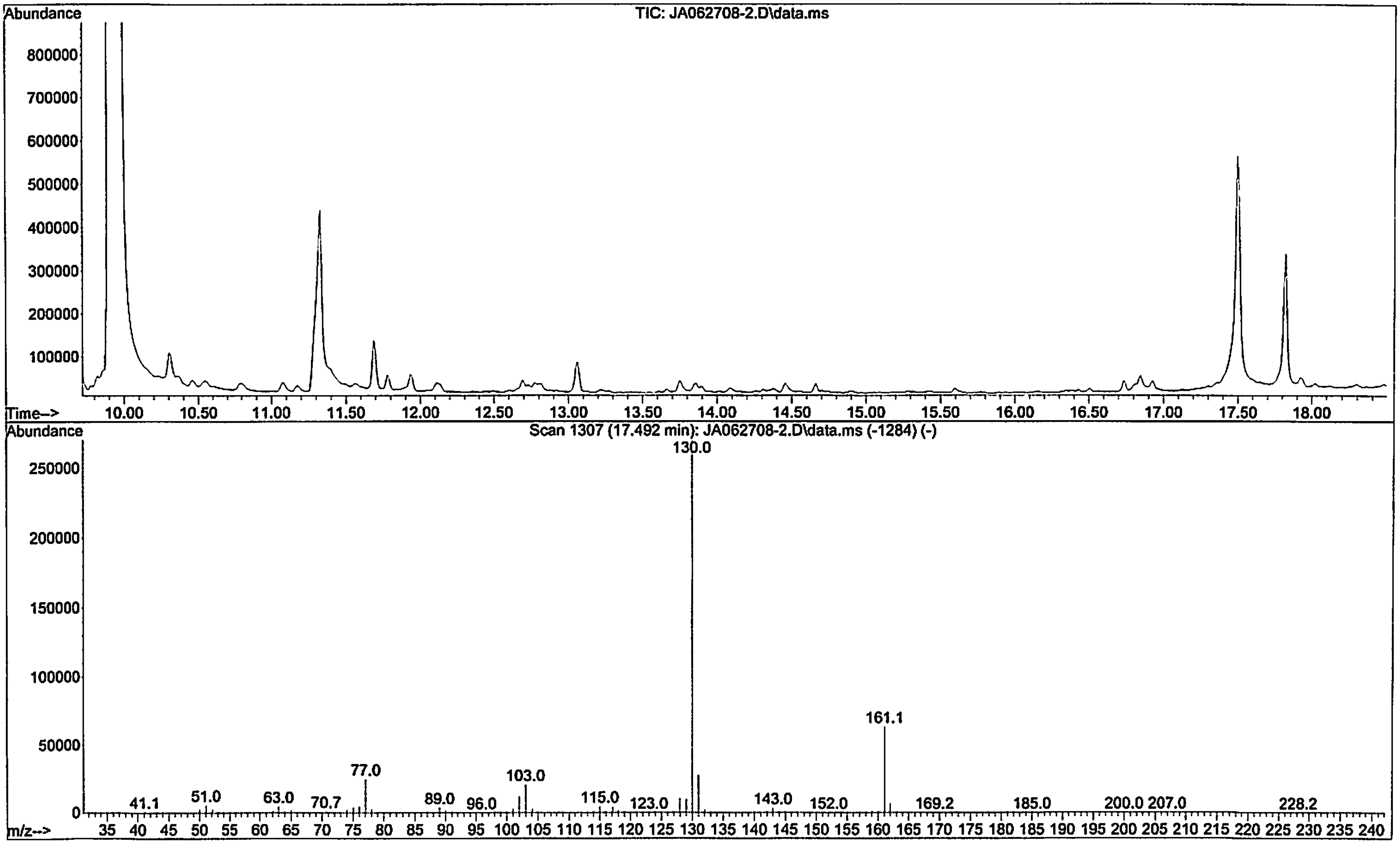

Library Searched : D:\DATABASE\W8N05ST.L
Quality : 91
ID : 1H-Indole-3-ethanol $ Indole-3-ethanol $ .beta.-(3-Indole)ethanol $ Ethanol, 2-indol-3-yl- $ Indoleethanol $ IEA $ Tryptophol $ 2-(3-Indolylethanol $ 3-(.beta.-Hydroxyethyl)indole $ 3-(2-Hydroxyethyl)indole $ 3-Indoleethanol $ dl-Tryptophanol $ Ethanol, 3

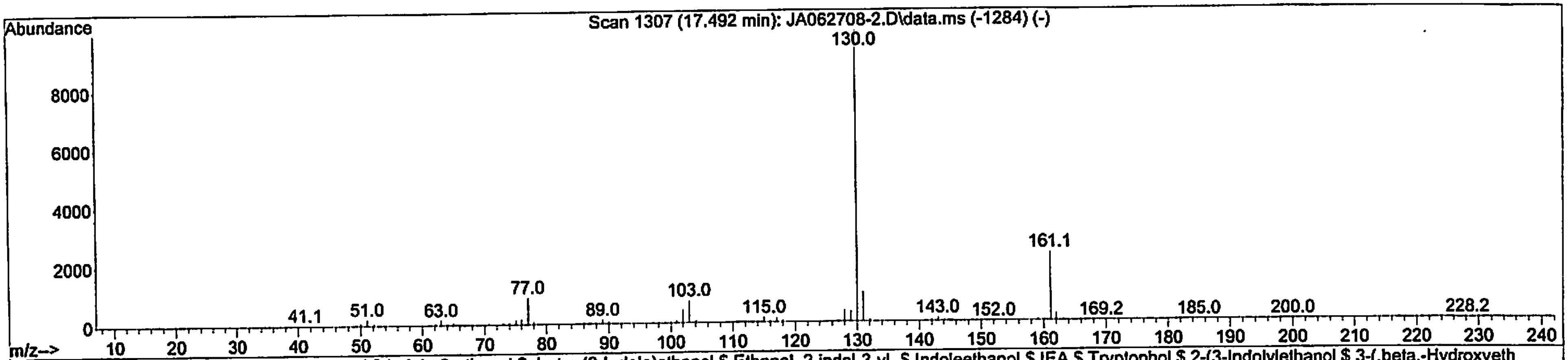

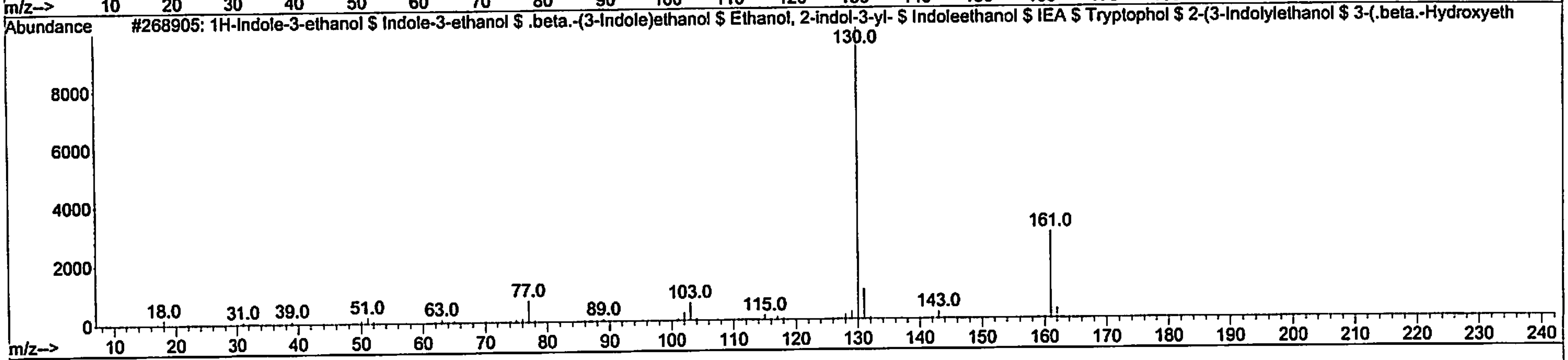

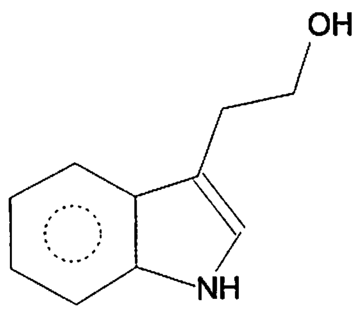

```
File        :D:\ALDRICH\Snapshot\JA062708-2.D
Operator    : Aldrich
Acquired    : 27 Jun 2008  12:39     using AcqMethod JA-50-280LESS.M
Instrument :    Buba
Sample Name: 8 lab-reared C.oculata male abdomen/CH2Cl2
Misc Info   : GC run JA0627_1.D;fed aphids, 3-8 days old
Vial Number: 1
```

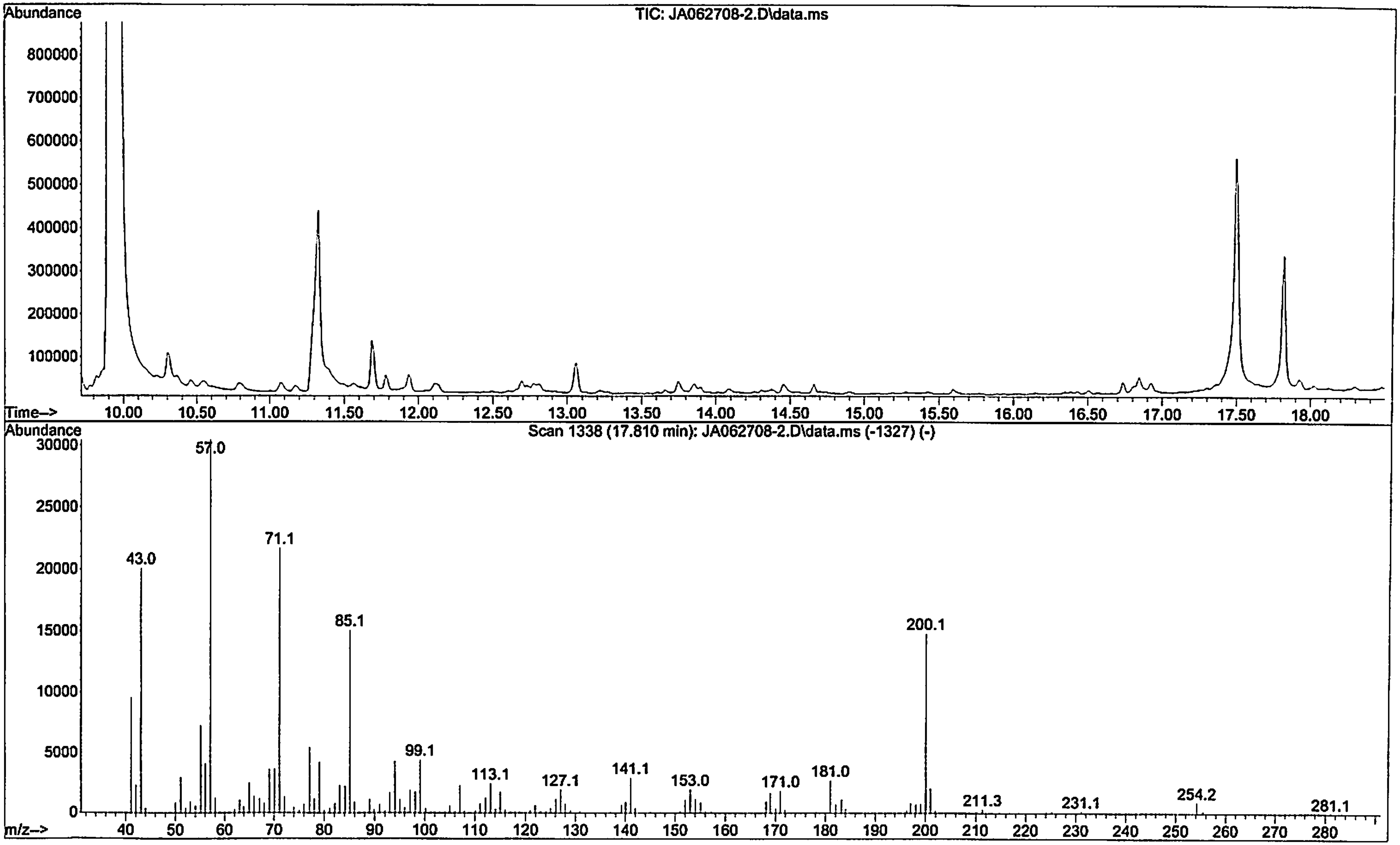

```
File        :D:\ALDRICH\Snapshot\JA062708-2.D
Operator    : Aldrich
Acquired    : 27 Jun 2008  12:39     using AcqMethod JA-50-280LESS.M
Instrument  :    Buba
Sample Name: 8 lab-reared C.oculata male abdomen/CH2Cl2
Misc Info   : GC run JA0627_1.D;fed aphids, 3-8 days old
Vial Number: 1
```

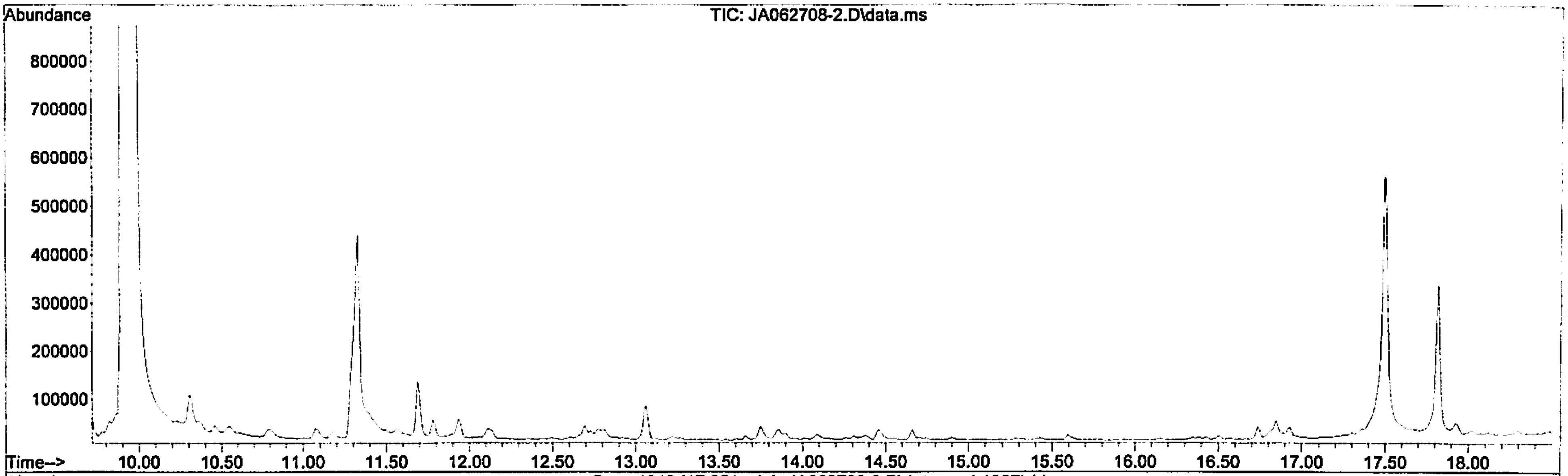

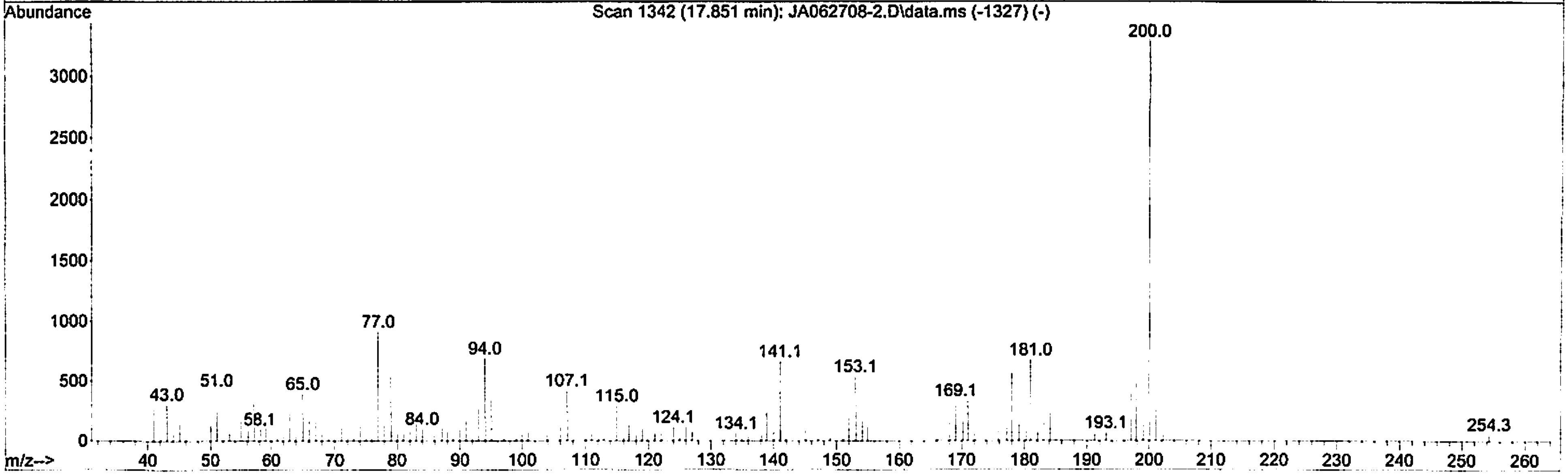

Library Searched : D:\DATABASE\W8N05ST.L
Quality : 93
ID : BENZENEMETHANOL, 3-PHENOXY- $ (3-PHENOXYPHENYL)METHANOL $ 3-(HYDROXYMETHYL)DIPHENYL ETHER $ 3-PHENOXYBENZENEMETHANOL $ 3-PHENOXYBENZYL ALCOHOL $ 3-PHENOXYBENZYLALCOHOL $ 3-PHENOXYBENZYLIC ALCOHOL $ BENZYL ALCOHOL, M-PHENOXY- $ BRN 0475312 $ EINECS 237-525

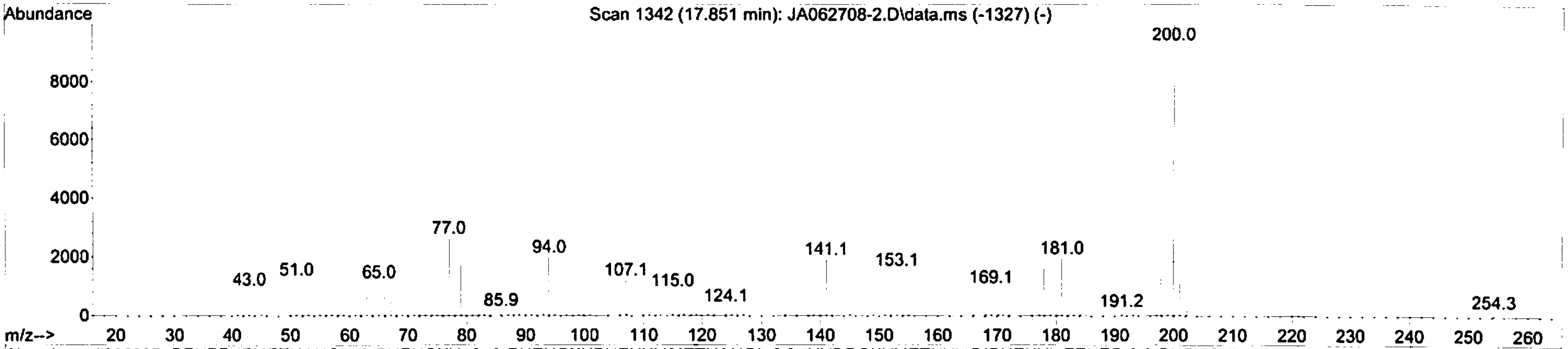

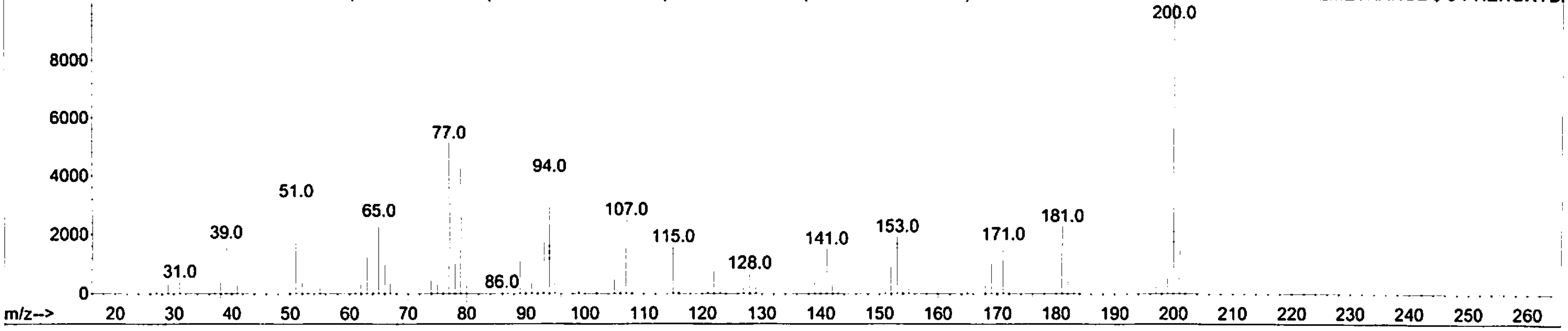

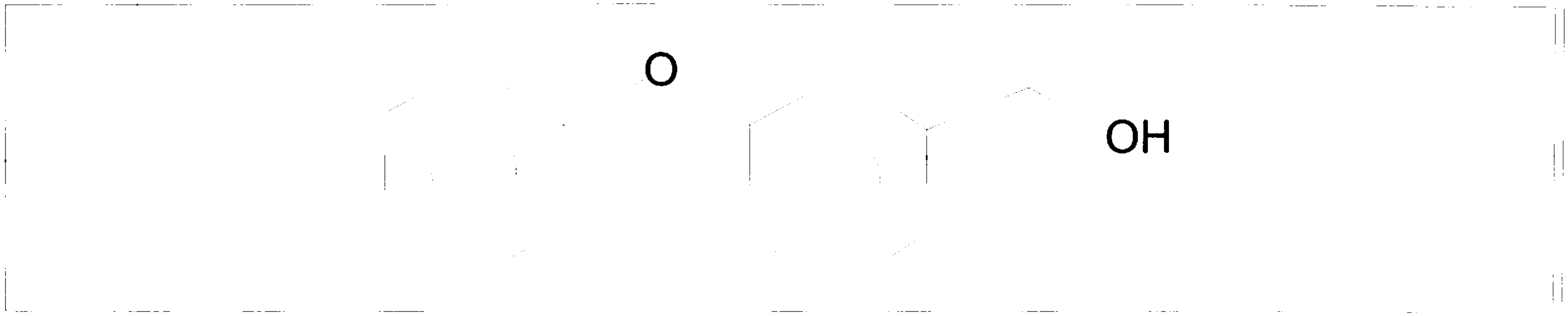

File :D:\ALDRICH\Snapshot\JA062708-2.D
Operator : Aldrich
Acquired : 27 Jun 2008 12:39 using AcqMethod JA-50-280LESS.M
Instrument : Buba
Sample Name: 8 lab-reared C.oculata male abdomen/CH2Cl2
Misc Info : GC run JA0627_1.D;fed aphids, 3-8 days old
Vial Number: 1

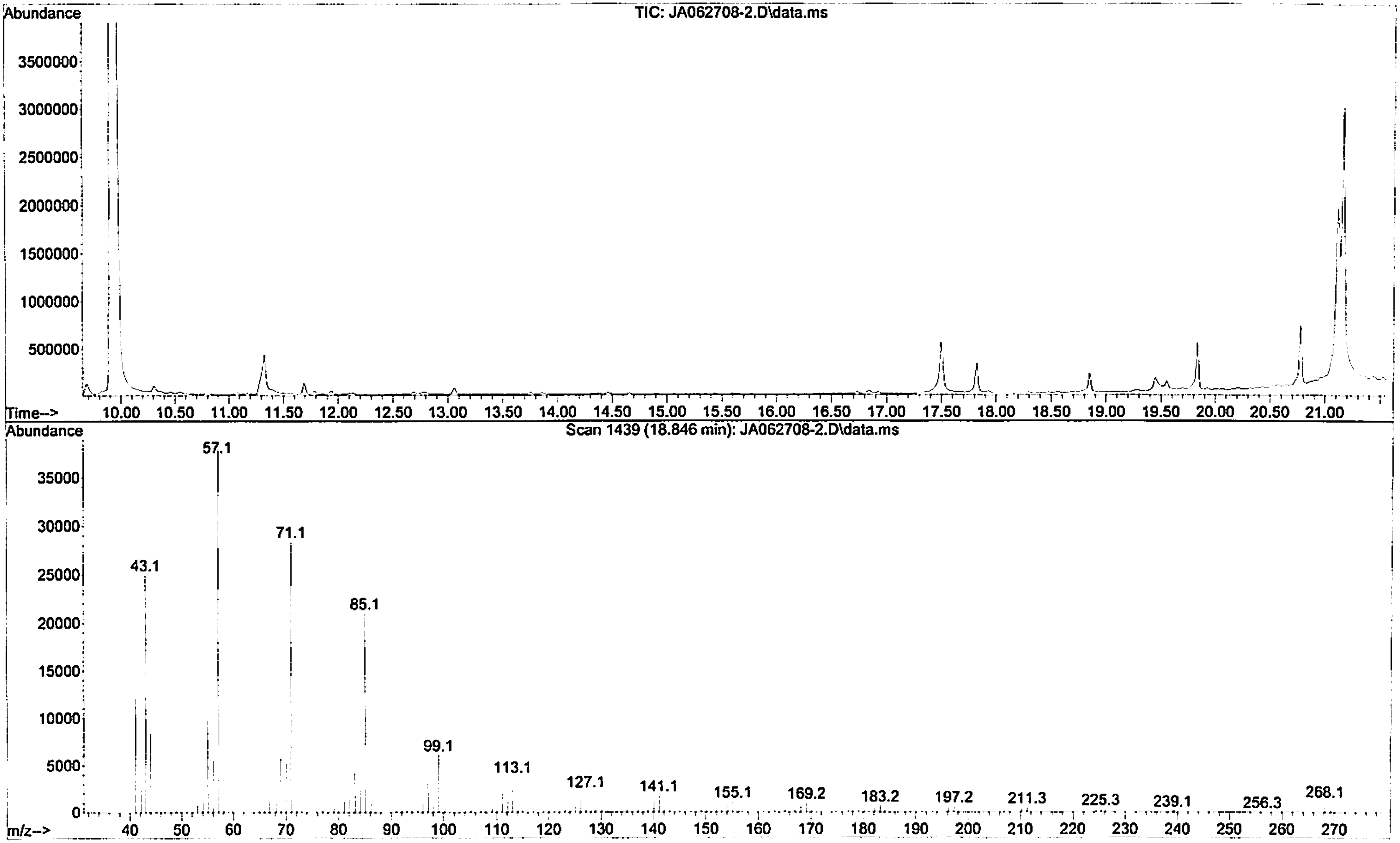

```
File        :D:\ALDRICH\Snapshot\JA062708-2.D
Operator    : Aldrich
Acquired    : 27 Jun 2008  12:39     using AcqMethod JA-50-280LESS.M
Instrument  :    Buba
Sample Name: 8 lab-reared C.oculata male abdomen/CH2Cl2
Misc Info   : GC run JA0627_1.D;fed aphids, 3-8 days old
Vial Number: 1
```

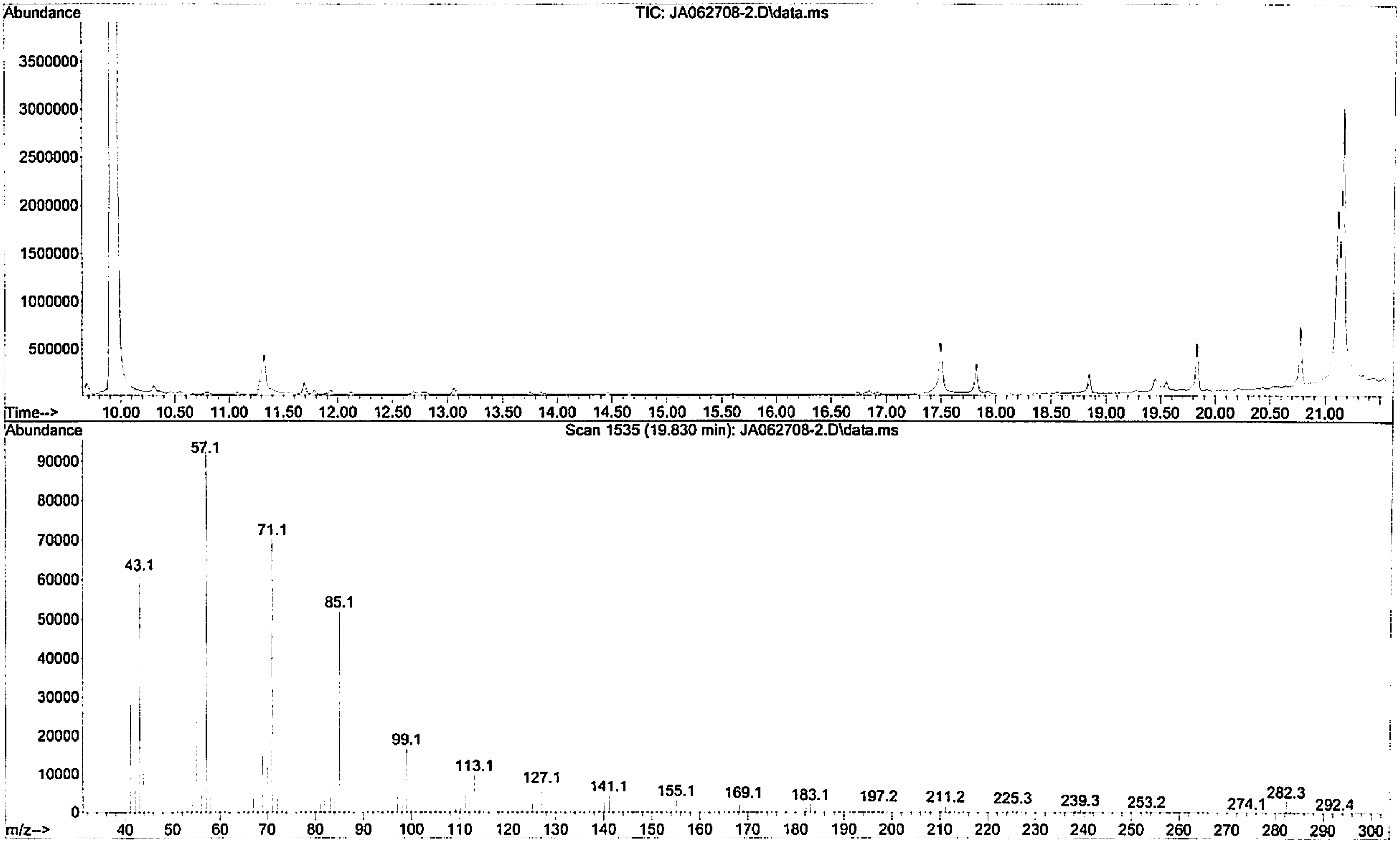

File   :D:\ALDRICH\Snapshot\JA062708-2.D
Operator   : Aldrich
Acquired   : 27 Jun 2008 12:39   using AcqMethod JA-50-280LESS.M
Instrument :   Buba
Sample Name: 8 lab-reared C.oculata male abdomen/CH2Cl2
Misc Info   : GC run JA0627_1.D;fed aphids, 3-8 days old
Vial Number: 1

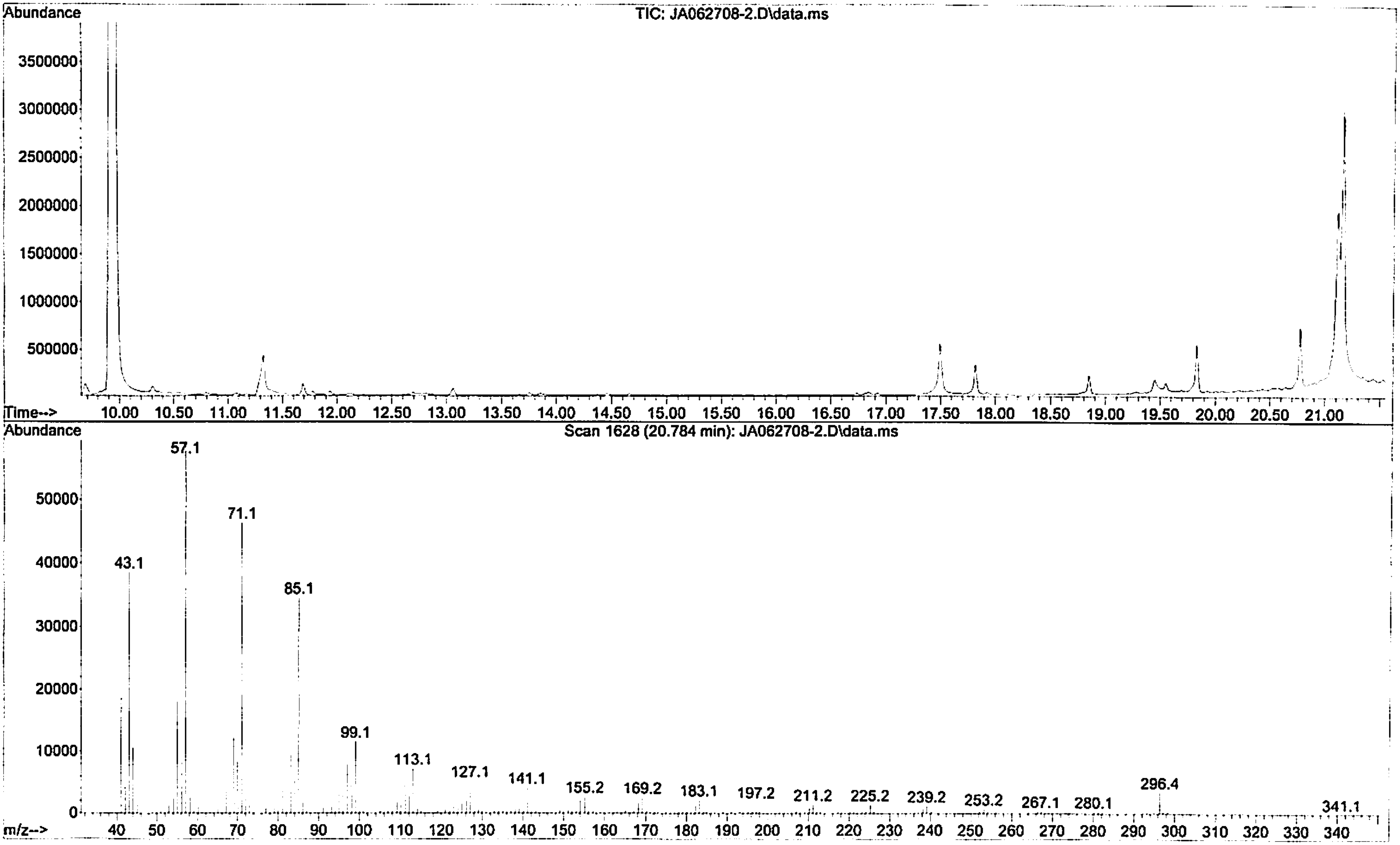

File   :D:\ALDRICH\Snapshot\JA062708-2.D
Operator   : Aldrich
Acquired   : 27 Jun 2008 12:39   using AcqMethod JA-50-280LESS.M
Instrument :   Buba
Sample Name: 8 lab-reared C.oculata male abdomen/CH2Cl2
Misc Info : GC run JA0627_1.D;fed aphids, 3-8 days old
Vial Number: 1

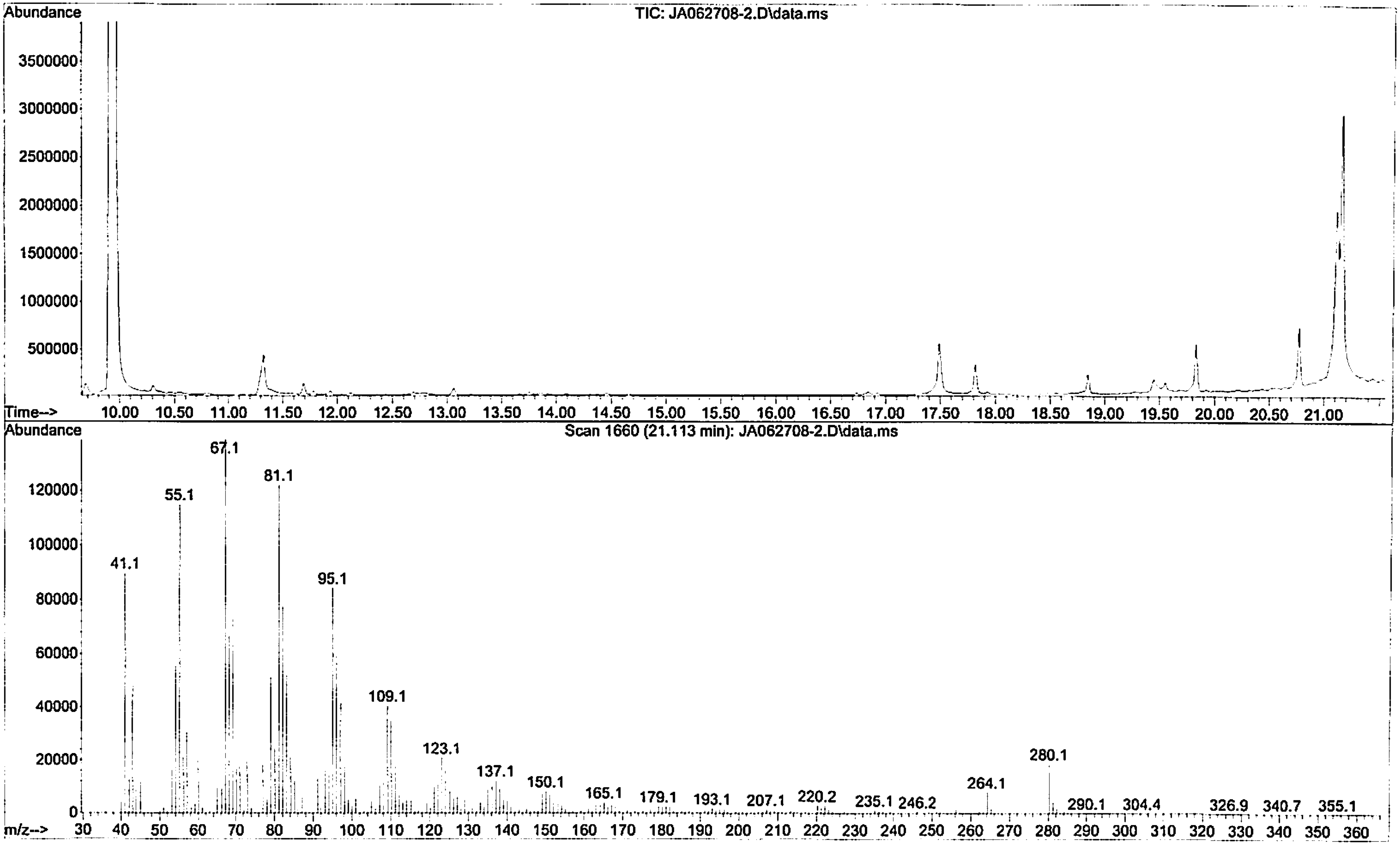

```
Library Searched : D:\DATABASE\W8N05ST.L
Quality          : 99
ID               : 9,12-OCTADECADIENOIC ACID (Z,Z)- $ (9E,12E)-9,12-OCTADECADIENOIC ACID # $ (9E,1
                   2E)-9,12-OCTADECADIENOIC ACID $ (9Z,12Z)-OCTADECADIENOIC ACID $ (Z,Z)-9,12-OCTA
                   DECADIENOIC ACID $ (Z,Z)-OCTADECA-9, 12-DIENOIC ACID $ 9, 12-OCTADECADIENOIC AC
                   ID $ 9,12-LINOLEIC
```

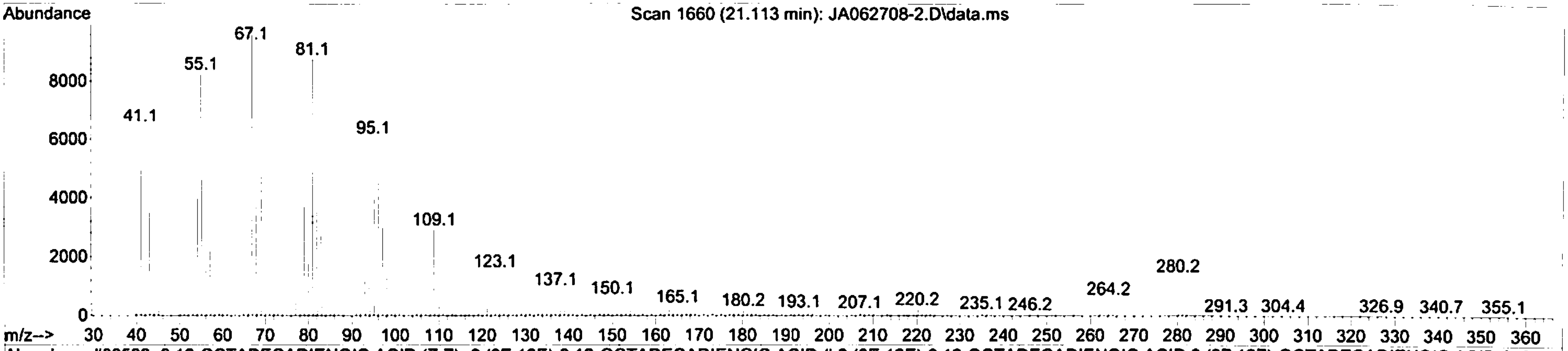

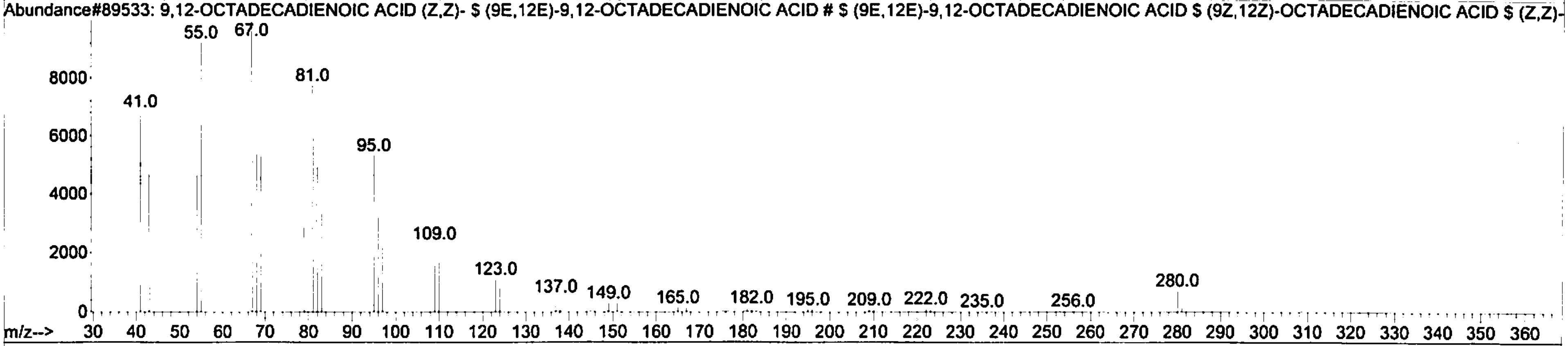

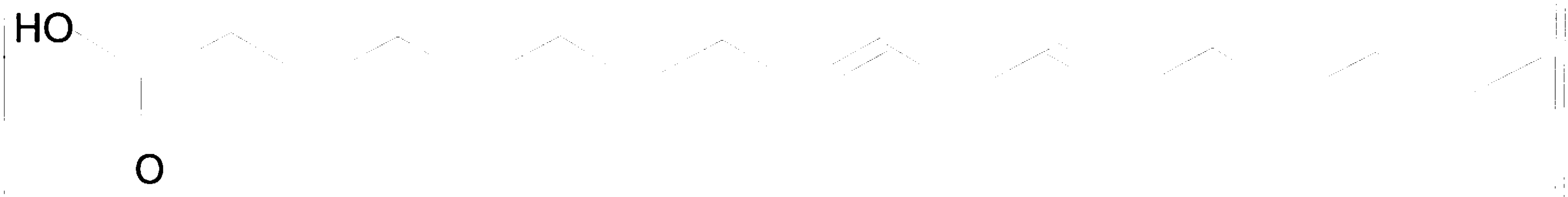

```
File        :D:\ALDRICH\Snapshot\JA062708-2.D
Operator    : Aldrich
Acquired    : 27 Jun 2008  12:39     using AcqMethod JA-50-280LESS.M
Instrument  :    Buba
Sample Name: 8 lab-reared C.oculata male abdomen/CH2Cl2
Misc Info   : GC run JA0627_1.D;fed aphids, 3-8 days old
Vial Number: 1
```

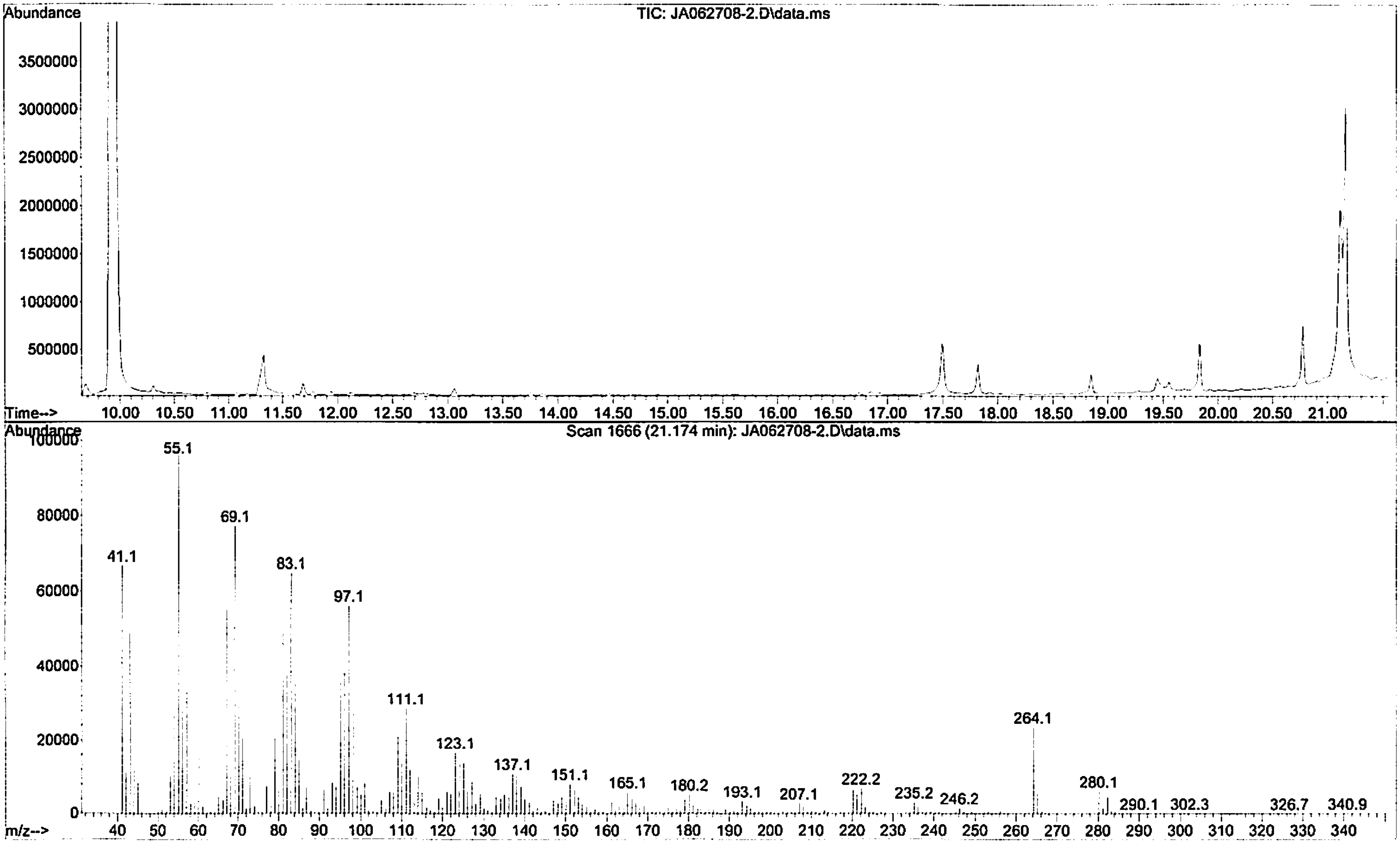

```
Library Searched : D:\DATABASE\W8N05ST.L
Quality          : 99
ID               : 9-OCTADECENOIC ACID (Z)- $ OCTADEC-9-ENOIC ACID $ (9E)-9-OCTADECENOIC ACID # $
                   (9E)-9-OCTADECENOIC ACID (COMPUTER-GENERATED NAME) $ (9Z)-9-OCTADECENOIC ACID $
                    (9Z)-OCTADECENOIC ACID $ (Z)-9-OCTADECANOIC ACID $ (Z)-9-OCTADECENOIC ACID $ (
                   Z)-OCTADEC-9-ENOIC
```

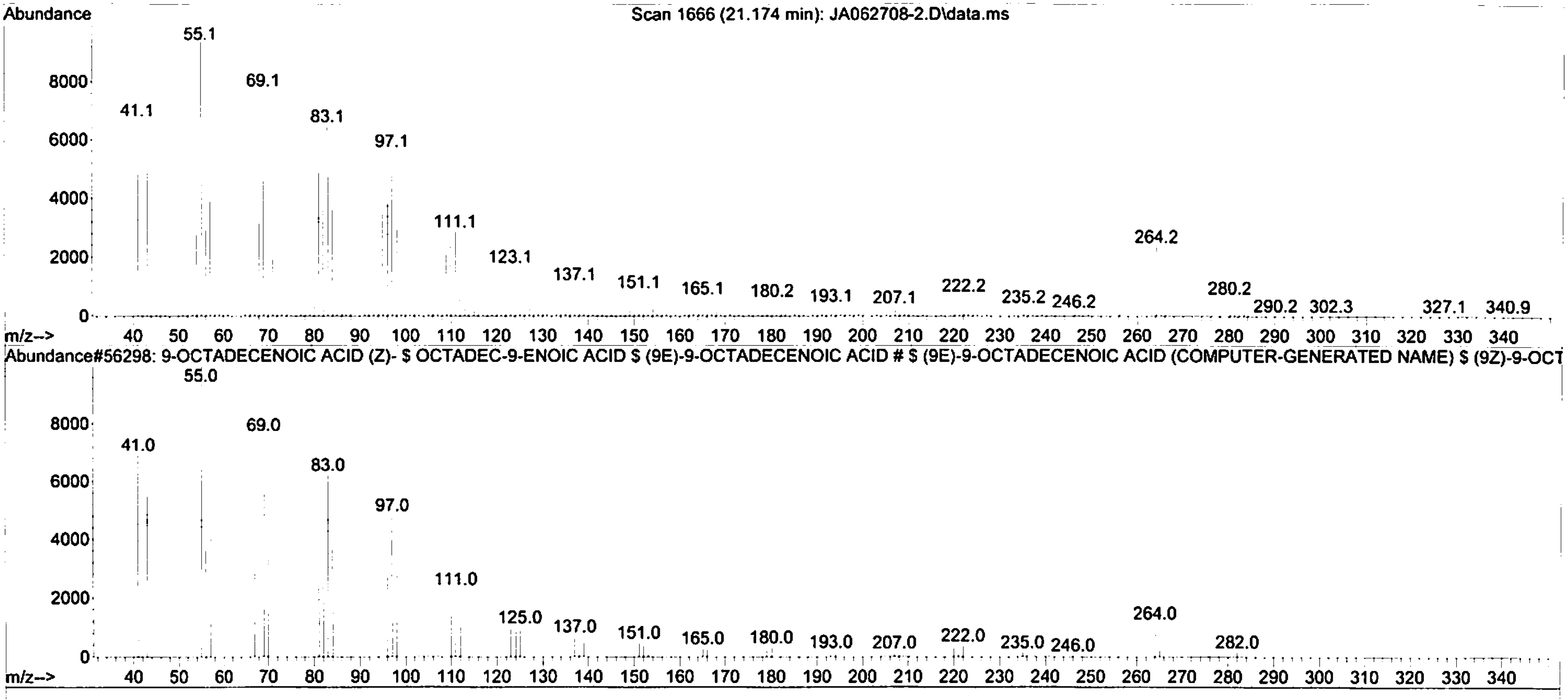

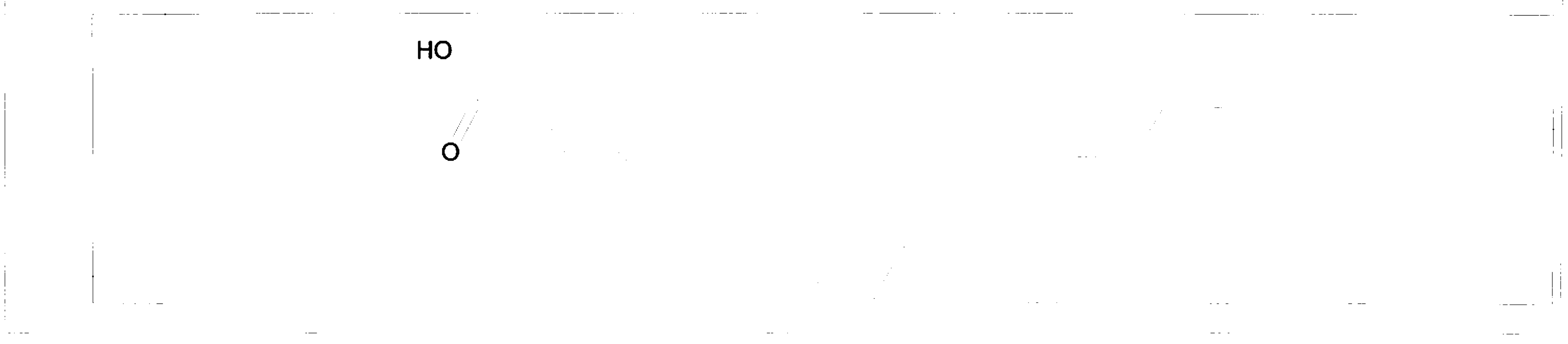

File :D:\ALDRICH\Snapshot\JA062708-2.D
Operator : Aldrich
Acquired : 27 Jun 2008 12:39 using AcqMethod JA-50-280LESS.M
Instrument : Buba
Sample Name: 8 lab-reared C.oculata male abdomen/CH2Cl2
Misc Info : GC run JA0627_1.D;fed aphids, 3-8 days old
Vial Number: 1

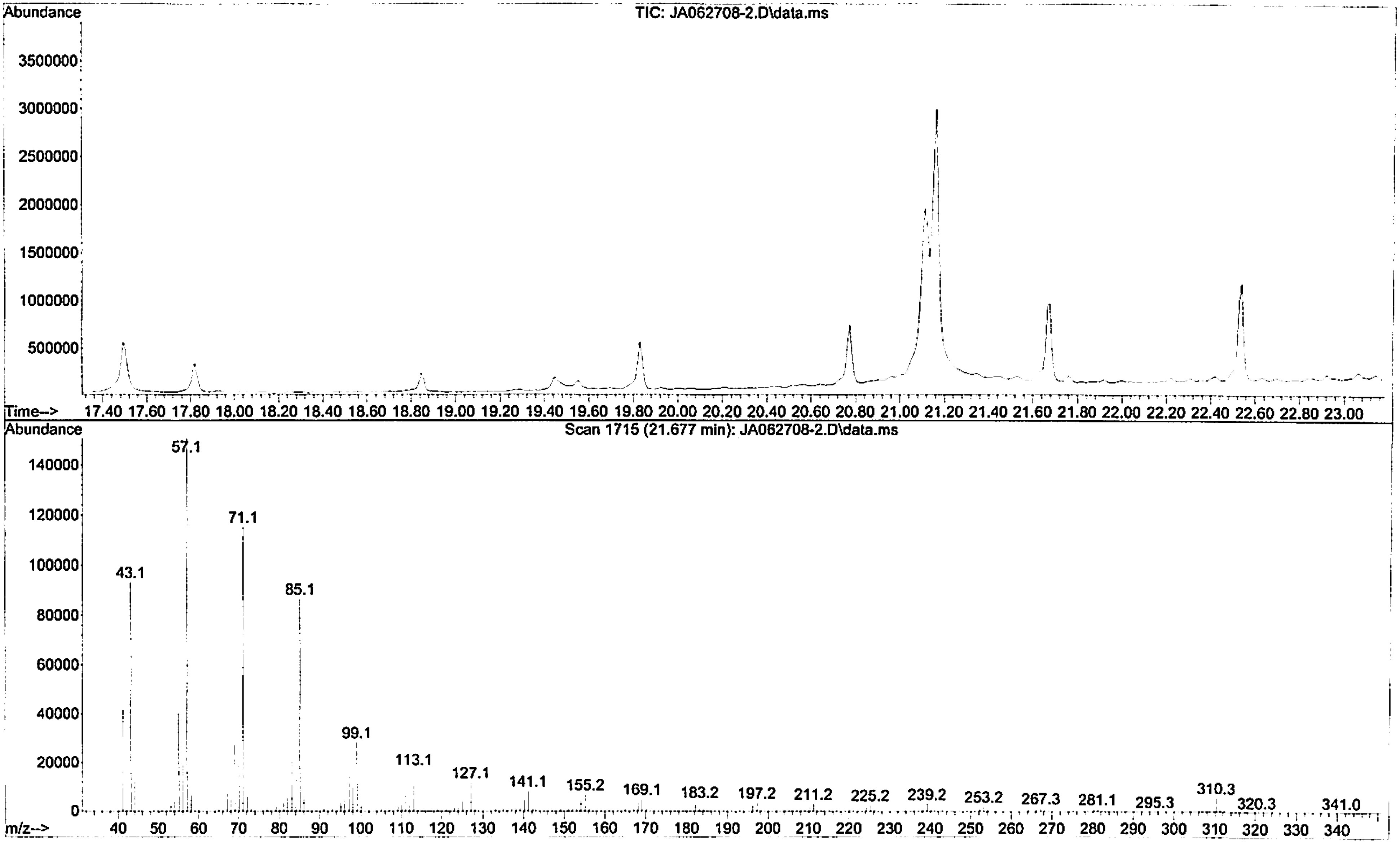

File : D:\ALDRICH\Snapshot\JA062708-2.D
Operator : Aldrich
Acquired : 27 Jun 2008 12:39 using AcqMethod JA-50-280LESS.M
Instrument : Buba
Sample Name: 8 lab-reared C.oculata male abdomen/CH2Cl2
Misc Info : GC run JA0627_1.D;fed aphids, 3-8 days old
Vial Number: 1

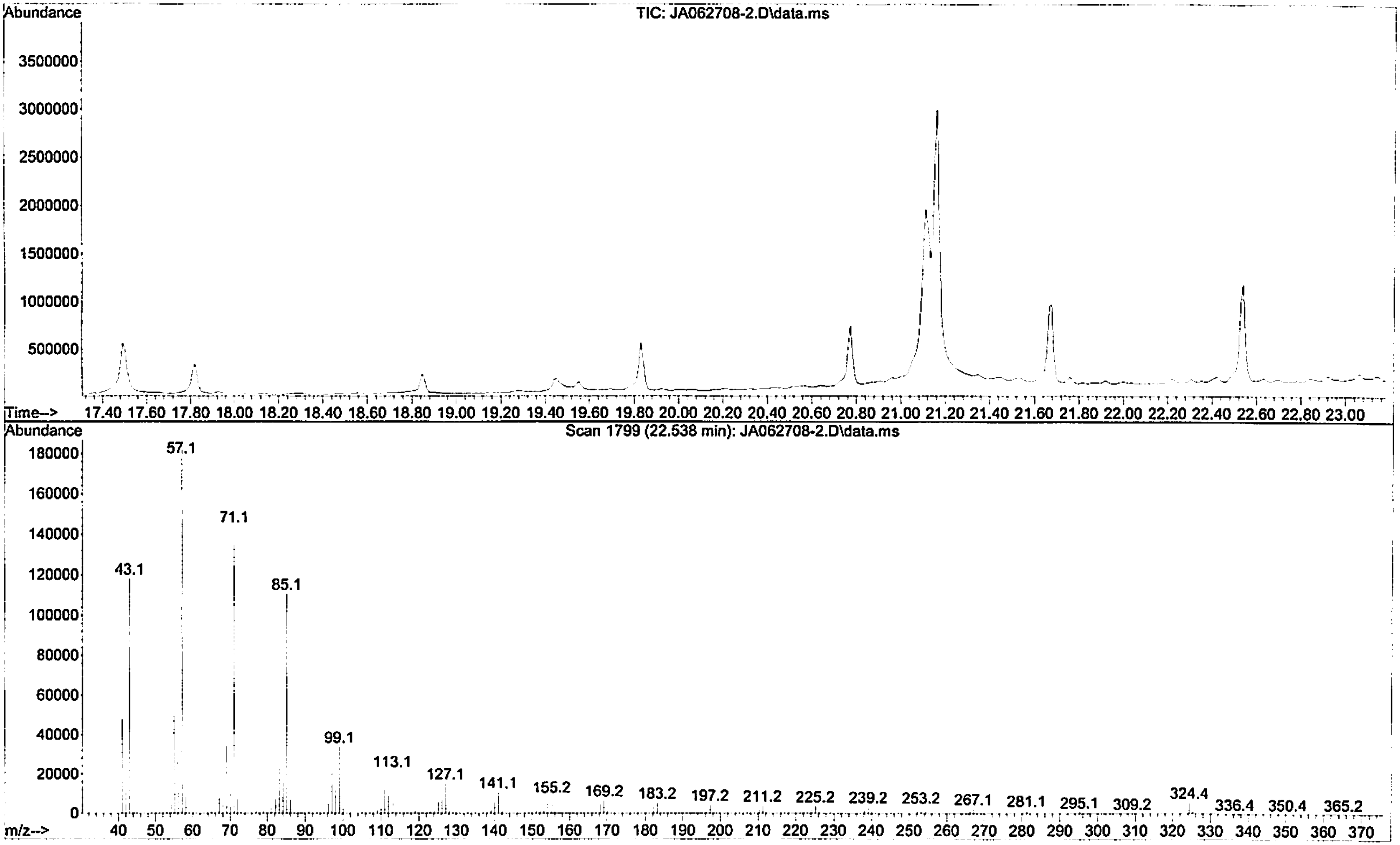

File :D:\ALDRICH\Snapshot\JA062708-2.D
Operator : Aldrich
Acquired : 27 Jun 2008 12:39 using AcqMethod JA-50-280LESS.M
Instrument : Buba
Sample Name: 8 lab-reared C.oculata male abdomen/CH2Cl2
Misc Info : GC run JA0627_1.D;fed aphids, 3-8 days old
Vial Number: 1

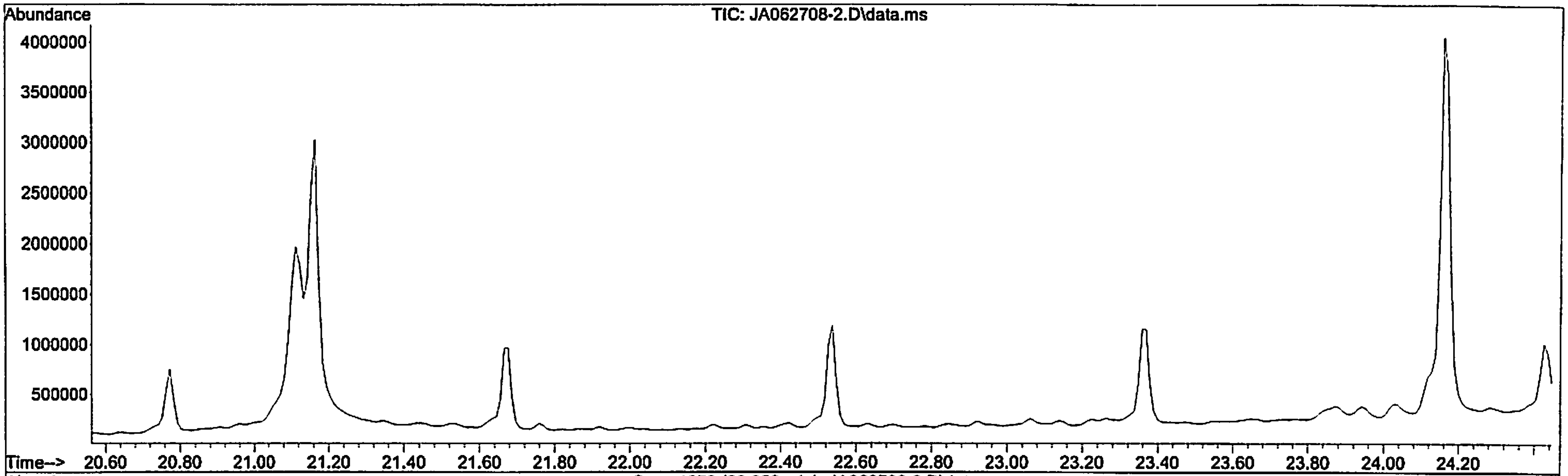

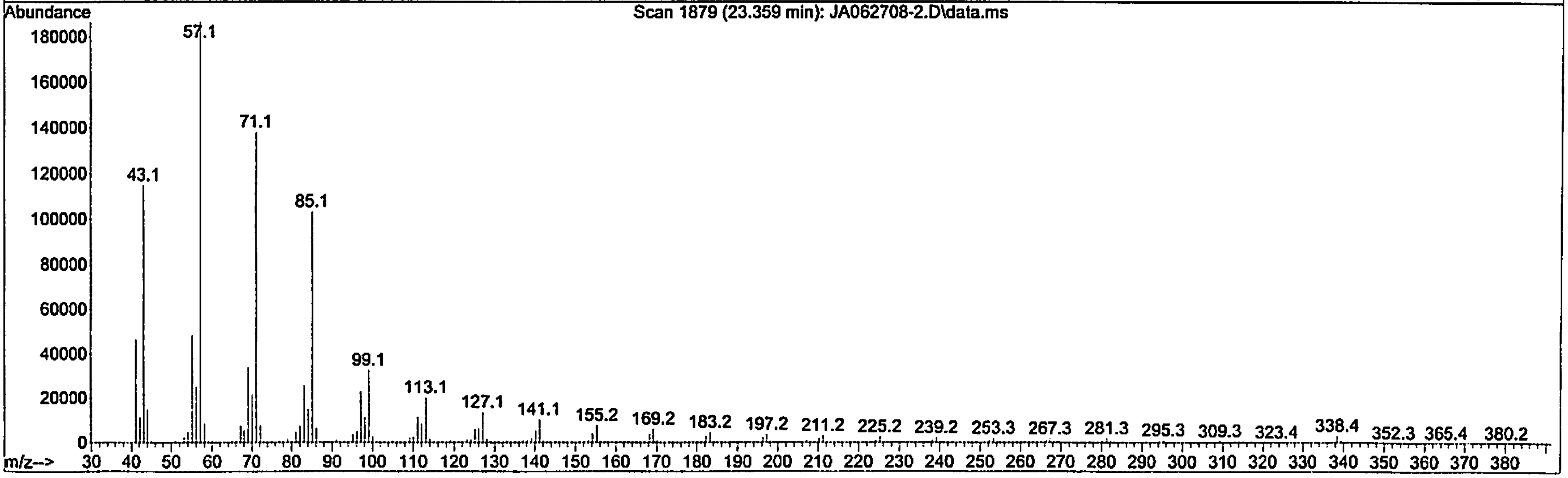

```
File        :D:\ALDRICH\Snapshot\JA062708-2.D
Operator    : Aldrich
Acquired    : 27 Jun 2008  12:39     using AcqMethod JA-50-280LESS.M
Instrument  :    Buba
Sample Name: 8 lab-reared C.oculata male abdomen/CH2Cl2
Misc Info   : GC run JA0627_1.D;fed aphids, 3-8 days old
Vial Number: 1
```

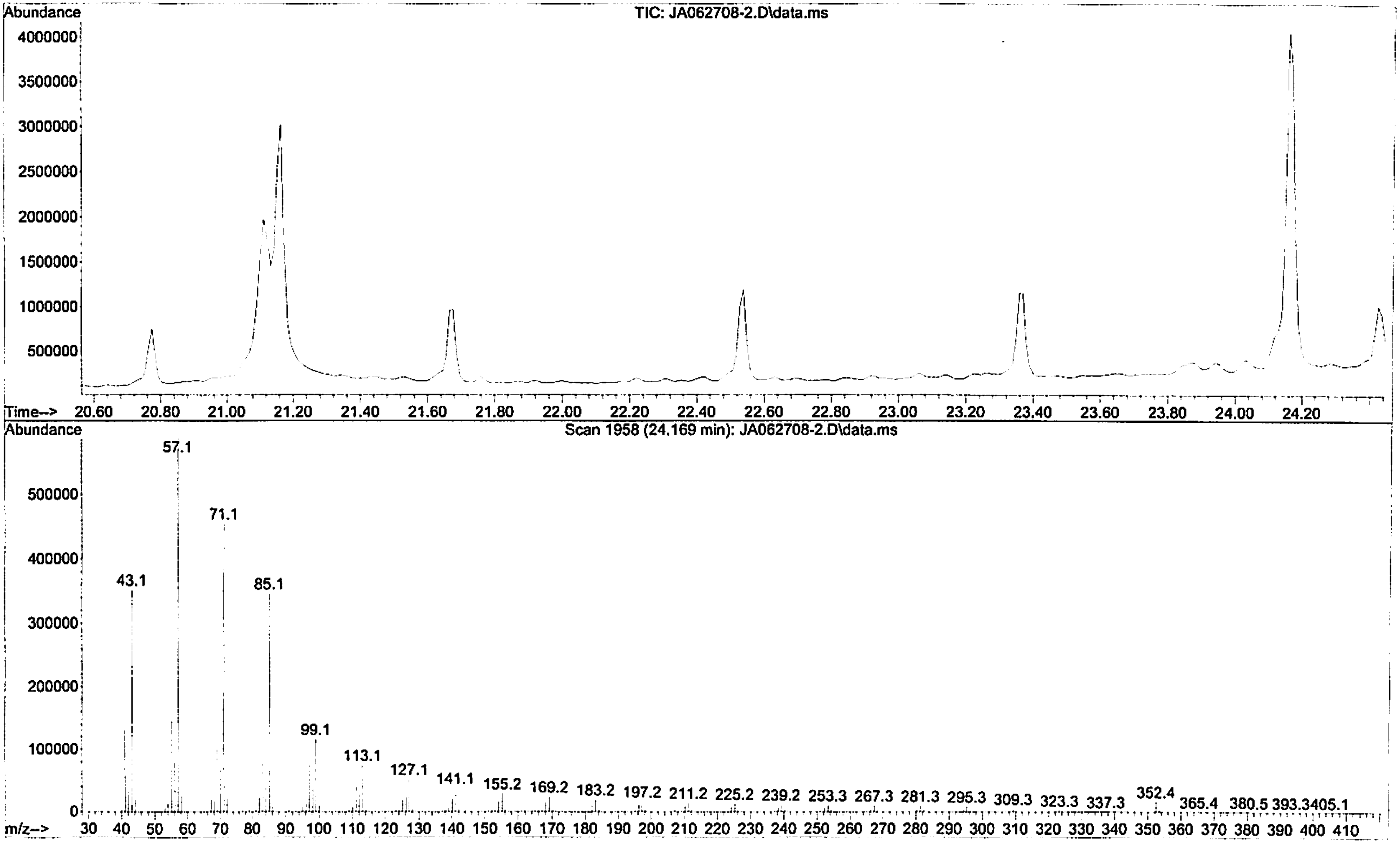

File   :D:\ALDRICH\Snapshot\JA062708-2.D
Operator   : Aldrich
Acquired   : 27 Jun 2008 12:39   using AcqMethod JA-50-280LESS.M
Instrument :   Buba
Sample Name: 8 lab-reared C.oculata male abdomen/CH2Cl2
Misc Info   : GC run JA0627_1.D;fed aphids, 3-8 days old
Vial Number: 1

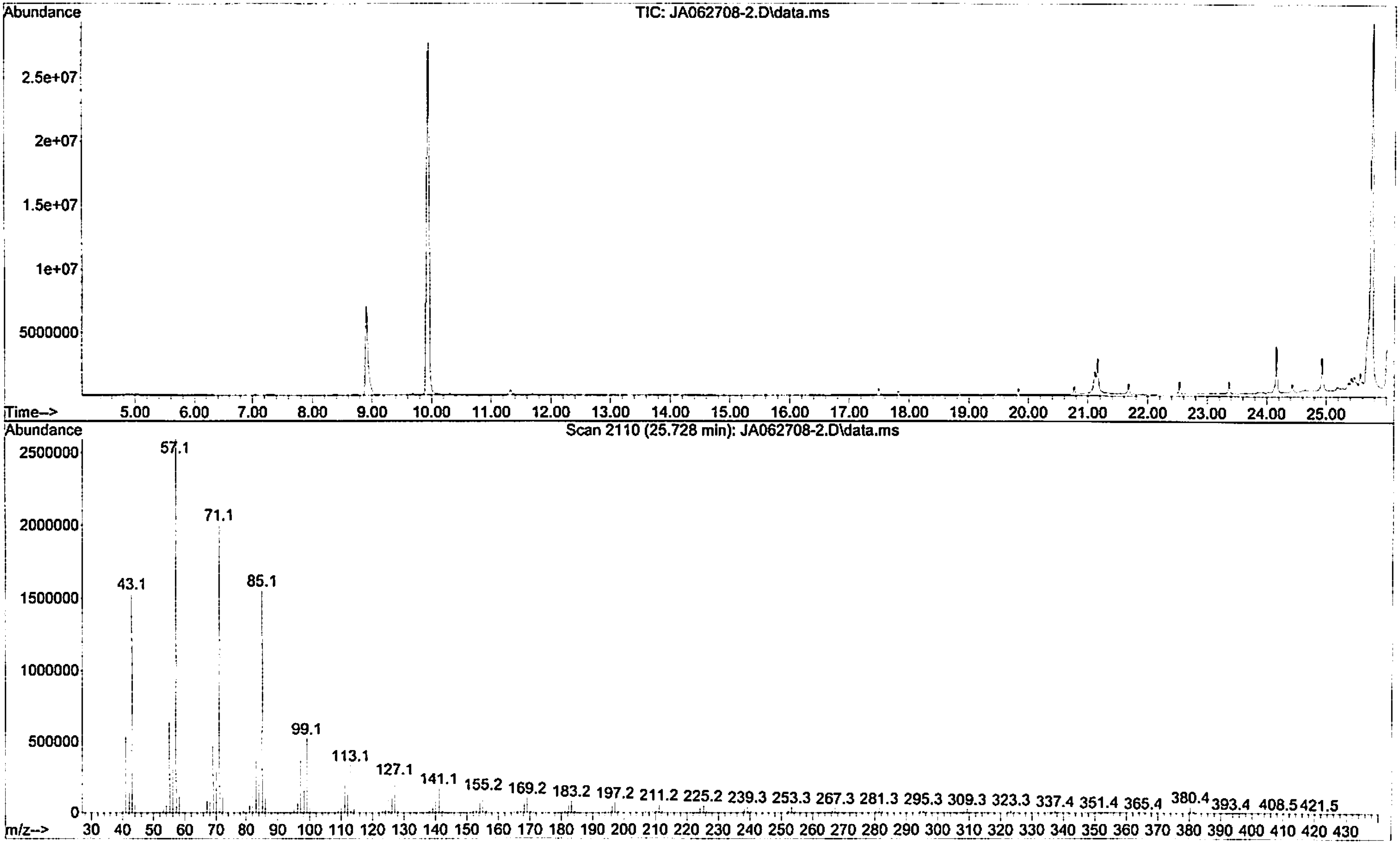

```
File          :D:\ALDRICH\Snapshot\JA062708-2.D
Operator      : Aldrich
Acquired      : 27 Jun 2008  12:39     using AcqMethod JA-50-280LESS.M
Instrument :     Buba
Sample Name: 8 lab-reared C.oculata male abdomen/CH2Cl2
Misc Info  : GC run JA0627_1.D;fed aphids, 3-8 days old
Vial Number: 1
```

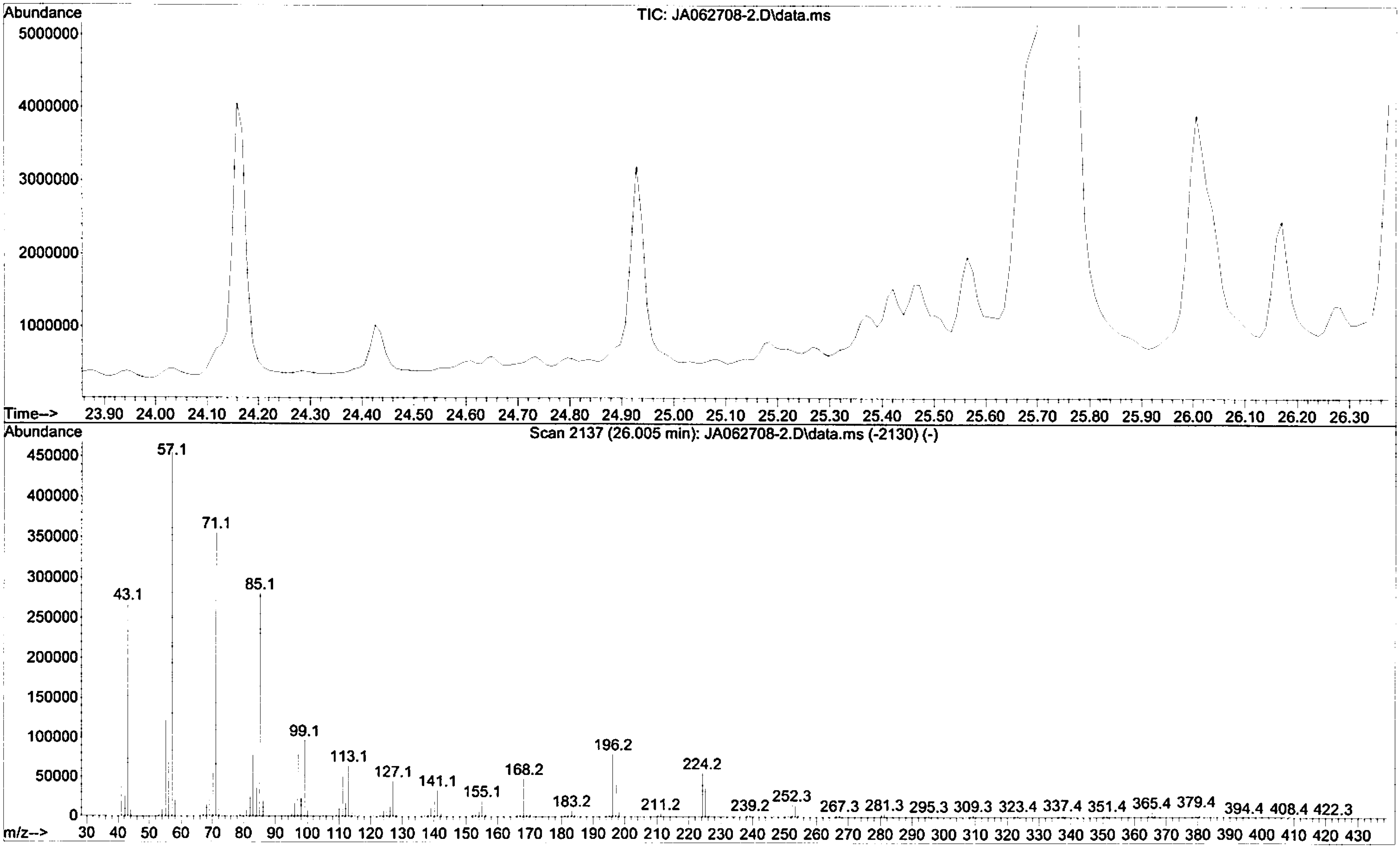

File :D:\ALDRICH\Snapshot\JA062708-2.D
Operator : Aldrich
Acquired : 27 Jun 2008 12:39 using AcqMethod JA-50-280LESS.M
Instrument : Buba
Sample Name: 8 lab-reared C.oculata male abdomen/CH2Cl2
Misc Info : GC run JA0627_1.D;fed aphids, 3-8 days old
Vial Number: 1

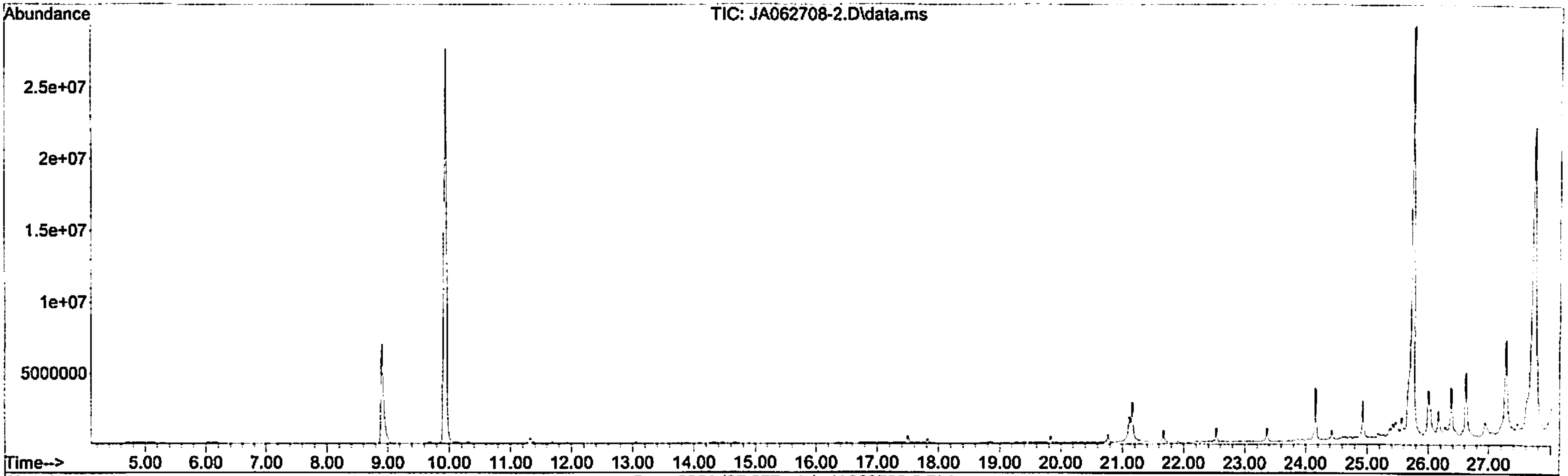

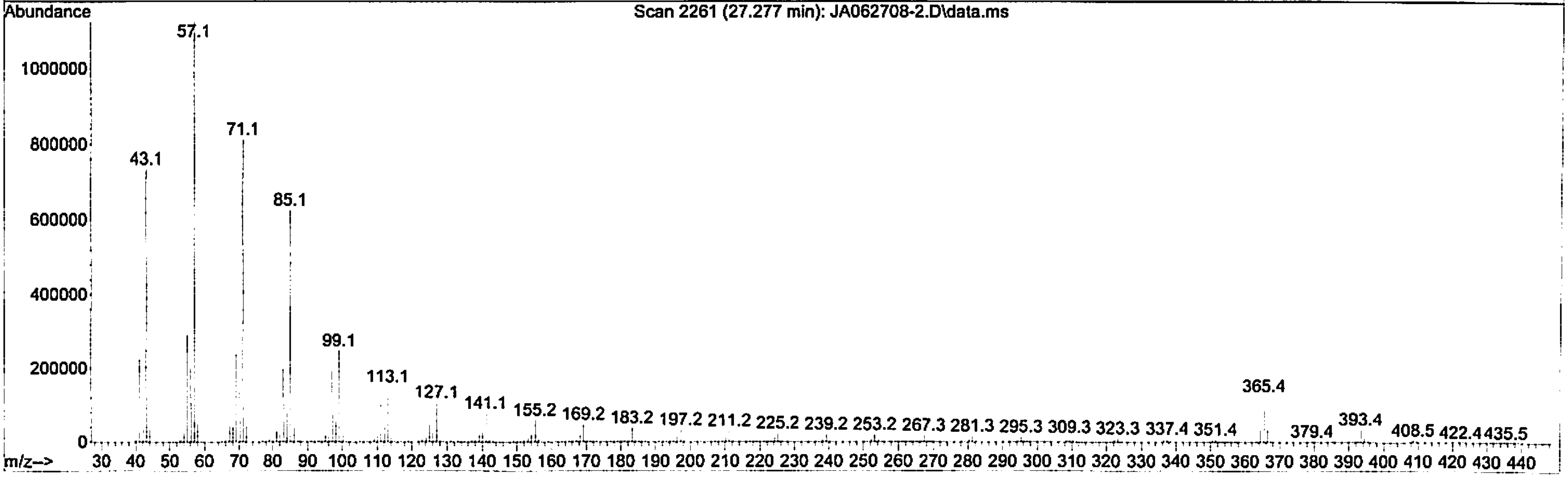

File   :D:\ALDRICH\Snapshot\JA062708-2.D
Operator   : Aldrich
Acquired   : 27 Jun 2008 12:39   using AcqMethod JA-50-280LESS.M
Instrument :   Buba
Sample Name: 8 lab-reared C.oculata male abdomen/CH2Cl2
Misc Info   : GC run JA0627_1.D;fed aphids, 3-8 days old
Vial Number: 1

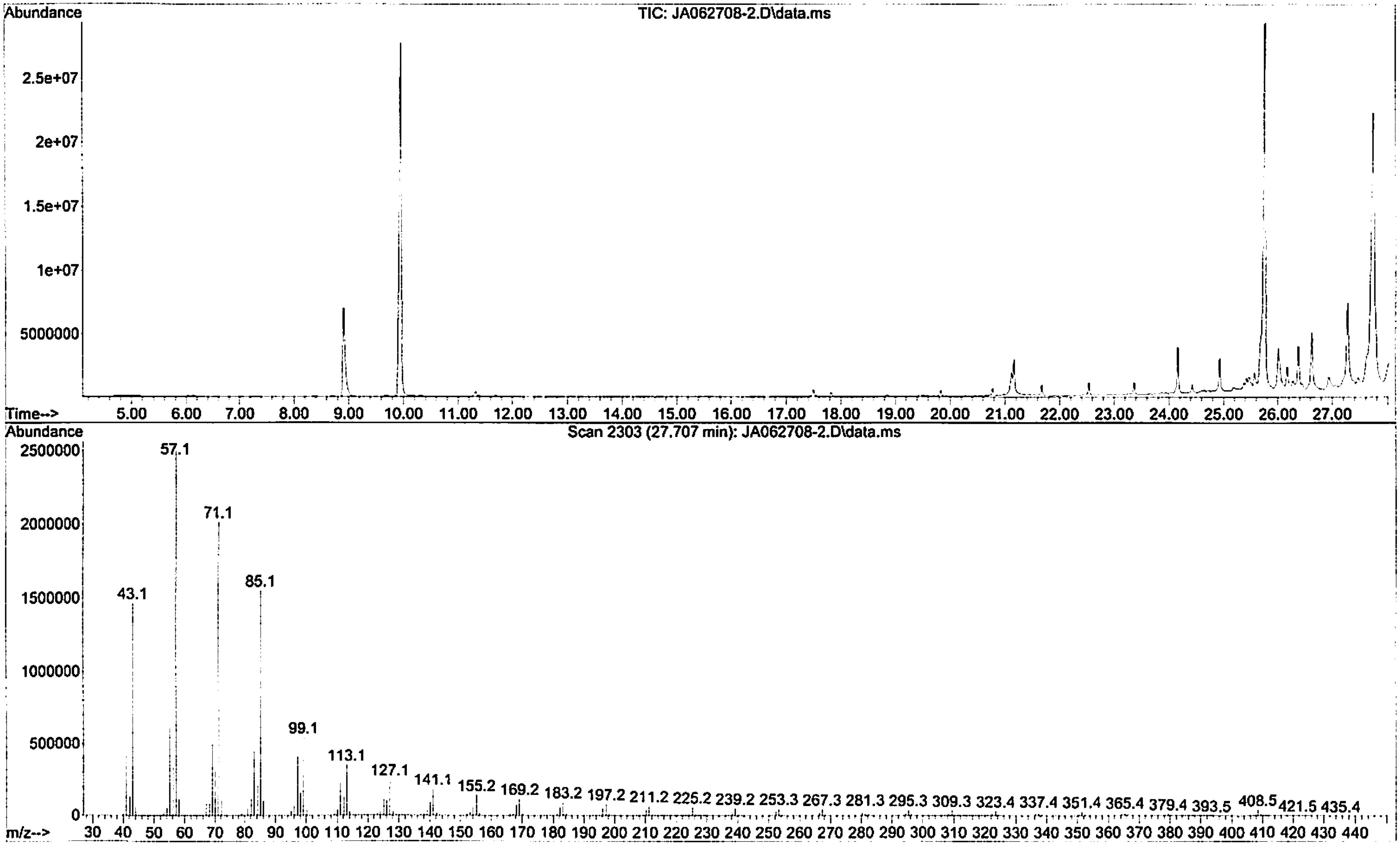

inj. 1ul abdominal cuticle extract of 5 lab-reared male
s 20-29-days-old without access to plants; fed pea aphi
d, Sitotroga eggs & honey water, & dissected 8/13 (5 ma
les /40ul CH2Cl2 conc. to ca. 5ul), HP-5.

===

Injection Date : 8/13/2008 12:16:15 PM
Sample Name : lab 5M C.ocu abd Location : -
Acq. Operator : Aldrich Inj : 1
Inj Volume : Manually
Method : C:\HPCHEM\1\METHODS\DBLESS08.M
Last changed : 8/12/2008 11:55:44 AM by Aldrich
6/11/08; editing new method for desired output

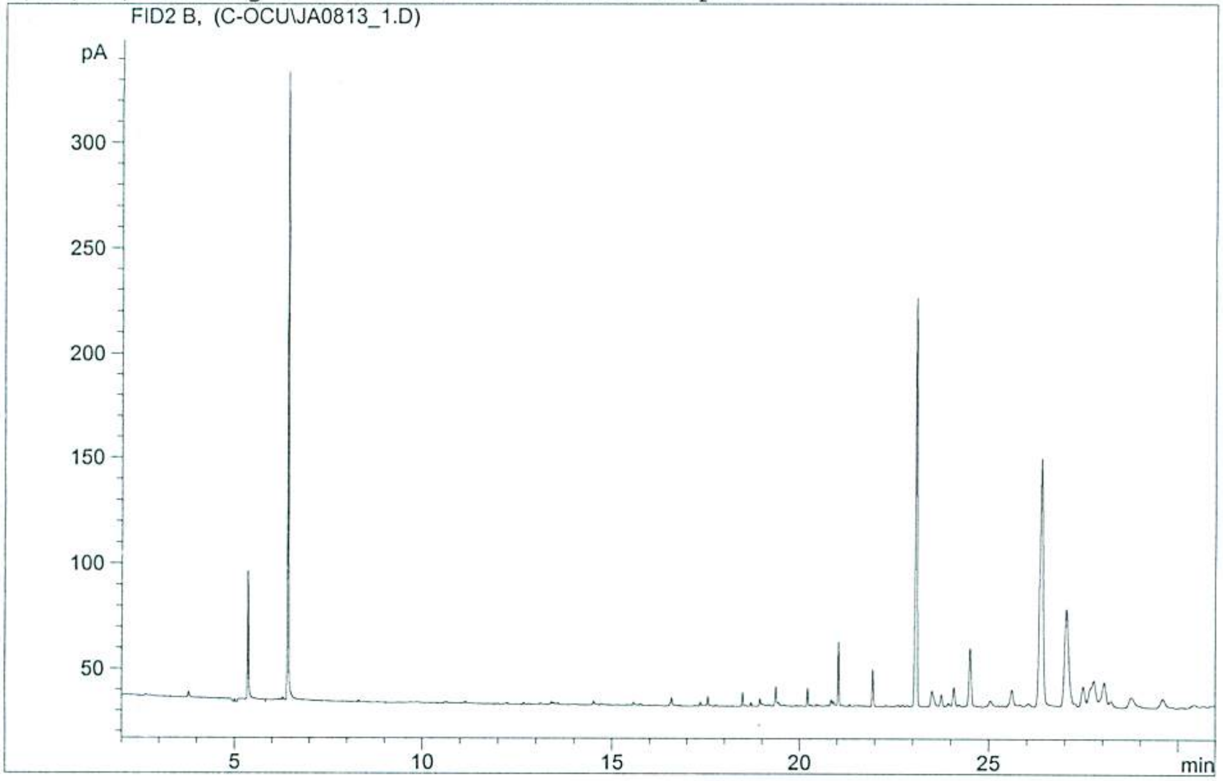

===

Area Percent Report

===

Sorted By : Signal
Multiplier : 1.0000
Dilution : 1.0000

Signal 1: FID2 B,

Peak #	RetTime [min]	Type	Width [min]	Area [pA*s]	Height [pA]	Area %
1	0.467	PP	0.0146	11.00343	11.05655	0.00042
2	0.533	VV S	0.0158	1.49228e5	1.78474e5	5.64316
3	0.580	VV S	0.1004	2.49193e6	4.13768e5	94.23391
4	1.460	VB S	0.0150	94.79893	105.41685	0.00358
5	1.607	BV	0.0346	3.69842	1.82972	0.00014
6	1.705	VV	0.0500	13.28734	3.69150	0.00050
7	1.808	VB	0.0391	8.82340	3.31357	0.00033
8	3.779	BB	0.0424	7.50409	2.69936	0.00028
9	5.000	PP	0.0199	1.56996	1.31328	5.937e-5
10	5.350	PB	0.0319	124.22678	60.51726	0.00470
11	6.423	PB	0.0291	560.39868	295.85358	0.02119
12	14.517	PP	0.0367	3.98305	1.67948	0.00015

Peak #	RetTime [min]	Type	Width [min]	Area [pA*s]	Height [pA]	Area %
13	15.573	PB	0.0323	2.98141	1.43181	0.00011
14	16.582	BB	0.0399	9.70355	3.54346	0.00037
15	17.346	PB	0.0380	4.34248	1.80657	0.00016
16	17.548	BB	0.0316	8.73558	4.31165	0.00033
17	18.474	PB	0.0305	12.21688	6.34637	0.00046
18	18.698	PP	0.0316	3.95342	1.95121	0.00015
19	18.937	PB	0.0366	8.24157	3.24932	0.00031
20	19.363	PB	0.0374	21.85039	8.65924	0.00083
21	20.219	PP	0.0305	16.78219	8.69974	0.00063
22	20.841	PV	0.0381	7.63631	3.05681	0.00029
23	20.905	VB	0.0408	6.59770	2.34588	0.00025
24	21.042	BB	0.0321	61.60423	29.81875	0.00233
25	21.952	BB	0.0384	42.66174	17.52662	0.00161
26	23.097	BB	0.0503	635.12579	193.42932	0.02402
27	23.514	PB	0.0719	40.07027	7.20589	0.00152
28	23.759	BP	0.0534	18.85434	5.44812	0.00071
29	24.090	PP	0.0583	31.83170	8.57640	0.00120
30	24.514	BP	0.0645	111.57770	27.38947	0.00422
31	25.609	BB	0.0803	39.63595	7.41917	0.00150
32	26.385	BB	0.0864	692.35138	116.42314	0.02618
33	27.046	BB	0.1021	337.83240	45.27579	0.01278
34	27.486	PV	0.0771	59.37449	9.47800	0.00225
35	27.771	VV	0.1364	135.91484	12.25323	0.00514
36	28.044	VB	0.1226	111.49355	11.55581	0.00422
Totals :				2.64441e6	5.93267e5	

Results obtained with enhanced integrator!

==

*** End of Report ***

```
File        :D:\Aldrich\JA112408-1.D
Operator    :
Acquired    : 24 Nov 2008  15:36     using AcqMethod JA-50-280LESS.M
Instrument :   Buba
Sample Name: 7M grouped C.oculata, abd. cuticle/CH2Cl2
Misc Info   : noGC; lab reared in grp. of 15; 17-21day-old
Vial Number: 1
```

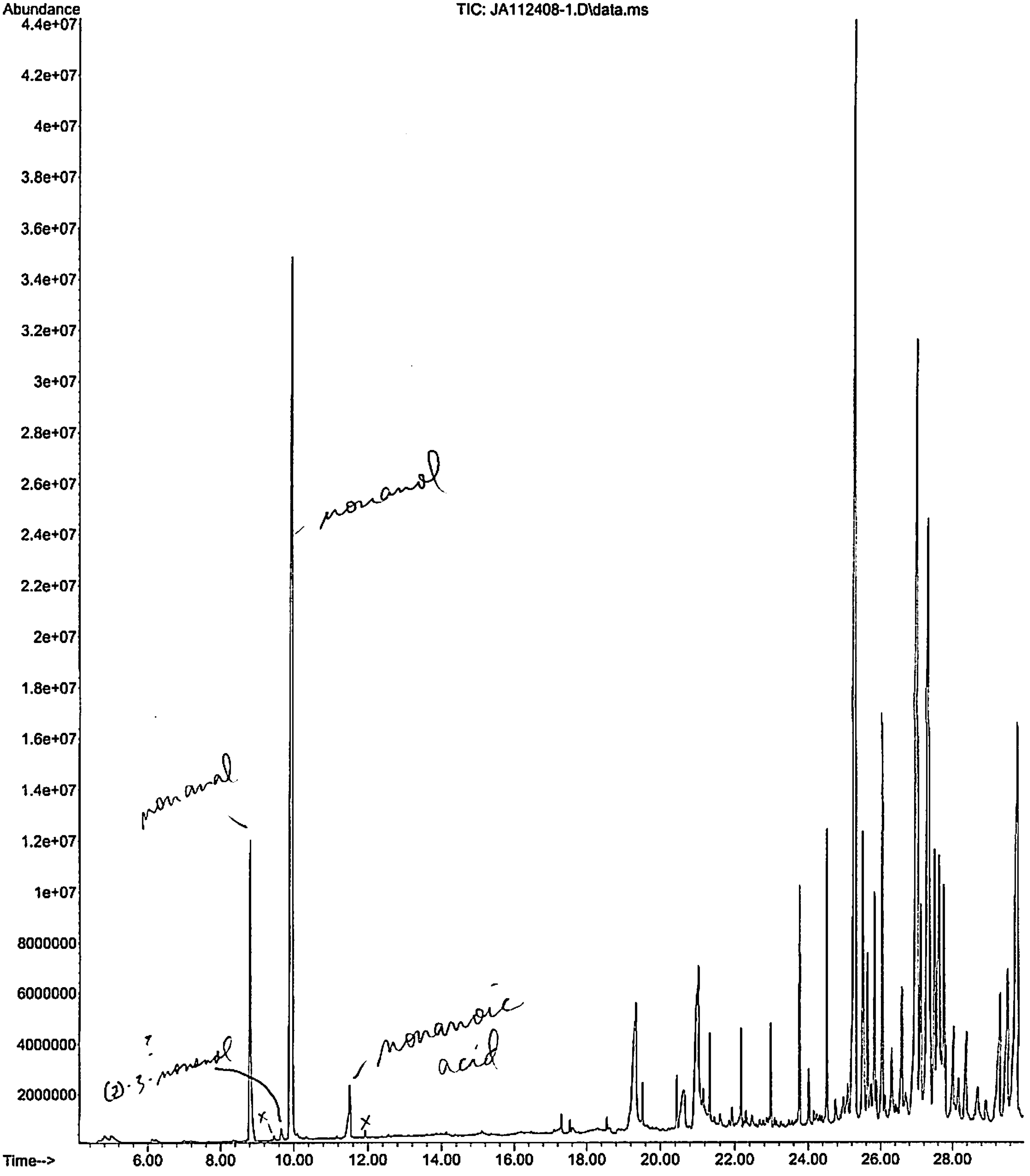

```
File        :D:\Aldrich\JA112408-1.D
Operator    :
Acquired    : 24 Nov 2008  15:36     using AcqMethod JA-50-280LESS.M
Instrument  :   Buba
Sample Name: 7M grouped C.oculata, abd. cuticle/CH2C12
Misc Info   : noGC; lab reared in grp. of 15; 17-21day-old
Vial Number: 1
```

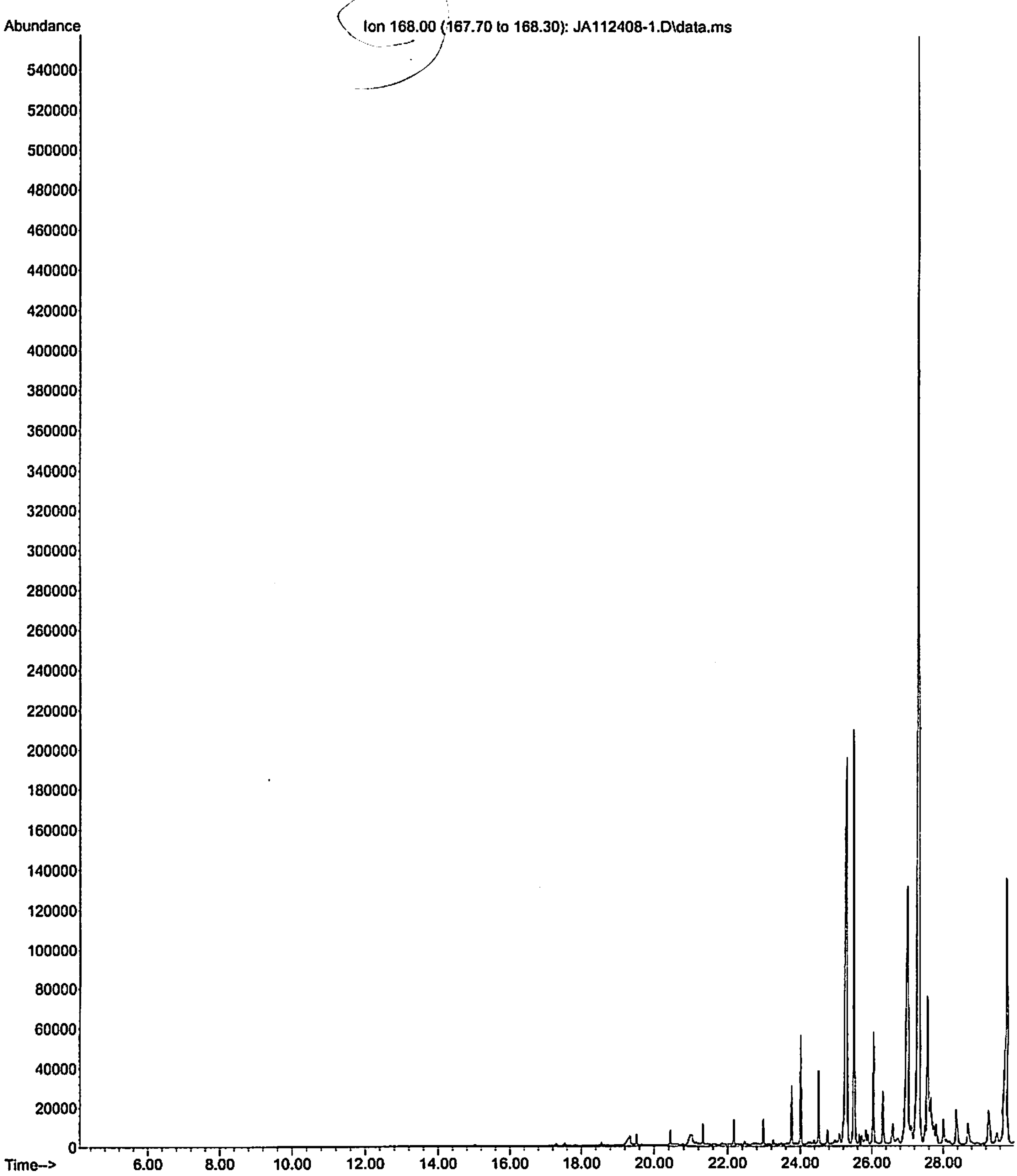

File   :D:\ALDRICH\Snapshot\JA112408-1.D
Operator :
Acquired : 24 Nov 2008 15:36   using AcqMethod JA-50-280LESS.M
Instrument :   Buba
Sample Name: 7M grouped C.oculata, abd. cuticle/CH2Cl2
Misc Info : noGC; lab reared in grp. of 15; 17-21day-old
Vial Number: 1

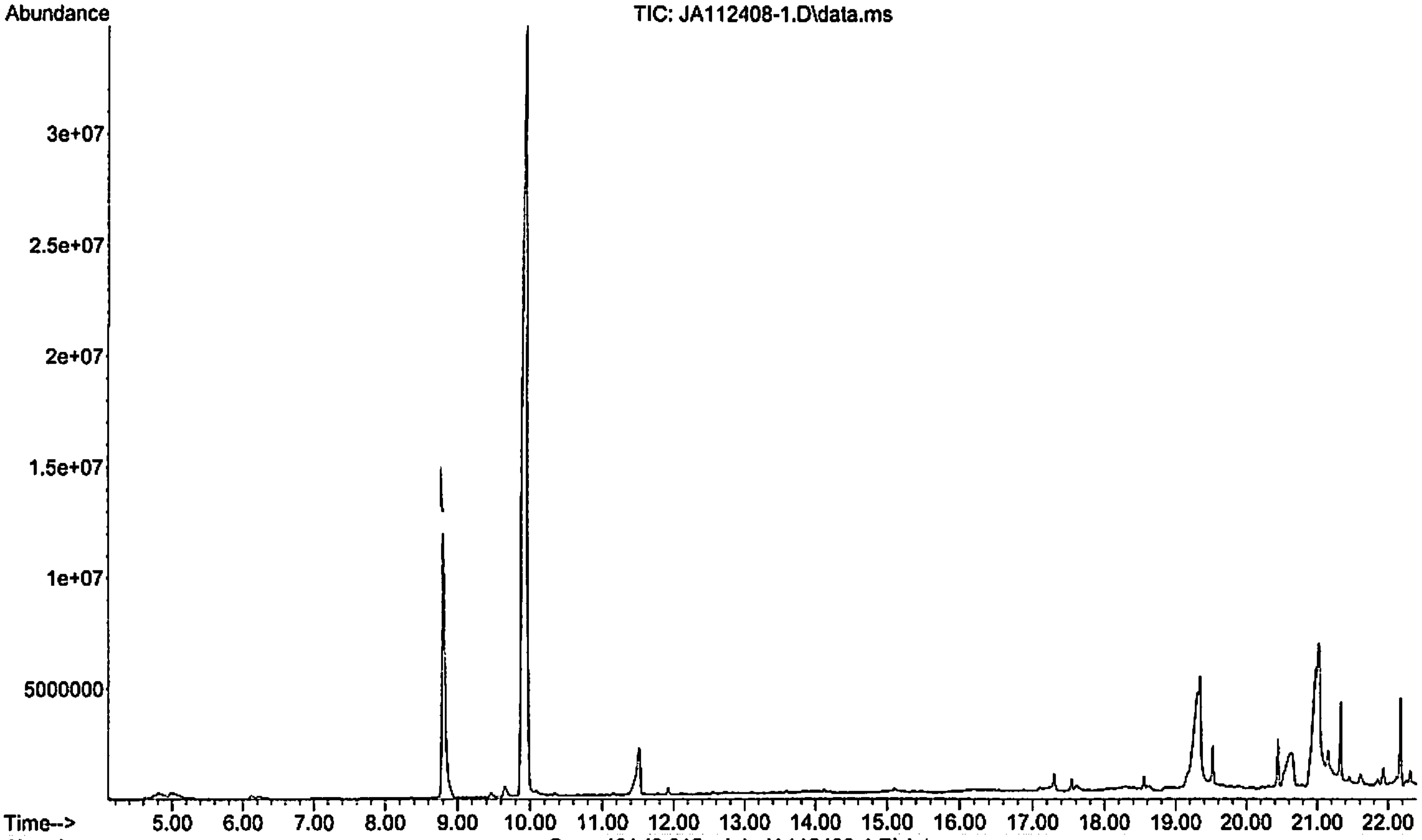

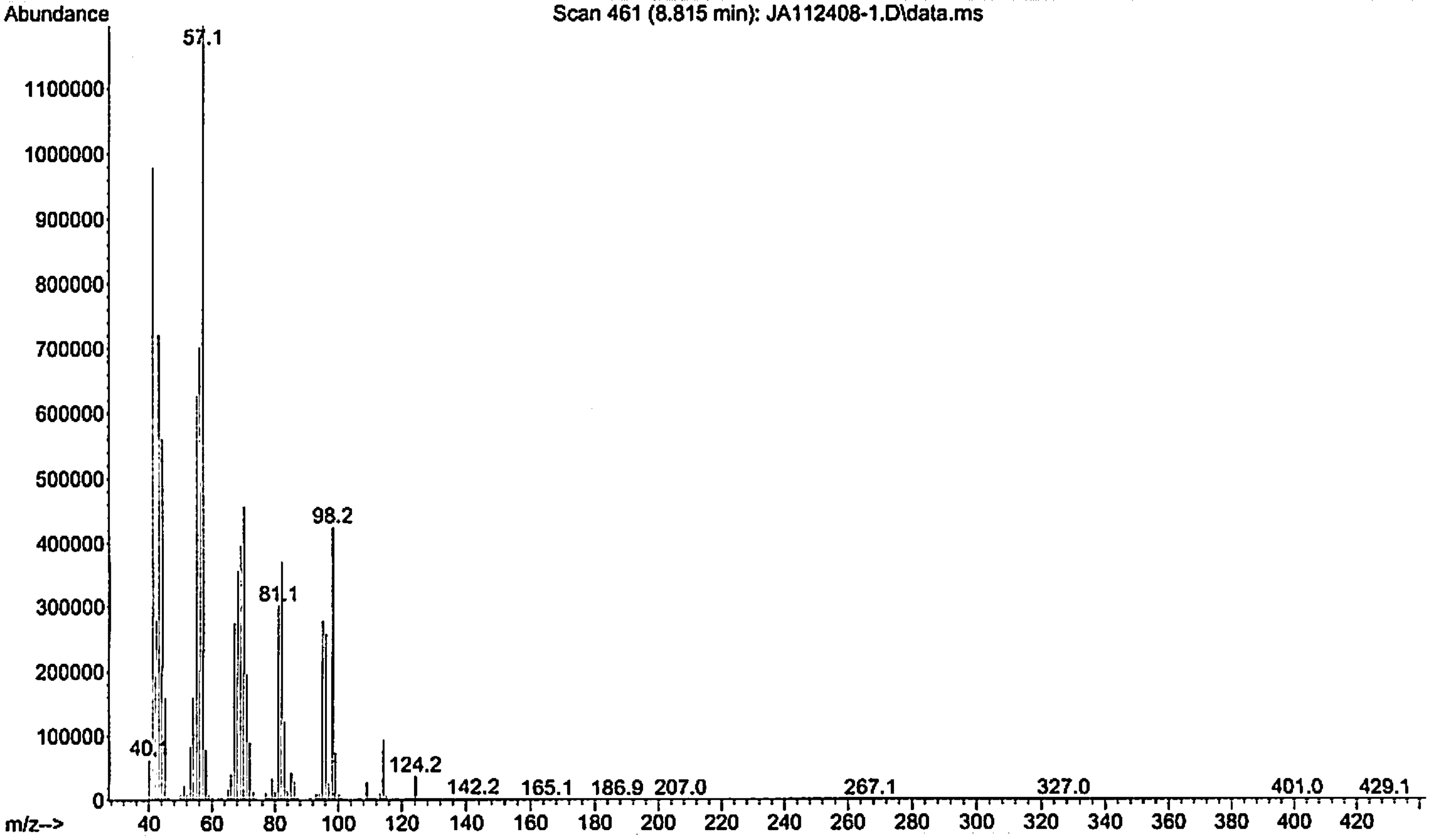

Library Searched : C:\Database\W8N05ST.L
Quality : 93
ID : NONANAL $ PELARGONALDEHYDE $ 1-NONALDEHYDE $ 1-NONANAL $ 1-NONYL ALDEHYDE $ AI3-04859 $ ALDEHYDE C-9 $ BRN 1236701 $ C-9 ALDEHYDE $ CCRIS 664 $ EINECS 204-688-5 $ FEMA NO. 2782 $ HSDB 7229 $ N-NONALDEHYDE $ N-NONAN-1-AL $ N-NONANAL $ N-NONYLALDEHYDE $ NCI

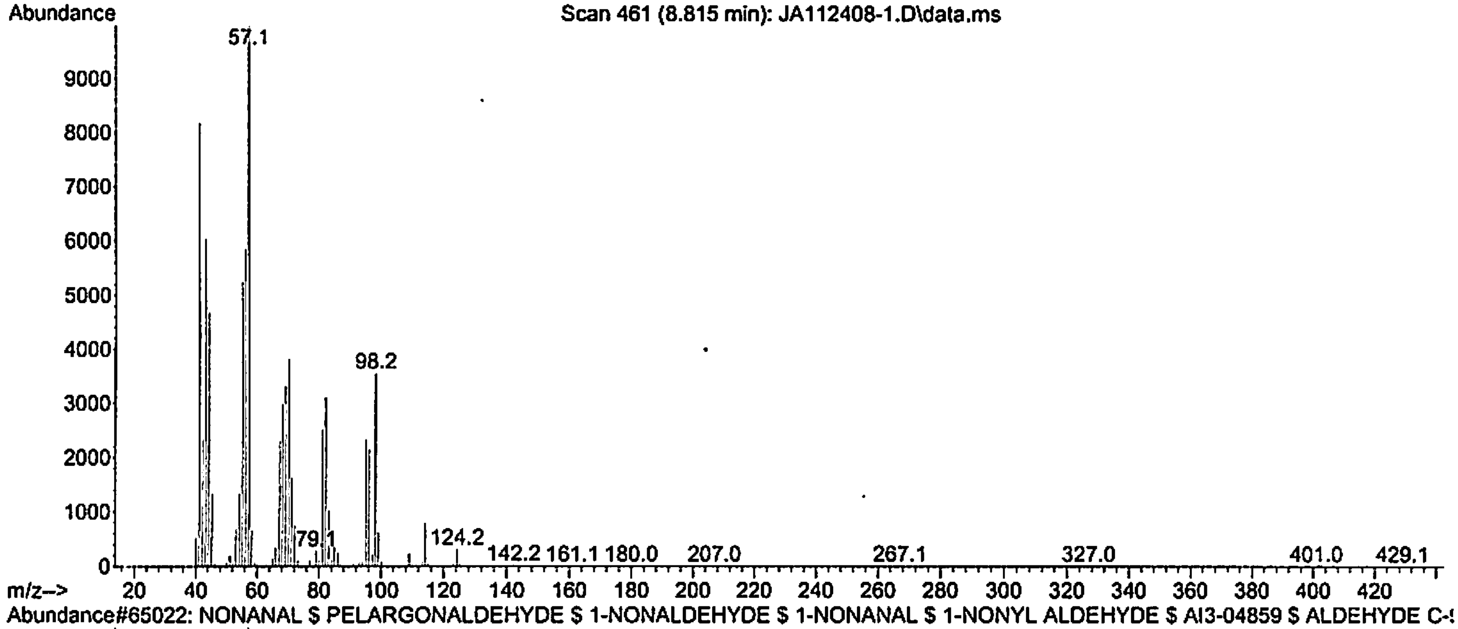

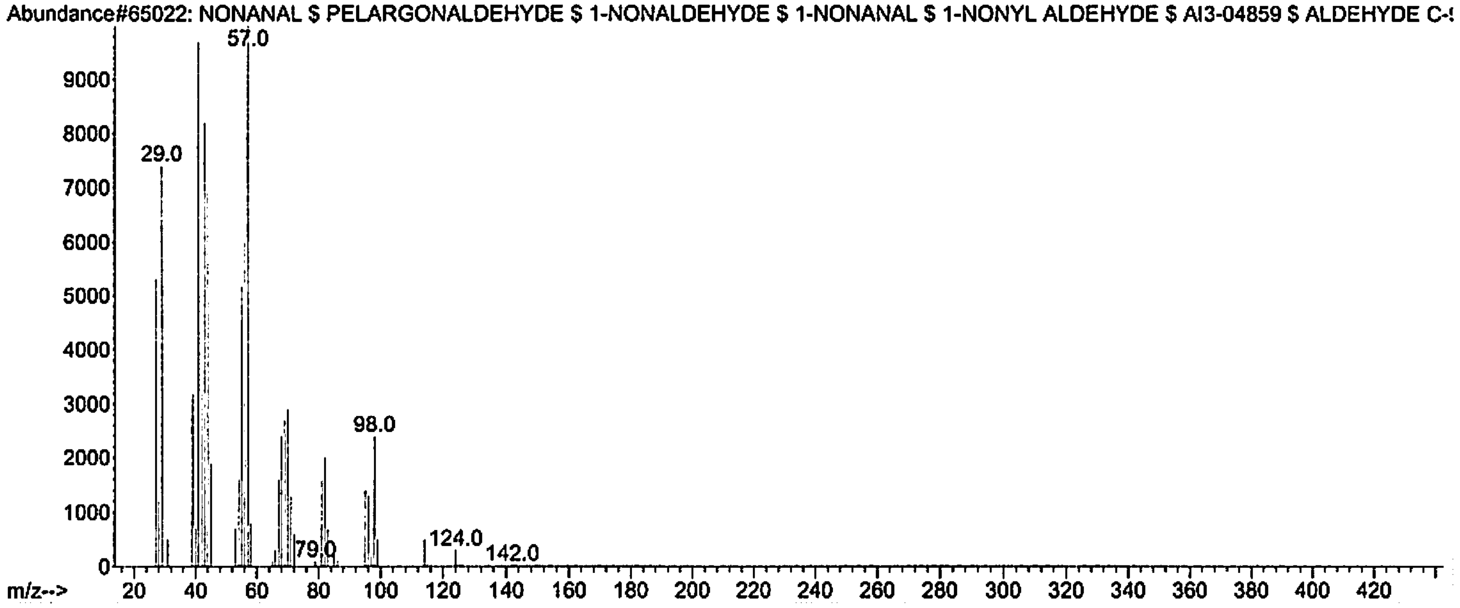

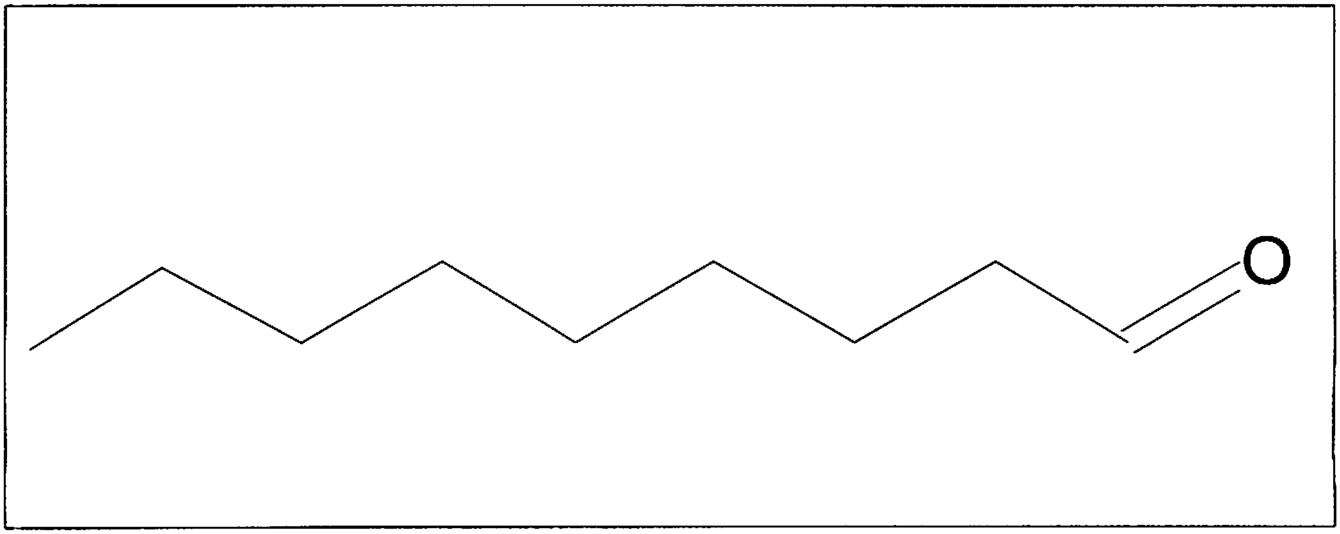

File :D:\ALDRICH\Snapshot\JA112408-1.D
Operator :
Acquired : 24 Nov 2008 15:36 using AcqMethod JA-50-280LESS.M
Instrument : Buba
Sample Name: 7M grouped C.oculata, abd. cuticle/CH2Cl2
Misc Info : noGC; lab reared in grp. of 15; 17-21day-old
Vial Number: 1

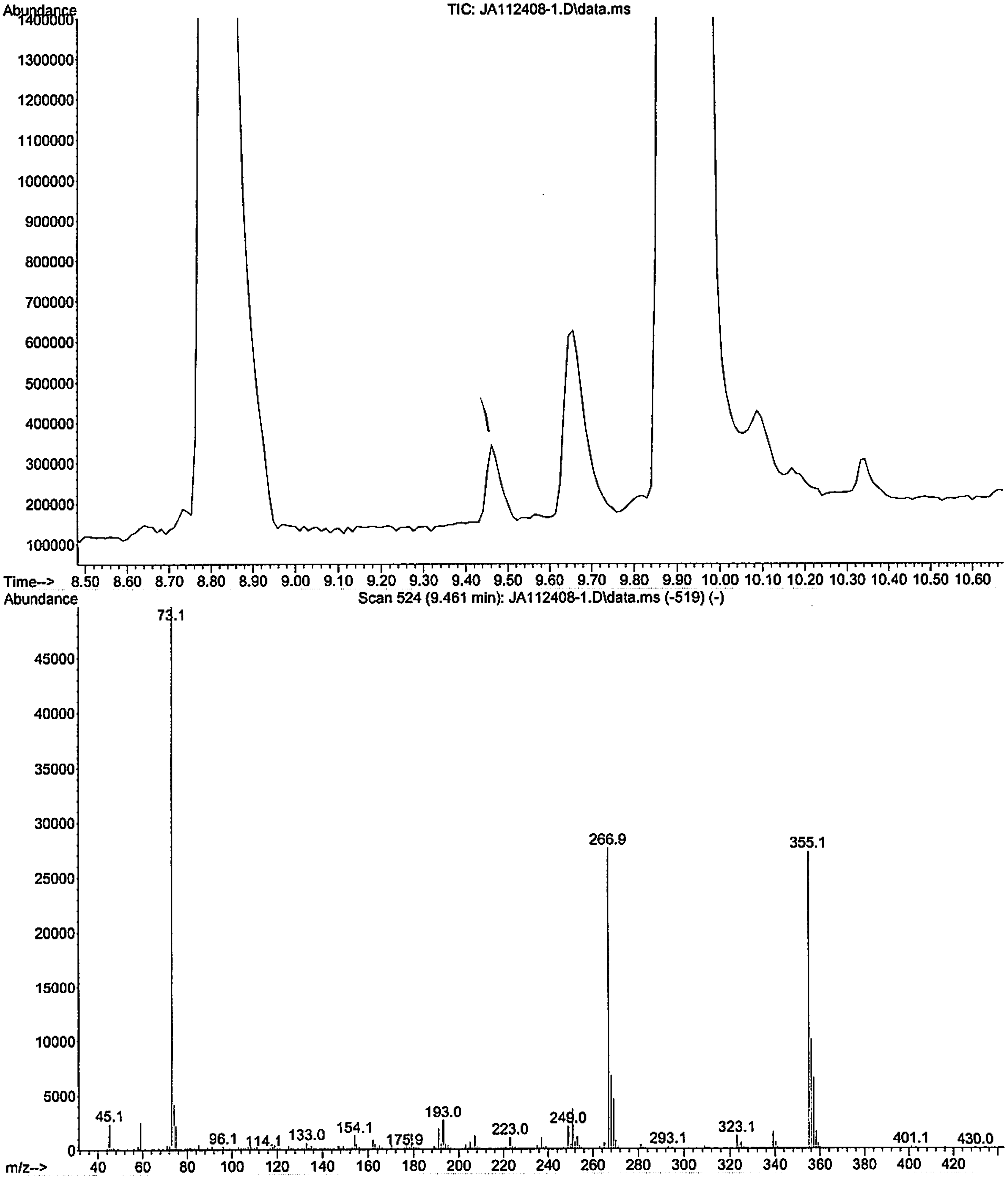

File   :D:\ALDRICH\Snapshot\JA112408-1.D
Operator :
Acquired : 24 Nov 2008 15:36   using AcqMethod JA-50-280LESS.M
Instrument :   Buba
Sample Name: 7M grouped C.oculata, abd. cuticle/CH2Cl2
Misc Info : noGC; lab reared in grp. of 15; 17-21day-old
Vial Number: 1

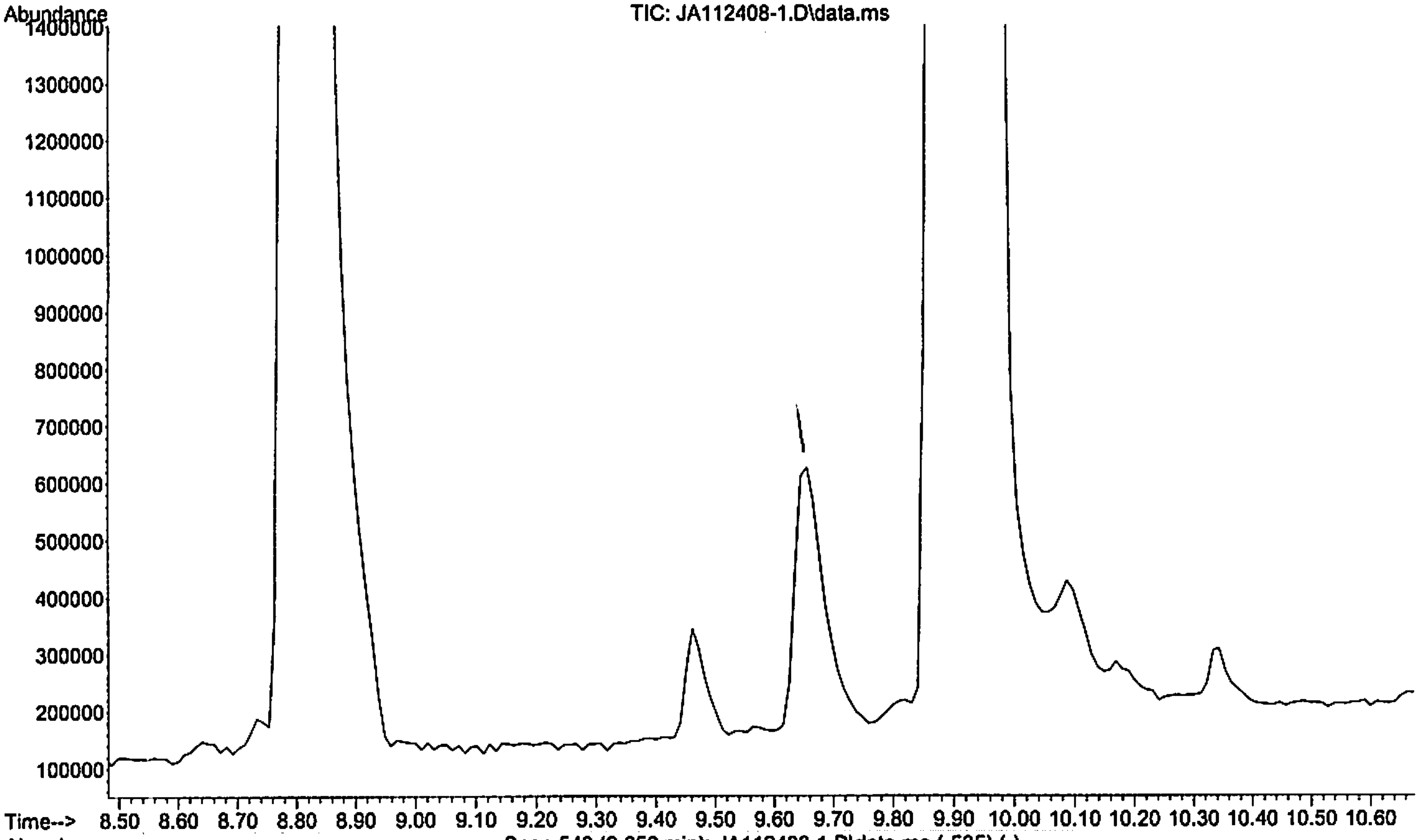

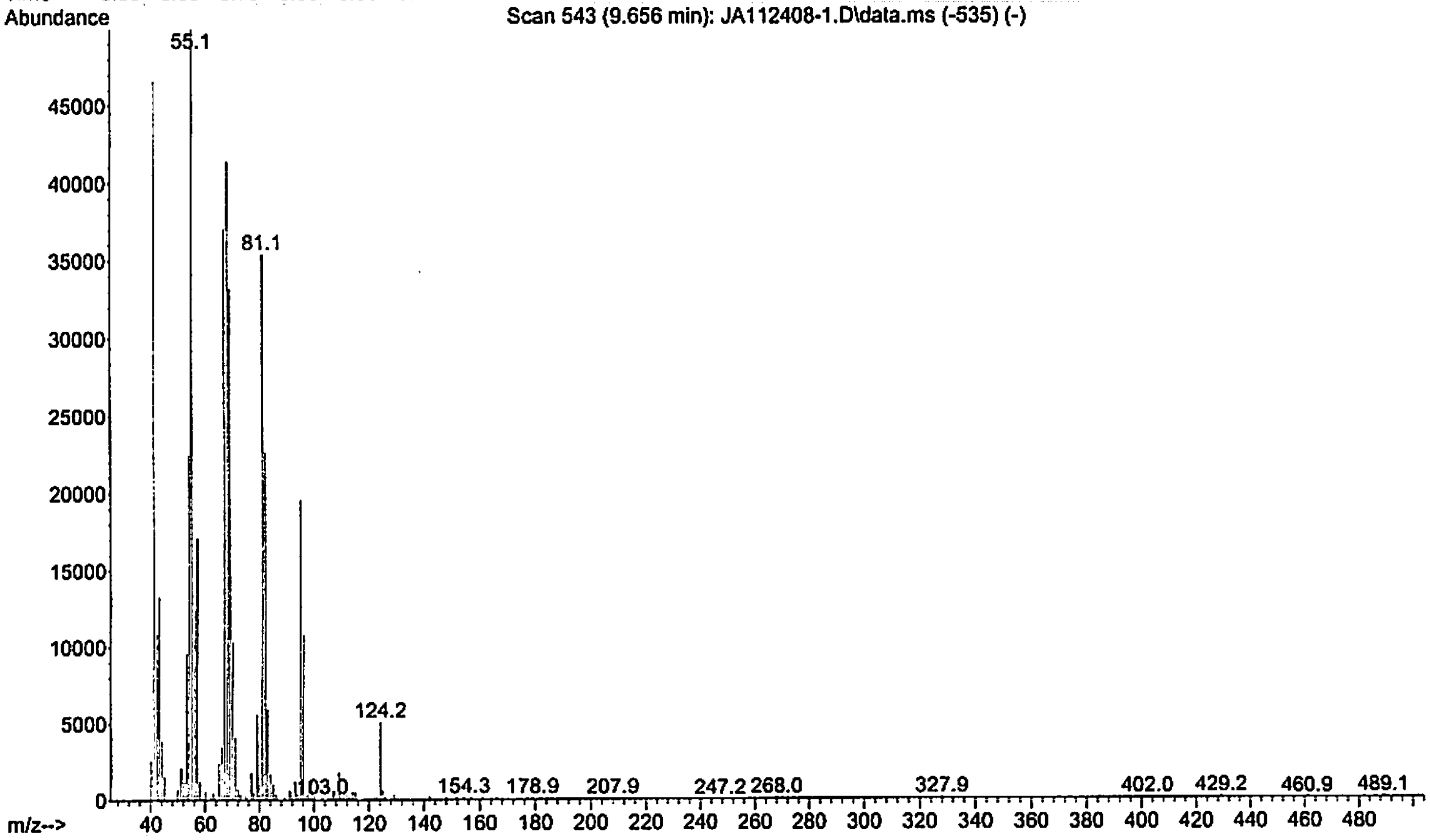

```
Library Searched : C:\Database\W8N05ST.L
Quality          : 91
ID               : 3-Nonen-1-ol, (Z)- $ cis-3-Nonen-1-Ol $ (3Z)-3-Nonen-1-ol #
```

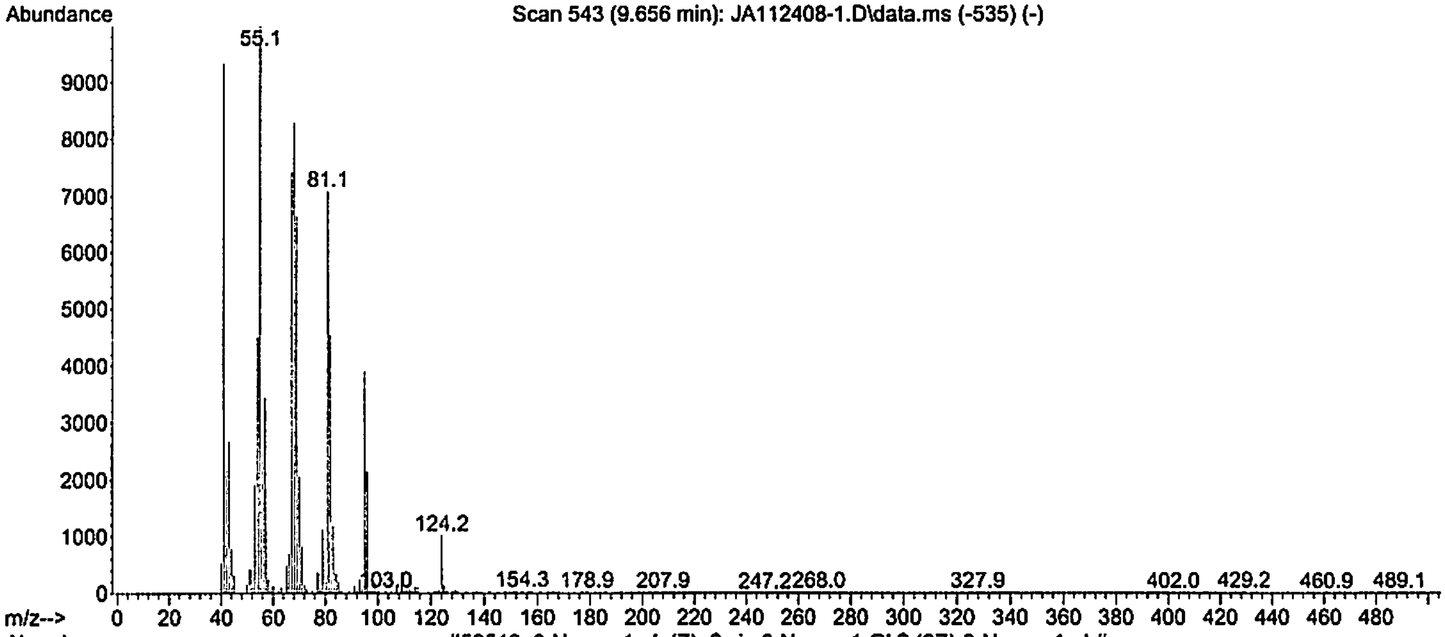

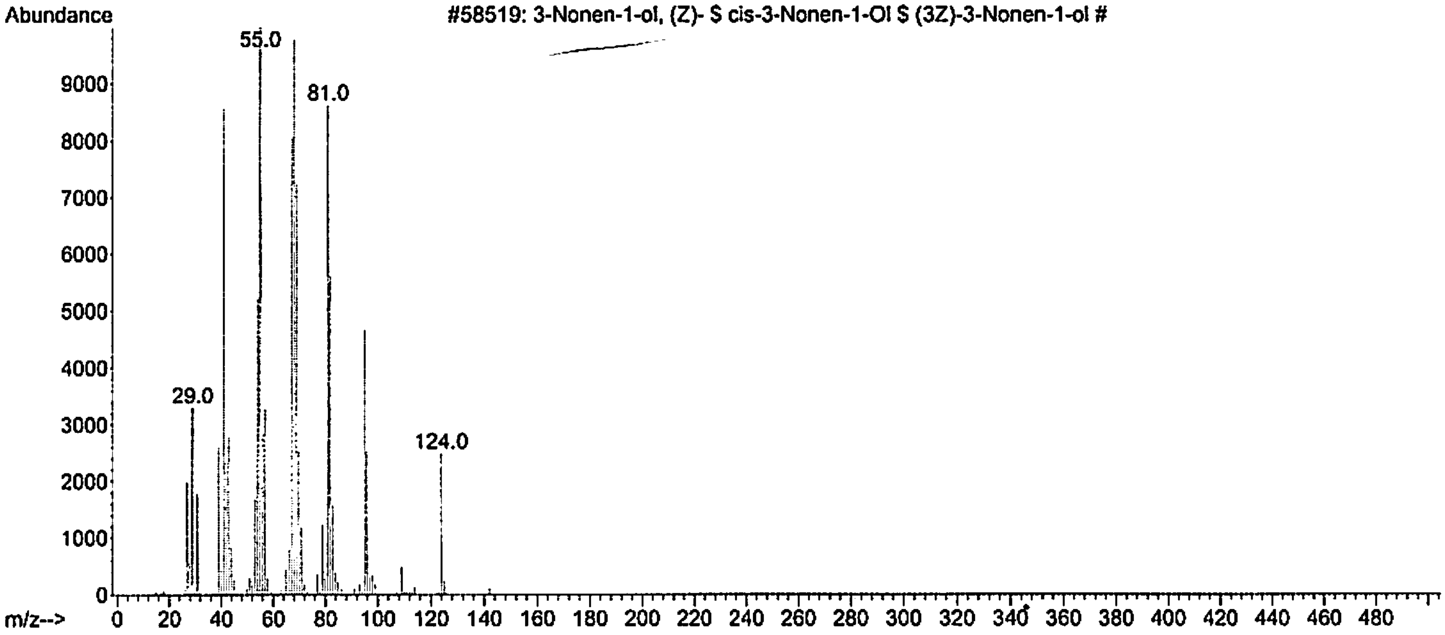

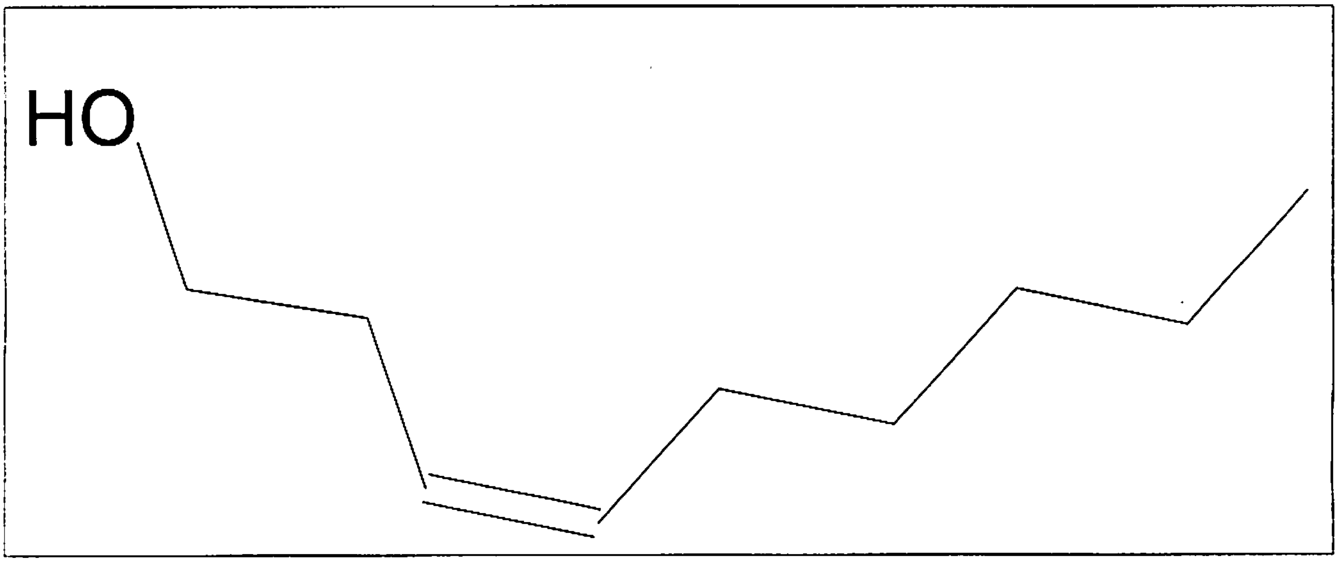

Library Searched : C:\Database\W8N05ST.L
Quality : 64
ID : 4-NONEN-1-OL, (Z)- $ (Z)-4-NONEN-1-OL $ (Z)-NON-4-EN-1-OL

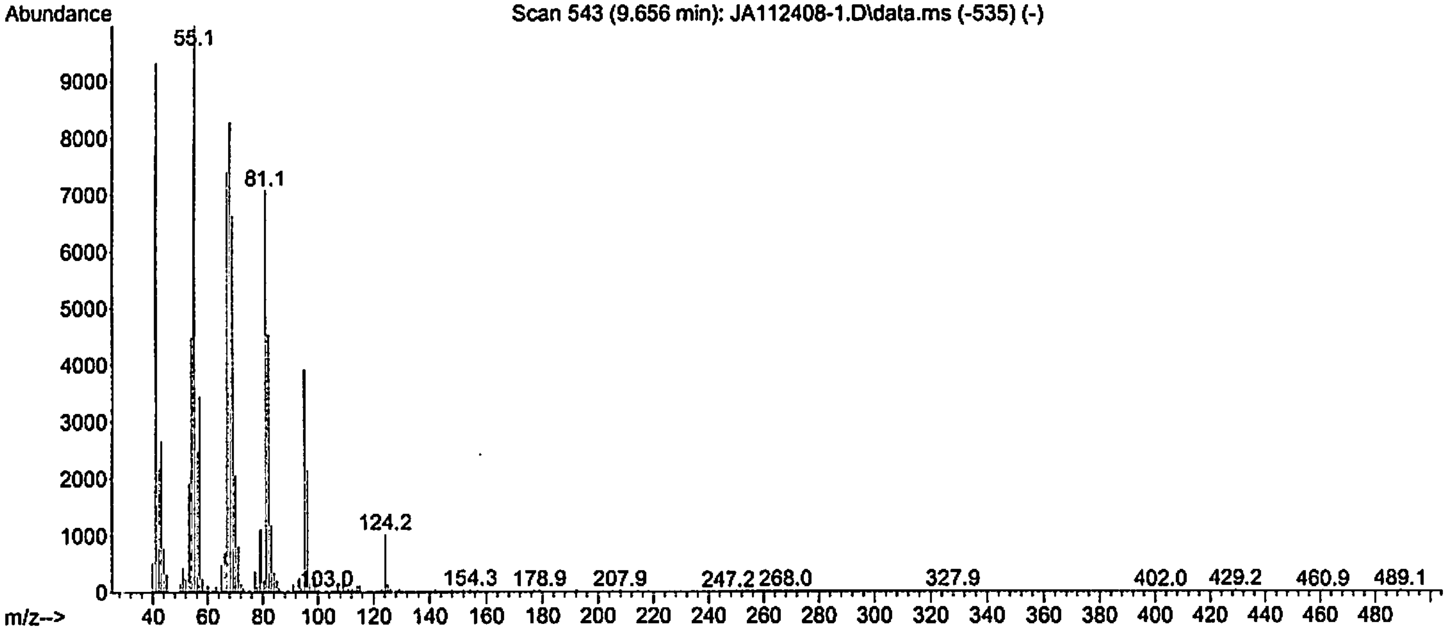

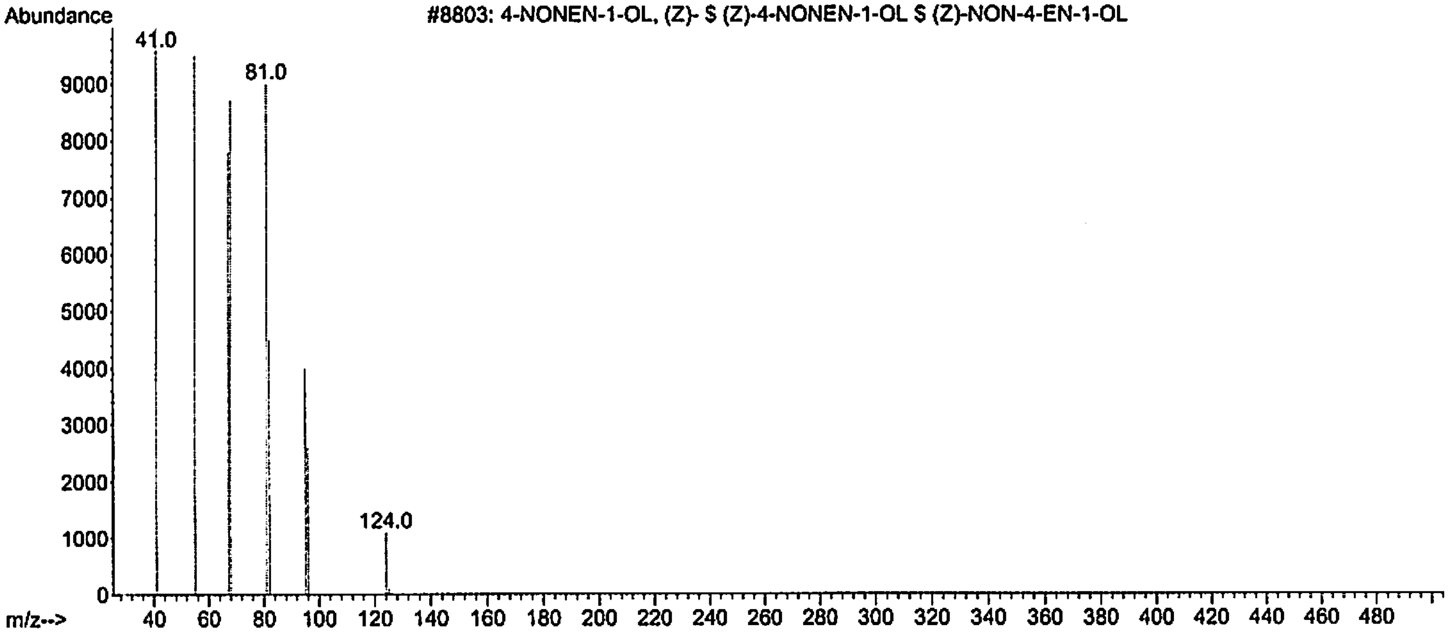

File : D:\ALDRICH\Snapshot\JA112408-1.D
Operator :
Acquired : 24 Nov 2008 15:36 using AcqMethod JA-50-280LESS.M
Instrument : Buba
Sample Name: 7M grouped C.oculata, abd. cuticle/CH2Cl2
Misc Info : noGC; lab reared in grp. of 15; 17-21day-old
Vial Number: 1

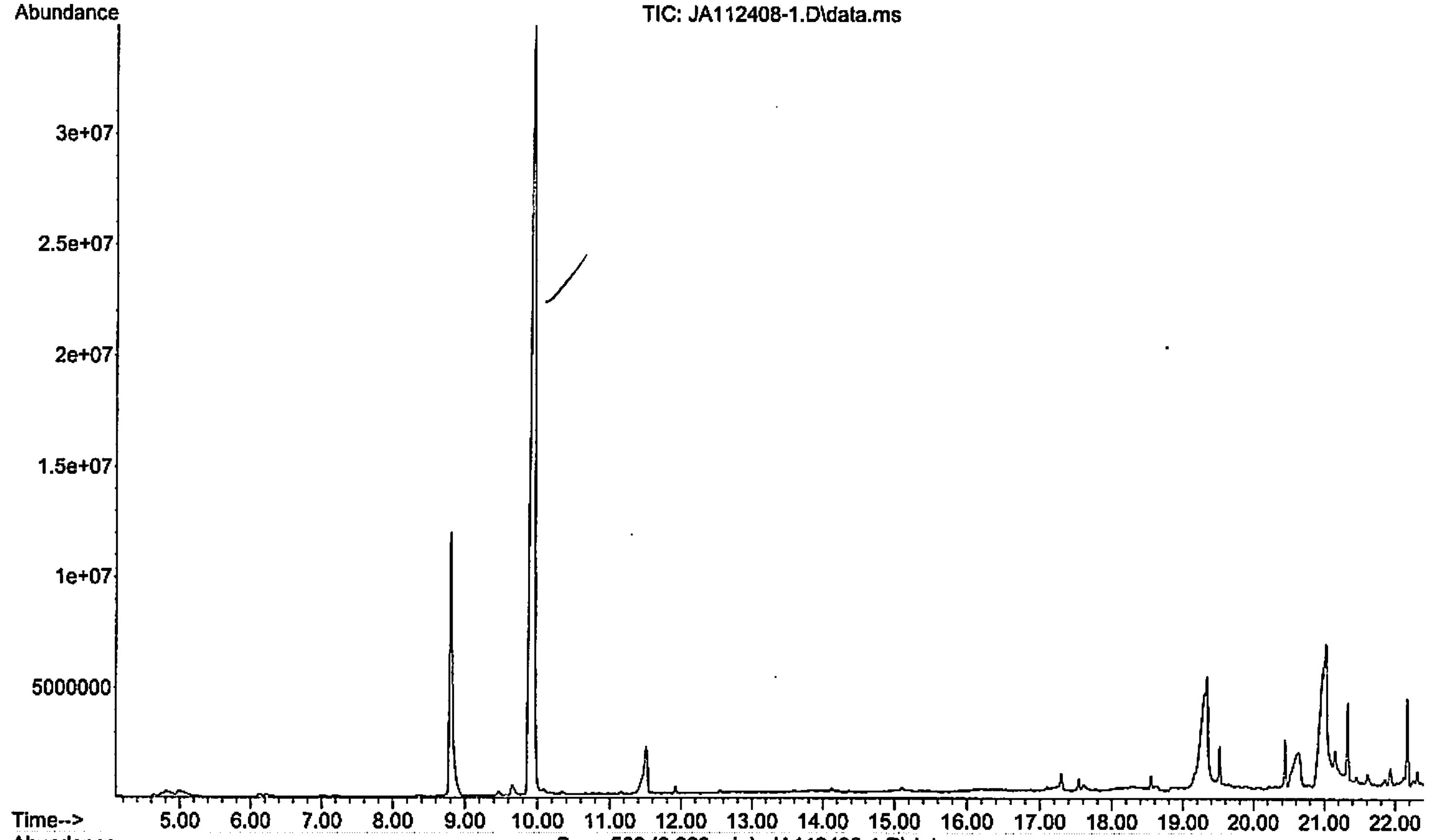

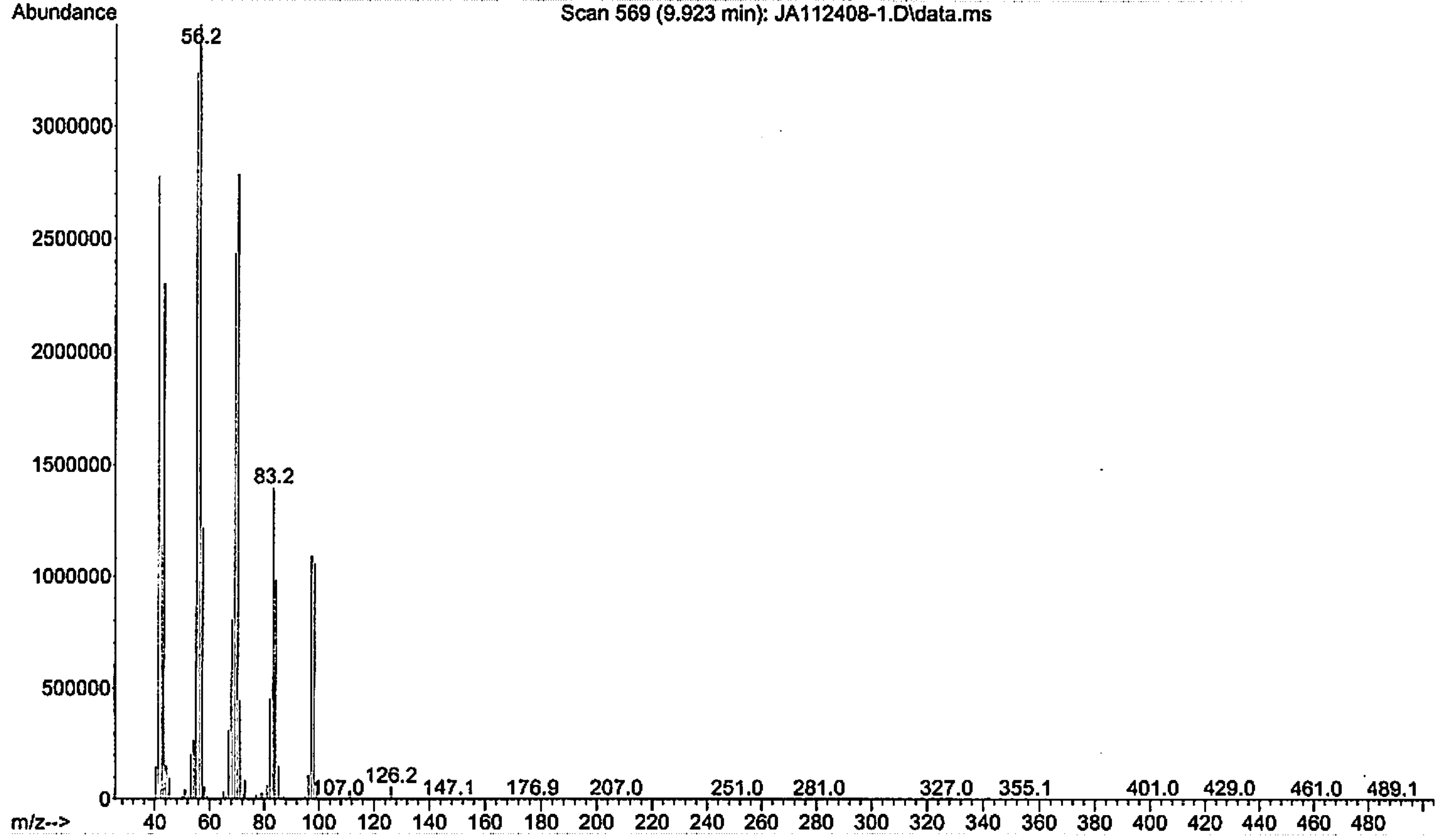

```
Library Searched : C:\Database\W8N05ST.L
Quality          : 91
ID               : 1-NONANOL $ NONANOL $ NONAN-1-OL $ 1-HYDROXYNONANE $ 1-NONANO $ AI3-03
                   962 $ ALCOHOL C-9 $ BRN 0969213 $ C9 ALCOHOL $ EINECS 205-583-7 $ FATT
                   Y ALCOHOL(C9) $ FEMA NO. 2789 $ HSDB 5145 $ N-NONAN-1-OL $ N-NONANOL $
                    N-NONYL ALCOHOL $ NONALOL $ NONANOL-(1) $ NO
```

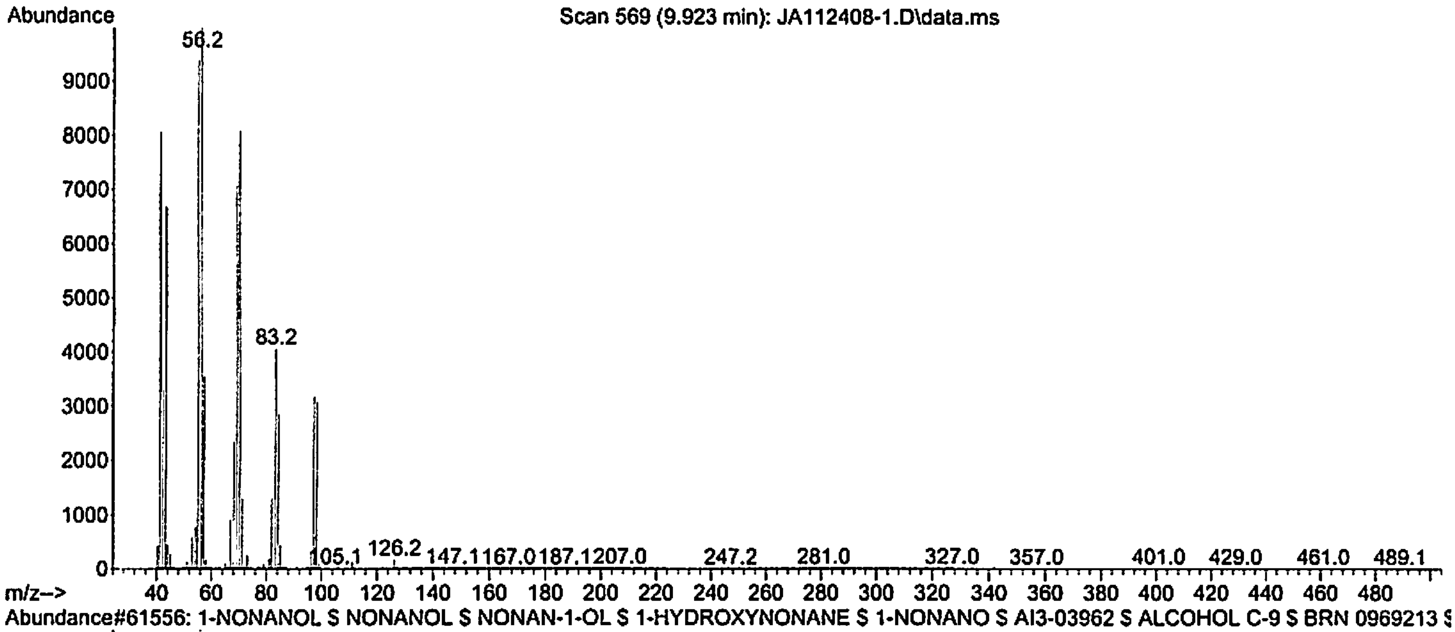

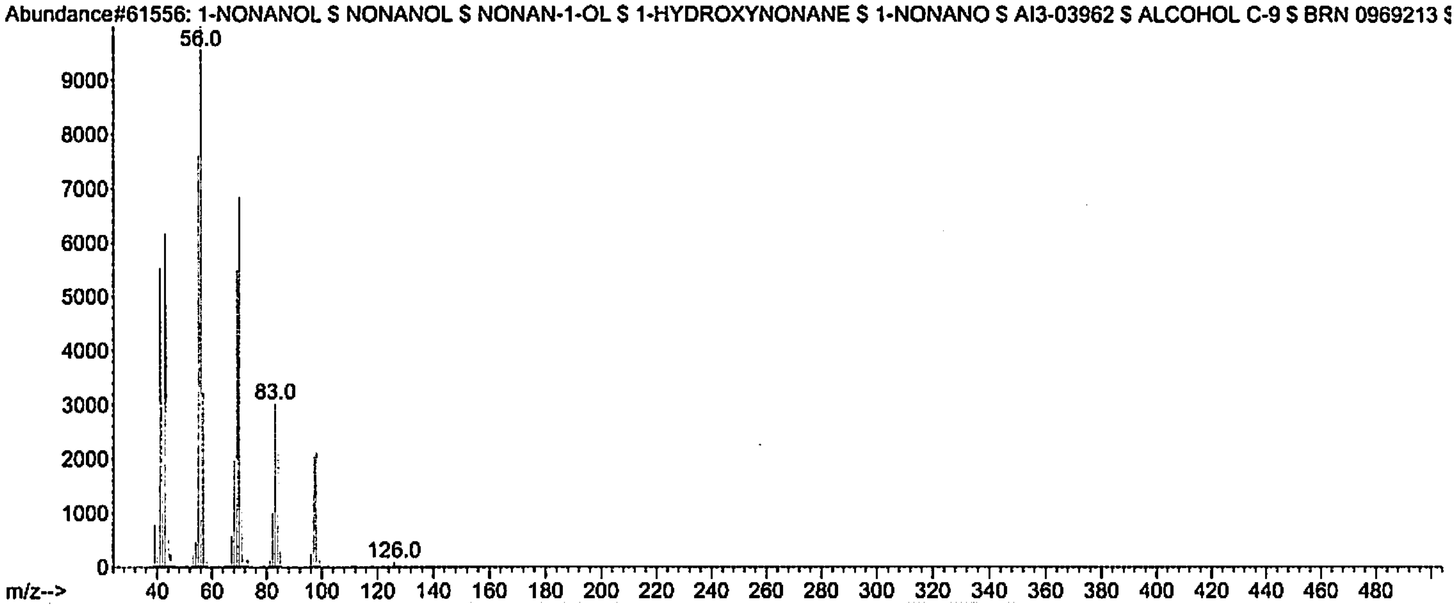

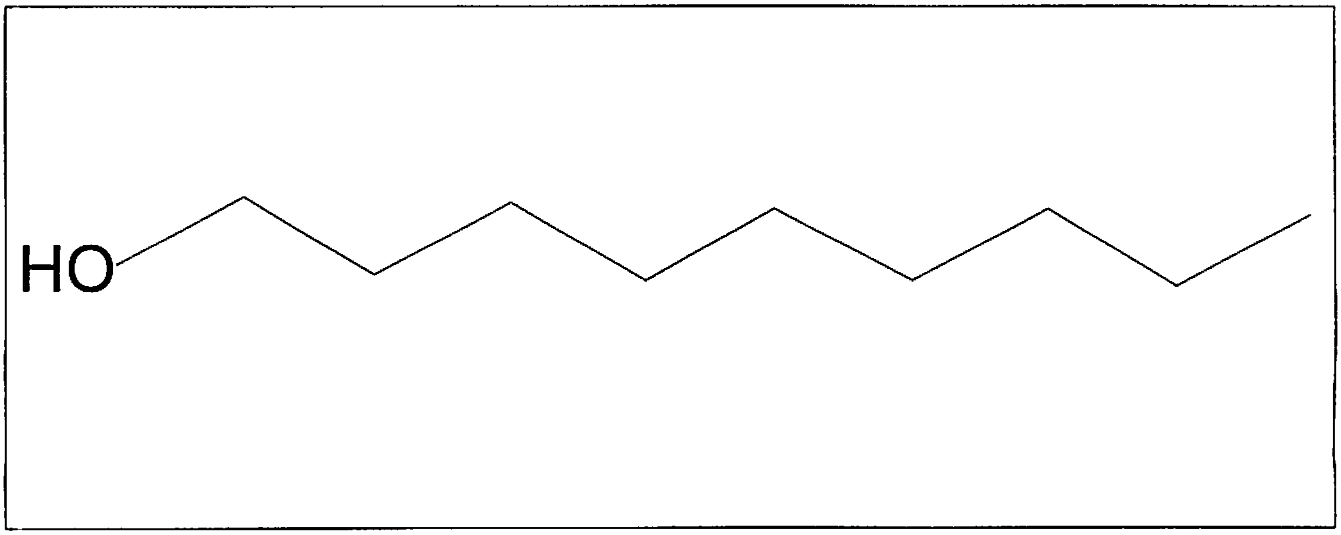

Library Searched : C:\Database\W8N05ST.L
Quality : 94
ID : DECANAL $ CAPRALDEHYDE $ 1-DECANAL $ 1-DECANAL (MIXED ISOMERS) $ 1-DECANAL(MIXED ISOMERS) $ 1-DECYL ALDEHYDE $ 3,4-DIHYDRO-5,7-DIHYDROXY-2H-1-BENZOPYRAN-3-YL 3,4-DIHYDROXYBENZOATE $ AI3-04860 $ AIDS-112312 $ ALDEHYDE C10 $ BRN 1362530 $ C-10 ALDEHYDE $ CA

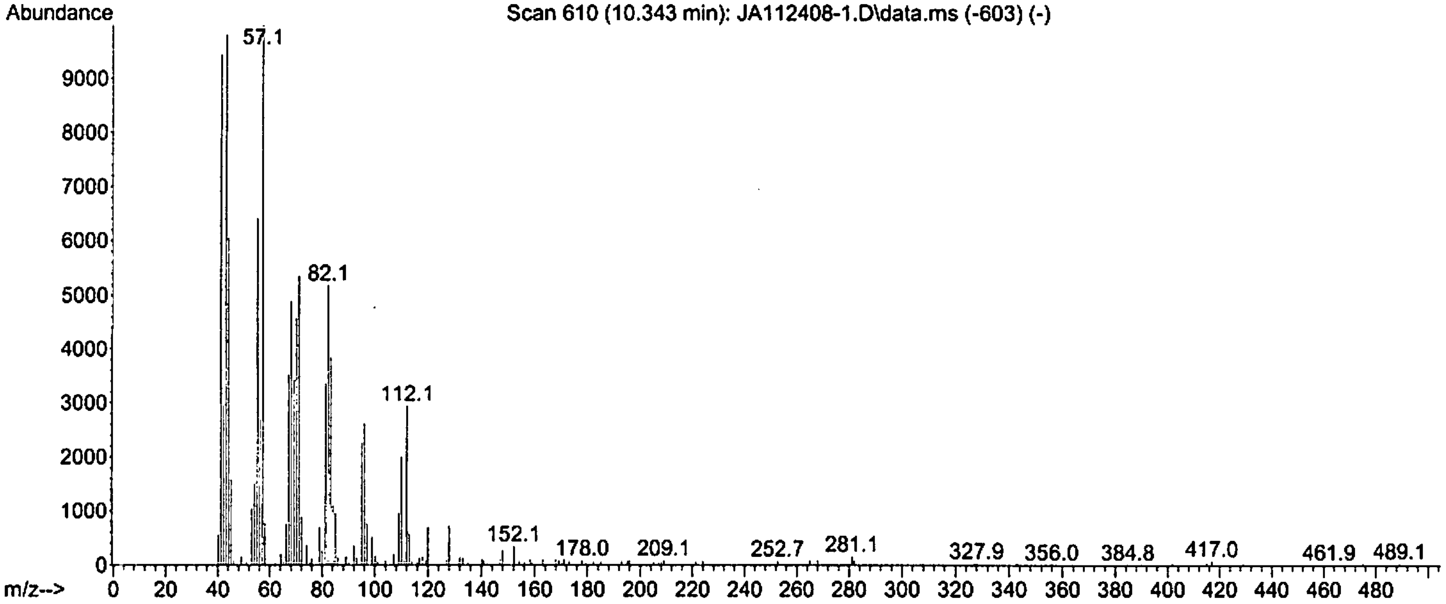

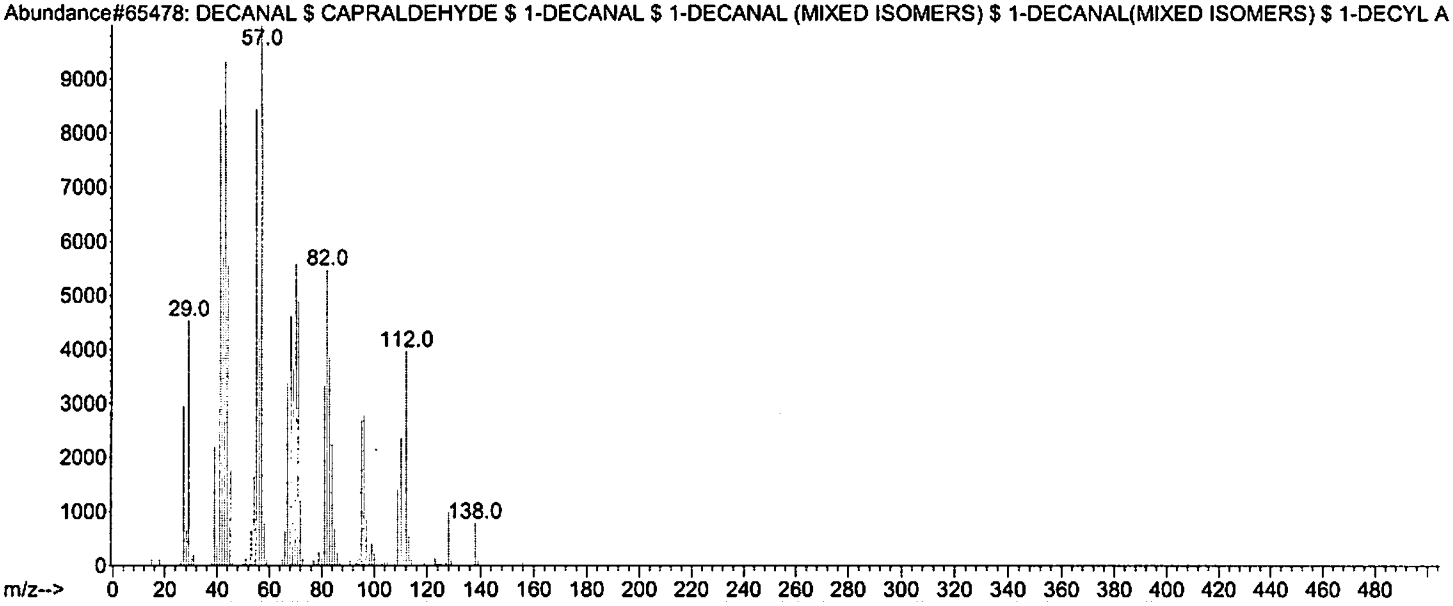

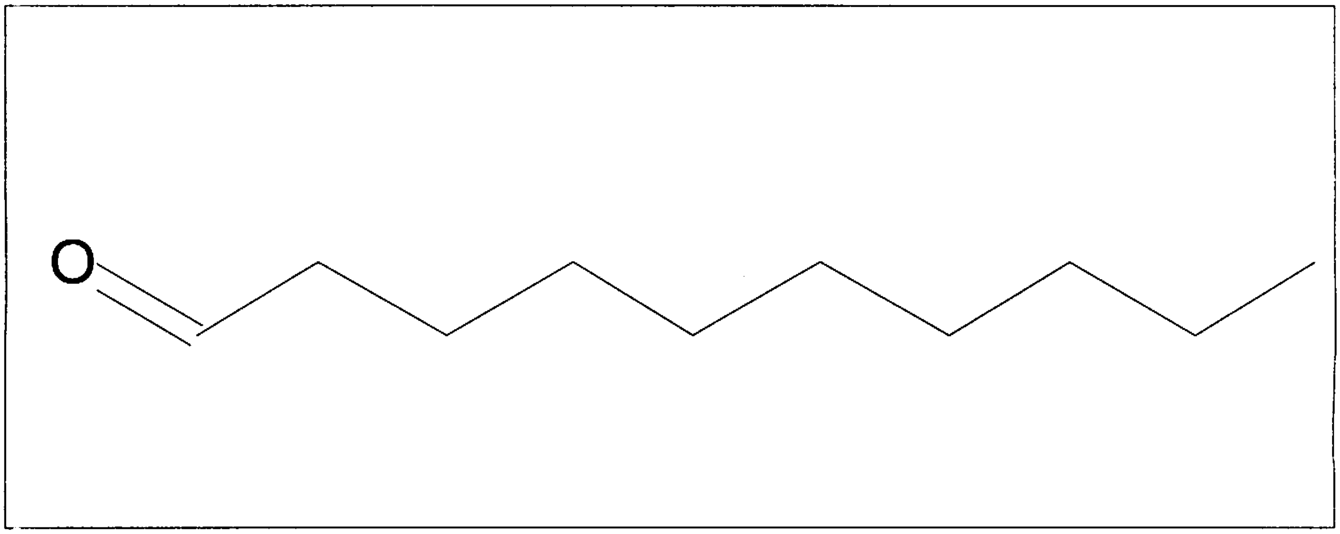

```
Library Searched : C:\Database\W8N05ST.L
Quality          : 92
ID               : 2-DECENAL, (E)- $ (2E)-2-DECENAL # $ (2E)-2-DECENAL $ (2E)-2-DECENAL (
                   COMPUTER-GENERATED NAME) $ (E)-2-DECENAL $ (E)-2-DECENOL $ (E)-DEC-2-E
                   N-1-AL $ (E)-DEC-2-ENAL $ 2-DECENAL $ 2-DECENAL (E) $ 2-DECENAL, (2E)-
                    $ EINECS 223-474-2 $ FEMA NO. 2366 $ TRANS-2
```

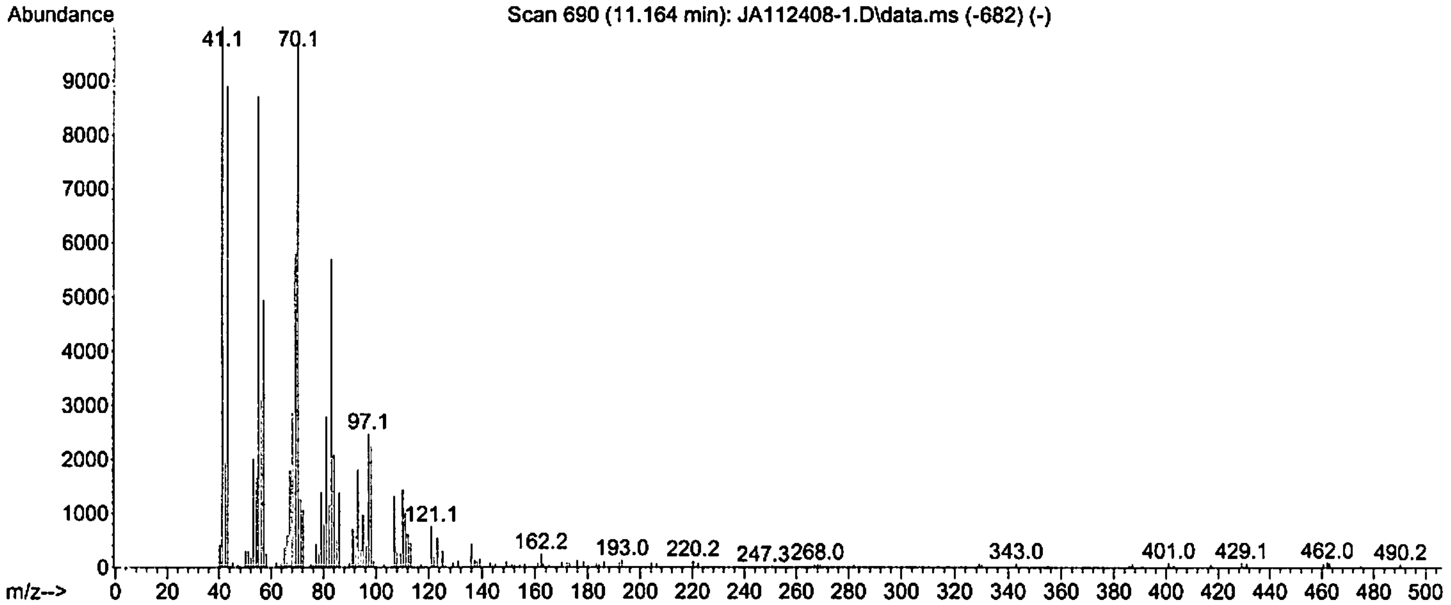

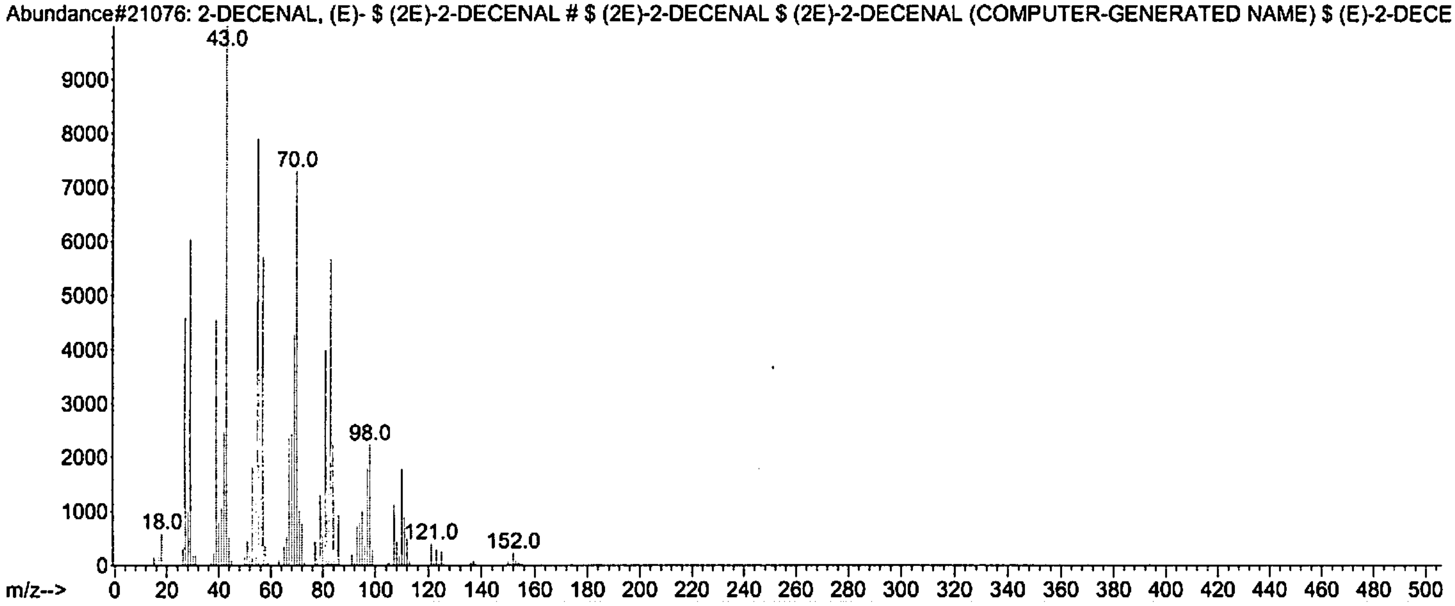

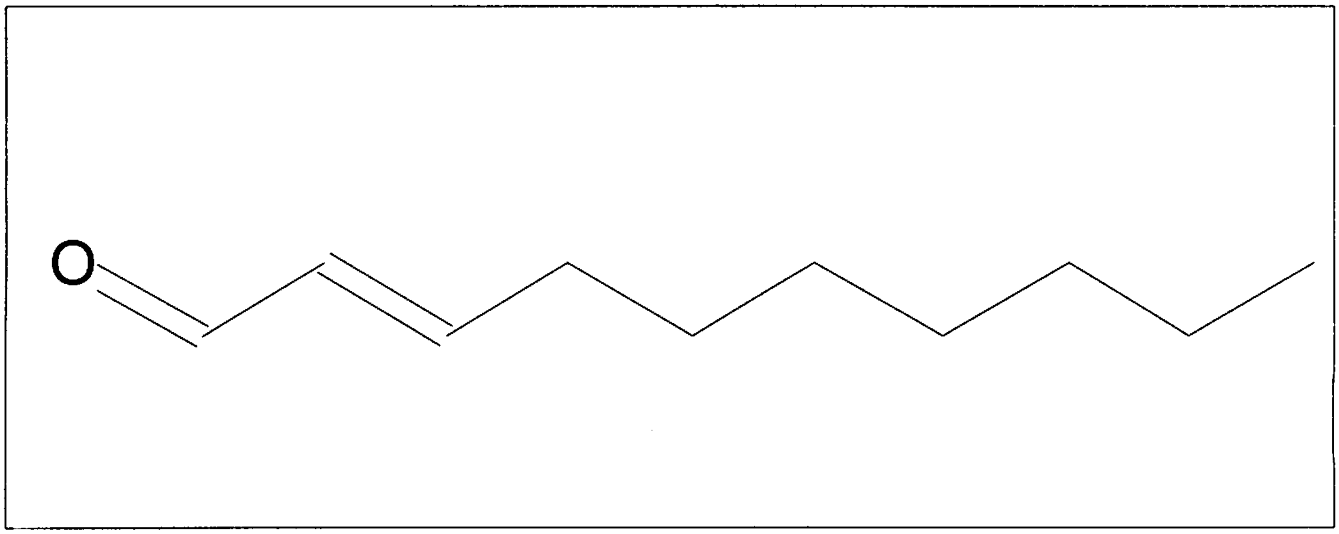

```
File        :D:\ALDRICH\Snapshot\JA112408-1.D
Operator    :
Acquired    : 24 Nov 2008  15:36     using AcqMethod JA-50-280LESS.M
Instrument  :    Buba
Sample Name: 7M grouped C.oculata, abd. cuticle/CH2Cl2
Misc Info   : noGC; lab reared in grp. of 15; 17-21day-old
Vial Number: 1
```

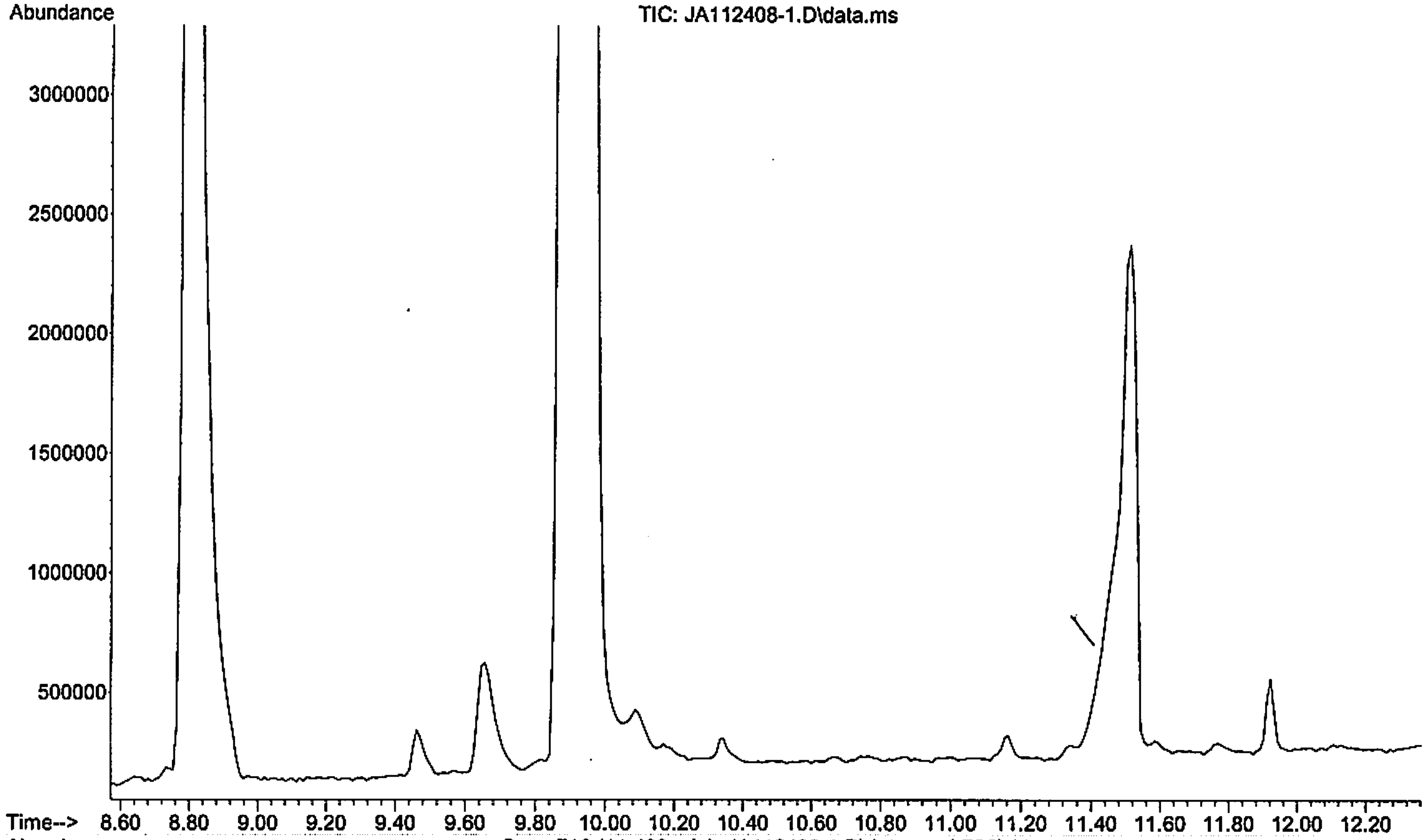

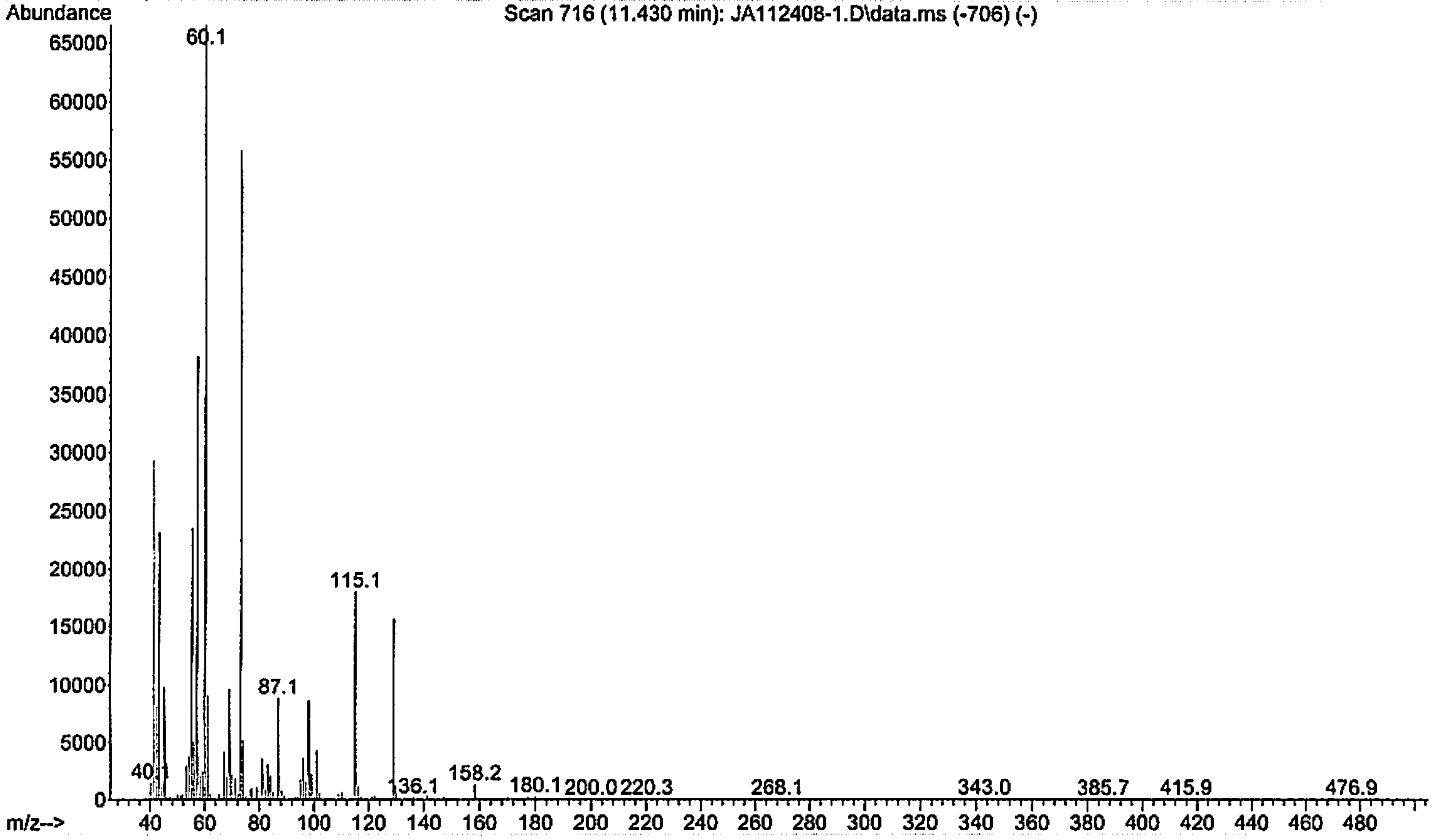

```
File     :D:\ALDRICH\Snapshot\JA112408-1.D
Operator :
Acquired : 24 Nov 2008  15:36     using AcqMethod JA-50-280LESS.M
Instrument :    Buba
Sample Name: 7M grouped C.oculata, abd. cuticle/CH2Cl2
Misc Info  : noGC; lab reared in grp. of 15; 17-21day-old
Vial Number: 1
```

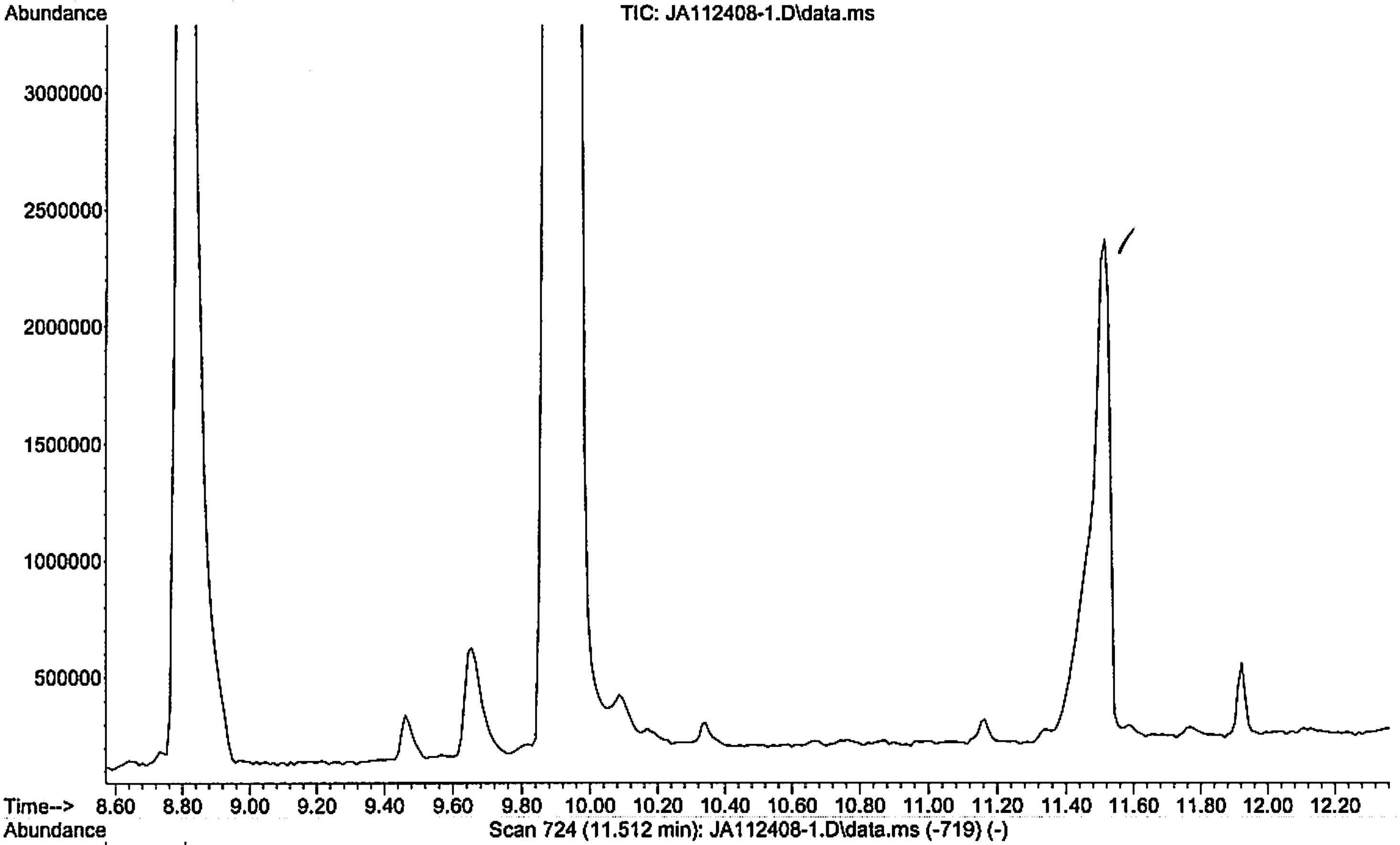

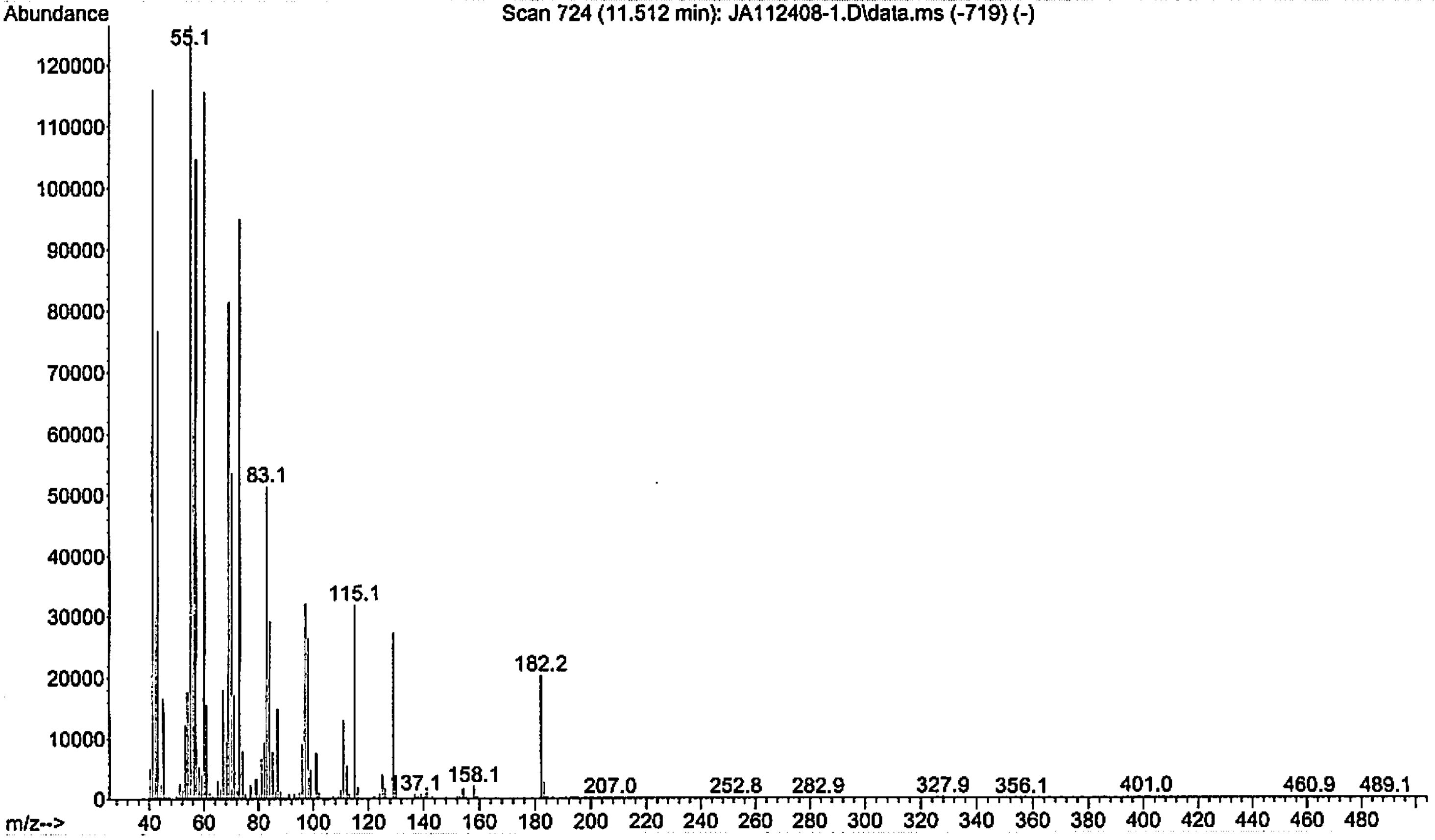

File : D:\ALDRICH\Snapshot\JA112408-1.D
Operator :
Acquired : 24 Nov 2008 15:36 using AcqMethod JA-50-280LESS.M
Instrument : Buba
Sample Name: 7M grouped C.oculata, abd. cuticle/CH2Cl2
Misc Info : noGC; lab reared in grp. of 15; 17-21day-old
Vial Number: 1

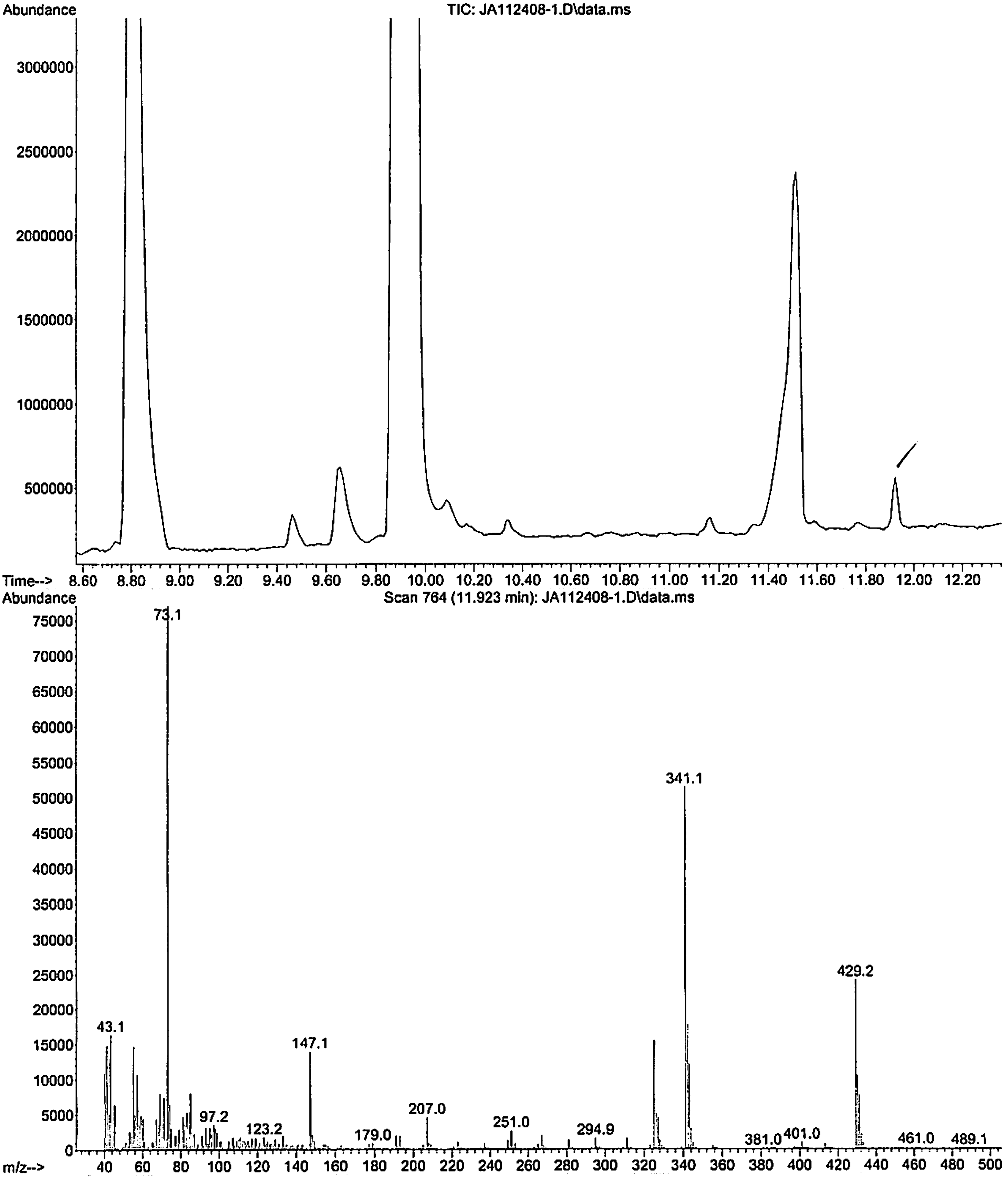

```
File        :D:\Aldrich\JA112408-2.D
Operator    :
Acquired    : 24 Nov 2008  16:20     using AcqMethod JA-50-280LESS.M
Instrument  :  Buba
Sample Name: 6M single C.oculata, abd. cuticle/CH2Cl2
Misc Info   : noGC; reared singly; 17-21day-old; mold probl
Vial Number: 1
```

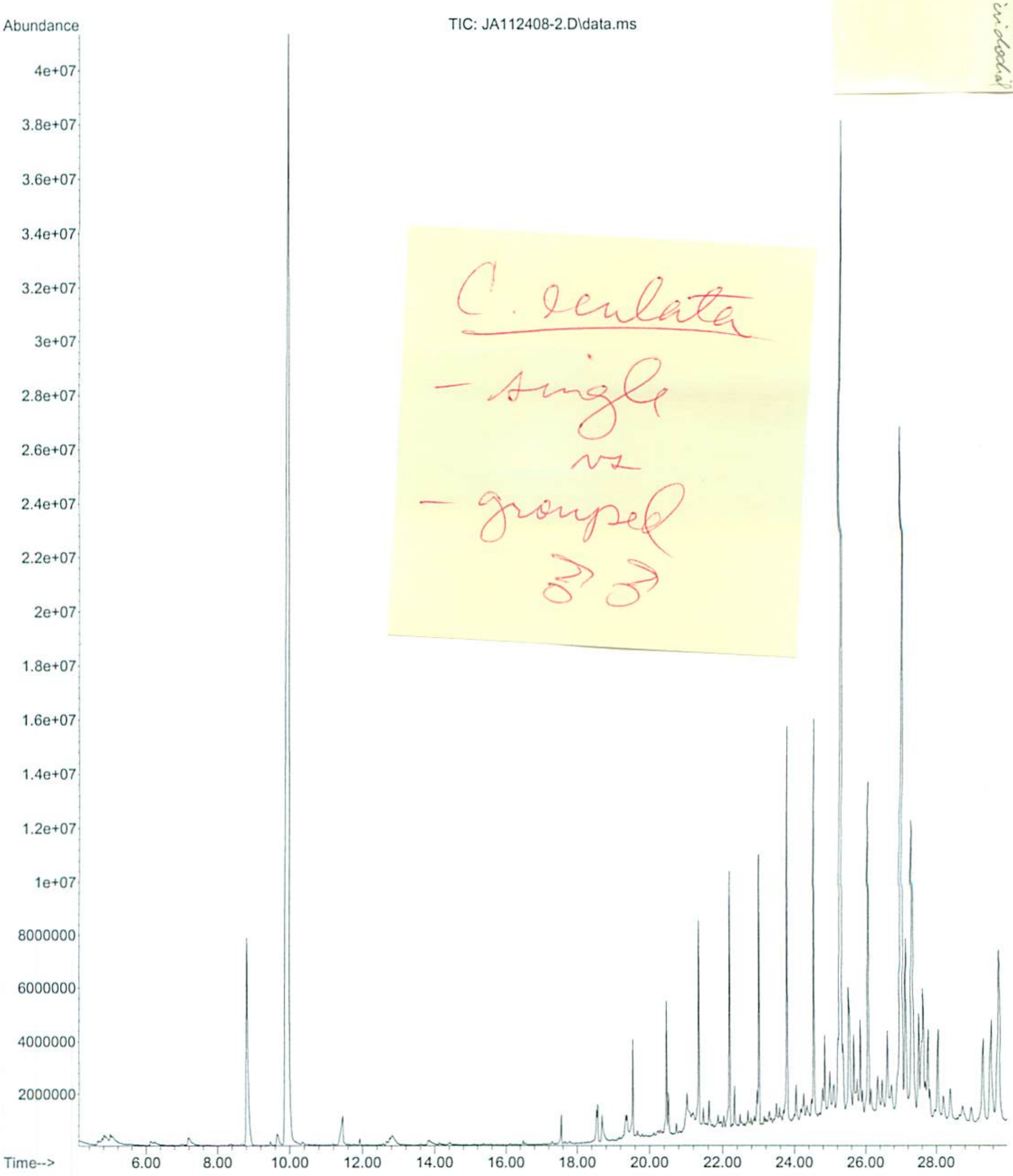

single vs. grouped
lab ♂♂ - no [illegible]

```
File        :D:\Aldrich\JA112408-2.D
Operator    :
Acquired    : 24 Nov 2008  16:20     using AcqMethod JA-50-280LESS.M
Instrument  :    Buba
Sample Name: 6M single C.oculata, abd. cuticle/CH2Cl2
Misc Info   : noGC; reared singly; 17-21day-old; mold probl
Vial Number: 1
```

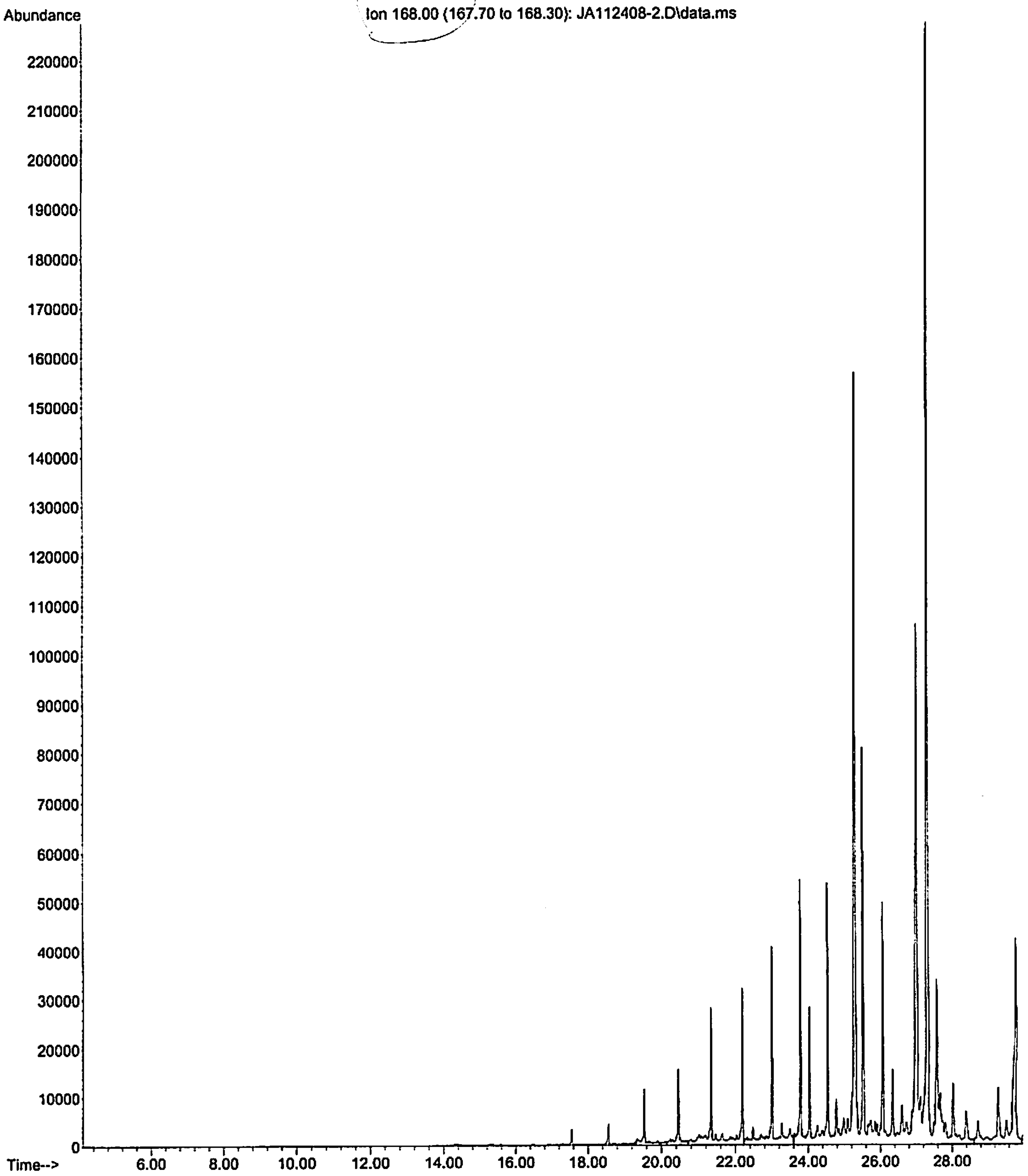

File :D:\Aldrich\JA112408-2.D
Operator :
Acquired : 24 Nov 2008 16:20 using AcqMethod JA-50-280LESS.M
Instrument : Buba
Sample Name: 6M single C.oculata, abd. cuticle/CH2Cl2
Misc Info : noGC; reared singly; 17-21day-old; mold probl
Vial Number: 1

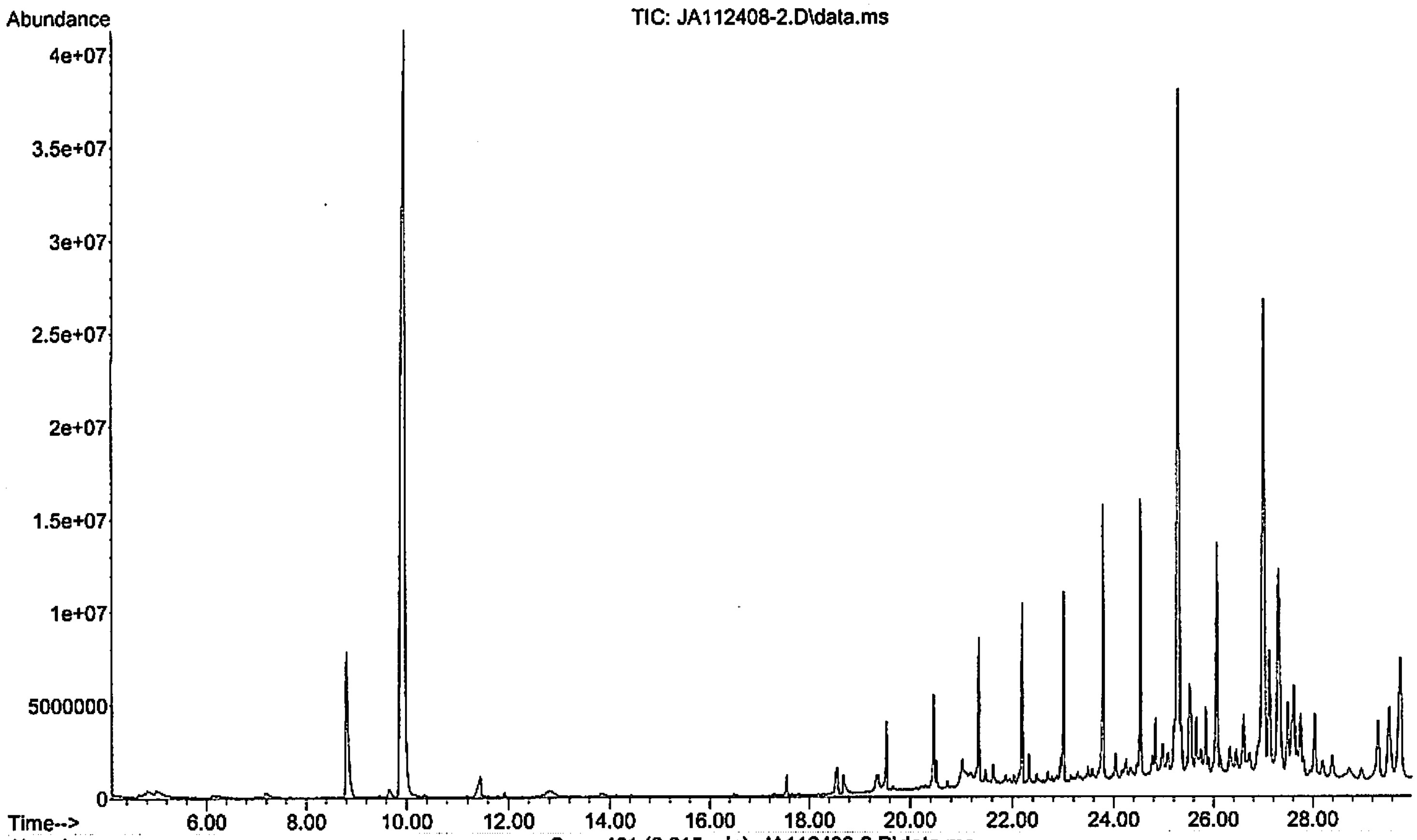

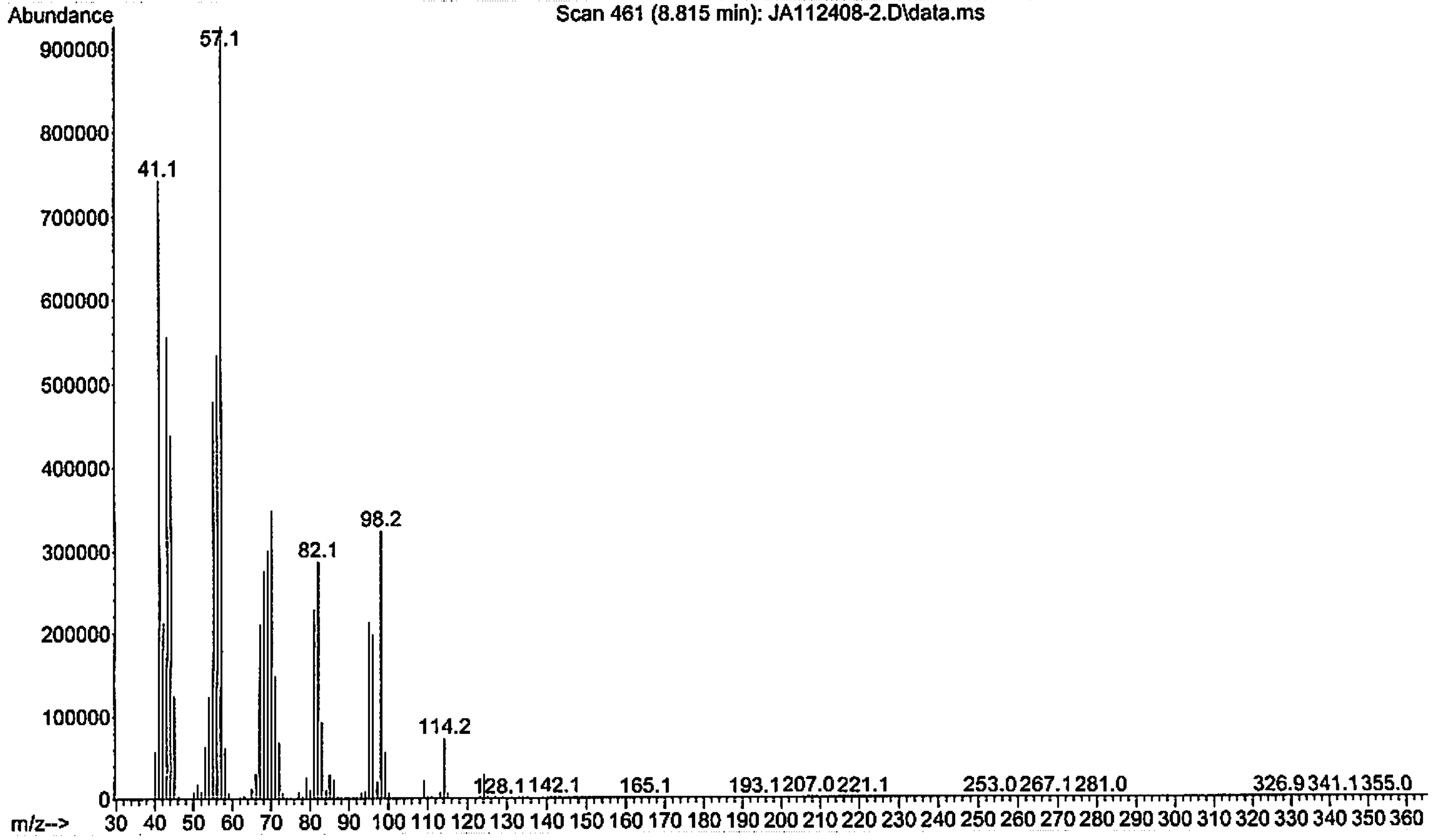

```
Library Searched : C:\Database\W8N05ST.L
Quality          : 93
ID               : NONANAL $ PELARGONALDEHYDE $ 1-NONALDEHYDE $ 1-NONANAL $ 1-NONYL ALDEH
                   YDE $ AI3-04859 $ ALDEHYDE C-9 $ BRN 1236701 $ C-9 ALDEHYDE $ CCRIS 66
                   4 $ EINECS 204-688-5 $ FEMA NO. 2782 $ HSDB 7229 $ N-NONALDEHYDE $ N-N
                   ONAN-1-AL $ N-NONANAL $ N-NONYLALDEHYDE $ NCI
```

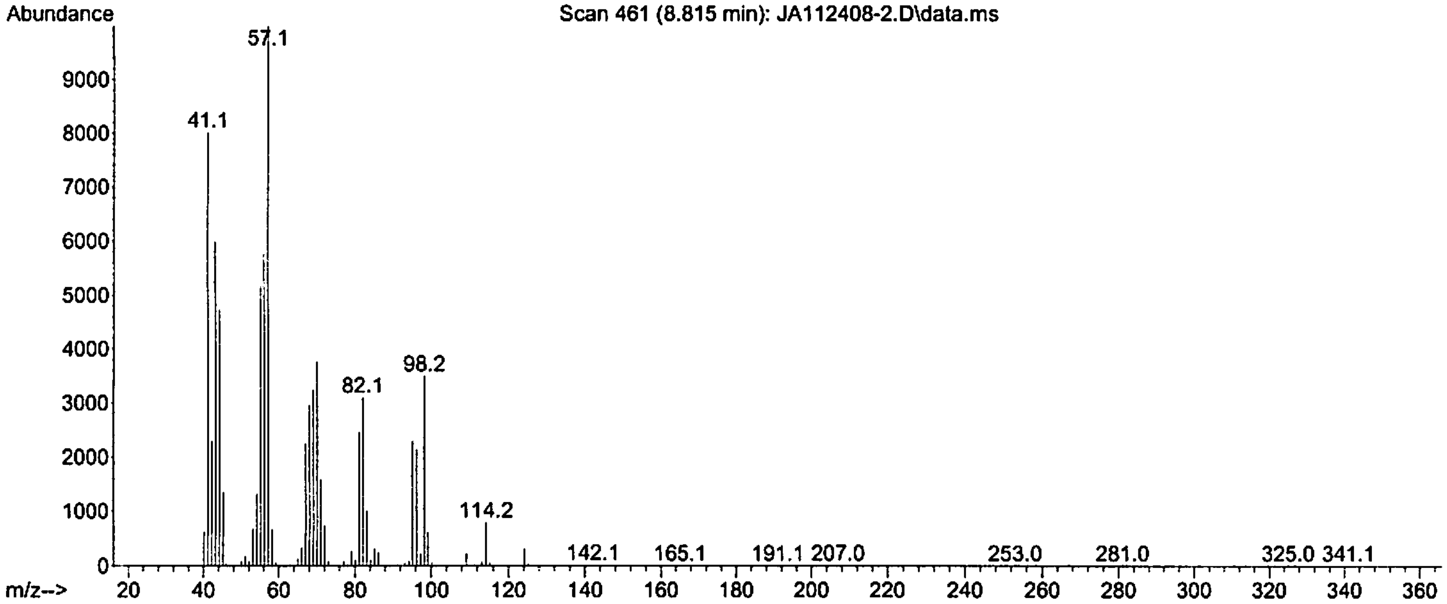

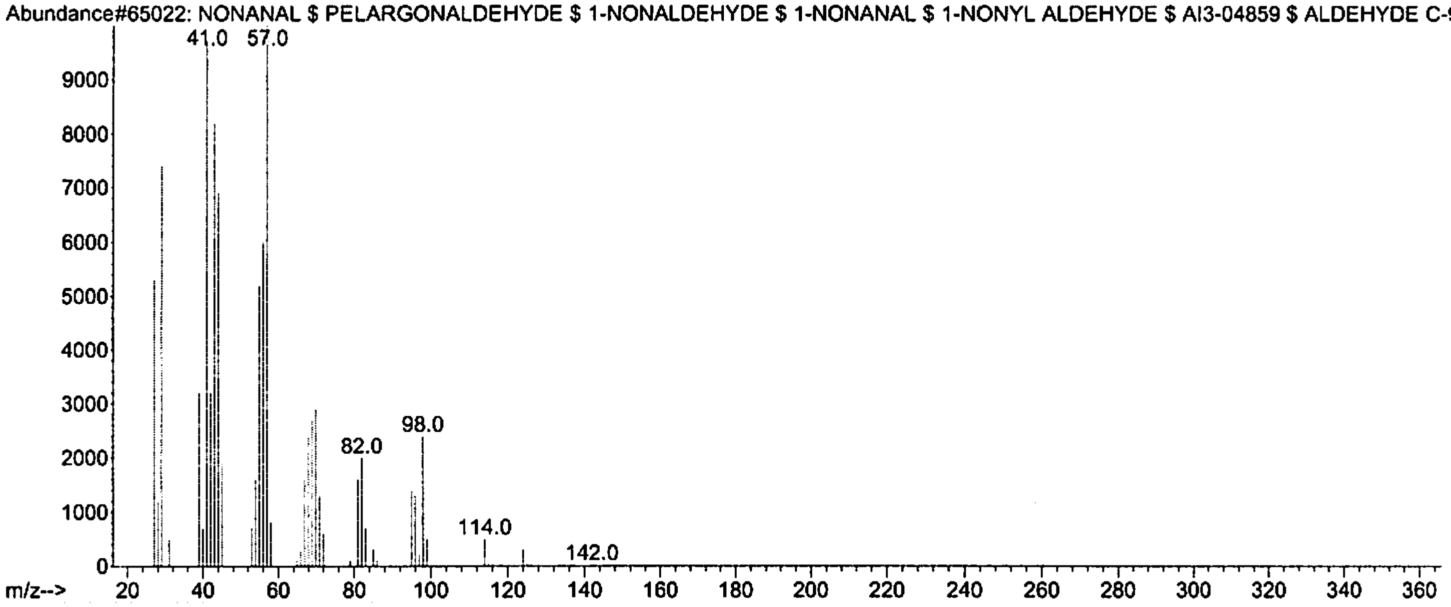

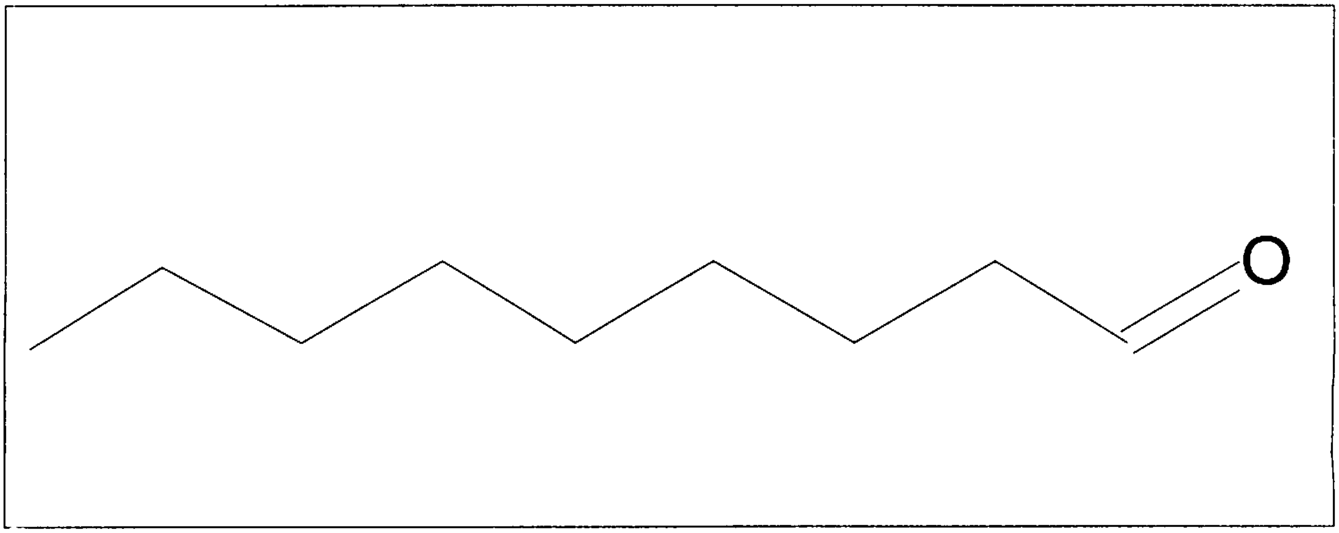

```
File        :D:\Aldrich\JA112408-2.D
Operator    :
Acquired    : 24 Nov 2008  16:20     using AcqMethod JA-50-280LESS.M
Instrument :     Buba
Sample Name: 6M single C.oculata, abd. cuticle/CH2Cl2
Misc Info   : noGC; reared singly; 17-21day-old; mold probl
Vial Number: 1
```

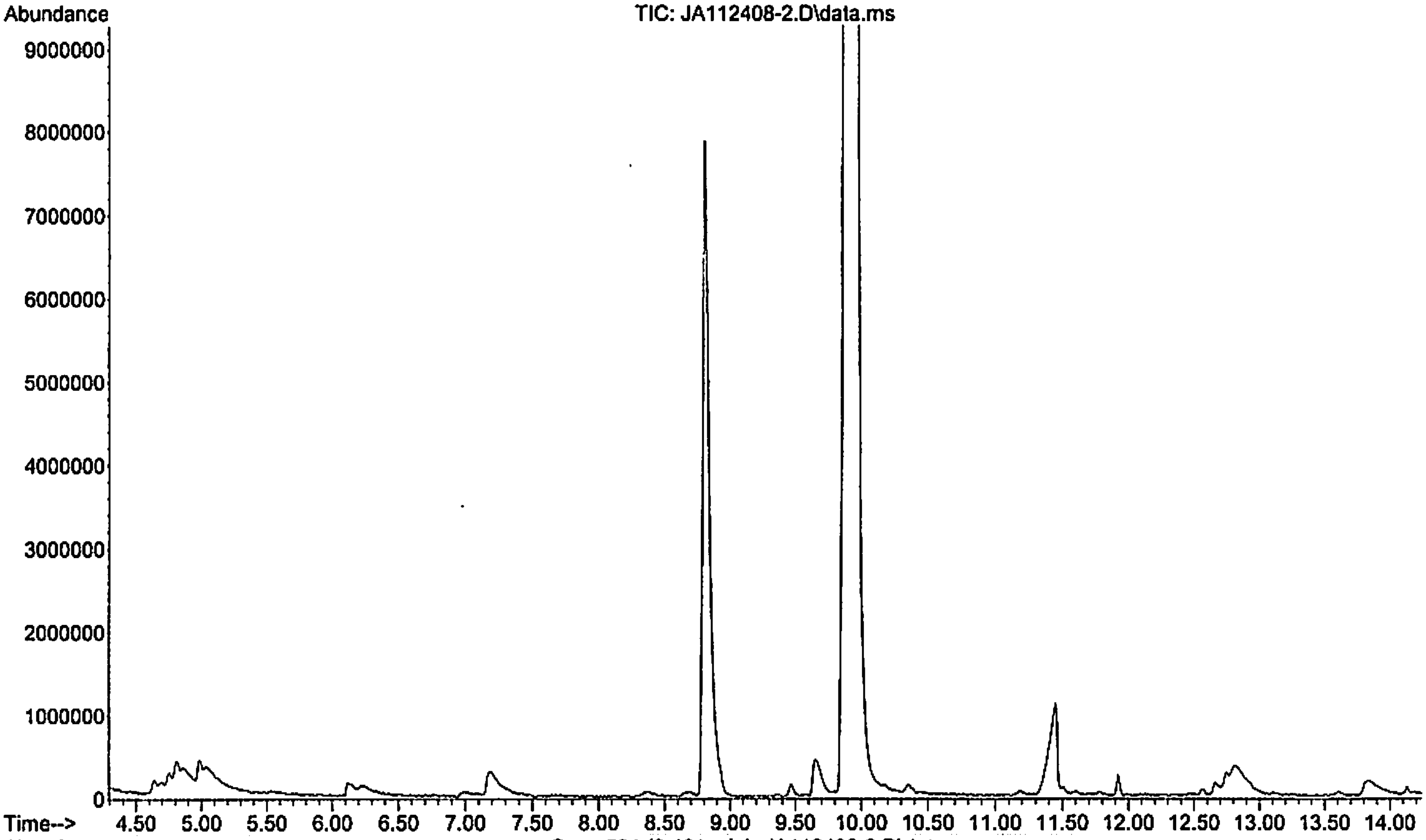

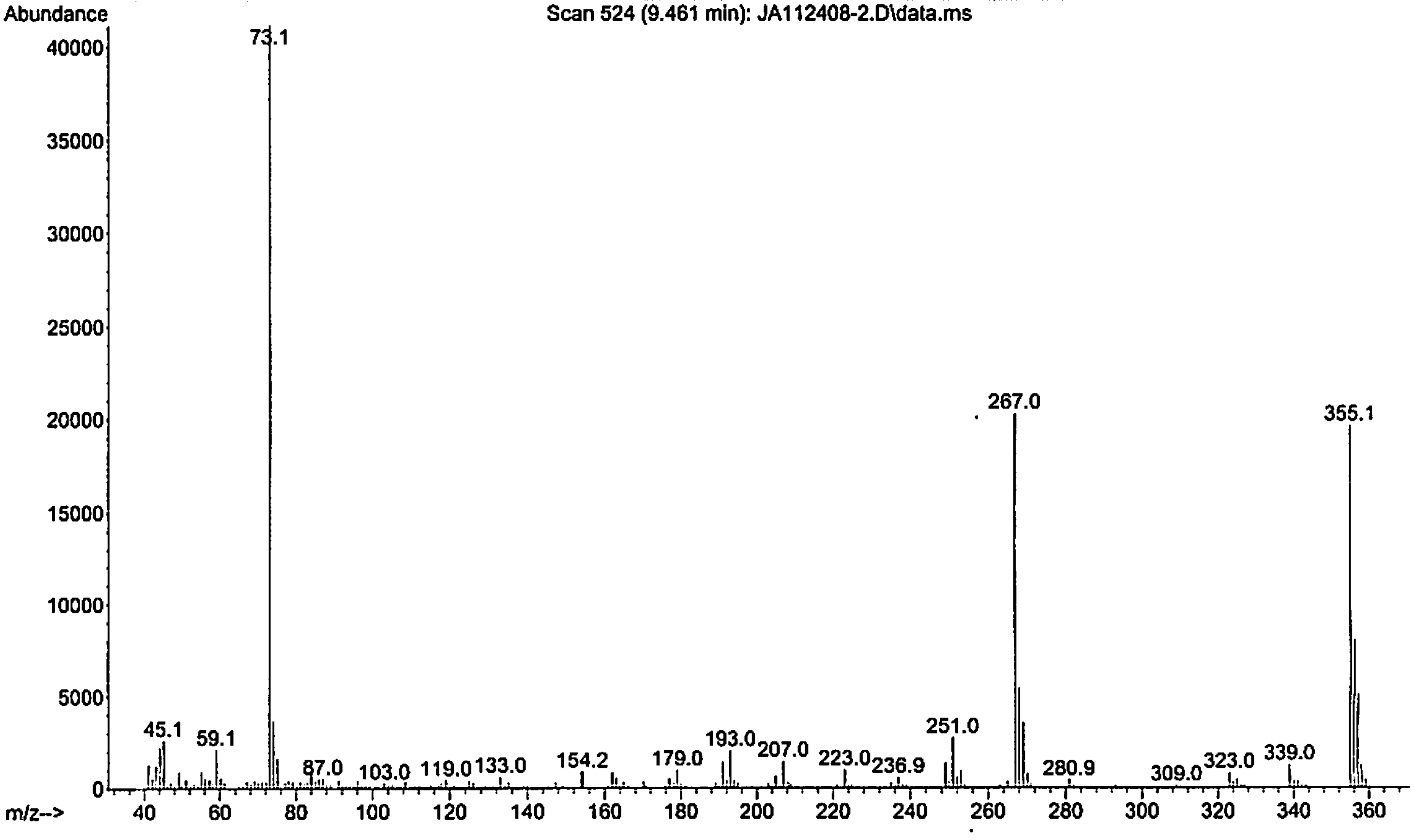

```
File        :D:\Aldrich\JA112408-2.D
Operator    :
Acquired    : 24 Nov 2008  16:20     using AcqMethod JA-50-280LESS.M
Instrument :    Buba
Sample Name: 6M single C.oculata, abd. cuticle/CH2Cl2
Misc Info   : noGC; reared singly; 17-21day-old; mold probl
Vial Number: 1
```

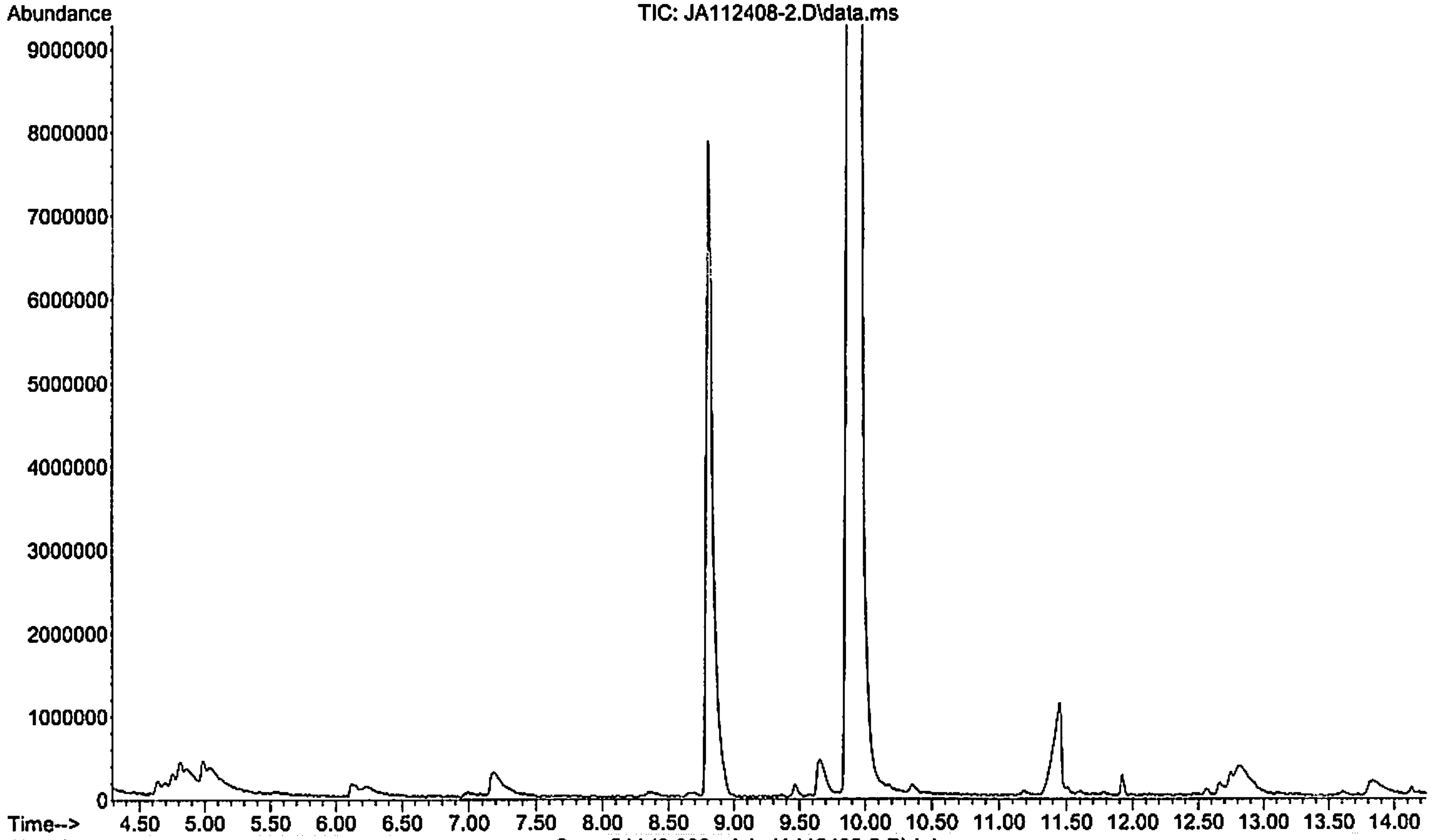

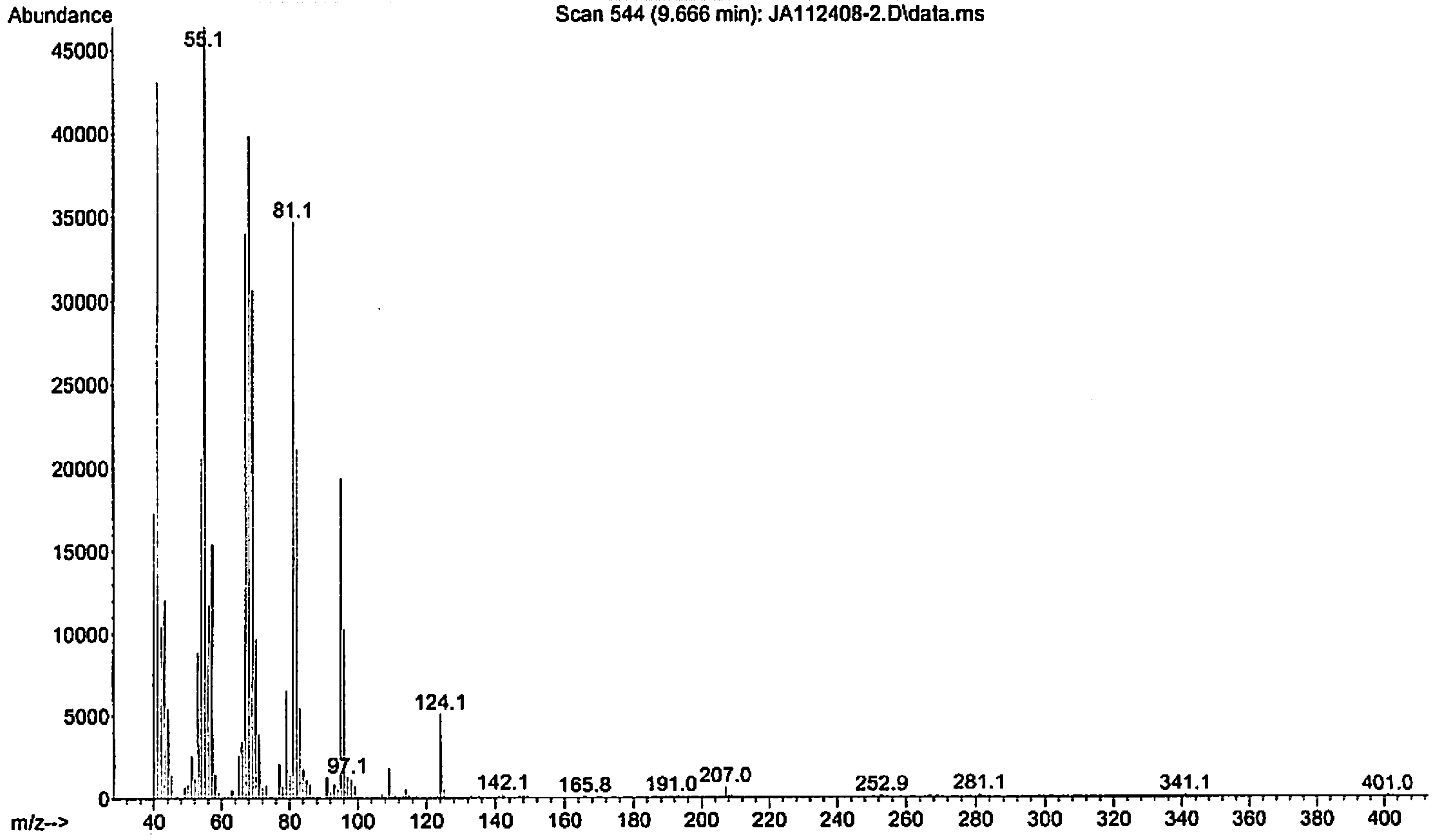

```
File        :D:\Aldrich\JA112408-2.D
Operator    :
Acquired    : 24 Nov 2008  16:20     using AcqMethod JA-50-280LESS.M
Instrument  :    Buba
Sample Name: 6M single C.oculata, abd. cuticle/CH2Cl2
Misc Info   : noGC; reared singly; 17-21day-old; mold probl
Vial Number: 1
```

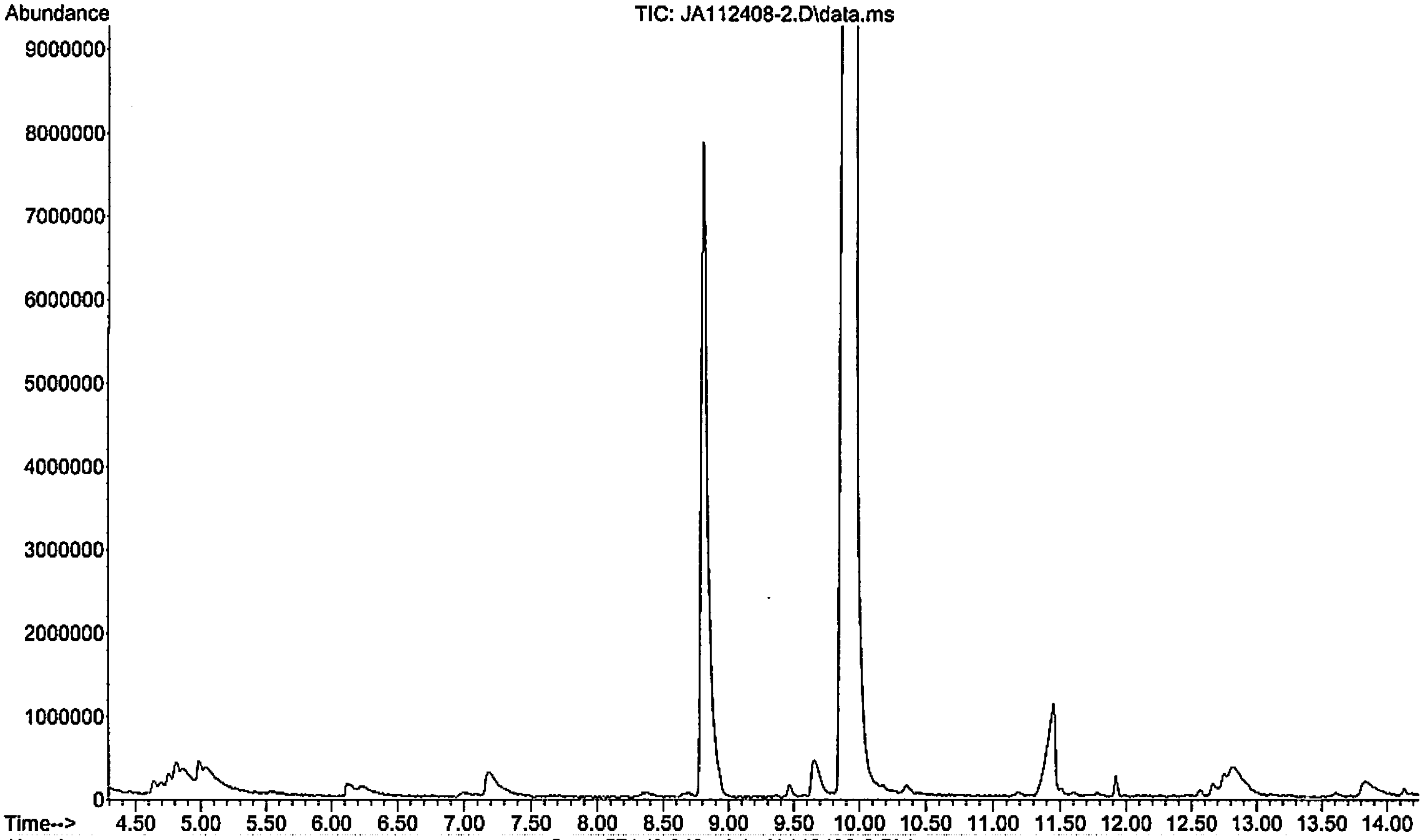

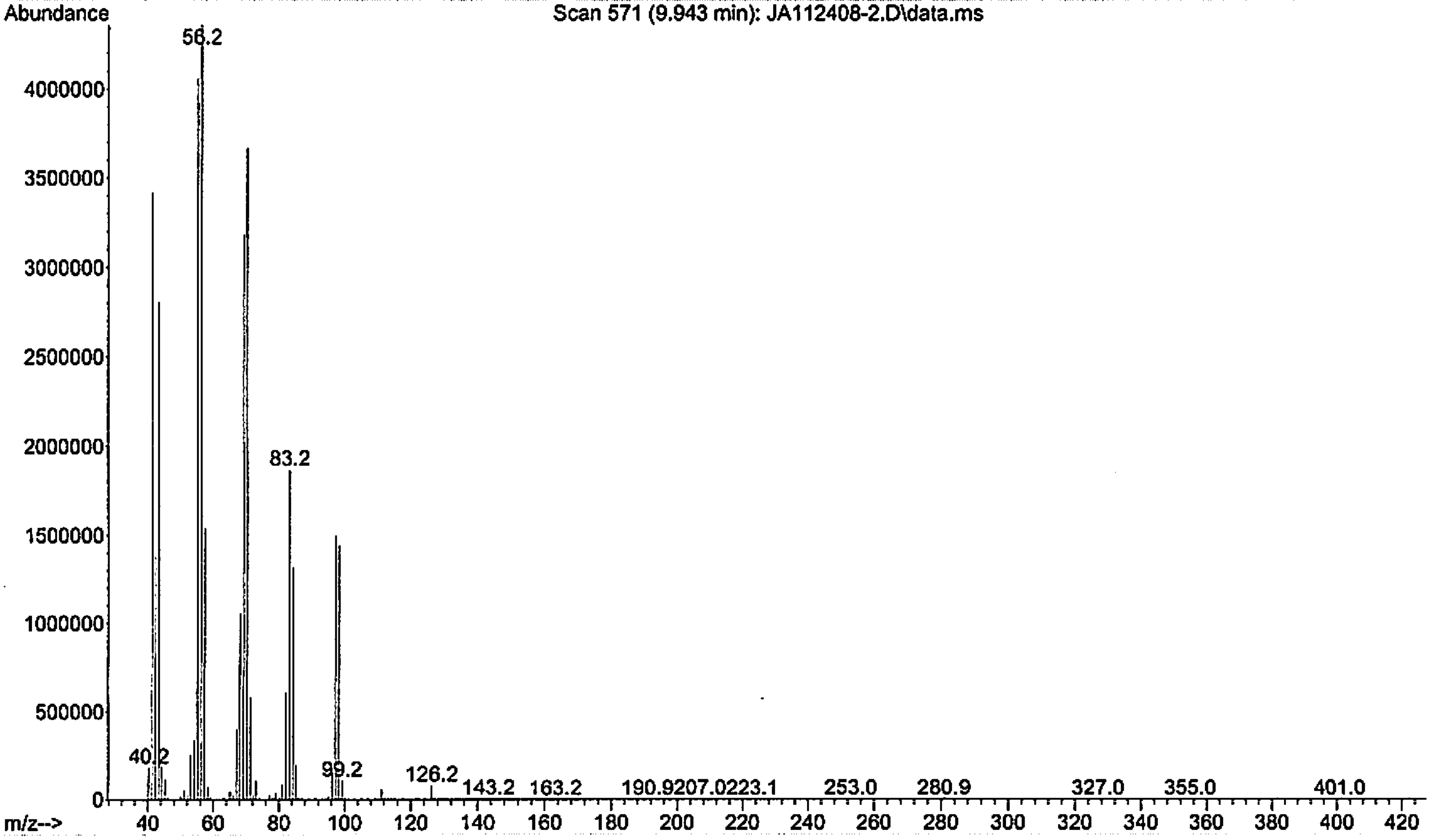

Library Searched : C:\Database\W8N05ST.L
Quality : 91
ID : 1-NONANOL $ NONANOL $ NONAN-1-OL $ 1-HYDROXYNONANE $ 1-NONANO $ AI3-03962 $ ALCOHOL C-9 $ BRN 0969213 $ C9 ALCOHOL $ EINECS 205-583-7 $ FATTY ALCOHOL(C9) $ FEMA NO. 2789 $ HSDB 5145 $ N-NONAN-1-OL $ N-NONANOL $ N-NONYL ALCOHOL $ NONALOL $ NONANOL-(1) $ NO

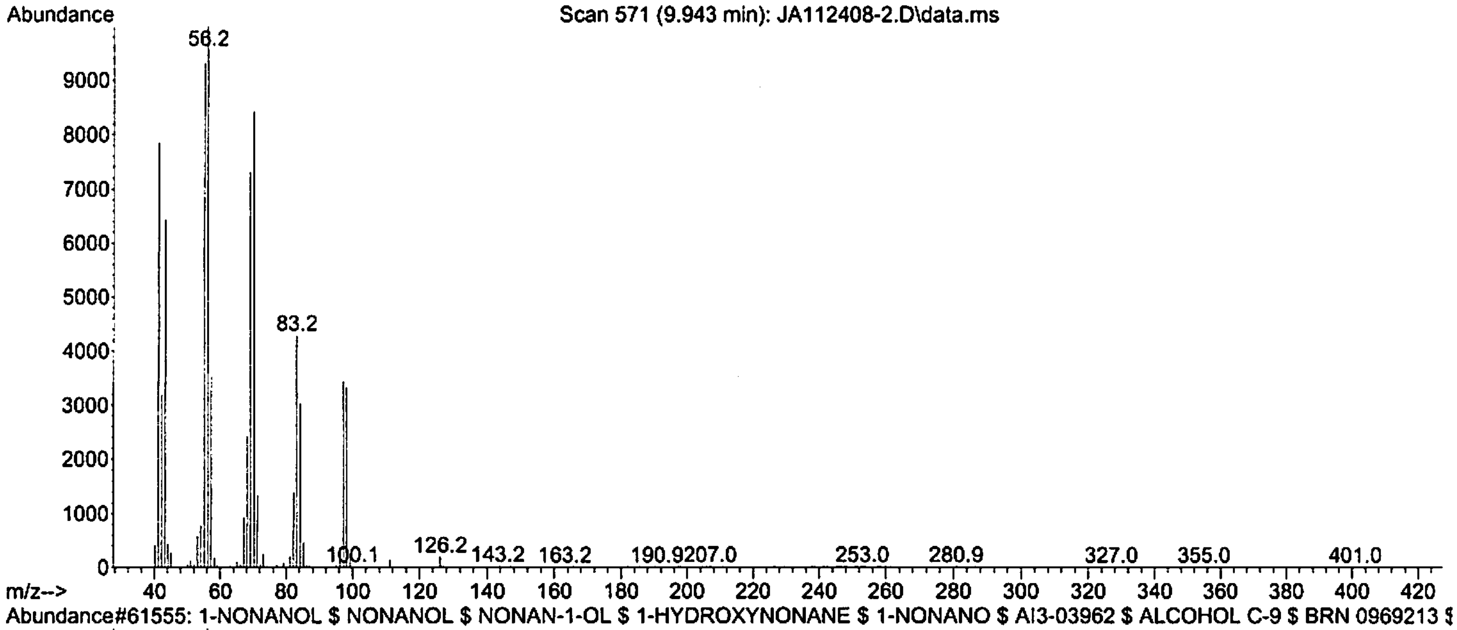

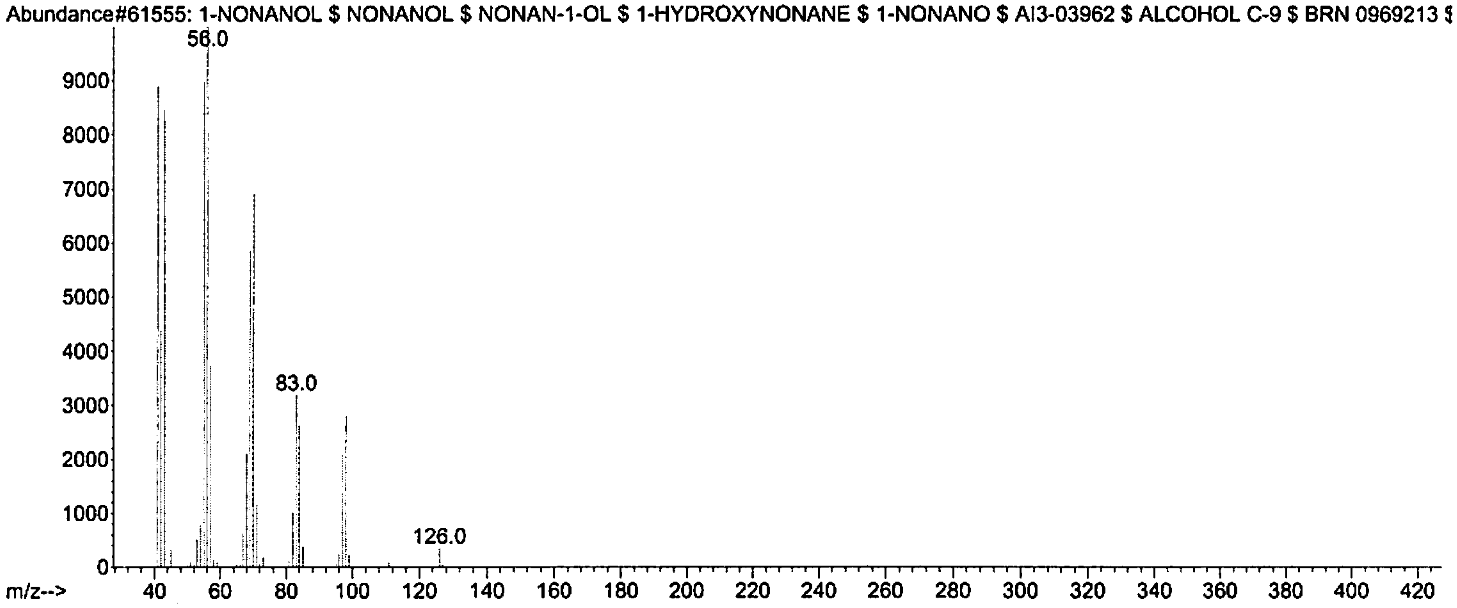

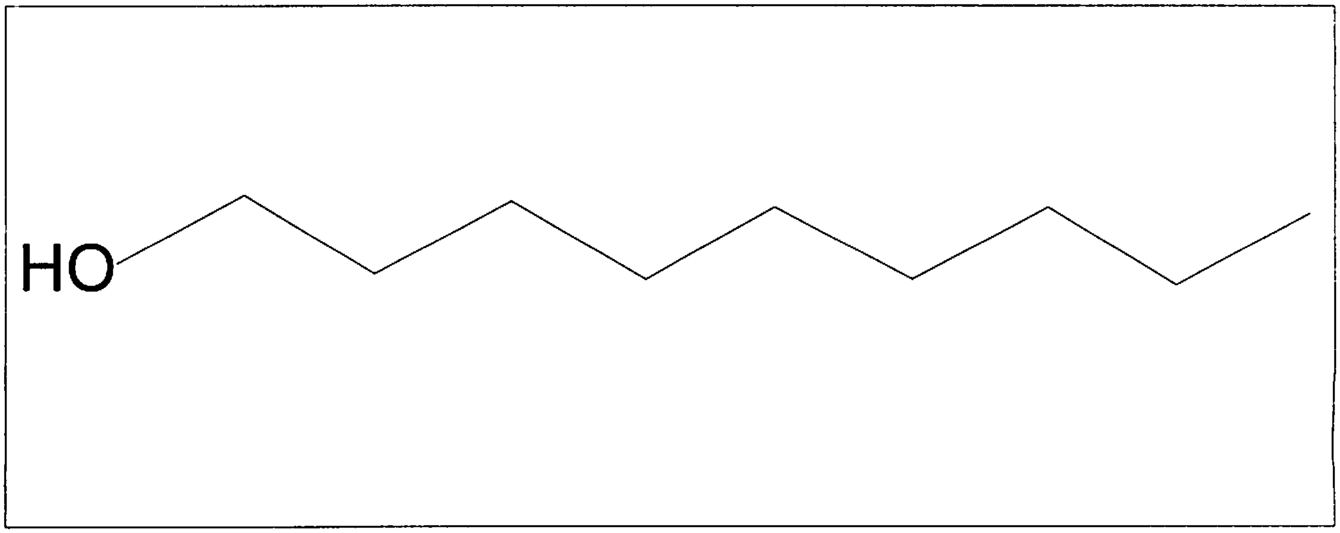

File :D:\Aldrich\JA112408-2.D
Operator :
Acquired : 24 Nov 2008 16:20 using AcqMethod JA-50-280LESS.M
Instrument : Buba
Sample Name: 6M single C.oculata, abd. cuticle/CH2Cl2
Misc Info : noGC; reared singly; 17-21day-old; mold probl
Vial Number: 1

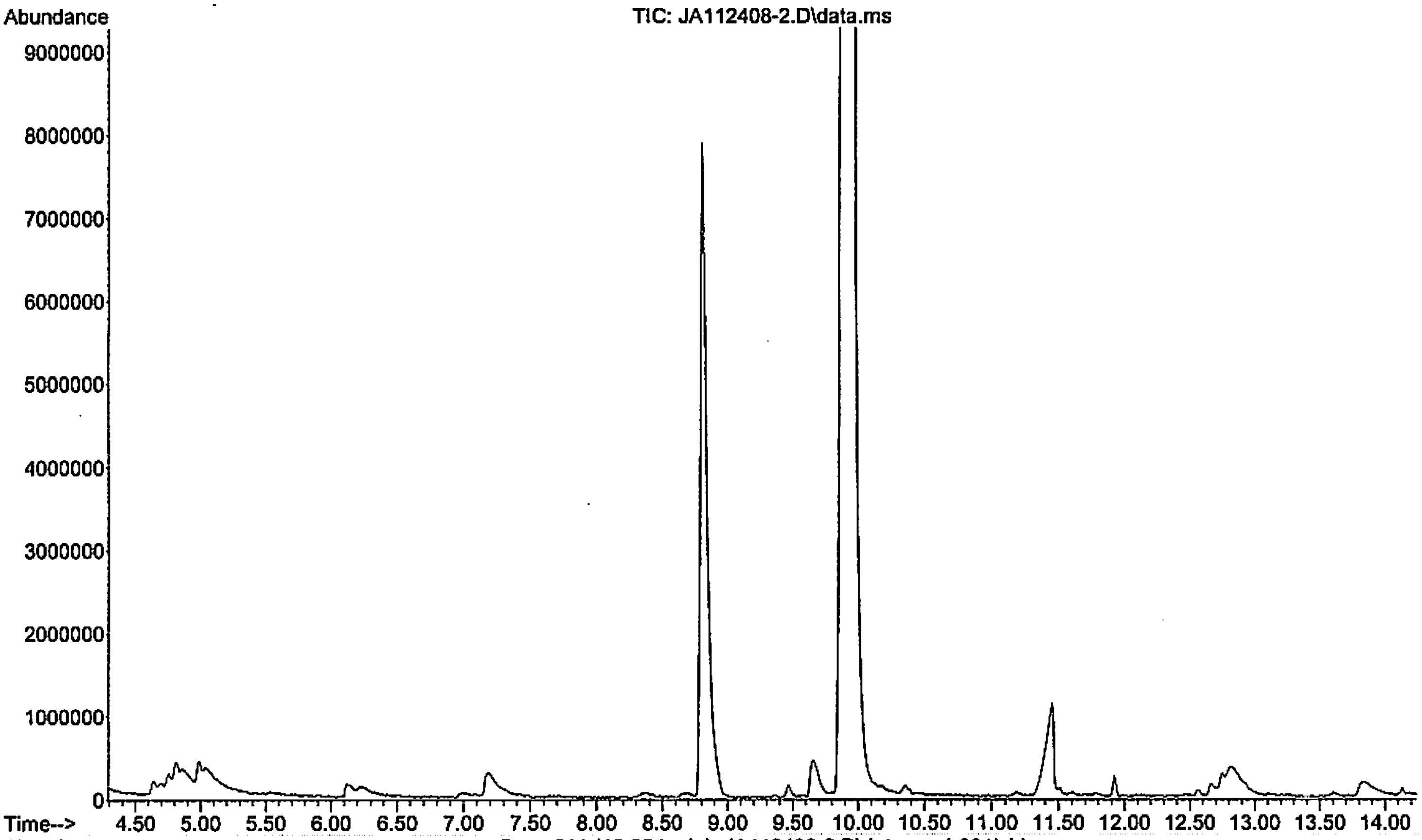

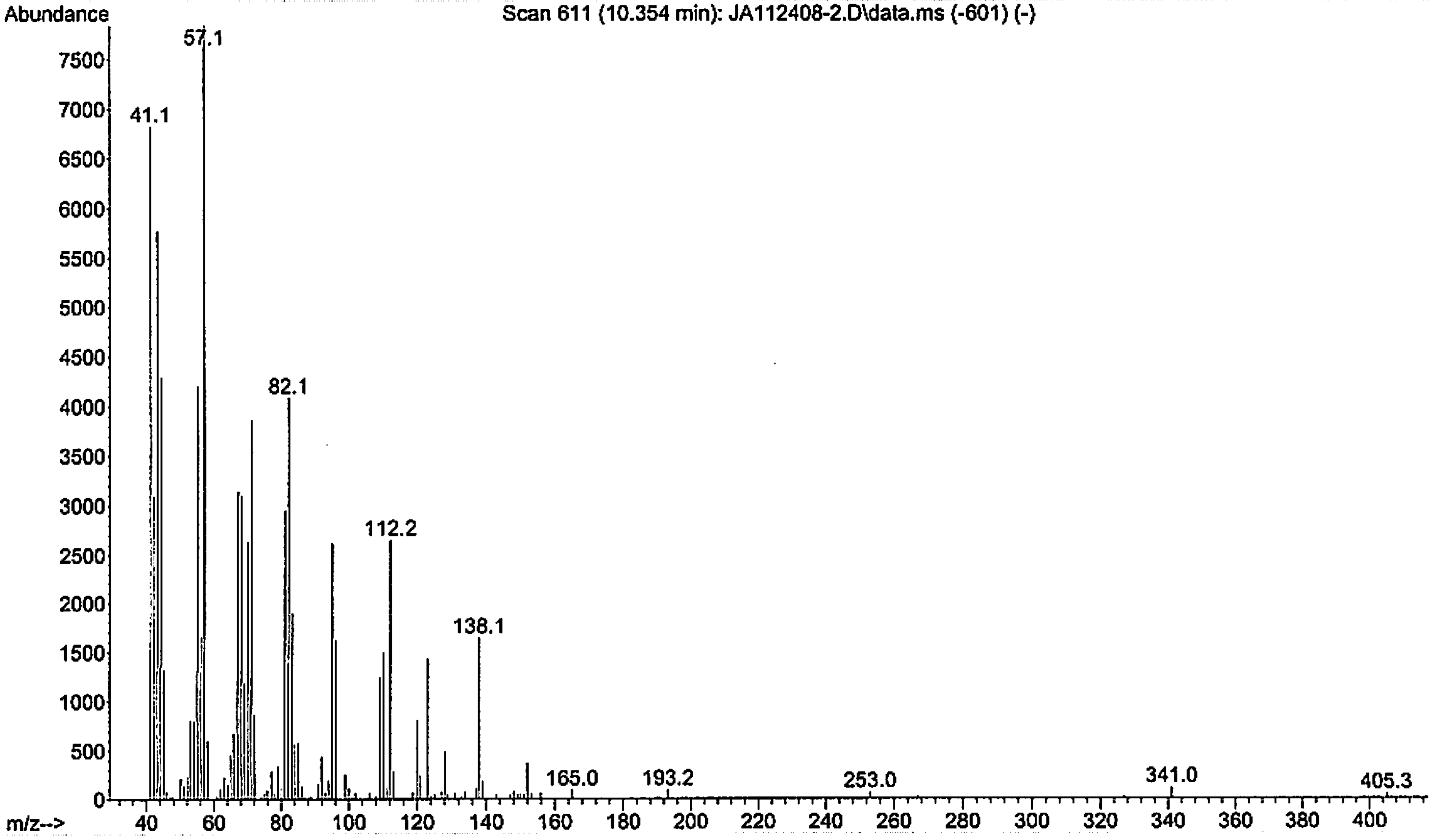

```
File        :D:\Aldrich\JA112408-2.D
Operator    :
Acquired    : 24 Nov 2008  16:20     using AcqMethod JA-50-280LESS.M
Instrument  :    Buba
Sample Name: 6M single C.oculata, abd. cuticle/CH2Cl2
Misc Info   : noGC; reared singly; 17-21day-old; mold probl
Vial Number: 1
```

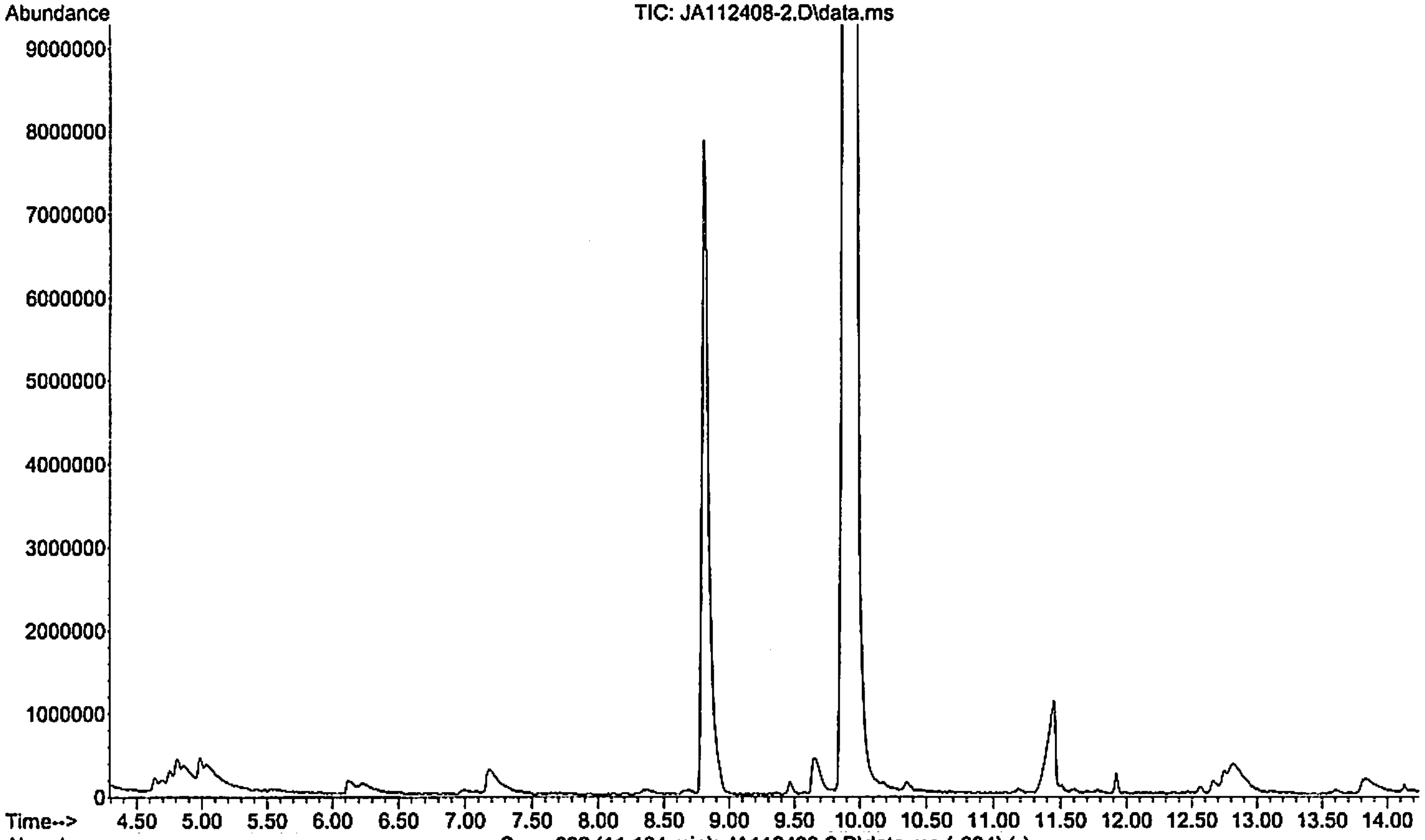

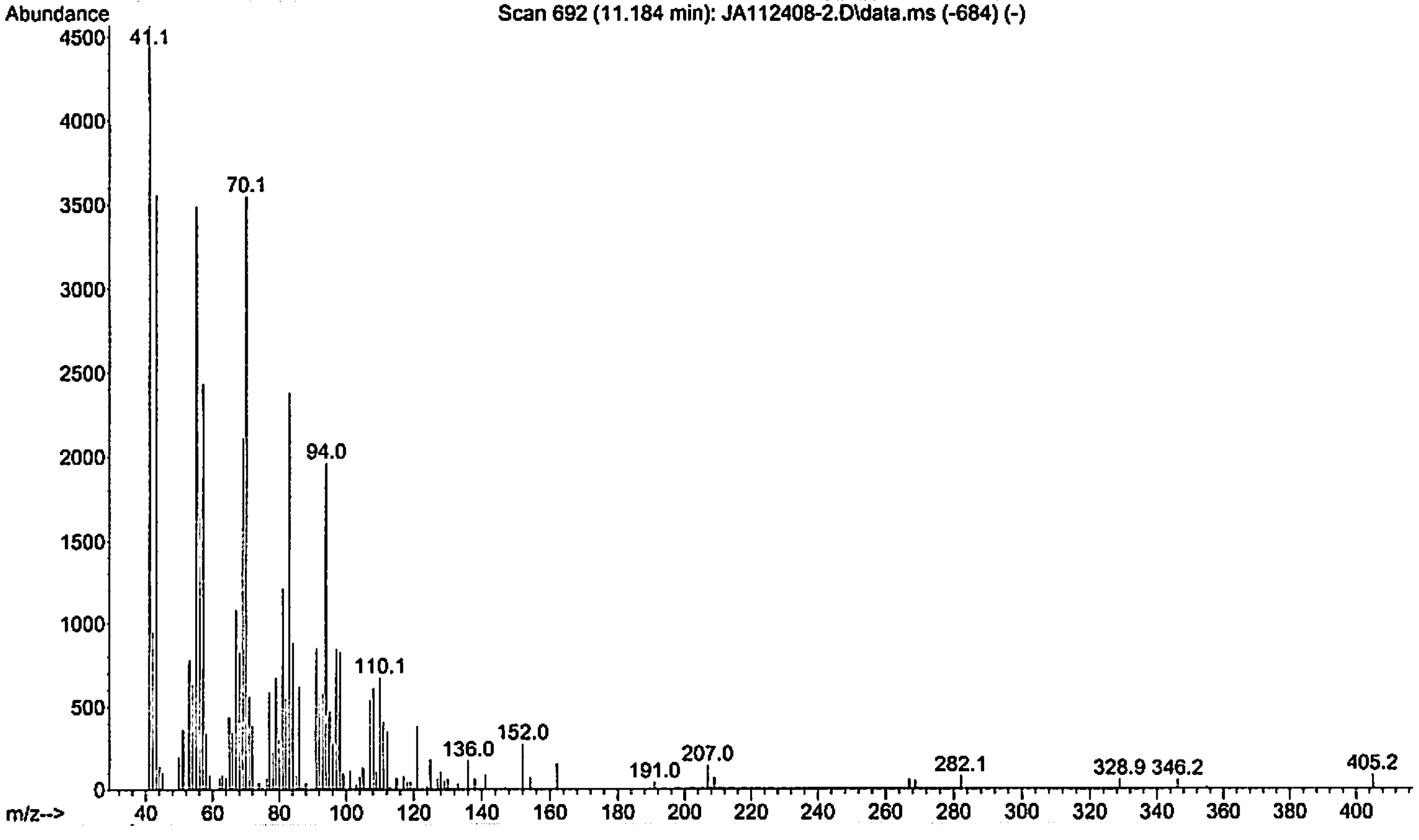

```
File        :D:\Aldrich\JA112408-2.D
Operator    :
Acquired    : 24 Nov 2008  16:20     using AcqMethod JA-50-280LESS.M
Instrument  :    Buba
Sample Name: 6M single C.oculata, abd. cuticle/CH2Cl2
Misc Info   : noGC; reared singly; 17-21day-old; mold probl
Vial Number: 1
```

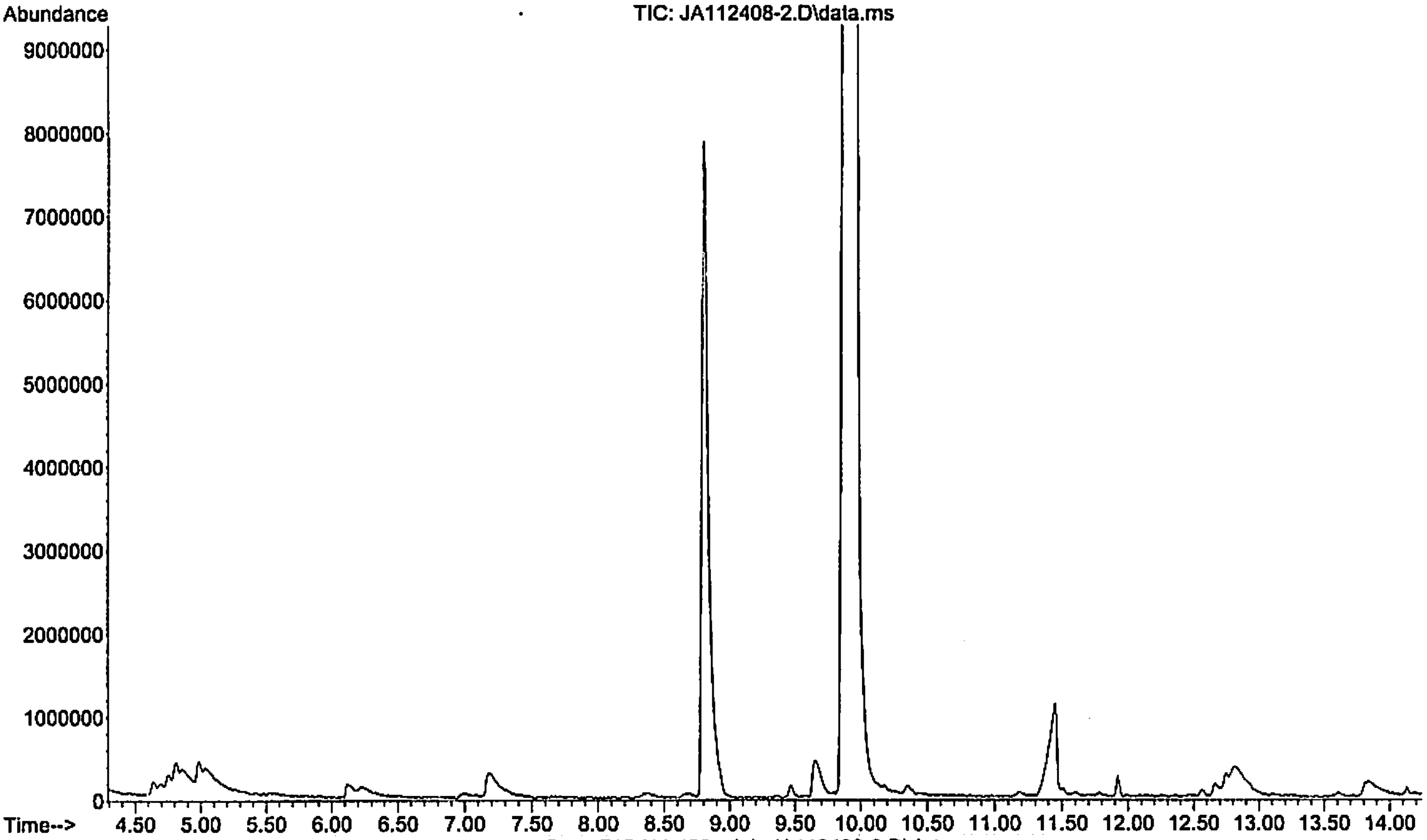

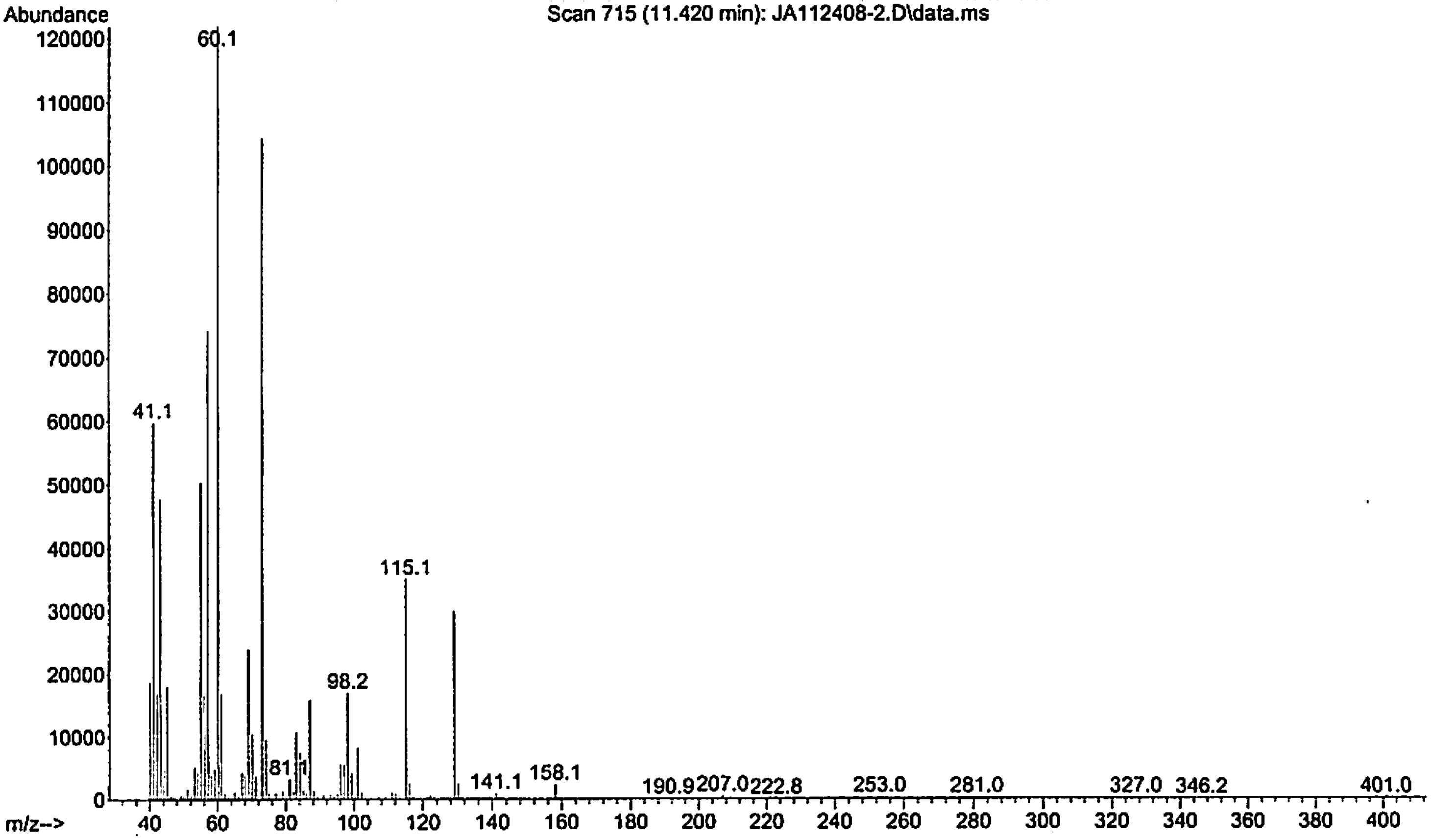

```
File        :D:\Aldrich\JA112408-2.D
Operator    :
Acquired    : 24 Nov 2008  16:20     using AcqMethod JA-50-280LESS.M
Instrument :     Buba
Sample Name: 6M single C.oculata, abd. cuticle/CH2Cl2
Misc Info   : noGC; reared singly; 17-21day-old; mold probl
Vial Number: 1
```

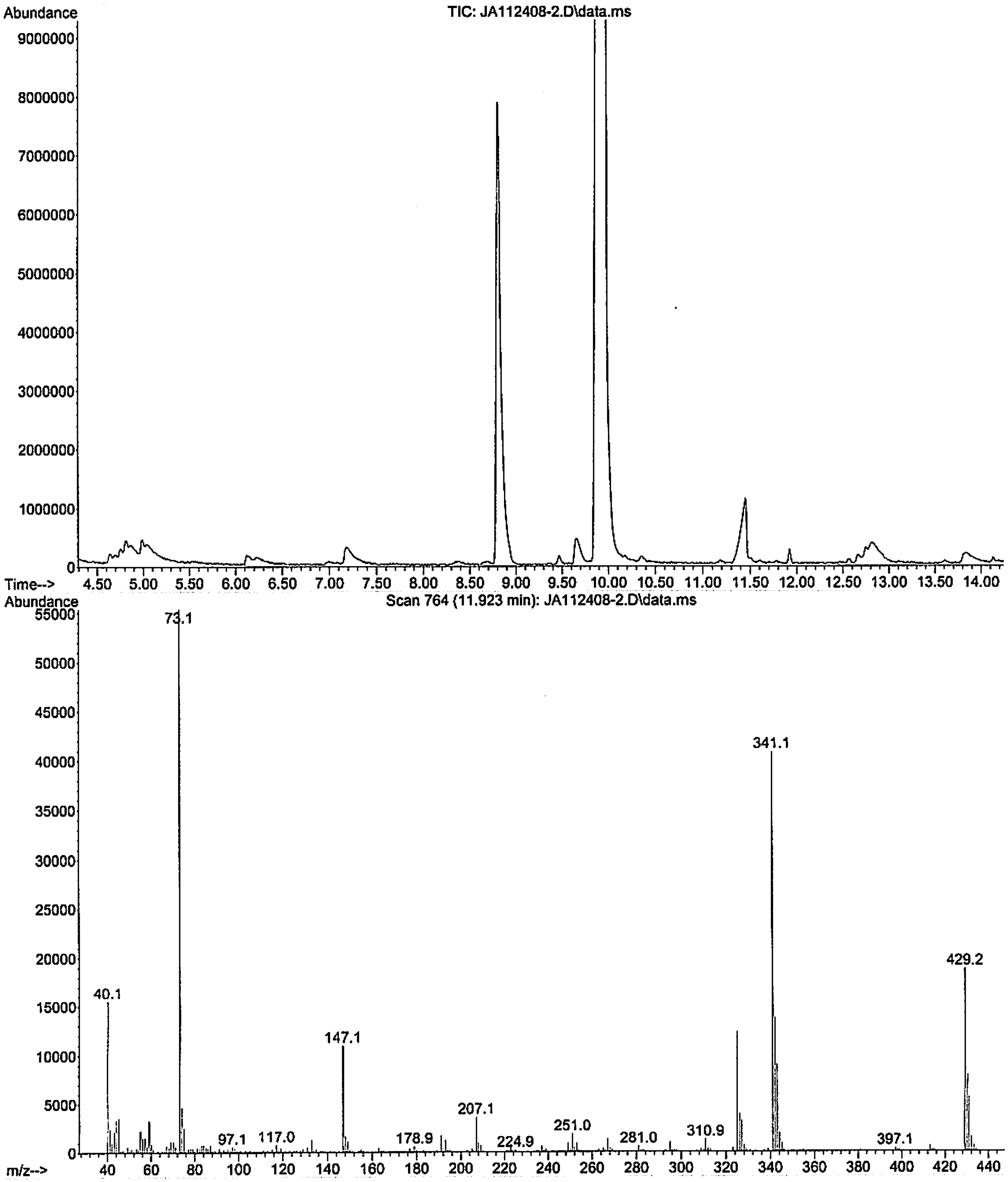

```
File         :D:\Aldrich\JA112408-2.D
Operator     :
Acquired     : 24 Nov 2008  16:20     using AcqMethod JA-50-280LESS.M
Instrument :     Buba
Sample Name: 6M single C.oculata, abd. cuticle/CH2Cl2
Misc Info    : noGC; reared singly; 17-21day-old; mold probl
Vial Number: 1
```

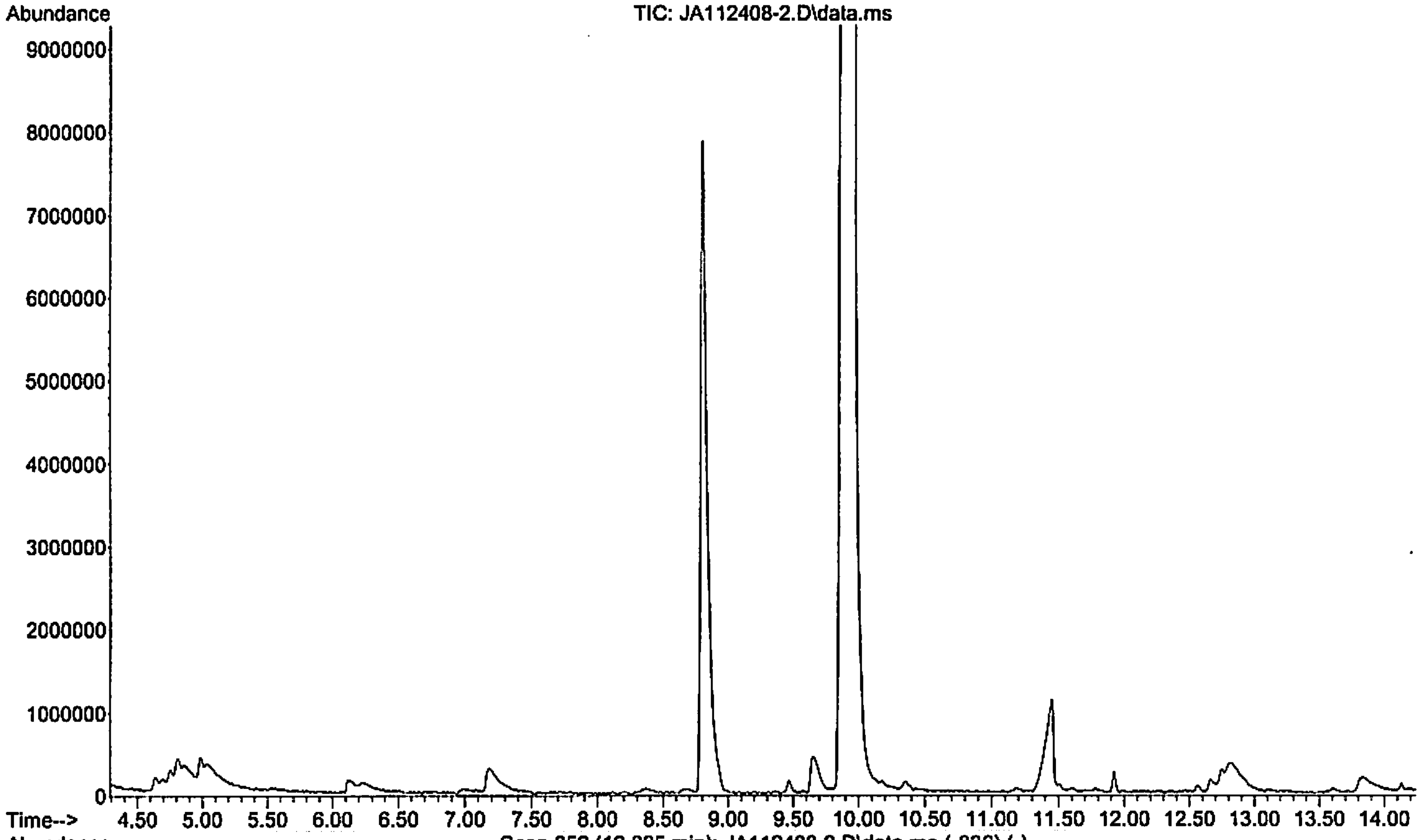

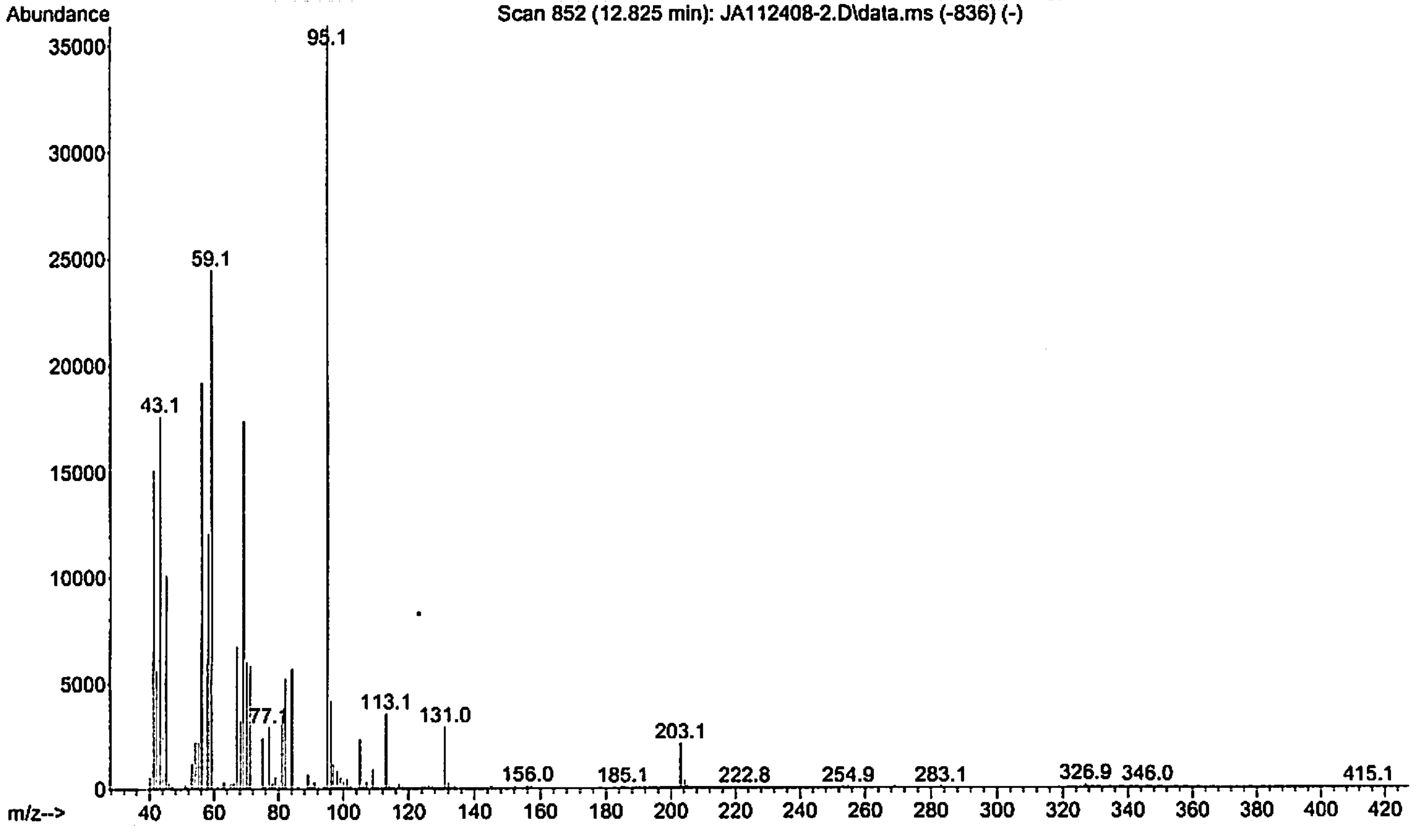

File :D:\Aldrich\JA112408-2.D
Operator :
Acquired : 24 Nov 2008 16:20 using AcqMethod JA-50-280LESS.M
Instrument : Buba
Sample Name: 6M single C.oculata, abd. cuticle/CH2Cl2
Misc Info : noGC; reared singly; 17-21day-old; mold probl
Vial Number: 1

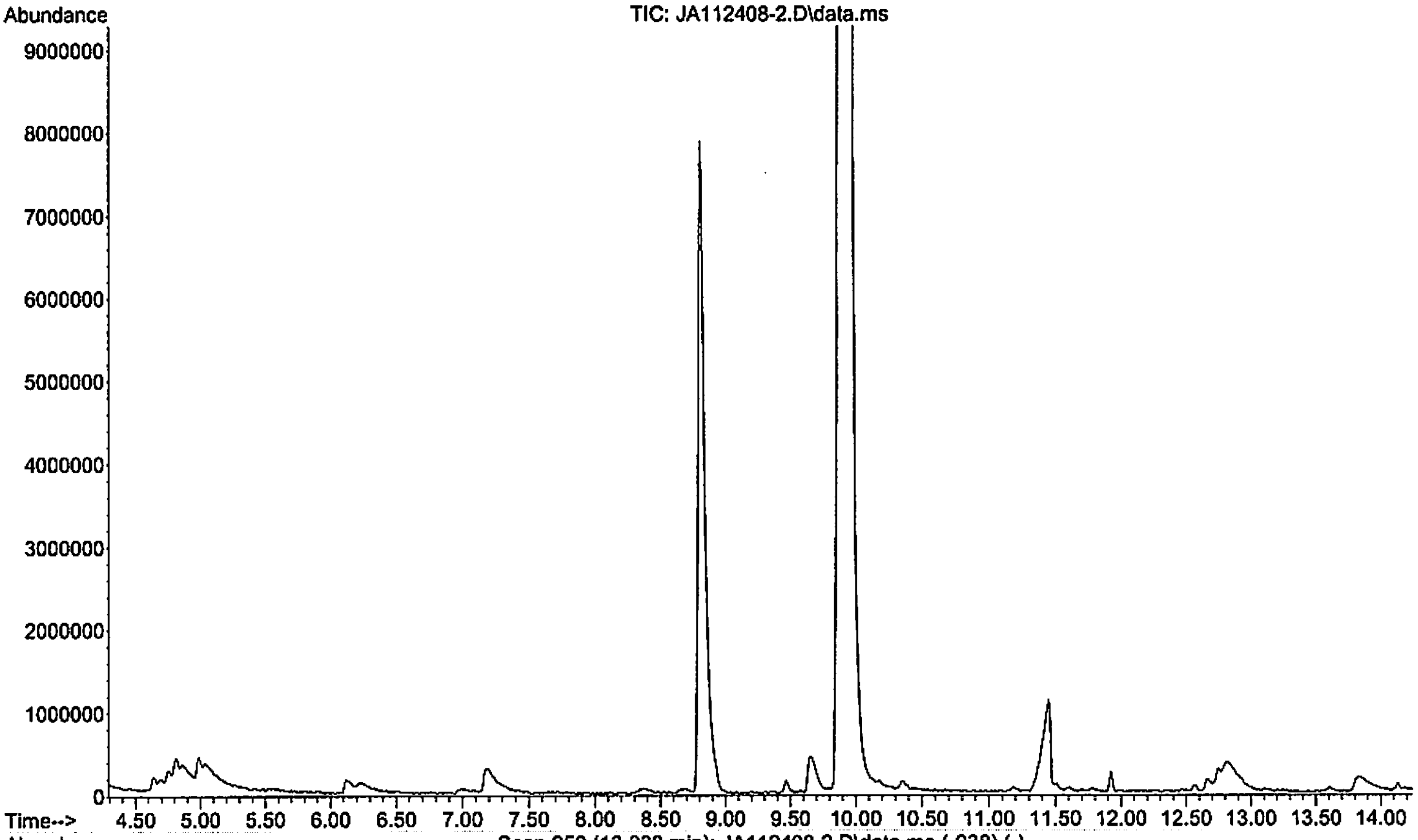

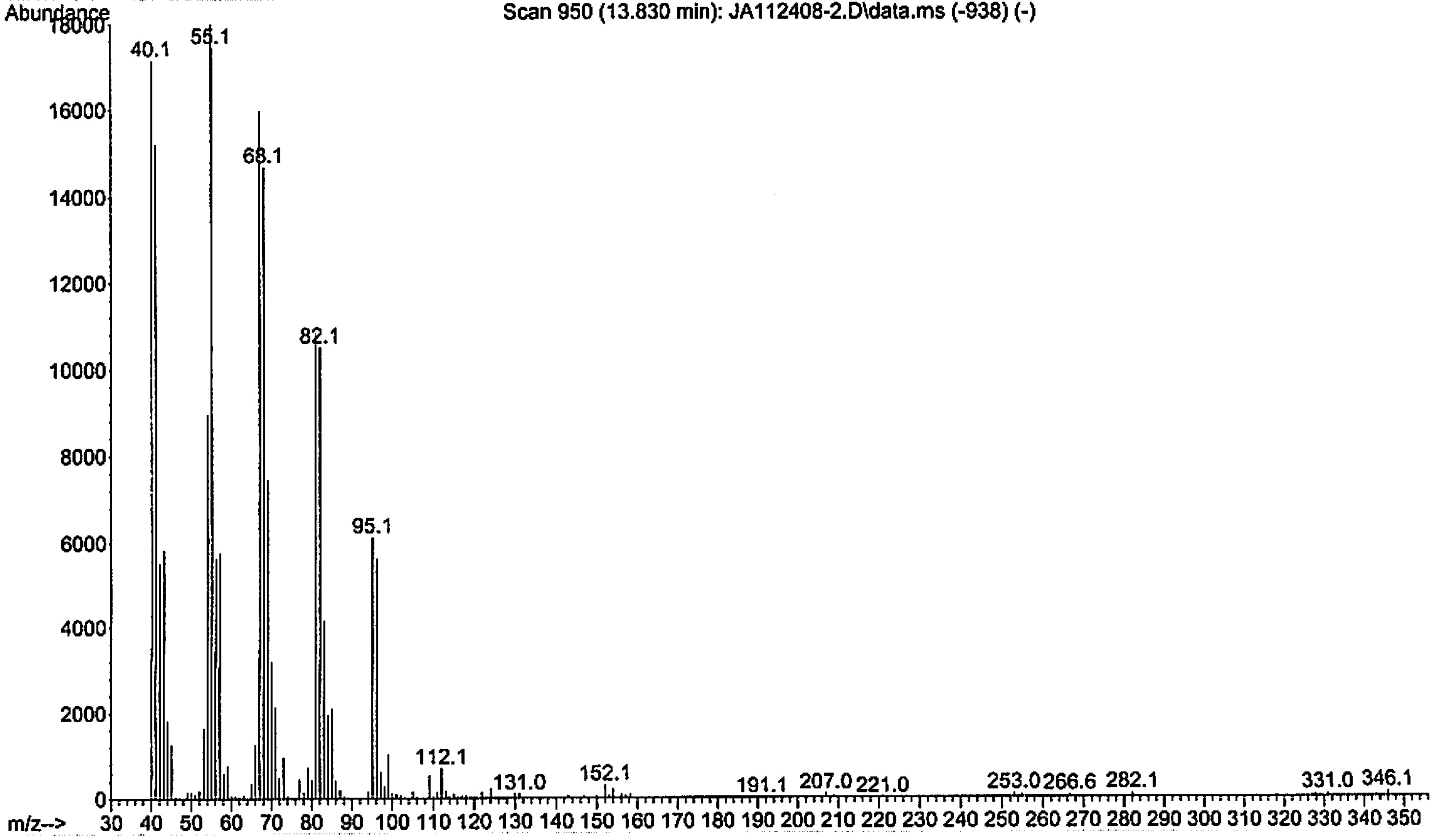

```
Library Searched : C:\Database\W8N05ST.L
Quality          : 91
ID               : 1,9-Nonanediol $ .alpha.,.omega.-Nonanediol
```

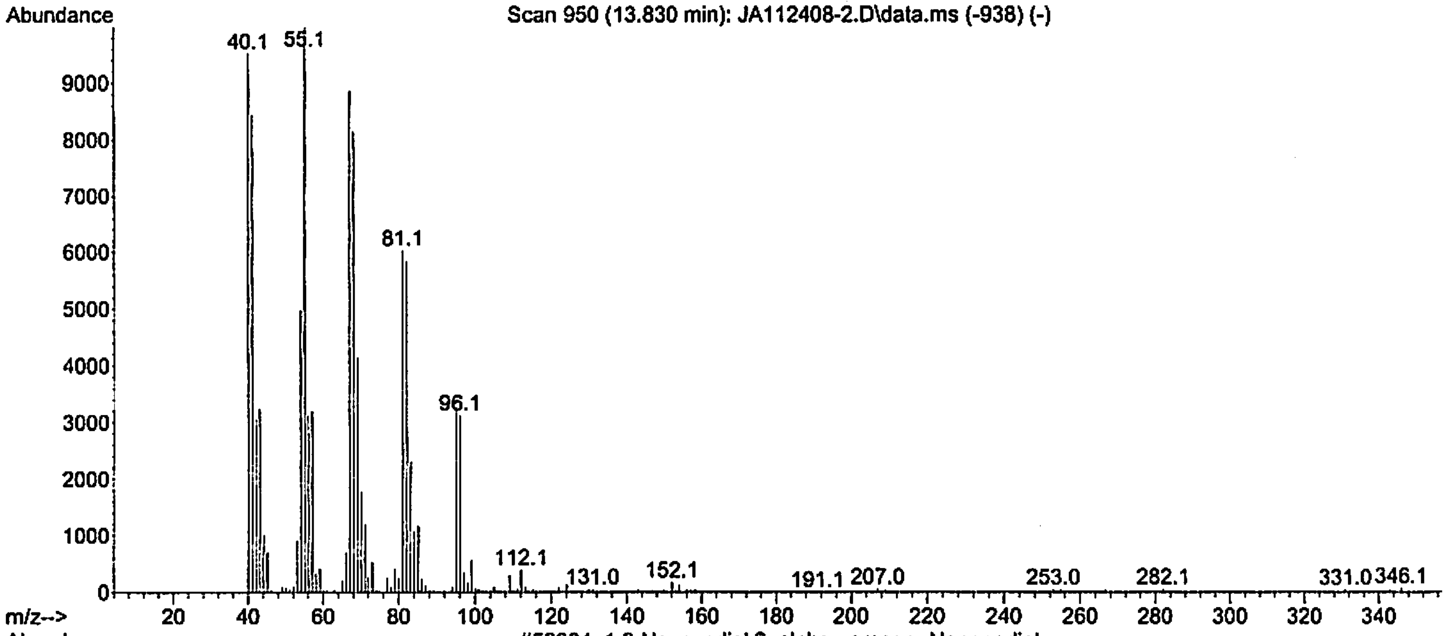

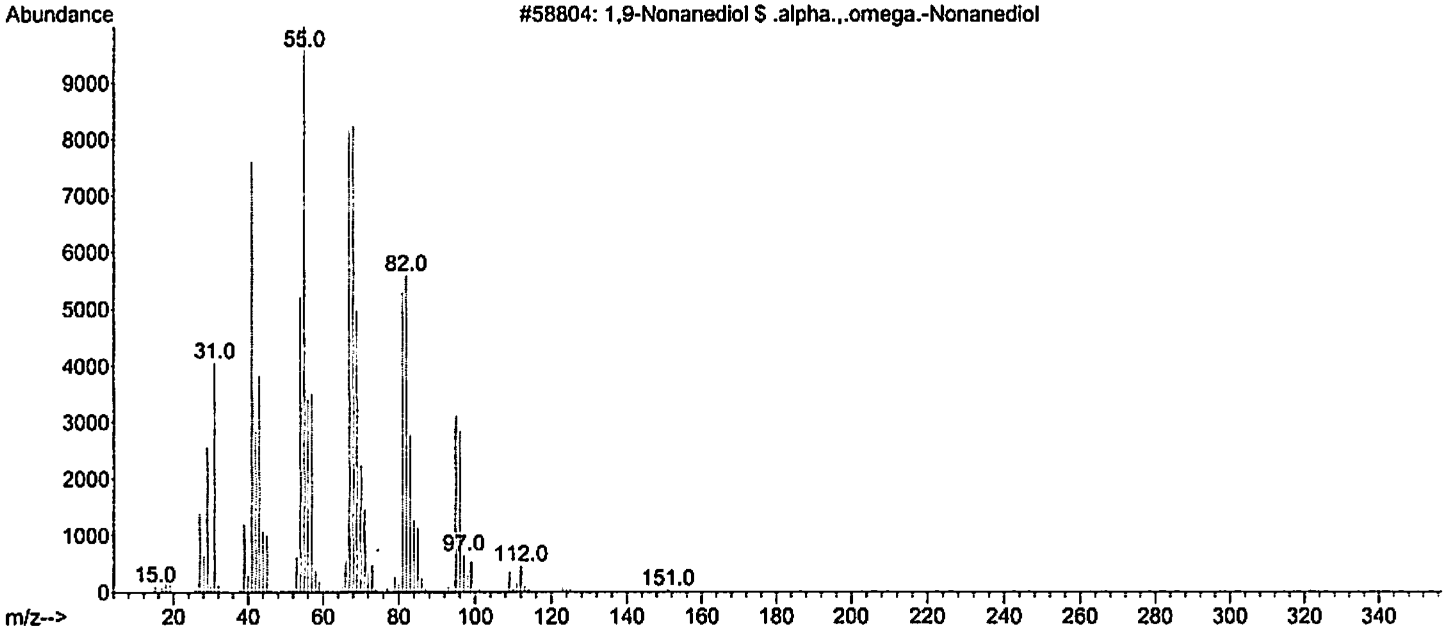

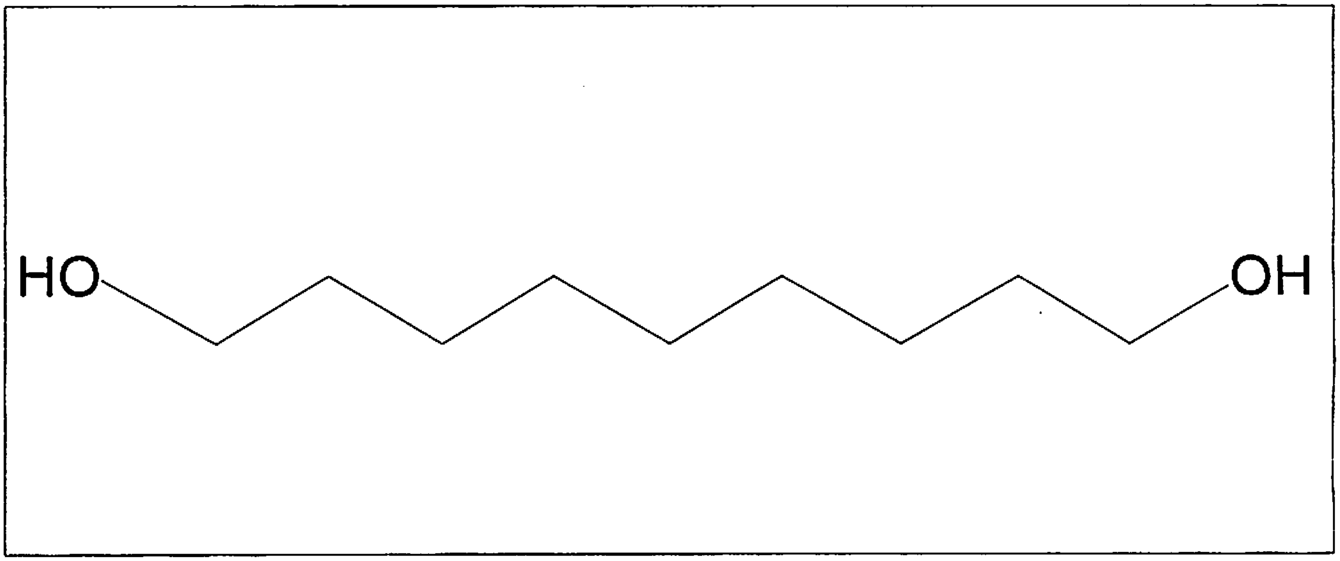

Supplement: Data S6 [file peerj-04-1564-s011.pdf]
