# Supplementary material for: Pharmacophagy in green lacewings (Neuroptera: Chrysopidae: Chrysopa spp.)?
Source: PeerJ. 2016 Jan 18;4:e1564. doi: 10.7717/peerj.1564 (PMC4727961; doi:10.7717/peerj.1564)

DB-XCB

File        :D:\DATA\Aldrich\JA-09\JA010509-2.D
Operator    : Aldrich
Acquired    :  5 Jan 2009  14:37     using AcqMethod JA-WAX08.M
Instrument :   Instrument #1
Sample Name: 8 male C.oculata abd.sternites/CH2Cl2
Misc Info  : w/ lug linalool/ul water/6 days; 2nd half
Vial Number: 1

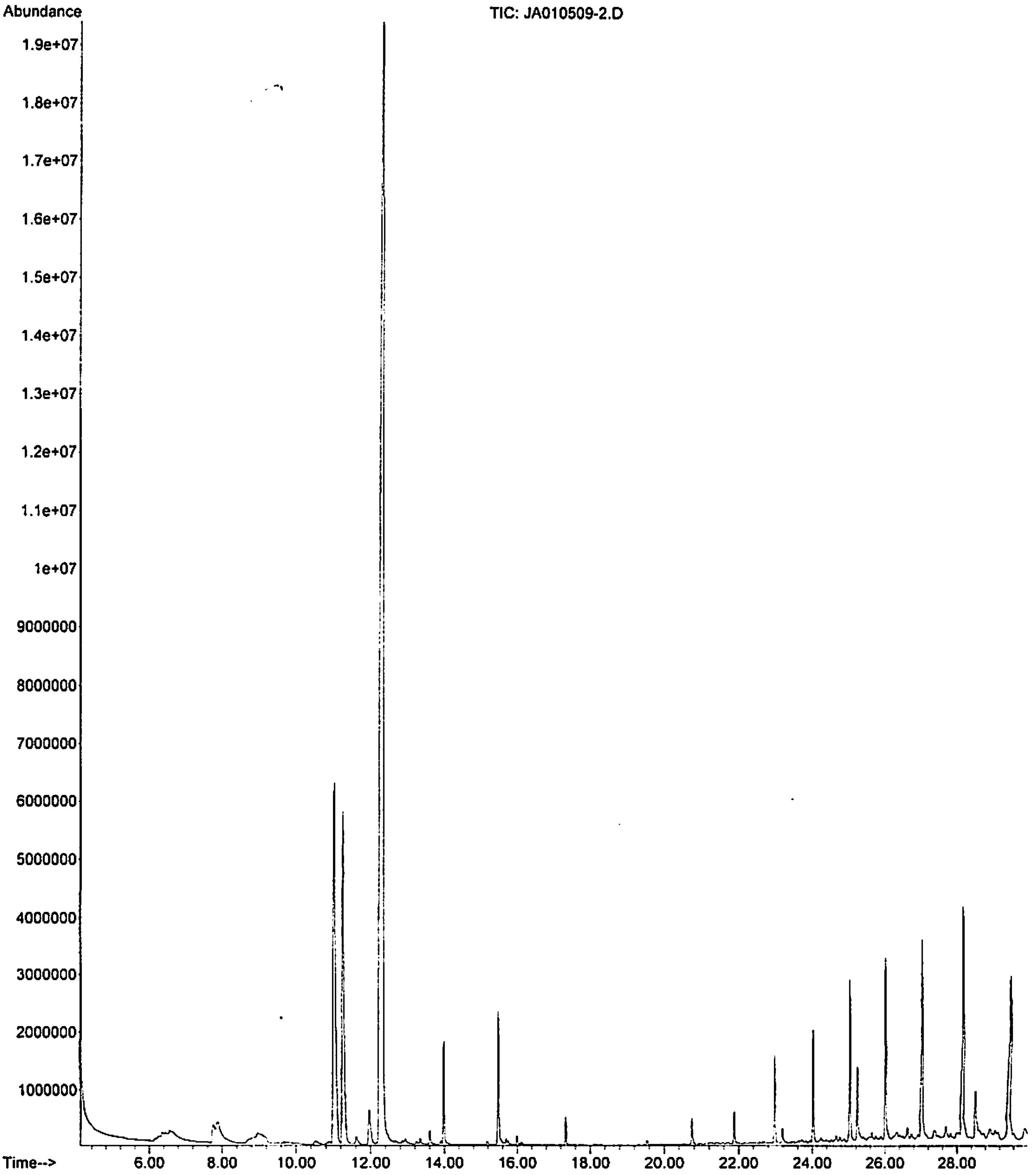

```
File        :D:\DATA\Aldrich\JA-09\JA010509-2.D
Operator    : Aldrich
Acquired    :  5 Jan 2009  14:37     using AcqMethod JA-WAX08.M
Instrument  :    Instrument #1
Sample Name: 8 male C.oculata abd.sternites/CH2Cl2
Misc Info   : w/ 1ug linalool/ul water/6 days; 2nd half
Vial Number: 1
```

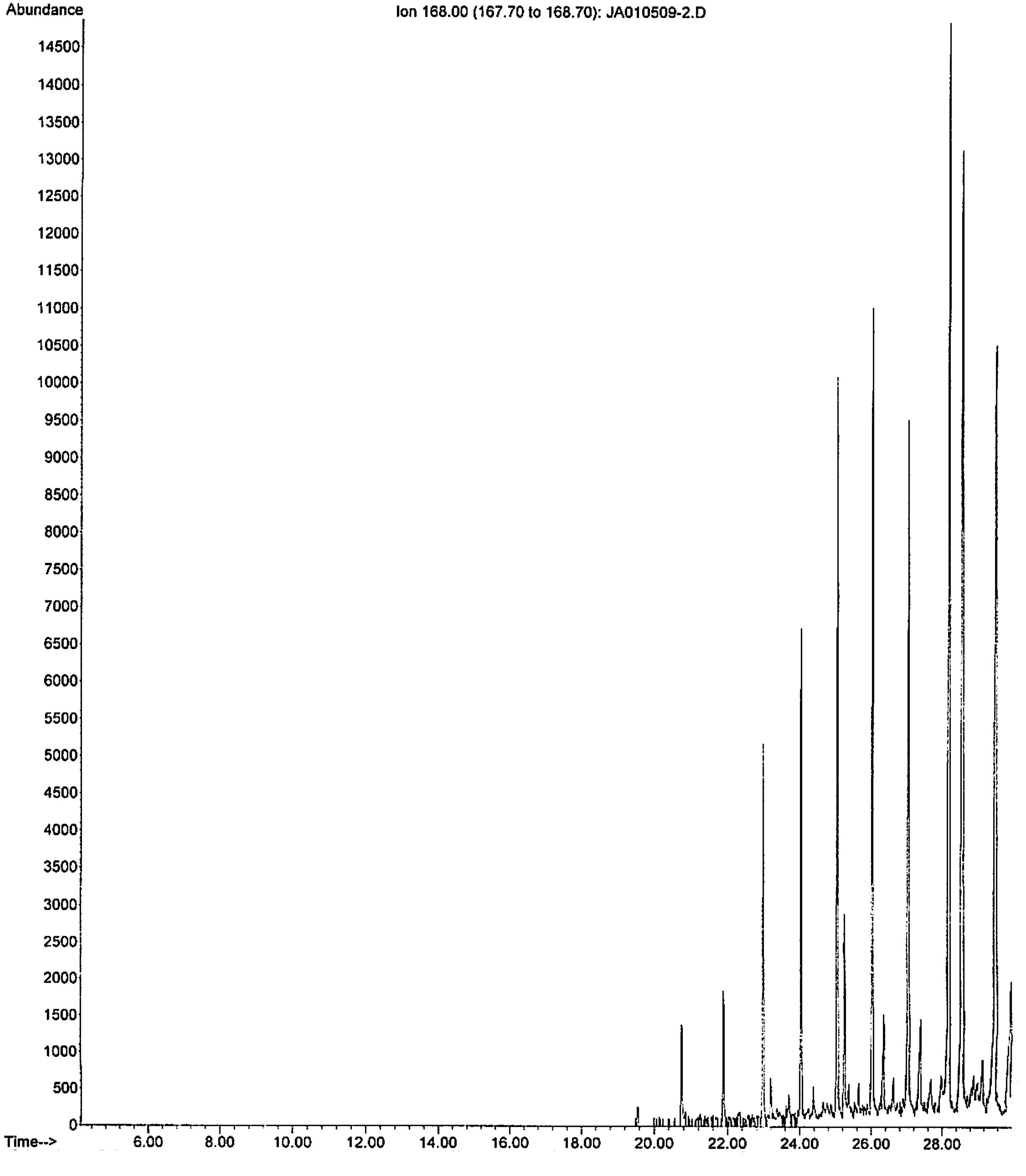

```
File         :D:\DATA\Aldrich\JA-09\JA010509-2.D
Operator     : Aldrich
Acquired     :  5 Jan 2009  14:37     using AcqMethod JA-WAX08.M
Instrument   :    Instrument #1
Sample Name: 8 male C.oculata abd.sternites/CH2Cl2
Misc Info    : w/ 1ug linalool/ul water/6 days; 2nd half
Vial Number: 1
```

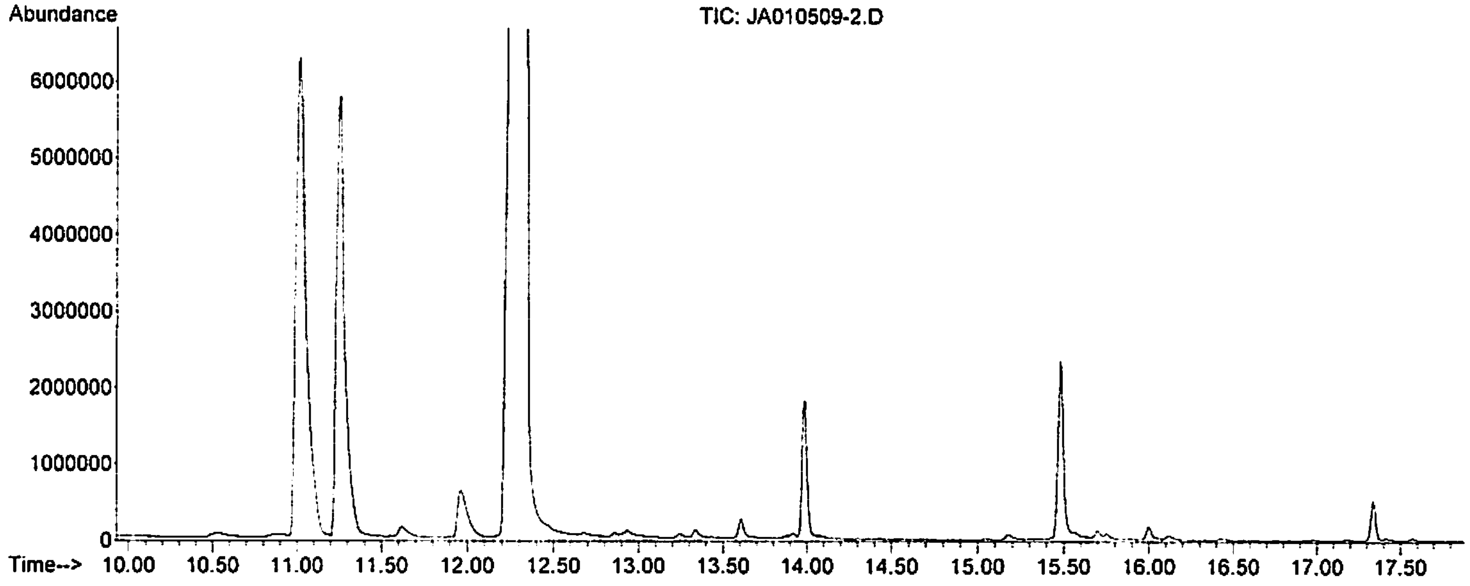


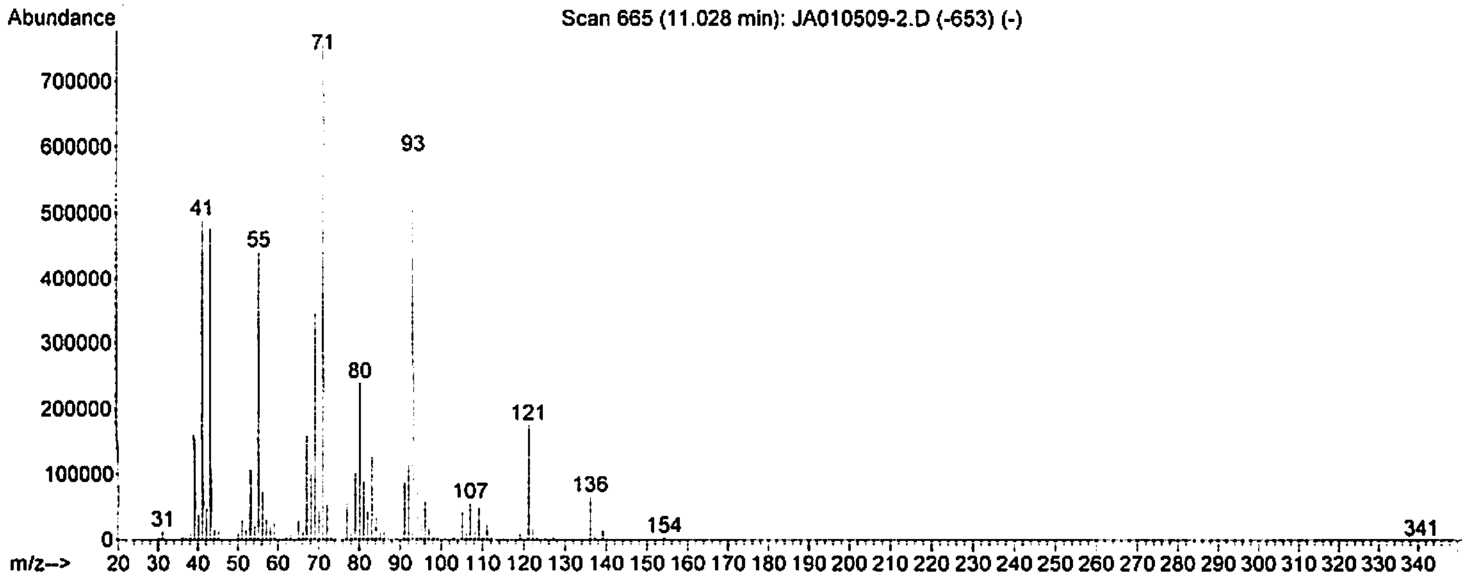

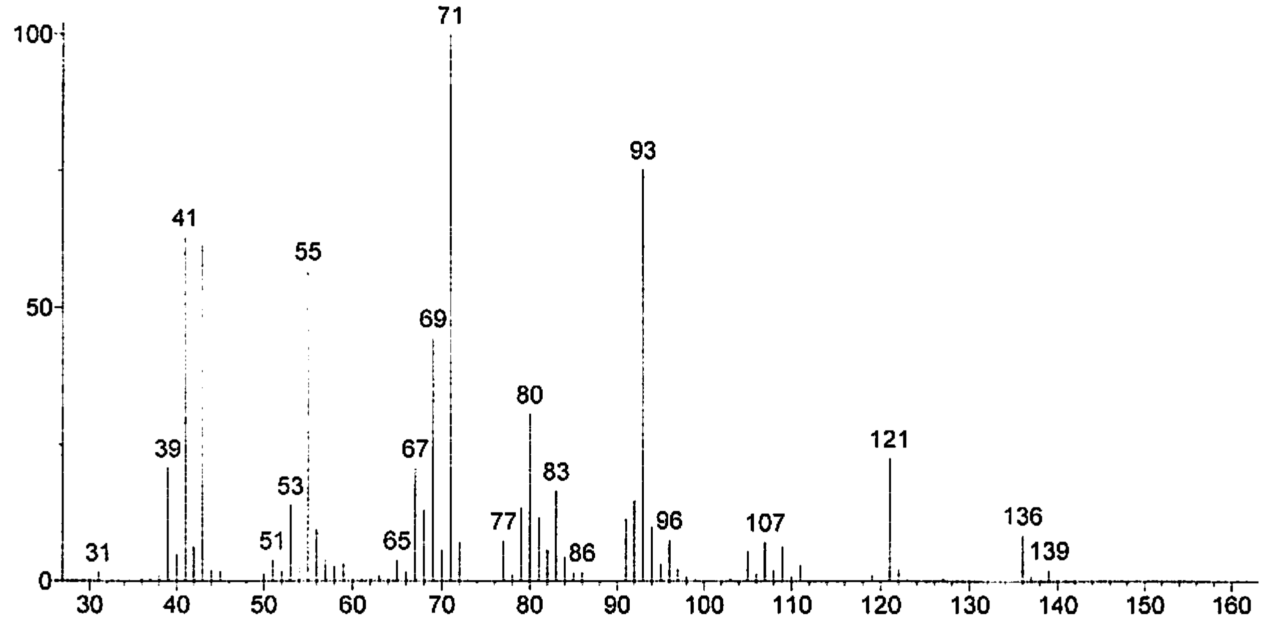


(Text File) Scan 665 (11.028 min): JA010509-2.D (-653)

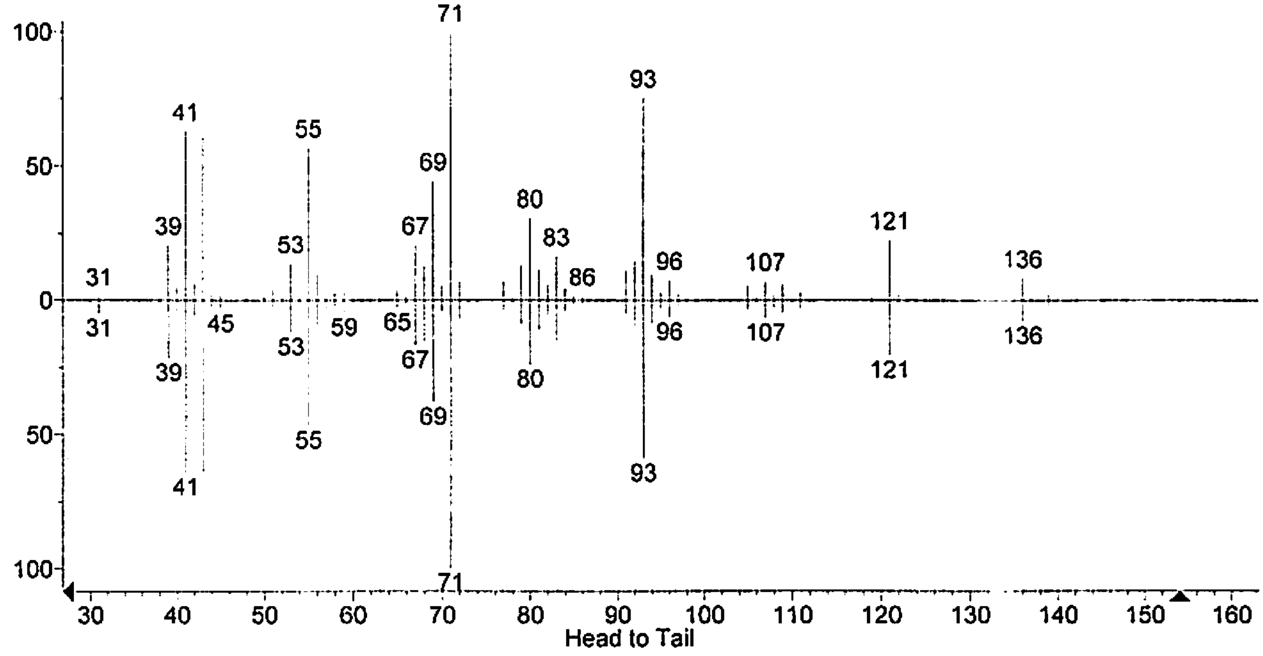


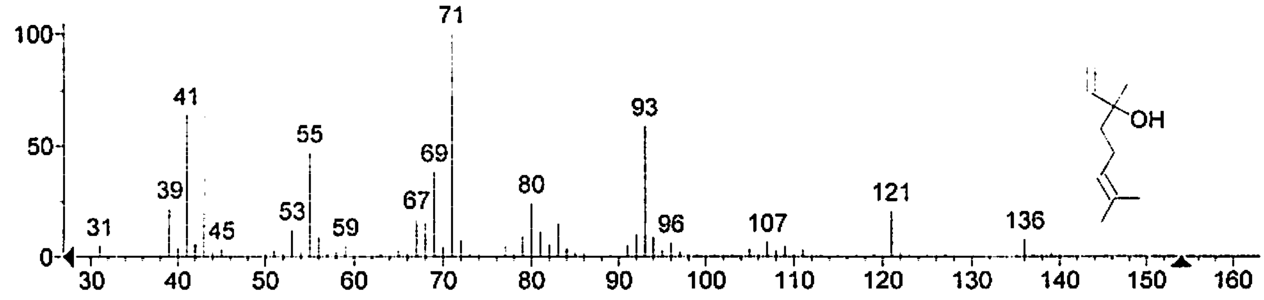


(mainlib) 1,6-Octadien-3-ol, 3,7-dimethyl-

```
File      :D:\DATA\Aldrich\JA-09\JA010509-2.D
Operator  : Aldrich
Acquired  :  5 Jan 2009  14:37     using AcqMethod JA-WAX08.M
Instrument :    Instrument #1
Sample Name: 8 male C.oculata abd.sternites/CH2Cl2
Misc Info  : w/ 1ug linalool/ul water/6 days; 2nd half
Vial Number: 1
```

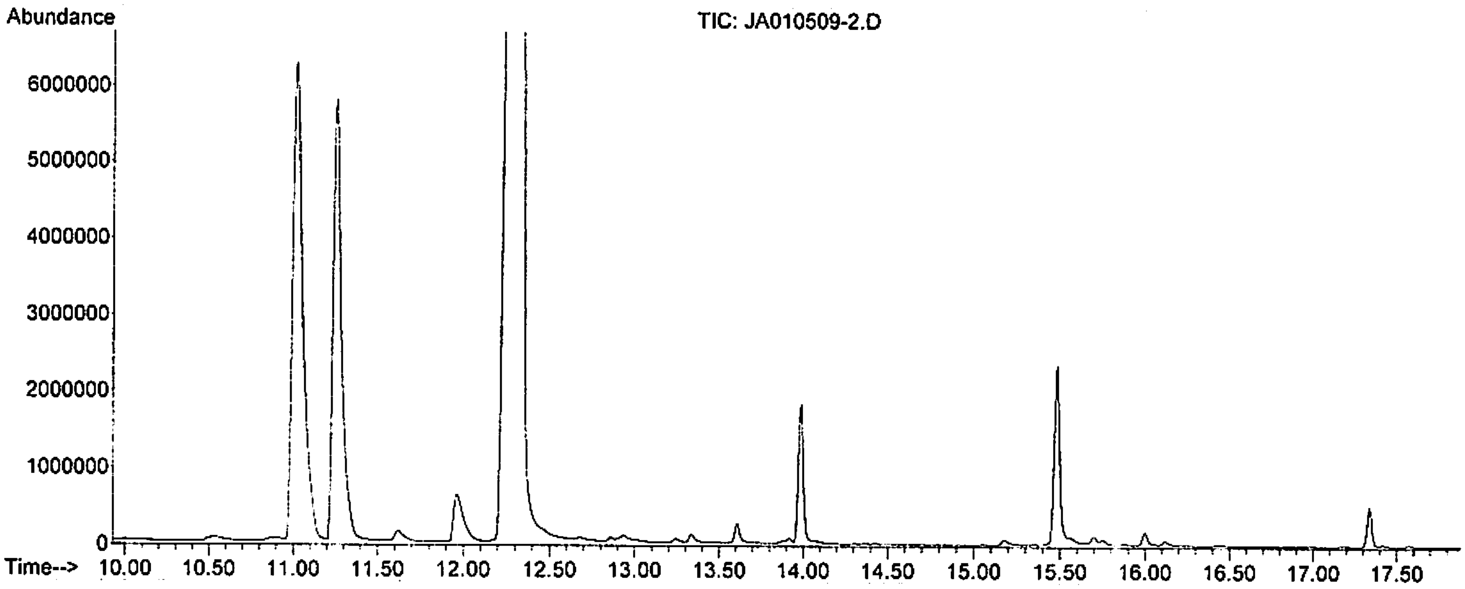


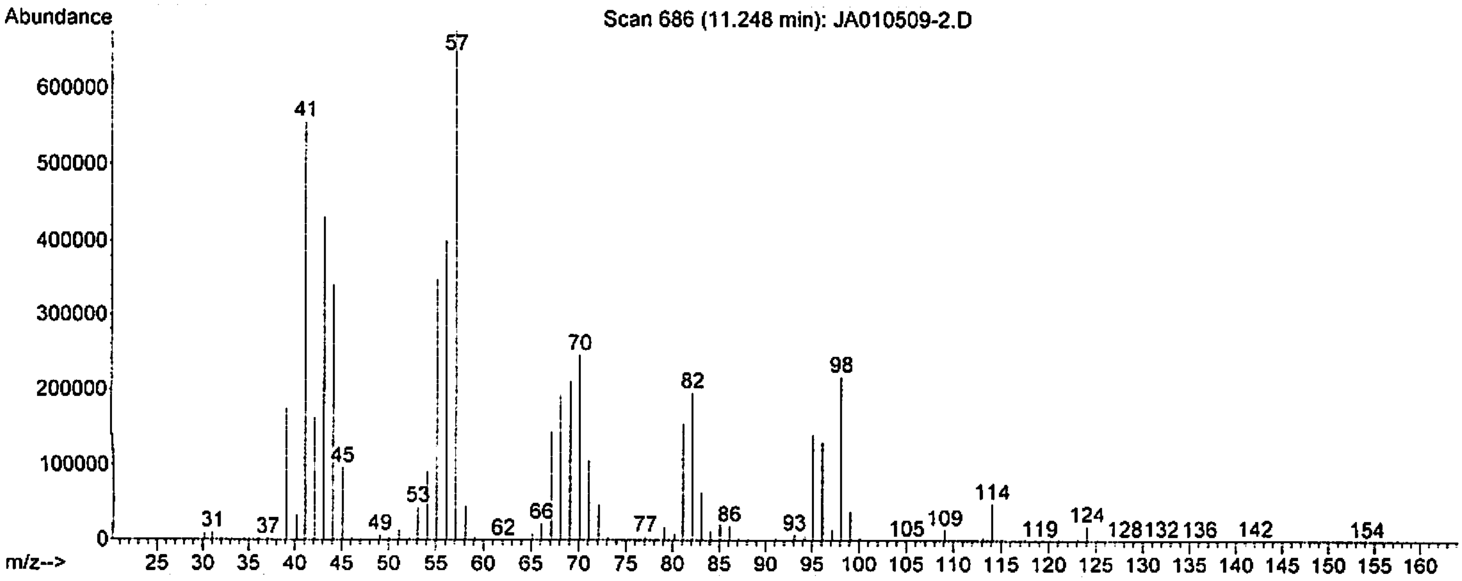

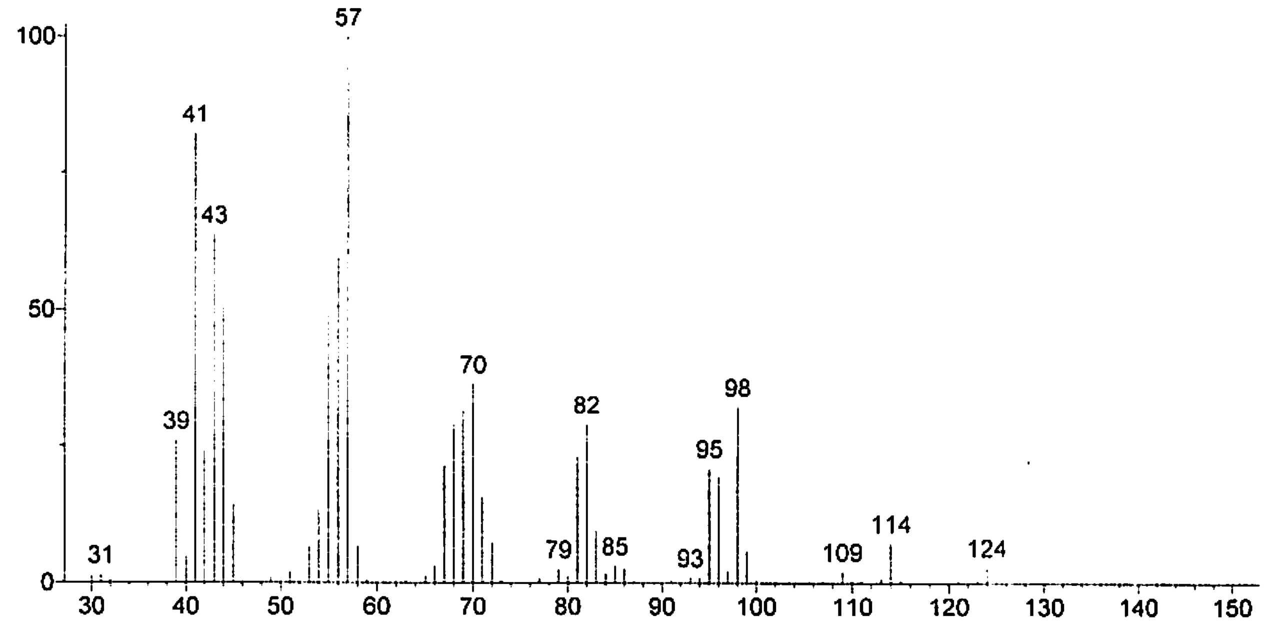


(Text File) Scan 686 (11.248 min): JA010509-2.D

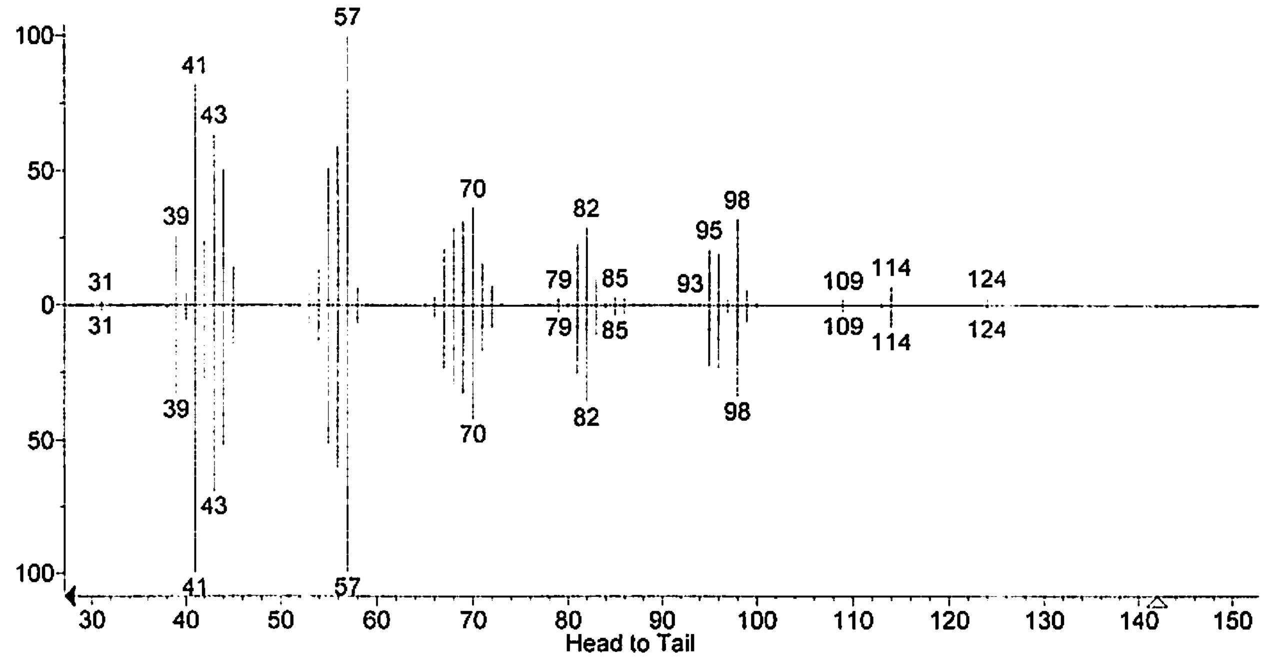


Head to Tail

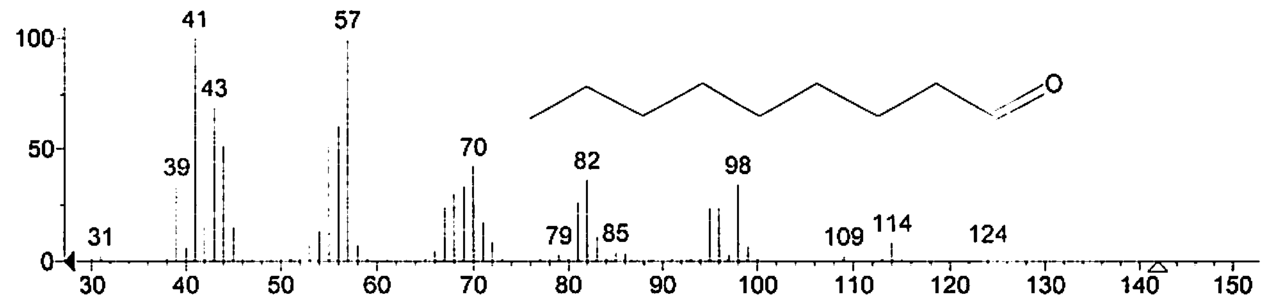


(replib) Nonanal

File : D:\DATA\Aldrich\JA-09\JA010509-2.D
Operator : Aldrich
Acquired : 5 Jan 2009 14:37 using AcqMethod JA-WAX08.M
Instrument : Instrument #1
Sample Name: 8 male C.oculata abd.sternites/CH2Cl2
Misc Info : w/ 1ug linalool/ul water/6 days; 2nd half
Vial Number: 1

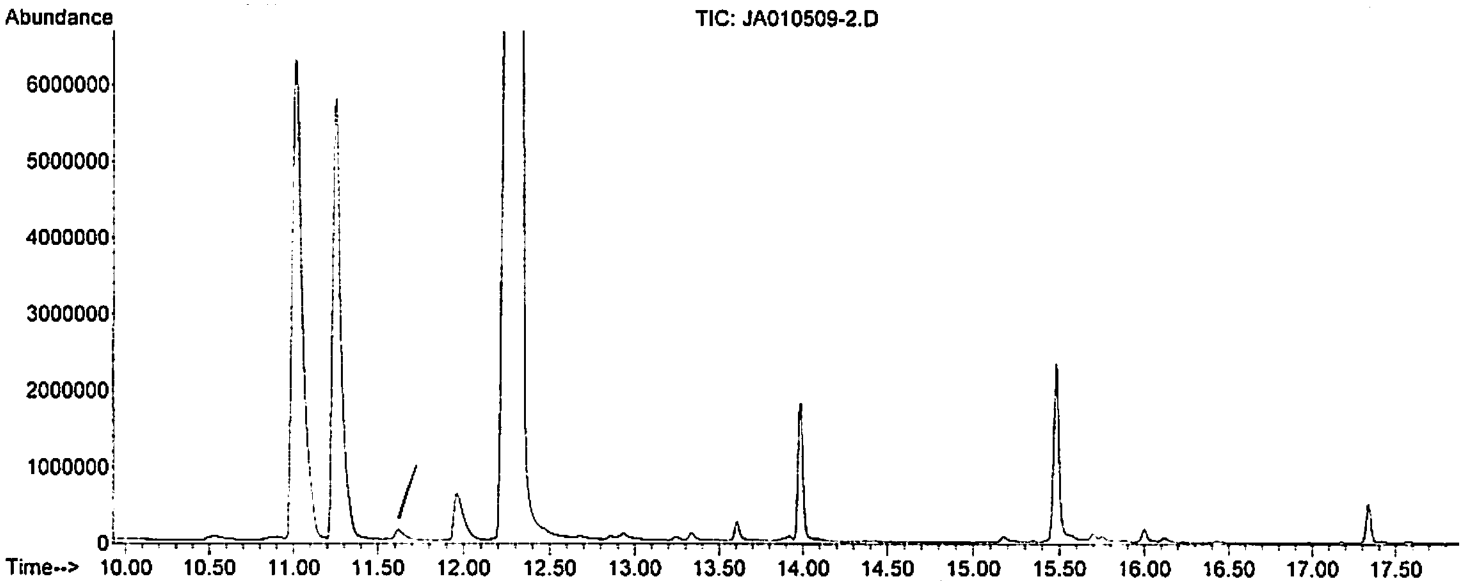


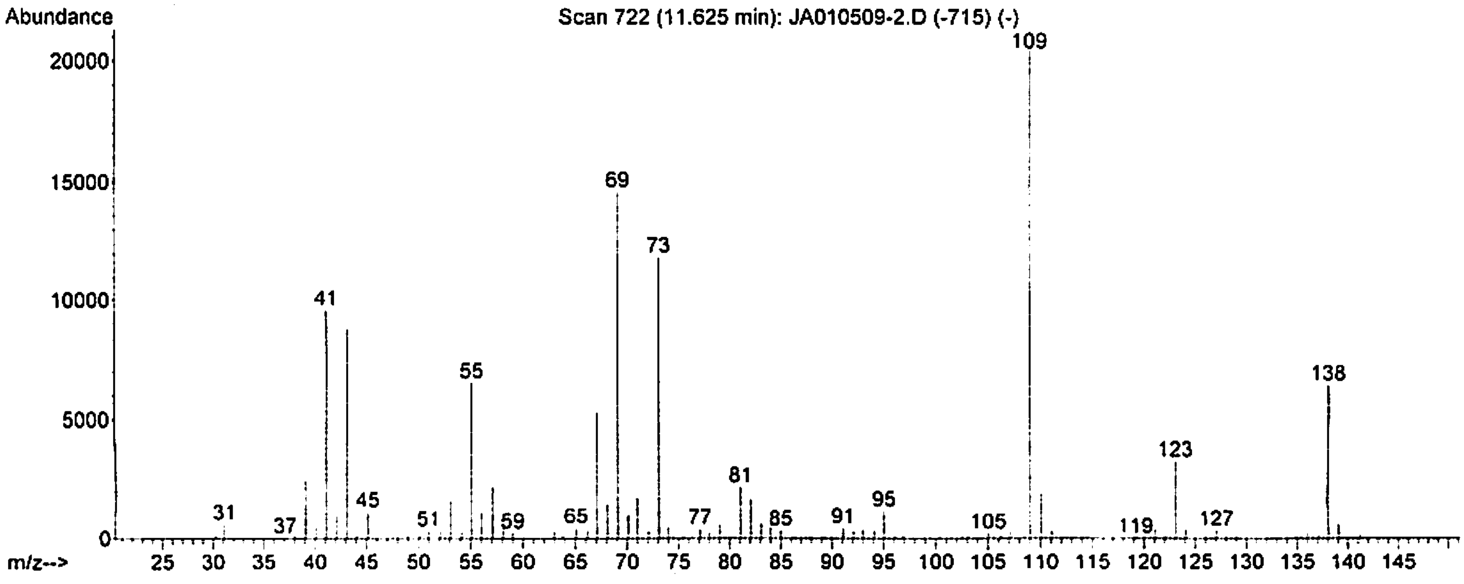

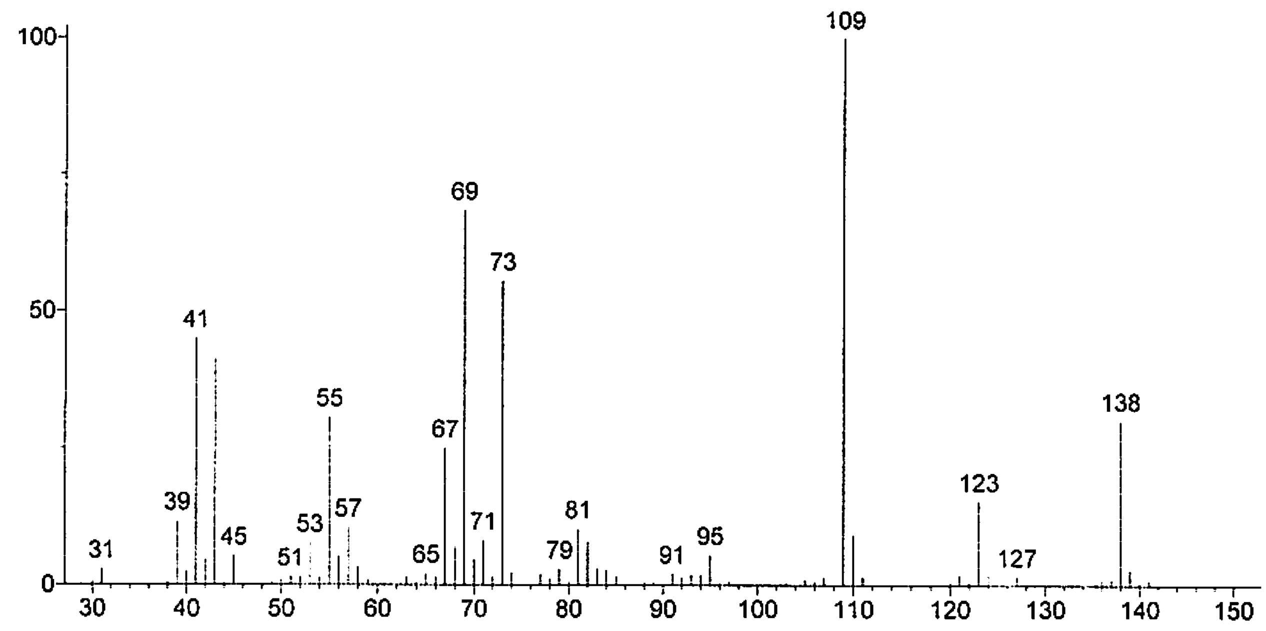

(Text File) Scan 722 (11.625 min): JA010509-2.D (-715)

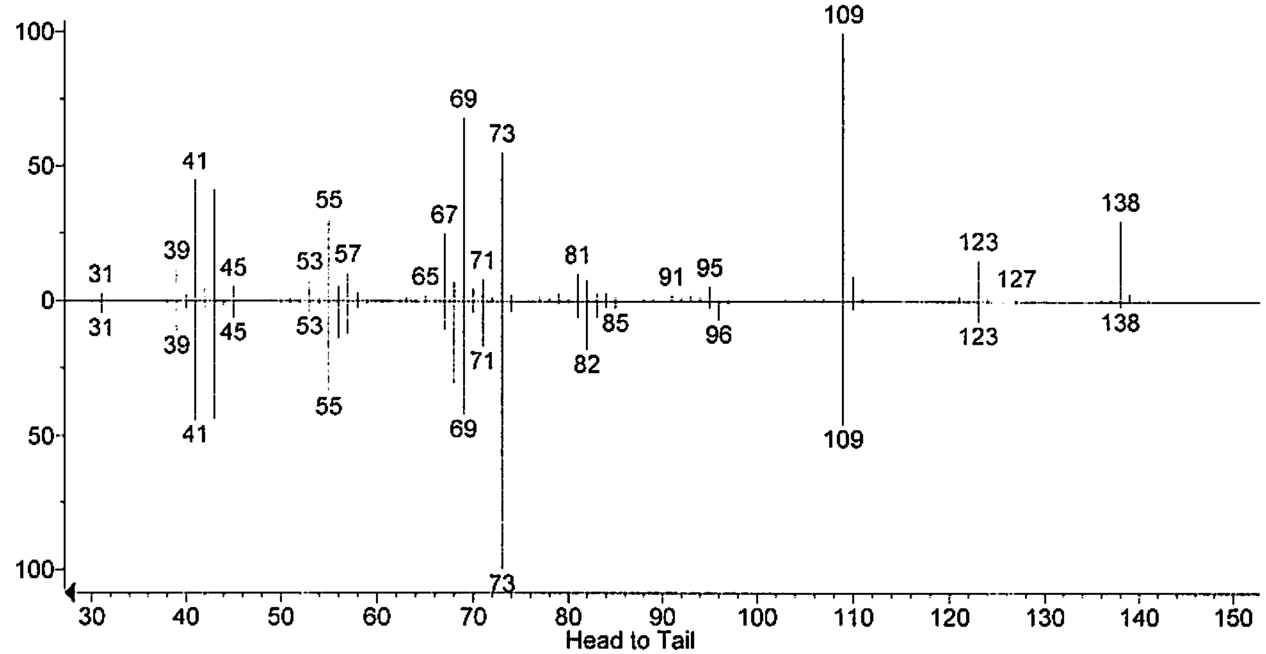

Head to Tail

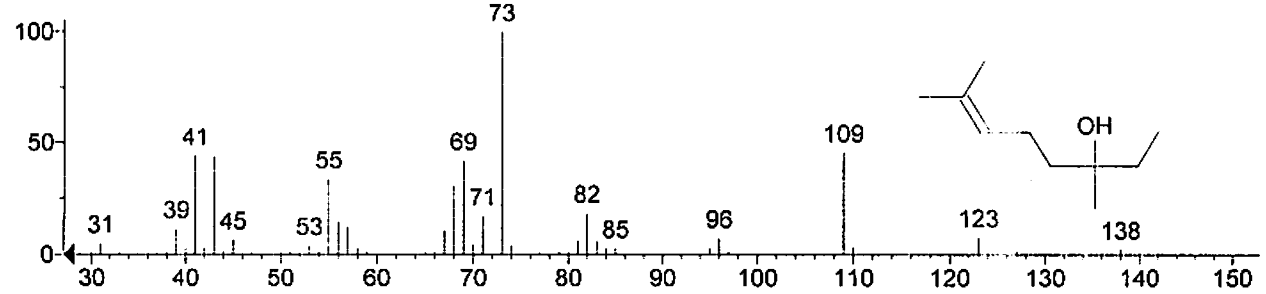

(mainlib) 1,2-Dihydrolinalool

File : D:\DATA\Aldrich\JA-09\JA010509-2.D
Operator : Aldrich
Acquired : 5 Jan 2009 14:37 using AcqMethod JA-WAX08.M
Instrument : Instrument #1
Sample Name: 8 male C.oculata abd.sternites/CH2Cl2
Misc Info : w/ 1ug linalool/ul water/6 days; 2nd half
Vial Number: 1

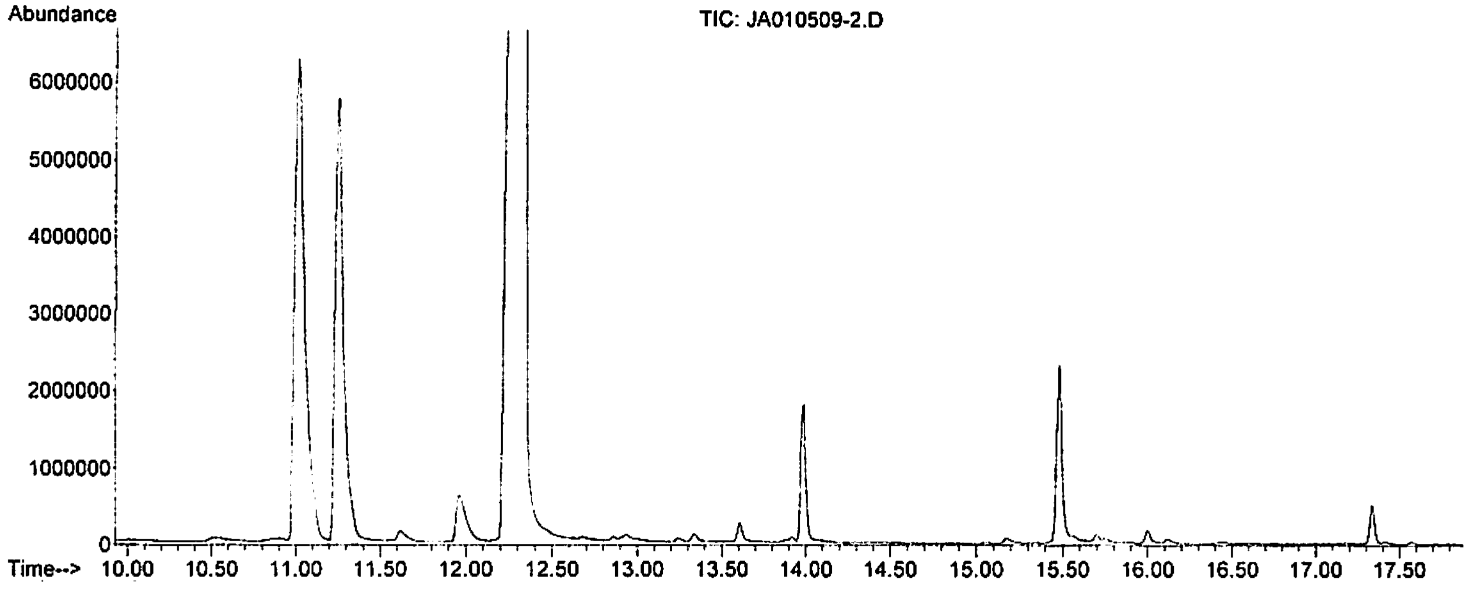


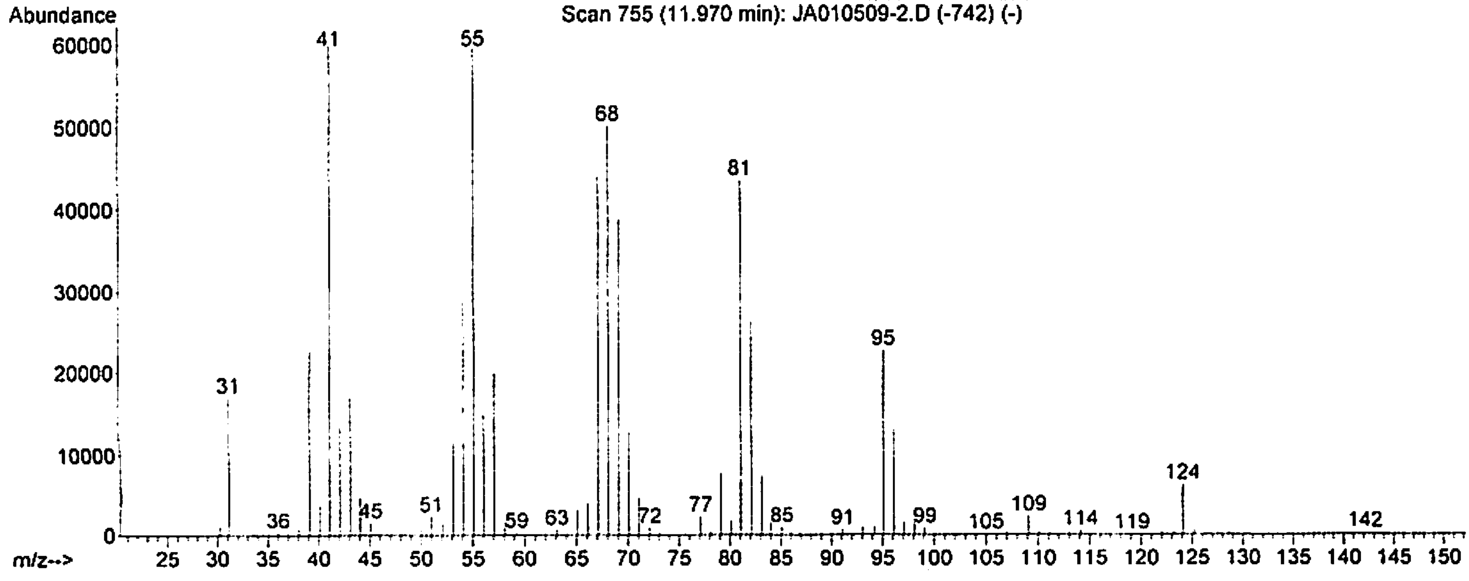

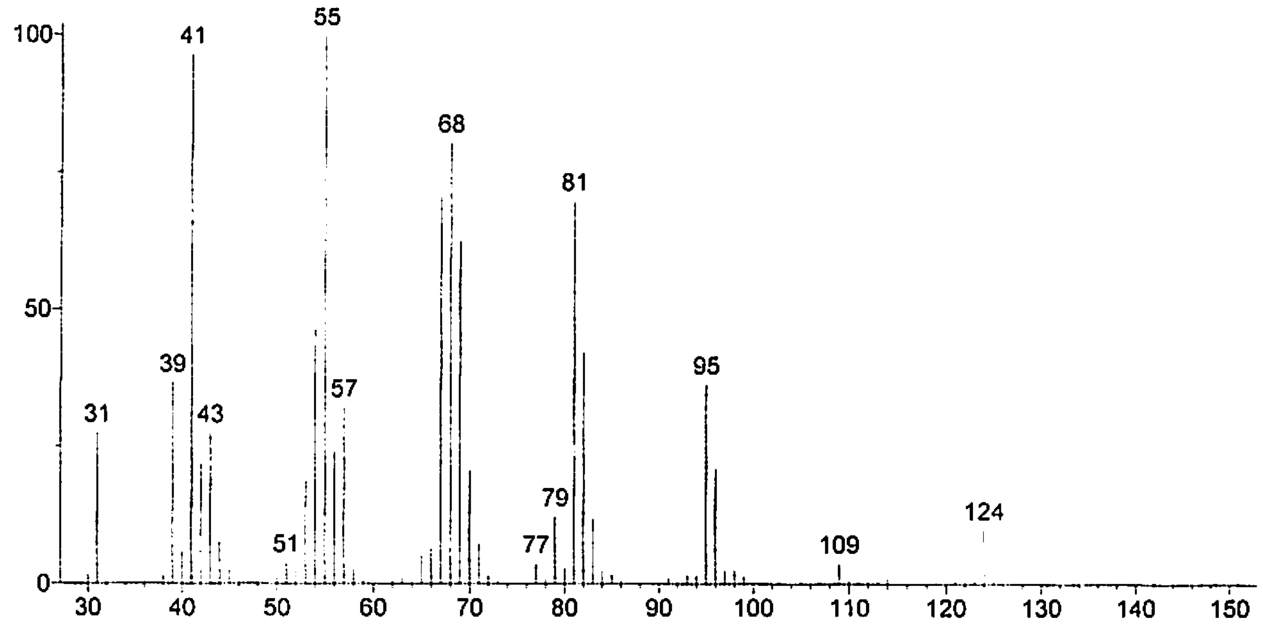


(Text File) Scan 755 (11.970 min): JA010509-2.D (-742)

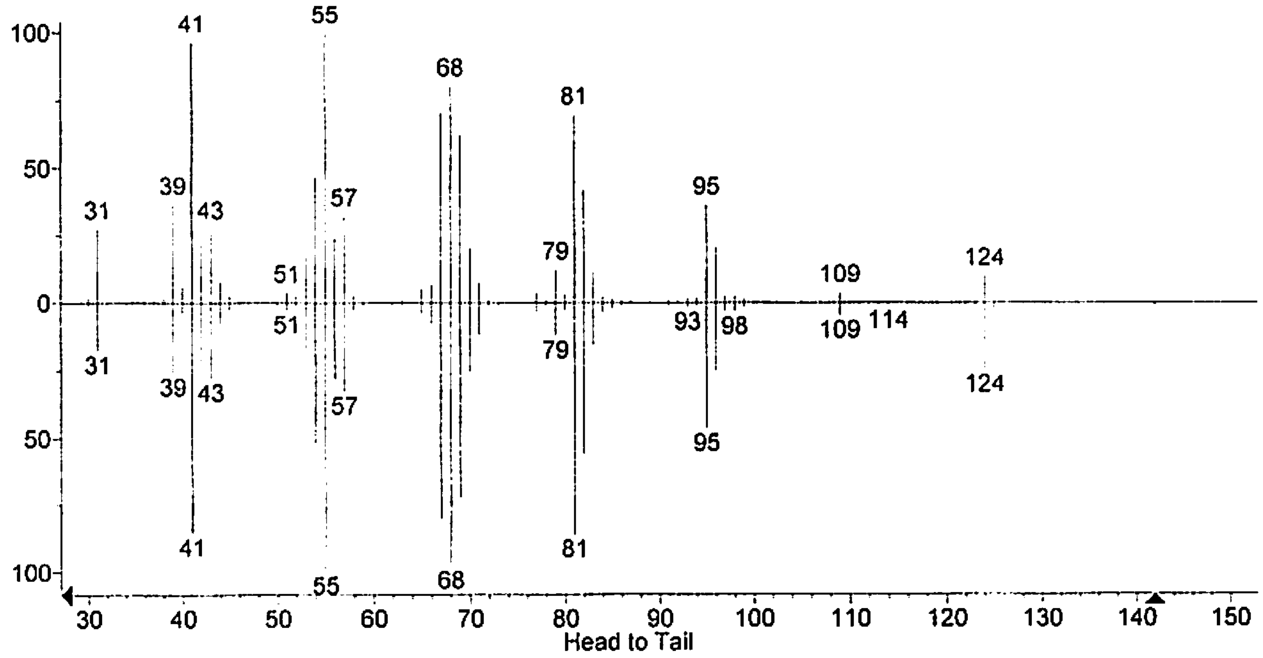


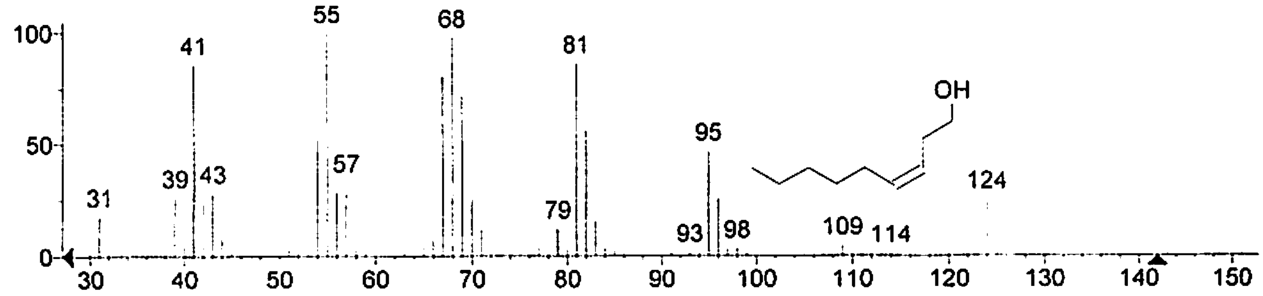


(mainlib) 3-Nonen-1-ol, (Z)-

File : D:\DATA\Aldrich\JA-09\JA010509-2.D
Operator : Aldrich
Acquired : 5 Jan 2009 14:37 using AcqMethod JA-WAX08.M
Instrument : Instrument #1
Sample Name: 8 male C.oculata abd.sternites/CH2Cl2
Misc Info : w/ 1ug linalool/ul water/6 days; 2nd half
Vial Number: 1

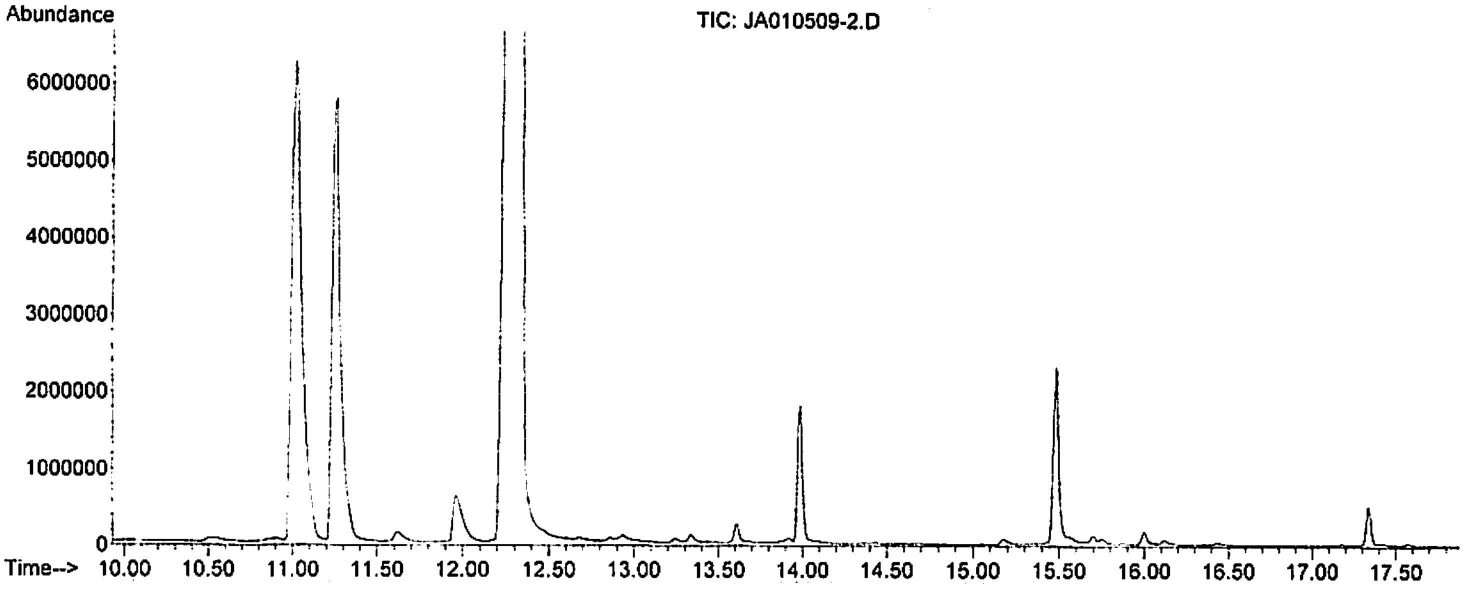


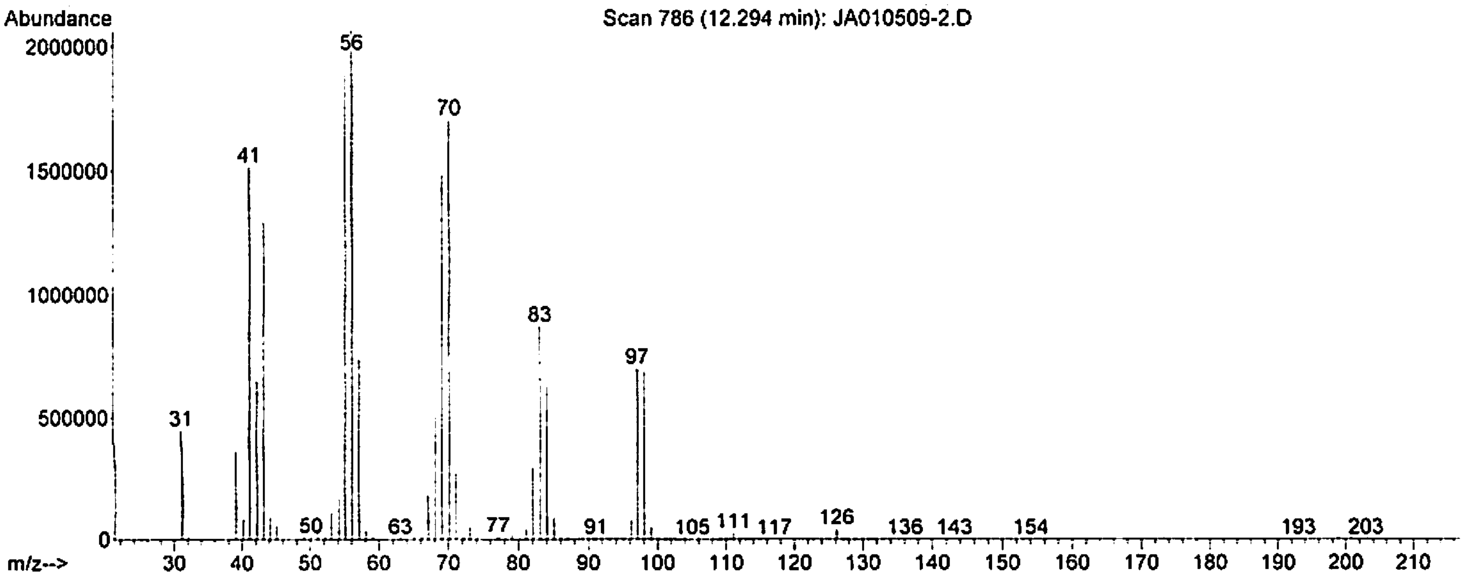

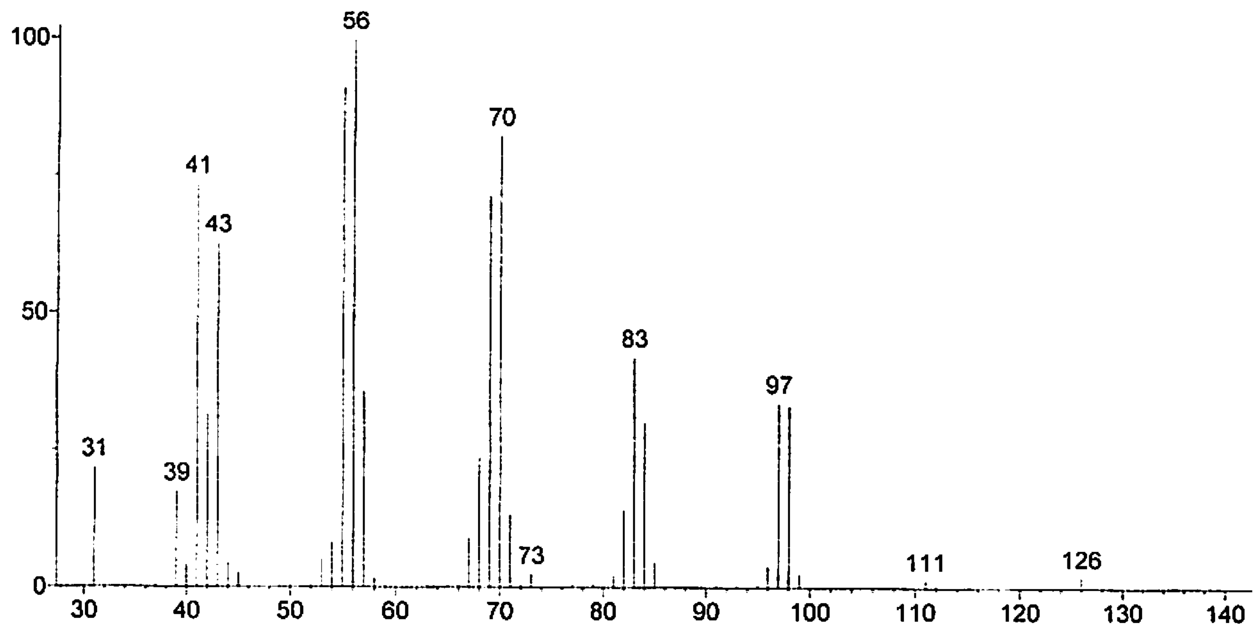


(Text File) Scan 786 (12.294 min): JA010509-2.D

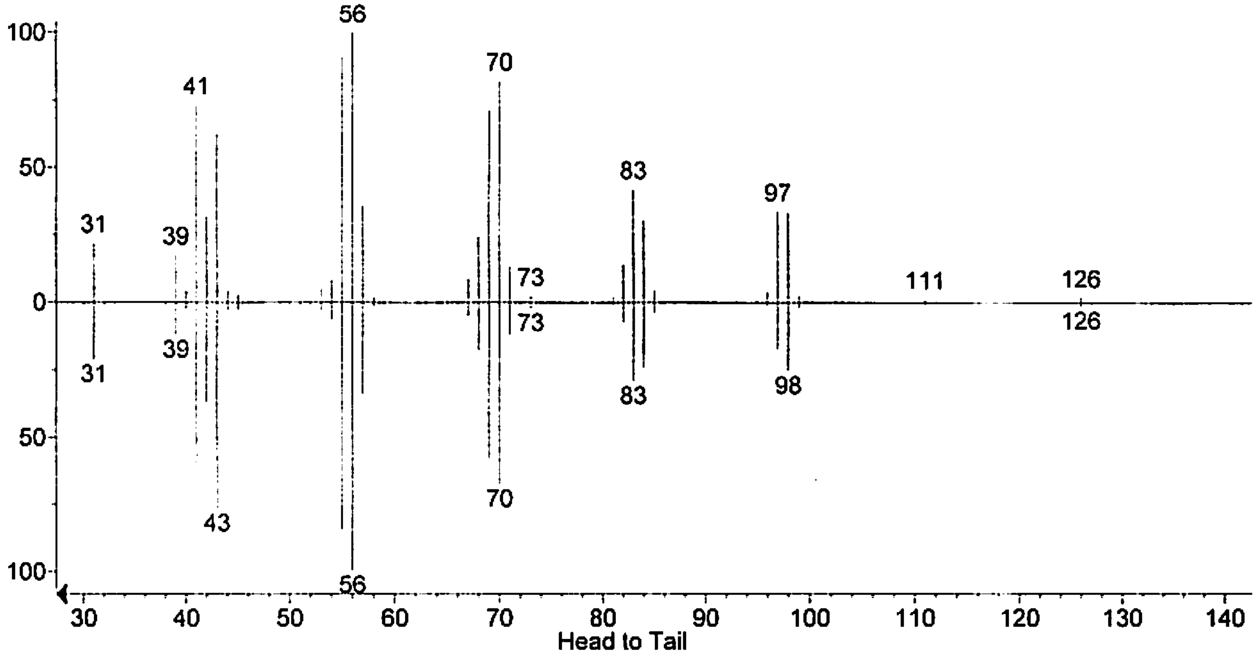


Head to Tail

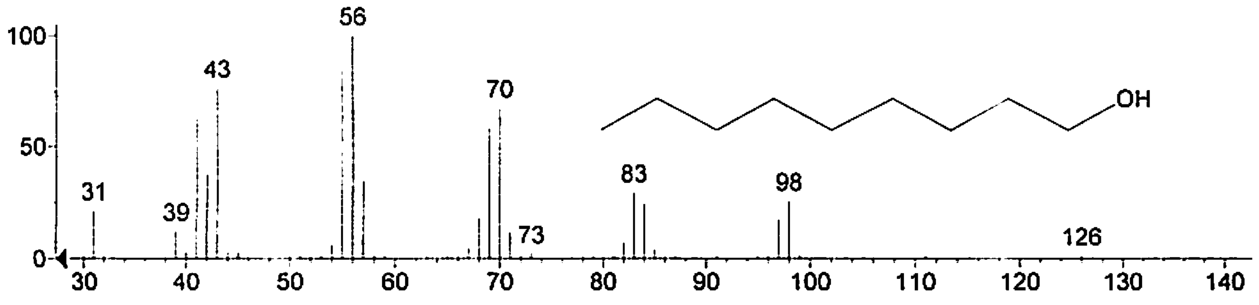


(mainlib) 1-Nonanol

File      :D:\DATA\Aldrich\JA-09\JA010509-2.D
Operator    : Aldrich
Acquired    :  5 Jan 2009  14:37     using AcqMethod JA-WAX08.M
Instrument :    Instrument #1
Sample Name: 8 male C.oculata abd.sternites/CH2Cl2
Misc Info   : w/ 1ug linalool/ul water/6 days; 2nd half
Vial Number: 1

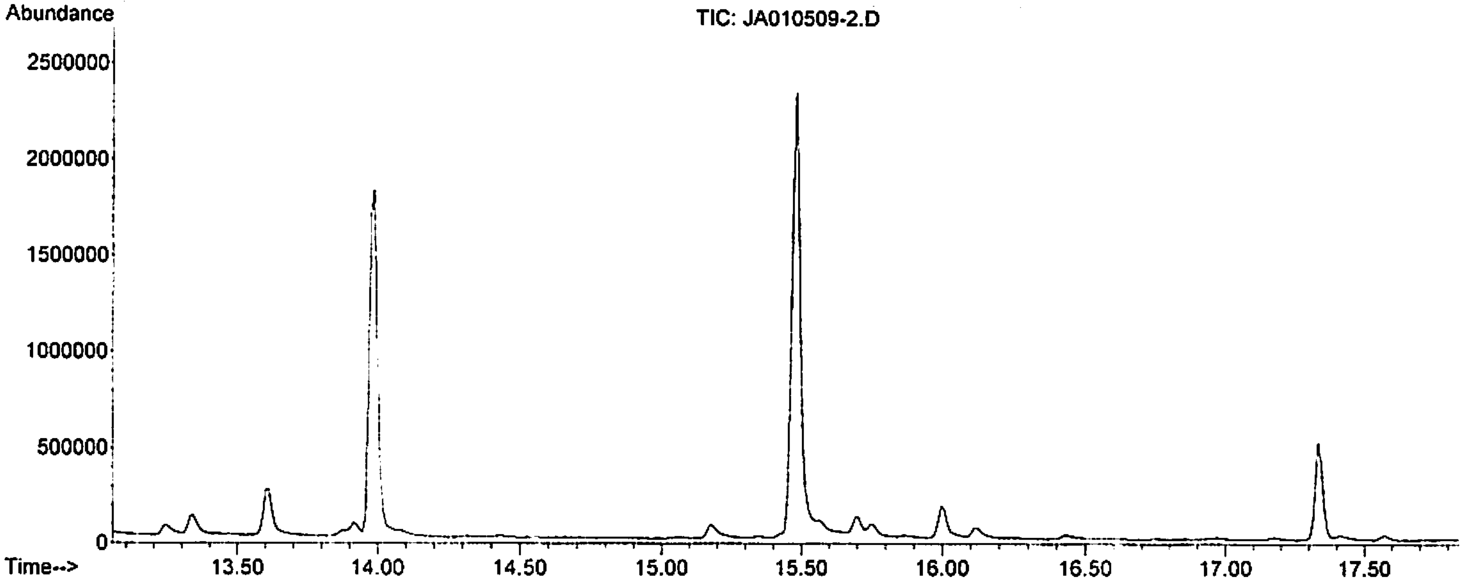


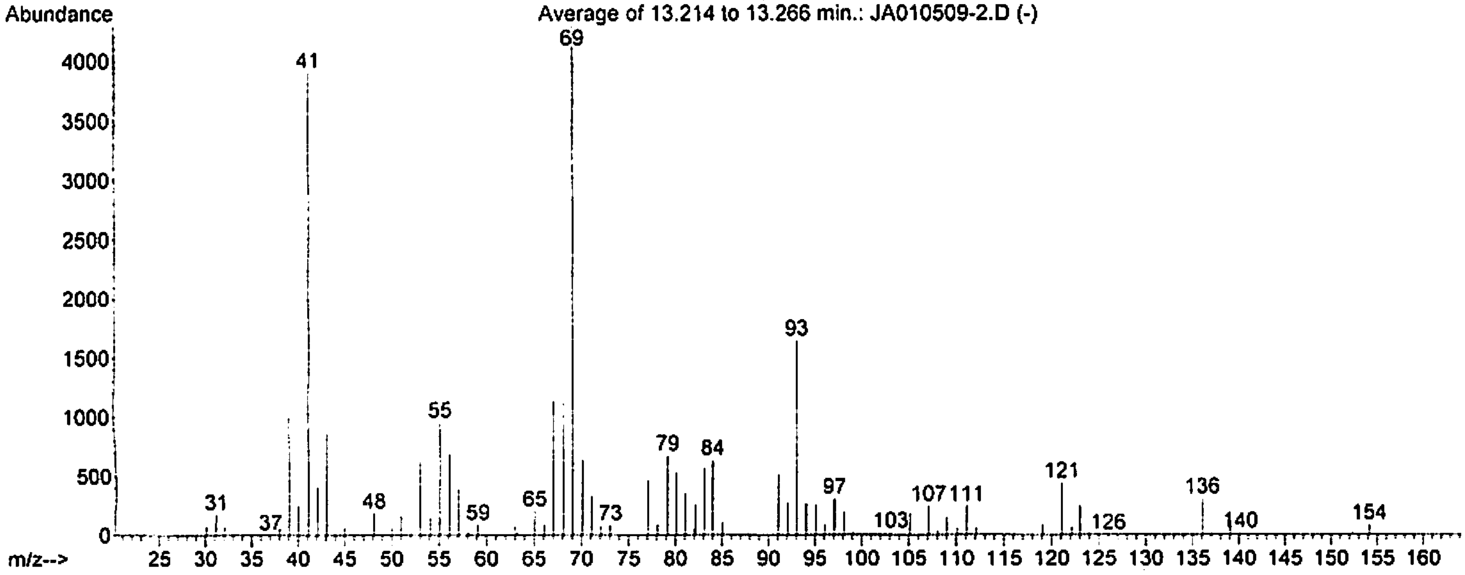

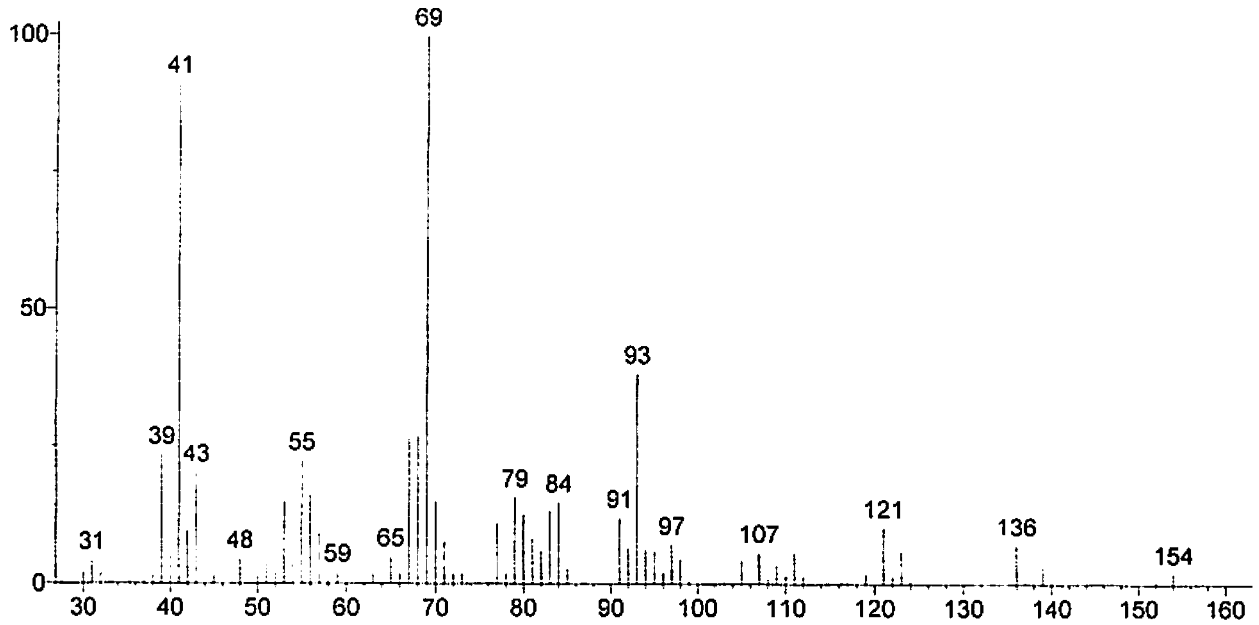


(Text File) Average of 13.214 to 13.266 min.: JA010509-2.D

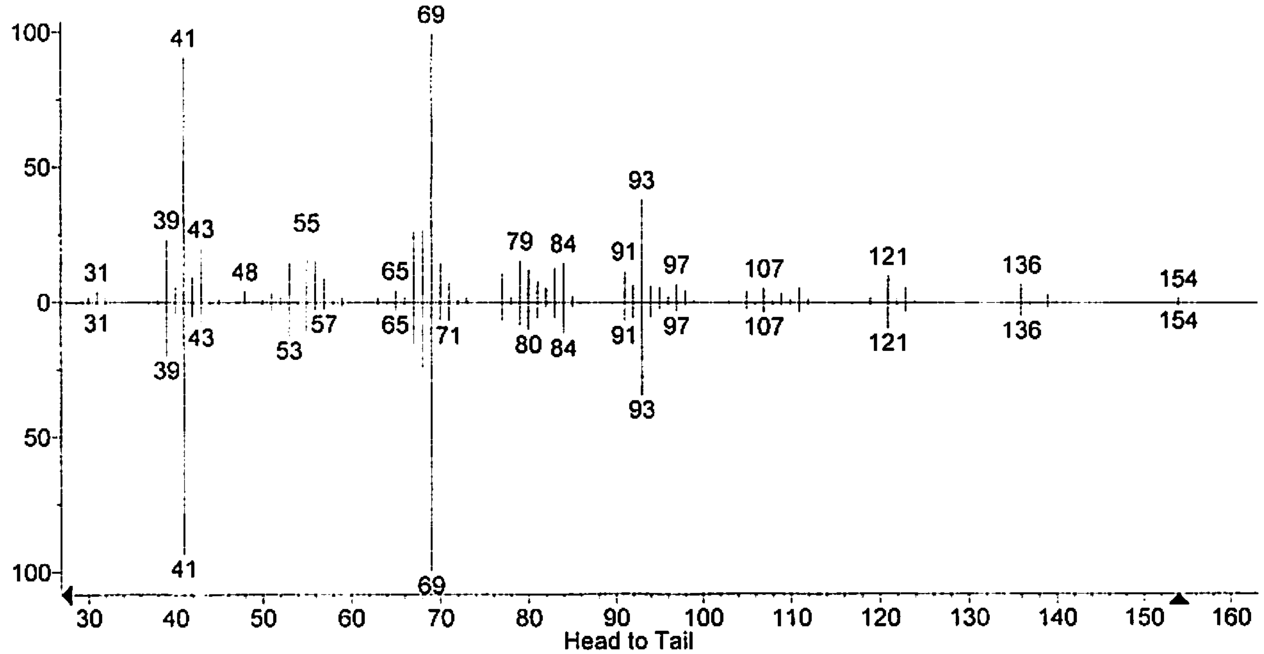


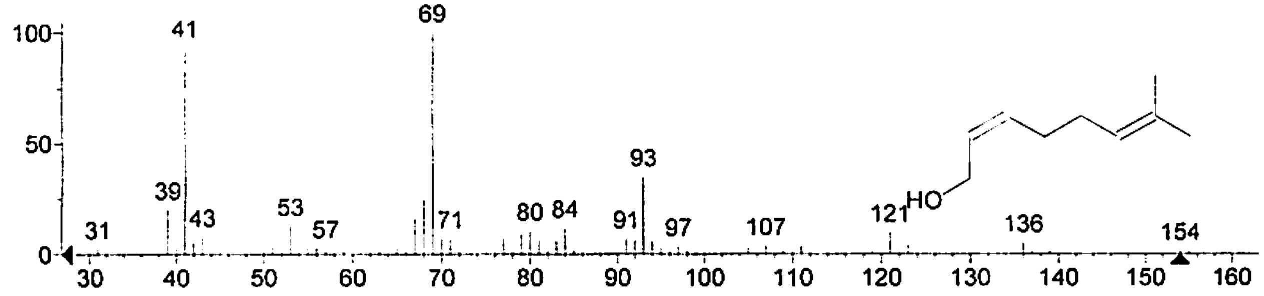


(mainlib) 2,6-Octadien-1-ol, 3,7-dimethyl-, (Z)-

File : D:\DATA\Aldrich\JA-09\JA010509-2.D
Operator : Aldrich
Acquired : 5 Jan 2009 14:37 using AcqMethod JA-WAX08.M
Instrument : Instrument #1
Sample Name: 8 male C.oculata abd.sternites/CH2Cl2
Misc Info : w/ 1ug linalool/ul water/6 days; 2nd half
Vial Number: 1

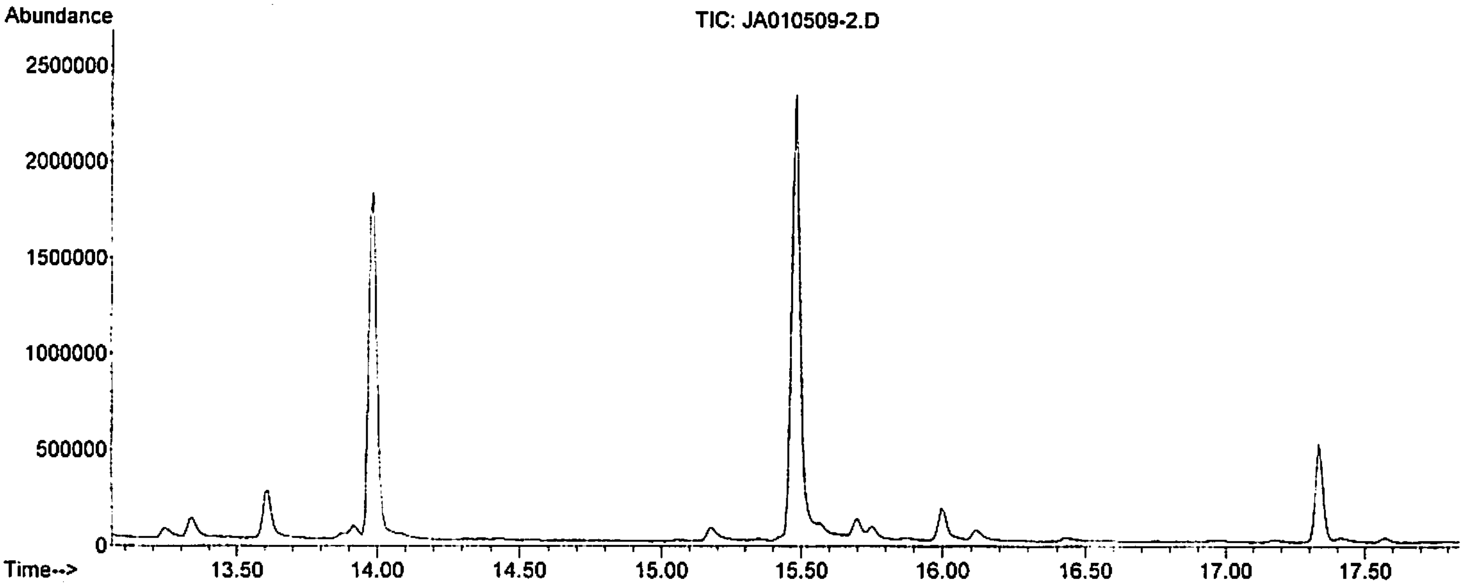


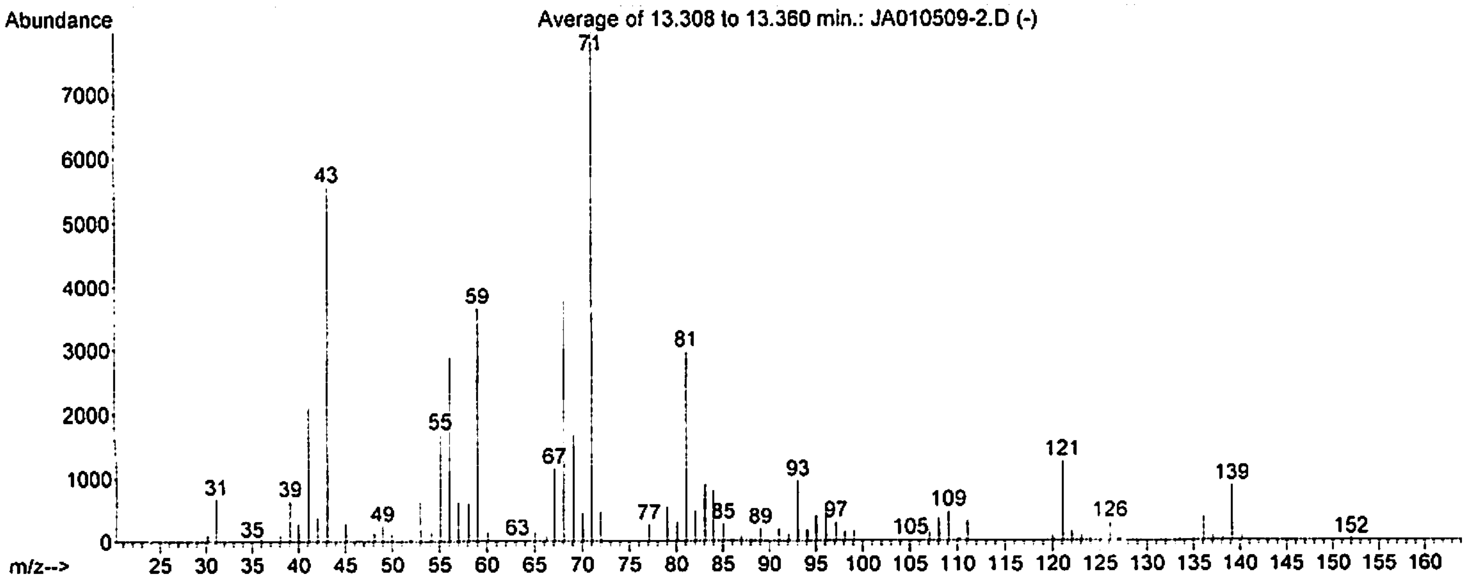

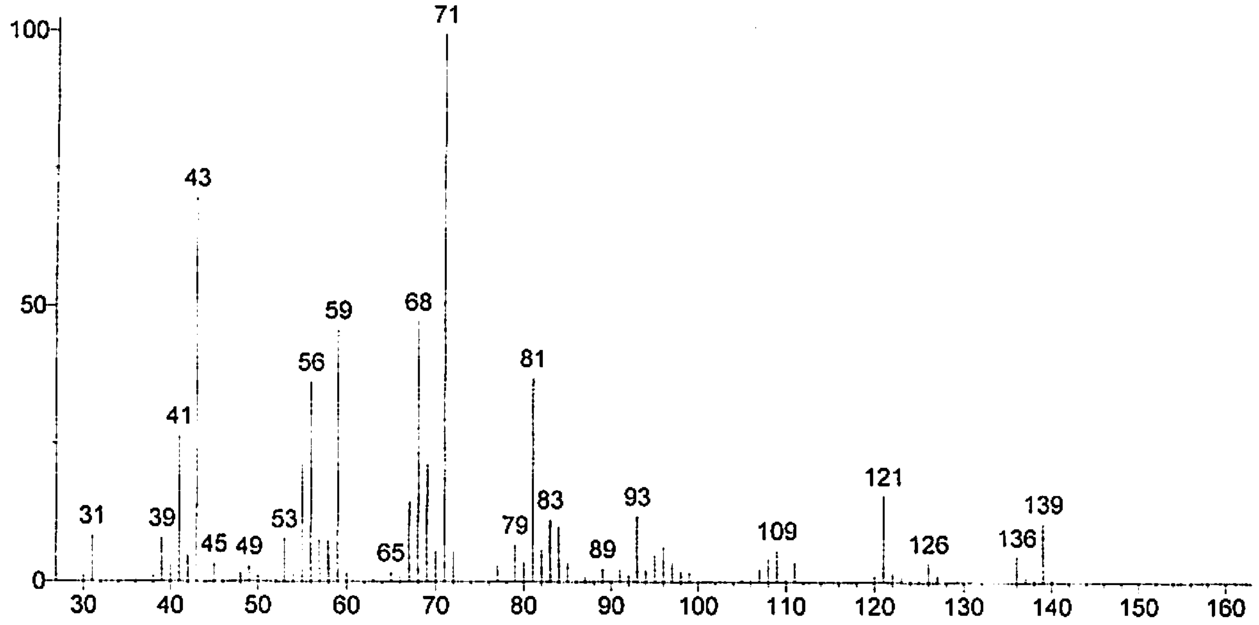


(Text File) Average of 13.308 to 13.360 min.: JA010509-2.D

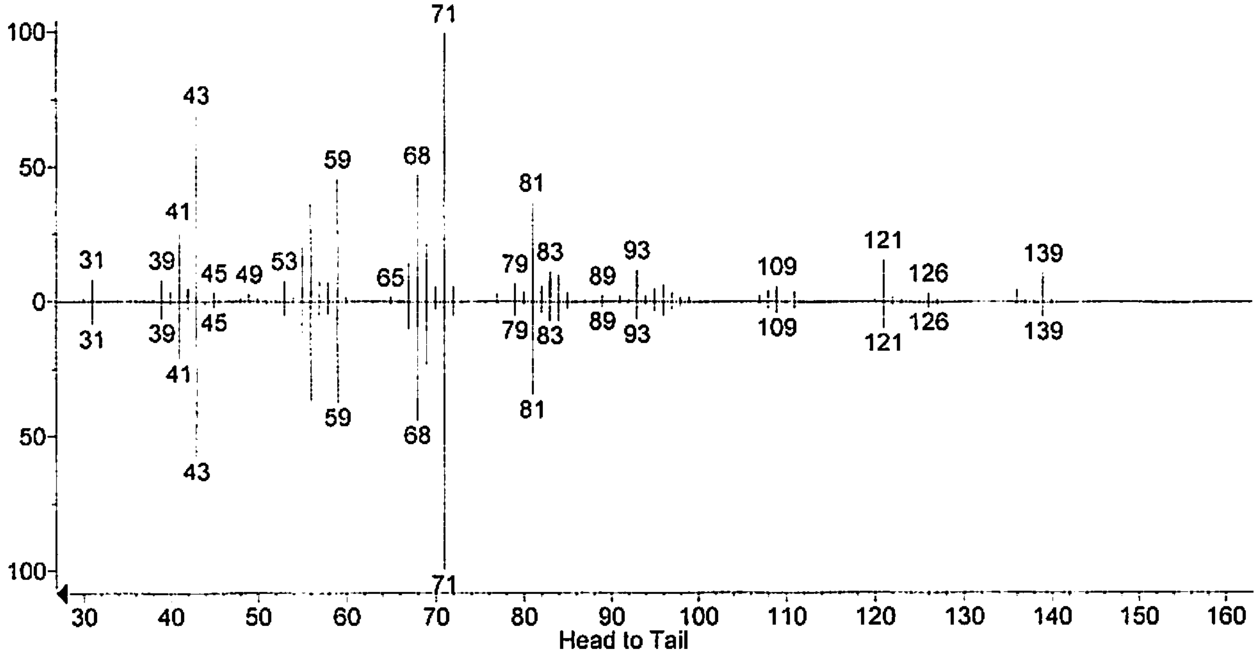


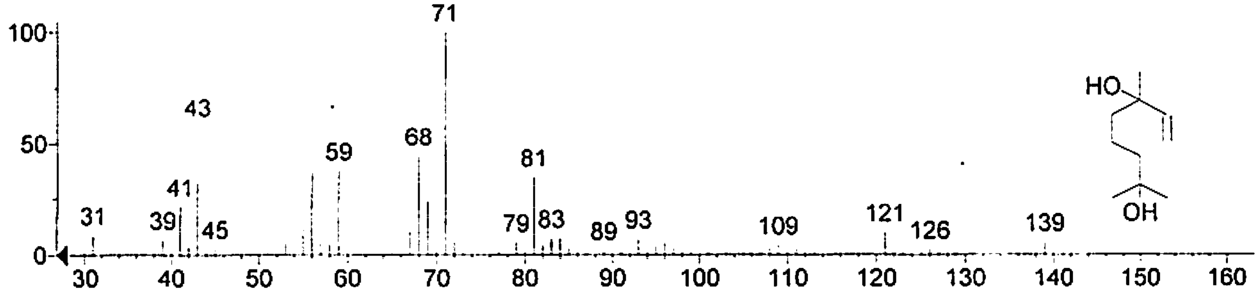


(mainlib) 7-Octene-2,6-diol, 2,6-dimethyl-

File :D:\DATA\Aldrich\JA-09\JA010509-2.D
Operator : Aldrich
Acquired : 5 Jan 2009 14:37 using AcqMethod JA-WAX08.M
Instrument : Instrument #1
Sample Name: 8 male C.oculata abd.sternites/CH2Cl2
Misc Info : w/ lug linalool/ul water/6 days; 2nd half
Vial Number: 1

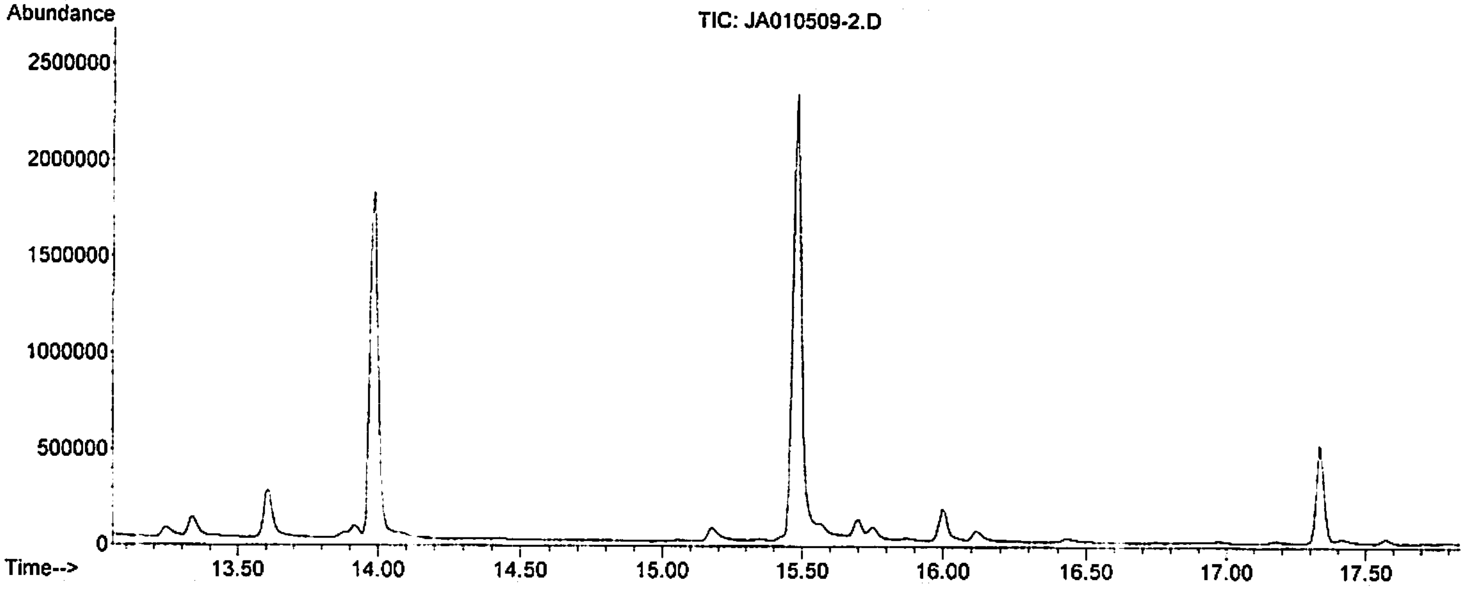


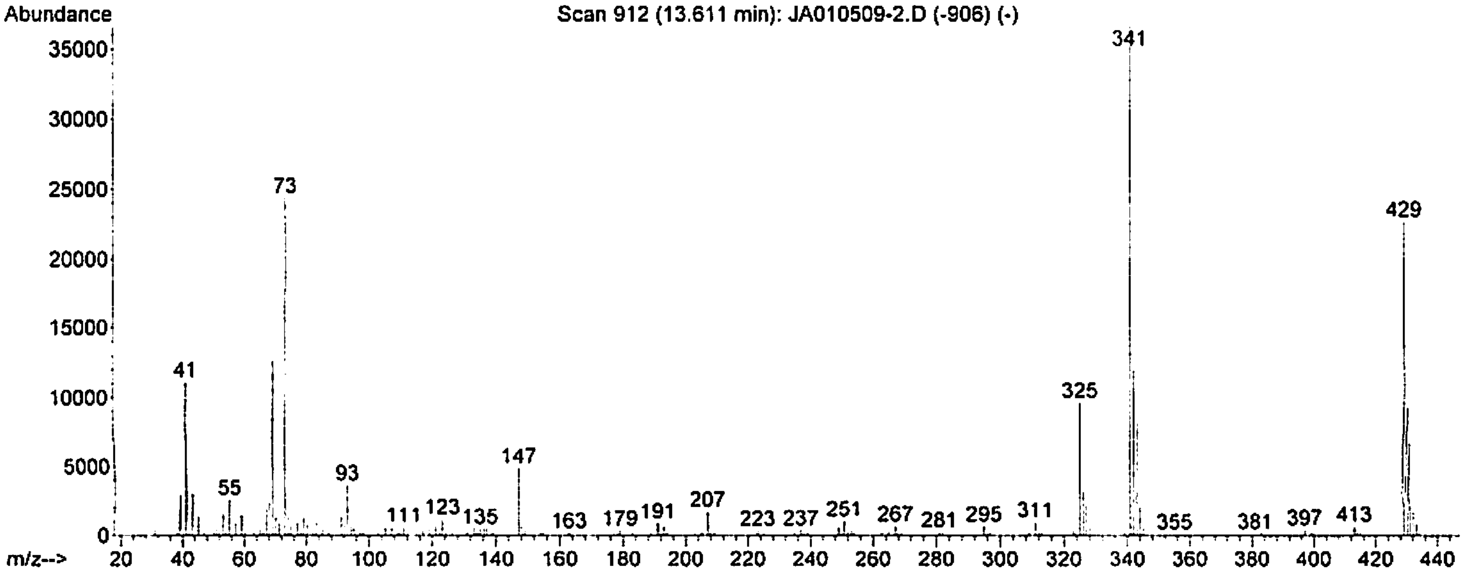

File : D:\DATA\Aldrich\JA-09\JA010509-2.D
Operator : Aldrich
Acquired : 5 Jan 2009 14:37 using AcqMethod JA-WAX08.M
Instrument : Instrument #1
Sample Name: 8 male C.oculata abd.sternites/CH2Cl2
Misc Info : w/ 1ug linalool/ul water/6 days; 2nd half
Vial Number: 1

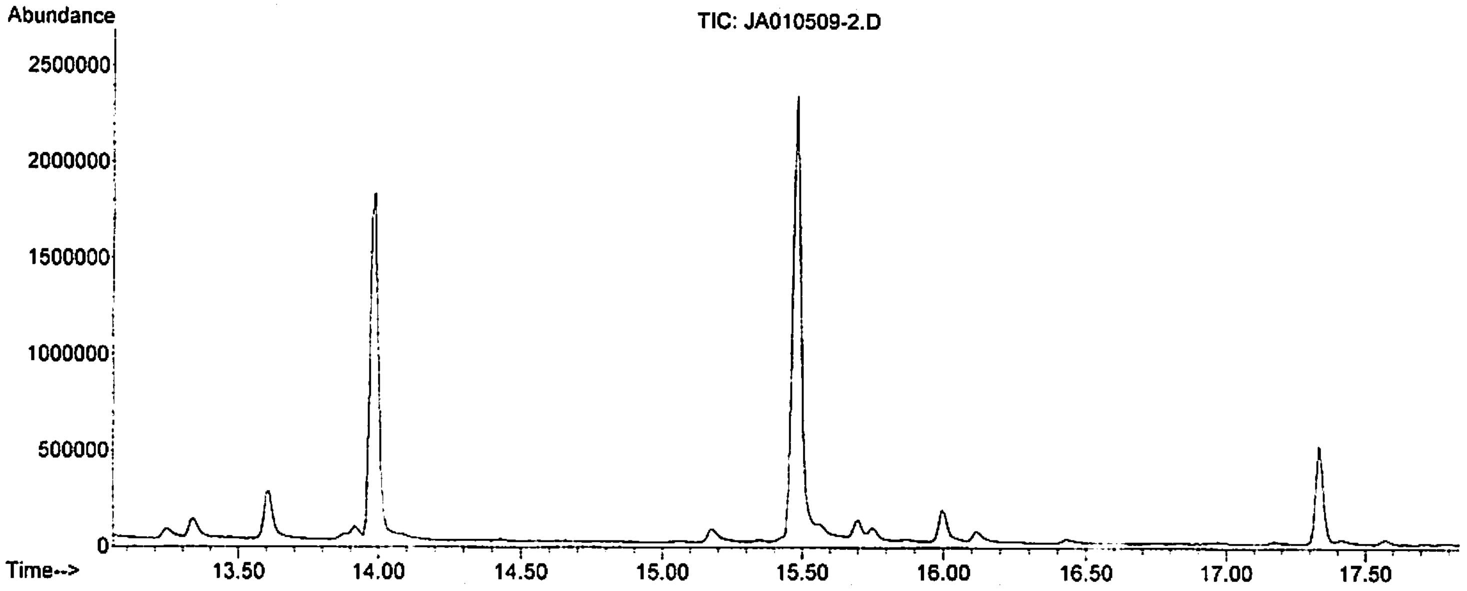


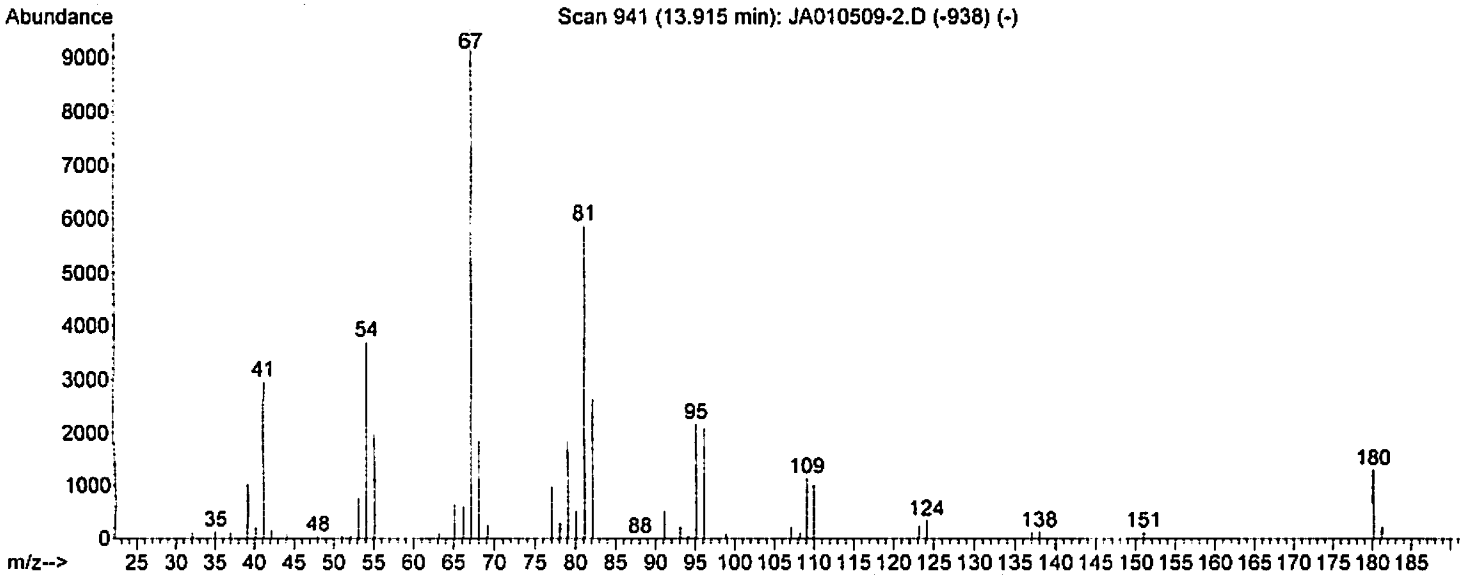

File : D:\DATA\Aldrich\JA-09\JA010509-2.D
Operator : Aldrich
Acquired : 5 Jan 2009 14:37 using AcqMethod JA-WAX08.M
Instrument : Instrument #1
Sample Name: 8 male C.oculata abd.sternites/CH2Cl2
Misc Info : w/ 1ug linalool/ul water/6 days; 2nd half
Vial Number: 1

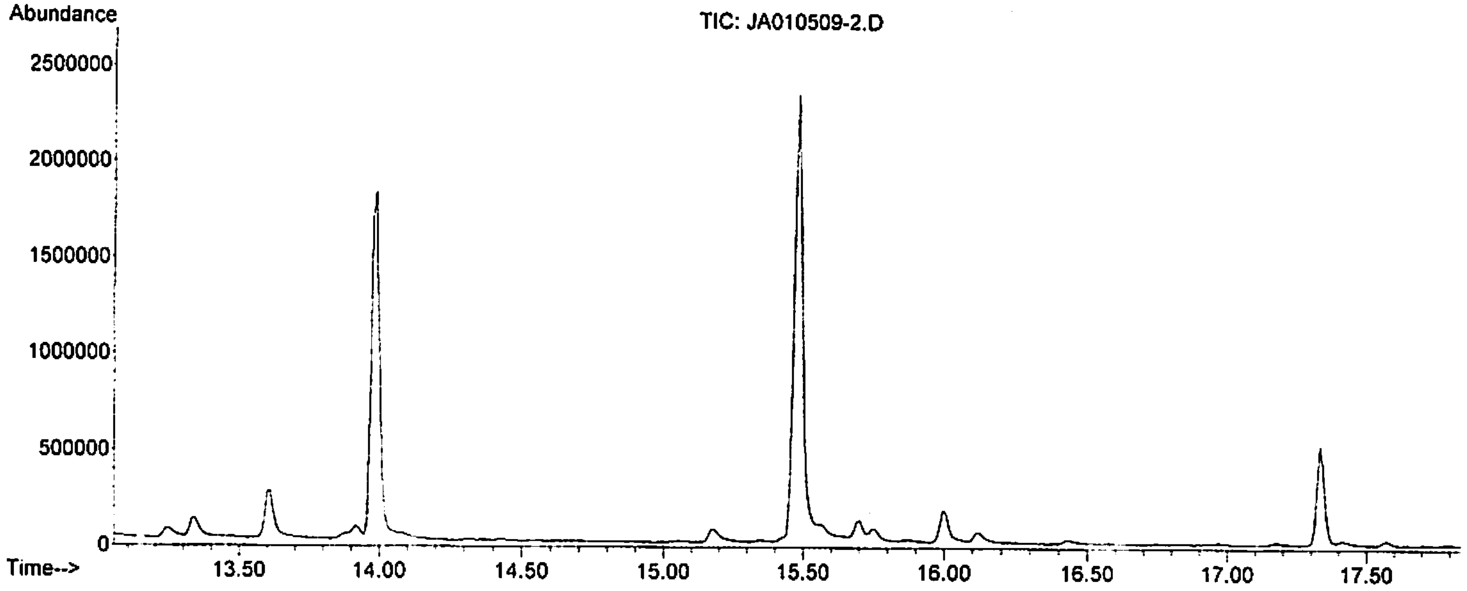


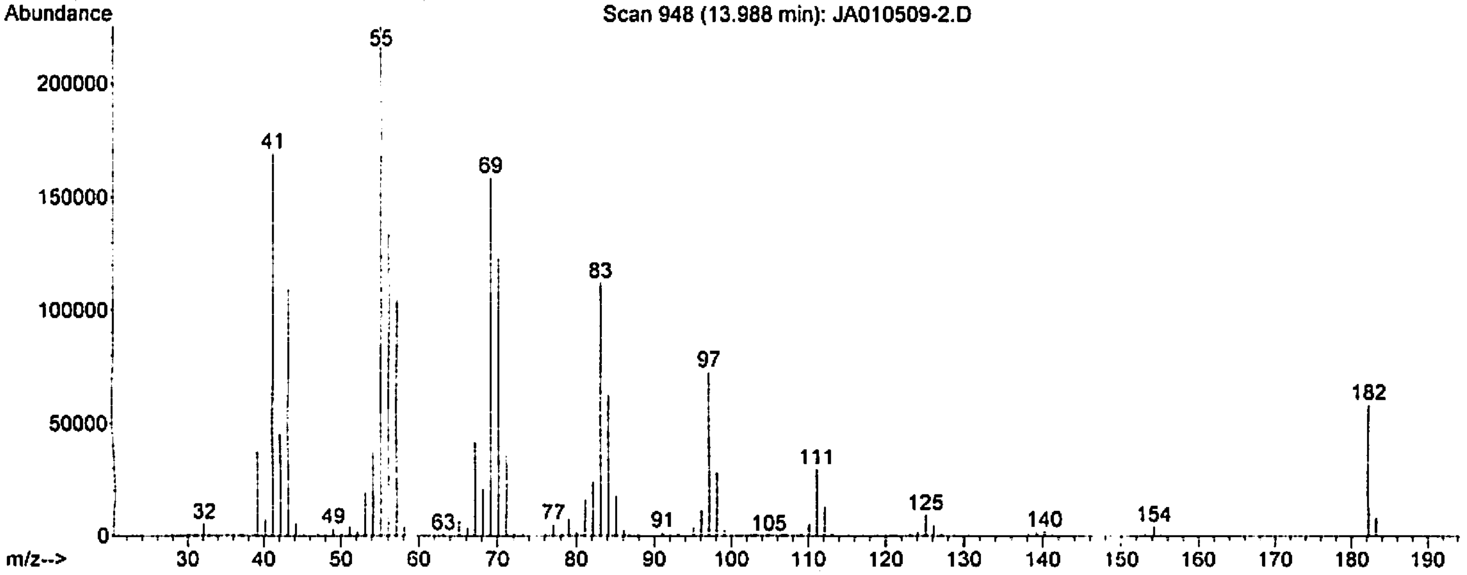

```
File      :D:\DATA\Aldrich\JA-09\JA010509-2.D
Operator    : Aldrich
Acquired    :  5 Jan 2009  14:37     using AcqMethod JA-WAX08.M
Instrument :    Instrument #1
Sample Name: 8 male C.oculata abd.sternites/CH2Cl2
Misc Info  : w/ 1ug linalool/ul water/6 days; 2nd half
Vial Number: 1
```

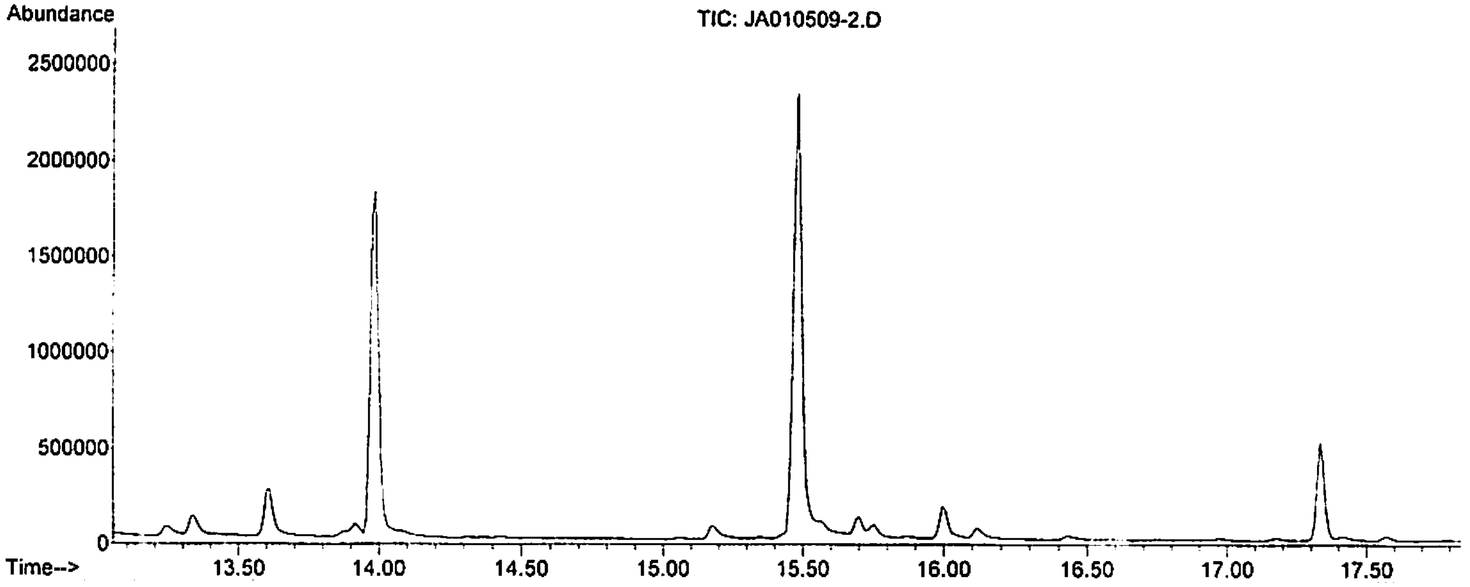


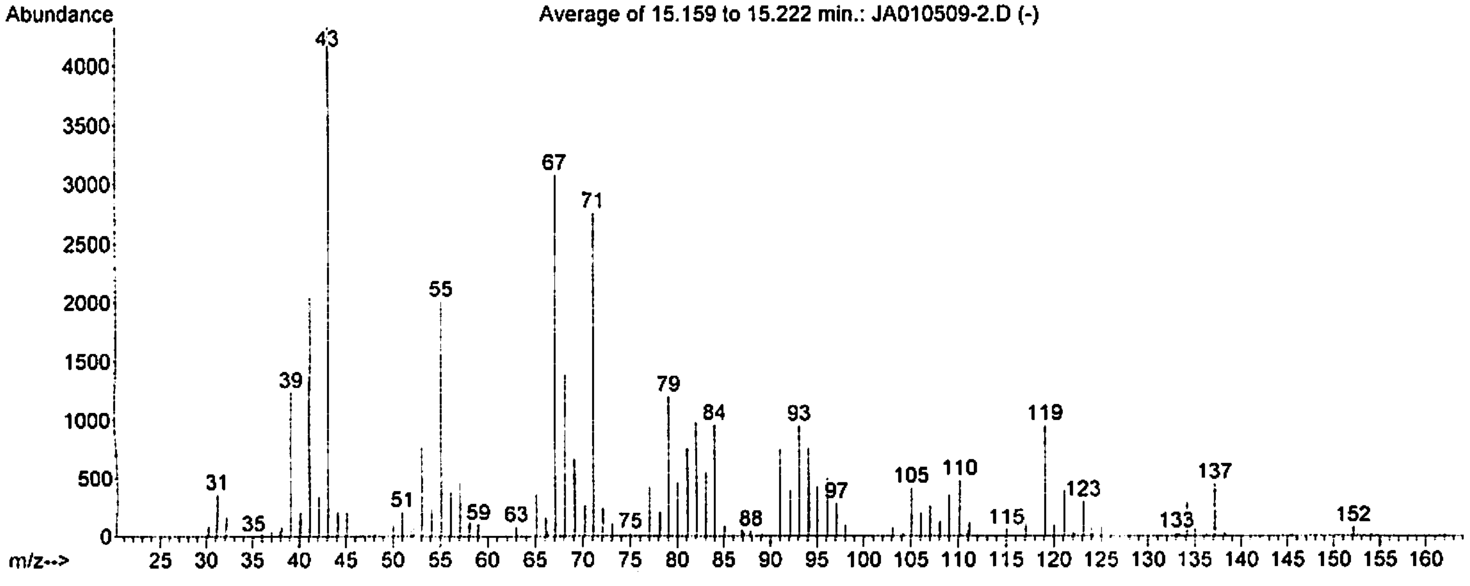

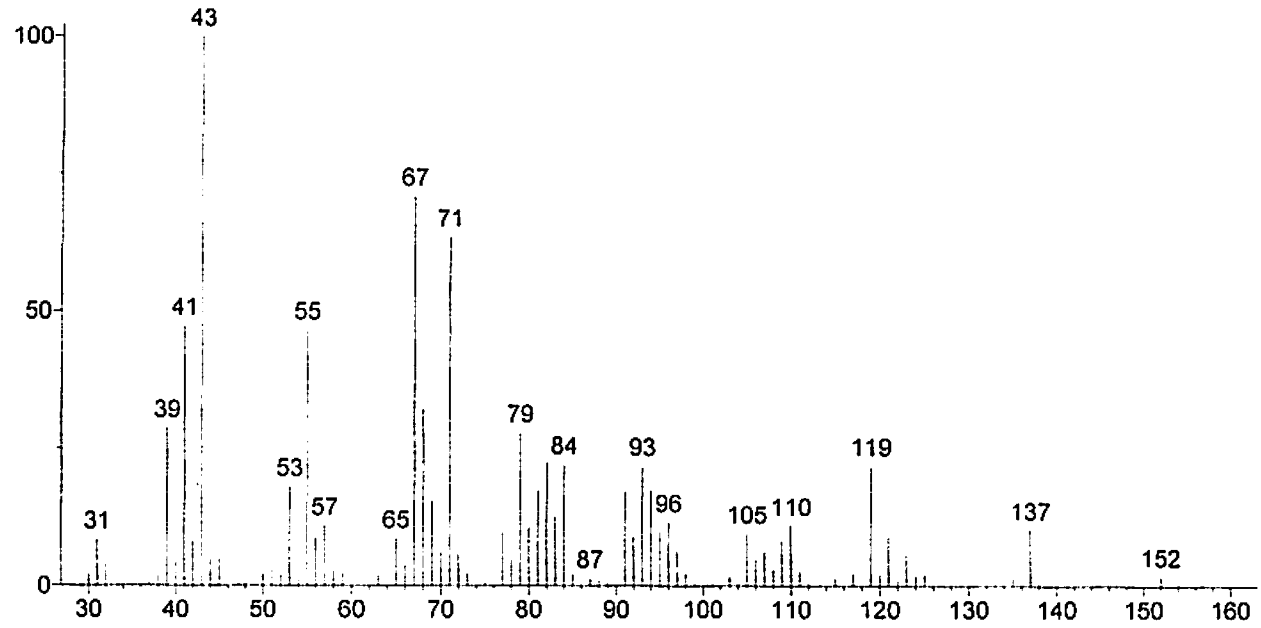


(Text File) Average of 15.159 to 15.222 min.: JA010509-2.D

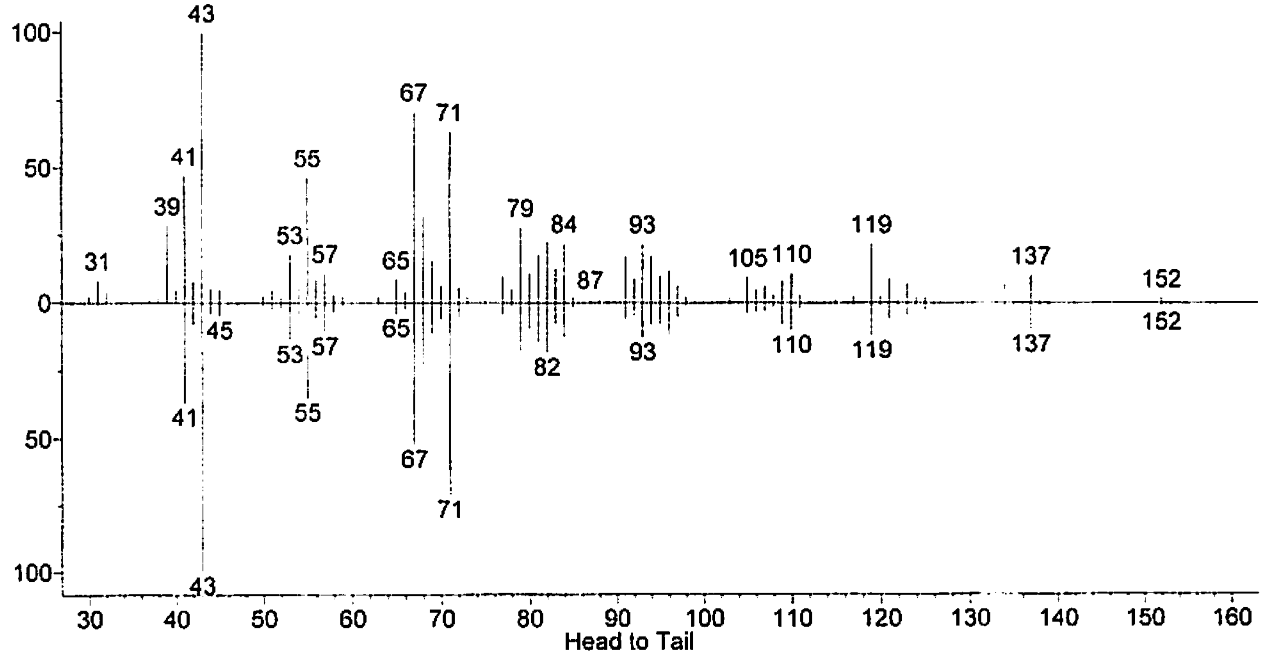


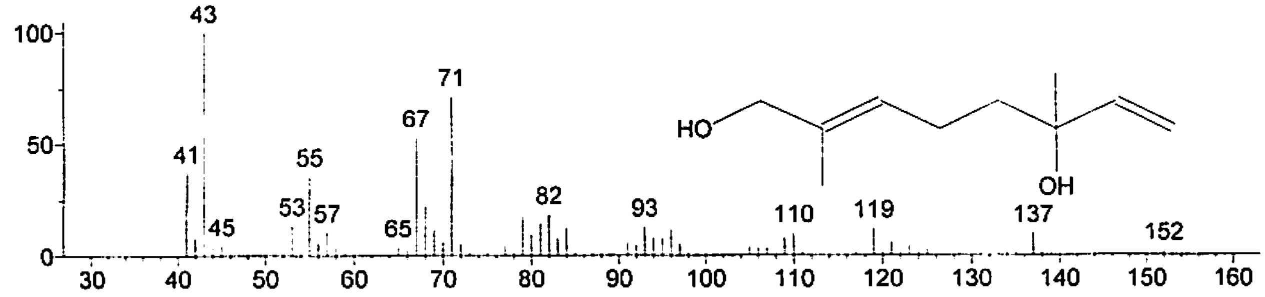


(mainlib) 2,7-Octadiene-1,6-diol, 2,6-dimethyl-

File : D:\DATA\Aldrich\JA-09\JA010509-2.D
Operator : Aldrich
Acquired : 5 Jan 2009 14:37 using AcqMethod JA-WAX08.M
Instrument : Instrument #1
Sample Name: 8 male C.oculata abd.sternites/CH2Cl2
Misc Info : w/ 1ug linalool/ul water/6 days; 2nd half
Vial Number: 1

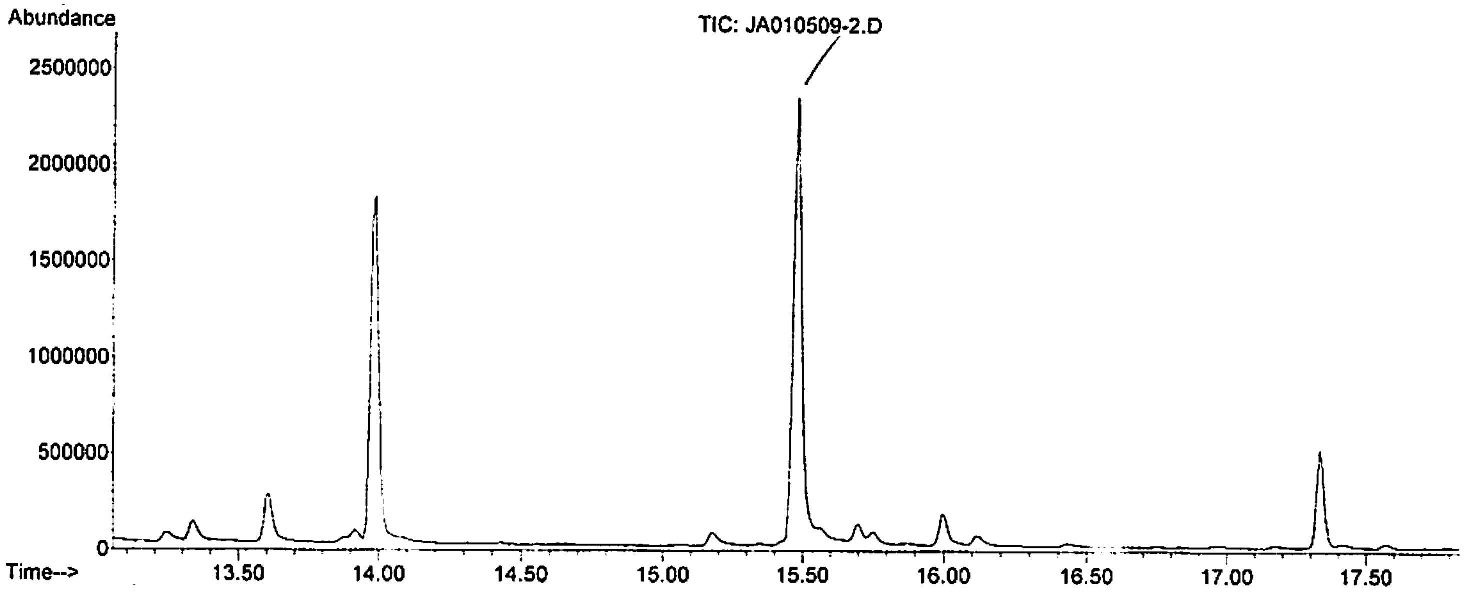


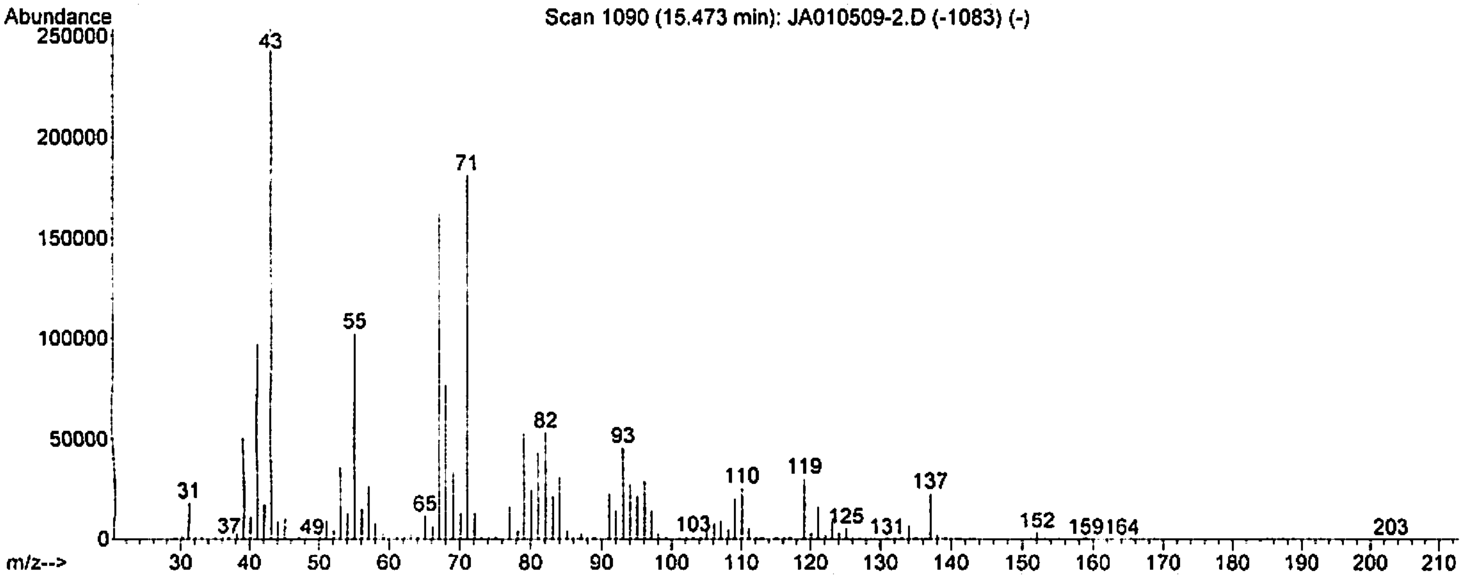

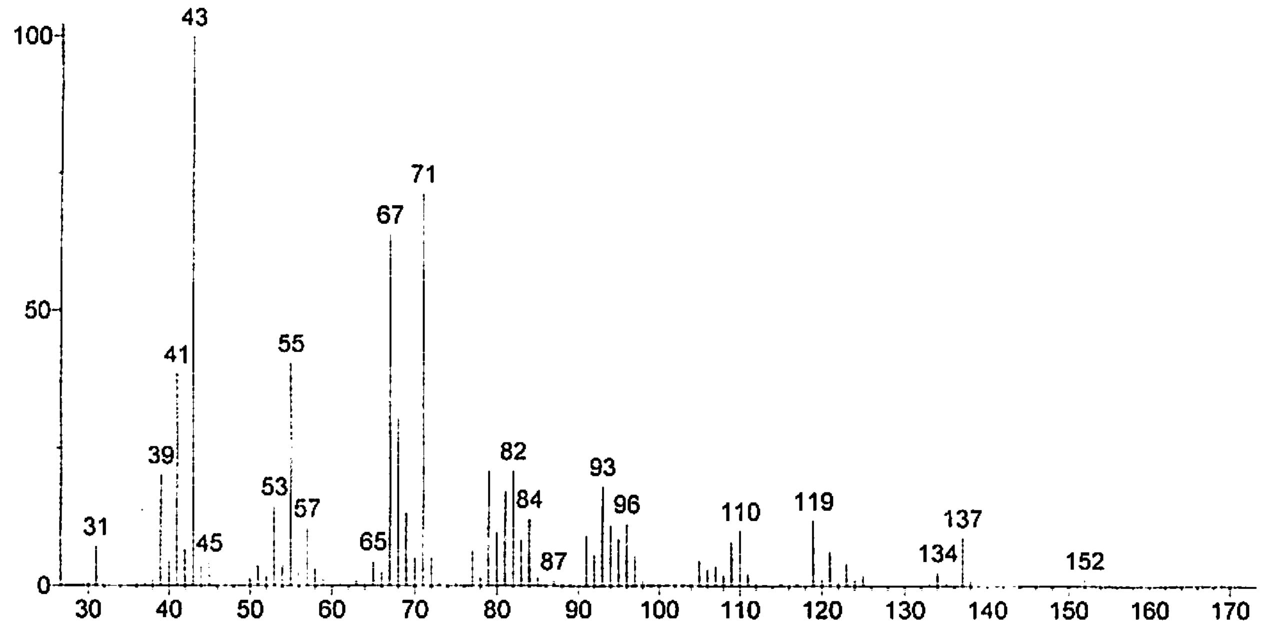


(Text File) Scan 1090 (15.473 min): JA010509-2.D (-1083)

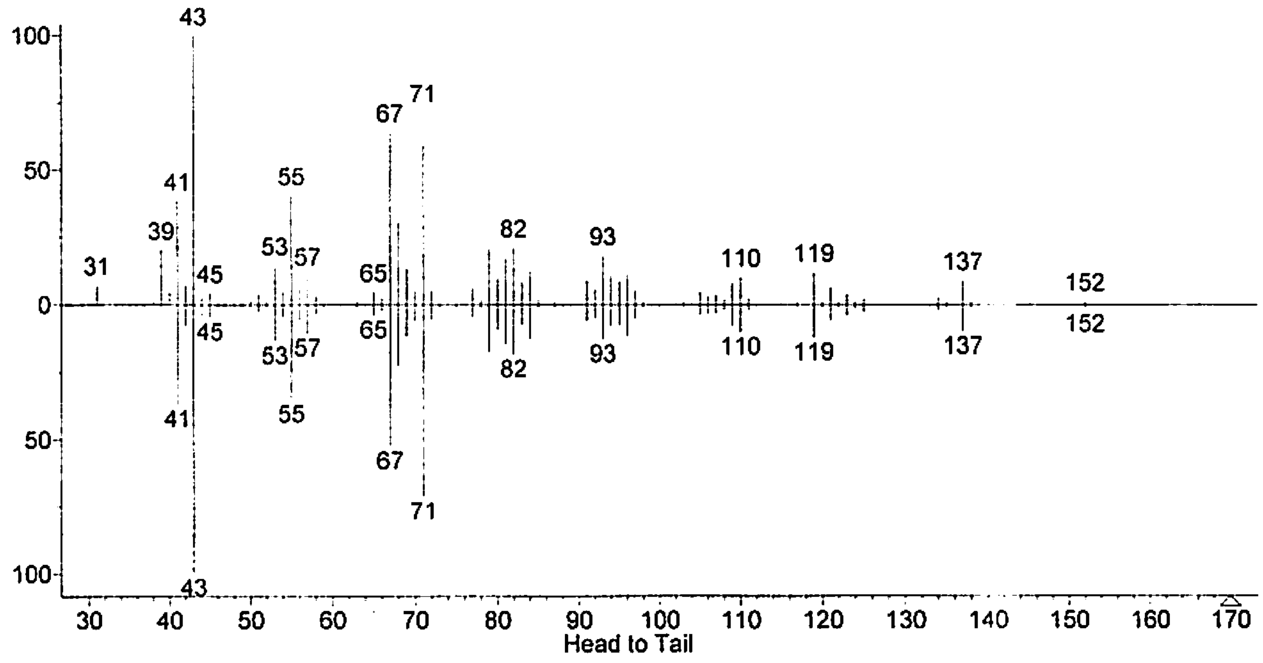


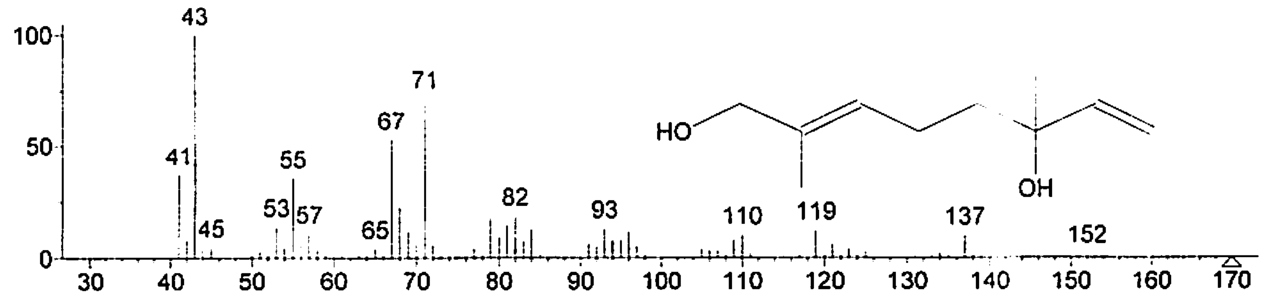


(mainlib) 2,7-Octadiene-1,6-diol, 2,6-dimethyl-

```
File         :D:\DATA\Aldrich\JA-09\JA010509-2.D
Operator     : Aldrich
Acquired     :  5 Jan 2009  14:37      using AcqMethod JA-WAX08.M
Instrument   :     Instrument #1
Sample Name: 8 male C.oculata abd.sternites/CH2Cl2
Misc Info    : w/ lug linalool/ul water/6 days; 2nd half
Vial Number: 1
```

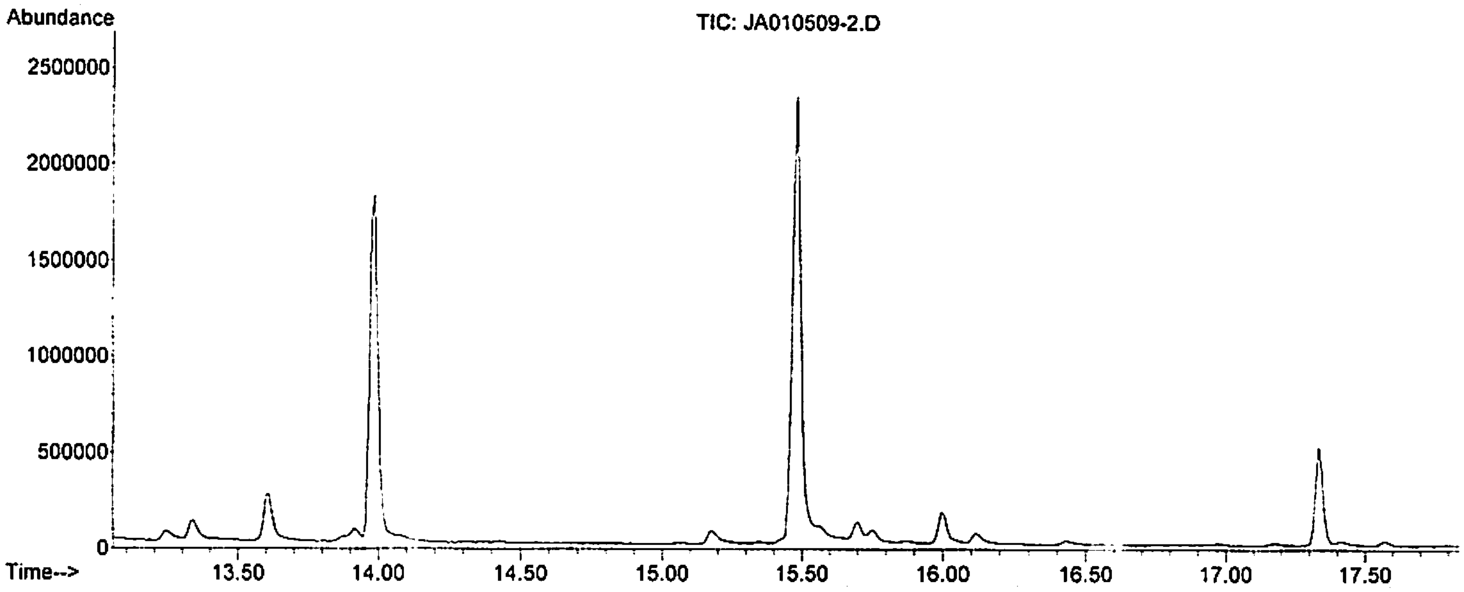


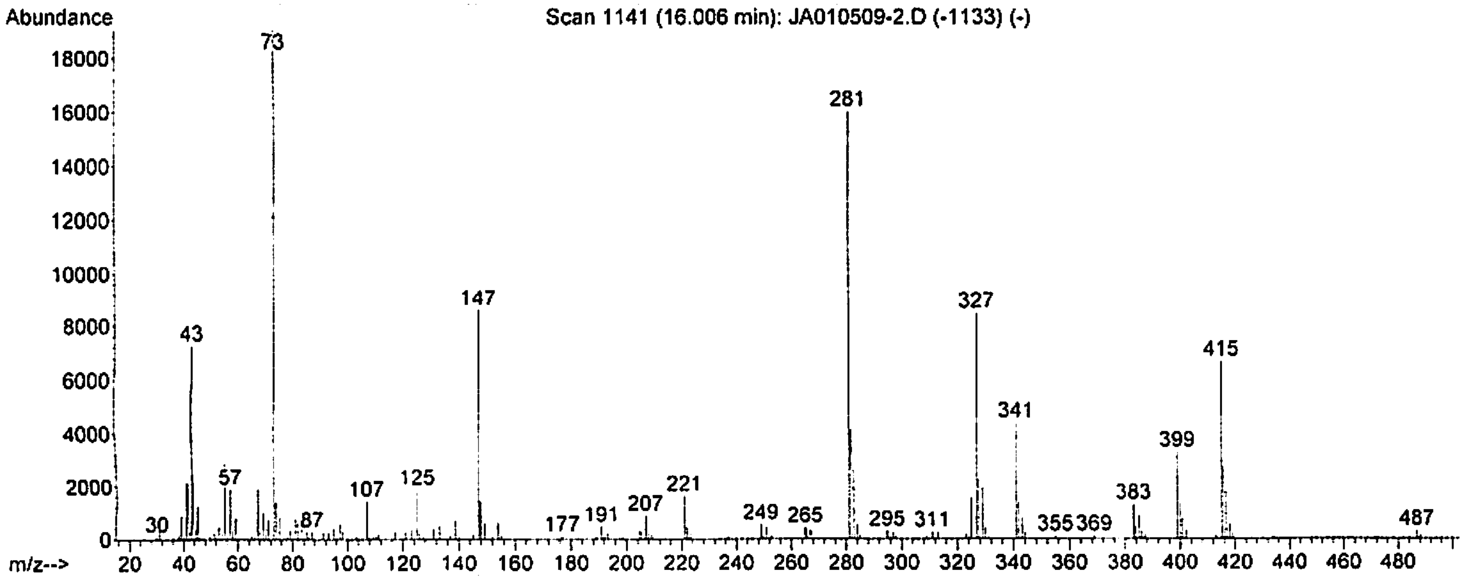

File : D:\DATA\Aldrich\JA-09\JA010509-2.D
Operator : Aldrich
Acquired : 5 Jan 2009 14:37 using AcqMethod JA-WAX08.M
Instrument : Instrument #1
Sample Name: 8 male C.oculata abd.sternites/CH2Cl2
Misc Info : w/ 1ug linalool/ul water/6 days; 2nd half
Vial Number: 1

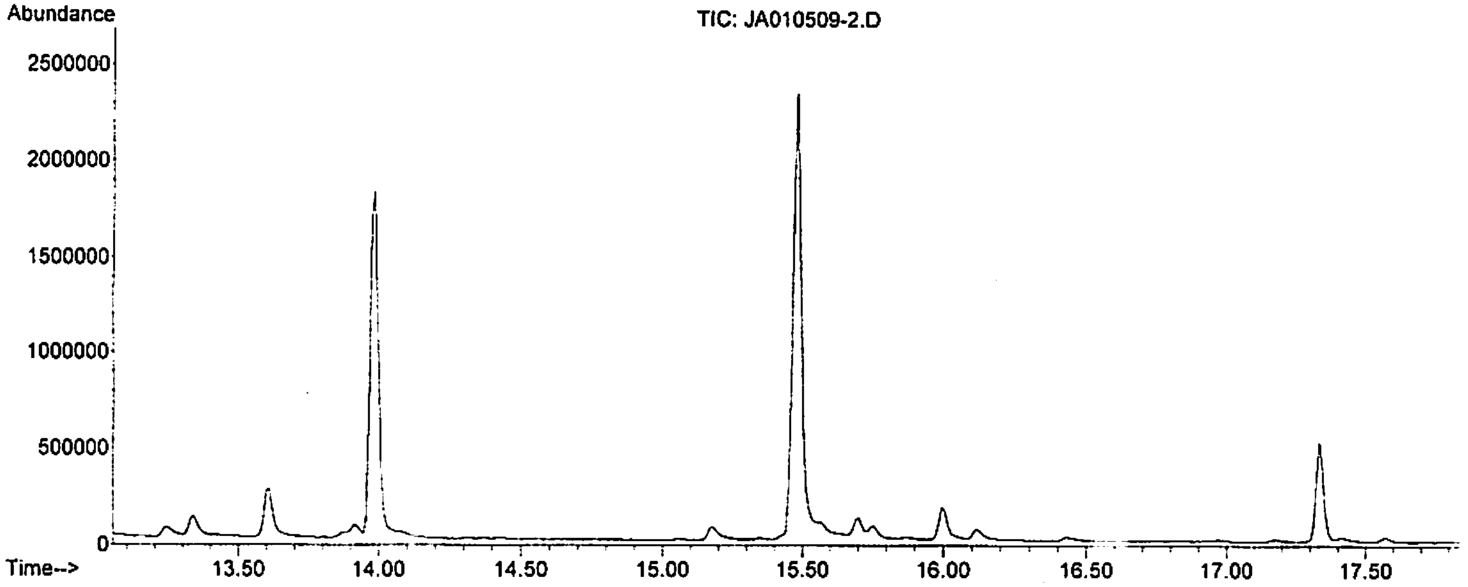


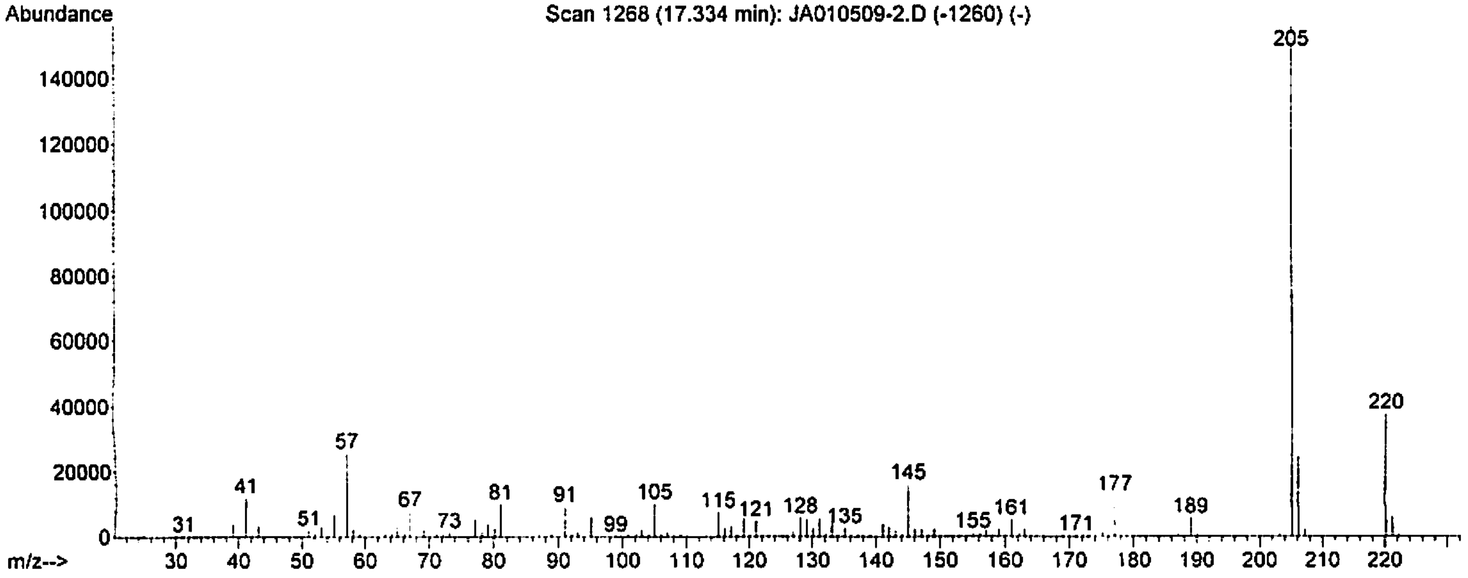

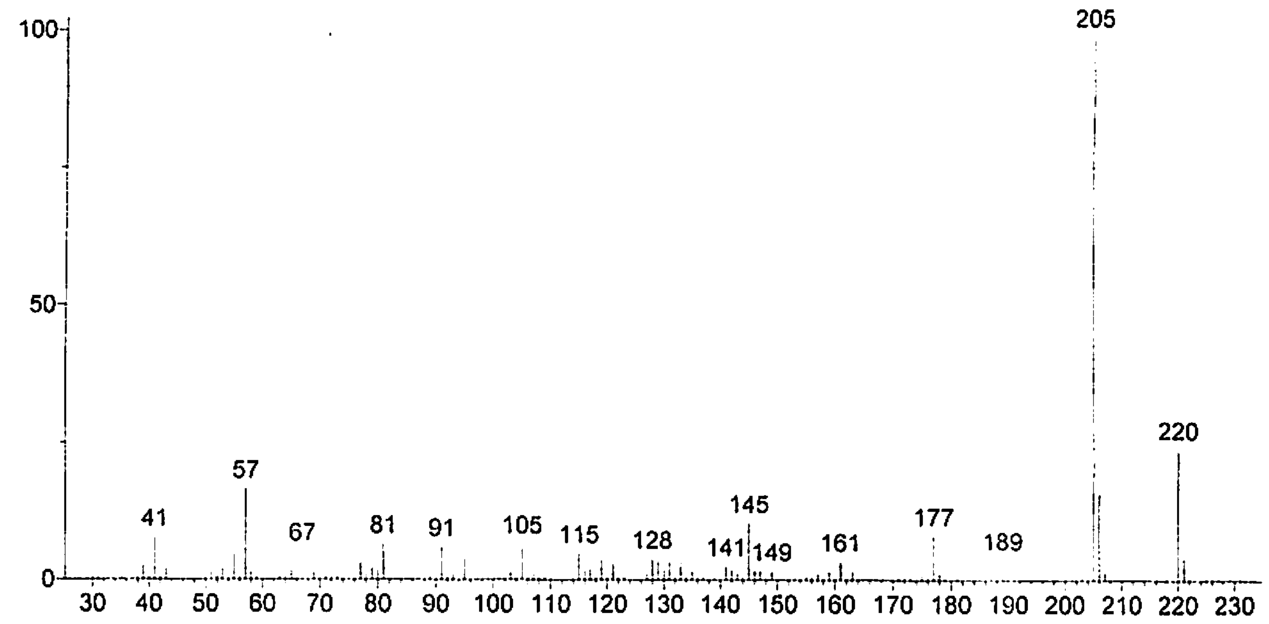


(Text File) Scan 1268 (17.334 min): JA010509-2.D (-1260)

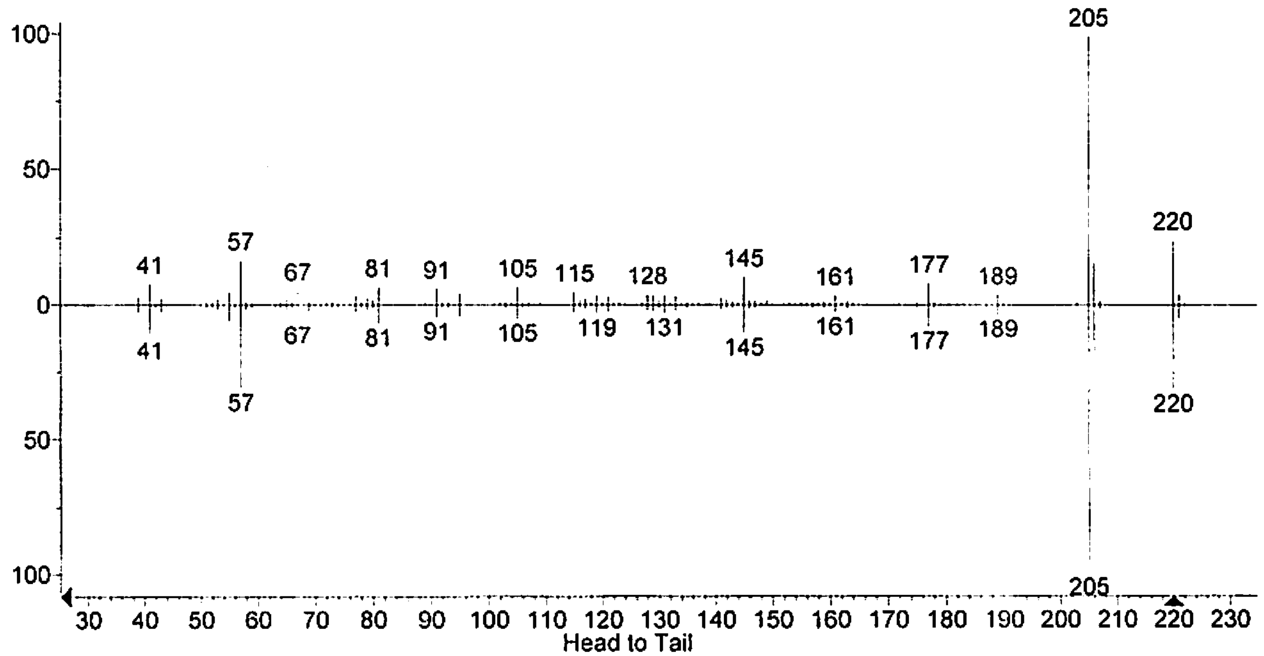


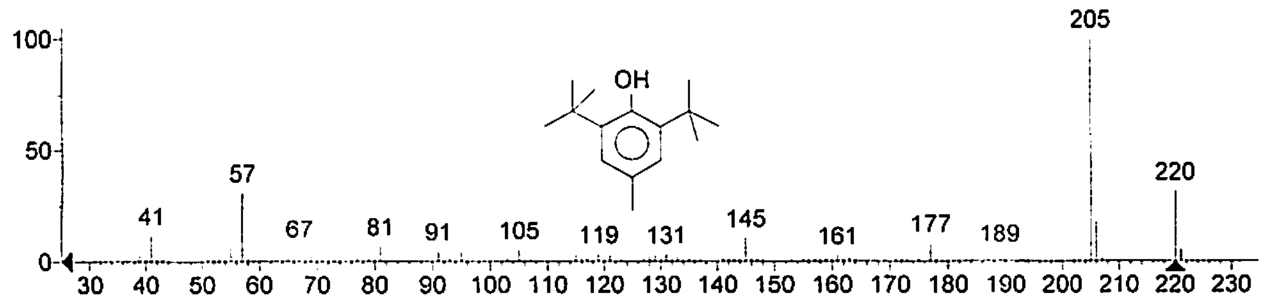


(replib) Butylated Hydroxytoluene

File :D:\DATA\Aldrich\JA-09\JA010509-2.D
Operator : Aldrich
Acquired : 5 Jan 2009 14:37 using AcqMethod JA-WAX08.M
Instrument : Instrument #1
Sample Name: 8 male C.oculata abd.sternites/CH2Cl2
Misc Info : w/ 1ug linalool/ul water/6 days; 2nd half
Vial Number: 1

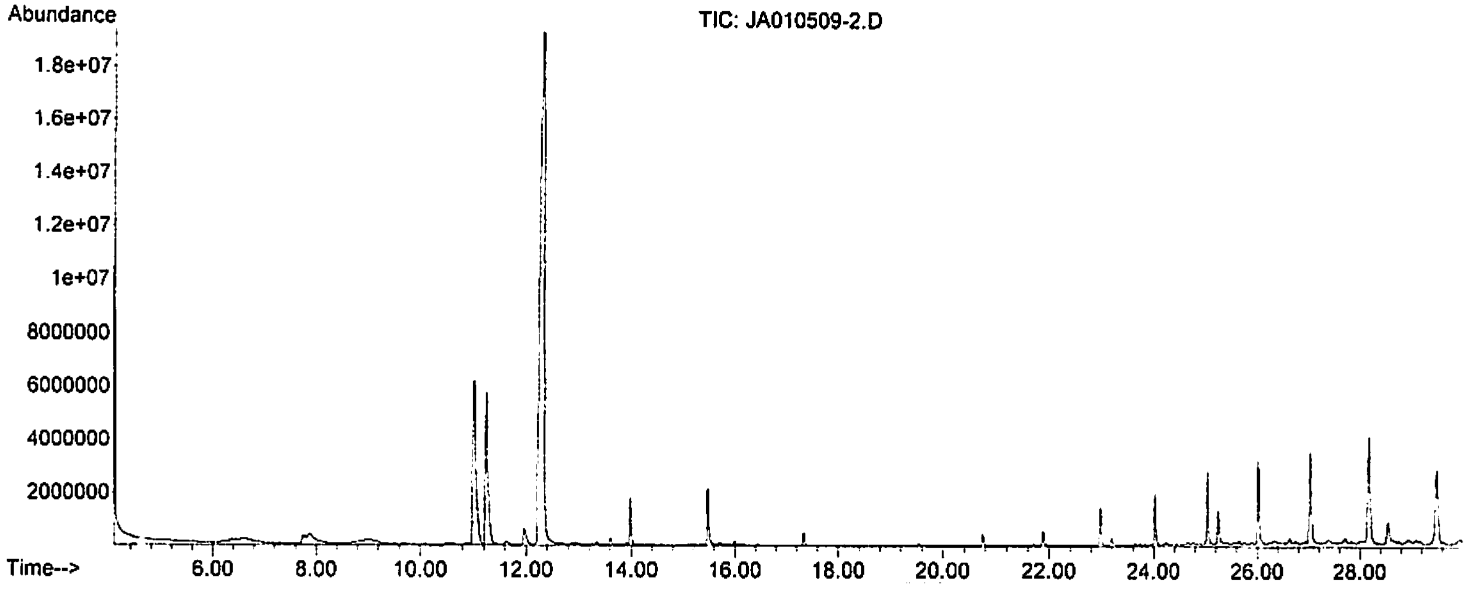


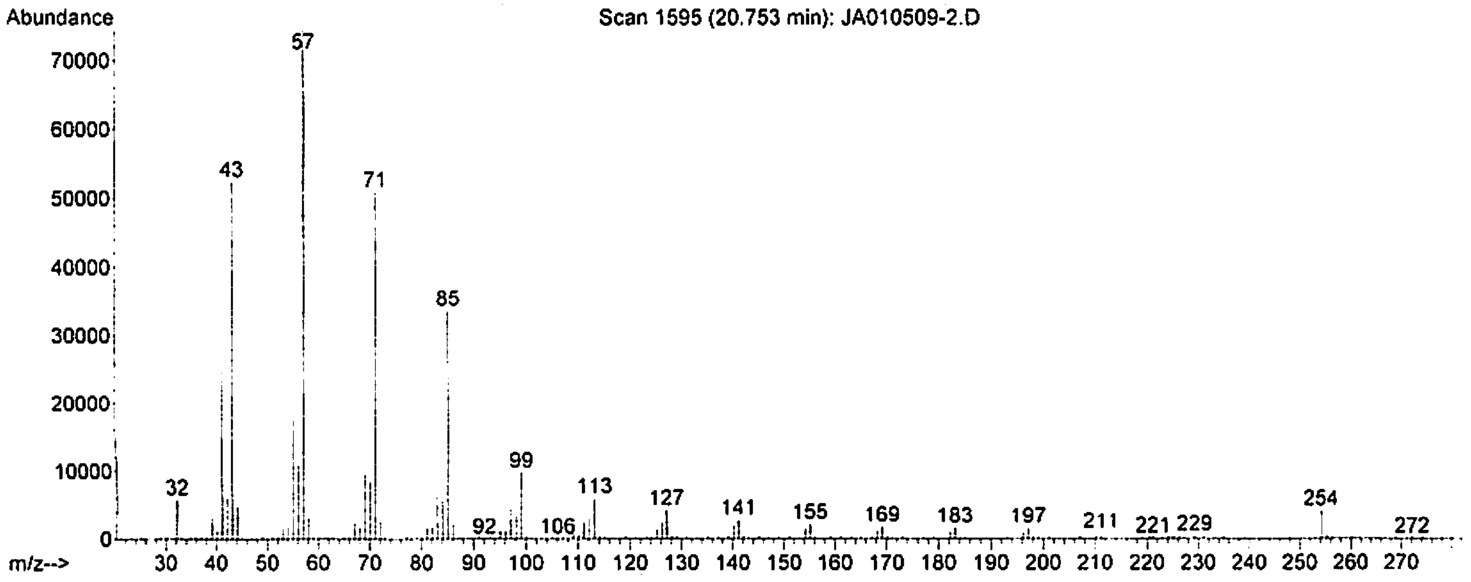

File :D:\DATA\Aldrich\JA-09\JA010509-2.D
Operator : Aldrich
Acquired : 5 Jan 2009 14:37 using AcqMethod JA-WAX08.M
Instrument : Instrument #1
Sample Name: 8 male C.oculata abd.sternites/CH2Cl2
Misc Info : w/ 1ug linalool/ul water/6 days; 2nd half
Vial Number: 1

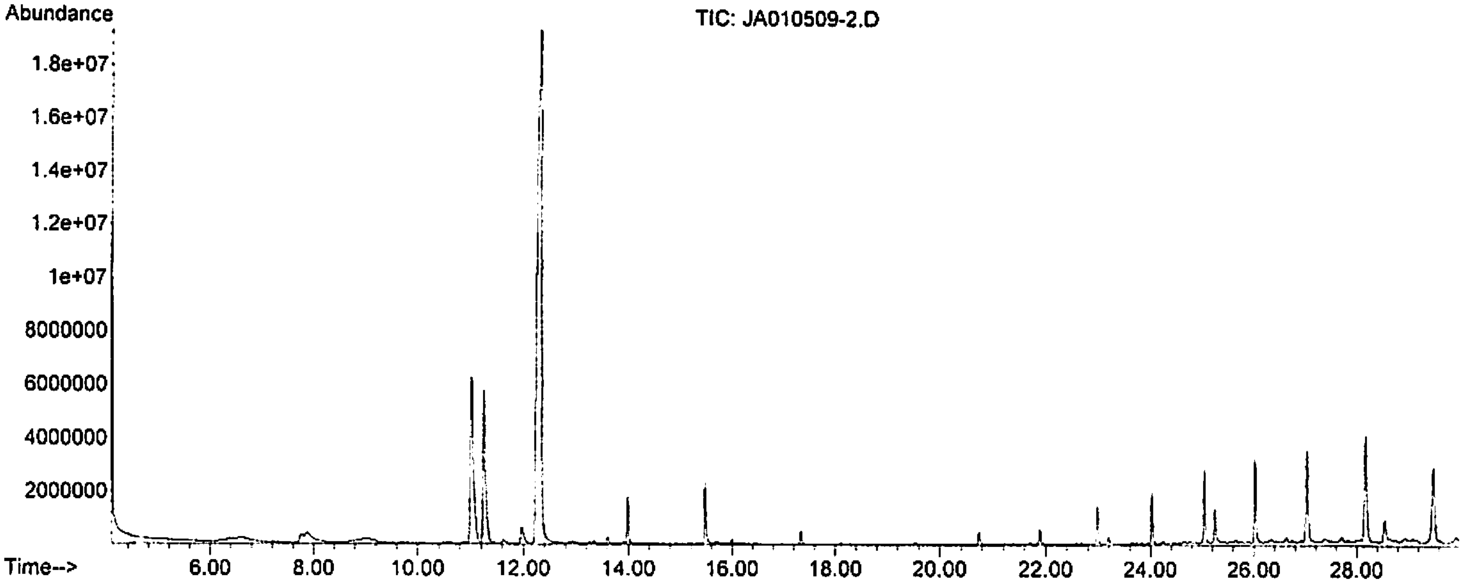


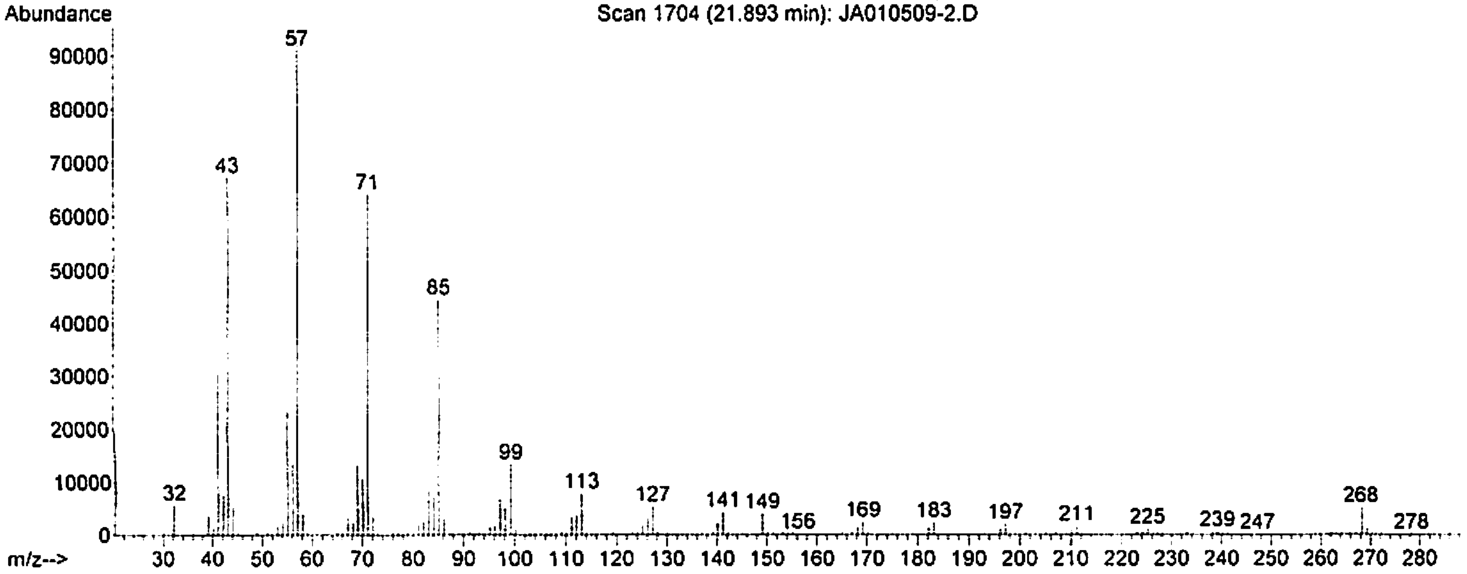

[illegible]le . :D:\DATA\Aldrich\JA-09\JA010509-2.D
[illegible]erator : Aldrich
[illegible]quired : 5 Jan 2009 14:37 using AcqMethod JA-WAX08.M
[illegible]strument : Instrument #1
[illegible]mple Name: 8 male C.oculata abd.sternites/CH2C12
[illegible]sc Info : w/ lug linalool/ul water/6 days; 2nd half
[illegible]al Number: 1

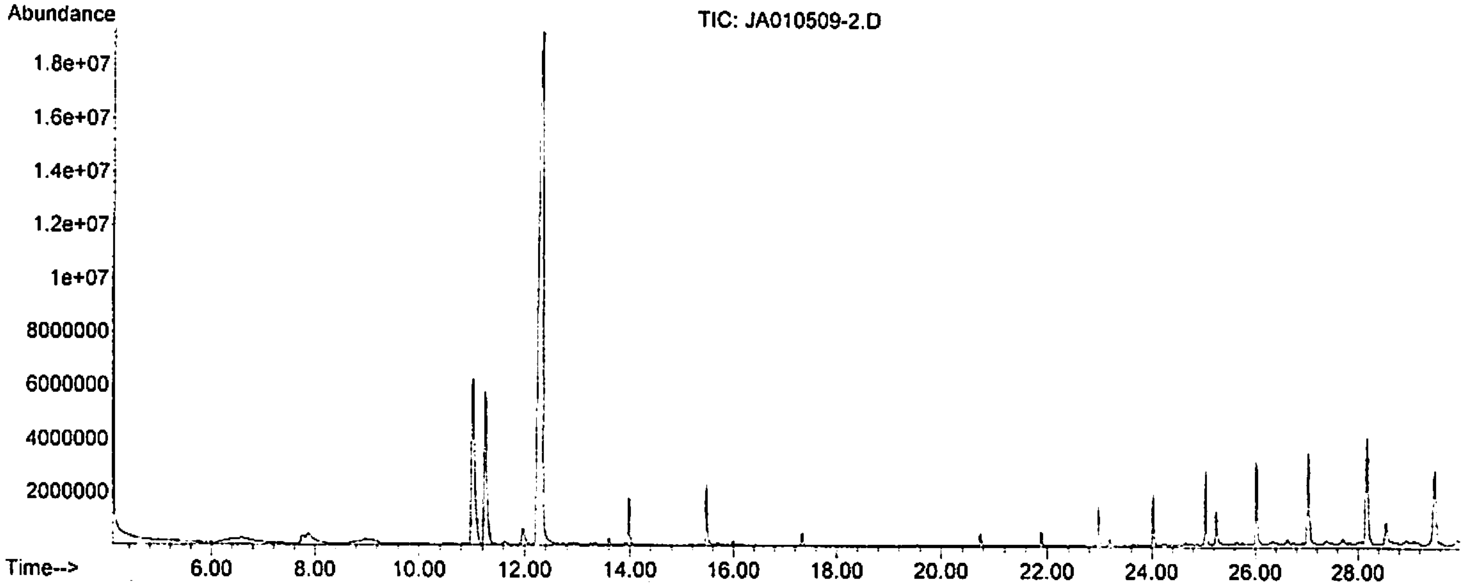


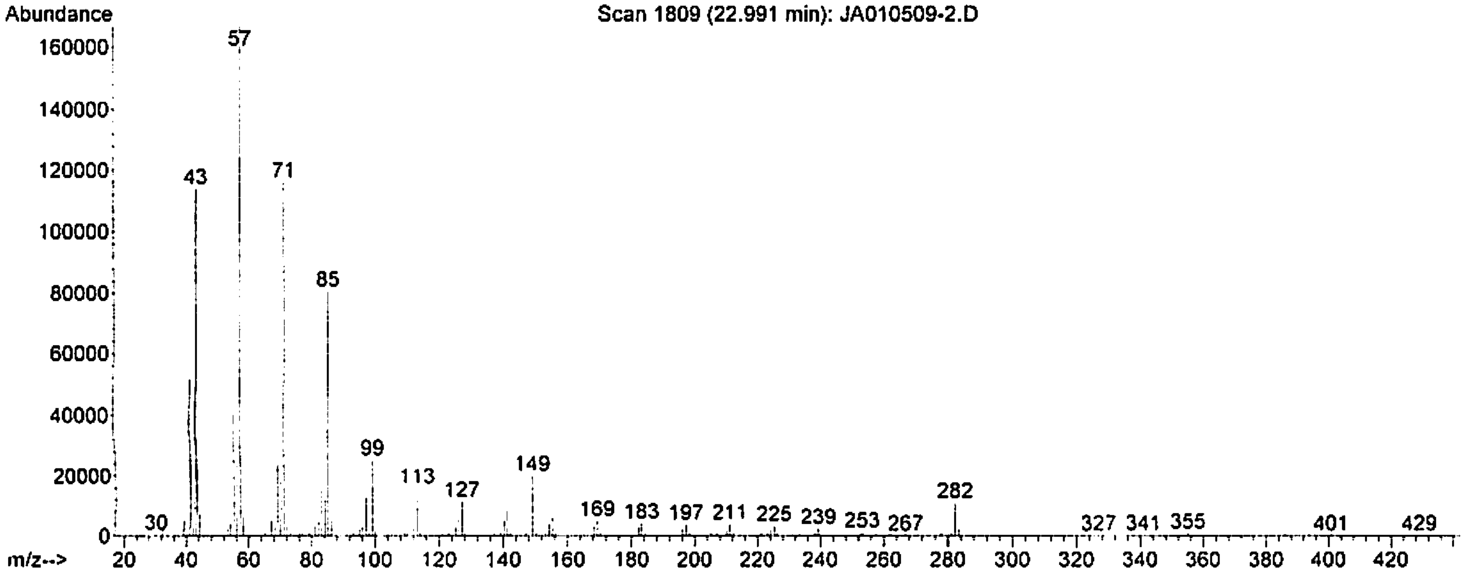

```
[illegible]le    :D:\DATA\Aldrich\JA-09\JA010509-2.D
[illegible]erator    : Aldrich
[illegible]quired    :  5 Jan 2009  14:37    using AcqMethod JA-WAX08.M
[illegible]strument :    Instrument #1
[illegible]mple Name: 8 male C.oculata abd.sternites/CH2Cl2
[illegible]sc Info  : w/ 1ug linalool/ul water/6 days; 2nd half
[illegible]al Number: 1
```

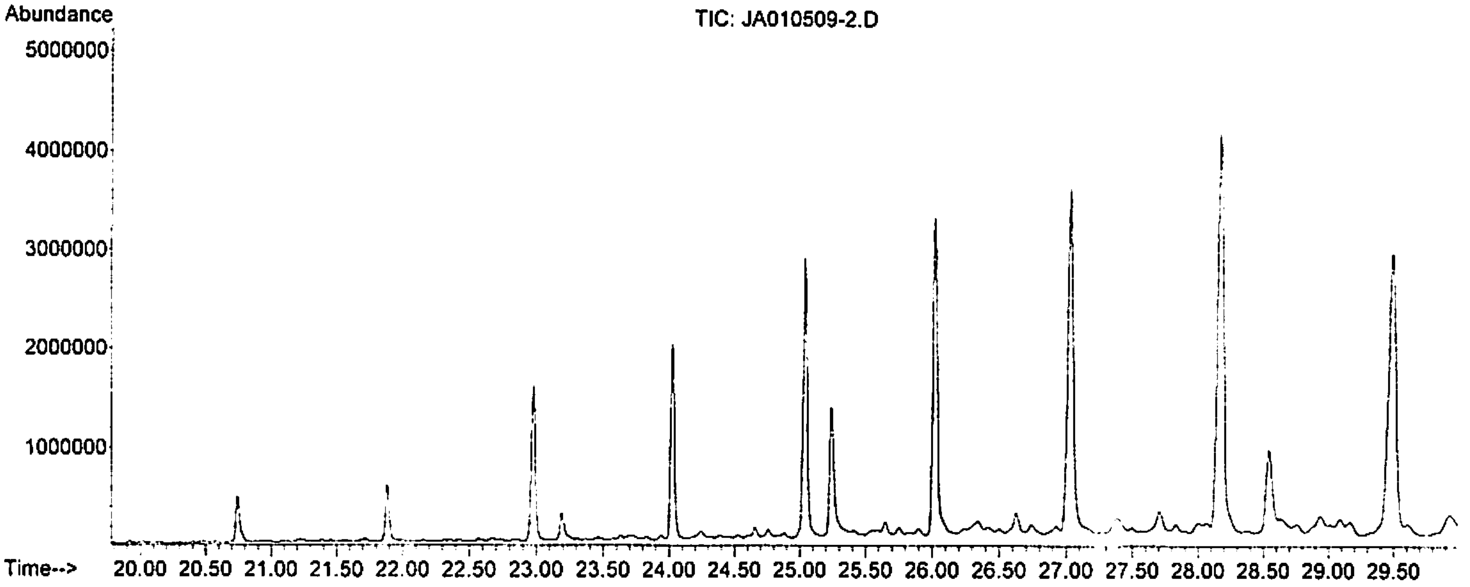


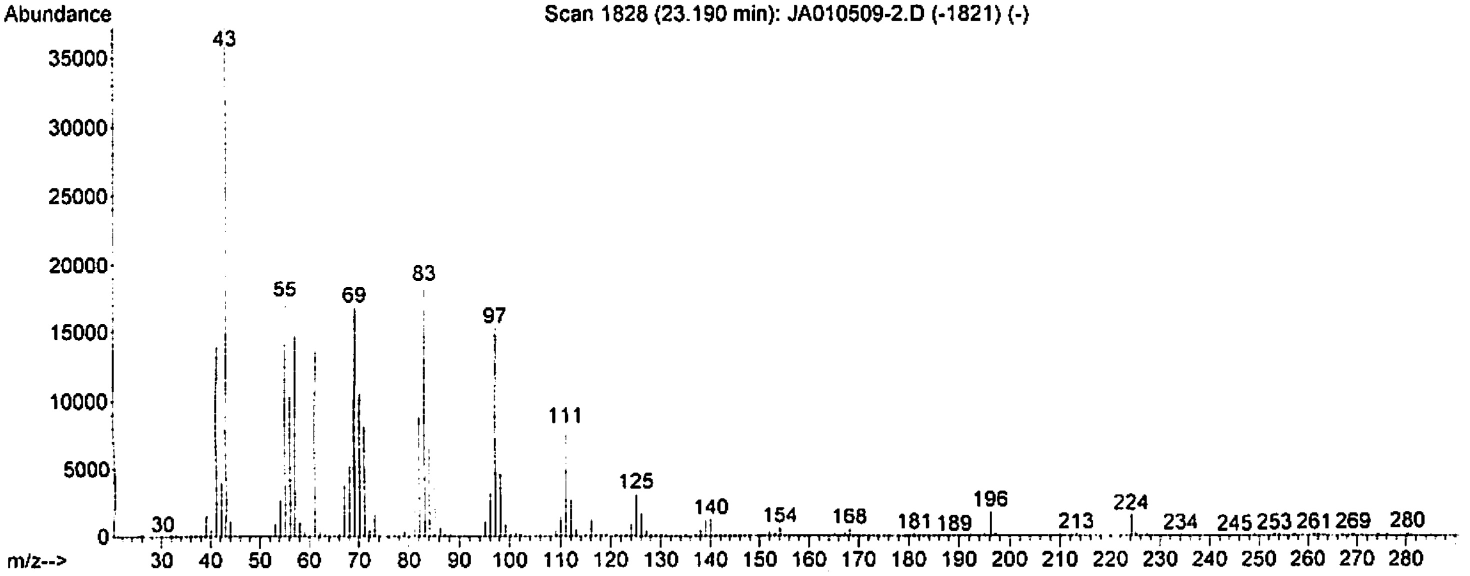

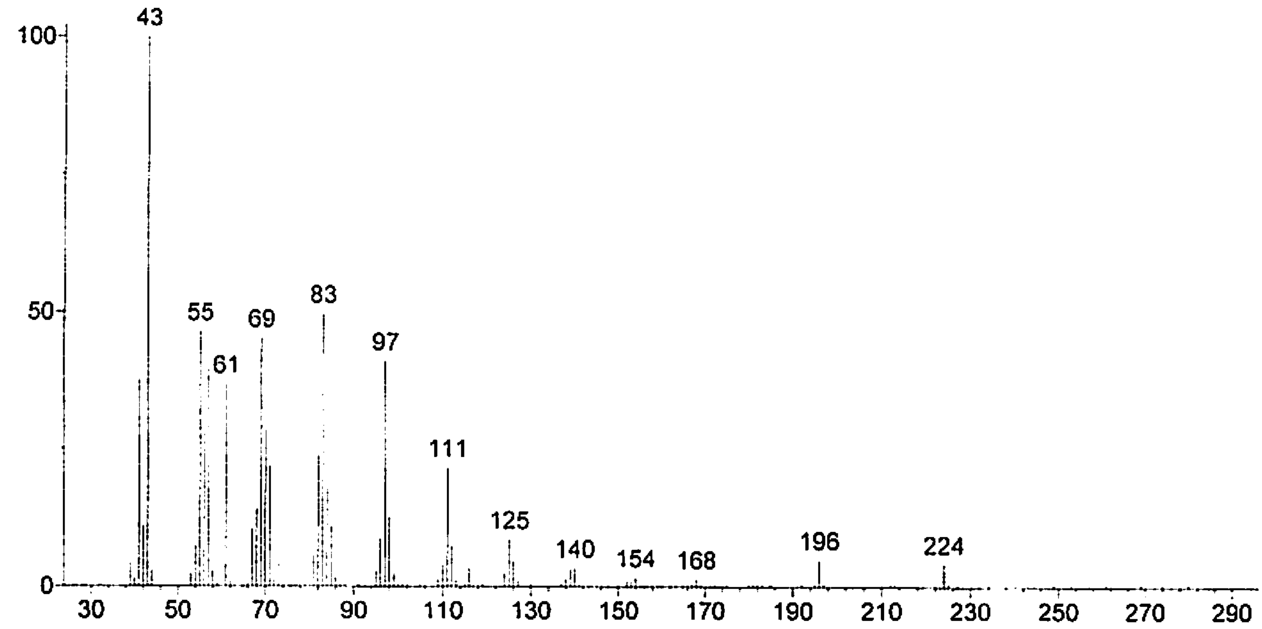


(Text File) Scan 1828 (23.190 min): JA010509-2.D (-1821)

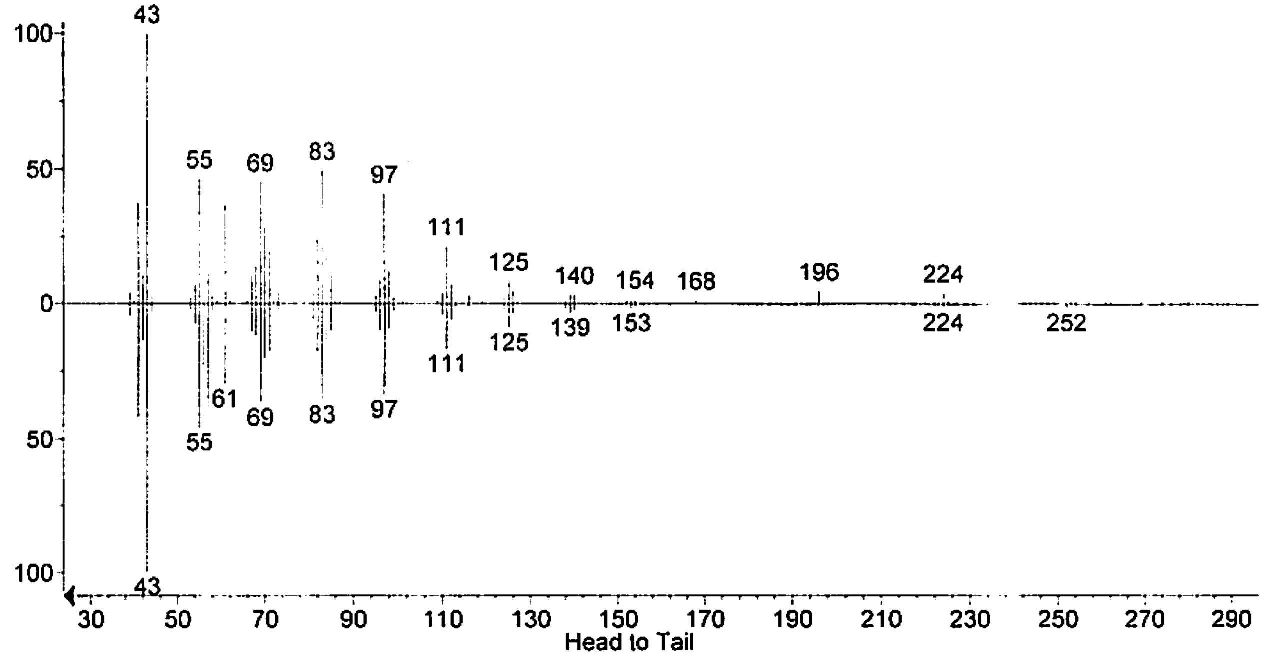


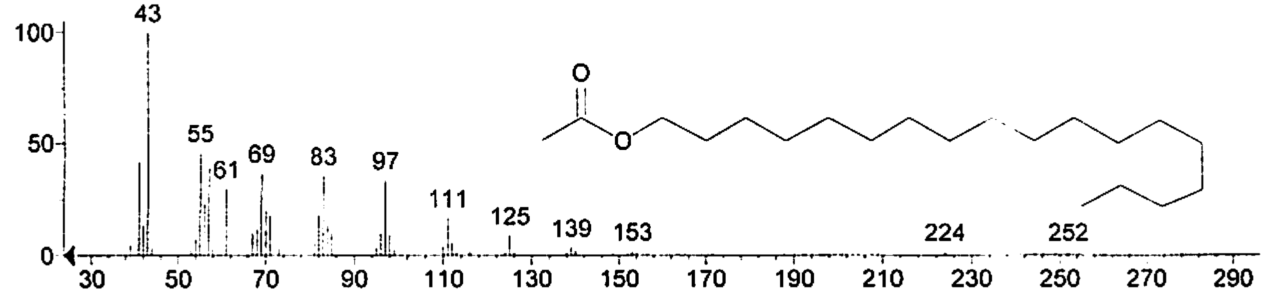


(mainlib) Acetic acid, octadecyl ester

[illegible]le : D:\DATA\Aldrich\JA-09\JA010509-2.D
[illegible]erator : Aldrich
[illegible]quired : 5 Jan 2009 14:37 using AcqMethod JA-WAX08.M
[illegible]strument : Instrument #1
[illegible]mple Name: 8 male C.oculata abd.sternites/CH2Cl2
[illegible]sc Info : w/ 1ug linalool/ul water/6 days; 2nd half
[illegible]al Number: 1

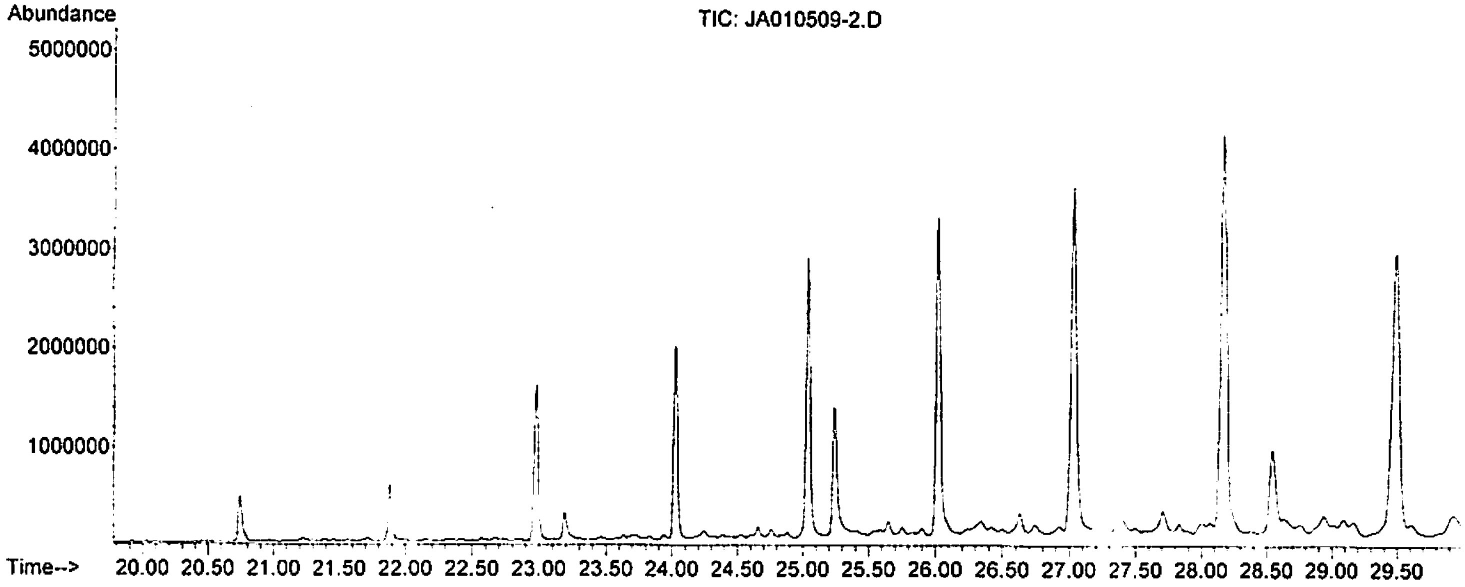


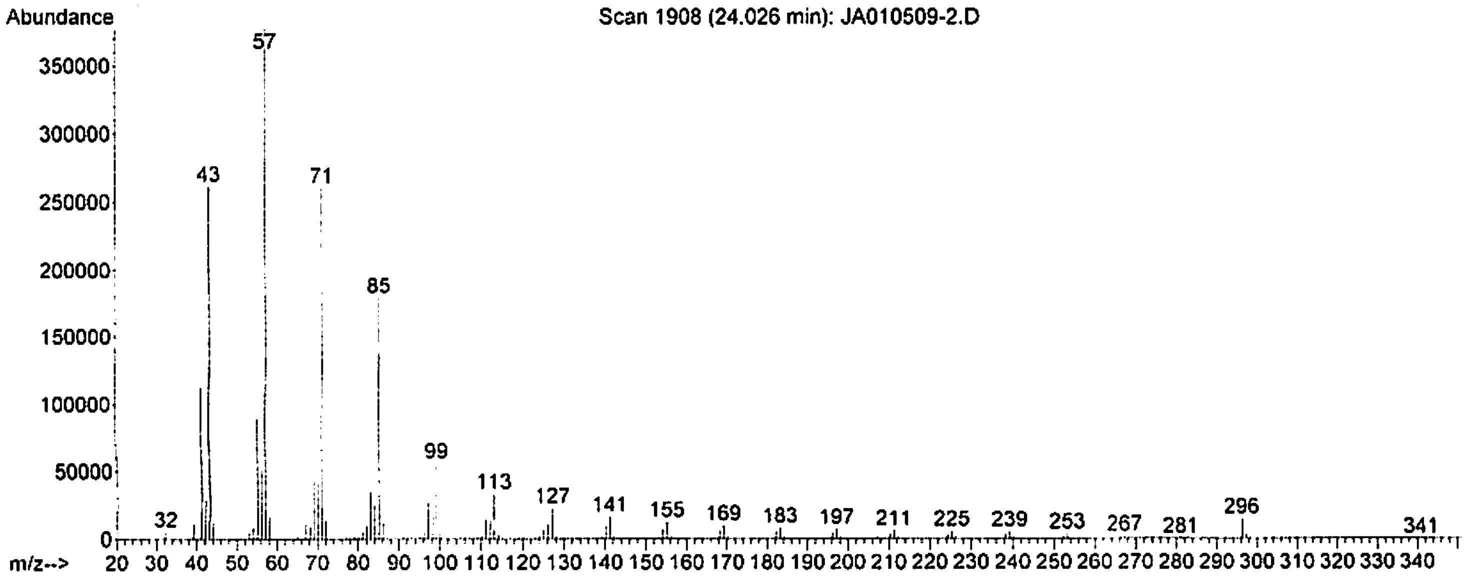

[illegible]le : D:\DATA\Aldrich\JA-09\JA010509-2.D
[illegible]perator : Aldrich
[illegible]quired : 5 Jan 2009 14:37 using AcqMethod JA-WAX08.M
[illegible]nstrument : Instrument #1
[illegible]mple Name: 8 male C.oculata abd.sternites/CH2Cl2
[illegible]isc Info : w/ 1ug linalool/ul water/6 days; 2nd half
[illegible]al Number: 1

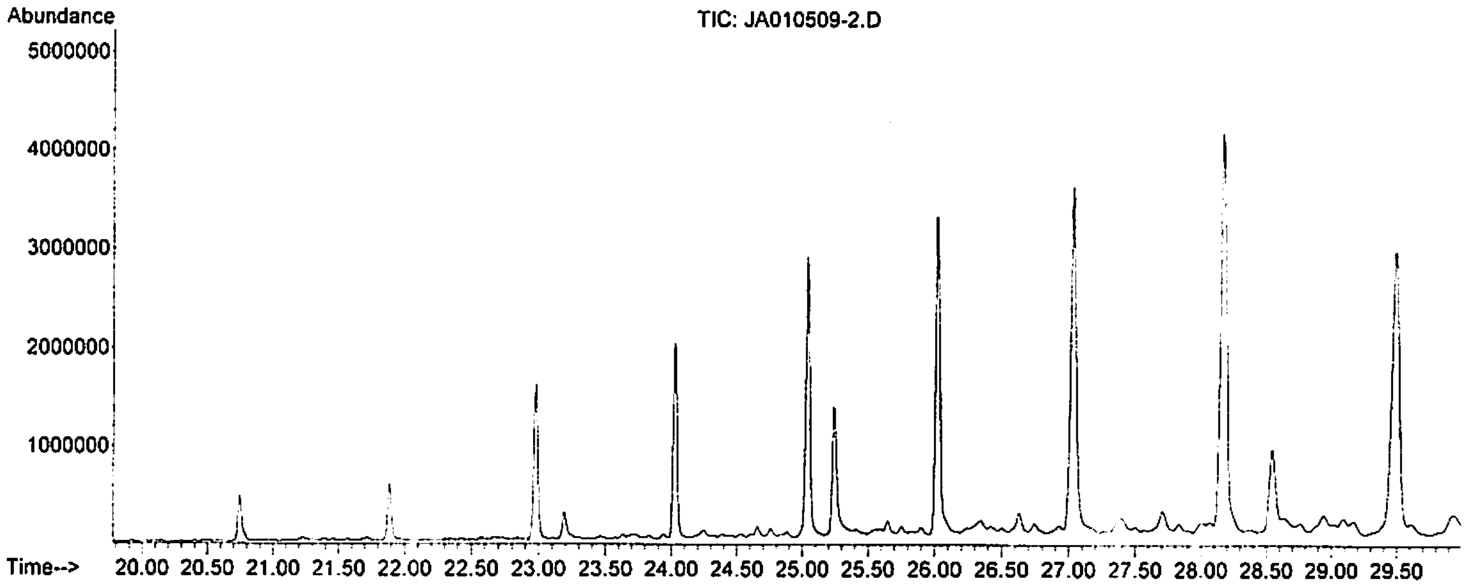


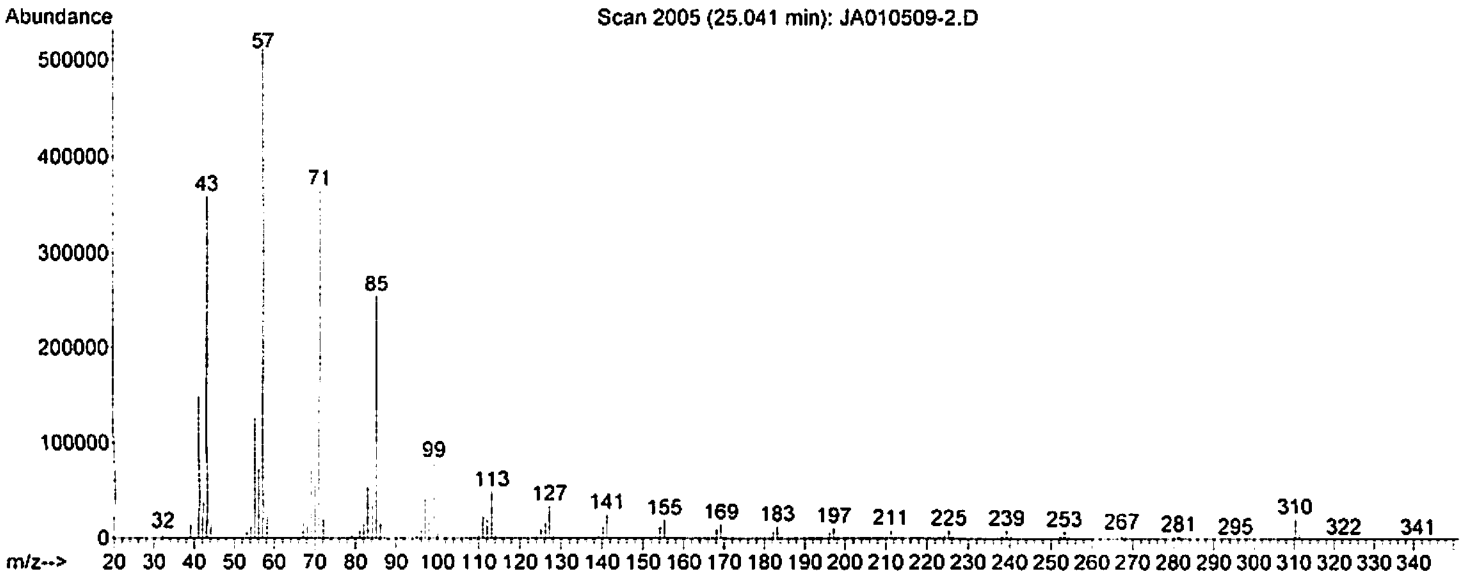

[illegible]le : D:\DATA\Aldrich\JA-09\JA010509-2.D
[illegible]erator : Aldrich
[illegible]quired : 5 Jan 2009 14:37 using AcqMethod JA-WAX08.M
[illegible]strument : Instrument #1
[illegible]mple Name: 8 male C.oculata abd.sternites/CH2Cl2
[illegible]sc Info : w/ 1ug linalool/ul water/6 days; 2nd half
[illegible]al Number: 1

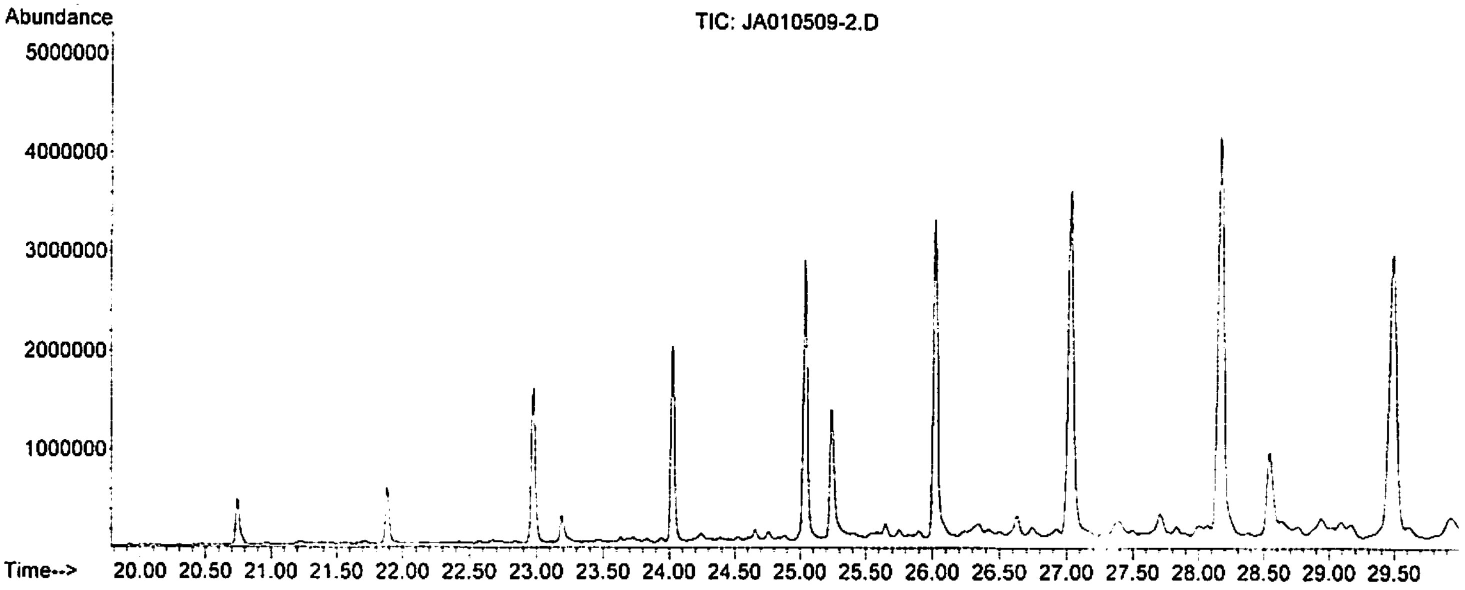


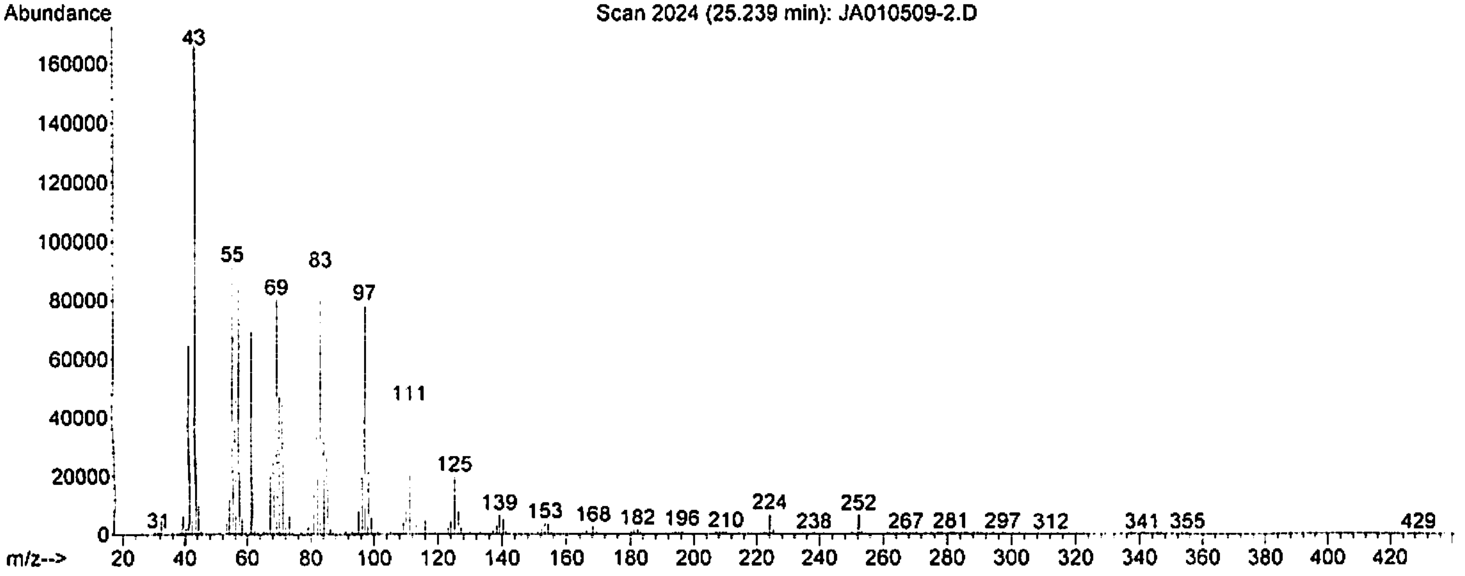

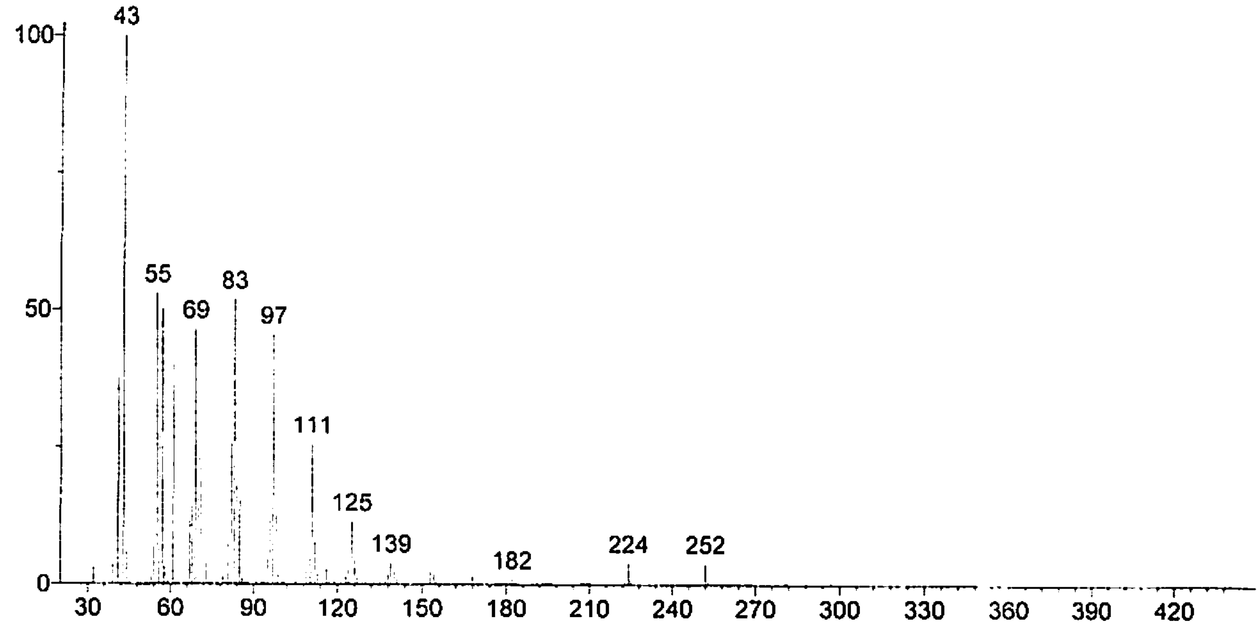

(Text File) Scan 2024 (25.239 min): JA010509-2.D

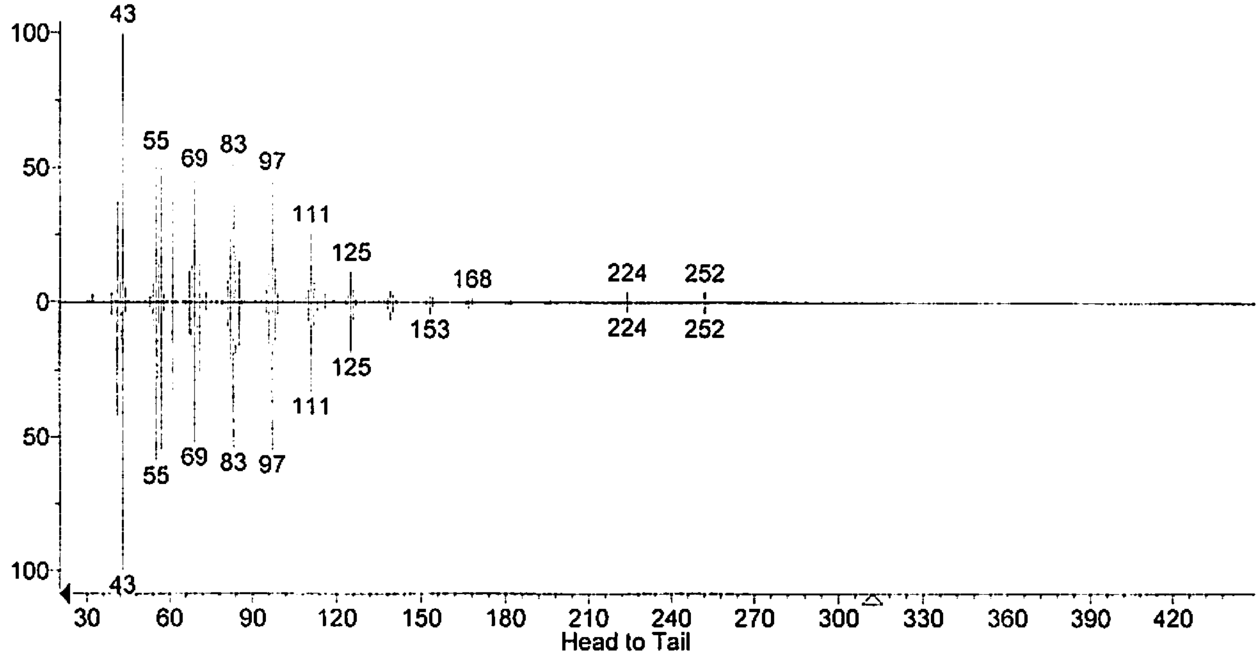


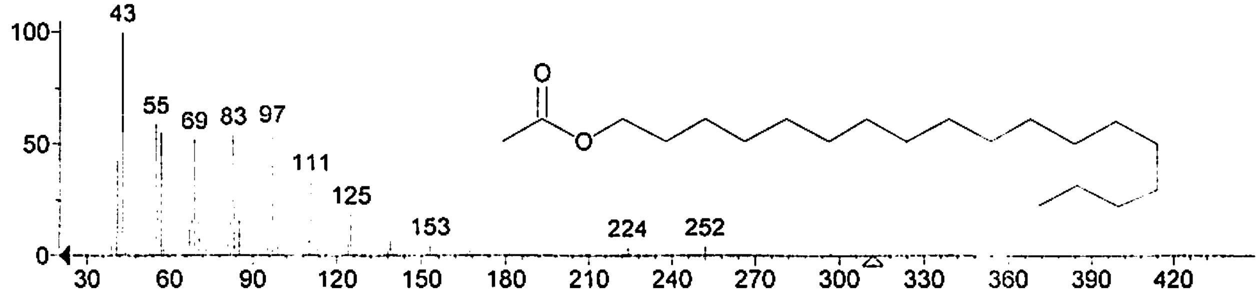

(replib) Acetic acid, octadecyl ester

File : D:\DATA\Aldrich\JA-09\JA010509-2.D
Operator : Aldrich
Acquired : 5 Jan 2009 14:37 using AcqMethod JA-WAX08.M
Instrument : Instrument #1
Sample Name: 8 male C.oculata abd.sternites/CH2Cl2
Misc Info : w/ 1ug linalool/ul water/6 days; 2nd half
Vial Number: 1

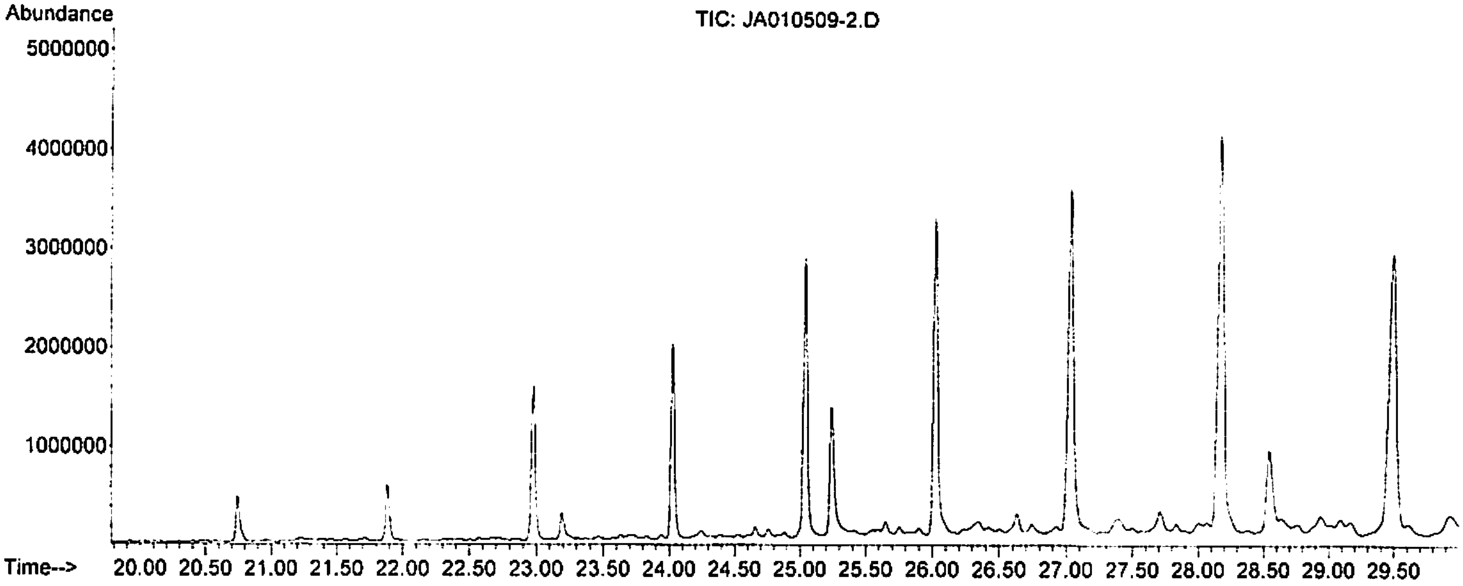


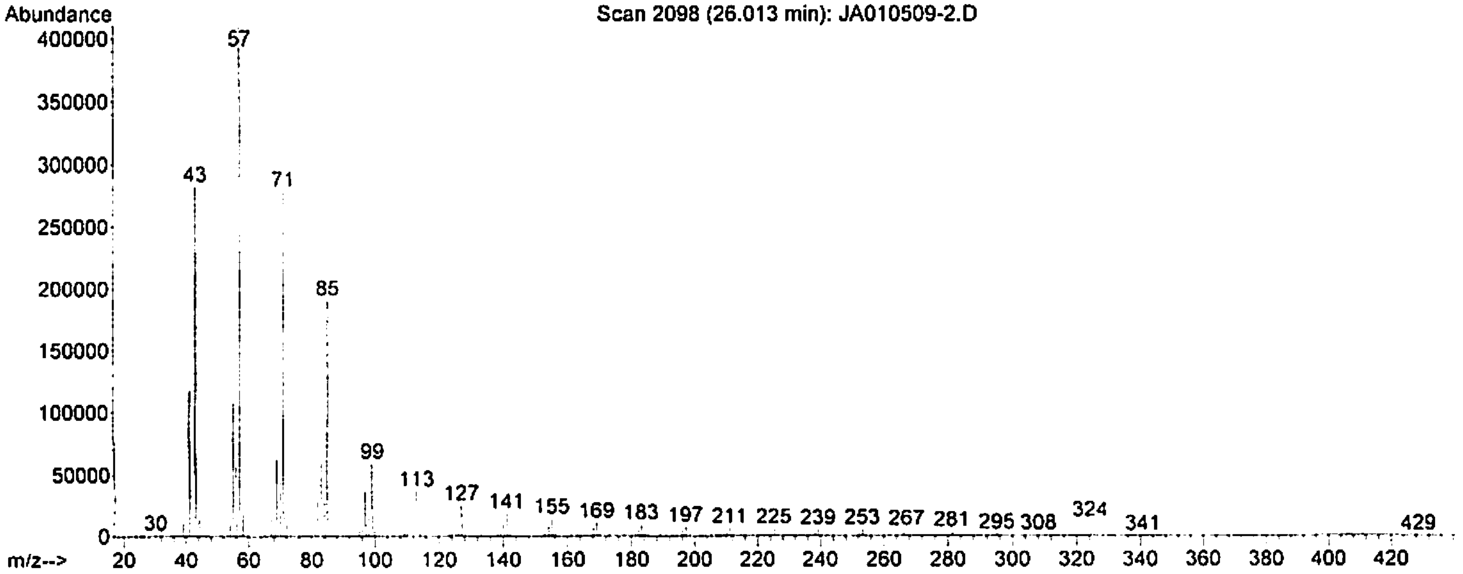

File : D:\DATA\Aldrich\JA-09\JA010509-2.D
Operator : Aldrich
Acquired : 5 Jan 2009 14:37 using AcqMethod JA-WAX08.M
Instrument : Instrument #1
Sample Name: 8 male C.oculata abd.sternites/CH2Cl2
Misc Info : w/ 1ug linalool/ul water/6 days; 2nd half
Vial Number: 1

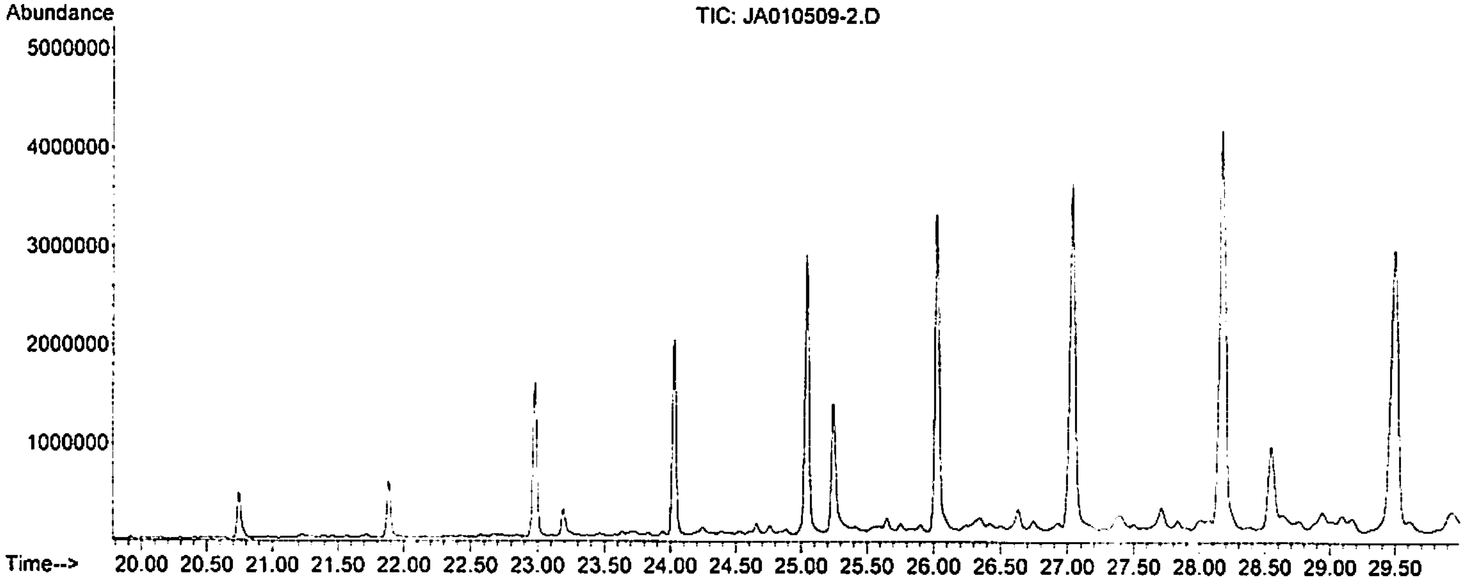


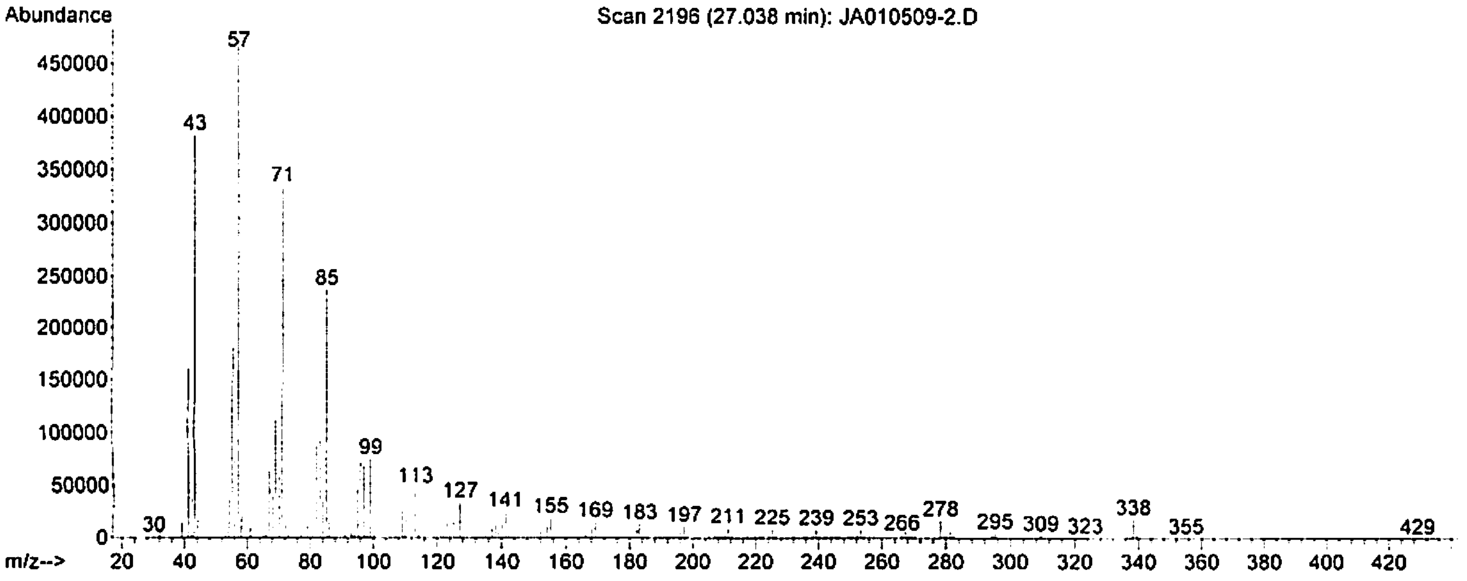

[illegible]le :D:\DATA\Aldrich\JA-09\JA010509-2.D
[illegible]erator : Aldrich
[illegible]quired : 5 Jan 2009 14:37 using AcqMethod JA-WAX08.M
[illegible]strument : Instrument #1
[illegible]mple Name: 8 male C.oculata abd.sternites/CH2Cl2
[illegible]sc Info : w/ 1ug linalool/ul water/6 days; 2nd half
[illegible]al Number: 1

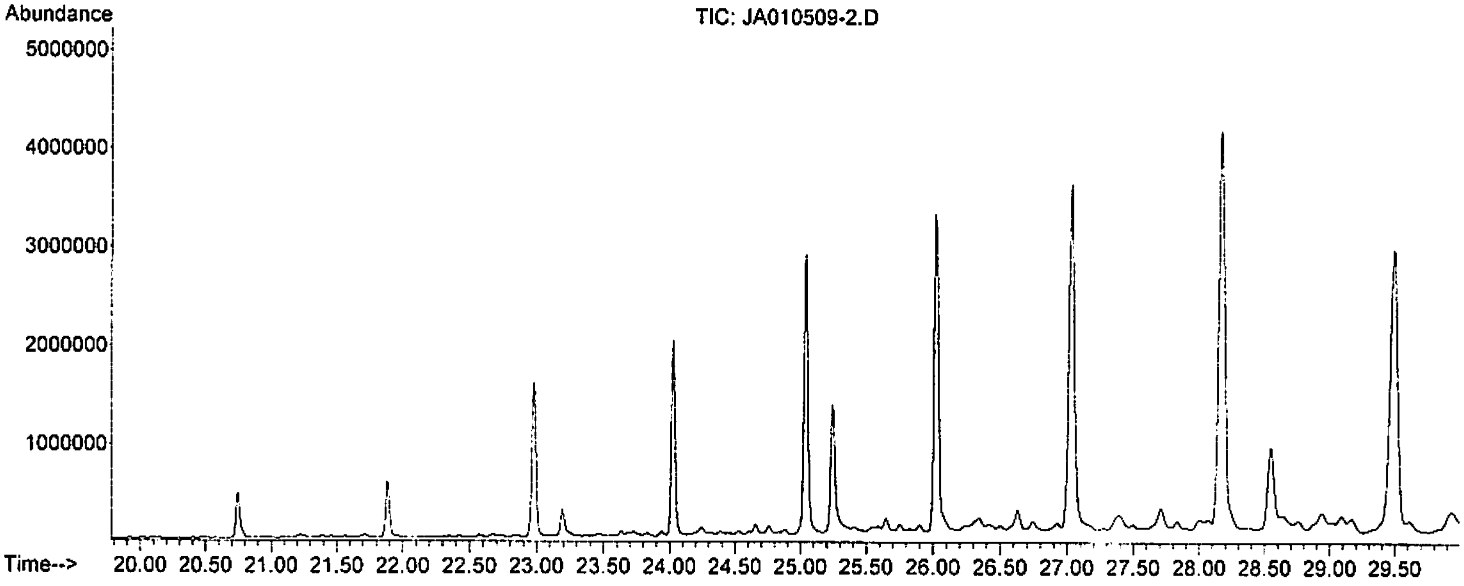


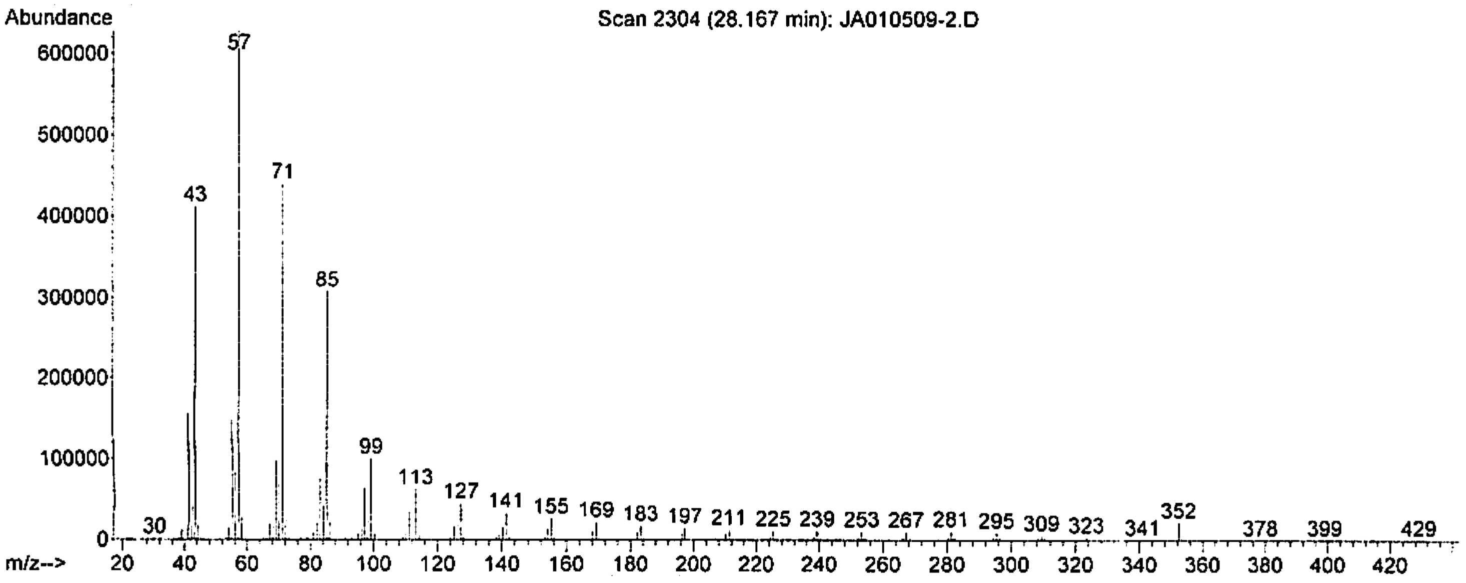

File :D:\DATA\Aldrich\JA-09\JA010509-2.D
Operator : Aldrich
Acquired : 5 Jan 2009 14:37 using AcqMethod JA-WAX08.M
Instrument : Instrument #1
Sample Name: 8 male C.oculata abd.sternites/CH2Cl2
Misc Info : w/ 1ug linalool/ul water/6 days; 2nd half
Vial Number: 1

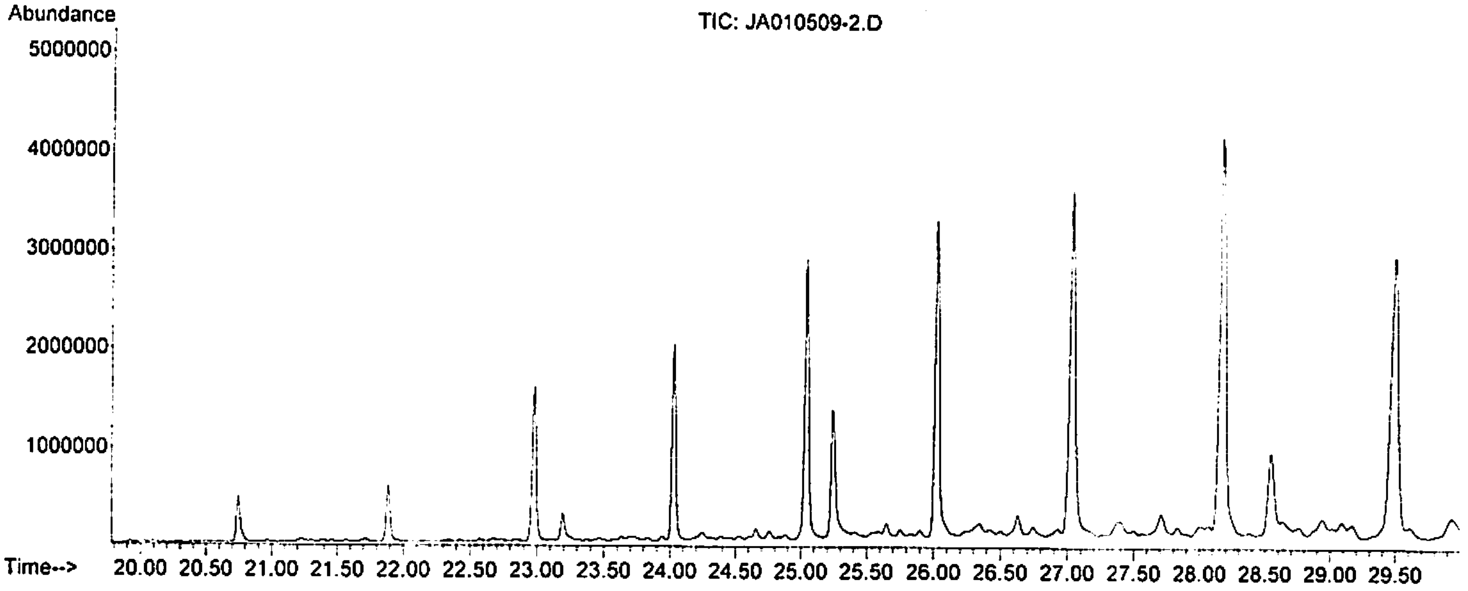


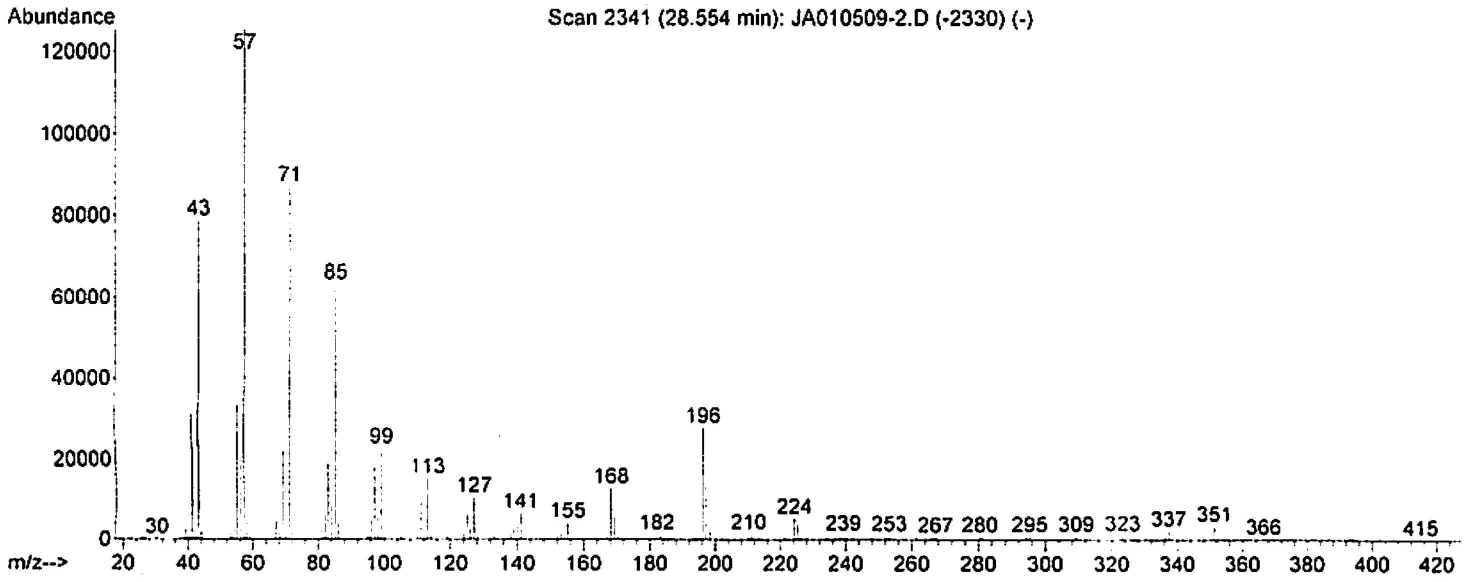

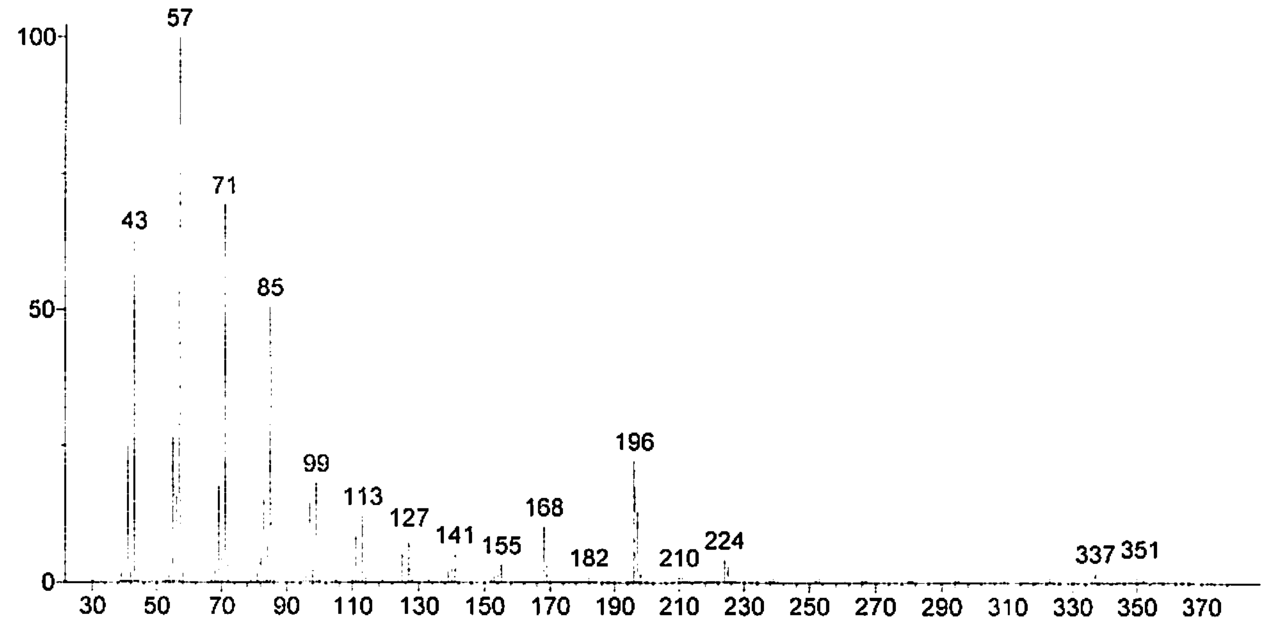


(Text File) Scan 2341 (28.554 min): JA010509-2.D (-2330)

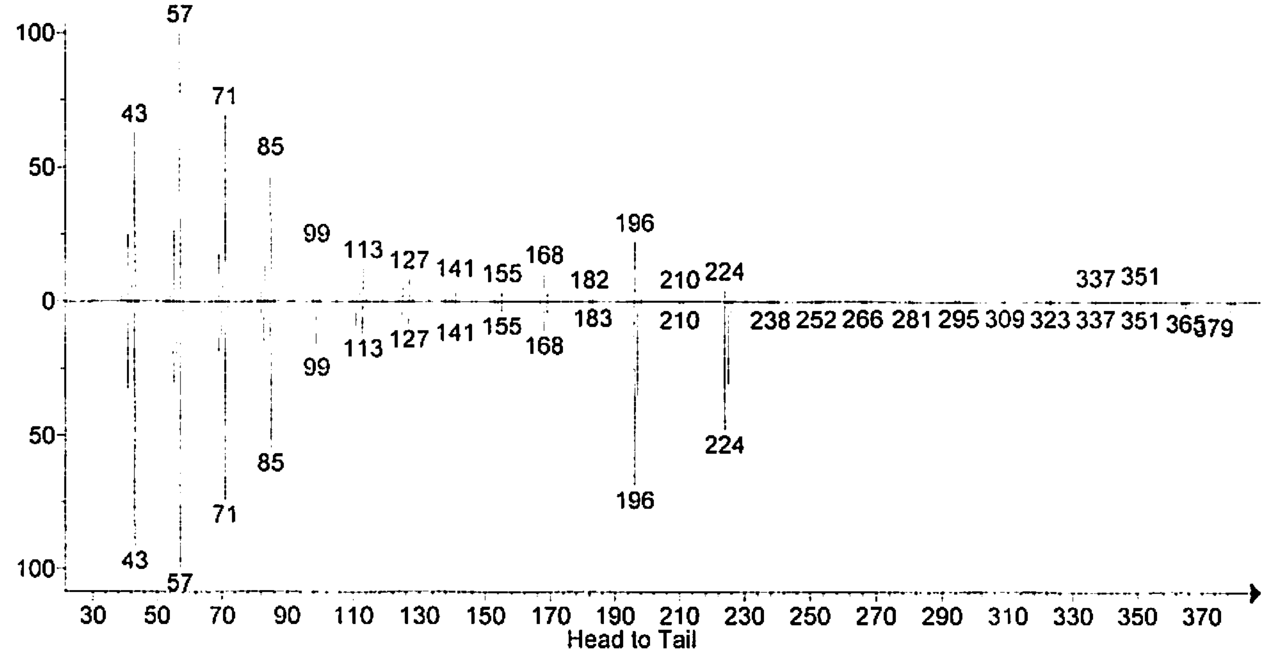


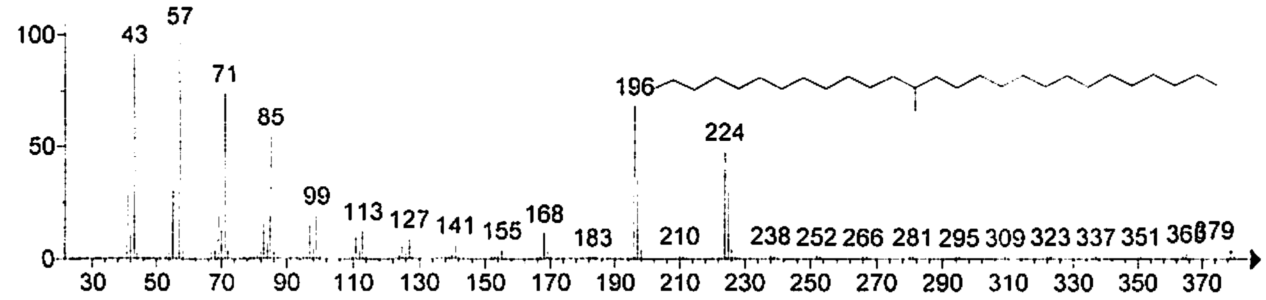


(mainlib) 13-Methylheptacosane

File : D:\DATA\Aldrich\JA-09\JA010509-2.D
Operator : Aldrich
Acquired : 5 Jan 2009 14:37 using AcqMethod JA-WAX08.M
Instrument : Instrument #1
Sample Name: 8 male C.oculata abd.sternites/CH2Cl2
Misc Info : w/ 1ug linalool/ul water/6 days; 2nd half
Vial Number: 1

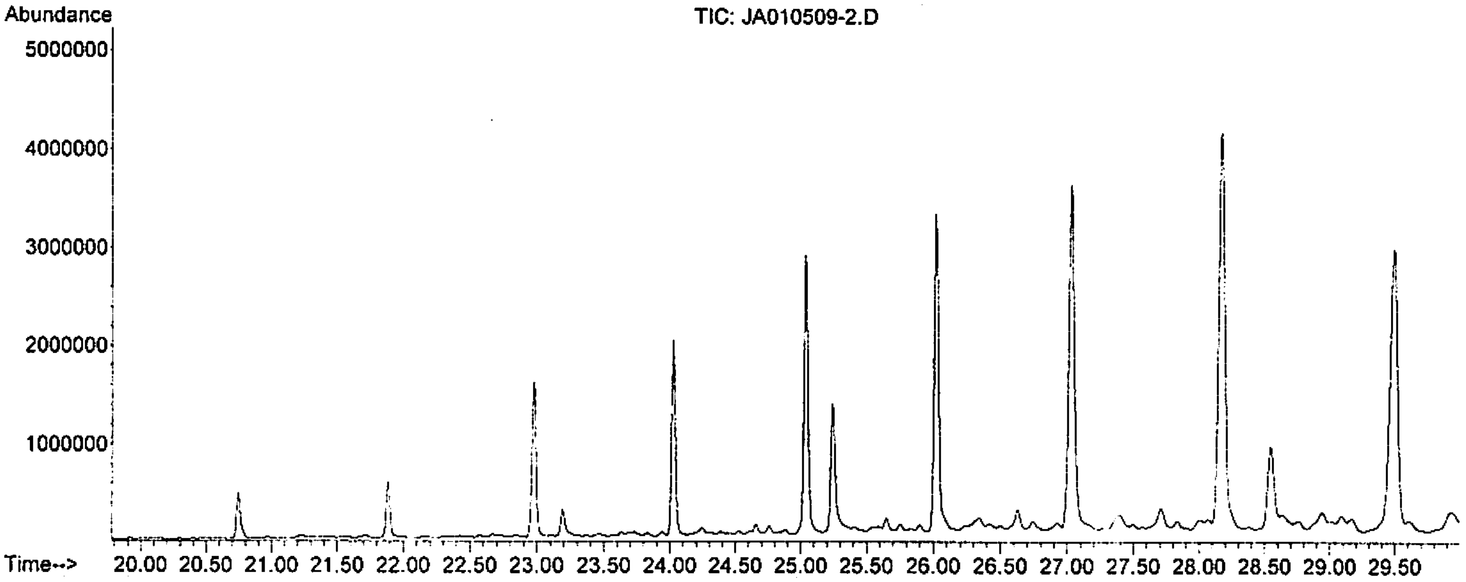


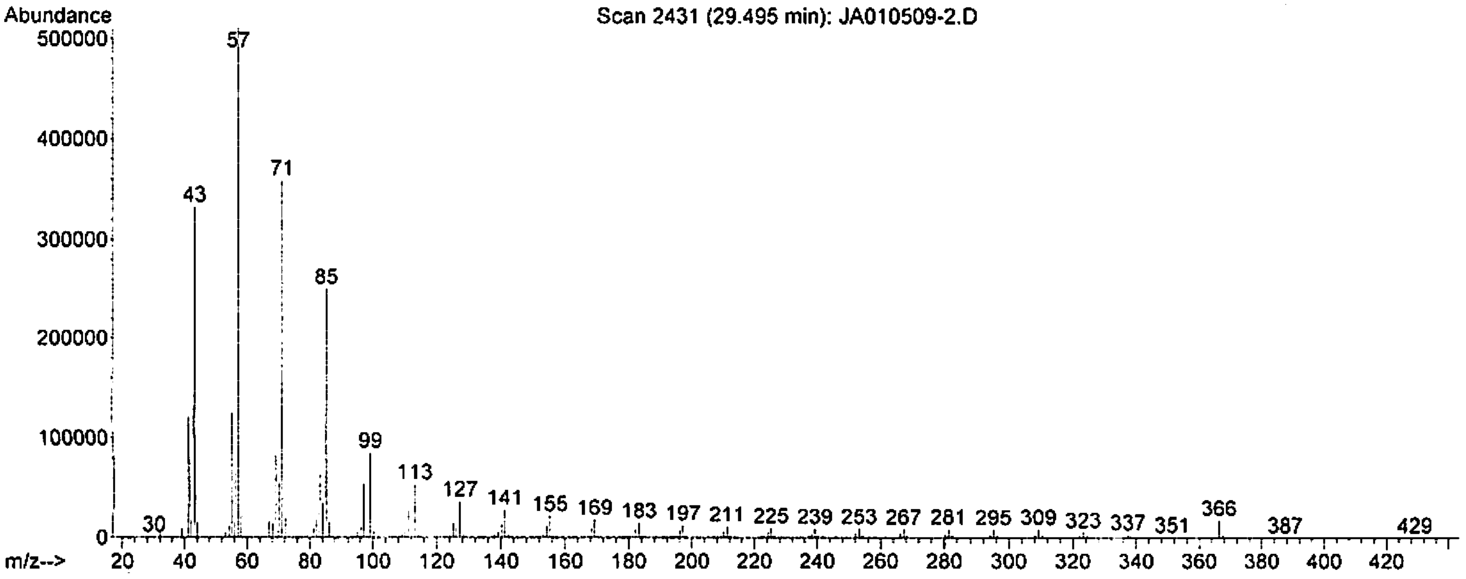

```
File        :D:\DATA\Aldrich\JA-09\JA071010-2.D
Operator    : Aldrich
Acquired    : 10 Jul 2009  17:18     using AcqMethod JA-WAX09.M
Instrument :    Instrument #1
Sample Name: 10 lab male 2wk-old fed nepetalactol 7/1-10
Misc Info   : JA071009-1 & here C.oculata; rev.diss order
Vial Number: 1
```

emerged: 06/15-17/09 & fed 7/1 & diss. 7/10 }
23-25 days old

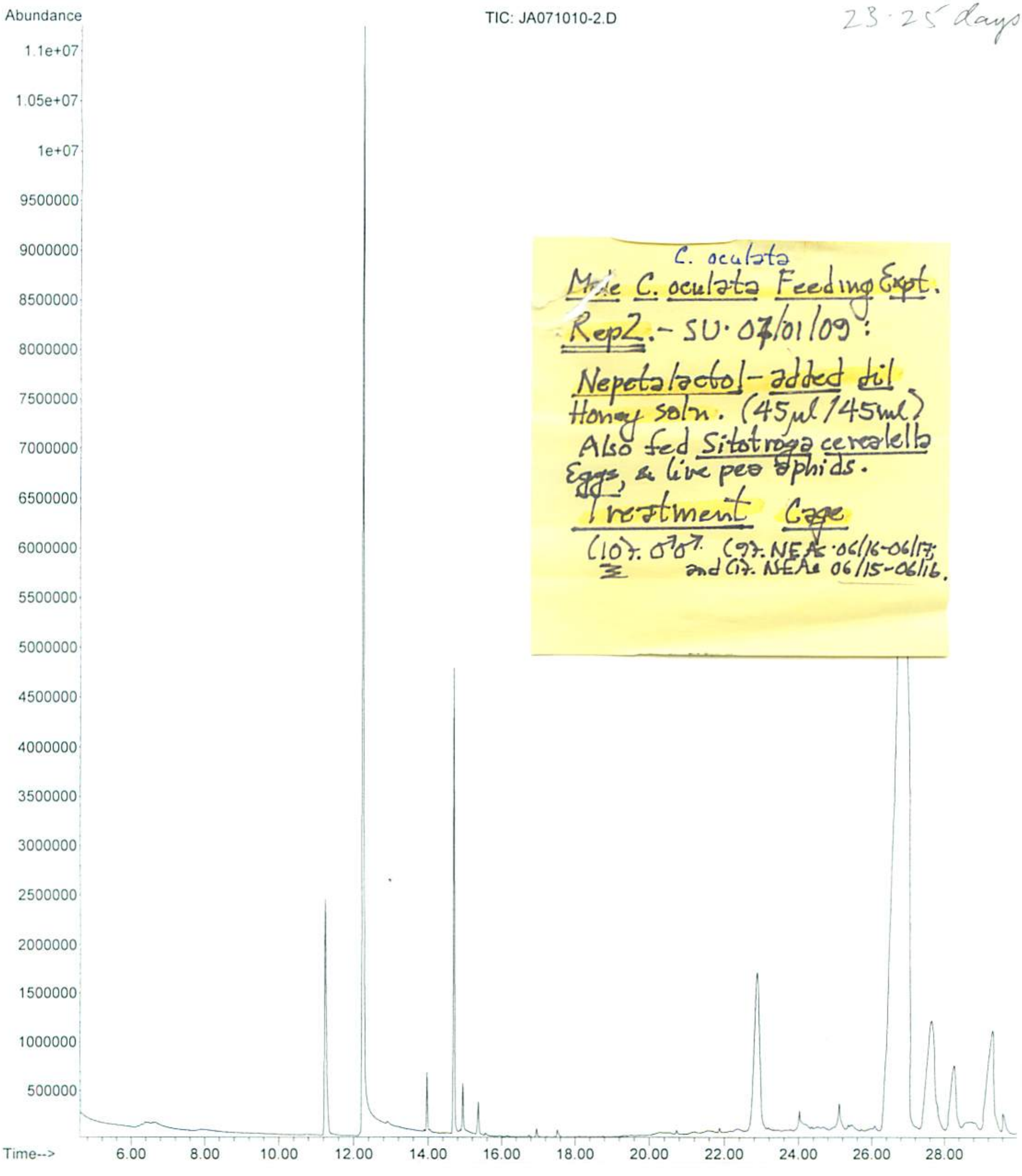

File :D:\DATA\ALDRICH\JA-09\Snapshot\JA071010-2.D
Operator : Aldrich
Acquired : 10 Jul 2009 17:18 using AcqMethod JA-WAX09.M
Instrument : Instrument #1
Sample Name: 10 lab male 2wk-old fed nepetalactol 7/1-10
Misc Info : JA071009-1 & here C.oculata; rev.diss order
Vial Number: 1

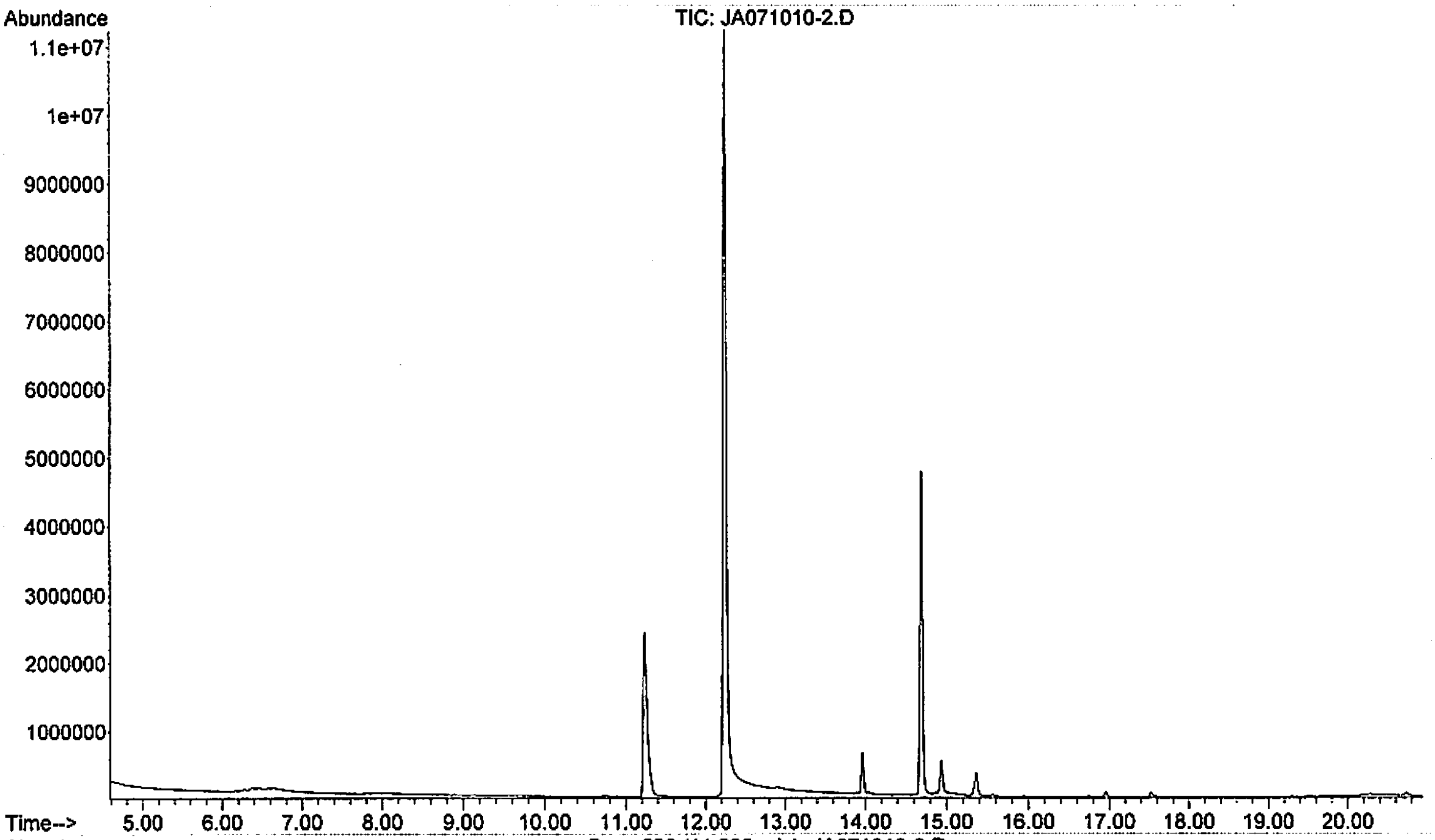


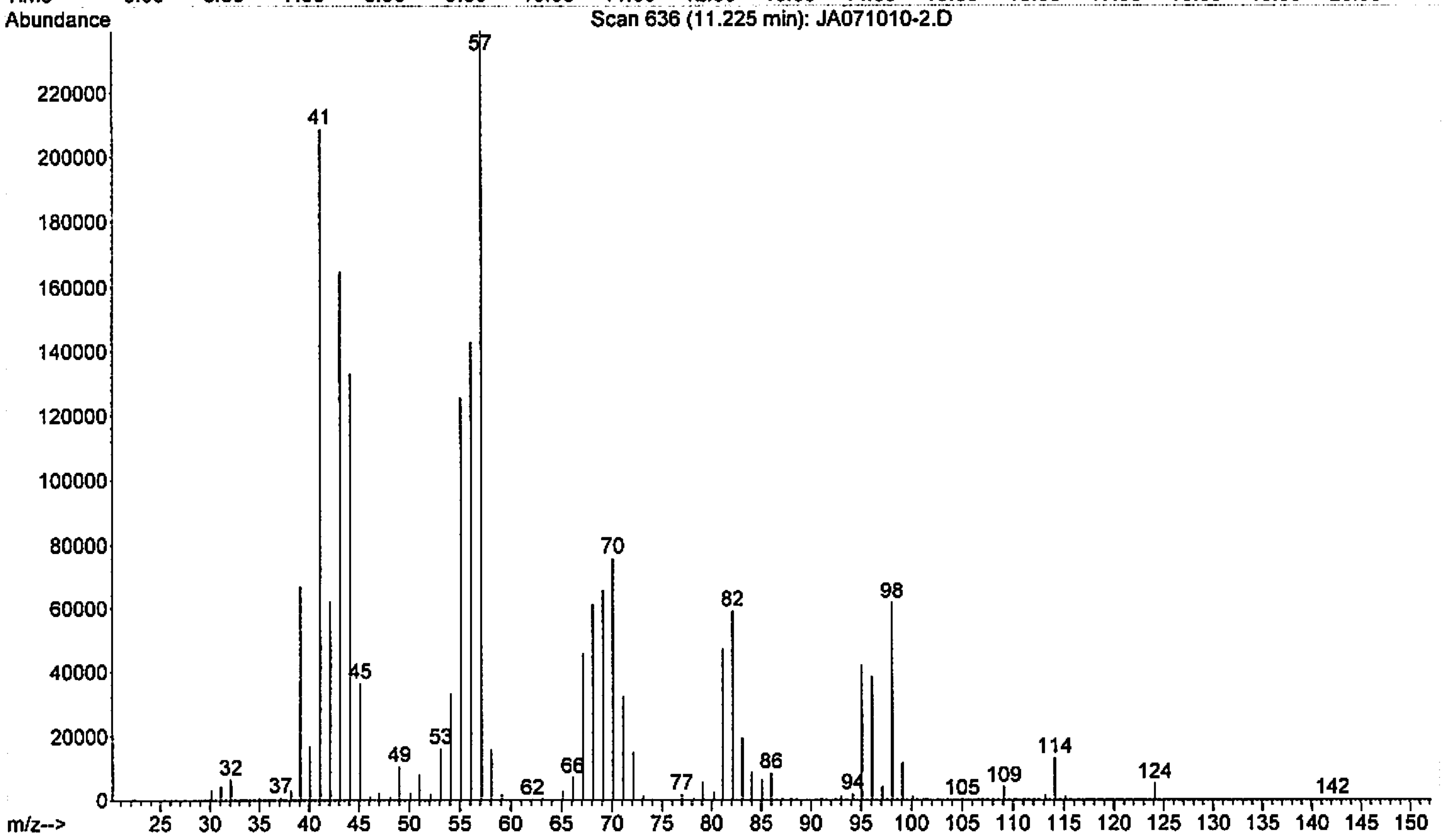

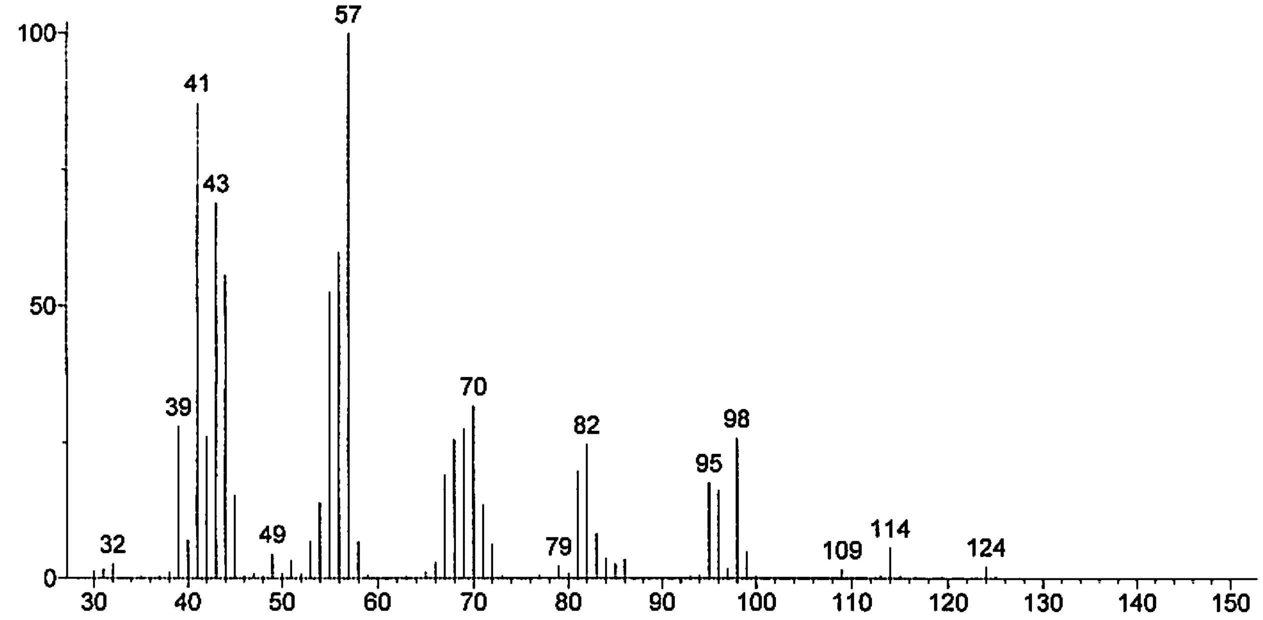

(Text File) Scan 636 (11.225 min): JA071010-2.D

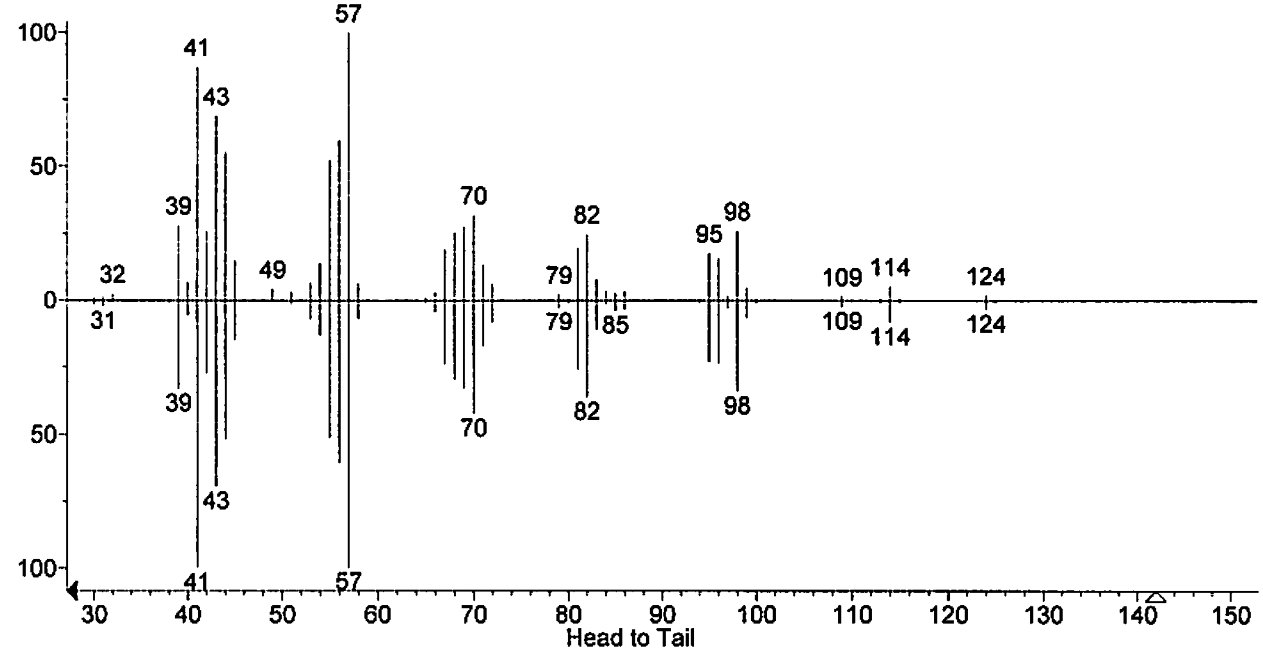


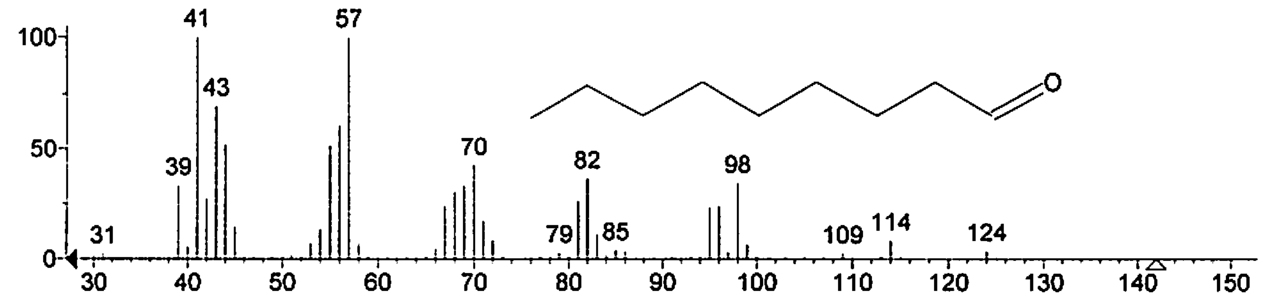

(replib) Nonanal

File       :D:\DATA\ALDRICH\JA-09\Snapshot\JA071010-2.D
Operator   : Aldrich
Acquired   : 10 Jul 2009  17:18      using AcqMethod JA-WAX09.M
Instrument :    Instrument #1
Sample Name: 10 lab male 2wk-old fed nepetalactol 7/1-10
Misc Info  : JA071009-1 & here C.oculata; rev.diss order
Vial Number: 1

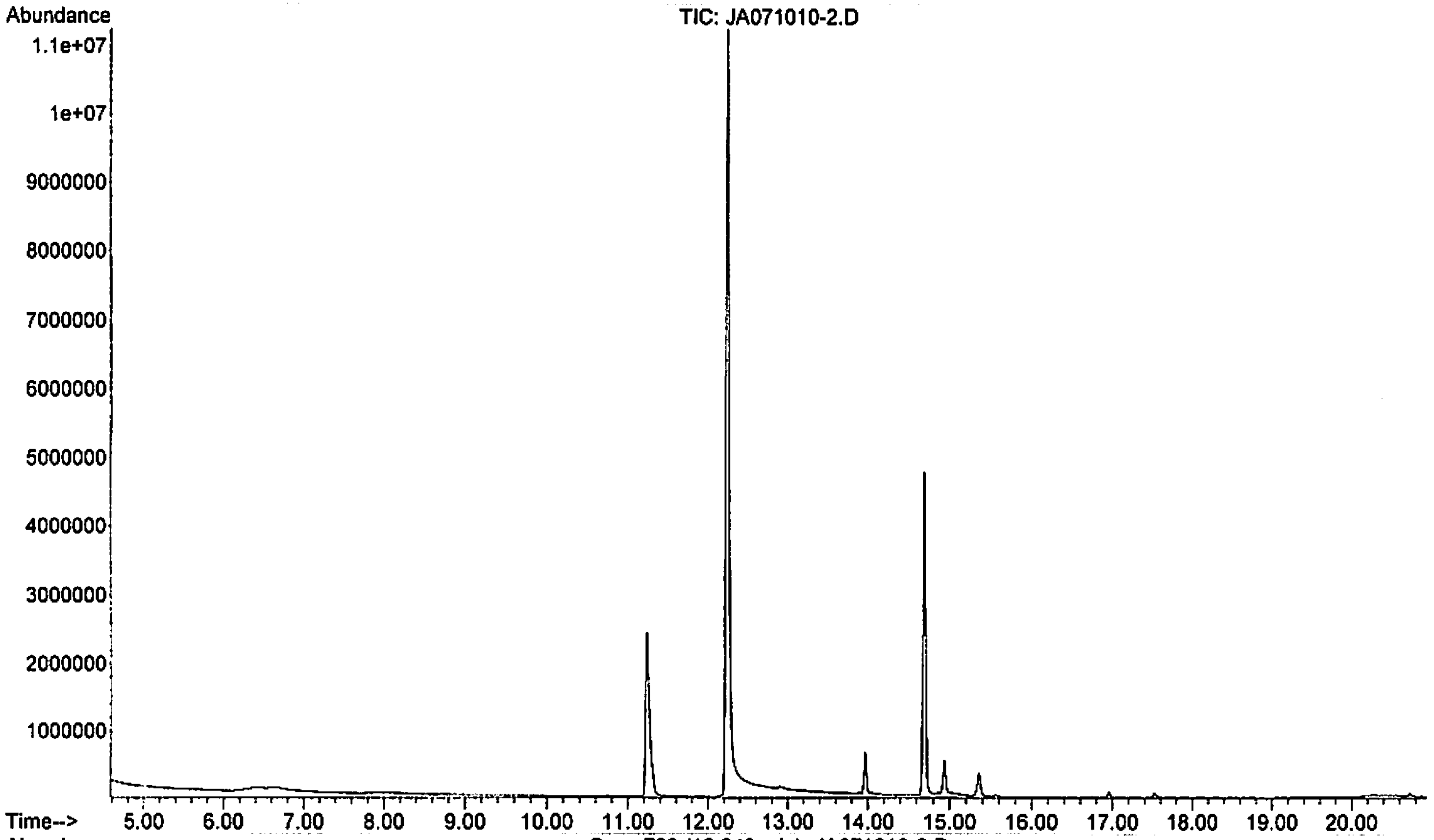


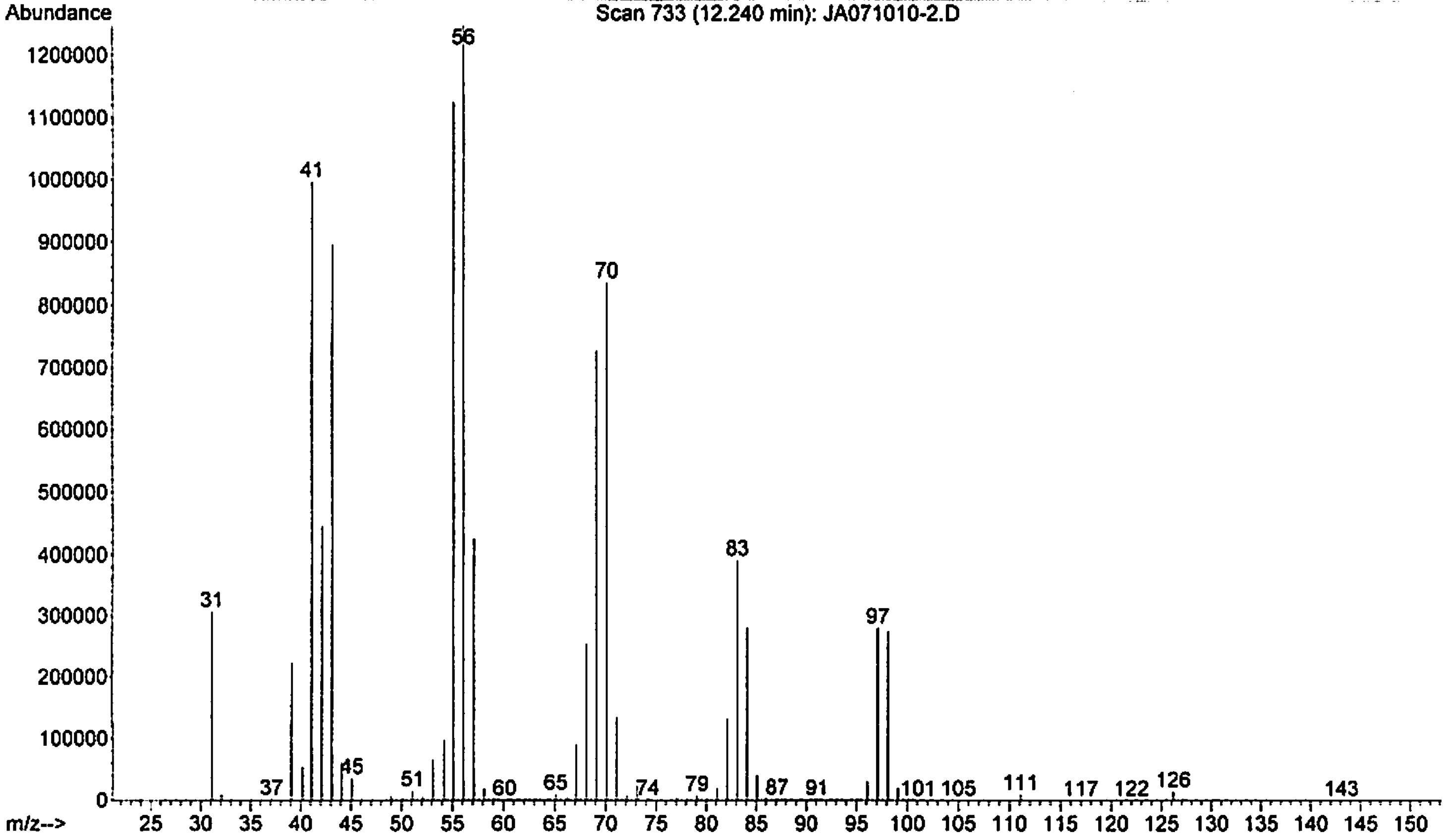

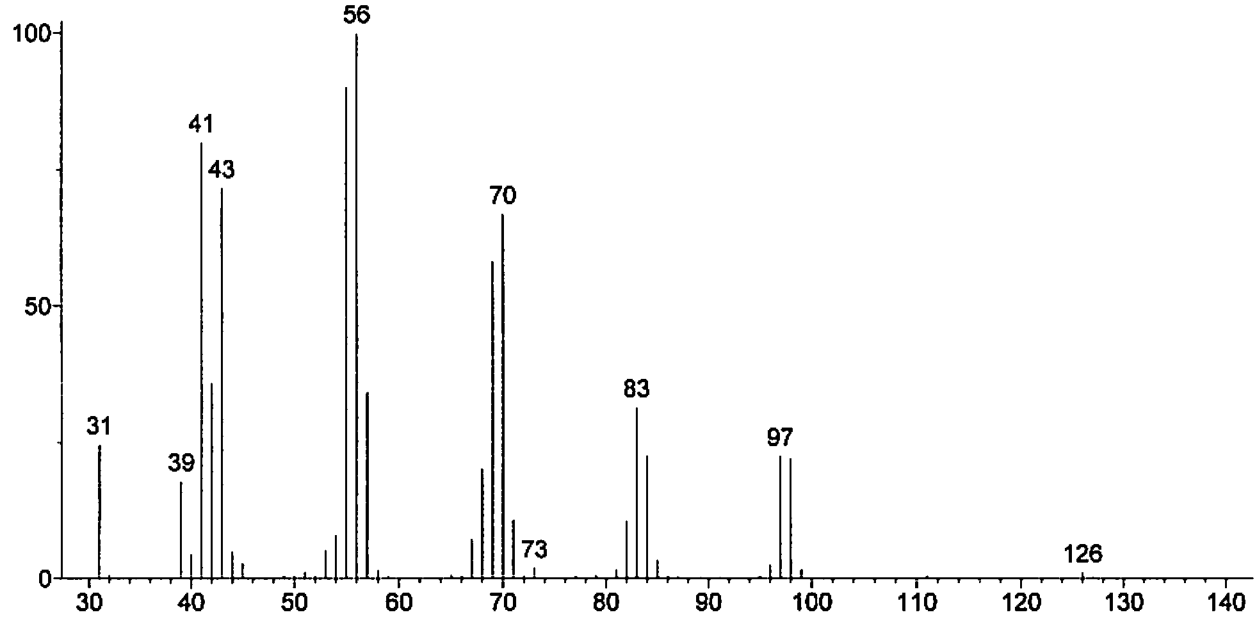


(Text File) Scan 733 (12.240 min): JA071010-2.D

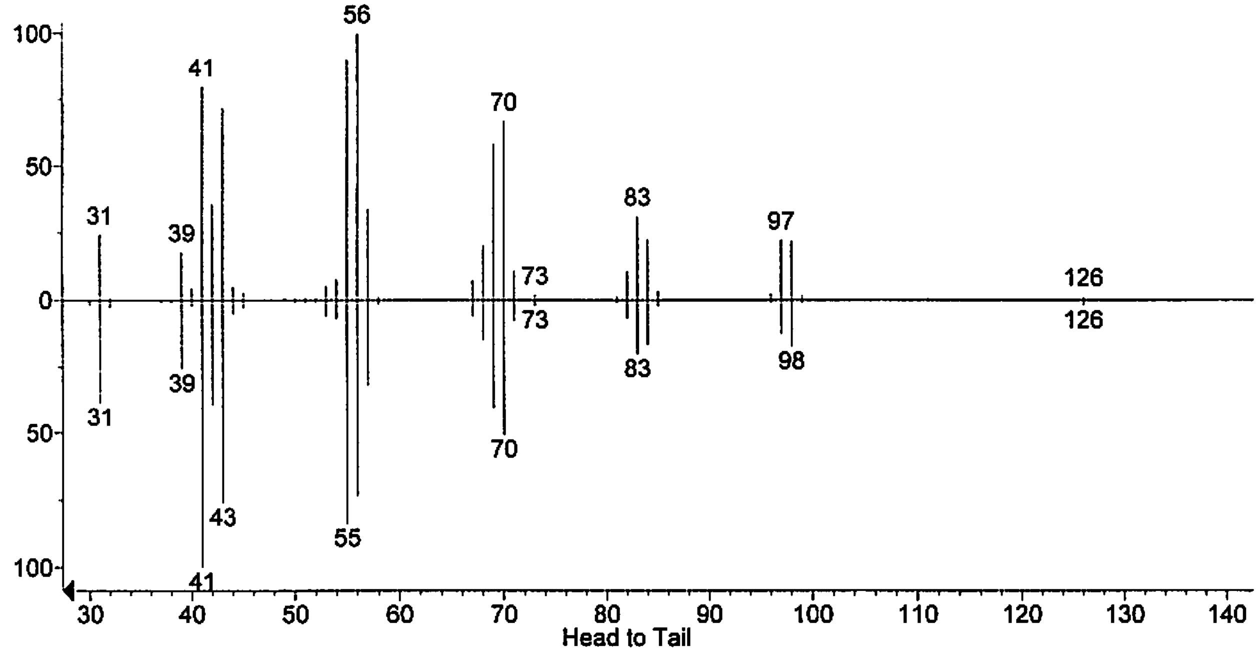


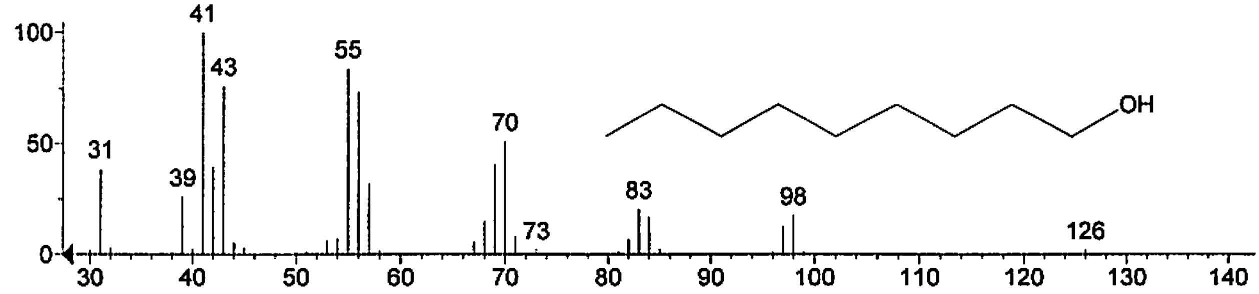


(replib) 1-Nonanol

File :D:\DATA\ALDRICH\JA-09\Snapshot\JA071010-2.D
Operator : Aldrich
Acquired : 10 Jul 2009 17:18 using AcqMethod JA-WAX09.M
Instrument : Instrument #1
Sample Name: 10 lab male 2wk-old fed nepetalactol 7/1-10
Misc Info : JA071009-1 & here C.oculata; rev.diss order
Vial Number: 1

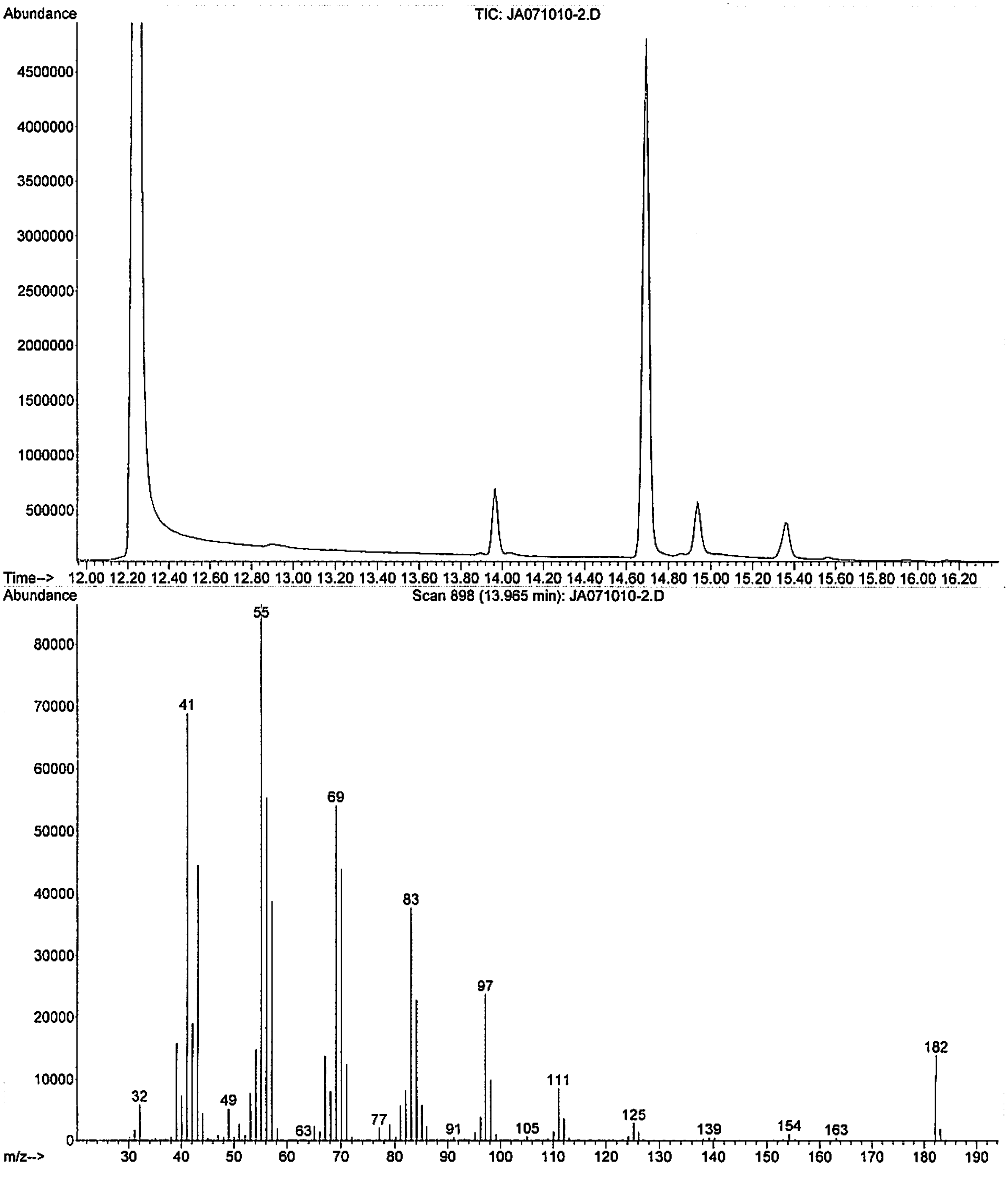

```
File        :D:\DATA\ALDRICH\JA-09\Snapshot\JA071010-2.D
Operator    : Aldrich
Acquired    : 10 Jul 2009  17:18     using AcqMethod JA-WAX09.M
Instrument  :    Instrument #1
Sample Name: 10 lab male 2wk-old fed nepetalactol 7/1-10
Misc Info   : JA071009-1 & here C.oculata; rev.diss order
Vial Number: 1
```

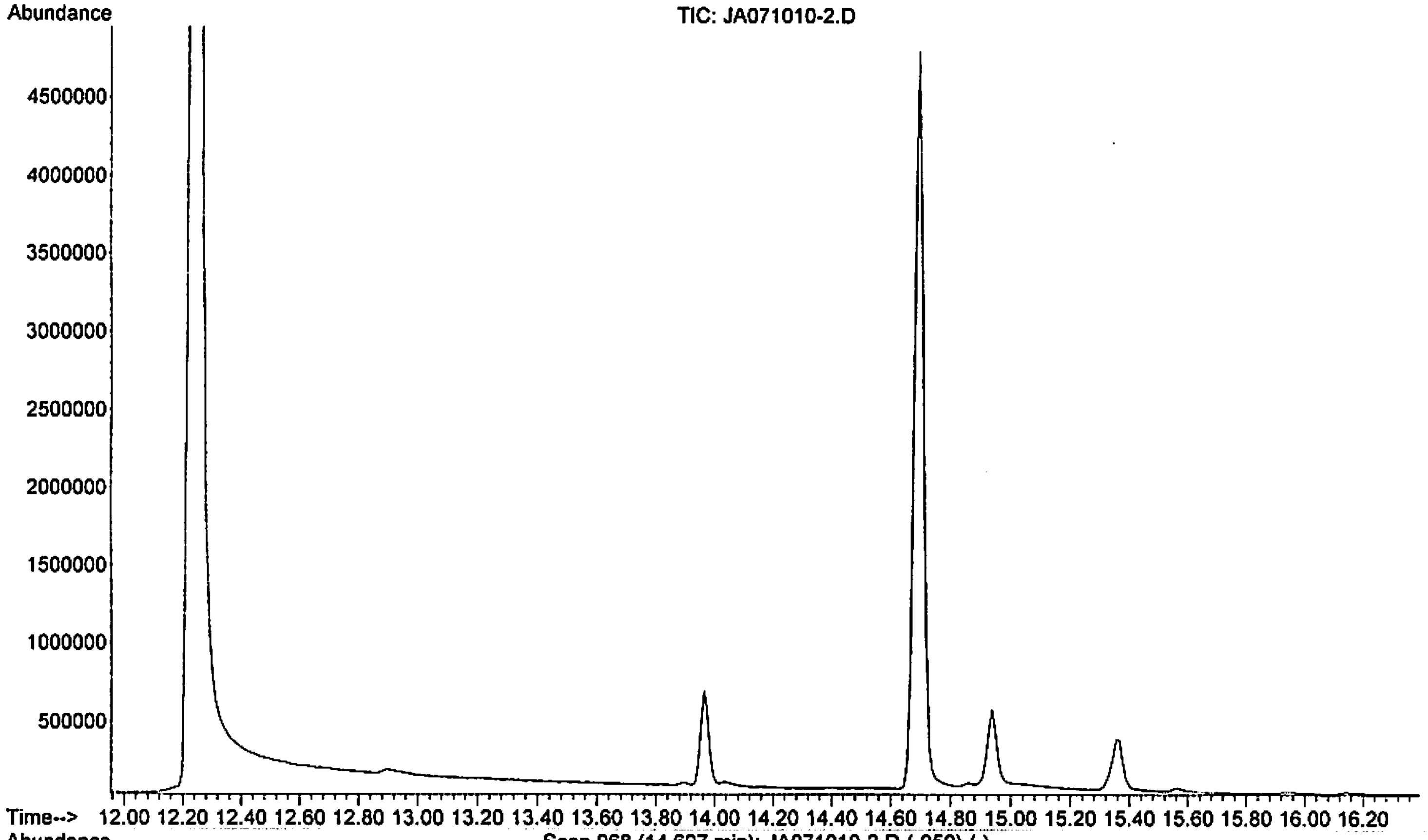


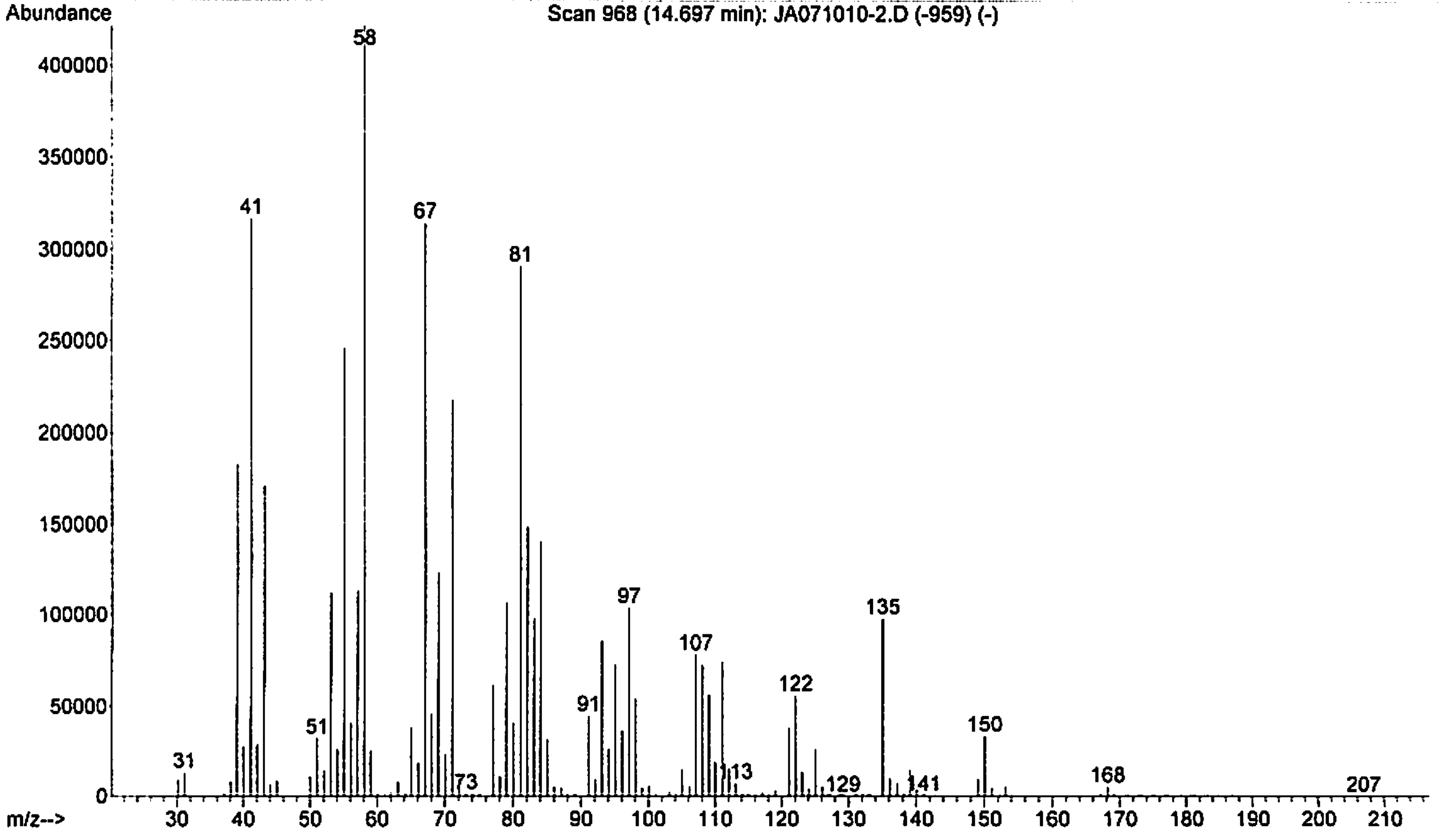

```
File         :D:\DATA\ALDRICH\JA-09\Snapshot\JA071010-2.D
Operator     : Aldrich
Acquired     : 10 Jul 2009  17:18      using AcqMethod JA-WAX09.M
Instrument :     Instrument #1
Sample Name: 10 lab male 2wk-old fed nepetalactol 7/1-10
Misc Info    : JA071009-1 & here C.oculata; rev.diss order
Vial Number: 1
```

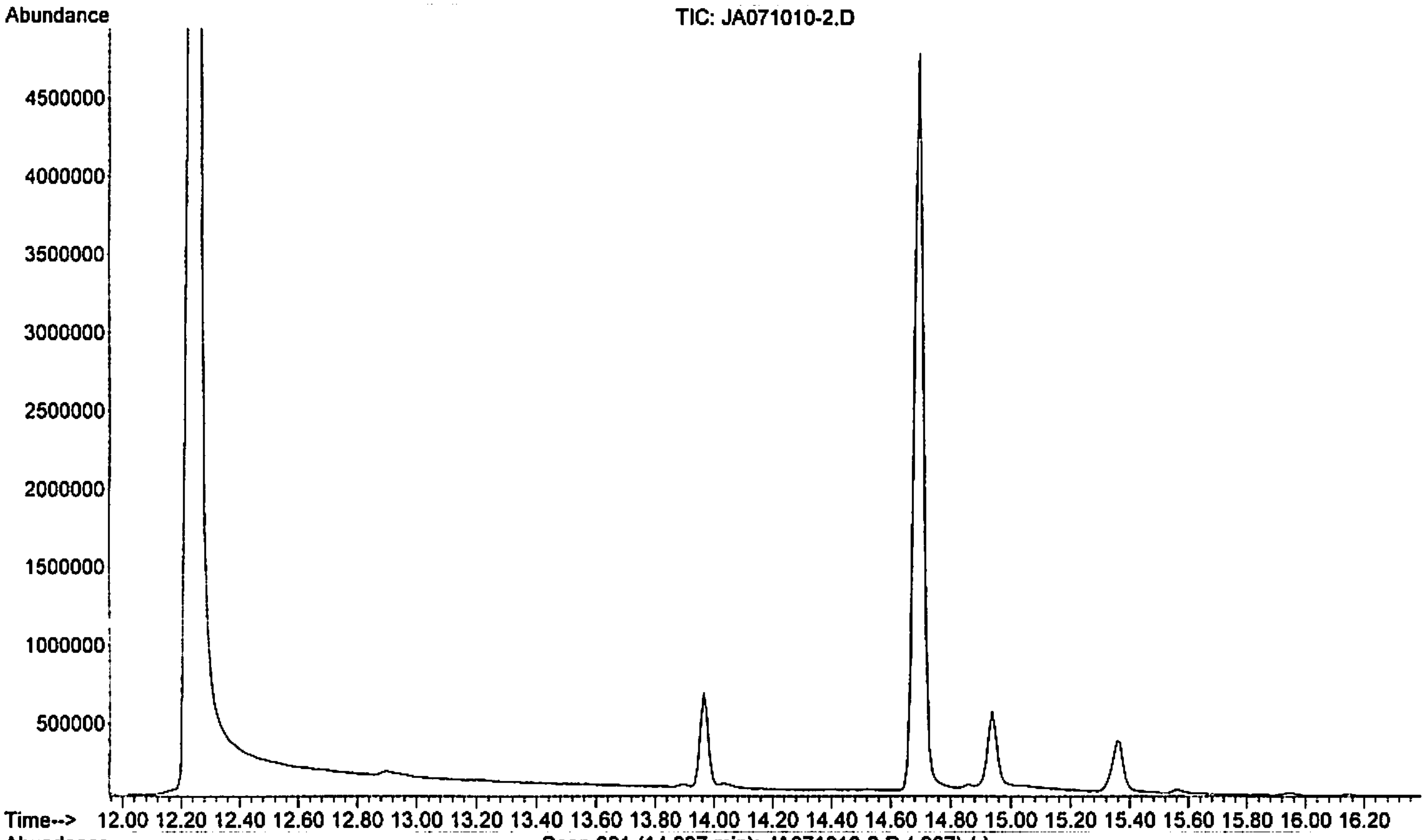


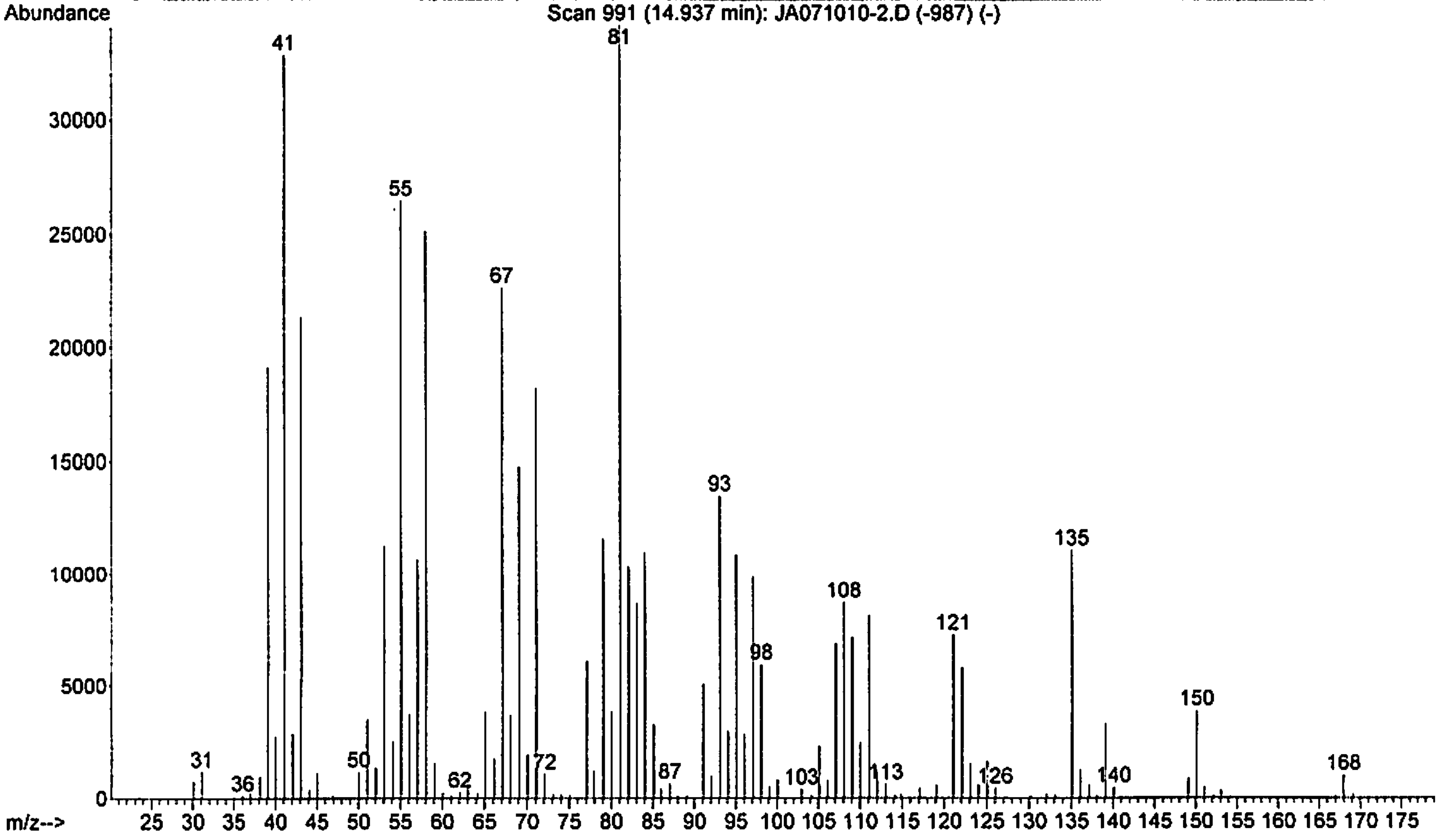

```
File         :D:\DATA\ALDRICH\JA-09\Snapshot\JA071010-2.D
Operator     : Aldrich
Acquired     : 10 Jul 2009  17:18     using AcqMethod JA-WAX09.M
Instrument :     Instrument #1
Sample Name: 10 lab male 2wk-old fed nepetalactol 7/1-10
Misc Info    : JA071009-1 & here C.oculata; rev.diss order
Vial Number: 1
```

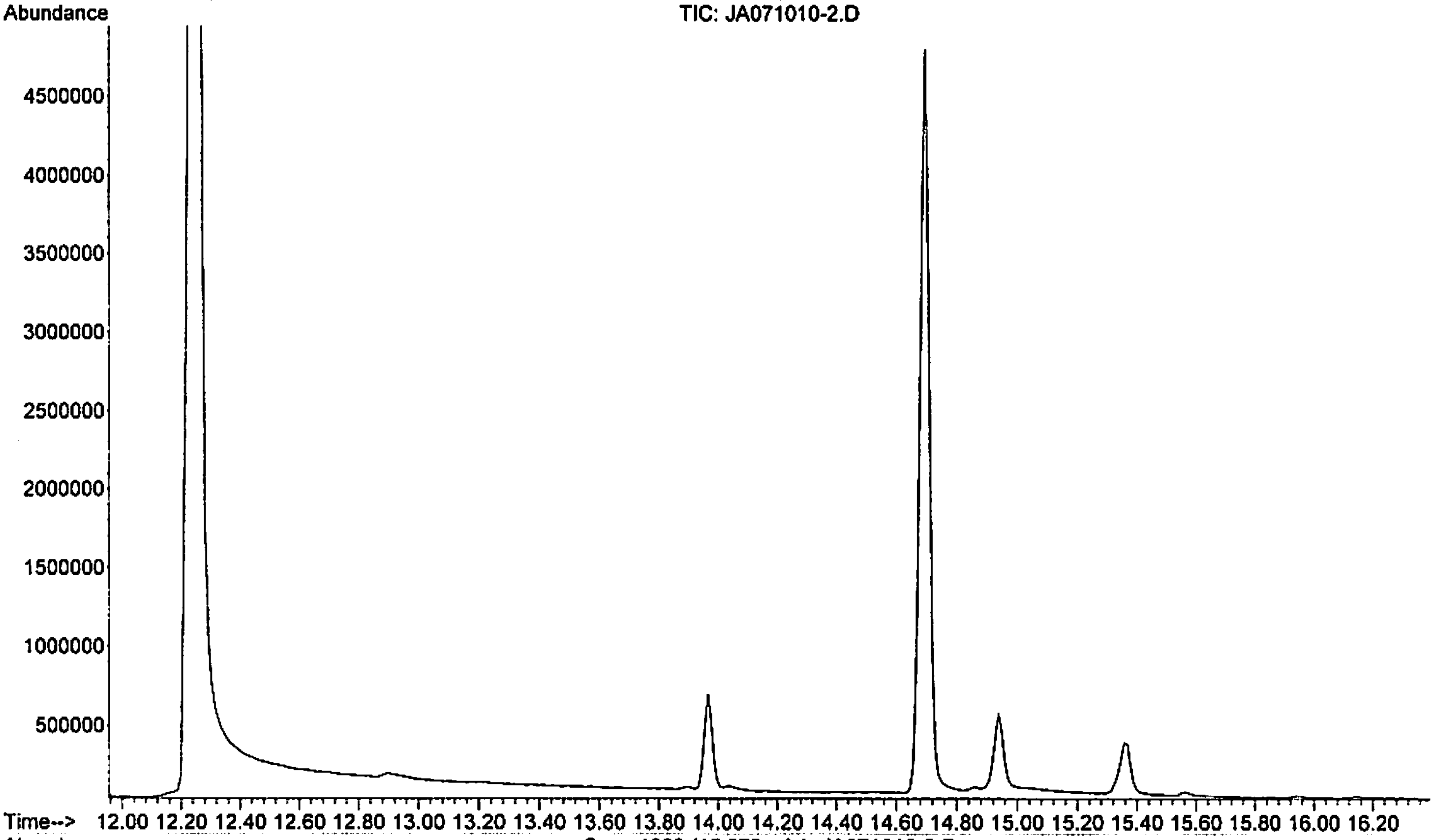


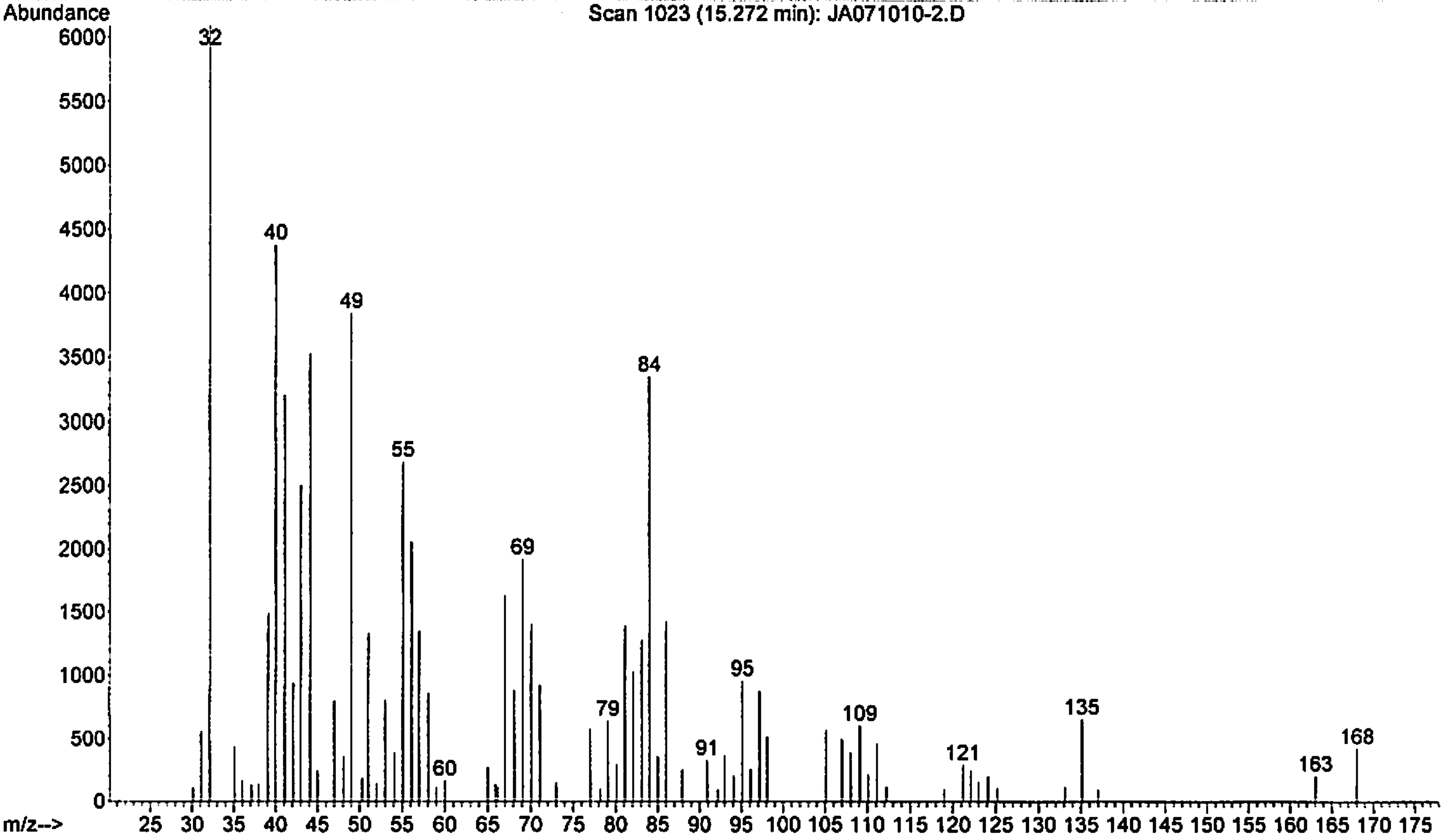

```
File        :D:\DATA\ALDRICH\JA-09\Snapshot\JA071010-2.D
Operator    : Aldrich
Acquired    : 10 Jul 2009  17:18     using AcqMethod JA-WAX09.M
Instrument  :    Instrument #1
Sample Name: 10 lab male 2wk-old fed nepetalactol 7/1-10
Misc Info   : JA071009-1 & here C.oculata; rev.diss order
Vial Number: 1
```

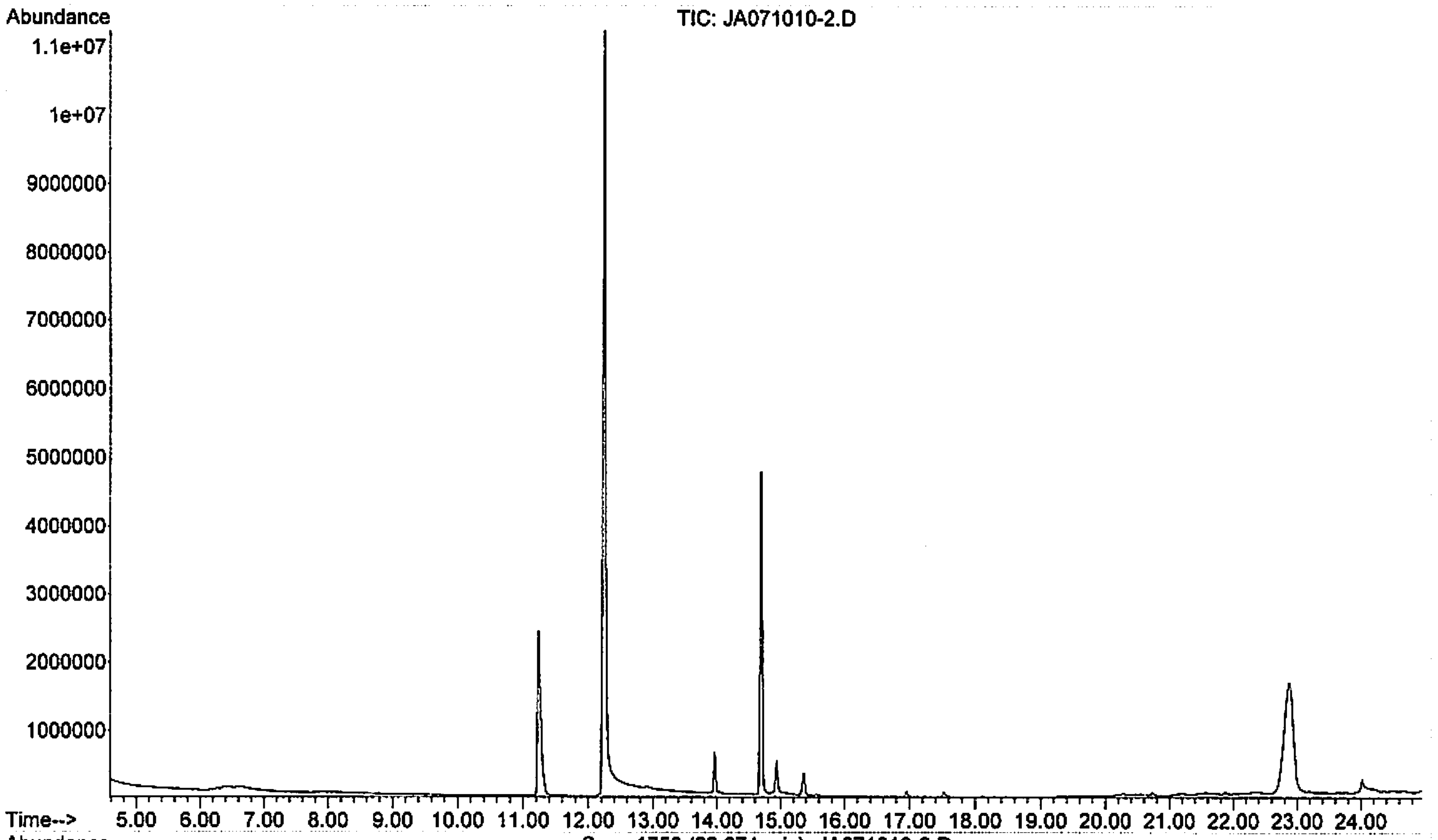


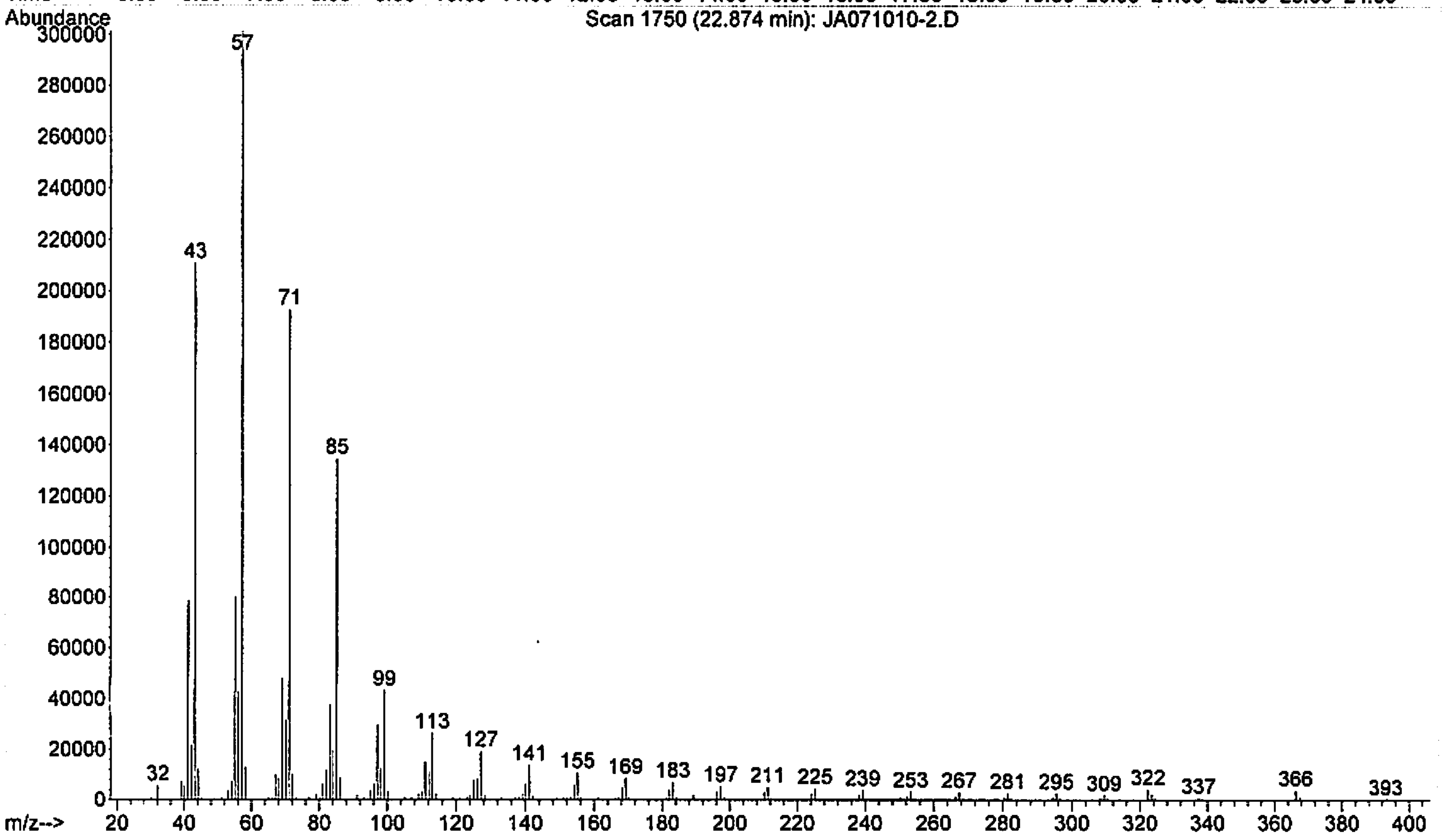

File          :D:\DATA\ALDRICH\JA-09\Snapshot\JA071010-2.D
Operator      : Aldrich
Acquired      : 10 Jul 2009   17:18      using AcqMethod JA-WAX09.M
Instrument :     Instrument #1
Sample Name: 10 lab male 2wk-old fed nepetalactol 7/1-10
Misc Info     : JA071009-1 & here C.oculata; rev.diss order
Vial Number: 1

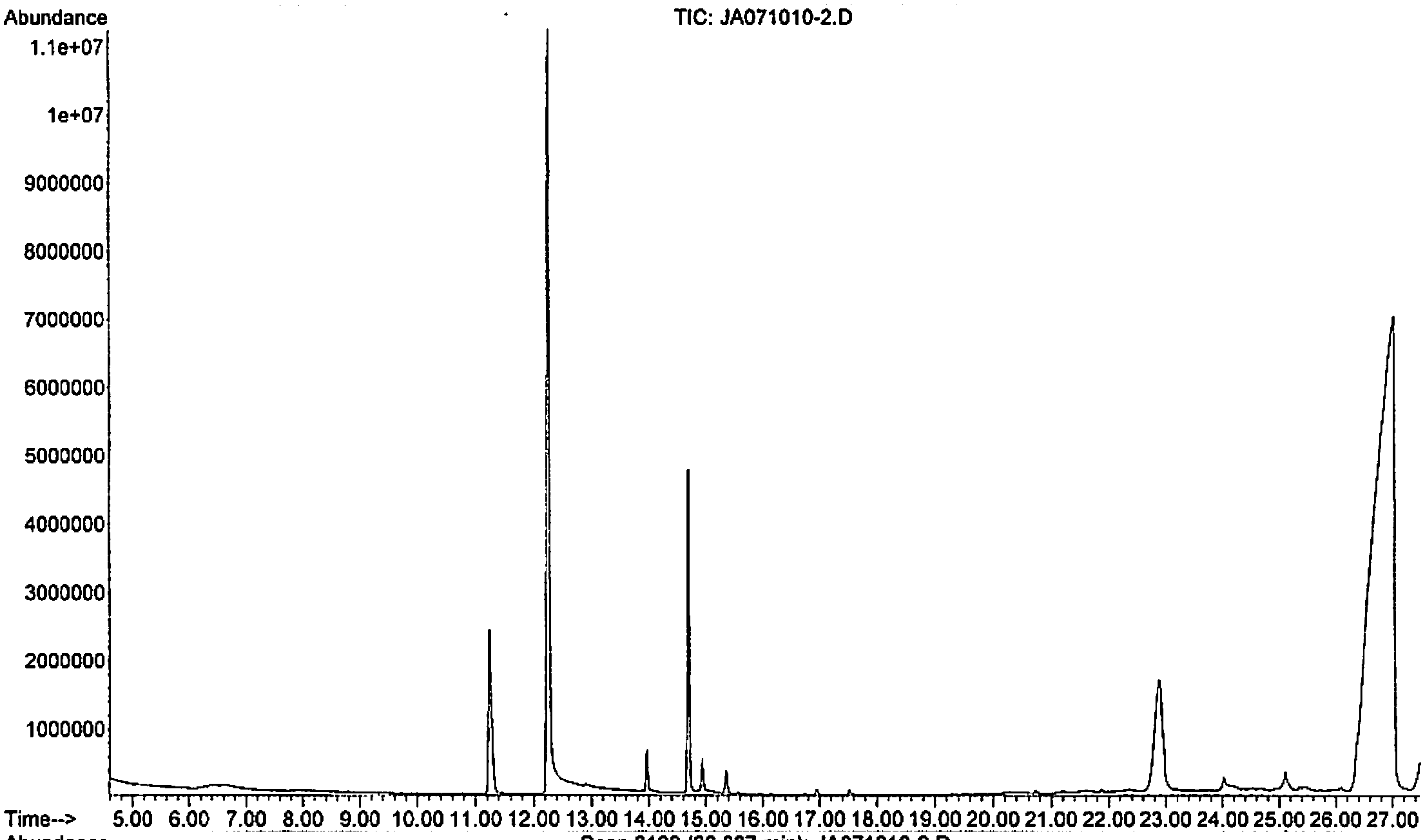


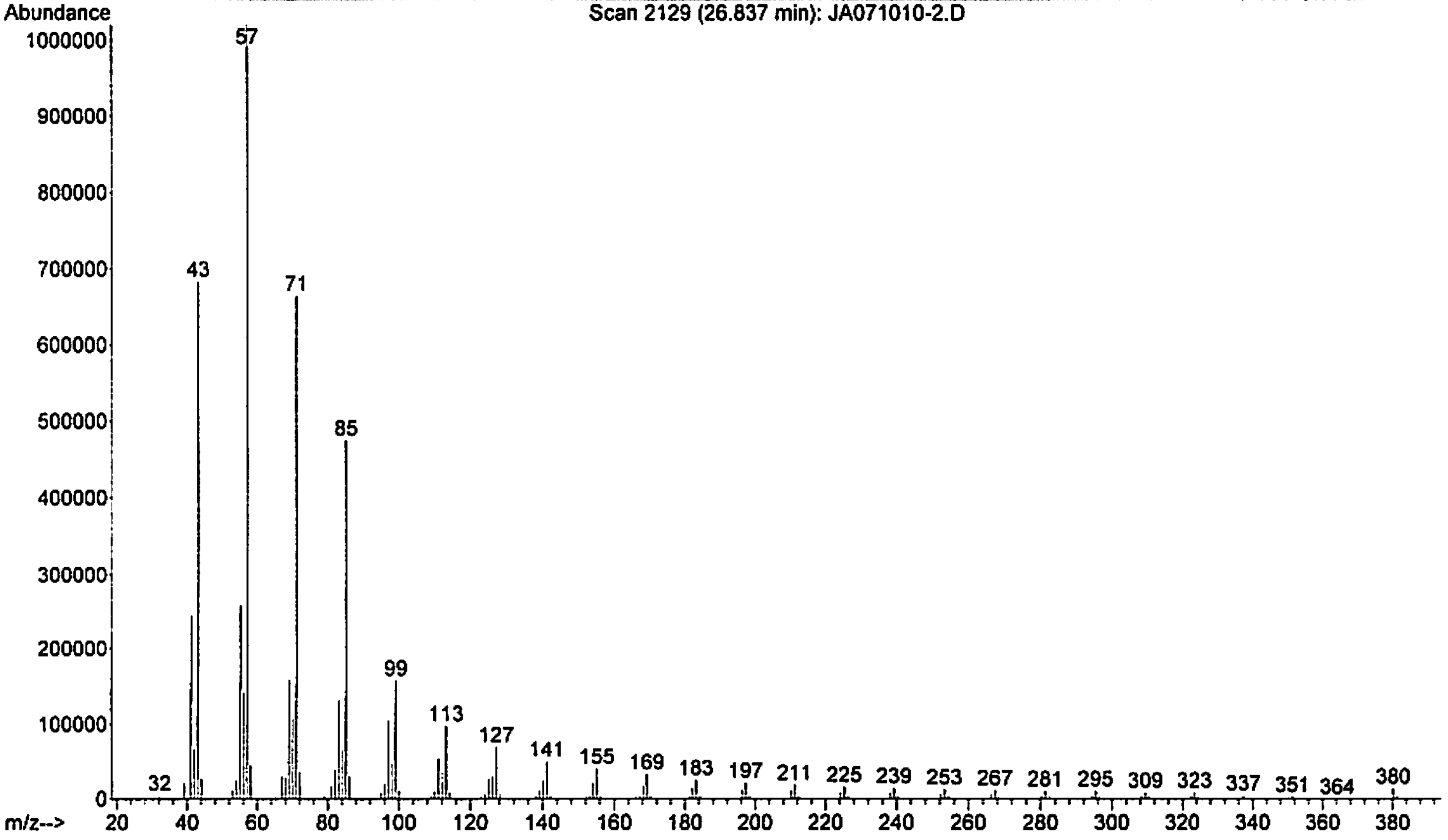

```
File        :D:\DATA\ALDRICH\JA-09\Snapshot\JA071010-2.D
Operator    : Aldrich
Acquired    : 10 Jul 2009  17:18     using AcqMethod JA-WAX09.M
Instrument  :     Instrument #1
Sample Name: 10 lab male 2wk-old fed nepetalactol 7/1-10
Misc Info   : JA071009-1 & here C.oculata; rev.diss order
Vial Number: 1
```

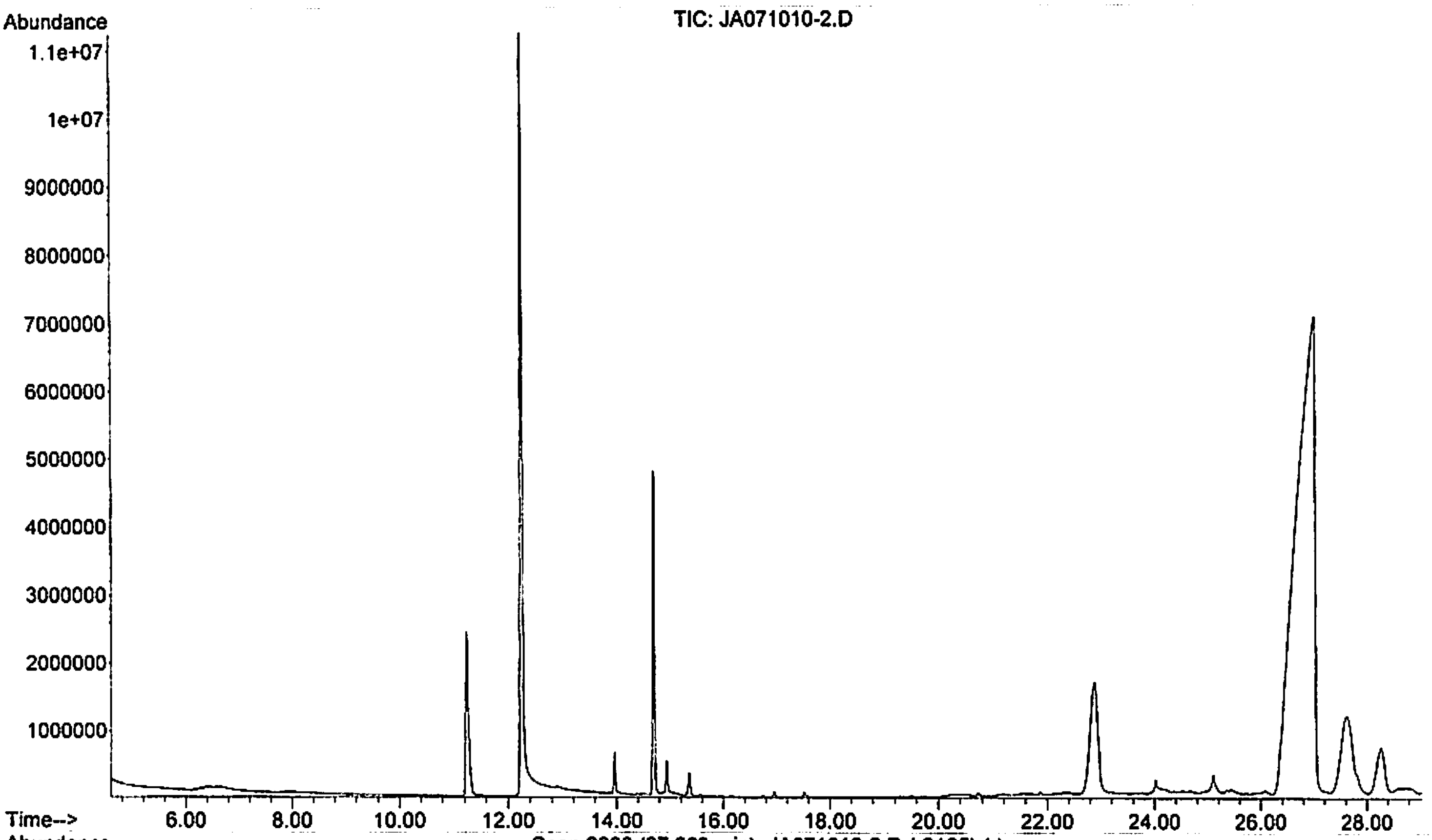


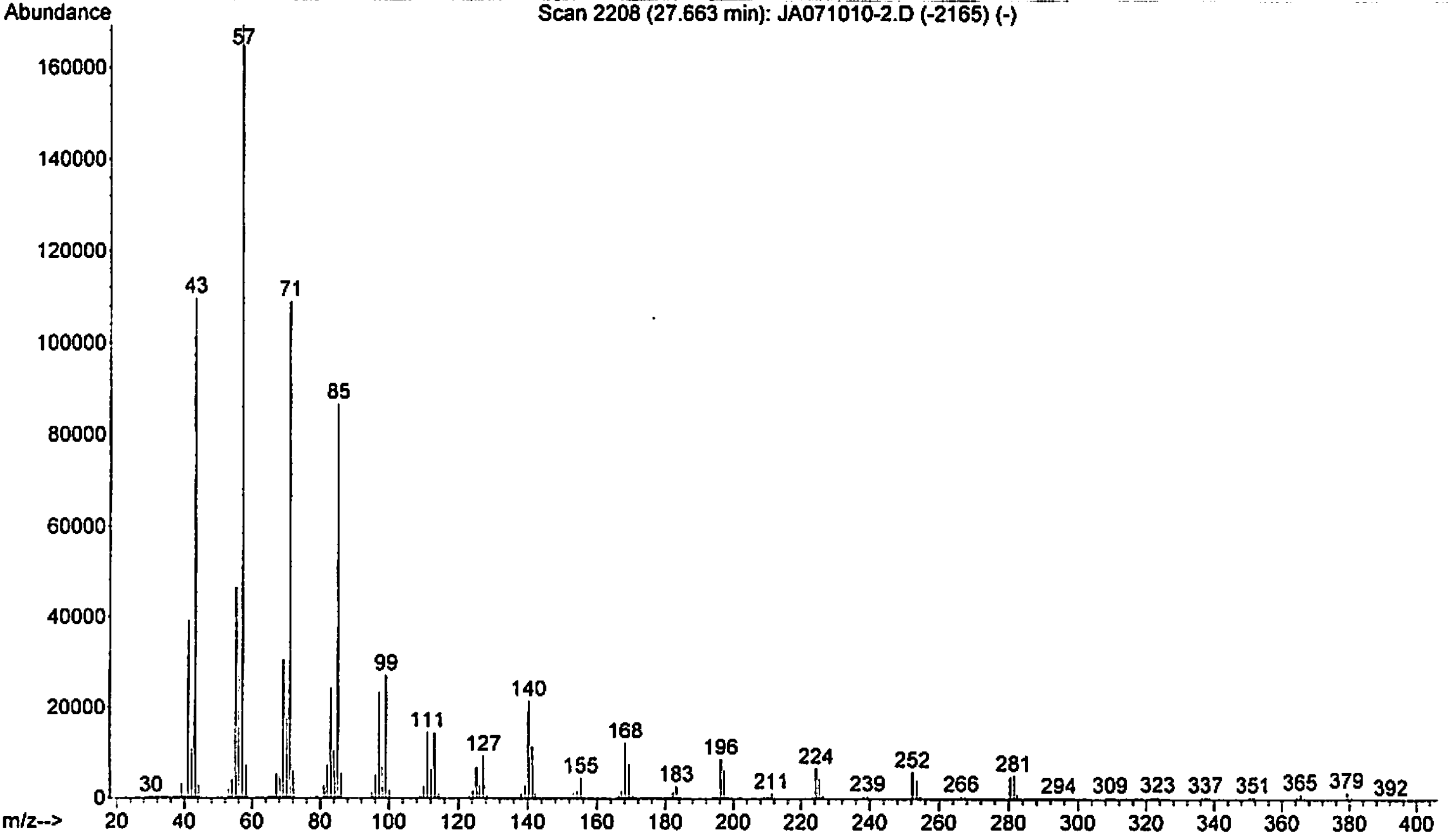

```
File         :D:\DATA\ALDRICH\JA-09\Snapshot\JA071010-2.D
Operator     : Aldrich
Acquired     : 10 Jul 2009  17:18     using AcqMethod JA-WAX09.M
Instrument :      Instrument #1
Sample Name: 10 lab male 2wk-old fed nepetalactol 7/1-10
Misc Info    : JA071009-1 & here C.oculata; rev.diss order
Vial Number: 1
```

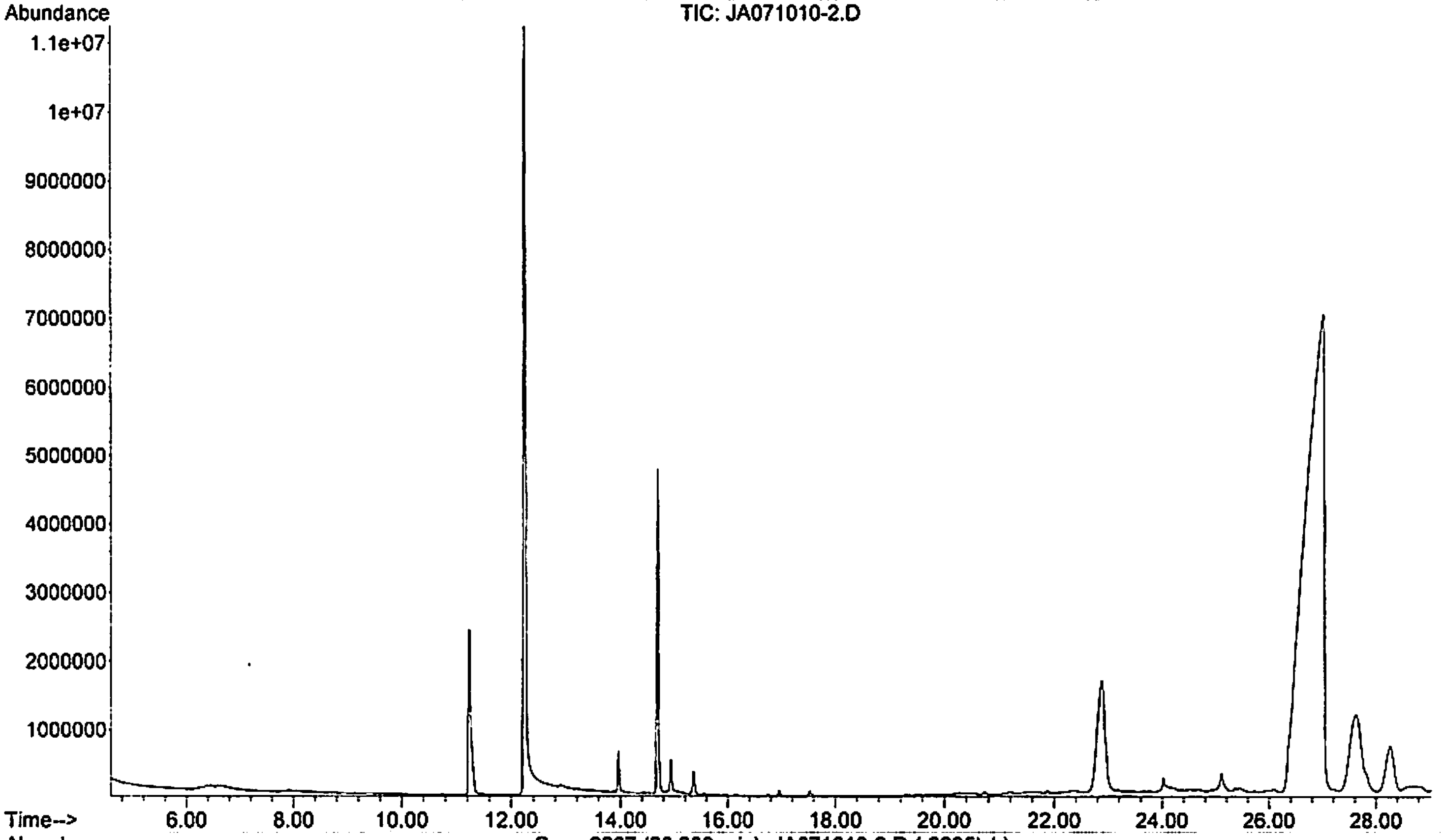


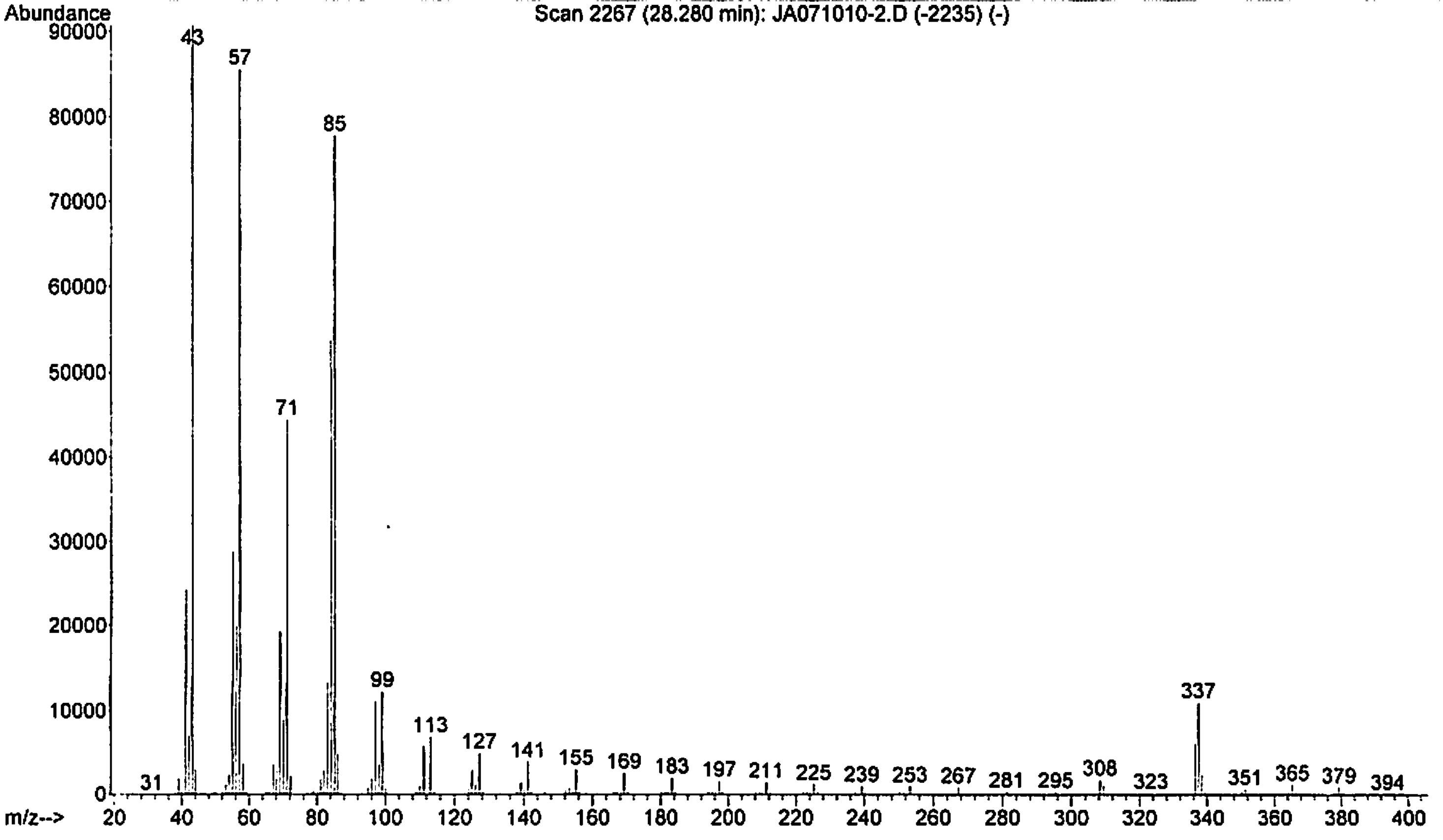

File :D:\DATA\Aldrich\JA-09\JA070709-5.D
Operator : Aldrich
Acquired : 7 Jul 2009 16:07 using AcqMethod JA-WAX09.M
Instrument : Instrument #1
Sample Name: 9 lab male C.oculata fed nepetalactol abd.
Misc Info : adult 6/12-16; fed lactol 6/25-7/7;100 to10ul
Vial Number: 1

emerged: 6/12-16/09 & fed 06/25 & diss. 7/7 } 21 - 25 days old

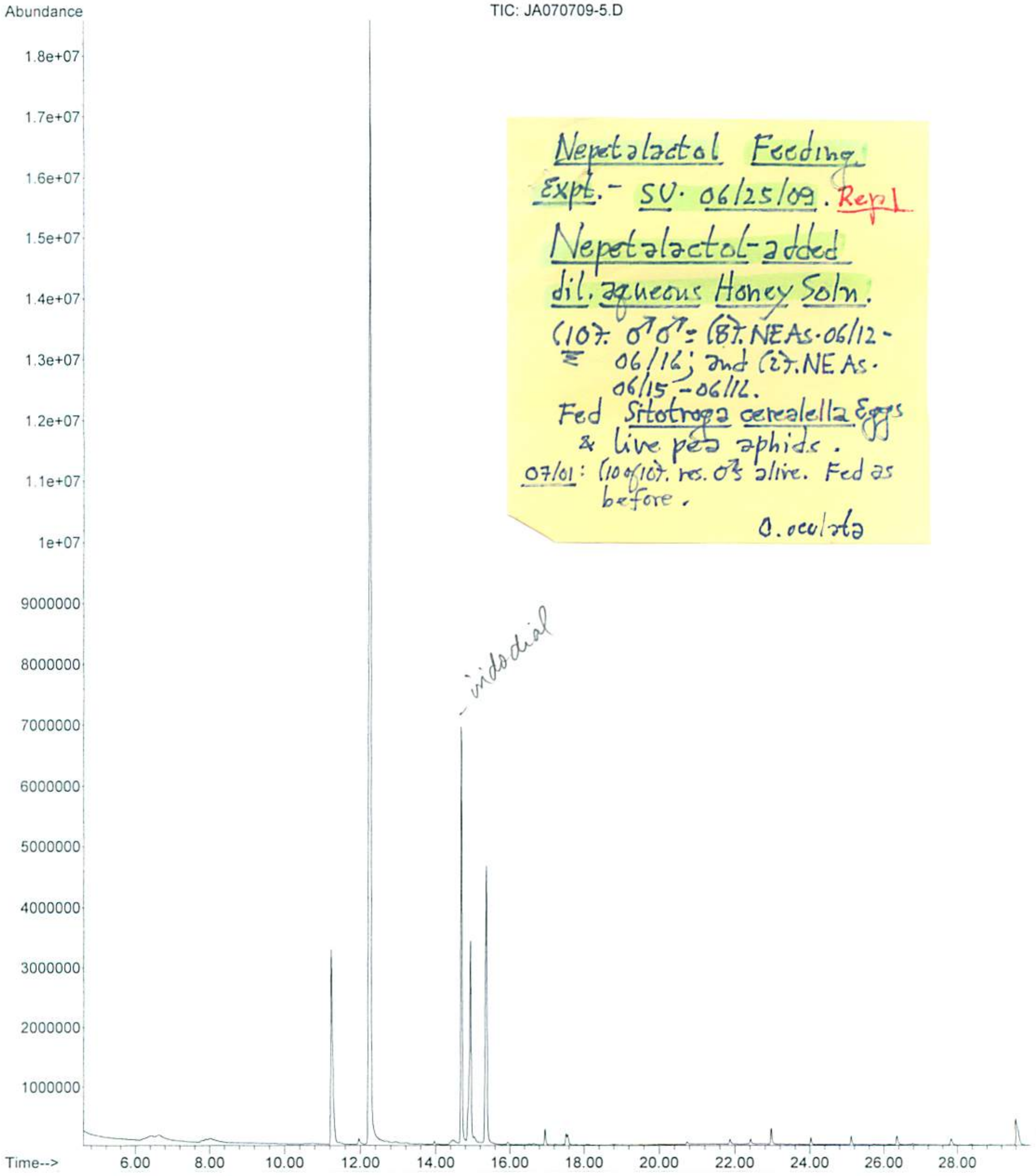


Nepetalactol Feeding. Expt. - SU. 06/25/09. Rep1

Nepetalactol-added dil. aqueous Honey Soln.

(10). ♂♂ = (8). NEAs. 06/12 - 06/16; and (2). NE As. 06/15 - 06/16.

Fed Sitotroga cerealella Eggs & live pea aphids.

07/01: (10 of 10). res. ♂s alive. Fed as before.

C. oculata

File : D:\DATA\KAMAL\KC061610B.D
Operator : kamal
Acquired : 16 Jun 2009 12:51 using AcqMethod JA-WAX09.M
Instrument : Instrument #1
Sample Name: ze lactol fr 3
Misc Info : NABH4 redn of ze lactone in meoh
Vial Number: 1

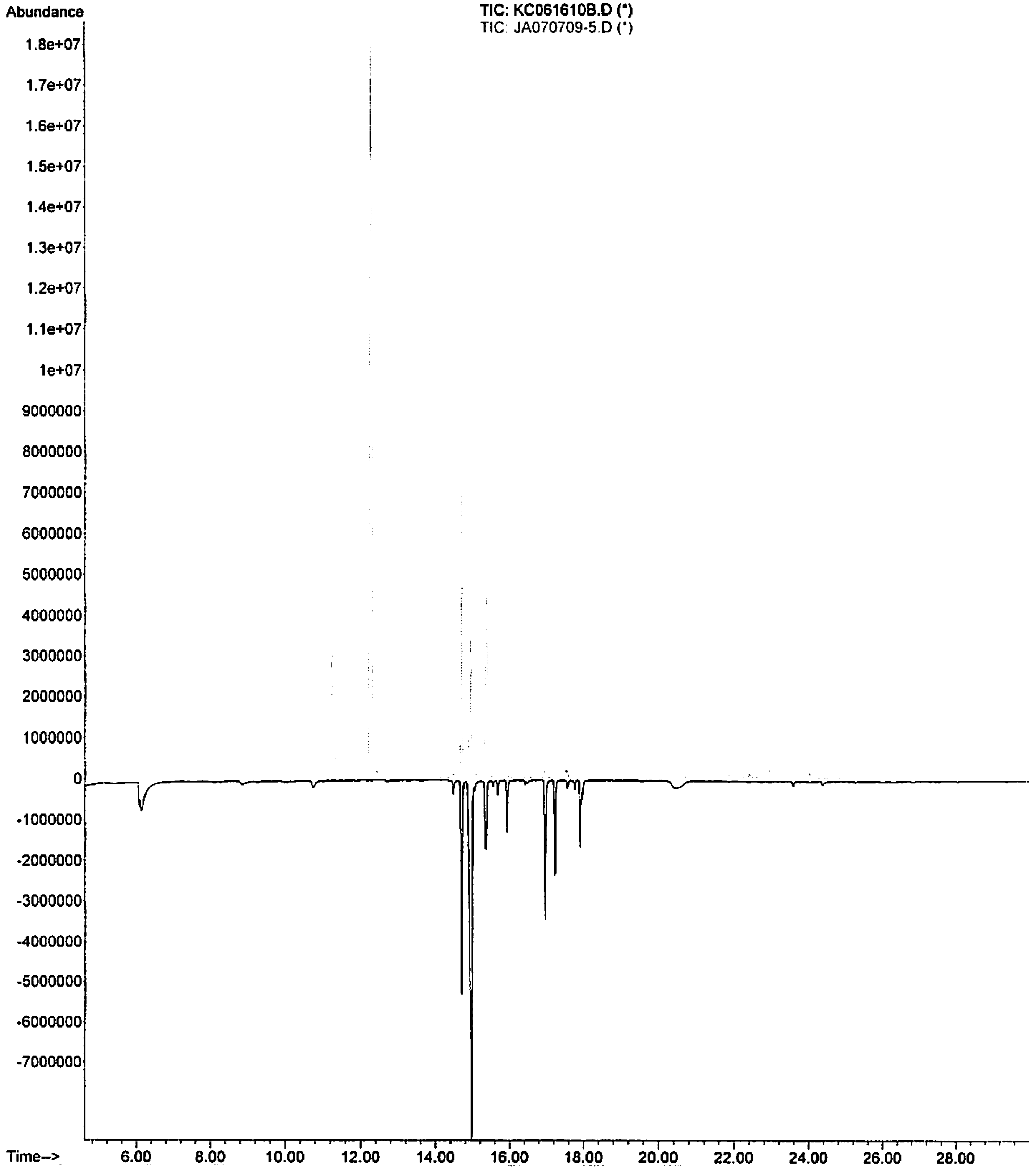

```
File       :D:\DATA\ALDRICH\JA-09\Snapshot\JA070709-5.D
Operator   : Aldrich
Acquired   :  7 Jul 2009  16:07     using AcqMethod JA-WAX09.M
Instrument :    Instrument #1
Sample Name: 9 lab male C.oculata fed nepetalactol abd.
Misc Info  : adult 6/12-16; fed lactol 6/25-7/7;100 tol0ul
Vial Number: 1
```

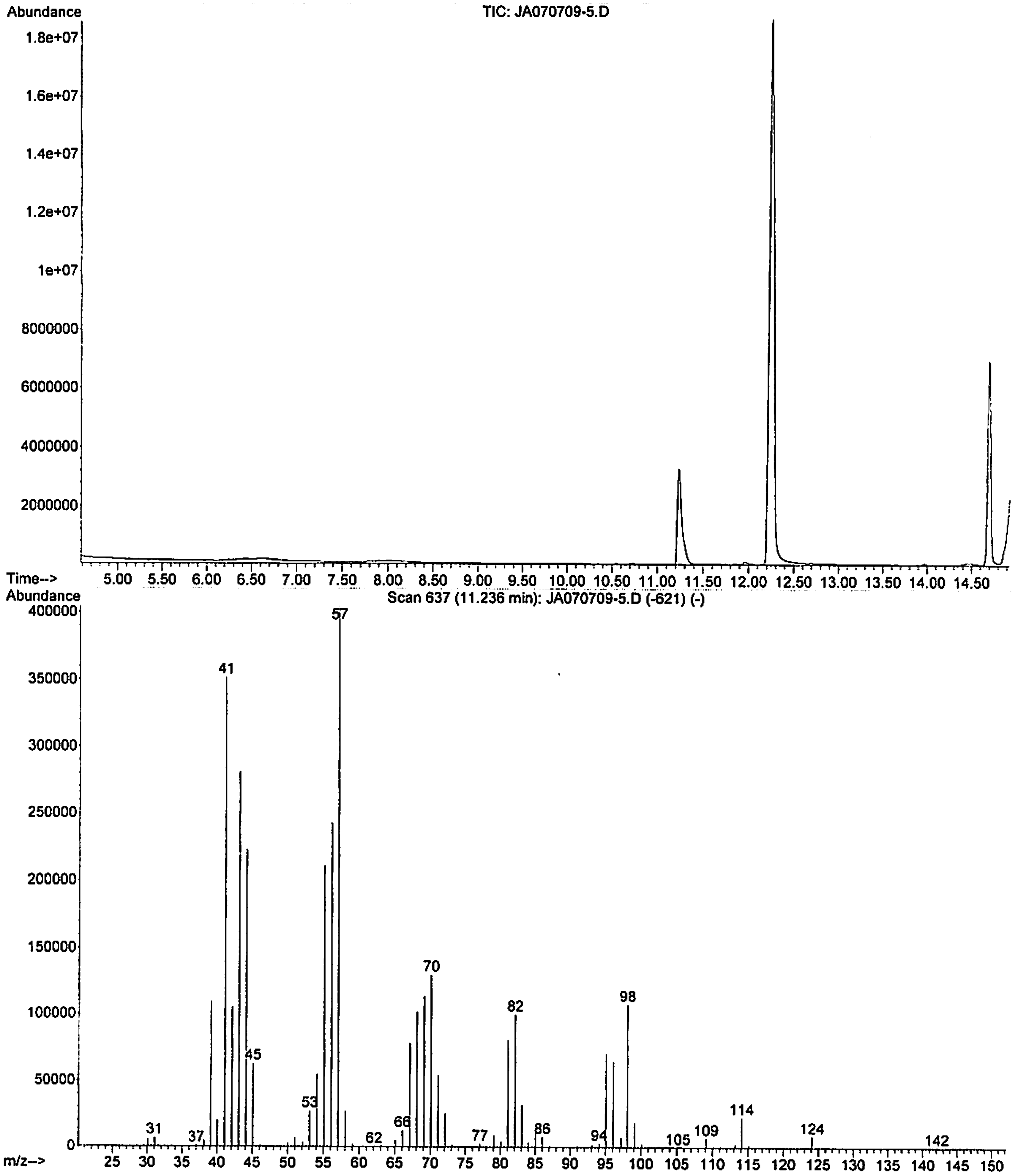

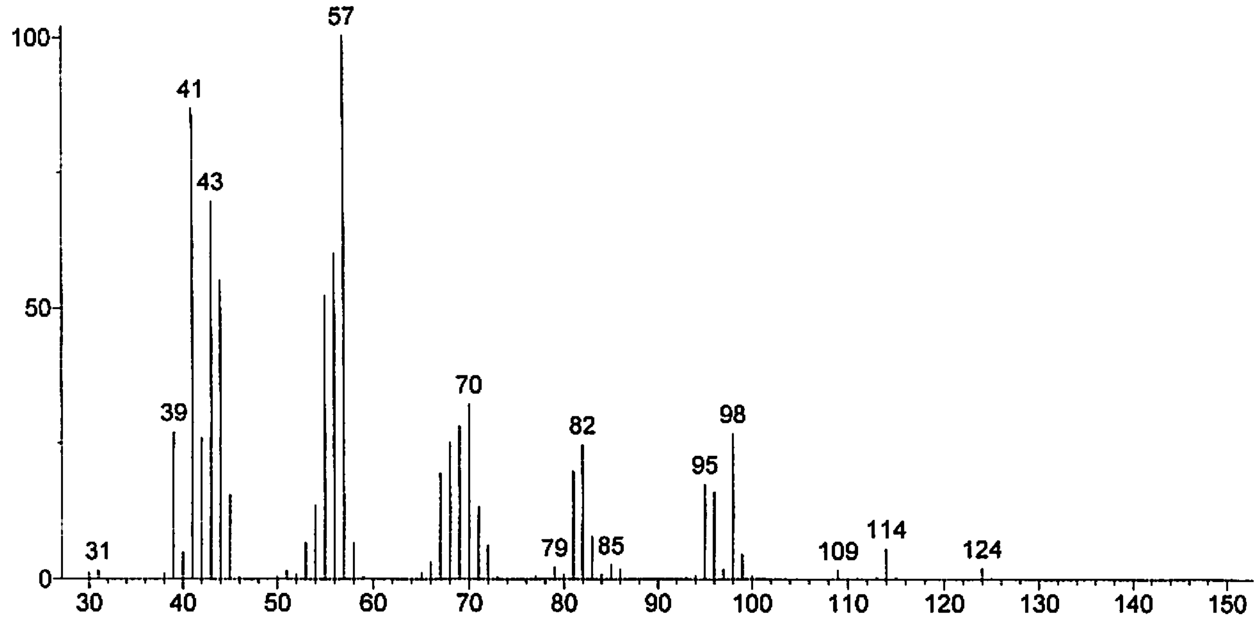


(Text File) Scan 637 (11.236 min): JA070709-5.D (-621)

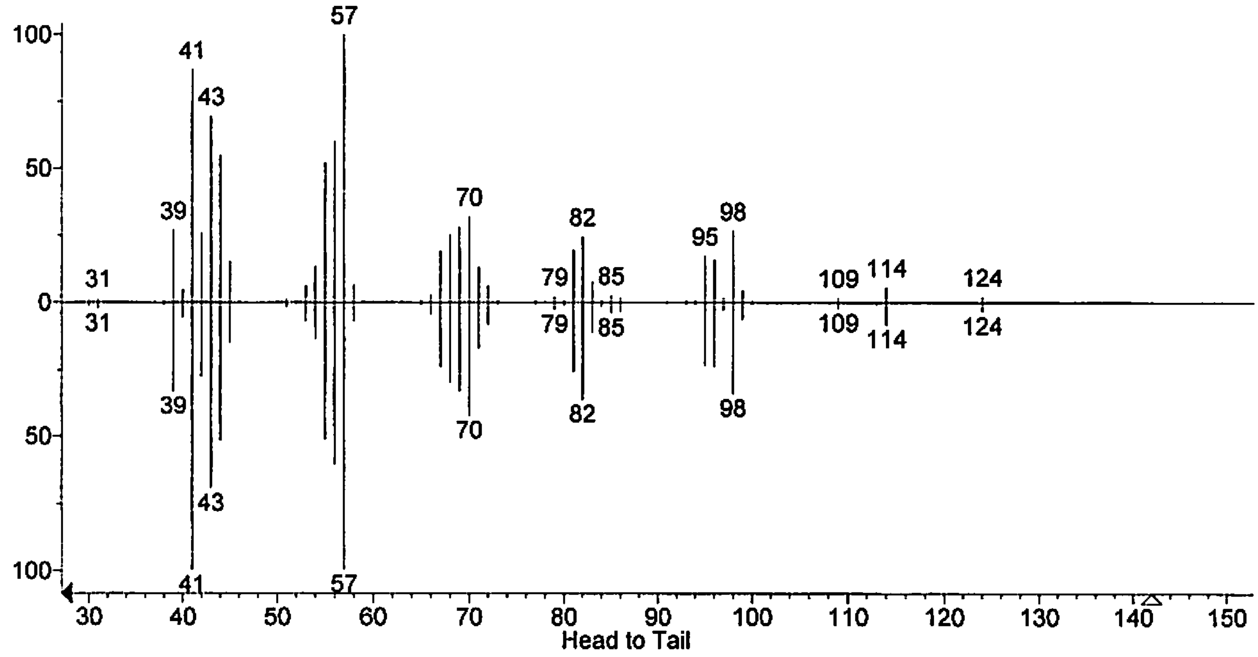


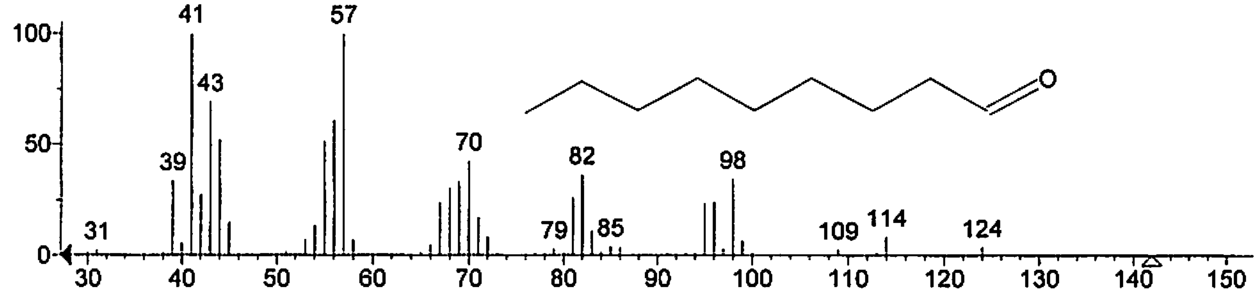


(replib) Nonanal

```
File        :D:\DATA\ALDRICH\JA-09\Snapshot\JA070709-5.D
Operator    : Aldrich
Acquired    :  7 Jul 2009  16:07     using AcqMethod JA-WAX09.M
Instrument  :     Instrument #1
Sample Name: 9 lab male C.oculata fed nepetalactol abd.
Misc Info   : adult 6/12-16; fed lactol 6/25-7/7;100 tol0ul
Vial Number: 1
```

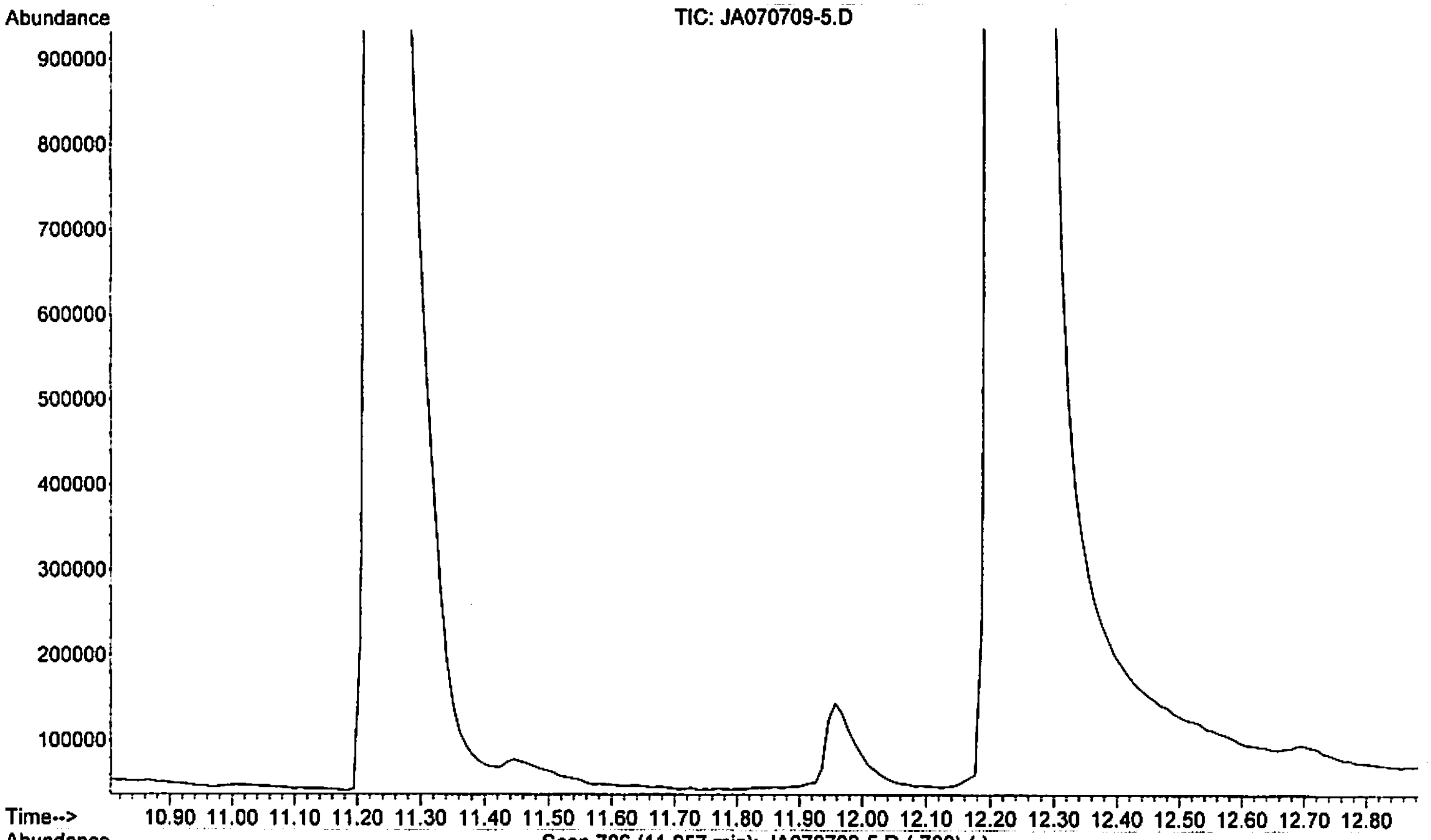


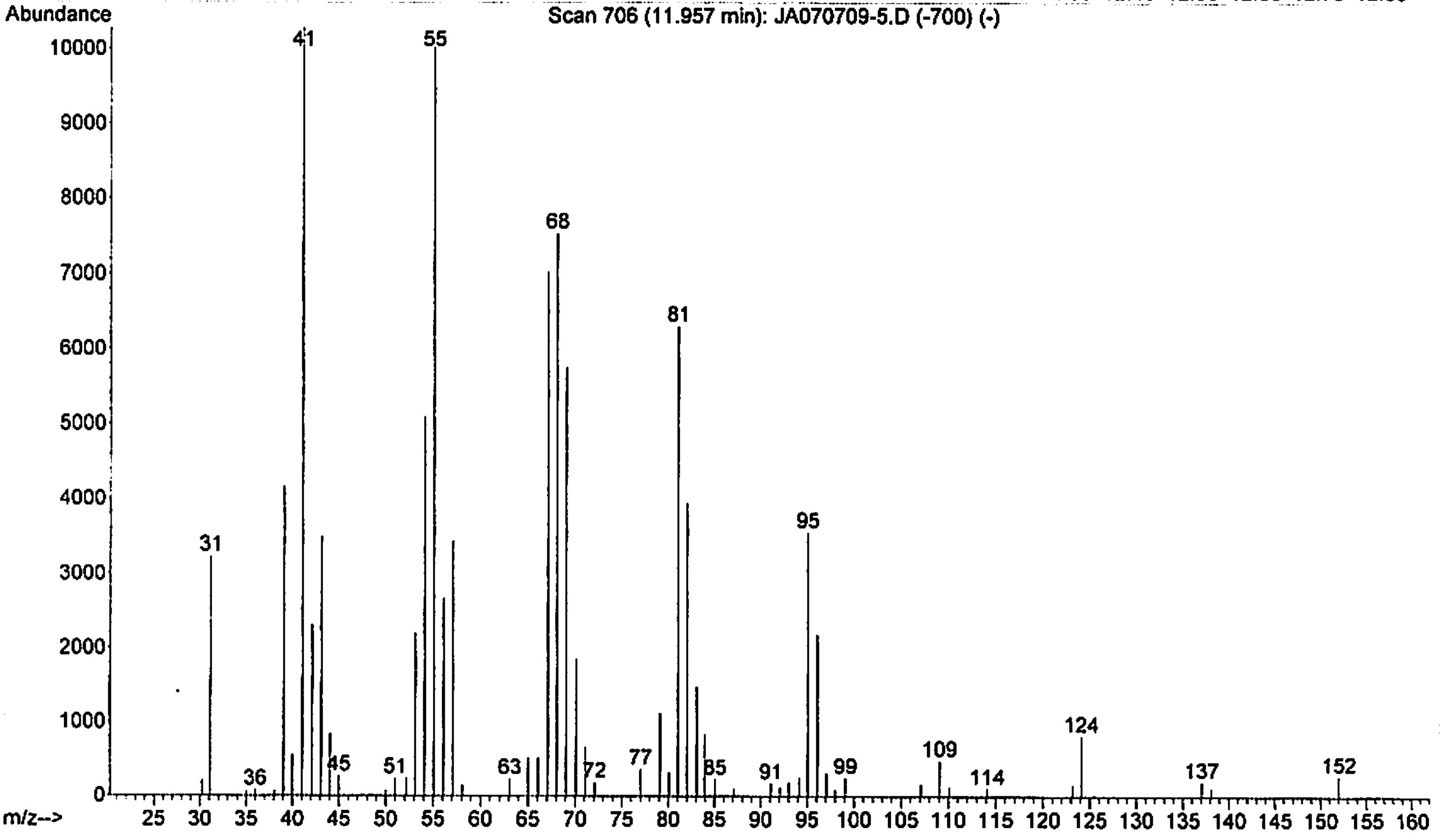

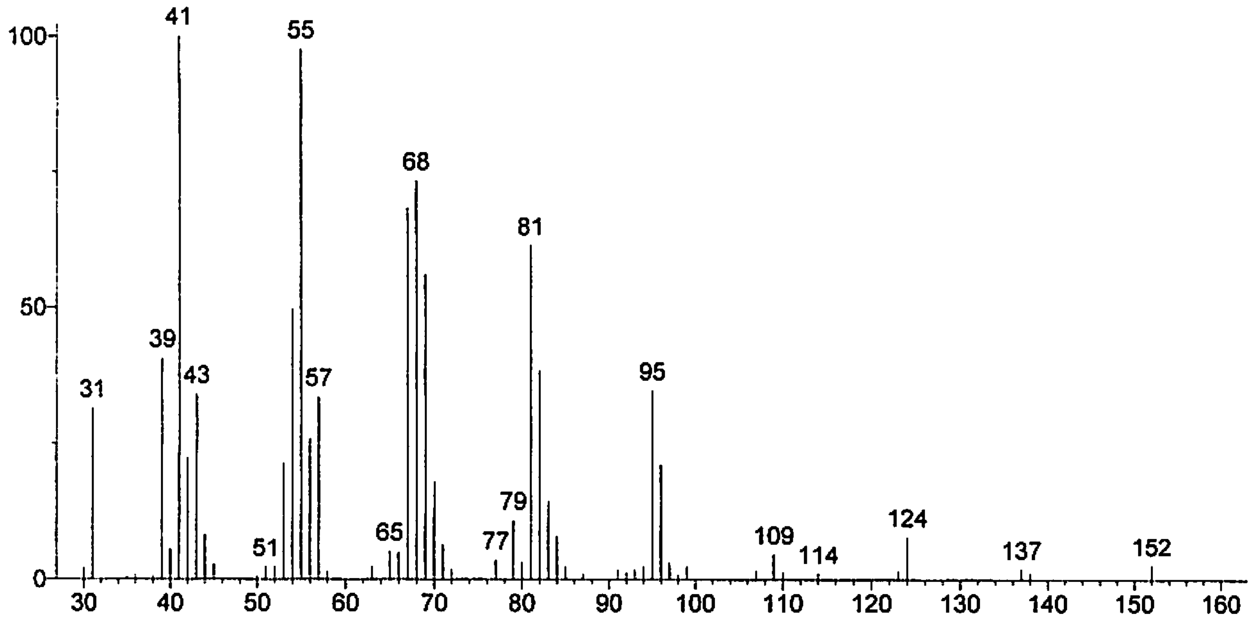


(Text File) Scan 706 (11.957 min): JA070709-5.D (-700)

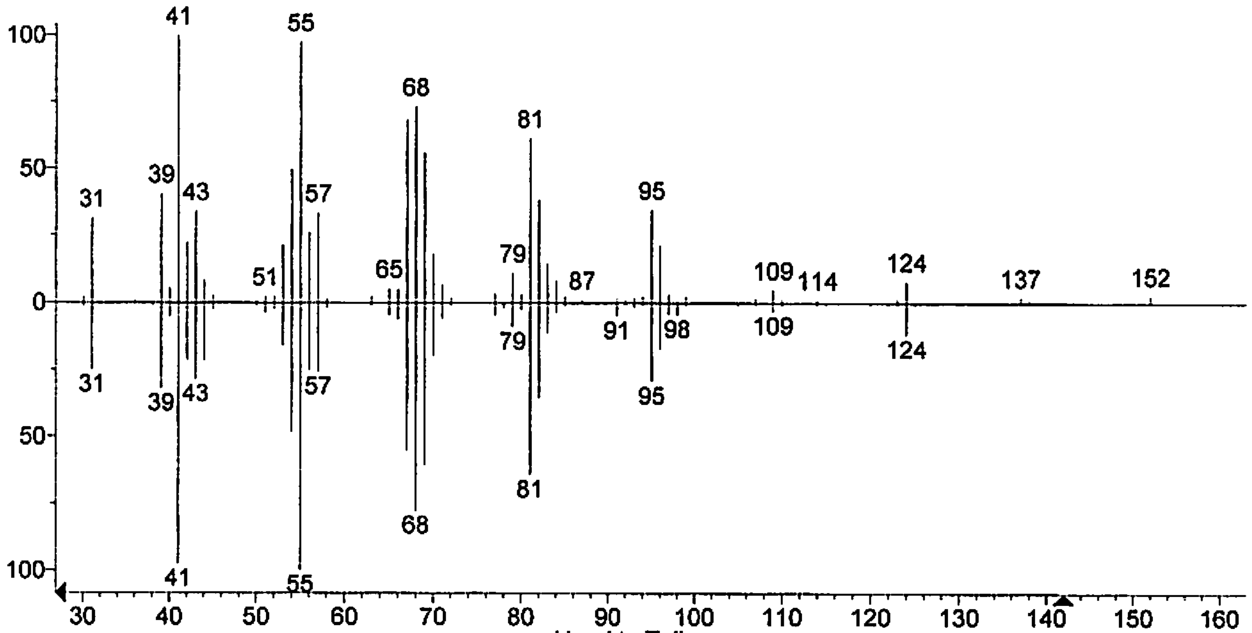


Head to Tail

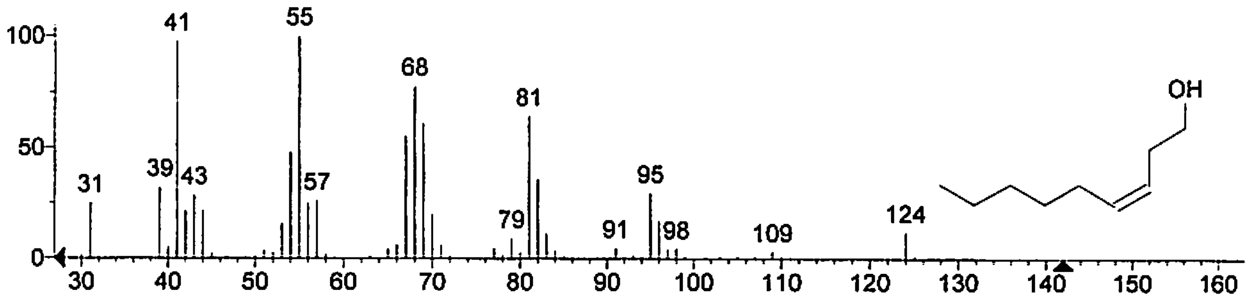


(replib) 3-Nonen-1-ol, (Z)-

```
File          :D:\DATA\ALDRICH\JA-09\Snapshot\JA070709-5.D
Operator      : Aldrich
Acquired      :  7 Jul 2009  16:07     using AcqMethod JA-WAX09.M
Instrument :     Instrument #1
Sample Name: 9 lab male C.oculata fed nepetalactol abd.
Misc Info   : adult 6/12-16; fed lactol 6/25-7/7;100 tol0ul
Vial Number: 1
```

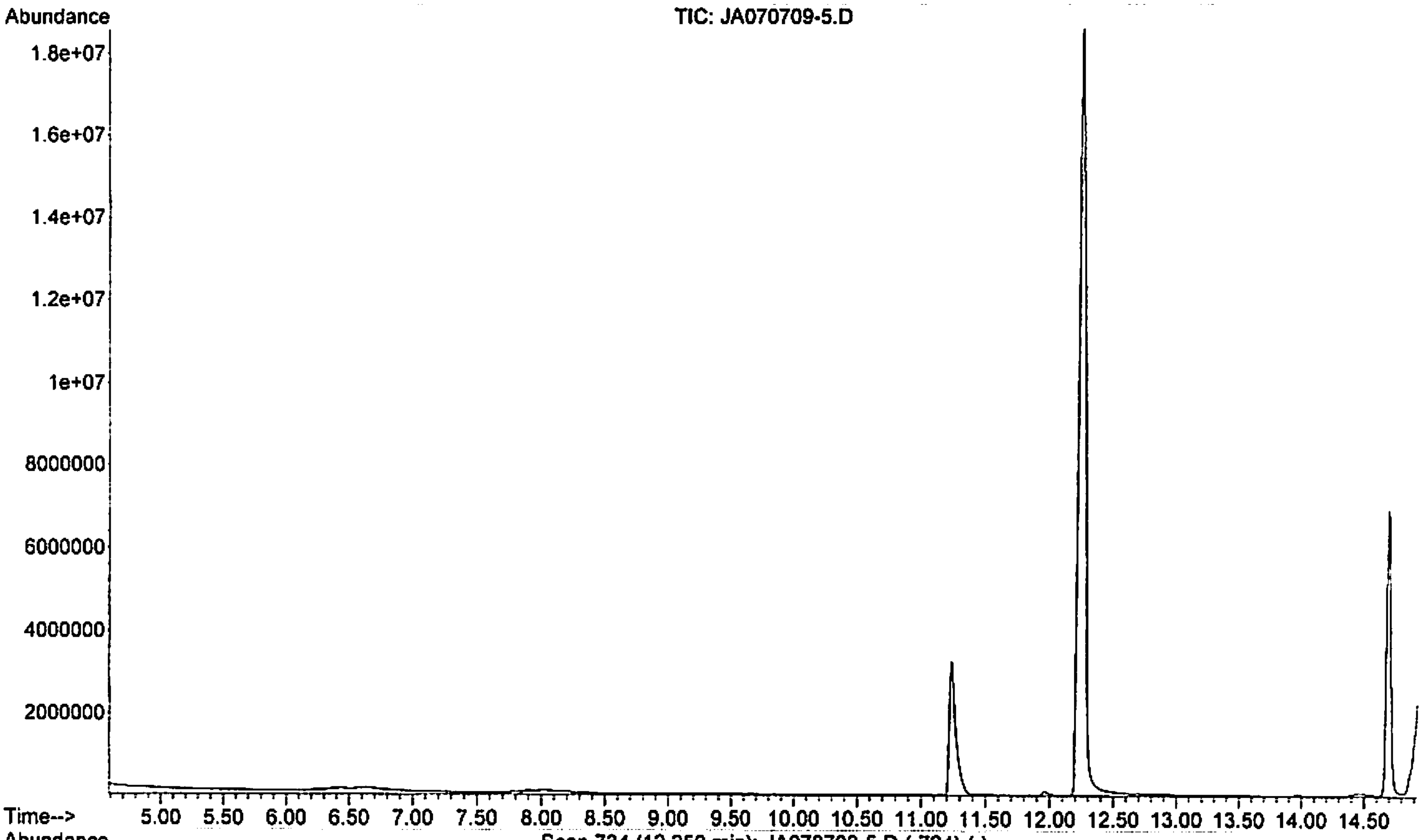


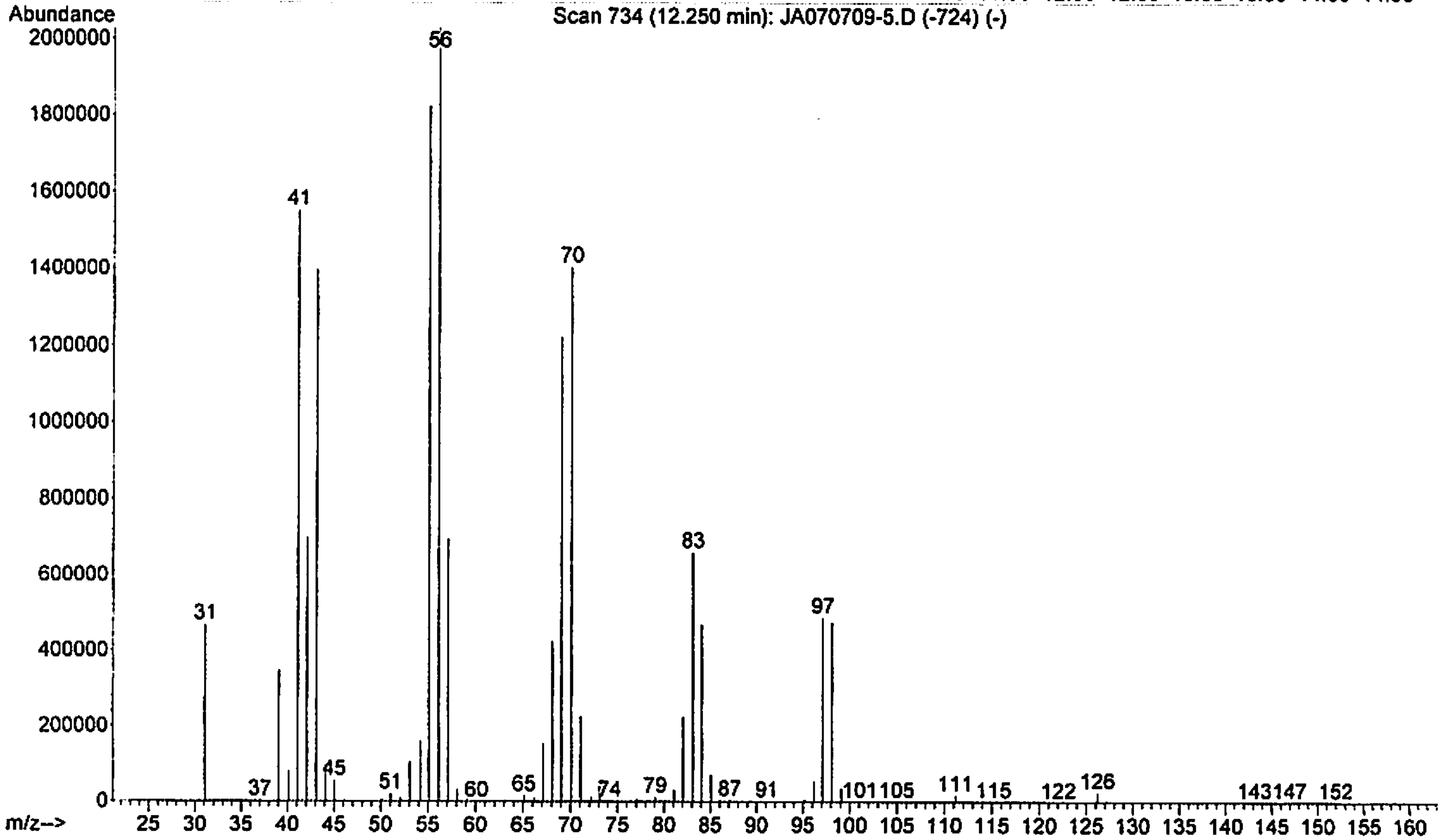

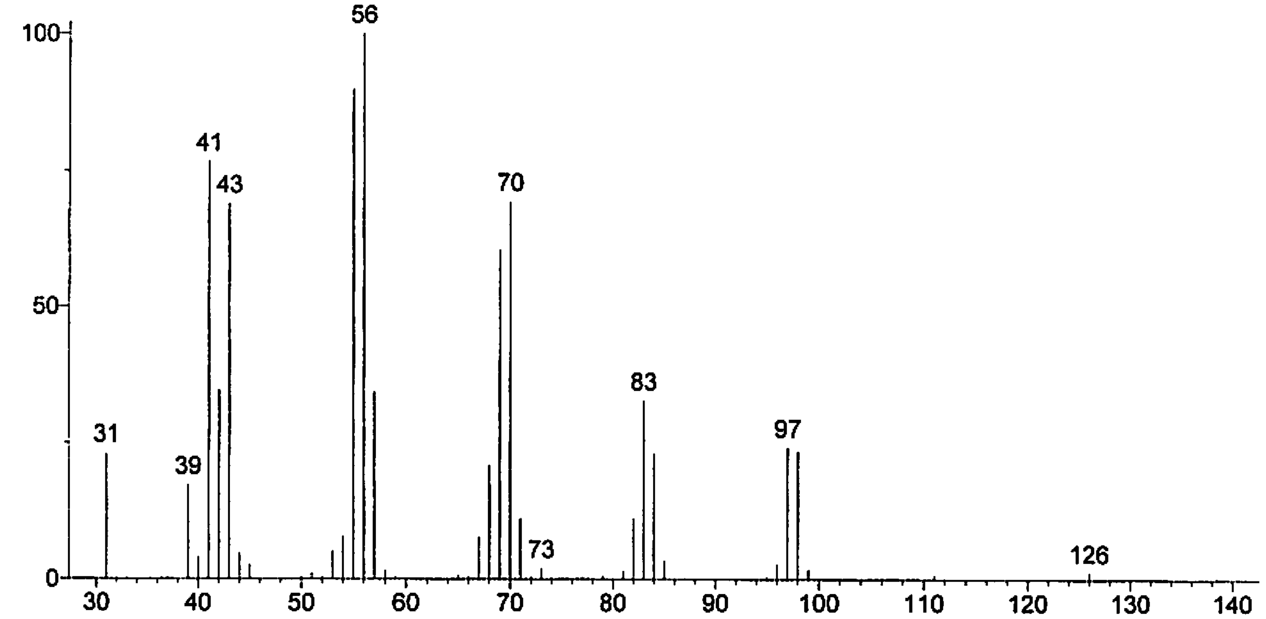


(Text File) Scan 734 (12.250 min): JA070709-5.D (-724)

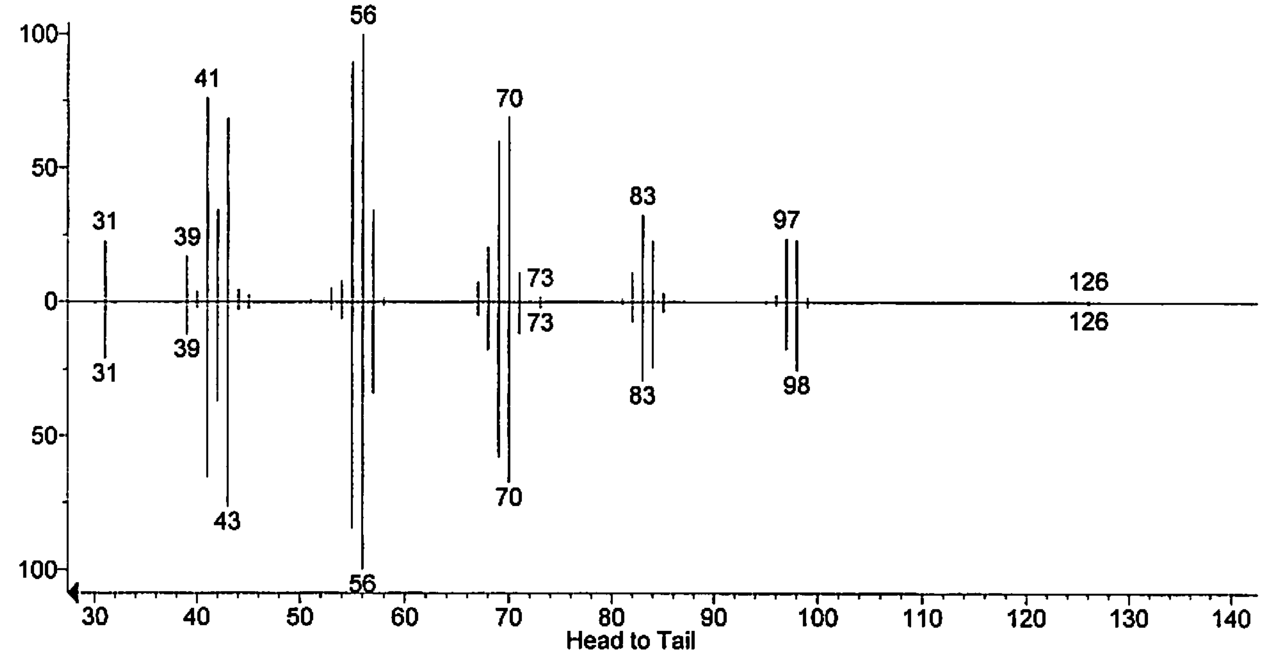


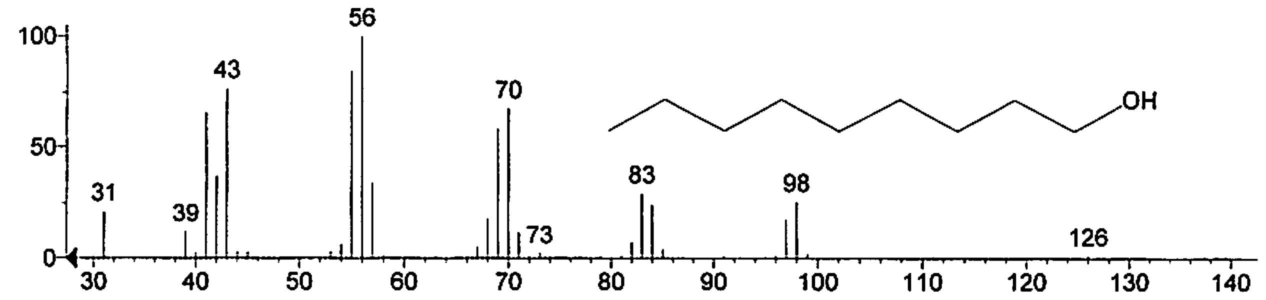


(mainlib) 1-Nonanol

File        :D:\DATA\ALDRICH\JA-09\Snapshot\JA070709-5.D
Operator    : Aldrich
Acquired    :  7 Jul 2009  16:07      using AcqMethod JA-WAX09.M
Instrument :     Instrument #1
Sample Name: 9 lab male C.oculata fed nepetalactol abd.
Misc Info   : adult 6/12-16; fed lactol 6/25-7/7;100 tol0ul
Vial Number: 1

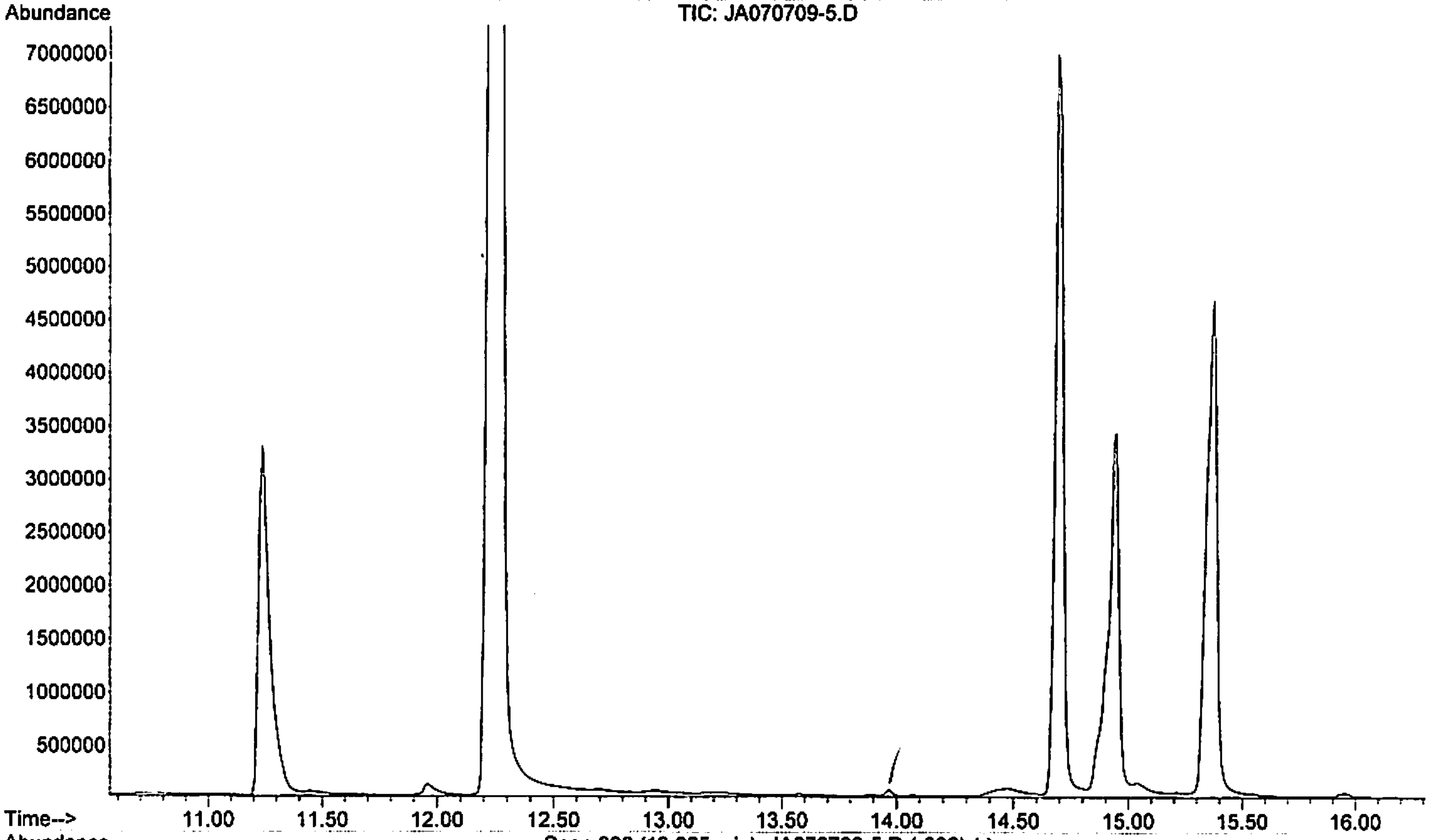


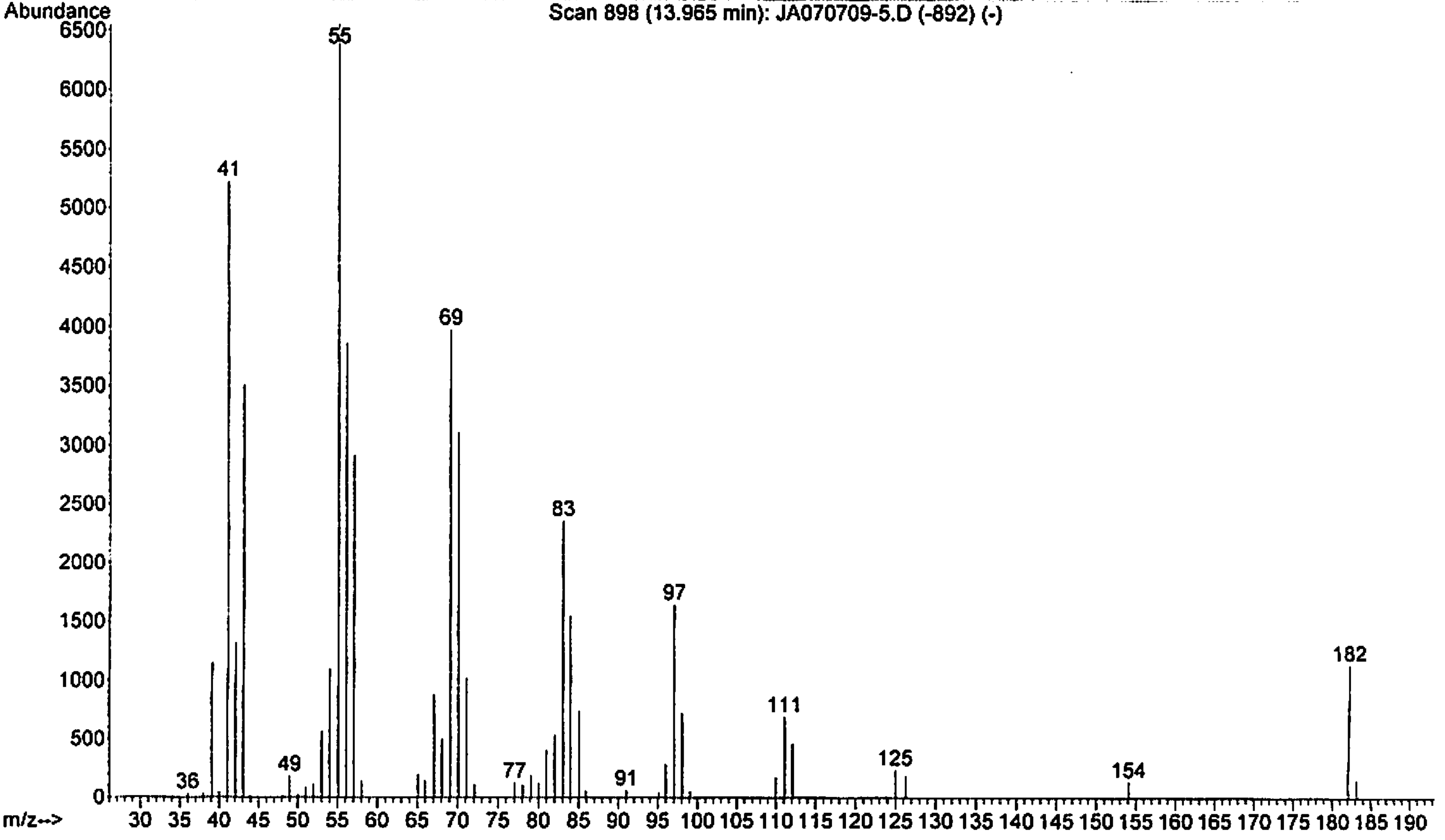

```
File        :D:\DATA\ALDRICH\JA-09\Snapshot\JA070709-5.D
Operator    : Aldrich
Acquired    :  7 Jul 2009  16:07     using AcqMethod JA-WAX09.M
Instrument  :    Instrument #1
Sample Name: 9 lab male C.oculata fed nepetalactol abd.
Misc Info   : adult 6/12-16; fed lactol 6/25-7/7;100 tol0ul
Vial Number: 1
```

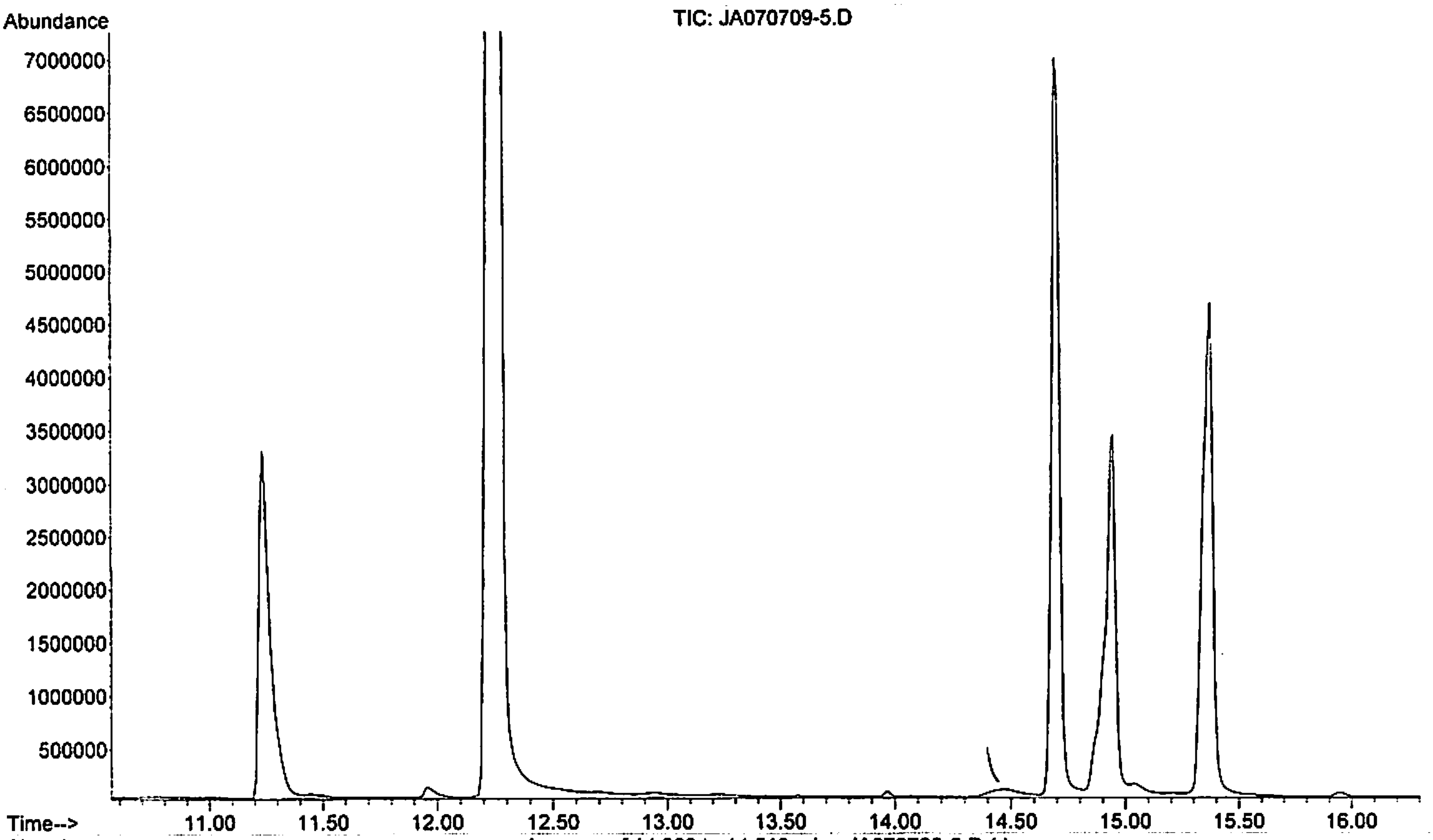


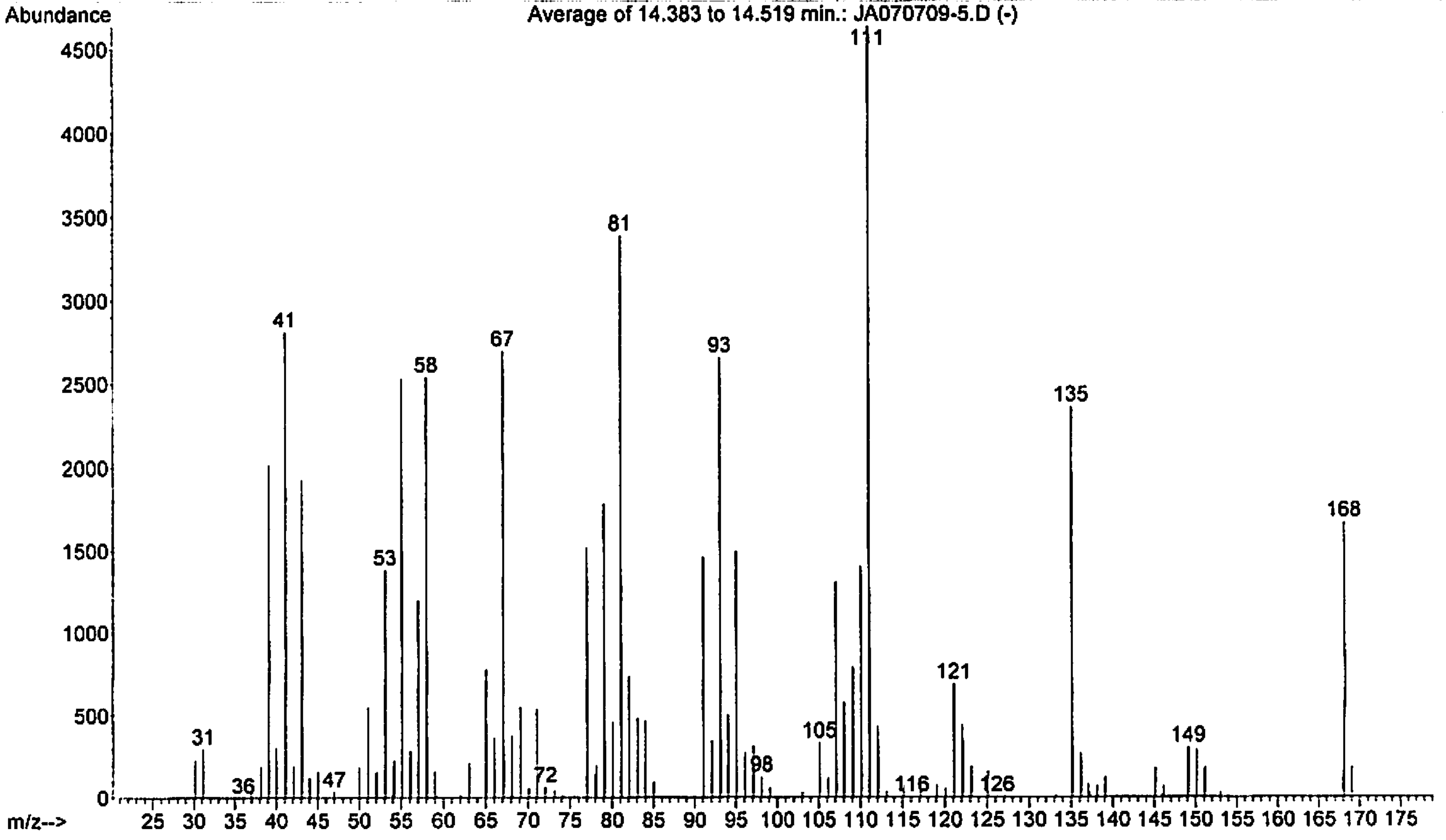

File        :D:\DATA\ALDRICH\JA-09\Snapshot\JA070709-5.D
Operator    : Aldrich
Acquired    :  7 Jul 2009  16:07     using AcqMethod JA-WAX09.M
Instrument :     Instrument #1
Sample Name: 9 lab male C.oculata fed nepetalactol abd.
Misc Info   : adult 6/12-16; fed lactol 6/25-7/7;100 to10ul
Vial Number: 1

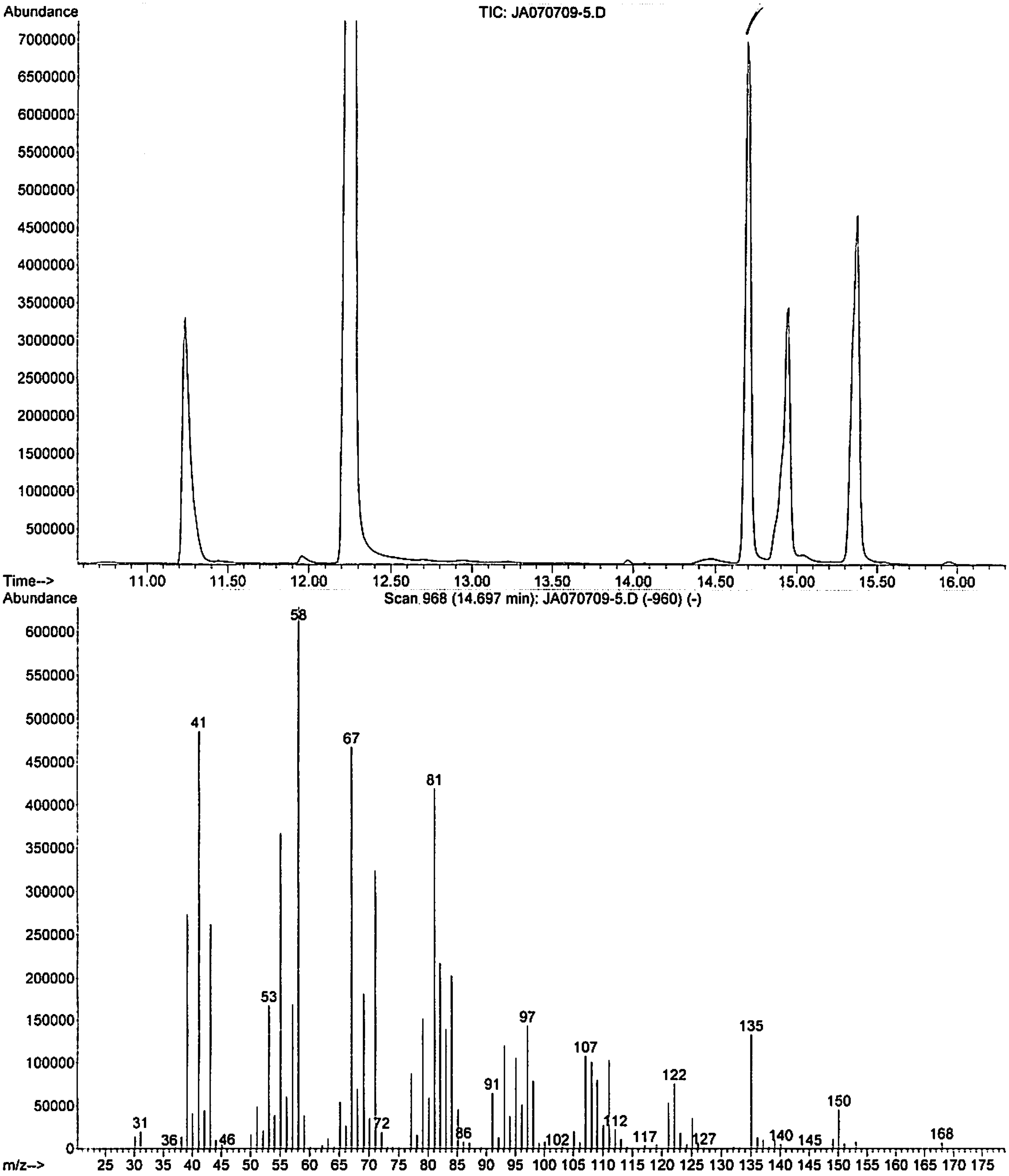

```
File        :D:\DATA\ALDRICH\JA-09\Snapshot\JA070709-5.D
Operator    : Aldrich
Acquired    :  7 Jul 2009  16:07     using AcqMethod JA-WAX09.M
Instrument  :    Instrument #1
Sample Name: 9 lab male C.oculata fed nepetalactol abd.
Misc Info   : adult 6/12-16; fed lactol 6/25-7/7;100 tol0ul
Vial Number: 1
```

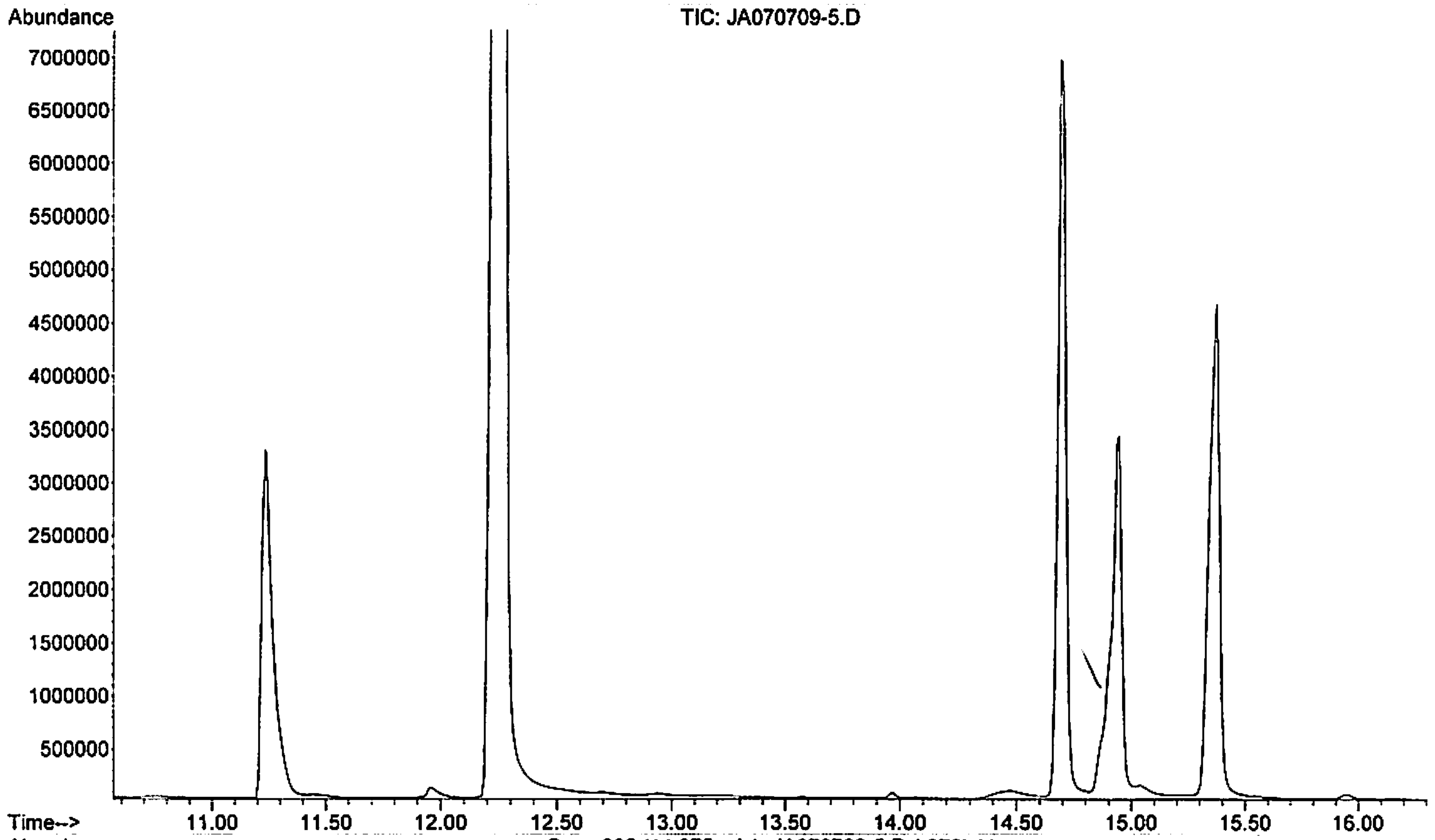


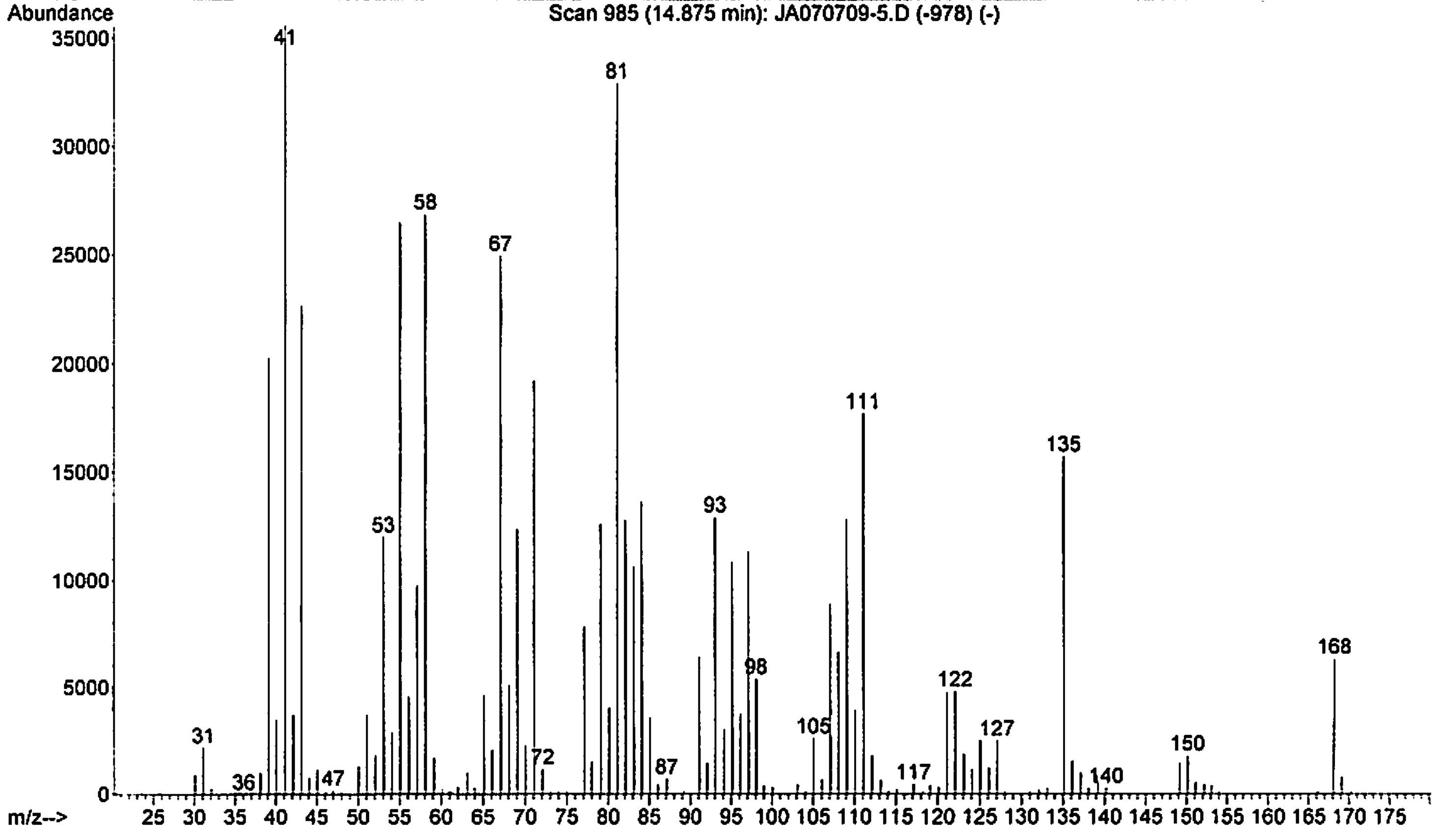

File        :D:\DATA\ALDRICH\JA-09\Snapshot\JA070709-5.D
Operator    : Aldrich
Acquired    :  7 Jul 2009  16:07     using AcqMethod JA-WAX09.M
Instrument :      Instrument #1
Sample Name: 9 lab male C.oculata fed nepetalactol abd.
Misc Info   : adult 6/12-16; fed lactol 6/25-7/7;100 tol0ul
Vial Number: 1

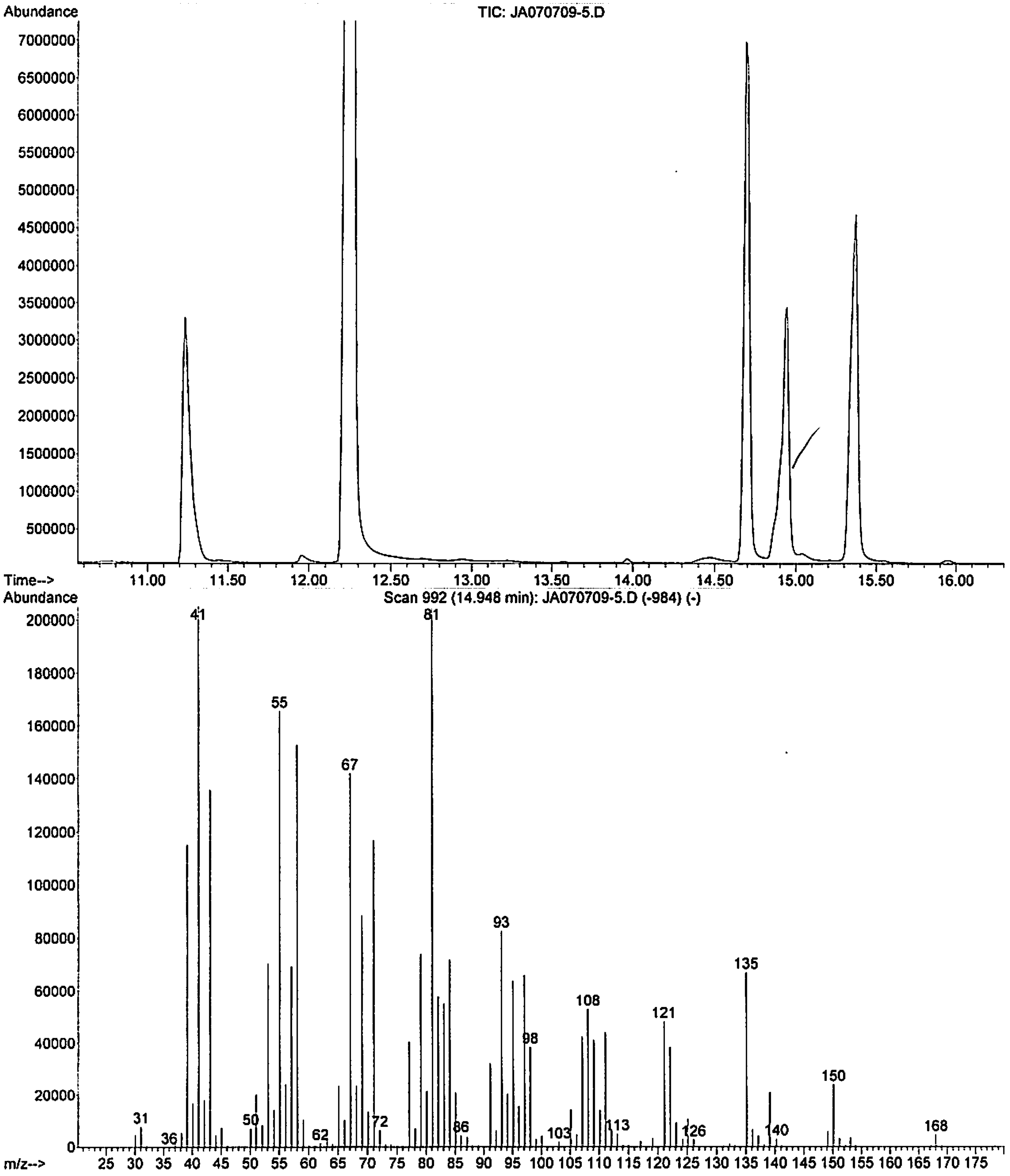

File       :D:\DATA\ALDRICH\JA-09\Snapshot\JA070709-5.D
Operator   : Aldrich
Acquired   :  7 Jul 2009  16:07     using AcqMethod JA-WAX09.M
Instrument :     Instrument #1
Sample Name: 9 lab male C.oculata fed nepetalactol abd.
Misc Info  : adult 6/12-16; fed lactol 6/25-7/7;100 to10ul
Vial Number: 1

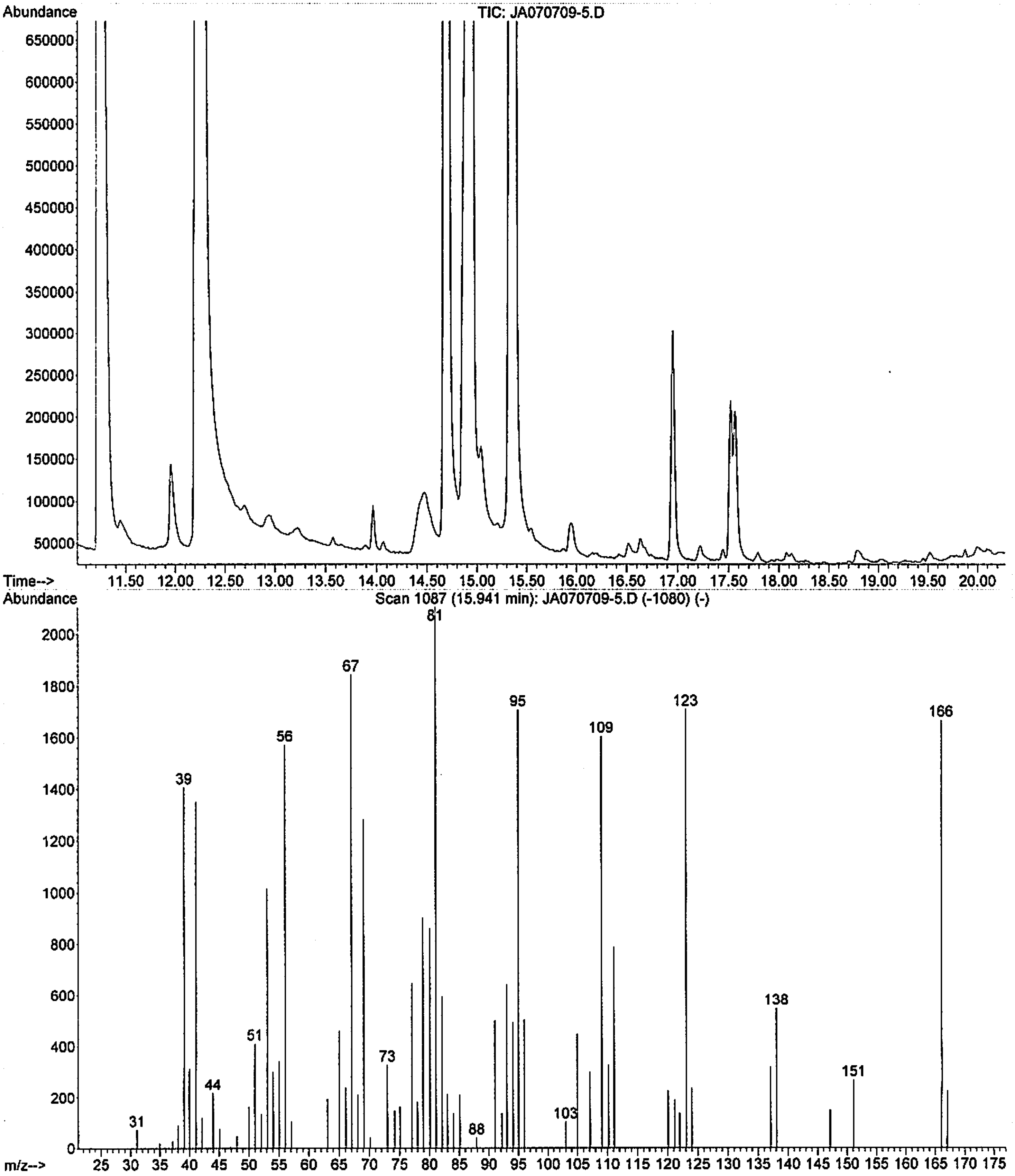

```
File.         :D:\DATA\ALDRICH\JA-09\Snapshot\JA070709-5.D
Operator      : Aldrich
Acquired      :  7 Jul 2009  16:07     using AcqMethod JA-WAX09.M
Instrument :     Instrument #1
Sample Name: 9 lab male C.oculata fed nepetalactol abd.
Misc Info     : adult 6/12-16; fed lactol 6/25-7/7;100 to10ul
Vial Number: 1
```

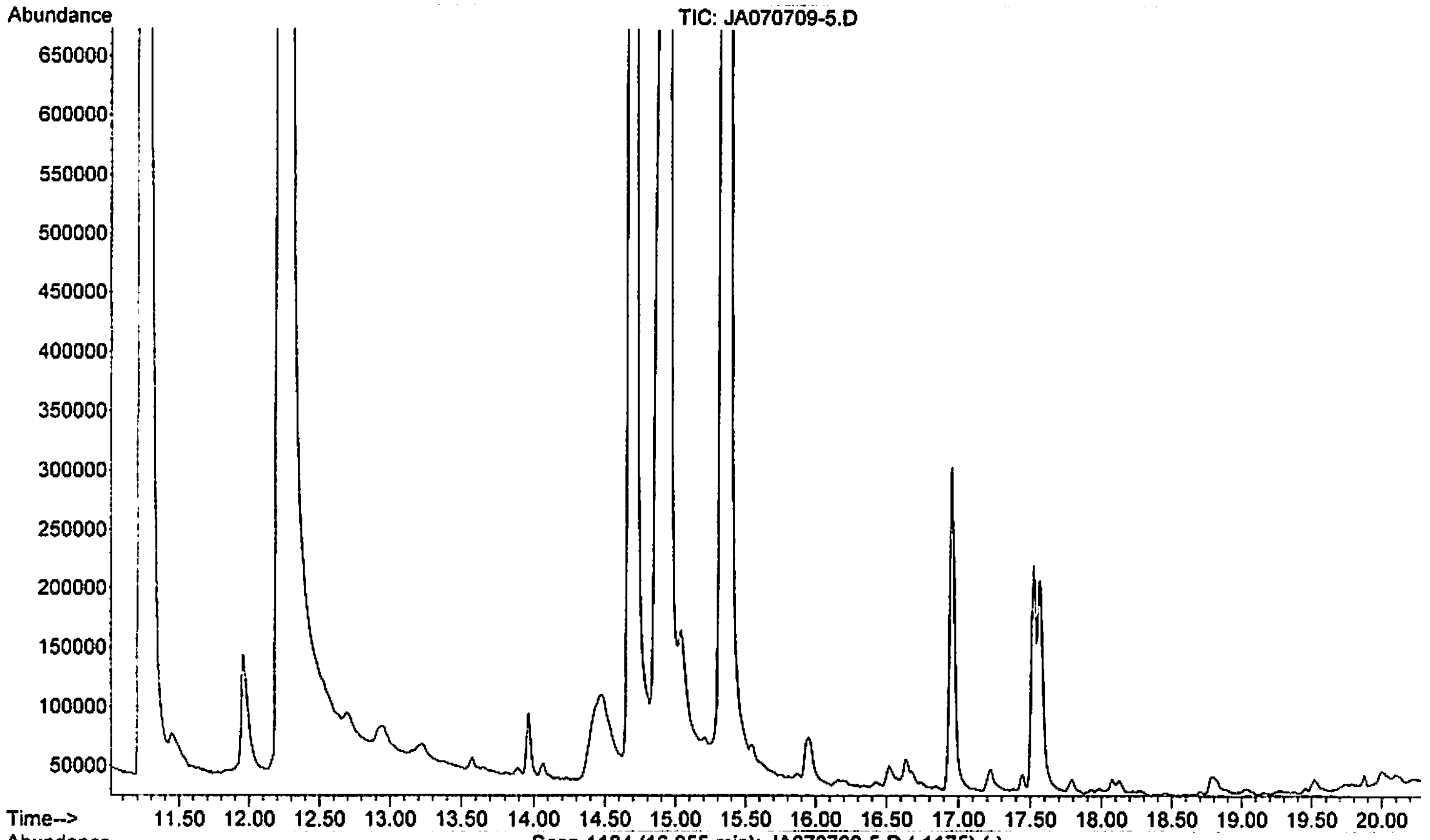


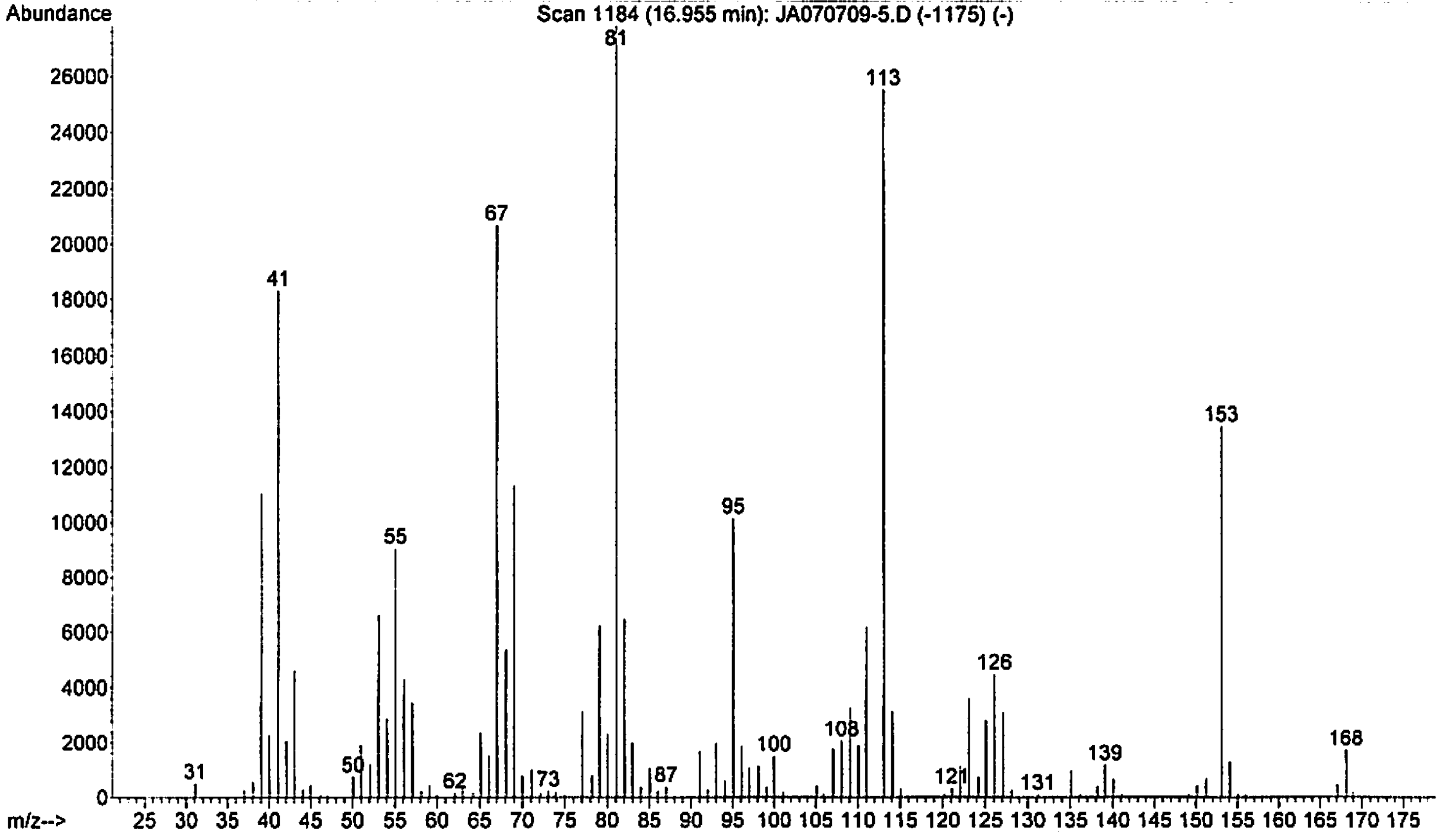

```
File        :D:\DATA\ALDRICH\JA-09\Snapshot\JA070709-5.D
Operator    : Aldrich
Acquired    :  7 Jul 2009  16:07     using AcqMethod JA-WAX09.M
Instrument  :    Instrument #1
Sample Name: 9 lab male C.oculata fed nepetalactol abd.
Misc Info   : adult 6/12-16; fed lactol 6/25-7/7;100 tol0ul
Vial Number: 1
```

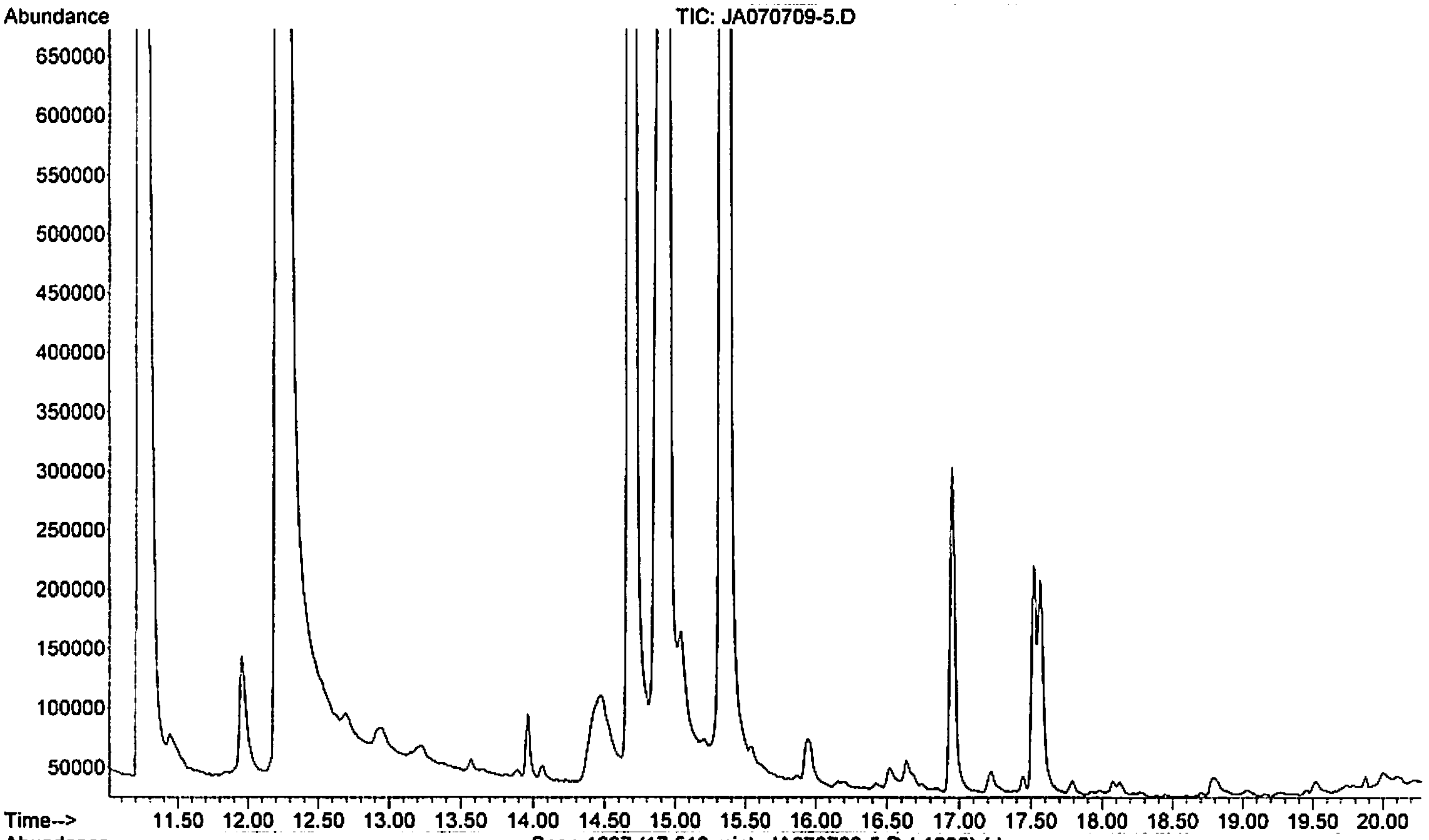


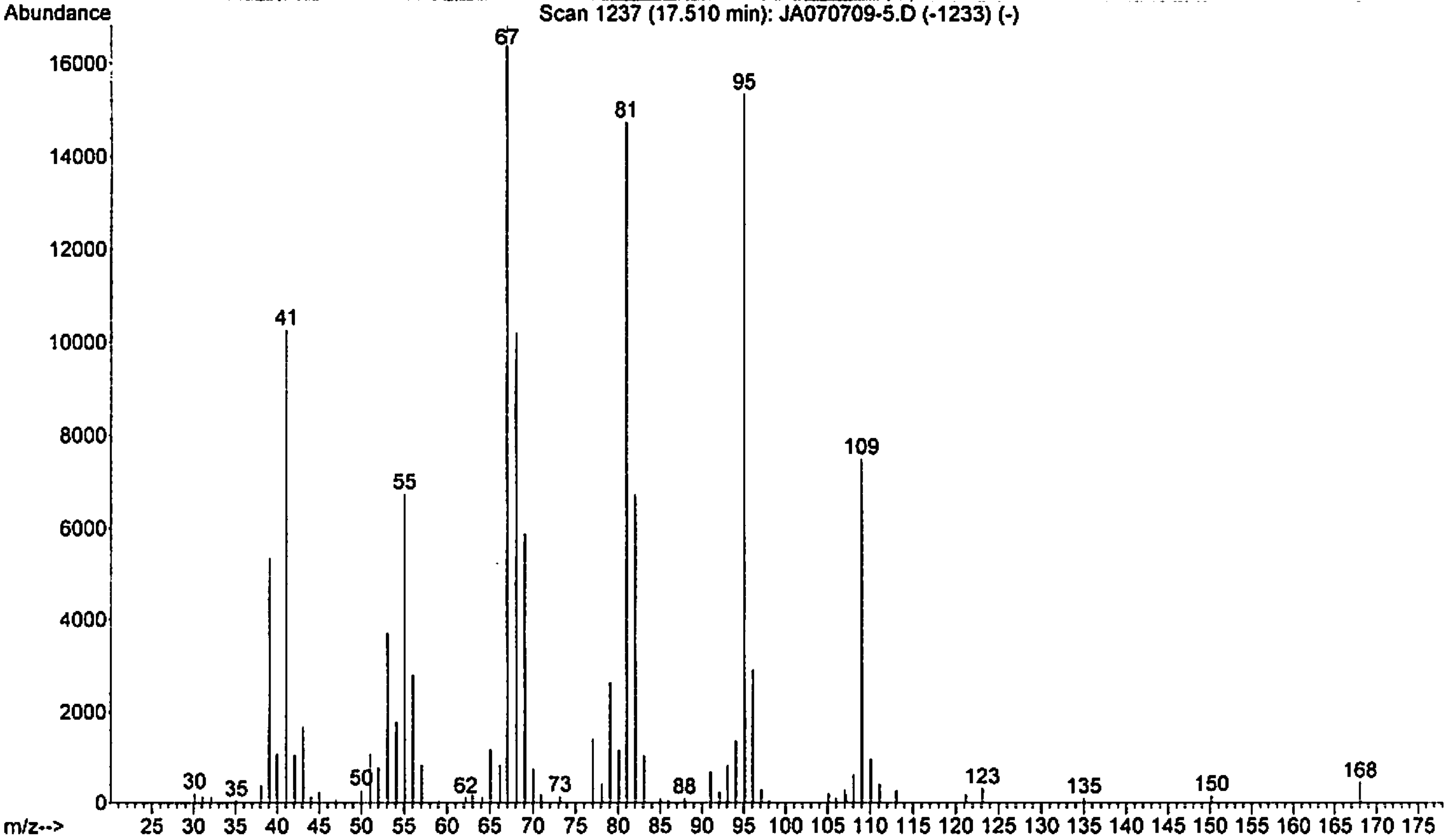

```
File         :D:\DATA\ALDRICH\JA-09\Snapshot\JA070709-5.D
Operator     : Aldrich
Acquired     :  7 Jul 2009  16:07     using AcqMethod JA-WAX09.M
Instrument   :    Instrument #1
Sample Name: 9 lab male C.oculata fed nepetalactol abd.
Misc Info    : adult 6/12-16; fed lactol 6/25-7/7;100 to10ul
Vial Number: 1
```

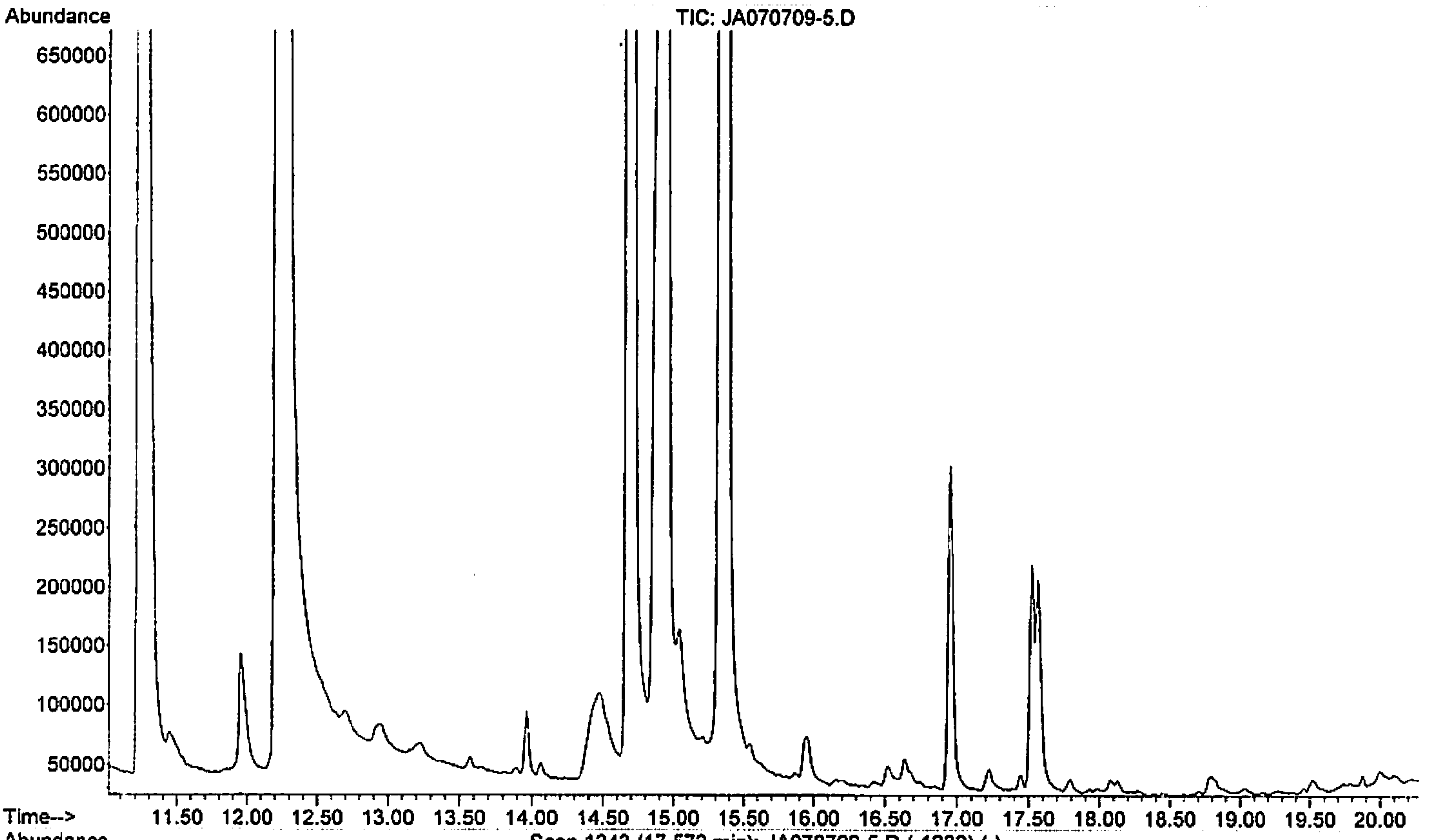


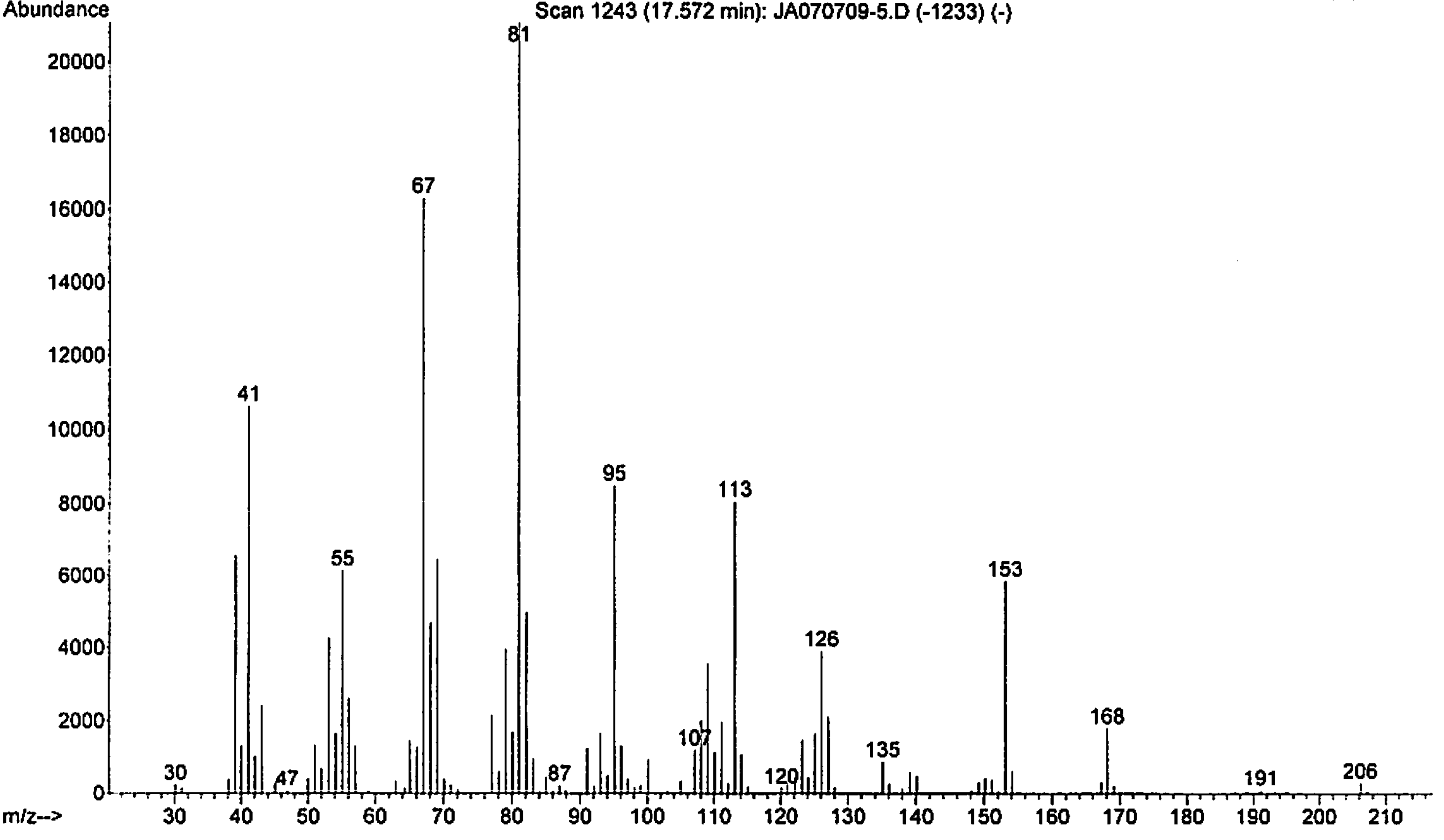

```
File        :D:\Aldrich\JA-11\JA030211-1.D
Operator    :
Acquired    :  2 Mar 2011  16:08     using AcqMethod JA-50-280LESS.M
Instrument :   Buba; IIBBL's magical mass spect
Sample Name: 4M 13-14d-old C.ocu. abdb.stern./5ul CH2Cl2
Misc Info  : +cont. JA021711-1;adults fed nepetalactol/4d
Vial Number: 1
```

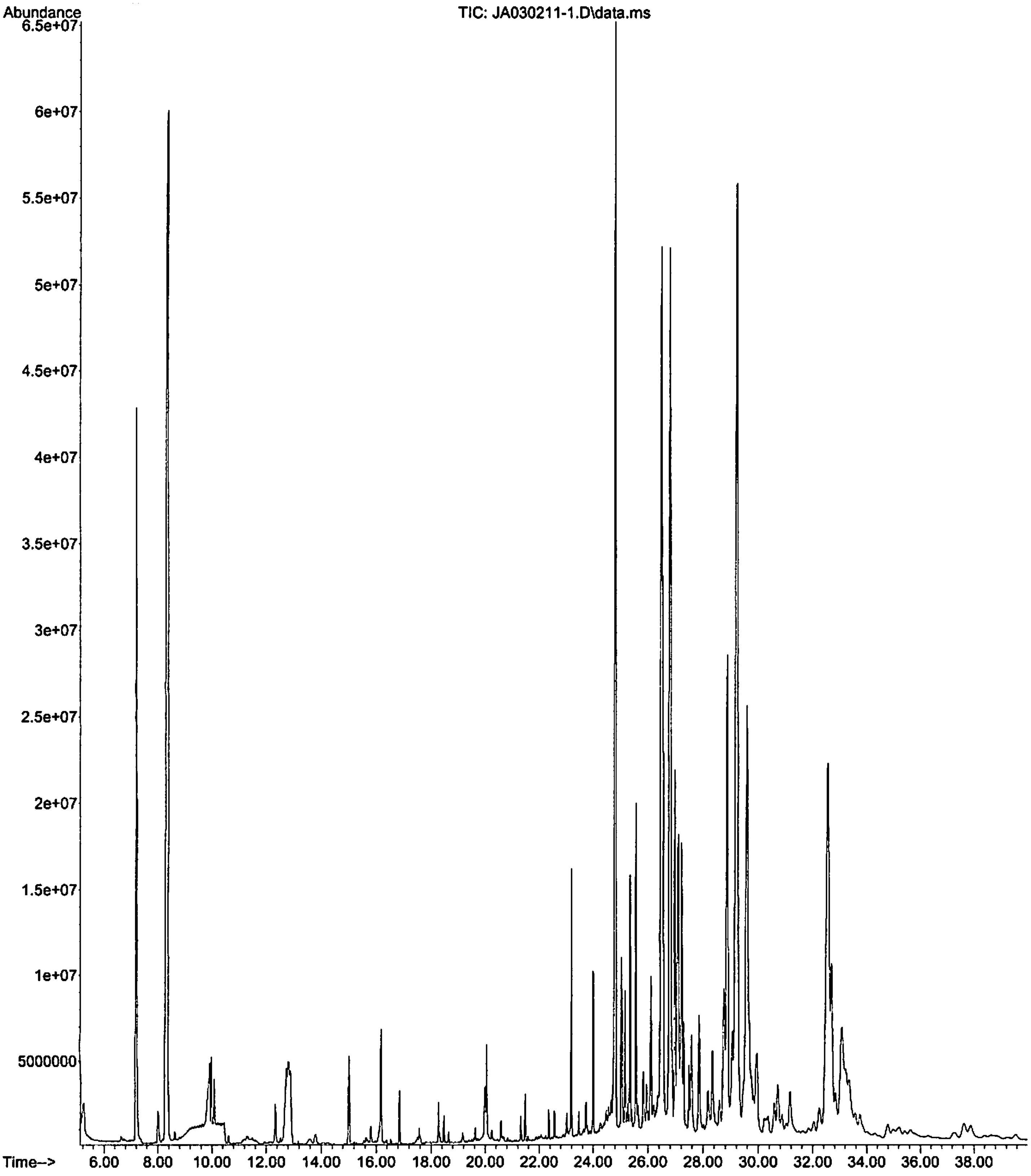

```
File        :D:\Aldrich\JA-11\JA030211-1.D
Operator    :
Acquired    :  2 Mar 2011  16:08     using AcqMethod JA-50-280LESS.M
Instrument :   Buba; IIBBL's magical mass spect
Sample Name: 4M 13-14d-old C.ocu. abdb.stern./5ul CH2Cl2
Misc Info   : +cont. JA021711-1;adults fed nepetalactol/4d
Vial Number: 1
```

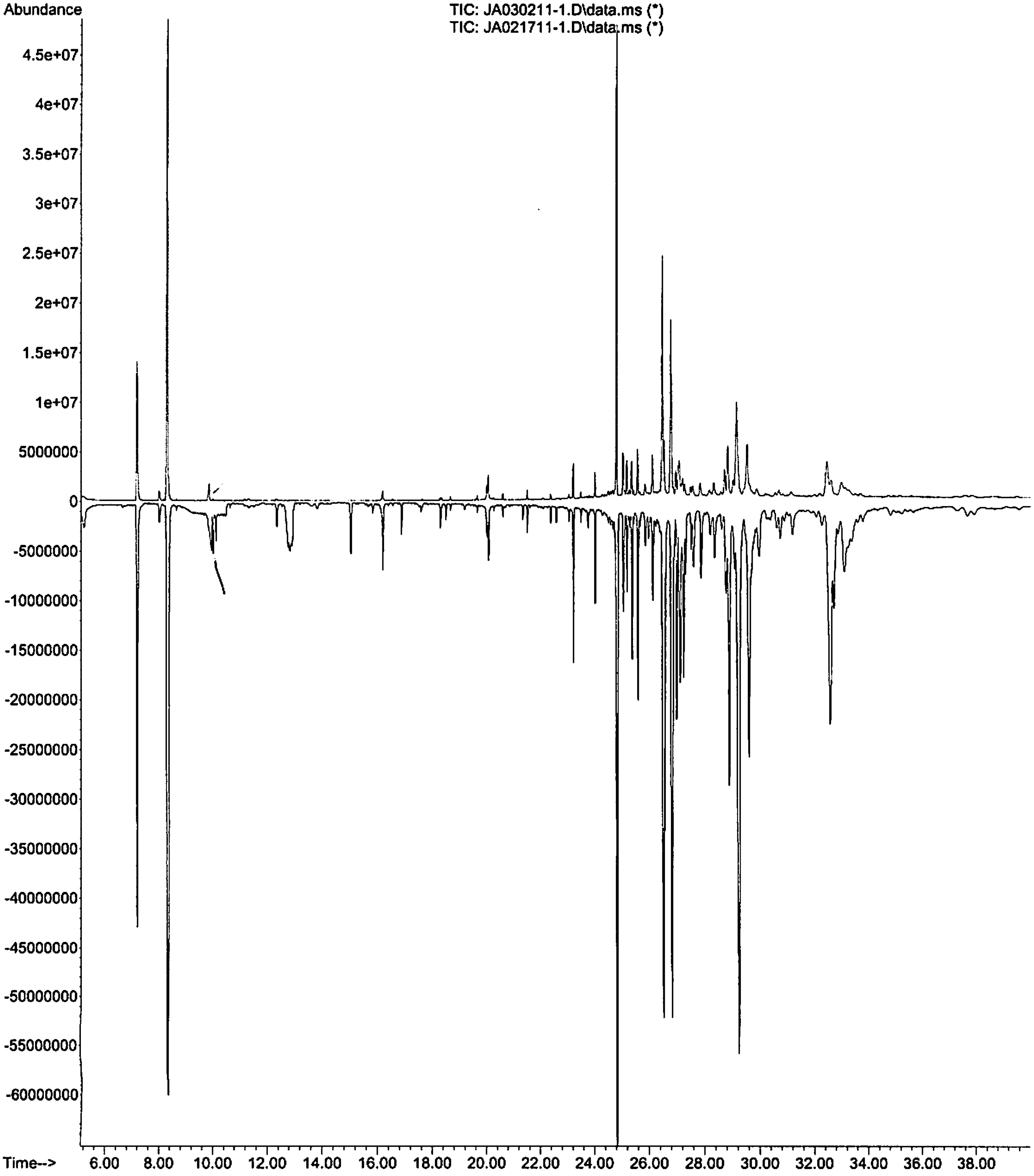

File         :D:\Aldrich\JA-11\JA030211-1.D
Operator     :
Acquired     :  2 Mar 2011  16:08     using AcqMethod JA-50-280LESS.M
Instrument :     Buba; IIBBL's magical mass spect
Sample Name: 4M 13-14d-old C.ocu. abdb.stern./5ul CH2Cl2
Misc Info   : +cont. JA021711-1;adults fed nepetalactol/4d
Vial Number: 1

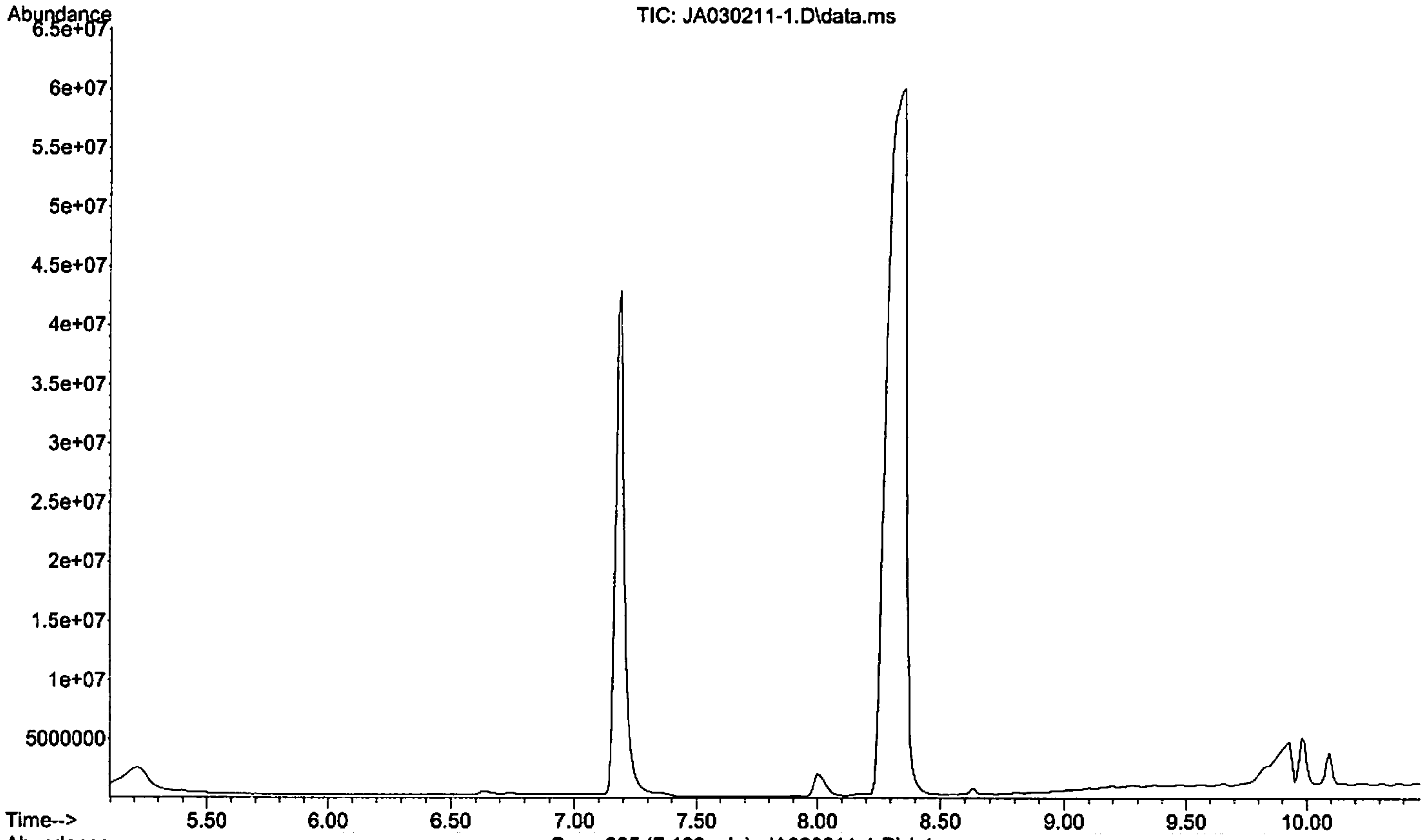


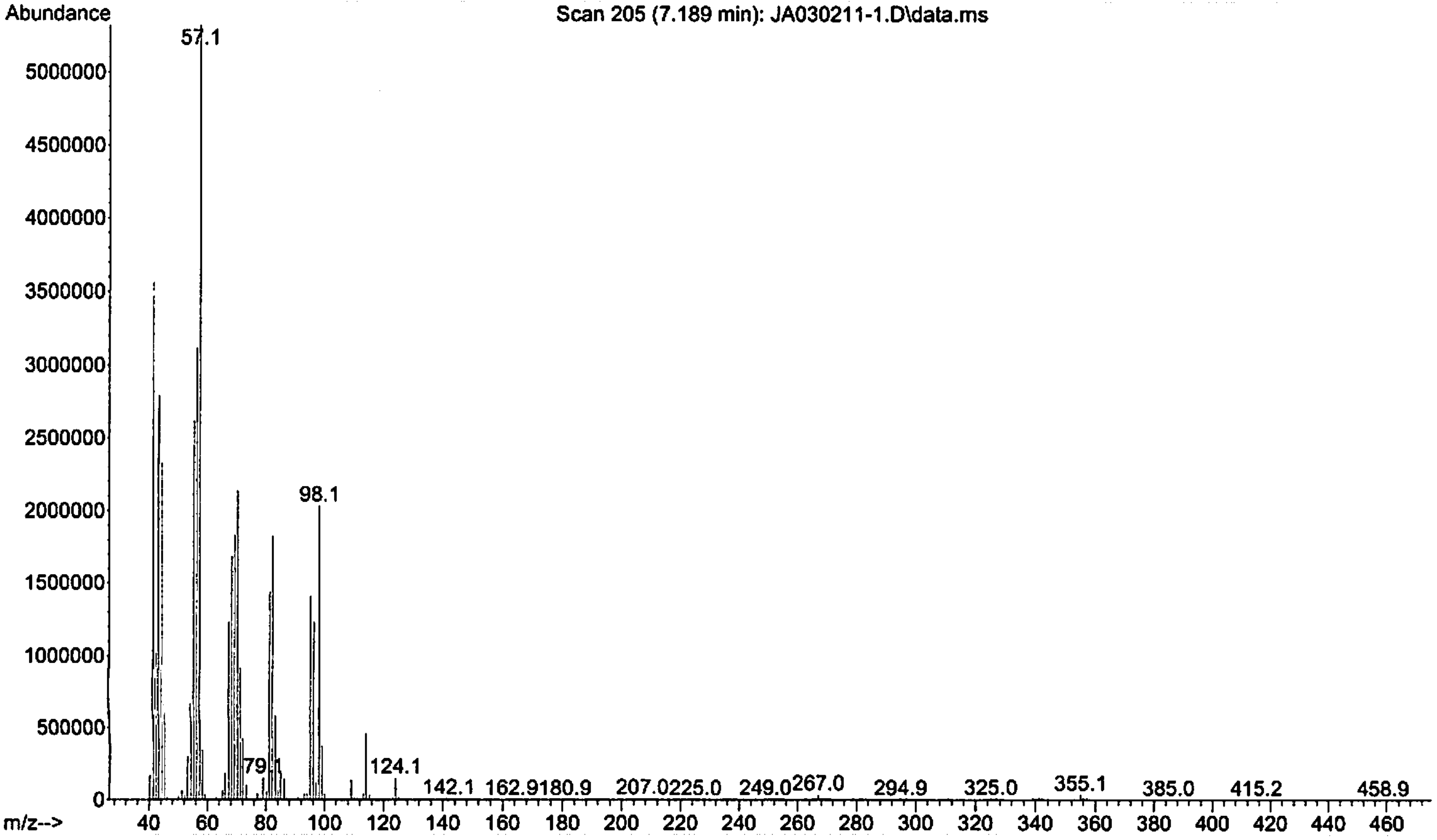

File :D:\Aldrich\JA-11\JA030211-1.D
Operator :
Acquired : 2 Mar 2011 16:08 using AcqMethod JA-50-280LESS.M
Instrument : Buba; IIBBL's magical mass spect
Sample Name: 4M 13-14d-old C.ocu. abdb.stern./5ul CH2Cl2
Misc Info : +cont. JA021711-1;adults fed nepetalactol/4d
Vial Number: 1

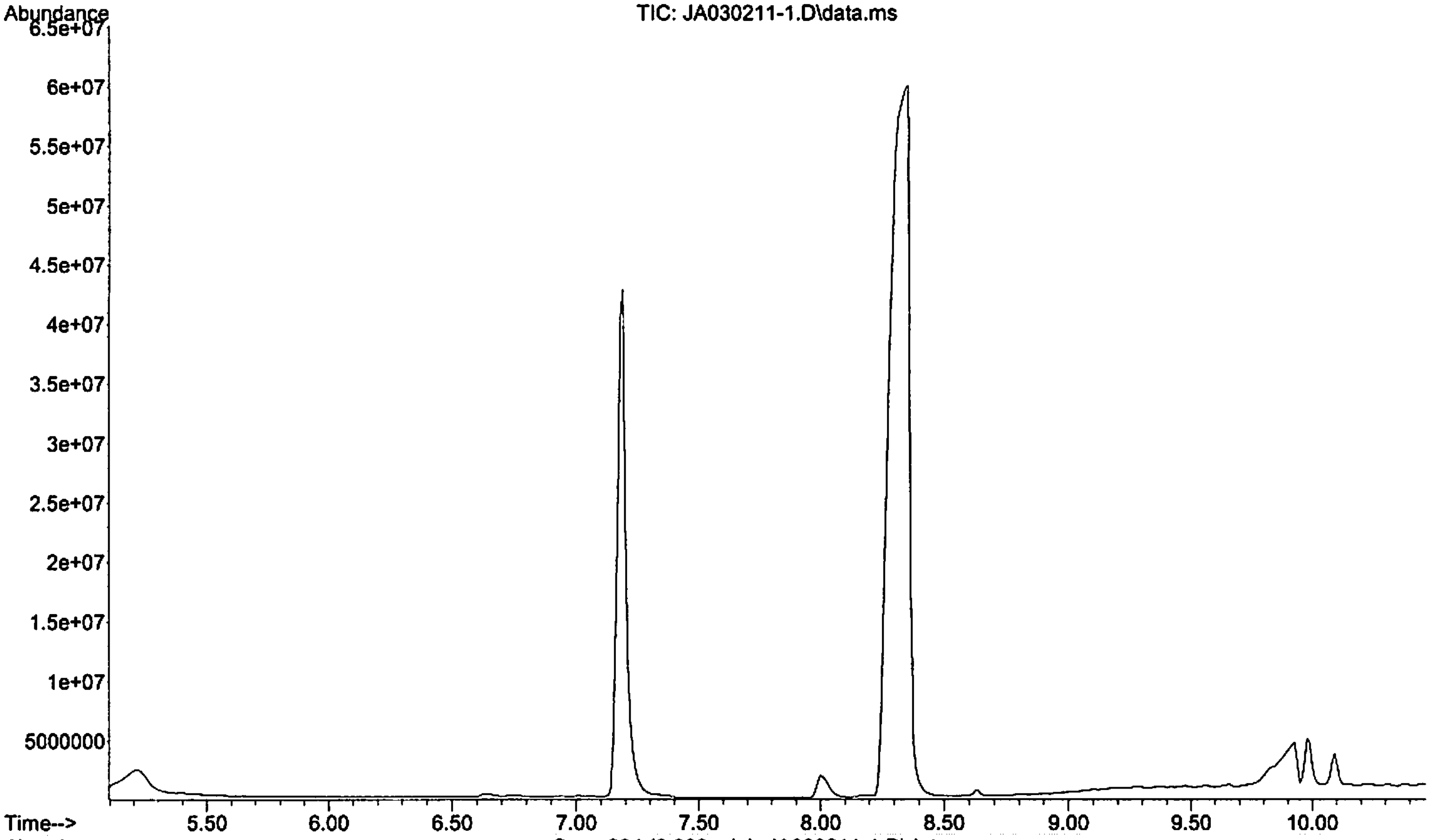


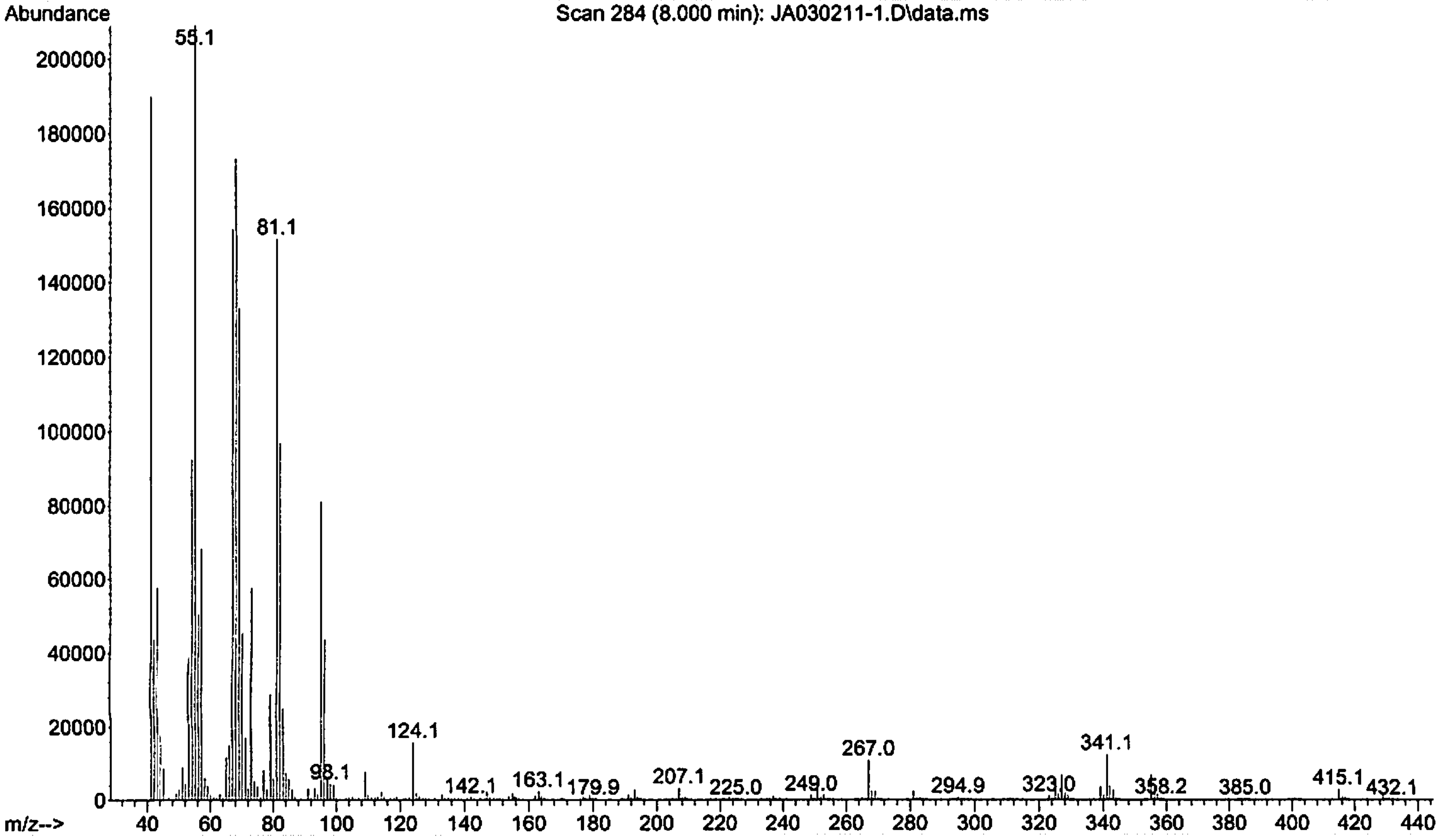

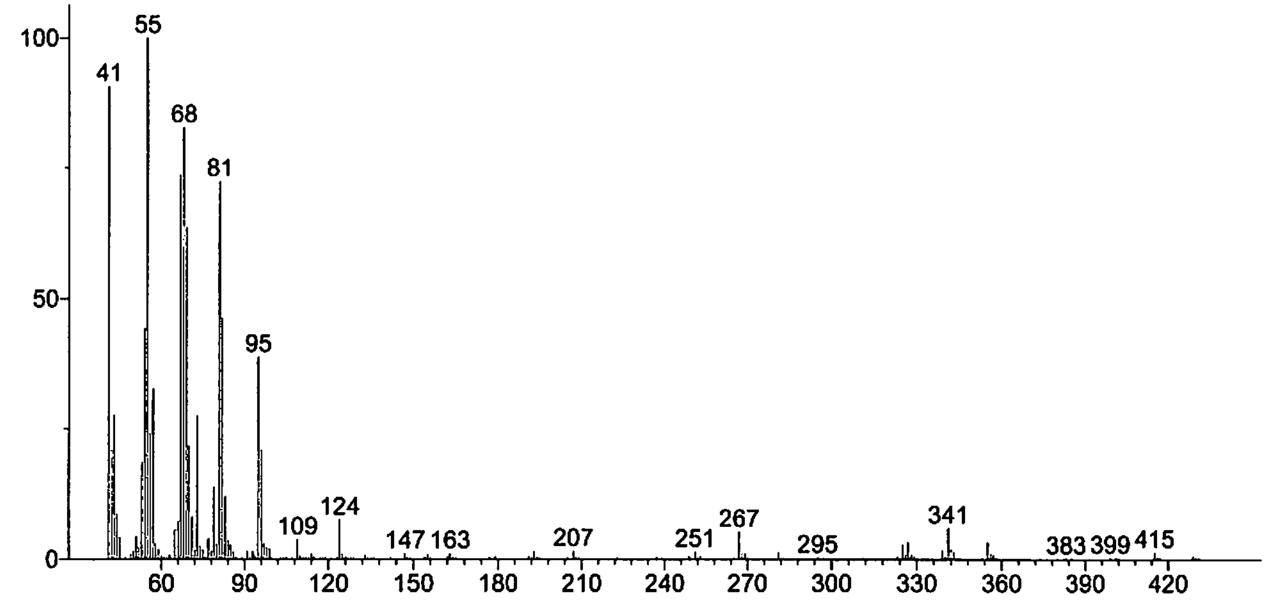


(Text File) Scan 284 (8.000 min): JA030211-1.D\data.ms

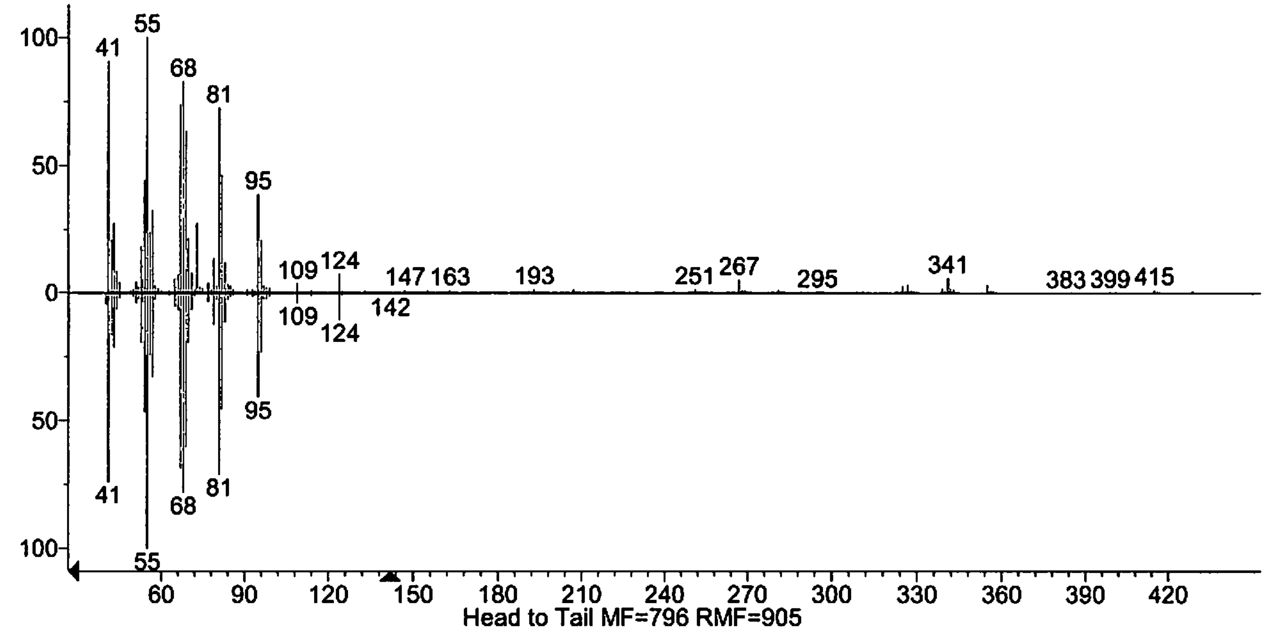


Head to Tail MF=796 RMF=905

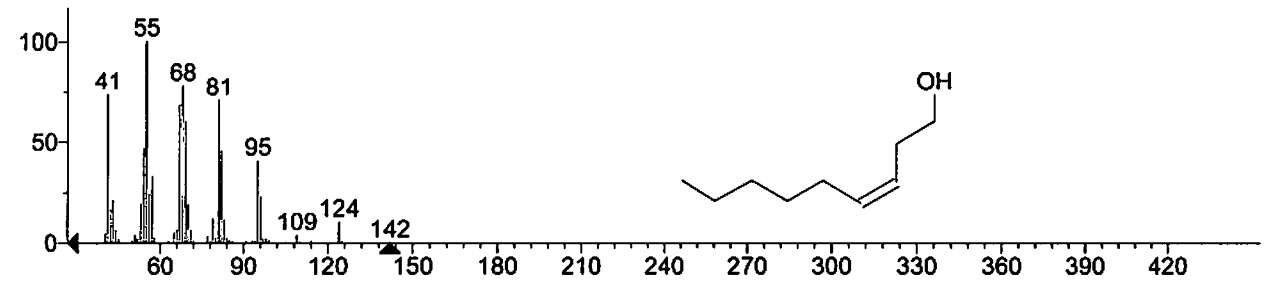


(mainlib) 3-Nonen-1-ol, (Z)-

```
File        :D:\Aldrich\JA-11\JA030211-1.D
Operator    :
Acquired    :  2 Mar 2011  16:08     using AcqMethod JA-50-280LESS.M
Instrument :    Buba; IIBBL's magical mass spect
Sample Name: 4M 13-14d-old C.ocu. abdb.stern./5ul CH2Cl2
Misc Info  : +cont. JA021711-1;adults fed nepetalactol/4d
Vial Number: 1
```

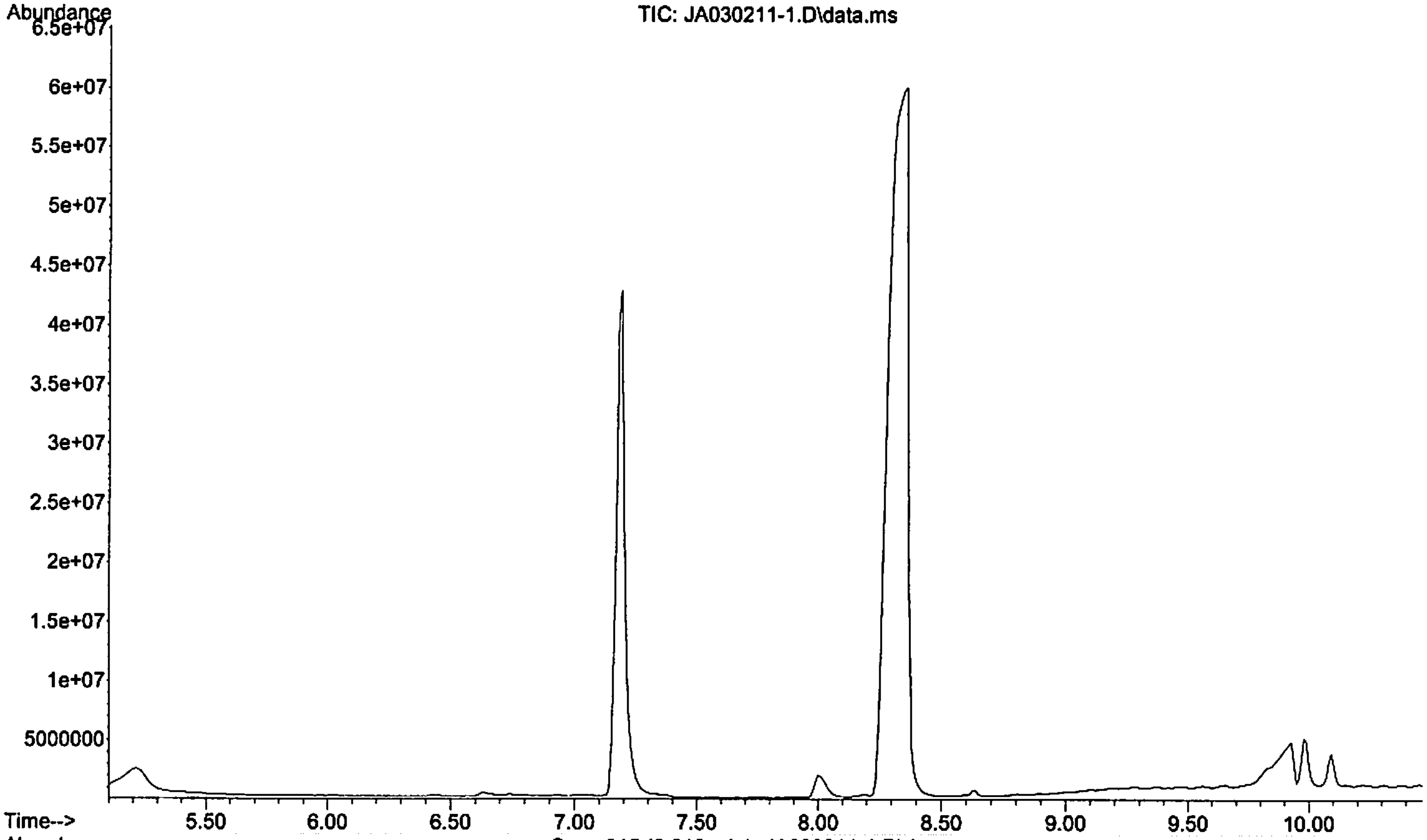


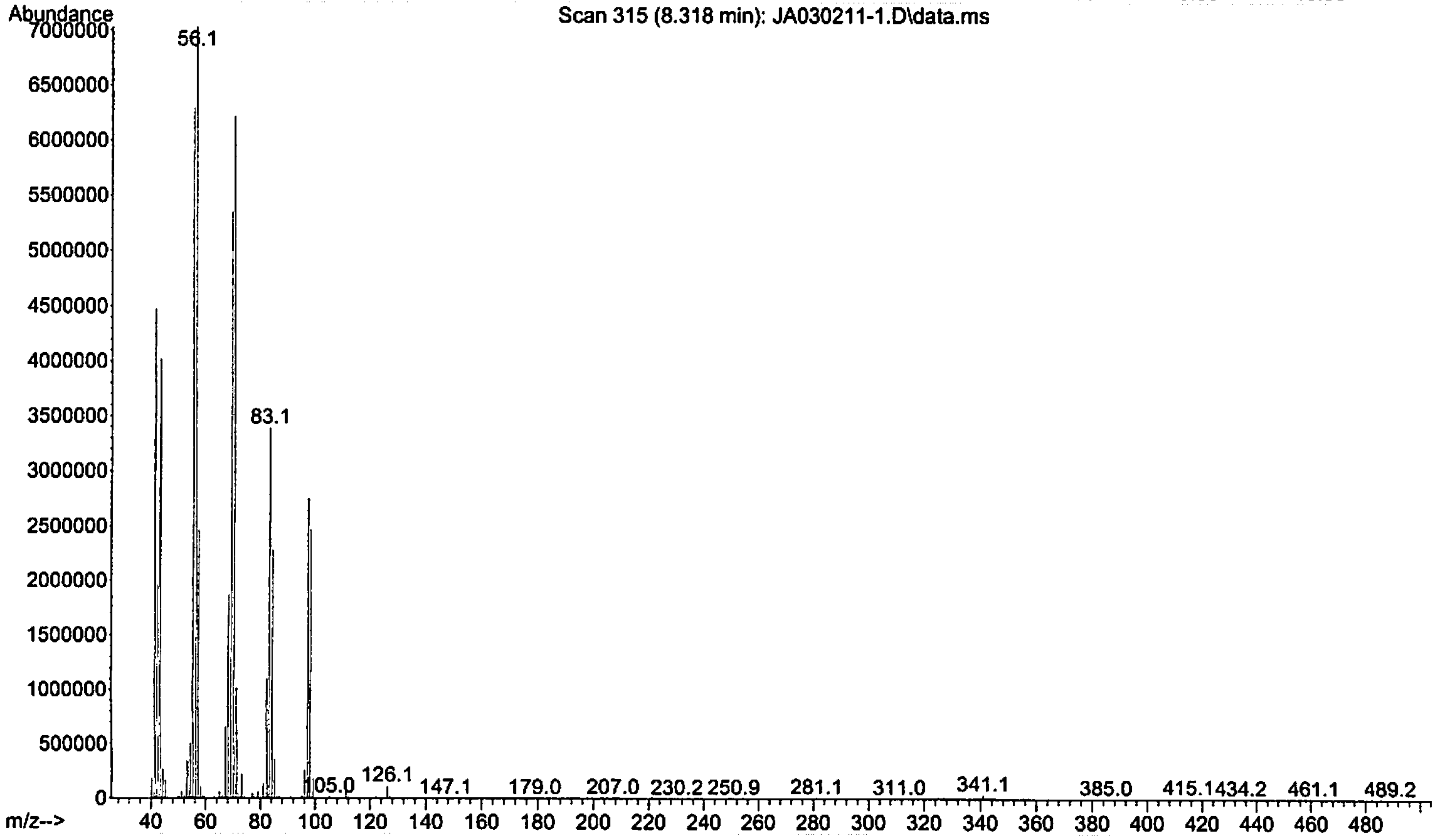

File        :D:\Aldrich\JA-11\JA030211-1.D
Operator    :
Acquired    :  2 Mar 2011  16:08     using AcqMethod JA-50-280LESS.M
Instrument :    Buba; IIBBL's magical mass spect
Sample Name: 4M 13-14d-old C.ocu. abdb.stern./5ul CH2Cl2
Misc Info  : +cont. JA021711-1;adults fed nepetalactol/4d
Vial Number: 1

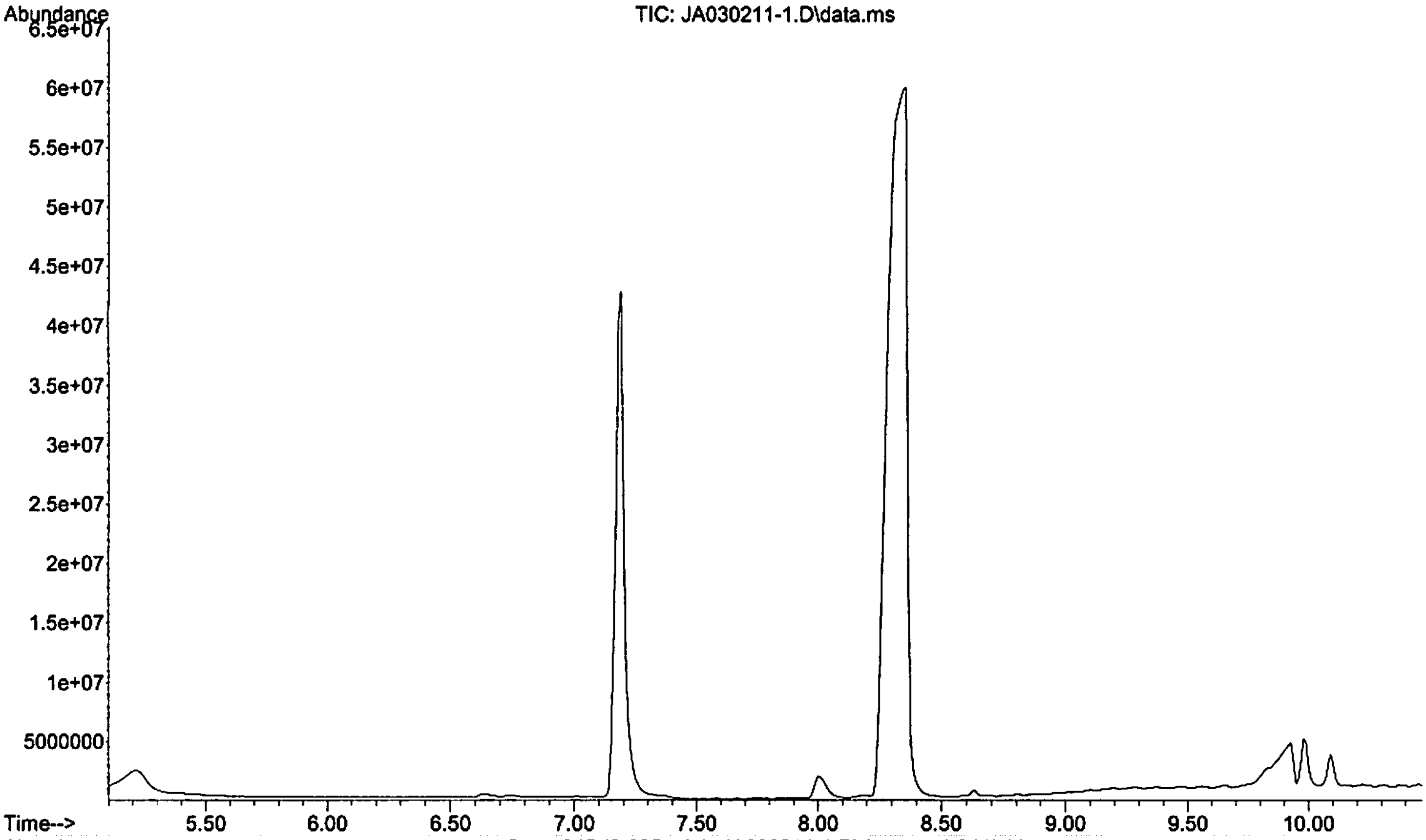


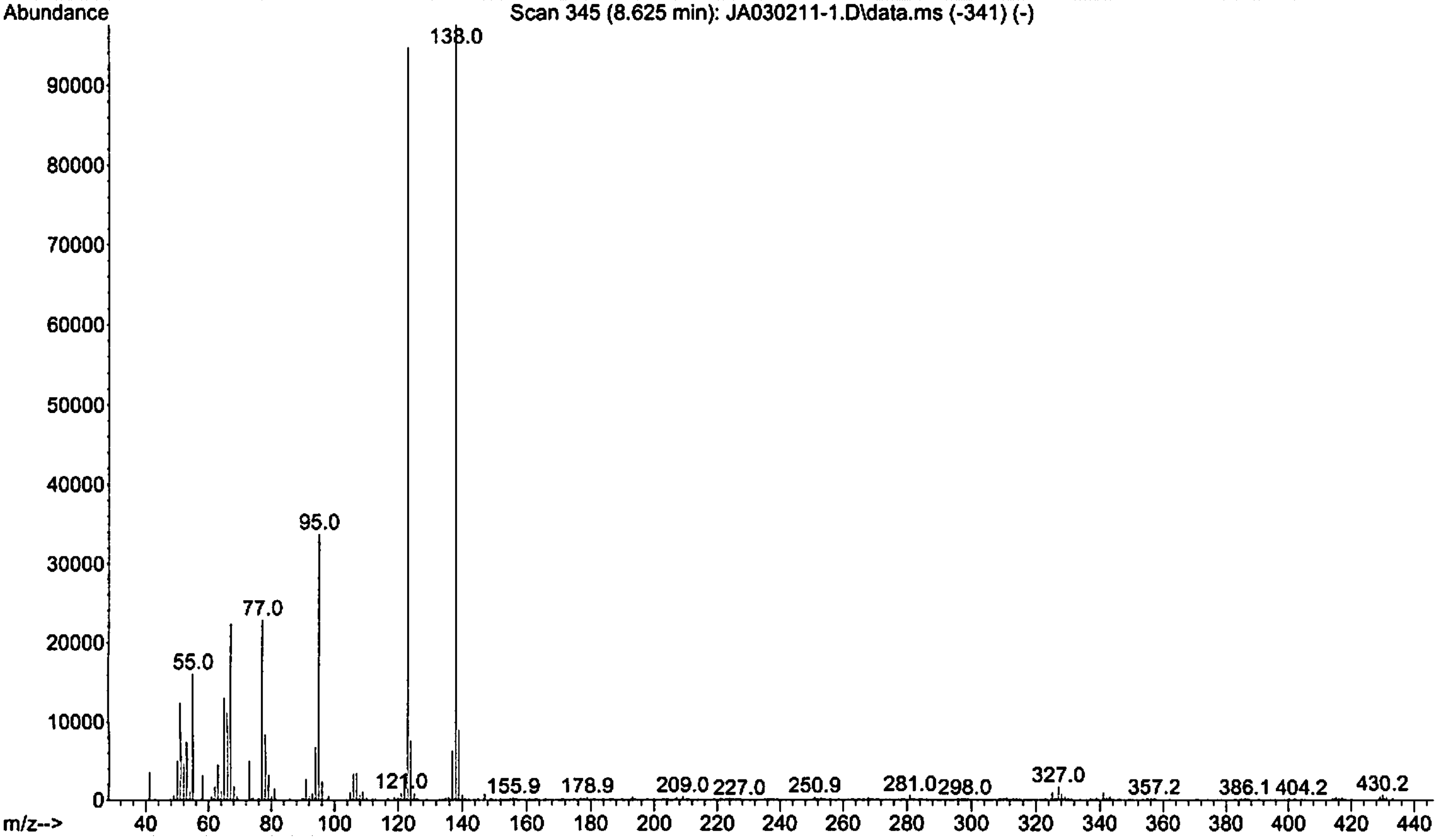

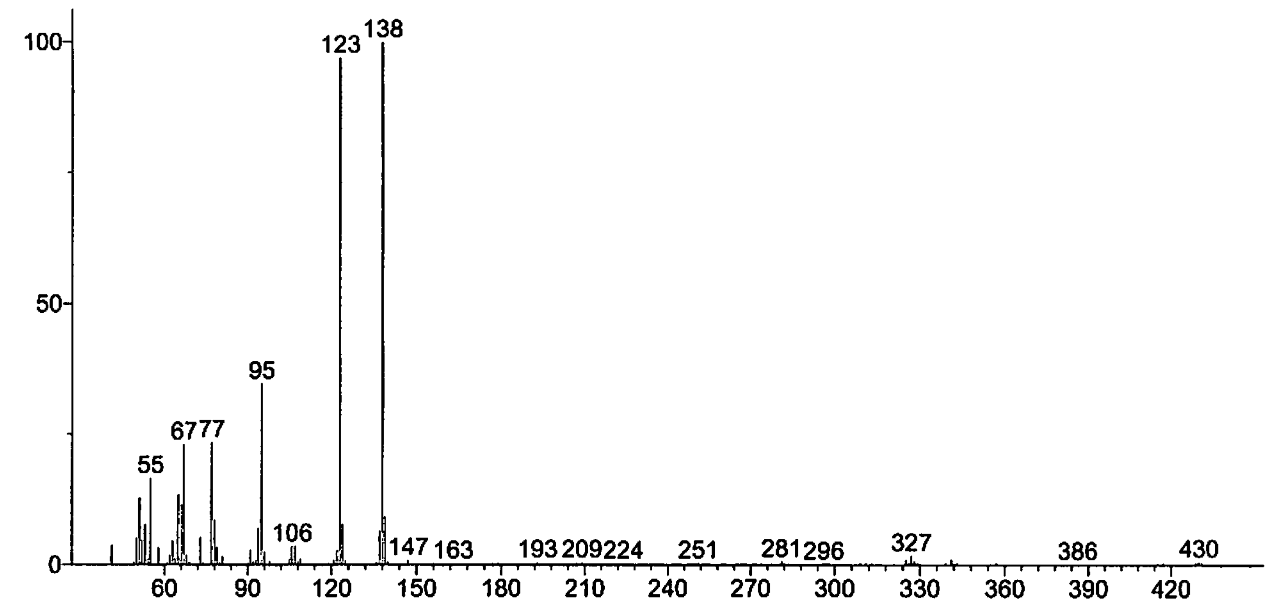


(Text File) Scan 345 (8.625 min): JA030211-1.D\data.ms (-341)

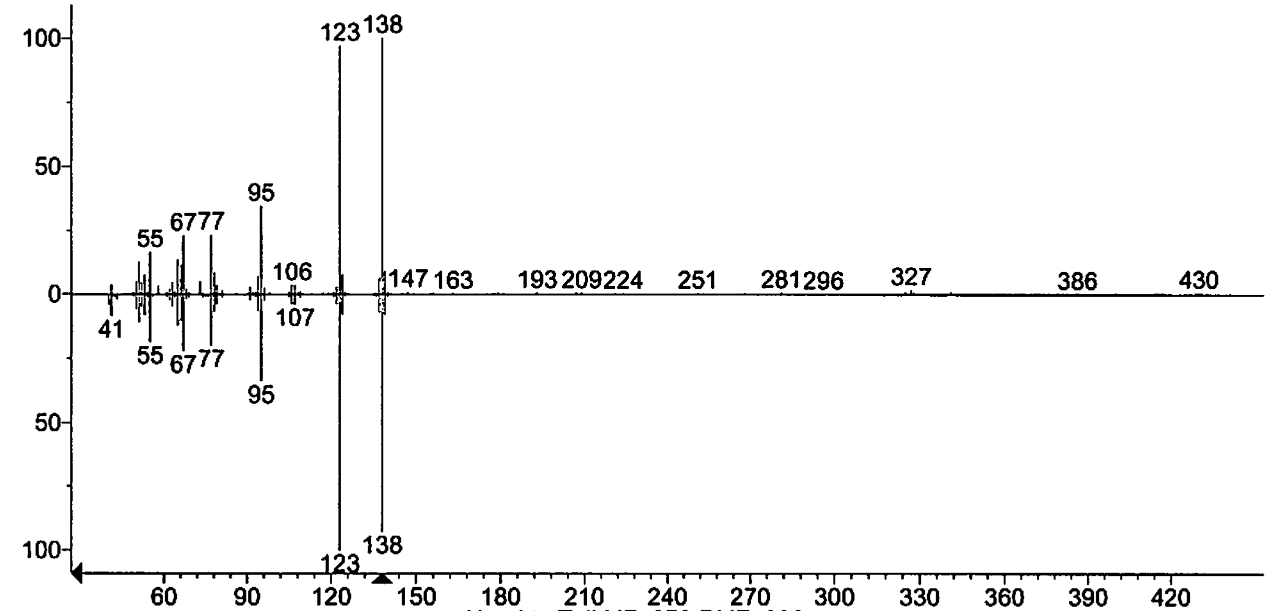


Head to Tail MF=878 RMF=923

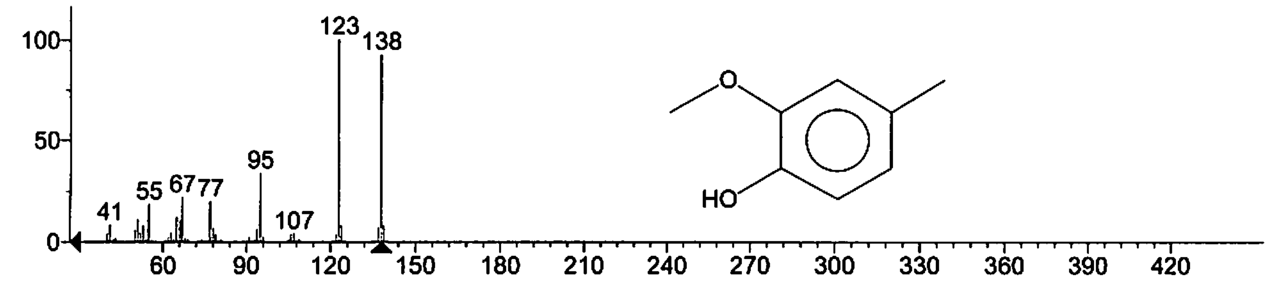


(replib) Phenol, 2-methoxy-4-methyl-

```
File        :D:\Aldrich\JA-11\JA030211-1.D
Operator    :
Acquired    :  2 Mar 2011  16:08     using AcqMethod JA-50-280LESS.M
Instrument  :     Buba; IIBBL's magical mass spect
Sample Name: 4M 13-14d-old C.ocu. abdb.stern./5ul CH2Cl2
Misc Info   : +cont. JA021711-1;adults fed nepetalactol/4d
Vial Number: 1
```

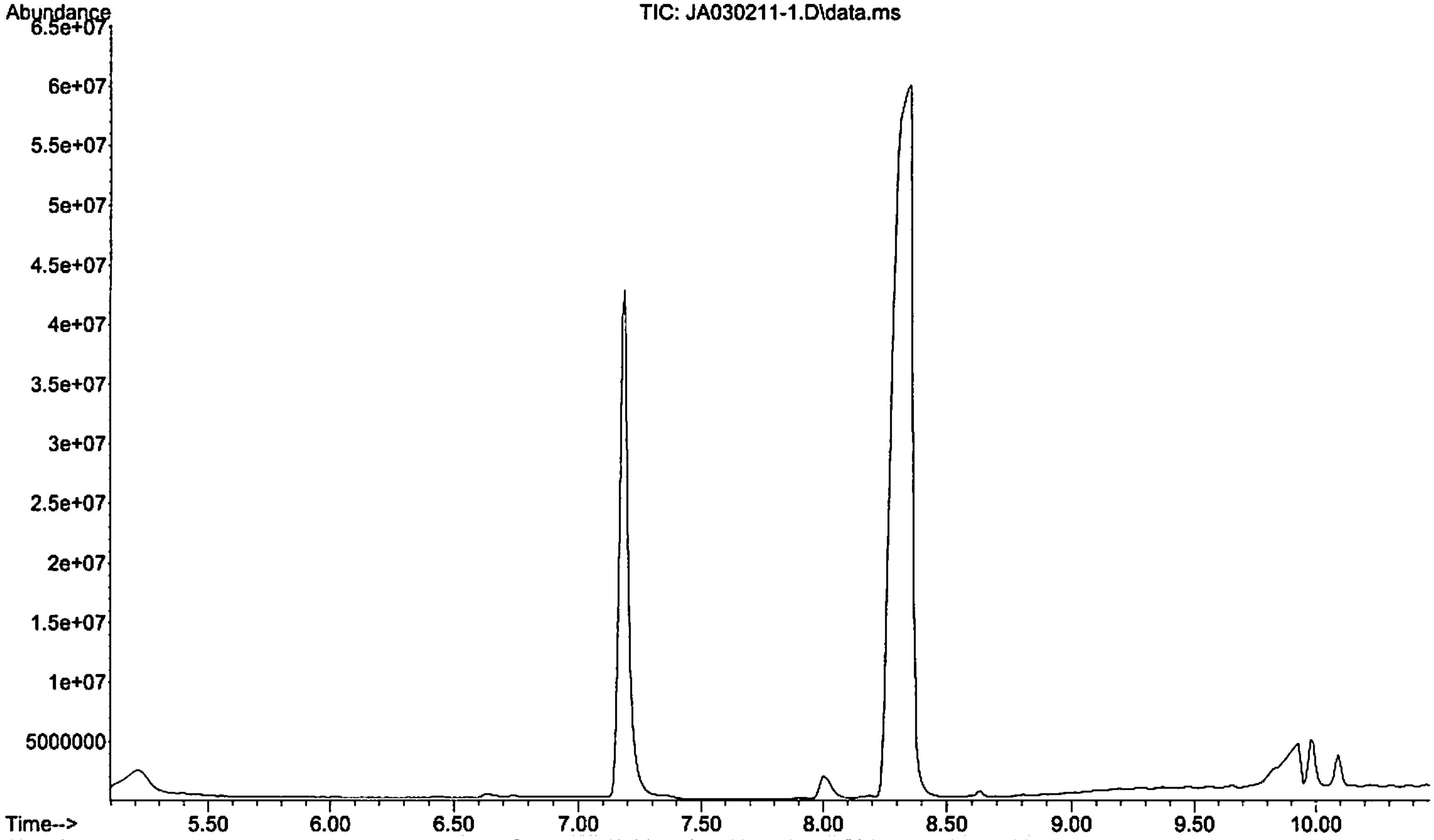


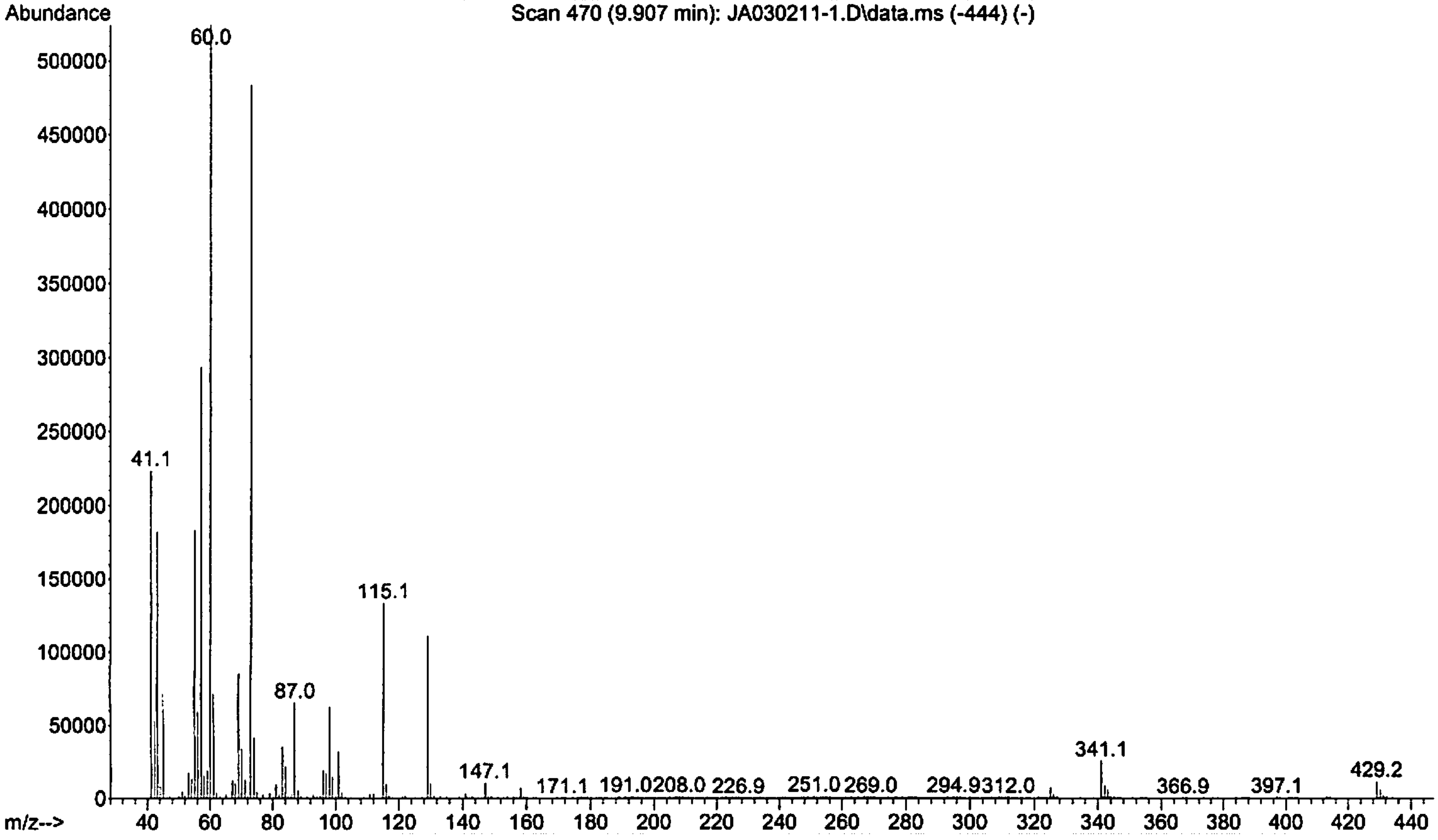

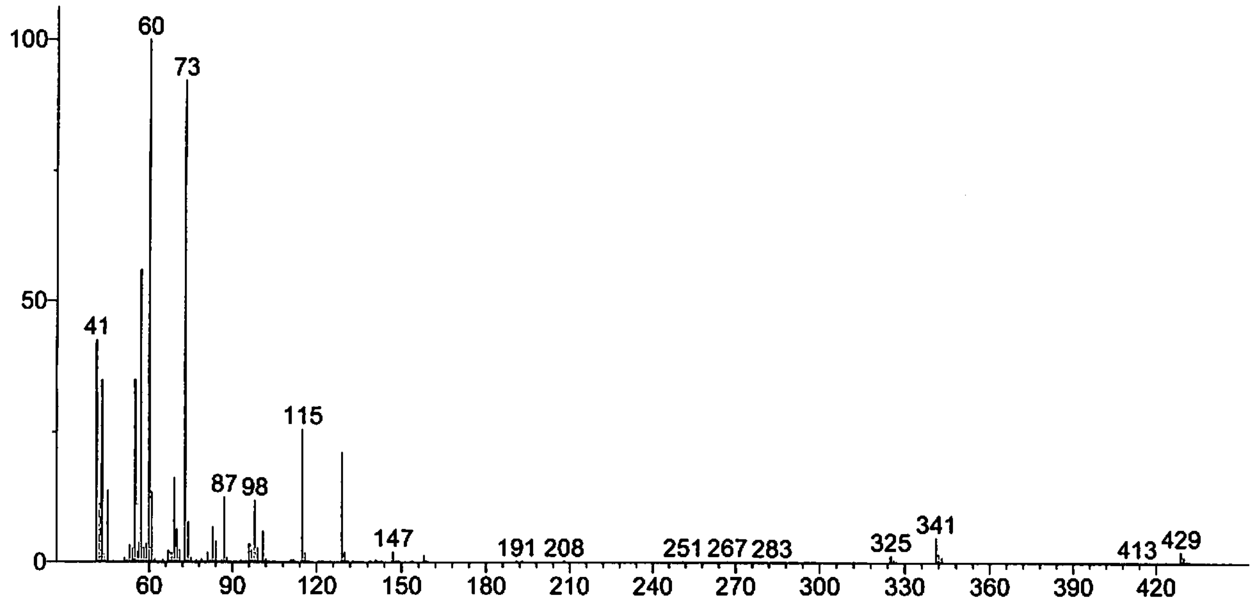


(Text File) Scan 470 (9.907 min): JA030211-1.D\data.ms (-444)

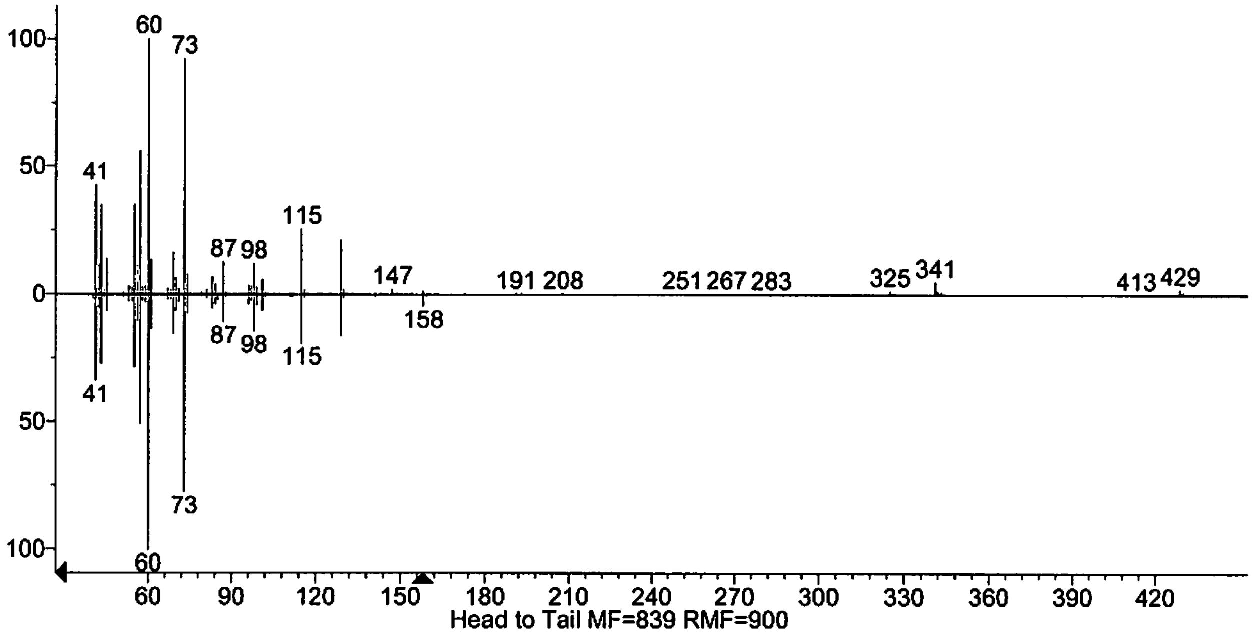


Head to Tail MF=839 RMF=900

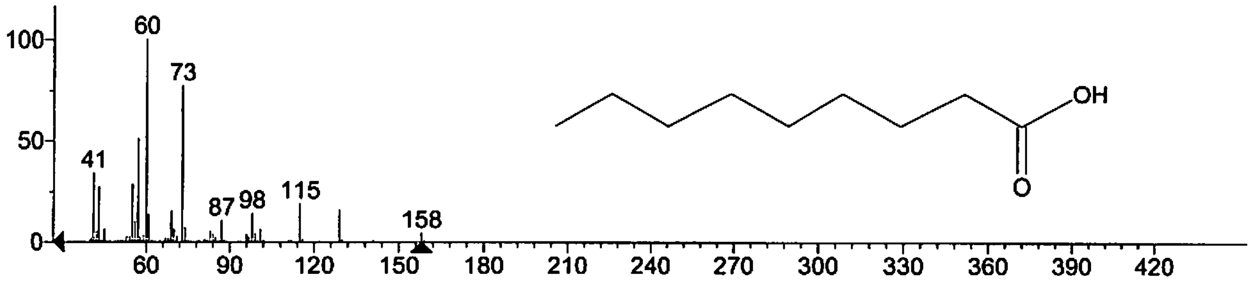


(mainlib) Nonanoic acid

```
File        :D:\Aldrich\JA-11\JA030211-1.D
Operator    :
Acquired    :  2 Mar 2011  16:08     using AcqMethod JA-50-280LESS.M
Instrument  :    Buba; IIBBL's magical mass spect
Sample Name: 4M 13-14d-old C.ocu. abdb.stern./5ul CH2Cl2
Misc Info   : +cont. JA021711-1;adults fed nepetalactol/4d
Vial Number: 1
```

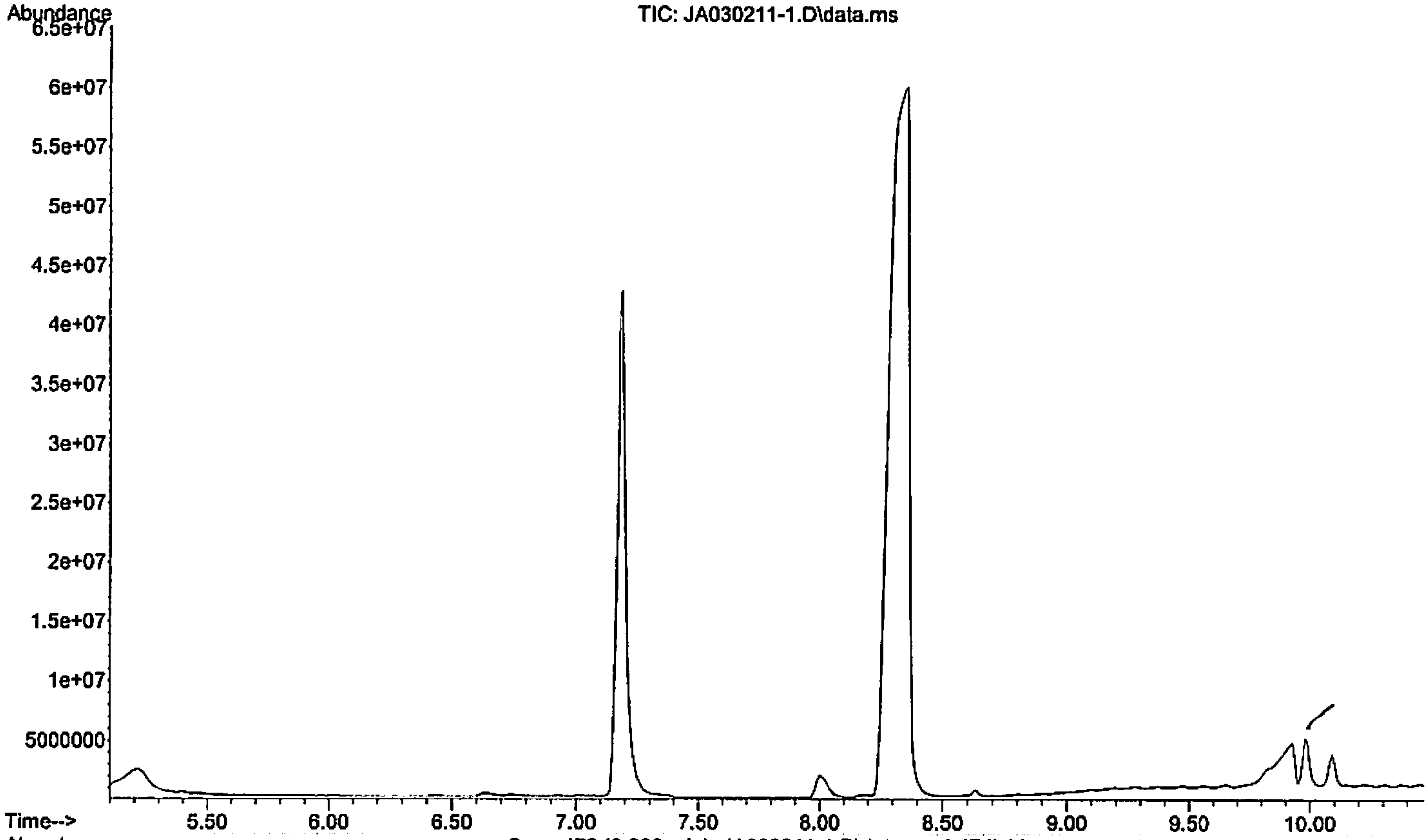


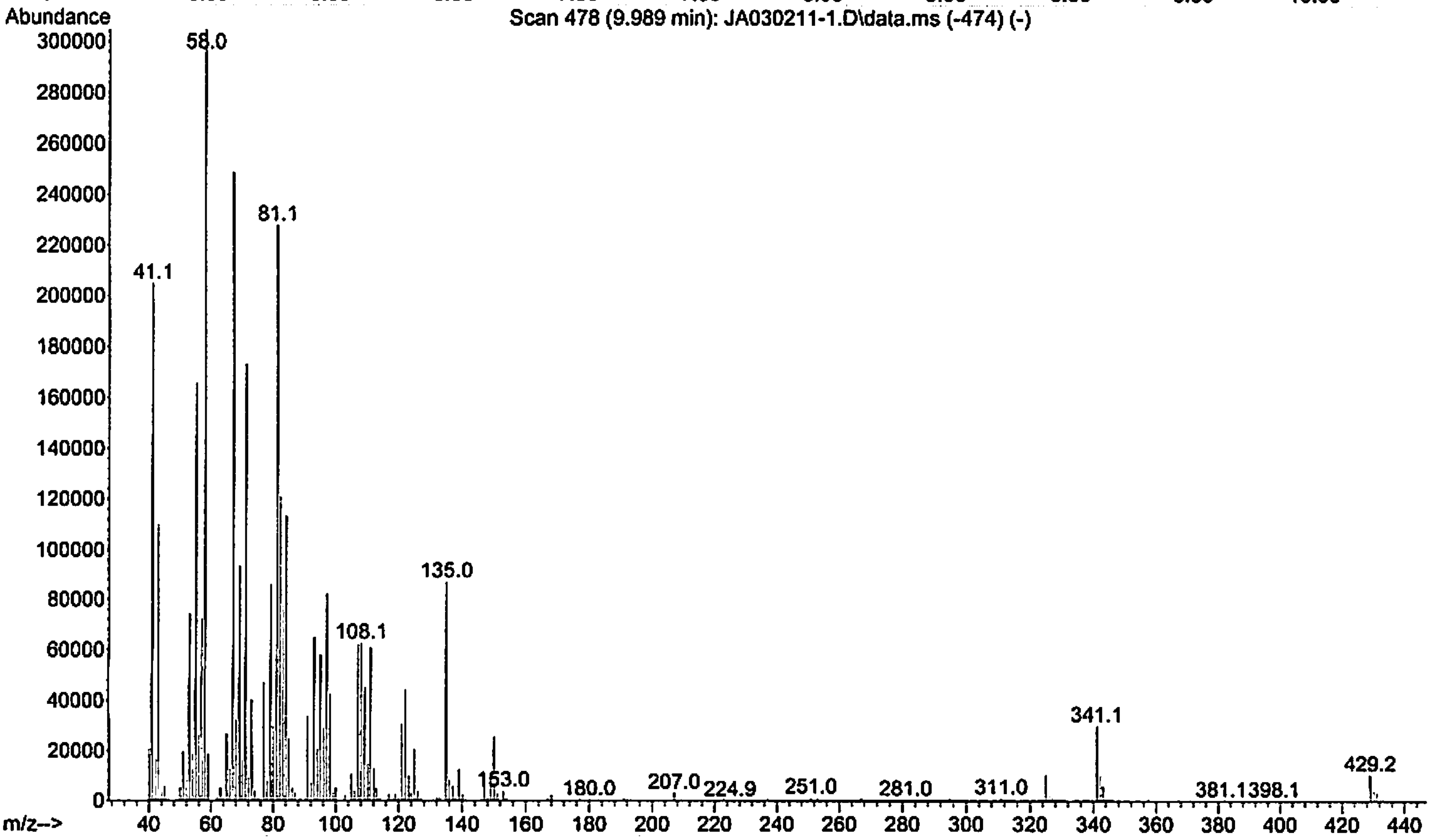

File        :D:\Aldrich\JA-11\JA030211-1.D
Operator    :
Acquired    :  2 Mar 2011  16:08     using AcqMethod JA-50-280LESS.M
Instrument  :    Buba; IIBBL's magical mass spect
Sample Name: 4M 13-14d-old C.ocu. abdb.stern./5ul CH2Cl2
Misc Info   : +cont. JA021711-1;adults fed nepetalactol/4d
Vial Number: 1

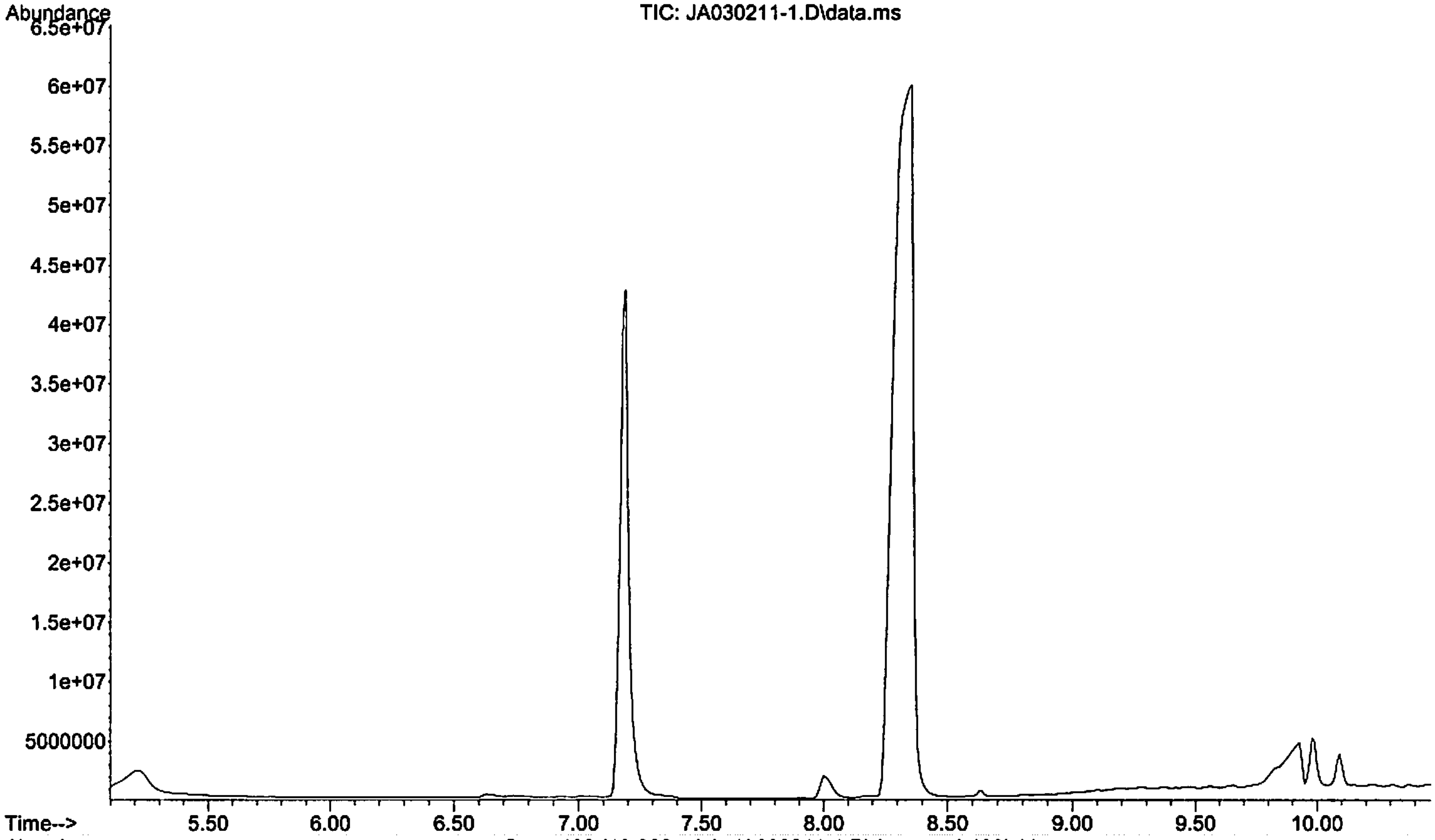


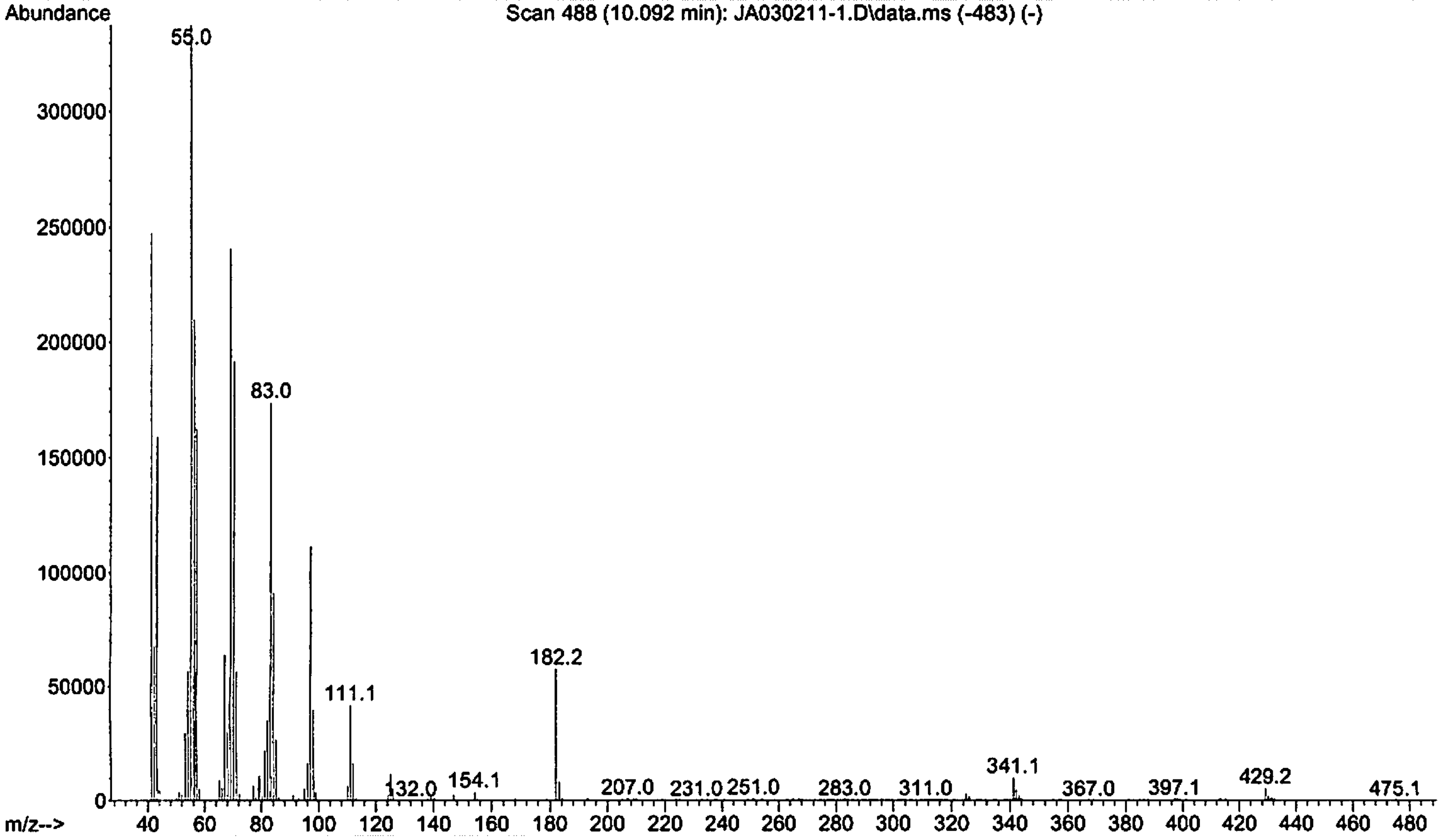

File        :D:\Aldrich\JA-11\JA030211-1.D
Operator    :
Acquired    :  2 Mar 2011  16:08     using AcqMethod JA-50-280LESS.M
Instrument :    Buba; IIBBL's magical mass spect
Sample Name: 4M 13-14d-old C.ocu. abdb.stern./5ul CH2Cl2
Misc Info   : +cont. JA021711-1;adults fed nepetalactol/4d
Vial Number: 1

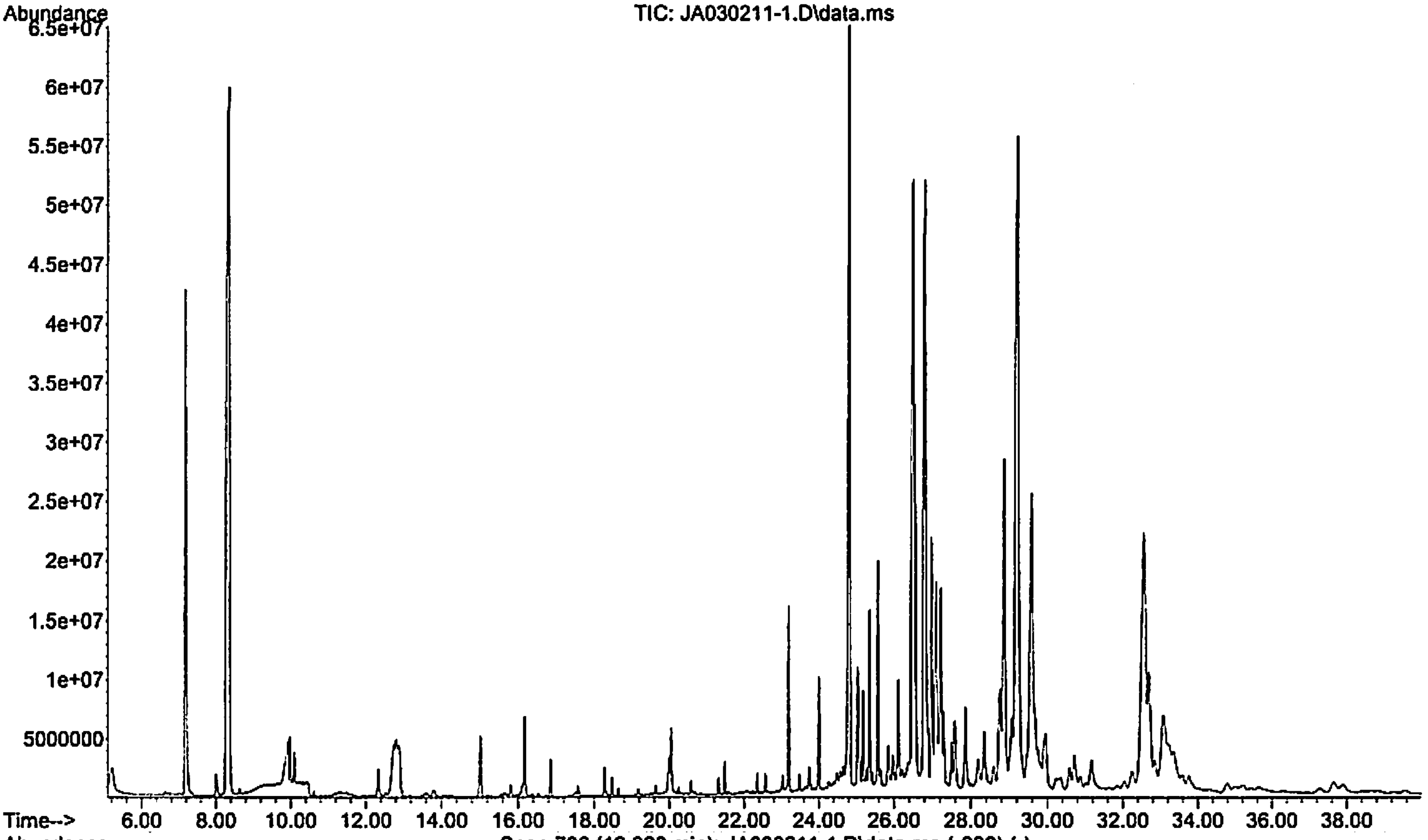


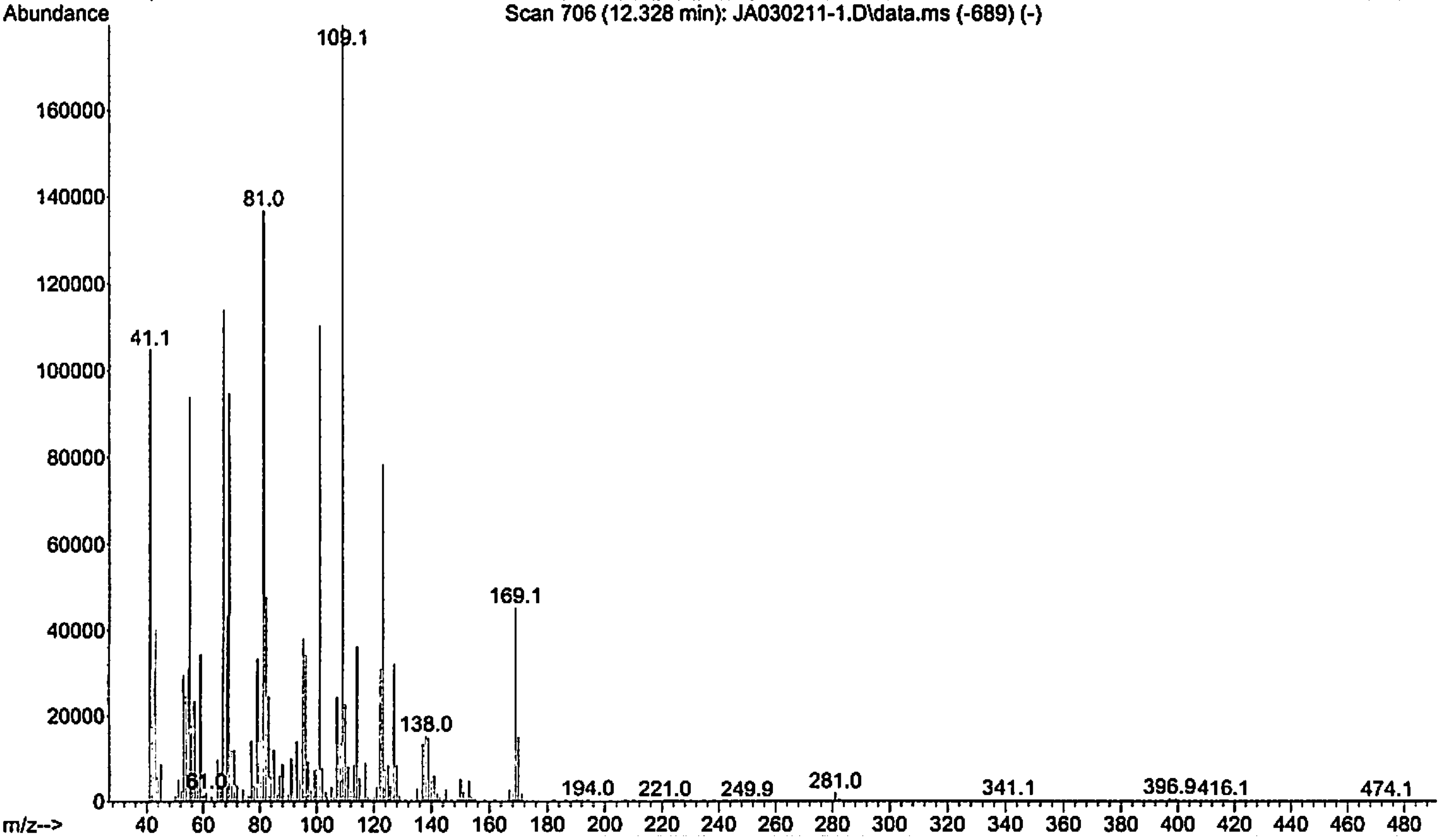

File :D:\Aldrich\JA-11\JA030211-1.D
Operator :
Acquired : 2 Mar 2011 16:08 using AcqMethod JA-50-280LESS.M
Instrument : Buba; IIBBL's magical mass spect
Sample Name: 4M 13-14d-old C.ocu. abdb.stern./5ul CH2Cl2
Misc Info : +cont. JA021711-1;adults fed nepetalactol/4d
Vial Number: 1

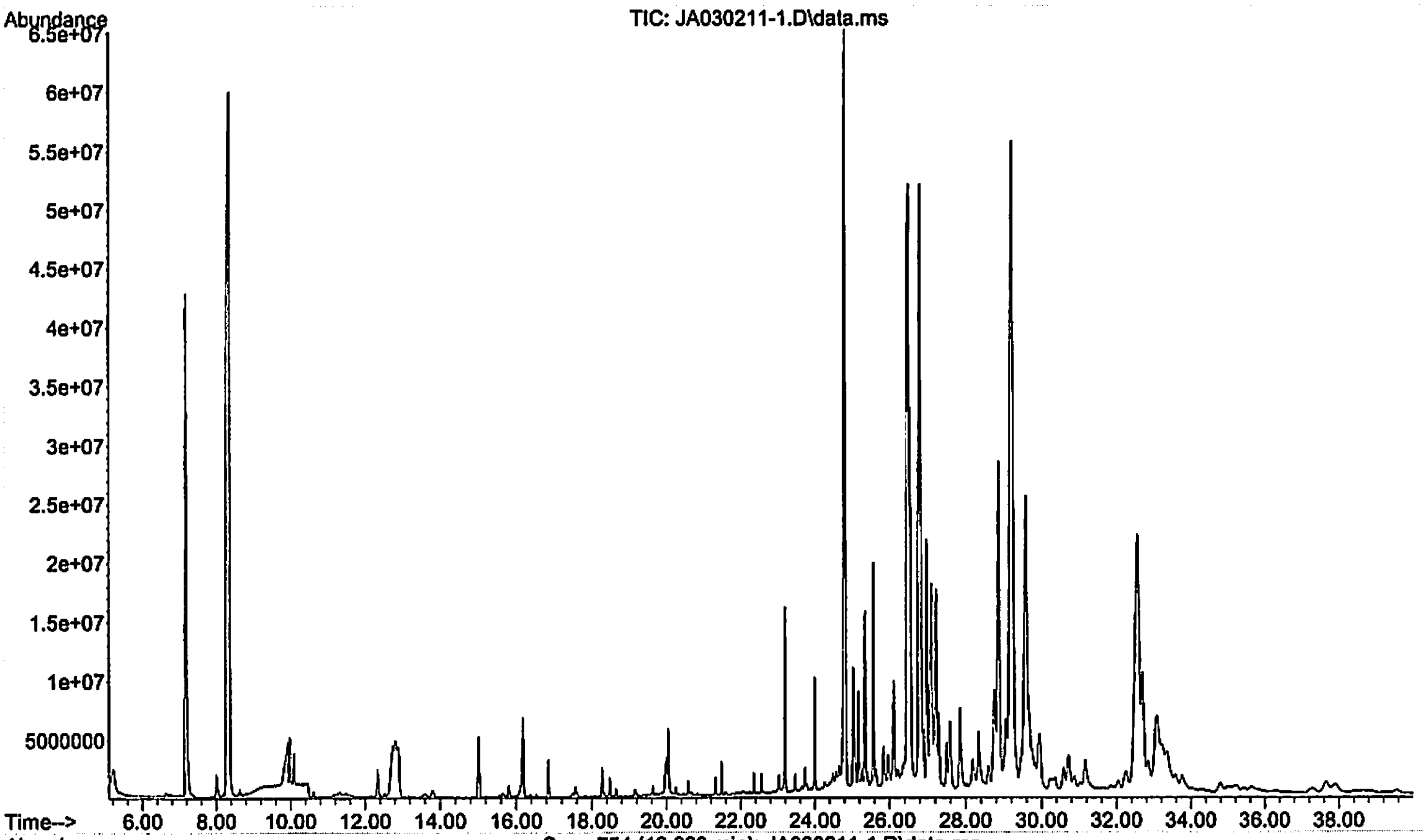


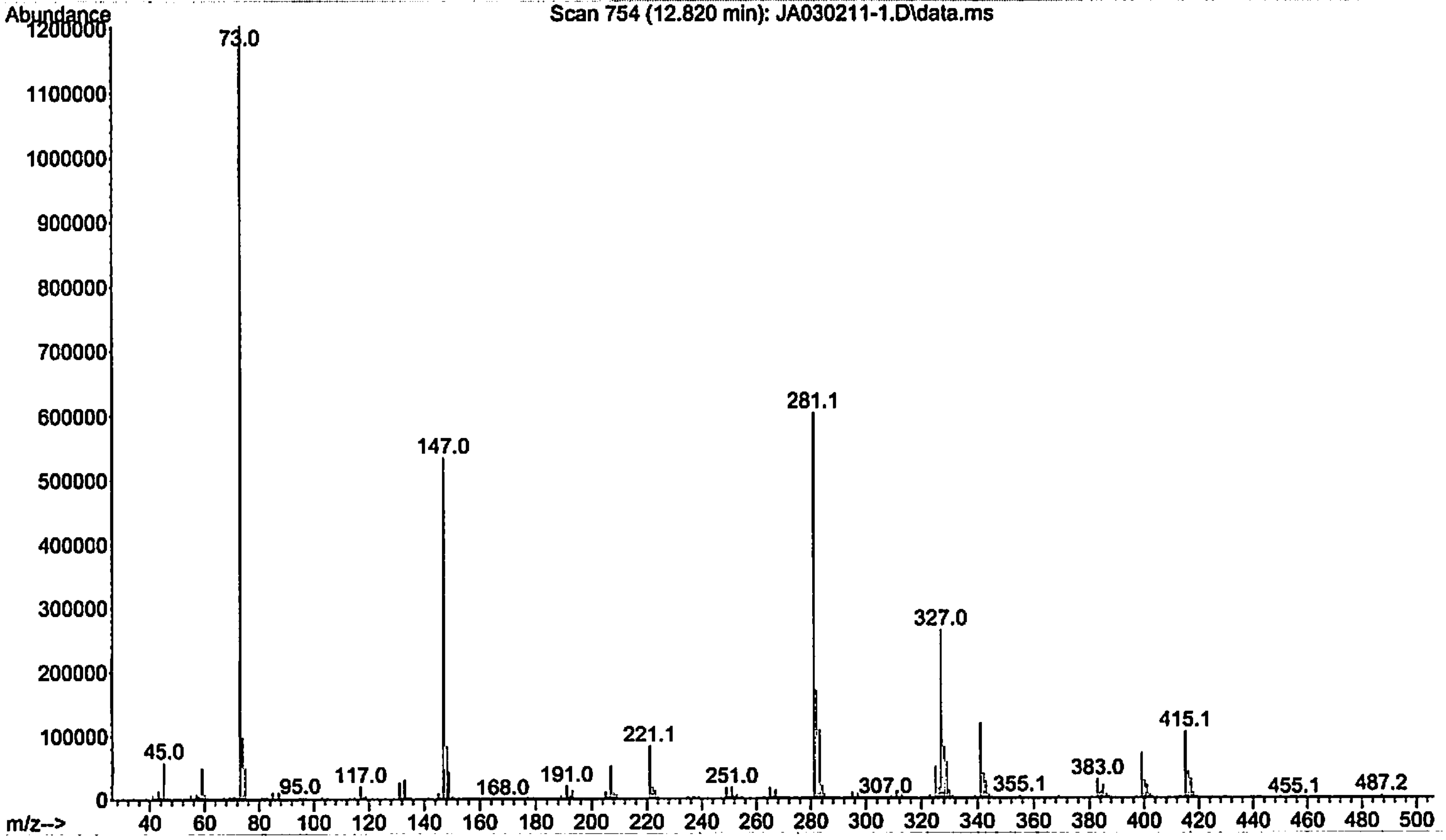

File        :D:\Aldrich\JA-11\JA030211-1.D
Operator    :
Acquired    :  2 Mar 2011  16:08     using AcqMethod JA-50-280LESS.M
Instrument :    Buba; IIBBL's magical mass spect
Sample Name: 4M 13-14d-old C.ocu. abdb.stern./5ul CH2Cl2
Misc Info   : +cont. JA021711-1;adults fed nepetalactol/4d
Vial Number: 1

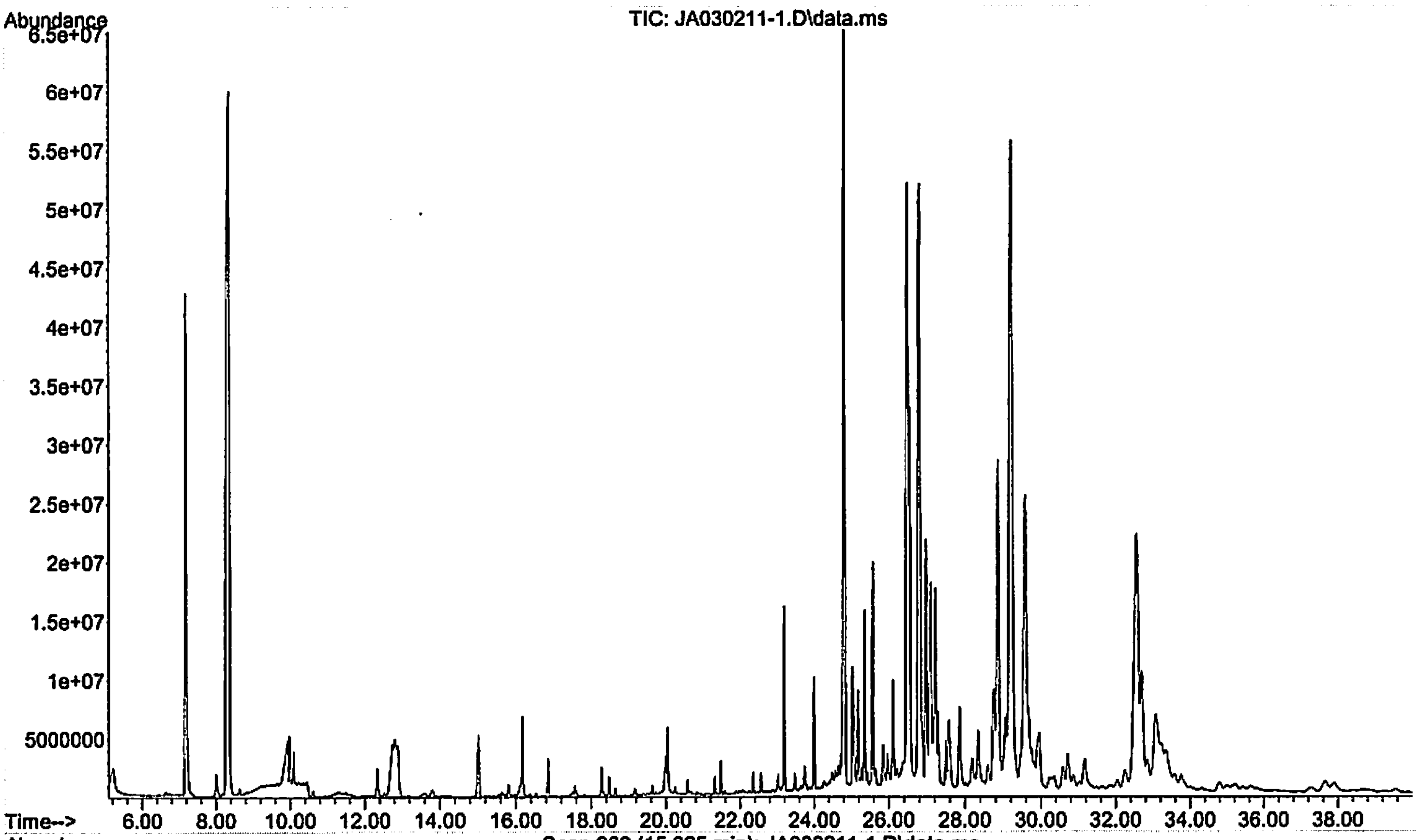


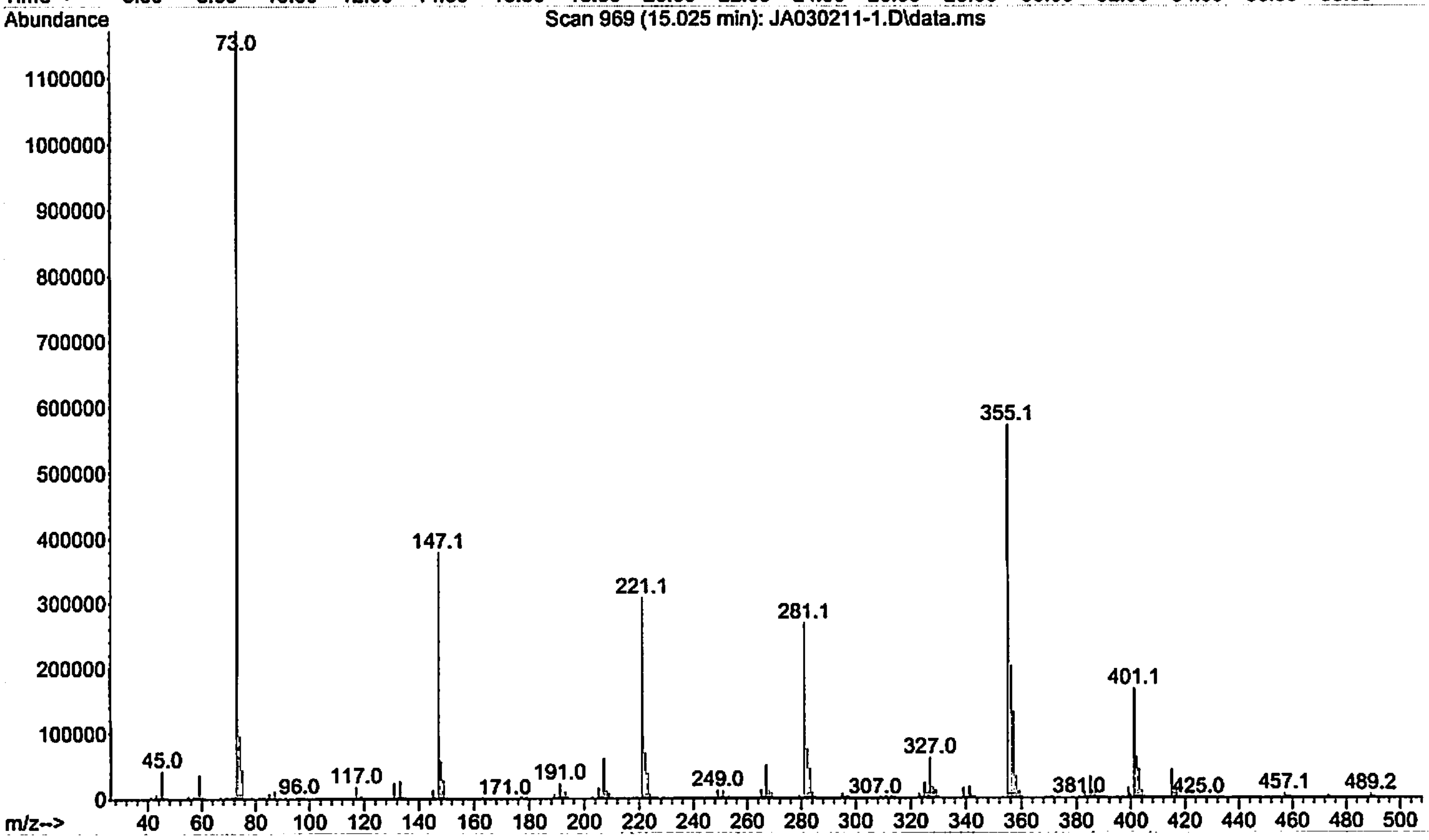

```
File        :D:\Aldrich\JA-11\JA030211-1.D
Operator    :
Acquired    :  2 Mar 2011  16:08     using AcqMethod JA-50-280LESS.M
Instrument :    Buba; IIBBL's magical mass spect
Sample Name: 4M 13-14d-old C.ocu. abdb.stern./5ul CH2Cl2
Misc Info   : +cont. JA021711-1;adults fed nepetalactol/4d
Vial Number: 1
```

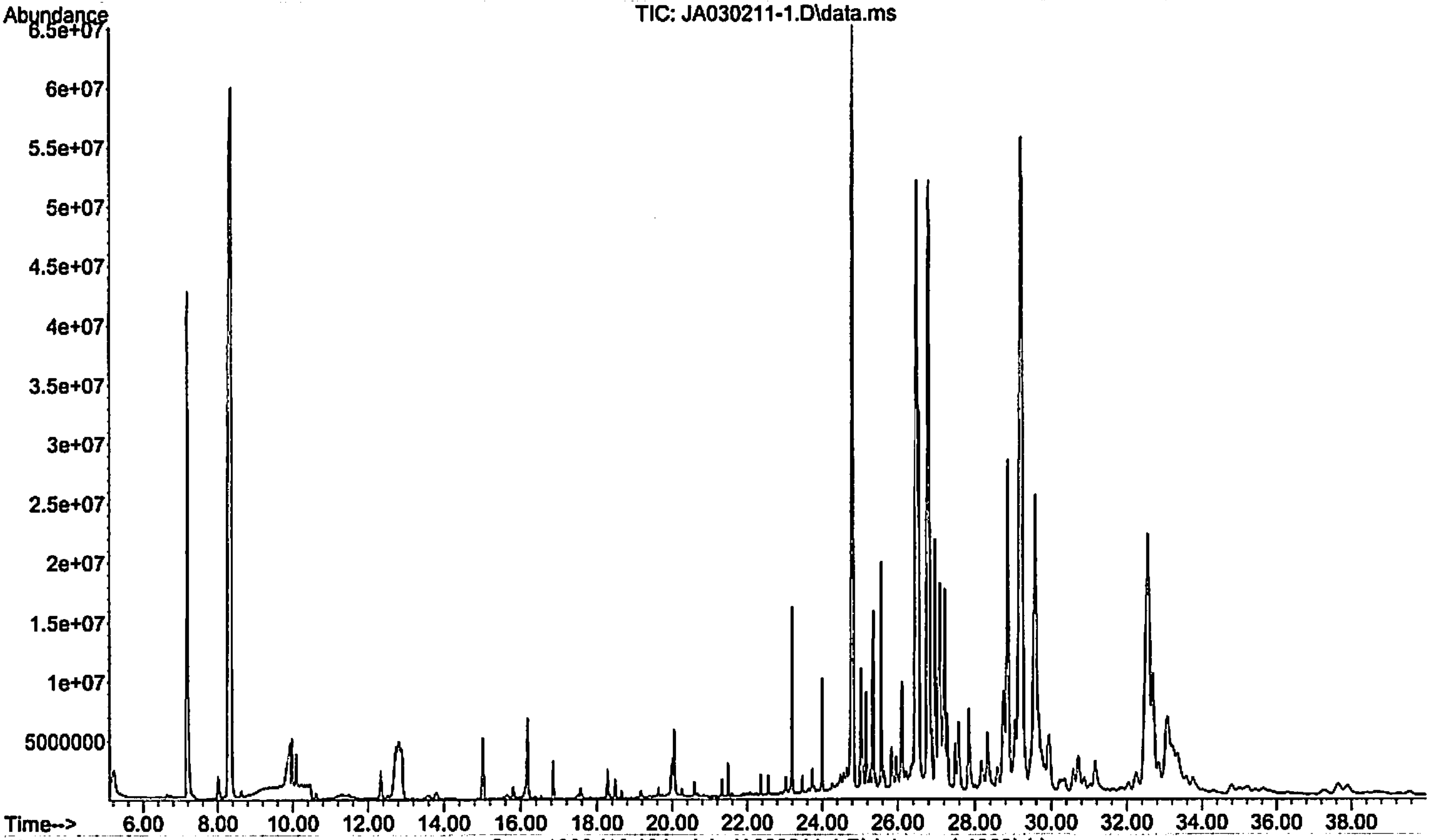


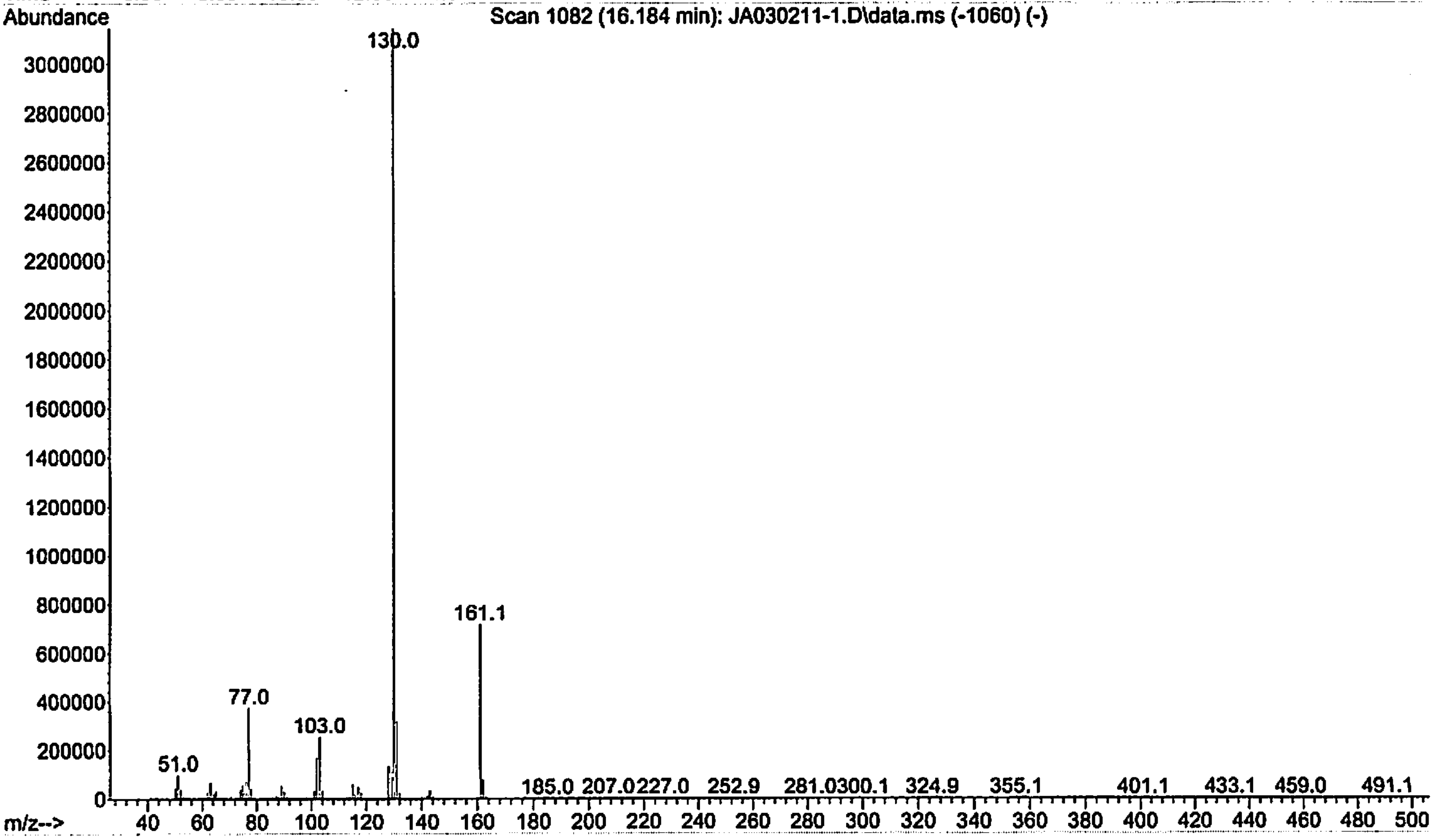

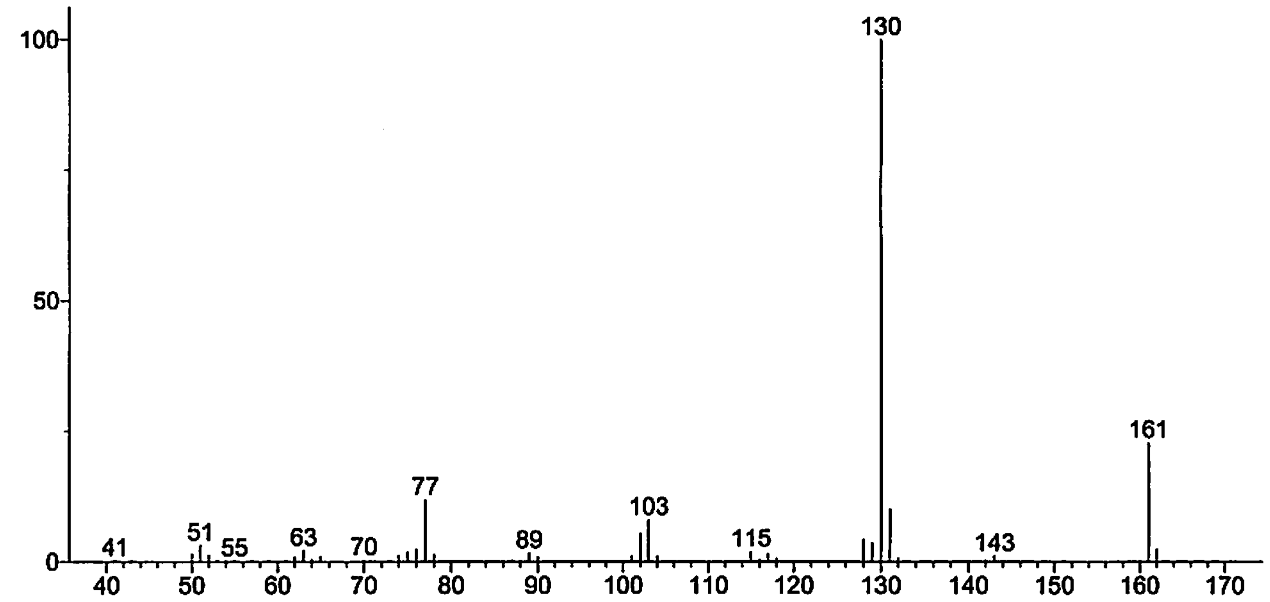


(Text File) Scan 1082 (16.184 min): JA030211-1.D\data.ms (-1060)

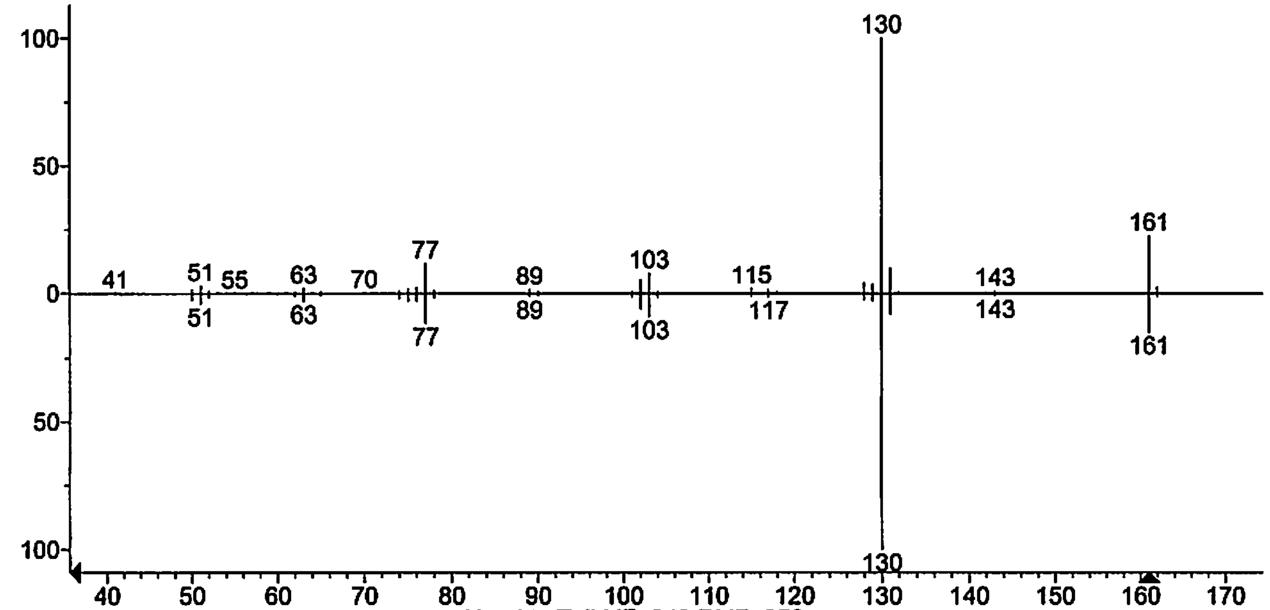


Head to Tail MF=943 RMF=958

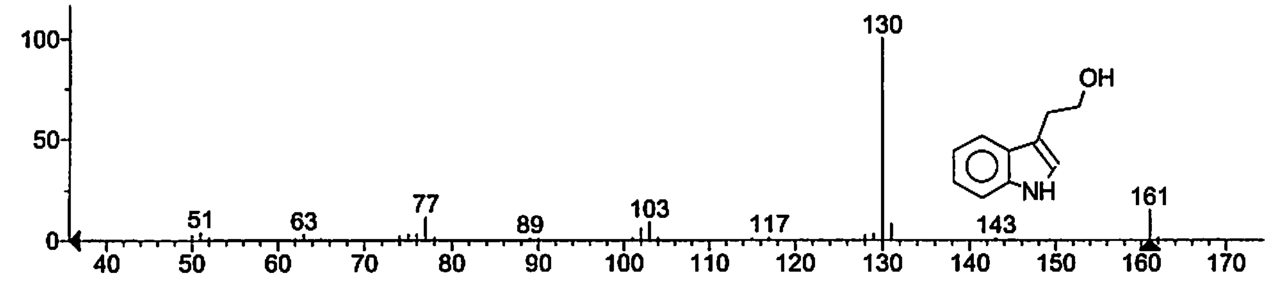


(replib) 1H-Indole-3-ethanol

File :D:\Aldrich\JA-11\JA030211-1.D
Operator :
Acquired : 2 Mar 2011 16:08 using AcqMethod JA-50-280LESS.M
Instrument : Buba; IIBBL's magical mass spect
Sample Name: 4M 13-14d-old C.ocu. abdb.stern./5ul CH2Cl2
Misc Info : +cont. JA021711-1;adults fed nepetalactol/4d
Vial Number: 1

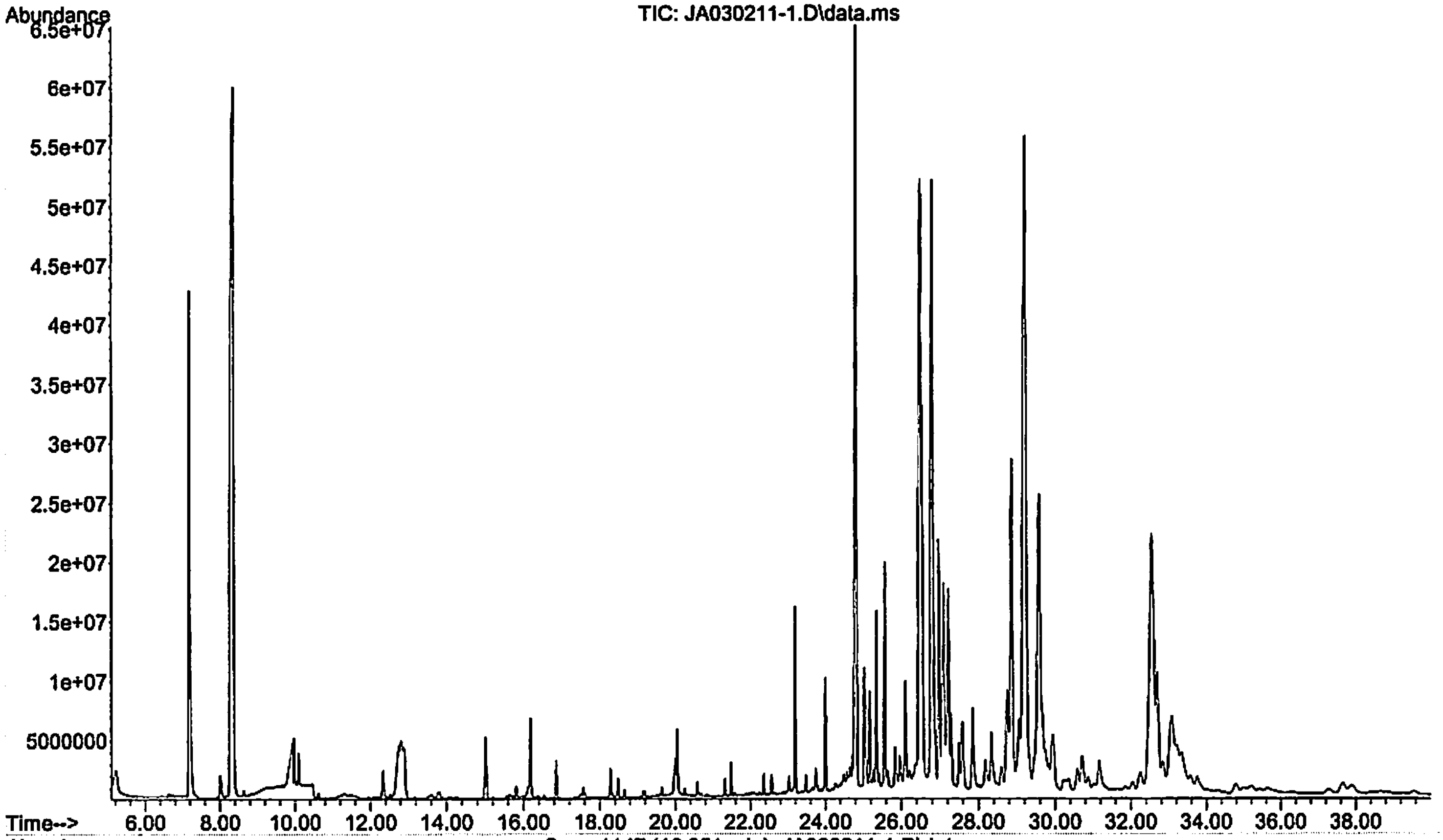


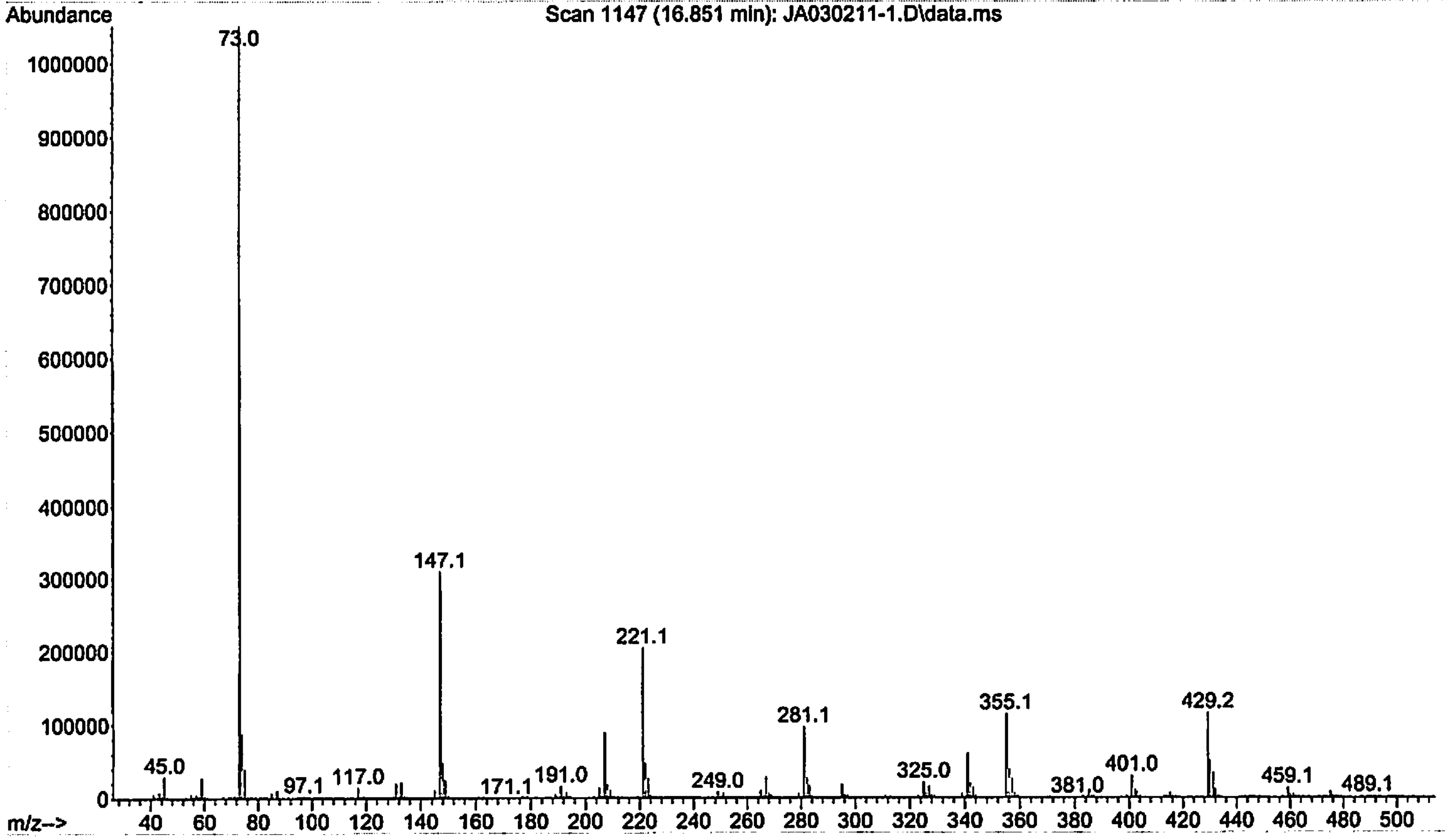

File : D:\DATA\Aldrich\JA-09\JA011509-2.D
Operator : Aldrich
Acquired : 15 Jan 2009 12:17 using AcqMethod JA-WAX08.M
Instrument : Instrument #1
Sample Name: 12 male C.oculata abd.ster./5ul CH2Cl2
Misc Info : ca. 1-week-old; fed. ug/ul citral/water/6days
Vial Number: 1

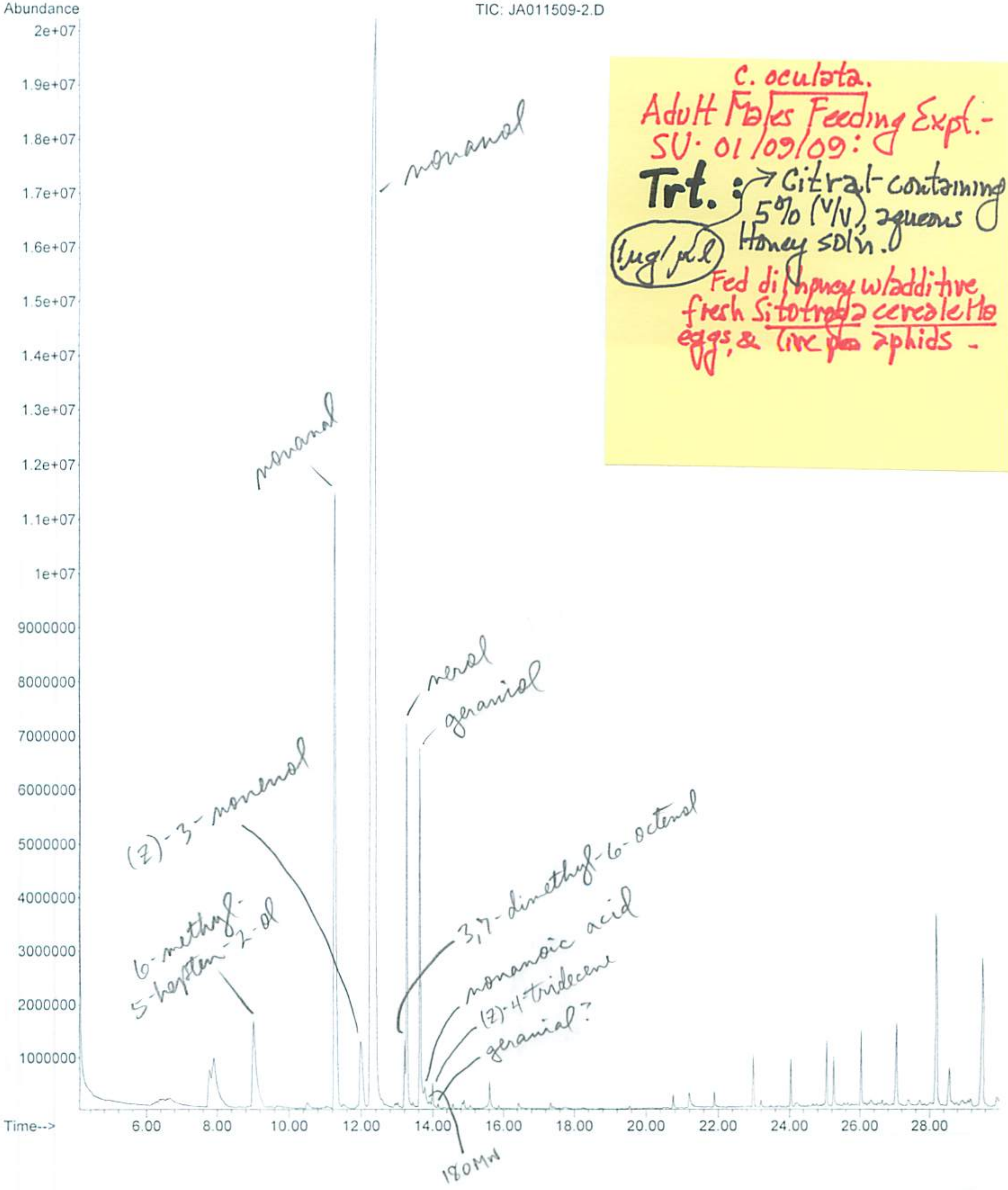


C. oculata.
Adult Males Feeding Expt.-
SU· 01/09/09:
Trt.: → Citral-containing 5% (v/v), aqueous Honey soln. (1ug/ul)
Fed di[illegible] honey w/additive fresh Sitotroga cerealella eggs, & live pea aphids -

File :D:\DATA\Aldrich\JA-09\JA011509-2.D
Operator : Aldrich
Acquired : 15 Jan 2009 12:17 using AcqMethod JA-WAX08.M
Instrument : Instrument #1
Sample Name: 12 male C.oculata abd.ster./5ul CH2Cl2
Misc Info : ca. 1-week-old; fed. ug/ul citral/water/6days
Vial Number: 1

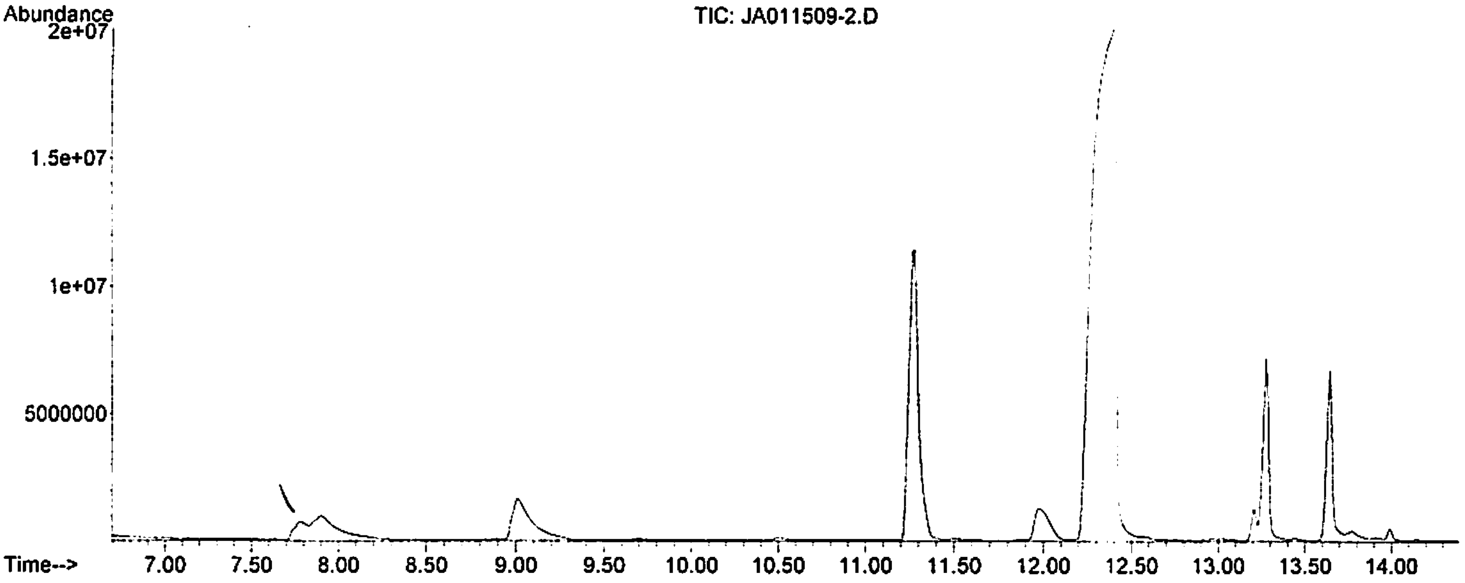


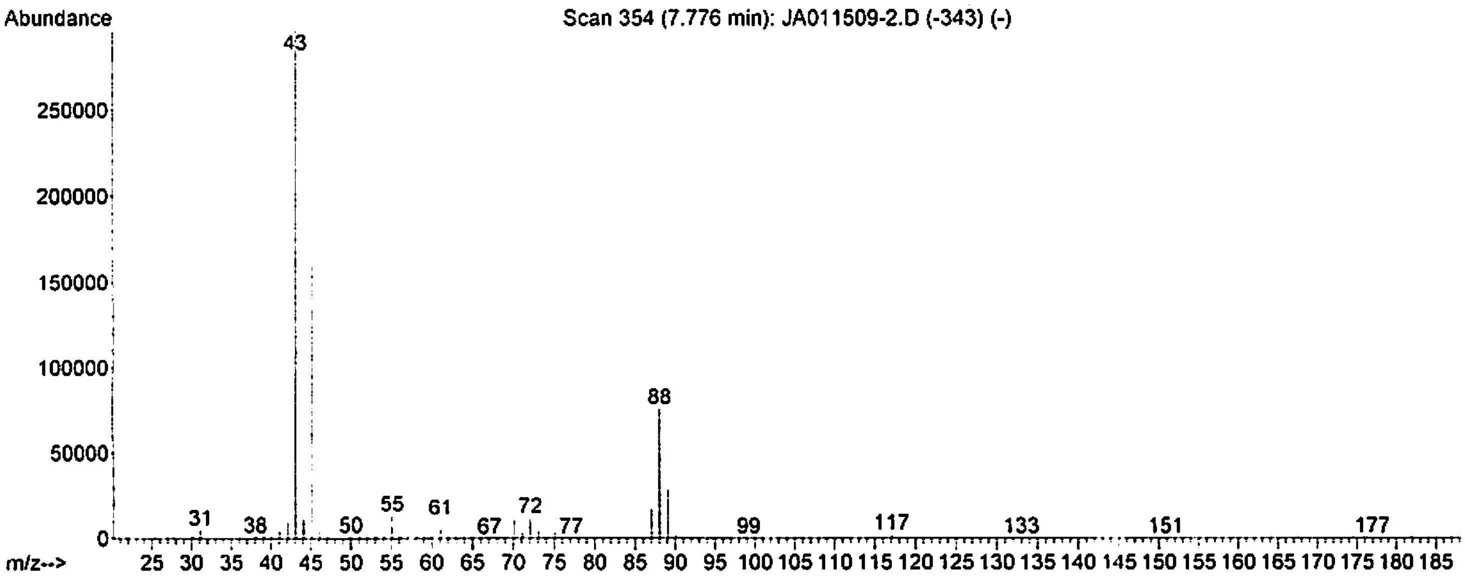

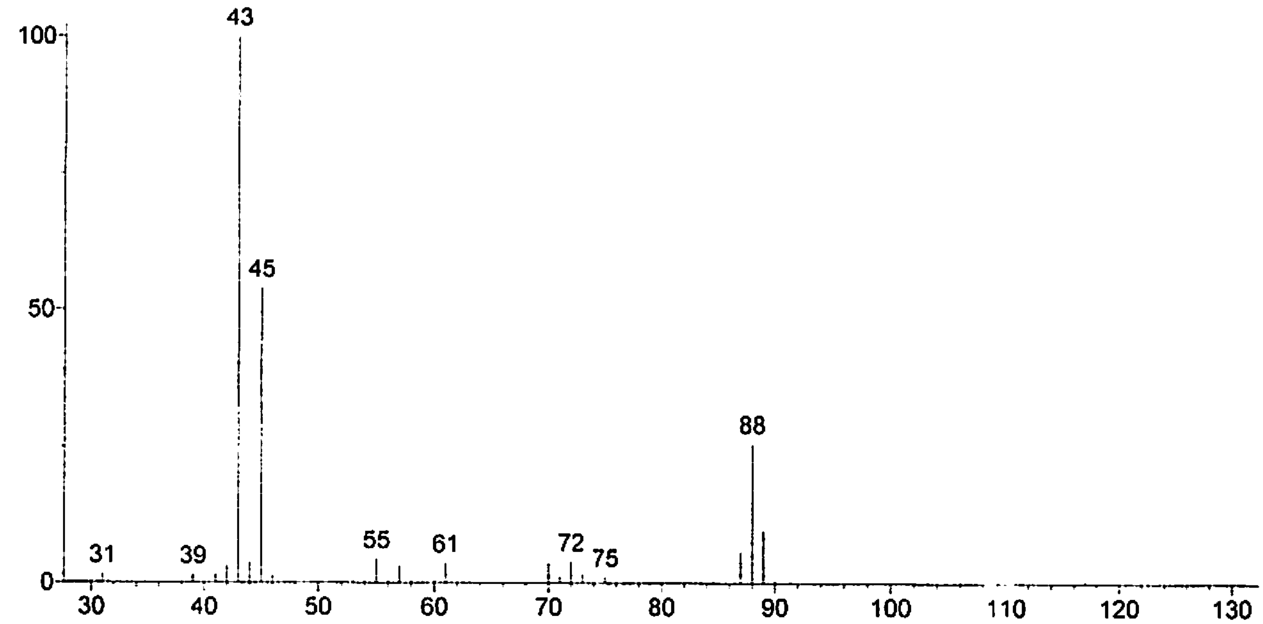


(Text File) Scan 354 (7.776 min): JA011509-2.D (-343)

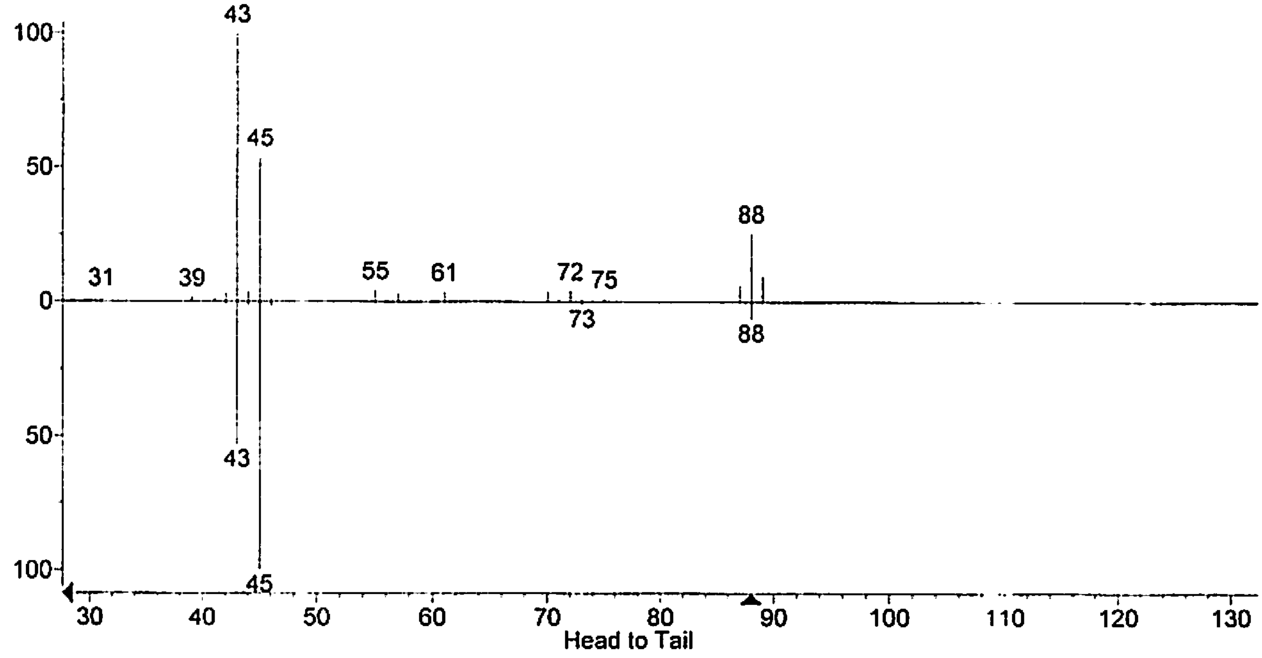


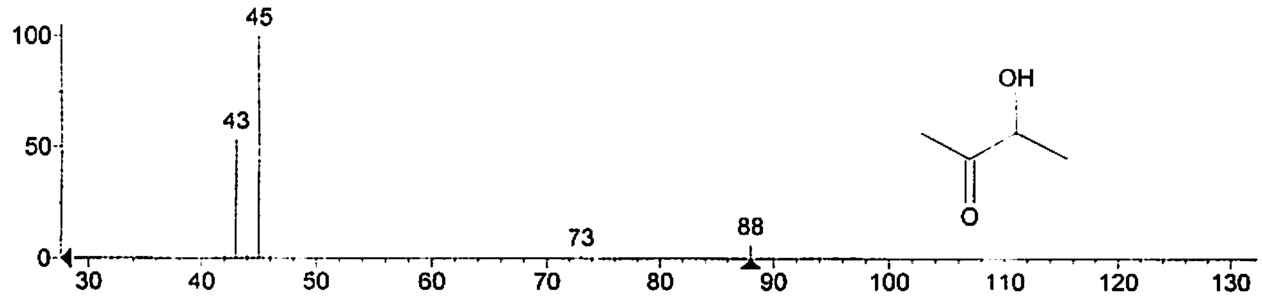


(replib) 2-Butanone, 3-hydroxy-

File :D:\DATA\Aldrich\JA-09\JA011509-2.D
Operator : Aldrich
Acquired : 15 Jan 2009 12:17 using AcqMethod JA-WAX08.M
Instrument : Instrument #1
Sample Name: 12 male C.oculata abd.ster./5ul CH2Cl2
Misc Info : ca. 1-week-old; fed. ug/ul citral/water/6days
Vial Number: 1

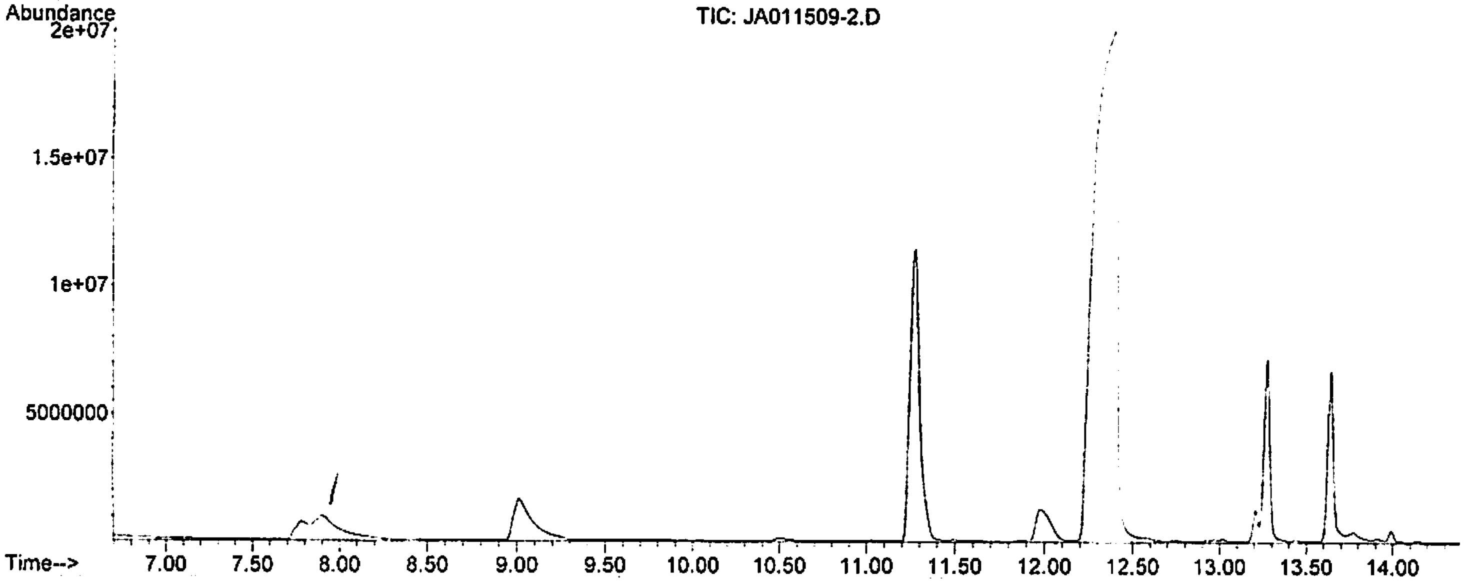


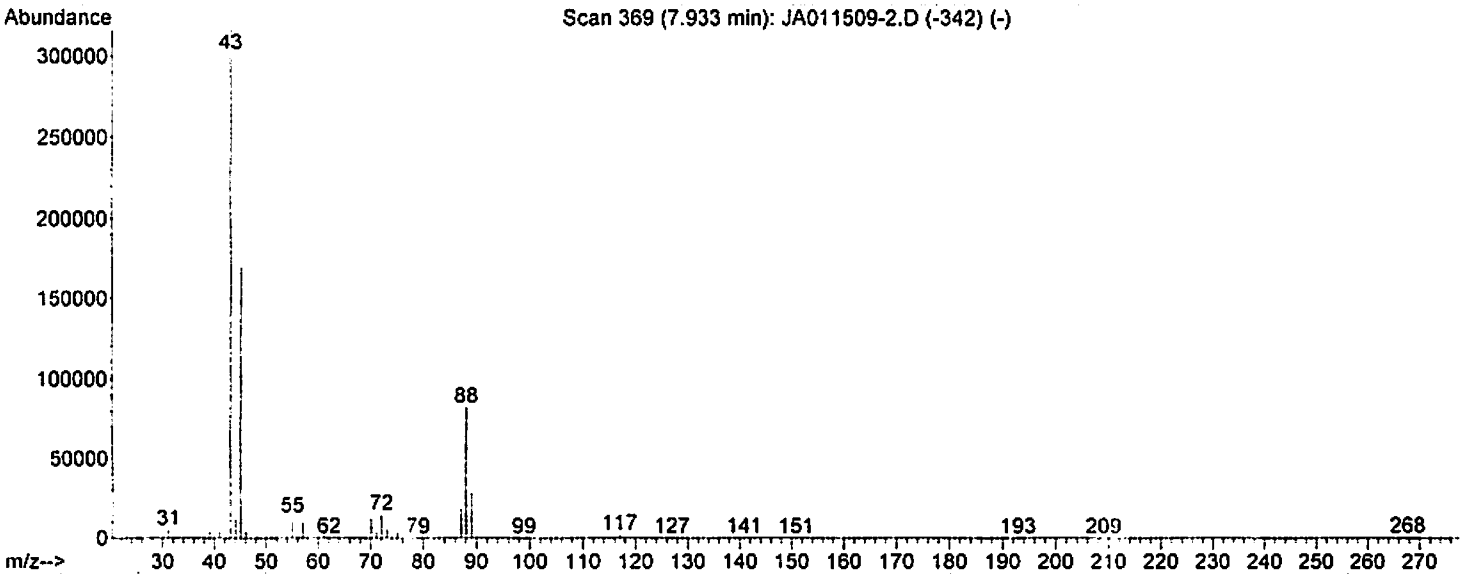

File :D:\DATA\Aldrich\JA-09\JA011509-2.D
Operator : Aldrich
Acquired : 15 Jan 2009 12:17 using AcqMethod JA-WAX08.M
Instrument : Instrument #1
Sample Name: 12 male C.oculata abd.ster./5ul CH2Cl2
Misc Info : ca. 1-week-old; fed. ug/ul citral/water/6days
Vial Number: 1

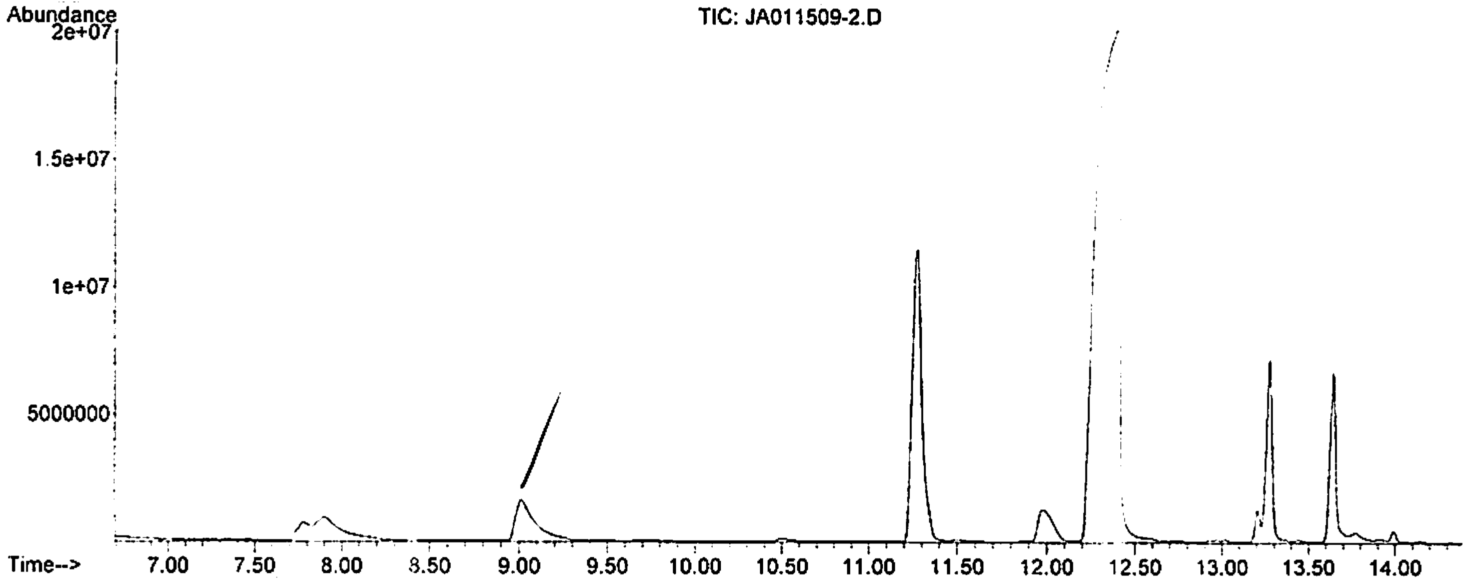


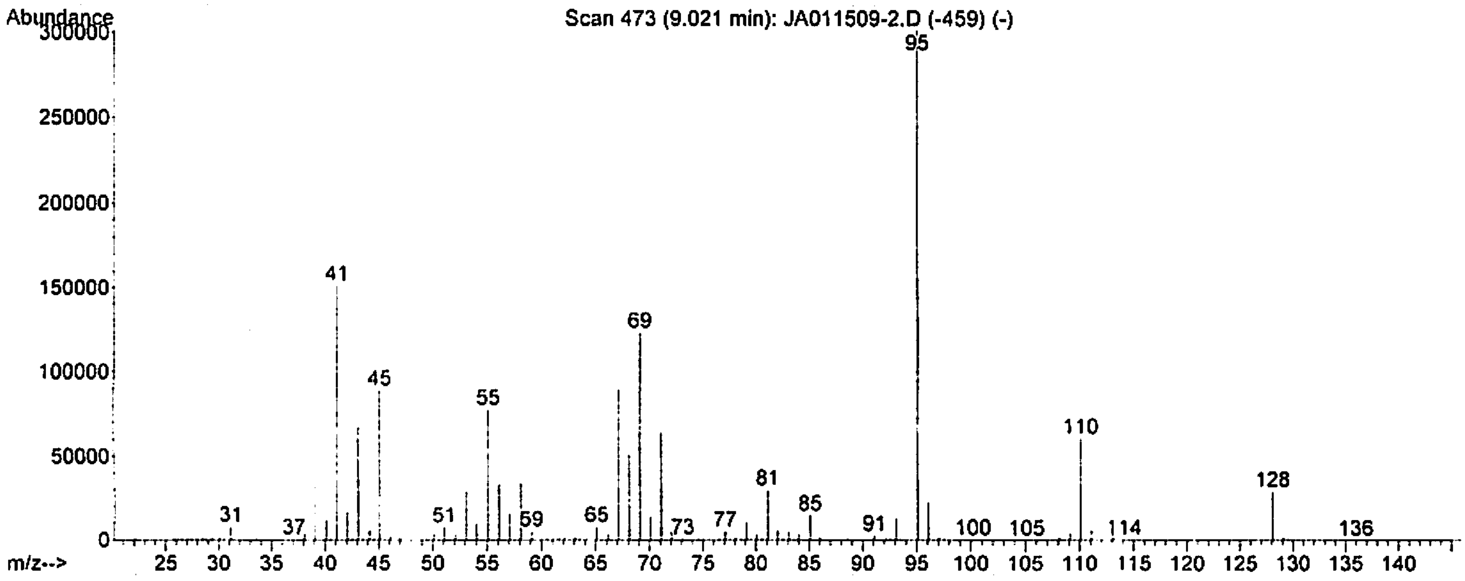

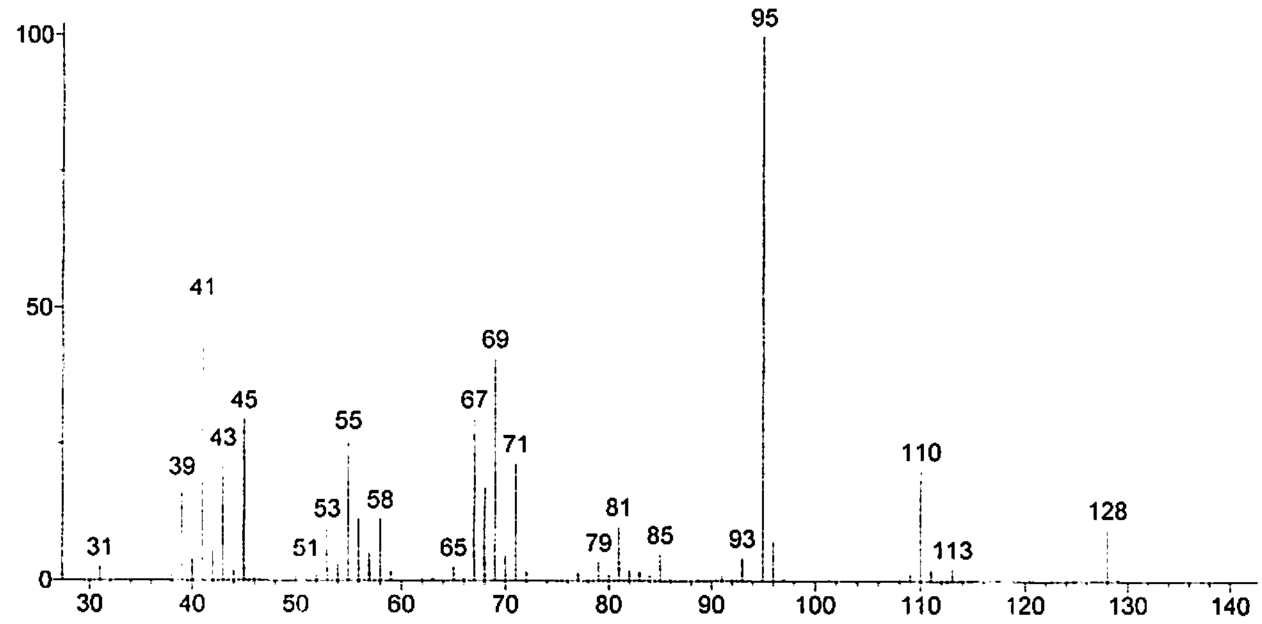


(Text File) Scan 473 (9.021 min): JA011509-2.D (-459)

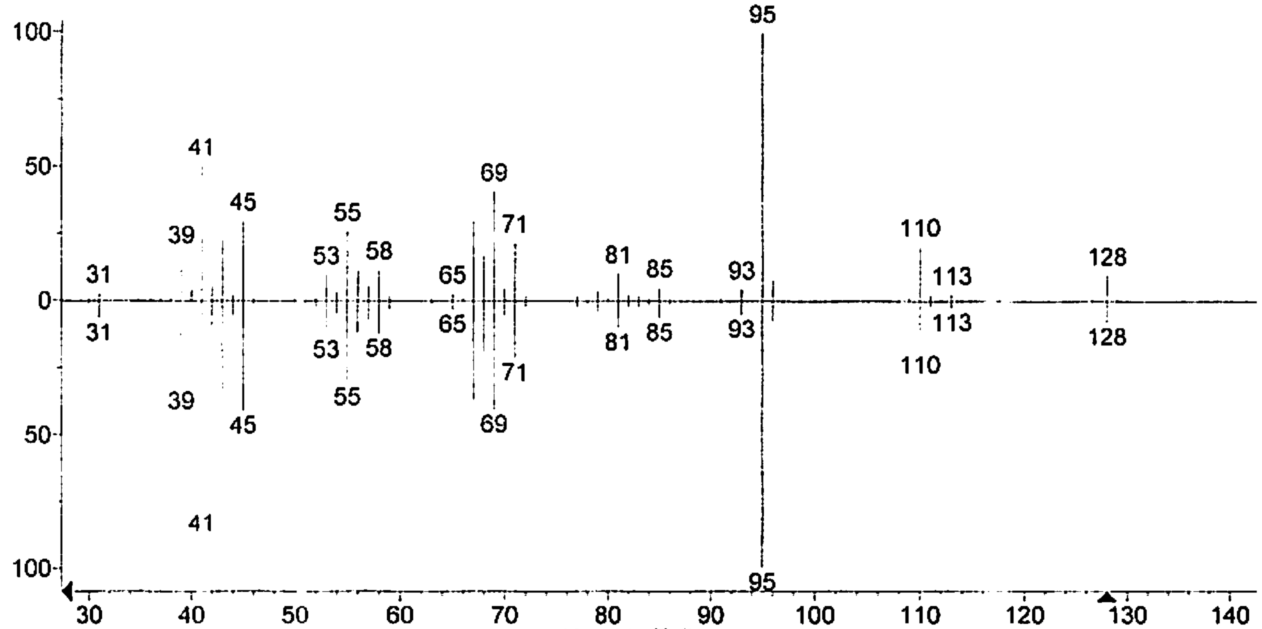


Head to Tail

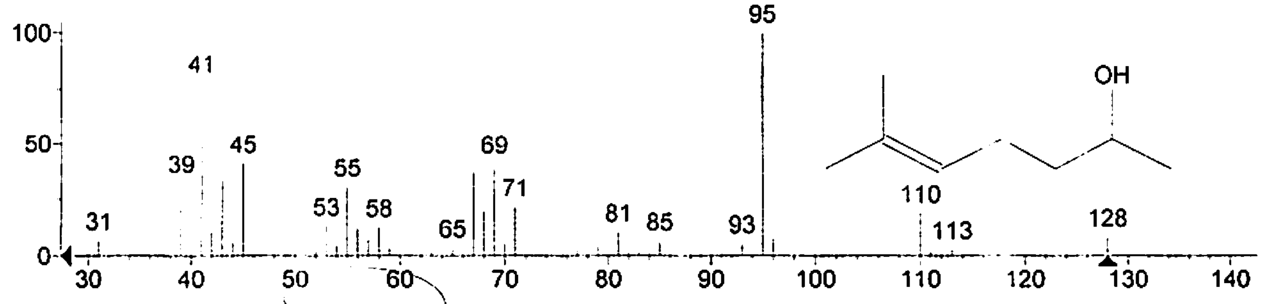


(mainlib) dl-6-Methyl-5-hepten-2-ol

File :D:\DATA\Aldrich\JA-09\JA011509-2.D
Operator : Aldrich
Acquired : 15 Jan 2009 12:17 using AcqMethod JA-WAX08.M
Instrument : Instrument #1
Sample Name: 12 male C.oculata abd.ster./5ul CH2Cl2
Misc Info : ca. 1-week-old; fed. ug/ul citral/water/6days
Vial Number: 1

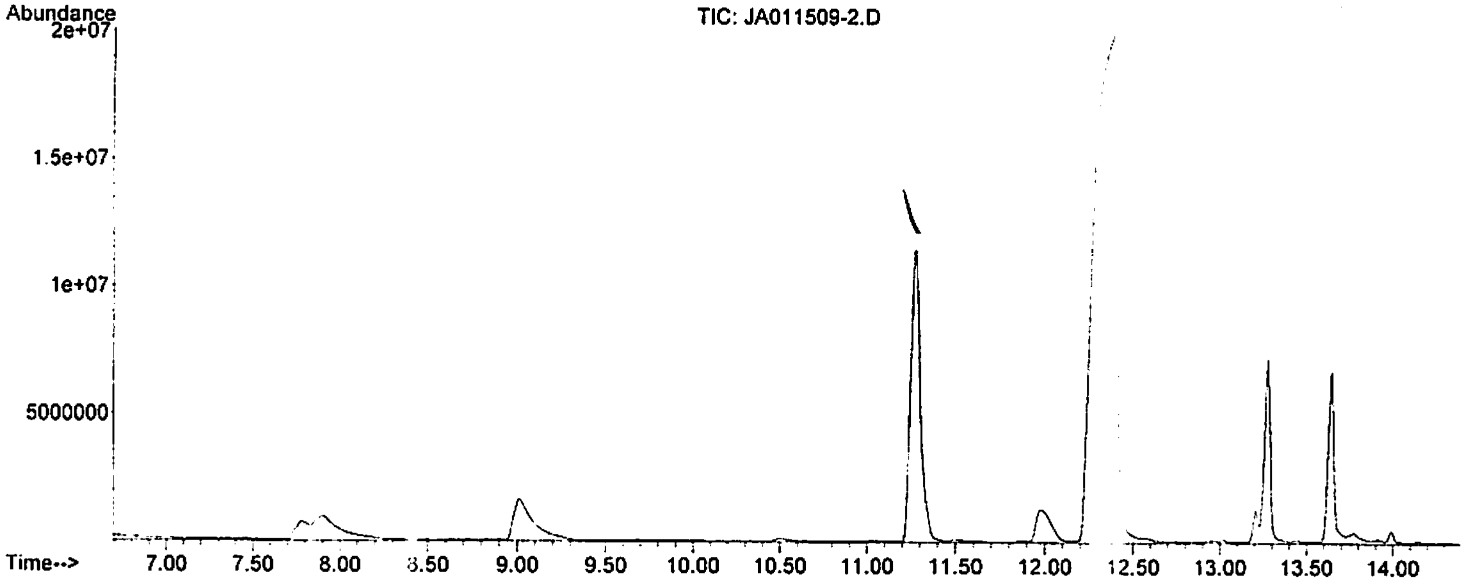


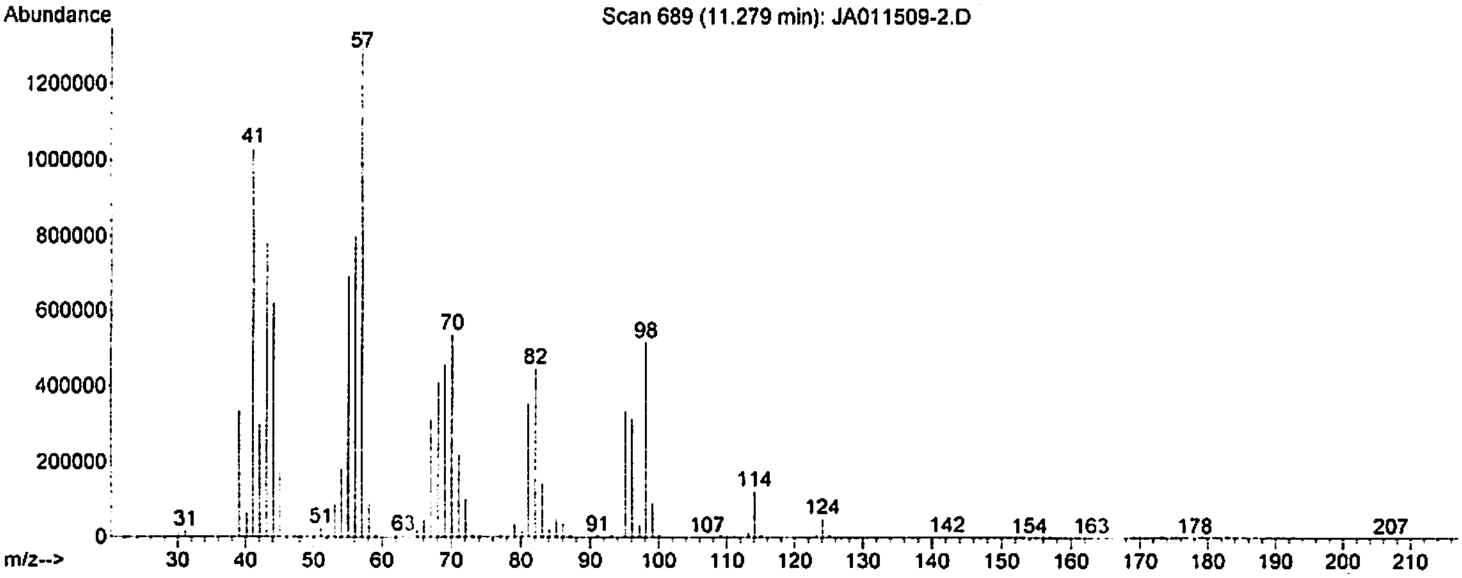

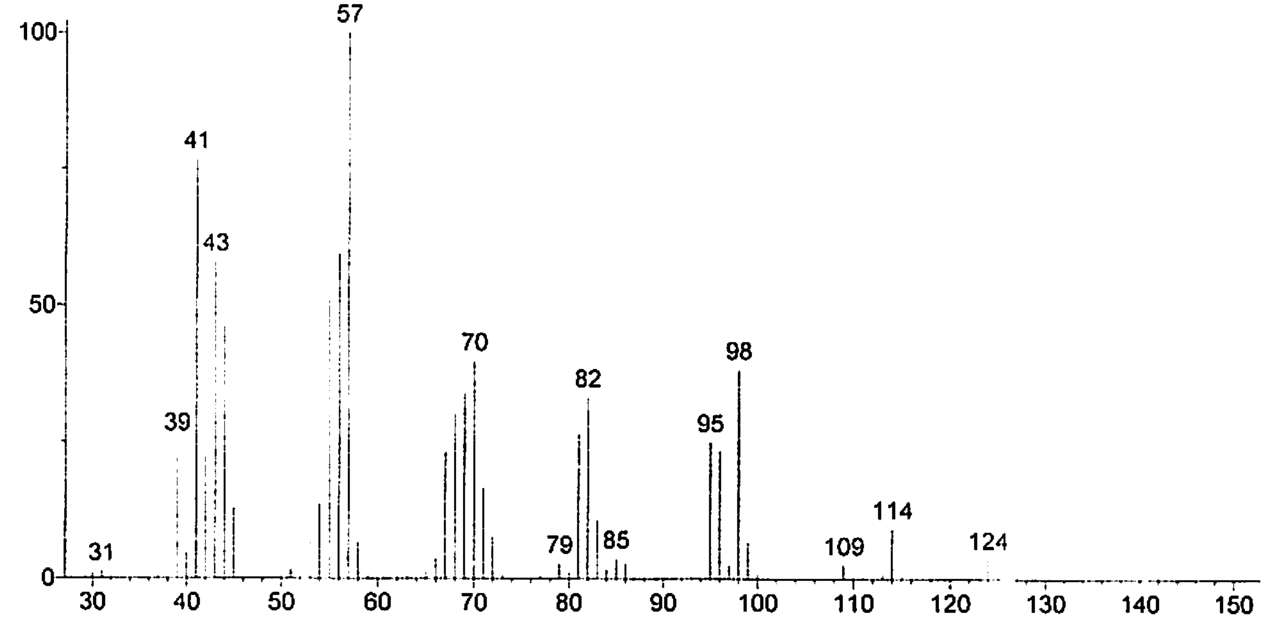


(Text File) Scan 689 (11.279 min): JA011509-2.D

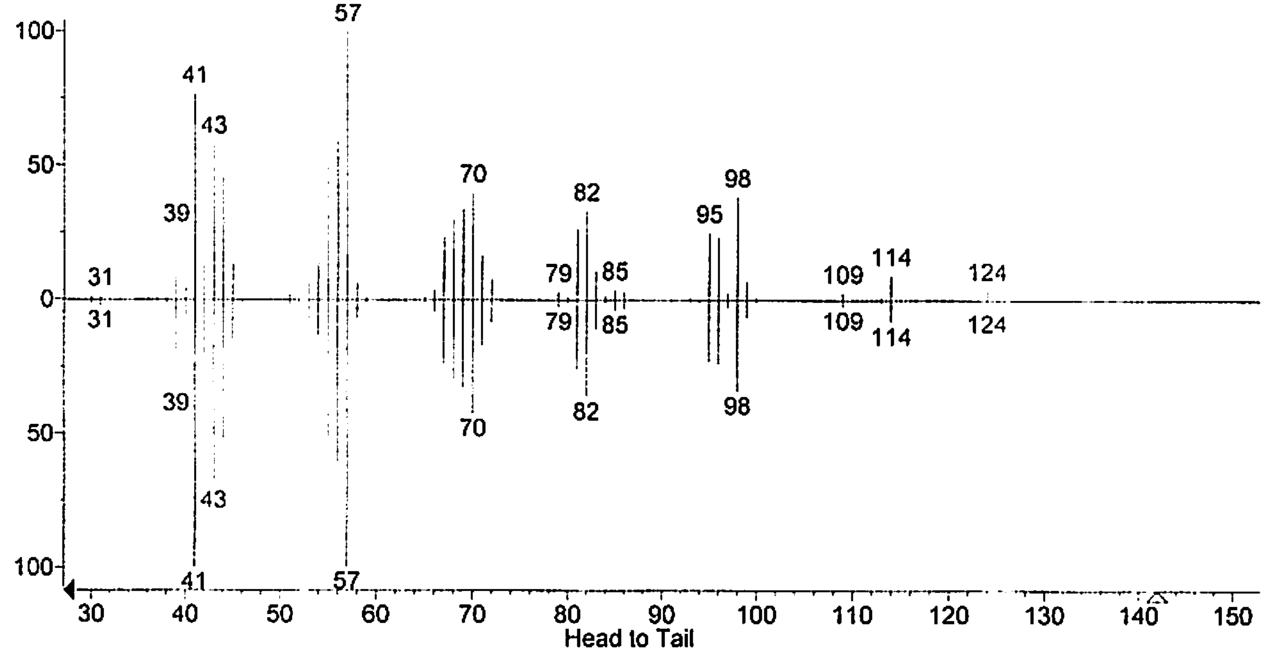


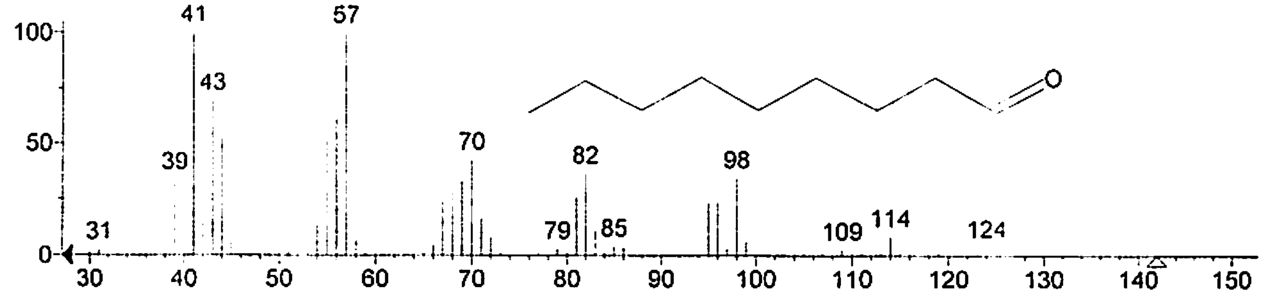


(replib) Nonanal

File :D:\DATA\Aldrich\JA-09\JA011509-2.D
Operator : Aldrich
Acquired : 15 Jan 2009 12:17 using AcqMethod JA-WAX08.M
Instrument : Instrument #1
Sample Name: 12 male C.oculata abd.ster./5ul CH2Cl2
Misc Info : ca. 1-week-old; fed. ug/ul citral/water/6days
Vial Number: 1

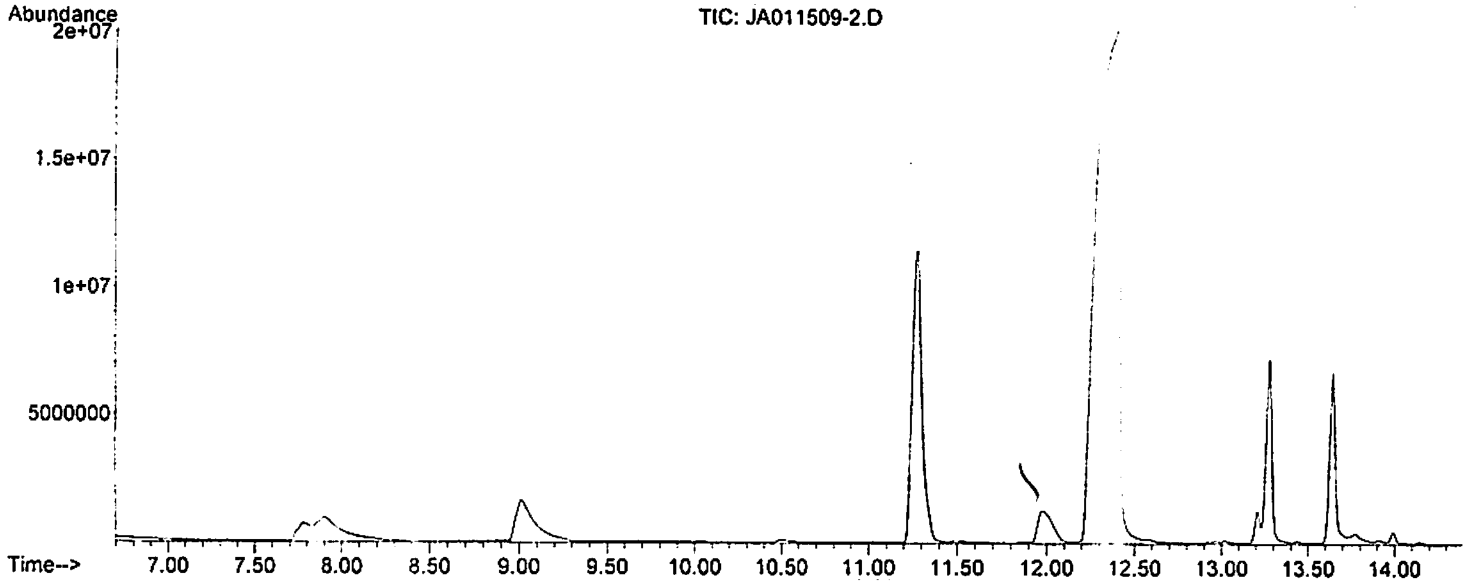


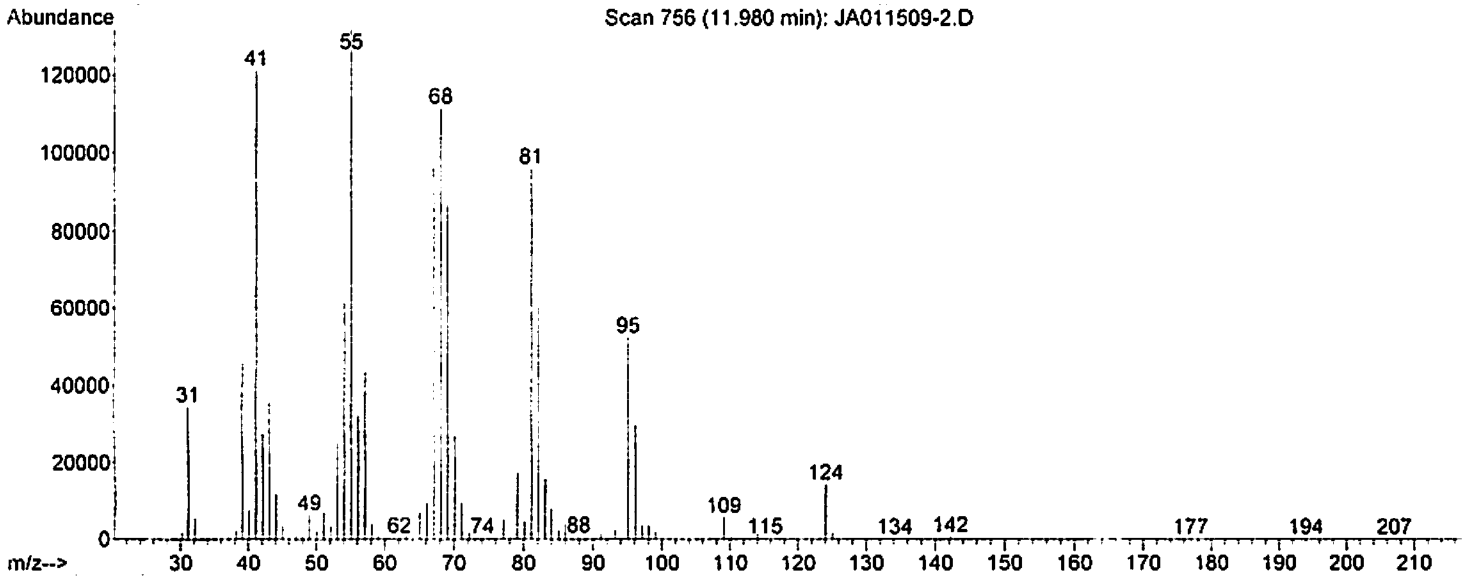

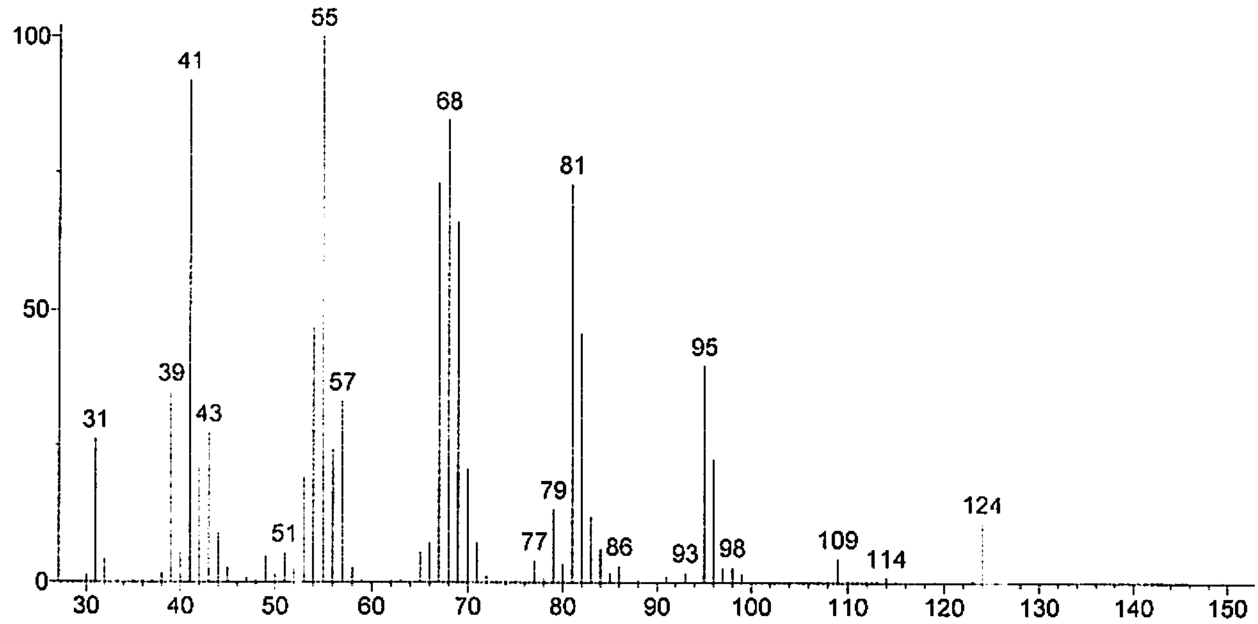


(Text File) Scan 756 (11.980 min): JA011509-2.D

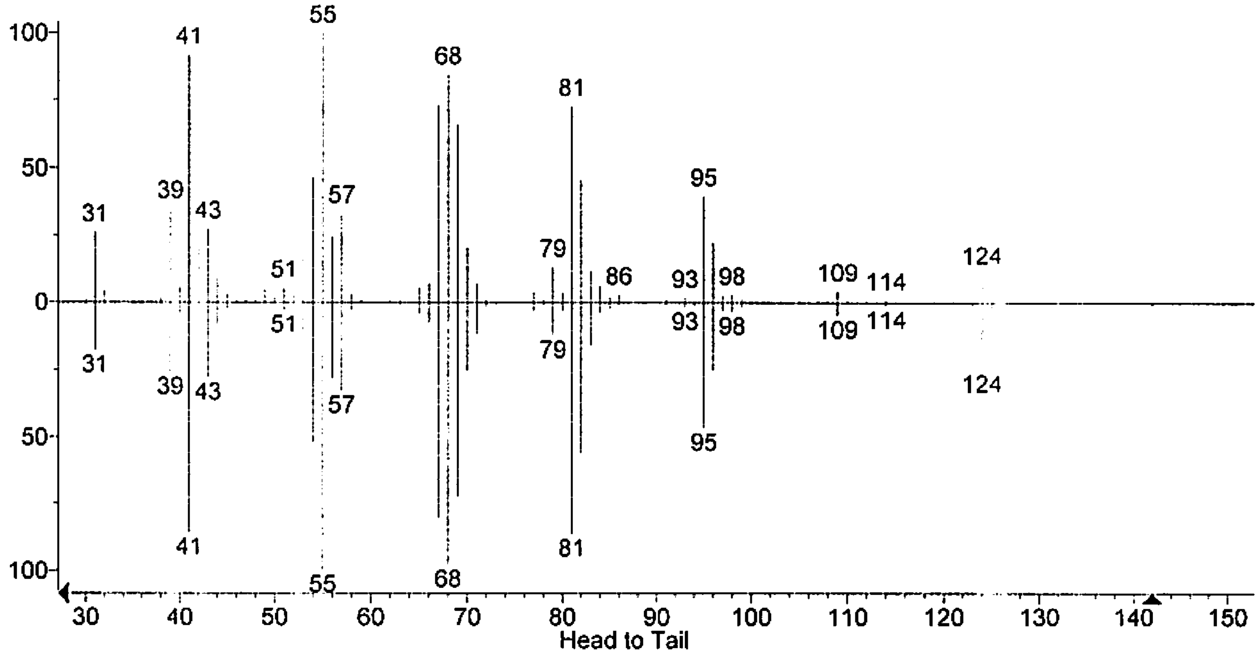


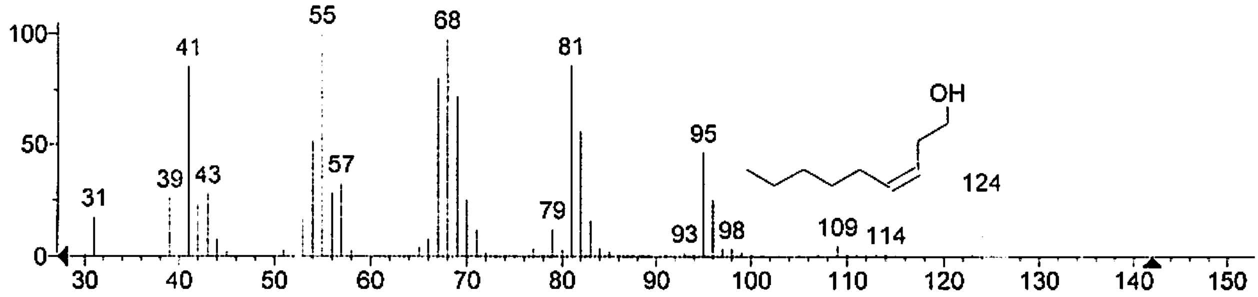


(mainlib) 3-Nonen-1-ol, (Z)-

```
File       :D:\DATA\Aldrich\JA-09\JA011509-2.D
Operator   : Aldrich
Acquired   : 15 Jan 2009   12:17      using AcqMethod JA-WAX08.M
Instrument :     Instrument #1
Sample Name: 12 male C.oculata abd.ster./5ul CH2Cl2
Misc Info  : ca. 1-week-old; fed. ug/ul citral/water/6days
Vial Number: 1
```

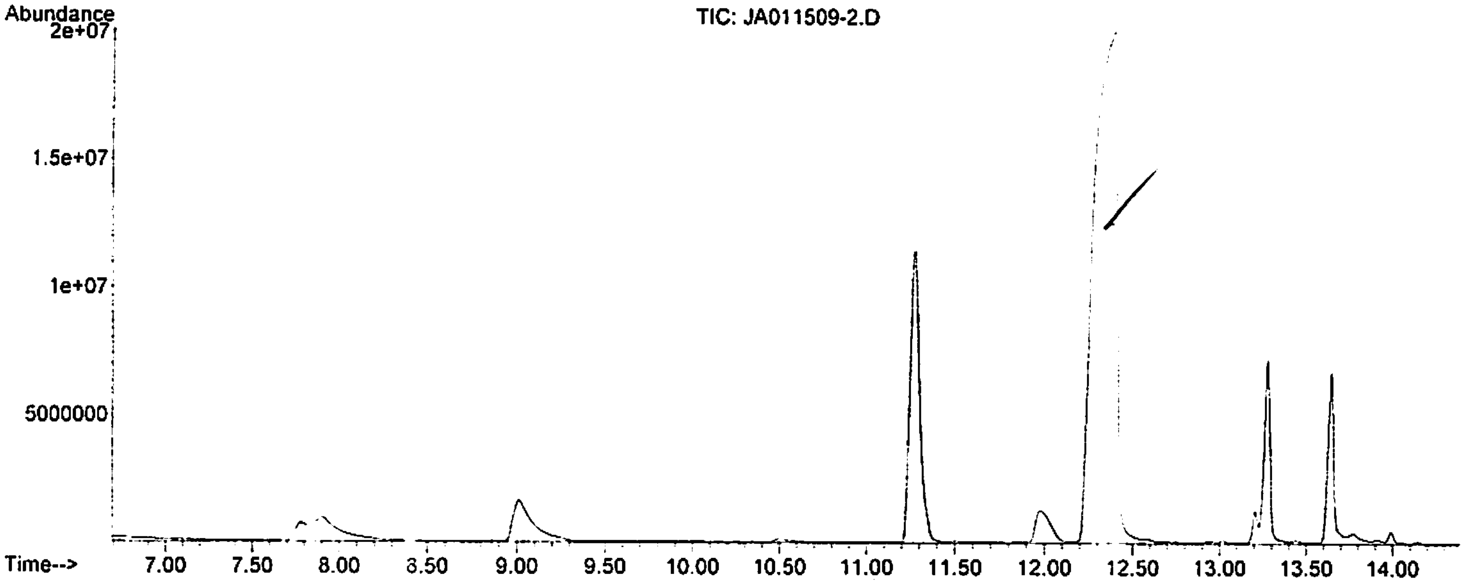


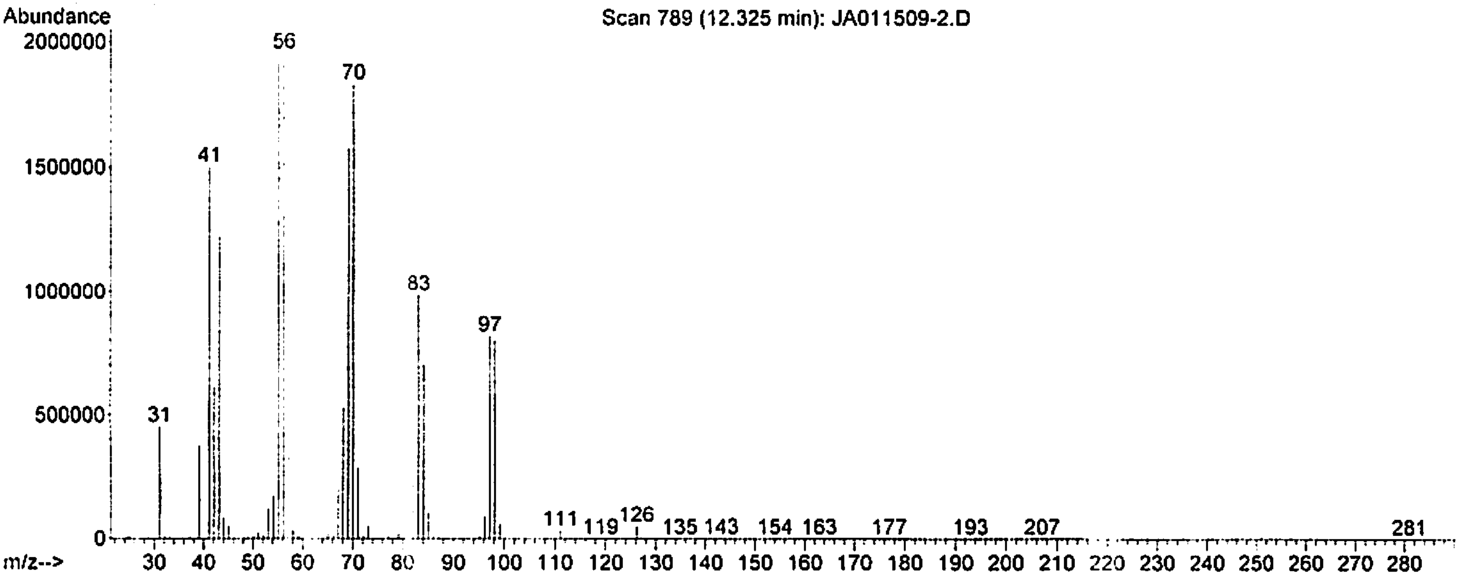

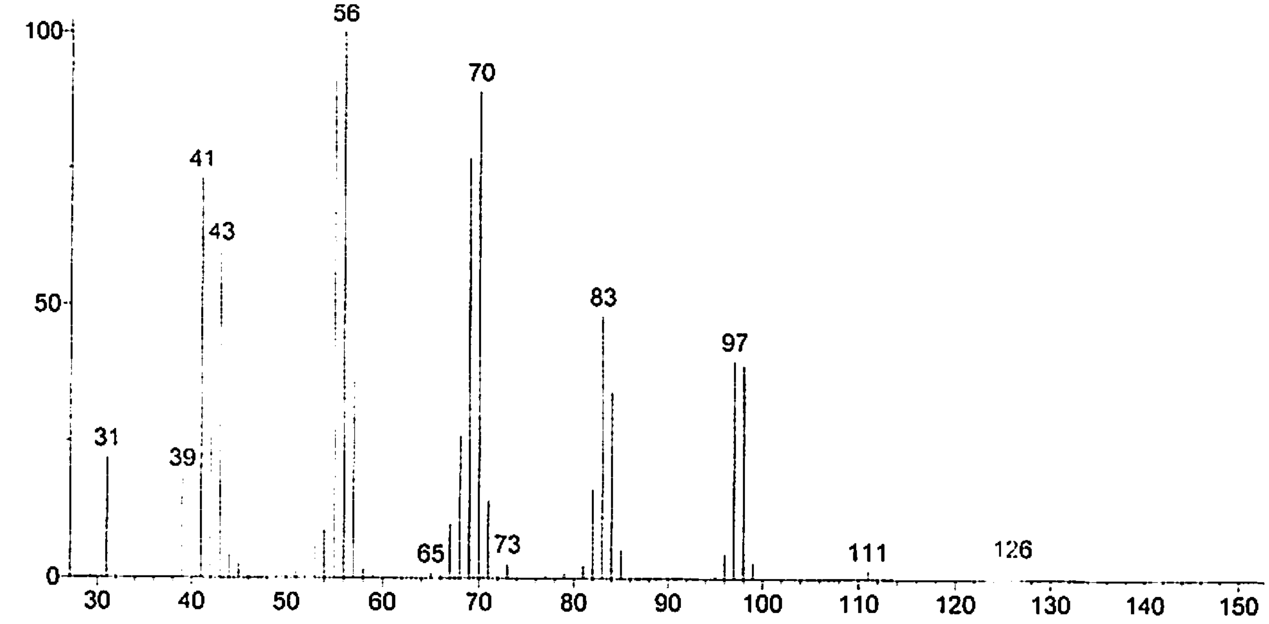


(Text File) Scan 789 (12.325 min): JA011509-2.D

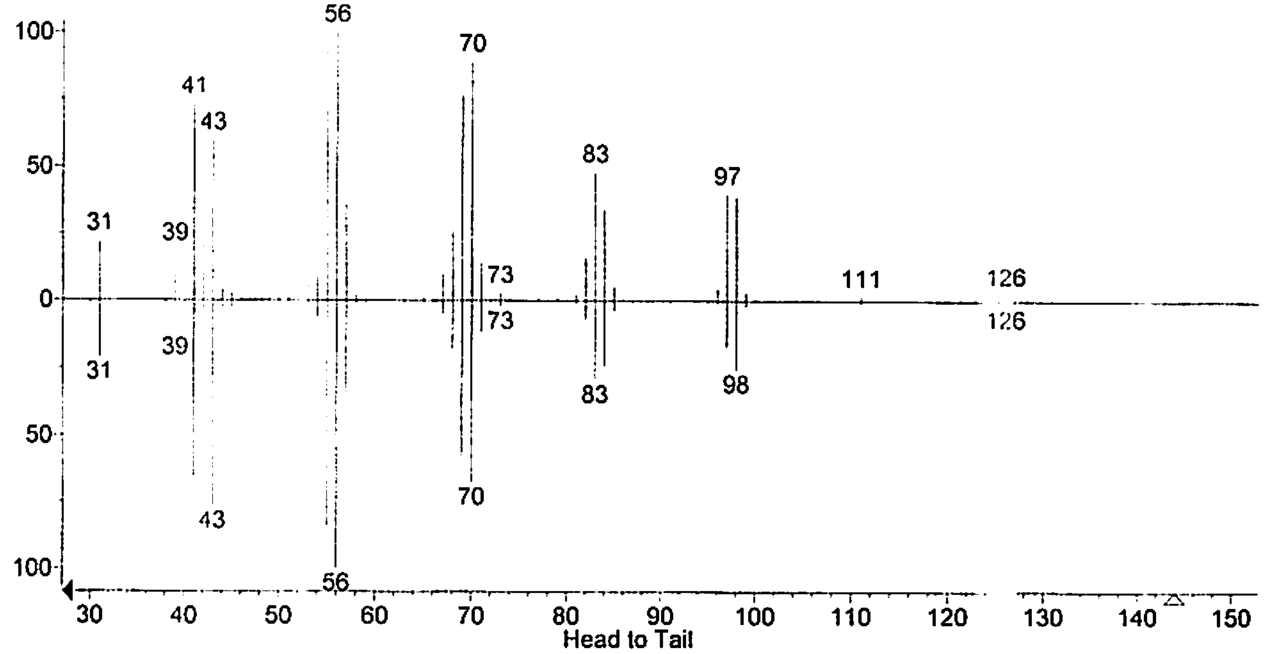


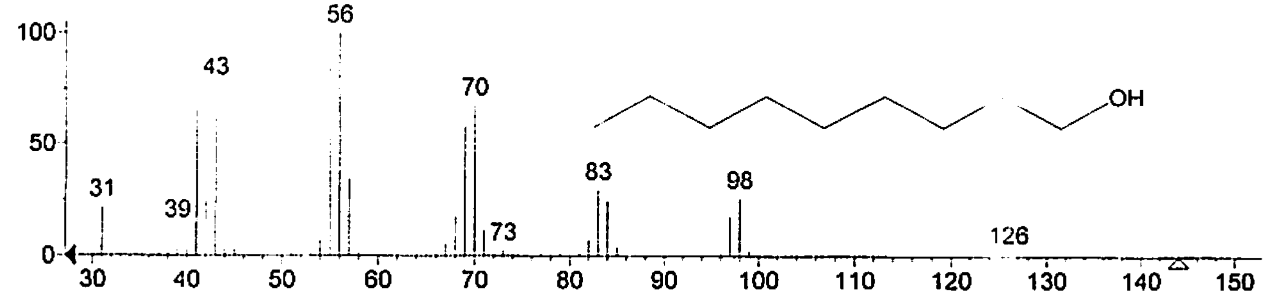


(mainlib) 1-Nonanol

File : D:\DATA\Aldrich\JA-09\JA011509-2.D
Operator : Aldrich
Acquired : 15 Jan 2009 12:17 using AcqMethod JA-WAX08.M
Instrument : Instrument #1
Sample Name: 12 male C.oculata abd.ster./5ul CH2Cl2
Misc Info : ca. 1-week-old; fed. ug/ul citral/water/6days
Vial Number: 1

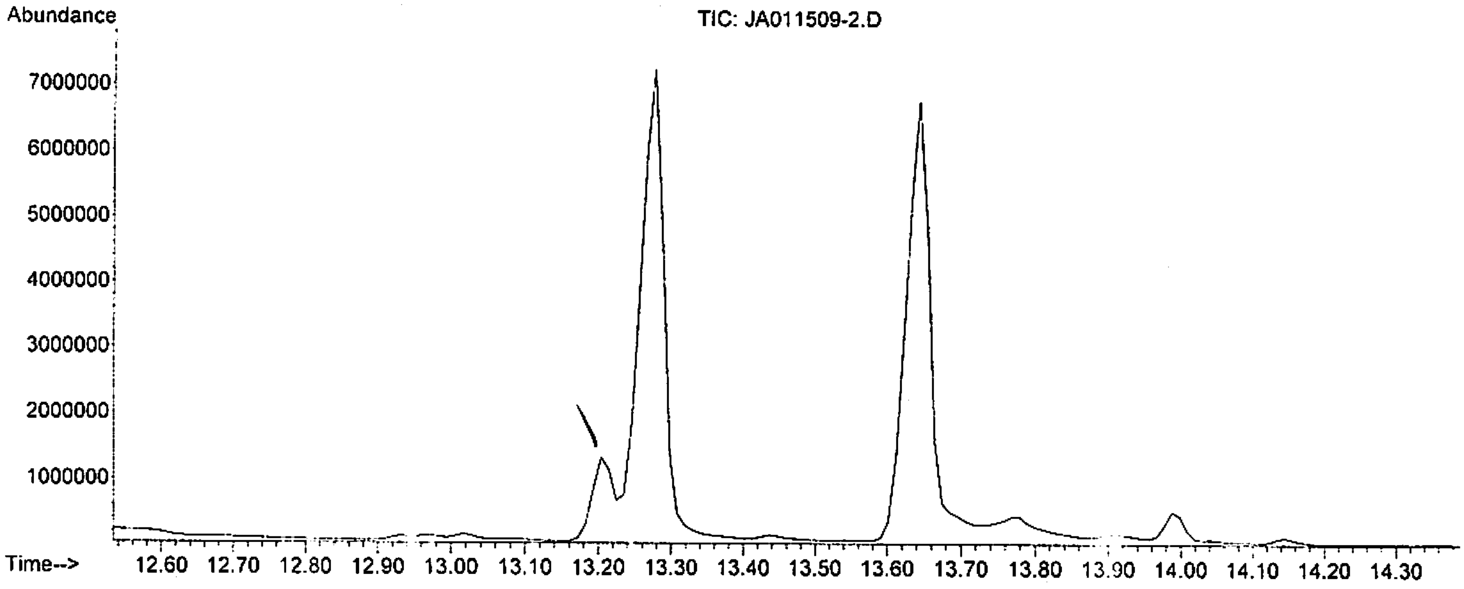


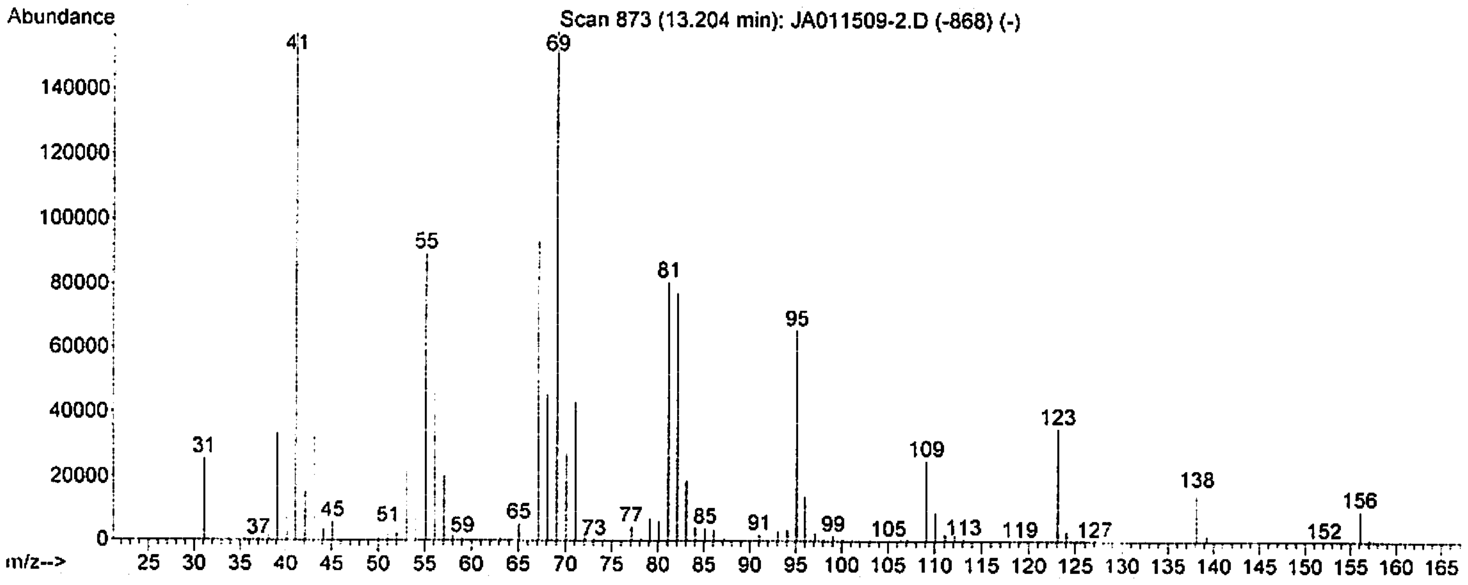

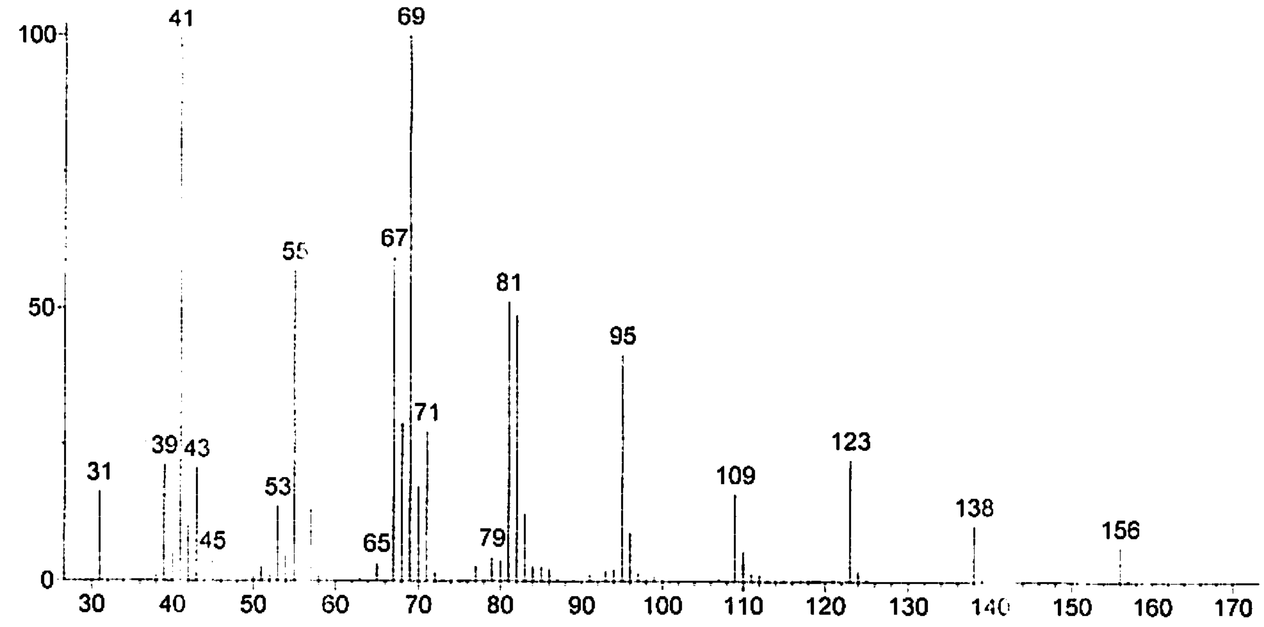


(Text File) Scan 873 (13.204 min): JA011509-2.D (-868)

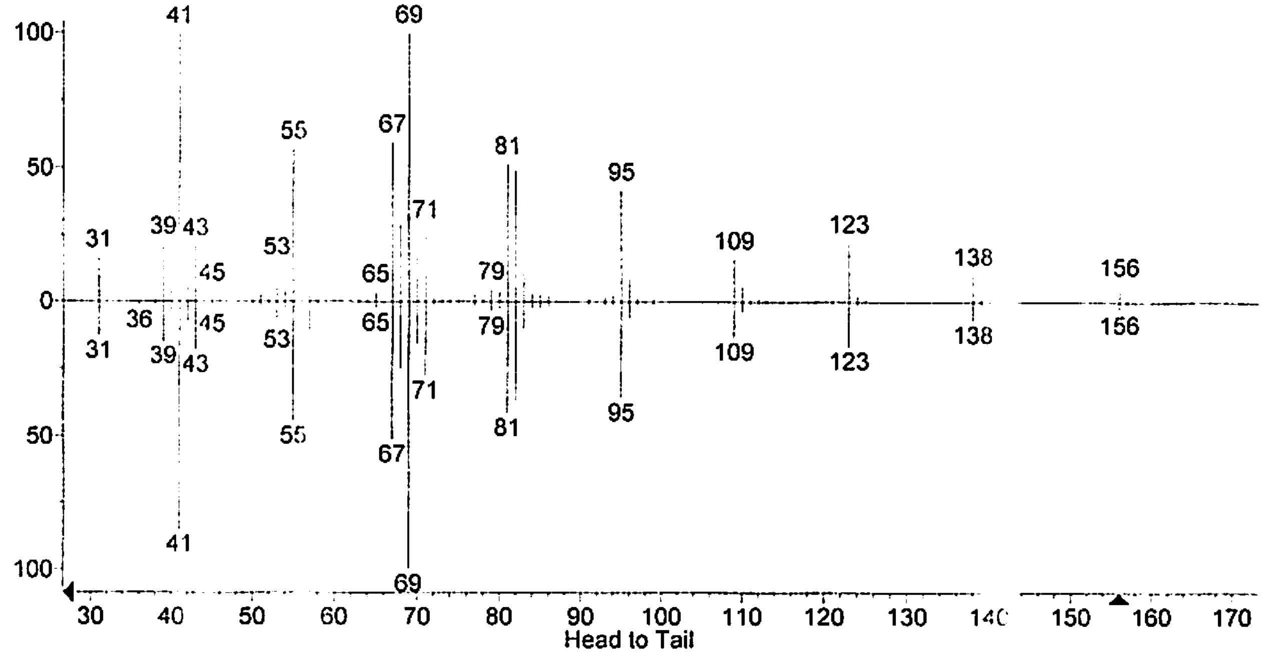


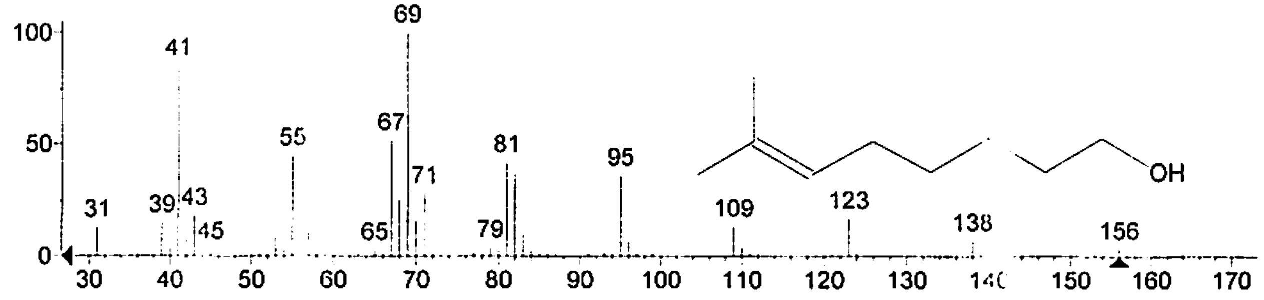


(mainlib) 6-Octen-1-ol, 3,7-dimethyl-, (R)-

File :D:\DATA\Aldrich\JA-09\JA011509-2.D
Operator : Aldrich
Acquired : 15 Jan 2009 12:17 using AcqMethod JA-WAX08.M
Instrument : Instrument #1
Sample Name: 12 male C.oculata abd.ster./5ul CH2Cl2
Misc Info : ca. 1-week-old; fed. ug/ul citral/water/6days
Vial Number: 1

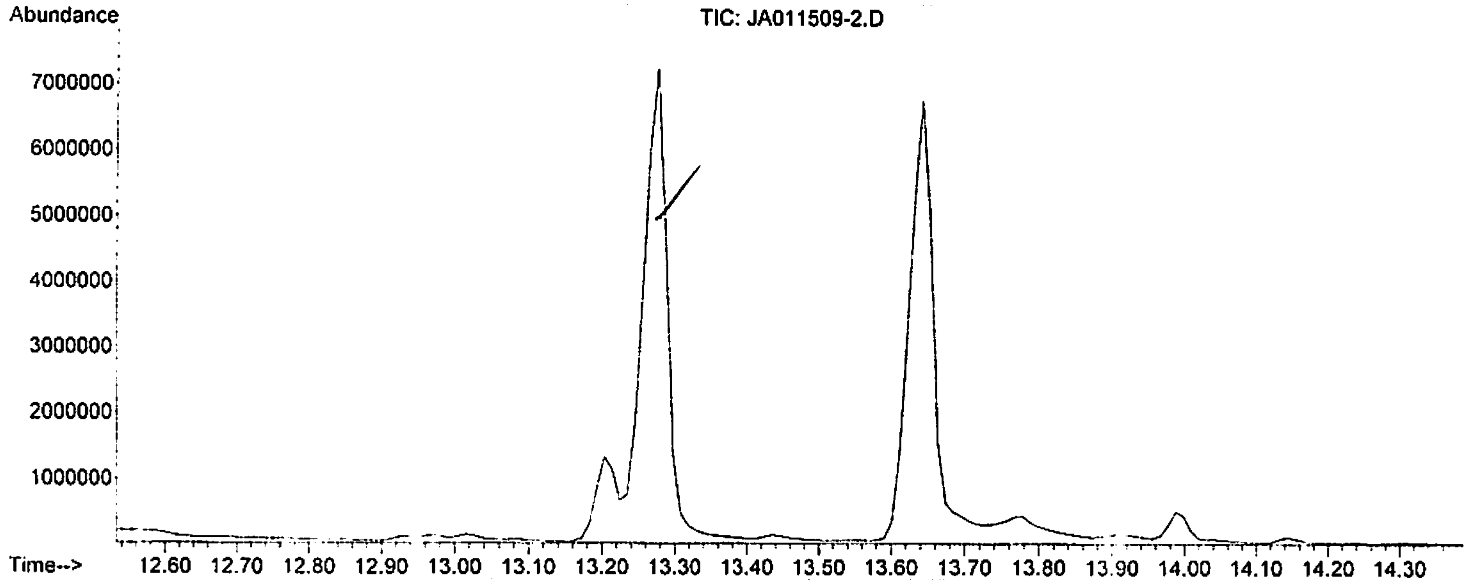


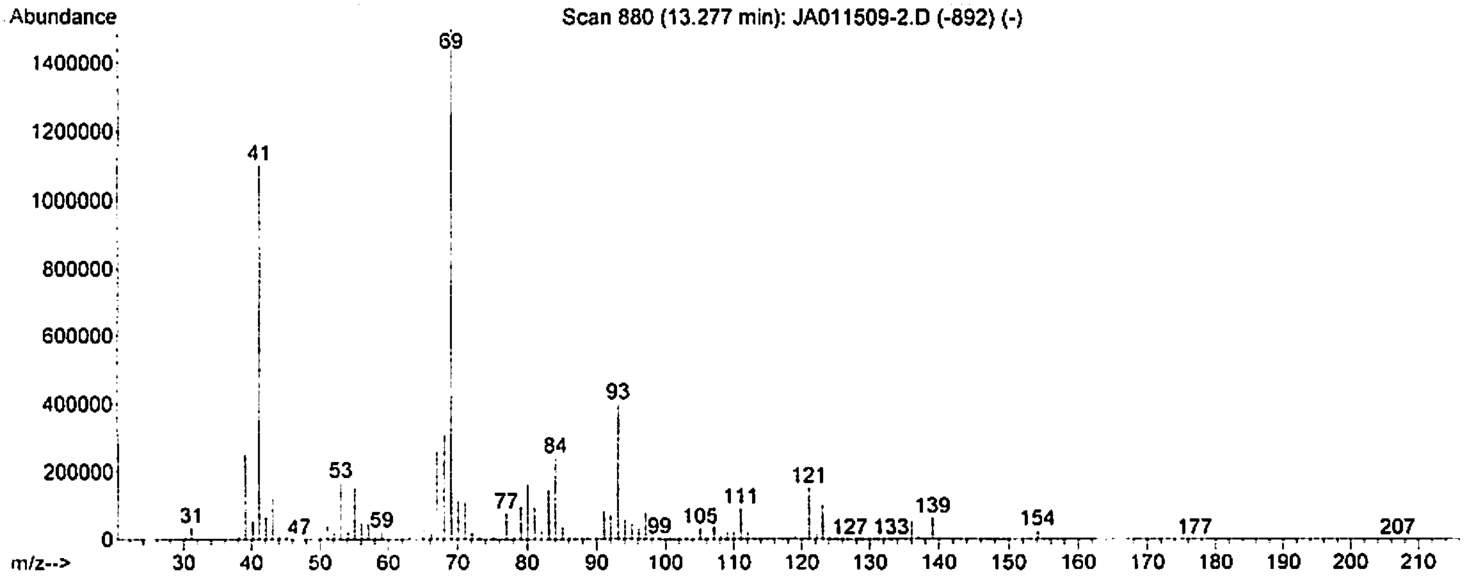

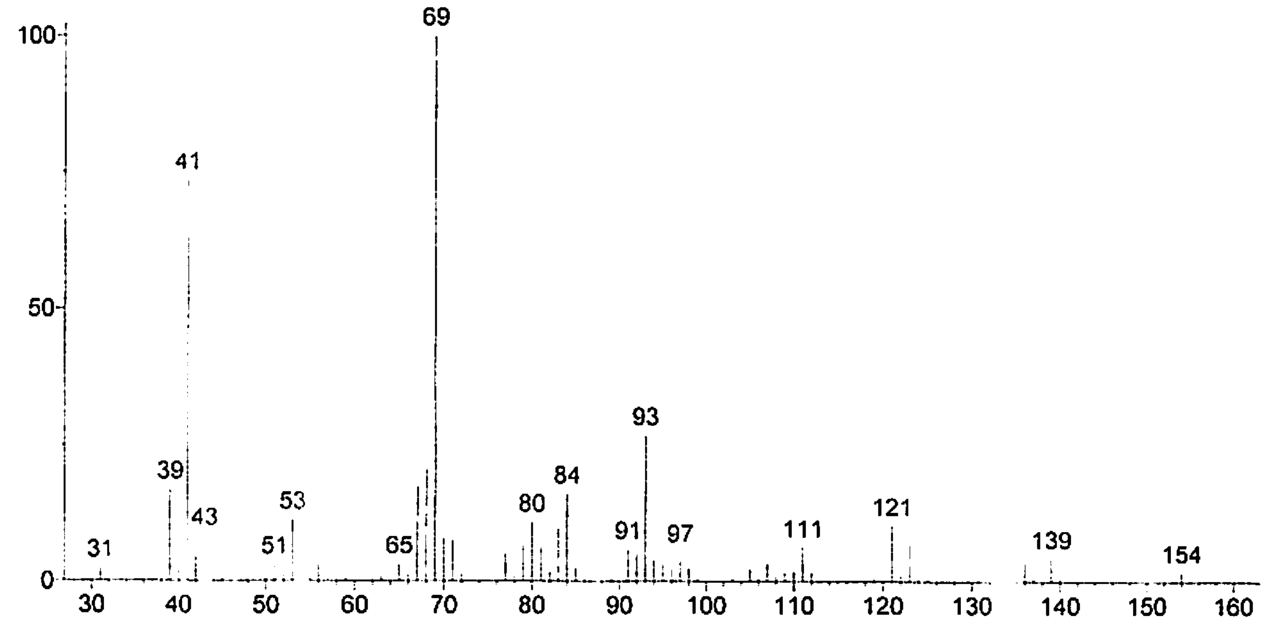

(Text File) Scan 880 (13.27? min): JA011509-2.D (-892)

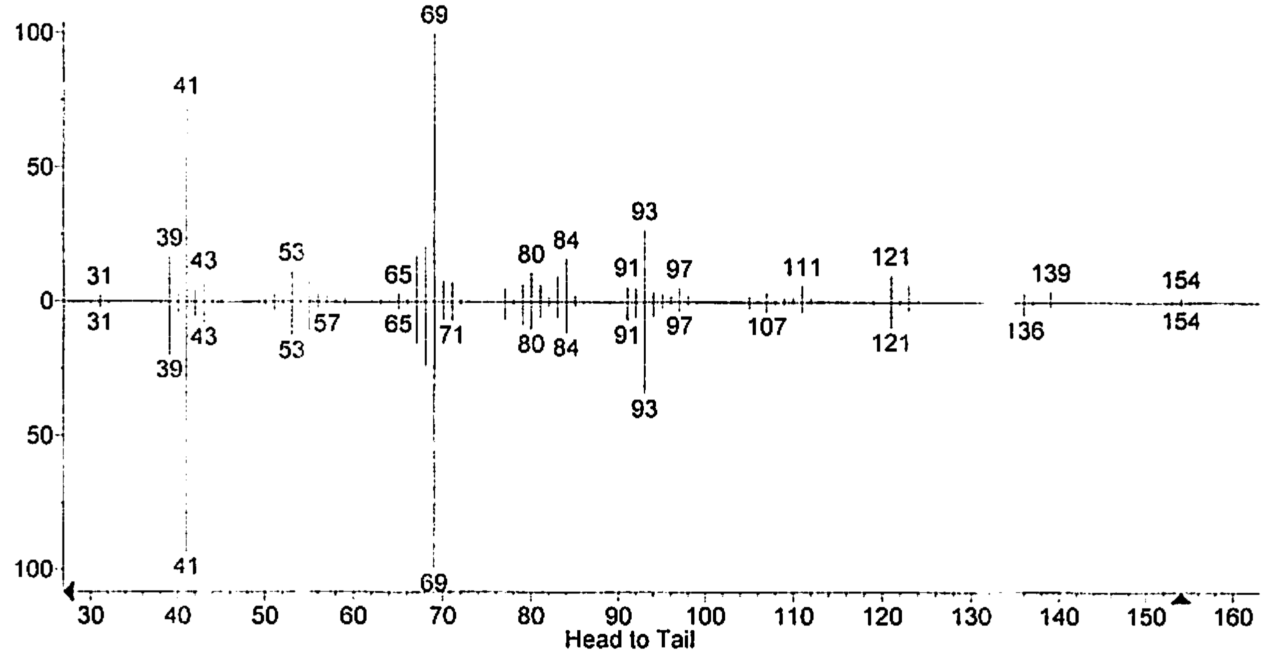


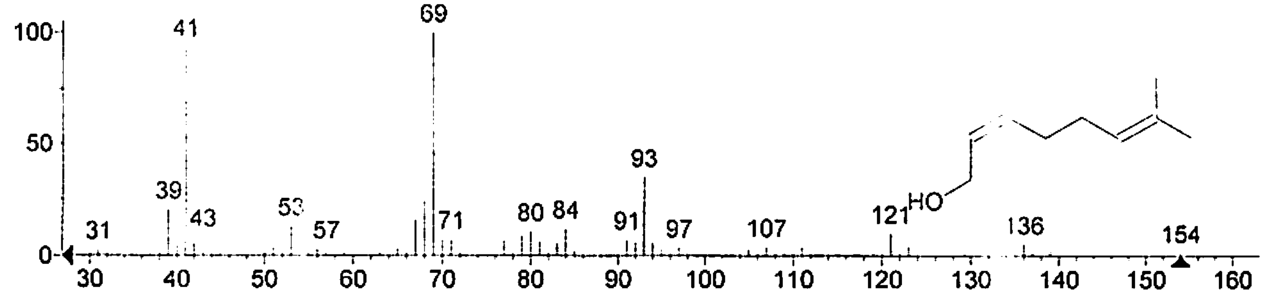

(mainlib) 2,6-Octadien-1-ol, 3,7-dimethyl-, (Z)-

File : D:\DATA\Aldrich\JA-09\JA011509-2.D
Operator : Aldrich
Acquired : 15 Jan 2009 12:17 using AcqMethod JA-WAX08.M
Instrument : Instrument #1
Sample Name: 12 male C.oculata abd.ster./5ul CH2Cl2
Misc Info : ca. 1-week-old; fed. ug/ul citral/water/6days
Vial Number: 1

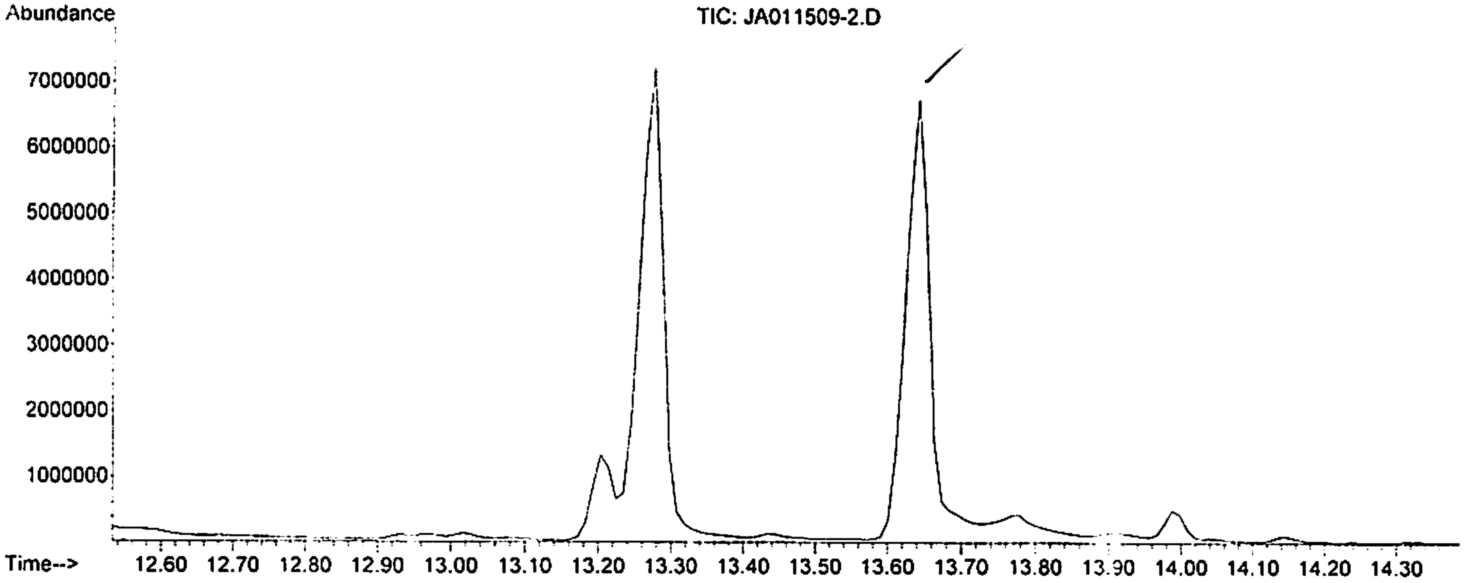


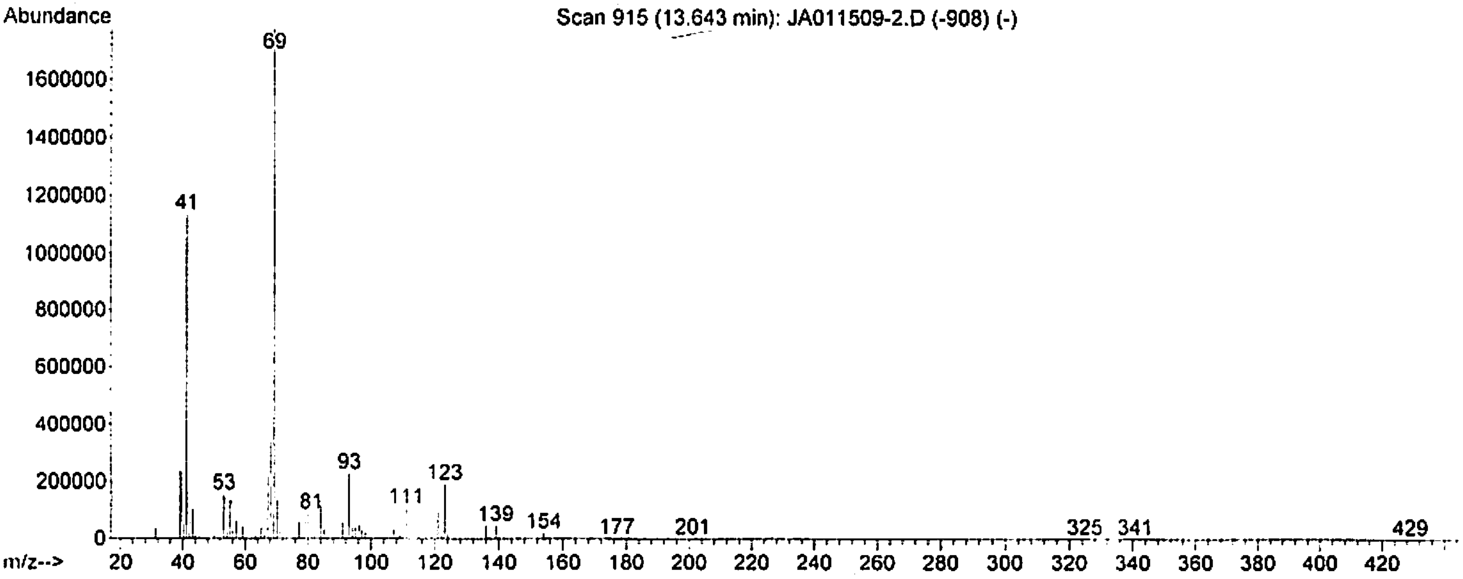

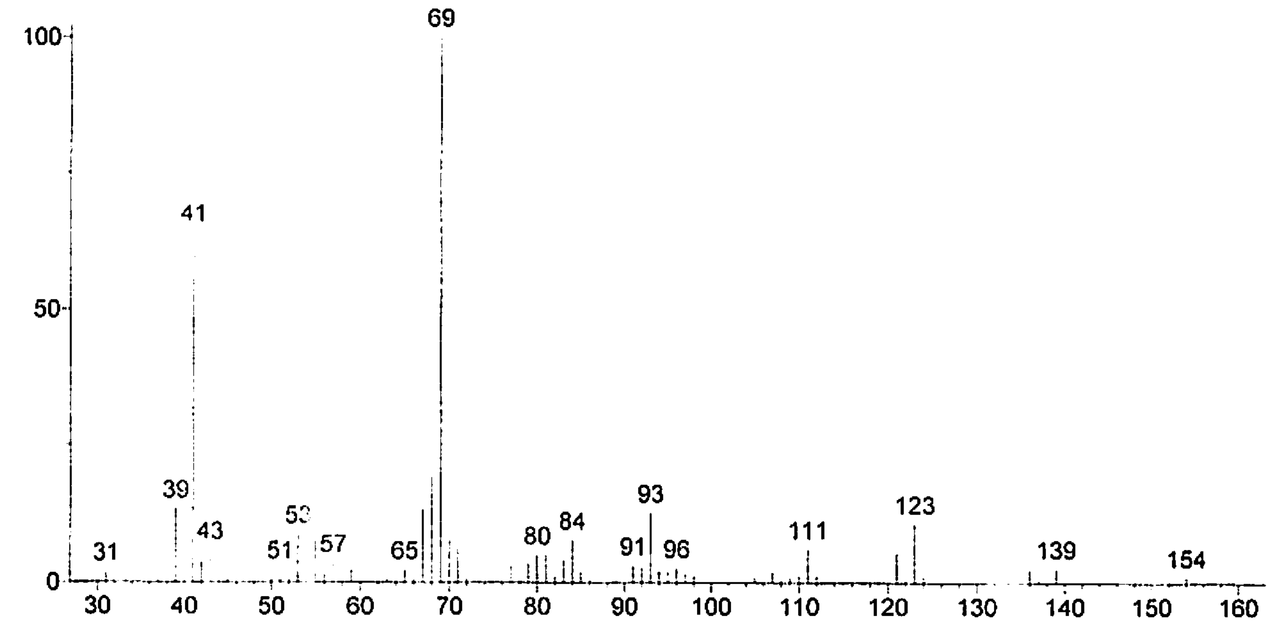


(Text File) Scan 915 (13.643 min): JA011509-2.D (-908)

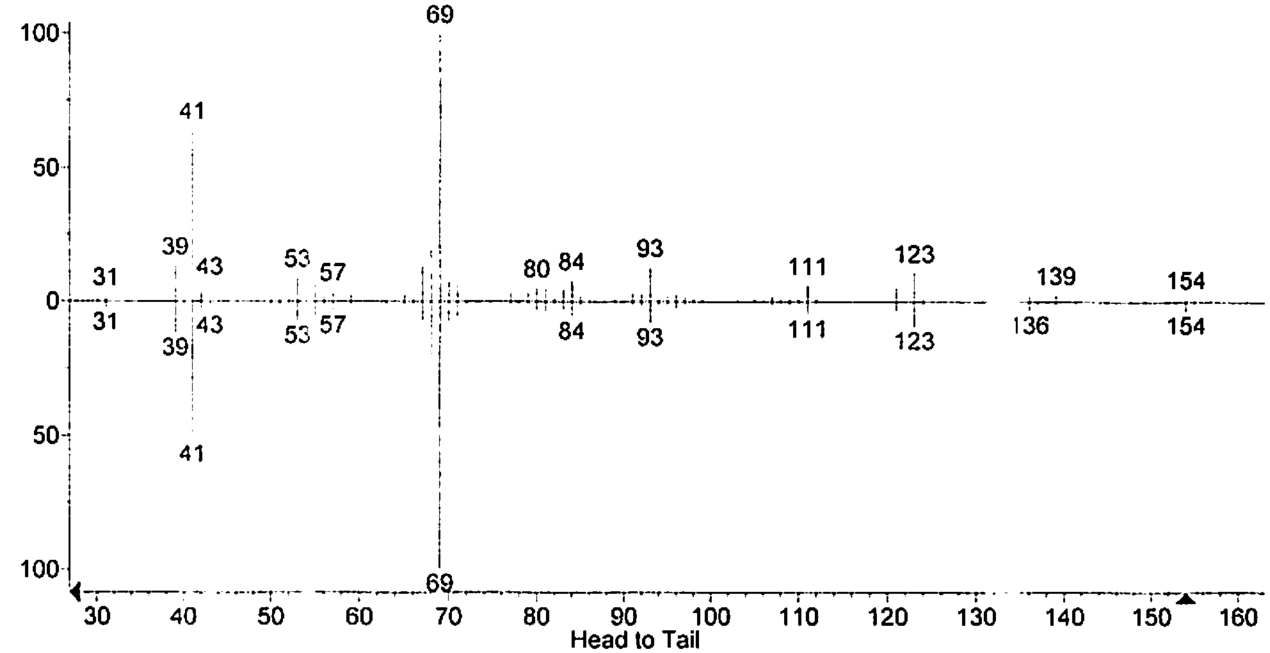


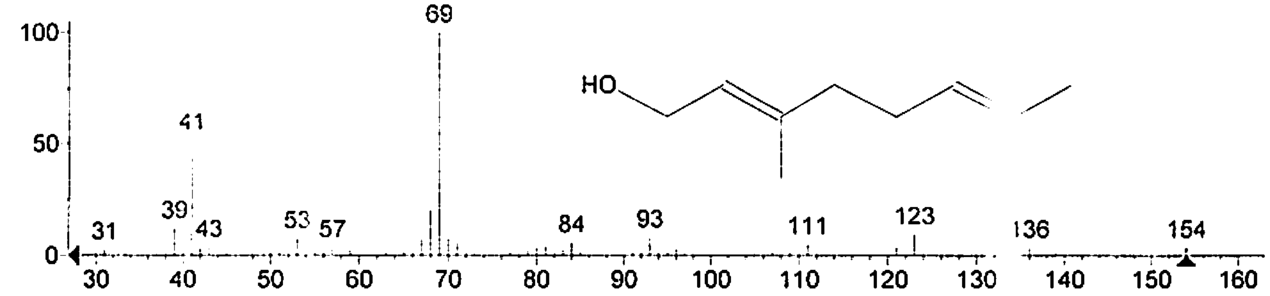


(mainlib) 2,6-Octadien-1-ol, 3,7-dimethyl-

[illegible]le : D:\DATA\Aldr[illegible]ch\JA-09\JA011509-2.D
[illegible]erator : Aldrich
[illegible]quired : 15 Jan 2009 12:17 using AcqMethod JA-WAX08.M
[illegible]strument : Instrument #1
[illegible]mple Name: 12 male C.oculata abd.ster./5ul CH2Cl2
[illegible] Info : ca. 1-week-old; fed. ug/ul citral/water/6days
[illegible]al Number: 1

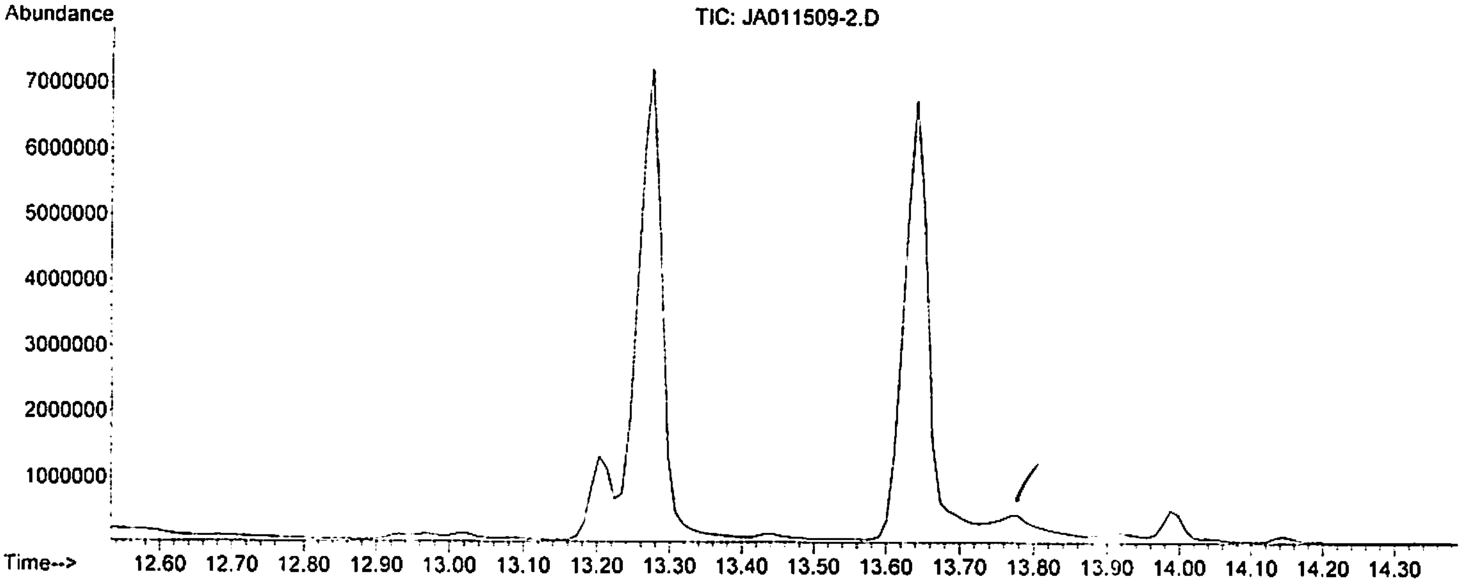


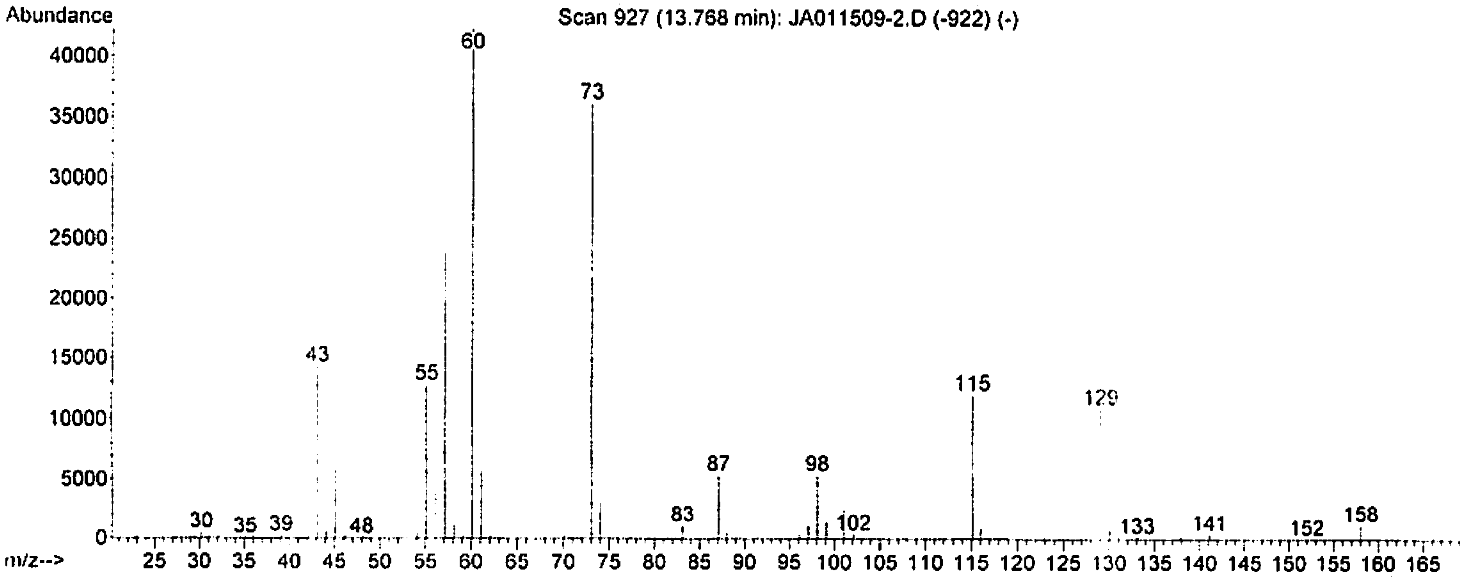

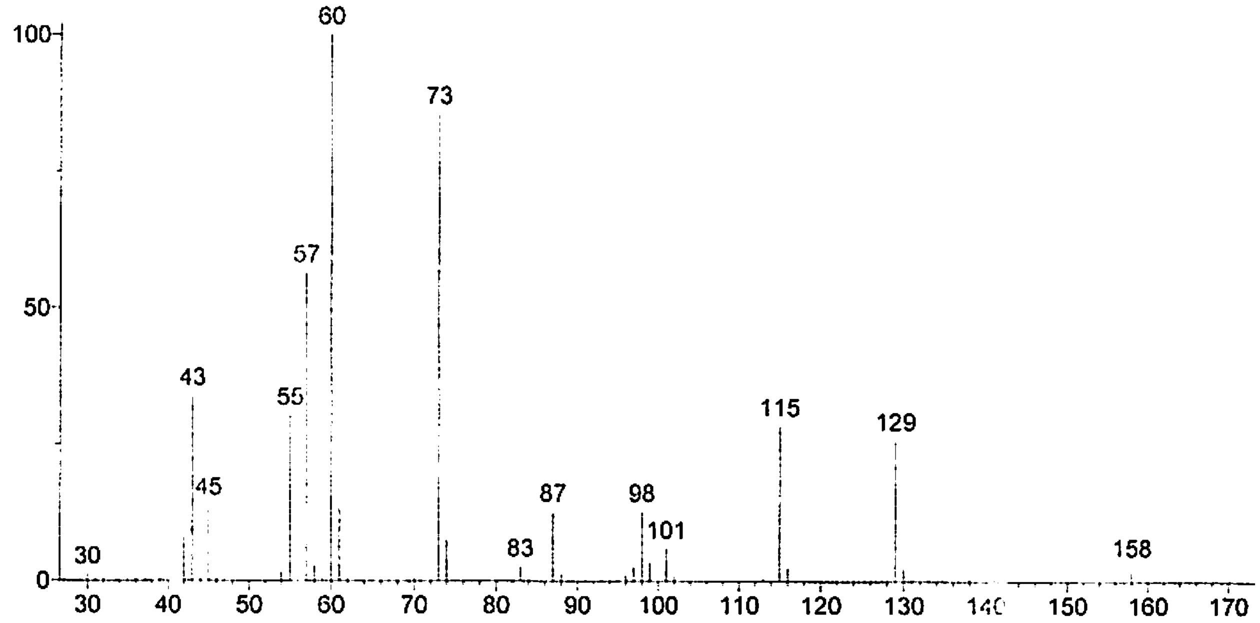


(Text File) Scan 927 (13.768 min): JA011509-2.D (-922)

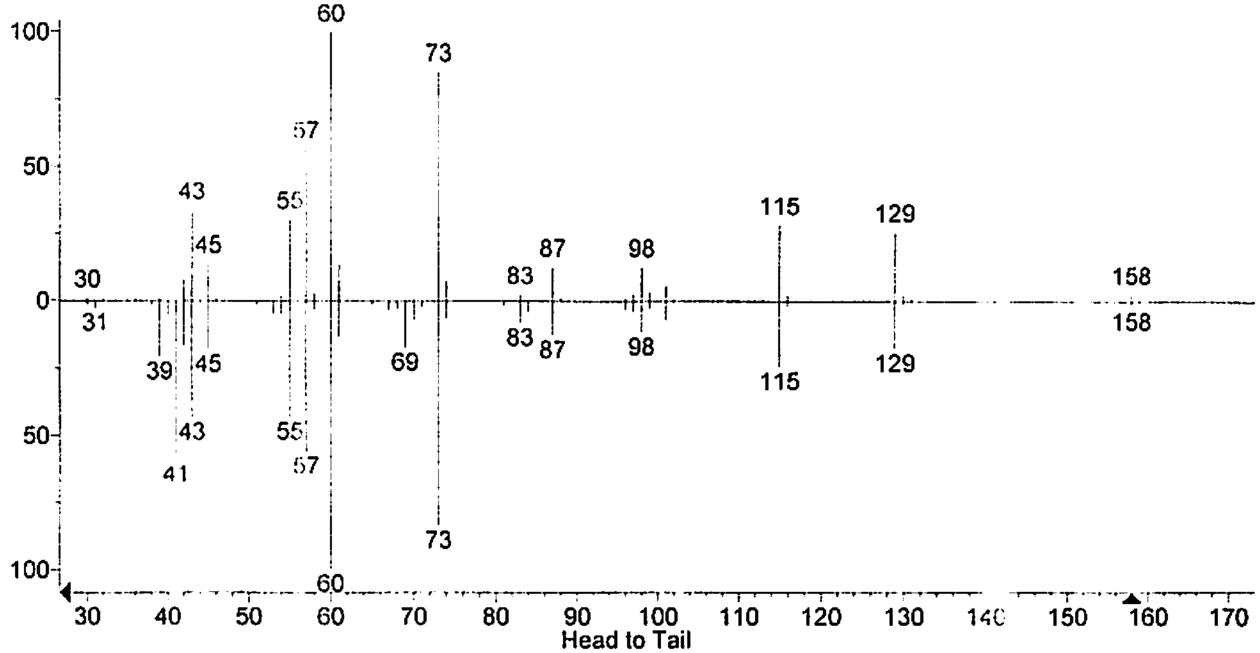


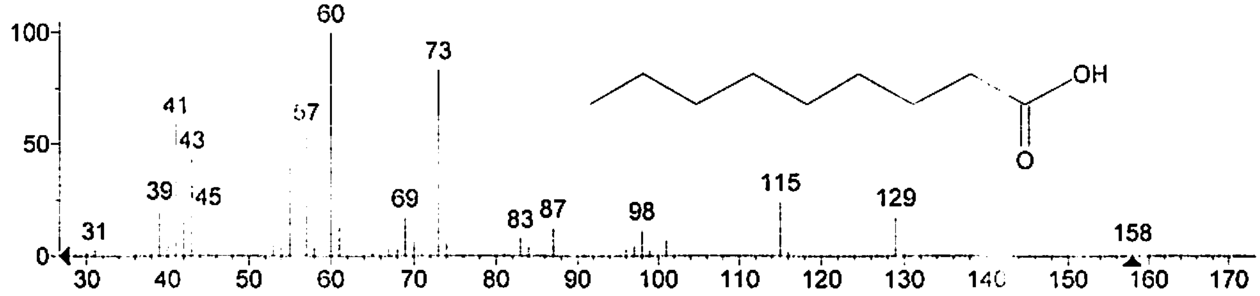


(replib) Nonanoic acid

[illegible]le : D:\DATA\Aldr[illegible]ch\JA-09\JA011509-2.D
[illegible]erator : Aldrich
[illegible]quired : 15 Jan 2009 12:17 using AcqMethod JA-WAX08.[illegible]
[illegible]strument : Instrument #1
[illegible]mple Name: 12 male C.oculata abd.ster./5ul CH2Cl2
[illegible]isc Info : ca. 1-week-old; fed. ug/ul citral/water/6days
[illegible]al Number: 1

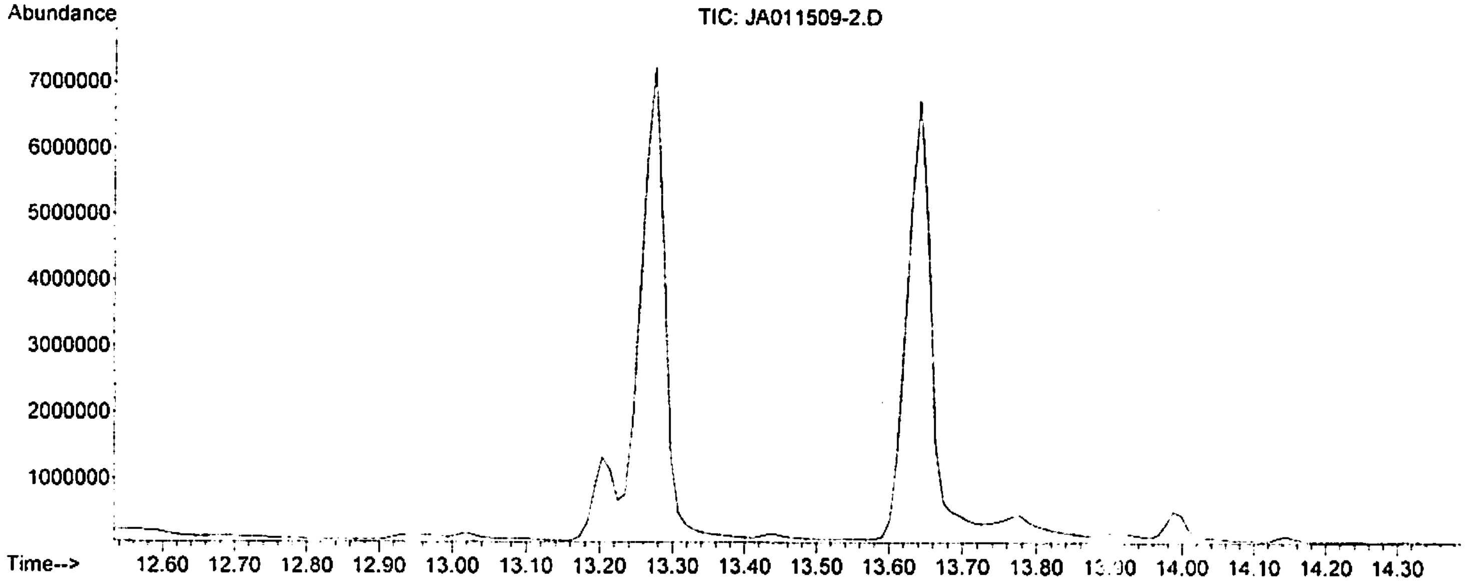


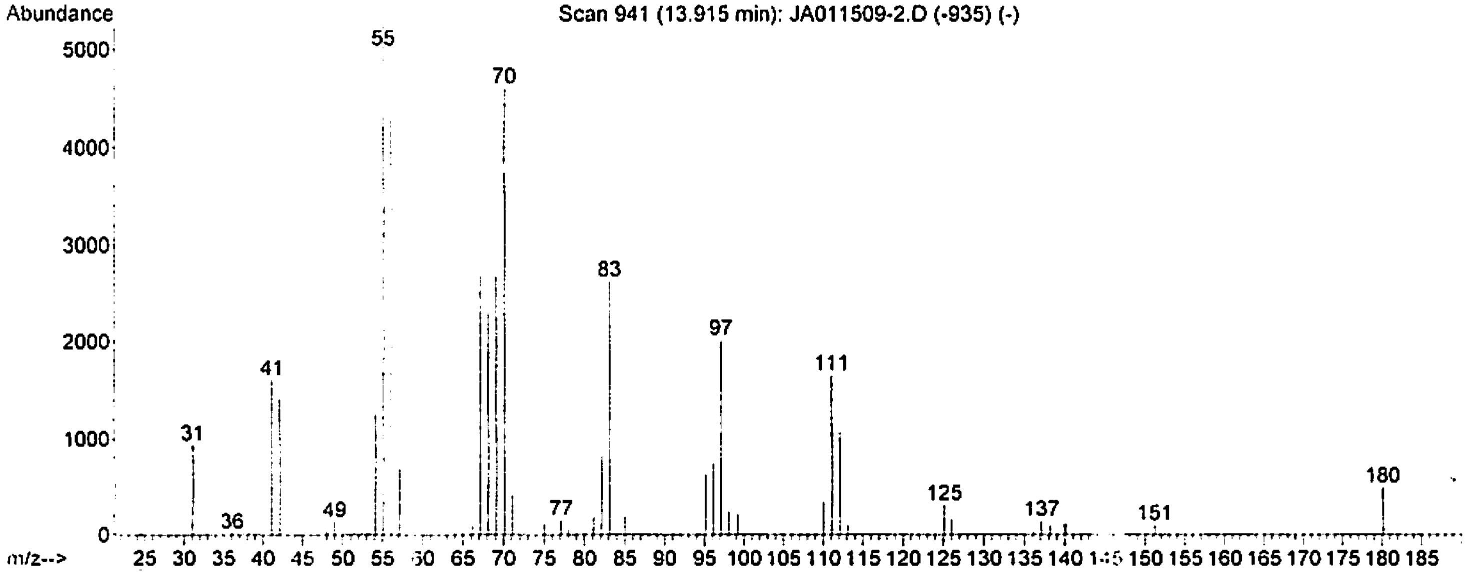

[illegible] :D:\DATA\Aldr ch\JA-09\JA011509-2.D
[illegible]rator : Aldrich
[illegible]quired : 15 Jan 2009 12:17 using AcqMethod JA-WAX08.4
[illegible]strument : Instrument #1
[illegible]mple Name: 12 male C.oculata abd.ster./5ul CH2Cl2
[illegible]sc Info : ca. 1-week-old; fed. ug/ul citral/water/6days
[illegible]al Number: 1

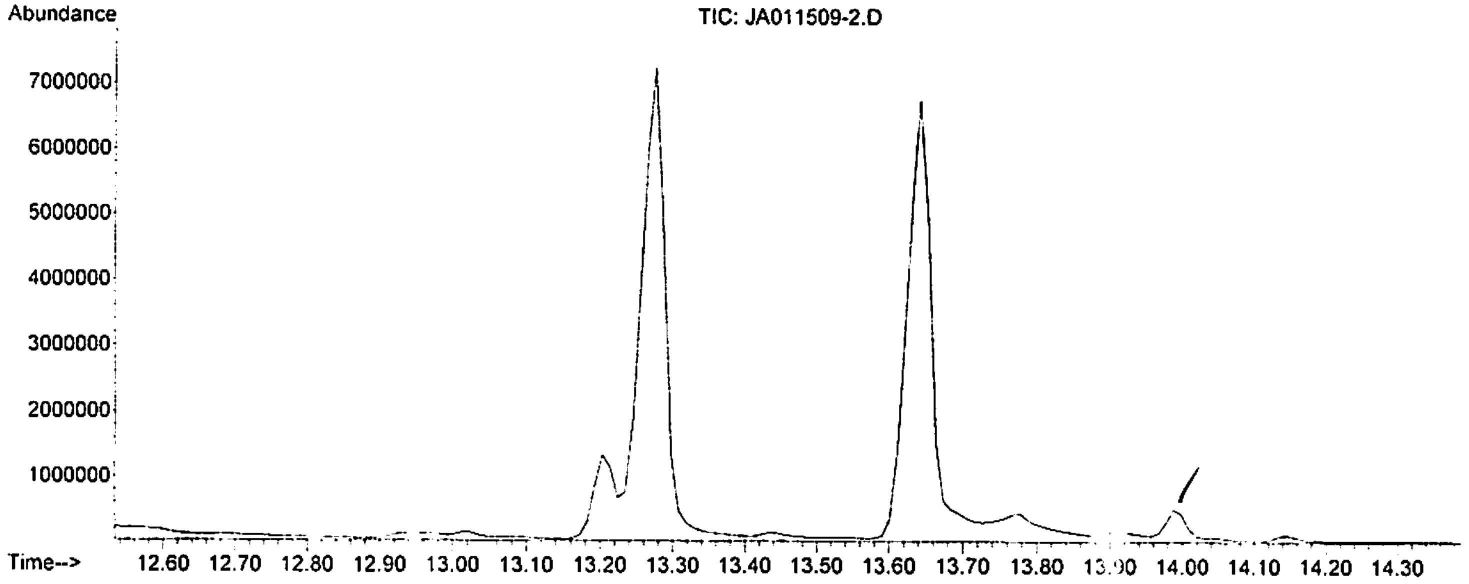


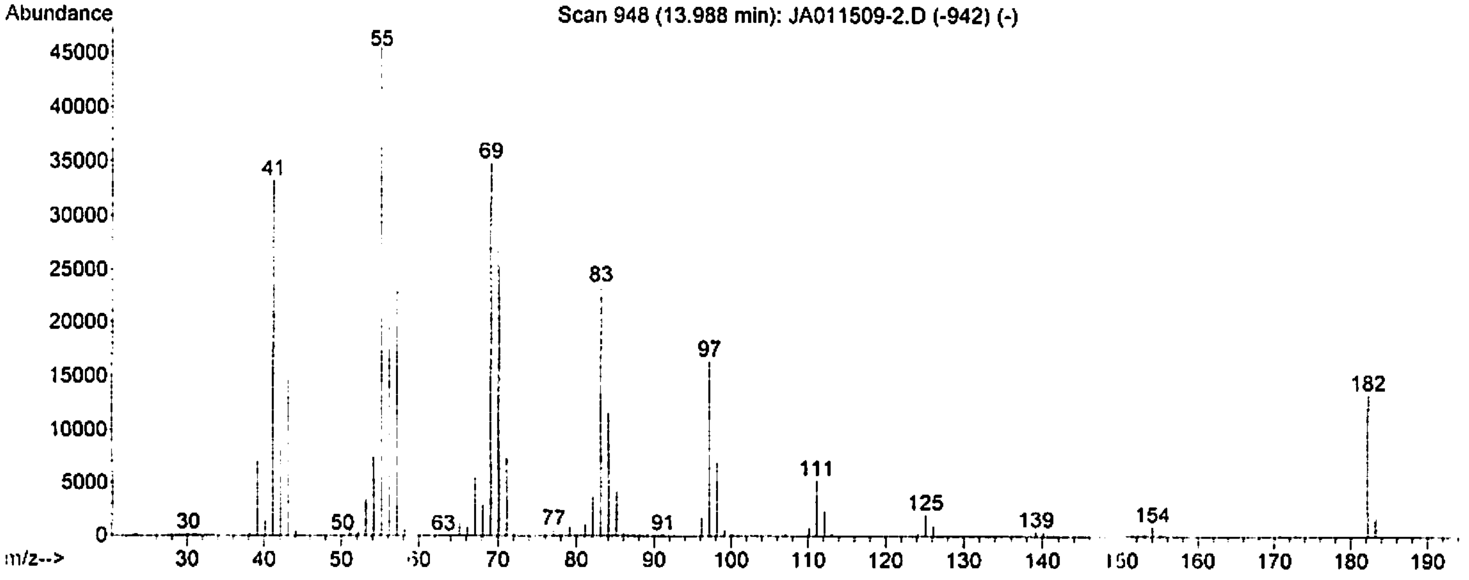

[illegible] :D:\DATA\Aldr ch\JA-09\JA011509-2.D
[illegible]rator : Aldrich
[illegible]uired : 15 Jan 2009 12:17 using AcqMethod JA-WAX08.M
[illegible]trument : Instrument #1
[illegible]mple Name: 12 male C.oculata abd.ster./5ul CH2Cl2
[illegible] Info : ca. 1-week-old; fed. ug/ul citral/water/6days
[illegible]l Number: 1

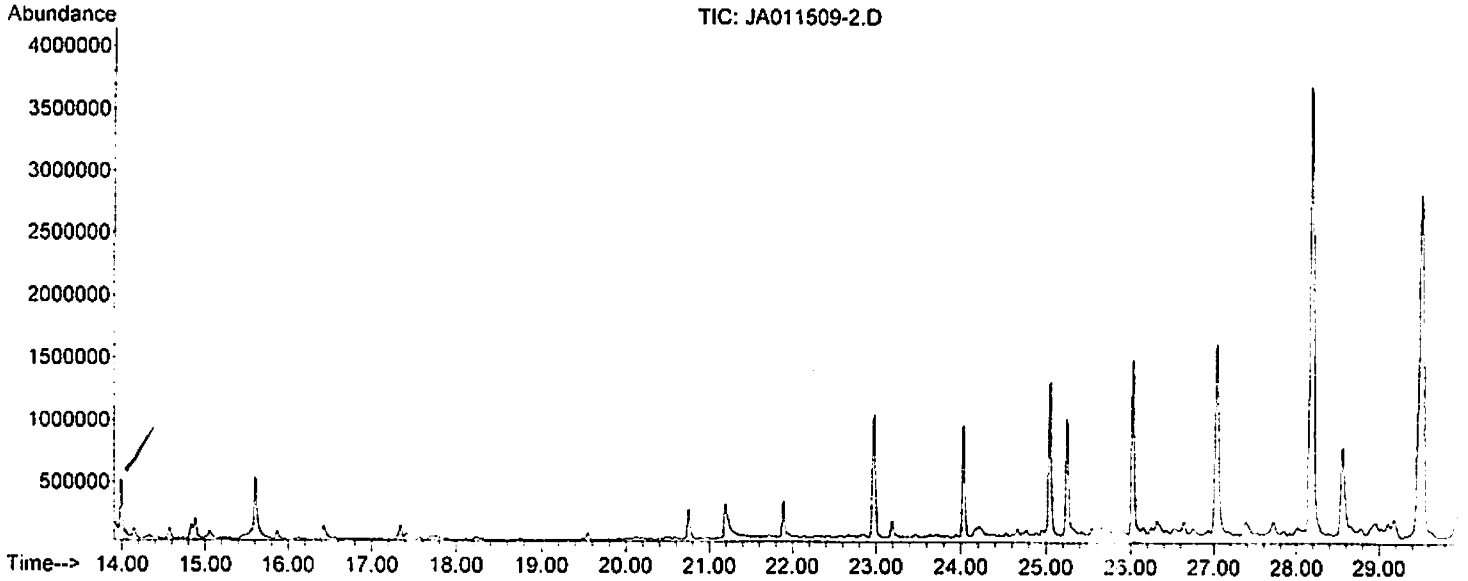


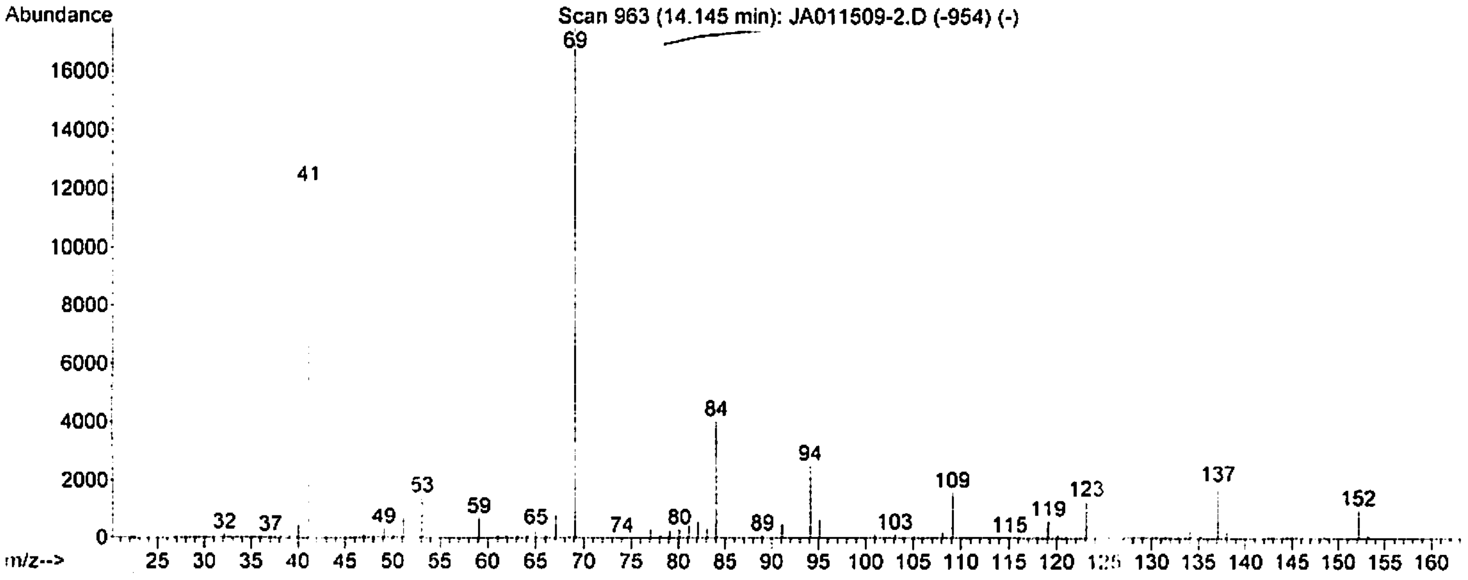

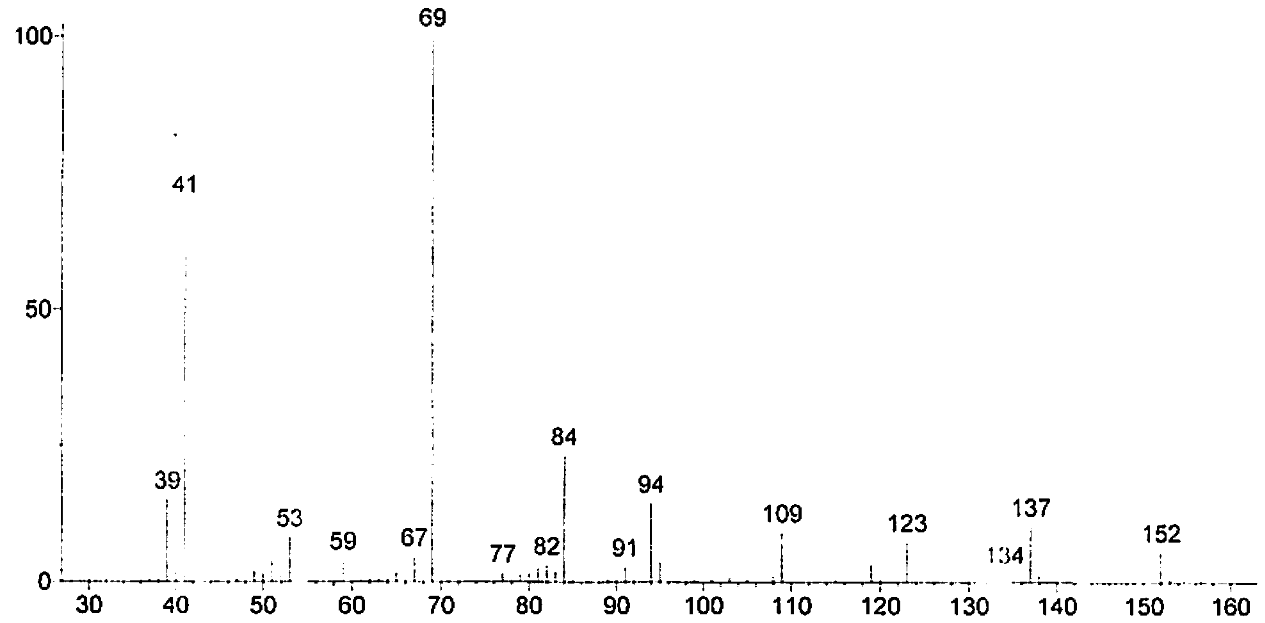


(Text File) Scan 963 (14.145 min): JA011509-2.D (-954)

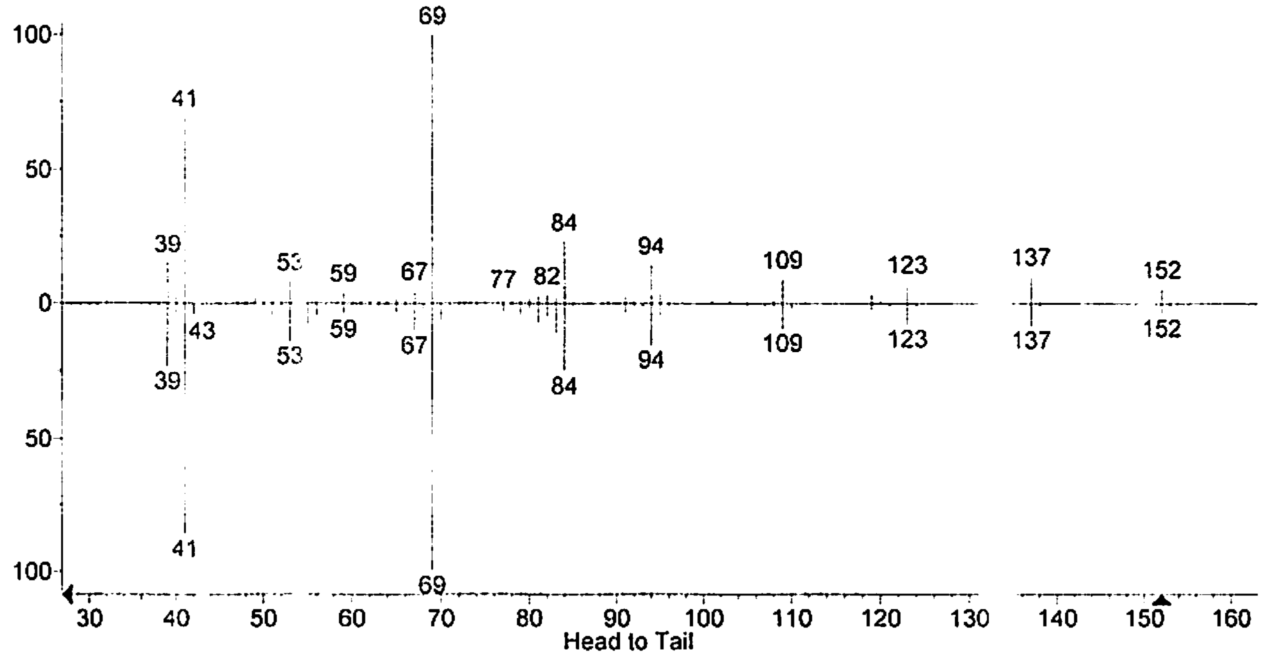


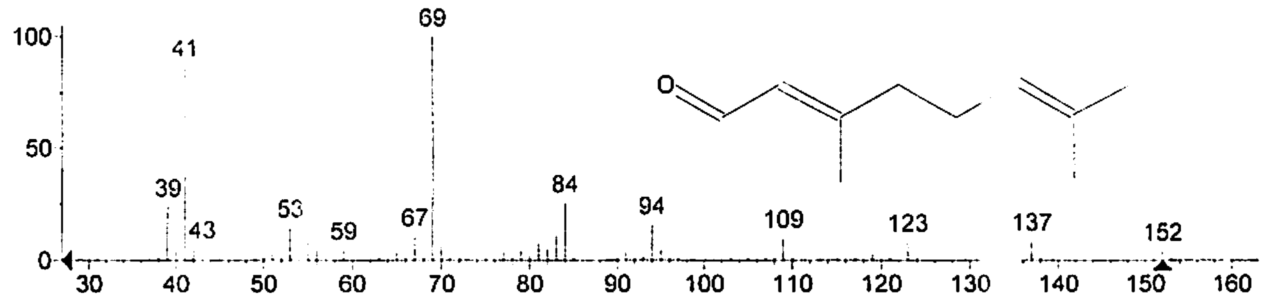

(replib) 2,6-Octadienal, 3,7-dimethyl-, (E)-

```
[illegible]e [illegible]    :D:\DATA\Aldr ch\JA-09\JA011509-2.D
[illegible]rator    : Aldrich
[illegible]quired   : 15 Jan 2009   12:17      using AcqMethod JA-WAX08.M
[illegible]strument :     Instrument #1
[illegible]mple Name: 12 male C.oculata abd.ster./5ul CH2Cl2
[illegible]sc Info  : ca. 1-week-old; fed. ug/ul citral/water/6days
[illegible]al Number: 1
```

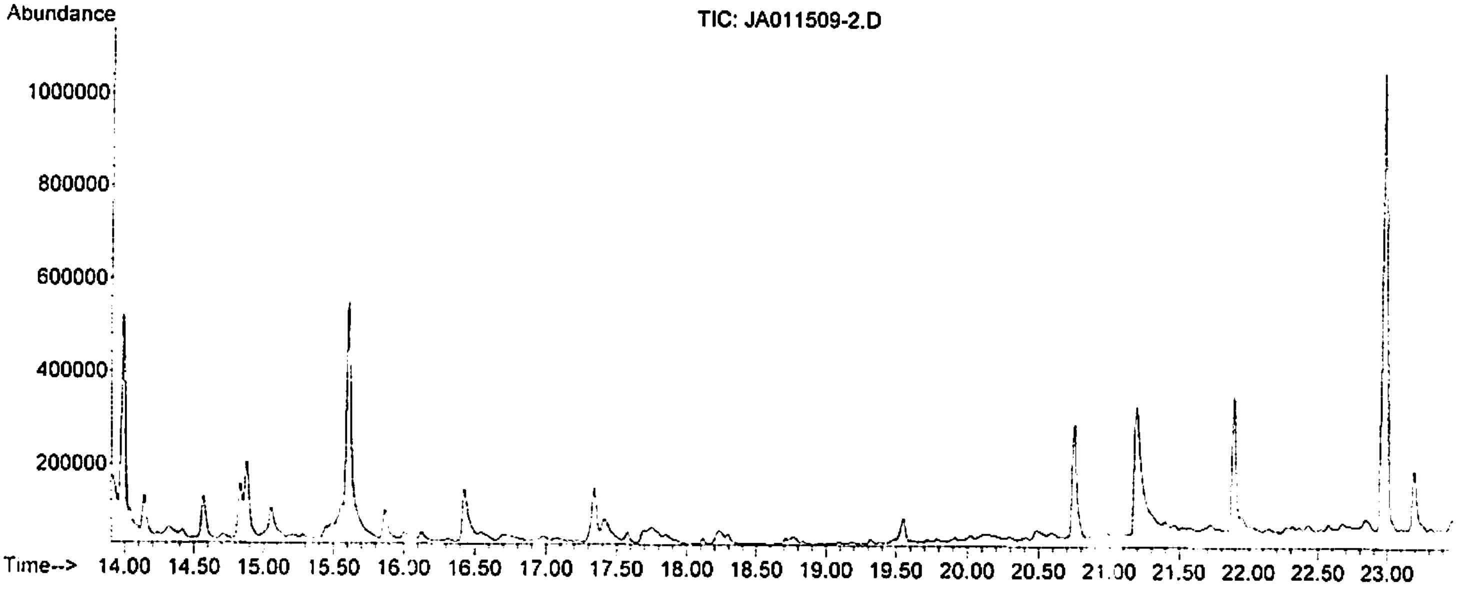


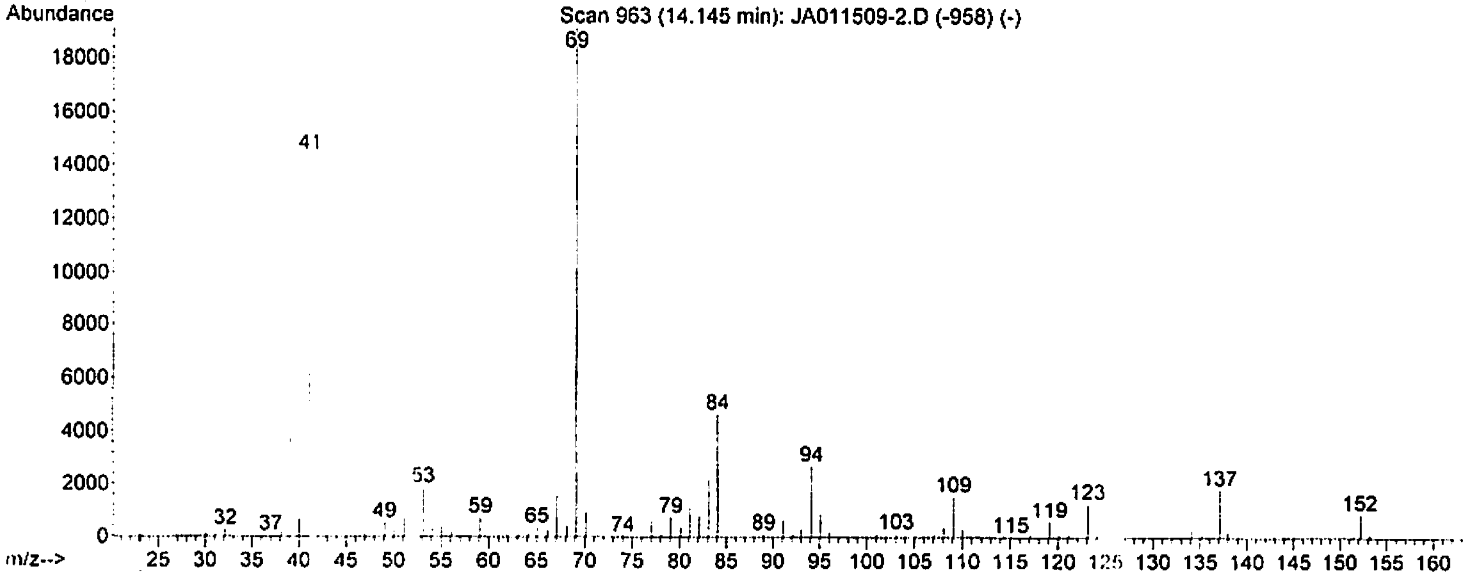

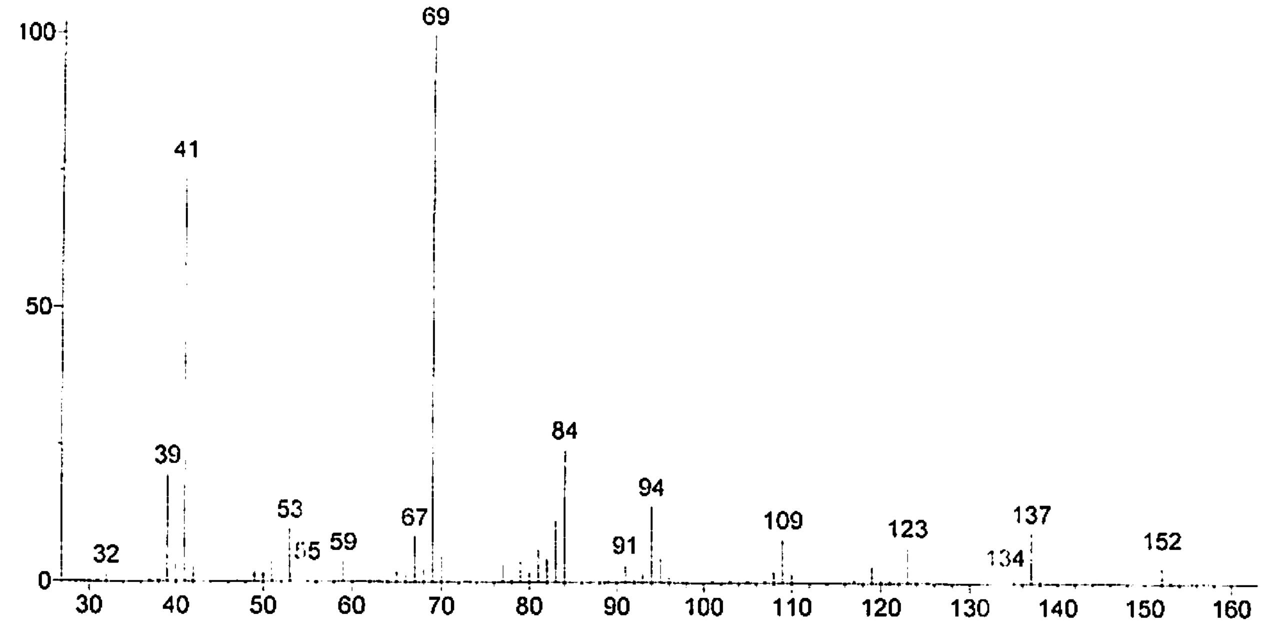


(Text File) Scan 963 (14.145 min): JA011509-2.D (-958)

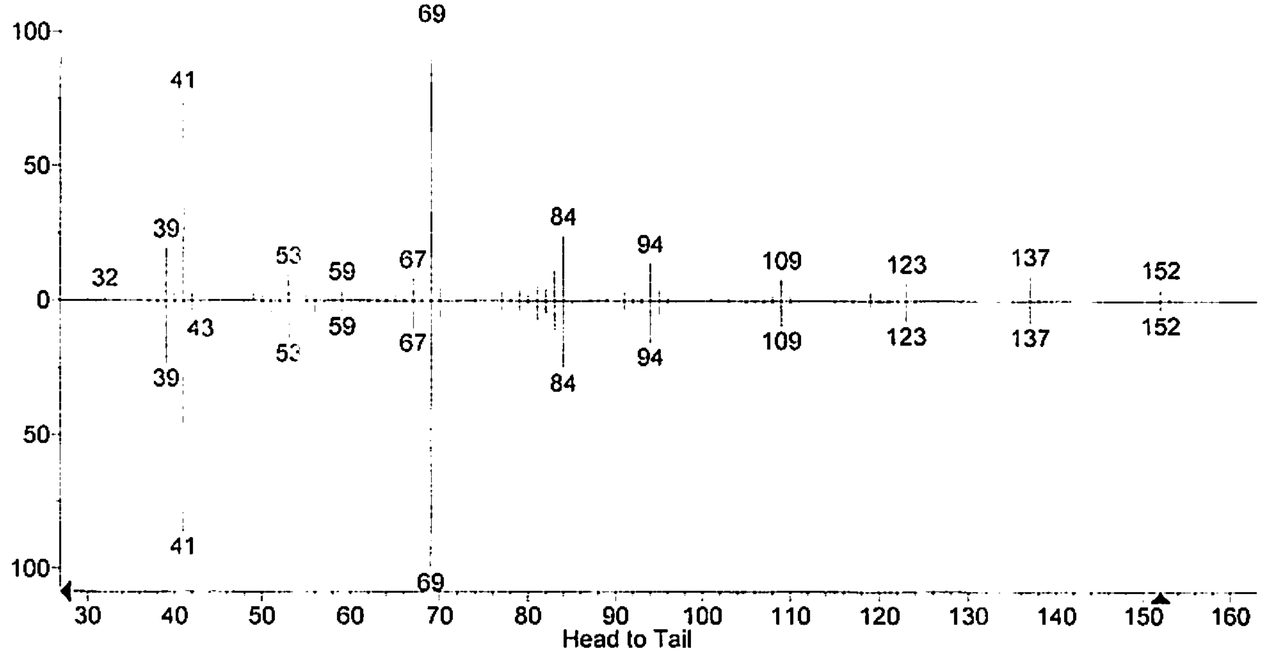


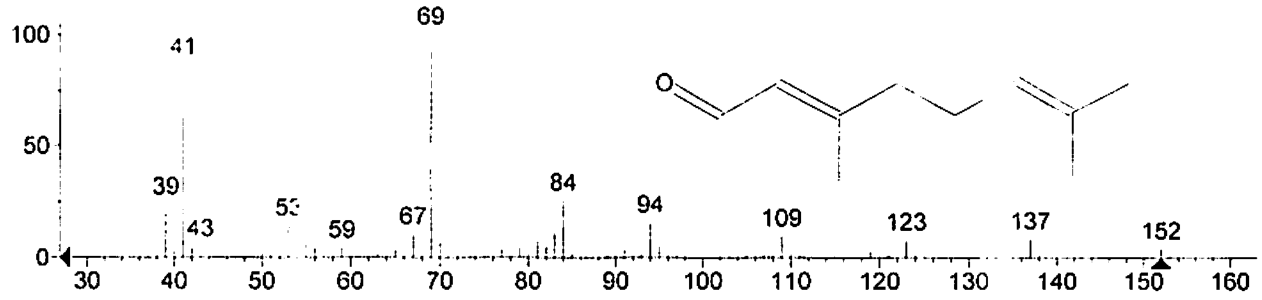


(replib) 2,6-Octadienal, 3,7-dimethyl-, (E)-

[illegible]le : D:\DATA\Aldr[illegible]ch\JA-09\JA011509-2.D
[illegible]perator : Aldrich
[illegible]quired : 15 Jan 2009 12:17 using AcqMethod JA-WAX08.M
[illegible]strument : Instrument #1
[illegible]mple Name: 12 male C.oculata abd.ster./5ul CH2Cl2
[illegible]isc Info : ca. 1-week-old; fed. ug/ul citral/water/6days
[illegible]al Number: 1

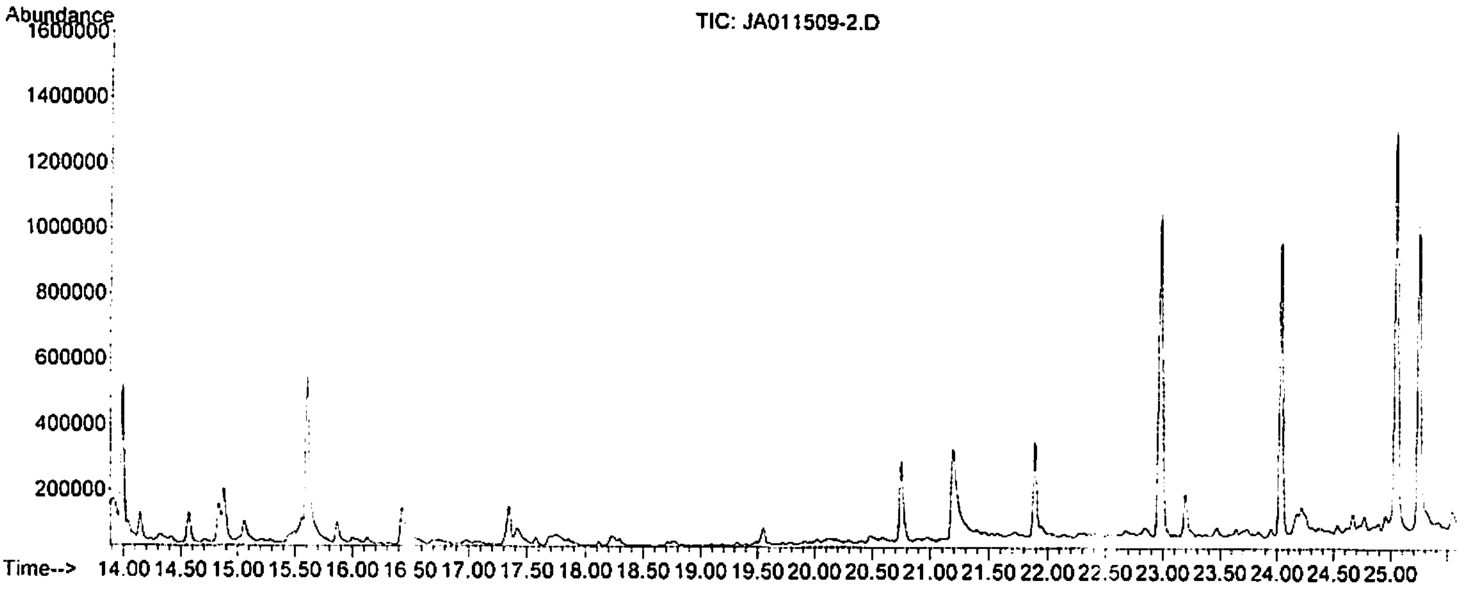


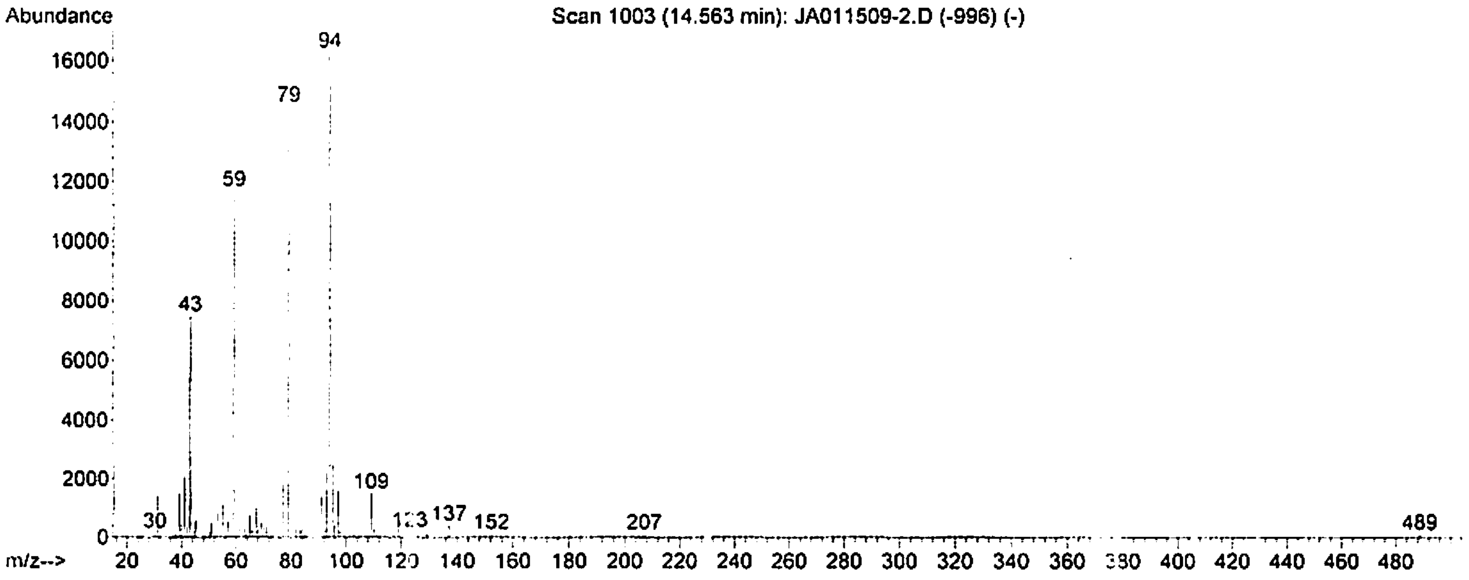

[illegible]le : D:\DATA\Aldr[illegible]ch\JA-09\JA011509-2.D
[illegible]erator : Aldrich
[illegible]quired : 15 Jan 2009 12:17 using AcqMethod JA-WAX08.M
[illegible]strument : Instrument #1
[illegible]mple Name: 12 male C.oculata abd.ster./5ul CH2Cl2
[illegible]isc Info : ca. 1-week-old; fed. ug/ul citral/water/6days
[illegible]ial Number: 1

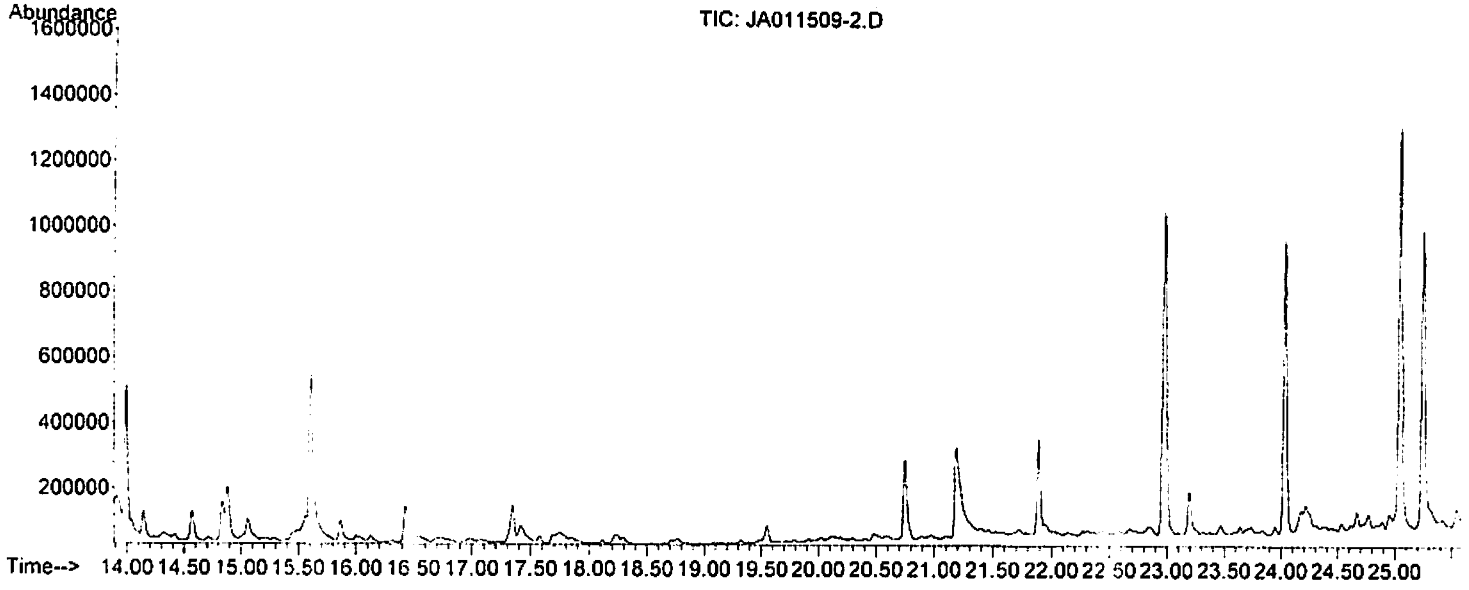


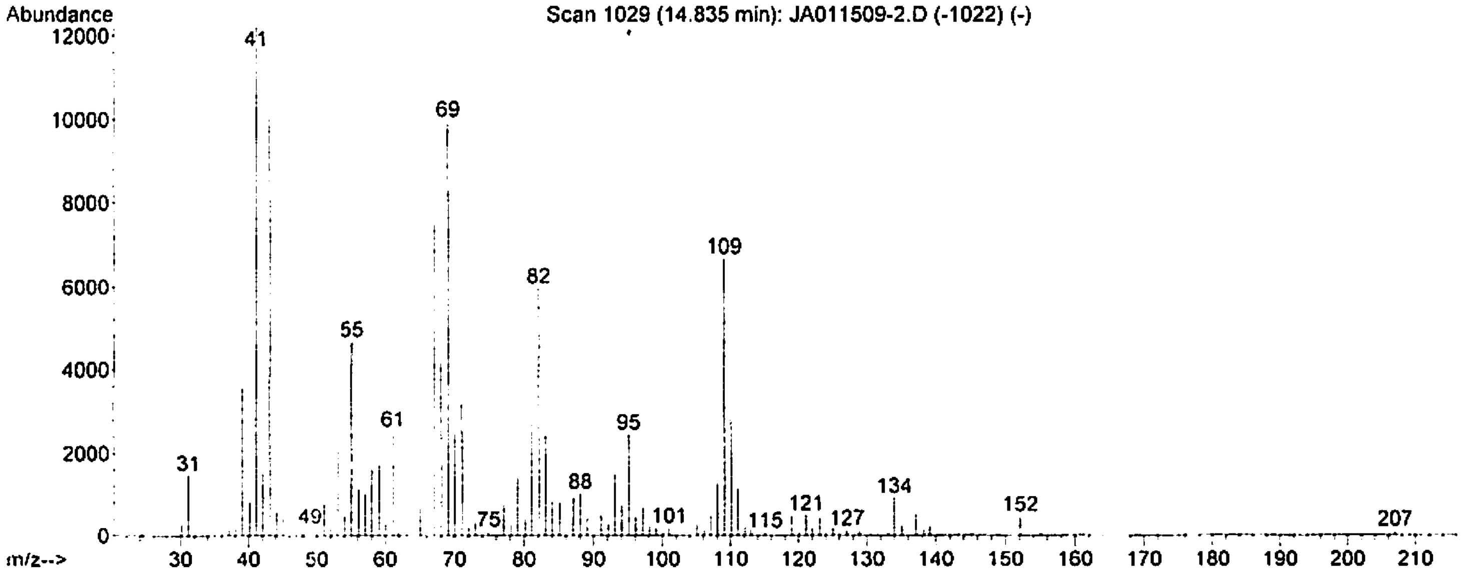

File :D:\DATA\Aldr ch\JA-09\JA011509-2.D
Operator : Aldrich
Acquired : 15 Jan 2009 12:17 using AcqMethod JA-WAX08.M
Instrument : Instrument #1
Sample Name: 12 male C.o[illegible]ulata abd.ster./5ul CH2Cl2
Misc Info : ca. 1-week-old; fed. ug/ul citral/water/6days
Vial Number: 1

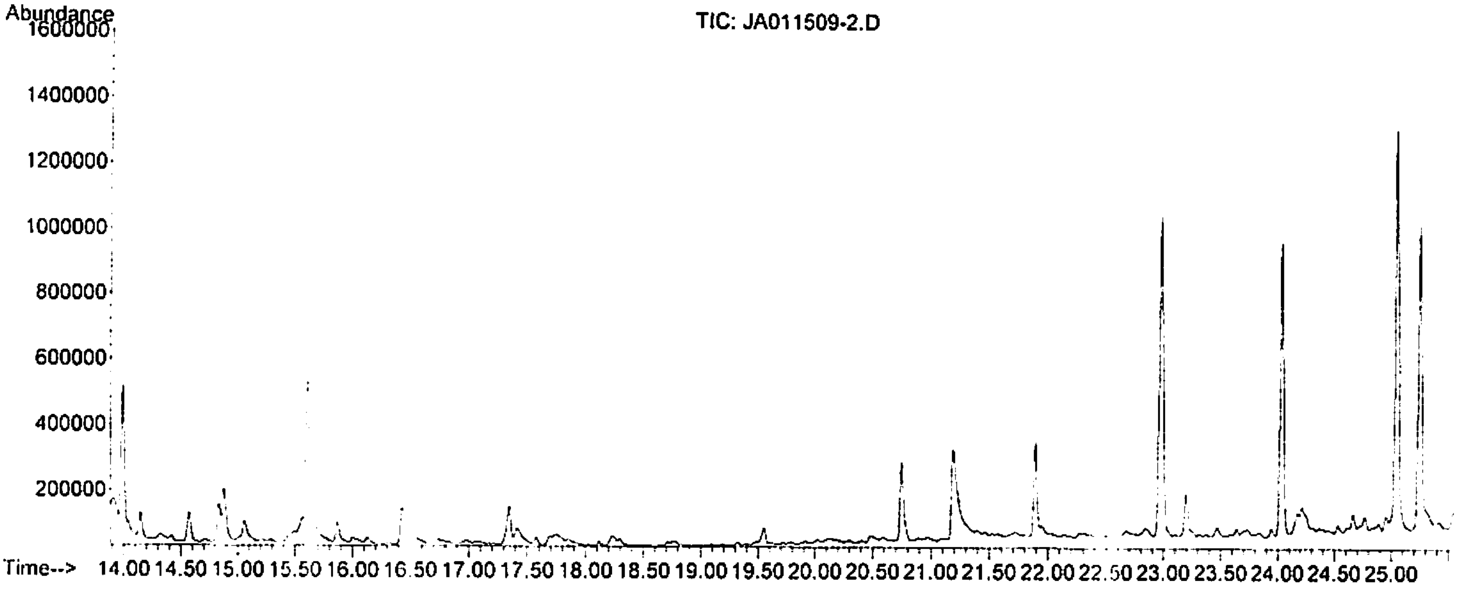


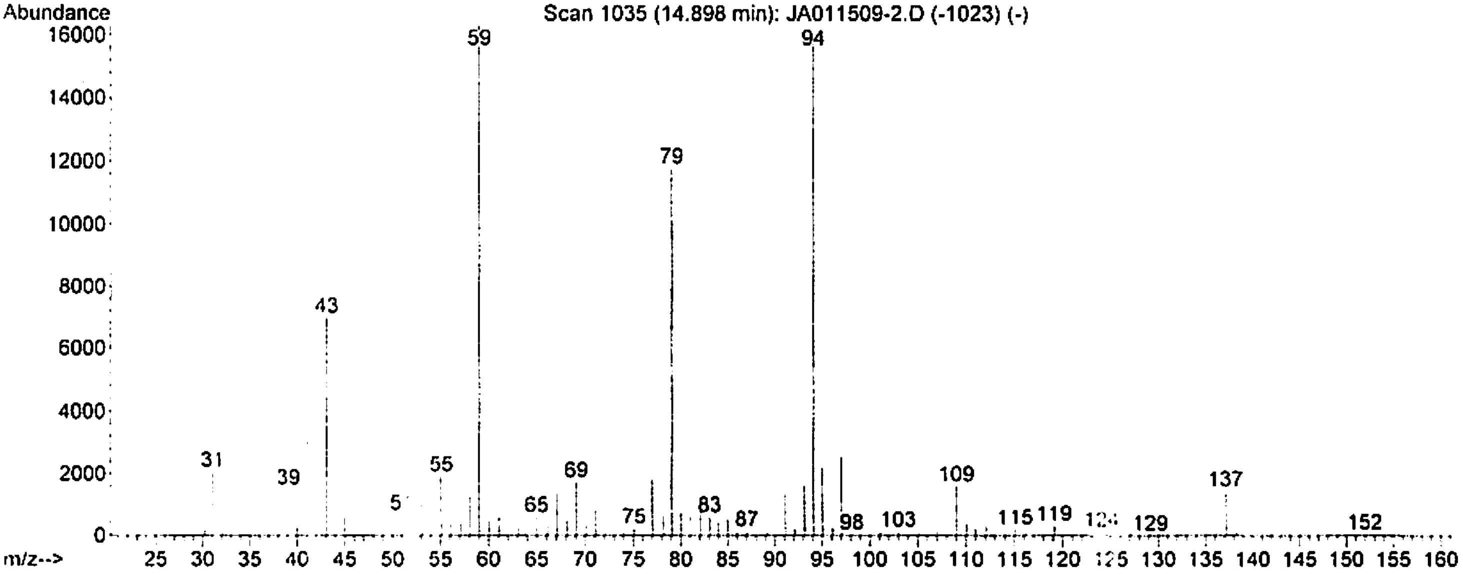

```
[illegible]le        :D:\DATA\Aldr ch\JA-09\JA011509-2.D
[illegible]erator    : Aldrich
[illegible]quired    : 15 Jan 2009   12:17      using AcqMethod JA-WAX08.M
[illegible]strument  :     Instrument #1
[illegible]mple Name: 12 male C.o[illegible]ulata abd.ster./5ul CH2Cl2
[illegible]sc Info   : ca. 1-week-old; fed. ug/ul citral/water/6days
[illegible]al Number: 1
```

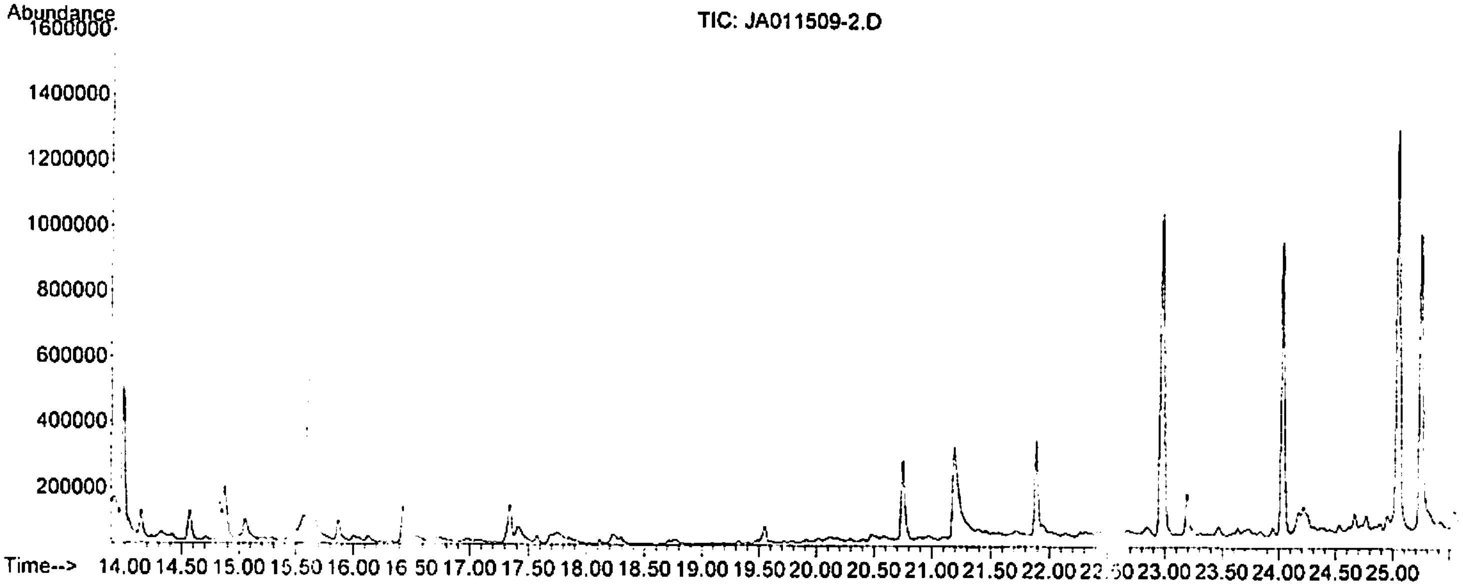


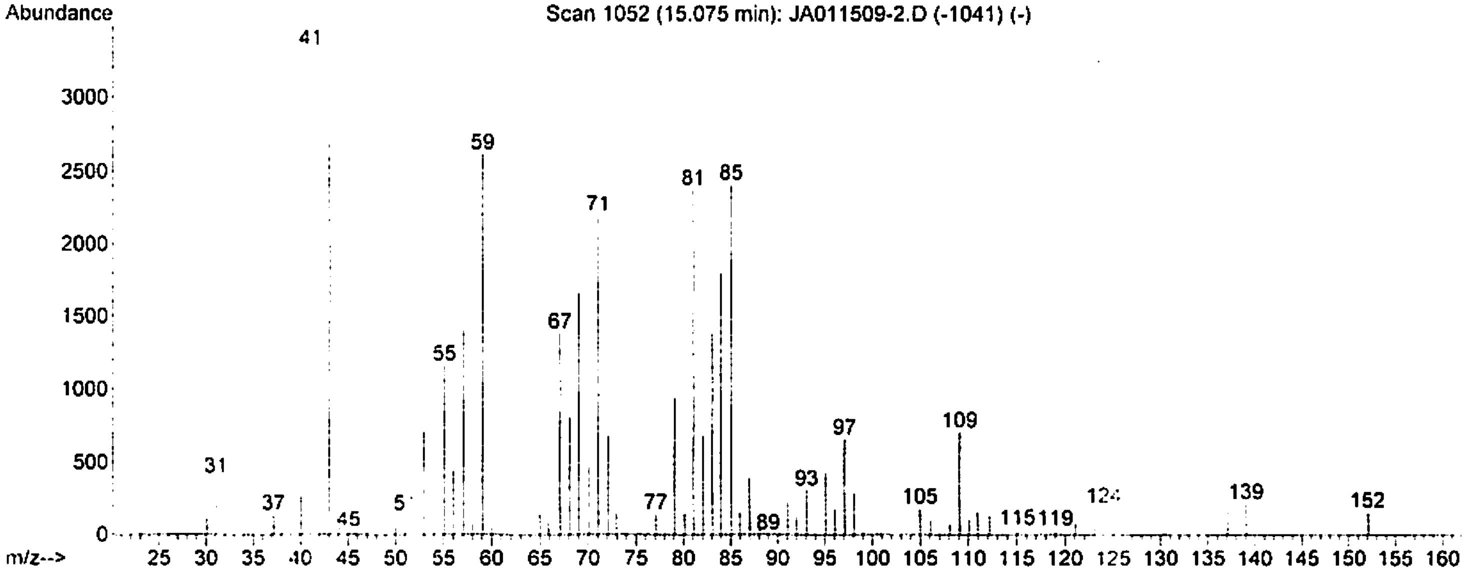

File :D:\DATA\Aldrich\JA-09\JA011509-2.D
Operator : Aldrich
Acquired : 15 Jan 2009 12:17 using AcqMethod JA-WAX08.4
Instrument : Instrument #1
Sample Name: 12 male C.oculata abd.ster./5ul CH2Cl2
Misc Info : ca. 1-week-old; fed. ug/ul citral/water/6days
Vial Number: 1

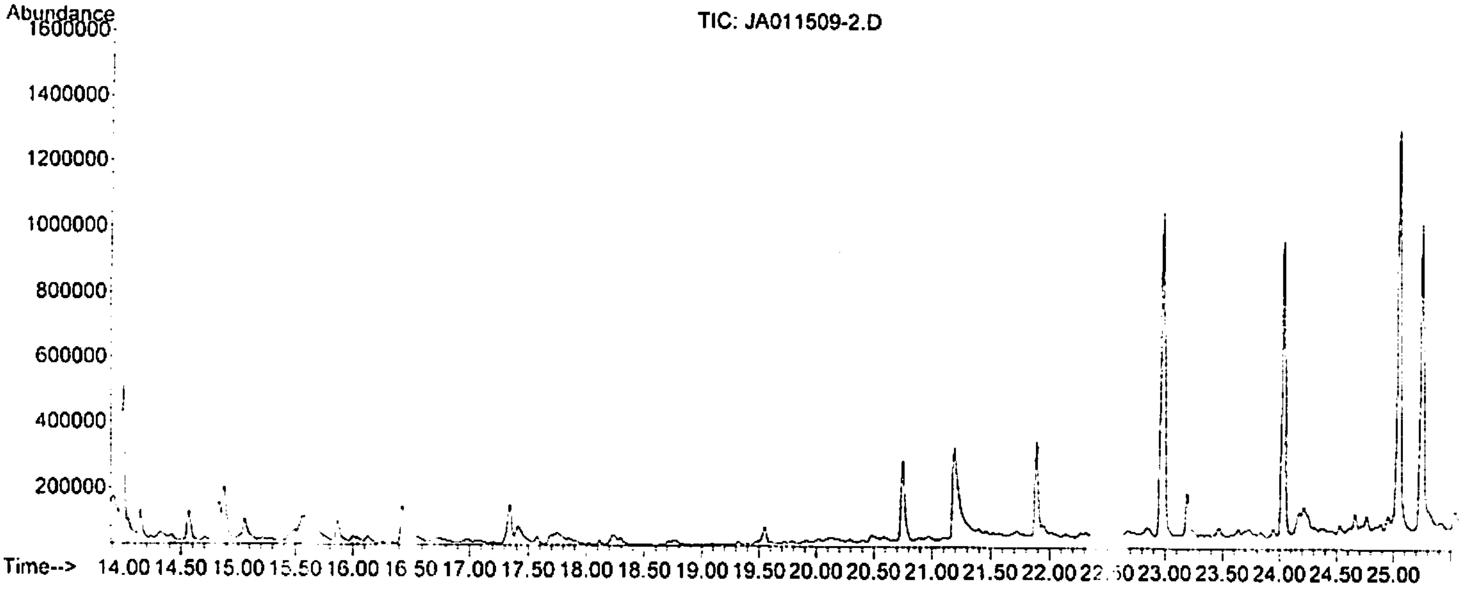


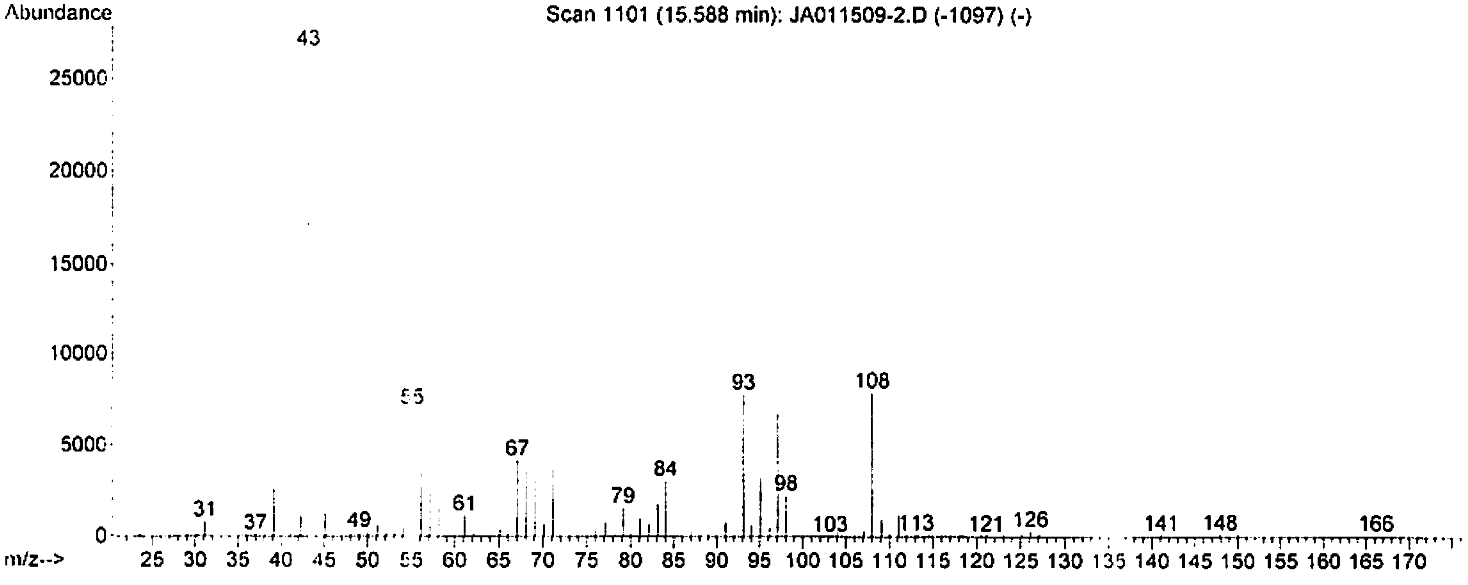

File :D:\DATA\Aldrich\JA-09\JA011509-2.D
Operator : Aldrich
Acquired : 15 Jan 2009 12:17 using AcqMethod JA-WAX08.4
Instrument : Instrument #1
Sample Name: 12 male C.oculata abd.ster./5ul CH2Cl2
Misc Info : ca. 1-week-old; fed. ug/ul citral/water/6days
Vial Number: 1

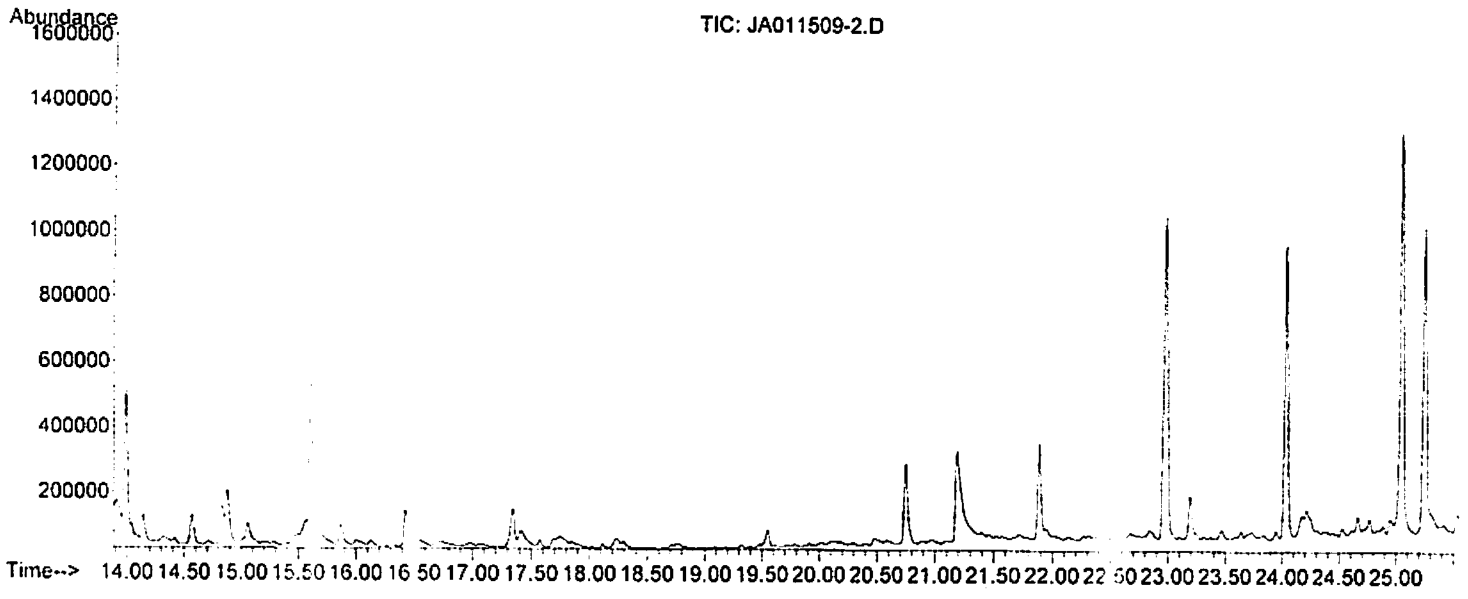


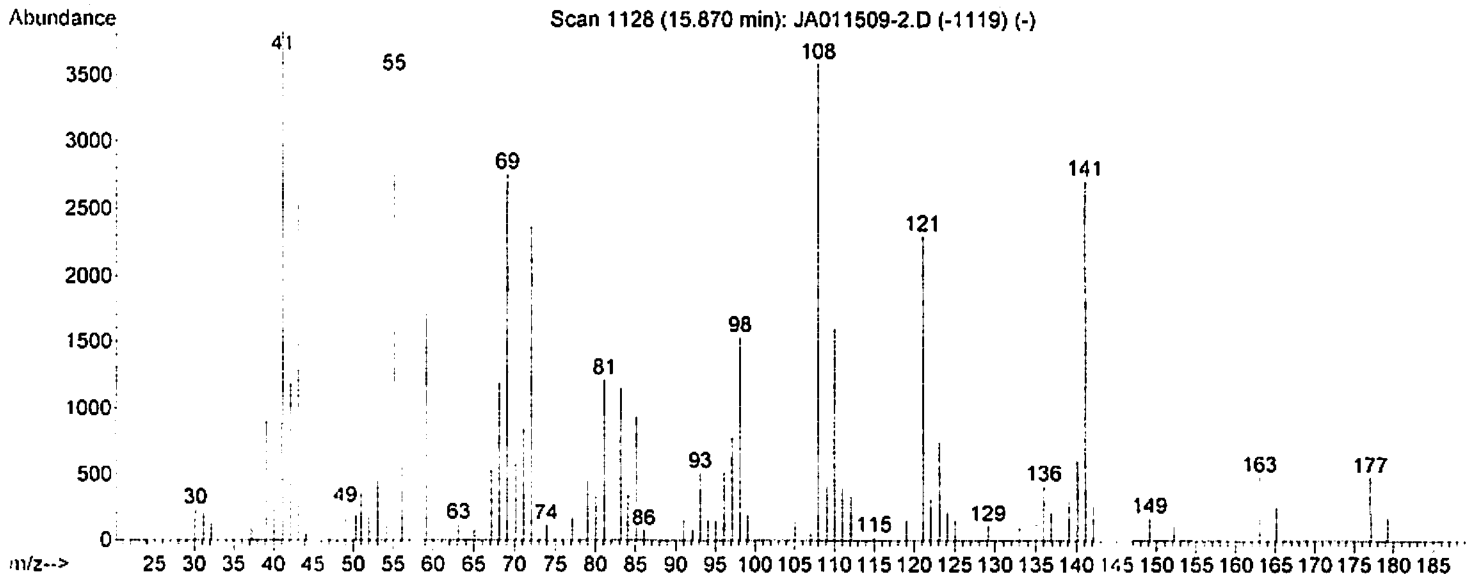

[illegible] : D:\DATA\Aldr ch\JA-09\JA011509-2.D
[illegible]ator : Aldri[illegible]
[illegible]quired : 15 Jan 2009 12:17 using AcqMethod JA-WAX08.[illegible]
[illegible]strument : Instrument #1
[illegible]ample Name: 12 male C.oculata abd.ster./5ul CH2Cl2
Misc Info : ca. 1-week-old; fed. ug/ul citral/water/6days
Vial Number: 1

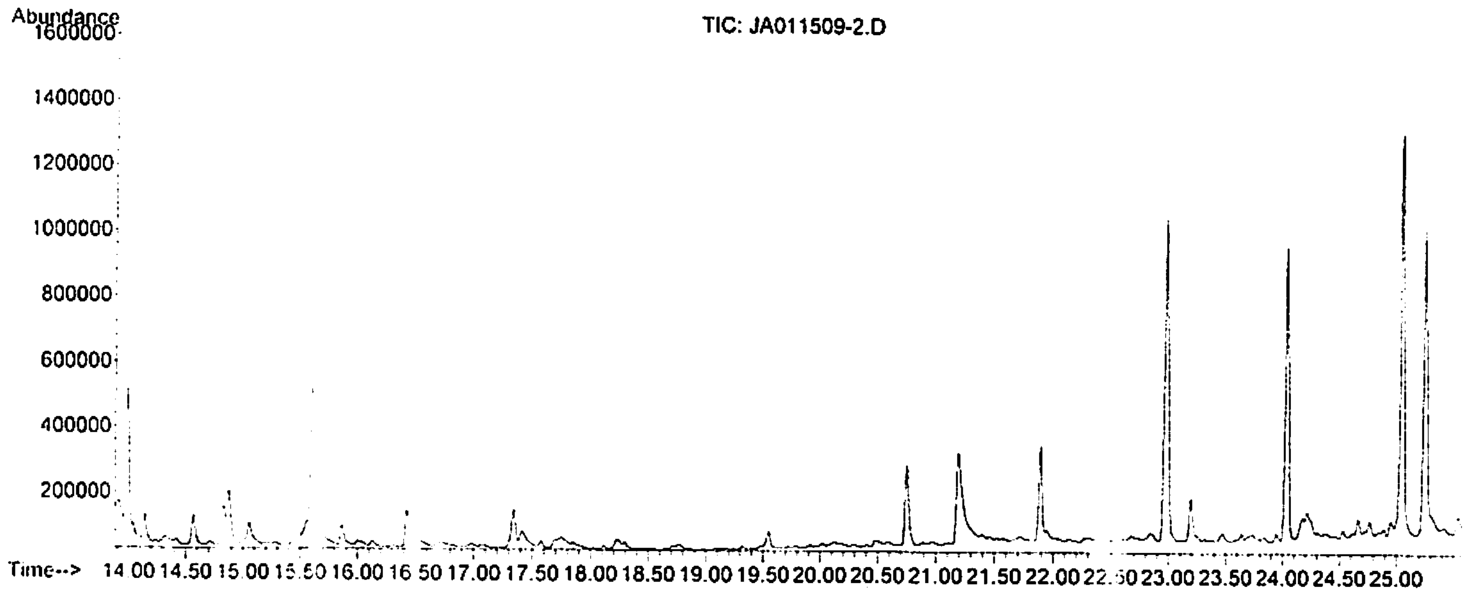


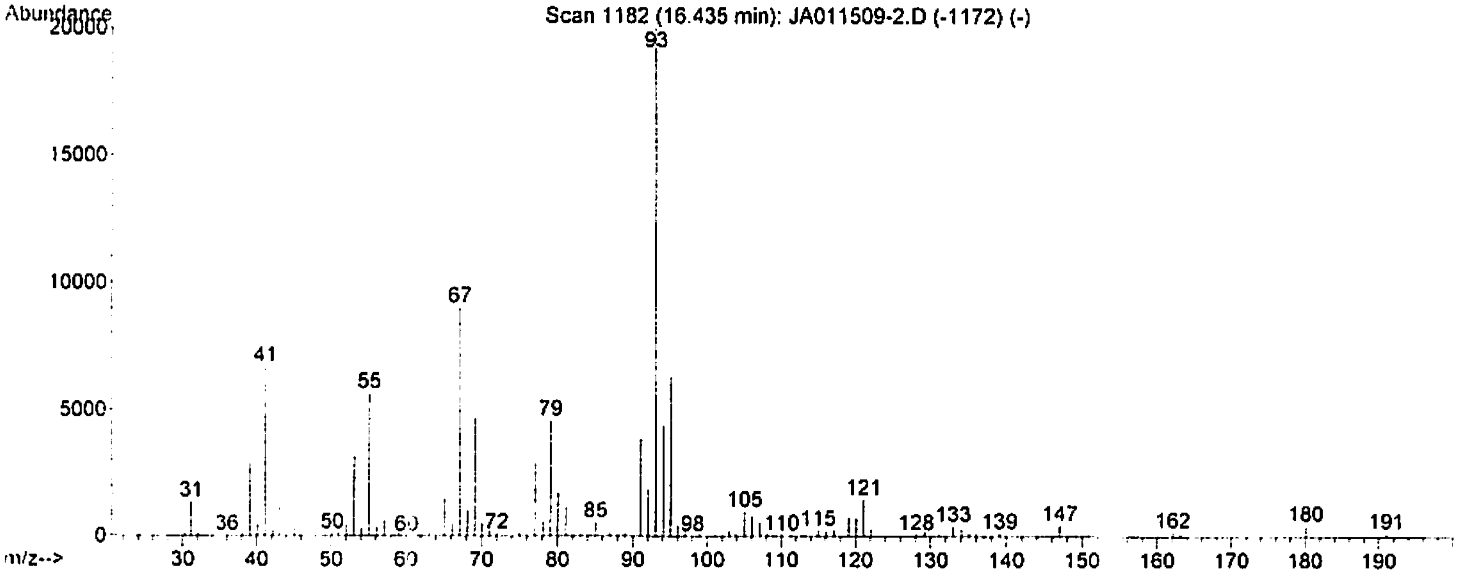

```
[illegible]    :D:\DATA\Aldr ch\JA-09\JA011509-2.D
erator    : Aldrich
quired    : 15 Jan 2009   12:17      using AcqMethod JA-WAX08.4
strument  :     Instrument #1
mple Name: 12 male C.oculata abd.ster./5ul CH2Cl2
isc Info  : ca. 1-week-old; fed. ug/ul citral/water/6days
al Number: 1
```

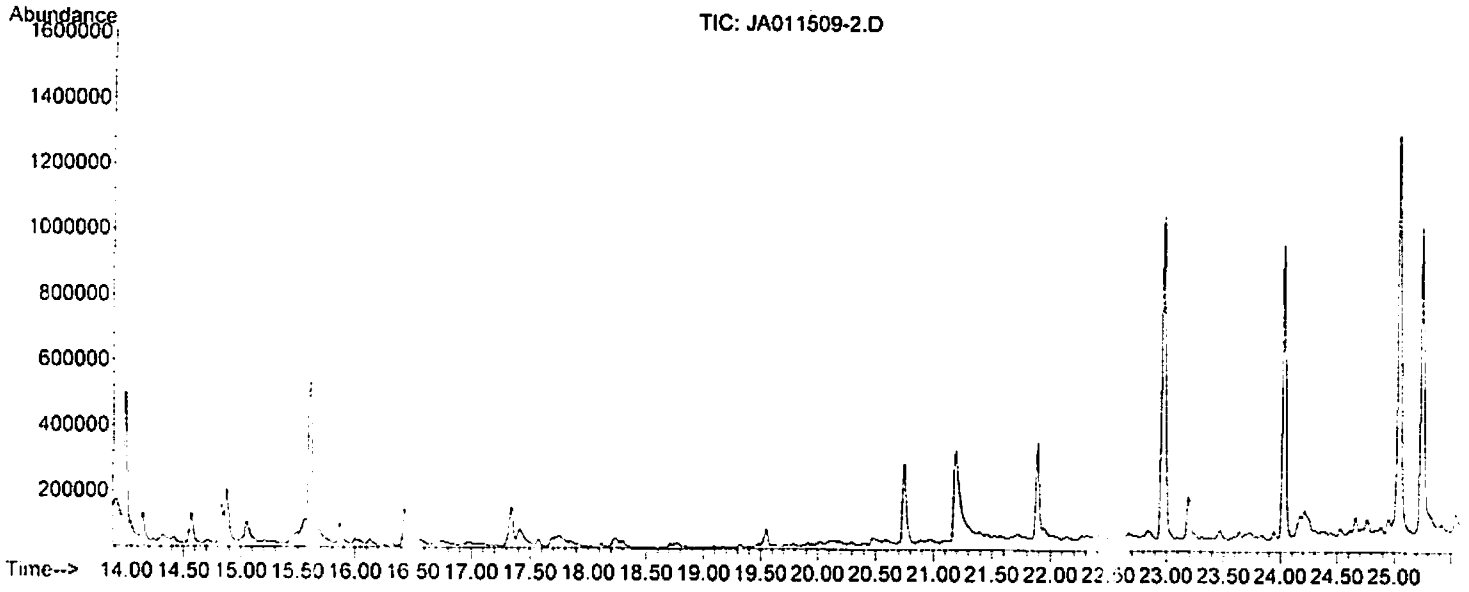


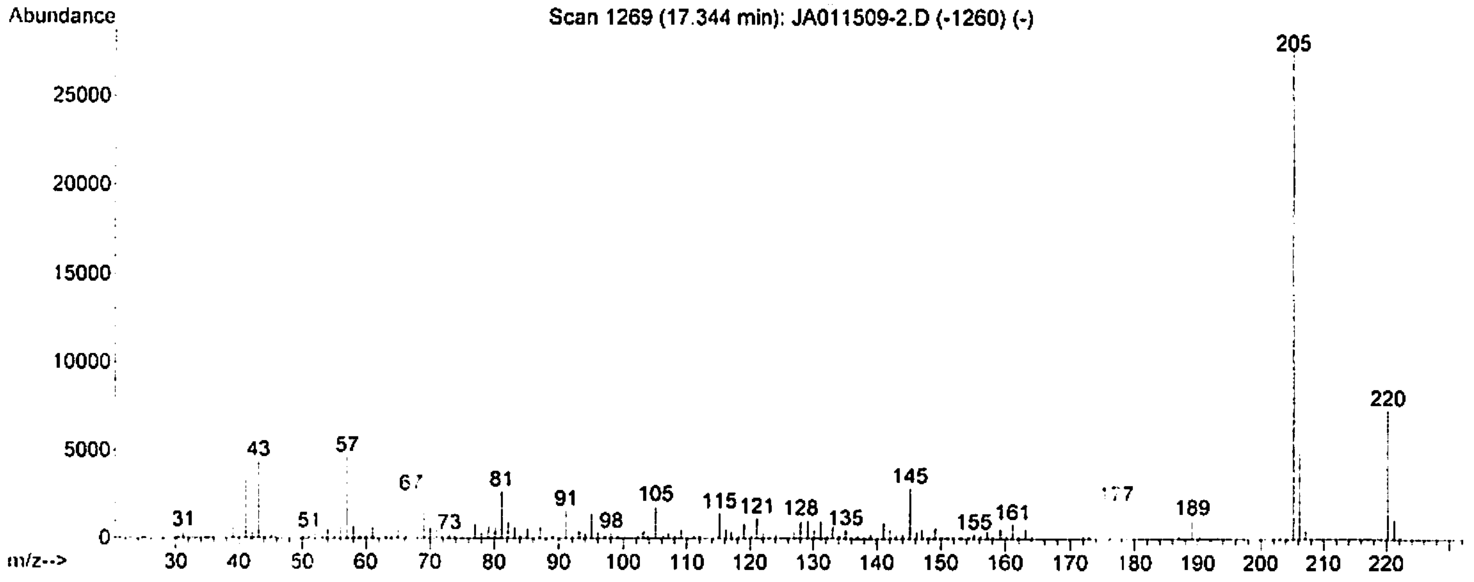

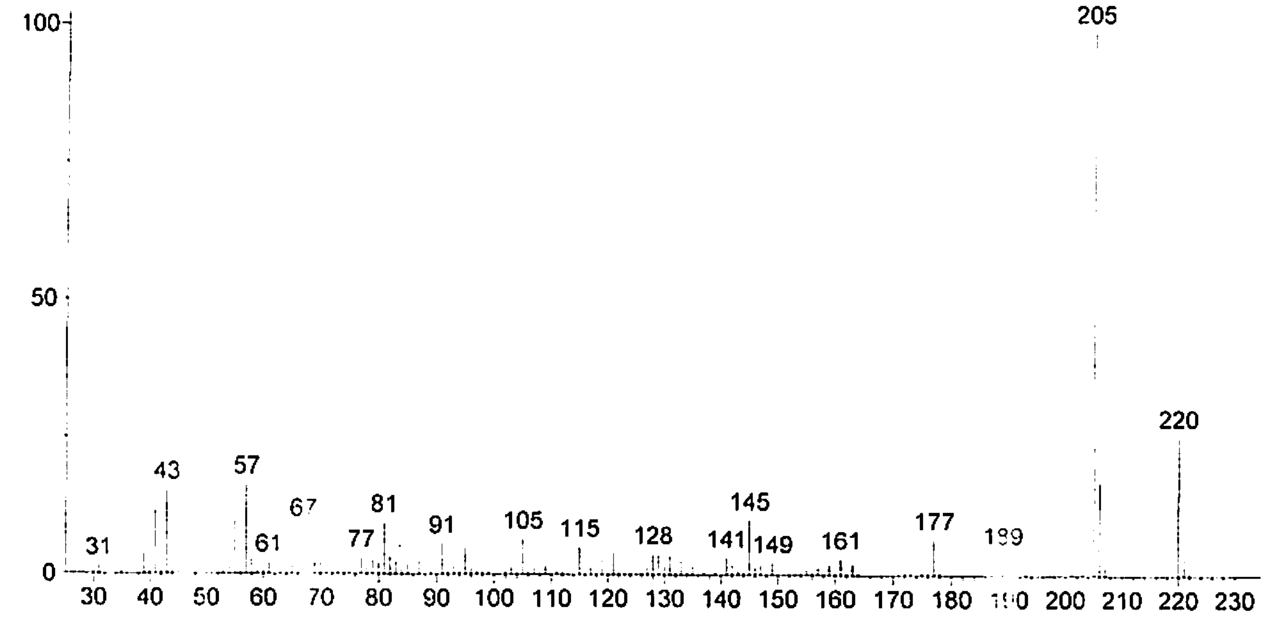


(Text File) Scan 1269 (17.344 min): JA011509-2.D (-1260)

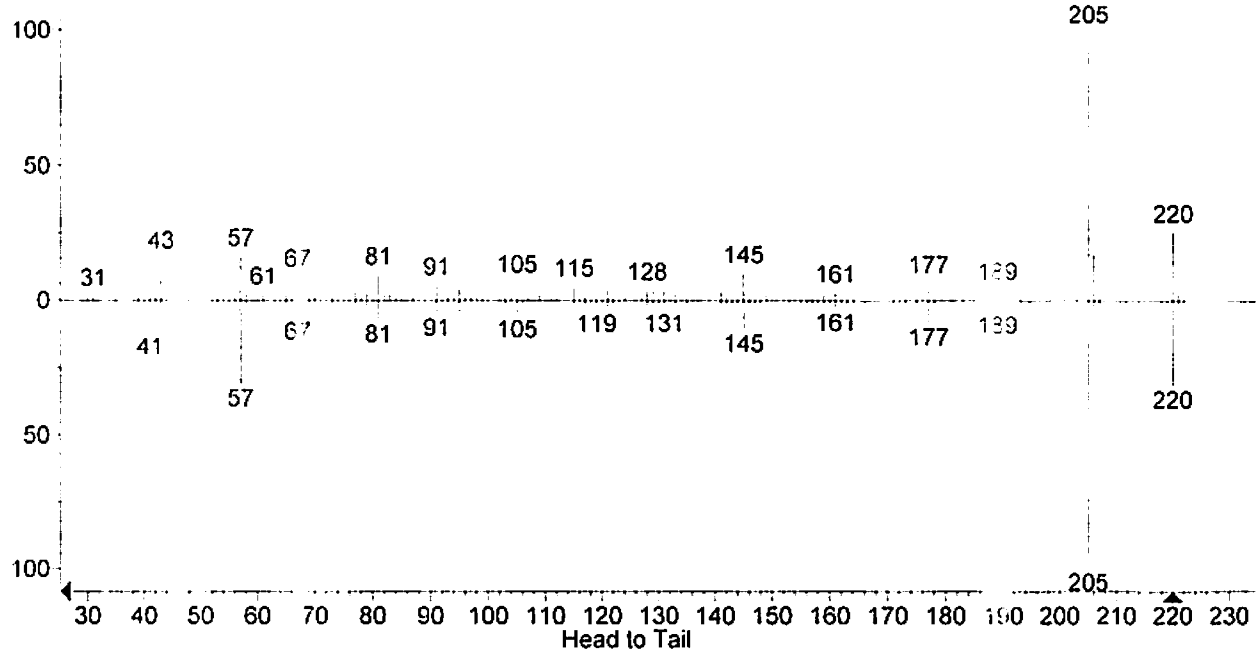


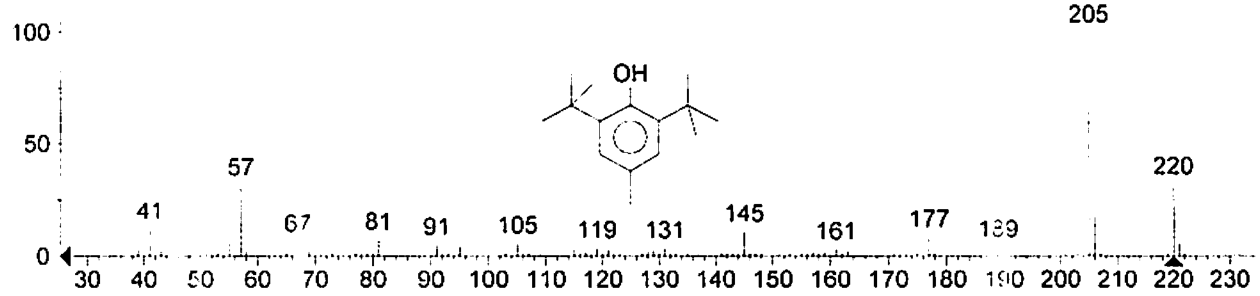


(replib) Butylated Hydroxytoluene

File :D:\DATA\Aldrich\JA-09\JA011509-2.D
Operator : Aldrich
Acquired : 15 Jan 2009 12:17 using AcqMethod JA-WAX08.M
Instrument : Instrument #1
Sample Name: 12 male C.oculata abd.ster./5ul CH2Cl2
Misc Info : ca. 1-week-old; fed. ug/ul citral/water/6days
Vial Number: 1

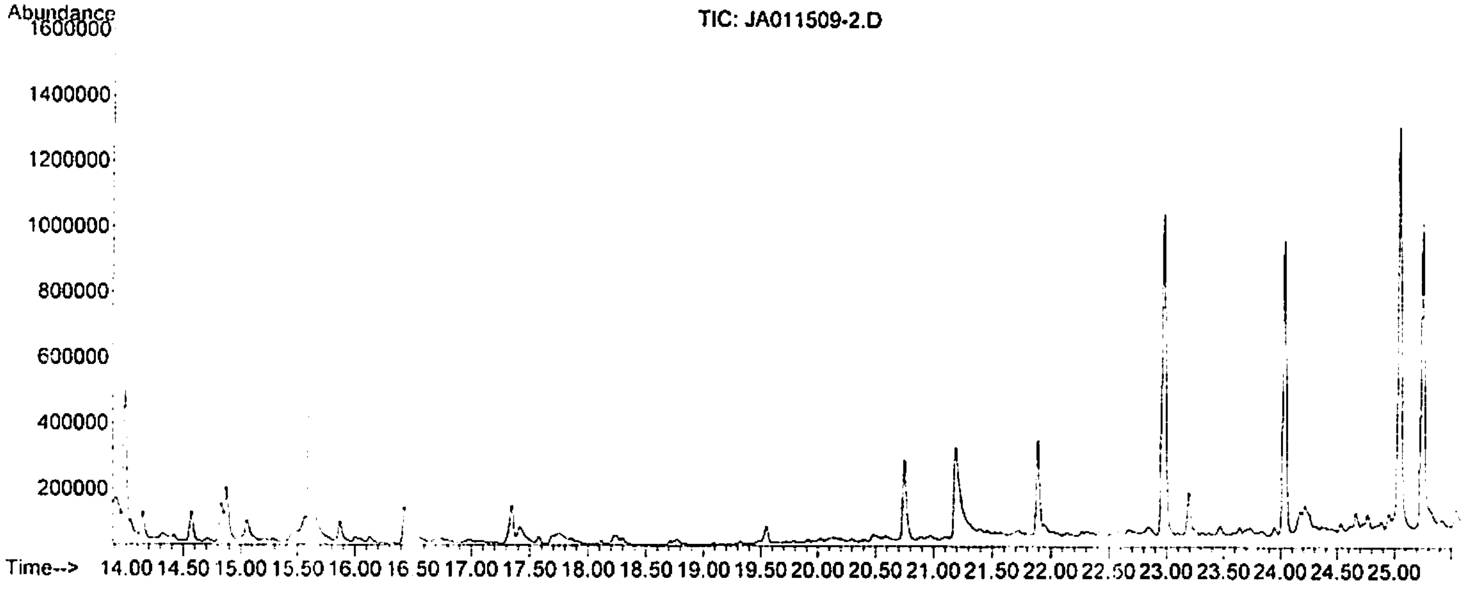


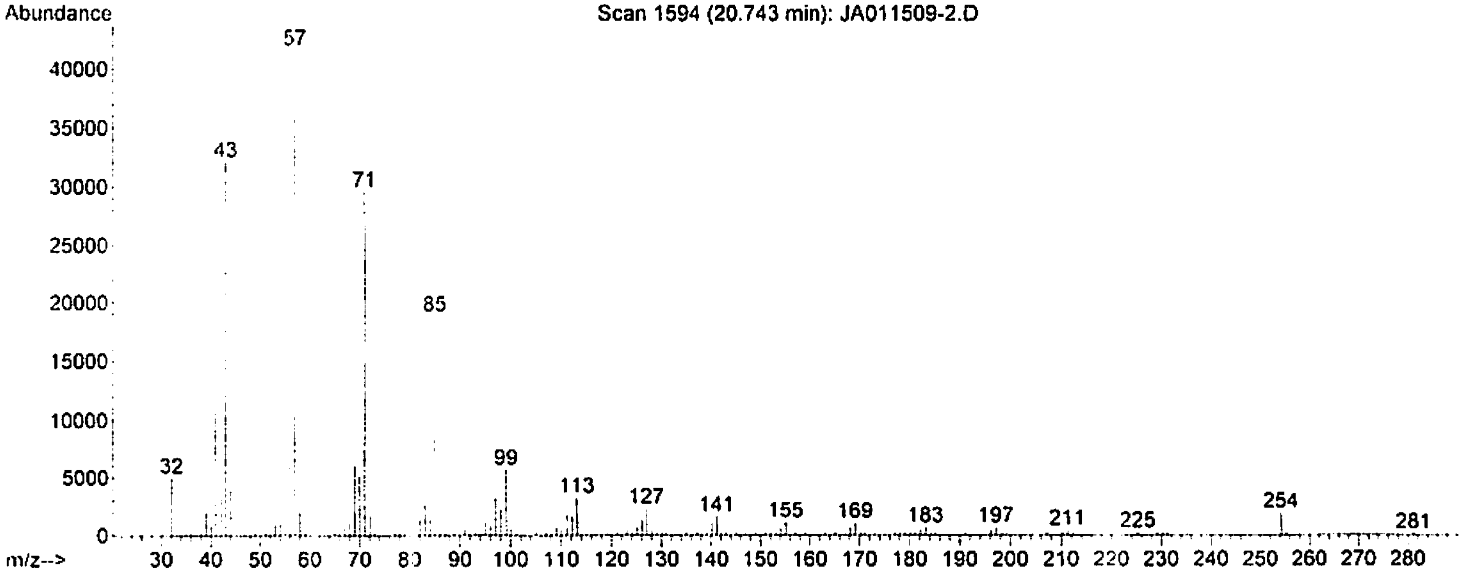

File : D:\DATA\Aldrich\JA-09\JA011509-2.D
Operator : Aldrich
Acquired : 15 Jan 2009 12:17 using AcqMethod JA-WAX08.M
Instrument : Instrument #1
Sample Name: 12 male C.oculata abd.ster./5ul CH2Cl2
Misc Info : ca. 1-week-old; fed. ug/ul citral/water/6days
Vial Number: 1

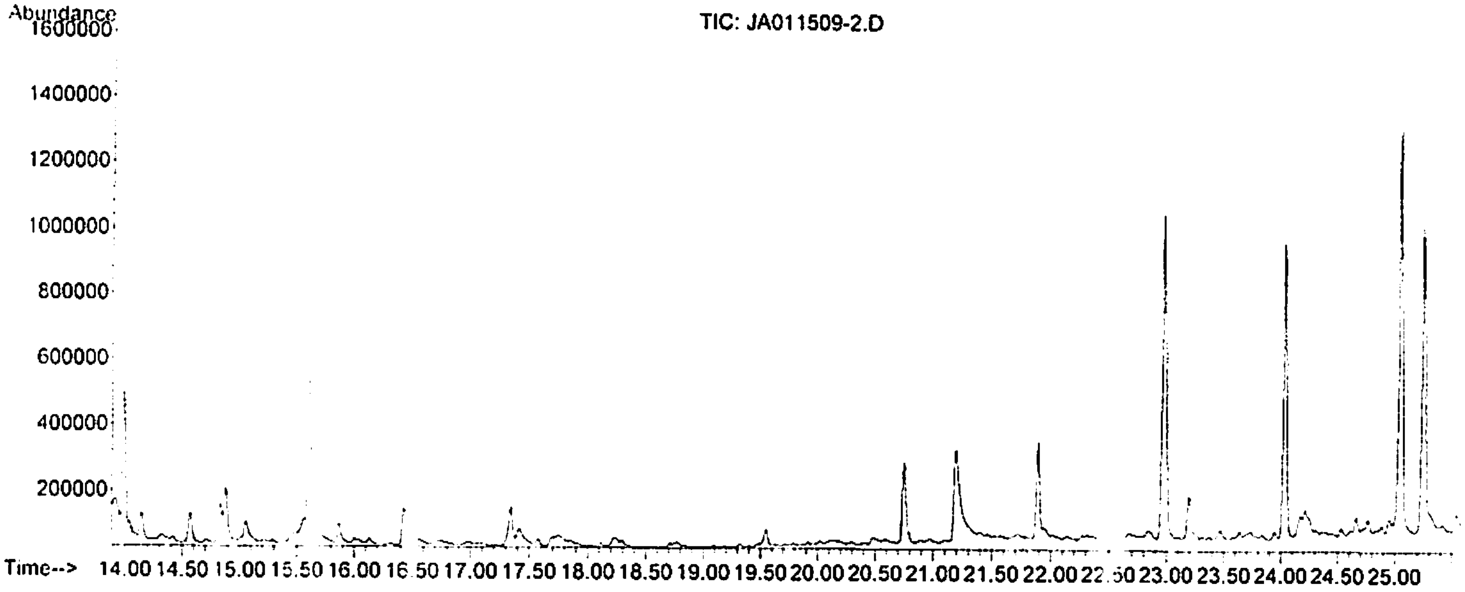


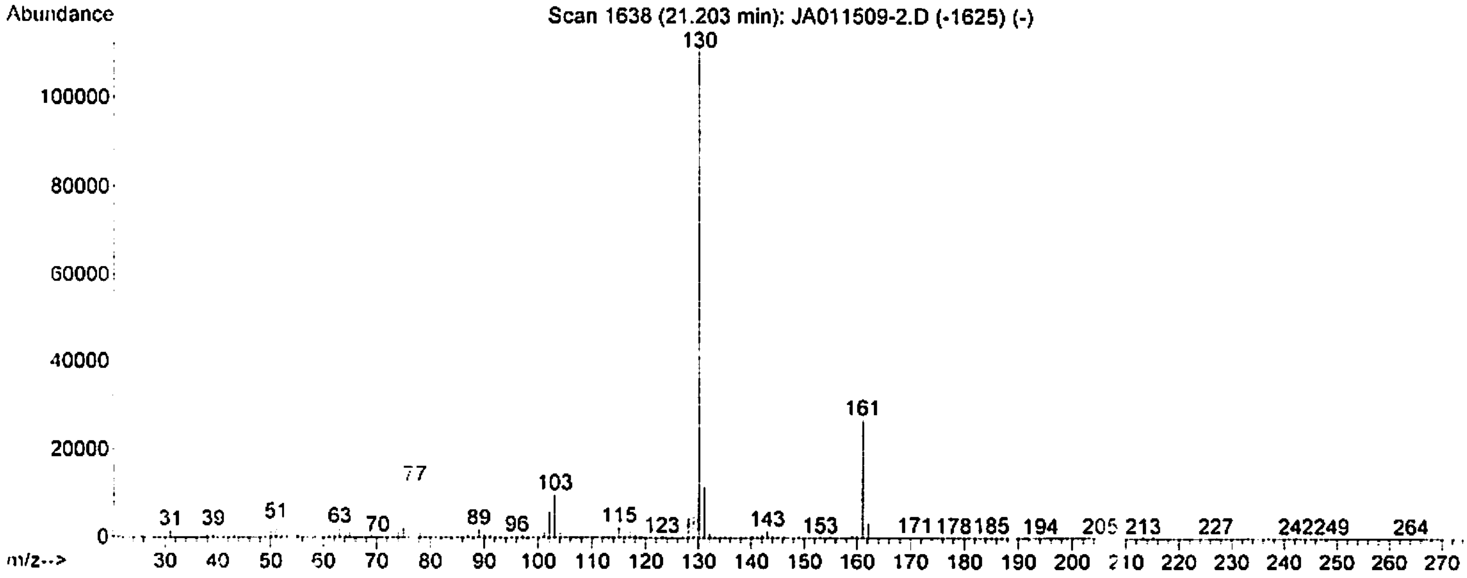

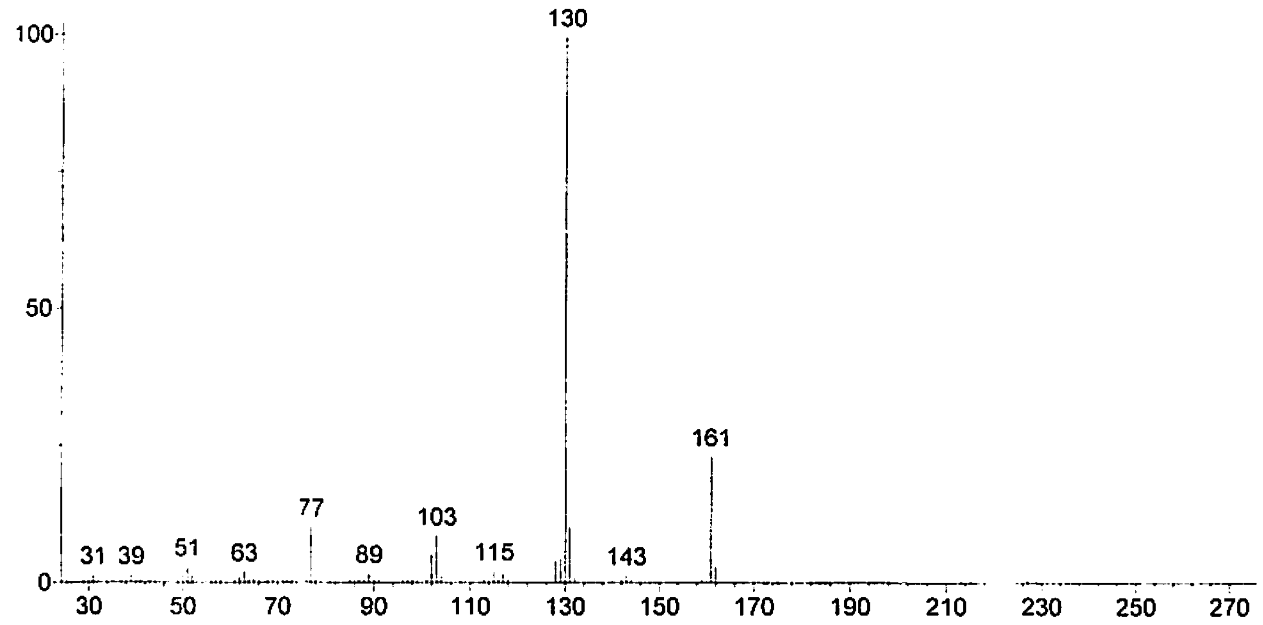


(Text File) Scan 1638 (21.203 min): JA011509-2.D (-1625)

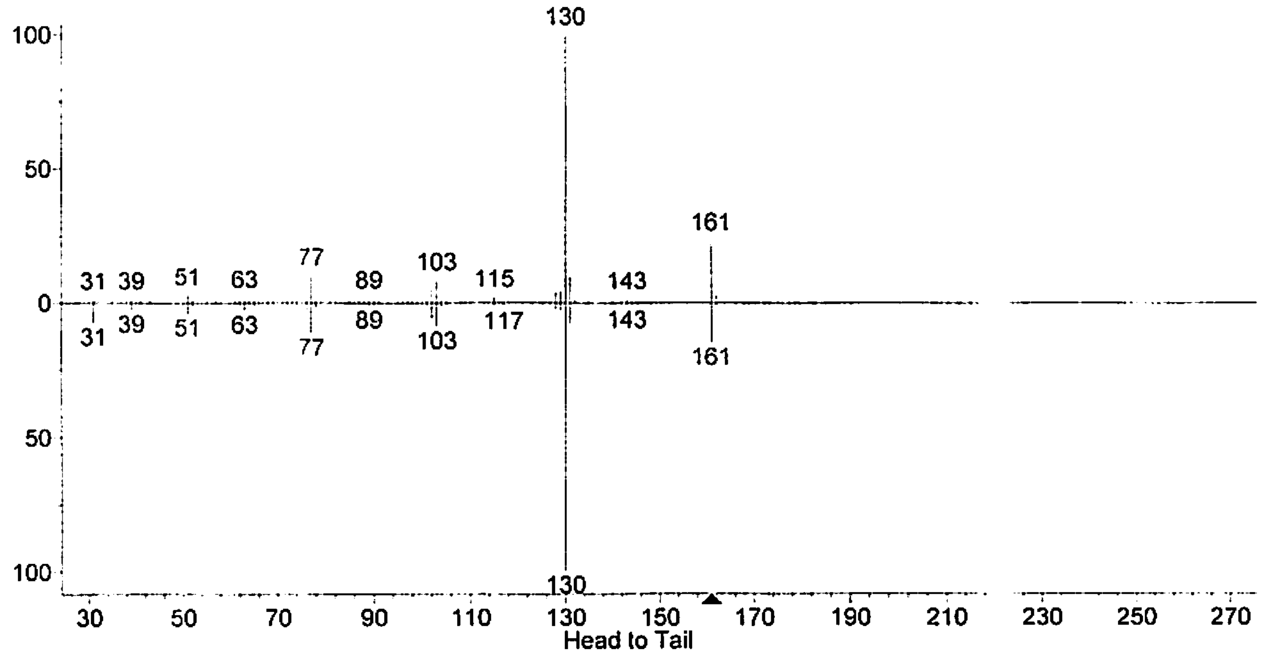


Head to Tail

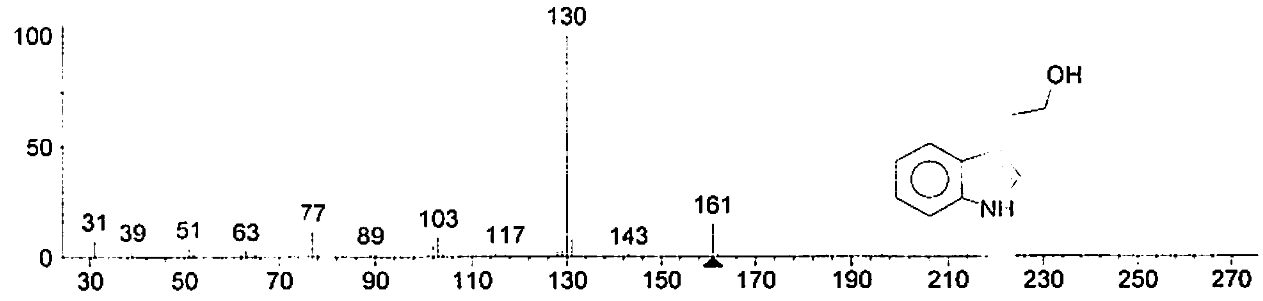


(replib) 1H-Indole-3-ethanol

File :D:\DATA\Aldrich\JA-09\JA011509-2.D
Operator : Aldrich
Acquired : 15 Jan 2009 12:17 using AcqMethod JA-WAX08.M
Instrument : Instrument #1
Sample Name: 12 male C.oculata abd.ster./5ul CH2C12
Misc Info : ca. 1-week-old; fed. ug/ul citral/water/6days
Vial Number: 1

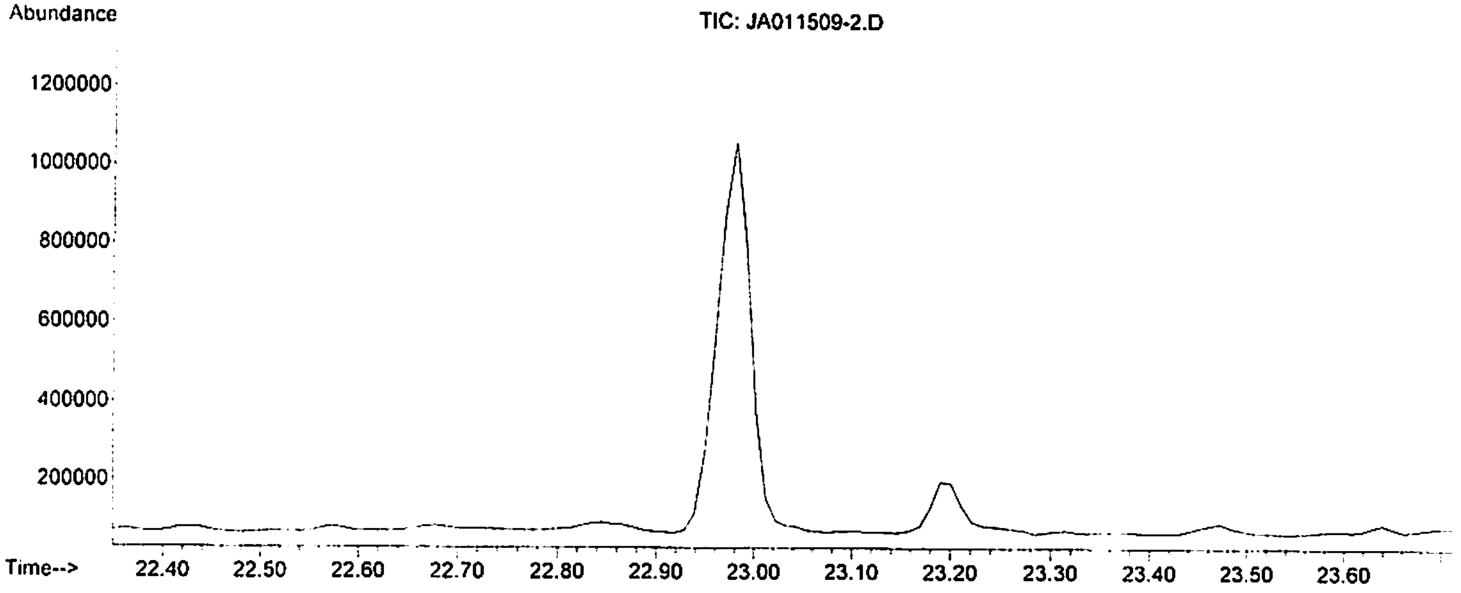


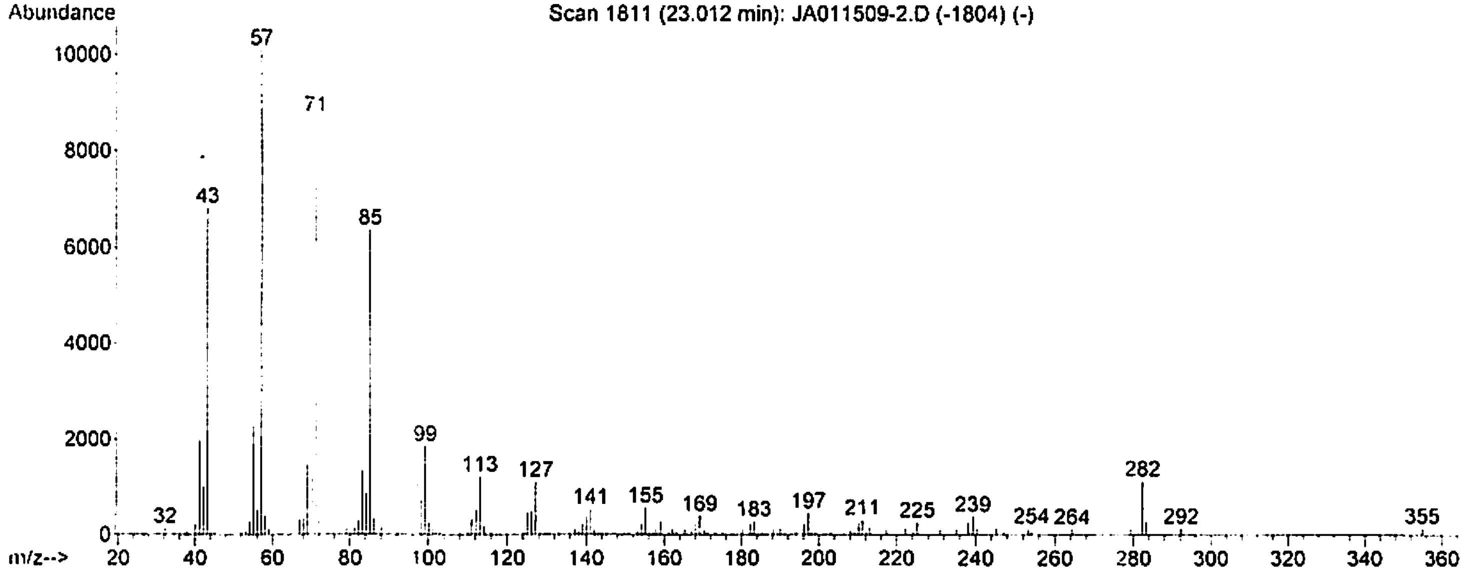

File : D:\DATA\Aldr ch\JA-09\JA011509-2.D
Operator : Aldrich
Acquired : 15 Jan 2009 12:17 using AcqMethod JA-WAX08.M
Instrument : Instrument #1
Sample Name: 12 male C.oculata abd.ster./5ul CH2Cl2
Misc Info : ca. 1-week-old; fed. ug/ul citral/water/6days
Vial Number: 1

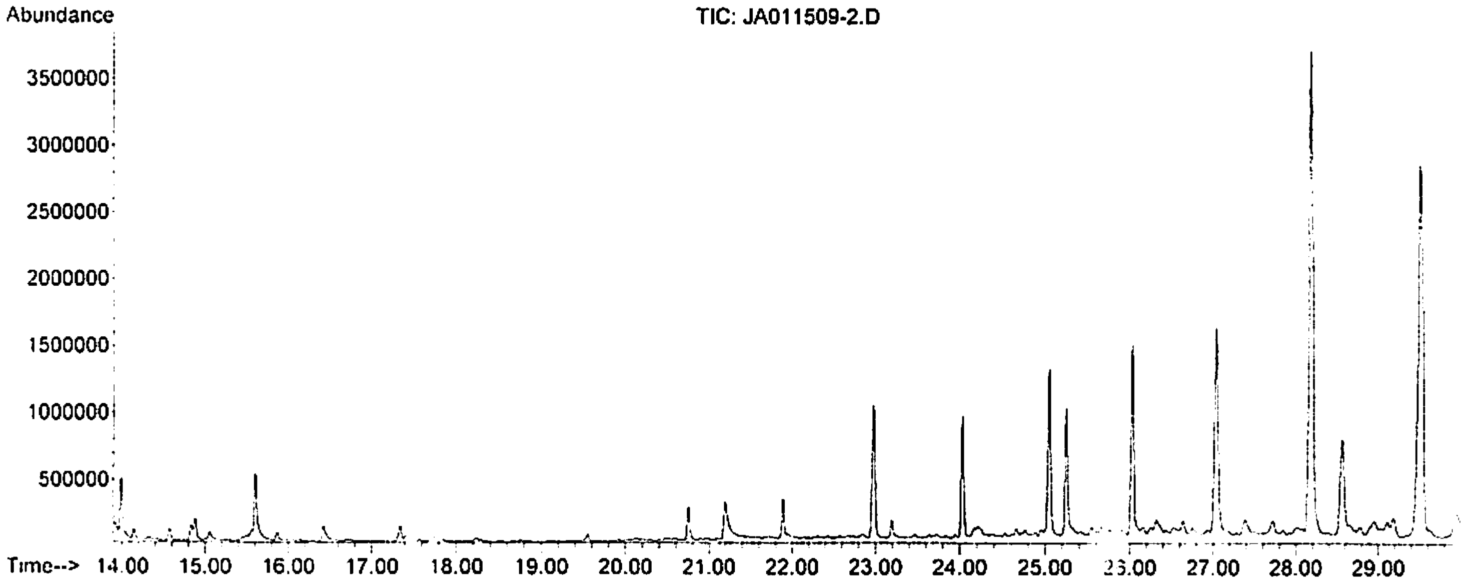


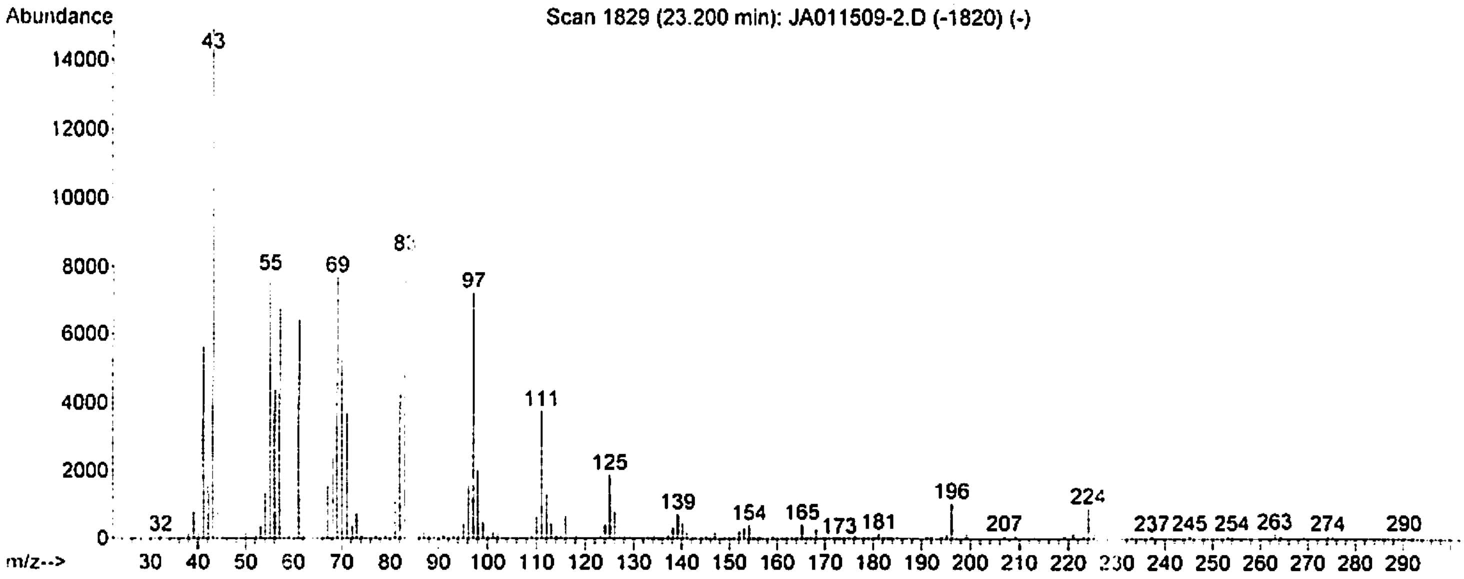

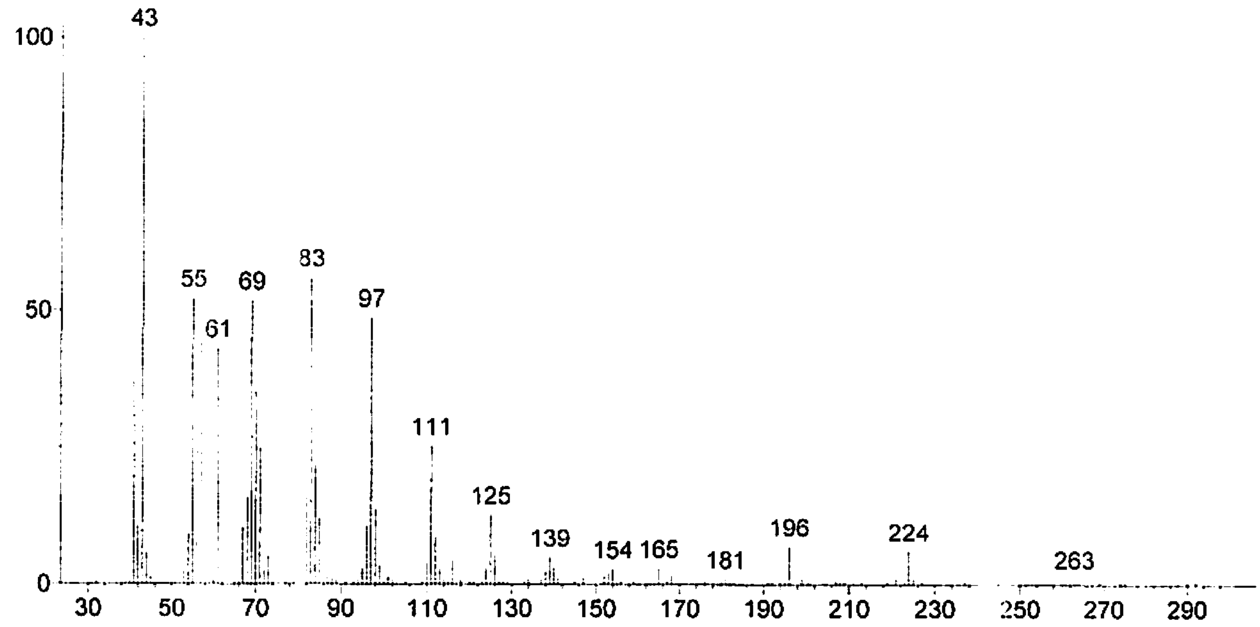

(Text File) Scan 1829 (23.2( 0 min): JA011509-2.D (-1820)

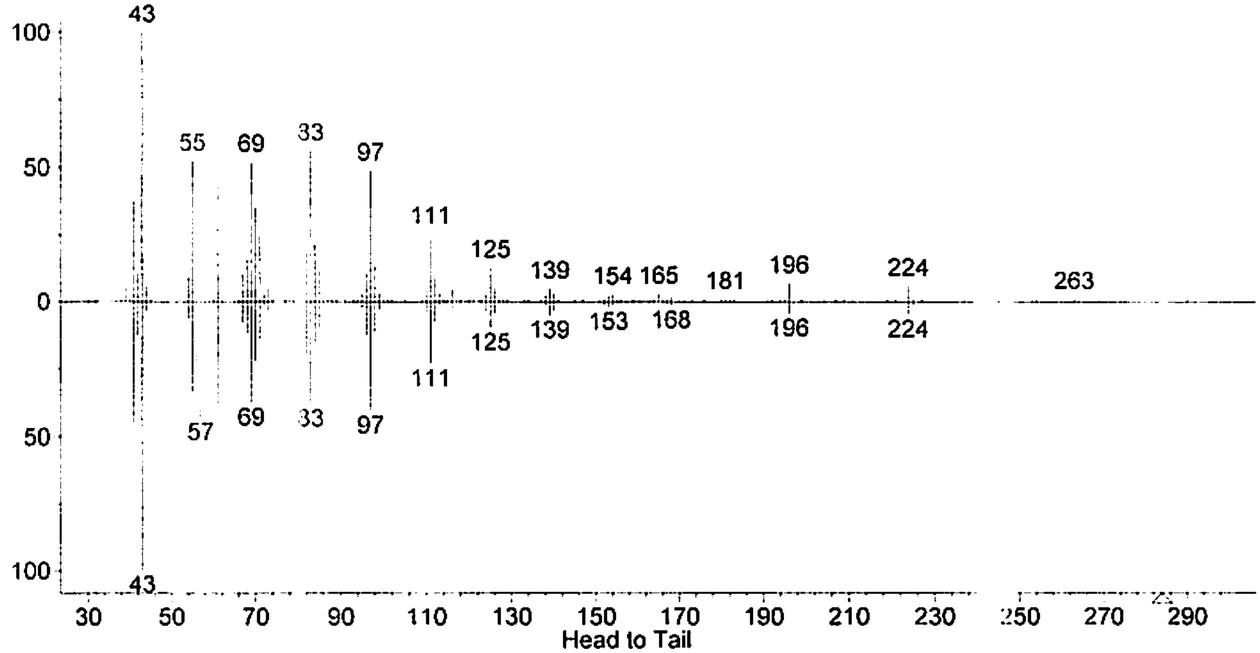

Head to Tail

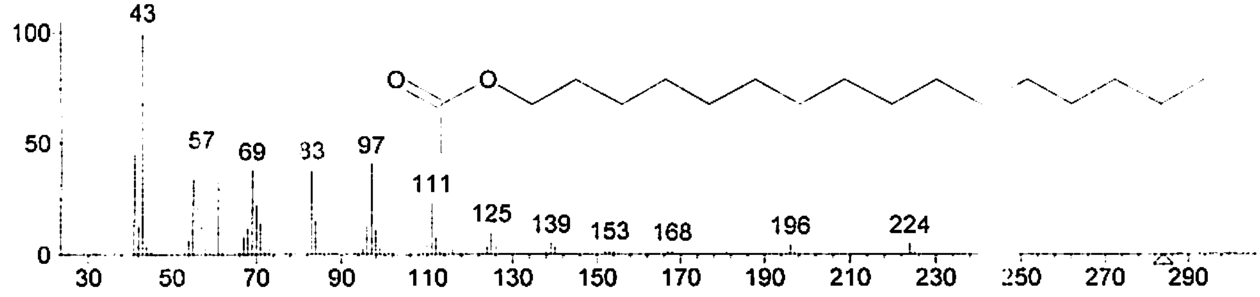

(mainlib) 1-Hexadecanol, acetate

File :D:\DATA\Aldrich\JA-09\JA011509-2.D
Operator : Aldrich
Acquired : 15 Jan 2009 12:17 using AcqMethod JA-WAX08.4
Instrument : Instrument #1
Sample Name: 12 male C.oculata abd.ster./5ul CH2Cl2
Misc Info : ca. 1-week-old; fed. ug/ul citral/water/6days
Vial Number: 1

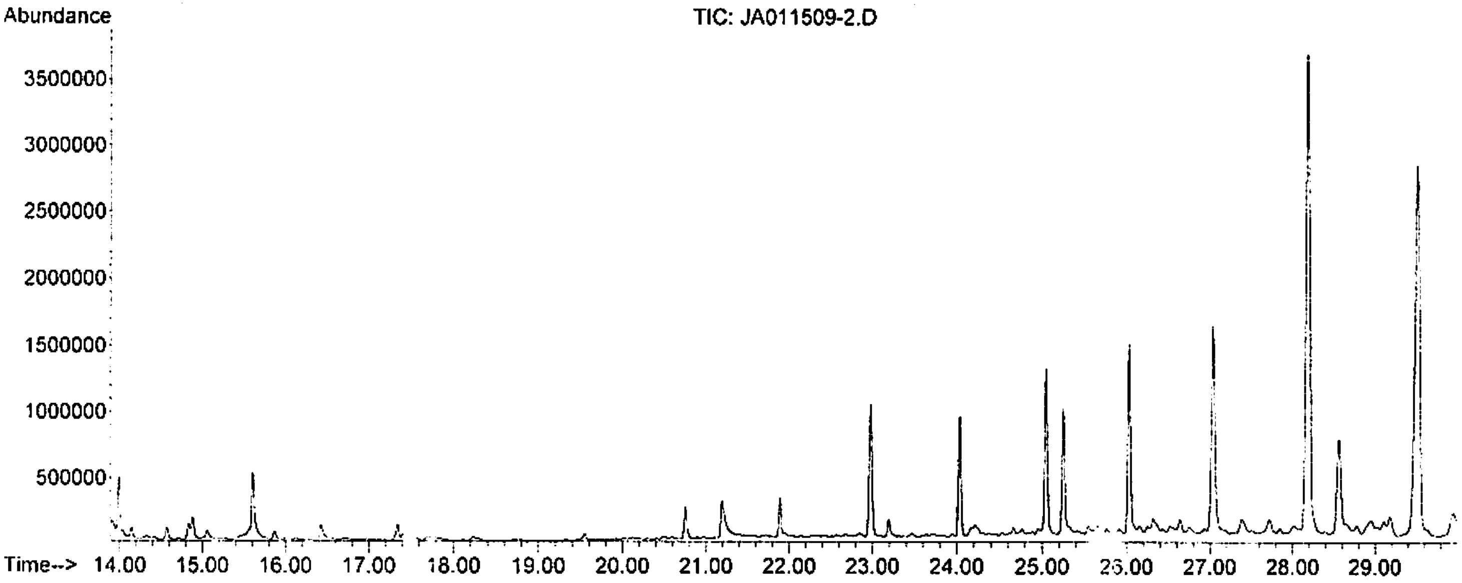


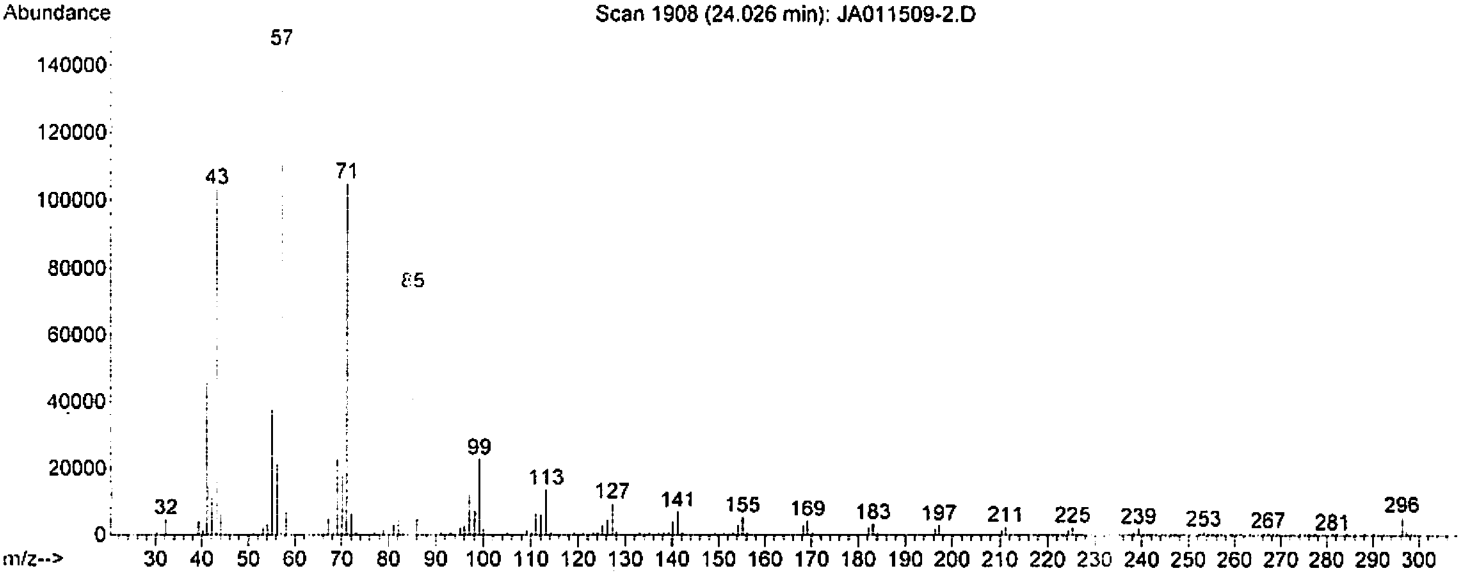

File :D:\DATA\Aldrich\JA-09\JA011509-2.D
Operator : Aldrich
Acquired : 15 Jan 2009 12:17 using AcqMethod JA-WAX08.M
Instrument : Instrument #1
Sample Name: 12 male C.oculata abd.ster./5ul CH2Cl2
Misc Info : ca. 1-week-old; fed. ug/ul citral/water/6days
Vial Number: 1

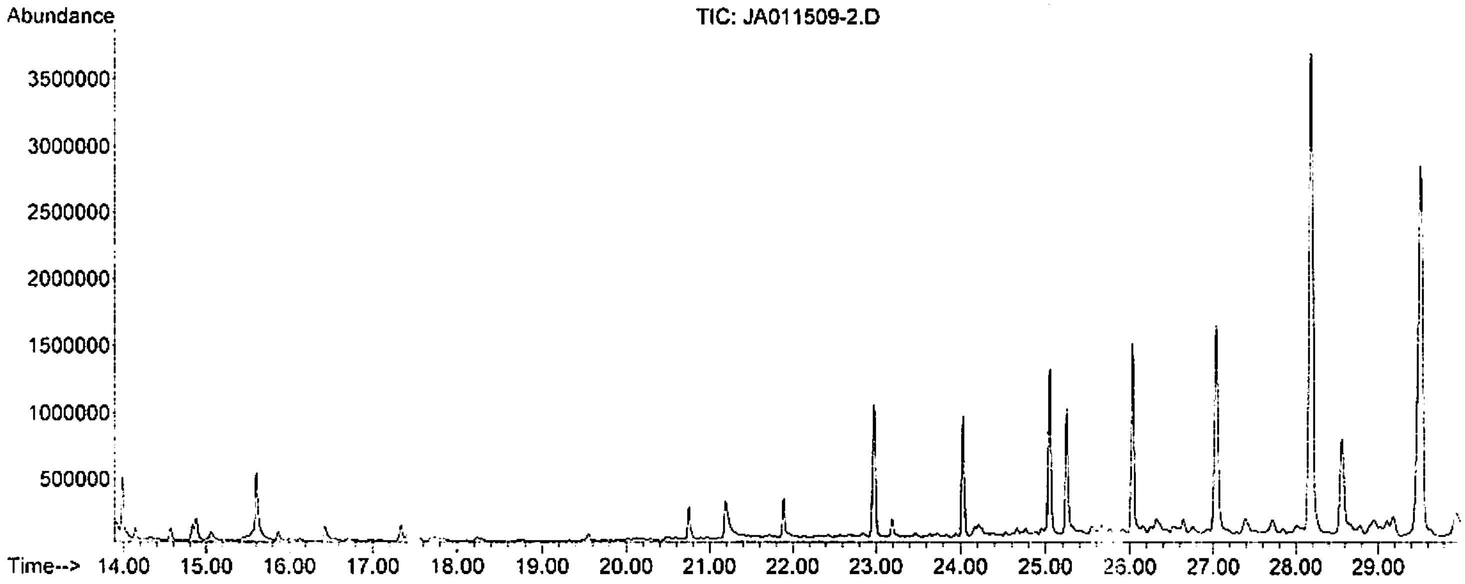


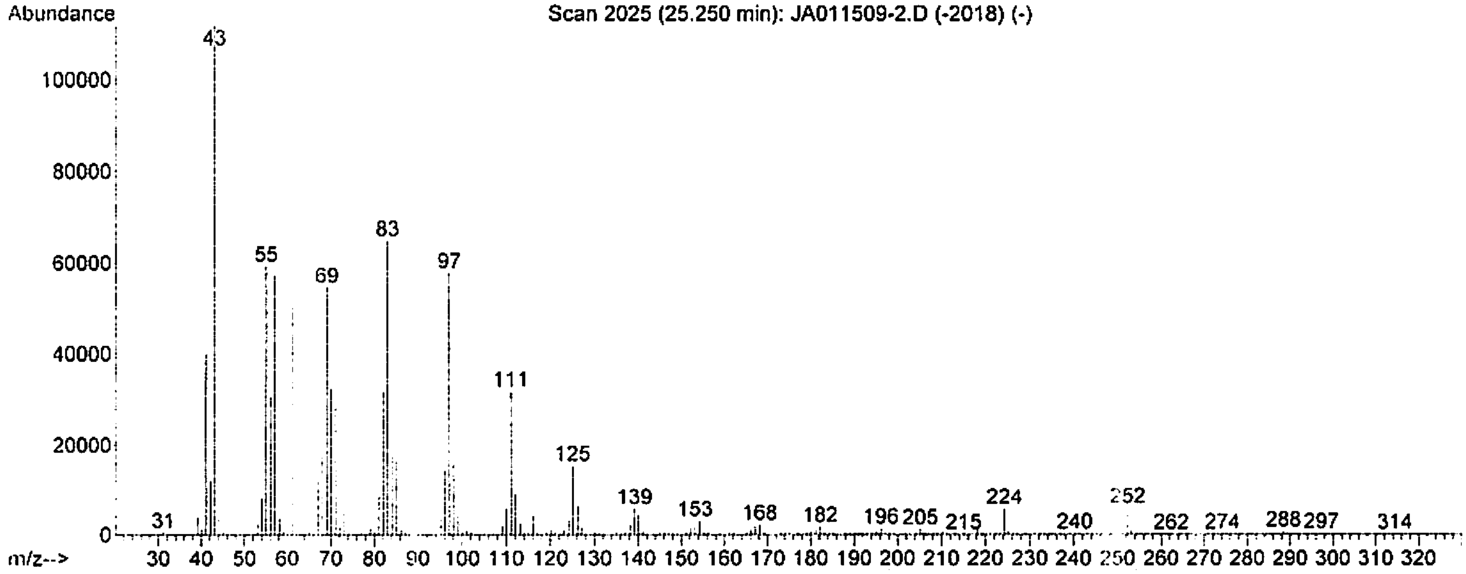

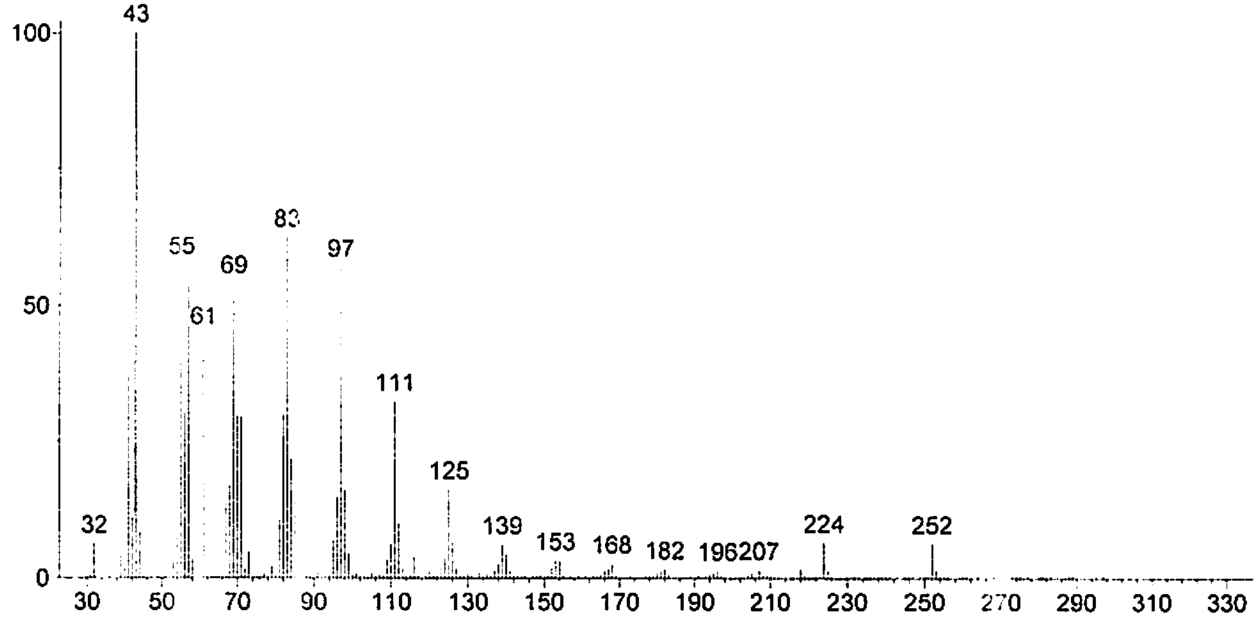


(Text File) Scan 2026 (25.200 min): JA011509-2.D

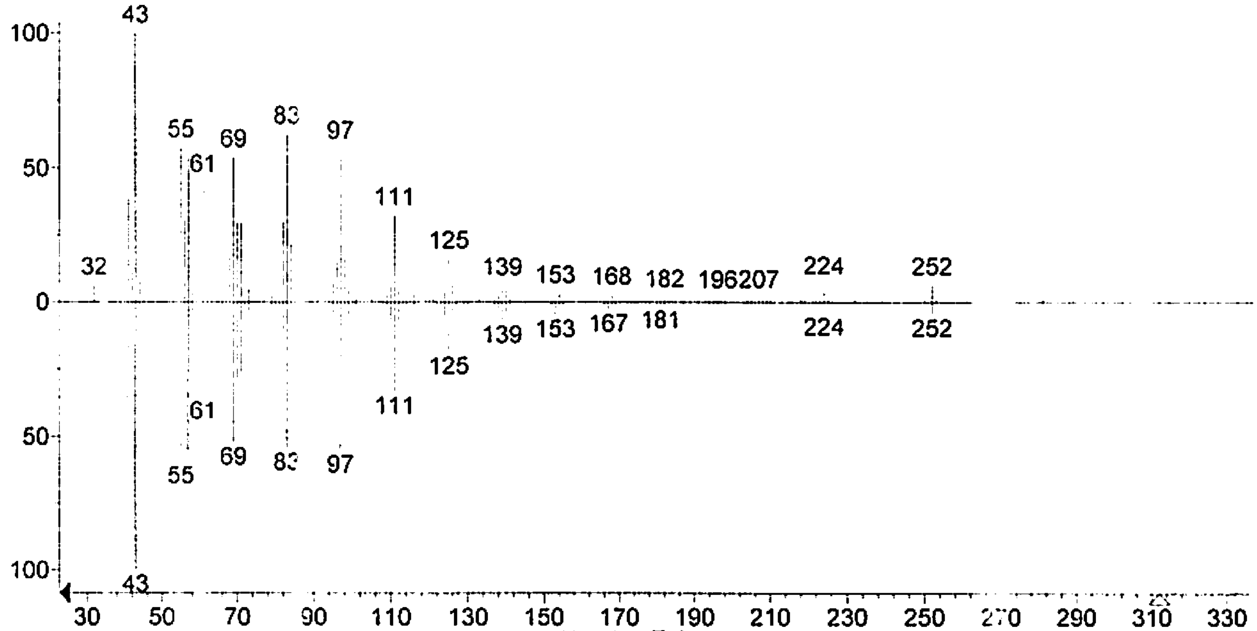


Head to Tail

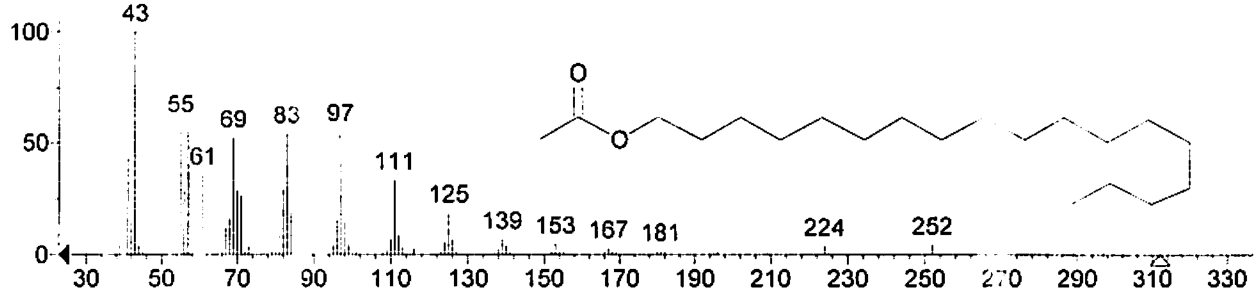


(replib) Acetic acid, octadecyl ester

[illegible]e :D:\DATA\Aldr.ch\JA-09\JA011509-2.D
[illegible]erator : Aldrich
A[illegible]quired : 15 Jan 2009 12:17 using AcqMethod JA-WAX08.M
[illegible]strument : Instrument #1
[illegible]mple Name: 12 male C.oculata abd.ster./5ul CH2Cl2
[illegible]isc Info : ca. 1-week-old; fed. ug/ul citral/water/6days
[illegible]al Number: 1

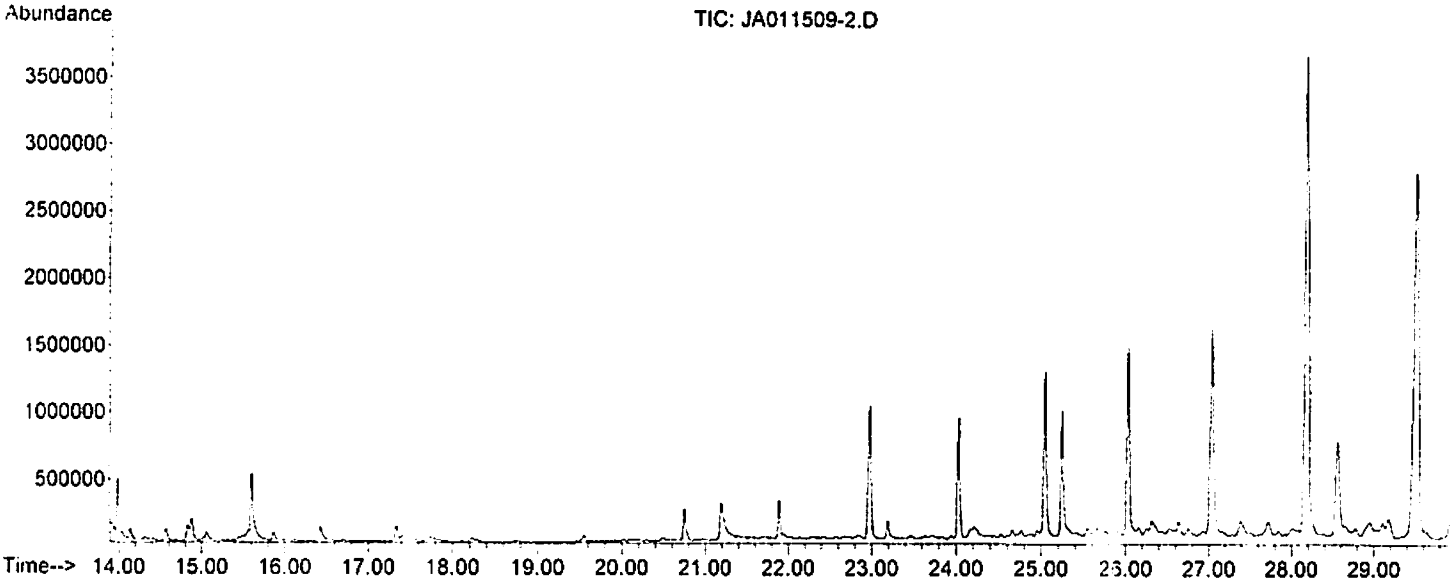


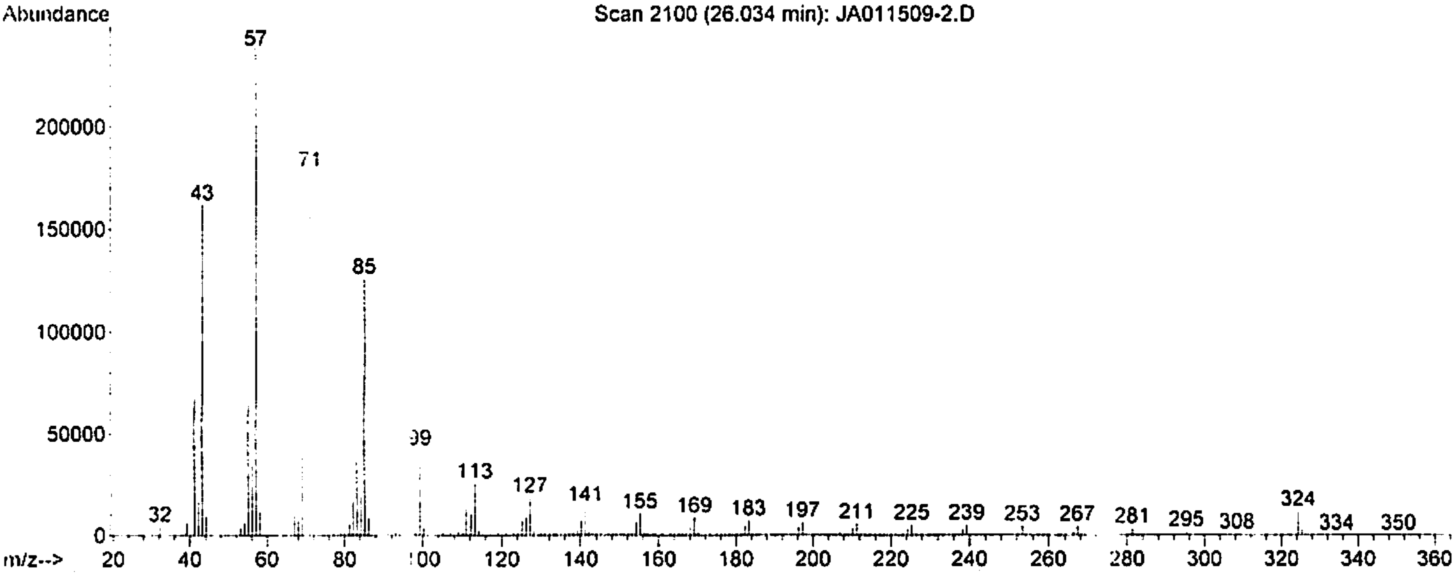

[illegible]le :D:\DATA\Aldr[illegible]ch\JA-09\JA011509-2.D
[illegible]erator : Aldrich
[illegible]quired : 15 Jan 2009 12:17 using AcqMethod JA-WAX08.[illegible]
[illegible]strument : Instrument #1
[illegible]mple Name: 12 male C.oculata abd.ster./5ul CH2Cl2
[illegible]sc Info : ca. 1-week-old; fed. ug/ul citral/water/6days
[illegible]al Number: 1

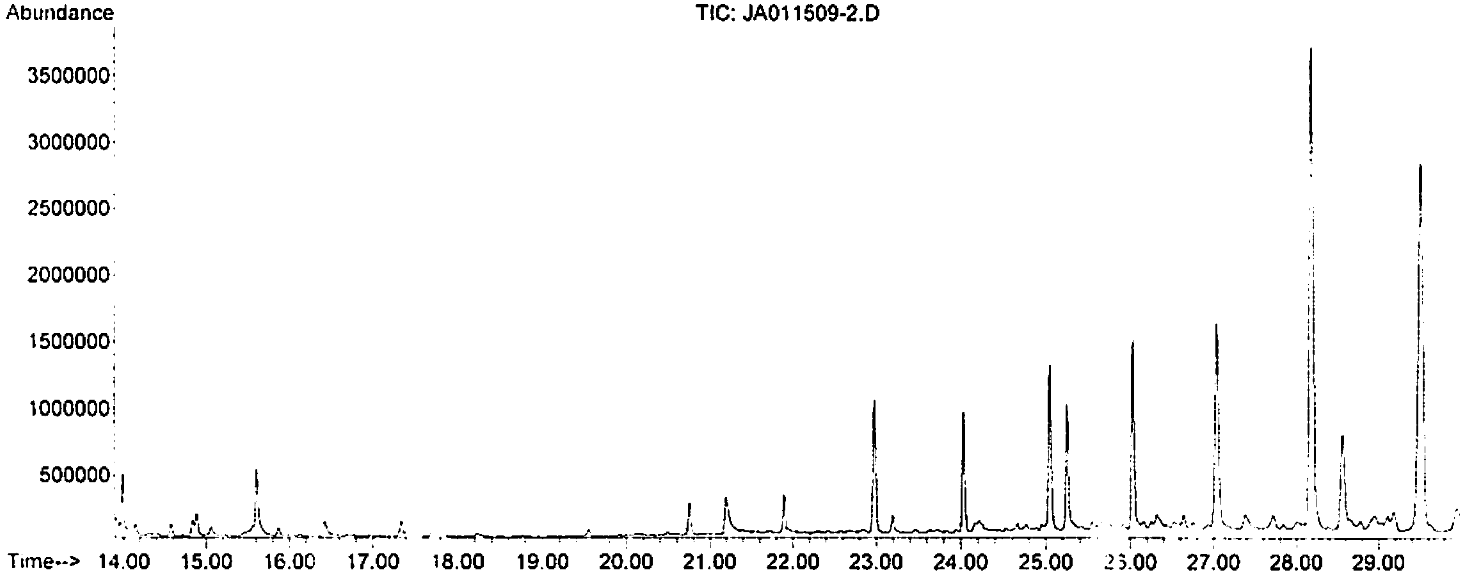


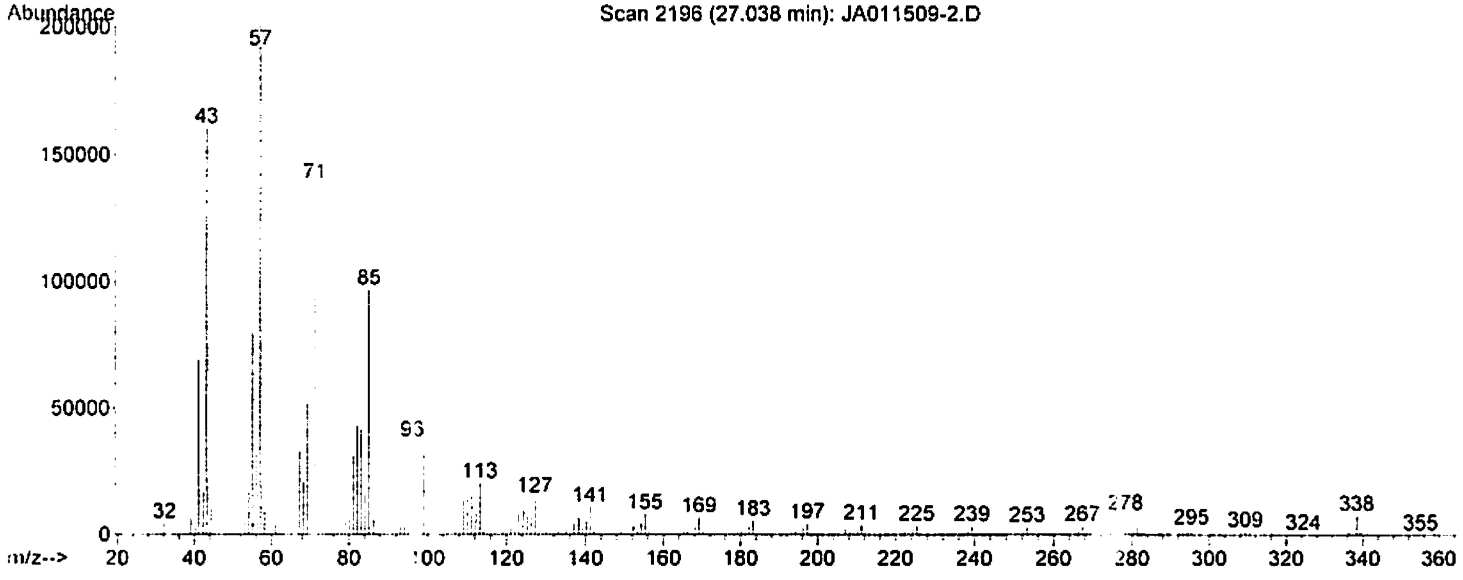

[illegible] :D:\DATA\Aldr[illegible]ch\JA-09\JA011509-2.D
[illegible]rator : Aldri[illegible]
[illegible]uired : 15 Jan 2009 12:17 using AcqMethod JA-WAX08.[illegible]
[illegible]trument : Instrument #1
[illegible]mple Name: 12 male C.oculata abd.ster./5ul CH2Cl2
[illegible]sc Info : ca. 1-week-old; fed. ug/ul citral/water/6days
[illegible]al Number: 1

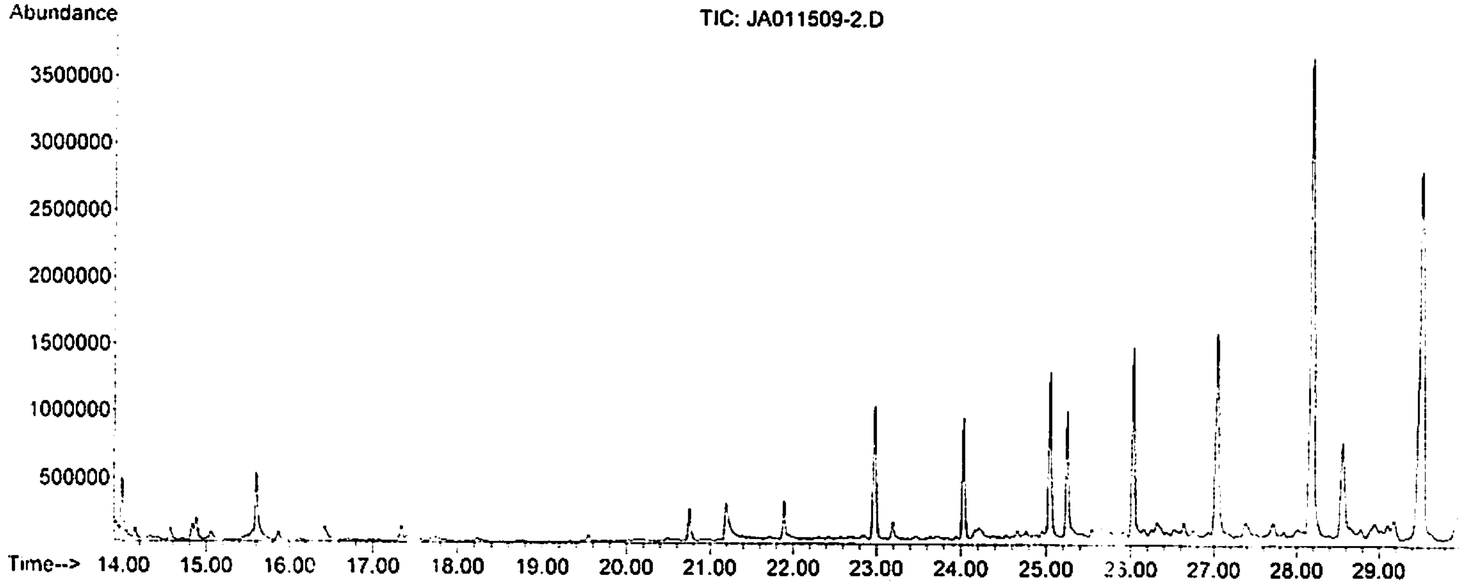


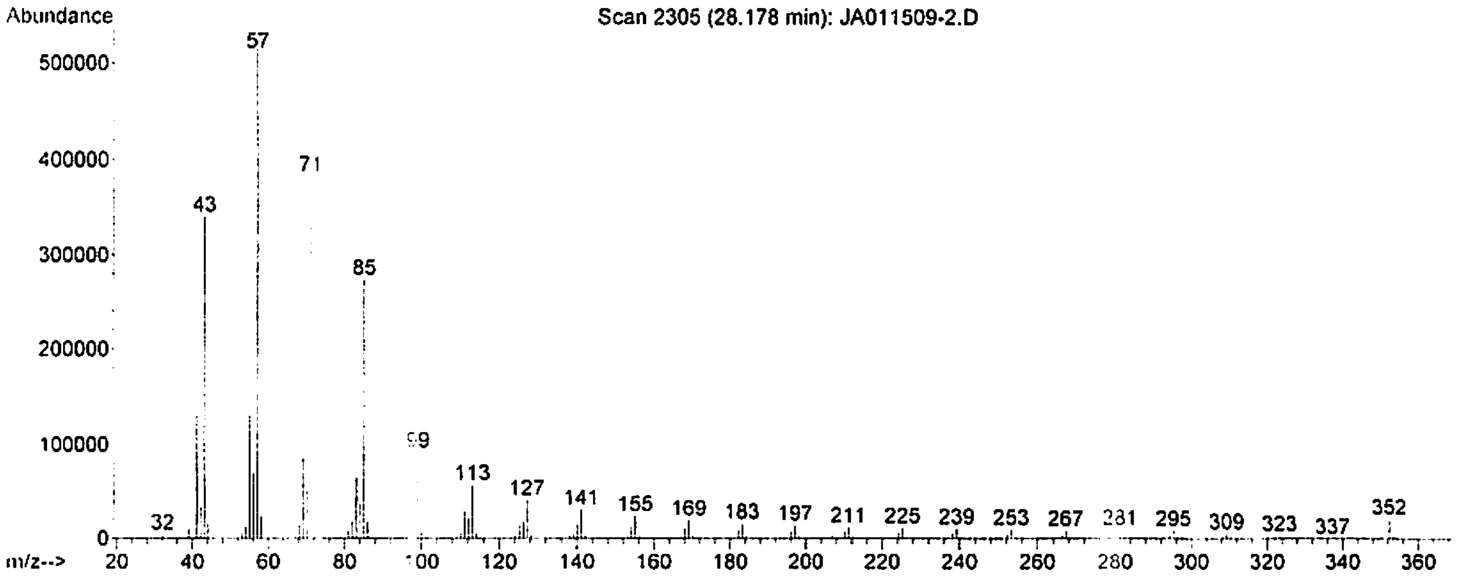

```
File        :D:\DATA\Aldr ch\JA-09\JA011509-2.D
Operator    : Aldrich
Acquired    : 15 Jan 2009  12:17      using AcqMethod JA-WAX08.4
Instrument  :    Instrument #1
Sample Name: 12 male C.oculata abd.ster./5ul CH2C12
Misc Info   : ca. 1-week-old; fed. ug/ul citral/water/6days
Vial Number: 1
```

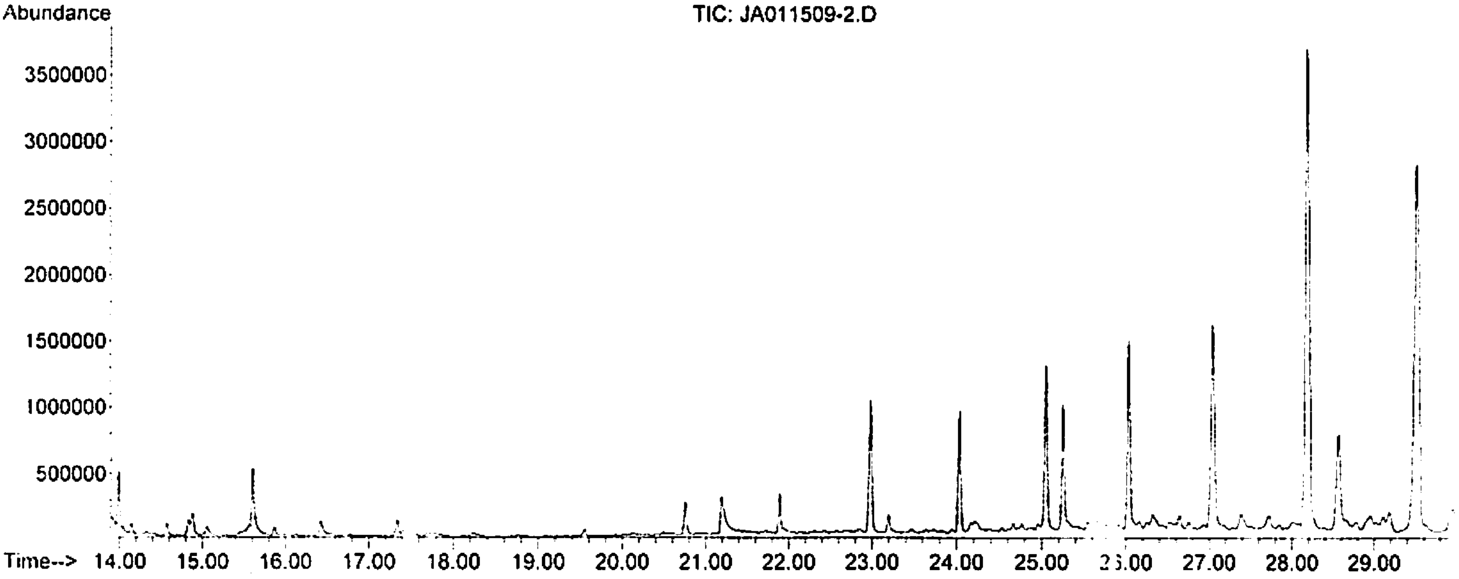


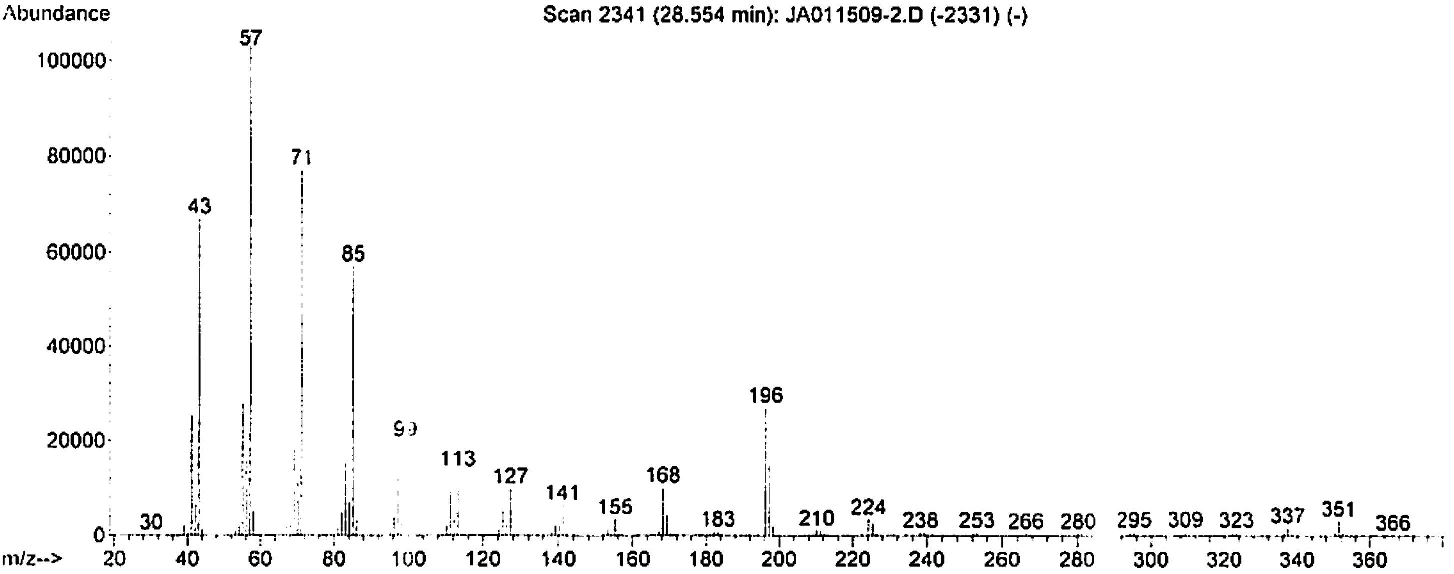

[illegible] :D:\DATA\Aldr ch\JA-09\JA011509-2.D
[illegible]rator : Aldrich
[illegible]quired : 15 Jan 2009 12:17 using AcqMethod JA-WAX08.M
[illegible]strument : Instrument #1
[illegible]mple Name: 12 male C.oculata abd.ster./5ul CH2Cl2
[illegible]sc Info : ca. 1-week-old; fed. ug/ul citral/water/6days
[illegible]al Number: 1

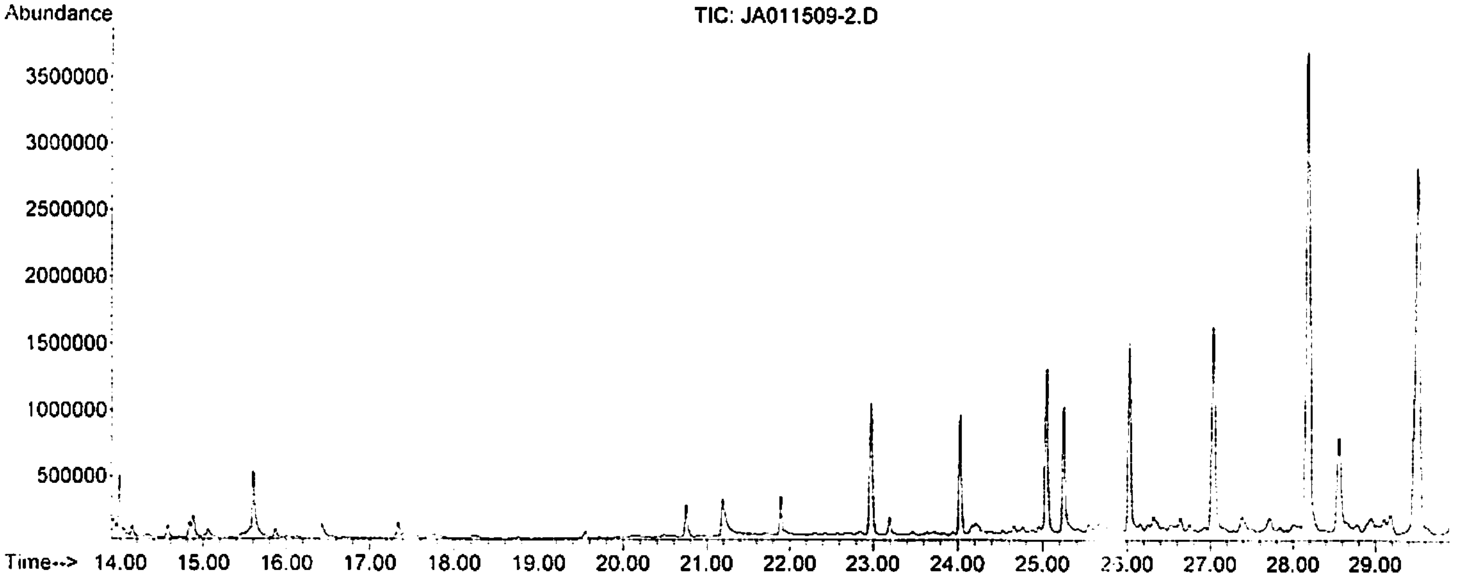


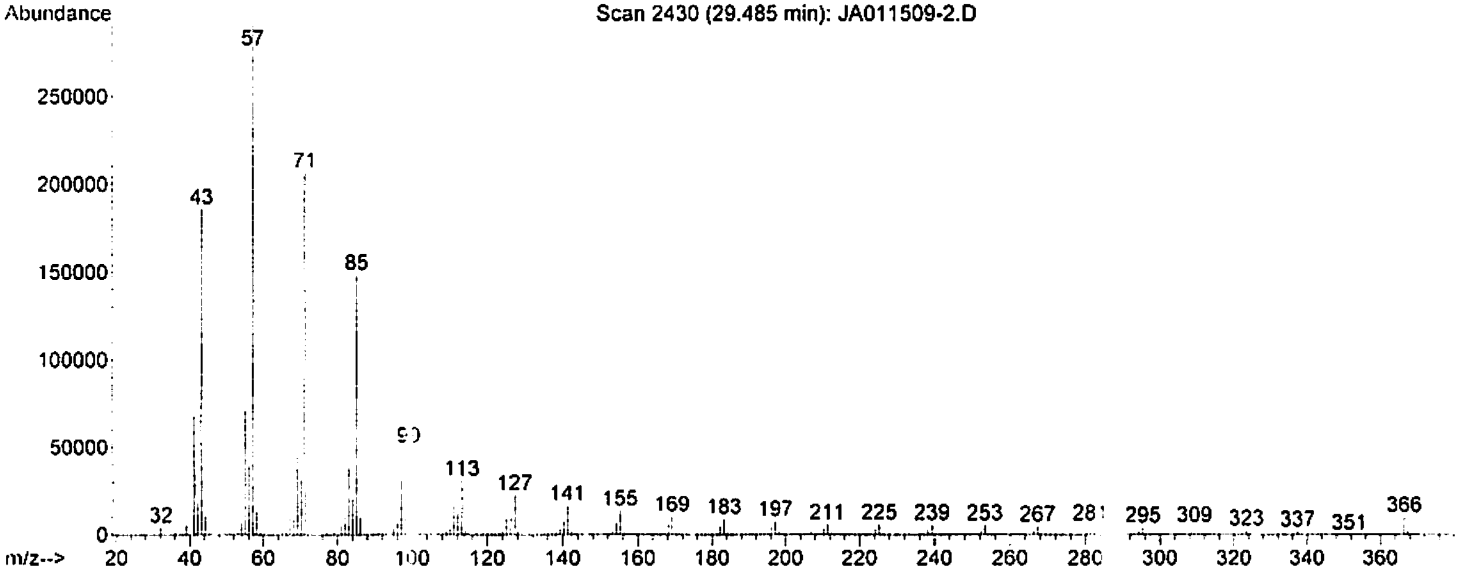

```
File          :D:\DATA\Aldrich\JA-09\JA012209-2.D
Operator      : Aldrich
Acquired      : 22 Jan 2009   11:14     using AcqMethod JA-WAX08.M
Instrument :      Instrument #1
Sample Name: ♂ male C.oculata abd.sternites/5ul CH2Cl2
Misc Info     : 3-8-day-old; fed 1 wk 6-CH3-5-hepten-2-one
Vial Number: 1
```

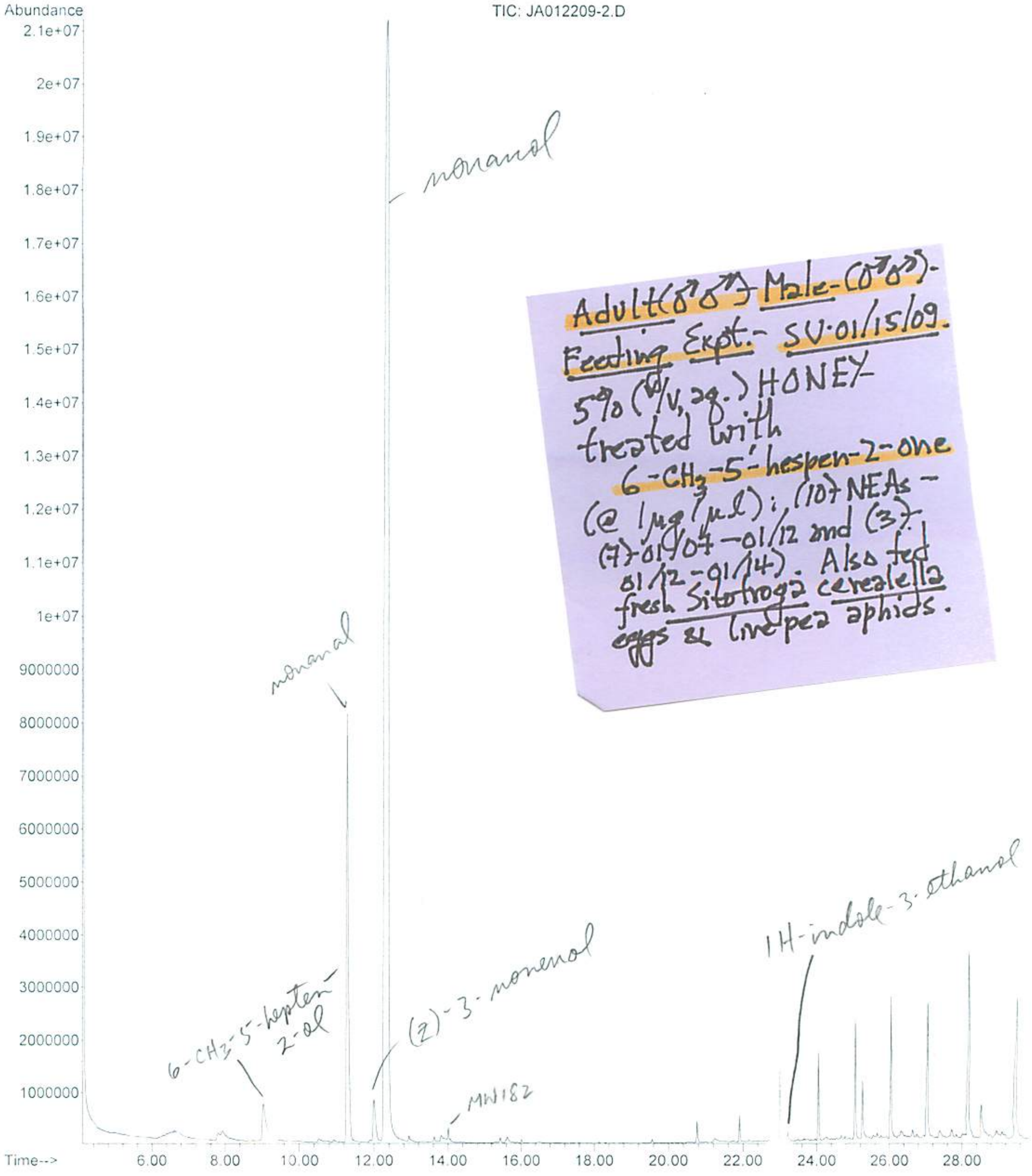

File :D:\DATA\ALDRICH\JA-09\Snapshot\JA012209-2.D
Operator : Aldrich
Acquired : 22 Jan 2009 11:14 using AcqMethod JA-WAX08.M
Instrument : Instrument #1
Sample Name: 9 male C.oculata abd.sternites/5ul CH2Cl2
Misc Info : 3-8-day-old; fed 1 wk 6-CH3-5-hepten-2-one
Vial Number: 1

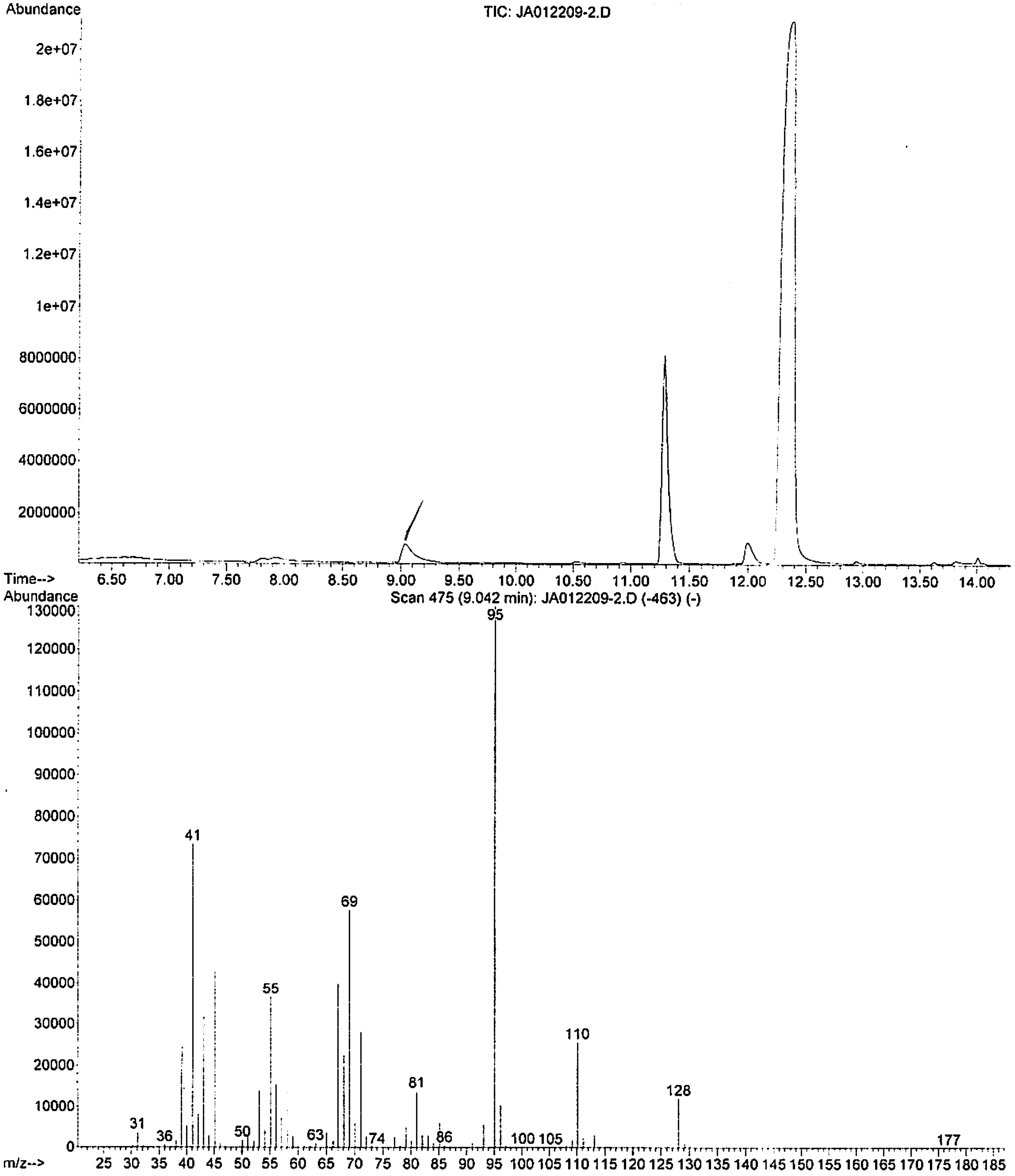

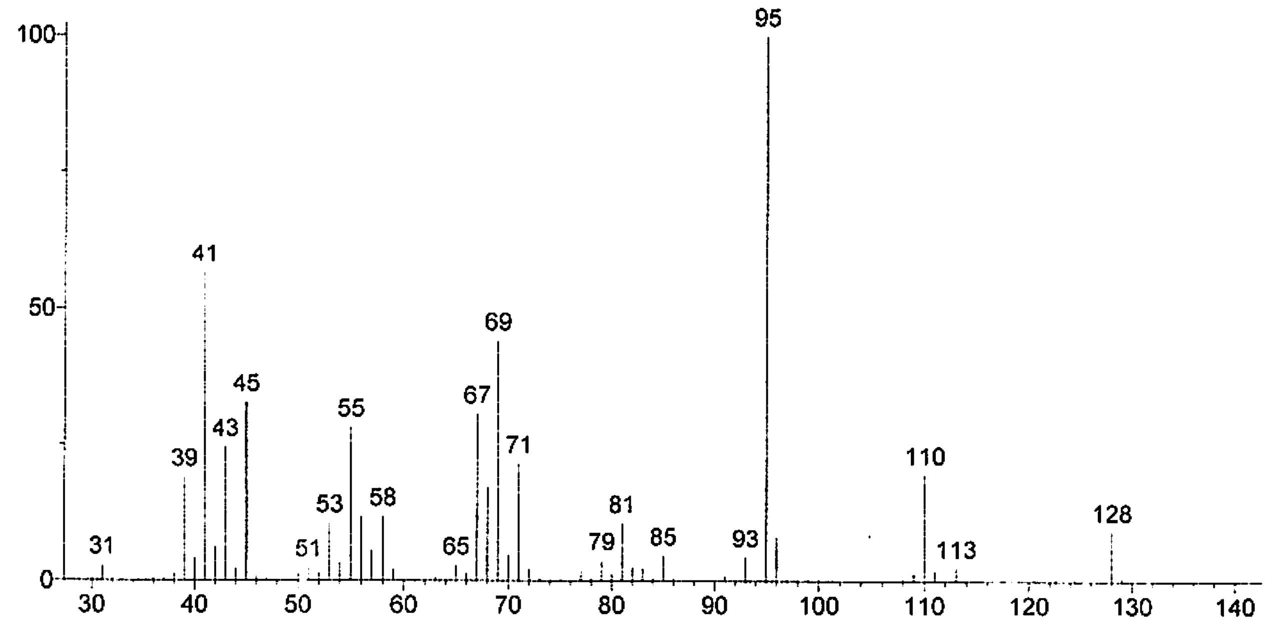

(Text File) Scan 475 (9.042 min): JA012209-2.D (-463)

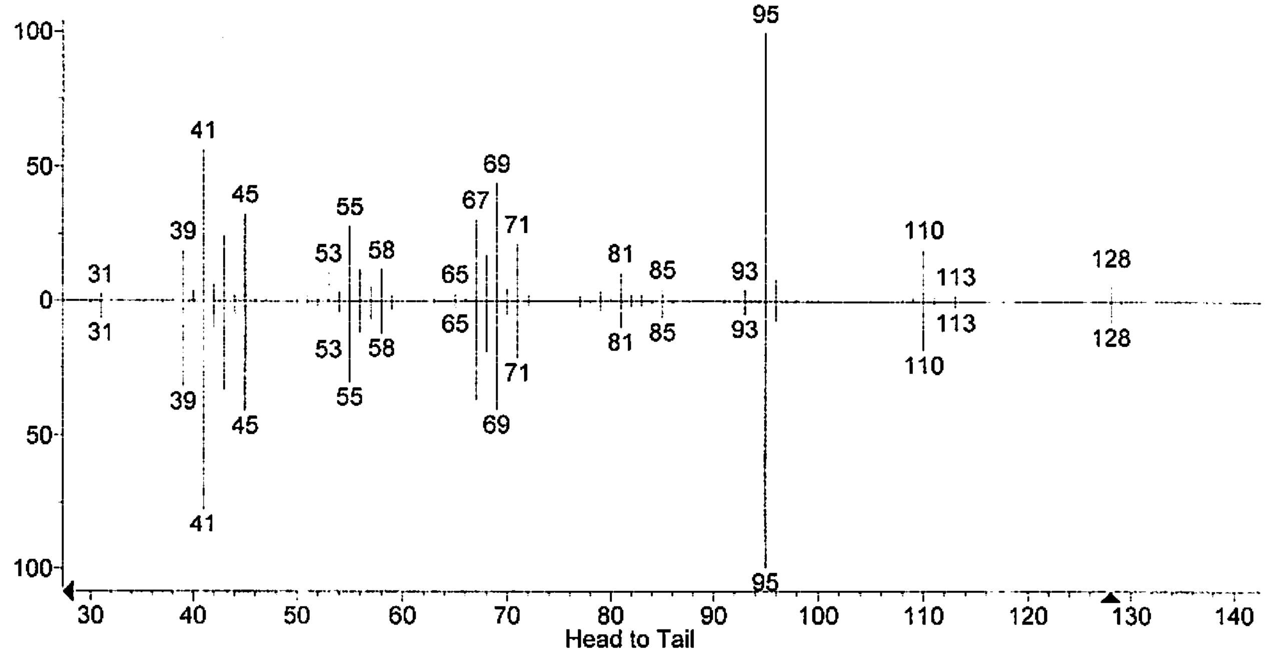

Head to Tail

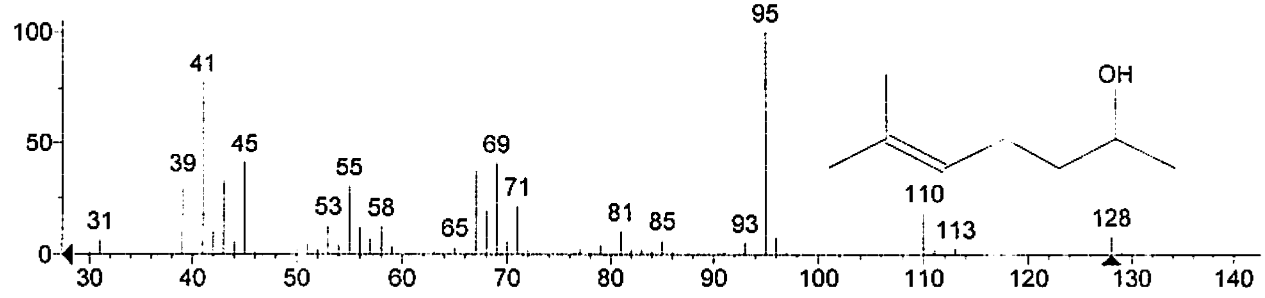

(mainlib) dl-6-Methyl-5-hepten-2-ol

[illegible]le • :D:\DATA\ALDRICH\JA-09\Snapshot\JA012209-2.D
[illegible]erator : Aldrich
[illegible]quired : 22 Jan 2009 11:14 using AcqMethod JA-WAX08.M
[illegible]strument : Instrument #1
[illegible]mple Name: 9 male C.oculata abd.sternites/5ul CH2Cl2
[illegible]sc Info : 3-8-day-old; fed 1 wk 6-CH3-5-hepten-2-one
[illegible]al Number: 1

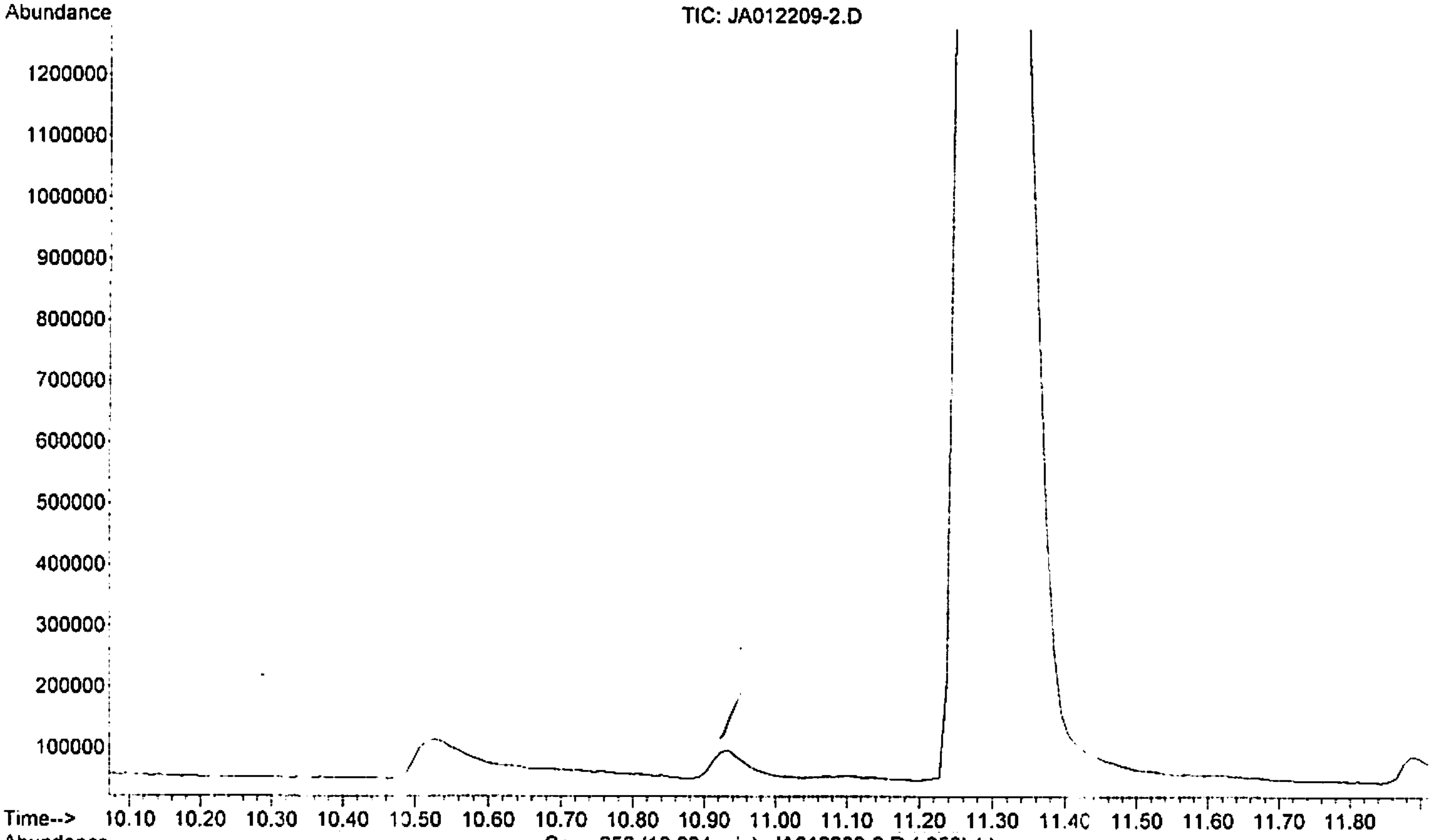


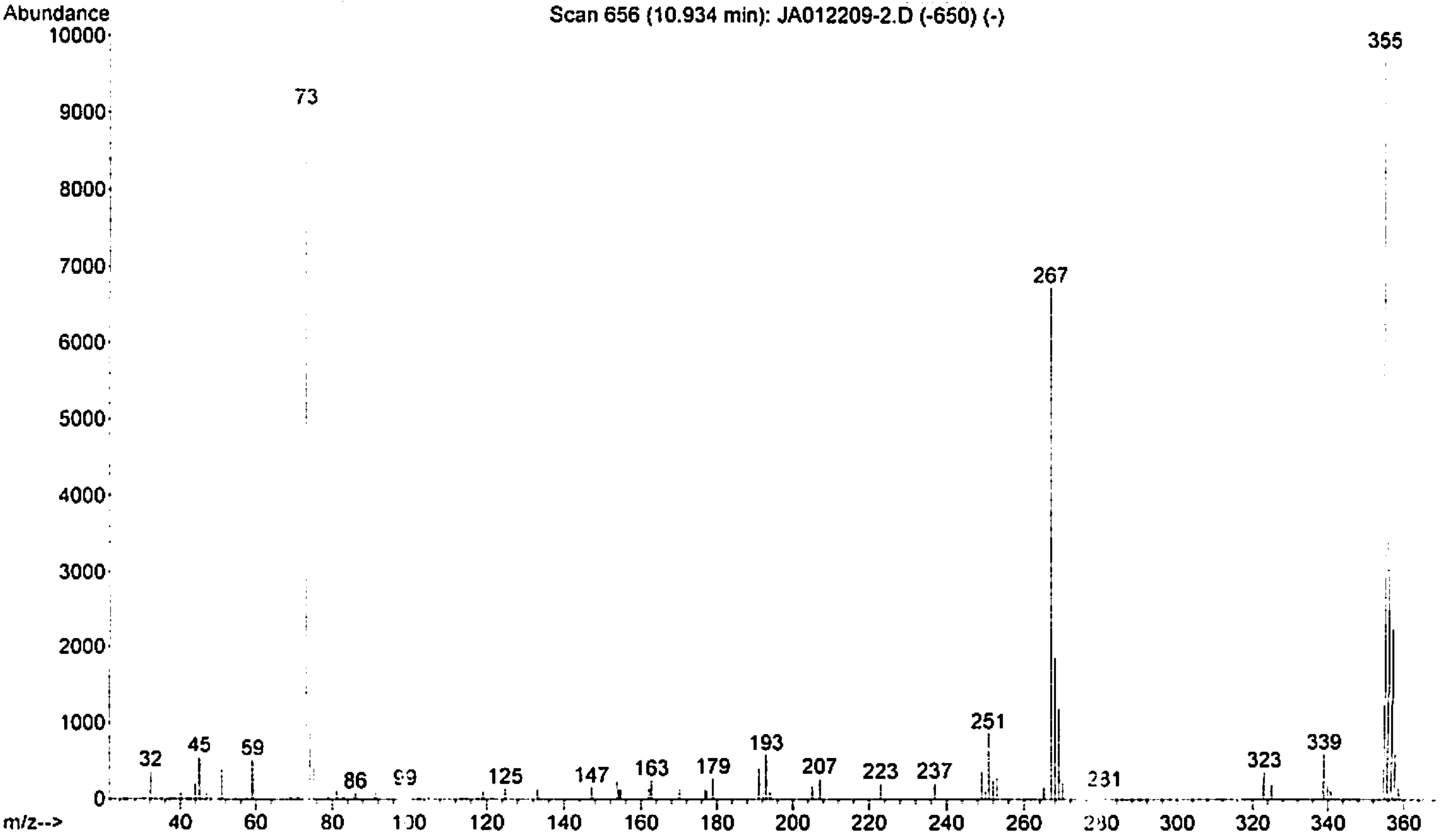

File : D:\DATA\ALDRICH\JA-09\Snapshot\JA012209-2.D
Operator : Aldrich
Acquired : 22 Jan 2009 11:14 using AcqMethod JA-WAX08.M
Instrument : Instrument #1
Sample Name: 9 male C.oculata abd.sternites/5ul CH2Cl2
Misc Info : 3-8-day-old; fed 1 wk 6-CH3-5-hepten-2-one
Vial Number: 1

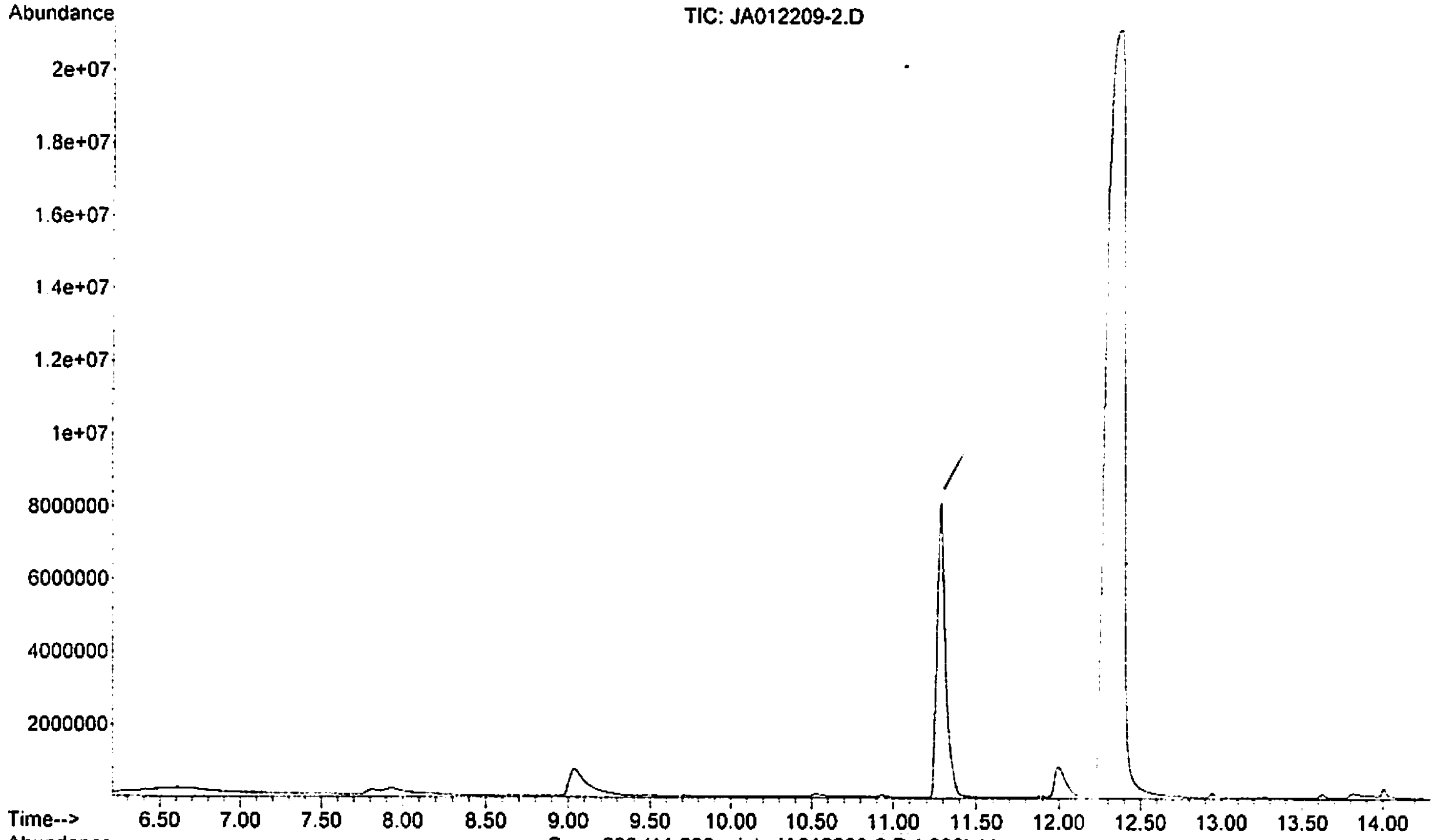


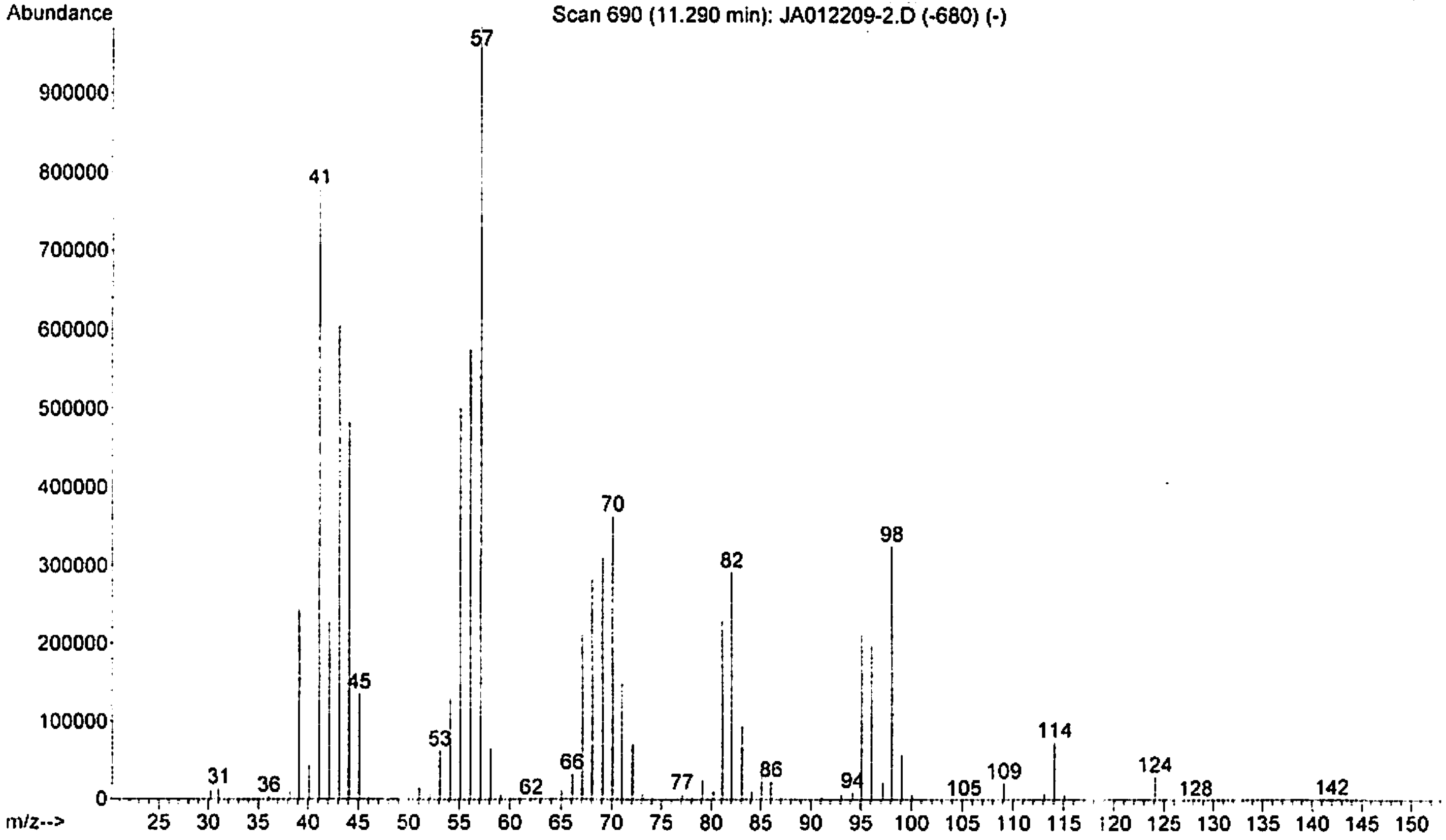

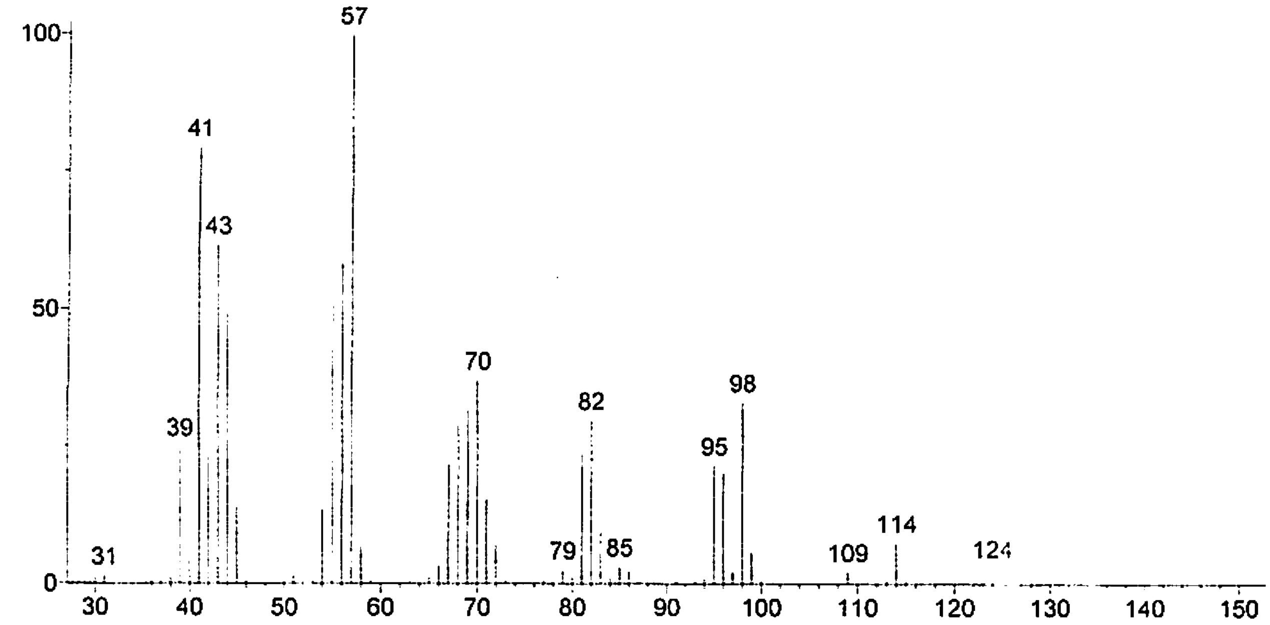


(Text File) Scan 690 (11.290 min): JA012209-2.D (-680)

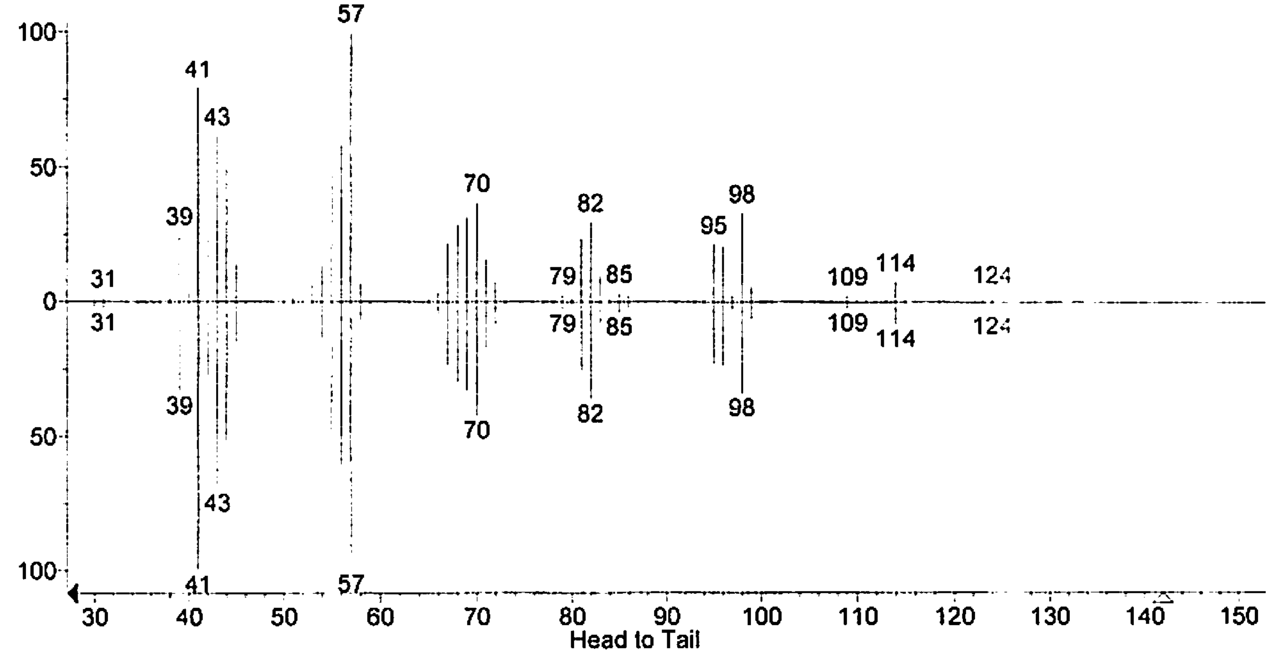


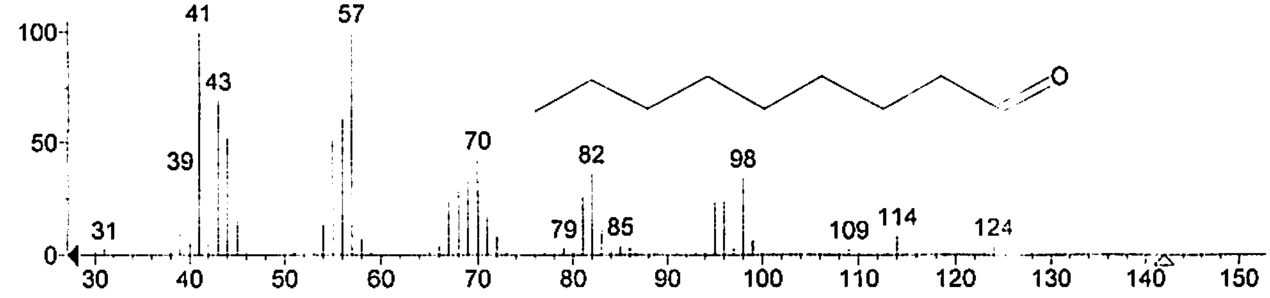


(replib) Nonanal

File :D:\DATA\ALDRICH\JA-09\Snapshot\JA012209-2.D
Operator : Aldrich
Acquired : 22 Jan 2009 11:14 using AcqMethod JA-WAX08.M
Instrument : Instrument #1
Sample Name: 9 male C.oculata abd.sternites/5ul CH2Cl2
Misc Info : 3-8-day-old; fed 1 wk 6-CH3-5-hepten-2-one
Vial Number: 1

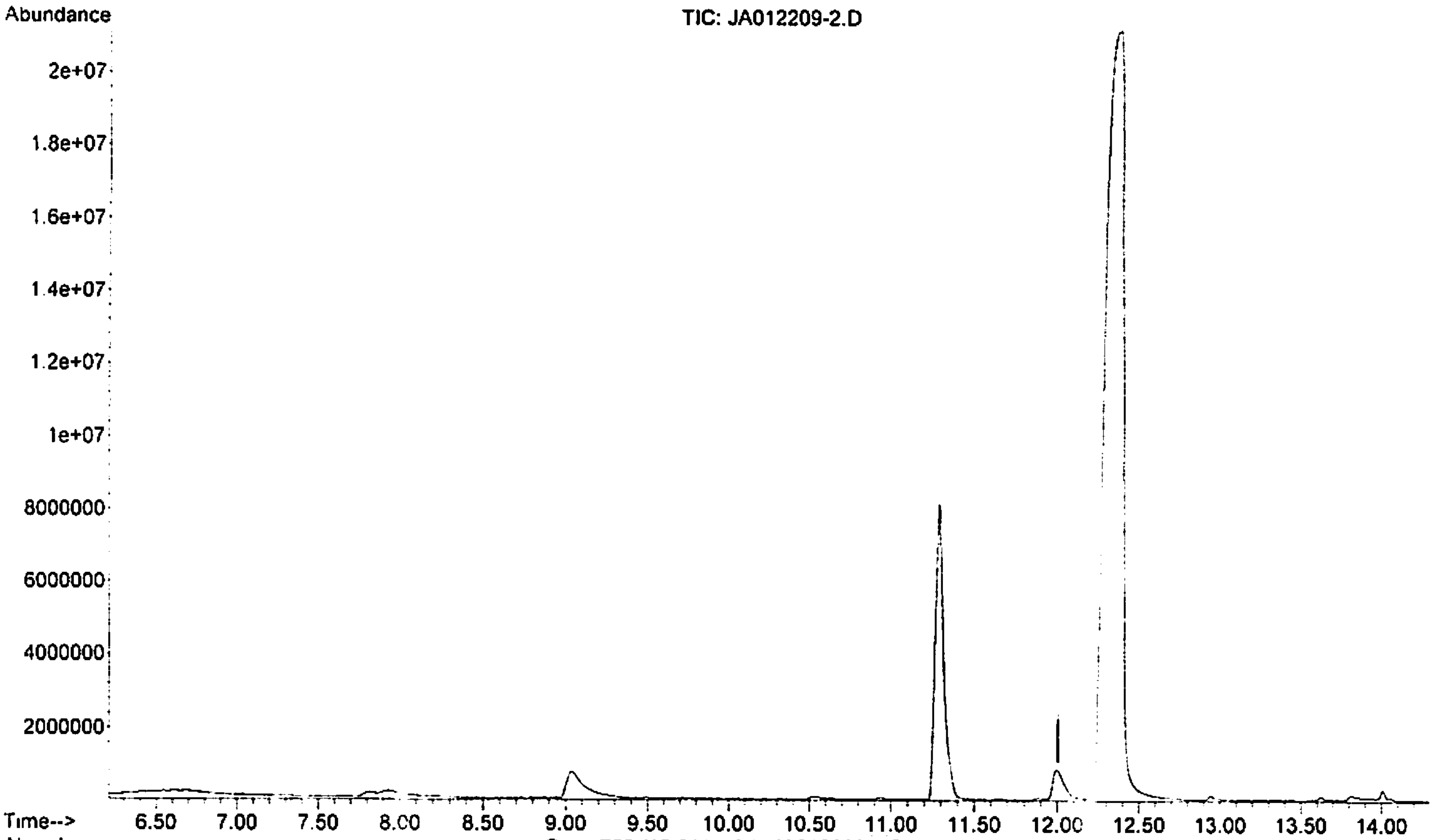


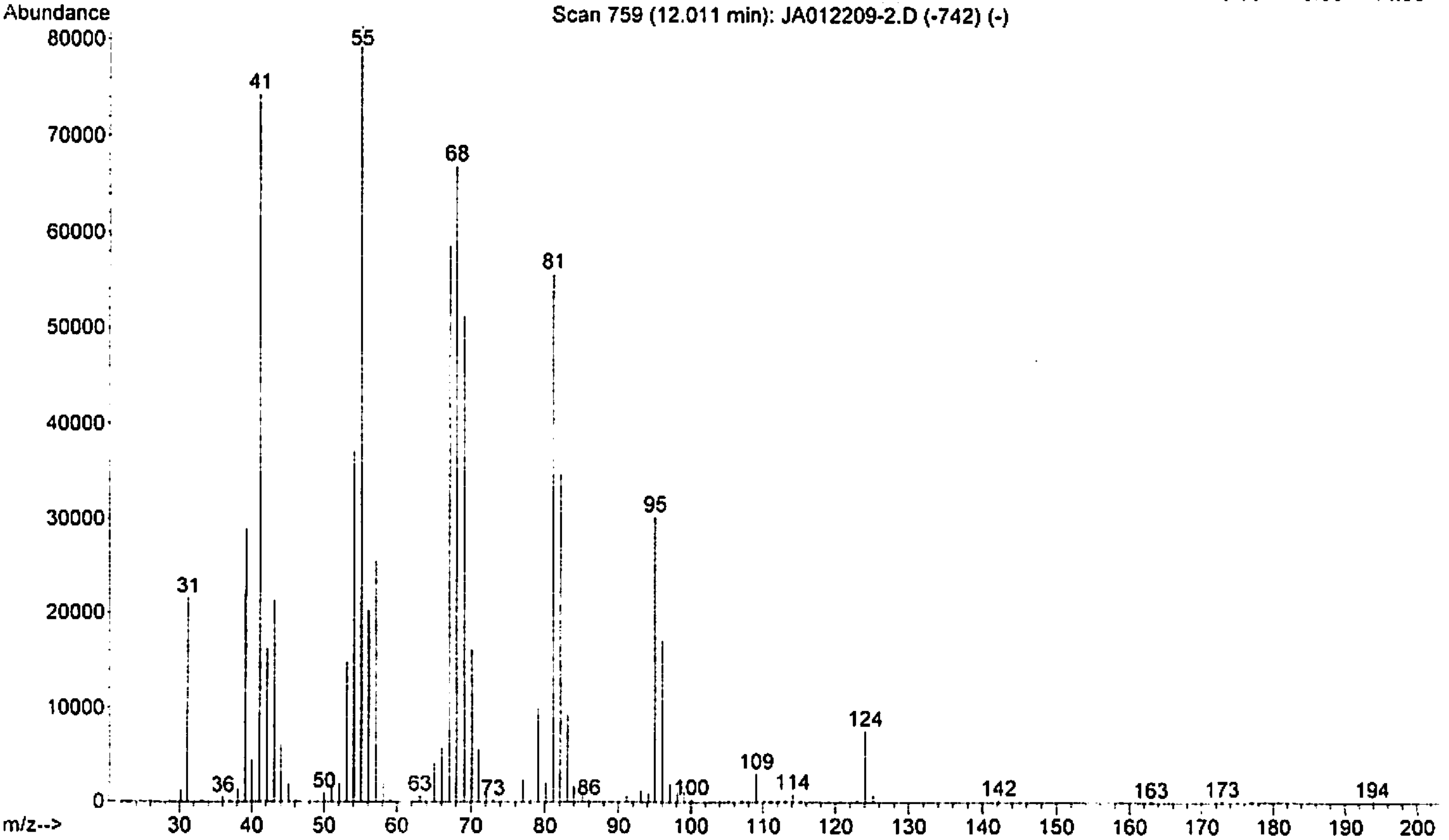

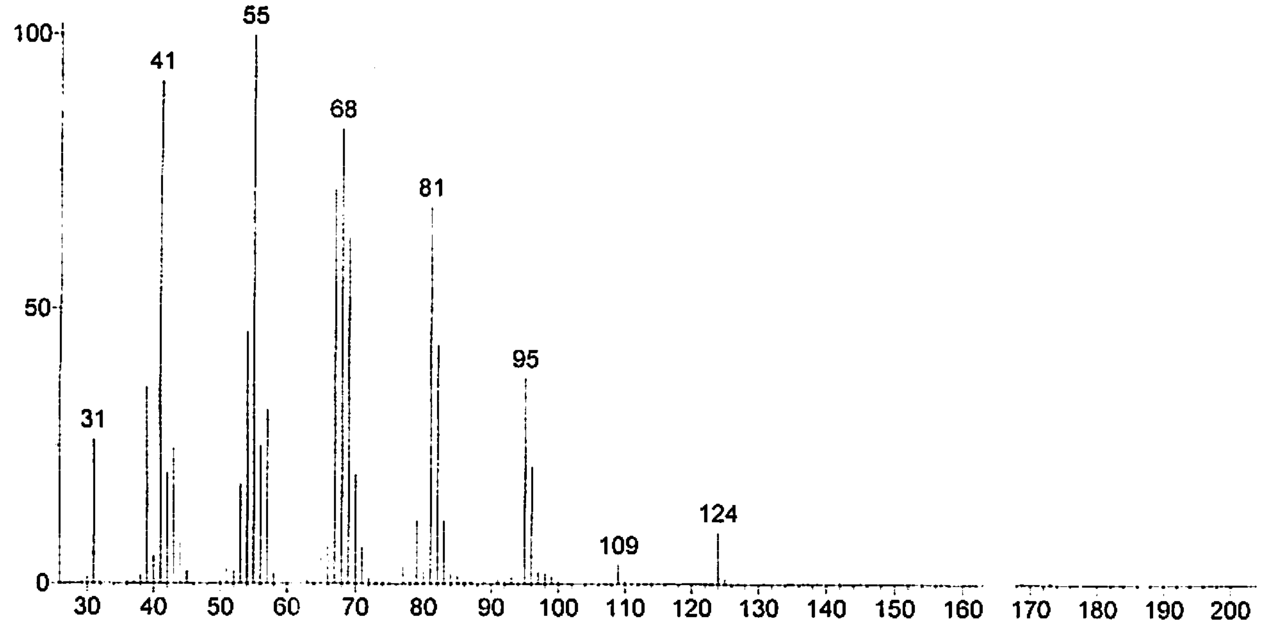


(Text File) Scan 759 (12.011 min): JA012209-2.D (-750)

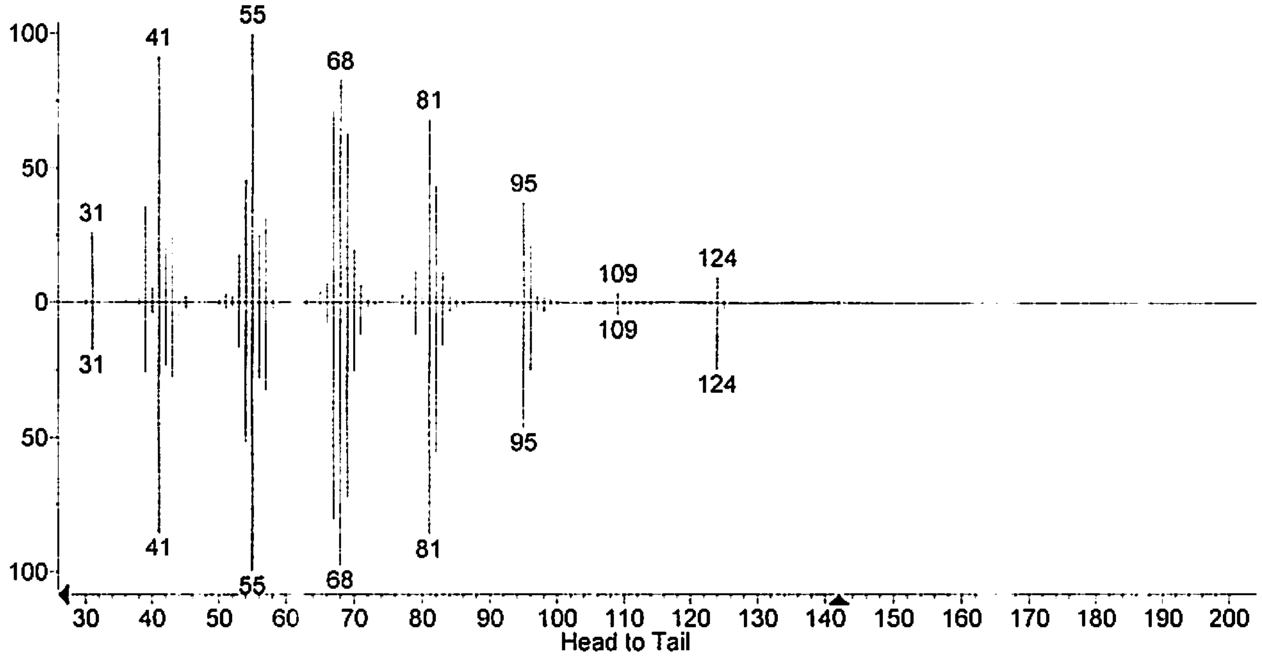


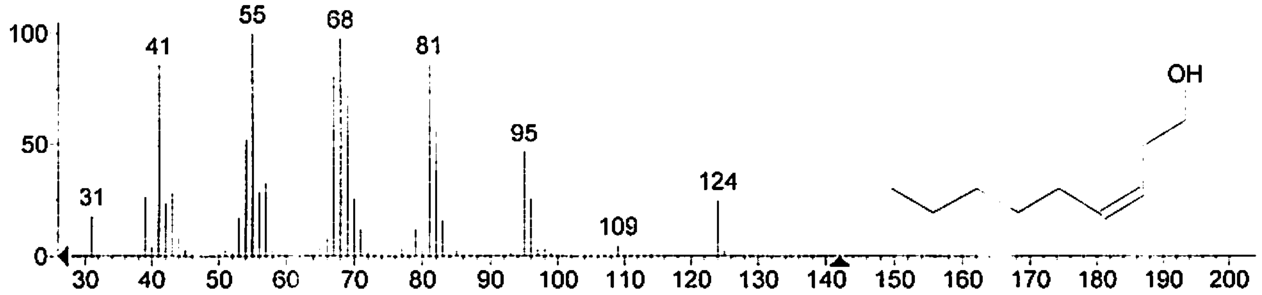


(mainlib) 3-Nonen-1-ol, (Z)-

[illegible] :D:\DATA\ALDR CH\JA-09\Snapshot\JA012209-2.D
[illegible]rator : Aldrich
[illegible]quired : 22 Jan 2009 11:14 using AcqMethod JA-WAX08.M
[illegible]trument : Instrument #1
[illegible]ple Name: 9 male C.oculata abd.sternites/5ul CH2Cl2
[illegible] Info : 3-8-day-old; fed 1 wk 6-CH3-5-hepten-2-one
[illegible]al Number: 1

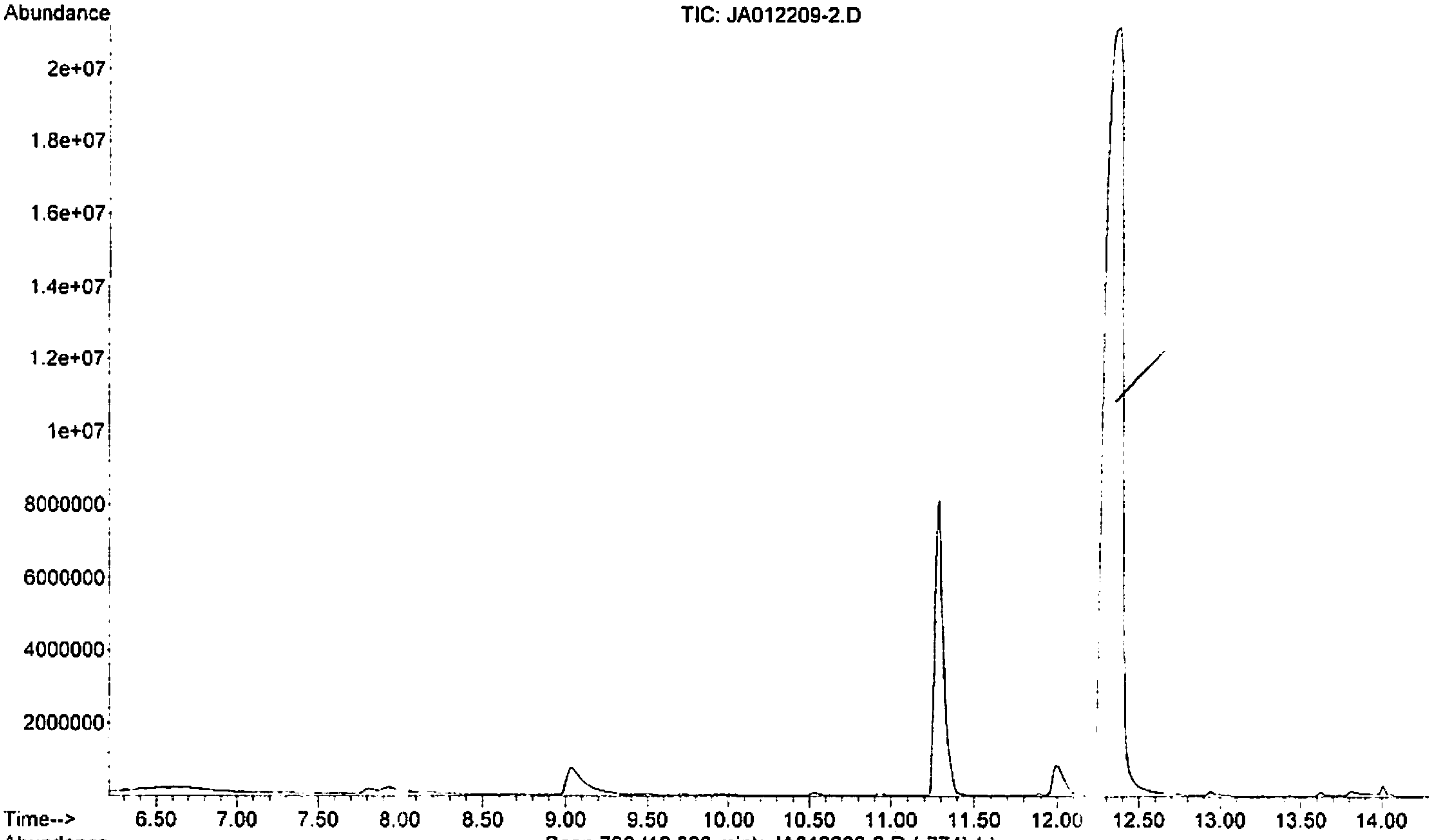


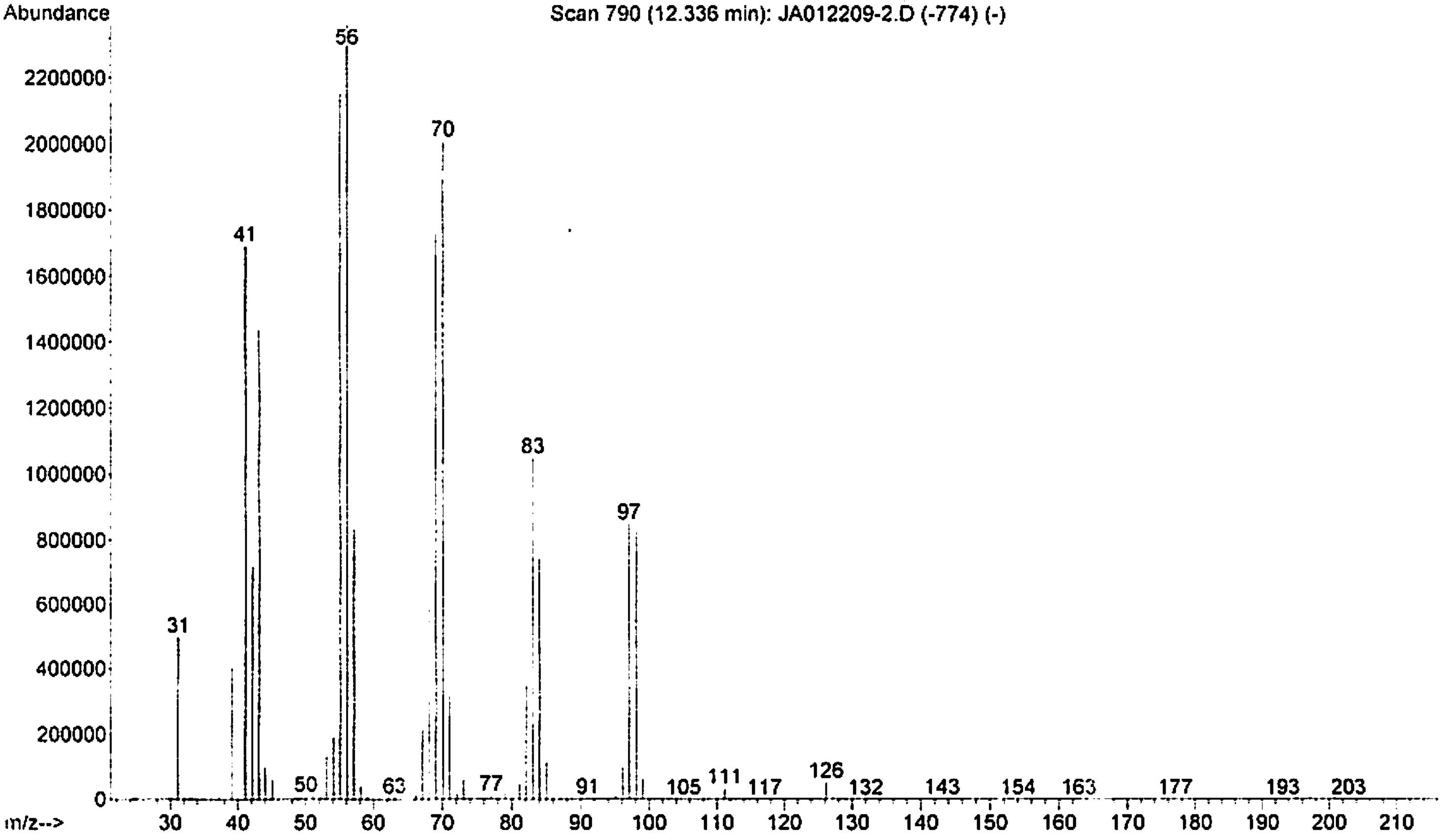

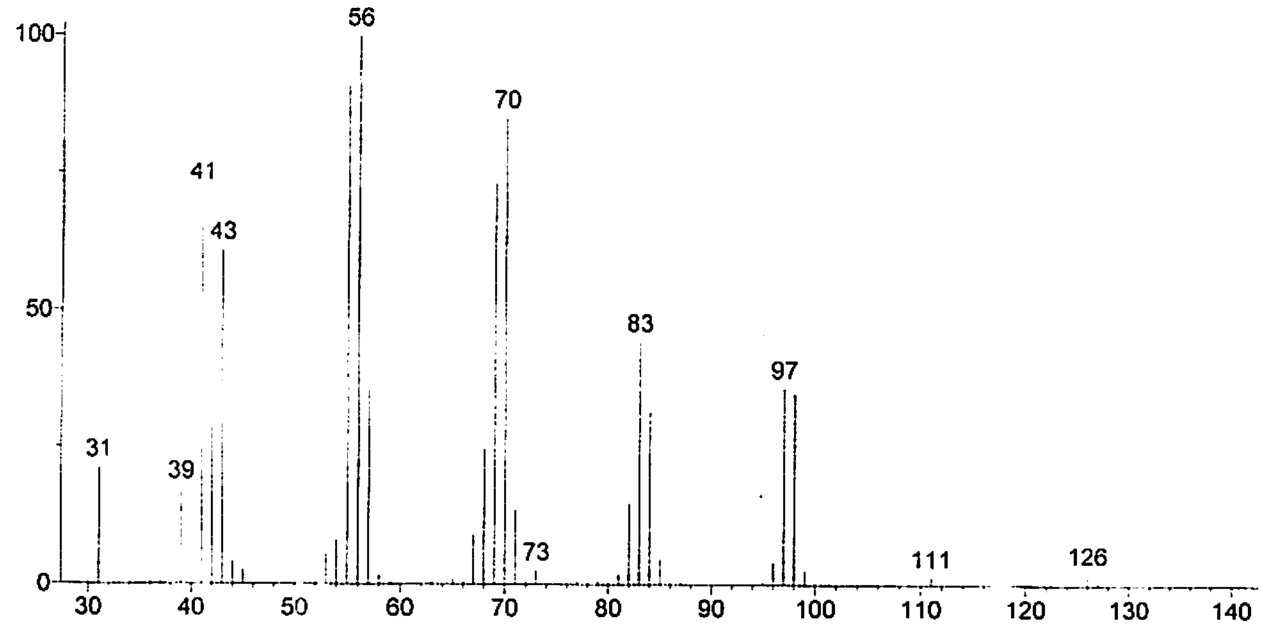

(Text File) Scan 790 (12.336 min): JA012209-2.D (-774)

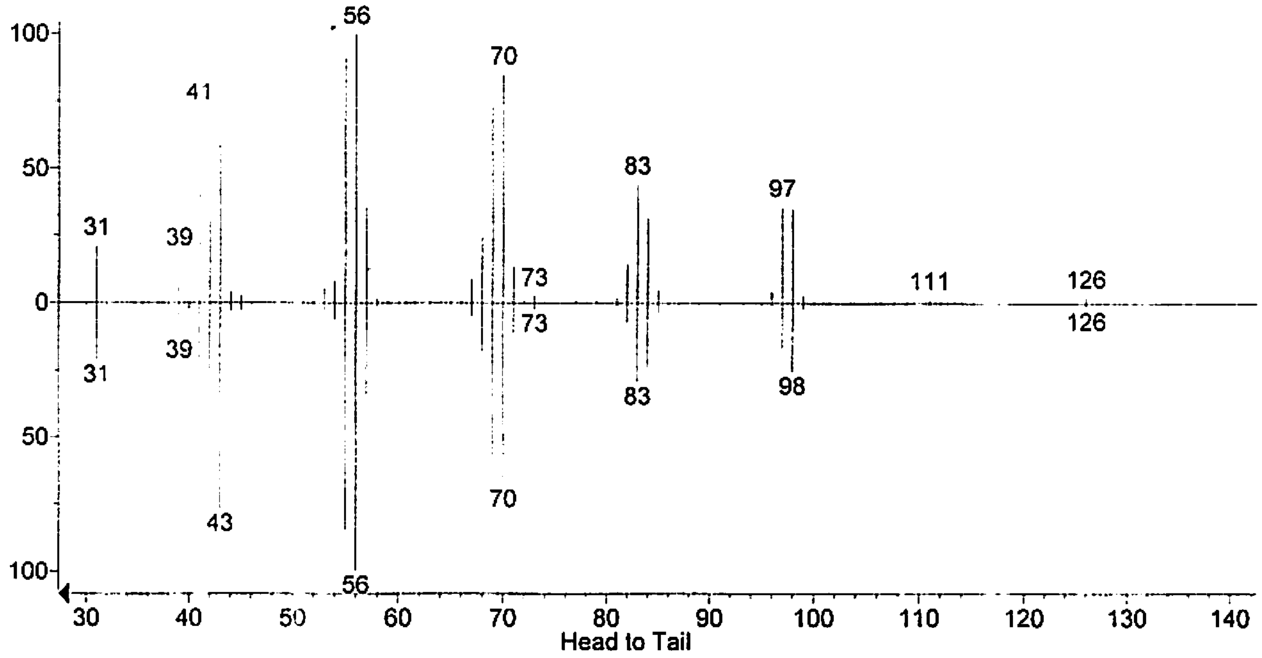


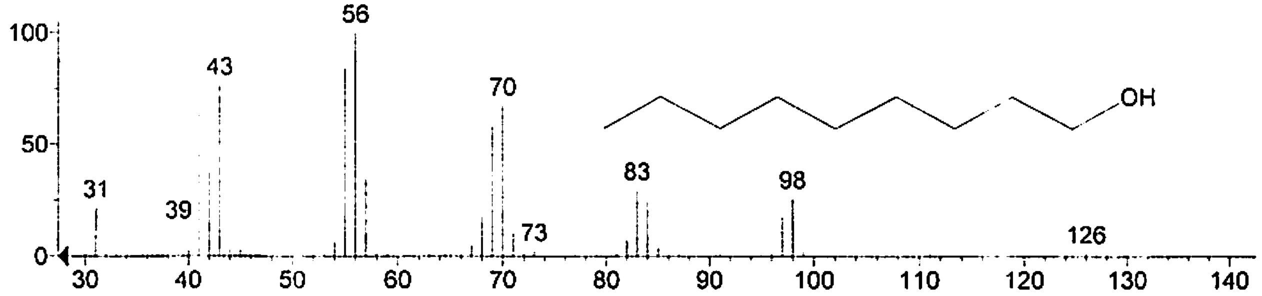

(mainlib) 1-Nonanol

```
[illegible]      :D:\DATA\ALDR[illegible]CH\JA-09\Snapshot\JA012209-2.D
[illegible]rator    : Aldrich
[illegible]uired    : 22 Jan 2009   11:14      using AcqMethod JA-WAX08.M
[illegible]trument  :     Instrument #1
[illegible]mple Name: 9 male C.oculata abd.sternites/5ul CH2Cl2
[illegible] Info    : 3-8-day-old; fed 1 wk 6-CH3-5-hepten-2-one
[illegible] Number: 1
```

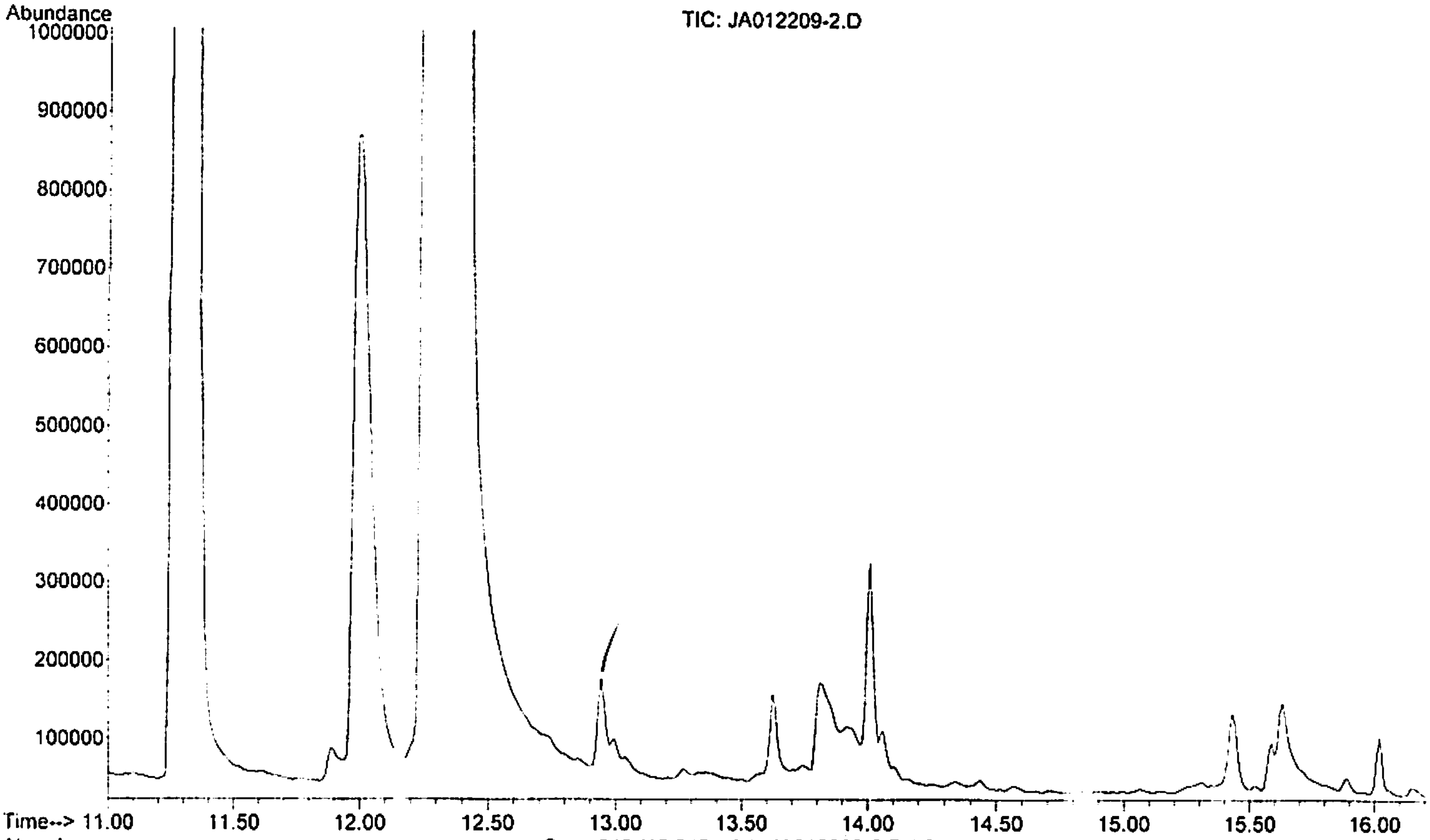


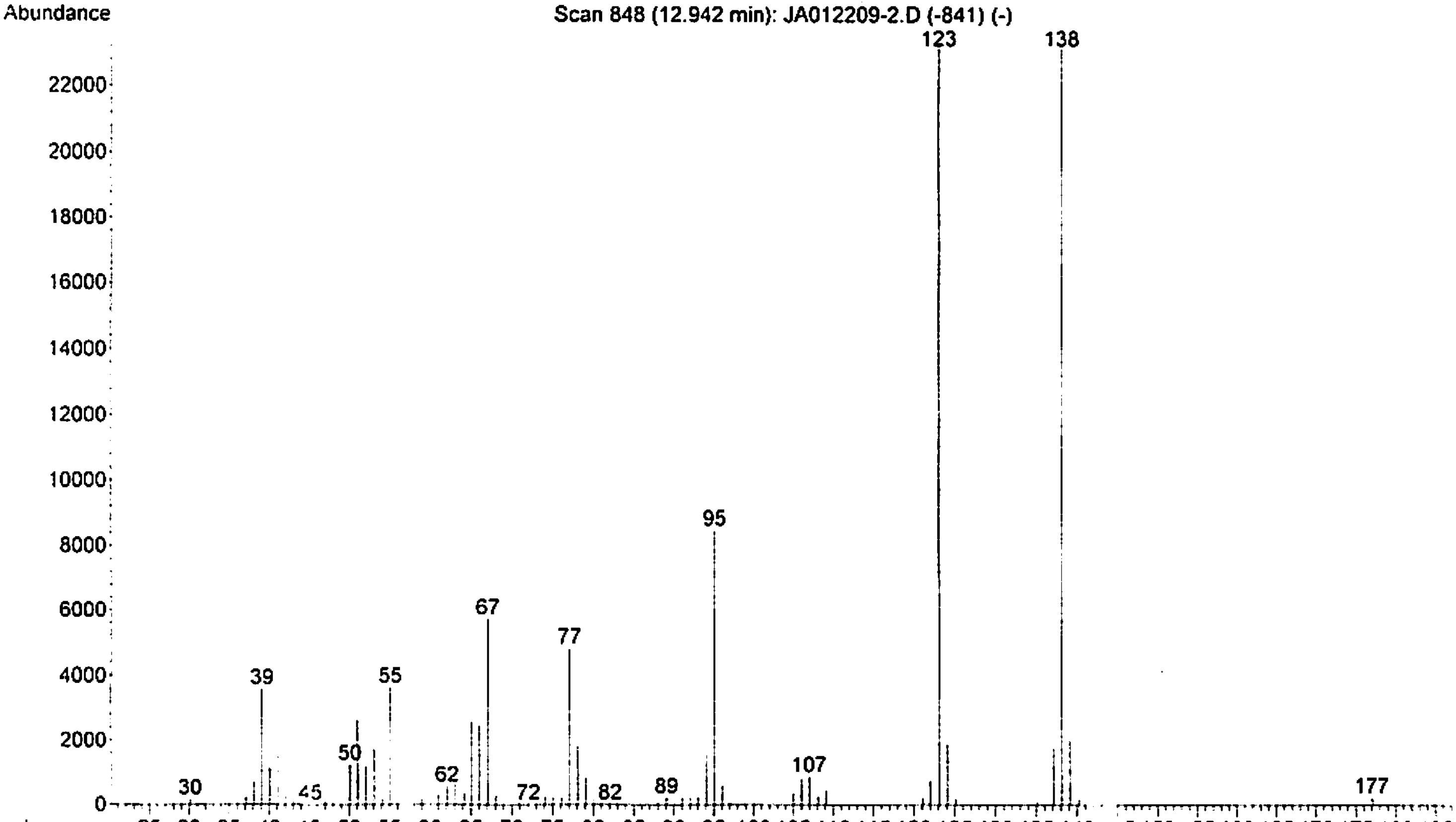

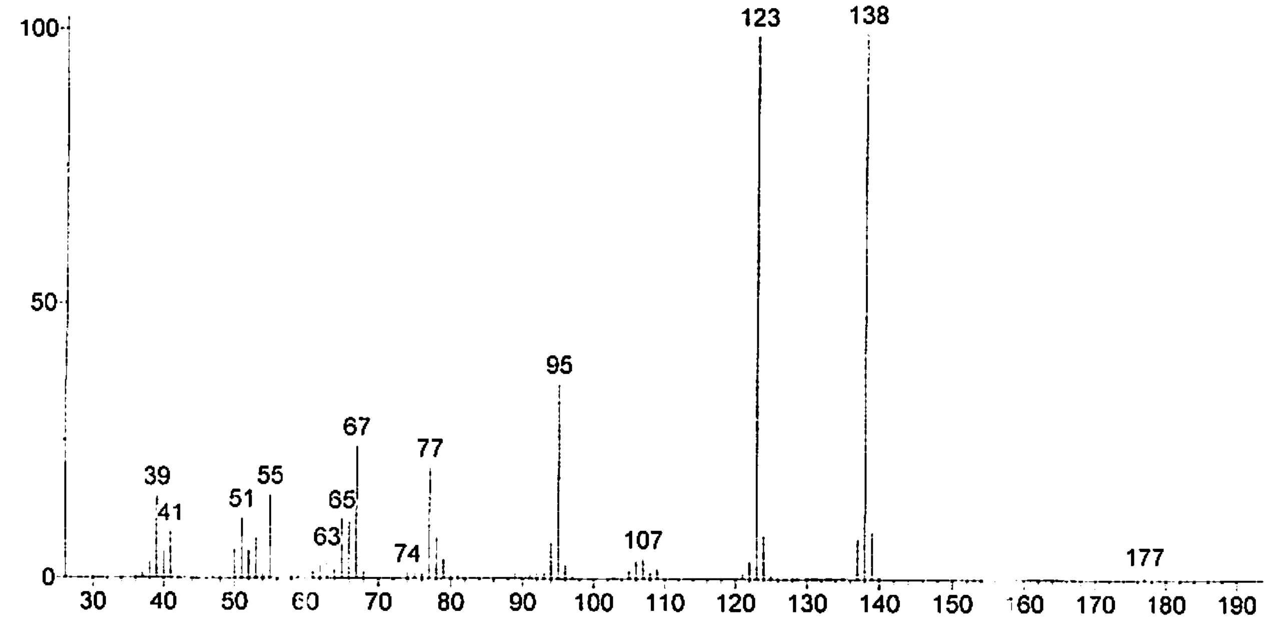


(Text File) Scan 848 (12.942 min): JA012209-2.D (-841)

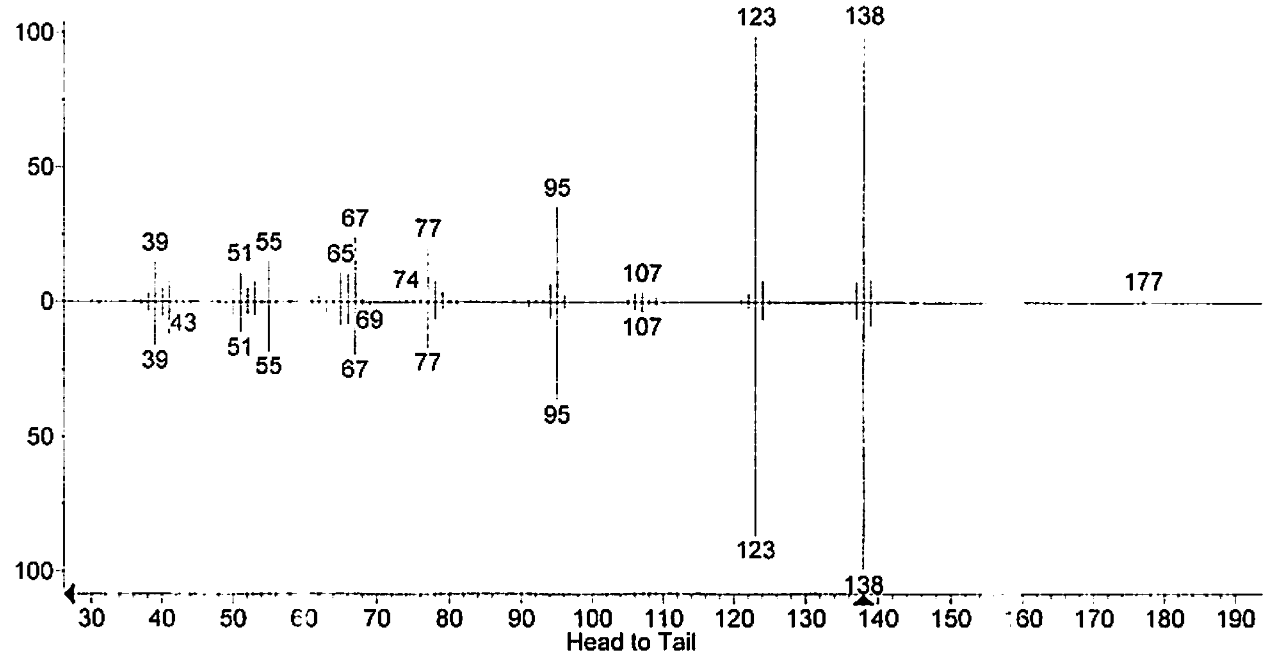


Head to Tail

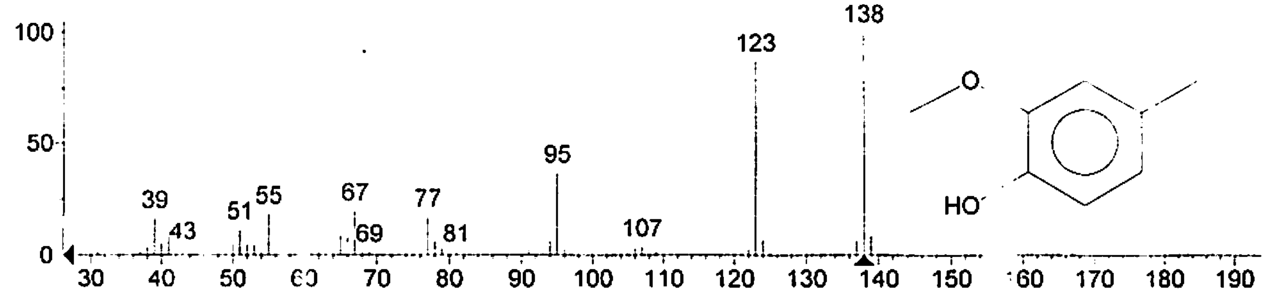


(mainlib) Phenol, 2-methoxy-4-methyl-

:D:\DATA\ALDRICH\JA-09\Snapshot\JA012209-2.D
rator : Aldrich
quired : 22 Jan 2009 11:14 using AcqMethod JA-WAX08.M
strument : Instrument #1
mple Name: 9 male C.oculata abd.sternites/5ul CH2Cl2
sc Info : 3-8-day-old; fed 1 wk 6-CH3-5-hepten-2-one
al Number: 1

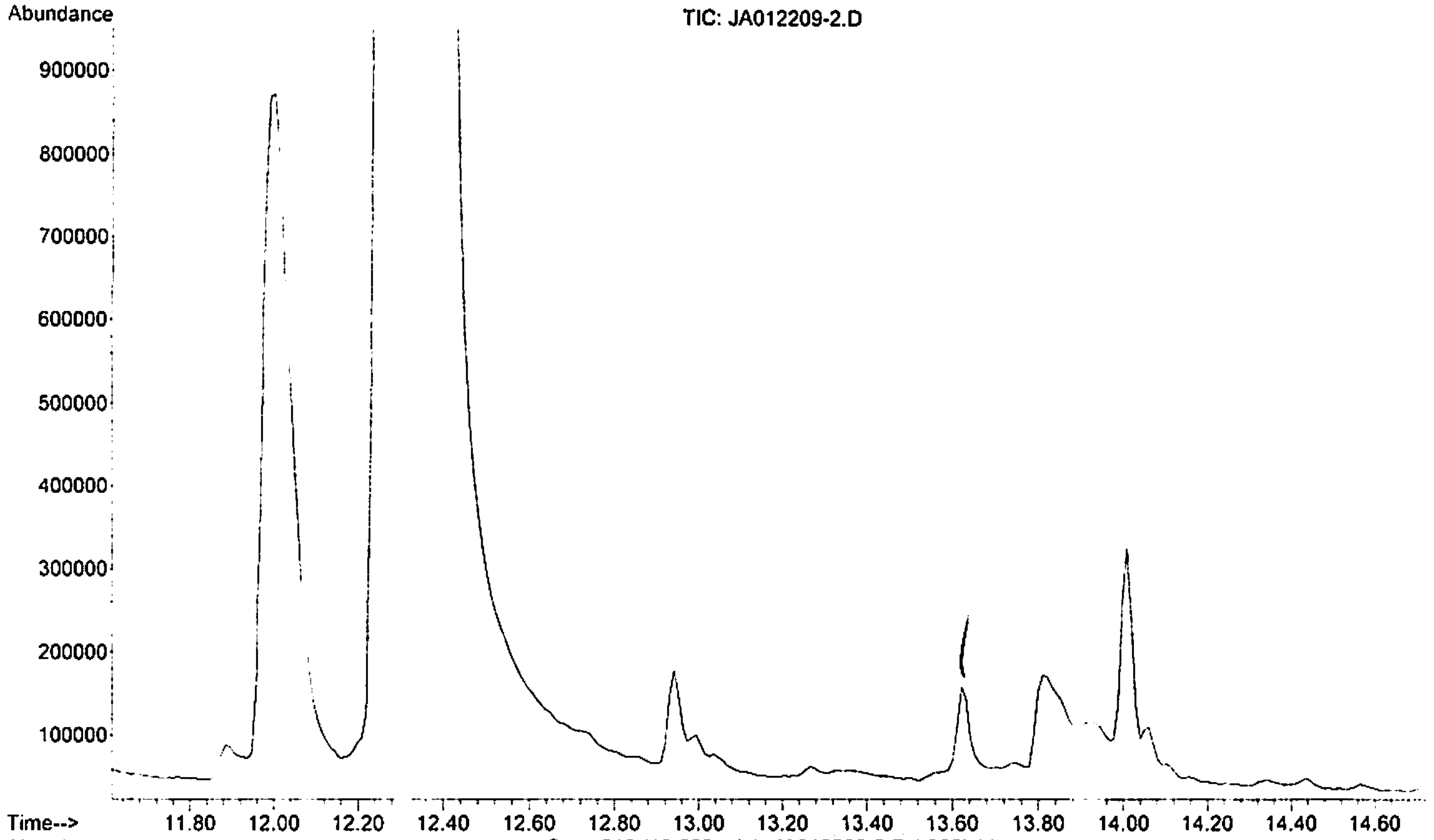


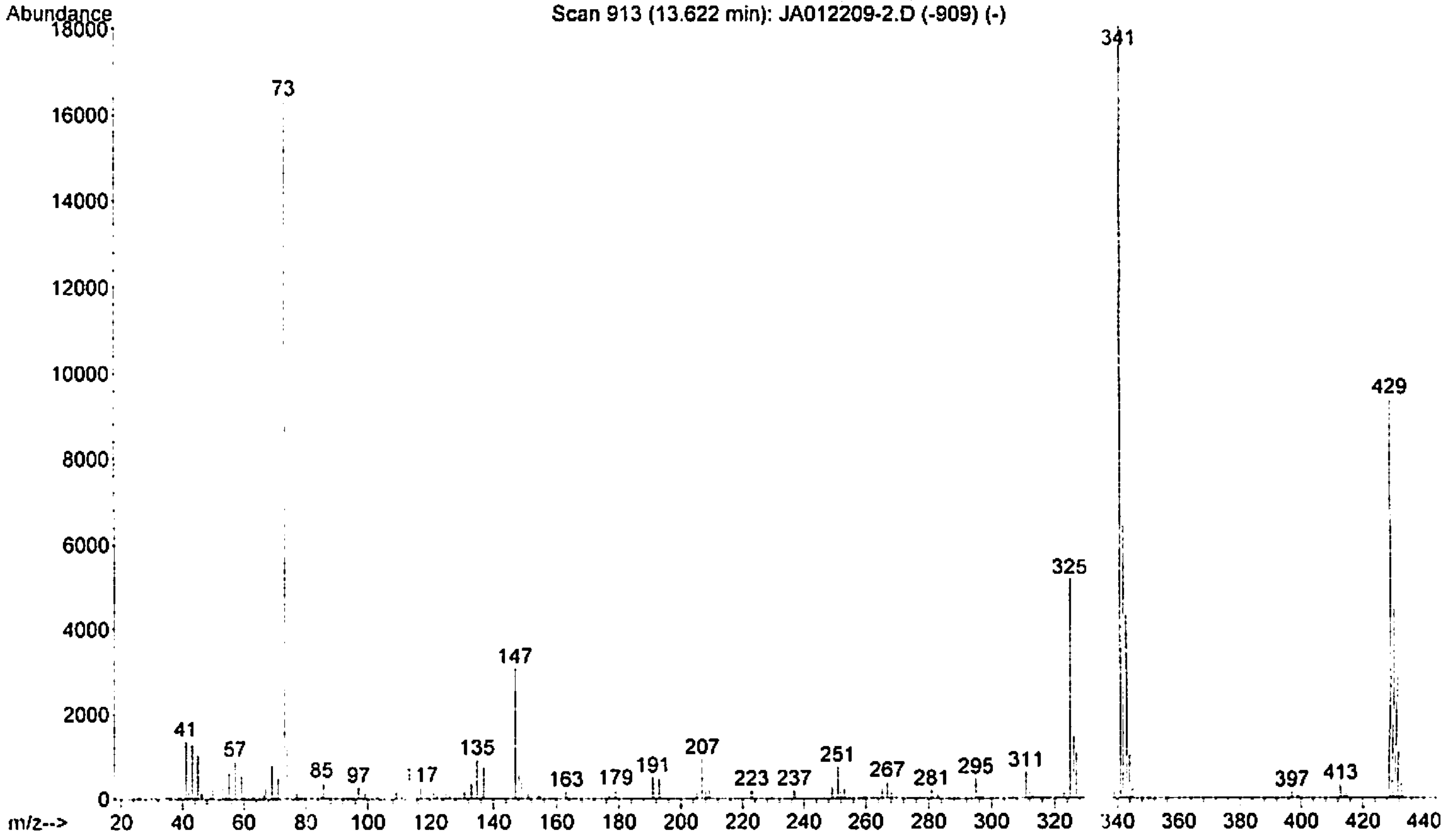

[illegible]e : :D:\DATA\ALDR.CH\JA-09\Snapshot\JA012209-2.D
[illegible]rator : Aldrich
[illegible]quired : 22 Jan 2009 11:14 using AcqMethod JA-WAX08.M
[illegible]strument : Instrument #1
[illegible]mple Name: 9 male C.oculata abd.sternites/5ul CH2Cl2
[illegible]sc Info : 3-8-day-old; fed 1 wk 6-CH3-5-hepten-2-one
[illegible]ial Number: 1

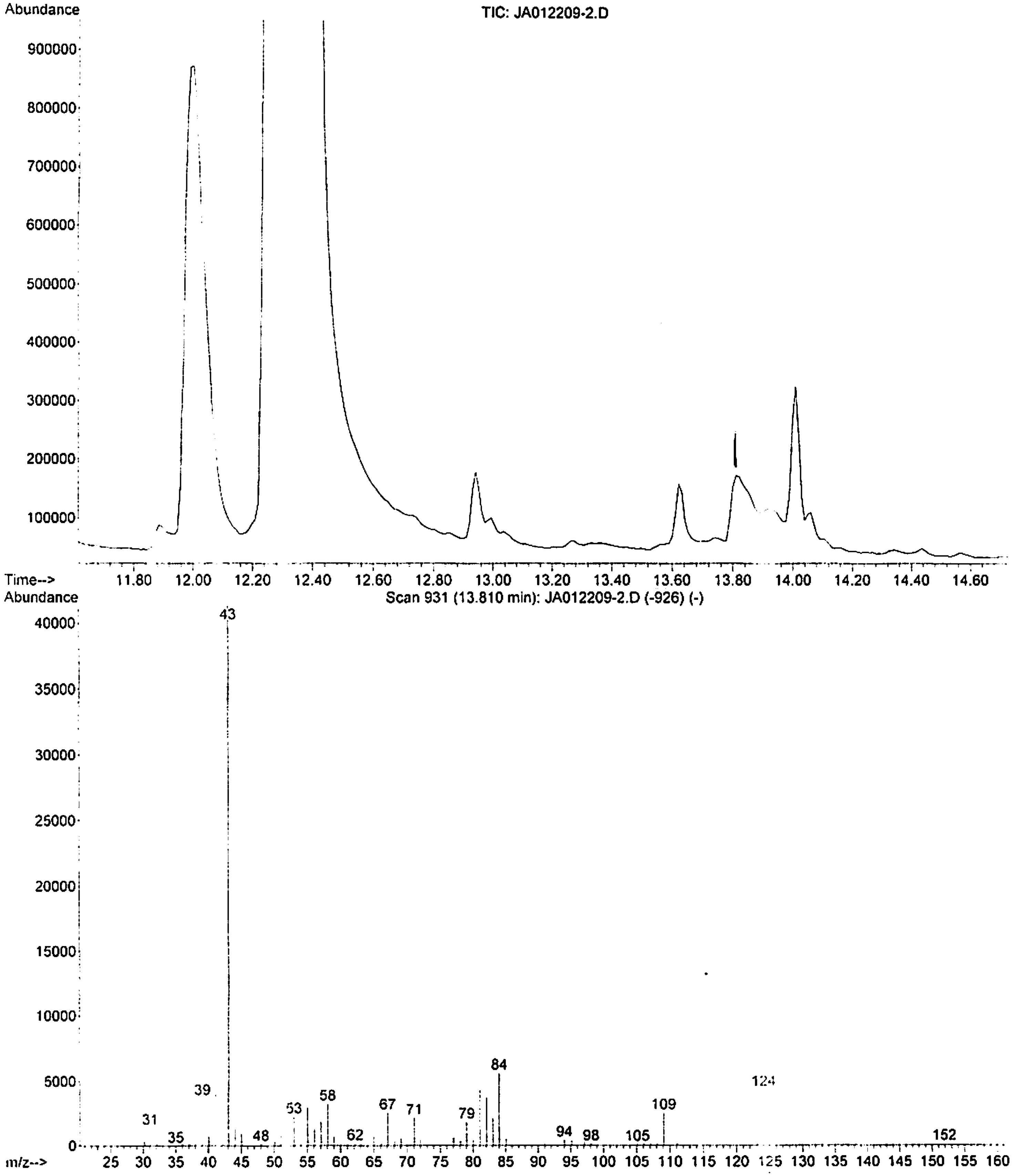

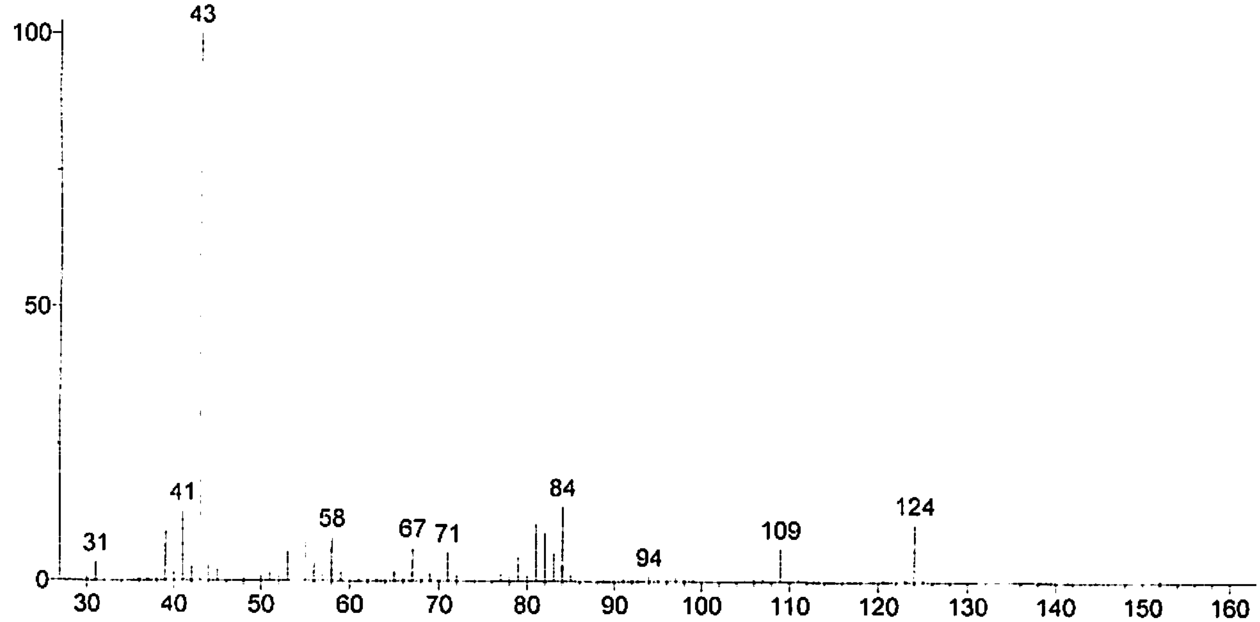


(Text File) Scan 931 (13.81( min): JA012209-2.D (-926)

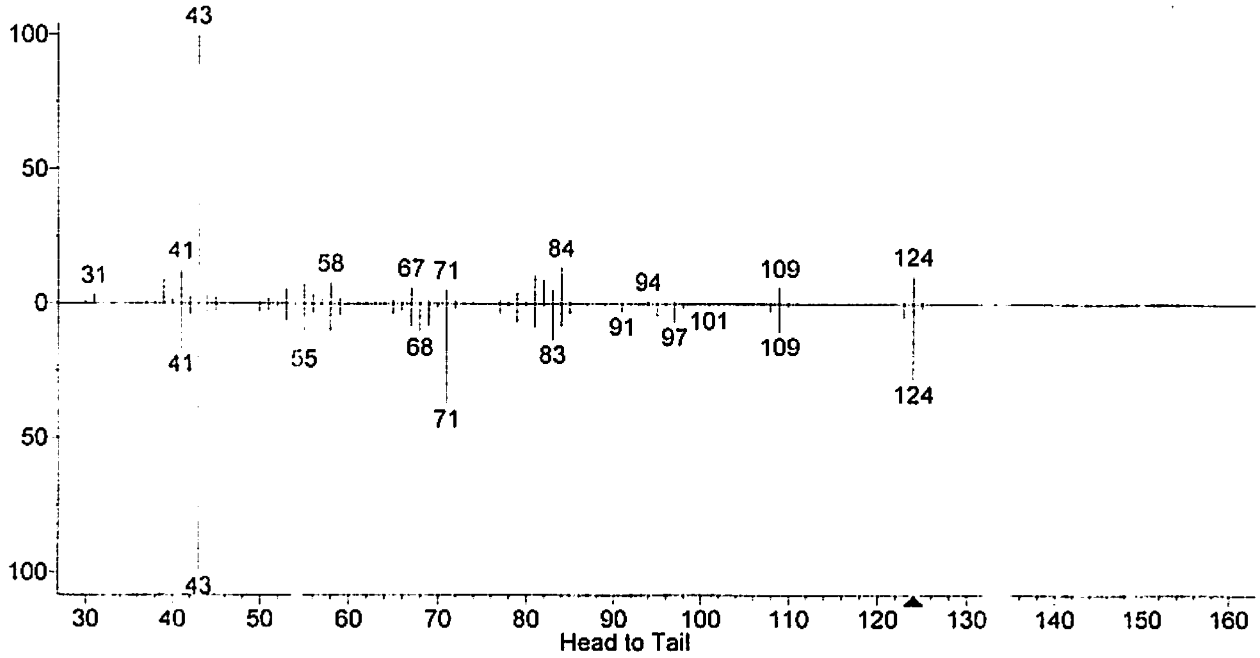


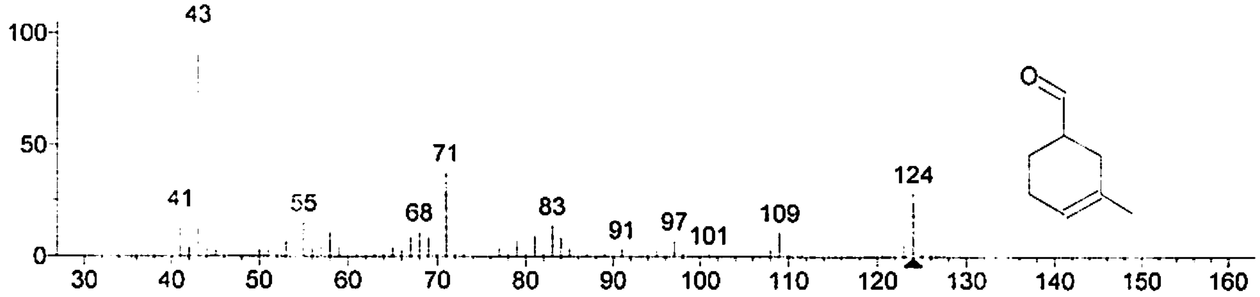


(mainlib) 3-Cyclohexen-1-carboxaldehyde, 3-methyl-

[illegible] :D:\DATA\ALDR[illegible]CH\JA-09\Snapshot\JA012209-2.D
[illegible]erator : Aldrich
[illegible]quired : 22 Jan 2009 11:14 using AcqMethod JA-WAX08.M
[illegible]strument : Instrument #1
[illegible]mple Name: 9 male C.oculata abd.sternites/5ul CH2Cl2
[illegible] Info : 3-8-day-old; fed 1 wk 6-CH3-5-hepten-2-one
[illegible] Number: 1

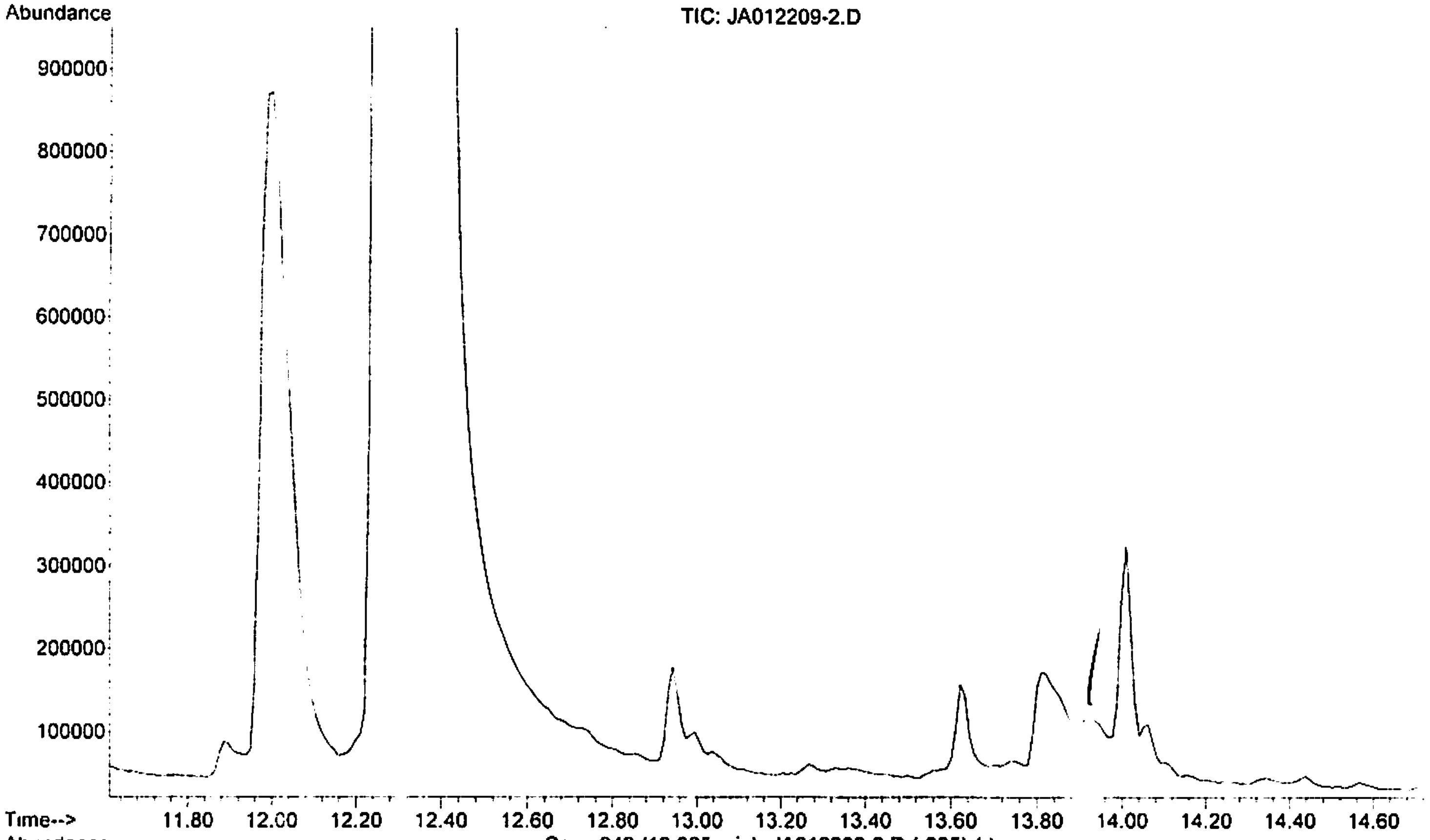


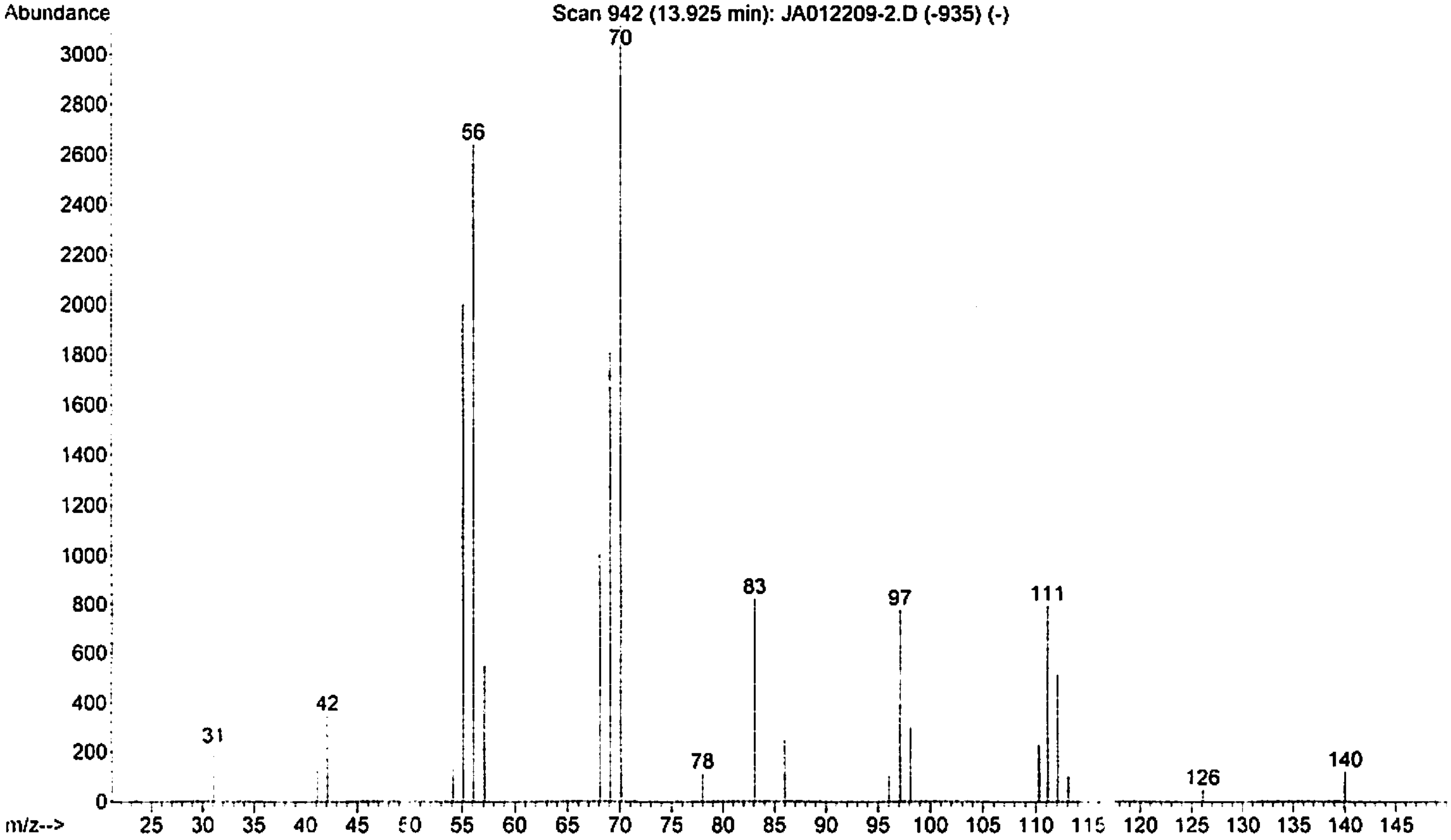

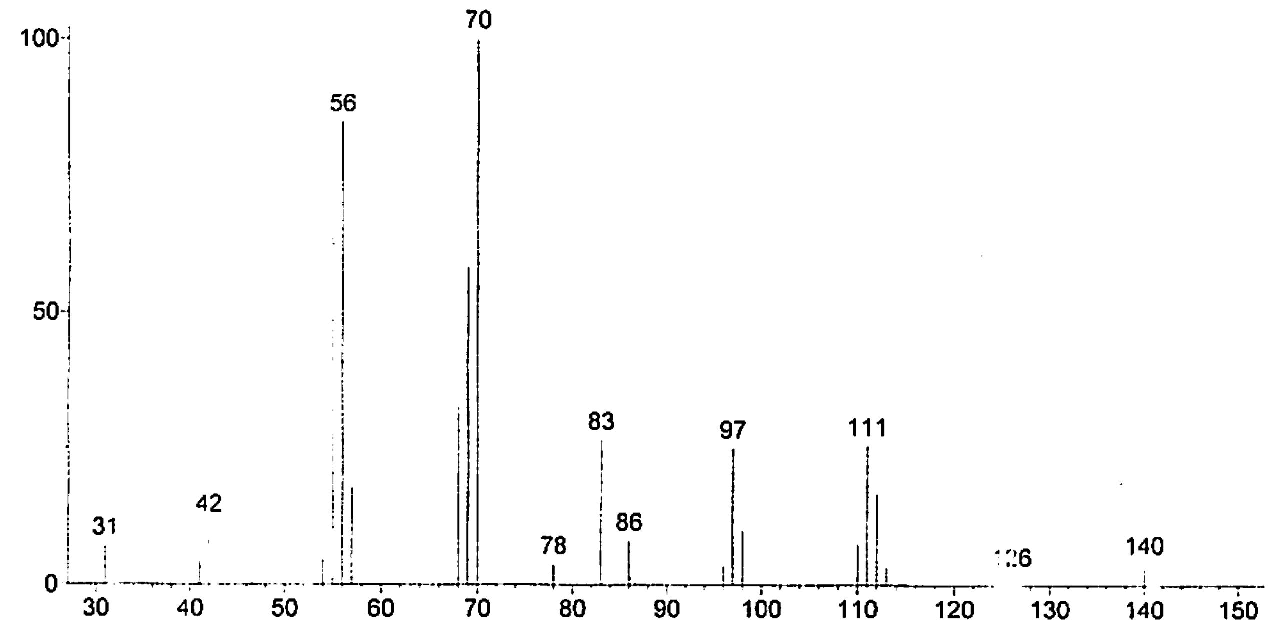


(Text File) Scan 942 (13.925 min): JA012209-2.D (-935)

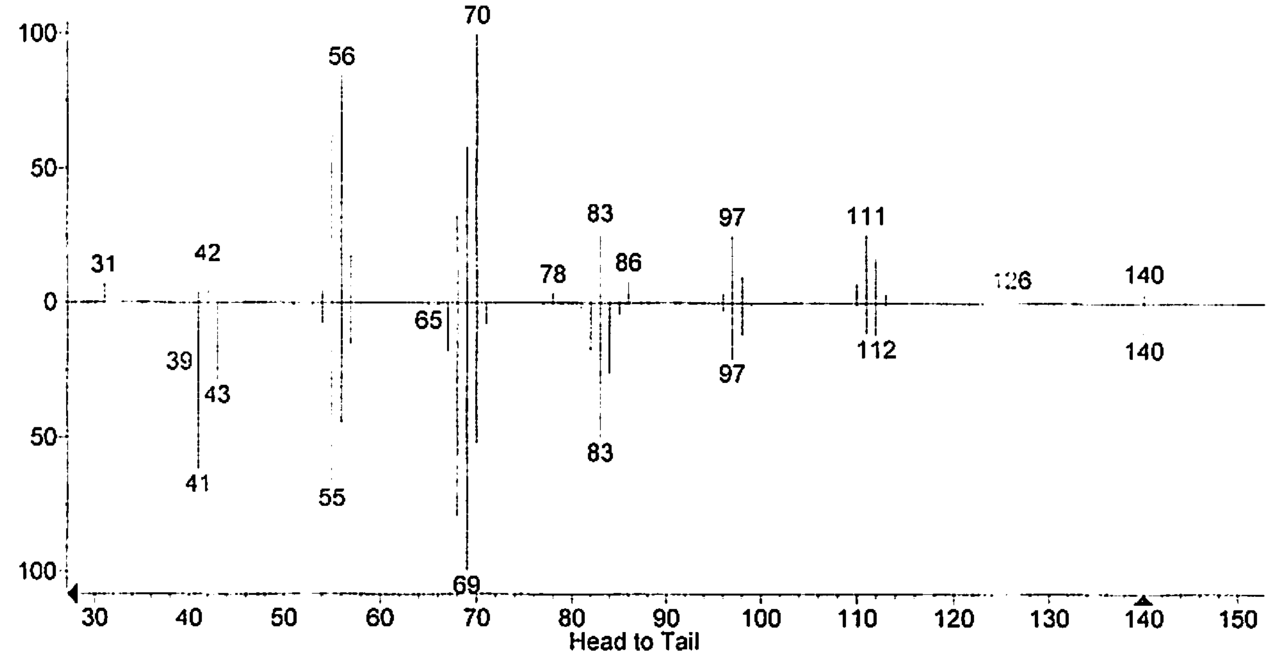


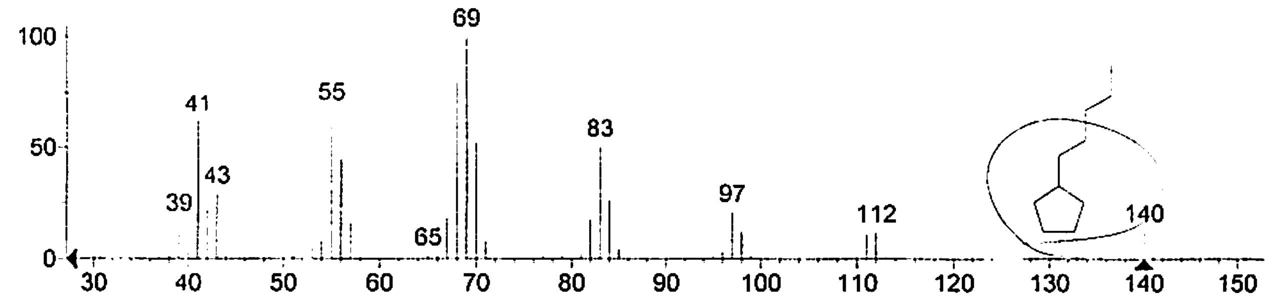


(replib) Cyclopentane, pentyl-

[illegible]le [illegible] :D:\DATA\ALDR[illegible]CH\JA-09\Snapshot\JA012209-2.D
[illegible]erator : Aldrich
[illegible]quired : 22 Jan 2009 11:14 using AcqMethod JA-WAX08.M
[illegible]strument : Instrument #1
[illegible]mple Name: 9 male C.oculata abd.sternites/5ul CH2Cl2
[illegible] Info : 3-8-day-old; fed 1 wk 6-CH3-5-hepten-2-one
[illegible] Number: 1

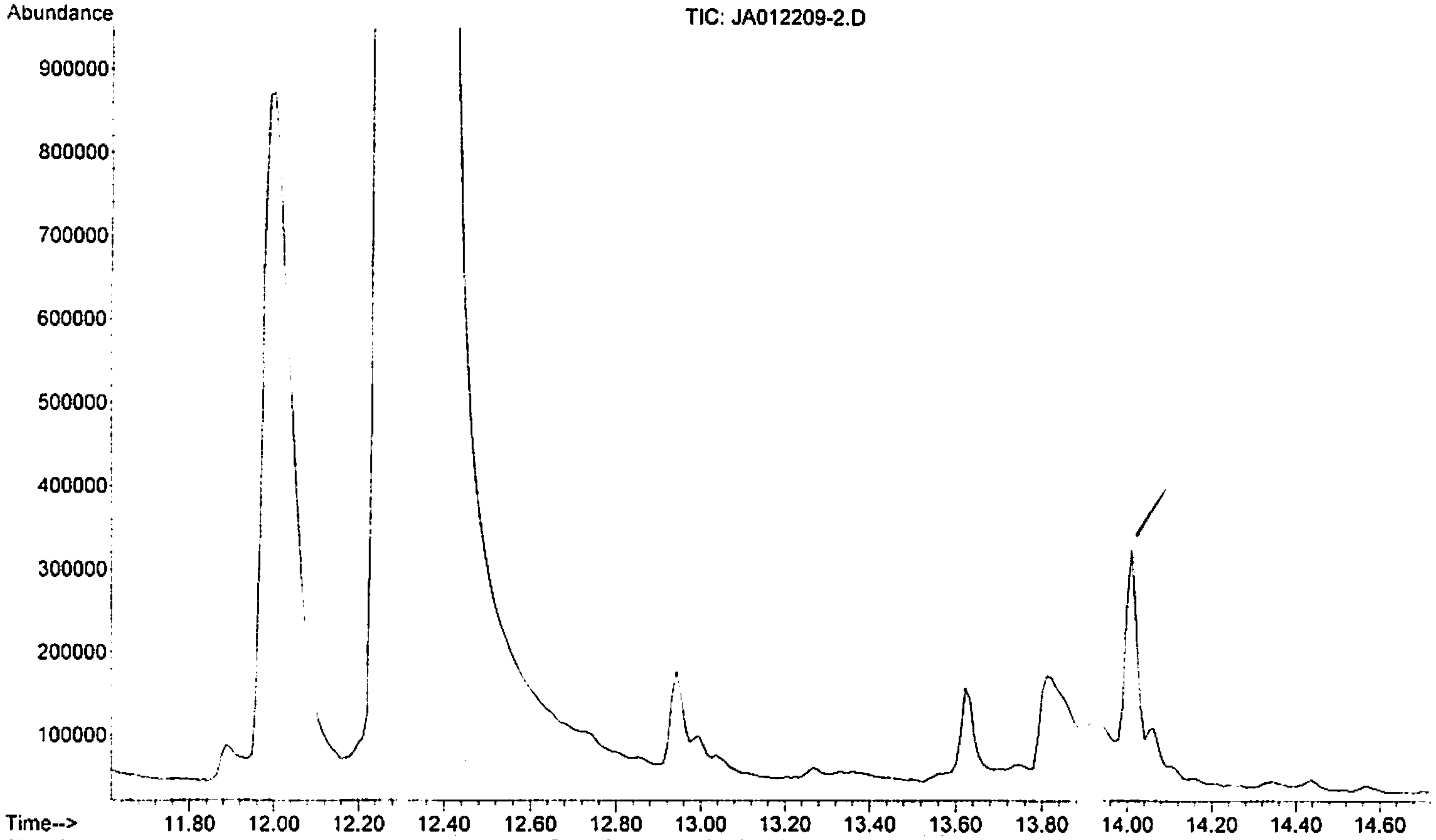


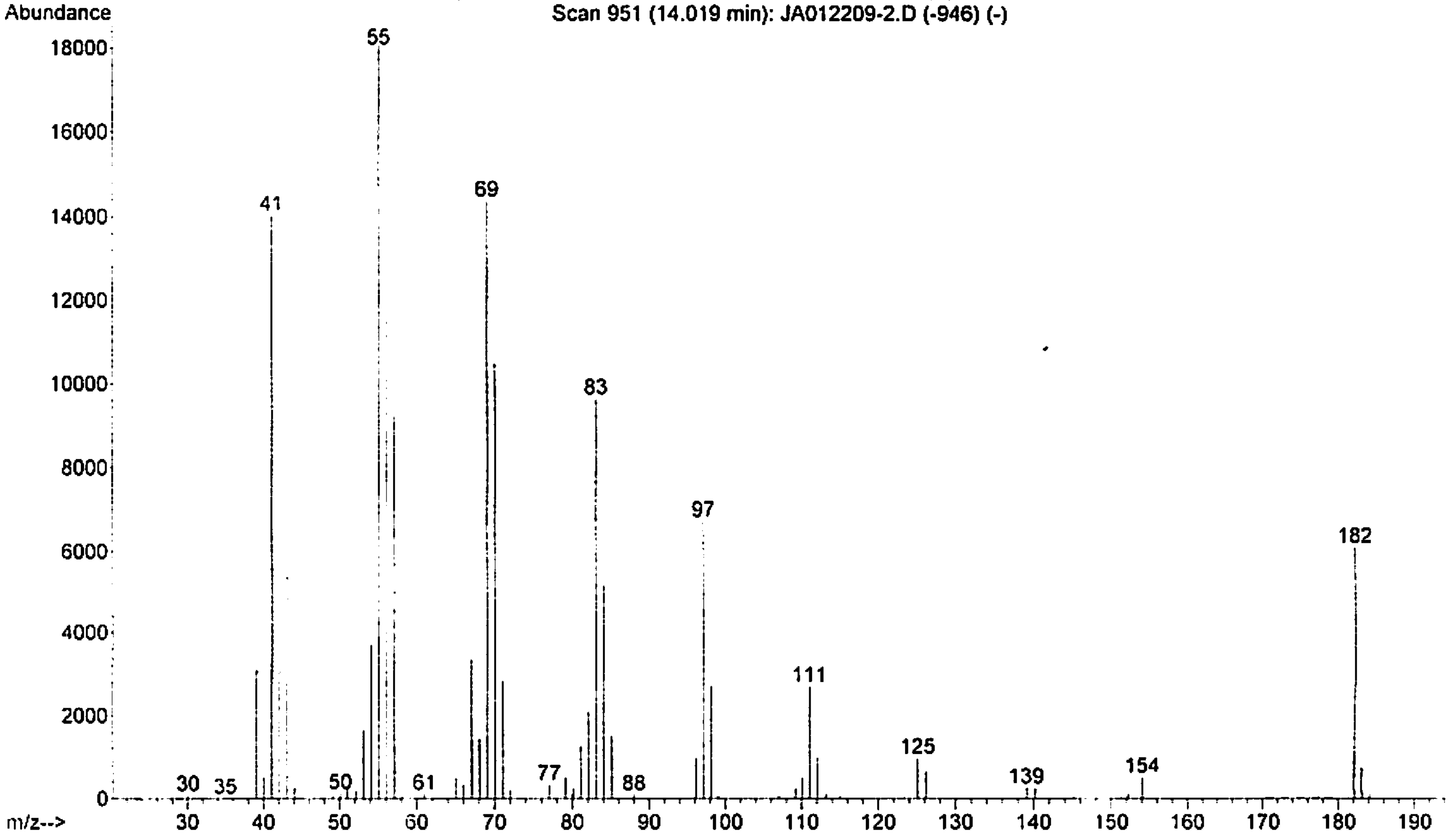

[illegible] :D:\DATA\ALDR CH\JA-09\Snapshot\JA012209-2.D
[illegible]rator : Aldrich
[illegible]ired : 22 Jan 2009 11:14 using AcqMethod JA-WAX08.4
[illegible]strument : Instrument #1
[illegible]mple Name: 9 male C.oculata abd.sternites/5ul CH2Cl2
[illegible]isc Info : 3-8-day-old; fed 1 wk 6-CH3-5-hepten-2-one
[illegible]al Number: 1

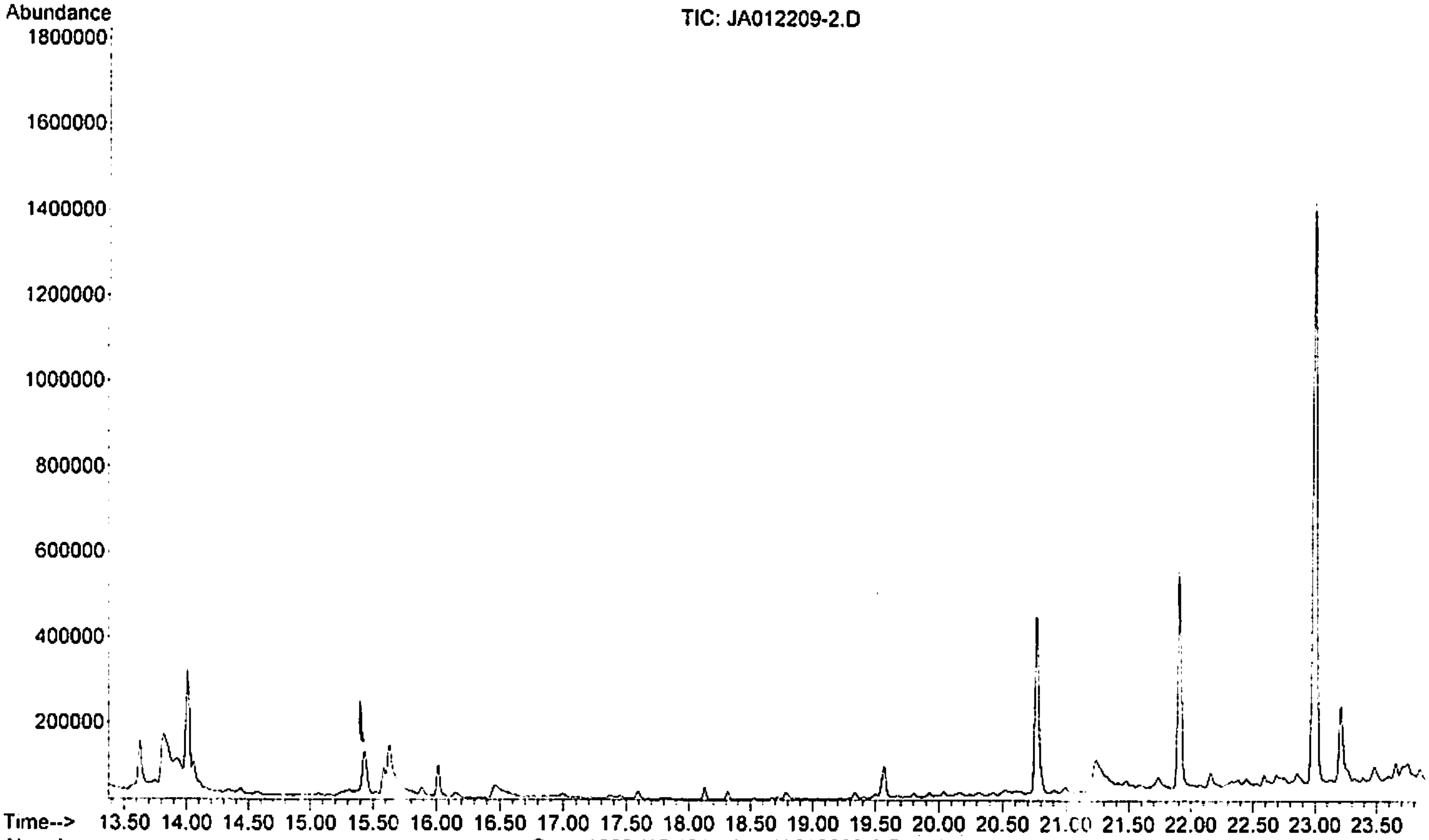


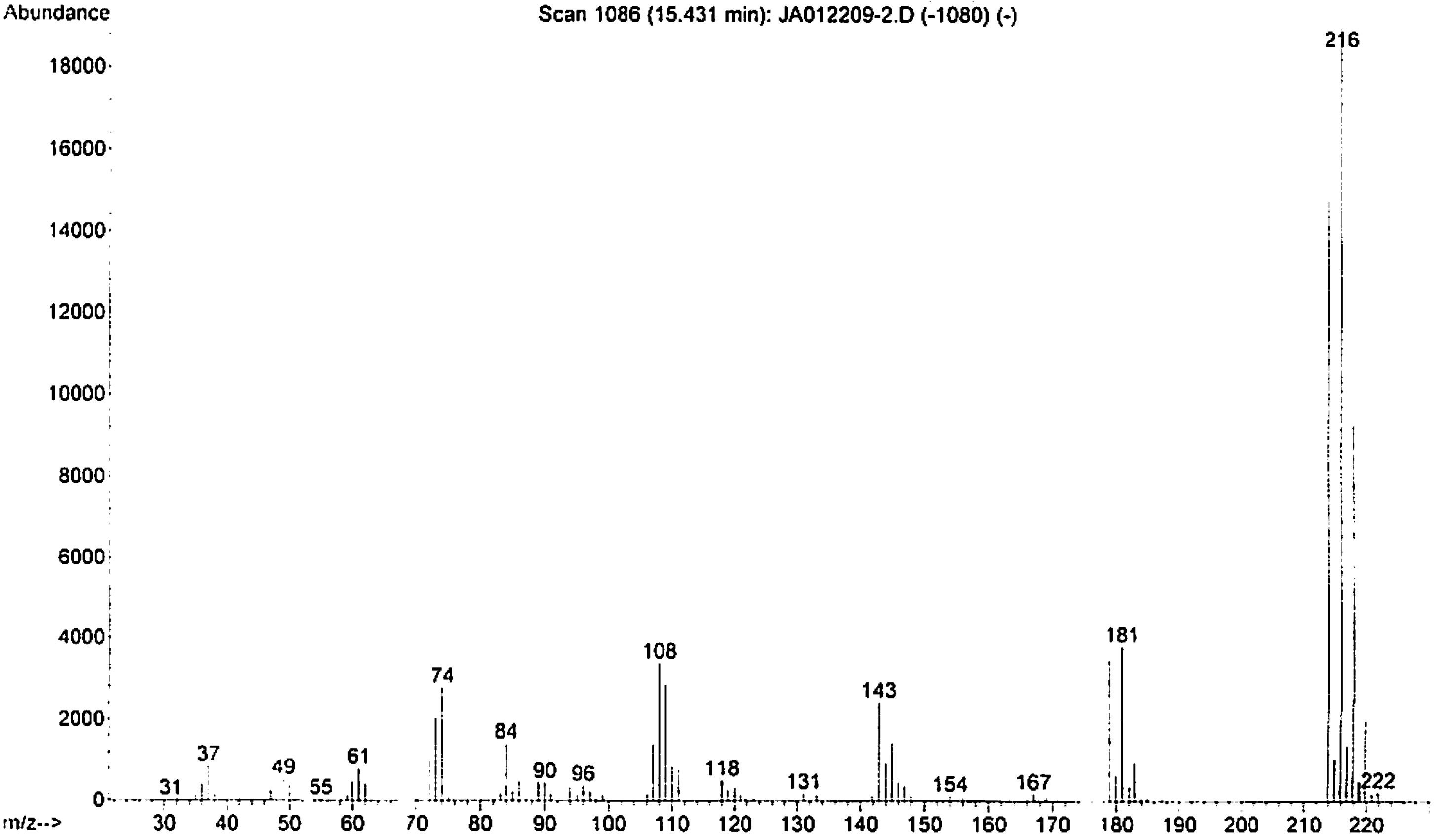

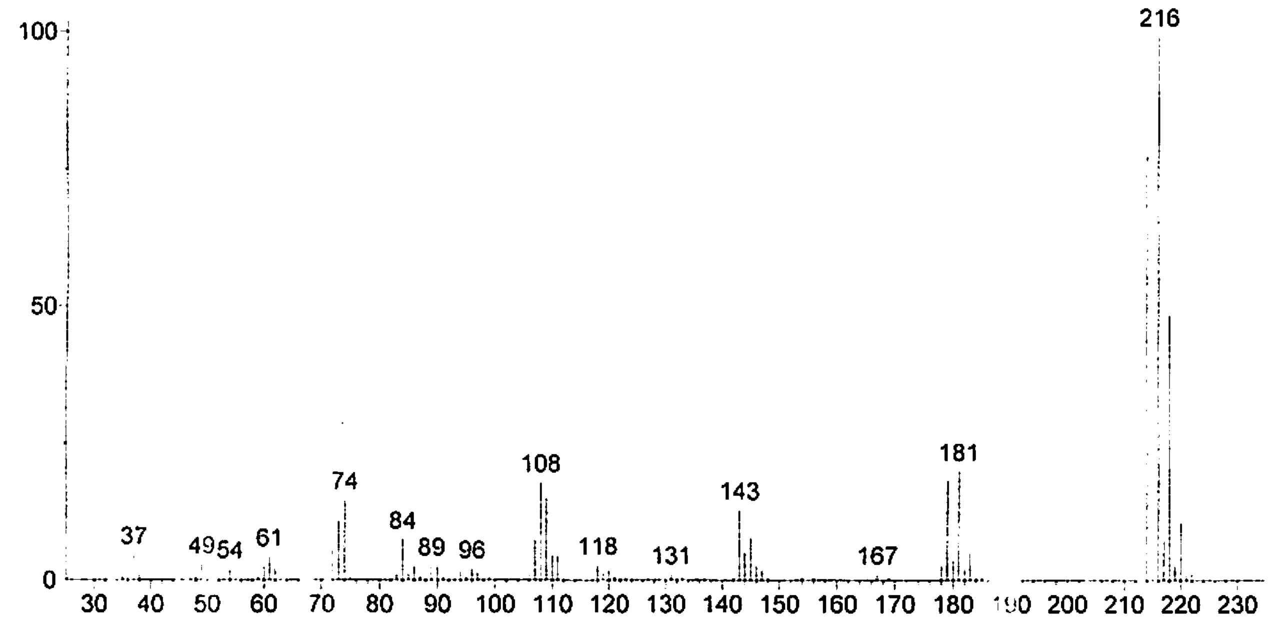


(Text File) Scan 1086 (15.431 min): JA012209-2.D (-1080)

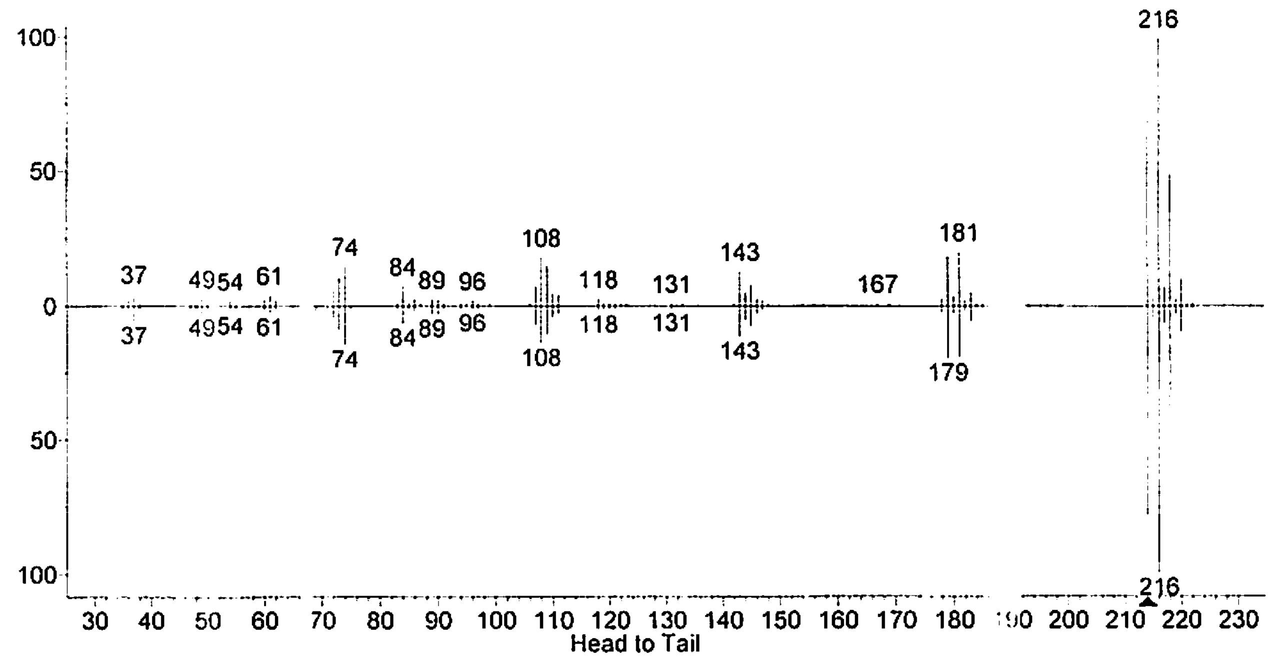


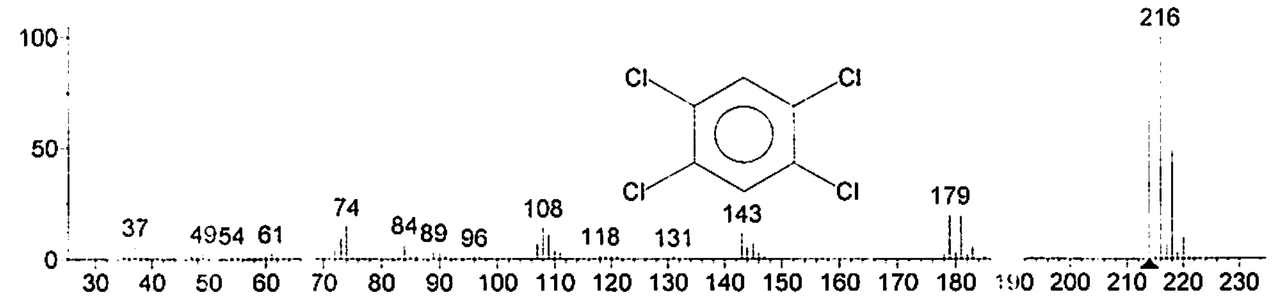


(replib) Benzene, 1,2,4,5-tetrachloro-

File : D:\DATA\ALDRICH\JA-09\Snapshot\JA012209-2.D
Operator : Aldrich
Acquired : 22 Jan 2009 11:14 using AcqMethod JA-WAX08.M
Instrument : Instrument #1
Sample Name: 9 male C.oculata abd.sternites/5ul CH2Cl2
Misc Info : 3-8-day-old; fed 1 wk 6-CH3-5-hepten-2-one
Vial Number: 1

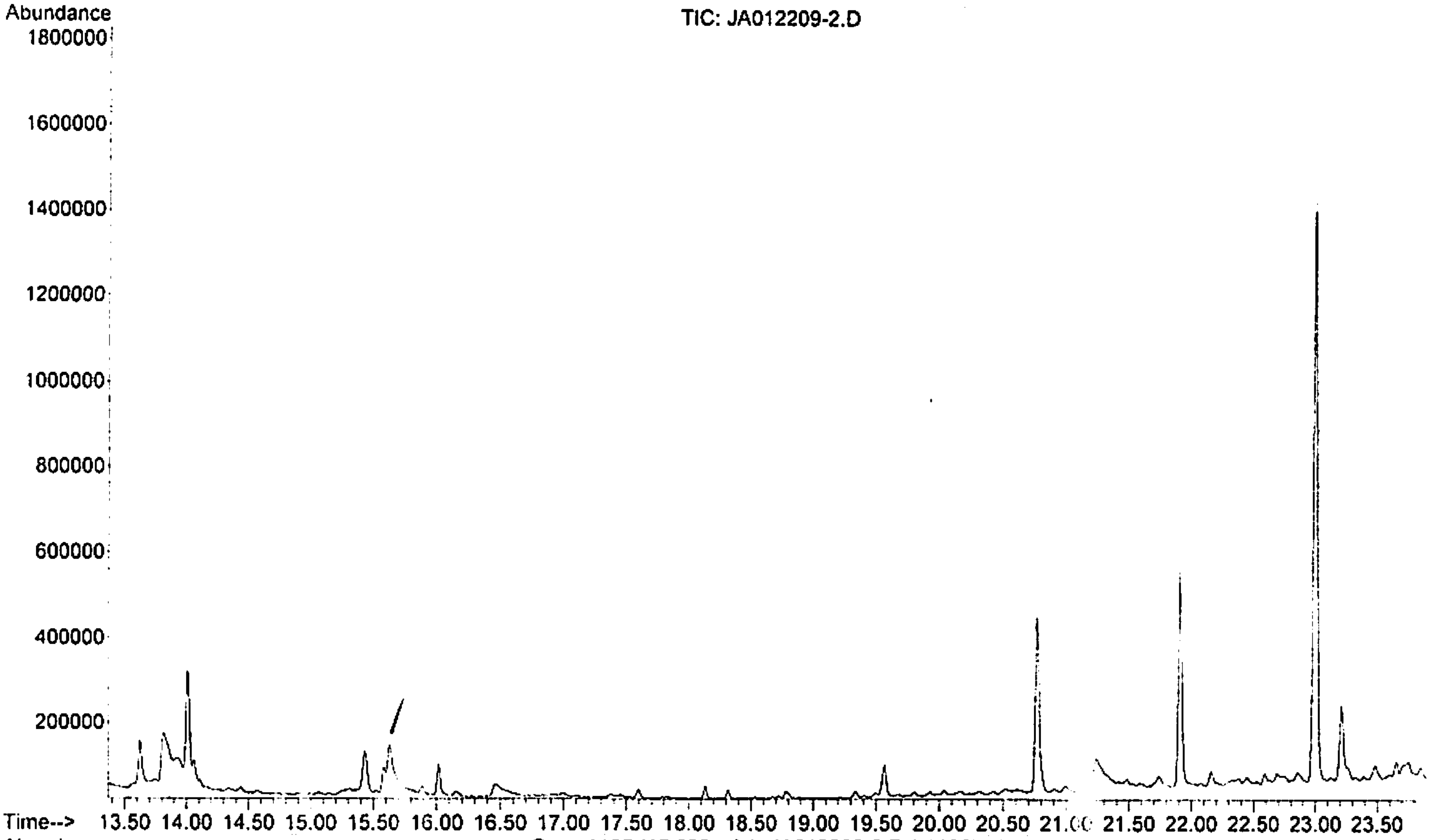


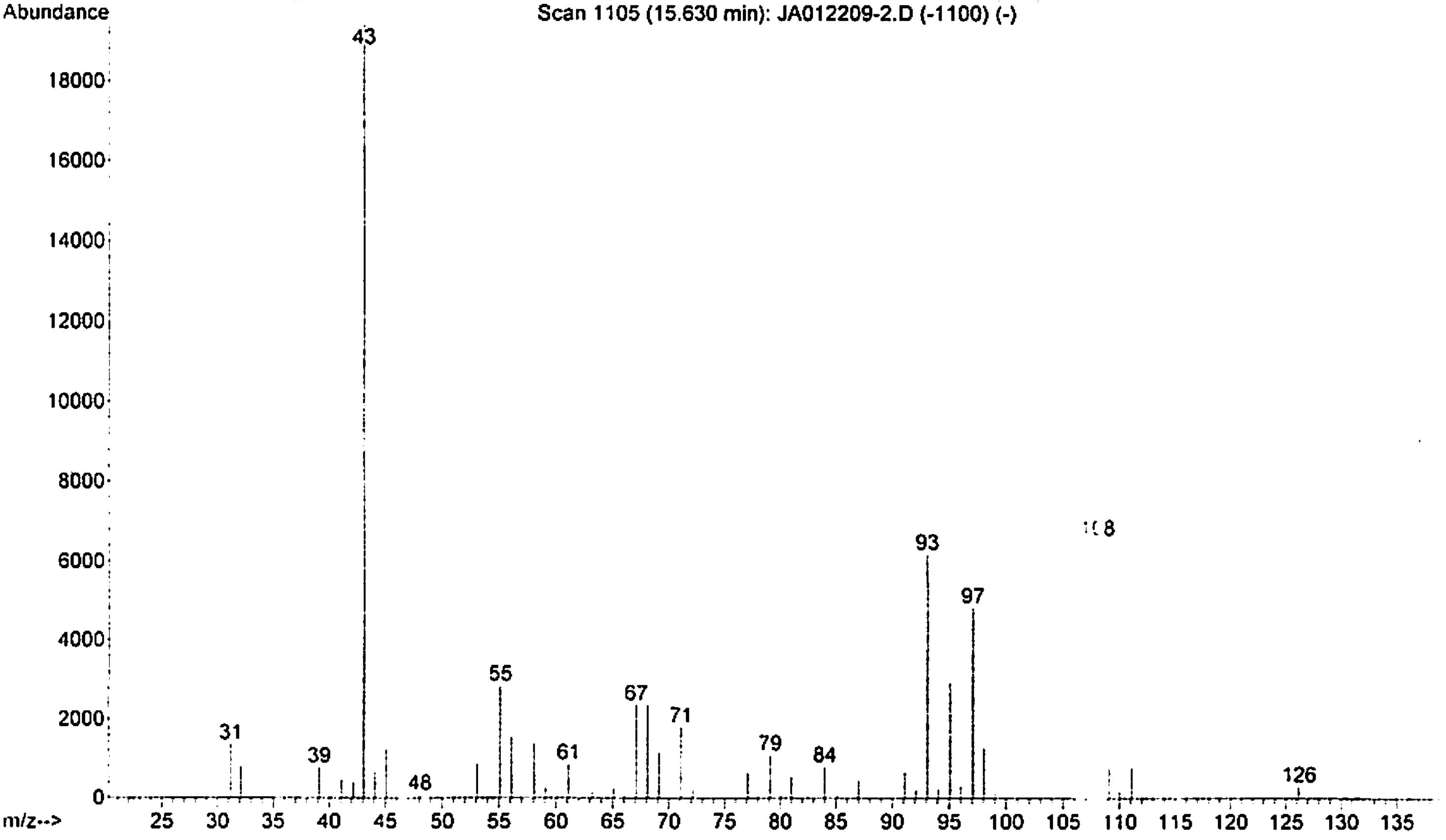

File : D:\DATA\ALDRICH\JA-09\Snapshot\JA012209-2.D
Operator : Aldrich
Acquired : 22 Jan 2009 11:14 using AcqMethod JA-WAX08.M
Instrument : Instrument #1
Sample Name: 9 male C.oculata abd.sternites/5ul CH2C12
Misc Info : 3-8-day-old; fed 1 wk 6-CH3-5-hepten-2-one
Vial Number: 1

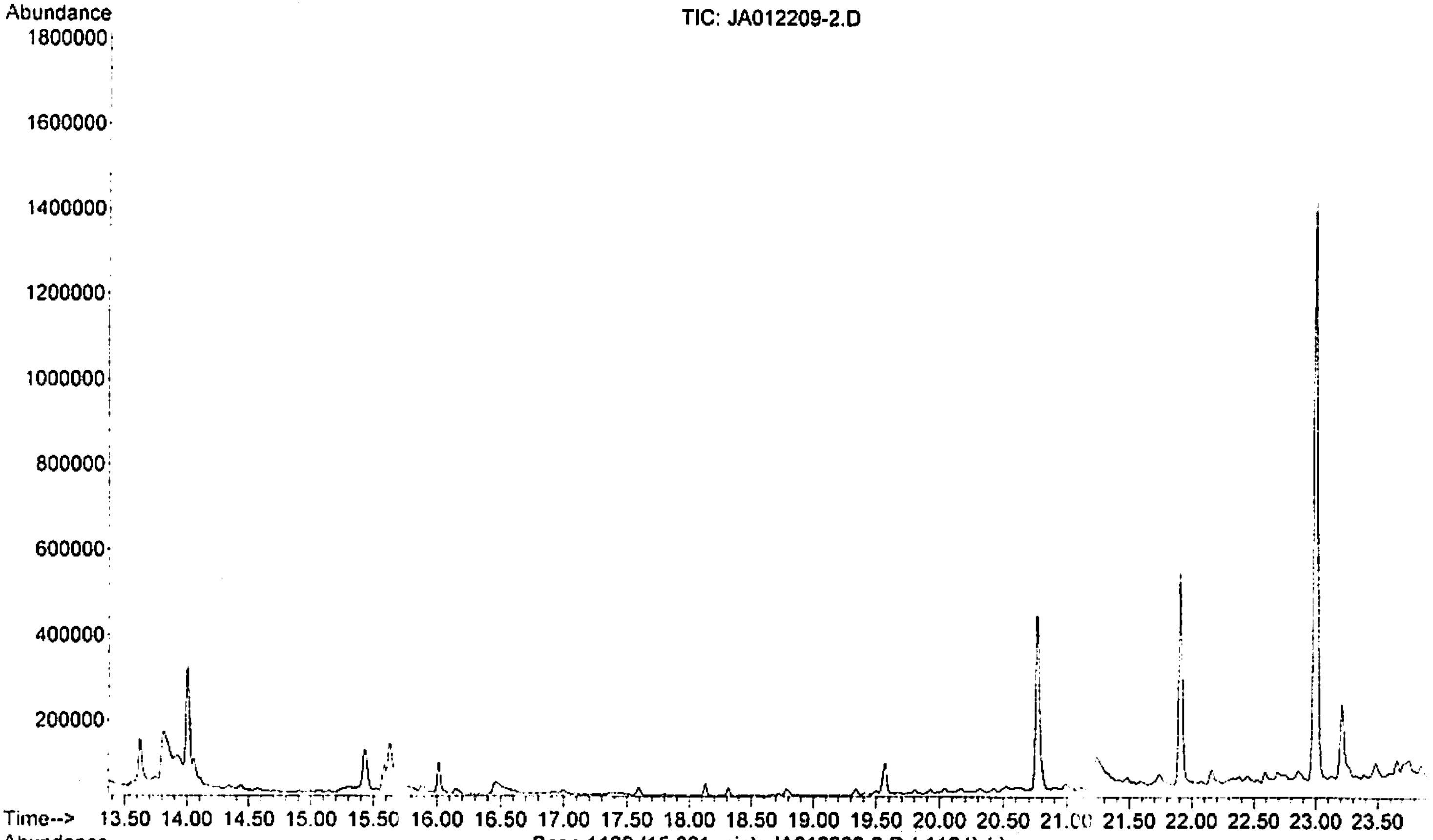


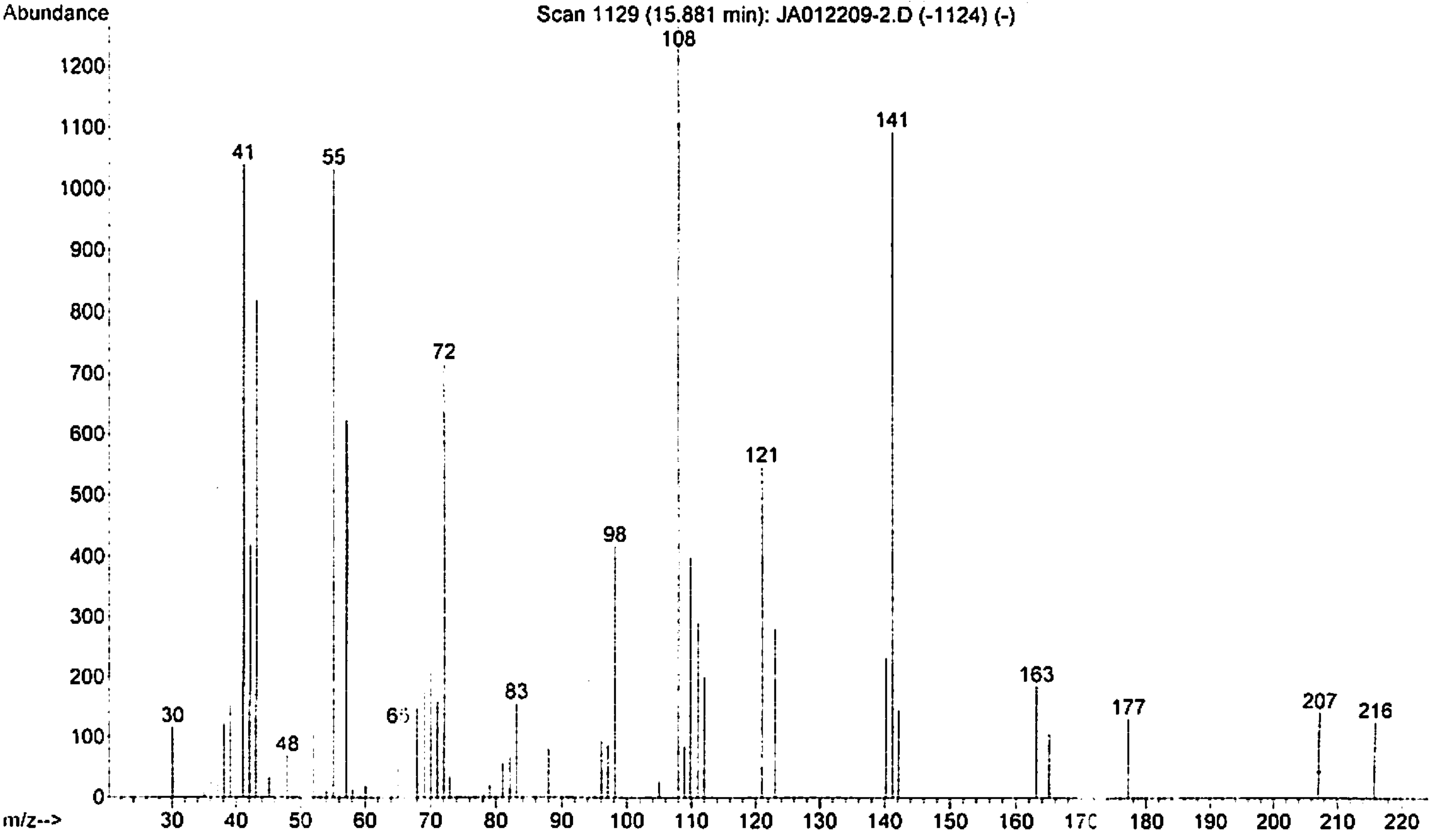

[illegible] : D:\DATA\ALDR[illegible]CH\JA-09\Snapshot\JA012209-2.D
[illegible]rator : Aldrich
[illegible]quired : 22 Jan 2009 11:14 using AcqMethod JA-WAX08.M
[illegible]strument : Instrument #1
[illegible]ple Name: 9 male C.oculata abd.sternites/5ul CH2Cl2
[illegible] Info : 3-8-day-old; fed 1 wk 6-CH3-5-hepten-2-one
[illegible] Number: 1

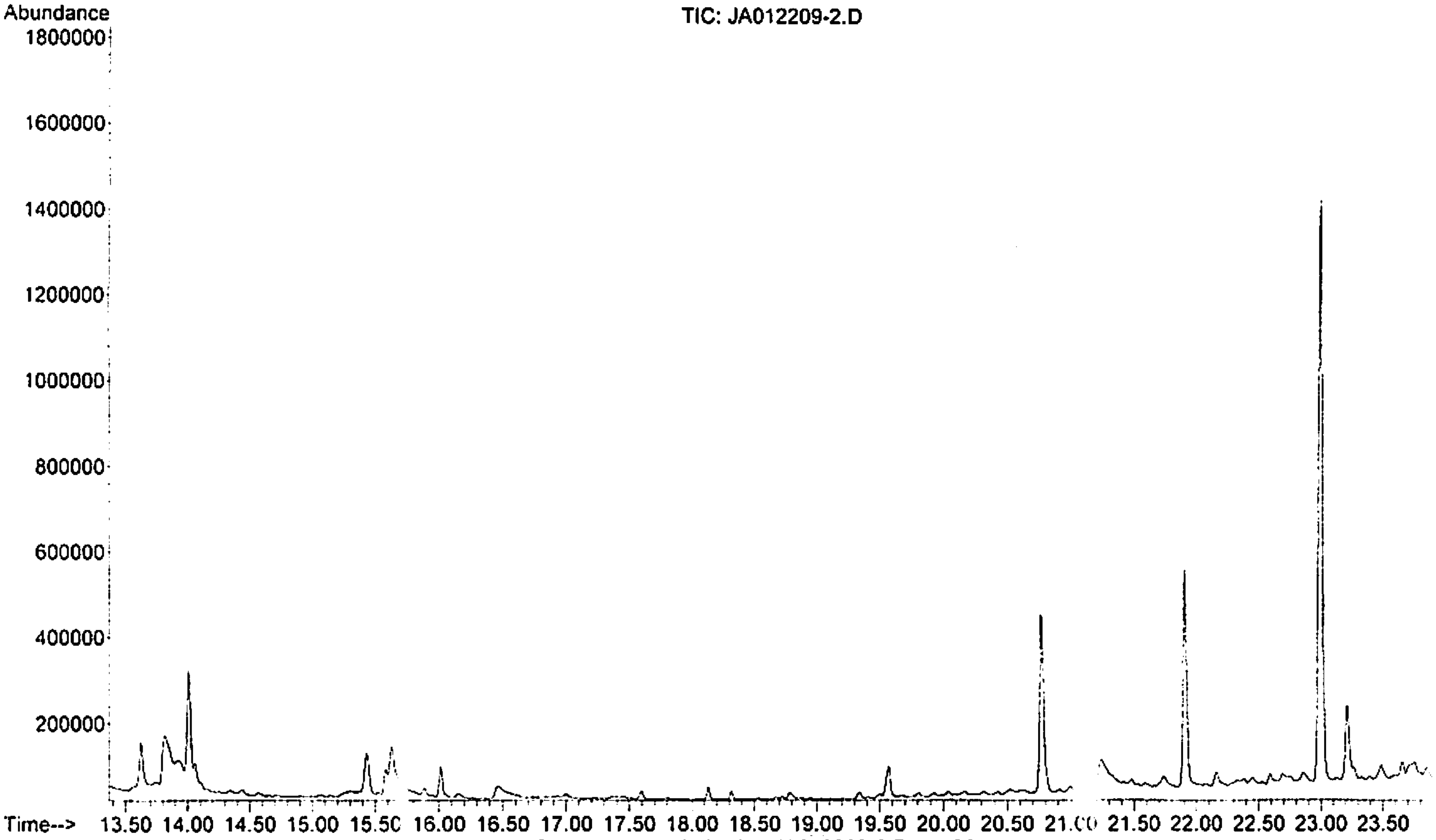


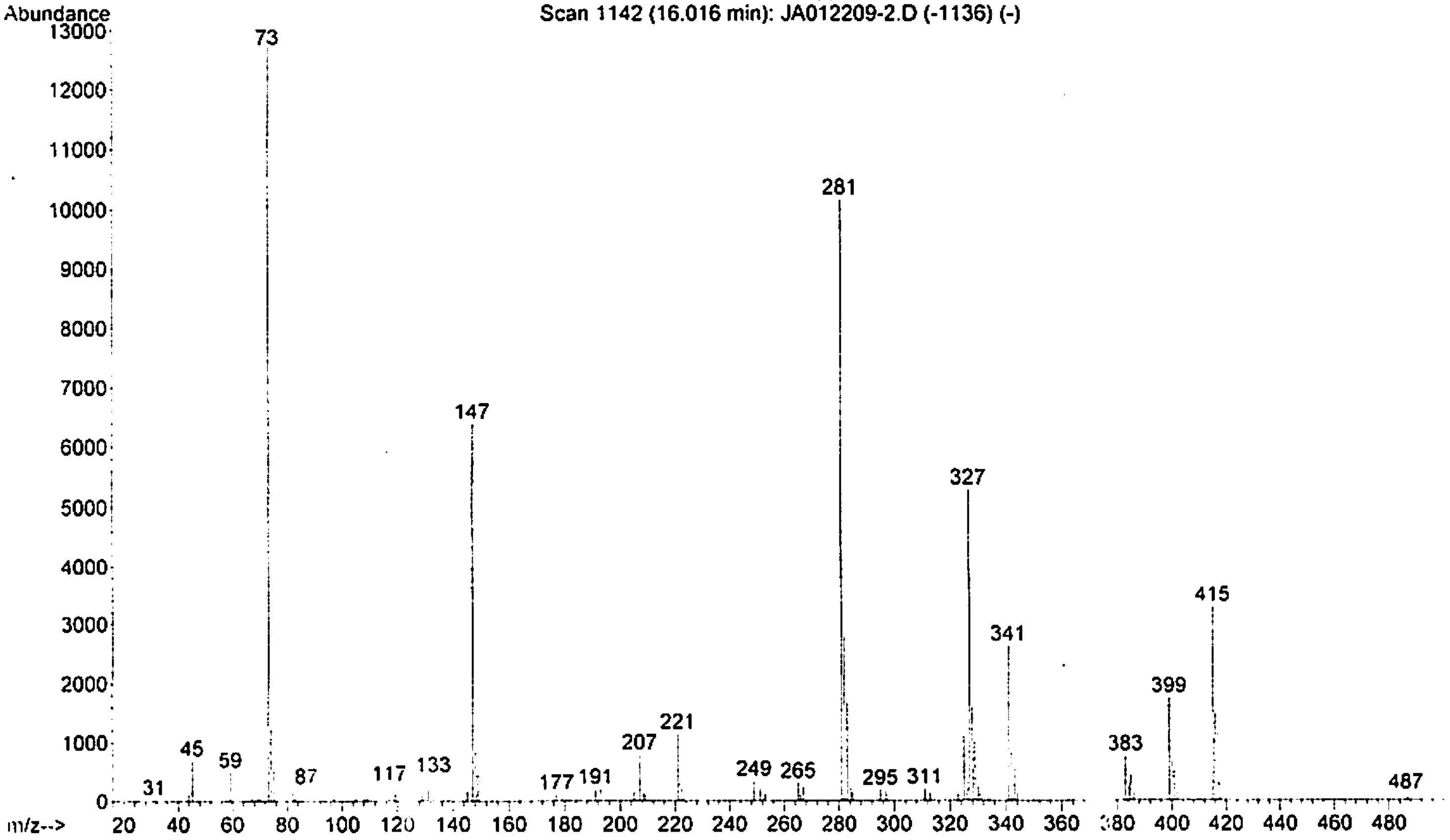

[illegible] : D:\DATA\ALDR CH\JA-09\Snapshot\JA012209-2.D
[illegible]rator : Aldrich
[illegible]uired : 22 Jan 2009 11:14 using AcqMethod JA-WAX08.4
[illegible]strument : Instrument #1
[illegible]mple Name: 9 male C.oculata abd.sternites/5ul CH2Cl2
[illegible]sc Info : 3-8-day-old; fed 1 wk 6-CH3-5-hepten-2-one
[illegible]al Number: 1

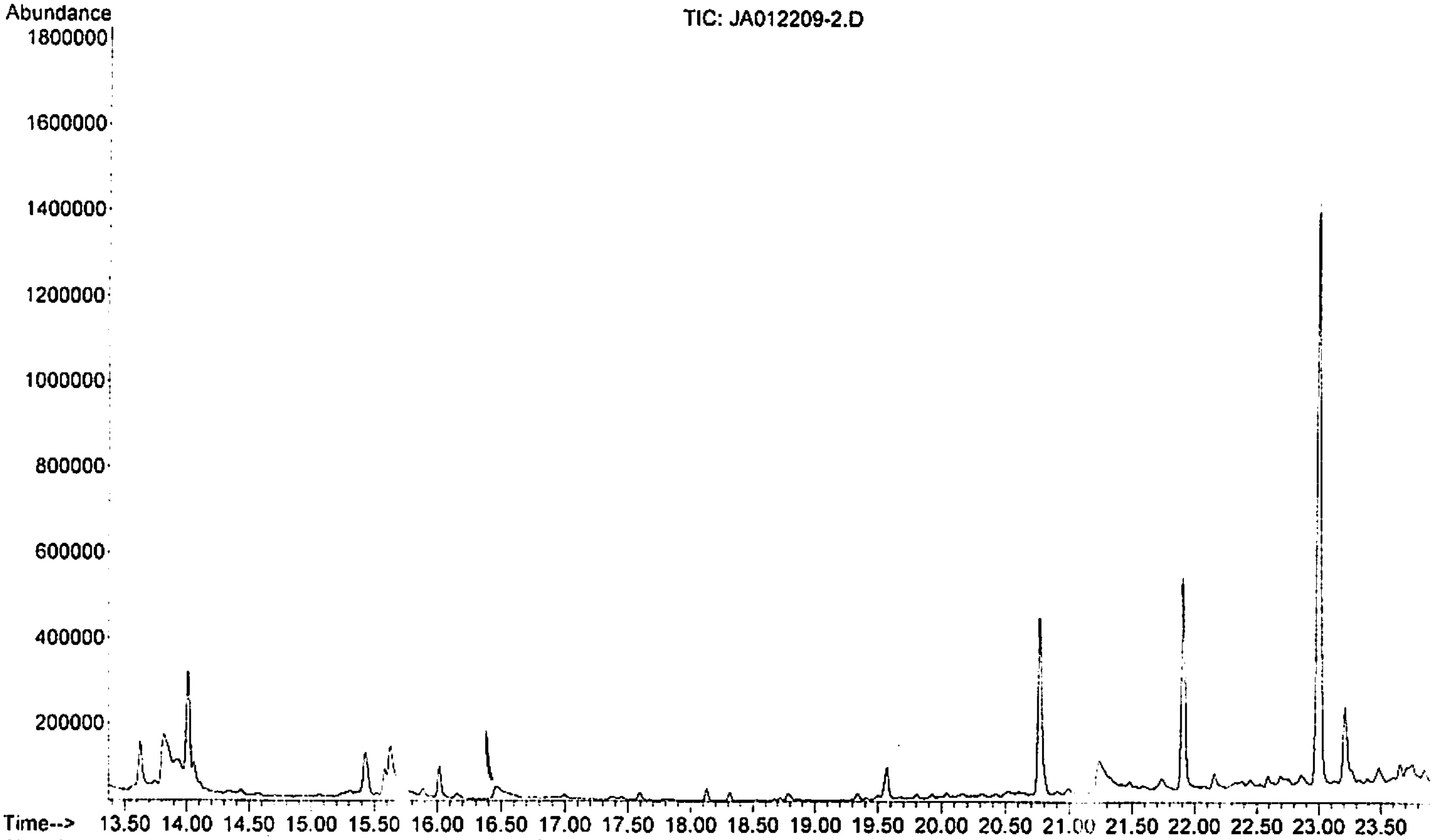


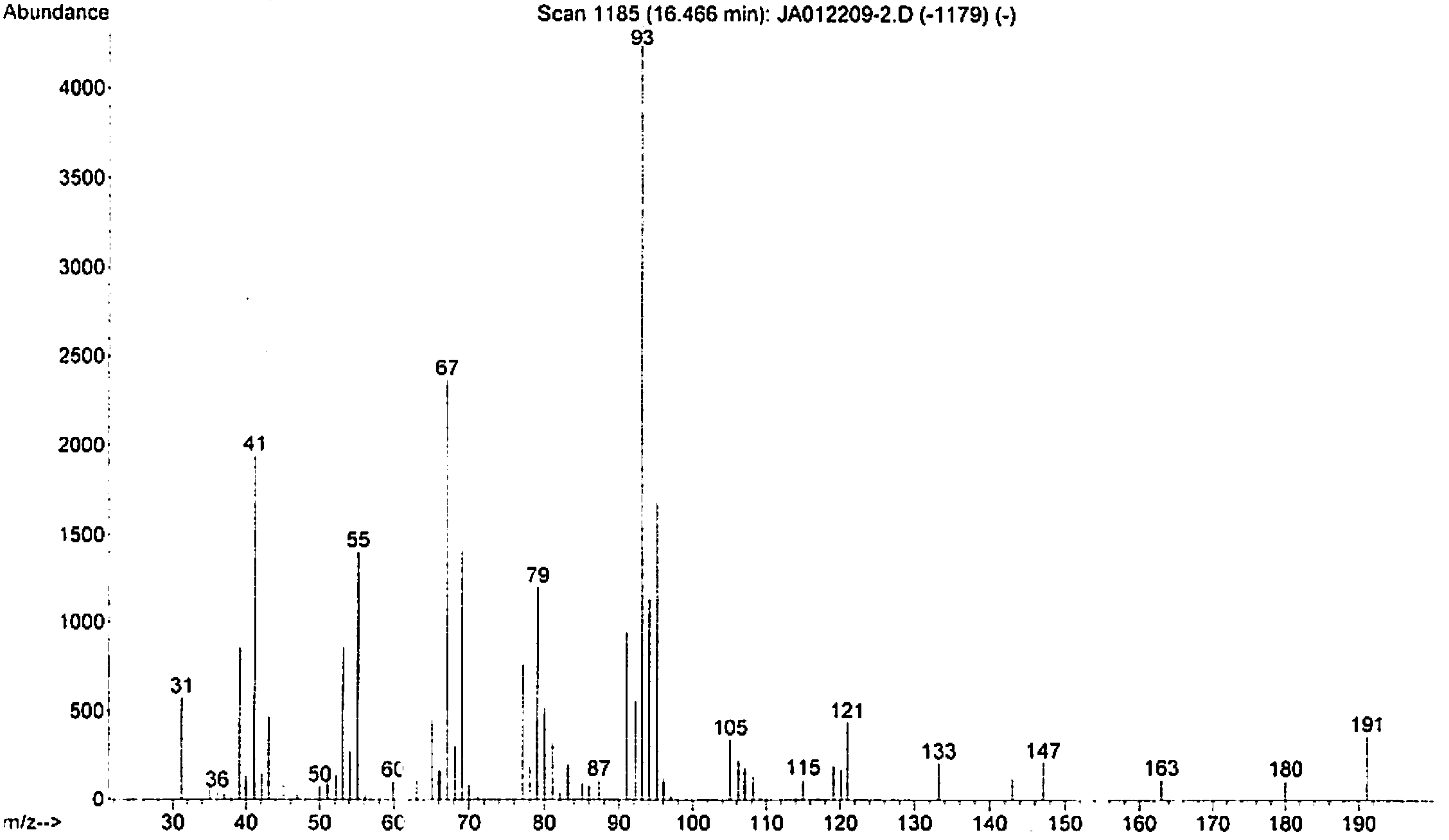

File :D:\DATA\ALDRICH\JA-09\Snapshot\JA012209-2.D
Operator : Aldrich
Acquired : 22 Jan 2009 11:14 using AcqMethod JA-WAX08.4
Instrument : Instrument #1
Sample Name: 9 male C.oculata abd.sternites/5ul CH2Cl2
Misc Info : 3-8-day-old, fed 1 wk 6-CH3-5-hepten-2-one
Vial Number: 1

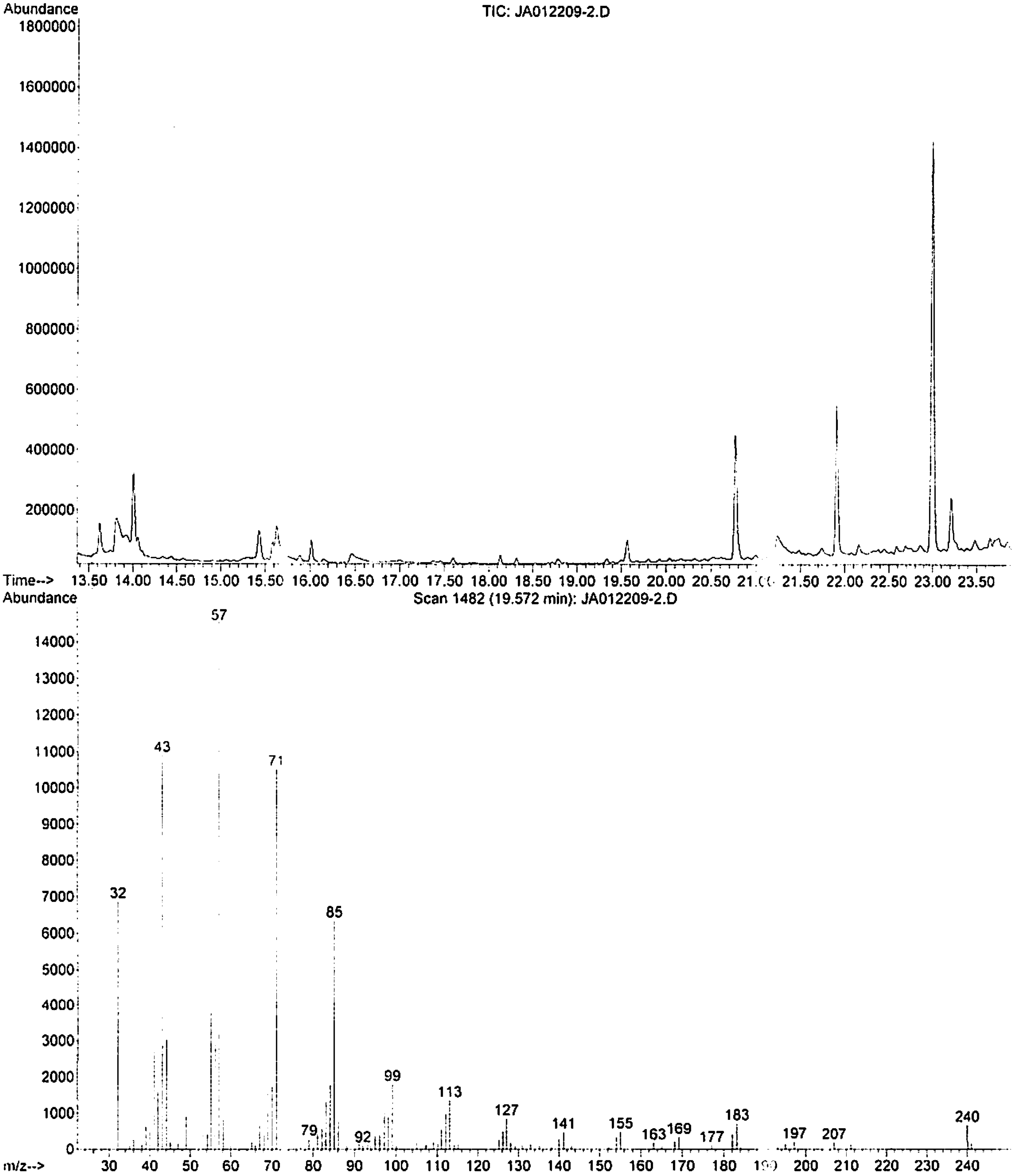

[illegible]e :D:\DATA\ALDR CH\JA-09\Snapshot\JA012209-2.D
[illegible]erator : Aldrich
[illegible]quired : 22 Jan 2009 11:14 using AcqMethod JA-WAX08.[illegible]
[illegible]strument : Instrument #1
[illegible]mple Name: 9 male C.oculata abd.sternites/5ul CH2Cl2
[illegible] Info : 3-8-day-old; fed 1 wk 6-CH3-5-hepten-2-one
[illegible]al Number: 1

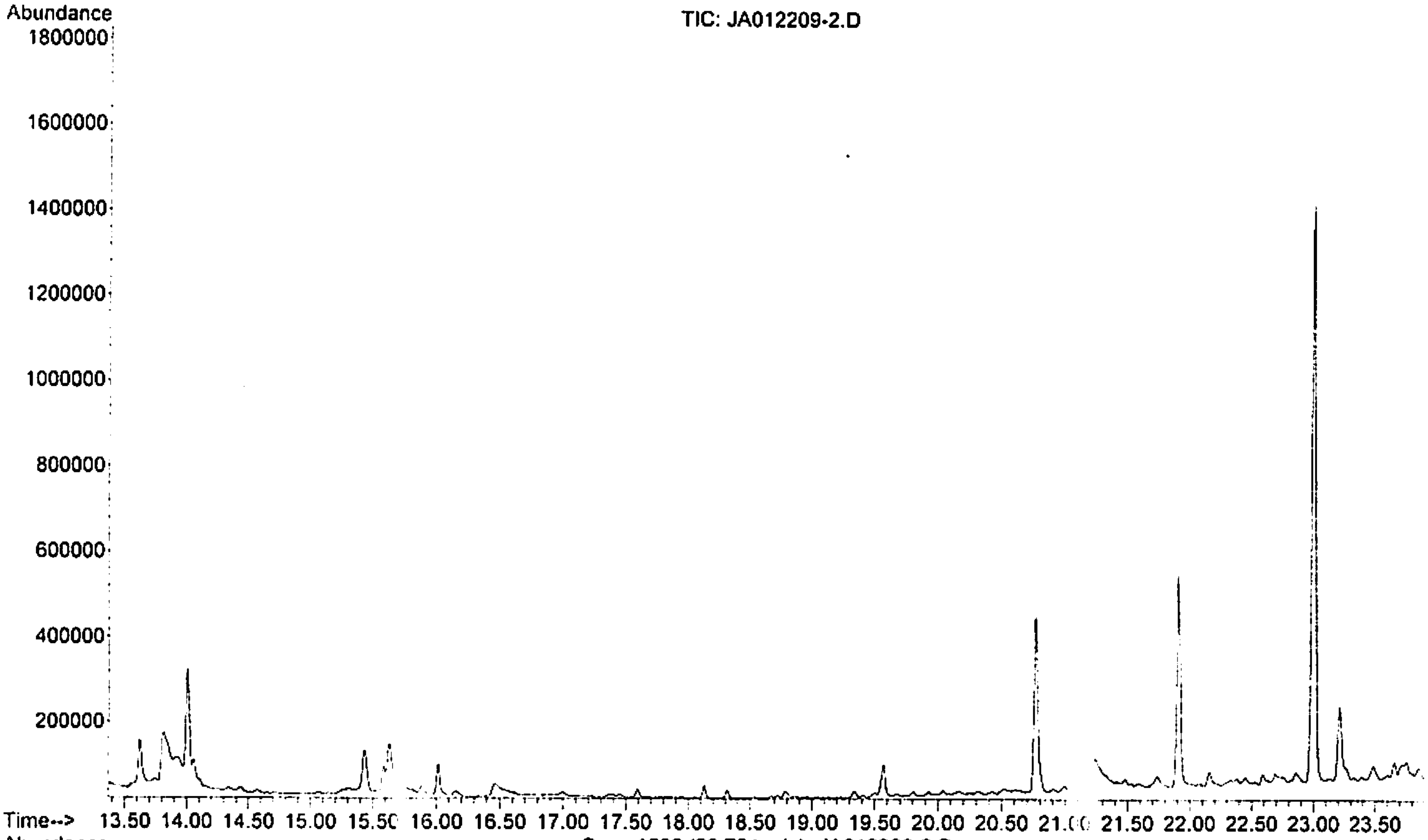


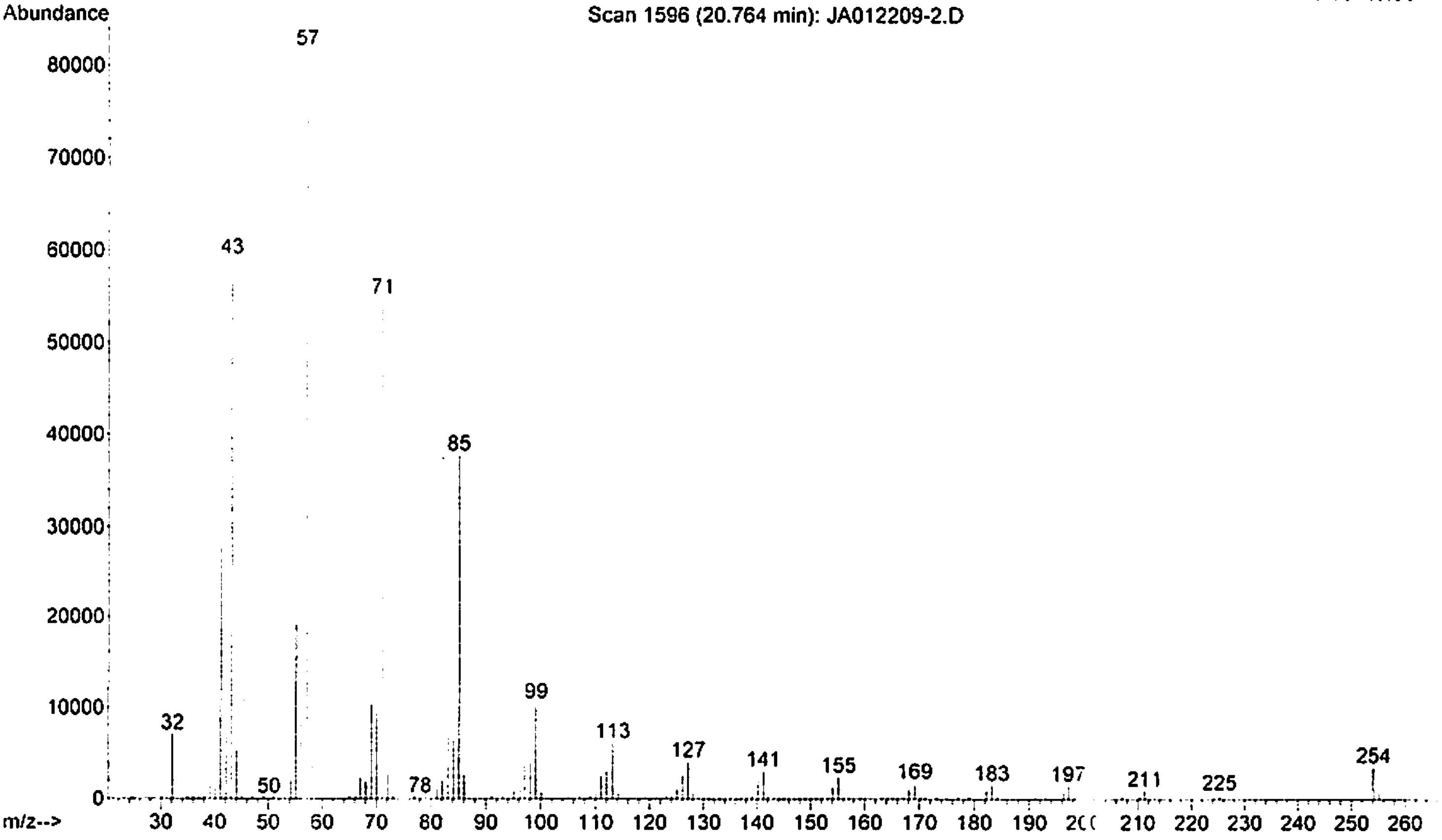

File : D:\DATA\ALDRICH\JA-09\Snapshot\JA012209-2.D
Operator : Aldrich
Acquired : 22 Jan 2009 11:14 using AcqMethod JA-WAX08.M
Instrument : Instrument #1
Sample Name: 9 male C.oculata abd.sternites/5ul CH2Cl2
Misc Info : 3-8-day-old; fed 1 wk 6-CH3-5-hepten-2-one
Vial Number: 1

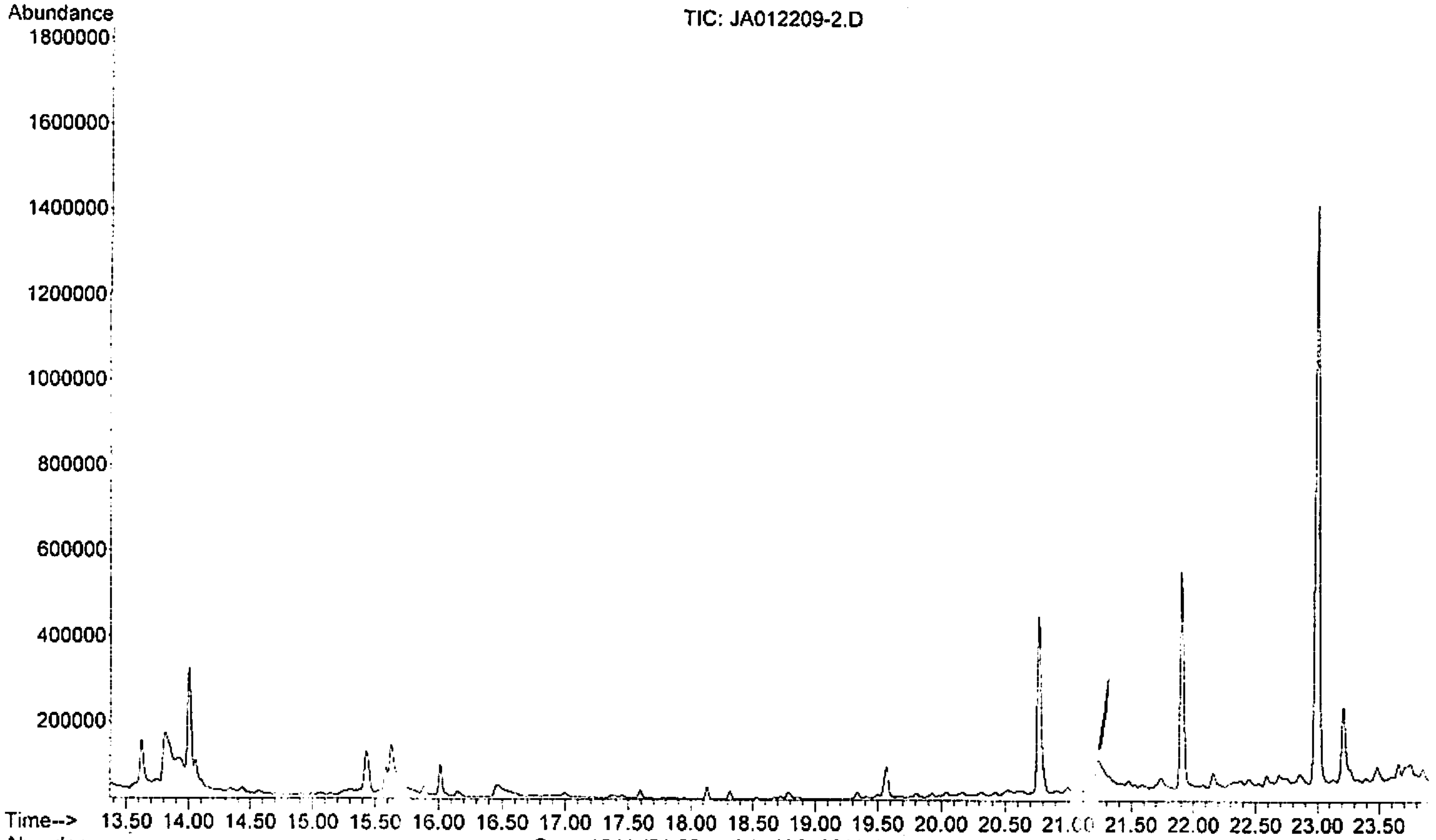


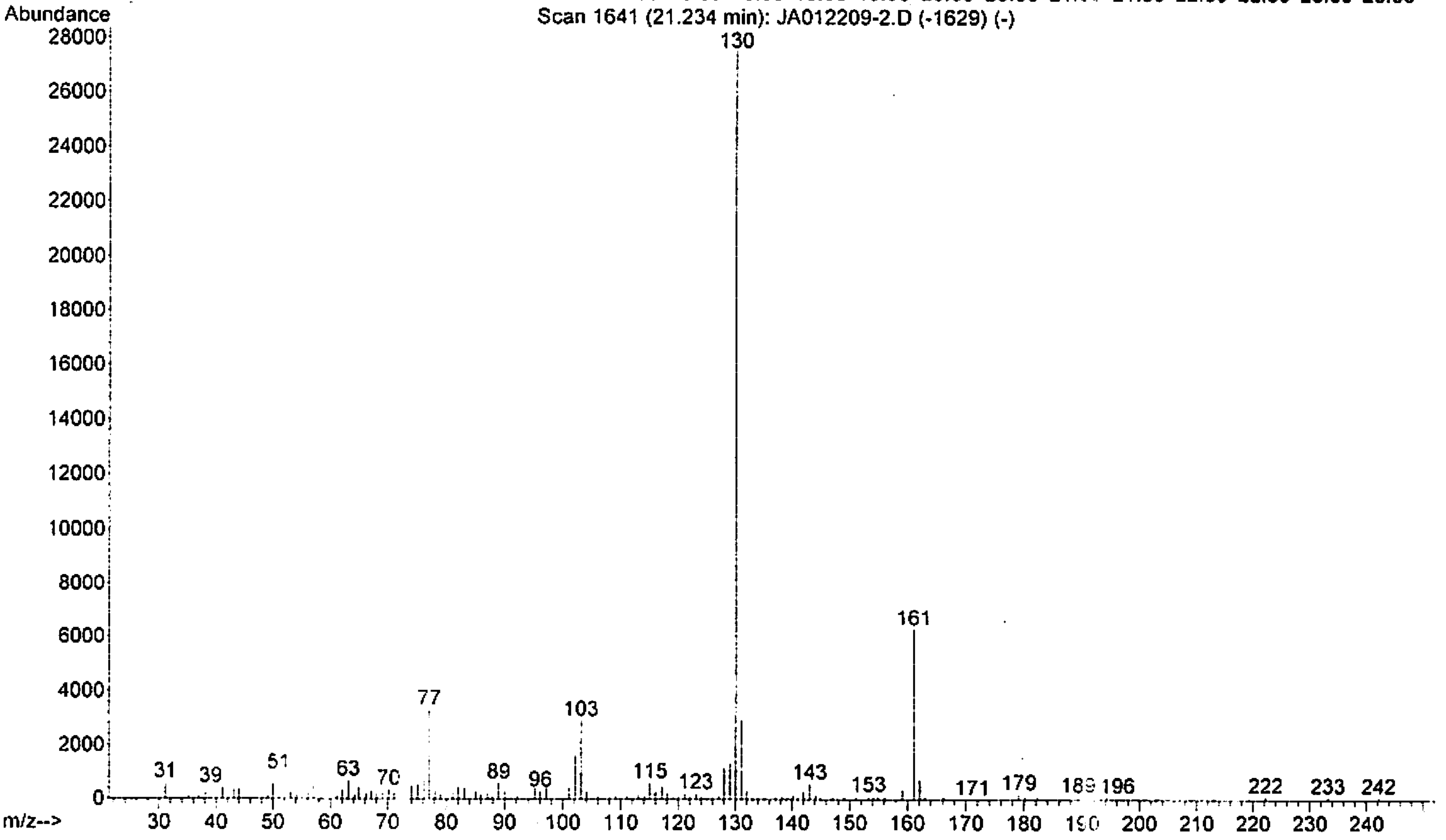

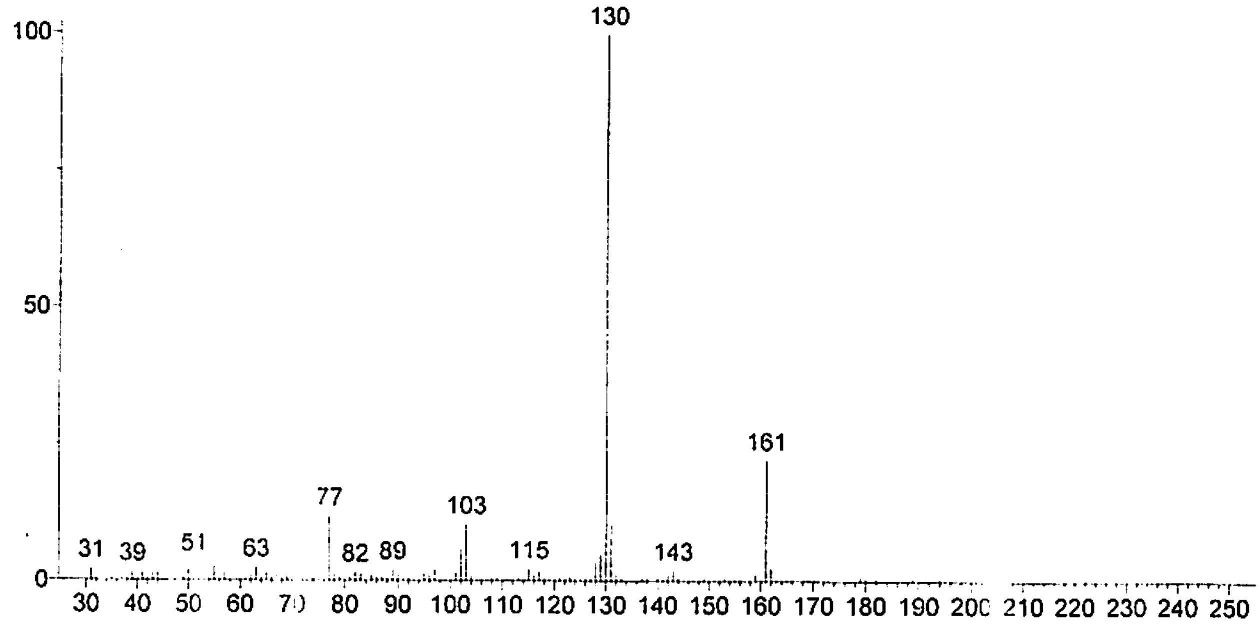


(Text File) Scan 1641 (21.2[illegible]4 min): JA012209-2.D (-1629)

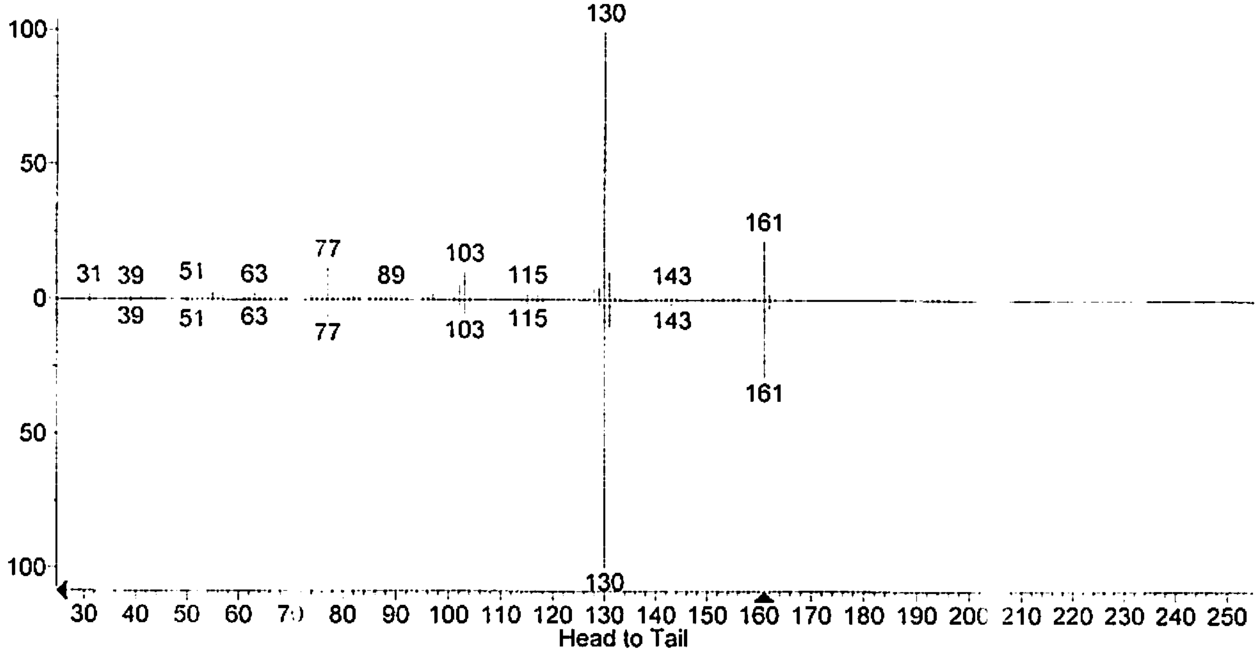


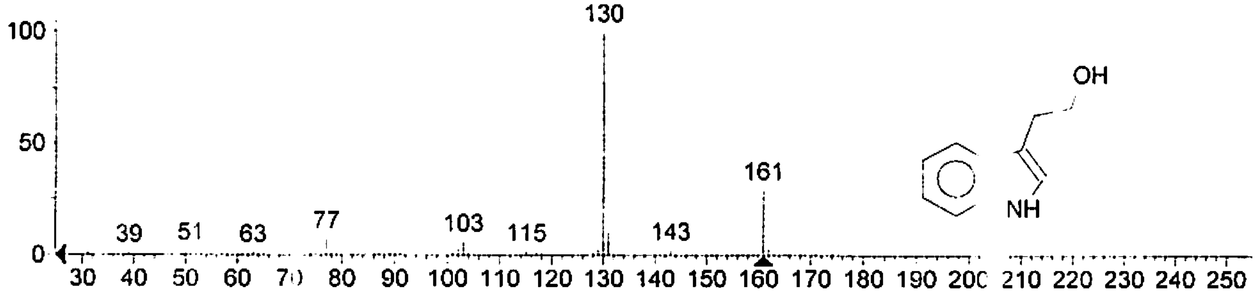


(mainlib) 1H-Indole-3-ethanol

[illegible]le : D:\DATA\ALDR CH\JA-09\Snapshot\JA012209-2.D
[illegible]rator : Aldrich
[illegible]quired : 22 Jan 2009 11:14 using AcqMethod JA-WAX08 4
[illegible]strument : Instrument #1
[illegible]mple Name: 9 male C.oculata abd.sternites/5ul CH2Cl2
[illegible]sc Info : 3-8-day-old; fed 1 wk 6-CH3-5-hepten-2-one
[illegible]al Number: 1

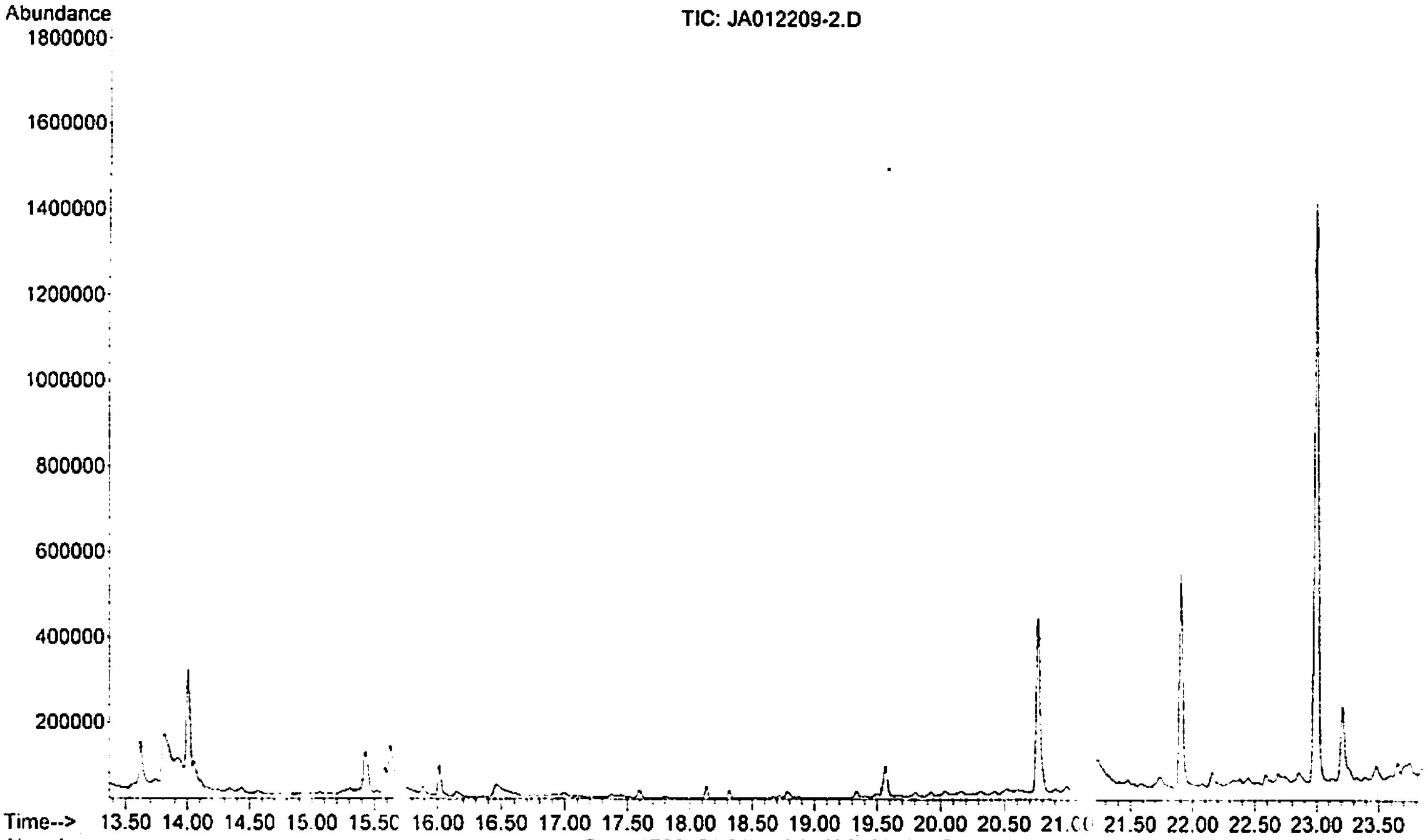


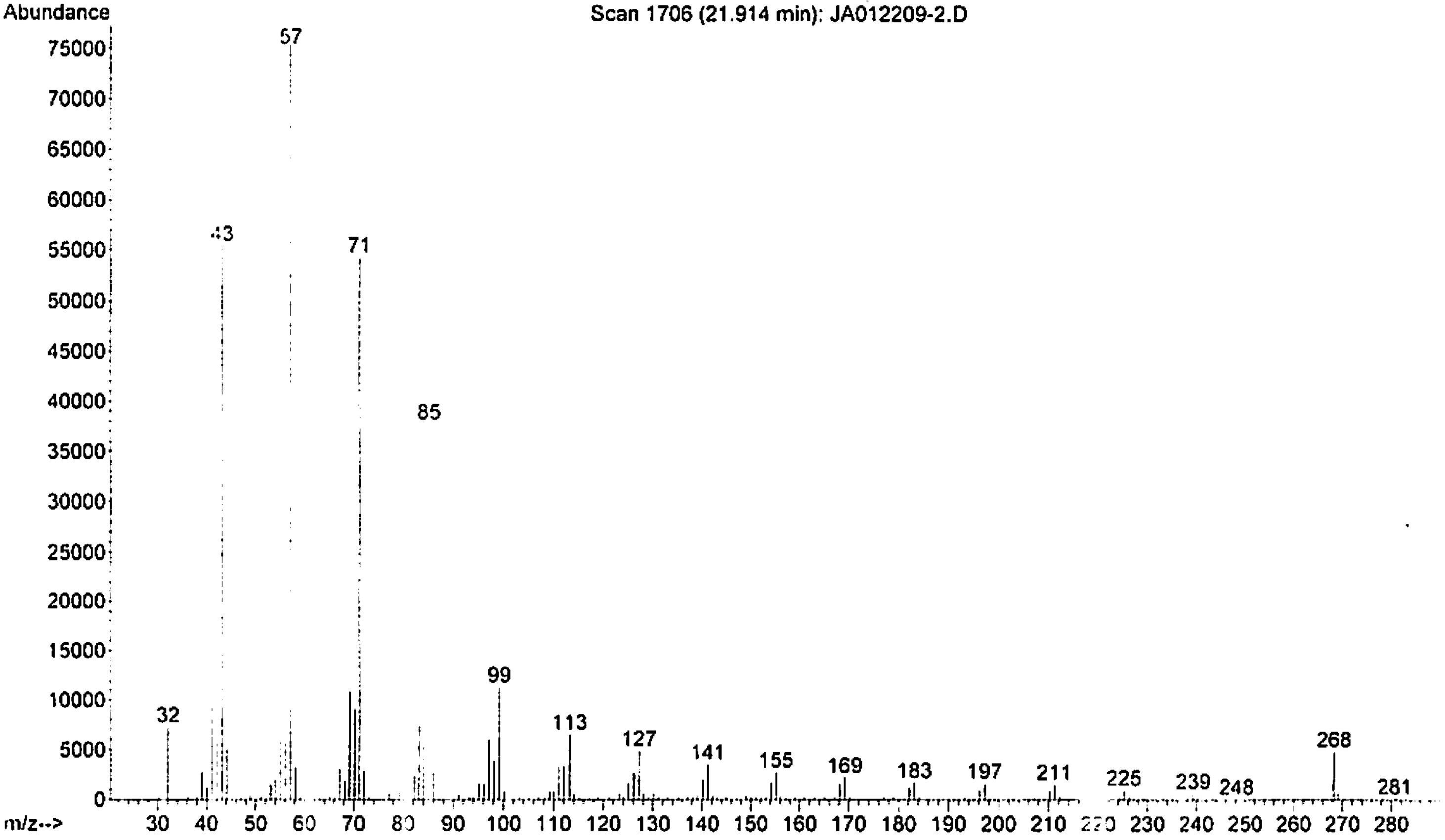

File : D:\DATA\ALDRICH\JA-09\Snapshot\JA012209-2.D
Operator : Aldrich
Acquired : 22 Jan 2009 11:14 using AcqMethod JA-WAX08.4
Instrument : Instrument #1
Sample Name: 9 male C.oculata abd.sternites/5ul CH2Cl2
Misc Info : 3-8-day-old; fed 1 wk 6-CH3-5-hepten-2-one
Vial Number: 1

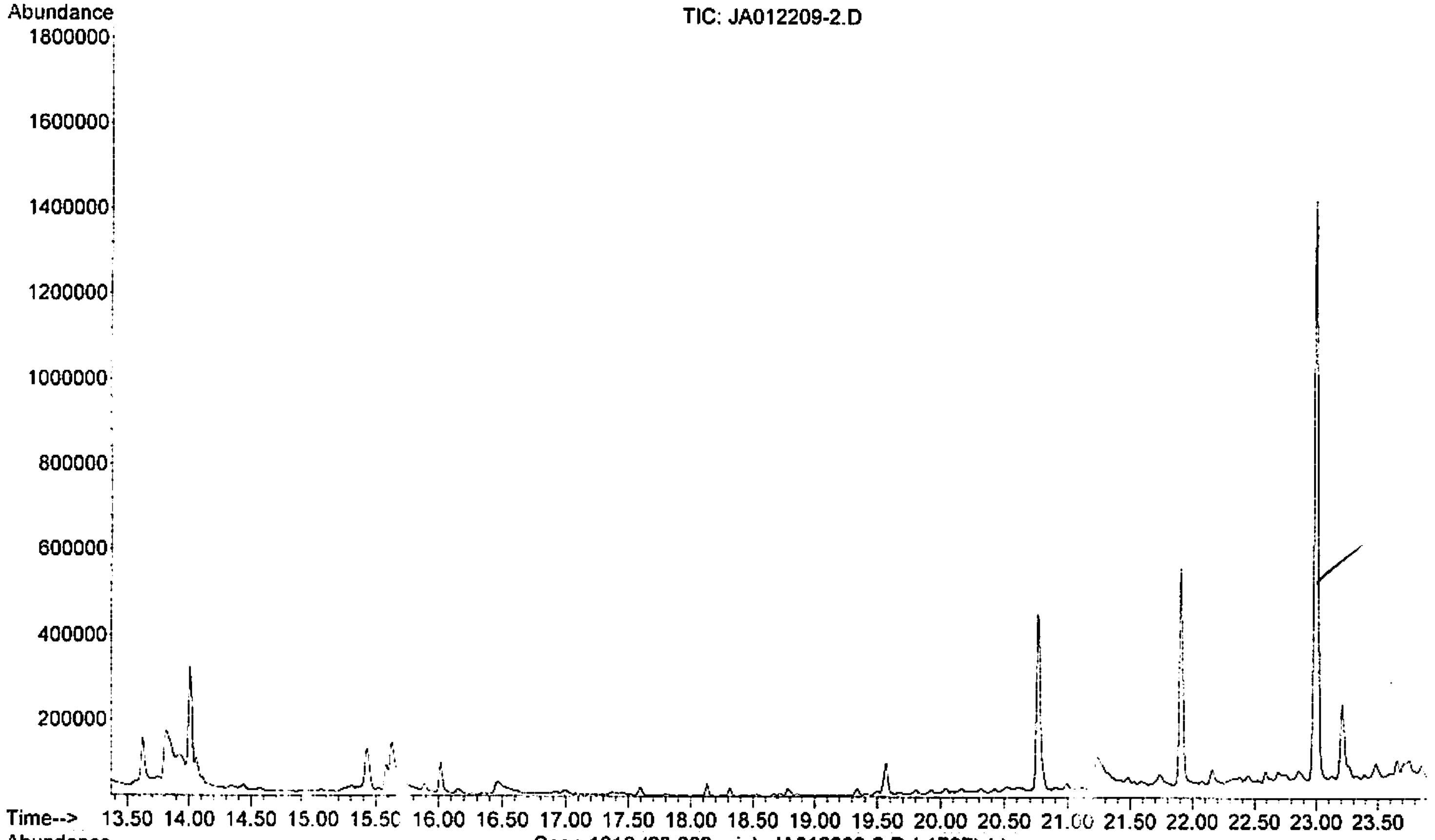


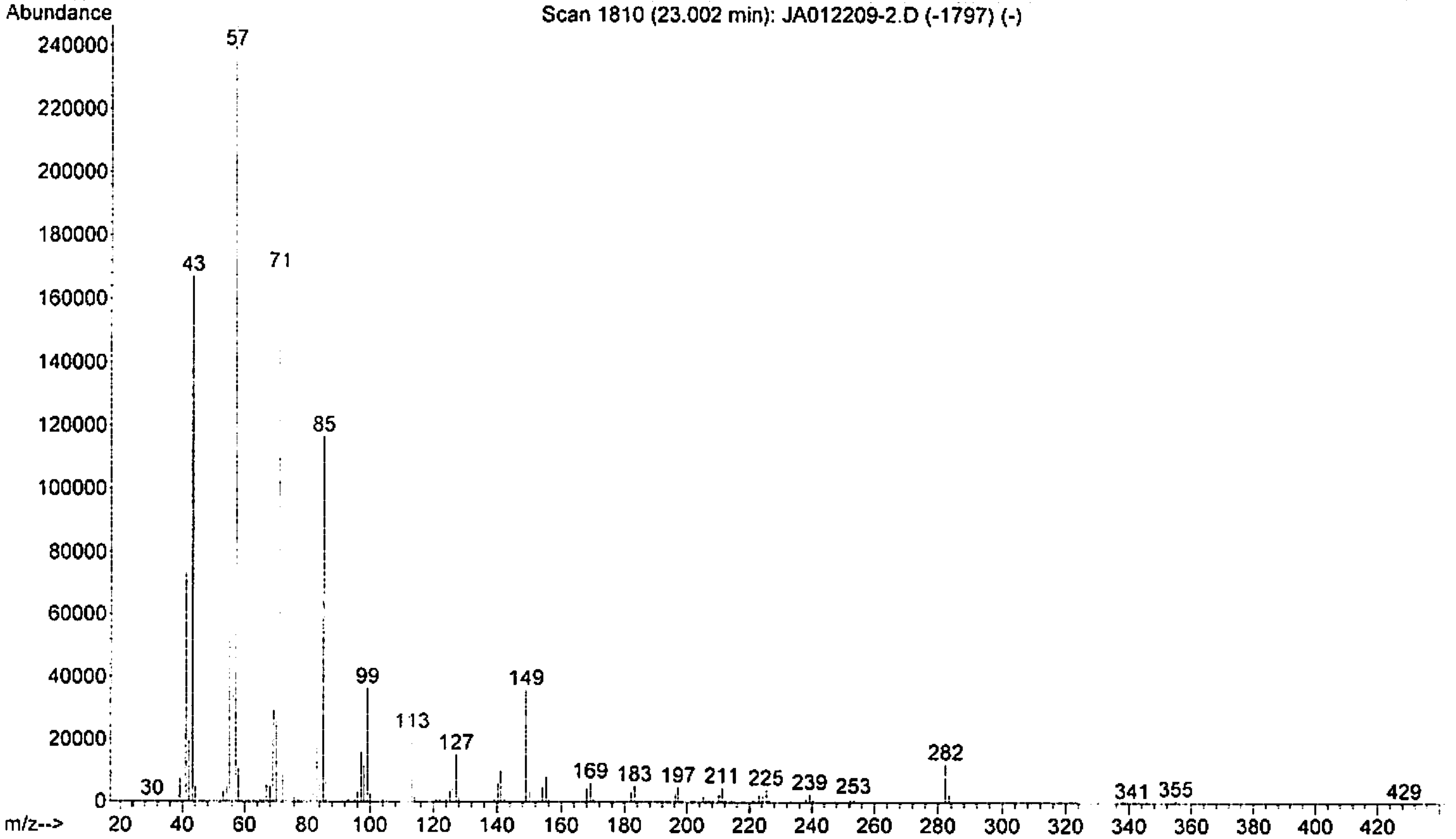

```
File      :D:\DATA\ALDR CH\JA-09\Snapshot\JA012209-2.D
Operator  : Aldrich
Acquired  : 22 Jan 2009  11:14     using AcqMethod JA-WAX08.4
Instrument :     Instrument #1
Sample Name: 9 male C.oculata abd.sternites/5ul CH2Cl2
Misc Info  : 3-8-day-old; fed 1 wk 6-CH3-5-hepten-2-one
Vial Number: 1
```

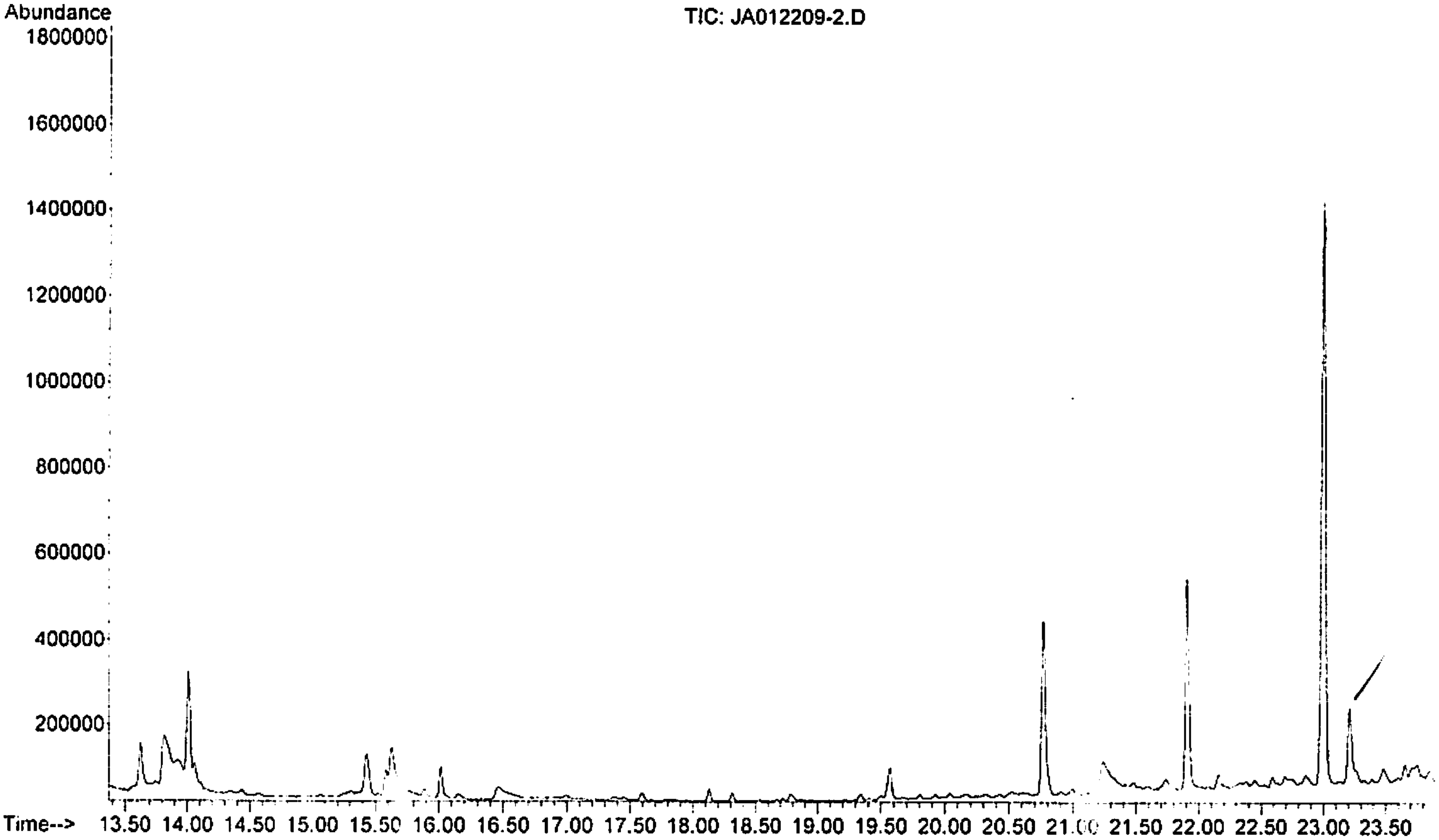


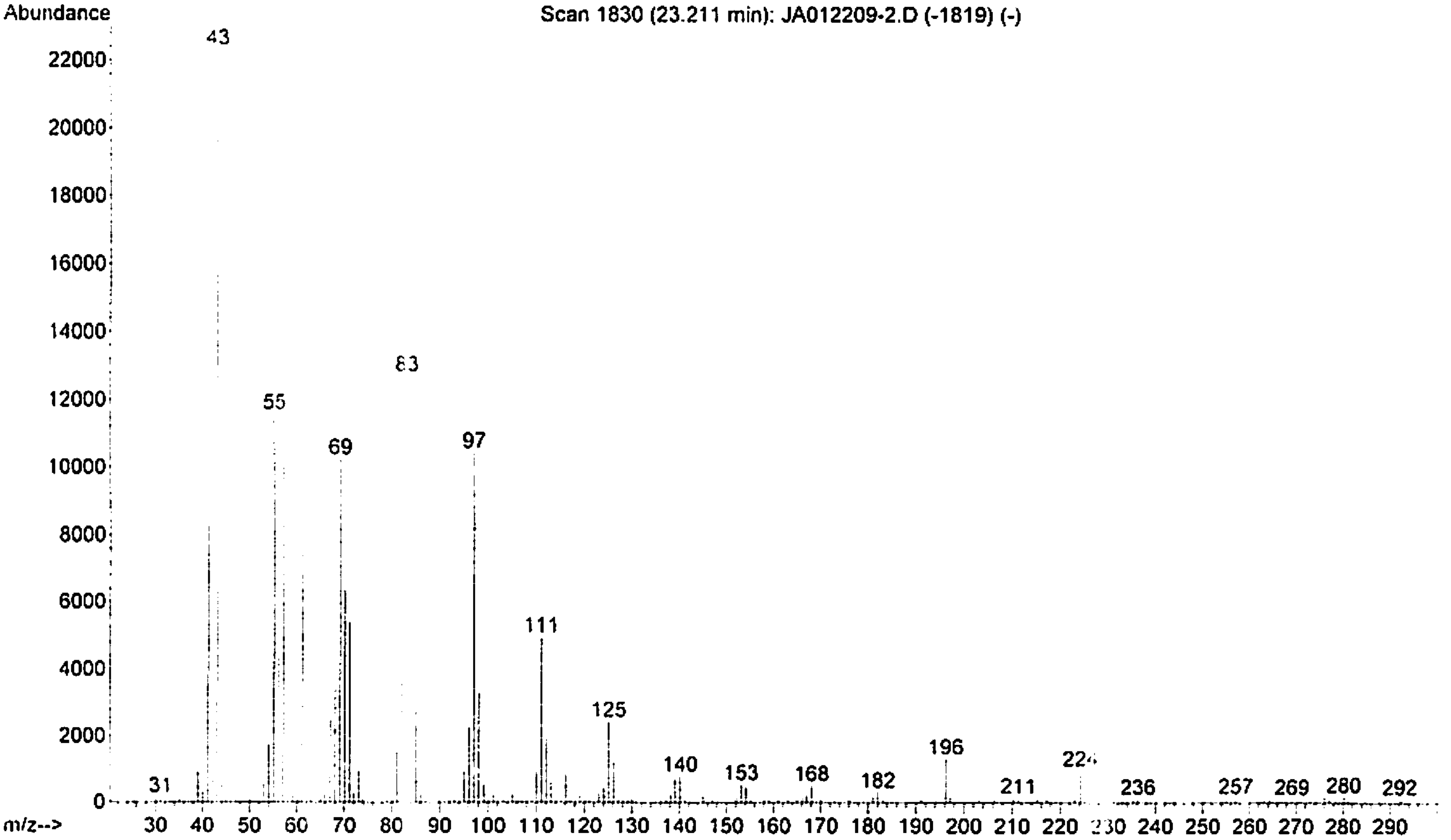

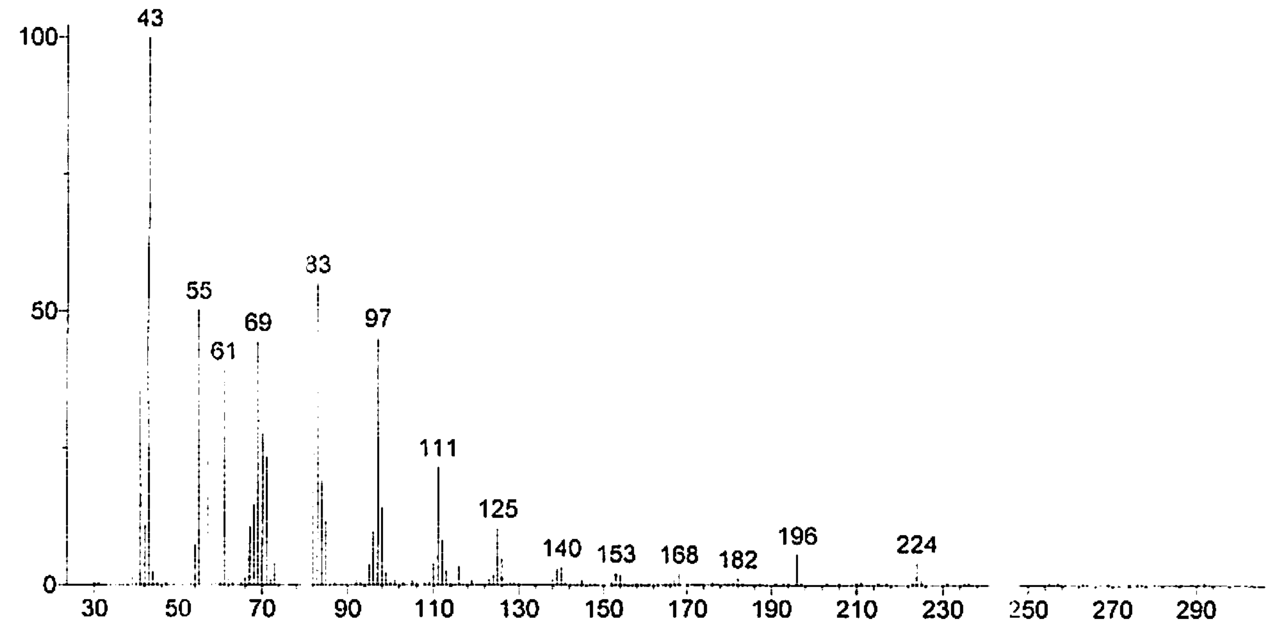


(Text File) Scan 1830 (23.2[illegible]1 min): JA012209-2.D (-1819)

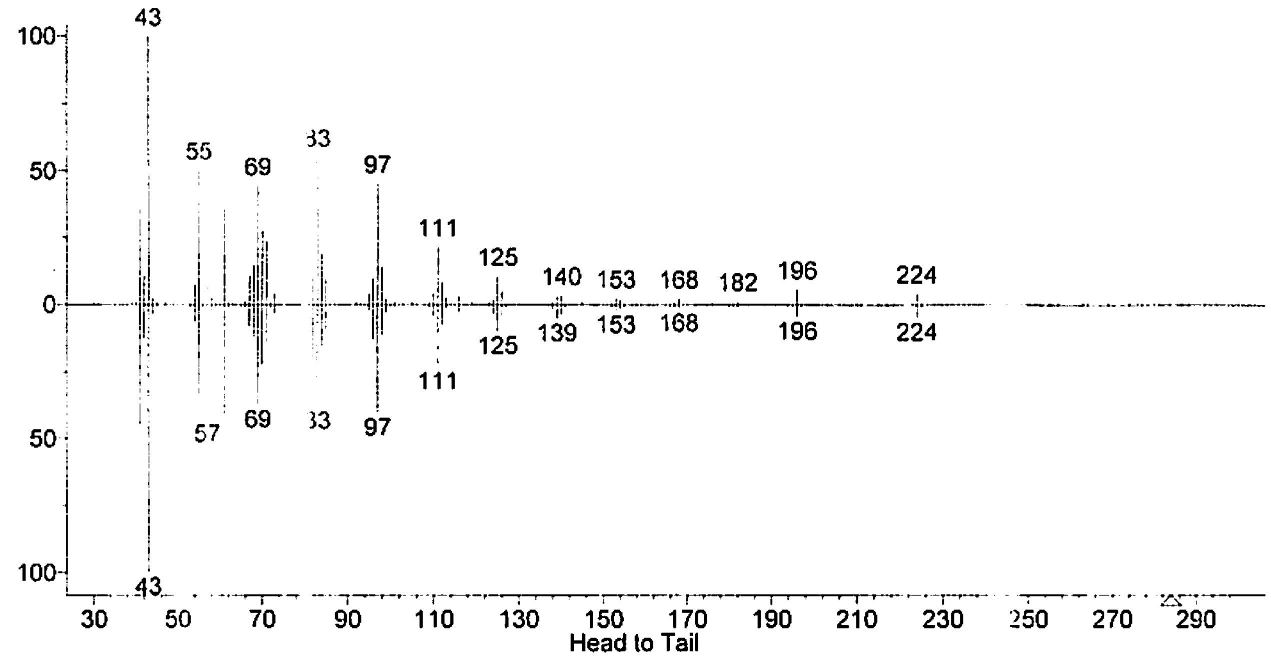


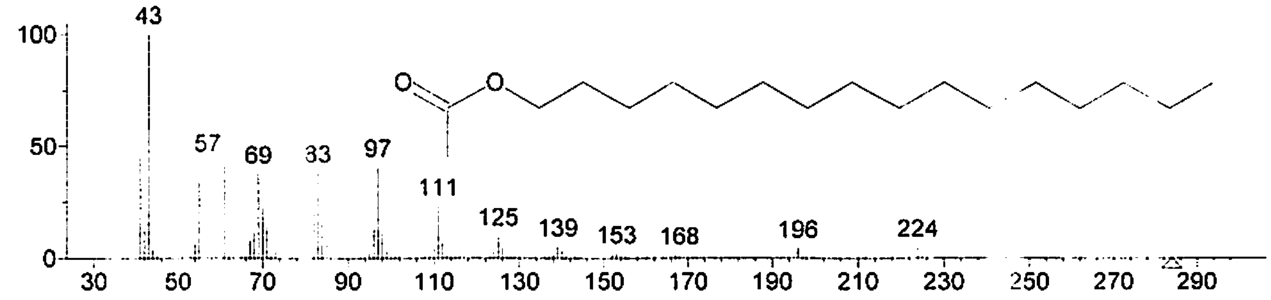


(mainlib) 1-Hexadecanol, acetate

[illegible]le , :D:\DATA\Aldr[illegible]ch\JA-09\JA012209-2.D
[illegible]erator : Aldrich
[illegible]uired : 22 Jan 2009 11:14 using AcqMethod JA-WAX08.4
[illegible]trument : Instrument #1
[illegible]mple Name: 9 male C.oc[illegible]lata abd.sternites/5ul CH2Cl2
[illegible]sc Info : 3-8-day-old; fed 1 wk 6-CH3-5-hepten-2-one
V[illegible]al Number: 1

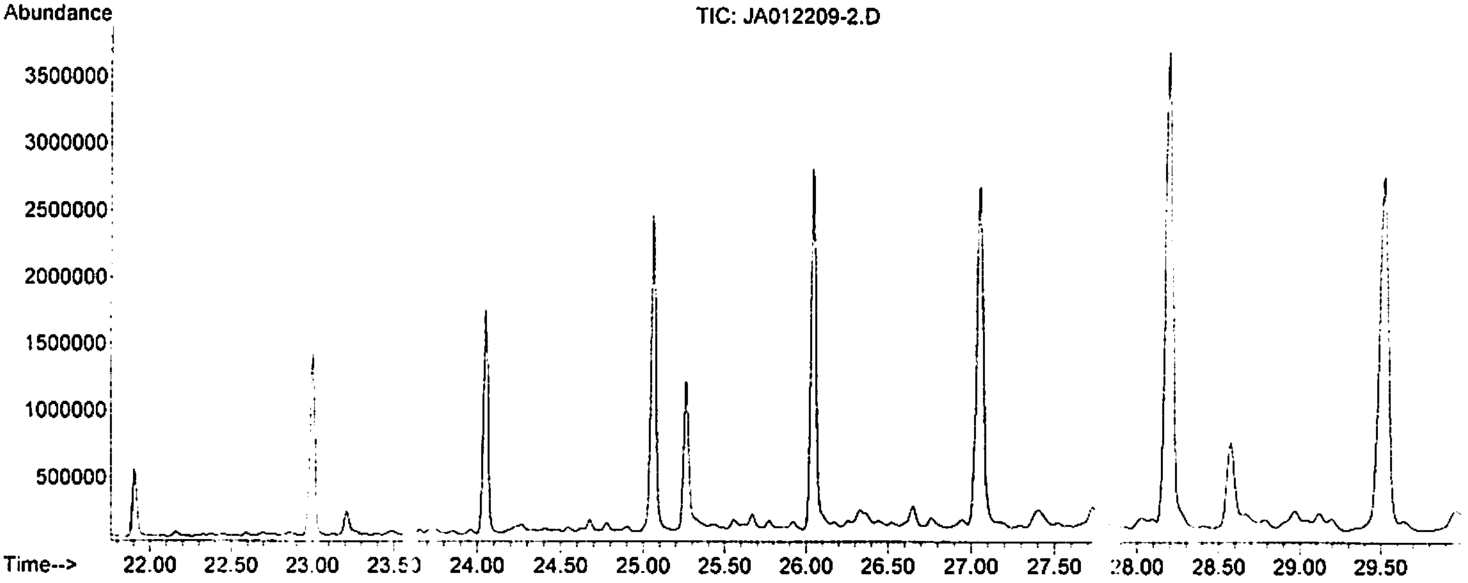


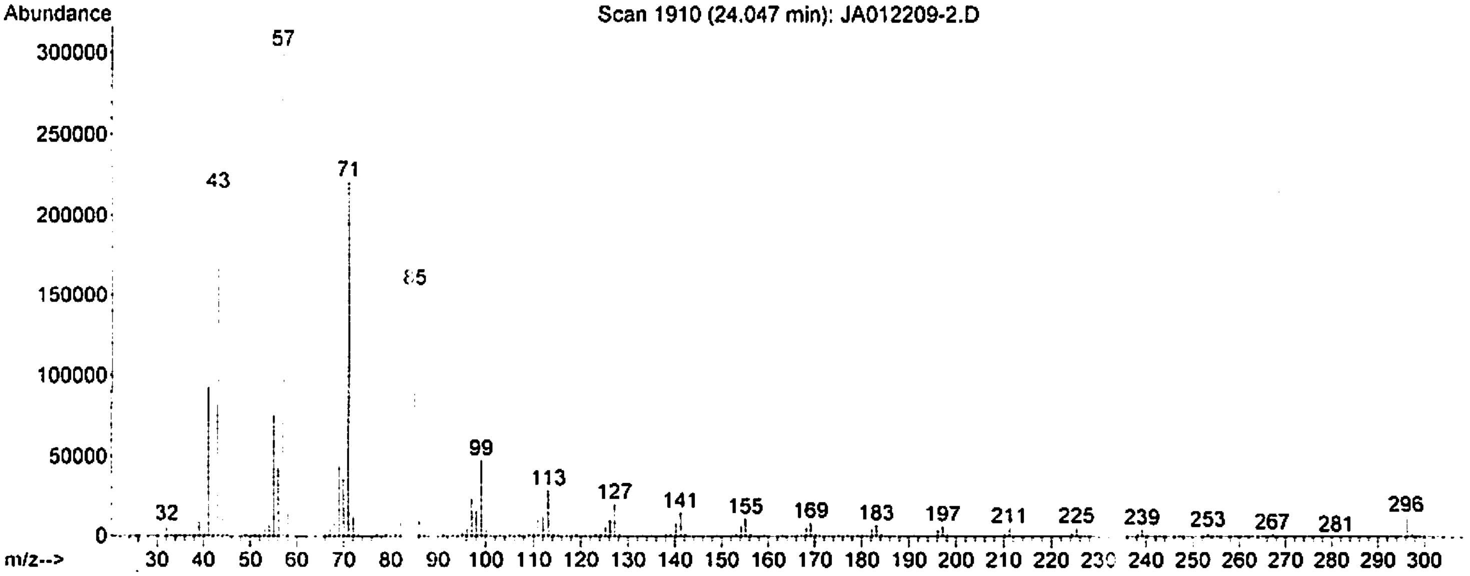

[illegible]le : D:\DATA\Aldr ch\JA-09\JA012209-2.D
[illegible]erator : Aldrich
[illegible]quired : 22 Jan 2009 11:14 using AcqMethod JA-WAX08.[illegible]
[illegible]strument : Instrume[illegible] #1
[illegible]mple Name: 9 male C.oculata abd.sternites/5ul CH2Cl2
[illegible]sc Info : 3-8-day-old; fed 1 wk 6-CH3-5-hepten-2-one
[illegible]al Number: 1

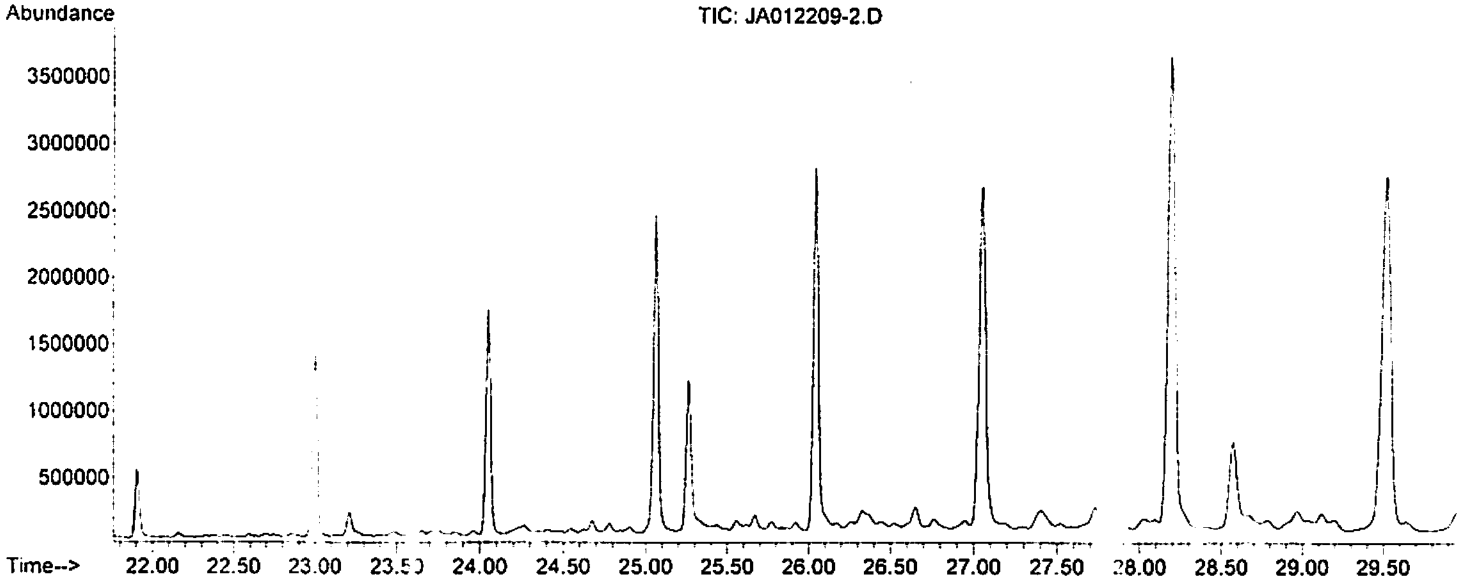


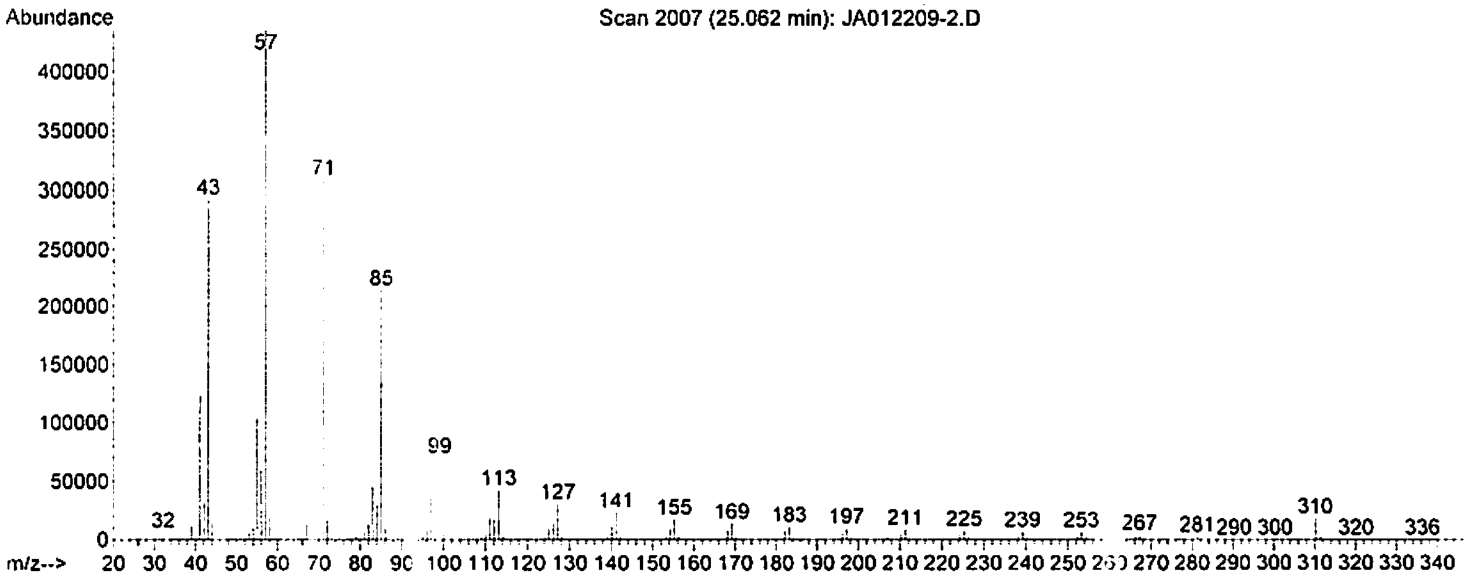

File : D:\DATA\Aldrich\JA-09\JA012209-2.D
Operator : Aldrich
Acquired : 22 Jan 2009 11:14 using AcqMethod JA-WAX08.M
Instrument : Instrument #1
Sample Name: 9 male C.oculata abd.sternites/5ul CH2Cl2
Misc Info : 3-8-day-old; fed 1 wk 6-CH3-5-hepten-2-one
Vial Number: 1

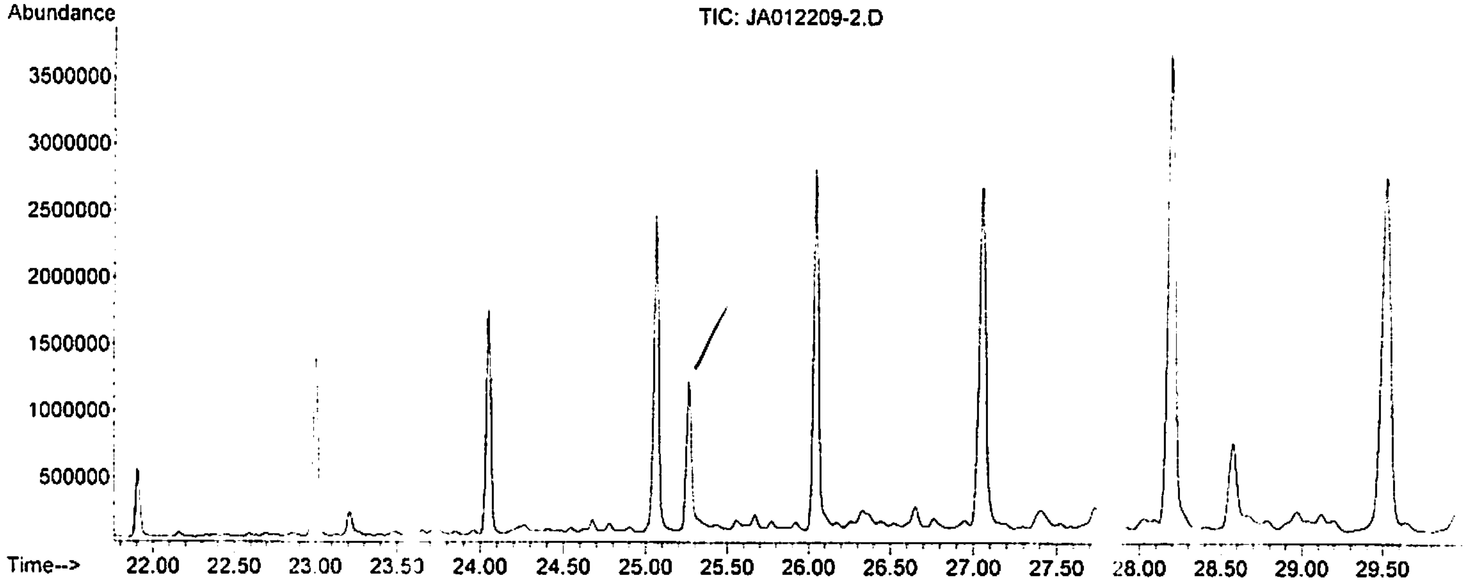


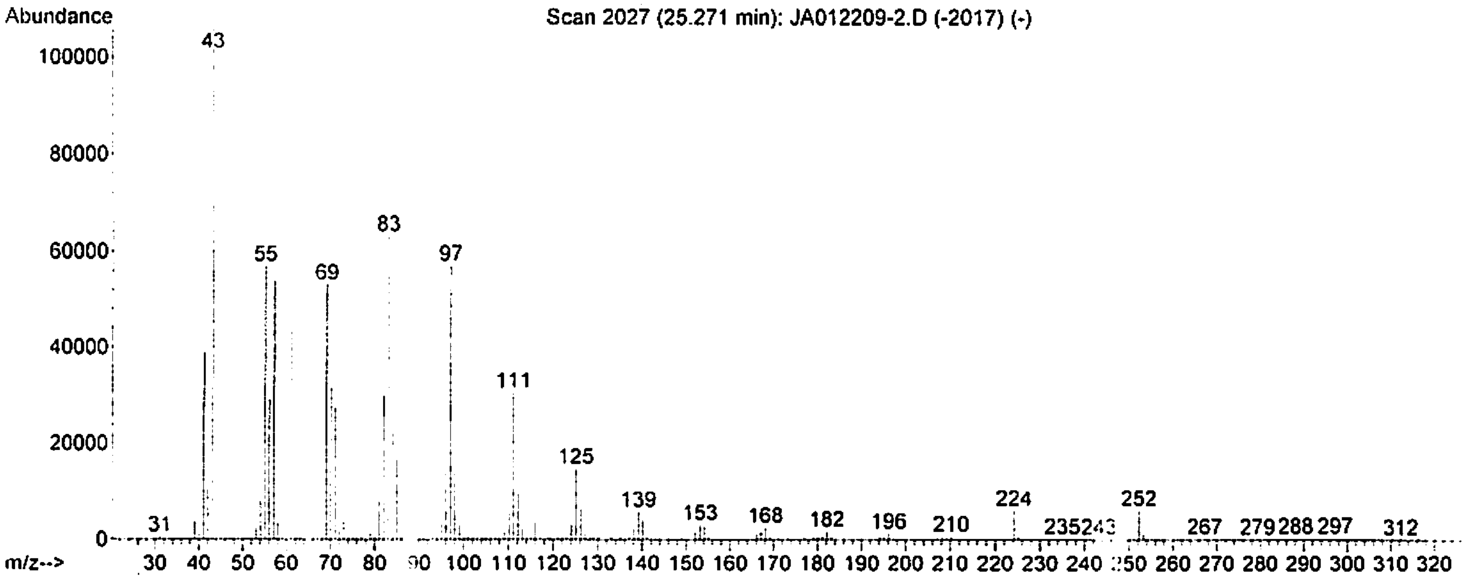

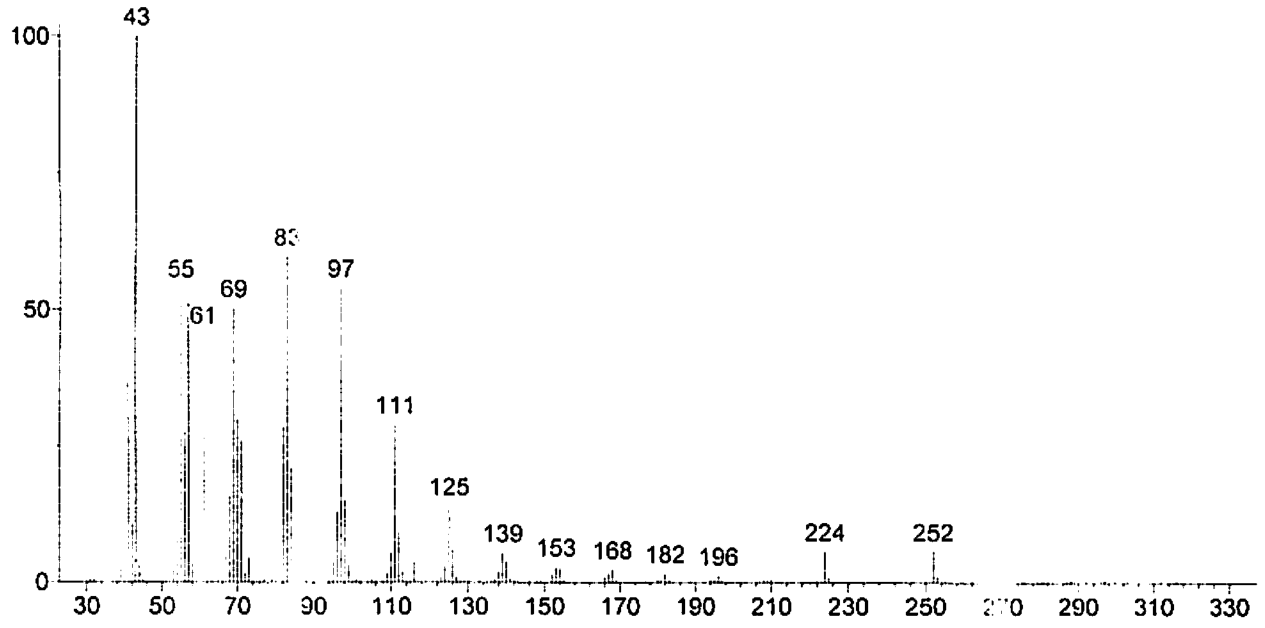

(Text File) Scan 2027 (25.2[illegible]1 min): JA012209-2.D (-2017)

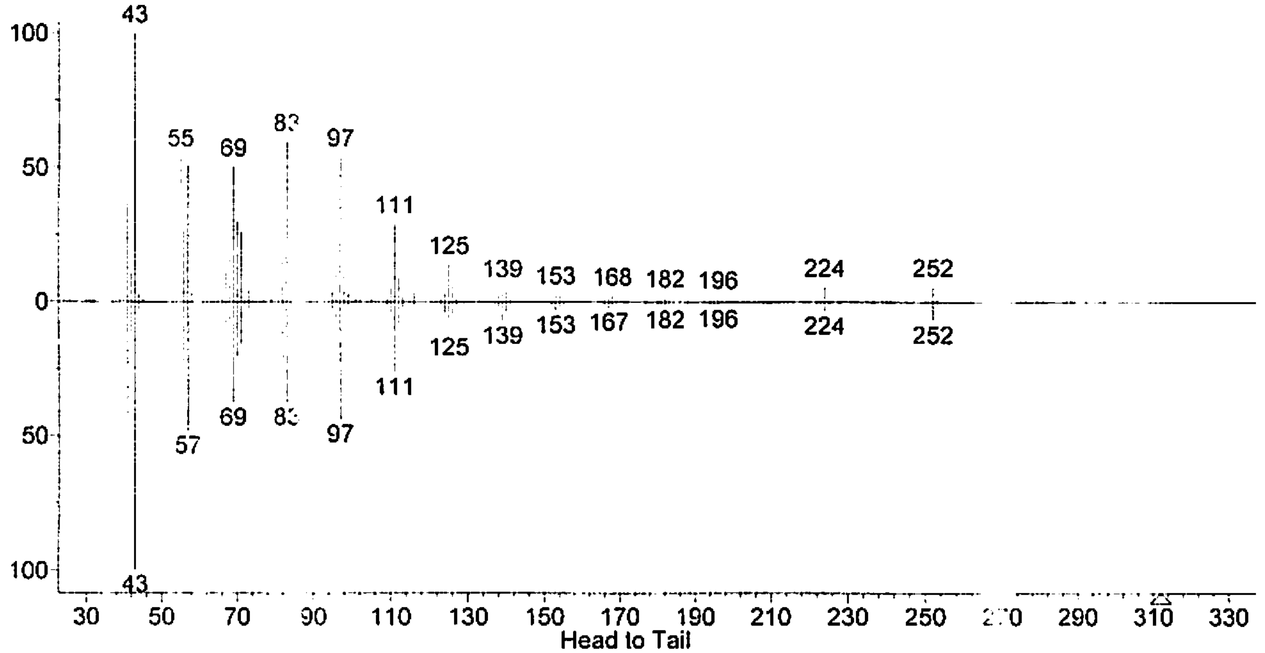

Head to Tail

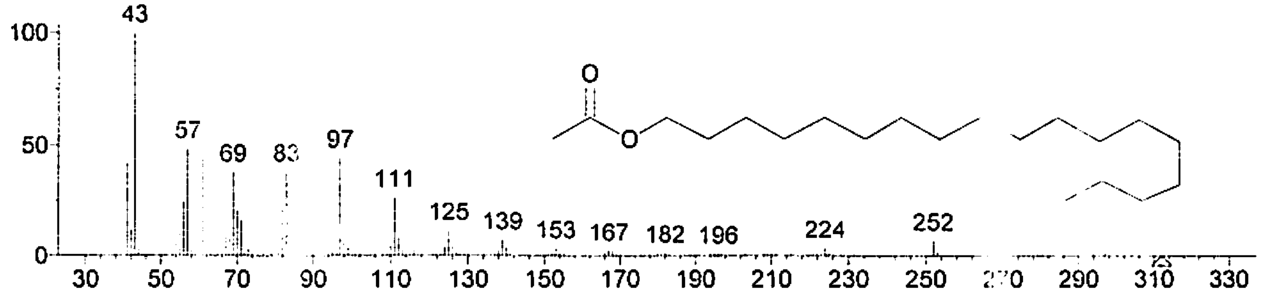

(replib) Acetic acid, octadec[illegible]l ester

:D:\DATA\Aldr ch\JA-09\JA012209-2.D
rator : Aldrich
uired : 22 Jan 2009 11:14 using AcqMethod JA-WAX08.M
strument : Instrument #1
mple Name: 9 male C.oculata abd.sternites/5ul CH2Cl2
isc Info : 3-8-day-old, fed 1 wk 6-CH3-5-hepten-2-one
ial Number: 1

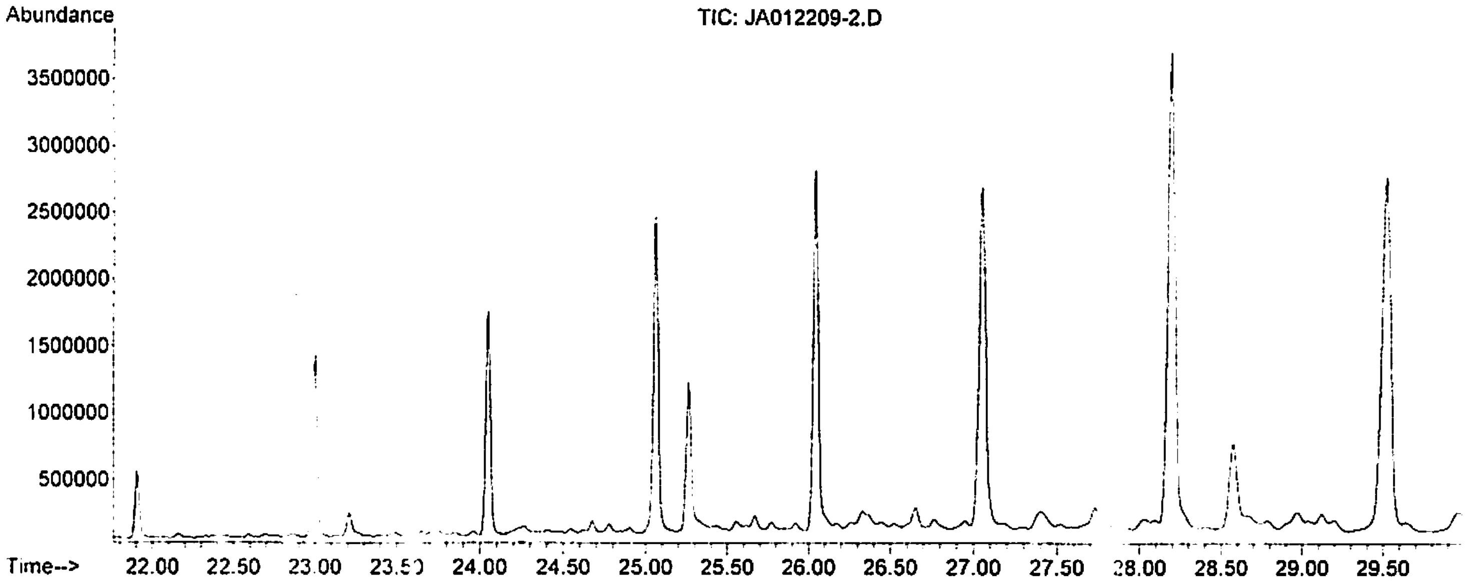


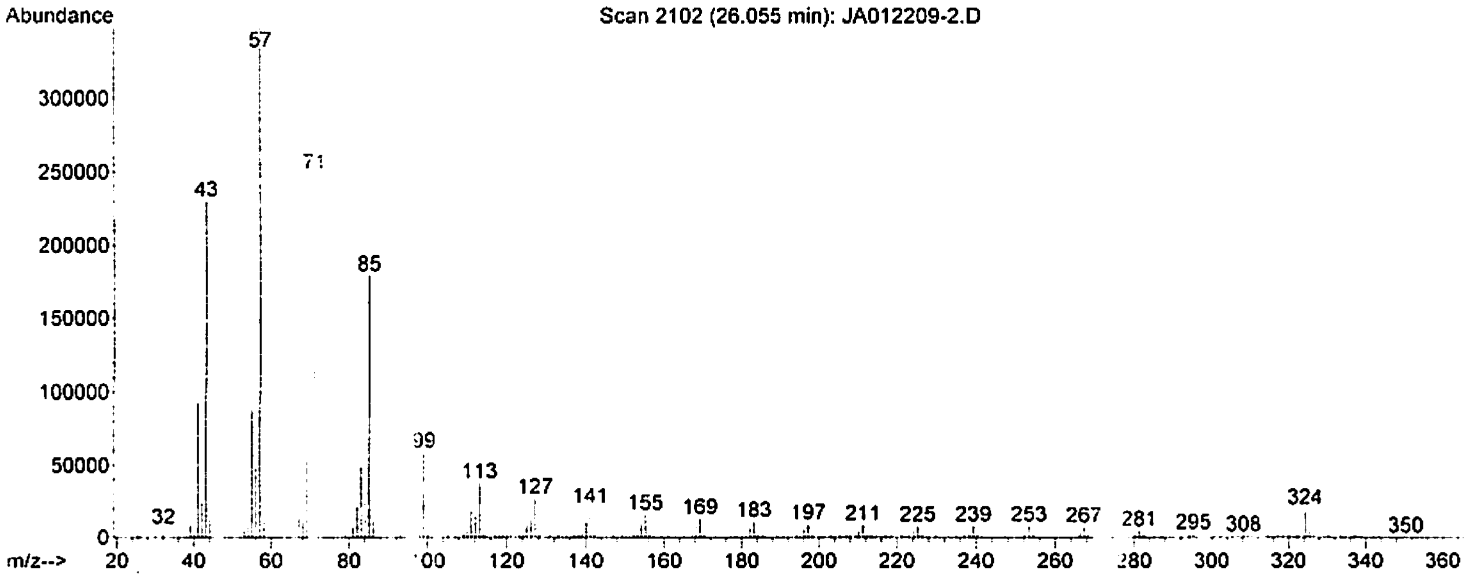

File : :D:\DATA\Aldrich\JA-09\JA012209-2.D
Operator : Aldrich
Acquired : 22 Jan 2009 11:14 using AcqMethod JA-WAX08.M
Instrument : Instrument #1
Sample Name: 9 male C.oculata abd.sternites/5ul CH2Cl2
Misc Info : 3-8-day-old; fed 1 wk 6-CH3-5-hepten-2-one
Vial Number: 1

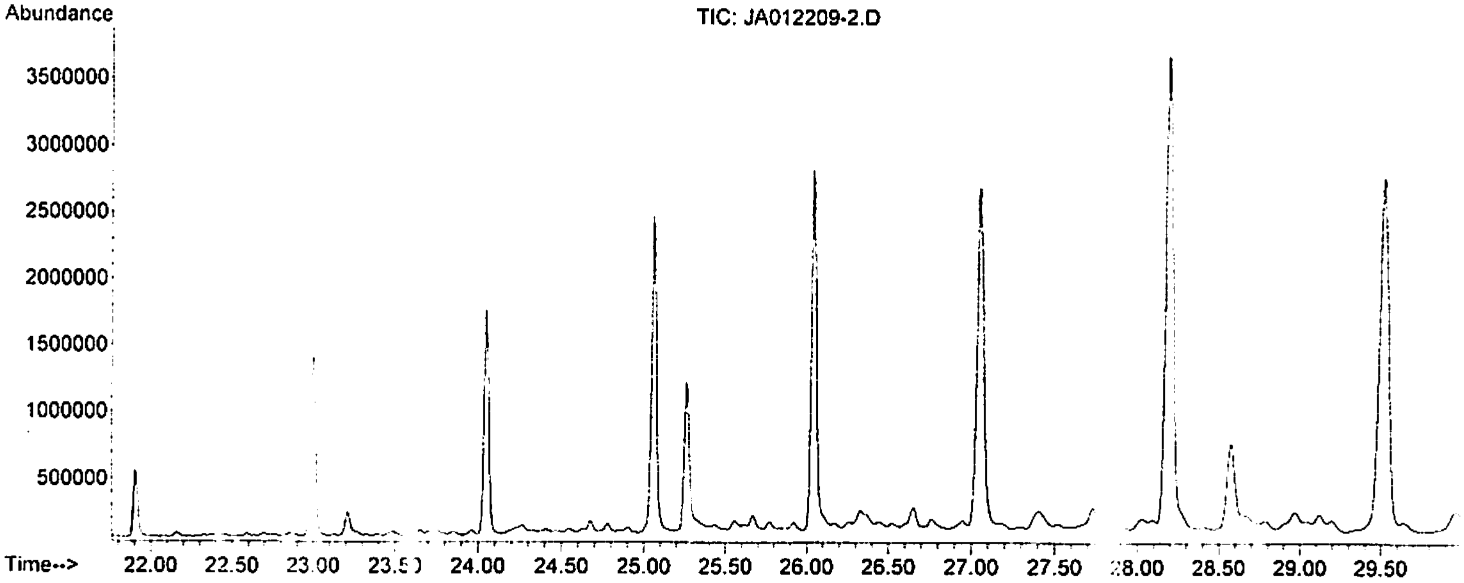


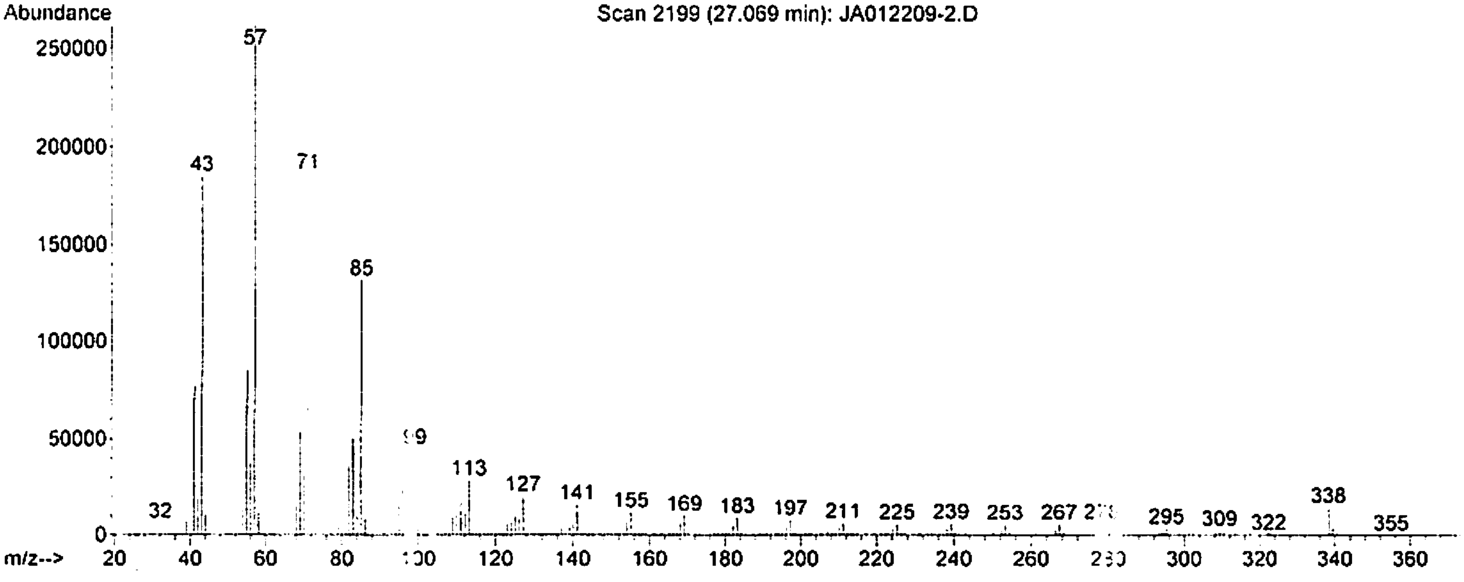

```
File         :D:\DATA\Aldrich\JA-09\JA012209-2.D
Operator     : Aldrich
Acquired     : 22 Jan 2009   11:14      using AcqMethod JA-WAX08.M
Instrument   :     Instrument #1
Sample Name: 9 male C.oculata abd.sternites/5ul CH2Cl2
Misc Info    : 3-8-day-old; fed 1 wk 6-CH3-5-hepten-2-one
Vial Number: 1
```

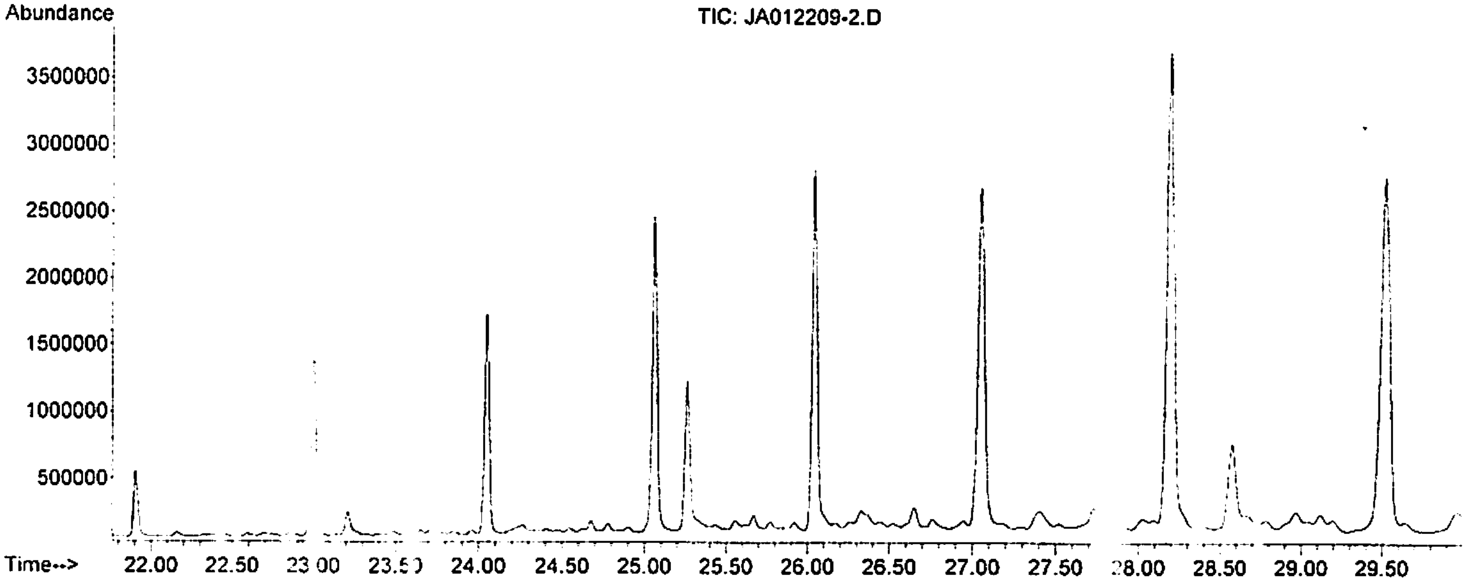


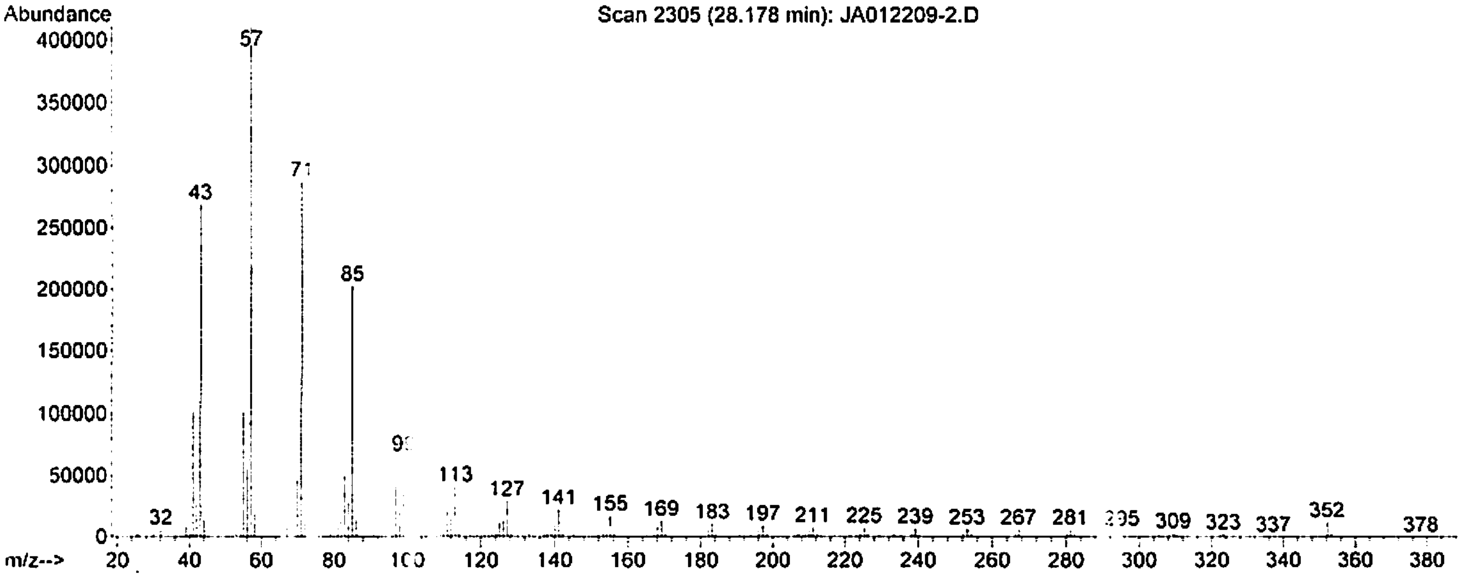

[illegible]e : D:\DATA\Aldr[illegible]ch\JA-09\JA012209-2.D
[illegible]rator : Aldric[illegible]
[illegible]uired : 22 Jan 2009 11:14 using AcqMethod JA-WAX08.[illegible]
[illegible]strument : Instrument #1
[illegible]mple Name: 9 male C.oculata abd.sternites/5ul CH2Cl2
[illegible]sc Info : 3-8-day-old; fed 1 wk 6-CH3-5-hepten-2-one
[illegible]al Number: 1

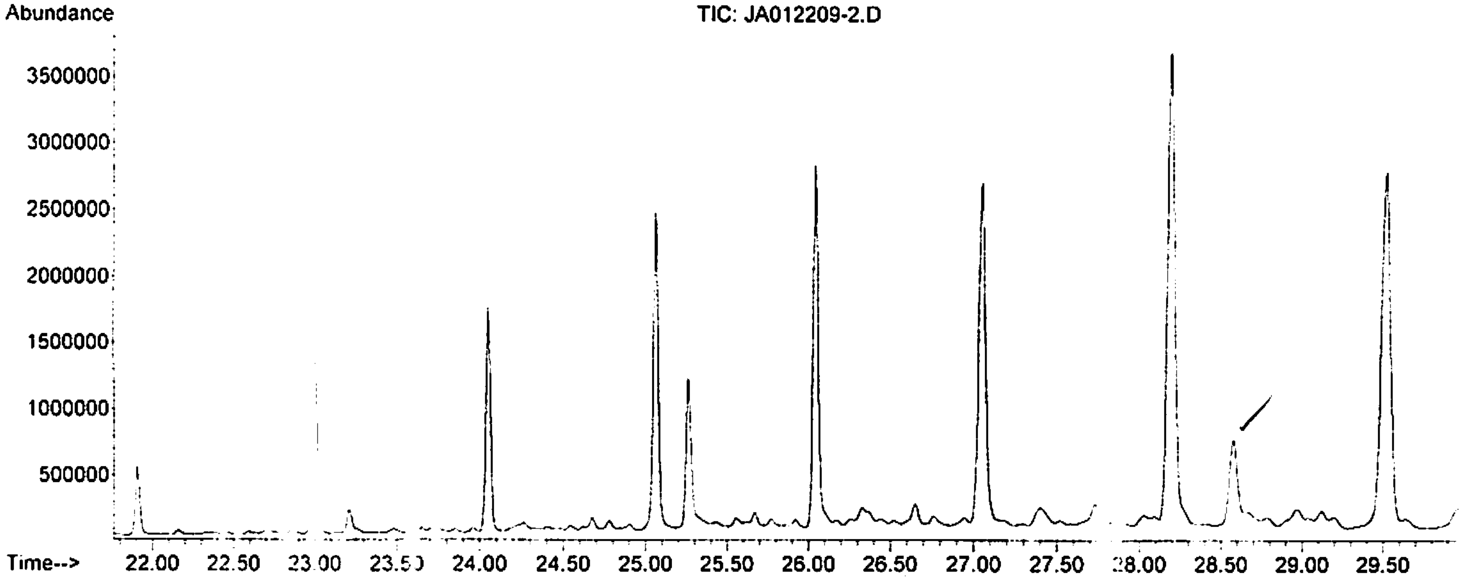


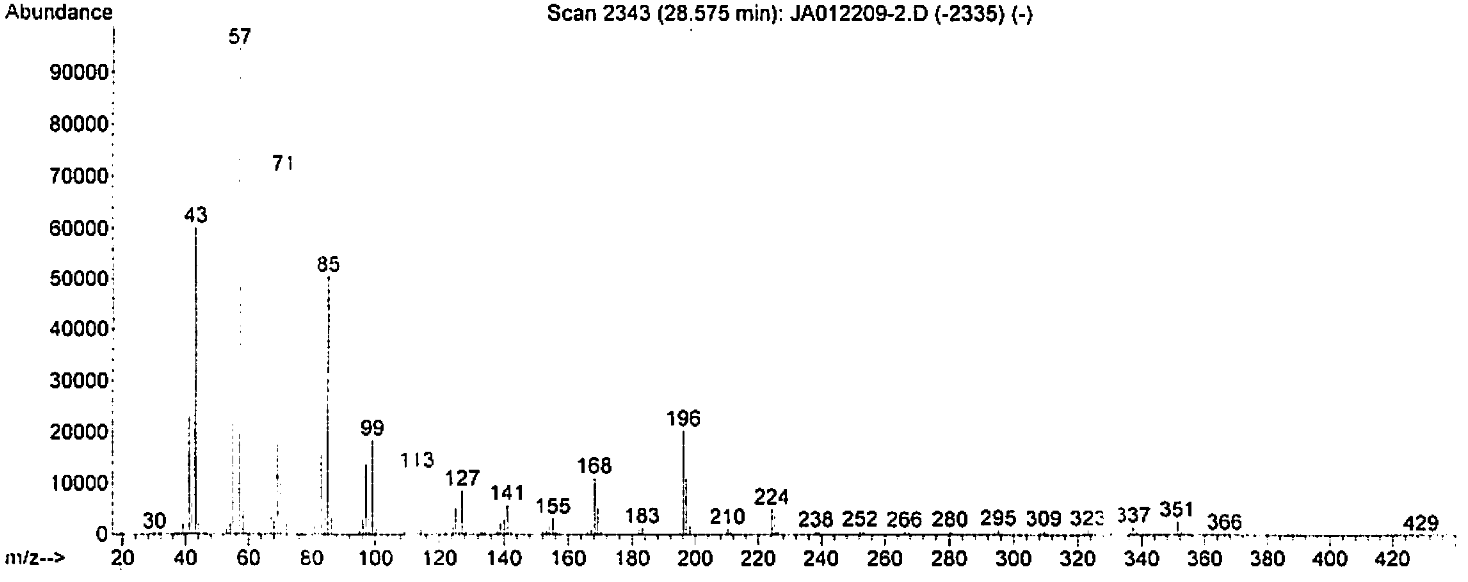

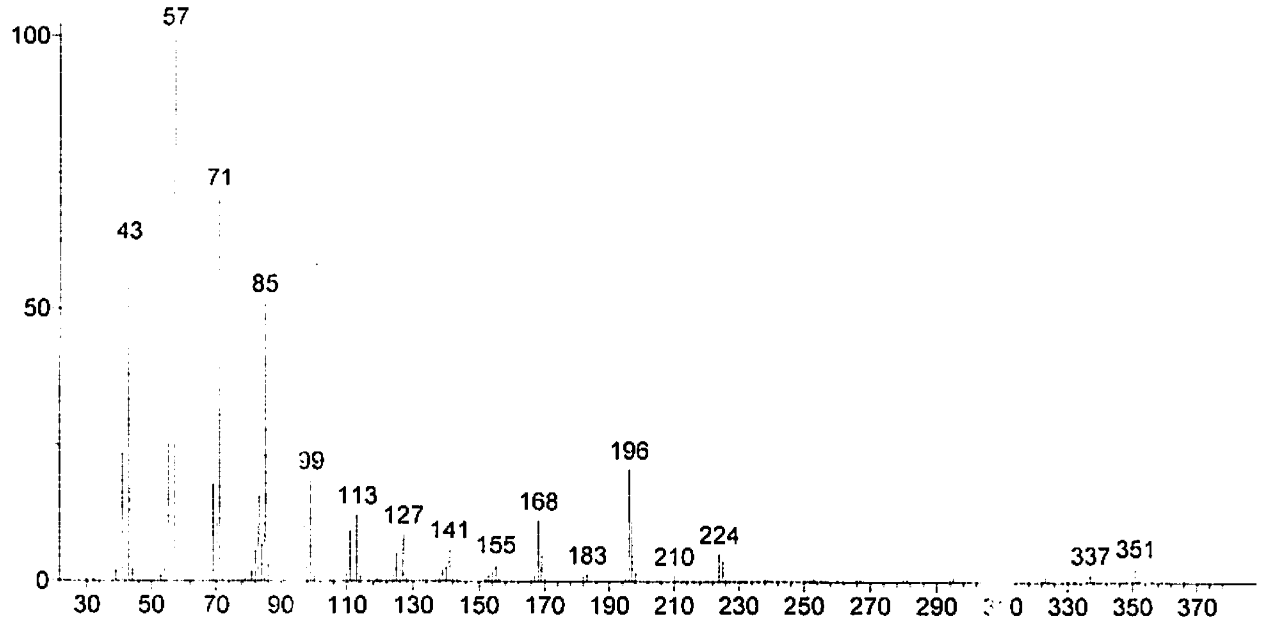


(Text File) Scan 2343 (28.5`5 min): JA012209-2.D (-2335)

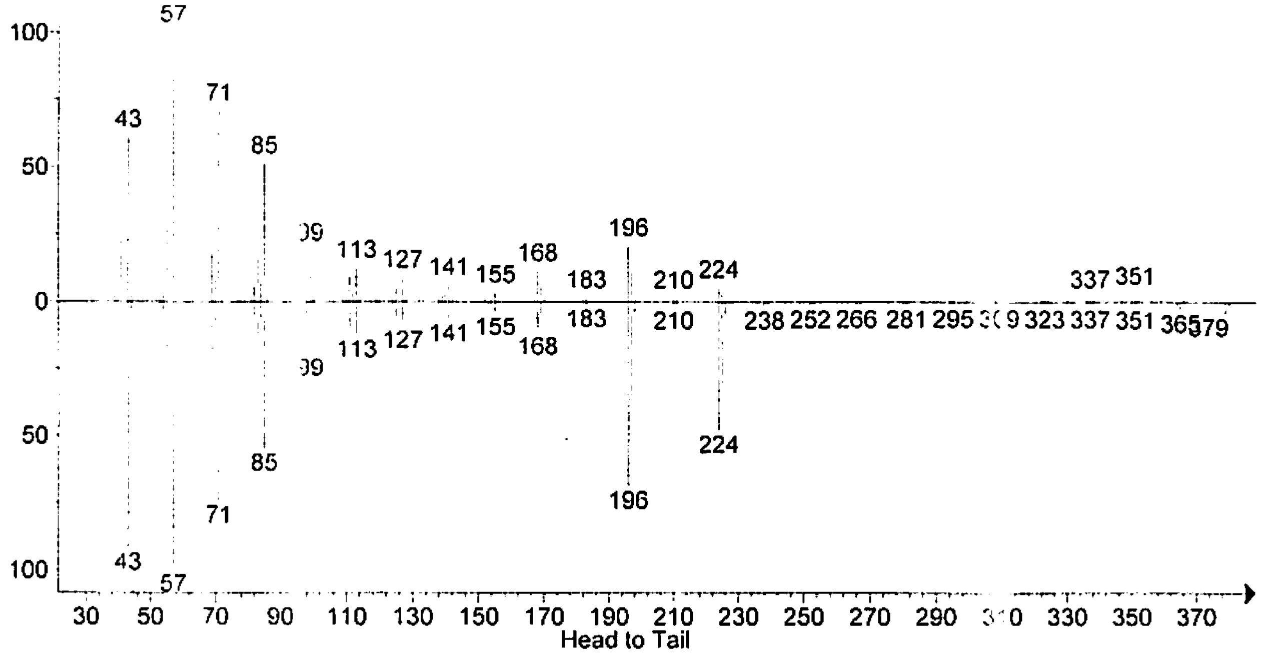


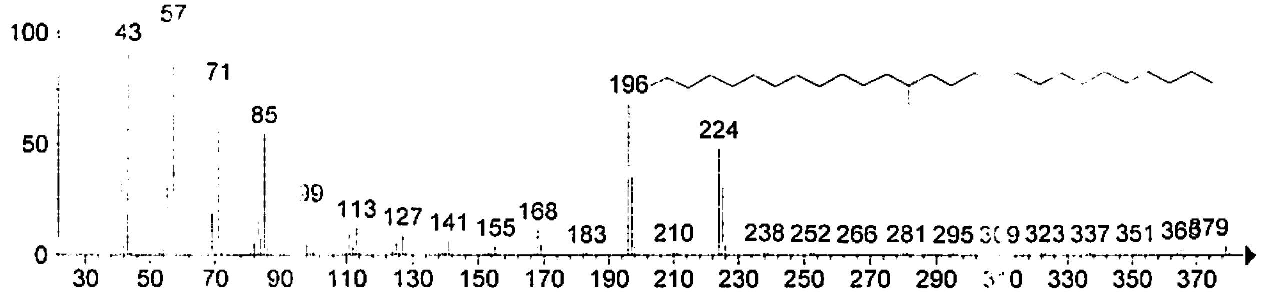


(mainlib) 13-Methylheptacosane

File :D:\DATA\Aldrich\JA-09\JA012209-2.D
Operator : Aldrich
Acquired : 22 Jan 2009 11:14 using AcqMethod JA-WAX08.M
Instrument : Instrument #1
Sample Name: 9 male C.oculata abd.sternites/5ul CH2Cl2
Misc Info : 3-8-day-old; fed 1 wk 6-CH3-5-hepten-2-one
Vial Number: 1

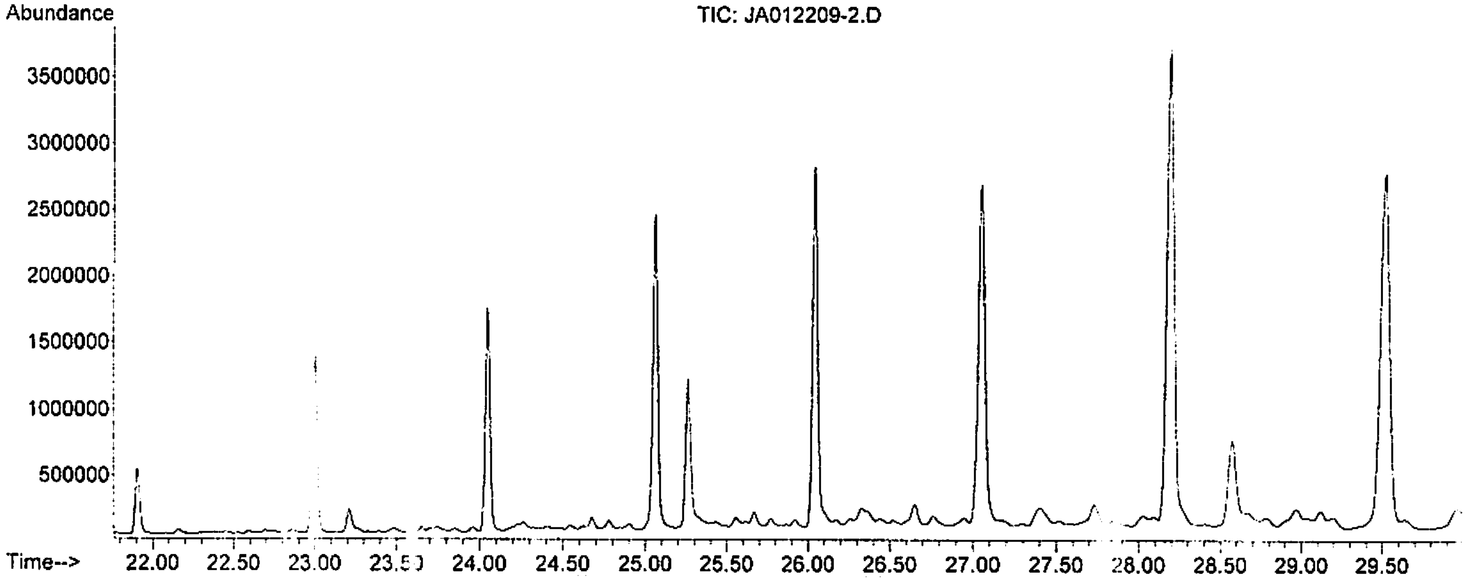


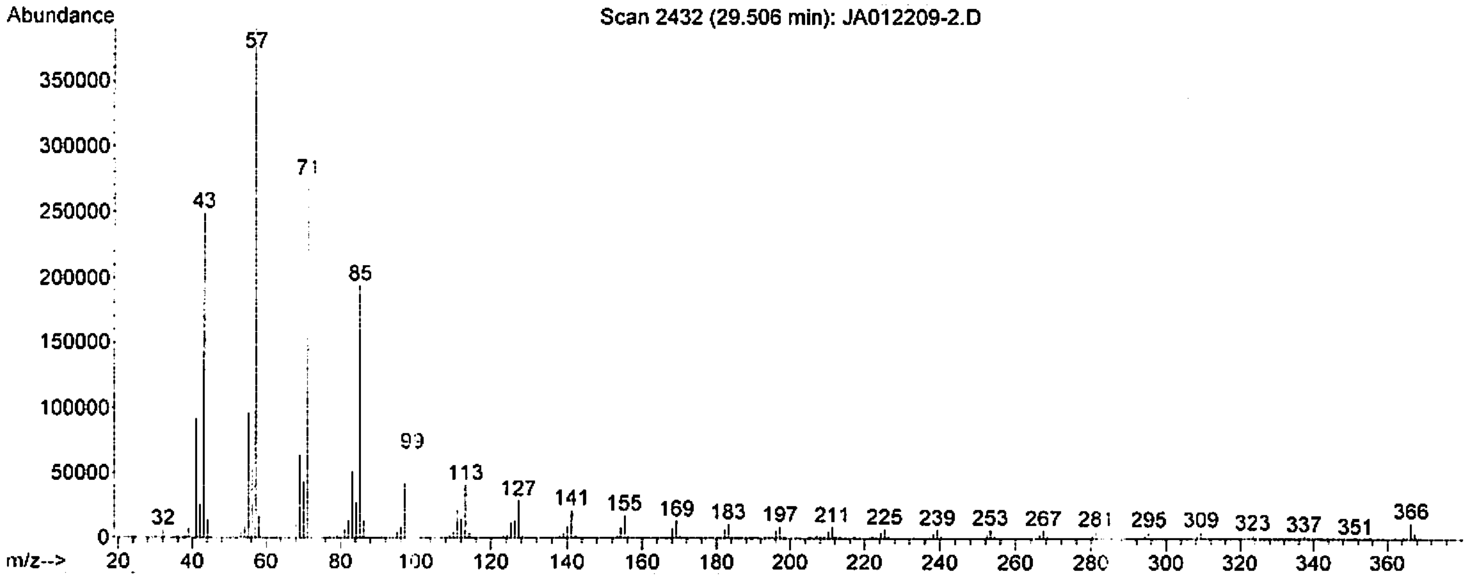

```
File        :D:\DATA\Aldrich\JA-09\JA012309-2.D
Operator    : Aldrich
Acquired    : 23 Jan 2009   14:32      using AcqMethod JA-WAX08.M
Instrument  :    Instrument #1
Sample Name: 10 male C.oculata abd.ster./5ul CH2Cl2
Misc Info   : 8-14-day-old; fed (+/-)-citronellal 7days
Vial Number: 1
```

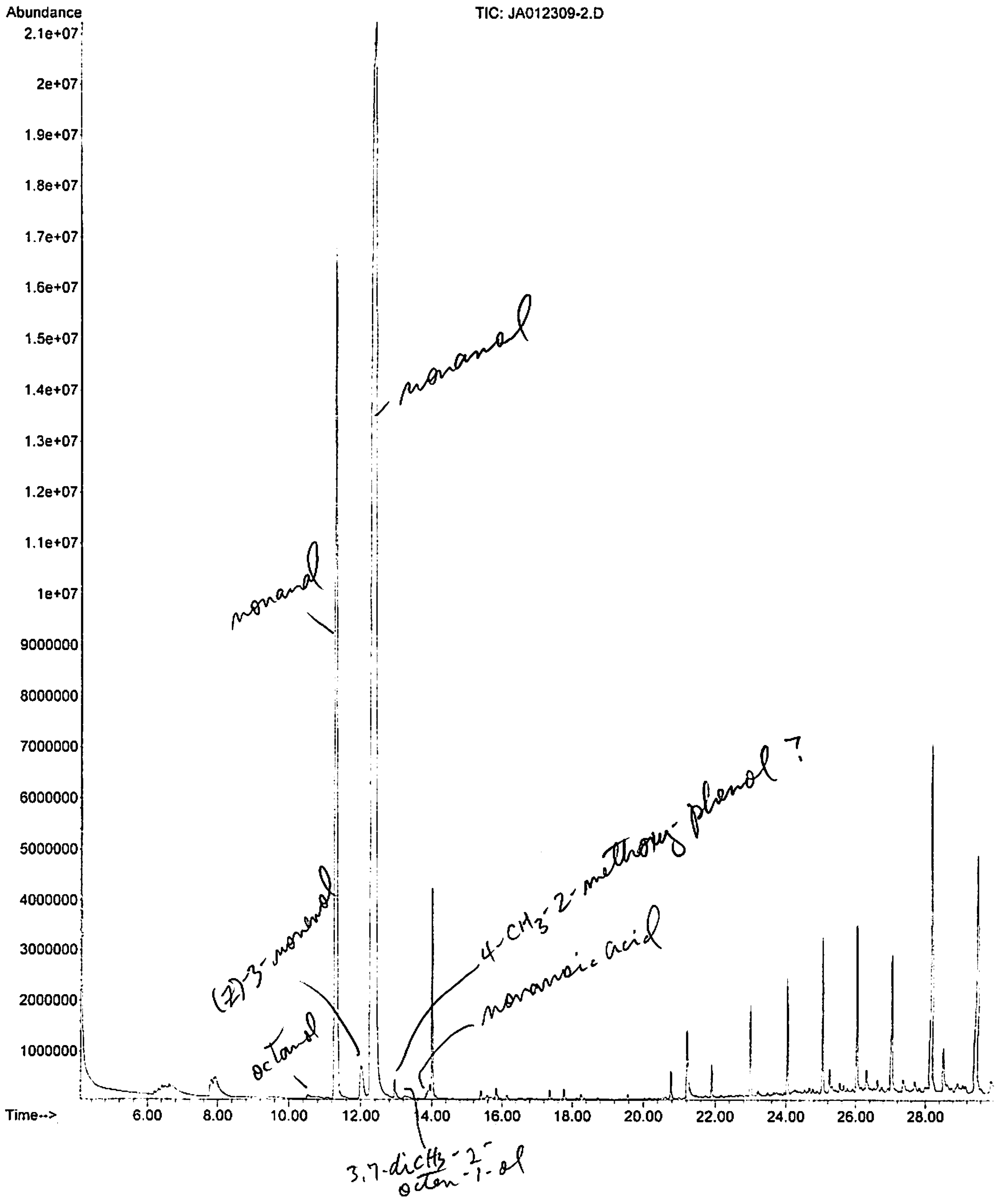

File : D:\DATA\Aldrich\JA-09\JA012309-2.D
Operator : Aldrich
Acquired : 23 Jan 2009 14:32 using AcqMethod JA-WAX08.M
Instrument : Instrument #1
Sample Name: 10 male C.oculata abd.ster./5ul CH2Cl2
Misc Info : 8-14-day-old; fed (+/-)-citronellal 7days
Vial Number: 1

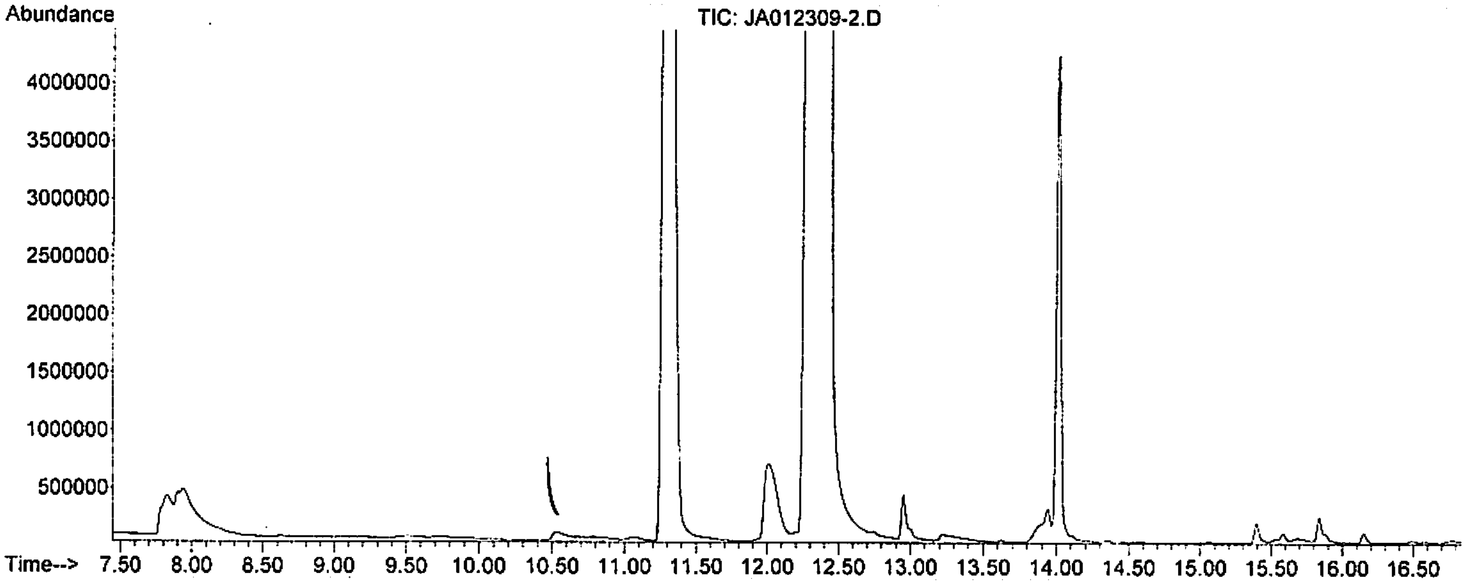


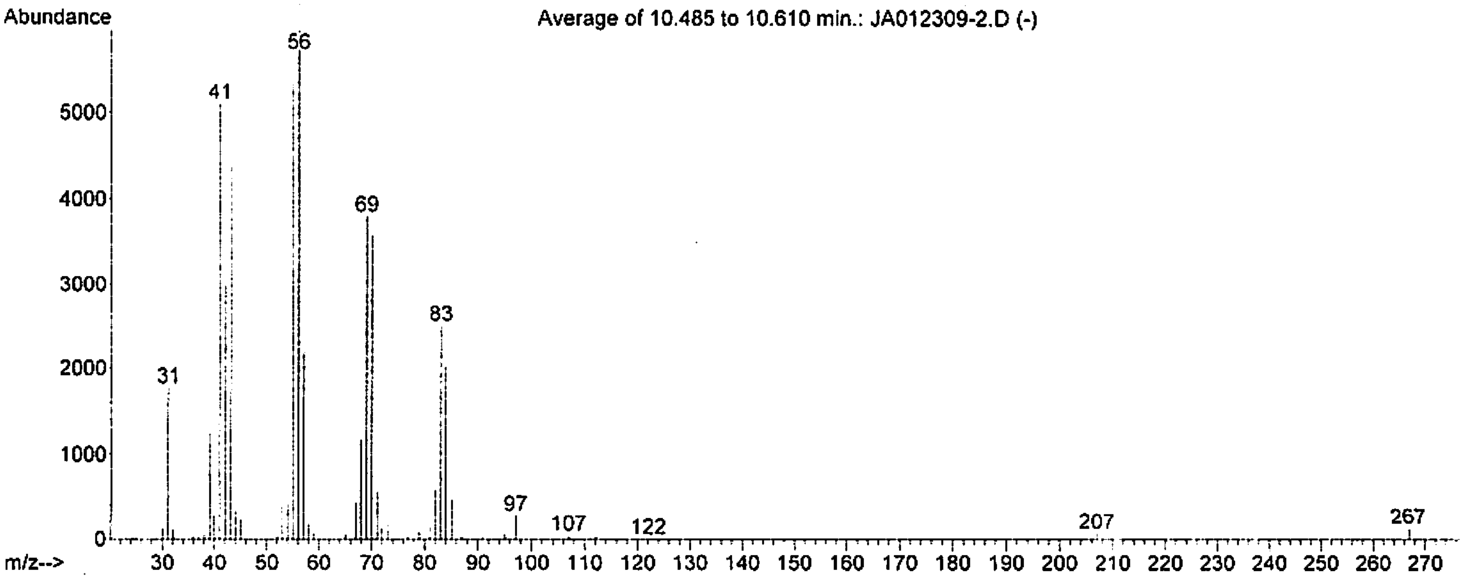

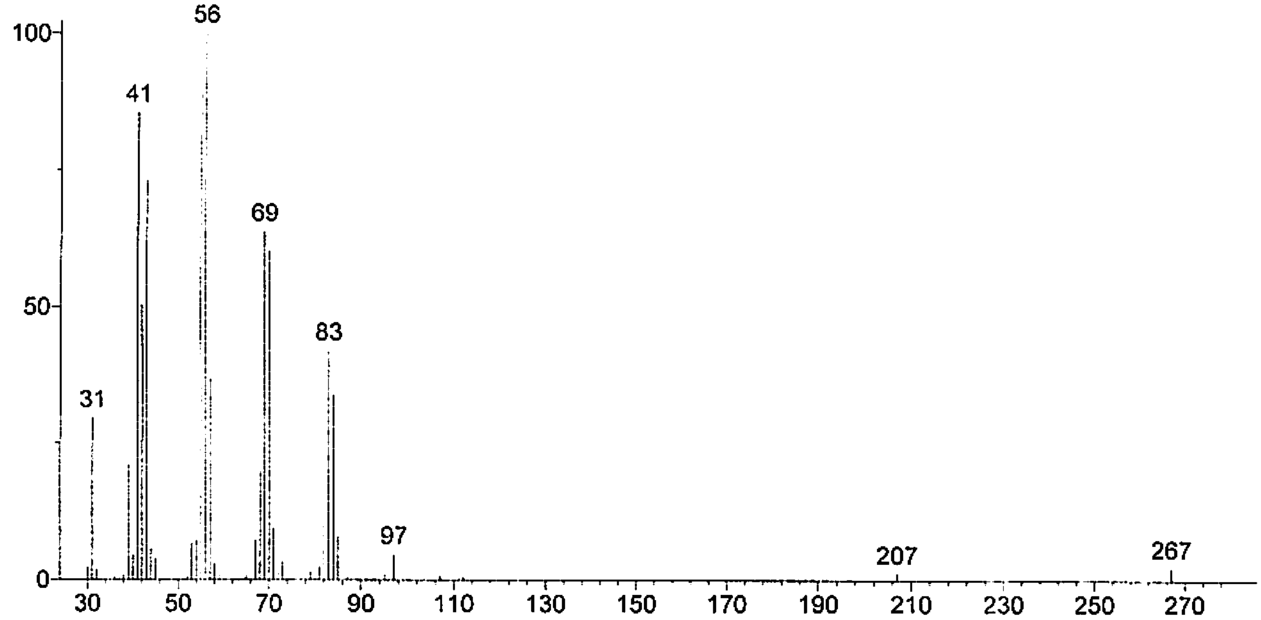


(Text File) Average of 10.485 to 10.610 min.: JA012309-2.D

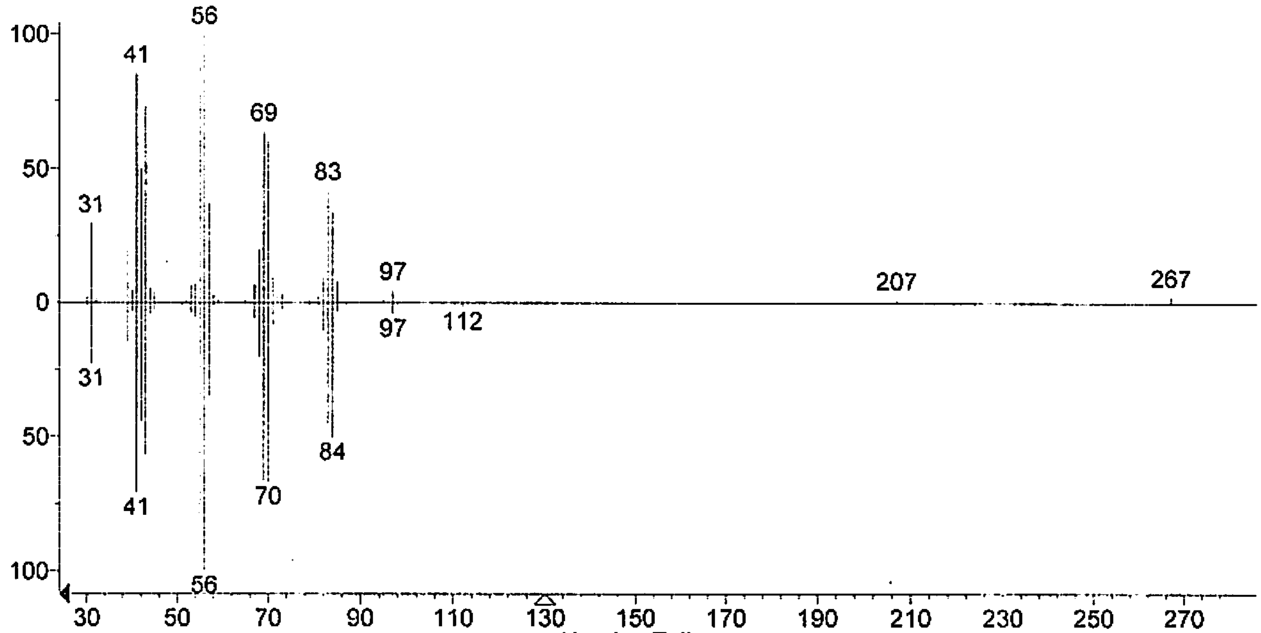


Head to Tail

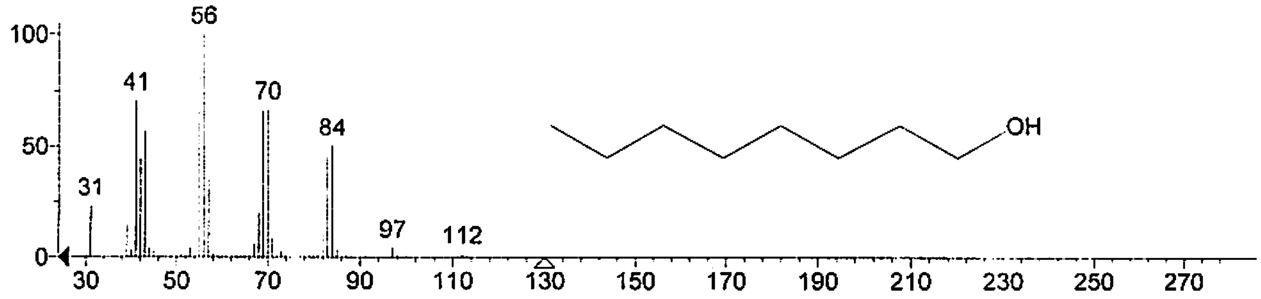


(replib) 1-Octanol

```
File .        : D:\DATA\Aldrich\JA-09\JA012309-2.D
Operator      : Aldrich
Acquired      : 23 Jan 2009  14:32      using AcqMethod JA-WAX08.M
Instrument    :    Instrument #1
Sample Name: 10 male C.oculata abd.ster./5ul CH2Cl2
Misc Info     : 8-14-day-old; fed (+/-)-citronellal 7days
Vial Number: 1
```

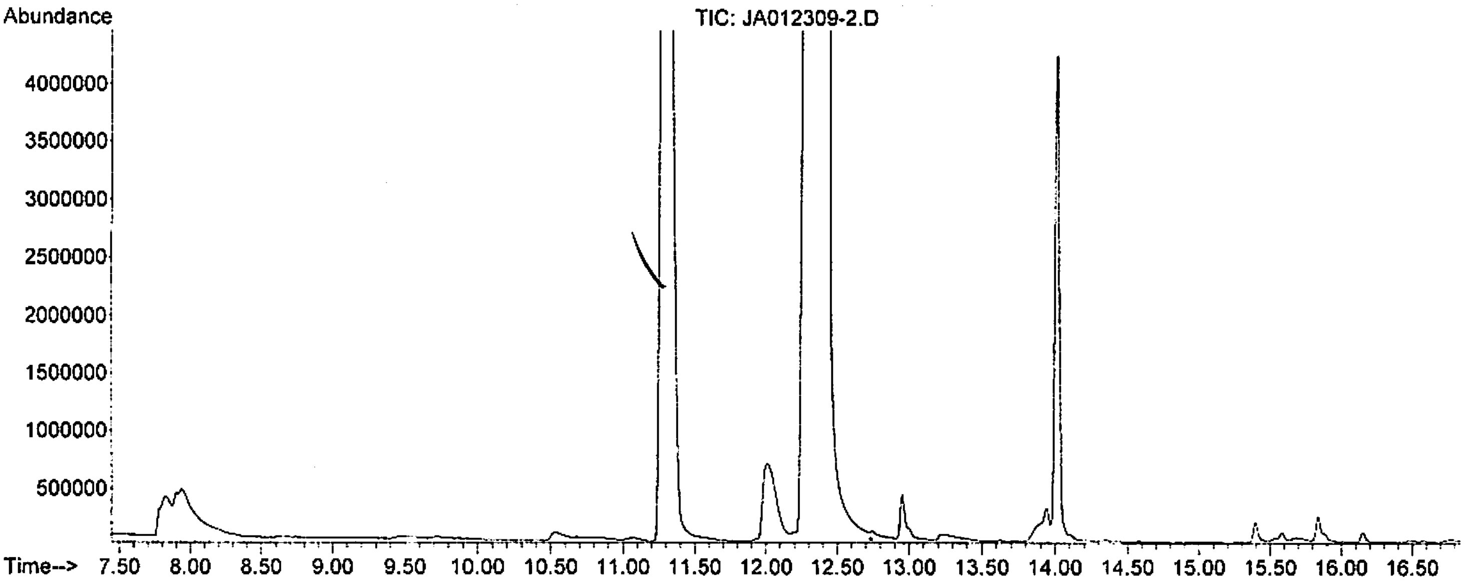


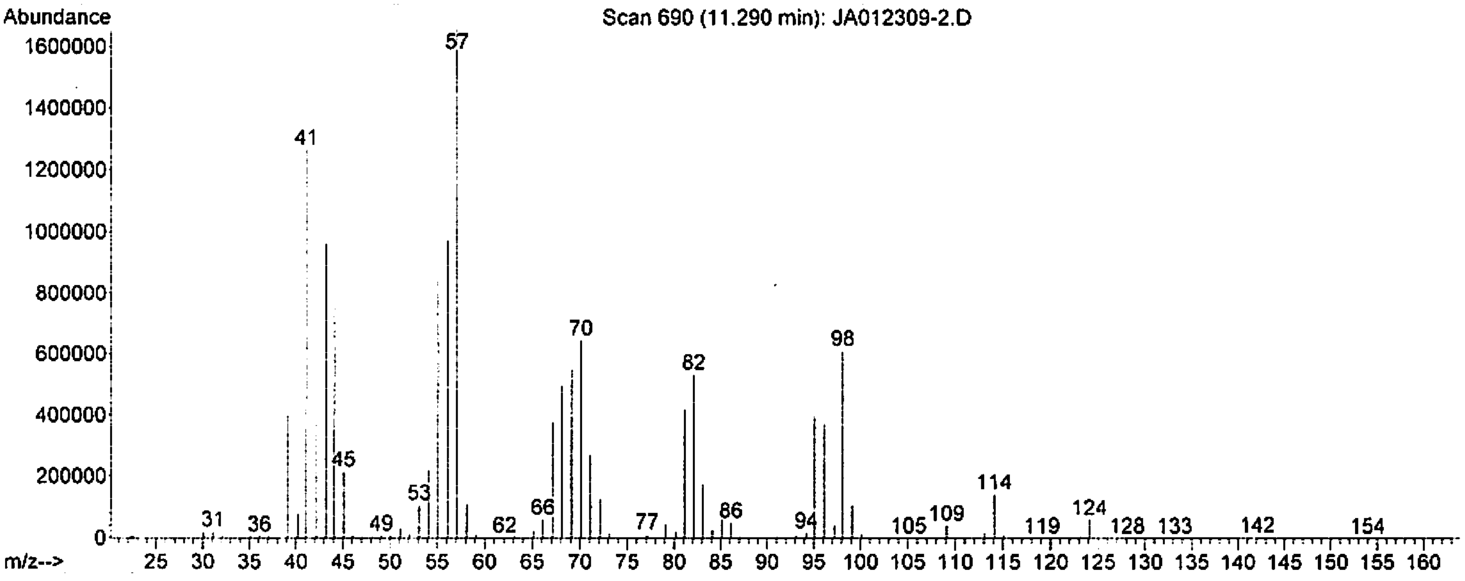

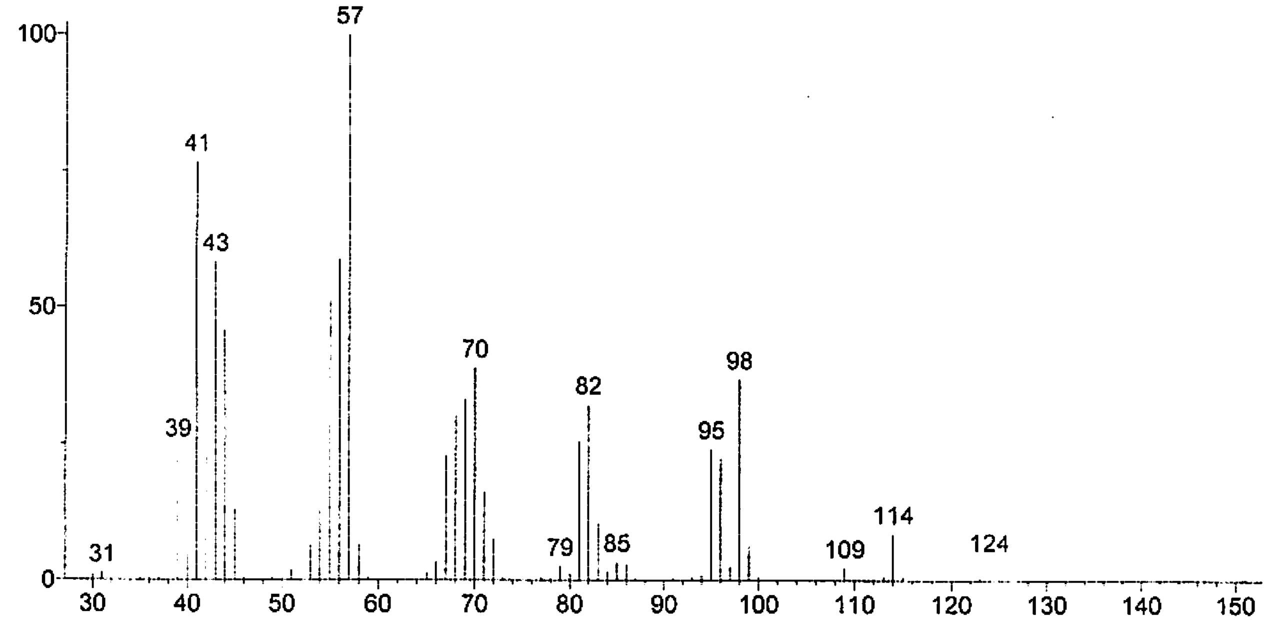


(Text File) Scan 690 (11.290 min): JA012309-2.D

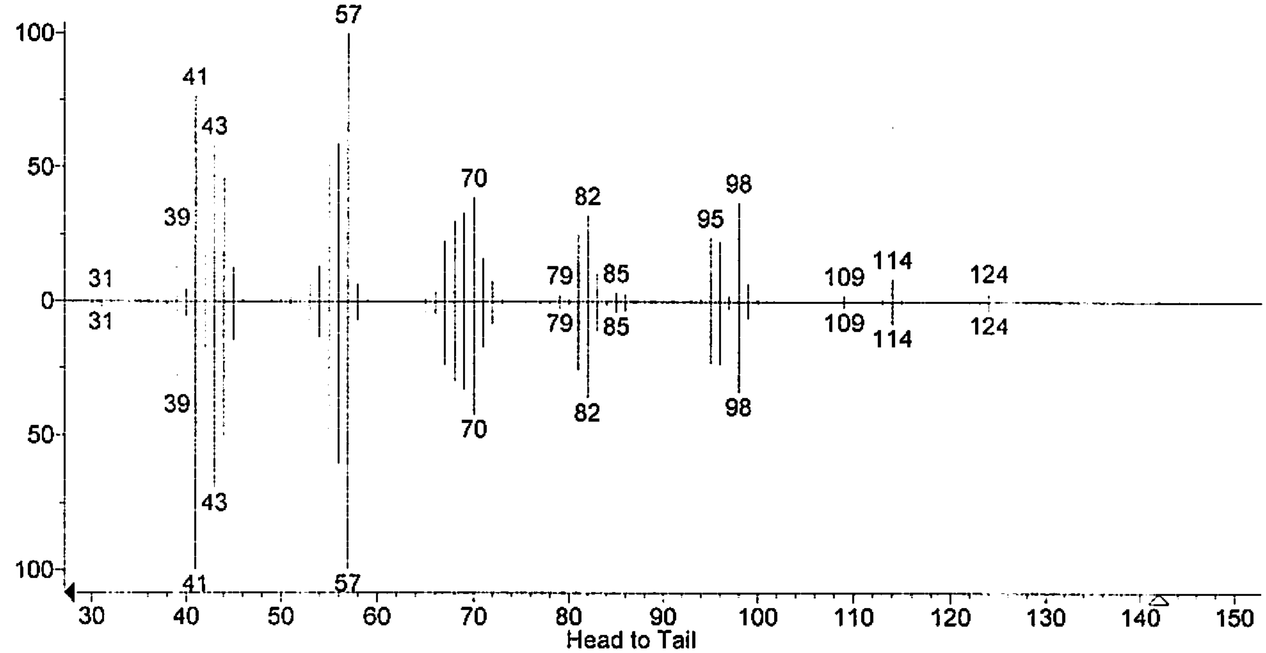


Head to Tail

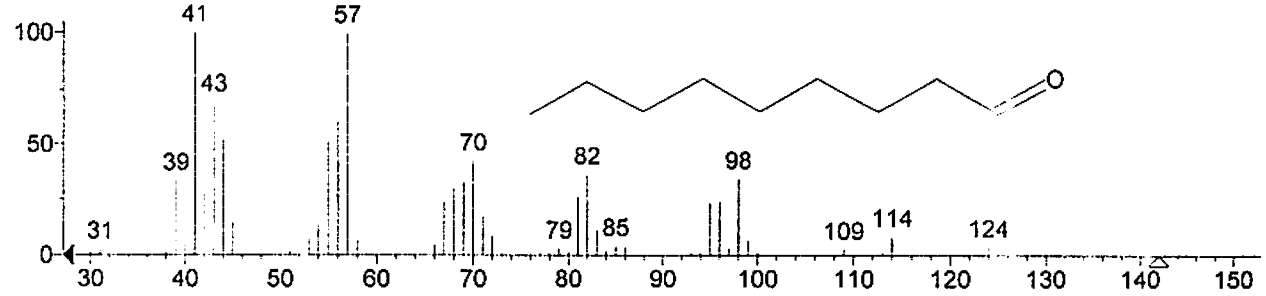


(replib) Nonanal

File : D:\DATA\Aldrich\JA-09\JA012309-2.D
Operator : Aldrich
Acquired : 23 Jan 2009 14:32 using AcqMethod JA-WAX08.M
Instrument : Instrument #1
Sample Name: 10 male C.oculata abd.ster./5ul CH2Cl2
Misc Info : 8-14-day-old; fed (+/-)-citronellal 7days
Vial Number: 1

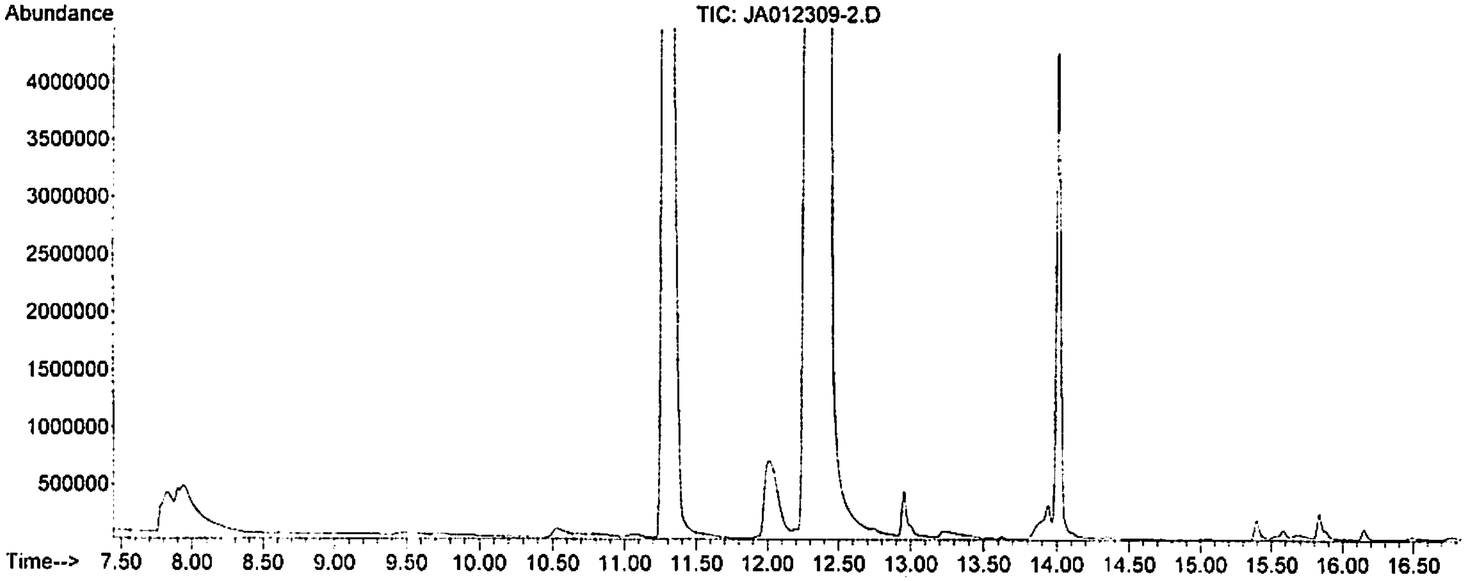


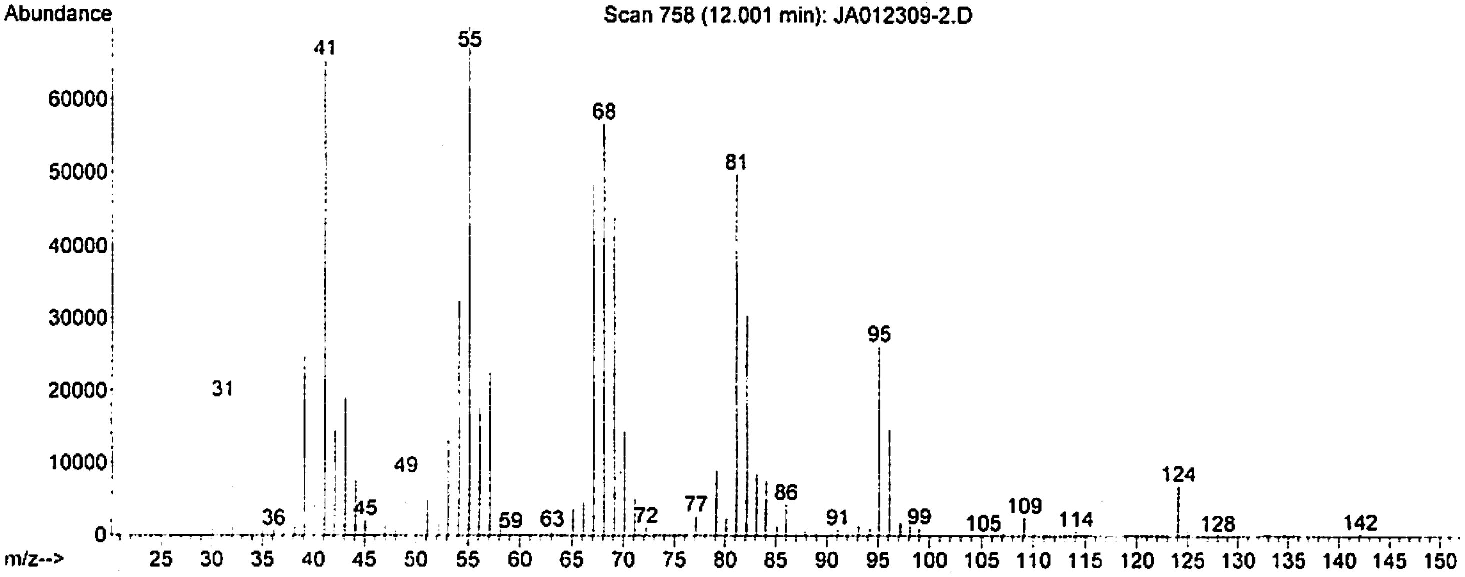

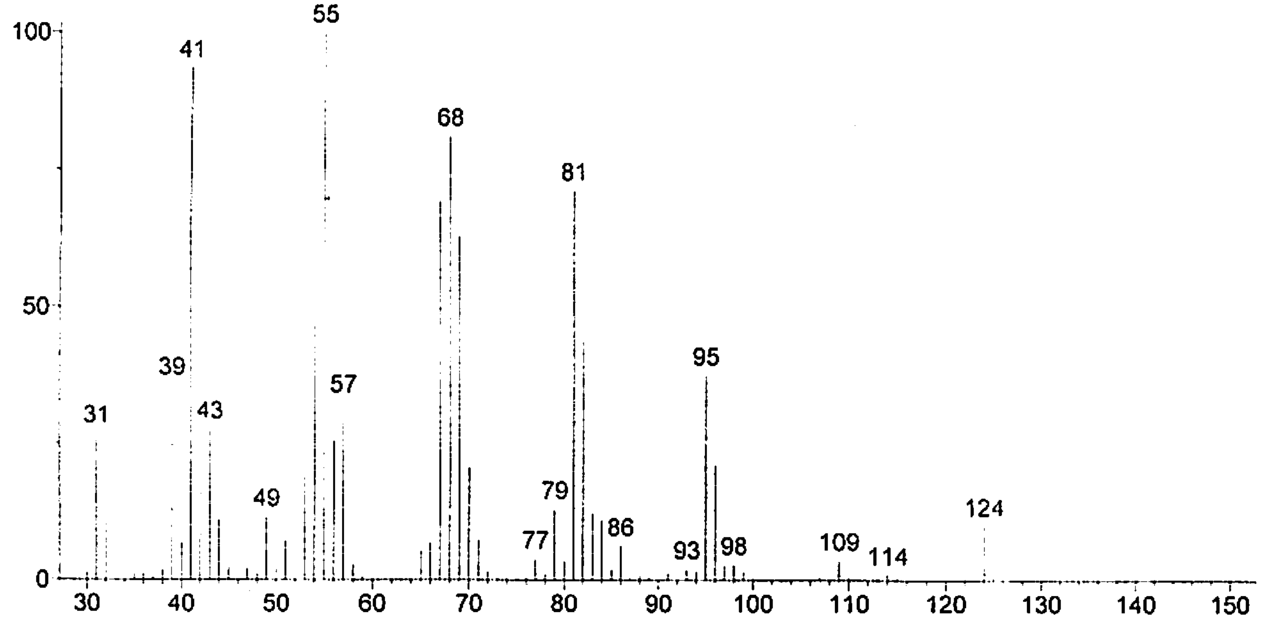


(Text File) Scan 758 (12.001 min): JA012309-2.D

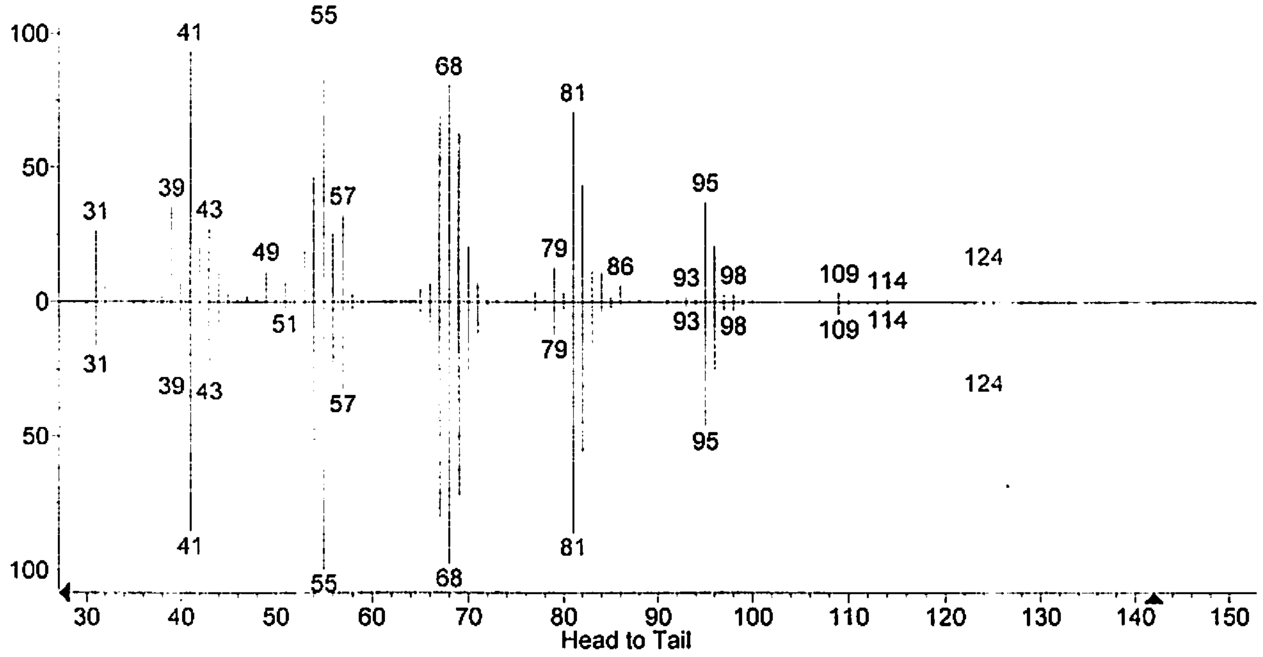


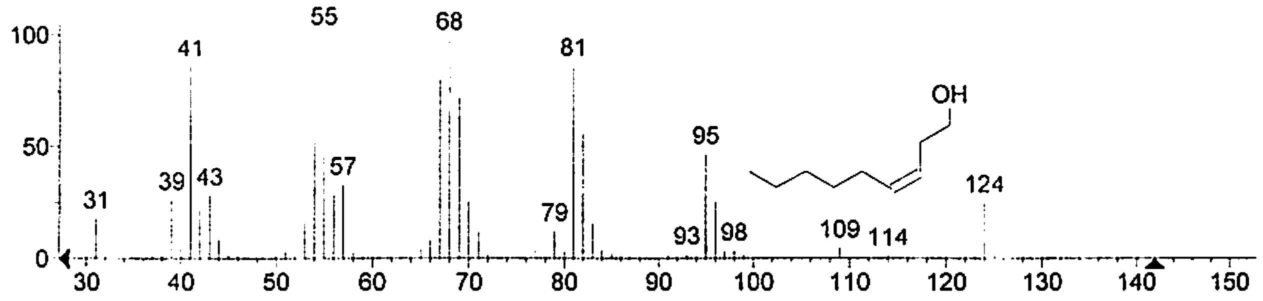


(mainlib) 3-Nonen-1-ol, (Z)-

File : D:\DATA\Aldrich\JA-09\JA012309-2.D
Operator : Aldrich
Acquired : 23 Jan 2009 14:32 using AcqMethod JA-WAX08.M
Instrument : Instrument #1
Sample Name: 10 male C.oculata abd.ster./5ul CH2Cl2
Misc Info : 8-14-day-old; fed (+/-)-citronellal 7days
Vial Number: 1

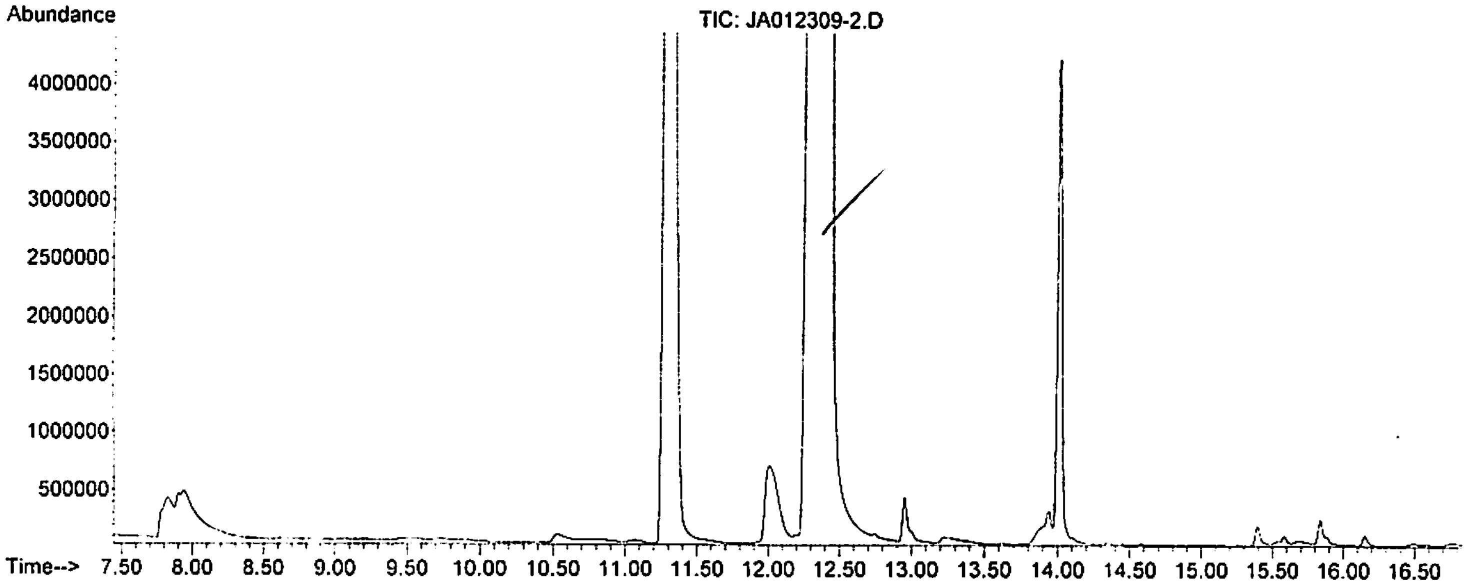


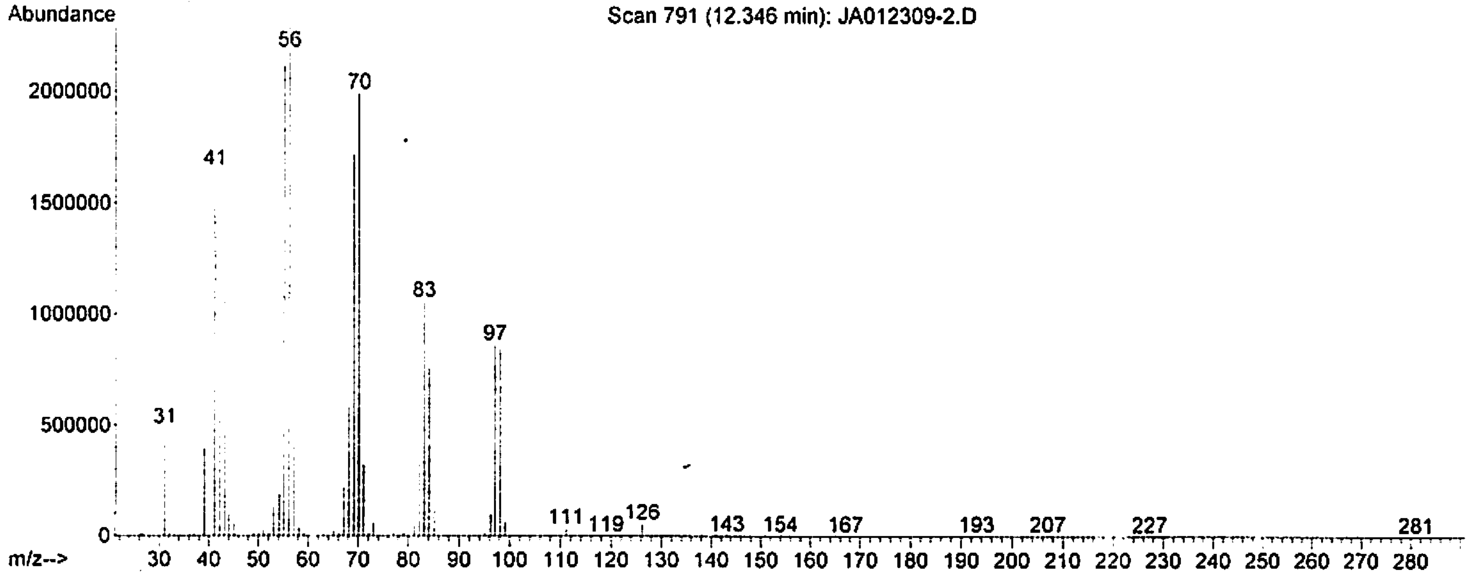

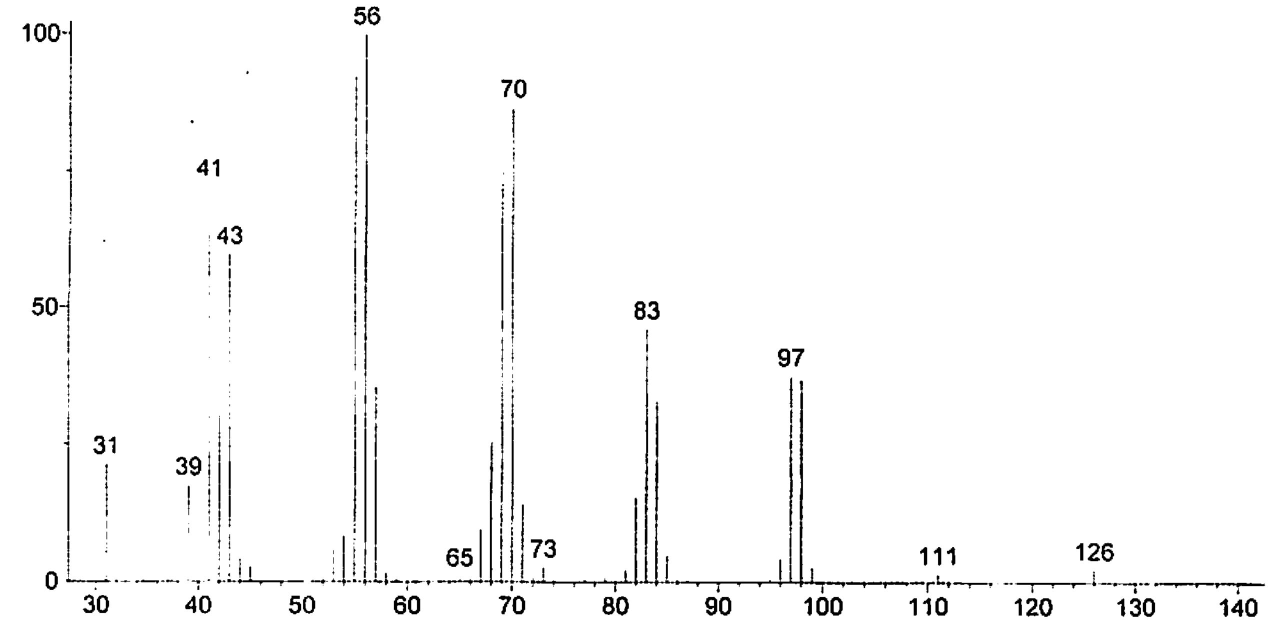


(Text File) Scan 791 (12.346 min): JA012309-2.D

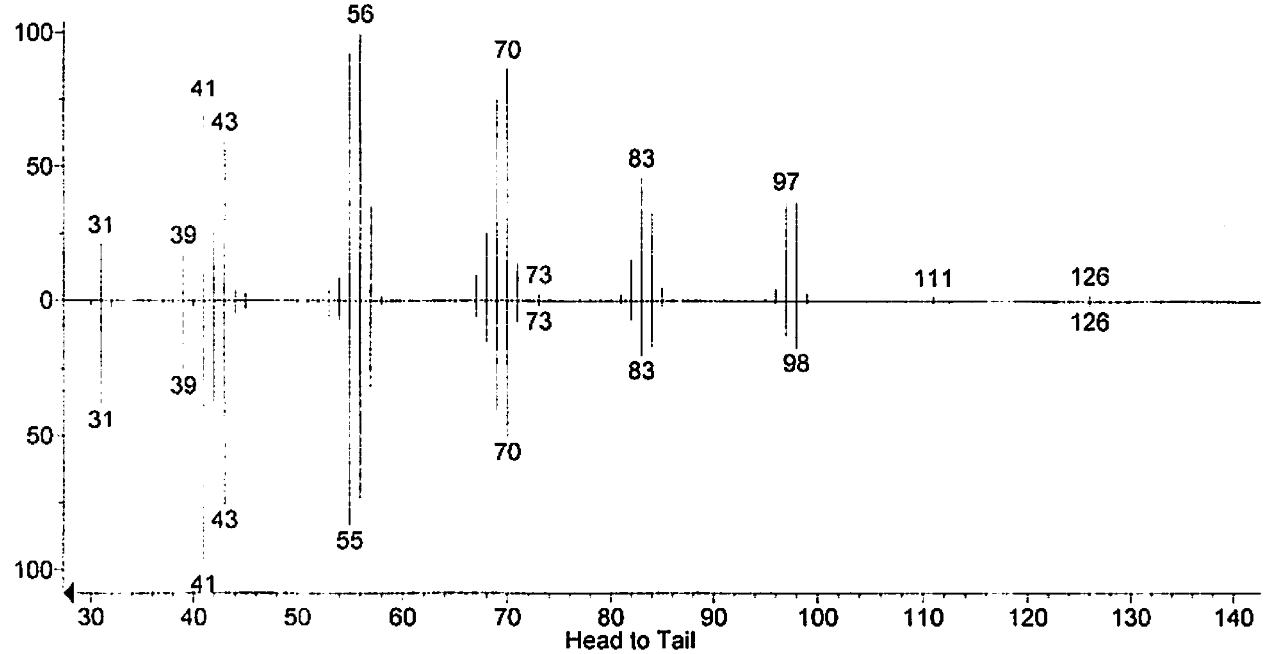


Head to Tail

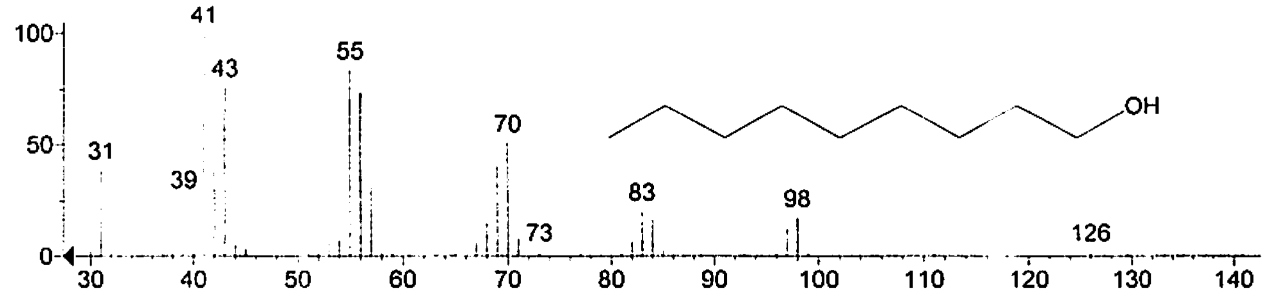


(replib) 1-Nonanol

```
File          :D:\DATA\Aldrich\JA-09\JA012309-2.D
Operator      : Aldrich
Acquired      : 23 Jan 2009  14:32      using AcqMethod JA-WAX08.M
Instrument    :    Instrument #1
Sample Name: 10 male C.oculata abd.ster./5ul CH2Cl2
Misc Info     : 8-14-day-old; fed (+/-)-citronellal 7days
Vial Number: 1
```

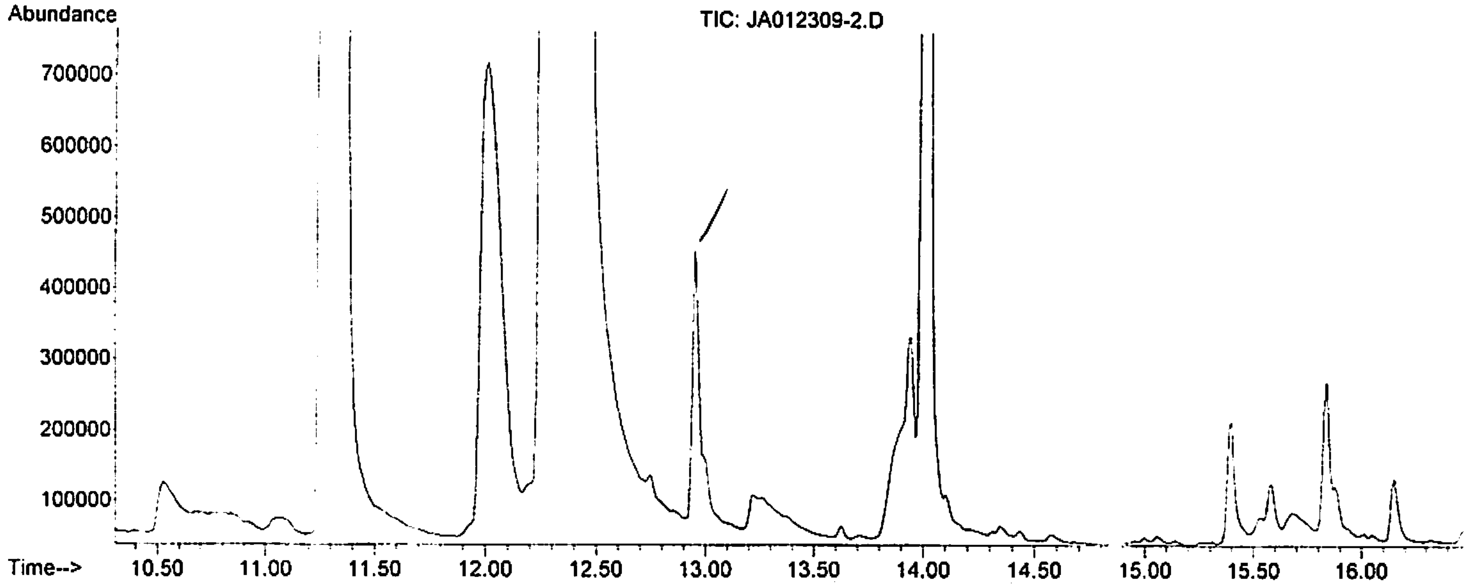


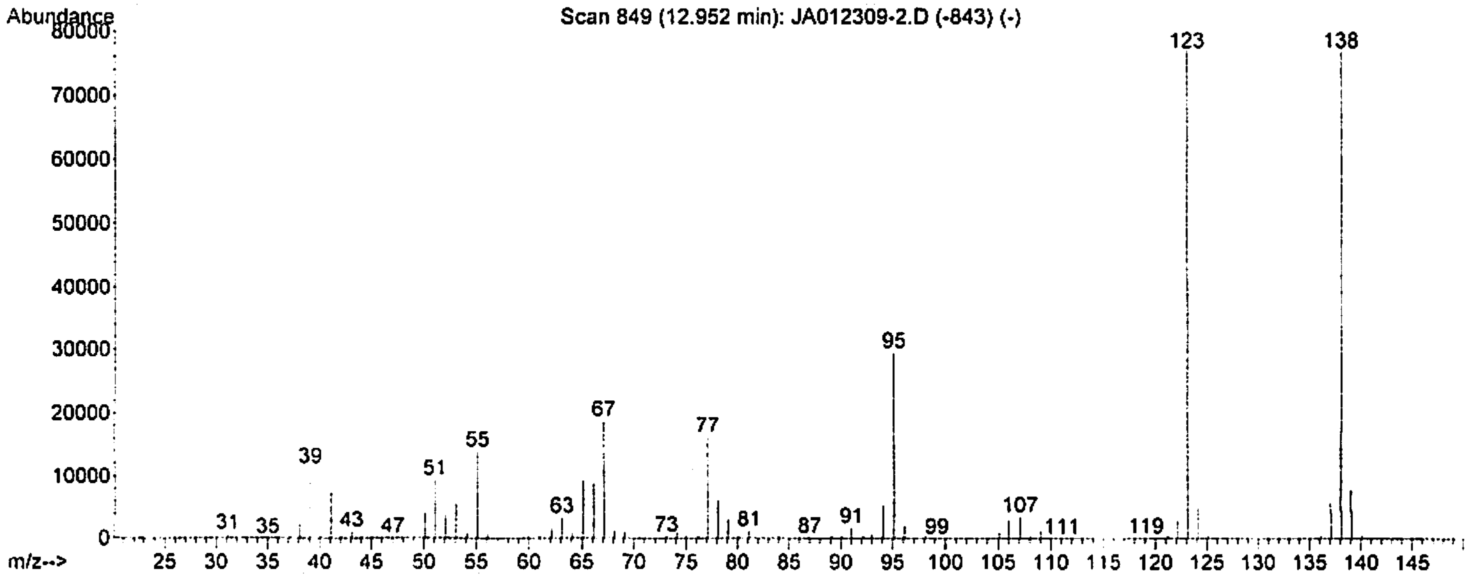

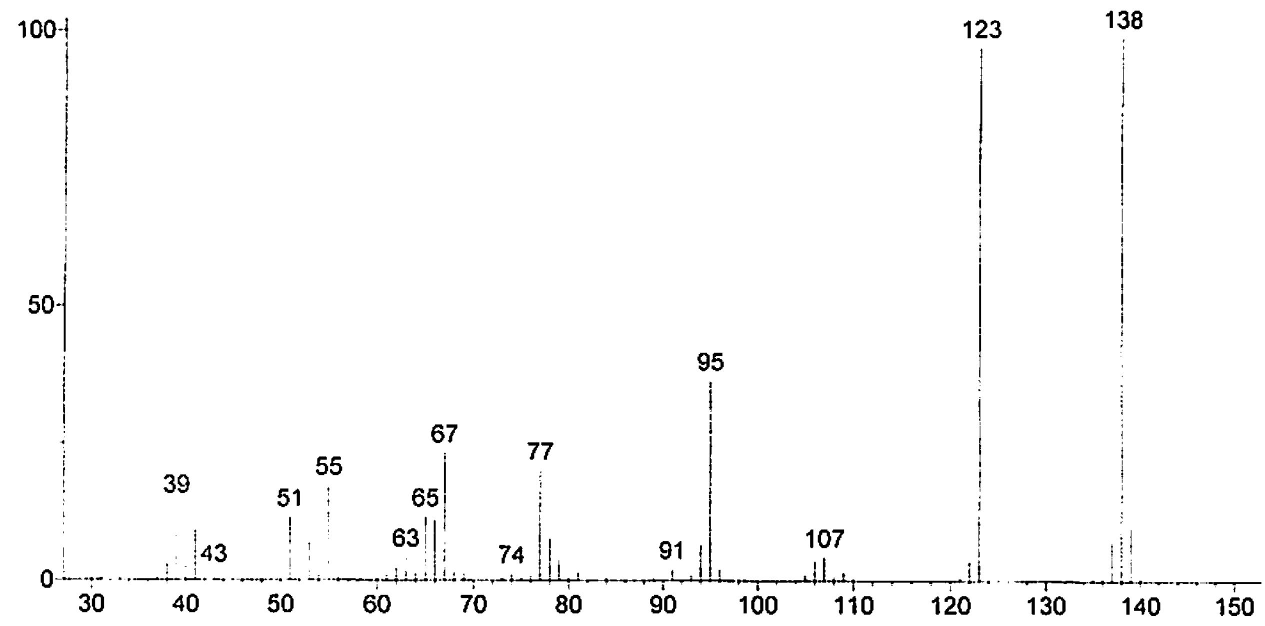

(Text File) Scan 849 (12.952 min): JA012309-2.D (-843)

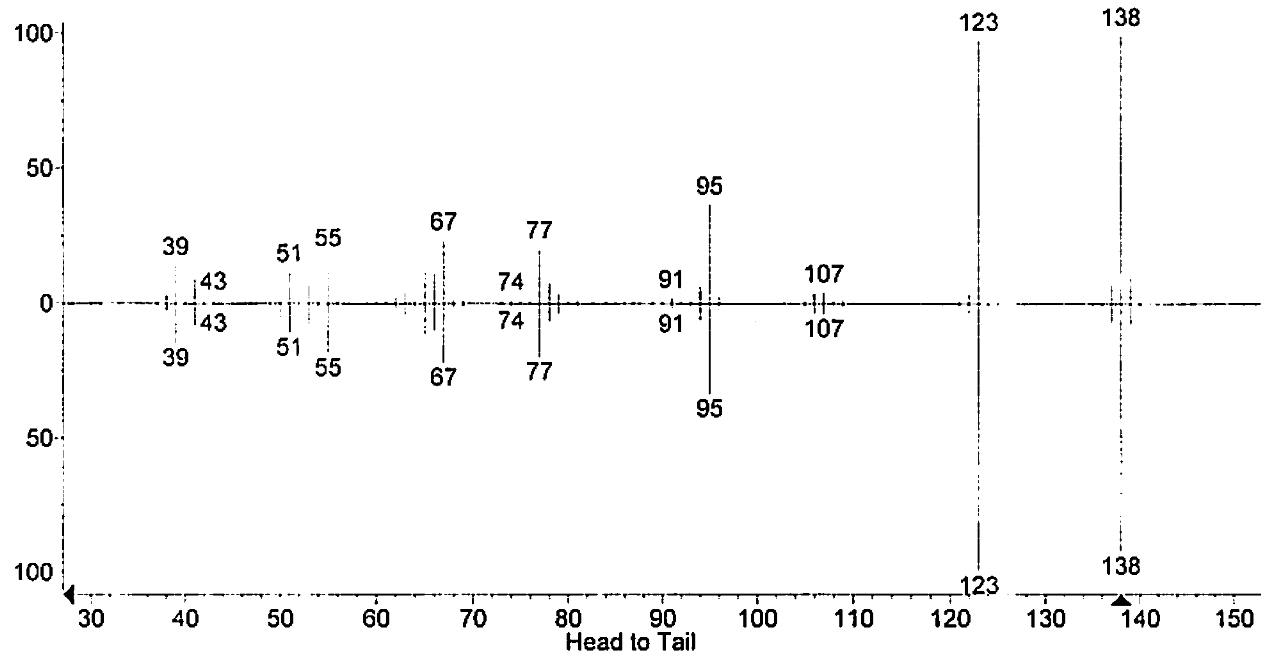


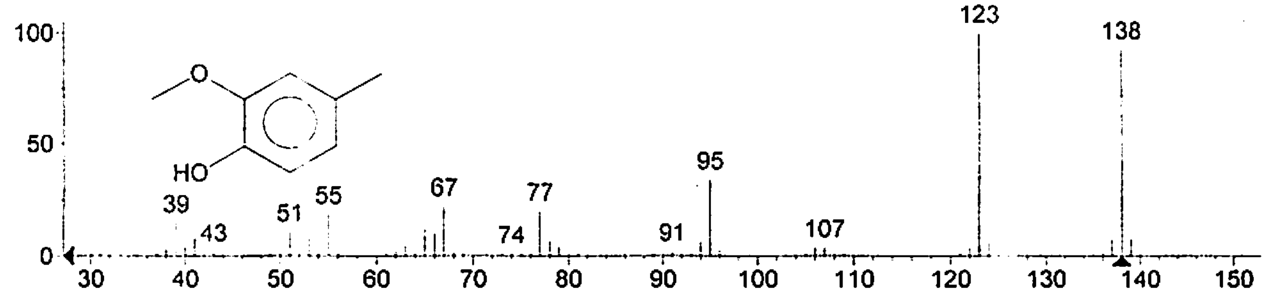

(replib) Phenol, 2-methoxy-4-methyl-

```
File          :D:\DATA\Aldrich\JA-09\JA012309-2.D
Operator      : Aldrich
Acquired      : 23 Jan 2009  14:32     using AcqMethod JA-WAX08.M
Instrument    :     Instrument #1
Sample Name:  10 male C.oculata abd.ster./5ul CH2Cl2
Misc Info     : 8-14-day-old; fed (+/-)-citronellal 7days
Vial Number:  1
```

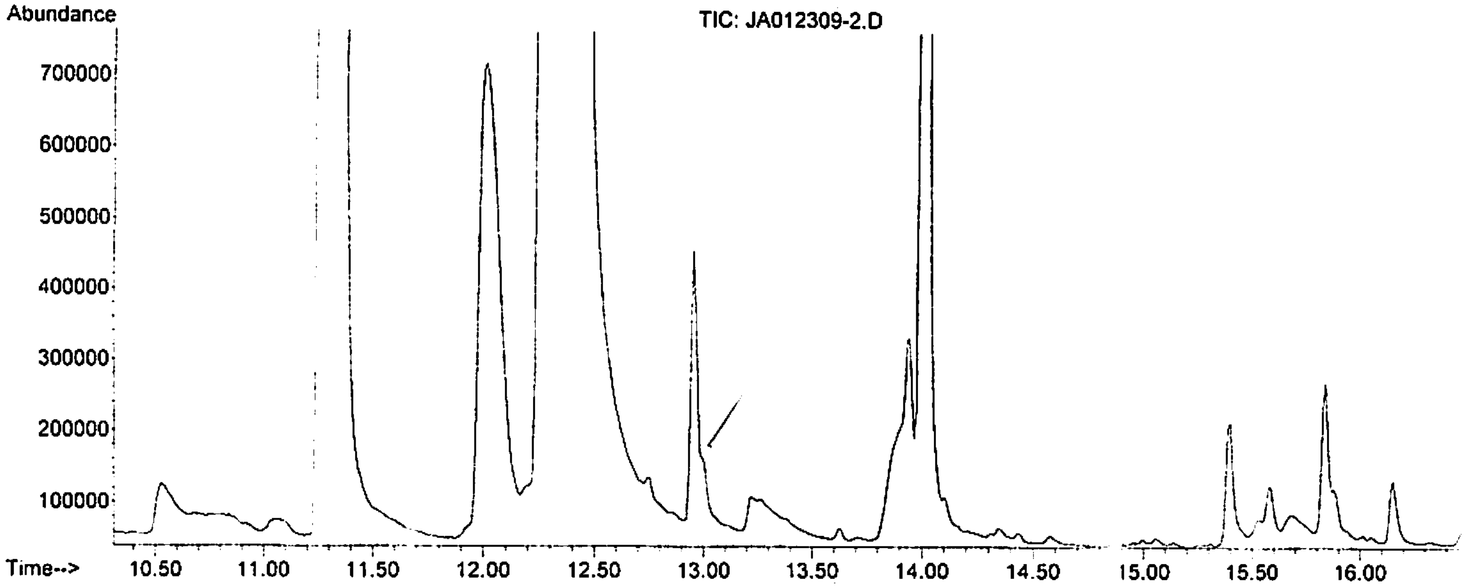


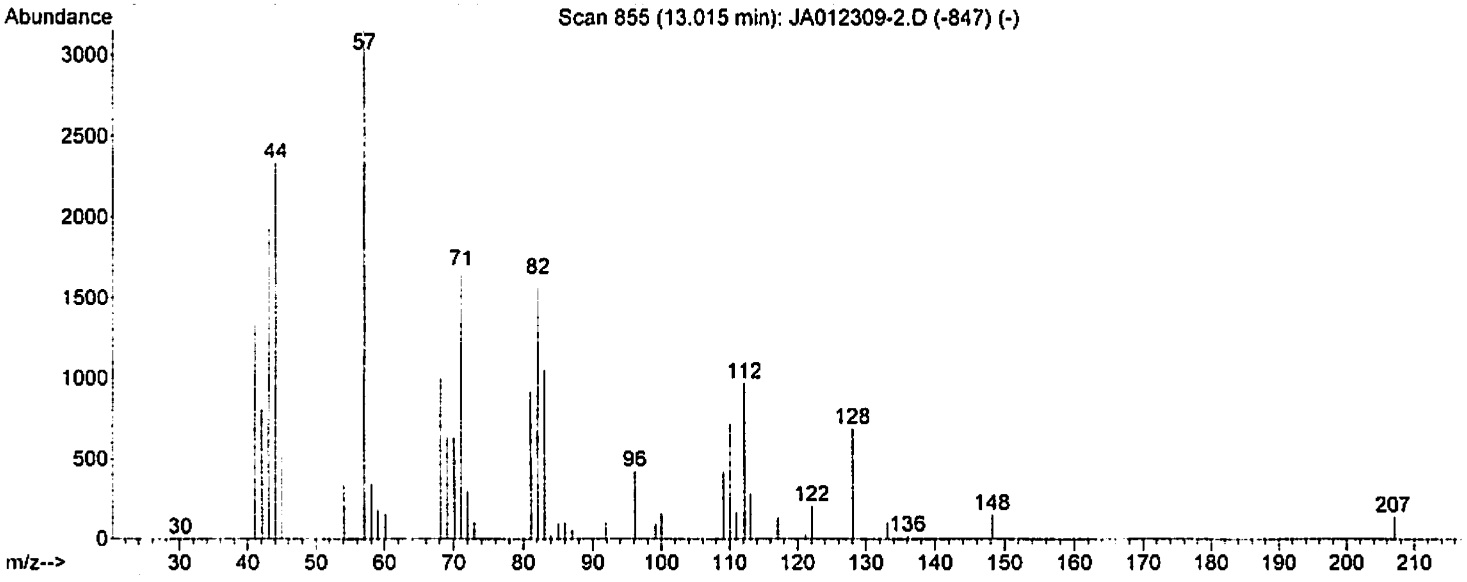

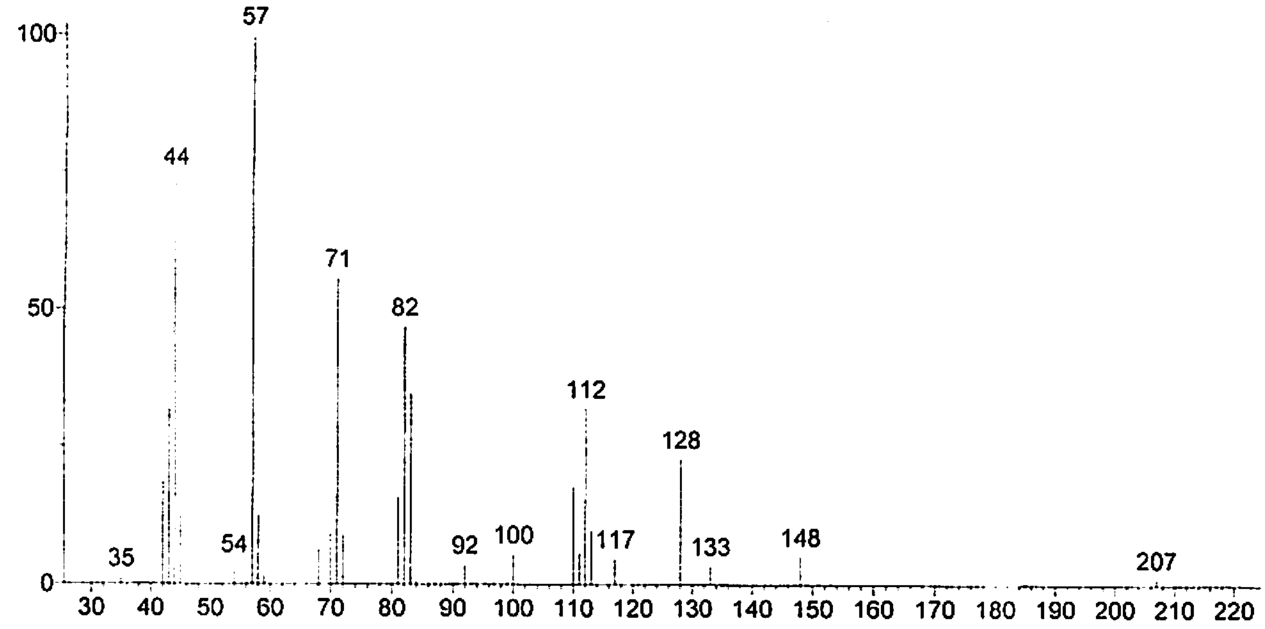


(Text File) Scan 855 (13.015 min): JA012309-2.D (-848)

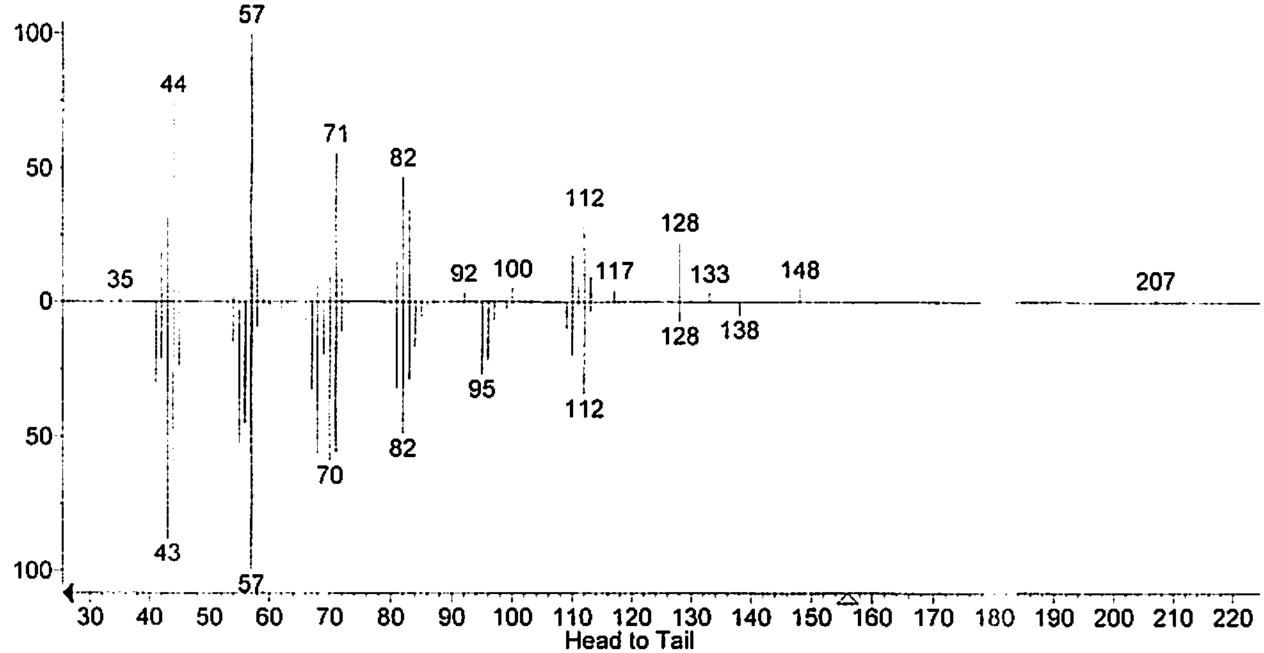


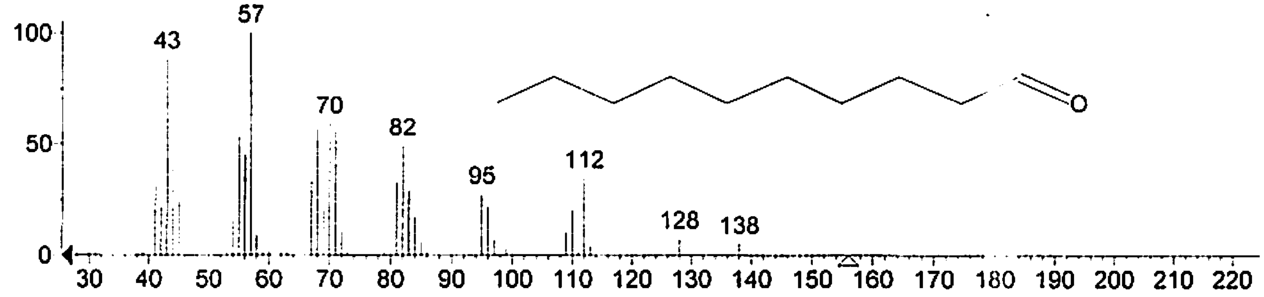


(replib) Decanal

File          :D:\DATA\Aldrich\JA-09\JA012309-2.D
Operator      : Aldrich
Acquired      : 23 Jan 2009  14:32     using AcqMethod JA-WAX08.M
Instrument :       Instrument #1
Sample Name: 10 male C.oculata abd.ster./5ul CH2Cl2
Misc Info     : 8-14-day-old; fed (+/-)-citronellal 7days
Vial Number: 1

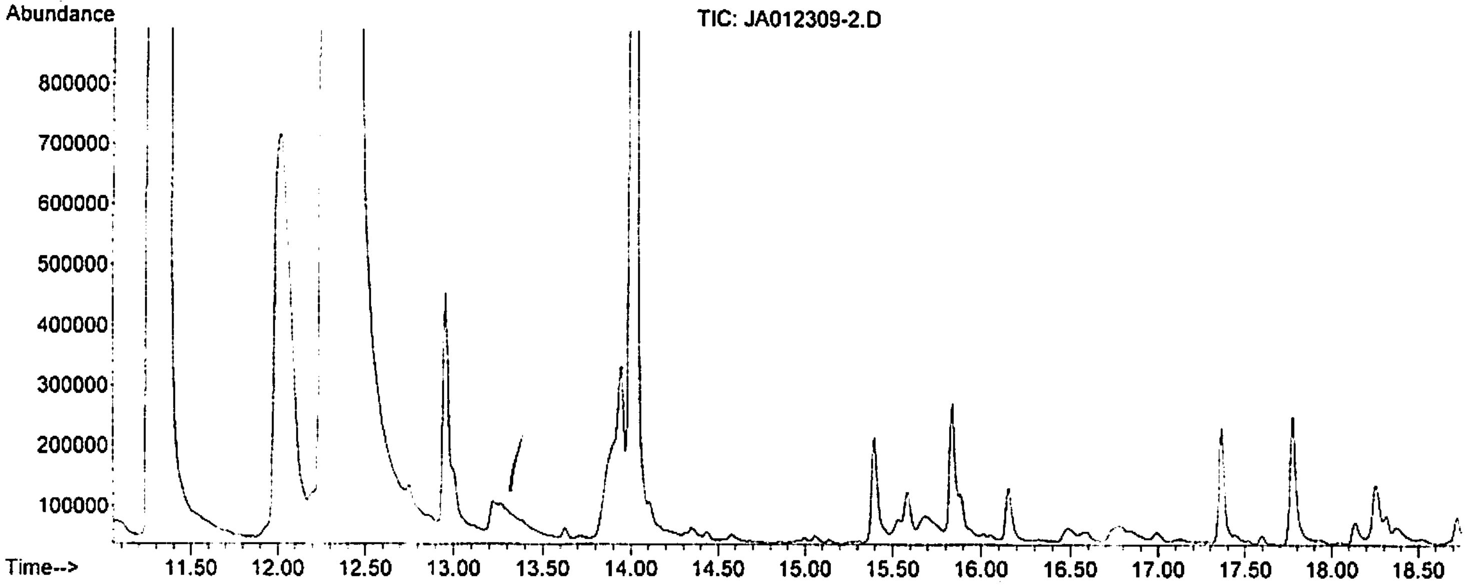


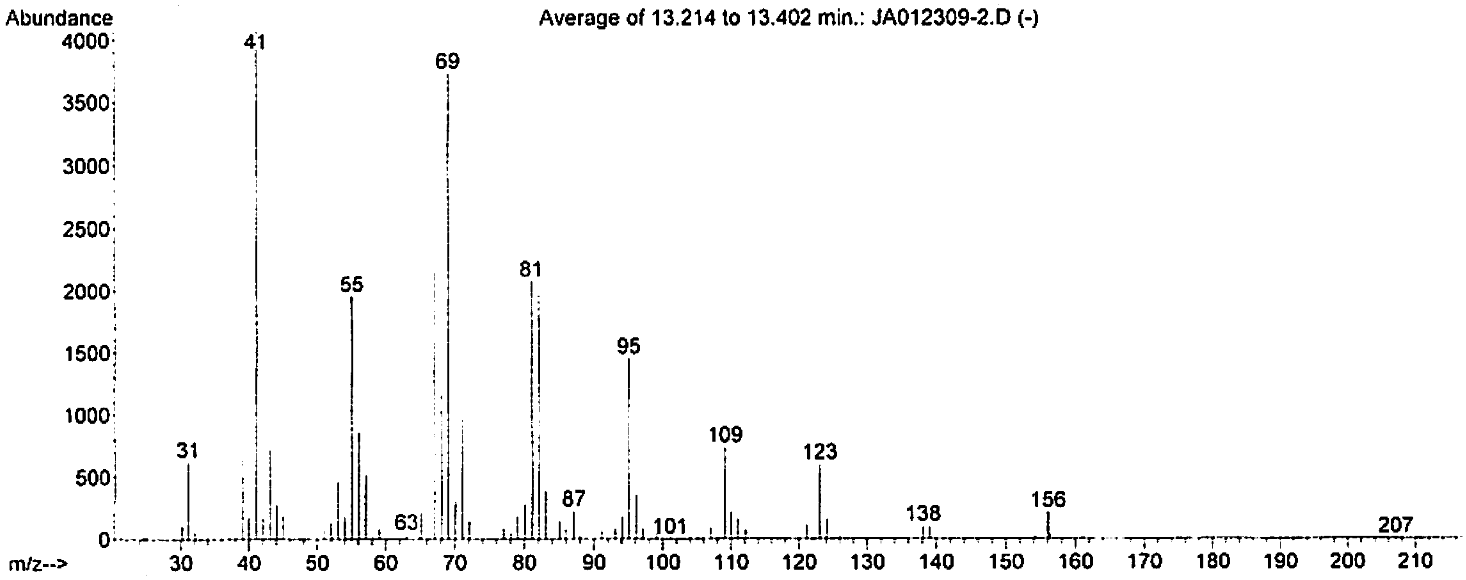

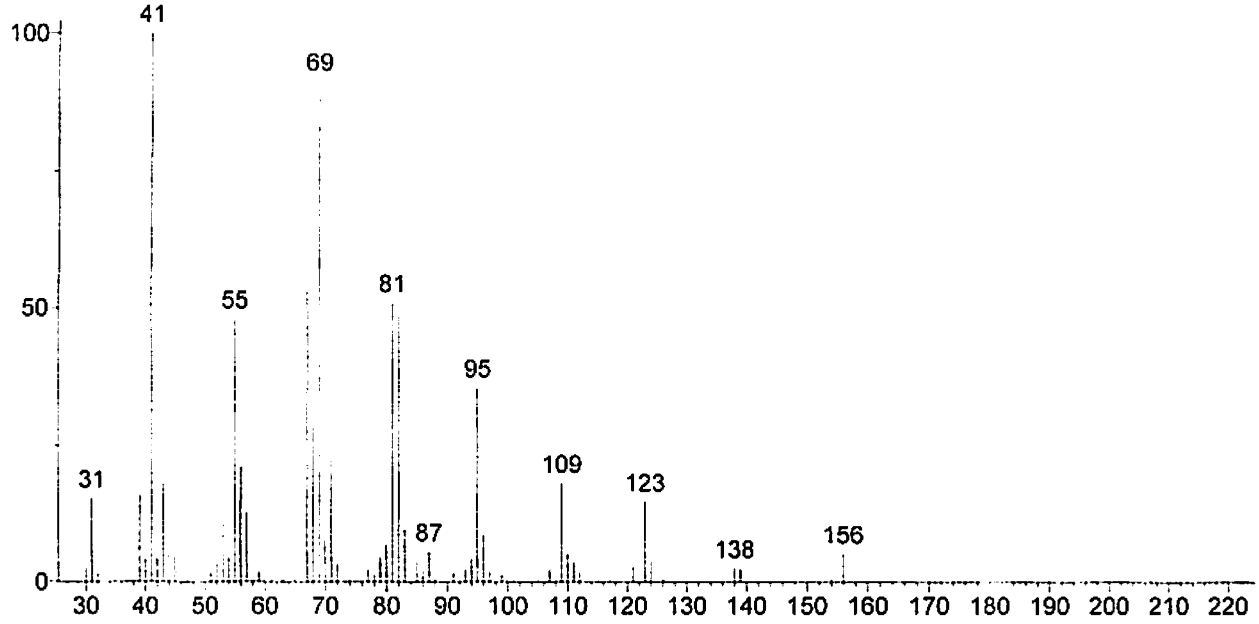


(Text File) Average of 13.214 to 13.402 min.: JA012309-2.D

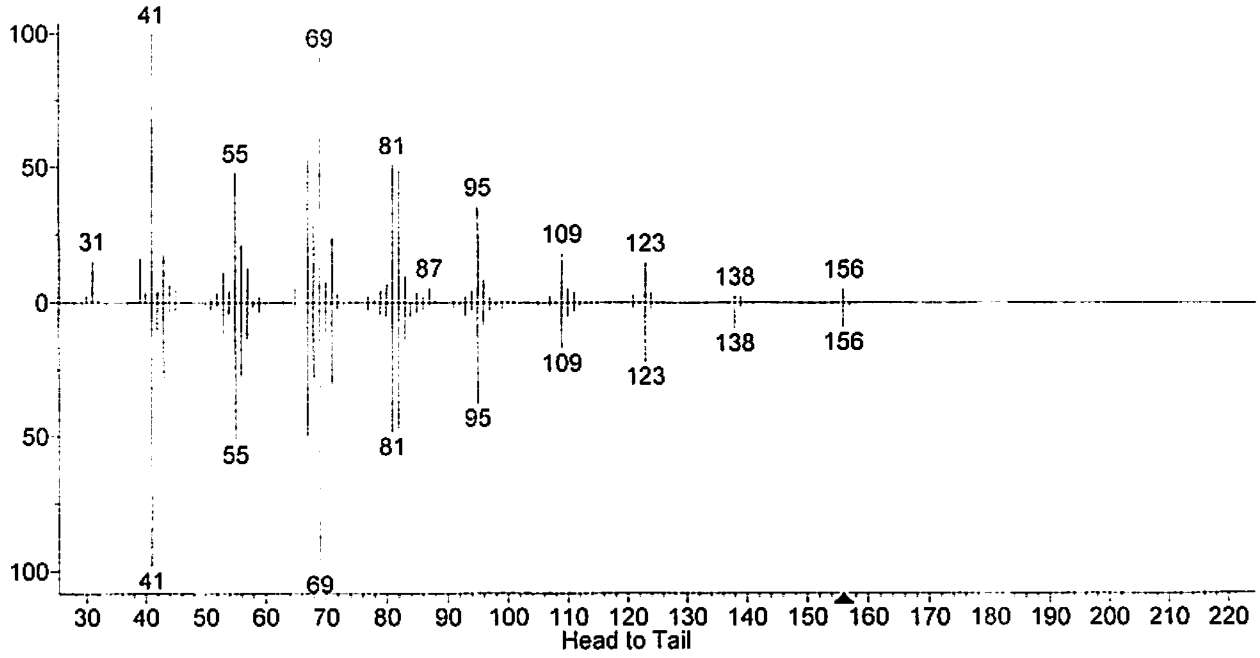


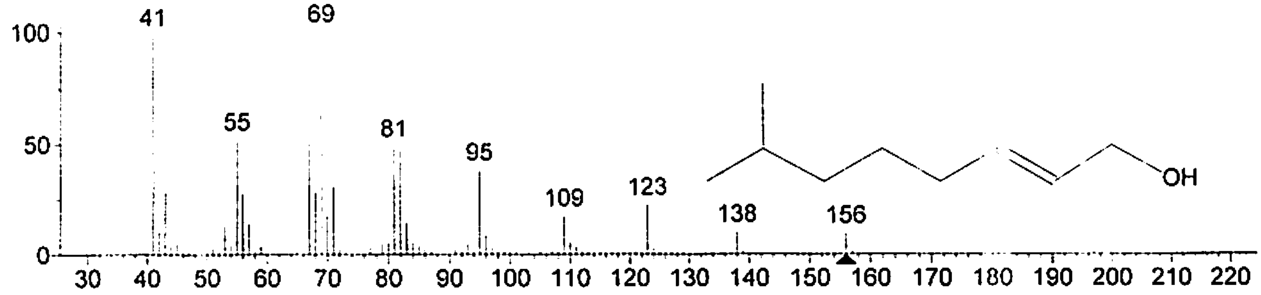


(mainlib) 2-Octen-1-ol, 3,7-dimethyl-

File       :D:\DATA\Aldrich\JA-09\JA012309-2.D
Operator   : Aldrich
Acquired   : 23 Jan 2009  14:32    using AcqMethod JA-WAX08.M
Instrument :    Instrument #1
Sample Name: 10 male C.oculata abd.ster./5ul CH2Cl2
Misc Info  : 8-14-day-old; fed (+/-)-citronellal 7days
Vial Number: 1

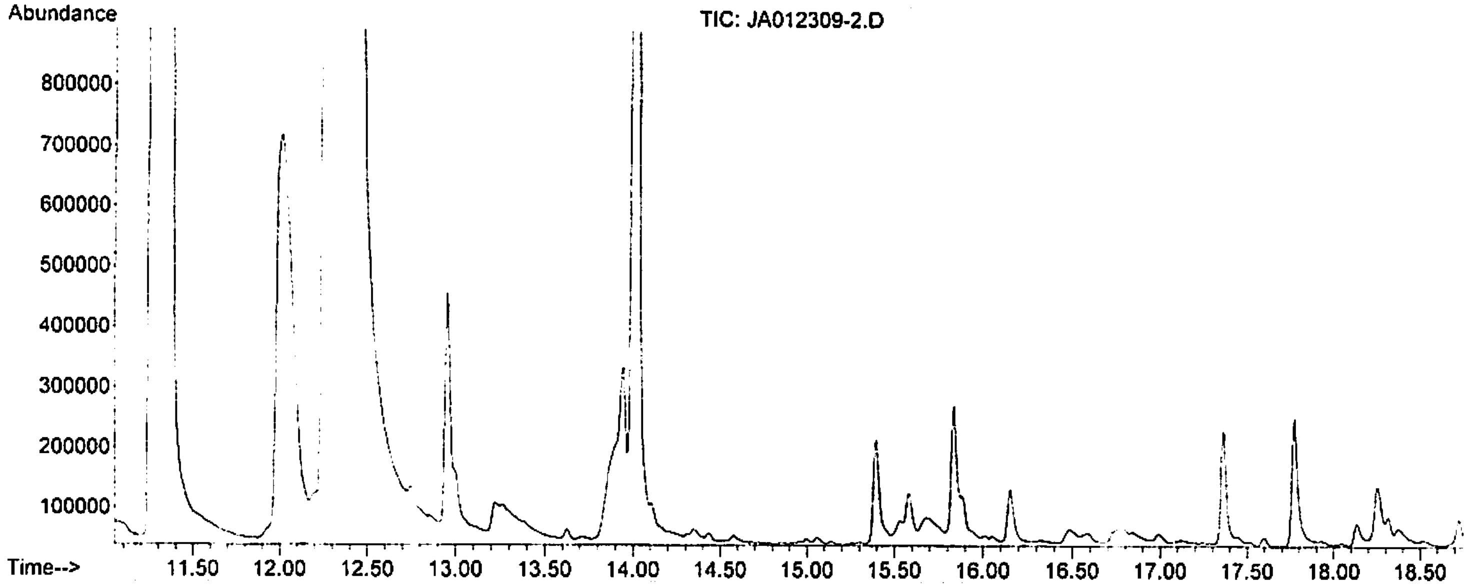


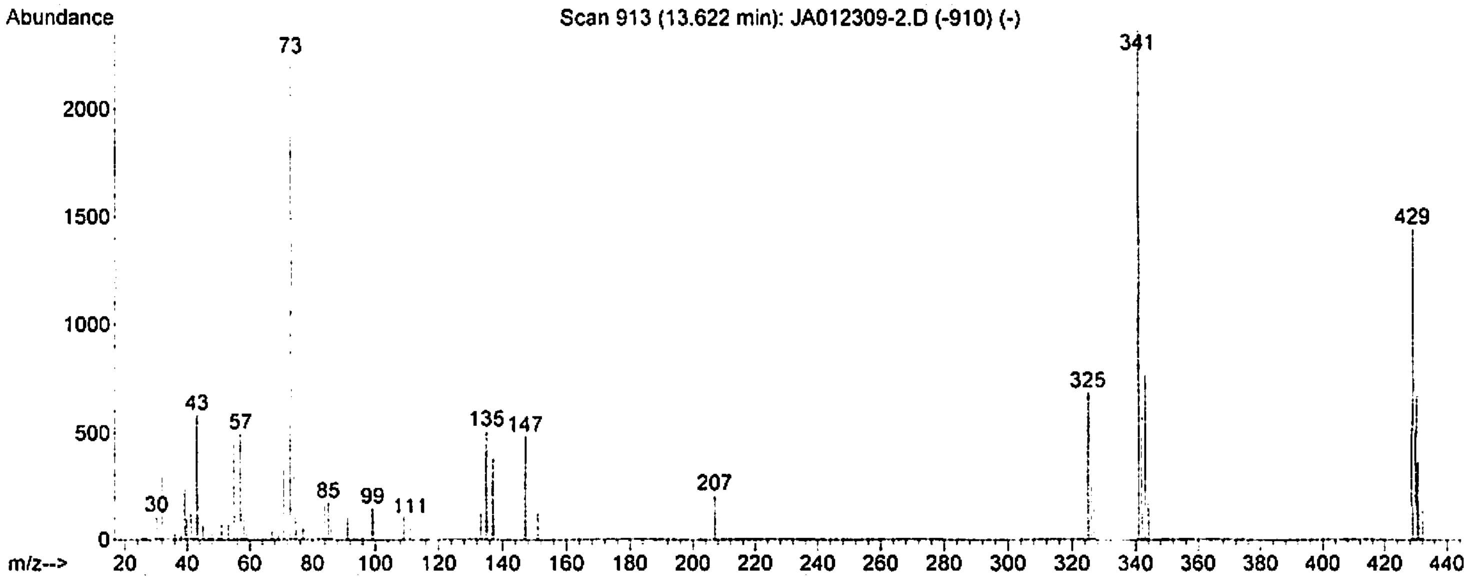

[illegible]le : D:\DATA\Aldrich\JA-09\JA012309-2.D
[illegible]perator : Aldrich
A[illegible]quired : 23 Jan 2009 14:32 using AcqMethod JA-WAX08.M
[illegible]strument : Instrument #1
[illegible]ample Name: 10 male C.oculata abd.ster./5ul CH2Cl2
[illegible]isc Info : 8-14-day-old; fed (+/-)-citronellal 7days
[illegible]ial Number: 1

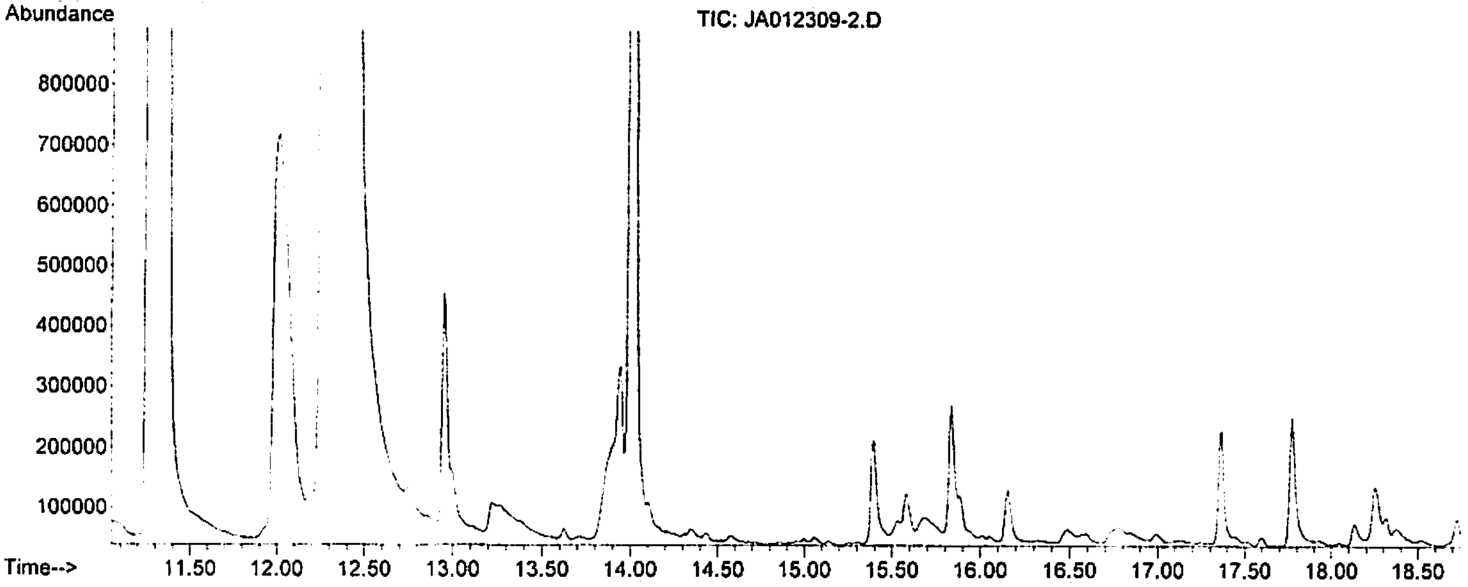


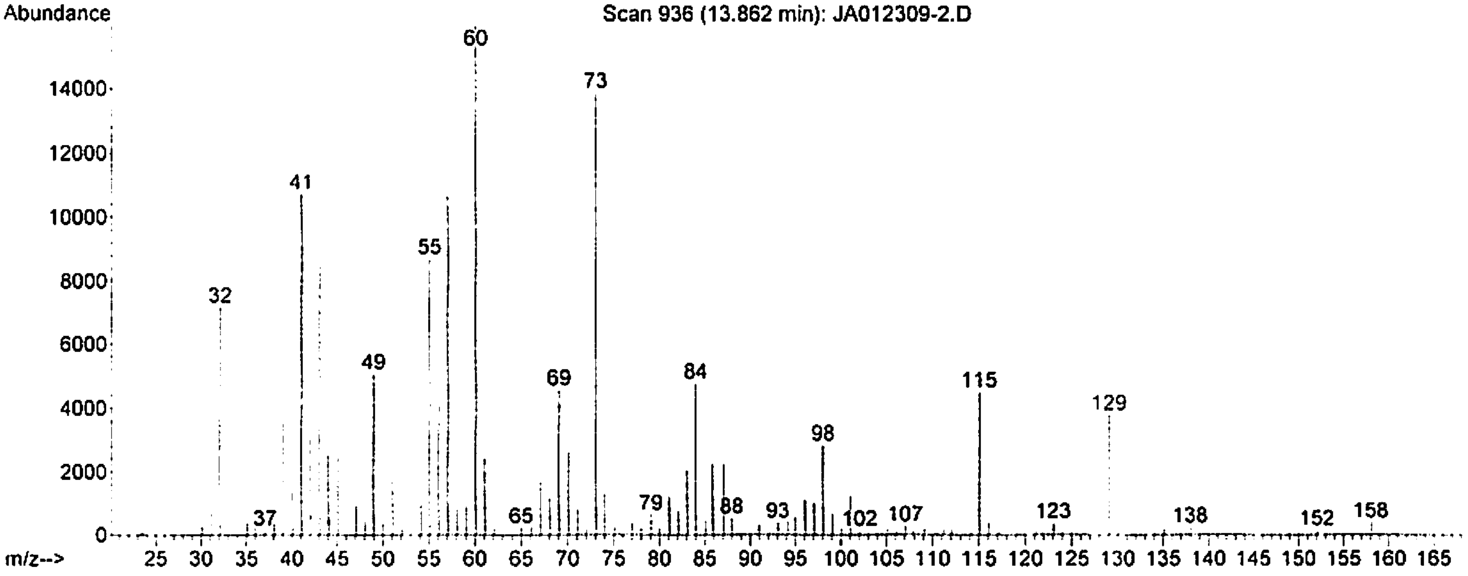

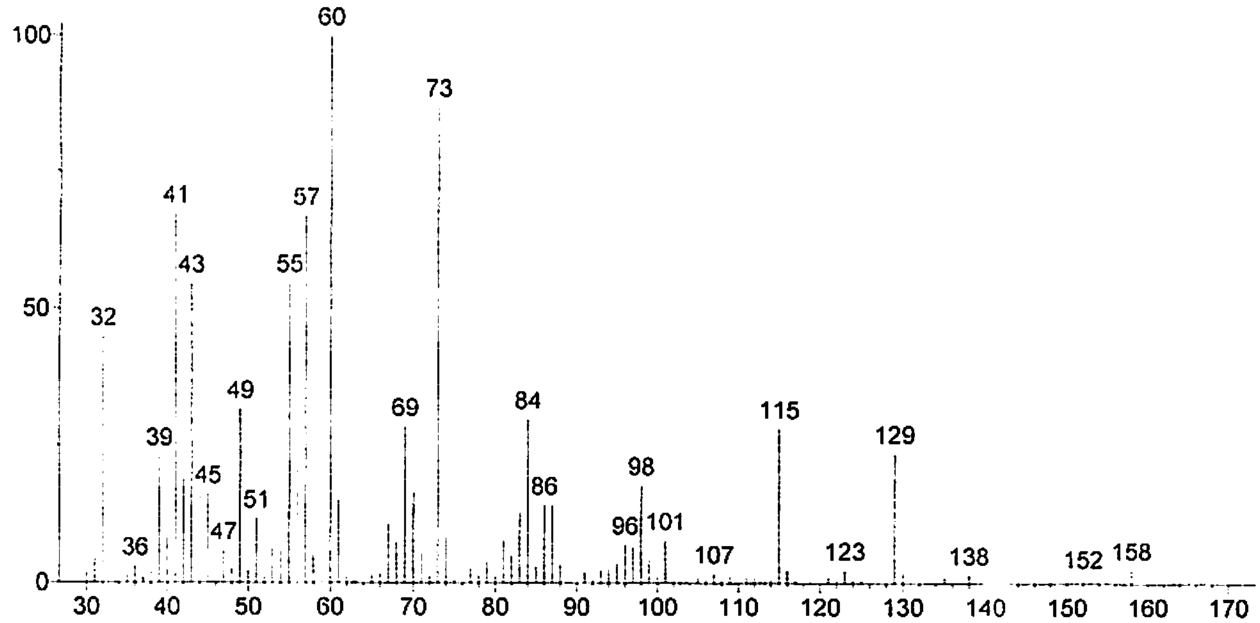


(Text File) Scan 936 (13.862 min): JA012309-2.D

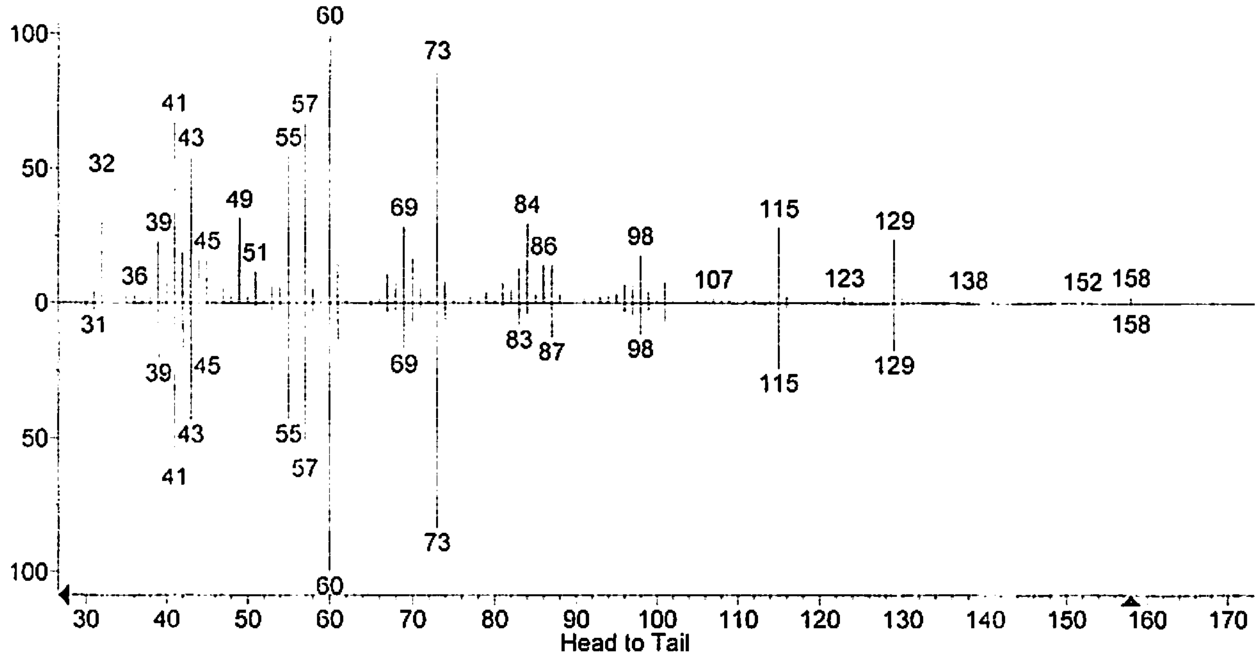


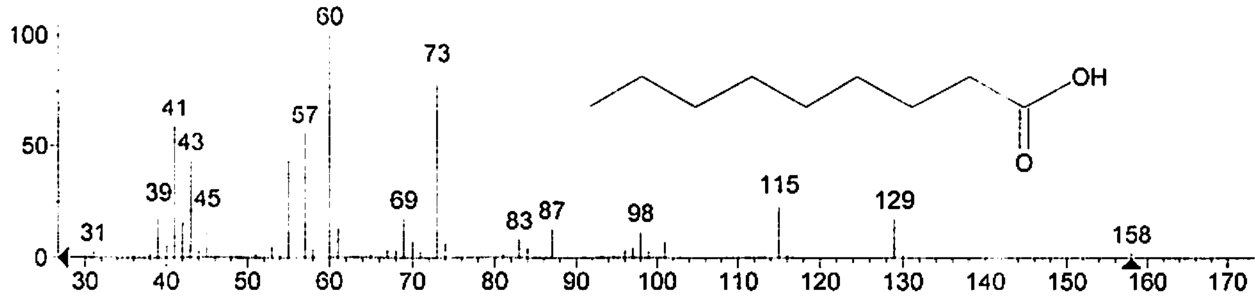


(replib) Nonanoic acid

File : D:\DATA\Aldrich\JA-09\JA012309-2.D
Operator : Aldrich
Acquired : 23 Jan 2009 14:32 using AcqMethod JA-WAX08.M
Instrument : Instrument #1
Sample Name: 10 male C.oculata abd.ster./5ul CH2Cl2
Misc Info : 8-14-day-old; fed (+/-)-citronellal 7days
Vial Number: 1

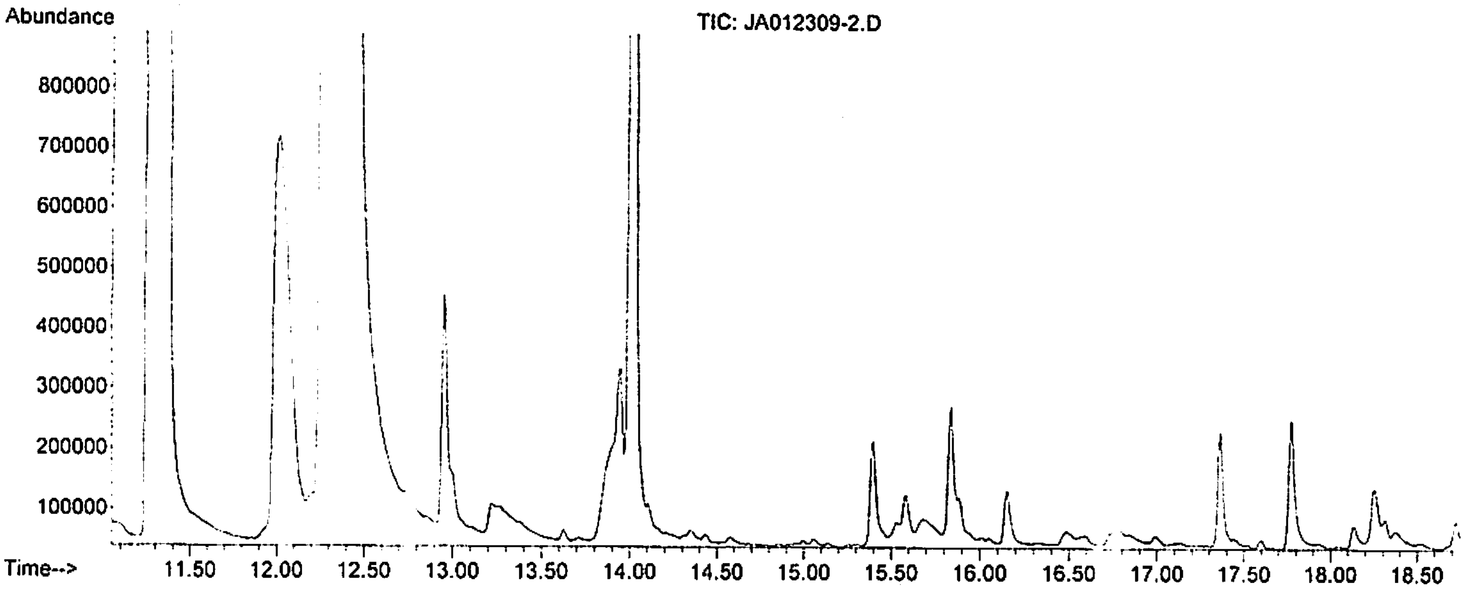


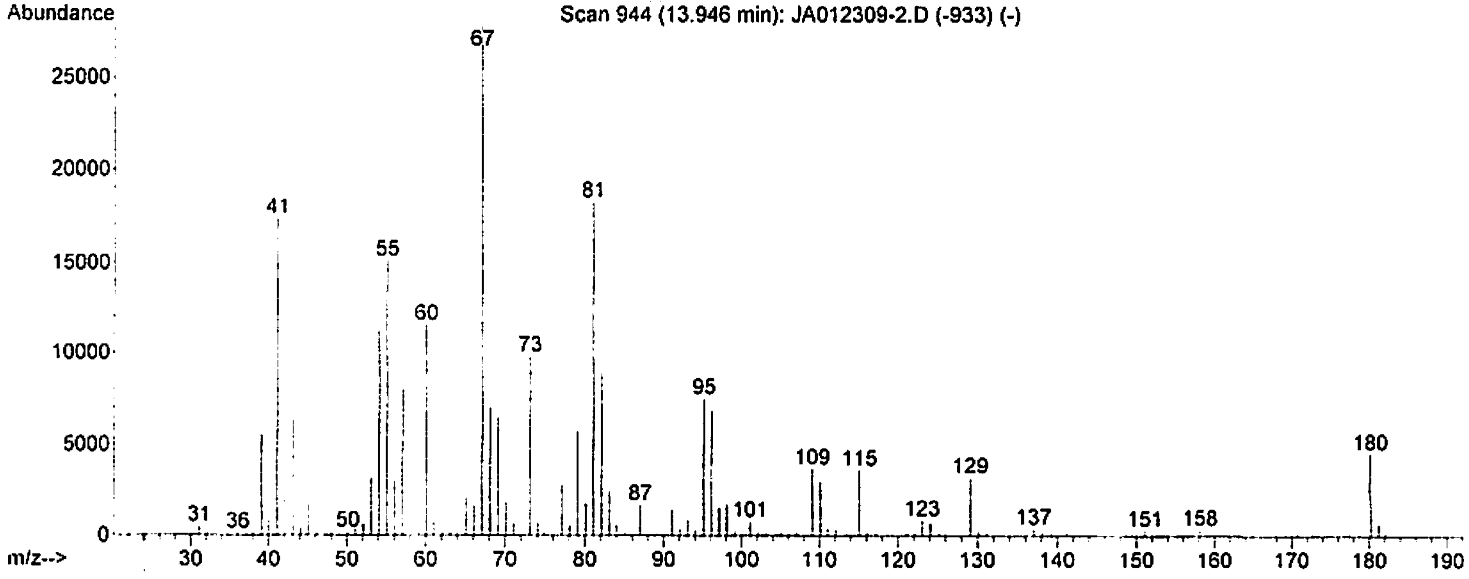

File :D:\DATA\Aldrich\JA-09\JA012309-2.D
Operator : Aldrich
Acquired : 23 Jan 2009 14:32 using AcqMethod JA-WAX08.M
Instrument : Instrument #1
Sample Name: 10 male C.oculata abd.ster./5ul CH2Cl2
Misc Info : 8-14-day-old; fed (+/-)-citronellal 7days
Vial Number: 1

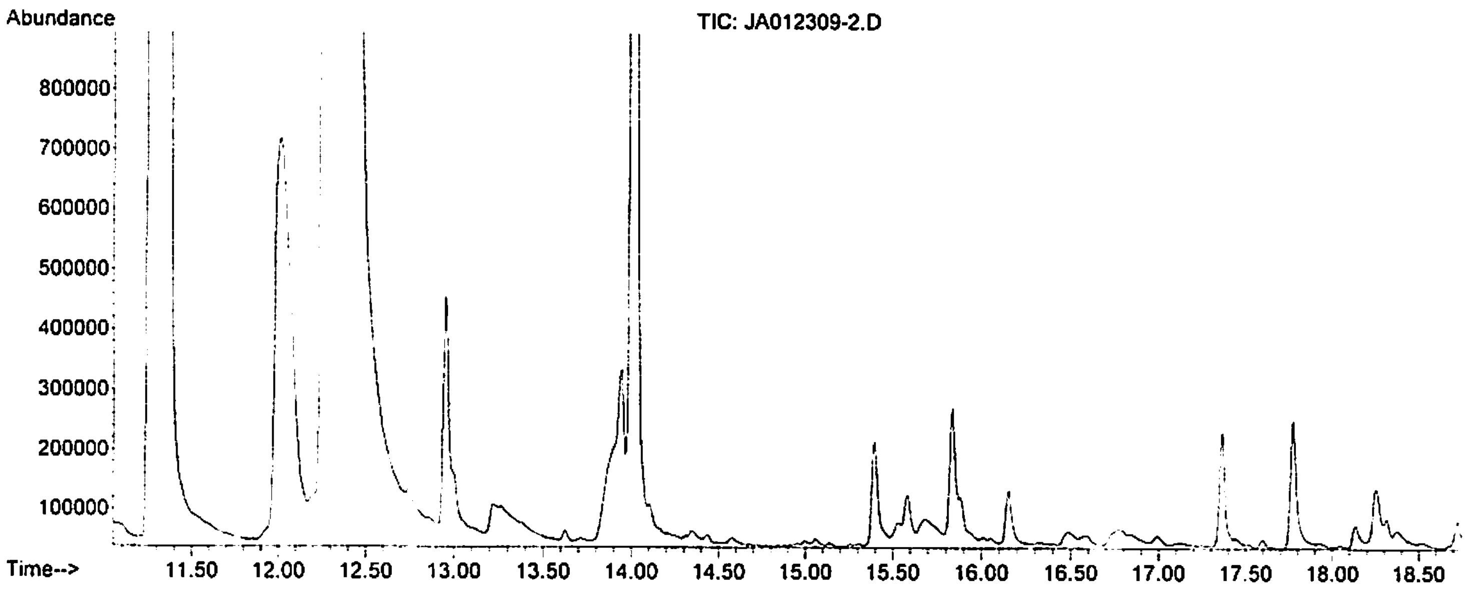


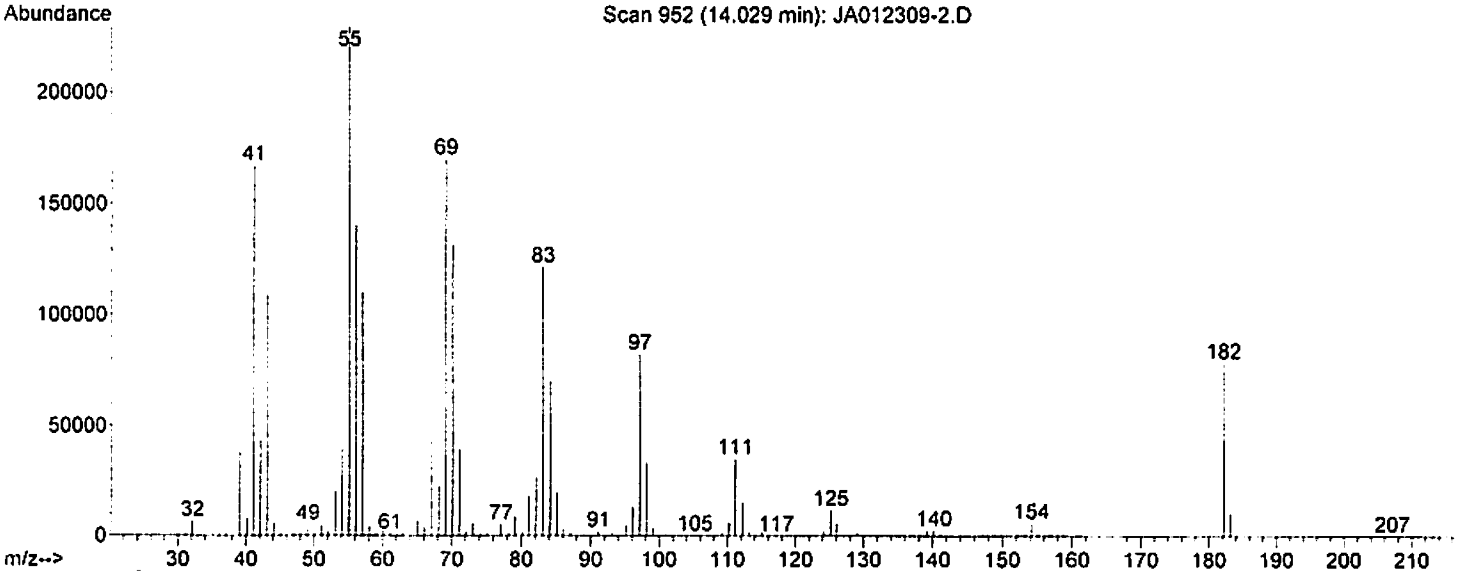

File : D:\DATA\Aldrich\JA-09\JA012309-2.D
Operator : Aldrich
Acquired : 23 Jan 2009 14:32 using AcqMethod JA-WAX08.M
Instrument : Instrument #1
Sample Name: 10 male C.oculata abd.ster./5ul CH2Cl2
Misc Info : 8-14-day-old; fed (+/-)-citronellal 7days
Vial Number: 1

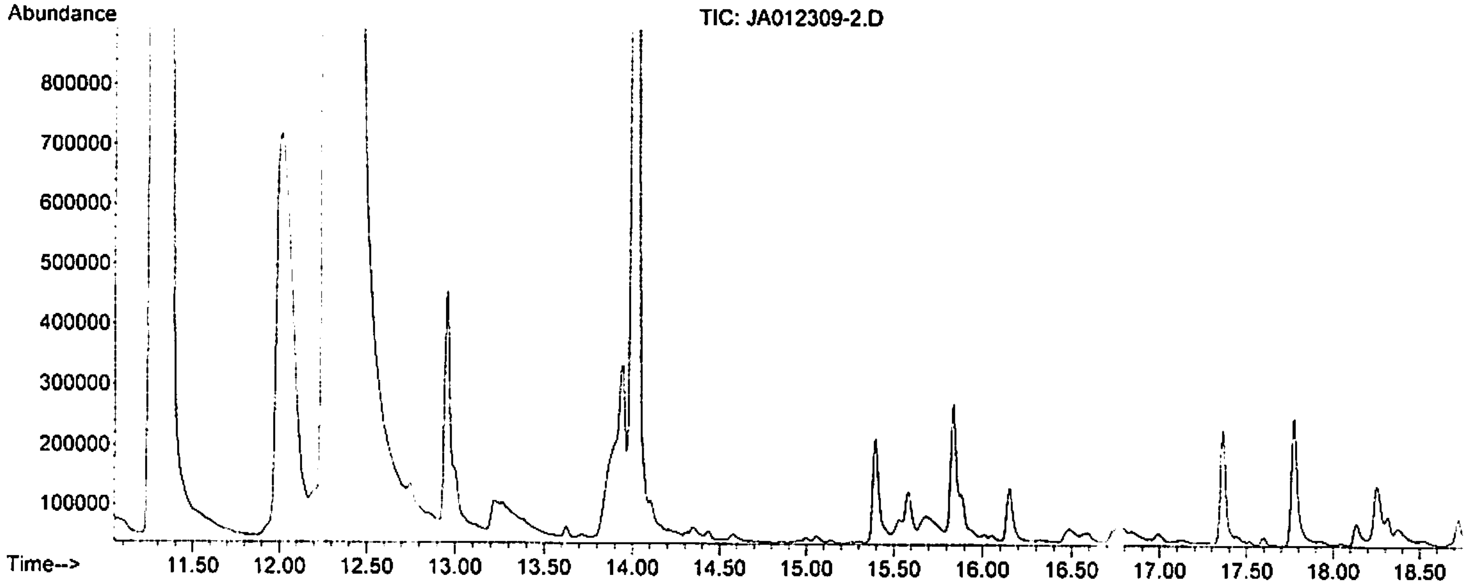


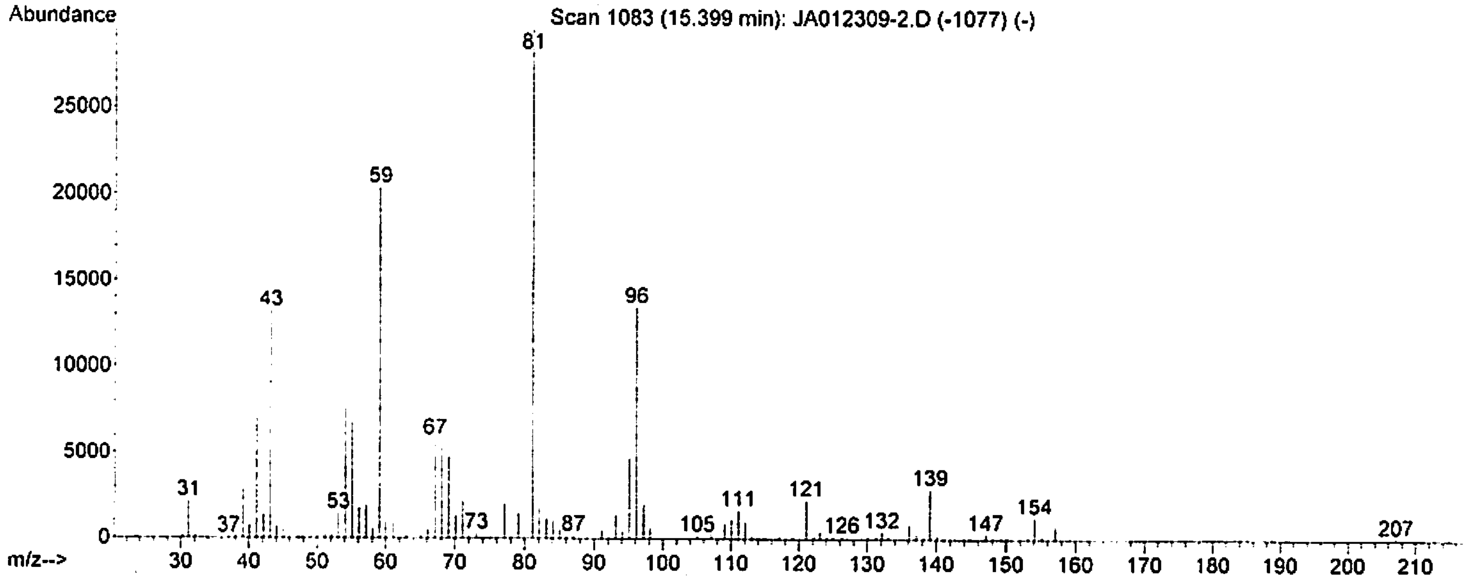

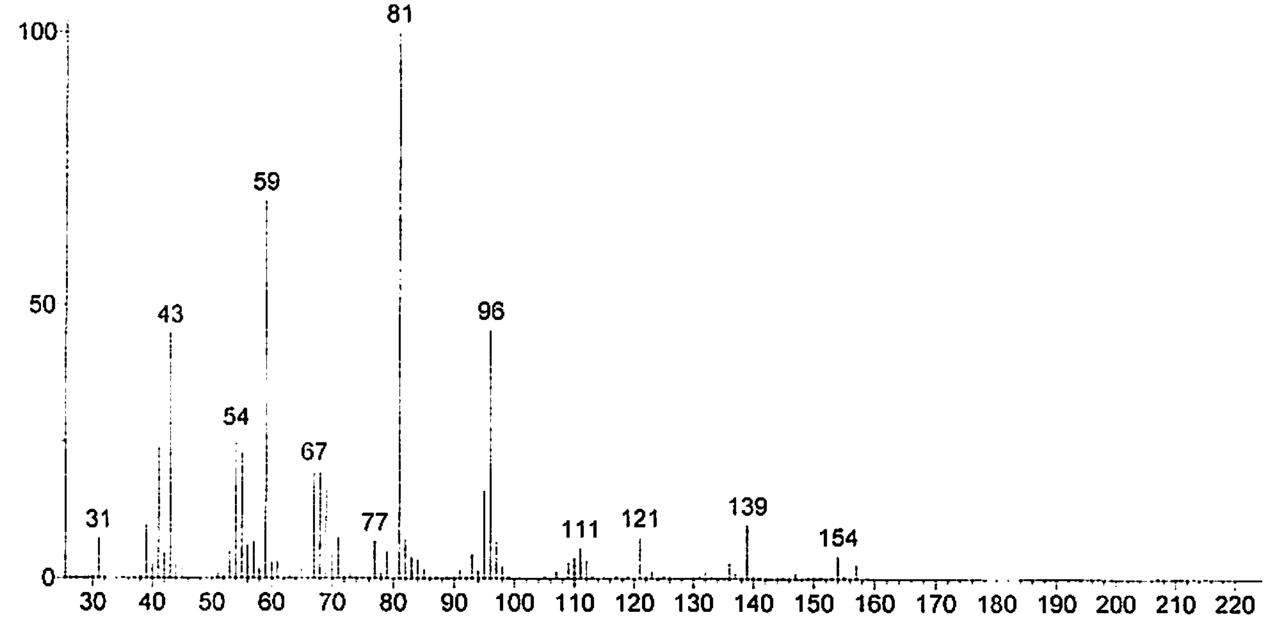


(Text File) Scan 1083 (15.399 min): JA012309-2.D (-1077)

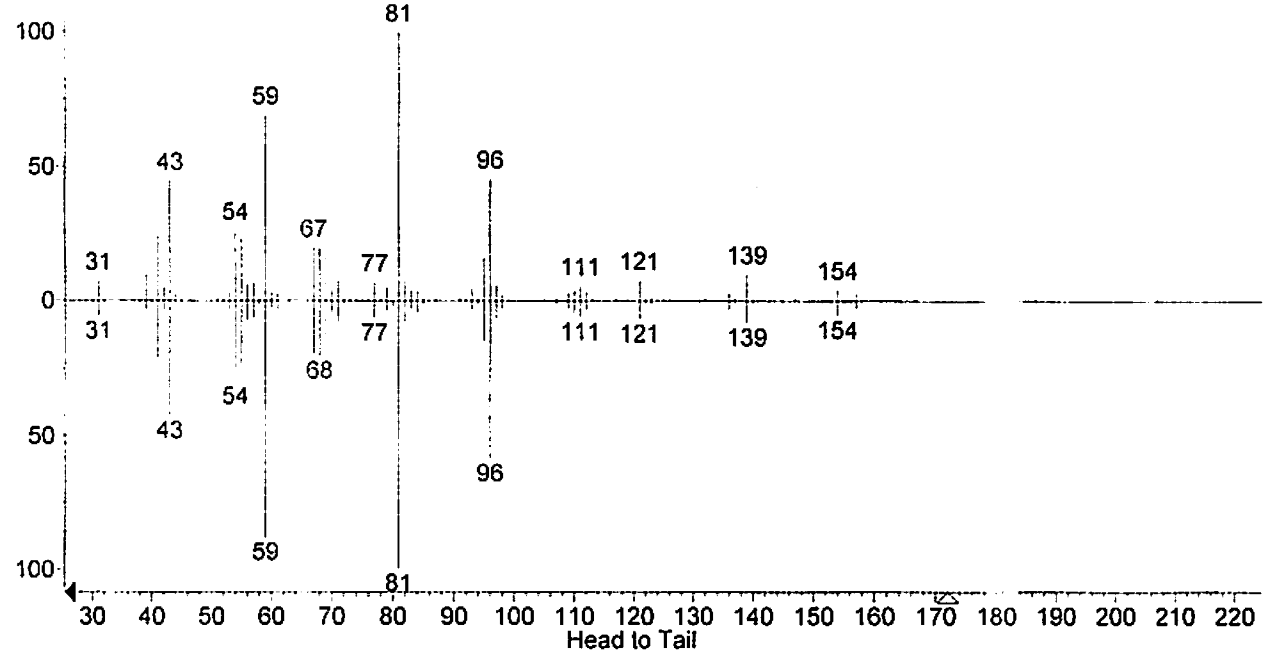


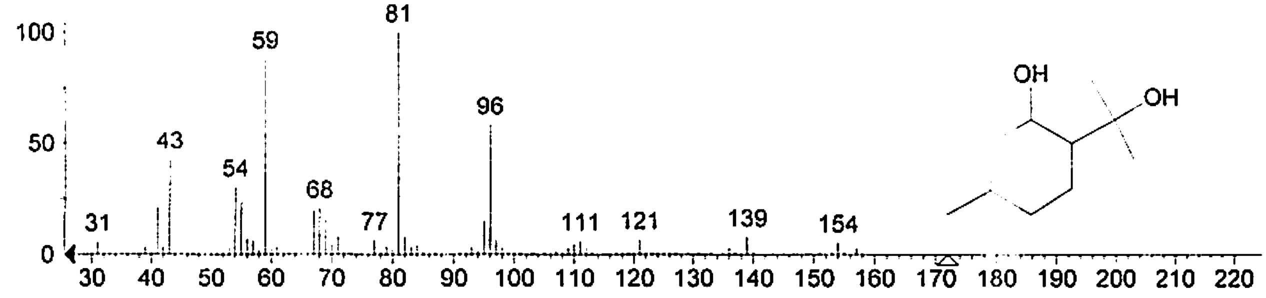


(mainlib) Cyclohexanol, 2-(2-hydroxy-2-propyl)-5-methyl-

```
File        : D:\DATA\Aldrich\JA-09\JA012309-2.D
Operator    : Aldrich
Acquired    : 23 Jan 2009  14:32     using AcqMethod JA-WAX08.M
Instrument  :      Instrument #1
Sample Name: 10 male C.oculata abd.ster./5ul CH2Cl2
Misc Info   : 8-14-day-old; fed (+/-)-citronellal 7days
Vial Number: 1
```

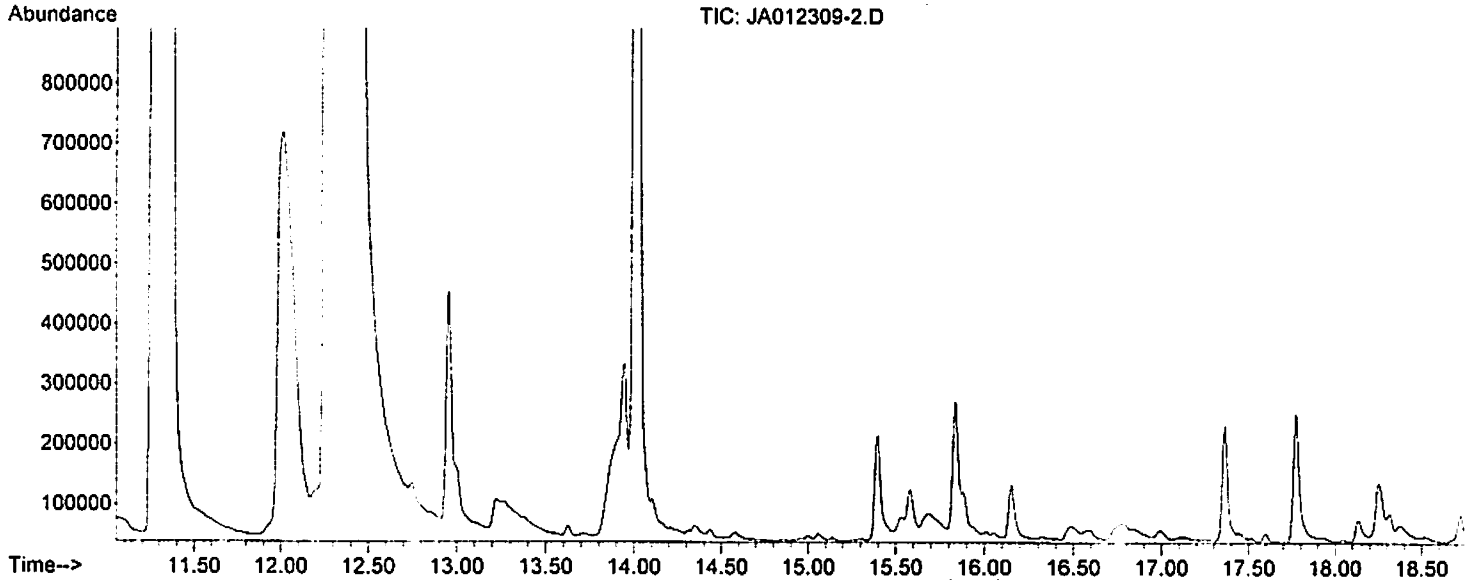


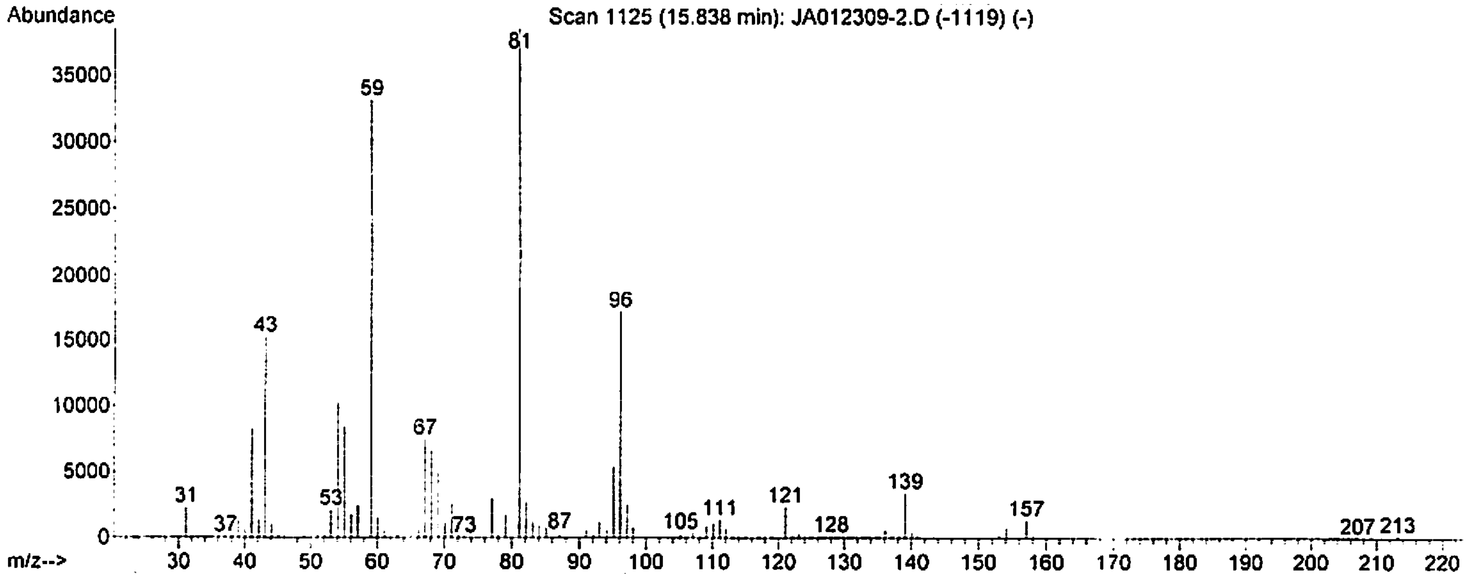

```
File         : D:\DATA\Aldrich\JA-09\JA012309-2.D
Operator     : Aldrich
Acquired     : 23 Jan 2009  14:32      using AcqMethod JA-WAX08.M
Instrument   :    Instrument #1
Sample Name: 10 male C.oculata abd.ster./5ul CH2Cl2
Misc Info    : 8-14-day-old; fed (+/-)-citronellal 7days
Vial Number: 1
```

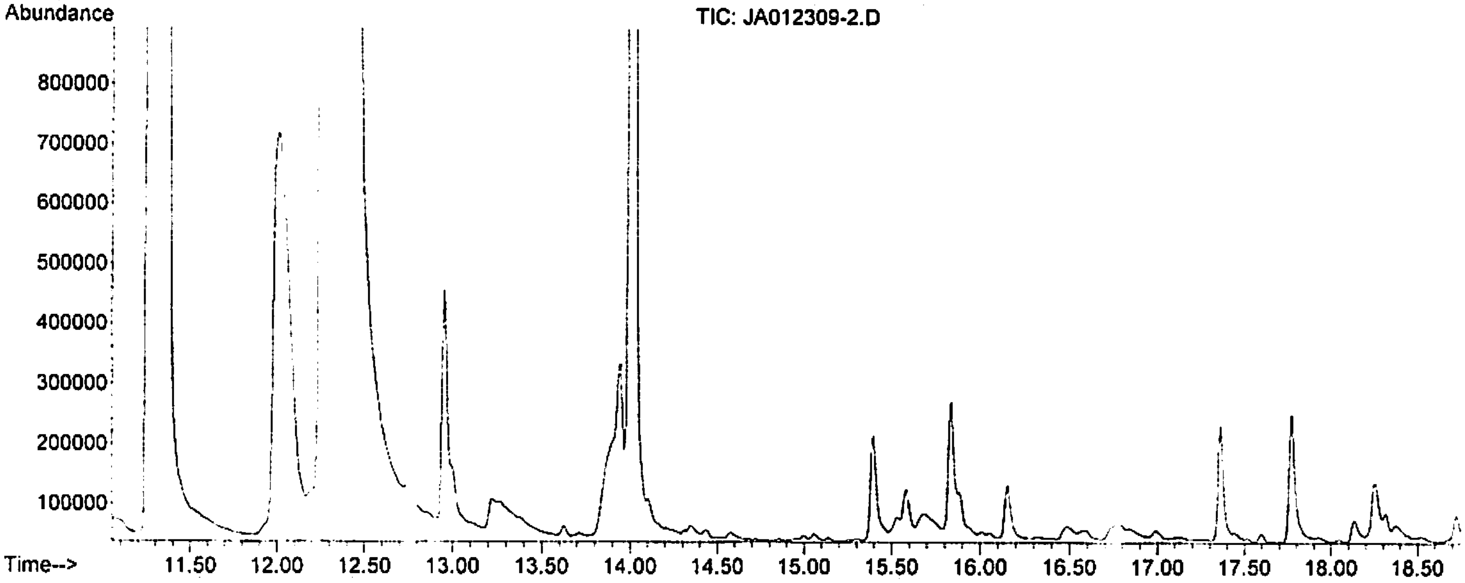


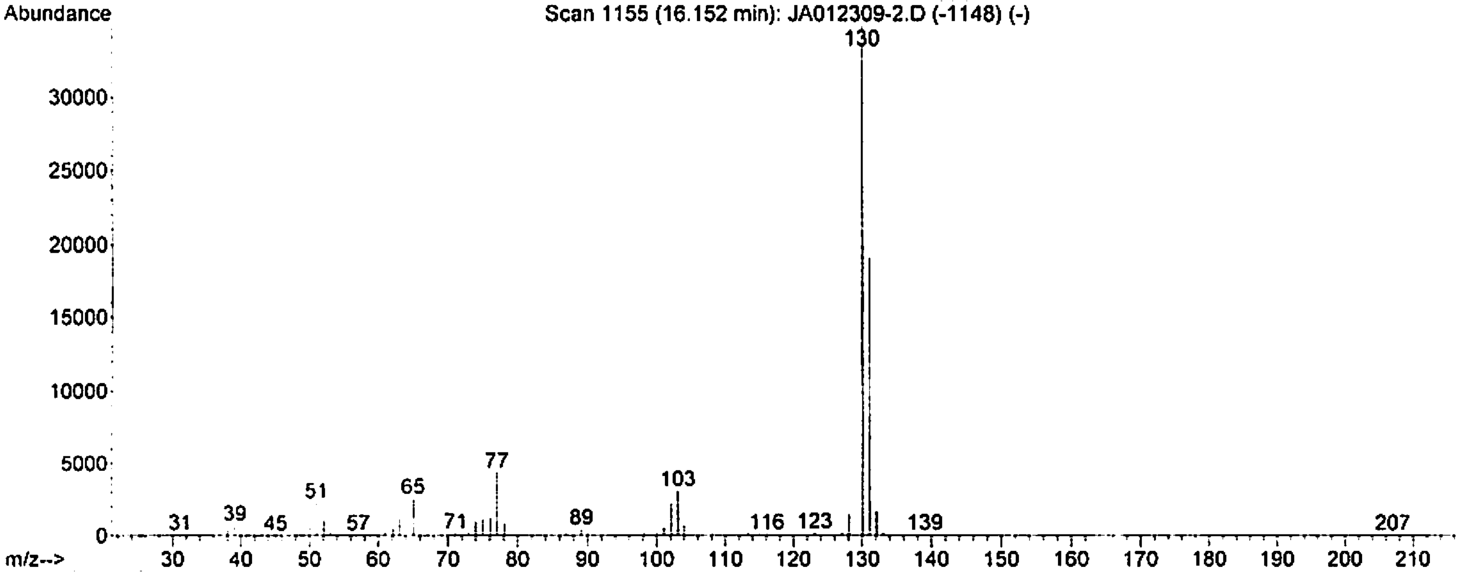

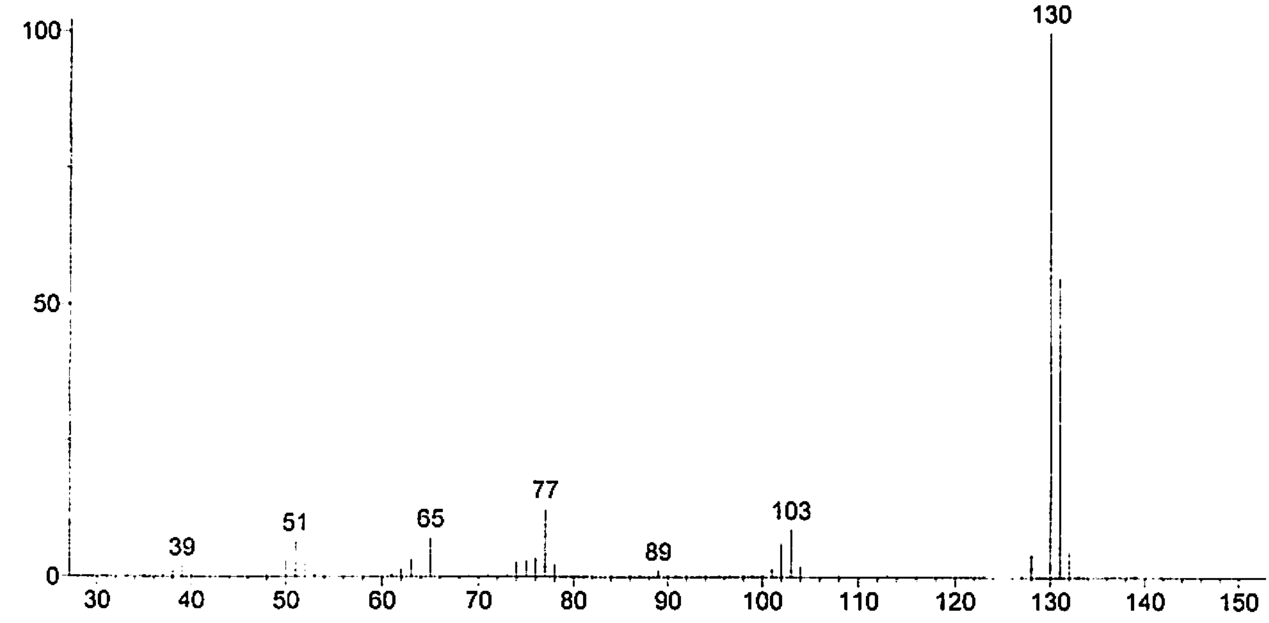


(Text File) Scan 1155 (16.152 min): JA012309-2.D (-1148)

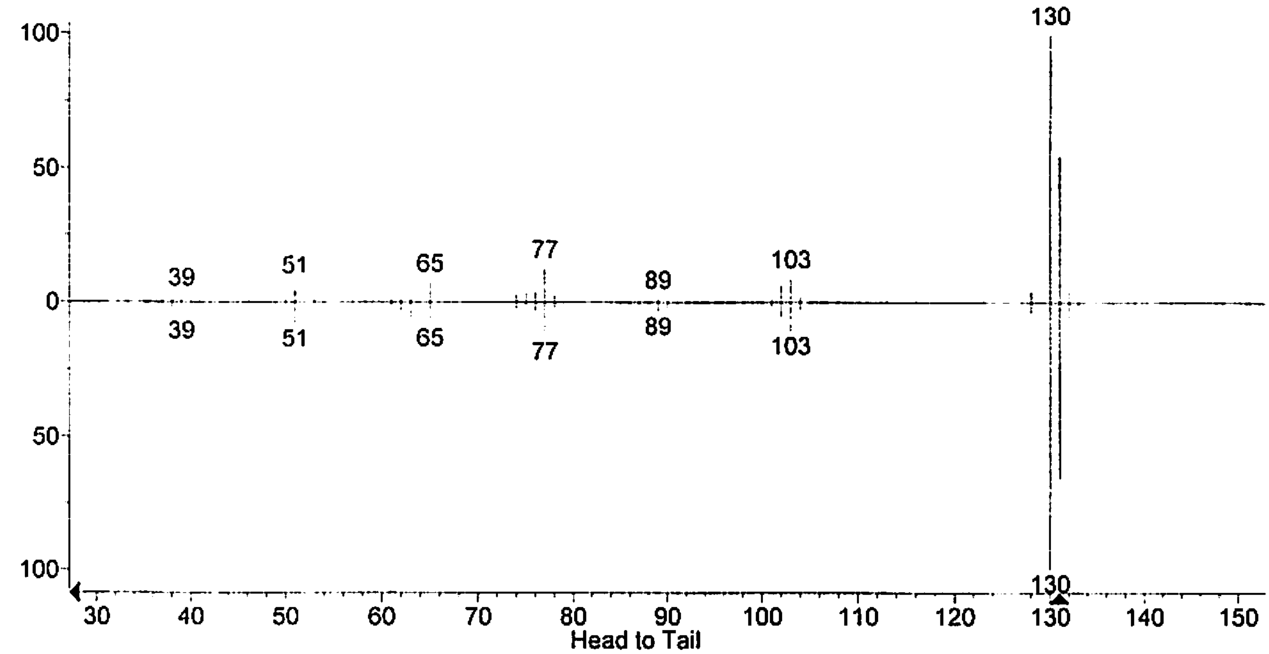


Head to Tail

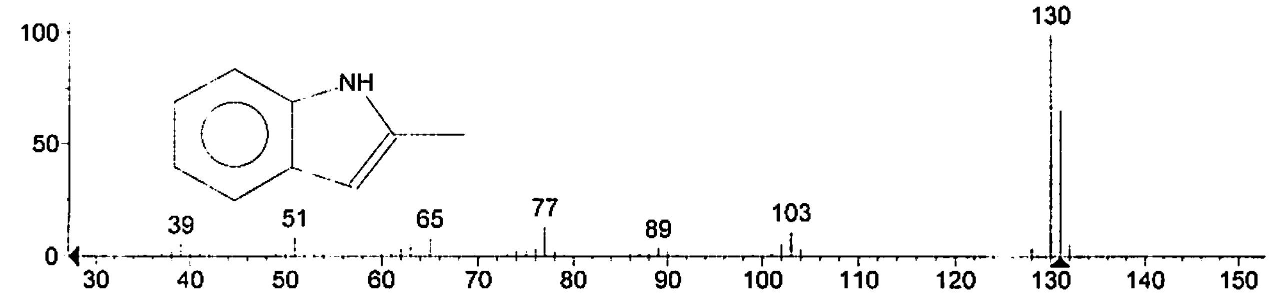


(mainlib) 1H-Indole, 2-methyl-

File :D:\DATA\Aldrich\JA-09\JA012309-2.D
Operator : Aldrich
Acquired : 23 Jan 2009 14:32 using AcqMethod JA-WAX08.M
Instrument : Instrument #1
Sample Name: 10 male C.oculata abd.ster./5ul CH2Cl2
Misc Info : 8-14-day-old; fed (+/-)-citronellal 7days
Vial Number: 1

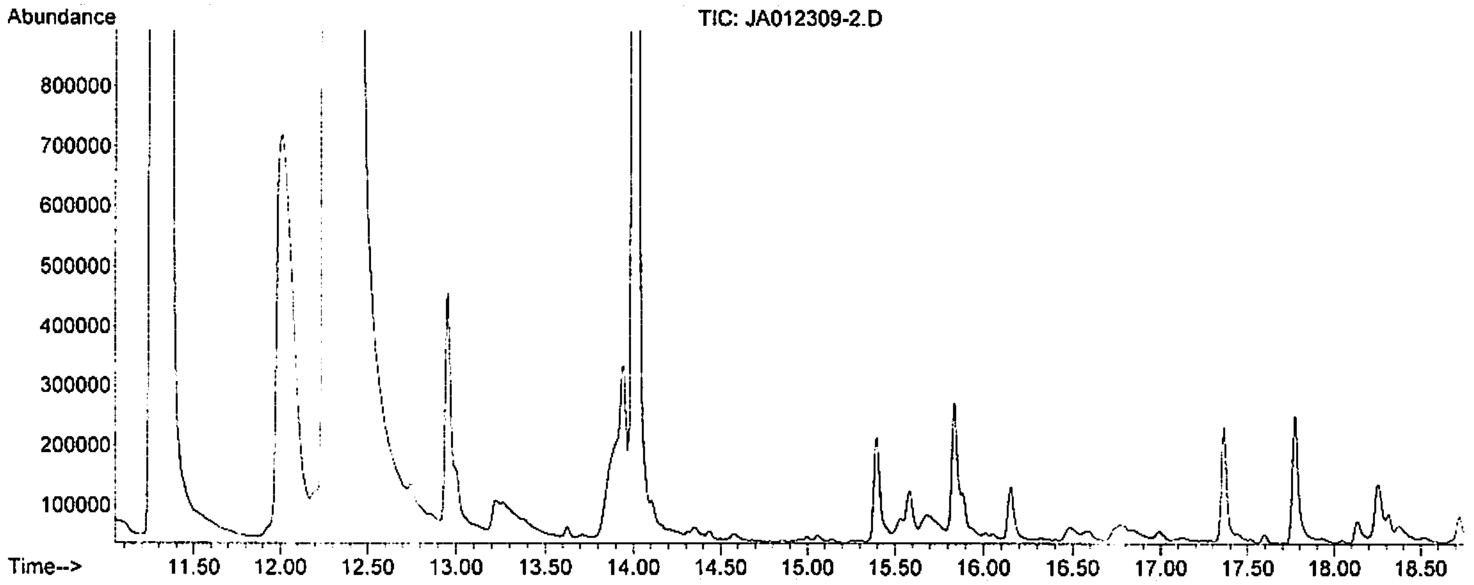


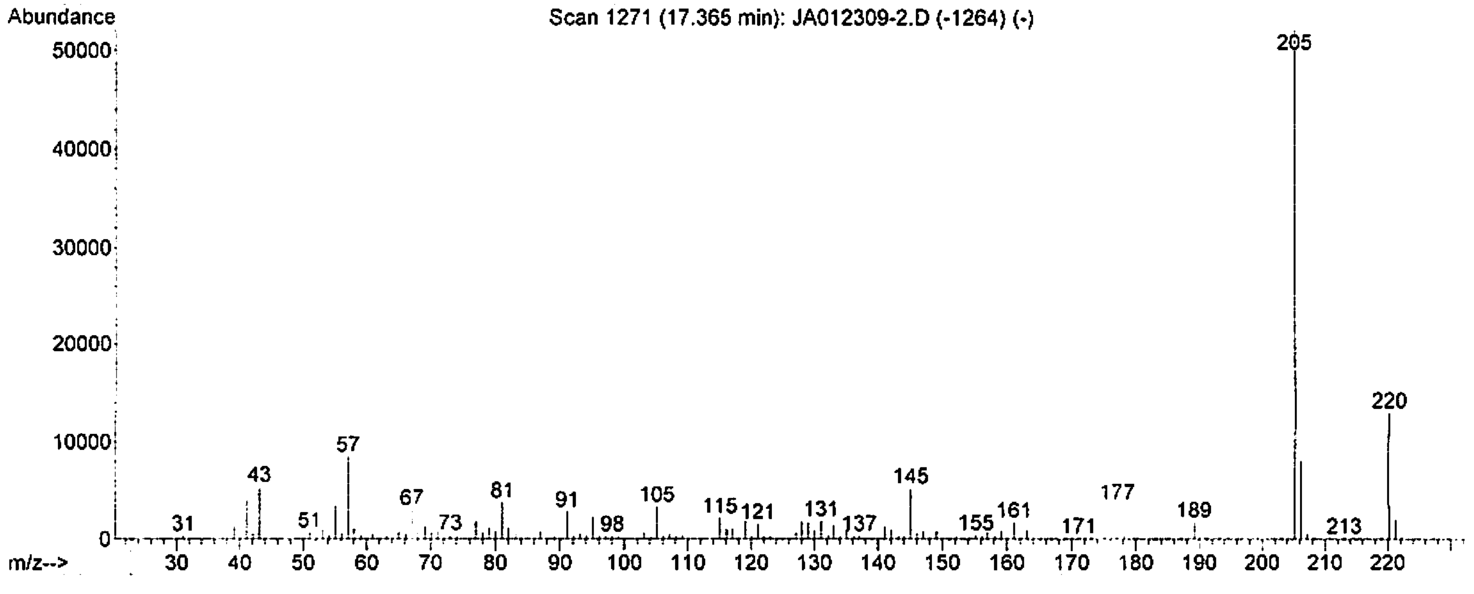

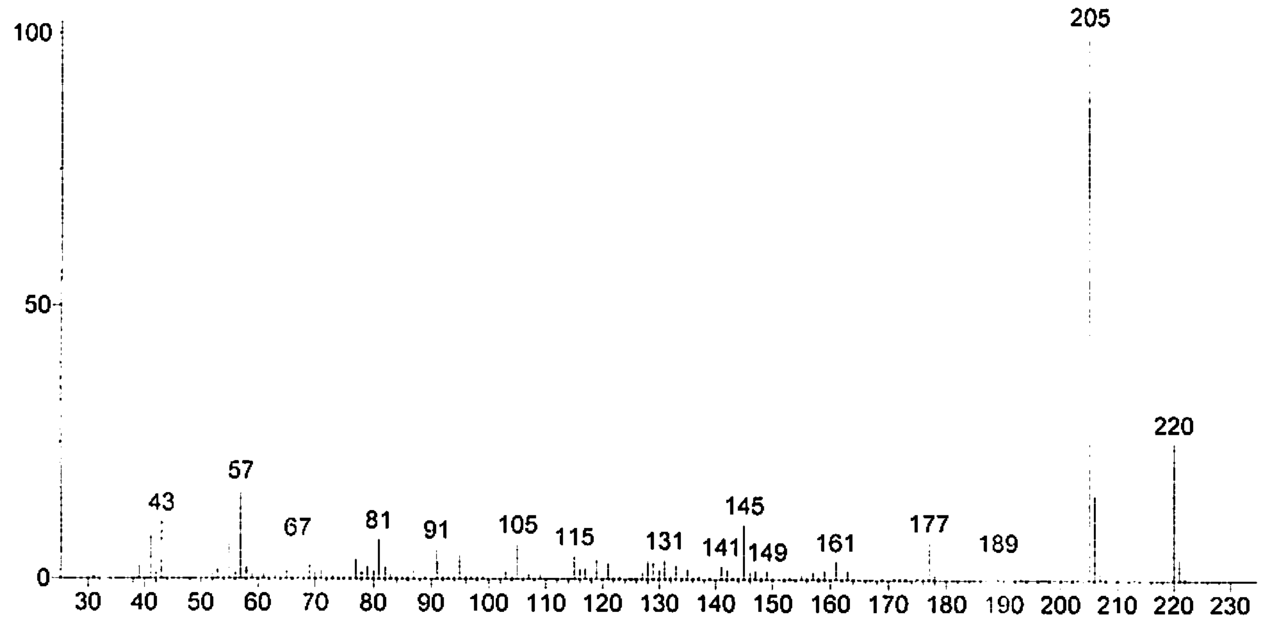


(Text File) Scan 1271 (17.365 min): JA012309-2.D (-1264)

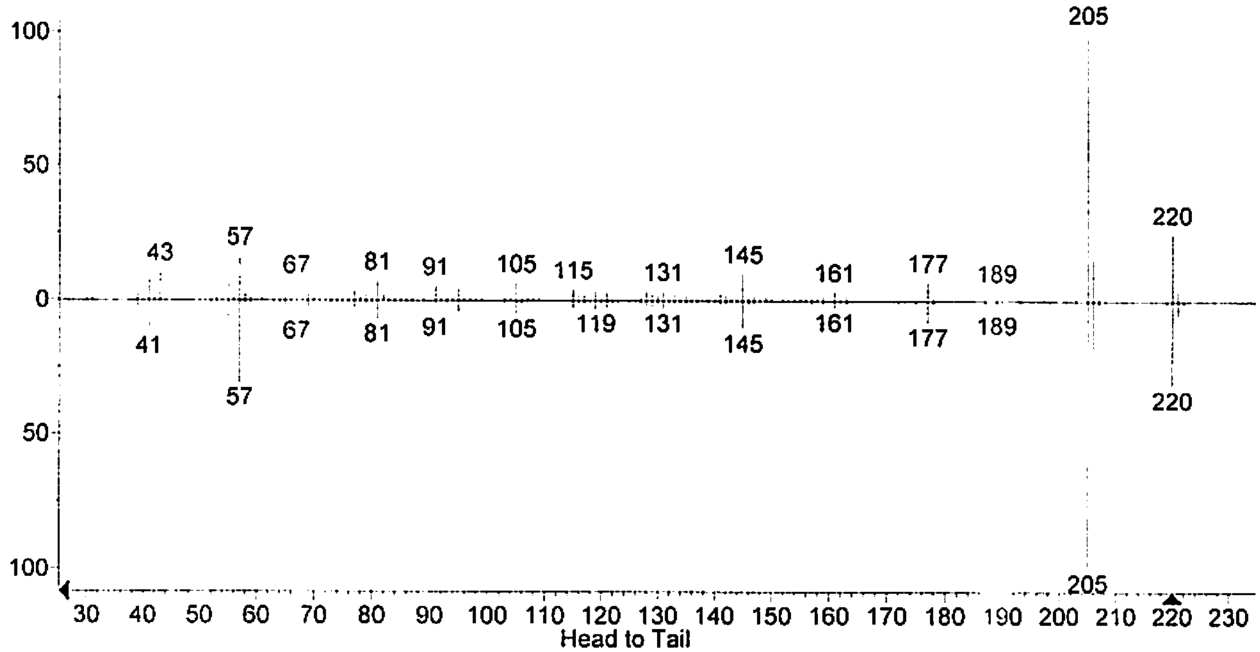


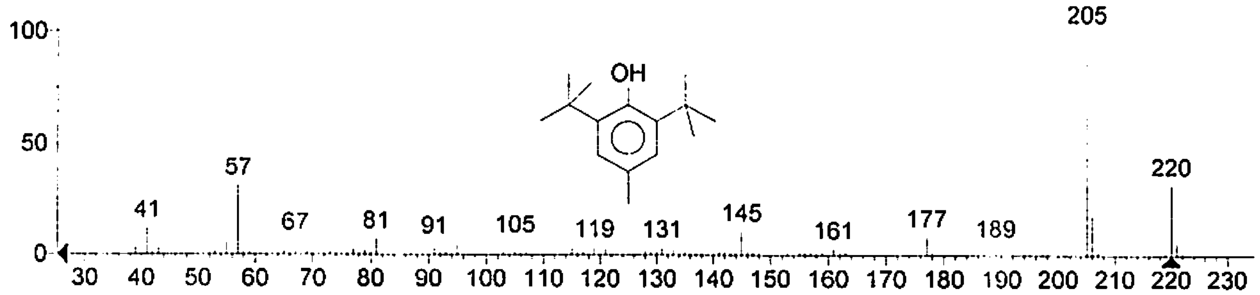


(replib) Butylated Hydroxytoluene

File :D:\DATA\Aldrich\JA-09\JA012309-2.D
Operator : Aldrich
Acquired : 23 Jan 2009 14:32 using AcqMethod JA-WAX08.M
Instrument : Instrument #1
Sample Name: 10 male C.oculata abd.ster./5ul CH2Cl2
Misc Info : 8-14-day-old; fed (+/-)-citronellal 7days
Vial Number: 1

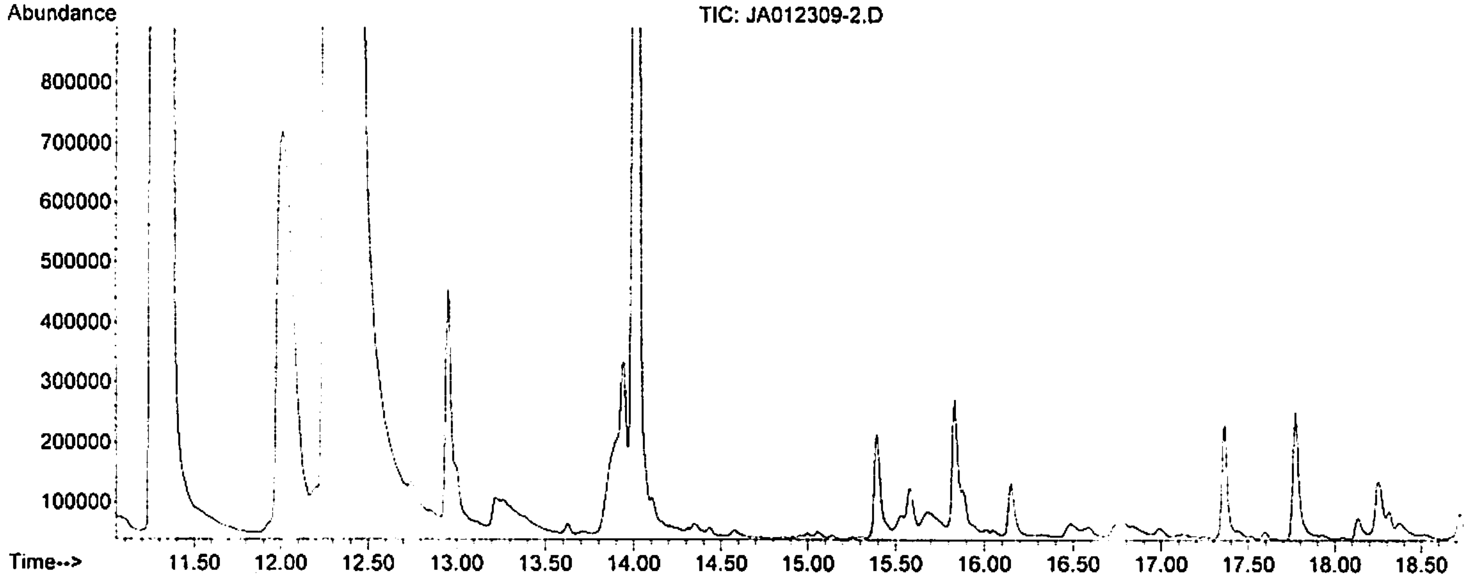


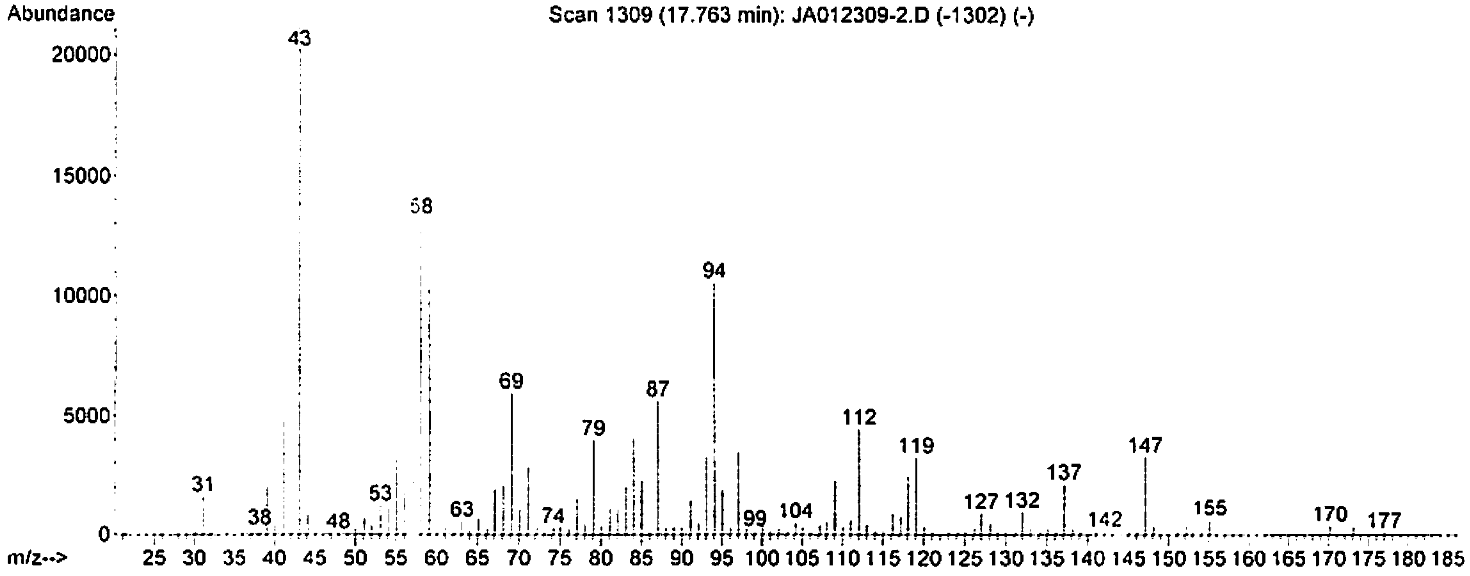

```
File       :D:\Aldrich\JA102308-2.D
Operator   :
Acquired   : 23 Oct 2008  14:31     using AcqMethod JA-50-280LESS.M
Instrument :   Buba
Sample Name: 7M C.oculata abdominal cuticle/10 ul CH2Cl2
Misc Info  : 10/8-14; 6 days w/ 1ug/ul citronellol in H2O
Vial Number: 1
```

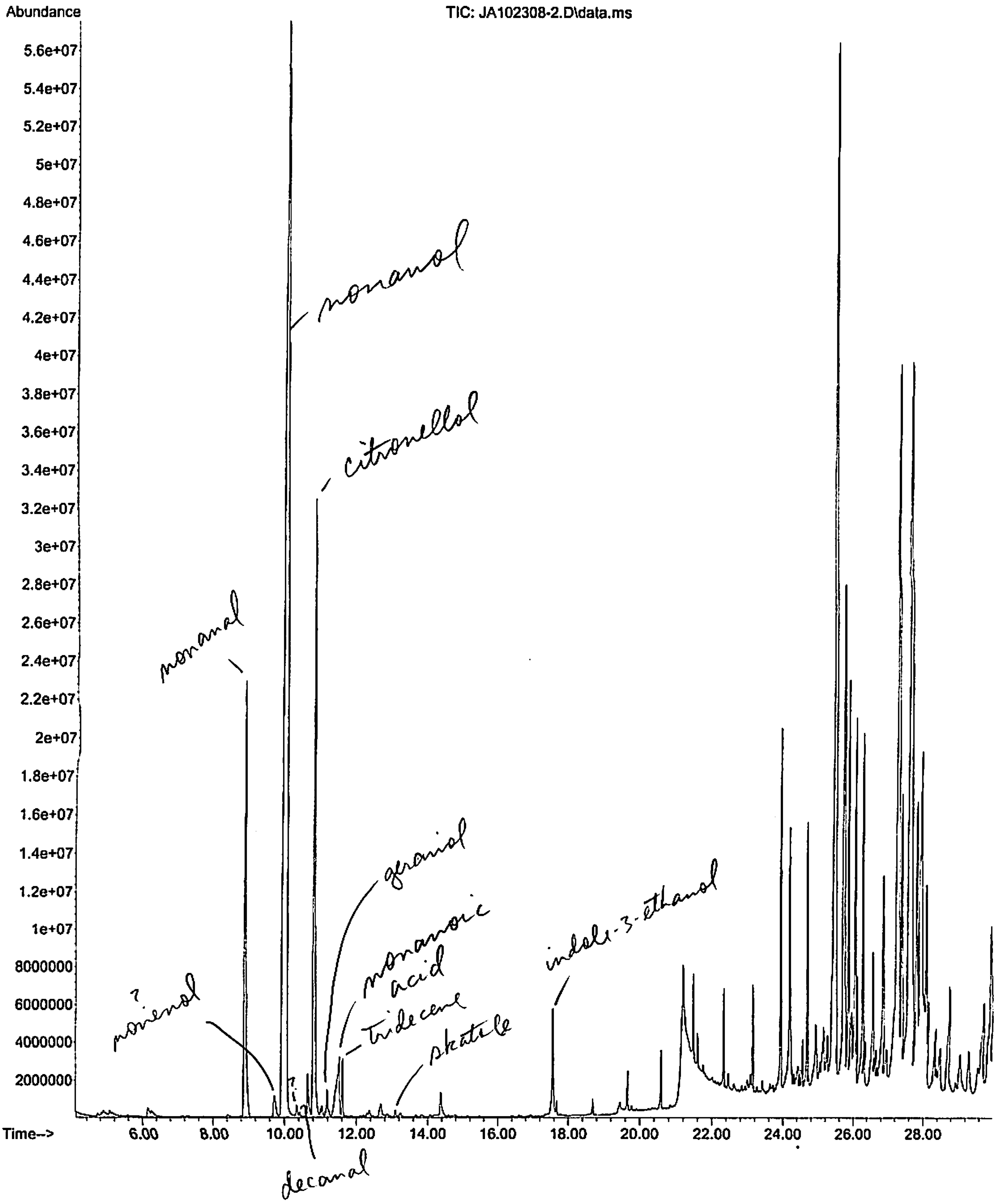

File :D:\Aldrich\JA102308-2.D
Operator :
Acquired : 23 Oct 2008 14:31 using AcqMethod JA-50-280LESS.M
Instrument : Buba
Sample Name: 7M C.oculata abdominal cuticle/10 ul CH2Cl2
Misc Info : 10/8-14; 6 days w/ 1ug/ul citronellol in H2O
Vial Number: 1

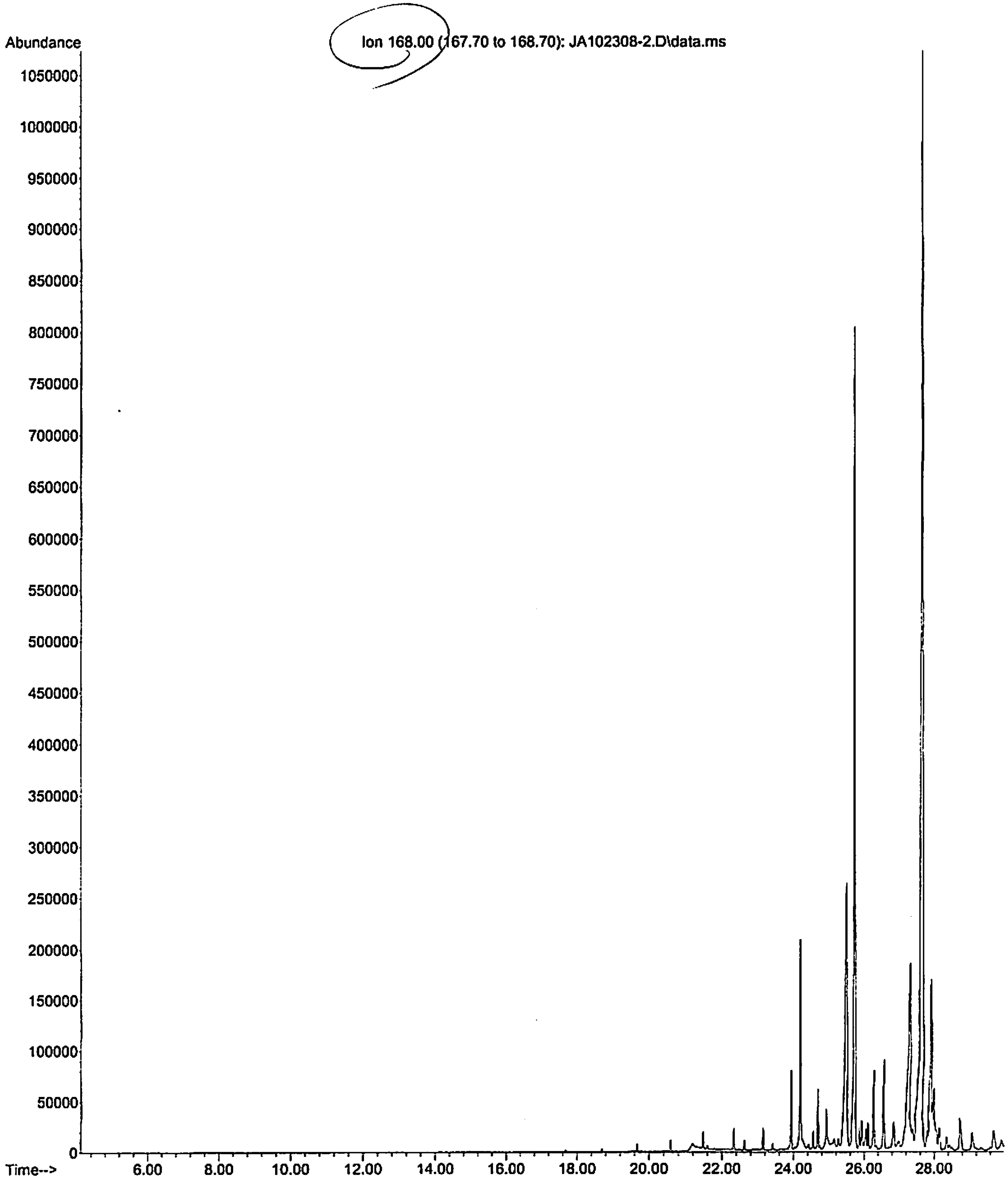

```
File        :D:\Aldrich\JA102308-2.D
Operator    :
Acquired    : 23 Oct 2008  14:31     using AcqMethod JA-50-280LESS.M
Instrument :     Buba
Sample Name: 7M C.oculata abdominal cuticle/10 ul CH2Cl2
Misc Info  : 10/8-14; 6 days w/ 1ug/ul citronellol in H2O
Vial Number: 1
```

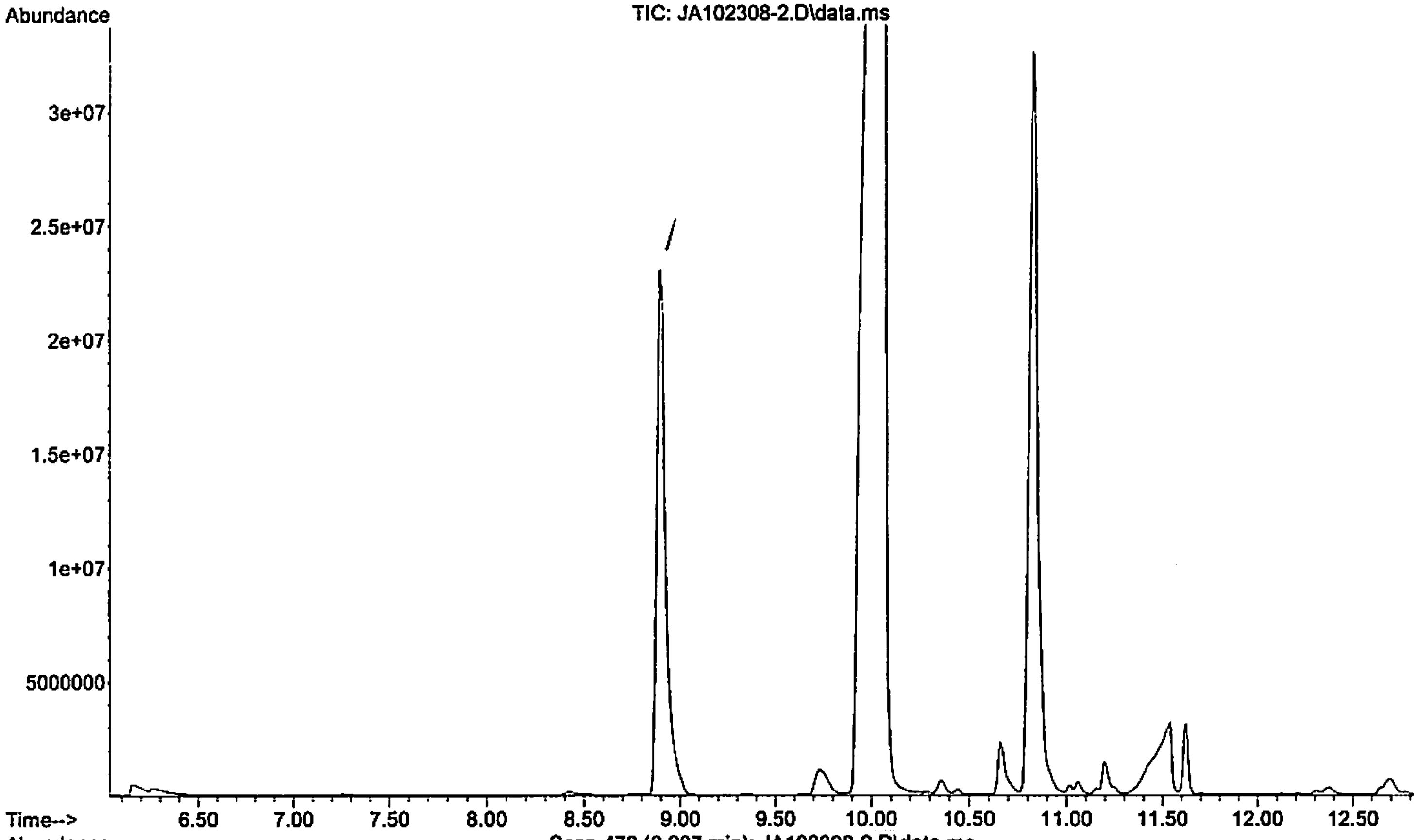


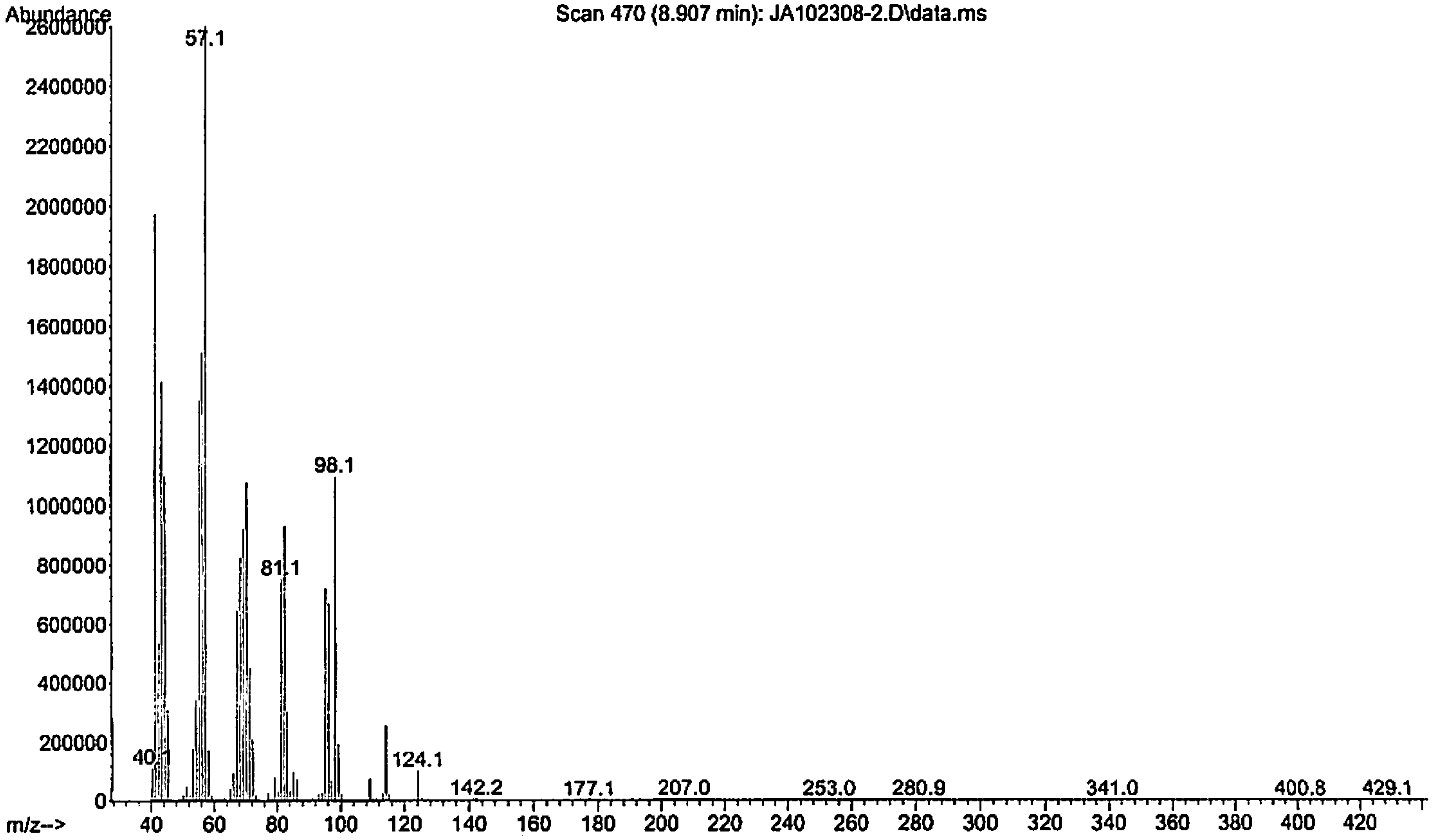

```
Library Searched : C:\Database\W8N05ST.L
Quality          : 91
ID               : NONANAL $ PELARGONALDEHYDE $ 1-NONALDEHYDE $ 1-NONANAL $ 1-NONYL ALDEH
                   YDE $ AI3-04859 $ ALDEHYDE C-9 $ BRN 1236701 $ C-9 ALDEHYDE $ CCRIS 66
                   4 $ EINECS 204-688-5 $ FEMA NO. 2782 $ HSDB 7229 $ N-NONALDEHYDE $ N-N
                   ONAN-1-AL $ N-NONANAL $ N-NONYLALDEHYDE $ NCI
```

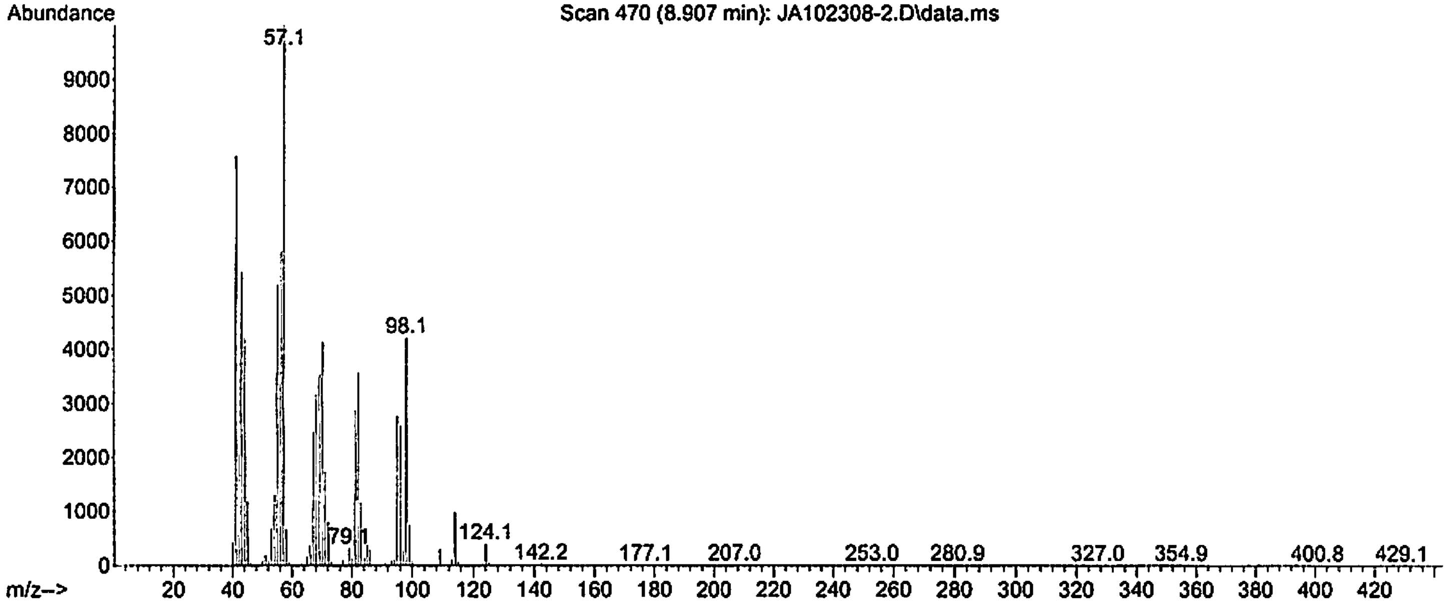


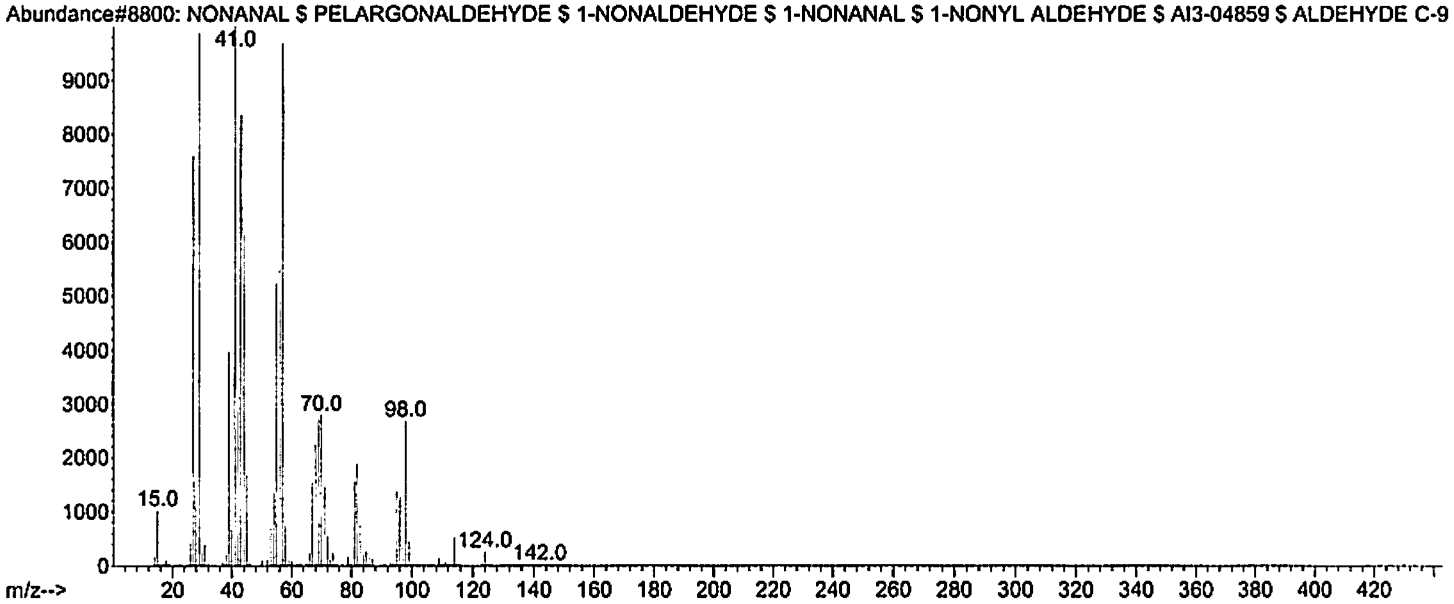


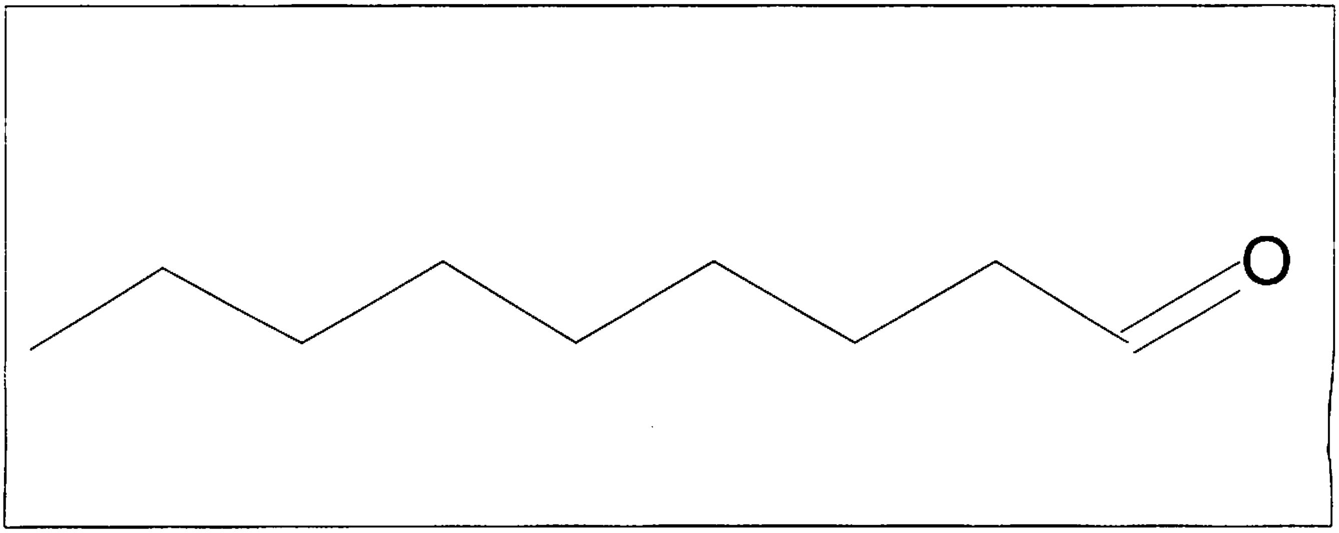

File       :D:\Aldrich\JA102308-2.D
Operator   :
Acquired   : 23 Oct 2008  14:31     using AcqMethod JA-50-280LESS.M
Instrument :    Buba
Sample Name: 7M C.oculata abdominal cuticle/10 ul CH2Cl2
Misc Info  : 10/8-14; 6 days w/ 1ug/ul citronellol in H2O
Vial Number: 1

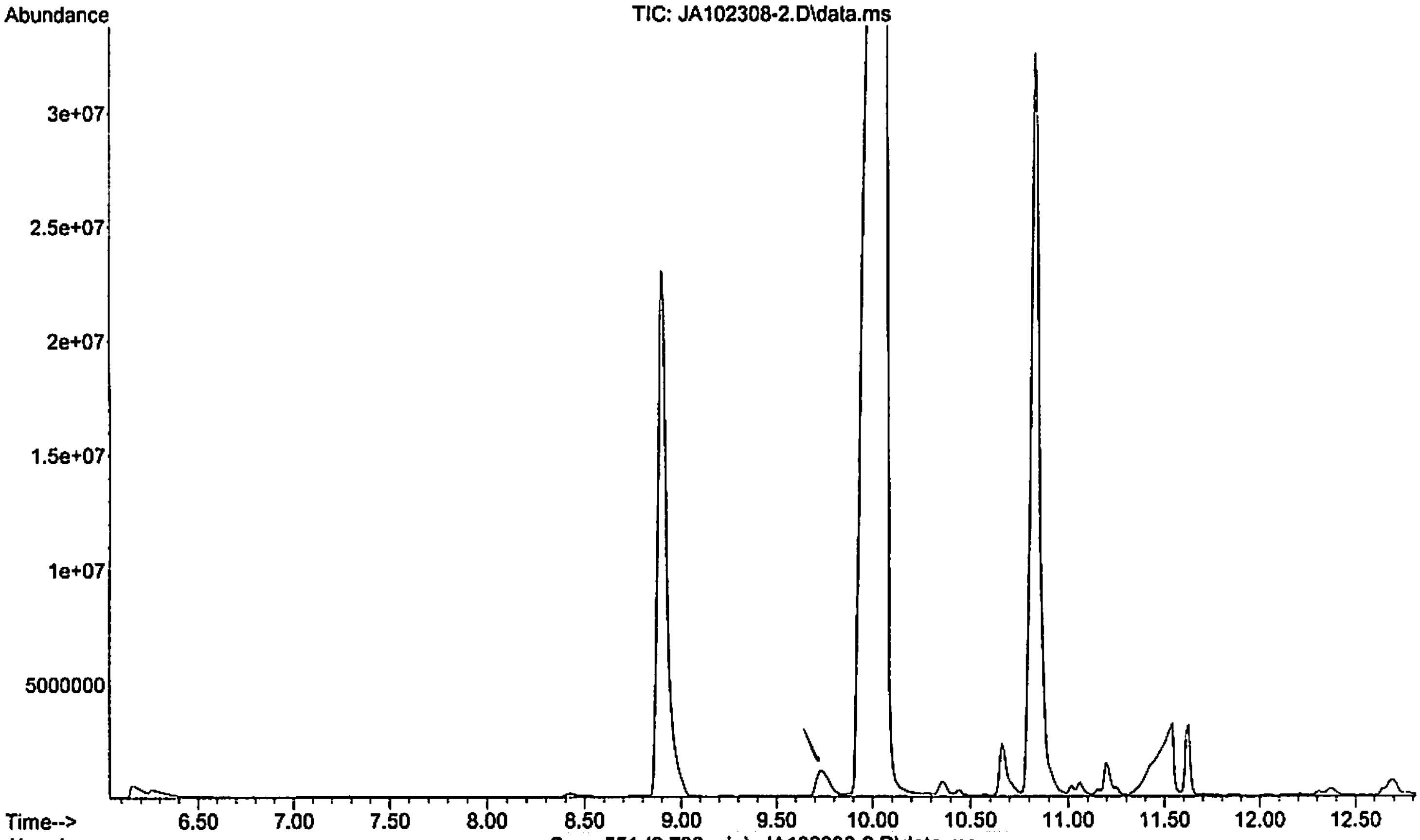


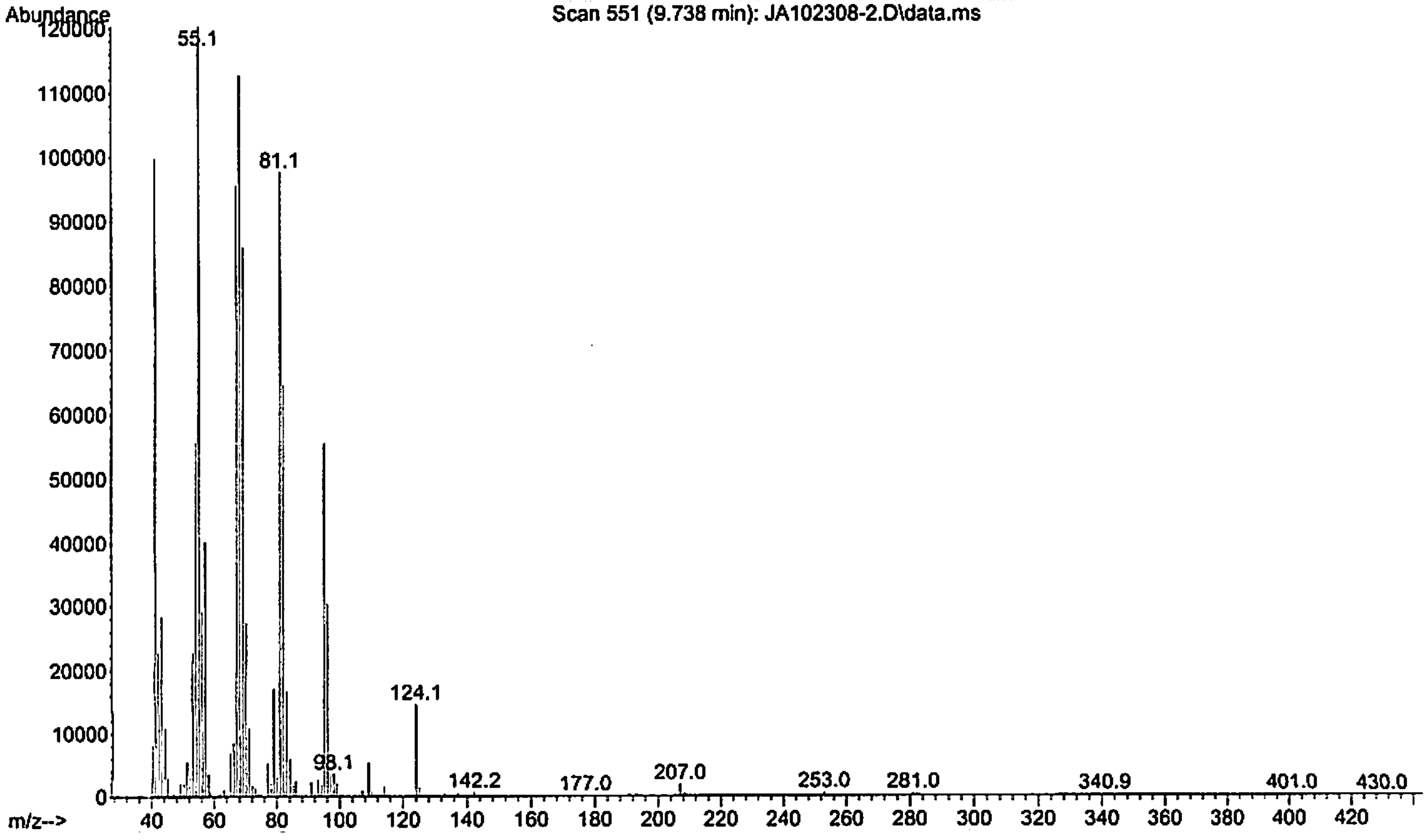

Library Searched : C:\Database\W8N05ST.L
Quality : 91
ID : 3-Nonen-1-ol, (Z)- $ cis-3-Nonen-1-Ol $ (3Z)-3-Nonen-1-ol #

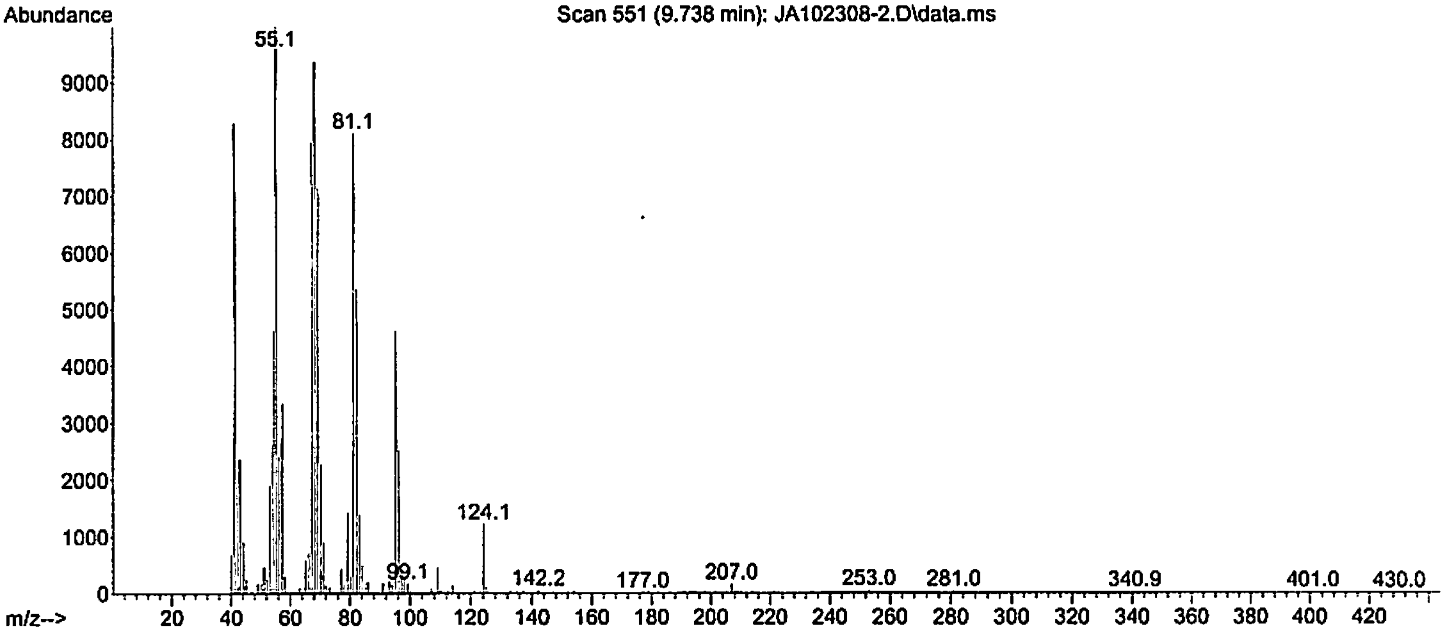


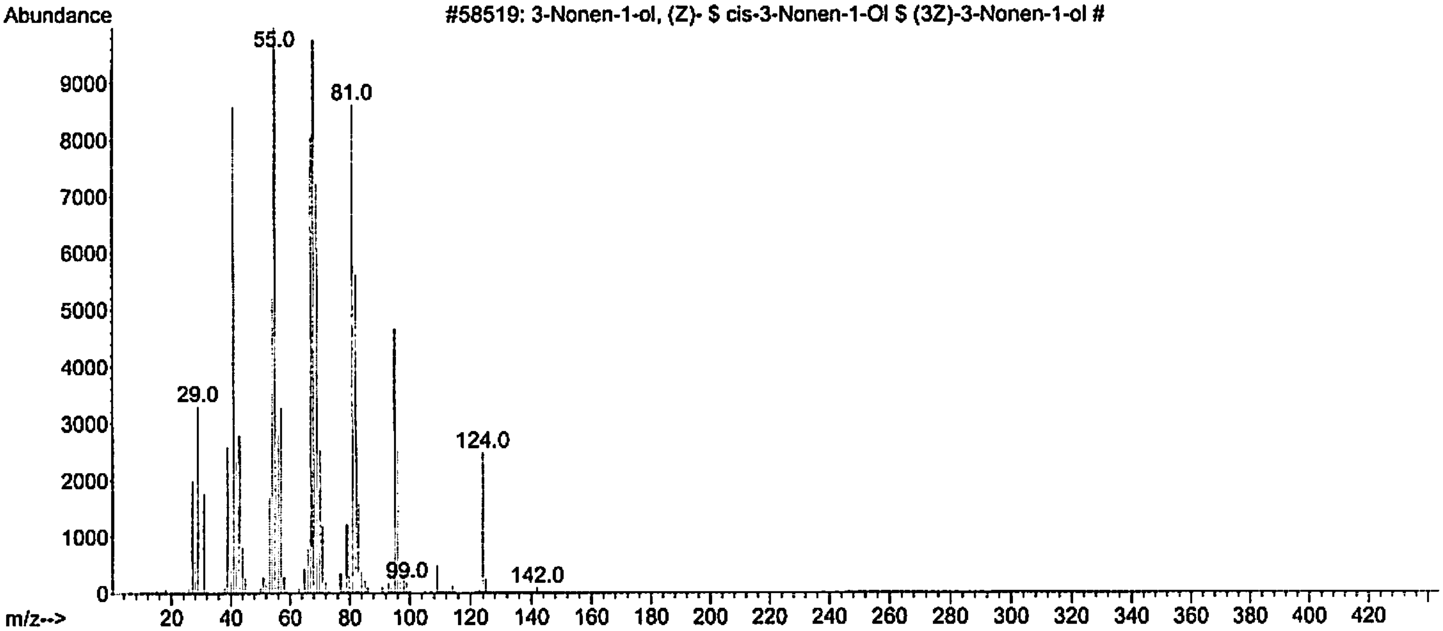


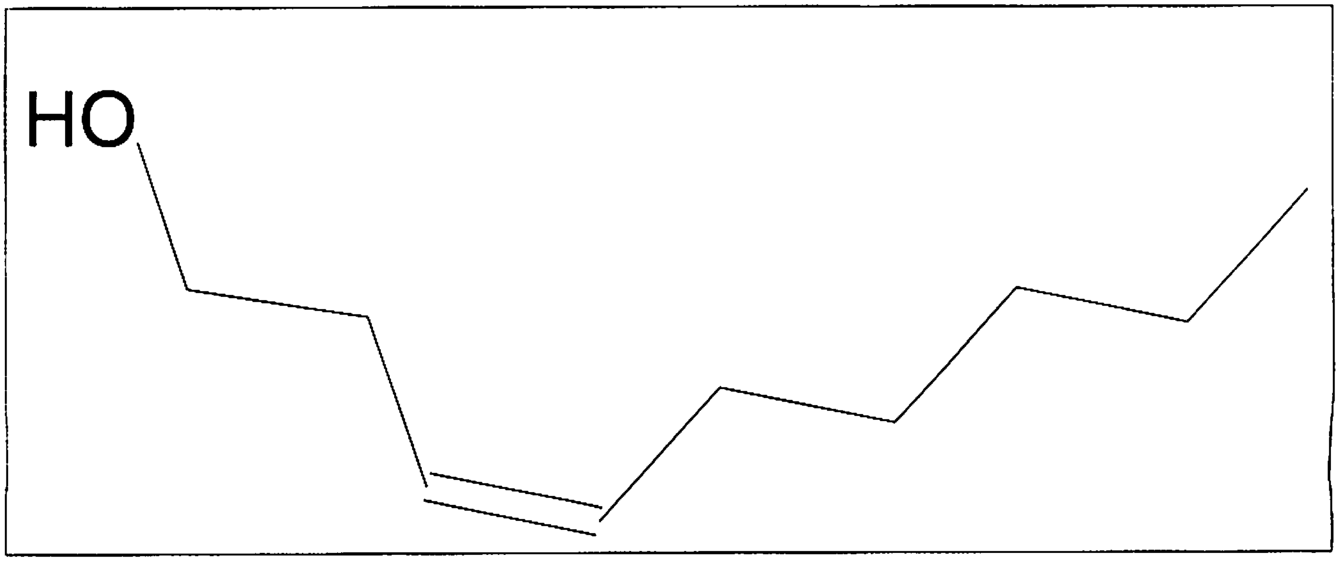

```
File        :D:\Aldrich\JA102308-2.D
Operator    :
Acquired    : 23 Oct 2008  14:31     using AcqMethod JA-50-280LESS.M
Instrument  :    Buba
Sample Name: 7M C.oculata abdominal cuticle/10 ul CH2Cl2
Misc Info   : 10/8-14; 6 days w/ 1ug/ul citronellol in H2O
Vial Number: 1
```

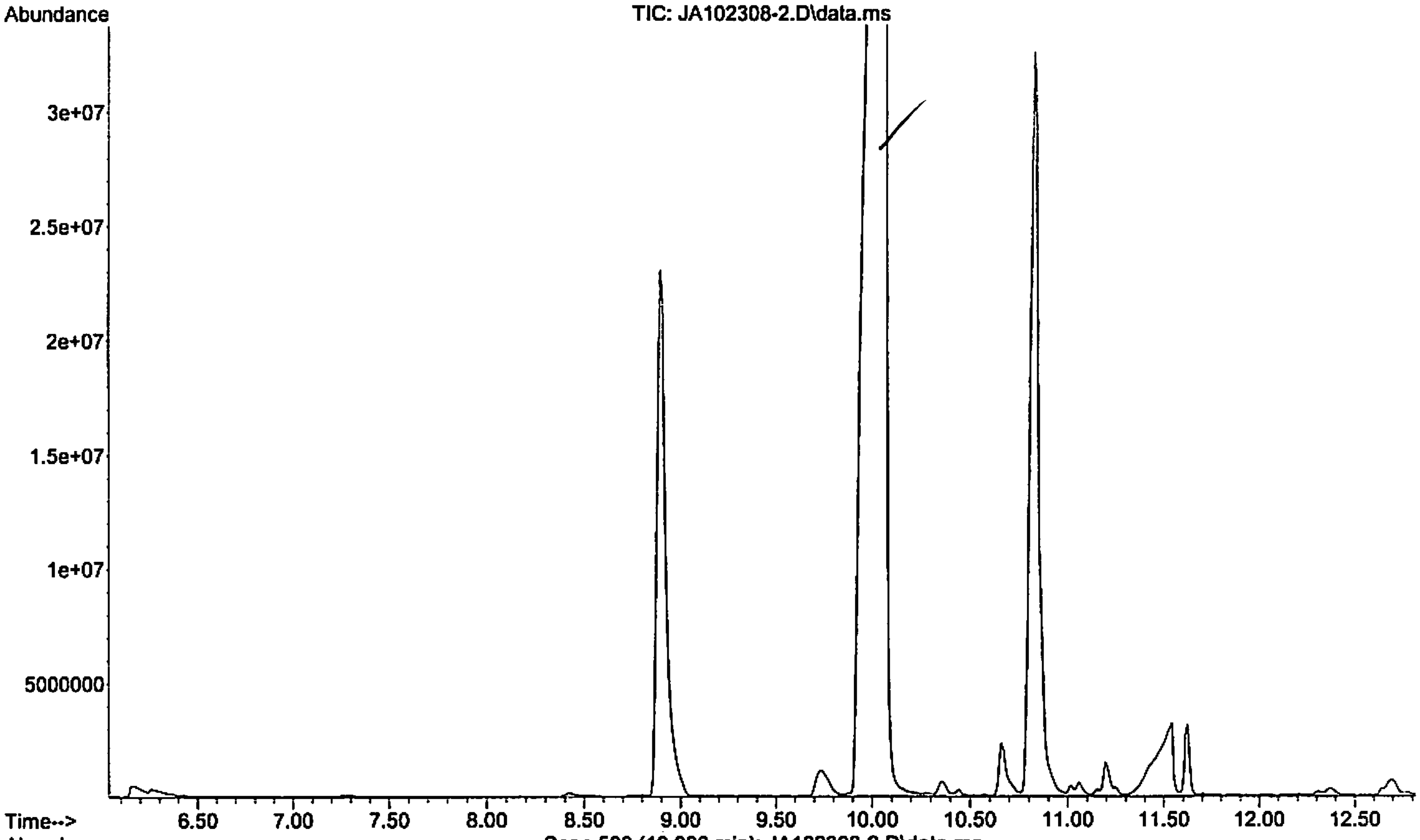


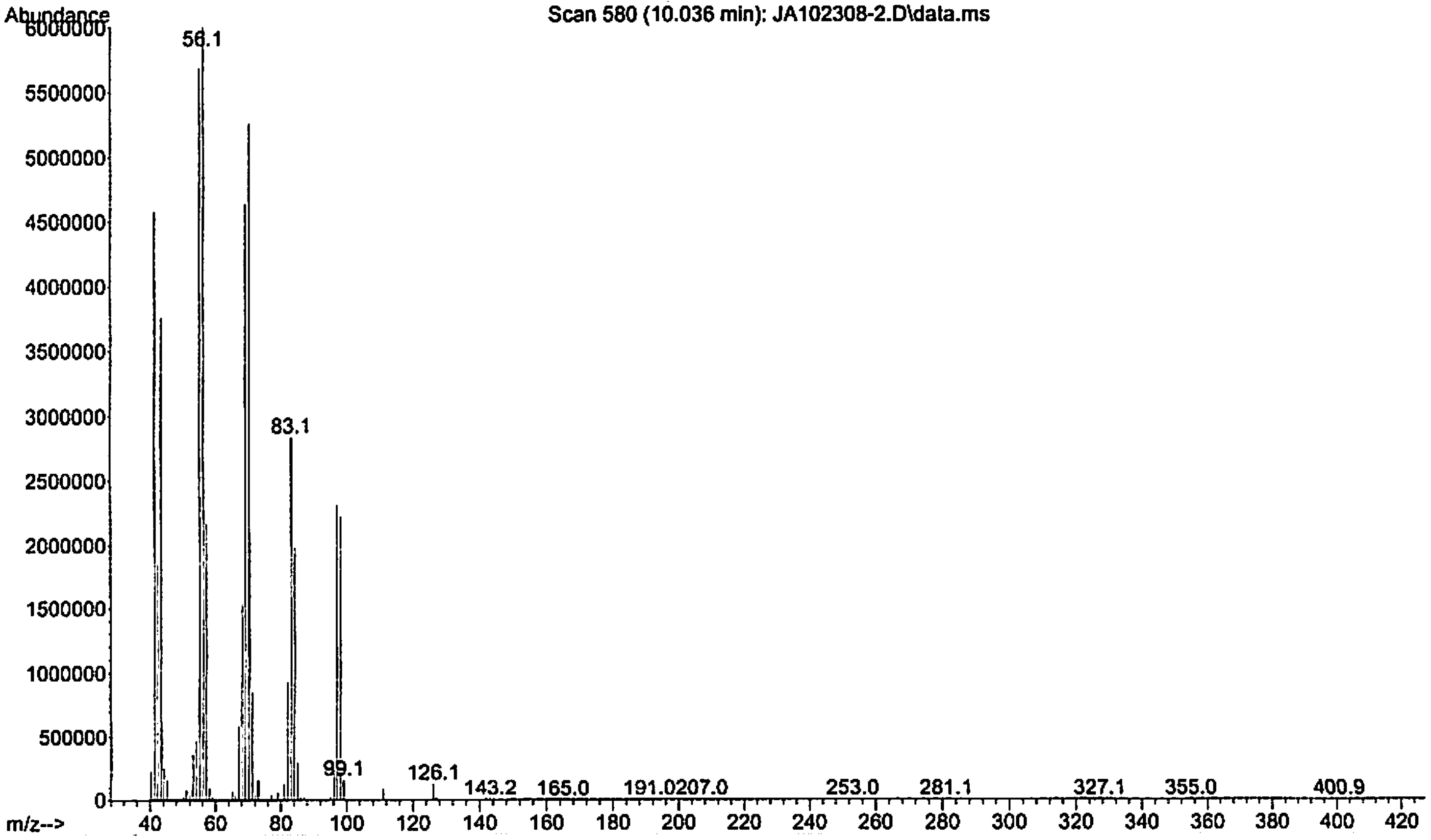

```
Library Searched : C:\Database\W8N05ST.L
Quality          : 91
ID               : 1-NONANOL $ NONANOL $ NONAN-1-OL $ 1-HYDROXYNONANE $ 1-NONANO $ AI3-03
                   962 $ ALCOHOL C-9 $ BRN 0969213 $ C9 ALCOHOL $ EINECS 205-583-7 $ FATT
                   Y ALCOHOL(C9) $ FEMA NO. 2789 $ HSDB 5145 $ N-NONAN-1-OL $ N-NONANOL $
                    N-NONYL ALCOHOL $ NONALOL $ NONANOL-(1) $ NO
```

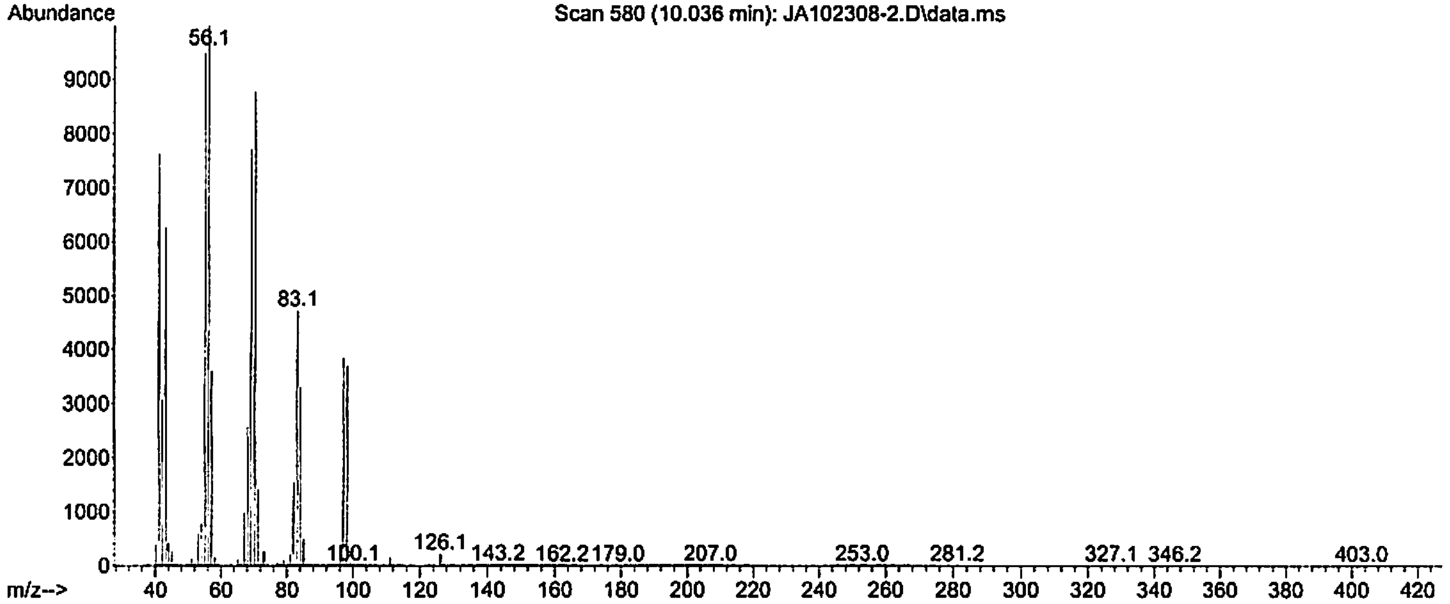


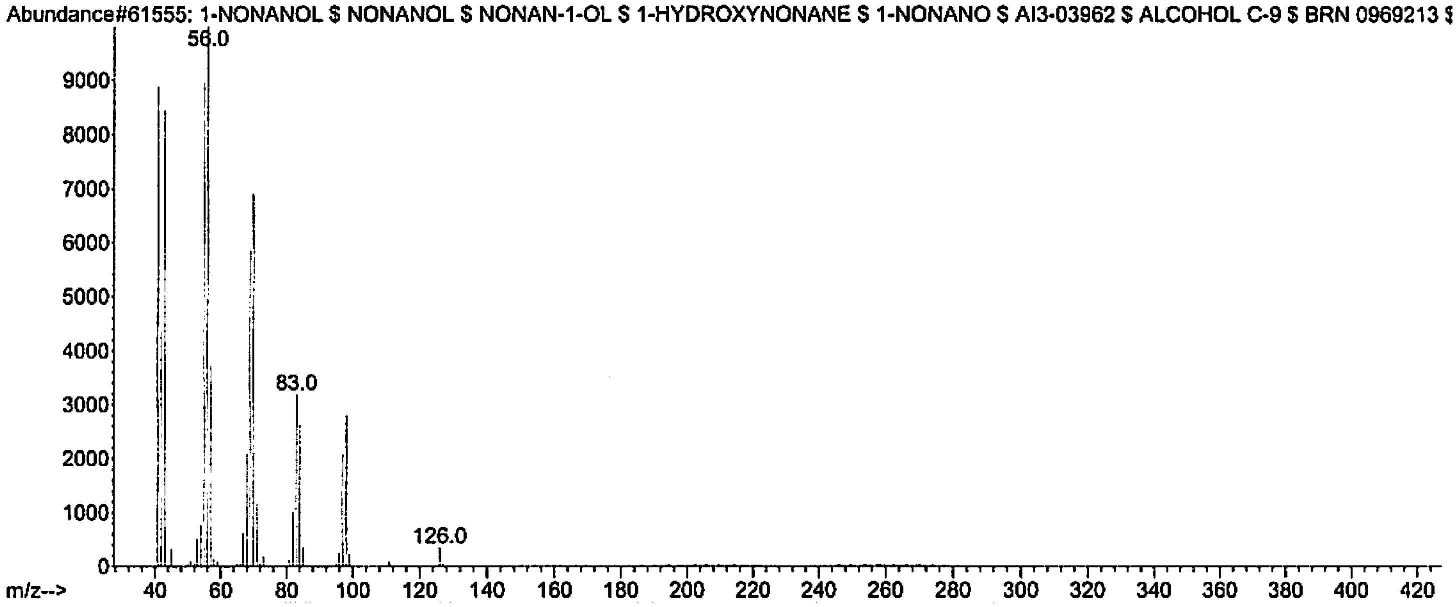


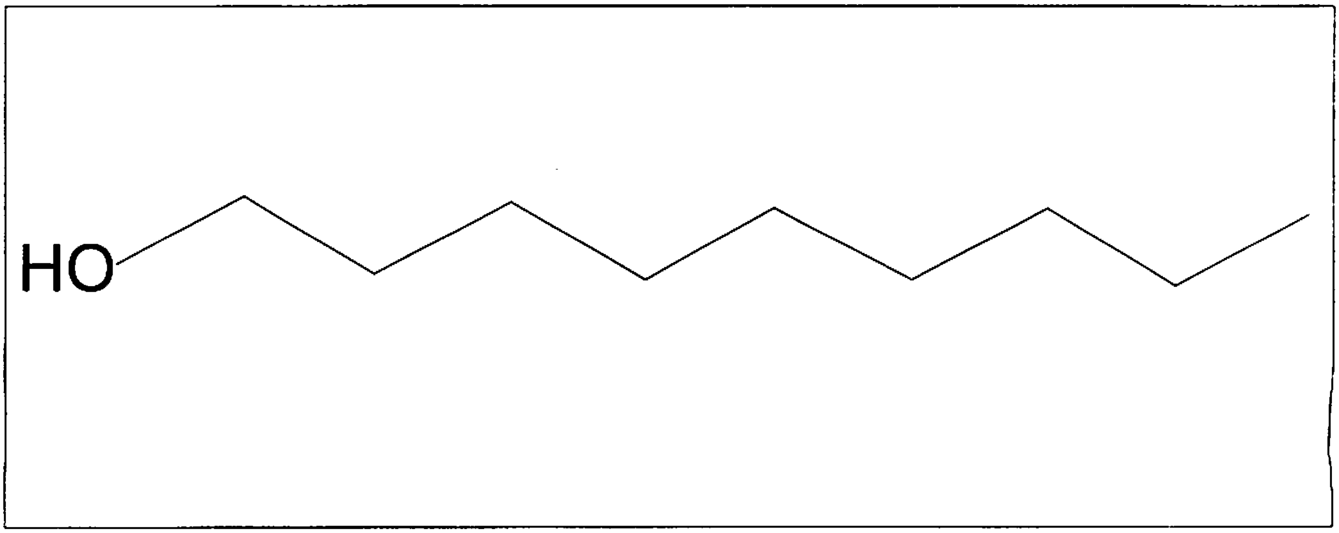

```
File        :D:\Aldrich\JA102308-2.D
Operator    :
Acquired    : 23 Oct 2008  14:31     using AcqMethod JA-50-280LESS.M
Instrument :    Buba
Sample Name: 7M C.oculata abdominal cuticle/10 ul CH2Cl2
Misc Info  : 10/8-14; 6 days w/ 1ug/ul citronellol in H2O
Vial Number: 1
```

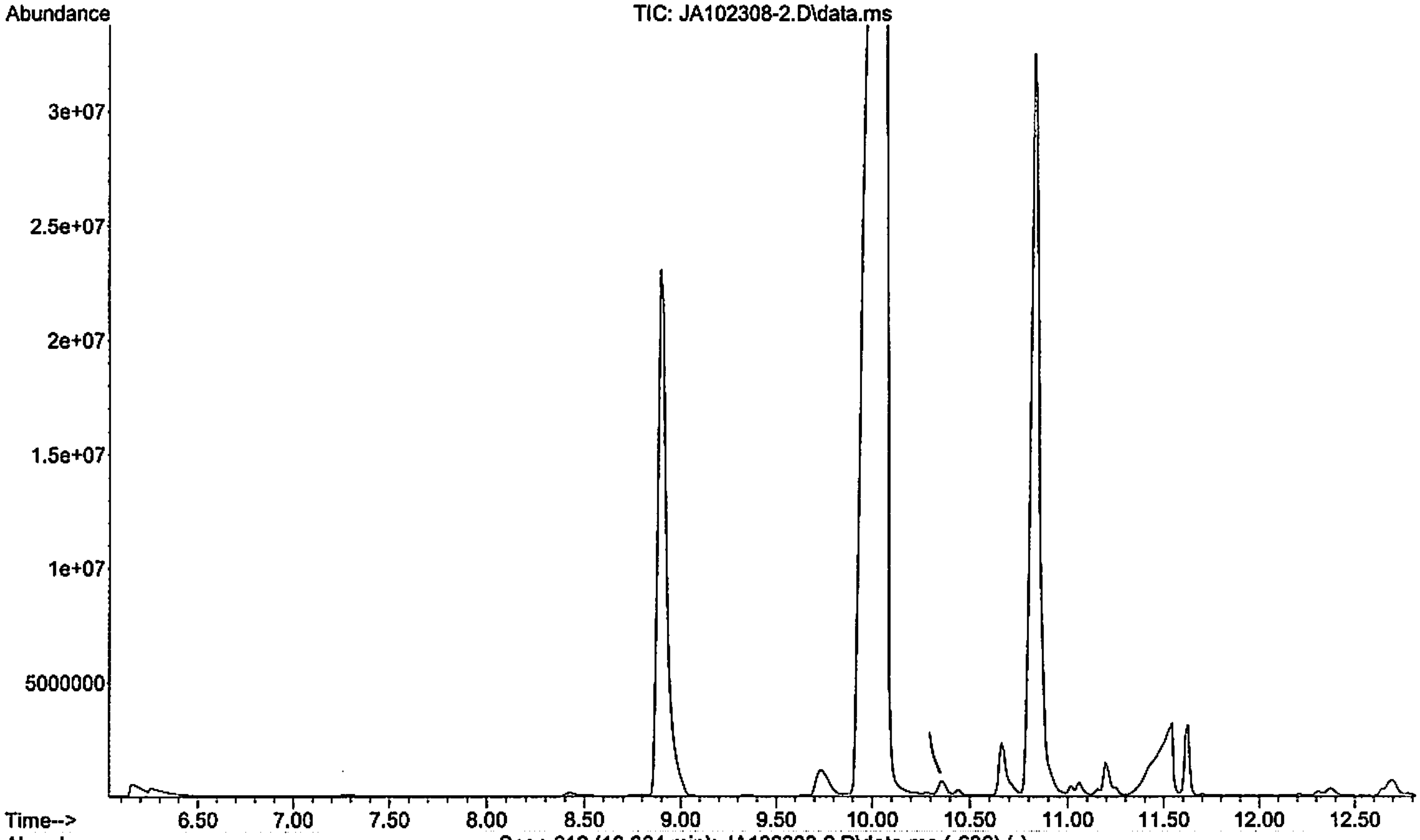


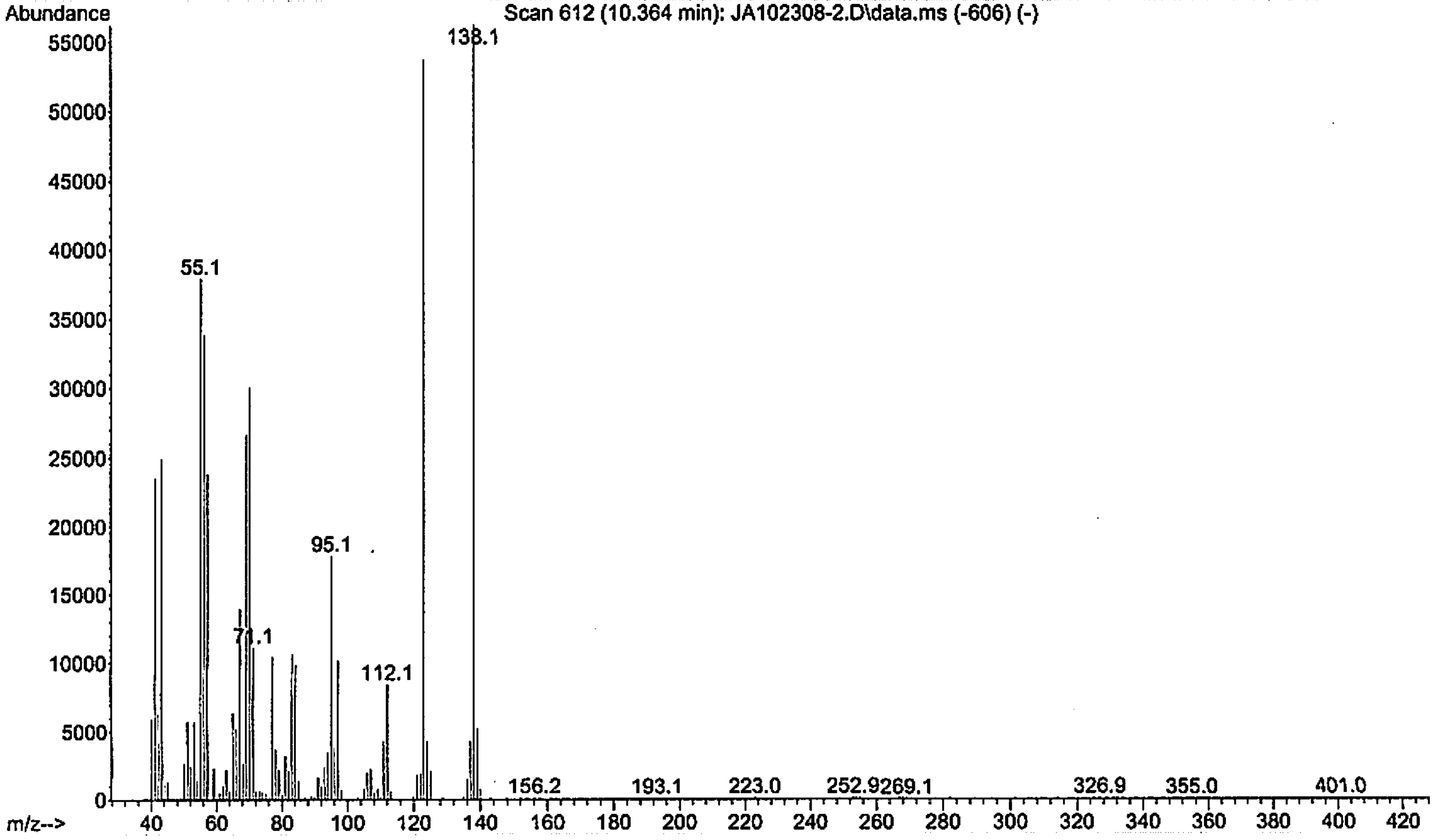

File :D:\Aldrich\JA102308-2.D
Operator :
Acquired : 23 Oct 2008 14:31 using AcqMethod JA-50-280LESS.M
Instrument : Buba
Sample Name: 7M C.oculata abdominal cuticle/10 ul CH2Cl2
Misc Info : 10/8-14; 6 days w/ 1ug/ul citronellol in H2O
Vial Number: 1

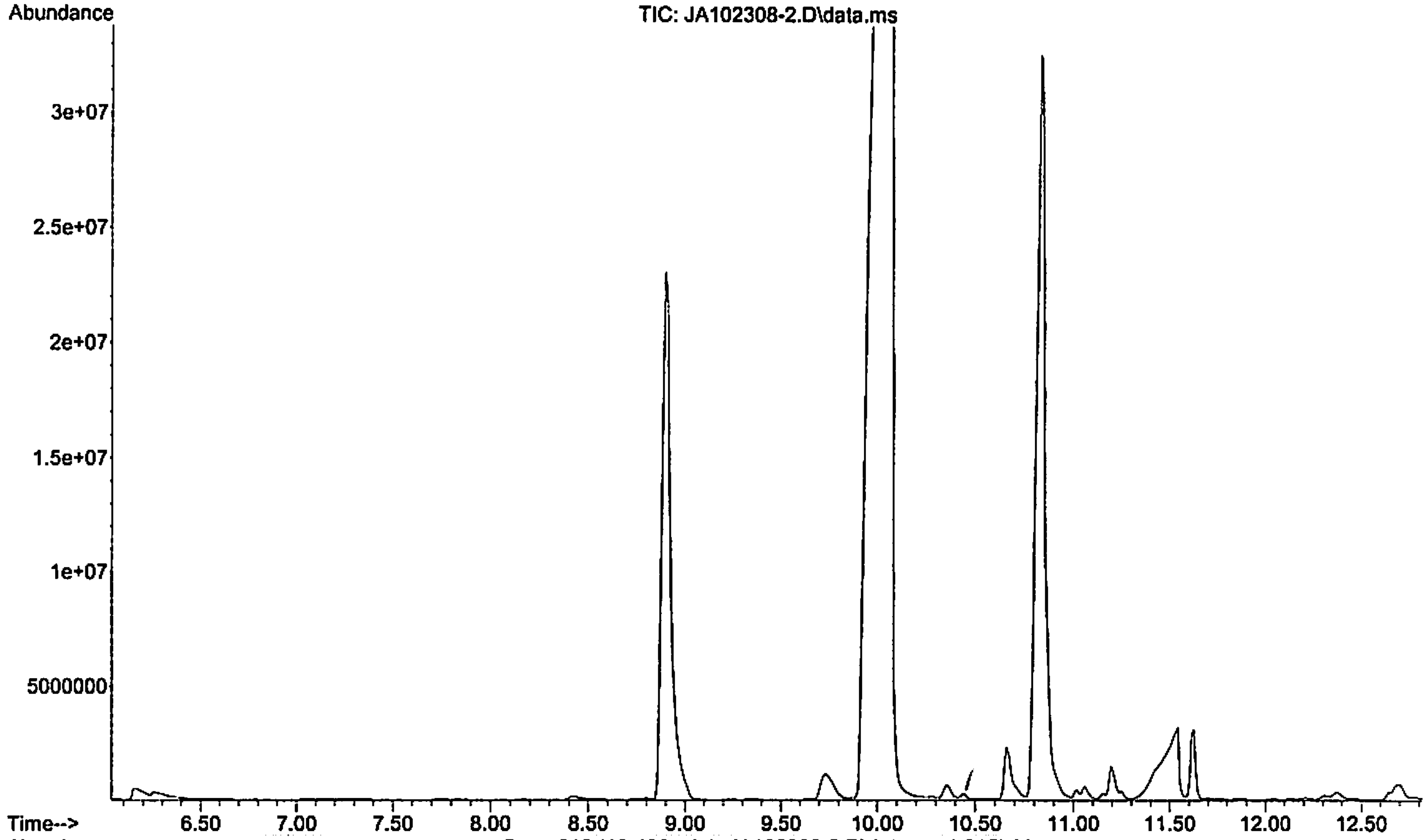


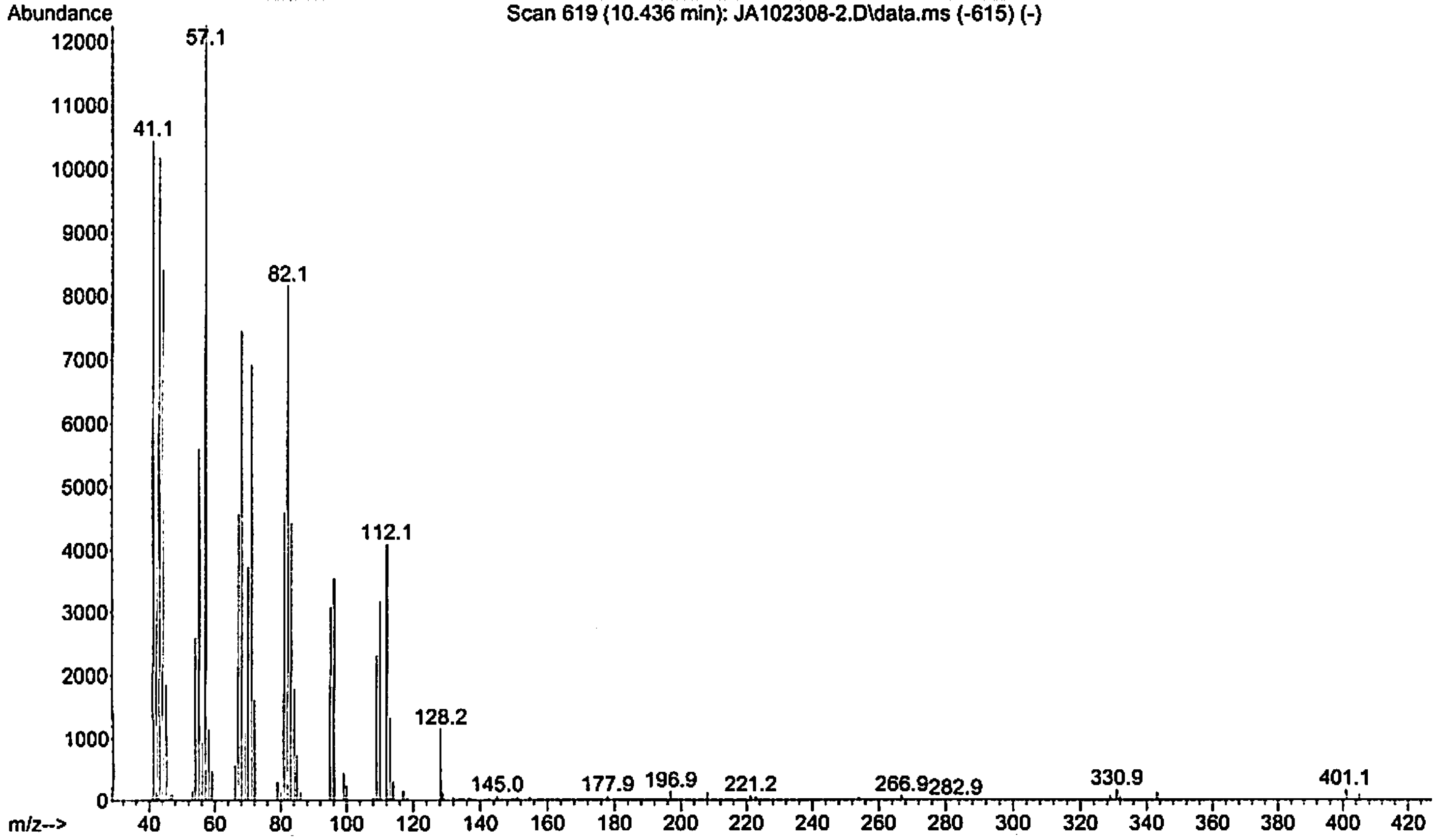

```
Library Searched : C:\Database\W8N05ST.L
Quality          : 90
ID               : DECANAL $ CAPRALDEHYDE $ 1-DECANAL $ 1-DECANAL (MIXED ISOMERS) $ 1-DEC
                   ANAL(MIXED ISOMERS) $ 1-DECYL ALDEHYDE $ 3,4-DIHYDRO-5,7-DIHYDROXY-2H-
                   1-BENZOPYRAN-3-YL 3,4-DIHYDROXYBENZOATE $ AI3-04860 $ AIDS-112312 $ AL
                   DEHYDE C10 $ BRN 1362530 $ C-10 ALDEHYDE $ CA
```

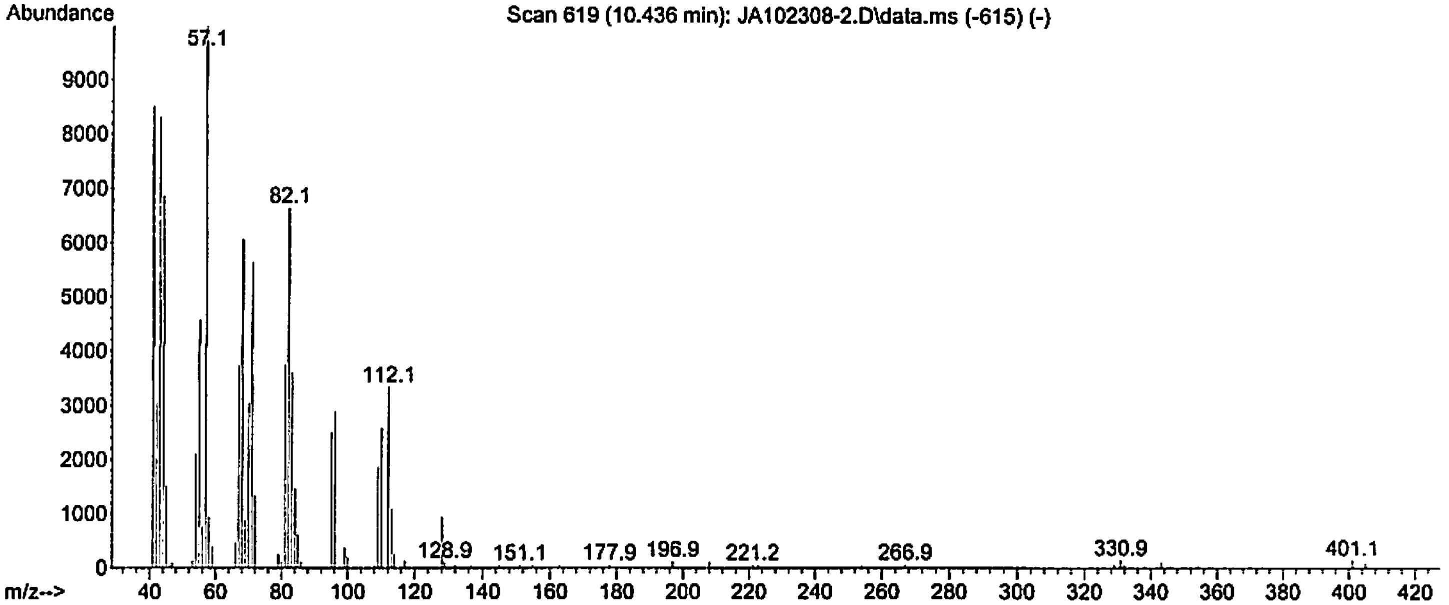


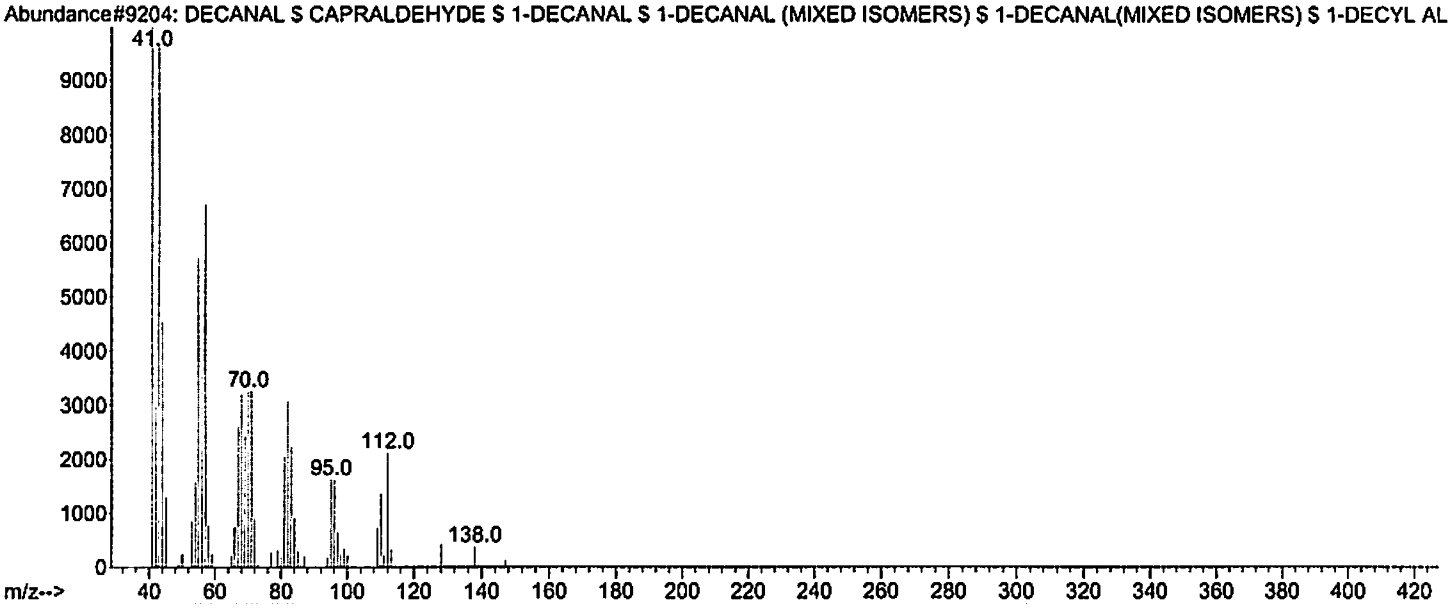


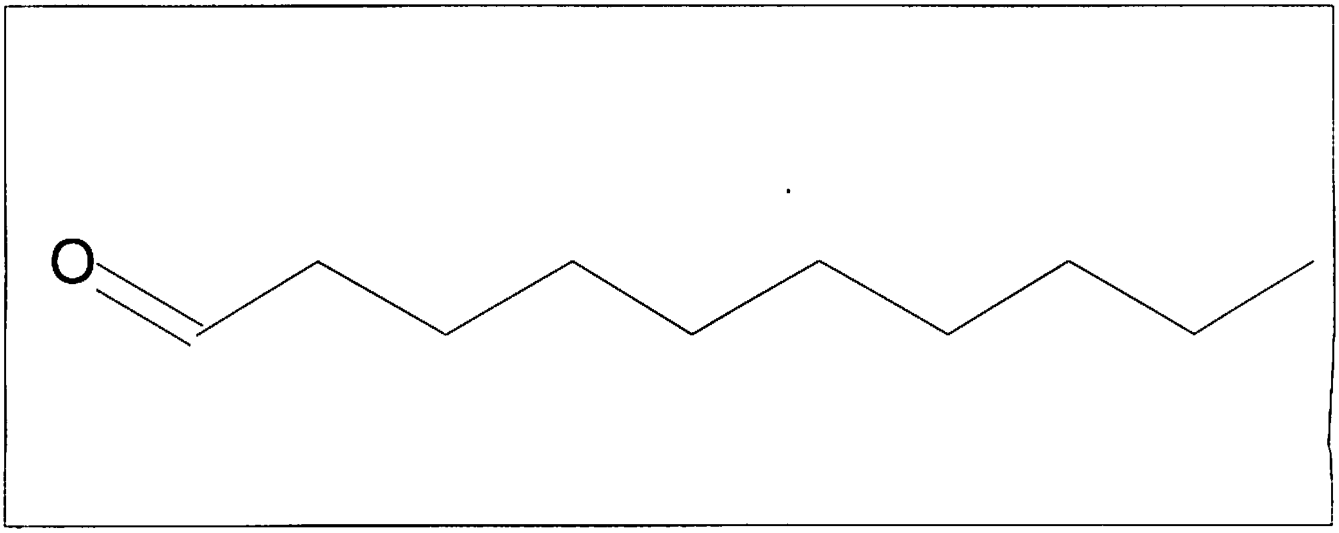

```
File        :D:\Aldrich\JA102308-2.D
Operator    :
Acquired    : 23 Oct 2008  14:31     using AcqMethod JA-50-280LESS.M
Instrument :     Buba
Sample Name: 7M C.oculata abdominal cuticle/10 ul CH2Cl2
Misc Info   : 10/8-14; 6 days w/ 1ug/ul citronellol in H2O
Vial Number: 1
```

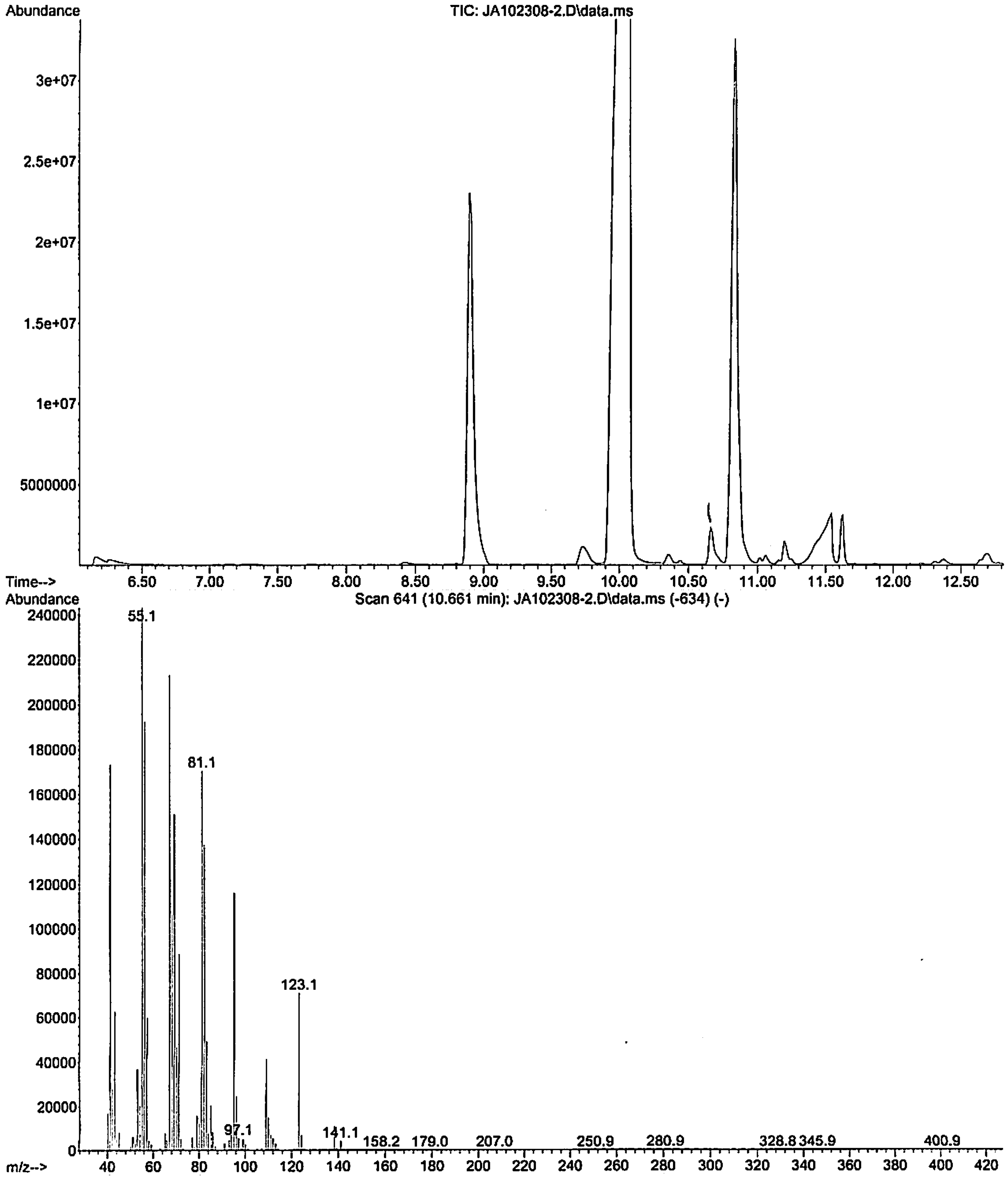

```
File        :D:\Aldrich\JA102308-2.D
Operator    :
Acquired    : 23 Oct 2008  14:31     using AcqMethod JA-50-280LESS.M
Instrument :     Buba
Sample Name: 7M C.oculata abdominal cuticle/10 ul CH2Cl2
Misc Info   : 10/8-14; 6 days w/ 1ug/ul citronellol in H2O
Vial Number: 1
```

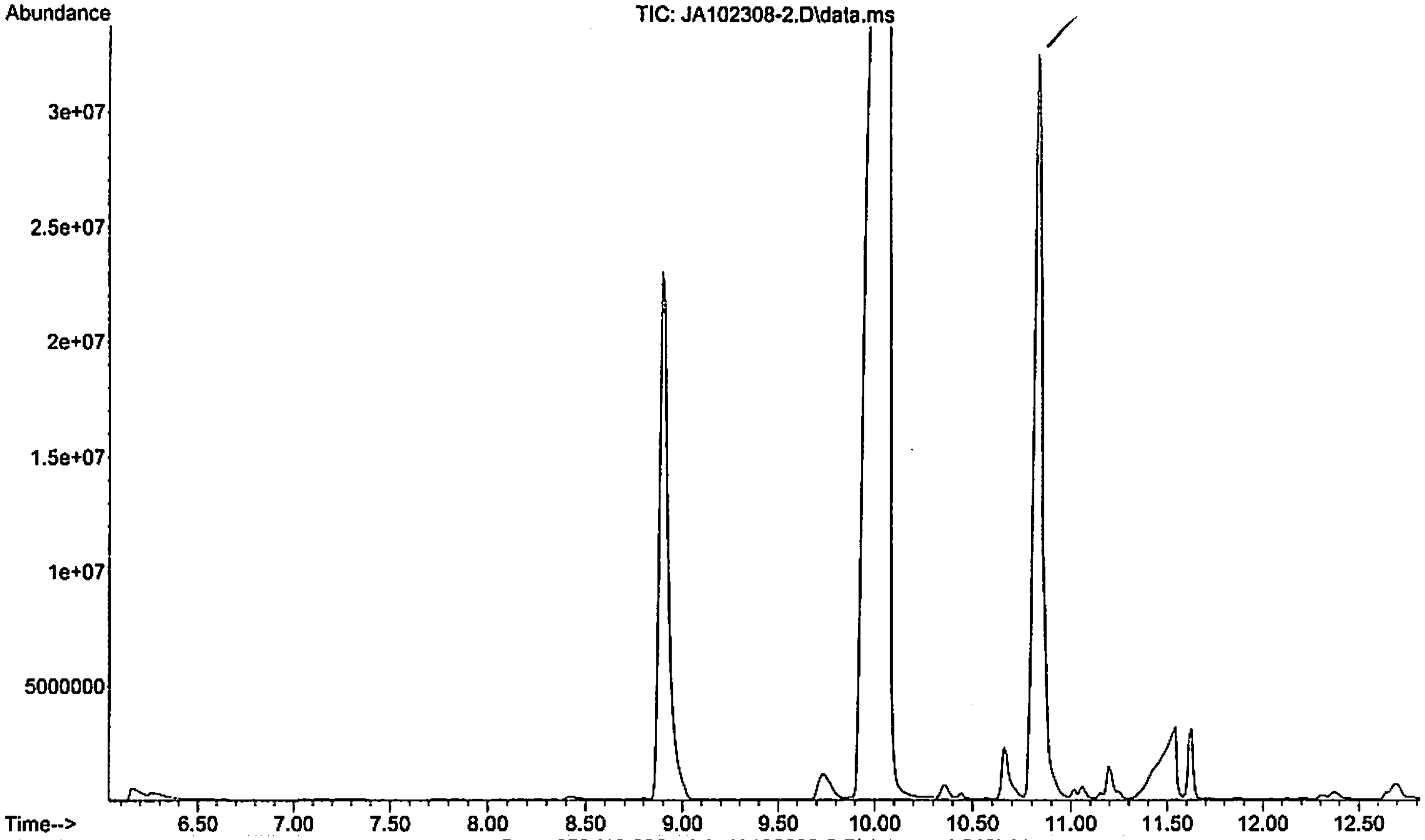


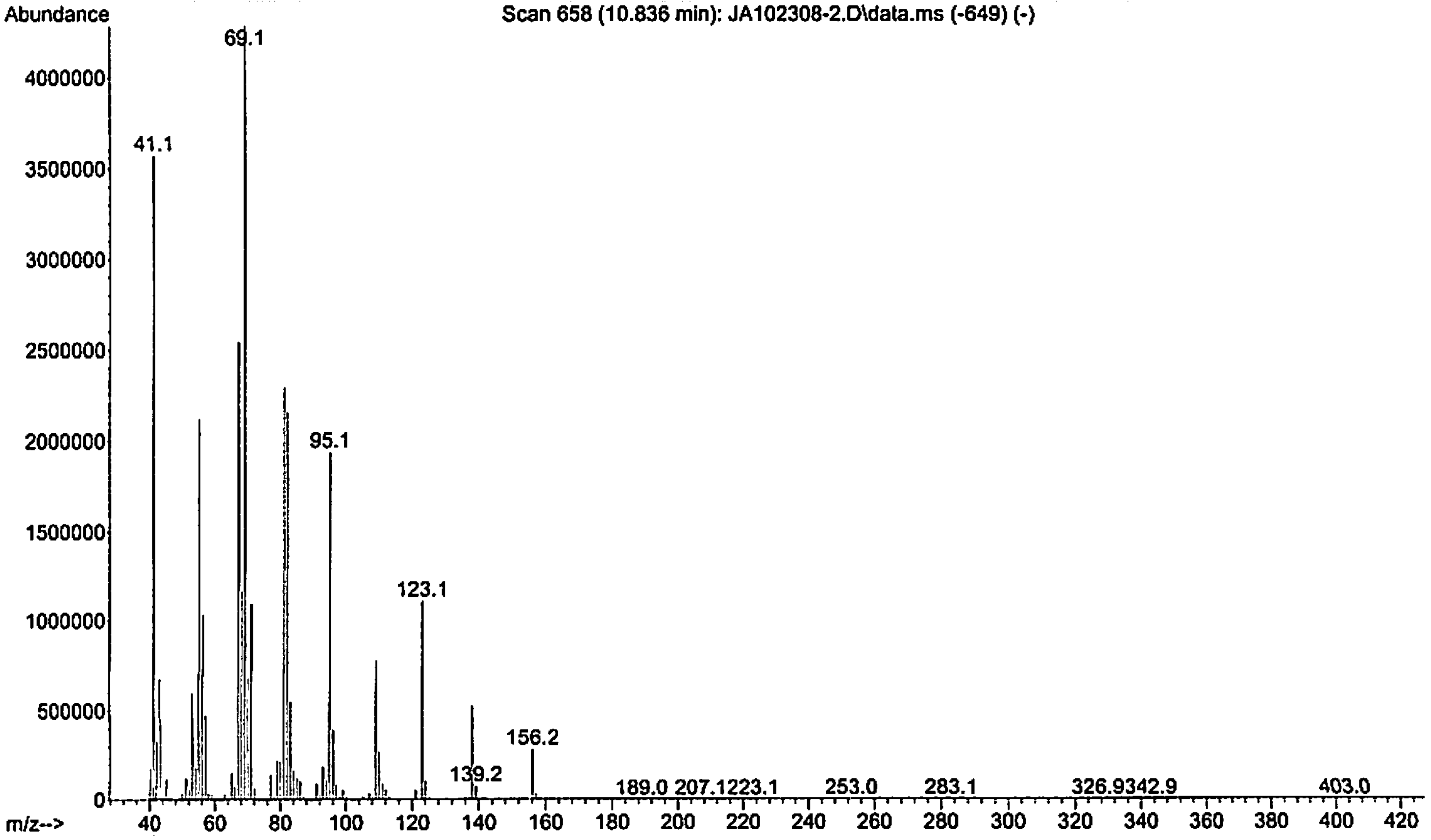

```
Library Searched : C:\Database\W8N05ST.L
Quality          : 98
ID               : 6-OCTEN-1-OL, 3,7-DIMETHYL- $ (R)-(+)-CITRONELLOL $ .BETA.-CITRONELLOL
                    $ 2,3-DIHYDROGERANIOL $ 2,6-DIMETHYL-2-OCTEN-8-OL $ 3, 7-DIMETHYL-6-O
                   CTEN-1-OL $ 3,7-DIMETHYL-6-OCTEN-1-OL $ AI3-25080 $ AIDS-032322 $ BETA
                   -CITRONELLOL $ BRN 1721507 $ CCRIS 7452 $ CEP
```

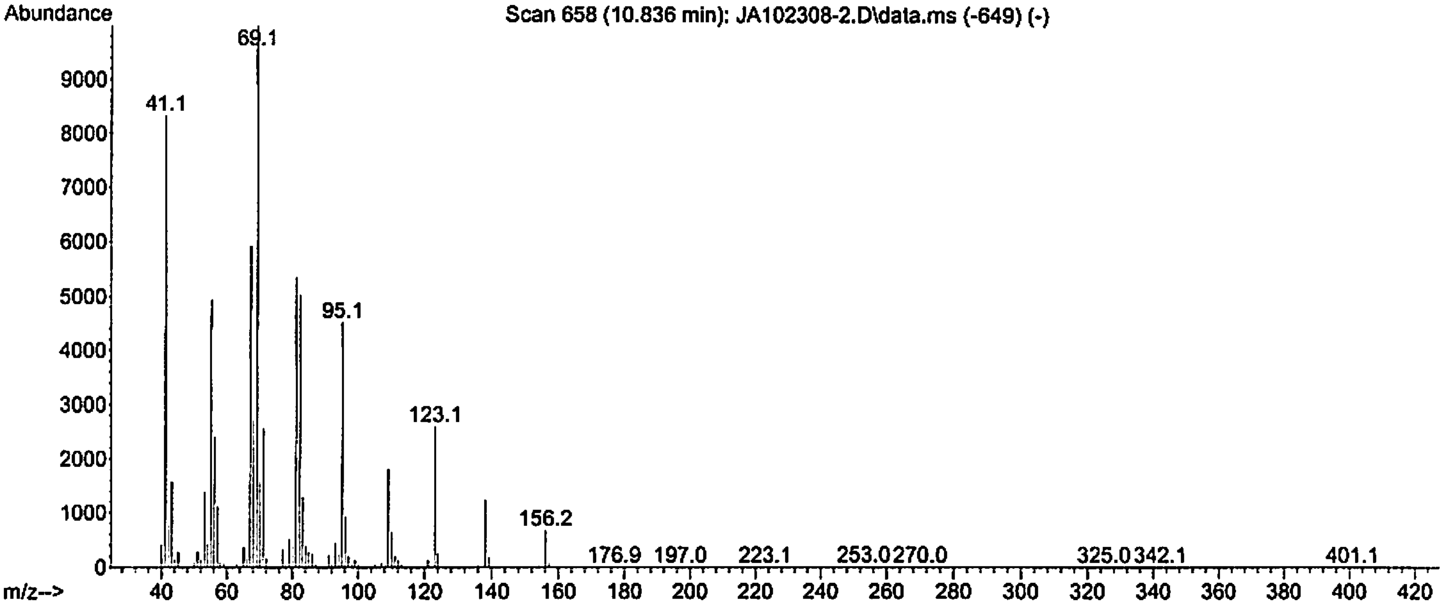


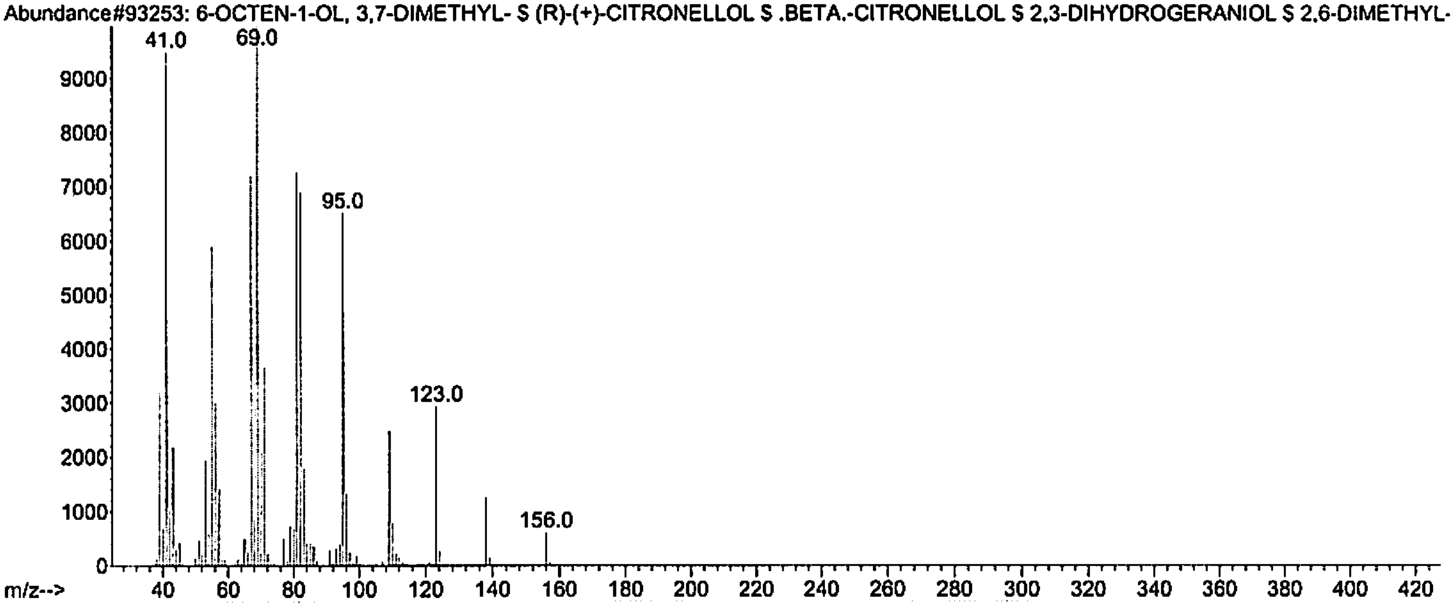


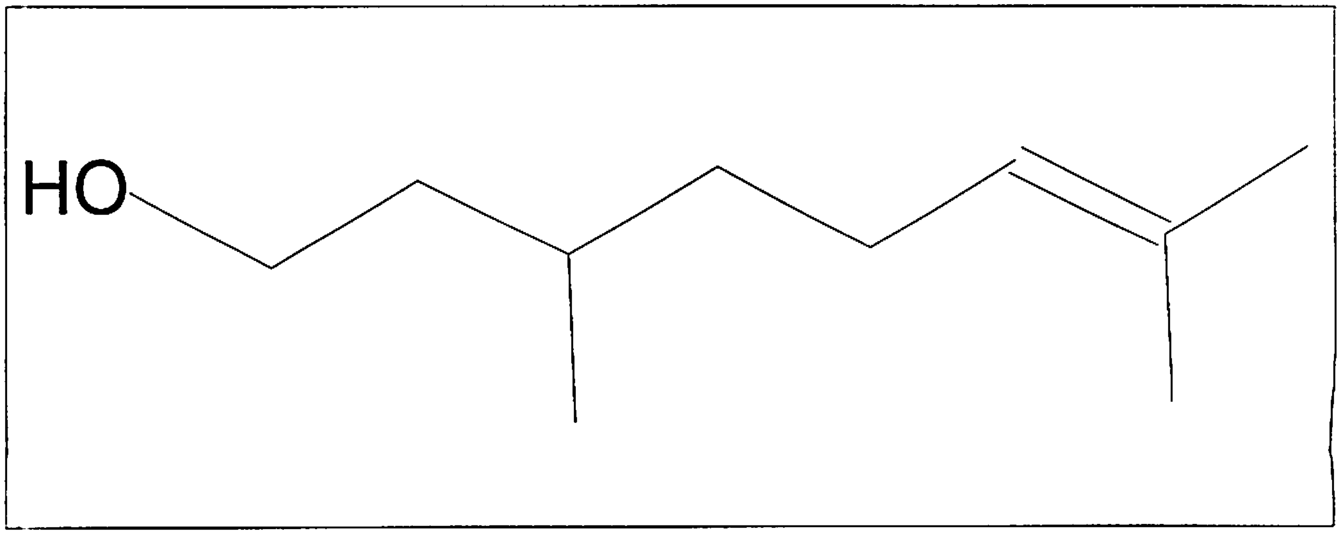

File :D:\Aldrich\JA102308-2.D
Operator :
Acquired : 23 Oct 2008 14:31 using AcqMethod JA-50-280LESS.M
Instrument : Buba
Sample Name: 7M C.oculata abdominal cuticle/10 ul CH2Cl2
Misc Info : 10/8-14; 6 days w/ 1ug/ul citronellol in H2O
Vial Number: 1

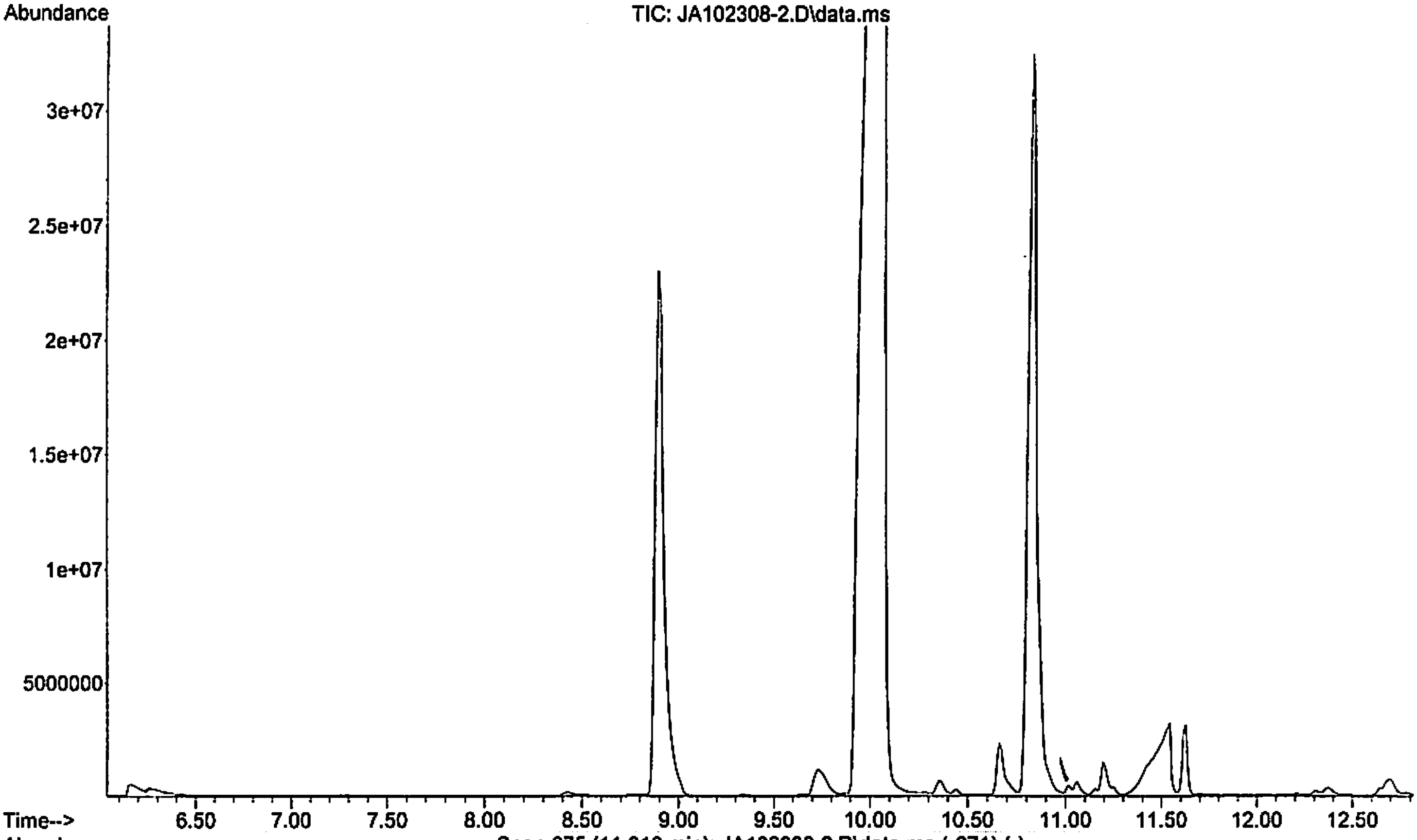


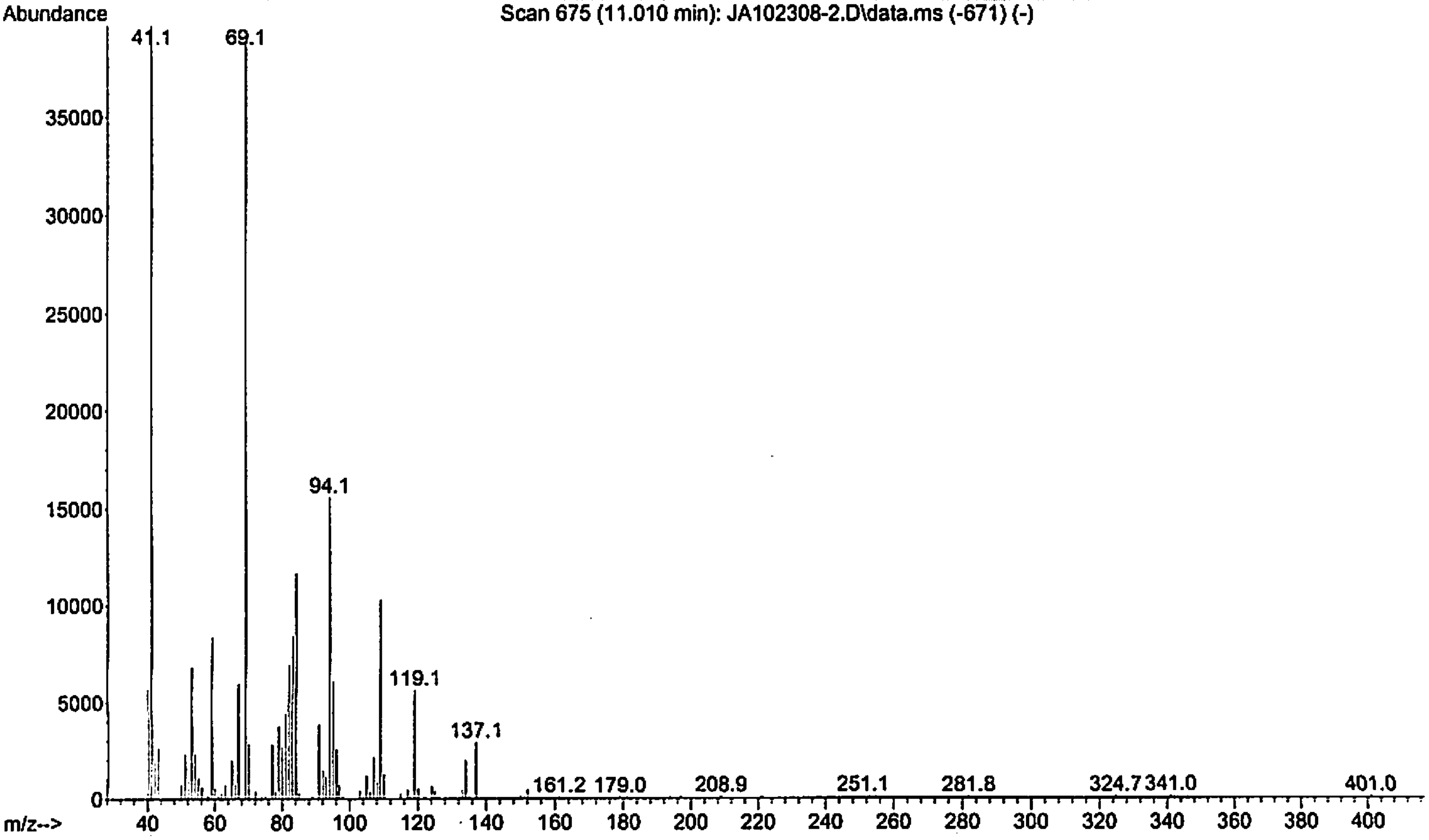

```
Library Searched : C:\Database\W8N05ST.L
Quality          : 87
ID               : 2,6-Octadienal, 3,7-dimethyl-, (Z)- $ .beta.-Citral $ cis-Citral $ cis
                   -3,7-Dimethyl-2,6-octadienal $ Citral b $ Neral $ Z-Citral $ (Z)-3,7-D
                   imethyl-2,6-octadienal $ Neral (Z-citral orcitral B) $ (2E)-3,7-Dimeth
                   yl-2,6-octadienal #
```

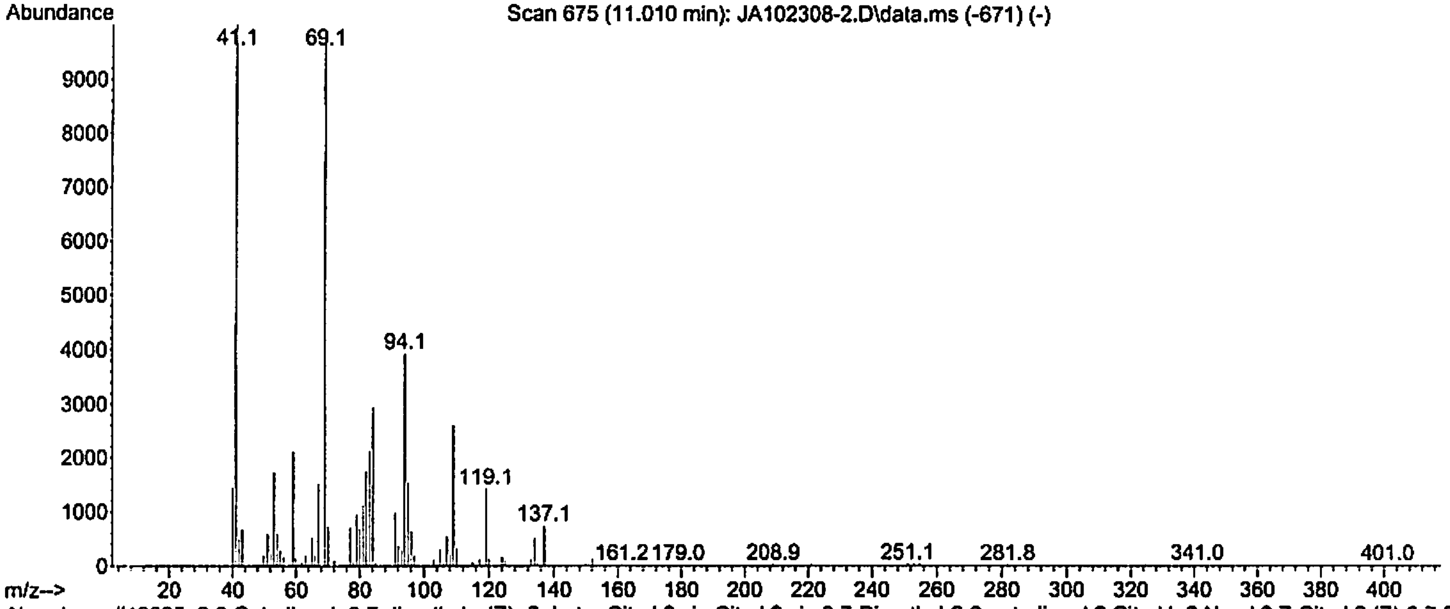


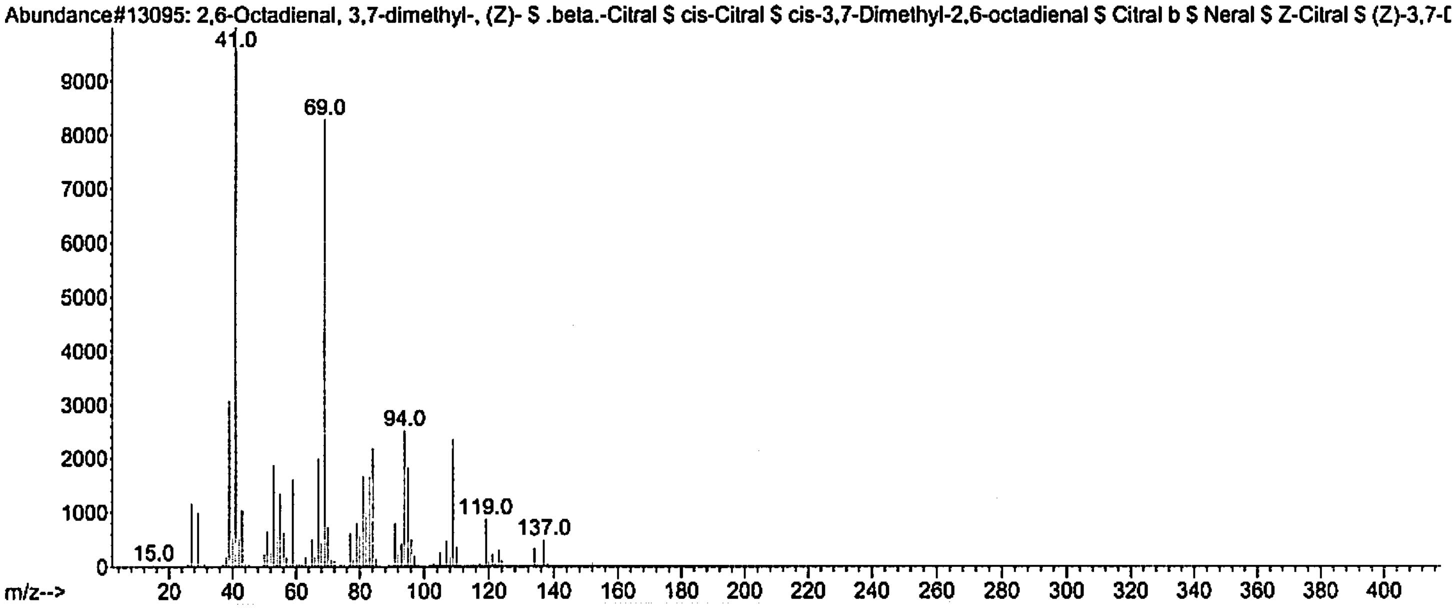


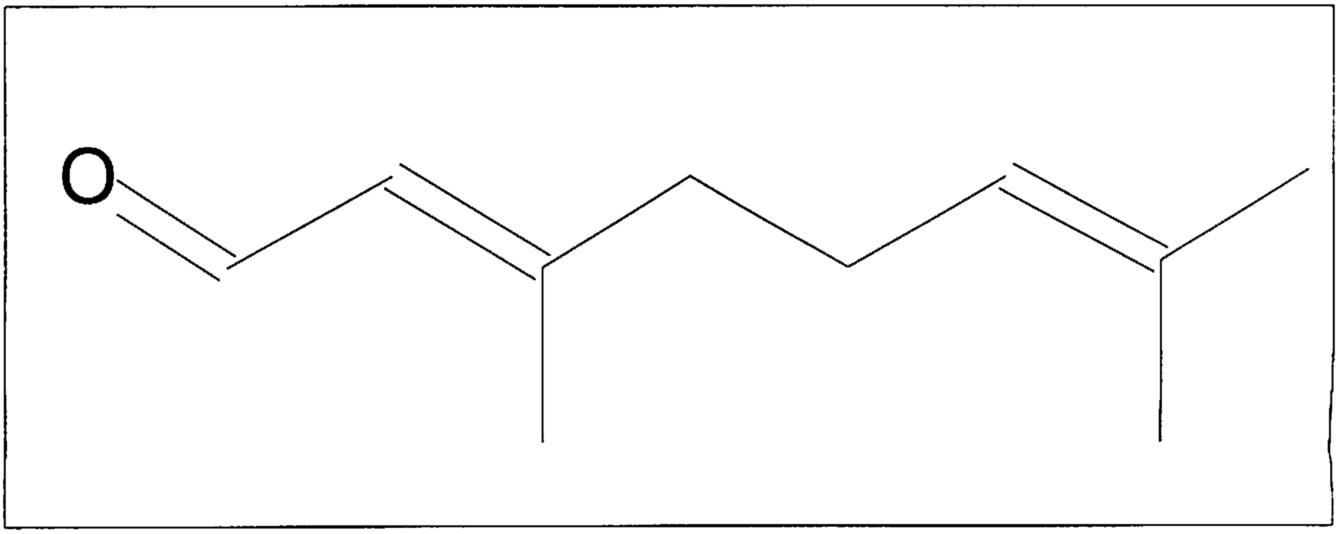

File :D:\Aldrich\JA102308-2.D
Operator :
Acquired : 23 Oct 2008 14:31 using AcqMethod JA-50-280LESS.M
Instrument : Buba
Sample Name: 7M C.oculata abdominal cuticle/10 ul CH2Cl2
Misc Info : 10/8-14; 6 days w/ 1ug/ul citronellol in H2O
Vial Number: 1

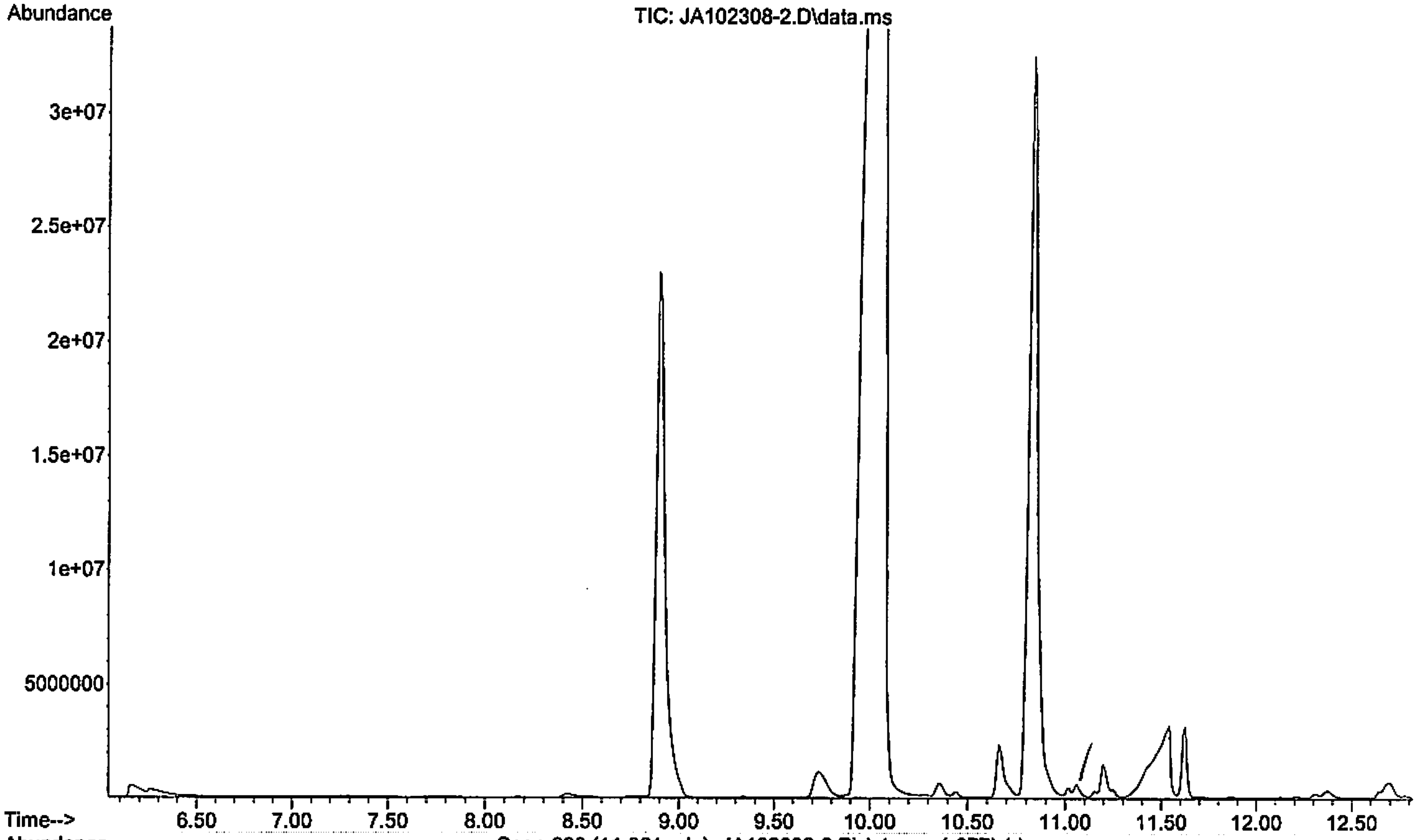


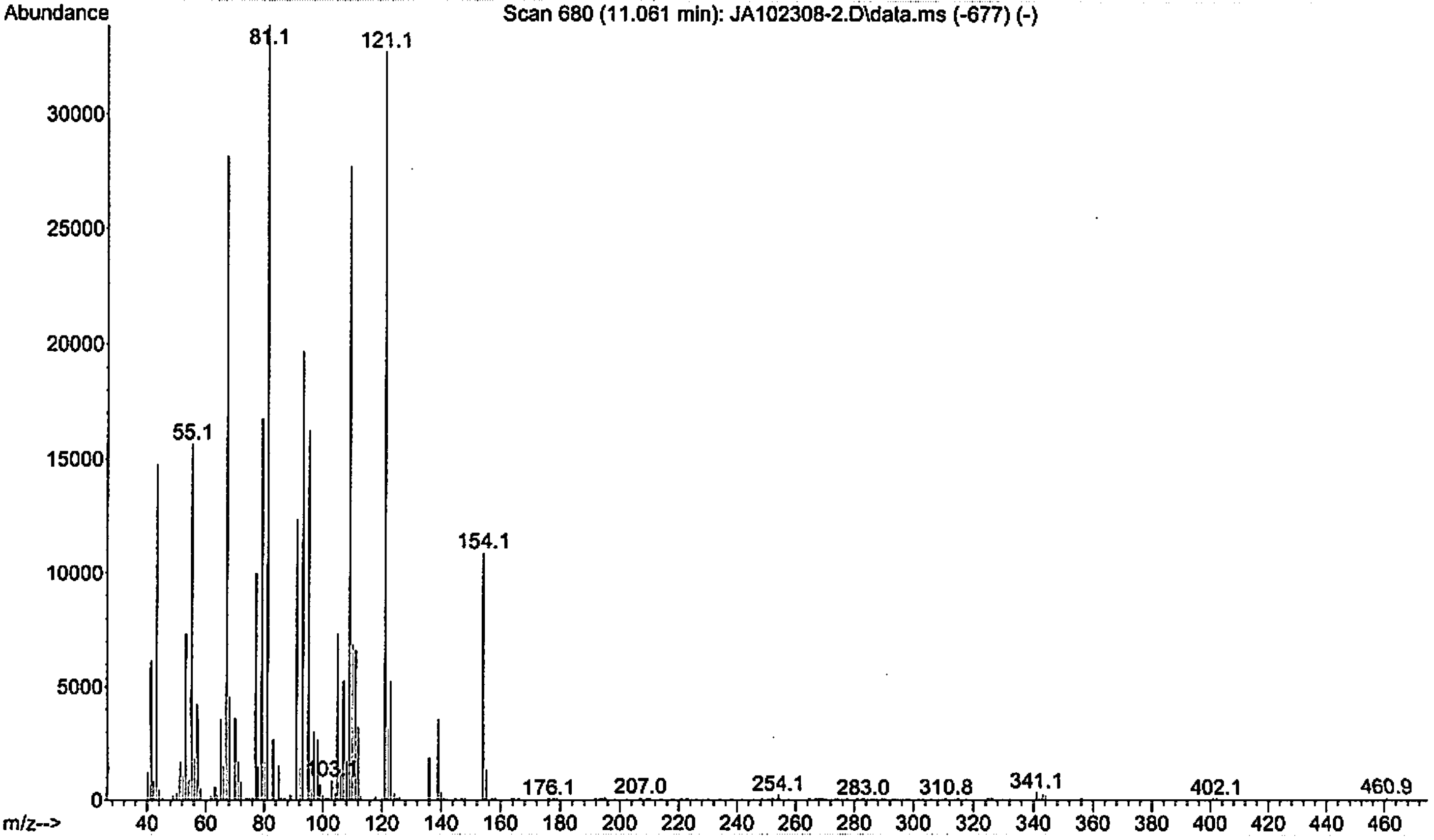

```
File        :D:\Aldrich\JA102308-2.D
Operator    :
Acquired    : 23 Oct 2008  14:31     using AcqMethod JA-50-280LESS.M
Instrument :    Buba
Sample Name: 7M C.oculata abdominal cuticle/10 ul CH2Cl2
Misc Info   : 10/8-14; 6 days w/ 1ug/ul citronellol in H2O
Vial Number: 1
```

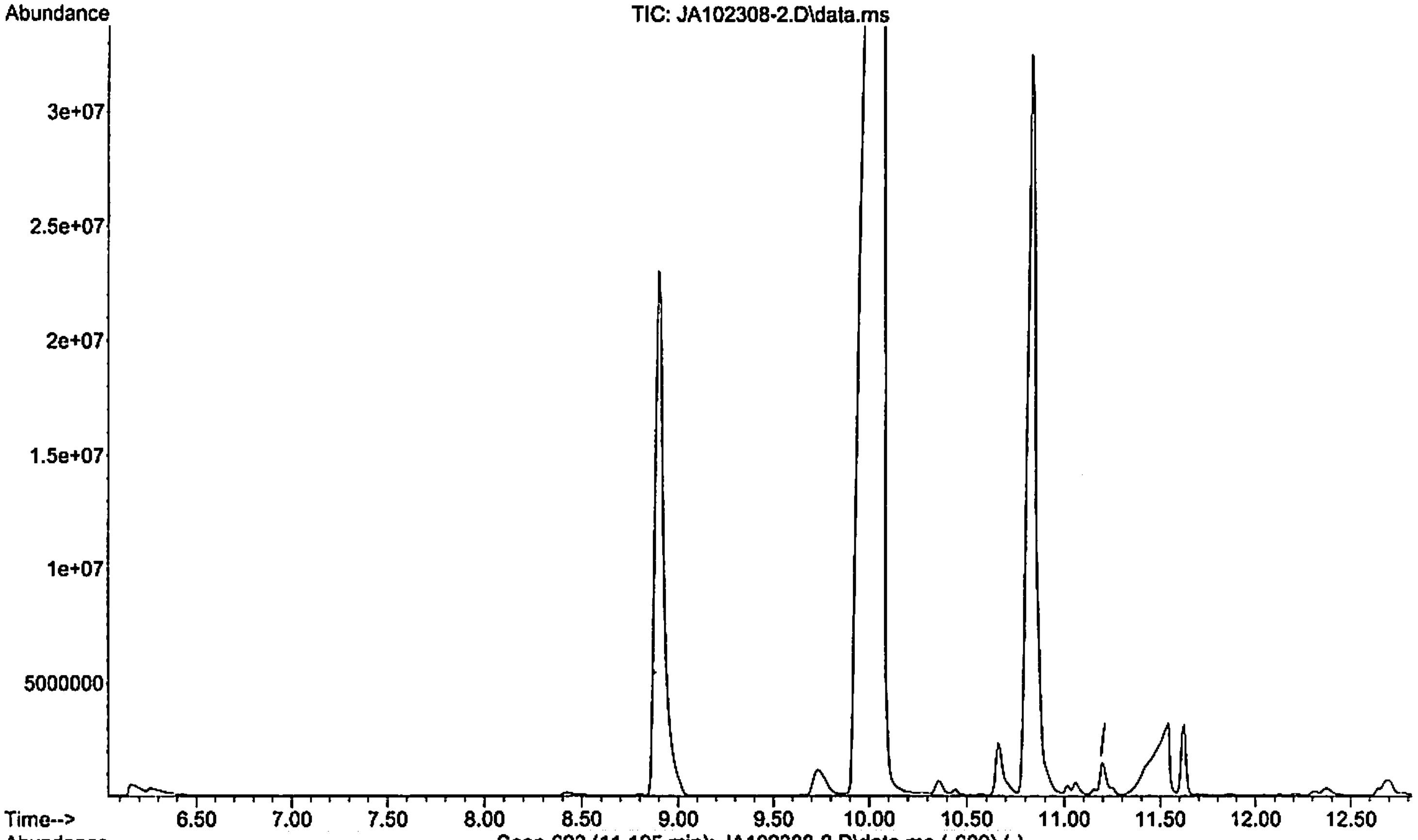


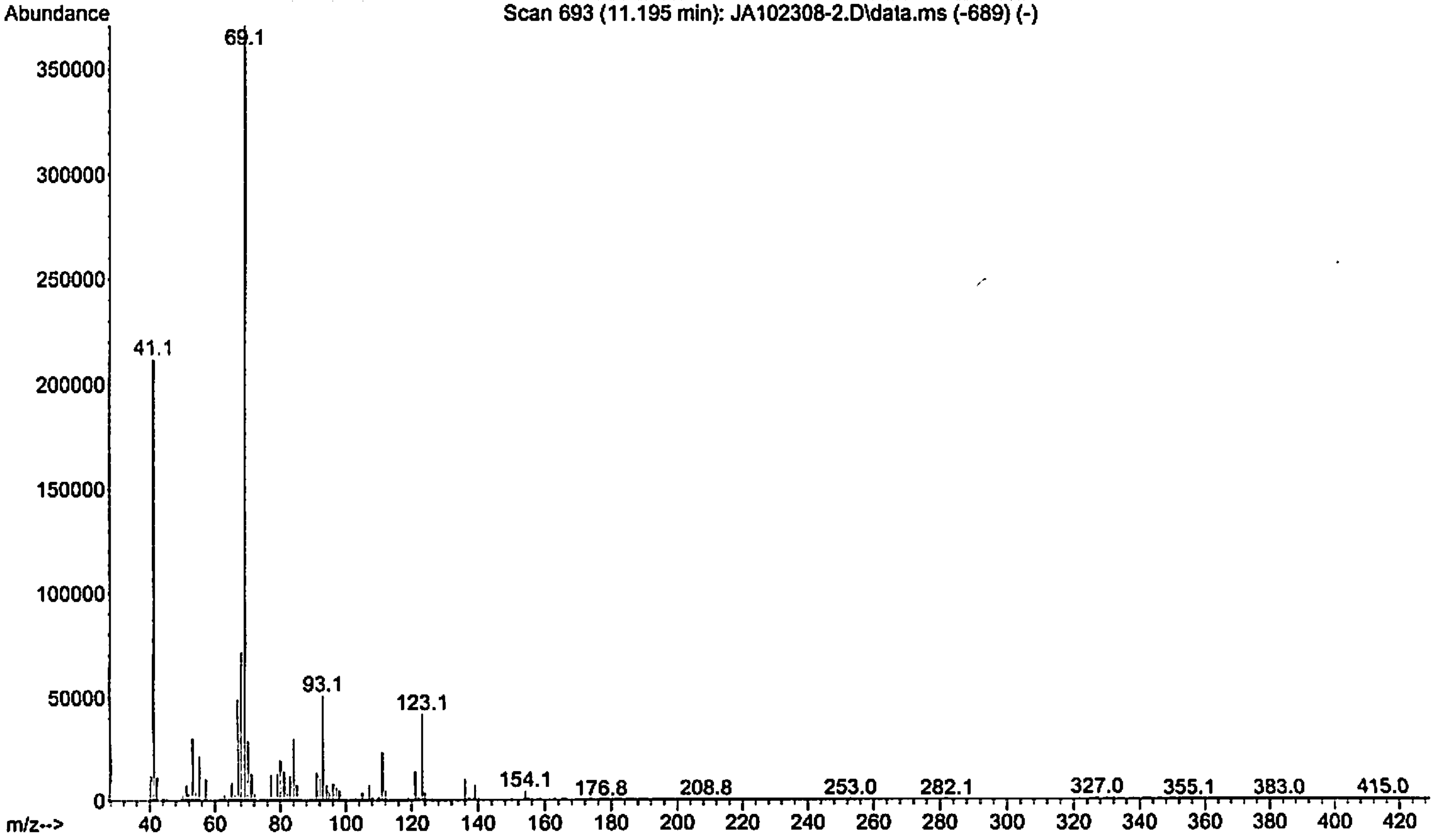

```
Library Searched : C:\Database\W8N05ST.L
Quality          : 94
ID               : 2,6-OCTADIEN-1-OL, 3,7-DIMETHYL-, (E)- $ 3,7-DIMETHYLOCTA-2,6-DIEN-1-O
                   L $ (2E)-3,7-DIMETHYL-2,6-OCTADIEN-1-OL # $ (2E)-3,7-DIMETHYL-2,6-OCTA
                   DIEN-1-OL $ (2E)-3,7-DIMETHYL-2,6-OCTADIEN-1-OL (COMPUTER-GENERATED NA
                   ME) $ (E)-3,7-DIMETHYL-2,6-OCTADIEN-1-OL $ (E
```

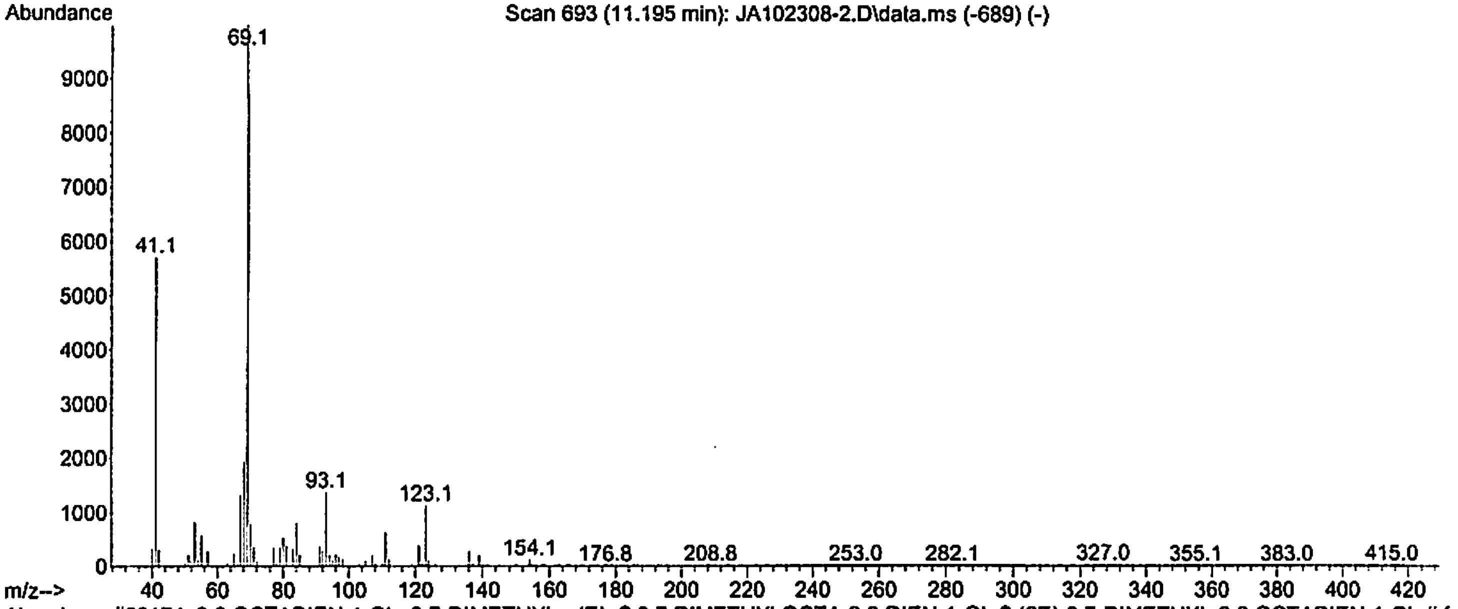


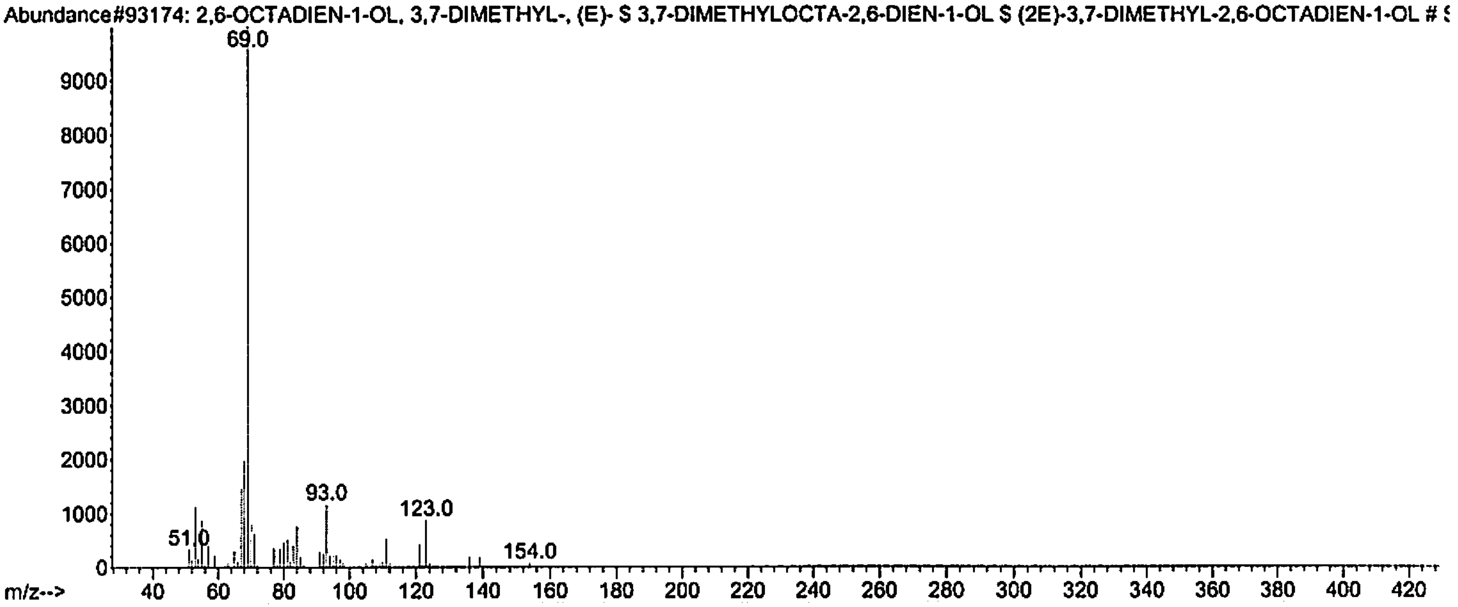


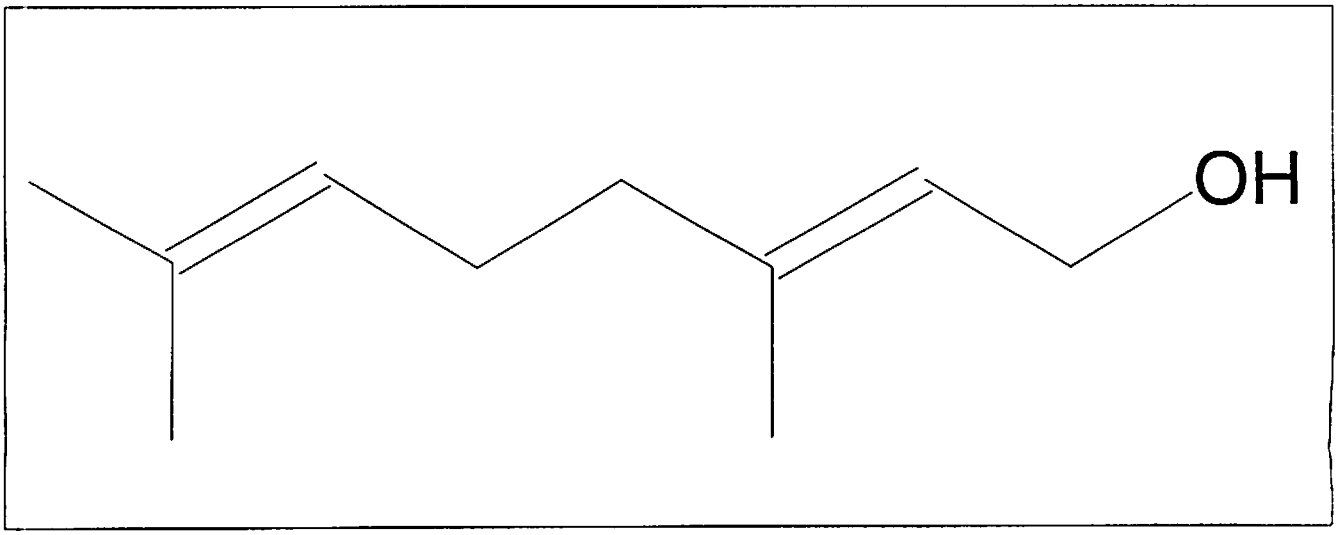

File        :D:\Aldrich\JA102308-2.D
Operator    :
Acquired    : 23 Oct 2008  14:31     using AcqMethod JA-50-280LESS.M
Instrument :    Buba
Sample Name: 7M C.oculata abdominal cuticle/10 ul CH2Cl2
Misc Info  : 10/8-14; 6 days w/ 1ug/ul citronellol in H2O
Vial Number: 1

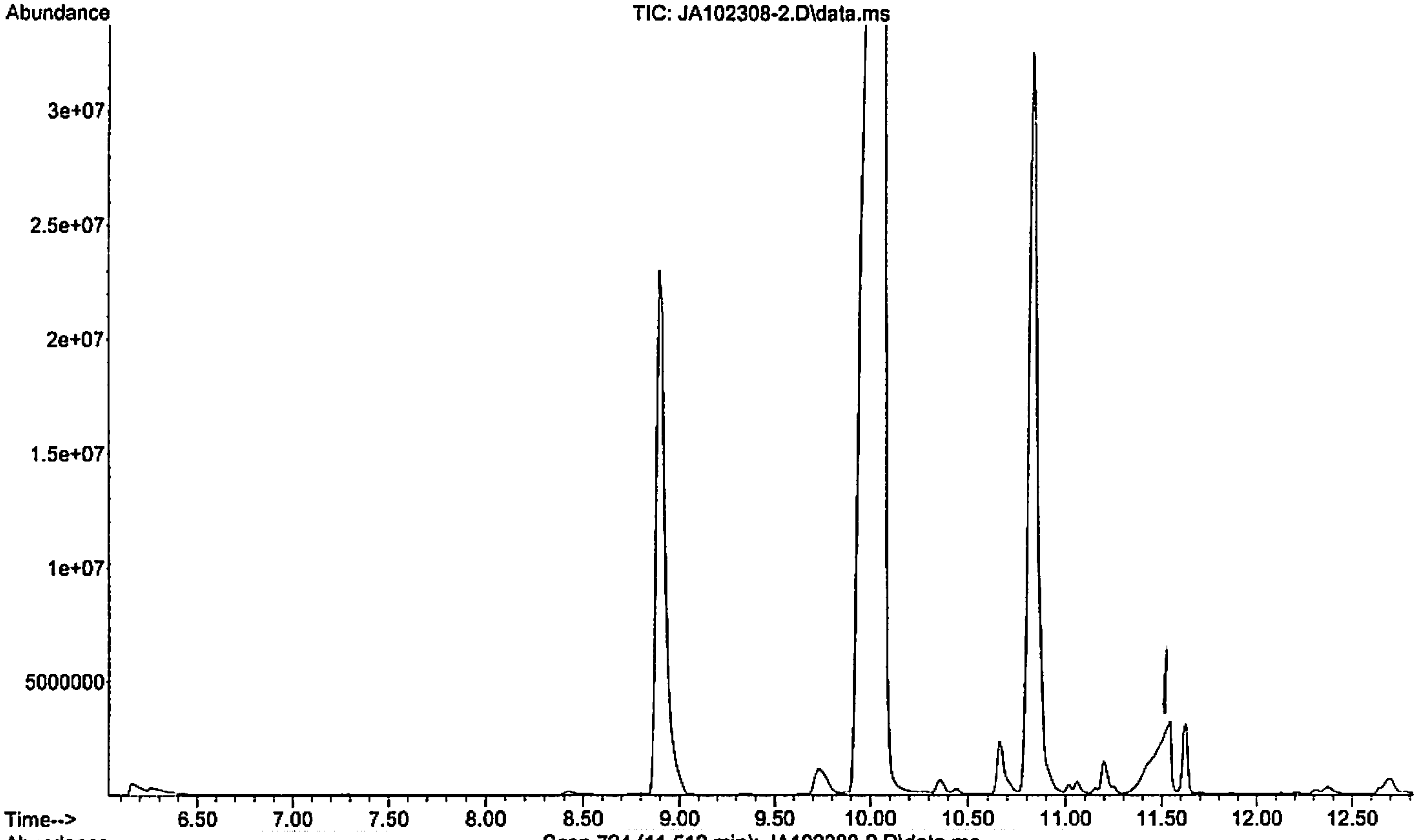


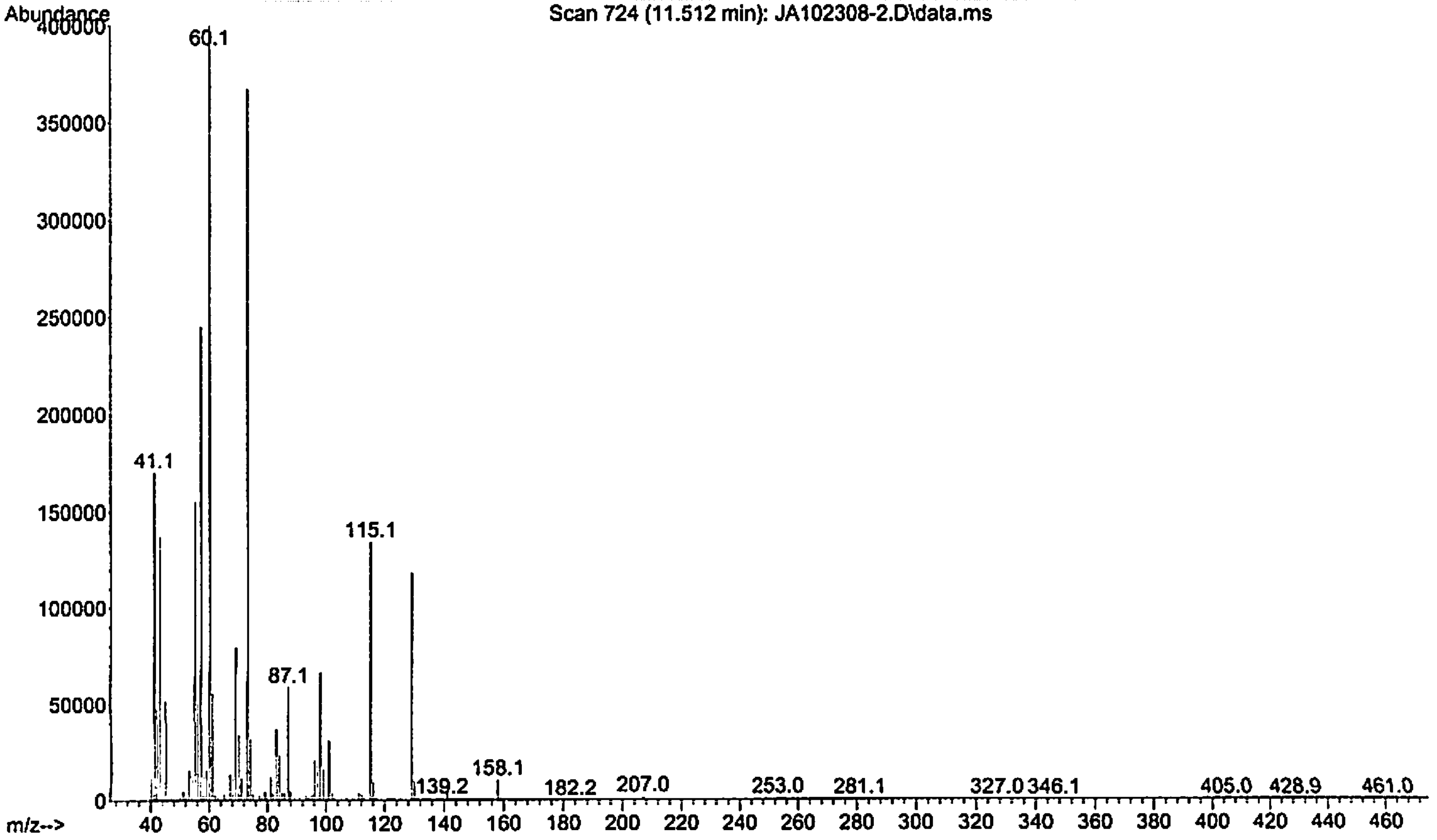

```
Library Searched : C:\Database\W8N05ST.L
Quality          : 95
ID               : Nonanoic acid $ n-Nonanoic acid $ n-Nonoic acid $ n-Nonylic acid $ Non
                   oic acid $ Nonylic acid $ Pelargic acid $ Pelargonic acid $ 1-Octaneca
                   rboxylic acid $ Cirrasol 185a $ Emfac 1202 $ Hexacid C-9 $ Pelargon $
                   Emery's L-114 $ Emery 1202
```

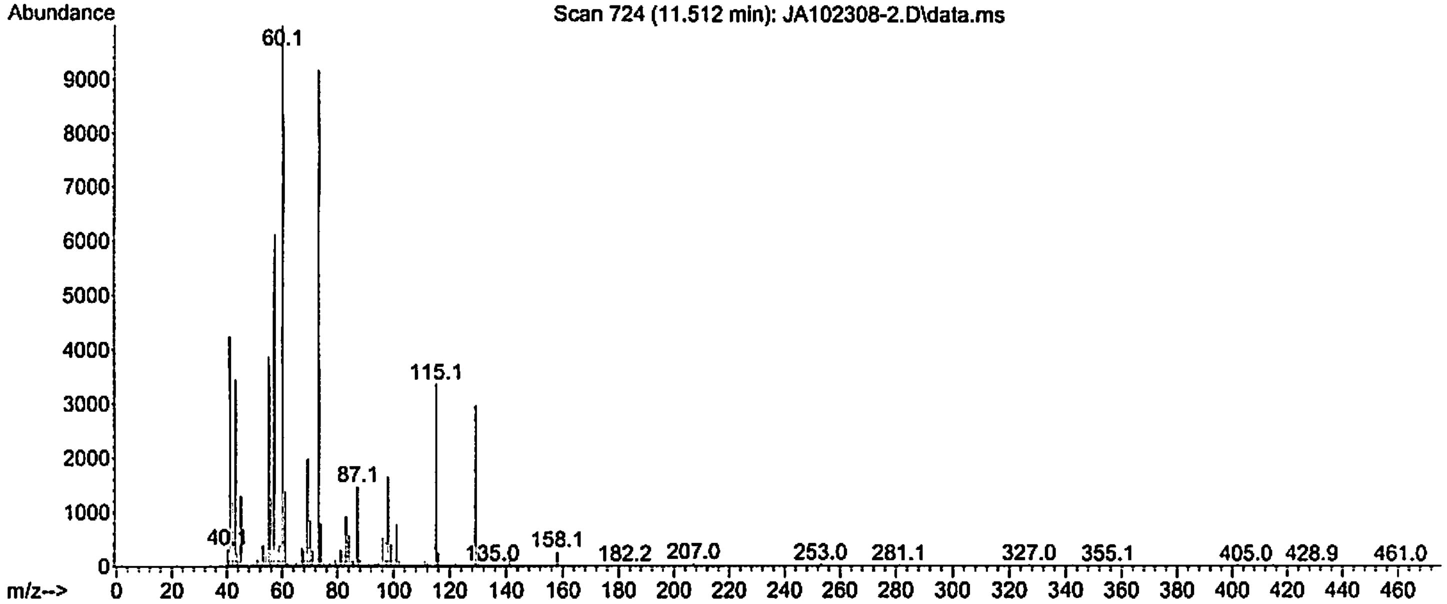


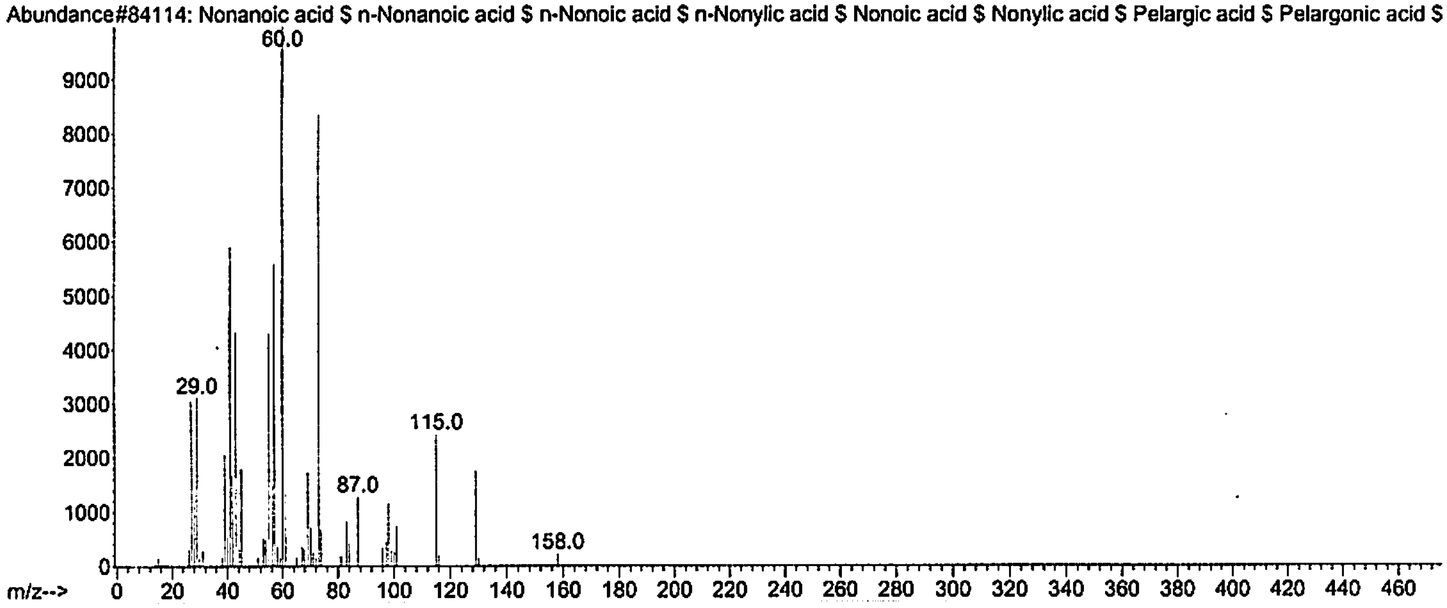


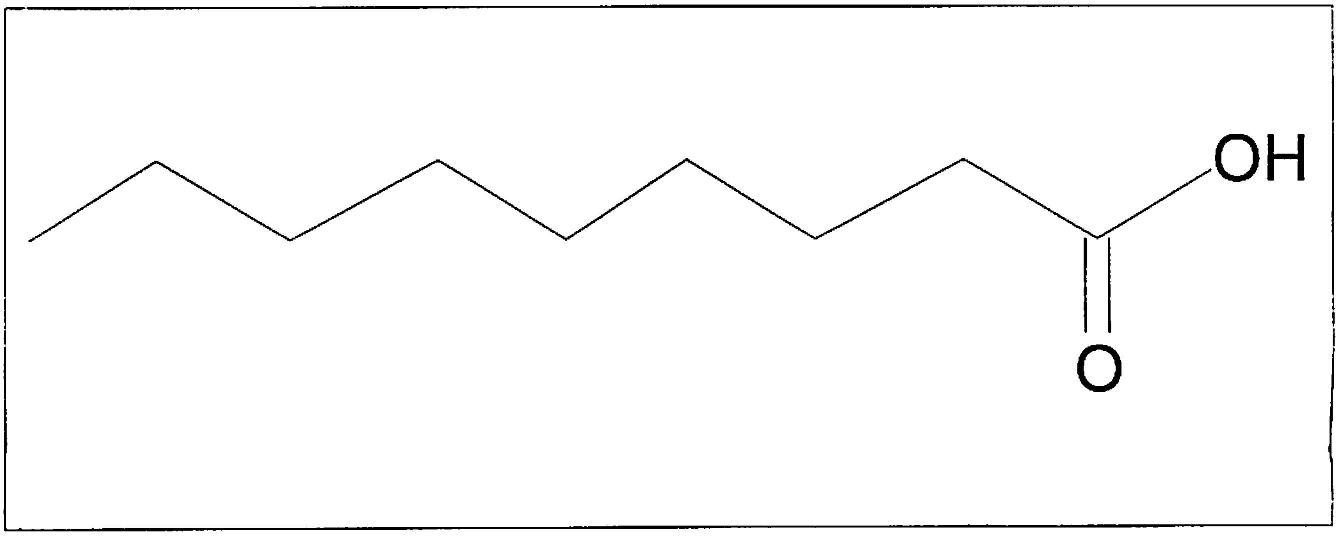

```
File        :D:\Aldrich\JA102308-2.D
Operator    :
Acquired    : 23 Oct 2008  14:31     using AcqMethod JA-50-280LESS.M
Instrument  :    Buba
Sample Name: 7M C.oculata abdominal cuticle/10 ul CH2Cl2
Misc Info  : 10/8-14; 6 days w/ 1ug/ul citronellol in H2O
Vial Number: 1
```

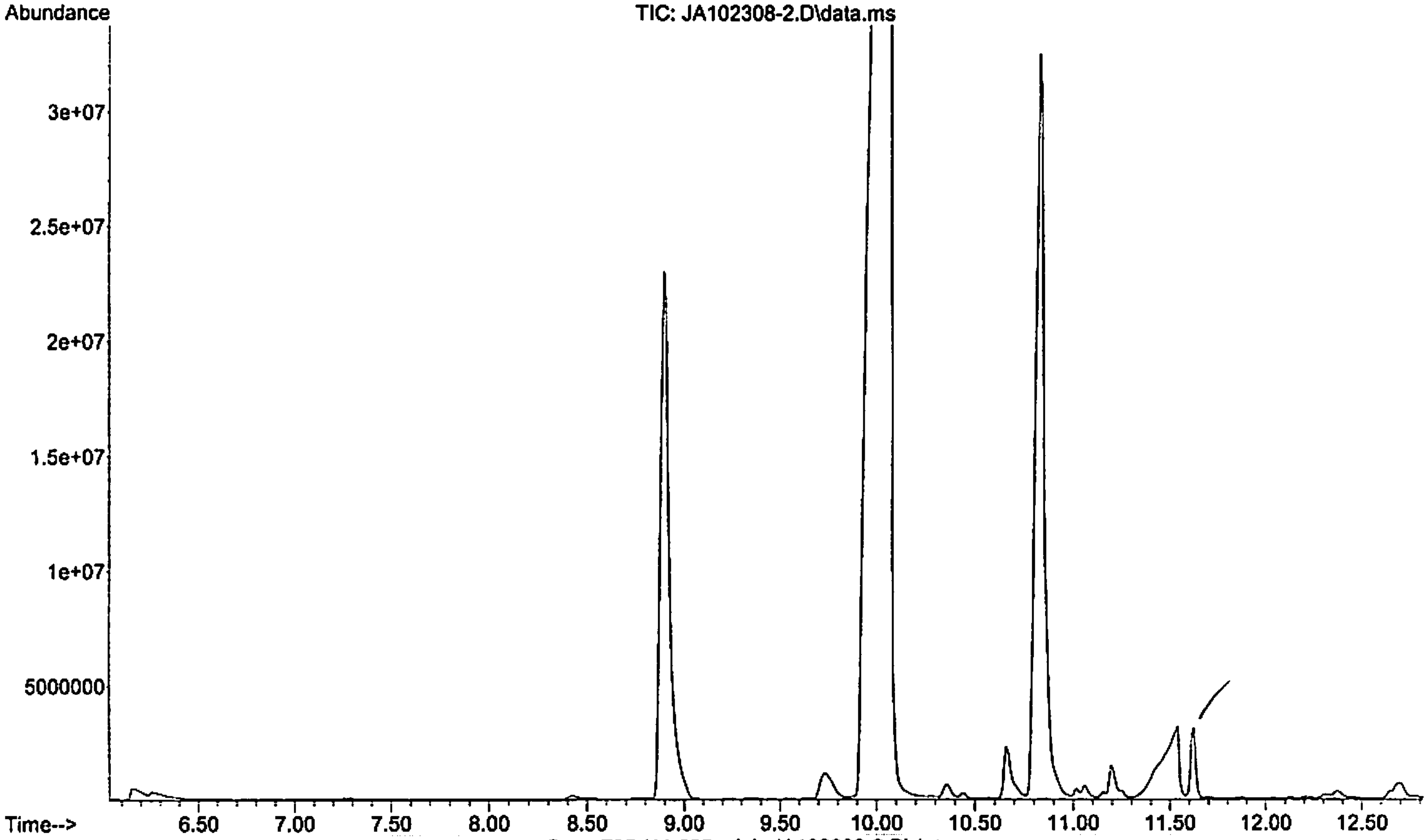


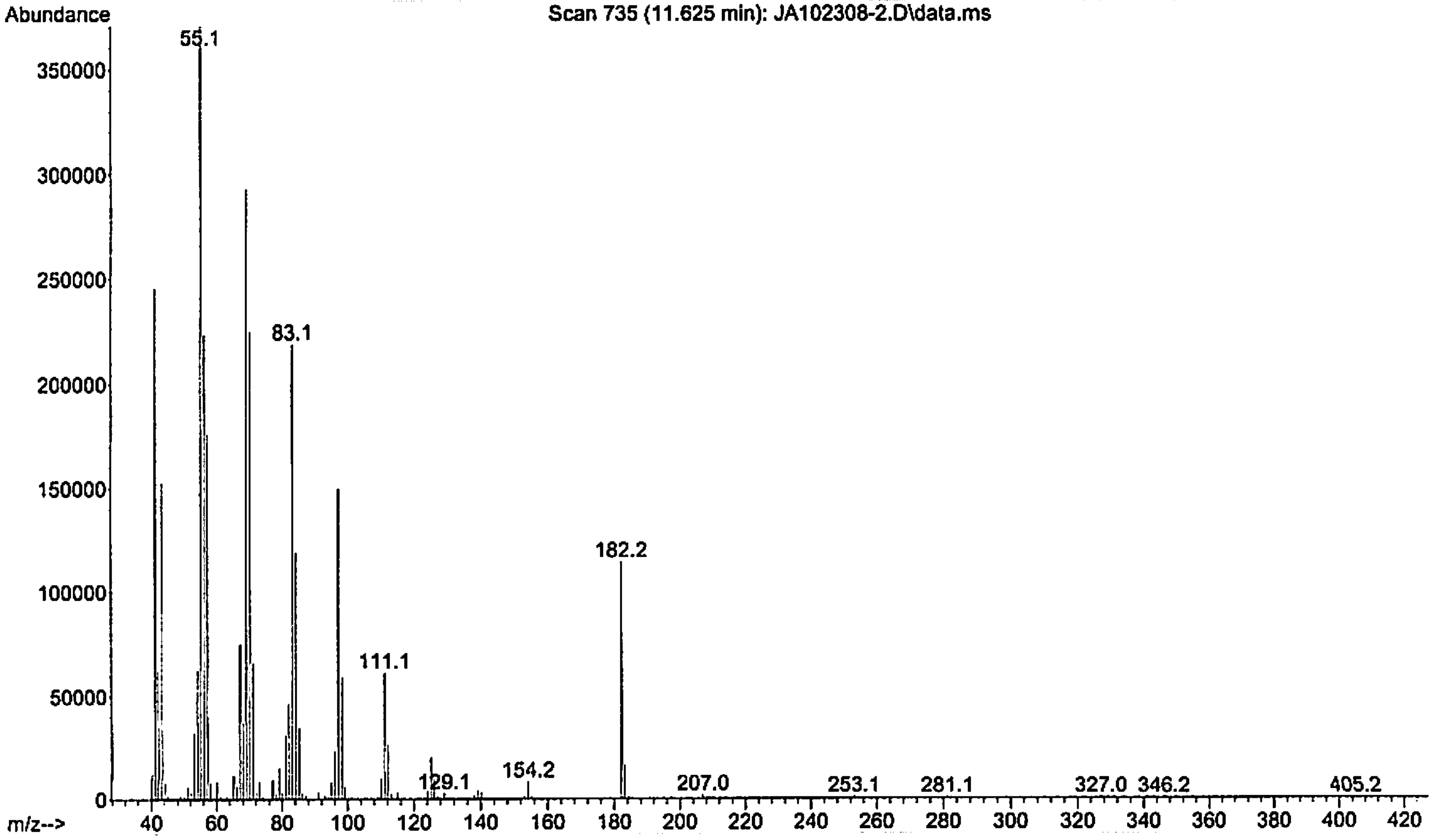

```
File        :D:\Aldrich\JA102308-2.D
Operator    :
Acquired    : 23 Oct 2008  14:31     using AcqMethod JA-50-280LESS.M
Instrument  :    Buba
Sample Name: 7M C.oculata abdominal cuticle/10 ul CH2Cl2
Misc Info   : 10/8-14; 6 days w/ 1ug/ul citronellol in H2O
Vial Number: 1
```

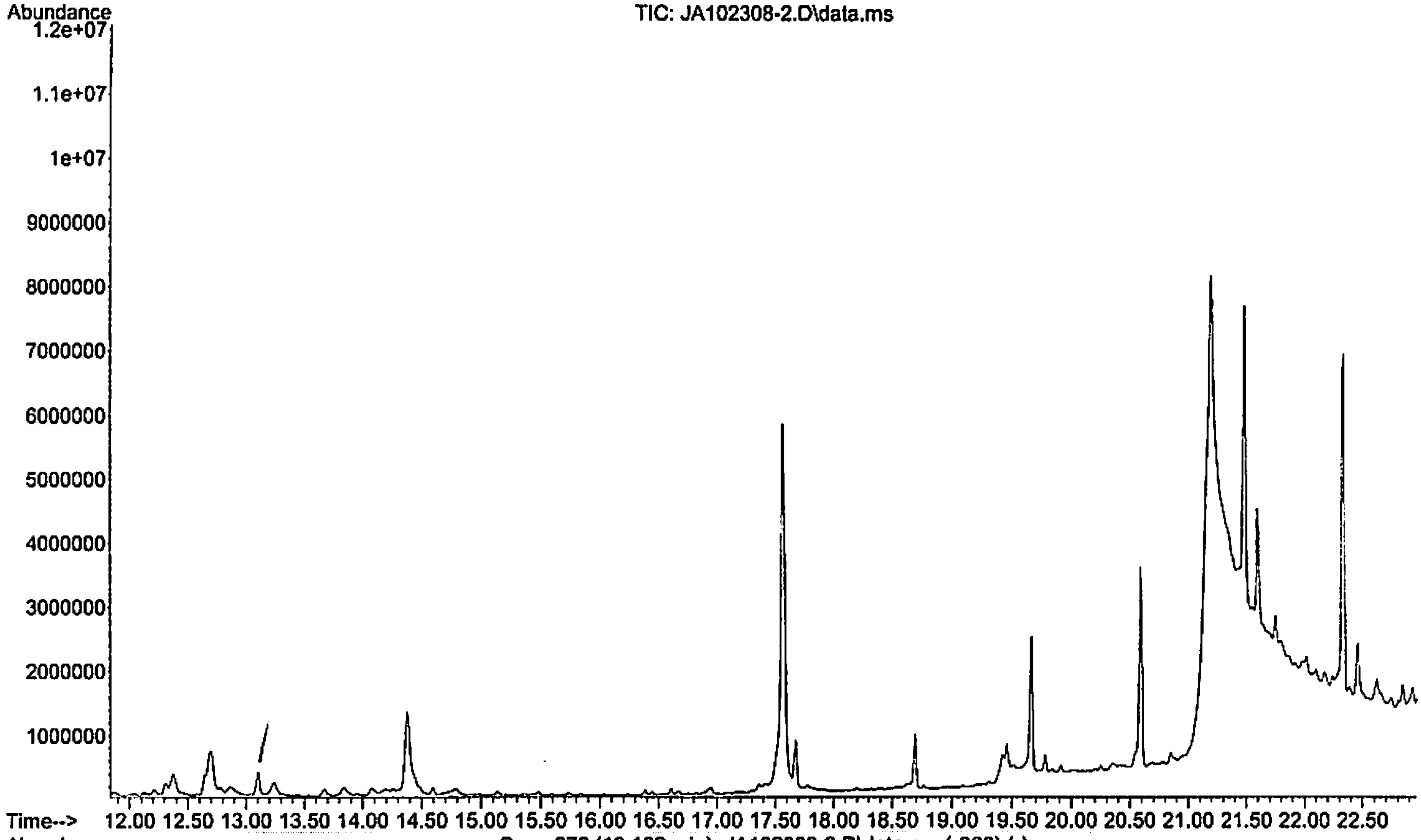


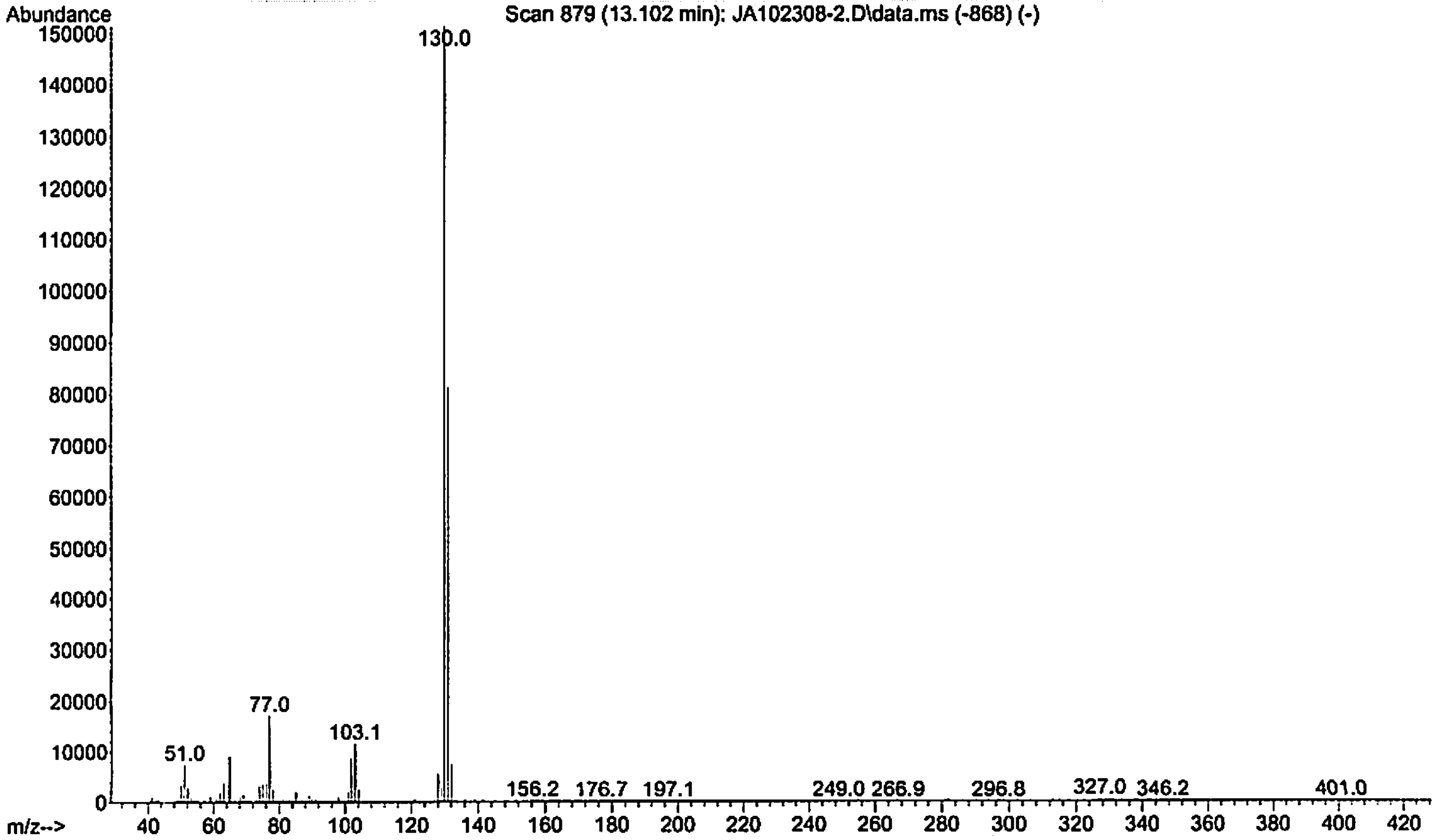

```
Library Searched : C:\Database\W8N05ST.L
Quality          : 95
ID               : 1H-Indole, 3-methyl- $ Indole, 3-methyl- $ .beta.-Methylindole $ Scato
                   le $ Skatol $ Skatole $ 3-Methylindole $ 3-MI $ 3-Methyl-1H-indole
```

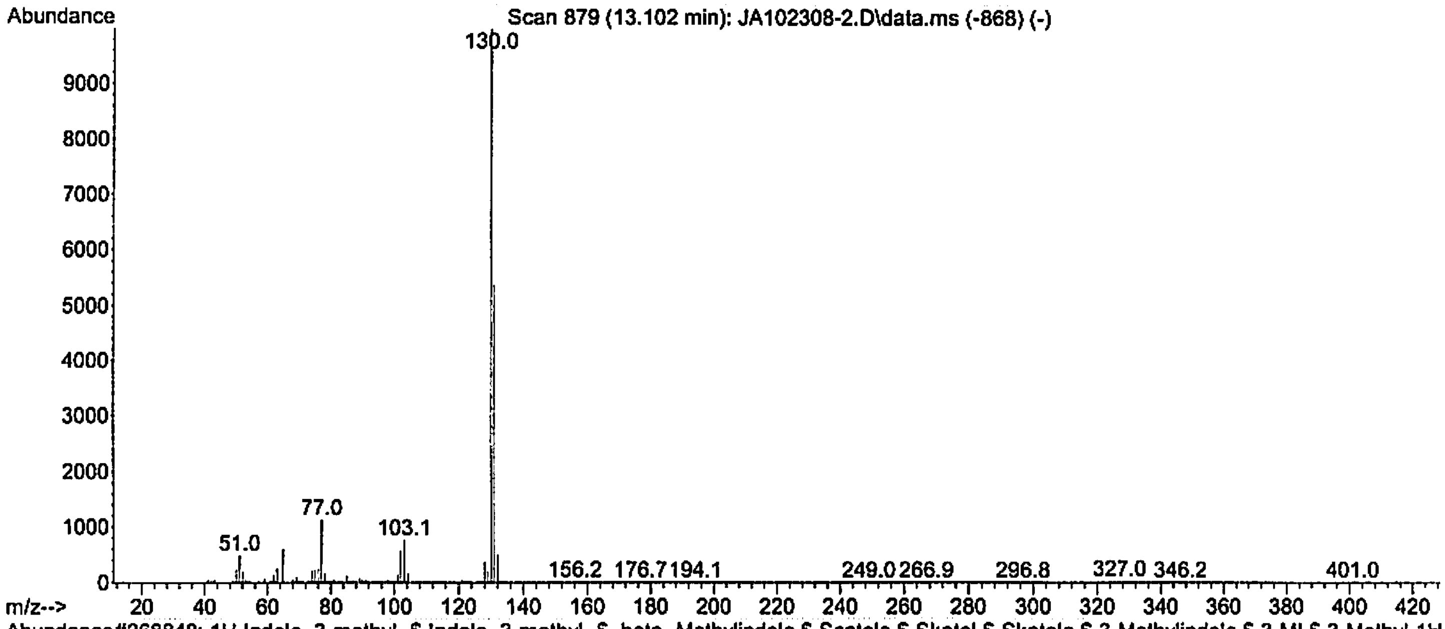


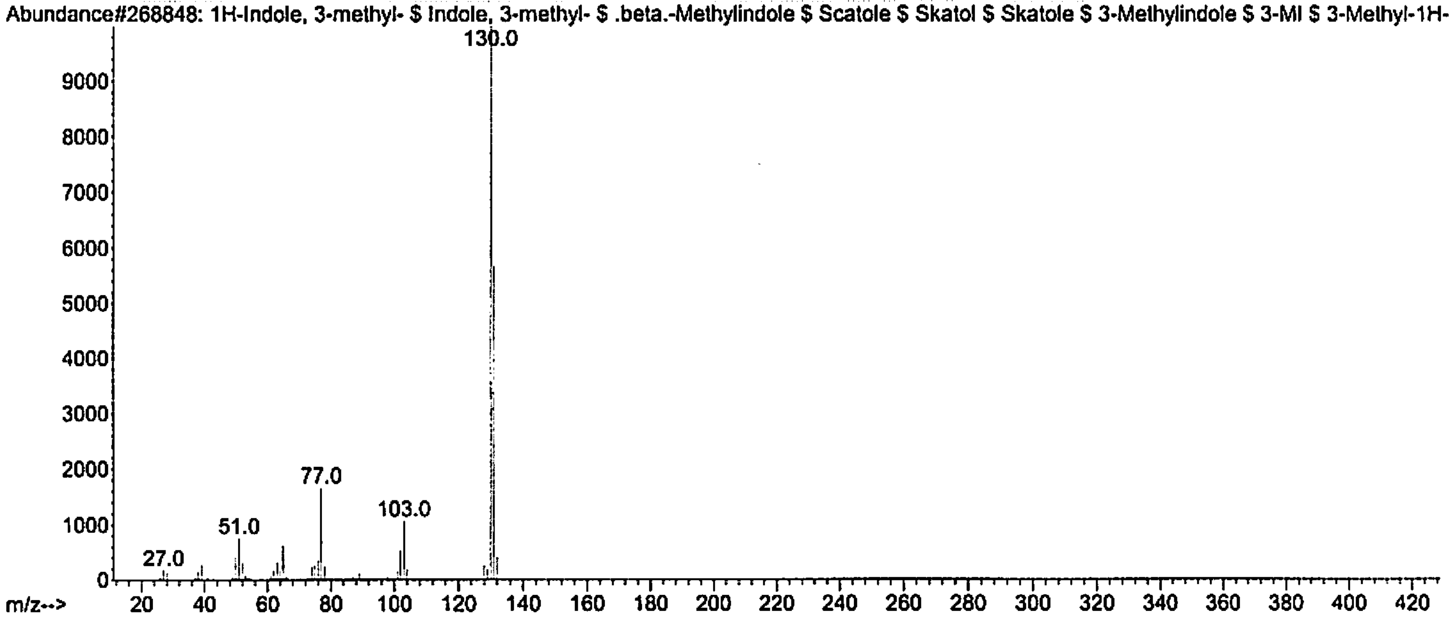


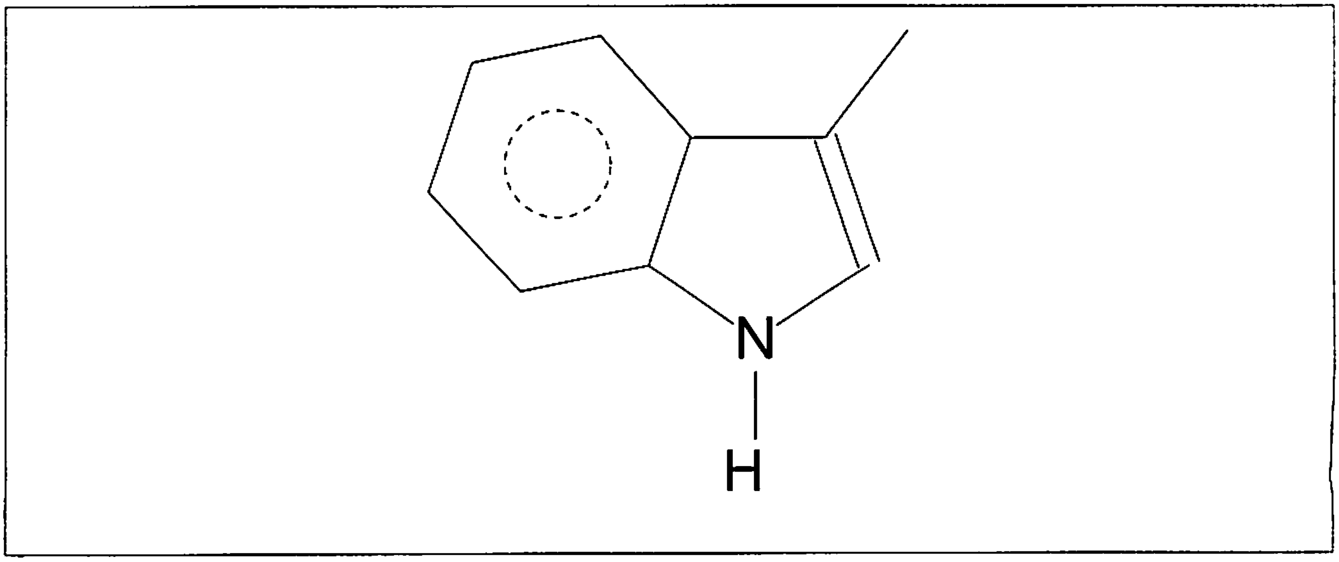

```
File        :D:\Aldrich\JA102308-2.D
Operator    :
Acquired    : 23 Oct 2008  14:31     using AcqMethod JA-50-280LESS.M
Instrument :    Buba
Sample Name: 7M C.oculata abdominal cuticle/10 ul CH2Cl2
Misc Info  : 10/8-14; 6 days w/ 1ug/ul citronellol in H2O
Vial Number: 1
```

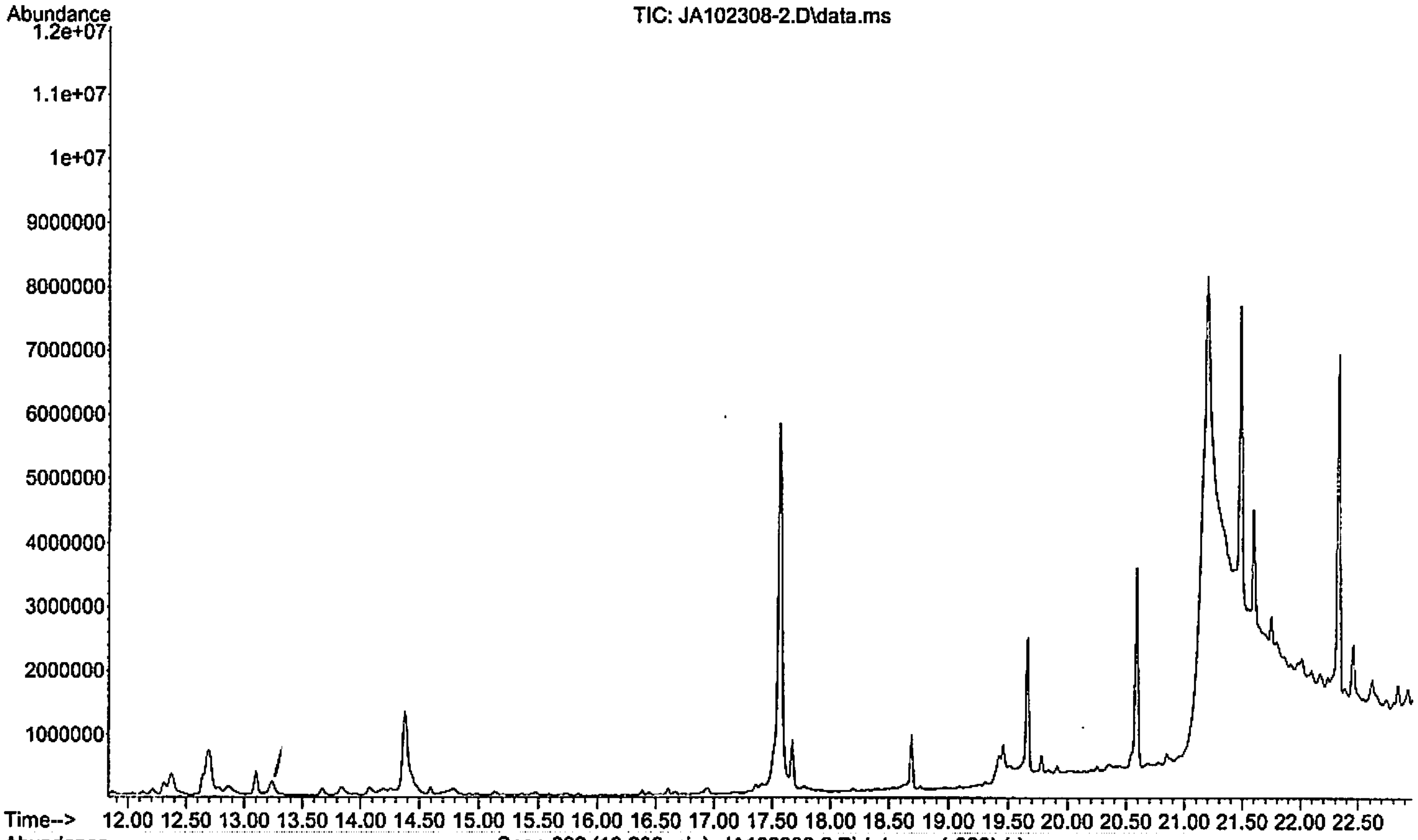


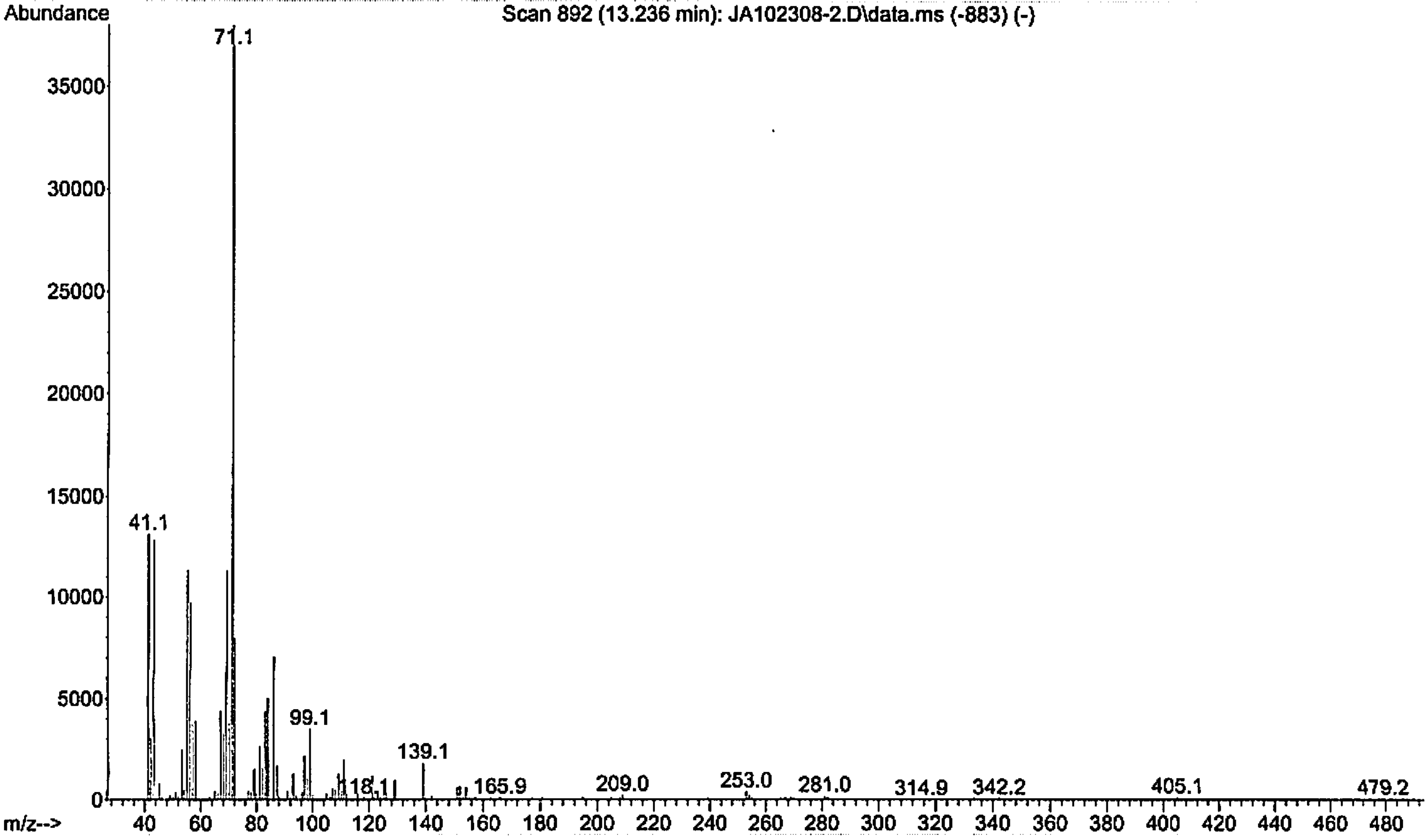

File :D:\Aldrich\JA102308-2.D
Operator :
Acquired : 23 Oct 2008 14:31 using AcqMethod JA-50-280LESS.M
Instrument : Buba
Sample Name: 7M C.oculata abdominal cuticle/10 ul CH2Cl2
Misc Info : 10/8-14; 6 days w/ 1ug/ul citronellol in H2O
Vial Number: 1

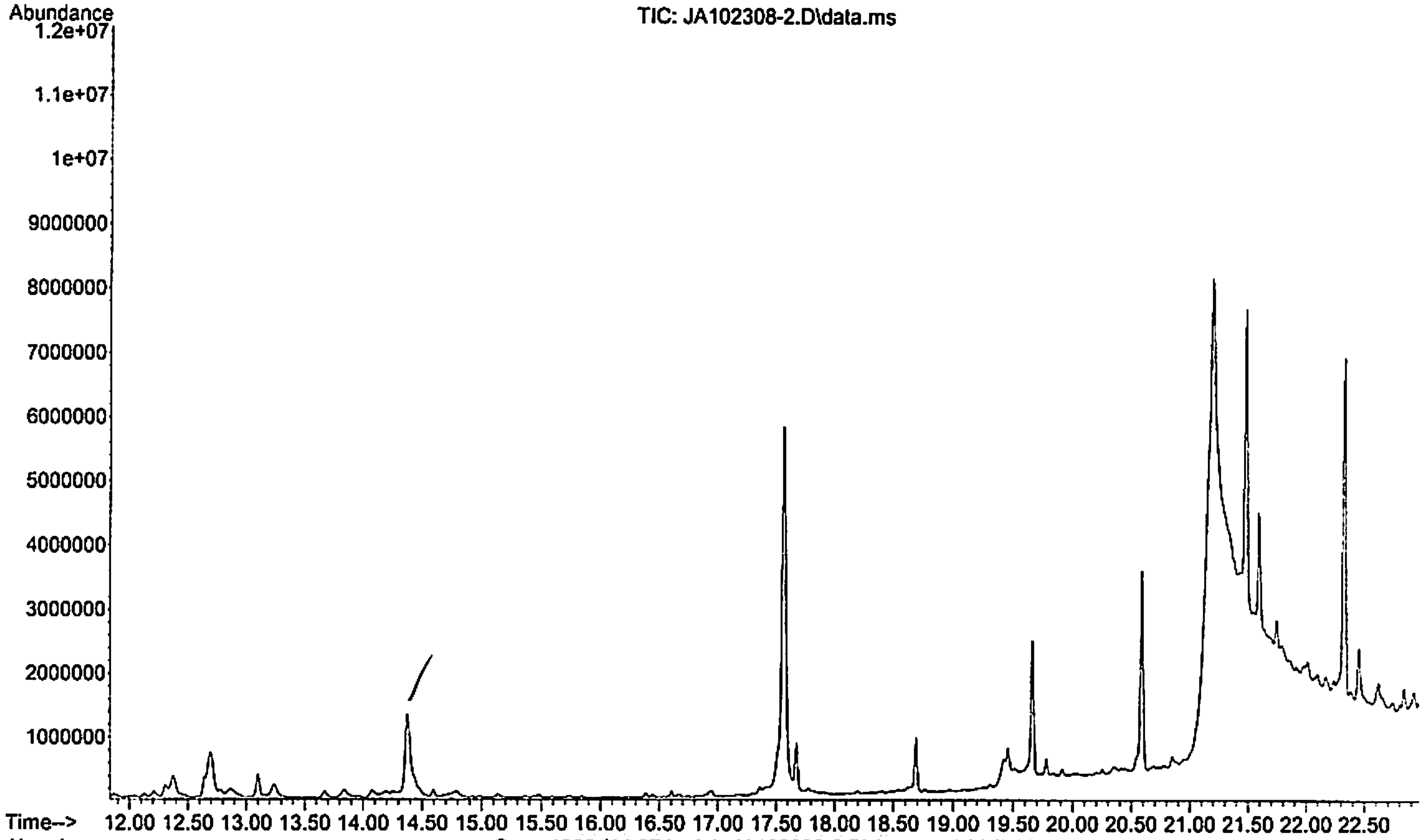


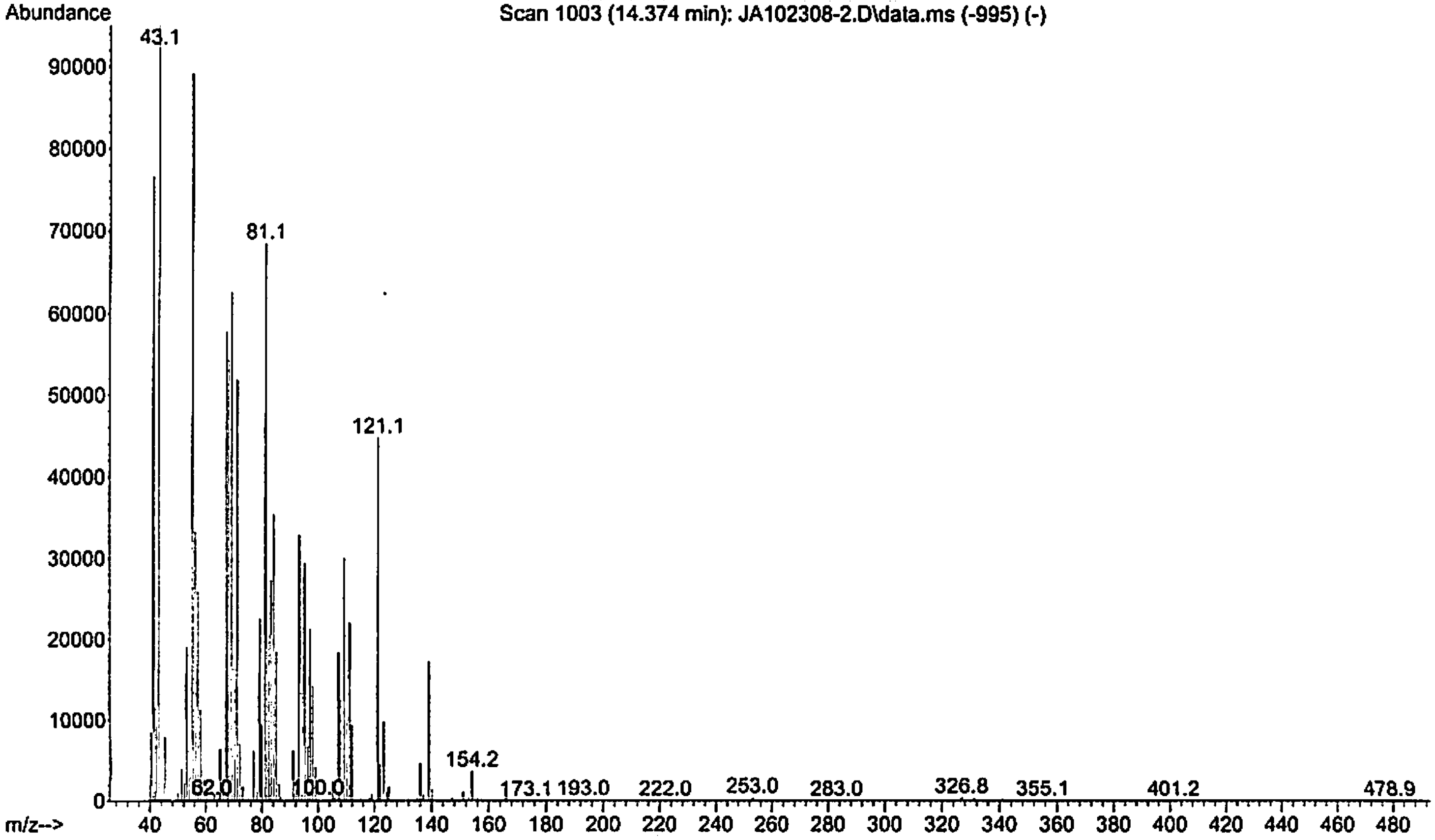

File        :D:\Aldrich\JA102308-2.D
Operator    :
Acquired    : 23 Oct 2008  14:31     using AcqMethod JA-50-280LESS.M
Instrument :    Buba
Sample Name: 7M C.oculata abdominal cuticle/10 ul CH2Cl2
Misc Info   : 10/8-14; 6 days w/ 1ug/ul citronellol in H2O
Vial Number: 1

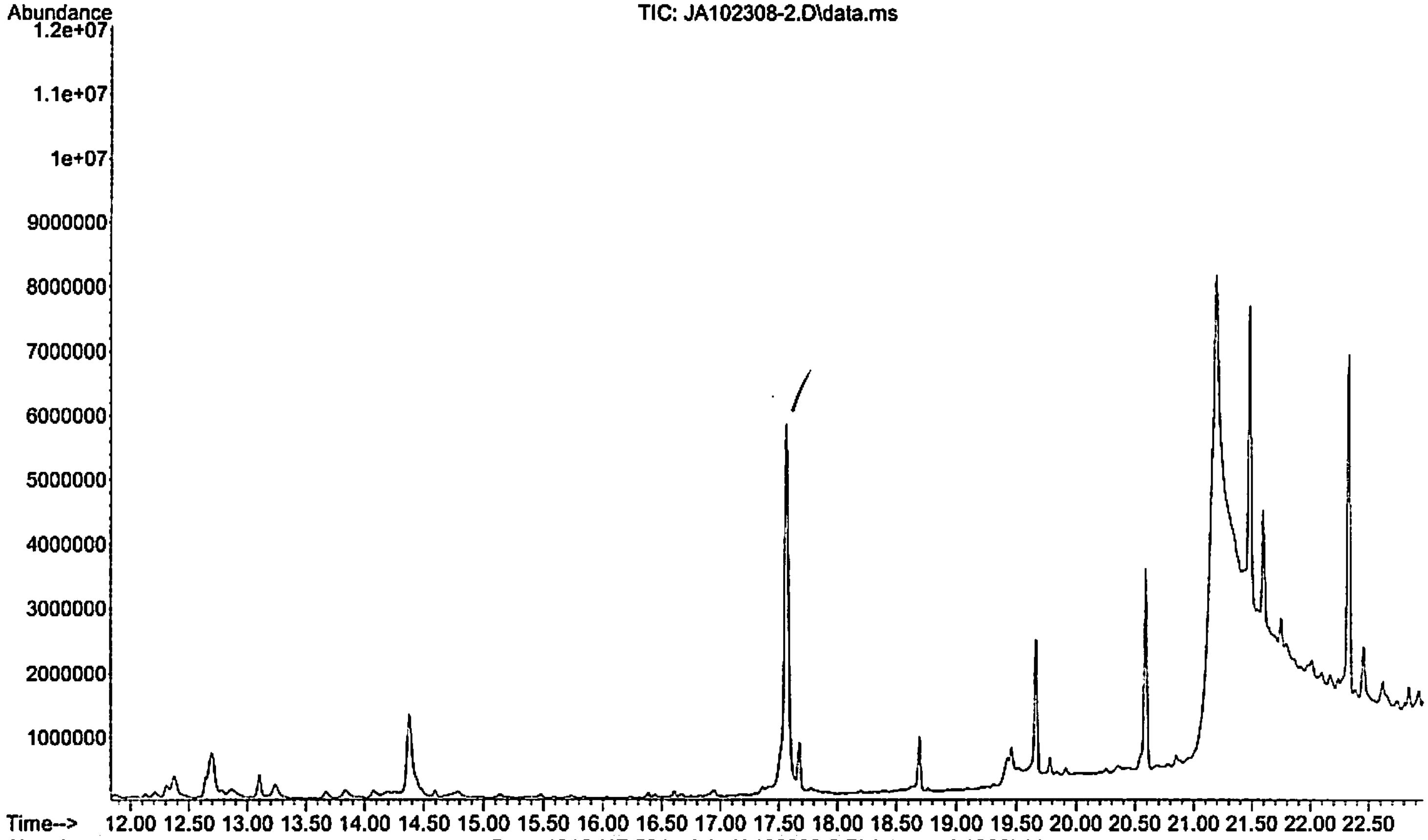


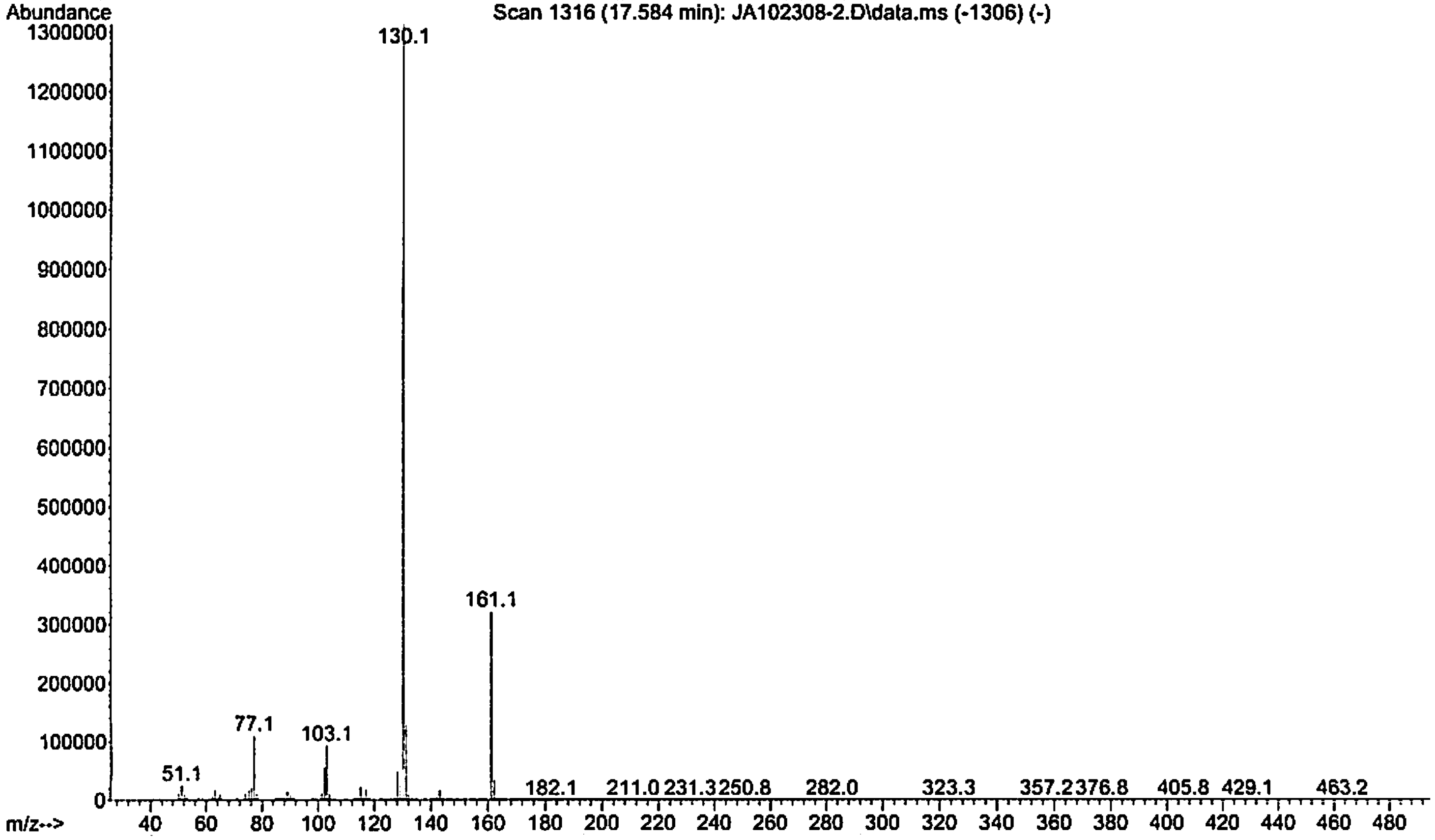

```
Library Searched : C:\Database\W8N05ST.L
Quality          : 91
ID               : 1H-INDOLE-3-ETHANOL $ 2-(1H-INDOL-3-YL)ETHANOL $ 2-(1H-INDOL-3-YL)ETHA
                   NOL # $ .BETA.-(3-INDOLE)ETHANOL $ 1H-INDOLYL-3-ETHANOL $ 2-(1H-INDOL-
                   3-YL)-ETHANOL $ 2-(1H-INDOL-3-YL)ETHANOL (COMPUTER-GENERATED NAME) $ 2
                   -(3-INDOLYL)ETHANOL $ 2-(3-INDOLYLETHANOL $ 3
```

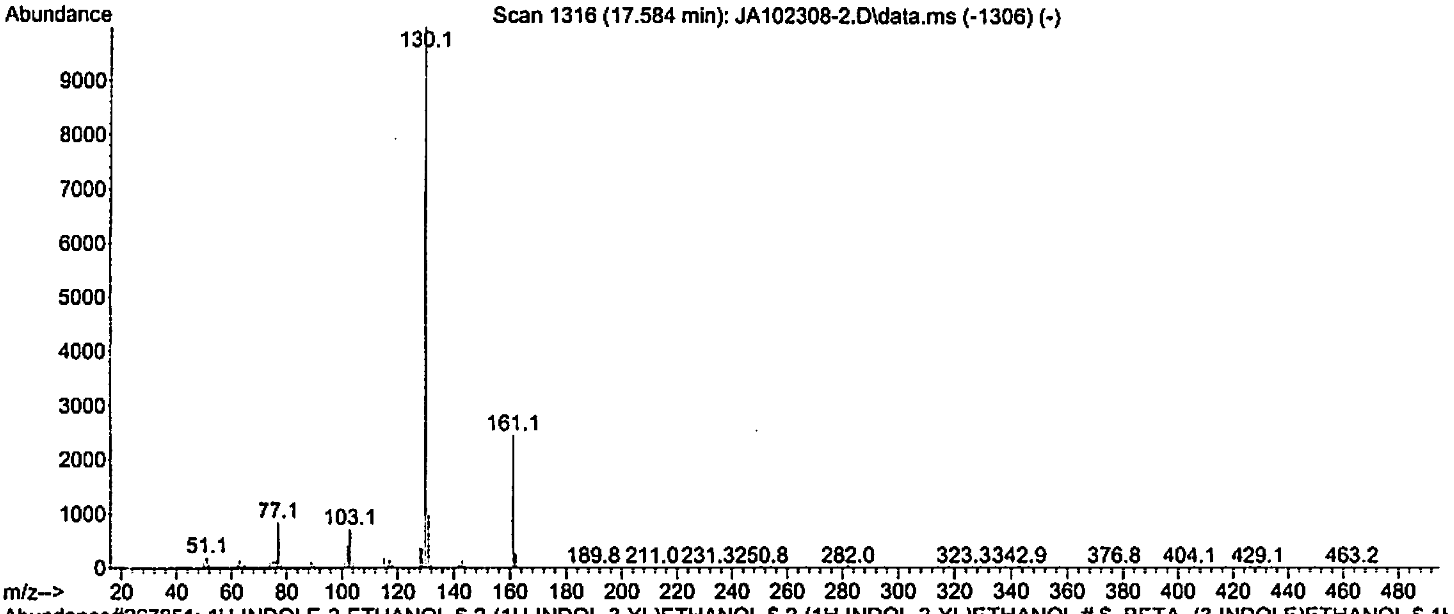


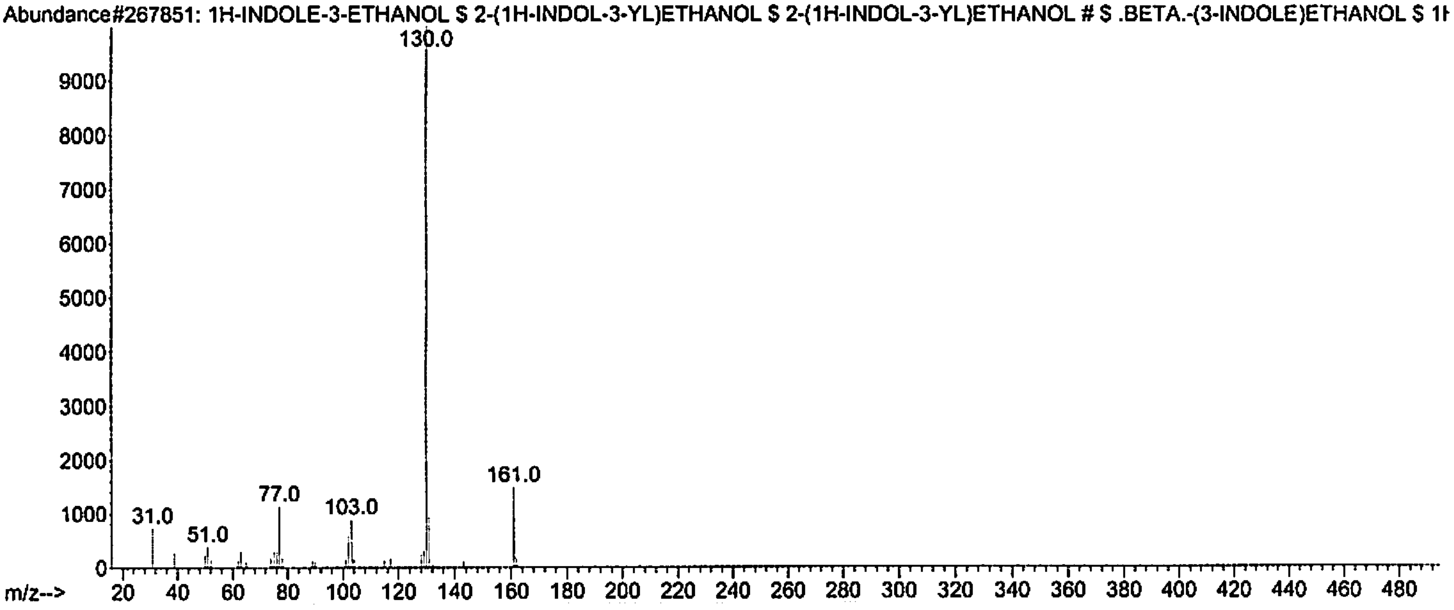


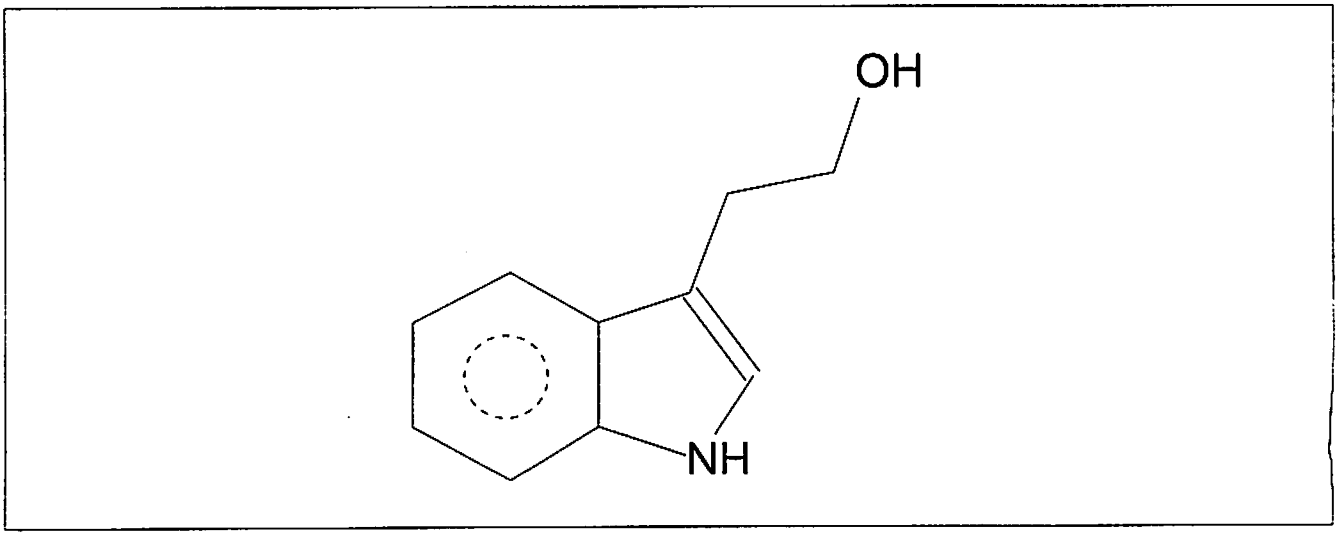

```
File       :D:\Aldrich\JA102308-2.D
Operator   :
Acquired   : 23 Oct 2008  14:31     using AcqMethod JA-50-280LESS.M
Instrument :     Buba
Sample Name: 7M C.oculata abdominal cuticle/10 ul CH2Cl2
Misc Info  : 10/8-14; 6 days w/ 1ug/ul citronellol in H2O
Vial Number: 1
```

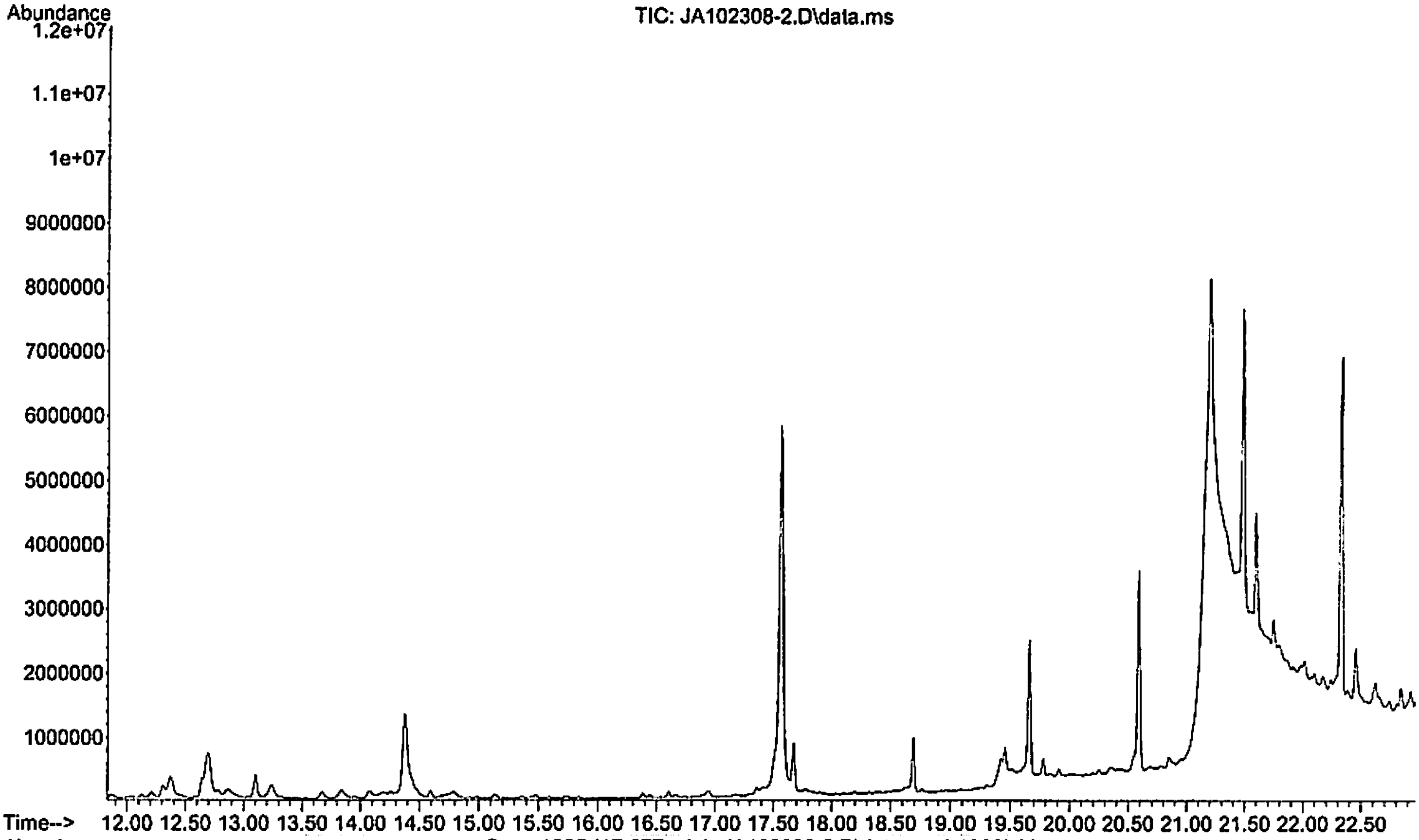


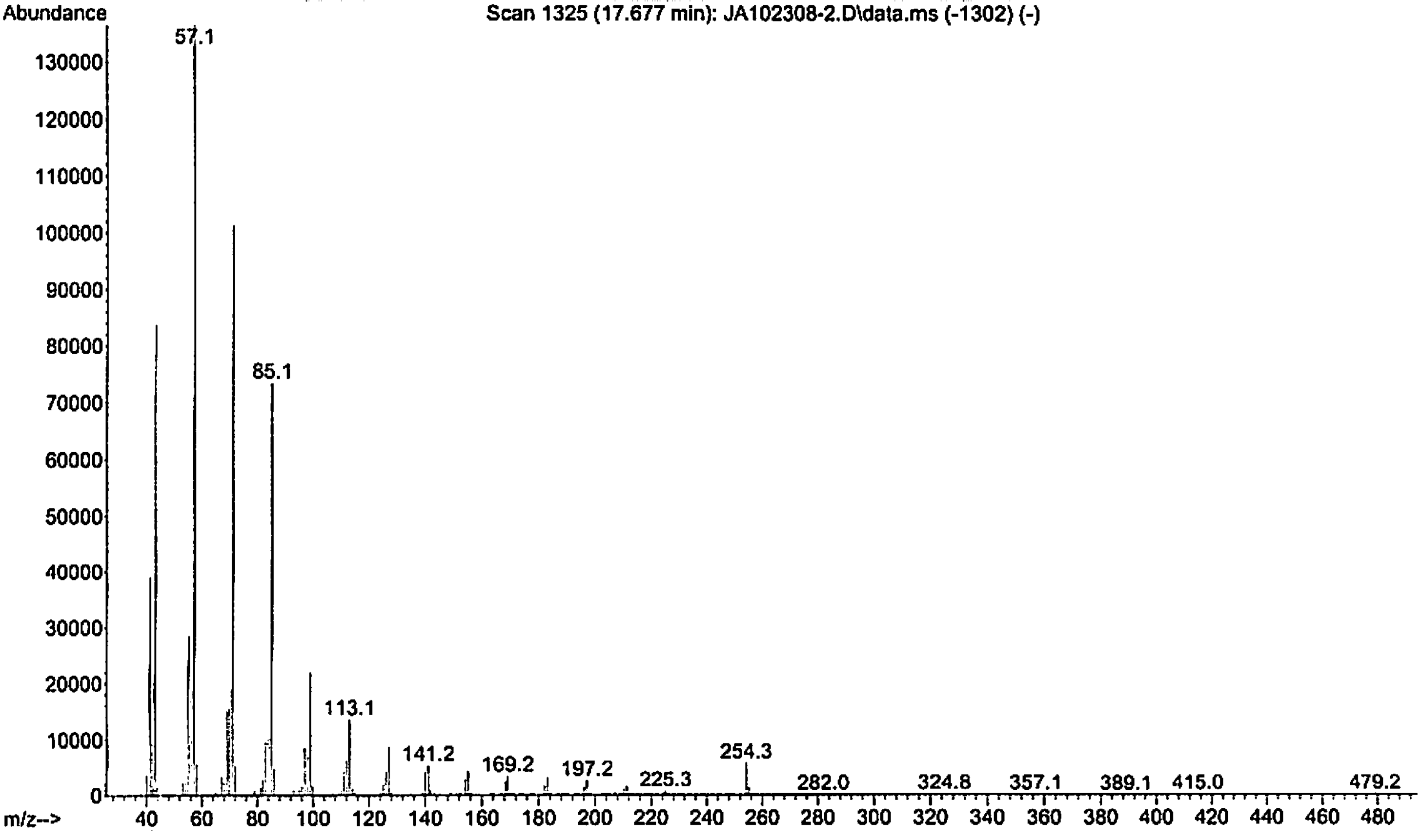

File :D:\Aldrich\JA102308-2.D
Operator :
Acquired : 23 Oct 2008 14:31 using AcqMethod JA-50-280LESS.M
Instrument : Buba
Sample Name: 7M C.oculata abdominal cuticle/10 ul CH2Cl2
Misc Info : 10/8-14; 6 days w/ 1ug/ul citronellol in H2O
Vial Number: 1

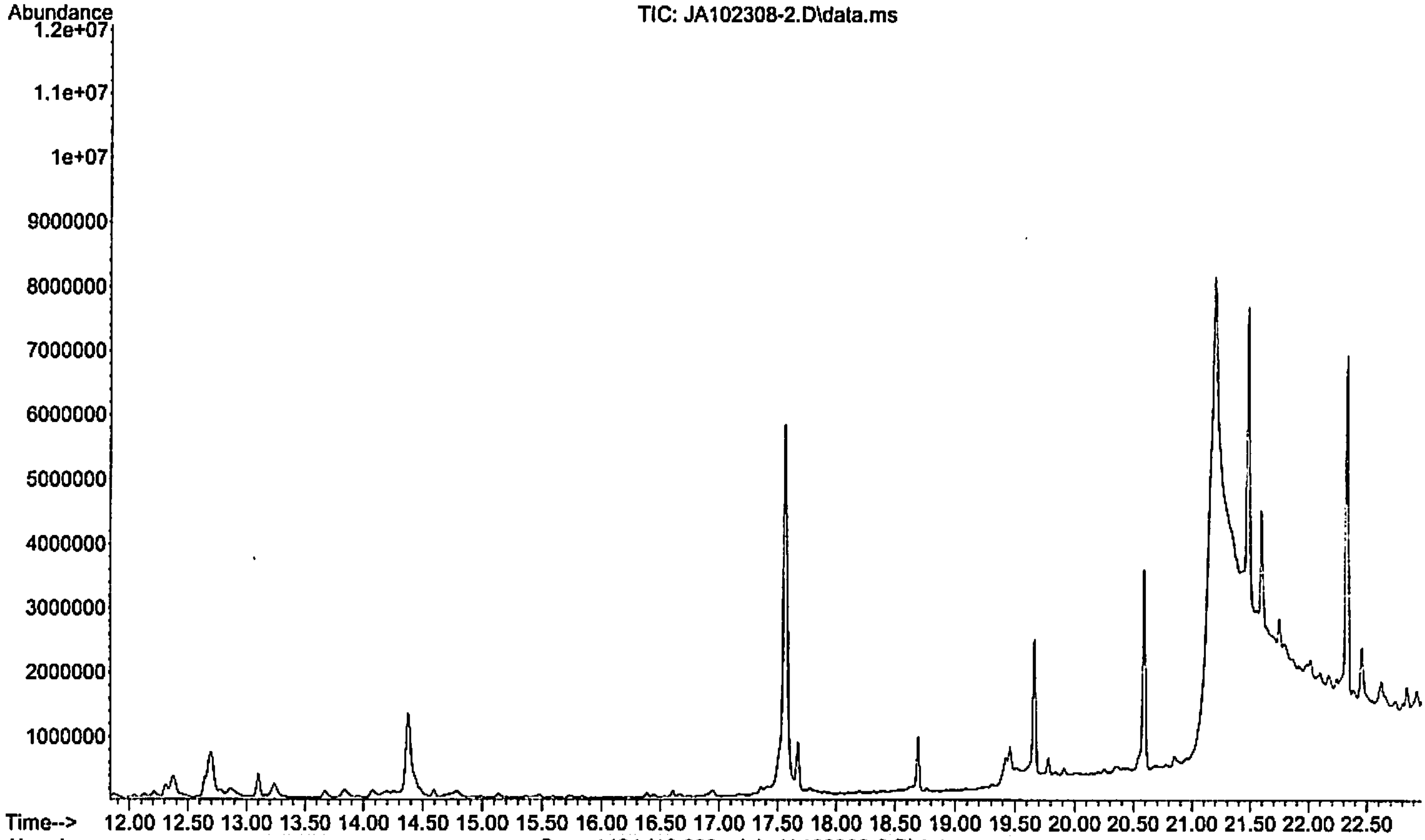


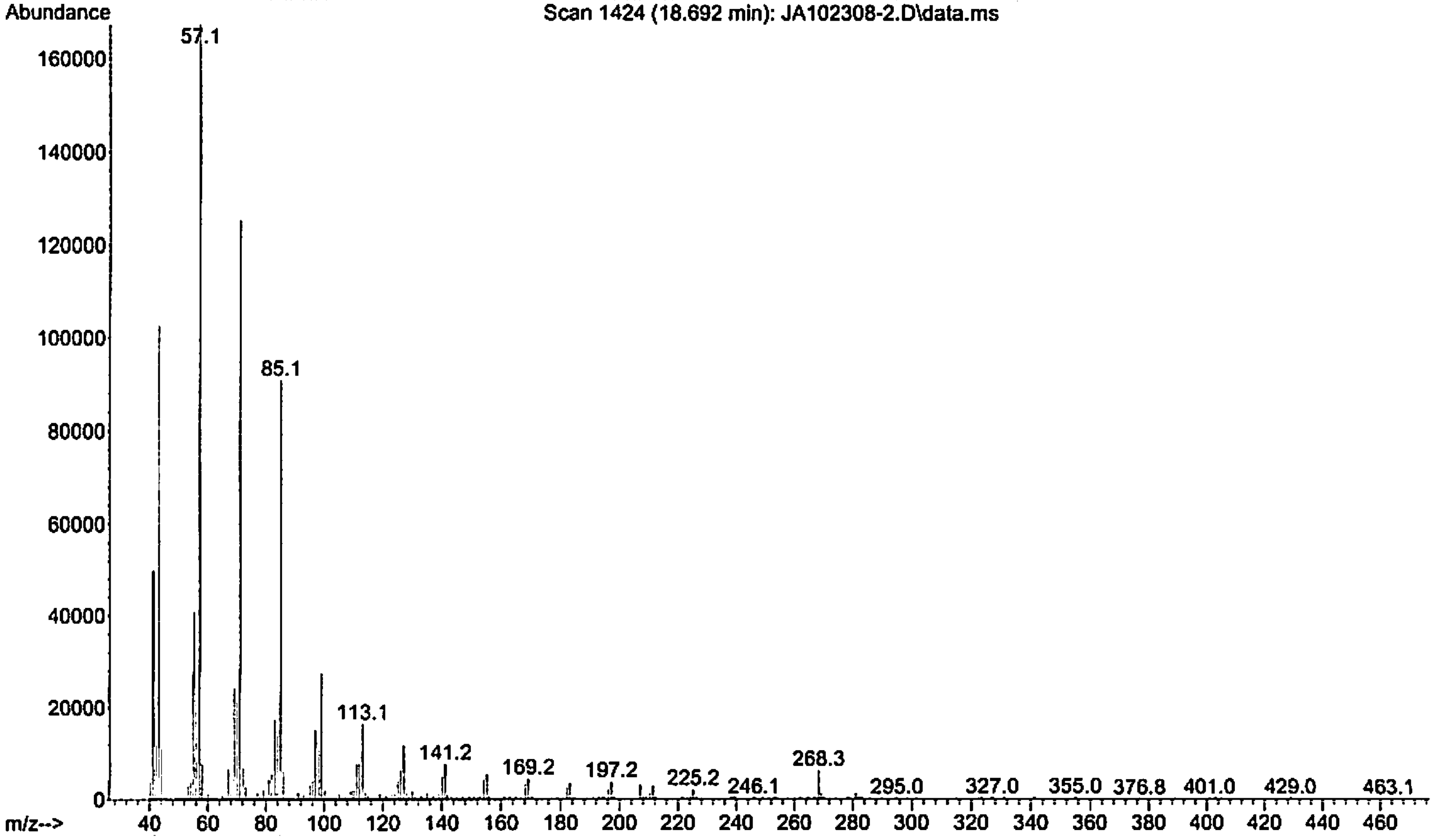

```
File        :D:\Aldrich\JA102308-2.D
Operator    :
Acquired    : 23 Oct 2008  14:31     using AcqMethod JA-50-280LESS.M
Instrument  :     Buba
Sample Name: 7M C.oculata abdominal cuticle/10 ul CH2Cl2
Misc Info   : 10/8-14; 6 days w/ 1ug/ul citronellol in H2O
Vial Number: 1
```

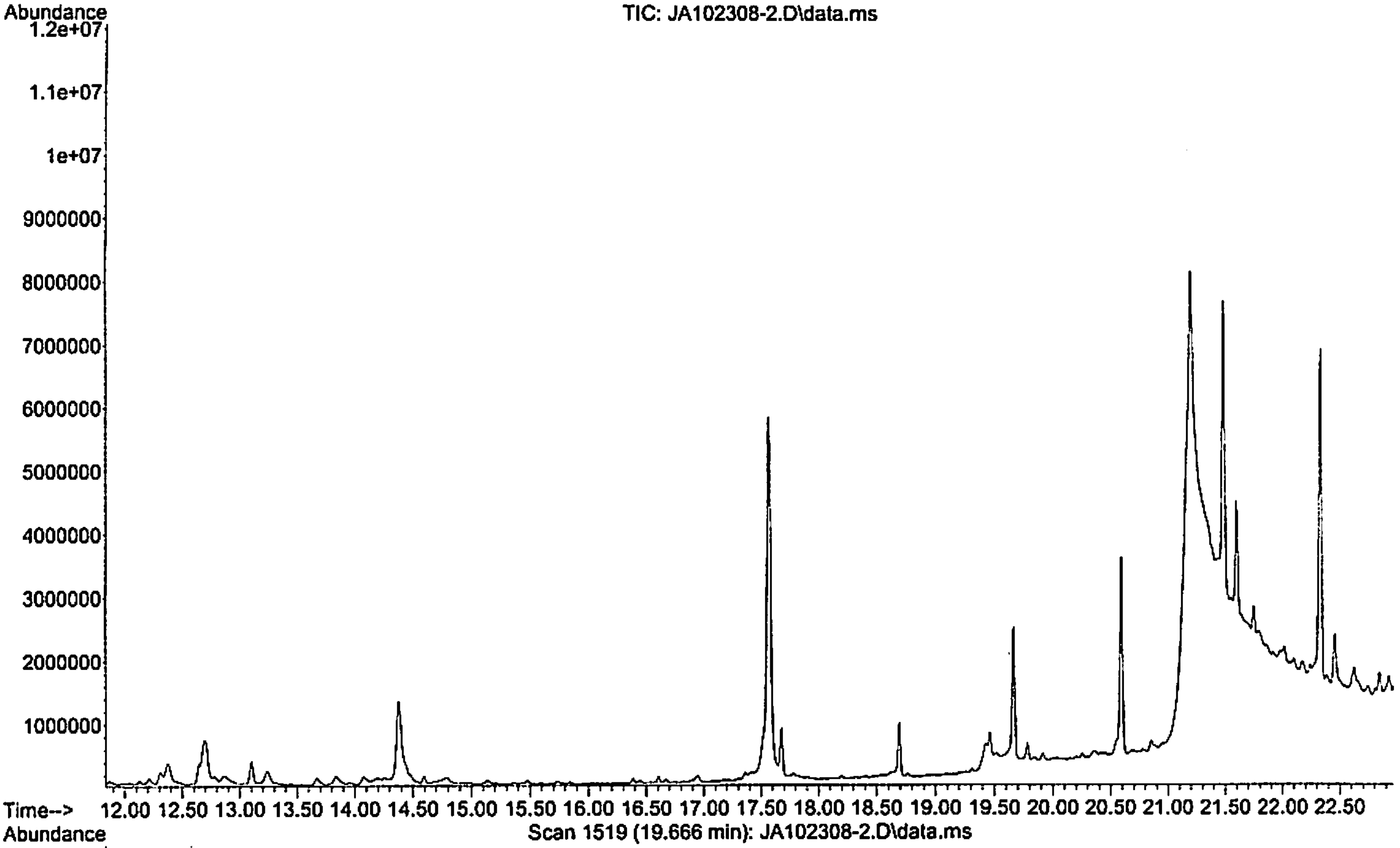


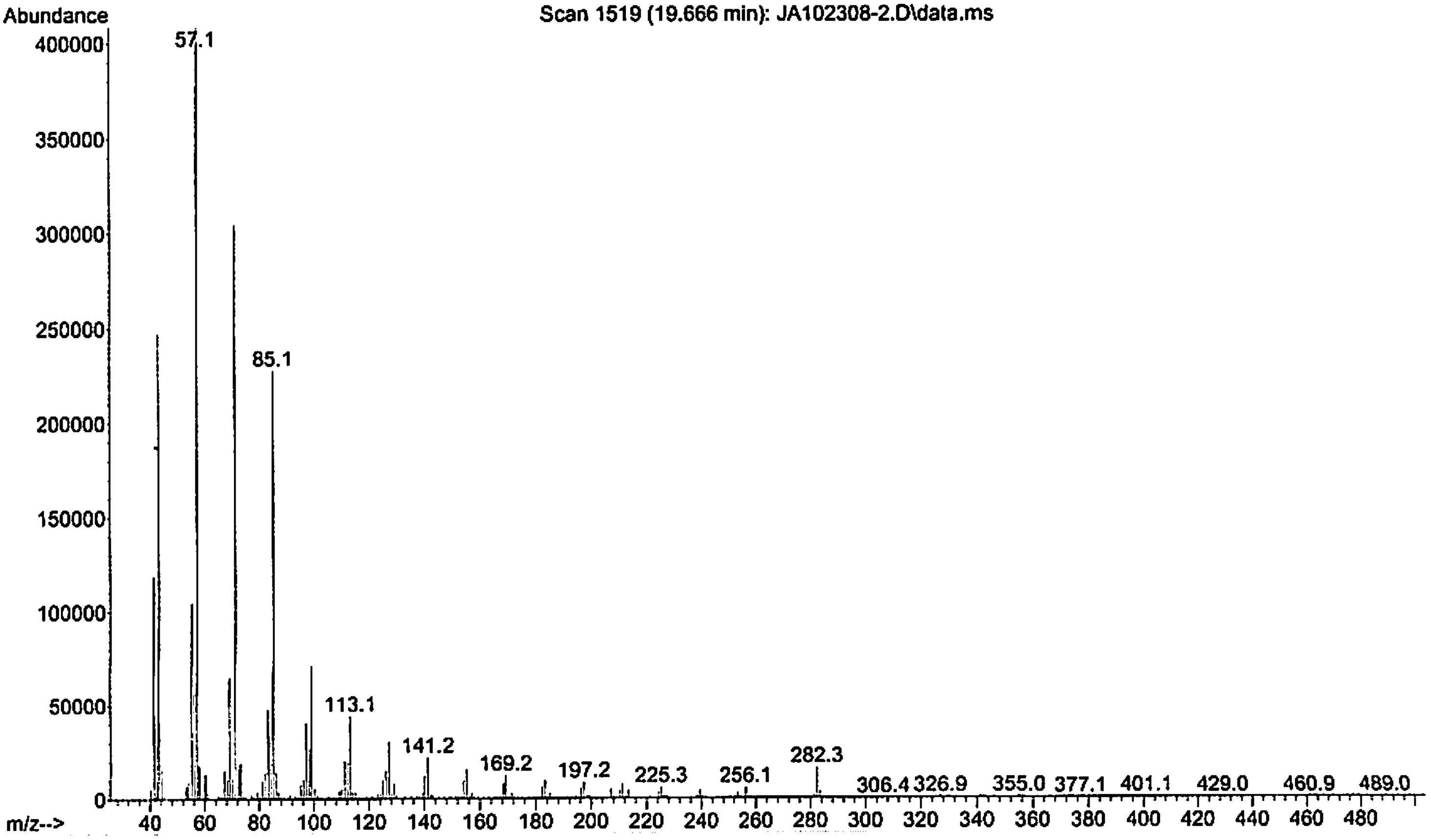

```
File        :D:\Aldrich\JA102308-2.D
Operator    :
Acquired    : 23 Oct 2008  14:31     using AcqMethod JA-50-280LESS.M
Instrument  :    Buba
Sample Name: 7M C.oculata abdominal cuticle/10 ul CH2Cl2
Misc Info   : 10/8-14; 6 days w/ 1ug/ul citronellol in H2O
Vial Number: 1
```

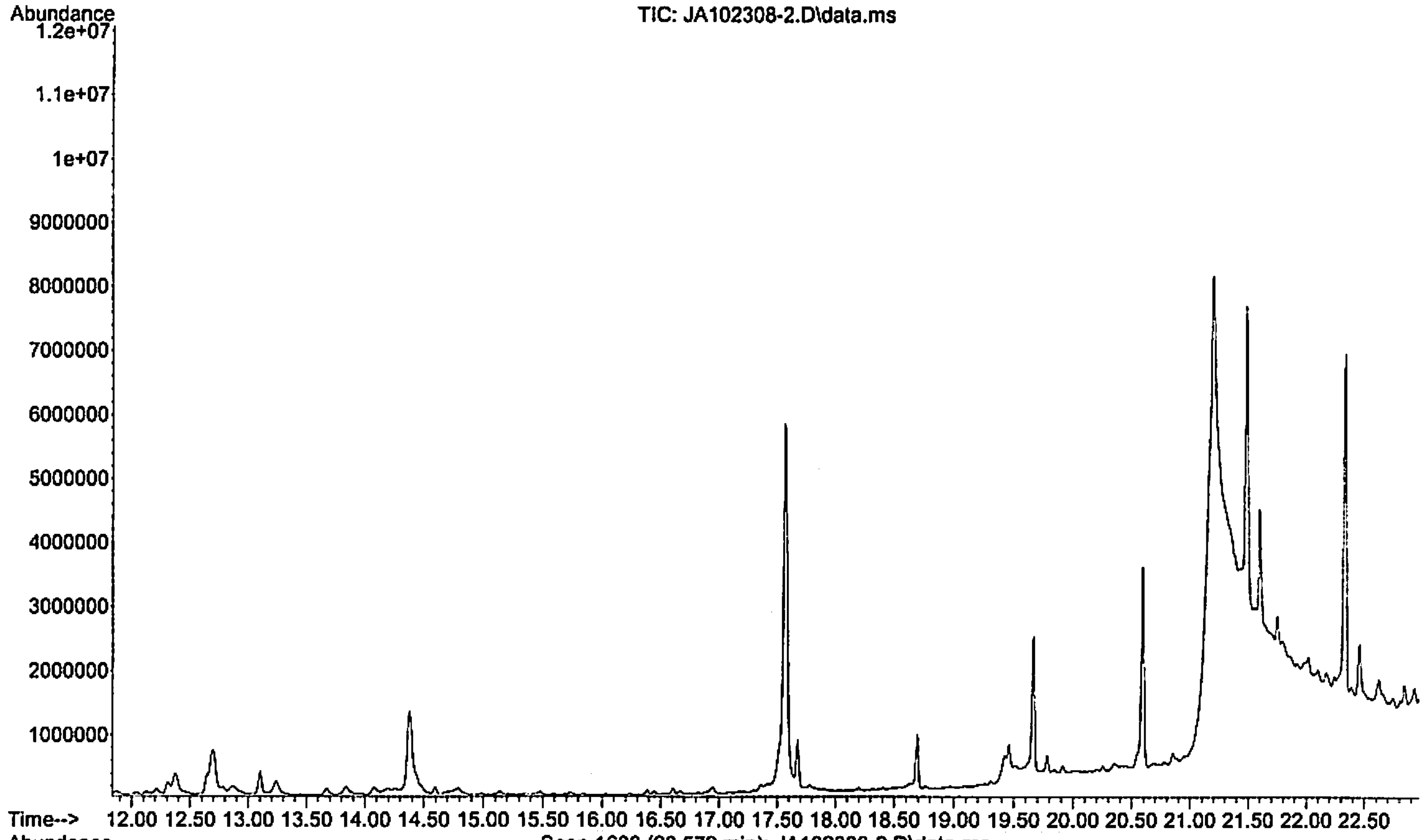


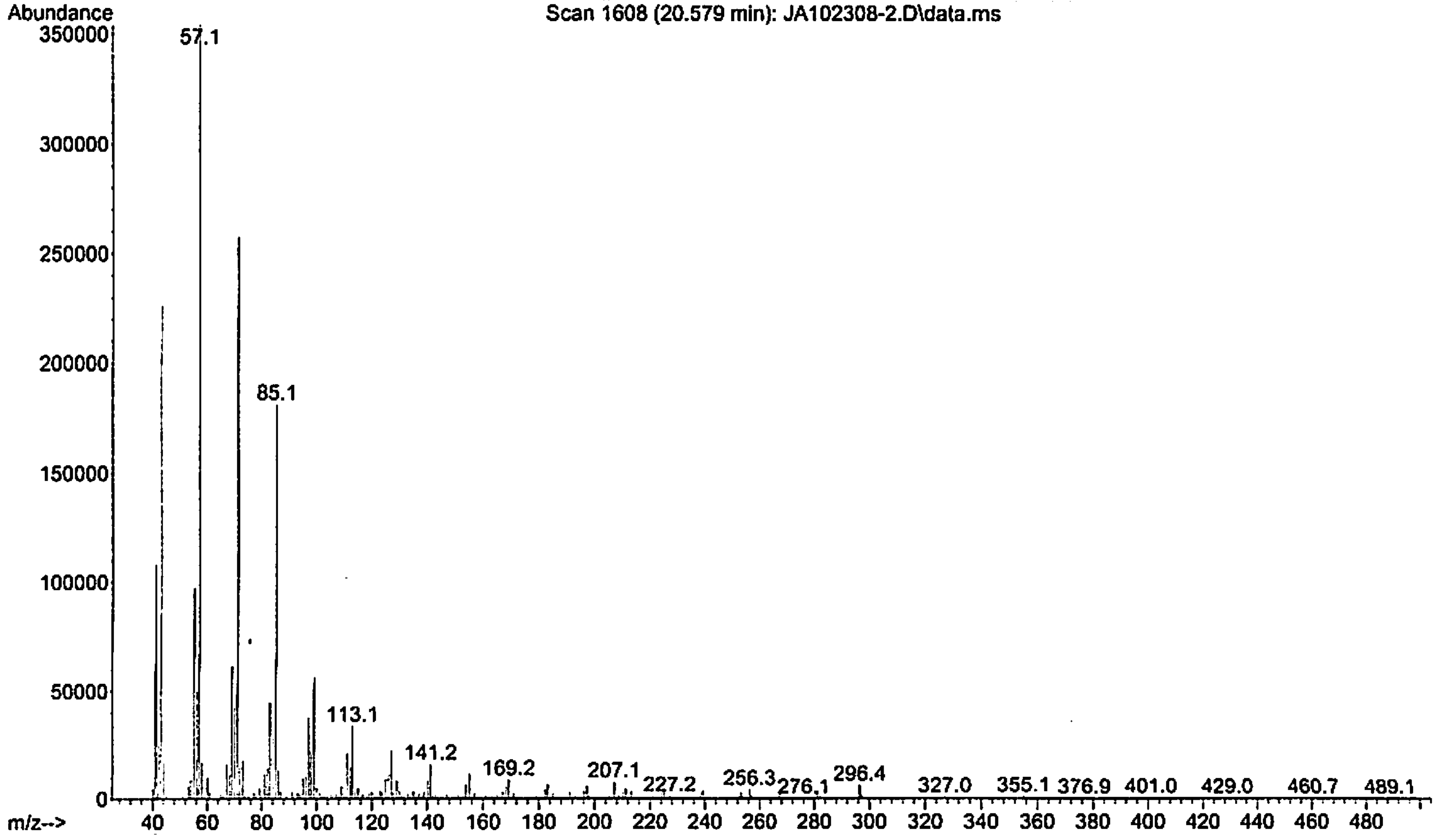

File :D:\Aldrich\JA102308-2.D
Operator :
Acquired : 23 Oct 2008 14:31 using AcqMethod JA-50-280LESS.M
Instrument : Buba
Sample Name: 7M C.oculata abdominal cuticle/10 ul CH2Cl2
Misc Info : 10/8-14; 6 days w/ 1ug/ul citronellol in H2O
Vial Number: 1

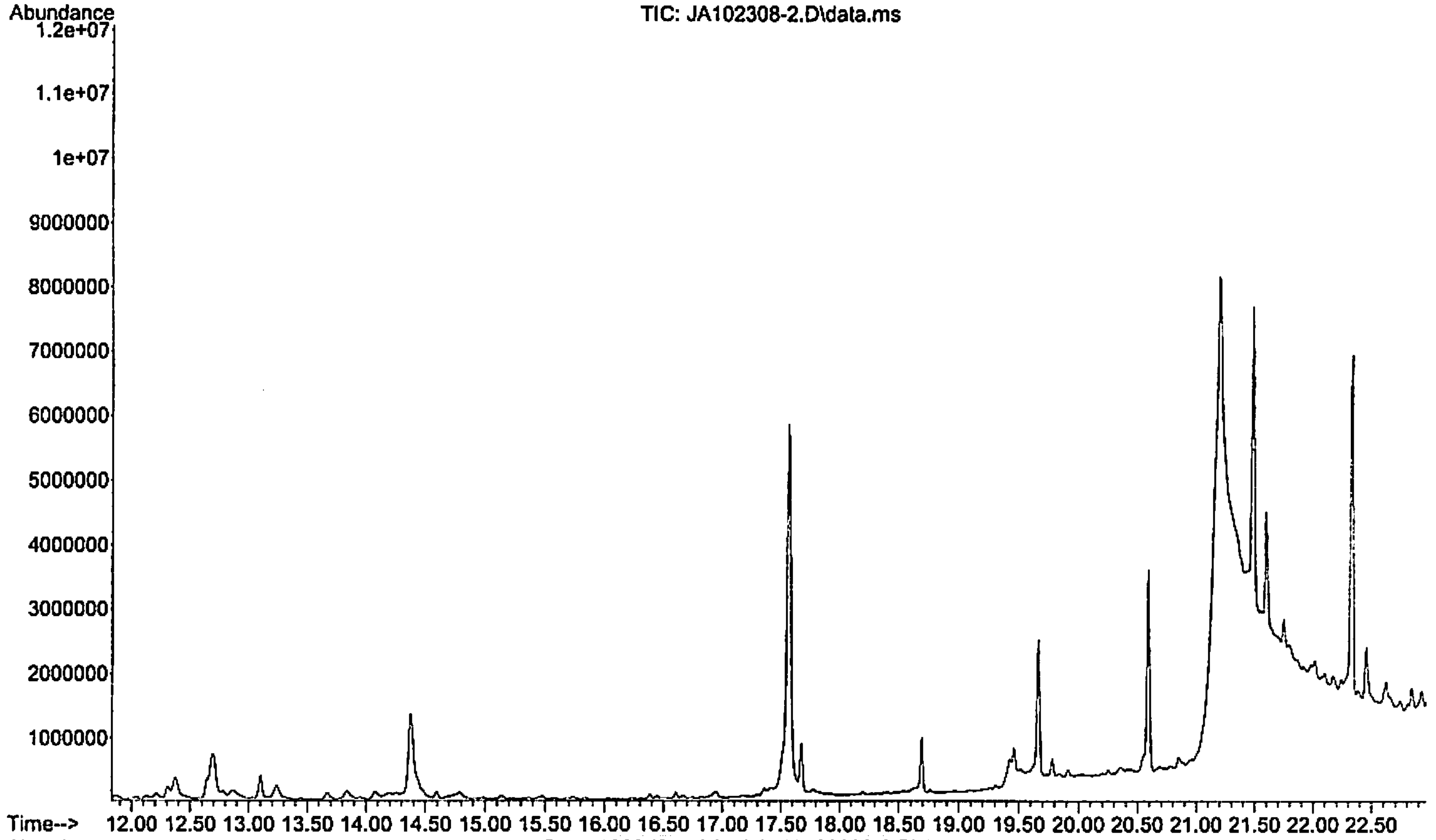


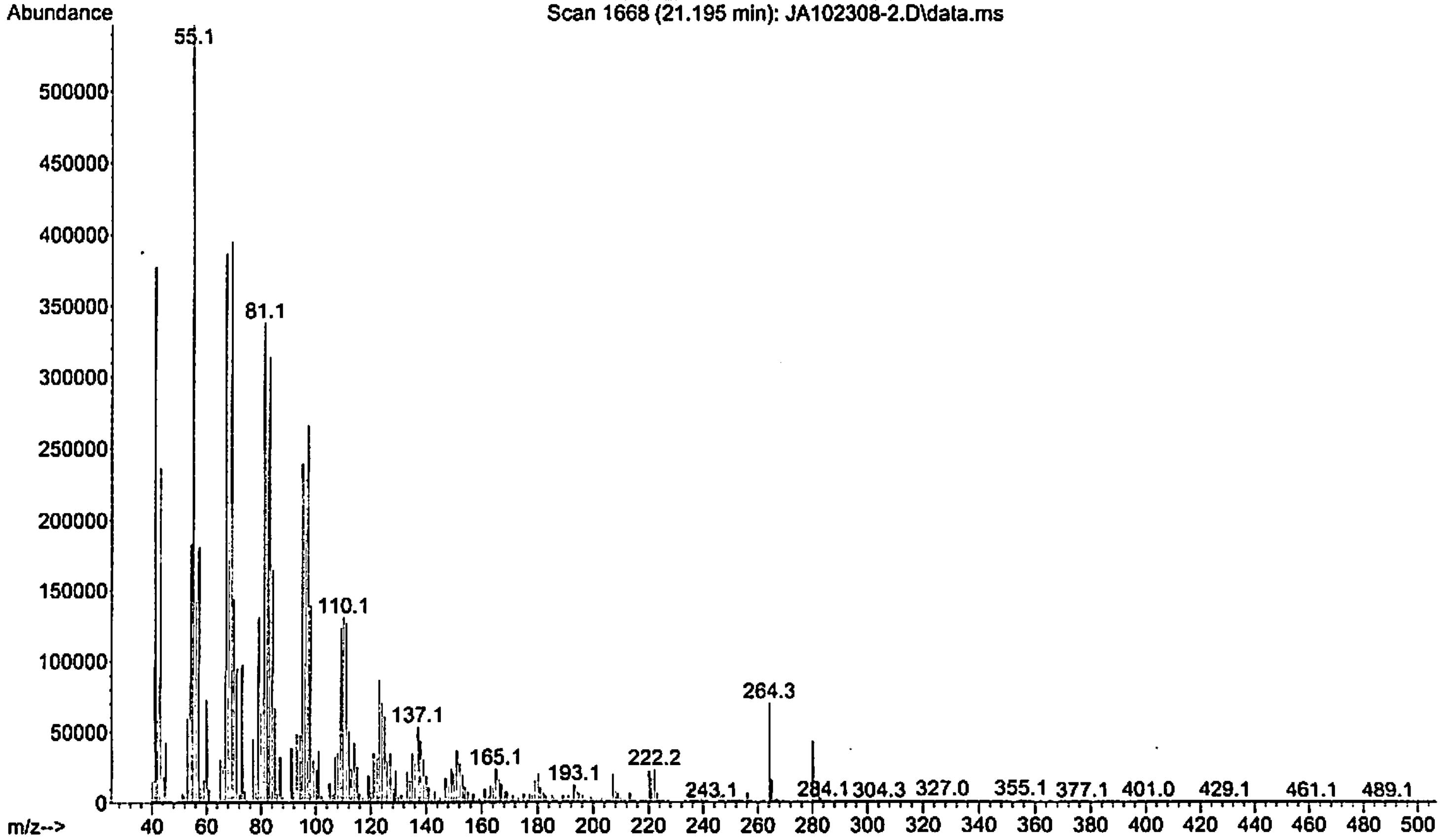

Library Searched : C:\Database\W8N05ST.L
Quality : 98
ID : 9-OCTADECENOIC ACID (Z)- $ OCTADEC-9-ENOIC ACID $ (9E)-9-OCTADECENOIC ACID # $ (9E)-9-OCTADECENOIC ACID (COMPUTER-GENERATED NAME) $ (9Z)-9-OCTADECENOIC ACID $ (9Z)-OCTADECENOIC ACID $ (Z)-9-OCTADECANOIC ACID $ (Z)-9-OCTADECENOIC ACID $ (Z)-OCTADEC-9-ENOIC

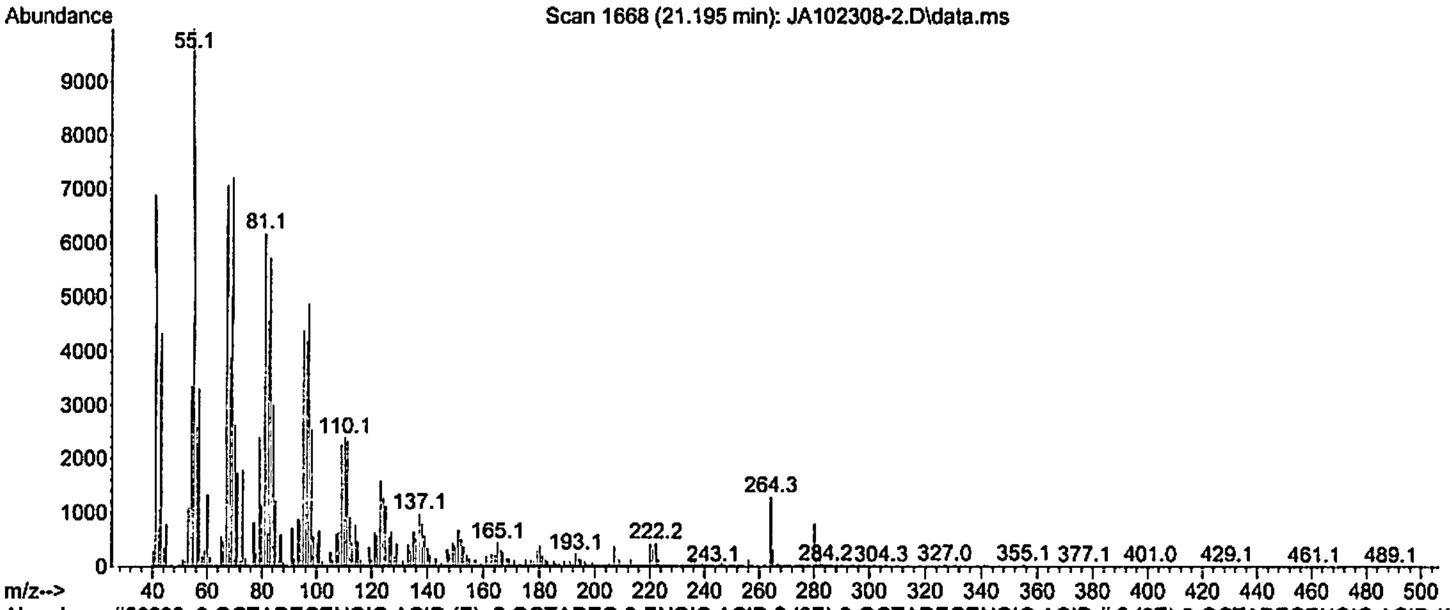


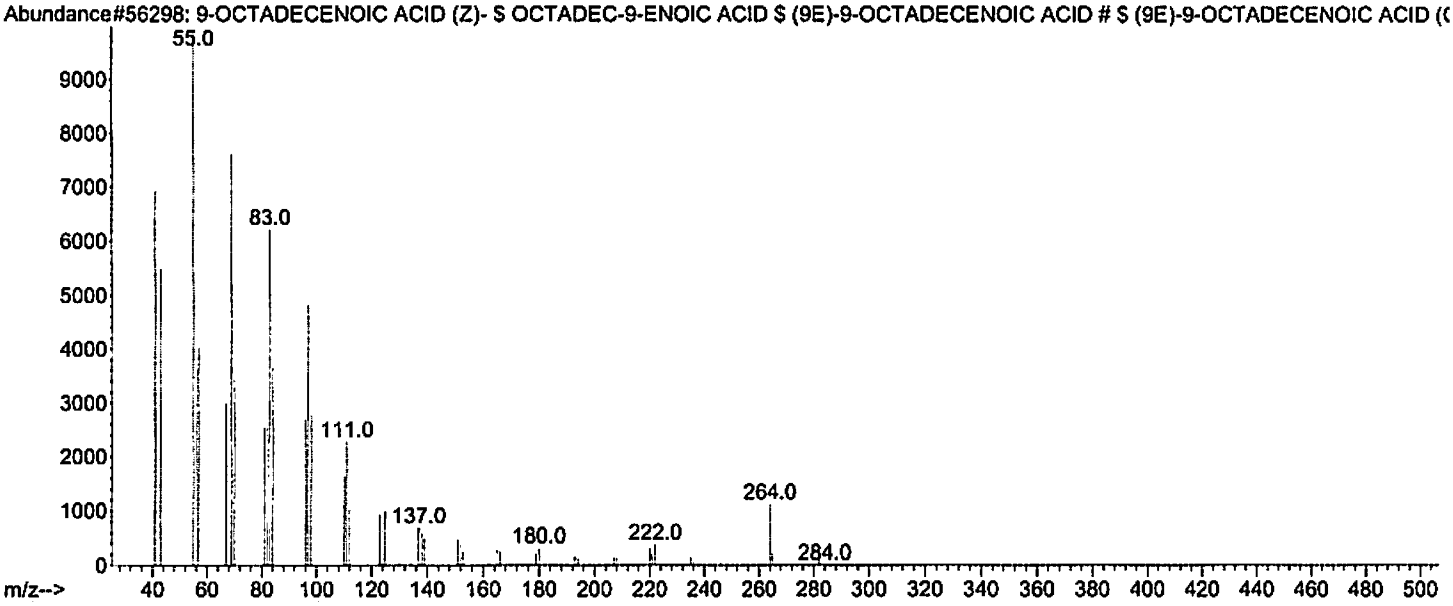


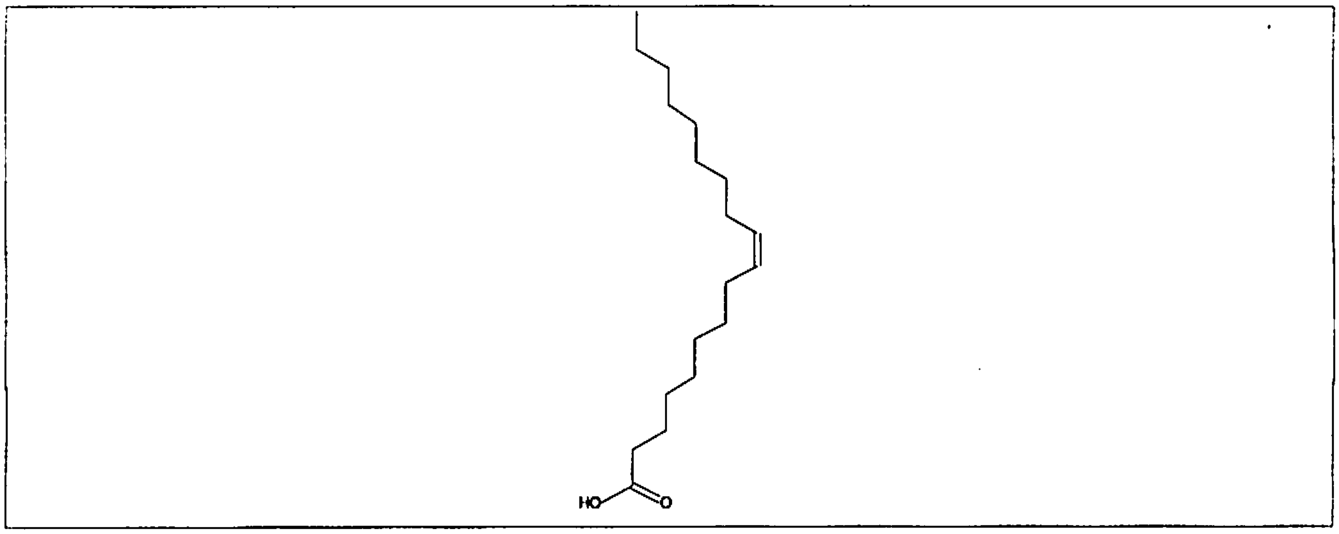

```
File        :D:\Aldrich\JA102408-3.D
Operator    :
Acquired    : 24 Oct 2008  16:27     using AcqMethod JA-50-280LESS.M
Instrument :   Buba
Sample Name: 5M C.oculata abd. cuticle/10ul CH2Cl2
Misc Info  : 7days w/geraniol; emerged 9/23; no GC
Vial Number: 1
```

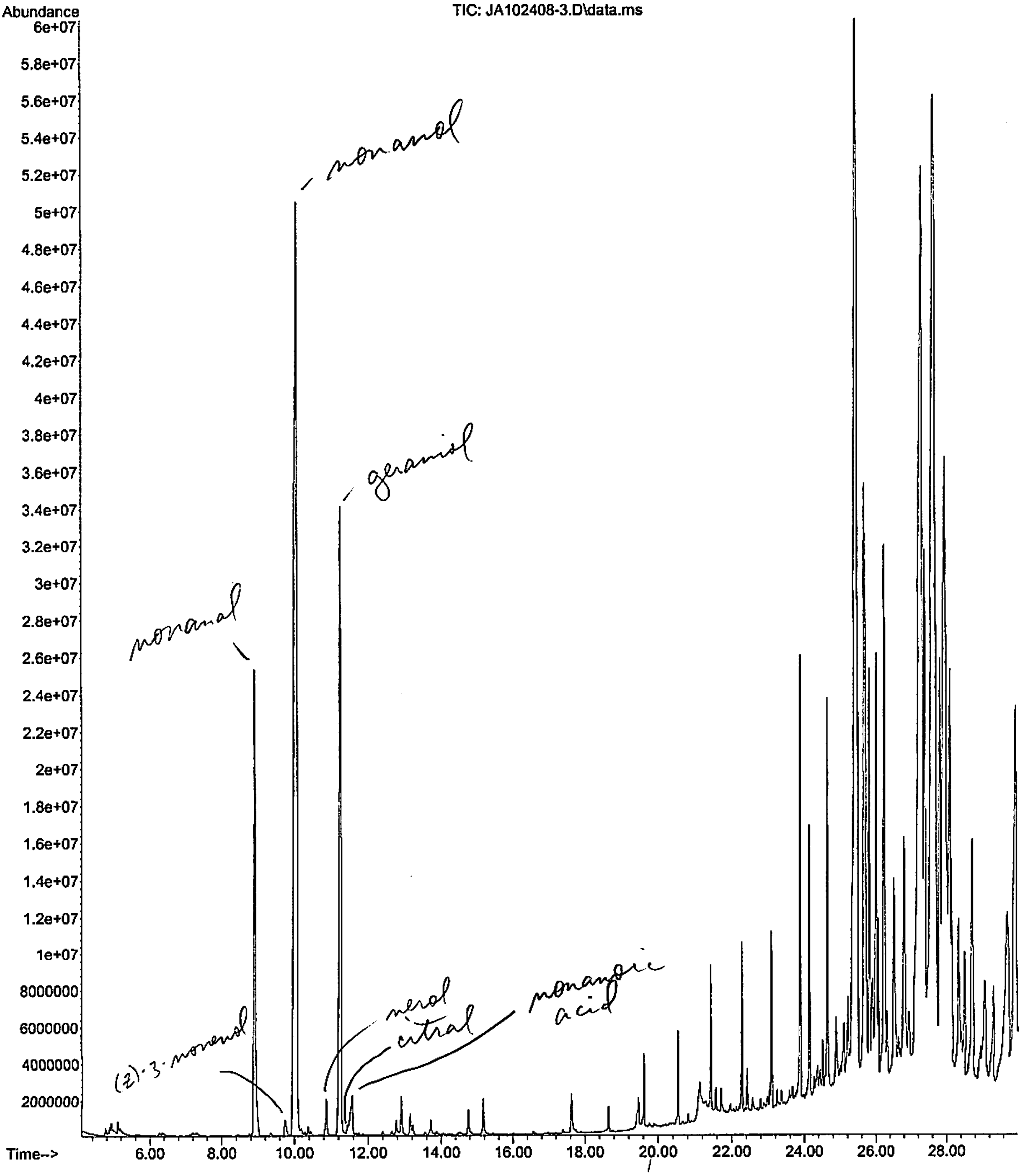

```
File        :D:\ALDRICH\Snapshot\JA102408-3.D
Operator    :
Acquired    : 24 Oct 2008  16:27     using AcqMethod JA-50-280LESS.M
Instrument  :    Buba
Sample Name: 5M C.oculata abd. cuticle/10ul CH2Cl2
Misc Info   : 7days w/geraniol; emerged 9/23; no GC
Vial Number: 1
```

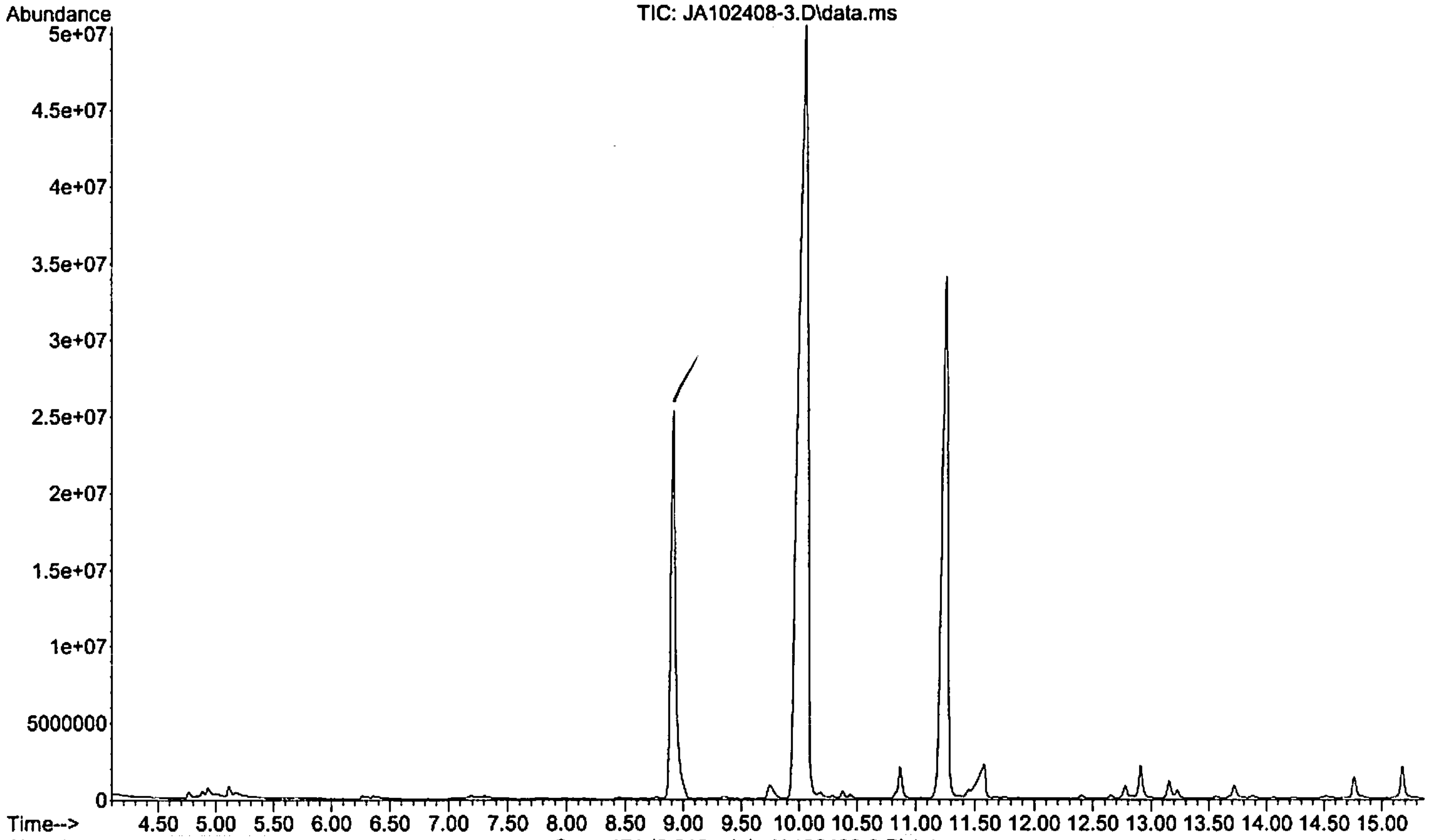


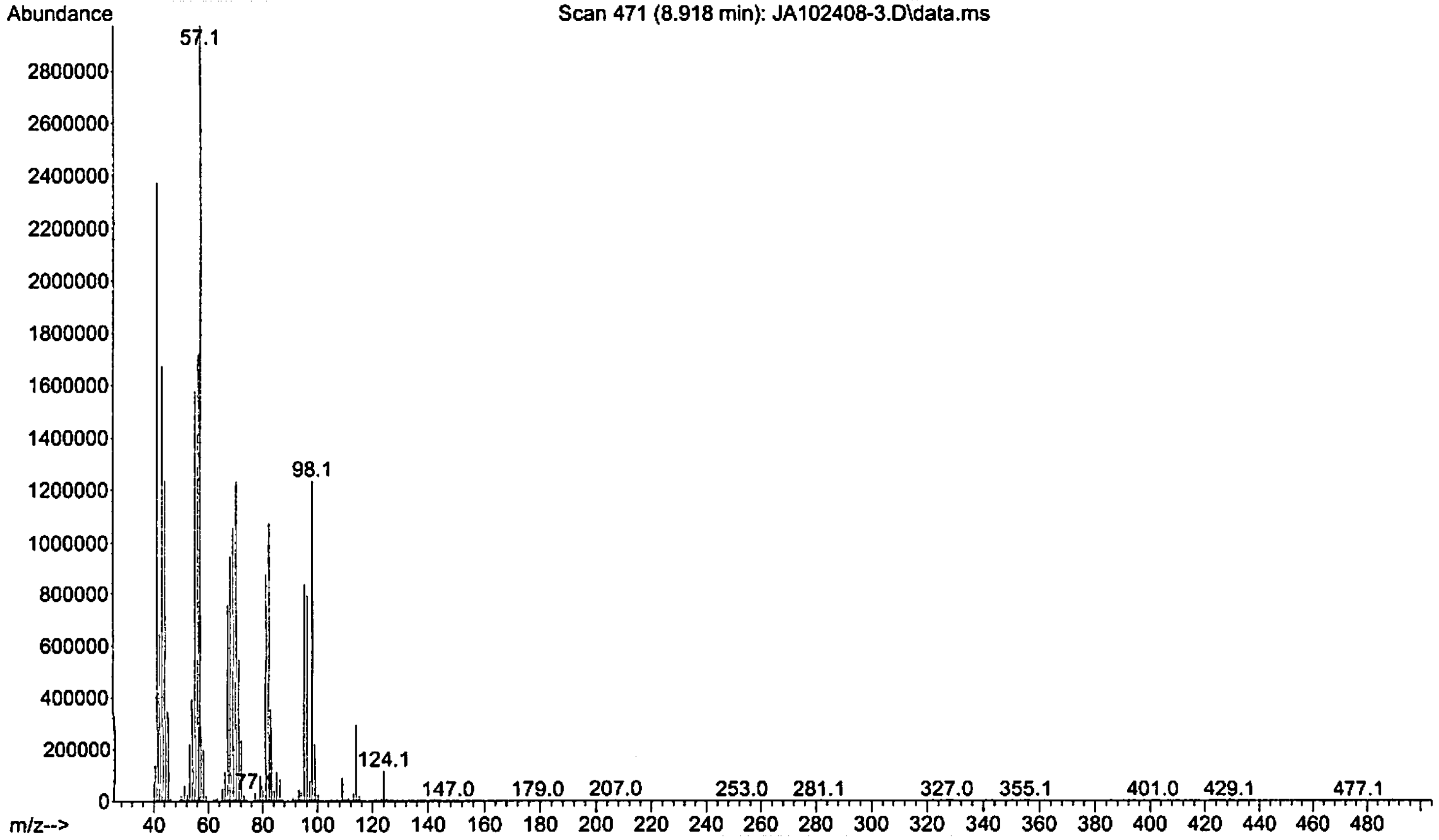

Library Searched : C:\Database\W8N05ST.L
Quality : 91
ID : NONANAL $ PELARGONALDEHYDE $ 1-NONALDEHYDE $ 1-NONANAL $ 1-NONYL ALDEHYDE $ AI3-04859 $ ALDEHYDE C-9 $ BRN 1236701 $ C-9 ALDEHYDE $ CCRIS 664 $ EINECS 204-688-5 $ FEMA NO. 2782 $ HSDB 7229 $ N-NONALDEHYDE $ N-NONAN-1-AL $ N-NONANAL $ N-NONYLALDEHYDE $ NCI

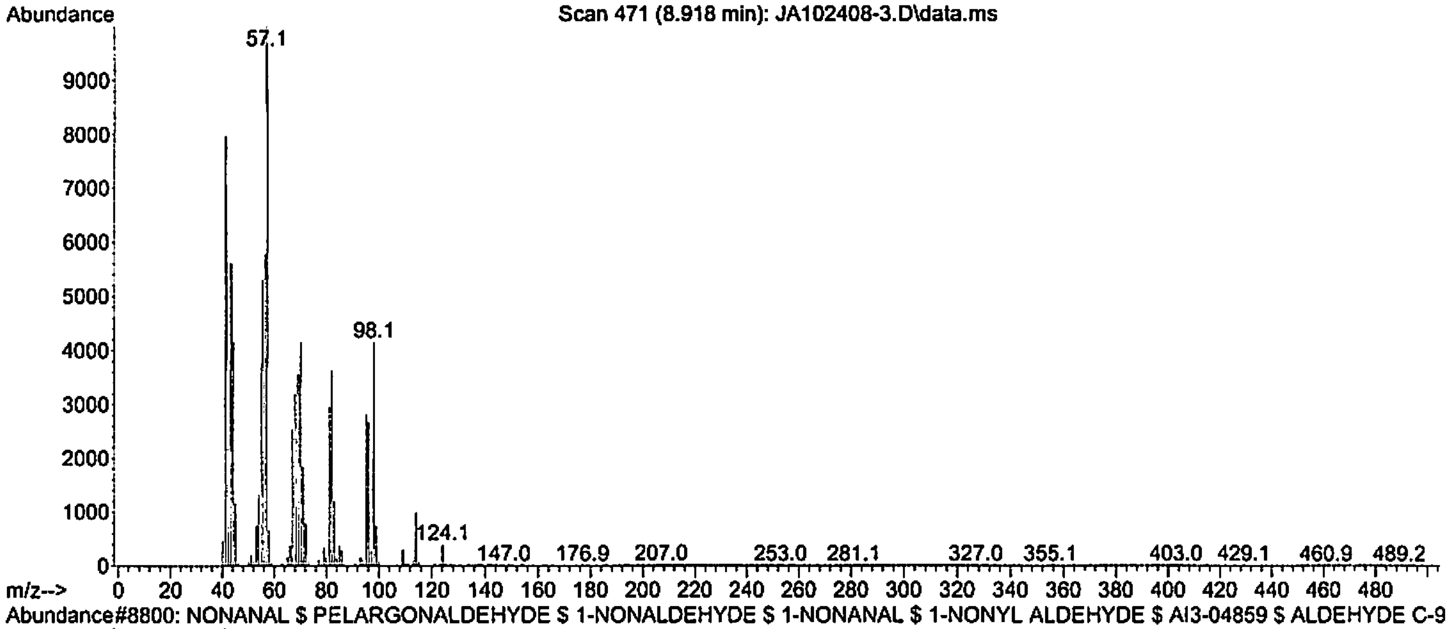


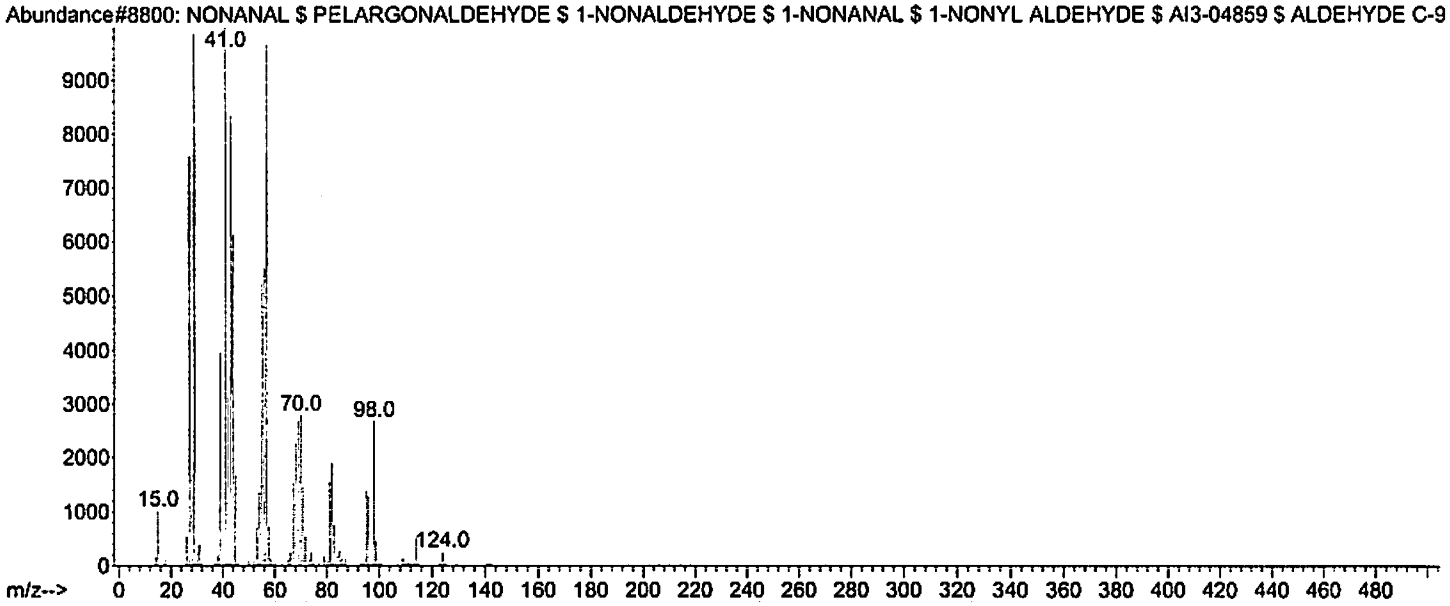


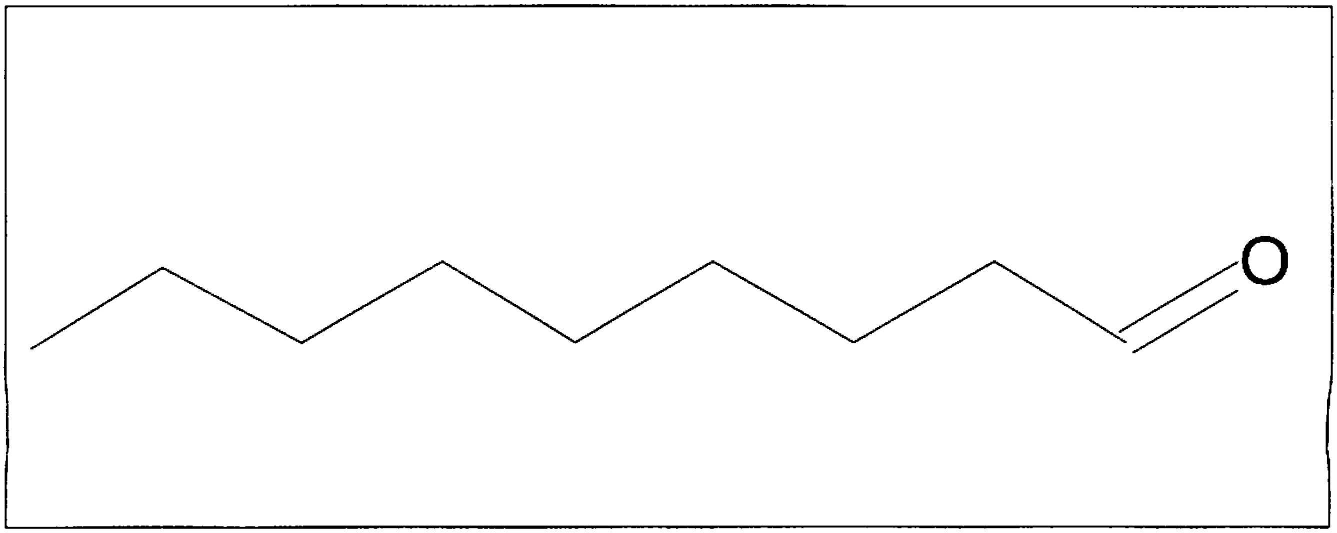

```
File        :D:\ALDRICH\Snapshot\JA102408-3.D
Operator    :
Acquired    : 24 Oct 2008  16:27     using AcqMethod JA-50-280LESS.M
Instrument  :    Buba
Sample Name: 5M C.oculata abd. cuticle/10ul CH2Cl2
Misc Info   : 7days w/geraniol; emerged 9/23; no GC
Vial Number: 1
```

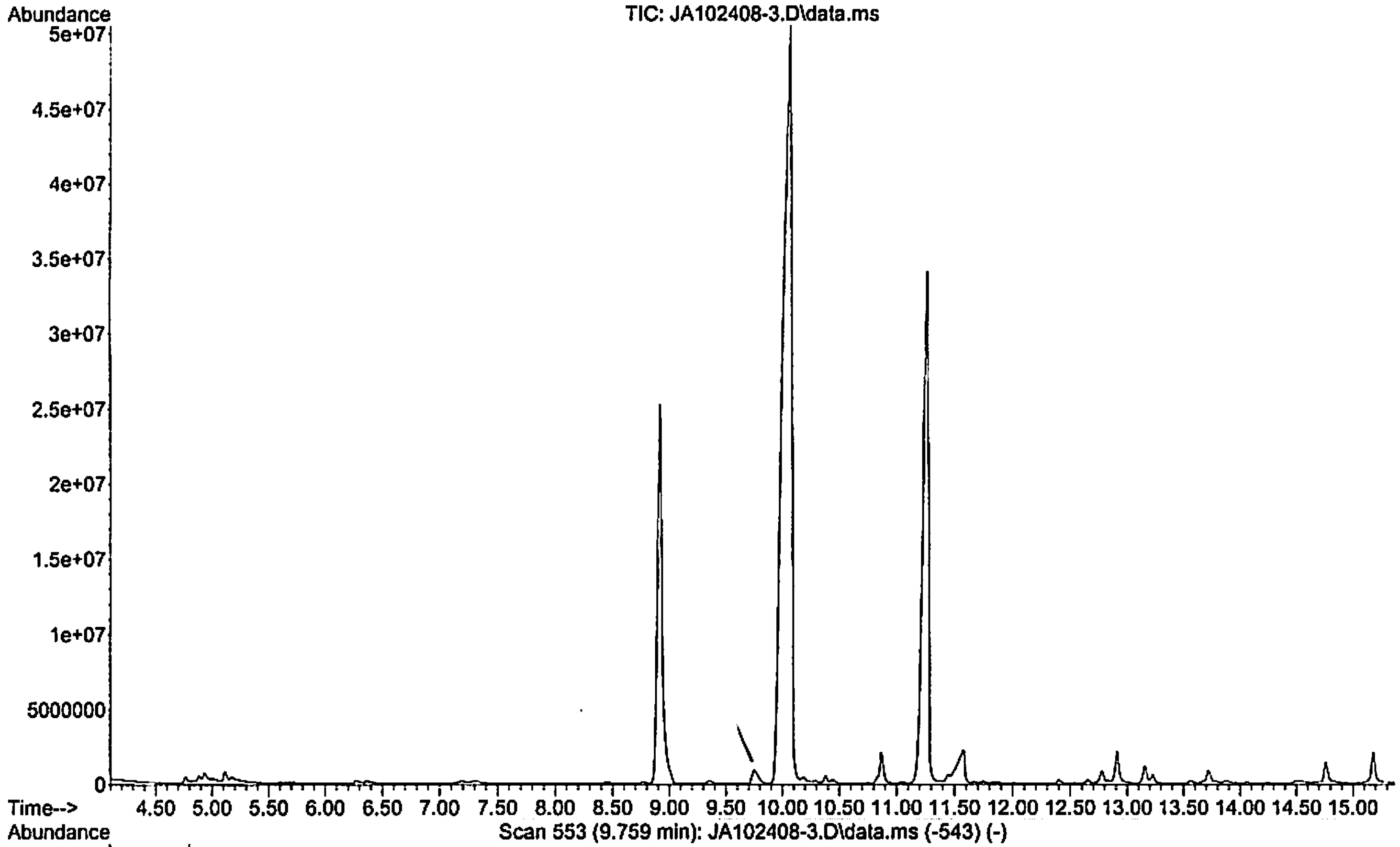


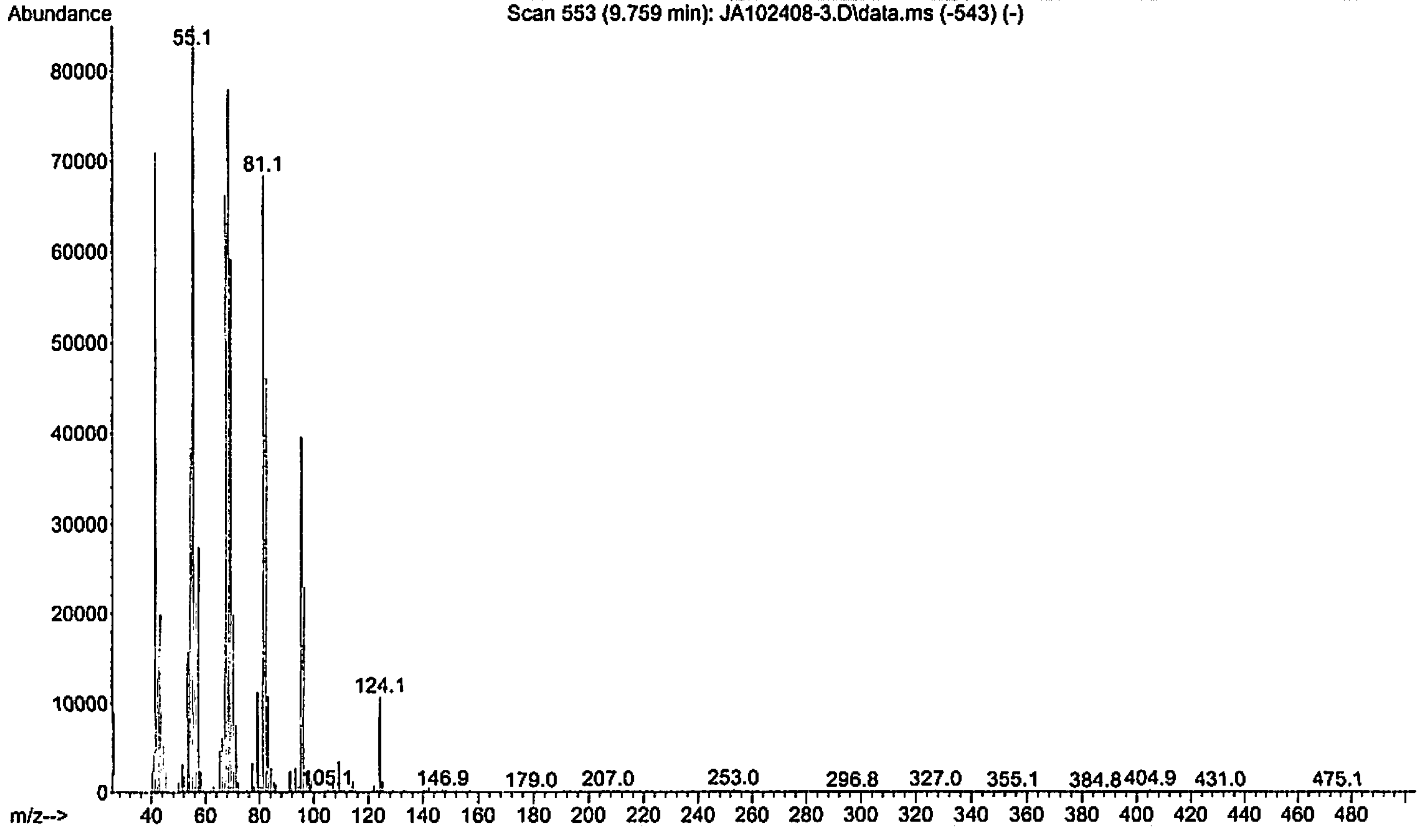

```
Library Searched : C:\Database\W8N05ST.L
Quality          : 91
ID               : 3-Nonen-1-ol, (Z)- $ cis-3-Nonen-1-Ol $ (3Z)-3-Nonen-1-ol #
```

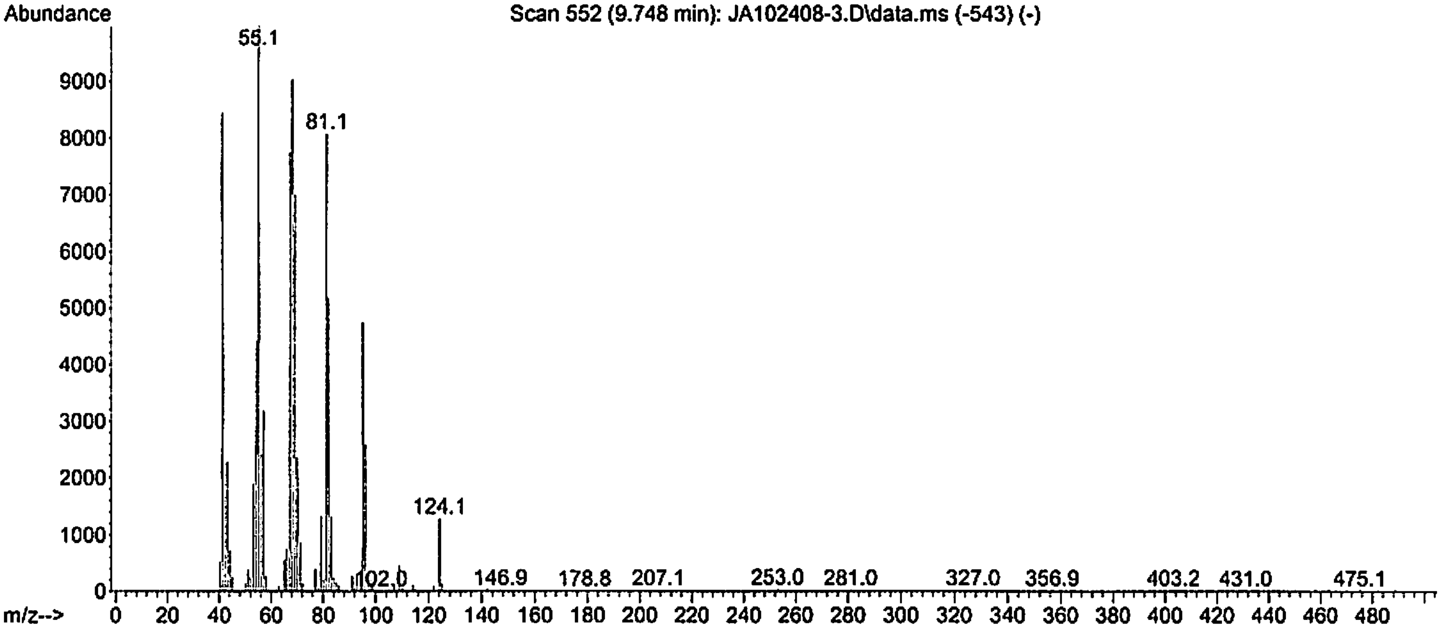


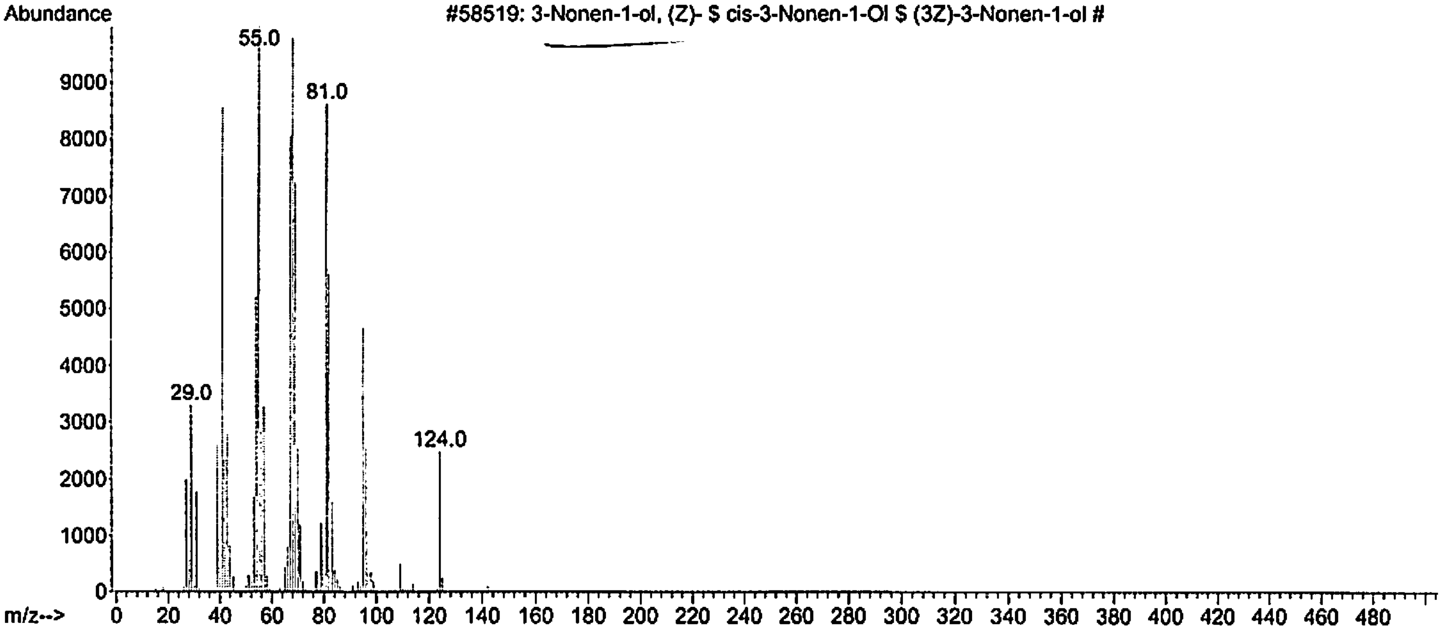


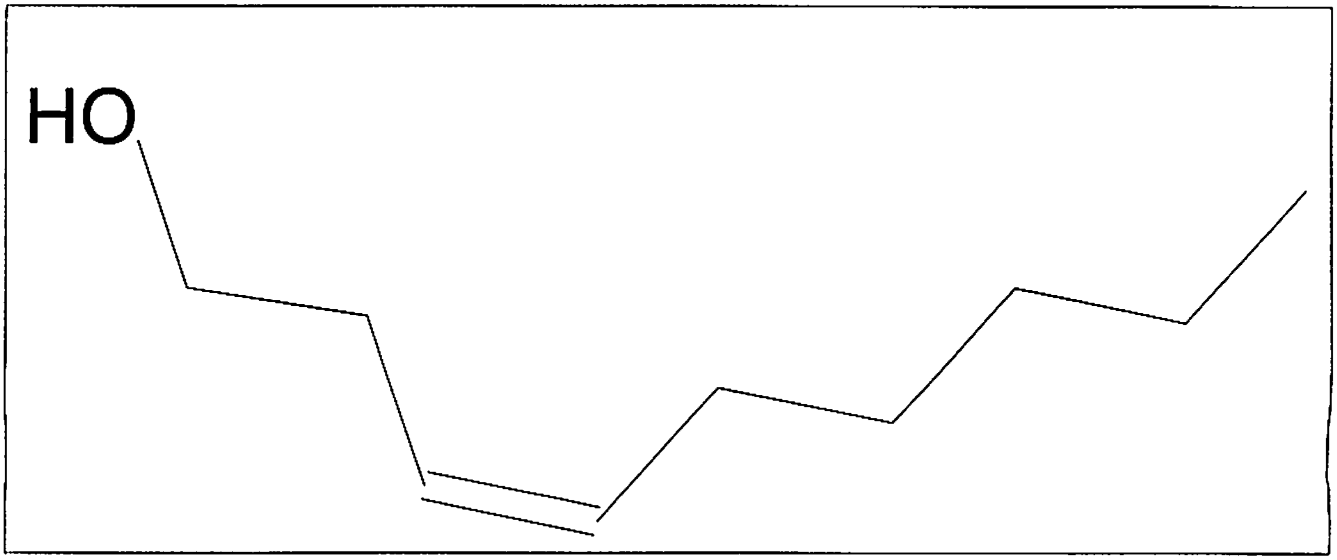

```
File        :D:\ALDRICH\Snapshot\JA102408-3.D
Operator    :
Acquired    : 24 Oct 2008  16:27     using AcqMethod JA-50-280LESS.M
Instrument :    Buba
Sample Name: 5M C.oculata abd. cuticle/10ul CH2Cl2
Misc Info   : 7days w/geraniol; emerged 9/23; no GC
Vial Number: 1
```

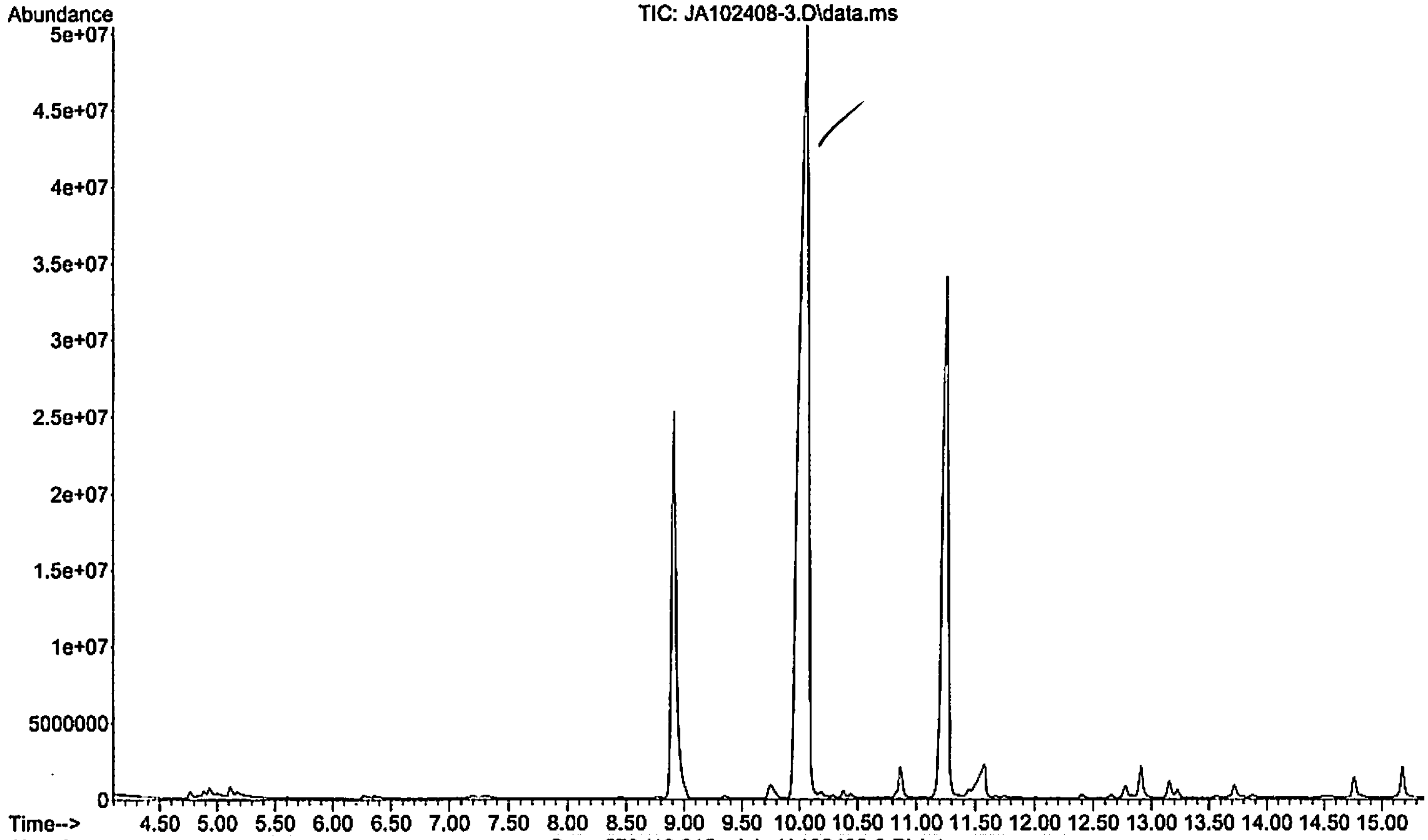


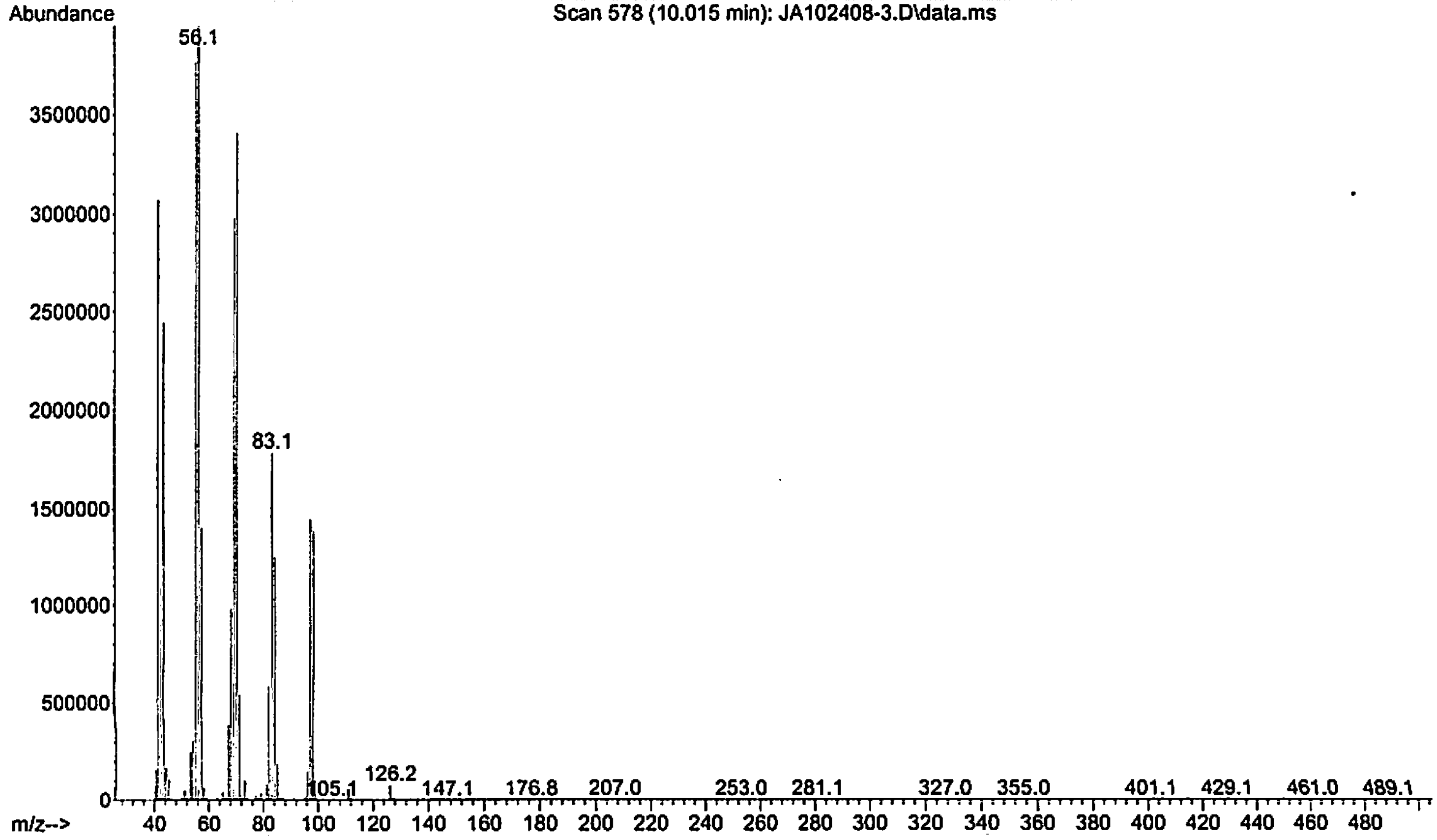

Library Searched : C:\Database\W8N05ST.L
Quality : 91
ID : 1-NONANOL $ NONANOL $ NONAN-1-OL $ 1-HYDROXYNONANE $ 1-NONANO $ AI3-03962 $ ALCOHOL C-9 $ BRN 0969213 $ C9 ALCOHOL $ EINECS 205-583-7 $ FATTY ALCOHOL(C9) $ FEMA NO. 2789 $ HSDB 5145 $ N-NONAN-1-OL $ N-NONANOL $ N-NONYL ALCOHOL $ NONALOL $ NONANOL-(1) $ NO

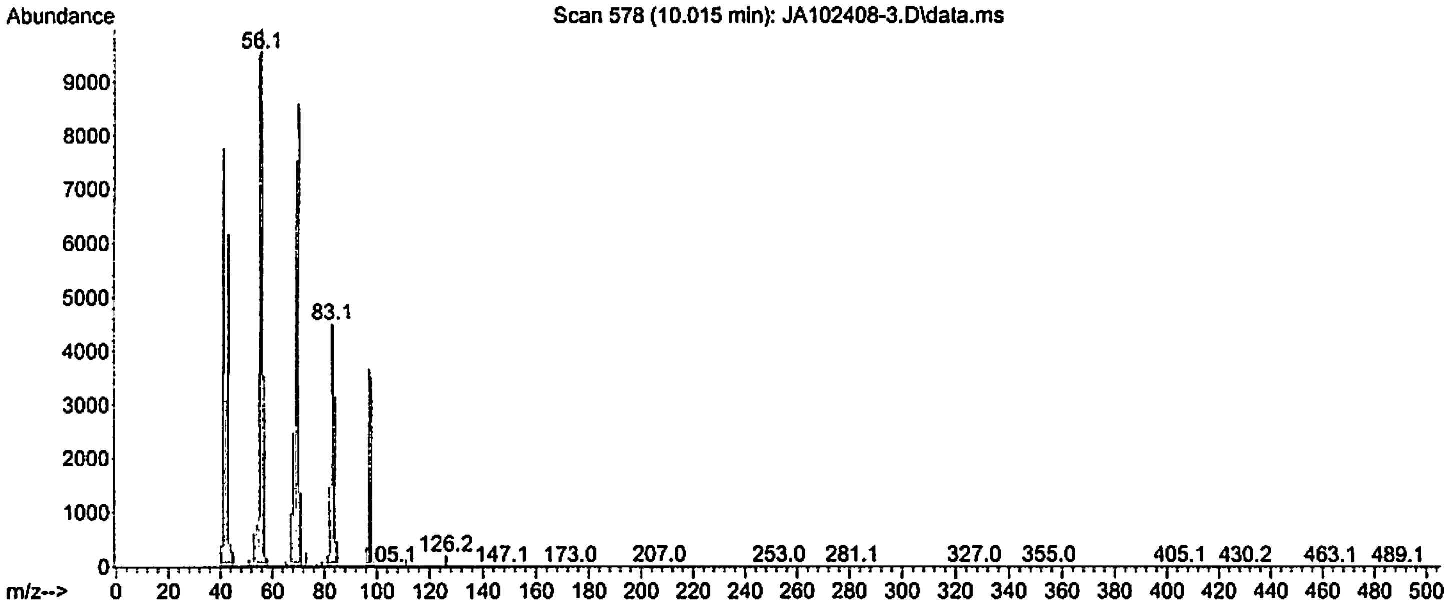


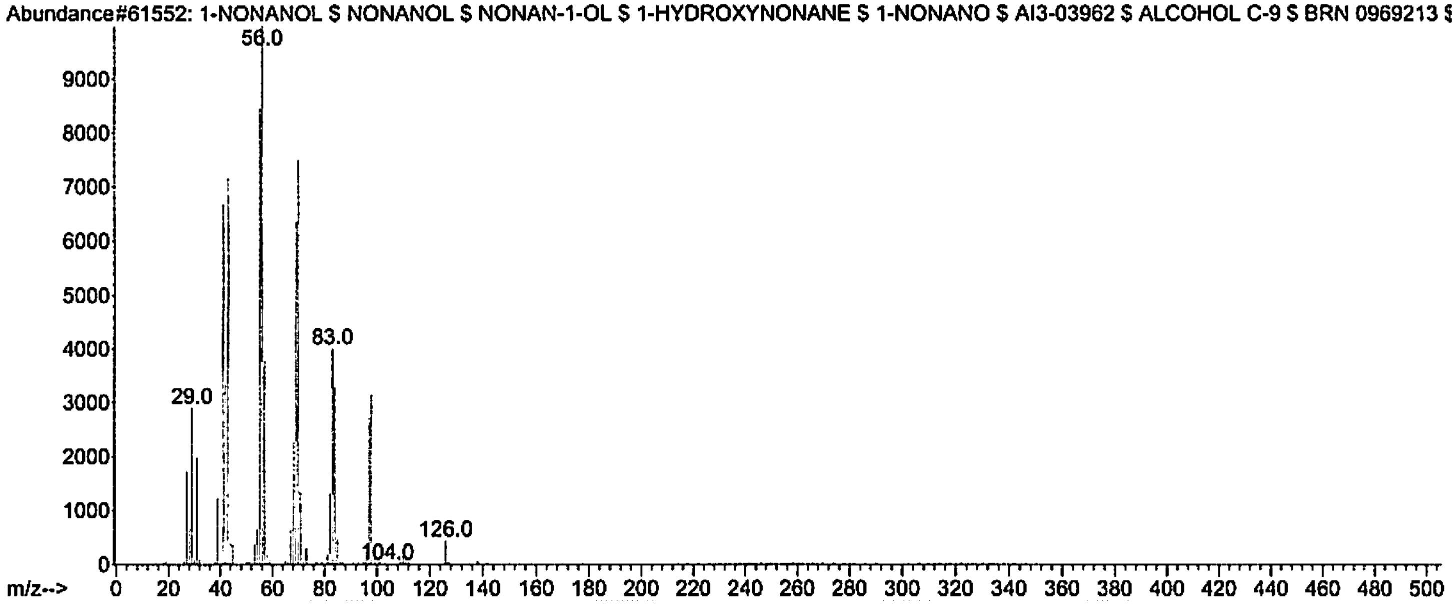


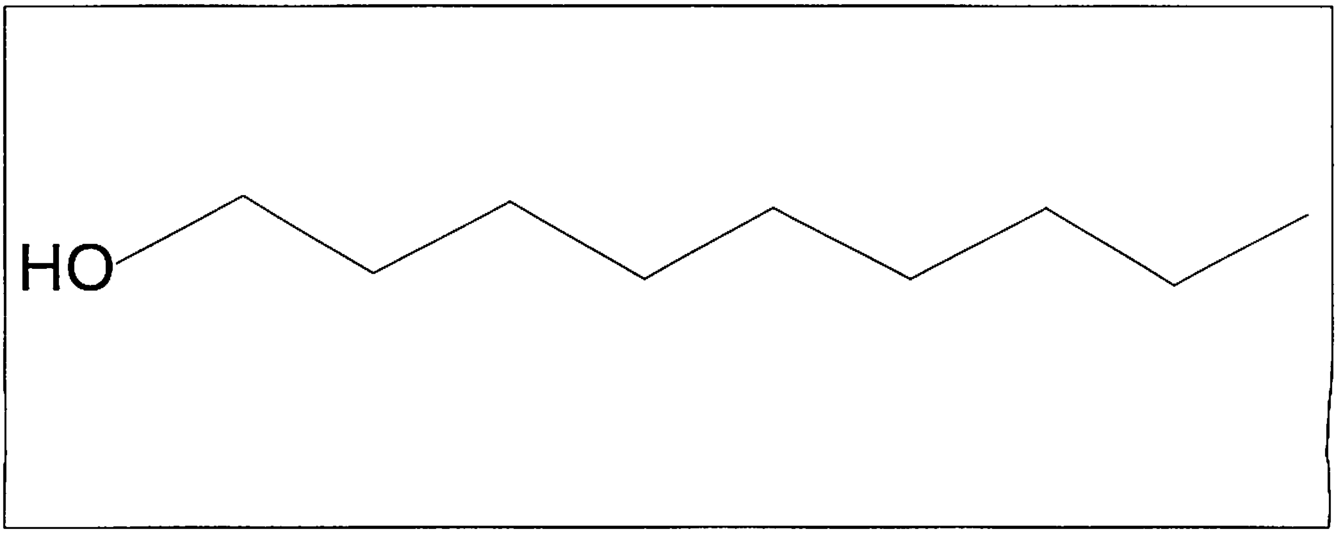

```
File        :D:\ALDRICH\Snapshot\JA102408-3.D
Operator    :
Acquired    : 24 Oct 2008  16:27     using AcqMethod JA-50-280LESS.M
Instrument :     Buba
Sample Name: 5M C.oculata abd. cuticle/10ul CH2Cl2
Misc Info   : 7days w/geraniol; emerged 9/23; no GC
Vial Number: 1
```

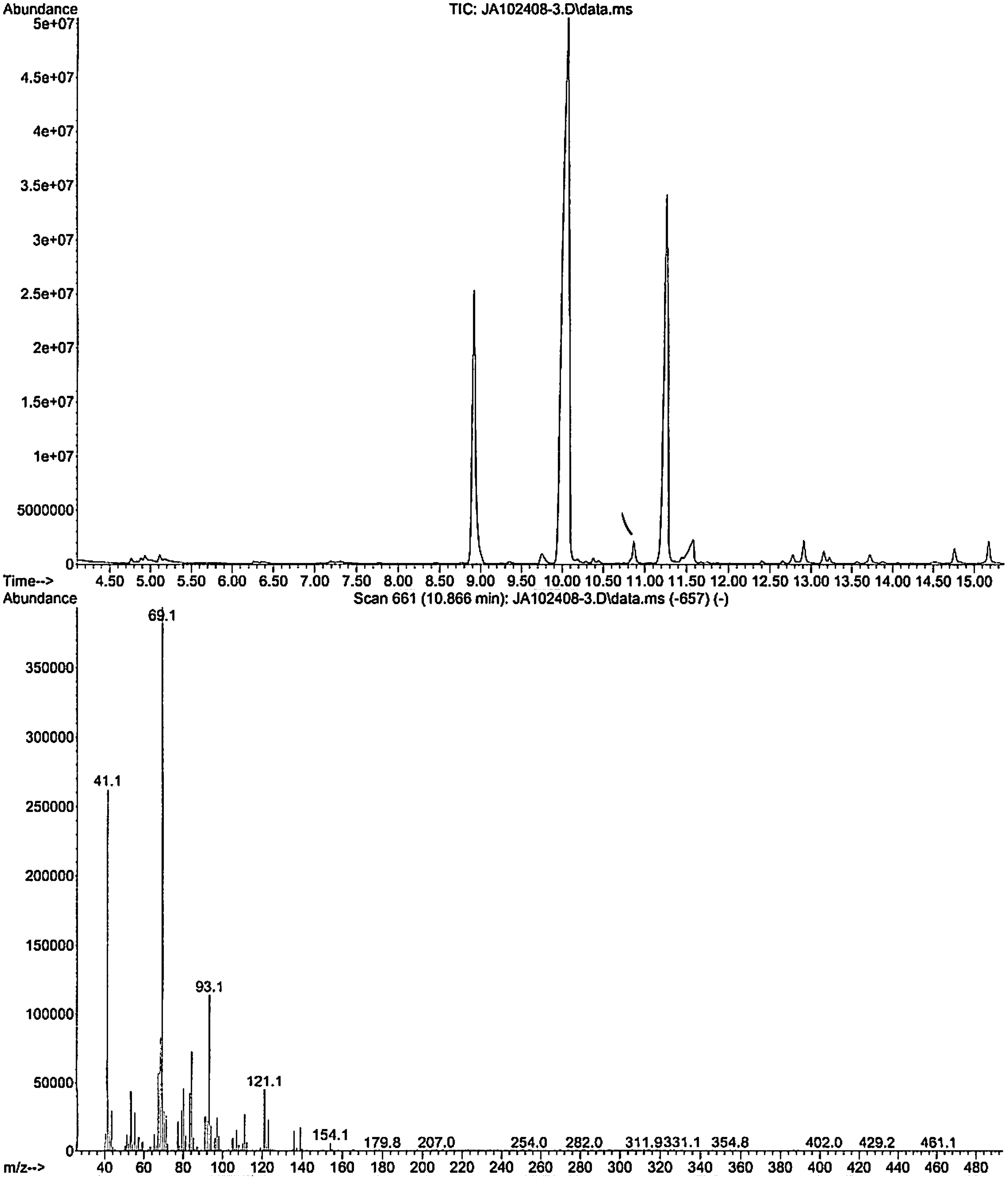

```
Library Searched : C:\Database\W8N05ST.L
Quality          : 95
ID               : 2,6-Octadien-1-ol, 3,7-dimethyl-, (Z)- $ cis-Geraniol $ cis-3,7-Dimeth
                   yl-2,6-octadien-1-ol $ Nerol $ Neryl alcohol $ 2-cis-3,7-Dimethyl-2,6-
                   octadien-1-ol $ 2,6-Dimethyl-2,6-octadien-8-ol $ 3,7-Dimethyl-2,6-octa
                   dien-1-ol $ Vernol $ (2Z)-3,7-Dimethyl-2,6-oc
```

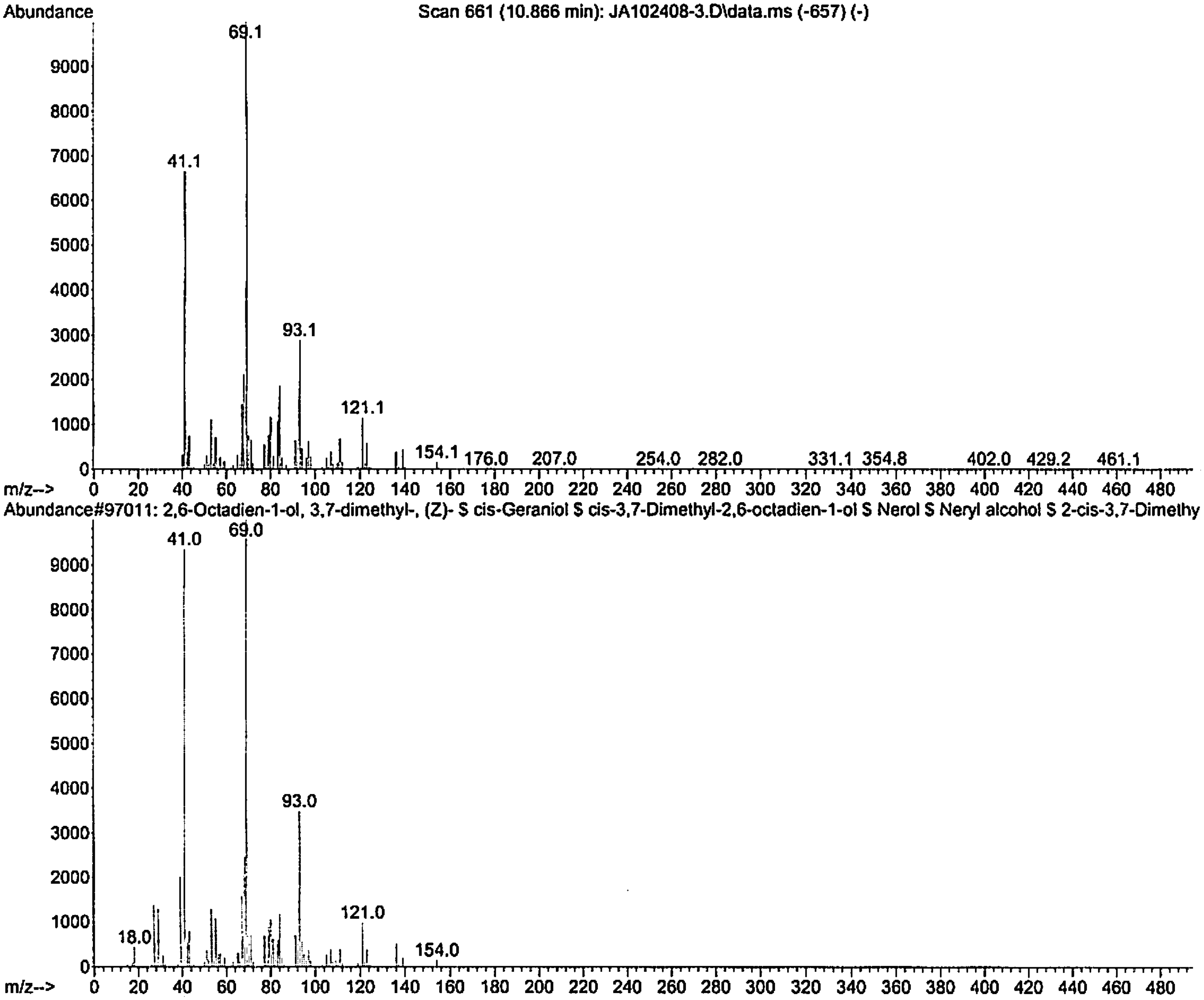


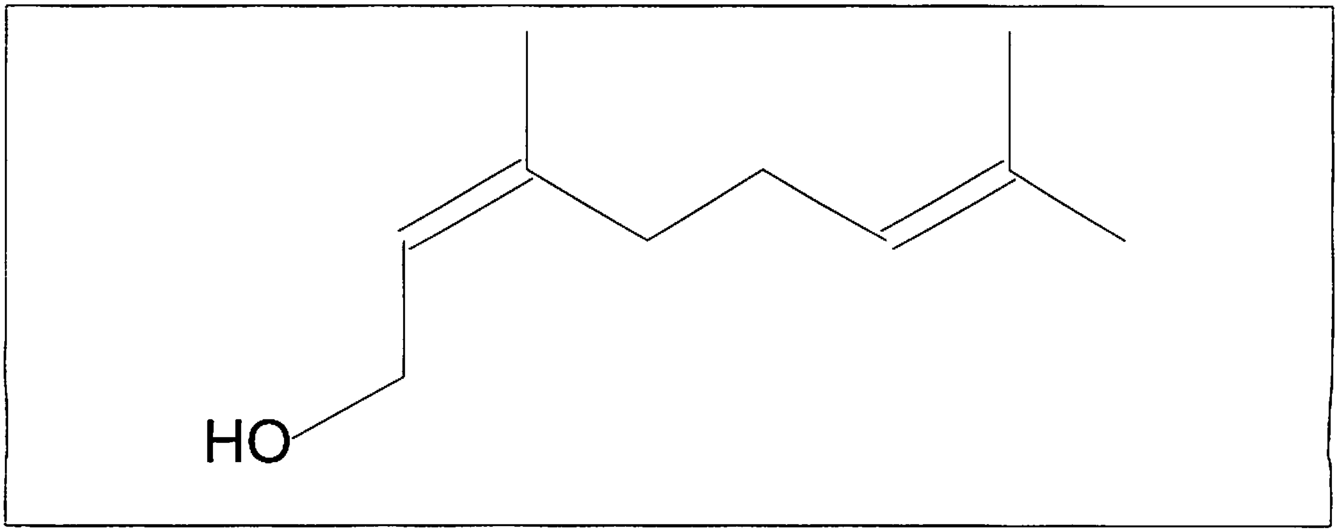

```
File        :D:\ALDRICH\Snapshot\JA102408-3.D
Operator    :
Acquired    : 24 Oct 2008  16:27     using AcqMethod JA-50-280LESS.M
Instrument  :    Buba
Sample Name: 5M C.oculata abd. cuticle/10ul CH2Cl2
Misc Info   : 7days w/geraniol; emerged 9/23; no GC
Vial Number: 1
```

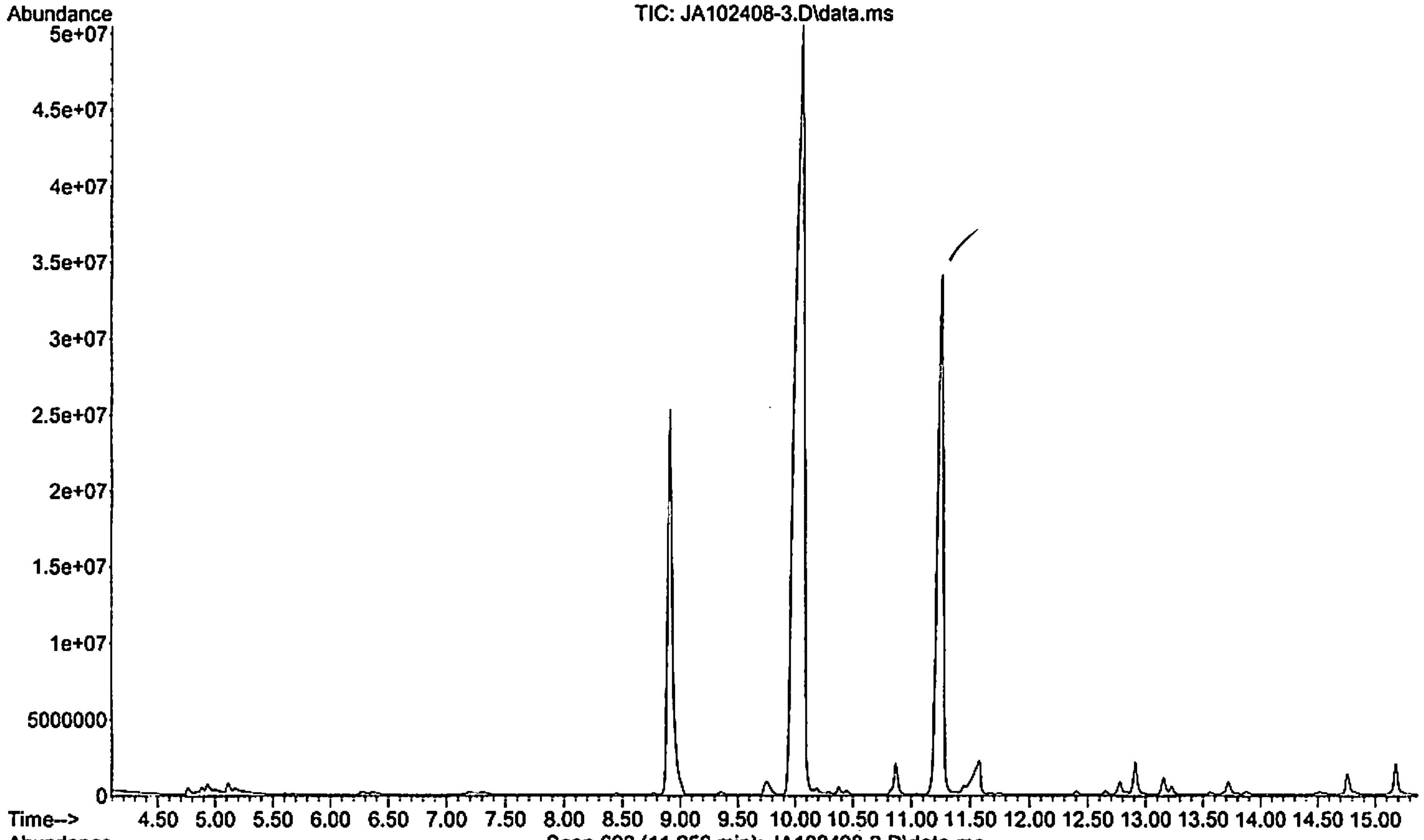


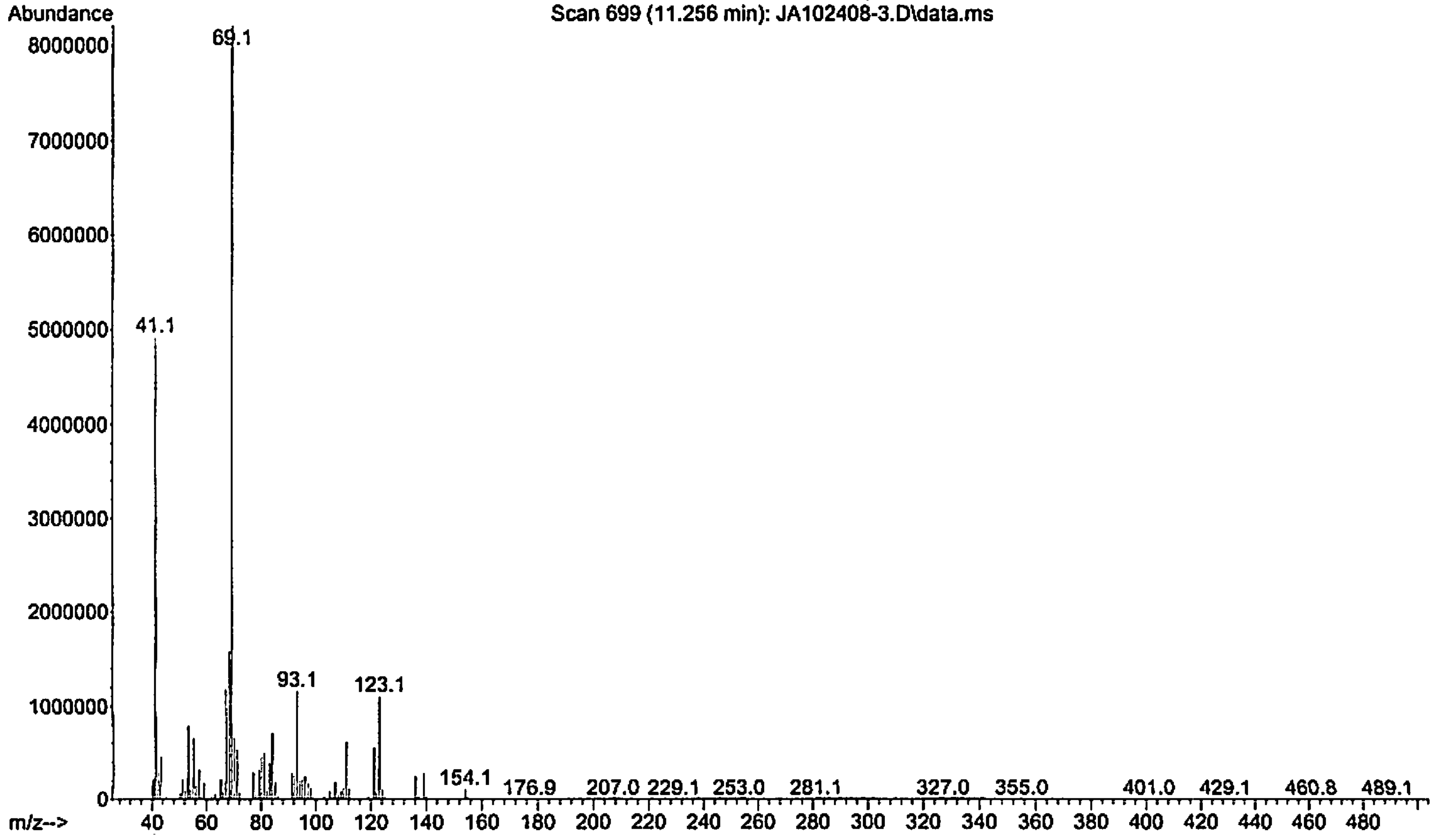

```
Library Searched : C:\Database\W8N05ST.L
Quality          : 96
ID               : 2,6-OCTADIEN-1-OL, 3,7-DIMETHYL-, (E)- $ 3,7-DIMETHYLOCTA-2,6-DIEN-1-O
                   L $ (2E)-3,7-DIMETHYL-2,6-OCTADIEN-1-OL # $ (2E)-3,7-DIMETHYL-2,6-OCTA
                   DIEN-1-OL $ (2E)-3,7-DIMETHYL-2,6-OCTADIEN-1-OL (COMPUTER-GENERATED NA
                   ME) $ (E)-3,7-DIMETHYL-2,6-OCTADIEN-1-OL $ (E
```

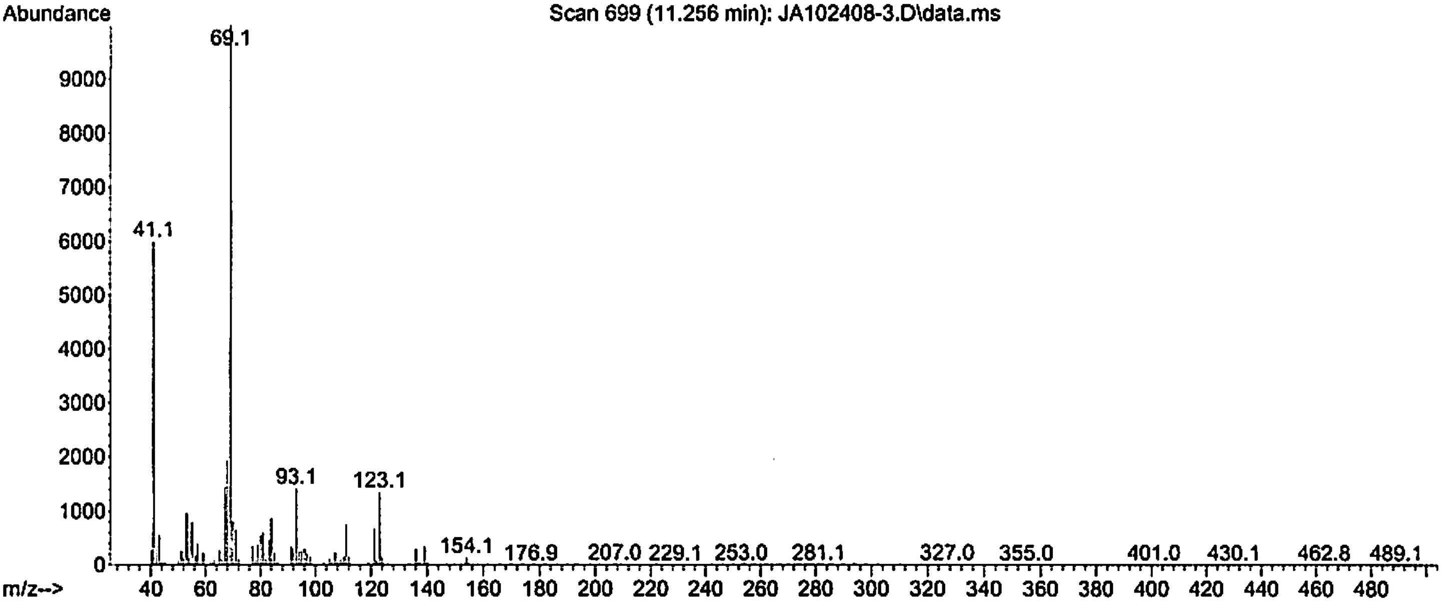


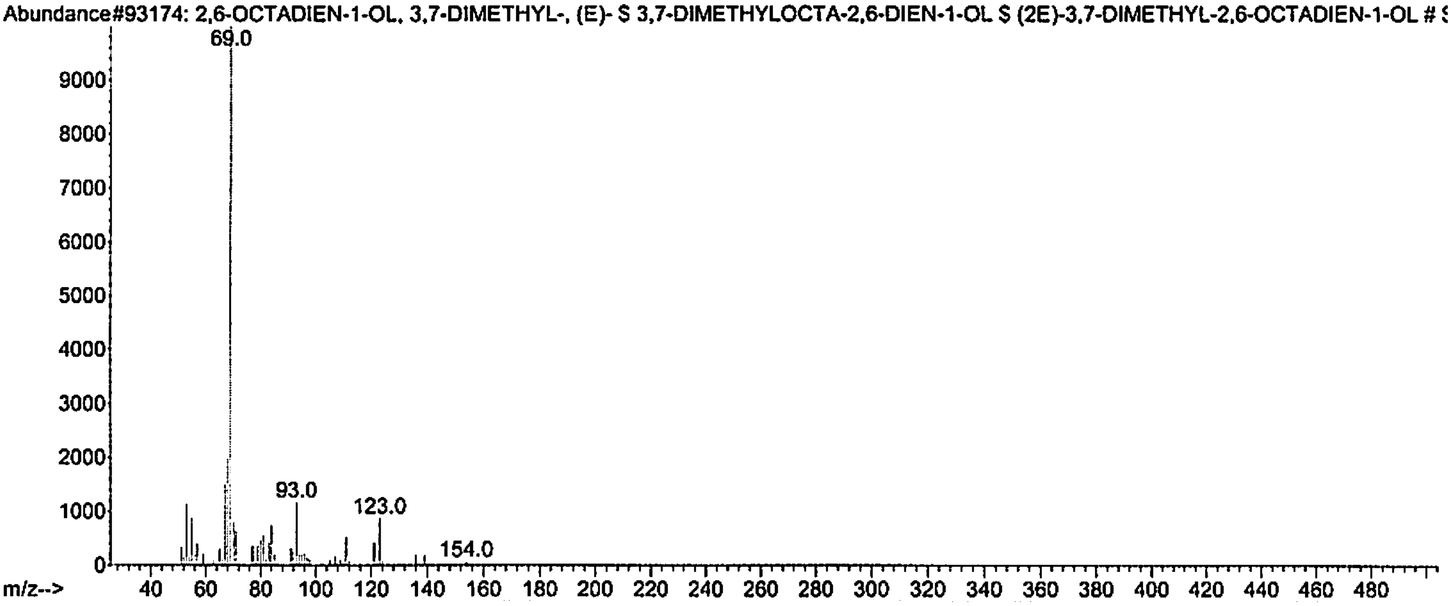


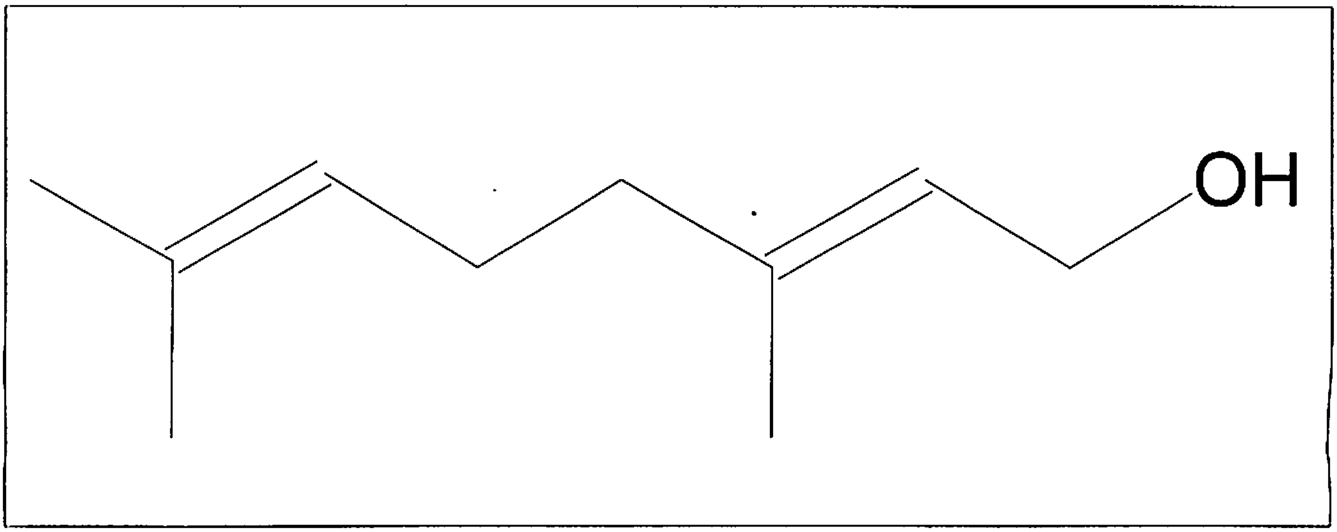

```
File        :D:\ALDRICH\Snapshot\JA102408-3.D
Operator    :
Acquired    : 24 Oct 2008  16:27     using AcqMethod JA-50-280LESS.M
Instrument :    Buba
Sample Name: 5M C.oculata abd. cuticle/10ul CH2Cl2
Misc Info   : 7days w/geraniol; emerged 9/23; no GC
Vial Number: 1
```

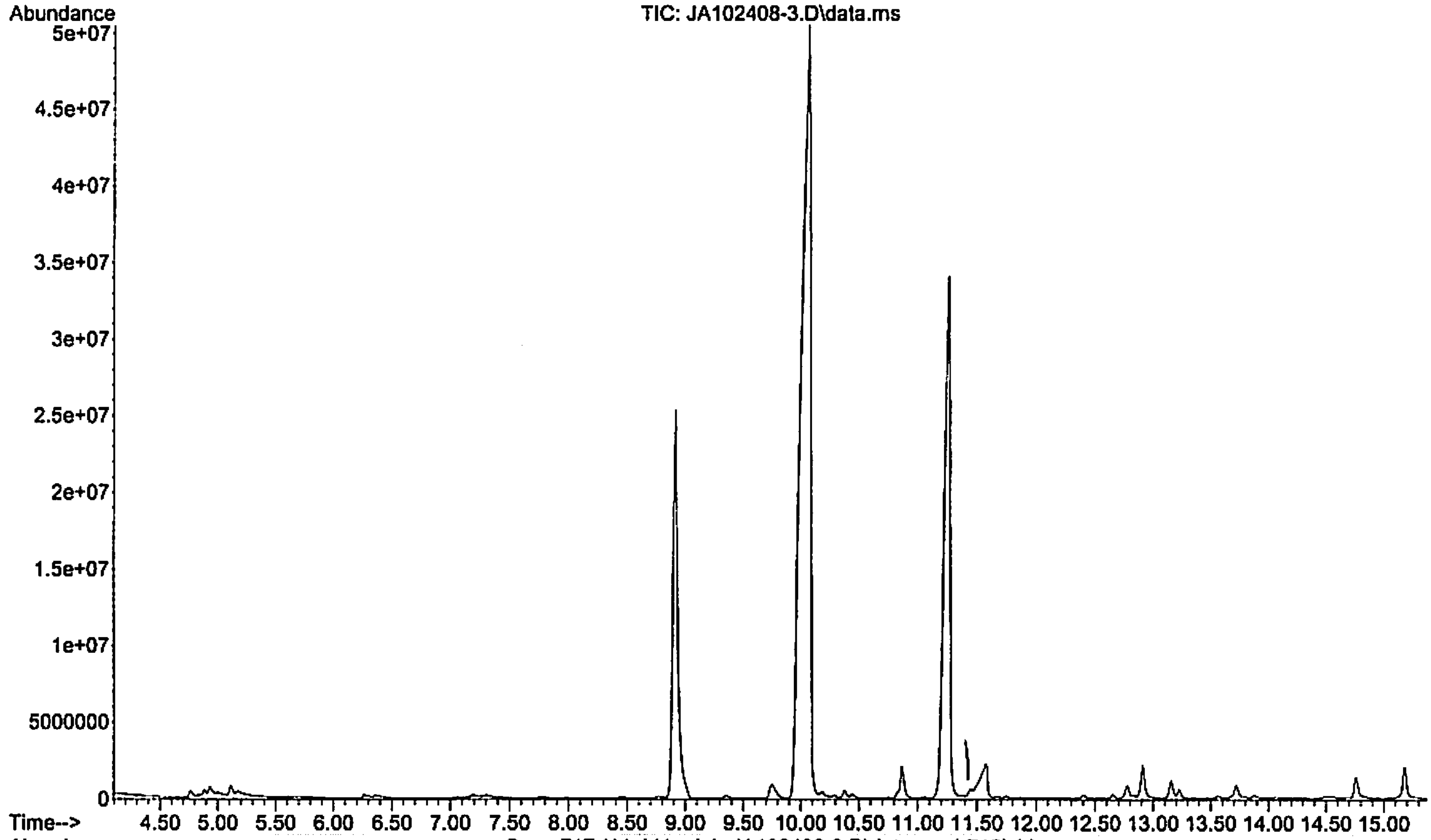


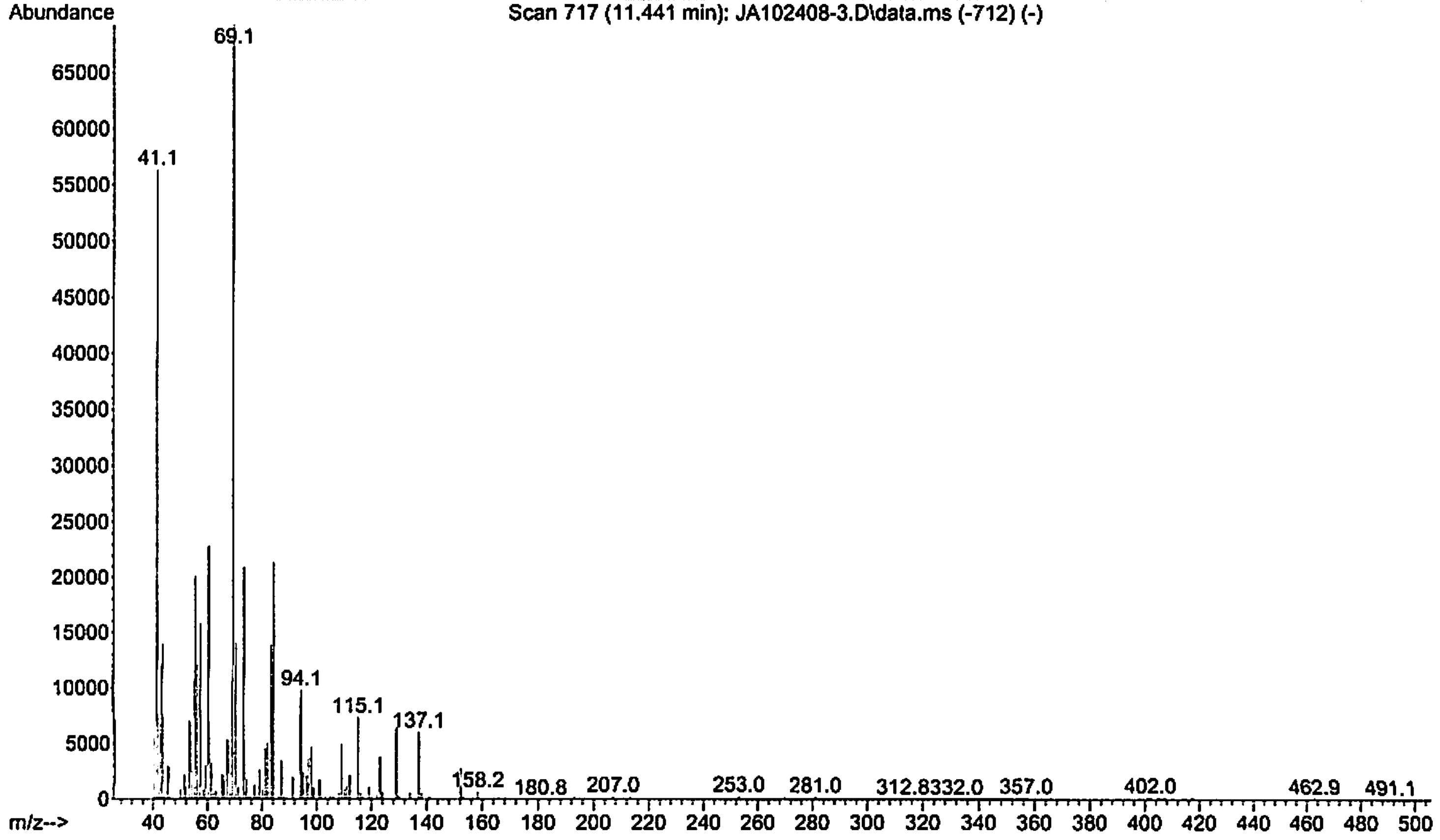

```
Library Searched : C:\Database\W8N05ST.L
Quality          : 86
ID               : 2,6-Octadienal, 3,7-dimethyl-, (E)- $ .alpha.-Citral $ (E)-Citral $ tr
                   ans-Citral $ trans-3,7-Dimethyl-2,6-octadienal $ Citral a $ Geranaldeh
                   yde $ Geranial $ Citral .alpha. $ (E)-3,7-Dimethyl-2,6-octadienal $ (2
                   E)-3,7-Dimethyl-2,6-octadienal #
```

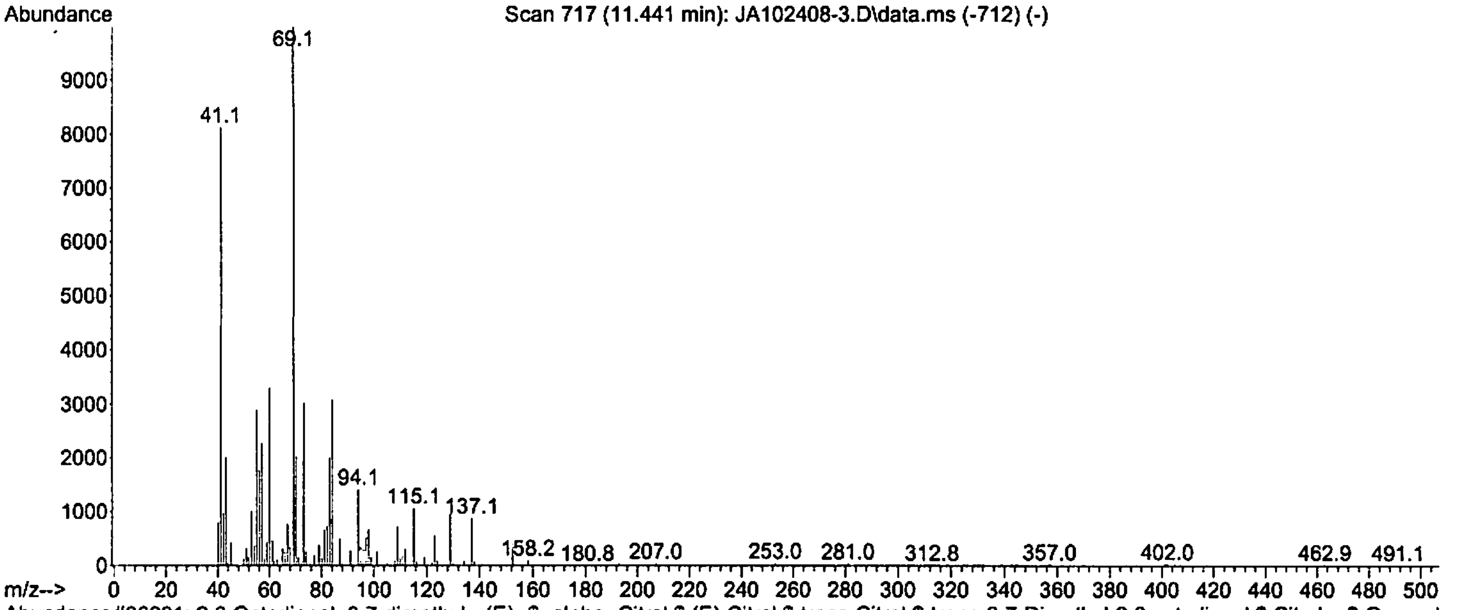


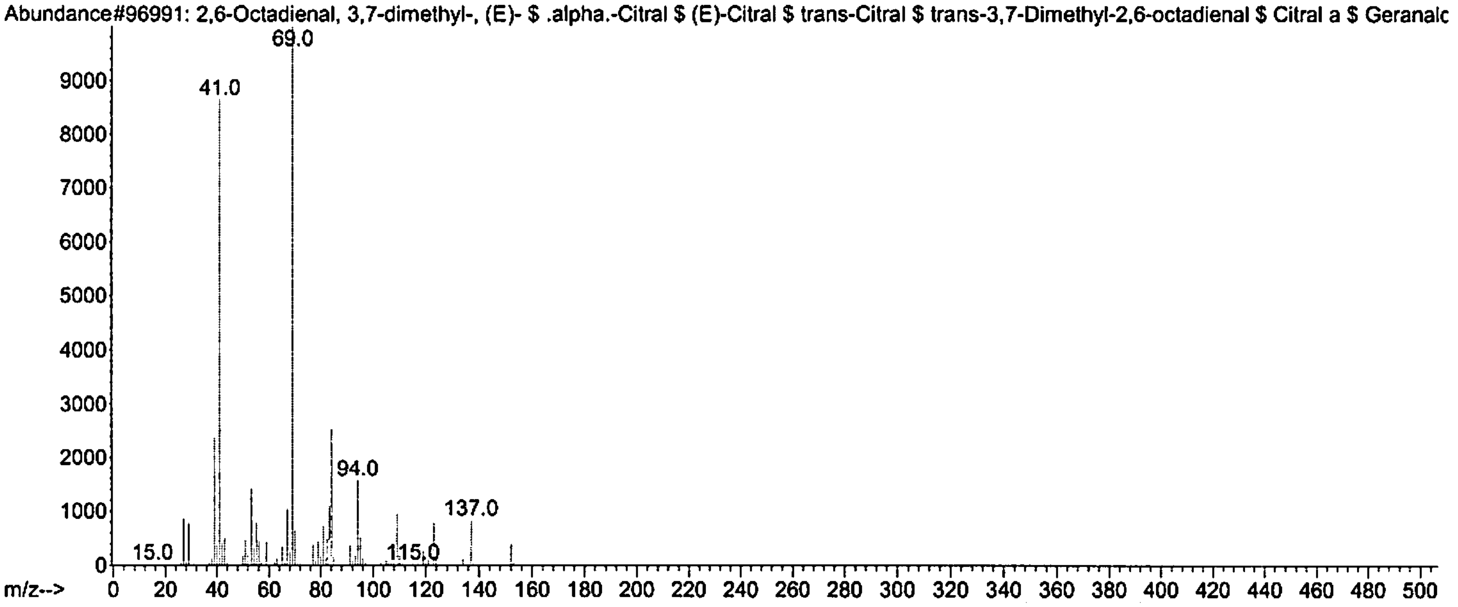


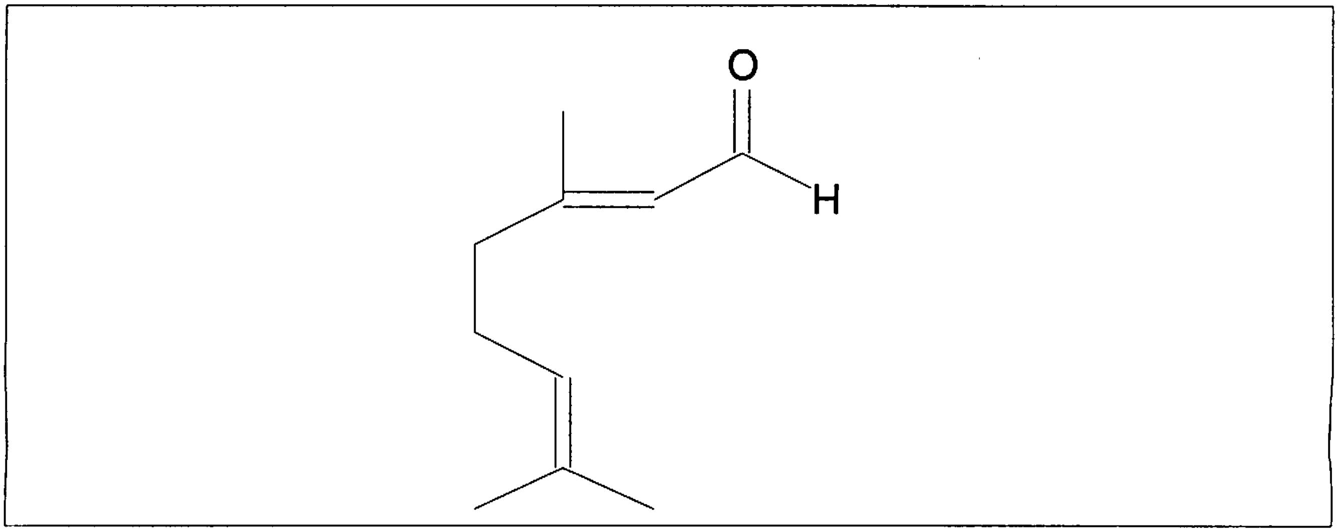

```
File        :D:\ALDRICH\Snapshot\JA102408-3.D
Operator    :
Acquired    : 24 Oct 2008  16:27     using AcqMethod JA-50-280LESS.M
Instrument  :     Buba
Sample Name: 5M C.oculata abd. cuticle/10ul CH2Cl2
Misc Info   : 7days w/geraniol; emerged 9/23; no GC
Vial Number: 1
```

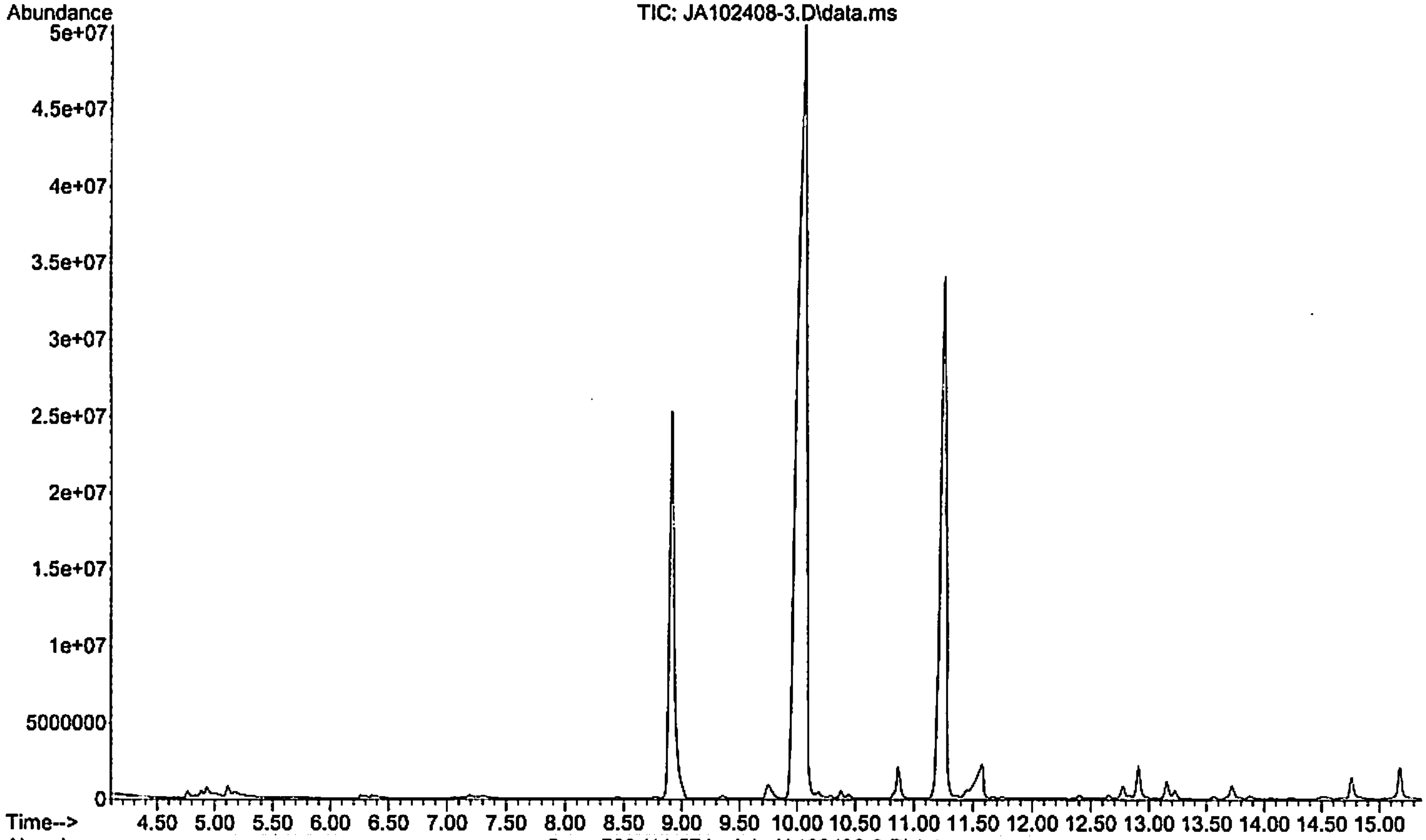


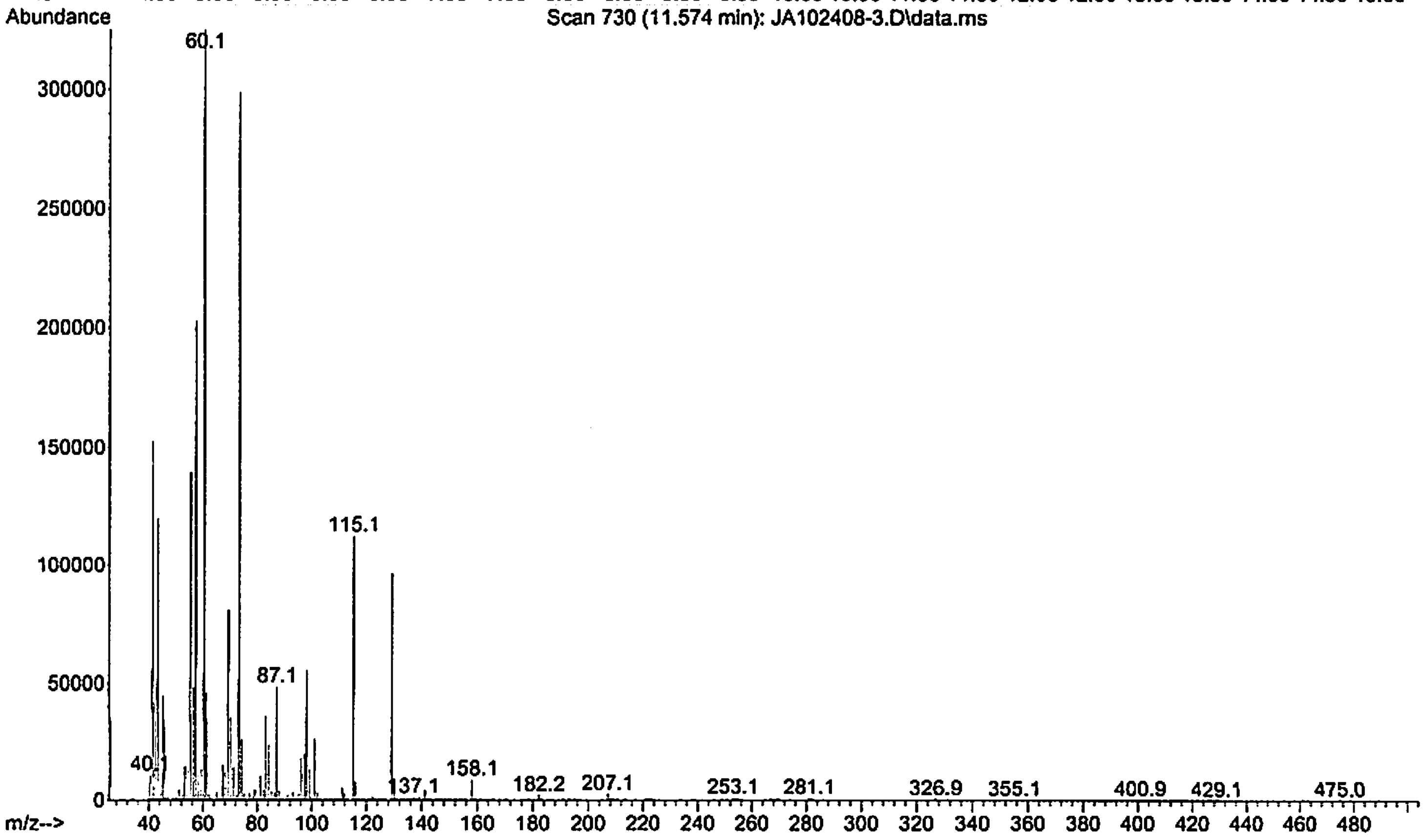

```
Library Searched : C:\Database\W8N05ST.L
Quality          : 95
ID               : NONANOIC ACID $ CALCIUM PELARGONATE $ PELARGONIC ACID $ POTASSIUM PELA
                   RGONATE $ 1-NONANOIC ACID $ 1-OCTANECARBOXYIC ACID $ 1-OCTANECARBOXYLI
                   C ACID $ AI3-04164 $ AIDS-017605 $ BRN 1752351 $ CADMIUM NONAN-1-OATE
                   $ CADMIUM PELARGONATE $ CALCIUM NONAN-1-OATE
```

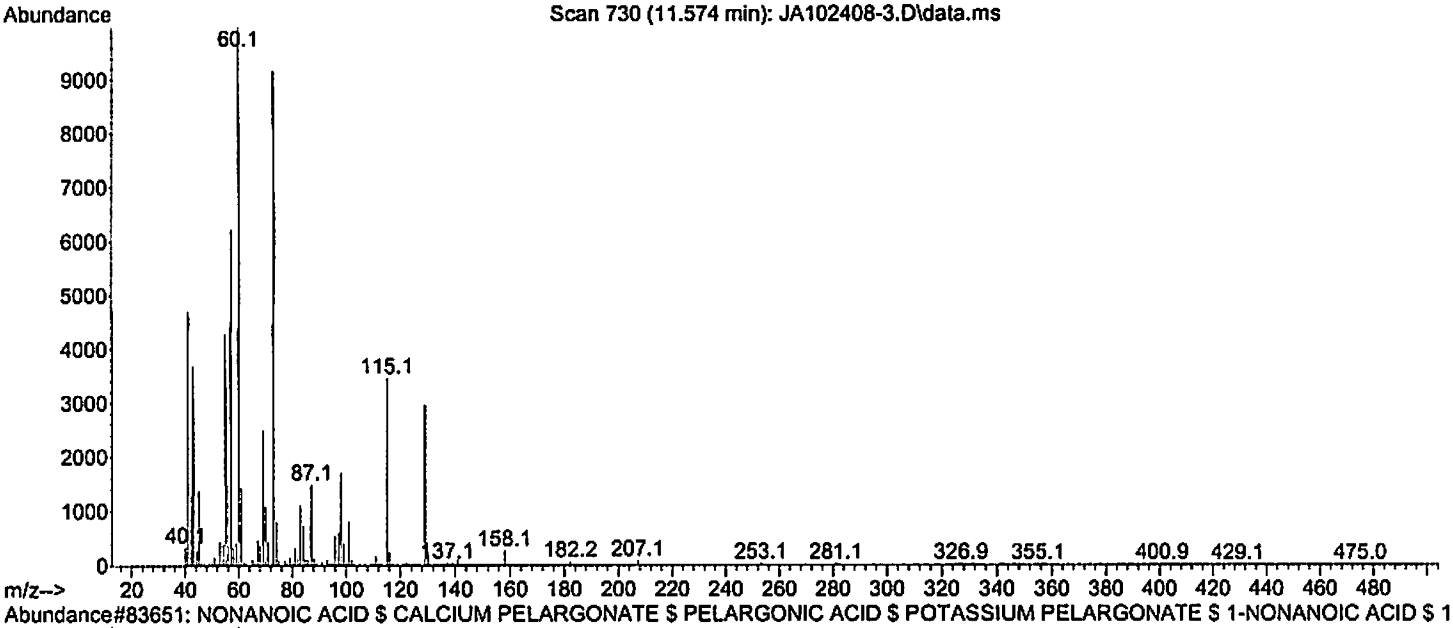


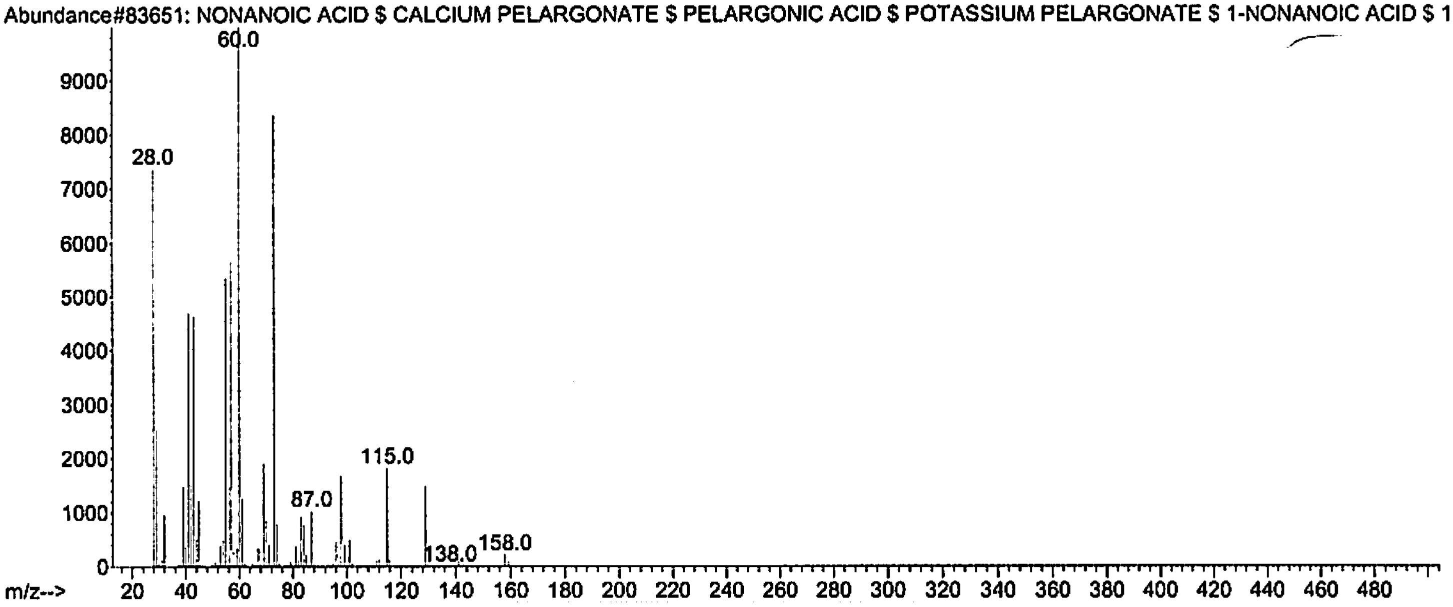


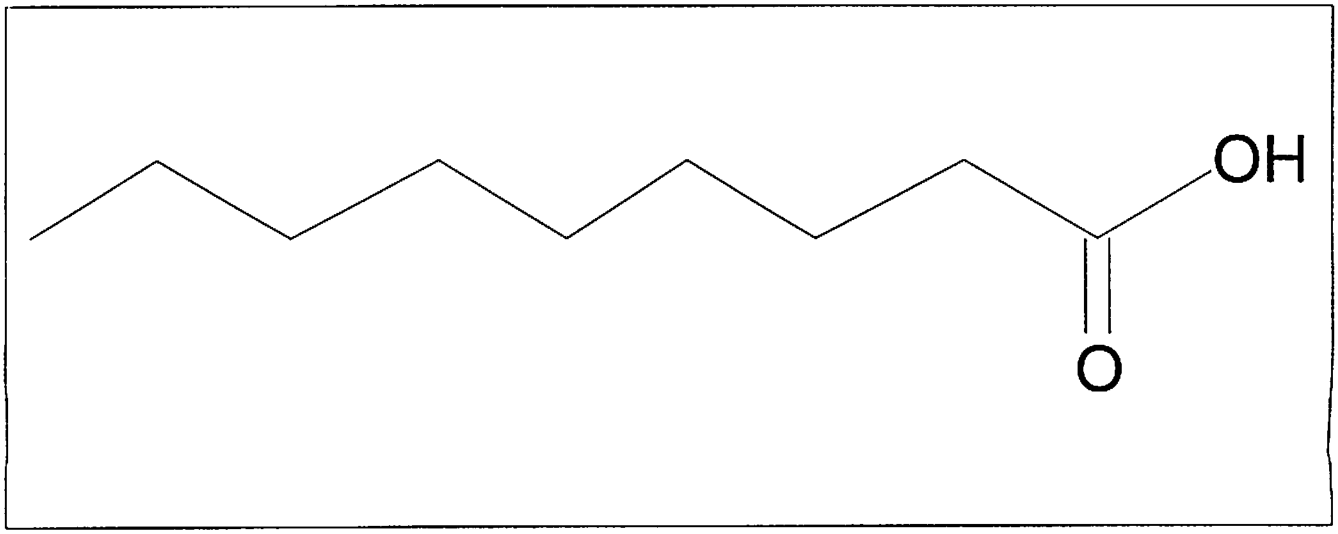

```
File        :D:\ALDRICH\Snapshot\JA102408-3.D
Operator    :
Acquired    : 24 Oct 2008  16:27     using AcqMethod JA-50-280LESS.M
Instrument :     Buba
Sample Name: 5M C.oculata abd. cuticle/10ul CH2Cl2
Misc Info   : 7days w/geraniol; emerged 9/23; no GC
Vial Number: 1
```

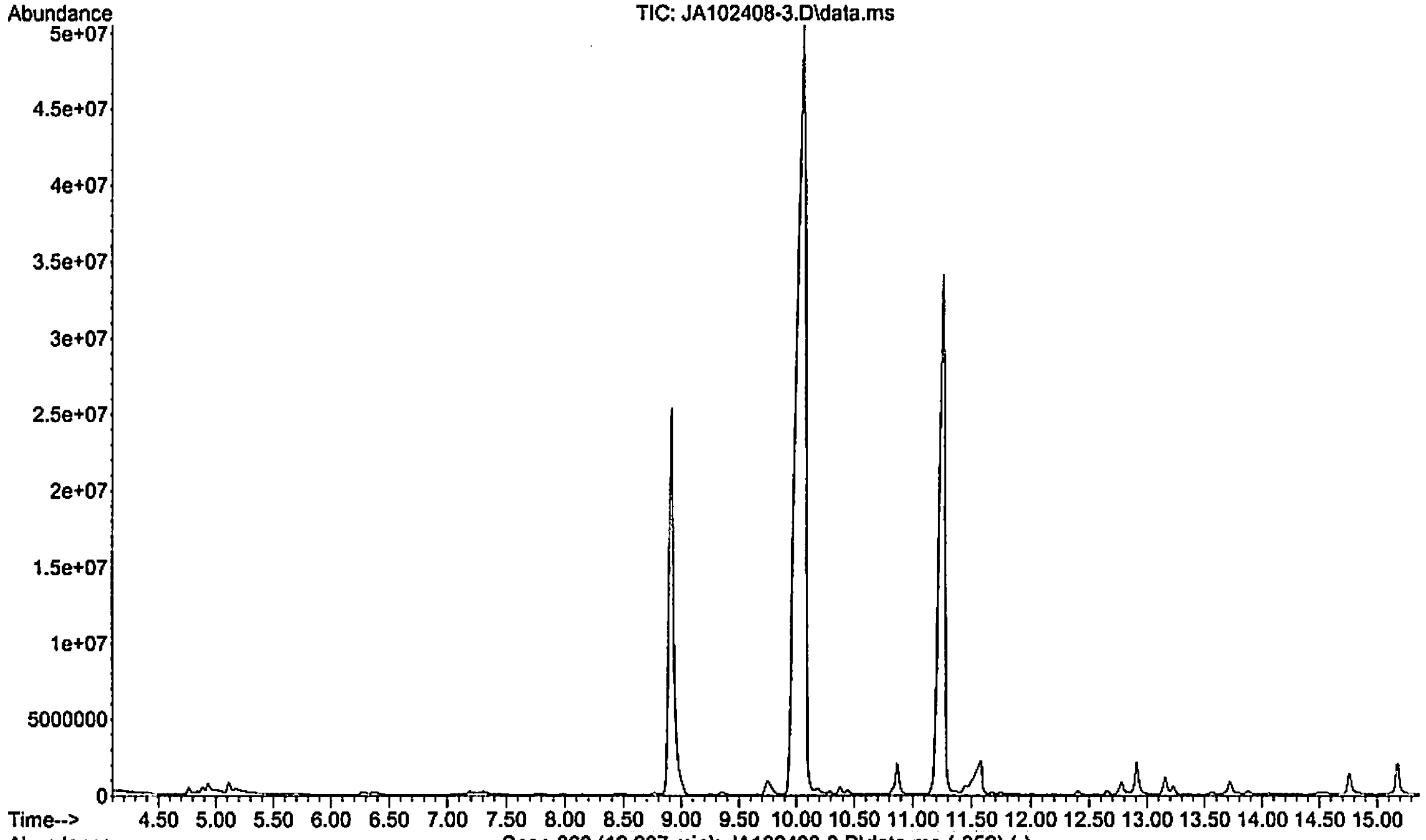


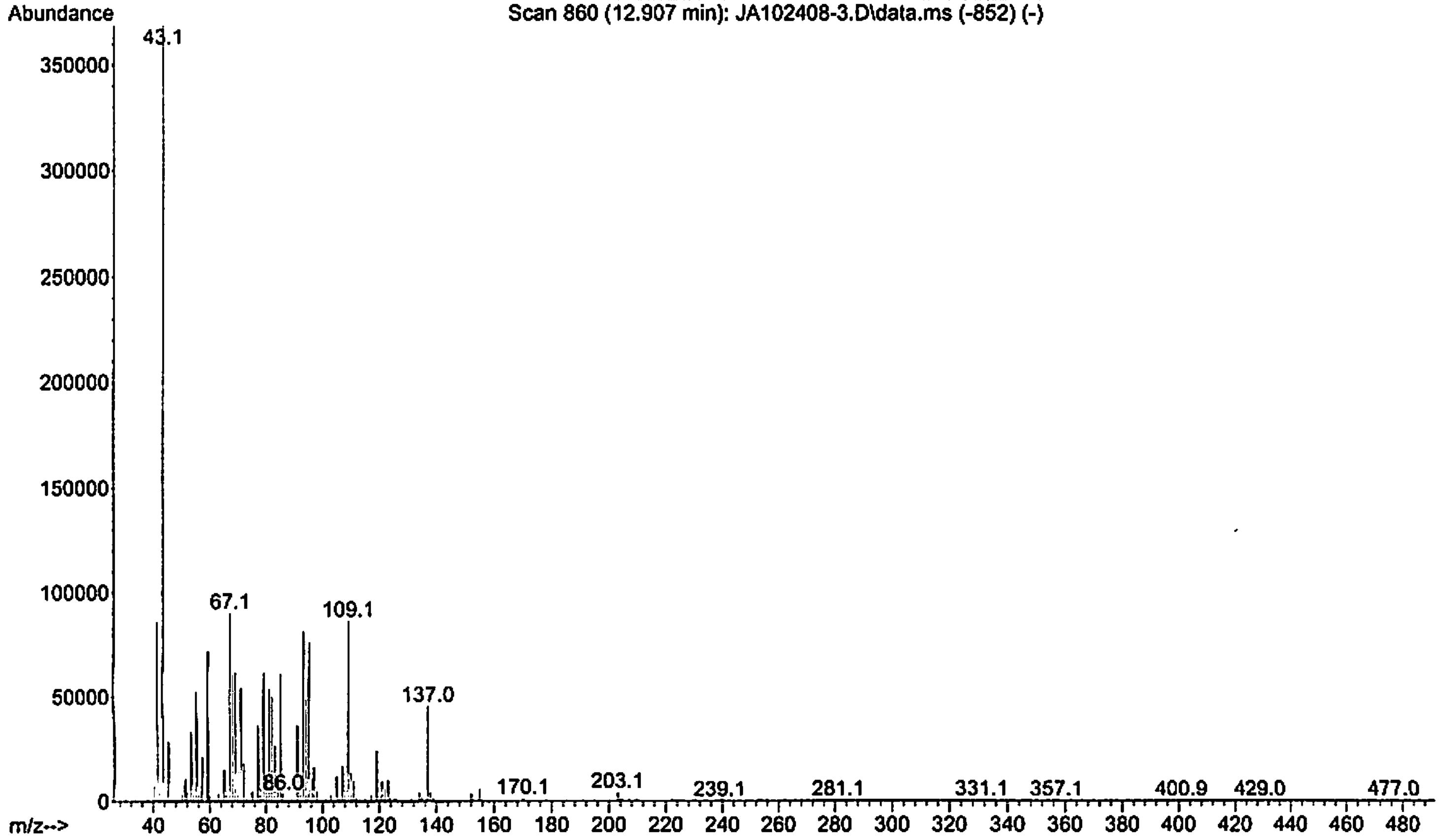

```
File        :D:\ALDRICH\Snapshot\JA102408-3.D
Operator    :
Acquired    : 24 Oct 2008  16:27     using AcqMethod JA-50-280LESS.M
Instrument  :    Buba
Sample Name: 5M C.oculata abd. cuticle/10ul CH2Cl2
Misc Info   : 7days w/geraniol; emerged 9/23; no GC
Vial Number: 1
```

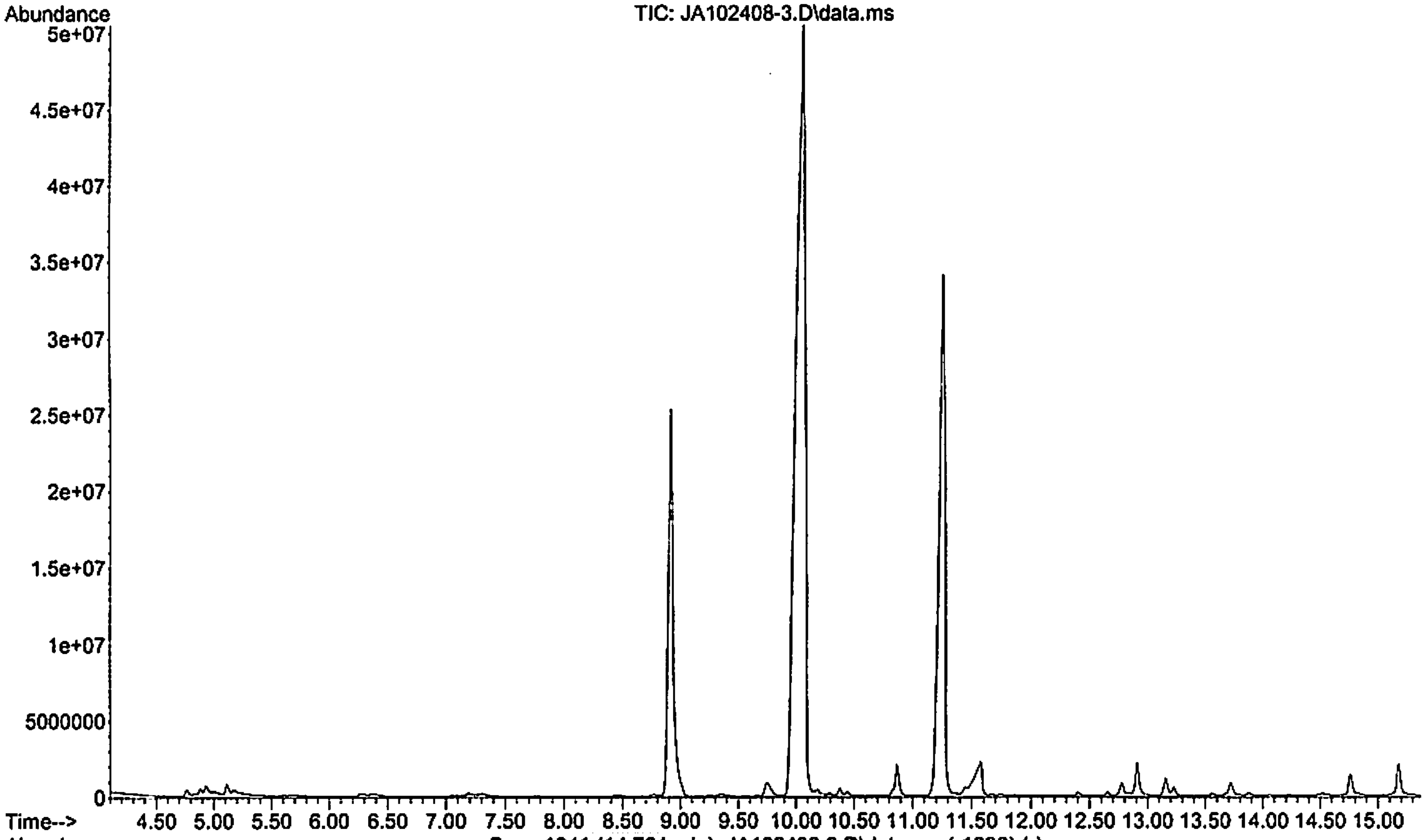


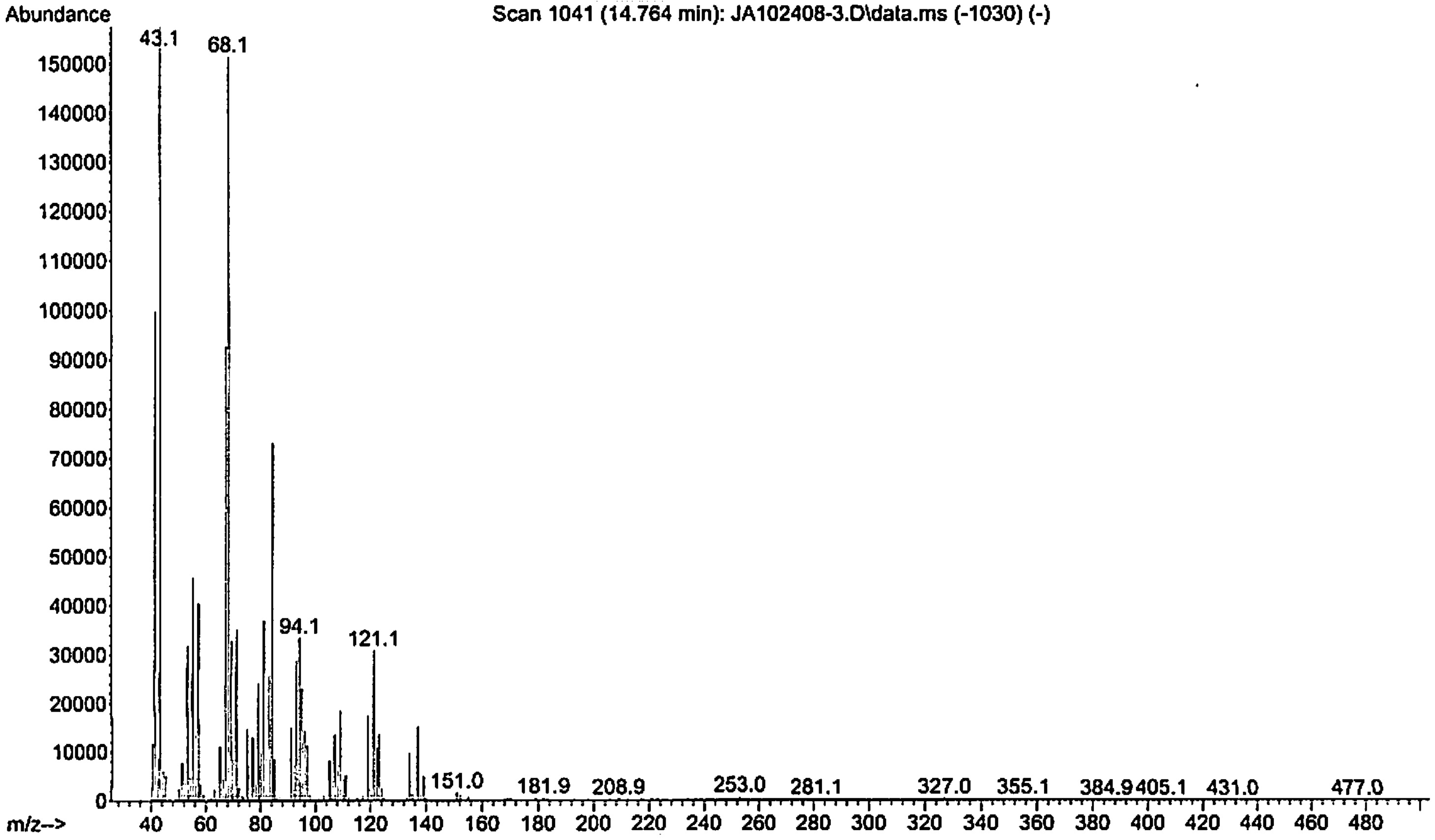

```
Library Searched : C:\Database\W8N05ST.L
Quality          : 83
ID               : trans,trans-2,6-Dimethyl-2,6-octadiene-1,8-diol $ (2E,6E)-2,6-Dimethyl
                   -2,6-octadiene-1,8-diol #
```

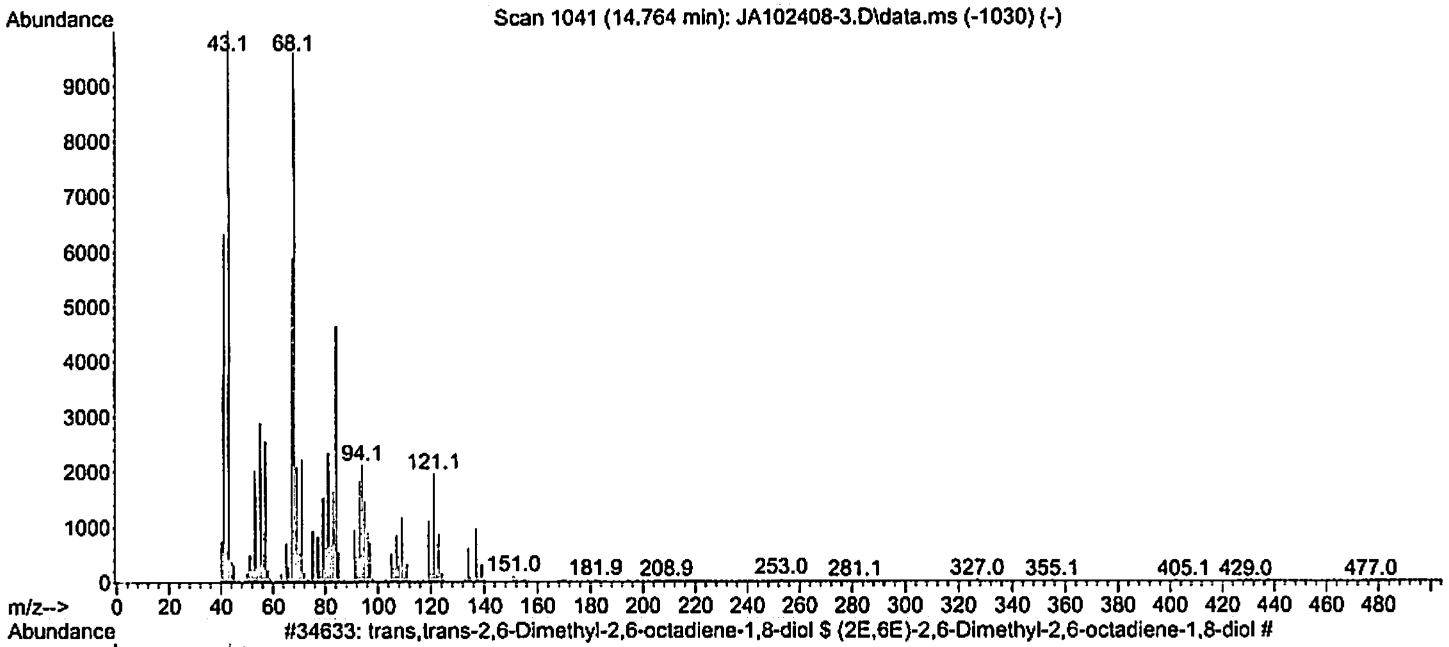


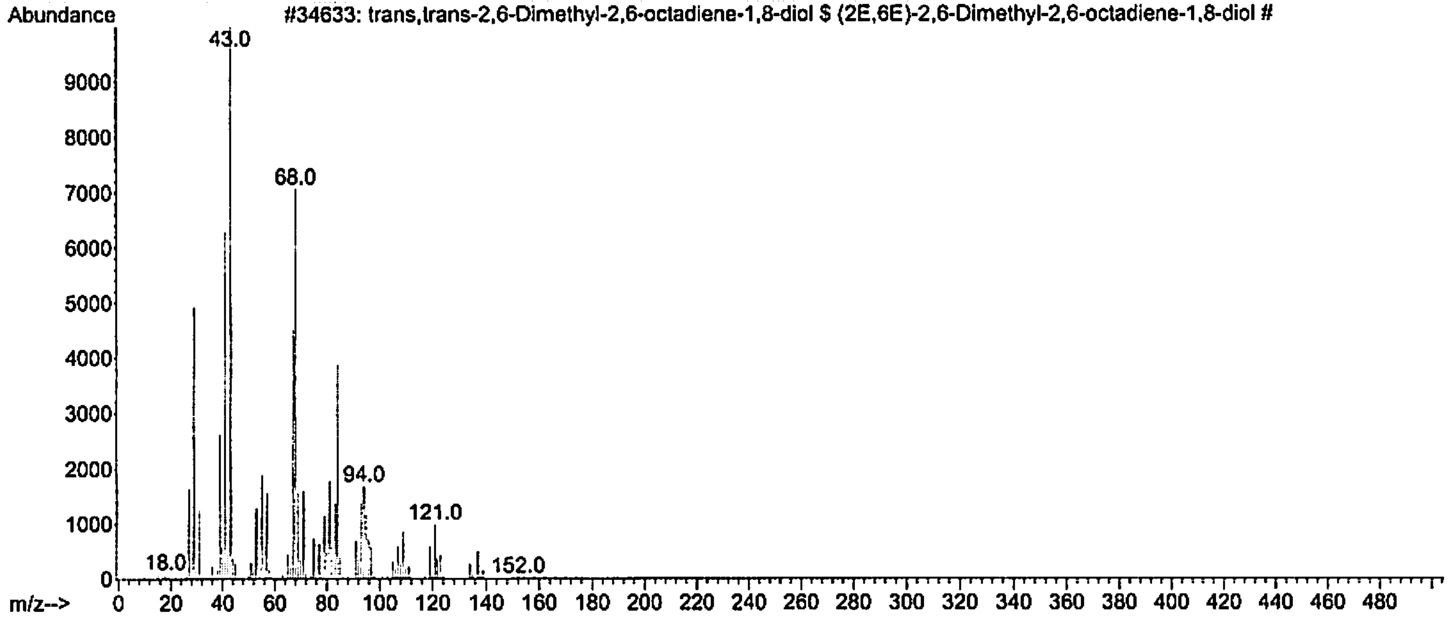


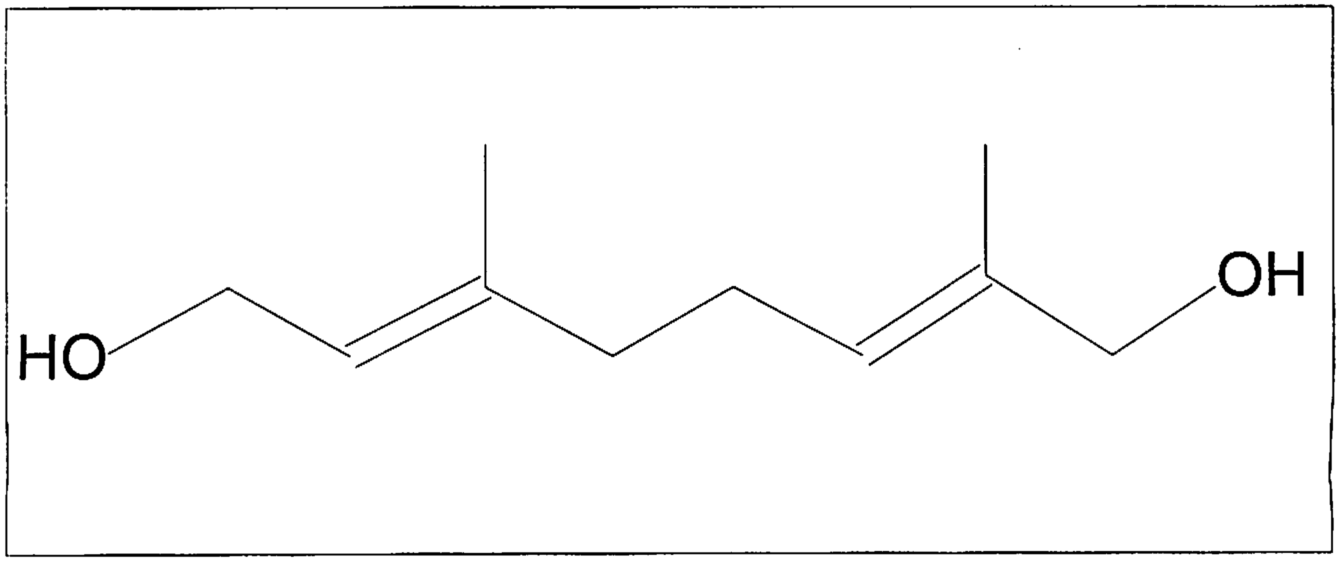

File        :D:\ALDRICH\Snapshot\JA102408-3.D
Operator    :
Acquired    : 24 Oct 2008  16:27     using AcqMethod JA-50-280LESS.M
Instrument  :    Buba
Sample Name: 5M C.oculata abd. cuticle/10ul CH2Cl2
Misc Info   : 7days w/geraniol; emerged 9/23; no GC
Vial Number: 1

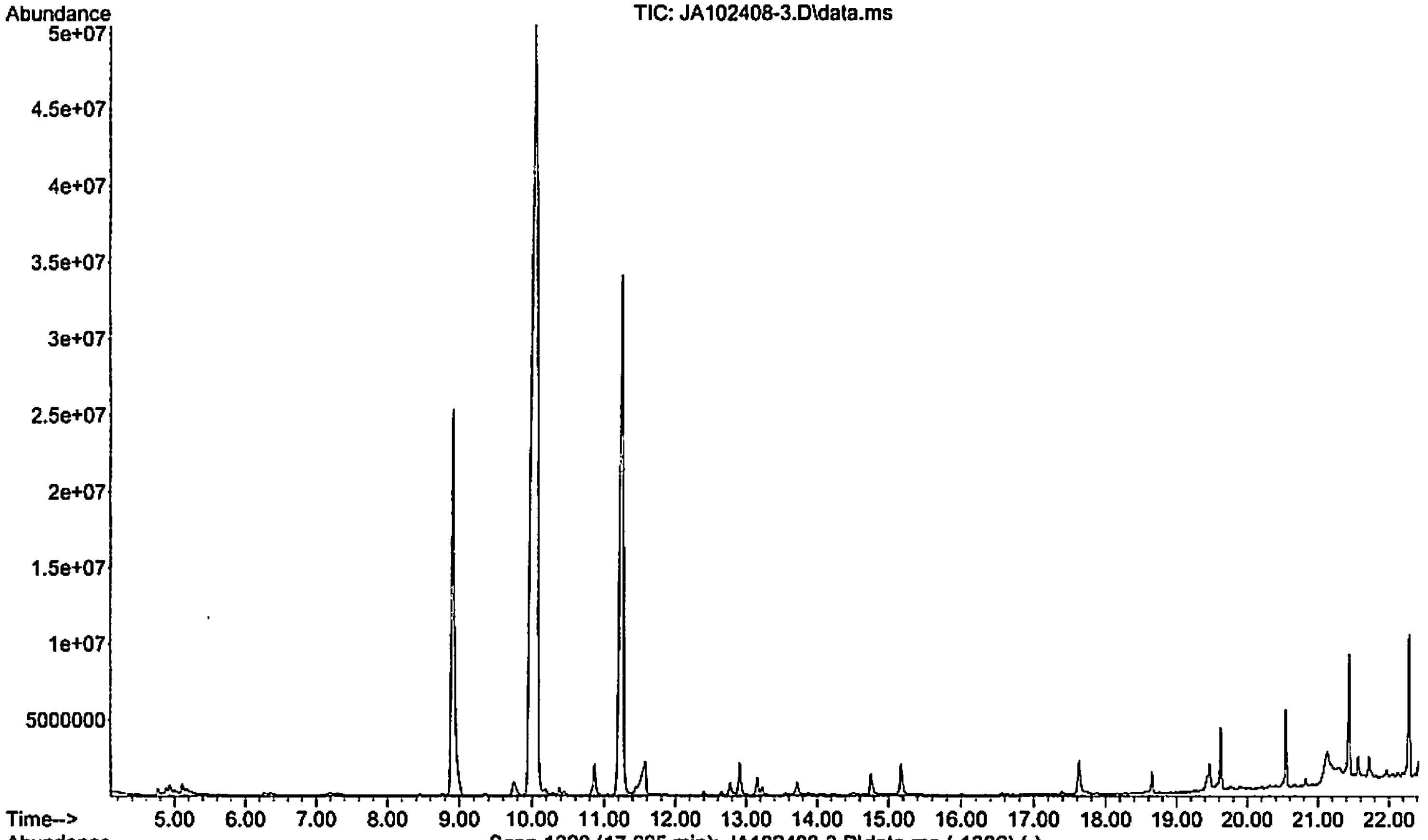


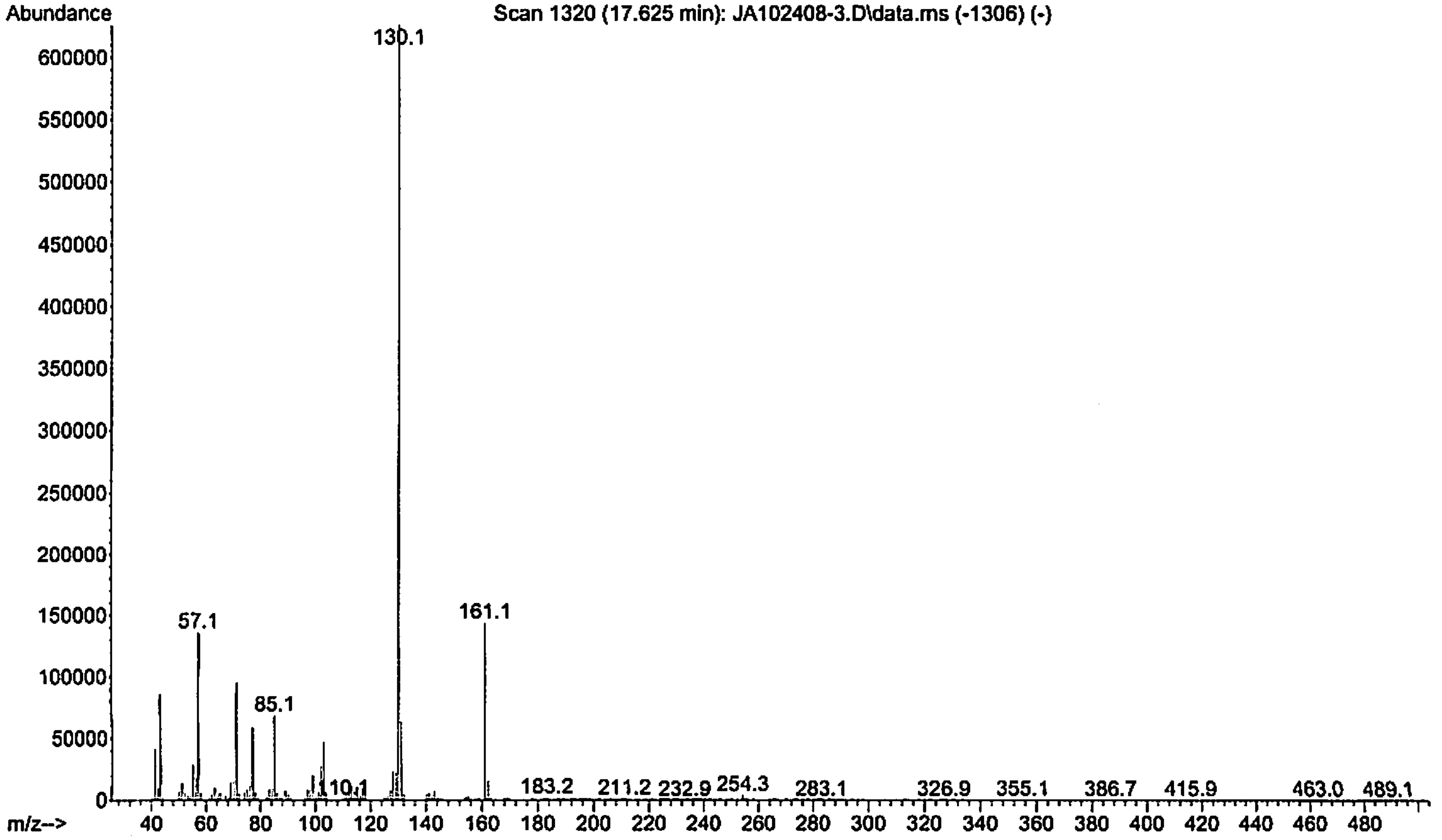

```
Library Searched : C:\Database\W8N05ST.L
Quality          : 87
ID               : 1H-Indole-3-ethanol $ Indole-3-ethanol $ .beta.-(3-Indole)ethanol $ Et
                   hanol, 2-indol-3-yl- $ Indoleethanol $ IEA $ Tryptophol $ 2-(3-Indolyl
                   ethanol $ 3-(.beta.-Hydroxyethyl)indole $ 3-(2-Hydroxyethyl)indole $ 3
                   -Indoleethanol $ dl-Tryptophanol $ Ethanol, 3
```

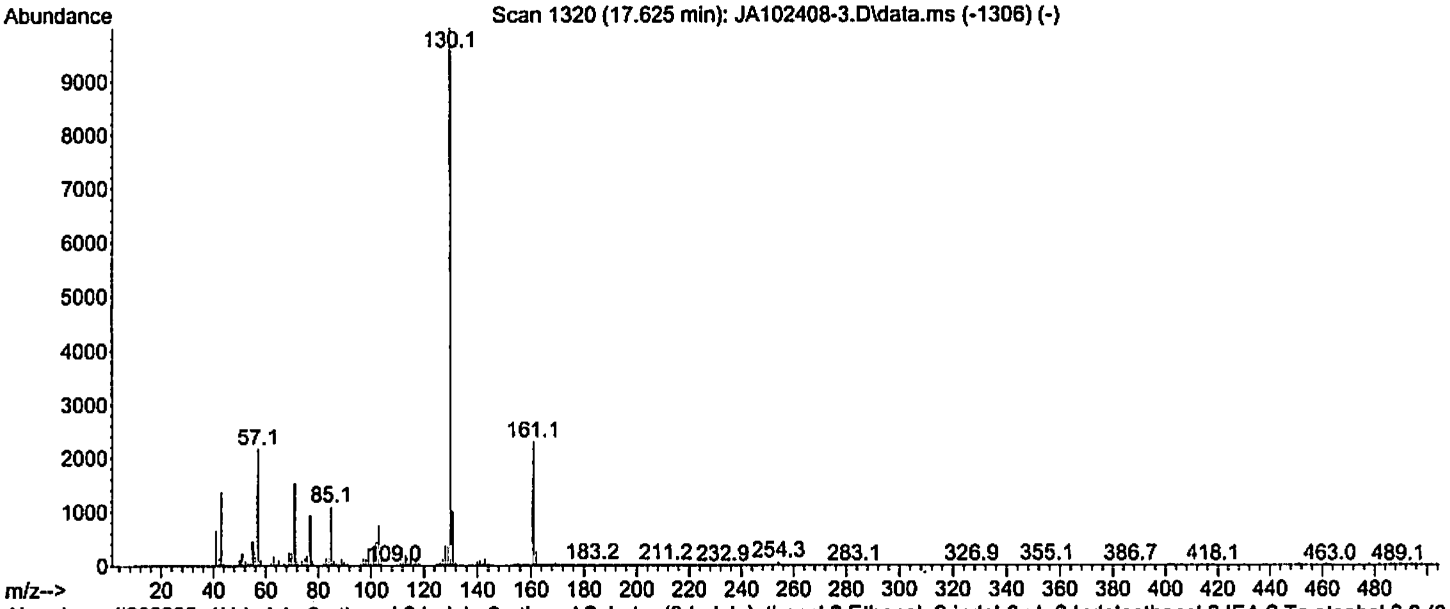


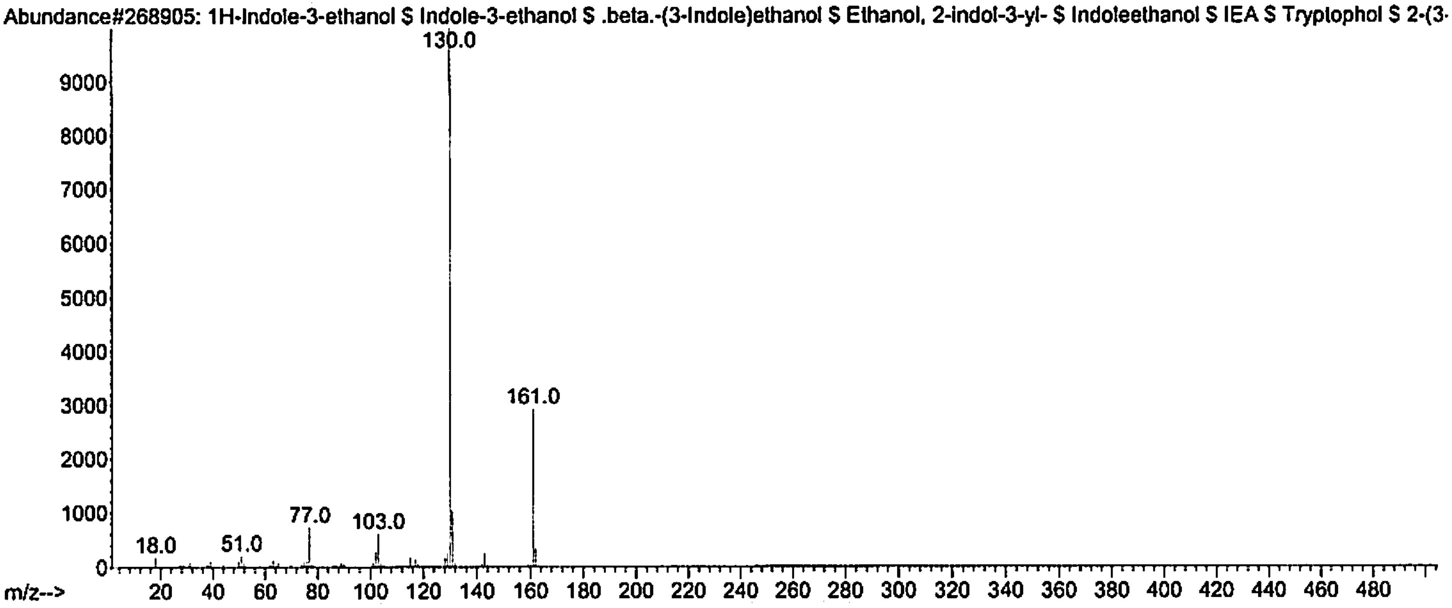


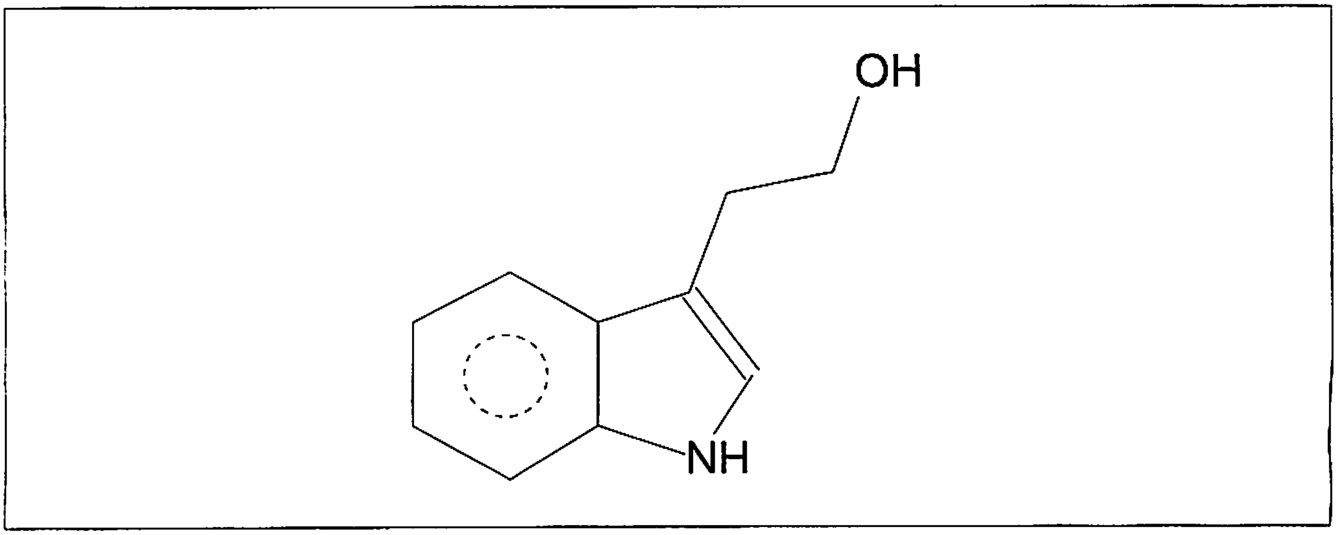

```
File        :D:\ALDRICH\Snapshot\JA102408-3.D
Operator    :
Acquired    : 24 Oct 2008  16:27     using AcqMethod JA-50-280LESS.M
Instrument :    Buba
Sample Name: 5M C.oculata abd. cuticle/10ul CH2Cl2
Misc Info   : 7days w/geraniol; emerged 9/23; no GC
Vial Number: 1
```

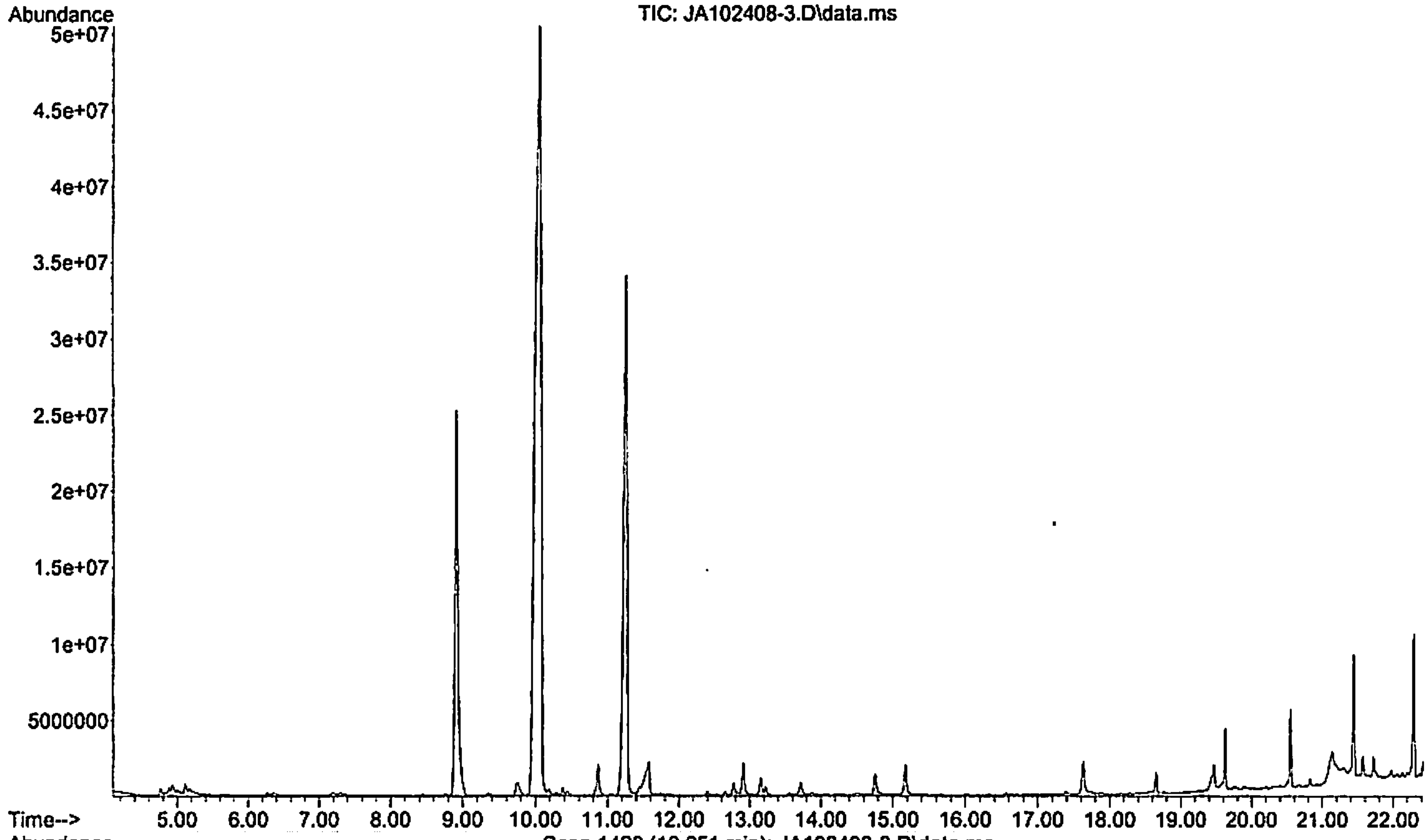


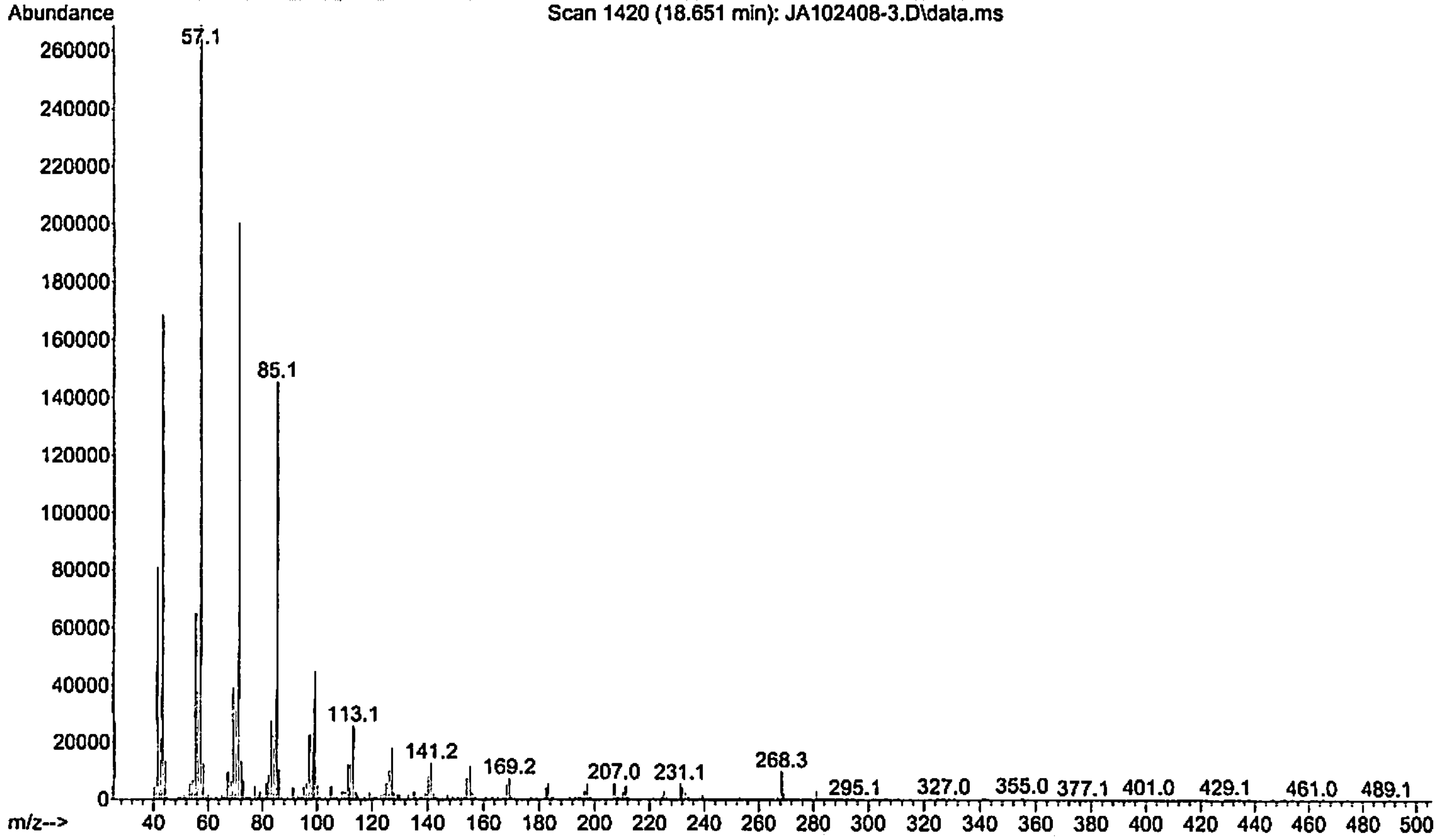

File        :D:\Aldrich\JA-09\JA112009-4.D
Operator    :
Acquired    : 20 Nov 2009  16:25     using AcqMethod JA-50-280LESS.M
Instrument  :    Buba
Sample Name: 15 M C.oculata abd.-fed 8-OH-citronellal
Misc Info   : 3-10d-old; fed 1wk; 100ul conc.to 5ulCH2Cl2
Vial Number: 1

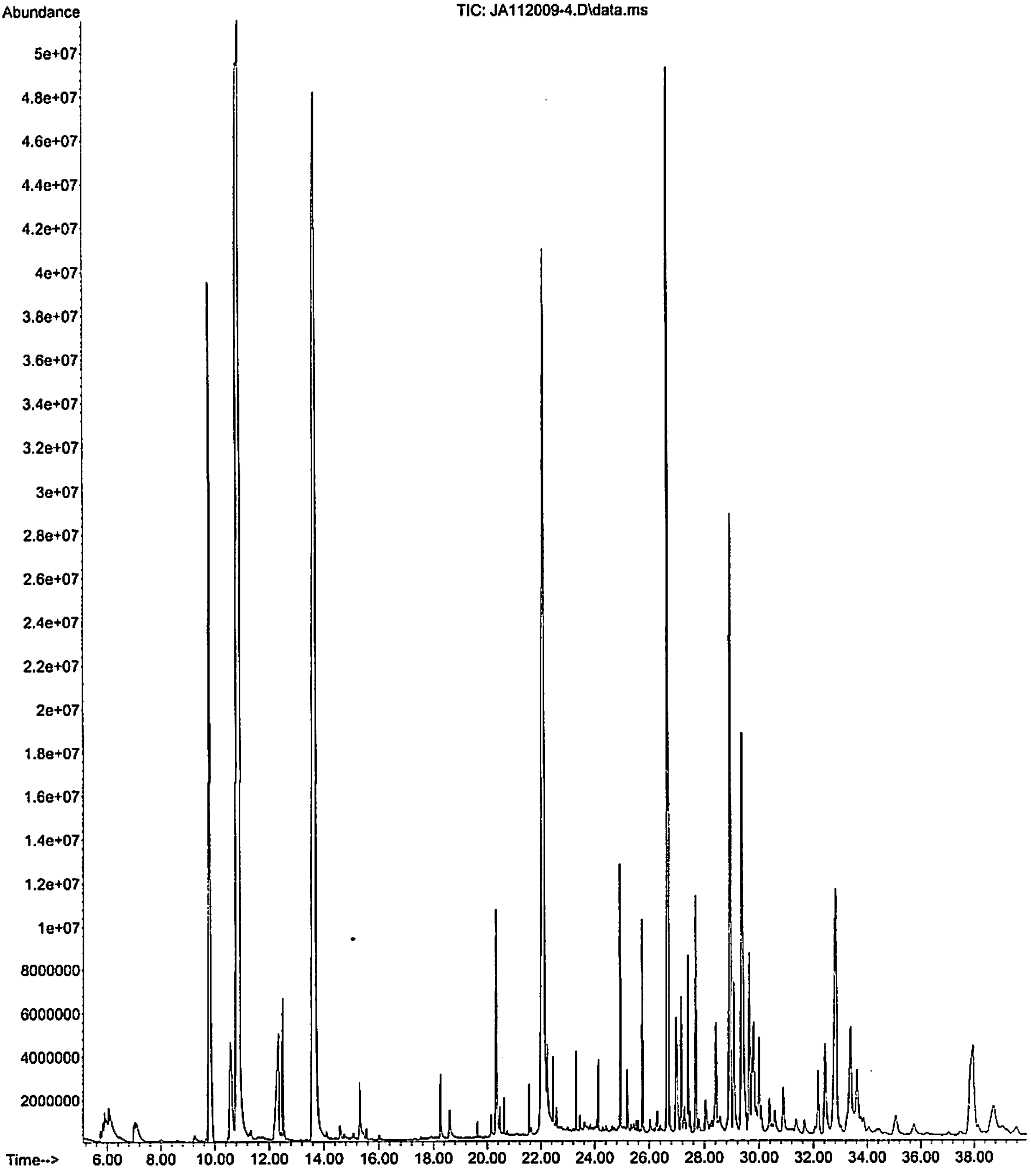

* C. oculata
Adult (♂♂). Male · Feeding ·
Expt. – SU · 11/13/09:
(15). ♂s

8-Hydroxy-citronellal
(aldehyde) Treatment.
(1). ♂ · NEA · 11/03–11/05 (M-71)
(3). ♂ · NEAs · 11/05–11/06 (M-72)
(6). ♂ · NEAs · 11/06–11/09 (M-73
& M-74)
11/19: All (15). res. ♂s · alive.
Fed · live · pea · aphids ·

```
File        :D:\ALDRICH\JA-09\Snapshot\JA112009-4.D
Operator    :
Acquired    : 20 Nov 2009  16:25     using AcqMethod JA-50-280LESS.M
Instrument  :    Buba
Sample Name: 15 M C.oculata abd.-fed 8-OH-citronellal
Misc Info   : 3-10d-old; fed 1wk; 100ul conc.to 5ulCH2Cl2
Vial Number: 1
```

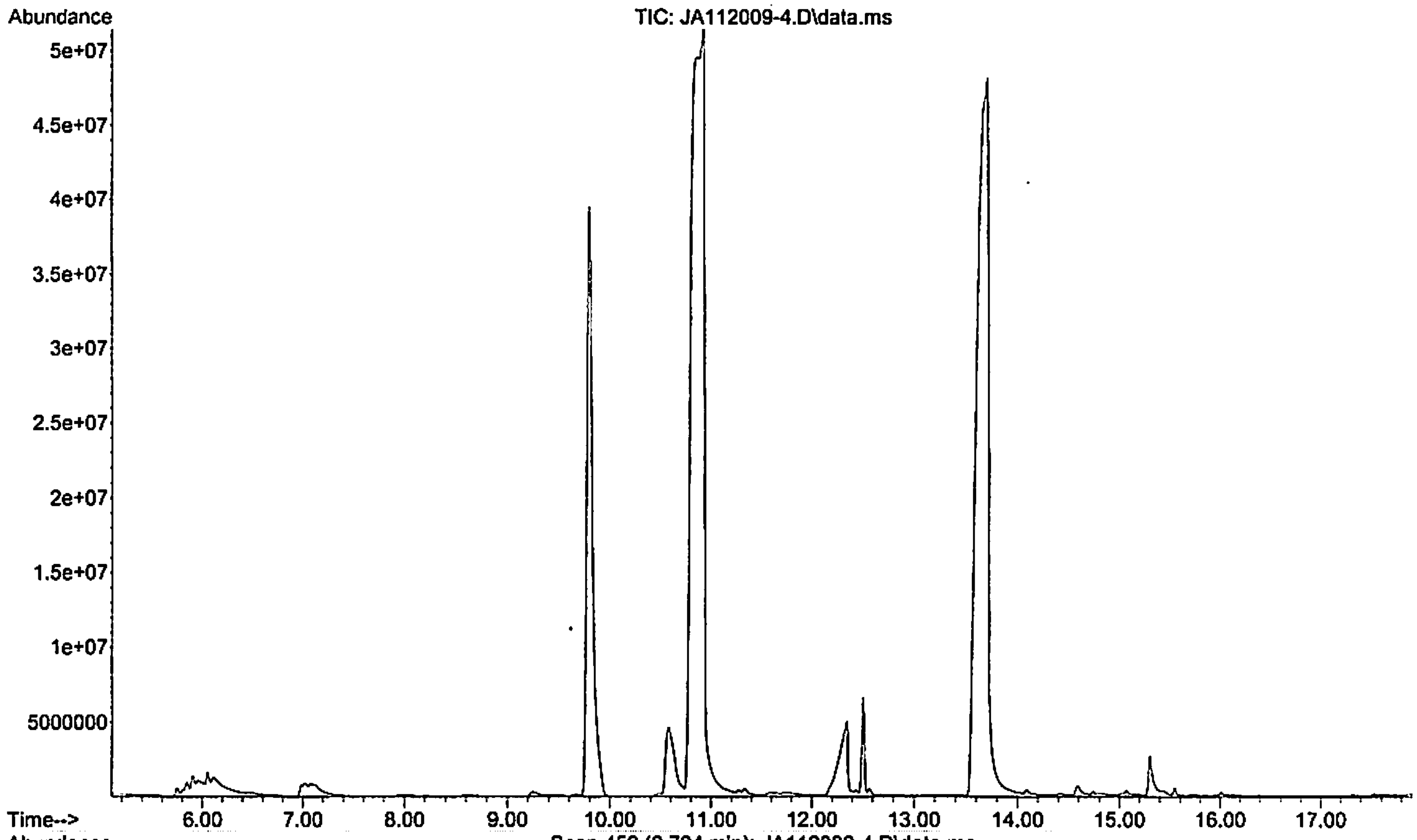


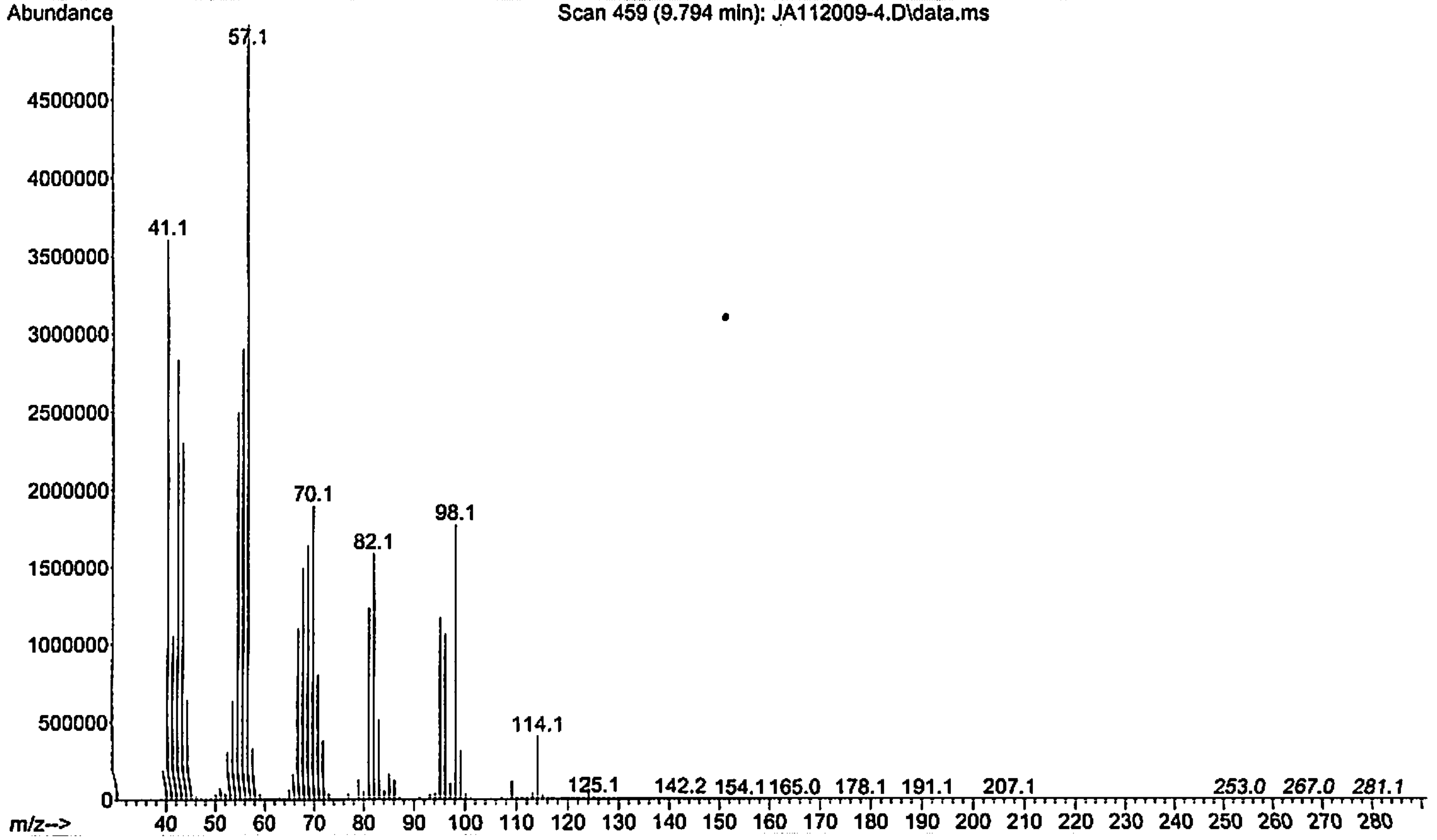

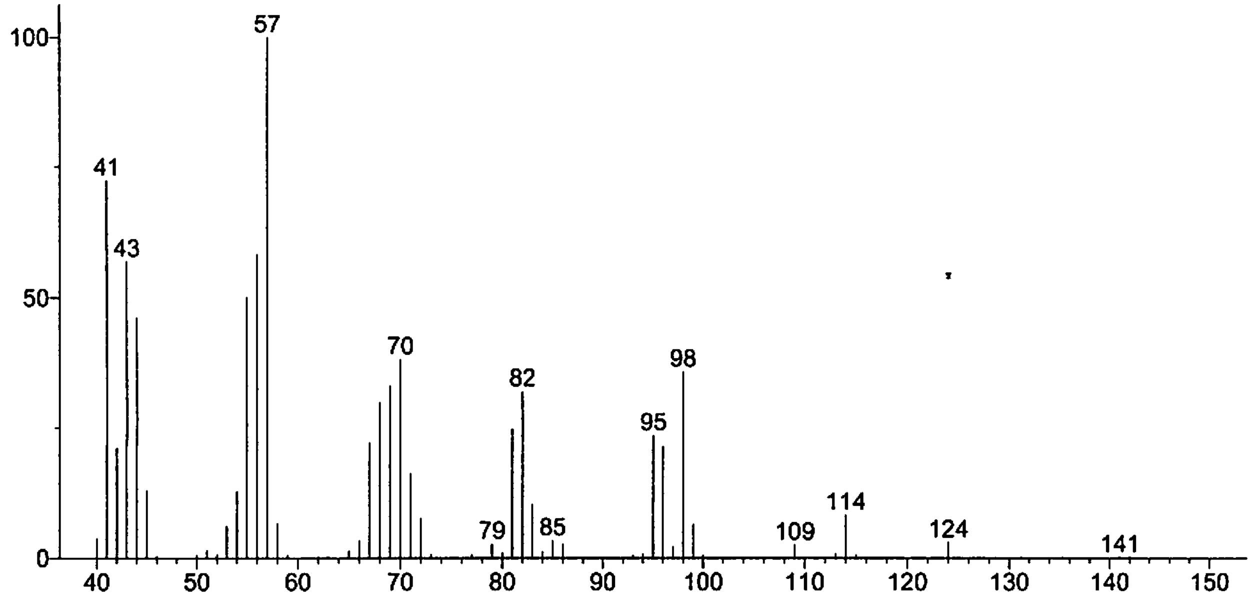

(Text File) Scan 459 (9.794 min): JA112009-4.D\data.ms

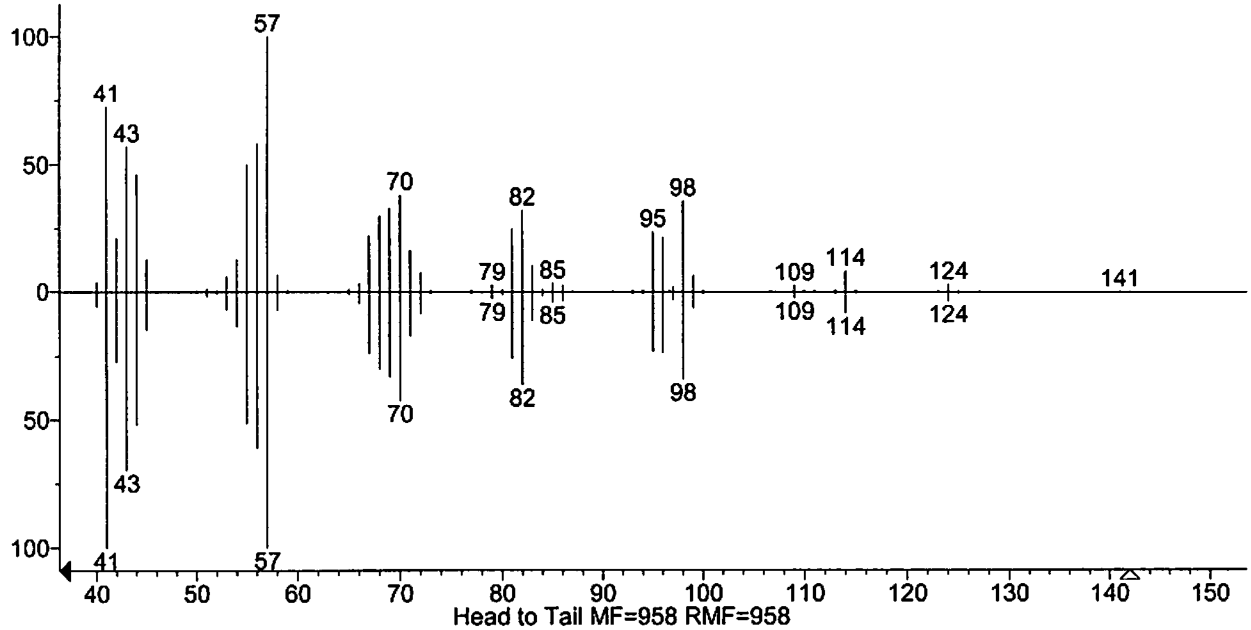

Head to Tail MF=958 RMF=958

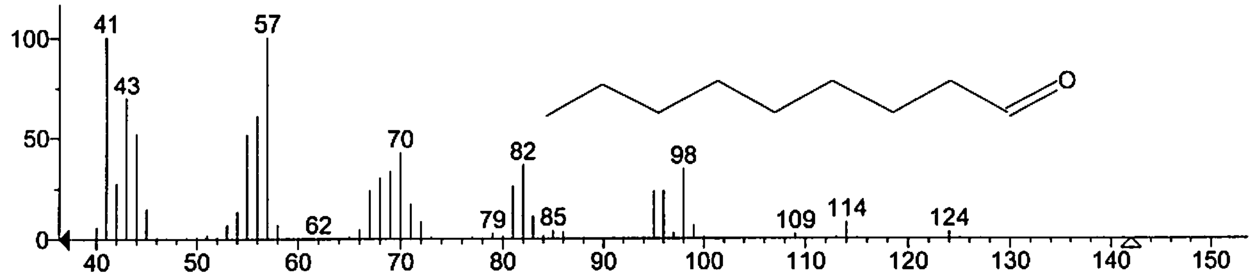

(replib) Nonanal

```
File        :D:\ALDRICH\JA-09\Snapshot\JA112009-4.D
Operator    :
Acquired    : 20 Nov 2009  16:25     using AcqMethod JA-50-280LESS.M
Instrument :    Buba
Sample Name: 15 M C.oculata abd.-fed 8-OH-citronellal
Misc Info   : 3-10d-old; fed 1wk; 100ul conc.to 5ulCH2Cl2
Vial Number: 1
```

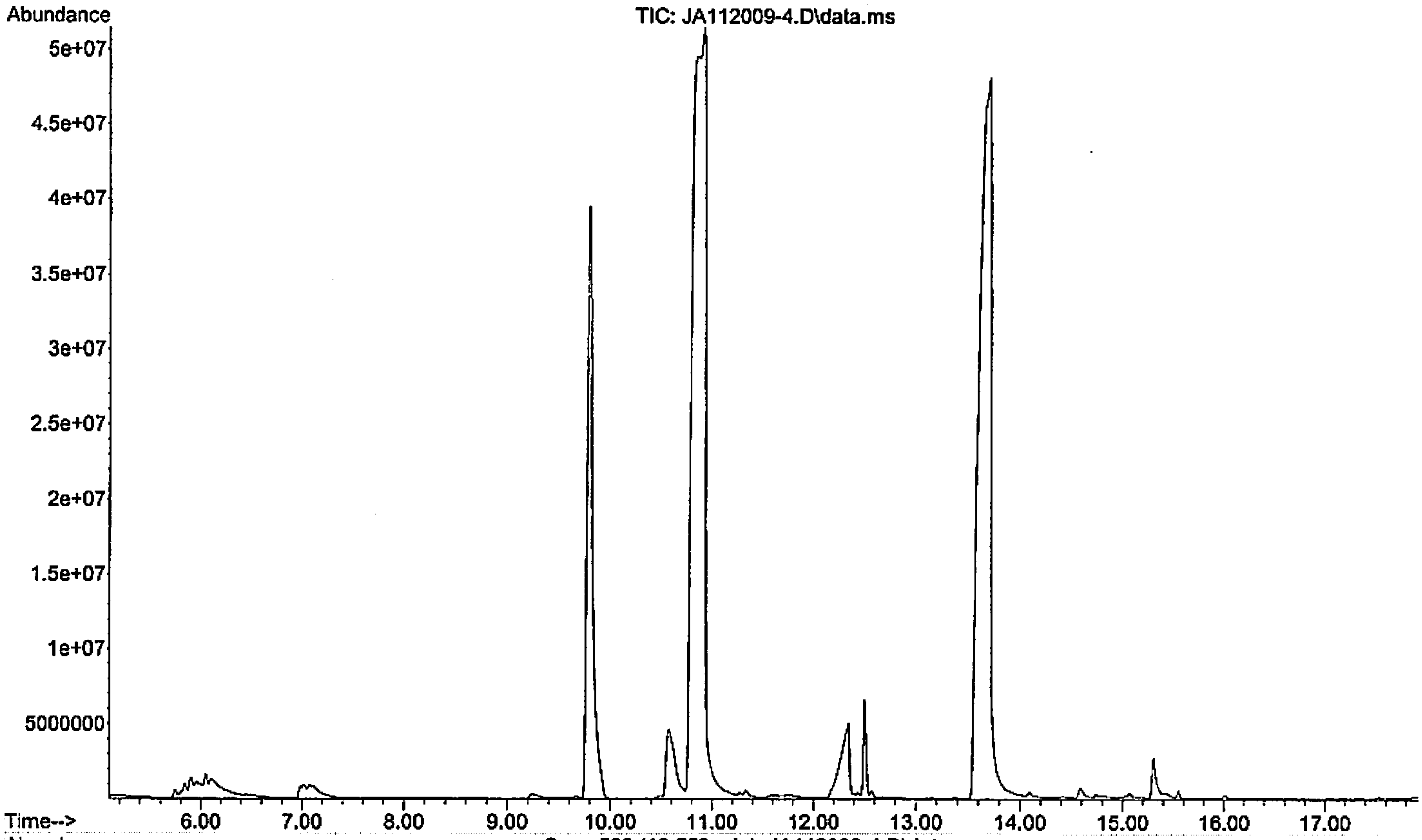


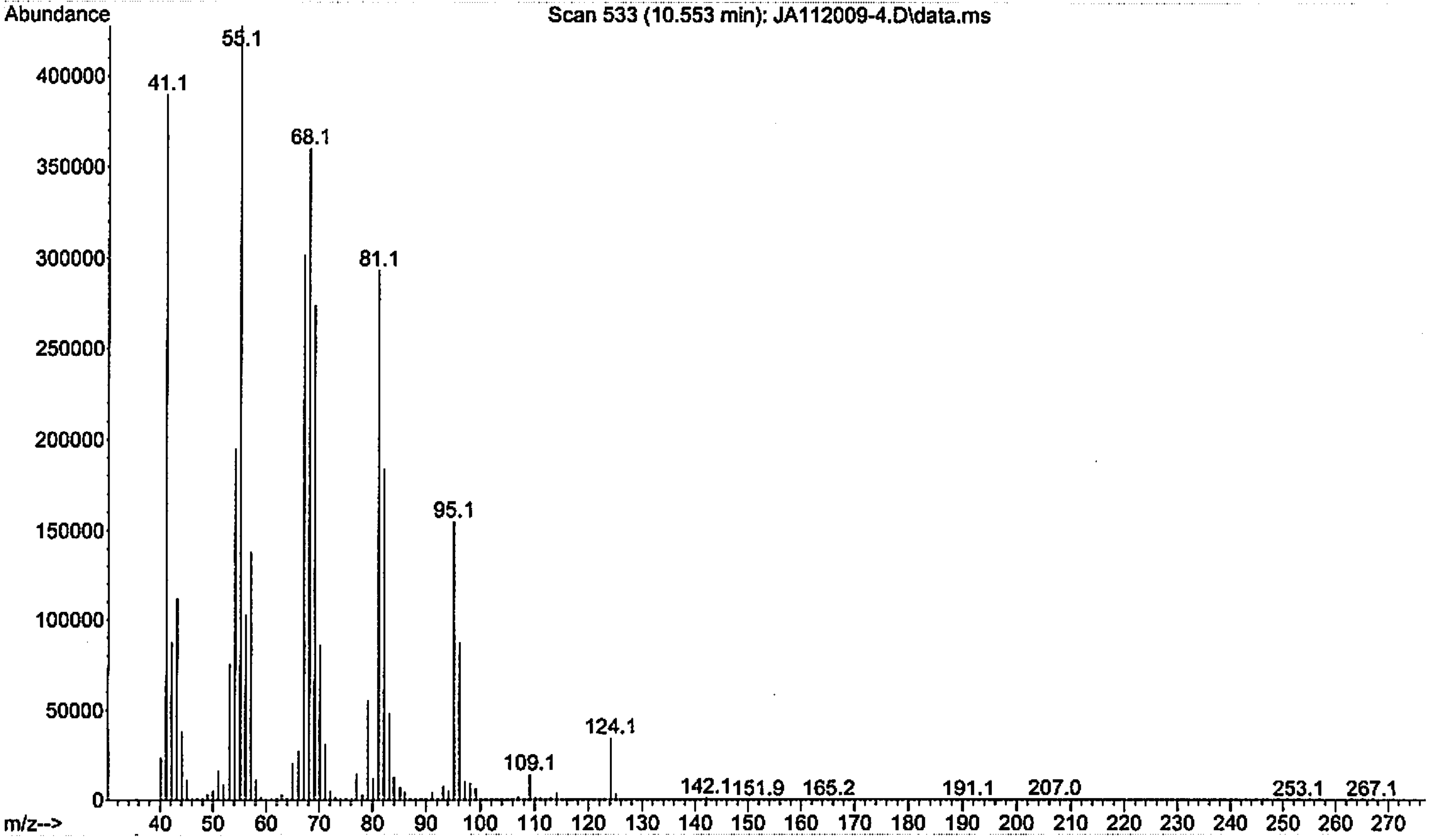

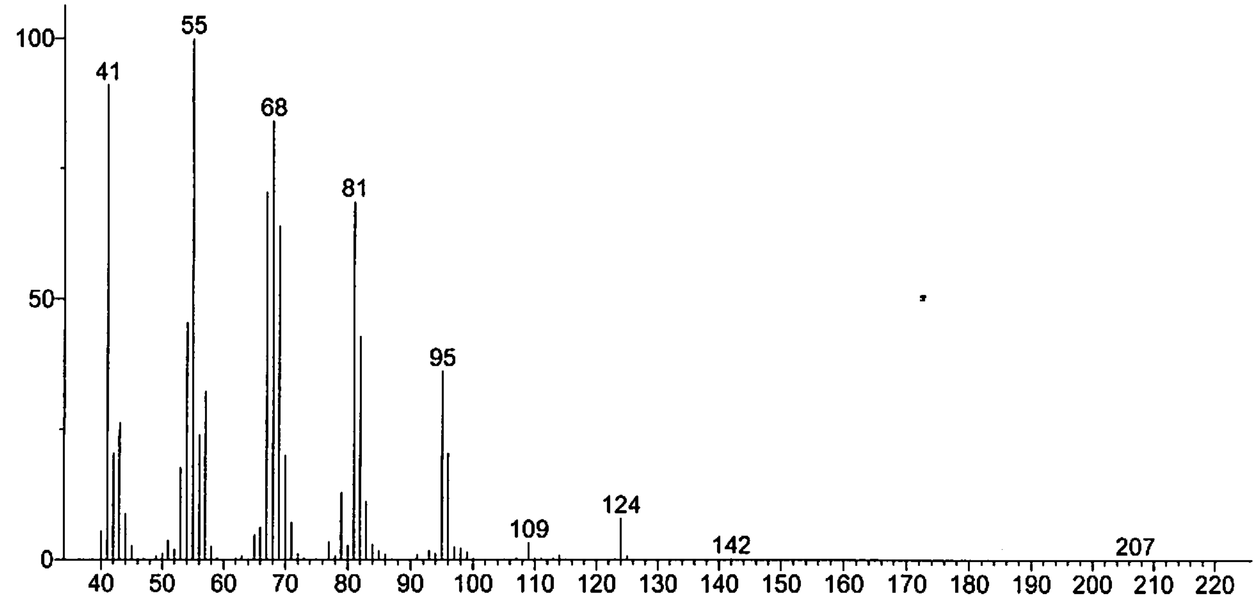


(Text File) Scan 533 (10.553 min): JA112009-4.D\data.ms

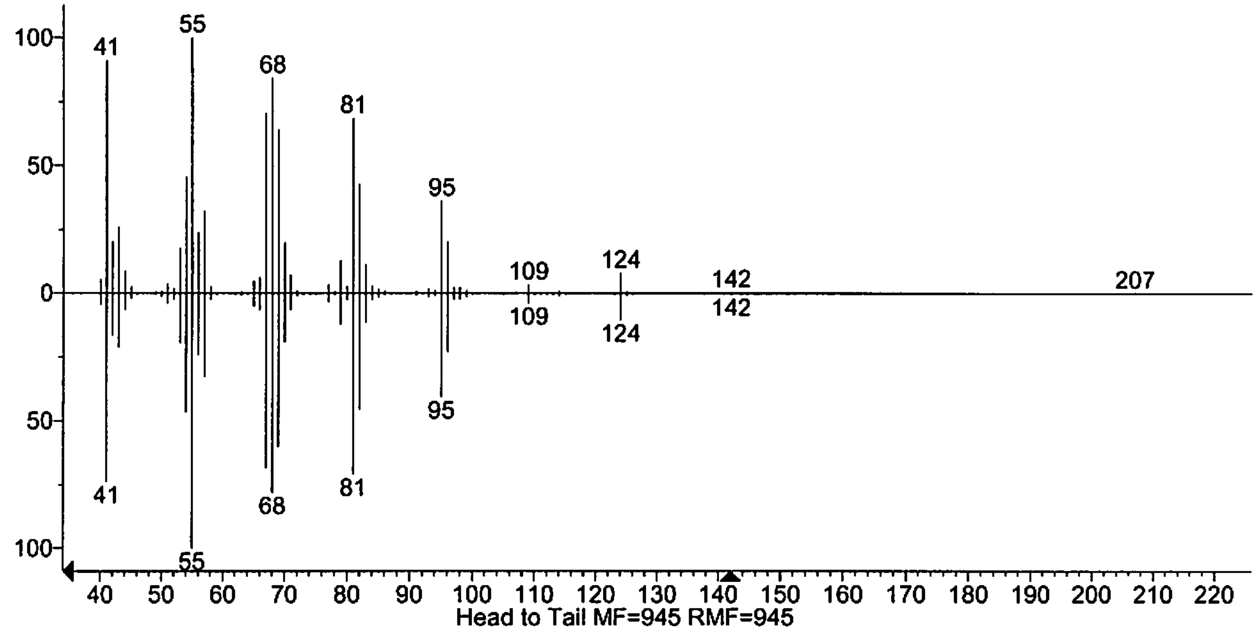


Head to Tail MF=945 RMF=945

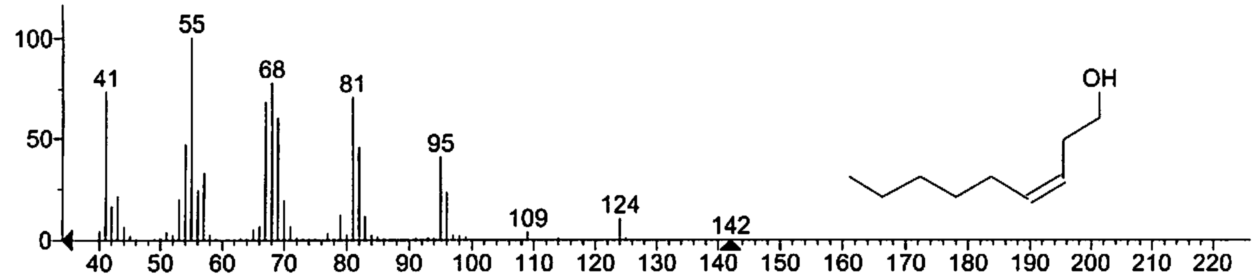


(mainlib) 3-Nonen-1-ol, (Z)-

```
File       :D:\ALDRICH\JA-09\Snapshot\JA112009-4.D
Operator   :
Acquired   : 20 Nov 2009  16:25     using AcqMethod JA-50-280LESS.M
Instrument :    Buba
Sample Name: 15 M C.oculata abd.-fed 8-OH-citronellal
Misc Info  : 3-10d-old; fed 1wk; 100ul conc.to 5ulCH2Cl2
Vial Number: 1
```

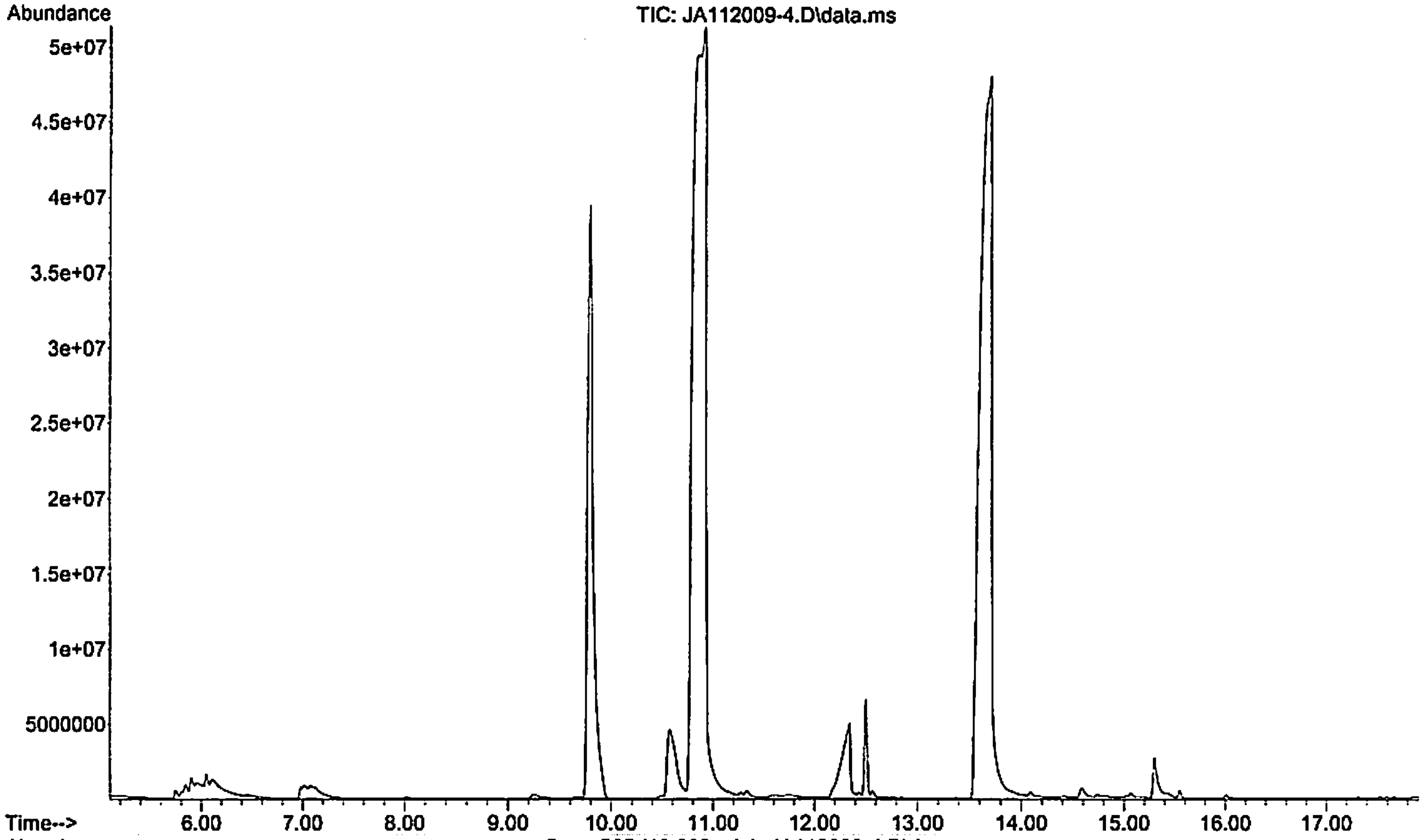


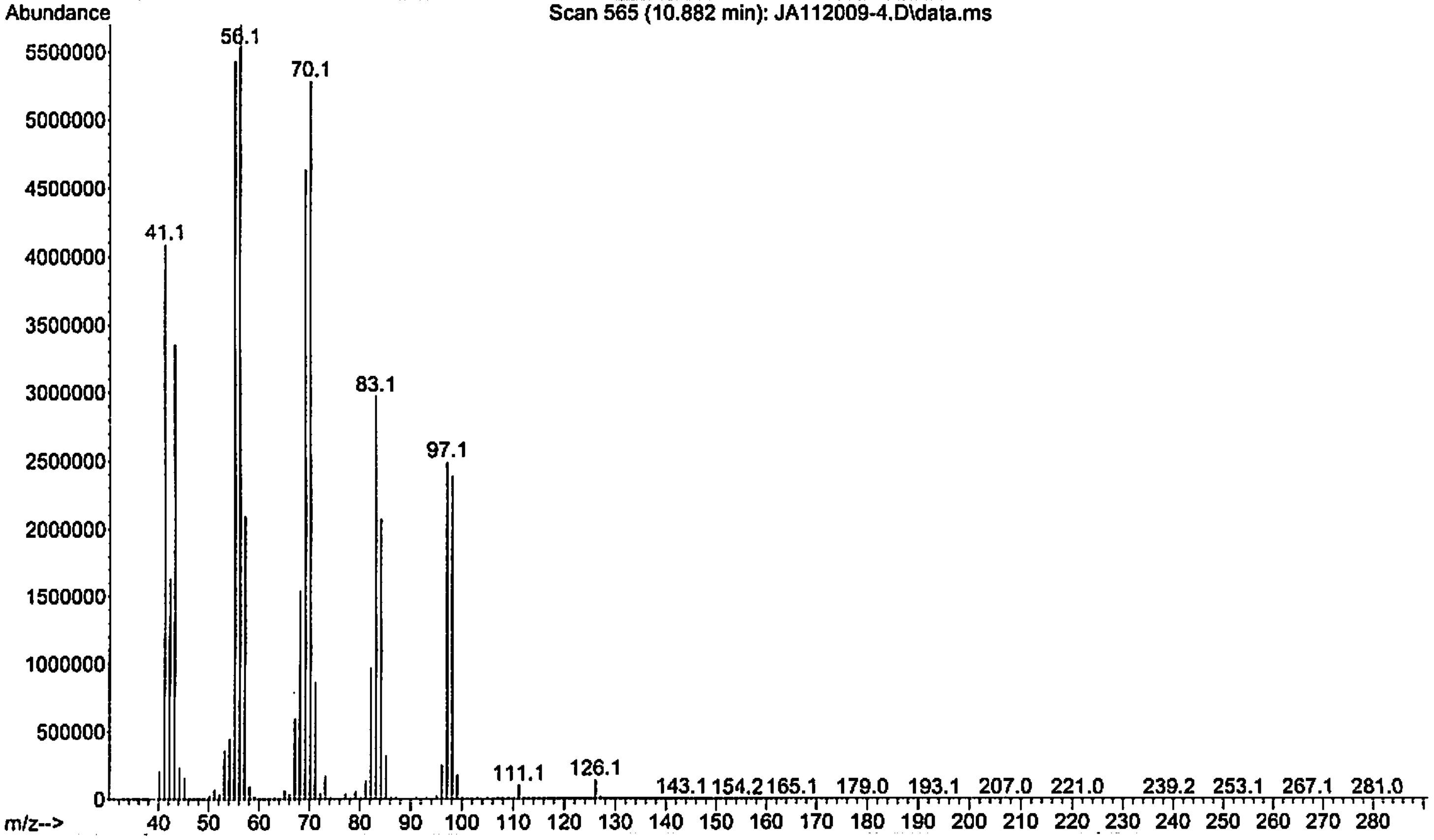

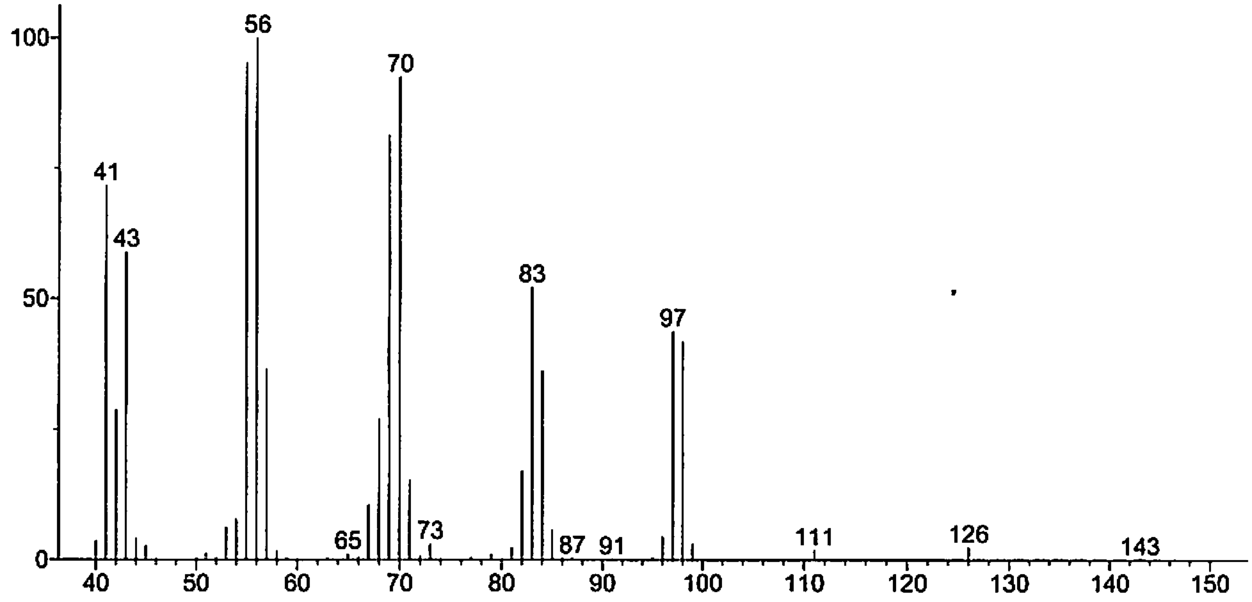


(Text File) Scan 565 (10.882 min): JA112009-4.D\data.ms

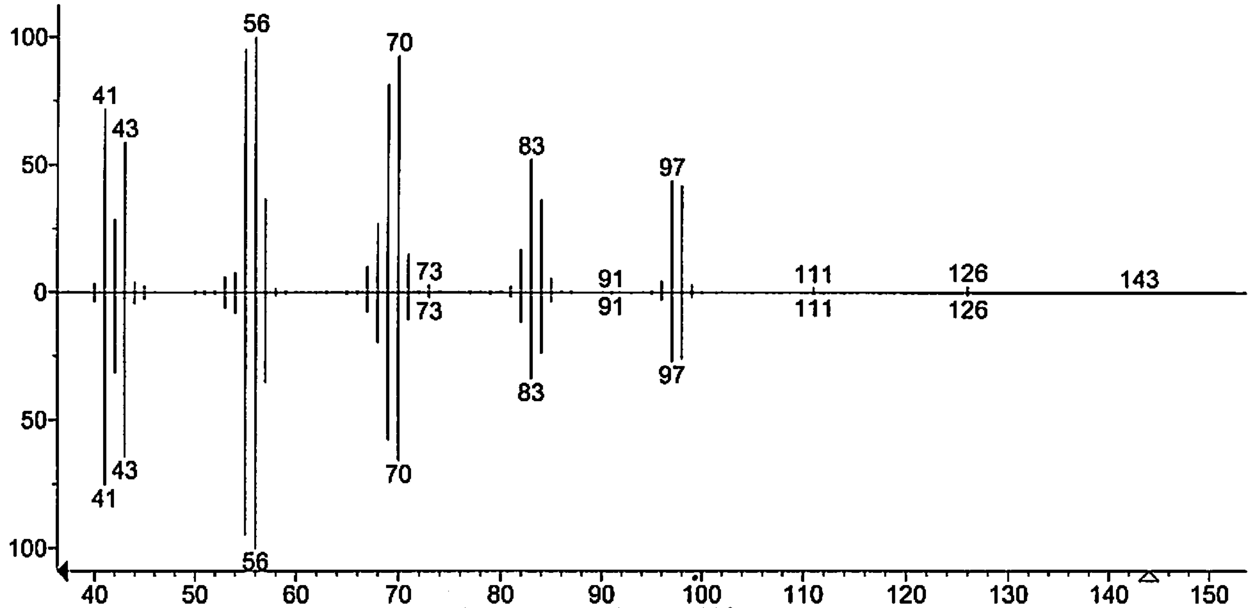

Head to Tail MF=967 RMF=967

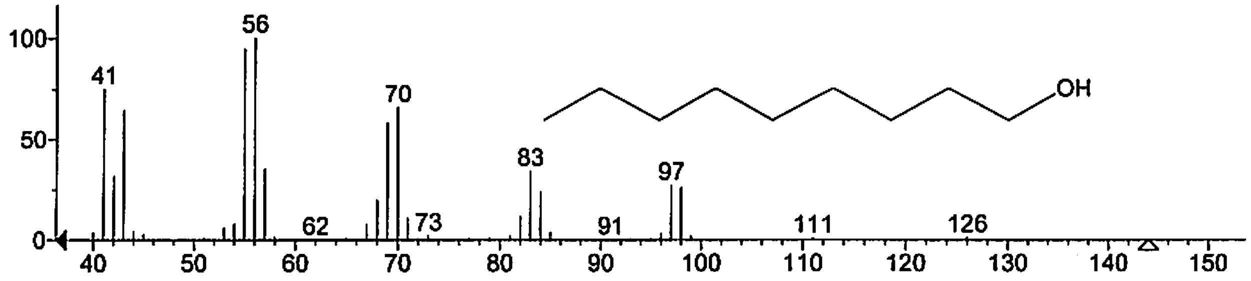


(replib) 1-Nonanol

```
File        :D:\ALDRICH\JA-09\Snapshot\JA112009-4.D
Operator    :
Acquired    : 20 Nov 2009  16:25     using AcqMethod JA-50-280LESS.M
Instrument  :    Buba
Sample Name: 15 M C.oculata abd.-fed 8-OH-citronellal
Misc Info   : 3-10d-old; fed 1wk; 100ul conc.to 5ulCH2Cl2
Vial Number: 1
```

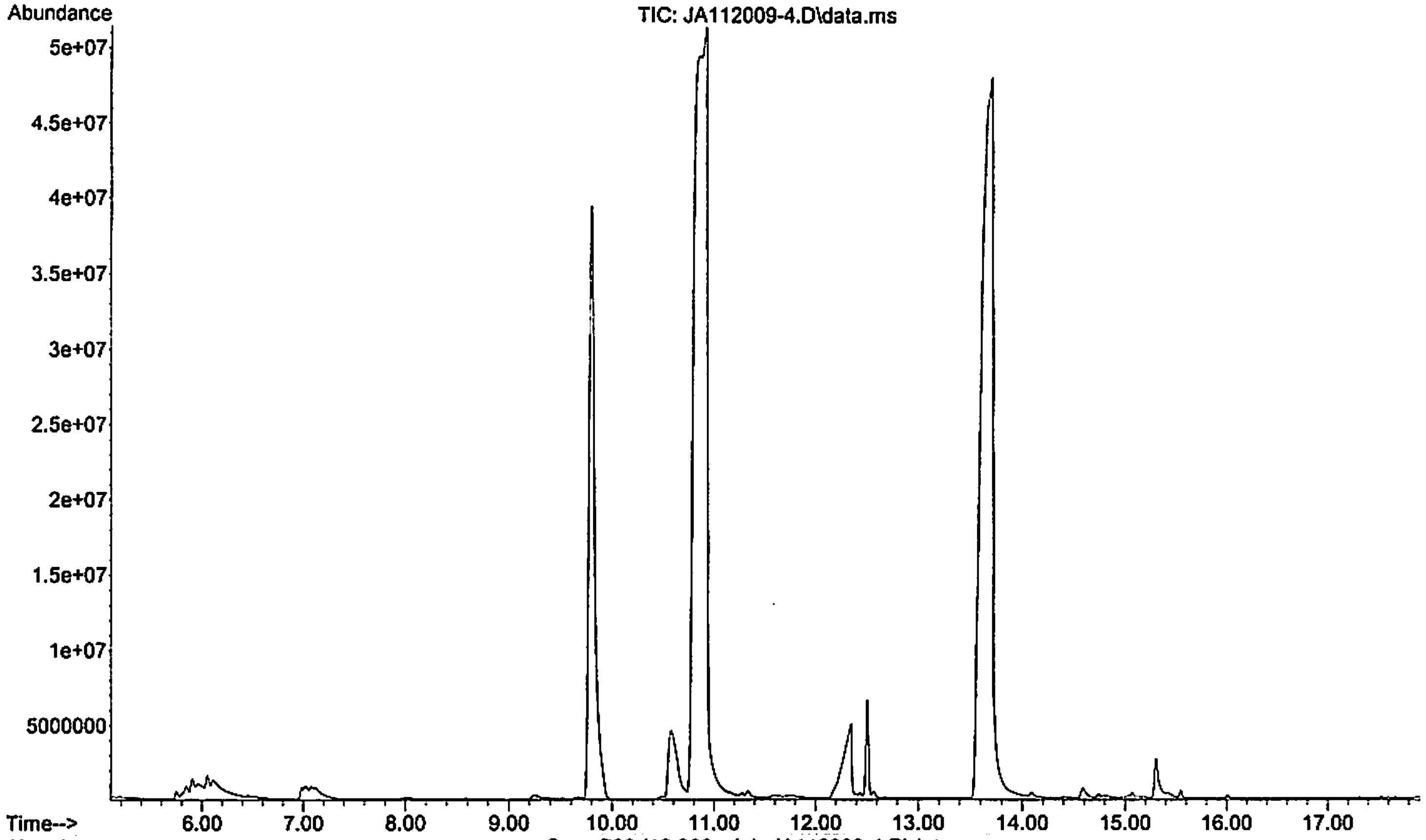


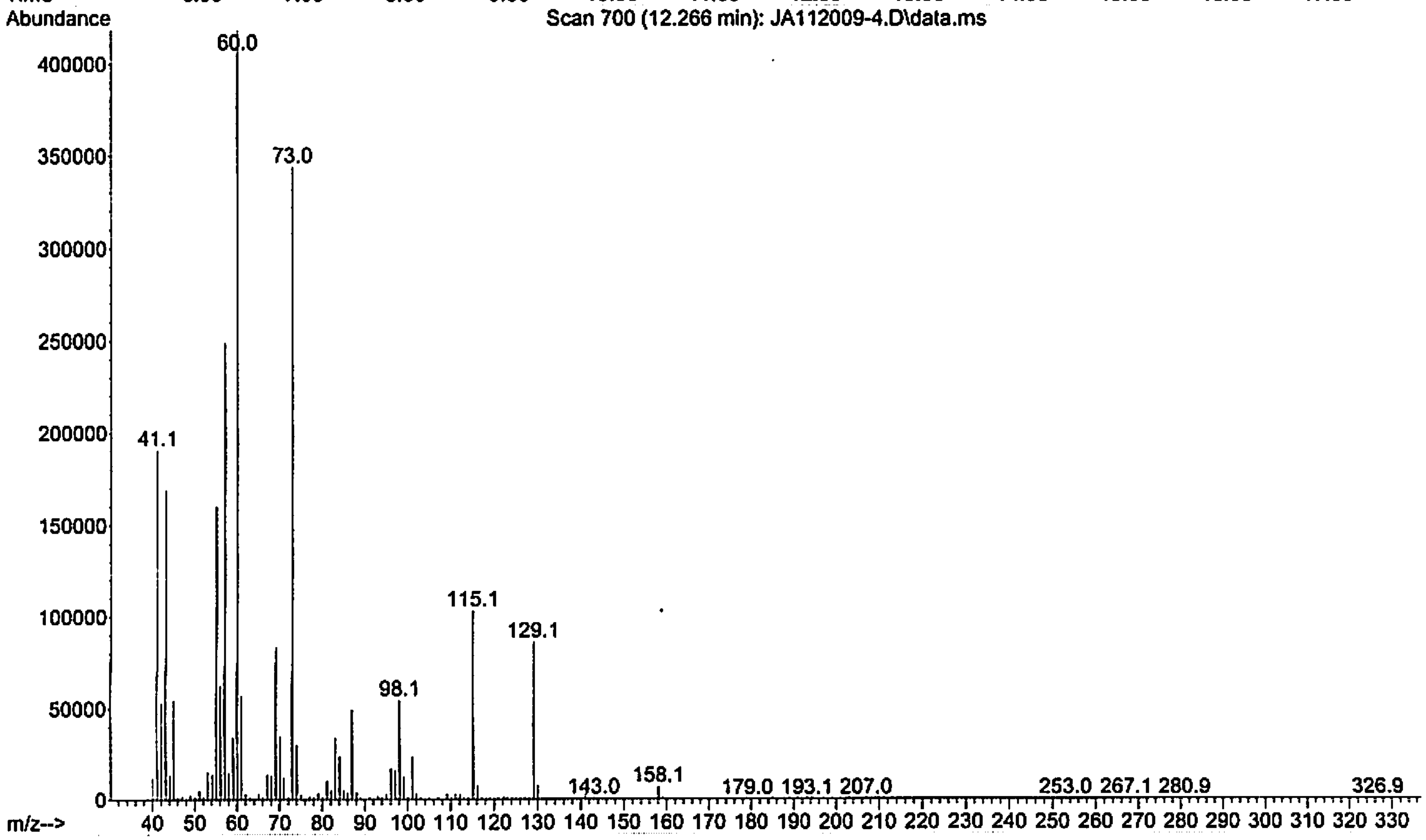

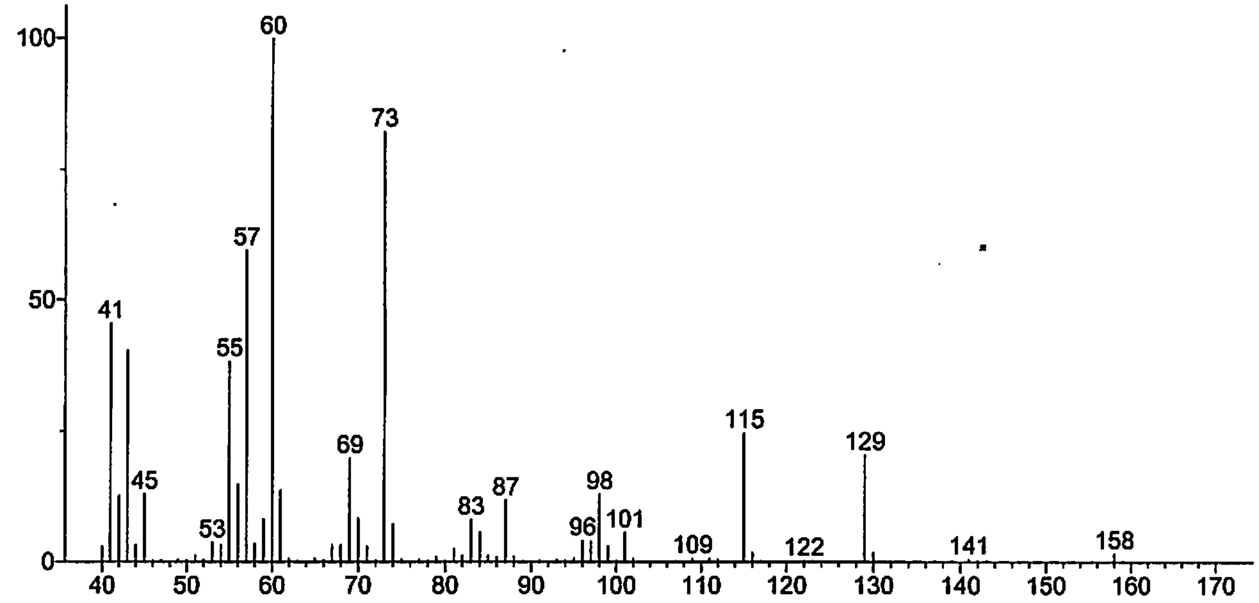


(Text File) Scan 700 (12.266 min): JA112009-4.D\data.ms

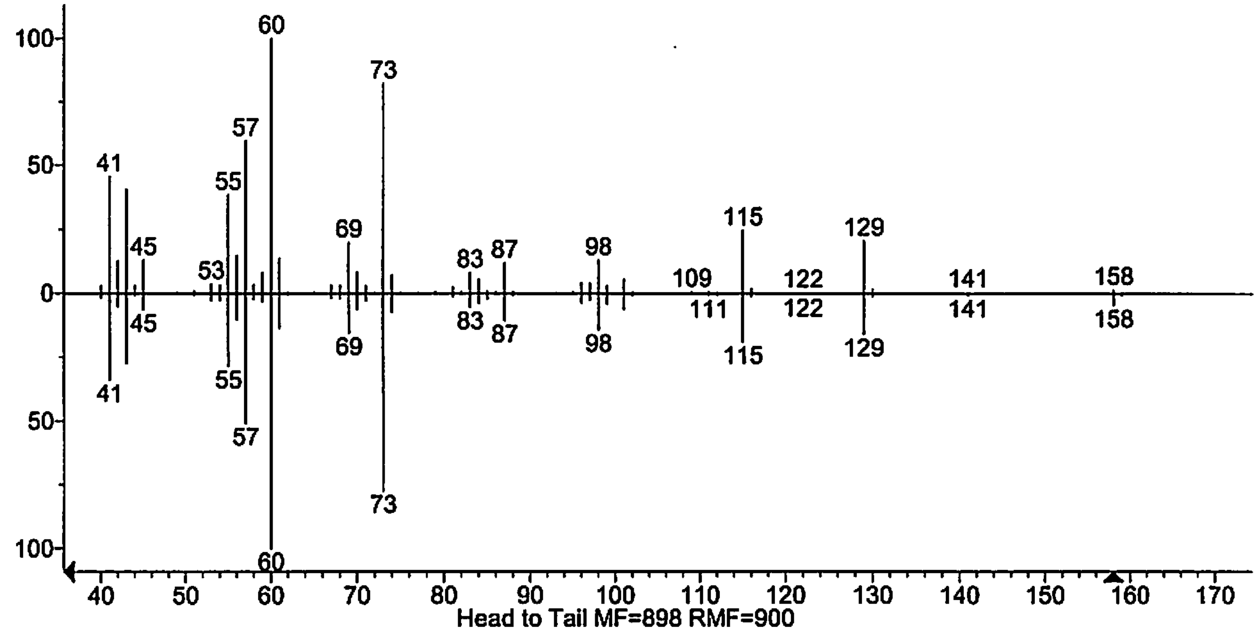


Head to Tail MF=898 RMF=900

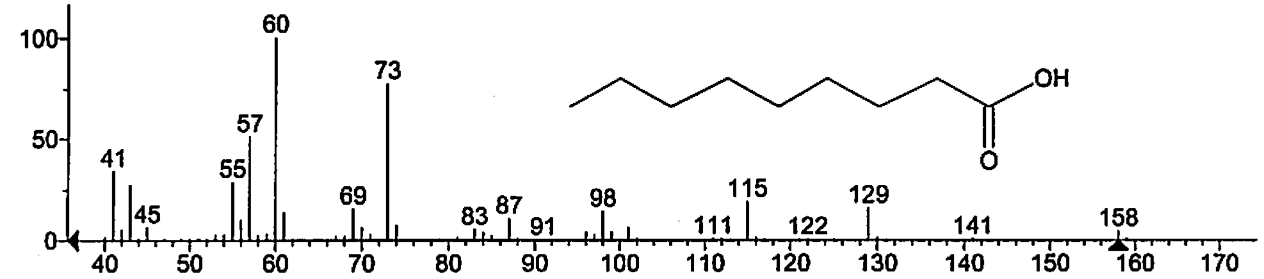


(mainlib) Nonanoic acid

File : D:\ALDRICH\JA-09\Snapshot\JA112009-4.D
Operator :
Acquired : 20 Nov 2009 16:25 using AcqMethod JA-50-280LESS.M
Instrument : Buba
Sample Name: 15 M C.oculata abd.-fed 8-OH-citronellal
Misc Info : 3-10d-old; fed 1wk; 100ul conc.to 5ulCH2Cl2
Vial Number: 1

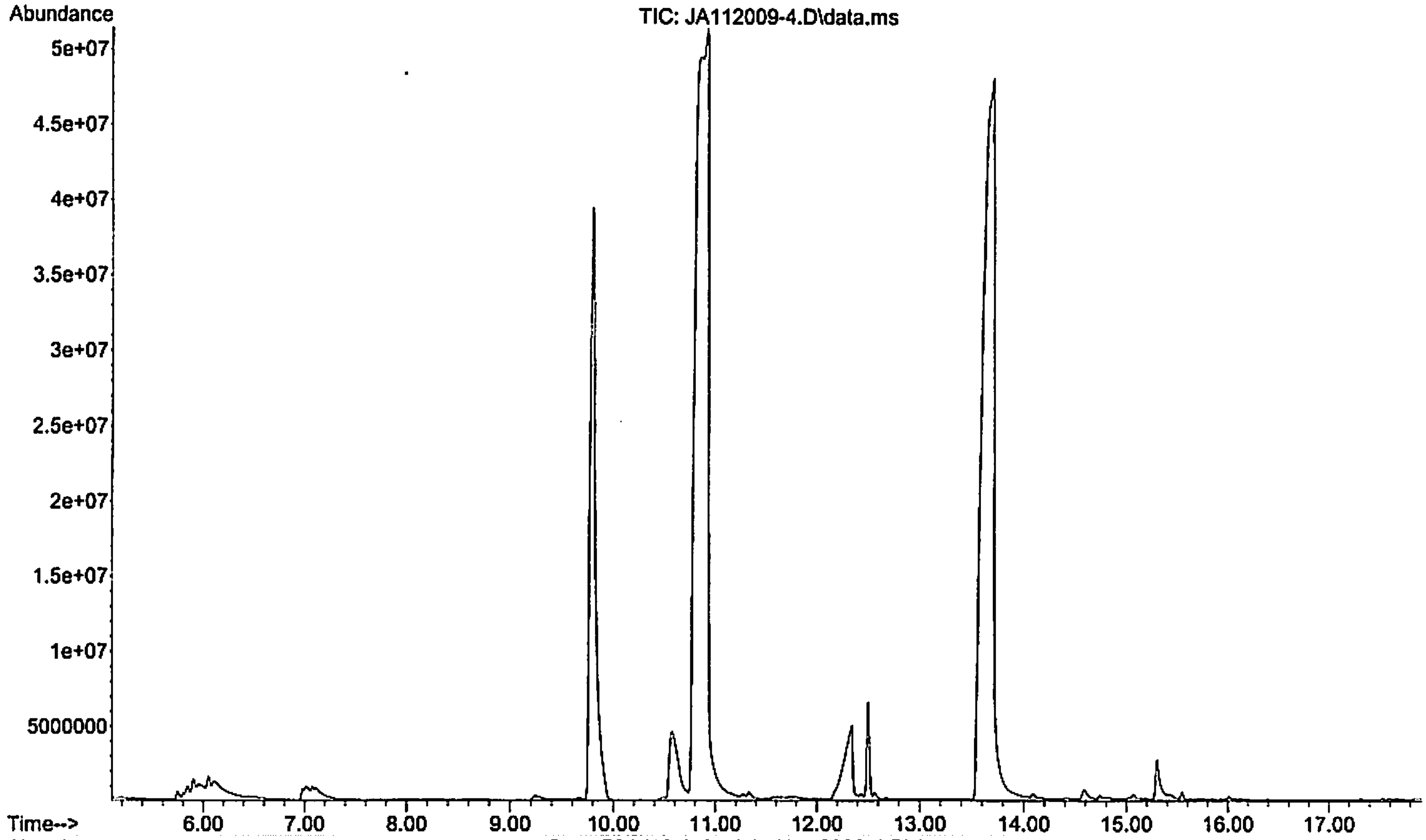


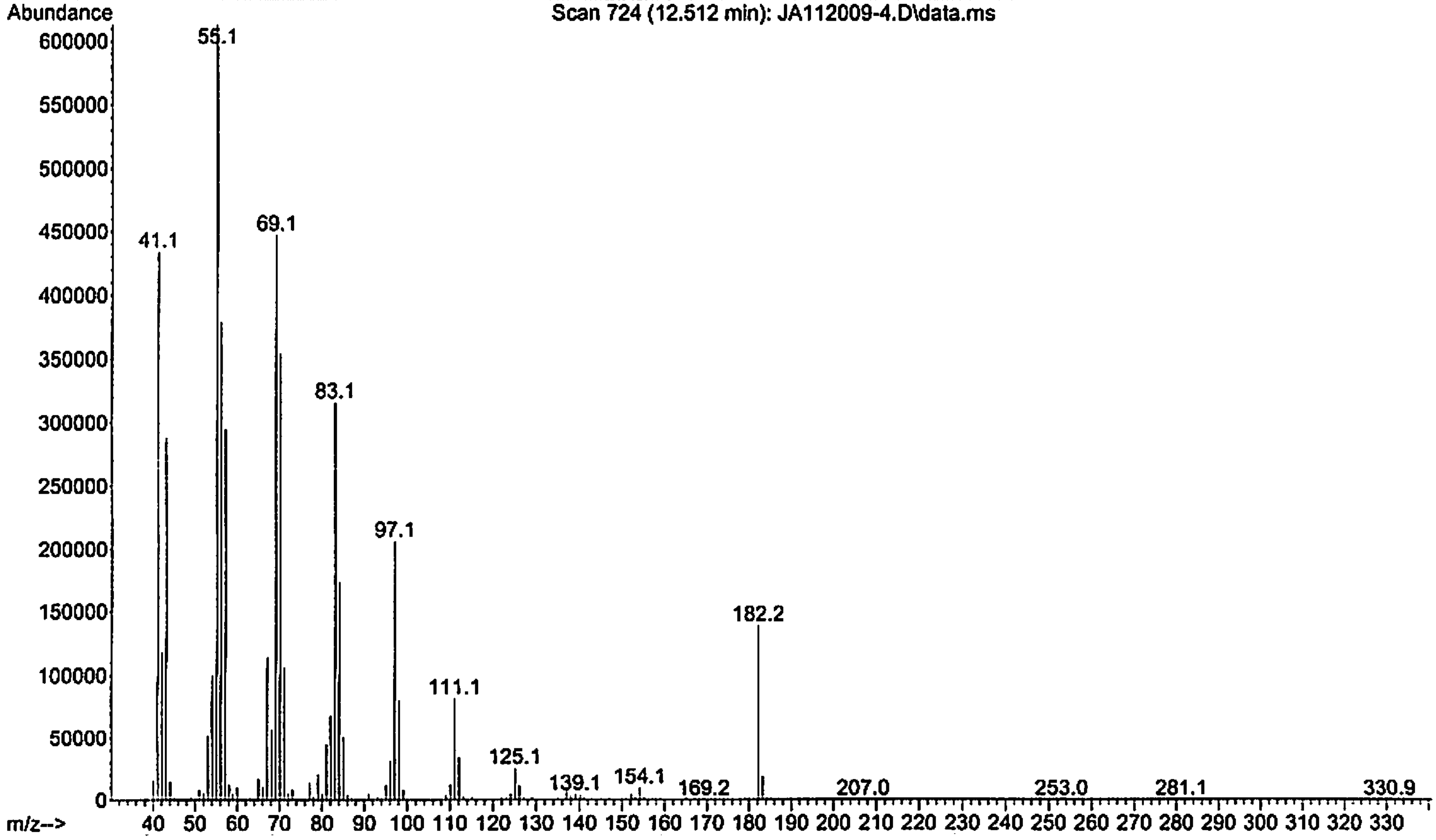

File         :D:\ALDRICH\JA-09\Snapshot\JA112009-4.D
Operator     :
Acquired     : 20 Nov 2009  16:25    using AcqMethod JA-50-280LESS.M
Instrument :    Buba
Sample Name: 15 M C.oculata abd.-fed 8-OH-citronellal
Misc Info  : 3-10d-old; fed 1wk; 100ul conc.to 5ulCH2Cl2
Vial Number: 1

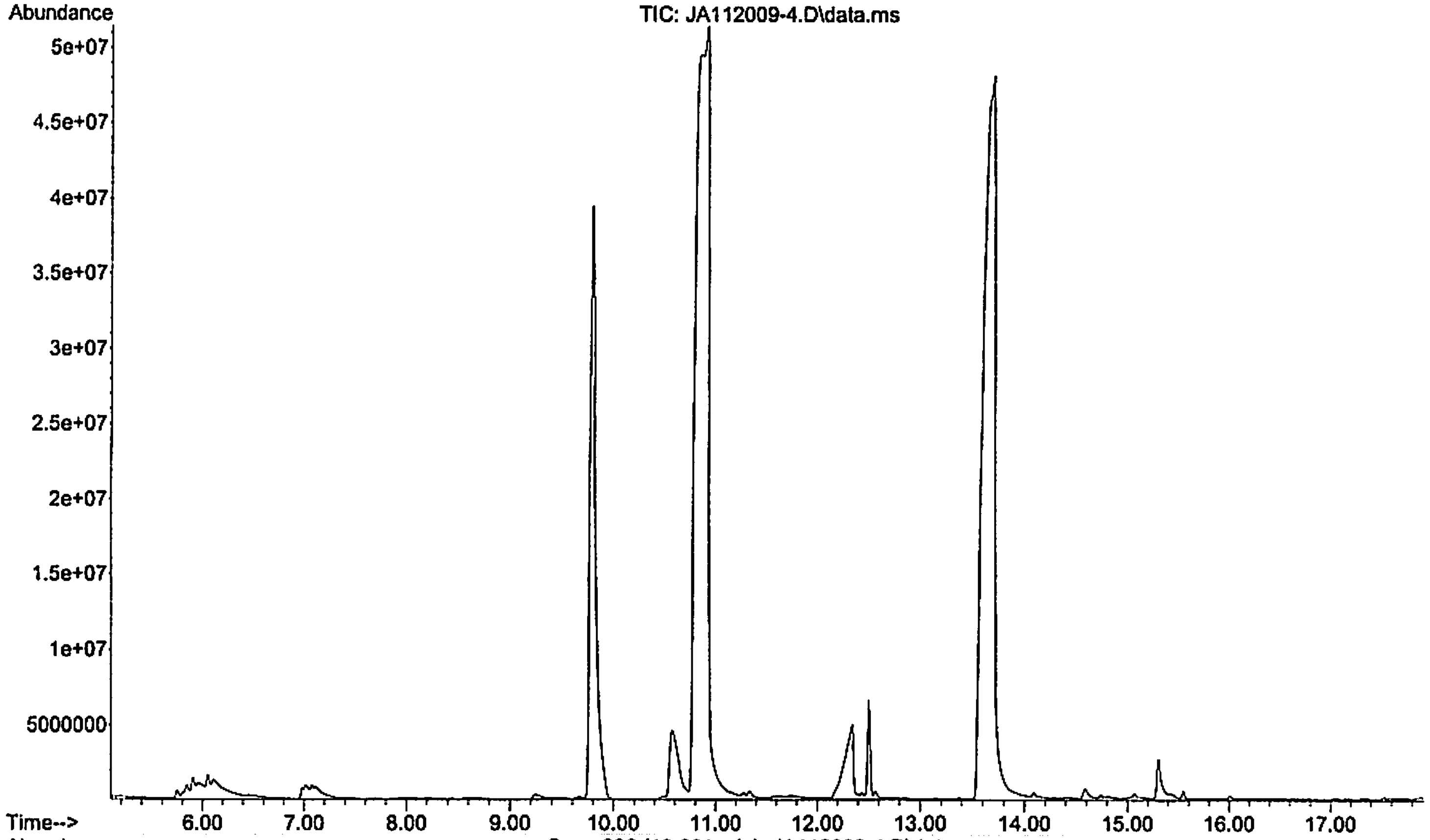


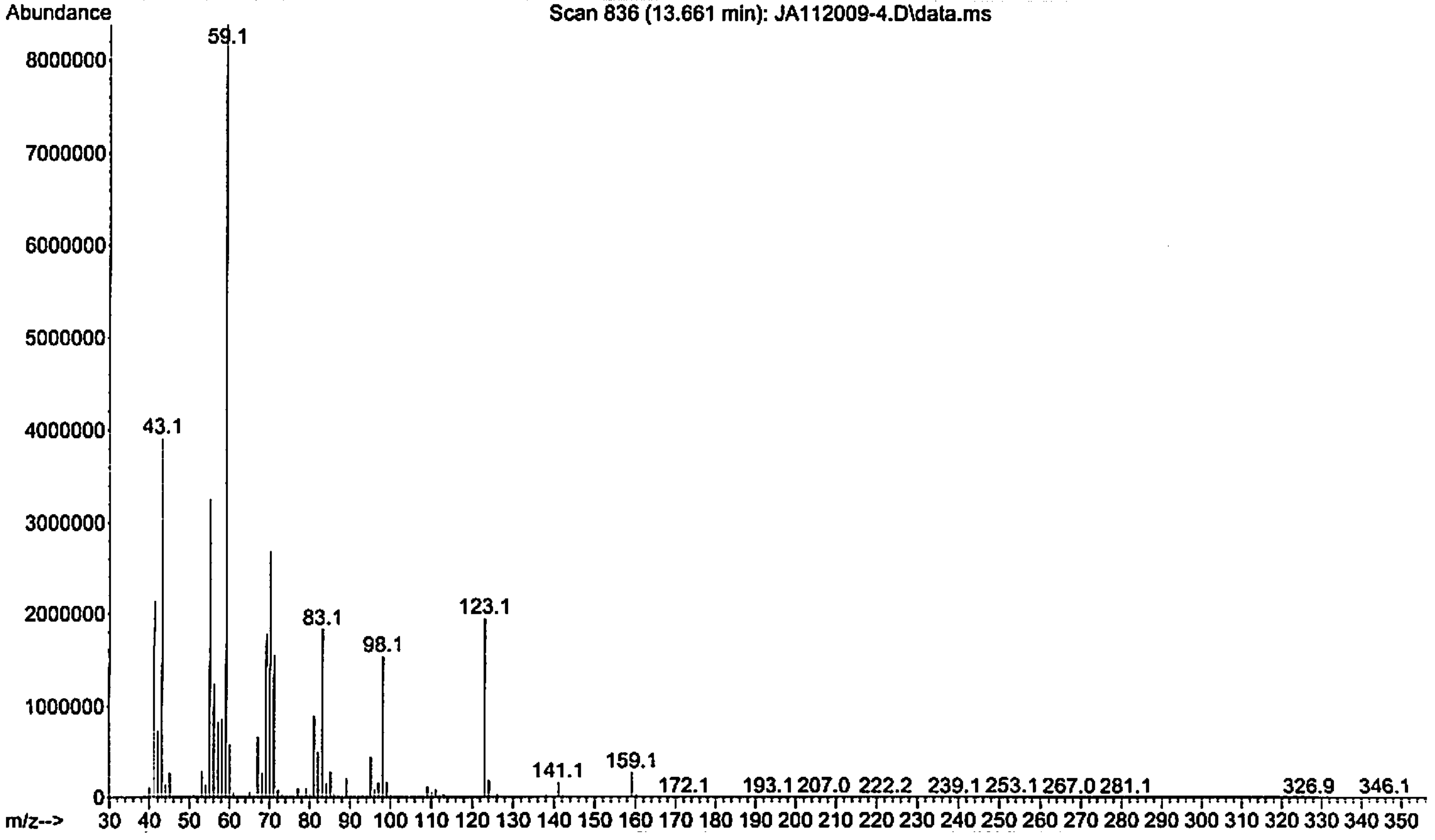

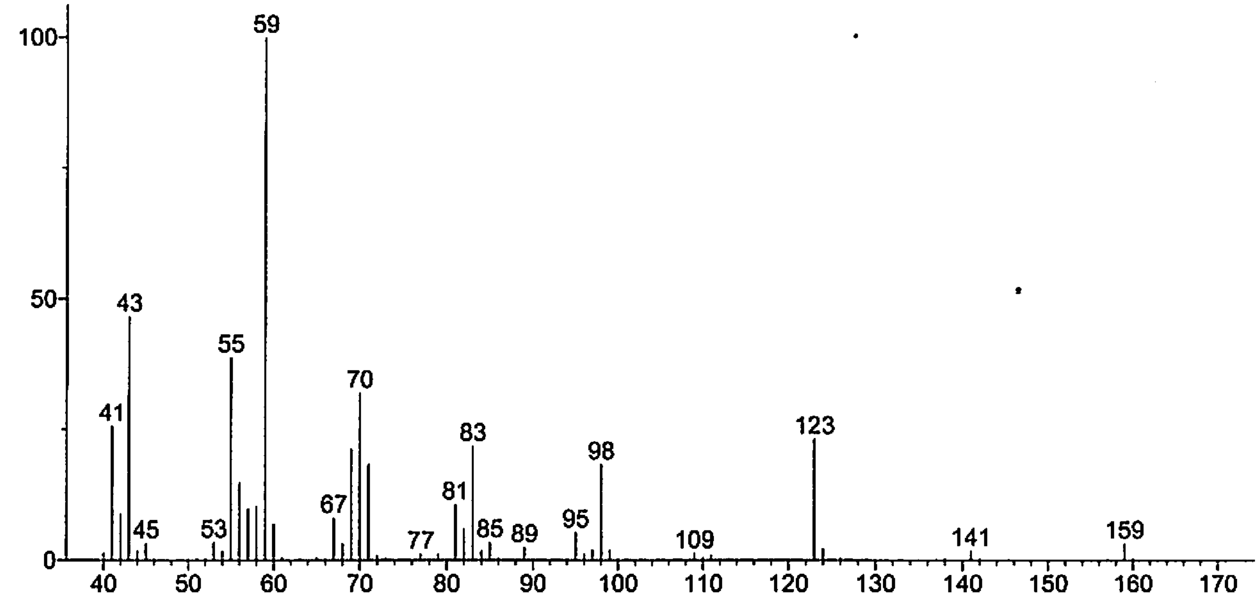


(Text File) Scan 836 (13.661 min): JA112009-4.D\data.ms

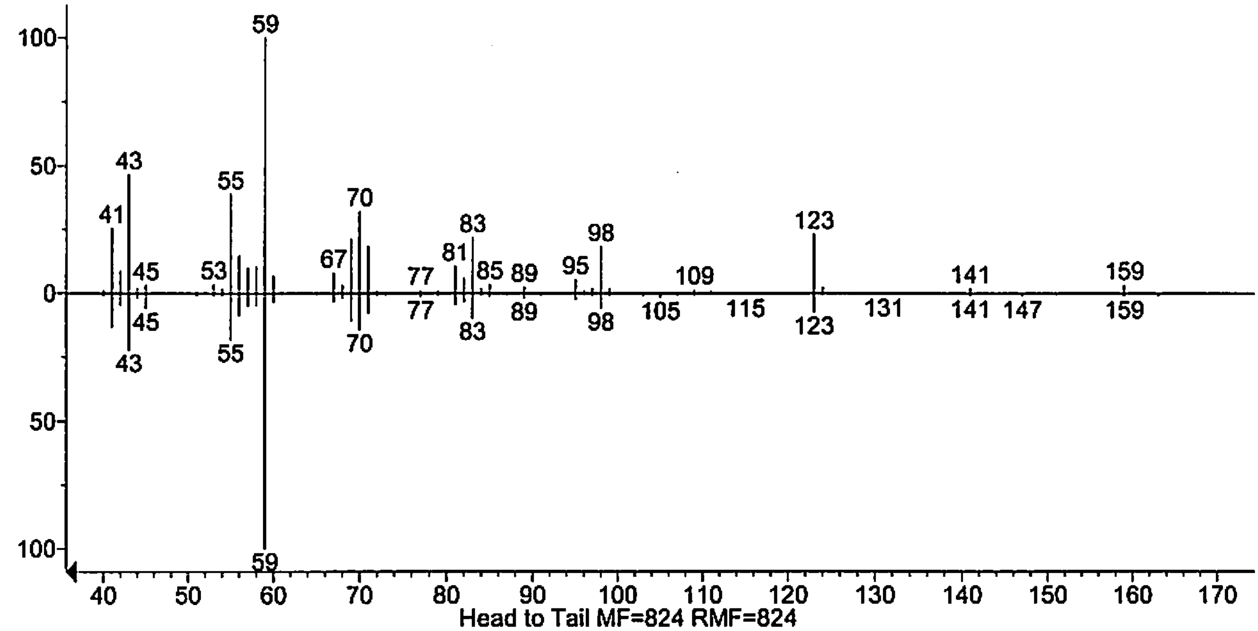


Head to Tail MF=824 RMF=824

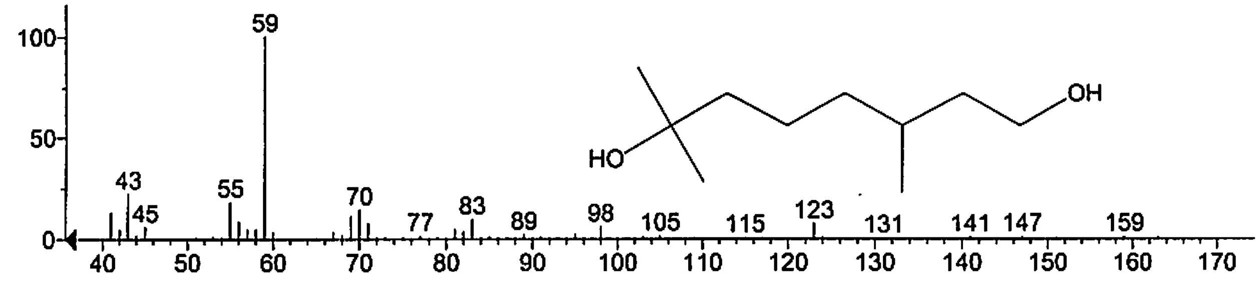


(replib) 1,7-Octanediol, 3,7-dimethyl-

File        :D:\ALDRICH\JA-09\Snapshot\JA112009-4.D
Operator    :
Acquired    : 20 Nov 2009  16:25     using AcqMethod JA-50-280LESS.M
Instrument :     Buba
Sample Name: 15 M C.oculata abd.-fed 8-OH-citronellal
Misc Info   : 3-10d-old; fed 1wk; 100ul conc.to 5ulCH2Cl2
Vial Number: 1

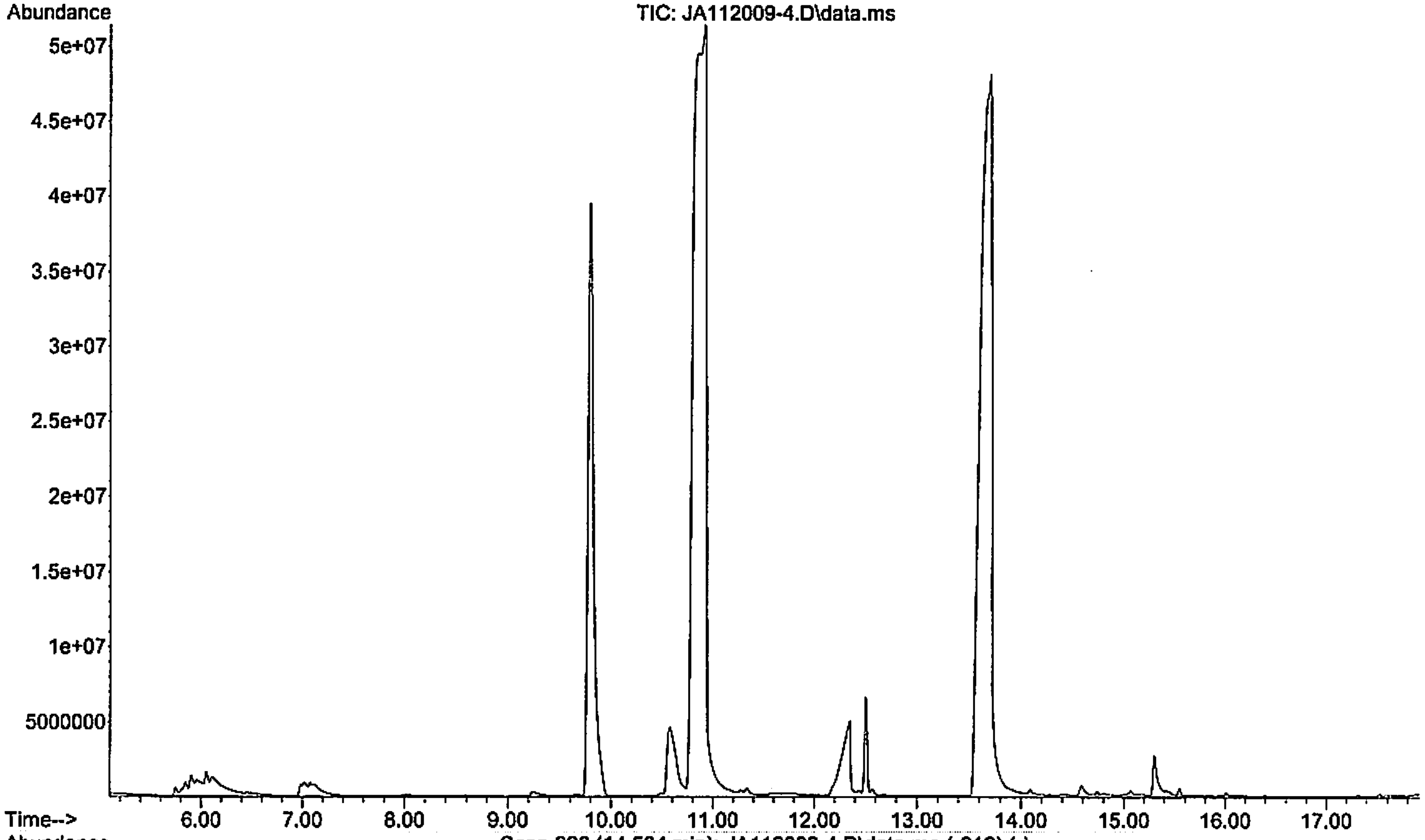


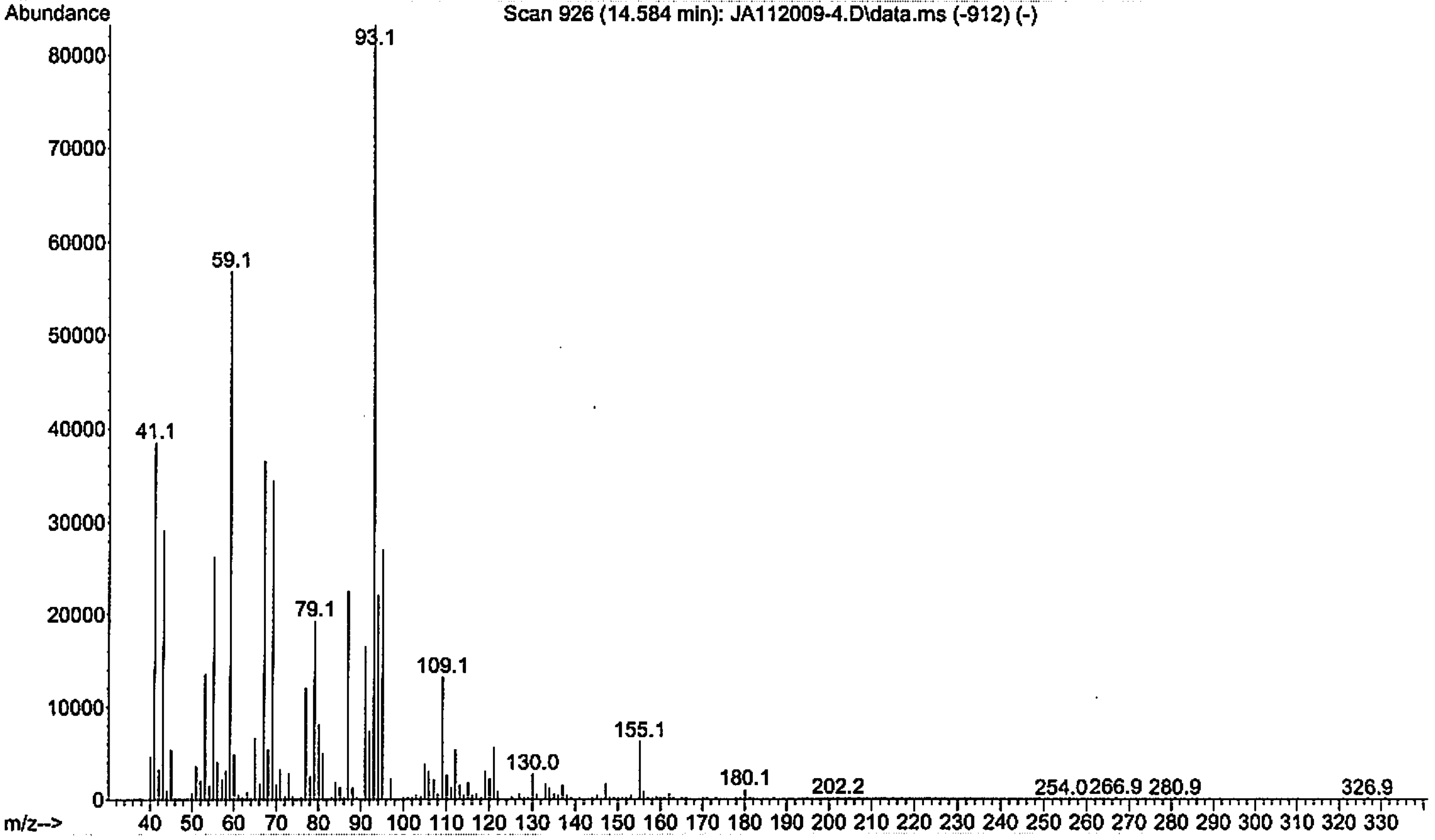

```
File        :D:\ALDRICH\JA-09\Snapshot\JA112009-4.D
Operator    :
Acquired    : 20 Nov 2009  16:25     using AcqMethod JA-50-280LESS.M
Instrument  :     Buba
Sample Name: 15 M C.oculata abd.-fed 8-OH-citronellal
Misc Info   : 3-10d-old; fed 1wk; 100ul conc.to 5ulCH2Cl2
Vial Number: 1
```

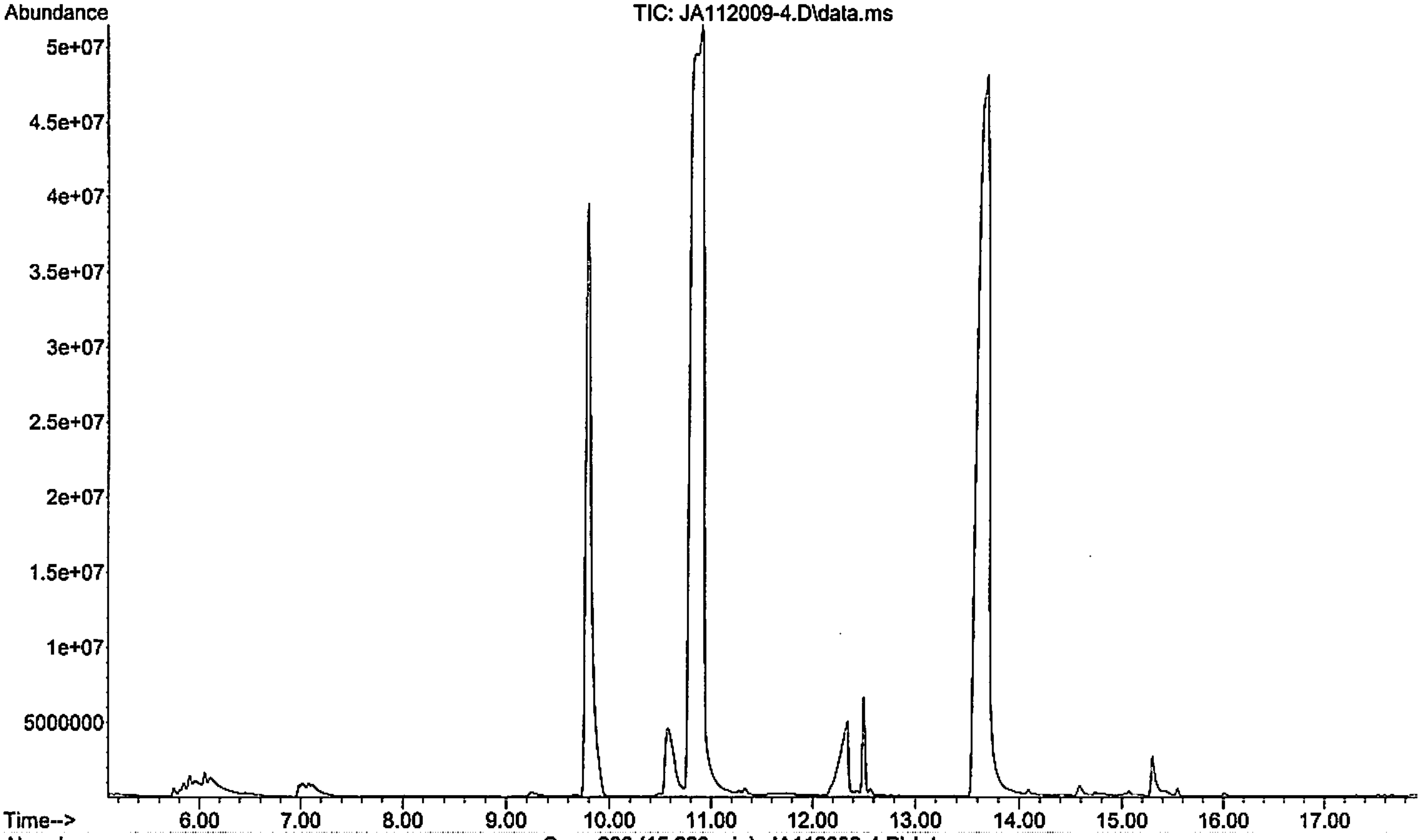


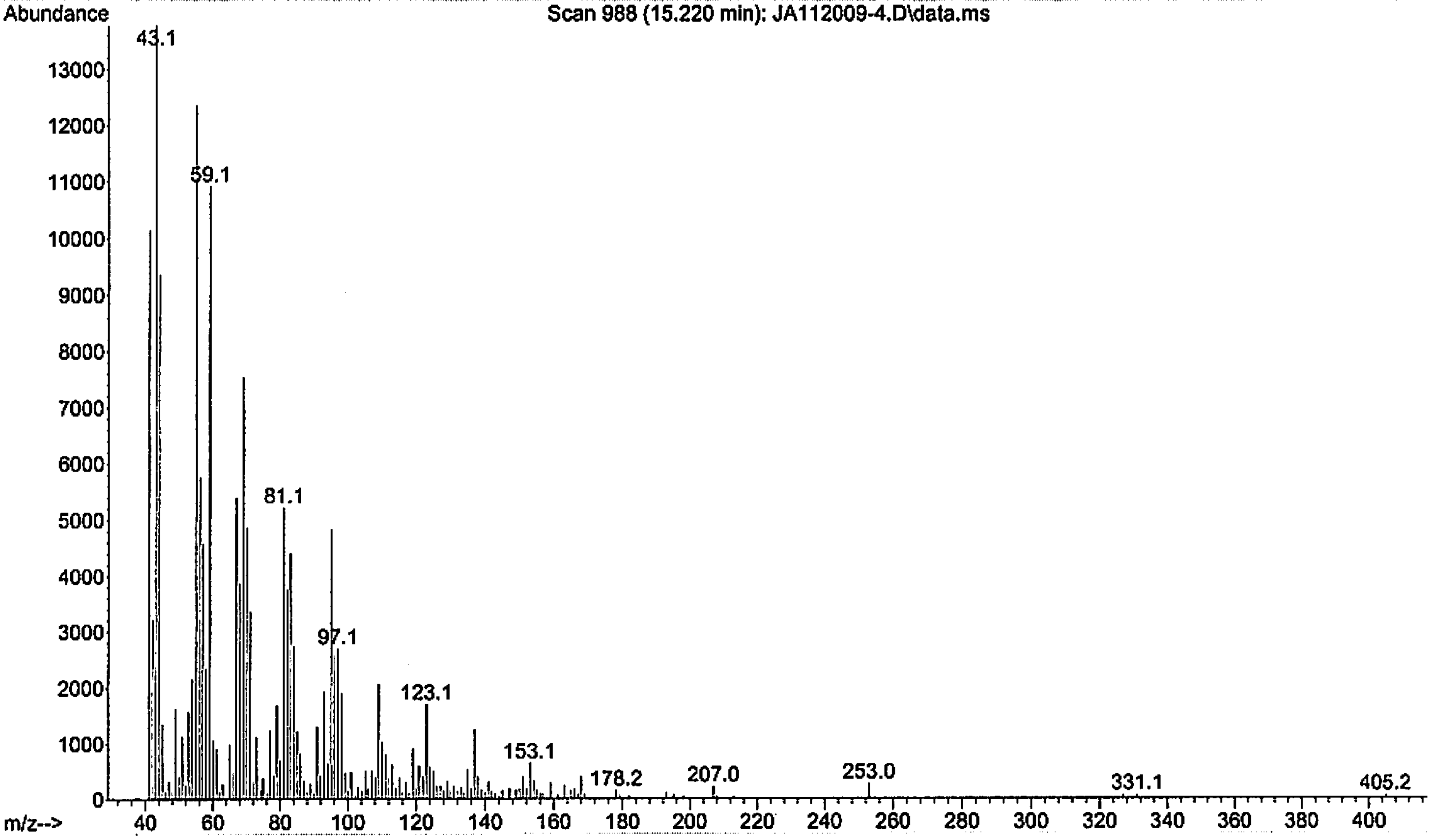

File :D:\ALDRICH\JA-09\Snapshot\JA112009-4.D
Operator :
Acquired : 20 Nov 2009 16:25 using AcqMethod JA-50-280LESS.M
Instrument : Buba
Sample Name: 15 M C.oculata abd.-fed 8-OH-citronellal
Misc Info : 3-10d-old; fed 1wk; 100ul conc.to 5ulCH2Cl2
Vial Number: 1

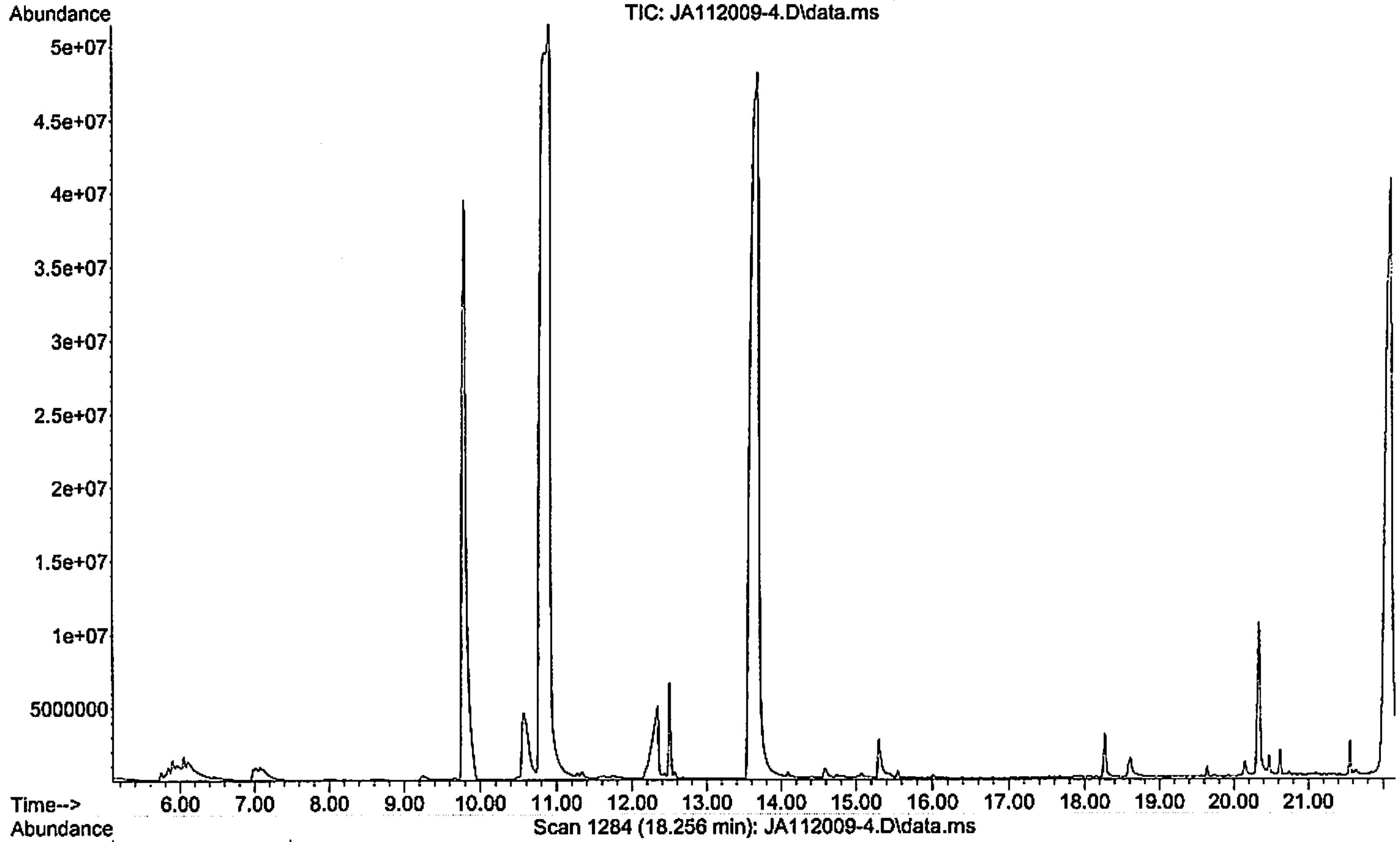


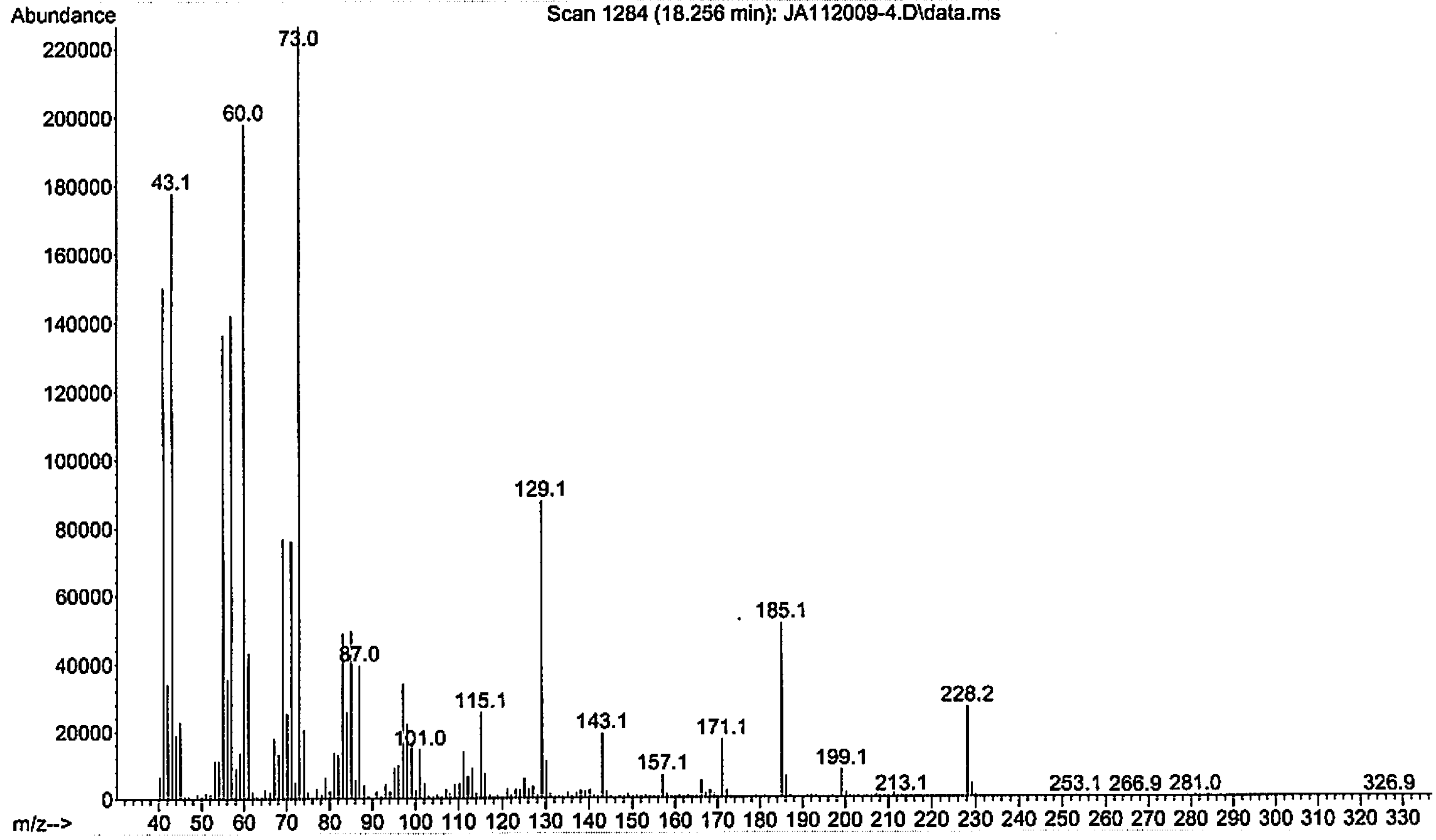

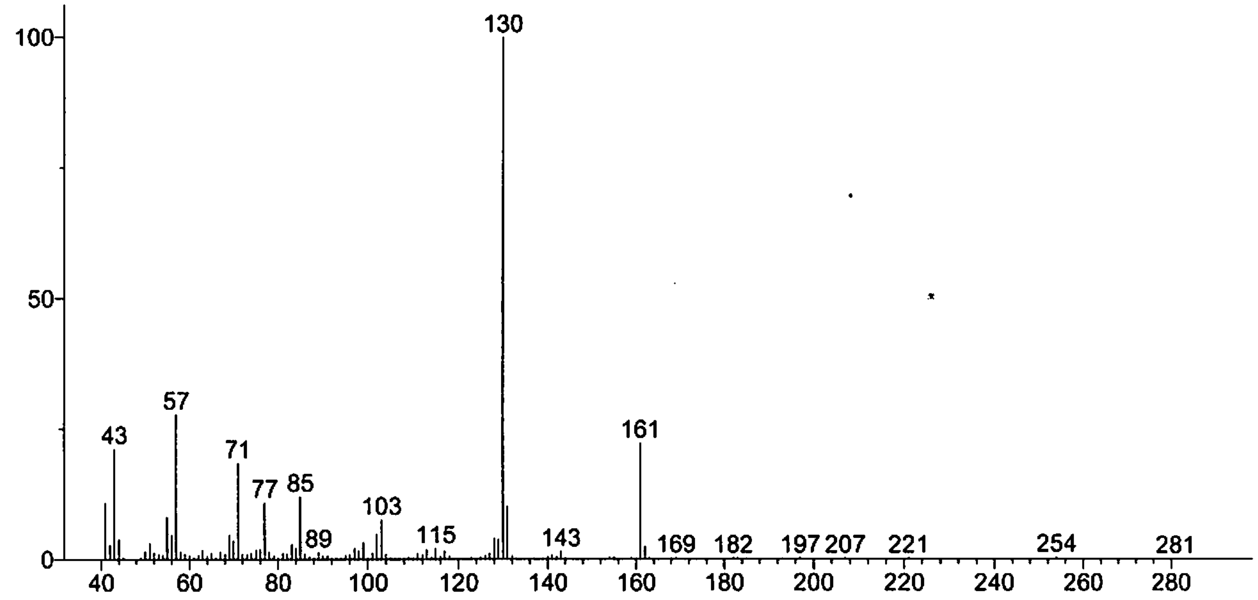


(Text File) Scan 1318 (18.605 min): JA112009-4.D\data.ms

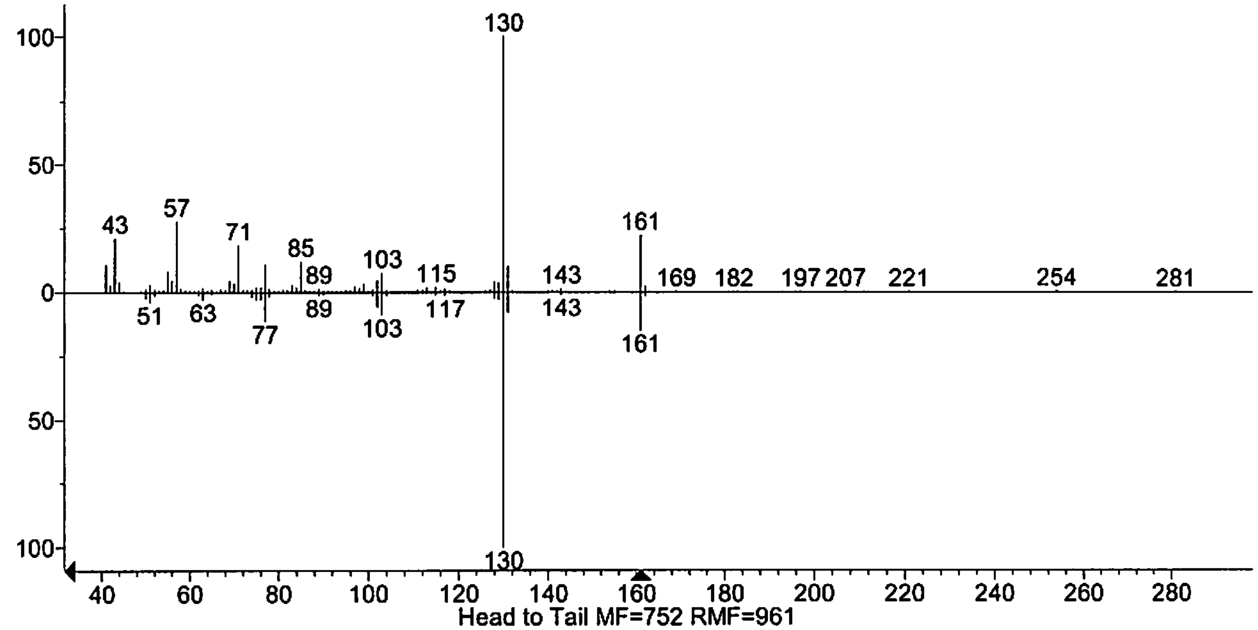


Head to Tail MF=752 RMF=961

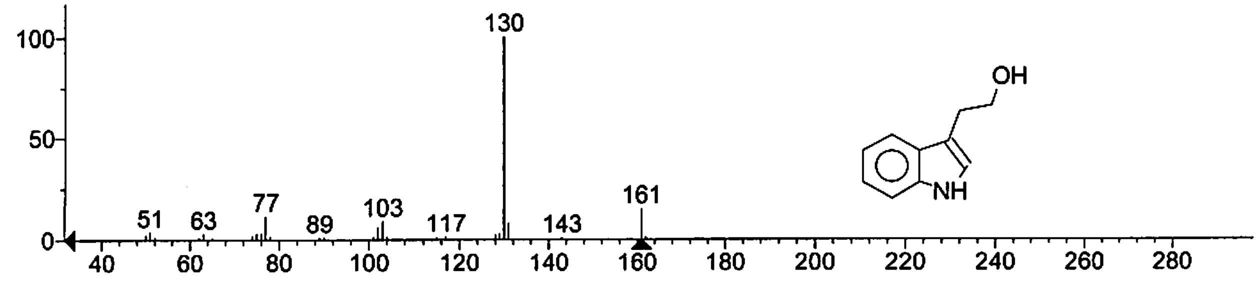


(replib) 1H-Indole-3-ethanol

File        :D:\ALDRICH\JA-09\Snapshot\JA112009-4.D
Operator    :
Acquired    : 20 Nov 2009  16:25     using AcqMethod JA-50-280LESS.M
Instrument :    Buba
Sample Name: 15 M C.oculata abd.-fed 8-OH-citronellal
Misc Info   : 3-10d-old; fed 1wk; 100ul conc.to 5ulCH2Cl2
Vial Number: 1

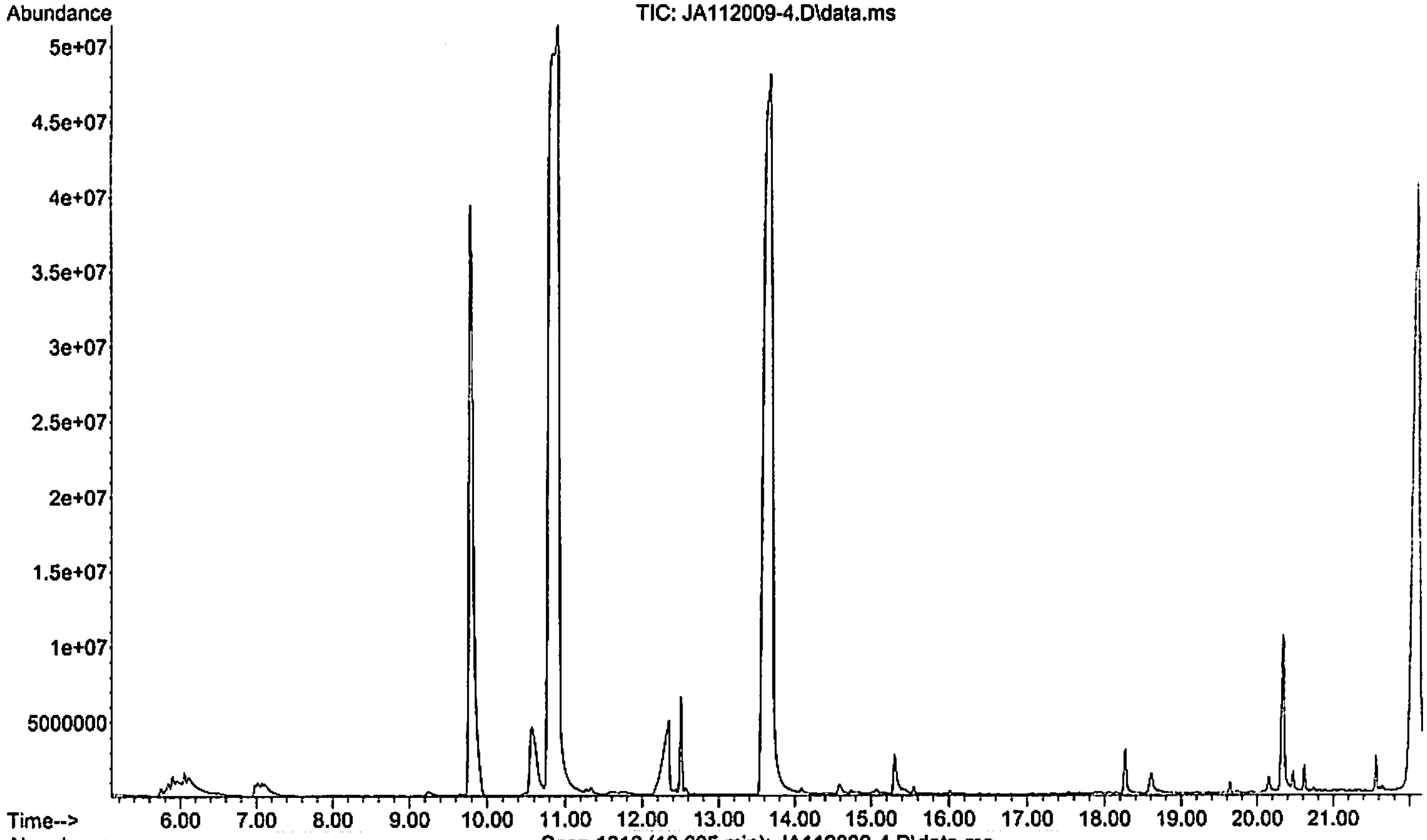


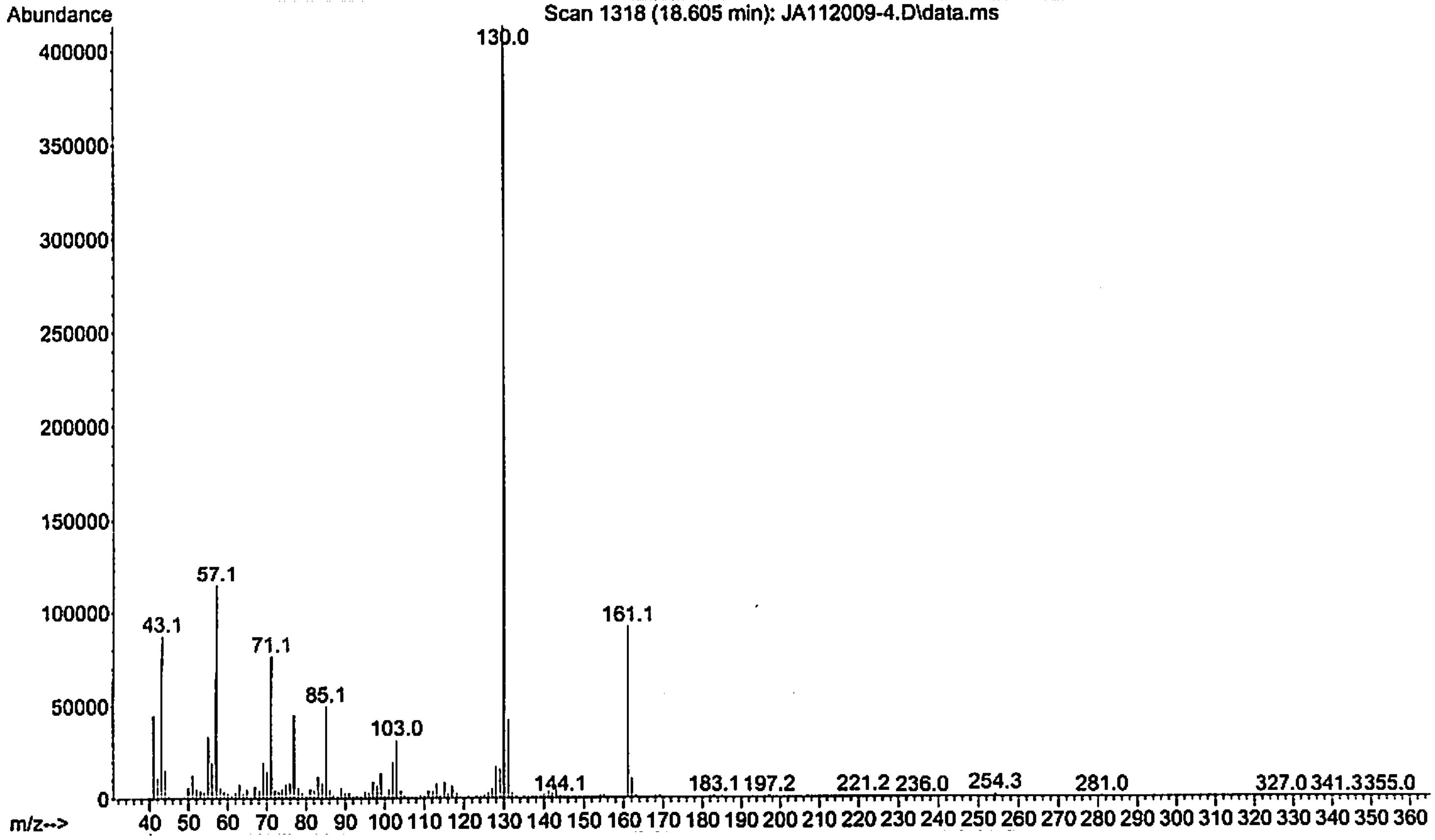

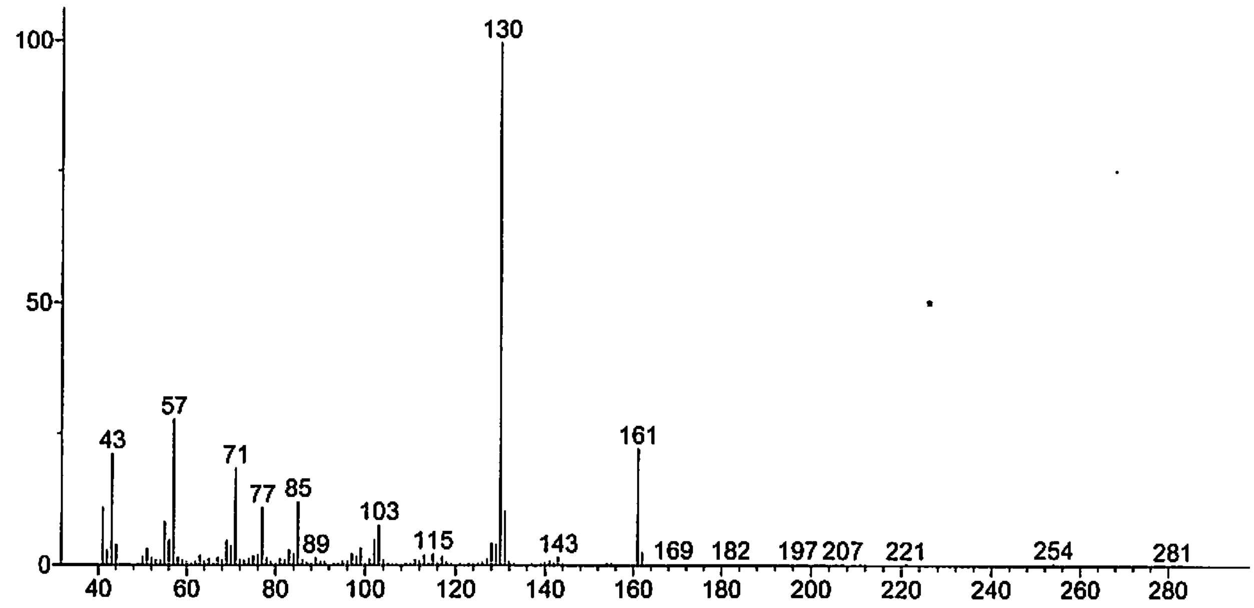


(Text File) Scan 1318 (18.605 min): JA112009-4.D\data.ms

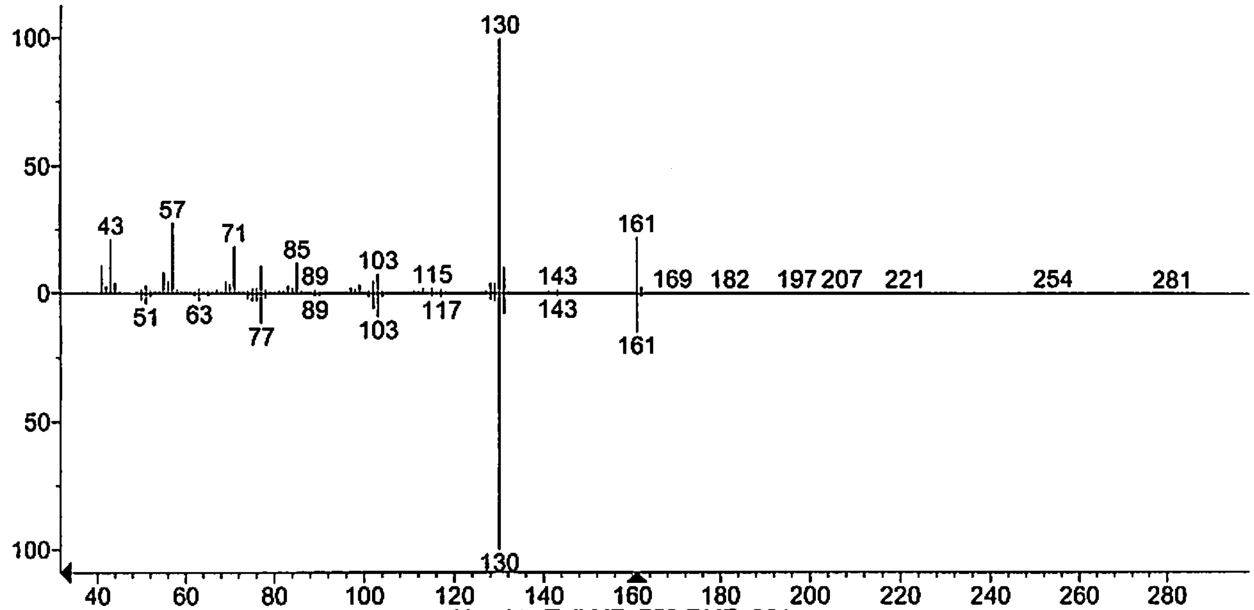


Head to Tail MF=752 RMF=961

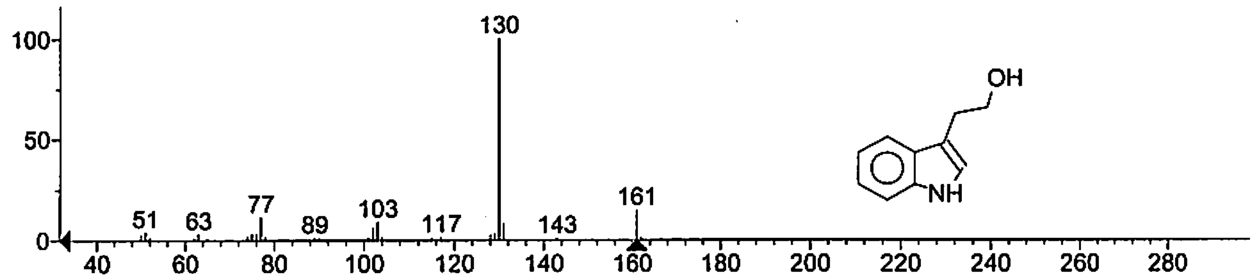


(replib) 1H-Indole-3-ethanol

```
File        :D:\ALDRICH\JA-09\Snapshot\JA112009-4.D
Operator    :
Acquired    : 20 Nov 2009  16:25     using AcqMethod JA-50-280LESS.M
Instrument :    Buba
Sample Name: 15 M C.oculata abd.-fed 8-OH-citronellal
Misc Info   : 3-10d-old; fed 1wk; 100ul conc.to 5ulCH2Cl2
Vial Number: 1
```

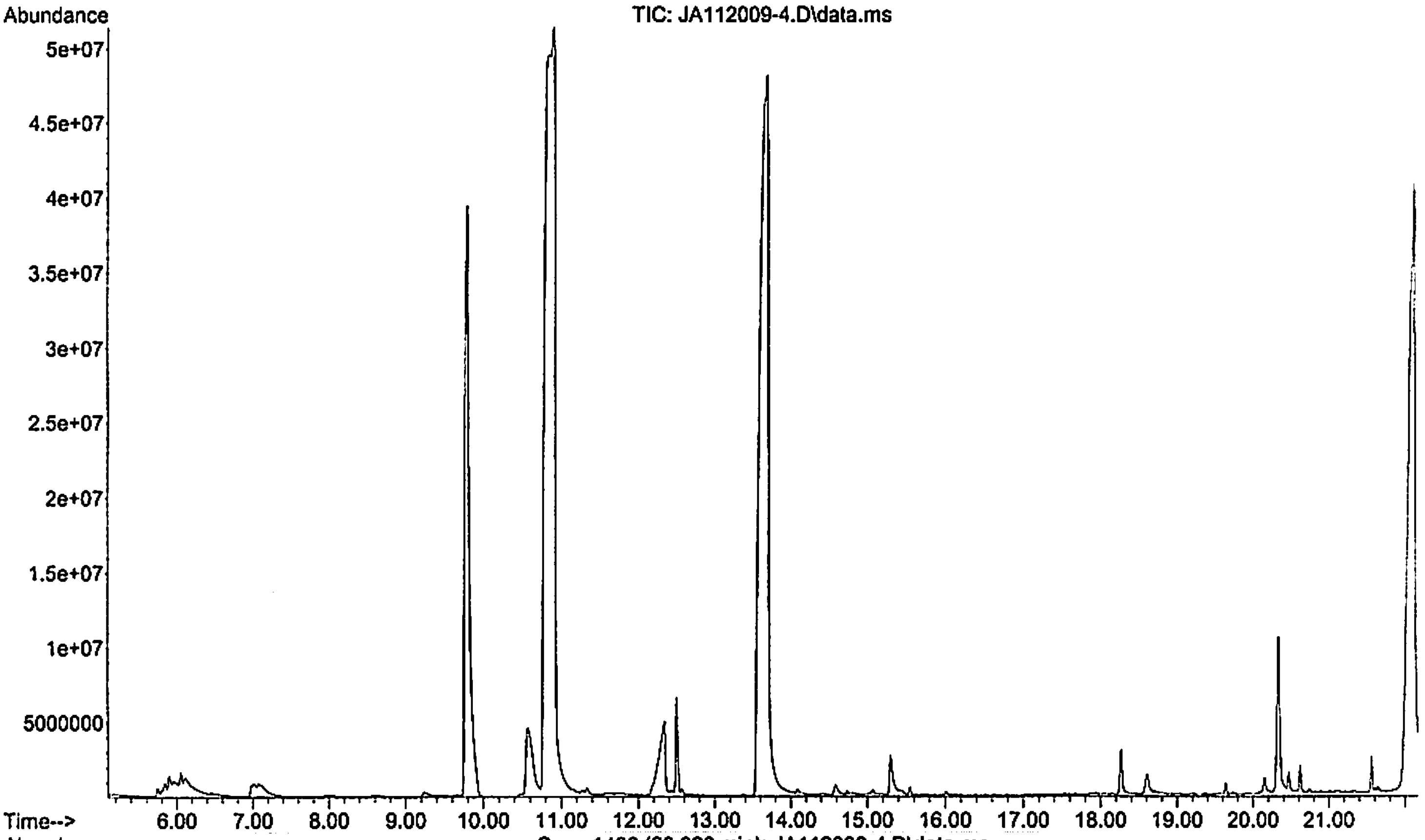


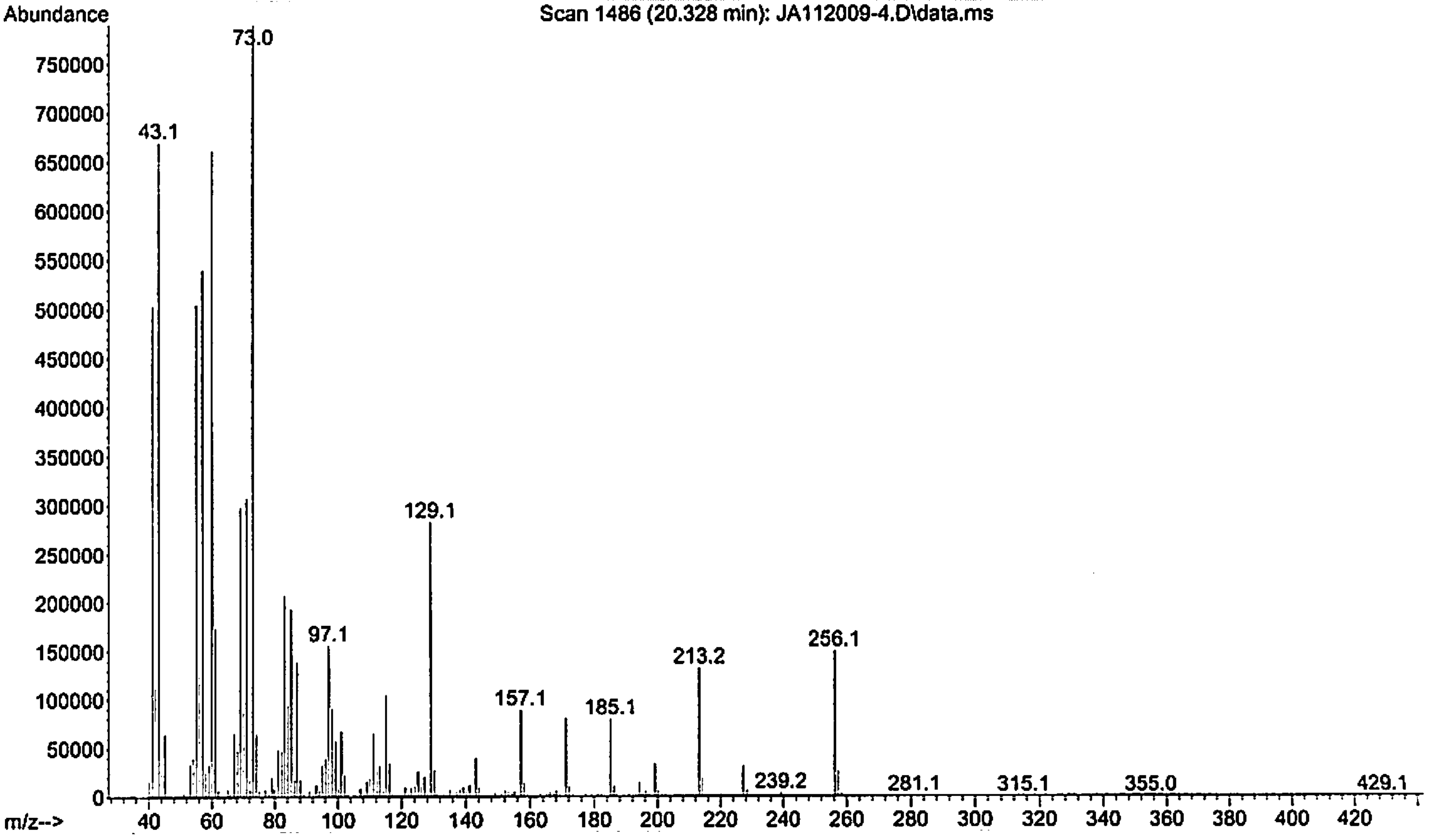

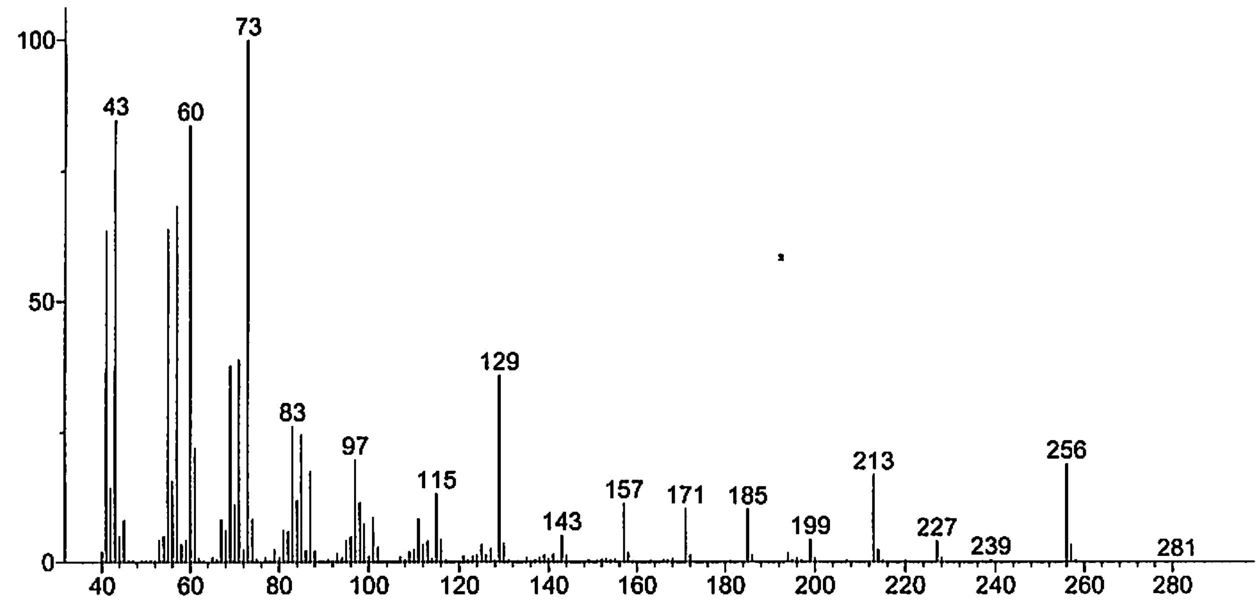


(Text File) Scan 1486 (20.328 min): JA112009-4.D\data.ms

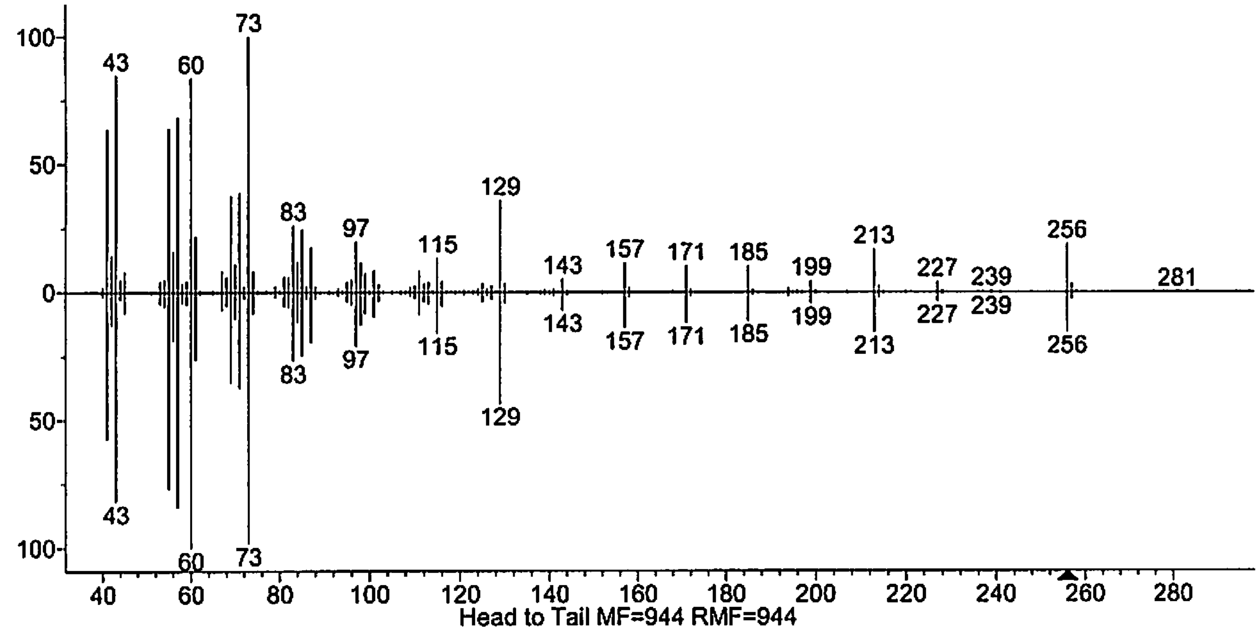


Head to Tail MF=944 RMF=944

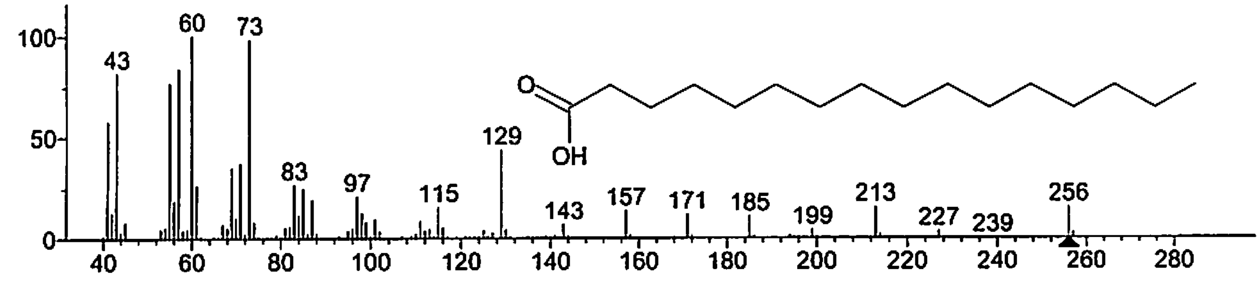


(replib) n-Hexadecanoic acid

```
File        :D:\ALDRICH\JA-09\Snapshot\JA112009-4.D
Operator    :
Acquired    : 20 Nov 2009  16:25     using AcqMethod JA-50-280LESS.M
Instrument :    Buba
Sample Name: 15 M C.oculata abd.-fed 8-OH-citronellal
Misc Info  : 3-10d-old; fed 1wk; 100ul conc.to 5ulCH2Cl2
Vial Number: 1
```

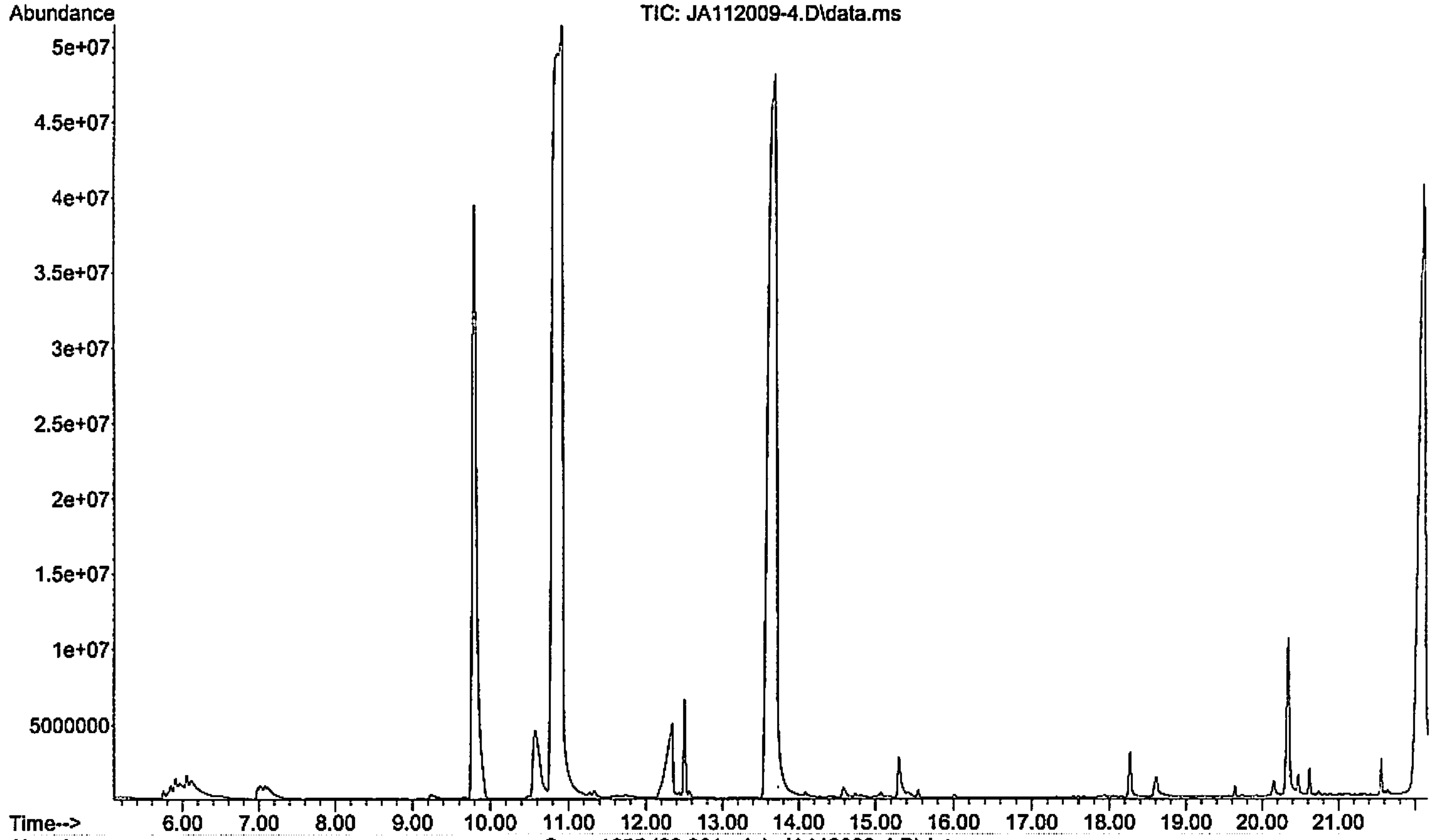


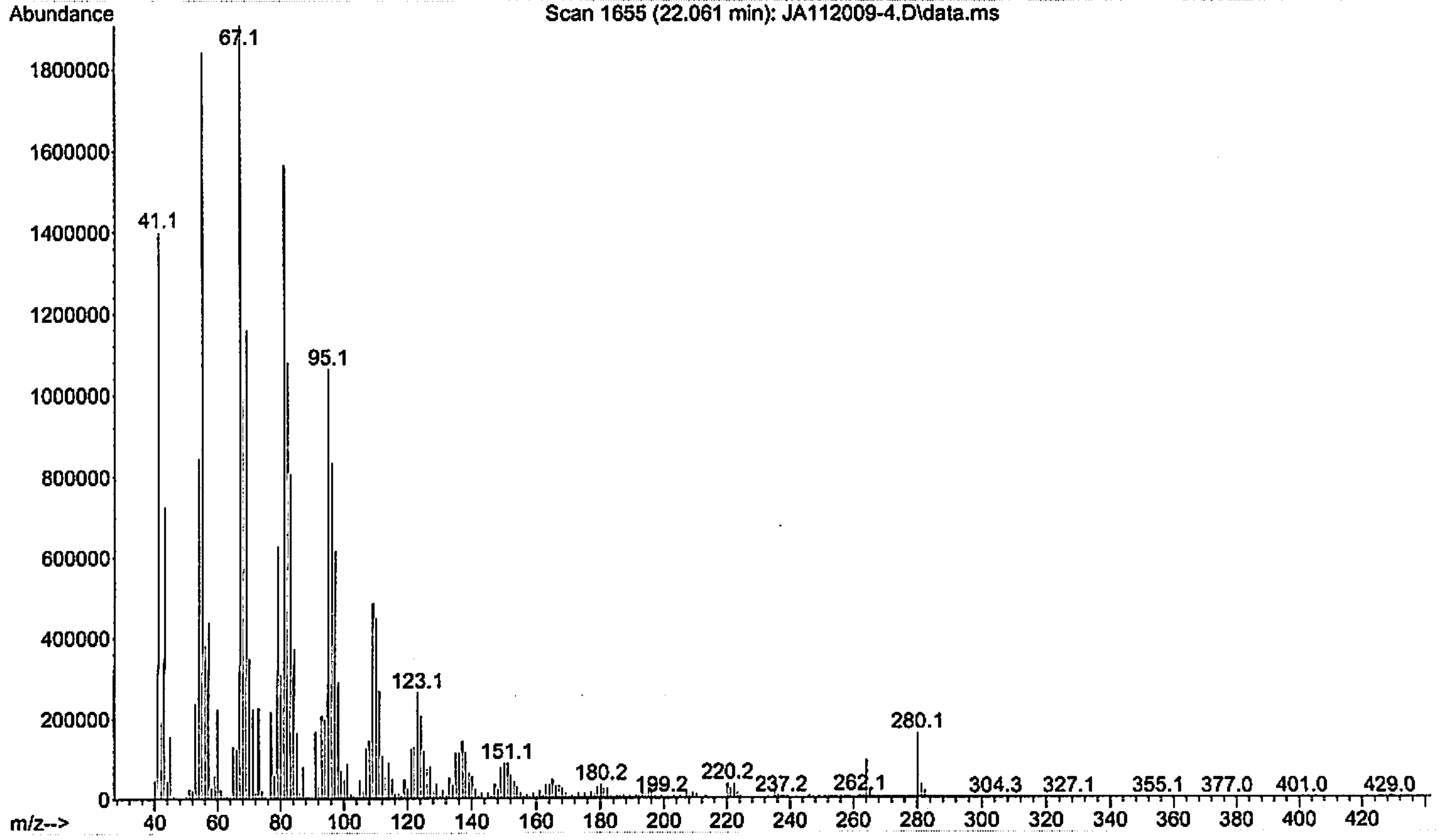

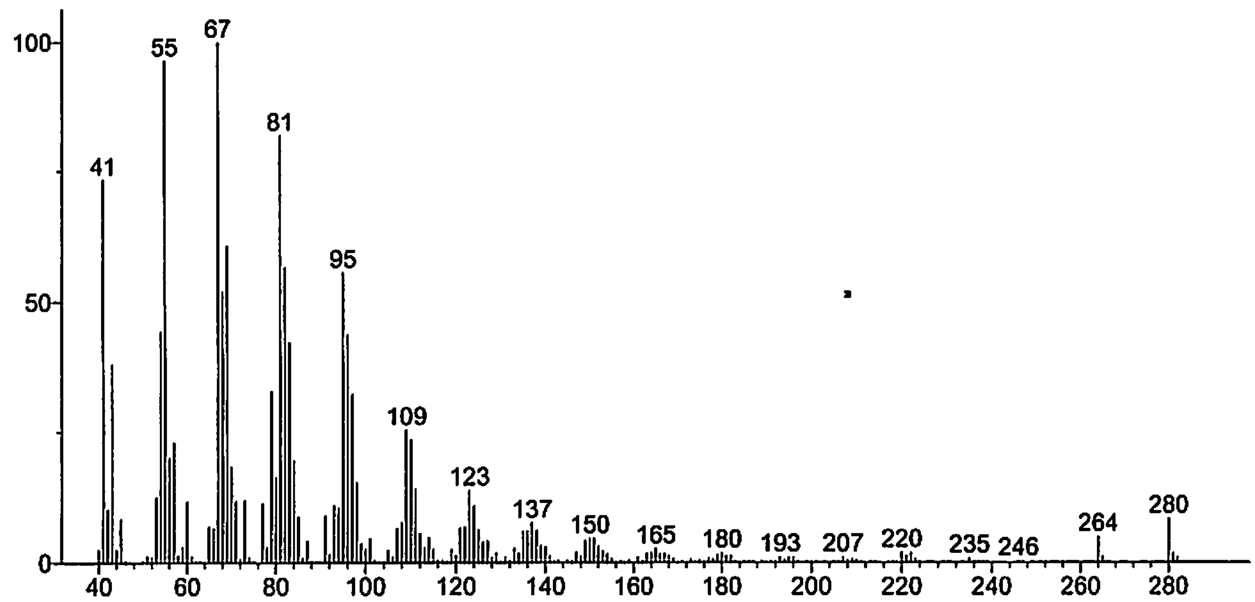

(Text File) Scan 1655 (22.061 min): JA112009-4.D\data.ms

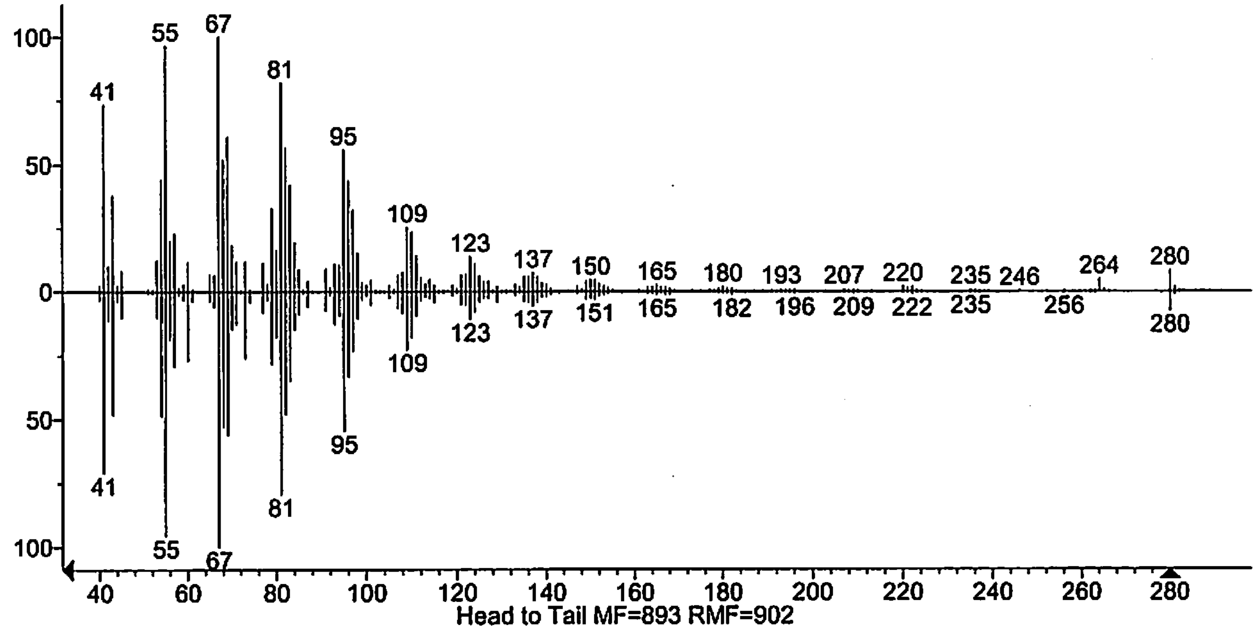

Head to Tail MF=893 RMF=902

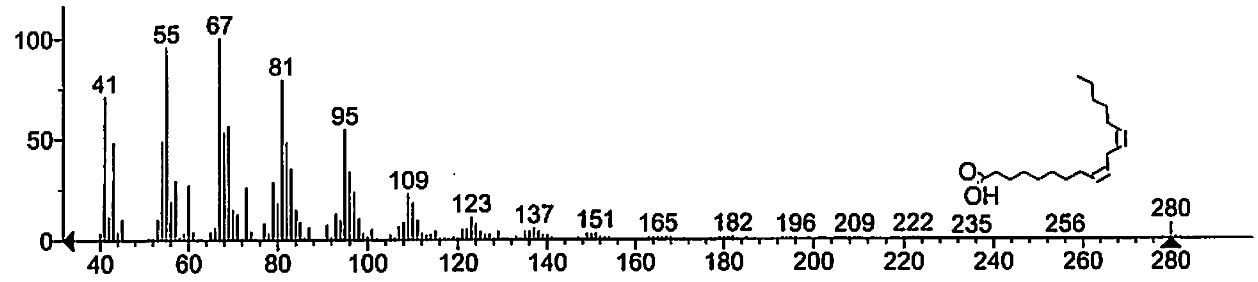

(replib) 9,12-Octadecadienoic acid (Z,Z)-

```
File        :D:\Aldrich\JA-09\JA112009-3.D
Operator    :
Acquired    : 20 Nov 2009  15:24     using AcqMethod JA-50-280LESS.M
Instrument :   Buba
Sample Name: 15 M C.oculata abd.-fed 8-OH-citronellol
Misc Info  : 3-10d-old; fed 1wk; 100ul conc.to 5ulCH2Cl2
Vial Number: 1
```

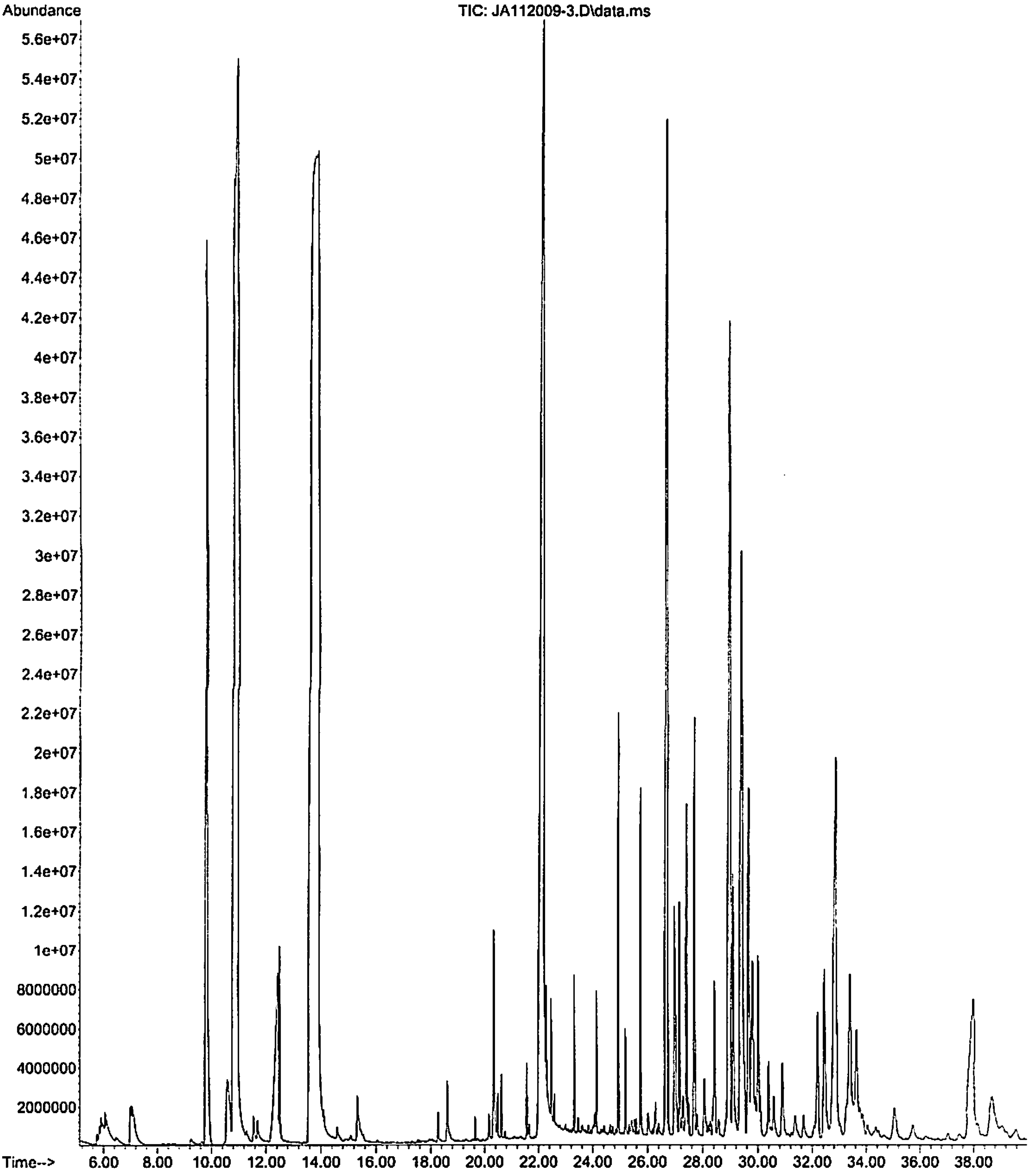

* *C. oculata*

Adult Male (♂♂). Feeding. Expt. - SU. 11/13/09:

(15). ♂s.

8-Hydroxy-citronellol (alcohol) - Treatment.

(1). ♂. NEA. 11/03-11/05 (M-71)

(3). ♂. NEAs. 11/05-11/06 (M-72)

(6). ♂. NEAs. 11/06-11/09 (M-73 & M-74)

11/19: All (15). res. ♂s. alive. Fed live pea aphids.

```
File        :D:\Aldrich\JA-09\JA112009-3.D
Operator    :
Acquired    : 20 Nov 2009  15:24     using AcqMethod JA-50-280LESS.M
Instrument :   Buba
Sample Name: 15 M C.oculata abd.-fed 8-OH-citronellol
Misc Info  : 3-10d-old; fed 1wk; 100ul conc.to 5ulCH2Cl2
Vial Number: 1
```

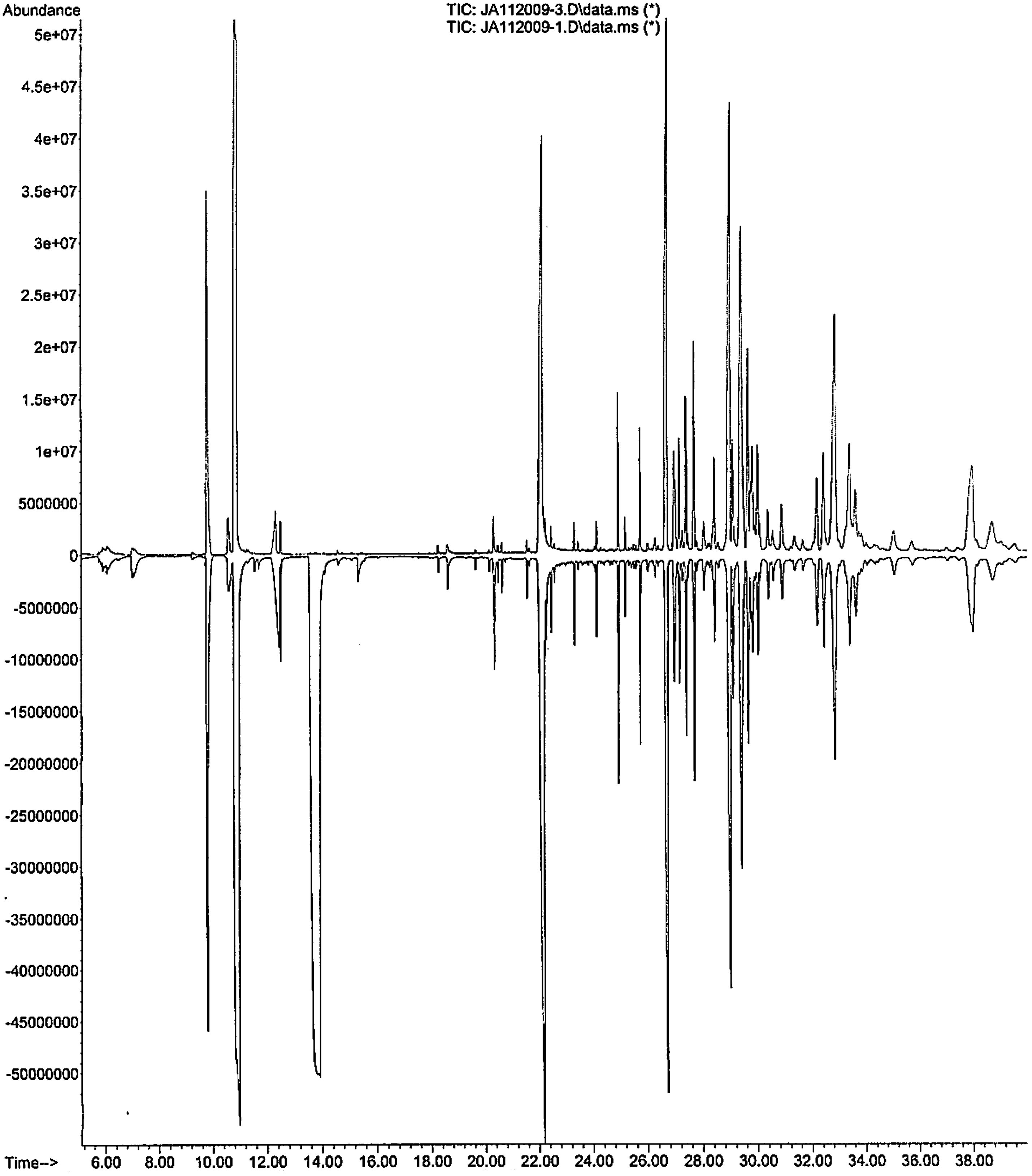

```
File        :D:\Aldrich\JA-09\JA112009-3.D
Operator    :
Acquired    : 20 Nov 2009  15:24     using AcqMethod JA-50-280LESS.M
Instrument :   Buba
Sample Name: 15 M C.oculata abd.-fed 8-OH-citronellol
Misc Info  : 3-10d-old; fed 1wk; 100ul conc.to 5ulCH2Cl2
Vial Number: 1
```

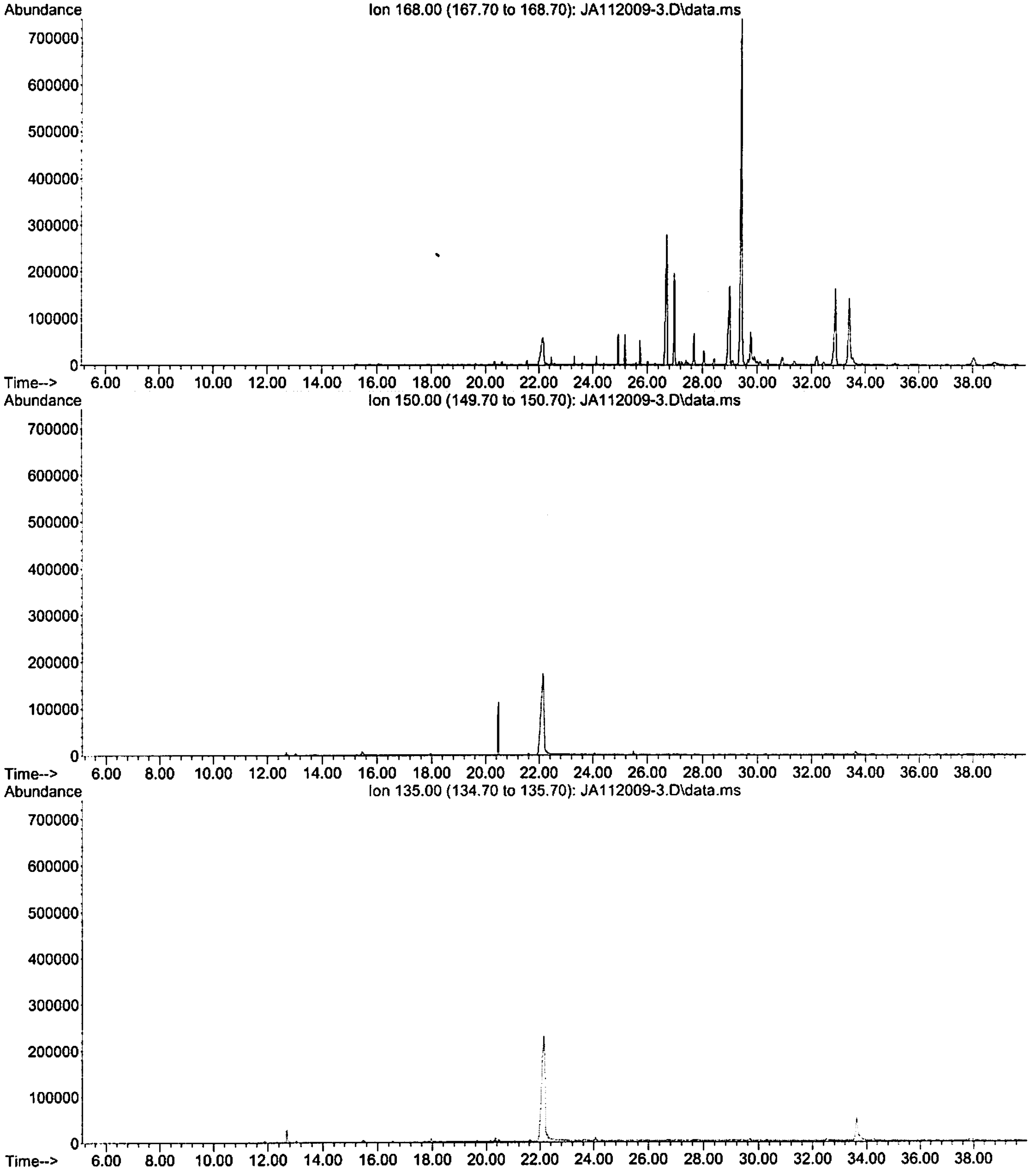

```
File       :D:\Aldrich\JA-09\JA112009-3.D
Operator   :
Acquired   : 20 Nov 2009  15:24     using AcqMethod JA-50-280LESS.M
Instrument :    Buba
Sample Name: 15 M C.oculata abd.-fed 8-OH-citronellol
Misc Info  : 3-10d-old; fed 1wk; 100ul conc.to 5ulCH2Cl2
Vial Number: 1
```

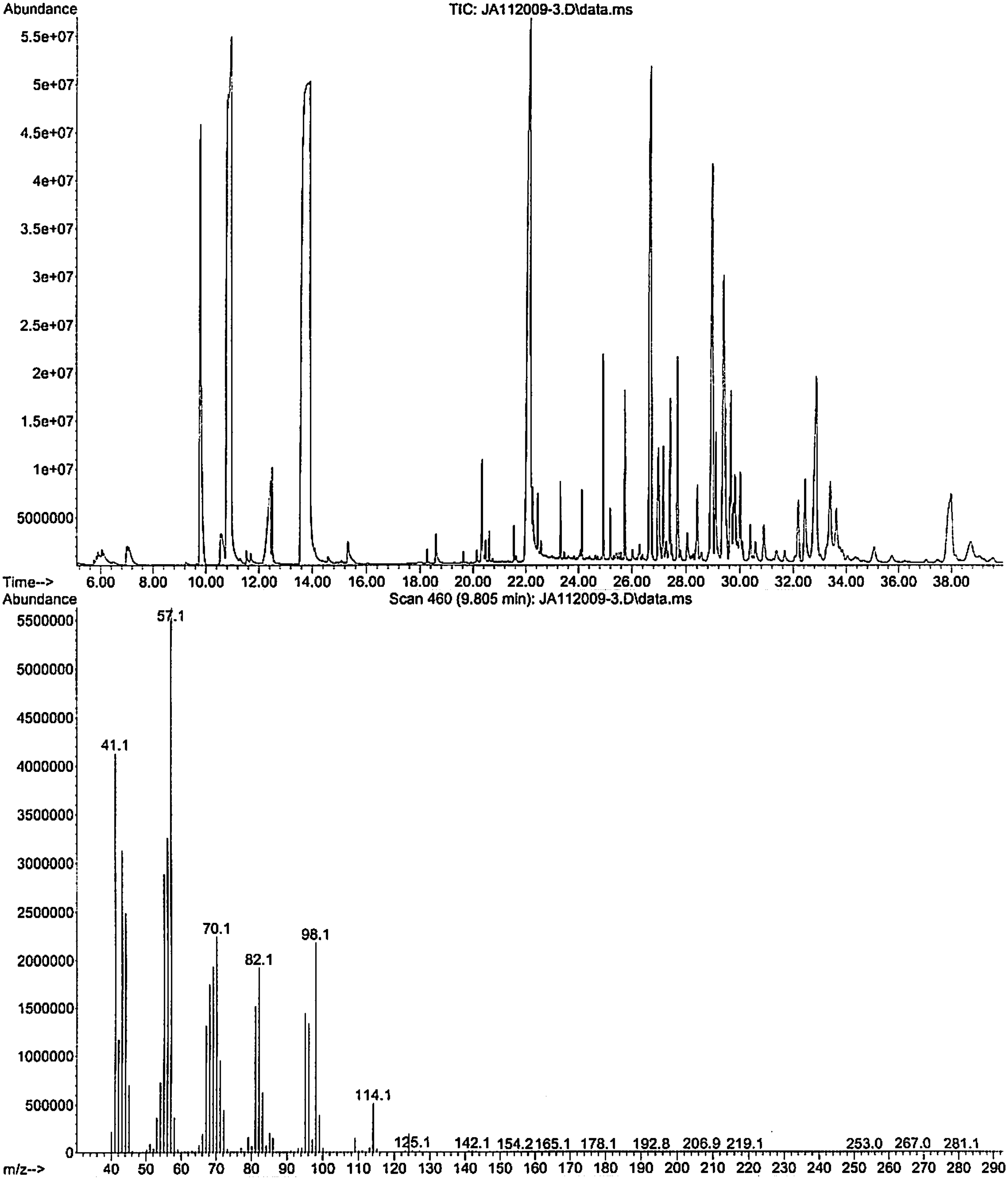

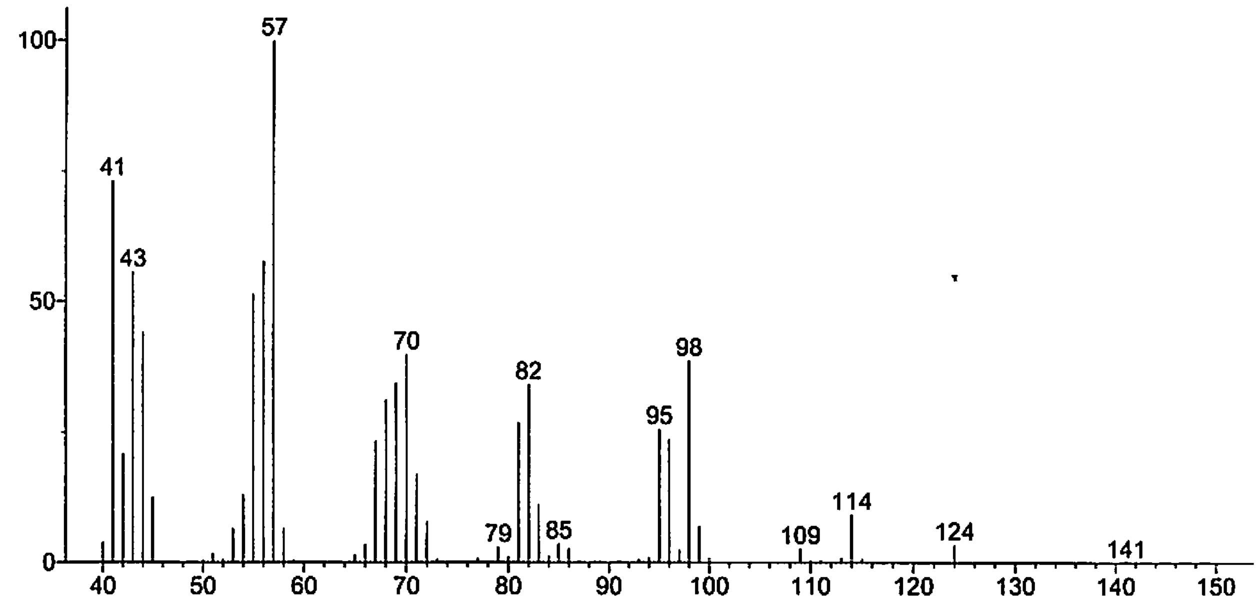


(Text File) Scan 460 (9.805 min): JA112009-3.D\data.ms

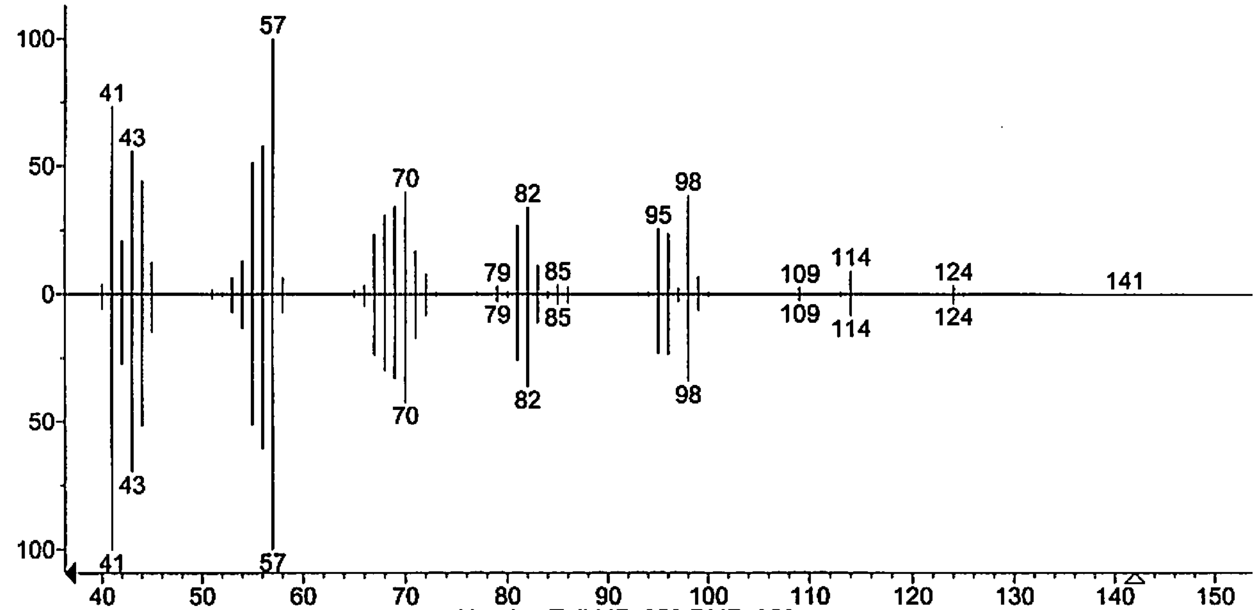


Head to Tail MF=952 RMF=953

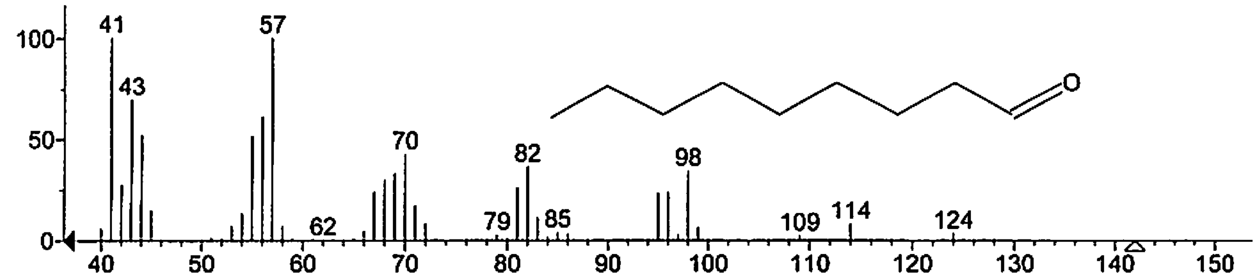


(replib) Nonanal

File :D:\Aldrich\JA-09\JA112009-3.D
Operator :
Acquired : 20 Nov 2009 15:24 using AcqMethod JA-50-280LESS.M
Instrument : Buba
Sample Name: 15 M C.oculata abd.-fed 8-OH-citronellol
Misc Info : 3-10d-old; fed 1wk; 100ul conc.to 5ulCH2Cl2
Vial Number: 1

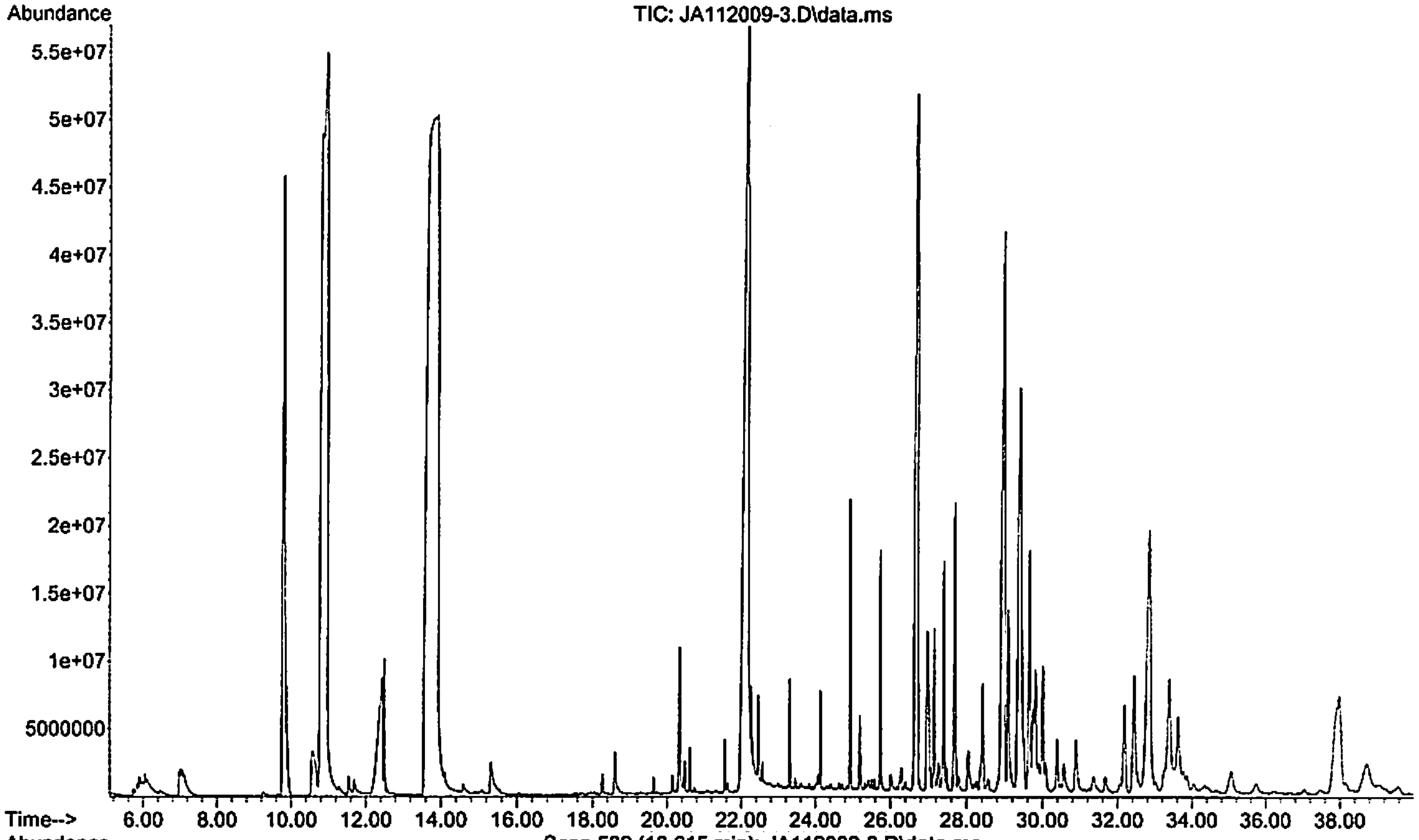


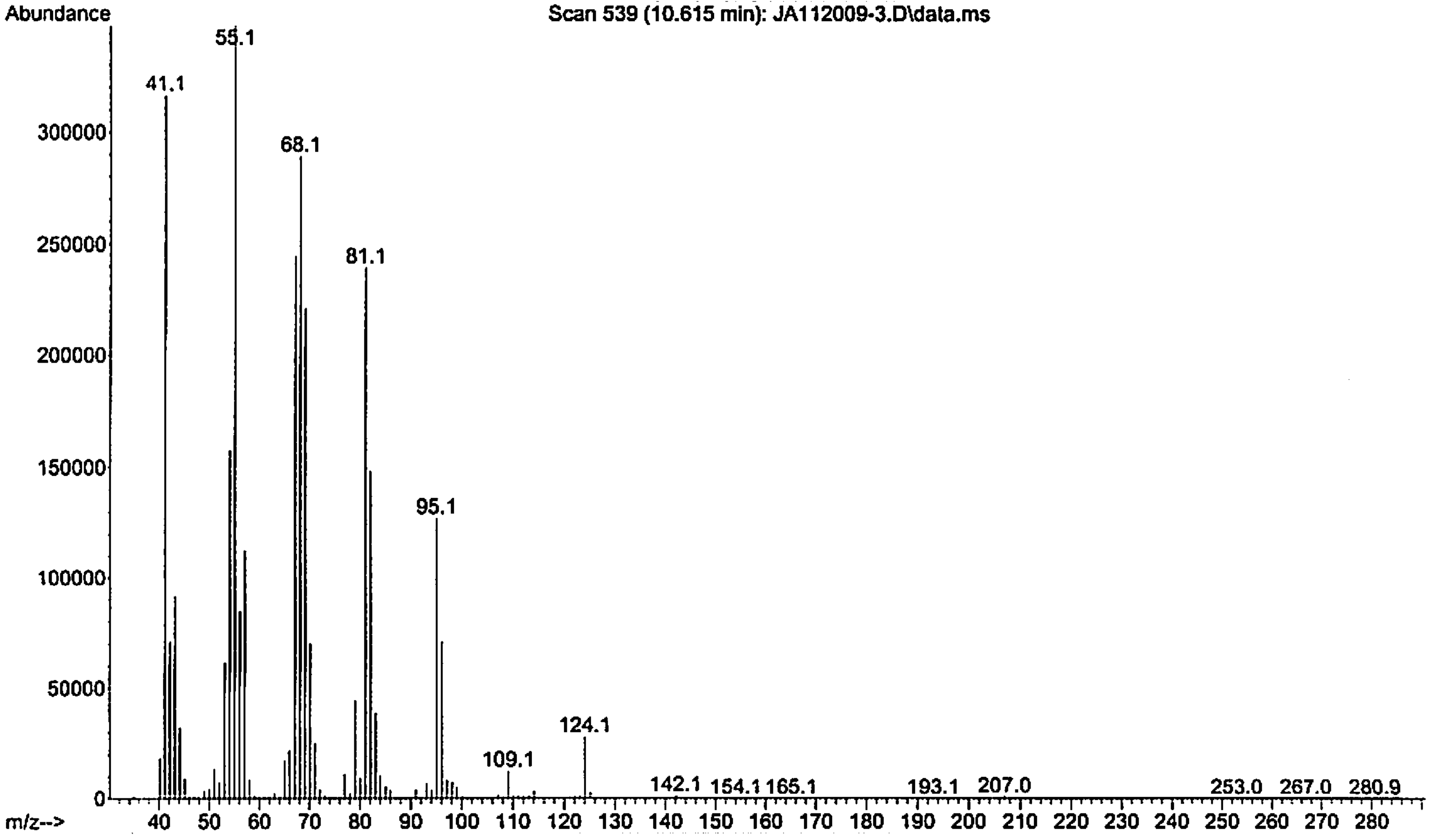

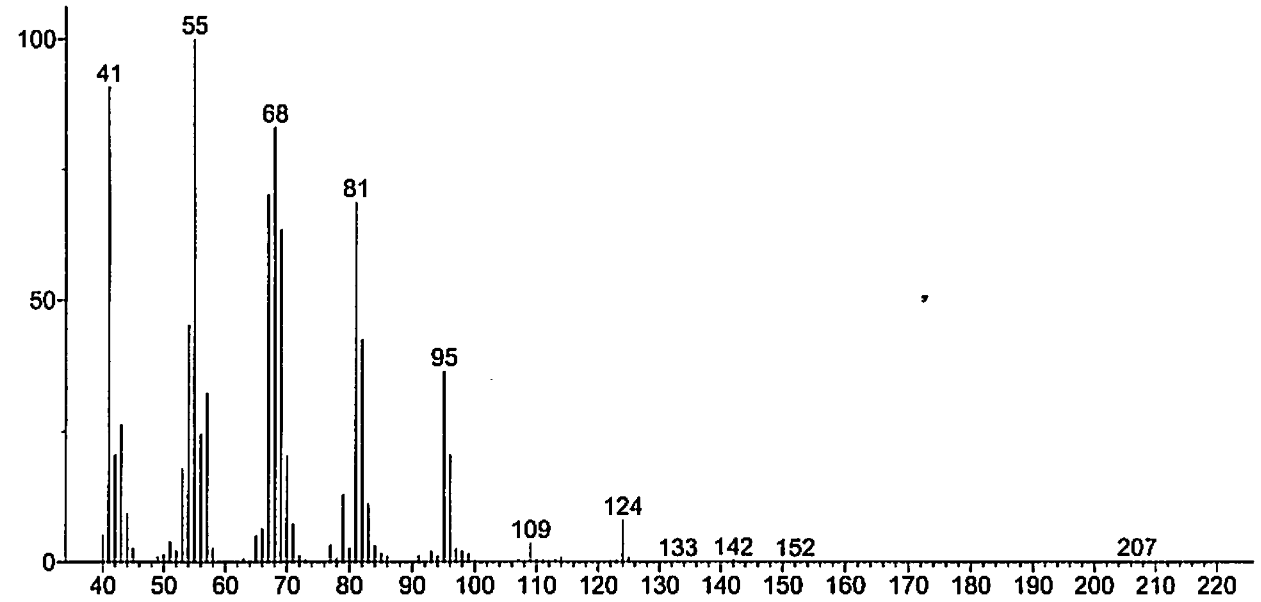


(Text File) Scan 539 (10.615 min): JA112009-3.D\data.ms

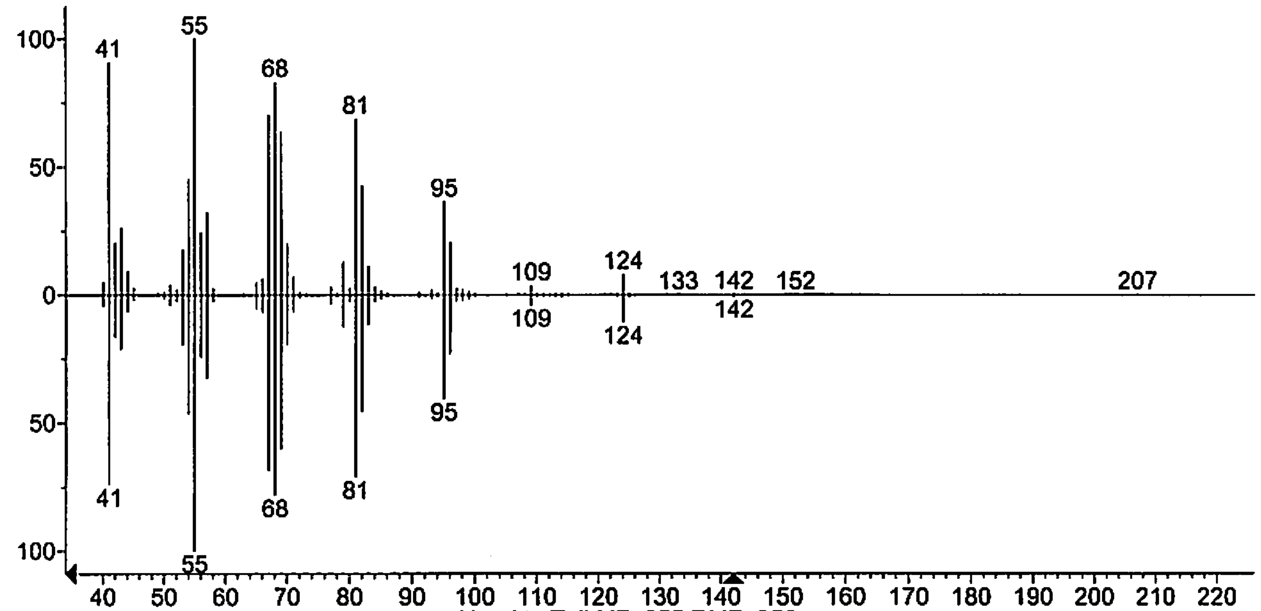


Head to Tail MF=955 RMF=956

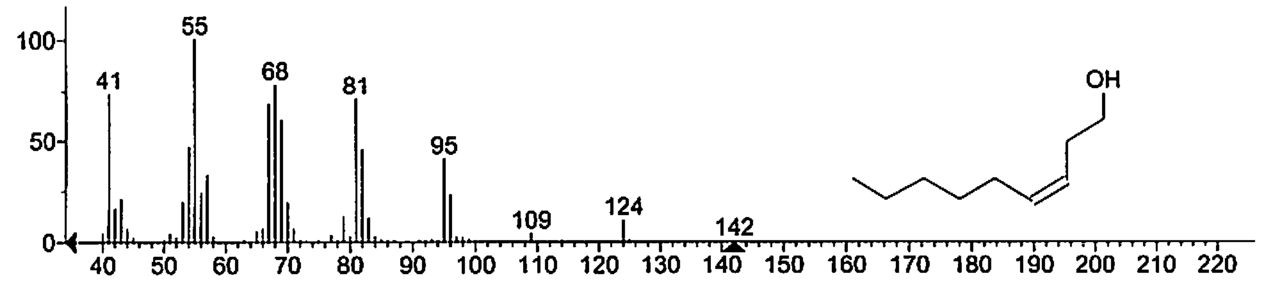


(mainlib) 3-Nonen-1-ol, (Z)-

File        :D:\Aldrich\JA-09\JA112009-3.D
Operator    :
Acquired    : 20 Nov 2009  15:24     using AcqMethod JA-50-280LESS.M
Instrument :    Buba
Sample Name: 15 M C.oculata abd.-fed 8-OH-citronellol
Misc Info   : 3-10d-old; fed 1wk; 100ul conc.to 5ulCH2Cl2
Vial Number: 1

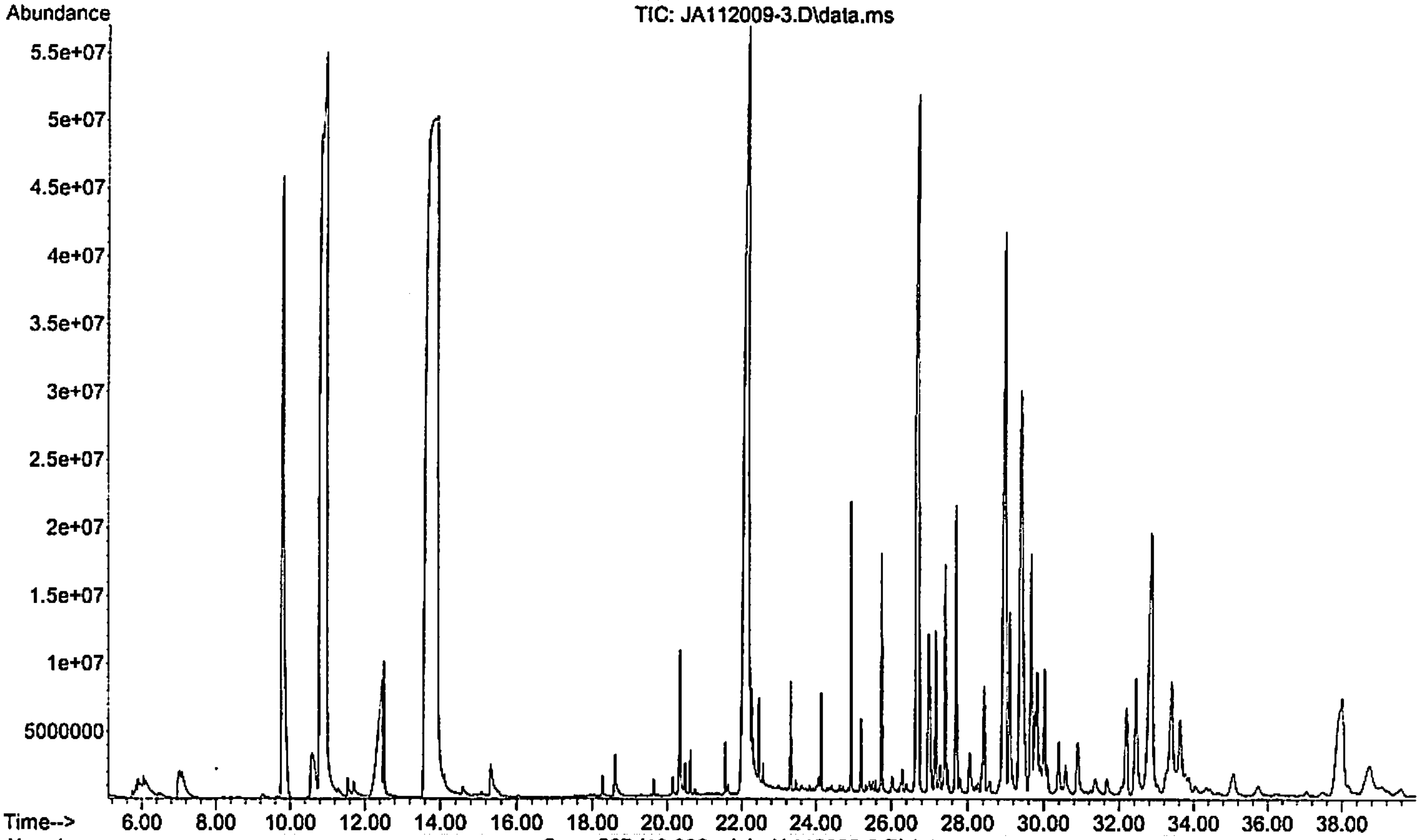


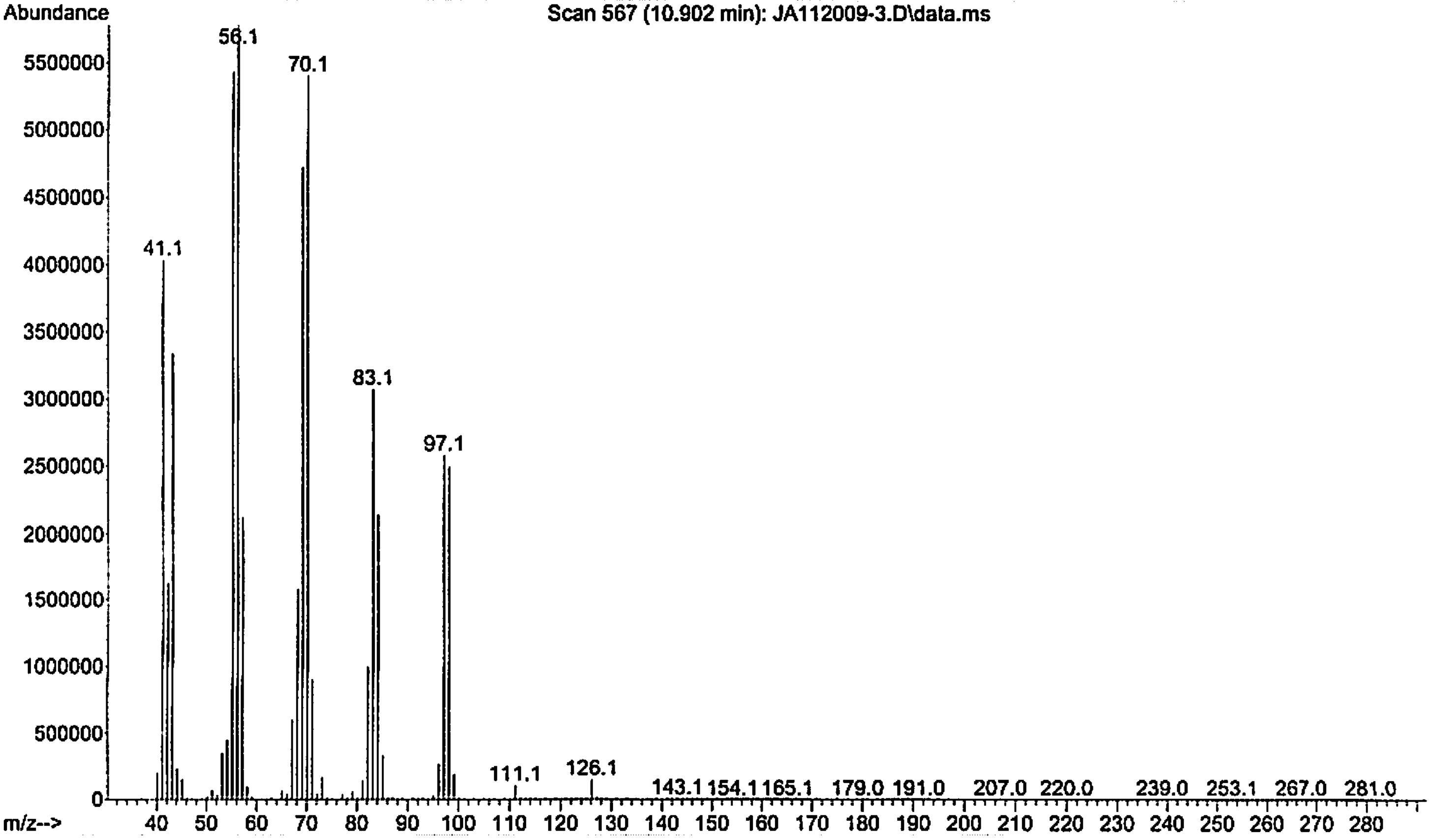

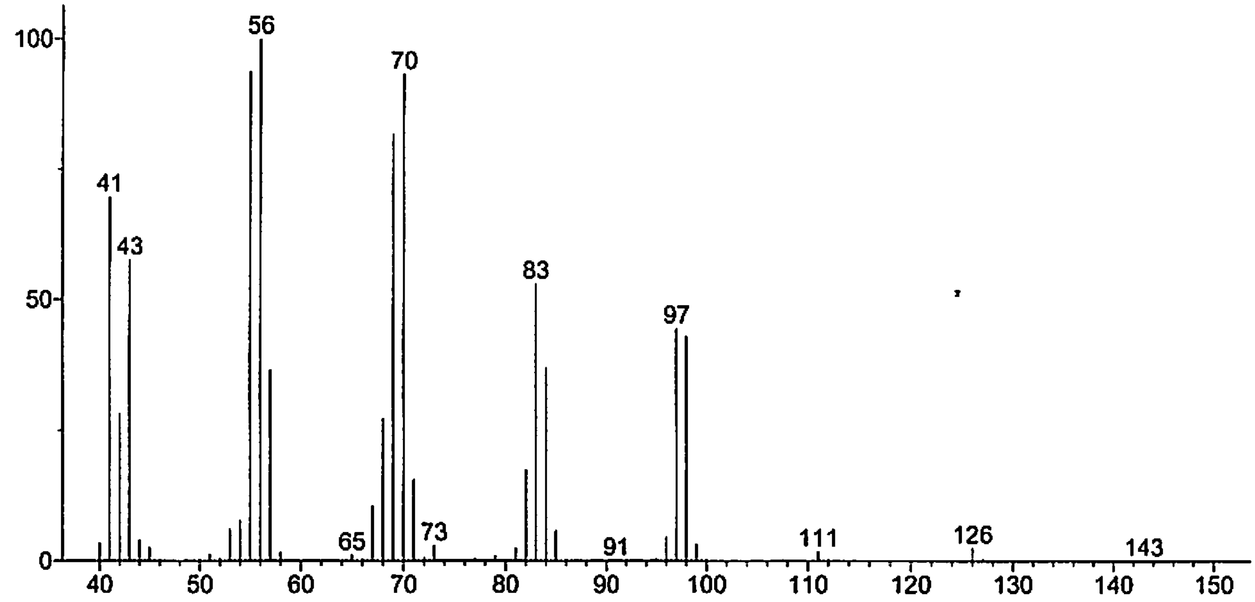


(Text File) Scan 567 (10.902 min): JA112009-3.D\data.ms

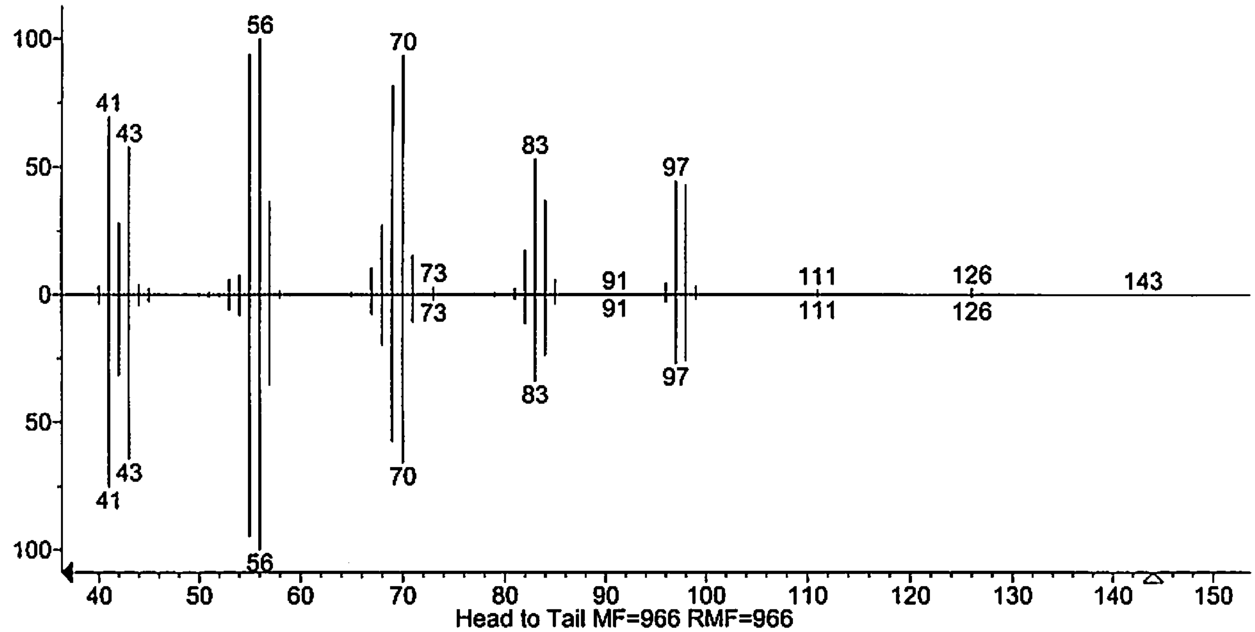


Head to Tail MF=966 RMF=966

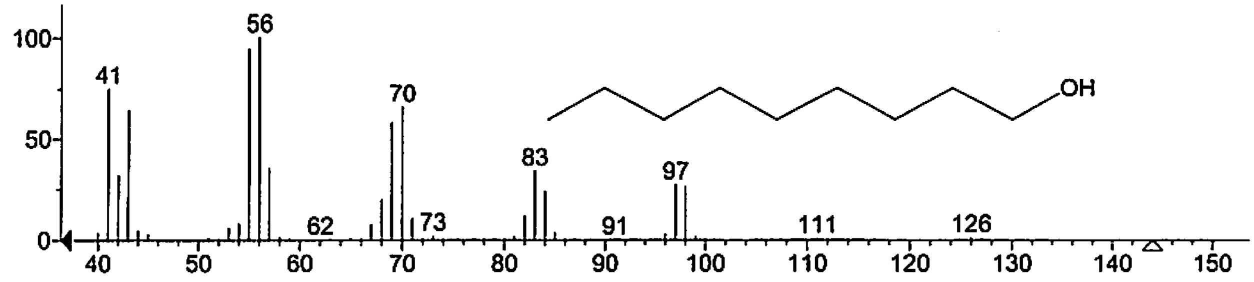


(replib) 1-Nonanol

```
File        :D:\Aldrich\JA-09\JA112009-3.D
Operator    :
Acquired    : 20 Nov 2009  15:24     using AcqMethod JA-50-280LESS.M
Instrument  :    Buba
Sample Name: 15 M C.oculata abd.-fed 8-OH-citronellol
Misc Info   : 3-10d-old; fed 1wk; 100ul conc.to 5ulCH2Cl2
Vial Number: 1
```

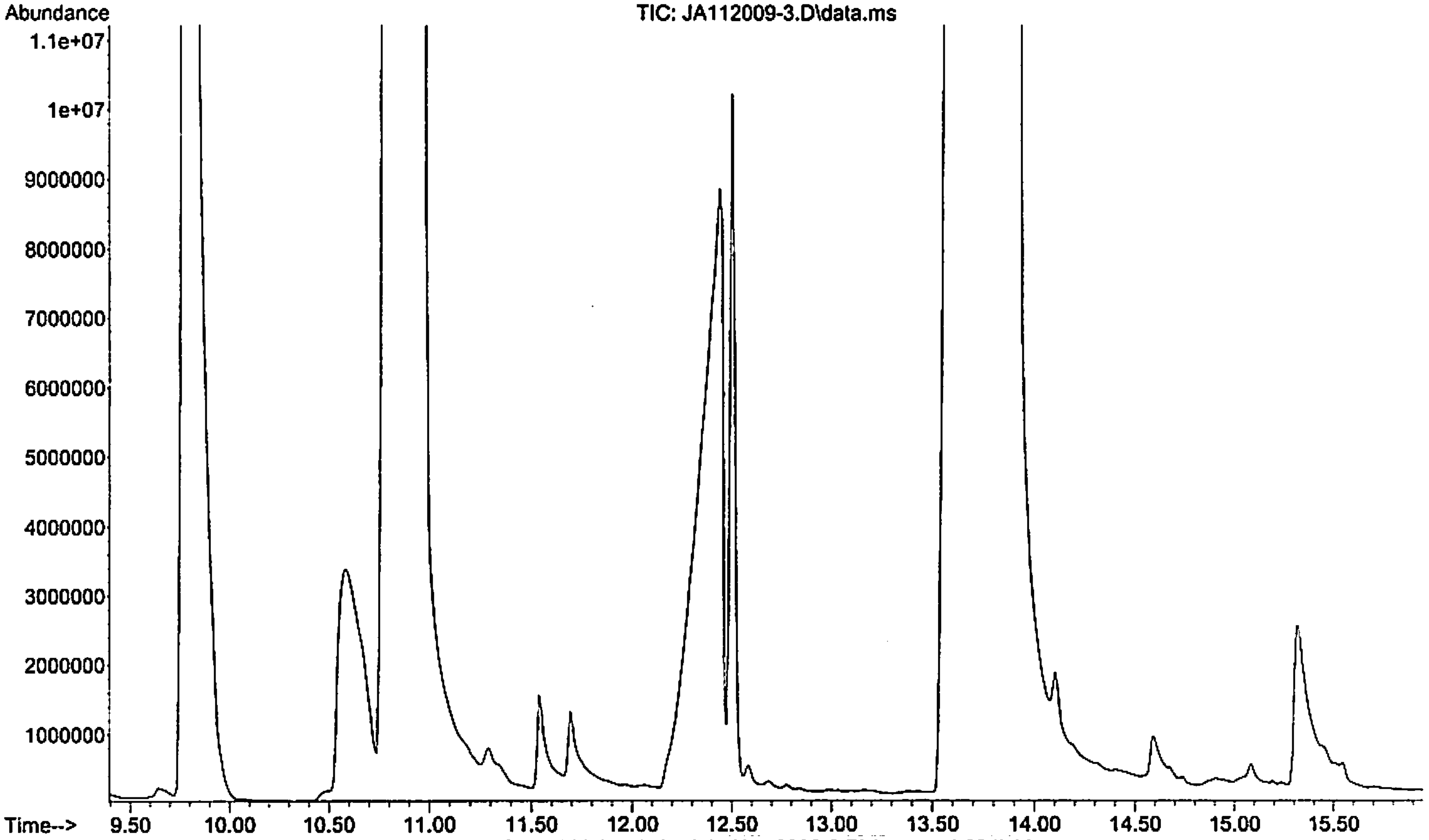


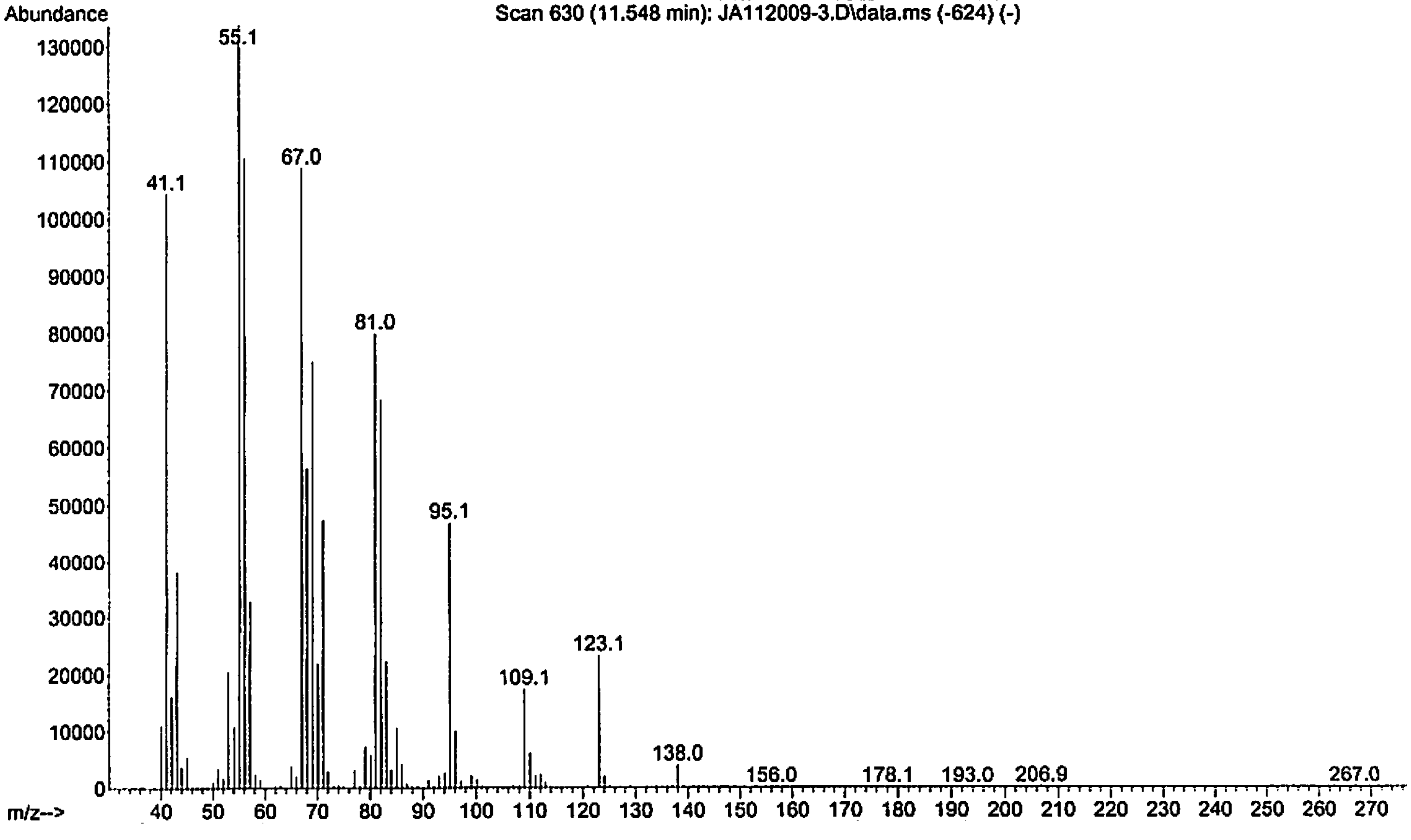

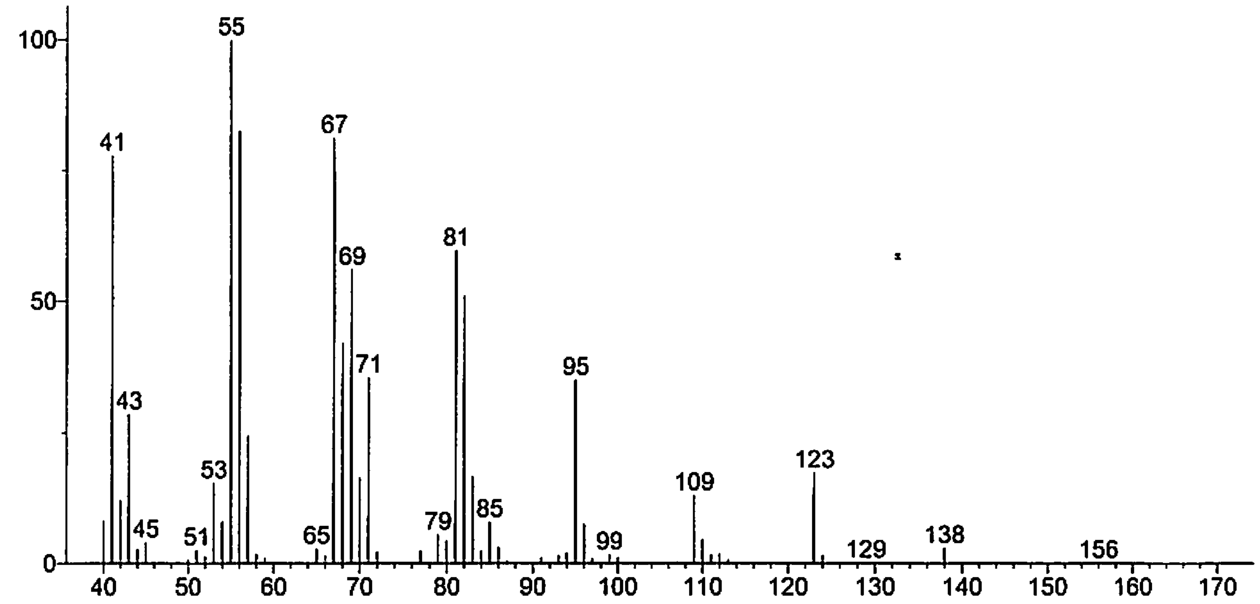


(Text File) Scan 630 (11.548 min): JA112009-3.D\data.ms (-624)

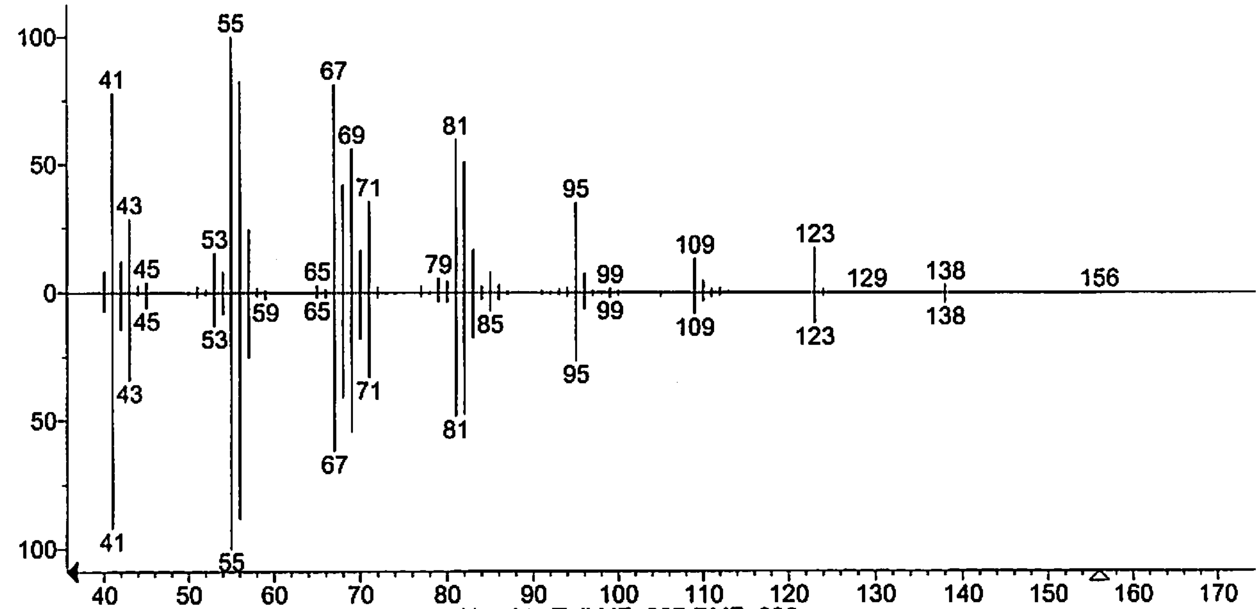


Head to Tail MF=897 RMF=898

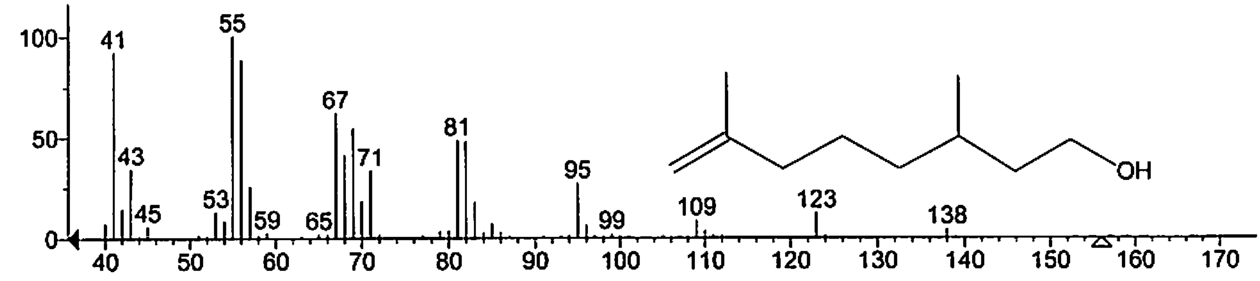


(mainlib) 7-Octen-1-ol, 3,7-dimethyl-, (S)-

File :D:\Aldrich\JA-09\JA112009-3.D
Operator :
Acquired : 20 Nov 2009 15:24 using AcqMethod JA-50-280LESS.M
Instrument : Buba
Sample Name: 15 M C.oculata abd.-fed 8-OH-citronellol
Misc Info : 3-10d-old; fed 1wk; 100ul conc.to 5ulCH2Cl2
Vial Number: 1

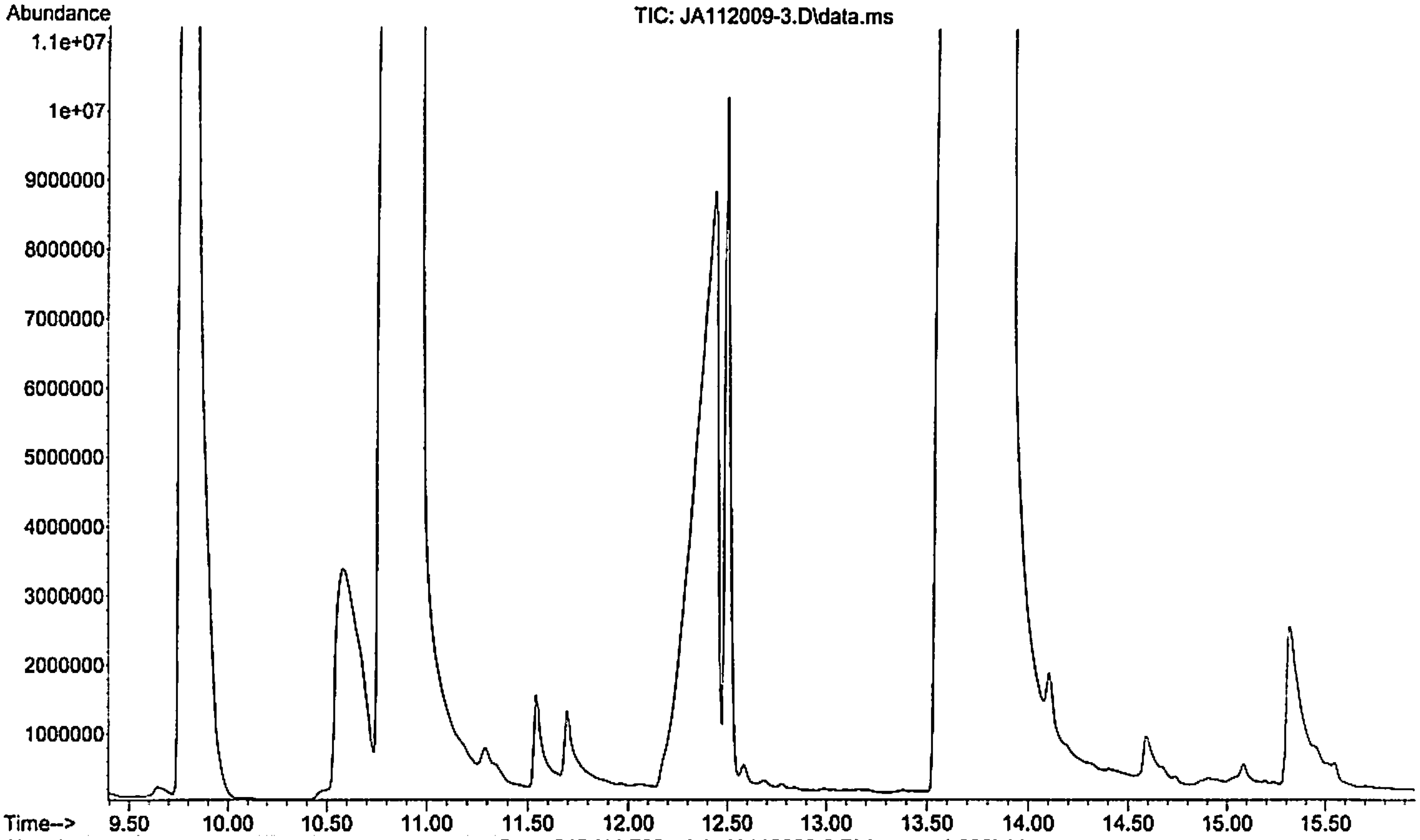


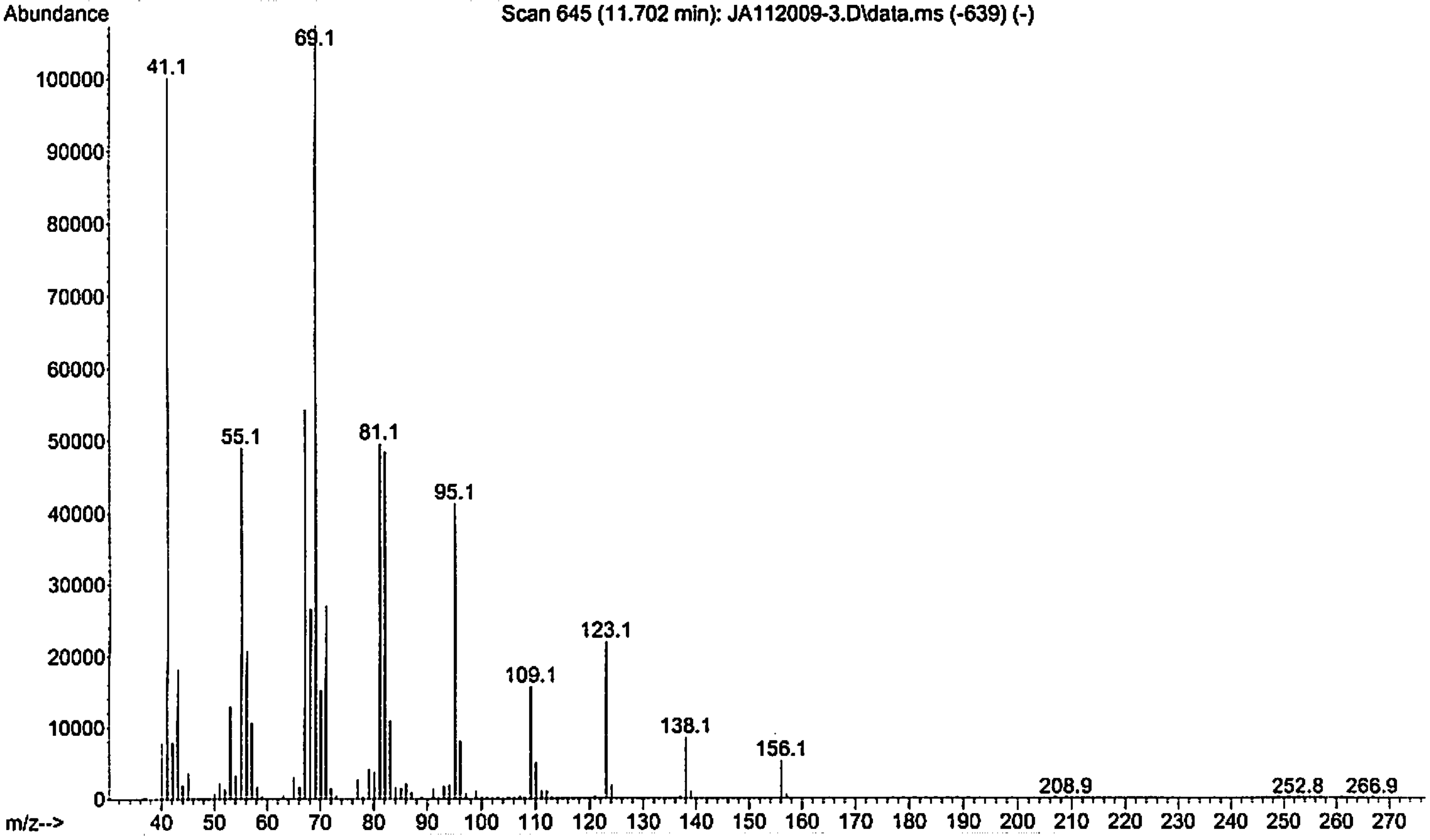

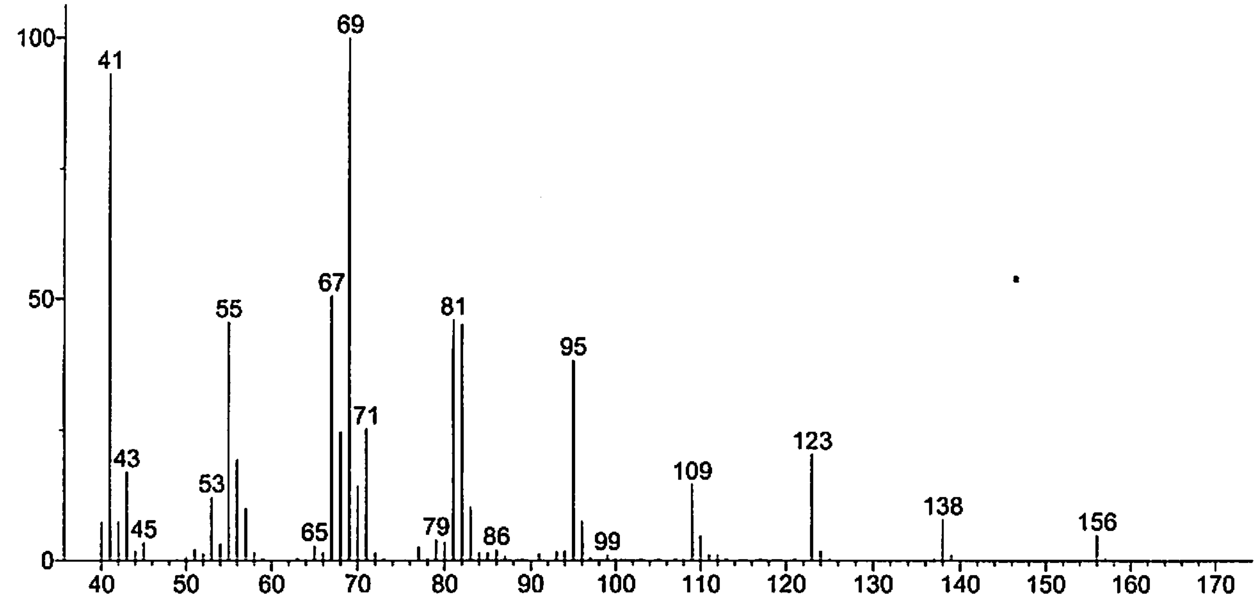

(Text File) Scan 645 (11.702 min): JA112009-3.D\data.ms (-639)

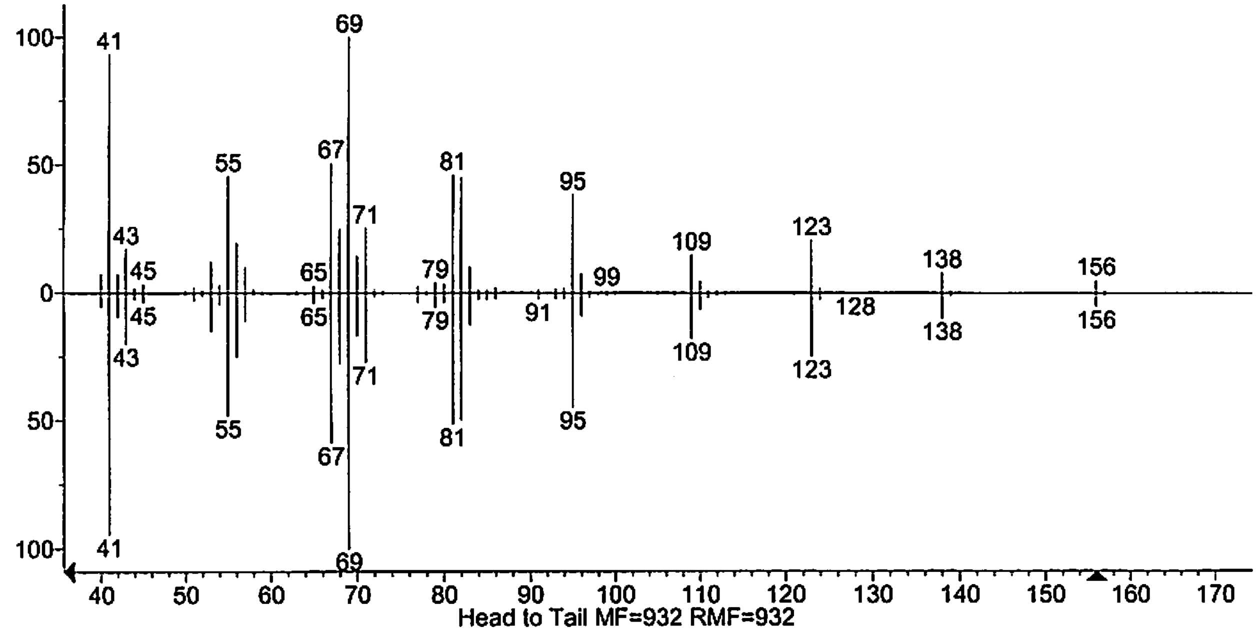


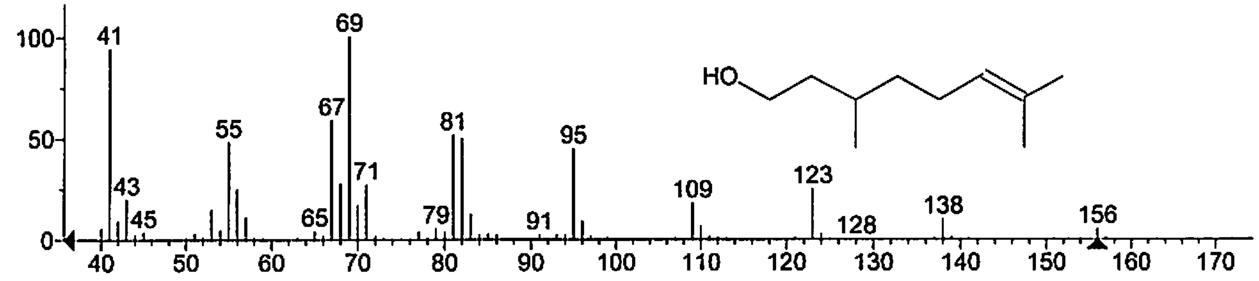

(mainlib) 6-Octen-1-ol, 3,7-dimethyl-

```
File          :D:\Aldrich\JA-09\JA112009-3.D
Operator      :
Acquired      : 20 Nov 2009  15:24     using AcqMethod JA-50-280LESS.M
Instrument :      Buba
Sample Name: 15 M C.oculata abd.-fed 8-OH-citronellol
Misc Info   : 3-10d-old; fed 1wk; 100ul conc.to 5ulCH2Cl2
Vial Number: 1
```

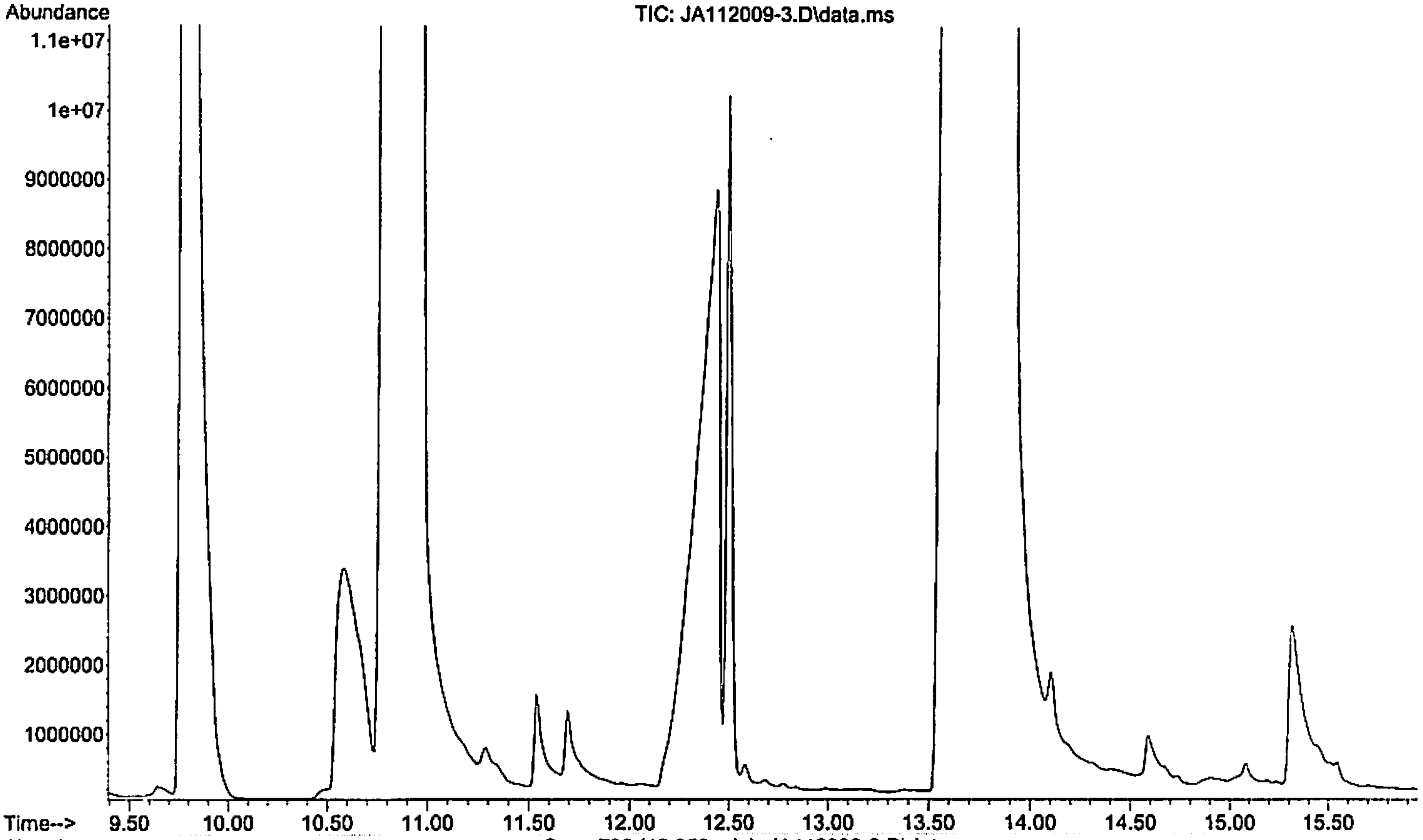


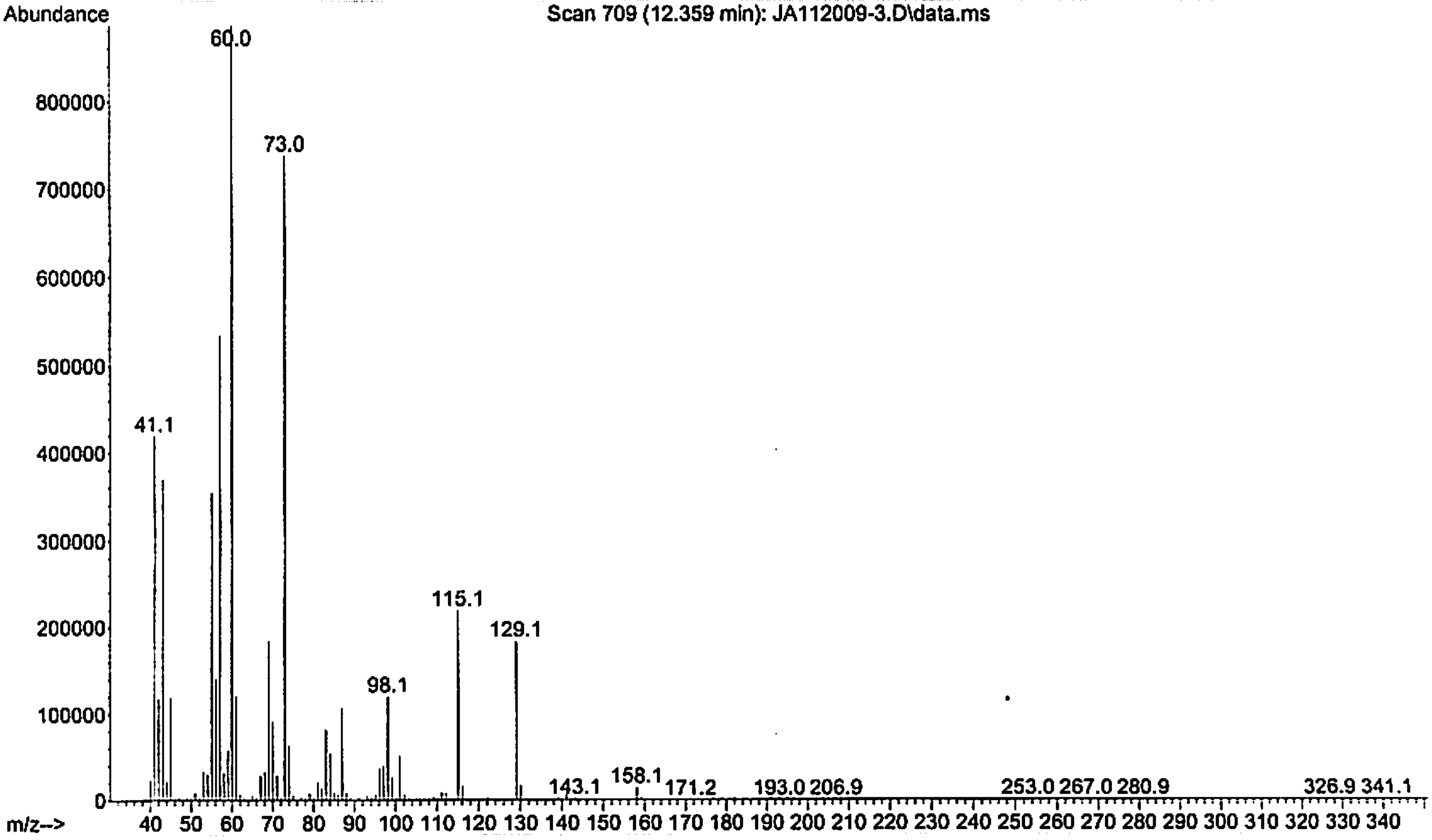

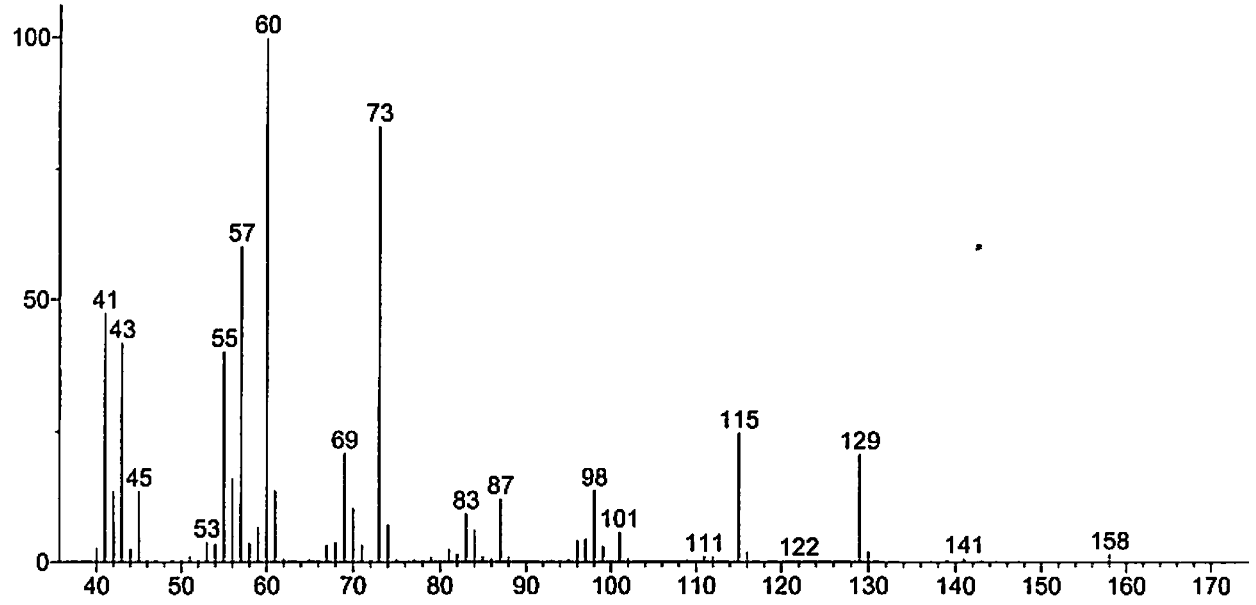


(Text File) Scan 709 (12.359 min): JA112009-3.D\data.ms

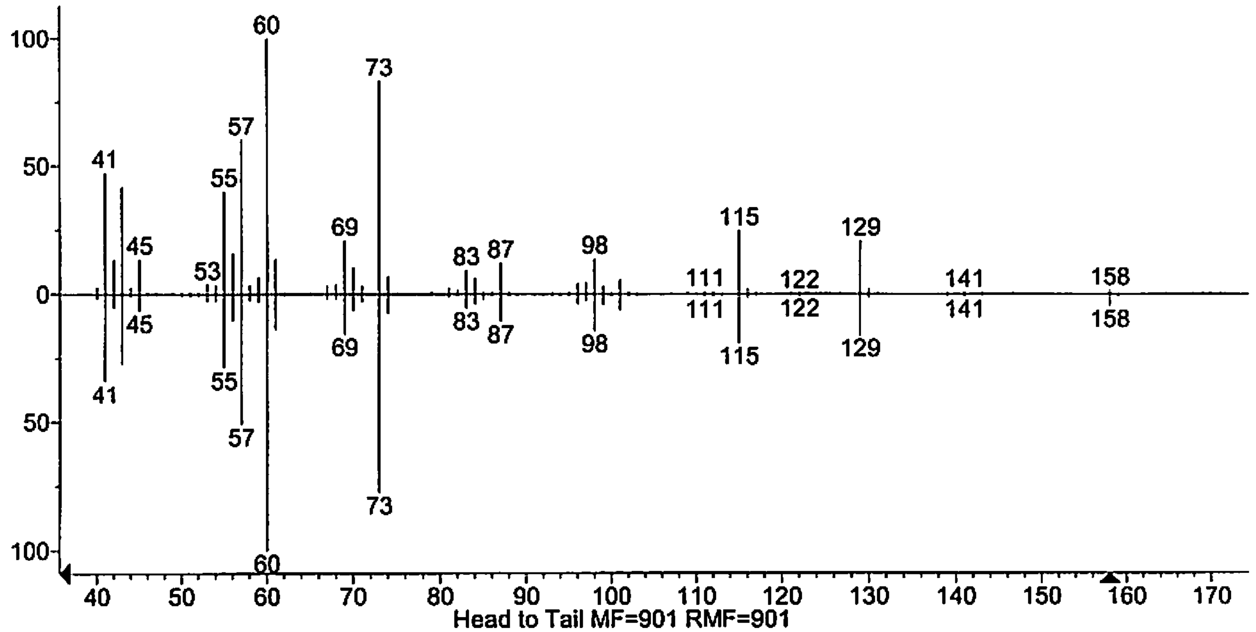


Head to Tail MF=901 RMF=901

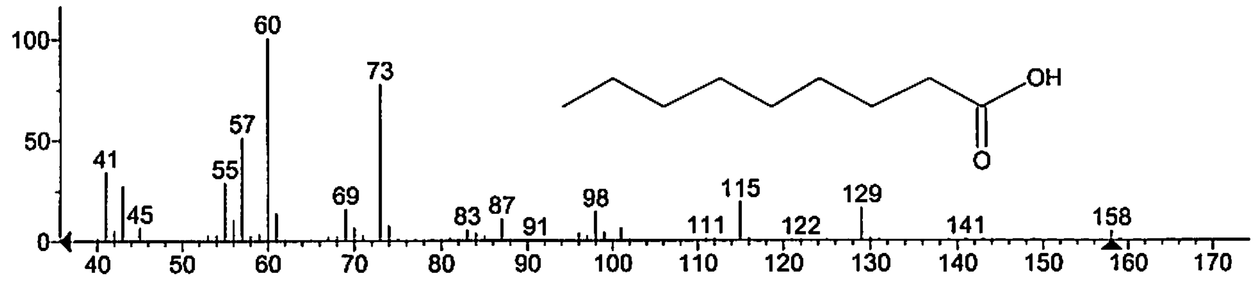


(mainlib) Nonanoic acid

```
File        :D:\Aldrich\JA-09\JA112009-3.D
Operator    :
Acquired    : 20 Nov 2009  15:24     using AcqMethod JA-50-280LESS.M
Instrument :    Buba
Sample Name: 15 M C.oculata abd.-fed 8-OH-citronellol
Misc Info  : 3-10d-old; fed 1wk; 100ul conc.to 5ulCH2Cl2
Vial Number: 1
```

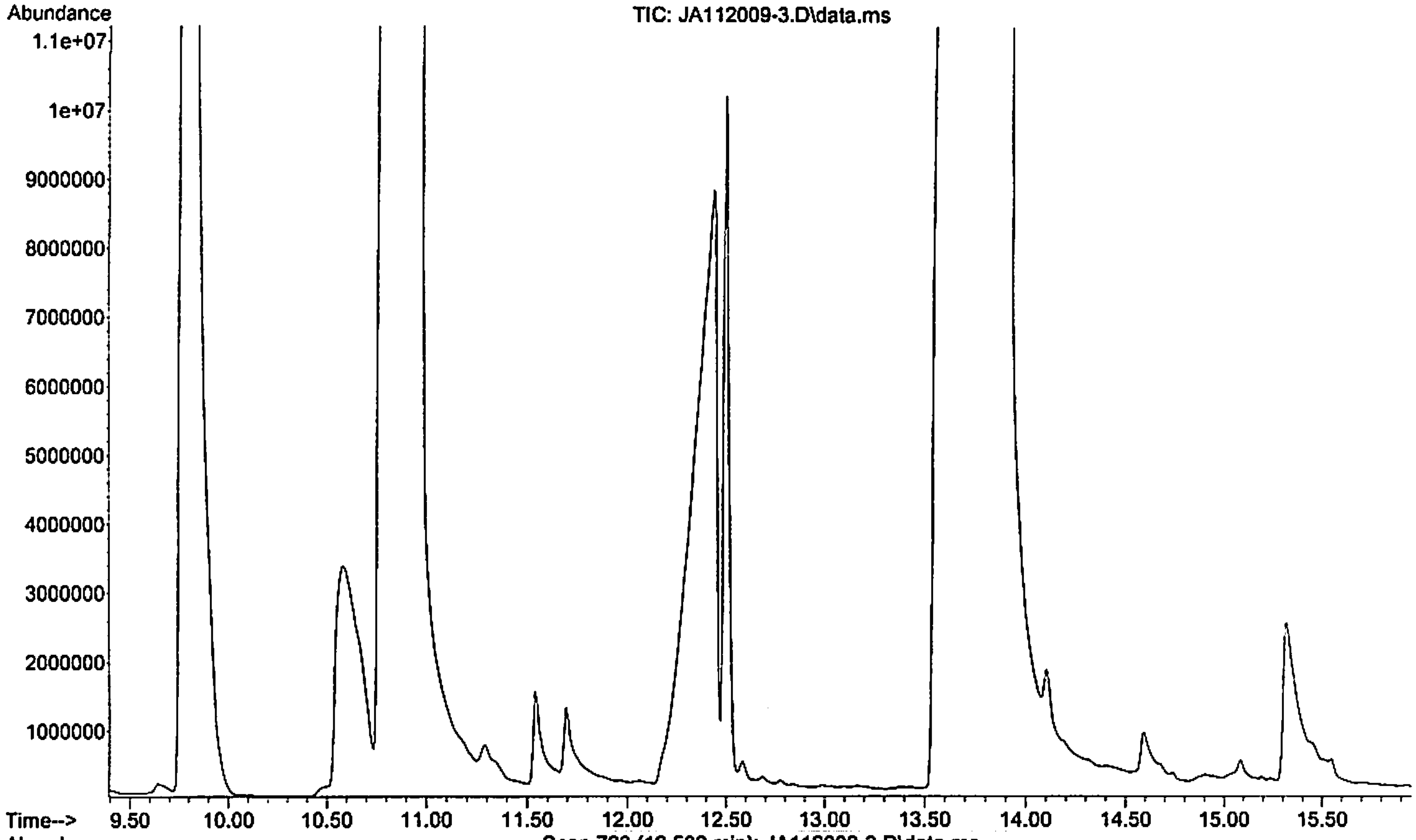


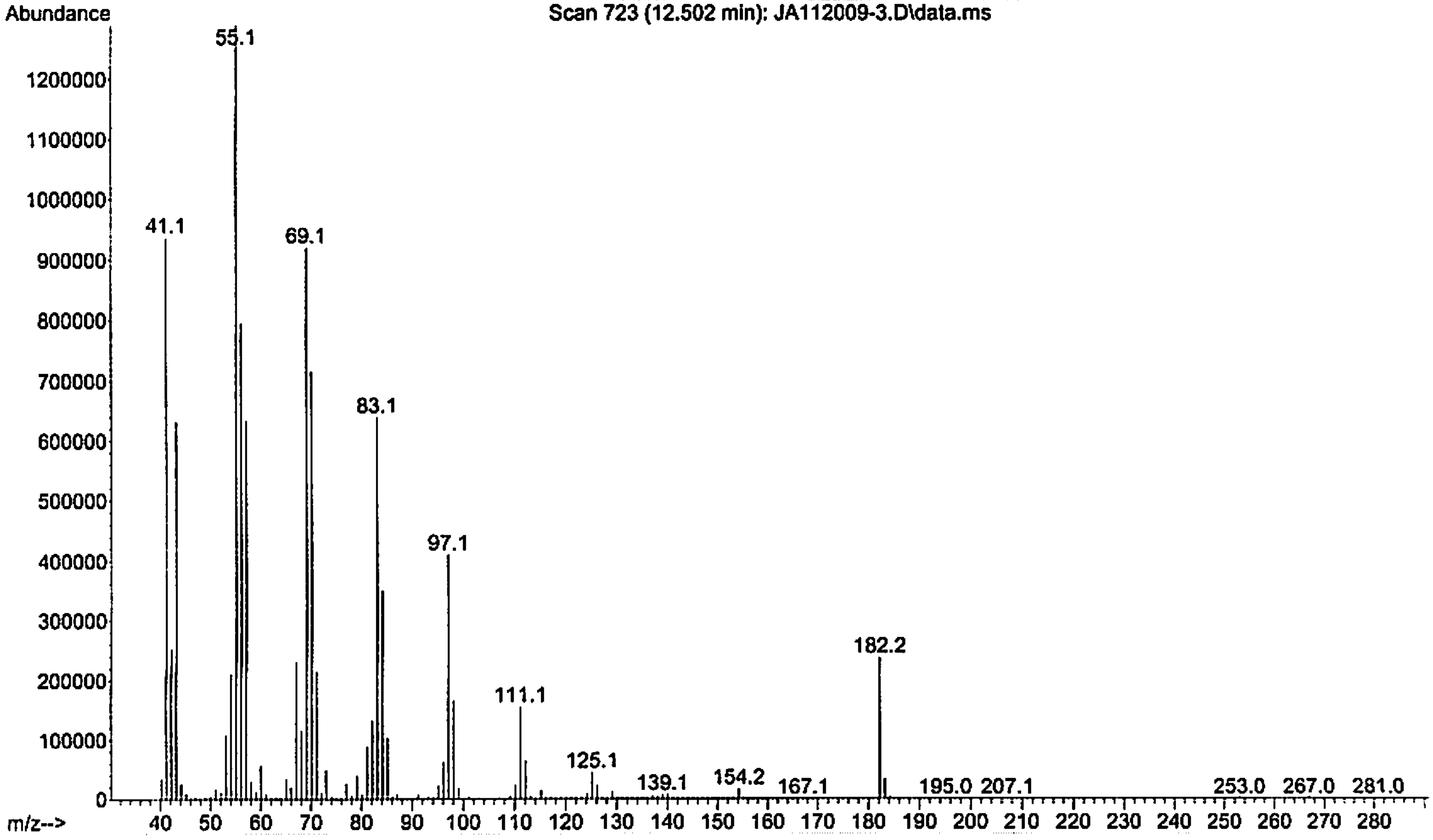

```
File        :D:\Aldrich\JA-09\JA112009-3.D
Operator    :
Acquired    : 20 Nov 2009  15:24     using AcqMethod JA-50-280LESS.M
Instrument :    Buba
Sample Name: 15 M C.oculata abd.-fed 8-OH-citronellol
Misc Info   : 3-10d-old; fed 1wk; 100ul conc.to 5ulCH2Cl2
Vial Number: 1
```

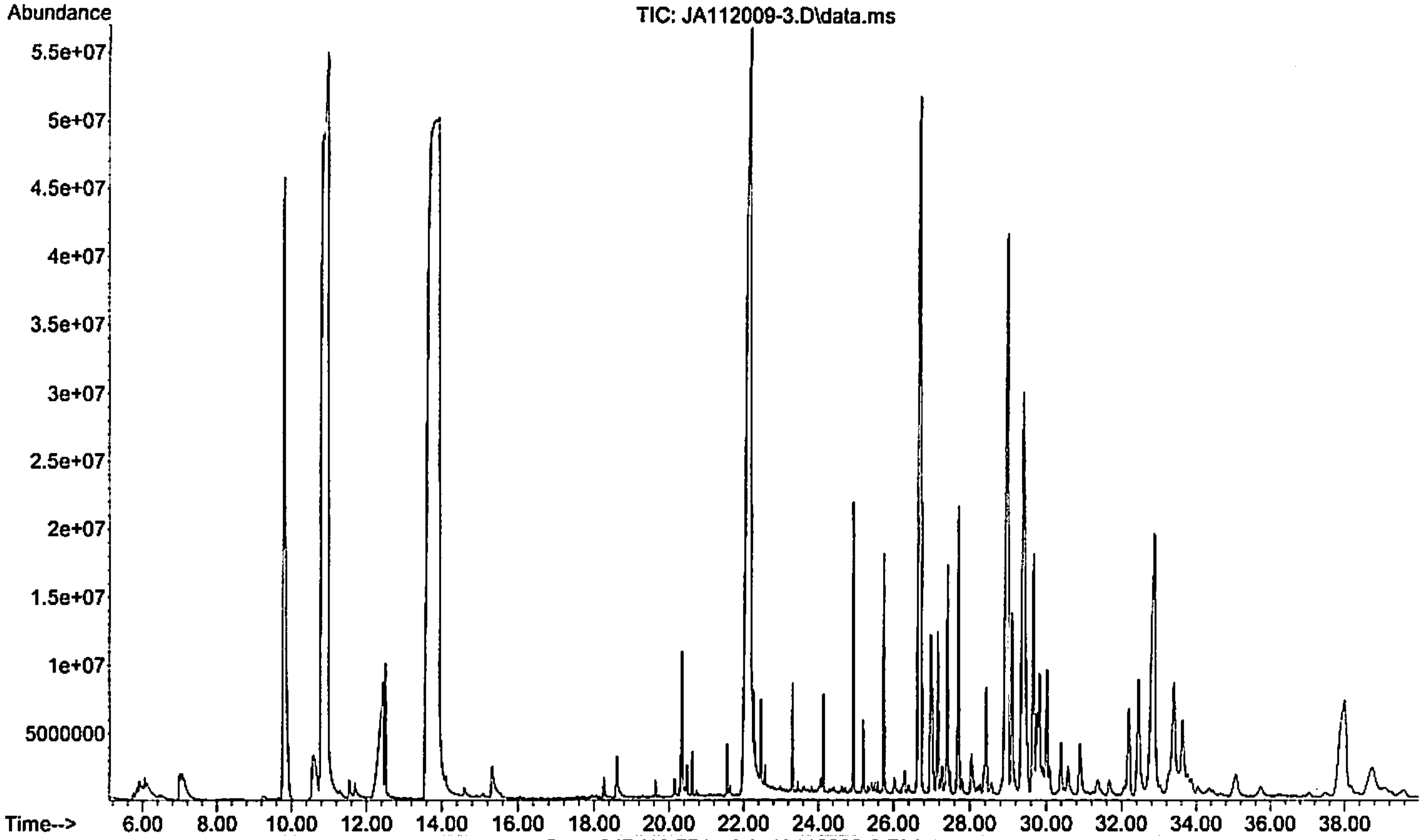


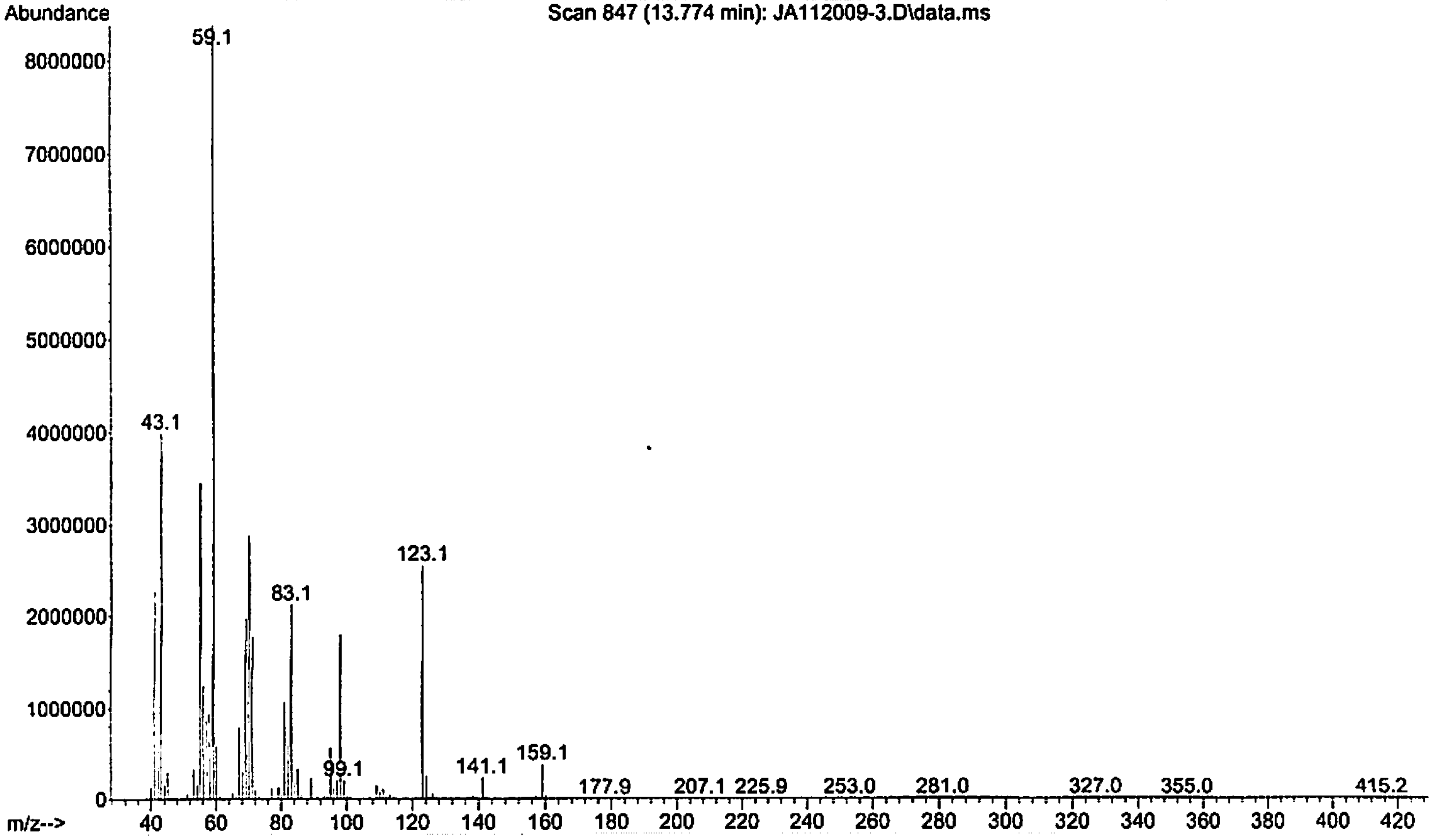

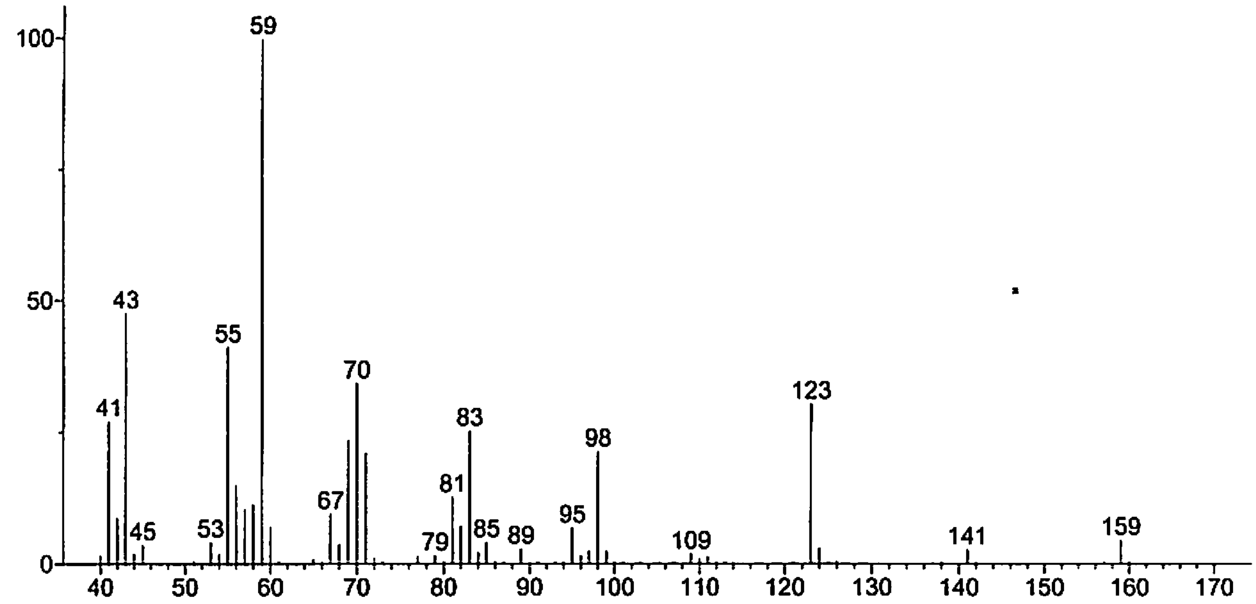


(Text File) Scan 847 (13.774 min): JA112009-3.D\data.ms

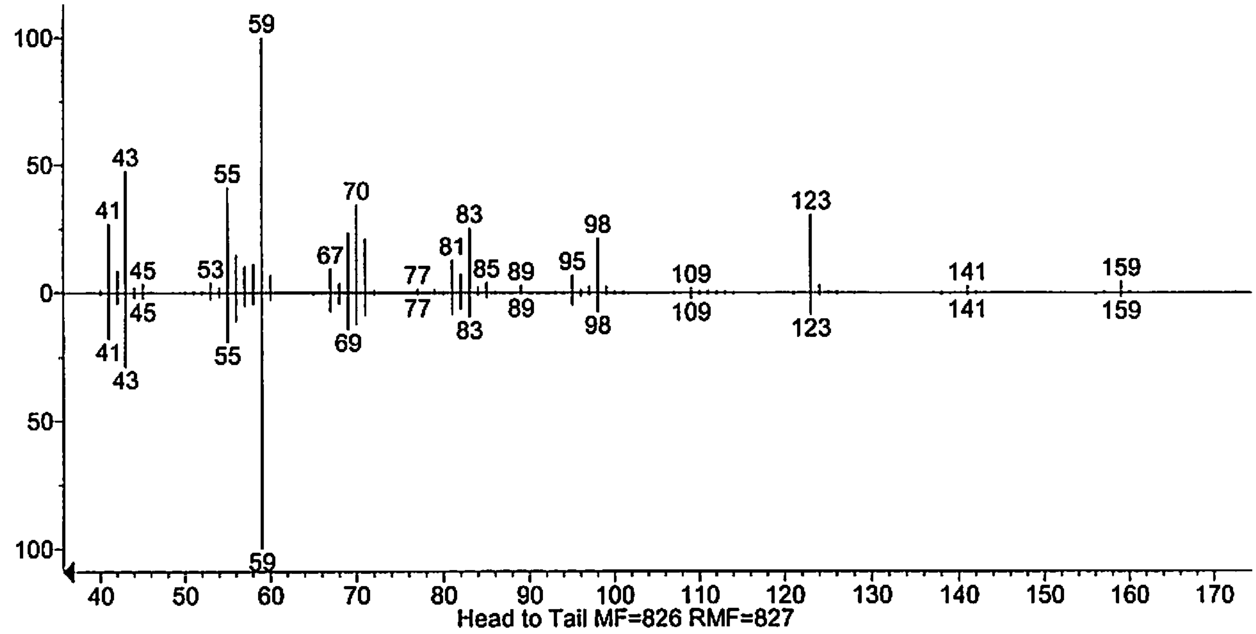


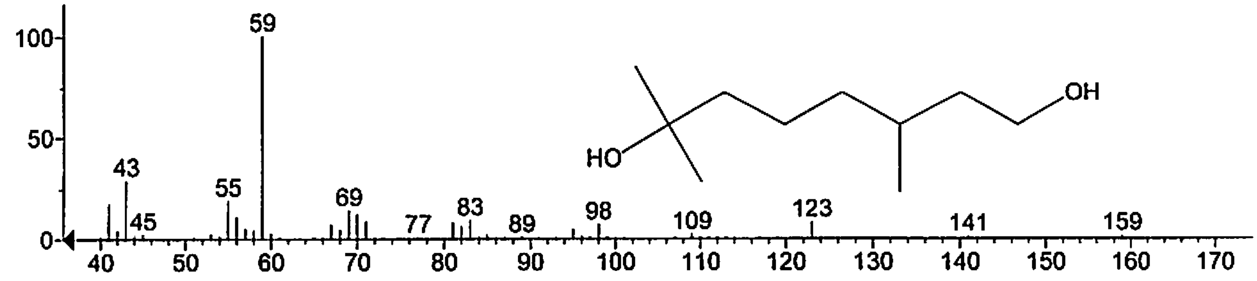


(mainlib) 1,7-Octanediol, 3,7-dimethyl-

```
File        :D:\Aldrich\JA-09\JA112009-3.D
Operator    :
Acquired    : 20 Nov 2009  15:24     using AcqMethod JA-50-280LESS.M
Instrument  :    Buba
Sample Name: 15 M C.oculata abd.-fed 8-OH-citronellol
Misc Info   : 3-10d-old; fed 1wk; 100ul conc.to 5ulCH2Cl2
Vial Number: 1
```

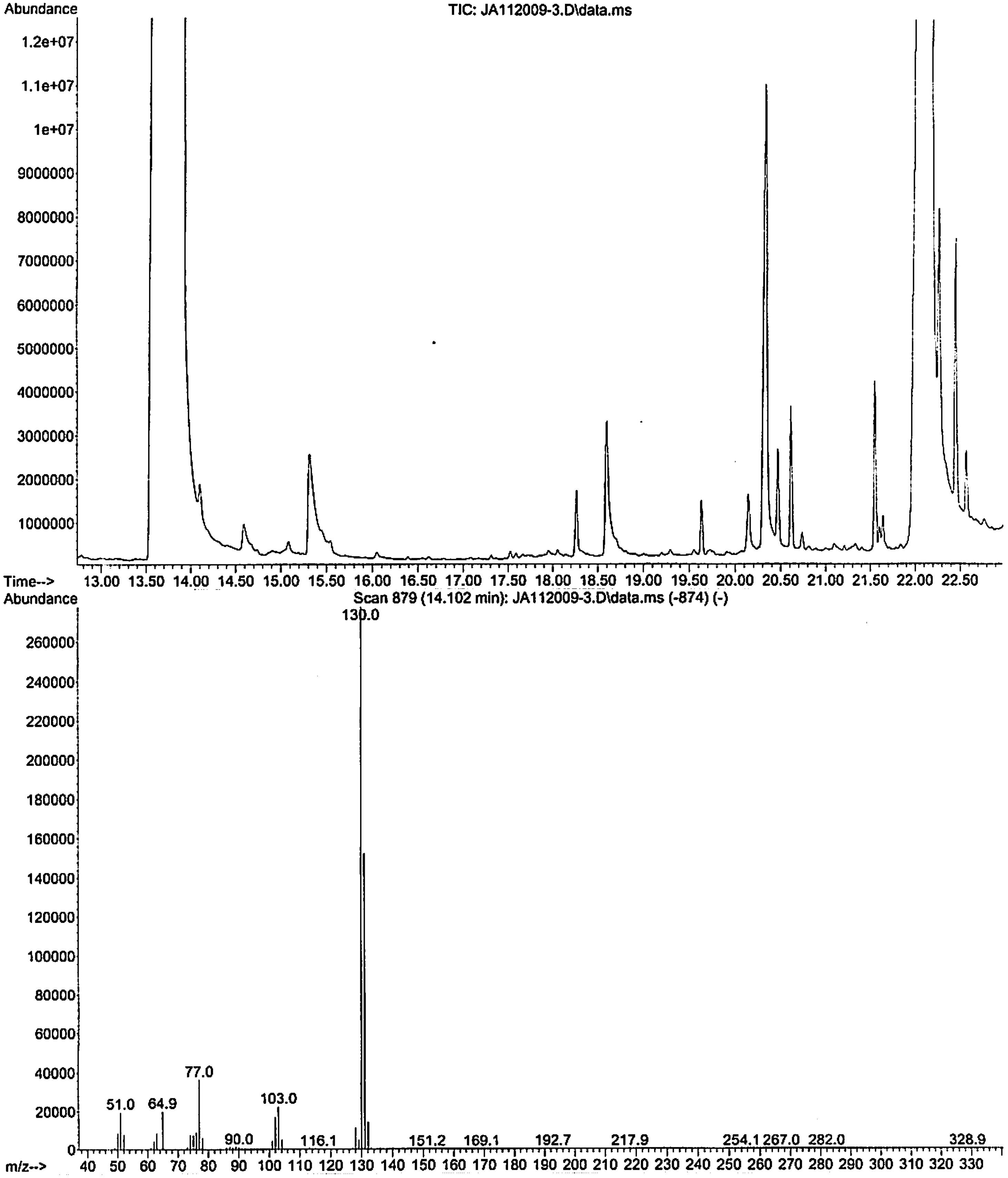

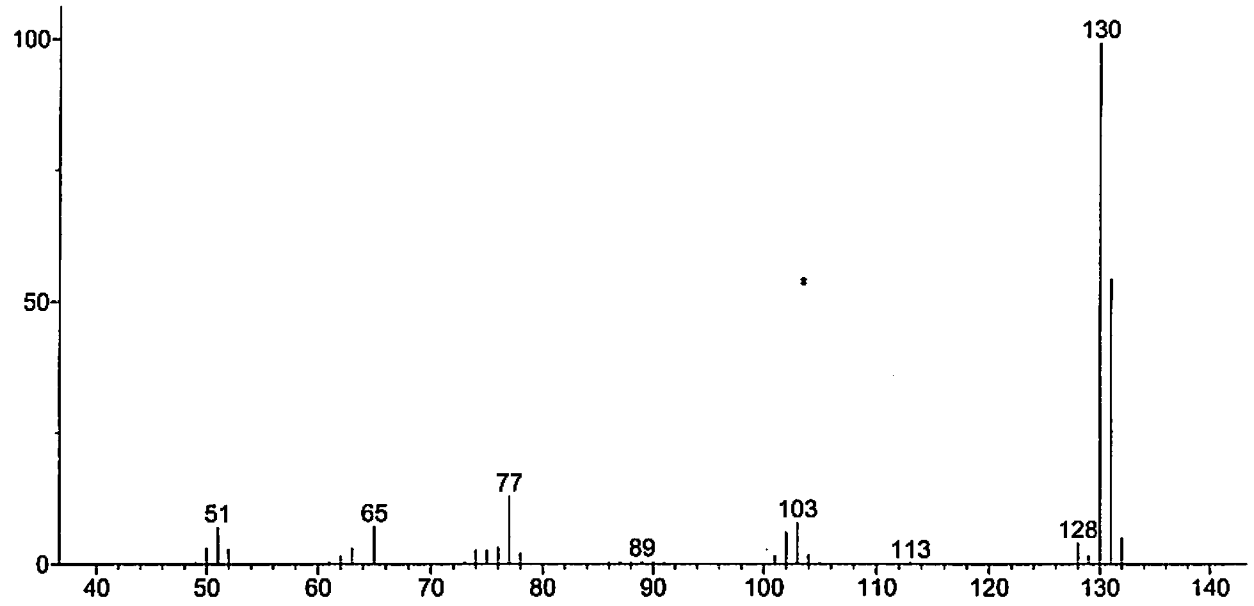


(Text File) Scan 879 (14.102 min): JA112009-3.D\data.ms (-874)

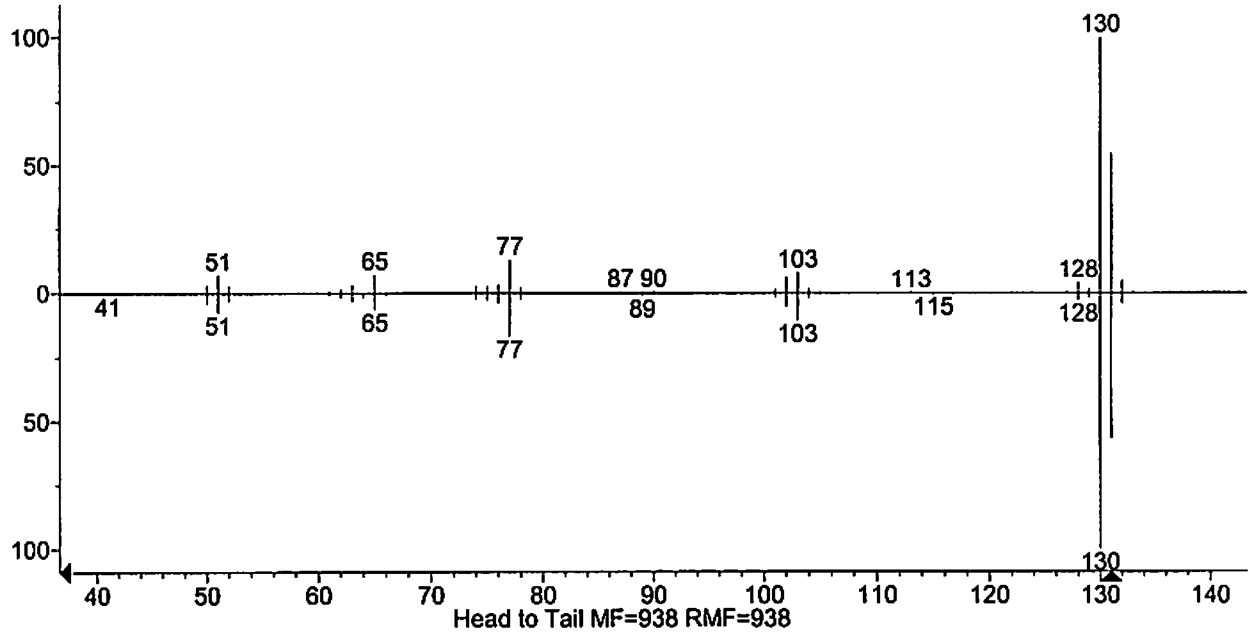


Head to Tail MF=938 RMF=938

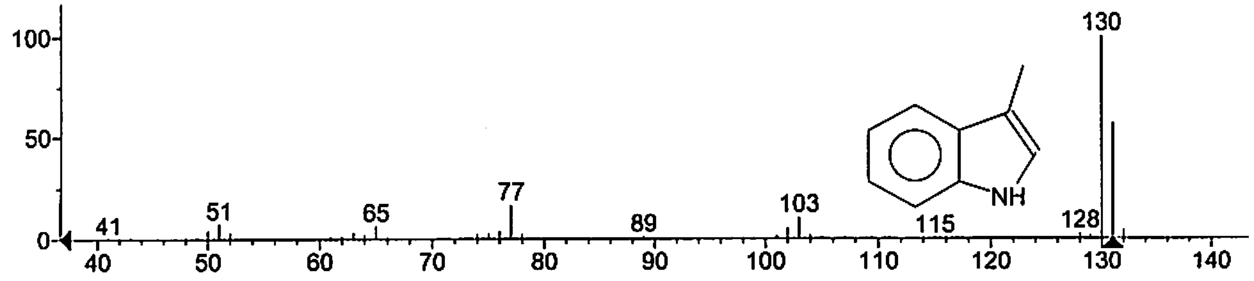


(replib) 1H-Indole, 3-methyl-

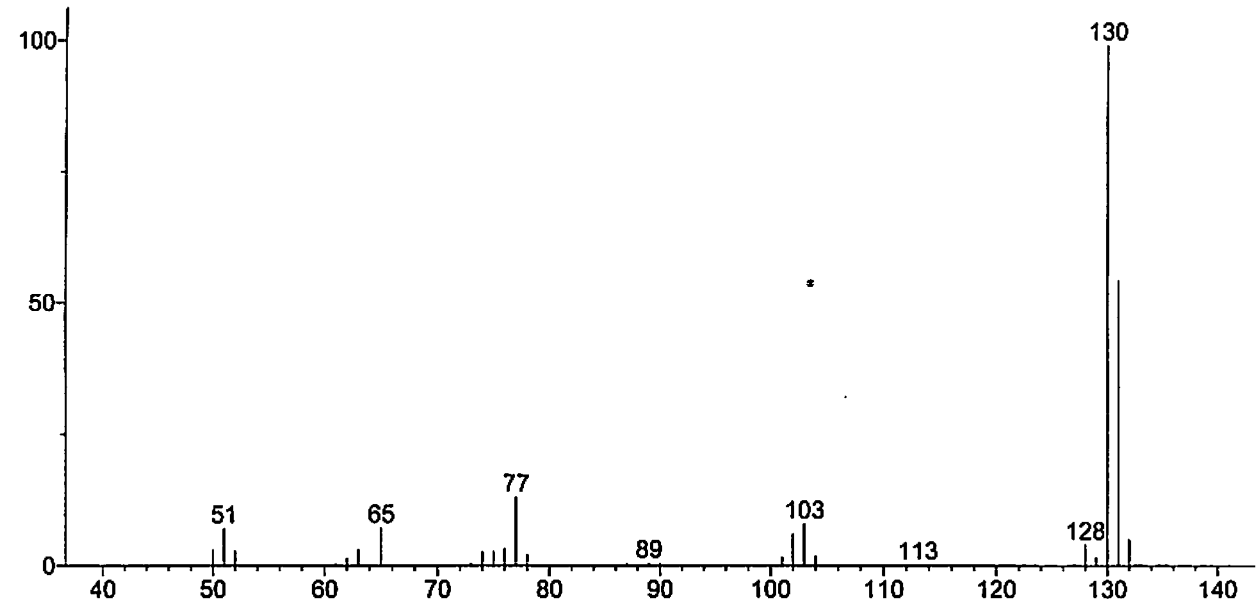


(Text File) Scan 879 (14.102 min): JA112009-3.D\data.ms (-874)

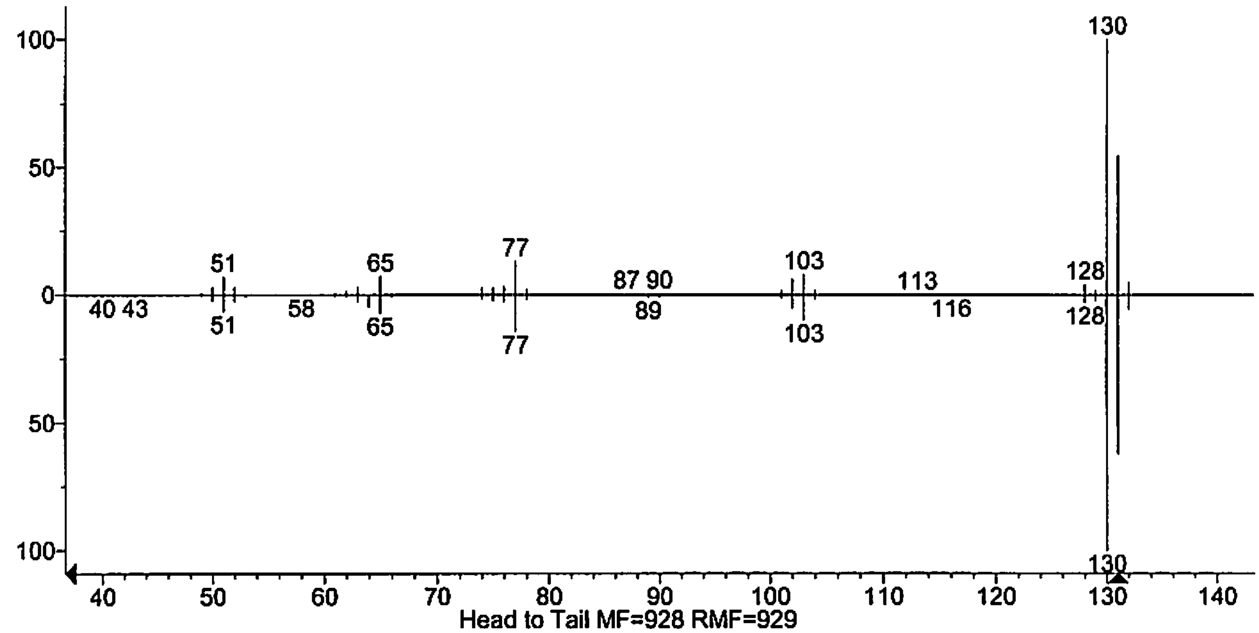


Head to Tail MF=928 RMF=929

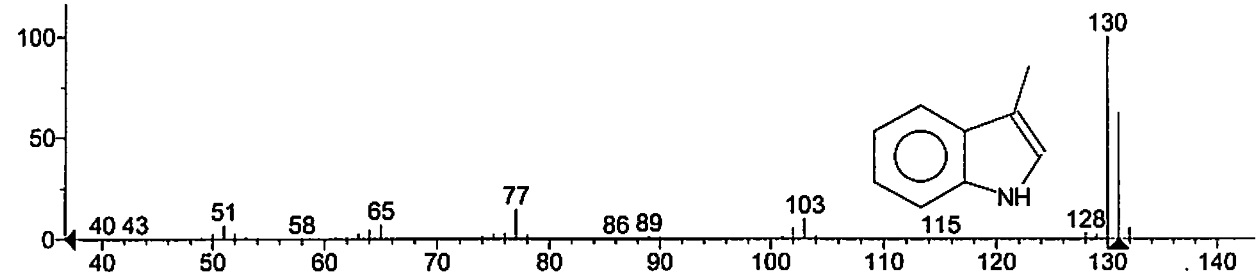


(mainlib) 1H-Indole, 3-methyl-

```
File          :D:\Aldrich\JA-09\JA112009-3.D
Operator      :
Acquired      : 20 Nov 2009  15:24     using AcqMethod JA-50-280LESS.M
Instrument :     Buba
Sample Name: 15 M C.oculata abd.-fed 8-OH-citronellol
Misc Info    : 3-10d-old; fed 1wk; 100ul conc.to 5ulCH2Cl2
Vial Number: 1
```

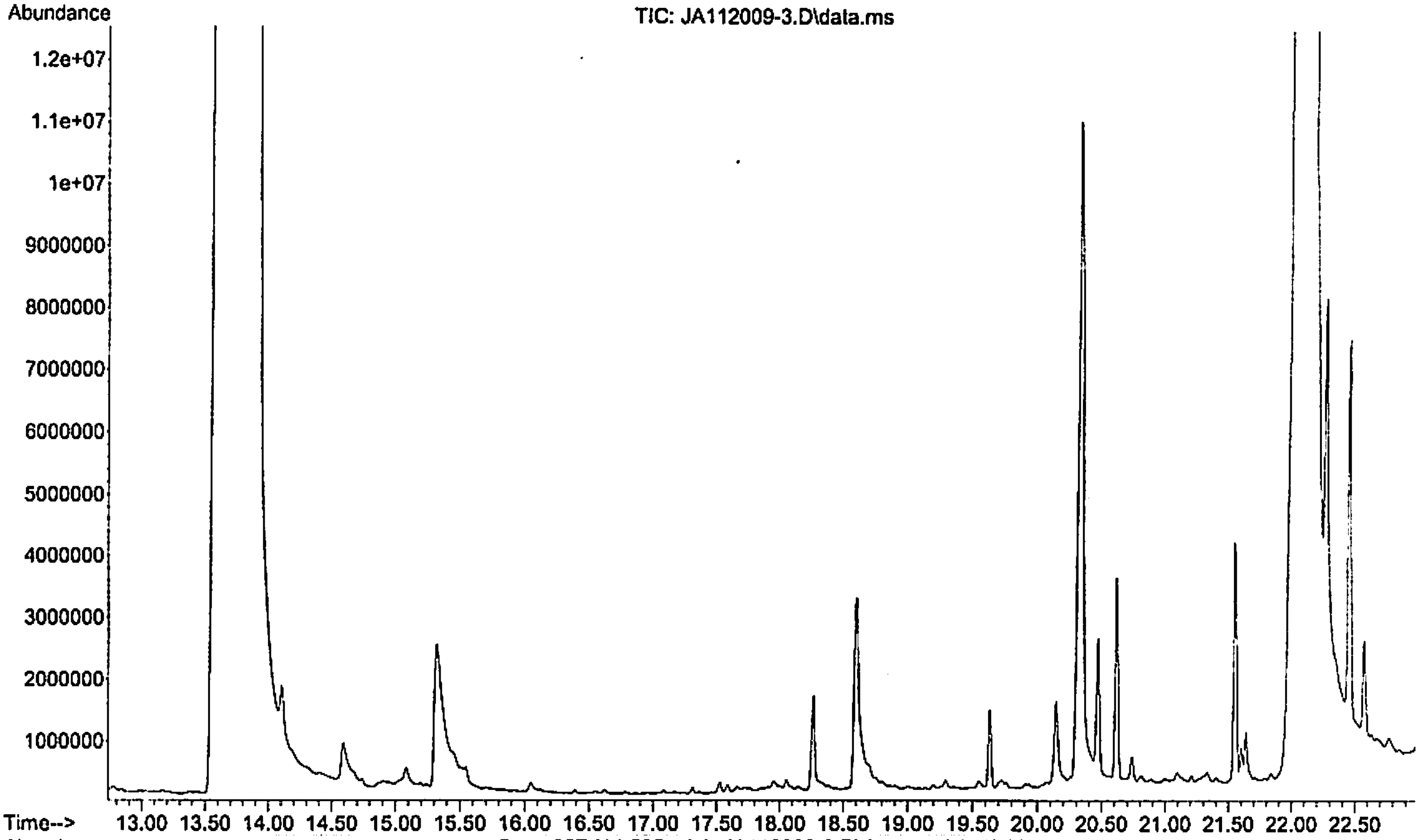


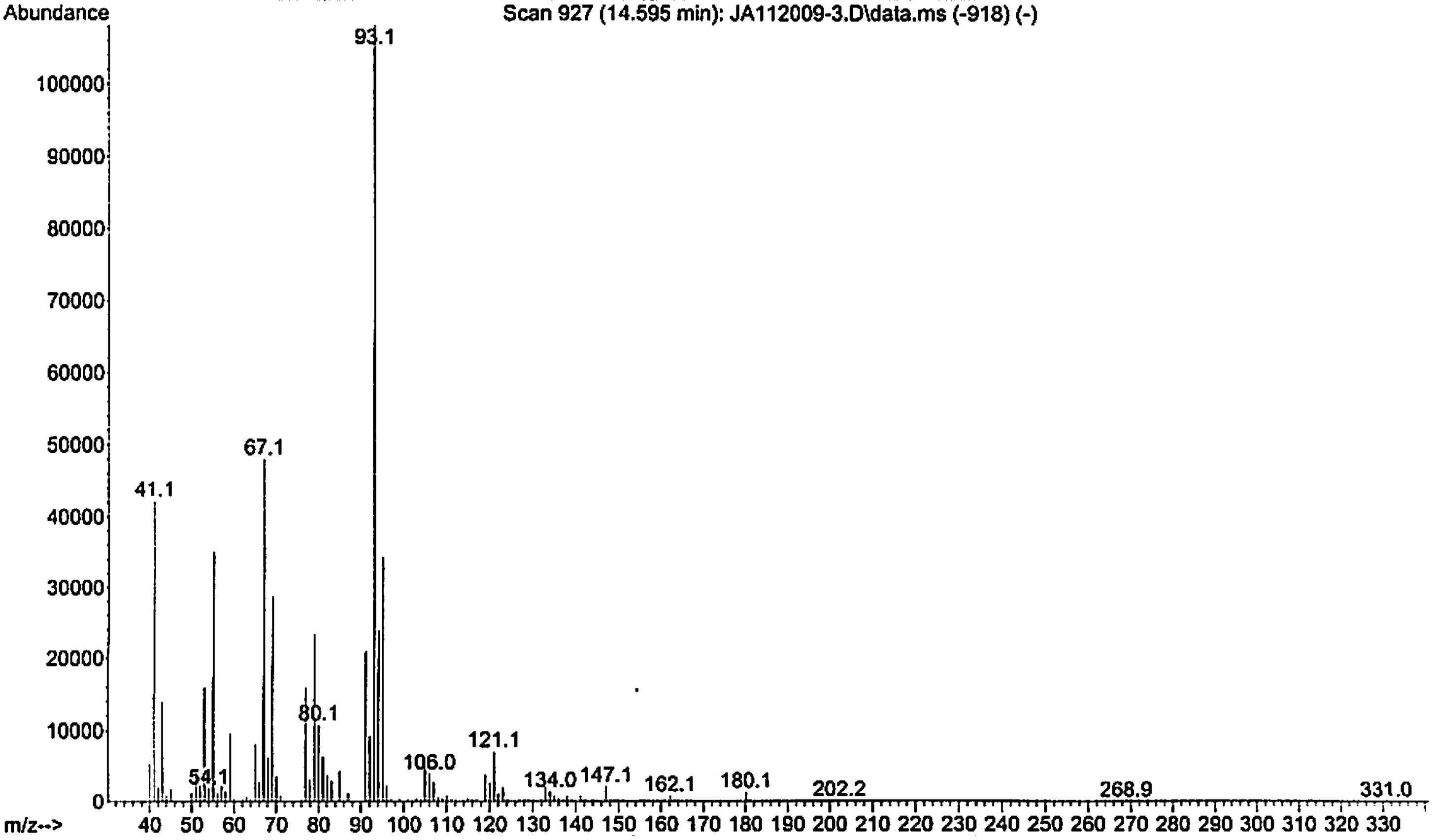

```
File        :D:\Aldrich\JA-09\JA112009-3.D
Operator    :
Acquired    : 20 Nov 2009  15:24     using AcqMethod JA-50-280LESS.M
Instrument :    Buba
Sample Name: 15 M C.oculata abd.-fed 8-OH-citronellol
Misc Info  : 3-10d-old; fed 1wk; 100ul conc.to 5ulCH2Cl2
Vial Number: 1
```

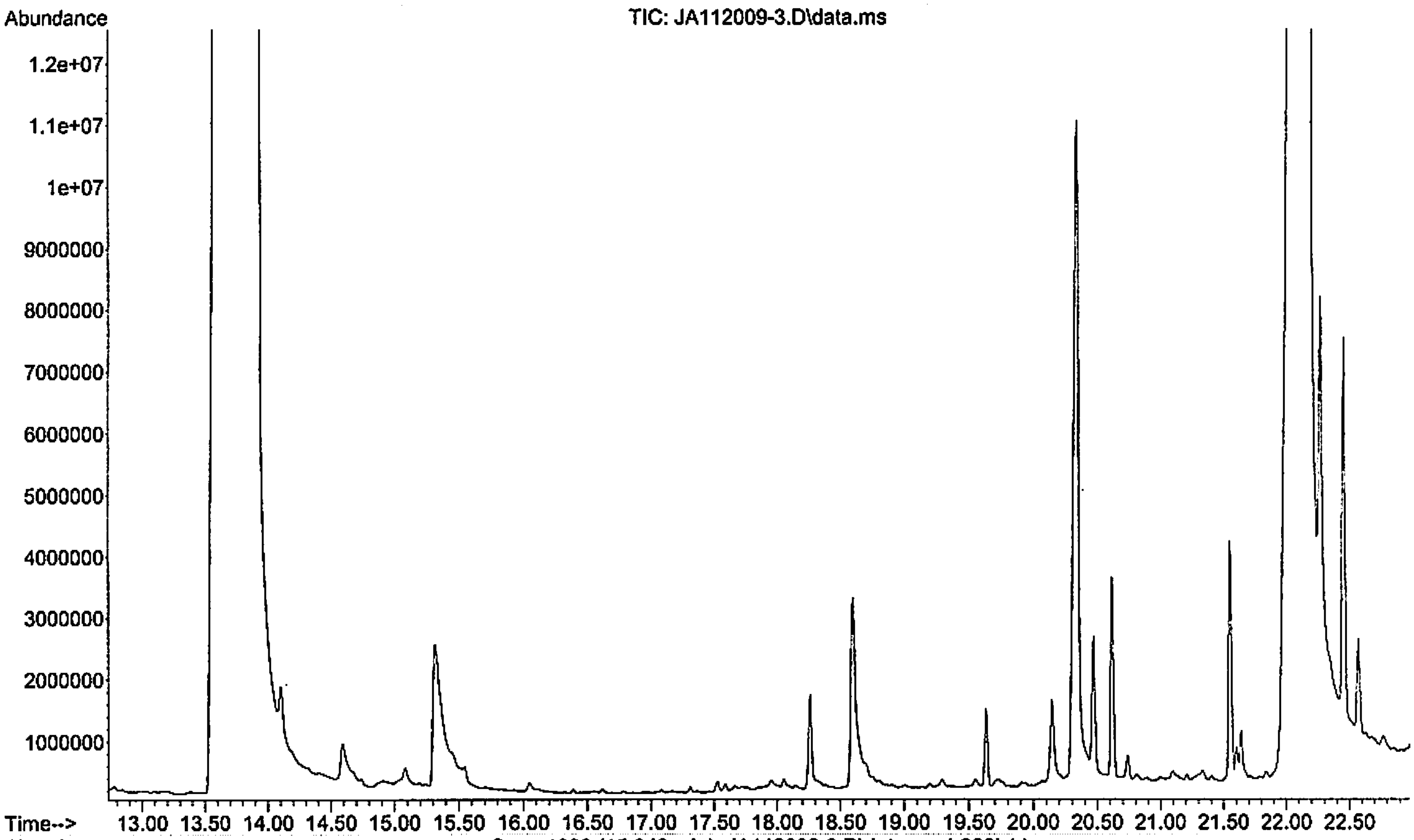


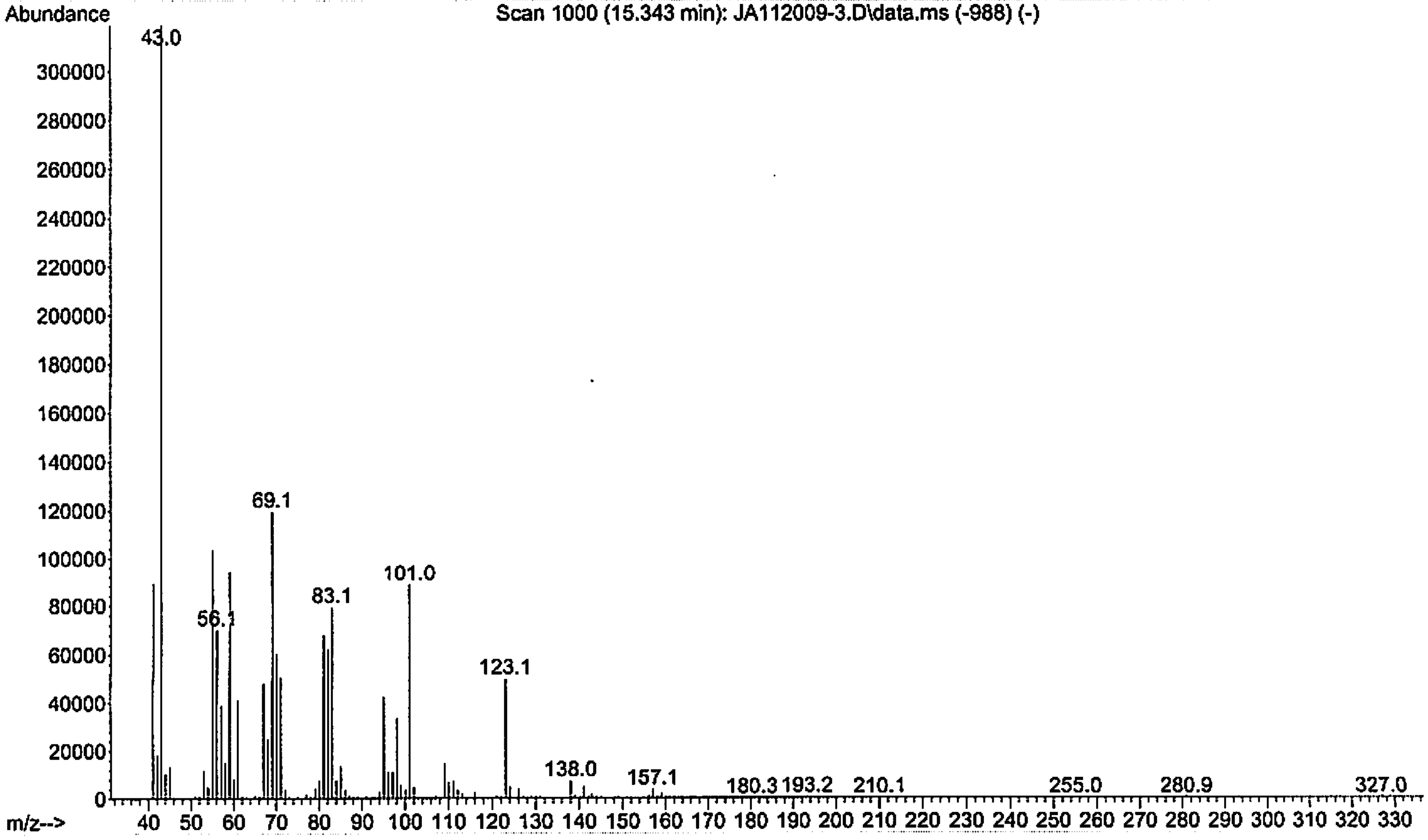

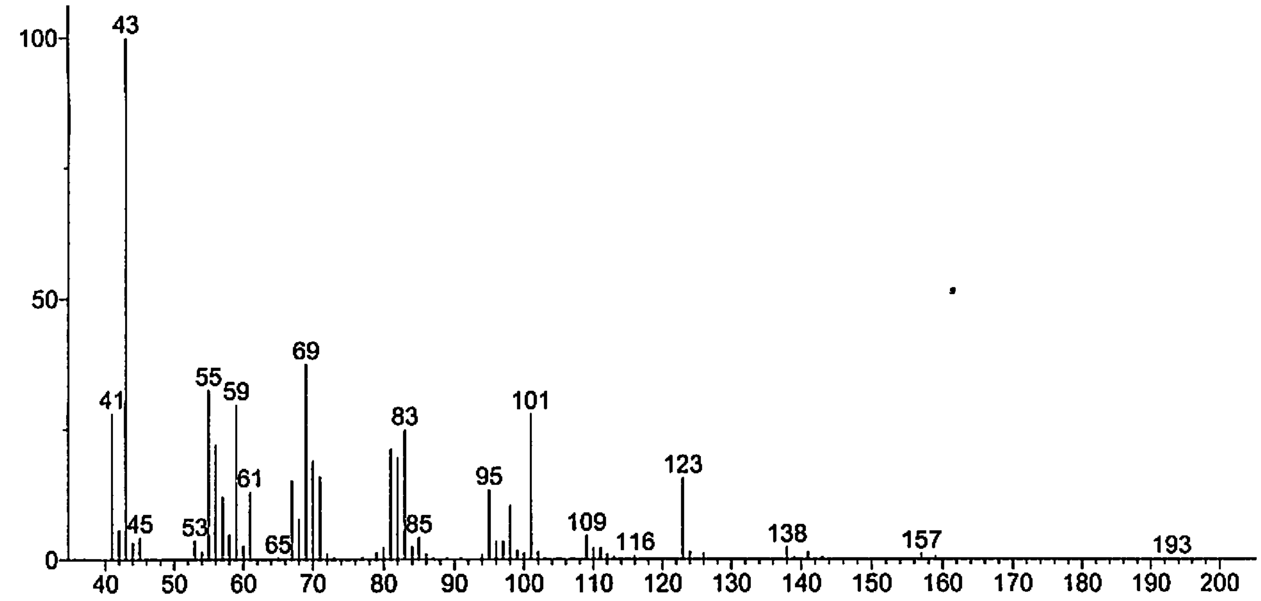


(Text File) Scan 1000 (15.343 min): JA112009-3.D\data.ms (-988)

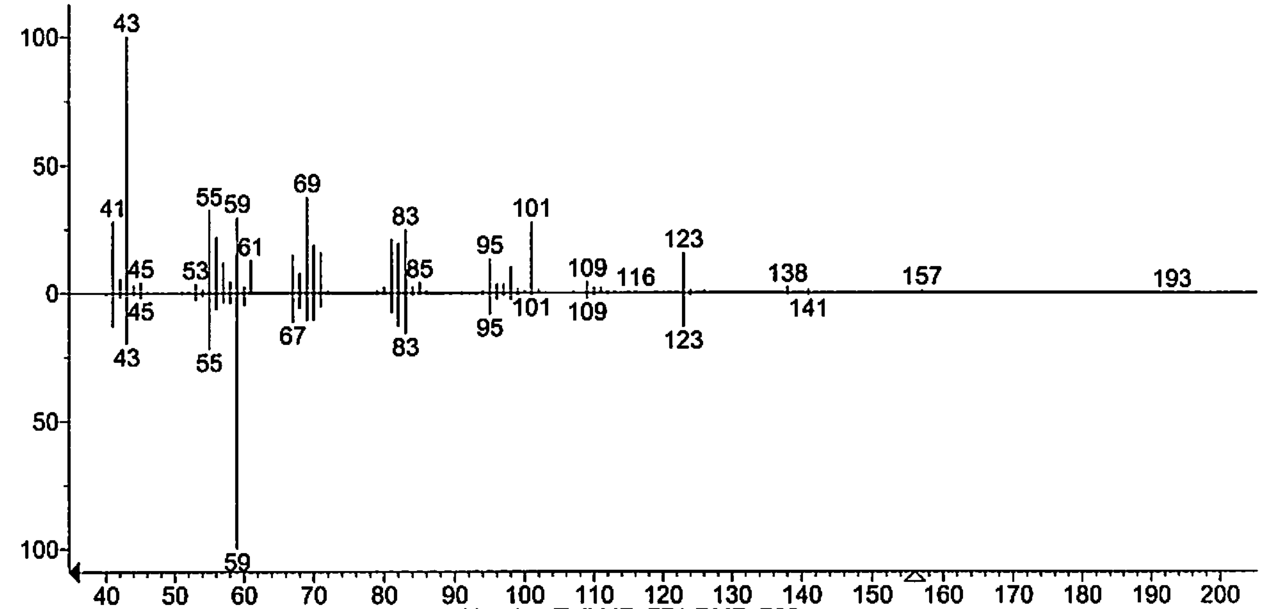


Head to Tail MF=771 RMF=782

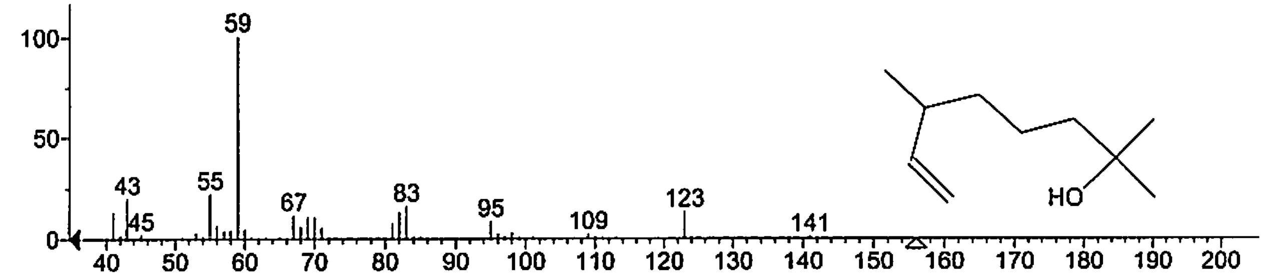


(replib) 7-Octen-2-ol, 2,6-dimethyl-

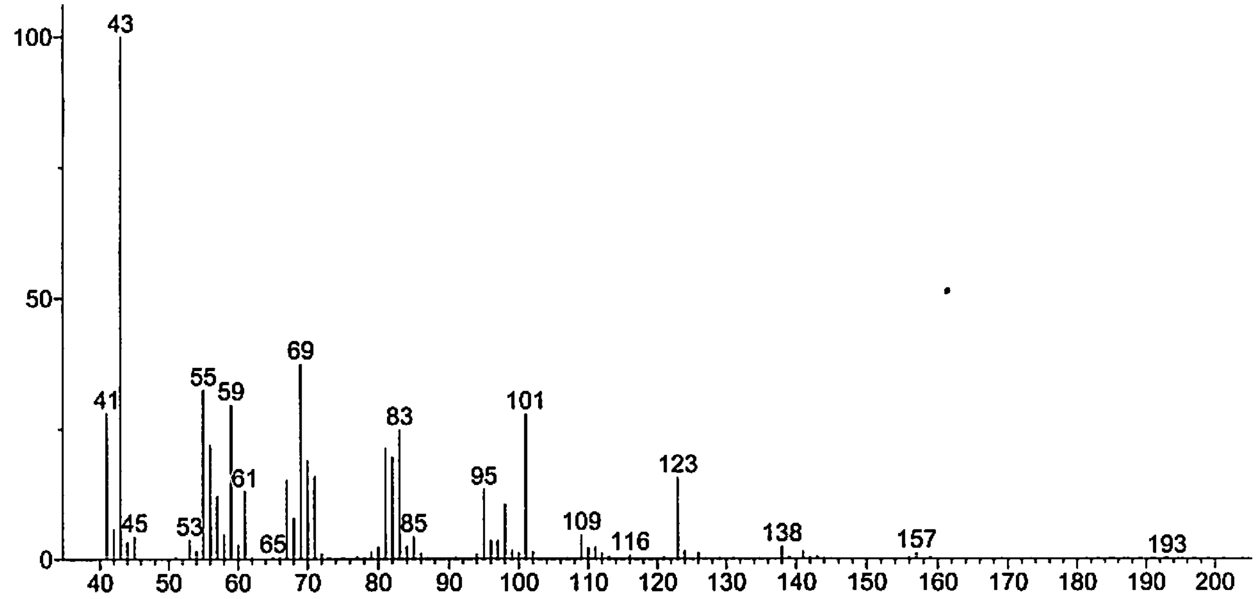


(Text File) Scan 1000 (15.343 min): JA112009-3.D\data.ms (-988)

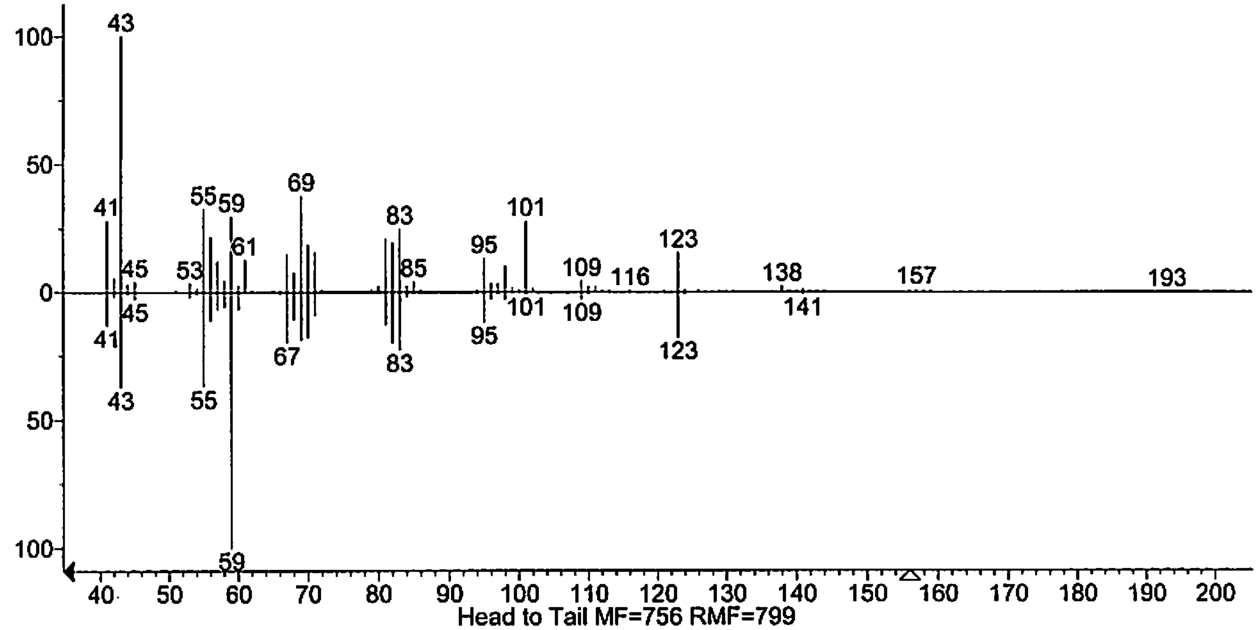


Head to Tail MF=756 RMF=799

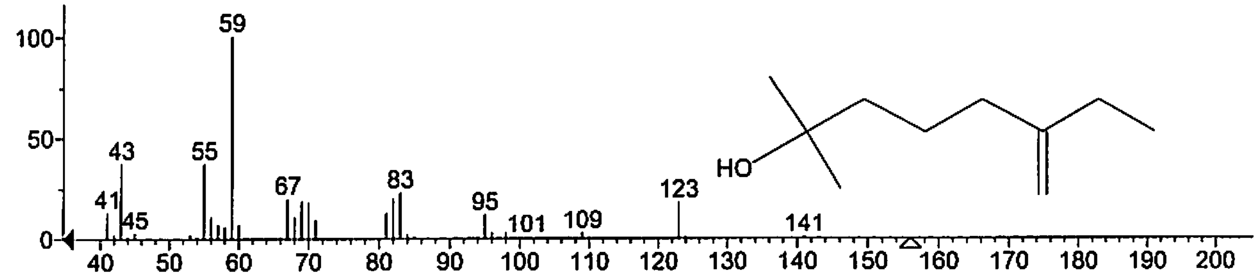


(replib) 2-Octanol, 2-methyl-6-methylene-

File          :D:\Aldrich\JA-09\JA112009-3.D
Operator      :
Acquired      : 20 Nov 2009  15:24     using AcqMethod JA-50-280LESS.M
Instrument :     Buba
Sample Name: 15 M C.oculata abd.-fed 8-OH-citronellol
Misc Info   : 3-10d-old; fed 1wk; 100ul conc.to 5ulCH2Cl2
Vial Number: 1

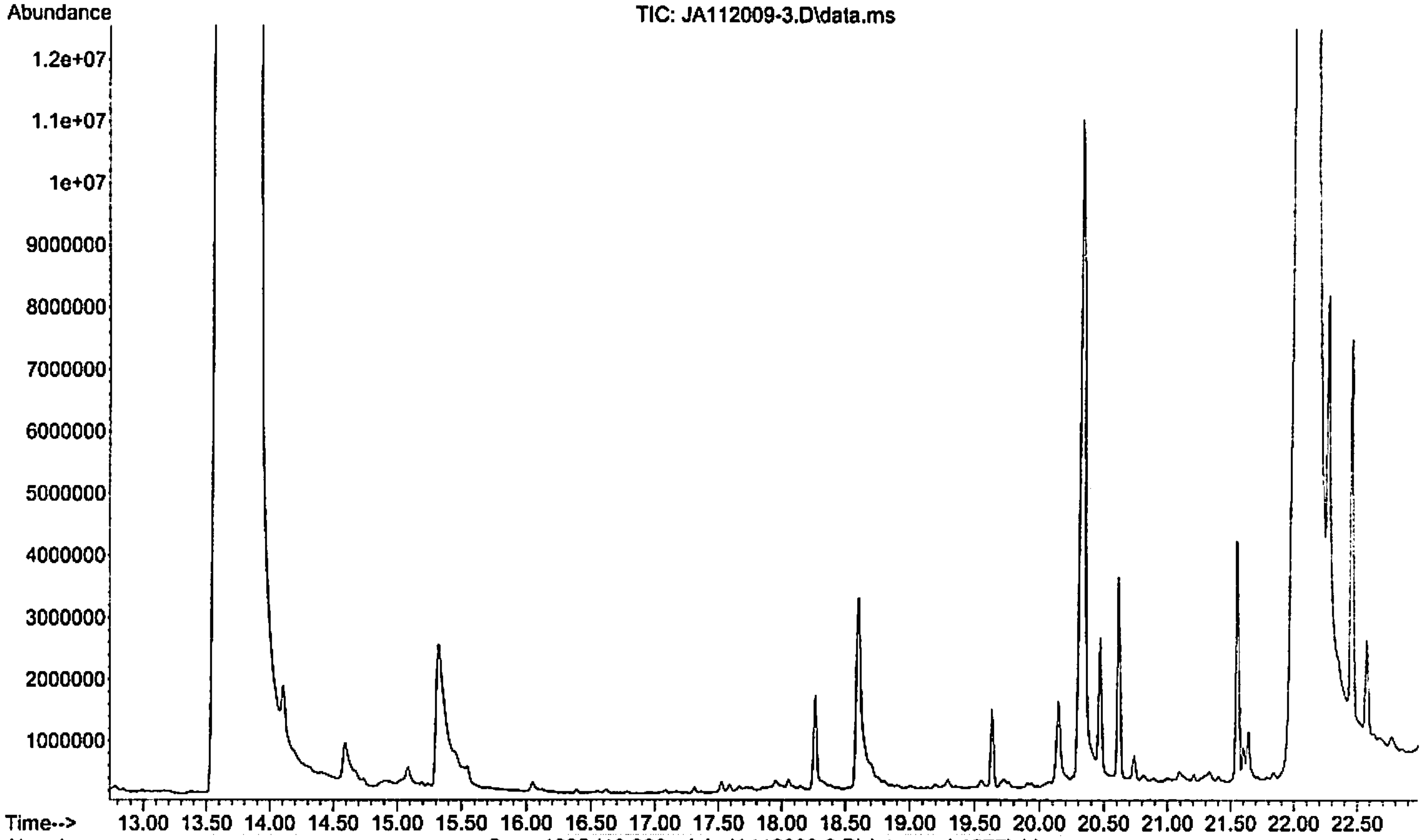


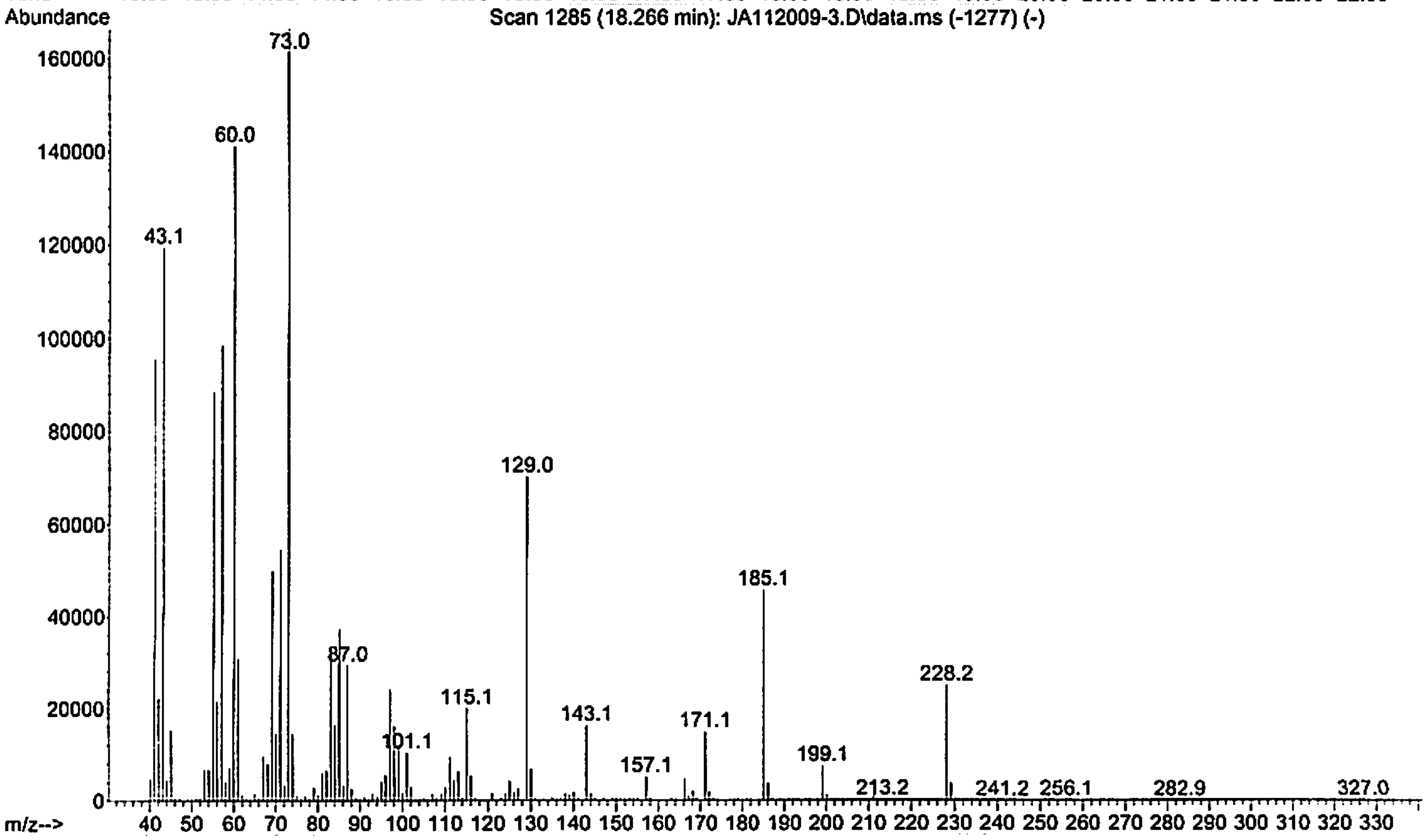

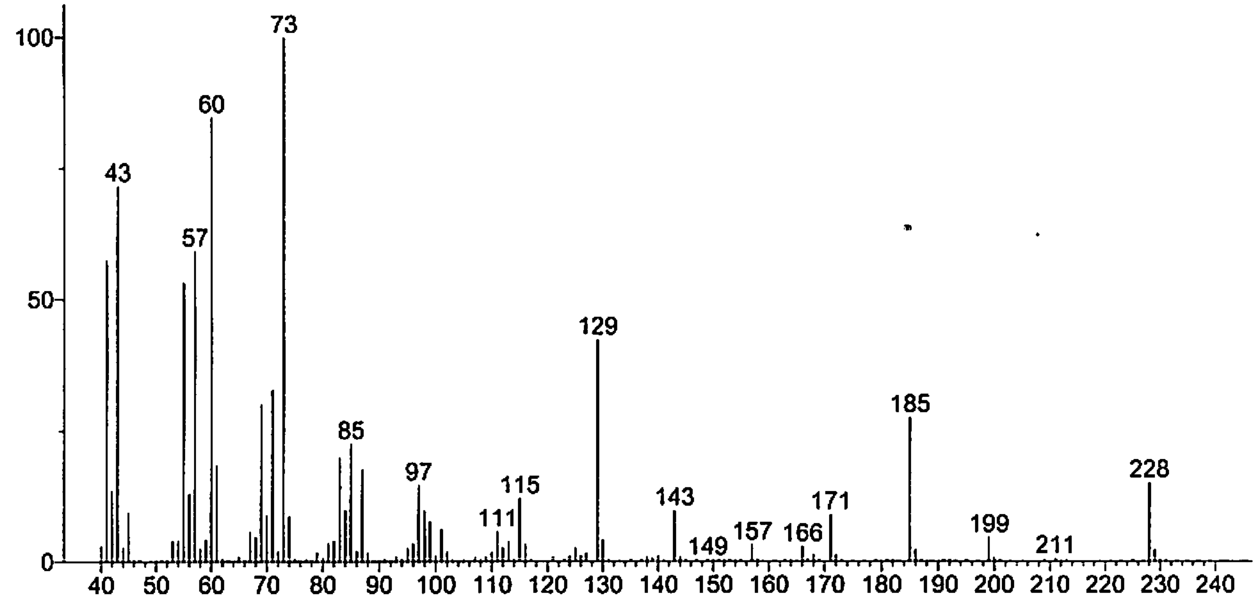

(Text File) Scan 1285 (18.266 min): JA112009-3.D\data.ms (-1277)

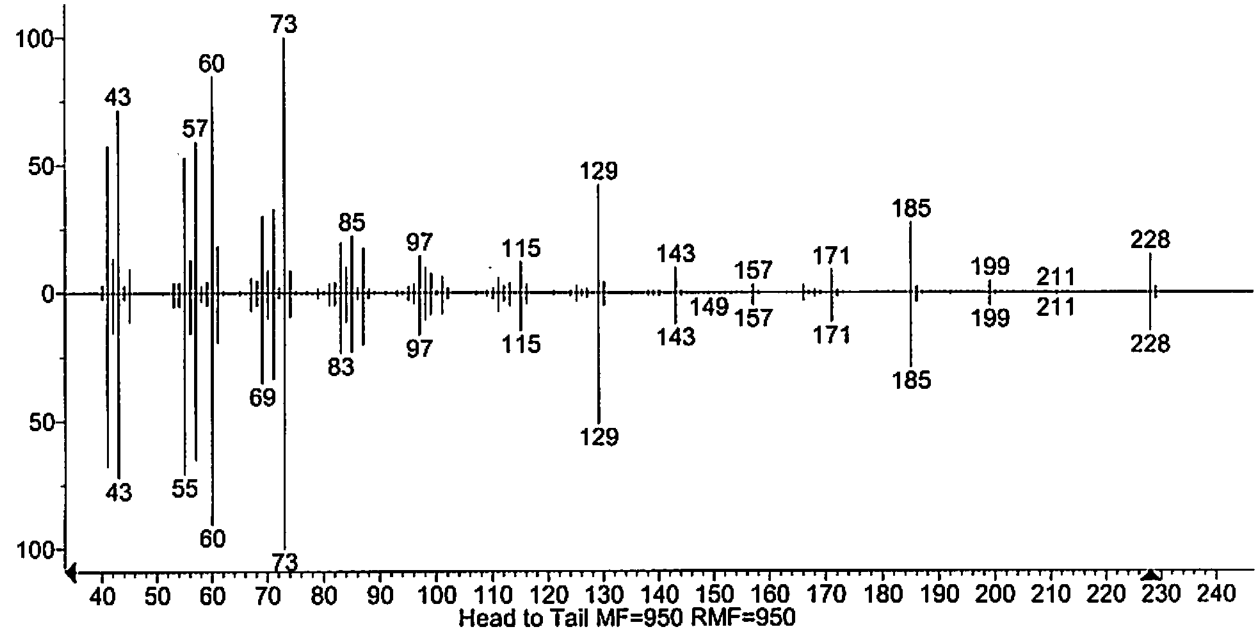

Head to Tail MF=950 RMF=950

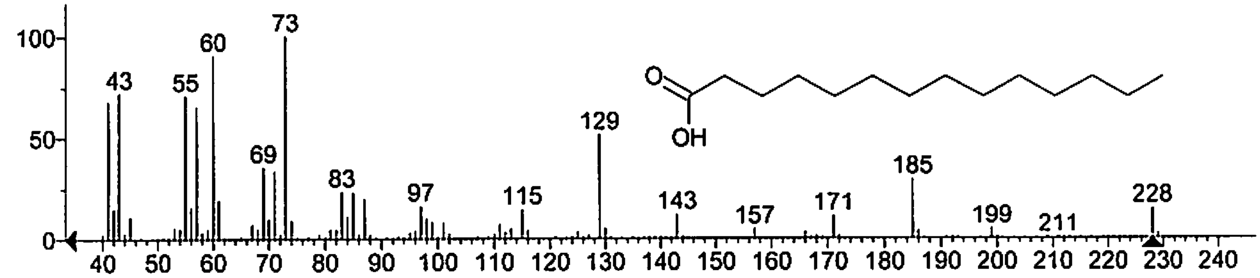


(mainlib) Tetradecanoic acid

File :D:\Aldrich\JA-09\JA112009-3.D
Operator :
Acquired : 20 Nov 2009 15:24 using AcqMethod JA-50-280LESS.M
Instrument : Buba
Sample Name: 15 M C.oculata abd.-fed 8-OH-citronellol
Misc Info : 3-10d-old; fed 1wk; 100ul conc.to 5ulCH2Cl2
Vial Number: 1

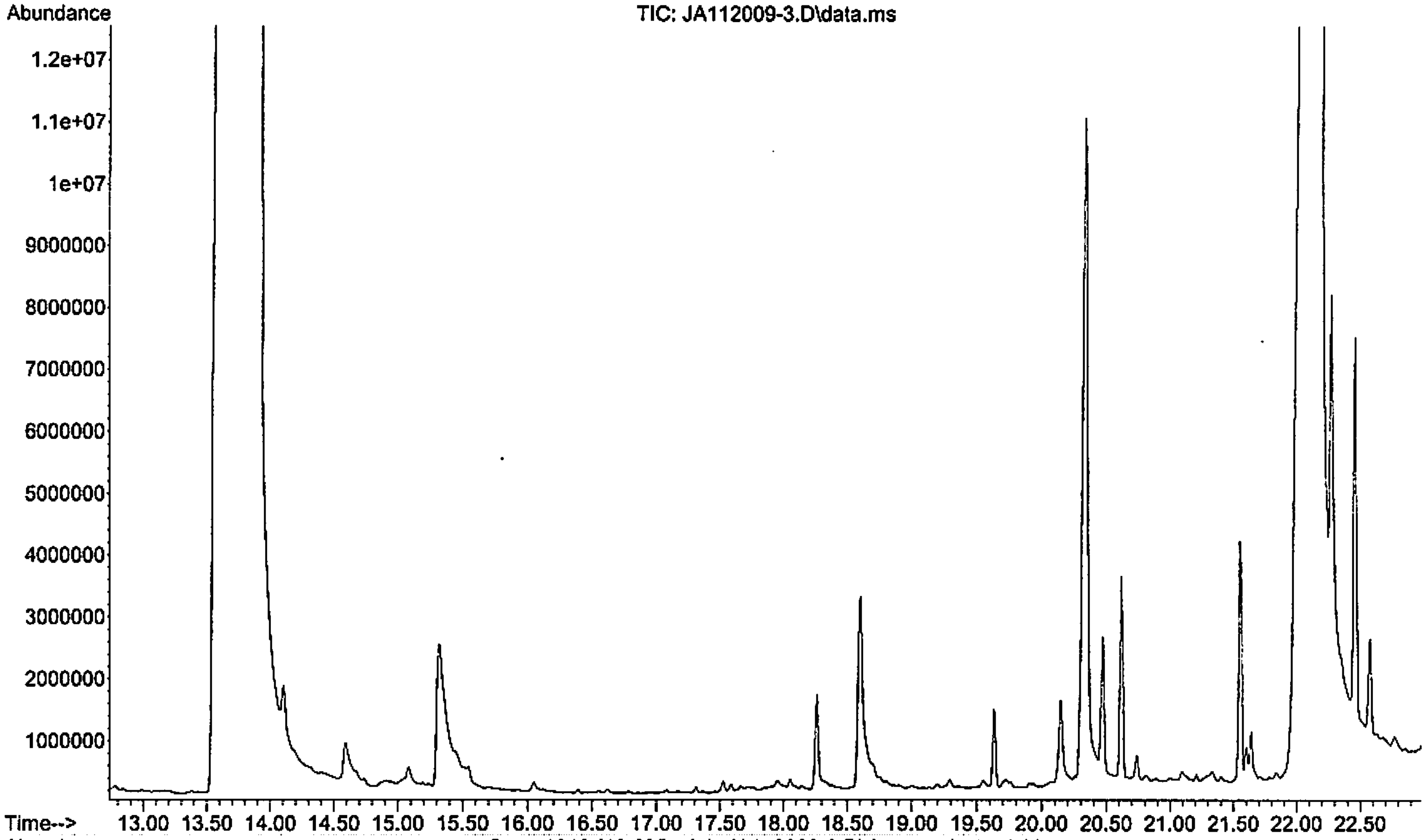


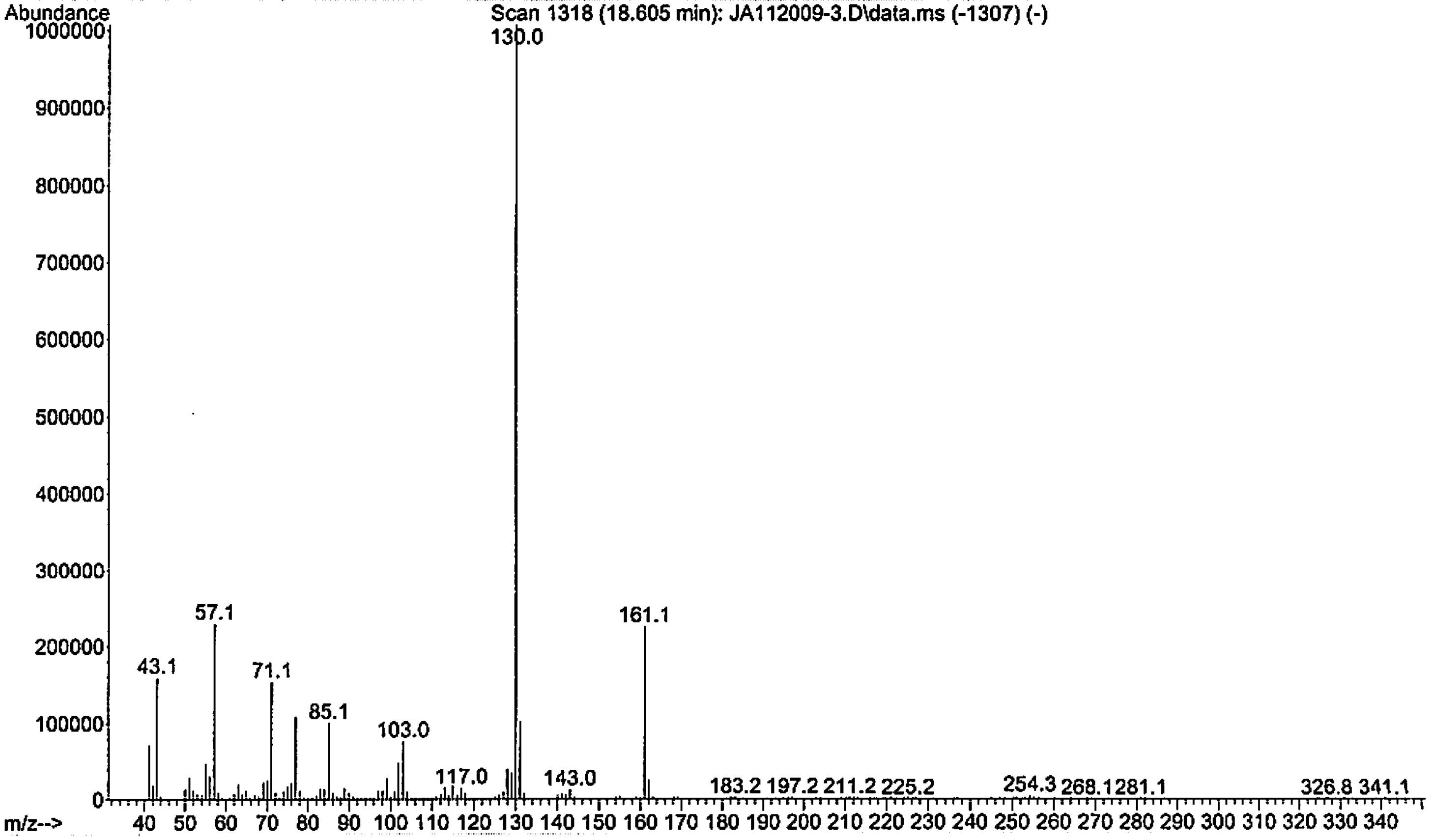

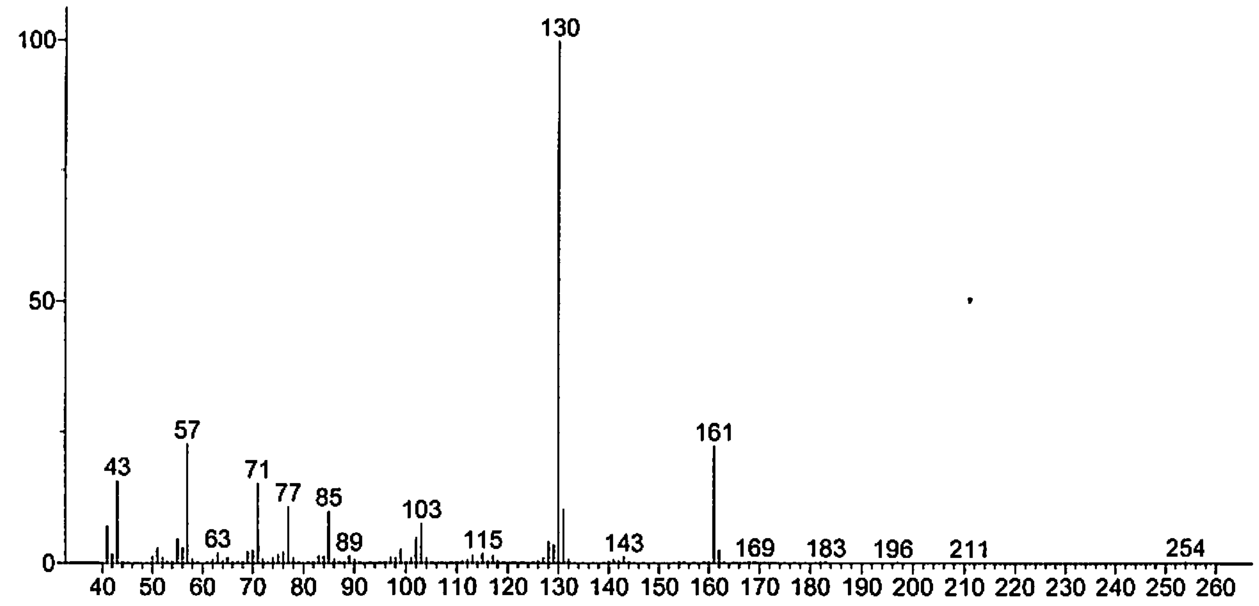


(Text File) Scan 1318 (18.605 min): JA112009-3.D\data.ms (-1307)

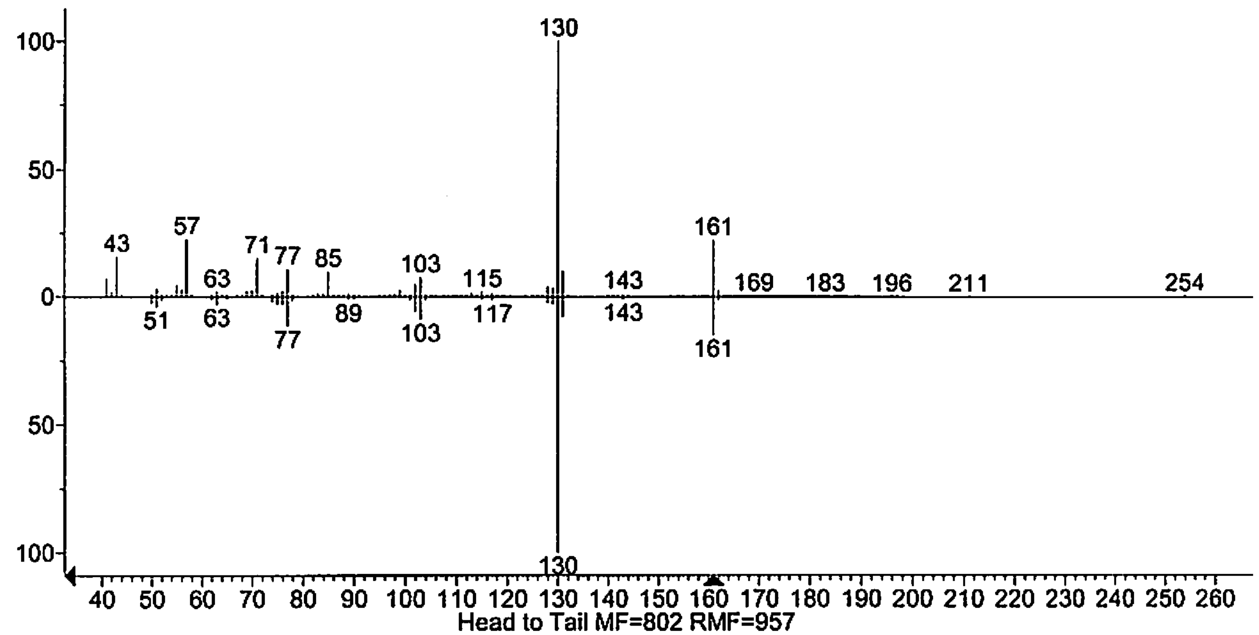


Head to Tail MF=802 RMF=957

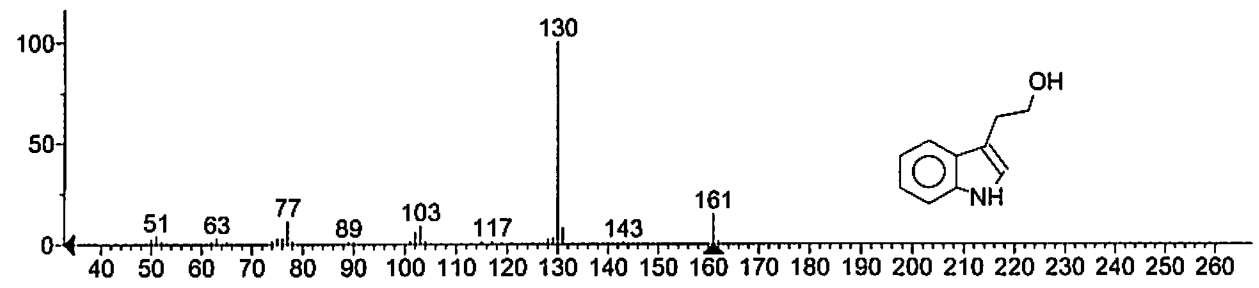


(replib) 1H-Indole-3-ethanol

```
File        :D:\Aldrich\JA-09\JA112009-3.D
Operator    :
Acquired    : 20 Nov 2009  15:24     using AcqMethod JA-50-280LESS.M
Instrument  :    Buba
Sample Name: 15 M C.oculata abd.-fed 8-OH-citronellol
Misc Info   : 3-10d-old; fed 1wk; 100ul conc.to 5ulCH2Cl2
Vial Number: 1
```

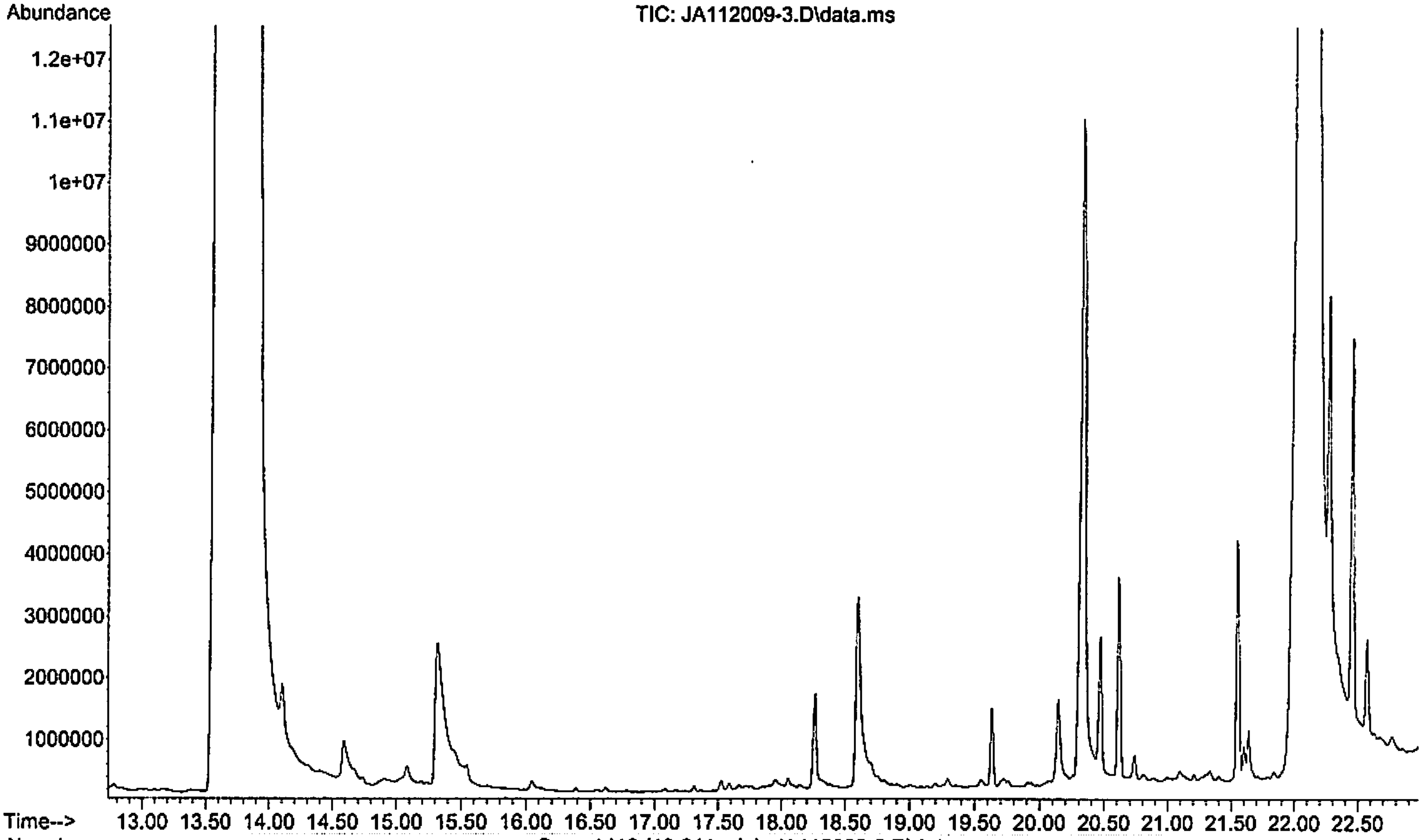


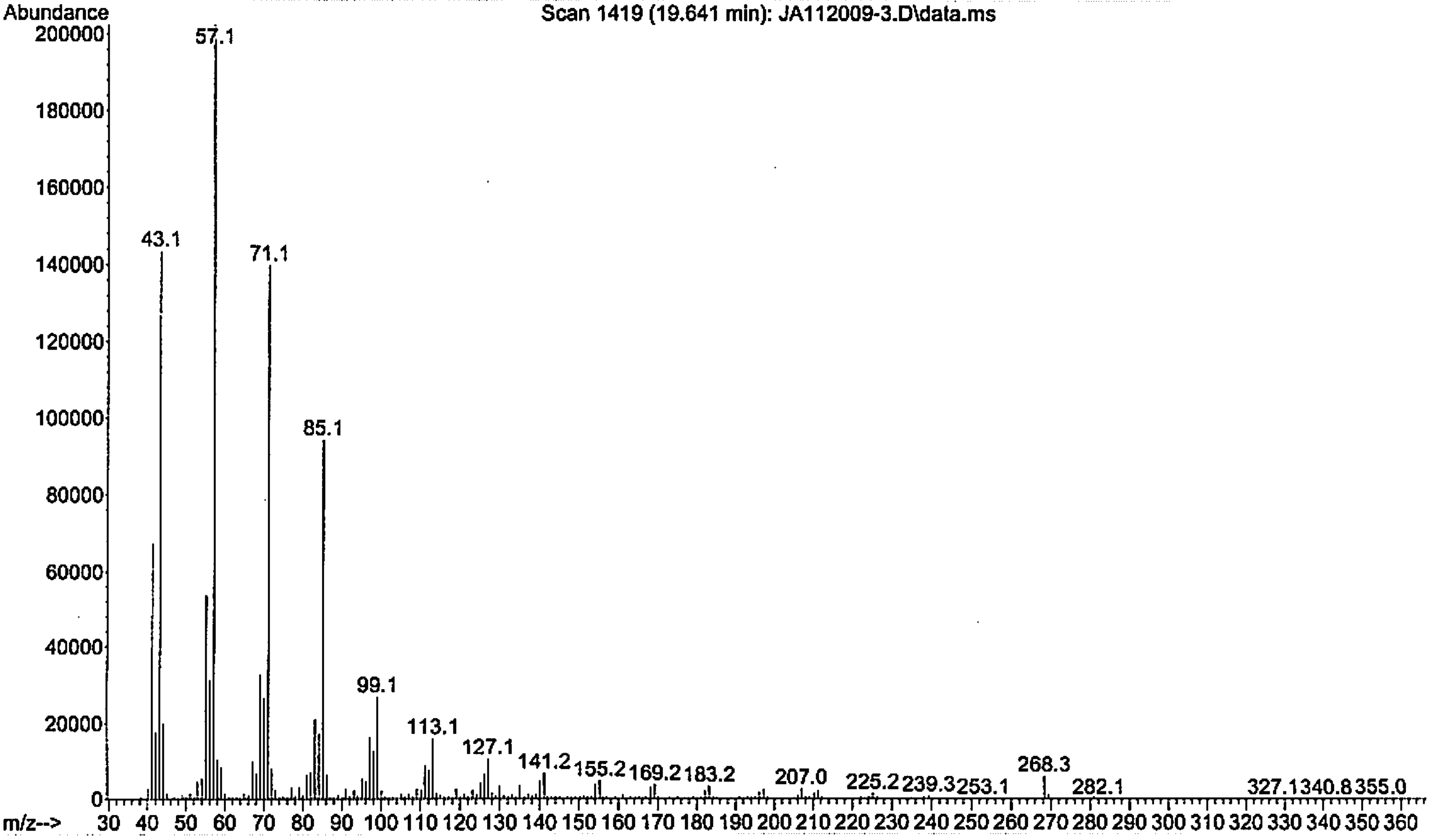

```
File        :D:\Aldrich\JA-09\JA112009-3.D
Operator    :
Acquired    : 20 Nov 2009  15:24     using AcqMethod JA-50-280LESS.M
Instrument  :    Buba
Sample Name: 15 M C.oculata abd.-fed 8-OH-citronellol
Misc Info   : 3-10d-old; fed 1wk; 100ul conc.to 5ulCH2Cl2
Vial Number: 1
```

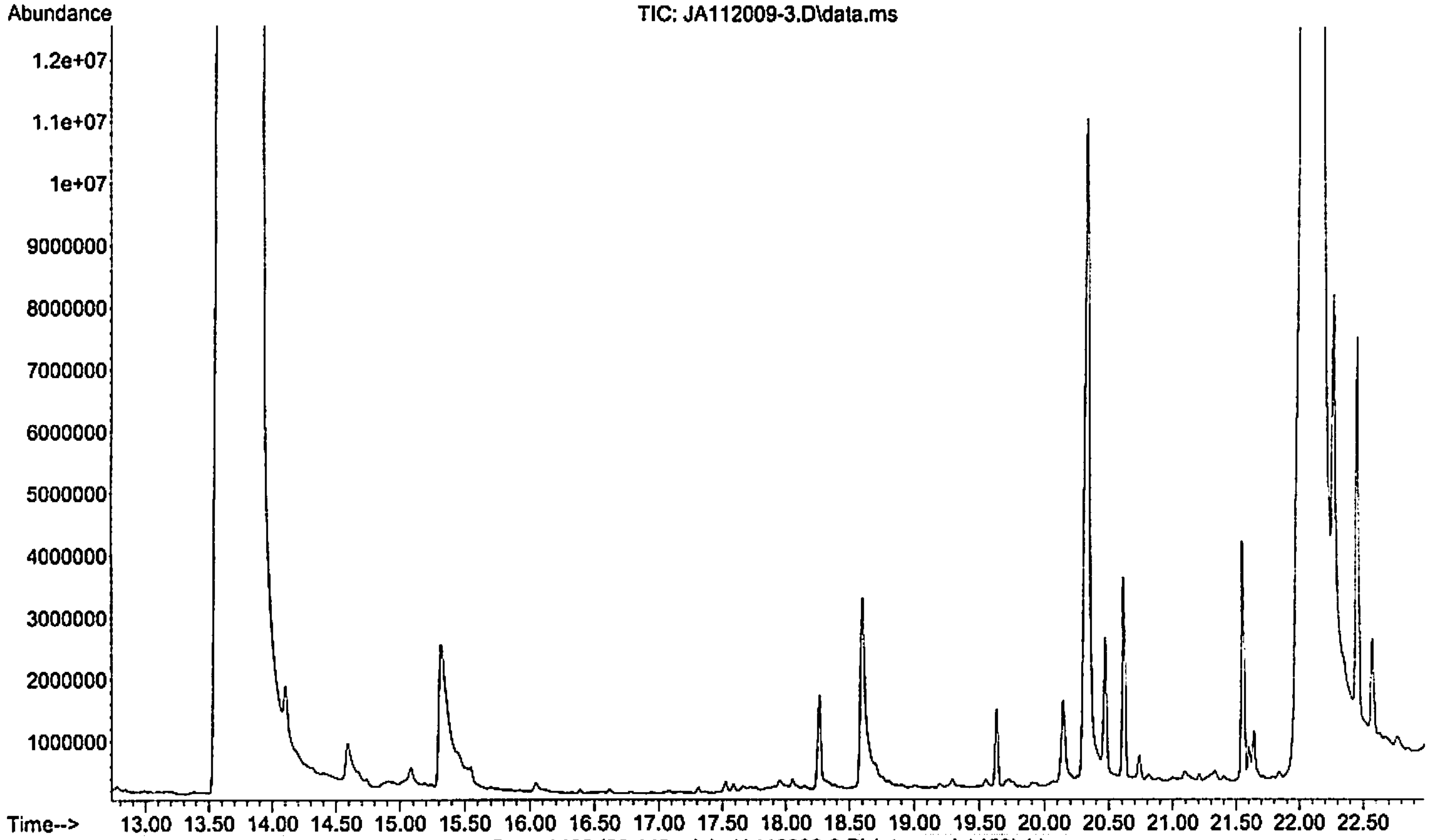


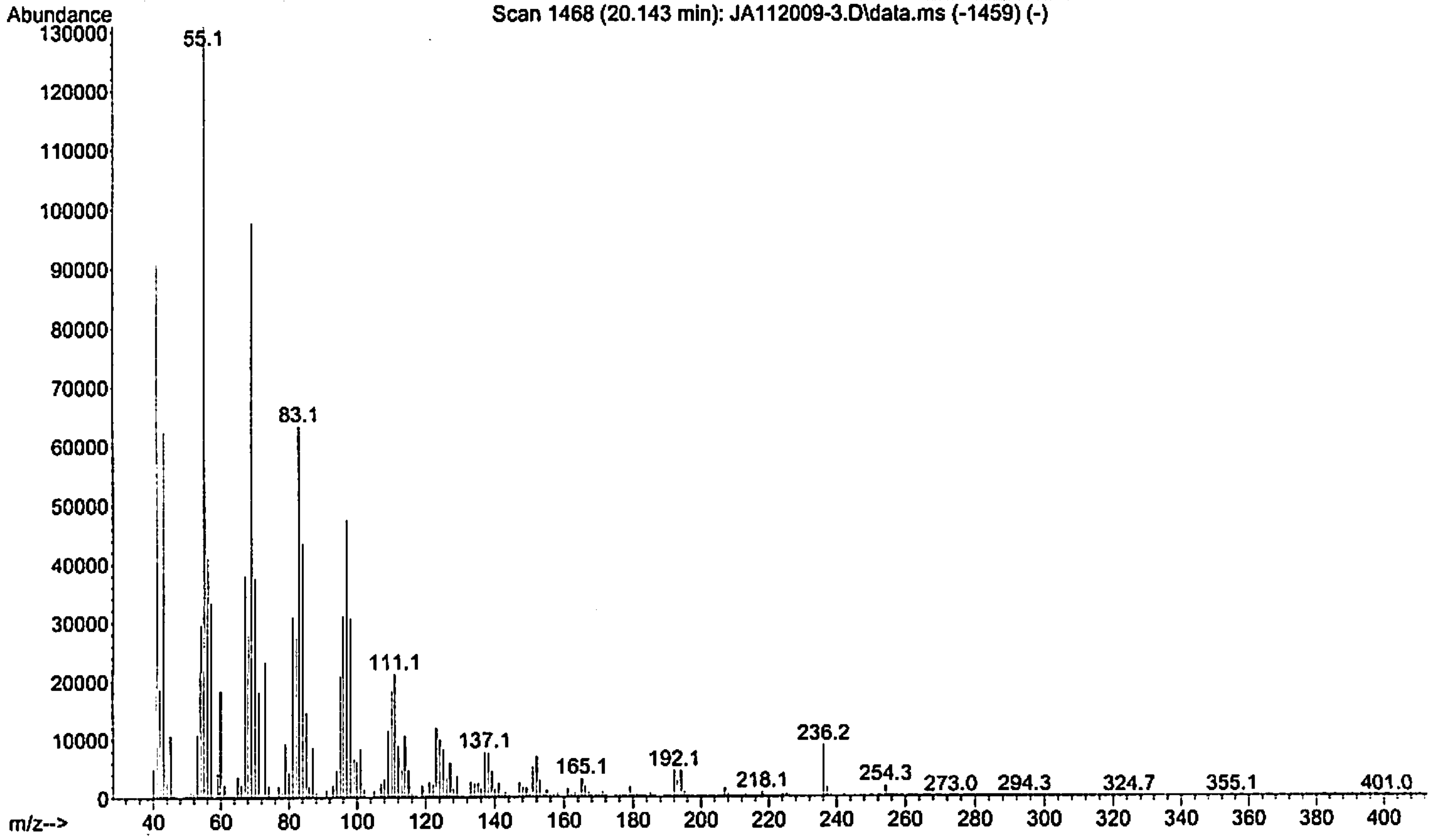

```
File        :D:\Aldrich\JA-09\JA112009-3.D
Operator    :
Acquired    : 20 Nov 2009  15:24     using AcqMethod JA-50-280LESS.M
Instrument :    Buba
Sample Name: 15 M C.oculata abd.-fed 8-OH-citronellol
Misc Info   : 3-10d-old; fed 1wk; 100ul conc.to 5ulCH2Cl2
Vial Number: 1
```

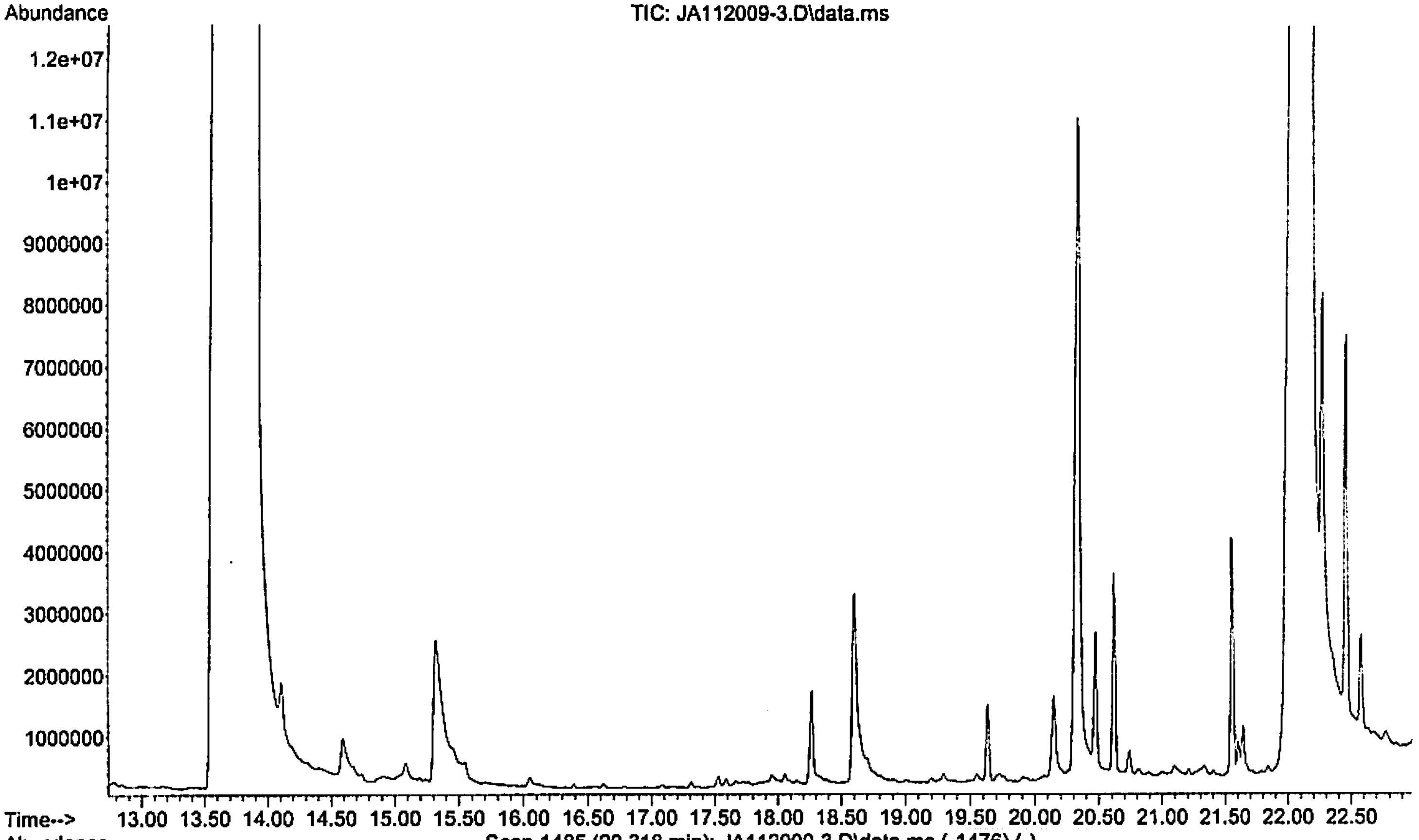


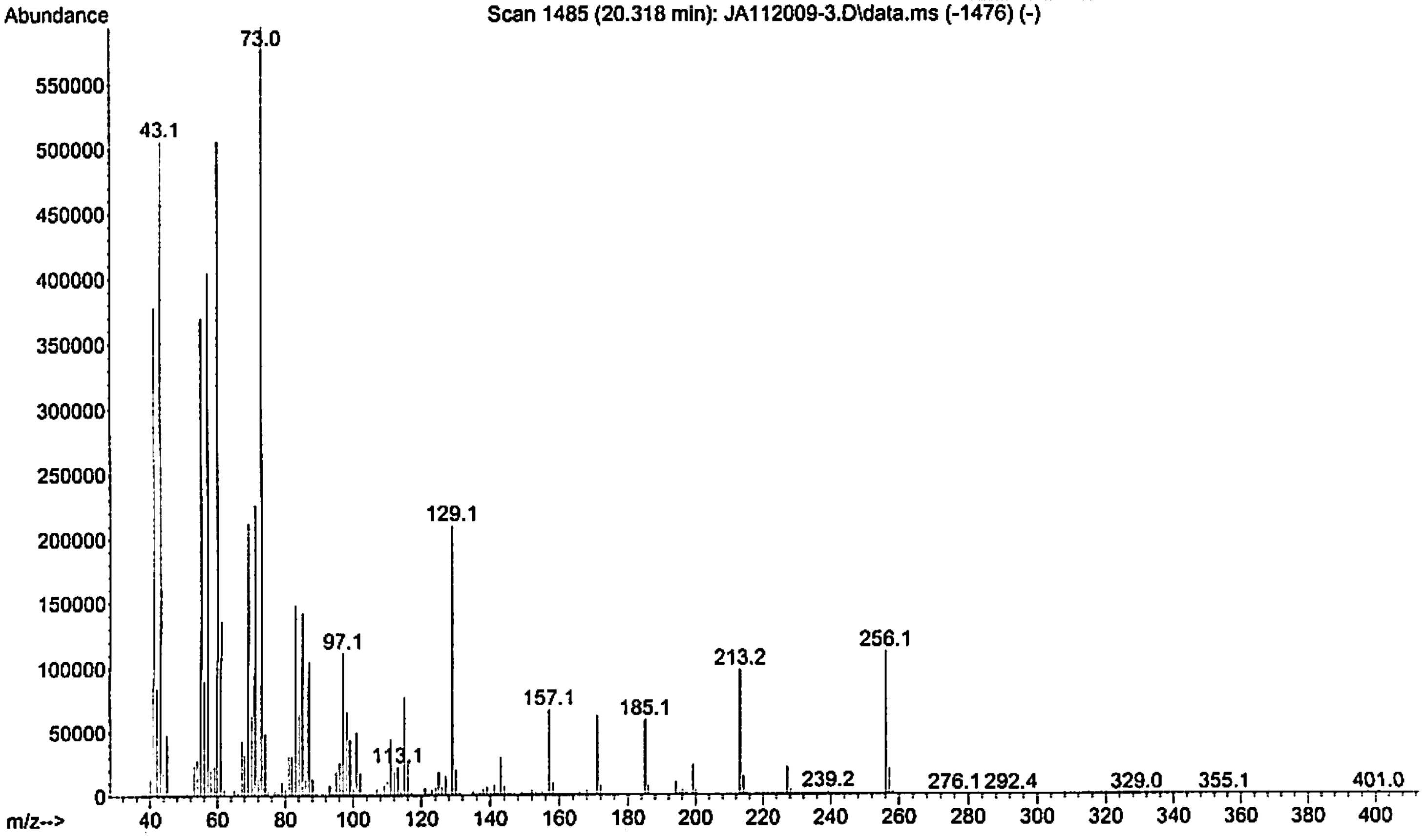

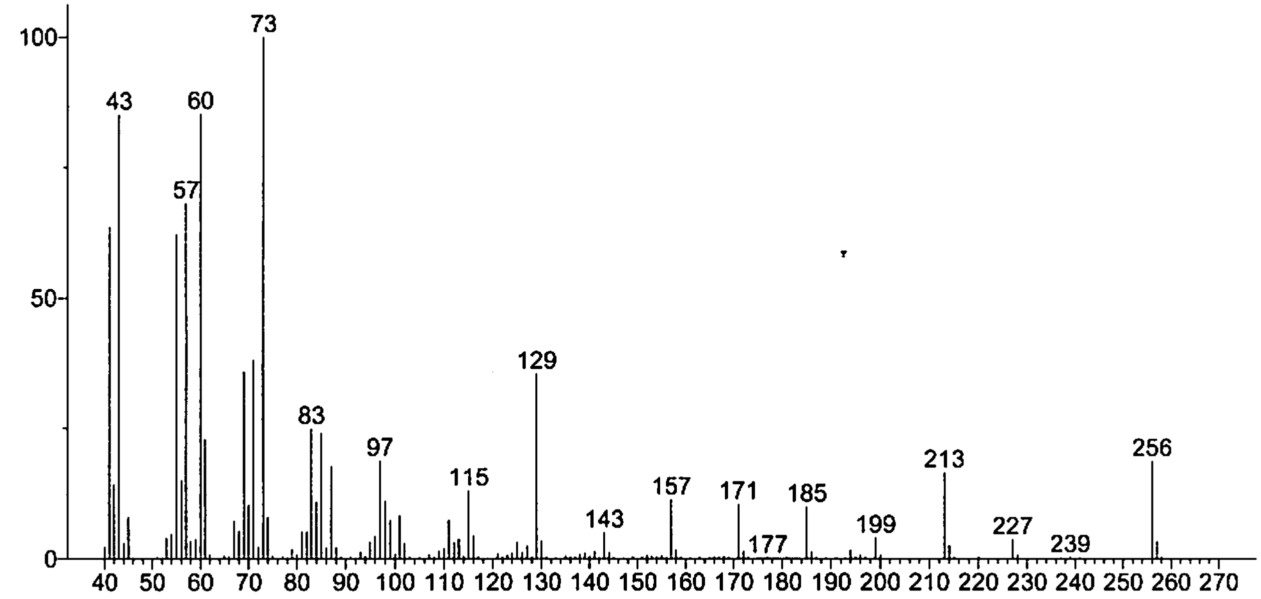


(Text File) Scan 1485 (20.318 min): JA112009-3.D\data.ms (-1476)

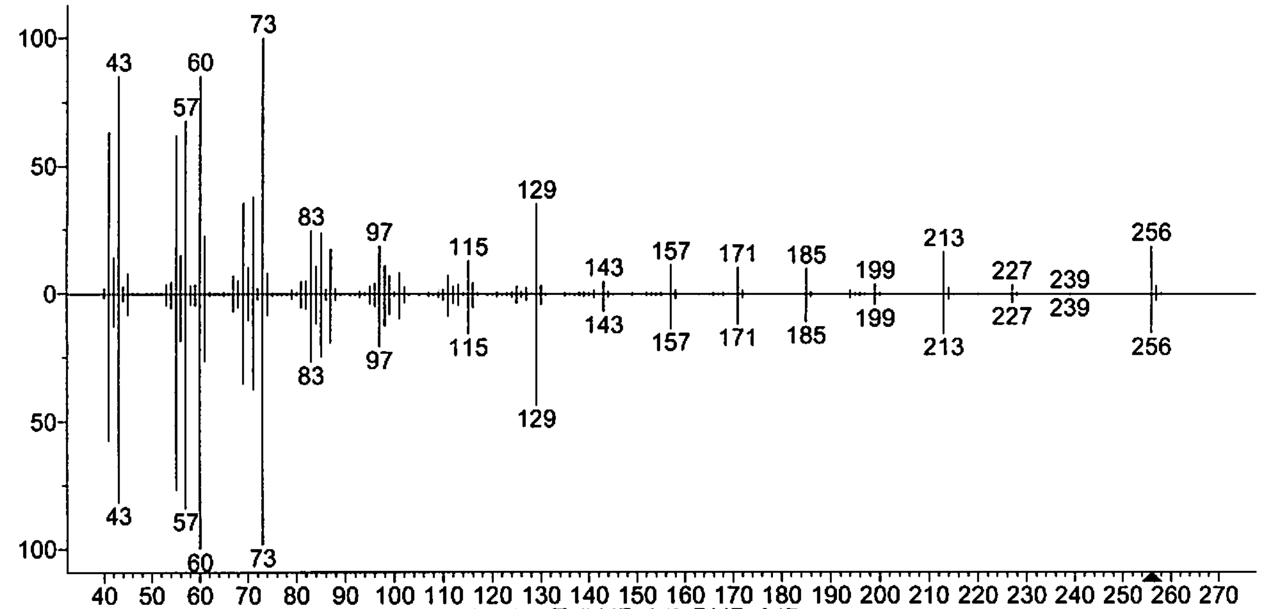


Head to Tail MF=947 RMF=947

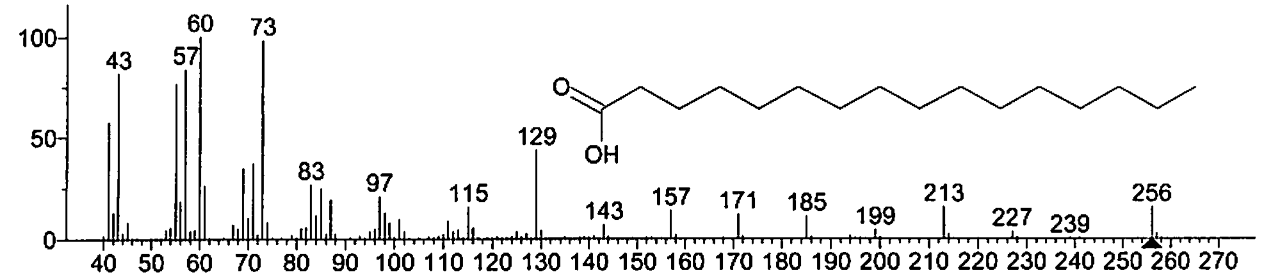


(replib) n-Hexadecanoic acid

File :D:\Aldrich\JA-09\JA112009-3.D
Operator :
Acquired : 20 Nov 2009 15:24 using AcqMethod JA-50-280LESS.M
Instrument : Buba
Sample Name: 15 M C.oculata abd.-fed 8-OH-citronellol
Misc Info : 3-10d-old; fed 1wk; 100ul conc.to 5ulCH2Cl2
Vial Number: 1

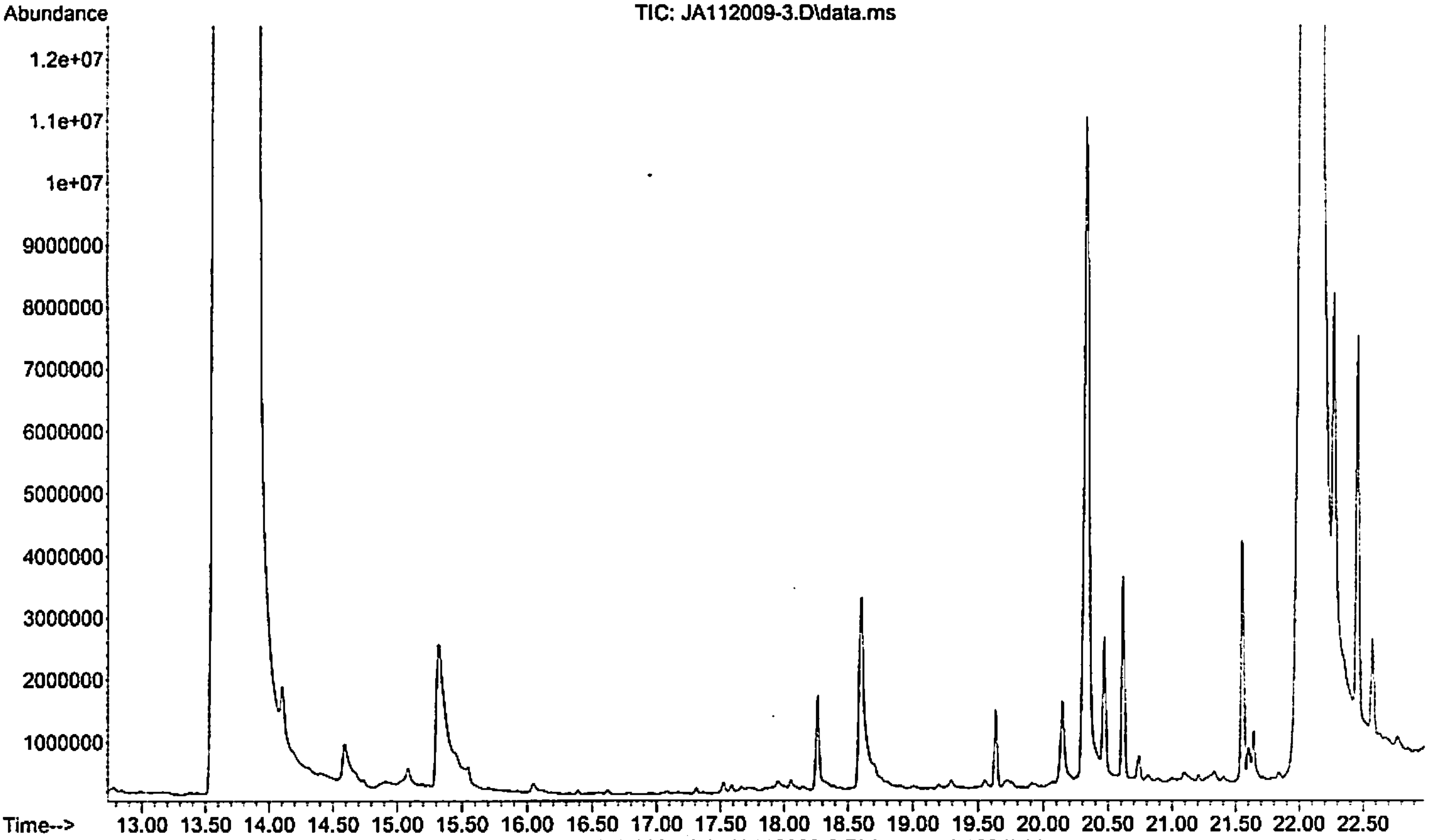


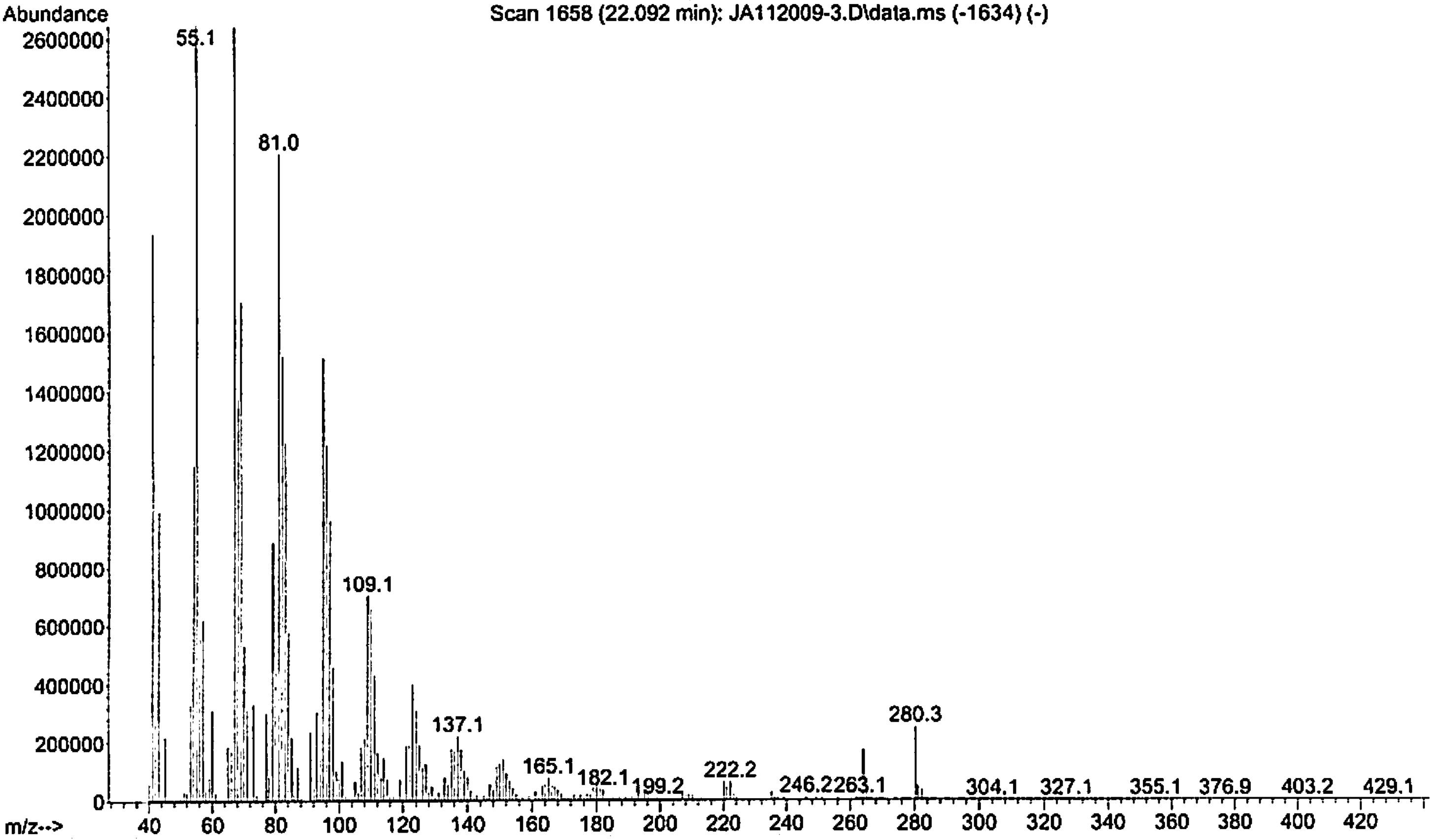

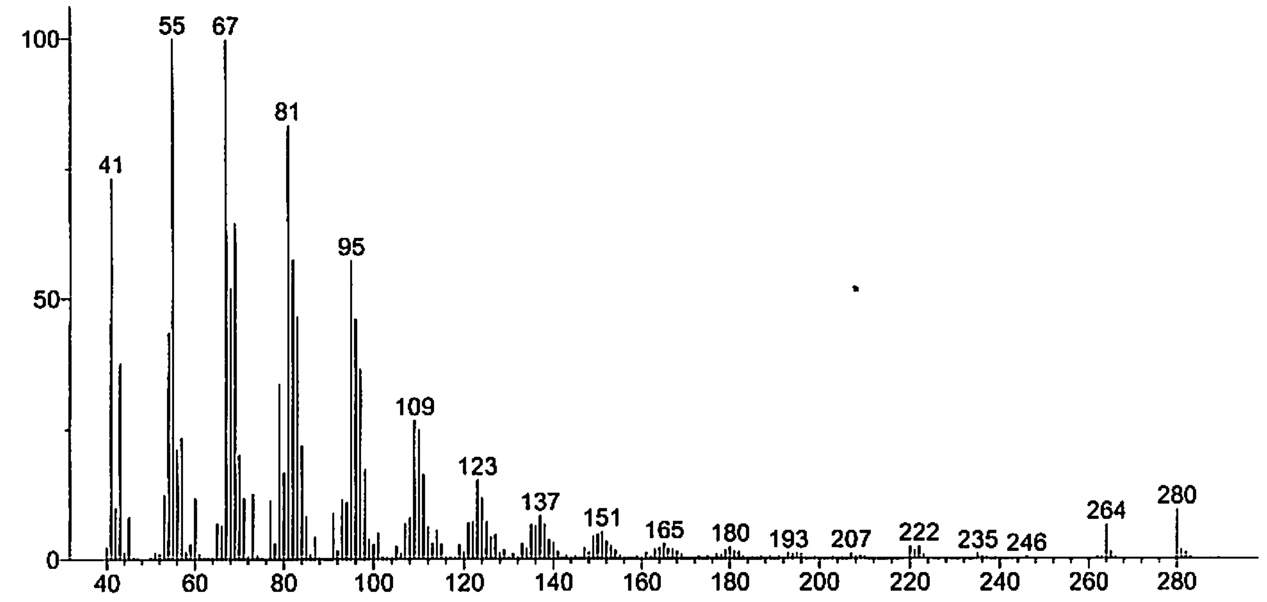


(Text File) Scan 1658 (22.092 min): JA112009-3.D\data.ms (-1634)

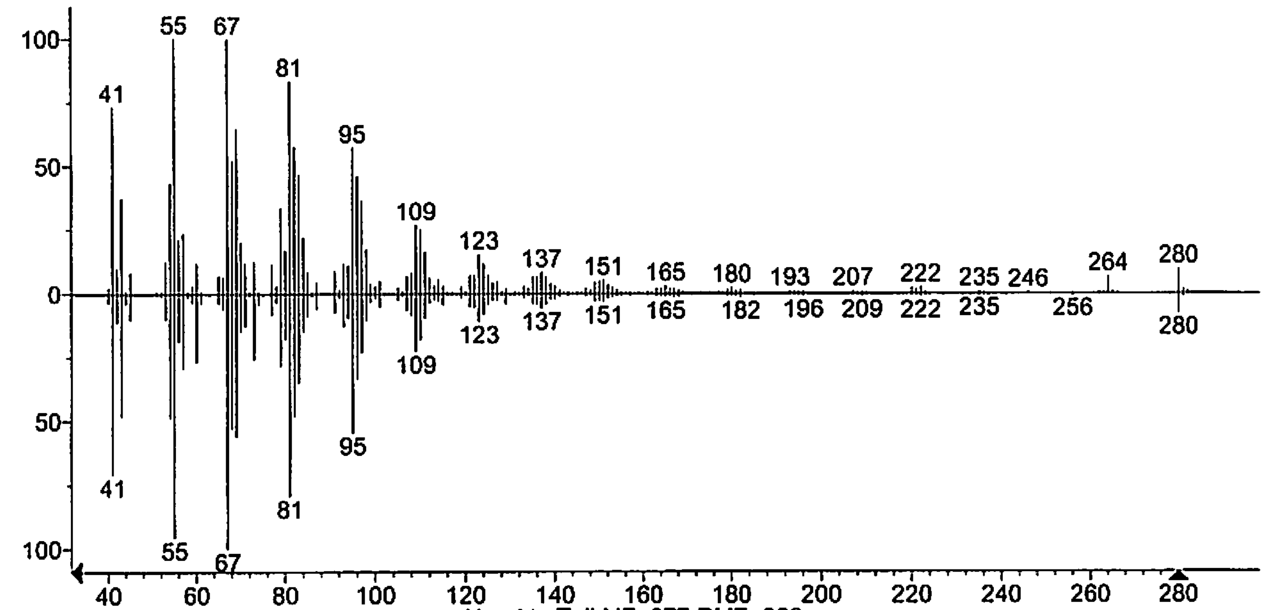


Head to Tail MF=877 RMF=888

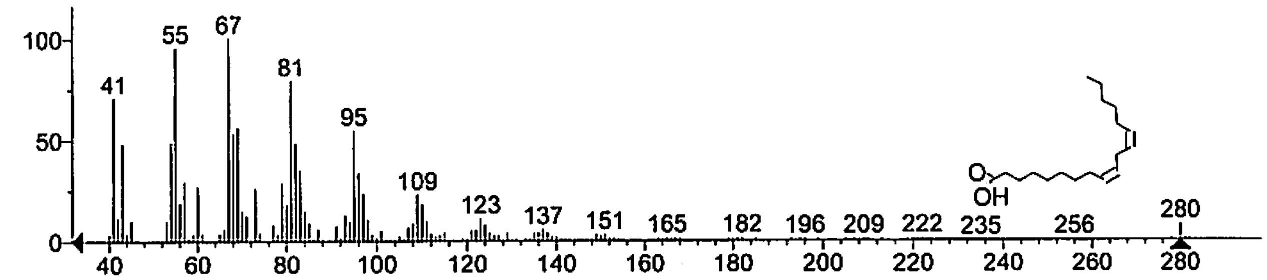


(replib) 9,12-Octadecadienoic acid (Z,Z)-

File :D:\DATA\Aldrich\JA-09\JA021009-2.D
Operator : Aldrich
Acquired : 10 Feb 2009 15:36 using AcqMethod JA-WAX08.M
Instrument : Instrument #1
Sample Name: 12 male 5-18-d C.oculata abd.ster./5ul CH2Cl2
Misc Info : fed 1ug/ul Z,E-nepetalactone 7 days; DB-WAX
Vial Number: 1

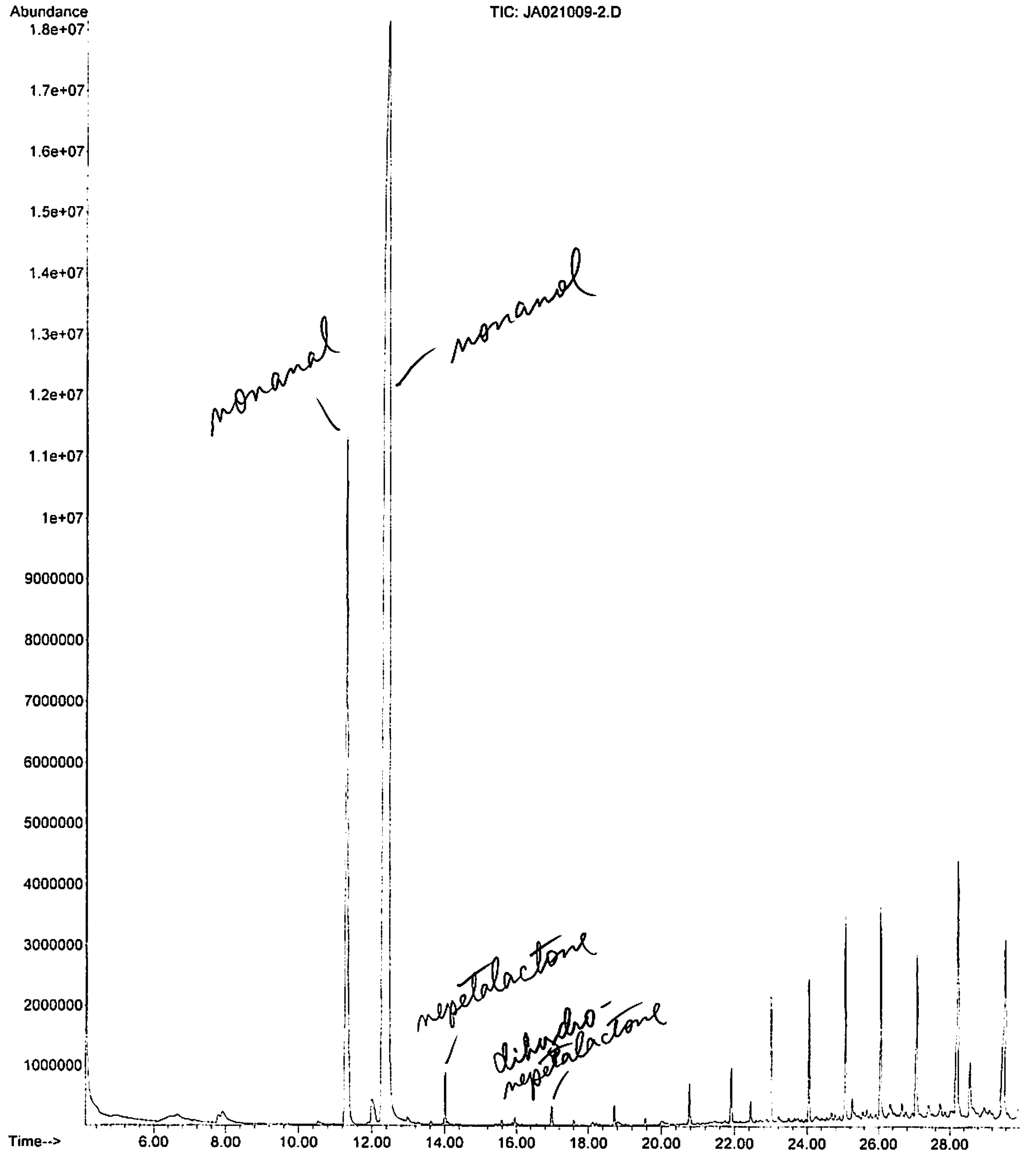

File :D:\DATA\ALDRICH\JA-09\Snapshot\JA021009-2.D
Operator : Aldrich
Acquired : 10 Feb 2009 15:36 using AcqMethod JA-WAX08.M
Instrument : Instrument #1
Sample Name: 12 male 5-18-d C.oculata abd.ster./5ul CH2Cl2
Misc Info : fed 1ug/ul Z,E-nepetalactone 7 days; DB-WAX
Vial Number: 1

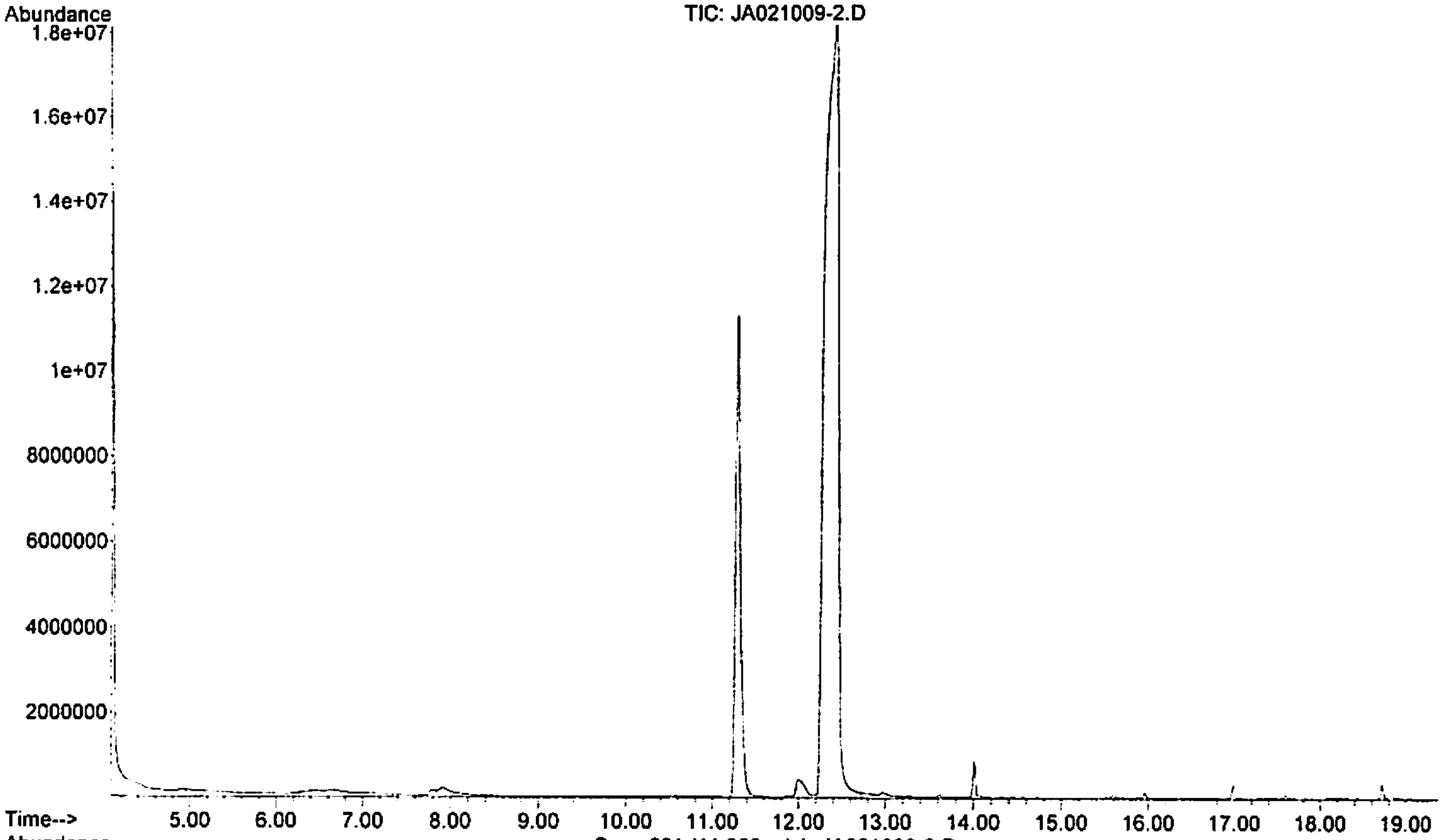


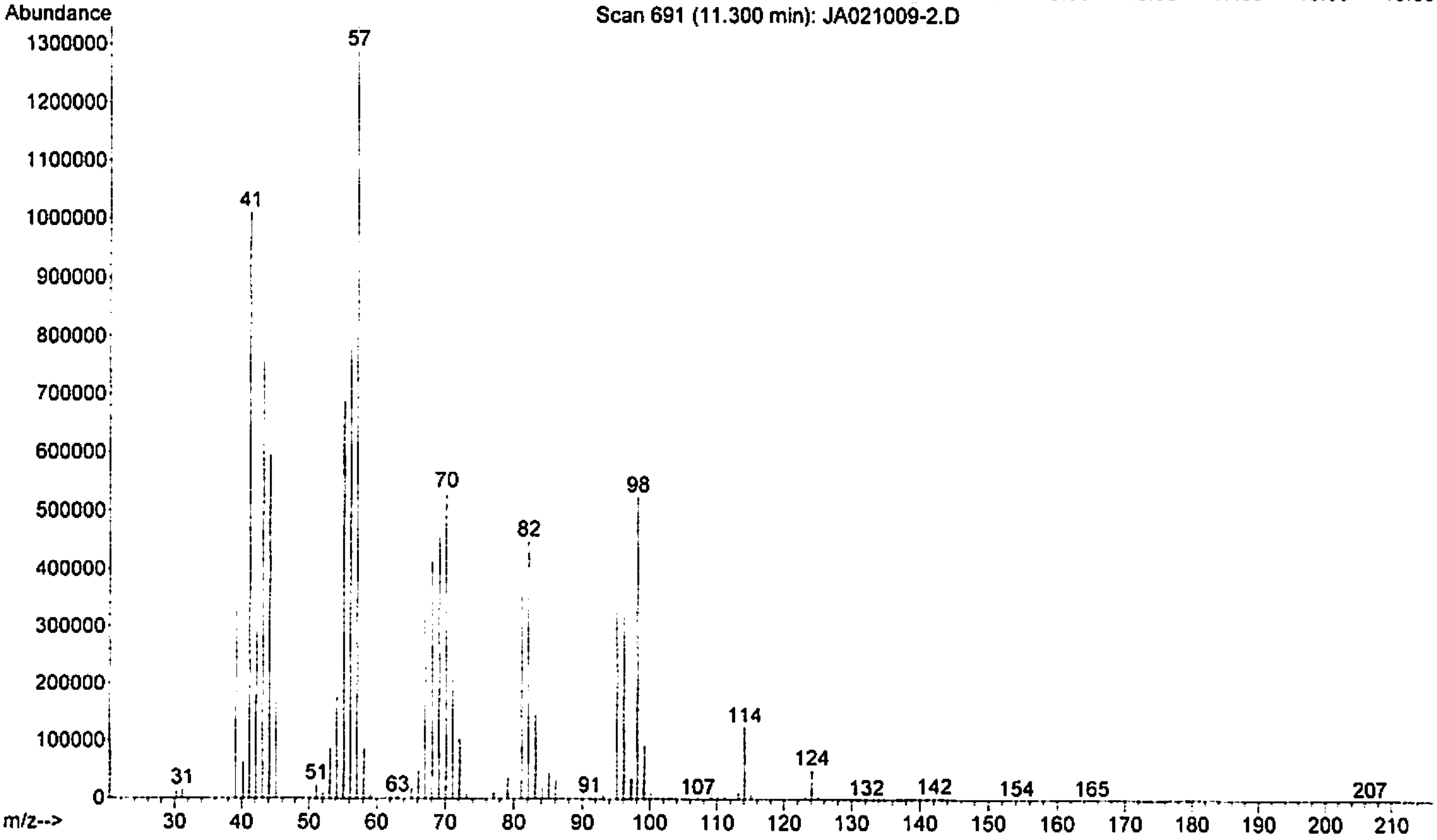

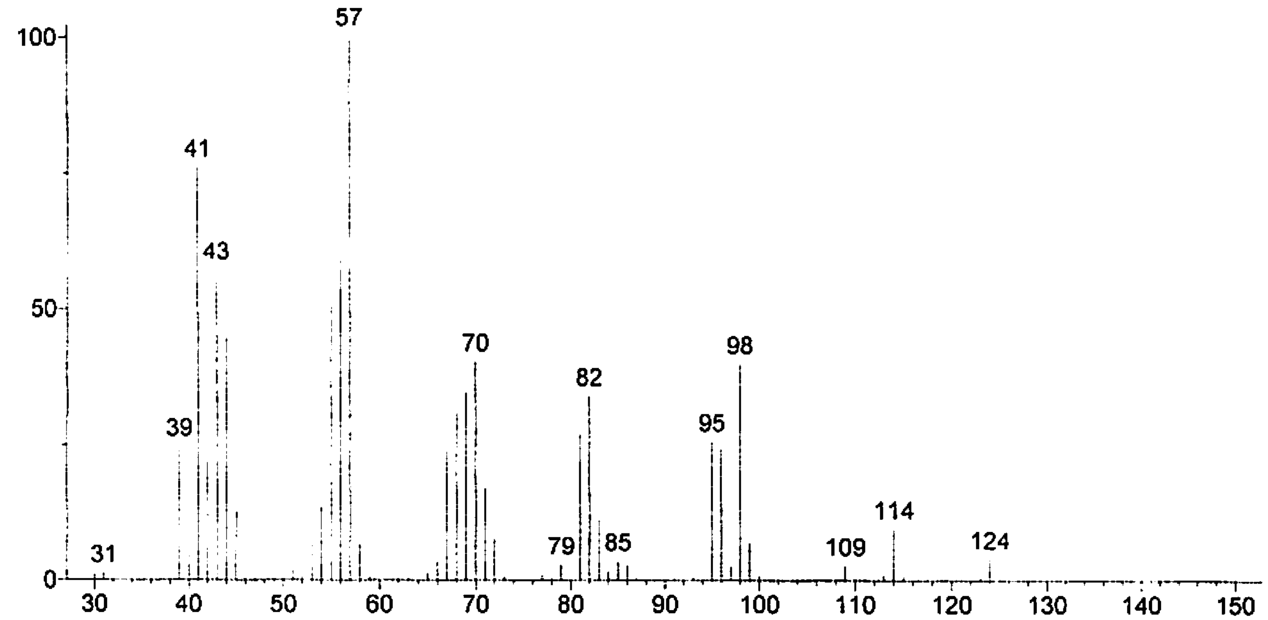


(Text File) Scan 691 (11.300 min): JA021009-2.D

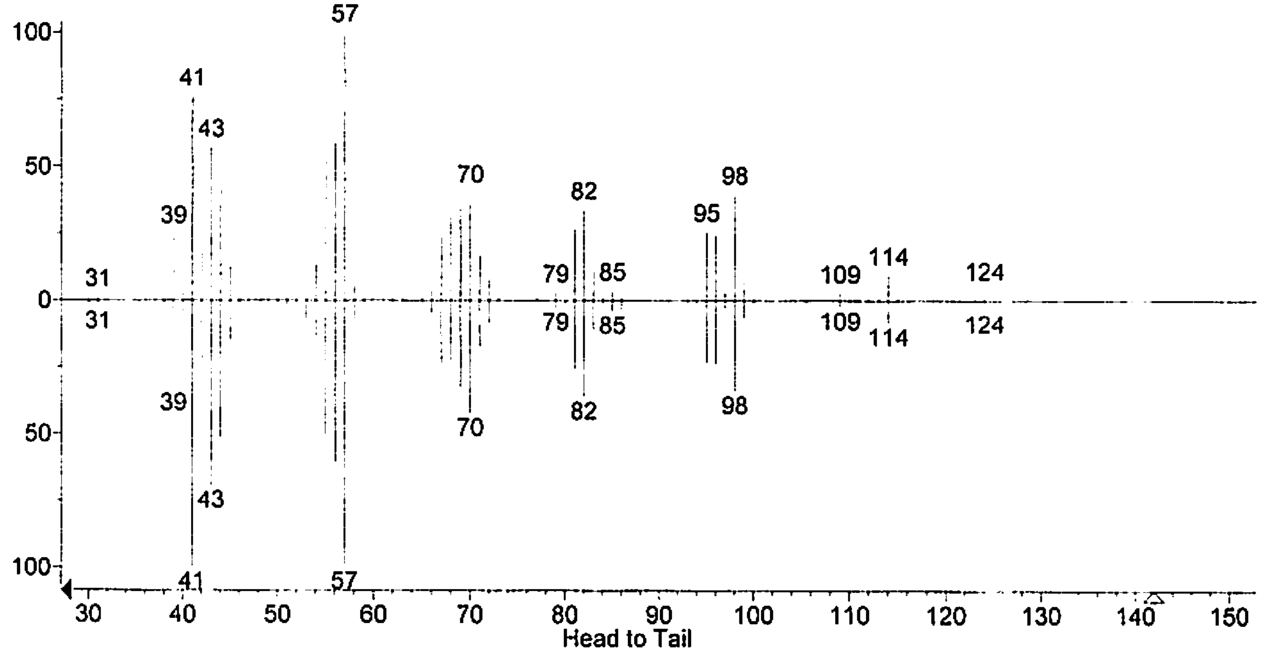


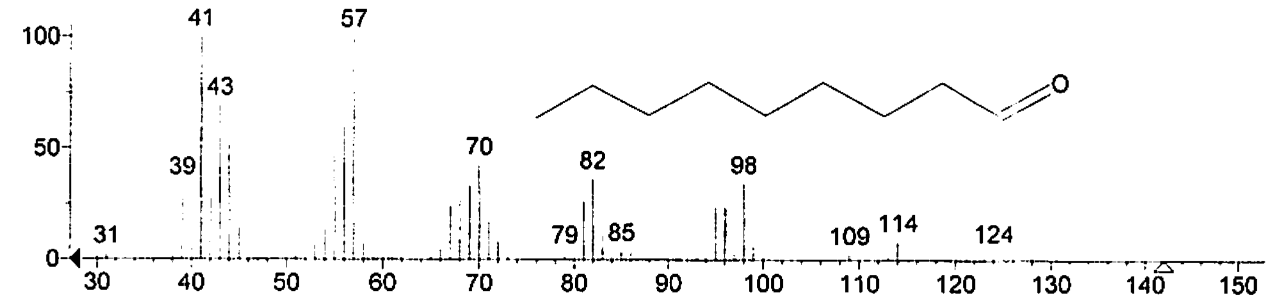


(replib) Nonanal

```
File        : D:\DATA\ALDRICH\JA-09\Snapshot\JA021009-2.D
Operator    : Aldrich
Acquired    : 10 Feb 2009  15:36     using AcqMethod JA-WAX08.M
Instrument  :    Instrument #1
Sample Name: 12 male 5-18-d C.oculata abd.ster./5ul CH2C12
Misc Info   : fed 1ug/ul Z,E-nepetalactone 7 days; DB-WAX
Vial Number: 1
```

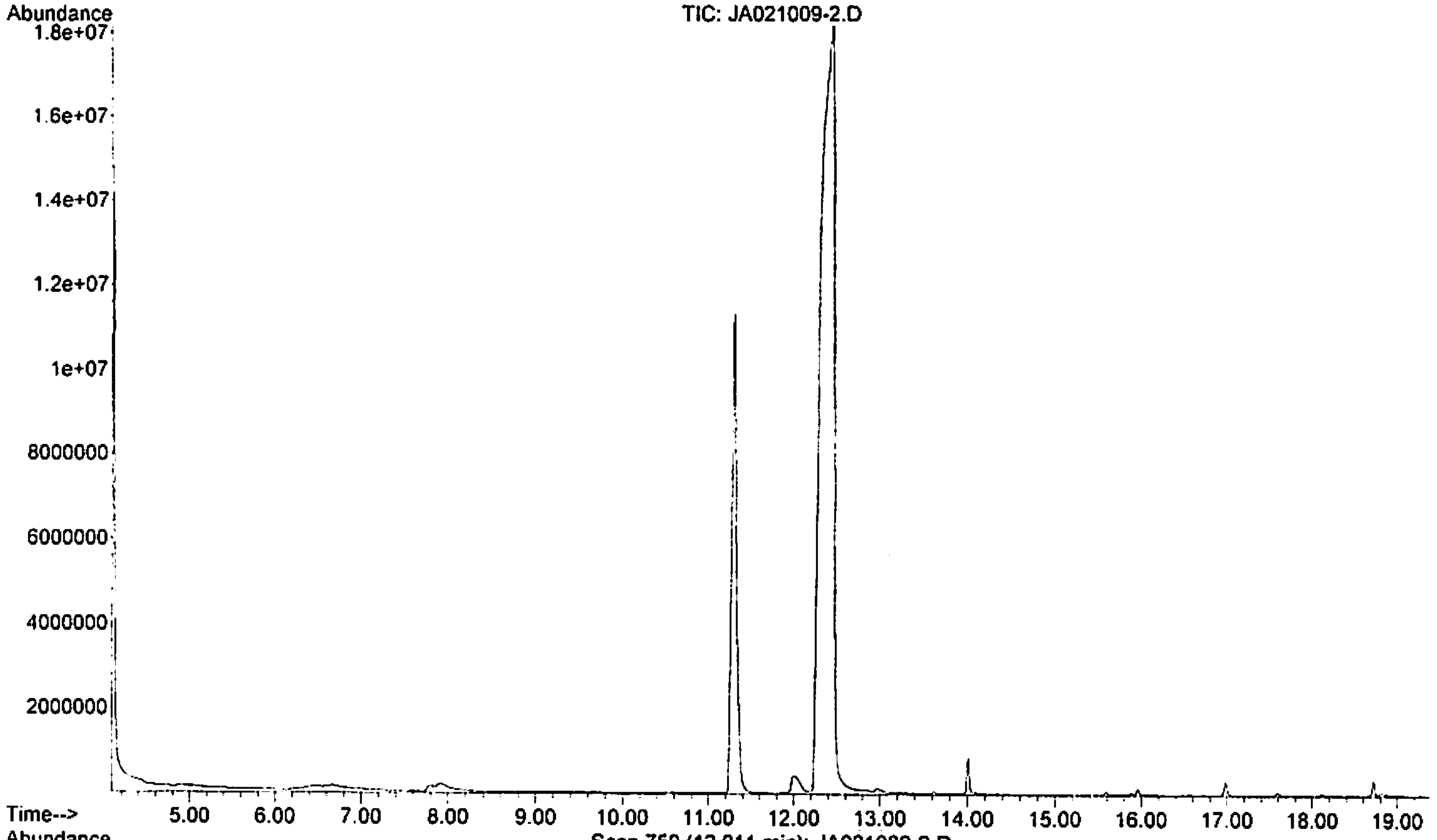


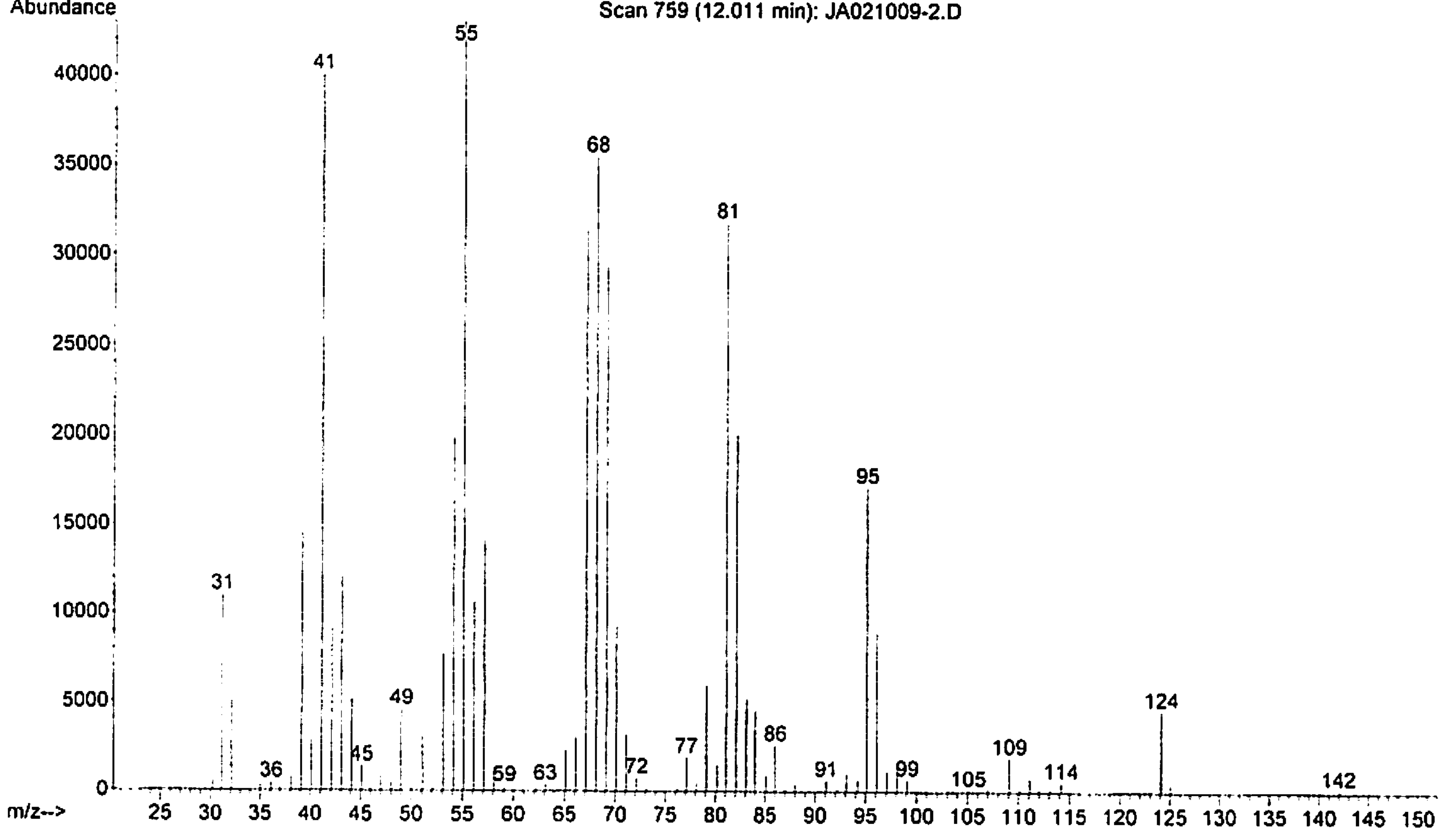

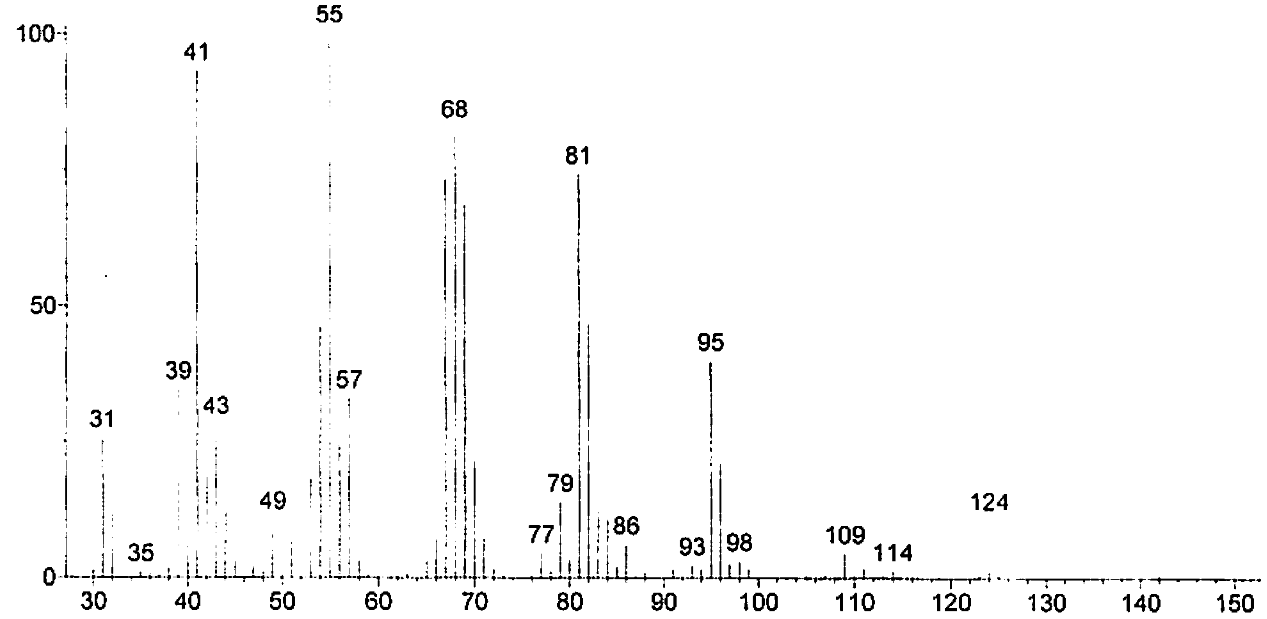


(Text File) Scan 759 (12.011 min): JA021009-2.D

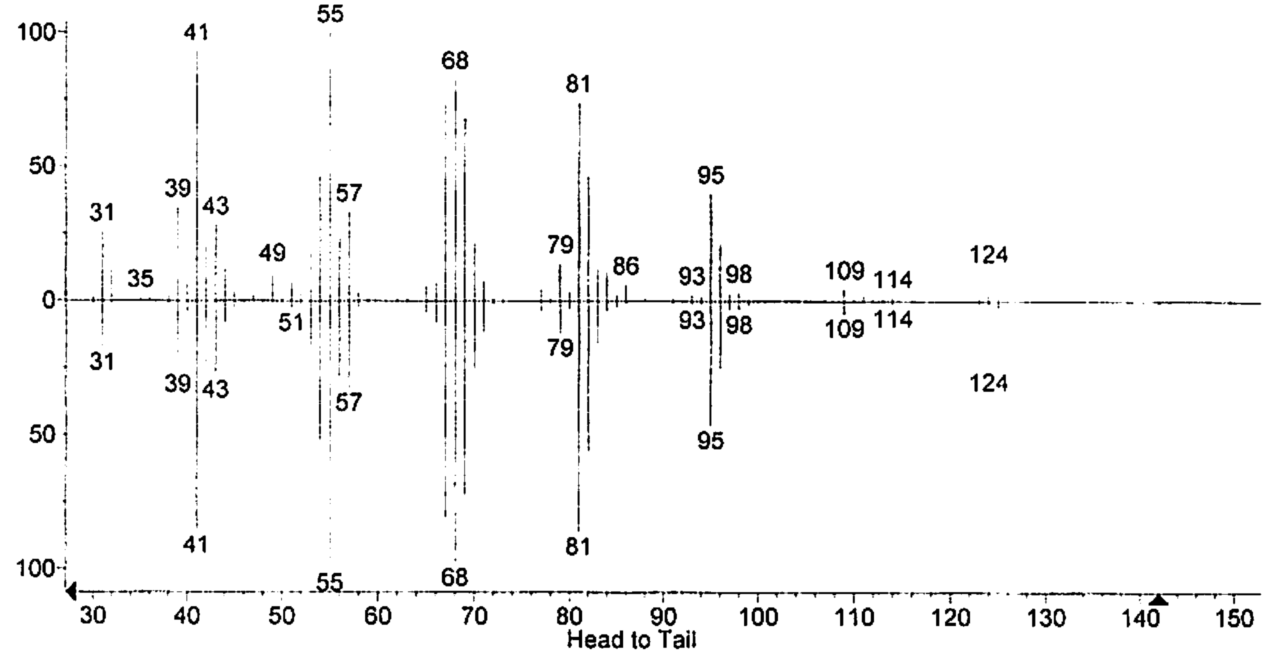


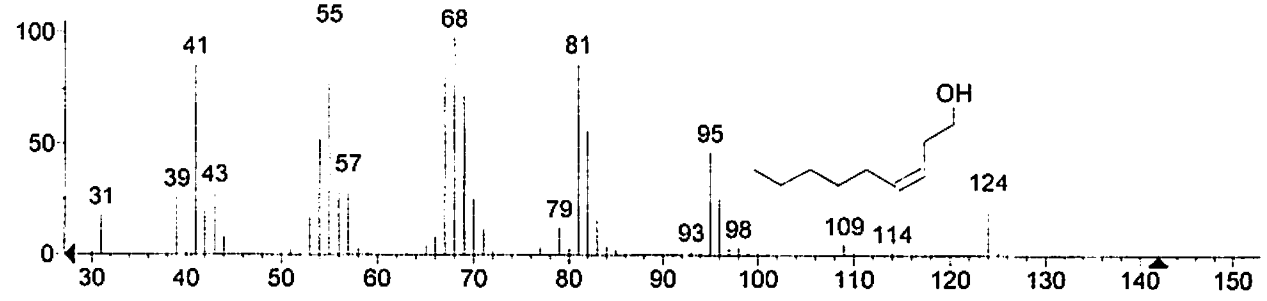


(mainlib) 3-Nonen-1-ol, (Z)-

File :D:\DATA\ALDRICH\JA-09\Snapshot\JA021009-2.D
Operator : Aldrich
Acquired : 10 Feb 2009 15:36 using AcqMethod JA-WAX08.M
Instrument : Instrument #1
Sample Name: 12 male 5-18-d C.oculata abd.ster./5ul CH2Cl2
Misc Info : fed 1ug/ul Z,E-nepetalactone 7 days; DB-WAX
Vial Number: 1

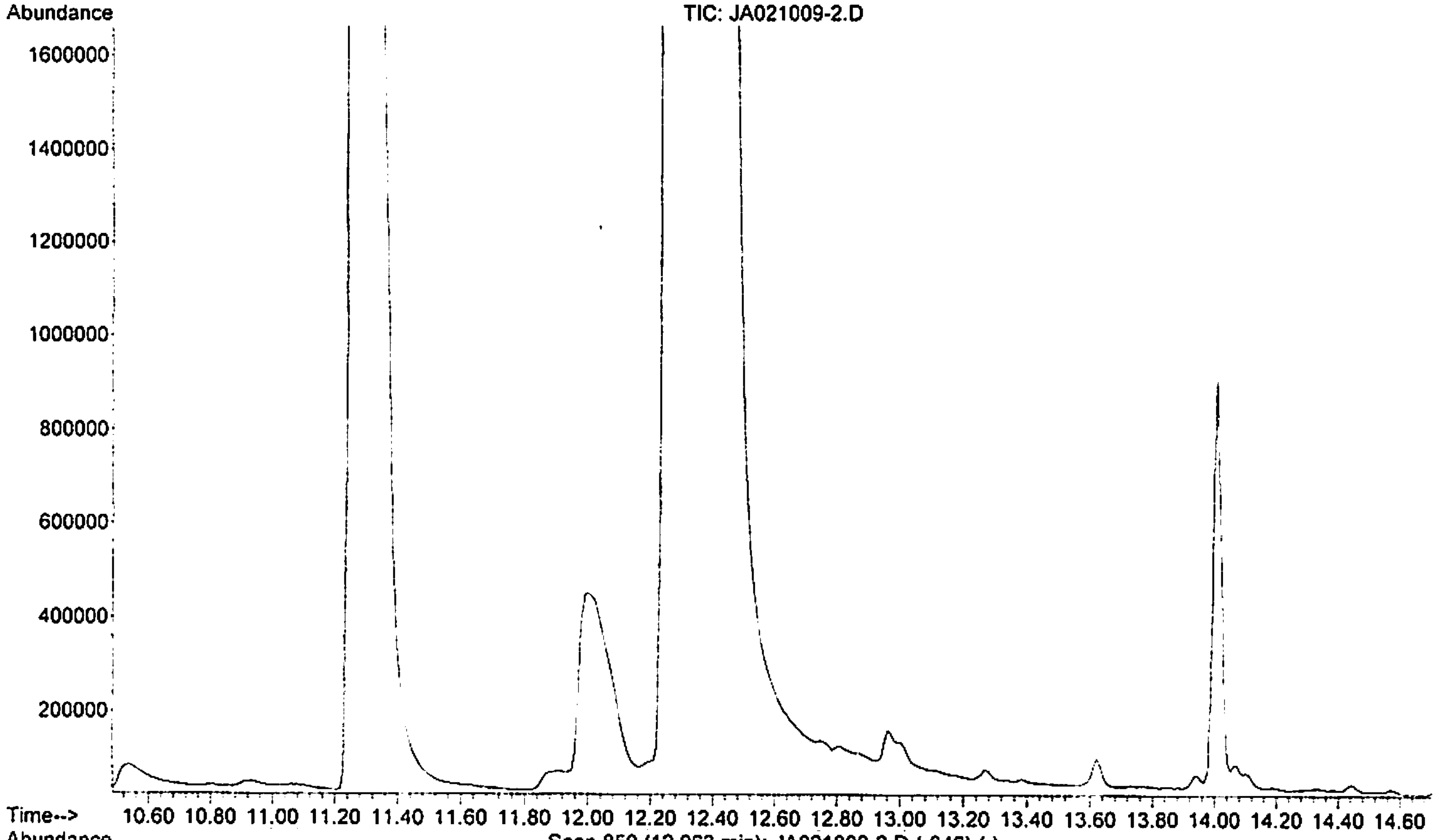


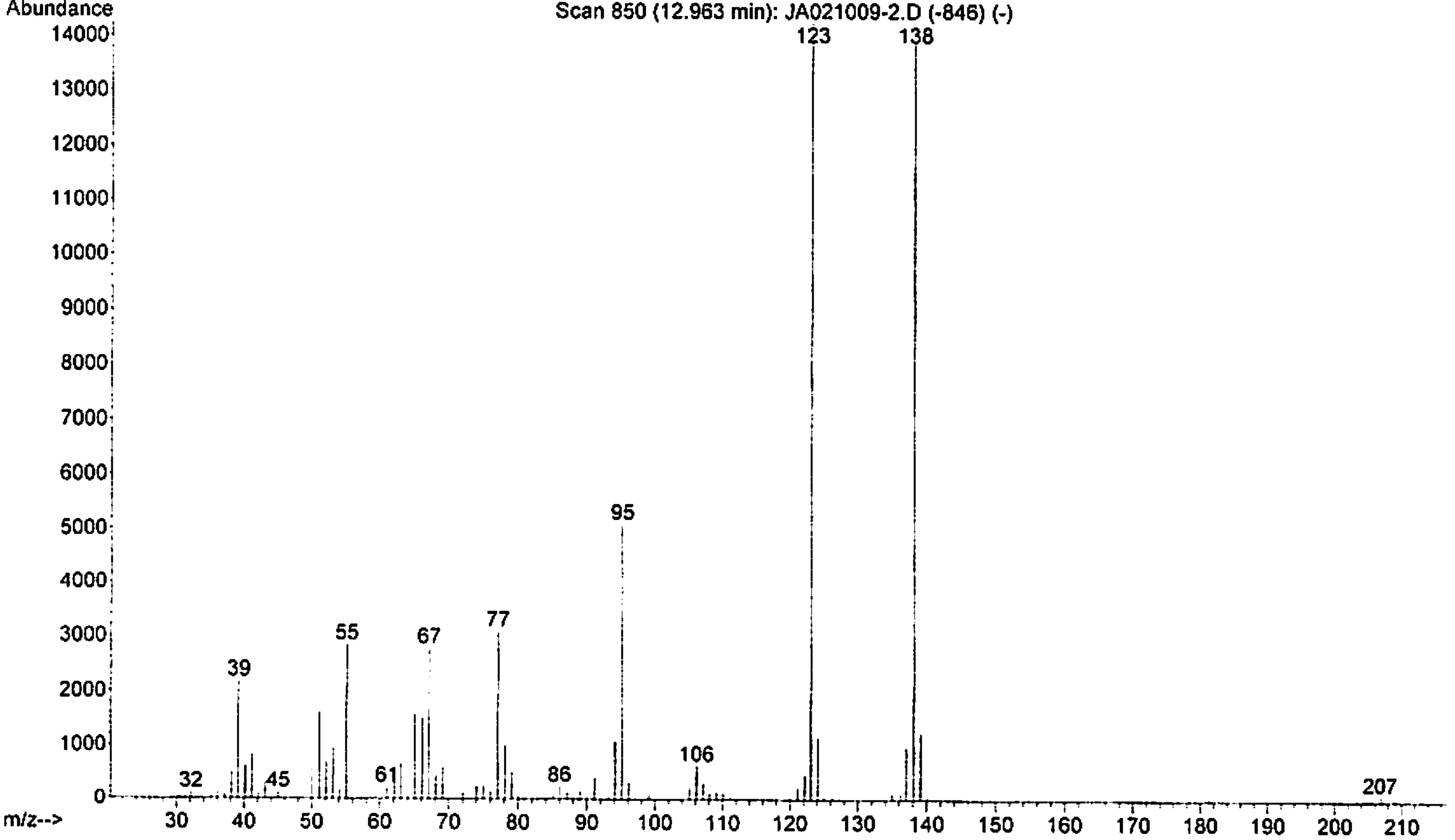

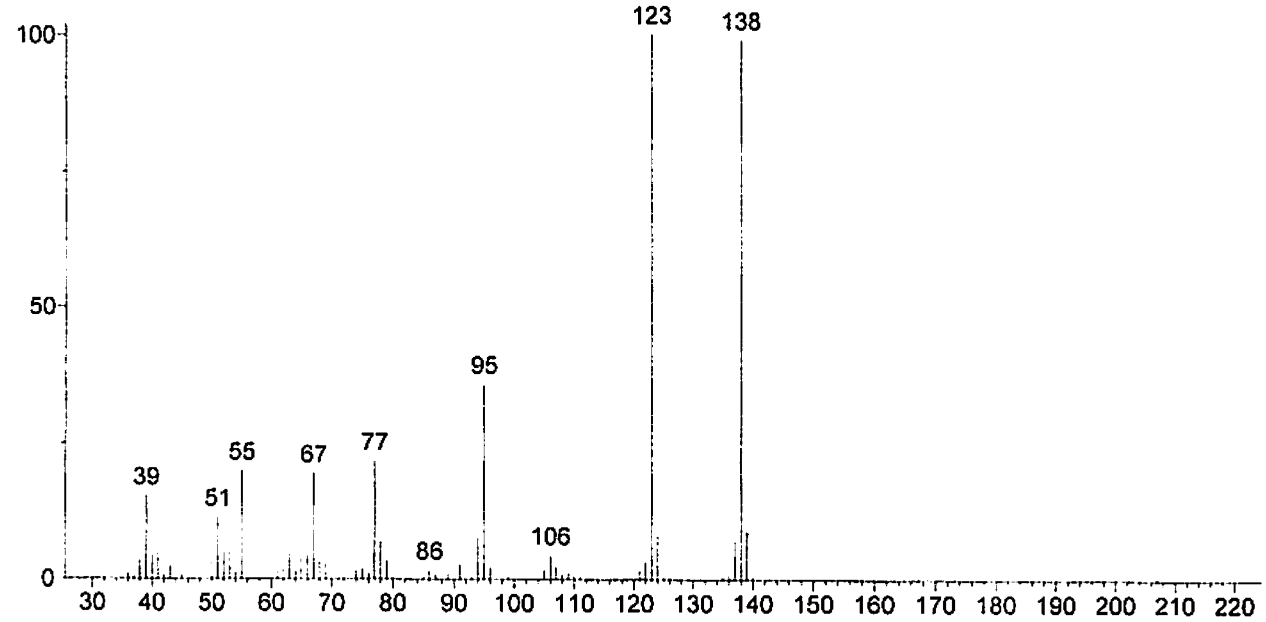


(Text File) Scan 850 (12.963 min): JA021009-2.D (-846)

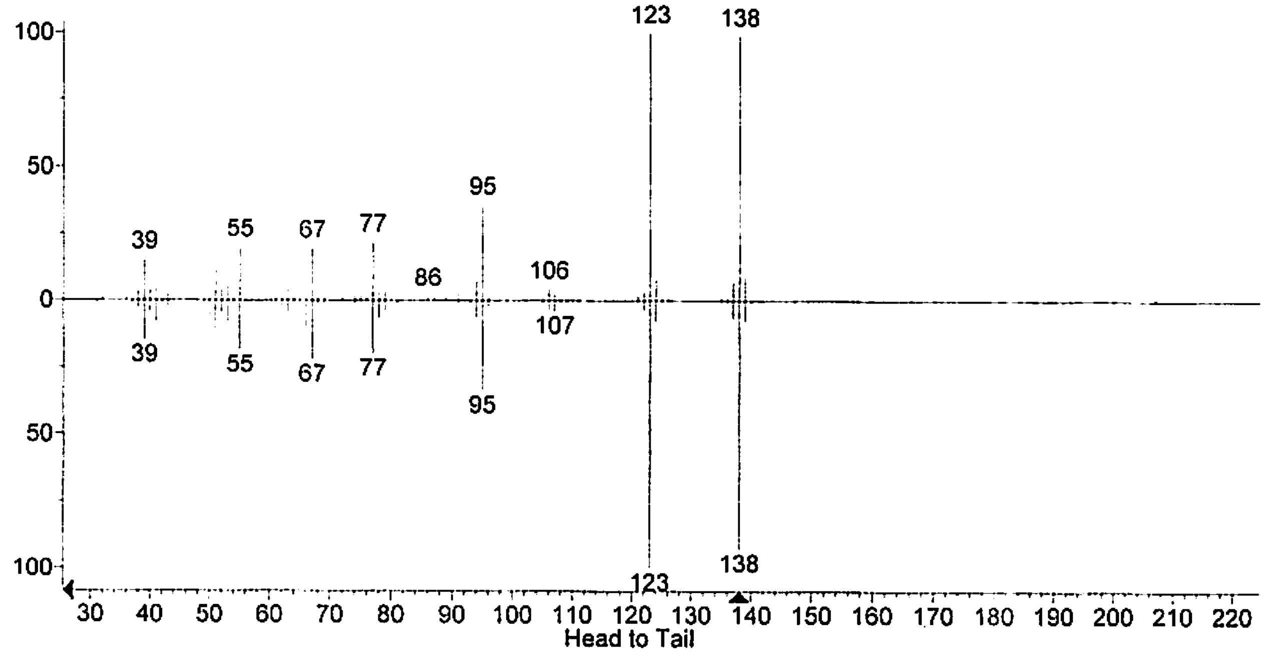


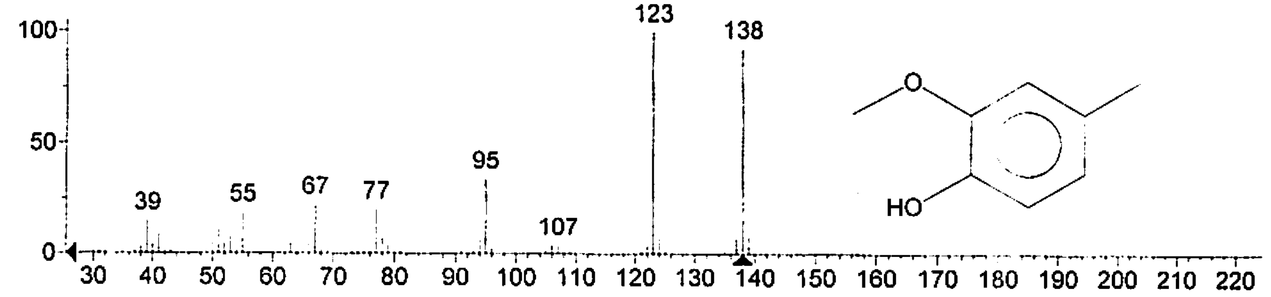


(replib) Phenol, 2-methoxy-4-methyl-

File        :D:\DATA\ALDRICH\JA-09\Snapshot\JA021009-2.D
Operator    : Aldrich
Acquired    : 10 Feb 2009  15:36     using AcqMethod JA-WAX08.M
Instrument :     Instrument #1
Sample Name: 12 male 5-18-d C.oculata abd.ster./5ul CH2Cl2
Misc Info   : fed 1ug/ul Z,E-nepetalactone 7 days; DB-WAX
Vial Number: 1

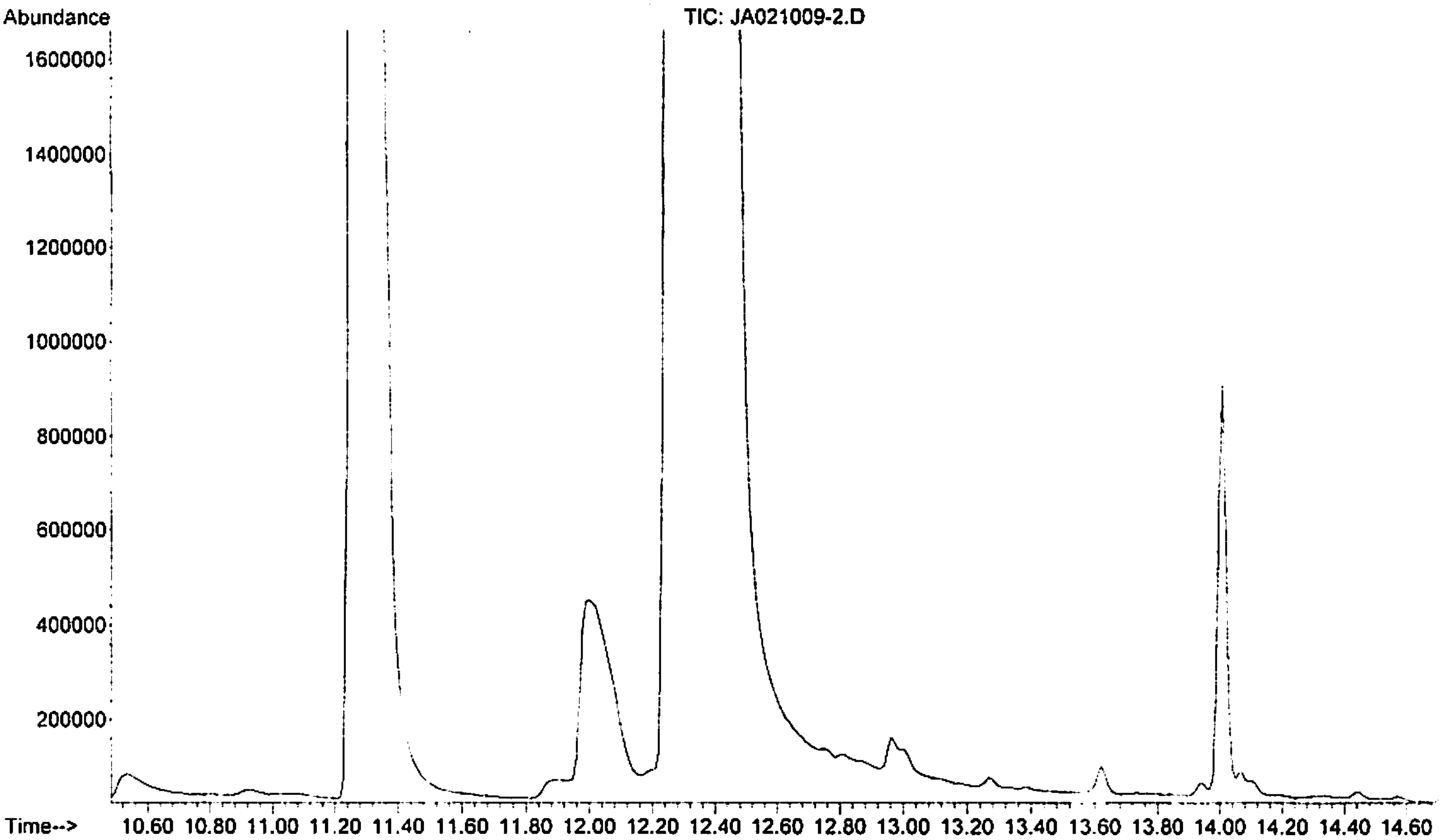


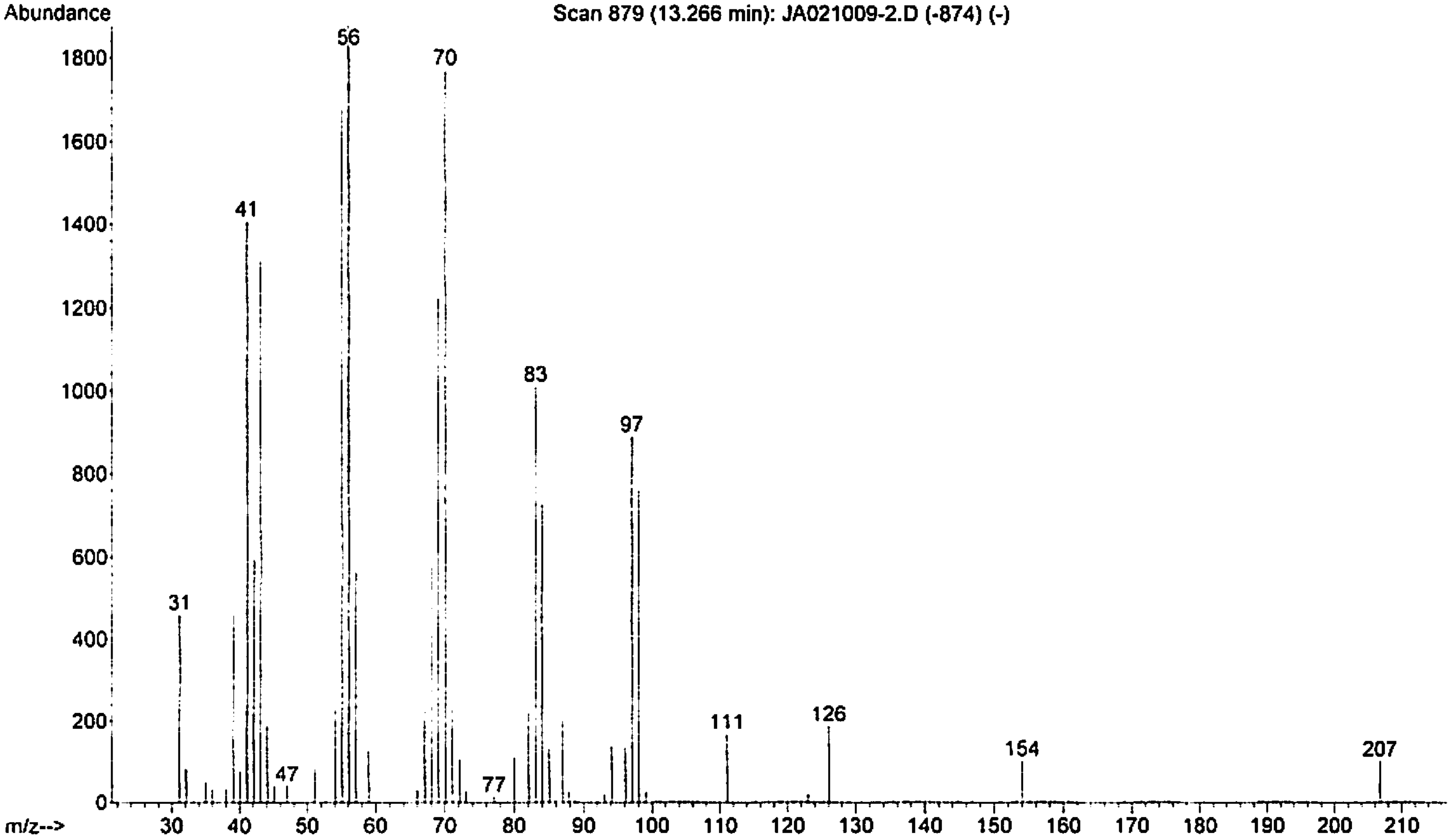

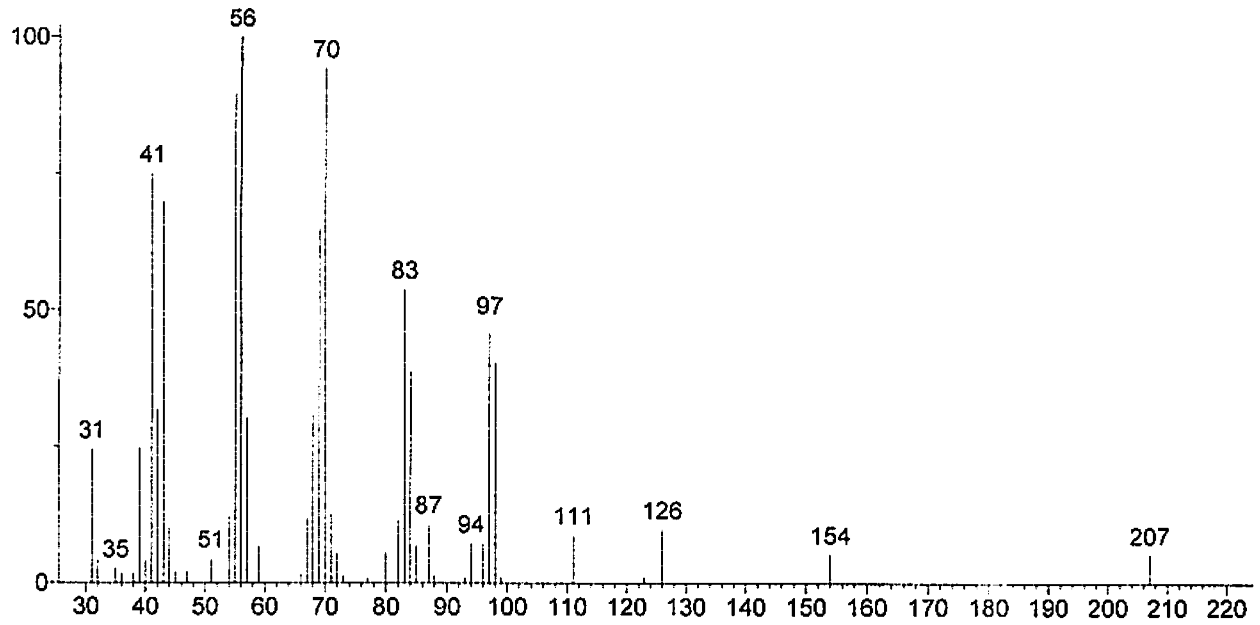


(Text File) Scan 879 (13.266 min): JA021009-2.D (-874)

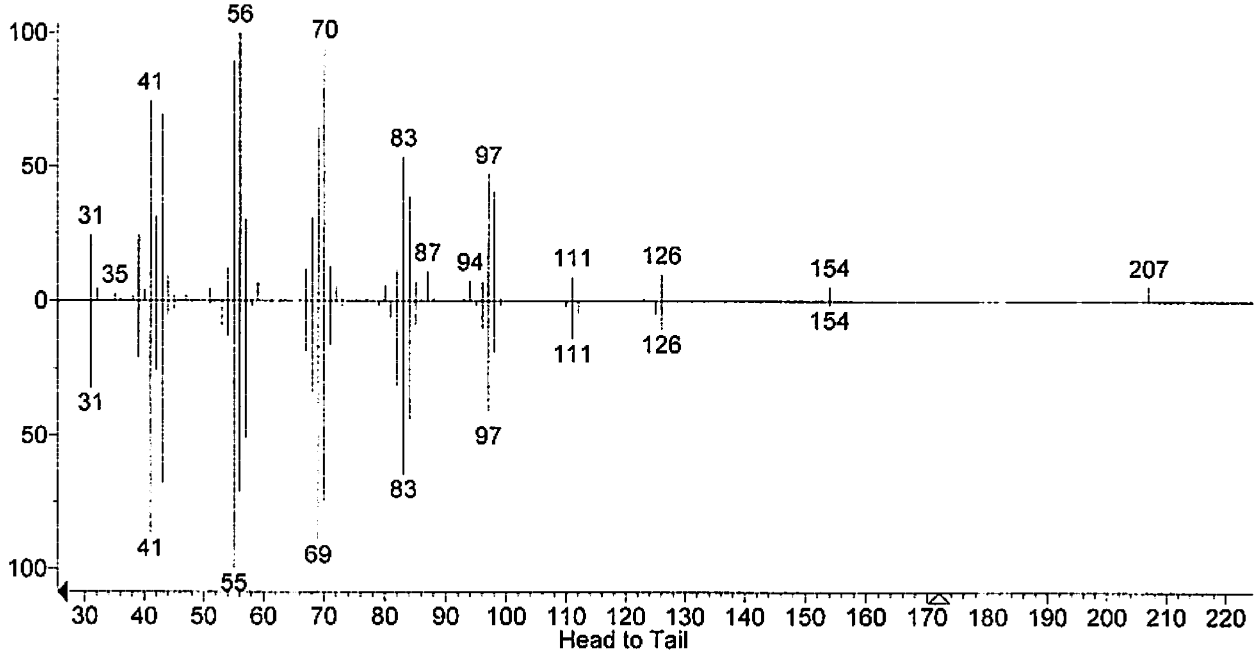


Head to Tail

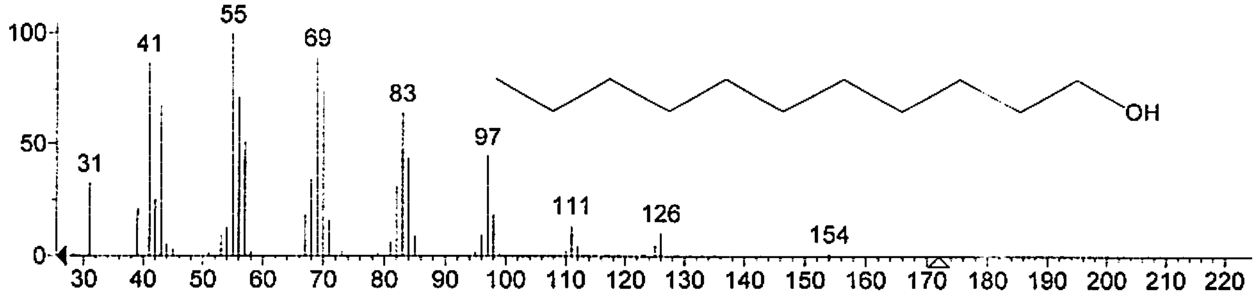


(mainlib) 1-Undecanol

```
File        :D:\DATA\ALDRICH\JA-09\Snapshot\JA021009-2.D
Operator    : Aldrich
Acquired    : 10 Feb 2009   15:36      using AcqMethod JA-WAX08.M
Instrument  :     Instrument #1
Sample Name: 12 male 5-18-d C.oculata abd.ster./5ul CH2Cl2
Misc Info   : fed 1ug/ul Z,E-nepetalactone 7 days; DB-WAX
Vial Number: 1
```

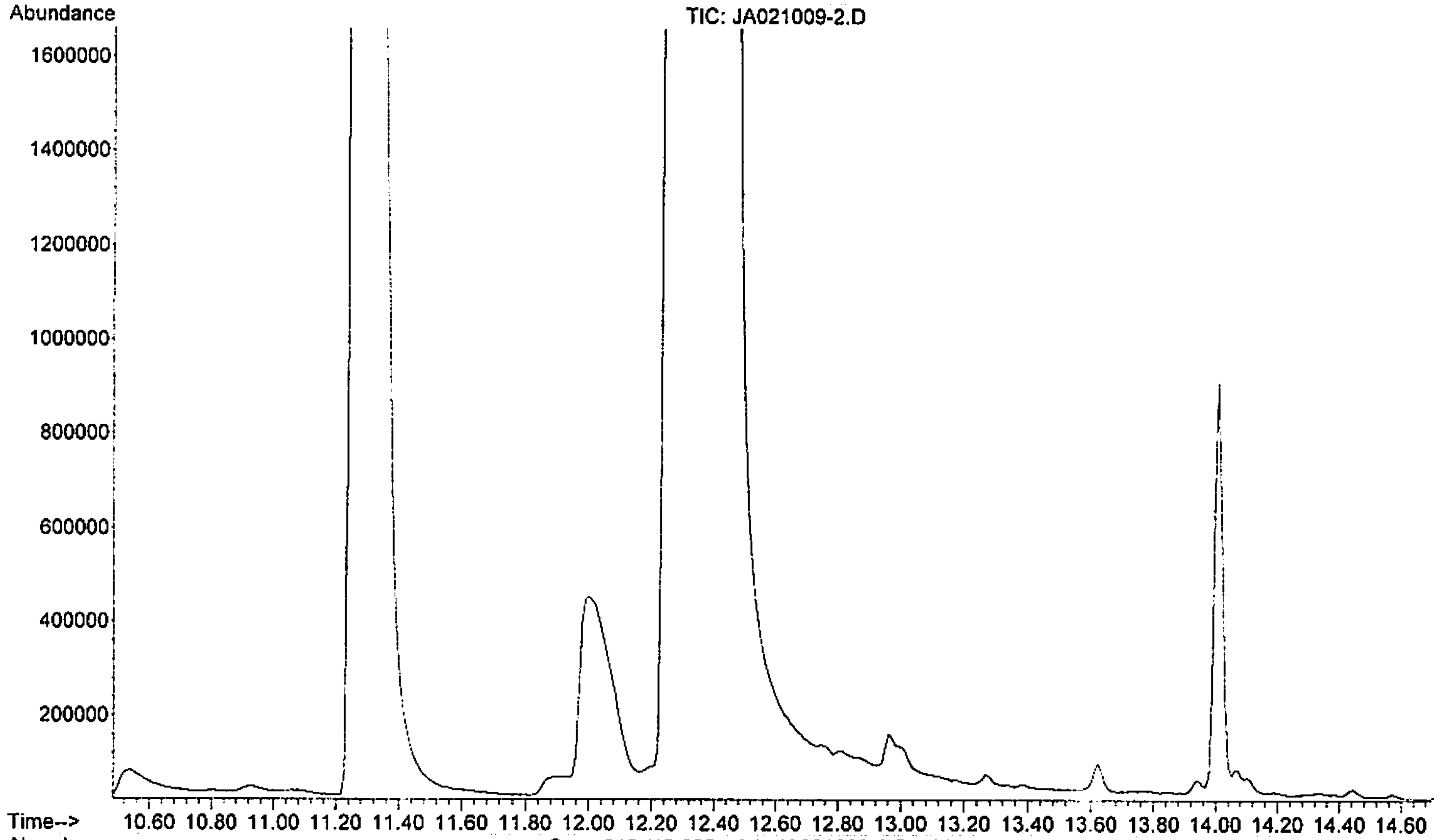


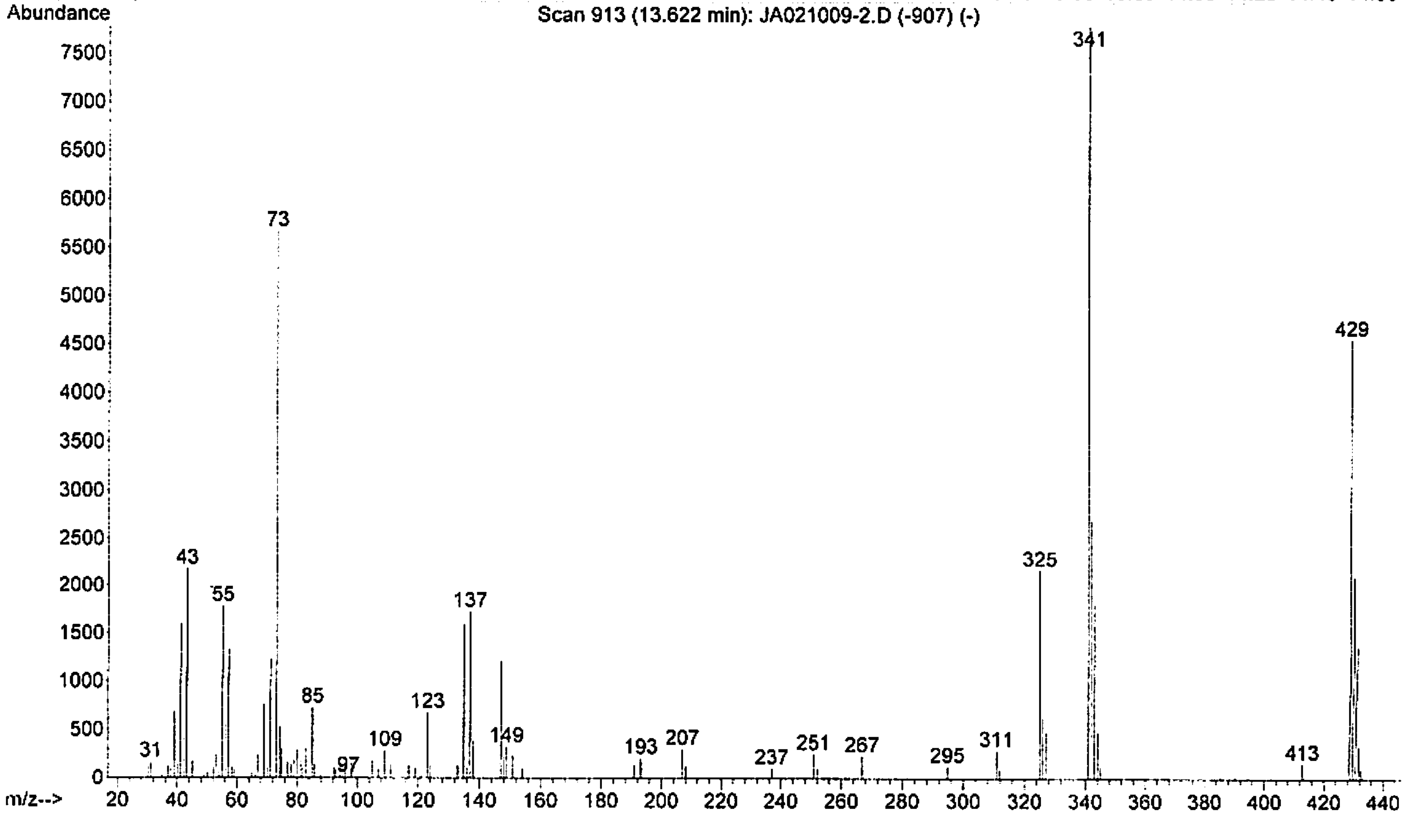

```
File        :D:\DATA\ALDRICH\JA-09\Snapshot\JA021009-2.D
Operator    : Aldrich
Acquired    : 10 Feb 2009  15:36     using AcqMethod JA-WAX08.M
Instrument  :    Instrument #1
Sample Name: 12 male 5-18-d C.oculata abd.ster./5ul CH2Cl2
Misc Info   : fed 1ug/ul Z,E-nepetalactone 7 days; DB-WAX
Vial Number: 1
```

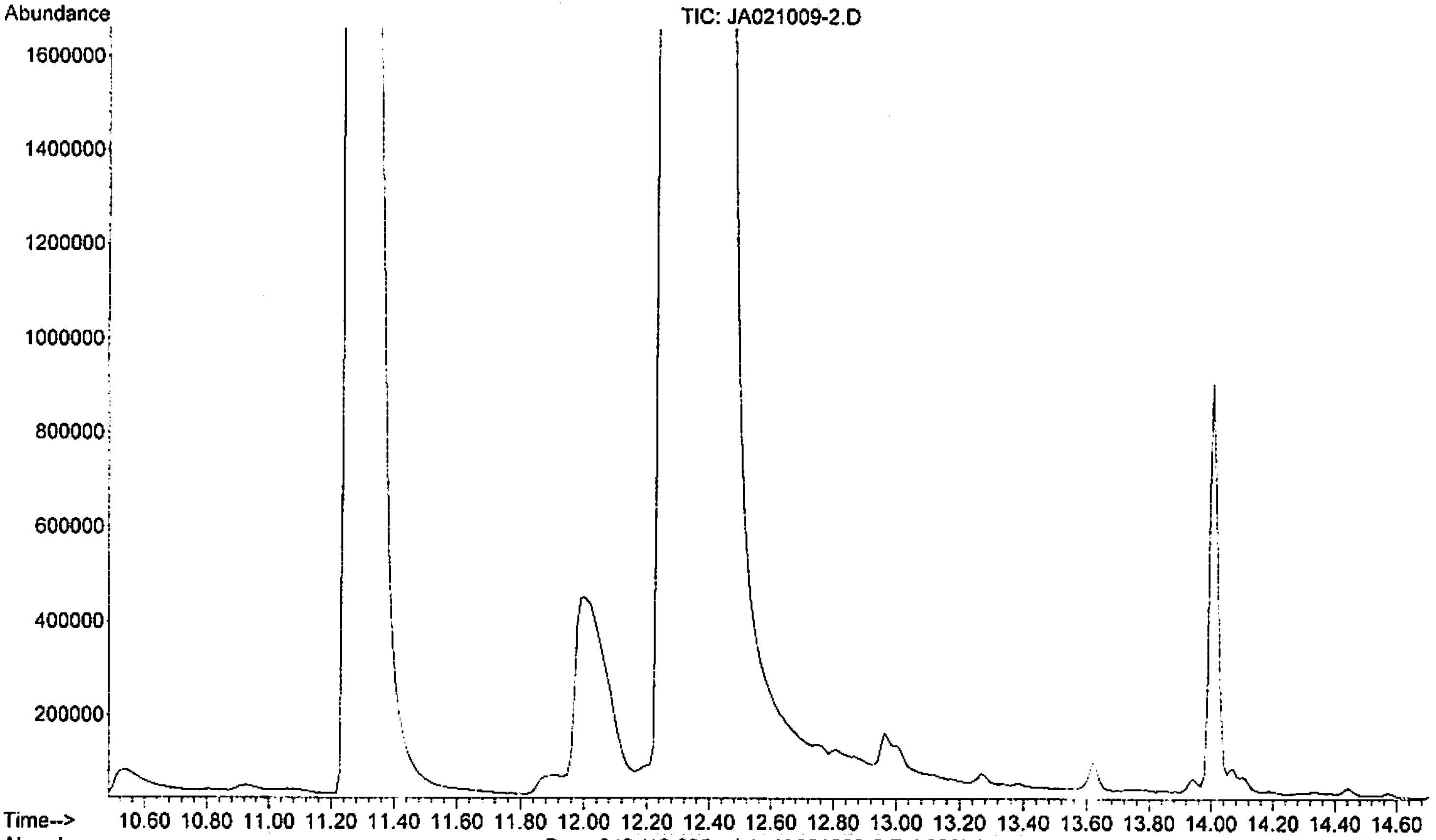


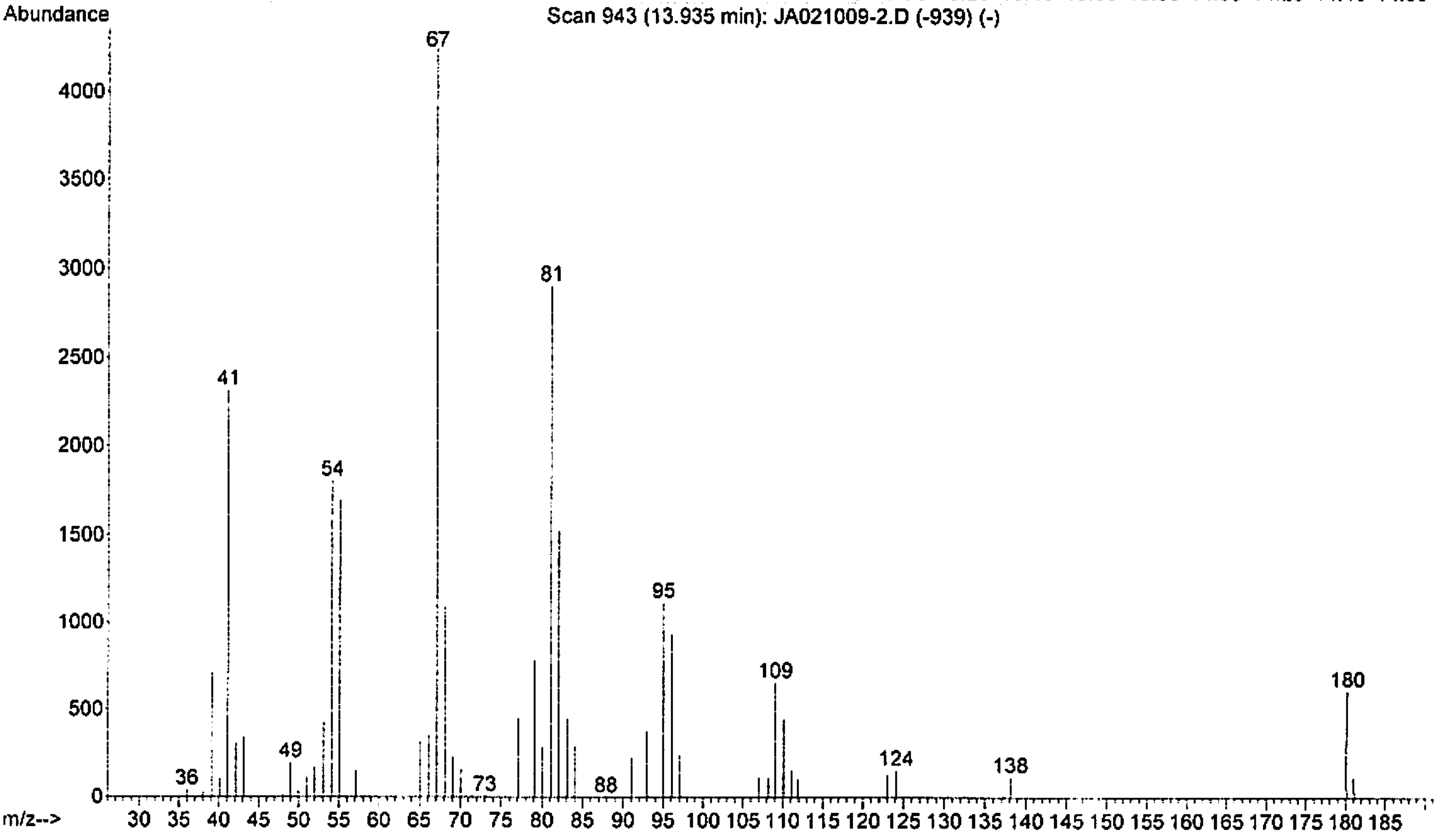

File :D:\DATA\ALDRICH\JA-09\Snapshot\JA021009-2.D
Operator : Aldrich
Acquired : 10 Feb 2009 15:36 using AcqMethod JA-WAX08.M
Instrument : Instrument #1
Sample Name: 12 male 5-18-d C.oculata abd.ster./5ul CH2Cl2
Misc Info : fed 1ug/ul Z,E-nepetalactone 7 days; DB-WAX
Vial Number: 1

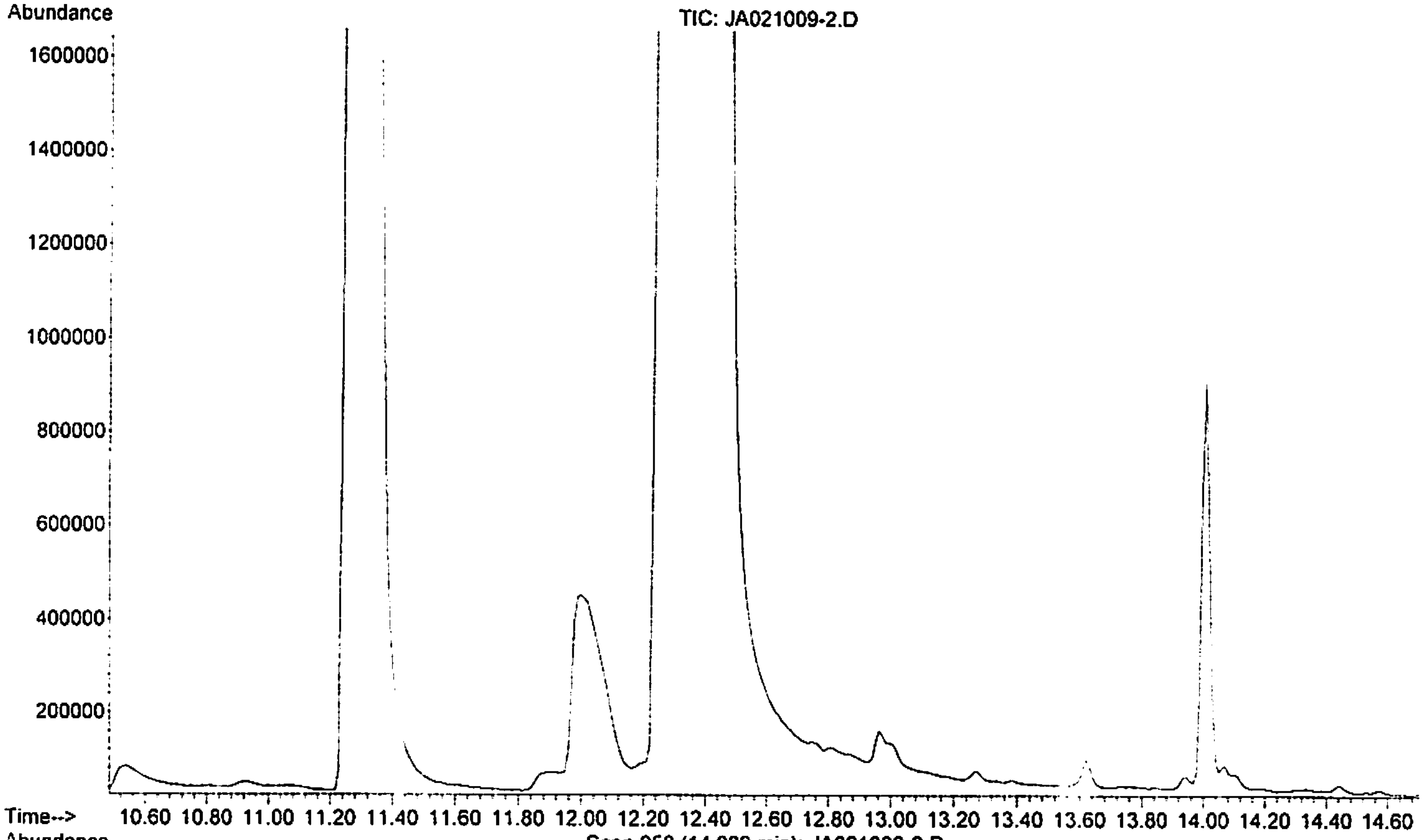


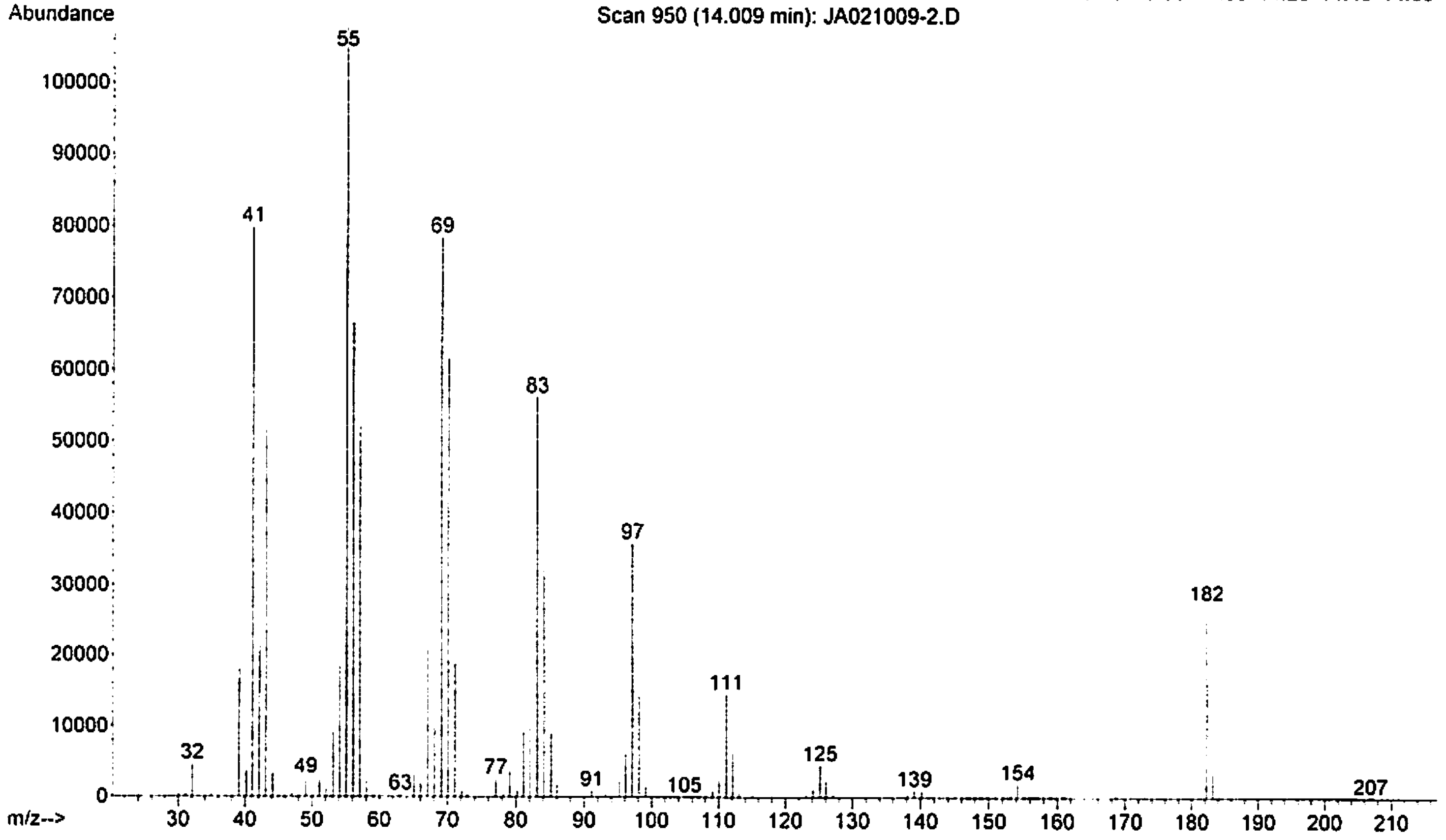

```
File          :D:\DATA\Aldrich\JA-09\JA021009-2.D
Operator      : Aldrich
Acquired      : 10 Feb 2009   15:36      using AcqMethod JA-WAX08.M
Instrument    :      Instrument #1
Sample Name: 12 male 5-18-d C.oculata abd.ster./5ul CH2Cl2
Misc Info     : fed 1ug/ul Z,E-nepetalactone 7 days; DB-WAX
Vial Number: 1
```

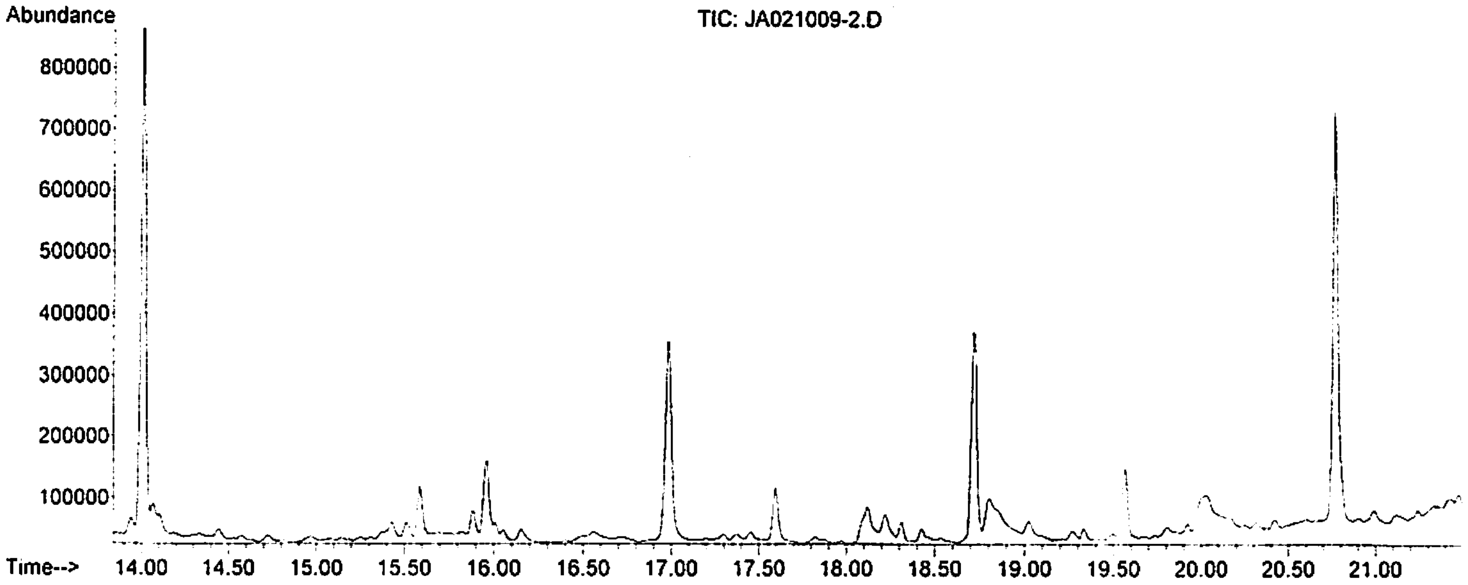


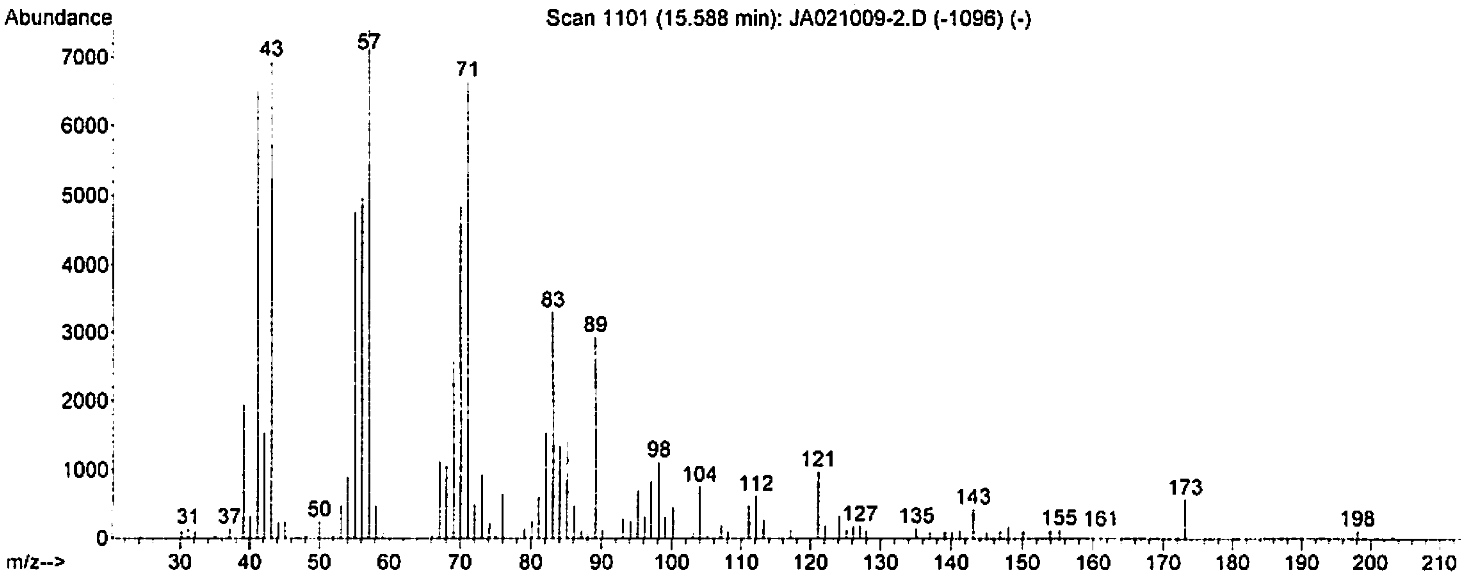

```
[illegible]le         :D:\DATA\Aldrich\JA-09\JA021009-2.D
[illegible]erator     : Aldrich
A[illegible]quired    : 10 Feb 2009  15:36     using AcqMethod JA-WAX08.M
[illegible]strument   :    Instrument #1
S[illegible]mple Name: 12 male 5-18-d C.oculata abd.ster./5ul CH2Cl2
M[illegible] Info     : fed 1ug/ul Z,E-nepetalactone 7 days; DB-WAX
[illegible] Number: 1
```

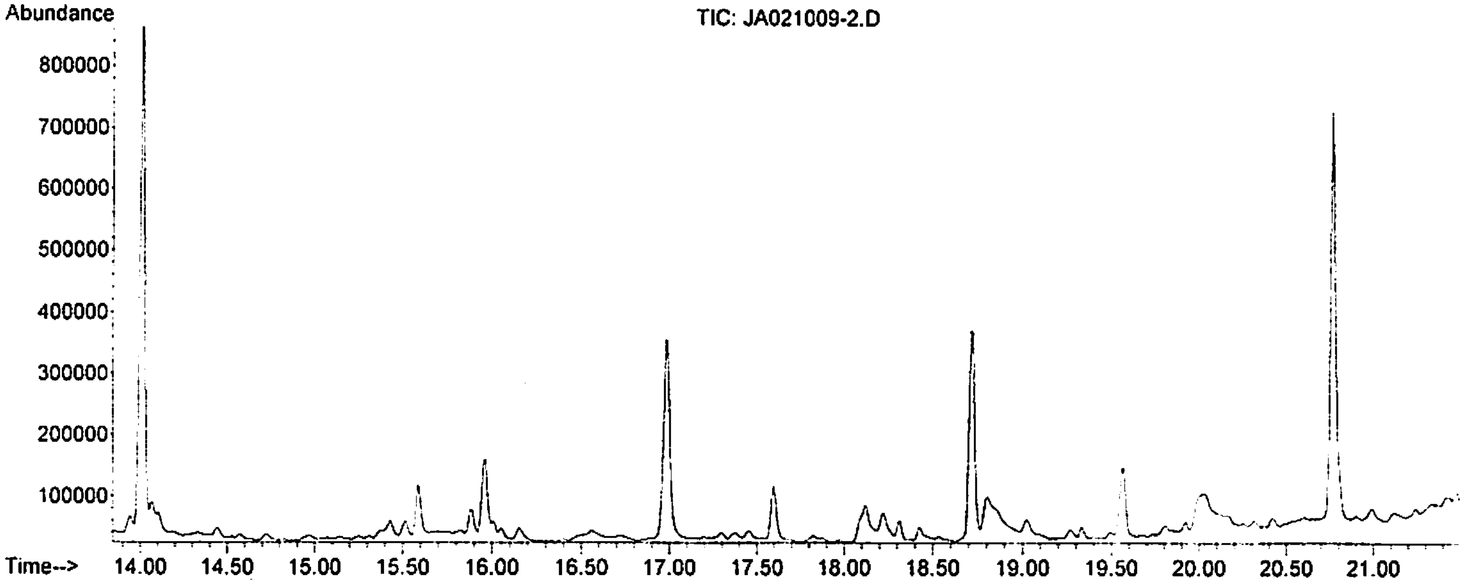


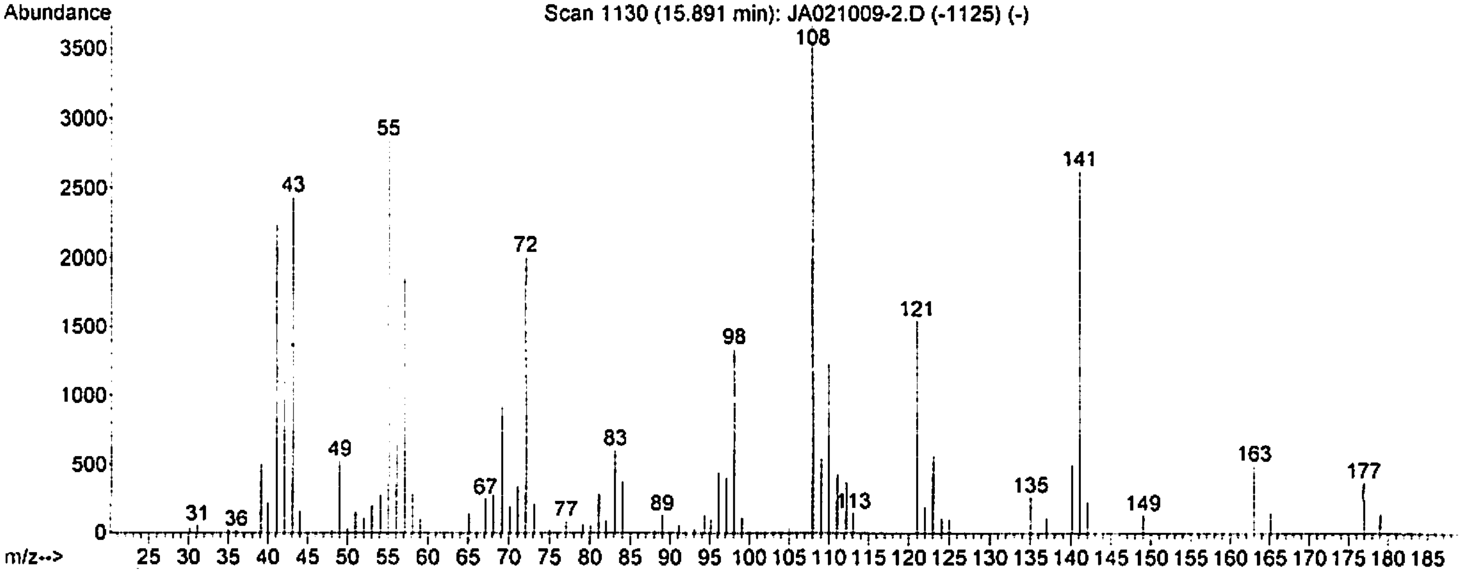

[illegible]e :D:\DATA\Aldrich\JA-09\JA021009-2.D
[illegible]rator : Aldrich
A[illegible]quired : 10 Feb 2009 15:36 using AcqMethod JA-WAX08.M
[illegible]strument : Instrument #1
[illegible]mple Name: 12 male 5-18-d C.oculata abd.ster./5ul CH2Cl2
[illegible]isc Info : fed 1ug/ul Z,E-nepetalactone 7 days; DB-WAX
[illegible]al Number: 1

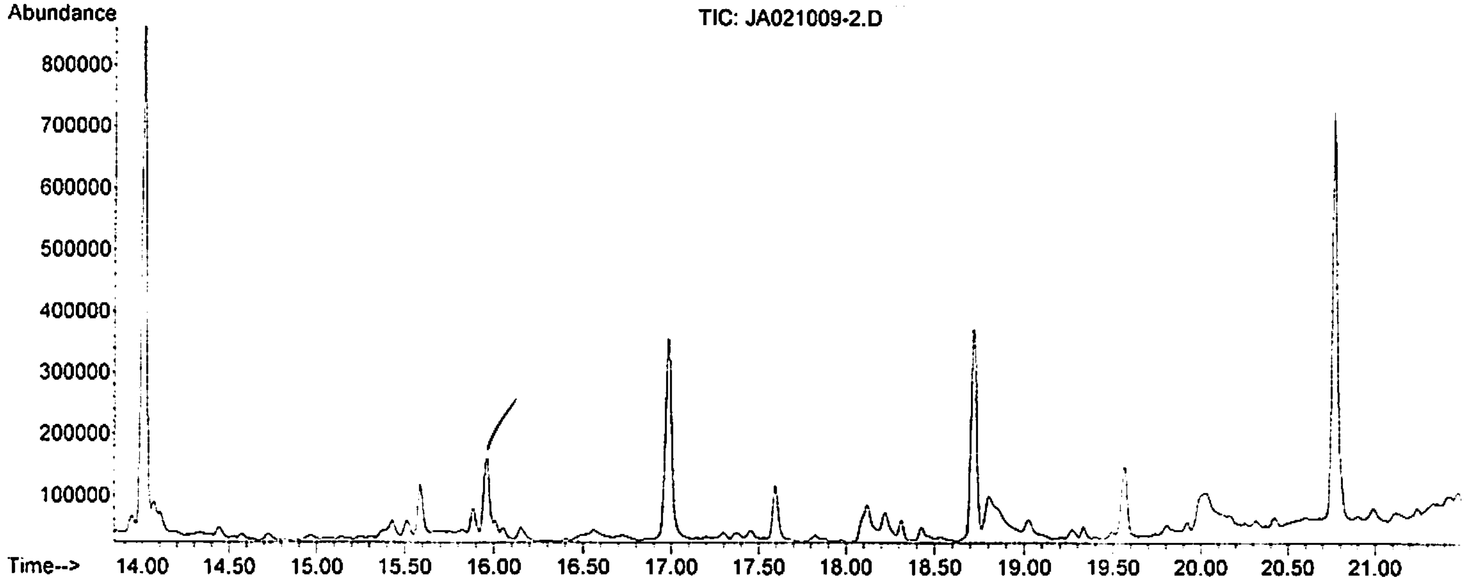


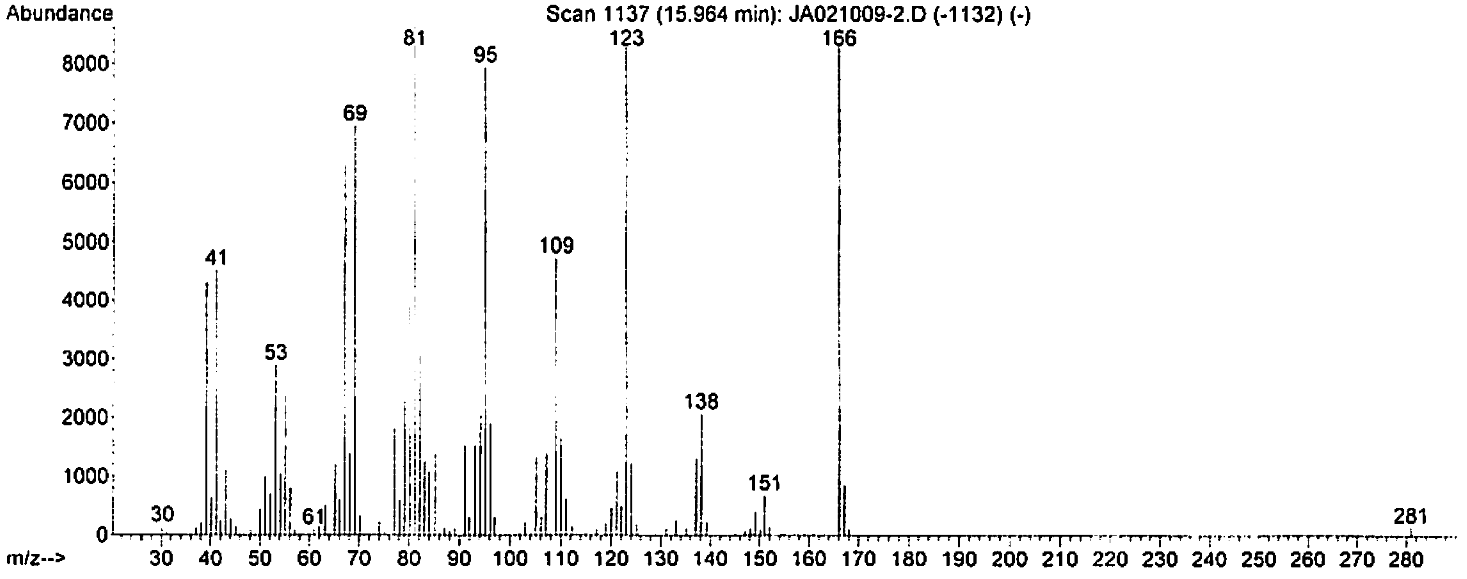

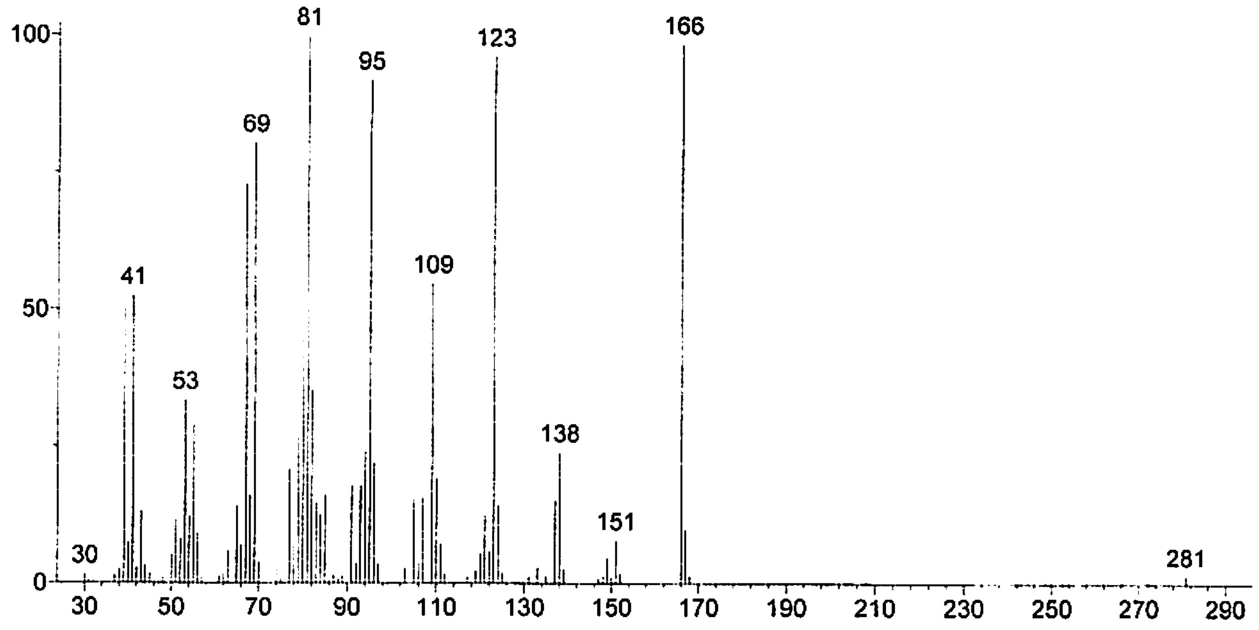


(Text File) Scan 1137 (15.964 min): JA021009-2.D (-1132)

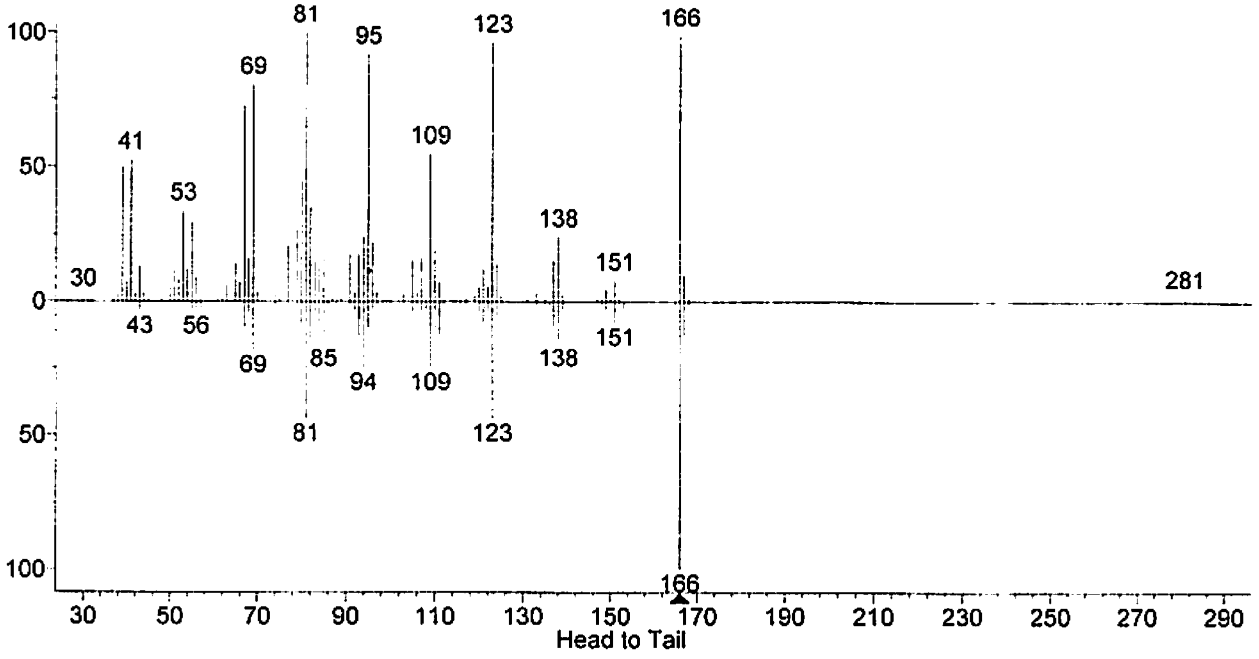


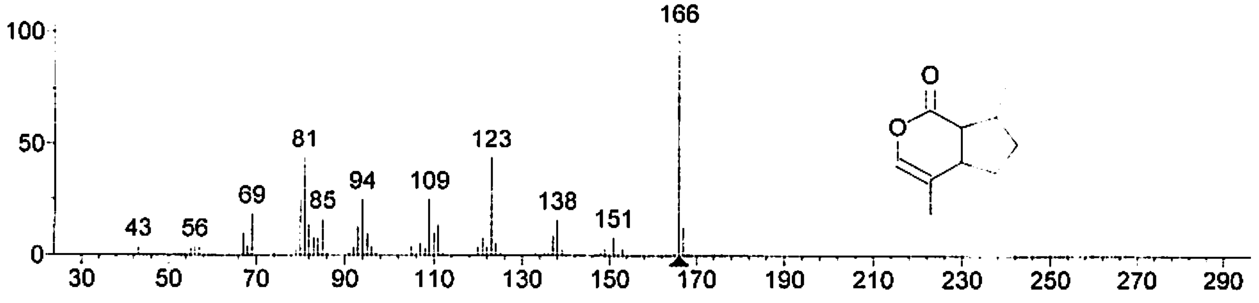


(mainlib) Nepetalactone

File :D:\DATA\Aldrich\JA-09\JA021009-2.D
Operator : Aldrich
Acquired : 10 Feb 2009 15:36 using AcqMethod JA-WAX08.M
Instrument : Instrument #1
Sample Name: 12 male 5-18-d C.oculata abd.ster./5ul CH2Cl2
Misc Info : fed 1ug/ul Z,E-nepetalactone 7 days; DB-WAX
Vial Number: 1

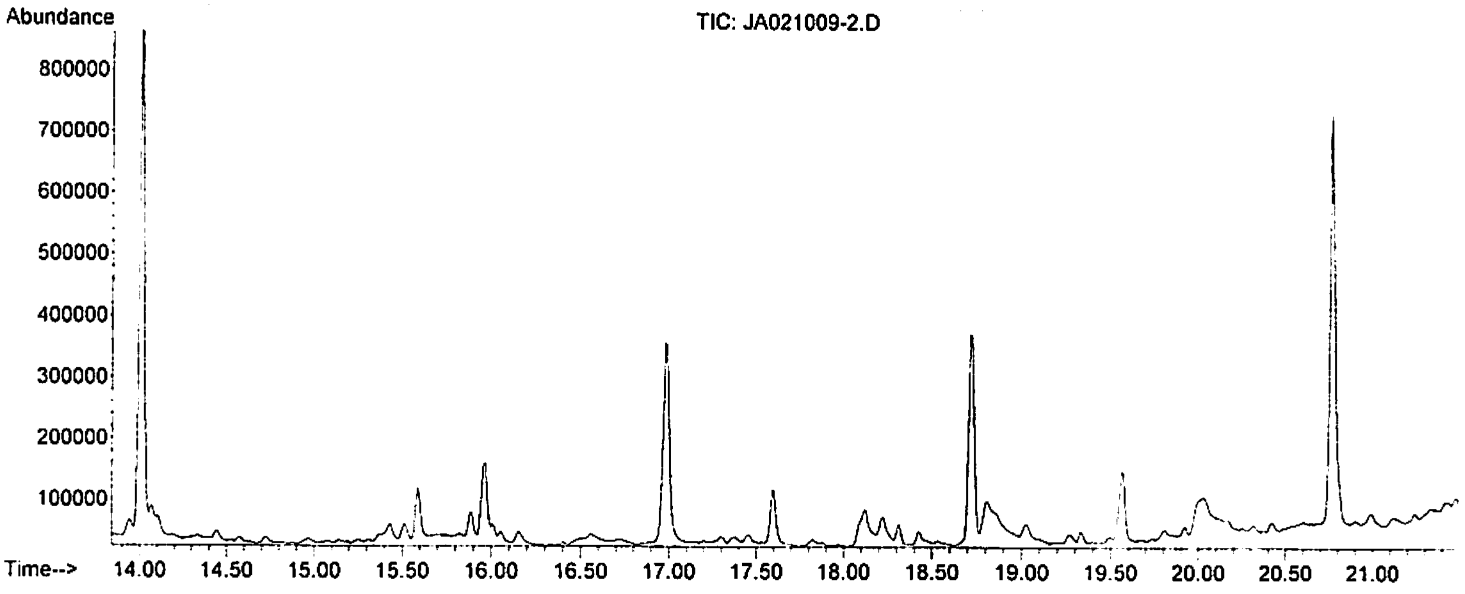


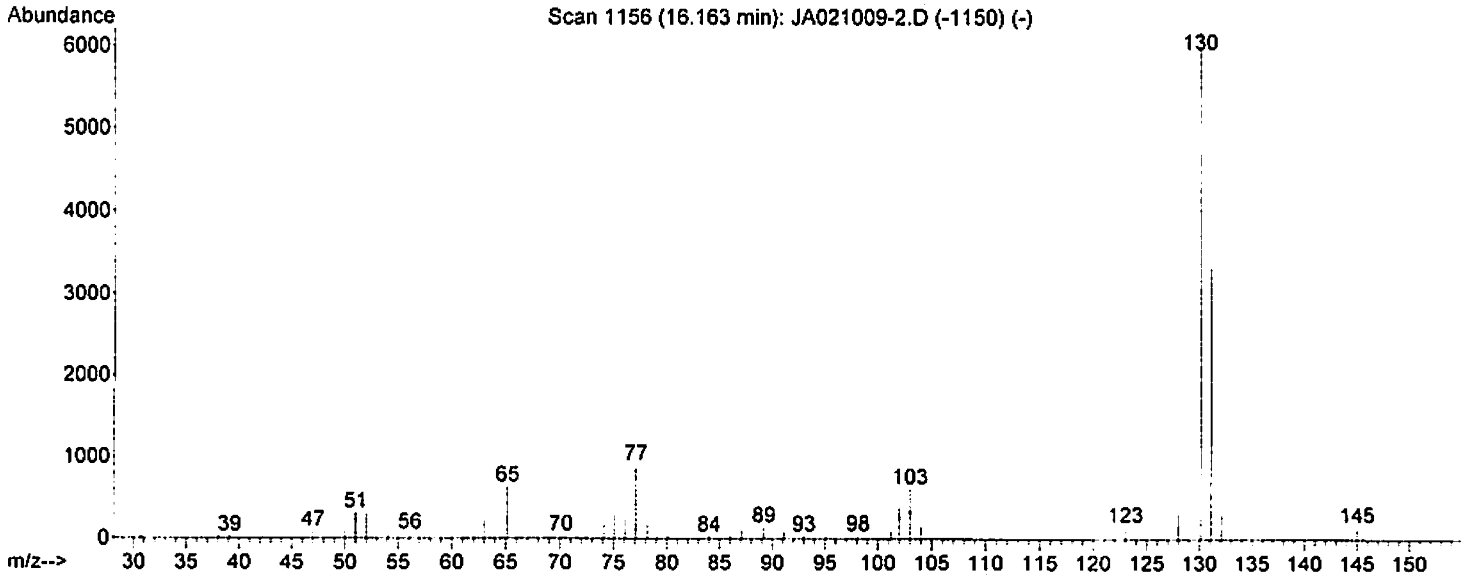

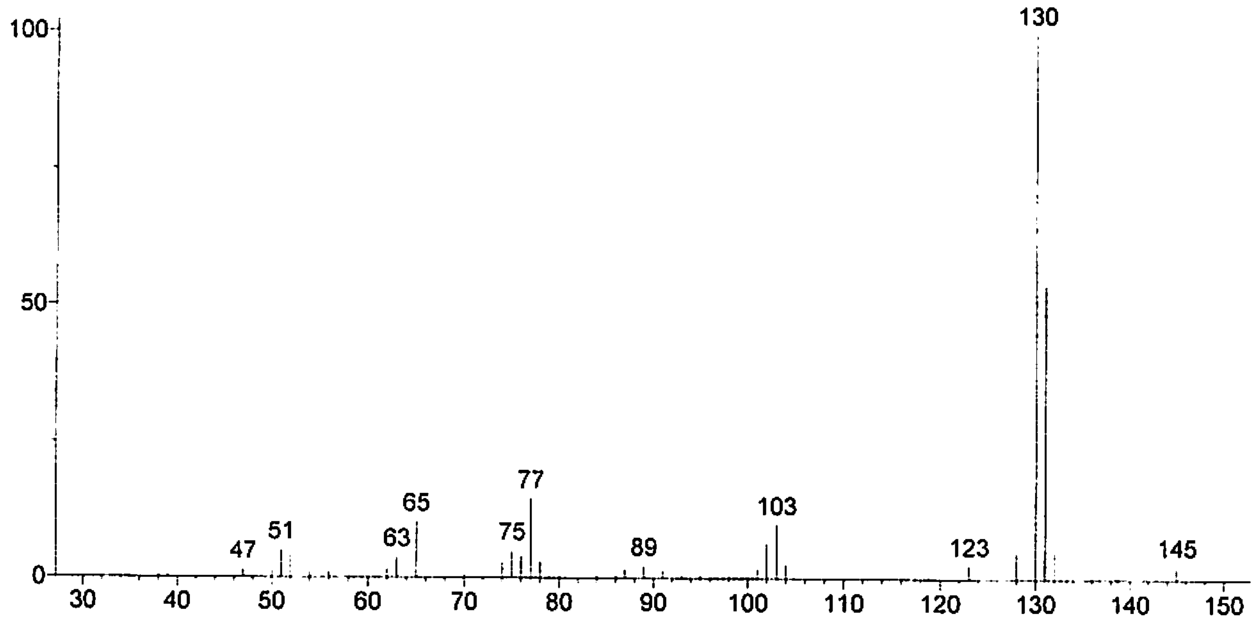


(Text File) Scan 1156 (16.163 min): JA021009-2.D (-1150)

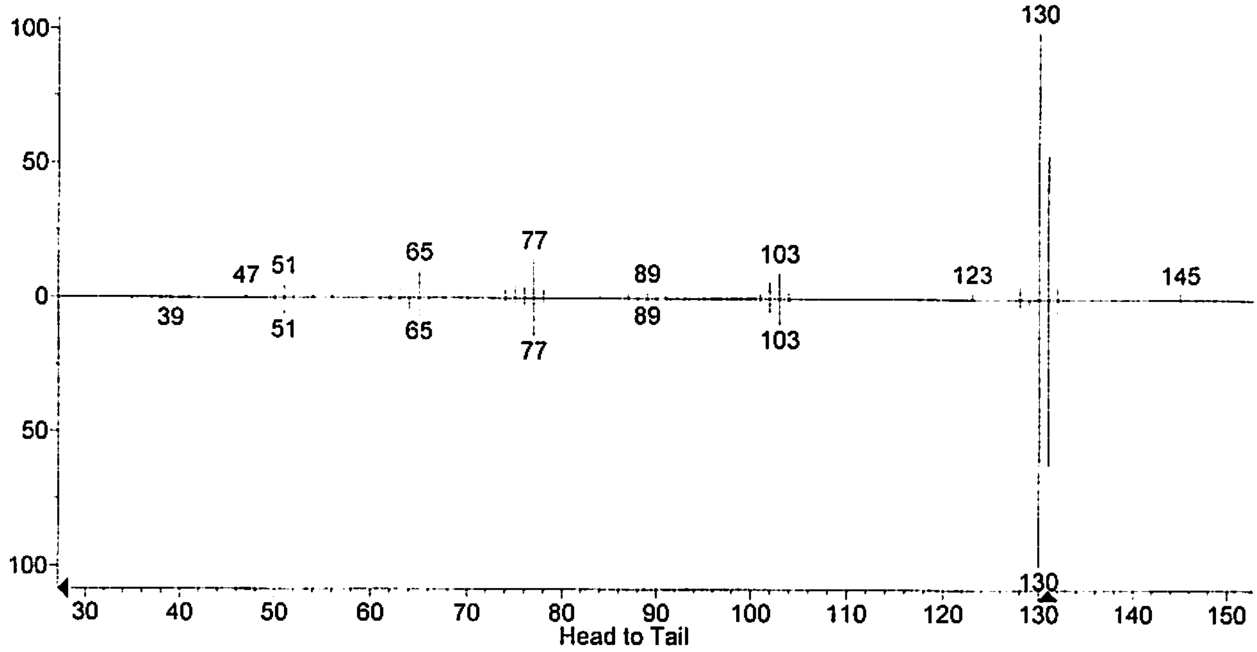


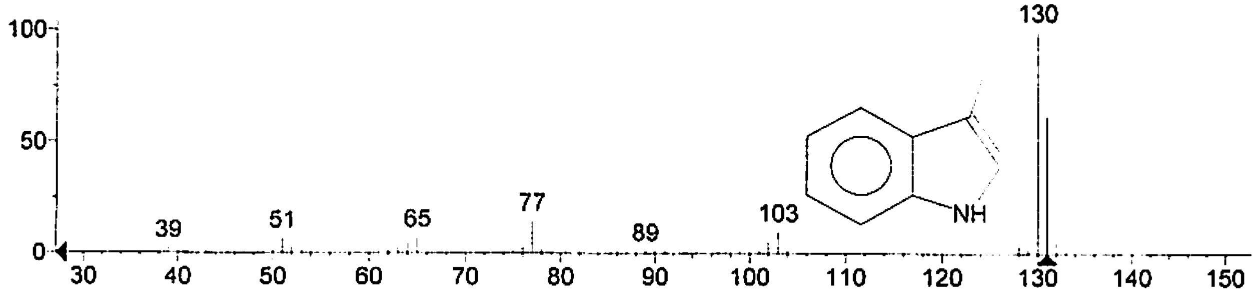


(mainlib) 1H-Indole, 3-methyl-

[illegible]le : D:\DATA\Aldrich\JA-09\JA021009-2.D
[illegible]rator : Aldrich
[illegible]quired : 10 Feb 2009 15:36 using AcqMethod JA-WAX08.M
[illegible]strument : Instrument #1
[illegible]mple Name: 12 male 5-18-d C.oculata abd.ster./5ul CH2Cl2
[illegible]sc Info : fed 1ug/ul Z,E-nepetalactone 7 days; DB-WAX
[illegible]al Number: 1

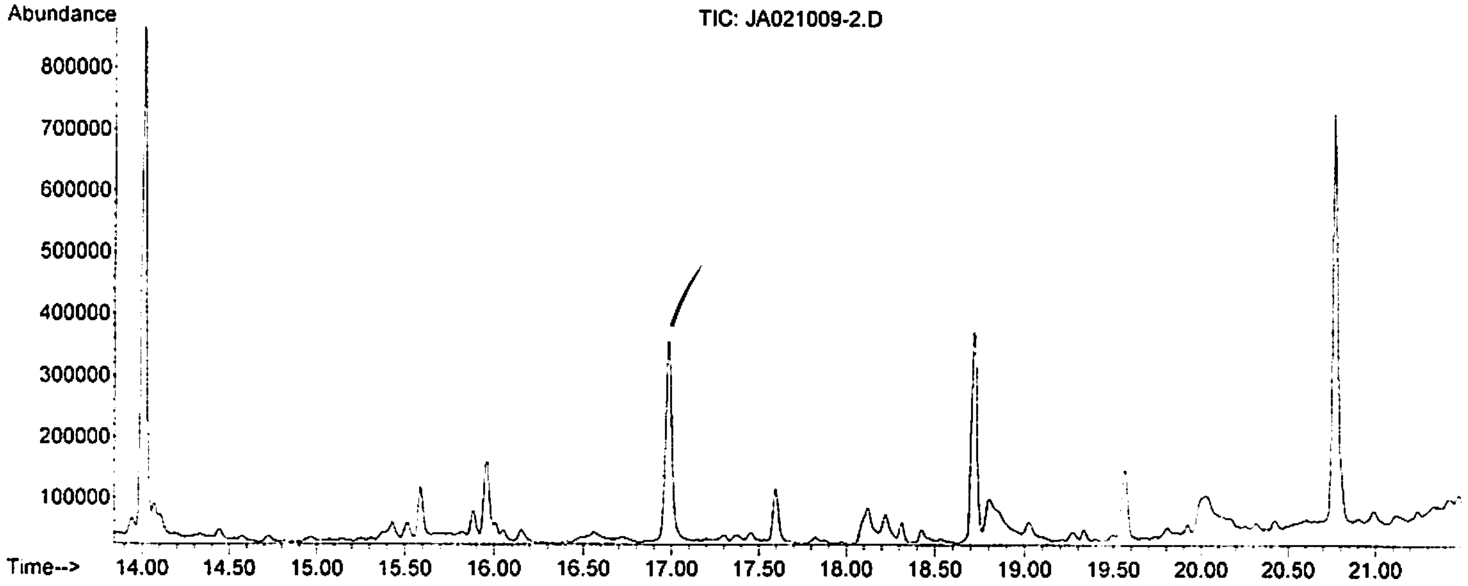


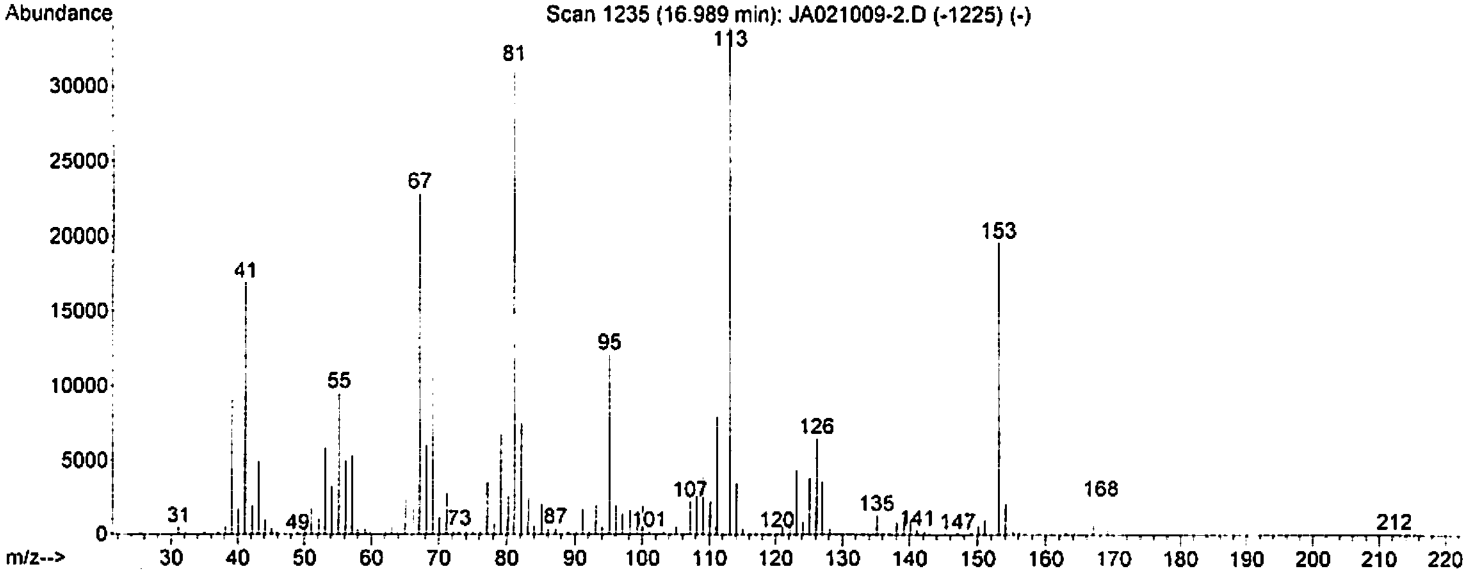

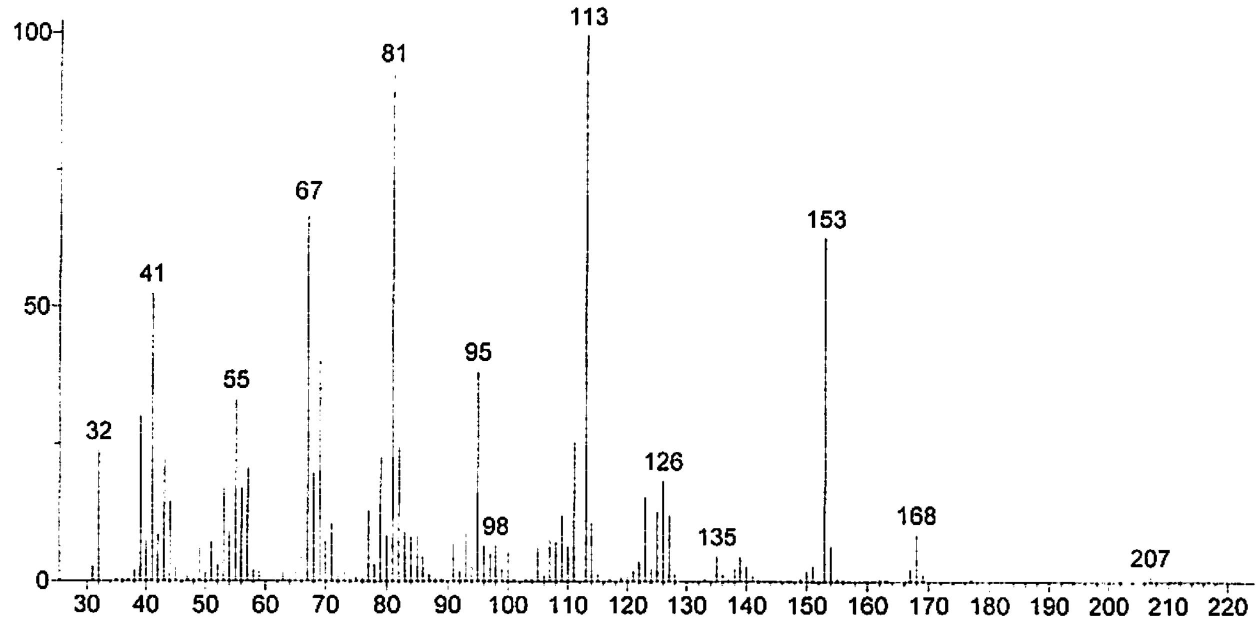


(Text File) Scan 1236 (16.999 min): JA021009-2.D

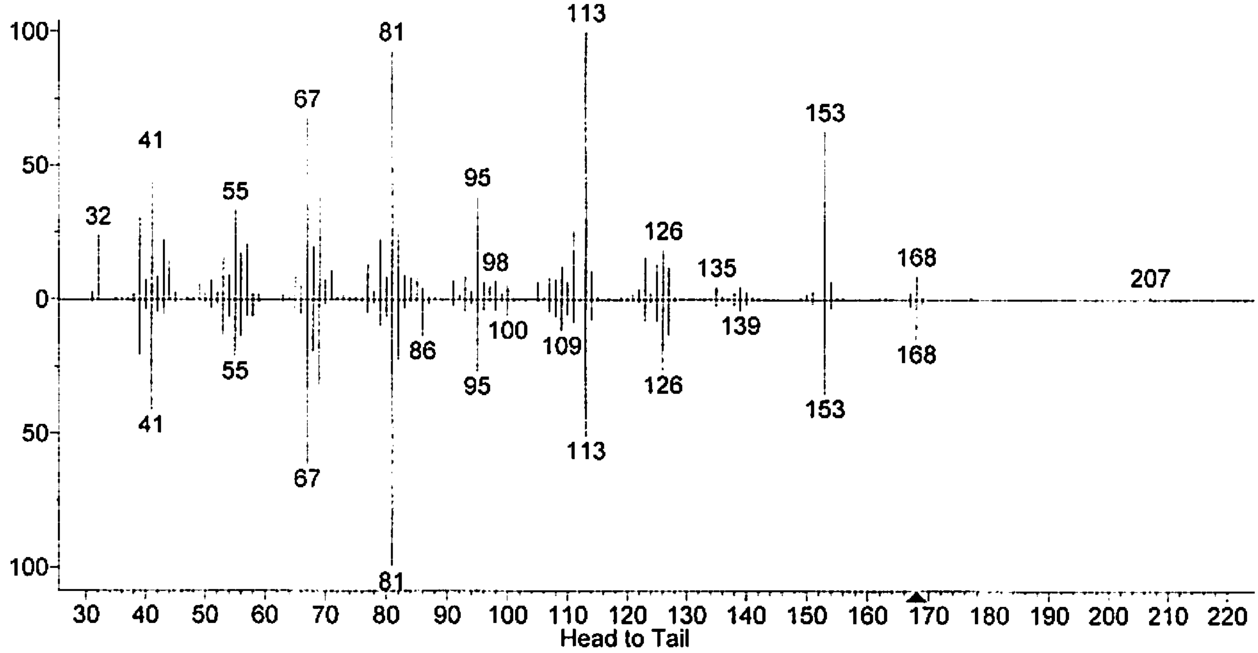


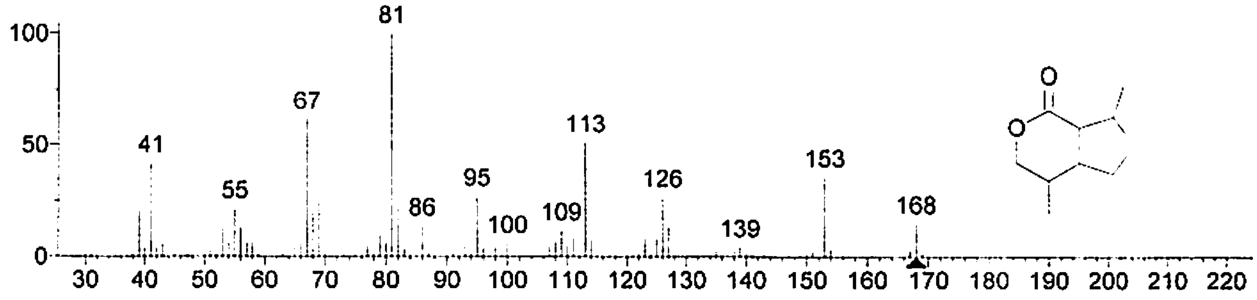


(mainlib) Cyclopenta[c]pyran-1(3H)-one, hexahydro-4,7-dimethyl-, (4.alpha.,4a.alpha.,7.alpha.,7a.alpha.)-

File :D:\DATA\Aldrich\JA-09\JA021009-2.D
Operator : Aldrich
Acquired : 10 Feb 2009 15:36 using AcqMethod JA-WAX08.M
Instrument : Instrument #1
Sample Name: 12 male 5-18-d C.oculata abd.ster./5ul CH2Cl2
Misc Info : fed 1ug/ul Z,E-nepetalactone 7 days; DB-WAX
Vial Number: 1

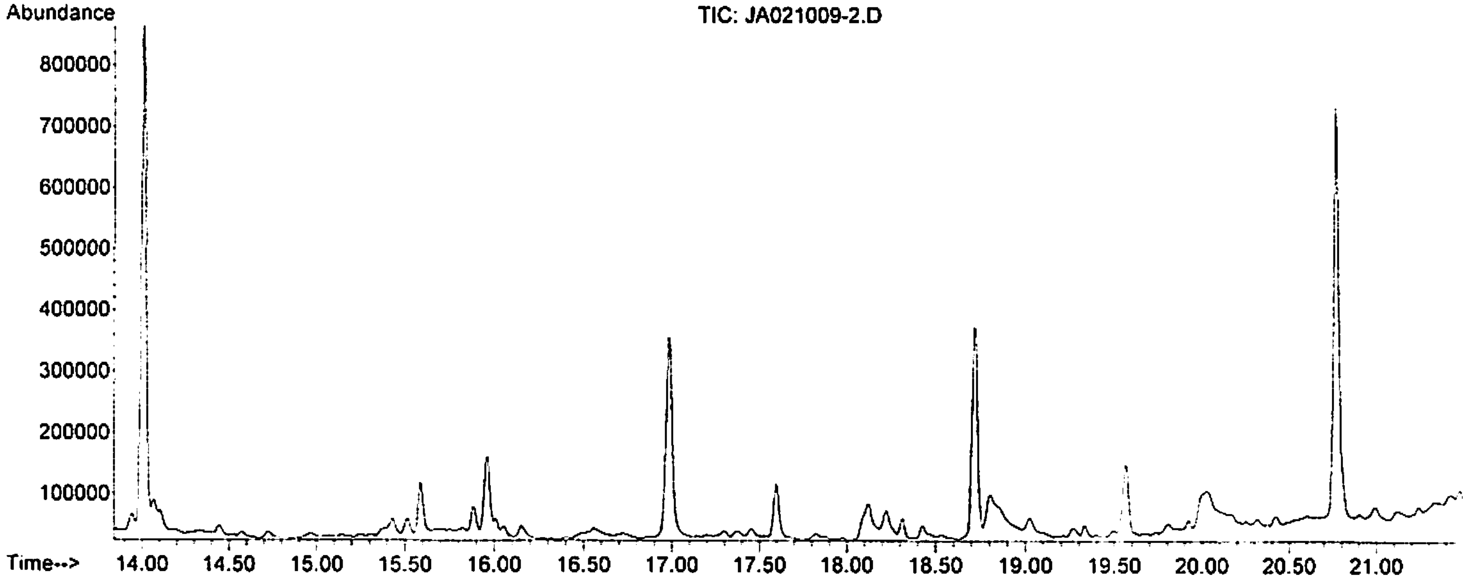


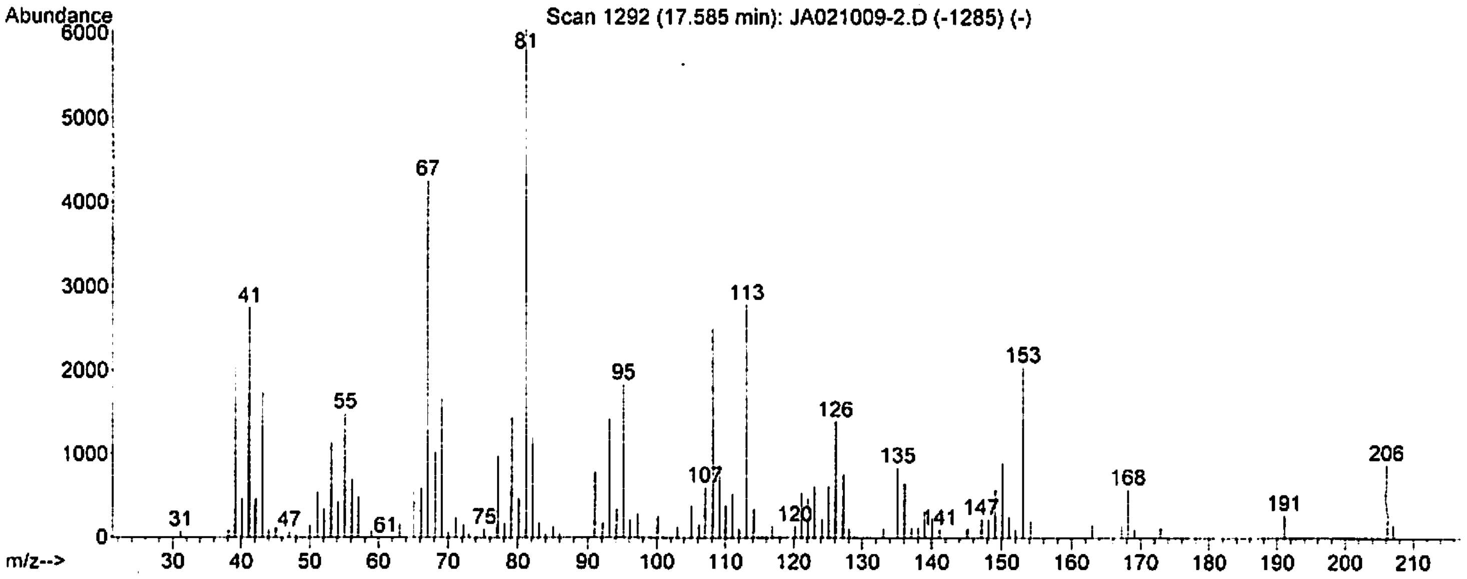

[illegible]le :D:\DATA\Aldrich\JA-09\JA021009-2.D
[illegible]erator : Aldrich
[illegible]quired : 10 Feb 2009 15:36 using AcqMethod JA-WAX08.M
[illegible]strument : Instrument #1
[illegible]mple Name: 12 male 5-18-d C.oculata abd.ster./5ul CH2Cl2
[illegible]sc Info : fed 1ug/ul Z,E-nepetalactone 7 days; DB-WAX
[illegible]al Number: 1

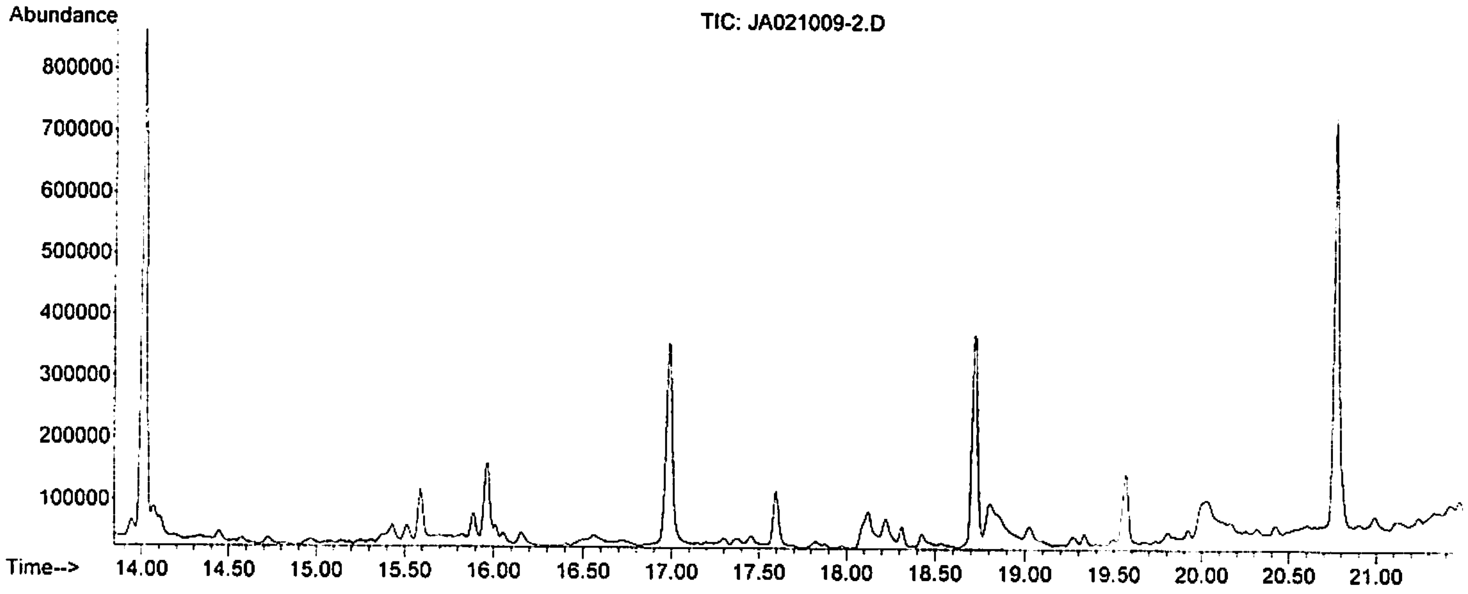


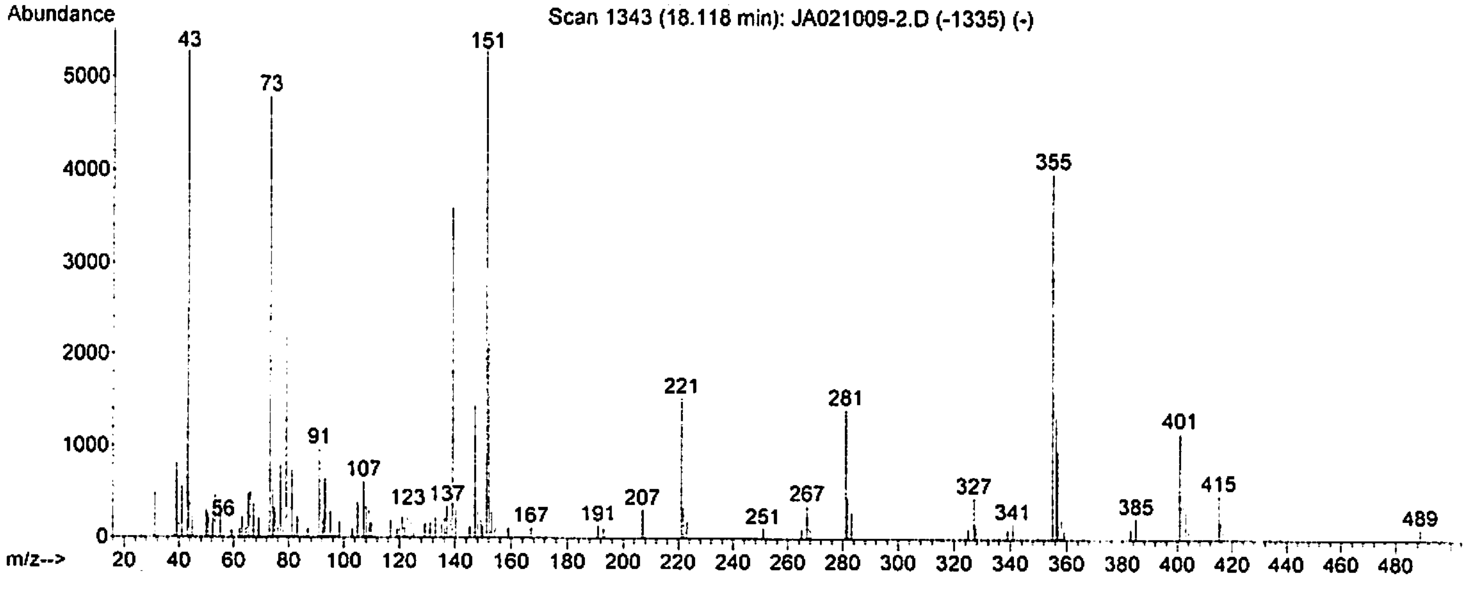

File :D:\DATA\Aldrich\JA-09\JA021009-2.D
Operator : Aldrich
Acquired : 10 Feb 2009 15:36 using AcqMethod JA-WAX08.M
Instrument : Instrument #1
Sample Name: 12 male 5-18-d C.oculata abd.ster./5ul CH2Cl2
Misc Info : fed 1ug/ul Z,E-nepetalactone 7 days; DB-WAX
Vial Number: 1

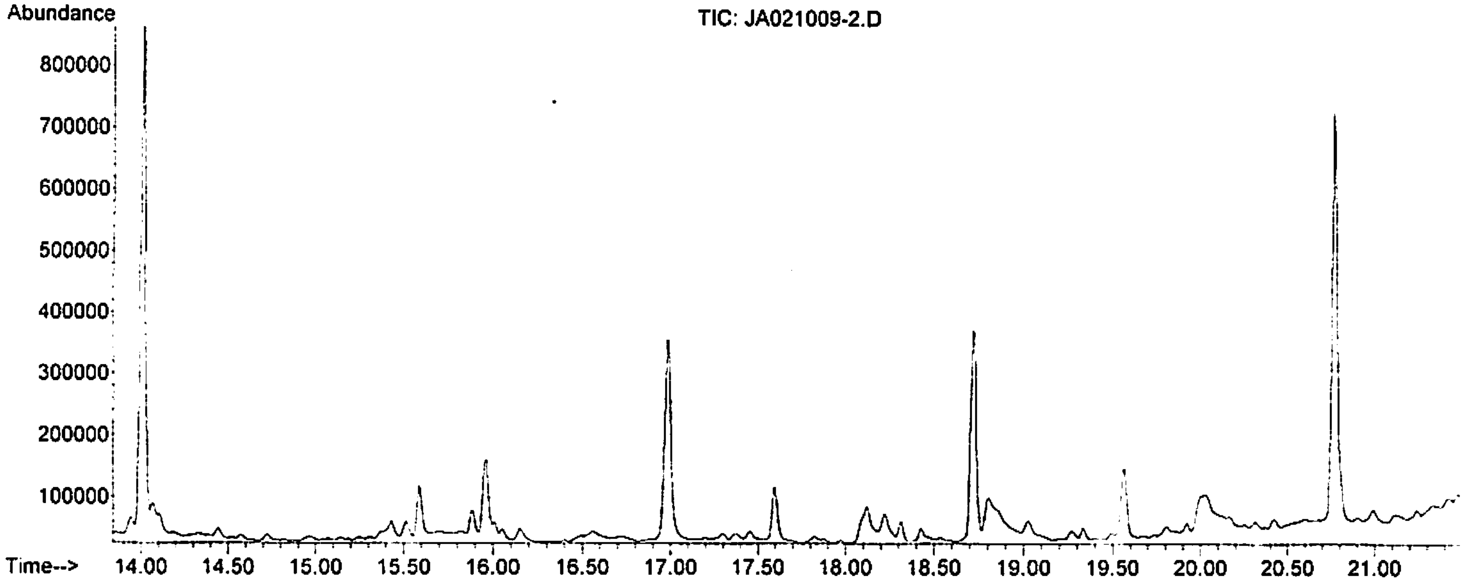


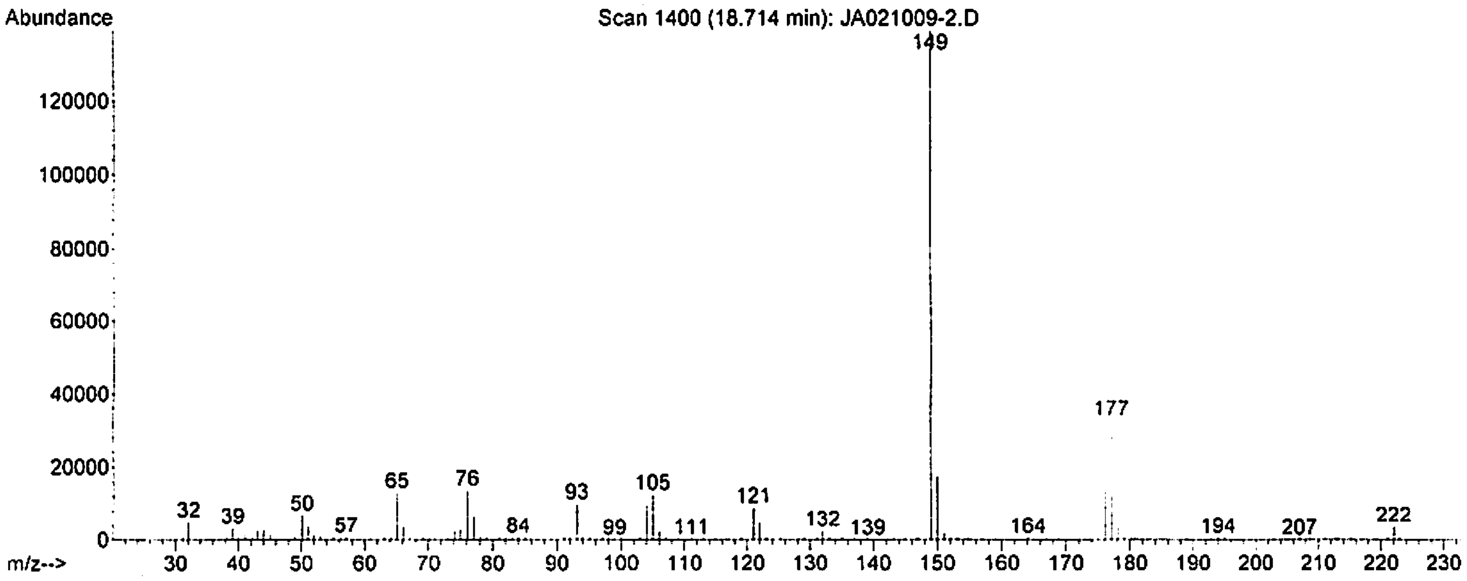

[illegible] :D:\DATA\Aldrich\JA-09\JA021009-2.D
[illegible]rator : Aldrich
A[illegible]quired : 10 Feb 2009 15:36 using AcqMethod JA-WAX08.M
[illegible]nstrument : Instrument #1
[illegible]mple Name: 12 male 5-18-d C.oculata abd.ster./5ul CH2Cl2
[illegible]sc Info : fed 1ug/ul Z,E-nepetalactone 7 days; DB-WAX
[illegible]al Number: 1

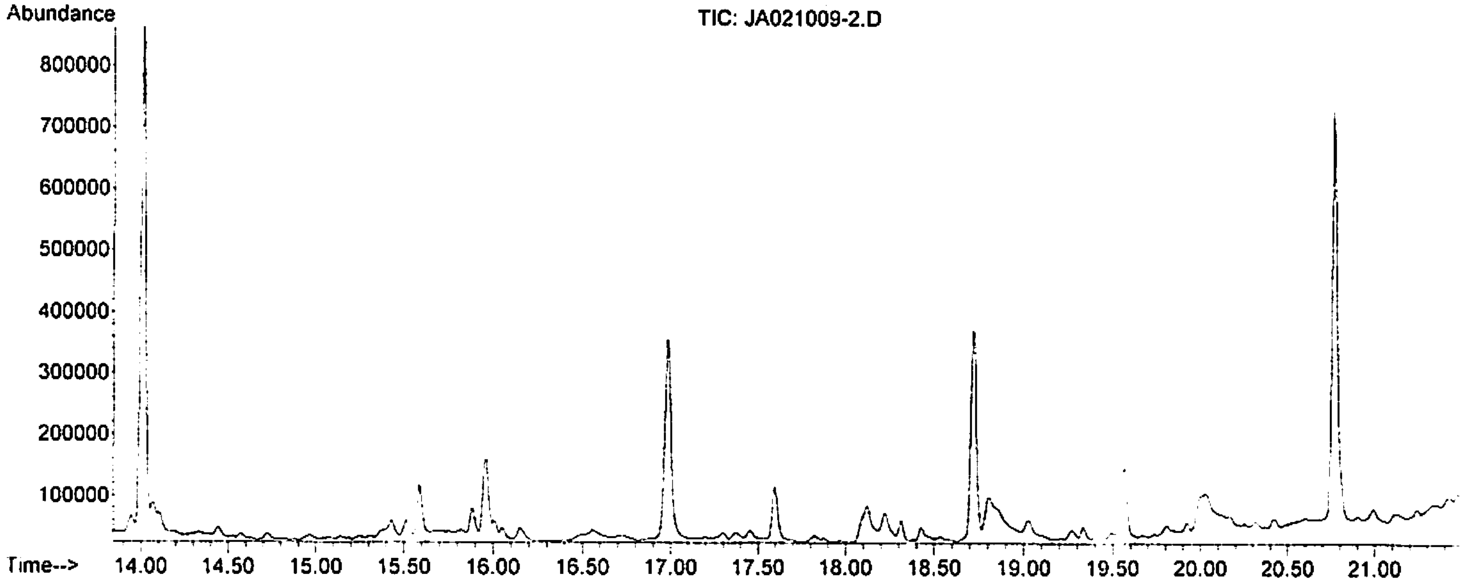


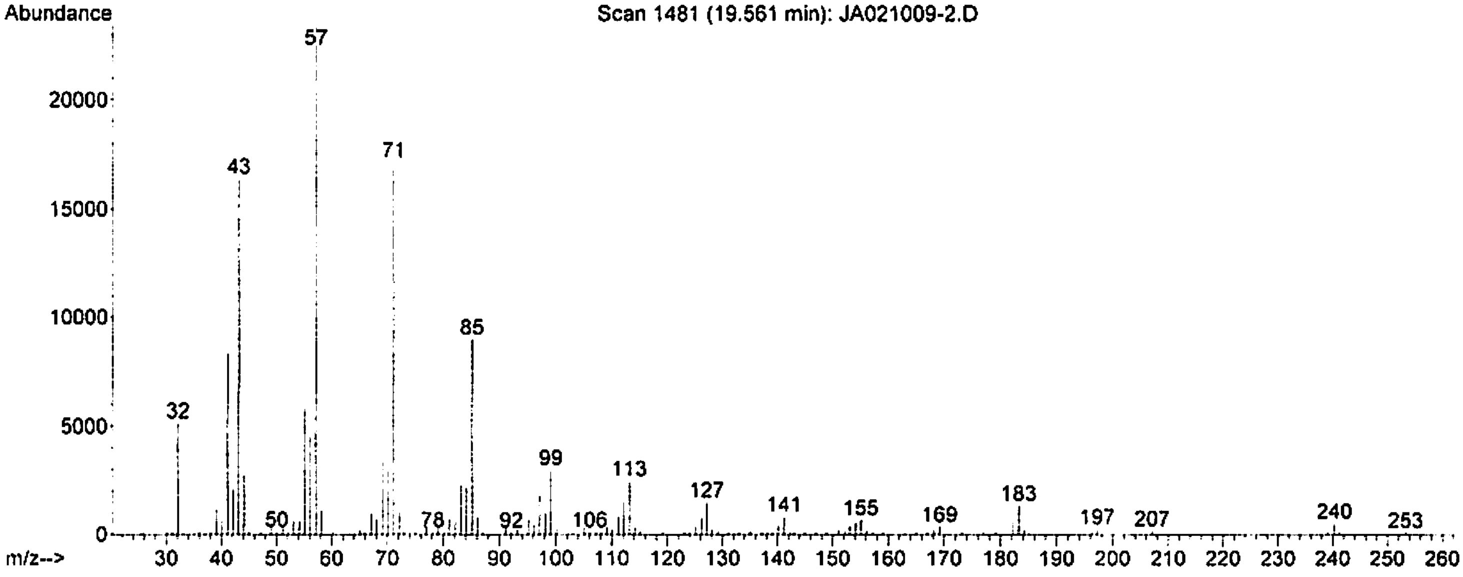

File : D:\DATA\Aldrich\JA-09\JA021009-2.D
Operator : Aldrich
Acquired : 10 Feb 2009 15:36 using AcqMethod JA-WAX08.M
Instrument : Instrument #1
Sample Name: 12 male 5-18-d C.oculata abd.ster./5ul CH2Cl2
Misc Info : fed 1ug/ul Z,E-nepetalactone 7 days; DB-WAX
Vial Number: 1

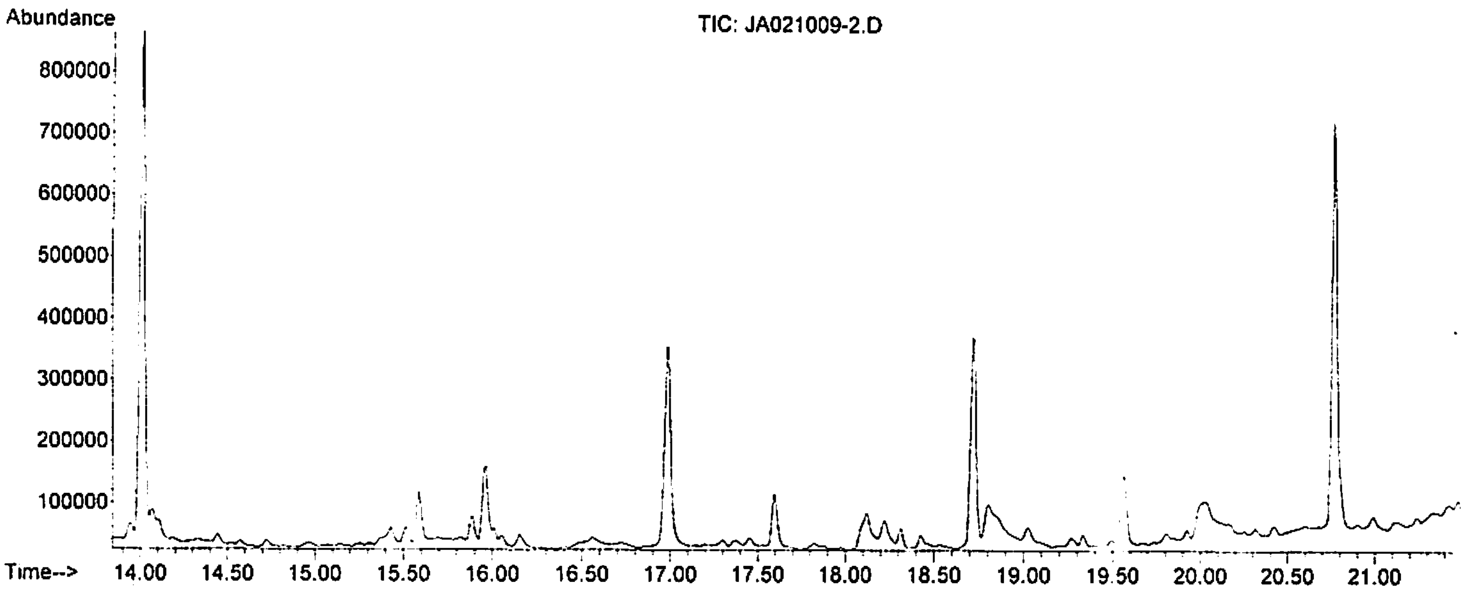


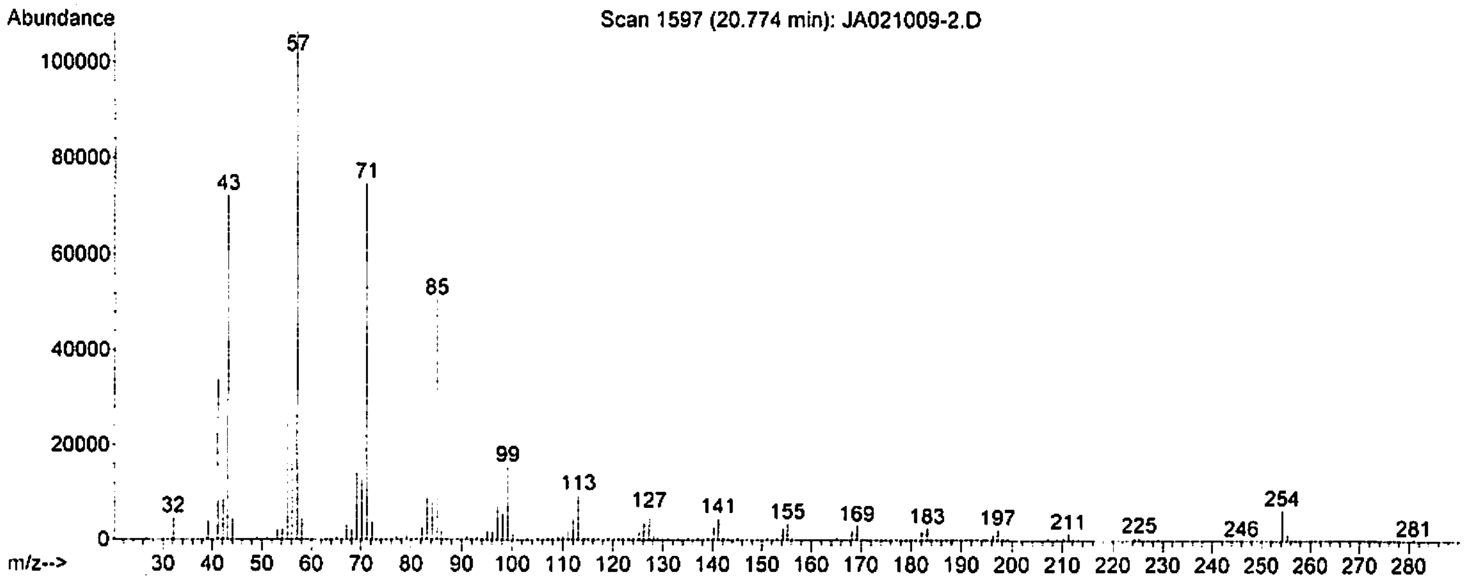

File : D:\DATA\Aldrich\JA-09\JA021009-2.D
Operator : Aldrich
Acquired : 10 Feb 2009 15:36 using AcqMethod JA-WAX08.M
Instrument : Instrument #1
Sample Name: 12 male 5-18-d C.oculata abd.ster./5ul CH2Cl2
Misc Info : fed 1ug/ul Z,E-nepetalactone 7 days; DB-WAX
Vial Number: 1

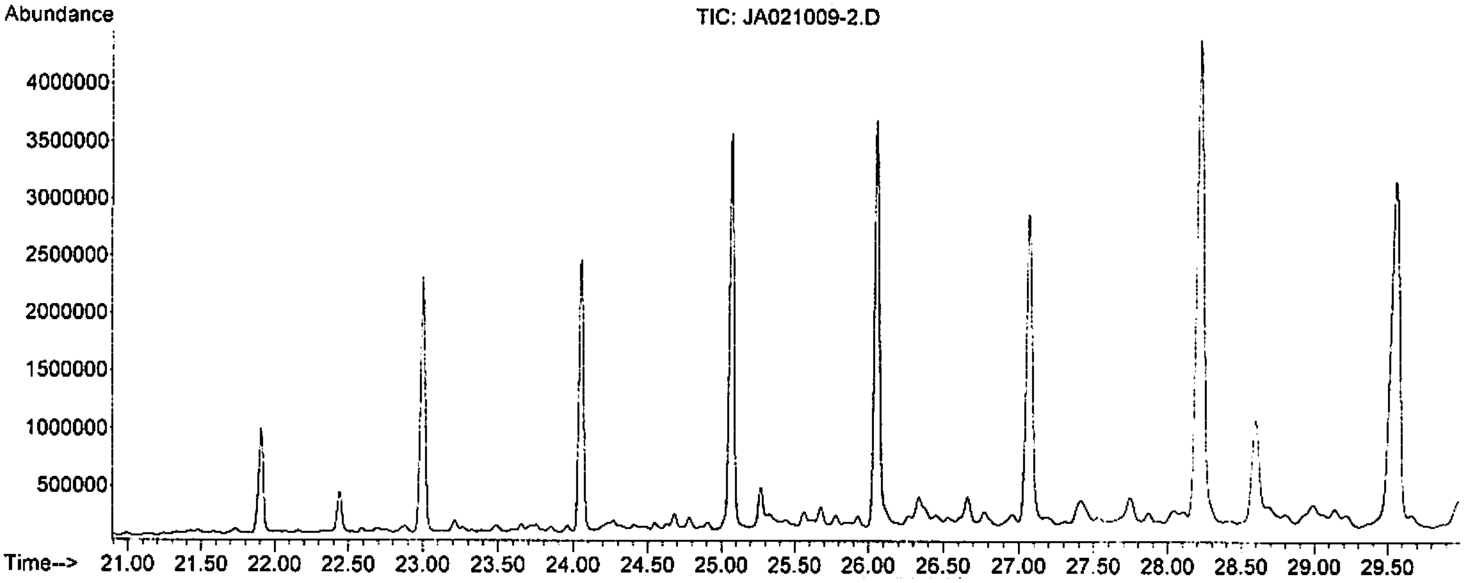


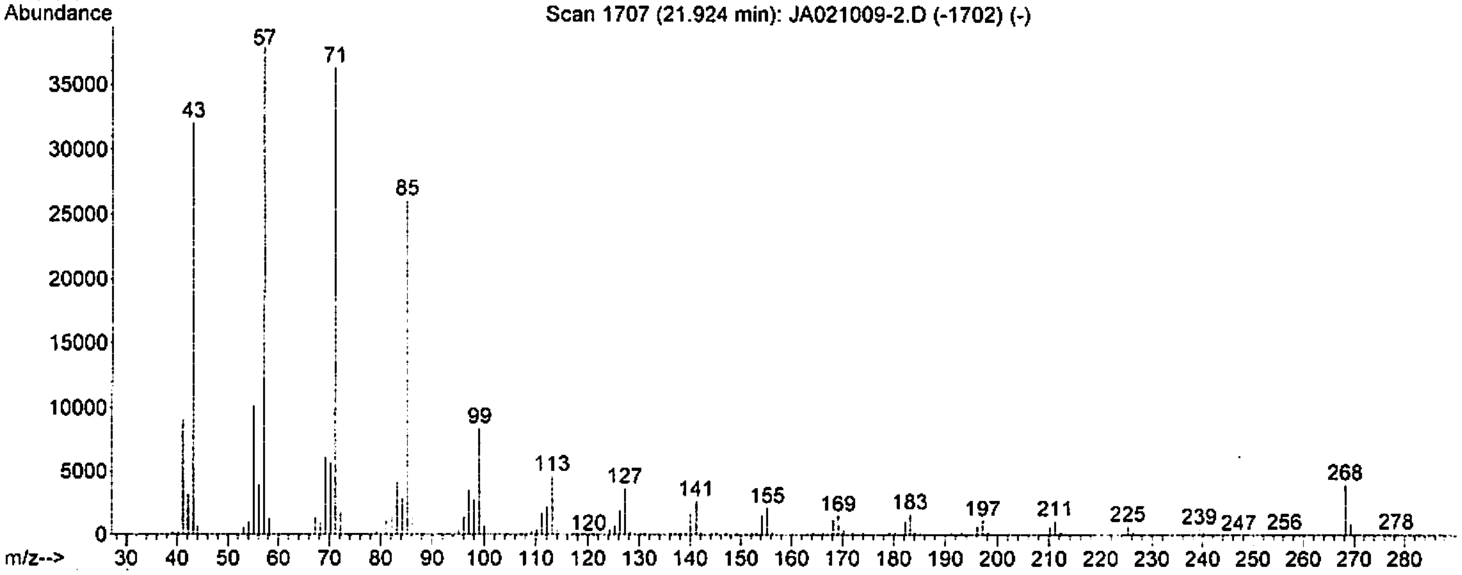

File : D:\DATA\Aldrich\JA-09\JA021009-2.D
Operator : Aldrich
Acquired : 10 Feb 2009 15:36 using AcqMethod JA-WAX08.M
Instrument : Instrument #1
Sample Name: 12 male 5-18-d C.oculata abd.ster./5ul CH2Cl2
Misc Info : fed 1ug/ul Z,E-nepetalactone 7 days; DB-WAX
Vial Number: 1

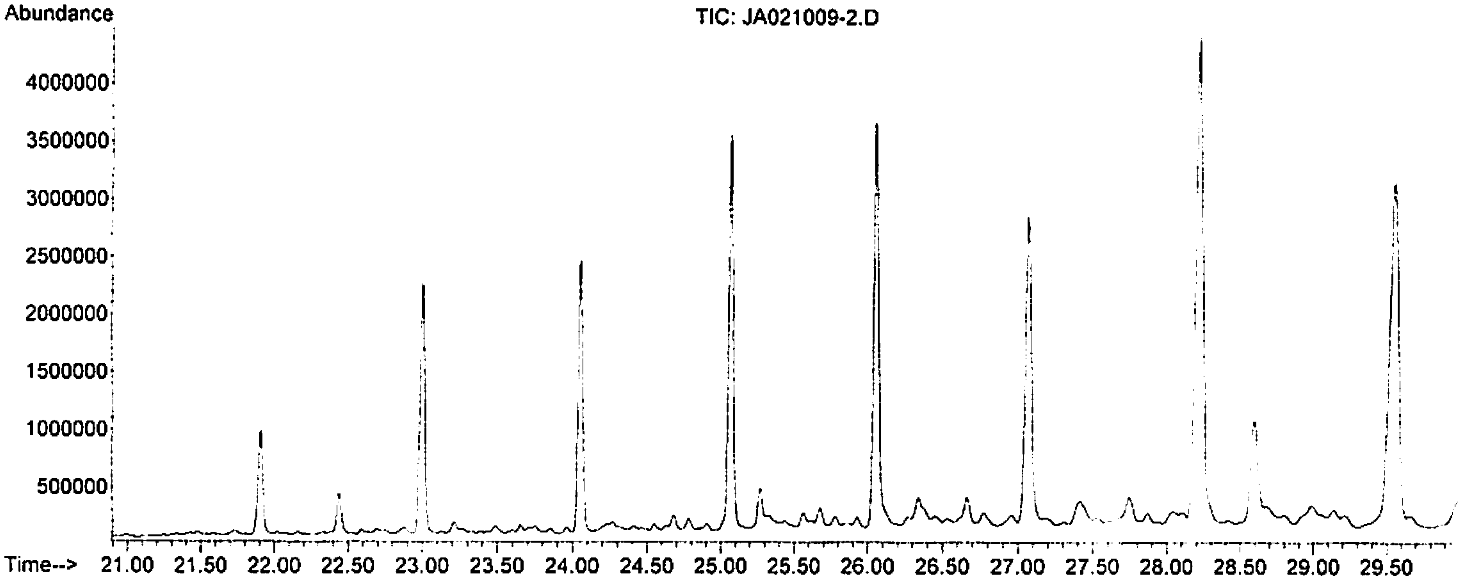


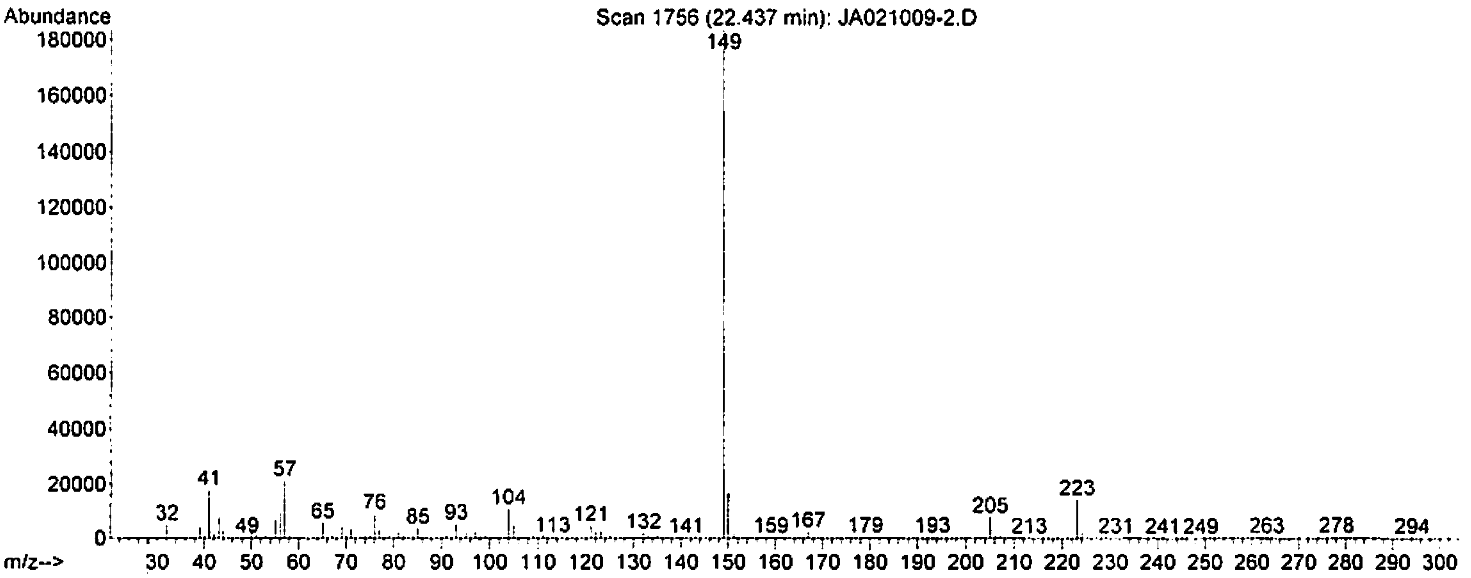

```
File        :D:\DATA\Aldrich\JA-09\JA021009-2.D
Operator    : Aldrich
Acquired    : 10 Feb 2009  15:36     using AcqMethod JA-WAX08.M
Instrument  :     Instrument #1
Sample Name: 12 male 5-18-d C.oculata abd.ster./5ul CH2Cl2
Misc Info   : fed 1ug/ul Z,E-nepetalactone 7 days; DB-WAX
Vial Number: 1
```

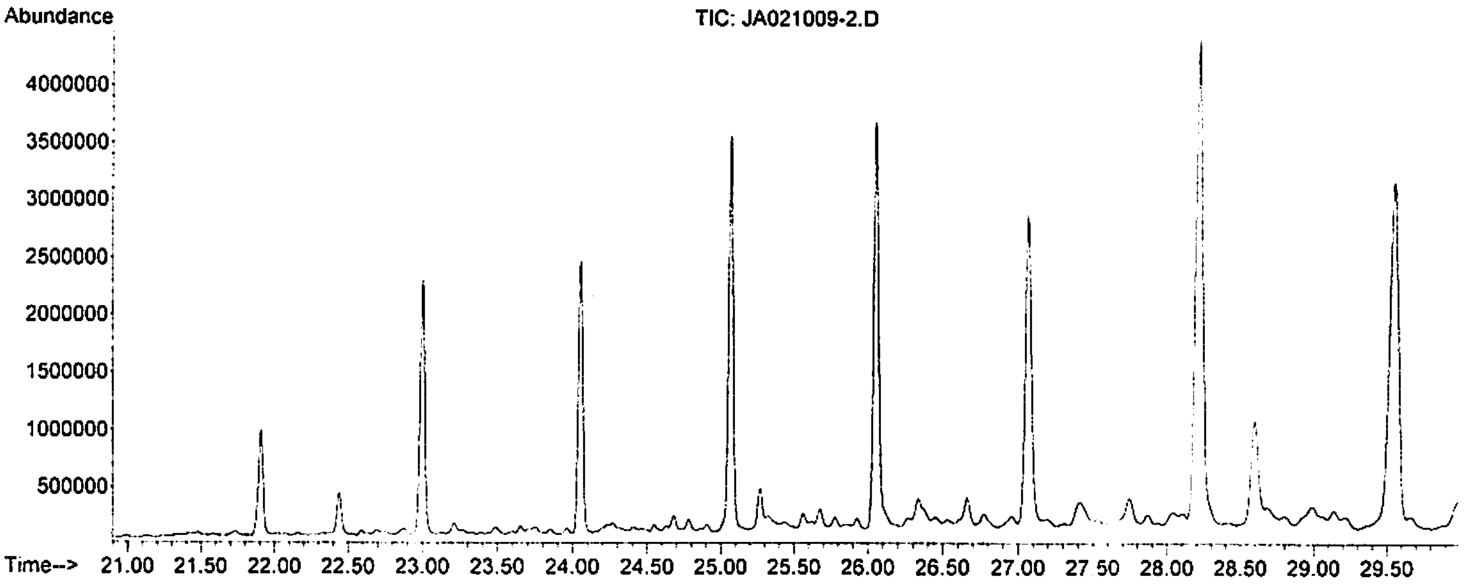


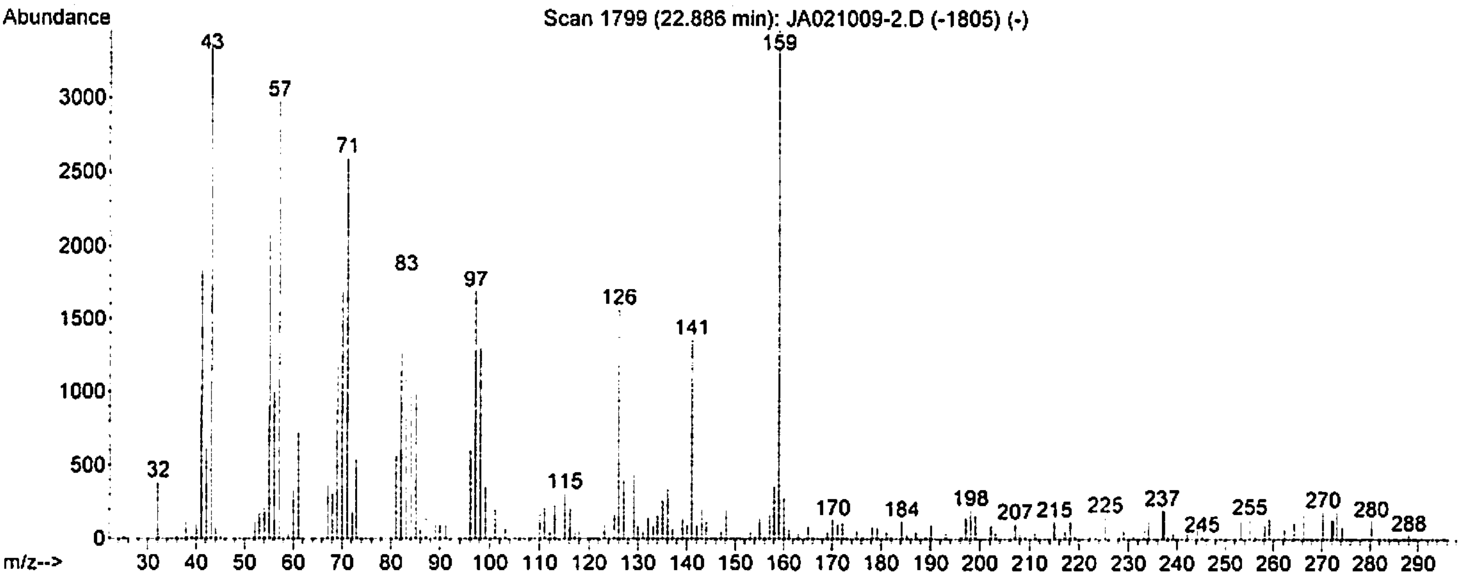

Supplement: Data S7 [file peerj-04-1564-s012.pdf]
