# Supplementary material for: Pharmacophagy in green lacewings (Neuroptera: Chrysopidae: Chrysopa spp.)?
Source: PeerJ. 2016 Jan 18;4:e1564. doi: 10.7717/peerj.1564 (PMC4727961; doi:10.7717/peerj.1564)
Supplement: Data S8 [file peerj-04-1564-s013.pdf]

Male #4 Chrysopa oculata abdominal cuticle + 1ul of Z-3
-octen-1-ol (50ng/ul)(/50ul conc. to 3ul CH2Cl2), DB-5.
Note: Caught 5/22 sweep netting; actually, all samples
run today I injected the entire extract of ~3ul.

===

Injection Date : 5/23/2008 12:36:09 PM
Sample Name : M4 C.ocu. abd+IS
Vial : -
Acq. Operator : Aldrich
Inj : 1
Inj Volume : Manually
Method : C:\HPCHEM\1\METHODS\DBLESS1.M
Last changed : 2/29/2008 10:02:00 AM by Aldrich
2/29/07; editing new method for desired output

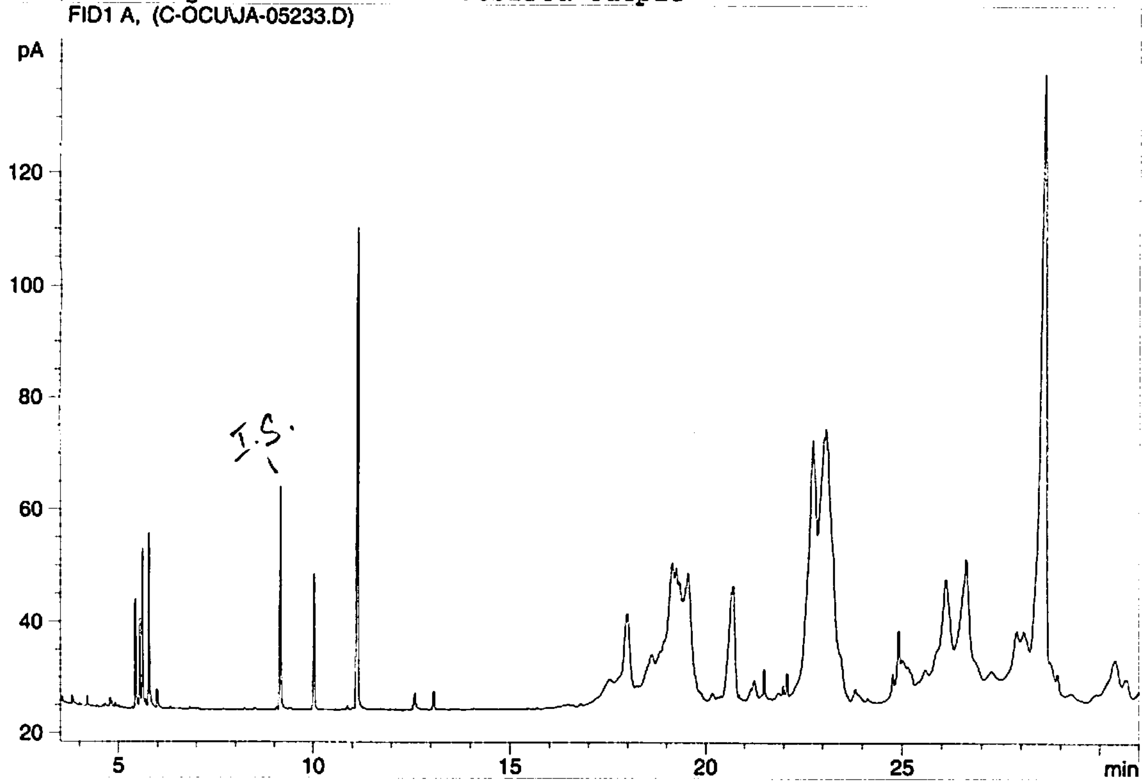

===

Area Percent Report

===

Sorted By : Signal
Multiplier : 1.0000
Dilution : 1.0000

Signal 1: FID1 A,

Peak #	RetTime [min]	Type	Width [min]	Area [pA*s]	Height [pA]	Area %
1	4.190	PB	0.0209	2.43881	1.79256	0.06000
2	4.773	PB	0.0316	3.33710	1.58547	0.08210
3	5.426	PB	0.0248	31.64631	19.60218	0.77856
4	5.534	BV	0.0244	24.31507	15.44477	0.59820
5	5.607	VB	0.0241	42.85784	27.56833	1.05438
6	5.772	PB	0.0235	48.48442	30.59177	1.19281
7	5.984	PB	0.0371	7.55877	3.25789	0.18596
8	9.153	BB	0.0345	86.14058	39.41009	2.11922
9	10.020	BB	0.0333	54.28423	24.08843	1.33550
10	11.128	BB	0.0356	207.42331	84.74079	5.10301
11	12.590	BB	0.0424	8.35635	2.83435	0.20558
12	13.068	BB	0.0342	7.36902	3.27691	0.18129
13	17.989	BB	0.1092	108.64110	12.28714	2.67278
14	19.134	BV	0.0919	83.22655	11.17811	2.04753
15	19.241	VP	0.0942	63.74343	8.62394	1.56821

Peak #	RetTime [min]	Type	Width [min]	Area [pA*s]	Height [pA]	Area %
16	19.545	BP	0.0827	50.87923	8.39119	1.25173
17	20.704	BB	0.1306	220.83432	20.35255	5.43294
18	21.497	BB	0.0334	10.86104	4.97960	0.26720
19	21.989	BB	0.0322	3.62505	1.74567	0.08918
20	22.084	BP	0.0337	8.52734	4.02046	0.20979
21	22.737	BV	0.1664	579.81201	43.52345	14.26448
22	23.067	VB	0.2636	1013.38995	46.07997	24.93132
23	24.759	PV	0.0438	7.97411	2.83406	0.19618
24	24.906	VB	0.0630	37.94100	8.35325	0.93342
25	26.092	BP	0.1254	124.45312	12.46958	3.06178
26	26.599	BB	0.1354	170.18707	16.35074	4.18693
27	28.581	BP	0.1366	1056.41882	105.72103	25.98992

Totals : 4064.72596 561.10429

Results obtained with enhanced integrator!

===

*** End of Report ***

inj. 1.0 ul Z-3-octen-1-ol (Bedoukian) (1ul/2.0 ml CH2C
12), DB-5. Note: Stock soln. for internal standard in C
hrysopa abdominal cuticle extracts.

===

Injection Date : 5/19/2008 1:22:46 PM
Sample Name : Z-3-octen-1-ol Vial : -
Acq. Operator : Aldrich Inj : 1
Inj Volume : Manually
Method : C:\HPCHEM\1\METHODS\DBLESS1.M
Last changed : 2/29/2008 10:02:00 AM by Aldrich
2/29/07; editing new method for desired output

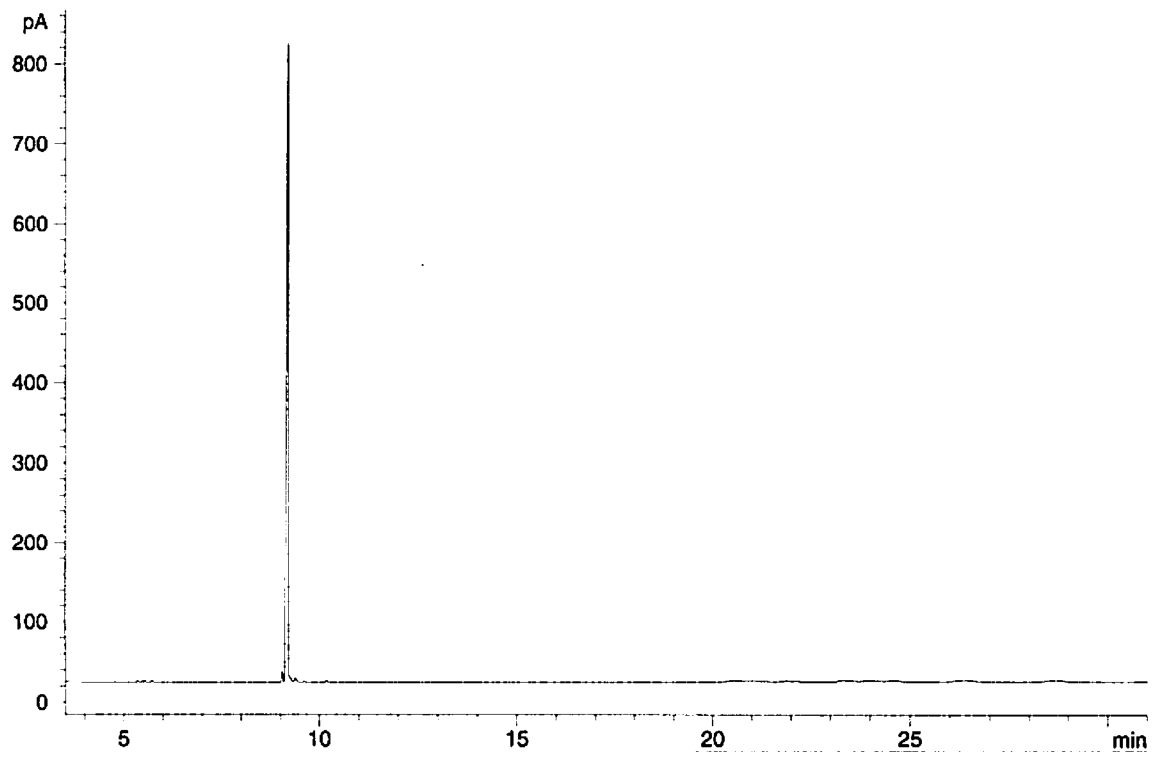

===

Area Percent Report

===

Sorted By : Signal
Multiplier : 1.0000
Dilution : 1.0000

Signal 1: FID1 A,

Peak #	RetTime [min]	Type	Width [min]	Area [pA*s]	Height [pA]	Area %
1	5.347	PB	0.0250	3.09408	1.90125	0.12868
2	5.462	PB	0.0246	2.30108	1.44156	0.09570
3	5.533	BB	0.0246	3.94501	2.47249	0.16407
4	5.707	PB	0.0250	4.05019	2.48694	0.16844
5	9.045	PV	0.0345	29.46025	13.46882	1.22520
6	9.192	VB	0.0424	2342.83228	794.80481	97.43449
7	9.369	PV	0.0236	6.92703	4.60414	0.28808
8	9.402	VB	0.0269	7.09688	3.95494	0.29515
9	10.175	PB	0.0275	4.81379	2.73970	0.20020

Totals : 2404.52058 827.87465

Results obtained with enhanced integrator!

===

*** End of Report ***

inj. 1.0 ul Z-3-octen-1-ol (Bedoukian) (50ng/ul CH2Cl2)
, DB-5. Note: Dilute soln. for internal standard in Chr
ysopa abdominal cuticle extracts.

===

Injection Date : 5/19/2008 2:20:58 PM
Sample Name : Z-3-octen-1-ol Vial : -
Acq. Operator : Aldrich Inj : 1
Inj Volume : Manually
Method : C:\HPCHEM\1\METHODS\DBLESS1.M
Last changed : 2/29/2008 10:02:00 AM by Aldrich
2/29/07; editing new method for desired output

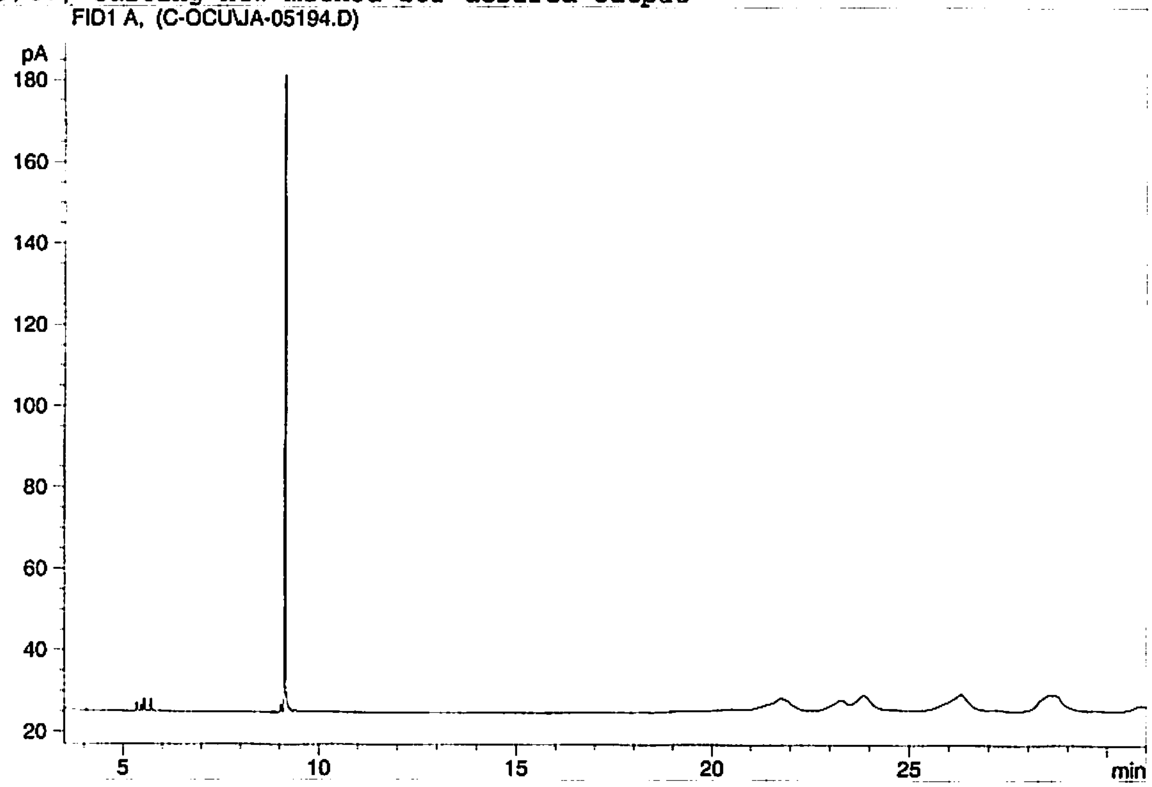

===

Area Percent Report

===

Sorted By : Signal
Multiplier : 1.0000
Dilution : 1.0000

Signal 1: FID1 A,

Peak #	RetTime [min]	Type	Width [min]	Area [pA*s]	Height [pA]	Area %
1	5.355	PB	0.0233	3.67373	2.33758	1.27797
2	5.470	PB	0.0243	2.86948	1.82610	0.99820
3	5.541	BB	0.0244	4.85035	3.08039	1.68727
4	5.715	PB	0.0247	4.99391	3.11310	1.73721
5	9.045	PP	0.0260	2.96520	1.81831	1.03150
6	9.146	BB	0.0271	268.11383	155.64771	93.26785

Totals : 287.46650 167.82319

Results obtained with enhanced integrator!

===

*** End of Report ***

Data File C:\HPCHEM\1\DATA\C-OCU\JA-05196.D Sample Name: M C.ocu abd.

inj. abdominal cuticle extract + Z-3-octene-1-ol as int
ernal standard (1ul of 50 ng/ul CH2Cl2) of 1 male Chrys
opa oculata caught this morning (5/19) sweep netting at
North Farm and sampled by SPME (GC run JA-05195.D) at
3:30pm (/20ul), DB-5.

===

Injection Date : 5/19/2008 3:44:31 PM
Sample Name : M C.ocu abd. Vial : -
Acq. Operator : Aldrich Inj : 1
Inj Volume : Manually
Method : C:\HPCHEM\1\METHODS\DBLESS1.M
Last changed : 2/29/2008 10:02:00 AM by Aldrich
2/29/07; editing new method for desired output

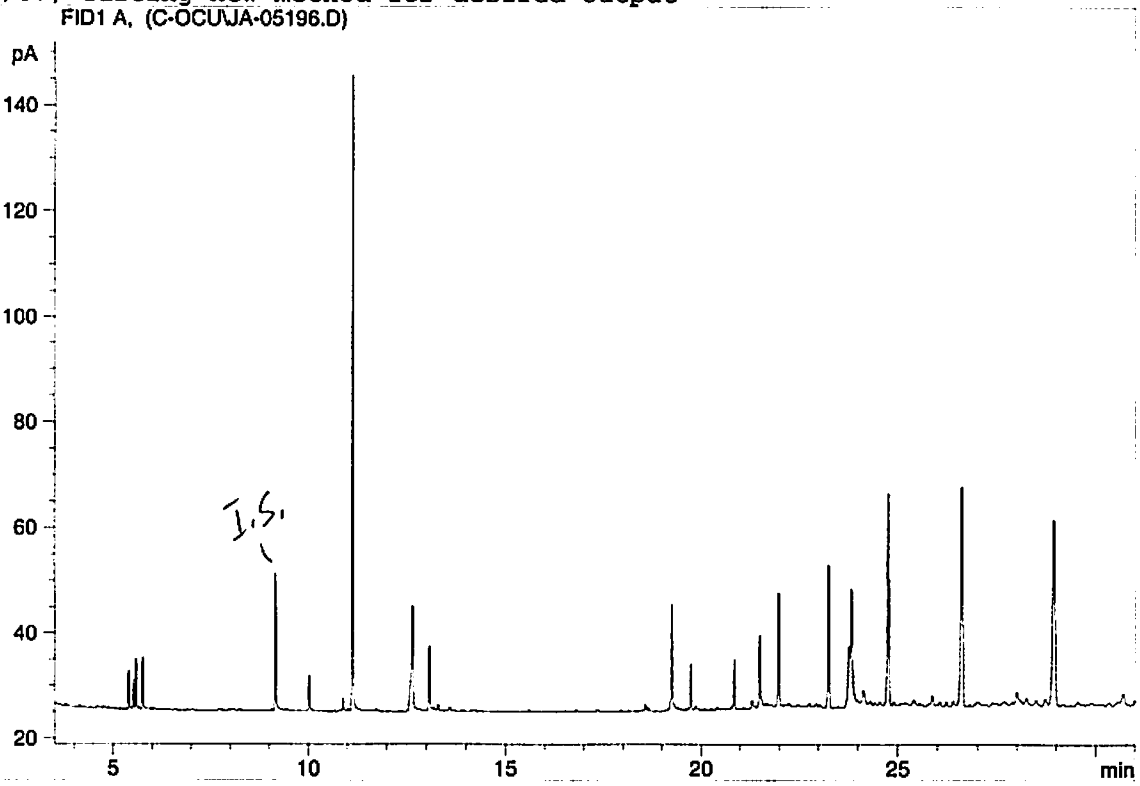

===

Area Percent Report

===

Sorted By : Signal
Multiplier : 1.0000
Dilution : 1.0000

Signal 1: FID1 A,

Peak #	RetTime [min]	Type	Width [min]	Area [pA*s]	Height [pA]	Area %	
1	5.403	PB	0.0231	10.88953	7.43829	0.92855	
2	5.514	PB	0.0237	8.57382	5.66322	0.73109	
3	5.586	BB	0.0232	14.21646	9.63817	1.21224	
4	5.756	PB	0.0234	14.74641	9.90678	1.25743	
I.S – 5	9.158	PB	0.0275	45.38021	25.77915	3.86959	
6	10.026	PB	0.0279	12.18851	6.81218	1.03932	– nonanal
7	10.882	PB	0.0288	4.65137	2.49382	0.39662	
8	11.135	BB	0.0276	211.38060	119.47237	18.02450	– nonanol
9	12.651	BB	0.0485	72.68665	19.94898	6.19802	
10	13.070	BB	0.0312	24.14357	12.14698	2.05873	– [illegible]
11	18.566	PP	0.0268	1.91516	1.18775	0.16331	
12	19.247	BB	0.0343	44.42618	19.67569	3.78824	
13	19.738	BP	0.0288	16.21338	8.69434	1.38252	
14	20.853	BB	0.0302	17.77602	9.36144	1.51577	

Peak #	RetTime [min]	Type	Width [min]	Area [pA*s]	Height [pA]	Area %
15	21.502	PB	0.0330	29.81712	13.35783	2.54252
16	21.987	BP	0.0342	46.51374	21.56734	3.96624
17	23.257	BP	0.0389	67.46822	27.19983	5.75304
18	23.768	BV	0.0463	33.63115	10.81431	2.86774
19	23.831	VB	0.0441	66.69970	21.53813	5.68751
20	24.135	PP	0.0585	9.64375	2.36792	0.82233
21	24.758	BP	0.0454	115.41588	40.27539	9.84155
22	26.600	BP	0.0540	145.66301	41.42872	12.42074
23	28.927	BP	0.0708	158.70012	35.07139	13.53242
Totals :				1172.74055	471.84003	

Results obtained with enhanced integrator!

===

*** End of Report ***

inj. 1ul of male Chrysopa oculata abdominal cuticle + 1 ul of Z-3-octen-1-ol (50ng/ul)(/20ul conc. to 10ul CH2C 12), DB-5. Note: Caught 5/21 sweep netting.

===

Injection Date : 5/22/2008 11:35:37 AM
Sample Name : M C.ocu. abd+IS Vial : -
Acq. Operator : Aldrich Inj : 1
Inj Volume : Manually
Method : C:\HPCHEM\1\METHODS\DBLESS1.M
Last changed : 2/29/2008 10:02:00 AM by Aldrich
2/29/07; editing new method for desired output

FID1 A, (C-OCU\JA-05222.D)

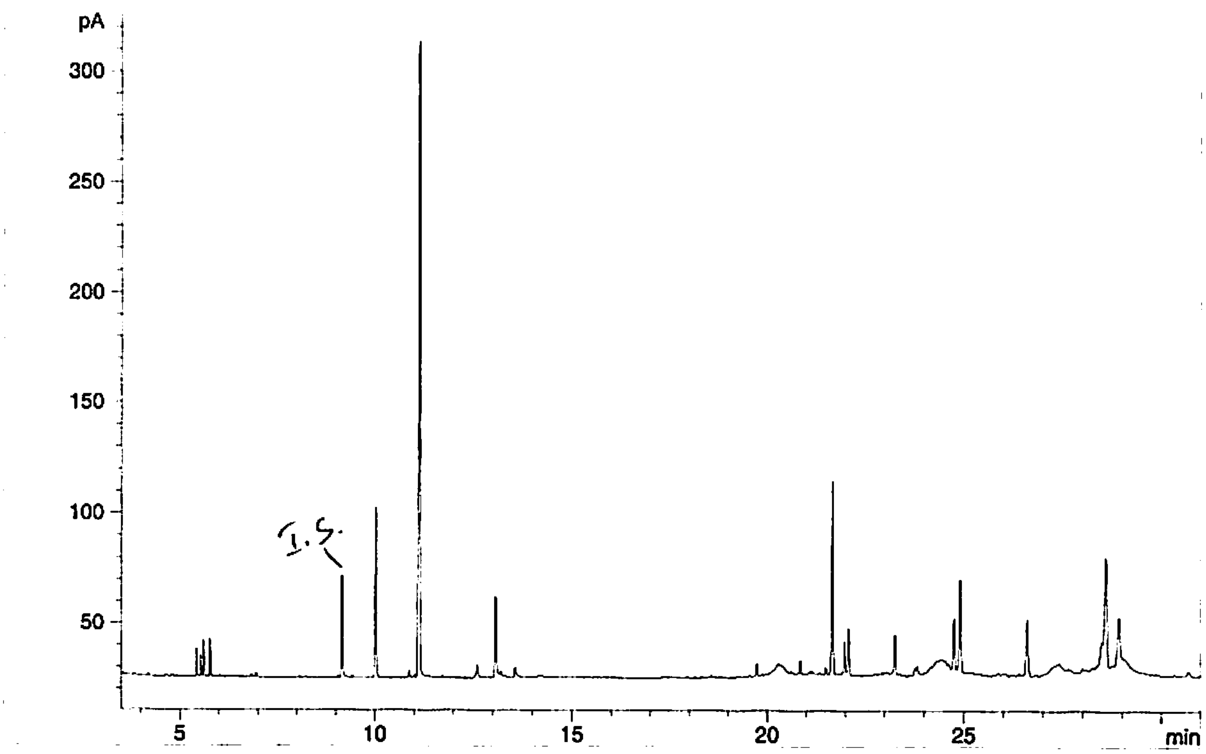

===

Area Percent Report

===

Sorted By : Signal
Multiplier : 1.0000
Dilution : 1.0000

Signal 1: FID1 A,

Peak #	RetTime [min]	Type	Width [min]	Area [pA*s]	Height [pA]	Area %
1	4.192	PB	0.0197	1.34418	1.06841	0.06011
2	5.429	PB	0.0214	18.27618	13.03830	0.81730
3	5.538	PB	0.0229	14.54416	10.06528	0.65041
4	5.609	BB	0.0231	24.20767	16.49281	1.08255
5	5.776	PB	0.0220	24.99474	17.17603	1.11775
6	9.158	BB	0.0321	95.33658	46.13399	4.26340
7	10.025	BB	0.0341	172.41638	77.20683	7.71036
8	10.877	BP	0.0312	6.59841	3.18703	0.29508
9	11.143	BB	0.0394	766.72314	285.32941	34.28742
10	12.603	BB	0.0437	17.52394	5.88805	0.78366
11	13.069	BB	0.0345	81.67187	35.97081	3.65232
12	13.558	PB	0.0408	11.85255	4.35411	0.53004
13	19.737	BP	0.0268	10.09000	5.66960	0.45122
14	20.852	BB	0.0289	11.23600	5.99714	0.50247
15	21.497	PB	0.0322	6.78371	3.27358	0.30336
16	21.679	BB	0.0343	204.56680	87.54313	9.14811

Peak #	RetTime [min]	Type	Width [min]	Area [pA*s]	Height [pA]	Area %
17	21.984	BV	0.0332	32.14594	14.87027	1.43755
18	22.081	VP	0.0333	45.38967	20.93574	2.02980
19	23.255	BP	0.0375	43.23876	17.71983	1.93361
20	23.814	PP	0.0766	22.77348	4.05215	1.01842
21	24.754	PV	0.0414	63.22416	22.76738	2.82735
22	24.904	VP	0.0447	120.82863	41.83409	5.40339
23	26.593	BP	0.0560	88.95678	25.30288	3.97810
24	28.583	BB	0.0758	255.52908	49.06987	11.42711
25	28.916	BB	0.0691	95.91190	20.29109	4.28912

Totals : 2236.16472 835.23780

Results obtained with enhanced integrator!

==

*** End of Report ***

Male #1 Chrysopa oculata abdominal cuticle + 1ul of Z-3
-octen-1-ol (50ng/ul)(/50ul conc. to 10ul CH2Cl2), DB-5
. Note: Caught 5/22 sweep netting.

===

Injection Date : 5/23/2008 9:39:53 AM
Sample Name : M1 C.ocu. abd+IS Vial : -
Acq. Operator : Aldrich Inj : 1
Inj Volume : Manually
Method : C:\HPCHEM\1\METHODS\DBLESS1.M
Last changed : 2/29/2008 10:02:00 AM by Aldrich
2/29/07; editing new method for desired output

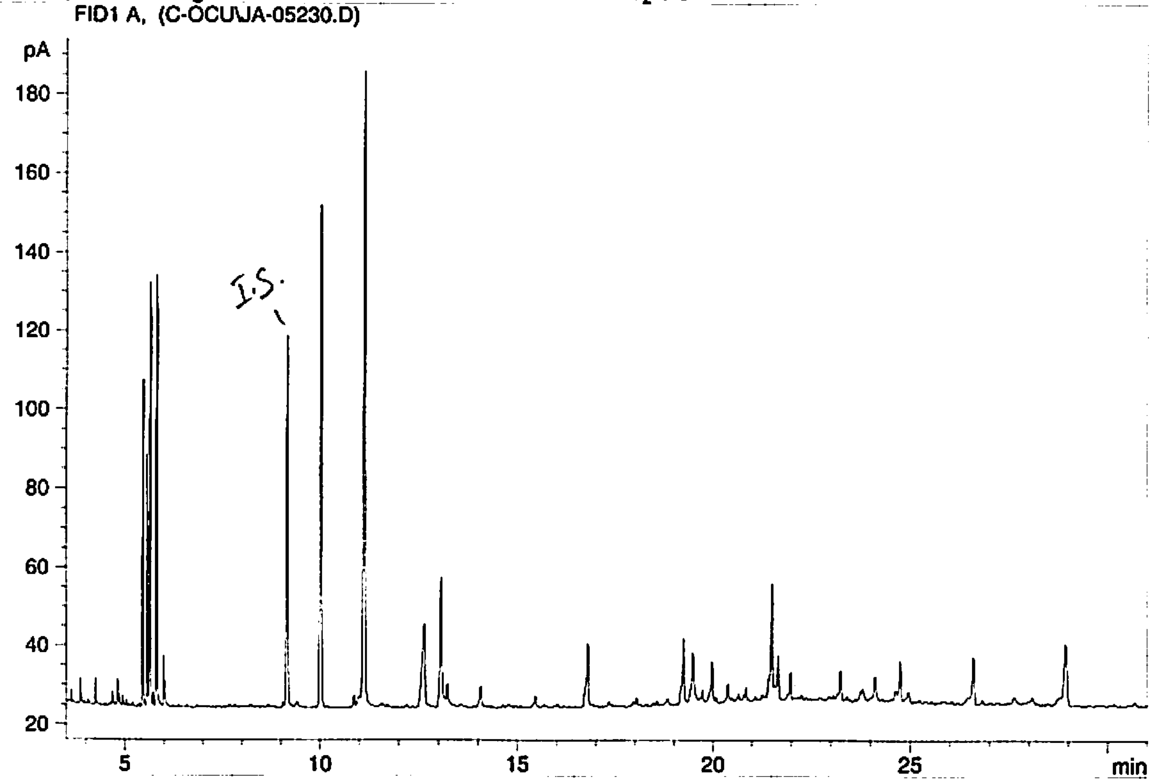

===

Area Percent Report

===

Sorted By : Signal
Multiplier : 1.0000
Dilution : 1.0000

Signal 1: FID1 A,

Peak #	RetTime [min]	Type	Width [min]	Area [pA*s]	Height [pA]	Area %
1	3.623	BP	0.0164	3.49991	3.29205	0.14079
2	3.856	PB	0.0183	7.82840	6.39621	0.31490
3	4.245	BB	0.0203	8.52106	6.48478	0.34277
4	4.682	BP	0.0222	4.87462	3.29772	0.19608
5	4.811	PV	0.0249	11.09469	6.50251	0.44629
6	4.854	VB	0.0216	3.73134	2.61824	0.15010
7	4.942	PB	0.0217	3.70444	2.58796	0.14901
8	5.032	BB	0.0285	2.96809	1.53823	0.11939
9	5.455	BB	0.0210	113.51548	82.60843	4.56624
10	5.562	BV	0.0216	90.95159	63.90150	3.65859
11	5.636	VB	0.0237	161.60429	106.67710	6.50065
12	5.721	BP	0.0240	5.36209	3.46788	0.21569
13	5.800	VB	0.0238	166.44795	109.05338	6.69548
14	5.987	PB	0.0292	25.98585	12.53267	1.04530
15	9.152	BB	0.0368	232.22421	94.13593	9.34138
16	10.018	BB	0.0417	346.98367	127.87128	13.95766

Peak #	RetTime [min]	Type	Width [min]	Area [pA*s]	Height [pA]	Area %
17	10.865	PP	0.0295	3.88721	2.32112	0.15637
18	11.125	BB	0.0452	478.16995	158.68205	19.23472
19	12.642	BB	0.0703	115.72092	21.31719	4.65495
20	13.066	BB	0.0493	110.43622	32.77827	4.44237
21	13.219	BB	0.0431	16.18824	5.37767	0.65118
22	14.064	BB	0.0448	16.09375	5.10729	0.64738
23	15.450	BB	0.0355	6.46340	2.64198	0.25999
24	16.787	BB	0.0503	59.92676	15.76798	2.41060
25	18.032	BB	0.0339	3.66788	1.78805	0.14754
26	19.240	BB	0.0471	55.21139	16.46107	2.22092
27	19.473	BB	0.0611	51.91311	12.09971	2.08824
28	19.731	BP	0.0274	4.77105	2.72222	0.19192
29	19.967	BP	0.0385	24.98255	9.54307	1.00494
30	20.379	BB	0.0361	9.84408	4.09355	0.39598
31	20.654	BP	0.0326	3.22216	1.58979	0.12961
32	20.848	BB	0.0288	5.21946	2.92119	0.20996
33	21.504	PB	0.0421	82.48651	27.42952	3.31808
34	21.666	BP	0.0332	19.21054	9.25652	0.77276
35	21.980	BP	0.0335	14.27465	6.53635	0.57421
36	23.245	BP	0.0416	19.05029	7.03327	0.76631
37	23.807	BP	0.0865	18.89989	2.95848	0.76026
38	24.115	BB	0.0513	20.02472	5.66263	0.80551
39	24.744	BP	0.0581	39.67739	10.04692	1.59605
40	26.581	BP	0.0566	40.54175	11.36360	1.63082
41	28.916	BP	0.0692	76.79160	15.13776	3.08900

Totals : 2485.97313 1023.60311

Results obtained with enhanced integrator!

===

*** End of Report ***

Male #2 Chrysopa oculata abdominal cuticle + 1ul of Z-3 -octen-1-ol (50ng/ul)(/50ul conc. to 10ul CH2Cl2), DB-5 . Note: Caught 5/22 sweep netting.

===

Injection Date : 5/23/2008 10:20:04 AM
Sample Name : M2 C.ocu. abd+IS Vial : -
Acq. Operator : Aldrich Inj : 1
Inj Volume : Manually
Method : C:\HPCHEM\1\METHODS\DBLESS1.M
Last changed : 2/29/2008 10:02:00 AM by Aldrich
2/29/07; editing new method for desired output

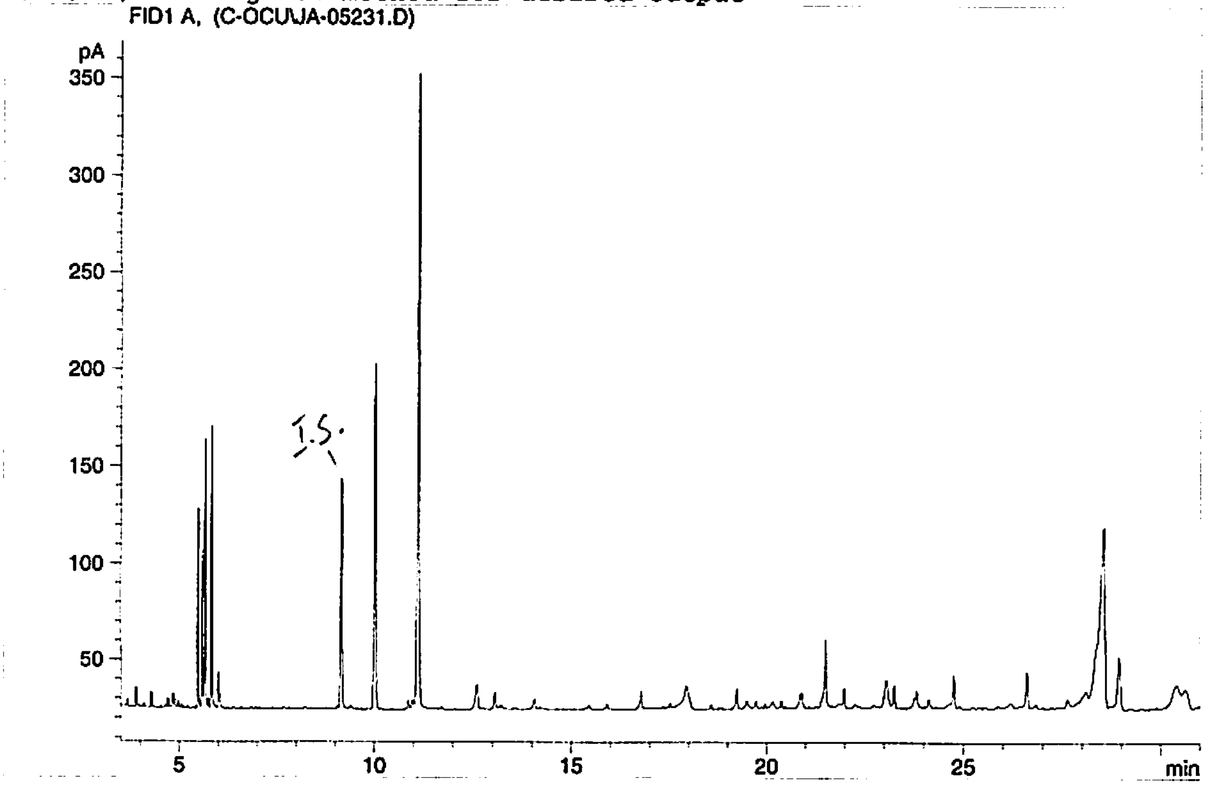

===

Area Percent Report

===

Sorted By : Signal
Multiplier : 1.0000
Dilution : 1.0000

Signal 1: FID1 A,

Peak #	RetTime [min]	Type	Width [min]	Area [pA*s]	Height [pA]	Area %
1	3.671	BP	0.0165	4.37498	4.09745	0.09208
2	3.893	PB	0.0183	12.90329	10.54579	0.27158
3	4.092	PB	0.0211	3.14564	2.28211	0.06621
4	4.285	PB	0.0209	10.79726	7.91162	0.22726
5	4.712	BP	0.0270	8.48638	4.50350	0.17862
6	4.843	PV	0.0273	13.25498	6.93642	0.27898
7	4.883	VP	0.0225	6.10125	4.31632	0.12842
8	4.971	BB	0.0224	5.26514	3.75182	0.11082
9	5.059	BB	0.0273	3.73197	2.04162	0.07855
10	5.481	BB	0.0232	151.87527	103.12519	3.19660
11	5.587	BV	0.0232	121.45475	82.59444	2.55632
12	5.662	VB	0.0245	219.24463	138.18460	4.61455
13	5.744	BP	0.0241	7.42349	4.77827	0.15625
14	5.825	VB	0.0243	227.02118	144.69940	4.77823
15	6.003	PV	0.0214	25.46510	18.14264	0.53598
16	6.038	VB	0.0232	10.40217	6.67088	0.21894

Peak #	RetTime [min]	Type	Width [min]	Area [pA*s]	Height [pA]	Area %
17	9.159	BB	0.0411	317.35965	118.96738	6.67963
18	10.023	BB	0.0447	514.13873	178.07433	10.82134
19	10.868	PV	0.0445	11.39856	4.09479	0.23991
20	10.974	VV	0.0425	12.66193	4.28455	0.26650
21	11.134	VB	0.0458	1004.42444	326.98627	21.14064
22	12.615	BB	0.0644	59.31108	12.49237	1.24835
23	13.067	BP	0.0429	23.74396	8.16636	0.49975
24	13.214	BB	0.0349	3.19761	1.43885	0.06730
25	14.064	BP	0.0455	15.83606	4.79302	0.33331
26	15.905	BP	0.0399	6.57981	2.40927	0.13849
27	16.779	BB	0.0421	26.48727	8.80390	0.55749
28	17.519	BP	0.0283	3.81540	2.09196	0.08030
29	17.934	BP	0.1112	83.31512	10.31358	1.75358
30	18.570	BB	0.0296	4.51012	2.43277	0.09493
31	19.236	BB	0.0363	25.55595	10.17758	0.53789
32	19.489	BB	0.0634	15.26641	3.60514	0.32132
33	19.735	BP	0.0336	7.72351	3.51771	0.16256
34	19.962	PP	0.0288	3.87798	2.07803	0.08162
35	20.378	BP	0.0344	7.88613	3.62103	0.16598
36	20.852	BV	0.0390	18.10093	6.80157	0.38098
37	20.900	VB	0.0350	18.27067	7.61660	0.38455
38	21.508	BB	0.0473	116.61505	34.62526	2.45446
39	21.983	BP	0.0329	20.68983	9.69575	0.43547
40	23.050	BV	0.0860	91.08951	14.16271	1.91721
41	23.253	VP	0.0412	32.52708	11.78891	0.68461
42	23.753	BV	0.0460	14.76727	4.92019	0.31081
43	23.813	VB	0.0494	26.93747	8.19465	0.56697
44	24.120	BB	0.0477	13.33077	4.23378	0.28058
45	24.748	BP	0.0435	47.86983	16.17009	1.00754
46	26.590	BP	0.0547	64.53616	18.48457	1.35833
47	27.623	PB	0.0754	18.90382	3.84009	0.39788
48	28.093	BV	0.1131	59.37616	6.78152	1.24972
49	28.538	VB	0.1390	997.31305	93.10902	20.99096
50	28.927	BP	0.0705	135.10269	26.94696	2.84358
51	30.405	BB	0.1552	97.68717	7.53681	2.05607
Totals :				4751.15467	1527.83943	

Results obtained with enhanced integrator!

==

*** End of Report ***

Male #3 Chrysopa oculata abdominal cuticle + 1ul of Z-3
-octen-1-ol (50ng/ul)(/50ul conc. to 3ul CH2Cl2), DB-5.
Note: Caught 5/22 sweep netting; actually, all samples
run today I injected the entire extract of ~3ul.

===

Injection Date : 5/23/2008 11:00:06 AM
Sample Name : M3 C.ocu. abd+IS Vial : -
Acq. Operator : Aldrich Inj : 1
Inj Volume : Manually
Method : C:\HPCHEM\1\METHODS\DBLESS1.M
Last changed : 2/29/2008 10:02:00 AM by Aldrich
2/29/07; editing new method for desired output

FID1 A, (C-OCU\JA-05232.D)

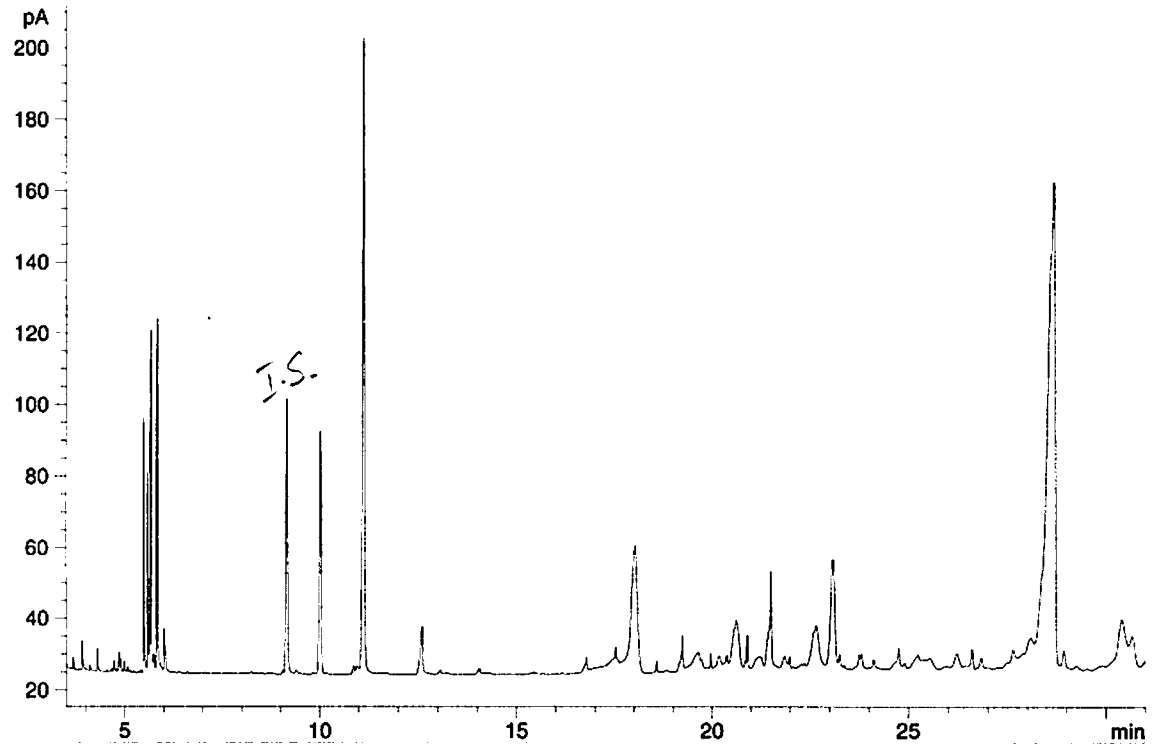

===

Area Percent Report

===

Sorted By : Signal
Multiplier : 1.0000
Dilution : 1.0000

Signal 1: FID1 A,

Peak #	RetTime [min]	Type	Width [min]	Area [pA*s]	Height [pA]	Area %
1	3.681	BP	0.0174	3.40957	3.20237	0.07333
2	3.906	PB	0.0186	10.15518	8.13391	0.21841
3	4.104	PB	0.0206	2.28383	1.60031	0.04912
4	4.294	PB	0.0209	8.35513	6.14767	0.17970
5	4.721	BP	0.0227	4.35555	2.87100	0.09368
6	4.852	PV	0.0263	9.63492	5.26835	0.20722
7	4.892	VB	0.0215	4.87399	3.43764	0.10483
8	4.979	BB	0.0209	4.12017	3.01604	0.08861
9	5.067	BB	0.0300	3.07631	1.49369	0.06616
10	5.487	BB	0.0235	105.83727	70.68427	2.27628
11	5.592	BV	0.0235	83.12566	55.33455	1.78781
12	5.666	VB	0.0237	151.62958	94.62186	3.26114
13	5.749	BP	0.0249	6.58540	4.07491	0.14163
14	5.828	VB	0.0246	155.98544	97.76833	3.35483
15	6.011	PB	0.0294	23.87917	11.42490	0.51358

Peak #	RetTime [min]	Type	Width [min]	Area [pA*s]	Height [pA]	Area %
16	9.159	BB	0.0469	229.86876	76.79199	4.94386
17	10.018	BP	0.0499	221.20992	68.10526	4.75763
18	10.866	PB	0.0449	4.52753	1.60411	0.09737
19	11.125	BB	0.0519	604.12512	176.47798	12.99311
20	12.606	BB	0.0586	58.55051	13.22761	1.25926
21	16.777	BB	0.0370	9.21157	3.58389	0.19812
22	17.522	BB	0.0337	8.93091	3.90476	0.19208
23	17.995	BV	0.0683	174.34375	32.66431	3.74967
24	18.034	VB	0.0631	168.57390	34.44708	3.62557
25	18.575	BB	0.0284	5.70264	3.10356	0.12265
26	19.238	BP	0.0455	30.51427	9.25400	0.65628
27	19.965	PP	0.0290	7.23748	4.02052	0.15566
28	20.380	BB	0.0370	6.02888	2.42942	0.12966
29	20.623	BP	0.1314	131.61337	12.75397	2.83065
30	20.905	VP	0.0350	21.77728	9.08747	0.46837
31	21.507	PB	0.0567	106.07313	25.96566	2.28135
32	21.987	BP	0.0289	4.29034	2.51317	0.09227
33	22.663	BB	0.1364	117.73811	10.77833	2.53223
34	23.087	BV	0.0958	219.51840	29.85352	4.72125
35	23.257	VB	0.0393	8.44585	3.24763	0.18165
36	23.754	BV	0.0436	10.96383	3.59306	0.23580
37	23.814	VB	0.0421	11.60955	3.85543	0.24969
38	24.120	BB	0.0445	6.97202	2.22982	0.14995
39	24.755	BP	0.0466	15.11654	4.82515	0.32512
40	26.596	BP	0.0517	16.94486	5.10879	0.36444
41	26.825	BB	0.0615	11.33118	2.78515	0.24370
42	28.673	BP	0.1655	1767.99390	134.41023	38.02479
43	28.922	VB	0.0599	19.46003	4.55020	0.41853
44	30.398	PB	0.1093	73.60098	8.55917	1.58296

< RT 13.066 ; area = 3.3

Totals : 4649.58175 1062.81104

Results obtained with enhanced integrator!

===

*** End of Report ***

Male #1 Chrysopa oculata abdominal cuticle + 1ul of Z-3 -octen-1-ol (50ng/ul)(/50ul conc. to 3ul CH2Cl2), DB-5. Note: Injected the entire extract of ~3ul; coll. sweeping 5/23.

===

Injection Date : 5/24/2008 1:33:54 PM
Sample Name : M1 C.ocu. abd+IS
Acq. Operator : Aldrich
Vial : -
Inj : 1
Inj Volume : Manually

Method : C:\HPCHEM\1\METHODS\DBLESS1.M
Last changed : 2/29/2008 10:02:00 AM by Aldrich
2/29/07; editing new method for desired output

FID1 A, (C-OCU\JA-05240.D)

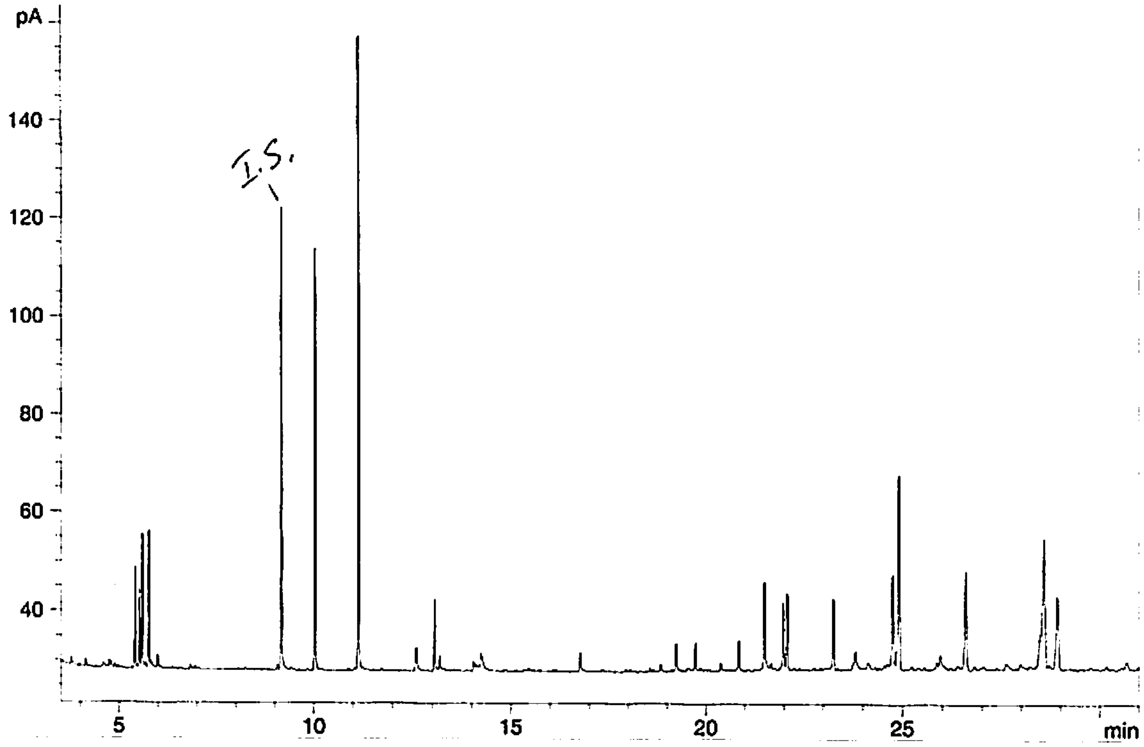

===

Area Percent Report

===

Sorted By : Signal
Multiplier : 1.0000
Dilution : 1.0000

Signal 1: FID1 A,

Peak #	RetTime [min]	Type	Width [min]	Area [pA*s]	Height [pA]	Area %	
1	4.137	PB	0.0207	2.01945	1.50348	0.14953	
2	4.739	PB	0.0249	2.54360	1.49018	0.18834	
3	5.400	PB	0.0230	29.60558	20.36728	2.19208	
4	5.511	PV	0.0231	23.20639	15.85104	1.71827	
5	5.584	VB	0.0230	38.74371	26.51748	2.86870	
6	5.754	PP	0.0234	40.46590	27.20241	2.99621	
7	5.982	PB	0.0259	4.68111	2.61534	0.34660	
8	9.158	BB	0.0271	160.16942	93.08414	11.85940	
9	10.024	BB	0.0261	146.60625	85.24084	10.85515	nonanal
10	11.133	BB	0.0264	224.29924	128.18231	16.60776	nonanol
11	12.605	PB	0.0471	14.57211	4.70538	1.07896	
12	13.067	BB	0.0309	27.61856	14.10098	2.04496	iridodial
13	13.196	PP	0.0297	5.40557	2.77300	0.40024	
14	14.059	PB	0.0273	2.91305	1.59888	0.21569	
15	14.238	PB	0.0416	8.32976	2.80496	0.61676	

Peak #	RetTime [min]	Type	Width [min]	Area [pA*s]	Height [pA]	Area %
16	16.776	BB	0.0319	8.08591	3.78156	0.59870
17	19.234	BB	0.0317	11.00860	5.42841	0.81511
18	19.735	BP	0.0283	10.04833	5.49625	0.74401
19	20.850	BB	0.0287	11.32750	6.08638	0.83872
20	21.502	BB	0.0338	37.92960	17.15381	2.80842
21	21.984	BV	0.0333	28.73876	13.26942	2.12790
22	22.080	VP	0.0332	33.09731	15.33829	2.45062
23	23.253	BP	0.0385	34.62203	14.15492	2.56351
24	23.812	BB	0.0498	11.15984	3.27125	0.82631
25	24.751	BB	0.0435	53.23800	18.55546	3.94189
26	24.899	BB	0.0443	110.94831	38.84958	8.21493
27	25.945	BB	0.0521	7.83572	2.27737	0.58018
28	26.588	BP	0.0475	65.99481	19.47974	4.88645
29	28.572	BB	0.0748	132.78416	25.46851	9.83172
30	28.910	PP	0.0676	62.57025	14.41719	4.63288

Totals : 1350.56883 631.06584

Results obtained with enhanced integrator!

==

*** End of Report ***

Male #2 Chrysopa oculata abdominal cuticle + 1ul of Z-3
-octen-1-ol (50ng/ul)(/50ul conc. to 3ul CH2Cl2), DB-5.
Note: Injected the entire extract of ~3ul; coll sweep
ing 5/23.

===

Injection Date	: 5/24/2008 2:15:59 PM		
Sample Name	: M2 C.ocu. abd+IS	Vial :	-
Acq. Operator	: Aldrich	Inj :	1
		Inj Volume :	Manually
Method	: C:\HPCHEM\1\METHODS\DBLESS1.M		
Last changed	: 2/29/2008 10:02:00 AM by Aldrich		

2/29/07; editing new method for desired output

FID1 A, (C-OCU\JA-05241.D)

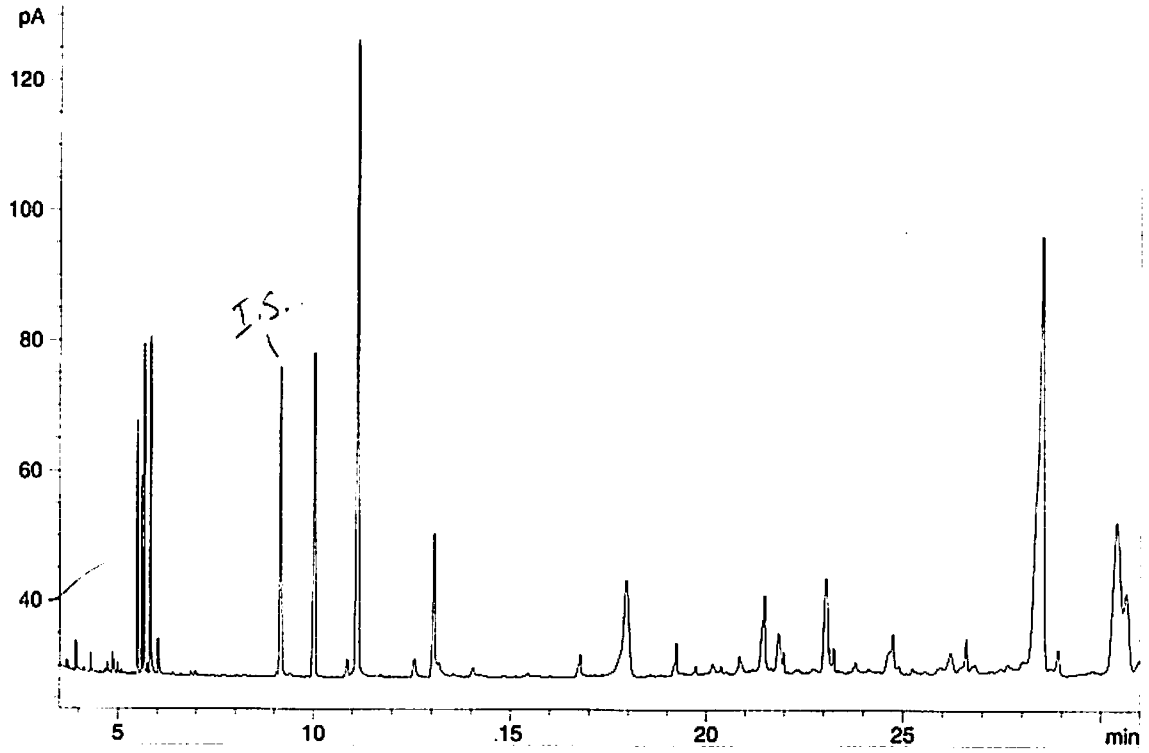

===

Area Percent Report

===

Sorted By	:	Signal
Multiplier	:	1.0000
Dilution	:	1.0000

Signal 1: FID1 A,

Peak #	RetTime [min]	Type	Width [min]	Area [pA*s]	Height [pA]	Area %	
1	3.689	PP	0.0161	1.54749	1.49178	0.06087	
2	3.919	PB	0.0188	5.87384	4.63134	0.23103	
3	4.301	PB	0.0201	3.86492	2.99211	0.15202	
4	4.732	BP	0.0230	2.44193	1.57847	0.09605	
5	4.858	PV	0.0240	5.34920	3.27983	0.21040	
6	4.899	VB	0.0213	2.79155	2.00018	0.10980	
7	4.985	PP	0.0202	2.33554	1.78926	0.09186	
8	5.489	BB	0.0219	53.07755	38.87034	2.08767	
9	5.595	BV	0.0213	42.51964	30.46233	1.67240	
10	5.666	VB	0.0224	71.34306	50.61925	2.80610	
11	5.754	BP	0.0245	2.79143	1.76262	0.10979	
12	5.827	VB	0.0234	76.91996	51.66477	3.02545	
13	6.020	PB	0.0303	12.04897	5.55793	0.47392	
14	9.157	BB	0.0492	147.03278	47.42173	5.78316	— I.S.
15	10.021	BB	0.0552	174.96461	49.50938	6.88179	nonanal

Peak #	RetTime [min]	Type	Width [min]	Area [pA*s]	Height [pA]	Area %	
16	10.867	PP	0.0512	7.84933	2.46212	0.30873	
17	11.116	BB	0.0486	362.68472	97.15283	14.26528	– nonanol
18	12.569	BB	0.0590	12.99440	2.70714	0.51110	– nonanoic acid
19	13.067	BB	0.0466	70.80232	20.83921	2.78483	– iridodial
20	16.775	BB	0.0386	8.42827	3.11294	0.33150	
21	17.946	BP	0.1079	118.23598	13.54045	4.65051	
22	19.234	BB	0.0348	11.20859	4.71082	0.44086	
23	21.447	BV	0.0553	30.98402	7.48593	1.21868	
24	21.496	VB	0.0377	29.73279	11.30565	1.16946	
25	21.850	BV	0.0762	35.42372	5.80534	1.39330	
26	21.984	VP	0.0349	7.38551	3.20647	0.29049	
27	23.055	PV	0.0839	97.92822	14.29927	3.85176	
28	23.254	VP	0.0462	11.08644	3.57724	0.43606	
29	24.749	BP	0.0525	16.03050	4.61817	0.63052	
30	26.591	BP	0.0527	16.14090	4.74510	0.63486	
31	28.499	BB	0.1335	680.10938	66.42564	26.75038	
32	28.908	BP	0.0671	14.75097	3.43490	0.58019	
33	30.404	BV	0.1617	297.37250	22.86571	11.69639	
34	30.657	VP	0.1200	108.37788	11.81621	4.26277	

Totals : 2542.42892 597.74245

Results obtained with enhanced integrator!

===

*** End of Report ***
