# Supplementary material for: Pharmacophagy in green lacewings (Neuroptera: Chrysopidae: Chrysopa spp.)?
Source: PeerJ. 2016 Jan 18;4:e1564. doi: 10.7717/peerj.1564 (PMC4727961; doi:10.7717/peerj.1564)

```
File          :D:\DATA\Aldrich\JA-09\JA021209-2.D
Operator      : Aldrich
Acquired      : 12 Feb 2009  15:15    using AcqMethod JA-WAX08.M
Instrument :    Instrument #1
Sample Name: 7 14-22-d-old male C.oc. abd. /5ulCH2Cl2
Misc Info   : larvae reared on aphids; different appearance
Vial Number: 1
```

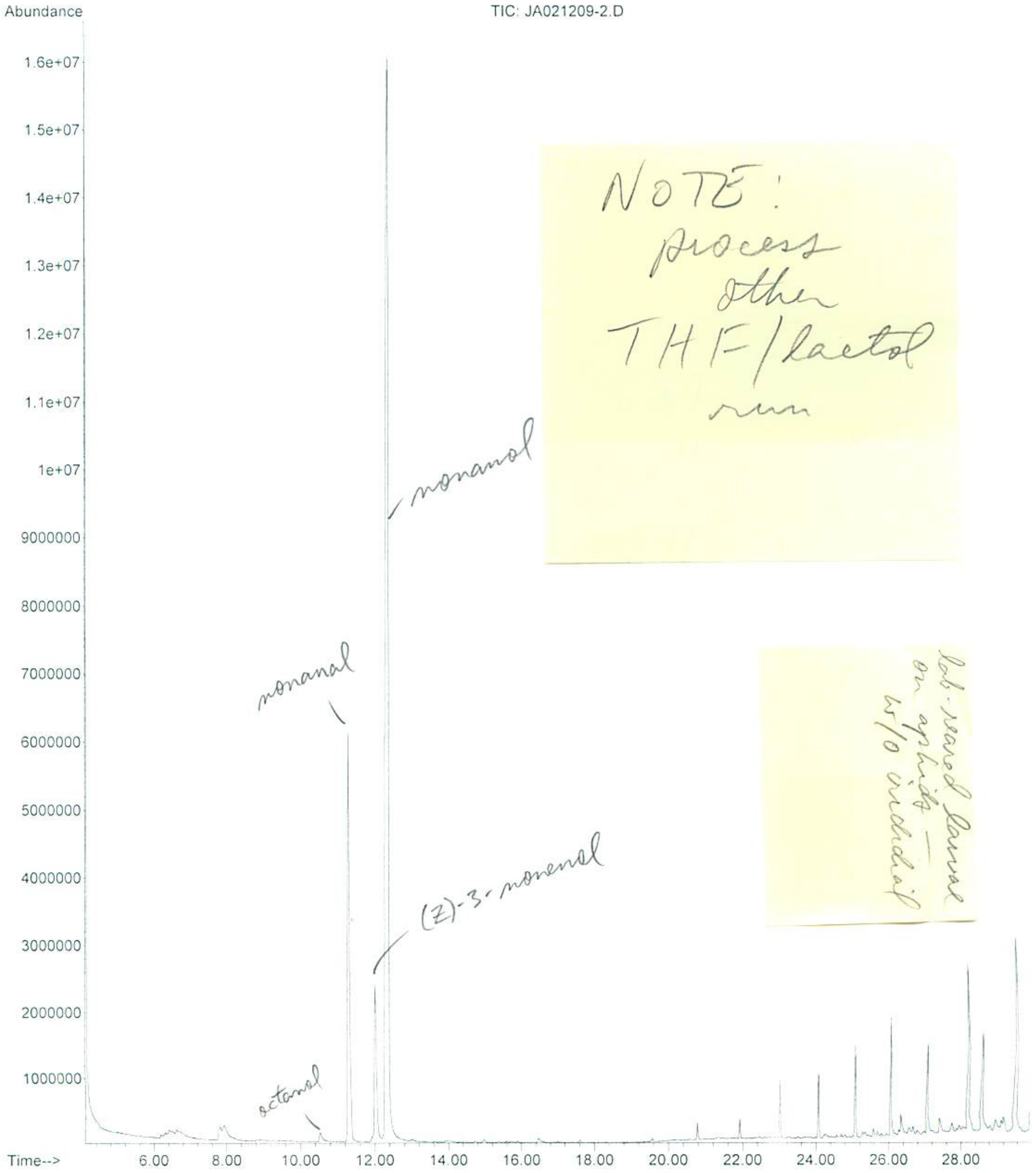

```
File      :D:\DATA\ALDRICH\JA-09\Snapshot\JA021209-2.D
Operator    : Aldrich
Acquired    : 12 Feb 2009  15:15     using AcqMethod JA-WAX08.M
Instrument :     Instrument #1
Sample Name: 7 14-22-d-old male C.oc. abd. /5ulCH2Cl2
Misc Info   : larvae reared on aphids; different appearance
Vial Number: 1
```

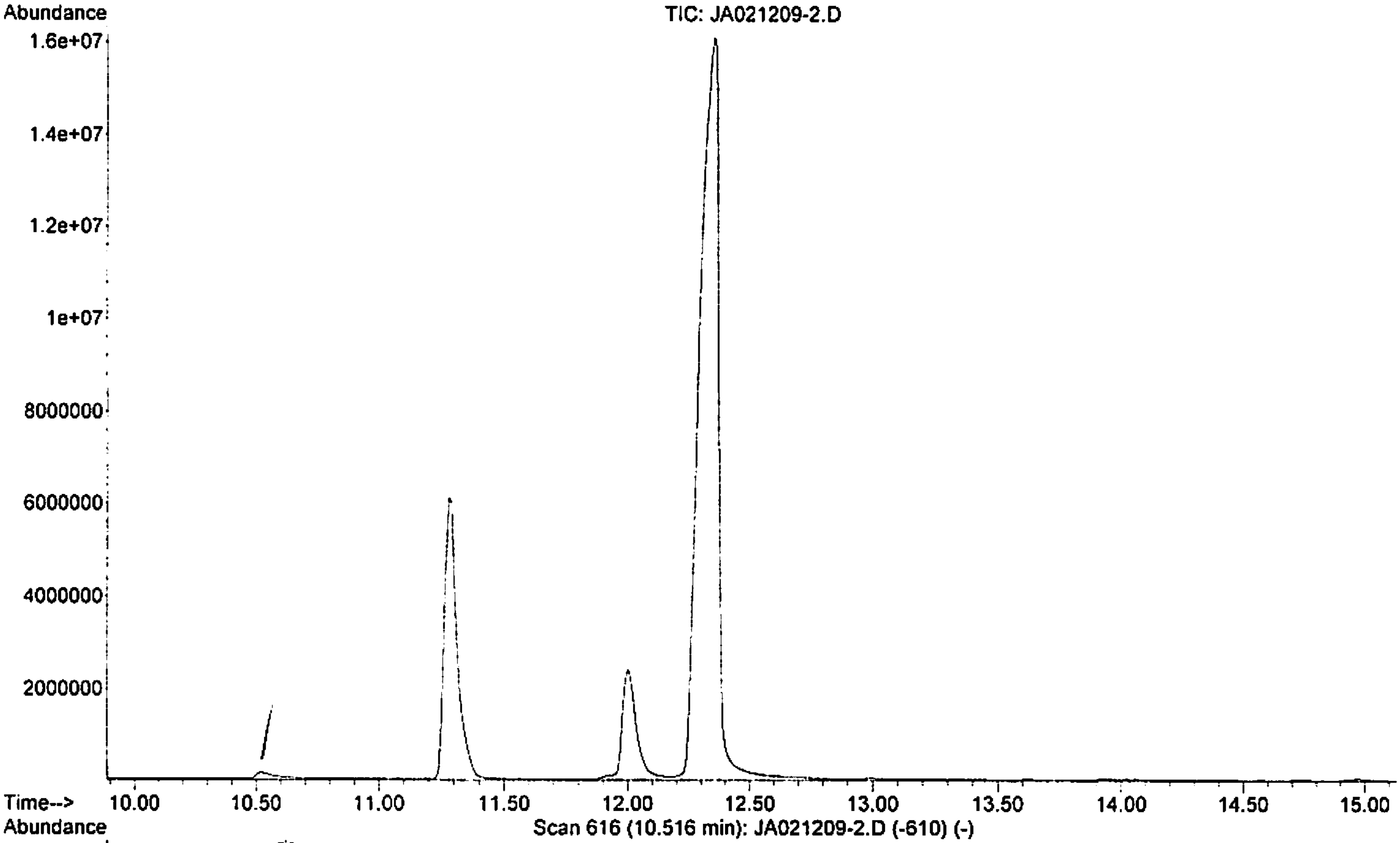


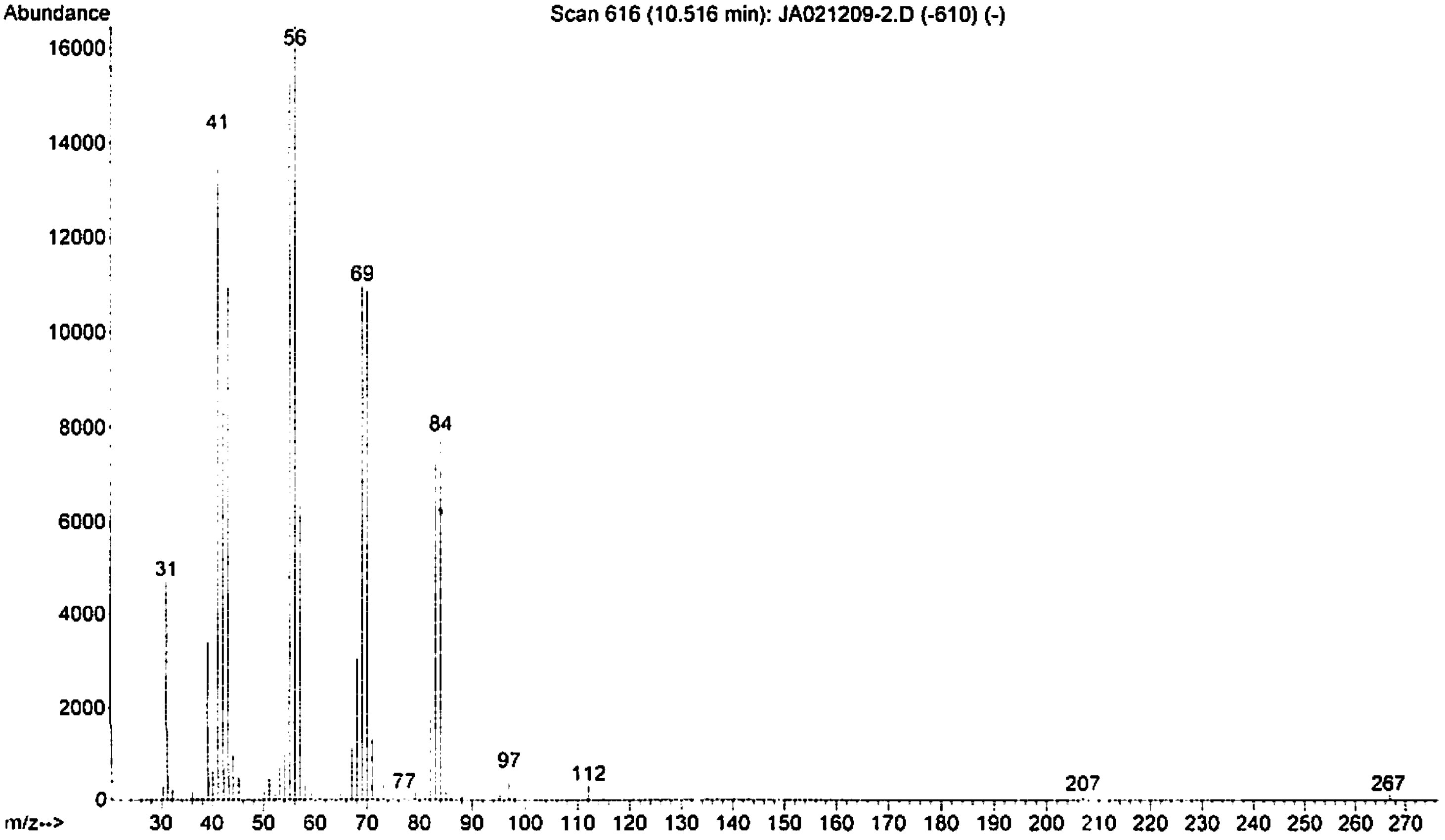

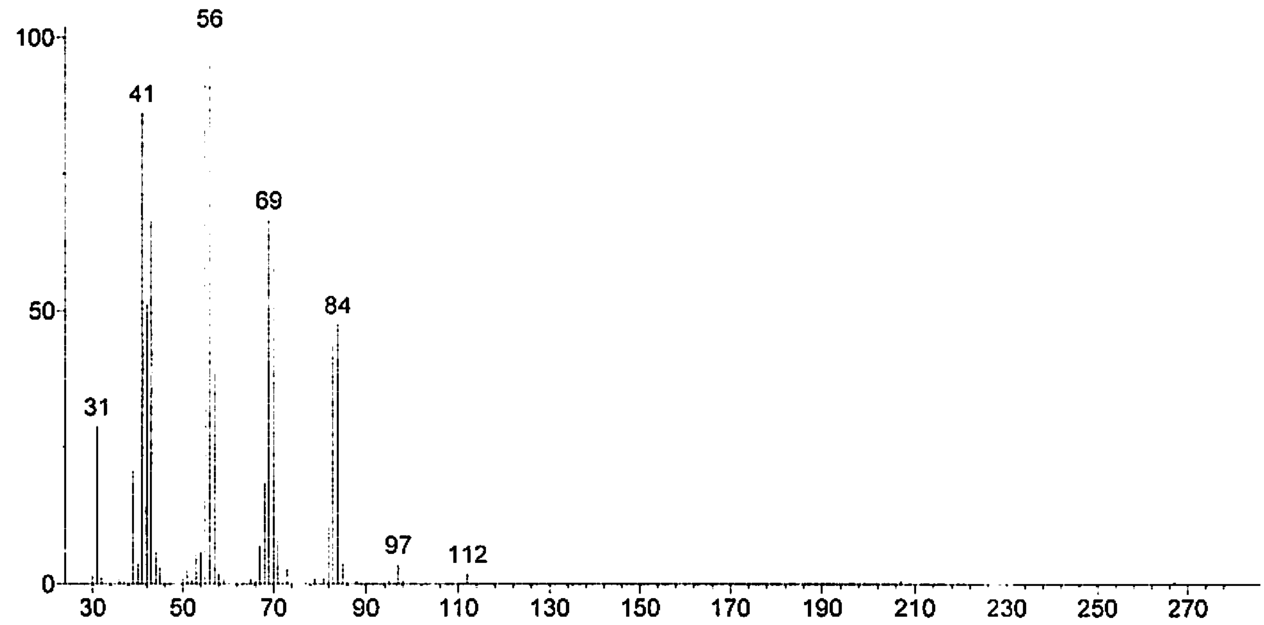


(Text File) Scan 616 (10.516 min): JA021209-2.D (-610)

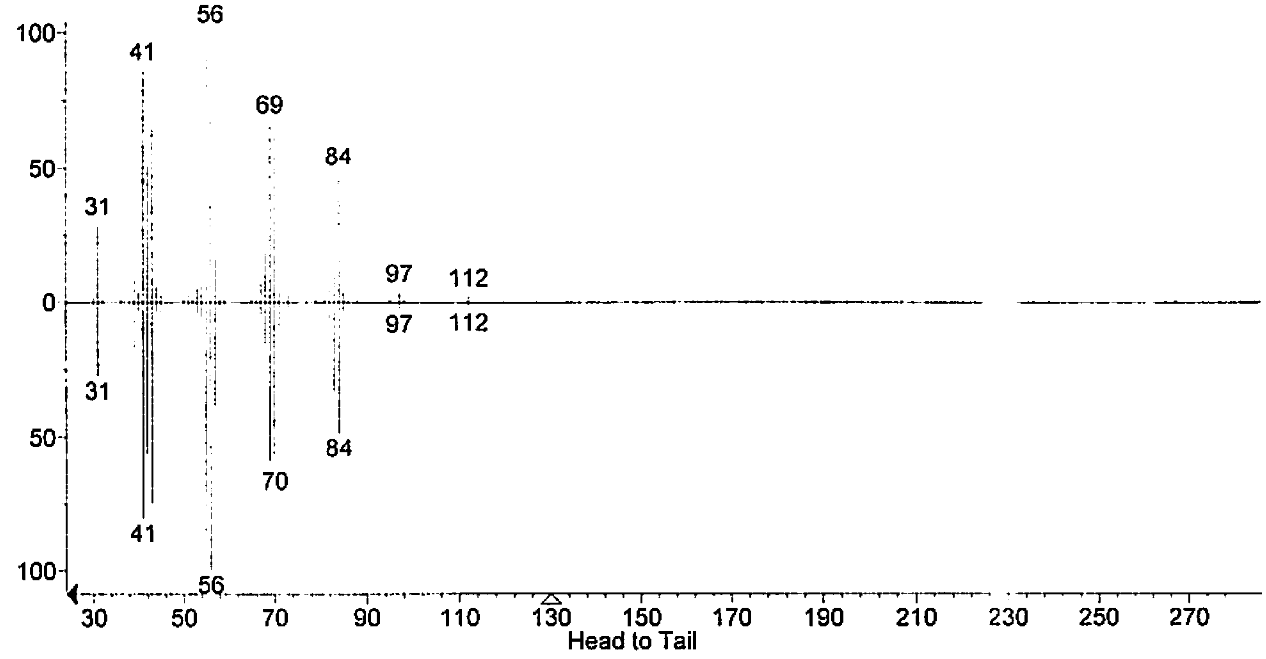


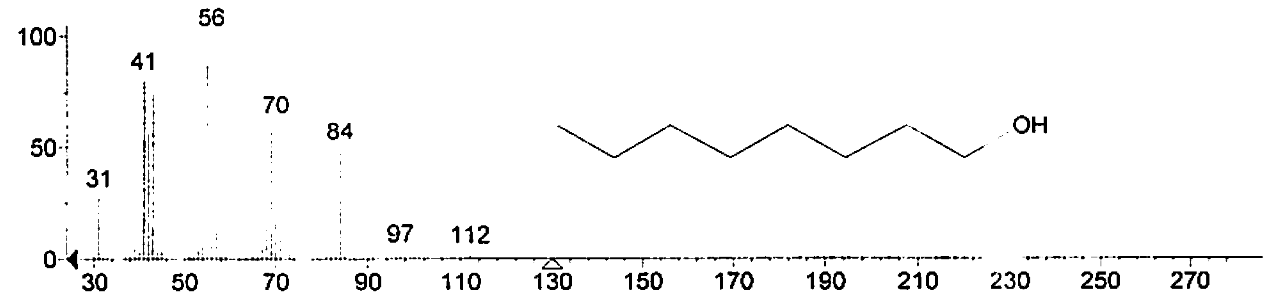


(mainlib) 1-Octanol

```
File        :D:\DATA\ALDRICH\JA-09\Snapshot\JA021209-2.D
Operator    : Aldrich
Acquired    : 12 Feb 2009  15:15      using AcqMethod JA-WAX08.M
Instrument  :     Instrument #1
Sample Name: 7 14-22-d-old male C.oc. abd. /5ulCH2Cl2
Misc Info   : larvae reared on aphids; different appearance
Vial Number: 1
```

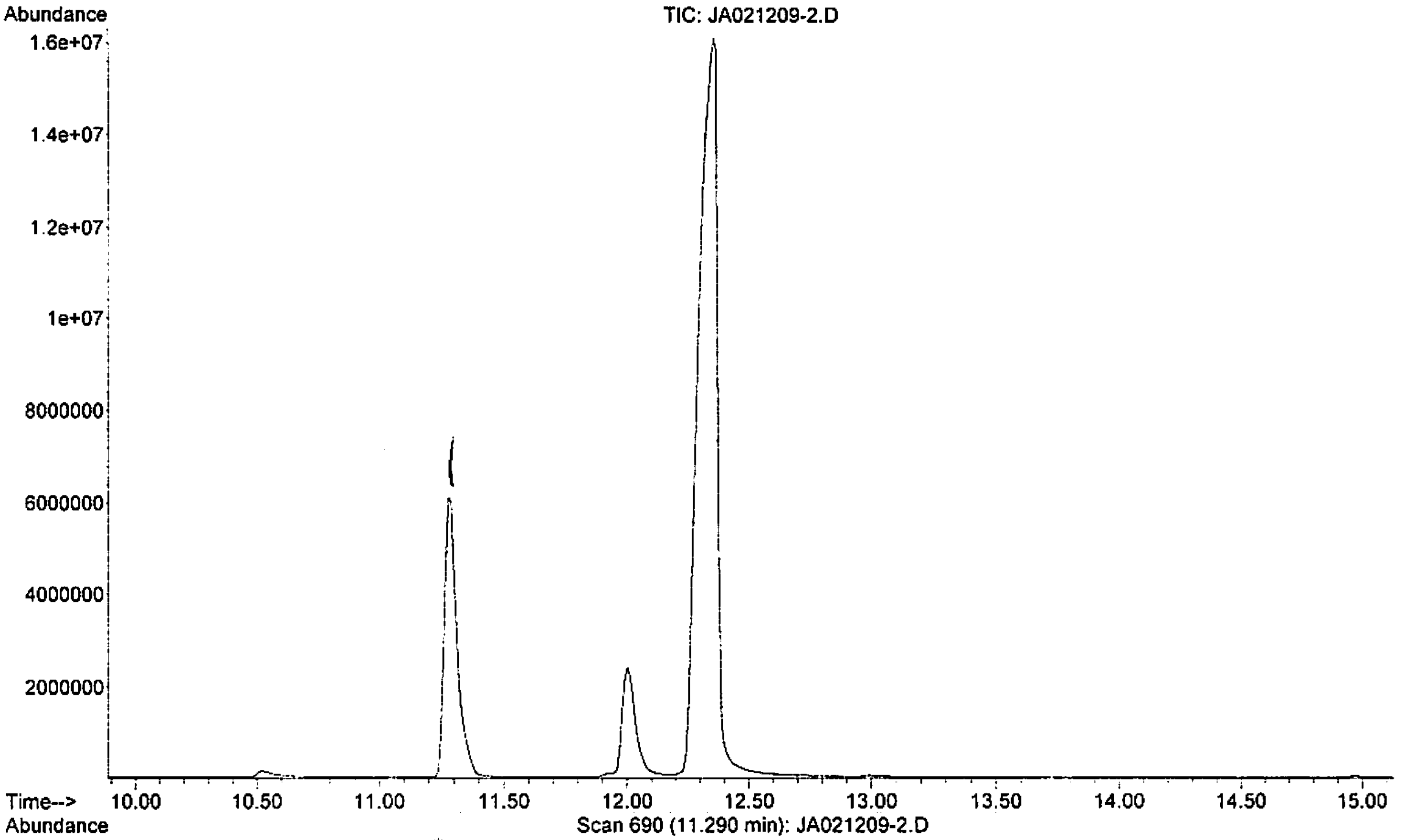


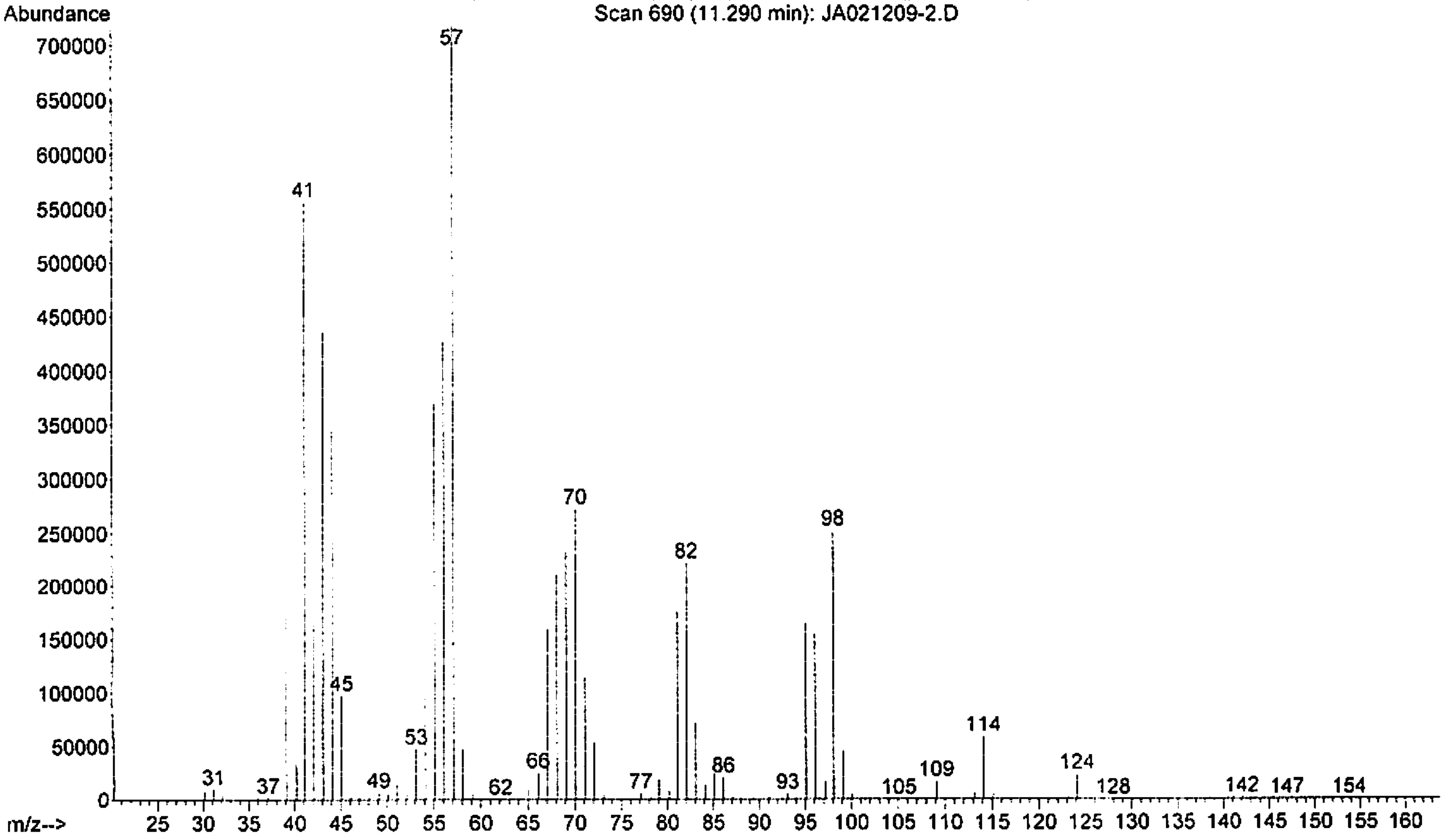

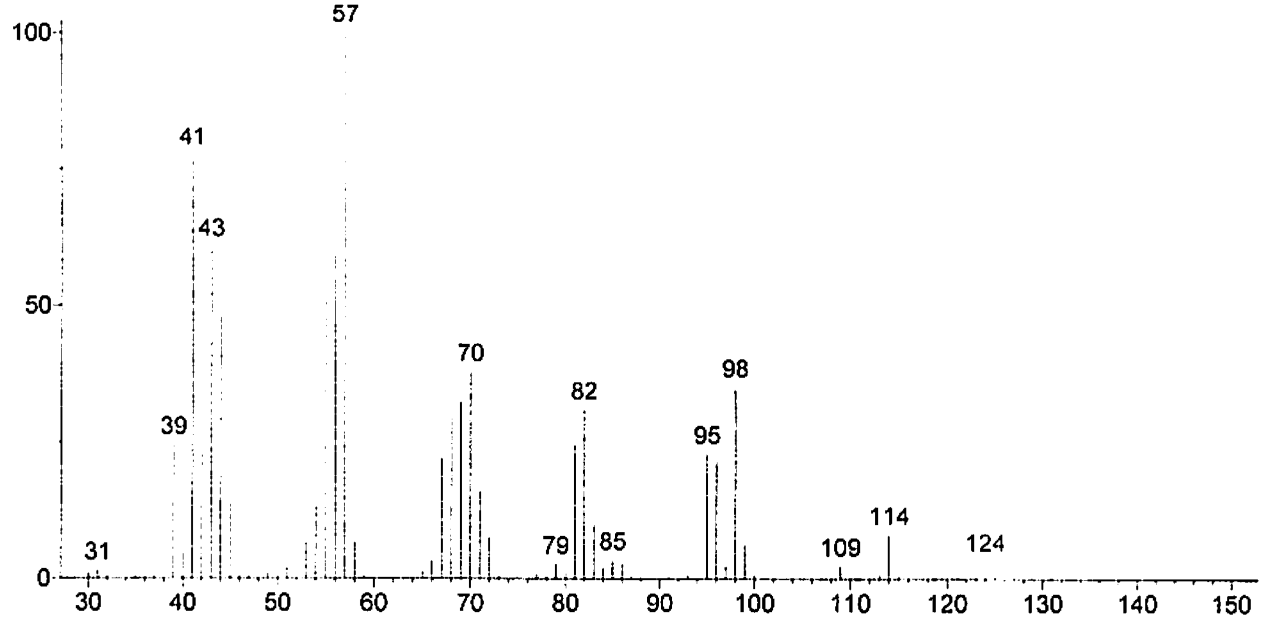


(Text File) Scan 690 (11.290 min): JA021209-2.D

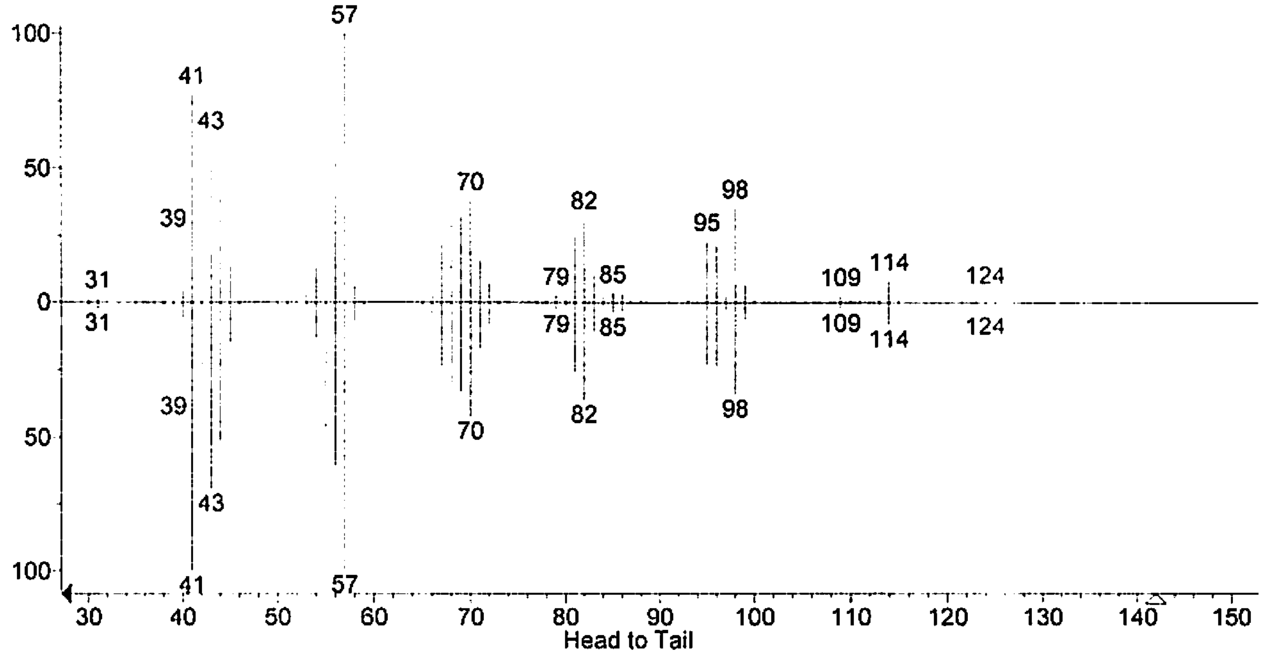


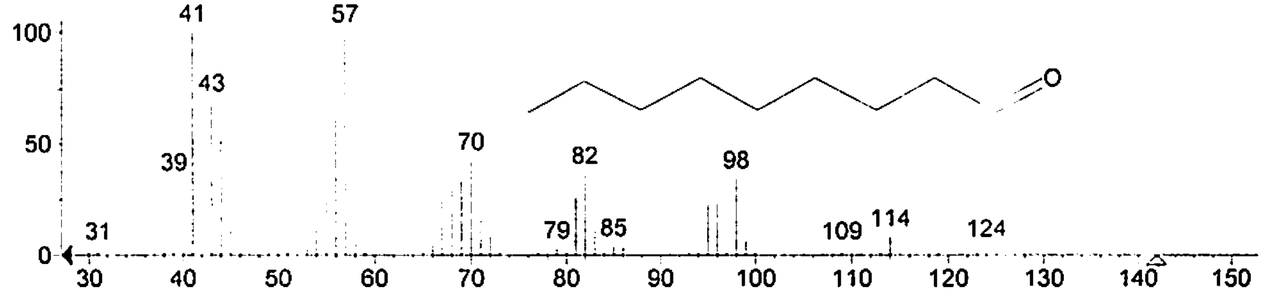


(replib) Nonanal

```
File ,        :D:\DATA\ALDRICH\JA-09\Snapshot\JA021209-2.D
Operator      : Aldrich
Acquired      : 12 Feb 2009  15:15      using AcqMethod JA-WAX08.M
Instrument :      Instrument #1
Sample Name: 7 14-22-d-old male C.oc. abd. /5ulCH2Cl2
Misc Info     : larvae reared on aphids; different appearance
Vial Number: 1
```

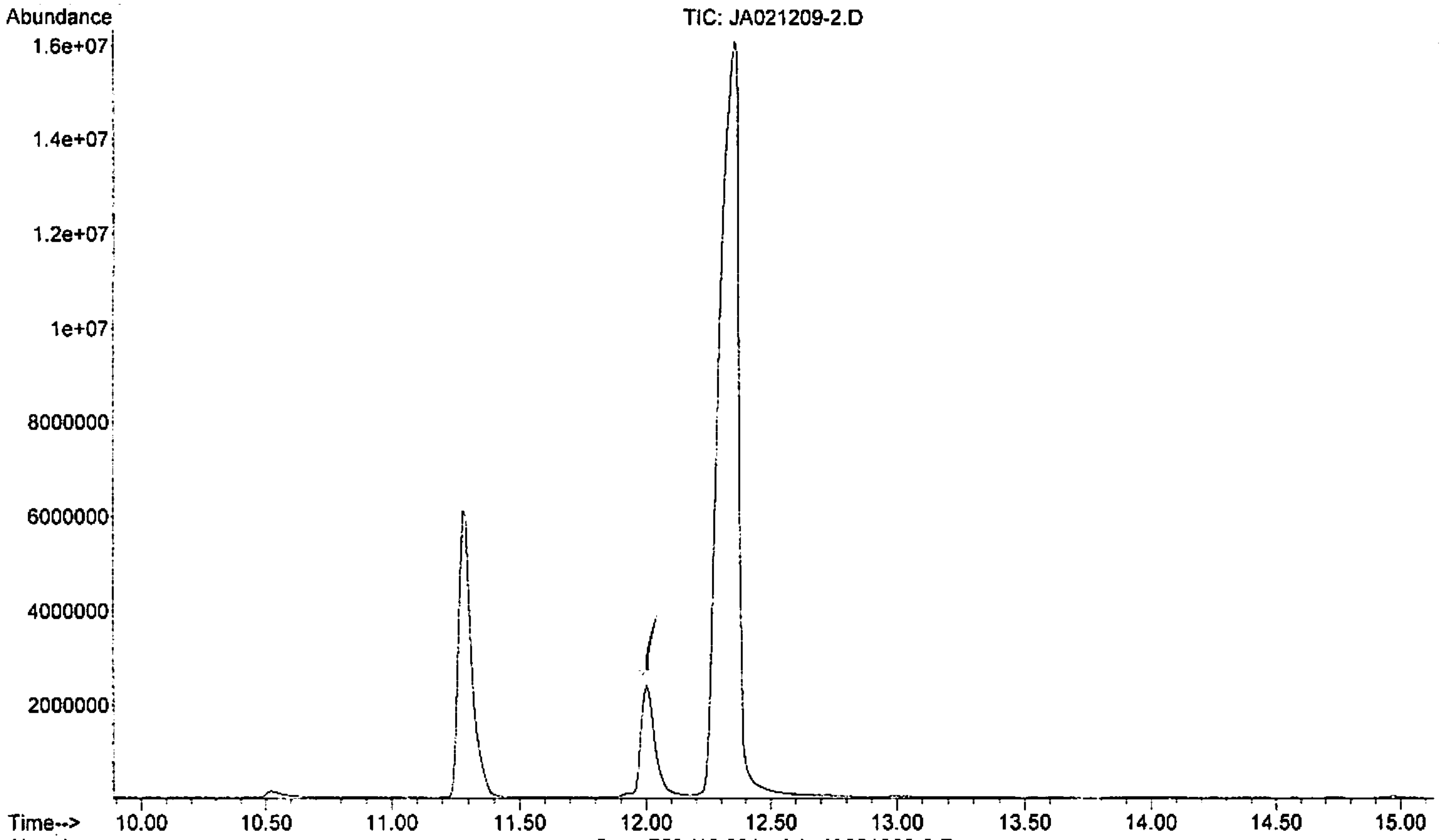


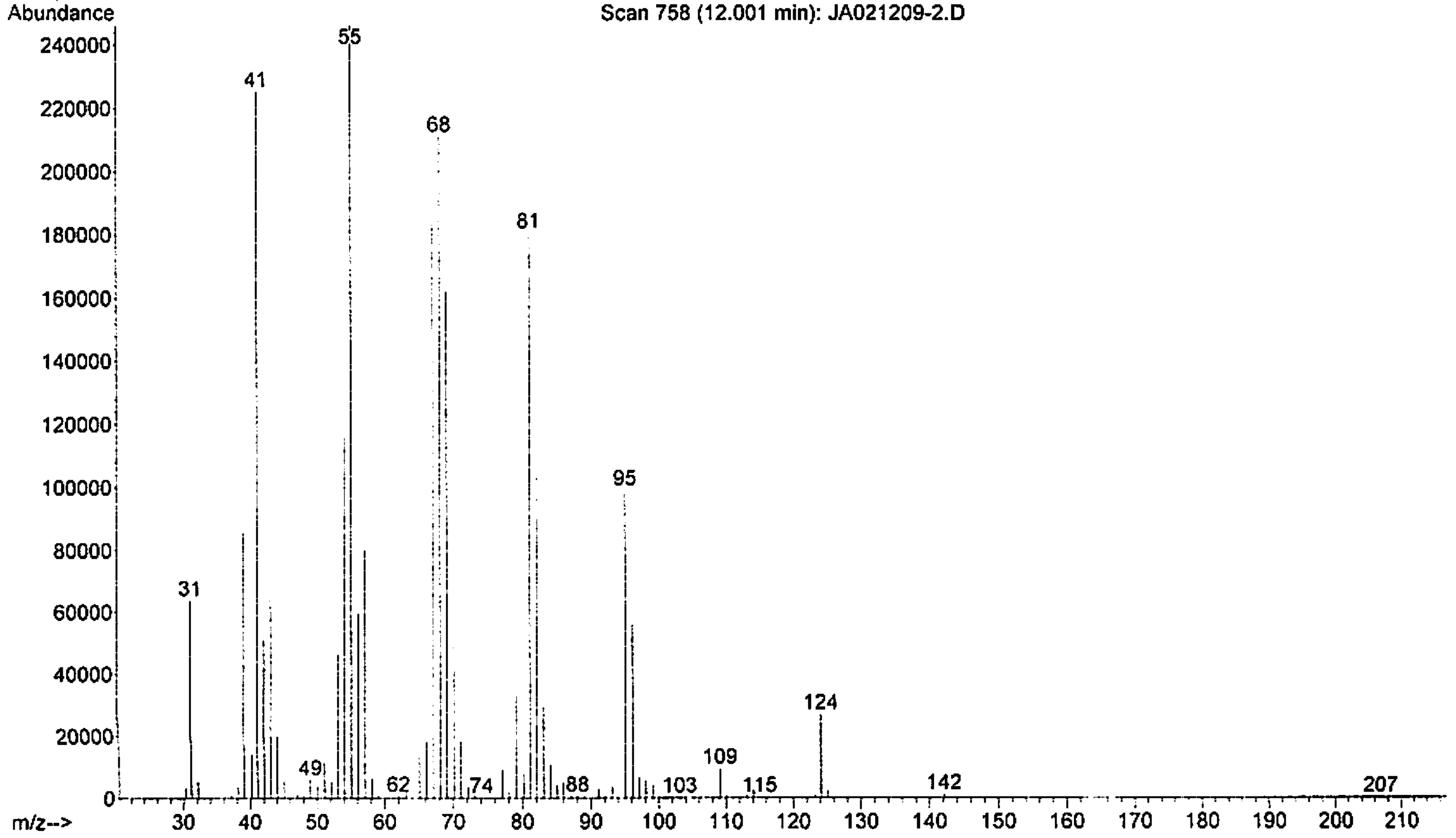

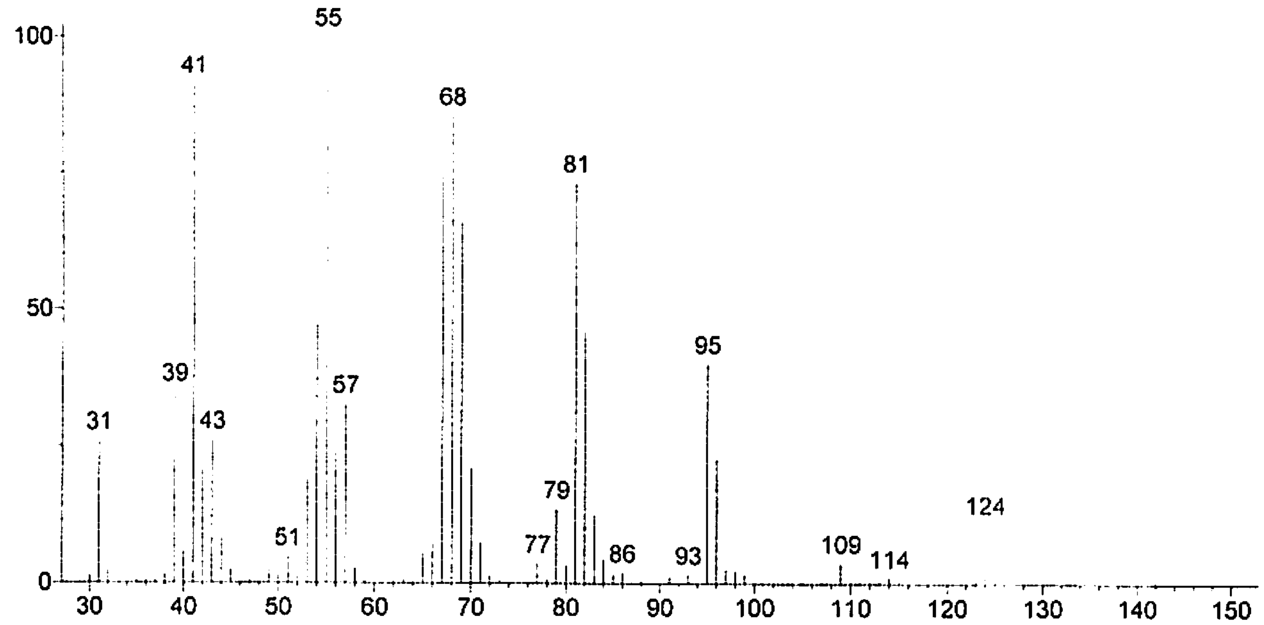

(Text File) Scan 758 (12.001 min): JA021209-2.D

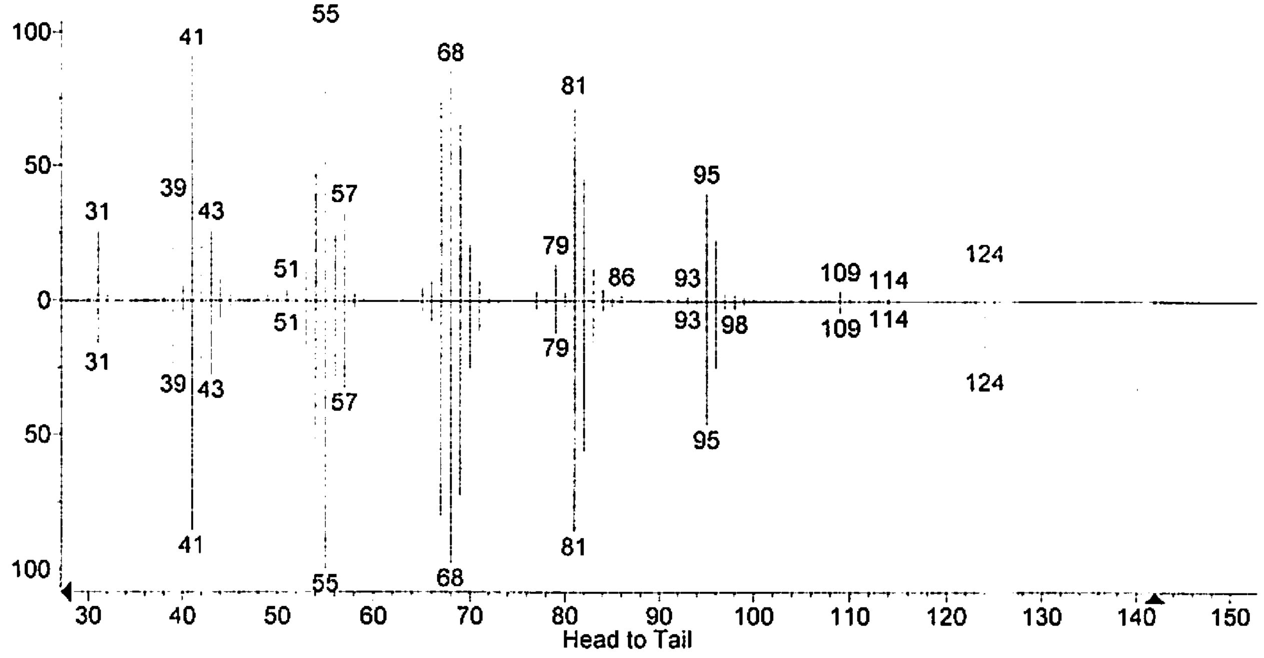


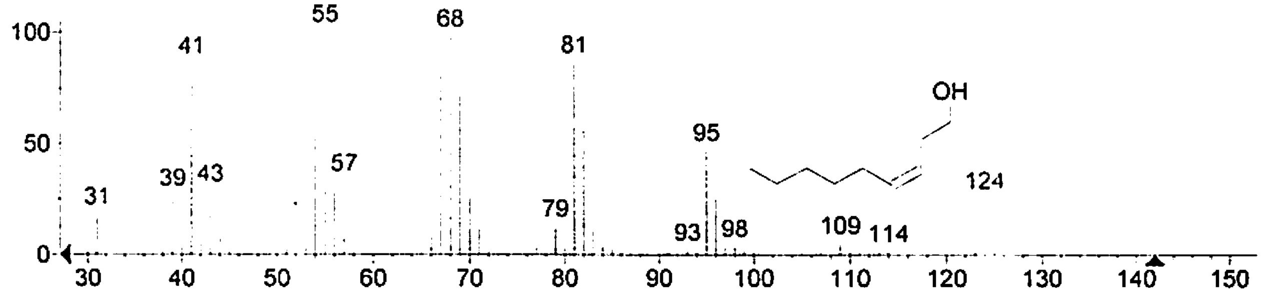

(mainlib) 3-Nonen-1-ol, (Z)-

```
File       :D:\DATA\ALDRICH\JA-09\Snapshot\JA021209-2.D
Operator   : Aldrich
Acquired   : 12 Feb 2009  15:15     using AcqMethod JA-WAX08.M
Instrument :     Instrument #1
Sample Name: 7 14-22-d-old male C.oc. abd. /5ulCH2C12
Misc Info  : larvae reared on aphids; different appearance
Vial Number: 1
```

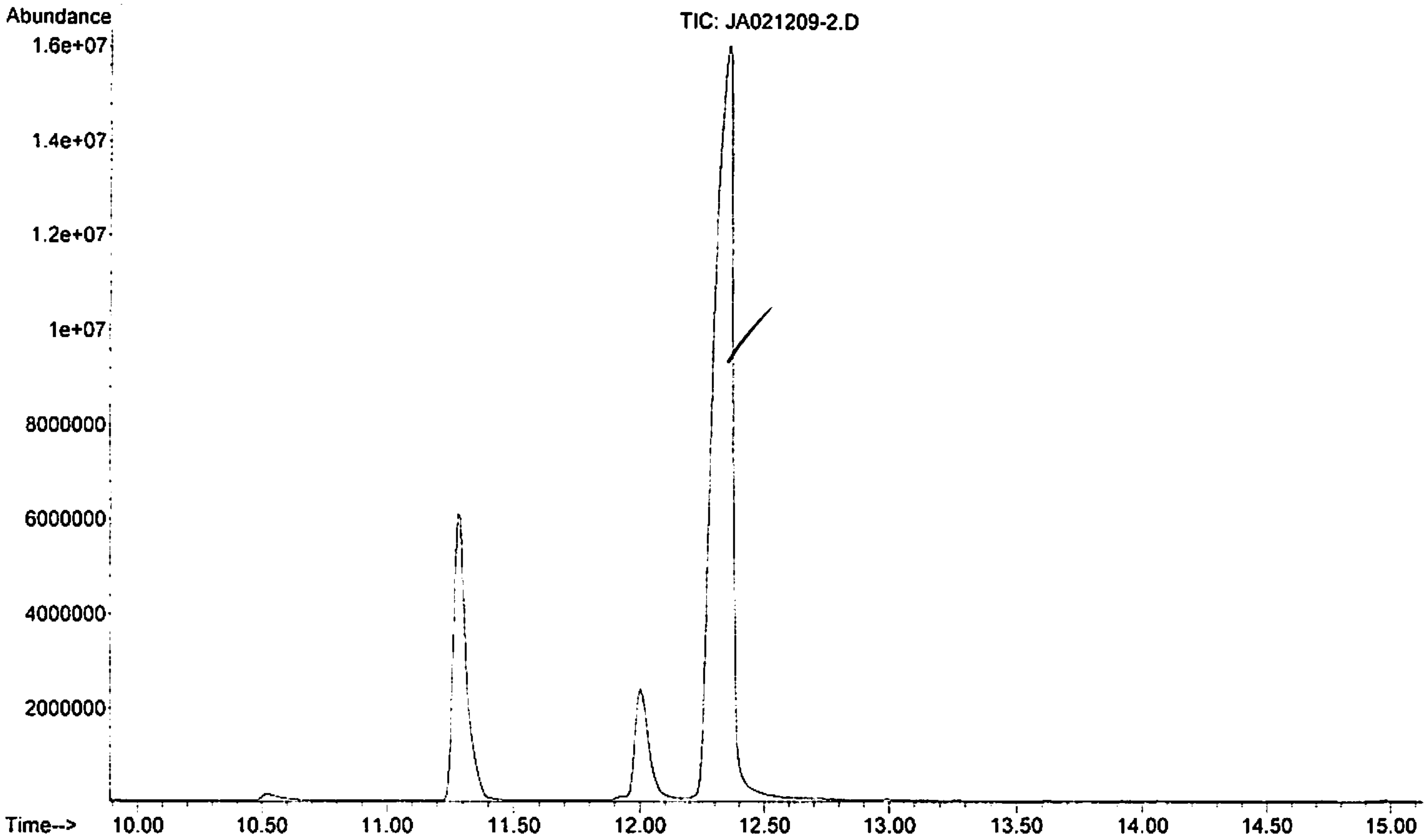


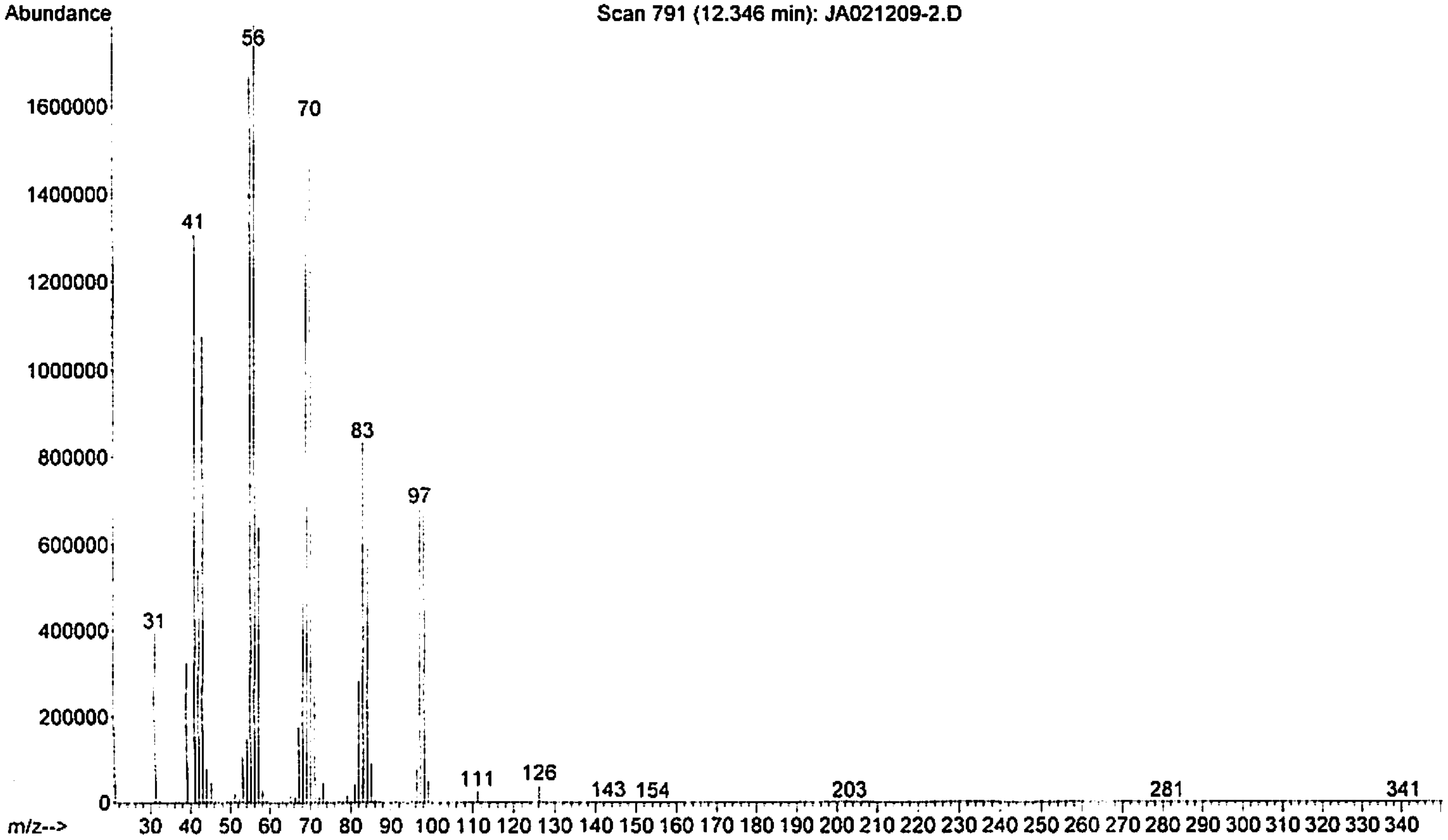

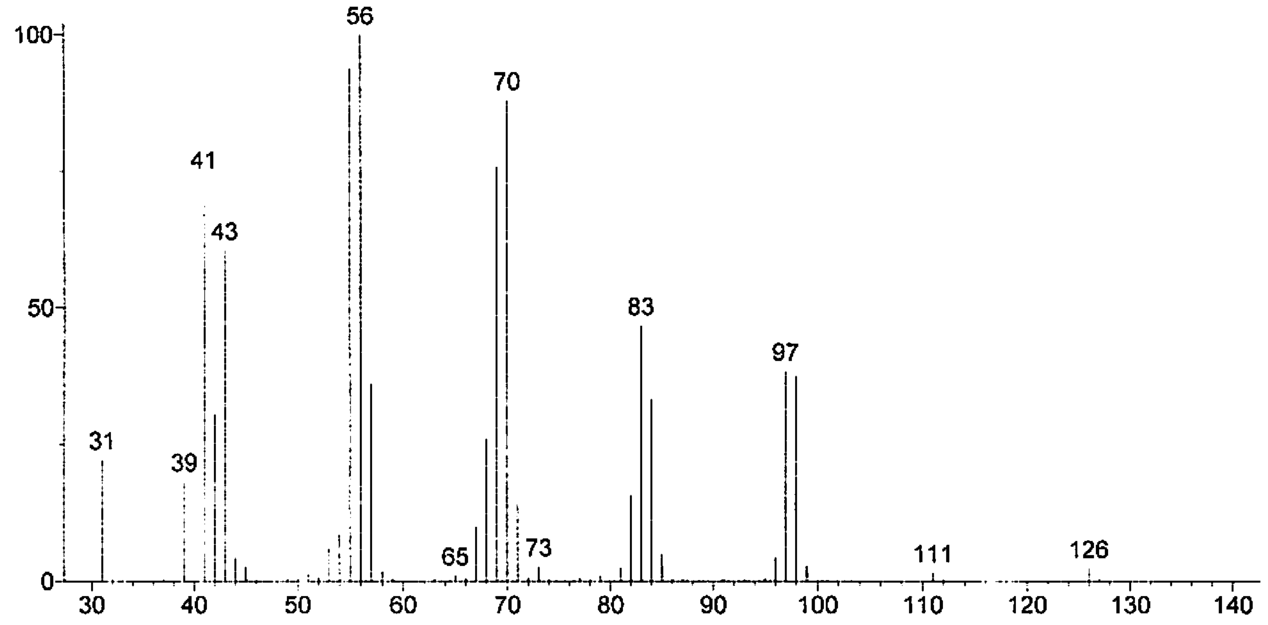


(Text File) Scan 791 (12.346 min): JA021209-2.D

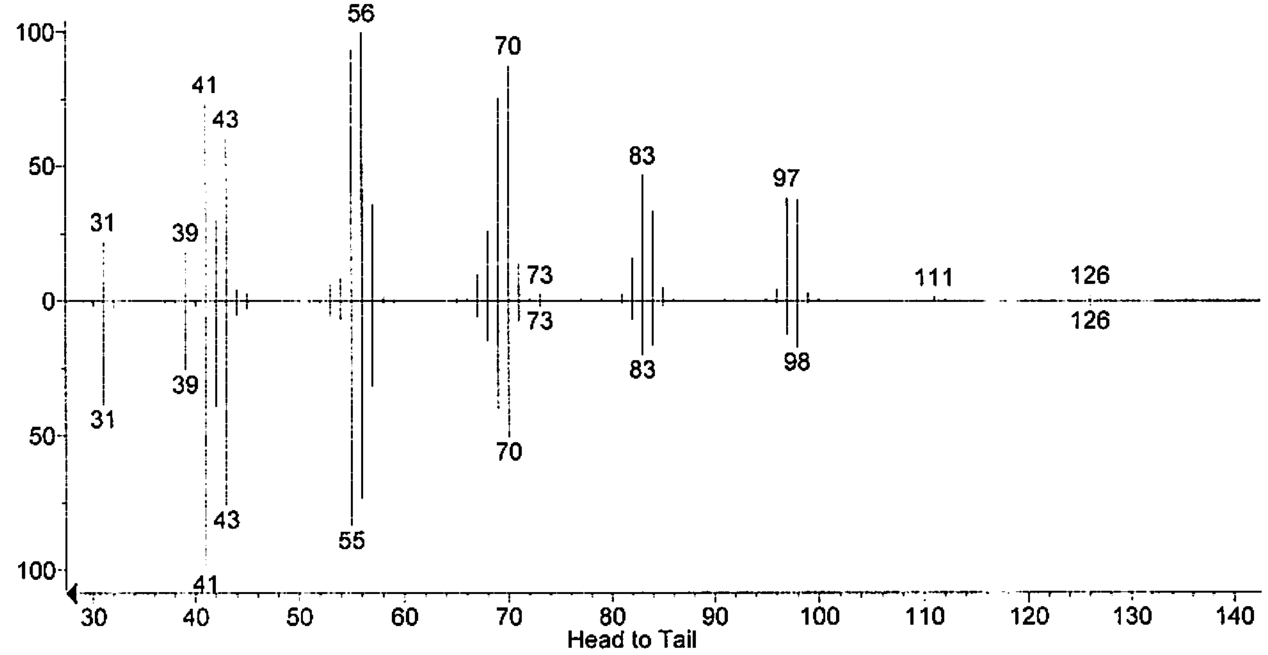


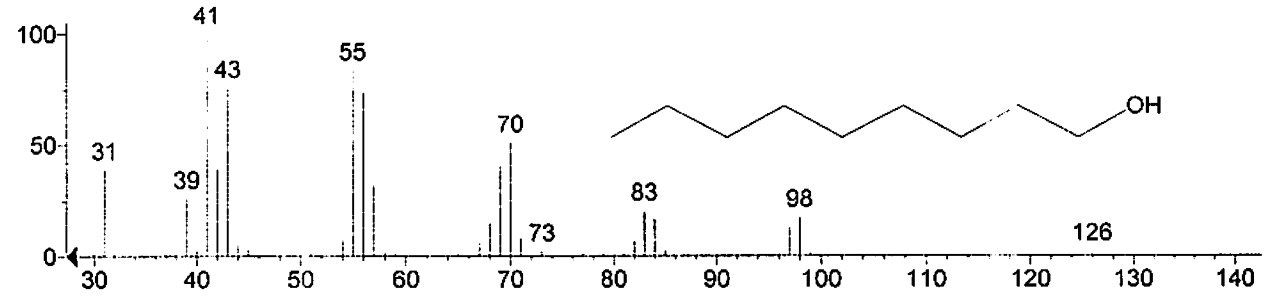


(replib) 1-Nonanol

File : D:\DATA\Aldrich\JA-09\JA021209-2.D
Operator : Aldrich
Acquired : 12 Feb 2009 15:15 using AcqMethod JA-WAX08.M
Instrument : Instrument #1
Sample Name: 7 14-22-d-old male C.oc. abd. /5ulCH2Cl2
Misc Info : larvae reared on aphids; different appearance
Vial Number: 1

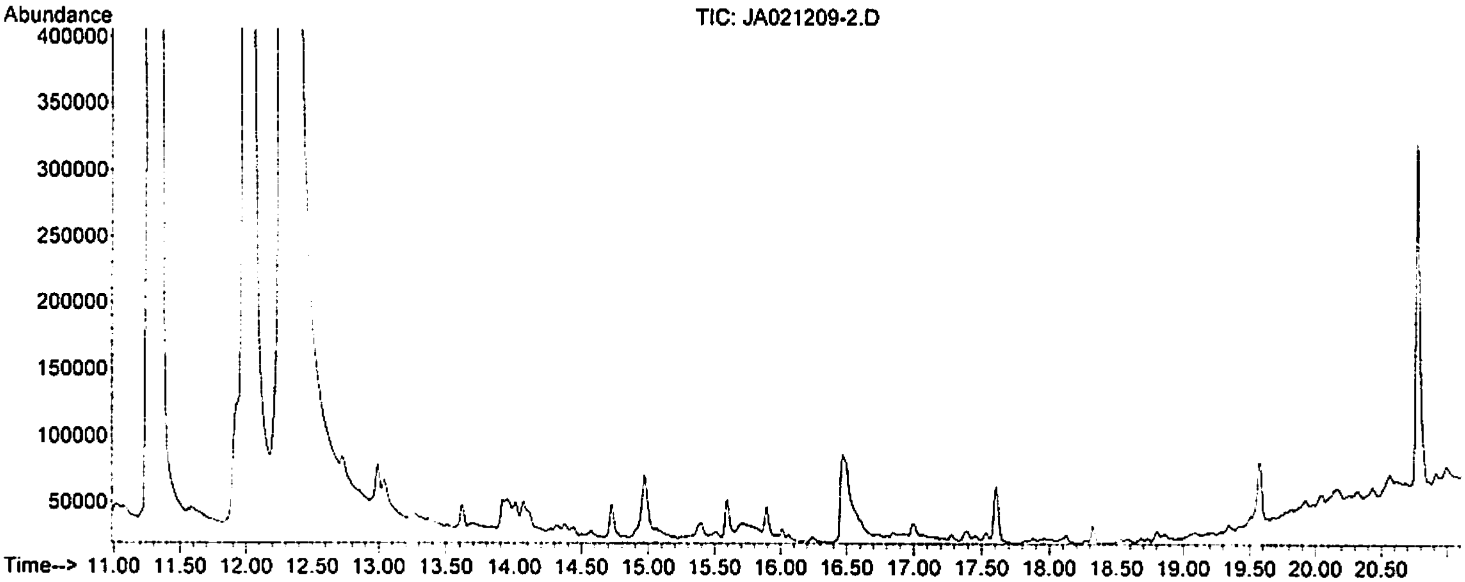


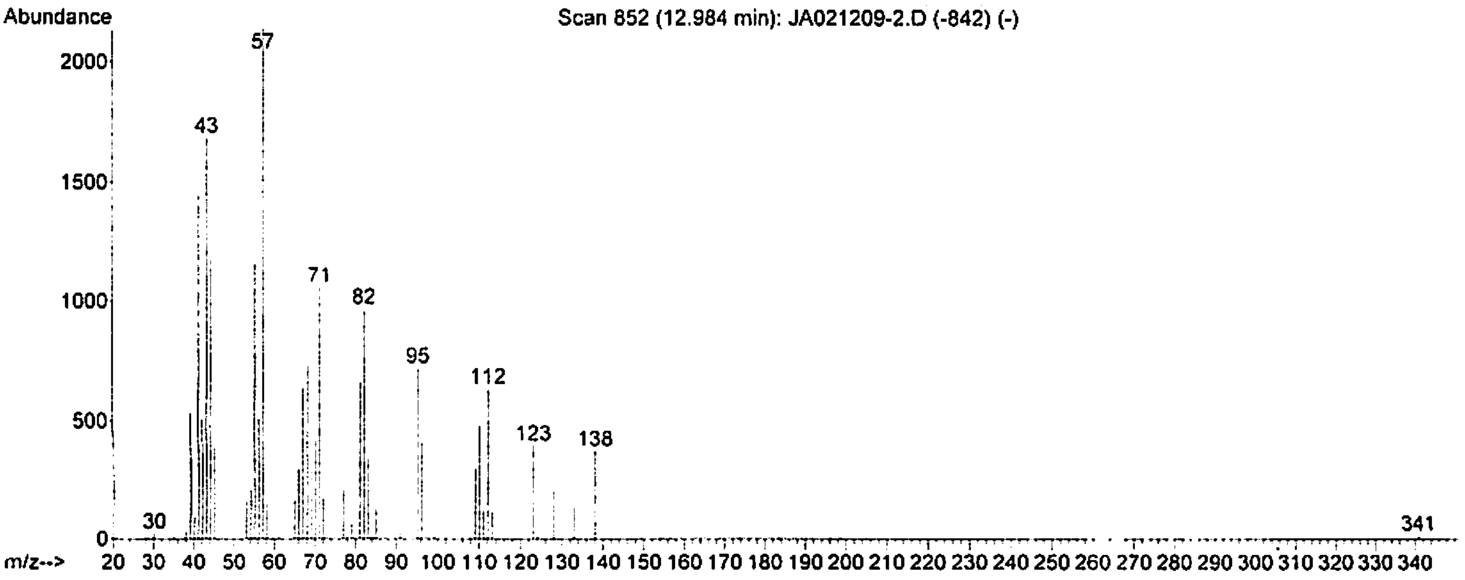

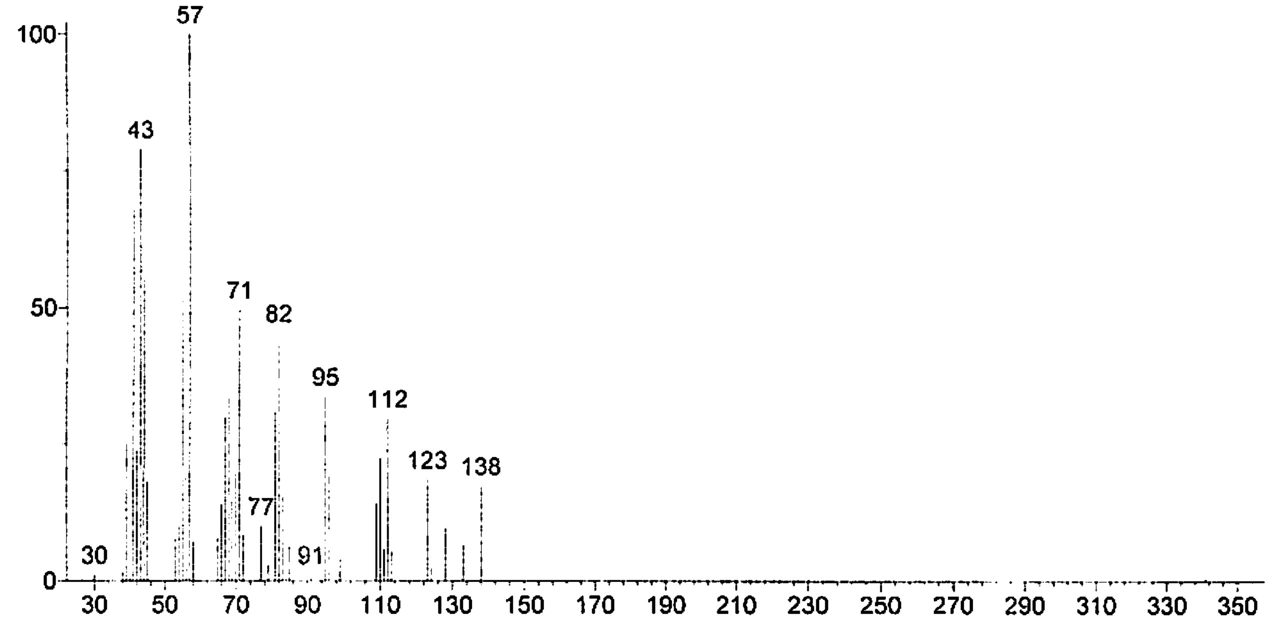


(Text File) Scan 852 (12.984 min): JA021209-2.D (-842)

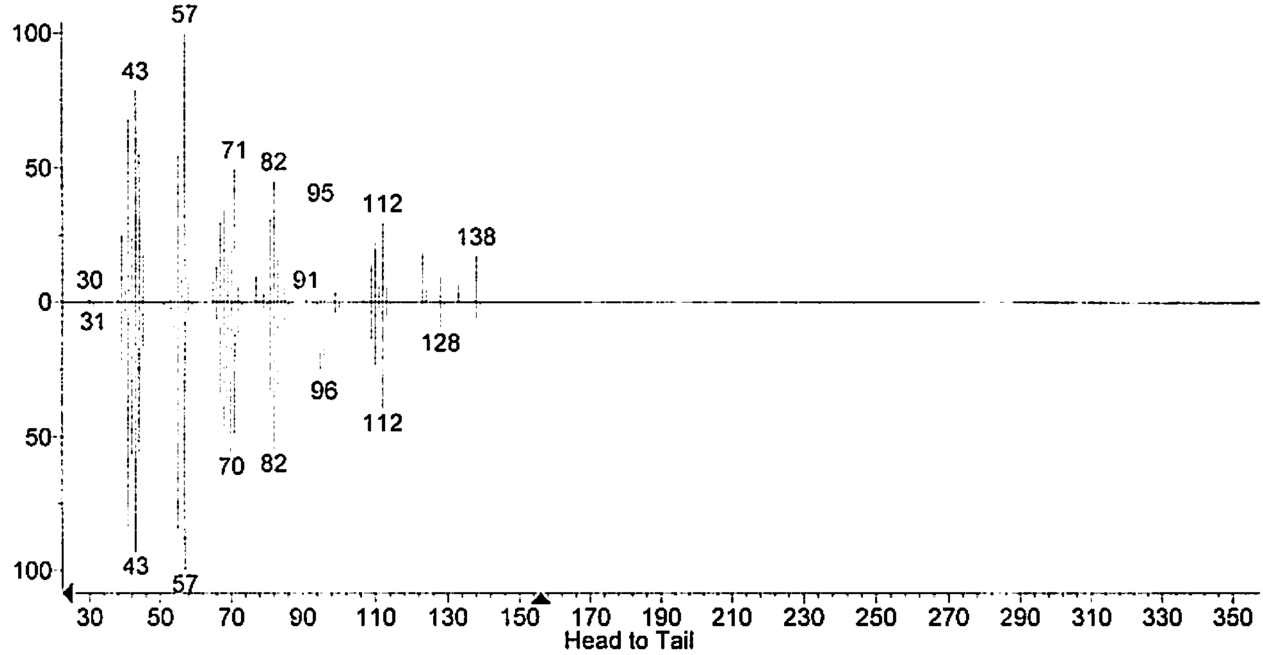


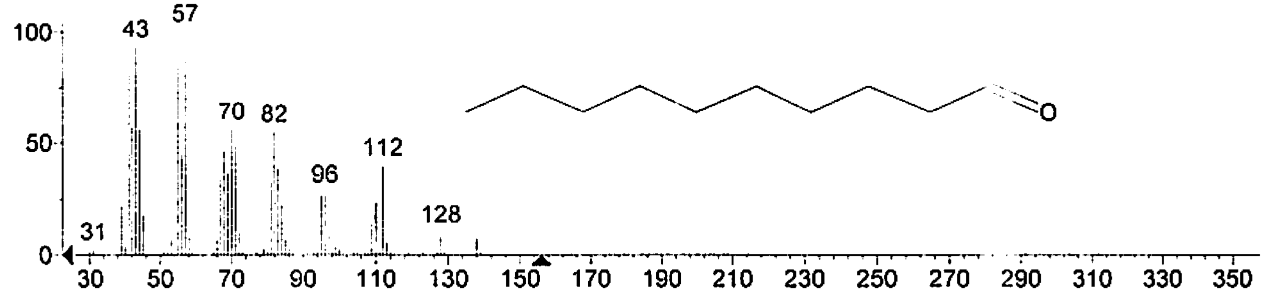


(replib) Decanal

```
File :        :D:\DATA\Aldrich\JA-09\JA021209-2.D
Operator    : Aldrich
Acquired    : 12 Feb 2009  15:15      using AcqMethod JA-WAX08.M
Instrument :     Instrument #1
Sample Name: 7 14-22-d-old male C.oc. abd. /5ulCH2Cl2
Misc Info   : larvae reared on aphids; different appearance
Vial Number: 1
```

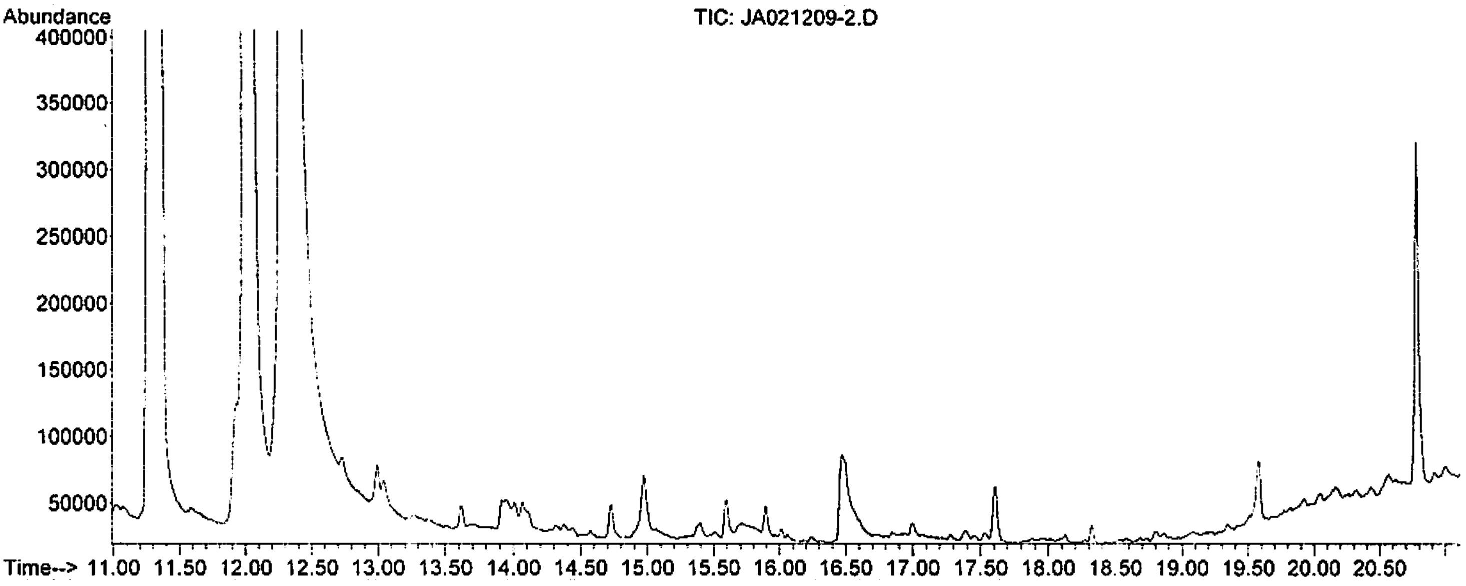


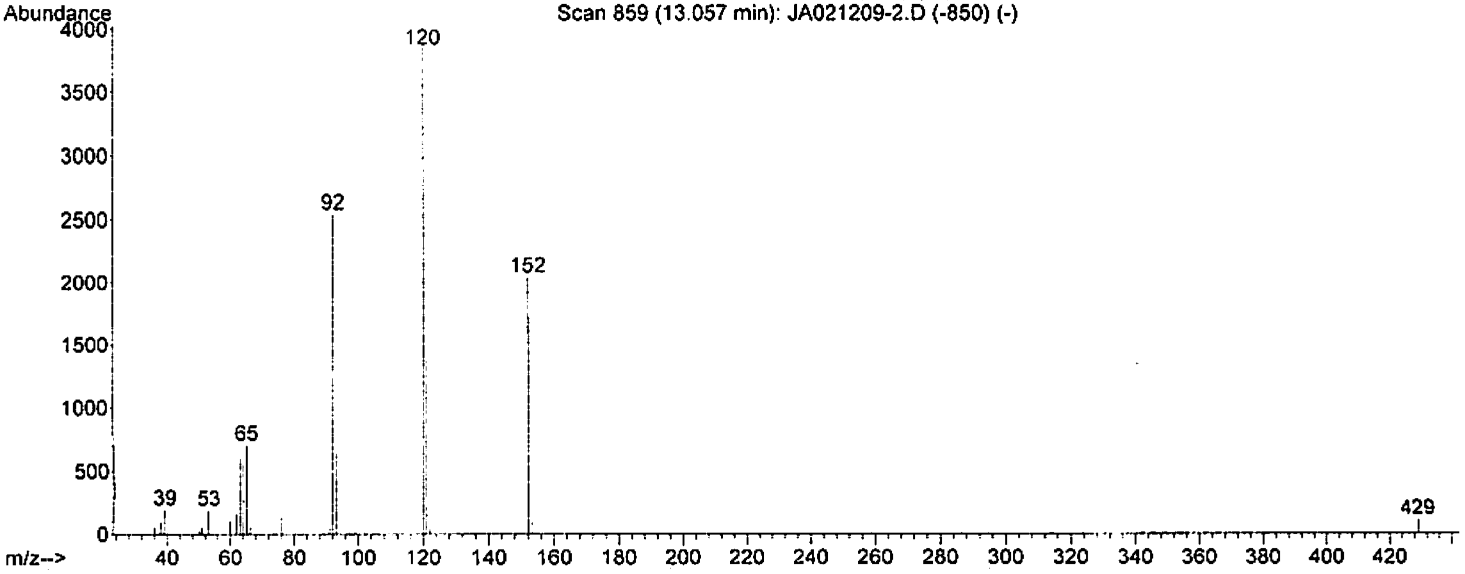

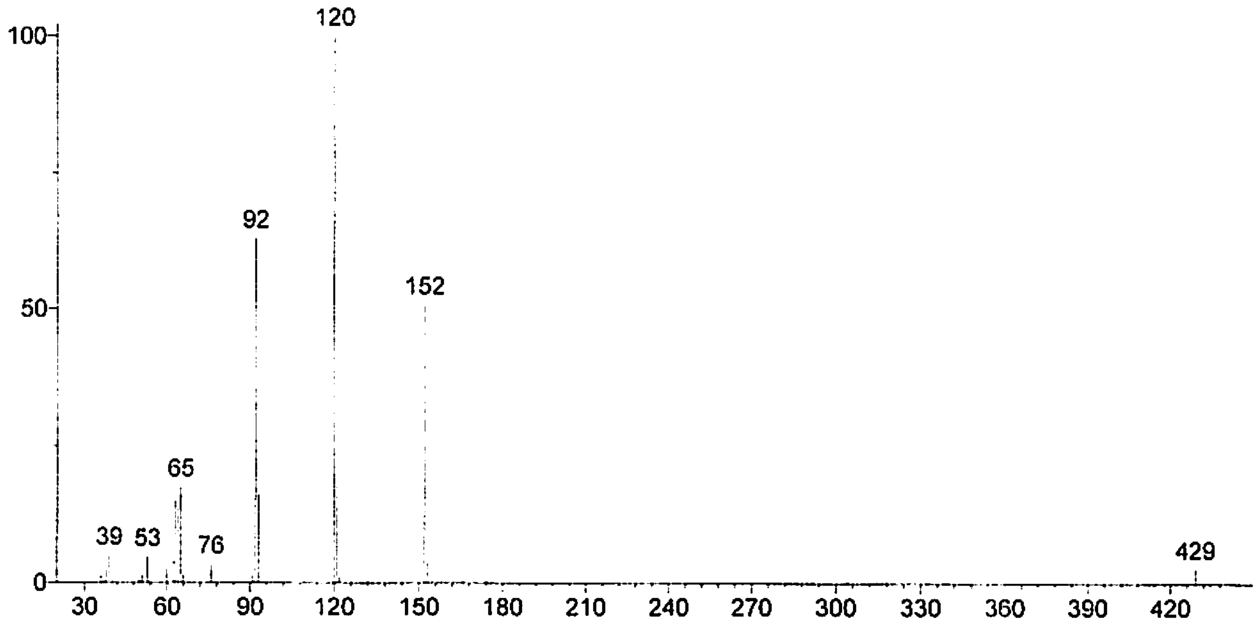


(Text File) Scan 859 (13.057 min): JA021209-2.D (-850)

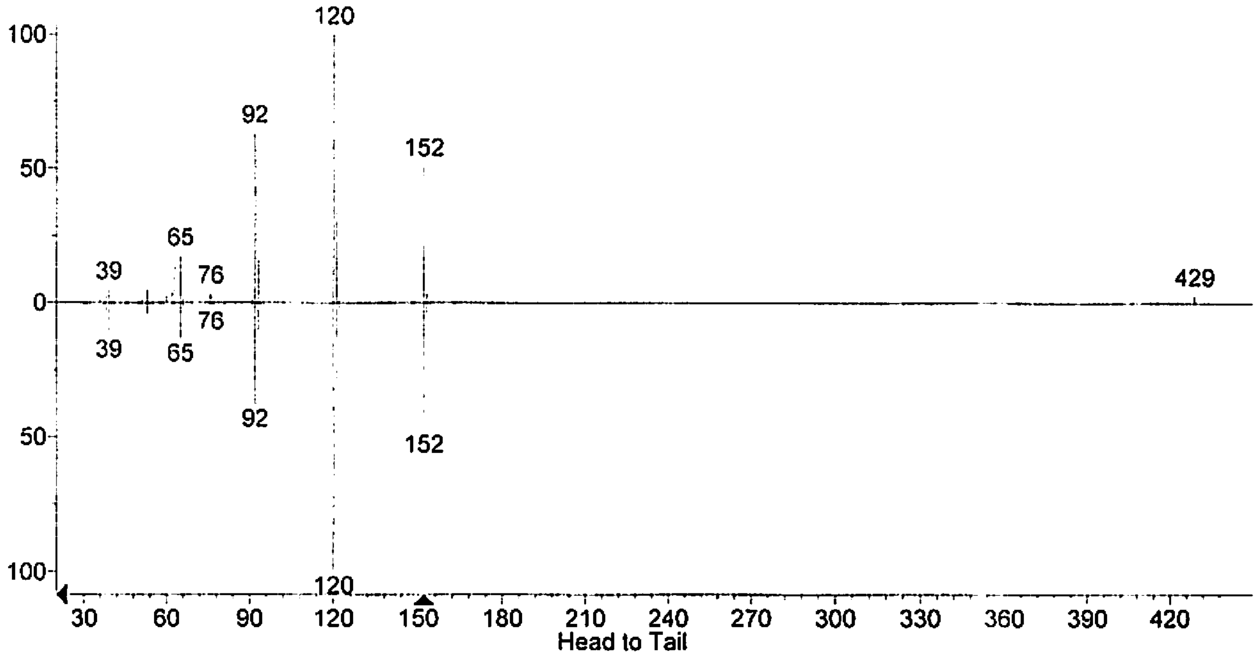


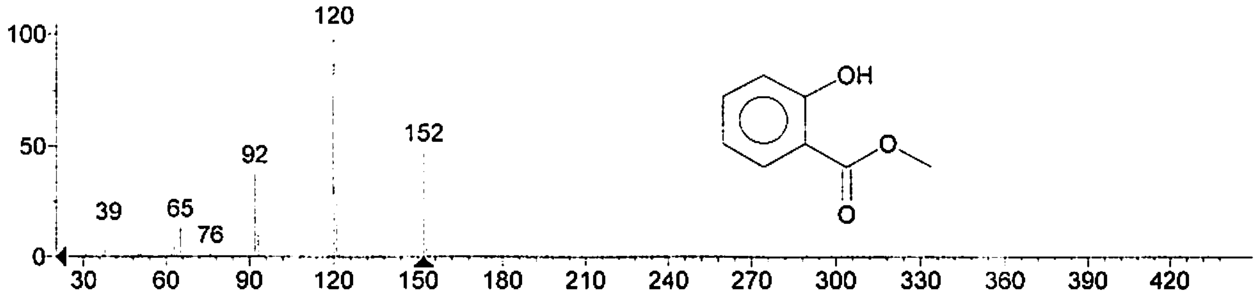


(replib) Methyl Salicylate

```
File :        :D:\DATA\Aldrich\JA-09\JA021209-2.D
Operator    : Aldrich
Acquired    : 12 Feb 2009  15:15     using AcqMethod JA-WAX08.M
Instrument :     Instrument #1
Sample Name: 7 14-22-d-old male C.oc. abd. /5ulCH2Cl2
Misc Info  : larvae reared on aphids; different appearance
Vial Number: 1
```

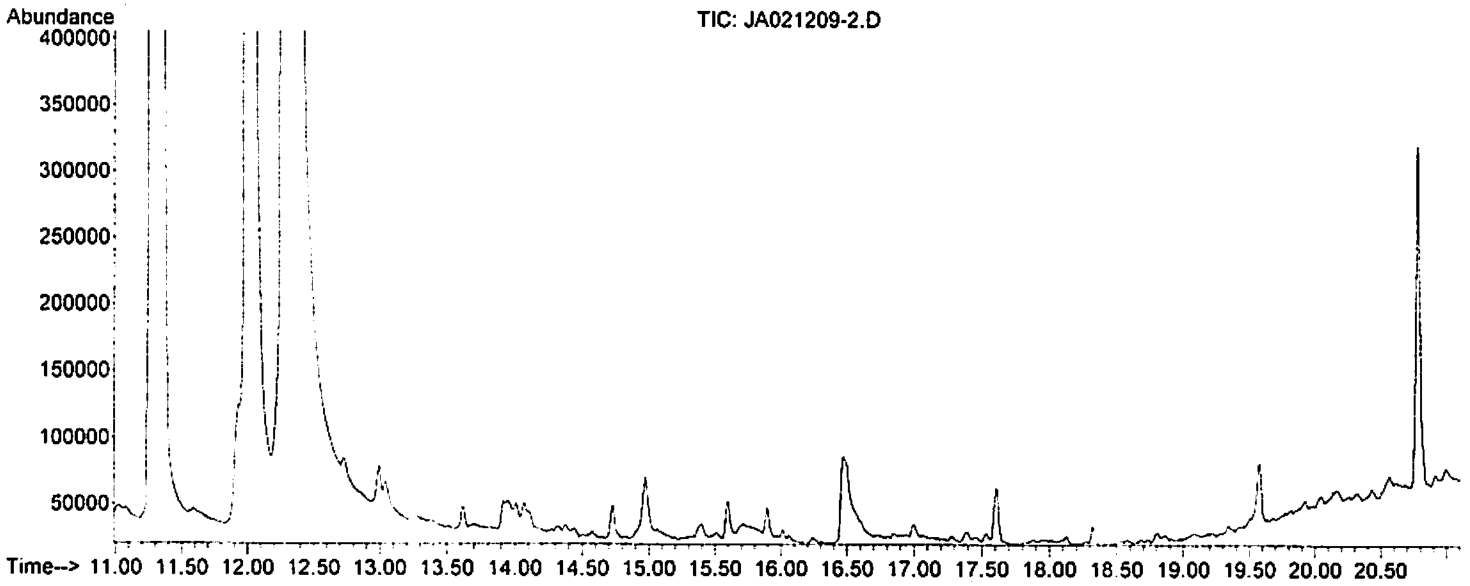


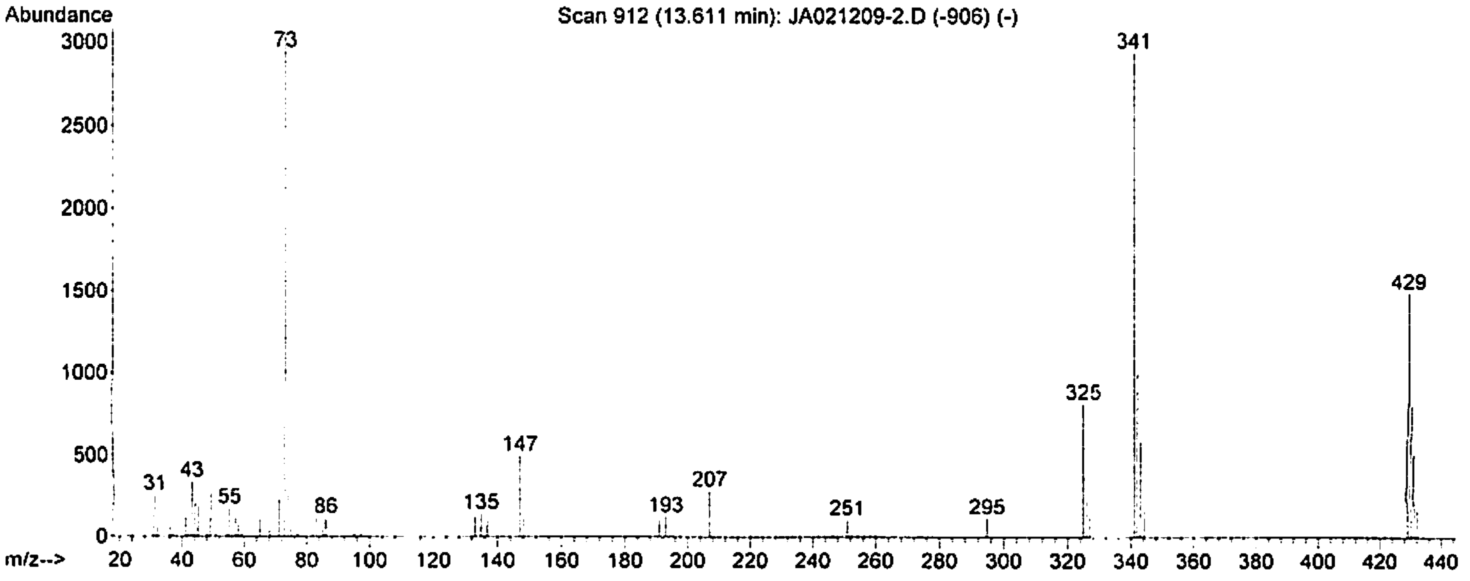

File , :D:\DATA\Aldrich\JA-09\JA021209-2.D
Operator : Aldrich
Acquired : 12 Feb 2009 15:15 using AcqMethod JA-WAX08.M
Instrument : Instrument #1
Sample Name: 7 14-22-d-old male C.oc. abd. /5ulCH2Cl2
Misc Info : larvae reared on aphids; different appearance
Vial Number: 1

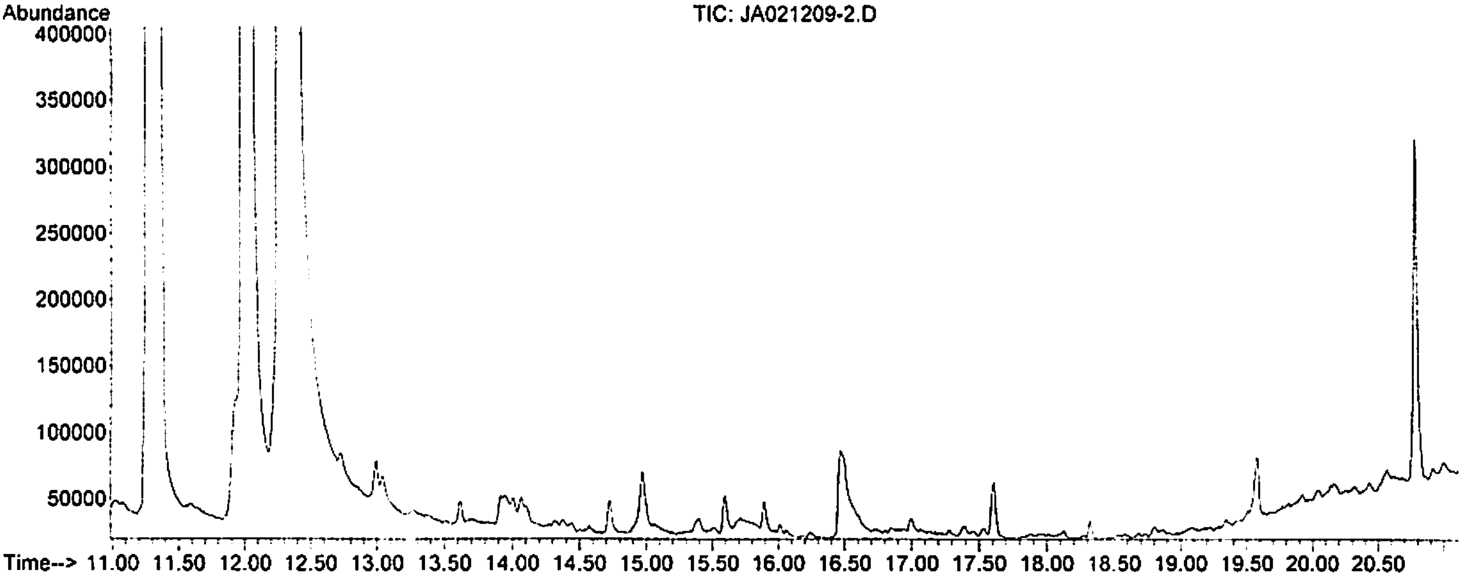


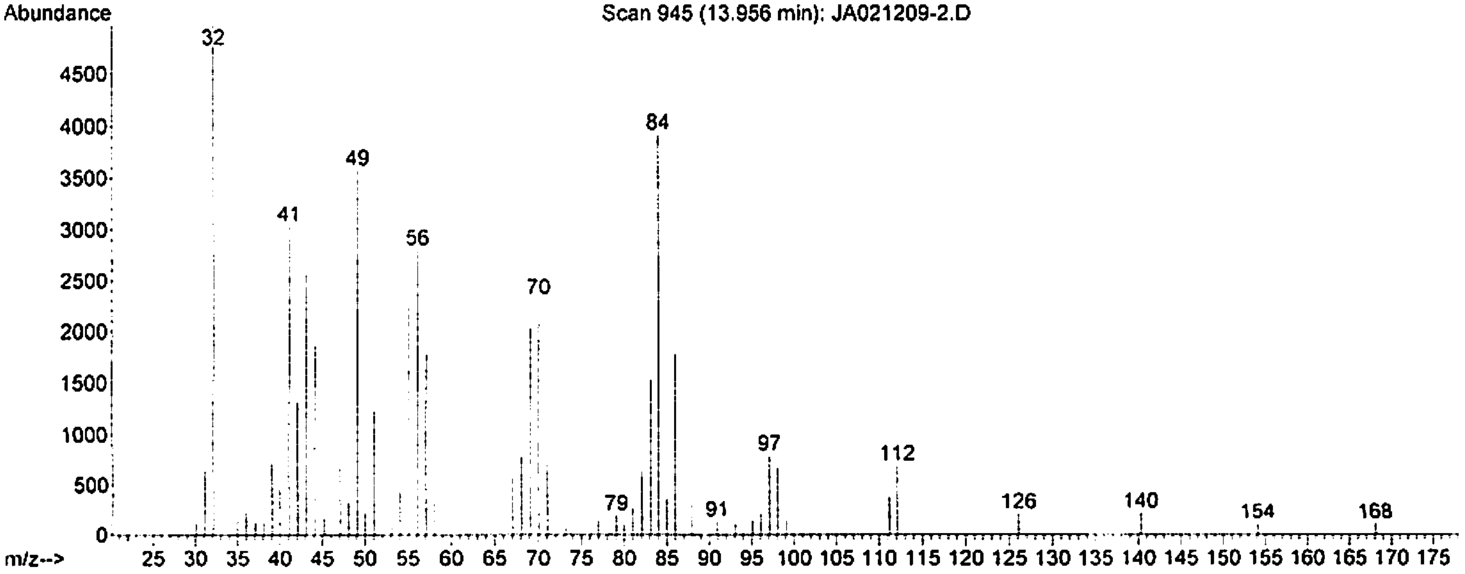

```
File          : D:\DATA\Aldrich\JA-09\JA021209-2.D
Operator      : Aldrich
Acquired      : 12 Feb 2009  15:15     using AcqMethod JA-WAX08.M
Instrument    :      Instrument #1
Sample Name: 7 14-22-d-old male C.oc. abd. /5ulCH2Cl2
Misc Info     : larvae reared on aphids; different appearance
Vial Number: 1
```

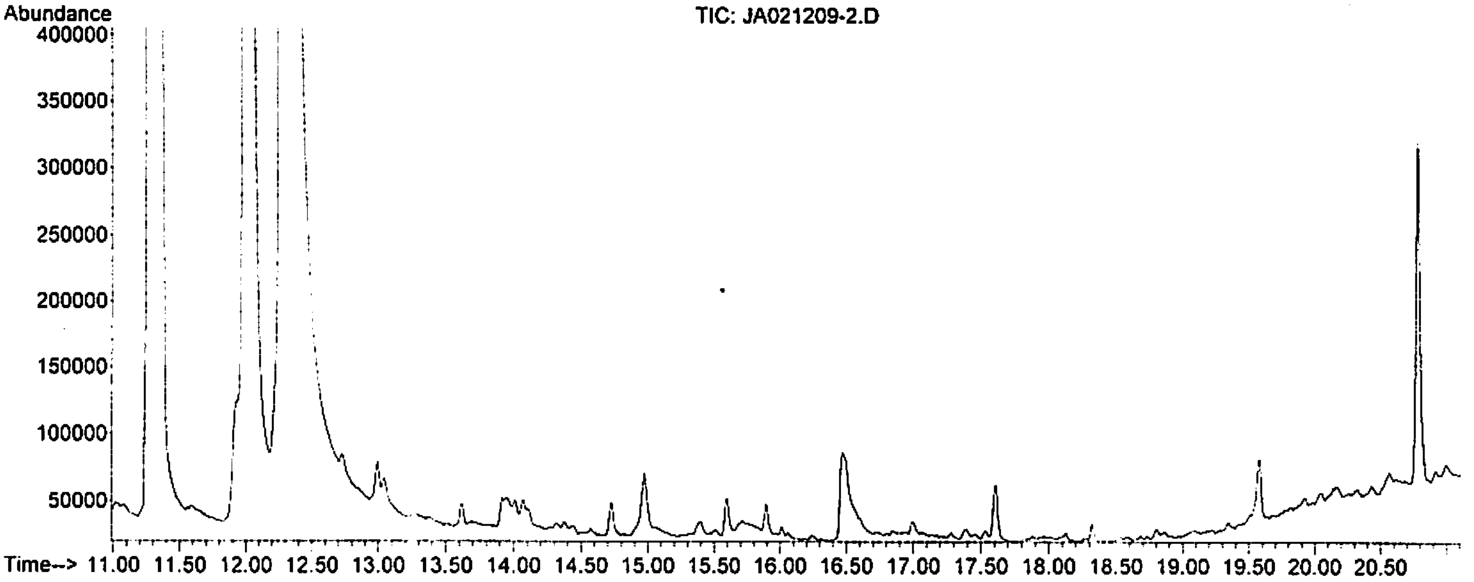


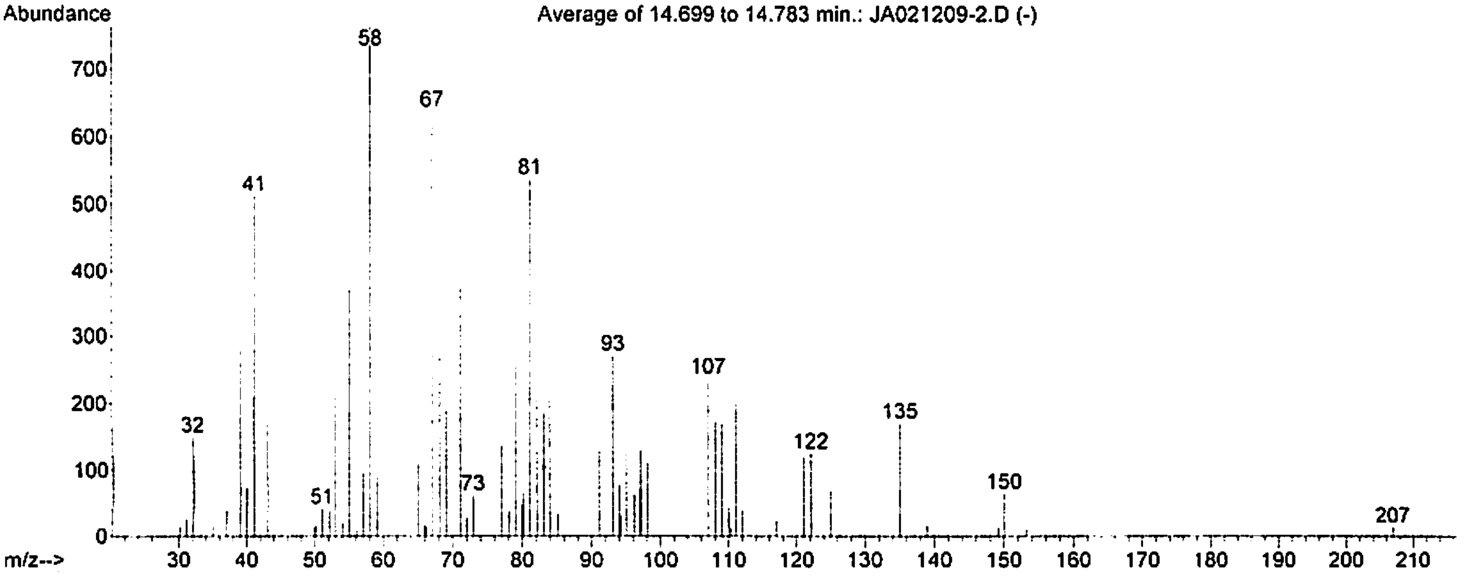

```
File        :D:\DATA\Aldrich\JA-09\JA021209-2.D
Operator    : Aldrich
Acquired    : 12 Feb 2009  15:15     using AcqMethod JA-WAX08.M
Instrument :     Instrument #1
Sample Name: 7 14-22-d-old male C.oc. abd. /5ulCH2Cl2
Misc Info   : larvae reared on aphids; different appearance
Vial Number: 1
```

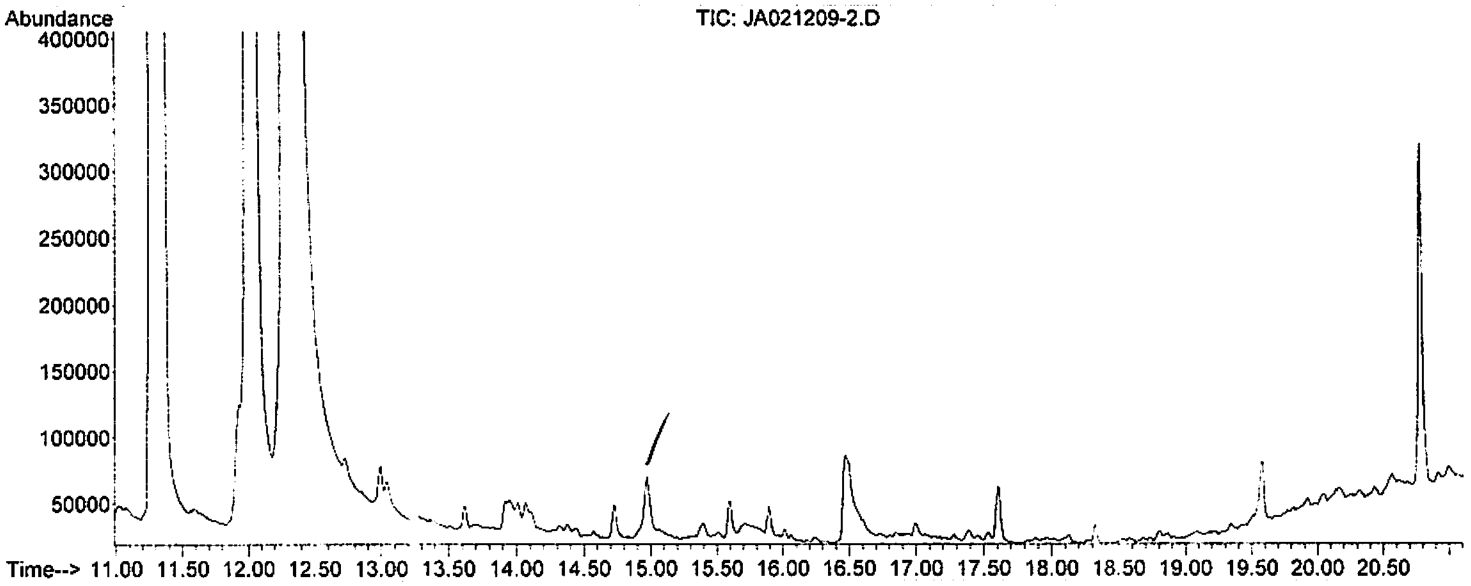


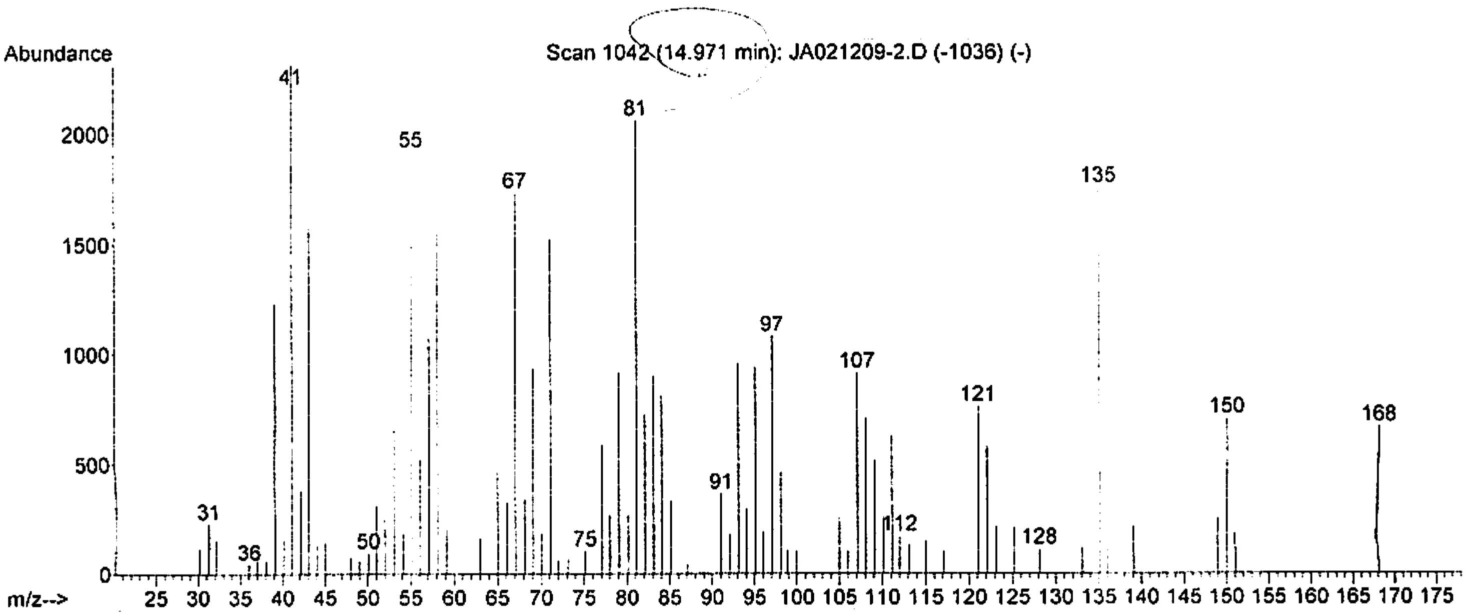

```
File          :D:\DATA\Aldrich\JA-09\JA021209-2.D
Operator      : Aldrich
Acquired      : 12 Feb 2009   15:15      using AcqMethod JA-WAX08.M
Instrument    :     Instrument #1
Sample Name: 7 14-22-d-old male C.oc. abd. /5ulCH2Cl2
Misc Info     : larvae reared on aphids; different appearance
Vial Number: 1
```

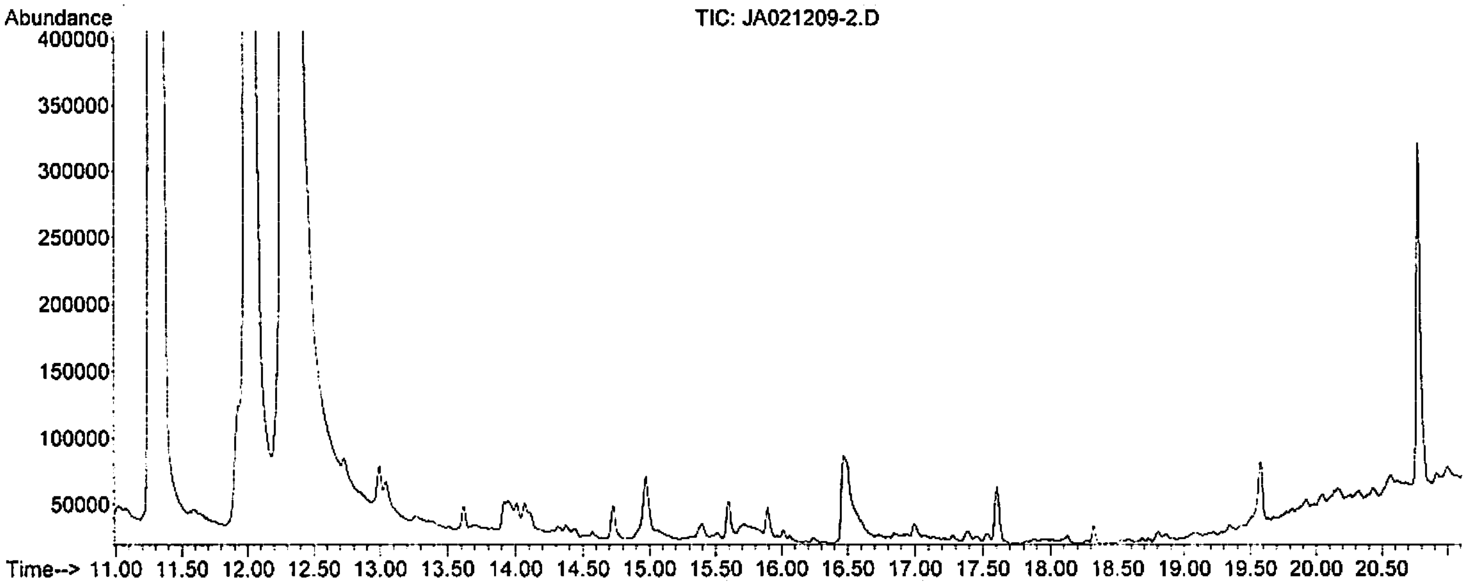


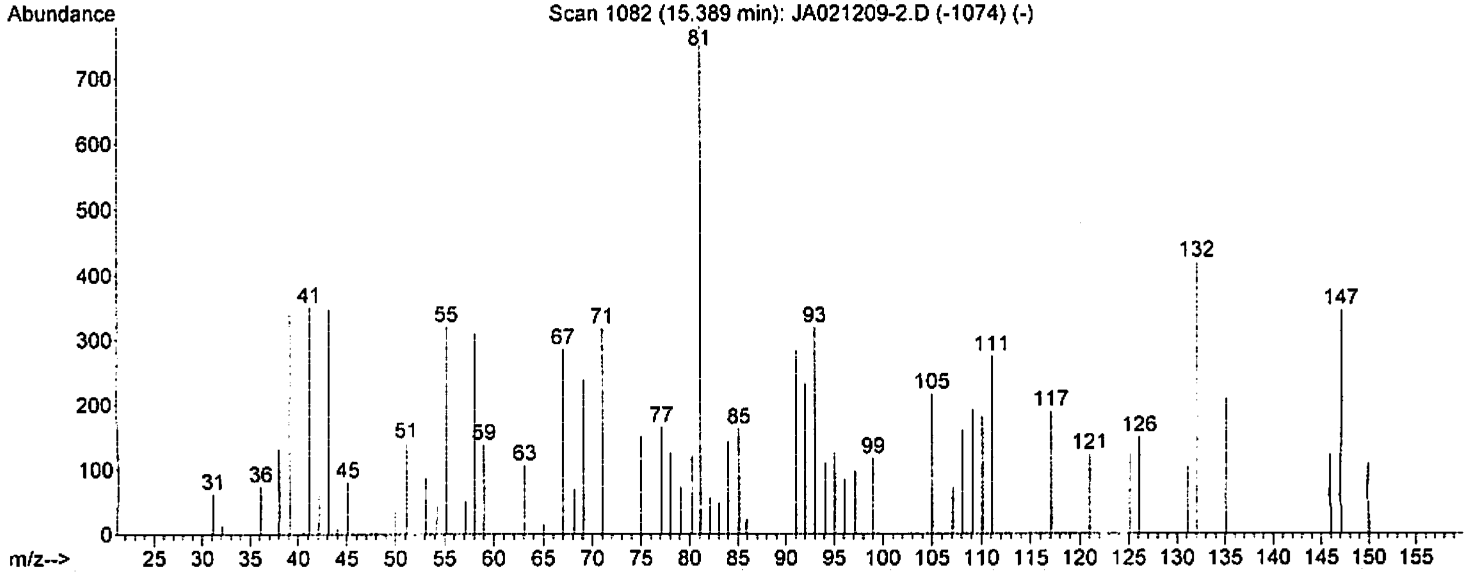

File : D:\DATA\Aldrich\JA-09\JA021209-2.D
Operator : Aldrich
Acquired : 12 Feb 2009 15:15 using AcqMethod JA-WAX08.M
Instrument : Instrument #1
Sample Name: 7 14-22-d-old male C.oc. abd. /5ulCH2Cl2
Misc Info : larvae reared on aphids; different appearance
Vial Number: 1

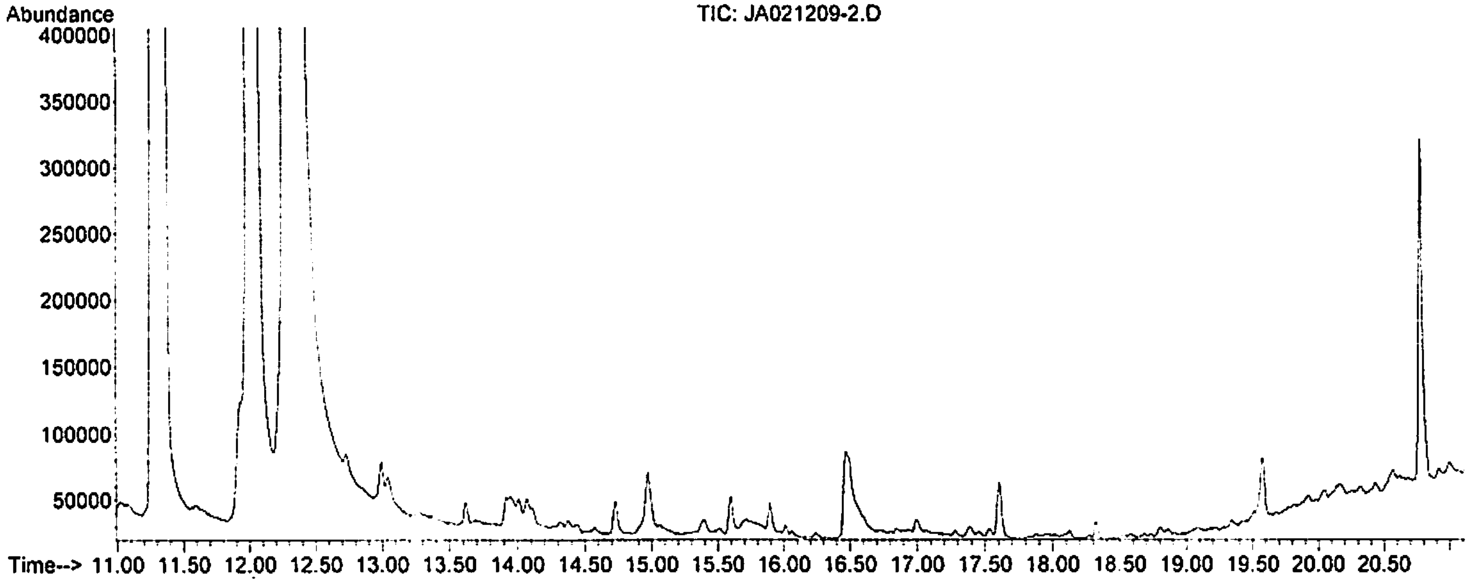


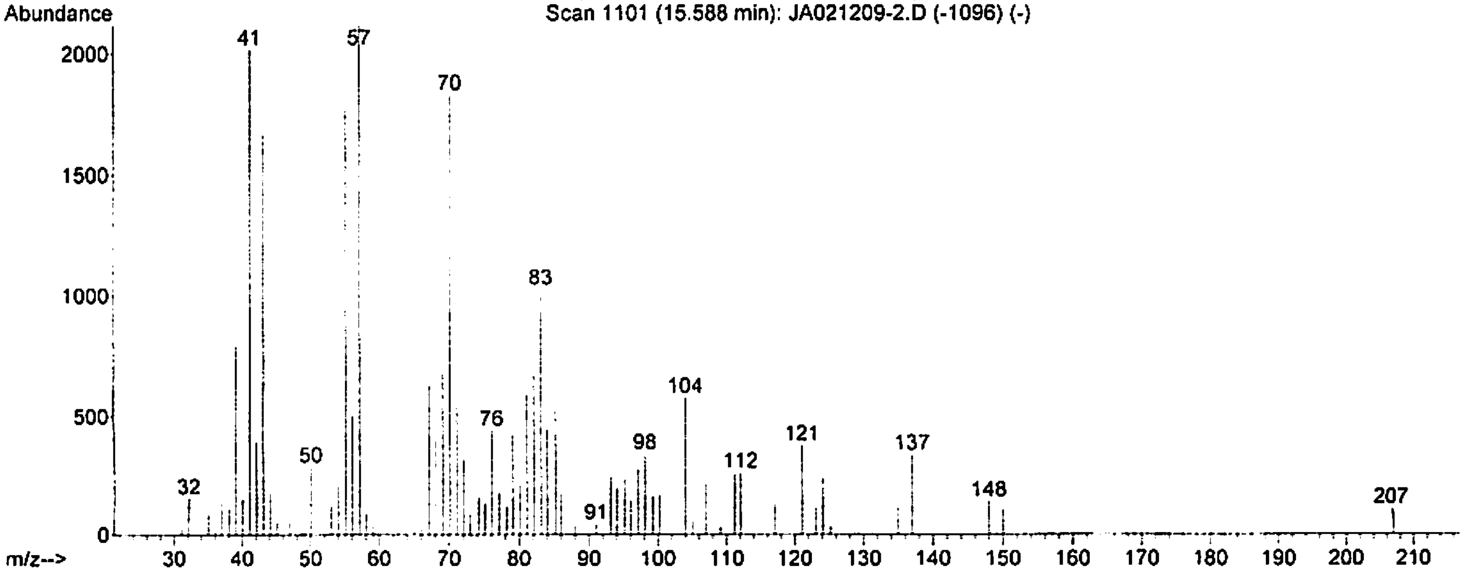

File : D:\DATA\Aldrich\JA-09\JA021209-2.D
Operator : Aldrich
Acquired : 12 Feb 2009 15:15 using AcqMethod JA-WAX08.M
Instrument : Instrument #1
Sample Name: 7 14-22-d-old male C.oc. abd. /5ulCH2Cl2
Misc Info : larvae reared on aphids; different appearance
Vial Number: 1

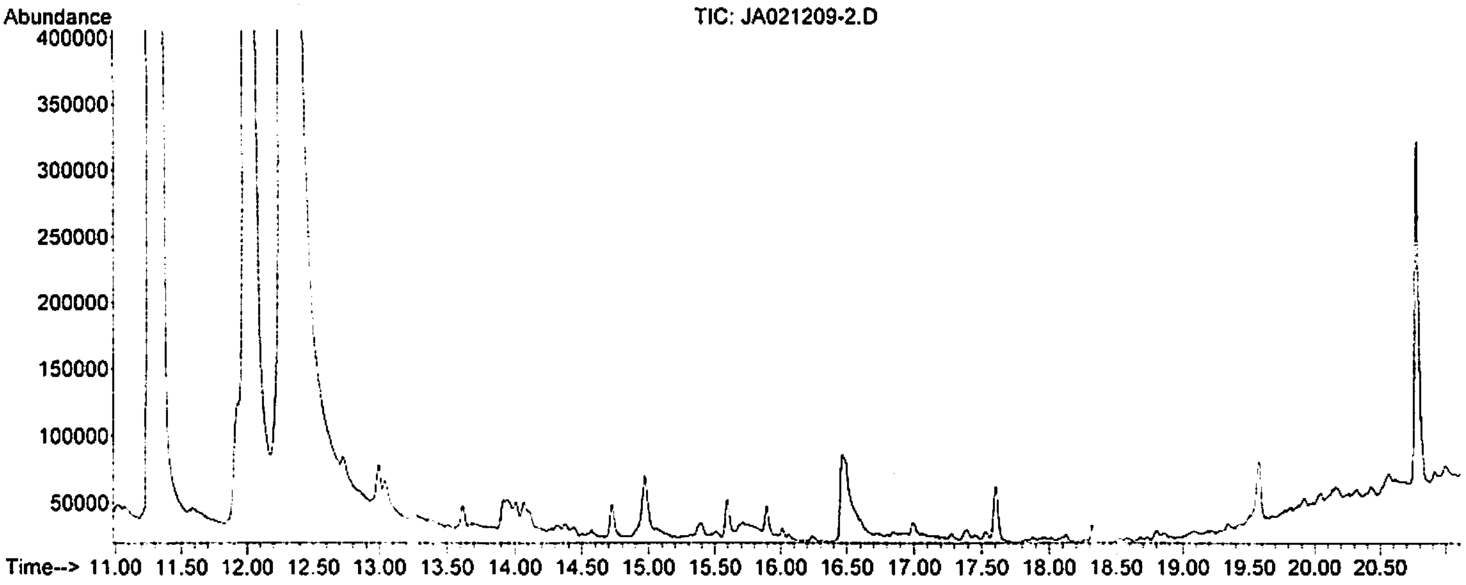


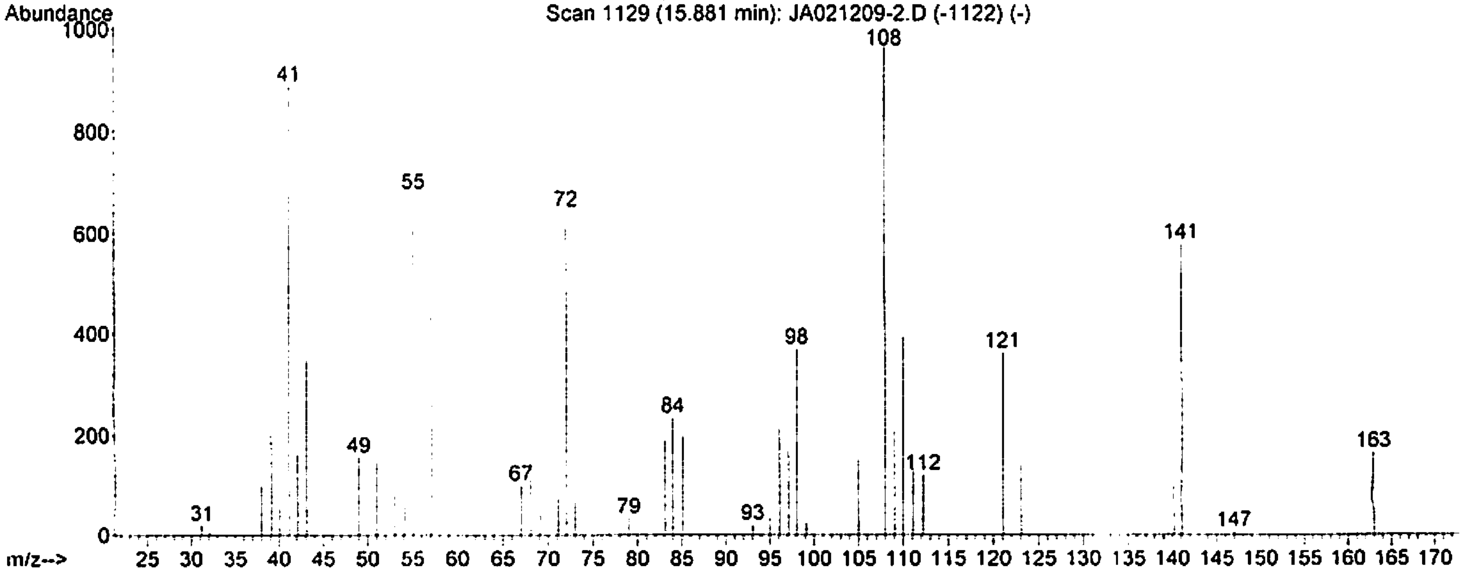

```
File        : D:\DATA\Aldrich\JA-09\JA021209-2.D
Operator    : Aldrich
Acquired    : 12 Feb 2009  15:15     using AcqMethod JA-WAX08.M
Instrument  :    Instrument #1
Sample Name: 7 14-22-d-old male C.oc. abd. /5ulCH2Cl2
Misc Info   : larvae reared on aphids; different appearance
Vial Number: 1
```

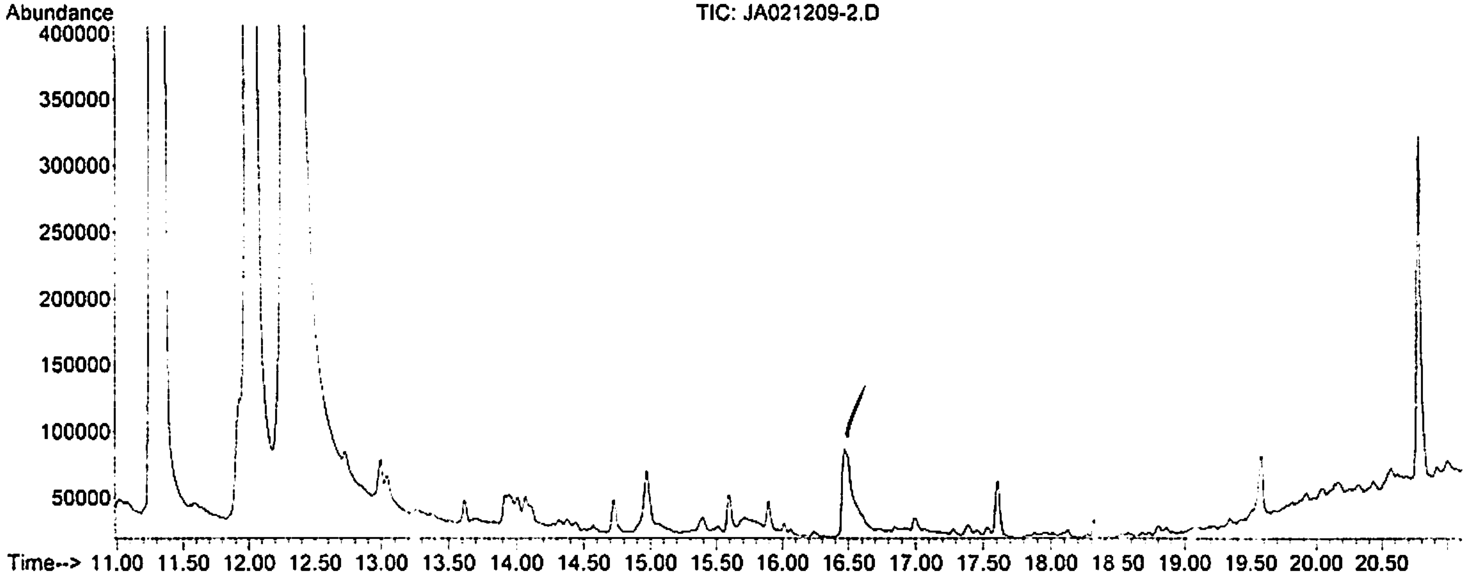


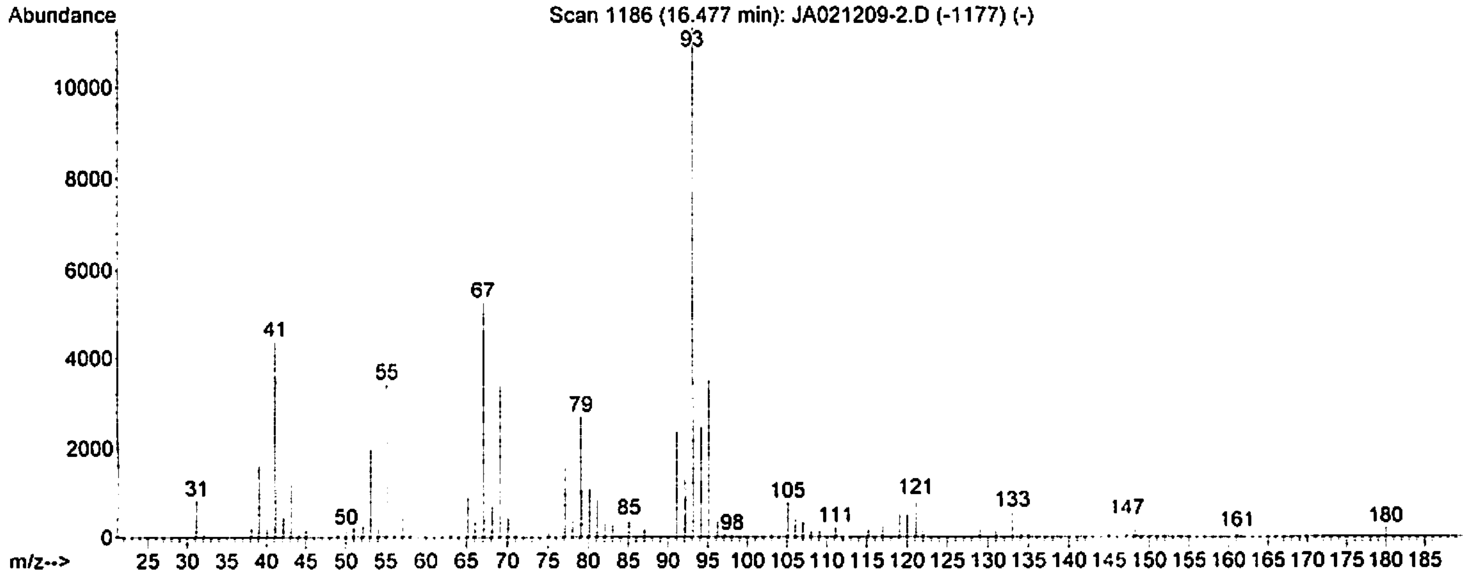

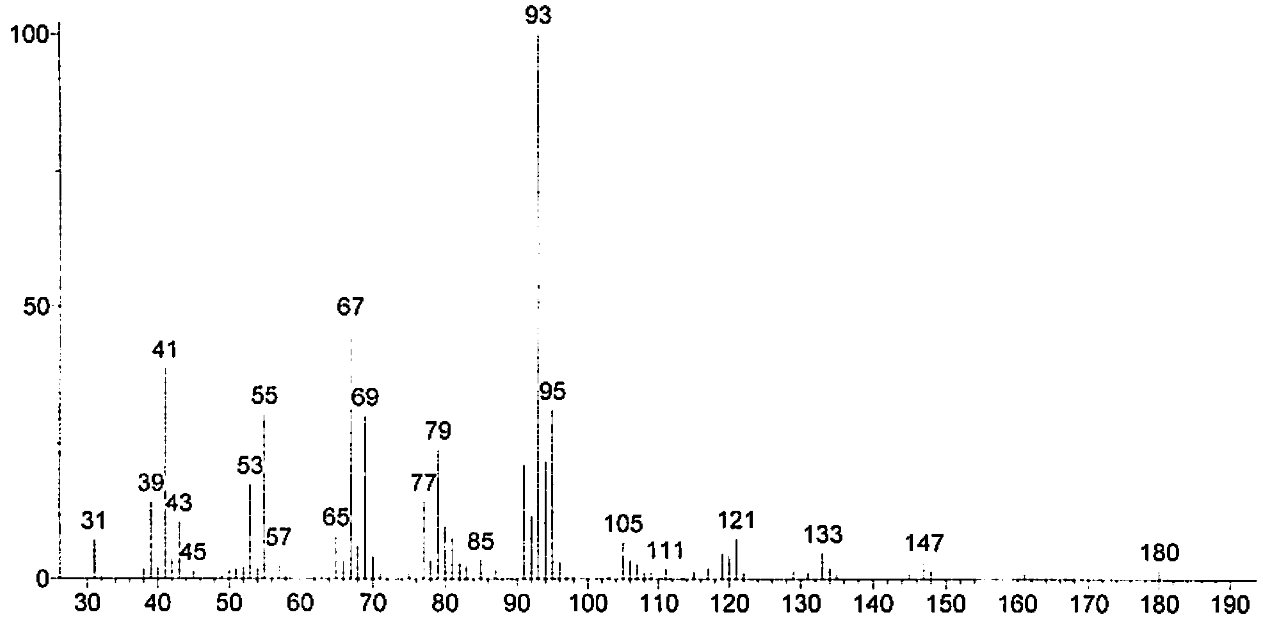


(Text File) Scan 1186 (16.477 min): JA021209-2.D (-1177)

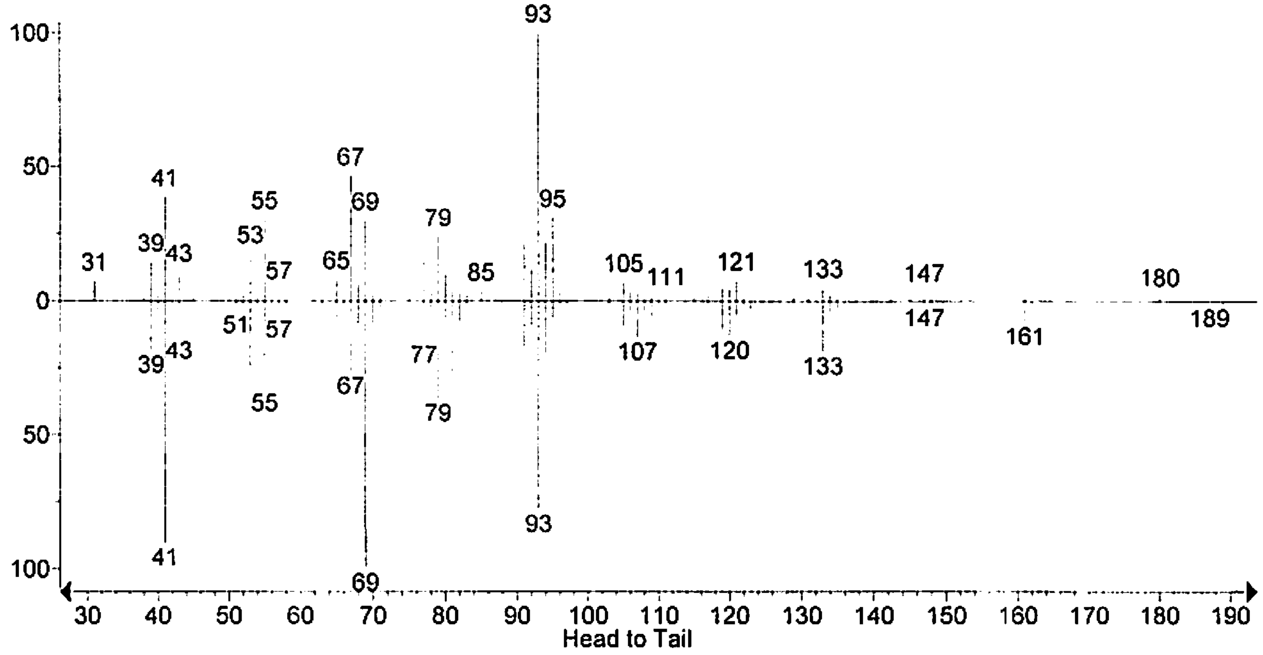


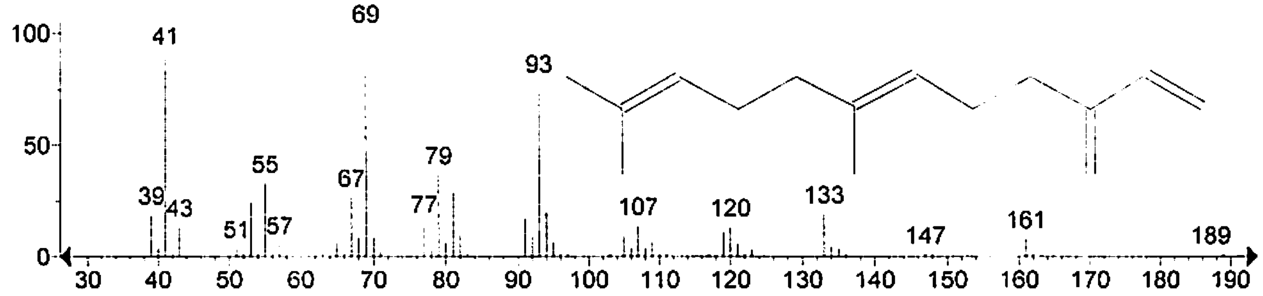


(replib) 1,6,10-Dodecatriene, 7,11-dimethyl-3-methylene-, (E)-

File : D:\DATA\Aldrich\JA-09\JA021209-2.D
Operator : Aldrich
Acquired : 12 Feb 2009 15:15 using AcqMethod JA-WAX08.M
Instrument : Instrument #1
Sample Name: 7 14-22-d-old male C.oc. abd. /5ulCH2Cl2
Misc Info : larvae reared on aphids; different appearance
Vial Number: 1

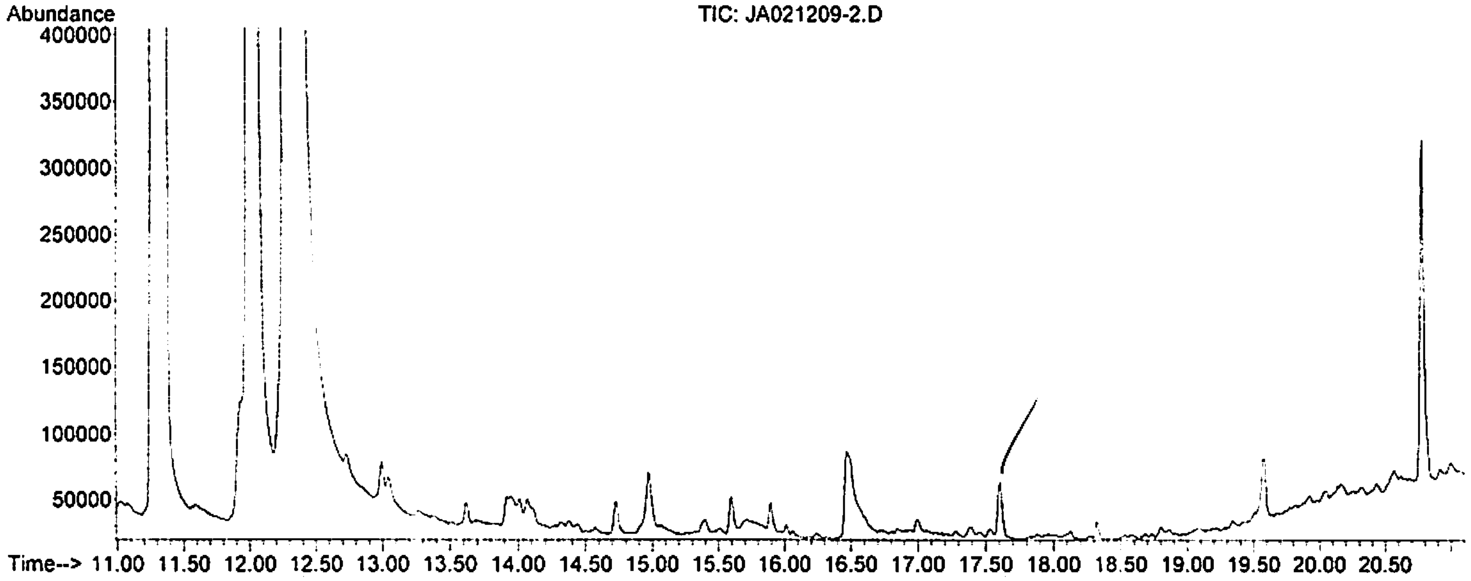


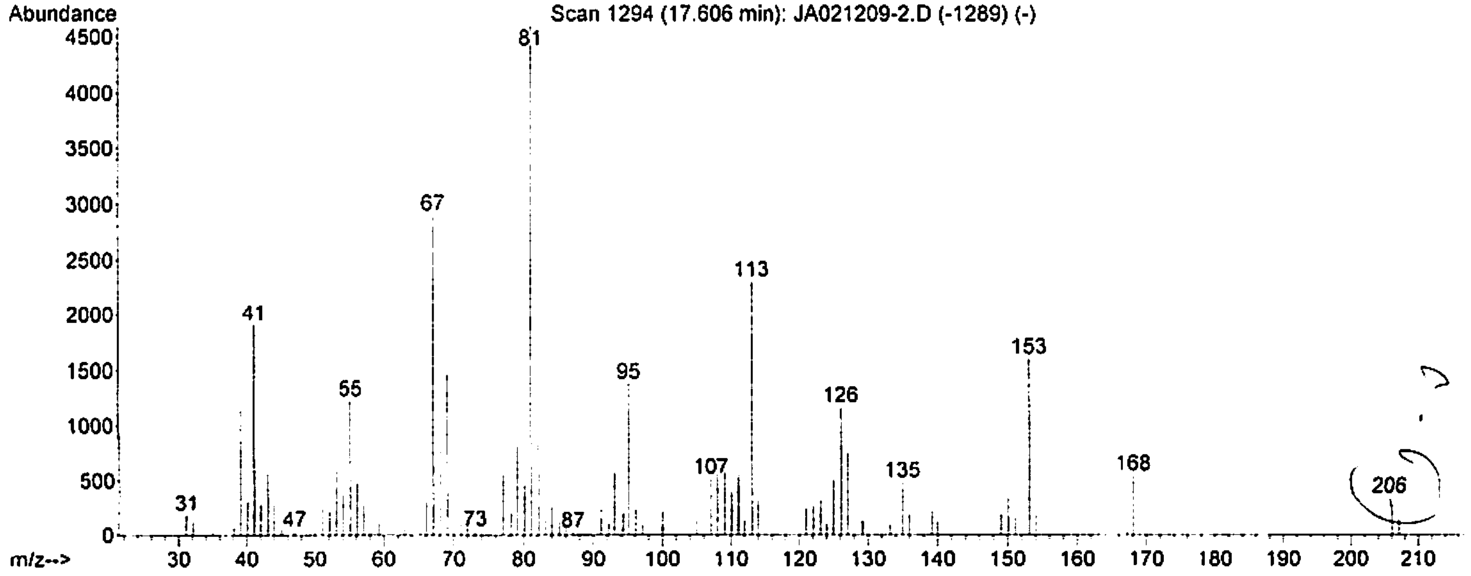

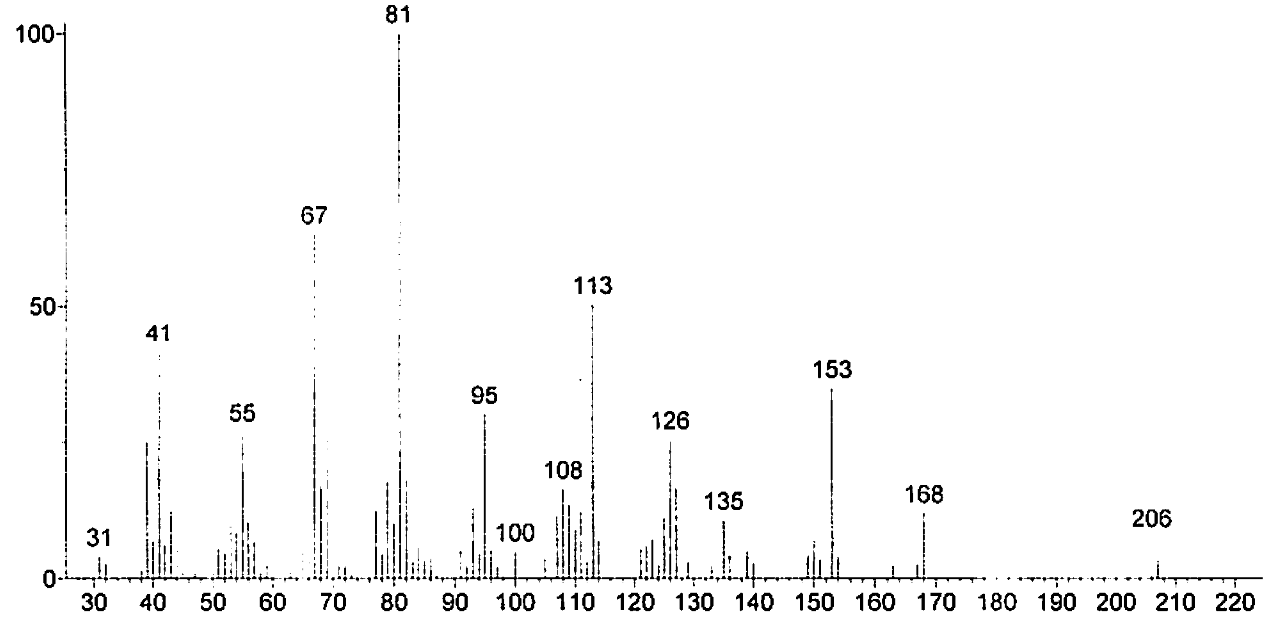


(Text File) Scan 1294 (17.606 min): JA021209-2.D (-1289)

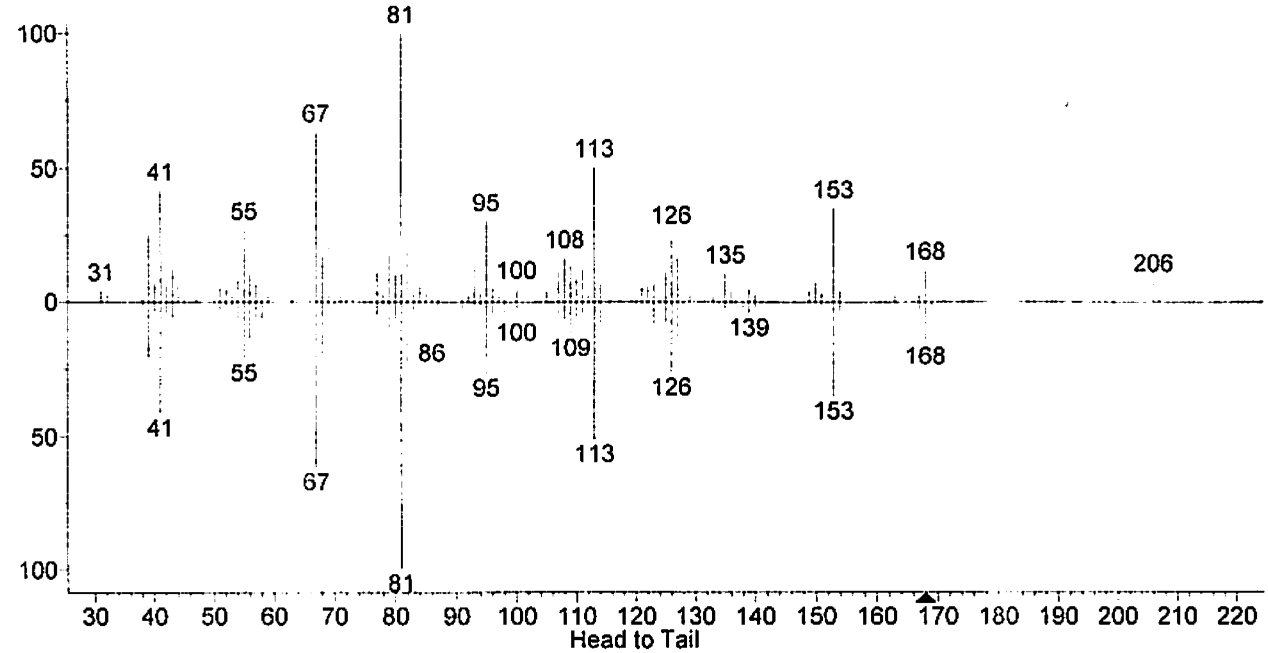


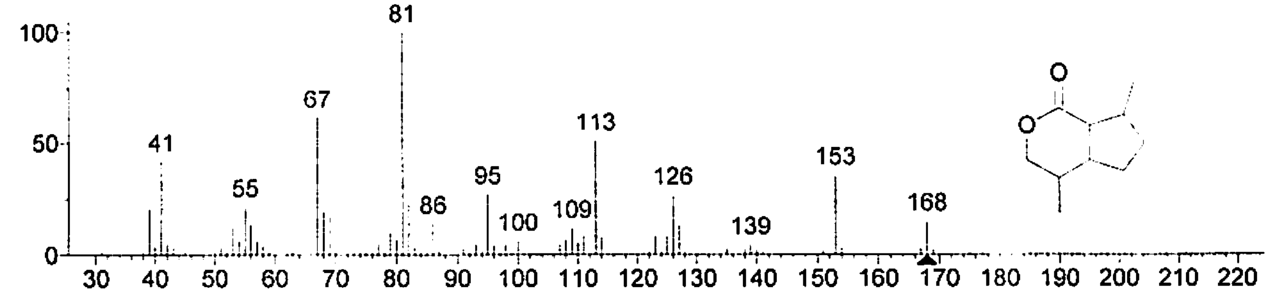


(mainlib) Cyclopenta[c]pyran-1(3H)-one, hexahydro-4,7-dimethyl-, (4.alpha.,4a.alpha.,7.alpha.,7a.alpha.)-

File : D:\DATA\Aldrich\JA-09\JA021209-2.D
Operator : Aldrich
Acquired : 12 Feb 2009 15:15 using AcqMethod JA-WAX08.M
Instrument : Instrument #1
Sample Name: 7 14-22-d-old male C.oc. abd. /5ulCH2Cl2
Misc Info : larvae reared on aphids; different appearance
Vial Number: 1

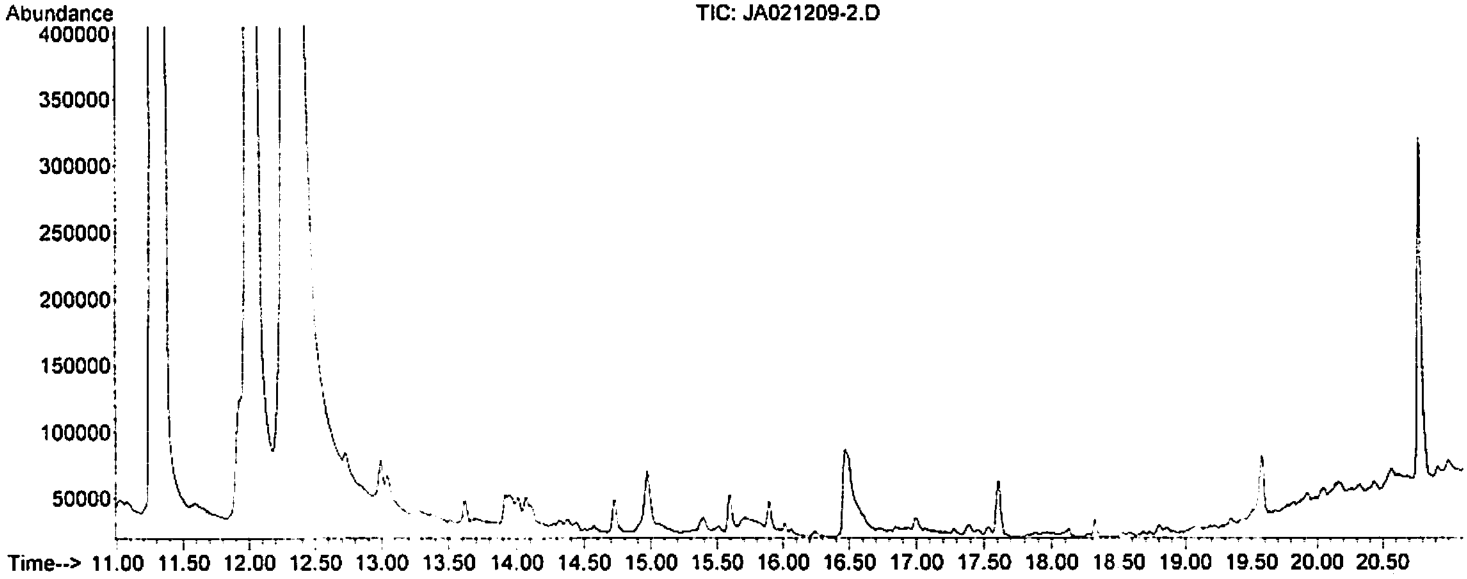


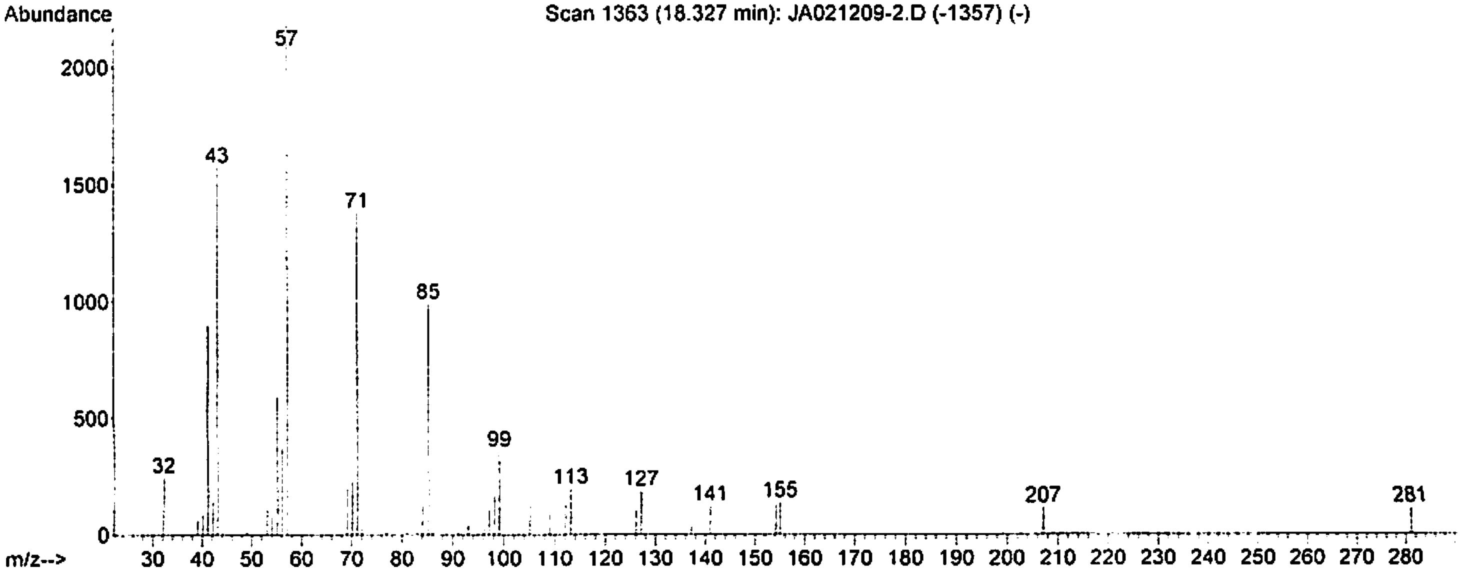

```
File          :D:\DATA\Aldrich\JA-09\JA021209-2.D
Operator      : Aldrich
Acquired      : 12 Feb 2009   15:15     using AcqMethod JA-WAX08.M
Instrument :      Instrument #1
Sample Name: 7 14-22-d-old male C.oc. abd. /5ulCH2Cl2
Misc Info    : larvae reared on aphids; different appearance
Vial Number: 1
```

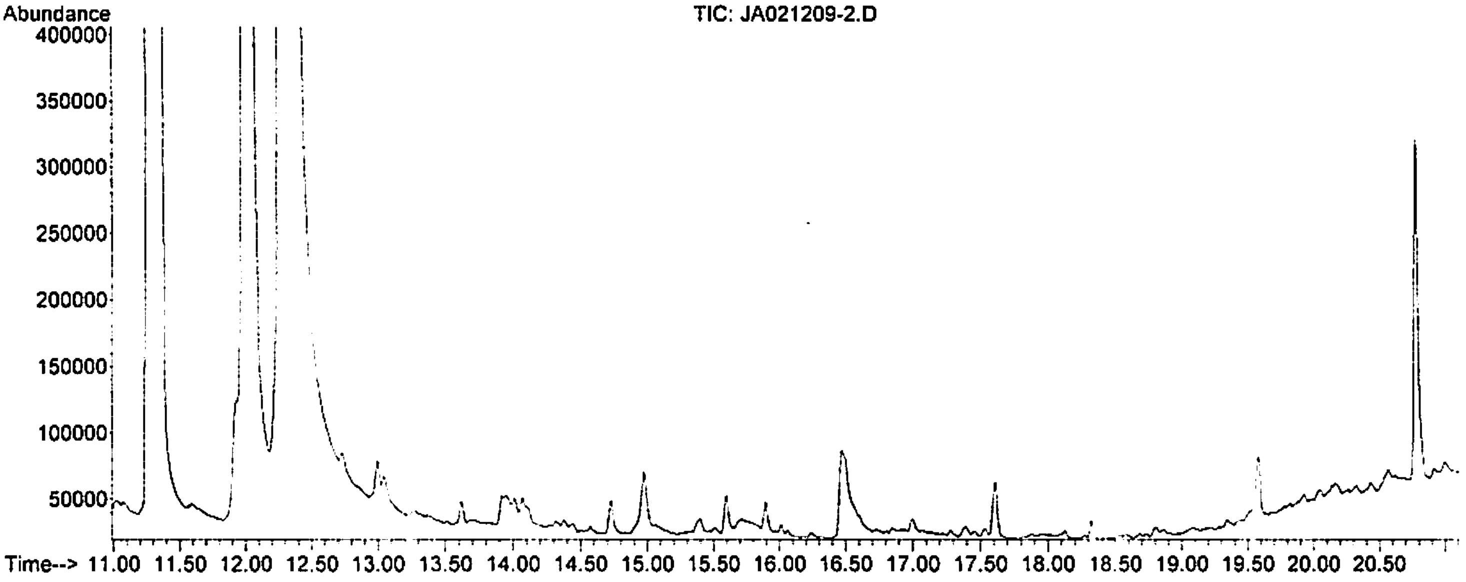


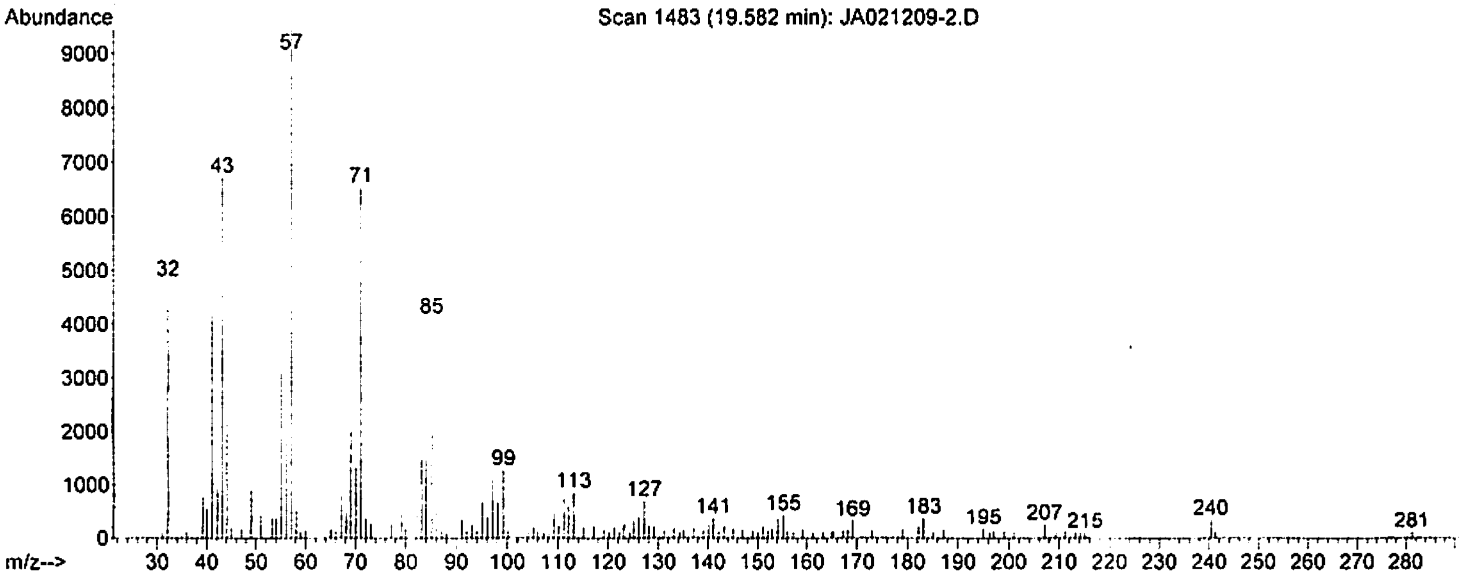

File : D:\DATA\Aldrich\JA-09\JA021209-2.D
Operator : Aldrich
Acquired : 12 Feb 2009 15:15 using AcqMethod JA-WAX08.M
Instrument : Instrument #1
Sample Name: 7 14-22-d-old male C.oc. abd. /5ulCH2Cl2
Misc Info : larvae reared on aphids; different appearance
Vial Number: 1

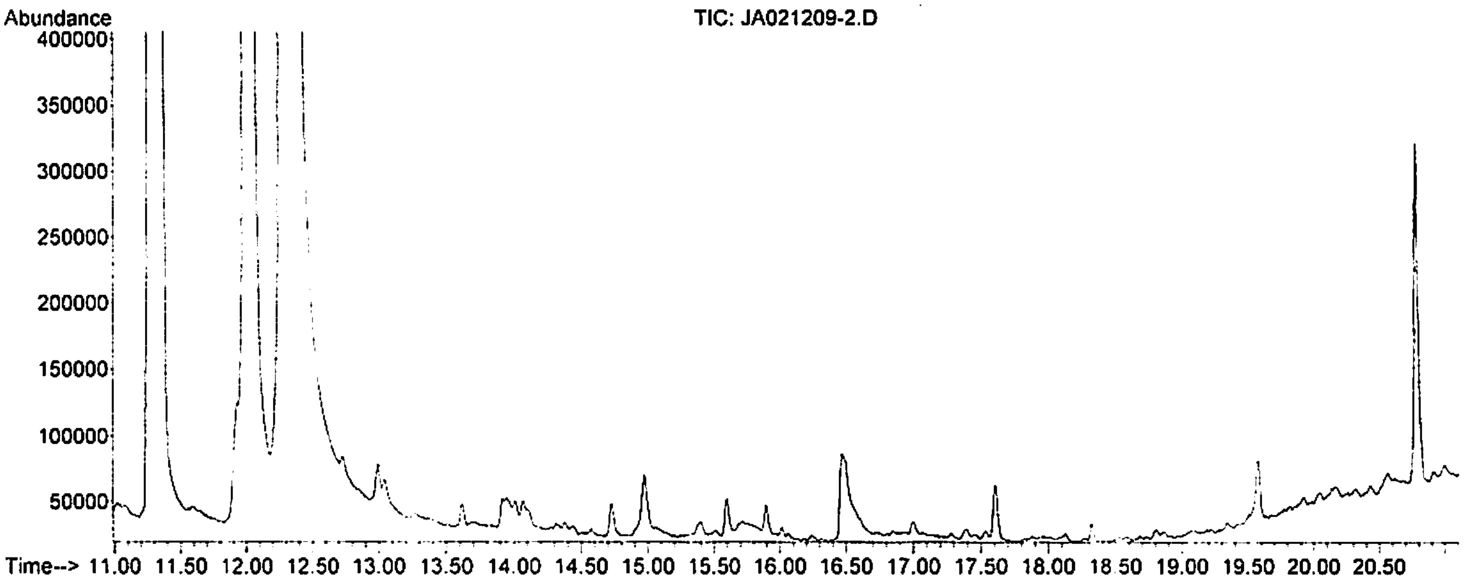


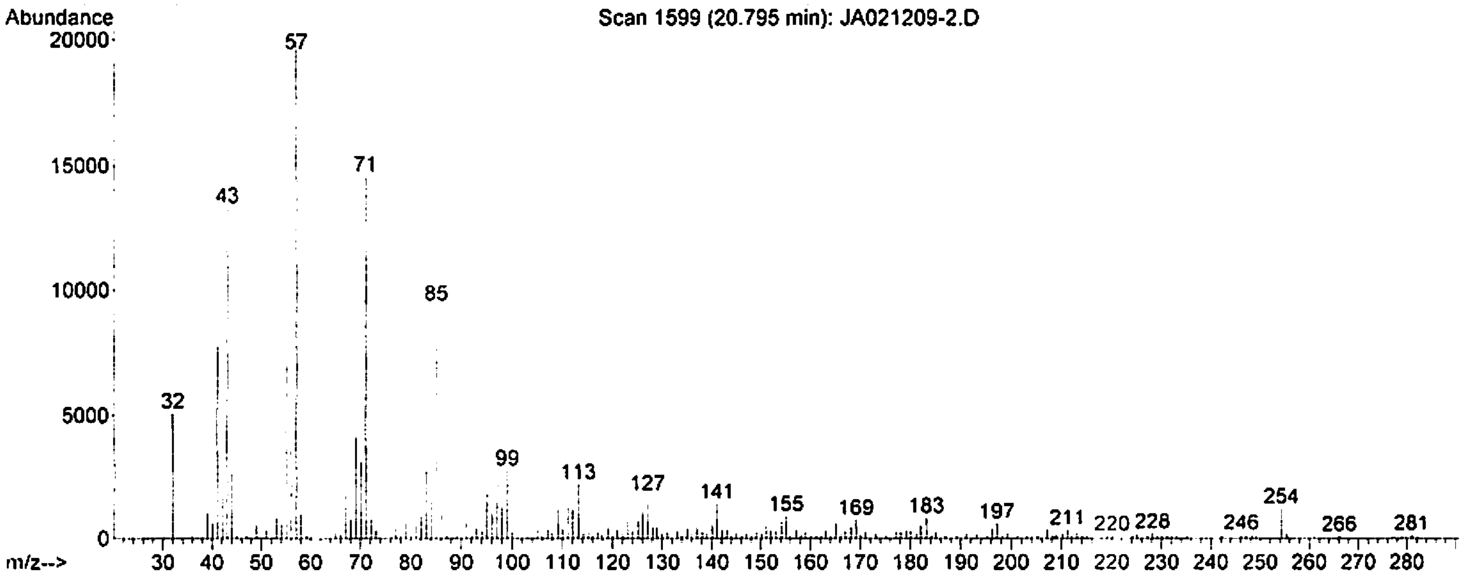

Supplement: Data S9 [file peerj-04-1564-s014.pdf]
