# Supplementary material for: Pharmacophagy in green lacewings (Neuroptera: Chrysopidae: Chrysopa spp.)?
Source: PeerJ. 2016 Jan 18;4:e1564. doi: 10.7717/peerj.1564 (PMC4727961; doi:10.7717/peerj.1564)

C. oculata · Larval · Feeding · Expt. involving Z, E-Nepetalactol (1µl/ml) contained in 10% (v/v) aqueous honey soln. (& Sitotroga cerealella eggs)

MJC. - SU · 02/09/11: (3?) ♂s.
NEAs · 02/08 - 02/09. Now fed Sitotroga eggs, live pea aphids, and 10% (v/v) aq. honey soln. 'sans additives'.

3

02/16: All (3?) res. ♂s alive.
Fed std. diet.

2/17/11
+ 1 ♂ from cage #2

Cf added honey water w/ 1 mg/ml nepetalactol (4:00 pm)

02/18:
Added live · pea · aphids & Sitotroga eggs.

File   :D:\Aldrich\JA-11\JA021711-1.D
Operator   :
Acquired   : 17 Feb 2011 17:20   using AcqMethod JA-50-280LESS.M
Instrument :   Buba; IIBBL's magical mass spect
Sample Name: 4M C. ocu. abd.sternites/5ul CH2Cl2;9-10 days
Misc Info   : larvae w/1ug/ul nepetalactol in honey soln.
Vial Number: 1

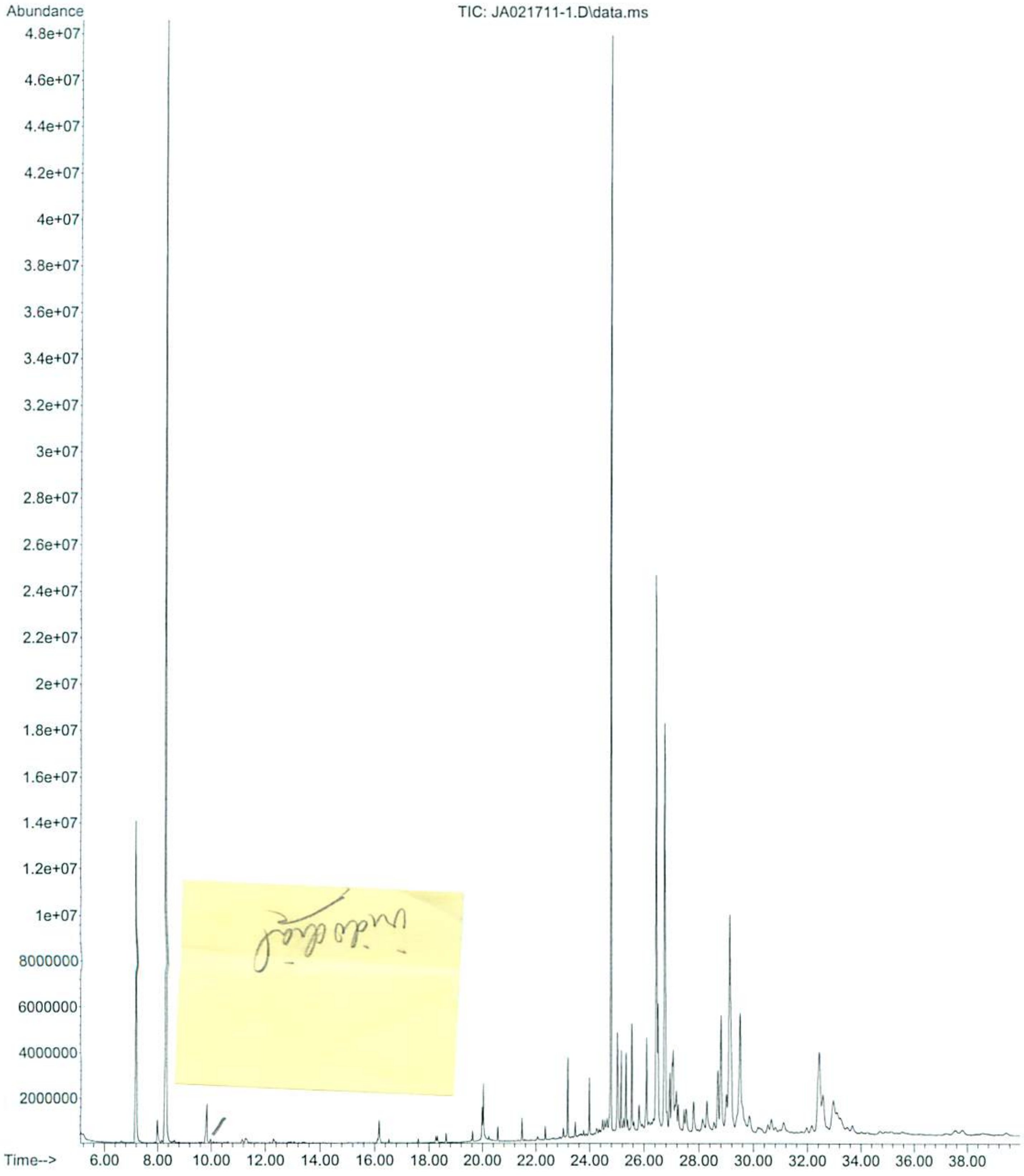

```
File        :D:\Aldrich\JA-11\JA021711-1.D
Operator    :
Acquired    : 17 Feb 2011  17:20     using AcqMethod JA-50-280LESS.M
Instrument :     Buba; IIBBL's magical mass spect
Sample Name: 4M C. ocu. abd.sternites/5ul CH2Cl2;9-10 days
Misc Info   : larvae w/1ug/ul nepetalactol in honey soln.
Vial Number: 1
```

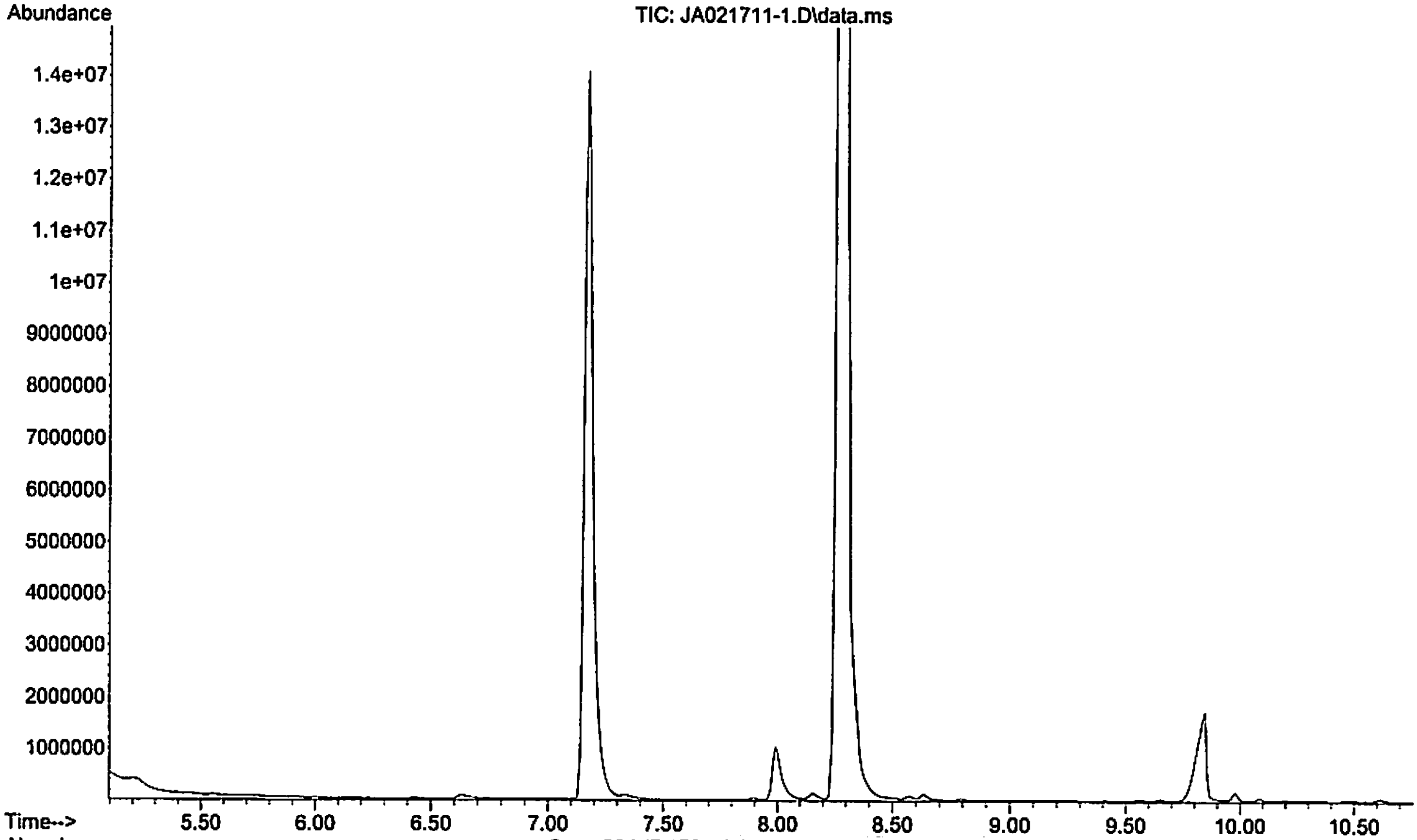

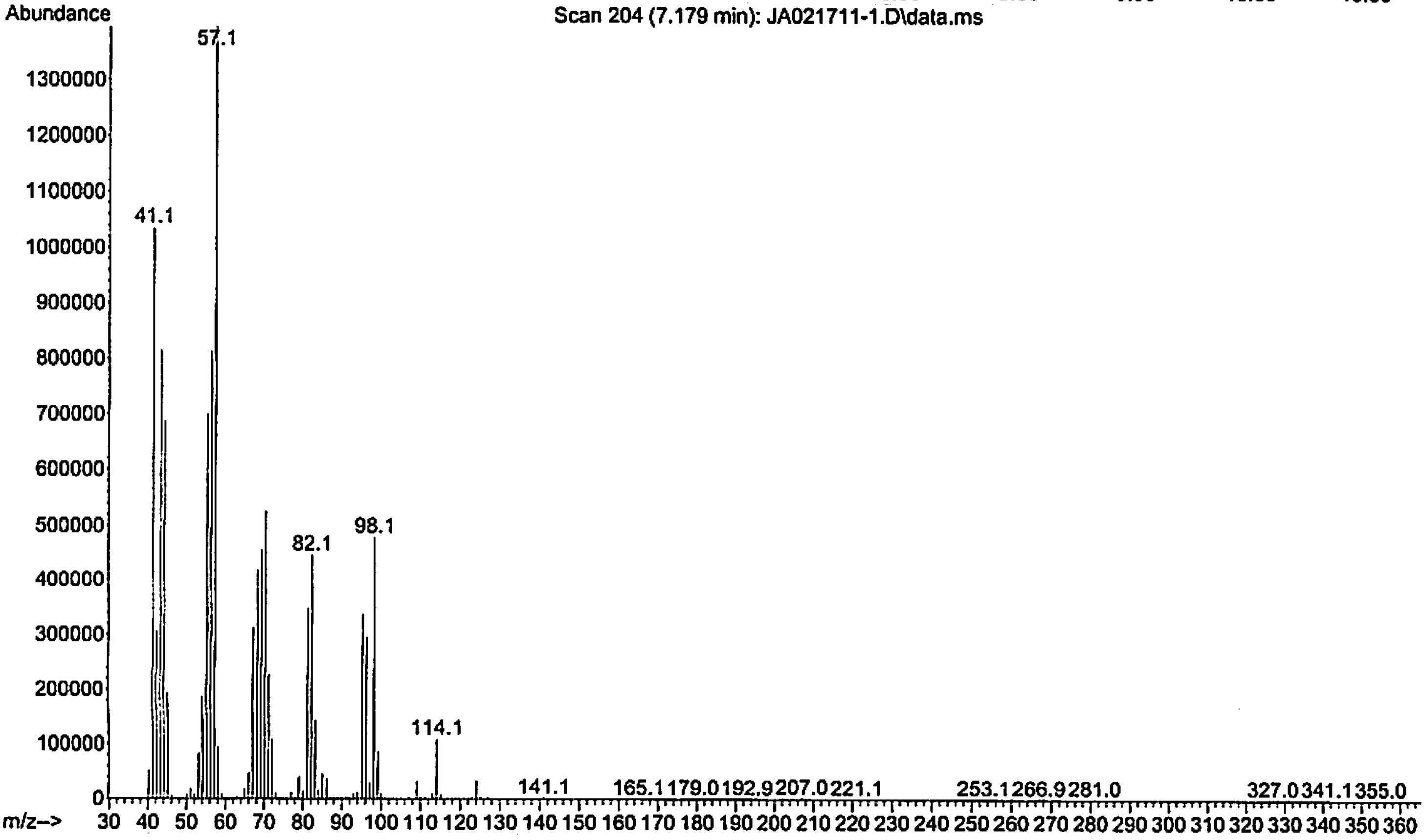

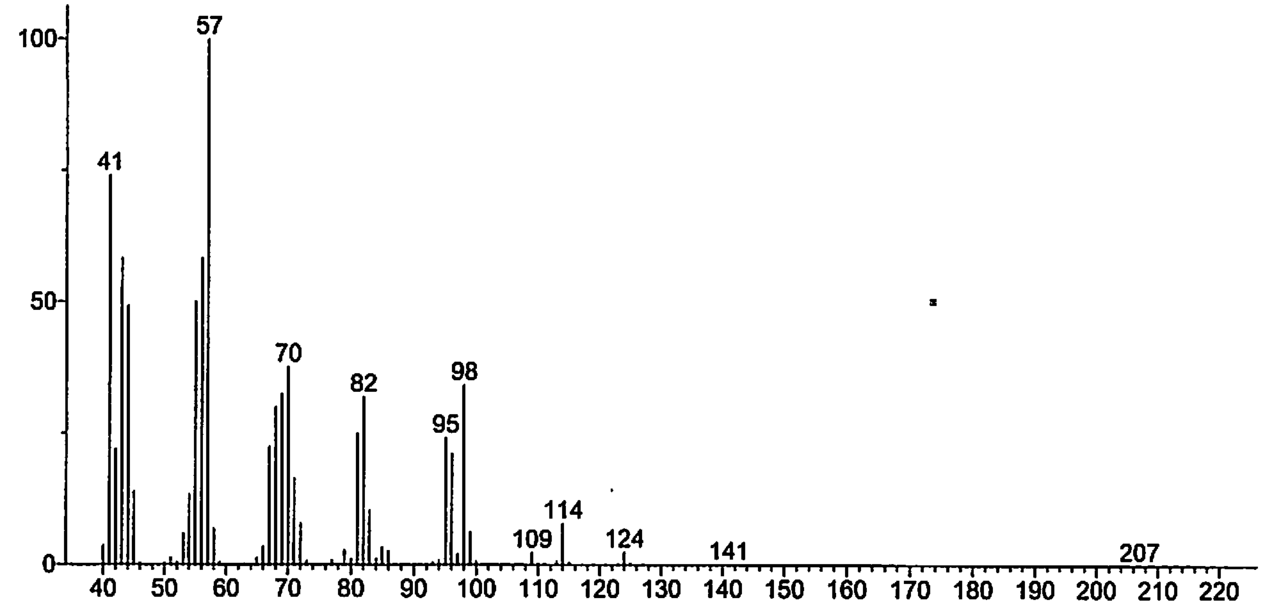

(Text File) Scan 204 (7.179 min): JA021711-1.D\data.ms

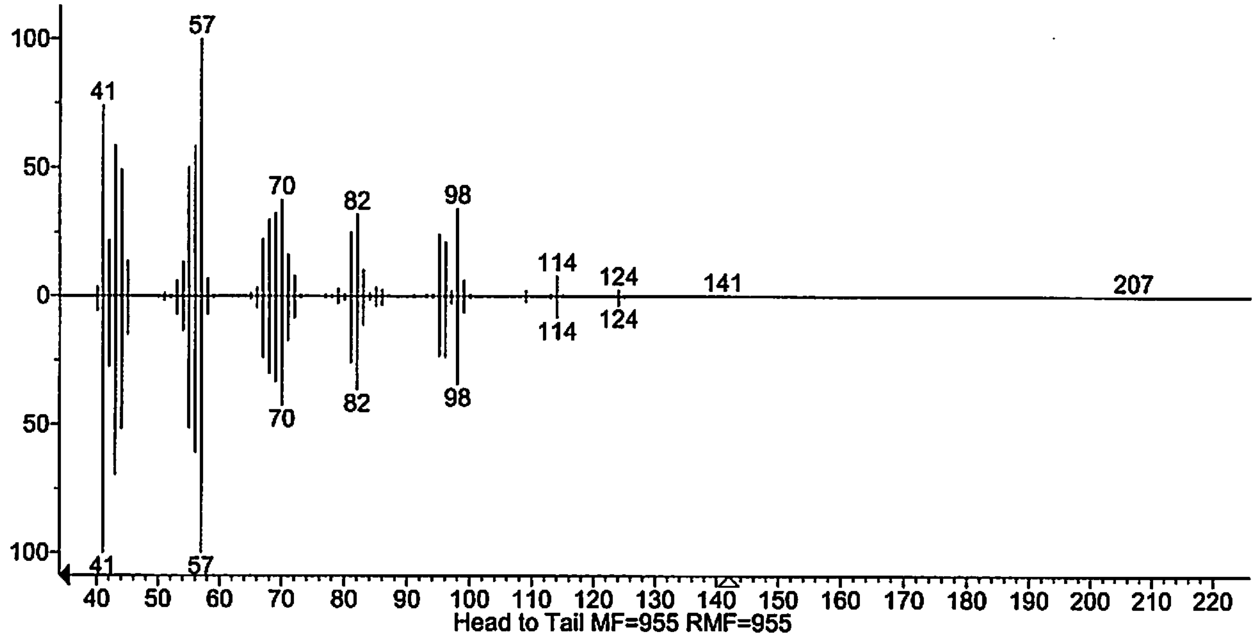

Head to Tail MF=955 RMF=955

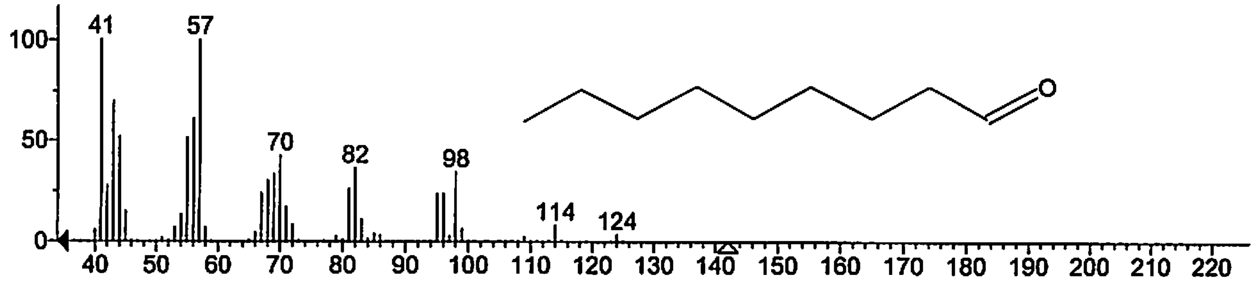

(replib) Nonanal

File :D:\Aldrich\JA-11\JA021711-1.D
Operator :
Acquired : 17 Feb 2011 17:20 using AcqMethod JA-50-280LESS.M
Instrument : Buba; IIBBL's magical mass spect
Sample Name: 4M C. ocu. abd.sternites/5ul CH2Cl2;9-10 days
Misc Info : larvae w/1ug/ul nepetalactol in honey soln.
Vial Number: 1

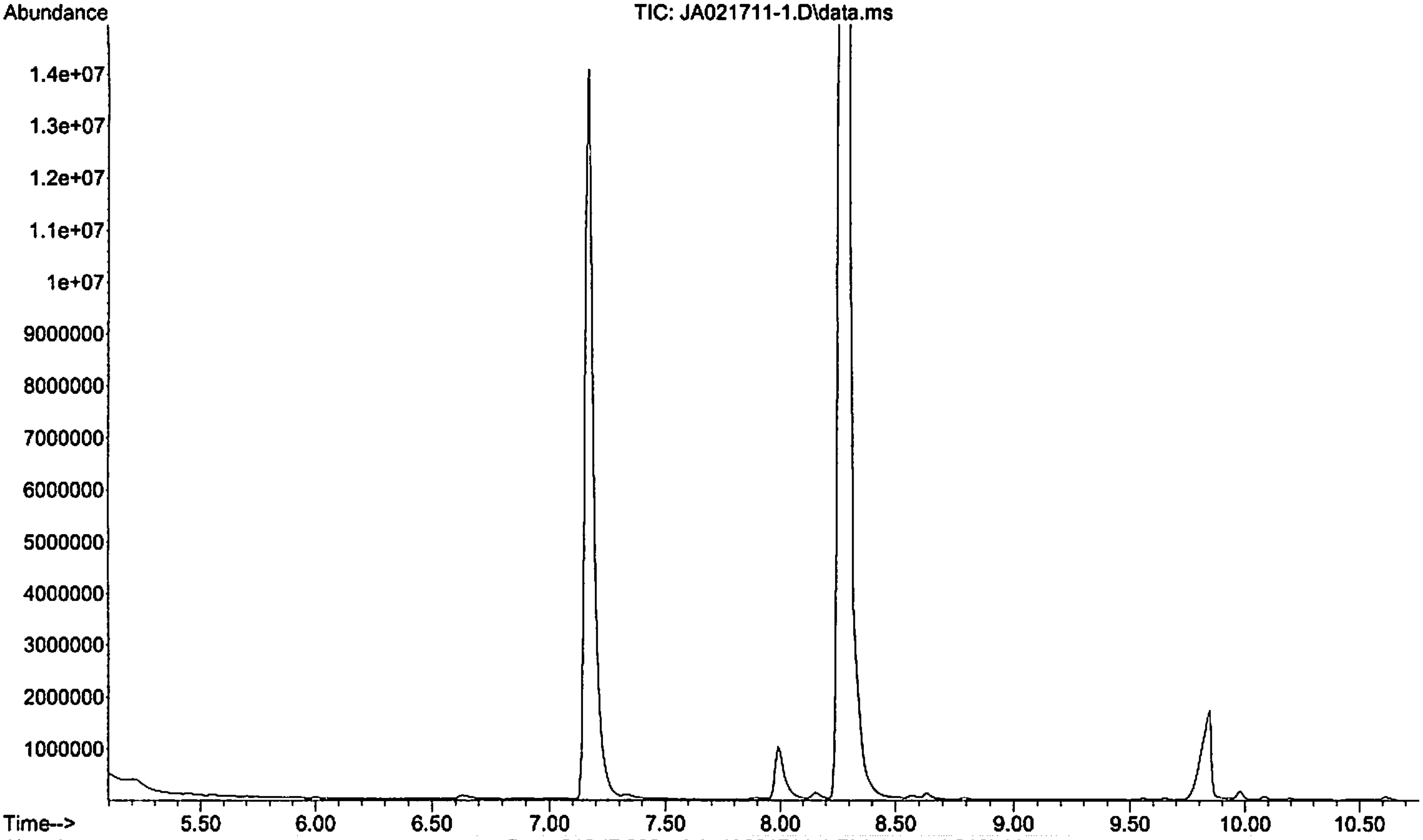

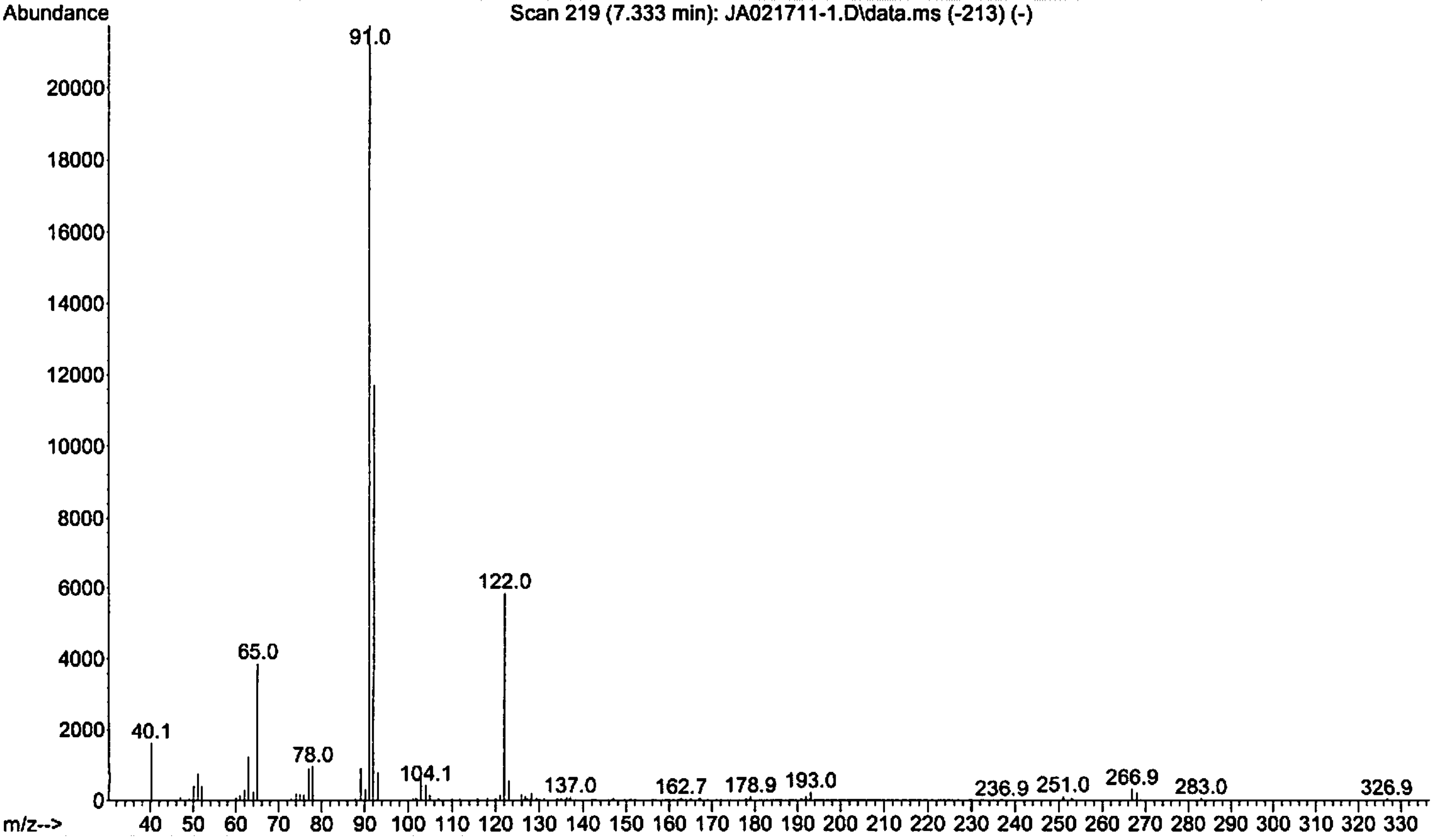

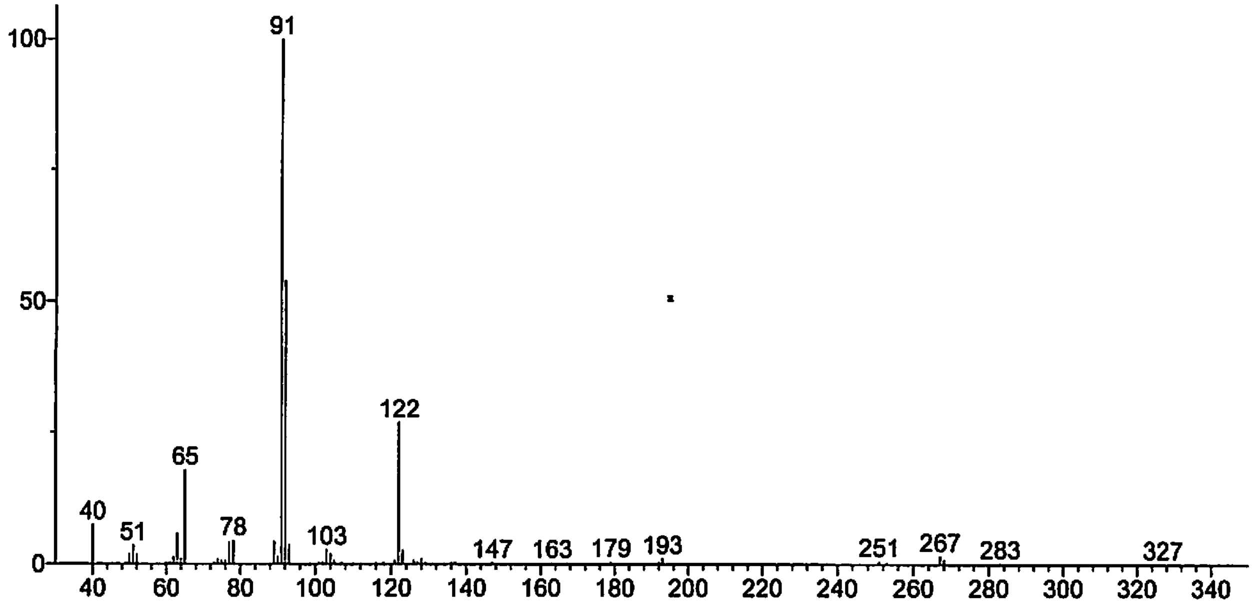

(Text File) Scan 219 (7.333 min): JA021711-1.D\data.ms (-213)

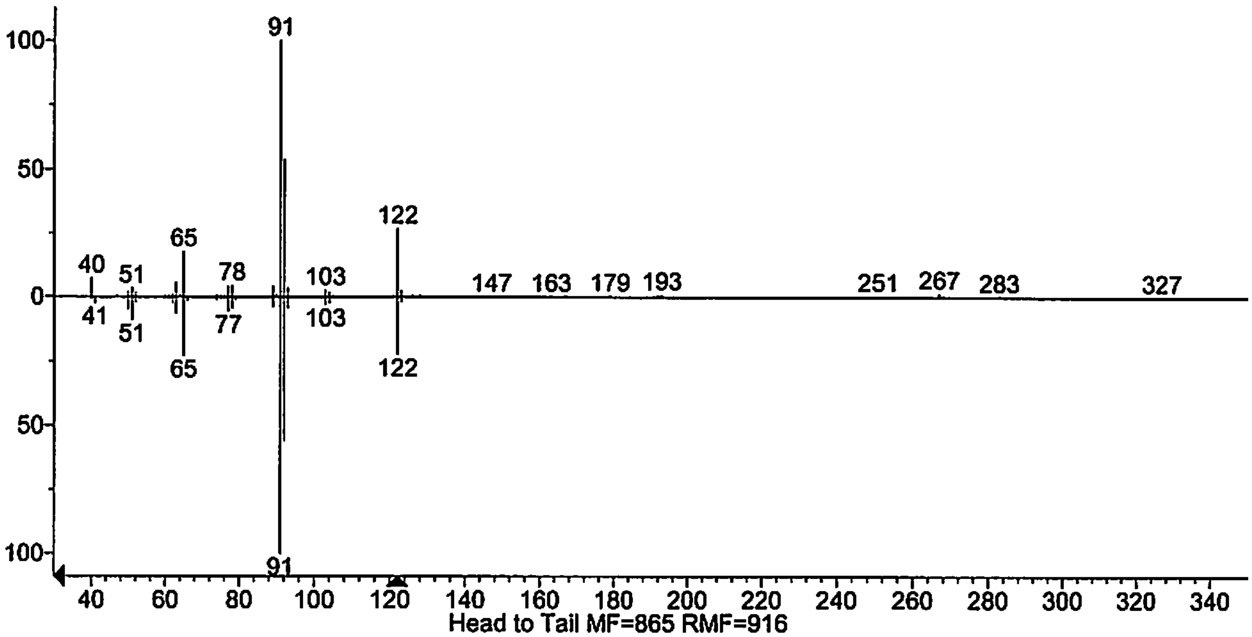

Head to Tail MF=865 RMF=916

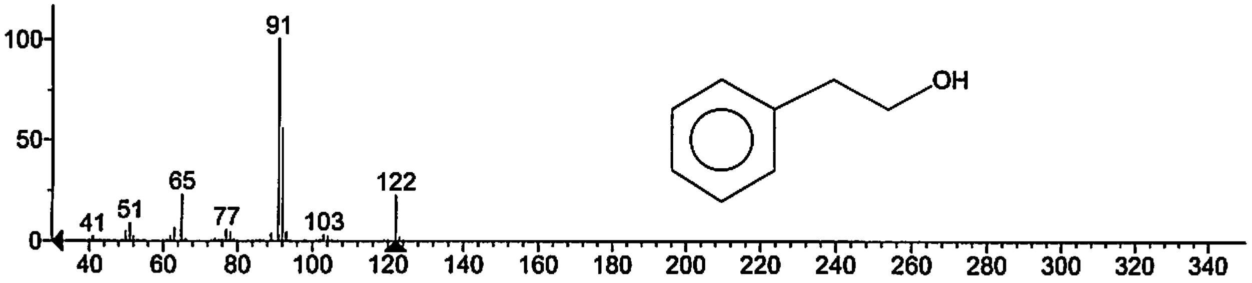

(mainlib) Phenylethyl Alcohol

File :D:\Aldrich\JA-11\JA021711-1.D
Operator :
Acquired : 17 Feb 2011 17:20 using AcqMethod JA-50-280LESS.M
Instrument : Buba; IIBBL's magical mass spect
Sample Name: 4M C. ocu. abd.sternites/5ul CH2Cl2;9-10 days
Misc Info : larvae w/1ug/ul nepetalactol in honey soln.
Vial Number: 1

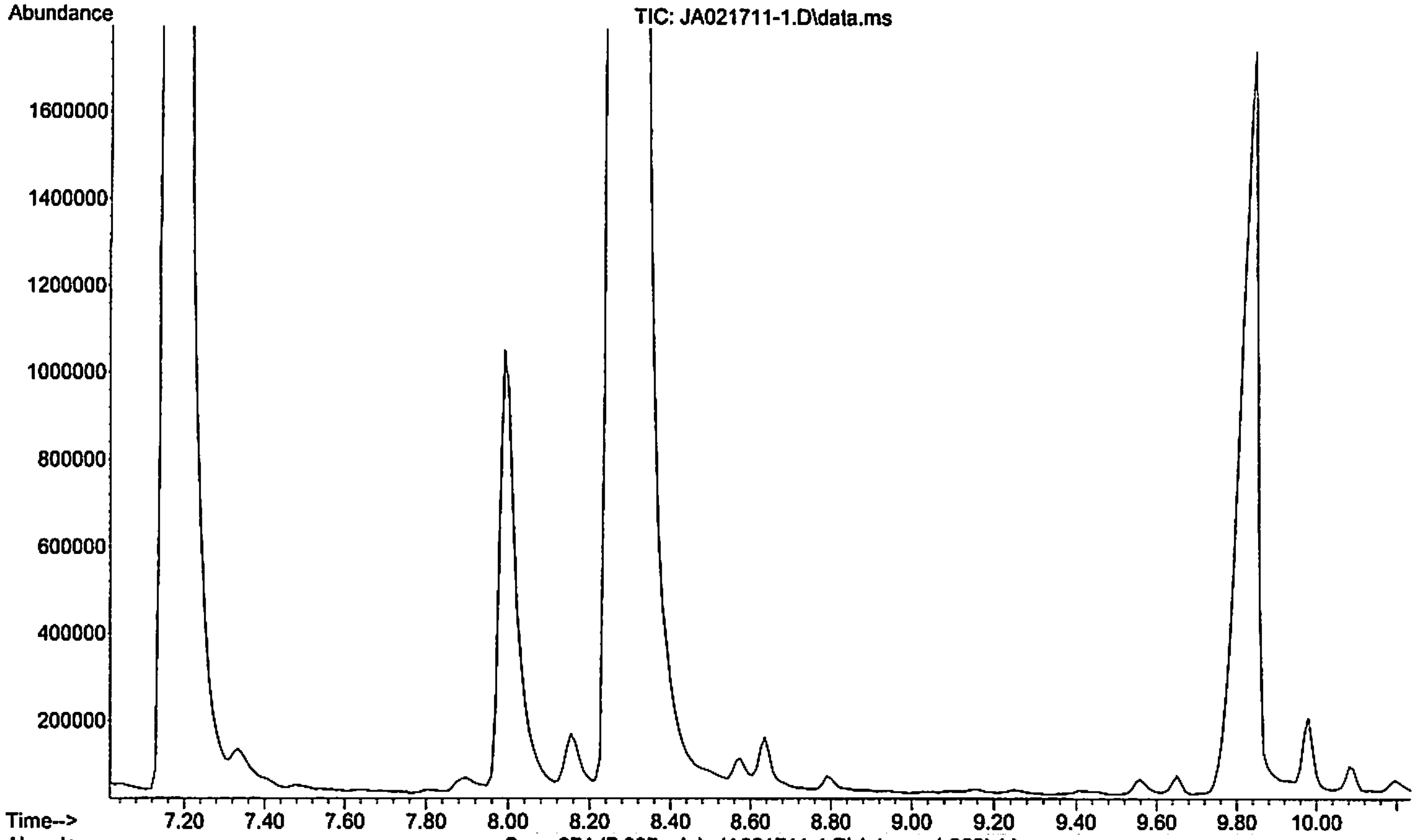

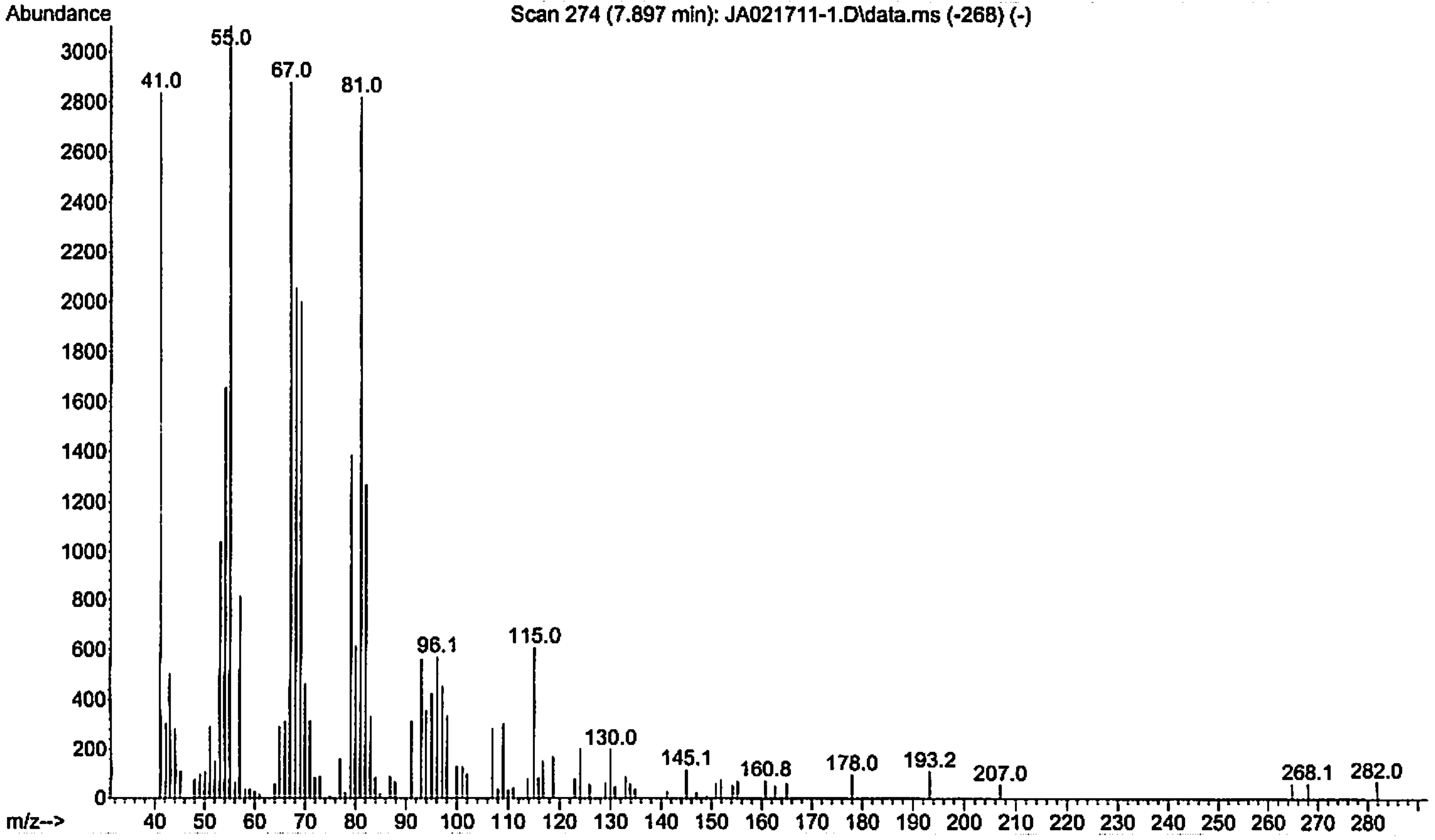

```
File        :D:\Aldrich\JA-11\JA021711-1.D
Operator    :
Acquired    : 17 Feb 2011  17:20     using AcqMethod JA-50-280LESS.M
Instrument  :    Buba; IIBBL's magical mass spect
Sample Name: 4M C. ocu. abd.sternites/5ul CH2Cl2;9-10 days
Misc Info   : larvae w/1ug/ul nepetalactol in honey soln.
Vial Number: 1
```

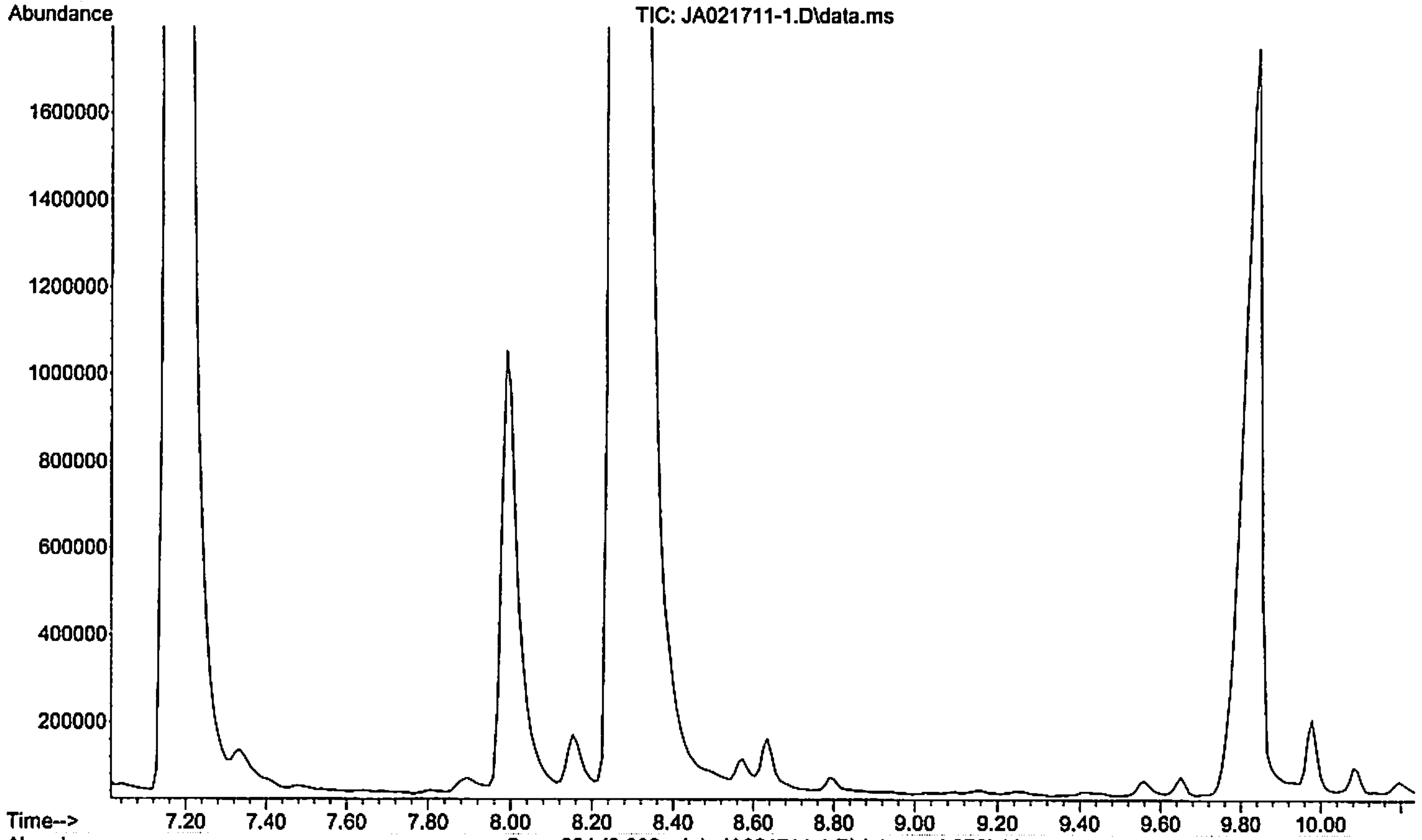

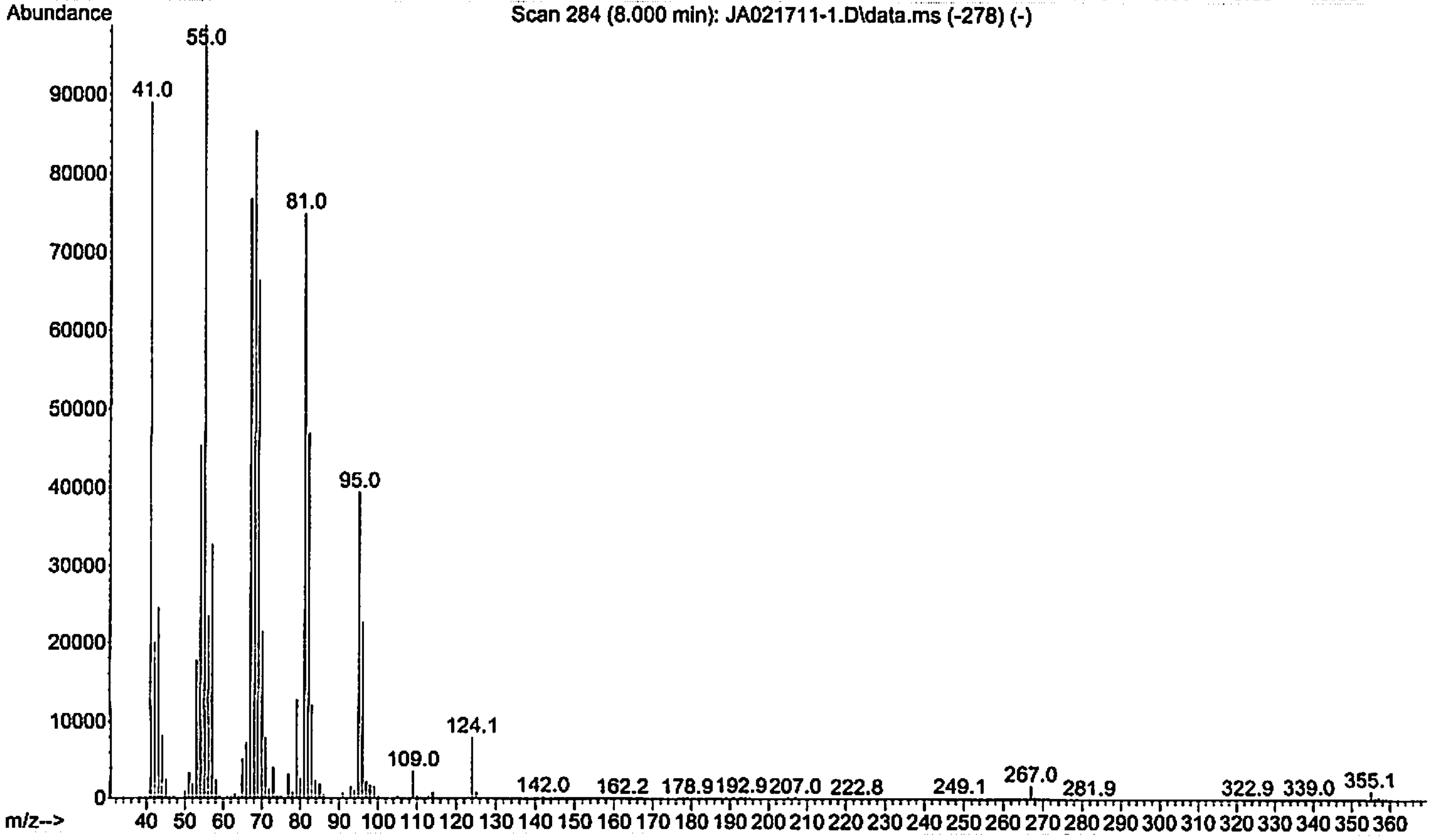

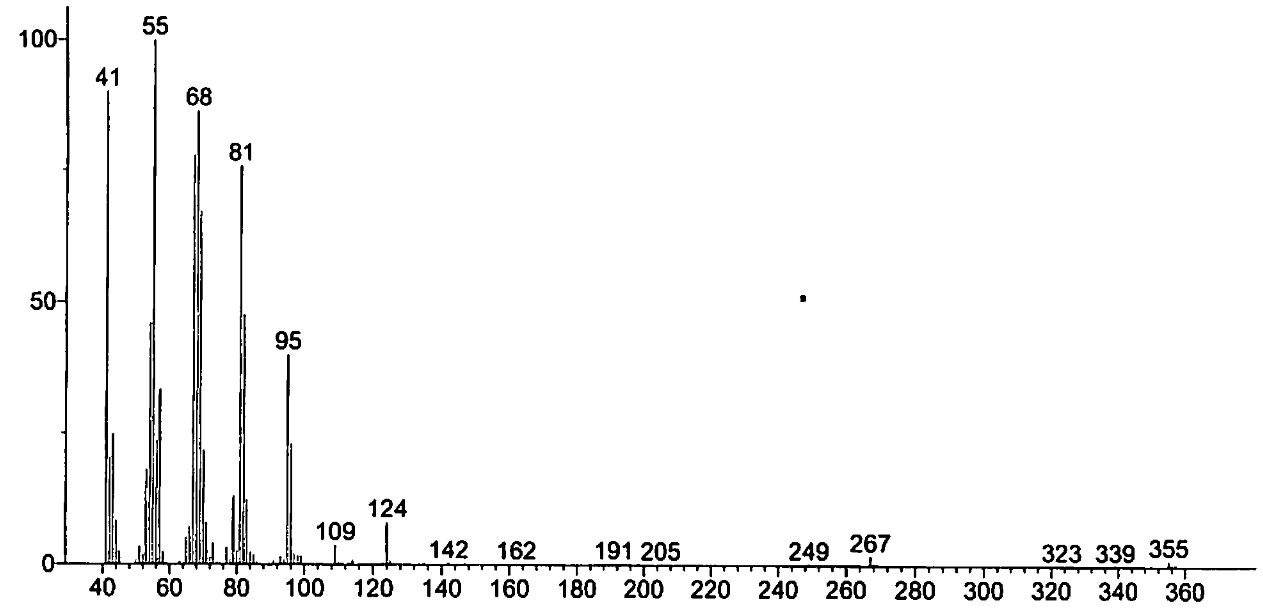

(Text File) Scan 284 (8.000 min): JA021711-1.D\data.ms (-278)

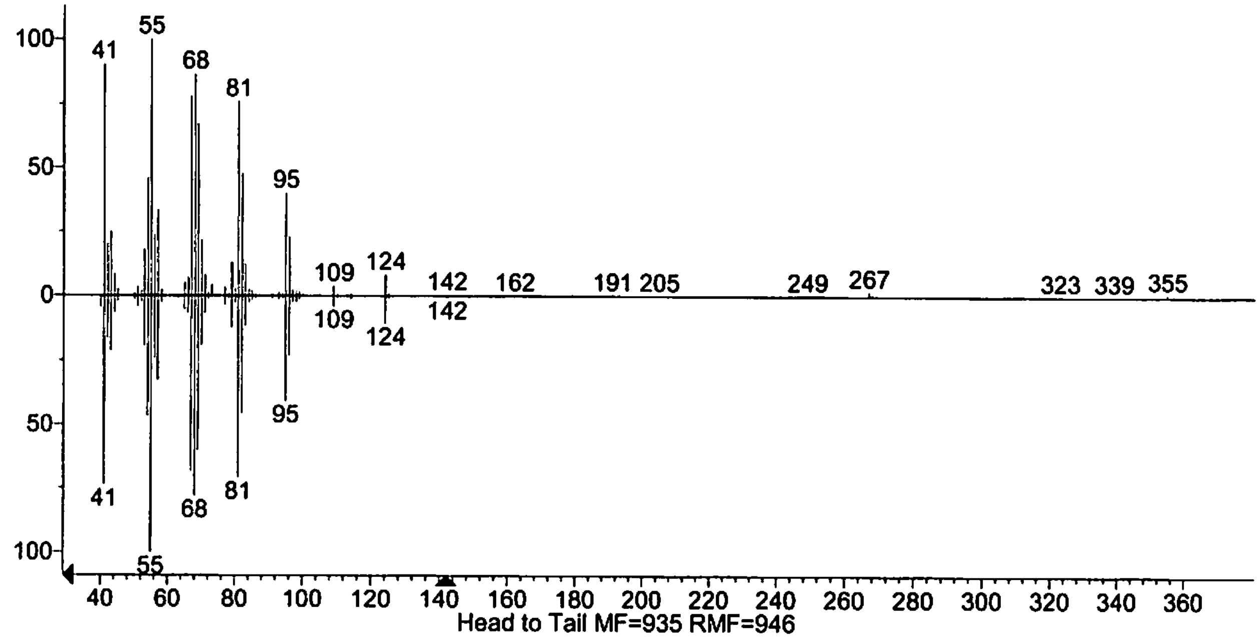

Head to Tail MF=935 RMF=946

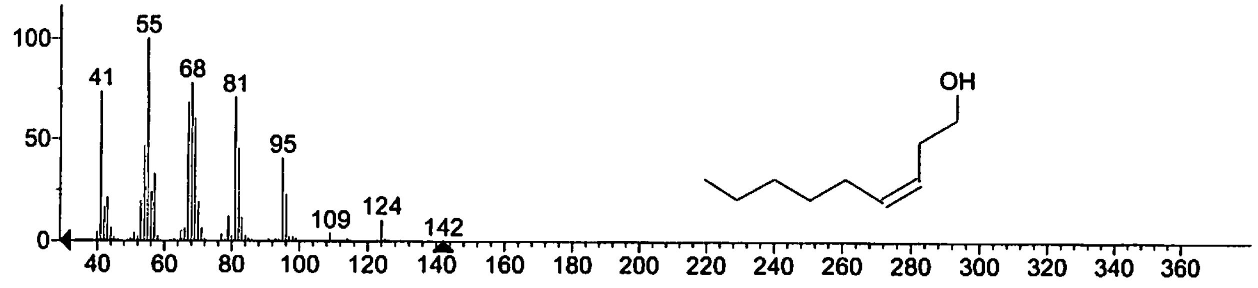

(mainlib) 3-Nonen-1-ol, (Z)-

```
File       ` :D:\Aldrich\JA-11\JA021711-1.D
Operator   :
Acquired   : 17 Feb 2011  17:20     using AcqMethod JA-50-280LESS.M
Instrument :    Buba; IIBBL's magical mass spect
Sample Name: 4M C. ocu. abd.sternites/5ul CH2Cl2;9-10 days
Misc Info  : larvae w/1ug/ul nepetalactol in honey soln.
Vial Number: 1
```

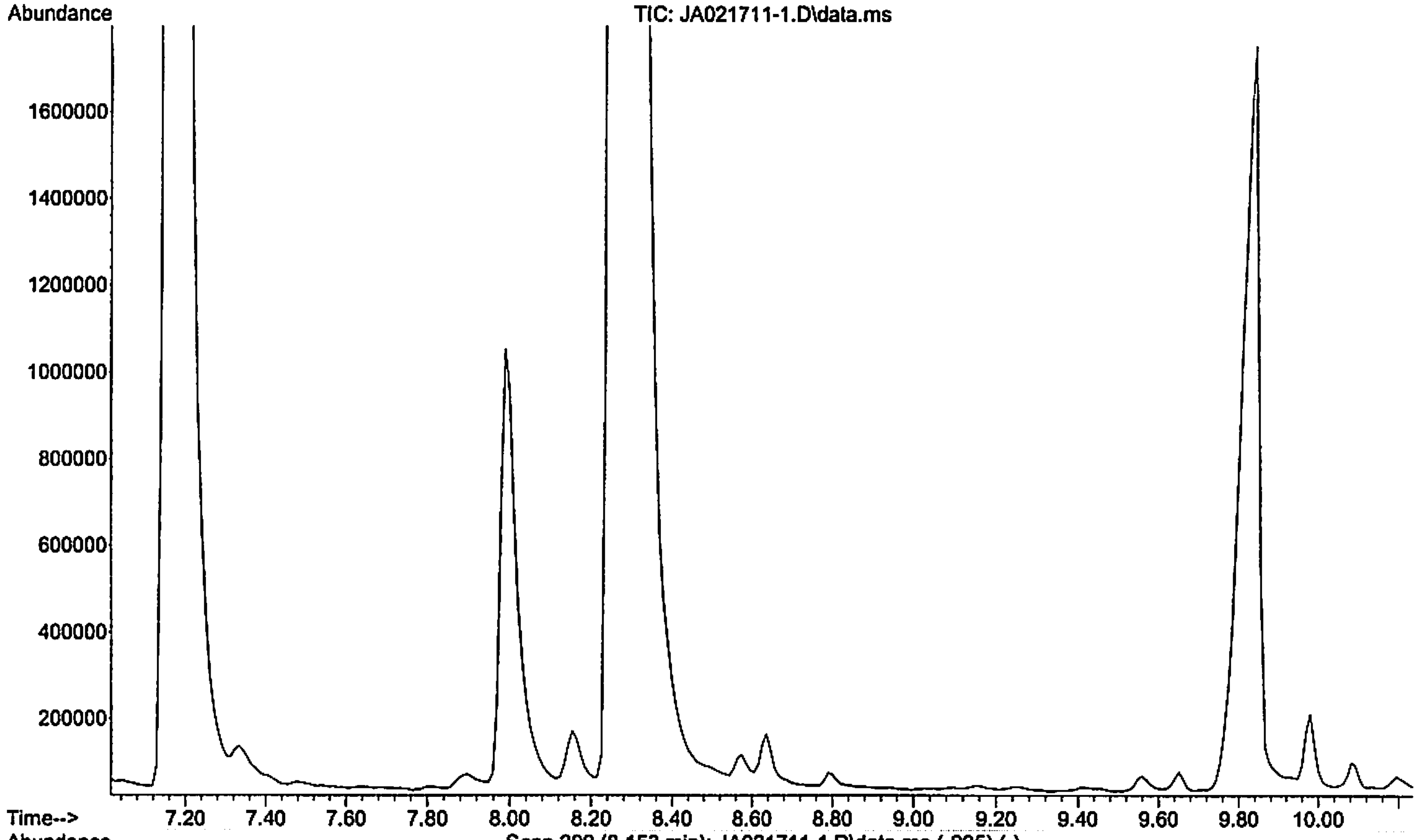

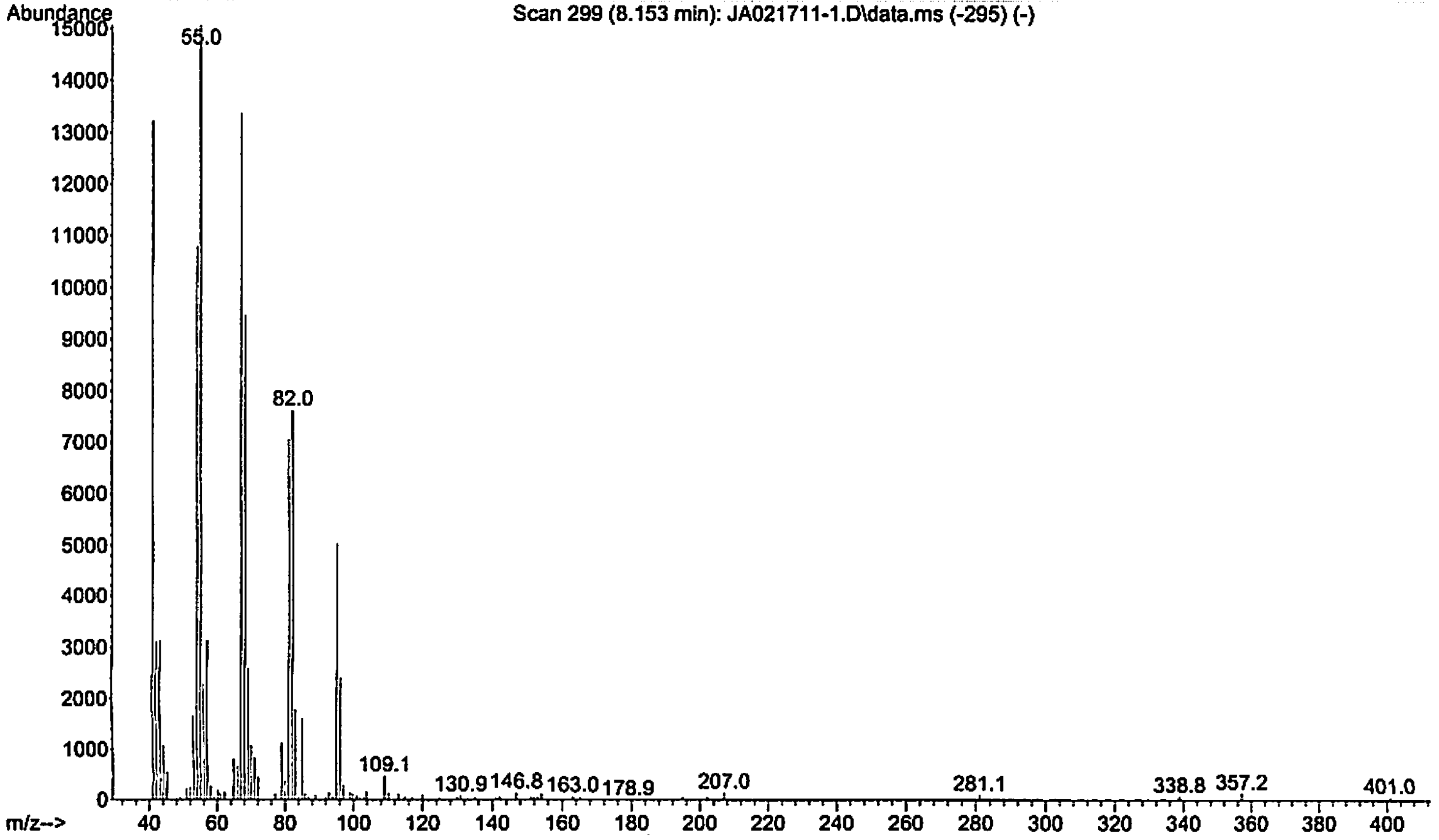

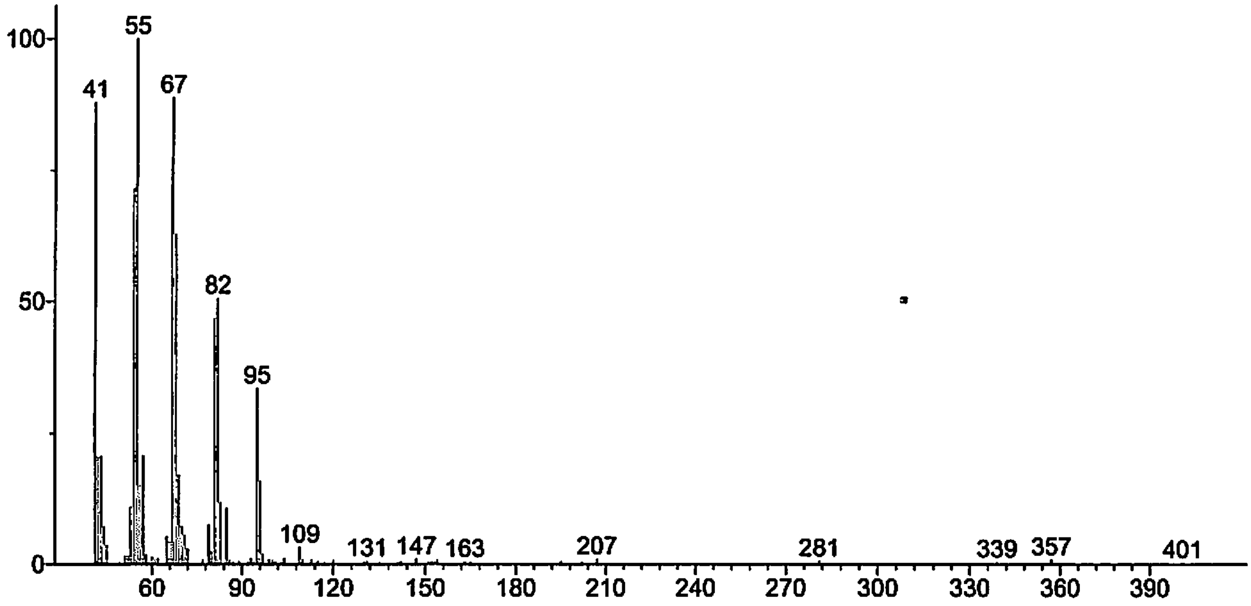

(Text File) Scan 299 (8.153 min): JA021711-1.D\data.ms (-295)

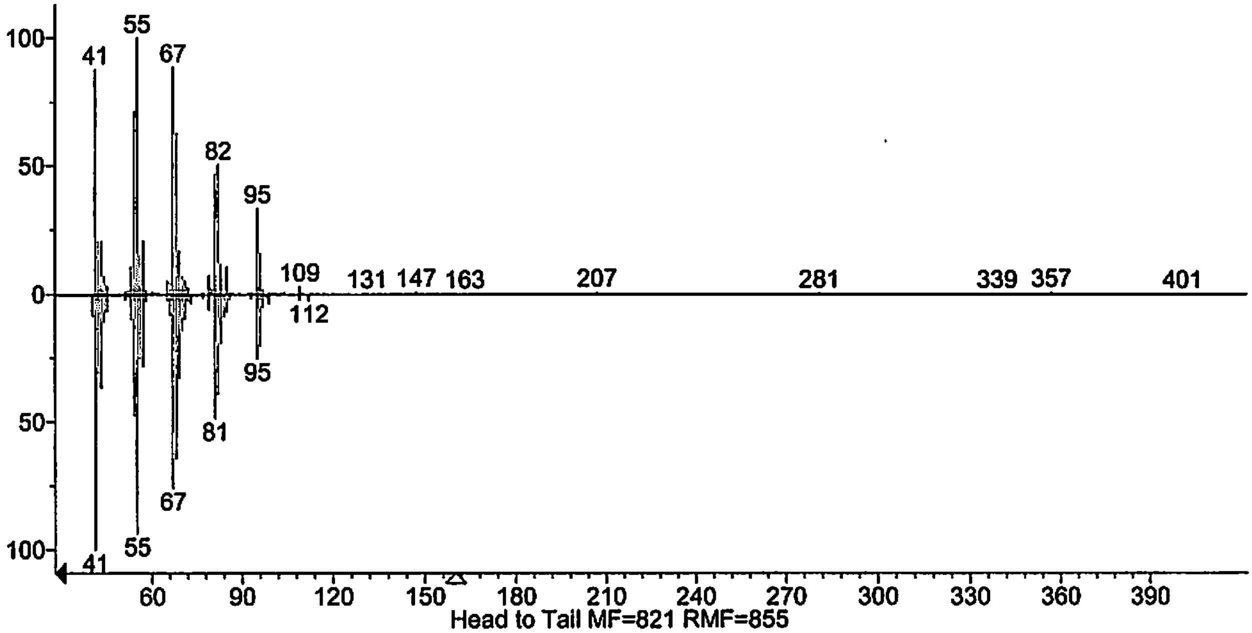

Head to Tail MF=821 RMF=855

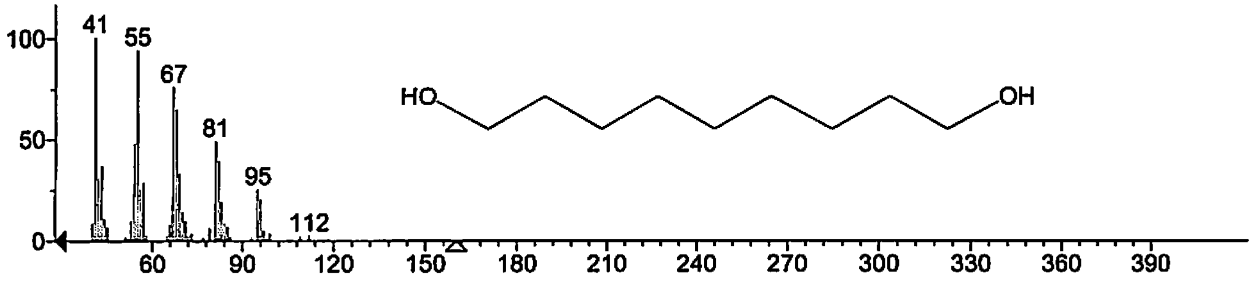

(replib) 1,9-Nonanediol

File   :D:\Aldrich\JA-11\JA021711-1.D
Operator :
Acquired : 17 Feb 2011 17:20   using AcqMethod JA-50-280LESS.M
Instrument :   Buba; IIBBL's magical mass spect
Sample Name: 4M C. ocu. abd.sternites/5ul CH2Cl2;9-10 days
Misc Info : larvae w/1ug/ul nepetalactol in honey soln.
Vial Number: 1

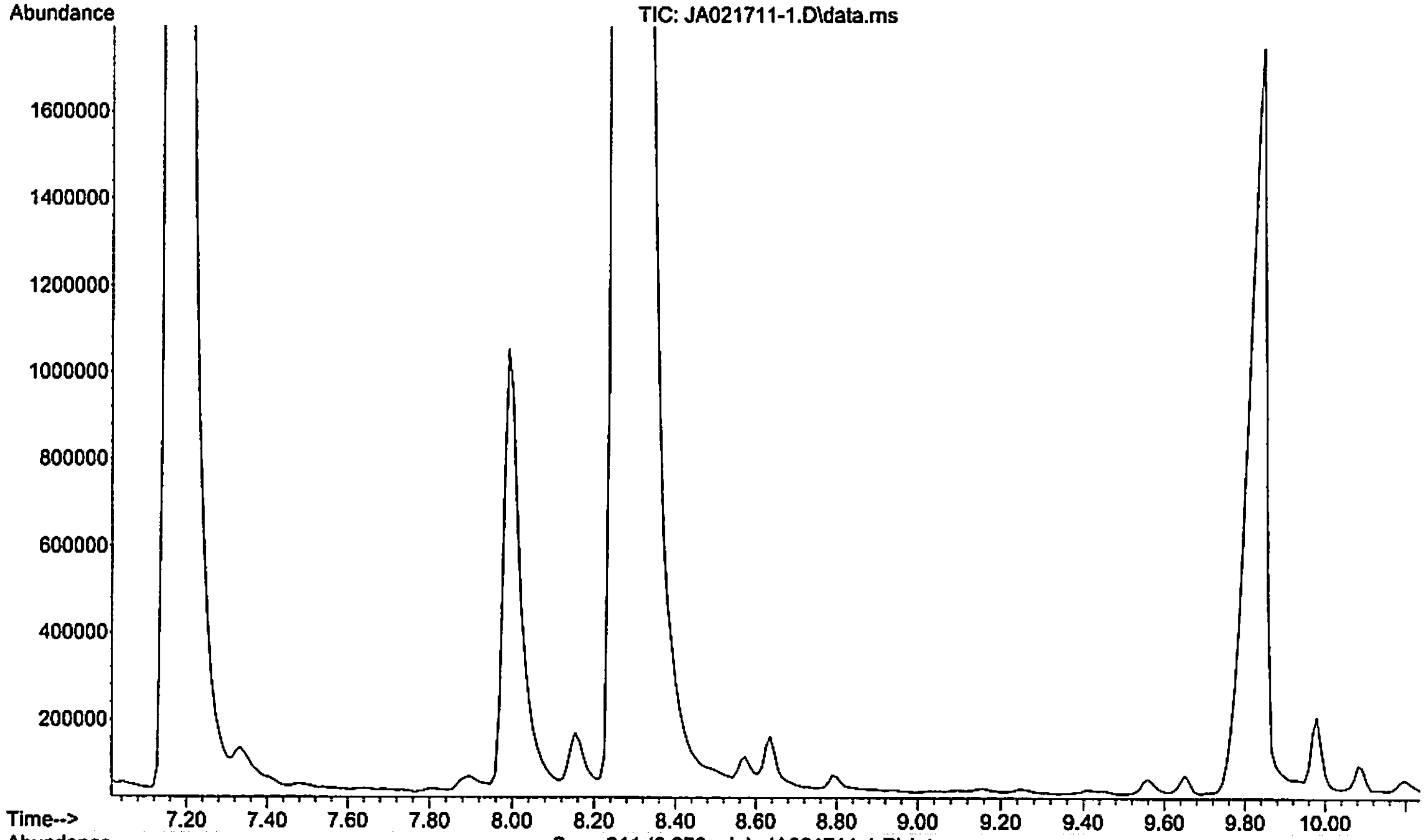

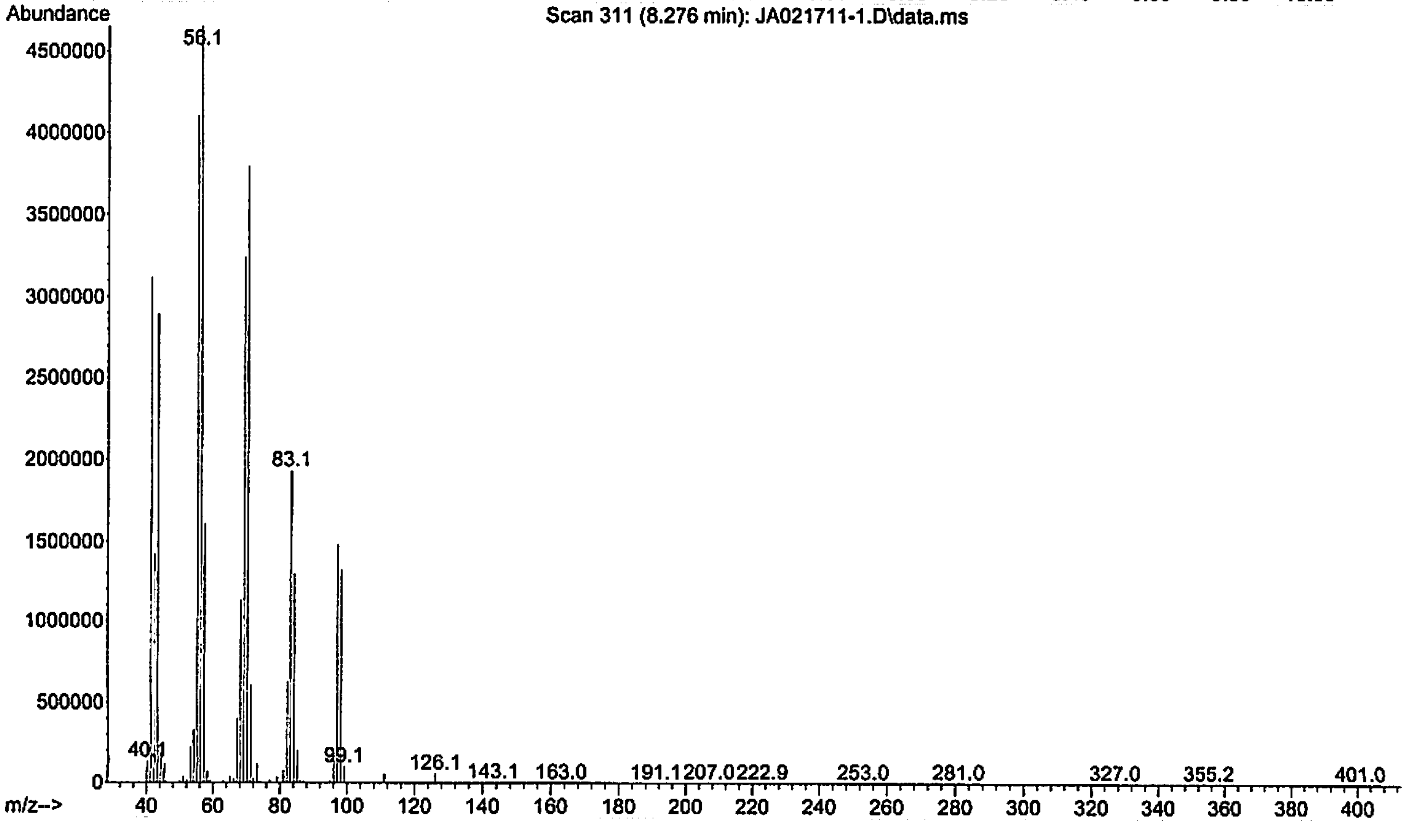

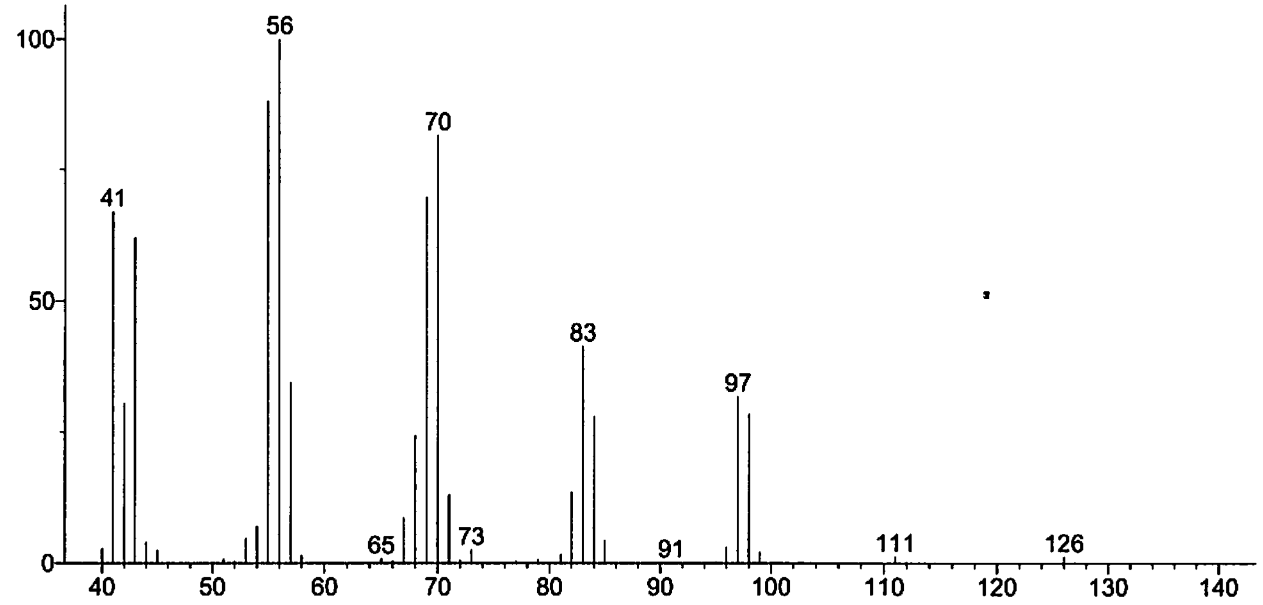

(Text File) Scan 311 (8.276 min): JA021711-1.D\data.ms

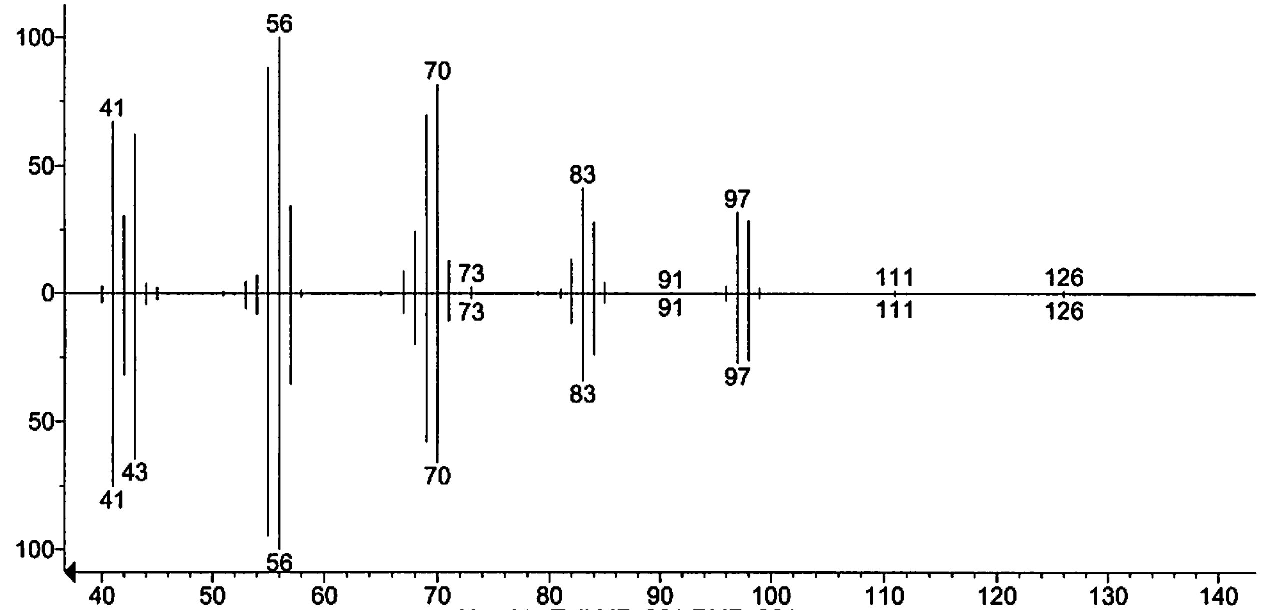

Head to Tail MF=961 RMF=961

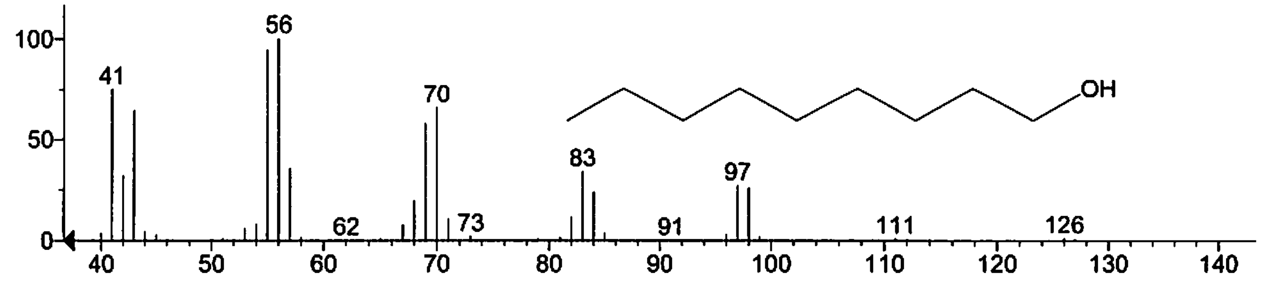

(replib) 1-Nonanol

File ` :D:\Aldrich\JA-11\JA021711-1.D
Operator :
Acquired : 17 Feb 2011 17:20 using AcqMethod JA-50-280LESS.M
Instrument : Buba; IIBBL's magical mass spect
Sample Name: 4M C. ocu. abd.sternites/5ul CH2Cl2;9-10 days
Misc Info : larvae w/1ug/ul nepetalactol in honey soln.
Vial Number: 1

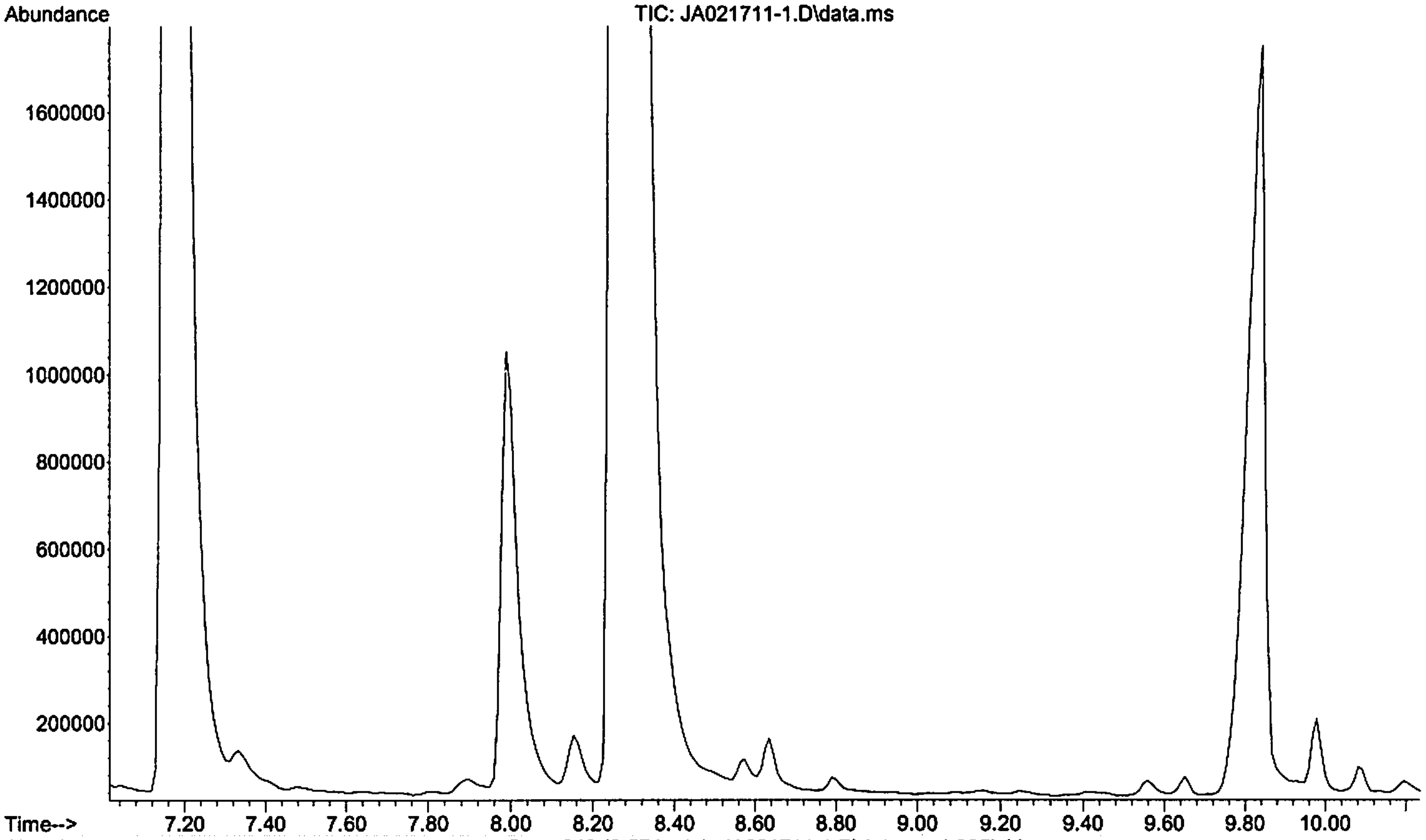

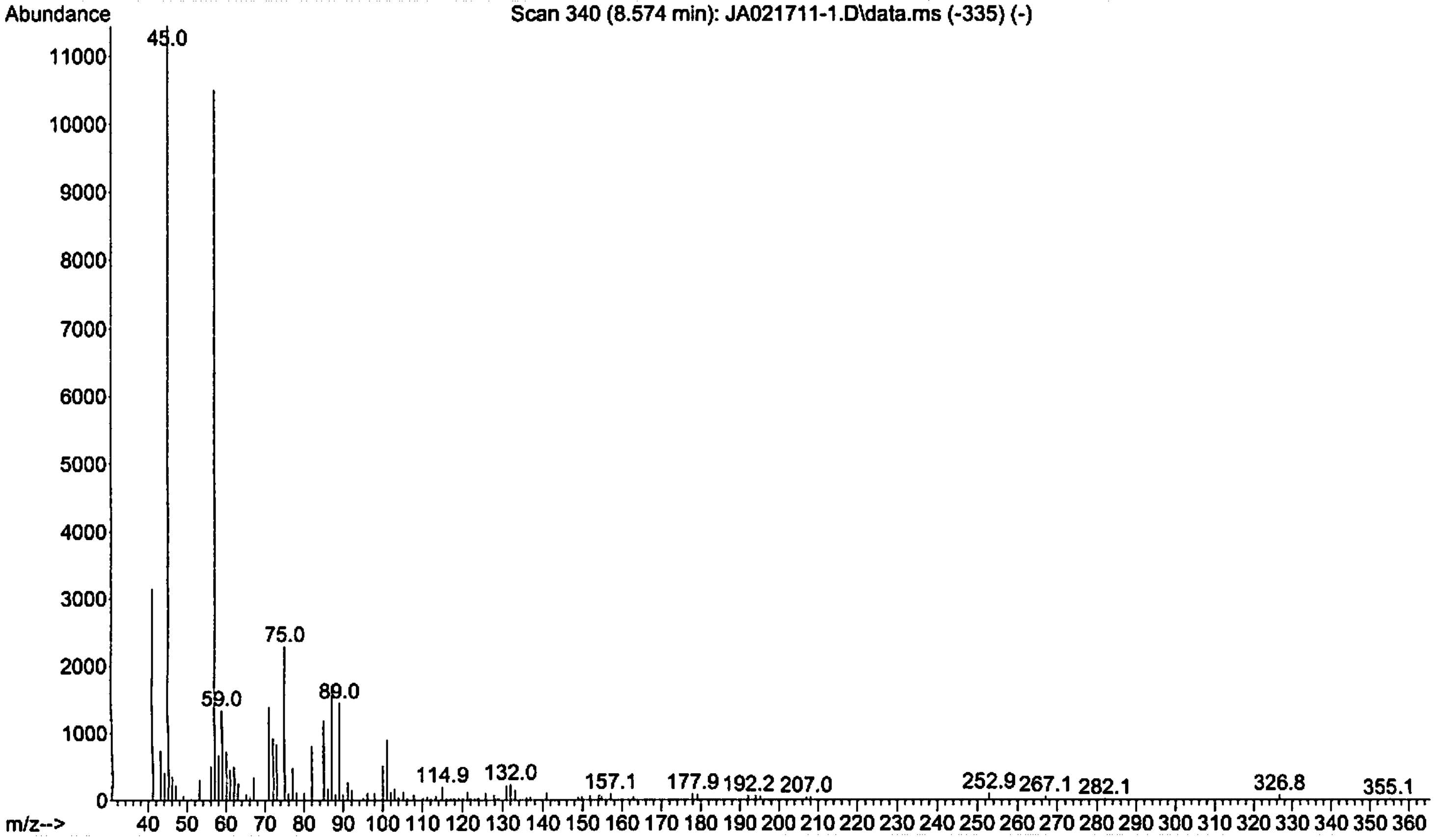

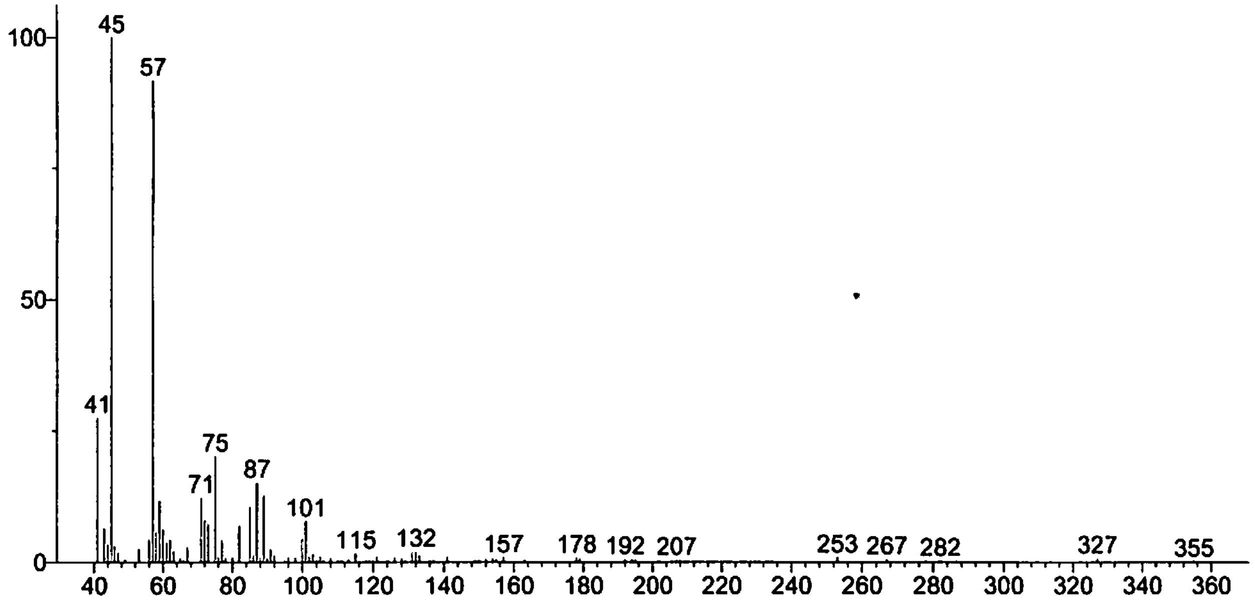

(Text File) Scan 340 (8.574 min): JA021711-1.D\data.ms (-335)

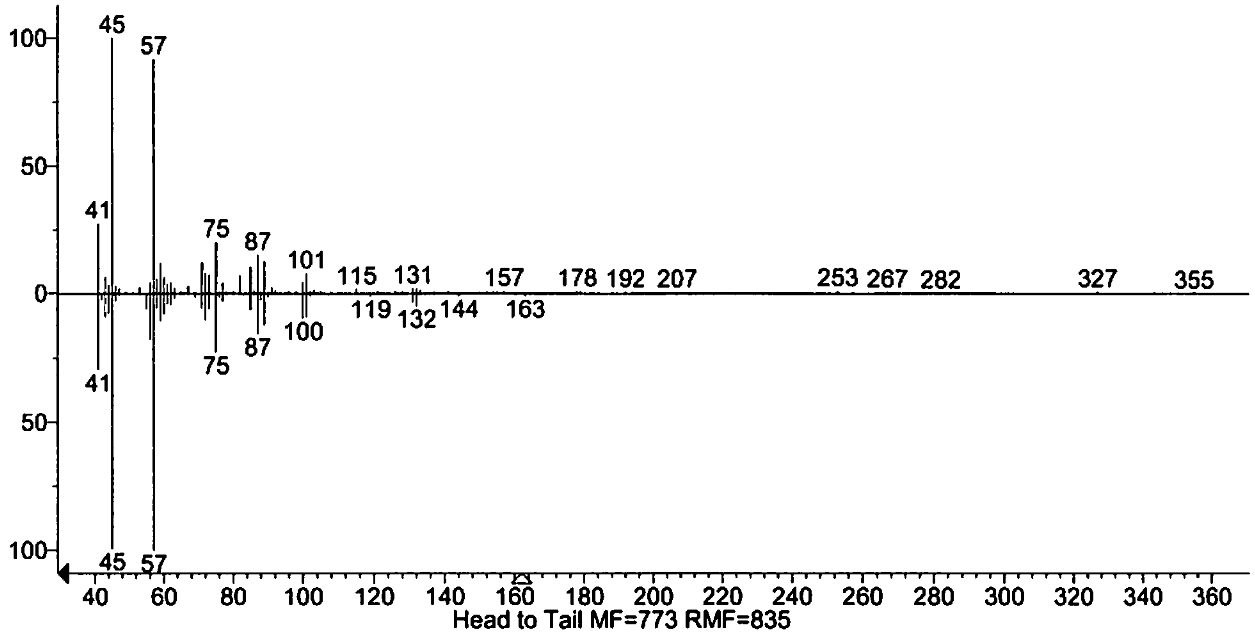

Head to Tail MF=773 RMF=835

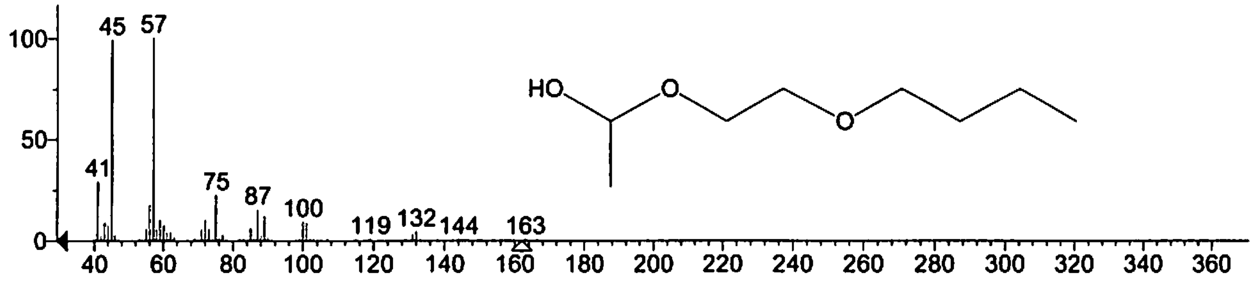

(mainlib) Ethanol, 1-(2-butoxyethoxy)-

```
File        :D:\Aldrich\JA-11\JA021711-1.D
Operator    :
Acquired    : 17 Feb 2011  17:20     using AcqMethod JA-50-280LESS.M
Instrument  :    Buba; IIBBL's magical mass spect
Sample Name: 4M C. ocu. abd.sternites/5ul CH2Cl2;9-10 days
Misc Info   : larvae w/1ug/ul nepetalactol in honey soln.
Vial Number: 1
```

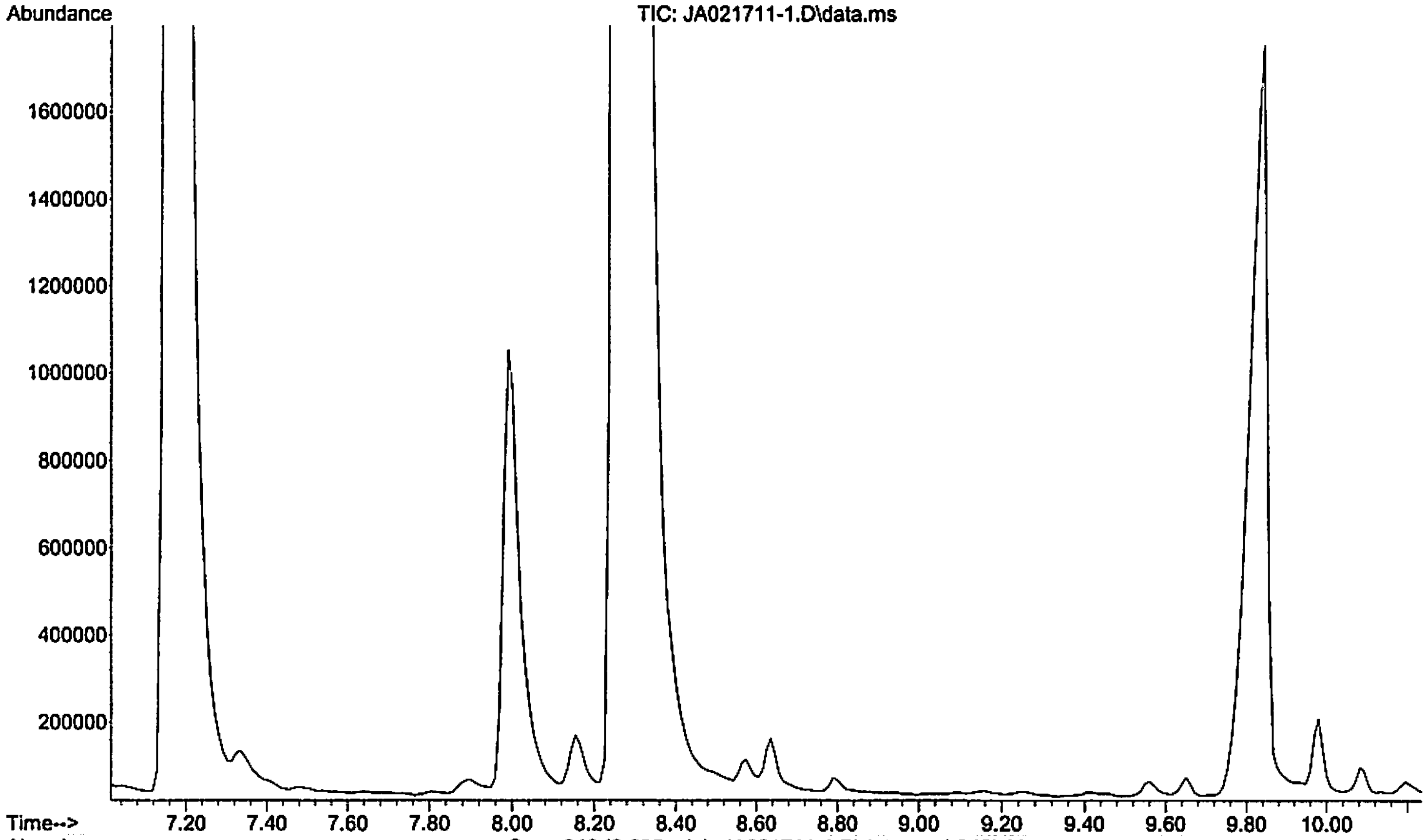

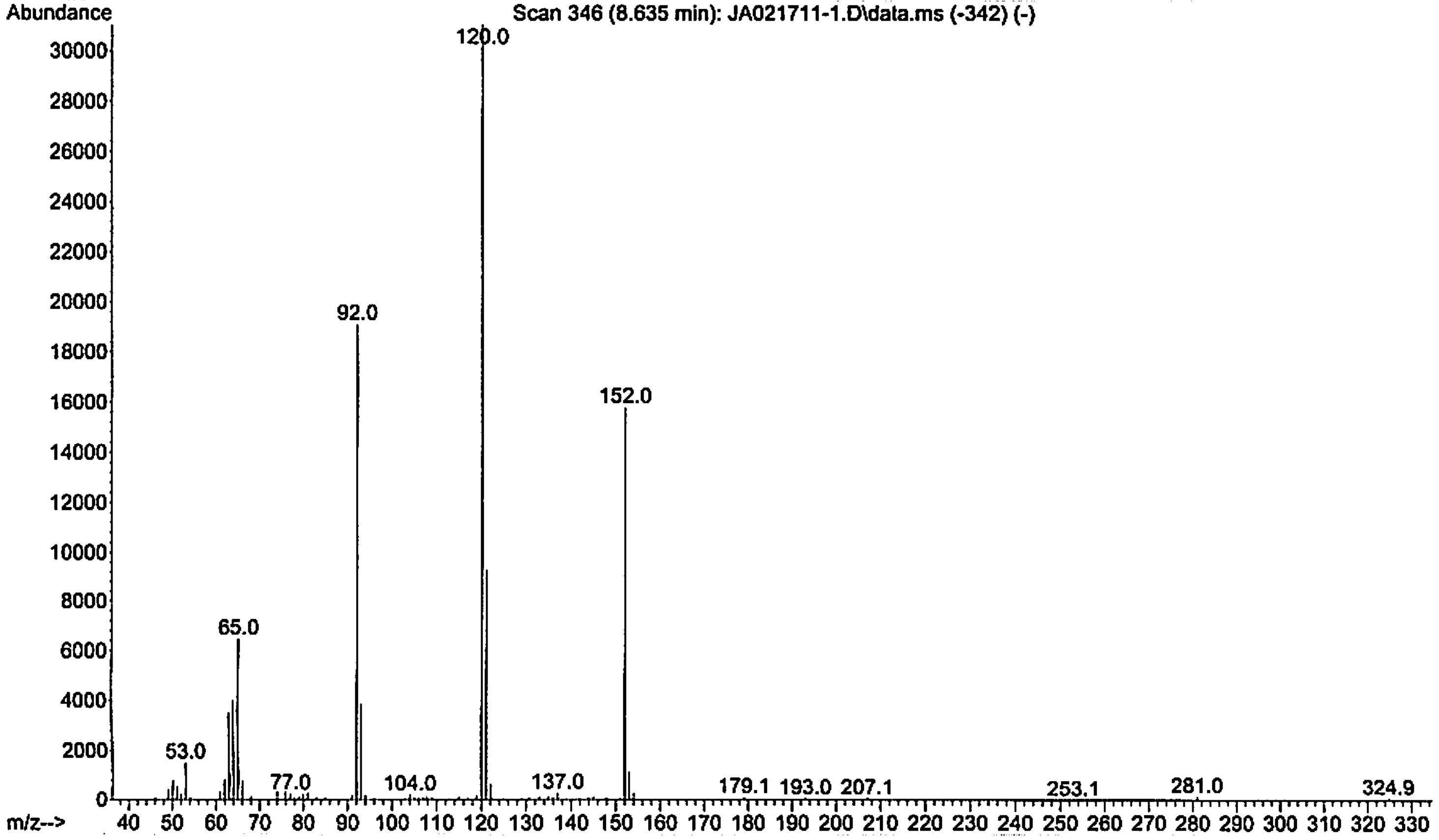

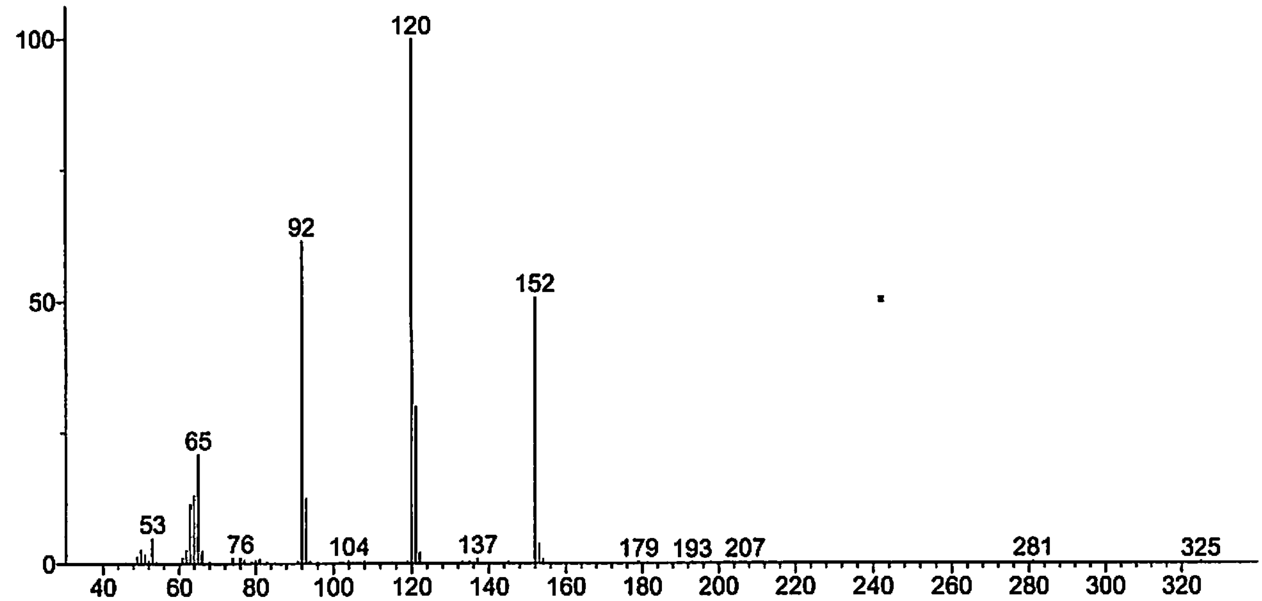

(Text File) Scan 346 (8.635 min): JA021711-1.D\data.ms (-342)

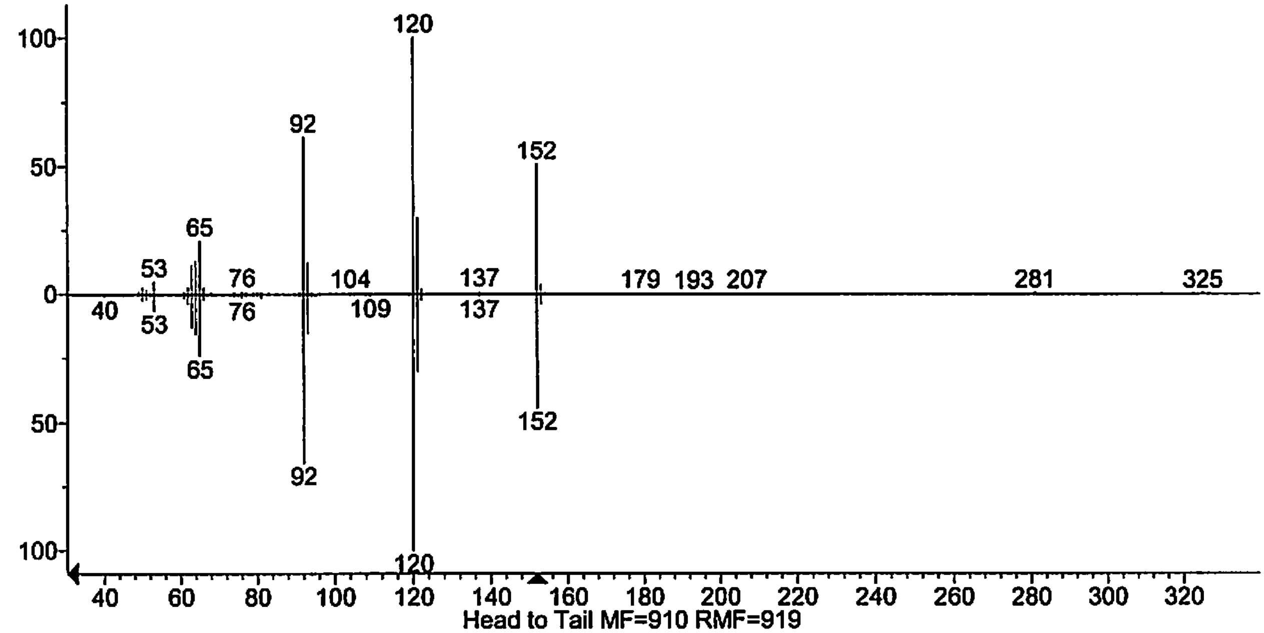

Head to Tail MF=910 RMF=919

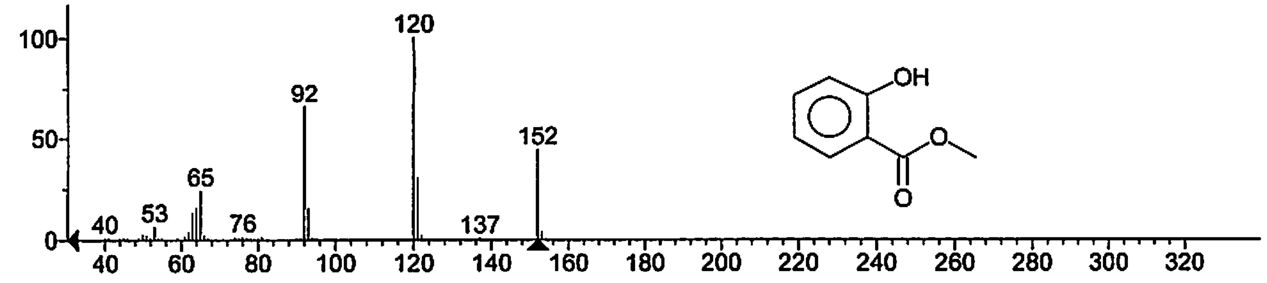

(replib) Methyl salicylate

```
File      ' :D:\Aldrich\JA-11\JA021711-1.D
Operator  :
Acquired  : 17 Feb 2011  17:20     using AcqMethod JA-50-280LESS.M
Instrument :    Buba; IIBBL's magical mass spect
Sample Name: 4M C. ocu. abd.sternites/5ul CH2Cl2;9-10 days
Misc Info  : larvae w/1ug/ul nepetalactol in honey soln.
Vial Number: 1
```

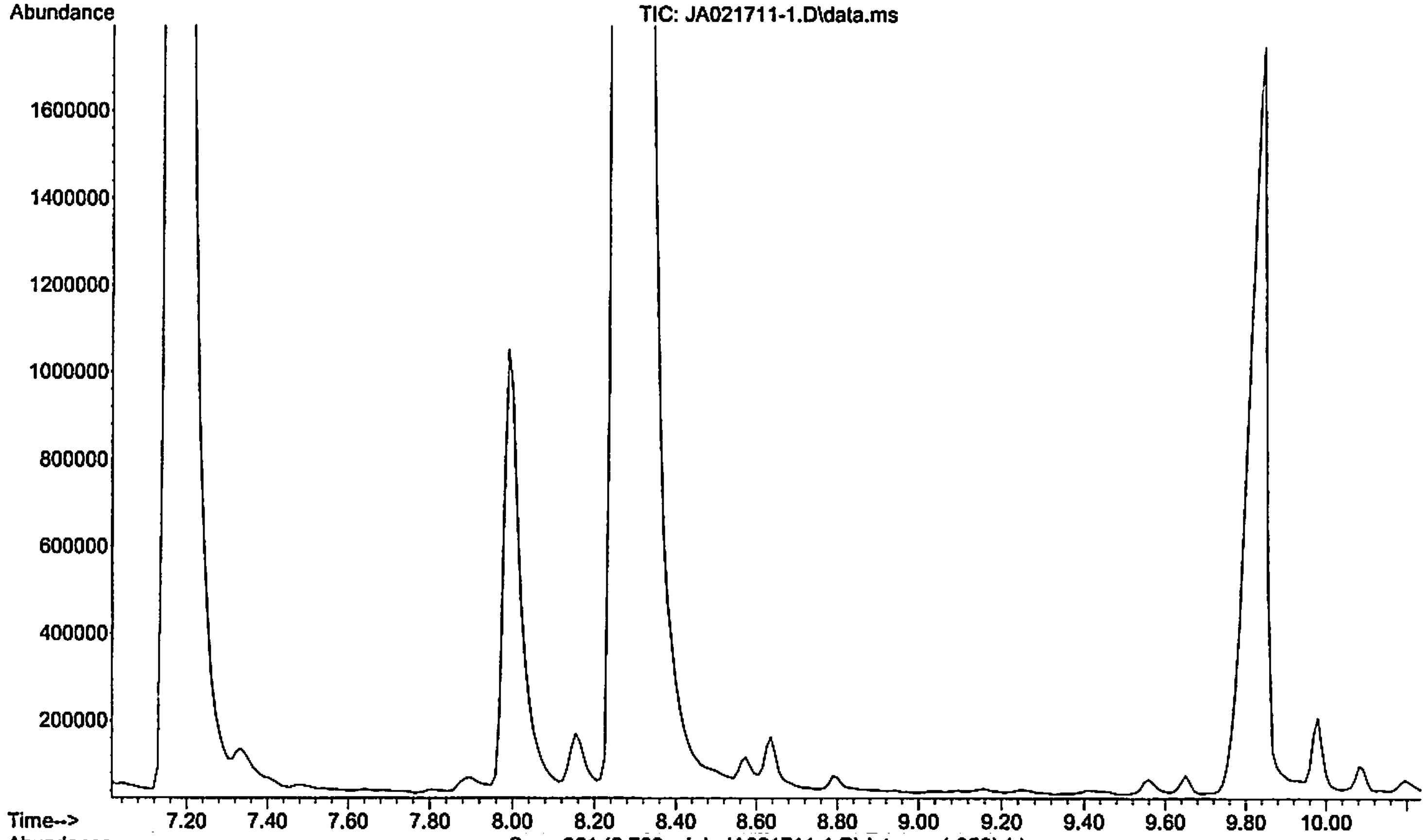

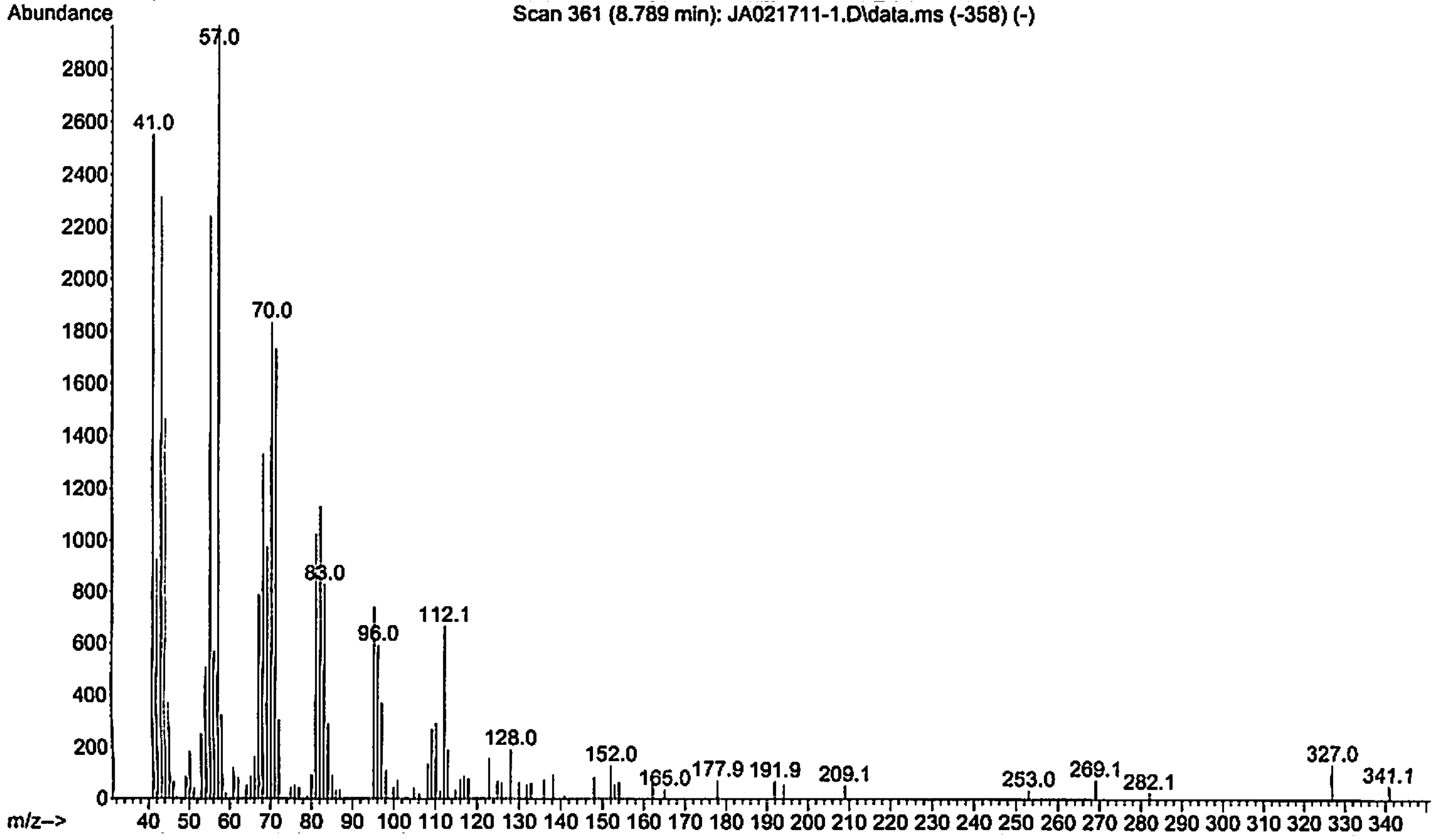

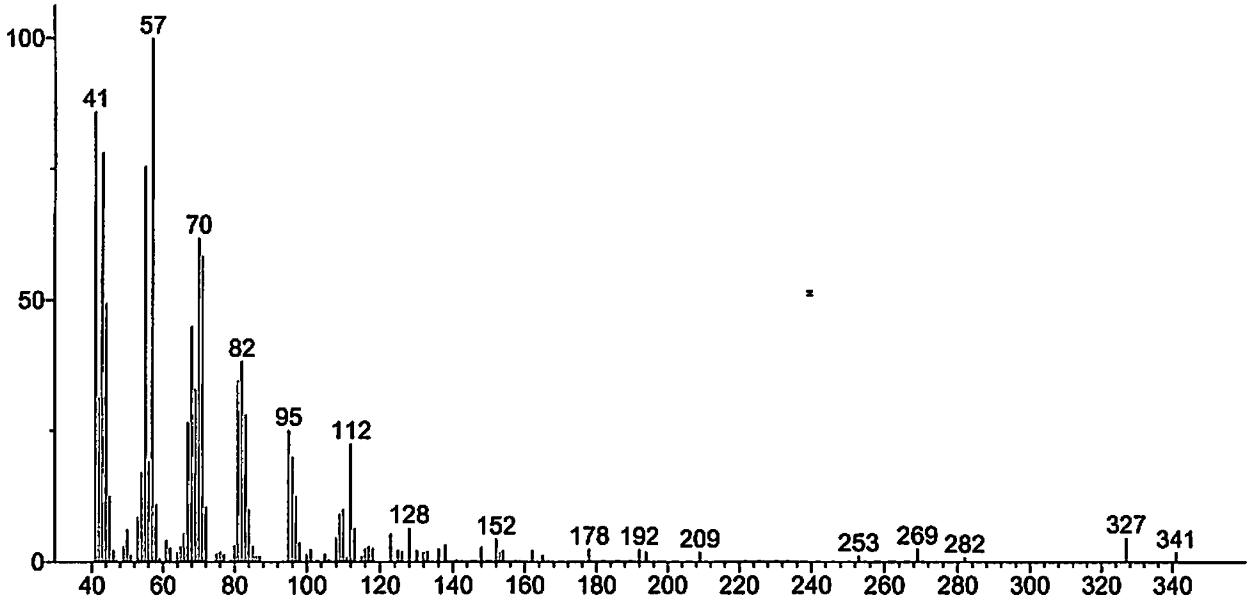

(Text File) Scan 361 (8.789 min): JA021711-1.D\data.ms (-358)

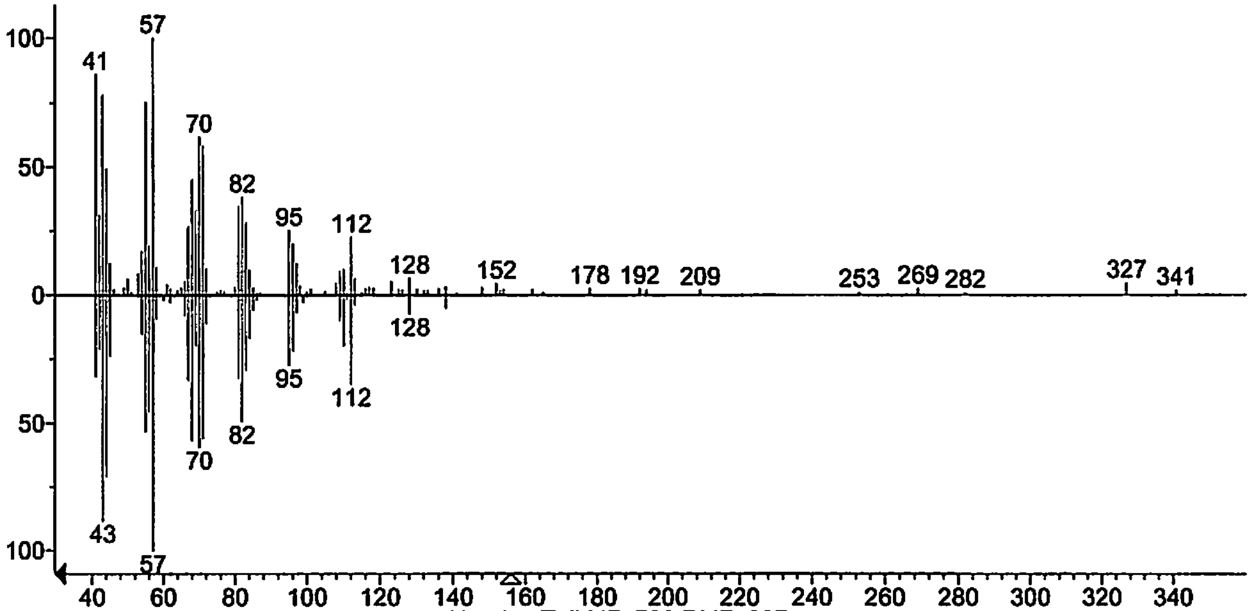

Head to Tail MF=790 RMF=887

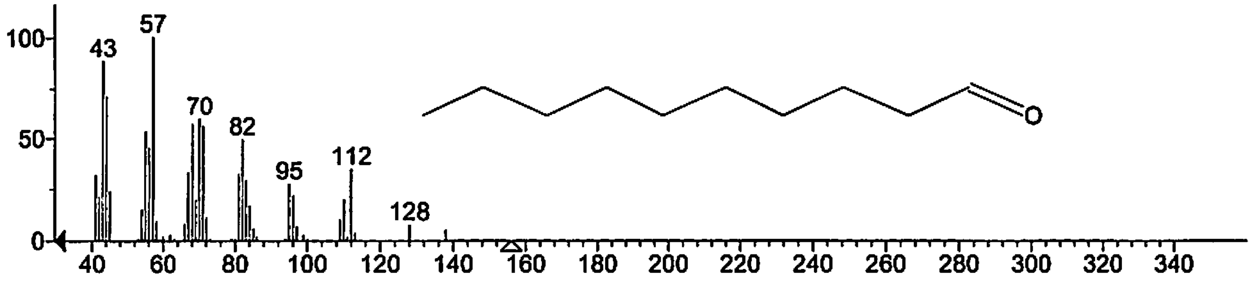

(replib) Decanal

```
File        :D:\Aldrich\JA-11\JA021711-1.D
Operator    :
Acquired    : 17 Feb 2011  17:20     using AcqMethod JA-50-280LESS.M
Instrument :    Buba; IIBBL's magical mass spect
Sample Name: 4M C. ocu. abd.sternites/5ul CH2Cl2;9-10 days
Misc Info   : larvae w/1ug/ul nepetalactol in honey soln.
Vial Number: 1
```

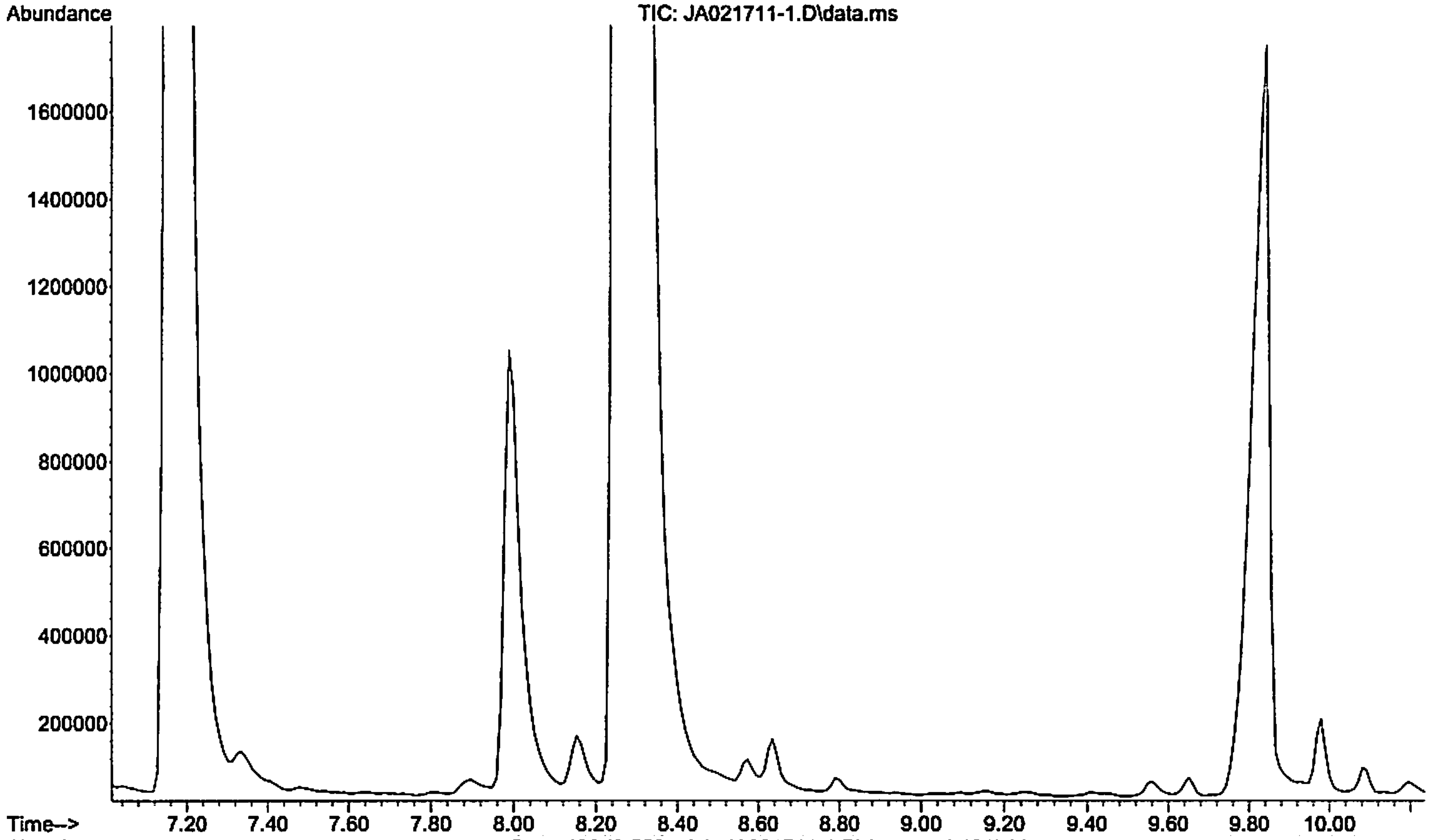

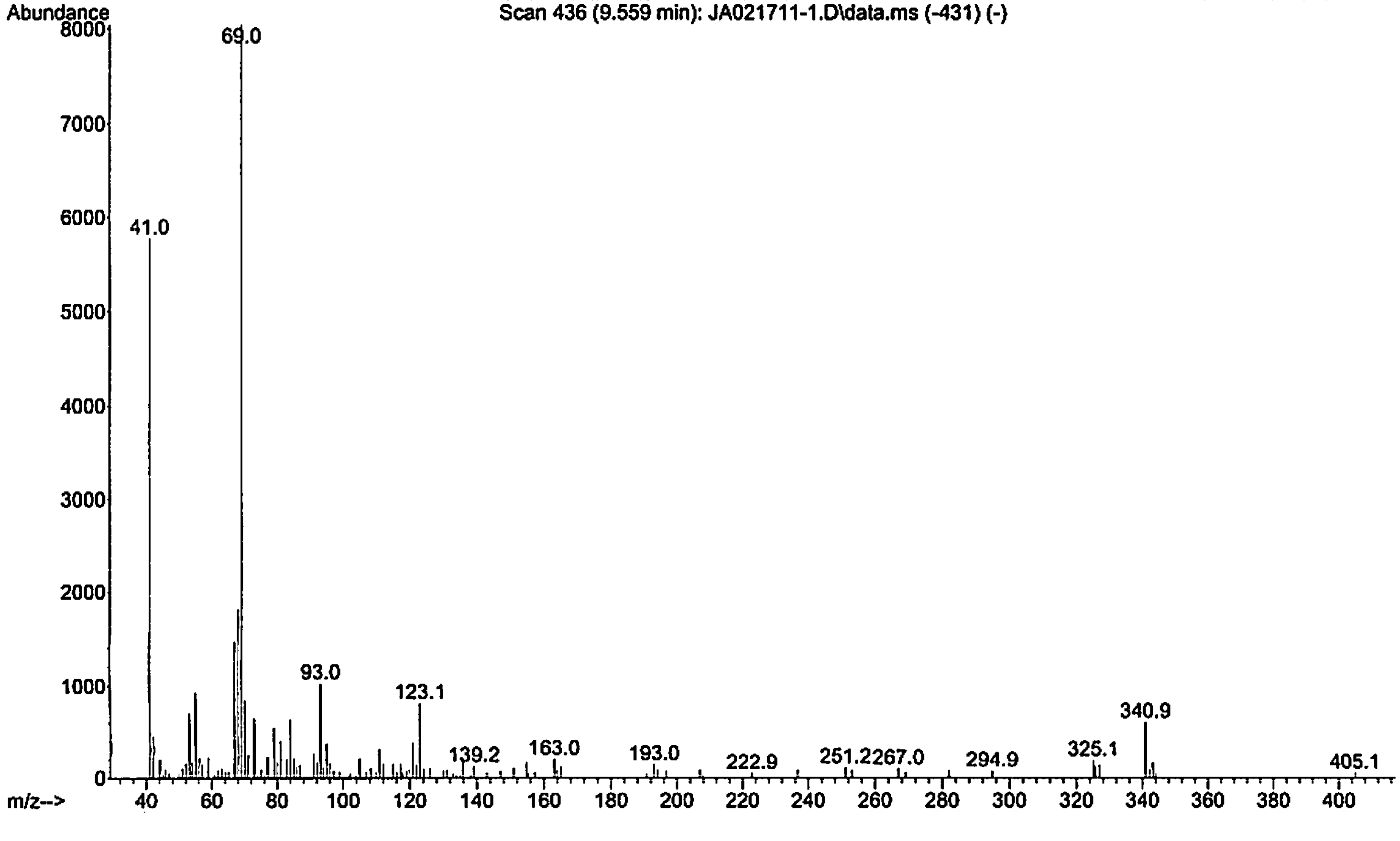

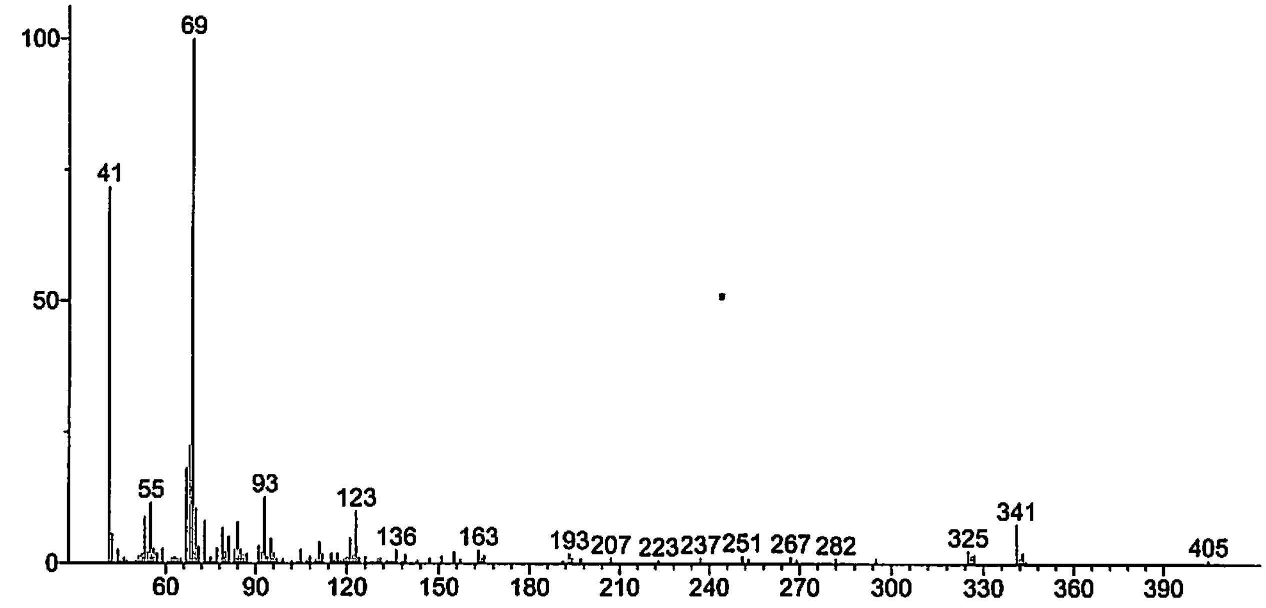

(Text File) Scan 436 (9.559 min): JA021711-1.D\data.ms (-431)

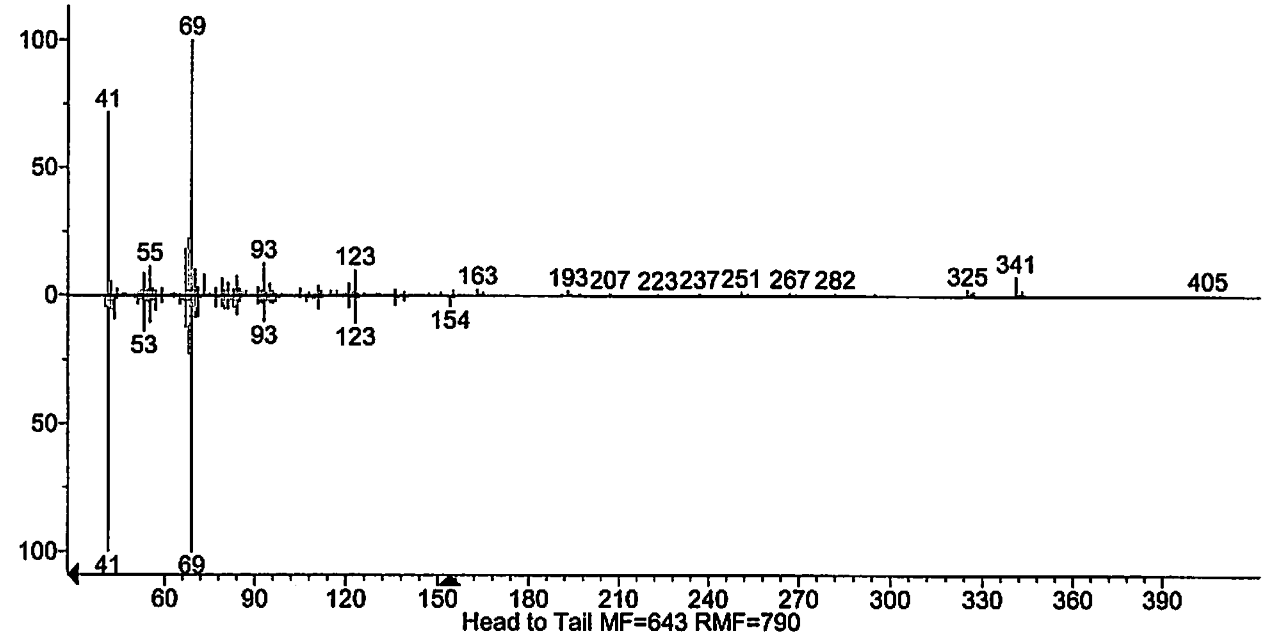

Head to Tail MF=643 RMF=790

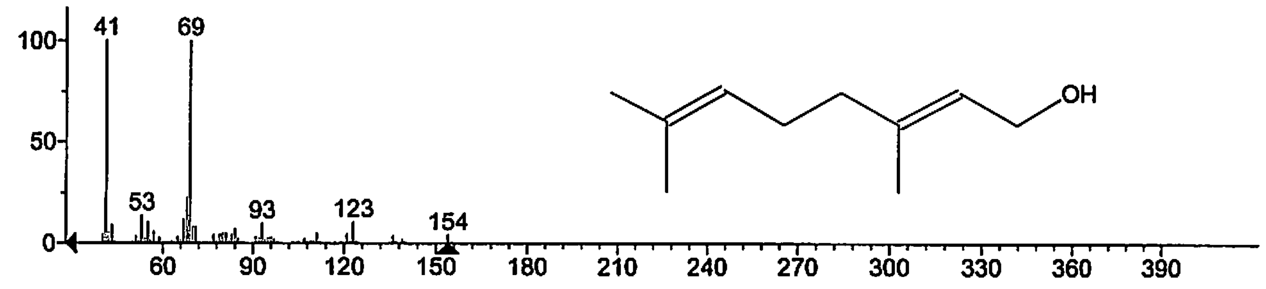

(replib) 2,6-Octadien-1-ol, 3,7-dimethyl-, (E)-

```
File       :D:\Aldrich\JA-11\JA021711-1.D
Operator   :
Acquired   : 17 Feb 2011  17:20     using AcqMethod JA-50-280LESS.M
Instrument :    Buba; IIBBL's magical mass spect
Sample Name: 4M C. ocu. abd.sternites/5ul CH2Cl2;9-10 days
Misc Info  : larvae w/1ug/ul nepetalactol in honey soln.
Vial Number: 1
```

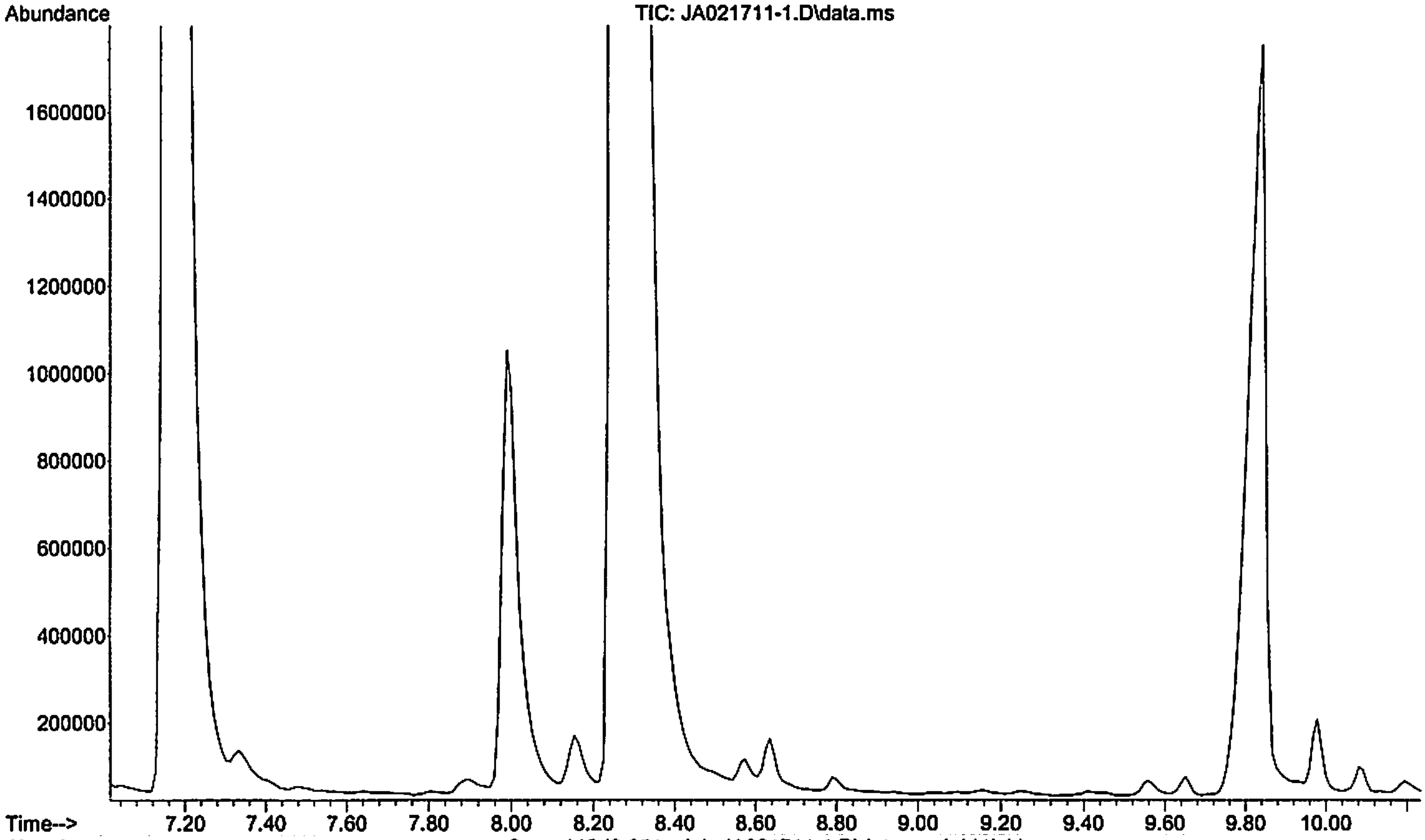

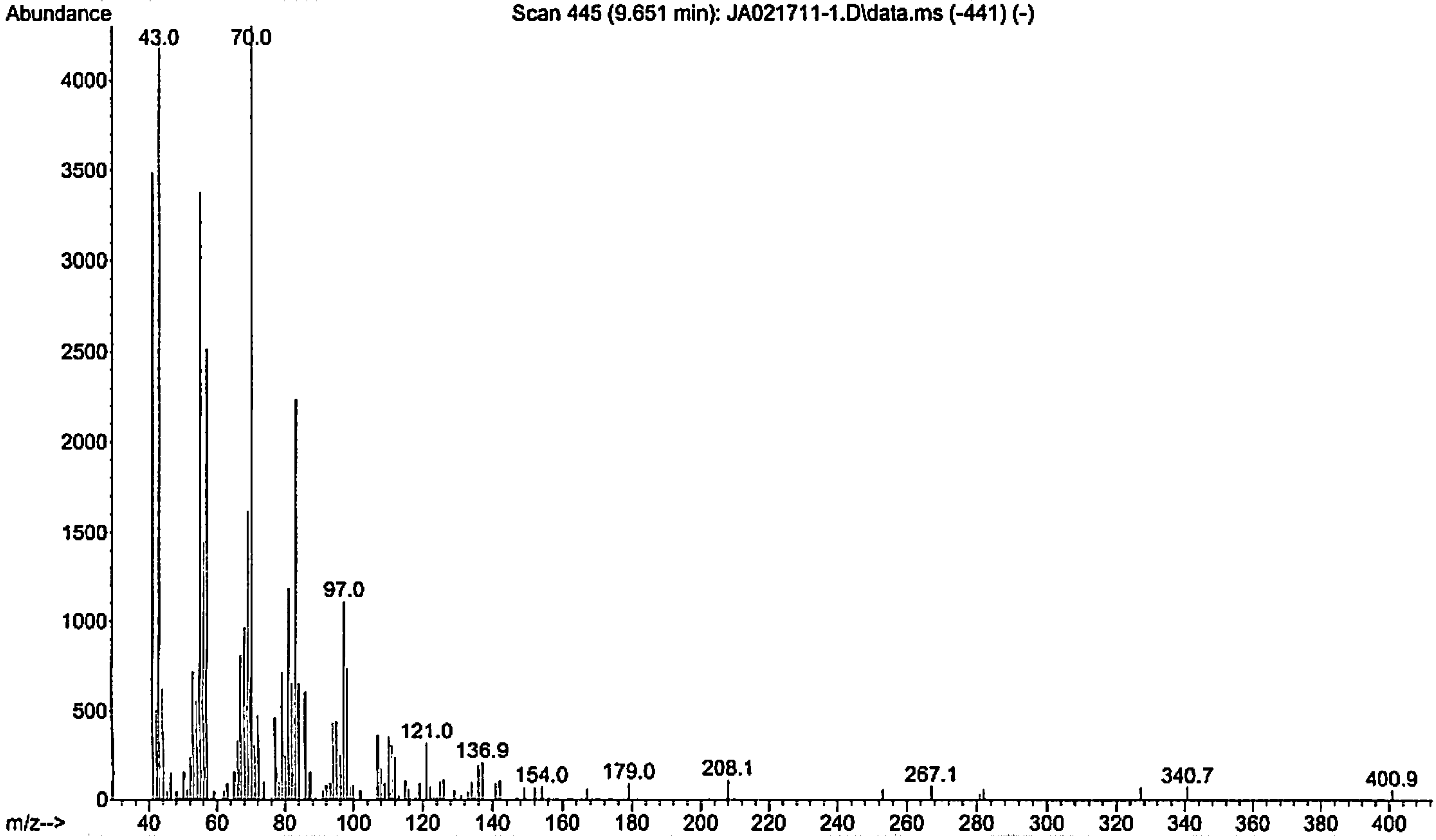

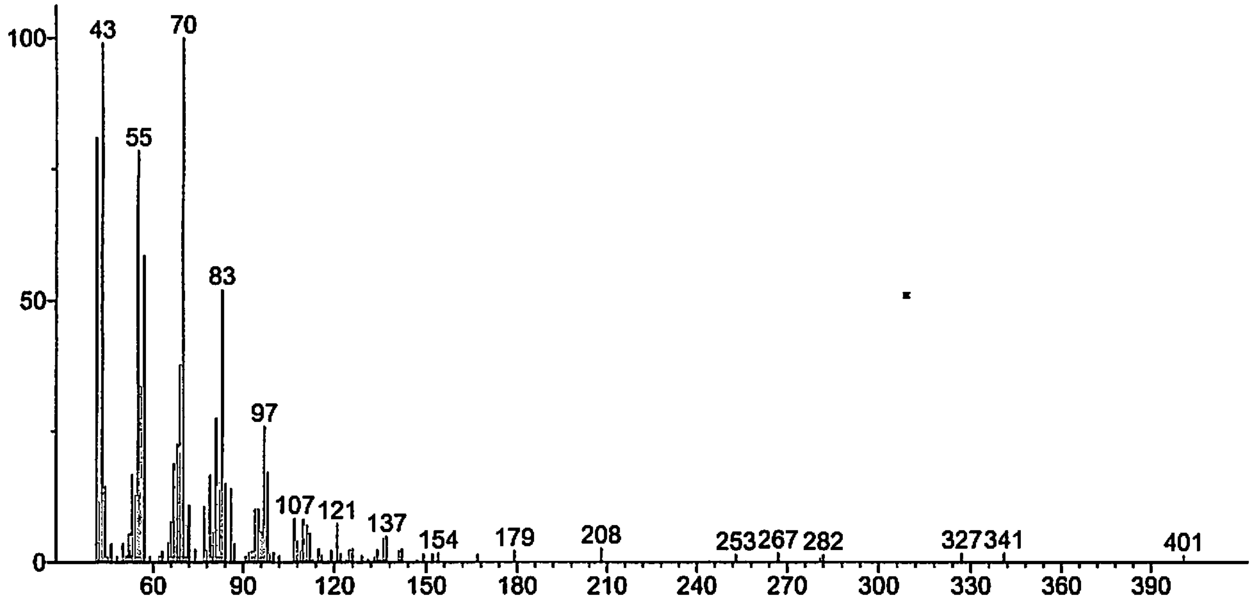

(Text File) Scan 445 (9.651 min): JA021711-1.D\data.ms (-441)

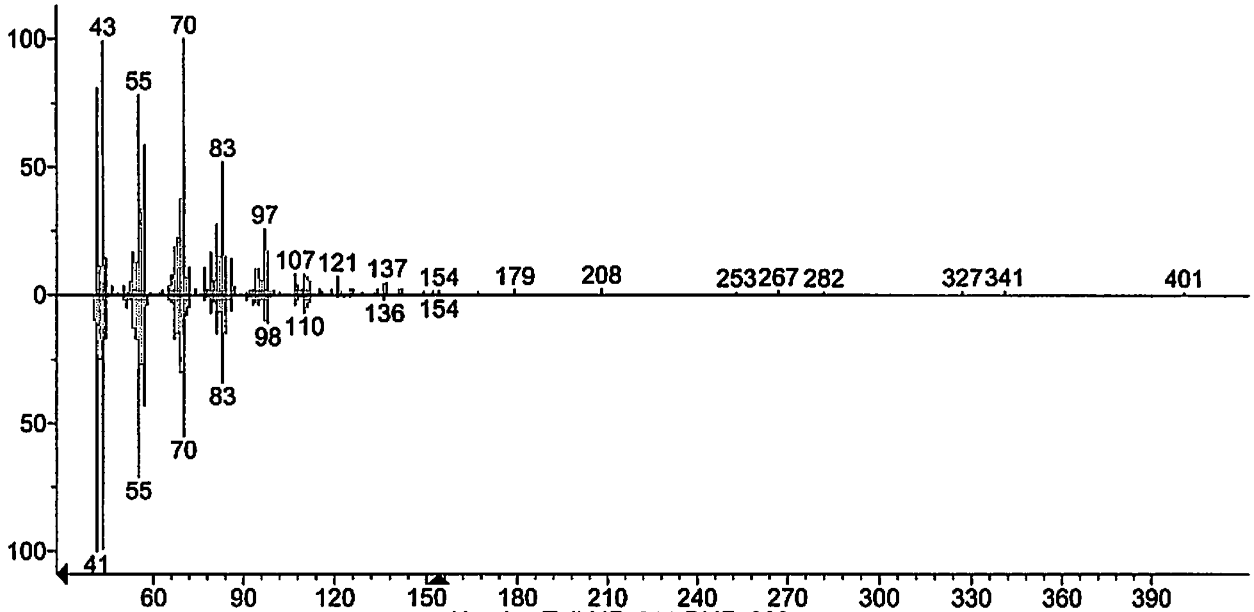

Head to Tail MF=811 RMF=882

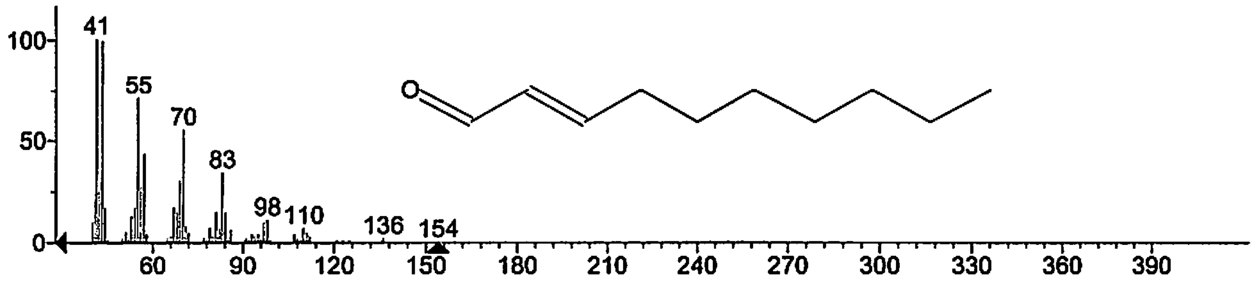

(replib) 2-Decenal, (E)-

```
File        :D:\Aldrich\JA-11\JA021711-1.D
Operator    :
Acquired    : 17 Feb 2011  17:20     using AcqMethod JA-50-280LESS.M
Instrument :     Buba; IIBBL's magical mass spect
Sample Name: 4M C. ocu. abd.sternites/5ul CH2Cl2;9-10 days
Misc Info   : larvae w/1ug/ul nepetalactol in honey soln.
Vial Number: 1
```

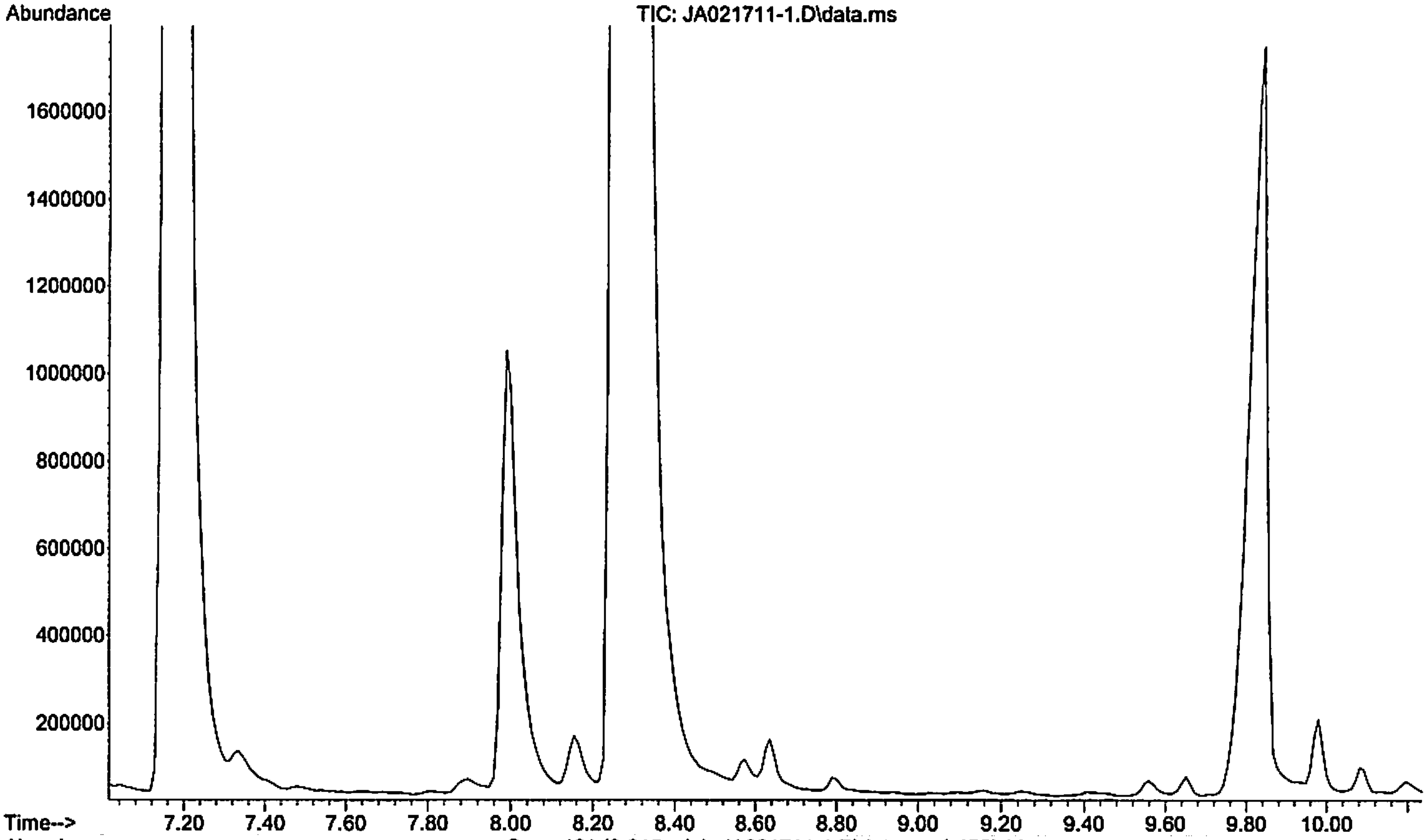

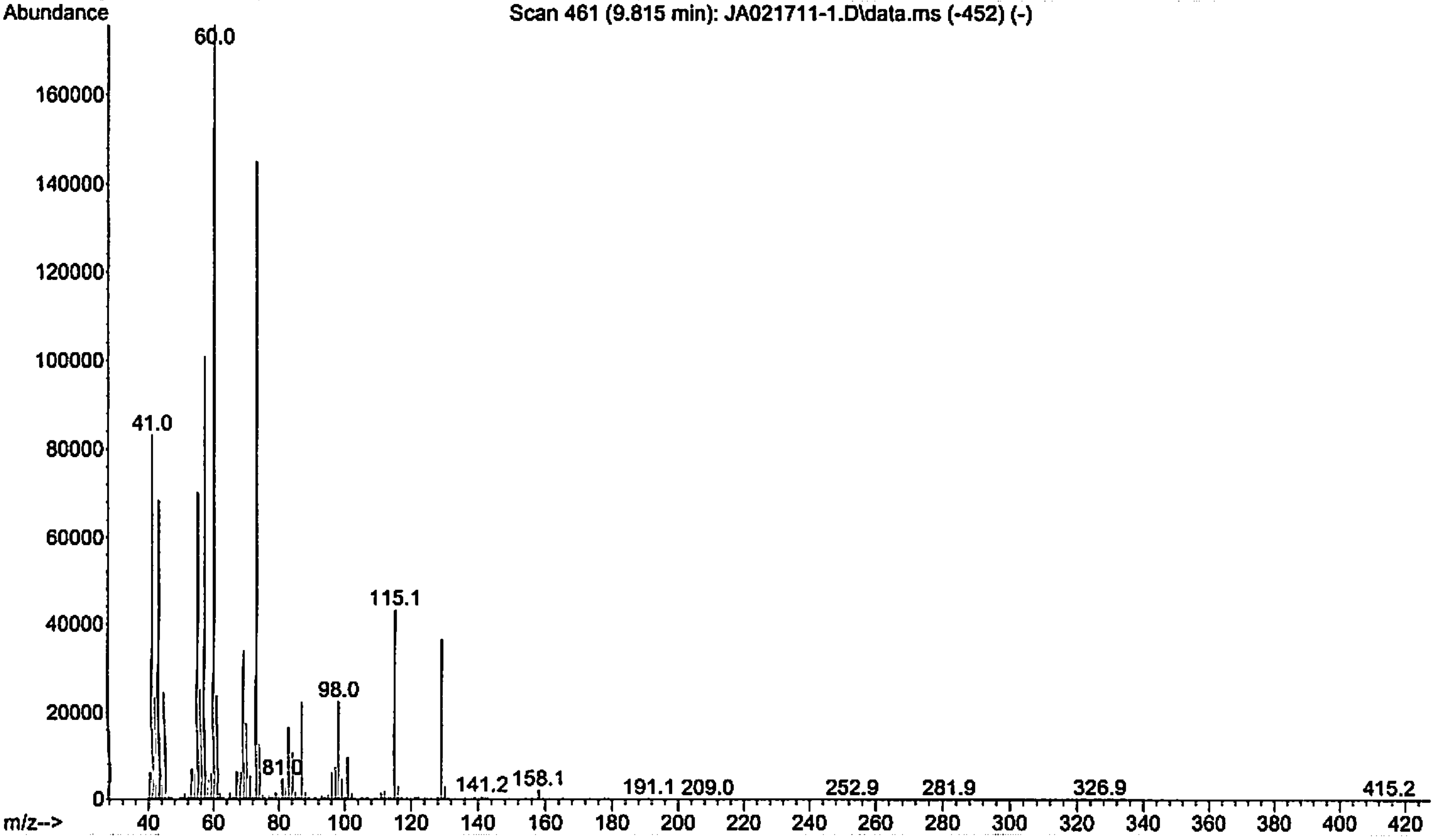

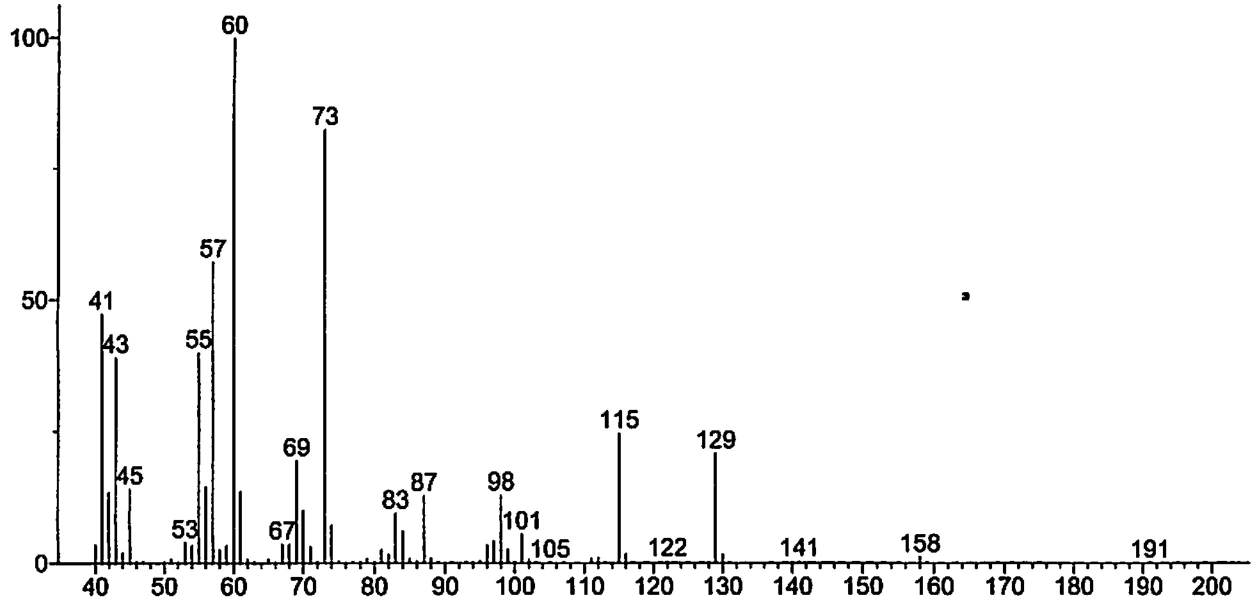

(Text File) Scan 461 (9.815 min): JA021711-1.D\data.ms (-452)

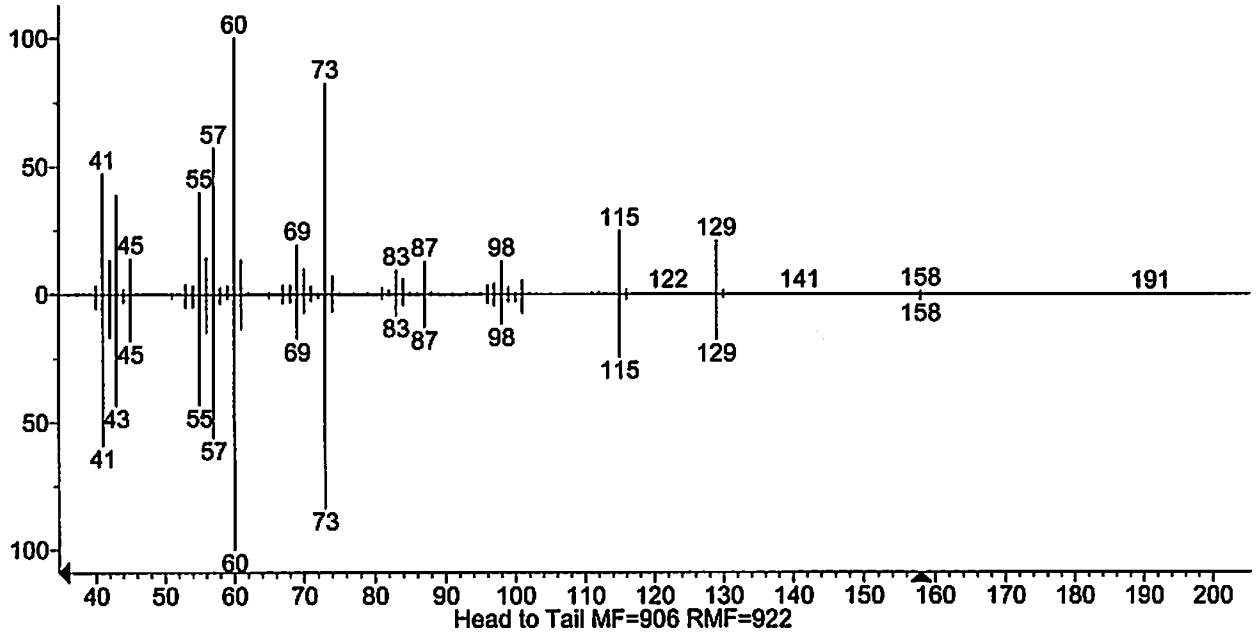

Head to Tail MF=906 RMF=922

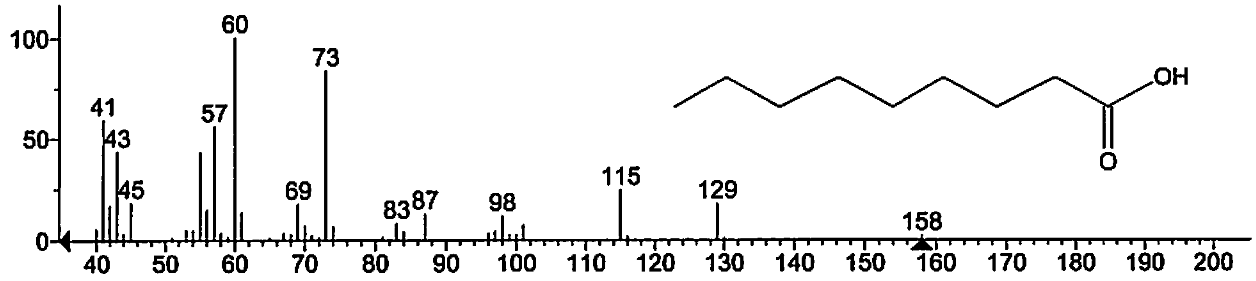

(replib) Nonanoic acid

```
File      :D:\Aldrich\JA-11\JA021711-1.D
Operator  :
Acquired  : 17 Feb 2011  17:20     using AcqMethod JA-50-280LESS.M
Instrument :    Buba; IIBBL's magical mass spect
Sample Name: 4M C. ocu. abd.sternites/5ul CH2Cl2;9-10 days
Misc Info  : larvae w/1ug/ul nepetalactol in honey soln.
Vial Number: 1
```

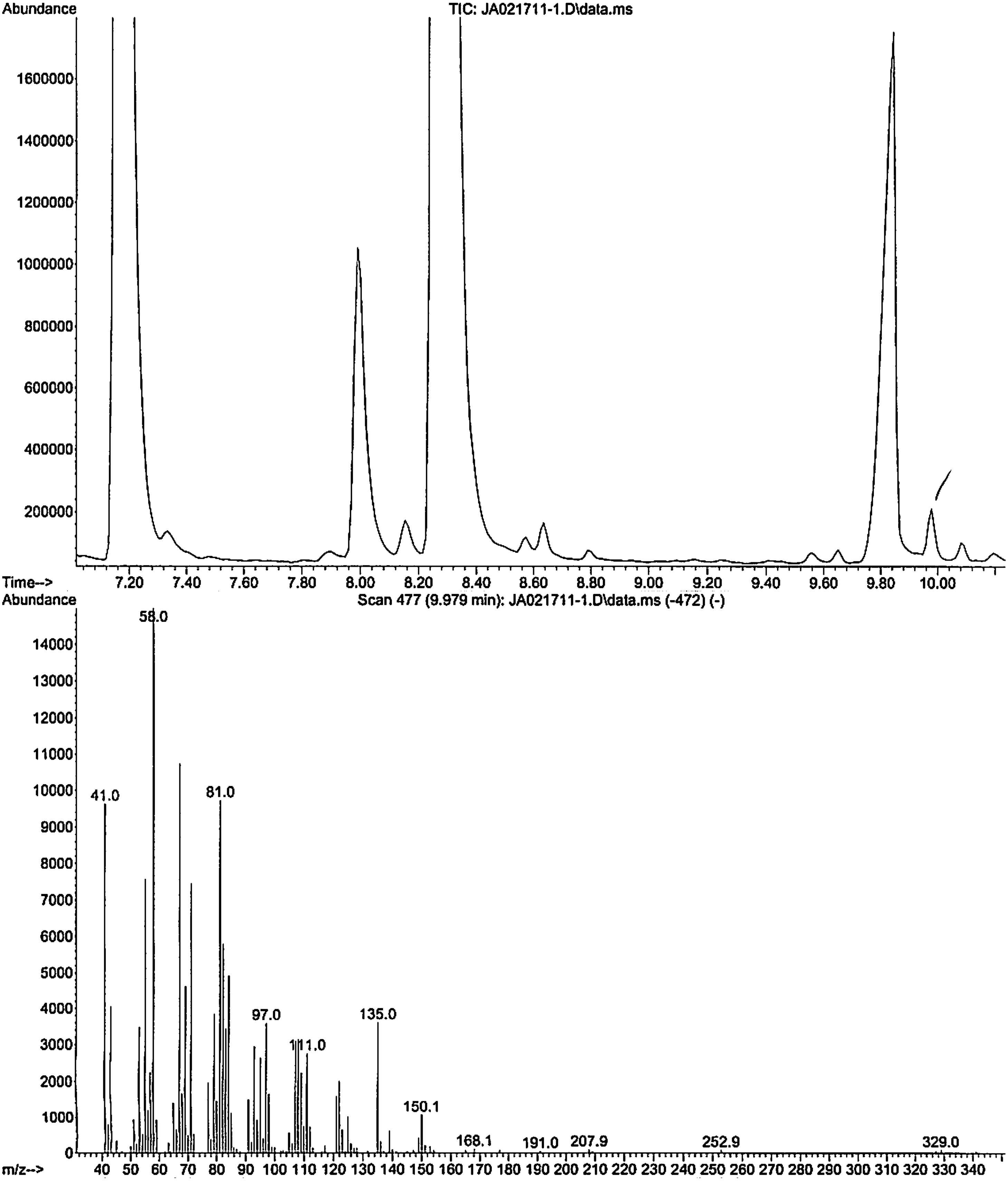

```
File      ` :D:\Aldrich\JA-11\JA021711-1.D
Operator   :
Acquired   : 17 Feb 2011  17:20     using AcqMethod JA-50-280LESS.M
Instrument :    Buba; IIBBL's magical mass spect
Sample Name: 4M C. ocu. abd.sternites/5ul CH2Cl2;9-10 days
Misc Info  : larvae w/1ug/ul nepetalactol in honey soln.
Vial Number: 1
```

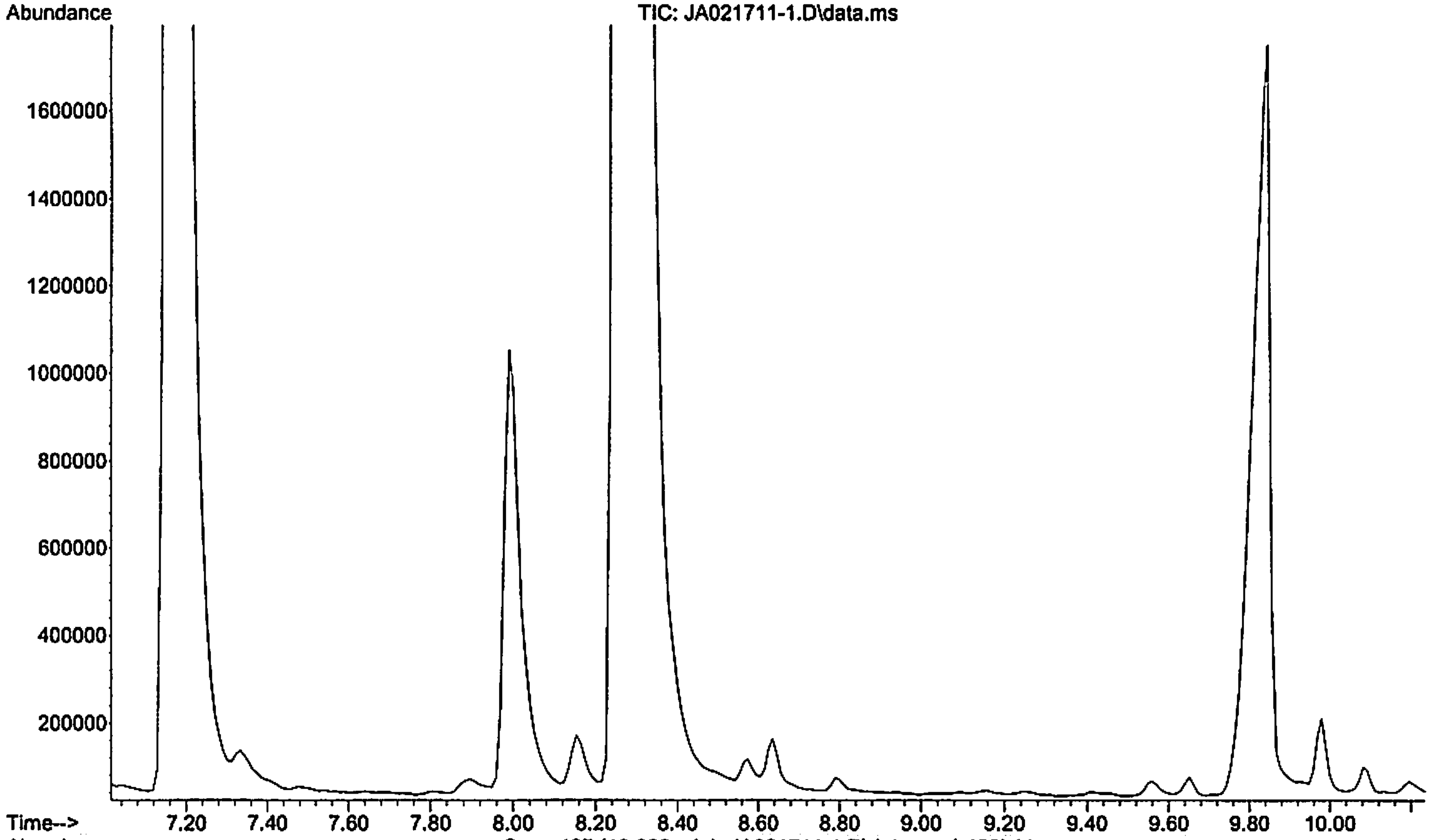

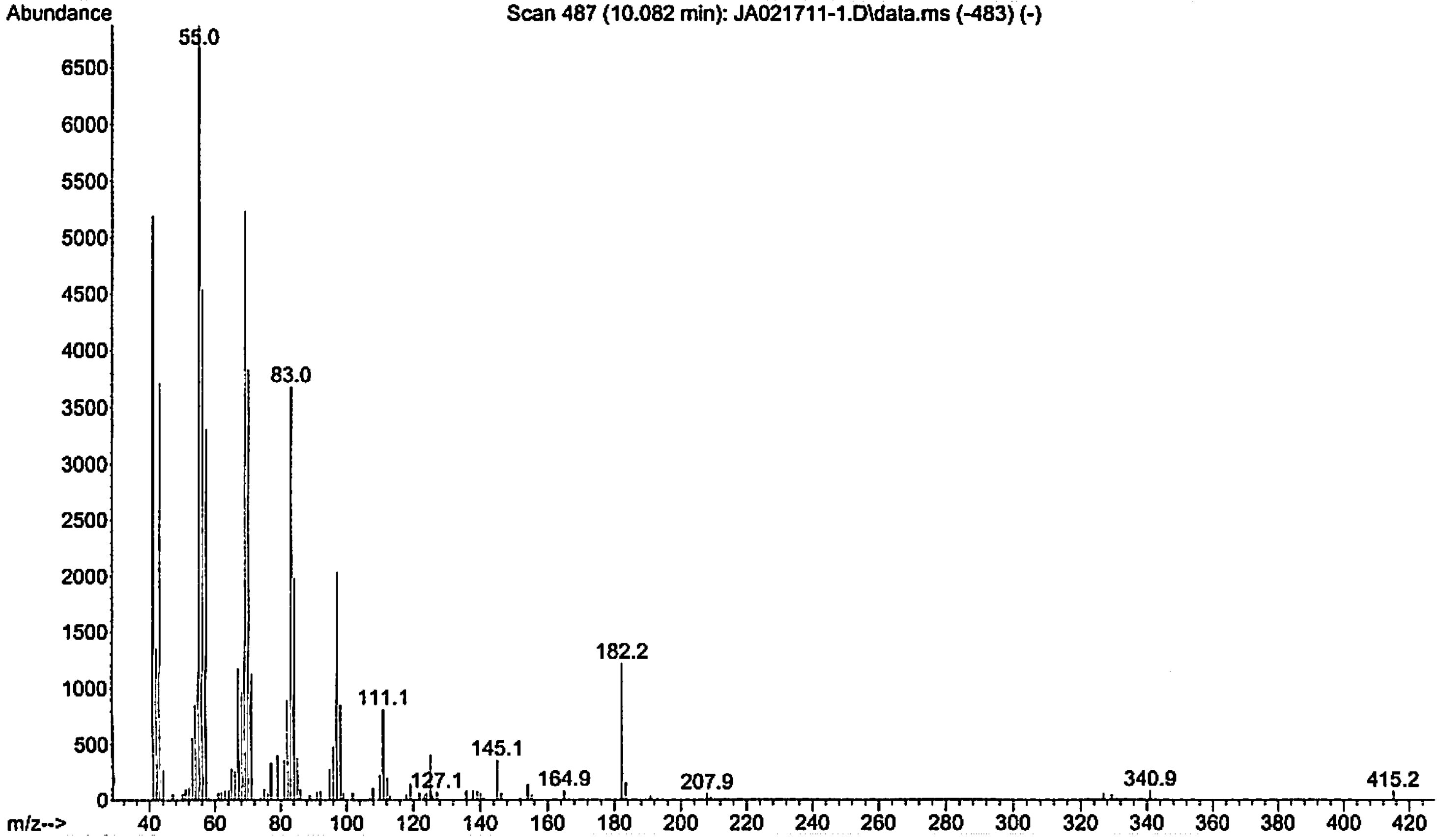

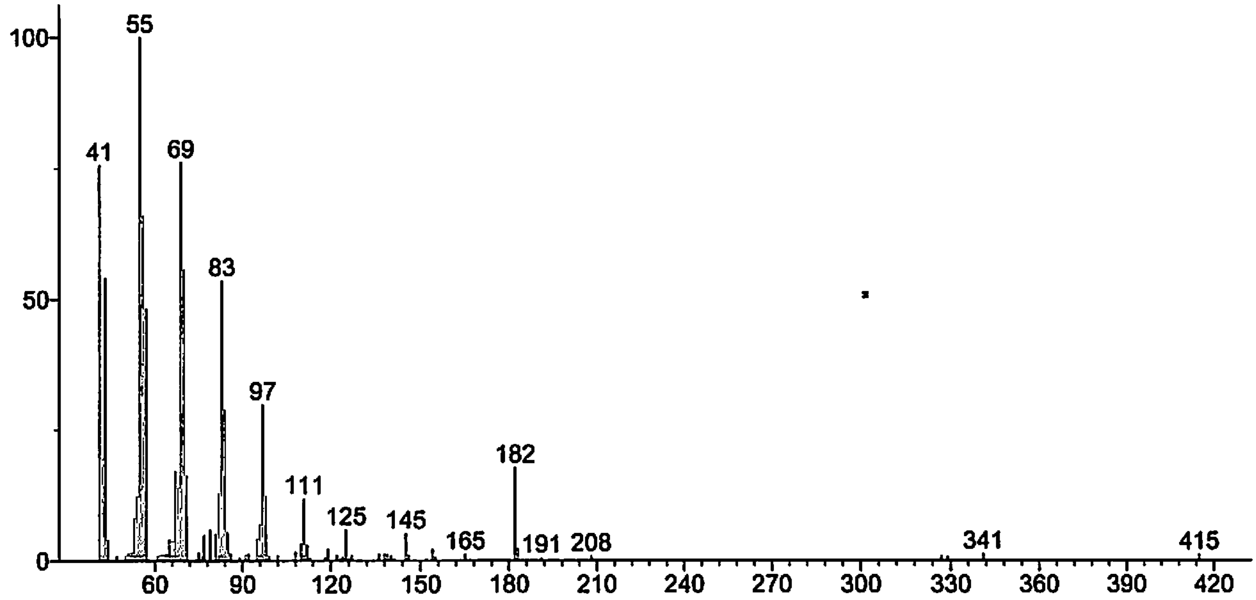

(Text File) Scan 487 (10.082 min): JA021711-1.D\data.ms (-483)

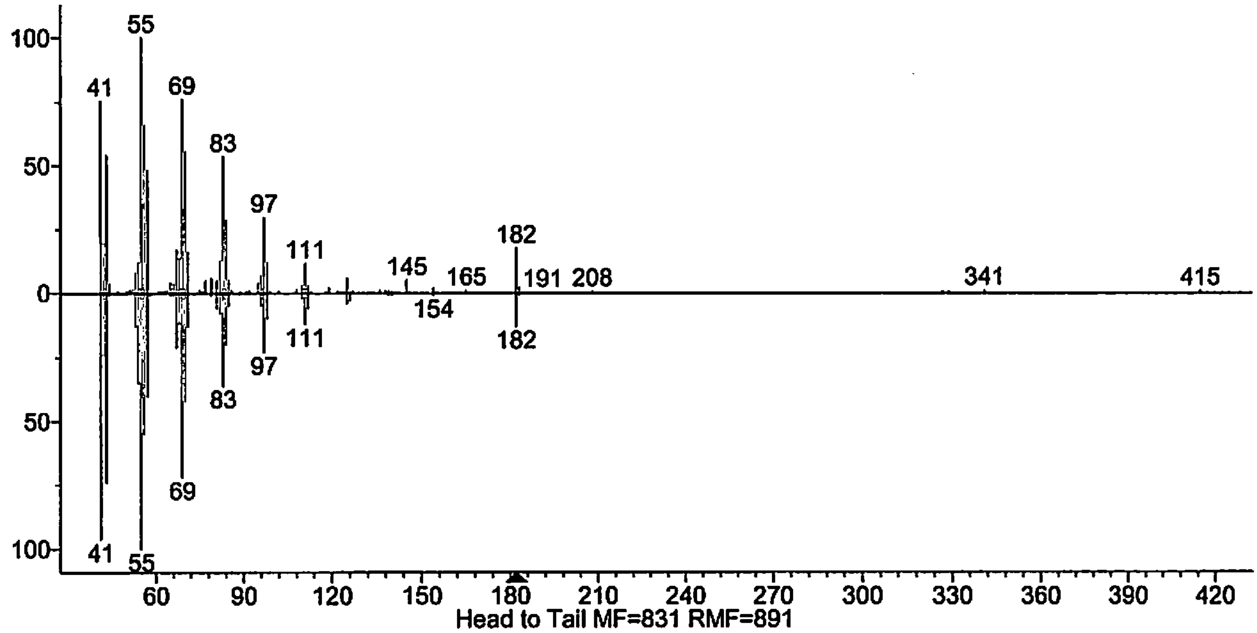

Head to Tail MF=831 RMF=891

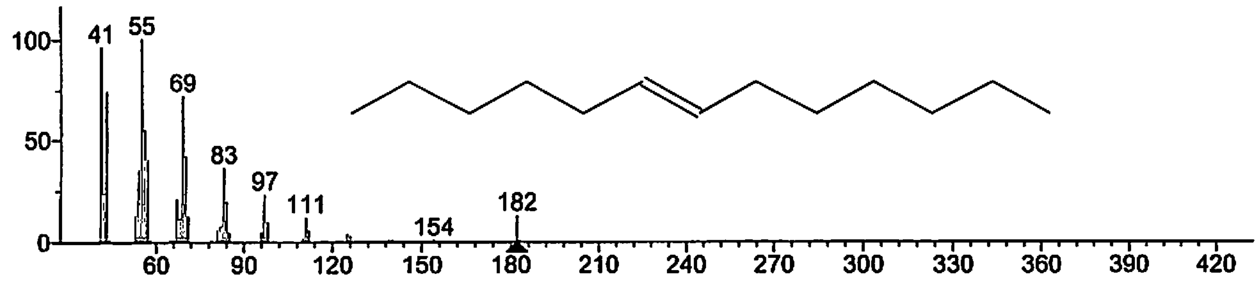

(mainlib) 6-Tridecene

File   ' :D:\Aldrich\JA-11\JA021711-1.D
Operator   :
Acquired   : 17 Feb 2011 17:20   using AcqMethod JA-50-280LESS.M
Instrument :   Buba; IIBBL's magical mass spect
Sample Name: 4M C. ocu. abd.sternites/5ul CH2Cl2;9-10 days
Misc Info   : larvae w/1ug/ul nepetalactol in honey soln.
Vial Number: 1

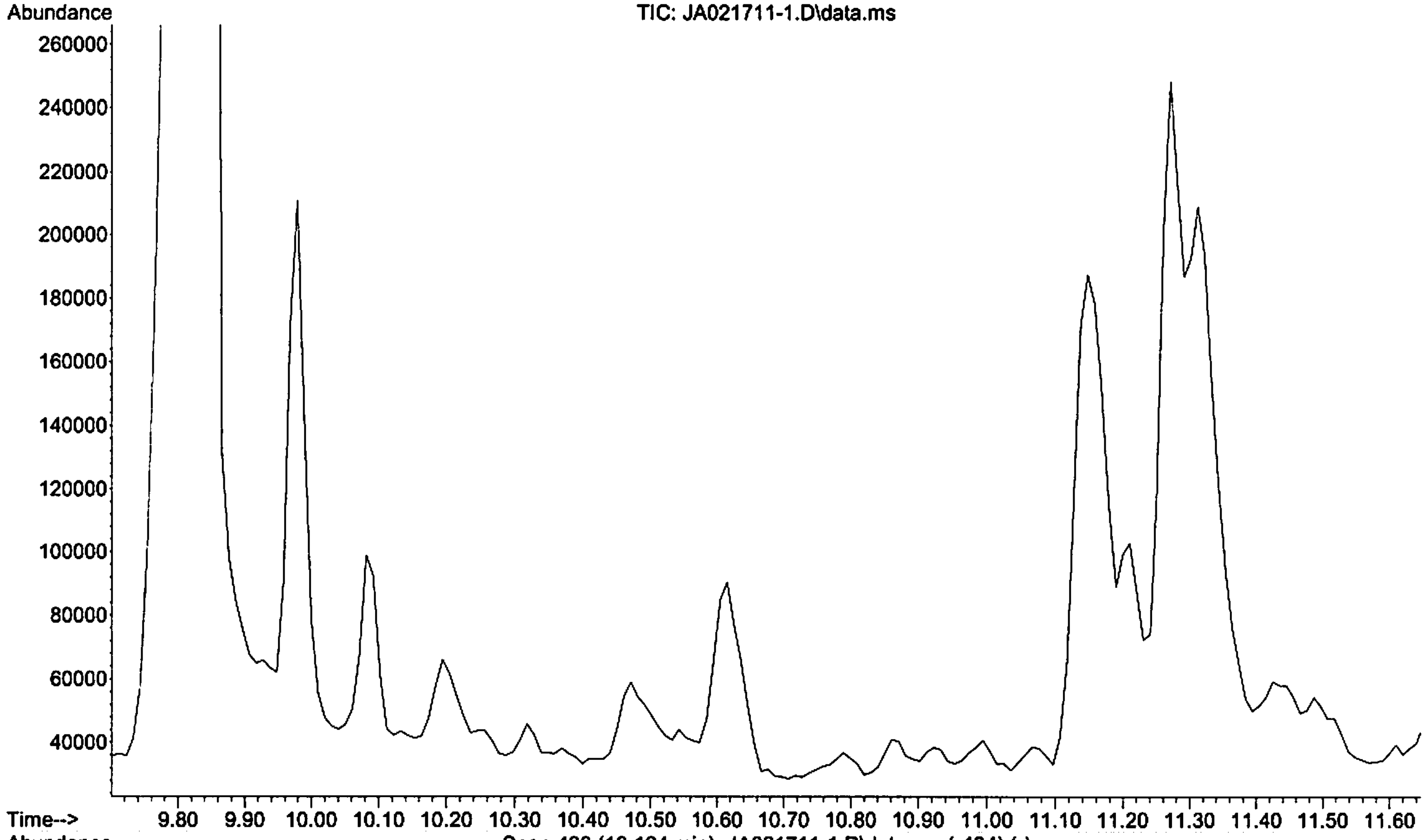

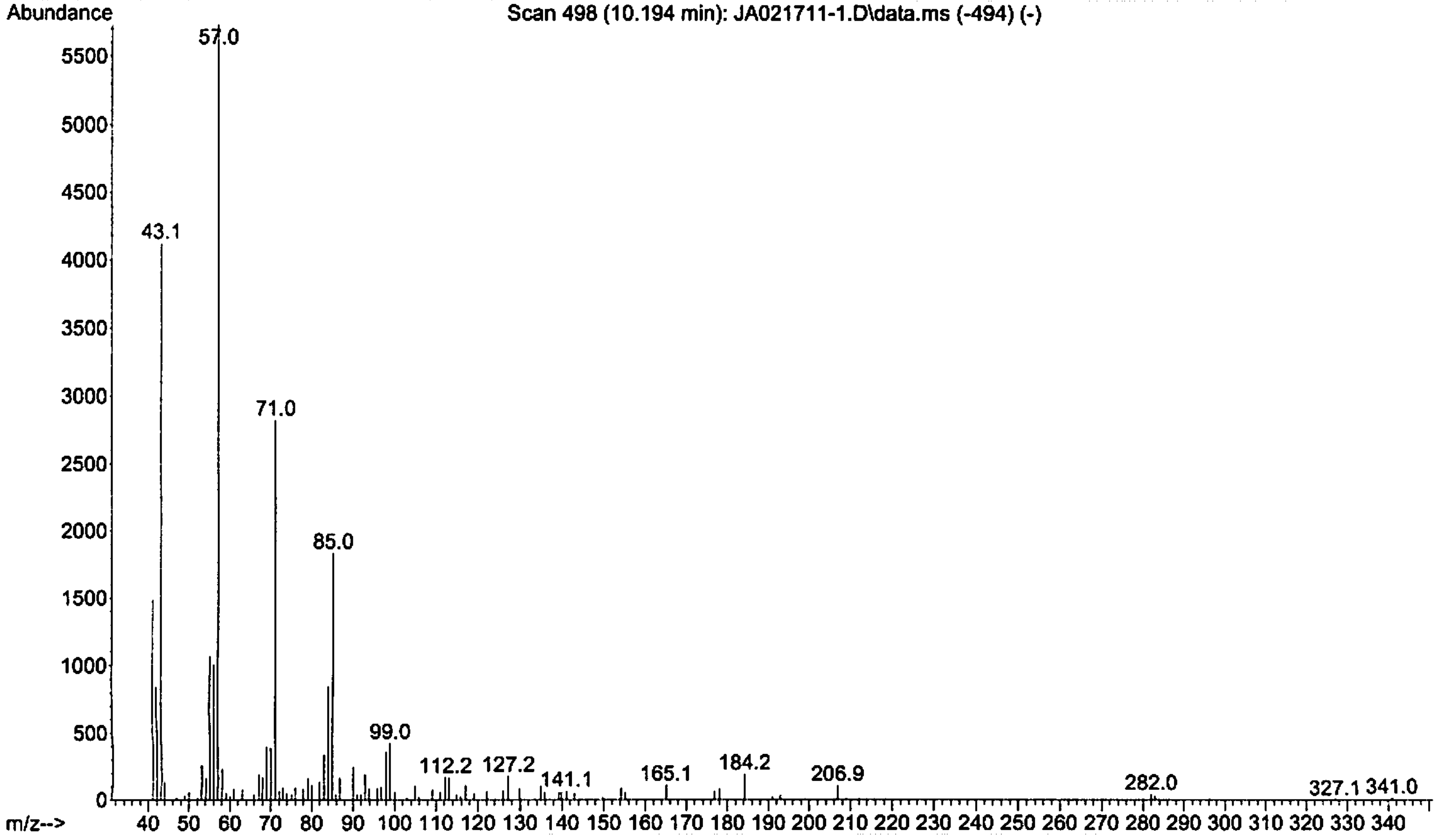

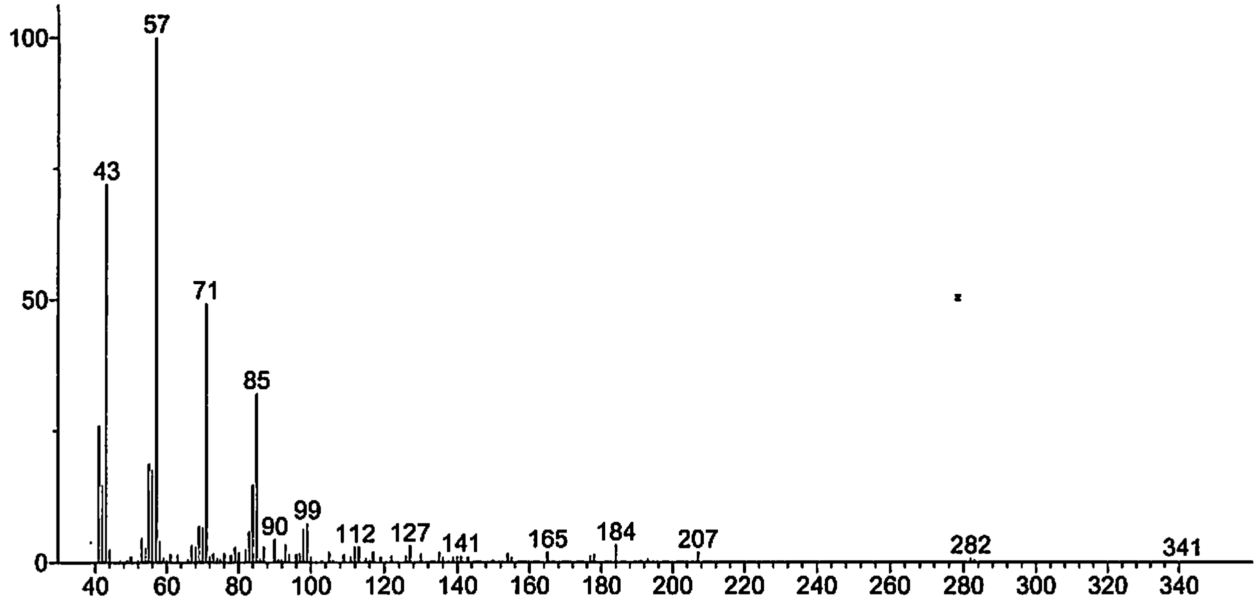

(Text File) Scan 498 (10.194 min): JA021711-1.D\data.ms (-494)

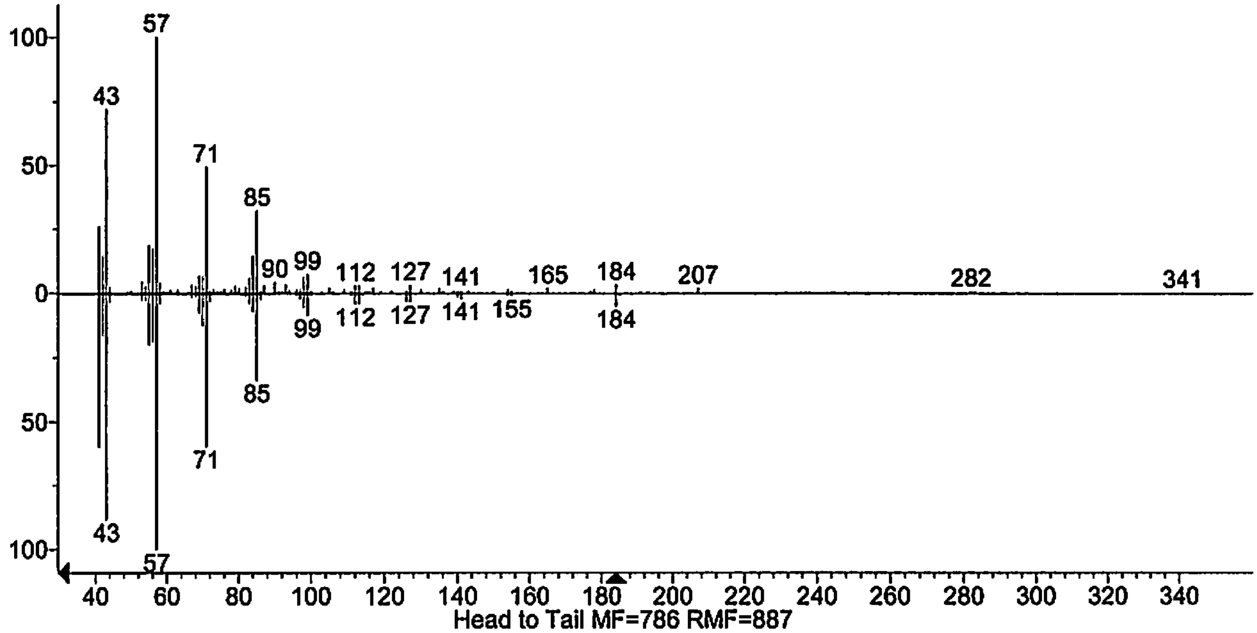

Head to Tail MF=786 RMF=887

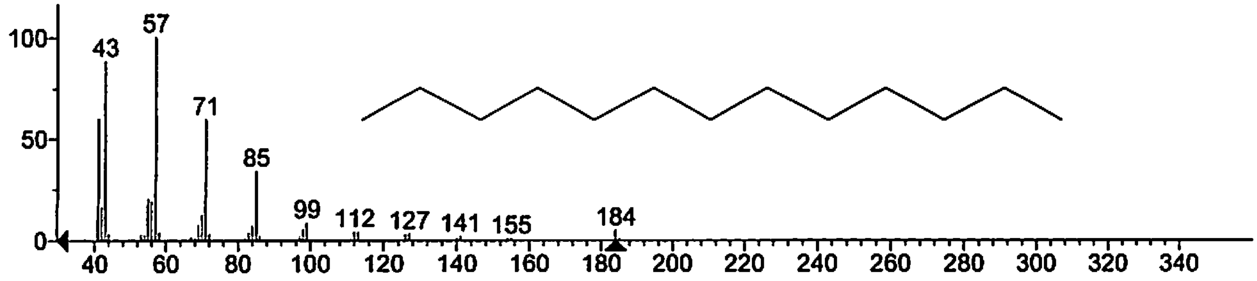

(replib) Tridecane

```
File      :D:\Aldrich\JA-11\JA021711-1.D
Operator  :
Acquired  : 17 Feb 2011  17:20     using AcqMethod JA-50-280LESS.M
Instrument :    Buba; IIBBL's magical mass spect
Sample Name: 4M C. ocu. abd.sternites/5ul CH2Cl2;9-10 days
Misc Info  : larvae w/1ug/ul nepetalactol in honey soln.
Vial Number: 1
```

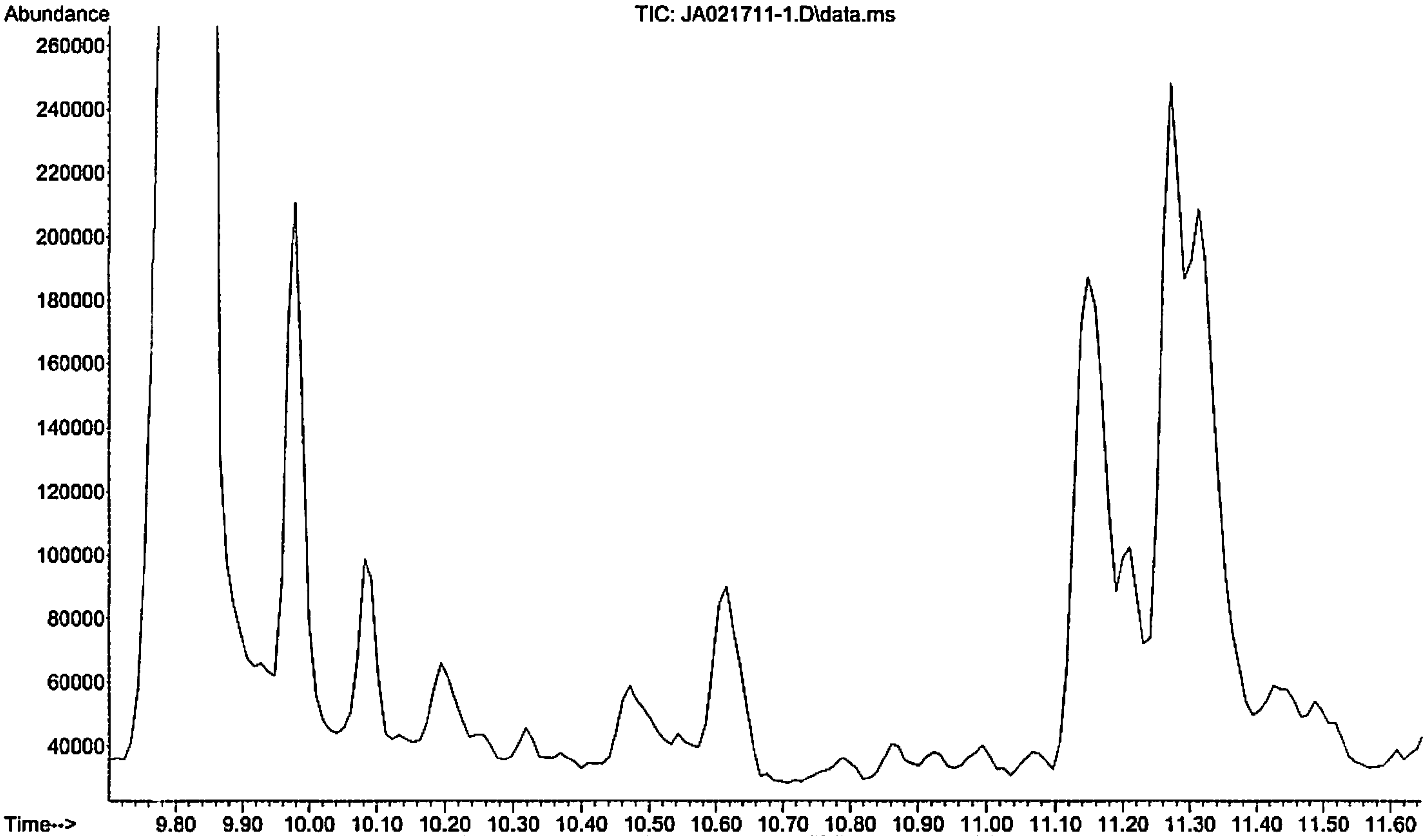

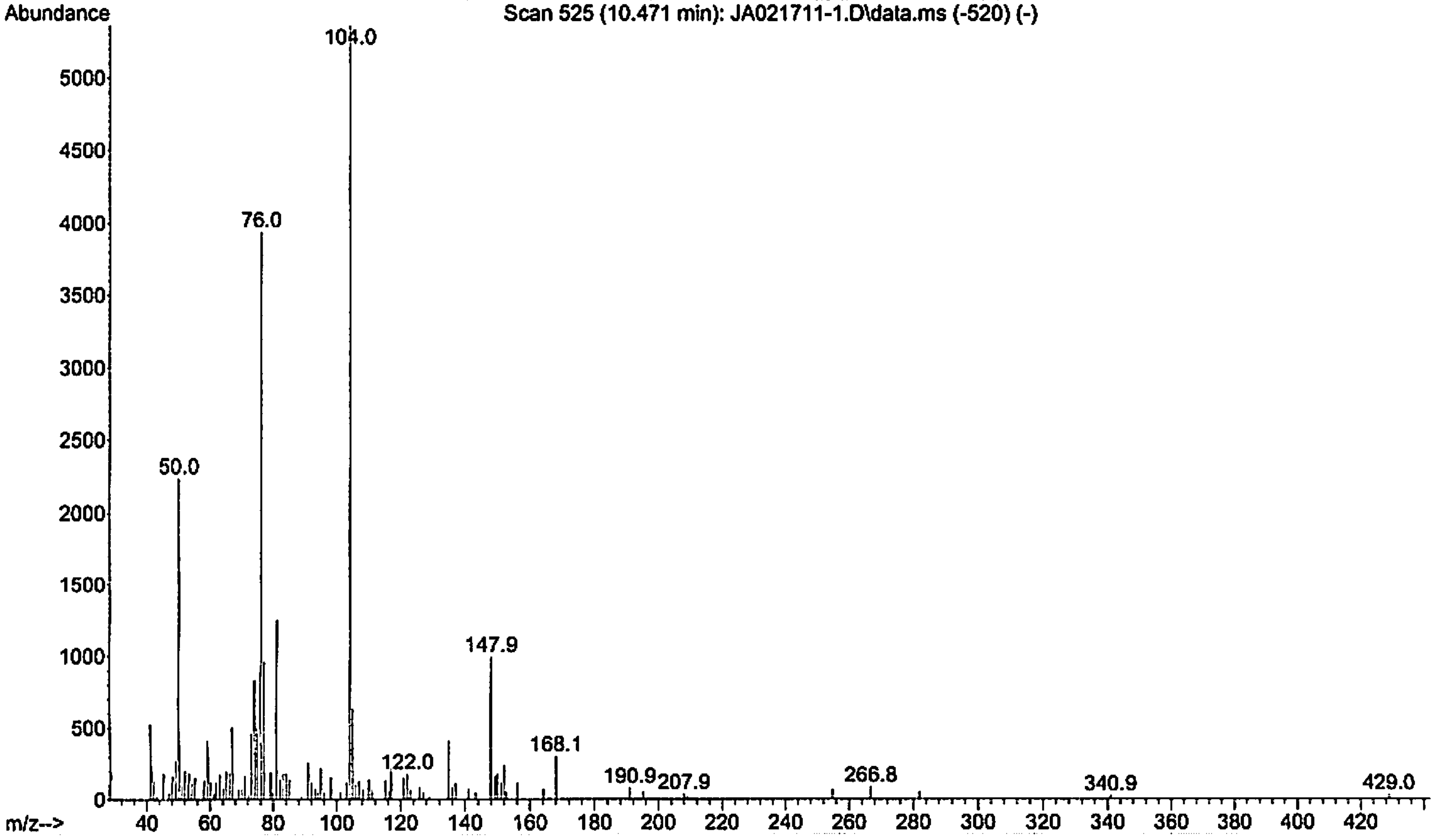

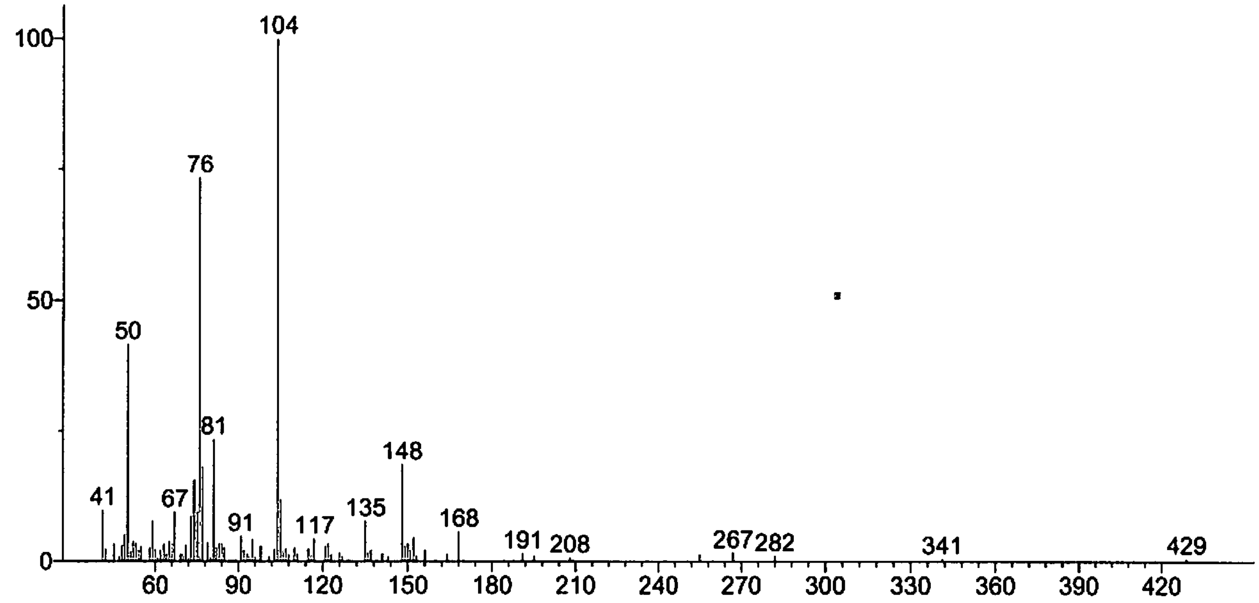

(Text File) Scan 525 (10.471 min): JA021711-1.D\data.ms (-520)

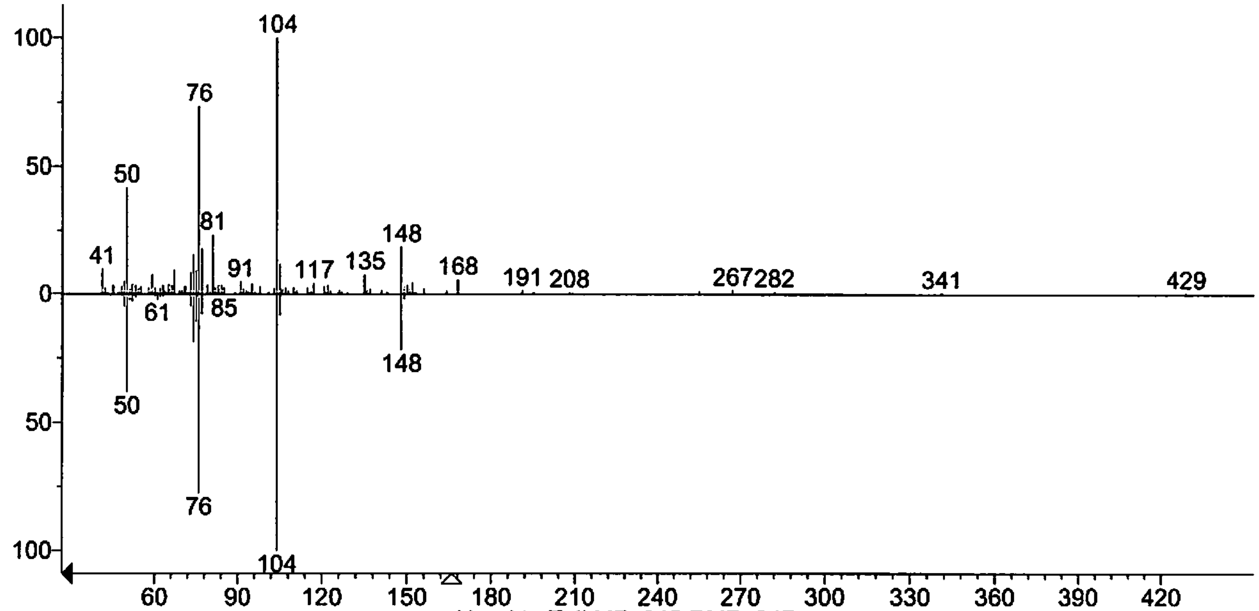

Head to Tail MF=645 RMF=847

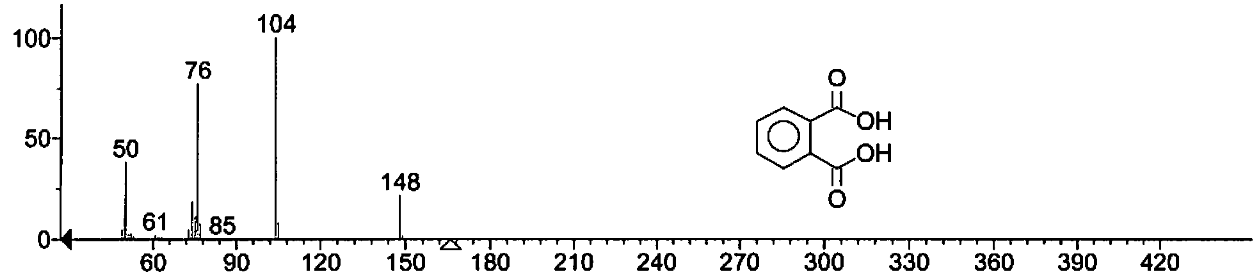

(replib) 1,2-Benzenedicarboxylic acid

```
File       :D:\Aldrich\JA-11\JA021711-1.D
Operator   :
Acquired   : 17 Feb 2011  17:20     using AcqMethod JA-50-280LESS.M
Instrument :    Buba; IIBBL's magical mass spect
Sample Name: 4M C. ocu. abd.sternites/5ul CH2Cl2;9-10 days
Misc Info  : larvae w/1ug/ul nepetalactol in honey soln.
Vial Number: 1
```

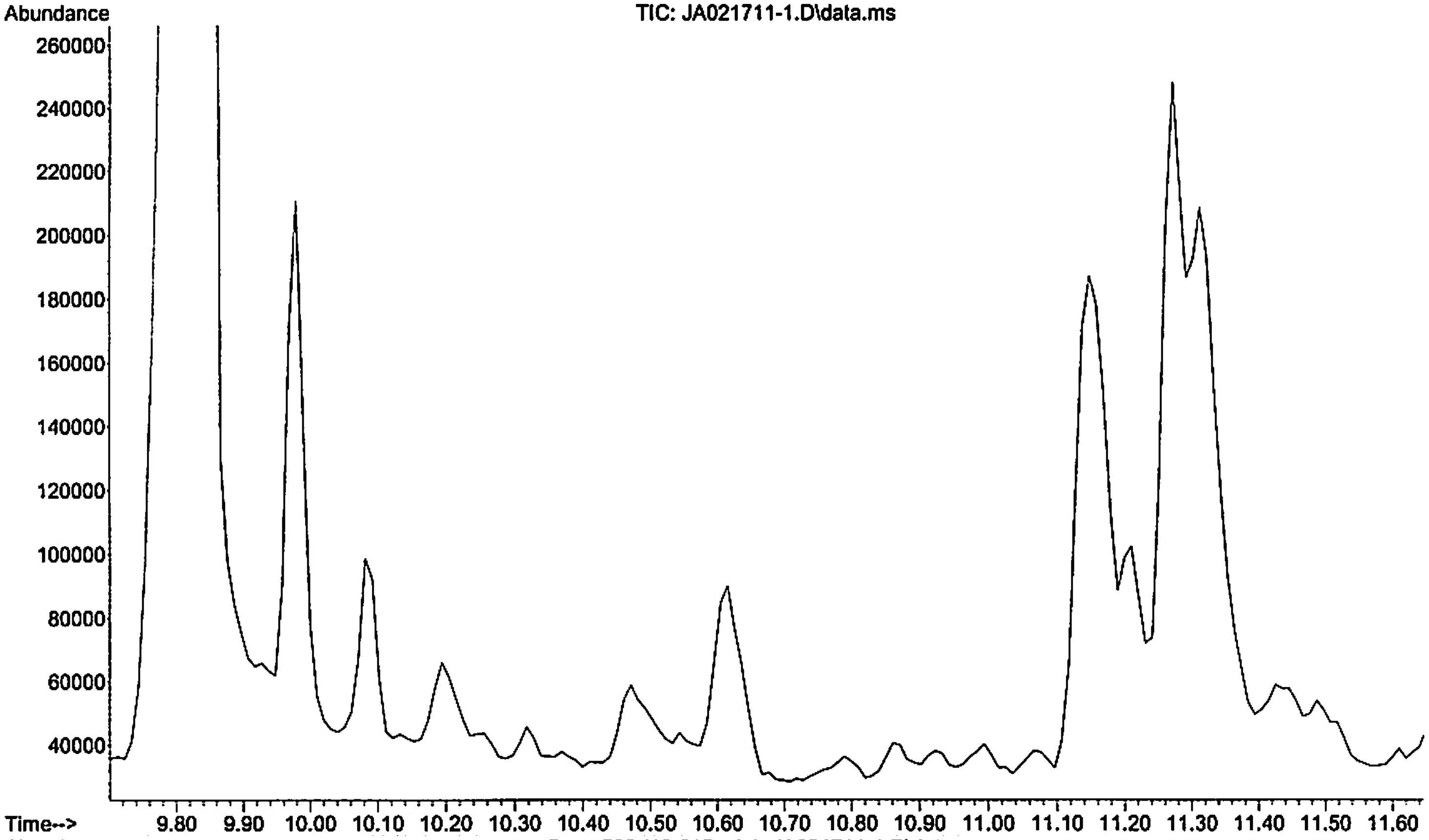

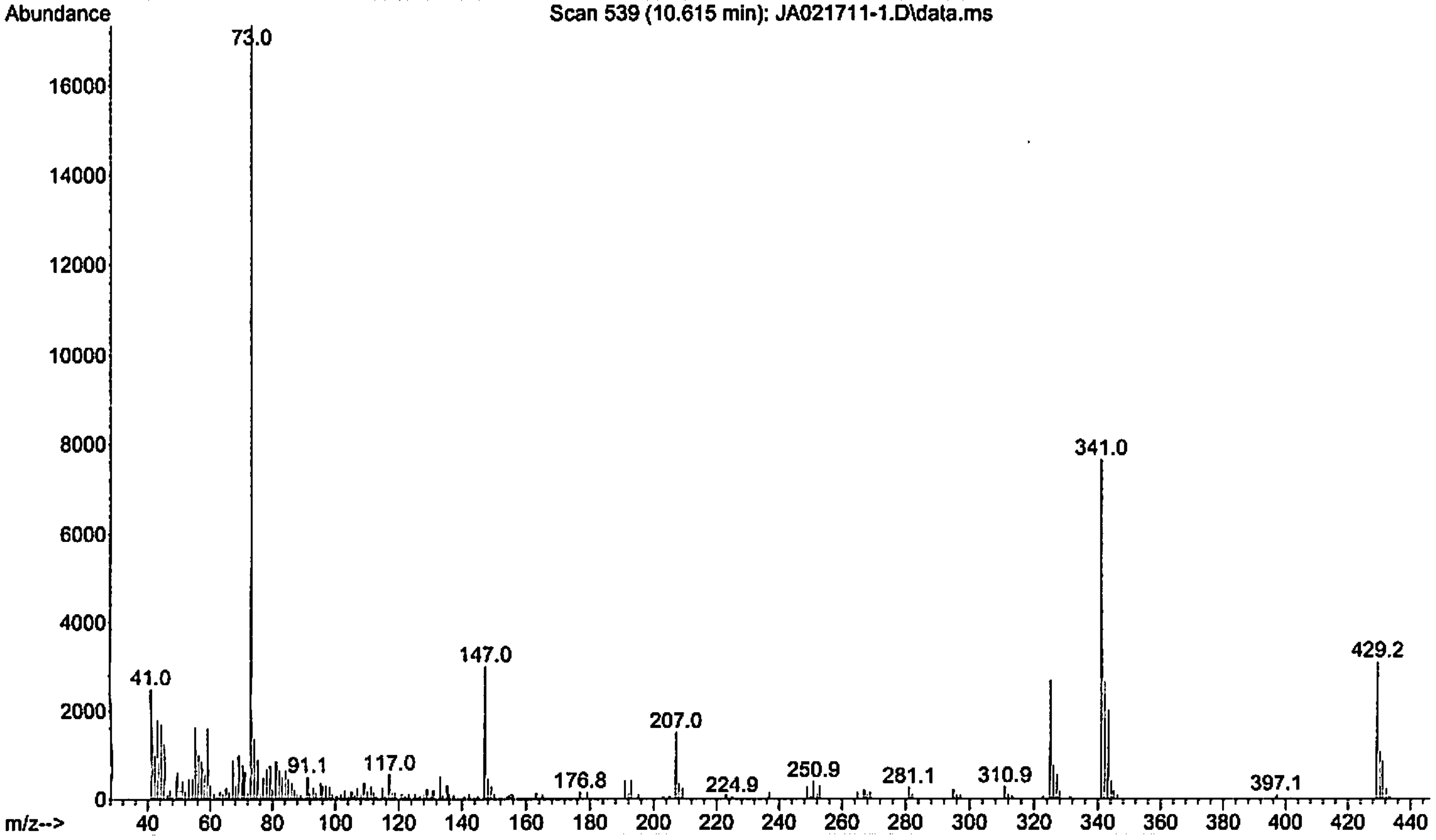

```
File       :D:\Aldrich\JA-11\JA021711-1.D
Operator   :
Acquired   : 17 Feb 2011  17:20     using AcqMethod JA-50-280LESS.M
Instrument :    Buba; IIBBL's magical mass spect
Sample Name: 4M C. ocu. abd.sternites/5ul CH2Cl2;9-10 days
Misc Info  : larvae w/1ug/ul nepetalactol in honey soln.
Vial Number: 1
```

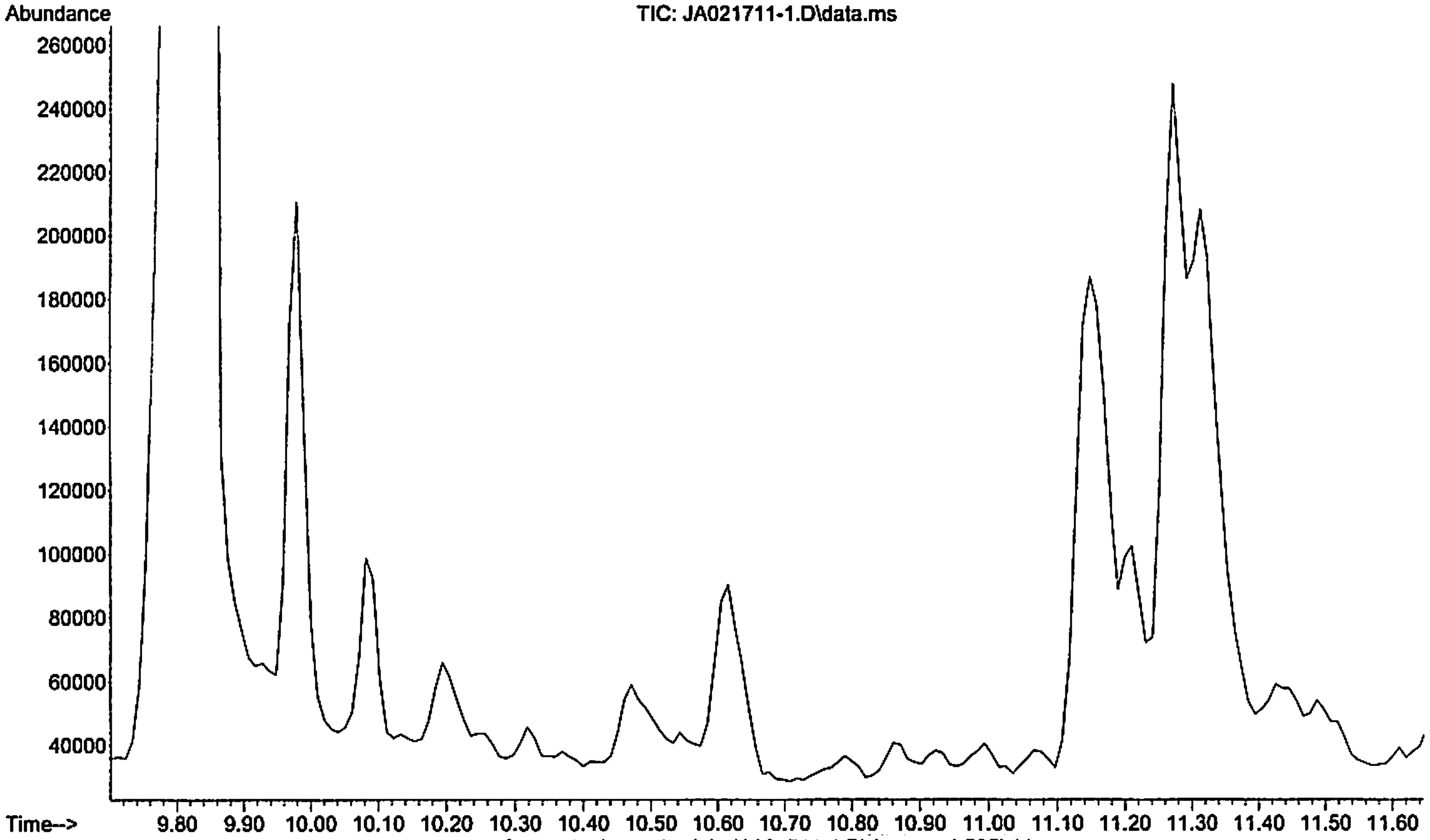

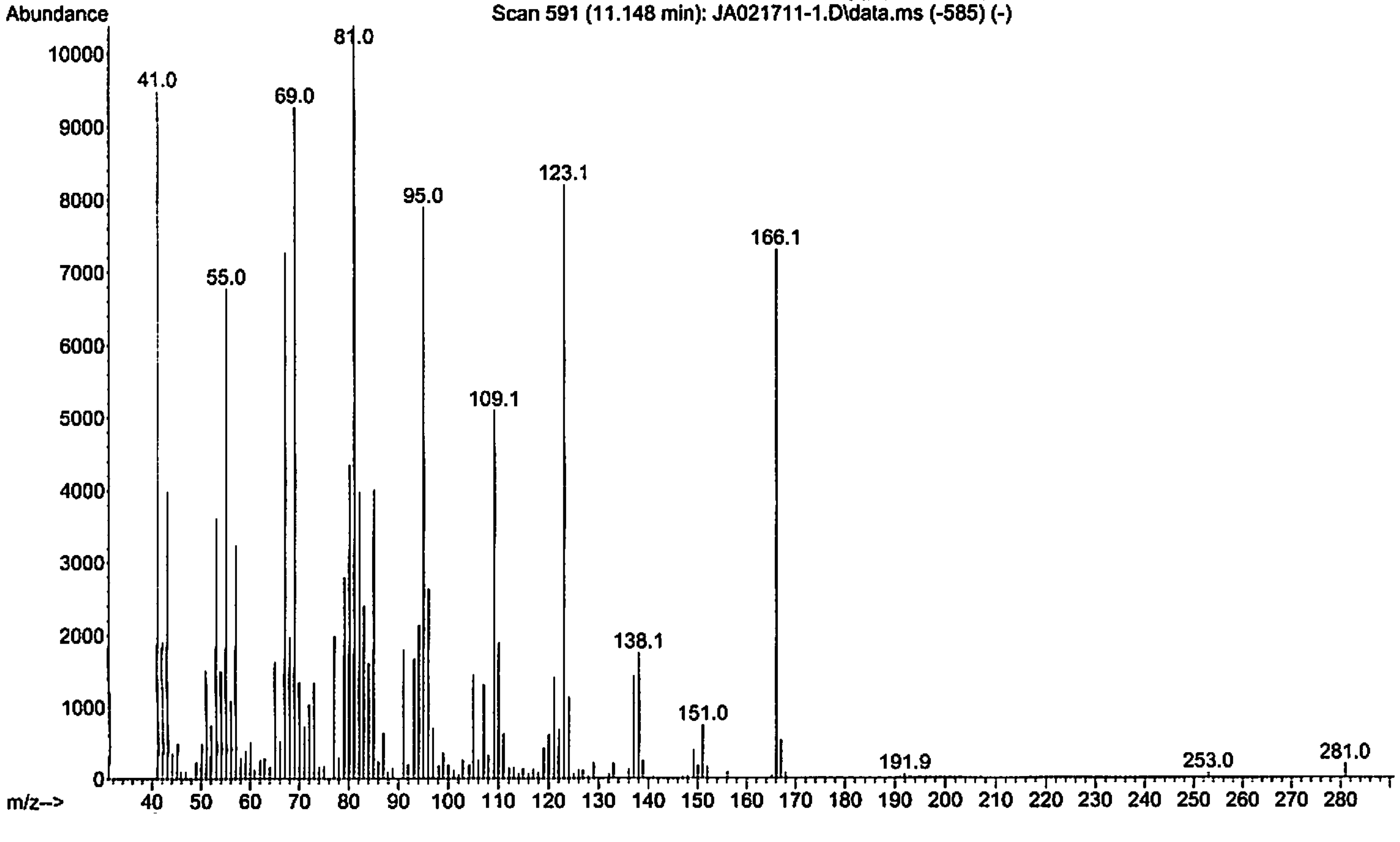

File   :D:\Aldrich\JA-11\JA021711-1.D
Operator   :
Acquired   : 17 Feb 2011 17:20   using AcqMethod JA-50-280LESS.M
Instrument :   Buba; IIBBL's magical mass spect
Sample Name: 4M C. ocu. abd.sternites/5ul CH2Cl2;9-10 days
Misc Info   : larvae w/1ug/ul nepetalactol in honey soln.
Vial Number: 1

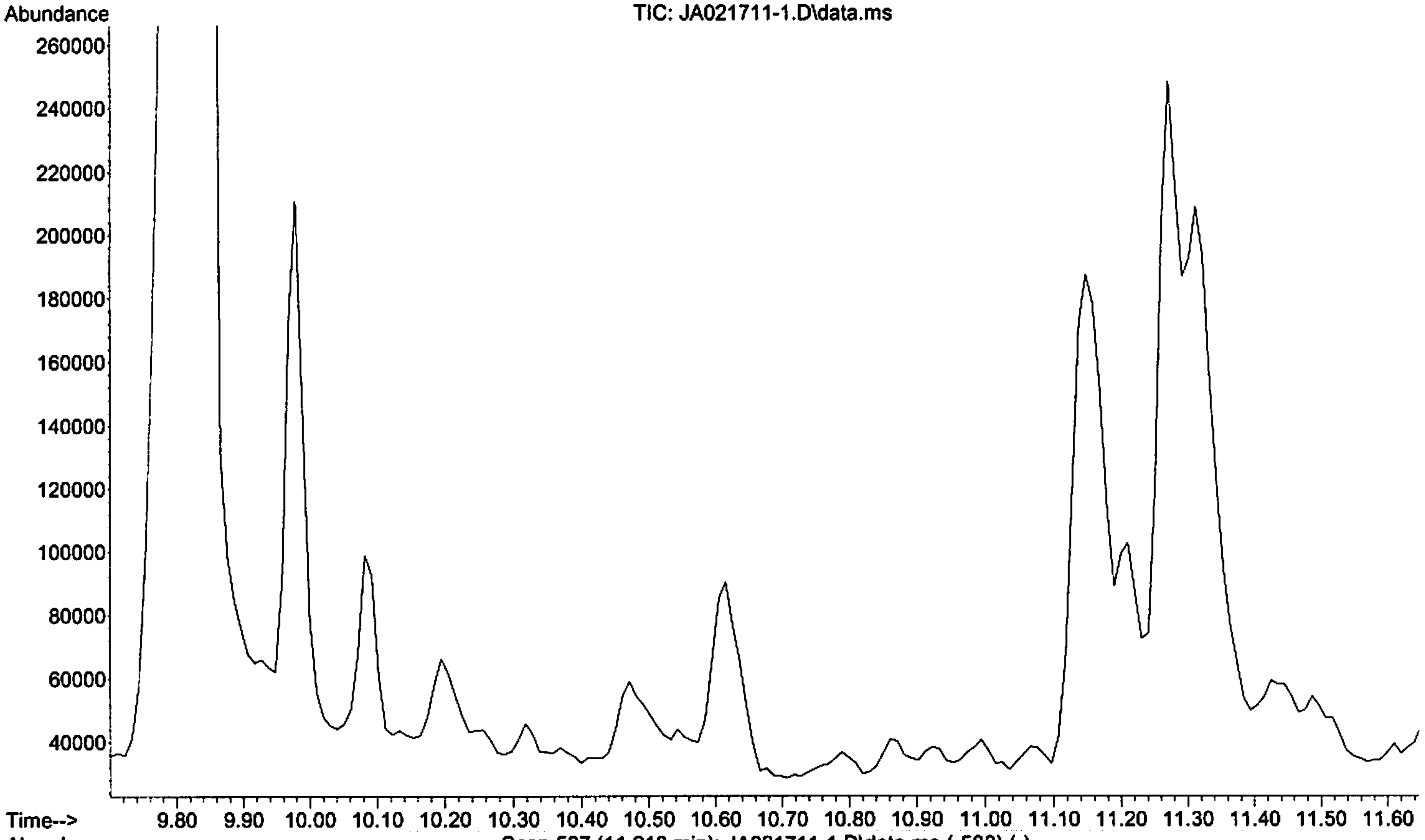

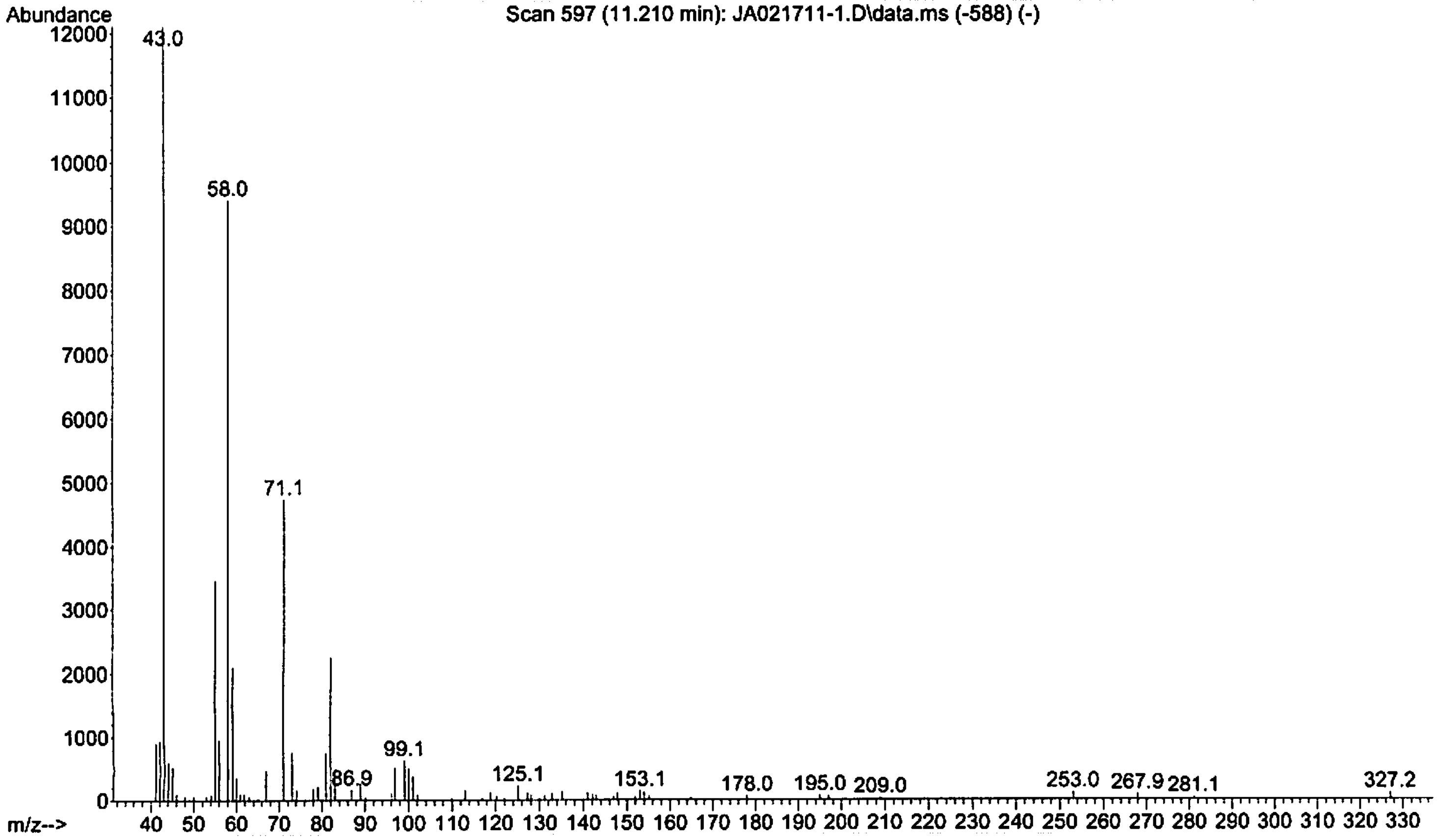

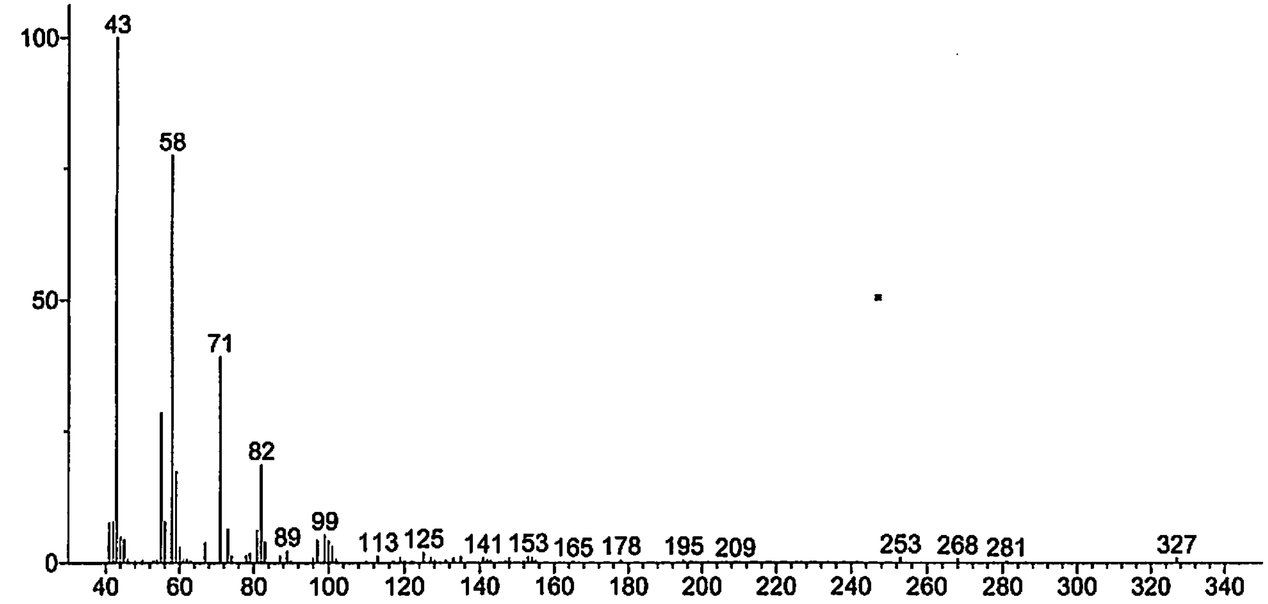

(Text File) Scan 597 (11.210 min): JA021711-1.D\data.ms (-588)

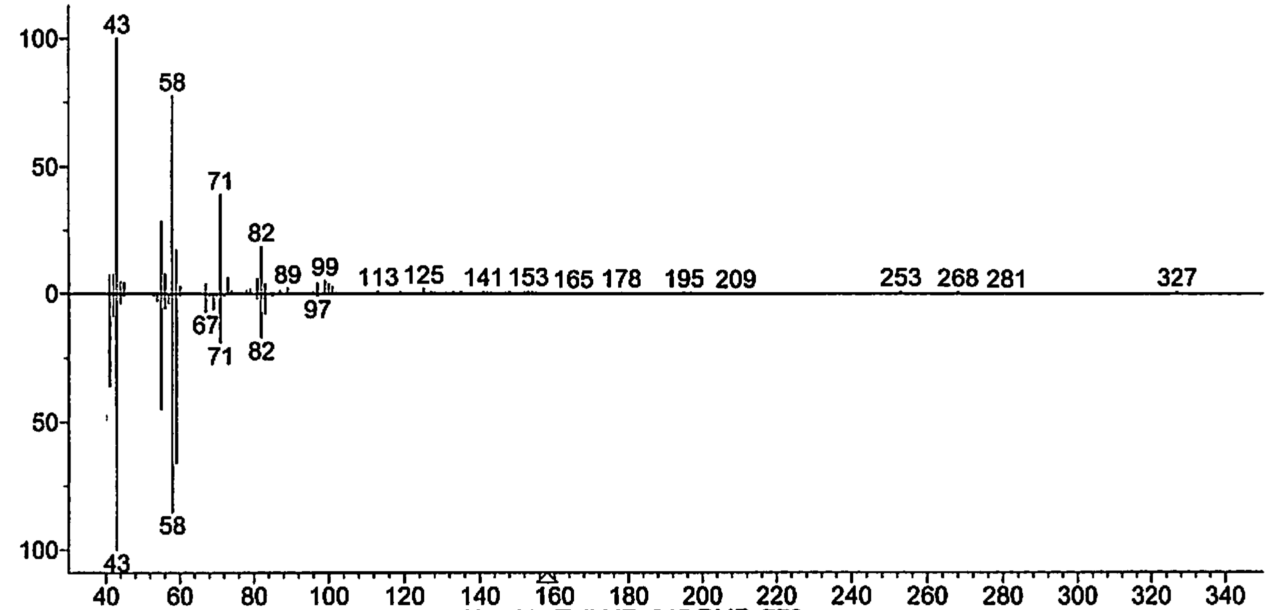

Head to Tail MF=645 RMF=772

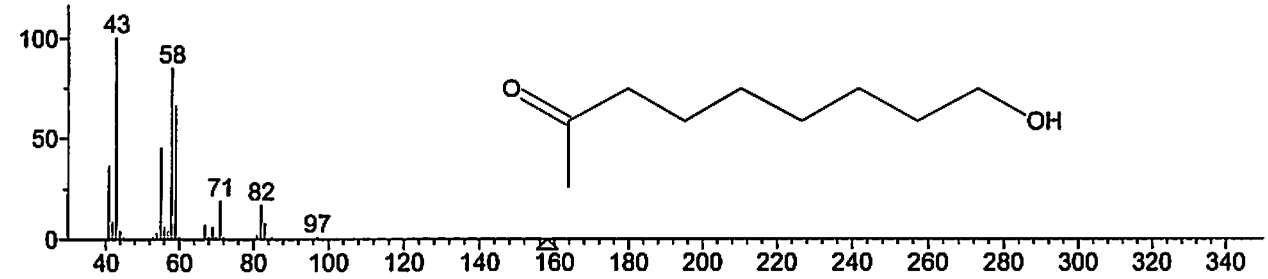

(mainlib) 2-Nonanone, 9-hydroxy-

```
File        :D:\Aldrich\JA-11\JA021711-1.D
Operator    :
Acquired    : 17 Feb 2011  17:20     using AcqMethod JA-50-280LESS.M
Instrument  :    Buba; IIBBL's magical mass spect
Sample Name: 4M C. ocu. abd.sternites/5ul CH2Cl2;9-10 days
Misc Info   : larvae w/1ug/ul nepetalactol in honey soln.
Vial Number: 1
```

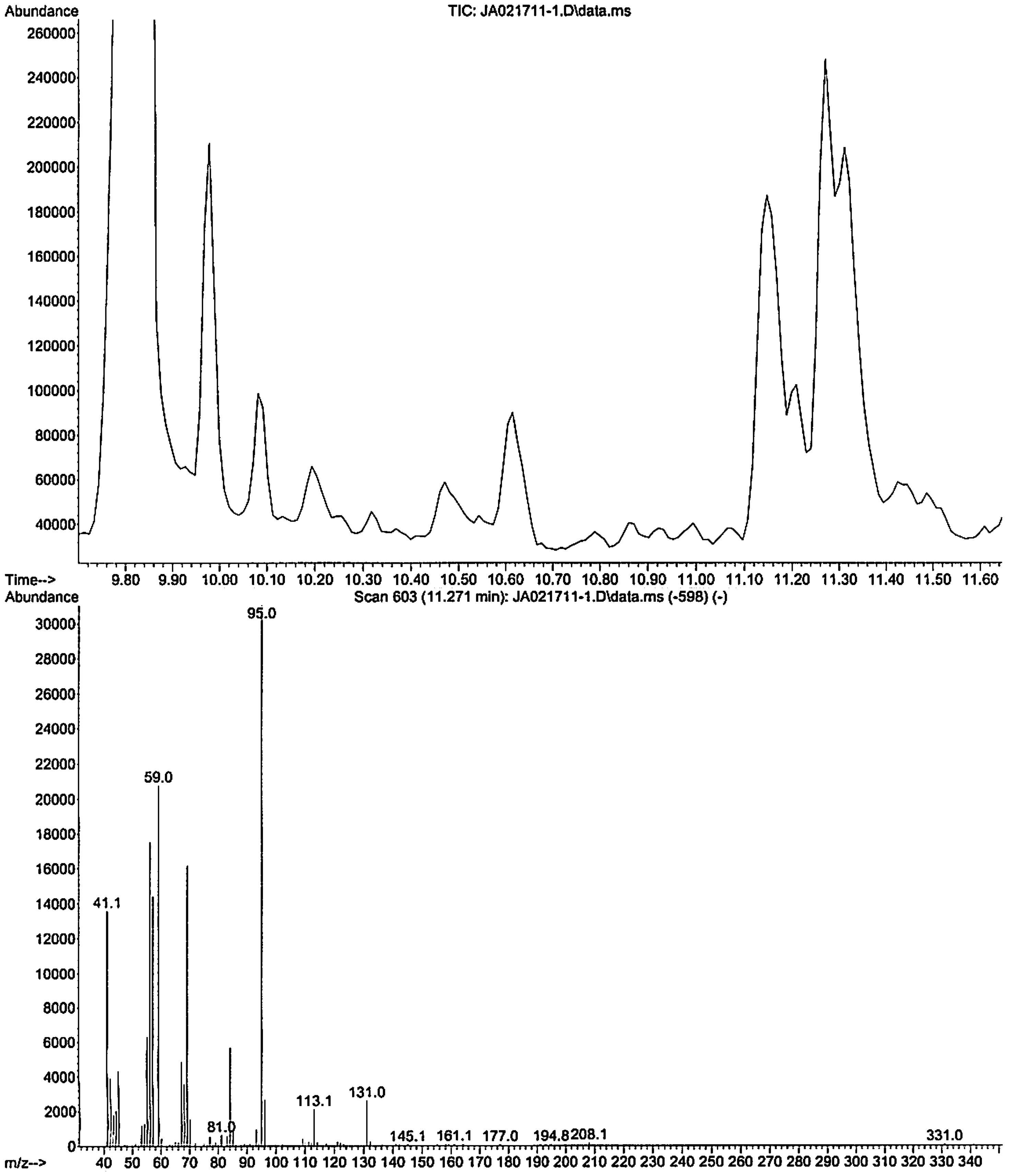

```
File        :D:\Aldrich\JA-11\JA021711-1.D
Operator    :
Acquired    : 17 Feb 2011  17:20     using AcqMethod JA-50-280LESS.M
Instrument :     Buba; IIBBL's magical mass spect
Sample Name: 4M C. ocu. abd.sternites/5ul CH2Cl2;9-10 days
Misc Info   : larvae w/1ug/ul nepetalactol in honey soln.
Vial Number: 1
```

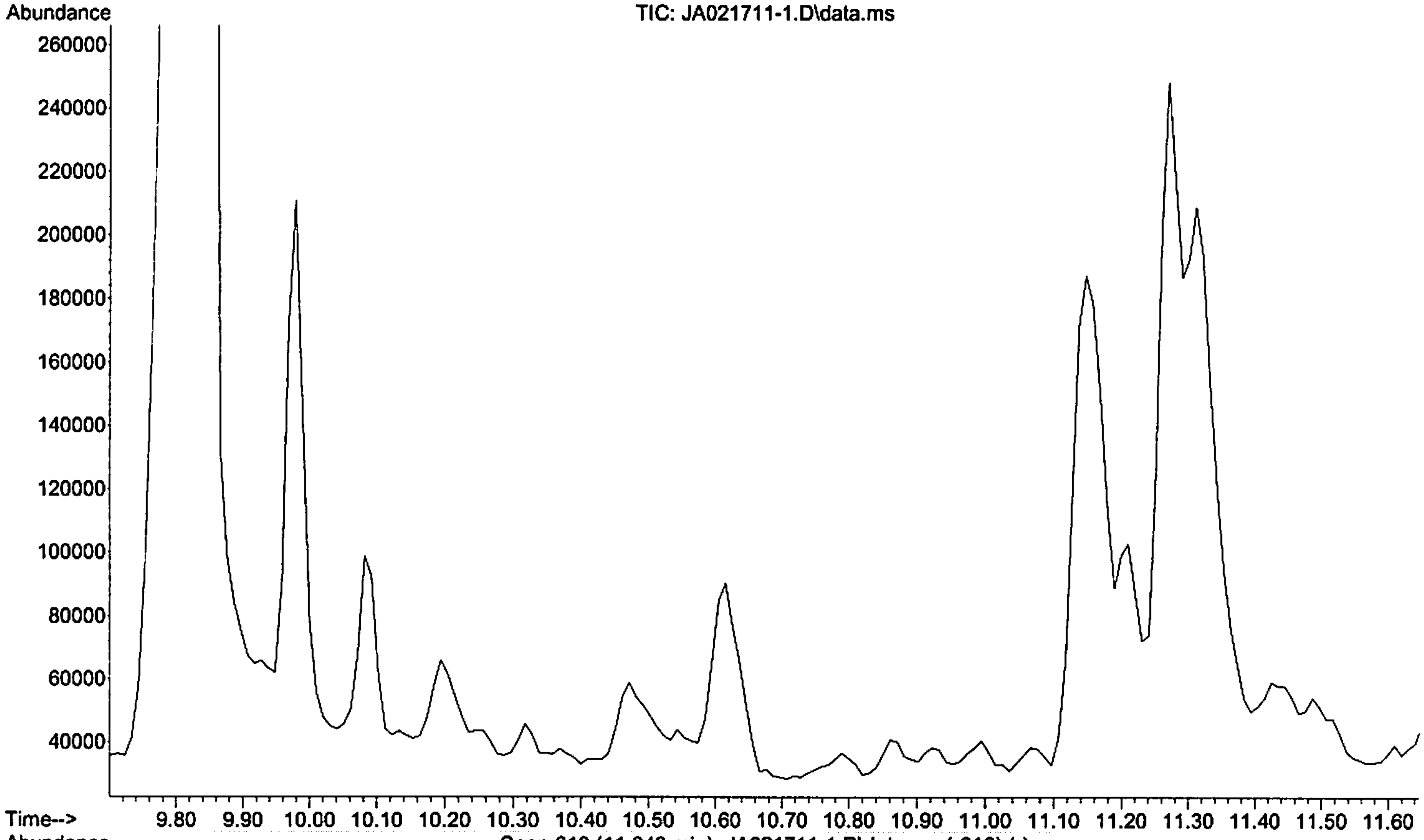

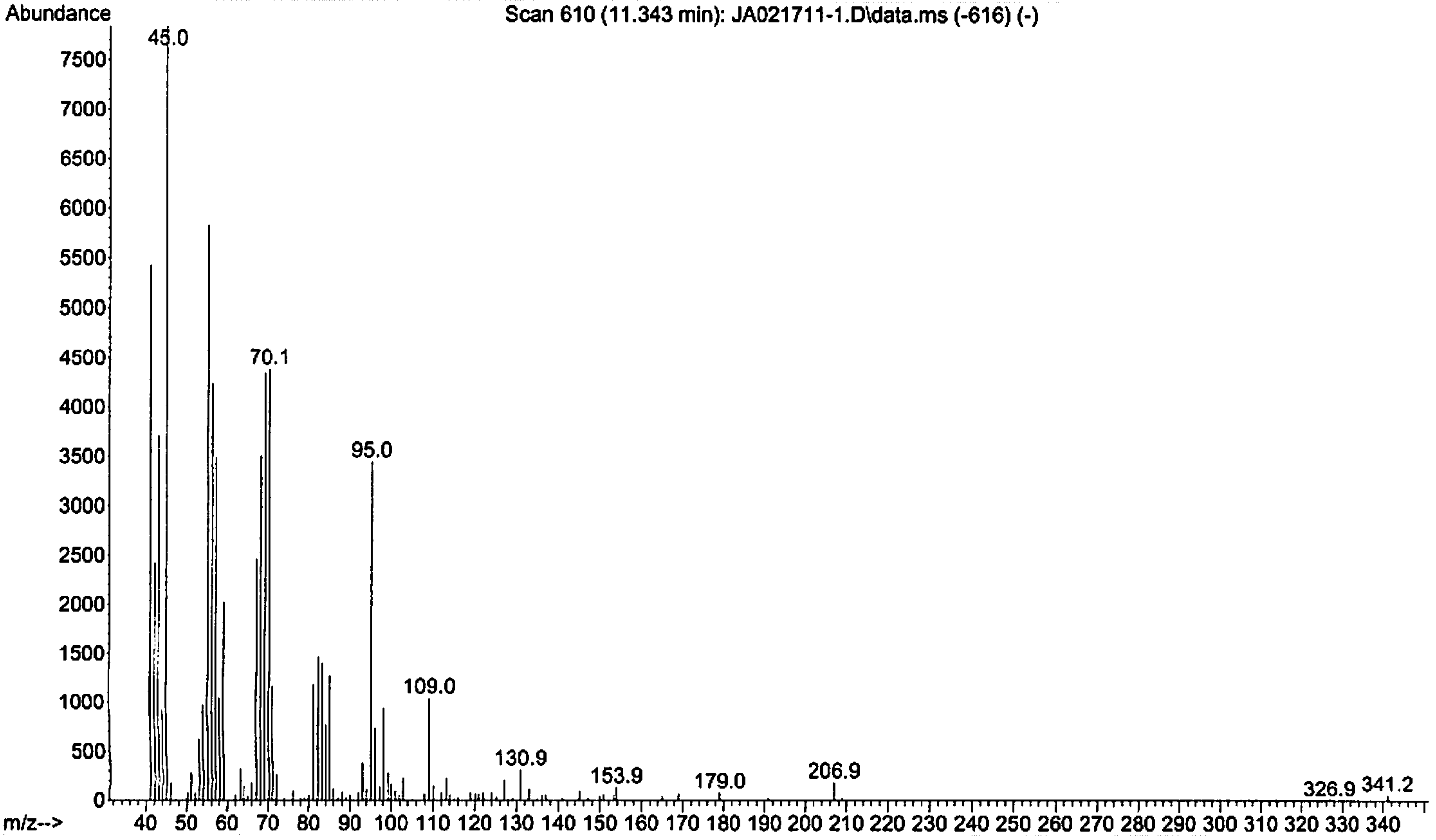

File ` :D:\Aldrich\JA-11\JA021711-1.D
Operator :
Acquired : 17 Feb 2011 17:20 using AcqMethod JA-50-280LESS.M
Instrument : Buba; IIBBL's magical mass spect
Sample Name: 4M C. ocu. abd.sternites/5ul CH2Cl2;9-10 days
Misc Info : larvae w/1ug/ul nepetalactol in honey soln.
Vial Number: 1

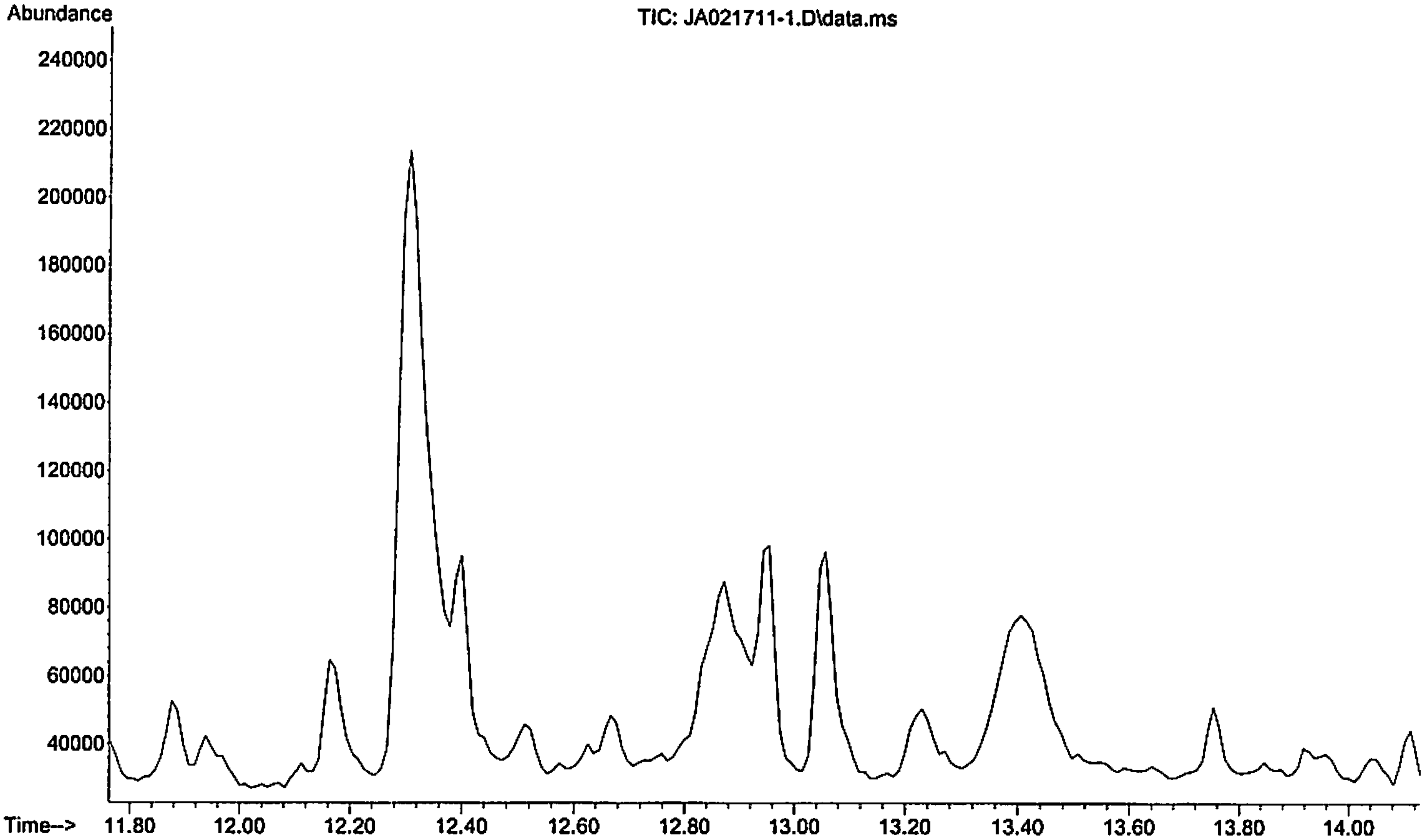

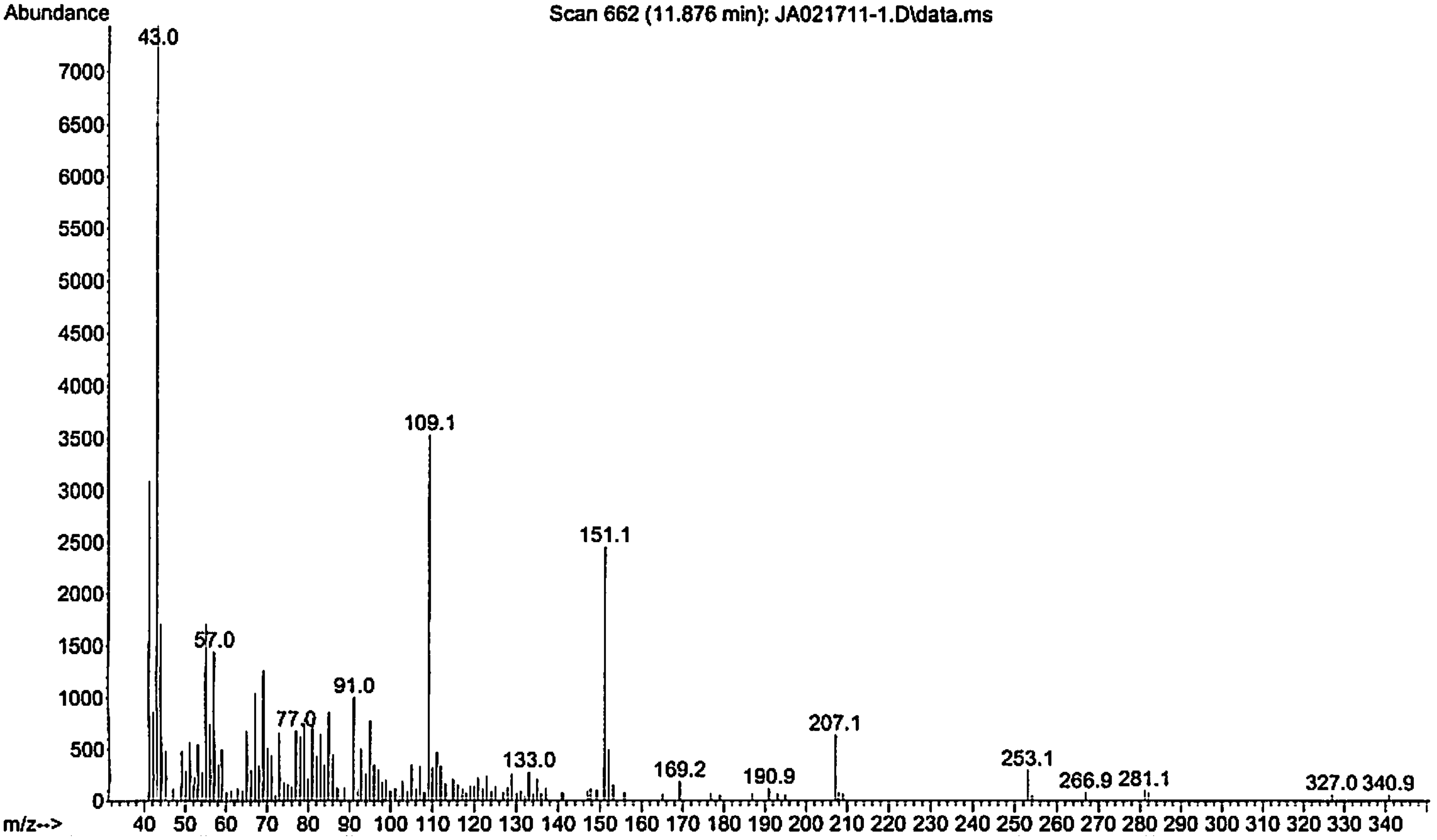

```
File      ` :D:\Aldrich\JA-11\JA021711-1.D
Operator    :
Acquired    : 17 Feb 2011  17:20     using AcqMethod JA-50-280LESS.M
Instrument  :    Buba; IIBBL's magical mass spect
Sample Name: 4M C. ocu. abd.sternites/5ul CH2Cl2;9-10 days
Misc Info   : larvae w/1ug/ul nepetalactol in honey soln.
Vial Number: 1
```

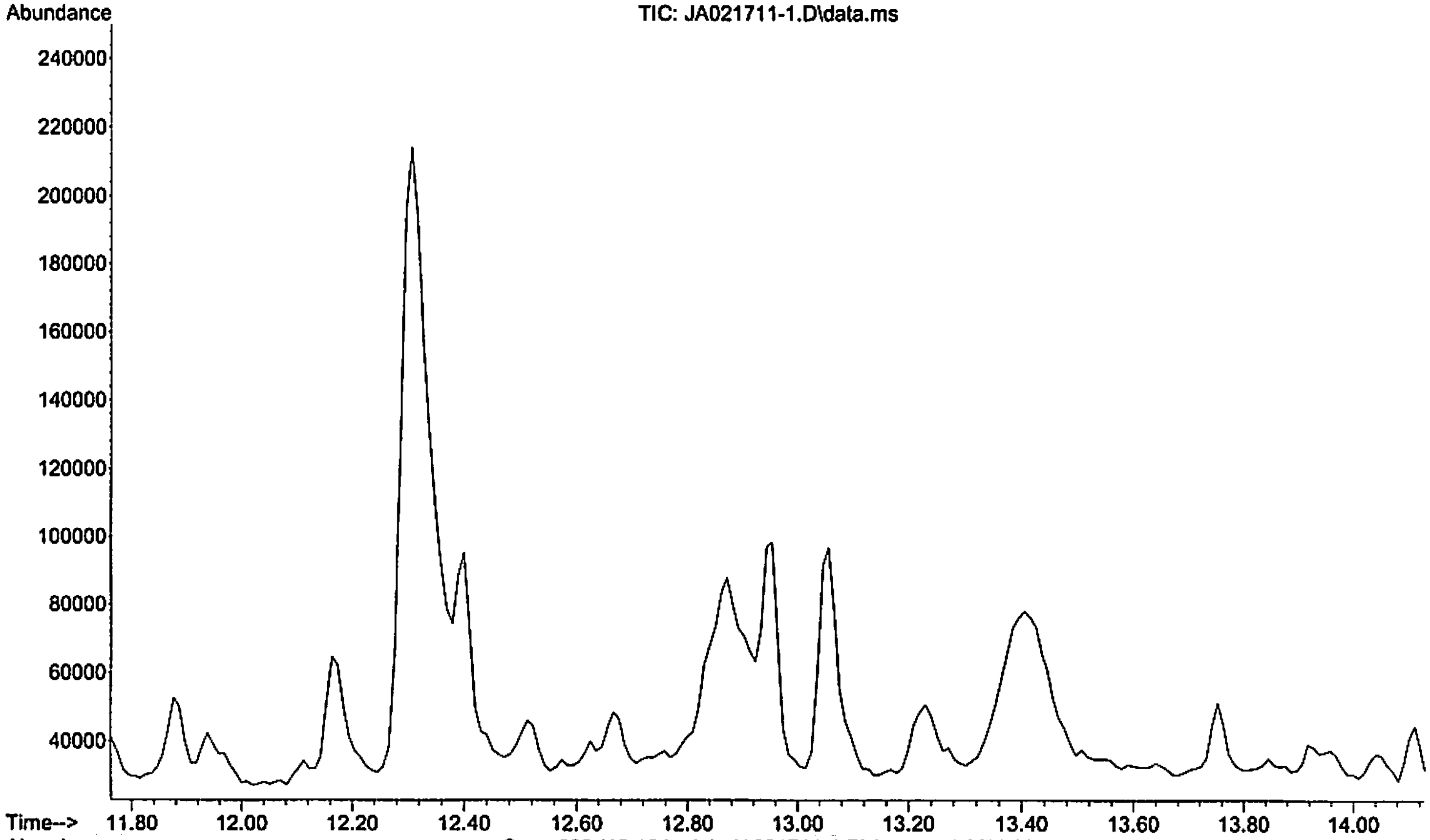

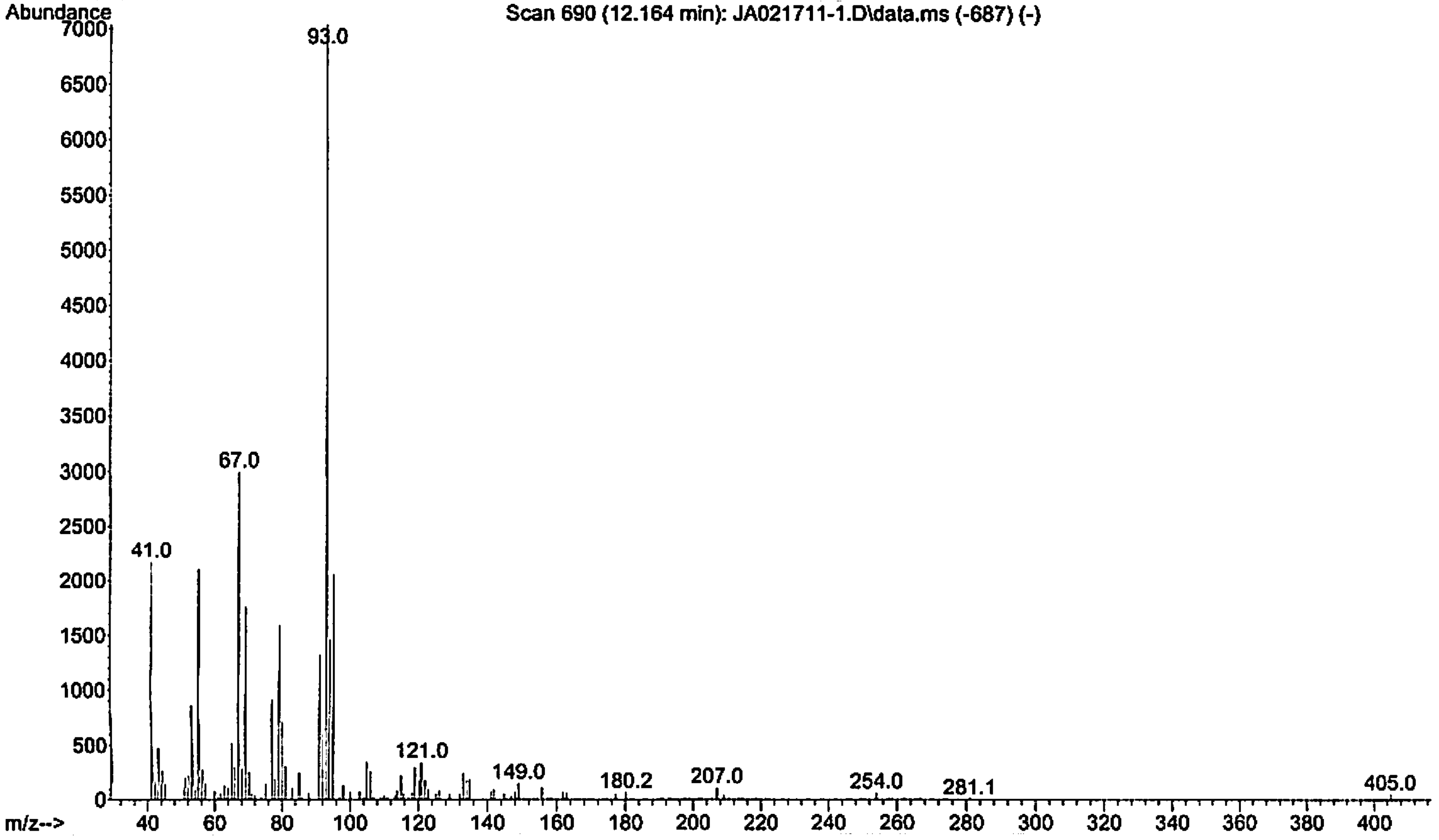

```
File       :D:\Aldrich\JA-11\JA021711-1.D
Operator   :
Acquired   : 17 Feb 2011  17:20     using AcqMethod JA-50-280LESS.M
Instrument :    Buba; IIBBL's magical mass spect
Sample Name: 4M C. ocu. abd.sternites/5ul CH2Cl2;9-10 days
Misc Info  : larvae w/1ug/ul nepetalactol in honey soln.
Vial Number: 1
```

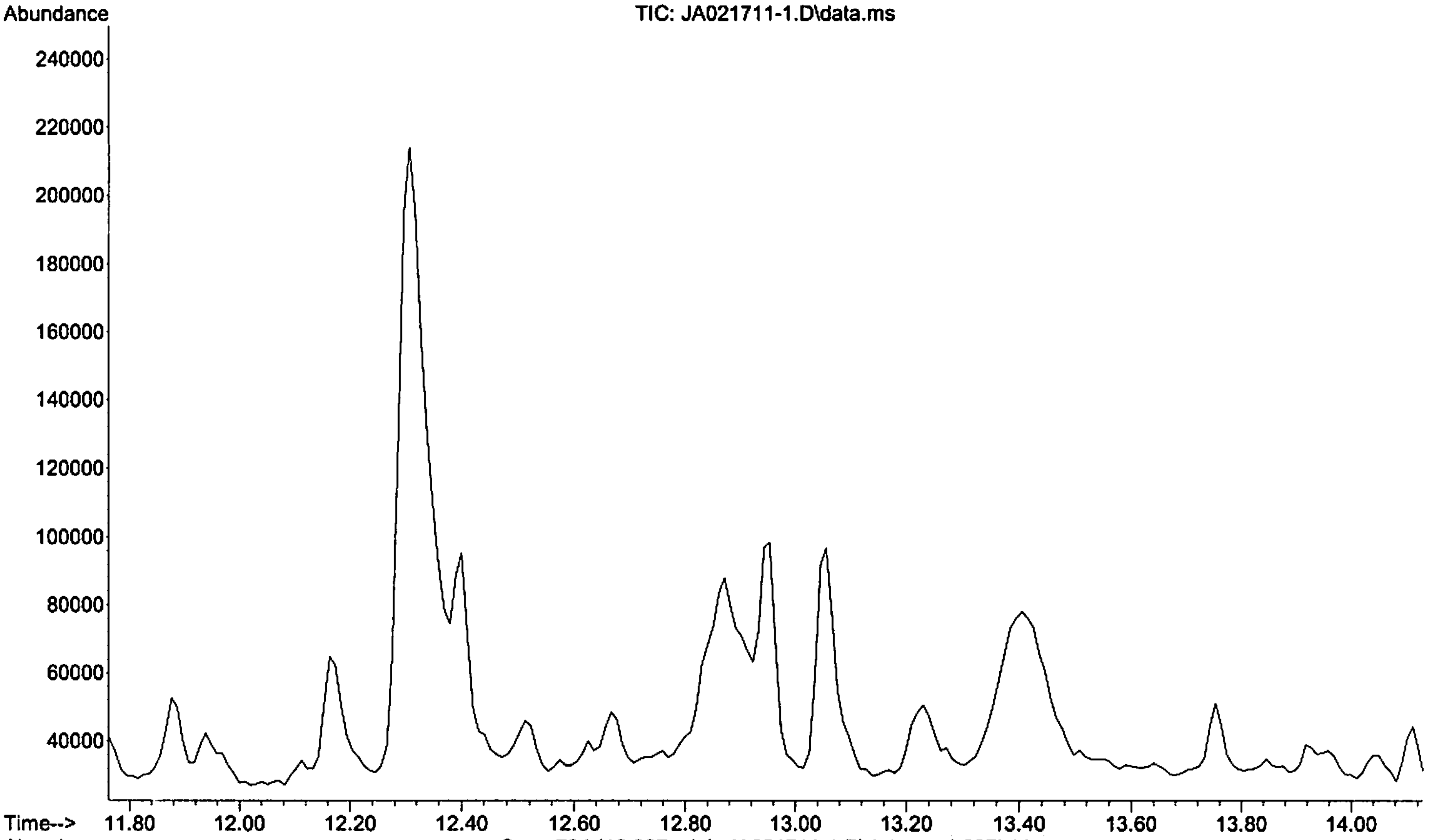

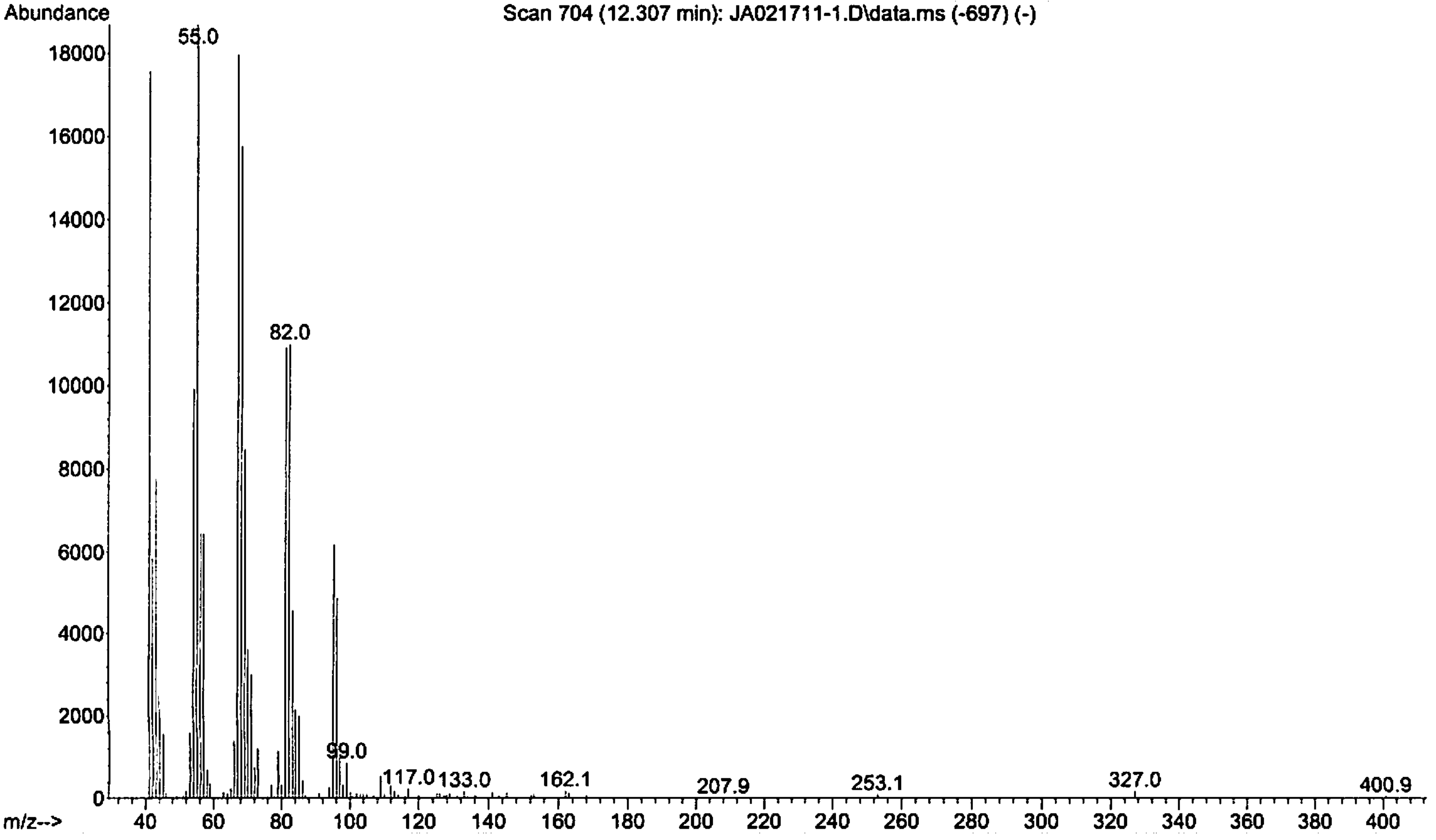

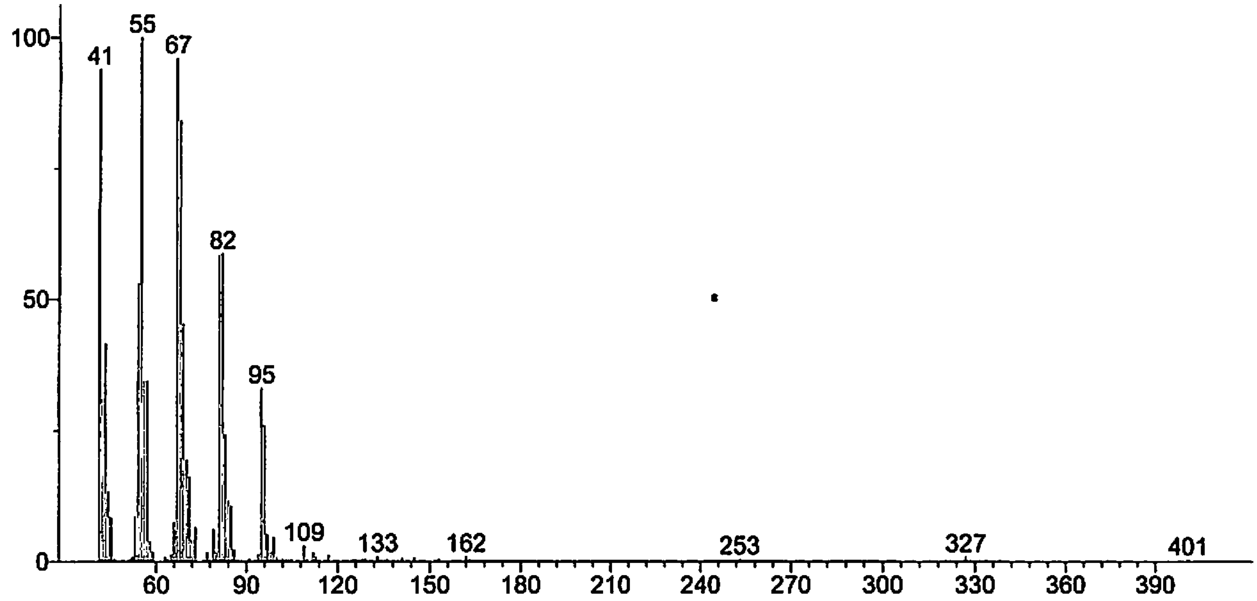

(Text File) Scan 704 (12.307 min): JA021711-1.D\data.ms (-697)

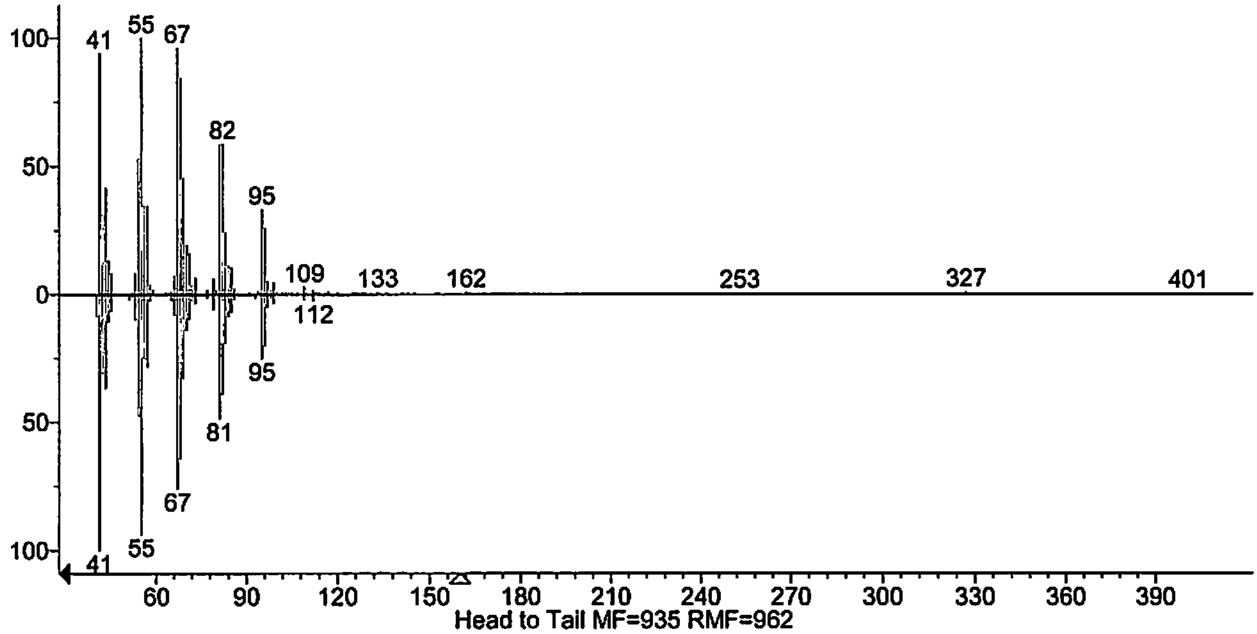

Head to Tail MF=935 RMF=962

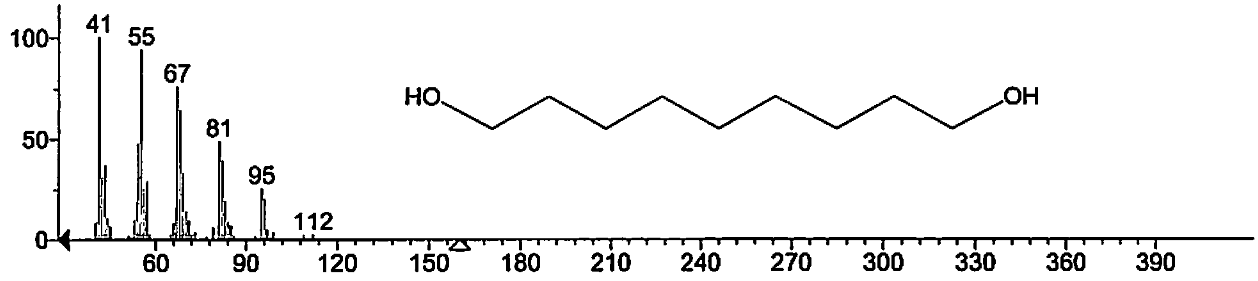

(replib) 1,9-Nonanediol

```
File        :D:\Aldrich\JA-11\JA021711-1.D
Operator    :
Acquired    : 17 Feb 2011  17:20     using AcqMethod JA-50-280LESS.M
Instrument :     Buba; IIBBL's magical mass spect
Sample Name: 4M C. ocu. abd.sternites/5ul CH2Cl2;9-10 days
Misc Info   : larvae w/1ug/ul nepetalactol in honey soln.
Vial Number: 1
```

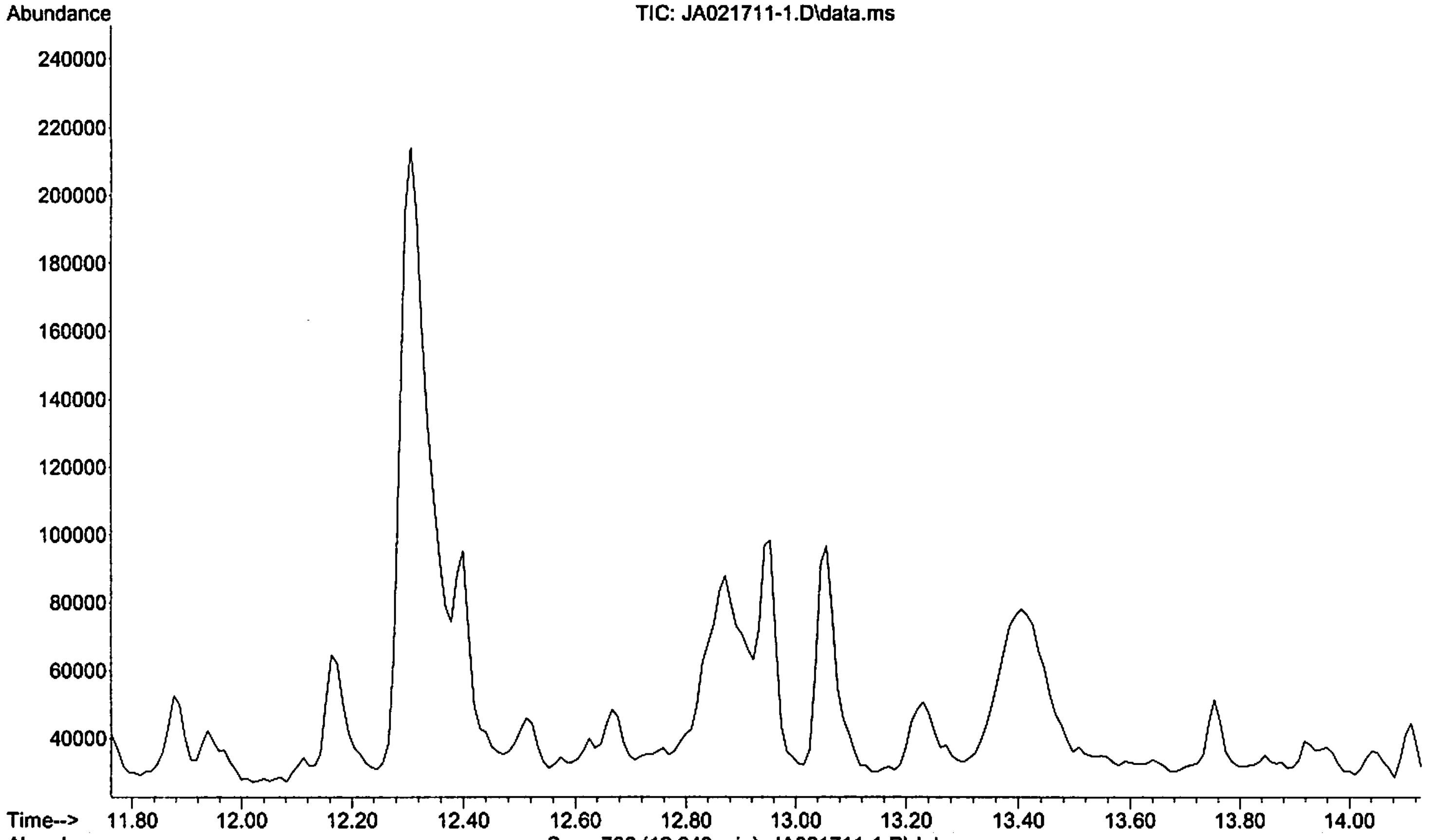

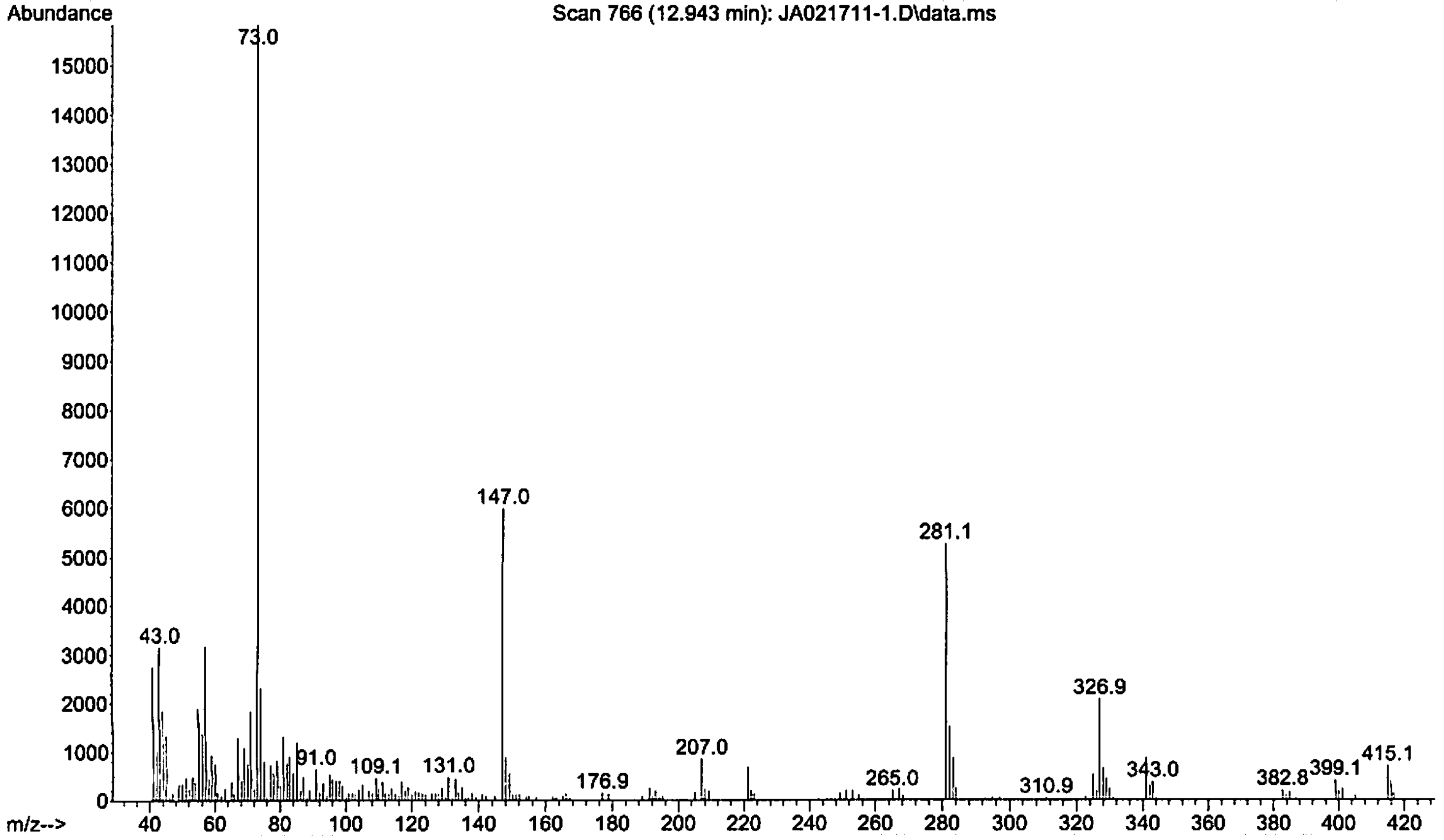

```
File        :D:\Aldrich\JA-11\JA021711-1.D
Operator    :
Acquired    : 17 Feb 2011  17:20     using AcqMethod JA-50-280LESS.M
Instrument :    Buba; IIBBL's magical mass spect
Sample Name: 4M C. ocu. abd.sternites/5ul CH2Cl2;9-10 days
Misc Info   : larvae w/1ug/ul nepetalactol in honey soln.
Vial Number: 1
```

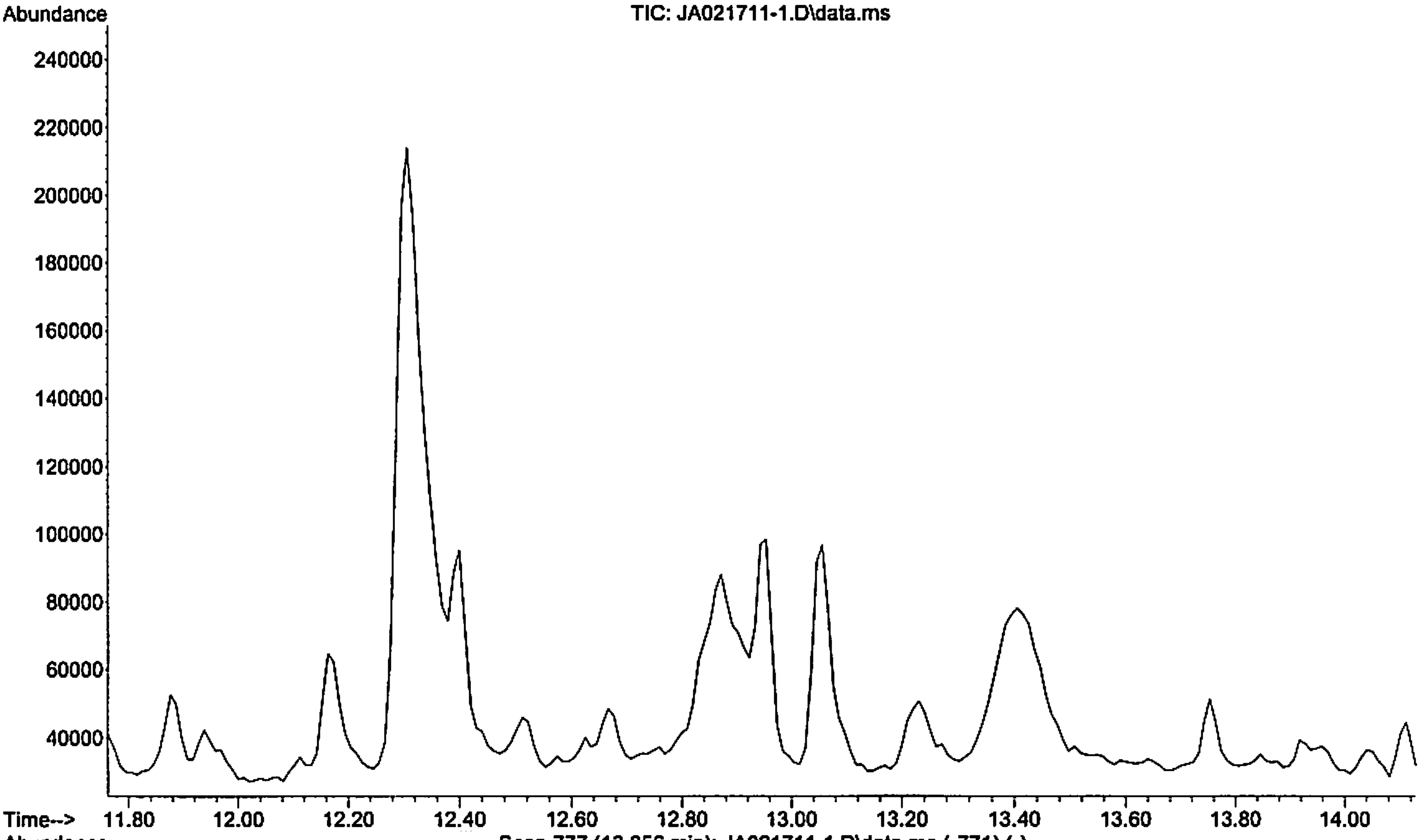

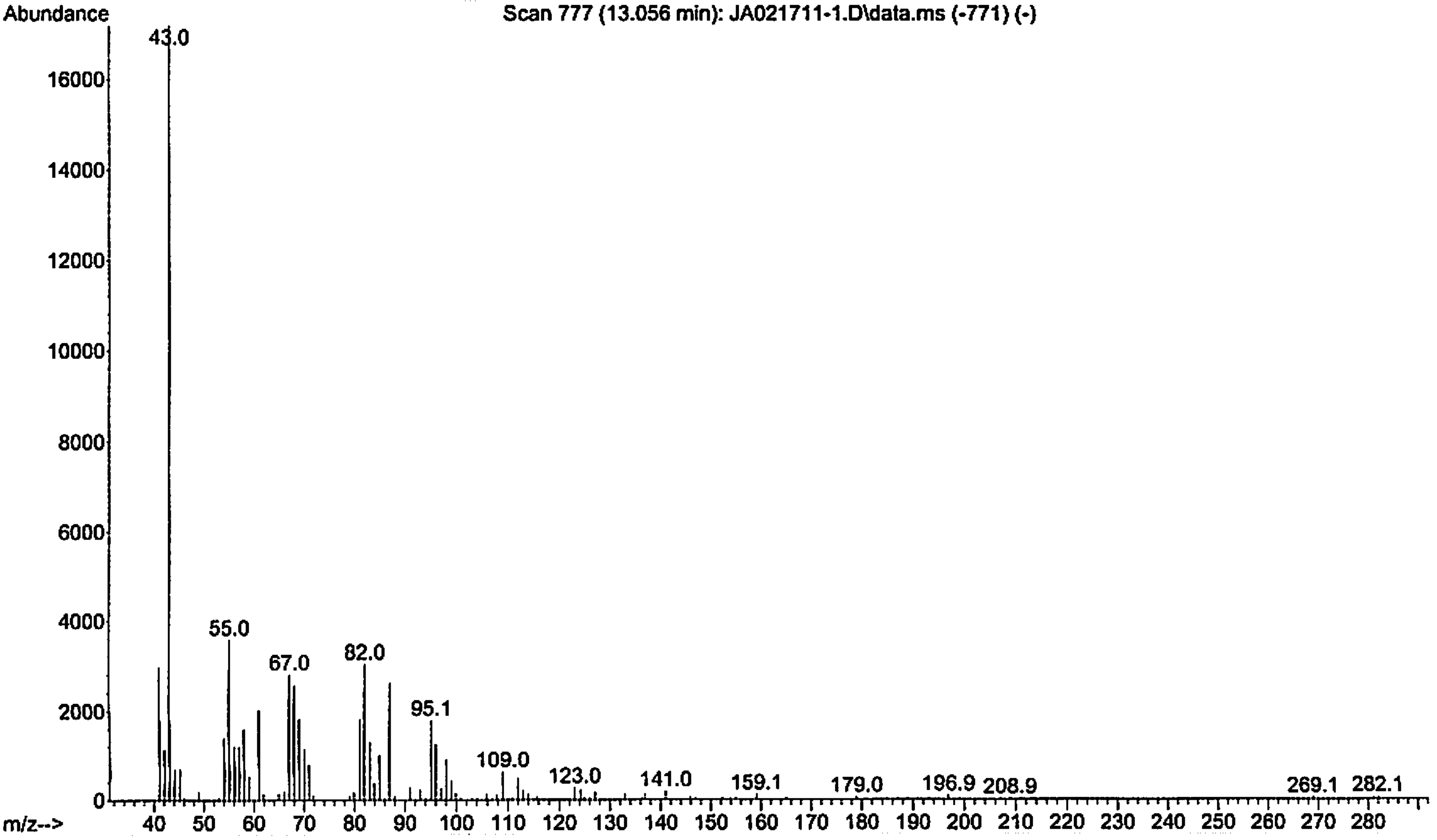

File   :D:\Aldrich\JA-11\JA021711-1.D
Operator   :
Acquired   : 17 Feb 2011 17:20   using AcqMethod JA-50-280LESS.M
Instrument :   Buba; IIBBL's magical mass spect
Sample Name: 4M C. ocu. abd.sternites/5ul CH2Cl2;9-10 days
Misc Info   : larvae w/1ug/ul nepetalactol in honey soln.
Vial Number: 1

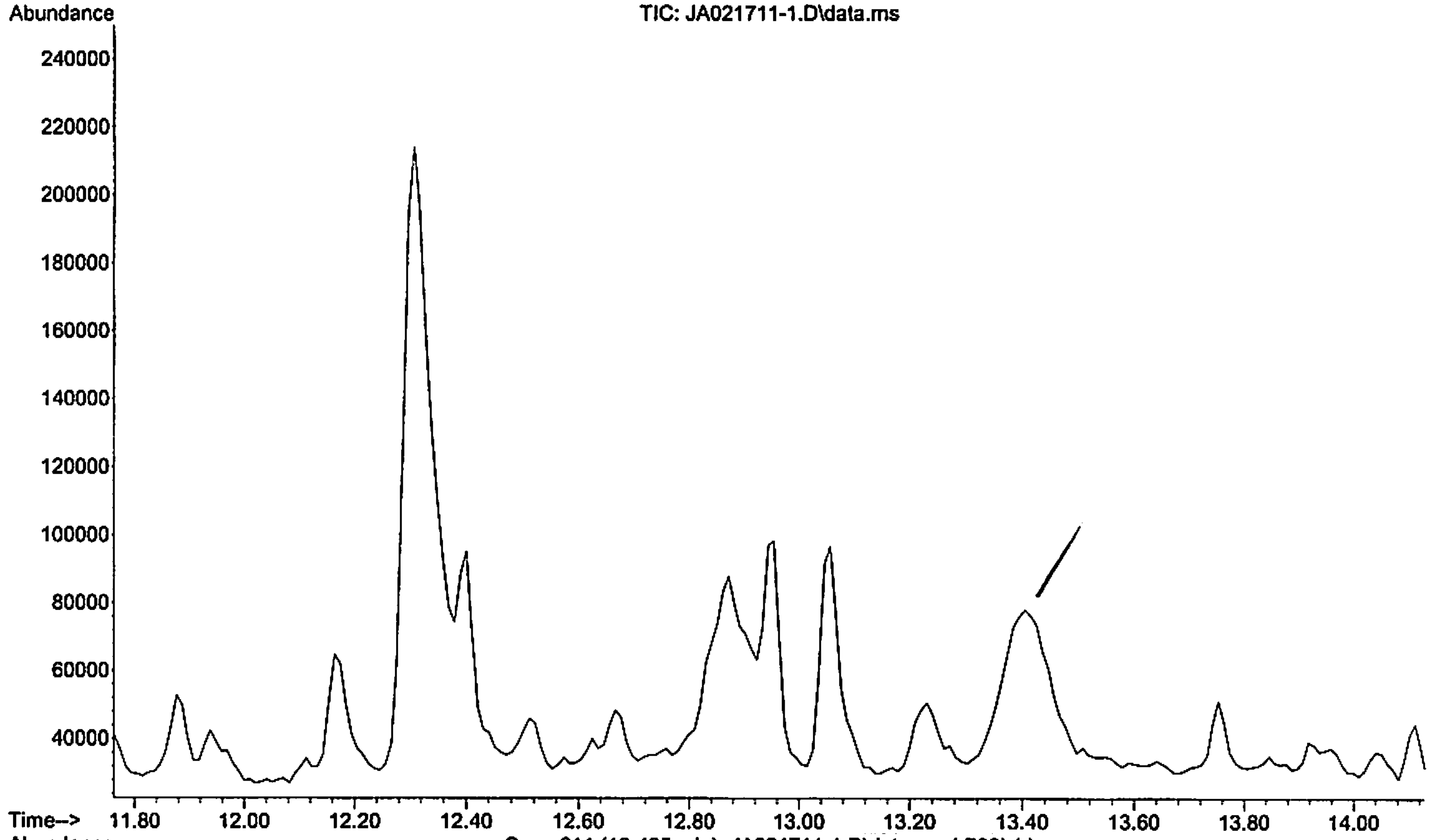

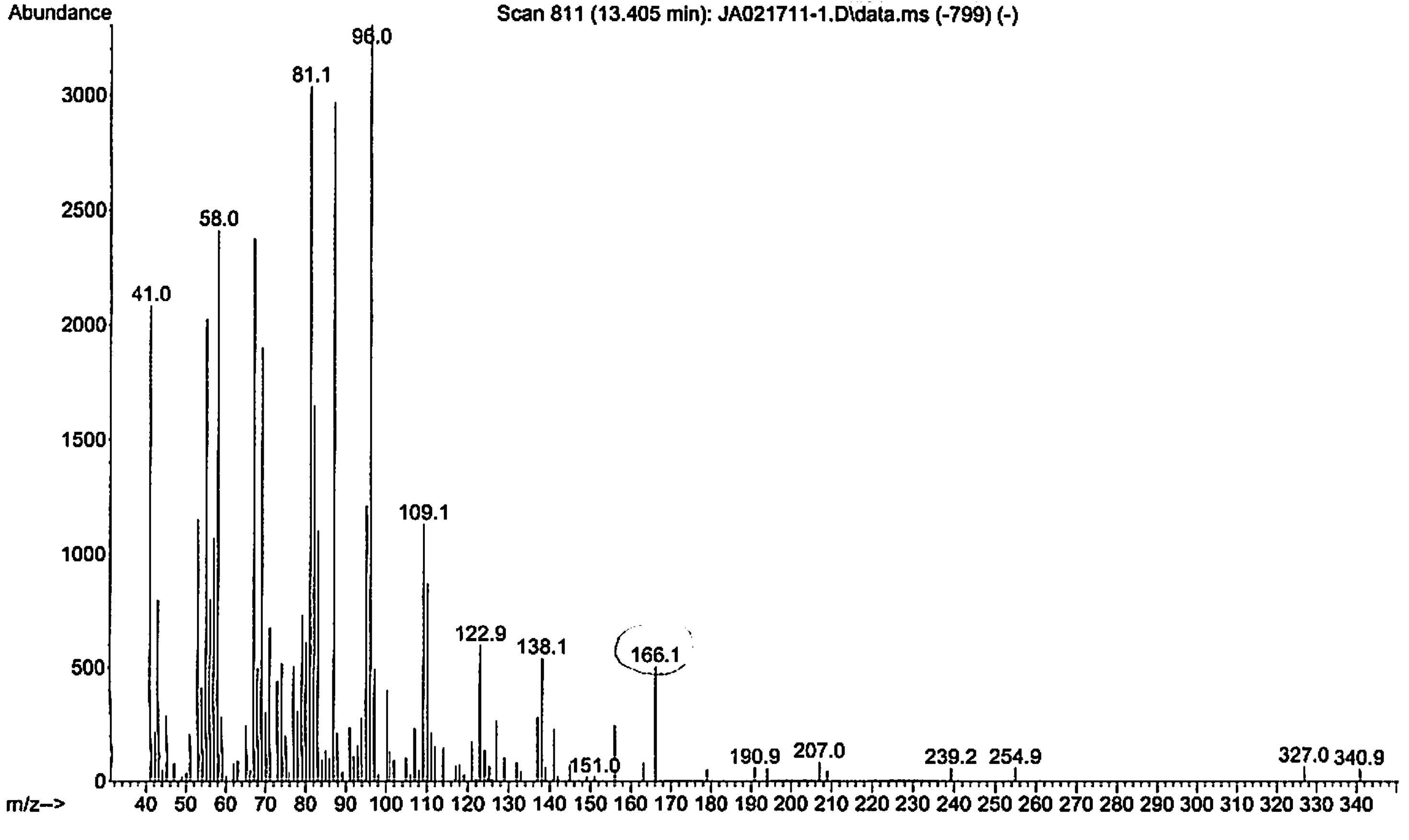

```
File        :D:\Aldrich\JA-11\JA021711-1.D
Operator    :
Acquired    : 17 Feb 2011  17:20     using AcqMethod JA-50-280LESS.M
Instrument :    Buba; IIBBL's magical mass spect
Sample Name: 4M C. ocu. abd.sternites/5ul CH2Cl2;9-10 days
Misc Info   : larvae w/1ug/ul nepetalactol in honey soln.
Vial Number: 1
```

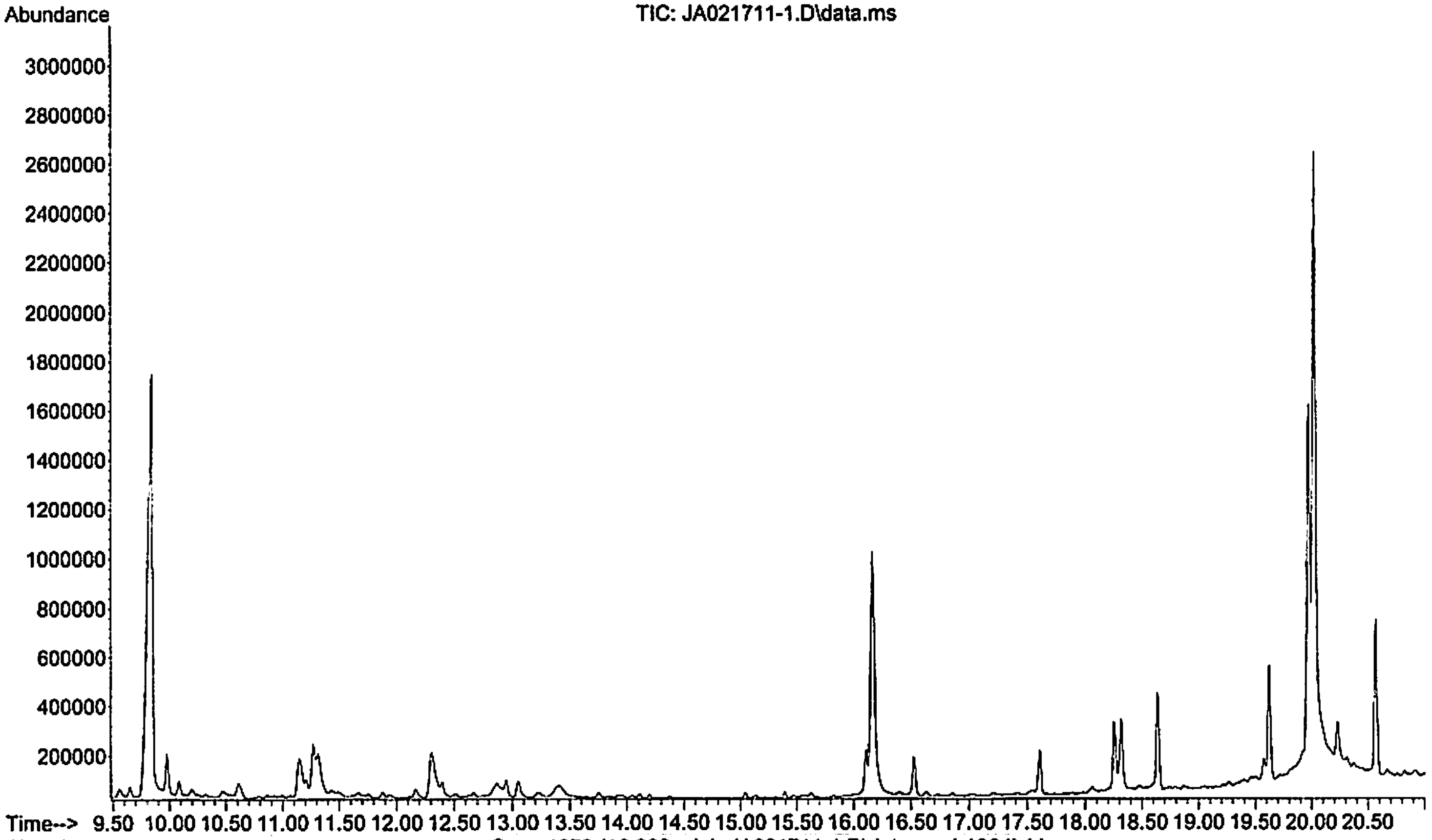

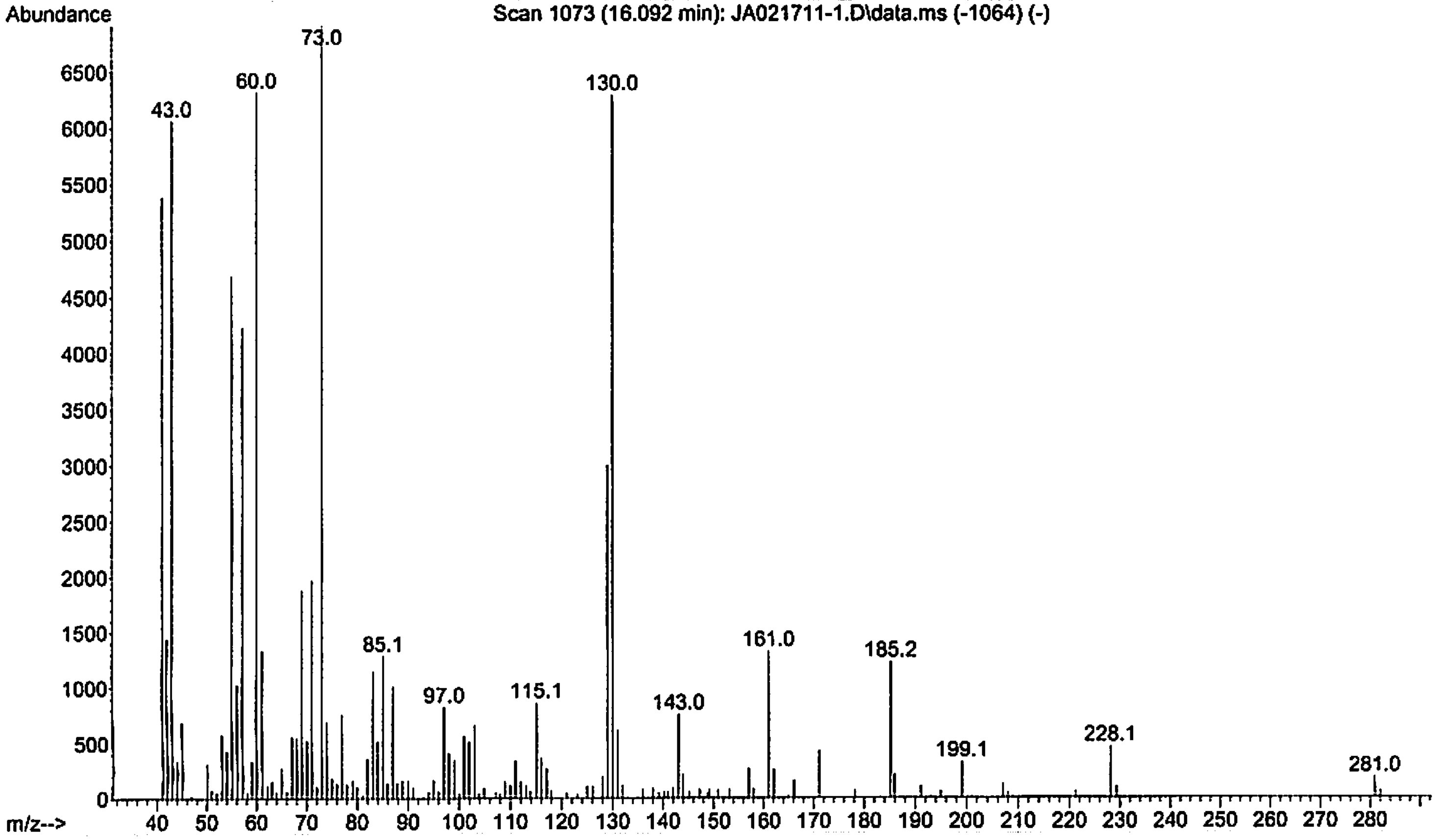

File :D:\Aldrich\JA-11\JA021711-1.D
Operator :
Acquired : 17 Feb 2011 17:20 using AcqMethod JA-50-280LESS.M
Instrument : Buba; IIBBL's magical mass spect
Sample Name: 4M C. ocu. abd.sternites/5ul CH2Cl2;9-10 days
Misc Info : larvae w/1ug/ul nepetalactol in honey soln.
Vial Number: 1

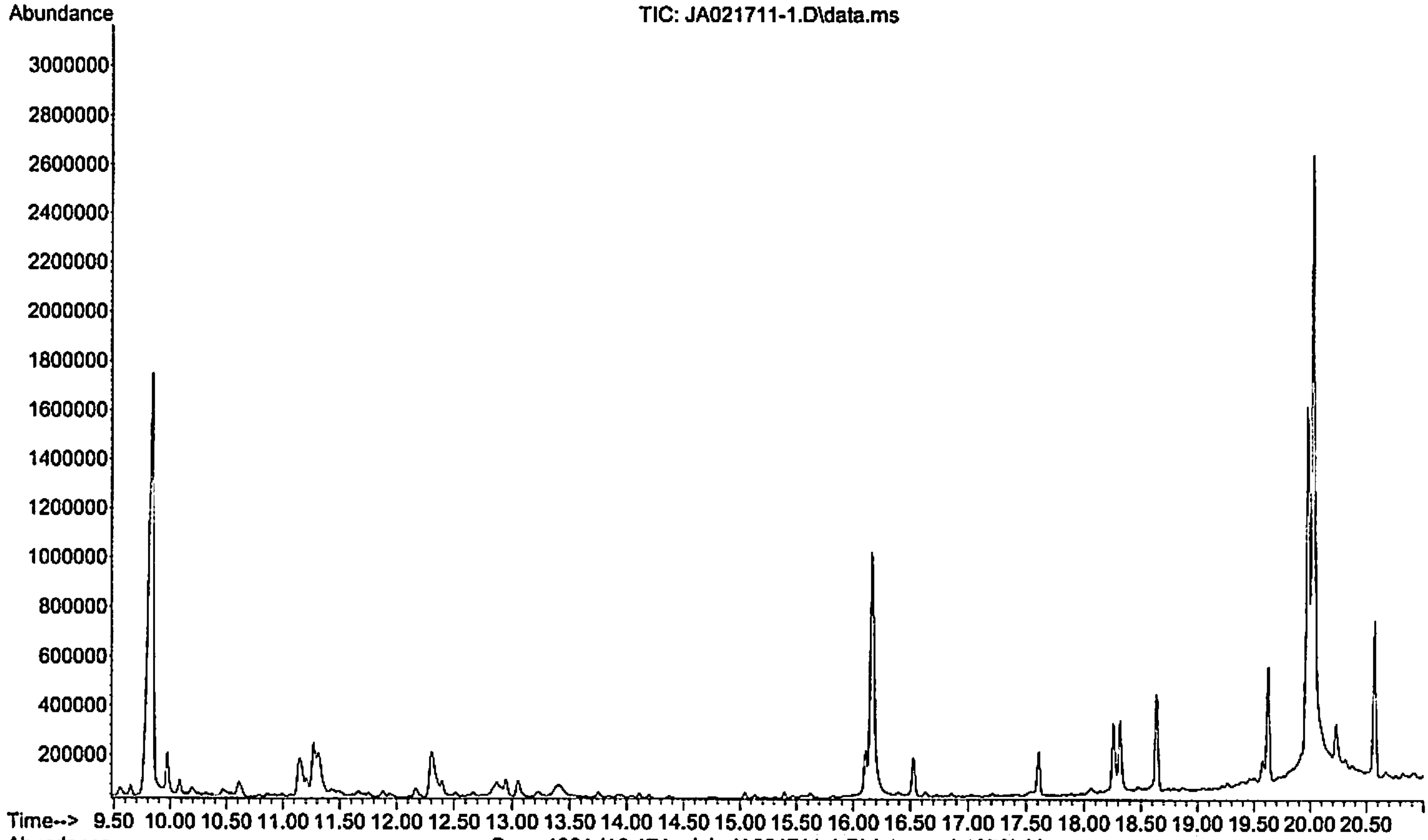

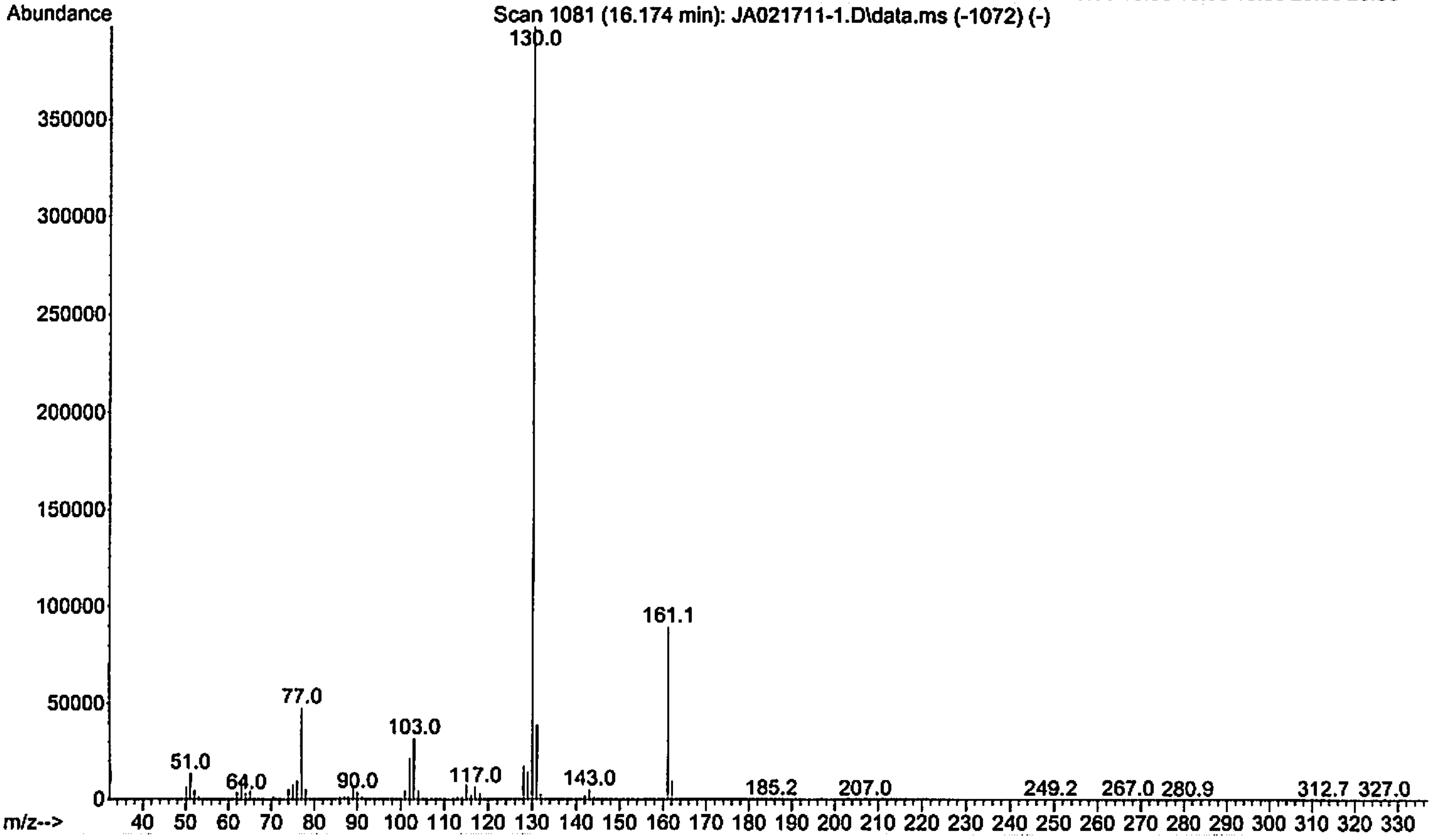

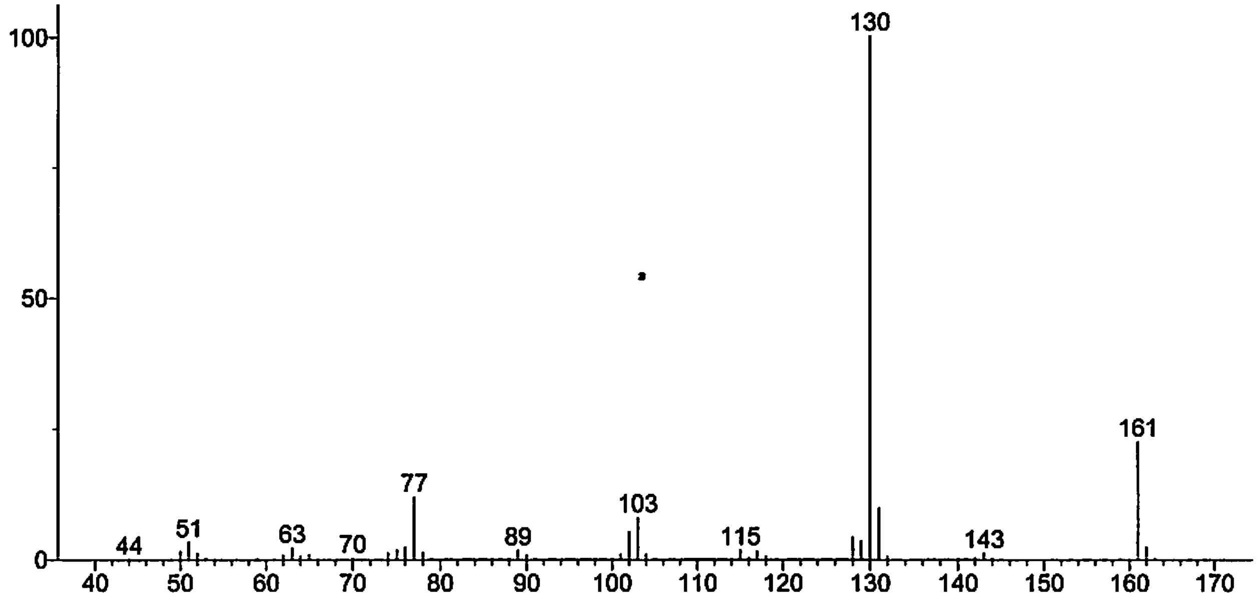

(Text File) Scan 1081 (16.174 min): JA021711-1.D\data.ms (-1072)

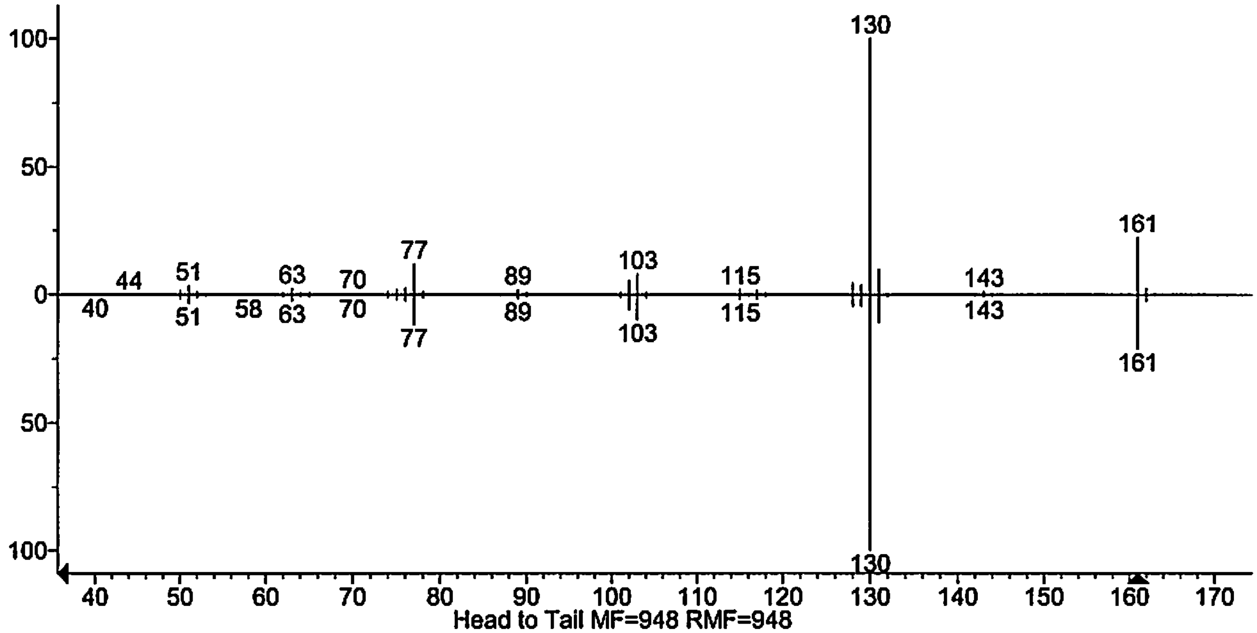

Head to Tail MF=948 RMF=948

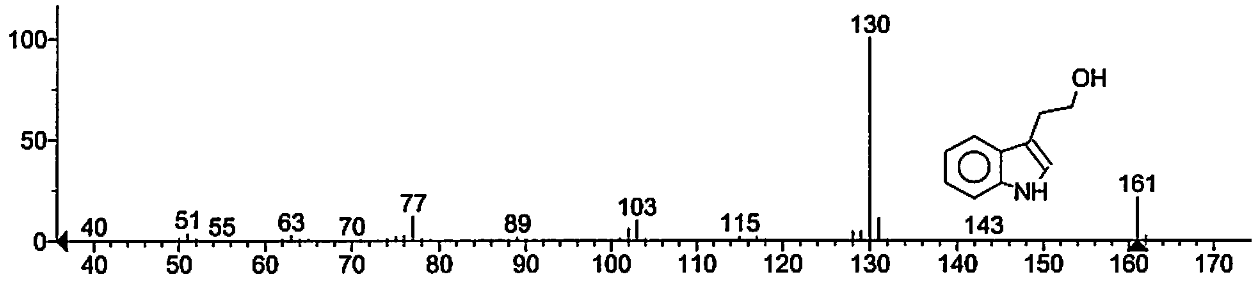

(mainlib) 1H-Indole-3-ethanol

```
File        :D:\Aldrich\JA-11\JA021711-1.D
Operator    :
Acquired    : 17 Feb 2011  17:20     using AcqMethod JA-50-280LESS.M
Instrument :     Buba; IIBBL's magical mass spect
Sample Name: 4M C. ocu. abd.sternites/5ul CH2Cl2;9-10 days
Misc Info   : larvae w/1ug/ul nepetalactol in honey soln.
Vial Number: 1
```

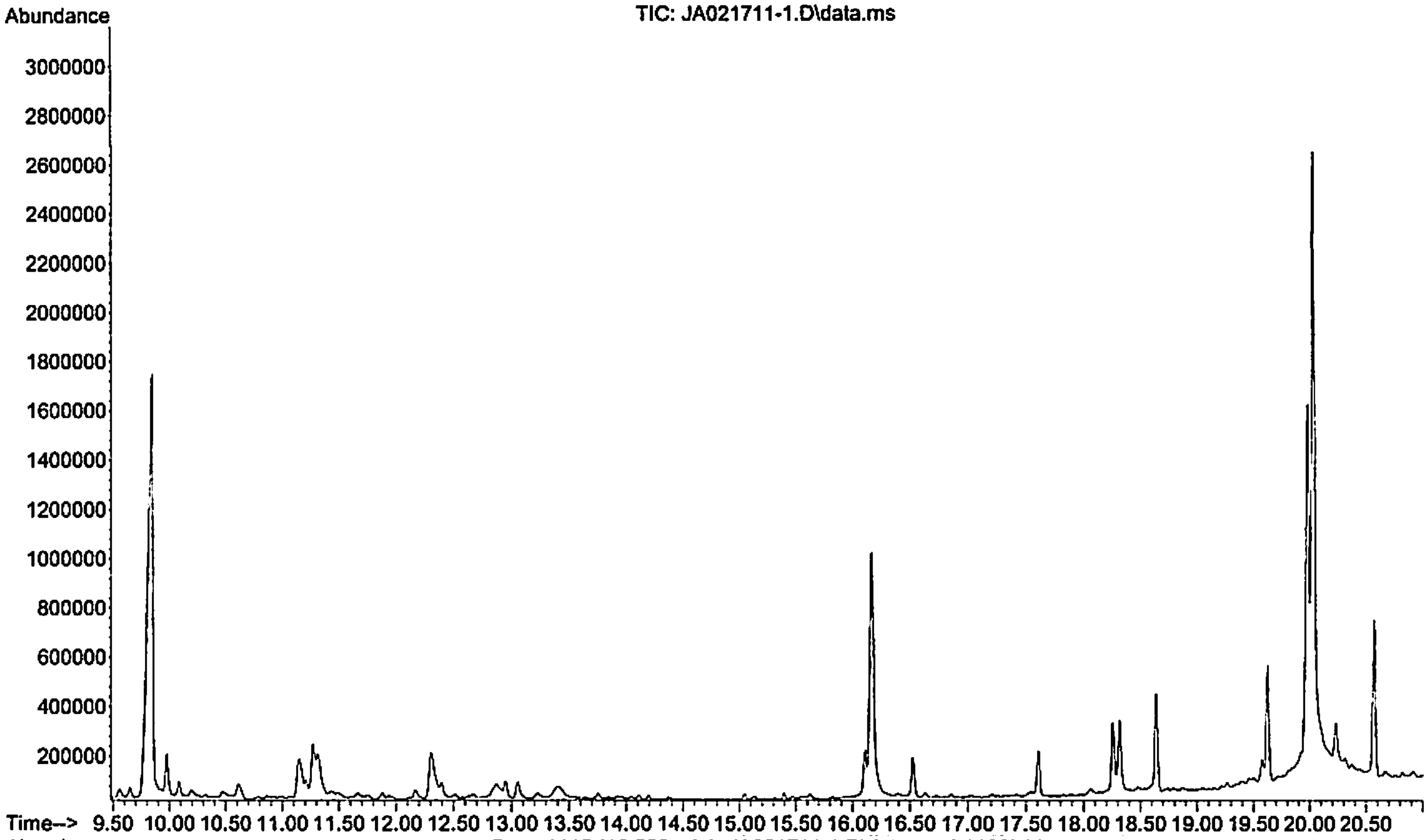

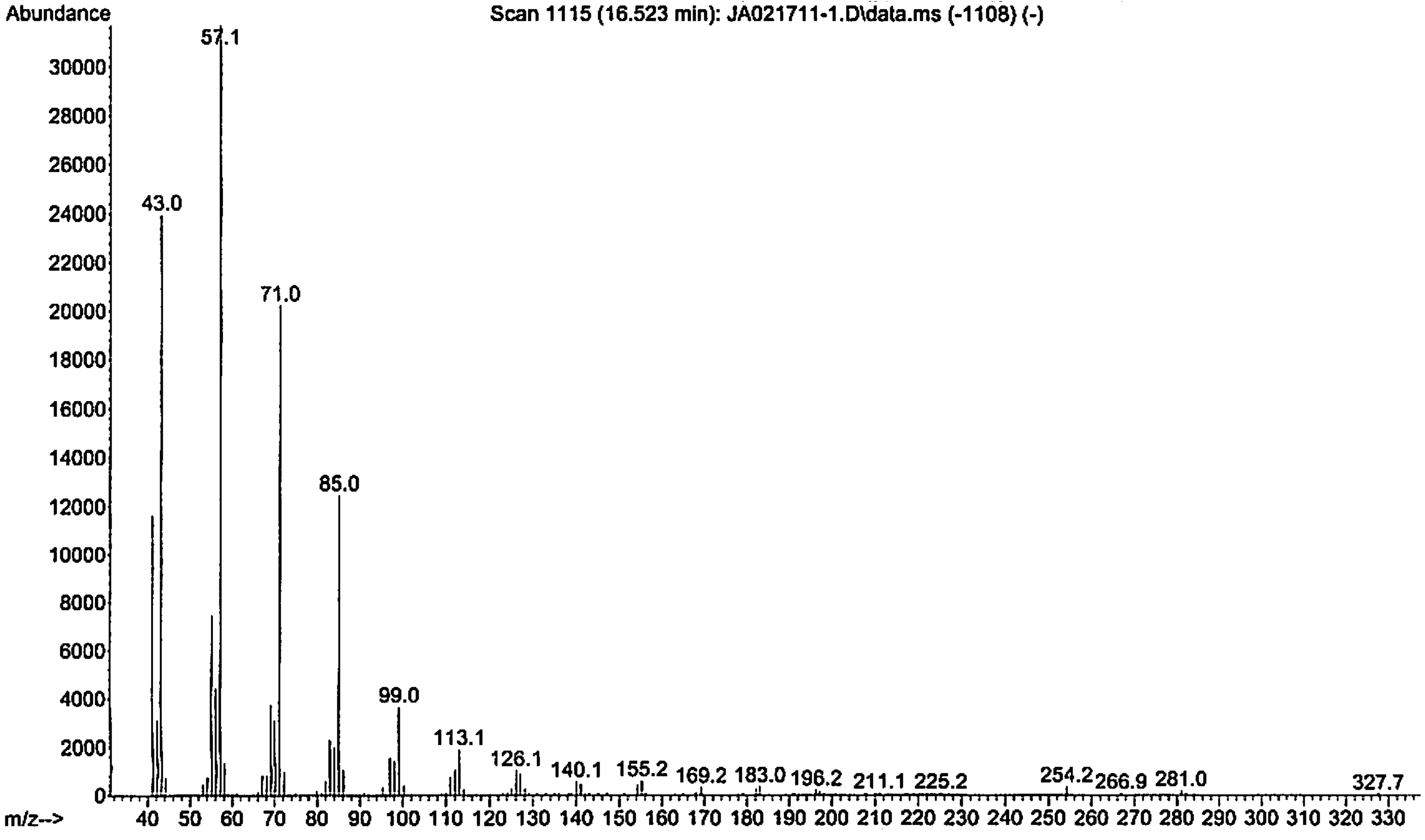

File : D:\Aldrich\JA-11\JA021711-1.D
Operator :
Acquired : 17 Feb 2011 17:20 using AcqMethod JA-50-280LESS.M
Instrument : Buba; IIBBL's magical mass spect
Sample Name: 4M C. ocu. abd.sternites/5ul CH2Cl2;9-10 days
Misc Info : larvae w/1ug/ul nepetalactol in honey soln.
Vial Number: 1

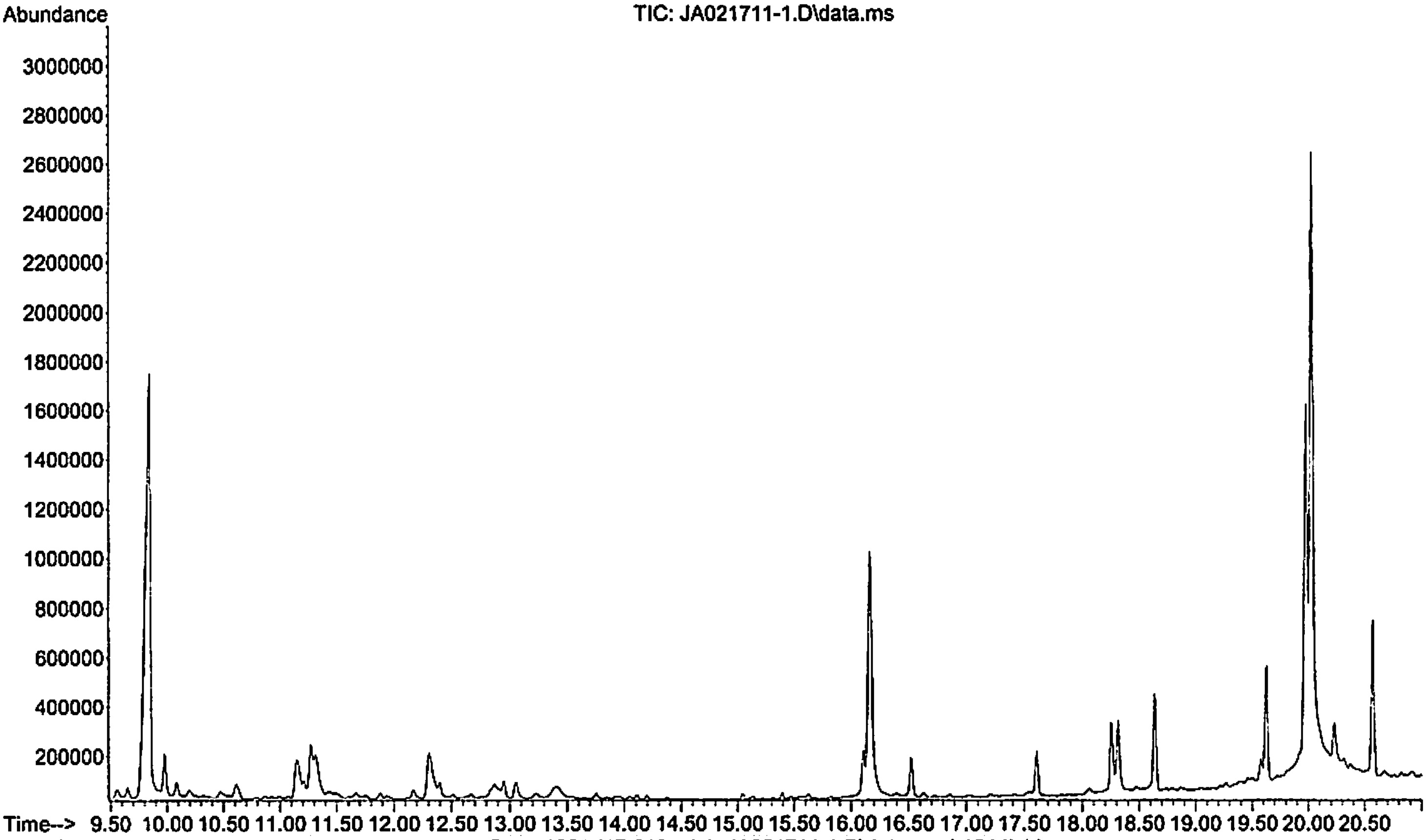

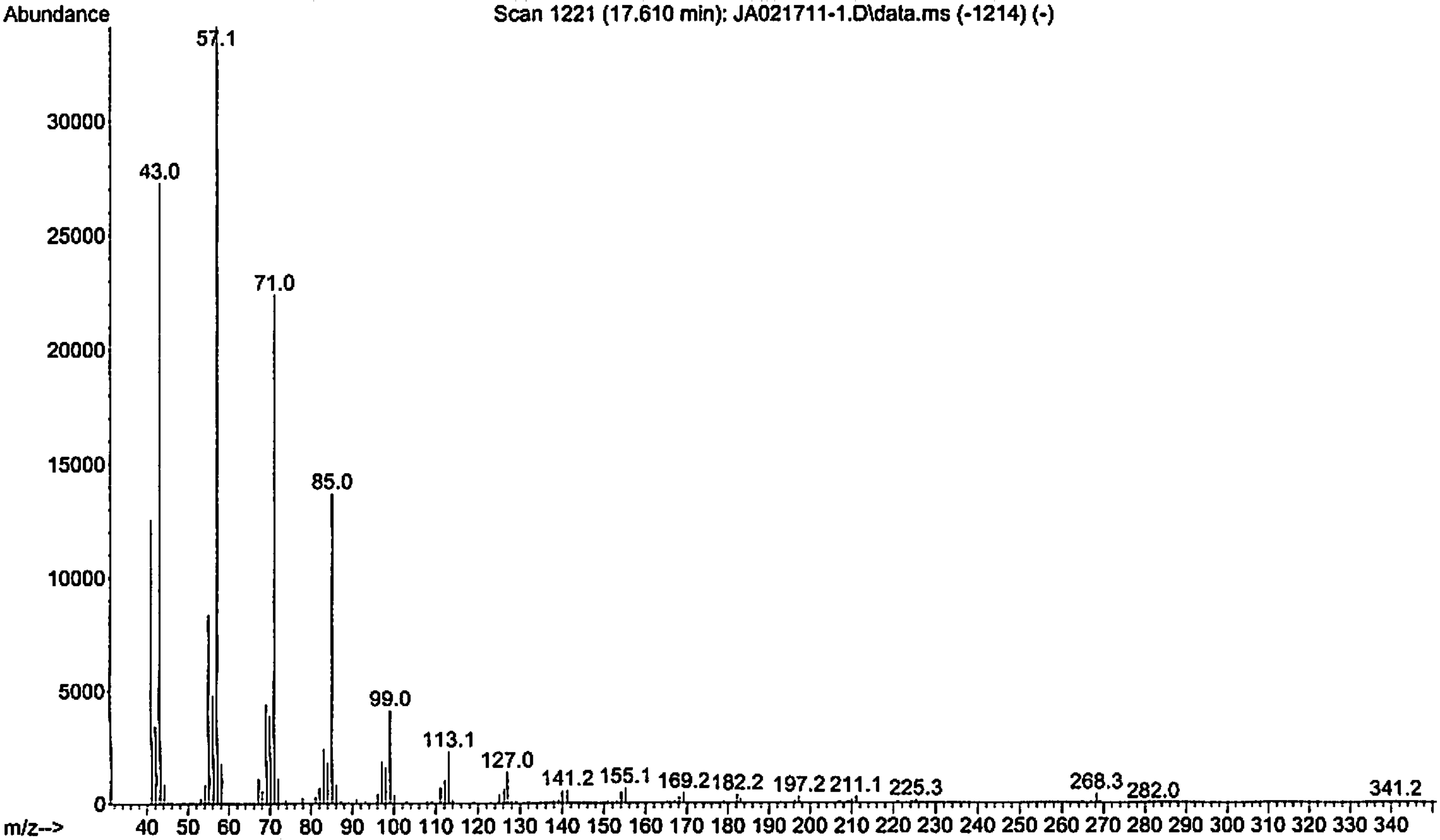

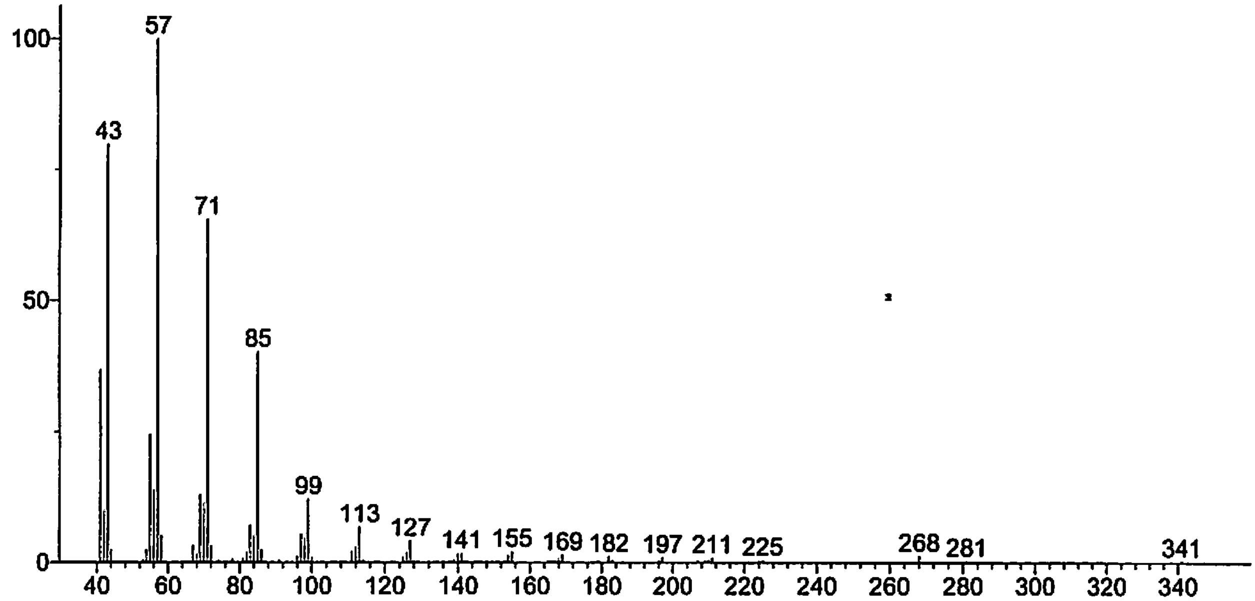

(Text File) Scan 1221 (17.610 min): JA021711-1.D\data.ms (-1214)

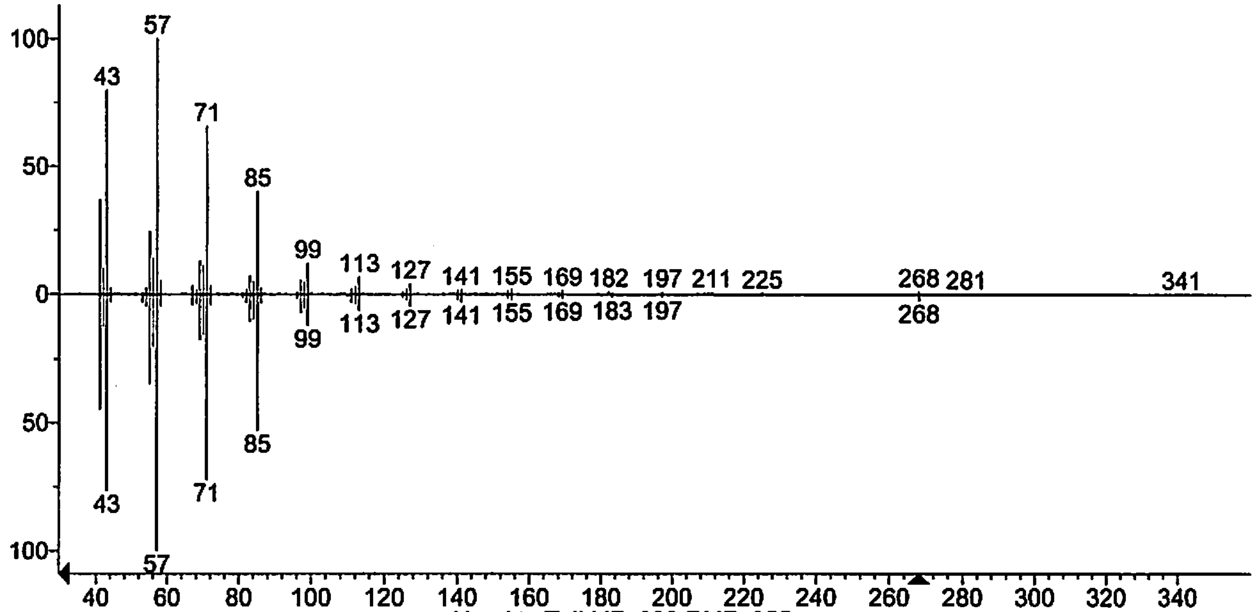

Head to Tail MF=922 RMF=955

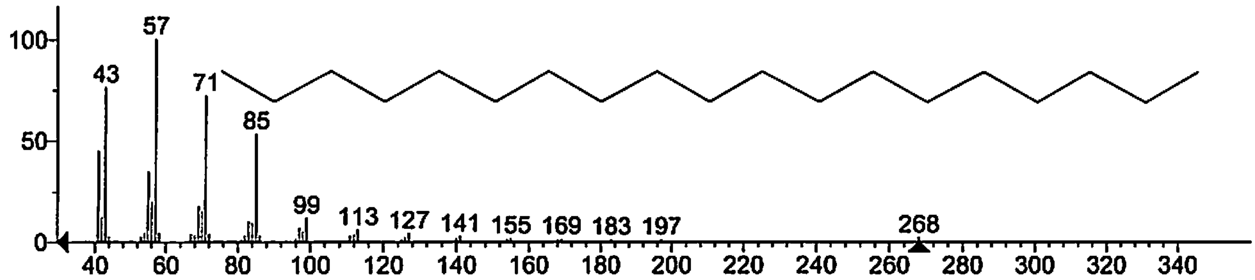

(replib) Nonadecane

```
File       :D:\Aldrich\JA-11\JA021711-1.D
Operator   :
Acquired   : 17 Feb 2011  17:20     using AcqMethod JA-50-280LESS.M
Instrument :    Buba; IIBBL's magical mass spect
Sample Name: 4M C. ocu. abd.sternites/5ul CH2Cl2;9-10 days
Misc Info  : larvae w/1ug/ul nepetalactol in honey soln.
Vial Number: 1
```

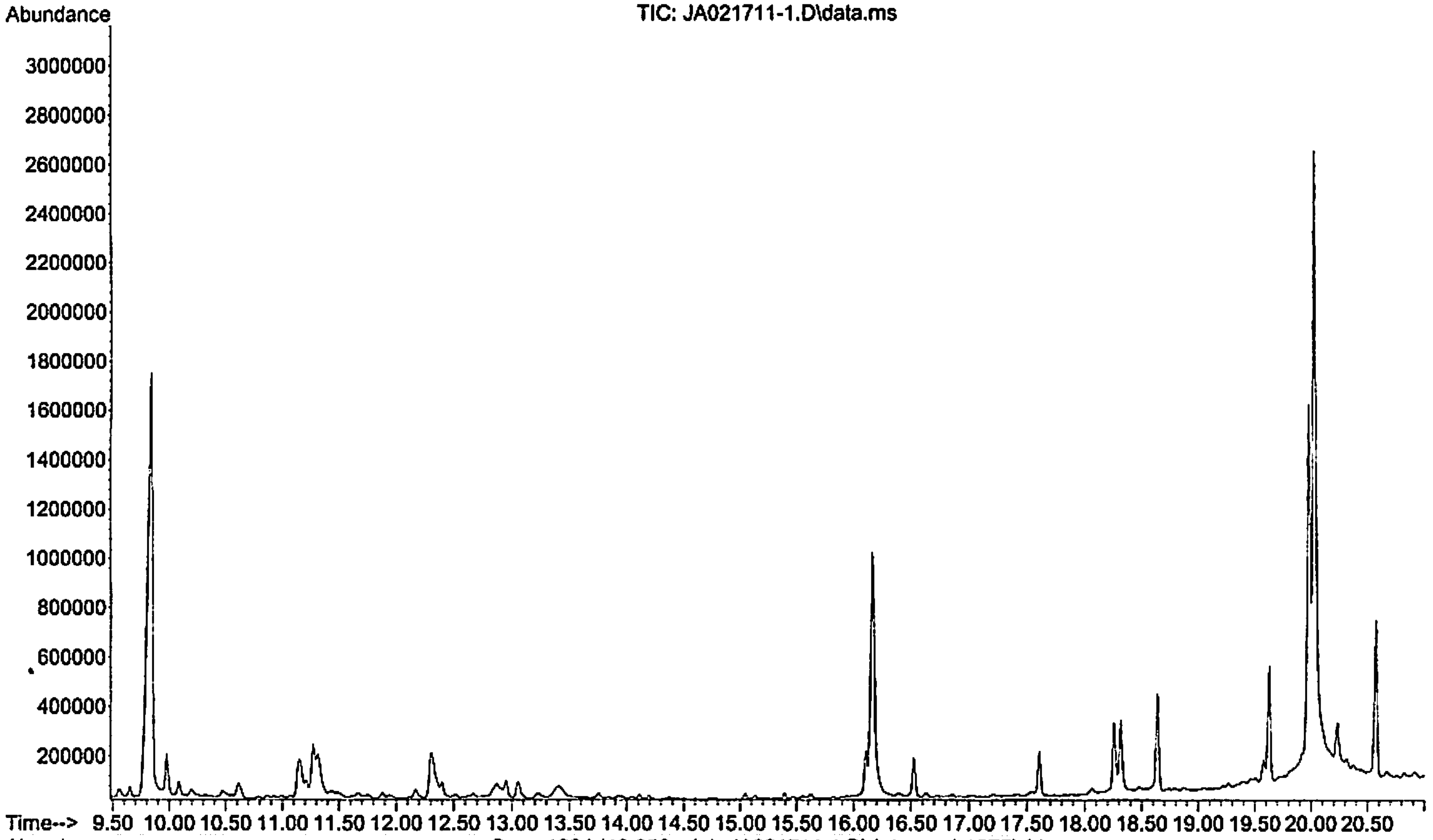

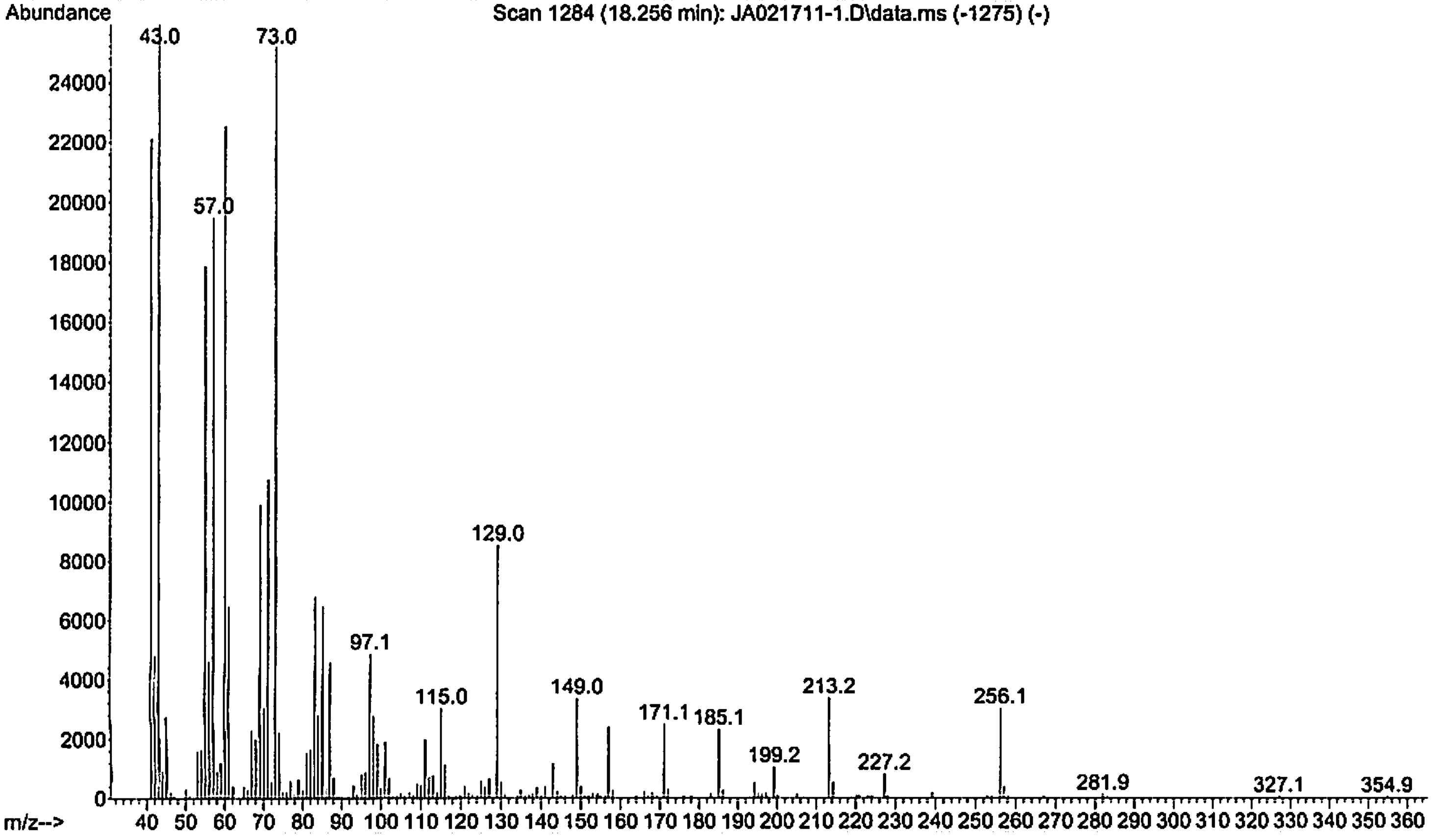

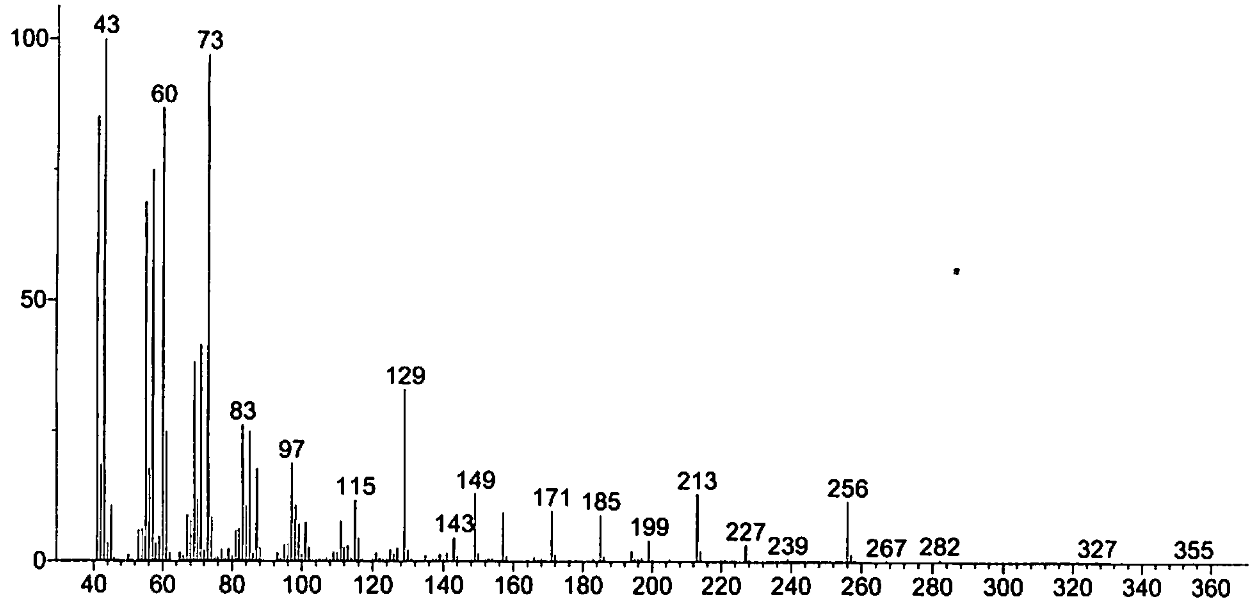

(Text File) Scan 1284 (18.256 min): JA021711-1.D\data.ms (-1275)

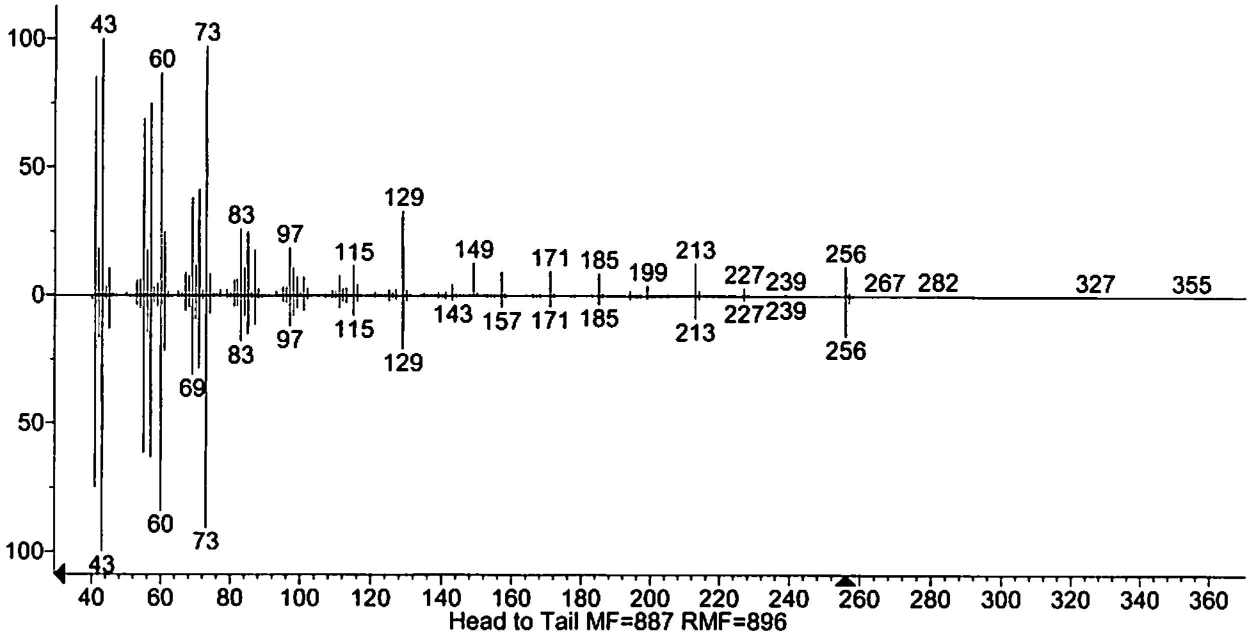

Head to Tail MF=887 RMF=896

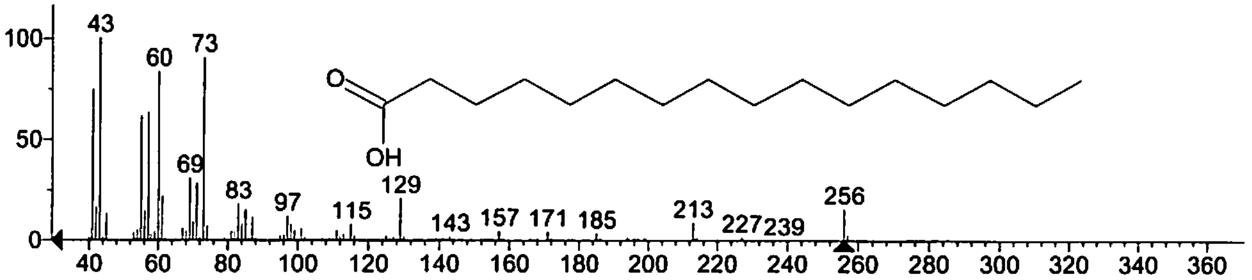

(mainlib) n-Hexadecanoic acid

```
File        :D:\Aldrich\JA-11\JA021711-1.D
Operator    :
Acquired    : 17 Feb 2011  17:20     using AcqMethod JA-50-280LESS.M
Instrument  :    Buba; IIBBL's magical mass spect
Sample Name: 4M C. ocu. abd.sternites/5ul CH2Cl2;9-10 days
Misc Info   : larvae w/1ug/ul nepetalactol in honey soln.
Vial Number: 1
```

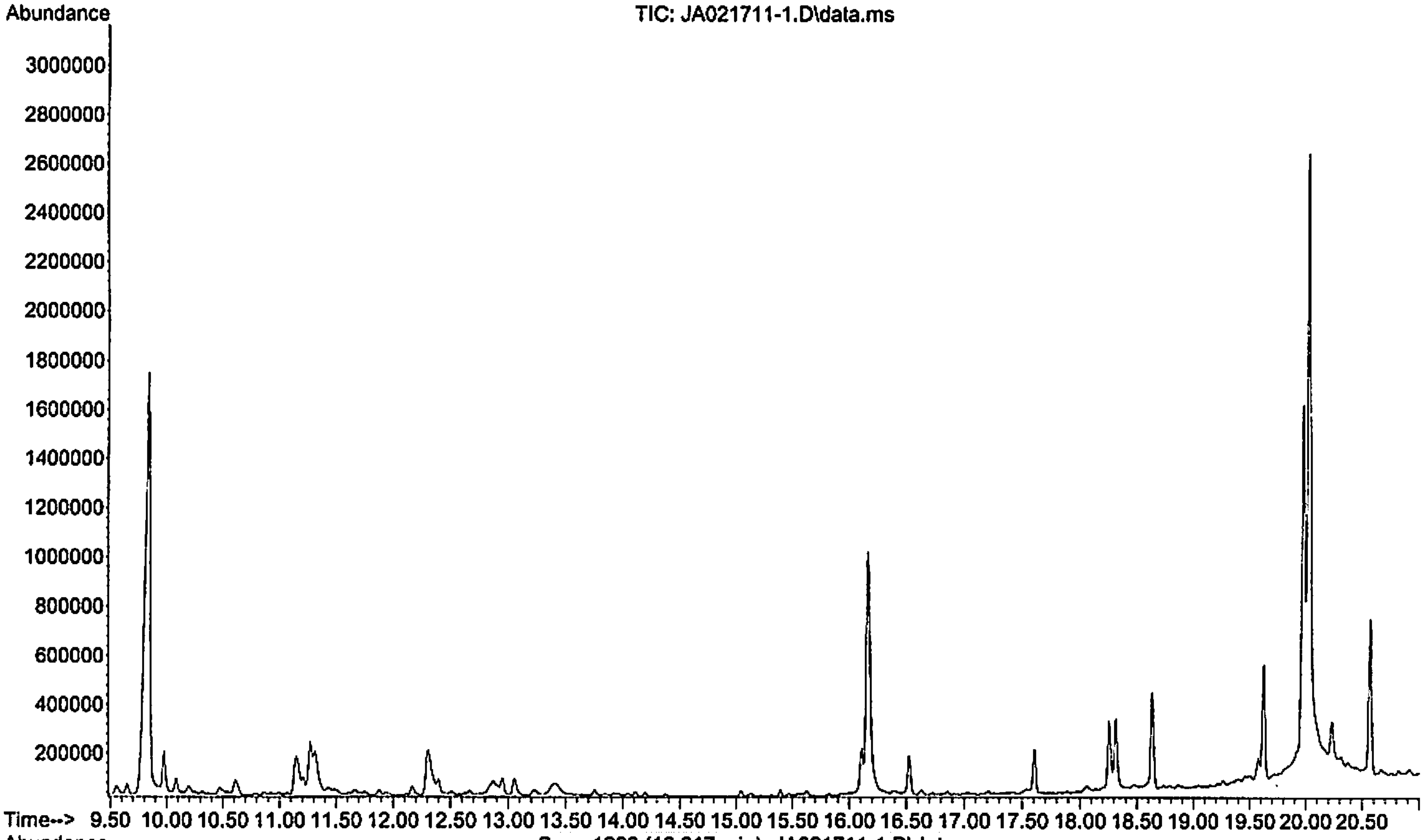

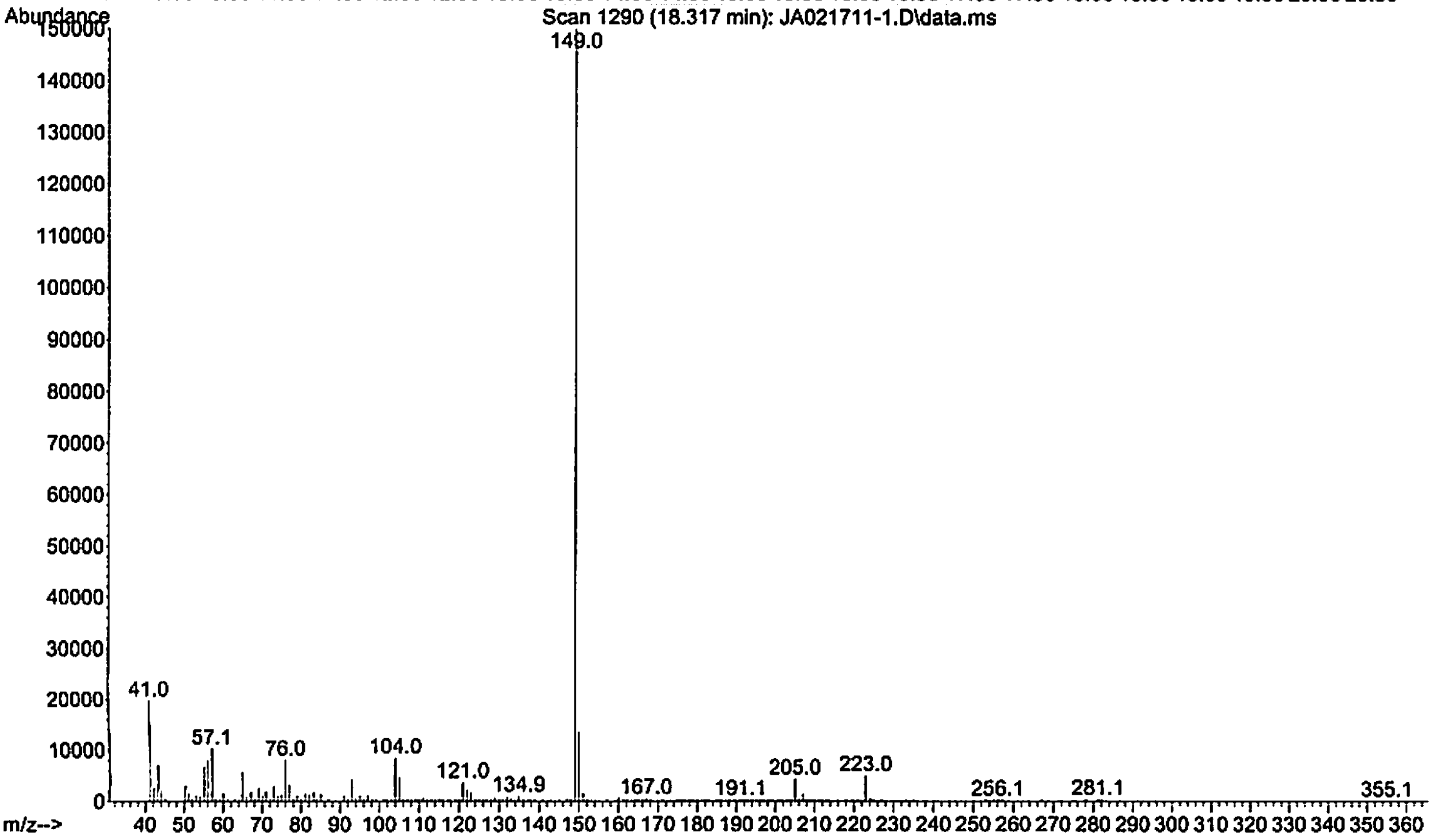

```
File        :D:\Aldrich\JA-11\JA021711-1.D
Operator    :
Acquired    : 17 Feb 2011  17:20     using AcqMethod JA-50-280LESS.M
Instrument :    Buba; IIBBL's magical mass spect
Sample Name: 4M C. ocu. abd.sternites/5ul CH2Cl2;9-10 days
Misc Info   : larvae w/1ug/ul nepetalactol in honey soln.
Vial Number: 1
```

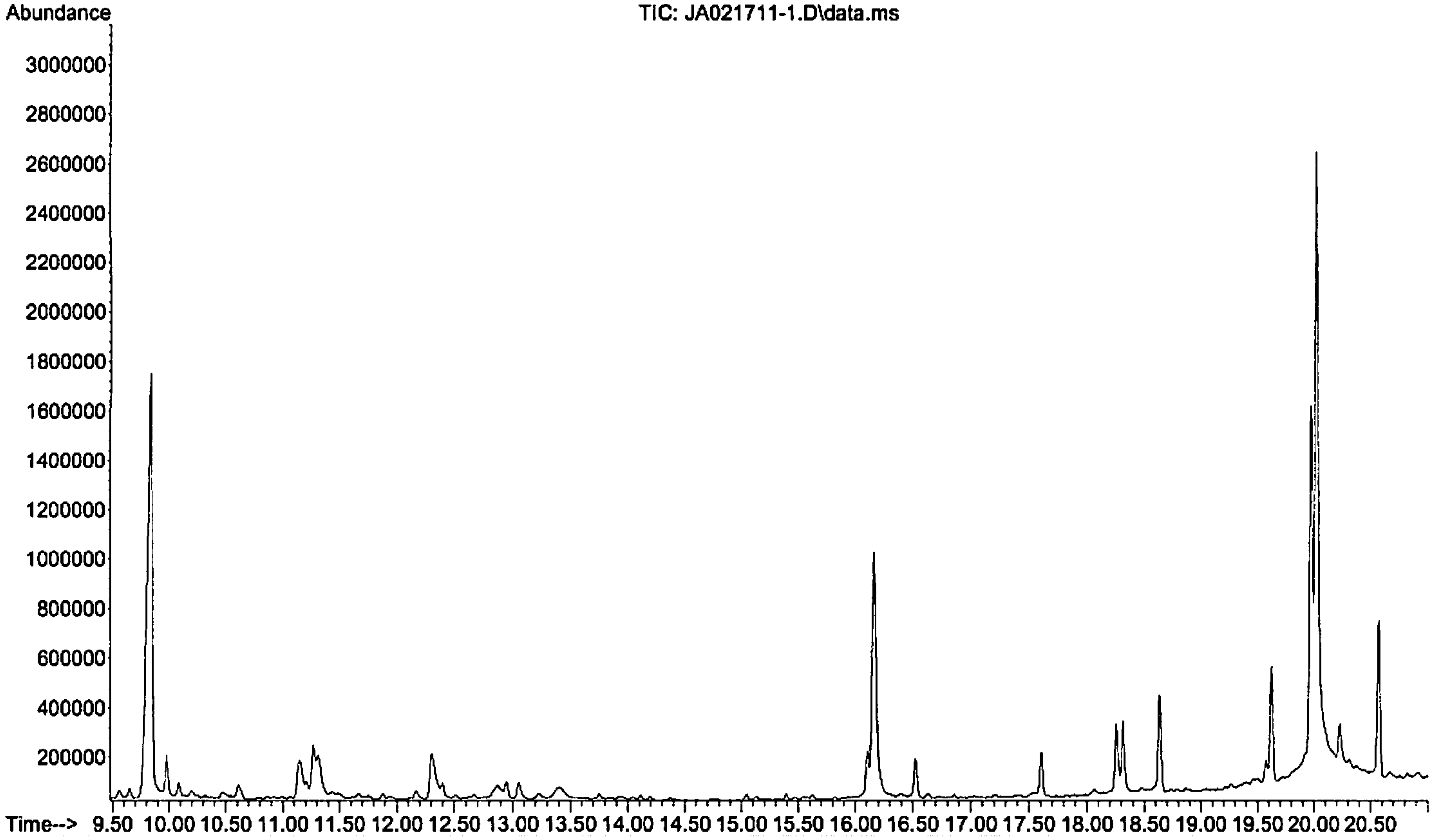

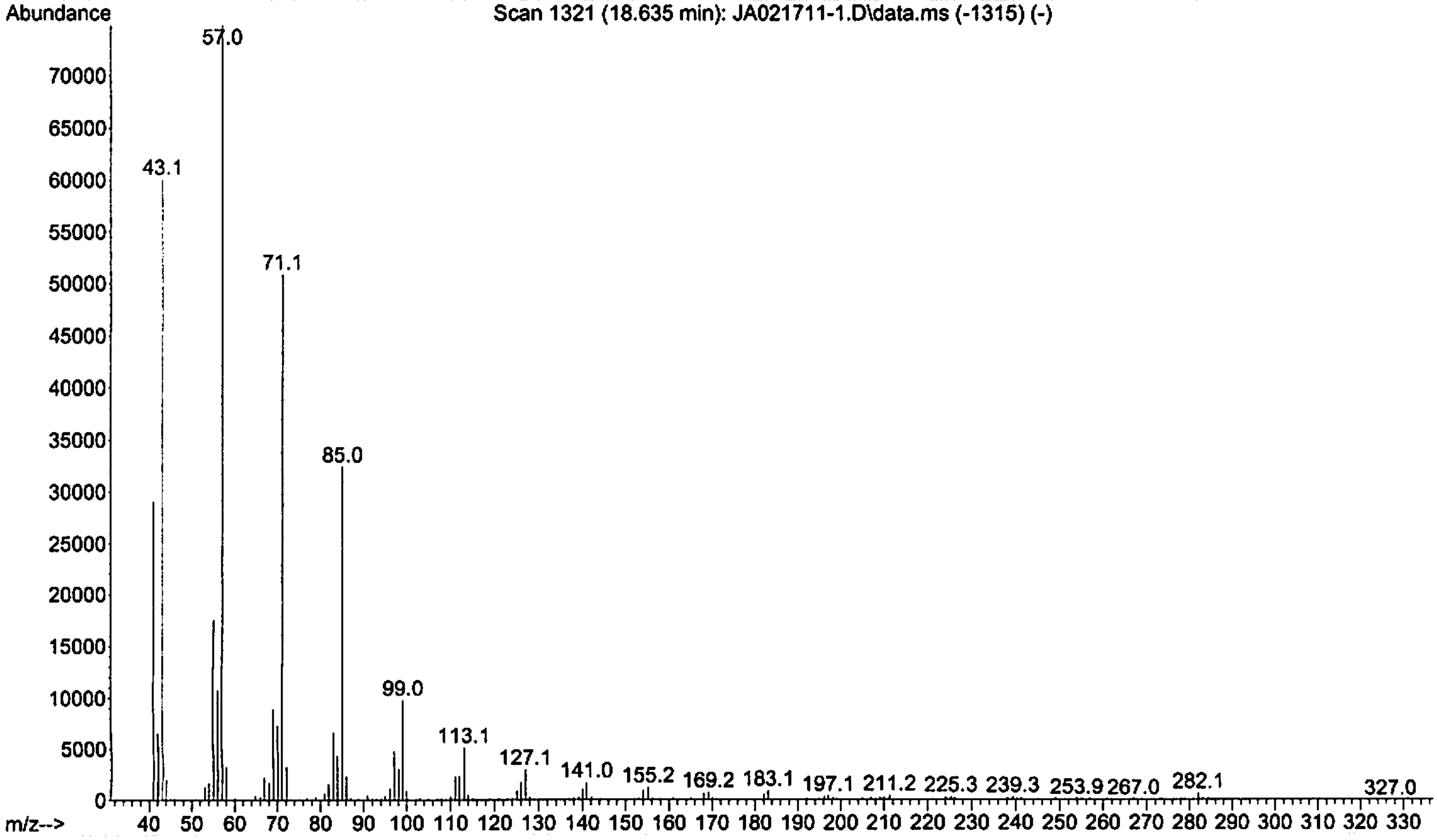

File :D:\Aldrich\JA-11\JA021711-1.D
Operator :
Acquired : 17 Feb 2011 17:20 using AcqMethod JA-50-280LESS.M
Instrument : Buba; IIBBL's magical mass spect
Sample Name: 4M C. ocu. abd.sternites/5ul CH2Cl2;9-10 days
Misc Info : larvae w/1ug/ul nepetalactol in honey soln.
Vial Number: 1

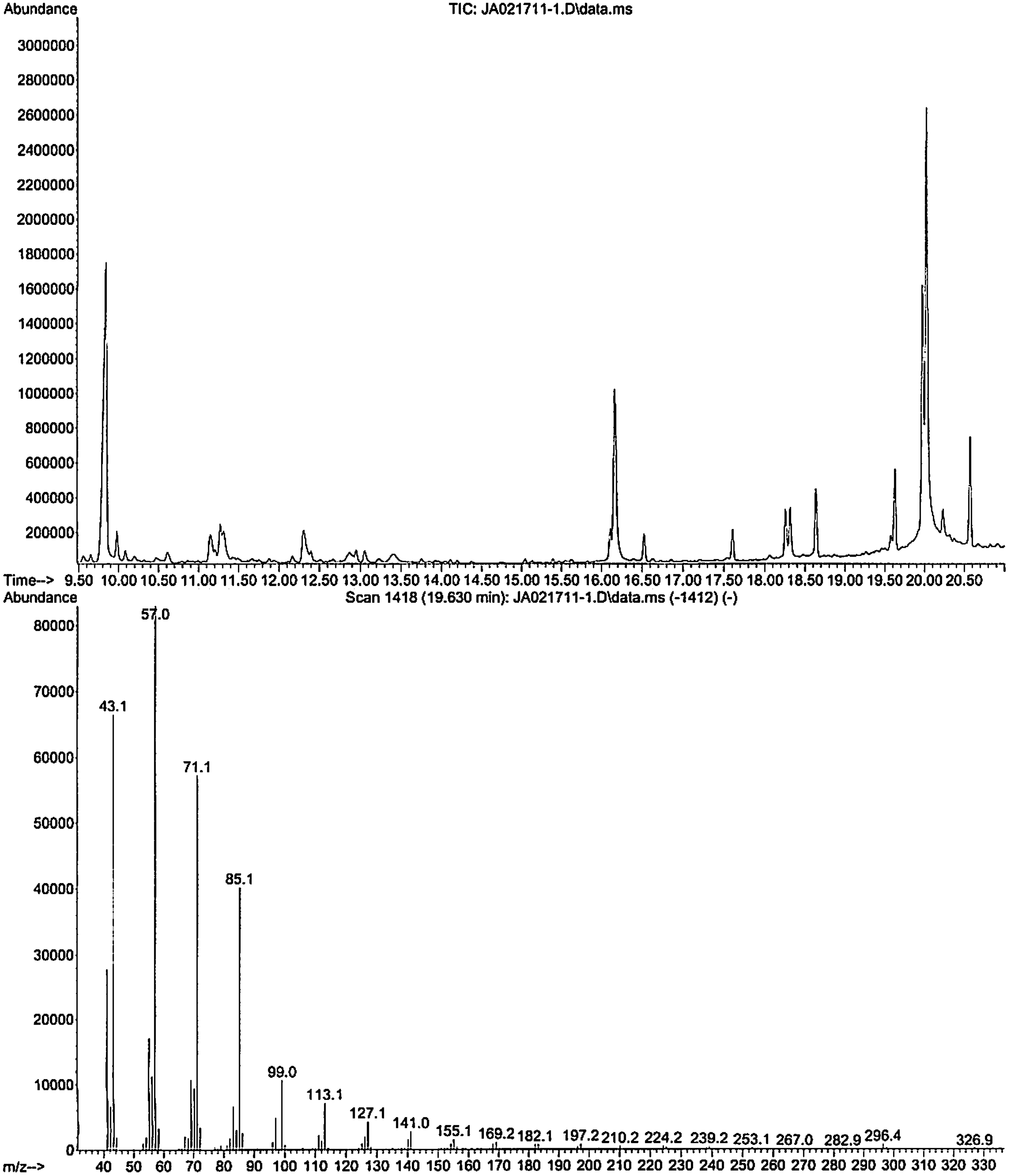

```
File        :D:\Aldrich\JA-11\JA021711-1.D
Operator    :
Acquired    : 17 Feb 2011  17:20     using AcqMethod JA-50-280LESS.M
Instrument :    Buba; IIBBL's magical mass spect
Sample Name: 4M C. ocu. abd.sternites/5ul CH2Cl2;9-10 days
Misc Info   : larvae w/1ug/ul nepetalactol in honey soln.
Vial Number: 1
```

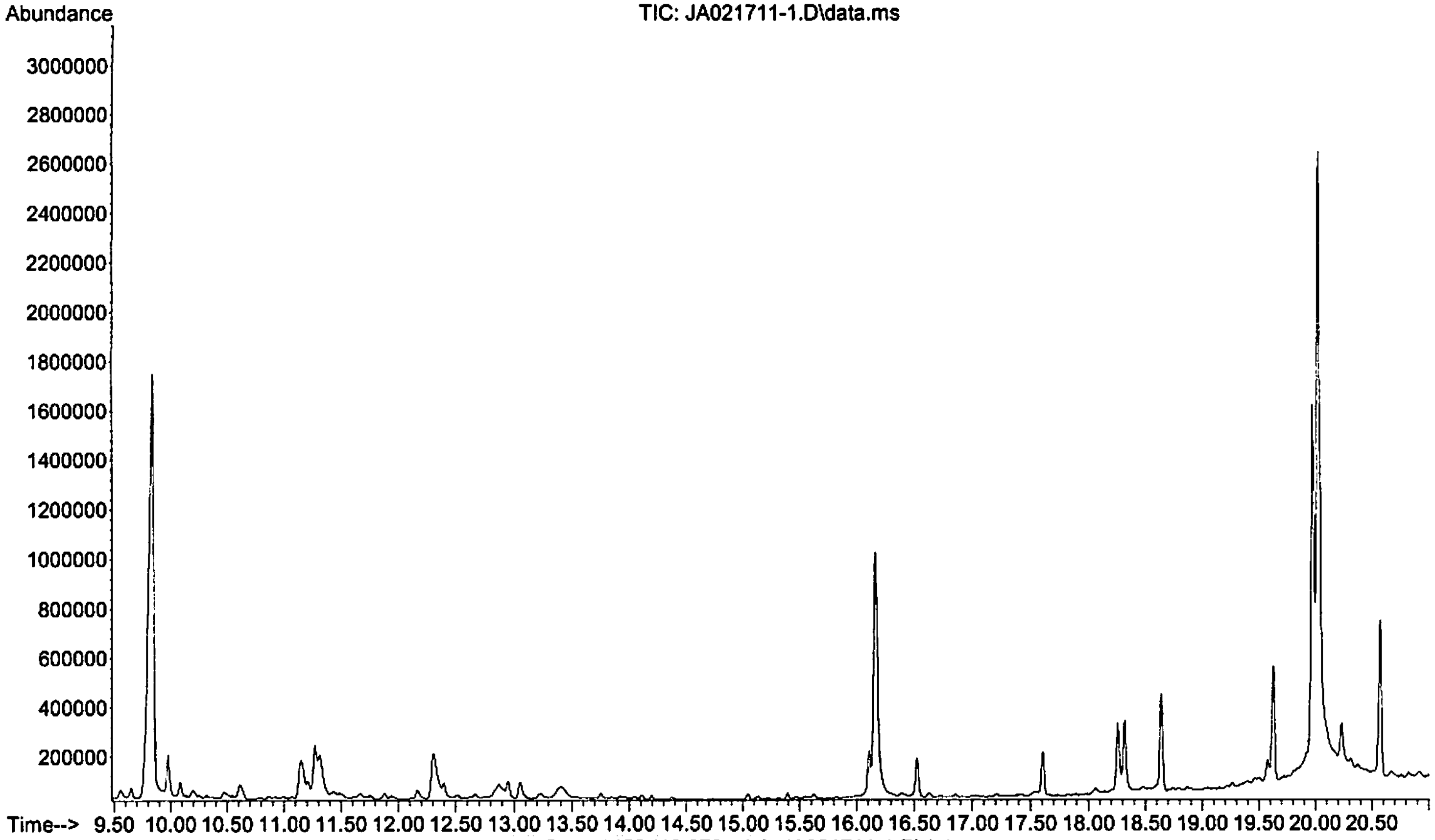

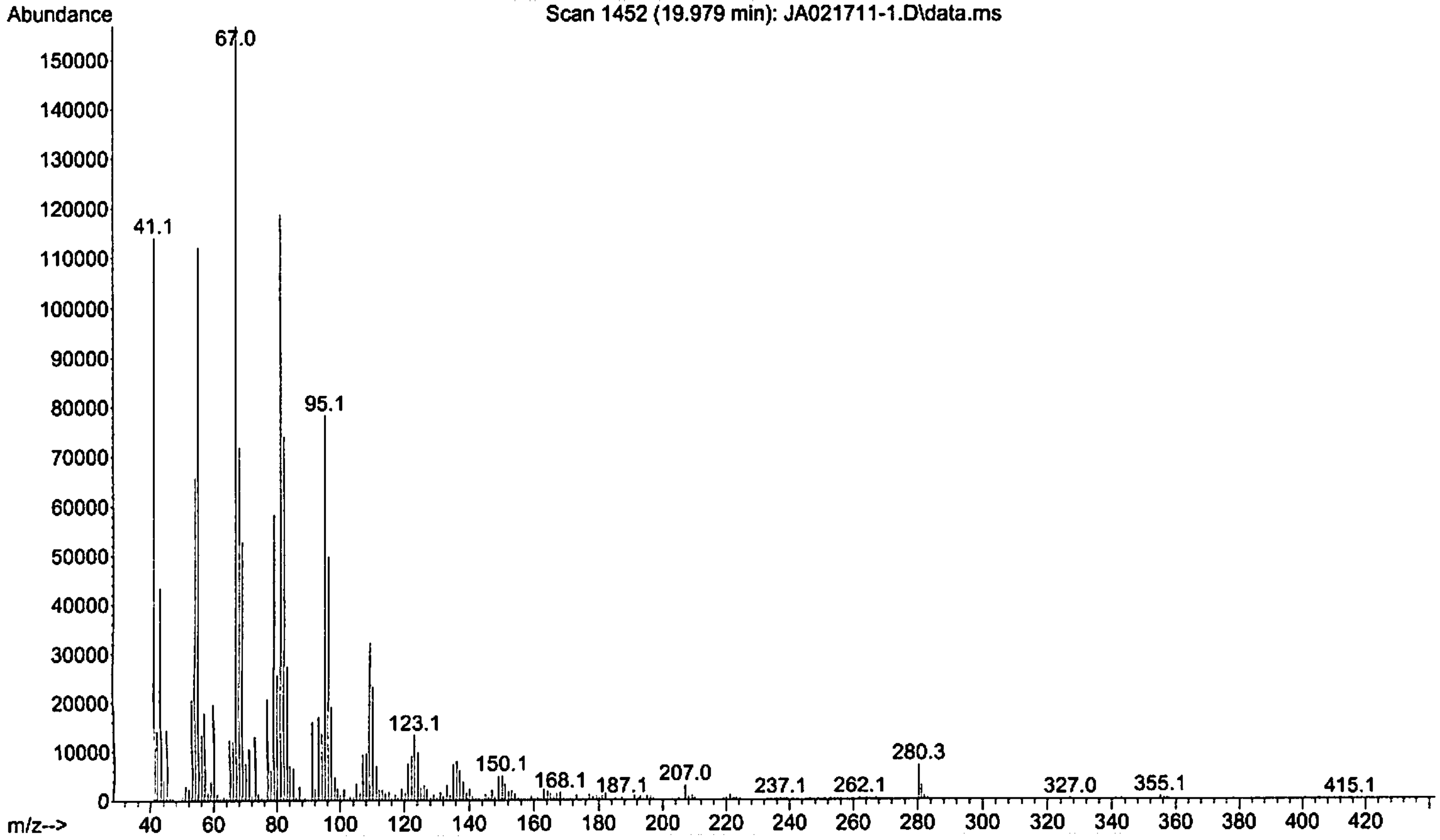

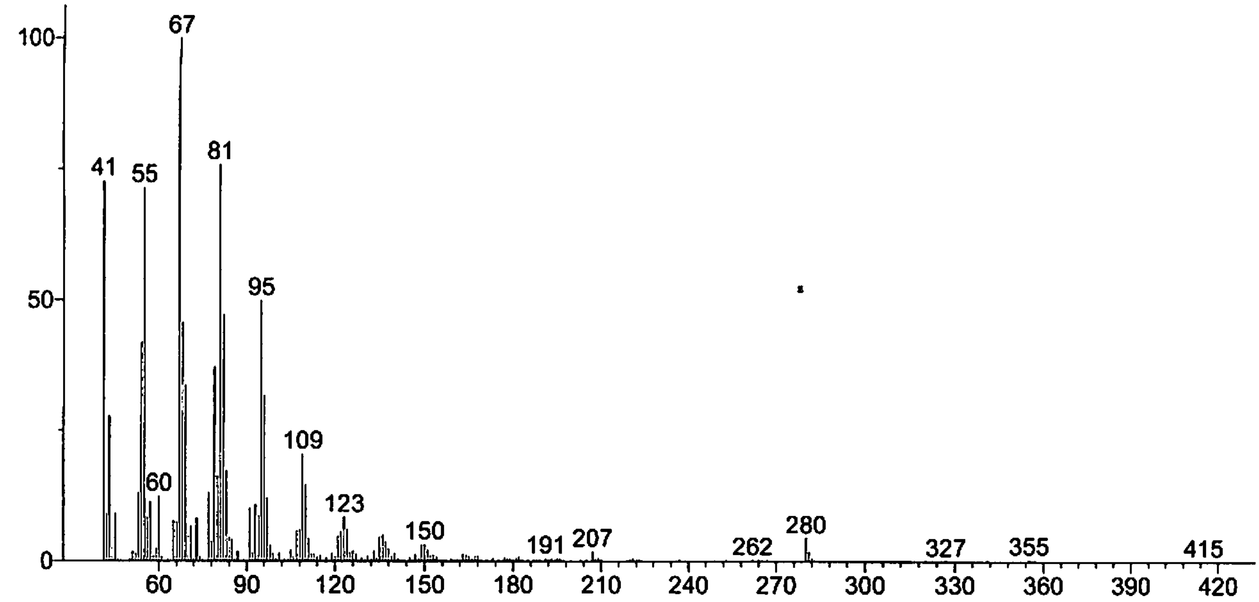

(Text File) Scan 1452 (19.979 min): JA021711-1.D\data.ms

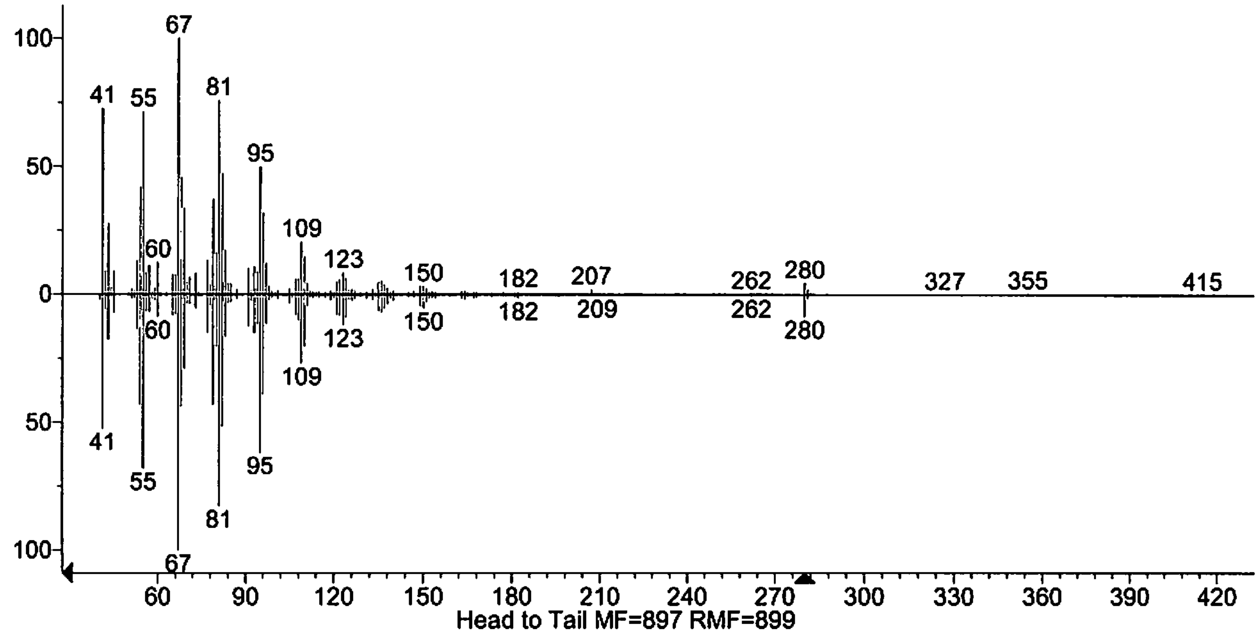

Head to Tail MF=897 RMF=899

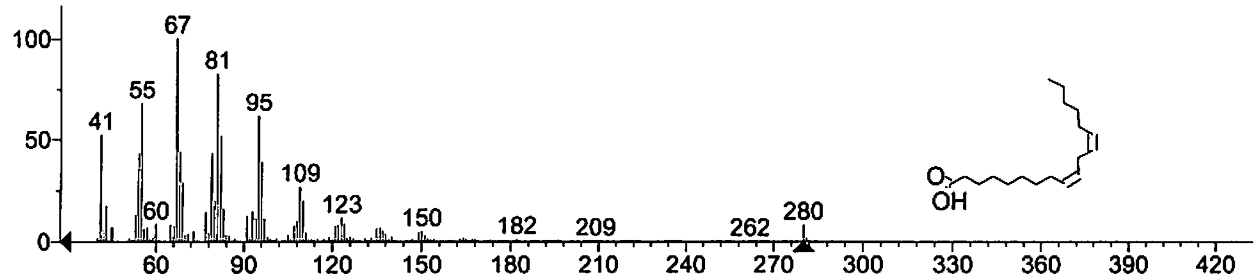

(replib) 9,12-Octadecadienoic acid (Z,Z)-

```
File        :D:\Aldrich\JA-11\JA021711-1.D
Operator    :
Acquired    : 17 Feb 2011  17:20     using AcqMethod JA-50-280LESS.M
Instrument  :    Buba; IIBBL's magical mass spect
Sample Name: 4M C. ocu. abd.sternites/5ul CH2Cl2;9-10 days
Misc Info   : larvae w/1ug/ul nepetalactol in honey soln.
Vial Number: 1
```

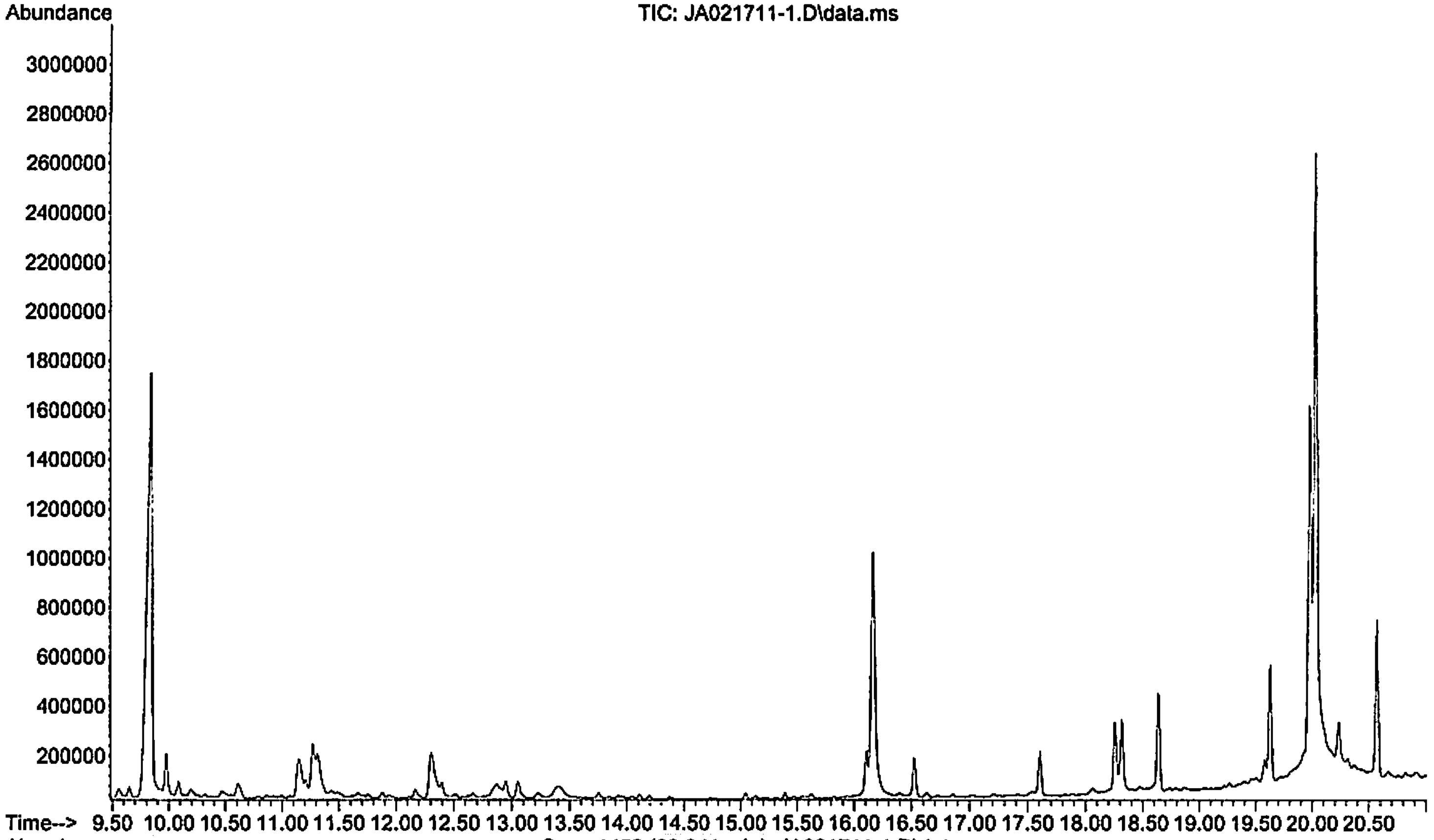

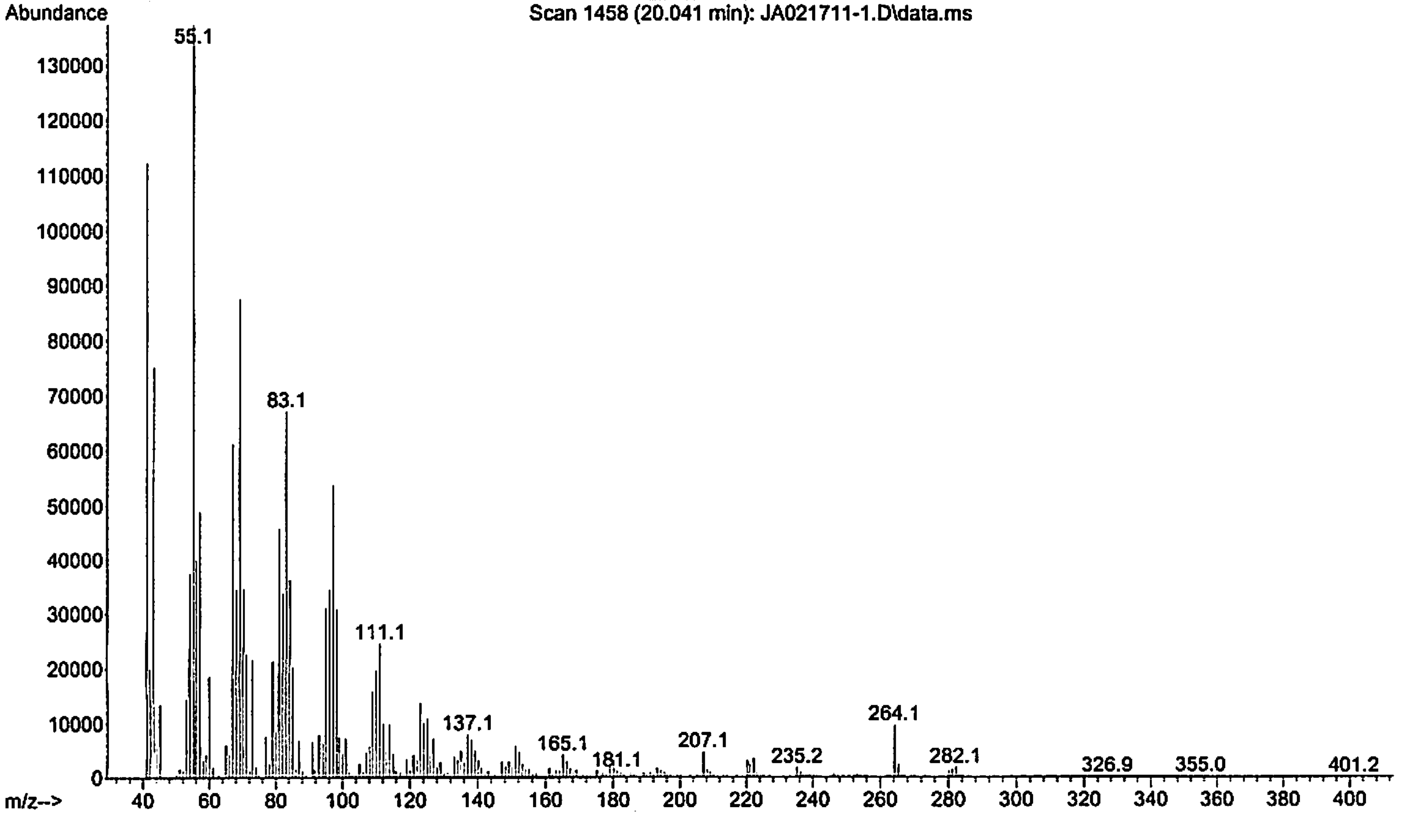

File :D:\Aldrich\JA-11\JA021711-1.D
Operator :
Acquired : 17 Feb 2011 17:20 using AcqMethod JA-50-280LESS.M
Instrument : Buba; IIBBL's magical mass spect
Sample Name: 4M C. ocu. abd.sternites/5ul CH2Cl2;9-10 days
Misc Info : larvae w/1ug/ul nepetalactol in honey soln.
Vial Number: 1

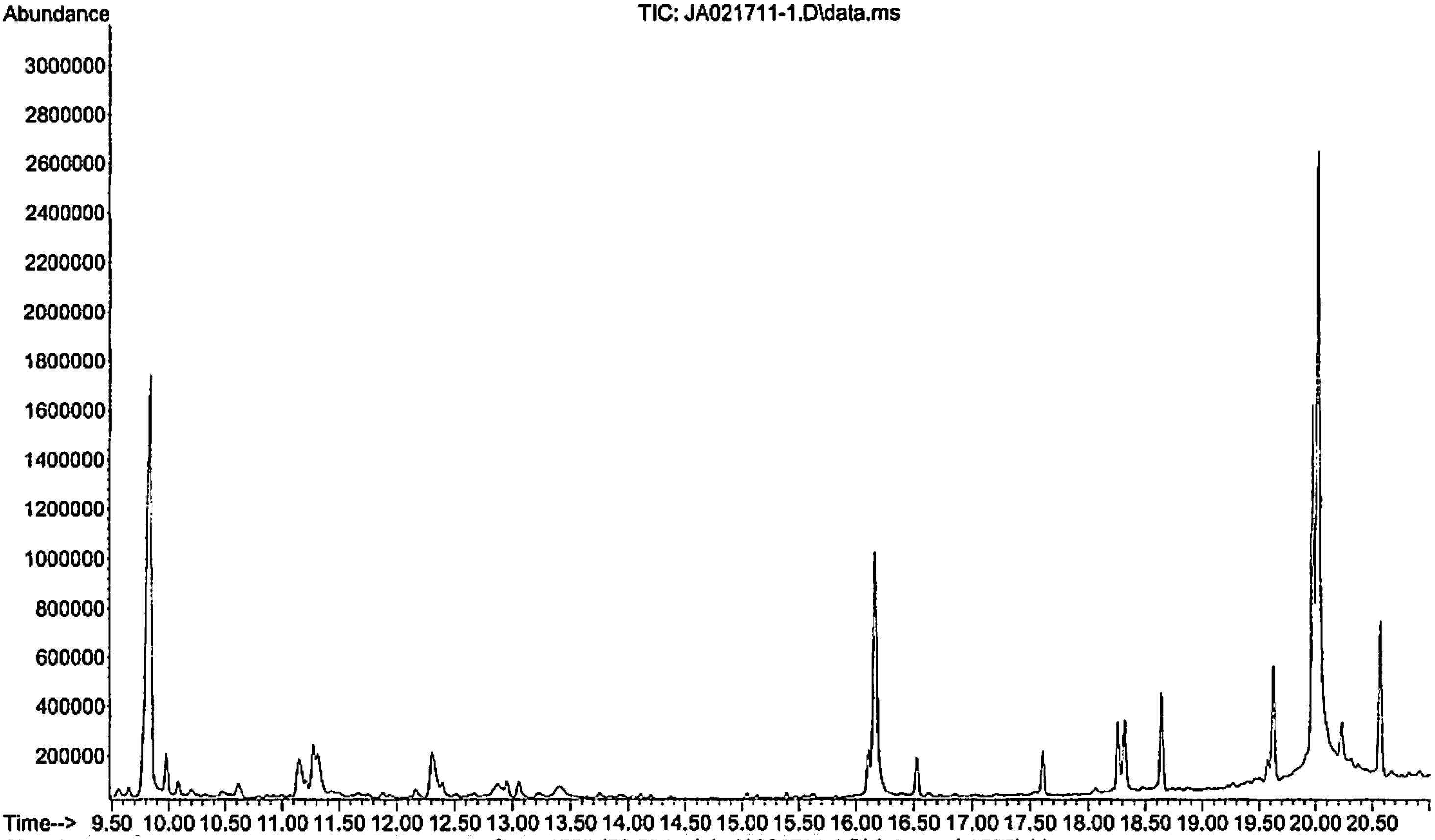

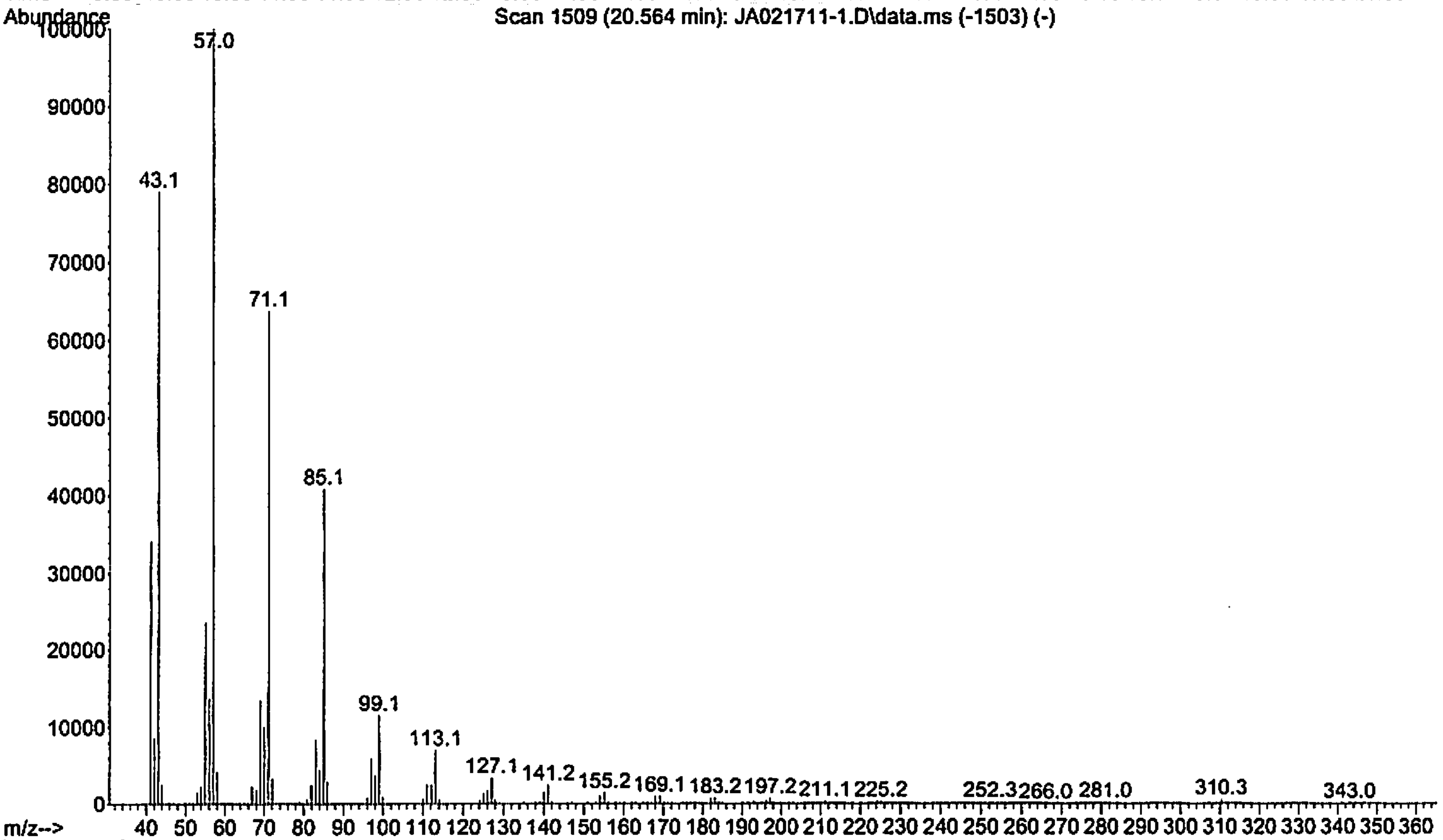

Supplement: Data S10 [file peerj-04-1564-s015.pdf]
