# Supplementary material for: Pharmacophagy in green lacewings (Neuroptera: Chrysopidae: Chrysopa spp.)?
Source: PeerJ. 2016 Jan 18;4:e1564. doi: 10.7717/peerj.1564 (PMC4727961; doi:10.7717/peerj.1564)

Status of C. oculata Larval Feeding Expt on 06/27/11.

Farnesyl pyrophosphate reared ♂s:

(2). NEAs · 06/09 - 06/10.
(2). NEAs · 06/10 - 06/13.
(3). NEAs · 06/17 - 06/21.

7 total ♂s

Geranyl pyrophosphate-reared ♂s:

(3). NEAs · 06/09 - 06/10.
(2). NEAs · 06/06 - 06/09.
(1). NEAs · 06/17 - 06/21.

6 total ♂s

Negative Control reared ♂s:

(1). NEA · 06/21 - 06/27.
(1). NEA · 06/09 - 06/10.
(2). NEAs · 06/10 - 06/13.
(2). NEAs · 06/17 - 06/21.

6 total ♂s

```
File        :D:\Aldrich\JA-11\JA070711-5.D
Operator    :
Acquired    :  7 Jul 2011  17:42     using AcqMethod JA-50-280LESS.M
Instrument  :   Buba; IIBBL's magical mass spect
Sample Name: 3M C.oculata abd.stern./ca.10ul CH2Cl2
Misc Info   : larvae fed geranylPO4; [125ul]
Vial Number: 1
```

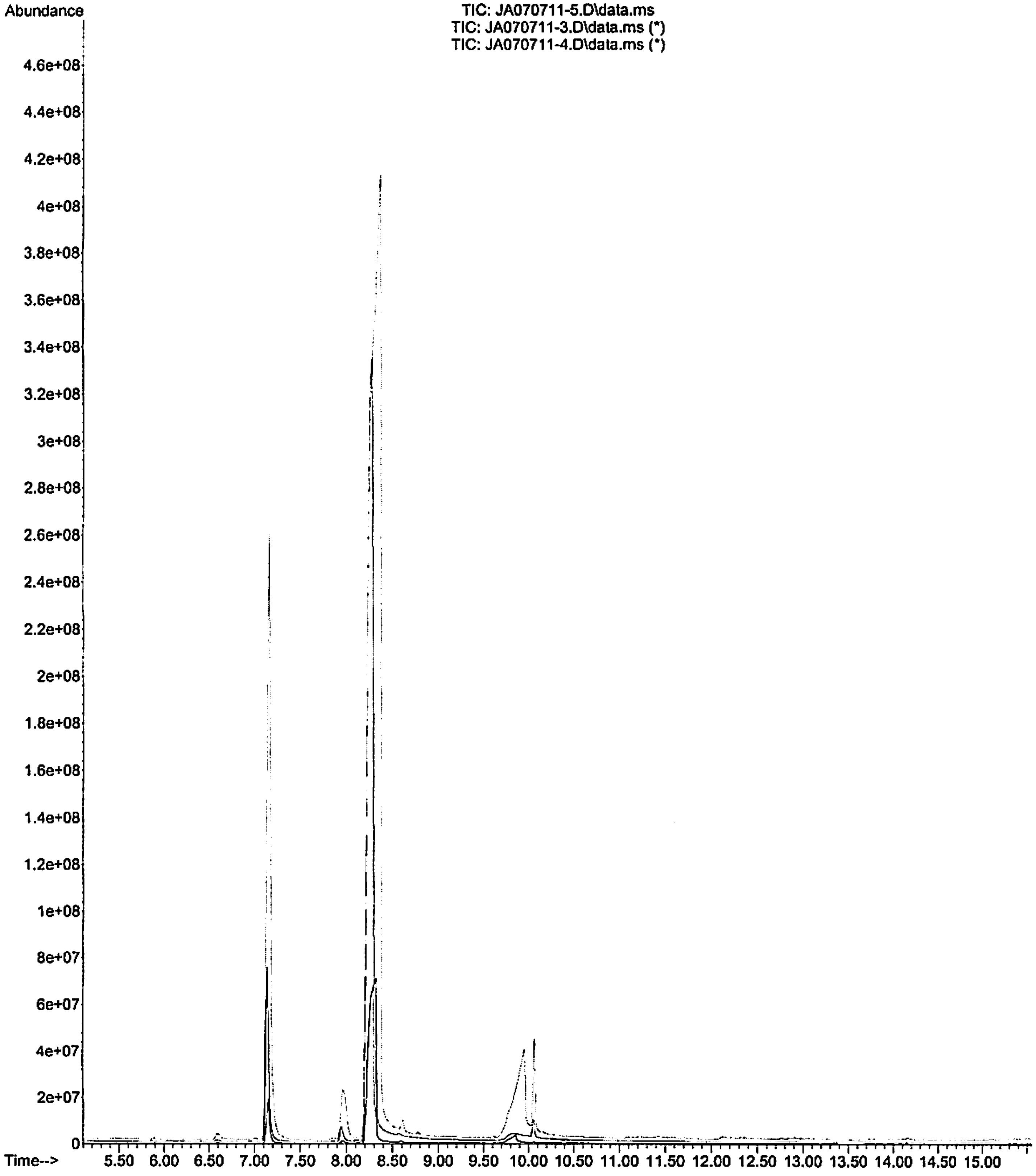

```
File        :D:\Aldrich\JA-11\JA070711-3.D
Operator    :
Acquired    :  7 Jul 2011  15:49     using AcqMethod JA-50-280LESS.M
Instrument :   Buba; IIBBL's magical mass spect
Sample Name: 4M C.oculata abd.stern./ca.10ul CH2Cl2
Misc Info   : from control larvae reared; conc. from 125ul
Vial Number: 1
```

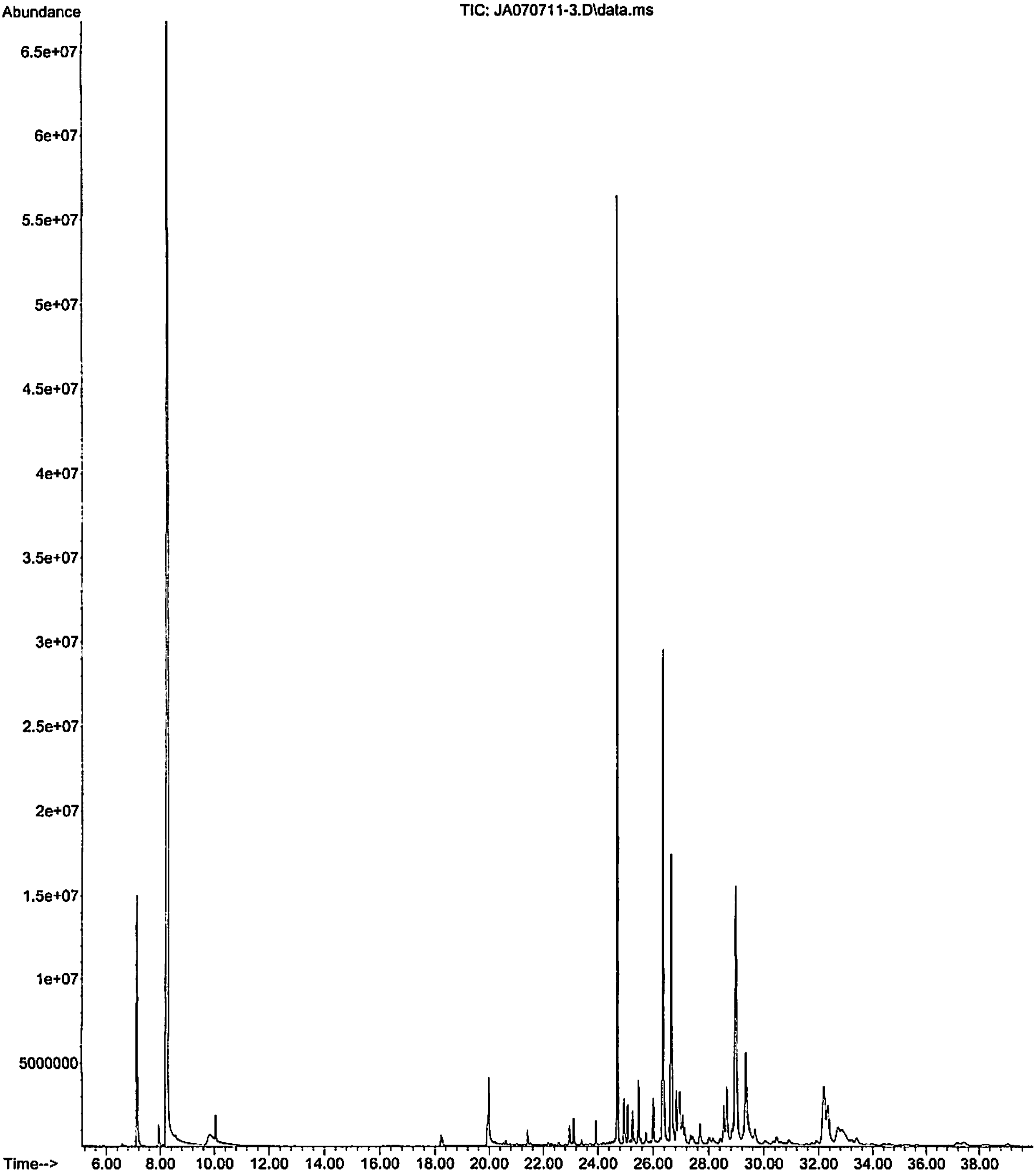

```
File        :D:\Aldrich\JA-11\JA070711-4.D
Operator    :
Acquired    :  7 Jul 2011  16:55     using AcqMethod JA-50-280LESS.M
Instrument  :   Buba; IIBBL's magical mass spect
Sample Name: 5M C.oculata abd.stern./ca.10ul CH2Cl2
Misc Info   : larvae fed geranylfarnesylPO4; [125ul]
Vial Number: 1
```

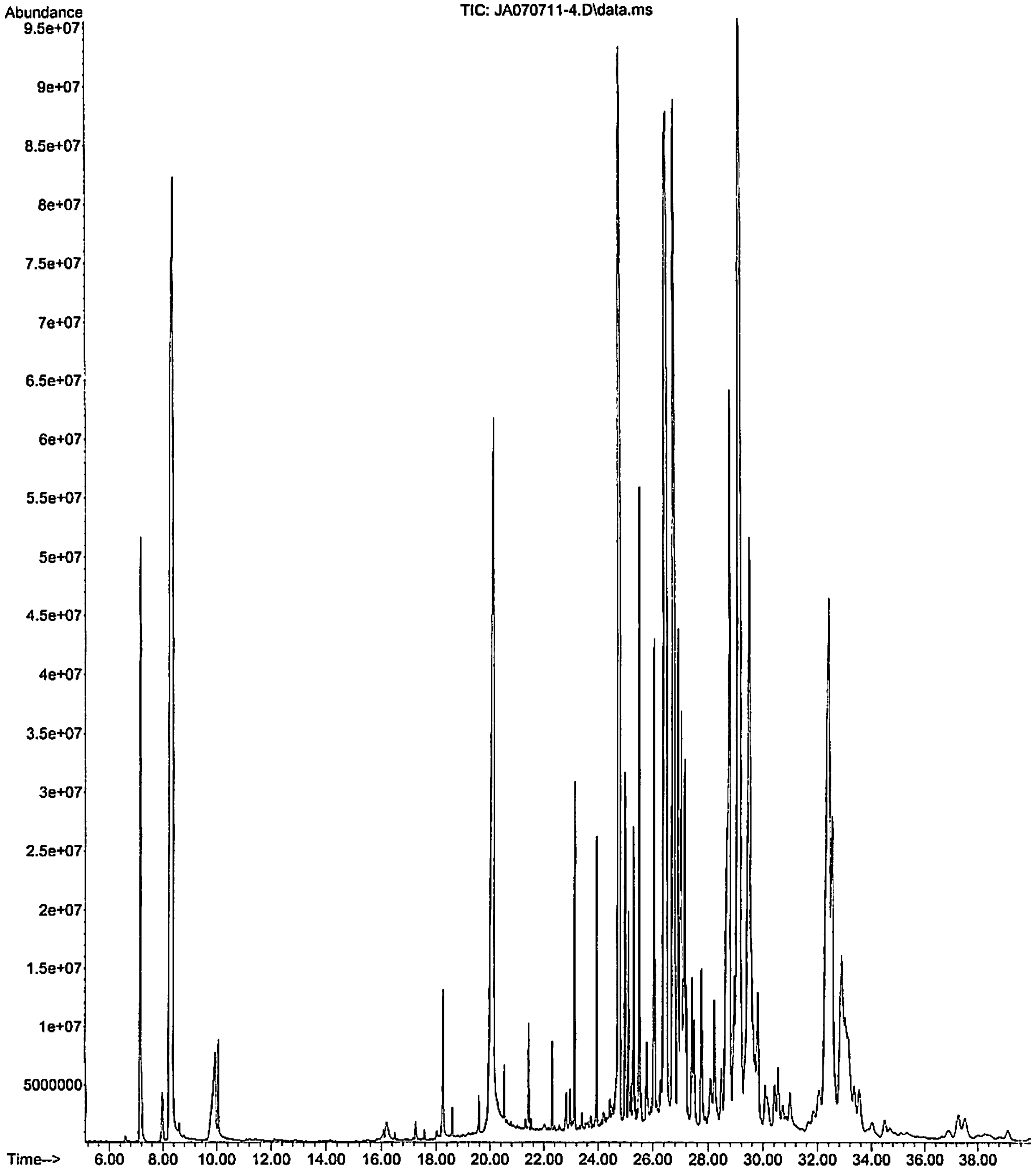

```
File       :D:\Aldrich\JA-11\JA070711-5.D
Operator   :
Acquired   :  7 Jul 2011  17:42     using AcqMethod JA-50-280LESS.M
Instrument :   Buba; IIBBL's magical mass spect
Sample Name: 3M C.oculata abd.stern./ca.10ul CH2Cl2
Misc Info  : larvae fed geranylPO4; [125ul]
Vial Number: 1
```

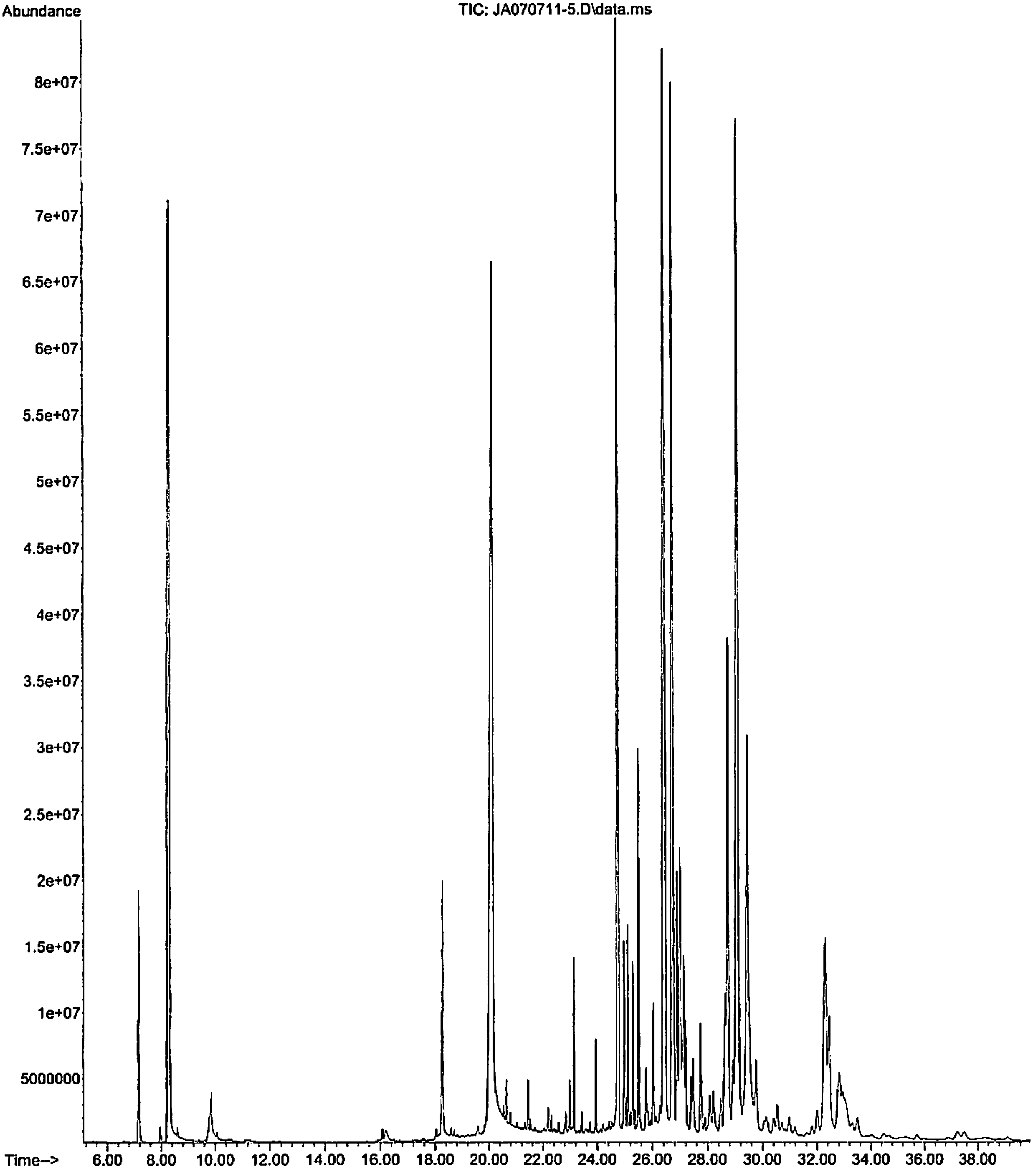

Supplement: Data S11 [file peerj-04-1564-s016.pdf]
